# Proteomics of extracellular vesicles in plasma reveals the characteristics and residual traces of COVID-19 patients without underlying diseases after 3 months of recovery

**DOI:** 10.1038/s41419-021-03816-3

**Published:** 2021-05-25

**Authors:** Kaimin Mao, Qi Tan, Yanling Ma, Sufei Wang, Hua Zhong, Yuhan Liao, Qi Huang, Wenjing Xiao, Hui Xia, Xueyun Tan, Ping Luo, Juanjuan Xu, Danling Long, Yang Jin

**Affiliations:** 1 Department of Respiratory and Critical Care Medicine, NHC Key Laboratory of Pulmonary Diseases, Union Hospital, Tongji Medical College, Huazhong University of Science and Technology, 1277 Jiefang Avenue, Wuhan, 430022 Hubei China; 2 Department of Critical Care Medicine, Renji Hospital, School of Medicine, Shanghai Jiaotong University, Shanghai, 200127 China; 3 College of Life Sciences, Wuhan University, Wuhan, Hubei province China 430072; 4 Center for Translational Medicine, Union Hospital, Tongji Medical College, Huazhong University of Science and Technology, Wuhan, Hubei China; 5 Department of stomatology, Taihe Hospital, Hubei University of Medicine, Shiyan, 442000 China

**Keywords:** Proteomics, Proteomics

## Abstract

More and more patients suffered from Coronavirus disease 2019 (COVID-19) have got recovery gradually due to suitable intervention. Increasing data mainly studies the clinical characteristics of recovered COVID-19 patients, and their molecular changes especially proteome changes also play the same important role in understanding of biological characteristics of recovered COVID-19 patients as clinical characteristics do. In our study, we reported the whole lung-ground glass-CT value-average of mild/severe recovered patients 3 months after discharge without underlying diseases was significantly lower than that of healthy subjects. Then we isolated the extracellular vesicles (EVs) of plasma from 19 healthy subjects and 67 recovered COVID-19 patients. Mass Spectrometry was used to catalogue the proteins of these EVs compared to a defined group of controls. Identified 174 proteins were differentially expressed in the EVs of COVID-19 patients compared with healthy subjects, which involved in lipid metabolic process, response to cellular, and response to stress oxygen-containing compound. Besides, we identified several protein of plasma EVs in recovered patients associated with coagulation activity, inflammatory reaction, immune response, and low organ function. In addition, proteins correlating with clinical index such as alkaline phosphatase (ALP) and alanine aminotransferase (ALT) were also detected. Moreover, we also identified many unique or characteristic associations found in the recovered COVID-19 patients, which especially involved the kidney, serum electrolyte levels, and inflammation functions. This finding suggests that monitoring the situation of recovered patients might be useful, especially the indexes of coagulation, inflammation, immunity, and organ function, which can prevent bleeding, reinfection and organ dysfunction.

## Introduction

Coronavirus disease 2019 (COVID-19) swept the world and did great damage, which was an emergent public health event to be solved^1^. Therefore, study on this disease has already become present research focus. COVID-19 has been extensively studied in its epidemiological and clinical characteristics, the molecular mechanism of virus infection, and antiviral therapy from the outbreak to the present^2,3^. Due to the prompt intervention, more and more patients have got recovery gradually. In another word, recovered patients would receive concern because their clinical characteristics and molecular changes are closely related to prognosis and recurrence risk.

Proteomics studies of COVID-19 began to research in the early stage of the epidemic situation, which has advanced our understanding of molecular biological features of this disease and has given some insights into and suggestions for the treatments of COVID-19. Yanchang Li et al. analyzed proteomic of urine samples from healthy control individuals and patients with COVID-19 and suggested the potential role of differential protein molecular characteristics between COVID-19 patients and healthy control individuals in the severity determination and treatment of COVID-19^4^. Denisa Bojkova et al. established infection model using human cells infected with a clinical isolate of SARS-CoV-2 and identified proteome proteomics changes at different times after infection, which revealed that the upregulation of cellular pathways such as translation, nucleic acid metabolism, carbon metabolism, and proteostasis in the SARS-CoV-2 infected cells. Targeting these pathways would be the potential therapies for the treatment of COVID-19^5^. Bo Shen et al. identified platelet degranulation in severe COVID-19 patients compared to non-severe patients through analyzing the proteomics of sera, suggesting the potential role of monitoring changes of platelets in the period of treatment^6^. However, there are few studies on proteomics of recovered COVID-19 patients.

Extracellular vesicles (EVs) act as communication signals between donor cells and recipient target cells^7,8^. Accumulating evidence indicates that EVs have applications as circulating biomarkers because they are widely distributed in various body fluids and easily isolated from these fluids^9,10^. Jia-Tao Zhang et al. analyzed transcriptomics of EVs from plasma of patients with solitary pulmonary nodules and revealed the potential role of EVs-miRNAs in prognostic factors in lung cancer^11^. Daniela Osti et al. identified that differentially expressed EV-associated proteins from the plasma of patients with Glioblastoma through analysis of proteomics of EVs, which was associated with complement, coagulation cascade and iron metabolism, suggesting that these differential proteins would be the potential biomarkers for Glioblastoma^12^. Capello et al.^13^ applied proteomic technology to investigate profiling of pancreatic ductal adenocarcinoma cell-derived exosomes and identified the evidence that exosomes derived from tumor cells present repertoire of tumor antigen to induce autoantibodies. Recently, the elevation of GM3-enriched exosomes was observed in the sera of serve COVID-19 patients, suggesting that it was related to the pathogenesis and pathological process of COVID-19^14^. In a word, study on the molecular characteristics of exosomes isolated from plasma of recovered COVID-19 patients may have important clinical value.

In our study, we isolated EVs from plasma samples of 19 healthy subjects and 67 recovered COVID-19 patients without underlying disease, then analyzed and compared their proteome properties to study the difference between asymptomatic, moderate, and severe and critical COVID-19 patients discharged for 3 months, which helps to further the understanding of biological states of recovered COVID-19 patients.

## Materials and methods

### Subjects and study design

All participants were from the Union Hospital of Huazhong University of Science and Technology and Wuhan Lung Hospital (Wuhan, China). Finally, we collected a total of 86 plasma samples from 19 healthy people (C), 19 recovered asymptomatic patients (A), 24 recovered mild patients (M/RM) and 24 recovered severe patients (S/RS). All patients were diagnosed according to COVID-19 clinical guidelines (New Coronavirus Pneumonia Prevention and Control Program (the 7th edition)). Our selected mild and severe subjects were tested for proteomics three months after discharge. According to the COVID-19 clinical guidelines, only those with fever and respiratory symptoms and with possibly characteristic imaging findings of pneumonia are mild patients, if (1) resting respiration rate (RR) ≥ 30/min; (2) resting blood oxygen saturation <93%; (3) PaO_2_/FiO_2_ ≤ 300 mmHg; (4) More than 50% aggravation of imaging signs within 1–2 days; (5) Shock, respiratory failure or other organ/system failure and ICU needs are regarded as severe patients. Healthy controls and asymptomatic infections came from outpatient physical examination reports.

### Plasma collection and isolation of EVs

All the selected subjects strictly fasted for 12 h to limit their fluid intake, and prohibited any drugs within 48 h before the collection of plasma samples. In the early morning, 10 ml of venous blood was collected with a sodium heparin anticoagulation tube, centrifuged at 4 °C (2000rpm, 10 min) to obtain plasma, and all plasma samples were stored in a refrigerator at −80 °C until analysis. We use the Life Technologies Corporation kit to process the specimens and extract exosomes (Catalog Number: 4484450). Take the plasma specimens from the −80 °C refrigerator and place them in a water bath at 25 °C to 37 °C to melt them until the specimens are completely liquid, and then immediately put them on ice until needed. Centrifuge the plasma sample (2000 × *g*, 20 min) at room temperature to remove cells and debris. The supernatant was then transferred to a new tube and placed on ice until the separation of EVs.

Transfer the required volume of plasma supernatant to a new tube, add 0.5 volume of 1 × PBS, and mix the sample thoroughly. Subsequently, 0.2 volume of EVs precipitation reagent was added to the sample and mixed thoroughly. The sample was incubated at room temperature for 10 min, and then centrifuged at room temperature (10,000 × *g*, 5 min) to obtain the supernatant and precipitate. Aspirate the supernatant with a pipette and discard, and then centrifuge the tube at room temperature (10,000 × g, 30 seconds) again. Then use a pipette to aspirate and discard the remaining supernatant, and hereafter add 1 × PBS to resuspend the EVs for subsequent analysis.

### Electron microscopy imaging

Drop 3 μl of the resuspended sample onto the discharge formvar-carbon coated grid, and incubate on ice for 5 min until analysis by transmission electron microscopy (TEM). After the filter paper absorbs the excess liquid, the grid is negatively stained with 10 μl of 2% phosphotungstic acid (Servicebio, Woburn, MA, USA). Use filter paper to absorb the excess dye solution and wash off the excess dye solution with a drop of double-distilled water. The grid was then air-dried in a dark environment at room temperature. After completing the above steps, analyze the sample using the HT 7700 transmission electron microscope (Hitachi, Tokyo, Japan) at an acceleration voltage of 80 kV.

### Nanoparticle tracking analysis of EVs

The resuspended samples were analyzed for the purity, concentration, and particle size of EVs using the Nanosight NS300 nanoparticle tracking analysis (NTA) function (NanoSight Ltd., Minton Park, UK). The PBS solution used to dilute the sample has previously been ultracentrifuged to remove the noise of the nanoparticles, thereby improving the accuracy of the analysis. At room temperature, control camera level of 14, visibility 0.91 cP, frames per second 25, measurement time 60 s and detection threshold 6. Then use NanoSight NTA version 3.2 software to analyze the data.

### Western blotting

The EVs pellet sample was lysed with RIPA buffer and centrifuged at 4 °C (11,000 × *g*, 15 min) to obtain the supernatant for western blot analysis. Transfer the supernatant to a new tube and perform quantitative analysis according to the bicinchoninic acid (BCA) Protein Assay kit (Beyotime, Shanghai, China) procedure. The samples were separated by electrophoresis and transferred to PVDF membrane (Millipore, Bedford, MA, USA). The PVDF membrane was blocked with 5% skimmed milk powder at 4 °C overnight, and then the primary antibody was added. Primary antibodies include CD9 (Abcam, ab92726, 1:500), CD81 (Abcam, ab109201, 1:1000), CD63 (Abcam, ab134045, 1: 1000), Tsg101 (Abcam, ab125011, 1:2000), GM130 (Abcam, ab52649, 1:500) and Calnexin (Abcam, ab92573, 1:1000). After washing thoroughly with PBS buffer, the PVDF membrane was incubated with the secondary antibody at room temperature for 1 h. Then washed three times and use ECL chemiluminescence staining assay kit (Bio-Rad) to display protein bands and quantitatively analyze protein concentration.

### Total protein extraction

The samples were suspended in protein lysis buffer (8 M urea, 1% SDS) which included an appropriate protease inhibitor to inhibit protease activity, then the mixture and incubated on ice for 30 min. After centrifugation at 12,000 × *g* at 4 °C for 30 min, the concentration of protein supernatant was determined by BCA method by BCA Protein Assay Kit (Thermo Scientific). Protein quantification was performed according to the kit protocol.

### Protein digestion

100 μg proteins re-suspended with triethylammonium bicarbonate buffer (TEAB) and the final concentration of it was 100 mM. The mixture was reduced with tris (2-carboxyethyl) phosphine (TCEP) at 37 °C for 60 min and the final concentration was 10 mM. Then the mixture was alkylated with iodoacetamide (IAM) at room temperature for 40 min in the darkness whose final concentration was 40 mM. After centrifugation at 4 °C for 20 min, the proteins were collected, which re-suspended with 100 μl TEAB which with the final concentration of 100 mM. Trypsin was added at 1:50 trypsin-to-protein mass ratio and incubated at 37 °C overnight.

### High pH RP-UPLC separation

Take an amount of the trypsin-digested peptides of each sample pool and vacuum dried, then re-suspended with UPLC loading buffer (Phase A: 5 mM Ammonium hydroxide solution containing 2% acetonitrile, pH 10). The mixed peptides were fractionated into fractions by Vanquish Flex binary UHPLC chromatography (Thermo, USA) with ACQUITY UPLC BEH C18 Column (1.7 μm, 2.1 mm × 150 mm, Waters, USA) to increase sequencing depth. Briefly, peptides were first separated with a gradient of elution (Phase B: 5 mM Ammonium hydroxide solution containing 80% acetonitrile, pH 10) over 47 min at a flowrate of 200 μl/min. The elution gradient: 0–16 min, 0%–0% B; 16–17 min, 0%–3.8% B; 17–34 min, 3.8–24% B; 34–37 min, 24%–30% B; 37–38 min, 30%–43% B; 38–39 min, 43%–100%B; 39–44 min, 100%–0% B; 44–47 min, maintain 0% B. Forty fractions were collected from the mixed sample, which was subsequently pooled.

### Liquid chromatography-mass spectrometry analysis

The peptides were redissolved in spectrometry loading buffer (2% ACN with 0.1% formic acid) which included appropriate iRT peptide which was used to calibrate retention time and analyzed by online nano flow liquid chromatography-tandem mass spectrometry performed on an EASY-nLC system (Thermo, USA) connected to a Q Exactive HF-X quadrupole orbitrap mass spectrometer (Thermo, USA) through a nanoelectrospray ion source. Briefly, the C18-reversed-phase column (75 μm × 25 cm, Thermo, USA) as equilibrated with solvent A (A:2% ACN with 0.1% formic acid) and solvent B (B: 80% ACN with 0.1% formic acid). The peptides were eluted using the following gradient: 0–70 min, 5%–23% B; 70–90 min, 23%–29% B; 90–100 min, 29%–38% B; 100–102 min,38%–48% B; 102–103 min, 48%–100% B; 103–110 min, maintain100% B, 110–120 min, 100%–0% B. The tryptic peptides were separated at a flow rate of 300 nL/min.

The Q Exactive HF-X instrument was operated in the data-independent acquisition mode (DIA) to automatically switch between full scan MS and MS/MS acquisition. The survey of full scan MS spectra (*m/z* 350–1500) was acquired in the Orbitrap with 60,000 resolution. Then all precursor ions were selected into collision cell for fragmentation by higher-energy collision dissociation (HCD), which was 28. The MS/MS resolution was set at 30,000, the automatic gain control (AGC) target at 1e4, the maximum fill time at automatic, DIA was performed with a variable isolation window, each window overlapped by 1 *m/z*, there were 40 windows in total.

### Protein identification and quantification

The MS/MS search criteria were as follows: Mass tolerance of 10 ppm for MS and 0.05 Da for MS/MS Tolorance, trypsin as the enzyme with two missed cleavage allowed, carbamido methylation of cysteine as fixed modification, and oxidation and Acetyl as dynamic modifications, respectively. High confidence peptides were used for protein identifications by setting a target false discovery rate (FDR) threshold of 1% at the peptide level. Only the proteins which has at least one unique peptide were used for protein identifications.

The quality of proteomic data was also ensured. A mixture of all peptide samples was generated by taking a small volume of each sample to serve as a technical replicate that was run multiple times throughout the experiment. The DIA data files were analyzed using Spectronaut (Biognosys AG, Version 14) with the default settings, and the retention time prediction type was set to dynamic iRT. Spectronaut determined the ideal extraction window dynamically on the basis of iRT calibration and gradient stability. A *Q*-value (FDR) cutoff of 1% was applied at the precursor and protein levels. Quantification was performed using the intensities of the six most intense peptides. In this study. The thresholds of fold change > 1.2 and *P*-value < 0.05 were used to identify differentially expressed proteins (DEPs).

### Functional enrichment analysis

The DEPs were further assigned to the GO (gene ontology) annotations using Blast2GO software, where the proteins were divided into biological process (BP), cellular component (CC), and molecular function (MF) three main categories. Pathway enrichment analysis was conducted using the KEGG (Kyoto Encyclopedia of genes and genomes) database (http://www.genome.jp/kegg/pathway.html). Differences were considered to be statistically significant as *p*-value < 0.05.

### Statistical analysis

A student’s *T*-test was used to compare metabolite or gene expression between two distinct treatments. Spearman correlation analyses among the clinical parameters and differential proteins were implemented using R language, and its networks were displayed by Cytoscape software (version 3.6.). The heatmap of differential protein alterations were generated by ‘pheatmap’ R package. The correlation plots of plasma lipids with clinical Indices were implemented by ‘corrplot’ R package. Differences were considered to be statistically significant as *p*-value < 0.05.

## Results

### Flow chart and clinical characteristics of COVID-19 patients without underlying diseases after 3 months of recovery

**Fig. 1:**
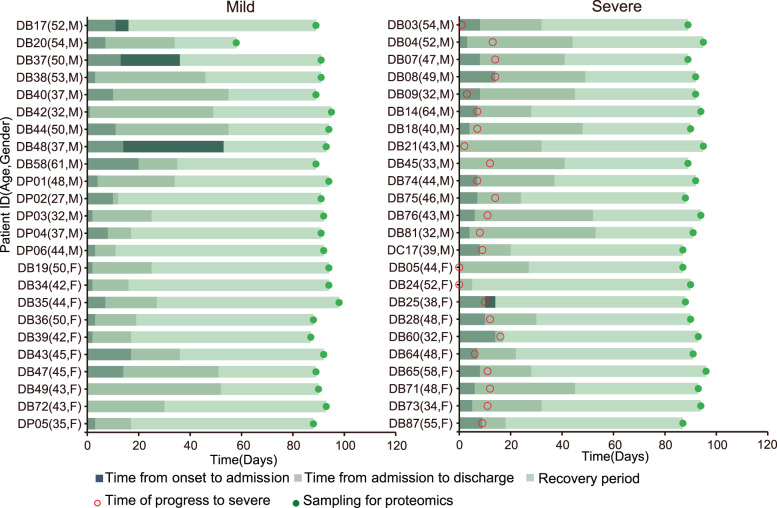
Summary of COVID-19 patients, including mild (*n* = 24) and severe (*n* = 24) patients with more details in Table 1 and Fig. S1. The 0 point on the *X* axis represents the time point of the onset of the patient, and the *Y* axis contains the patient number, age and gender.

Figure S1 showed the conditions for the selection of patients in the recovery period of COVID-19 and healthy controls, and then Fig. 1 specifically implicated the specific time for the collection of samples for the mild and severe recovery patients of COVID-19. Through statistical analysis of the basic demographic information, laboratory test results and CT test data results of 86 samples, we listed the relevant data of all samples in Table 1. We found that the inflammation-related indicators of AM (admission mild) and AS (admission severe) were significantly higher than C (control), such as CRP (C-reactive protein). Compared with AM, the number of lymphocytes in AS group decreased. And AS monocytes, eosinophils and basophils were all decreased compared with C. In liver and kidney function tests, AM and AS had abnormal changes in AST (Aspartate aminotransferase), ALT, LDH, Uric acid, and other indicators compared to C. In addition, the α-HBDH of AM was significantly higher than that of C and AS. In our samples, the coagulation function of AM and AS did not differ from that of C or RA(recovered asymptomatic). Subsequently, in lung imaging, the whole lung-ground glass-CT value-average of RM (recovered mild) and RS (recovered severe) was significantly lower than that of C and RA.

**Table 1:** Clinical and laboratory findings of controls and COVID-19 patients.

Items		C	A	Mild patients		Severe patients	
				At admission	RM/M	At admission	RS/S
*Demographics features*							
Age (year)		45.00 (38.00, 54.00)	46.00 (38.00, 54.00)	44.00 (37.00, 50.00)	44.00 (37.00, 50.00)	46.00 (39.00, 52.00)	45.00 (38.25, 51.25)
Gender							
Female, *n* (%)		8 (42.11)	8 (42.11)	10 (41.67)	10 (41.67)	10 (41.67)	10 (41.67)
Male, *n* (%)		11 (57.89)	11 (57.89)	14 (58.33)	14 (58.33)	14 (58.33)	14 (58.33)
BMI (kg/m^2^)		23.60 (21.67, 25.60)	24.44 (22.96, 25.50)	22.22 (20.99, 24.06)	22.22 (20.99, 24.06)	23.60 (22.15, 25.21)	23.57 (22.11, 25.11)
*Antibody test for SARS-CoV-2 nucleic acid*							
IgG (+), *n* (%)		0 (100)	17 (89.47)	–	18 (75.00)	–	23 (95.83)
IgM (+), *n* (%)		0 (100)	2 (10.53)	–	9 (37.50)	–	4 (16.67)
*Blood routine*							
hsCPR (mg/L)		0.72 (0.43, 2.51)	0.62 (0.08, 1.29)	3.01 (0.18, 4.66)*	0.43 (0.08, 1.25)**	11.75 (2.16, 26.71)*^,†^	0.69 (0.37, 1.36)***
WBC (×10^9^/L)		5.31 (4.79, 6.17)	5.21 (4.78, 5.72)	5.89 (4.15, 7.82)	4.89 (4.20, 6.10)	5.31 (3.79, 6.81)	4.97 (3.99, 6.01)
RBC (×10^12^/L)		4.87 (4.67, 5.25)	4.80 (4.37, 5.34)	4.38 (4.09, 4.75)*	4.81 (4.54, 5.13)**	4.53 (3.75, 4.87)*	4.92 (4.35, 5.31)***
Haemoglobin (g/L)		155.00 (143.00,162.00)	150.00 (132.00, 162.00)	133.50 (123.25, 145.75)*	152.00 (140.25, 158.75)**	136.00 (116.00, 149.00)*	152.00 (135.75, 165.00)***
HCT (L/L)		45.30 (42.10, 48.50)	44.40 (41.10, 47.80)	39.70 (37.73, 42.85)*	45.25 (42.23, 47.25)**	40.40 (34.80, 44.30)*	45.75 (41.50, 49.18)***
MCV (fl)		93 (89.7, 95.7)	93.40 (89.70, 96.20)	91.55 (89.18, 93.90)	92.45 (89.28, 95.90)	91.80 (90.00, 93.60)	92.40 (90.20, 93.65)
MCH (pg)		31 (30.1, 32.7)	31.40 (29.80, 33.00)	30.35 (29.83, 31.70)	30.80 (30.05,31.58)	30.70 (30.10, 31.30)	30.90 (30.25, 31.30)
MCHC (g/L)		336 (332, 343)	340.00 (333.00, 345.00)	332.00 (326.25, 336.00)*	335.50 (328.25, 338.75)	335.00 (331.00, 338.00)	334.00 (330.00, 338.75)
Platelets (×10^9^/L)		224 (185, 264)	193.00 (181.00, 221.00)	201.00 (169.50, 259.25)	211.50 (174.00, 236.75)	228.00 (165.00, 260.00)	212.00 (182.25, 228.00)
Neutrophils (%)		56 (52.3, 62.9)	65.10 (53.90, 67.90)	55.35 (49.53, 64.05)	55.00 (50.53, 63.70)	62.20 (55.10, 72.00)	59.55 (51.73, 63.78)
Lymphocytes (%)		34.3 (26.6, 39.3)	29.10 (25.90, 38.80)	35.45 (26.28, 39.28)	35.80 (29.75, 40.98)	26.60 (20.60, 33.30)	32.65 (27.40, 38.95)
Monocytes (%)		4.6 (4.4, 5.1)	5.50 (4.50, 6.10)	7.00 (6.10, 8.78)*	5.30 (4.80, 6.75)*^,^**	7.80 (4.50, 11.10)	5.05 (4.43, 6.13)
Eosinophils (%)		2.5 (0.9, 3.9)	1.30 (0.90, 1.90)	1.80 (0.85, 3.45)	2.05 (1.23, 3.55)	0.70 (0.00, 1.90)*	2.10 (1.30, 3.40)***
Basophils (%)		0.1 (0.1, 0.3)	0.10 (0.10, 0.30)	0.30 (0.13, 0.50)*	0.20 (0.10, 0.30)**	0.30 (0.20, 0.40)*	0.20 (0.00, 0.30)
Neutrophils (×10^9^/L)		3.15 (2.7, 3.38)	3.28 (2.69, 3.56)	3.22 (2.17, 4.68)	2.70 (2.02, 3.55)	3.31 (2.12,4.57)	2.99 (2.42, 3.30)
Lymphocytes (×10^9^/L)		1.85 (1.45, 2.48)	1.48 (1.26, 2.02)	1.62 (1.39, 2.15)	1.73 (1.42, 2.06)	1.24 (0.91,1.56)^†^	1.51(1.35, 1.73)***
Monocytes (×10^9^/L)		0.26 (0.21, 0.30)	0.29 (0.21, 0.31)	0.39 (0.32, 0.58)	0.31 (0.22, 0.35)	0.39 (0.29, 0.43)*	0.27 (0.18, 0.32)***
Eosinophils (×10^9^/L)		0.13 (0.06, 0.21)	0.07 (0.04, 0.10)	0.10 (0.05, 0.29)	0.10 (0.06, 0.18)	0.04 (0.01, 0.10)*^,†^	0.10 (0.06, 0.16)***
Basophils (×10^9^/L)		0.01 (0.00, 0.01)	0.01 (0.00, 0.01)	0.02 (0.01, 0.03)*	0.01 (0.00, 0.02)**	0.01 (0.01, 0.03)*	0.01 (0.00, 0.01)***
RDW		12.3 (11.9, 12.9)	12.20 (12.00, 12.70)	12.70 (12.30, 13.28)*	12.50 (11.95, 12.90)	12.30 (11.80, 12.60)^†^	12.50 (11.92, 12.70)
PDW		16.1 (15.9, 16.3)	16.20 (15.90, 16.40)	16.10 (15.80, 16.30)	16.15 (15.83, 16.30)	16.30 (16.00, 16.50)^†^	16.10 (15.83, 16.30)
MPV (fl)		9.6 (9.00, 10.20)	10.10 (9.30, 11.20)	9.60 (8.93, 10.90)	9.60 (9.05, 10.45)	9.90 (9.00, 10.40)	9.75 (9.15, 10.55)
PCT		0.21 (0.19, 0.24)	0.20 (0.18, 0.22)	0.21 (0.17, 0.22)	0.21 (0.18, 0.23)	0.21 (0.16, 0.26)	0.19 (0.18, 0.23)
*Liver and kidney functions*							
AST (U/L)		22.00 (20.00, 26.00)	25.00 (22.00, 30.00)*	26.50 (21.30, 39.25)	19.50 (15.75, 26.25)**	24.00 (21.00, 42.00)	23.00 (20.25, 29.00)
ALT (U/L)		21.00 (16.00, 31.00)	26.00 (17.00, 55.00)	34.50 (26.25, 65.00)*	24.00 (16.50, 33.50)**	33.00 (14.00, 52.00)	22.00 (18.25, 45.75)
AST/ALT		1.00 (0.78, 1.25)	1.04 (0.55, 1.38)	0.63 (0.41, 0.98)*	0.78 (0.69, 1.08)*	0.90 (0.67, 1.27)^†^	1.00 (0.68, 1.17)
ALP (U/L)		68.00 (56.00, 86.00)	54.00 (48.00, 75.00)	50.00 (42.25, 67.75)	57.00 (44.50, 68.75)	52.00 (44.00, 74.00)*	66.00 (51.25, 72.75)
LDH (U/L)		184.00 (150.00, 205.00)	183.00 (168.00, 205.00)	182.50 (154.50, 224.75)*	181.50 (157.75, 200.50)	230.00 (188.00, 283.00)*^,^^†^	179.00 (166.00, 203.25)***
GGT (U/L)		20.00 (13.00, 25.00)	23.00 (17.00, 61.00)	27.00 (14.50, 47.75)	19.50 (13.00, 35.50)	27.00 (19.00, 42.00)	20.00 (13.75, 29.50)
TBil (μmol/L)		12.30 (10.50, 18.70)	14.70 (13.10, 18.20)	9.30 (6.58, 10.45)	12.25 (9.43, 15.08)	9.10 (7.70, 11.50)*	13.90 (10.78, 16.45)***
DBil (μmol/L)		2.90 (2.30, 4.80)	3.50 (2.80, 4.60)	2.35 (1.73, 3.28)*	3.20 (2.63, 4.28)	3.00 (2.40, 3.70)^†^	3.25 (2.83, 4.35)
IBil (μmol/L)		9.80 (7.90, 13.90)	11.60 (9.90, 13.80)	6.35 (5.10, 7.90)	8.90 (6.50, 10.73)**	6.80 (5.30, 8.50)*	10.15 (7.85, 12.75)***
Total protein (g/L)		76.50 (73.70, 79.70)	78.40 (77.00, 81.70)	67.00 (64.65, 71.08)*	75.20 (72.95, 79.03)**	64.50 (58.20, 67.90)*^,†^	76.30 (73.45, 78.73)***
Albumin (g/L)		47.60 (45.30, 48.30)	47.80 (45.20, 48.90)	41.20 (36.95, 43.00)	45.85 (44.23, 49.30)	33.70 (29.10, 36.60)*^,†^	47.65 (45.43, 50.10)***
Globin (g/L)		29.50 (26.80, 31.40)	31.00 (29.60, 34.00)	28.85 (26.15, 31.40)	29.35 (26.88, 31.78)	29.60 (27.10, 32.50)	28.50 (27.33, 31.13)
Albumin/Globin		1.60 (1.50, 1.70)	1.50 (1.40, 1.60)	1.40 (1.25, 1.70)*	1.60 (1.43, 1.78)**	1.10 (1.00, 1.30)*^,†^	1.70 (1.60, 1.78)***
BUN (mmol/L)		5.28 (4.88, 5.79)	4.69 (4.22, 5.34)	4.20 (3.27, 5.16)	4.23 (3.88, 4.75)*	3.62 (3.02, 4.37)*	4.73 (4.05, 5.69)***
Creatinine (μmol/L)		71.80 (64.30, 76.90)	74.30 (57.90, 80.70)	64.50 (52.80, 72.15)	66.40 (56.58, 79.25)	71.80 (55.10, 86.10)	62.55 (56.70, 77.83)
Uric acid (μmol/L)		335.10 (271.60, 386.10)	377.90 (256.00, 451.60)	300.40 (231.23, 341.93)	322.10 (247.10, 412.78)	229.20 (204.80, 311.80)*	372.80 (306.18, 452.13)***
Glucose (mmol/L)		5.15 (4.98, 5.29)	5.19 (5.02, 5.61)	5.25 (4.96, 5.56)	5.02 (4.82, 5.41)	5.69 (5.16, 6.35)*	5.23 (4.73, 5.62)***
Mg (mmol/L)		0.89 (0.87, 0.94)	0.92 (0.89, 0.95)	0.88 (0.84, 0.92)	0.88 (0.83, 0.90)*	0.84 (0.76, 0.89)	0.87 (0.83, 0.89)
P (mmol/L)		0.92 (0.77, 1.04)	0.97 (0.86, 1.08)	1.22 (1.02, 1.34)*	0.94 (0.85, 1.07)**	1.05 (0.92, 1.27)*	0.94 (0.82, 1.11)
Ca (mmol/L)		2.18 (2.14, 2.26)	2.18 (2.14, 2.30)	2.16 (2.06, 2.21)	2.23 (2.15, 2.33)	1.99 (1.93, 2.04)*^,†^	2.21 (2.16, 2.26)***
Creatinine kinase (U/L)		98.00 (81.00, 167.00)	96.00 (80.00, 144.00)	76.00 (45.00, 141.75)*	101.50 (114.25, 140.75)	69.00 (52.00, 94.00)*	108.50 (83.50, 130.50)***
CK-MB activity (U/L)		13.00 (11.00, 16.00)	13.00 (10.00, 15.00)	10.50 (9.00, 15.75)	9.50 (8.25, 12.00)	11.00 (8.00, 16.00)	11.00 (8.00, 13.00)
α-HBDH (U/L)		145.00 (120.00, 170.00)	145.00 (129.00, 156.00)	137.00 (118.75, 156.75)	133.50 (114.25, 140.75)	191.00 (153.00, 299.00)*^,^^†^	141.00 (126.75, 160.75)***
CO_2_ (mmol/L)		24.30 (22.90, 26.80)	24.50 (22.80, 27.30)	27.60 (25.83, 29.50)*	24.65 (23.73, 26.60)**	26.10 (22.70, 29.00)	25.95 (24.03, 27.18)
*Coagulation function*							
PT (s)		11.70 (11.50, 12.10)	11.80 (11.60, 12.70)	12.70 (12.40,13.20)	12.15 (11.63, 12.93)	13.00 (12.40, 13.90)	12.15 (11.60, 12.95)***
INR		0.87 (0.84, 0.91)	0.88 (0.86, 0.97)	0.97 (0.94, 1.02)	0.92 (0.88, 1.01)	1.01 (0.94, 1.10)	0.92 (0.86, 1.00)***
APTT (s)		35.60 (33.70, 37.80)	35.50 (33.50, 39.50)	37.75 (35.18, 40.58)	36.95 (34.13, 39.33)	35.40 (33.70, 37.80)	38.25 (36.33, 39.15)*^,^***
FIB (g/L)		3.04 (2.82, 3.27)	3.02 (2.82, 3.29)	3.26 (3.05, 3.98)	2.83 (2.69, 3.35)	3.68 (3.15, 4.58)	2.96 (2.75,3.20)
TT (s)		16.10 (15.50, 16.20)	16.10 (15.50, 16.40)	15.75 (15.15, 16.68)	16.20 (15.93, 16.80)	15.00 (14.60, 15.70)	16.20 (15.73, 16.85)***
CT value							
Whole lung-lesion-CT value-mean		0.00 (−408.91, 0.00)	0.00 (−578.23, 0.00)	–	−257.37 (−534.04, 0.00)	–	−278.10(−650.52, 0.00)
Whole lung-ground glass-CT value-average		0.00 (−432.88, 0.00)	0.00 (−654.33, 0.00)	–	−506.37 (−606.51, 0.00)*	–	−424.16 (−688.77, 0.00)*
Whole lung-consolidation shadow-CT value-mean		0.00 (−59.48, 0.00)	0.00 (−11.02, 0.00)	–	−1.88 (−101.058, 0.00)	–	−36.39 (−94.10, 0.00)

Data were shown as median (interquartile range, IQR) or *n* (%).

Categorical variables were compared between two groups by using *χ*^2^ test.

Comparisons were made in terms of continuous variables between two groups by using *t* test for variables with a normal distribution and Mann–Whitney *U* test for variables with a non-normal distribution.

*C* control, *A* recovered asymptomatic, *M* mild, *S* severe, *RM* recovered mild, *RS* recovered severe, *BMI* body mass index, *CPR* C-reactive protein, *WBC* white blood cell, *RBC* red blood cell, *HCT* hematocrit, *MCV* mean corpuscular volume, *MCH* mean corpuscular hemoglobin, *MCHC* mean corpuscular hemoglobin concentration, *RDW* red cell volume distribution width, *PDW* platelet distribution width, *MPV* mean platelet volume, *PCT* plateletcrit, *AST* aspartate aminotransferase, *ALT* alanine aminotransferase, *ALP* alkaline phosphatase, *LDH* lactate dehydrogenase, *GGT* γ-glutamyl transpeptidase, *TBil* total bilirubin, *DBil* direct bilirubin, *IBil* indirect bilirubin, *BUN* blood urea nitrogen, *Mg* magnesium, *P* phosphorus, *Ca* calcium, *CK-MB* creatine kinase–myocardial band, *α-HBDH* α-hydroxybutyrate dehydrogenase, *PT* prothrombin time, *INR* international normalized ratio, *APTT* activated partial thromboplastin time, *FIB* fibrinogen, *TT* thrombin time, *CT* computed tomography.

*P < 0.05, C versus M or S or A or RM or RS; **P < 0.05, M versus RM; ***P < 0.05, S versus RS; ^†^*P* < 0.05, M vs S.

### Characterization and identification of EVs

**Fig. 2:**
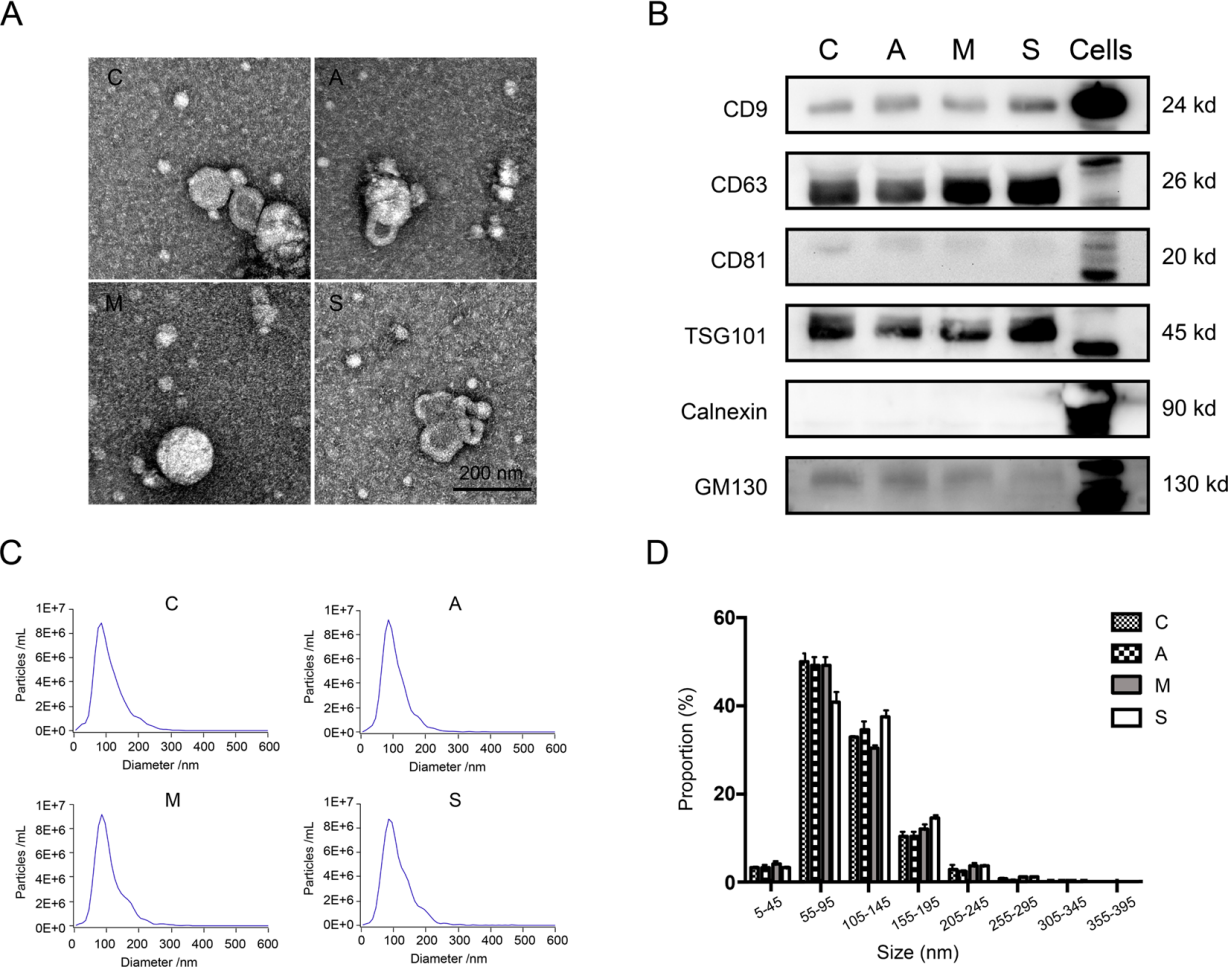
Verification of EVs sample quality. **A** The typical circular morphology of EVs was observed under electron microscope (EM) from groups C, A, M, and S. **B** Western blot was used to analyze the EV marker proteins CD9, CD64 and CD81, TSG101, non-EV marker Calnexin and GM130 in groups C, A, M, S, and Cells. Each well was loaded with 20 μg protein. **C**, **D** The size distribution curve and distribution proportion of EVs in plasma of C, A, M, and S were shown by NTA analysis. The diameter of EVs in these four groups was mostly concentrated in the interval of 55–145 nm, and the data were shown with mean + SD. There was no significant difference in the proportion of groups C, A, M, and S in each distribution interval (*P* > 0.05).

In the results of TEM, we observed the typical round morphology of EVs, and their size was <200 nm (Fig. 2A). The results of NTA analysis showed that the diameters of EVs separated from the four groups of C, A, M, and S samples were mostly distributed around 100 nm, and they were all in the 50–150 nm interval (Fig. 2C). TEM and NTA results showed that EVs from plasma samples of COVID-19 patients were isolated successfully. In addition, through NTA statistical analysis, there was no significant differences were found in the size of isolated EVs among C, A, M, and S groups (Fig. 2D). Next, we analyzed the four groups membrane proteins CD9, CD63, CD81, TSG101, Calnexin and GM130 by Western blotting. The results showed that the membrane proteins of EVs obtained from these four groups of samples were not significantly different (Fig. 2B). The above results verified that we separated EVs from C, A, M, and S plasma samples through rigorous and scientifically detailed experimental methods.

### Proteomic profiling of plasma EVs in COVID-19 and non-COVID-19 patients

The quantitative proteomic approach was implemented to obtain insights into the molecular differences between the recovered patients (A, M, and S) and healthy control (C). In total, 328763 spectra were generated. Then, 394 proteins were identified in 86 specimens based on the spectral data (Table. S1).

**Fig. 3:**
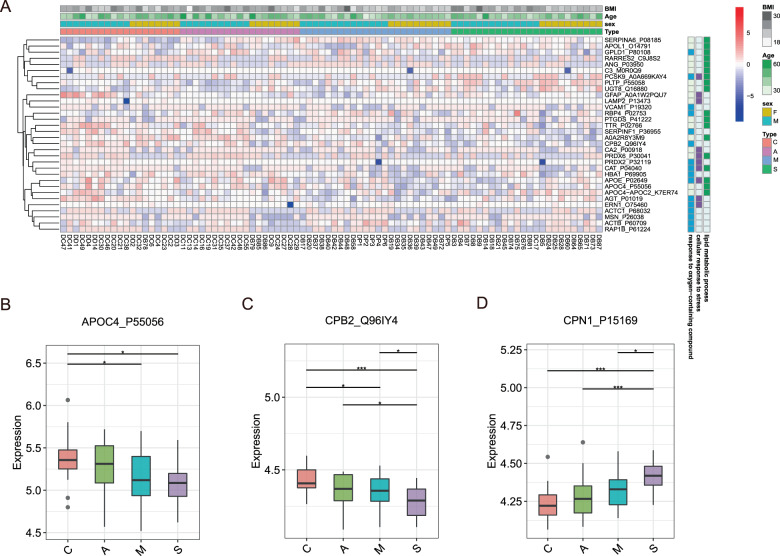
Dysregulated proteins in recovered COVID-19 patients. **A** Heatmap of selected proteins whose regulation concentrated on three enriched pathways. The expression level change of APOL1_O14791 (**B**), APOC4-APOC2_K7ER74 (**C**), and CPN1_P15168 (**D**) with significant difference between non-severe and severe cases. Asterisks indicate statistical significance based on *t* test. *P*-value: *<0.05; ***<0.001.

We found that 174 proteins were differentially expressed in the EVs of COVID-19 patients, but not in the non-COVID-19 patients. Of these, 32 proteins were found belonged to three major GO functional enrichments, lipid metabolic process, response to cellular, and response to stress oxygen-containing compound (Fig. 3A). Activated acute phase proteins (APPs) are involved in the early stages of immune responses to virus infection. Multiple apolipoproteins are associated with macrophage functions and were downregulated. Thus, we detected the expression profiles of APPs and apolipoproteins. Our data manifested that APOC4_P55056 and CPB2_Q96IY4 were significantly downregulated while CPN1_P15169 was distinctly more upregulated in the EVs of the severe COVID-19 patients than the control group (Fig. 3B–D). As a major contributor to acute phase response, the complement system plays a crucial role in eliminating invading pathogens in the early stage of infection. Carboxypeptidase N catalytic chain (CPN1) is a regulator of the complement system.

**Fig. 4:**
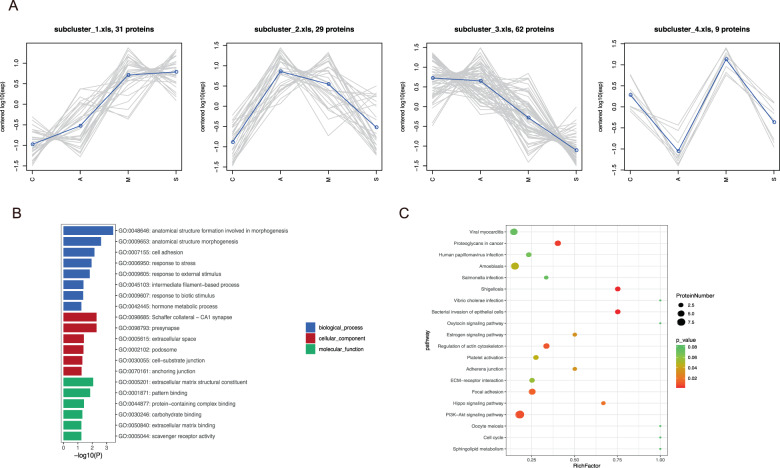
Identification of proteins in the first four specific clusters. **A** The first four subcluster 1–4 of proteins. Top 20 terms of GO functional (**B**) and KEGG pathway (**C**) enrichment analyses for 31 proteins in subcluster 1.

After correlating their expression with clinical disease severity, 174 proteins were clustered into eight significant discrete subclusters to illustrate the relative expression changes of the proteomics data (Fig. 4A and Fig. S2A). 31 proteins in subcluster 1 showed a specific upregulated pattern in COVID-19 patients compared to healthy control (Fig. 4A). Then, we performed KEGG pathway and GO terms enrichment analyses of these 31 DEPs. The results showed that the top 20 significantly GO terms were enriched in response to external stimulus (BP), Schaffer collateral−CA1 synapse (CC), and extracellular matrix structural constituent (MF) (Fig. 3B). Correspondingly, top 20 KEGG pathways were mainly involved in PI3K–Akt signaling pathway, Focal adhesion, etc (Fig. 4C). In addition, modulation in subcluster3 was reduced as the disease progresses (Fig. 4A). Functional enrichment and pathway analyses of the 62 DEPs implicated that these proteins were associated with the immune effector process (GO) and complement and coagulation cascades (KEGG) pathway (Fig. S2B, C).

### Differential protein expression and functional enrichment between the COVID-19 patients and health control

**Fig. 5:**
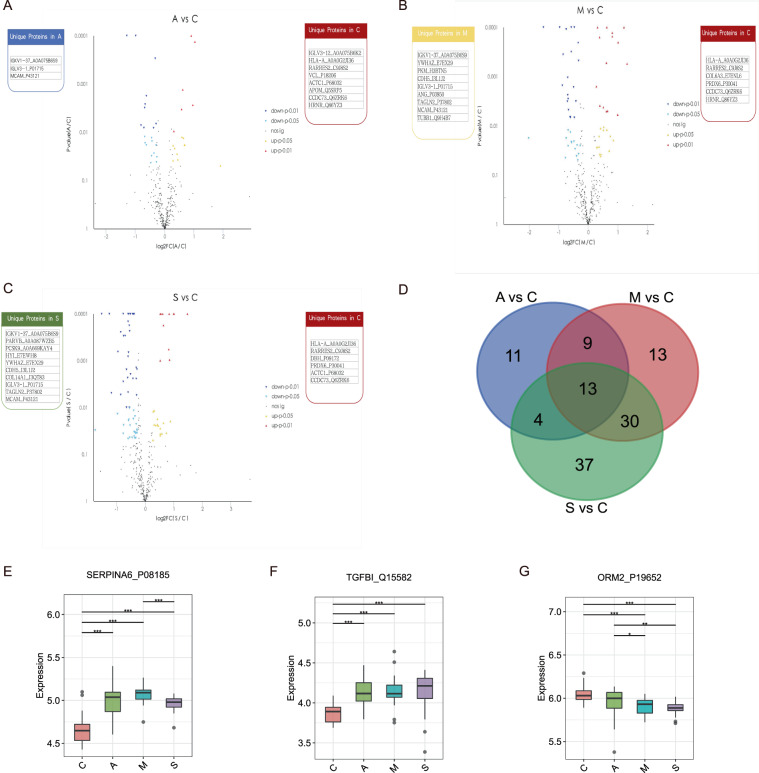
DEPs identification between the recovered patients and control. Volcano plots of DEPs in A vs C (**A**), M vs C (**B**), and S vs C (**C**) treatments. The *x*-axis presents the log2FC and the y-axis presents −log10 (*p*-value). The warm color triangles indicate significantly upregulated genes (red: *p*-value < 0.01; yellow: *p*-value < 0.05). The blue inverted triangles indicate downregulated genes (navy: *p*-value < 0.01; lightblue: *p*-value < 0.05), and black points indicate non-significantly different genes. The red, blue, yellow, and green boxes on either side of each volcano plot represent unique proteins in C, A, M, and S groups, successively. **D** Venn plot for identified DEPs between the recovered patients and control. The red, blue, and green circles represent A vs C, M vs C, and S vs C groups, respectively. Box plots for SERPINA6_P08185 (**E**) TGFBI_Q15582 (**F**) and ORM2_P19652 (**G**) expression levels in C, A, M, and S treatments.

Consequently, we found 37, 65, and 83 proteins were differentially expressed in A, M, and S patients compared with the control. Among them, 16, 29, and 26 proteins were differentially upregulated, respectively (Fig. 5A–C and Table S2). KEGG analysis of DEPs was then performed. As shown in Table S3, the DEPs in the A vs C group were classified into 67 pathways with a majority of the proteins focused on phagosome, Natural killer cell-mediated cytotoxicity, and PI3K-Akt signaling pathway.

Likewise, DEPs in M vs C and S vs C groups were found enriched for several KEGG terms such as complement and coagulation cascades and pancreatic secretion (Table S3). Furthermore, GO annotation analysis displayed that DEPs in A vs C treatment were involved in immune system process (BP), response to stimulus (BP), cell part (CC), and binding (MF). Of these, upregulated DEPs had relative enrichment in membrane, response to stimulus, biological regulation, and binding (Fig. S3). Meanwhile, DEPs in M vs C and S vs C treatments were mainly enriched in similar biological annotations (Fig. S2). The PLS-DA score plot of the four groups was shown in Fig. S4. We noted that there were 13 putative proteins validated simultaneously in A vs C, M vs C, and S vs C comparisons (Fig. 5D). Especially, SERPINA6_P08185 and TGFBI_Q15582 expressions were elevated in M vs C compared to A vs C, but barely lower than S vs C (Fig. 5E, F). SERPINA6_P08185 is a kind of corticosteroid-binding globulin precursor. TGFBI_Q15582 is a transforming growth factor-beta-induced protein ig-h3 precursor. On the contrary, we noticed that the expression levels of ORM2_P19652 declined gradually in A, M, and S compared to C (Fig. 5G). ORM2_P19652 is an Alpha-1-acid glycoprotein 2.

### Differential protein expression and functional enrichment among the diverse recovered patients groups

To exploit biologically meaningful proteins and to better understand the molecular mechanism of COIVD-19, we further tested the protein expression patterns among the diverse recovered patients groups (A, M, and S). Among 394 identified proteins, a total of 40, 71, and 31 proteins were differentially expressed in M vs A, S vs A, and S vs M groups, respectively (Table S4).

In M vs A treatment, DEPs were significantly enriched in regulation of actin.cytoskeleton and Leukocyte transendothelial migration in KEGG pathways while cell junction (CC), antioxidant activity (MF), and detoxification (BP) in GO functional analysis. As for S vs A group, DEPs were tightly involved in nitrogen metabolism and mineral absorption pathways as well as binding (MF) and biological regulation (BP) functions. Then DEPs in S vs M group were annotated in multi-organism process (BP) and catalytic activity (MF) which focused on leukocyte transendothelial migration and phospholipase D signaling pathways (Tables S5 and S6).

**Fig. 6:**
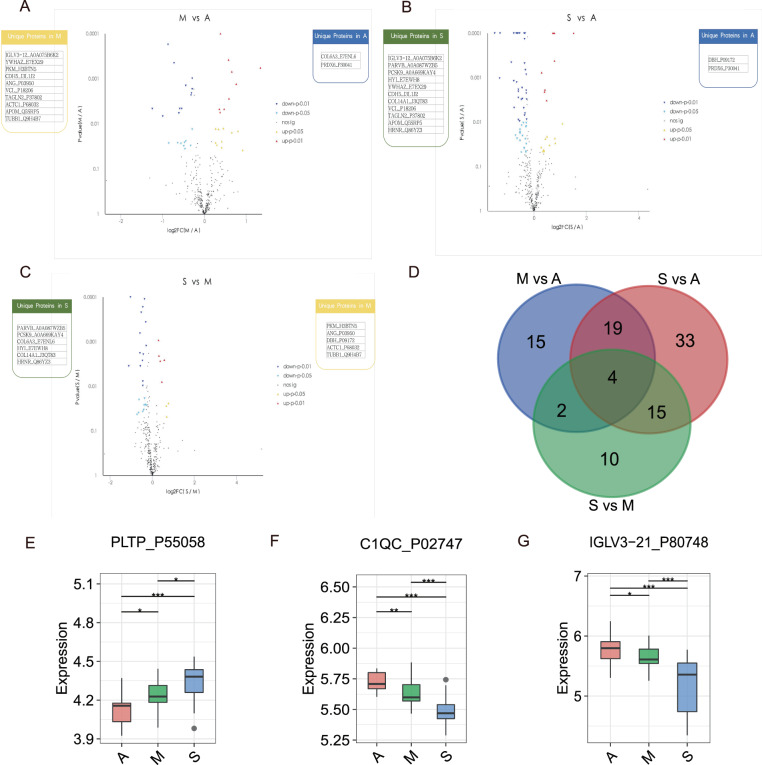
DEPs identification among the diverse recovered patients groups. Volcano plots of DEPs in M vs A (**A**), S vs A (**B**), and S vs M (**C**) treatments. The *x*-axis presents the log2FC and the *y*-axis presents −log10 (*p* value). The warm color triangles indicate significantly upregulated genes (red: *p*-value < 0.01; yellow: *p*-value < 0.05). The blue inverted triangles indicate downregulated genes (navy: *p*-value < 0.01; lightblue: *p*-value < 0.05), and black points indicate non-significantly different genes. The blue, yellow, and green boxes on either side of each volcano plot represent unique proteins in A, M, and S groups, successively. **D** Venn plot for identified DEPs among the diverse recovered patients groups. The blue, red, and green circles represent M vs A, S vs A, and S vs M groups, respectively. Box plots for PLTP_P55058 (**E**), C1QC_P02747 (**F**), IGLV3-21_P80748 (**G**) expression levels in A, M, and S treatments.

Ultimately, expression profiles of 19, 19, and 8 proteins were enhanced in M vs A, S vs A, and S vs M comparisons (Fig. 6A–C), while four putative proteins were determined simultaneously (Fig. 6D). Thereinto, one protein of four common proteins in three groups was PLTP_P55058 (Phospholipid Transfer Protein), whose expression was increased in S vs A comparing to M vs A, but decreased in S vs M (Fig. 6E). PLTP_P55058 is a phospholipid transfer protein. Inversely, C1QC_P02747 (Complement C1q subcomponent subunit C) and IGLV3-21_P80748 (Immunoglobulin lambda variable 3–21) expression were reduced successively in A, M, and S groups (Fig. 6F, G). C1QC_P02747 is known as Complement C1q subcomponent subunit C. IGLV3-21_P80748 is Immunoglobulin lambda variable 3–21.

### Association of plasma lipids with pathologically relevant clinical indices

**Fig. 7:**
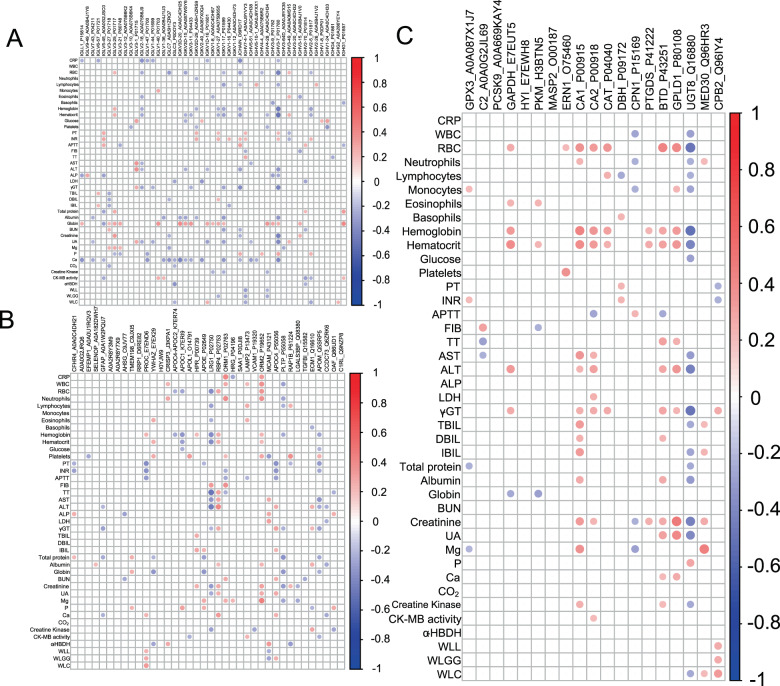
Correlation of plasma lipids with clinical Indices. Correlation plots illustrate spearman correlations between clinical indices with immune-related DEPs (**A**), proteinase (**B**), and the rest (**C**). Only correlations with *p*-value < 0.05 were indicated with colored circles. Negative correlations were shown in red and positive correlations were shown in blue, with sizes of circles representing the magnitude of the correlations.

We then evaluated if altered plasma proteins in COVID-19 were significantly correlated with relevant clinical indices. Spearman correlations were performed and only correlations with *p*-value < 0.05 were indicated as colored circles on the correlation plots (Fig. 7). We observed that DEPs in at least one group could be divided into three classes, namely immune-related, proteinase, and the rest. As for immune-related DEPs, the IGLV1-40_P01703 (Immunoglobulin lambda variable 1–40) was significantly and negatively associated with clinical indicators of systemic inflammation (Monocytes and gliobin), while, Immunoglobulin lambda variable (IGLV3-10_A0A075B6K4, IGHV4-28_A0A0C4DH34, and IGHV1-24_A0A0C4DH33) and Immunoglobulin kappa variable (IGKV2D-29_A0A5H1ZRS9) displayed negative correlations with (Fig. 7A). As shown in Fig. 7B, reductions in plasma LRG1_P02750 (Leucine-rich alpha-2-glycoprotein) and VCAM1_P19320 (Vascular cell adhesion protein 1) were associated with aggravated Creatine Kinase. Corroborating these observations in Fig. 7C, it was shown that Carboxypeptidase B2 (CPB2_Q96IY4) was positive indicators of lung function (WLL, WLGG, and WLC) and negative indicators of PT (Prothrombin Time) and INR (international normalized ratio).

### Multiscale embedded correlation networks between DEPs and clinical indices

**Fig. 8:**
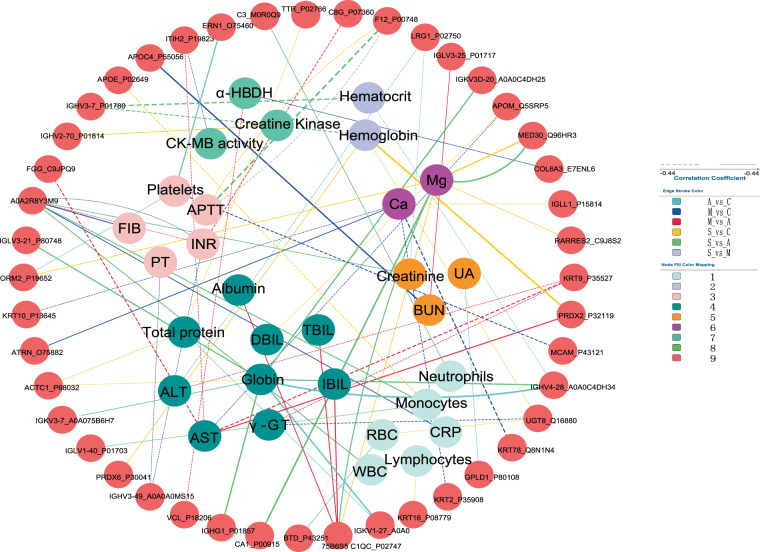
Network of correlations between clinical indexes and DEPs. Network is based on the Spearman correlations analysis between the levels of clinical indexes and differential proteins found in A, M, and S patients as compared to C. Each dot represents a differential protein or a clinical index, respectively. The colors of lines indicate different relationships in the C, A, M, and S patients. Solid and dash lines show positive and negative relationships separately. The thickness of lines was positively linked with the correlations coefficients. This picture shows the total interactions between connected clinical indexes and DEPs in C, A, M, and S patients. Only |correlation coefficients| > 0.5 and *p*-value < 0.05 were displayed in the network of interactions.

Co-regulated genes often display similar patterns of gene expression, which translates to strong correlations between their gene expression levels. To understand the mechanism of the protein changes in recovered COVID-19 patients, the relationships between these DEPs and clinical parameters were investigated by correlation analysis in the C, A, M, and S patients. Correlations with *p*-value < 0.05 and |correlation coefficients| > 0.3 were regarded as significant interactions for further analysis (Table S7). As expected, the level of clinical indexes bore complex relationships with the level of differential proteins (Fig. 8). For example, the level of ALP (alkaline phosphatase) and ALT (alanine aminotransferase) were significantly correlated with the level of 31 and 40 DEPs, separately. In addition, most of altered proteins, such as SERPINA1_P01009 and PRDX6_P30041(Peroxiredoxin 6) were closely associated with these clinical indexes. It was worth noting that many of these relationships significantly differed between health control and recovered COVID-19 patients.

#### There are weak or inverse relationships between DEPs and clinical indices in the recovered COVID-19 patients

We found some DEPs had a strong relationship with clinical indexes in the C group, whereas these correlations were quite weak in recovered COVID-19 patients. For example, the expression profiles of IGHV4-28_A0A0C4DH34 were remarkably positively related (|correlation coefficients| > 0.5) with the level of clinical indexes of Hematocrit, or Globin in the A vs C group. Other similar significant patterns were also observed between ERN1_O75460 (Endoplasmic Reticulum To Nucleus Signaling 1) and Platelets, between BTD_P43251 (Biotinidase) and RBC (red blood cell), and between RBCGPLD1_P80108 and UA (uric acid) in the A vs C group (from Figs. S5 to S9). On the other hand, no such associations were found in the M vs C and S vs C groups.

In addition, 48 strong associations between DEPs and clinical indices such as those between O00187 and BUN (blood urea nitrogen), between A0A087X0Q4 and Lymphocytes, and between H3BTN5 and CRP, were clearly negative in S vs M, but not found in S vs A and M vs A treatments. The finding delineated these inverse relationships were specific in the severe recovered COVID-19 patients (M and S groups). Moreover, RARRES2_C9J8S2 (Retinoic Acid Receptor Responder 2) associated with clinical indexes of liver (ALP) positively and with coagulation (INR and PT) negatively in the C group, indicating it might play pivotal roles during these physiological processes. However, these relationships were not observed in these recovered COVID-19 patients, which implied that the status of these recovered COVID-19 patients may not restore to reach the level of the C group, even their clinical indicators were within the normal range.

#### There are unique relationships between differential proteins and clinical indexes in the recovered COVID-19 patients

Many unique or characteristic associations were found in the recovered COVID-19 patients, and they especially involved the kidney, serum electrolyte levels, and inflammation functions. For example, in the S patients, COL14A1_J3QT83 (Collagen Type XIV Alpha 1 Chain) was negatively correlated with Creatinine and Mg. Meanwhile, PARVB_A0A087WZB5 (Parvin Beta) was positively associated with Eosinophils (from Figs. S8–S10). In the M patients, ANG_P03950 (Angiogenin) showed a strong relationship with Creatinine and UA (Fig. S9).

#### Abnormal clinical indexes are correlated with differential proteins in the S patients

Notably, the Creatine Kinase was lower in the S patients at admission, whereas it was higher in the S patients when compared with C group. Correlation analysis revealed that the increased level of Creatine Kinase was obviously correlated with the expression of IGLV1-36_A0A0B4J1U3, IGHV2-70_P01814, and COL6A3_E7ENL6. Other heart-related clinical index α-HBDH (α-hydroxybutyrate-dehydrogenase) was higher in the S patients at admission, which was associated tightly with MCAM_P43121 (Melanoma Cell Adhesion Molecule), COL6A3_E7ENL6, and IGLC3_P0DOY3 (Immunoglobulin Lambda Constant 3) (Fig. S11). What is more, the WLGG was decreased in the M and S patients compared to C group in the infection period. The relationships with this index and PARVB_A0A087WZB5, HYI_E7EWH8 (Hydroxypyruvate Isomerase), and COL14A1_J3QT83 were weak in the S (Fig. S12). Previous studies revealed that the SARS-CoV-2 may inflict a systemic attack, thereby damaging kidney or lymph nodes, especially in the S patients. Therefore, these relationships between novel abnormal clinical indexes and differential proteins in the recovered COVID-19 patients might be present right from the viral attacks during the infection, suggesting that damages are ongoing in the recovered COVID-19 patients.

## Discussion

Previous study focus on patients during the disease revealed DEPs between COVID-19 patients and healthy control. In that study, Elettra Barberis et al. found that DEPs were mainly belonged to three functional enrichments, inflammatory, immune response, and coagulation, while we found the DEPs between recovered COVID-19 patients and healthy subjects mainly focus on lipid metabolic process, response to cellular, and response to stress oxygen-containing compound, suggesting that different biological characteristics also occurred between recovered COVID-19 patients and patients during the disease^15^. Through analyzing DEPs, we found that some acute phase associated proteins decreased in severe recovered patients such as apolipoprotein APOC4 and CPB1 protein, while the regulator of complement system CPN1 protein was elevated. In previous study, there is some acute phase associated proteins such as Alpha 1-antichymotrypsin and CRP protein increased in EVs from severe patients during the disease^15^. In other word, APOC4 and CPB1 proteins were damaged, which may be associated with the degree of infection. The elevation of CPN1protein suggested that severe recovered patients may be in the state of inflammatory activity. Similarly, multiple proteins were found to show a specific upregulated pattern in COVID-19 patients with the development of the severity of clinical disease. Among these proteins, SERPINA6 (Serpin Family A Member 6), TGFBI (Transforming Growth Factor Beta Induced) and ORM2 (Orosomucoid 2) were brought to our attention. SERPINA6 was corticosteroid-binding globulin and it served as the circulatory transport protein through binding cortisol^16^. In other words, it played an important role both in the storage of cortisol and transporting cortisol to targeted tissues. The anti-inflammatory effect of cortisol was proved by previous report^16,17^. The elevation of SERPINA6 was an indirect appearance of increased cortisol, which is common under the conditions of infections. In addiction, severe patients during the disease have a higher level of SERPINA6 than non-severe patients, while severe recovered patients have a lower level of SERPINA6 than non-severe patients, suggesting that SERPINA6 was consumed in severe recovered patients^15^. As an important protein, TGFBI was widely studied in malignant progression, which was suggested to play an important role both in immune response and tumor immune microenvironment^18–20^. Previous work has described that the relationship between the upregulation of TGFBI in M2 macrophages and acute inflammation processes, suggesting the elevation of TGFBI would be a potential marker sensitively reflecting the conditions of infections^21^. ORM2 as acute-phase protein was associated with inflammation^22^. There was a positive correlation between the levels of ORM2 and disease activity of rheumatoid arthritis, which was a common inflammatory diseases^23^. Han-Zhang Zhu et al. found ORM2 was mainly expressed in liver tissues, suggesting that it was associated with regulation of liver function^24^. Previous studies also reported a similar phenomenon that ORM2 was specifically elevated in hepatocytes under the condition of infections^25,26^. In other words, the elevation of ORM2 from plasma would be the marker of the status of infections. Moreover, we found that the IGLV1-40_P01703 (Immunoglobulin lambda variable 1-40) was significantly and negatively associated with clinical indicators of systemic inflammation (Monocytes and globin) in recovered COVID-19 patients. In other words, there were unique relationships between differential proteins and clinical indexes especially inflammatory indices found in the recovered COVID-19 patients. On the basis of the above-mentioned results, it seemed that recovered COVID-19 patients were still under the condition of inflammation due to the effect of SARS-COV-2 infection, which showed a positive correlation with clinical disease severity. However, serum inflammatory index hsCRP was in the normal range, which had no difference between healthy subjects and recovered COVID-19 patients. Considering the role of EVs in sensitively reflecting the change of biological status, it is very important that we should closely observe recovered COVID-19 patients’ symptoms and inflammatory clinical indices because whether or not the situations of these recovered COVID-19 patients got worsen, which was still unknown. Previous study has reported that 44% discharged patients felt their life quality got worsen^27^, suggesting that the prognosis of recovered COVID-19 patients was not optimistic. With the continuous development of the epidemic situation, in addition to COVID-19 patients of the acute stage, medical staff should also pay more attention to the treatment of recovered COVID-19 patients. Meanwhile, whether the protein from EVs we identified would be the potential markers for indicating the condition of inflammation in recovered COVID-19 patients or not needs further research, which would be focal point of our further study.

Previous study found that there is no difference of complement-related proteins C1QC between non-infected group and infected group. In this study, we found C1QC in recovered patients decreased with the degree of disease, which implied that this protein plays an important role in the course of SARS-CoV-2 infection. In addition, all fibrinogen components in EVs of patients with COVID-19 infection were downregulated, indicating that the coagulation activity had changed, which may reflect the compensatory response to potential thrombosis. This change trend also exists in our recovered patients, and with the severity of the disease down. Similar studies have also been found in EVs secreted by macrophages infected with influenza, suggesting the relationship between virus infection and coagulation^28^. So we need to be alert to the risk of bleeding in recovered patients, especially in mild/severe patients. Interestingly, protein-encoding antibody downregulated was found in severe patients at an early stage: IGHV1-2, IGHV3-15, IGHV3-23, IGHV3-9, IGHV4-28, IGHV4-38-2, IGKV1-5, and IGKV4-1, which was associated with humoral immune response. Similarly, we found IGLV3 decreased with the degree of disease, which suggested low humoral immune response may occur in severe recovered patients. Wen Wen et al. found that T cells decreased remarkably in the recovered COVID-19 patients by single-cell RNA sequencing technique, suggesting that the immunity of recovered COVID-19 patients were still vulnerable^29^.

Through analyzing the relationship between DEPs and clinical indexes, we inferred that recovered COVID-19 patients were suffered from declining of organ functions compared with healthy control although their clinical symptoms, CT image, and clinical indexes were improved. Previous studies have reported that organ dysfunction was observed in COVID-19 patients, especially in severe patients, which was induced by infection of SARS-CoV-2^30,31^. Our results found in healthy control, the correlations between DEPs and clinical indexes were related to normal physiological processes. For instance, RARRES2 was observed to have close associations with ALP, which was the important clinical index of the liver for reflecting the function of the liver because it was mainly produced by liver cells^32^. RARRES2 also known as Chemerin was a new discovery adipose cell cytokine^33^. It is highly expressed in liver tissues and promotes glucose transport and regulates immune and inflammatory response^34,35^. Therefore, the level of RARRES2 was associated with liver function under normal conditions. However, this correction was not identified in recovered COVID-19 patients in our study, which implied that the function of the liver was declining in recovered COVID-19 patients although their clinical symptoms and signs, radiological characteristics, and clinical indexes were improved. Considering the level of serum clinical indices of liver function including ALT, AST and ALP were in normal range, which suggesting that there were not liver cell damages ongoing in recovered COVID-19 patients. Besides, the recovered COVID-19 patients enrolled in our study were without underlying disease. In another word, the liver cells function was affected due to the effect of SARS-CoV-2 infection. Whether or not the function of liver cells was restored to the levels of normal needs further study. Thus, it might be useful for monitoring and estimating the function of liver cells.

However, the limitations of our study should not be neglected. Firstly, the sample size was relatively small in this single-center prospective study; Secondly, we could not exclude the impact of drugs in clinic therapy on proteomics change of EVs, especially in RM and RS groups.

## Conclusions

We reported the protein of plasma EVs in recovered patients and revealed the relationship between these proteins and inflammatory activity, immune response and organ function in recovered COVID-19 patients without underlying disease after 3 months discharge, which implied the important role for EVs involved in coagulation activity, inflammatory reaction, low immune response, and low organ function Therefore, we should pay close attention to the indexes of coagulation, inflammation, immunity and organ function for recovered patients and should alert to the risk of bleeding and reinfection for the severe recovered patients.

## Supplementary information

Supplementary Figure and Table Legends

Figure S1

Figure S2

Figure S3

Figure S4

Figure S5

Figure S6

Figure S7

Figure S8

Figure S9

Figure S10

Figure S11

Figure S12

Table S1

Table S2

Table S3

Table S4

Table S5

Table S6

Table S7
