# Supplementary material for: Proteomics of extracellular vesicles in plasma reveals the characteristics and residual traces of COVID-19 patients without underlying diseases after 3 months of recovery
Source: Cell Death Dis. 2021 May 25;12(6):541. doi: 10.1038/s41419-021-03816-3 (PMC8146187; doi:10.1038/s41419-021-03816-3)
Supplement: Supplementary file 1 — Supplementary Figure and Table Legends [file 41419_2021_3816_MOESM1_ESM.docx]

**Supplementary Figure and Table Legends**

Figure S1. Flow chart for the inclusion of covid-19 convalescent patients and healthy controls.

Figure S2. Identification of proteins in the latter four specific clusters. (A) The latter four subcluster 5-8 of proteins. Top 20 terms of GO functional (B) and KEGG pathway (C) enrichment analyses for 31 proteins in subcluster 1.

Figure S3. GO enrichment analyses in A vs C, M vs C, and S vs C treatments. GO annotation result of DEPs in A vs C (A), M vs C (B) and in S vs C (C) groups. The x-axis stands for GO terms and the y-axis stands for enriched DEPs numbers. Functional GO enrichment analysis of up-regulated DEPs in A vs C (D), M vs C (E) and in S vs C (F) groups.

Figure S4. The PLS-DA score plot of C, A, M and S groups.

Figure S5. Present the DEPs and clinical indexes related to the functions of blood glucose and red blood cell condition. Interactions with |correlation coefficients| > 0.3, and p-value < 0.05 were retained.

Figure S6. Present the DEPs and clinical indexes related to the functions of liver. Interactions with |correlation coefficients| > 0.3, and p-value < 0.05 were retained.

Figure S7. Present the DEPs and clinical indexes related to the functions of coagulation state. Interactions with |correlation coefficients| > 0.3, and p-value < 0.05 were retained.

Figure S8. Present the DEPs and clinical indexes related to the functions of inflammation. Interactions with |correlation coefficients| > 0.3, and p-value < 0.05 were retained.

Figure S9. Present the DEPs and clinical indexes related to the functions of kidney. Interactions with |correlation coefficients| > 0.3, and p-value < 0.05 were retained.

Figure S10. Present the DEPs and clinical indexes related to the functions of serum electrolyte levels. Interactions with |correlation coefficients| > 0.3, and p-value < 0.05 were retained.

Figure S11. Present the DEPs and clinical indexes related to the functions of heart. Interactions with |correlation coefficients| > 0.3, and p-value < 0.05 were retained.

Figure S12. Present the DEPs and clinical indexes related to the functions of lung. Interactions with |correlation coefficients| > 0.3, and p-value < 0.05 were retained.

Table S1. 394 proteins identified by DIA in 86 specimens.

Table S2. Proteins identified between the recovered patients and control.

Table S3. KEGG pathway enrichment of DEPs between the recovered patients and control.

Table S4. Proteins identified among the diverse recovered patients groups.

Table S5. KEGG pathway enrichment of DEPs among the diverse recovered patients groups.

Table S6. GO functional enrichment of DEPs among the diverse recovered patients groups.

Table S7. Relationships between these DEPs and clinical parameters among C, A, M, and S comparisons.
