# Supplementary figures and images for: Proteomics of extracellular vesicles in plasma reveals the characteristics and residual traces of COVID-19 patients without underlying diseases after 3 months of recovery
Source: Cell Death Dis. 2021 May 25;12(6):541. doi: 10.1038/s41419-021-03816-3 (PMC8146187; doi:10.1038/s41419-021-03816-3)

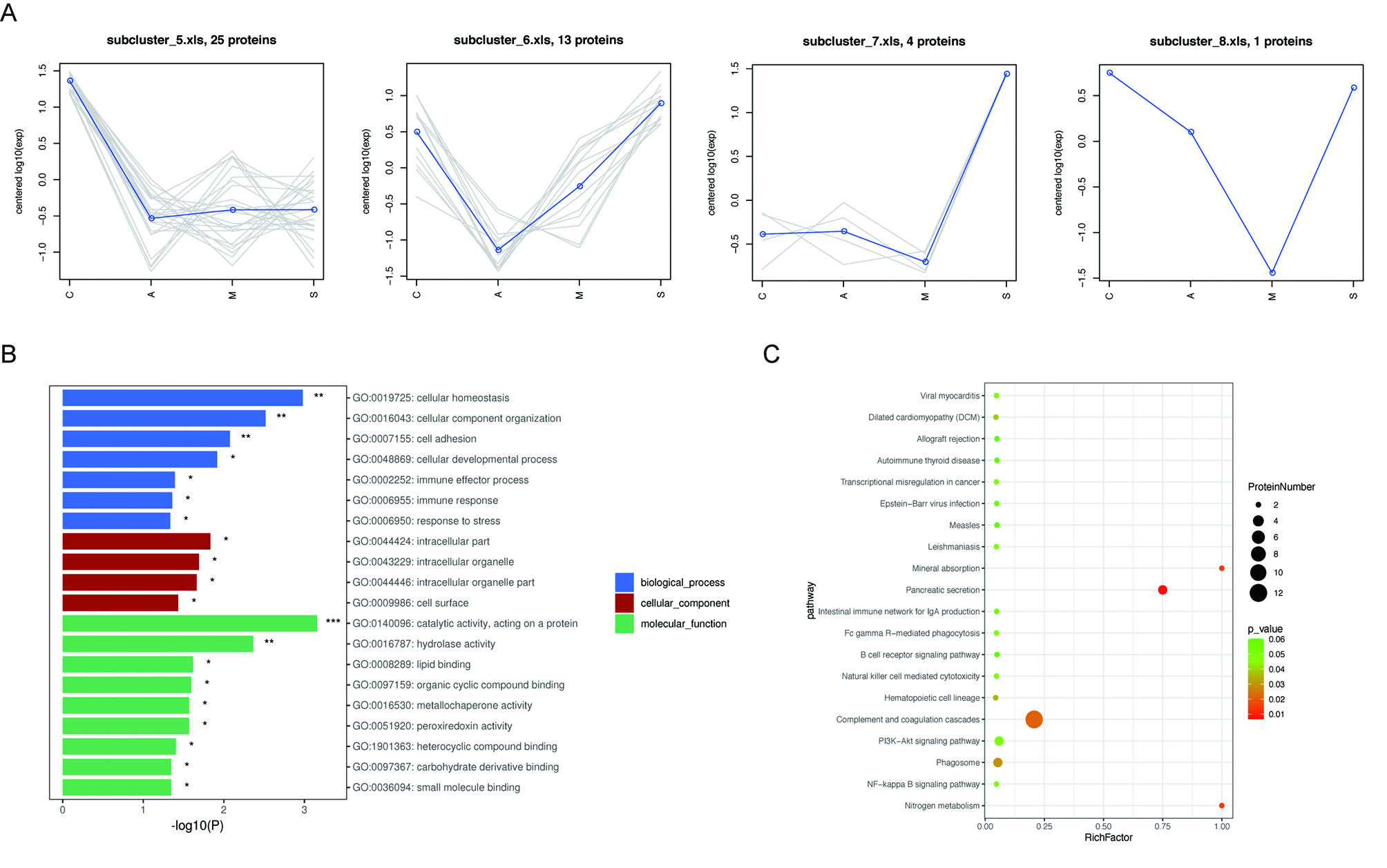

Supplement: Supplementary file 3 — Figure S2 [file 41419_2021_3816_MOESM3_ESM.tif]
