# Supplementary material for: Proteomics of extracellular vesicles in plasma reveals the characteristics and residual traces of COVID-19 patients without underlying diseases after 3 months of recovery
Source: Cell Death Dis. 2021 May 25;12(6):541. doi: 10.1038/s41419-021-03816-3 (PMC8146187; doi:10.1038/s41419-021-03816-3)
Supplement: Supplementary file 14 — Table S1 [file 41419_2021_3816_MOESM14_ESM.docx]

| Table S1 394 proteins identified by DIA in 86 specimens | | | | | | | | | | | | | | | | | | | | | | | | | | | | | | | | | | | | | | | | | | | | | | | | | | | | | | | | | | | | | | |
| --- | --- | --- | --- | --- | --- | --- | --- | --- | --- | --- | --- | --- | --- | --- | --- | --- | --- | --- | --- | --- | --- | --- | --- | --- | --- | --- | --- | --- | --- | --- | --- | --- | --- | --- | --- | --- | --- | --- | --- | --- | --- | --- | --- | --- | --- | --- | --- | --- | --- | --- | --- | --- | --- | --- | --- | --- | --- | --- | --- | --- | --- | --- |
| Accession | DB3 | DB4 | DB5 | DB7 | DB8 | DB9 | DB14 | DB17 | DB18 | DB19 | DB20 | DB21 | DB24 | DB25 | DB28 | DB34 | DB35 | DB36 | DB37 | DB38 | DB39 | DB40 | DB42 | DB43 | DB44 | DB45 | DB47 | DB48 | DB49 | DB58 | DB60 | DB64 | DB65 | DB71 | DB72 | DB73 | DB74 | DB75 | DB76 | DB78 | DB79 | DB81 | DB85 | DB86 | DB87 | DC2 | DC4 | DC6 | DC9 | DC11 | DC13 | DC14 | DC16 | DC17 | DC19 | DC20 | DC21 | DC23 | DC24 | DC27 | DC28 | DC29 |
| A0A075B6H7 | 240614.9375 | 152936.4375 | 164937.9375 | 82306.55469 | 108008.0469 | 116661.6563 | 149478.1719 | 168932.7969 | 122646.0156 | 103944.3828 | 186987.9688 | 168140.6563 | 85131.11719 | 92924.85938 | 138829.125 | 154751.8438 | 152412.1563 | 132986.8438 | 135960.5 | 149705.625 | 273853.9063 | 211792.9375 | 206070.5313 | 210181.8438 | 220090.375 | 79596.83594 | 188540.2813 | 120852.75 | 219599.0625 | 258127.8125 | 53601.67969 | 139704.8125 | 164477 | 75753.5 | 64130.42969 | 205651.7969 | 165826.6406 | 73115.78906 | 194040.0156 | 36827.35547 | 161753 | 227491.9375 | 139981.4688 | 304108.6563 | 241235.5469 | 273003.4375 | 152600.5156 | 105397.9063 | 98309.23438 | 168769.5938 | 181620.7188 | 309491 | 193913.8125 | 90868.21094 | 316876.5313 | 210361.1719 | 228402.1563 | 124119.75 | 209418.0313 | 90257.3125 | 141355.4688 | 209525.3438 |
| A0A075B6I0 | 514262.8438 | 387301.6875 | 958411.75 | 418103 | 479522.9688 | 336796.7813 | 691657 | 811123.4375 | 627424.1875 | 559732.75 | 456000 | 603640.9375 | 1084307.5 | 342281.7813 | 0 | 1065357.25 | 471205.2188 | 104263.5547 | 742075.9375 | 1080864.375 | 282495.2188 | 886006.625 | 850109.9375 | 870436.5625 | 6072.38916 | 379703.375 | 805979.25 | 402040.125 | 254565.5625 | 1046894.375 | 879580.3125 | 479391.8438 | 355196.7813 | 27687.78711 | 29037.11719 | 9967.932617 | 613059.25 | 0 | 593471.25 | 414770.375 | 960089.75 | 529332.5 | 0 | 613086.1875 | 9999.378906 | 645546 | 835640.25 | 155140.6719 | 0 | 312654.1875 | 672925.5 | 1900635.25 | 357161.7188 | 617713.3125 | 467621.3438 | 307622.0625 | 742851.9375 | 510958.2813 | 261736.8906 | 581413.5 | 751372.25 | 1248965.625 |
| A0A075B6J9 | 73643.13281 | 95355.90625 | 111719.1875 | 66375.82031 | 48681.96875 | 33920.41406 | 69929.40625 | 103450.4531 | 109656.2422 | 108042.9219 | 95212.73438 | 31991.31641 | 49050.23828 | 42415.26172 | 130385.2266 | 151130.3125 | 99684.01563 | 62535.69922 | 45690.98438 | 181630.1719 | 128654.7969 | 61566.60156 | 78578.72656 | 81993.67188 | 63471.17188 | 23990.69531 | 37133.24219 | 53535.38281 | 138701.5781 | 60295.52344 | 111786.7813 | 52949.00391 | 131320.5156 | 44401.52344 | 90858.84375 | 71941 | 128684.375 | 105047.8594 | 108737.8047 | 36222.64844 | 69487.0625 | 56149.48828 | 227579.7344 | 120059.875 | 84887.23438 | 106233.4922 | 113680.9688 | 136036.4375 | 96016.76563 | 65241.875 | 122008.1094 | 101411.5938 | 76081.20313 | 55682.70313 | 102014.0313 | 26673.90234 | 86766.80469 | 92460.99219 | 182105.8594 | 86074.71094 | 183049.9063 | 209957.5313 |
| A0A075B6K2 | 0 | 145455.9375 | 84438.77344 | 2020.391357 | 70625.92969 | 182936.2969 | 2551.573242 | 7259.161621 | 97262.1875 | 7585.192871 | 4218.563477 | 0 | 11388.49121 | 0 | 10740.02246 | 0 | 0 | 0 | 79445.57031 | 0 | 0 | 5287.617676 | 57597.59766 | 59598.35547 | 0 | 8417.513672 | 66858.96875 | 55576.07813 | 154865.4375 | 12703.29883 | 154642.0313 | 104578.8828 | 0 | 81506.96094 | 7066.582031 | 6385.440918 | 0 | 0 | 0 | 110923.6016 | 0 | 5045.243652 | 0 | 102330.1953 | 4424.731934 | 6062.056152 | 3981.654541 | 124285.1953 | 14044.84473 | 0 | 0 | 0 | 0 | 0 | 185806.2813 | 0 | 77866.10938 | 3912.923096 | 0 | 72266.1875 | 159915.6563 | 0 |
| A0A075B6K4 | 115733.5625 | 23258.00195 | 66825.3125 | 34415.64844 | 52660.25781 | 22596.7793 | 44032.79688 | 88066.73438 | 48139.99219 | 97685.52344 | 52658.39063 | 64987.05469 | 50987.60547 | 16923.70703 | 44976.02344 | 62378.05859 | 61913.73438 | 69646.42969 | 68603.61719 | 60577.53516 | 130177.1094 | 80876.53125 | 50216.57031 | 56642.14453 | 68562.10938 | 45576.39063 | 70078.01563 | 50338.71484 | 50719.40234 | 248143 | 44540.97266 | 87029.1875 | 90153.89844 | 37773.48047 | 45649.8125 | 55011.00781 | 36882.10156 | 34277.26953 | 31611.62695 | 60982.16016 | 29267.37891 | 64634.94141 | 87676.42969 | 73189.61719 | 56066.90234 | 104896.4375 | 55005.67188 | 49820.26172 | 55617.30469 | 48153.45313 | 45810.84375 | 60932.69531 | 129670.4219 | 24647.93555 | 101176.2813 | 52759.96484 | 44311.3125 | 109498.1563 | 75295.73438 | 46186.66797 | 117437.6875 | 60269.99609 |
| A0A075B6K5 | 116543.8281 | 0 | 16744.13281 | 0 | 10011.07813 | 0 | 8218.635742 | 20398.41016 | 13753.86816 | 0 | 13929.77734 | 8225.396484 | 4628.035156 | 0 | 8133.006348 | 5756.984375 | 10328.0791 | 25582.77734 | 9399.048828 | 119756.4531 | 8535.676758 | 22724.87109 | 6864.44873 | 6881.141602 | 19482.31641 | 31862.86719 | 6836.975586 | 11784.66113 | 8772.796875 | 0 | 8019.800781 | 8729.518555 | 12029.50098 | 5868.990723 | 8249.114258 | 29294.60156 | 0 | 0 | 0 | 0 | 0 | 37972.47656 | 0 | 11820.2373 | 0 | 19536.32617 | 11918.9375 | 33546.72266 | 5706.424805 | 30958.14648 | 0 | 0 | 20304.00195 | 0 | 68659.89063 | 9073.771484 | 12731.36035 | 0 | 8860.697266 | 9154.795898 | 14666.54883 | 51546.84766 |
| A0A075B6Q5 | 118416.4922 | 0 | 15660.11523 | 12405.24609 | 0 | 34188.80078 | 12628.85449 | 0 | 14018.40137 | 56557.46875 | 12349.375 | 14671.18945 | 0 | 10537.65918 | 14140.09375 | 23078.89453 | 103155.3984 | 26676.90234 | 74146.82813 | 93764.38281 | 94371.90625 | 70356.625 | 74300.17188 | 74926.42188 | 9198.211914 | 36807.13281 | 11865.16797 | 71472.08594 | 101450.7656 | 18539.17578 | 72027.76563 | 16152.06543 | 0 | 106240.7344 | 16115.98438 | 89422.74219 | 12309.19531 | 68499.67188 | 15578.94141 | 0 | 84155.15625 | 58651.98438 | 207992.8125 | 93902.63281 | 68883.25 | 23765.84961 | 34547.0625 | 22620.6875 | 63988.30469 | 119311.4922 | 29458.25781 | 85614.98438 | 31890.97461 | 59798.07422 | 31512.99219 | 29811.67383 | 46827.13281 | 63933.99219 | 97694.28906 | 12477.45996 | 23474.00781 | 32959.13281 |
| A0A075B6R2 | 181461.6406 | 230057.0625 | 245067.0469 | 137052.2344 | 102792.3203 | 183470.6406 | 154240.6875 | 9827.822266 | 253310.5781 | 35434.23828 | 206103.9219 | 125563.2422 | 283545.4063 | 206068.5625 | 165215.9688 | 60017.03125 | 247220.8906 | 153206.0625 | 247304.8438 | 29372.52539 | 461094.4688 | 221946.8438 | 213437.0156 | 271060.9688 | 117397.7344 | 117438.3828 | 277357.2188 | 116594.8594 | 205776.625 | 395949.3125 | 185211.1875 | 438921.5938 | 183971.9688 | 284501.5625 | 77619.71094 | 113108.2422 | 84121.51563 | 69748.16406 | 266742.75 | 122486.0234 | 292606.5938 | 71518.47656 | 77074.34375 | 387277.3438 | 196764.6406 | 270738.7188 | 221235.625 | 56393.44141 | 191087.4063 | 238649.9375 | 234730.3438 | 93839.71094 | 192413.5313 | 182511.4844 | 352894.25 | 74675.32031 | 26899.70313 | 96387.25781 | 375954.4375 | 258848 | 259119.9063 | 76826.27344 |
| A0A075B6R9 | 186400.2656 | 123908.2188 | 267535.1563 | 78778.26563 | 243504.1406 | 50805.76172 | 34419.92578 | 257875.4375 | 97373.46875 | 120222.0156 | 138215.2031 | 63753.24609 | 165829.1094 | 231212.2188 | 131159.7188 | 117658.2109 | 207554.4375 | 92510.45313 | 45474.95313 | 218115.625 | 178155.6094 | 129620.5469 | 112778.2969 | 144383.9531 | 236607.5469 | 58709.41406 | 124803.8672 | 74846.21875 | 97524.07813 | 157884 | 310862.75 | 105469.3203 | 75562.64844 | 103449.6719 | 89904.09375 | 76239.25781 | 224144.0781 | 196818.0156 | 173590.25 | 153318.6563 | 269002.5625 | 105800.4453 | 751954.3125 | 219303 | 224896.8438 | 196023.9375 | 491377.5313 | 295702.6563 | 128243.9375 | 85410.32813 | 116985.9297 | 316759.2188 | 95977.16406 | 97395.14844 | 142810.2656 | 121813.9453 | 184225.625 | 134872.1719 | 155873.6406 | 404283.1875 | 196886.5 | 473361.25 |
| A0A075B6S5 | 83959.39063 | 52725.21875 | 90866.72656 | 42125.16016 | 79413.21094 | 38867.85547 | 94571.875 | 64538.92578 | 44031.75391 | 49583.125 | 74200.28125 | 53454.25781 | 31898.95508 | 67751.11719 | 59138.68359 | 58091.76172 | 45669.45313 | 61245.17969 | 40522.62891 | 51260.78125 | 111954.4531 | 62467.50781 | 50134.57422 | 44729.22656 | 76782.07813 | 25799.67969 | 54905.16797 | 35175.54688 | 38955.91016 | 76528.35938 | 95647.40625 | 39770.80078 | 59073.95703 | 45015.92578 | 49313.22266 | 55619.75781 | 83767.375 | 49623.27734 | 40041.42578 | 21500.93945 | 82637.00781 | 24345.41016 | 73657.39844 | 46080.62891 | 45139.41797 | 74801.42969 | 85429.21875 | 34346.8125 | 43664.07031 | 49640.66797 | 71961.5625 | 101100.6719 | 90951.8125 | 30938.33984 | 47140.42188 | 21615.50195 | 61304.73047 | 54695.66797 | 83249.96094 | 66434.46094 | 99205.85156 | 131992.9844 |
| A0A075B6S9 | 5524.833496 | 7933.443848 | 3558.767822 | 23884.74805 | 4651.501465 | 2434.345703 | 0 | 4089.7229 | 7342.211914 | 0 | 0 | 2440.072998 | 0 | 1003.529358 | 2433.056152 | 0 | 0 | 5507.79541 | 0 | 2666.171631 | 8816.022461 | 0 | 0 | 4485.723145 | 3413.466553 | 0 | 2510.382324 | 6738.718262 | 1141.611938 | 7417.59375 | 3717.167969 | 0 | 0 | 7149.338867 | 1836.596313 | 0 | 1097.743042 | 6114.754883 | 0 | 0 | 0 | 0 | 0 | 36004.13672 | 3972.817627 | 13229.15527 | 3151.839355 | 0 | 13641.72949 | 1959.134766 | 4343.228027 | 840.5441284 | 0 | 0 | 0 | 0 | 6954.73291 | 0 | 1883.314941 | 0 | 3573.9021 | 11940.87891 |
| A0A075B7B8 | 687249.125 | 621235.625 | 18458.13086 | 901786.875 | 648107.3125 | 552659.375 | 1093788.75 | 2116371 | 665924.25 | 769367.5625 | 508941.75 | 778332.5 | 655104.375 | 716751.4375 | 712720.5625 | 754313 | 951632 | 1964968.625 | 695617.1875 | 955875.6875 | 1041988.875 | 629356.75 | 761491.375 | 1169305.125 | 1407134 | 636913.875 | 870834.9375 | 1664809.375 | 1291787.875 | 1159020.375 | 757823.5625 | 651001.4375 | 1257972.125 | 2061238.75 | 980780 | 1315750.25 | 941553.375 | 550523 | 842970.6875 | 1297281.625 | 961686.3125 | 605629.625 | 1314009.875 | 756490.875 | 1371810.625 | 3571121 | 940863.5 | 1285679 | 870019.375 | 862857.1875 | 990724.125 | 1423215.25 | 1010805.5 | 571057.625 | 882658.5 | 914968.0625 | 914117.25 | 843841.9375 | 788858.5 | 877191.75 | 1171629.25 | 1534860.125 |
| A0A075B7D0 | 401973.2813 | 508187.3438 | 117877.3906 | 134222.8281 | 389239.25 | 628966.125 | 169108.0781 | 175952.4531 | 426082 | 434181.7813 | 178015.3594 | 223484.2969 | 80152.22656 | 84586.0625 | 251618.9375 | 106335.2422 | 289977.875 | 389056.125 | 899434 | 180360.7813 | 543697.5625 | 170194.2813 | 780491.5625 | 166225.0313 | 68207.26563 | 267523.4063 | 324414.6563 | 470689.4063 | 513726.0313 | 10014.31641 | 645041.875 | 132345.2344 | 85978.29688 | 216363.4531 | 341305.75 | 301413.9375 | 244240.7813 | 420094.9688 | 189710.2344 | 258506.9219 | 211379.7031 | 229468.5 | 150638.4219 | 246421.8438 | 139672.75 | 167996.4844 | 245916.4688 | 353556.4375 | 275038.375 | 669910.3125 | 902668.3125 | 949647.75 | 96774.85938 | 440950.0313 | 278940.0625 | 74476.20313 | 147483.875 | 125999.0469 | 308231.8438 | 40722.71484 | 330385.5 | 412625.1875 |
| A0A075B7D8 | 0 | 0 | 0 | 0 | 0 | 0 | 0 | 0 | 0 | 0 | 0 | 31177.58594 | 0 | 0 | 0 | 0 | 0 | 0 | 0 | 0 | 0 | 0 | 0 | 0 | 0 | 0 | 0 | 0 | 0 | 0 | 0 | 0 | 0 | 0 | 19800.8418 | 0 | 0 | 0 | 0 | 13615.72949 | 0 | 0 | 0 | 0 | 0 | 0 | 0 | 0 | 0 | 0 | 0 | 0 | 0 | 0 | 0 | 0 | 0 | 0 | 0 | 0 | 0 | 0 |
| A0A075B7F0 | 39399.75781 | 31029.33984 | 23334.95313 | 7247.084961 | 30742.48633 | 20047.42578 | 20179.57422 | 92150.84375 | 17880.66797 | 49914.77734 | 19714.67969 | 20318.4043 | 22625.53516 | 44996.05859 | 12867.78223 | 26796.25 | 27169.55664 | 22375.83594 | 15187.42383 | 20233.625 | 24744.21484 | 22033.20898 | 14364.44922 | 27760.41406 | 68474.46875 | 12076.45801 | 17152.14648 | 18266.87305 | 20699.06641 | 29615.06445 | 29147.65234 | 9642.463867 | 15914.73926 | 11899.70508 | 31390.41406 | 23705.42969 | 17804.83203 | 34455.45313 | 30717.55078 | 13691.33984 | 28037.85547 | 111068.2188 | 63309.16406 | 26892.51953 | 38036.87891 | 20014.45313 | 17898.21875 | 13664.71875 | 18954.57813 | 22376.71875 | 35432.69531 | 15910.13672 | 19701.85938 | 18451.21289 | 27709.8418 | 18142.41016 | 19358.78516 | 13691.83984 | 16072.89551 | 12889.19336 | 0 | 25804.6875 |
| A0A087WSY4 | 36144.78516 | 25560.42383 | 34341.34766 | 0 | 14661.91602 | 17535.85938 | 22541.35352 | 0 | 22134.35742 | 57294.79688 | 18460.49219 | 0 | 0 | 22431.66797 | 0 | 20816.01563 | 0 | 37111.86719 | 37491.05078 | 0 | 31602.78906 | 44566.26953 | 14382.6416 | 30833.67969 | 21461.75977 | 0 | 0 | 12614.62793 | 0 | 18259.98047 | 27644.375 | 0 | 20884.64648 | 21095.87891 | 7510.979492 | 0 | 0 | 7187.152832 | 28953.45703 | 50536.48828 | 41857.36328 | 24457.56836 | 40789.23438 | 25485.41211 | 19526.22852 | 0 | 25591.70898 | 0 | 36603.88672 | 16519.31445 | 33474.90234 | 35126.07031 | 0 | 15170.38965 | 25664.03516 | 0 | 6164.199707 | 17512.86523 | 30865.62695 | 15569.49121 | 33489.70703 | 0 |
| A0A087WSY6 | 301724.5 | 395554 | 267495.5 | 116106.6016 | 108633.4922 | 114059.1641 | 141177.5313 | 237774.8125 | 46025.1875 | 362852.8438 | 110799.5156 | 100602.9063 | 126437.625 | 235266.7813 | 139970.6406 | 211399.9063 | 338699.1875 | 330391.0625 | 92258.45313 | 128836.2656 | 352130.6875 | 240957.0313 | 312962.4688 | 208791.125 | 560145 | 40111.35938 | 371597.25 | 363186.1563 | 318970.5938 | 78088.97656 | 629321.9375 | 118327.4844 | 166196.0625 | 110964.3984 | 322066.5625 | 197408.7813 | 321433.4688 | 157171.3438 | 291613.125 | 253007.8906 | 244736.9688 | 150648.5 | 523086 | 389136.5313 | 115370.8047 | 134490.1094 | 243645.5938 | 216171.0469 | 244988.2813 | 347948.6875 | 282569.4375 | 216262.0938 | 162218.875 | 212450.9688 | 220655.9219 | 165324.0469 | 132744.6719 | 240932.7031 | 349023.5 | 171995.125 | 401885.6875 | 403129.25 |
| A0A087WSZ0 | 7573.67334 | 35748.54297 | 48626.63281 | 8817.408203 | 32808.25391 | 27571.24219 | 21150.67578 | 65939.40625 | 131520.5313 | 23337.50586 | 5067.438477 | 23878.36328 | 22048.60742 | 24679.41211 | 155438.1406 | 20002.32813 | 48593.71094 | 33334.16406 | 8223.308594 | 25908.33984 | 47770.53125 | 256214.875 | 32811.50781 | 29076.44336 | 49144.39453 | 26342.08984 | 29583.14063 | 46066.21875 | 50117.78125 | 41882.31641 | 38458.60156 | 15736.68359 | 36936.22656 | 42906.61719 | 29113.4707 | 33790.23828 | 36080.18359 | 229244.9063 | 24308.08789 | 135671.4375 | 27553.37891 | 35641.4375 | 24954.66602 | 21965.91406 | 48670.03906 | 56711.52344 | 34777.21094 | 30954.77344 | 141007.5938 | 30716.66406 | 34179.40234 | 33785.12891 | 111270.2344 | 41282.1875 | 36800.86719 | 60381.46094 | 36132.63672 | 11283.8457 | 27357.50781 | 37066.375 | 25033.33789 | 31414.74023 |
| A0A087WWT3 | 38679.46094 | 63809.38281 | 82766.30469 | 67989.17188 | 62861.83203 | 53230.37109 | 85483.36719 | 57465.32031 | 77244.33594 | 39504.35938 | 46356.25 | 68293.78125 | 86113.75 | 70436.50781 | 61975.30078 | 64211.33594 | 76803.42188 | 74287.51563 | 102882.1719 | 94794.71875 | 69720.84375 | 90560.28906 | 51605.12891 | 29089.19922 | 77336.26563 | 55054.79688 | 55885.48438 | 65677.01563 | 53364.50781 | 99423.11719 | 70887.75781 | 71100.90625 | 89665.78906 | 101834.2344 | 62830.91797 | 62812.81641 | 60119.25391 | 124163.9688 | 70900.66406 | 60096.63281 | 54546.24609 | 67513.74219 | 81583.36719 | 64783.28125 | 73239 | 67089.28125 | 57690.8125 | 64813.07422 | 53310.23047 | 47289.93359 | 82786.63281 | 92903.78125 | 74803.17969 | 70427.60156 | 60172.25781 | 54272.27734 | 60550.97656 | 65483.9375 | 63388.92969 | 69036.14063 | 73559.77344 | 63326.875 |
| A0A087WWU8 | 0 | 6852.649902 | 0 | 0 | 0 | 0 | 0 | 0 | 0 | 0 | 3615.611816 | 0 | 0 | 0 | 0 | 0 | 0 | 0 | 0 | 2400.519775 | 0 | 0 | 2375.253174 | 0 | 0 | 0 | 0 | 0 | 0 | 786.571106 | 0 | 0 | 0 | 0 | 0 | 0 | 0 | 5805.578125 | 0 | 0 | 0 | 0 | 0 | 0 | 0 | 0 | 0 | 0 | 0 | 0 | 0 | 0 | 0 | 0 | 0 | 0 | 0 | 0 | 0 | 0 | 0 | 0 |
| A0A087WZB5 | 888.3394165 | 2728.716064 | 0 | 1300.116943 | 651.2648315 | 1132.504272 | 1407.964966 | 0 | 1064.108521 | 0 | 0 | 0 | 1489.747681 | 2012.192993 | 2945.780029 | 0 | 0 | 0 | 2097.473389 | 0 | 7212.917969 | 0 | 2270.157227 | 0 | 0 | 3802.164795 | 3869.930908 | 0 | 0 | 0 | 0 | 0 | 0 | 3567.514648 | 0 | 0 | 0 | 0 | 0 | 0 | 0 | 3777.506836 | 0 | 2783.10376 | 0 | 0 | 0 | 2741.959473 | 0 | 0 | 0 | 0 | 0 | 0 | 0 | 0 | 0 | 1094.513184 | 0 | 0 | 0 | 0 |
| A0A087X0Q4 | 144806.3125 | 142945.0781 | 89449.83594 | 139388.0781 | 66908.96875 | 67819.47656 | 112220.7969 | 85468.28906 | 149340.3281 | 54059.13281 | 99710.78906 | 107541.3438 | 104099.1563 | 86650.6875 | 78194.65625 | 109135.4141 | 206849.25 | 78493.29688 | 97259.48438 | 57243.98047 | 67686.96875 | 14064.65137 | 53823.93359 | 153925.6094 | 143340.5469 | 37334.22656 | 97130.82813 | 147902.4375 | 94487.25 | 50881.92969 | 101816.7188 | 171571.8125 | 124539.9609 | 209572.6094 | 81763.25 | 171127.1094 | 106402.9922 | 174507.2813 | 143002.0313 | 46416.02344 | 79829.82813 | 156743.2344 | 76845.77344 | 179417.4219 | 224783.9063 | 233104.1875 | 231210.4375 | 81429.72656 | 92350.57813 | 59103.91406 | 169790.8125 | 135448.0156 | 68644.65625 | 132921.2813 | 68556.25 | 68063.21094 | 117937.4063 | 91184.50781 | 98183.39844 | 209444.9219 | 107918.5938 | 282843.625 |
| A0A087X0S5 | 0 | 1598.689697 | 0 | 2043.00354 | 0 | 2610.222656 | 0 | 1368.20813 | 2541.703857 | 2511.656494 | 0 | 0 | 0 | 0 | 0 | 0 | 0 | 1546.143066 | 1812.608032 | 0 | 0 | 0 | 0 | 0 | 0 | 0 | 0 | 0 | 0 | 1579.08313 | 0 | 0 | 0 | 0 | 0 | 0 | 0 | 0 | 1543.050659 | 0 | 0 | 4457.782227 | 0 | 0 | 0 | 1996.061401 | 0 | 0 | 0 | 1444.496826 | 1335.810913 | 0 | 0 | 0 | 0 | 0 | 0 | 0 | 0 | 1093.408691 | 0 | 0 |
| A0A087X1J7 | 25958.26953 | 57864.51563 | 50356.26953 | 54696.45703 | 37848.9375 | 44634.74609 | 40958.23047 | 39372.86719 | 61291 | 35192.05469 | 39133.83594 | 59283.35547 | 29733.99023 | 25720.73828 | 20232.0293 | 60174.375 | 58720.67969 | 64061.89063 | 41858.05859 | 48290.28125 | 58022.18359 | 58409.61719 | 54004.47656 | 60073.30469 | 72273.51563 | 24407.4668 | 34202.00391 | 51153.39063 | 27437.38867 | 38513.64844 | 81230.67969 | 58722.45703 | 54123.08594 | 58118.51563 | 60566.60547 | 36331.04688 | 47938.16797 | 51707.39063 | 55161.51563 | 31133.85547 | 22122.0293 | 25901.08008 | 51565.91406 | 56593.41016 | 21890.54688 | 29817.2168 | 45254.63672 | 51133.94141 | 38271.83203 | 28874.92188 | 53592.45703 | 33146.14453 | 26015.21484 | 24834.01953 | 48490.60938 | 55386.35156 | 38709.49219 | 64110.01563 | 16195.5293 | 52858.12891 | 42619.01563 | 47634.51953 |
| A0A087X1L8 | 2169.131836 | 2377.90625 | 1544.878174 | 2919.93335 | 2184.299561 | 1731.853149 | 2751.827393 | 1547.019165 | 1904.702881 | 2506.186768 | 959.0308838 | 0 | 4055.632324 | 2874.03125 | 2404.414063 | 0 | 1443.804932 | 0 | 0 | 3547.01709 | 0 | 4010.933105 | 2111.817627 | 1400.627075 | 2111.465576 | 1914.21167 | 1371.844238 | 2800.359375 | 0 | 0 | 4213.277832 | 2075.649658 | 0 | 2777.839111 | 544.8818359 | 3924.914063 | 1397.834717 | 1574.158936 | 3217.963135 | 3011.611084 | 1045.972412 | 3961.294189 | 1563.287231 | 0 | 0 | 1283.877197 | 0 | 0 | 2597.972168 | 1080.535156 | 0 | 2278.796143 | 2244.264404 | 3455.586914 | 3046.823242 | 0 | 1379.306641 | 2852.47998 | 1843.907715 | 1175.576782 | 0 | 0 |
| A0A096LPE2 | 682565.375 | 259311.4844 | 656421.3125 | 416447.125 | 729287.25 | 954767 | 524917.9375 | 740609.6875 | 563200.4375 | 949723.625 | 673181.5 | 531029.375 | 811323.4375 | 729958.75 | 675688.625 | 523090.875 | 328971.1563 | 656194.875 | 970595.625 | 334133.4688 | 522764.2813 | 555060.75 | 961012 | 728592.375 | 353458.5938 | 463134.75 | 710205.75 | 804049.25 | 389913.7813 | 455587.25 | 712293.9375 | 520367.75 | 602871.5 | 896839.625 | 465831.0938 | 631879.25 | 475440.875 | 350900.9375 | 630710 | 421647.9688 | 333666.75 | 440727.4375 | 311112.375 | 481731.4063 | 465363.0313 | 409432.4375 | 449176.75 | 417479.2813 | 678195.8125 | 477519.4375 | 539703.75 | 242407.9375 | 737273.625 | 684087.5 | 451485.625 | 277950.125 | 325037.75 | 380130.7188 | 549272.3125 | 678319.75 | 558429.625 | 607683.625 |
| A0A0A0MRJ7 | 26034.87305 | 17369.34766 | 25774.25 | 37266.19531 | 25946.79297 | 17579.51563 | 15750.89844 | 26165.44141 | 27317.2207 | 31500.34375 | 19370.67188 | 14934.17676 | 26617.76172 | 15214.47266 | 36123.73828 | 30318.67969 | 17642.44727 | 11963.11621 | 25841.23047 | 16931.78711 | 24029.19727 | 21158.24219 | 12731.06348 | 13992.95605 | 19499.24219 | 20855.23438 | 20059.88672 | 14044.6582 | 18493.53125 | 12630.2002 | 16357.04883 | 14919.87012 | 24669.86719 | 18437.67188 | 24363.79297 | 26585.86719 | 20616.50586 | 18125.64453 | 29824.34375 | 18744.67969 | 27084.15039 | 29125.12109 | 28262.66797 | 28413.00391 | 33576.79297 | 19014.63281 | 9504.175781 | 24246.99414 | 37671.28906 | 19900.55664 | 14422.21191 | 15489.3623 | 19139.51367 | 22587.44141 | 17121.98633 | 21674.625 | 13397.10547 | 16483.43164 | 25261.41797 | 27064.80469 | 20975.3418 | 9163.011719 |
| A0A0A0MRZ9 | 0 | 0 | 0 | 0 | 5478.88916 | 0 | 0 | 0 | 0 | 0 | 5359.474121 | 0 | 7018.675293 | 0 | 0 | 0 | 0 | 0 | 0 | 0 | 0 | 0 | 0 | 0 | 0 | 0 | 0 | 0 | 0 | 0 | 0 | 0 | 0 | 0 | 1214.131226 | 0 | 0 | 0 | 0 | 0 | 6757.536621 | 3880.093262 | 0 | 0 | 0 | 0 | 0 | 0 | 0 | 0 | 0 | 0 | 0 | 0 | 0 | 4953.745605 | 0 | 0 | 0 | 0 | 0 | 0 |
| A0A0A0MS09 | 5052.041016 | 59859.58203 | 0 | 6594.005371 | 22397.19531 | 5697.591797 | 8531.366211 | 22152.67773 | 13200.76953 | 21061.44141 | 16340.63672 | 0 | 10923.69336 | 39934.96094 | 71239.58594 | 2887.272949 | 10656.07324 | 126793.9531 | 116285.5234 | 122172.9141 | 22710.49219 | 44693.82031 | 10533.93359 | 20405.83594 | 37865.71094 | 14446.13574 | 236886.6563 | 14450.66895 | 40403.53125 | 79686.32031 | 53864.09375 | 96994.45313 | 10695.88281 | 0 | 0 | 0 | 51067.69922 | 13261.47656 | 99462.85156 | 181826.5 | 6363.475586 | 10236.25 | 27070.31836 | 0 | 185574.2656 | 38895.73047 | 0 | 171543.6875 | 32624.91797 | 51275.62891 | 44721.22656 | 42746.85547 | 7910.921875 | 25058.43164 | 95855.17188 | 36095.09375 | 0 | 56329.71484 | 0 | 59194.54688 | 95442.61719 | 171300.1875 |
| A0A0A0MS14 | 0 | 17451.67969 | 0 | 0 | 0 | 0 | 16703.89453 | 0 | 0 | 0 | 0 | 0 | 0 | 0 | 0 | 0 | 0 | 24760.14648 | 0 | 0 | 0 | 0 | 0 | 0 | 0 | 0 | 0 | 0 | 0 | 0 | 0 | 0 | 0 | 0 | 0 | 8642.859375 | 0 | 0 | 0 | 0 | 0 | 0 | 0 | 0 | 25310.0293 | 0 | 0 | 0 | 0 | 0 | 0 | 0 | 0 | 0 | 0 | 0 | 0 | 0 | 0 | 0 | 0 | 0 |
| A0A0A0MS15 | 295756.2188 | 349536.2188 | 297604.5 | 266971.0938 | 65814.03125 | 57033.70703 | 126425 | 192696.4063 | 123414.5313 | 252612.0938 | 219111.375 | 39609.99219 | 240245.0469 | 232155.3438 | 206057.1875 | 80494.58594 | 300802.7188 | 150980.875 | 484527.4688 | 95896.14844 | 40259.22656 | 192876.25 | 227578.9375 | 77595.42969 | 360642.8125 | 123664.7969 | 341826.375 | 147052.9688 | 84415.25 | 218681.3594 | 201921.2188 | 211962.0781 | 159932.3438 | 271822.4688 | 76315.25 | 31675.31641 | 451446.1563 | 166332.7656 | 264312.4063 | 290321.8125 | 117207 | 219703.8906 | 44873.65625 | 87235.17188 | 140480.3438 | 201424.2188 | 178601.8125 | 440223.2813 | 393802.25 | 304411.25 | 50872.08594 | 868496.4375 | 282086.8125 | 350533.5625 | 195378.4531 | 241171.2344 | 275681.4063 | 209860.125 | 40025.91016 | 136265.9688 | 336275.9688 | 53820.27344 |
| A0A0A0MS51 | 169864.8906 | 292344.0313 | 256144.3438 | 265252.9688 | 262599.1563 | 229840.6563 | 279575.0313 | 281521.3125 | 239238.75 | 197380.8594 | 197609.8125 | 221037.0625 | 273398 | 266278.4063 | 208365.5938 | 125726.9375 | 339945.0625 | 319054.1563 | 198131.5469 | 267393.2813 | 257799.8125 | 371664.6875 | 247442.0469 | 211738.3438 | 334693.125 | 244734.8906 | 115021.3281 | 305673.5938 | 242709.0313 | 362953.0313 | 185793.3438 | 188021.375 | 317330.5 | 311776.375 | 271130.625 | 213202.2813 | 281989.0625 | 218379.4063 | 242991.0625 | 309677.6875 | 192130.125 | 214055.5469 | 278354.375 | 240485.5469 | 240215.625 | 322434.5938 | 257889.3438 | 236839.7969 | 237528.25 | 312687.75 | 340695.5625 | 401226 | 321359.875 | 287025.9063 | 336209.9688 | 325666.8125 | 268322.875 | 366476.25 | 222525.9844 | 234093.4063 | 247014.0156 | 229676.4375 |
| A0A0A0MT36 | 134246.0625 | 119636.5781 | 195883.3438 | 49076.35938 | 144828.1563 | 175418.9844 | 284745.875 | 139984.3906 | 166647.1563 | 84359.21094 | 42012.46094 | 442363.125 | 48023.55078 | 26454.29297 | 25424.08594 | 200464.6875 | 118677.4844 | 139853.75 | 136573.6719 | 48556.73047 | 192250.9219 | 292242.3438 | 143378.6563 | 33544.78516 | 121883.6563 | 116798.4922 | 136267.3438 | 763094.375 | 152303.875 | 143691.7031 | 269542.9063 | 112617.7344 | 74325.42969 | 105296.1484 | 115305.0156 | 131010.1641 | 17154.1582 | 41551.59375 | 166119.9531 | 15222.01758 | 34635.76172 | 115963.0234 | 47957.85156 | 79541.21875 | 106722.8047 | 159284.7031 | 94665.9375 | 41761 | 90474.875 | 99415.94531 | 281389.8125 | 115850.3672 | 422242.7188 | 42950.79688 | 123889.9844 | 59519.94531 | 77053.0625 | 104665.3438 | 173311.2344 | 27328.38672 | 98425.04688 | 73113.42188 |
| A0A0A0MTH3 | 0 | 9348.744141 | 0 | 0 | 0 | 0 | 0 | 0 | 0 | 0 | 0 | 0 | 2421.599854 | 2778.519775 | 5807.076172 | 0 | 0 | 0 | 3057.871582 | 0 | 1825.48938 | 0 | 4127.852051 | 0 | 0 | 2998.868408 | 6333.9375 | 0 | 0 | 0 | 0 | 0 | 795.0734253 | 2084.501465 | 0 | 0 | 0 | 5144.449707 | 0 | 0 | 0 | 0 | 2020.880493 | 0 | 0 | 0 | 0 | 1421.886719 | 0 | 0 | 0 | 0 | 0 | 0 | 1220.987549 | 0 | 0 | 0 | 0 | 0 | 0 | 0 |
| A0A0B4J1U3 | 12004.13965 | 0 | 12488.75879 | 12886.06738 | 18957.68555 | 30408.40625 | 8658.72168 | 41408.06641 | 25388.48828 | 21582.31836 | 12638.93066 | 6686.334473 | 27819.36719 | 0 | 17019.35547 | 16050.66504 | 0 | 28782.64648 | 13547.86035 | 31717.46094 | 79841.35156 | 30105.93359 | 25379.67188 | 49749.92578 | 31146.03711 | 31037.46289 | 35246.5 | 0 | 39041.50781 | 58398.32422 | 84080.95313 | 29786.91406 | 23675.08594 | 20775.93164 | 31286.05273 | 39884.39453 | 15613.00879 | 24549.48633 | 43621.41406 | 23118.58594 | 57846.71094 | 75835.41406 | 20825.88086 | 37079.03516 | 41782.14453 | 34429.88672 | 24464.02148 | 35673.05078 | 27437.30664 | 28735.61719 | 23654.45117 | 34993.09375 | 14882.34668 | 5509.444336 | 42564.08594 | 28673.03125 | 31286.44336 | 52002.50781 | 28921.92773 | 82915.9375 | 26578.47461 | 46248.71484 |
| A0A0B4J1U7 | 15862.03613 | 19017.30859 | 19428.75977 | 61815.04688 | 30572.26172 | 23659.82422 | 68535.53125 | 44881.95703 | 88105.64063 | 109135.1641 | 73327.07813 | 18068.11133 | 128574.4063 | 15033.24023 | 80637.44531 | 120030.8906 | 86391.71875 | 10273.07617 | 51974.80469 | 12841.02148 | 40403.86719 | 21859.04102 | 166292.375 | 70279.47656 | 44463.25 | 18026.08594 | 94390.09375 | 99677.49219 | 74394.4375 | 85629.85938 | 20706.4043 | 129314.4141 | 12022.56543 | 86071.79688 | 128910.2031 | 42039.15625 | 134990.4375 | 12608.0498 | 145656.5 | 86086.89844 | 28277.97266 | 87194.71875 | 38017.55859 | 34036.67188 | 28194.30273 | 130372.9453 | 31704.25781 | 53002.36719 | 41036.58203 | 17833.14453 | 125910.7656 | 73712.34375 | 104899.5703 | 92059.39063 | 17656.61914 | 72696.375 | 106573.0469 | 14753.05859 | 85131.78125 | 29169.33789 | 36402.14453 | 53366.55859 |
| A0A0B4J1V0 | 39280.89844 | 12190.67773 | 22629.92773 | 15217.9707 | 17179.8418 | 14709.93262 | 23133.63086 | 38325.63672 | 25605.75977 | 17752.03125 | 36793.66016 | 39844.48438 | 14517.62793 | 15248.4082 | 17533.91016 | 31278.36719 | 33697.34375 | 25620.97852 | 10378.37012 | 40087.26172 | 37279.54688 | 27425.06055 | 24085.14258 | 31914.41211 | 44087.74609 | 32322.83789 | 15548.96582 | 25446.1543 | 32561.52344 | 55128.42188 | 62447.07813 | 14807.06348 | 15462.3916 | 15602.09082 | 26656.11719 | 24159.50391 | 29038.47266 | 17256.31836 | 40334.28516 | 18268.29492 | 42041.53125 | 17425.23828 | 27839.57227 | 34934.13281 | 33292.69141 | 26135.9375 | 39401.09766 | 24108.60938 | 12271.57031 | 45241.36328 | 33863.75391 | 32820.64844 | 48531.80469 | 21907.125 | 41999.98438 | 30424.1543 | 33580.77734 | 61491.13672 | 27729.99609 | 22156.39063 | 26943.11523 | 45067.39063 |
| A0A0B4J1V1 | 87696.53125 | 0 | 104983 | 38045.04297 | 202566.1875 | 183850.4219 | 78539.57031 | 17157.23047 | 0 | 34891.51172 | 76467.42969 | 148754.5313 | 21004.40625 | 6979.876953 | 158456.3281 | 36396.71484 | 55766.87109 | 41285.94922 | 26062.64063 | 300291.8125 | 44272.83203 | 54485.53125 | 0 | 0 | 51528.07813 | 0 | 42691.19531 | 57571.00391 | 114394.8438 | 196939.125 | 32349.73242 | 47187.62891 | 49012.11328 | 173802.1719 | 62801.14063 | 52094.04297 | 51152.84766 | 0 | 60150.09375 | 271941.5938 | 42581.51953 | 18651.29297 | 56995.78906 | 13183.39648 | 41575.12891 | 66515.46094 | 0 | 9543.773438 | 19526.40625 | 18349.17578 | 17417.16016 | 91917.95313 | 43596.63672 | 16512.23047 | 44297.41797 | 3726.989014 | 17192.32031 | 83948.71094 | 65621.88281 | 26821.66992 | 36842.04688 | 58071.84375 |
| A0A0B4J1V2 | 21295.38281 | 3349.625977 | 3442.639404 | 0 | 4807.47998 | 8494.722656 | 3564.294189 | 3229.645264 | 8380.342773 | 9886.751953 | 7397.87207 | 1937.624512 | 0 | 14924.55469 | 6587.37793 | 2373.307129 | 3572.02832 | 5318.526855 | 2470.430664 | 6091.323242 | 3206.713867 | 21379.41016 | 4733.425293 | 0 | 5292.578125 | 6526.387695 | 9089.017578 | 8274.780273 | 6751.402344 | 8802.734375 | 12501.2959 | 50998.05078 | 2293.74292 | 3682.606201 | 1409.315186 | 1981.912109 | 3629.89502 | 3153.15625 | 7102.55127 | 15721.6543 | 5013.757324 | 8580.743164 | 6107.654785 | 3138.643799 | 2808.826416 | 8422.288086 | 69481.28125 | 6925.379883 | 3304.5271 | 5733.53418 | 5892.826172 | 0 | 3978.922607 | 2646.228027 | 17682.26172 | 32052.10352 | 2314.403564 | 3370.623535 | 5502.34668 | 4832.171387 | 69418.52344 | 5519.137695 |
| A0A0B4J1X5 | 77698.95313 | 44841.43359 | 51033.22656 | 81833.32813 | 43629.01563 | 67930.8125 | 87916.69531 | 50084.08984 | 35675.37109 | 55774.42969 | 69431.89063 | 41060.87891 | 35785.46094 | 50846.71875 | 22264.80078 | 39578.875 | 149220.375 | 50993.82422 | 63518.05859 | 106903.875 | 69338.64844 | 0 | 52407.66406 | 42743.68359 | 109093.0313 | 0 | 37755.3125 | 40473.16406 | 41500.76172 | 26743.40039 | 0 | 41314.1875 | 136190.1719 | 95652.58594 | 46772.85156 | 33171.81641 | 35906.33594 | 75859.57813 | 45442.68359 | 41938.16016 | 57536.07813 | 0 | 51833.06641 | 39036.11328 | 63406.30859 | 77746.59375 | 117685.6641 | 107973.2031 | 56708.83203 | 80533.83594 | 86290.9375 | 46139.23828 | 44958.01953 | 74022.24219 | 59384.66797 | 52448.08594 | 42922.99609 | 51831.38672 | 39983.67188 | 35780.03906 | 50025.38281 | 167184.2188 |
| A0A0B4J1X8 | 77086.96094 | 61065.80469 | 153674.6406 | 82760.6875 | 68097.71875 | 58337.85156 | 73204.76563 | 109231.3047 | 66435.60938 | 73104.22656 | 82116.60938 | 27108.63477 | 76801.14063 | 197773.9375 | 176937.4063 | 90771.84375 | 83486.30469 | 84691.16406 | 88277.25 | 84835.98438 | 74935.44531 | 78626.30469 | 57261.96875 | 93602.92188 | 57740.64844 | 37704.82031 | 42598.10938 | 90727.42969 | 121836.9609 | 154562.8438 | 39337.50781 | 56108.95313 | 82400.85938 | 81533.89844 | 78339.96875 | 129306.3984 | 177420.9688 | 52436.53906 | 116960.4922 | 201021.0469 | 103957.6016 | 79670.23438 | 138830.8594 | 178536.9375 | 184429.0313 | 128615.7188 | 76522.375 | 77887 | 86038.97656 | 124298.0391 | 100267.2656 | 92354.65625 | 110537.2656 | 101923.6406 | 131490.9219 | 48646.28125 | 54700.31641 | 76836.67969 | 67026.4375 | 48155.51563 | 102841.5859 | 140669.125 |
| A0A0B4J1Y8 | 64857.89063 | 438661.125 | 0 | 104134.7422 | 60802.80078 | 192388.6719 | 151691.0469 | 0 | 72879.98438 | 95469.58594 | 72018.25781 | 45222.59766 | 93477.34375 | 150015.1875 | 164305.9531 | 114038.9922 | 54663.06641 | 87800.50781 | 32490.75586 | 111765.6484 | 52781.73828 | 33813.05078 | 82258.59375 | 162230.6094 | 89717.02344 | 69305.64063 | 39339.01563 | 47462.36719 | 85426.55469 | 78341.03125 | 0 | 107985.2969 | 73837.61719 | 32995.75391 | 125275.4844 | 119500.875 | 156591.7344 | 32263.99219 | 158160.5781 | 130203.5469 | 258384.2031 | 208420.8906 | 0 | 76406.875 | 91615.82031 | 141517.2031 | 131664.5938 | 69203.10938 | 82582.04688 | 0 | 130366.625 | 117278.4375 | 0 | 146932.4375 | 183288.1719 | 46987.93359 | 77091.36719 | 174042.8125 | 0 | 0 | 49317.87109 | 35568.87109 |
| A0A0B4J231 | 10638459 | 14958653 | 17739932 | 12439384 | 8067932 | 14200050 | 11813375 | 8593449 | 11244798 | 12381658 | 21386996 | 12896119 | 14254743 | 10844515 | 19046500 | 8691962 | 8232626 | 6516789 | 15177407 | 28391.54297 | 9750843 | 10229064 | 7367531 | 19367998 | 16218084 | 5582485.5 | 7341142 | 12693713 | 17602246 | 17639280 | 17815208 | 6821926.5 | 2186022.5 | 16725665 | 22039.2207 | 12824266 | 16741862 | 14368570 | 18576430 | 12722718 | 12733531 | 18284508 | 18900552 | 11894257 | 19176008 | 10112519 | 19490908 | 11841657 | 13902752 | 8497646 | 16638618 | 19817972 | 17231636 | 7993573 | 14765792 | 4811517 | 7080799.5 | 13363775 | 7152107 | 6174574 | 9280309 | 13584246 |
| A0A0B4J2D9 | 16109.72461 | 10904.58496 | 24338.40234 | 19506.29688 | 15724.41699 | 12947.22168 | 11595.37891 | 17555.11719 | 0 | 18563.08594 | 7624.456543 | 10354.16797 | 16247.95703 | 16482.43945 | 12971.53906 | 17532.30664 | 20783.29492 | 25859.28906 | 19274.13281 | 17819.69922 | 26316.63672 | 18643.49805 | 10372.03223 | 6254.208984 | 22926.92578 | 0 | 19971.26758 | 16370.20508 | 17197.41211 | 13535.06738 | 7075.036133 | 15693.73535 | 11881.82422 | 13412.42383 | 10084.25293 | 15061.69531 | 22571.37109 | 10803.33691 | 9504.623047 | 9914.395508 | 10720.64746 | 1406.146973 | 39127.20703 | 35037.26953 | 16552.74414 | 29328.06836 | 40854.74219 | 5890.512695 | 20542.18359 | 0 | 16979.30273 | 2263.692627 | 10144.0625 | 15273.00879 | 14405.57031 | 0 | 9909.388672 | 10080.69238 | 15187.82715 | 13367.00293 | 18331.54688 | 23033.41602 |
| A0A0C4DGZ8 | 7411.321289 | 32438.75586 | 10859.80566 | 13023.56934 | 24298.01953 | 11358.69922 | 16209.72754 | 18825.89453 | 20633.85156 | 23307.34961 | 22958.36133 | 11194.93457 | 10705.0625 | 44736.81641 | 16971.24023 | 15073.75391 | 12978.5166 | 8587.40625 | 24402.02734 | 21928.1582 | 12539.9541 | 12392.85547 | 12064.7168 | 17768.81836 | 11111.42871 | 10004.28613 | 15349.68555 | 31755.52148 | 21985.34375 | 10384.10156 | 47321.35938 | 4217.249023 | 1347.7854 | 9206.120117 | 18613.52734 | 11218.94629 | 16142.86914 | 6699.348633 | 19924.96875 | 20103.48828 | 15030.27637 | 11041.78516 | 36029.51953 | 12606.33691 | 16298.50684 | 0 | 19600.83789 | 18603.57617 | 32897.04688 | 8166.077148 | 14319.29492 | 15119.12109 | 0 | 10056.41309 | 17368.57227 | 15496.6377 | 0 | 8747.949219 | 12512.32324 | 14142.22461 | 21531.21484 | 4942.484863 |
| A0A0C4DH21 | 59070.08984 | 24391.16797 | 22698.94531 | 56987.61328 | 25411.02148 | 40546.77344 | 34336.85547 | 41578.92969 | 31911.08984 | 45159.27734 | 115330 | 26848.61328 | 15170.66309 | 24866.42578 | 50790.01953 | 56634.37891 | 21356.375 | 27066.7793 | 61235.82422 | 26765.58789 | 24660.38477 | 22717.95898 | 40358.34766 | 29958.17188 | 47167.33594 | 54074.63281 | 27289.98242 | 48261.9375 | 39430.53516 | 71418.59375 | 42905.27734 | 72502.4375 | 61308.30078 | 38641.91406 | 32951.67969 | 33945.90625 | 40375.80859 | 19841.87891 | 35074.98438 | 62529.26172 | 28717.00586 | 29292.40625 | 71287.85156 | 15826.12402 | 37158.0625 | 50288.85547 | 54168.90234 | 42276.8125 | 24447.67578 | 22834.65234 | 63053.65625 | 44135.26172 | 31805.31641 | 31785.64063 | 57330.80078 | 27443.38672 | 23919.91992 | 50533.30078 | 33774.71484 | 30903.30859 | 46851.49219 | 24078.99219 |
| A0A0C4DH24 | 38592.65234 | 35441.9375 | 57985.31641 | 40261.95703 | 22462.65234 | 8403.744141 | 6124.579102 | 28626.6582 | 5837.708008 | 24483.17578 | 33482.17969 | 11948.39844 | 86480.47656 | 72150.92969 | 31587.45117 | 48529.44141 | 31165.97266 | 20681.65625 | 21077.80078 | 38595.5625 | 20865.25 | 17555.5918 | 78094.28906 | 0 | 47762.35938 | 4886.771484 | 36111.27344 | 18961.34766 | 11331.21387 | 55542.85156 | 32366.01953 | 17579.07813 | 6358.791992 | 29024.9375 | 21729.79688 | 12127.05371 | 82370.14844 | 47849.83984 | 21878.45313 | 65898.71875 | 50344.08984 | 28093.33984 | 95695.11719 | 67962.15625 | 90327.57031 | 28600.08594 | 32445.05859 | 33929.25 | 60947.79297 | 31940.71094 | 16877.125 | 34938.625 | 12284.16602 | 35827.71875 | 22098.42773 | 47335.71484 | 23713.79297 | 31522.48828 | 115866.6563 | 30540.63086 | 47088.85938 | 114619.9844 |
| A0A0C4DH25 | 566798.375 | 369341.7813 | 548729.75 | 200011.4844 | 339428.4375 | 436082.5313 | 222952.2813 | 425828.2813 | 413429.8125 | 338051.9688 | 371668 | 302241.6875 | 357199.5313 | 399500.0313 | 244336.6094 | 510652.8438 | 607016 | 460403.3125 | 338087.7188 | 336877.3438 | 715053.6875 | 345855.25 | 591307 | 471709.9375 | 1191085 | 365121.4063 | 780972.875 | 706091.6875 | 562423.5 | 977807.5625 | 747680.75 | 300467.625 | 280660.4375 | 499777.0625 | 689198.25 | 377687.9375 | 474601.0313 | 285327.625 | 321440.875 | 390316 | 471080.8438 | 524113.8438 | 795586.125 | 608416.25 | 637939.125 | 602503.125 | 827898.375 | 324614.1563 | 403173.4688 | 637864.9375 | 636480.3125 | 641922 | 657389.625 | 296251.4688 | 698796 | 473452.8438 | 594593.1875 | 639190.8125 | 520173.125 | 572152.125 | 517743.0938 | 646722.6875 |
| A0A0C4DH29 | 48697.72656 | 41944.89844 | 47181.84375 | 28448.89648 | 40850.71875 | 40012.01953 | 22257.66211 | 38542.15234 | 41434.89453 | 24474.8125 | 57428.66016 | 20541.42773 | 30951.9043 | 42833.82031 | 30445.50586 | 0 | 46181.75781 | 21862.19531 | 57846.67969 | 26613.08203 | 48812.62891 | 38795.42188 | 28400.85742 | 58483.16797 | 0 | 17505.06055 | 41090.62109 | 36304.09766 | 30911.32422 | 14517.42188 | 14797.68652 | 44394.07422 | 32333.08398 | 21464.05859 | 60974.87109 | 27047.61914 | 69389.5625 | 20414.71875 | 20372.60156 | 19779.86719 | 35394.95703 | 18687.84375 | 34604.25781 | 27852.47461 | 28967.65039 | 18900.12695 | 53711.16406 | 20395.2168 | 24774.13672 | 27325.97656 | 37117.62891 | 30571.27344 | 29105.16406 | 23921.68555 | 24621.76563 | 65189.83984 | 38462.40234 | 26725.36719 | 13519.12695 | 23159.9707 | 54413.76563 | 26890.82227 |
| A0A0C4DH31 | 41342.06641 | 39186.85938 | 21061.42188 | 37382.71875 | 28892.69336 | 34950.53516 | 21801.45703 | 32336.29102 | 46503.88672 | 25817.94141 | 29229.125 | 23615.30078 | 23846.74414 | 20168.13672 | 34245.90234 | 18444.31055 | 30865.48438 | 33823.90625 | 28686.0957 | 38036.32031 | 42124.60547 | 34978.01172 | 36314.49219 | 36233.68359 | 32576.35156 | 19745.11914 | 31340.85547 | 35082.17578 | 25844.50781 | 55625.67188 | 39937.33594 | 27146.49414 | 28331.39063 | 39308.43359 | 15403.73047 | 23404.83789 | 24890.69531 | 26195.42578 | 41100.83594 | 42490.42188 | 20250.80078 | 34001.875 | 47406.27344 | 42341.75391 | 45621.10156 | 23161.42383 | 19156.23633 | 16652.08594 | 17310.72266 | 39717.4375 | 34472.5625 | 21999.30078 | 30271.26563 | 23939.49414 | 23897.41211 | 24314.58789 | 23502.77734 | 29855.35156 | 23333.07227 | 18983.29688 | 17956.38672 | 24689.16406 |
| A0A0C4DH32 | 70127.1875 | 32942.34766 | 138746.8906 | 72507.73438 | 82195.04688 | 106149.1563 | 122301.625 | 35978.27734 | 30941.9082 | 88616.42969 | 29082.09766 | 38853.83594 | 85956.89844 | 57092.21875 | 65387.90625 | 81416.48438 | 85184.82813 | 66835.65625 | 35357.55469 | 90559.53906 | 89784.38281 | 65326.60156 | 39348.66406 | 82406.3125 | 56221.32422 | 84133.0625 | 0 | 65868.91406 | 19588.96484 | 0 | 97698.15625 | 39683.97266 | 97890.91406 | 128206.2734 | 23167.375 | 51820.06641 | 55107.02344 | 0 | 109368.7734 | 52649.27344 | 57548.42969 | 92347.27344 | 58940.13672 | 110352.5859 | 92658.89844 | 46835.72656 | 67622.26563 | 18929.41016 | 36683.26563 | 0 | 39593.15234 | 80004.16406 | 108694.7734 | 79135.77344 | 85683.42188 | 58271.12891 | 97753.39063 | 60845.3125 | 64120.67578 | 77887.46094 | 64105.85156 | 55169.03906 |
| A0A0C4DH33 | 9183.898438 | 6614.159668 | 6065.493652 | 25301.36328 | 16842.52344 | 8795.632813 | 4596.365723 | 17330.51953 | 22263.18359 | 6389.228516 | 10736.04492 | 4414.038574 | 0 | 2611.081299 | 6204.70752 | 22992.61914 | 8036.089844 | 11035.95801 | 7083.348145 | 8522.428711 | 7910.805176 | 10486.33496 | 3387.535889 | 11604.53516 | 3949.165771 | 2274.20874 | 7021.042969 | 29514.08594 | 4208.333008 | 17580.28516 | 20732.62109 | 12342.45801 | 19655.0293 | 7593.553711 | 7479.671875 | 4847.701172 | 6875.367188 | 9336.935547 | 4277.6875 | 0 | 5378.111328 | 16113.63086 | 3047.592285 | 9429.018555 | 24992.53711 | 12249.38281 | 0 | 4057.525879 | 4331.484863 | 11958.78027 | 15701.26855 | 17224.78125 | 14843.02832 | 6498.425293 | 10154.0957 | 13270.65039 | 11929.9541 | 10715.78613 | 15990.65723 | 3208.479492 | 12074.40039 | 9918.577148 |
| A0A0C4DH34 | 1605240.875 | 742919.9375 | 1517413.25 | 902144.3125 | 362664.3438 | 1115601.125 | 945412.9375 | 1128245 | 1229193.5 | 1531097.875 | 8040311.5 | 736052.5625 | 390364.6875 | 737862.125 | 125356.3203 | 1122296.375 | 1376563.875 | 541792.125 | 979319 | 1101207.625 | 2193101.5 | 1962796.5 | 1042035.813 | 84758.89063 | 1621839.375 | 713154.1875 | 1393792.25 | 845897.1875 | 1224412.875 | 1802972.125 | 1219873.125 | 1117105.5 | 804796.0625 | 791509.0625 | 1293018.875 | 272568.9063 | 1341054.625 | 1216876.375 | 163253.125 | 969407.8125 | 1495091.375 | 1462313.5 | 1909713.125 | 1558222.875 | 1086450.75 | 2109351 | 1077166.375 | 880799.25 | 1270949.25 | 1536409.75 | 1559942.375 | 2333952.5 | 1159428.25 | 1045820.5 | 1438293.875 | 161776.7188 | 1269290.5 | 1192104.875 | 1506996.5 | 903224.1875 | 1711854.625 | 1955667 |
| A0A0C4DH36 | 72535.03906 | 52917.88281 | 45124.46094 | 27053.4375 | 64749.73438 | 61513.20313 | 63246.45703 | 61183.42188 | 67898.66406 | 76817.8125 | 79299.35938 | 79358.20313 | 59599.03906 | 74706.1875 | 44906.44531 | 80195.74219 | 67752.26563 | 132965.7188 | 43136.57422 | 55859.74609 | 99809.03125 | 77545.61719 | 69069.59375 | 57055.42188 | 97634.40625 | 48601.21875 | 76872.67969 | 69025.17969 | 55544.28125 | 65777.25 | 95951.41406 | 56783.36719 | 68417.53125 | 60455.375 | 45657.23438 | 63622.82422 | 61314.90625 | 55570.89063 | 80271.15625 | 38737.16406 | 47234.25781 | 49449.99609 | 69467 | 63811.63281 | 93332.94531 | 45140.25781 | 57333.08594 | 36669.44531 | 51393.30469 | 37880.03906 | 58014.67188 | 87233.15625 | 46428.60156 | 50568.17188 | 44527.71094 | 46481.27344 | 52150.92969 | 55627.4375 | 37358.82031 | 51178.16797 | 37064.33984 | 74289.57813 |
| A0A0C4DH38 | 126684.4609 | 30613.40234 | 258958.75 | 91148.90625 | 81212.15625 | 90826.41406 | 109014.7344 | 223360.4219 | 20238.14258 | 98082.52344 | 98938.24219 | 83800.65625 | 78733.71094 | 110463.0391 | 188496.1875 | 129787.2969 | 183454.75 | 38941.42188 | 129172.8047 | 127206.1875 | 203031.6406 | 50918.58594 | 142574.375 | 121279.3984 | 155517.0625 | 75404.8125 | 135599.1875 | 152159.2188 | 190441.1563 | 146880.8906 | 115274.4688 | 174997.9688 | 36198.47266 | 73749.8125 | 128699.9531 | 60011.40625 | 158330.4844 | 64441.59766 | 100657.3906 | 134897.0625 | 99363.78906 | 181788.0625 | 175694.8438 | 38206.78516 | 113418.7188 | 16659.48438 | 250559.2656 | 106070.6094 | 136896.9688 | 107790.8047 | 149066.7813 | 53681.07031 | 163570.8594 | 23261.12305 | 91169.70313 | 156680 | 144890.9063 | 96312.11719 | 136631.7813 | 79659.28125 | 147479.6875 | 131312.1719 |
| A0A0C4DH39 | 100070.2969 | 57258.07031 | 17202.31641 | 46511.26172 | 5973.040039 | 13151.14844 | 41084.46094 | 86130.46094 | 18475.67578 | 28771.09961 | 32039.54297 | 4293.230469 | 12832.74609 | 29533.48242 | 38932.87109 | 35353.05469 | 12044.88672 | 7799.350586 | 42275.625 | 34949.28906 | 10509.34766 | 31116.17188 | 6609.680664 | 9774.541992 | 34703.09375 | 39732.53516 | 13168.70703 | 8699.607422 | 10037.29883 | 48140.49609 | 47721.13672 | 37879.79688 | 25902.91406 | 2372.004639 | 13951.94531 | 12512.06738 | 3467.317627 | 18995.54102 | 30631.85547 | 2698.875977 | 92480.25781 | 18696.09375 | 83816.75781 | 11963.97559 | 39676.88672 | 45474.82422 | 56643.3125 | 5027.557129 | 5448.766113 | 32122.16406 | 2376.857422 | 9533.915039 | 5086.110352 | 14463.50098 | 31365.5332 | 4768.550293 | 61340.89844 | 35556.16016 | 52742.25 | 23146.82031 | 16840.26367 | 77410.94531 |
| A0A0C4DH43 | 450016.875 | 463001.9688 | 38001.29297 | 56713.71484 | 601732.375 | 676018.9375 | 42904.01563 | 663705.3125 | 22826.65039 | 1356368.125 | 678391.3125 | 585206.0625 | 4674.413574 | 11882.24023 | 525342.9375 | 331322.6563 | 515367.1563 | 808302 | 1211966.75 | 506896.8125 | 535142.75 | 313090.7188 | 778882.375 | 498780.8438 | 74521.33594 | 366380.5 | 38381.74609 | 536547.3125 | 842071.25 | 143133.9063 | 845380.9375 | 32554.50586 | 18960.32227 | 423613.0625 | 342561.25 | 40019.25391 | 417207.4063 | 1007351.313 | 360348.75 | 66295.82813 | 603625.1875 | 642975.5 | 563268.0625 | 26594.60742 | 164966.0625 | 53412.53125 | 492571.1563 | 441397.75 | 287759.625 | 851794.4375 | 1648583.125 | 1731765.875 | 137821.875 | 903376.375 | 0 | 46377.44922 | 97642.20313 | 600570.5625 | 607733.1875 | 0 | 24524.10352 | 621645 |
| A0A0C4DH55 | 8715454 | 11229568 | 5489365 | 5785337.5 | 10924262 | 7702251.5 | 3516560.5 | 4187425 | 11658913 | 9146908 | 3499464.25 | 8350817.5 | 7387928 | 9211950 | 6675375 | 9621502 | 10707474 | 11427423 | 7264268 | 4043743 | 11440239 | 10232356 | 11100458 | 9628110 | 14314199 | 6943472.5 | 10869966 | 11852030 | 4104857.75 | 14818848 | 10955543 | 7374473 | 6214263 | 10869409 | 11157378 | 8058700.5 | 9720108 | 4388682.5 | 10258361 | 7369714 | 6455753.5 | 8419660 | 13354829 | 10612610 | 8204979.5 | 11597015 | 11866248 | 8068511 | 7797467 | 8797743 | 11869164 | 14110891 | 10787505 | 7277465.5 | 5357645 | 3332117 | 10406937 | 9853089 | 5139927 | 6958745.5 | 5616184.5 | 16672601 |
| A0A0C4DH67 | 0 | 0 | 25213.87109 | 7025.051758 | 32662.5957 | 14513.40039 | 49243.09766 | 10211.69434 | 24252.71484 | 0 | 27776.58203 | 31654.65039 | 10770.80762 | 3705.560303 | 2533.908447 | 0 | 20074.27344 | 16461.625 | 7665.630371 | 32006.54297 | 32693.05859 | 36262.66797 | 10246.54102 | 9903.676758 | 29298.58398 | 6257.223633 | 36891.48438 | 13712.75684 | 28789.40234 | 49649.04297 | 41244.75 | 6769.358887 | 6469.068848 | 10100.625 | 26986.98047 | 18517.48633 | 16729.80469 | 17211.0918 | 11890.32813 | 0 | 15245.89258 | 13461.3916 | 40534.70703 | 41856.74219 | 27060.98242 | 62928.71094 | 55536.04688 | 27984.57031 | 8715.522461 | 20972.42578 | 32881.14453 | 38811.21484 | 15831.36719 | 0 | 23313.40625 | 16951.43164 | 29902.90234 | 52530.07031 | 30692.10742 | 12003.49219 | 38288.04688 | 33989.24609 |
| A0A0C4DH73 | 133372.5156 | 67264.26563 | 71269.76563 | 83608.15625 | 140590.7813 | 161447.2969 | 65890.21094 | 117242.7188 | 46906.57422 | 120917.3125 | 89073.1875 | 86038.27344 | 74468.41406 | 114747.8906 | 71549.85938 | 71852.44531 | 113578.3125 | 135627.2188 | 45047.64063 | 87871.57031 | 90395.35938 | 93367.5625 | 39508.67188 | 33677.00781 | 166618.125 | 51563.30859 | 83165.69531 | 61699.8125 | 45618.30078 | 34097.40625 | 149687.1875 | 92973.52344 | 25967.52539 | 69857.07813 | 140505.1719 | 73960.3125 | 59761.85547 | 65224.51563 | 83930.78125 | 109897.9219 | 98055.4375 | 111558.9375 | 24068.57422 | 51899.26172 | 97963.45313 | 130681.9453 | 119034.8438 | 129823.3906 | 97199.71094 | 51803.6875 | 77342.59375 | 84034.42188 | 34761.46875 | 25396.65039 | 85539.73438 | 112447.125 | 126437.2266 | 172056.8125 | 49460.58984 | 68037.70313 | 58661.21484 | 82103.50781 |
| A0A0G2JI36 | 0 | 0 | 12481.95117 | 0 | 16528.54297 | 0 | 14292.53223 | 0 | 0 | 0 | 0 | 19334.80078 | 0 | 4262.873047 | 0 | 0 | 0 | 0 | 0 | 12217.45703 | 22645.14844 | 20370.6582 | 22058.20703 | 17064.17578 | 0 | 0 | 0 | 0 | 12081.88184 | 0 | 0 | 0 | 0 | 0 | 0 | 19759.44922 | 0 | 0 | 19006.91797 | 0 | 0 | 1675.701782 | 14741.72754 | 0 | 0 | 15237.62988 | 13699.89648 | 0 | 0 | 0 | 0 | 0 | 0 | 0 | 0 | 4298.947266 | 0 | 24885.66211 | 0 | 0 | 0 | 16439.36328 |
| A0A0G2JL69 | 24178.09961 | 24363.96875 | 39422.6875 | 35997.60156 | 24429.54883 | 28053.26172 | 41963.38672 | 37166.66016 | 30378.32031 | 21030.80078 | 44482.20313 | 32187.73633 | 33269.58594 | 27437.33203 | 41475.71094 | 35162.35938 | 34805.95703 | 39196.89844 | 47308.84375 | 35482.30469 | 33110.89844 | 33195.74609 | 27182.1582 | 33337.46094 | 36928.41406 | 28464.10938 | 40686.78906 | 44000.64063 | 34221.12891 | 32784.88281 | 35236.91797 | 33598.98438 | 30650.04688 | 28574.30078 | 37364.42969 | 22795.83203 | 37487.0625 | 38454.63281 | 28394.18359 | 46676.38281 | 35219.4375 | 29032.07422 | 44963.90625 | 38098.0625 | 19951.49805 | 48670.88672 | 34401.3125 | 45366.0625 | 44990.96875 | 23737.98438 | 44053.96875 | 25696.50781 | 46343.78906 | 29600.1875 | 37216.60938 | 13714.11621 | 31576.05078 | 38983.75 | 39277.02344 | 30508.23828 | 43234.0625 | 41723.375 |
| A0A0G2JMB2 | 2524202 | 1535753 | 3688775.5 | 1526564.5 | 3188154.5 | 1735711.375 | 1604480.5 | 3023491 | 3749750.5 | 2299404.75 | 6898225 | 2105084 | 2350532.25 | 1018697.375 | 3162637.5 | 1608376 | 2871121 | 3278489 | 5179965 | 4209178 | 2738735 | 2057094.25 | 2983997 | 1780923.75 | 2883735 | 1636608.375 | 4396996 | 1349005 | 2831573.5 | 3382357 | 2341017 | 2162465.75 | 1653746.75 | 3702584 | 3975019.75 | 2550787 | 2299059.5 | 3679242 | 5042567 | 2540626 | 2720710 | 2003457 | 2611150.5 | 1438598 | 2084215 | 2558186.25 | 1547085.75 | 1468169.75 | 3538386.75 | 3452785.5 | 2770287.25 | 5992814 | 3720377.25 | 2767959.5 | 2873566 | 1687626.25 | 1363731.75 | 1794638.5 | 2473394.25 | 2269013 | 3300262 | 3295867 |
| A0A0G2JMI3 | 0 | 0 | 0 | 0 | 0 | 0 | 0 | 0 | 0 | 0 | 0 | 0 | 0 | 0 | 0 | 0 | 0 | 0 | 0 | 0 | 0 | 0 | 0 | 0 | 0 | 0 | 15729.72559 | 0 | 0 | 0 | 0 | 0 | 0 | 0 | 0 | 0 | 0 | 0 | 0 | 0 | 4140.09668 | 0 | 0 | 0 | 0 | 0 | 0 | 0 | 0 | 0 | 0 | 0 | 0 | 0 | 0 | 0 | 0 | 0 | 0 | 0 | 0 | 0 |
| A0A0G2JPR0 | 258659.9688 | 1125680.25 | 541815.4375 | 321998.9063 | 194947.0938 | 393770.125 | 329325.2188 | 618592.75 | 583312.8125 | 761294.25 | 401328.625 | 343641.5313 | 226456.5625 | 336360.7188 | 499158.5938 | 318787.375 | 254981.125 | 556836.25 | 359889.3125 | 575132.125 | 364665.4375 | 570992 | 399341.875 | 255764.6719 | 283941.5938 | 505657.7813 | 343791.875 | 539776.4375 | 521999.0938 | 389176.1563 | 600546.0625 | 706017.4375 | 394327.6563 | 387234.125 | 300311.0625 | 207653.5781 | 310704.1875 | 451255.3125 | 318528.8125 | 328175.125 | 398625.3125 | 314723.4375 | 273213.2188 | 206734.8438 | 655034.25 | 313146.8125 | 472957.4063 | 467558.1875 | 411243.7188 | 174122.1094 | 302382.1875 | 738158.5625 | 462346.2813 | 578161.25 | 372571.2188 | 376633.8125 | 566035.6875 | 413195.3125 | 560346.75 | 348386.1563 | 769837.8125 | 535018.625 |
| A0A0G2JRQ6 | 252072.0469 | 158031.3906 | 200535.4688 | 123731.0234 | 242449.9219 | 244070.4219 | 139548.6719 | 291961.2813 | 216023.4531 | 161322.6094 | 299347.7813 | 131683.7344 | 296769.1875 | 145352.5938 | 294584.9375 | 375336.9063 | 429441.5 | 340188.4375 | 206141.875 | 392954.1875 | 203955.6563 | 188833.8438 | 413882.0938 | 282226.25 | 275401.5938 | 256389.0625 | 243233.6875 | 399404.8125 | 325216.4688 | 489376.9688 | 140181.2813 | 188233.5 | 163092.2656 | 381970.8438 | 223700.0938 | 295388.4688 | 306861.125 | 246443.7344 | 187185.1719 | 252925.3281 | 371040.2813 | 166210.2188 | 288757.9063 | 386570.875 | 201980.3906 | 282515.4063 | 376724.4063 | 380125.7813 | 442267.3125 | 343220.5313 | 589355 | 246350.9063 | 236083 | 319914.7813 | 348327.2188 | 242322.2969 | 256063.2656 | 143311.4688 | 177833.6563 | 344325.875 | 257630.2031 | 285588.5313 |
| A0A0G2JSC0 | 63427.74219 | 34234.10938 | 77899.01563 | 52056.27734 | 43319.64063 | 0 | 18725.17578 | 36798.66406 | 27911.54492 | 29714.08203 | 19001.34375 | 37716.07422 | 32298.66992 | 22383.96094 | 67885.6875 | 3141.934326 | 35571.89844 | 33118.47656 | 36158.02344 | 38903.10938 | 119301.8594 | 46439.66016 | 4334.977051 | 105870.8672 | 23073.42969 | 2966.88623 | 25100.78125 | 49016.93359 | 0 | 30311.49023 | 20760.57813 | 56642.38281 | 48612.76953 | 79477.64063 | 73362.44531 | 183790.8438 | 64976.71094 | 27280.09375 | 42752.37109 | 7416.61084 | 30838.27344 | 57560.41406 | 45083.5625 | 36644.60938 | 41089.89453 | 0 | 78203.60938 | 19543.33203 | 0 | 3287.526855 | 45929.26172 | 11232.08105 | 12504.66211 | 28064.83984 | 22297.08594 | 29548.18945 | 10031.21973 | 4824.660156 | 0 | 37956.73047 | 40556.60156 | 8004.708984 |
| A0A0J9YVY3 | 90663.95313 | 82978.59375 | 54372.86328 | 67215.39063 | 55482.08594 | 58026.00781 | 0 | 0 | 88351.42188 | 64209.5 | 98315.60938 | 0 | 25119.42773 | 30224.32422 | 0 | 0 | 46855.22656 | 0 | 92754.03125 | 0 | 51027.35938 | 108096.4688 | 0 | 53024.07031 | 0 | 0 | 61238.46484 | 53053.46094 | 43368.89453 | 100732.2656 | 0 | 33500.5 | 35419.04688 | 0 | 49528.60156 | 47880.74219 | 68736.60938 | 32815.70703 | 31372.49609 | 0 | 0 | 63899.55469 | 44025.64453 | 0 | 20936.47656 | 43719.11719 | 44570.09375 | 0 | 63197.30469 | 0 | 0 | 60906.97656 | 0 | 0 | 0 | 41477.02734 | 41607.48438 | 47702.82813 | 0 | 0 | 66916.74219 | 68075.125 |
| A0A0J9YW62 | 0 | 0 | 0 | 0 | 0 | 0 | 0 | 0 | 0 | 20888.69922 | 0 | 0 | 6214.317871 | 0 | 0 | 0 | 0 | 0 | 0 | 0 | 0 | 0 | 0 | 0 | 0 | 0 | 0 | 0 | 0 | 0 | 0 | 7447.223633 | 0 | 0 | 0 | 0 | 0 | 0 | 0 | 0 | 0 | 0 | 0 | 0 | 10386.36426 | 0 | 0 | 0 | 6803.441895 | 0 | 0 | 0 | 0 | 17612.63281 | 0 | 0 | 0 | 0 | 0 | 0 | 0 | 0 |
| A0A0J9YX35 | 57604.42188 | 64346.27734 | 29341.13672 | 0 | 73184.85156 | 50394.53125 | 45702.07813 | 83089.98438 | 42029.40625 | 102239.4297 | 16837.58984 | 26376.4082 | 0 | 55657.28906 | 51467.54688 | 3622.898926 | 14269.57031 | 102559.3438 | 18631.04492 | 7734.615234 | 80128.75 | 41063.53516 | 85974.34375 | 95427.94531 | 11385.12793 | 59603.02734 | 89583.10938 | 79449.83594 | 94977.60938 | 37956.83203 | 109514.8125 | 89394.78125 | 35218.30469 | 25759.79102 | 62454.6875 | 120614.8125 | 65749.05469 | 47475.42969 | 104275.4375 | 19895.29688 | 30722.05469 | 67463.95313 | 22241.85742 | 19490.8125 | 110212.1953 | 20552.95313 | 26240.07617 | 61796.26953 | 57647.5625 | 5050.211426 | 16009.41895 | 19773.92188 | 31437.82422 | 52980.54297 | 28942.69336 | 22380.38672 | 12837.1543 | 17229.12109 | 24137.77734 | 13657.58496 | 14590.69434 | 17181.92188 |
| A0A0J9YXX1 | 293705.5313 | 297594.4063 | 263524.1875 | 161552.5625 | 182576.5 | 224307.5781 | 368239.7188 | 250751.5938 | 186830.0469 | 157216.4375 | 250307.2188 | 126430.0078 | 115047.0859 | 140711.3125 | 123072.9063 | 212140.3438 | 272694.5 | 325344.4375 | 218391.25 | 87172.42969 | 373645.75 | 318216 | 174343.625 | 120518.5078 | 342249.7188 | 72399.8125 | 129596.4766 | 103437.1719 | 253446.9531 | 488042.6563 | 395189.7188 | 163032.7188 | 92370.5625 | 210222.8125 | 56520.36719 | 161541.75 | 178933.9375 | 169547.9531 | 95403.72656 | 264764.5 | 133194.125 | 230561.8125 | 252923.8594 | 159149.4219 | 291130.6875 | 232288.3438 | 157695.8125 | 65647.79688 | 133487.6875 | 395144.375 | 327619.875 | 713721.375 | 333783.5 | 199114.5625 | 141772.9844 | 324617.4063 | 322029.2188 | 211189.7031 | 201027.4844 | 110221.1797 | 225485.5938 | 348797.375 |
| A0A0J9YY99 | 1377116 | 34060.88281 | 59165.75391 | 434287.75 | 34967.10547 | 30340.15625 | 39547.46875 | 909376 | 46590.97656 | 23057.35742 | 743832.875 | 45827.95703 | 778108 | 18909.68359 | 805300.625 | 30667.11719 | 46545.43359 | 45258.24219 | 45832.35547 | 48062.28125 | 52958.76953 | 29078.06836 | 42944.45703 | 70434.4375 | 57397.58594 | 22980.83594 | 37964.74609 | 48938.36328 | 76965.09375 | 896581.625 | 30450.48047 | 58290.66016 | 7289.260254 | 48103.06641 | 84849.08594 | 81270.58594 | 971736 | 19509.58594 | 47032.01953 | 689477.4375 | 9659.958984 | 32172.36914 | 1856508 | 29458.37891 | 18016.30664 | 34174.08594 | 16546.98828 | 384551 | 651244.375 | 608415.125 | 40731.97266 | 61780.5625 | 12817.02246 | 56638.39844 | 34549.86719 | 25378.47461 | 31809.5 | 15369.67773 | 39663.78906 | 15987.59961 | 21517.13672 | 17808.76758 |
| A0A0S2Z4L3 | 216724.5625 | 149988.1563 | 210790.875 | 235461.625 | 301625.5625 | 257047.0938 | 171717.375 | 288219.125 | 207272.3125 | 252371.0938 | 176358.9063 | 189850.6875 | 208694.3594 | 190091.5 | 286275.875 | 167484.9375 | 159885.9063 | 141423.1094 | 362712.0625 | 150021.9063 | 257890.2188 | 128768.5938 | 161183.8125 | 179977.6875 | 140351 | 188693.9688 | 267802.625 | 172826.6875 | 296646.9688 | 107886.0469 | 152027.3125 | 159634.4688 | 182848.875 | 153373.625 | 206729.5313 | 173218.5156 | 191935.7344 | 161602.8281 | 236139.0625 | 178410.6563 | 302069.75 | 186274.7344 | 286309.2813 | 114915.8594 | 180862 | 172014.0469 | 124662.6953 | 253466.0156 | 367967.375 | 111148.4844 | 160779.9844 | 149576.25 | 220291.6406 | 186442.4063 | 135174.0156 | 167434.7031 | 126541.1875 | 206921.25 | 246970.6094 | 236138.0469 | 203930.5625 | 102385.2188 |
| A0A0U1RQV3 | 28359.10156 | 23567.10547 | 20176.17188 | 27337.64648 | 21157.96875 | 23971.2207 | 19241.7168 | 23623.69922 | 21165.0625 | 29954.99805 | 22843.12891 | 16870.04883 | 26009.62109 | 22915.46875 | 26223.29688 | 40649.26172 | 34544 | 35880.98438 | 28814.65625 | 34235.875 | 28330.38281 | 36162.60156 | 27555.97266 | 25072.12891 | 38584.23047 | 19909.375 | 27148.69922 | 27496.85547 | 23143.99219 | 38346.07031 | 14878.96191 | 28560 | 21386.01563 | 23974.87109 | 27488.15625 | 18980.55078 | 22086.33594 | 26522.25 | 27699.04297 | 36751.46094 | 31065.4043 | 23485.29102 | 25528.83984 | 26498.59766 | 34100.60547 | 32904.01563 | 27631.89453 | 28023.9707 | 24867.22266 | 41241.19141 | 27462.13867 | 39681.54688 | 22579.00586 | 22493.97461 | 33164.54297 | 23668.46094 | 24164.17969 | 31505.86719 | 26084.63281 | 20958.86914 | 26325.67578 | 21078.18555 |
| A0A140T8Y3 | 7211.742188 | 13574.53516 | 8039.529785 | 8097.422852 | 5165.595703 | 6724.018555 | 5994.951172 | 6076.937012 | 4979.307129 | 0 | 0 | 0 | 5028.324219 | 10499.18945 | 6611.585938 | 4345.866211 | 5952.938965 | 0 | 12471.66113 | 6950.643555 | 6760.447266 | 7928.670898 | 8296.084961 | 7077.449219 | 10826.90137 | 5103.279297 | 8003.057129 | 12451.8457 | 5335.764648 | 8523.039063 | 0 | 8308.060547 | 6549.28418 | 8757.199219 | 0 | 2773.939453 | 3993.001709 | 3229.12207 | 6137.586914 | 21013.54297 | 3592.348145 | 9683.09375 | 20660.43164 | 7454.576172 | 6879.495605 | 11245.1084 | 0 | 7750.515625 | 8305.628906 | 0 | 0 | 6741.131836 | 12559.09473 | 3510.356201 | 0 | 14379.08594 | 9161.470703 | 7830.305664 | 0 | 7480.949707 | 5317.233887 | 6020.701172 |
| A0A182DWH7 | 33899.17188 | 22604.69531 | 27184.10156 | 22900.10352 | 38029.39063 | 34325.32031 | 38747.34766 | 31796.04492 | 35546.71875 | 21212.63867 | 22115.62695 | 31049.20313 | 39507.75 | 27806.2168 | 34494.39844 | 37576.00781 | 31108.15234 | 42539.60938 | 33774.59375 | 38551.66406 | 25349.92773 | 30705.23438 | 30855.49805 | 27428.68164 | 18068.9375 | 21953.33203 | 29020.92773 | 34971.04688 | 29409.49805 | 28100.79688 | 50076.0625 | 31196.51172 | 31899.21289 | 31375.30078 | 26840.75781 | 34341.64453 | 37693.94922 | 36584.70703 | 32294.09766 | 55924.11328 | 24989.72656 | 42640.94531 | 42312.73438 | 16936.70898 | 23256.49805 | 42898.66406 | 15646.38477 | 34954.44922 | 27609.05273 | 24970.60742 | 37983.53516 | 15734.71094 | 21579.00195 | 31914.98828 | 37065.07422 | 41785.72656 | 16475.51367 | 44096.92188 | 14527.17969 | 16304.75 | 19133.28906 | 29981.16211 |
| A0A1W2PQU7 | 113612.9219 | 64139.02734 | 91062.6875 | 127331.4844 | 118698.6875 | 111066.8672 | 103202.3984 | 60970.14844 | 91572.91406 | 85164.125 | 70062.73438 | 66538.29688 | 83646.82031 | 89985.64844 | 79460.16406 | 113380.7031 | 124493.8906 | 96176.50781 | 56357.61328 | 40290.82031 | 133164.9531 | 78300.60938 | 65849.17969 | 107354.1016 | 68163.47656 | 68434.84375 | 45921.39063 | 36464.5 | 90581.28906 | 74411.17969 | 105271.9297 | 102445.6094 | 82996.45313 | 86950.40625 | 70521.28906 | 92588.39063 | 78274.08594 | 69319.53125 | 98371.14844 | 59801.62891 | 85733.875 | 63103.63672 | 80466.04688 | 76640.65625 | 57066.97266 | 119320.2188 | 89329.99219 | 77748.19531 | 66578.22656 | 104776.7344 | 98261.39063 | 78290.74219 | 78404.57813 | 65304.10156 | 85228.625 | 154978.2188 | 85887.35938 | 79842.58594 | 70846.88281 | 53098.49609 | 182573.9688 | 72991.75 |
| A0A286YEY1 | 7052752 | 2818960.5 | 4789335 | 4906438 | 9421022 | 4482410 | 1956347.875 | 3894840.25 | 4522259 | 5575731 | 8274903 | 2661252 | 3290148 | 1728002.375 | 9840616 | 5330167.5 | 9067913 | 10323506 | 5409254.5 | 5411268 | 4376215.5 | 2678656.5 | 4299251.5 | 2465798.5 | 4023010.5 | 1970301.625 | 13508702 | 2134485 | 9166368 | 4086839.75 | 7112189 | 5249570 | 4239225 | 4020286 | 11230820 | 3255771.75 | 3340233.5 | 3445043.5 | 12169628 | 2522134.25 | 7605567 | 2197064.25 | 7511577 | 4100643.75 | 3570973.25 | 4152457 | 4382699.5 | 1812940.25 | 4483751 | 3941696.5 | 3848750 | 7953190 | 4927389 | 7037293.5 | 9685365 | 3385953.5 | 1804434 | 4618305.5 | 2418156.75 | 2551969.75 | 4598313.5 | 9016414 |
| A0A286YEY4 | 952874 | 593154.4375 | 675848.875 | 423410.3125 | 603383.8125 | 156468.3125 | 400994.0625 | 685518.5625 | 499779.4063 | 567129.5625 | 268860.5625 | 497452.9375 | 398910.5625 | 547339.0625 | 416814.3438 | 707409.0625 | 576172.6875 | 593747.875 | 220681.5156 | 623082.0625 | 660380.25 | 227515.3125 | 714588.75 | 520564.125 | 456402 | 462520.0938 | 853655.8125 | 326322.5625 | 610043 | 905654.125 | 1025330.375 | 630630.0625 | 381163.6875 | 486342.1875 | 407403.4375 | 330798.9375 | 592536.125 | 492132.0938 | 521919.125 | 924517.625 | 355104.0313 | 631826.5625 | 840981.0625 | 705599.75 | 440032.3438 | 690191.4375 | 417511.125 | 492029 | 618004 | 503806.5 | 405877.5625 | 600282.625 | 1230544.625 | 718406.625 | 699733.3125 | 459114.7813 | 819727.1875 | 908562.75 | 475873.0938 | 827164.8125 | 600094.9375 | 459652.9063 |
| A0A2Q2TTZ9 | 130613.3906 | 283492.5 | 248868.3281 | 288381.4688 | 163375.2813 | 279337.3125 | 173555.125 | 660334.75 | 312403.5 | 40796.17578 | 250840.6719 | 118457.6719 | 293399.4063 | 379958.375 | 229288.9375 | 199435.4063 | 218714.5313 | 200966.9531 | 341671.4063 | 497134.4375 | 236930.5938 | 396563.625 | 228720.6563 | 323870.5938 | 37273.51953 | 163045 | 258245.0469 | 181421.625 | 220058.3906 | 321397.3438 | 365368.0313 | 261173.9219 | 178826.8125 | 216465.75 | 236905.1406 | 172394.0625 | 229968.5156 | 119646.3281 | 559021.9375 | 352386.7813 | 141018.6406 | 155318.7344 | 225745.4219 | 633899.75 | 629256.625 | 485185.0313 | 26060.46484 | 125017.0469 | 81998.8125 | 199516.7188 | 212476.6719 | 1294661.125 | 964229.375 | 195069.9375 | 57729.81641 | 224159.1094 | 160599.3438 | 116672.8125 | 246089.1719 | 650909.0625 | 377765.5938 | 277637.6563 |
| A0A2R8Y3M9 | 141111.3125 | 117860.5469 | 153734.6875 | 137192.25 | 140534.8125 | 187750.9063 | 186963.1719 | 113184.6484 | 142545.0625 | 173255.6094 | 141536.875 | 162451.875 | 133150.1719 | 140371.3125 | 133674.4531 | 200972.2188 | 135753.3281 | 202221.7188 | 193643.0781 | 154711.1875 | 124754.0625 | 140141.375 | 149535.2188 | 193817.2031 | 137458.4688 | 214445.7188 | 198877.6563 | 160403.2188 | 138002.2188 | 130950.7813 | 91243.32031 | 171895.75 | 143734.1719 | 111071.7969 | 166542.875 | 93242.875 | 159079.0625 | 132432.5313 | 154035.7969 | 205977.1875 | 179920.0625 | 131223.1094 | 153179.7813 | 139701.3125 | 159757.3125 | 248751.3125 | 140977.4688 | 221745.4531 | 171033.2813 | 169627.8125 | 160456.1875 | 191099.1406 | 206038.1563 | 125982.3047 | 197427.2813 | 171502.8438 | 190172.5 | 198645.5938 | 186603.625 | 214747.5781 | 125286.5 | 123775.0625 |
| A0A2R8Y5V9 | 468.6205444 | 13769.96484 | 0 | 4449.155273 | 793.0488281 | 0 | 0 | 0 | 0 | 0 | 1662.014893 | 0 | 0 | 4314.14209 | 2744.703857 | 0 | 0 | 0 | 4737.680664 | 0 | 1590.115845 | 0 | 5442.37207 | 0 | 0 | 4573.036133 | 7627.28418 | 0 | 0 | 0 | 0 | 0 | 0 | 4400.573242 | 0 | 0 | 0 | 2553.417969 | 0 | 0 | 0 | 0 | 2105.712158 | 0 | 0 | 0 | 0 | 1090.255737 | 0 | 0 | 0 | 0 | 961.7350464 | 0 | 0 | 3716.3396 | 0 | 0 | 0 | 0 | 0 | 0 |
| A0A2R8Y619 | 0 | 21705.46289 | 0 | 0 | 0 | 0 | 0 | 1511.366699 | 0 | 0 | 0 | 0 | 0 | 0 | 0 | 0 | 0 | 0 | 0 | 0 | 0 | 0 | 0 | 0 | 0 | 0 | 0 | 0 | 0 | 0 | 0 | 0 | 6397.057129 | 0 | 0 | 0 | 0 | 0 | 0 | 0 | 0 | 0 | 0 | 0 | 0 | 0 | 0 | 0 | 0 | 0 | 0 | 0 | 0 | 0 | 0 | 0 | 0 | 0 | 0 | 0 | 0 | 0 |
| A0A2R8Y6G6 | 0 | 5638.313965 | 0 | 2264 | 0 | 0 | 0 | 0 | 0 | 0 | 0 | 0 | 2284.025635 | 0 | 0 | 0 | 0 | 0 | 2253.380371 | 0 | 0 | 0 | 3754.586426 | 0 | 0 | 0 | 2933.467041 | 0 | 921.8513794 | 0 | 0 | 0 | 0 | 0 | 0 | 0 | 0 | 0 | 0 | 0 | 0 | 0 | 0 | 0 | 0 | 0 | 14013.23926 | 0 | 0 | 0 | 0 | 0 | 0 | 0 | 772.1977539 | 0 | 0 | 0 | 0 | 0 | 0 | 0 |
| A0A2R8Y7X9 | 49366.41797 | 51374.69922 | 16266.10156 | 15645.91602 | 50236.41406 | 148785.7188 | 44207.66797 | 24824.41797 | 32770.90625 | 40968.47266 | 0 | 29925.69531 | 32460.48242 | 48895.29297 | 21736.0625 | 33317.67188 | 31649.18945 | 65338.73828 | 10413.50977 | 12042.15625 | 21845.87891 | 6706.949219 | 6183.96582 | 73018.27344 | 15568.82715 | 89465.67188 | 64245.07031 | 49204.48047 | 22220.50781 | 12799.98047 | 33050.75391 | 17716.99609 | 37390.00781 | 49823.19531 | 20690.49609 | 16618.31641 | 17756.20508 | 16865.61523 | 70869.1875 | 17195.59961 | 56636.24609 | 29228.125 | 24956.39063 | 53950.27734 | 69859.97656 | 62573.14844 | 35738.93359 | 75433.54688 | 45214.59375 | 13976.52539 | 11050.84863 | 41841.25781 | 8361.704102 | 68152.5625 | 46866.92188 | 44972.77734 | 46891.39453 | 6955.424316 | 12573.30078 | 12410.93945 | 0 | 47766.64453 |
| A0A3B3ISR2 | 224181 | 215599.2813 | 249817.2188 | 240050.0625 | 281583.625 | 197168.5313 | 231976.3438 | 297503.9375 | 166767.125 | 344985.4063 | 224963.625 | 208836.2656 | 293239.3125 | 212182.4688 | 285792.1563 | 273666.875 | 210712.9375 | 198528.6875 | 276876.4688 | 241715.5938 | 233860.0625 | 332056.875 | 191388.9375 | 181412.7813 | 145521.4531 | 326277.5 | 326439.0938 | 361299.6875 | 272179.5313 | 162823.4531 | 266898.6875 | 234083.9219 | 306764 | 316772.9063 | 320563.0313 | 205982.125 | 443649.9688 | 197814.7188 | 383106.4375 | 264236.9375 | 359881.5938 | 499505.9375 | 274017.6875 | 258320.5156 | 381468.3125 | 245703.6875 | 223032.7813 | 325652.0313 | 495606.375 | 195597.75 | 247928.5938 | 255355.9688 | 236516.5156 | 290919.3125 | 295548.0313 | 353723.5313 | 244136.1563 | 329118 | 372075.875 | 368036.3438 | 339967.4375 | 229852.2813 |
| A0A4W8ZXM2 | 1776199.875 | 1036728.938 | 1074625.5 | 948298.3125 | 744669.4375 | 463443.9375 | 1136791.5 | 213905.6094 | 552707 | 843214.1875 | 993792.5 | 75893.91406 | 552170.8125 | 634582.9375 | 915532.375 | 663410.625 | 1268784.125 | 704527.5625 | 365183.4063 | 623548 | 1138029.875 | 512894.5625 | 1818608.625 | 691135.5 | 822110 | 846788.375 | 519290.5313 | 502361.9063 | 734599.3125 | 935274 | 1069934.875 | 973167.125 | 867205.6875 | 563647.375 | 772070.25 | 877033.3125 | 559113.8125 | 1134556.875 | 726909.875 | 1736611.125 | 662736.6875 | 164508.0625 | 1468244 | 1108240.375 | 881623.5625 | 1105159.5 | 1133165.375 | 88650.39063 | 957145 | 506494.7813 | 677684.6875 | 979454.5 | 427053.6563 | 855868.3125 | 132442.7188 | 306105.875 | 582747.625 | 537848.5625 | 367026.2188 | 755971.25 | 1827168.375 | 1140752.625 |
| A0A5H1ZRQ7 | 25577.125 | 8576.420898 | 11682.26855 | 26197.42188 | 20182.79297 | 12337.95996 | 12268.4248 | 21318.48438 | 20737.20508 | 17815.5957 | 15403.96973 | 0 | 19826.00586 | 19783.68359 | 33246.47656 | 24200.87109 | 16958.10156 | 30833.92188 | 0 | 9656.899414 | 25590.68359 | 8275.124023 | 0 | 24881.08984 | 10943.33887 | 11815.07813 | 9603.512695 | 0 | 24157.28125 | 9056.97168 | 21590.21094 | 29250.59961 | 38255.12891 | 10592.70703 | 15149.50488 | 0 | 0 | 16721.08789 | 17990.8418 | 19249.03516 | 6491.112793 | 11288.01953 | 14914.4248 | 0 | 32248.93945 | 16135.7002 | 36766.76563 | 10691.56641 | 21519.08398 | 21023.41992 | 11244.43652 | 0 | 2217.224609 | 15811.96875 | 4424.382813 | 19957.72852 | 24354.45508 | 11734.05664 | 0 | 10022.67285 | 0 | 21476.54688 |
| A0A5H1ZRS2 | 1575709 | 432889.4063 | 603570.1875 | 317347.4063 | 469377.1875 | 494894.75 | 363145.25 | 623605.0625 | 476887.4688 | 350512.25 | 367952.5625 | 401801.7813 | 1716229.875 | 518300.5 | 599229.1875 | 357719.5313 | 479112 | 335163.5 | 1317520.75 | 465793.4688 | 630730.3125 | 1854117.125 | 326618.25 | 534262.5625 | 740303.375 | 278938.3125 | 2825080.5 | 332640.4688 | 624020.5 | 562833.25 | 552225.9375 | 1978221.375 | 1504419.25 | 471774.1875 | 467106.0313 | 580246.6875 | 1583535.125 | 650780.875 | 578875.6875 | 1843052.5 | 518928.4688 | 501663.9063 | 3501977.5 | 702563.375 | 753715.5625 | 725264.5625 | 695306.6875 | 385305.5625 | 348382.6563 | 314349.6563 | 2832114.75 | 815082.3125 | 426591.5 | 524135.5938 | 375530 | 470317.0625 | 607420.8125 | 2435168 | 601026.75 | 764222.125 | 2925778.5 | 926103.4375 |
| A0A5H1ZRS9 | 144165.2813 | 108445.2578 | 118017.6563 | 74930.42969 | 63850.9375 | 146481.5625 | 118510.5078 | 99561.49219 | 130274.8594 | 124847.4219 | 76878.71094 | 130102.7578 | 65182.55469 | 73302.32031 | 75363.17969 | 123677.5469 | 131207.2344 | 124773.1797 | 57031.25781 | 83689.85156 | 180688.625 | 150328.3906 | 107914.2422 | 54729.30078 | 91563.17188 | 79496.20313 | 98644.25 | 137832.2969 | 140337.7813 | 200140.4375 | 132953.7813 | 146041.75 | 85616.19531 | 27848.49023 | 146114.0781 | 135730.0781 | 40948.25781 | 6400.352051 | 124567.2734 | 12290.16797 | 49965.53906 | 67679 | 66726.03906 | 80620.10156 | 172250.5938 | 29700.34961 | 101225.8984 | 40508.83203 | 33362.03125 | 37452.44922 | 136269.6406 | 137773.0781 | 126314.9219 | 27388.94727 | 37092.50391 | 37868.74609 | 72635.92969 | 101729.75 | 116687.0938 | 69082.85156 | 125903.9453 | 119477.4141 |
| A0A669KAY4 | 0 | 2263.741943 | 4519.15918 | 2155.786377 | 2112.675537 | 1970.767578 | 0 | 0 | 0 | 0 | 0 | 0 | 2647.271973 | 5055.308594 | 0 | 0 | 0 | 0 | 0 | 0 | 0 | 1347.270508 | 0 | 0 | 0 | 1896.116211 | 0 | 0 | 2416.235107 | 1433.447632 | 0 | 0 | 4112.154297 | 0 | 0 | 4443.283203 | 0 | 0 | 0 | 0 | 0 | 0 | 0 | 0 | 4948.417969 | 0 | 0 | 4767.284668 | 1408.676147 | 0 | 0 | 0 | 0 | 1978.443237 | 0 | 0 | 1972.053833 | 0 | 1250.579224 | 0 | 0 | 0 |
| A6XND0 | 8336.563477 | 16009.97266 | 41839.60938 | 18960.59375 | 32192.75586 | 44133.90625 | 50331.18359 | 41376.80469 | 25219.11133 | 54322.30859 | 48507.71875 | 27931.20313 | 19641.71289 | 30723.90234 | 33022.53125 | 32863.60547 | 19688.57813 | 23127.65234 | 36680.99219 | 41536.22266 | 22588.95508 | 19068.35547 | 32135.58594 | 20355.65234 | 37715.36328 | 30870.61914 | 32770.25391 | 32046.98828 | 32808.34375 | 23509.32813 | 25604.35156 | 16392.44531 | 28238.89063 | 36984.05859 | 28201.22461 | 22546.07031 | 29769.19922 | 49971.41016 | 28047.18555 | 29849.26563 | 14771.36426 | 31160.63281 | 24542.57617 | 29495.08594 | 19195.54102 | 20471.81836 | 16833.6543 | 34451.23828 | 39137.88672 | 15570.02441 | 33553.9375 | 38961.71875 | 29604.30859 | 34809.25 | 28314.17578 | 31497.37109 | 23415.57422 | 32694.26953 | 26236.625 | 28934.77344 | 26637.48828 | 25934.48633 |
| B0YIW2 | 176564.4531 | 1540137.5 | 2254339.5 | 3254215.25 | 587190.75 | 2540524.75 | 1758850.875 | 992683.625 | 1202086.375 | 178901.5469 | 2720792.75 | 1342542.75 | 4120404.5 | 195619.0313 | 2284176.75 | 1177190.5 | 810986.3125 | 1281601.125 | 3092109.5 | 2261343.5 | 1861475.625 | 2092568.625 | 2480855.75 | 133609.2188 | 2134548 | 201587.0938 | 2804042.5 | 4818771 | 1363103.625 | 745085.5 | 2194600.5 | 2952603 | 2395465.25 | 678391.6875 | 227536.4844 | 1898467.25 | 2121054.5 | 3021670.5 | 2783302.25 | 223619.4375 | 1894885.875 | 1424686.5 | 3452018.25 | 1597879.125 | 2094184.25 | 2927053 | 133392.9375 | 2486551 | 1246641.25 | 1830890.625 | 274456.1875 | 1638056.625 | 498693.5938 | 2203531.25 | 1834138.125 | 226027.5 | 2905474.25 | 2400473.25 | 650514.8125 | 6372980 | 1843131.75 | 1376665.875 |
| B1AHL2 | 21889.52344 | 13466.50586 | 10713.23047 | 13848.76074 | 12482.89355 | 21446.13672 | 13437.84277 | 0 | 20756.07422 | 0 | 0 | 19117.05078 | 12958.15527 | 0 | 9148.231445 | 12671.49805 | 21645.02539 | 0 | 16899.54297 | 10103.81836 | 21490.94727 | 15174.28711 | 23315.82422 | 15379.21094 | 0 | 13573.21387 | 11081.01953 | 0 | 13457.25391 | 11615.67871 | 12526.8457 | 17813.43359 | 17703.56055 | 11473.87207 | 11614.13184 | 7550.276855 | 13418.45898 | 16066.17676 | 10531.52051 | 0 | 19849.375 | 15384.52246 | 0 | 20123.96289 | 10320.8252 | 6389.72998 | 15524.21387 | 9283.24707 | 10360.00781 | 17046.62109 | 0 | 17276.08008 | 0 | 13576.34082 | 20447.84375 | 15522.2832 | 21826.09375 | 24534.09375 | 21306.52148 | 12519.09082 | 1966.276611 | 0 |
| B4E1Z4 | 492155.1875 | 501056.0938 | 397985.3438 | 535373.0625 | 437701.375 | 514522.5 | 744997.875 | 571477.625 | 560371 | 345468.5938 | 593940.6875 | 622028.375 | 435235.125 | 378633.1875 | 489408 | 565083.625 | 401252.75 | 561779.8125 | 678888.6875 | 582958.1875 | 447537.1875 | 456909.6563 | 439571.4375 | 520557.375 | 481500 | 543605.4375 | 565376.875 | 824344.75 | 502053.2188 | 442673.75 | 453412.25 | 604183.6875 | 307695.5 | 544141.75 | 652718.5625 | 404958.6875 | 614821.1875 | 603918.375 | 396866.4375 | 645580.125 | 667043 | 402192.625 | 384202.0625 | 501069.5625 | 346823.1563 | 629479.125 | 448812.125 | 552668.25 | 453690.1875 | 494486.375 | 482506.5938 | 400773.375 | 473933.4375 | 572051.125 | 325889.7188 | 539647.375 | 649773.1875 | 634745.875 | 484439 | 649128.8125 | 557073.625 | 444910.9063 |
| B7ZKJ8 | 450169.75 | 474723.125 | 490134.0938 | 454664.8125 | 418298.75 | 419455.125 | 660974.875 | 449681.1875 | 393099.125 | 498133.1875 | 449280.1875 | 551663.875 | 558257.75 | 501003.625 | 573667.75 | 534284.375 | 413765.8125 | 506231.1563 | 587870.6875 | 498403.8438 | 473390.625 | 466187.125 | 332205.125 | 589872.25 | 466116.125 | 429164.25 | 507020.125 | 536735.625 | 475264.5625 | 385887.3125 | 510332.1875 | 656169 | 503079.125 | 549840.1875 | 626600.5625 | 437025.3125 | 529002.375 | 400504.625 | 413973.5 | 548202.5625 | 566998.625 | 427429.2188 | 585356.9375 | 576495.25 | 248067.0938 | 577136.6875 | 526277.5625 | 545278.9375 | 594057.875 | 366169.75 | 530636.125 | 541580.25 | 674244.125 | 526356.5 | 587864.5 | 439325.9688 | 476053.25 | 606032.125 | 562264.625 | 470895.7188 | 603680.9375 | 586239.875 |
| C9J5S7 | 0 | 4829.22168 | 0 | 1784.437012 | 0 | 0 | 1055.566895 | 0 | 0 | 0 | 0 | 0 | 796.6590576 | 2393.338135 | 685.0136108 | 0 | 0 | 0 | 0 | 2012.181763 | 0 | 0 | 3726.733398 | 0 | 0 | 4683.317871 | 0 | 0 | 0 | 0 | 0 | 0 | 0 | 2423.917725 | 0 | 0 | 0 | 4025.034424 | 0 | 0 | 0 | 0 | 0 | 0 | 0 | 0 | 0 | 1976.7146 | 0 | 0 | 0 | 0 | 0 | 0 | 0 | 3038.299072 | 1733.178955 | 0 | 0 | 0 | 0 | 0 |
| C9J8S2 | 0 | 0 | 0 | 1436.536499 | 0 | 0 | 0 | 0 | 0 | 0 | 0 | 0 | 0 | 0 | 5638.041016 | 3683.653809 | 0 | 4425.925293 | 3279.386719 | 1345.936646 | 0 | 0 | 0 | 0 | 8130.850586 | 0 | 0 | 4490.168457 | 0 | 0 | 1882.359253 | 0 | 0 | 4503.721191 | 1835.035645 | 3249.090576 | 4599.300781 | 0 | 0 | 2241.803467 | 0 | 0 | 0 | 0 | 0 | 6260.94043 | 3372.387207 | 3636.381592 | 9007.986328 | 1729.509521 | 0 | 0 | 3731.60083 | 6117.195313 | 3322.006836 | 3452.391113 | 3331.676025 | 1865.508057 | 5298.206055 | 5098.925781 | 0 | 0 |
| C9JB55 | 3093.891113 | 10237.2793 | 13930.50781 | 0 | 11371.7832 | 9445.878906 | 11731.68262 | 17306.09766 | 9049.831055 | 10352.21777 | 13350.43945 | 17680.91406 | 4823.647461 | 12427.60059 | 8495.902344 | 18628.29297 | 16936.90234 | 14402.71191 | 12685.52734 | 23329.58984 | 14972.10352 | 12157.52637 | 15400.13281 | 8527.493164 | 15984.72852 | 8900.818359 | 11541.27246 | 20325.25781 | 10586.09863 | 17304.40625 | 5292.72168 | 0 | 9990.375 | 10295.7627 | 14372.98438 | 10123.88477 | 9201.320313 | 12780.74609 | 9863.806641 | 8638.808594 | 12560.4707 | 9120.204102 | 13843.26367 | 15950.9541 | 7104.934082 | 6783.782227 | 17326.09375 | 11724.02637 | 10322.03613 | 19082.20703 | 15234.62305 | 10948.30664 | 13898.73242 | 10888.36719 | 25319.59766 | 20348.81445 | 15433.85352 | 16332.39844 | 11575.37891 | 12877.59375 | 10929.91211 | 10497.41211 |
| C9JC84 | 7791626 | 4757485.5 | 5029996 | 4783628 | 6074574 | 6514117.5 | 6194151 | 4290855.5 | 4658224.5 | 6070907.5 | 4642477.5 | 4776942 | 5491472 | 4954341.5 | 5317808.5 | 6704173 | 4526866.5 | 5613779 | 8240507.5 | 4333262 | 5717079.5 | 4104150.25 | 3826330 | 5073756.5 | 3845821 | 4090967 | 6848147 | 5529105.5 | 6010185 | 4701263 | 4842406 | 5952684 | 6158182 | 2784201.25 | 5814990 | 3963486 | 5382888 | 4729550 | 4752872.5 | 4983322.5 | 6629781 | 4408345.5 | 5343961 | 5157550.5 | 5841101 | 6396652 | 4304144 | 5721811.5 | 4942694 | 6247537 | 5103347.5 | 3770798.25 | 3941427.5 | 4308832 | 4393289.5 | 4326994 | 4784189 | 4050770.5 | 5502550 | 4308796 | 4904312.5 | 3654205.5 |
| C9JD84 | 1095.135986 | 1310.476563 | 0 | 0 | 1992.164185 | 0 | 0 | 1137.189819 | 0 | 0 | 0 | 0 | 0 | 0 | 0 | 0 | 0 | 0 | 0 | 0 | 0 | 0 | 0 | 0 | 0 | 0 | 0 | 0 | 0 | 0 | 0 | 0 | 0 | 0 | 0 | 9096.332031 | 0 | 0 | 0 | 0 | 0 | 0 | 5753.328125 | 1104.046387 | 0 | 0 | 0 | 3682.731201 | 0 | 0 | 0 | 0 | 0 | 0 | 0 | 0 | 0 | 0 | 0 | 0 | 0 | 0 |
| C9JF17 | 481059.7188 | 817823.25 | 840823.5625 | 1041560.5 | 654634.4375 | 909192.875 | 571325.625 | 774042.5 | 789321.0625 | 913221.25 | 620886.8125 | 828165.5 | 834667.125 | 1247798 | 766427 | 631767.5 | 707678.875 | 581876 | 781102.375 | 544144.375 | 859937.4375 | 1190780.25 | 704465.375 | 804780.625 | 1013217.188 | 735853.5625 | 627174.125 | 723027.375 | 620823.75 | 1386885.375 | 1012715.5 | 869144.25 | 1994709.5 | 766388.875 | 772718.625 | 912901.625 | 1218981.375 | 905148.4375 | 1195882.75 | 714759.75 | 744457 | 888340.125 | 1199536.5 | 1221773.625 | 590828.75 | 713164.4375 | 789354.75 | 656609.6875 | 736420 | 1426886.75 | 961404.25 | 271565.6875 | 933378.375 | 1183094.5 | 752742.625 | 765946.75 | 692400.5 | 1208447 | 590567.3125 | 780838.25 | 610148.625 | 666905.6875 |
| C9JPQ9 | 3207068.25 | 2332482.5 | 2879777.25 | 2440905.75 | 3442742.5 | 2949616.5 | 2785830.75 | 1503477.875 | 1595235 | 2119006.25 | 1157445.25 | 1338022 | 3068640.25 | 1832432.5 | 2541993.25 | 2533446.5 | 2064318.25 | 1994548 | 3153122.5 | 1027994.813 | 3070233 | 1422264.375 | 1105507.75 | 986695.3125 | 1382316.625 | 1464363.125 | 4175818 | 2221326 | 3300952 | 1318403.625 | 1130835.5 | 2485233.5 | 2577936.75 | 1225257.75 | 2728886.25 | 1716870.25 | 2820150 | 1529067.25 | 2321787.5 | 2345484 | 2086084.375 | 1091675.5 | 2238186.25 | 1885863.5 | 3010780.25 | 3330919.25 | 1265179.625 | 2176101.5 | 2428328.75 | 1174091 | 1827387 | 1376654.25 | 1169673 | 1779600.5 | 970195 | 1035979.438 | 1303419.25 | 1418828.125 | 2606903 | 2026982.625 | 1982415.75 | 1610798.625 |
| C9JV77 | 2260058.25 | 2509718 | 2227322 | 2174964.5 | 1770050 | 1832984 | 2071455.25 | 1864987.125 | 2056107.75 | 2021878 | 2413961.5 | 1869065.5 | 1778197.5 | 2565582.5 | 2012944 | 1877034.5 | 2859812 | 2331662 | 1983393.625 | 2027967.25 | 1632398.75 | 2425640 | 2170119 | 2259909 | 2977214 | 2084313.5 | 2198110 | 3067231.25 | 1994024.25 | 2773670.75 | 2711240 | 2243888.5 | 1292242 | 2024717.25 | 2419162.25 | 1590519.125 | 2770578.5 | 1911406.125 | 1828314.5 | 2179940 | 1635836 | 2321316 | 1884278.75 | 2464725 | 1559901.5 | 1608646.5 | 1547587.375 | 1907589.75 | 1357012.5 | 2219183 | 1746163.75 | 2438550.5 | 2381809.5 | 1816370.875 | 2508373 | 2262749 | 1988954.75 | 1923881.75 | 1995766 | 2364053 | 2339240.25 | 2712357.5 |
| C9JXI5 | 177174.7344 | 231255.375 | 13755.55859 | 176101.5625 | 8733.072266 | 157411.4844 | 142877.8594 | 3450.37915 | 136046.9219 | 6402.747559 | 169196.8594 | 10516.97559 | 334664.3125 | 9670.22168 | 17280.49609 | 6728.661133 | 10432.10352 | 8156.929688 | 12049.29102 | 16513.10742 | 9362.150391 | 11770.60645 | 259939.5156 | 188440.625 | 14347.47168 | 12367.13574 | 10901.99316 | 10929.3457 | 222417.5 | 251202.3594 | 14935.65039 | 236485.3594 | 122933.8516 | 9745.40918 | 9315.079102 | 167390.4844 | 219318.0156 | 9551.746094 | 177302.5313 | 327886.375 | 176617.1563 | 9988.257813 | 238195.9375 | 124155.8516 | 7530.041016 | 235325.5313 | 257531.0156 | 249266 | 200400.5313 | 146248.75 | 264491.0625 | 20304.56055 | 190994.4688 | 8856.663086 | 194468.5313 | 30044.32227 | 189092.5938 | 261621.4219 | 193037.8125 | 16624.85352 | 245510.5625 | 188448.3281 |
| D6R934 | 146201.2188 | 88798.40625 | 100972.2188 | 82665.21875 | 154356.8281 | 94518.53906 | 96366.51563 | 97145.95313 | 66086.13281 | 95386.57031 | 122598.9219 | 97547 | 77040.34375 | 89376.5 | 87624.51563 | 154907.875 | 92915.01563 | 122275.4688 | 111409.2578 | 134872.1563 | 122568.0469 | 95755.14844 | 106573.3125 | 103290.2656 | 115579.2656 | 102493.8906 | 138189.2969 | 126006.9219 | 165539.7188 | 98901.80469 | 102404.0234 | 92243.14063 | 76388.33594 | 95516.69531 | 99525.16406 | 63937.65625 | 94588.34375 | 107003.9063 | 92953.76563 | 113669.5703 | 112460.6641 | 110113.4531 | 106527.8516 | 94669.85938 | 107410.0938 | 81421.85938 | 98439.61719 | 110158.0781 | 80486.51563 | 115850.2344 | 114313.0703 | 103471.7344 | 131810.2188 | 93175.04688 | 148427.7188 | 105194.5469 | 98161.89063 | 90595.67188 | 87298.60156 | 80952.88281 | 105356.2031 | 109822.8594 |
| D6RAR4 | 13751.84277 | 35550.67188 | 30628.27344 | 23784.28516 | 31337.99219 | 26732.45703 | 18280.85547 | 14430.55664 | 16987.39453 | 23273.33008 | 17466.95117 | 15294.02246 | 28575.03125 | 31187.48242 | 39879.77344 | 29338.12305 | 35505.69531 | 22249.83398 | 17023.52734 | 24563.41406 | 32723.74805 | 21347.53125 | 24413.98633 | 33886.79688 | 36642.73438 | 14859.4541 | 16510.74219 | 35806.85156 | 23334.69336 | 19521.0625 | 33949.125 | 25533.47656 | 35179.05469 | 27823.48242 | 37449.16016 | 22149.48242 | 19395.58398 | 25567.91211 | 21373.98633 | 50406.16406 | 20601.73047 | 27967.76172 | 30124.43164 | 24683.3125 | 32194.41797 | 39115.17578 | 32196.55859 | 36891.61328 | 33283.67188 | 24592.80859 | 18356.6582 | 28945.0293 | 32615.55078 | 22970.05078 | 29066.27344 | 20214.54883 | 33668.21094 | 42274.42188 | 23916.65625 | 22775.78125 | 20259.0625 | 15497.59473 |
| D6RD17 | 4344782.5 | 2417018.25 | 9100835 | 1761586 | 3952629 | 1346974.125 | 1432508.875 | 2706968.75 | 2106863.25 | 2572744.5 | 2661210 | 1828816.875 | 3056658.25 | 2945592 | 3495465.75 | 1677120.375 | 2630381.25 | 741107.5625 | 1350169.625 | 2744870.5 | 4563396.5 | 3562834.75 | 1421588.75 | 2414845.5 | 1710474.375 | 806728.5625 | 2065970.75 | 1093776 | 4287702.5 | 2516838 | 2282768.75 | 1930364.125 | 3285110 | 2231939.75 | 2026938.5 | 3065671.75 | 2844928 | 1889934.875 | 4687034 | 1245921.25 | 3421603.75 | 2259885 | 7595578.5 | 1904011.5 | 3475737.25 | 2709899.75 | 2369768.5 | 1765508.25 | 3356822.5 | 1812090.125 | 2526364.75 | 3486739.5 | 1315041.875 | 2218520 | 2028602.625 | 1081341.375 | 1337604 | 1213423.25 | 2801836 | 2481381.75 | 3033082.25 | 2632793.75 |
| D6RE82 | 1102826.125 | 23619.71875 | 76528.625 | 26537.49805 | 16170.18262 | 6155.973145 | 21907.58203 | 13523.81055 | 62752.30859 | 387356.75 | 21385.22461 | 15405.82715 | 69438.78906 | 57051.16016 | 83400.70313 | 45394.46875 | 29273.63477 | 39116.17578 | 175565.7031 | 98362.10938 | 22897.33594 | 121935.625 | 4601.936523 | 20244.5957 | 135526.8906 | 56019.86328 | 16412.04883 | 257445.6875 | 49129.40625 | 108432.8281 | 3906145.25 | 2270535.25 | 1545303 | 76687.48438 | 60212.86719 | 52715.79297 | 16588.28711 | 39293.83984 | 50317.77734 | 290139.4063 | 208625.5156 | 110849.7891 | 179766.9531 | 122590.6719 | 54275148 | 204036.125 | 163188.2188 | 165938.6875 | 86364.36719 | 5639.663086 | 142549.3281 | 129205.75 | 194937.6406 | 47111.83203 | 68806.29688 | 104067.875 | 336886.8125 | 177401.3594 | 263083.25 | 124656.0156 | 179101.0625 | 4342.047363 |
| D6RF35 | 1872142 | 2789452.75 | 2440526.5 | 2587862 | 1735583.75 | 2436560.5 | 3122805.25 | 2678737.5 | 2525548 | 2095177.25 | 2715526 | 2954572 | 2785298.5 | 2704504 | 2600376.75 | 2405361 | 2737545 | 2933424.25 | 2534117.75 | 2899328.75 | 2585758.75 | 2545860.75 | 2285632.75 | 1676586.75 | 1814214.75 | 2460415.5 | 2767494 | 3122690 | 2632330.5 | 2877932 | 1992604.625 | 2792757.25 | 2037911.75 | 2836012.5 | 3147754.5 | 2457344 | 3278123.25 | 2154410.25 | 2134404.5 | 3089833.5 | 2366380 | 2037919.5 | 2466275.75 | 2694882.25 | 2249995.25 | 2809779.75 | 2772487.5 | 2909378 | 2415515.75 | 2628533.5 | 2355868.5 | 2862172.5 | 3002889.5 | 2717929 | 2816943 | 2261570 | 2621534 | 3247285.75 | 3250242 | 2061959.5 | 2621572.25 | 3409596.25 |
| E7END6 | 13899.06152 | 17670.75781 | 9377.887695 | 14683.30859 | 23801.48047 | 15660.92773 | 18250.66211 | 23892.51172 | 15588.77051 | 13032.05664 | 21851.00195 | 17185.81445 | 17533.29102 | 15939.30078 | 19948.10938 | 18694.41211 | 16929.6543 | 21638.01172 | 21461.36133 | 19828.87305 | 16803.21484 | 13038.8877 | 15535.83594 | 14863.49609 | 19296.49219 | 19017.91797 | 15481.35156 | 19581.57227 | 20160.34766 | 13777.88086 | 17098.86719 | 16864.625 | 14571.76074 | 20764.30078 | 19069.90625 | 17452.03906 | 15683.35156 | 16586.95508 | 16992.77148 | 21272.84766 | 16386.15039 | 14872.57227 | 16221.53223 | 18473.68164 | 12720.25879 | 26743.21875 | 15341.52734 | 27747.66602 | 23001.51563 | 20887.03906 | 20428.79297 | 14770.81543 | 27536.68555 | 18808.25781 | 26126.86914 | 21134.39648 | 14869.82227 | 22925.10742 | 20747.58398 | 20824.51953 | 18757.50391 | 13289.16895 |
| E7ENL6 | 0 | 4472.297852 | 0 | 6026.260254 | 5196.842773 | 0 | 10090.98242 | 6651.032715 | 5661.013184 | 0 | 4358.645508 | 8487.261719 | 0 | 3689.040527 | 8753.313477 | 0 | 5537.630859 | 7249.771484 | 0 | 6949.533203 | 0 | 6163.917969 | 0 | 0 | 4482.72168 | 0 | 0 | 0 | 0 | 13711.79883 | 0 | 0 | 0 | 6257.160645 | 19626.78516 | 3656.022217 | 0 | 5661.349121 | 0 | 10528.70117 | 4441.38916 | 11225.90234 | 0 | 13933.2793 | 0 | 8115.282227 | 0 | 6431.413086 | 0 | 7875.228516 | 8601.803711 | 10674.71973 | 0 | 0 | 6848.145508 | 5213.506348 | 122109.8281 | 5322.317871 | 0 | 0 | 0 | 0 |
| E7EQB2 | 0 | 1947.010742 | 0 | 0 | 0 | 0 | 0 | 0 | 0 | 0 | 0 | 0 | 0 | 0 | 0 | 0 | 0 | 0 | 2849.856689 | 0 | 0 | 0 | 0 | 0 | 0 | 0 | 0 | 27756.14844 | 0 | 0 | 0 | 0 | 0 | 0 | 0 | 0 | 0 | 0 | 0 | 0 | 24560.60352 | 0 | 0 | 0 | 0 | 0 | 0 | 0 | 0 | 24059.22852 | 0 | 0 | 0 | 0 | 0 | 29376.25195 | 0 | 0 | 0 | 0 | 0 | 0 |
| E7EUT5 | 9669.4375 | 20738.09375 | 5367.898926 | 14639.59863 | 7052.024414 | 9544.892578 | 6760.154785 | 5924.449219 | 10027.5625 | 8586.395508 | 5403.347656 | 3027.911865 | 10851.3584 | 7750.132813 | 17832.21484 | 4600.618652 | 6973.282227 | 8463.695313 | 10108.43066 | 9193.973633 | 7369.652344 | 9199.618164 | 13651.07617 | 7105.381348 | 11751.41602 | 13787.02344 | 15631.41504 | 5542.253418 | 5013.14209 | 16793.91602 | 11427.02148 | 0 | 7011.208008 | 13757.32227 | 4792.483887 | 12141.40625 | 7608.741211 | 18681.82031 | 8519.946289 | 8410.209961 | 4984.700684 | 12964.26367 | 8277.868164 | 8239.514648 | 7567.68457 | 8381.779297 | 3179.715088 | 9247.538086 | 4299.237305 | 6190.694336 | 6567.282227 | 7930.396484 | 12201.06934 | 7542.322754 | 8385.456055 | 14062.74414 | 9824.658203 | 9183.827148 | 5259.494629 | 6688.80127 | 3011.318848 | 3319.831543 |
| E7EWH8 | 97329.52344 | 0 | 65298.39063 | 9131.192383 | 0 | 0 | 0 | 0 | 51010.14063 | 0 | 0 | 0 | 20256.01367 | 49995.27344 | 70439.07031 | 0 | 0 | 13705.46094 | 0 | 0 | 0 | 0 | 0 | 0 | 0 | 102041.0938 | 0 | 0 | 0 | 0 | 127155.7031 | 0 | 0 | 143557.0781 | 30056.17578 | 59616.66406 | 68760.72656 | 0 | 78793.29688 | 0 | 0 | 53168.94531 | 0 | 0 | 140361.5625 | 0 | 0 | 0 | 0 | 0 | 0 | 0 | 0 | 21018.58984 | 0 | 0 | 0 | 0 | 0 | 0 | 0 | 0 |
| E7EX29 | 1219.068726 | 9847.291992 | 1697.744751 | 4715.56543 | 2045.478882 | 1284.111572 | 0 | 0 | 0 | 0 | 478.3484192 | 0 | 3031.354492 | 3267.109131 | 3474.288086 | 0 | 0 | 0 | 4250.508301 | 3549.289307 | 2047.888794 | 1241.244629 | 6297.344727 | 0 | 3335.243164 | 4069.614502 | 5990.679688 | 0 | 0 | 2505.1521 | 1784.313843 | 0 | 761.1897583 | 3647.786621 | 0 | 1854.755005 | 0 | 2116.191162 | 0 | 0 | 0 | 1758.633789 | 1220.622192 | 477.8096924 | 0 | 0 | 0 | 1956.068726 | 0 | 0 | 0 | 0 | 0 | 950.925354 | 1681.687622 | 2762.942871 | 0 | 0 | 0 | 377.8262024 | 0 | 0 |
| E9PAQ1 | 26162.67578 | 22242.33008 | 25842.93945 | 26886.75781 | 30000.94531 | 33761.31641 | 29852.66406 | 33871.63281 | 30262.89063 | 29613.7168 | 42308.24609 | 24394.97266 | 28355.38672 | 20869.20703 | 27270.83008 | 22605.85938 | 23874.93359 | 23579.41016 | 35833.09766 | 28808.53516 | 26506.47656 | 20356.32031 | 27219.52344 | 23113.71484 | 21531.34375 | 15199.61426 | 32435.00391 | 28204.46094 | 32042.72852 | 22609.96484 | 22697.45703 | 32071.86328 | 24958.55859 | 28723.07031 | 18850.80273 | 24471.27734 | 35858.78125 | 26271.5332 | 34061.84766 | 20458.49609 | 23620 | 31535.45898 | 21135.72266 | 23073.02539 | 36601.63281 | 23431.58789 | 30086.76953 | 28696.96875 | 32695.29102 | 21159.94336 | 24604.83984 | 39436.91016 | 31328.44531 | 31196.21484 | 30843.18945 | 37975 | 26828.53516 | 28227.85938 | 28548.90039 | 22275.19922 | 35270.56641 | 30608.75 |
| E9PHK0 | 125364.3125 | 161363.5625 | 146583.3906 | 147706.7656 | 135453.5 | 118491.0156 | 154318.5938 | 131106.3906 | 161497.9219 | 124734.8594 | 89467.92188 | 142907.8438 | 155328.7813 | 164294.5625 | 123689.625 | 134476.7031 | 144645.9531 | 172094.1719 | 123570.2969 | 142677.0313 | 159259.3438 | 172976.2188 | 140447.3438 | 136659.8594 | 148158.9063 | 143890.9531 | 131795.9063 | 145777.5938 | 138644.8438 | 167682.0469 | 118289.1797 | 164365.2969 | 196994.5156 | 205082.6563 | 159656.4375 | 132419.7344 | 128248.875 | 157399.3125 | 115438.5313 | 155422.0313 | 95593.72656 | 162825.6875 | 147091.1563 | 170832.375 | 163916.1563 | 167562.8594 | 132346.1875 | 114205.5391 | 126079.7813 | 139841.9063 | 146235.2188 | 219440.0781 | 214891.6406 | 156666.2969 | 208792 | 146459.6094 | 152990.2813 | 169315.1406 | 161632.4375 | 124518.7734 | 145604.3594 | 124068 |
| E9PK25 | 0 | 12367.22461 | 0 | 0 | 0 | 0 | 0 | 0 | 0 | 0 | 0 | 0 | 0 | 7010.083984 | 6425.853027 | 0 | 0 | 0 | 0 | 3580.556885 | 0 | 0 | 8866.919922 | 0 | 0 | 7107.835449 | 5404.585449 | 0 | 0 | 0 | 0 | 0 | 0 | 3660.098389 | 0 | 0 | 0 | 0 | 0 | 0 | 0 | 2628.230469 | 0 | 0 | 0 | 0 | 0 | 0 | 0 | 0 | 0 | 0 | 0 | 0 | 0 | 2922.074951 | 0 | 0 | 0 | 0 | 0 | 0 |
| F5H8B0 | 4780.874512 | 8621.625 | 0 | 7106.915527 | 5512.998047 | 5026.394531 | 8263.69043 | 2473.705811 | 4141.40625 | 9401.792969 | 5442.647461 | 4031.716553 | 7476.506348 | 6370.330078 | 6733.84668 | 7696.304688 | 7913.401855 | 6850.044434 | 2624.358643 | 6336.708496 | 6900.452637 | 6773.733887 | 5957.932617 | 6510.169434 | 6231.466797 | 5536.196289 | 3954.944336 | 10567.93457 | 4288.312988 | 4466.331055 | 8659.954102 | 6840.444336 | 3979.426025 | 8713.548828 | 6349.428711 | 5025.836914 | 5150.051758 | 6822.604492 | 2406.400391 | 6510.381836 | 5596.305176 | 7220.918945 | 4552.54248 | 3766.056641 | 6038.510742 | 9181.632813 | 5979.191895 | 5361.504395 | 5021.743652 | 4695.472656 | 3884.818115 | 6337.945801 | 6740.748535 | 7579.6875 | 5917.981445 | 5123.580078 | 6047.629883 | 7028.643555 | 9220.942383 | 5134.010742 | 5024.678223 | 7312.256348 |
| F8W1S1 | 9560.558594 | 4202.304199 | 5466.364258 | 5987.447266 | 7469.641113 | 6483.217285 | 4851.026367 | 4218.516113 | 5645.625 | 5633.01123 | 0 | 4761.564941 | 5013.333496 | 0 | 5144.746094 | 6588.219727 | 10172.99609 | 8310.475586 | 0 | 0 | 8390.269531 | 4818.063477 | 4468.358887 | 6392.800781 | 4598.510254 | 6000.939453 | 3392.843994 | 0 | 8253.604492 | 4101.825195 | 6967.164063 | 7243.057129 | 4457.280762 | 5884.506348 | 6770.304199 | 4524.458496 | 6533.78125 | 2638.419434 | 5497.128418 | 4187.589355 | 5398.374512 | 3737.217529 | 0 | 4896.889648 | 4378.550293 | 12566.51953 | 4254.444824 | 5364.071777 | 4907.103516 | 5056.610352 | 7142.02002 | 3693.083008 | 0 | 4698.593262 | 3264.843506 | 6320.288574 | 0 | 5575.162598 | 0 | 0 | 12186.16992 | 4901.659668 |
| F8WF14 | 28689.05469 | 39332.98438 | 30108.57031 | 28266.07813 | 46812.8125 | 33838.57813 | 25476.19922 | 30851.11328 | 25780.26563 | 38635.71484 | 24750.13477 | 17524.24805 | 18922.99414 | 26340.44922 | 20174.08984 | 22272.2793 | 20469.13281 | 17903.31445 | 31793.74219 | 26447.89453 | 23368.27148 | 26349.93359 | 19616.52734 | 17231.12891 | 19493.05078 | 23843.26758 | 35364.89844 | 29576.45117 | 26027.62695 | 14205.03418 | 22756.55078 | 25119.13086 | 24082.94531 | 14785.07324 | 24789.2793 | 19438.09375 | 29820.09375 | 22236.72656 | 37065.61719 | 17475.66406 | 20495.11914 | 18735.38281 | 33914.21094 | 17946.83203 | 22690.10352 | 22293.07031 | 12158.19434 | 25850.61328 | 41703.23438 | 12935.96094 | 19457.16602 | 26596.9707 | 32804.76563 | 24030.54297 | 18766.57422 | 23145.60352 | 17642.00781 | 18843.89844 | 23912.00586 | 26070.44922 | 30711.30664 | 18303.38281 |
| G3V0E5 | 5214.556641 | 6896.581543 | 10887.2832 | 4337.584961 | 4331.263184 | 5900.104492 | 6351.155273 | 5849.15332 | 5844.342773 | 5840.983398 | 3578.067139 | 4670.24707 | 5073.281738 | 8039.501953 | 9379.679688 | 4168.004395 | 7823.063477 | 8664.927734 | 4440.442871 | 7440.828613 | 6685.101563 | 6951.004395 | 4055.413086 | 5207.724121 | 4813.657227 | 4853.326172 | 5284.820801 | 7653.881348 | 5253.213867 | 4930.010742 | 5612.139648 | 5533.71875 | 6108.832031 | 3490.846191 | 5041.593262 | 10425.1709 | 5516.21582 | 6486.256348 | 5122.43457 | 8525.232422 | 3598.527832 | 6832.138672 | 7594.926758 | 3350.18457 | 2677.162598 | 8262.678711 | 8600.274414 | 2531.121094 | 5581.378906 | 4029.995605 | 12802.17969 | 11045.34375 | 4579.609375 | 3017.831055 | 3991.170898 | 5367.913086 | 6704.072266 | 4433.163574 | 3250.14624 | 3629.760986 | 8827.875977 | 6232.321777 |
| G3V2W1 | 5887.166992 | 10308.0498 | 5730.830078 | 6883.703125 | 11967.76367 | 14363.69727 | 12680.02734 | 15709.17383 | 5401.023926 | 11810.53125 | 17786.5 | 12434.19043 | 6451.542969 | 11763 | 10644.30078 | 8787.561523 | 6561.558594 | 11496.32031 | 14674.08594 | 7635.99707 | 8274.637695 | 8902.907227 | 9483.255859 | 13905.39453 | 6387.352051 | 10391.92969 | 8969.667969 | 11386.03125 | 5549.039063 | 7848.271484 | 5994.455566 | 13080.52539 | 10483.31543 | 8026.089355 | 9911.675781 | 10476.37598 | 5622.787109 | 8960.708984 | 10892.97461 | 13202.16504 | 5360.668945 | 8456.78418 | 12347.02344 | 8675.591797 | 4996.129395 | 7730.282227 | 8013 | 9666.733398 | 14275.17383 | 6695.609375 | 10817.22754 | 7794.895508 | 26779.76563 | 7958.163574 | 9998.112305 | 9542.230469 | 9282.496094 | 11649.40723 | 7706.094727 | 9436.78125 | 7975.847656 | 2864.001953 |
| G3XAK1 | 11810.62305 | 14368.93164 | 14162.12305 | 17636.98633 | 12943.19238 | 11592.79297 | 14509.20508 | 16815.85547 | 13169.72949 | 15715.92188 | 17878.55469 | 14514.01367 | 15500.04688 | 13471.22168 | 14362.69336 | 18761.82422 | 18281.67188 | 15293.71387 | 23020.4043 | 21416.30078 | 18509.84766 | 16548.18359 | 11773.64453 | 21071.82227 | 11817.21582 | 19237.43555 | 15186.55273 | 28186.52734 | 13955.94727 | 23281.82813 | 10053.96973 | 17232.91992 | 16270.80664 | 20362.24219 | 17268.77148 | 18316.34375 | 22090.54688 | 14670.11914 | 17815.89453 | 29108.54297 | 12157.78125 | 13135.76563 | 11208.22754 | 13249.56934 | 19051.71875 | 21312.51563 | 12311.47461 | 21759.32031 | 15570.0957 | 20367.41992 | 16329.06738 | 13444.91406 | 22337.40234 | 17339.38867 | 15557.44629 | 14284.5166 | 16414.69141 | 19250.76758 | 16617.21289 | 19067.35352 | 17847.30078 | 17706.5 |
| G3XAP6 | 8829.885742 | 9321.490234 | 7574.886719 | 12627.12305 | 12812.3457 | 12203.33594 | 7014.973145 | 0 | 8089.624023 | 16064.49414 | 4694.632324 | 5568.978027 | 8765.488281 | 5554.447754 | 3733.617676 | 8763.064453 | 8592.921875 | 12582.94531 | 9666.046875 | 12717.80664 | 8454.522461 | 13912.57422 | 8223.783203 | 6459.616699 | 9732.939453 | 7154.416504 | 0 | 8863.546875 | 8773 | 10743.54199 | 4782.505859 | 11131.1377 | 9790.105469 | 11549.24023 | 5719.443359 | 9428.234375 | 12914.42188 | 7851.381348 | 8987.914063 | 0 | 9267.634766 | 16323.32031 | 0 | 9753.206055 | 11516.88965 | 14796.92969 | 0 | 0 | 6819.514648 | 13739.50293 | 8130.021973 | 12251.74609 | 15526.46191 | 7848.883789 | 10239.38086 | 11327.35352 | 7396.783691 | 11371.97852 | 7757.553711 | 5791.086914 | 4166.128906 | 5759.810547 |
| H0Y2Y8 | 0 | 10480.74707 | 0 | 7473.821289 | 0 | 0 | 0 | 0 | 0 | 0 | 0 | 0 | 6290.929199 | 8054.692383 | 6448.783691 | 0 | 0 | 0 | 9291.349609 | 9130.594727 | 0 | 0 | 13109.34375 | 0 | 10911.51855 | 8498.52832 | 12560.16797 | 0 | 0 | 0 | 0 | 0 | 0 | 6146.396484 | 0 | 4656.036621 | 0 | 2106.783203 | 0 | 0 | 0 | 0 | 7125.779785 | 0 | 0 | 0 | 0 | 7101.03125 | 0 | 0 | 0 | 0 | 0 | 0 | 0 | 7796.521973 | 4493.562012 | 0 | 0 | 0 | 0 | 0 |
| H0Y5E4 | 25508.24414 | 23458.33594 | 21684.80078 | 20260.82031 | 19963.18555 | 12332.13086 | 22597.88086 | 17172.13281 | 22810.04297 | 13391.65137 | 22965.82813 | 17391.76563 | 11068.22949 | 17662.10547 | 18260.66992 | 15450.89551 | 19080.5918 | 15338.44043 | 21397.64648 | 21958.36523 | 17795.03711 | 23032.27539 | 23931.33008 | 19811.85156 | 20920.39453 | 15043.35449 | 17486.0332 | 22408.66797 | 18643.38086 | 24860.95508 | 12640.58398 | 19069.08203 | 14361.72363 | 16227.46191 | 16436.8457 | 11518.55078 | 18091.66797 | 23866.2832 | 12790.77539 | 17594.12305 | 21103.07031 | 18407.64063 | 22849.09766 | 18759.87305 | 21832.21875 | 20554.16797 | 12922.83301 | 16557.375 | 23843.76758 | 24049.93945 | 17786.10547 | 23867.4668 | 24379.28906 | 19635.14453 | 22037.21484 | 16387.76953 | 12435.48828 | 17534.29883 | 18198.39844 | 17141.01563 | 22591.12891 | 12120.68555 |
| H0Y755 | 9440.064453 | 7492.646484 | 8988.173828 | 7151.035645 | 5346.280273 | 4825.551758 | 10282.97949 | 7305.191406 | 7809.237305 | 9631.806641 | 6403.0625 | 8439.893555 | 7282.981445 | 5832.565918 | 6119.334961 | 10123.79688 | 4729.918945 | 5497.407227 | 0 | 18560.85156 | 9009.626953 | 8861.826172 | 3968.427002 | 12461.3418 | 7523.210938 | 5561.008789 | 9082.419922 | 10406.46094 | 9479.841797 | 9058.750977 | 8938.988281 | 8030.885742 | 5418.77832 | 6421.017578 | 5812.535156 | 7753.602539 | 0 | 8010.712402 | 12379.65527 | 5569.148438 | 7029.716797 | 16329.15332 | 8922.080078 | 8131.723633 | 12904.74609 | 11287.37305 | 9411.428711 | 7945.599121 | 3936.200439 | 11087.5332 | 6179.969727 | 6842.789063 | 9146.219727 | 9079.885742 | 9936.591797 | 5857.78125 | 6726.570313 | 11104.93652 | 7690.847656 | 12100.94336 | 5213.057129 | 5052.808594 |
| H0YAC1 | 127880.3906 | 137214.1406 | 107910.6641 | 126108.2031 | 121577.2969 | 149095.0625 | 185619.0938 | 159610.5 | 124412.75 | 169424.3438 | 161722.4219 | 151605.0938 | 137034.5625 | 117260.4063 | 127237.4063 | 121147.25 | 169298.8438 | 161040.9844 | 226618.75 | 115428.6016 | 150095.7813 | 104073.5313 | 169830.7813 | 145841.1094 | 122171.125 | 120837.6875 | 94929.17188 | 200157.6406 | 129465.1406 | 112227.3906 | 136474.4375 | 116552.1875 | 110939.5 | 168474.7188 | 152312.9063 | 130563.6484 | 183558.6563 | 131527.3906 | 143071.5625 | 215553.7813 | 128737.4766 | 117764.5078 | 171159.375 | 129492.0313 | 104595.5469 | 185873.0938 | 136320.25 | 174518.2188 | 194759.4063 | 57548.42969 | 149358.7188 | 144707.2969 | 226073.1875 | 127901.7266 | 154446.0625 | 134858.4844 | 181998.625 | 191883.1719 | 149941.4375 | 138863.1875 | 133256.5938 | 152856.7813 |
| H0YCR7 | 0 | 0 | 0 | 0 | 0 | 4806.902344 | 0 | 0 | 4491.730957 | 0 | 0 | 0 | 5228.708496 | 0 | 0 | 0 | 0 | 0 | 0 | 0 | 0 | 973.7526245 | 0 | 0 | 0 | 3556.803223 | 0 | 7174.221191 | 0 | 0 | 0 | 3236.969971 | 0 | 0 | 2979.17334 | 0 | 0 | 2745.294189 | 0 | 0 | 0 | 0 | 0 | 4910.100586 | 0 | 0 | 4441.147461 | 61713.17578 | 0 | 5508.702637 | 0 | 0 | 0 | 0 | 13327.63281 | 6707.564941 | 4041.436279 | 0 | 6701.021973 | 5949.584473 | 0 | 0 |
| H0YGX7 | 0 | 0 | 0 | 0 | 0 | 0 | 0 | 0 | 0 | 0 | 0 | 0 | 10662.8623 | 0 | 0 | 0 | 0 | 0 | 0 | 0 | 0 | 0 | 16017.17285 | 0 | 0 | 9576.873047 | 4889.776367 | 0 | 0 | 4917.520996 | 0 | 0 | 0 | 8773.018555 | 0 | 0 | 0 | 0 | 0 | 0 | 0 | 0 | 0 | 0 | 0 | 0 | 0 | 0 | 0 | 0 | 0 | 0 | 0 | 0 | 0 | 0 | 0 | 0 | 0 | 0 | 0 | 0 |
| H0YJW9 | 713393.9375 | 1683279.25 | 783350.3125 | 1492981.625 | 86481.96875 | 791703.8125 | 1080211.125 | 704634.3125 | 661507.6875 | 247464.2031 | 826178.375 | 894539.25 | 1139185.25 | 747730.625 | 936250.875 | 721782.5 | 749817.0625 | 712276.8125 | 560020.625 | 802832.1875 | 827937.9375 | 604659.875 | 777875.625 | 721492.625 | 665335.25 | 1021058.563 | 1036995.063 | 1051538.125 | 703163 | 973923.5625 | 667020.6875 | 704506.0625 | 975582.25 | 797110 | 904450.5 | 638836.125 | 1080405.75 | 2204722 | 676794 | 683572.375 | 573133.4375 | 799649.1875 | 872687.125 | 750588.375 | 1268883.875 | 768598.625 | 550272.875 | 760272.0625 | 711914.1875 | 677977.75 | 523852.1875 | 650245.0625 | 860564.875 | 693969.4375 | 1444698.625 | 597238.75 | 557331.3125 | 541196.0625 | 1033118.5 | 715772.125 | 875921.625 | 693742.4375 |
| H3BTN5 | 0 | 5454.570313 | 0 | 6467.92627 | 0 | 0 | 0 | 0 | 0 | 0 | 16255.74609 | 0 | 0 | 0 | 7404.786621 | 5286.175781 | 4551.478027 | 0 | 5122.074707 | 7696.918457 | 4793.130859 | 537731.75 | 2188.947754 | 0 | 10230.17871 | 9426.685547 | 222.2299347 | 0 | 0 | 0 | 8670.944336 | 0 | 0 | 7860.366211 | 0 | 1803.64978 | 0 | 0 | 0 | 3294.064697 | 0 | 5334.849609 | 3425.93335 | 0 | 0 | 0 | 0 | 0 | 0 | 0 | 0 | 0 | 694.4683228 | 0 | 3162.271973 | 1638.991211 | 4630.966309 | 4690.289551 | 1030.095337 | 0 | 0 | 0 |
| H3BUA5 | 349831.9063 | 370004.5938 | 492156.6875 | 297924.4688 | 524251.6563 | 398144.8438 | 433201.5313 | 375556.6563 | 399024.5625 | 338459.5938 | 278434.7813 | 388501.0938 | 449962.9375 | 325269.8438 | 409126.875 | 349348.9688 | 369461.0313 | 402336.375 | 262961.875 | 296254.1875 | 715960.6875 | 379326.5313 | 517836.5625 | 442349.3438 | 759090.875 | 291223.9063 | 592981.875 | 268088.7813 | 352917.8125 | 670245.125 | 365994.8438 | 524508.5 | 383343.6563 | 522288.75 | 478388.9375 | 685353.375 | 422509.1875 | 313859.8438 | 358716.9063 | 317473.875 | 285604.0625 | 309378.7188 | 468470.2188 | 589806.5 | 297074.6563 | 896828.5625 | 334891.4063 | 417534.5 | 215849.75 | 294857.625 | 735137.875 | 853087.375 | 282307.4688 | 422533.1875 | 482494.9063 | 303319.125 | 613190.8125 | 288984.2188 | 367973.6563 | 321241.0313 | 422760.4375 | 501895.4688 |
| H9KV75 | 7059.634766 | 19834.33594 | 0 | 7729.000977 | 1744.365479 | 3313.124512 | 752.3317261 | 0 | 0 | 0 | 3881.11499 | 0 | 1687.696289 | 3975.262451 | 3406.296875 | 0 | 0 | 0 | 4958.756348 | 4752.37207 | 1632.626221 | 0 | 5875.78418 | 0 | 4301.491699 | 6174.57666 | 10076.99219 | 6146.976074 | 0 | 3790.554199 | 3917.546631 | 0 | 2290.467529 | 4924.527832 | 0 | 0 | 0 | 5377.258789 | 0 | 0 | 8790.407227 | 2280.763428 | 6624.226563 | 31592.91211 | 0 | 2237.35376 | 0 | 4492.13623 | 0 | 0 | 0 | 0 | 3029.850098 | 0 | 4632.497559 | 6873.080566 | 5106.619629 | 3041.433105 | 0 | 2840.702881 | 0 | 0 |
| I3L145 | 12859.64941 | 18466.20508 | 23451.91016 | 15681.16602 | 6145.517578 | 4654.64502 | 5375.015625 | 1848.722412 | 1914.428101 | 6742.684082 | 7702.602051 | 8765.691406 | 21225.23242 | 12427.2832 | 20561.98633 | 13814.27344 | 11799.88672 | 6386.016113 | 6972.45166 | 7712.721191 | 35877.63281 | 8805.075195 | 4183.527344 | 8654.841797 | 13429.43945 | 8873.232422 | 3879.901611 | 13392.80566 | 8611.237305 | 18938.75391 | 17488.12305 | 16101.84375 | 22406.77148 | 10193.47754 | 15741.21875 | 19895.58398 | 5714.470215 | 11981.98438 | 8207.945313 | 18838.33984 | 6189.306641 | 4915.027344 | 3137.965088 | 38143.72656 | 22483.04297 | 10811.18555 | 16686.80859 | 7800.053711 | 8368.754883 | 19627.02148 | 17995.11328 | 15513.52539 | 13785.37891 | 3600.847656 | 7583.15918 | 8543.155273 | 8837.613281 | 21799.27734 | 5935.87207 | 5701.80127 | 8733.336914 | 57771.14063 |
| I3L1J2 | 0 | 0 | 9208.087891 | 6635.908203 | 15544.76367 | 4724.629883 | 0 | 0 | 9128.295898 | 1291.412109 | 3556.038818 | 0 | 0 | 13482.72656 | 0 | 0 | 7053.259766 | 8607.121094 | 0 | 0 | 0 | 16210.86914 | 5749.305664 | 4611.822754 | 0 | 0 | 6004.318359 | 0 | 0 | 9345.00293 | 0 | 8009.218262 | 0 | 5647.242676 | 3547.507324 | 3703.193848 | 0 | 8494.499023 | 4999.287598 | 9110.535156 | 0 | 0 | 0 | 0 | 11487.05078 | 7504.768555 | 0 | 0 | 0 | 0 | 538.359314 | 0 | 4018.341064 | 18413.25195 | 14174.94531 | 0 | 0 | 0 | 7178.410645 | 0 | 0 | 0 |
| J3KNB4 | 23161.17969 | 11137.2334 | 11152.38184 | 15216.50488 | 22032.04102 | 12335.95313 | 15485.54004 | 28718.97656 | 17337.44727 | 24464.39063 | 8207.380859 | 8248.740234 | 19320.08008 | 9058.463867 | 8592.175781 | 4716.183594 | 5700.606445 | 8976.168945 | 6927.104004 | 15409.16309 | 11358.84766 | 7350.042969 | 23943.80664 | 17460.95508 | 13862.66699 | 10976.13965 | 16760.23633 | 16332.53613 | 7767.961426 | 8219.258789 | 14958.45898 | 21051.38867 | 14866.44141 | 19753.58398 | 19558.68945 | 19859.27344 | 14043.04297 | 10113.33301 | 16064.7998 | 9117.626953 | 8345.333984 | 12880.75293 | 11953.31543 | 12621.02148 | 9693.576172 | 12124.53809 | 16126.71387 | 11383.76465 | 26111.0293 | 7444.619629 | 14174.42383 | 9894.735352 | 20030.78125 | 9317.836914 | 9275.776367 | 8577.770508 | 9359.875 | 17697.57617 | 37280.50781 | 38241.00781 | 8387.0625 | 3709.910889 |
| J3KPA1 | 10811.55664 | 15834.82813 | 18279.16797 | 17013.90039 | 11918.4834 | 2125.875977 | 0 | 14021.97168 | 10745.3291 | 8853.958008 | 13214.35449 | 7466.270996 | 0 | 0 | 0 | 0 | 13475.42773 | 0 | 8309.493164 | 13693.66602 | 0 | 8923.199219 | 7930.25 | 11609.85254 | 0 | 4077.768799 | 10730.4043 | 14953.44727 | 11344.26367 | 15106.5 | 0 | 0 | 0 | 11963.66602 | 0 | 10356.4668 | 12659.03711 | 6965.532227 | 0 | 14893.83984 | 11137.57129 | 12432.06055 | 11852.79688 | 16708.44727 | 12571.93945 | 13992.55566 | 16526.52148 | 9971.162109 | 10357.00879 | 11156.46289 | 0 | 0 | 8676.487305 | 4856.546875 | 0 | 15228.33789 | 12867.31934 | 7857.62207 | 9765.461914 | 14622.58398 | 13617.71289 | 8385.827148 |
| J3KPS3 | 0 | 11247.93848 | 0 | 4231.104004 | 0 | 0 | 0 | 0 | 0 | 0 | 18079.23633 | 0 | 2941.138184 | 0 | 0 | 0 | 0 | 0 | 0 | 0 | 0 | 0 | 7217.133301 | 0 | 0 | 4068.220703 | 6597.937988 | 0 | 0 | 3515.089111 | 0 | 0 | 0 | 0 | 1653.002563 | 0 | 0 | 1347.495117 | 0 | 0 | 0 | 0 | 0 | 0 | 0 | 0 | 0 | 0 | 0 | 0 | 0 | 0 | 0 | 0 | 0 | 0 | 0 | 0 | 0 | 0 | 0 | 0 |
| J3KRP0 | 37252.62109 | 37371.46094 | 55113.89063 | 7443.650879 | 51117.4375 | 35306.52734 | 39773.17969 | 17296.08203 | 22664.28516 | 32592.51953 | 29531.17969 | 32633.01172 | 28622.90039 | 28923.96875 | 13433.30273 | 21403.63867 | 41579.10156 | 24913.36523 | 24310.41602 | 19914.59766 | 33395.91016 | 5007.759766 | 18543.15625 | 23631.04492 | 18484.67969 | 21598.87891 | 38865.66797 | 46622.80469 | 37542.00781 | 22108.9707 | 26159.94141 | 21009.58008 | 24999.0625 | 38493.17188 | 25373.95117 | 16456.10547 | 15770.64258 | 25362.33594 | 35471.6875 | 18961.37109 | 14635.62988 | 27513.67383 | 22146.57031 | 13391.27246 | 34444.44531 | 46260.45313 | 19208.66992 | 34689.67188 | 62742.5 | 18750.7168 | 23821.27734 | 38168.82031 | 36502.32031 | 36373.52734 | 23853.50391 | 29177.94141 | 41966.03125 | 56324.88281 | 26742.92969 | 35781.48047 | 17221.83594 | 27158.26563 |
| J3QRV5 | 210293.8125 | 316518.5 | 242738.4375 | 290633.9375 | 244046.4688 | 245847.4375 | 302340.8438 | 476450.7188 | 201625.875 | 276221.625 | 276362.4063 | 284058.375 | 303014.8125 | 456468.75 | 279954.6875 | 592129.0625 | 555194.5625 | 237945.4844 | 343245.2813 | 525935.625 | 588733.0625 | 474459.9688 | 189205.8906 | 395923.125 | 318024.9688 | 262610.875 | 260885.9375 | 628158.625 | 273164.8125 | 408044.9688 | 367377.5625 | 314250.875 | 260301.1406 | 284474.5 | 576289.4375 | 201876.875 | 504313.5625 | 217407.375 | 160541.7969 | 832395.125 | 334515.5625 | 252838.9219 | 500449.1563 | 313893.625 | 222235.5781 | 214894.5 | 518606.0938 | 265345.9063 | 317501.3438 | 254088.2969 | 221865.8438 | 380994.7813 | 773688.875 | 262511.75 | 430240.5625 | 441980.1875 | 445966.5625 | 235047.5625 | 426828.3438 | 580042.6875 | 193522.25 | 676865.375 |
| J3QT83 | 101123.1484 | 111260.875 | 109812.4688 | 83749.49219 | 83323.60938 | 86784.85938 | 0 | 0 | 103149.1328 | 221350.6563 | 0 | 69725.17188 | 70860.29688 | 127517.9453 | 210321.8281 | 180513.5625 | 146911.8125 | 173482.1094 | 0 | 0 | 115300.6406 | 0 | 0 | 91668.94531 | 0 | 119705.5078 | 61033.95313 | 0 | 0 | 0 | 238752.125 | 36662.01563 | 33377.59375 | 164808.2031 | 0 | 120255.3125 | 0 | 0 | 99366.52344 | 0 | 0 | 42237.48047 | 0 | 0 | 233092.3906 | 0 | 0 | 0 | 0 | 0 | 0 | 0 | 0 | 0 | 0 | 8652.125977 | 0 | 0 | 48448.64063 | 0 | 0 | 40721.21484 |
| K7ER74 | 172609.5469 | 796820.125 | 553791.25 | 1149494.25 | 135900.0156 | 127962.5313 | 210650.3594 | 382463.5313 | 777771.5 | 1725421.125 | 134258.8438 | 97620.67969 | 2162058.75 | 439508.6875 | 1049891.125 | 56027.28125 | 190321.375 | 595170.125 | 291768.4375 | 134977.0625 | 146100.4531 | 74440.96875 | 135389.7344 | 69035.65625 | 210889.4531 | 161521.9688 | 159020.8281 | 480837.5625 | 492602.7188 | 430697.9688 | 842003.3125 | 938456.625 | 763976.25 | 633398.625 | 775573.1875 | 970000.1875 | 1010858.25 | 390300.5625 | 1468492.625 | 328668 | 630365.25 | 125520.4219 | 386882.5625 | 39998.65625 | 644594 | 314851.4688 | 445701.875 | 289236.25 | 322556.25 | 143002.1406 | 1088095.25 | 179047.4688 | 436253.0938 | 995456.375 | 147200.9844 | 202986.9063 | 98296.5625 | 465793.9063 | 489388.1563 | 780339.25 | 198453.5313 | 633151.125 |
| K7ERG9 | 10245.35352 | 11349.99121 | 10201.45703 | 5545.12793 | 10517.25684 | 6261.680664 | 11027.70703 | 4237.450195 | 8863.039063 | 6455.208984 | 8815.663086 | 7333.860352 | 9611.335938 | 8663.865234 | 6621.543945 | 11409.58301 | 9612.805664 | 9626.914063 | 3095.989014 | 9920.591797 | 8541.335938 | 6529.84375 | 9521.953125 | 5636.199219 | 10174.38379 | 10360.90527 | 5788.652832 | 11330.07617 | 5285.384766 | 12078.43359 | 10995.20703 | 13859.83594 | 6281.373047 | 10233.99023 | 7376.92627 | 10655.03711 | 16117.42383 | 7108.304688 | 13545.00098 | 6401.558105 | 11143.33301 | 8523.894531 | 10631.53906 | 11535.65234 | 9961.9375 | 12893.82617 | 12702.63477 | 11992.47656 | 13892.41113 | 8274.06543 | 16370.97168 | 14071.66211 | 11653.02051 | 14388.11133 | 12777.83203 | 13904.85742 | 6105.903809 | 18718.83203 | 8538.878906 | 11226.06641 | 18229.64648 | 12504.19824 |
| K7ERI9 | 1762749.125 | 2074026.75 | 3382721.5 | 2439030 | 2456334 | 1883628.25 | 1531939.875 | 3421857.25 | 1662586 | 2513009.5 | 1725158.875 | 1781221.125 | 3436941.25 | 3290992.75 | 2113919.75 | 1540751.5 | 1961540.25 | 1902600.875 | 2705371 | 1509089 | 2439577.75 | 2298158 | 2346763.5 | 2485067.5 | 2641796.75 | 1660889.25 | 3652759.25 | 3209725.25 | 1540948 | 2042337.25 | 2329376.5 | 2398973.25 | 2875984.25 | 2450206.25 | 2072177.875 | 2373430.25 | 2103041.25 | 1652023.75 | 1685272.5 | 2992220 | 1782933.875 | 1100764.75 | 5086179.5 | 1674272.375 | 1762572.125 | 1730543.625 | 2203040.75 | 2108268.25 | 4374390.5 | 2247086 | 2788961.75 | 1993011.125 | 2554609.25 | 2090107.25 | 1860967.25 | 1700993.375 | 1251897.875 | 2107863.75 | 3563951.25 | 3567942 | 2004845 | 3235600.5 |
| M0R0Q9 | 5308.398438 | 5247.26709 | 6312.57373 | 7304.830566 | 7064.03125 | 5668.829102 | 7272.54834 | 8288.632813 | 5499.129883 | 7512.96582 | 6454.071289 | 4053.119141 | 4476.859863 | 3335.535889 | 4553.055176 | 6121.629395 | 3986.312256 | 0 | 5080.916016 | 4357.348633 | 7144.20459 | 5142.484863 | 5408.428223 | 6778.042969 | 5294.231445 | 4269.902344 | 6453.507813 | 8136.420898 | 5872.258789 | 4886.34082 | 0 | 6449.430176 | 3937.451172 | 4593.431641 | 6822.959473 | 4594.921875 | 6164.419434 | 4742.197754 | 5303.836914 | 5682.298828 | 5761.624023 | 5033.40918 | 5640.595215 | 6018.282715 | 3114.217773 | 5147.756836 | 4742.727051 | 6313.947754 | 7069.48291 | 3720.683838 | 5813.468262 | 5131.129883 | 7017.260742 | 5482.030273 | 6709.735352 | 4942.723145 | 6982.885742 | 5062.79248 | 7106.206055 | 6490.255859 | 8213.867188 | 6909.799805 |
| O00187 | 31960.41211 | 43798.50781 | 27847.77539 | 9255.133789 | 10040.46484 | 23639.03125 | 7145.029297 | 24687.04883 | 18868.66406 | 35869.46484 | 62089.57031 | 21432.03906 | 37814.97656 | 46521.25781 | 31429.83008 | 35837.88672 | 73126.82031 | 20726.04297 | 9374.291992 | 22993.06836 | 33409.6875 | 60175.375 | 23646.84961 | 16002.02832 | 39077.00781 | 9765.401367 | 33043.0625 | 37899.42578 | 37403.22656 | 31118.42773 | 55377.82422 | 21018.65039 | 17627.25781 | 21892.17188 | 31497.58789 | 12439.99707 | 24349.53711 | 6581.037109 | 34012.02734 | 44609.75391 | 41491.03125 | 48527.32031 | 13938.5918 | 50061.40625 | 8121.710938 | 25680.52734 | 0 | 27712.62109 | 14767.08301 | 25505.5293 | 23121.12695 | 41861.75781 | 46394.20313 | 21372.87305 | 46655.45313 | 52185.66406 | 48668.30469 | 35848.98438 | 33238.69531 | 44769.99609 | 19606.48047 | 51983.98438 |
| O00391 | 11337.66992 | 10214.66406 | 8501.294922 | 8811.509766 | 6438.463867 | 7265.411621 | 12168.90039 | 7824.558594 | 12042.03516 | 7802.086914 | 17197.08789 | 7592.998047 | 11010.125 | 9267.836914 | 9456.270508 | 5569.398926 | 10708.4043 | 9798.333984 | 10922.0293 | 6981.90625 | 8296.651367 | 10374.78809 | 12703.12207 | 10461.52441 | 19598.92578 | 6061.246094 | 10145.79102 | 11594.00098 | 8574.075195 | 13133.88867 | 9130.117188 | 9978.208008 | 9608.704102 | 10617.01367 | 10432.19629 | 7536.691406 | 9076.716797 | 11648.64453 | 10185.5625 | 15415.85645 | 12850.67188 | 11434.20508 | 15619.40723 | 10237.25098 | 10430.5459 | 10840.14453 | 11184.07422 | 5255.20459 | 7974.948242 | 5618.668457 | 9198.708008 | 22701.36523 | 8627.337891 | 9783.035156 | 9754.119141 | 6900.427734 | 8148.484375 | 7959.465332 | 8356.025391 | 13813.04883 | 9560.775391 | 9108.766602 |
| O14791 | 47987.4375 | 50025.39063 | 46903.67188 | 29380.875 | 78756.92188 | 54684.5625 | 43956.05469 | 52001.24219 | 41112.59375 | 27994.26172 | 31824.95703 | 48546.15625 | 43266.01563 | 40110.46484 | 37910.60156 | 31928.99219 | 47265.66406 | 30620.0293 | 31171.74609 | 26009.6875 | 61650.58594 | 17793.94141 | 20382.48828 | 43340.9375 | 17642.7793 | 39272.10938 | 48565.875 | 20356.55859 | 34399.33203 | 17617.10938 | 47330.66406 | 19325.81641 | 26564.83984 | 34411.57422 | 45052.43359 | 50441.32813 | 51693.86719 | 43620.71875 | 62270.49219 | 25486.45898 | 42775.80859 | 31059.50781 | 44703.48828 | 31452.13086 | 30383.72461 | 21192.13672 | 30758.75781 | 52081.99219 | 25566.90234 | 39963.96875 | 31905.96875 | 33200.05859 | 41028.90625 | 38836.29688 | 41634.43359 | 40993.18359 | 53733.77344 | 22265.44141 | 25083.74609 | 28956.04102 | 30293.48047 | 46913.59766 |
| O43866 | 283424.8438 | 229061.5313 | 356394.1875 | 130370.3438 | 175868.5625 | 115300.4453 | 64492.81641 | 143004.2344 | 132511.8281 | 155215.2969 | 162945.3438 | 146490.625 | 211869.6406 | 192363.1563 | 212202.9219 | 154870.2031 | 232335.9375 | 63343.23047 | 78052.28125 | 93100.82031 | 199780.1563 | 253571.2656 | 115488.8047 | 161588.5313 | 108423.6797 | 65216.78516 | 155492.3438 | 100473.3828 | 172911.8281 | 105196.0313 | 219547.5 | 122365.2266 | 148854 | 90301.1875 | 100833.6953 | 129845.7578 | 179961.0313 | 170066.6563 | 245083.1563 | 132256.5781 | 181842.8125 | 166541.6094 | 407290.125 | 108099.0625 | 206401.0938 | 162790.7031 | 142111.3281 | 124242.1484 | 128067.625 | 65338.6875 | 139064.375 | 282725.4063 | 84060.67188 | 112301.2813 | 89928.85938 | 66873.28906 | 98508.26563 | 93772.44531 | 189964.5625 | 112482.4922 | 226451.1563 | 146912.2188 |
| O75460 | 44364.64063 | 86807.375 | 140019.9219 | 40758.23047 | 117318.0078 | 258902.5469 | 180413.8438 | 156193.8906 | 115597.7109 | 238932.1094 | 261676.5156 | 120881.3984 | 78283.33594 | 248420.4844 | 158604.2344 | 197470.5625 | 75978.34375 | 134657.5938 | 51000.29297 | 12112.03613 | 134039.5938 | 69789.15625 | 113920.2578 | 135975.0781 | 45977.89844 | 175597.9844 | 132112.8906 | 93446.67969 | 100545.6484 | 6777.224609 | 117646.25 | 122840.3984 | 62050.44531 | 66654.57031 | 144832.1563 | 86679.47656 | 113550.2813 | 186903.4063 | 115237.6953 | 138904.4219 | 118858.6797 | 36081.33594 | 101602.2188 | 92733.125 | 73287.73438 | 67664.32031 | 71836.5625 | 235354.3906 | 120186.2813 | 68491.48438 | 94554.38281 | 75577.57031 | 36089.88672 | 146626.1563 | 105276.2969 | 127217.3828 | 184650.0469 | 78991.17969 | 81650.78906 | 110506.7578 | 0 | 54164.28906 |
| O75636 | 64395.78516 | 28481.20703 | 43128.83594 | 43946.07031 | 140978 | 93367.5625 | 46973.51563 | 80595.65625 | 51651.78125 | 66621.09375 | 62724.41797 | 43941.80469 | 77952.95313 | 60293.47656 | 58827.25781 | 35341.34375 | 34619.63672 | 59035.76172 | 74051.70313 | 52861.60547 | 50099.65234 | 40176.35938 | 57545.35156 | 32187.89258 | 62384.82031 | 83799.875 | 27883.95703 | 12782.95508 | 25290.69141 | 62897.50781 | 21948.84961 | 33191.99219 | 73240.70313 | 55201.03125 | 30425.05078 | 76739.07031 | 26037.63867 | 66117.19531 | 72117.76563 | 16805.91406 | 38564.20313 | 56397.85547 | 15480.91113 | 32027.2793 | 71314.52344 | 21849.60547 | 21506.20313 | 31553.20703 | 68652.59375 | 30976.66797 | 48239.41016 | 43539.01953 | 35036.27734 | 116526.0469 | 31856.06641 | 47073.82813 | 36846.58984 | 21750.47461 | 31678.08594 | 25670.69922 | 49181.40625 | 23225.46875 |
| O75882 | 44320.19922 | 56251.92969 | 40746.58594 | 44904.13281 | 72543.44531 | 57091.95313 | 50823.20703 | 59677.48438 | 47646.62109 | 58128.02344 | 60216.82031 | 47320.80859 | 28196.08008 | 32023.14063 | 53700.96875 | 42145.39063 | 44580.14453 | 38325.85938 | 68584.46875 | 51859.875 | 39101.625 | 45588.55469 | 37659.25 | 42436.875 | 43004.64063 | 50821.14844 | 50474.64844 | 62551.57031 | 38619.66016 | 46330.38281 | 28653.68359 | 40053.87109 | 29691.50586 | 43026.00391 | 43055.72656 | 27108.69141 | 44683.44531 | 43151.54688 | 52602.48438 | 42965.39844 | 38861.8125 | 37357.96484 | 49779.86328 | 40394.26953 | 47009.36719 | 37582.36328 | 31931.06641 | 33485.10156 | 68729.42969 | 45955.82031 | 45844.24219 | 26959.11719 | 36485.39063 | 47762.07031 | 25442.82617 | 49603.66406 | 53124.4375 | 37050.08594 | 50544.09375 | 53128.19141 | 40526.85938 | 32318.28516 |
| O95445 | 60273.89063 | 51643.01563 | 54189.67578 | 57737.24609 | 71624.04688 | 44778.61719 | 46539.17578 | 60592.85938 | 54276.26172 | 65371.66016 | 36667.87109 | 46252.80859 | 64644.61719 | 39547.63281 | 69194.10156 | 40434.48828 | 50941.53125 | 52072.91016 | 47390.24219 | 42780.1875 | 70410.67969 | 64040.33203 | 43329.10938 | 61819.84375 | 57566.80469 | 42688.73828 | 62051.00781 | 60755.83203 | 42855.23828 | 48824.86328 | 75977.89844 | 68780.26563 | 55869.40234 | 49779.10938 | 63212.89844 | 56100.6875 | 75998.27344 | 59557.375 | 63244.28516 | 71481.99219 | 60068.78125 | 47802.87109 | 70099.11719 | 49094.02344 | 49317.25781 | 46070.44922 | 36659.19922 | 48717.36328 | 71746.875 | 59596.73047 | 83483.28125 | 46096.35938 | 94112.28906 | 73090.77344 | 47429.41016 | 43130.10156 | 65821.23438 | 68688.125 | 77630.79688 | 67804.20313 | 53982.94531 | 79087.8125 |
| O95497 | 0 | 3802.591797 | 0 | 0 | 0 | 0 | 0 | 0 | 0 | 0 | 0 | 0 | 0 | 0 | 0 | 0 | 0 | 0 | 0 | 5981.969238 | 0 | 0 | 0 | 0 | 0 | 4654.585938 | 0 | 0 | 18319.04688 | 0 | 0 | 0 | 20257.41406 | 0 | 0 | 0 | 0 | 0 | 27523.39648 | 0 | 0 | 0 | 0 | 0 | 20276.22266 | 0 | 0 | 0 | 0 | 4591.338379 | 0 | 0 | 5272.637695 | 0 | 9420.253906 | 0 | 17965.89063 | 4943.825195 | 0 | 0 | 0 | 4728.068848 |
| P00450 | 459039.875 | 788446.5 | 829904.875 | 732534.9375 | 467503.875 | 632222.25 | 762172.8125 | 413484.5 | 571450.4375 | 654672.5 | 552854.5 | 830644.5625 | 748389.5 | 642515.125 | 549523.625 | 698466.0625 | 647636.3125 | 638324.75 | 646882.3125 | 681970 | 571477.625 | 635386.125 | 451502.9688 | 842315.25 | 485180.375 | 402192.6563 | 863830.125 | 771778 | 599008.625 | 545286.875 | 506155.0625 | 798342.9375 | 670647.125 | 478214.4375 | 838443 | 727553.8125 | 723373.875 | 528154.5 | 588505.6875 | 772842.375 | 637398.125 | 497411.4063 | 512754.3125 | 626544.75 | 609075 | 677930.625 | 579928.25 | 698637.25 | 685891.375 | 595365 | 665446.125 | 561420.375 | 581218.0625 | 567519.25 | 647190.5 | 495106 | 642561.125 | 854736.8125 | 991223 | 554982.3125 | 547715.125 | 1192987.5 |
| P00488 | 15080.20117 | 16787.57227 | 18908.78516 | 15585.97852 | 19909.40234 | 22727.29883 | 35414.23047 | 43183.52344 | 34978.62109 | 15293.4668 | 26537.85352 | 17209.24023 | 15484.45313 | 48392.5 | 13984.98438 | 46068.52734 | 14886.0166 | 5782.42334 | 28762.4707 | 23319.52539 | 18610.60547 | 19289.94336 | 9595.950195 | 12670.76074 | 51097.85547 | 15827.28613 | 19836.0625 | 30237.32422 | 18384.41992 | 19040.88867 | 42834.84766 | 39325.84766 | 19406.62305 | 16415.03906 | 46134.33984 | 10577.91113 | 6316.541992 | 11757.55762 | 33020.66797 | 19790.18555 | 31389 | 38048.5 | 25392.23047 | 21008.25391 | 8180.101563 | 26057.71484 | 20129.23828 | 27284.6543 | 12588.02051 | 19664.2207 | 18300.92578 | 17964.83203 | 13027.33594 | 13061.14063 | 20423.07813 | 20843.35742 | 22735.88086 | 32229.92969 | 33979.44922 | 46809.56641 | 48829.00781 | 21412.0625 |
| P00734 | 492754.9688 | 544728.875 | 454509.8125 | 496809.5 | 526186 | 623843.3125 | 584373.5 | 573533.0625 | 510209.75 | 599280.5 | 583238.875 | 571045.75 | 518942.7813 | 546515.6875 | 662545.4375 | 596159.5625 | 640360.5 | 725119.875 | 690668 | 584104.125 | 549677.75 | 490288.75 | 520325.875 | 597269.875 | 524724.75 | 572086 | 502168.5 | 578082.875 | 515891.5938 | 467564.625 | 592955.8125 | 613030.3125 | 596510.625 | 602211.9375 | 611102.6875 | 617747 | 639546.375 | 556813 | 467506.5938 | 694454.8125 | 451710.875 | 529029.125 | 529326.5 | 442894.6563 | 474525.3438 | 614168.4375 | 498382.7813 | 626152.3125 | 535045.875 | 448507.9063 | 497351.25 | 529508.375 | 731306.25 | 568838.9375 | 511020.5938 | 587061.625 | 508554.5938 | 627004.8125 | 639189.75 | 566157.875 | 526050.375 | 540927.8125 |
| P00738 | 3108001.75 | 9520076 | 15996346 | 2422395.5 | 9762618 | 21652468 | 13368466 | 9266251 | 13937104 | 14335218 | 22730154 | 13232330 | 8730554 | 18661620 | 13242736 | 15223662 | 8805300 | 10180312 | 6598796 | 1137378.375 | 11556354 | 7539347 | 10025636 | 14167481 | 3781725 | 17342936 | 18532806 | 11816020 | 15314978 | 819747 | 9622394 | 11439367 | 5940745 | 7621895.5 | 19366172 | 8316310 | 17179284 | 11583134 | 12891328 | 12973096 | 10826398 | 3346038.25 | 11093366 | 8903640 | 11260212 | 6924079 | 4601682 | 18032640 | 13803058 | 5760740.5 | 10438343 | 8576322 | 5365376 | 16990676 | 5854085 | 7392516 | 9165496 | 7685471 | 8898719 | 9962943 | 571907.75 | 6771246 |
| P00739 | 55068.00781 | 23010.04297 | 27266.75781 | 27798.9375 | 110748.7656 | 26922.80078 | 31281.6875 | 57712.20313 | 23114.05078 | 22819.38281 | 35849.89453 | 95154.75 | 39966.49219 | 24494.37109 | 21312.10938 | 43622.39063 | 92344.29688 | 36563.84766 | 17581.81641 | 27315.92773 | 31029.41406 | 39605.26563 | 27747.26953 | 26837.10547 | 78336.91406 | 29080.66797 | 21088.64258 | 35364.45313 | 20084.27148 | 41009.39844 | 46613.6875 | 30109.98438 | 37620.06641 | 28302.38672 | 23853.47656 | 31876.36719 | 63394.39453 | 55377.87891 | 62294.91406 | 25574.25 | 100486.9531 | 82091.22656 | 101789.25 | 23550.21875 | 18612.64844 | 21238.85156 | 30165.24609 | 22322.41016 | 37335.5625 | 99436.61719 | 59887.79688 | 28109.22266 | 24652.66016 | 23927.05078 | 62374.65625 | 60152.06641 | 62952.15625 | 46052.875 | 44707.95313 | 57023.0625 | 21201.28516 | 46921.85938 |
| P00740 | 31000.35547 | 30670.74414 | 23560.06445 | 30493.71094 | 29305.10156 | 34525.51563 | 42078.50391 | 35291.40625 | 31535.44531 | 34201.23047 | 43892.13281 | 34302.8125 | 29170.62305 | 27688.0293 | 32012.48828 | 31257.24609 | 32635.30859 | 37756.21484 | 41688.93359 | 36071.375 | 28095.63086 | 32324.46484 | 35701.21875 | 32280.25586 | 38713.71875 | 30736.58203 | 31998.62305 | 39448.88281 | 28925.5332 | 29937.71289 | 30070.52734 | 31722.35156 | 23735.66797 | 39287.33594 | 38023.53516 | 24784.13086 | 38272.38672 | 27700.79688 | 32477.75195 | 28209.87109 | 35166.92578 | 29345.38477 | 36112.80469 | 31133.83594 | 27988.48828 | 36861.30469 | 24980.43555 | 40215.71875 | 32516.62695 | 33114.35938 | 35373.08594 | 39577.16016 | 39913 | 33382.76563 | 33359.62109 | 29567.70703 | 33769.34375 | 32334.9043 | 43617.83594 | 32998.78516 | 29864.57227 | 35542.05469 |
| P00742 | 66485.11719 | 97795.77344 | 63191.375 | 51459.03906 | 107503.5156 | 93551.90625 | 96869.73438 | 78112.02344 | 75755.70313 | 75056.32031 | 76449.08594 | 85826.46875 | 60049.08203 | 77634.94531 | 91136.17188 | 101175.6406 | 79217.88281 | 103569.0234 | 109843.7266 | 68100.25 | 78993.14063 | 74630.8125 | 66049.13281 | 74314.15625 | 69922.53125 | 114711.9844 | 137314.1719 | 89474.9375 | 104882.1094 | 58326.55469 | 52215.22266 | 71867.125 | 75830.85938 | 83907.74219 | 88063.25 | 76260.48438 | 99661.10938 | 54391.09375 | 66889.28125 | 85837.76563 | 70836.24219 | 48590.39844 | 77573.83594 | 59590.71875 | 74208.77344 | 84758.375 | 64712.73438 | 87310.625 | 77407.24219 | 62571.21094 | 77432.49219 | 81588.98438 | 122590.1641 | 85922.23438 | 68433.57031 | 63313.76563 | 80825.42188 | 59823.55469 | 118907.5859 | 87359.0625 | 74005.86719 | 71223.39063 |
| P00747 | 1131433.75 | 1291011 | 1066643 | 1277132.125 | 1255403.5 | 1233463.75 | 1313993.5 | 977066.875 | 989671.875 | 1094734.25 | 1018277.75 | 1088224 | 1250305.5 | 1163016.5 | 1456413.125 | 1272257.25 | 1354565.25 | 1365570 | 1148746.625 | 1158317.5 | 1257829 | 1153541.75 | 1005478.75 | 728573 | 1226951.75 | 1179066 | 897974.75 | 1490179.375 | 995738.4375 | 1091321.125 | 889259.125 | 1165717.5 | 1147621.625 | 1196909.25 | 1124162.25 | 1095307.5 | 1334773 | 1302525.375 | 1055220.5 | 1213966.125 | 922501.25 | 792681.6875 | 1181638 | 1137152.875 | 1010629.625 | 1139060.5 | 983734.5 | 1142459.5 | 1071791.125 | 963928.875 | 961499.1875 | 1070000.375 | 1275993 | 1191068.375 | 942115.125 | 967794 | 1028918.375 | 1251601.5 | 1315901 | 1429938.75 | 1222408.875 | 1307851.75 |
| P00748 | 46777.66797 | 29882.91602 | 18022.8125 | 46862.8125 | 34219.11719 | 52286.0625 | 69409.53125 | 35499.34375 | 56207.22656 | 39205.73047 | 77460.1875 | 36096.48828 | 31679.74219 | 24878.29688 | 51426.66797 | 39688.05078 | 85378.04688 | 44094.67188 | 64406.24219 | 53639.85156 | 56138.73438 | 29163.08594 | 76890.46094 | 89324.75 | 43032.29297 | 82637.0625 | 45690.5 | 102816.0781 | 51008.3125 | 35823.61719 | 30875.98633 | 30816.42188 | 25124.26563 | 36817.56641 | 34021.61719 | 61330.27344 | 31675.02148 | 37910.47656 | 34135.60938 | 83868.48438 | 28879.58203 | 27734.22461 | 29453.83789 | 64185.3125 | 22683.13086 | 89190.96875 | 104346.3438 | 38344.28125 | 30037.90234 | 75946.33594 | 31934.64453 | 45766.56641 | 60563.94531 | 43003.125 | 46654.0625 | 44485.99219 | 90781.49219 | 133971.875 | 101178.5547 | 71414.45313 | 44245.99609 | 85195.64063 |
| P00915 | 8193.47168 | 30109.25 | 862.8479614 | 5858.149414 | 12320.16895 | 9253.296875 | 6964.486816 | 15480.77832 | 9680.183594 | 9390.56543 | 12483.71387 | 10253.89648 | 11812.66797 | 4421.275879 | 8279.018555 | 8134.407715 | 12636.82813 | 11022.36133 | 11892.11426 | 16212.00879 | 0 | 12108.18359 | 13836.11914 | 10563.50977 | 10627.99902 | 30540.8457 | 11153.5918 | 33222.51563 | 15005.74219 | 32438.32227 | 12074.10938 | 12363.3916 | 12943.47168 | 13250.00879 | 10772.99805 | 11772.66016 | 38299.11719 | 17182.49609 | 22934.38281 | 28419.50195 | 17983.91211 | 20803.67188 | 25242.4707 | 12577.69141 | 16864.98438 | 16096.80078 | 15392.42969 | 9007.172852 | 17776.23242 | 15296.21582 | 29348.95117 | 35063.30078 | 20620.81836 | 14885.1582 | 25489.0918 | 28227.99414 | 17165.29883 | 16322.28125 | 19767.13672 | 16430.23047 | 12185.25586 | 8547.182617 |
| P00918 | 0 | 13195.70703 | 0 | 4759.319824 | 4389.876465 | 3548.473633 | 5780.60498 | 0 | 4524.930664 | 0 | 0 | 4527.316895 | 0 | 0 | 6695.36084 | 3329.29834 | 5359.262695 | 0 | 4768.366699 | 5423.66748 | 0 | 7455.950195 | 6508.917969 | 0 | 0 | 7157.665039 | 7568.1875 | 5463.834961 | 8207.390625 | 17963.83594 | 1941.315308 | 6194.334473 | 5772.641113 | 6193.742676 | 1486.333252 | 4984.171875 | 11315.28125 | 7341.789551 | 10877.50684 | 0 | 7601.856445 | 10547.60156 | 8450.669922 | 8438.542969 | 0 | 10674.99414 | 0 | 13167.88379 | 5689.108887 | 6192.216797 | 0 | 17284.48828 | 16117.68555 | 6358.68457 | 16497.71289 | 8850.580078 | 7104.298828 | 9703.972656 | 5038.438965 | 5791.065918 | 0 | 9059.384766 |
| P01008 | 335641.375 | 467040.5 | 342433.4688 | 424668 | 342978.2188 | 327630.375 | 459866 | 423026.625 | 364826.5 | 364048.6875 | 438951.5 | 467398.8125 | 455815.25 | 418125.3125 | 520366 | 438018.625 | 576867.8125 | 532801.25 | 438451.625 | 569937.5 | 447549.4375 | 545305.75 | 437327.4688 | 422146 | 457277.9688 | 426365.1875 | 362529.625 | 564786.75 | 373537.2813 | 502185.8438 | 471752.875 | 424073.8438 | 245733.2656 | 421314.8125 | 384898.9375 | 320759.5625 | 590457.0625 | 414646.9688 | 315839.0625 | 706211.25 | 399174.9375 | 376591.5625 | 492685 | 436227.0938 | 397982.9063 | 438876.4688 | 584298 | 473384.125 | 406698.5 | 423215 | 588762.125 | 365314.0313 | 658240.625 | 417169.75 | 697706.875 | 471089.0625 | 418100.0938 | 670819.75 | 512471.25 | 528840.75 | 563186.375 | 519522.6563 |
| P01009 | 2981674.75 | 5548744.5 | 6438091 | 4995352 | 3179944 | 3413826.5 | 5361034 | 4497732 | 3966429.75 | 4051669.75 | 5157629 | 4923461 | 5488889 | 5058871 | 5127683 | 6370327 | 5425463.5 | 4785303 | 4944263 | 4166547.5 | 5531361.5 | 5585352.5 | 3949844.5 | 3689078.25 | 4906991 | 3214017.5 | 4415979.5 | 5797832 | 3833354 | 6063121 | 4214475 | 4027572.75 | 4320730 | 5186082 | 5515292 | 3410657.25 | 5579523 | 4156719 | 3684126.5 | 7443742.5 | 3769679 | 3917812.5 | 6363203.5 | 6439221.5 | 3927512.5 | 5530828 | 5548068 | 4719475 | 3902795.25 | 4727089 | 6390271.5 | 6314657 | 5354765 | 4140279 | 6229899 | 4562424.5 | 5292353 | 6059137.5 | 5914987 | 4991424.5 | 6164037.5 | 7051577.5 |
| P01011 | 590087.75 | 798548.25 | 887528.125 | 827776.5 | 558940.625 | 656681.0625 | 980631.1875 | 588998.5 | 662250.0625 | 680535.0625 | 625264.0625 | 848274.375 | 765833.9375 | 718222.0625 | 908108.625 | 913148.125 | 721613.5625 | 884670.75 | 761660.5 | 836744.5 | 848714.625 | 646290.125 | 619173 | 940747.875 | 370550.9688 | 586457.875 | 753653.625 | 789833.6875 | 617766.375 | 814419.1875 | 891220.625 | 882166 | 653413.125 | 721127.875 | 901161 | 713419.4375 | 741398.375 | 708447.1875 | 628642.1875 | 996462.375 | 714937.625 | 585401.25 | 798351 | 708982.6875 | 749871.875 | 859560.5625 | 721509.8125 | 900730.125 | 592143.5 | 558375.3125 | 673028 | 594096.25 | 638953.6875 | 883048.6875 | 723721.875 | 600972.4375 | 755264.5 | 884819.8125 | 940149.25 | 844741.8125 | 885254 | 713101.875 |
| P01019 | 115992.0313 | 178189.125 | 174491.25 | 187881.125 | 183129.5156 | 172374.8906 | 267247.0625 | 165740.7188 | 150193.3594 | 149200.8594 | 154292.4063 | 159544.75 | 210979.5313 | 171980.0156 | 255180.2656 | 115327.3438 | 153781.125 | 179550.3125 | 131890.8438 | 148130.4063 | 125485.6719 | 120601.3594 | 157680.6406 | 187680.25 | 169191.9688 | 203113.9688 | 175221.0469 | 192210.2813 | 146989.7188 | 101205.1719 | 187408.9688 | 211157.4063 | 186071.8438 | 237411.1875 | 162397.0781 | 233332.4375 | 213832.5 | 146846.8906 | 201964.5625 | 210482.9063 | 192093.2188 | 121663.3281 | 190297.9375 | 137718.5938 | 154427.1875 | 240426.8438 | 186420.8438 | 183457.7969 | 203936.375 | 161417.6875 | 153923.1094 | 153268.5938 | 231672.9375 | 166546.3125 | 155355.7813 | 202840.5625 | 218259.0625 | 190162 | 178250.2656 | 178328.9219 | 163301.2188 | 248846.0938 |
| P01023 | 8505148 | 7728336 | 14804771 | 12343053 | 8072818 | 5135668 | 3735374.75 | 7286159.5 | 5198919 | 9907680 | 6627780.5 | 5137403 | 11626154 | 9198285 | 11326044 | 8250653 | 7745938 | 4334964.5 | 6428147 | 5683185 | 13063201 | 12003060 | 4247165.5 | 9907038 | 7355148.5 | 3010949.25 | 7052500 | 5115922.5 | 10684776 | 9533663 | 5291680 | 5735201 | 8310246 | 4226994 | 7494599.5 | 6923789.5 | 5209919 | 3566448.5 | 12625590 | 9908644 | 11431187 | 7767598 | 12875306 | 10546080 | 14140154 | 10549597 | 6253160.5 | 11650632 | 13361473 | 10059882 | 10098318 | 7246780.5 | 5492277 | 6292378 | 5474268 | 4315404.5 | 4817433.5 | 11752464 | 7498207.5 | 10035898 | 8737023 | 7378655 |
| P01024 | 6282339.5 | 7054614 | 7872515 | 7182333 | 9610424 | 8886647 | 10257912 | 7969768 | 8481364 | 8959970 | 9508890 | 8217346 | 7625064 | 5837430 | 8593591 | 8494768 | 8386871 | 7074389 | 7631596 | 7289354 | 7482987 | 6836506 | 7455410 | 7411657 | 7834488.5 | 8446784 | 10668786 | 11089990 | 8766210 | 5451389 | 4673080 | 7214443 | 6751778.5 | 7877265.5 | 9565886 | 7983902.5 | 8270572 | 6996668 | 7707426 | 8619796 | 7251410 | 5436488.5 | 6868261 | 7392748 | 7247511 | 8080612 | 5774380.5 | 8000711 | 7937615 | 5093048 | 5976474 | 6262169 | 8804731 | 9899565 | 7790901 | 5748469 | 7204927 | 7687278.5 | 10030316 | 8650239 | 9840284 | 8886774 |
| P01031 | 170401.0156 | 236817.3438 | 252482.2188 | 199728.4375 | 184121.0625 | 230137.2813 | 270776.125 | 225086.7813 | 216386.5625 | 230249.375 | 230868.8906 | 260513.5469 | 249537.125 | 195346.75 | 298653.625 | 257782.8594 | 195665.0156 | 232219.25 | 231138.0469 | 154841.5156 | 223545.125 | 159065.3125 | 197344.3438 | 239294.9531 | 193861.4375 | 226641.8125 | 266581.875 | 286463.5 | 208436.6094 | 203484.0781 | 145876.4375 | 176303.3281 | 198913.5313 | 238357.0938 | 249606.4063 | 197667.25 | 251795.8125 | 206095.2813 | 253254.625 | 290623.375 | 169457.625 | 184383.4063 | 210421.9063 | 181639.25 | 187213.75 | 266955.125 | 221749.0313 | 280367.5 | 254699.3125 | 134183.2813 | 183236.0938 | 181973.4688 | 213068.7656 | 173343.5625 | 193994.5313 | 197073.25 | 176077.7188 | 270957.3438 | 304658.5313 | 195874.0625 | 215805.8594 | 229283.875 |
| P01034 | 0 | 10200.59766 | 9221.003906 | 12697.18848 | 8646.924805 | 10698.25 | 15791.85742 | 11581.47852 | 9782.382813 | 16288.03027 | 10780.5166 | 10251.64648 | 13040.32422 | 13345.73828 | 8296.414063 | 9381.177734 | 8911.458008 | 17520.05664 | 6625.353027 | 13299.18457 | 10342.65723 | 17404.43164 | 10152.42871 | 11584.65234 | 13285.12695 | 7335.414063 | 6094.859375 | 15638.16602 | 6015.895508 | 12520.52148 | 13152.6543 | 14641.80469 | 9831.642578 | 12200.50977 | 7741.803223 | 0 | 12004.24512 | 17940.0918 | 8693.813477 | 10747.69727 | 0 | 18607.67578 | 0 | 8004.70166 | 0 | 18518.89258 | 14047.77637 | 7511.882813 | 11049.8916 | 11711.90527 | 13495.25879 | 17275.95898 | 12654.92188 | 14435.64258 | 14613.52246 | 15035.88281 | 13912.37695 | 11013.46777 | 11833.04688 | 10811.06152 | 9145.016602 | 9672.118164 |
| P01042 | 965982.3125 | 1314353.75 | 1112545.75 | 942998.75 | 1303695.25 | 1212449.5 | 1086827.75 | 1223616.375 | 1042934.438 | 1244174.5 | 1128441.75 | 1125535 | 1468659.5 | 1094465.5 | 1422353.625 | 1185808.75 | 1303924.375 | 1213036.125 | 1502532 | 1148678.375 | 1158526 | 959586.5625 | 1139306.25 | 791982.625 | 1193707.625 | 1073242 | 1266881.5 | 1242159.125 | 1116112.375 | 1165805.25 | 832701.75 | 1169591.375 | 1170518.75 | 1405995.625 | 1198376.125 | 1044890.563 | 1401541 | 1197950 | 1237090.625 | 1469713.625 | 1025427.25 | 867103.875 | 1074287.5 | 1071879.125 | 987533 | 1328553.125 | 1021609.75 | 1278640.75 | 1254901.625 | 1061830.5 | 996056.125 | 1509115.5 | 1682823.5 | 1206706.5 | 1433157.5 | 1213882.75 | 1129702.25 | 1513151.75 | 1804135.875 | 1243032.5 | 1157880.875 | 1085803.75 |
| P01344 | 33283.89844 | 33238.82422 | 35941.97266 | 35467.75 | 47231.84375 | 42447.47266 | 64849.18359 | 55559.49609 | 34660.44141 | 44909.89063 | 60870.3125 | 43505.80469 | 43947.8125 | 43906.66797 | 41955.65625 | 43121.33984 | 38377.12891 | 42332.03125 | 52706.22266 | 35451.86328 | 47332.19922 | 39598.94141 | 51100.11719 | 46847.78125 | 19311.78711 | 41091.57031 | 42234.89453 | 66664.54688 | 46013.05469 | 38649.10938 | 43505.31641 | 40723.57813 | 40398.69922 | 46175.04688 | 30784.9707 | 44380.73438 | 38931.64063 | 59125.66406 | 38867.88672 | 59004.24219 | 32254.95898 | 35790.43359 | 58070.08203 | 45554.00391 | 30419.00391 | 34945.9375 | 50038.25391 | 54352.11328 | 45886.53906 | 28056.31641 | 43730.34375 | 67835.09375 | 34527.67578 | 42034.60156 | 37018.45703 | 46666.69531 | 41608.65625 | 54931.5 | 67573.09375 | 46890.23828 | 51526.58203 | 42105.58594 |
| P01597 | 45381.35938 | 38819.52344 | 58035.72266 | 35024.13281 | 44133.29688 | 41630.17969 | 43534.07422 | 51825.95313 | 39995.60938 | 41280.53125 | 27367.68359 | 44523.71484 | 31566.82813 | 29674.41406 | 29132.08984 | 24510.58203 | 41785.02344 | 54996.36719 | 31914.57031 | 38132.10156 | 54355.57813 | 50217.49219 | 27727.46484 | 49457.01172 | 51175.16406 | 24884.94727 | 52462.00781 | 39953.55078 | 39339.45313 | 54149.54688 | 52740.46094 | 40645.82813 | 36843.38672 | 41453.71094 | 54078.79688 | 47277.44531 | 33683.32031 | 47376.14844 | 32995.28906 | 38091.34766 | 29699.20898 | 61046.28125 | 40247.21484 | 62474.07813 | 56626.13281 | 64635.60156 | 44506.91797 | 36090.32813 | 19670.28516 | 40300.57031 | 37920.94531 | 58358.56641 | 28009.91406 | 29596.31641 | 39263.87891 | 47470.78906 | 42620.54688 | 48370.51953 | 56073.78906 | 19070.49609 | 57329.05469 | 49763.18359 |
| P01599 | 141309.125 | 98219.67188 | 136140.8125 | 99864.64063 | 140272.7969 | 59200.60156 | 51522.85156 | 138203 | 88223.63281 | 92349.94531 | 134659.125 | 73737.90625 | 168295.375 | 187523.75 | 101164.9531 | 131106.6719 | 172256.6094 | 183811.9375 | 38610.83984 | 114753.4766 | 244148.5156 | 66069.64844 | 98461.67188 | 103796.1875 | 231563.3438 | 11332.67773 | 214287.0313 | 136010.5 | 22055.05664 | 222613.7031 | 162810.0938 | 136488.2188 | 91675.84375 | 110967.6953 | 135421.4531 | 53795.28125 | 179846.4063 | 85702.32031 | 157480.2813 | 127985.8281 | 172636.5781 | 100365.8281 | 332175.3125 | 268231.5 | 142783.6875 | 217322.25 | 130819.3906 | 87664.72656 | 92337.21875 | 118110.25 | 113513.3906 | 238857.7188 | 116485.6719 | 80423.47656 | 121161.2813 | 75238.03125 | 180951 | 139975.9219 | 155792.8125 | 178676.7813 | 224744.4531 | 280540.5 |
| P01601 | 86056.71875 | 48515.37109 | 69846.76563 | 37231.27734 | 122418.3125 | 41940.24219 | 57675.83984 | 71100.30469 | 105637.1328 | 65367.70313 | 71798.50781 | 74396.16406 | 62383.02344 | 73407.72656 | 42154.17969 | 76530.24219 | 91599.25781 | 122093.3438 | 43578.9375 | 106107.7656 | 130429.3672 | 97910.54688 | 77147.73438 | 105336.2344 | 112755.0469 | 31150.20508 | 146507 | 74891.63281 | 108123.6563 | 135716.3594 | 70727.60938 | 72663.90625 | 23364.94141 | 67336.21094 | 71811.875 | 45538.6875 | 83393.94531 | 102464.5547 | 51785.37891 | 126914.9609 | 95448.53906 | 70134.61719 | 154373.5313 | 96321.54688 | 79401.92969 | 98106.07813 | 105490.5156 | 137754.75 | 79446.77344 | 68349.65625 | 123423.0625 | 119619.9844 | 116988.9063 | 58533.26172 | 118946.3984 | 99851.1875 | 127097.5625 | 103723.6484 | 114674.0859 | 121282.2109 | 126866.9844 | 134837.8906 |
| P01602 | 70619.84375 | 55122.50781 | 117629.3828 | 258530.2813 | 78096.125 | 60196.82031 | 22516.05078 | 180432.9063 | 584492 | 110931.8906 | 753187.875 | 73734.19531 | 69558.60156 | 898935.3125 | 527738 | 122535.5703 | 138220.6719 | 68377.32813 | 566489.5 | 79305.61719 | 110058.5625 | 115876.9766 | 94325.21875 | 65250.57422 | 122363.8359 | 60923.87109 | 225209.5781 | 35242.01953 | 733139.1875 | 160621.7344 | 83141.46875 | 831572.5625 | 60434.69922 | 81070.92969 | 836829.8125 | 114571.375 | 800258.4375 | 676278.8125 | 873684.5625 | 672903.6875 | 45642.25391 | 103905.4375 | 113780.1641 | 203407.4063 | 920598.5 | 177000.4844 | 101625.375 | 85716.21094 | 303121.625 | 74290.21094 | 27299.06836 | 92471.17188 | 78978.39063 | 100277.2031 | 470990.125 | 72049.63281 | 119469.9219 | 78945.38281 | 87411.95313 | 686022 | 139406.9219 | 107181.875 |
| P01619 | 4107963.25 | 3050603.5 | 4012127.25 | 2079345.625 | 1753660.875 | 3239403 | 2983858.25 | 3048408.25 | 3293326 | 2848474.75 | 2573228 | 2578013.75 | 2150452 | 2657490 | 2136499.5 | 2899532.25 | 3664026 | 4493483.5 | 2300592.75 | 2482086.75 | 5453413.5 | 3130382.5 | 3432784.25 | 3810582.25 | 4696791.5 | 2347007.75 | 3768867.25 | 3813809 | 3363872.5 | 4306572.5 | 4129921.25 | 3099374.25 | 2165084.5 | 2664154.5 | 3163996.25 | 3133408 | 3548206.25 | 2288862.5 | 3224124.5 | 3254766.25 | 3025117.25 | 3159349.25 | 4248913.5 | 5034946 | 3108702.25 | 4210561.5 | 2423877.5 | 3144056.75 | 2849265.75 | 3070296.25 | 3774934.5 | 14387661 | 5238425.5 | 2451568.25 | 1709420.625 | 2665915 | 4109743.75 | 4001349.75 | 3837172.75 | 3481939.5 | 3579506.5 | 4874594 |
| P01699 | 162079.5938 | 105841.2813 | 130869.3438 | 131977.3125 | 179897.9375 | 86384.67188 | 149173.0625 | 139506.9219 | 195374.625 | 149448.4531 | 125674.0156 | 84530.47656 | 101109.2031 | 92770.75781 | 146065.75 | 243825.5 | 123257.8672 | 145182.5781 | 102697.4063 | 152163.375 | 215004.2188 | 126512.5547 | 108356.2813 | 182330.4063 | 160097.9531 | 102676.5469 | 132098.0313 | 108240.4375 | 135203.1875 | 156424.0781 | 172730.2188 | 113027.0078 | 129106.3906 | 36002.92188 | 186733.8438 | 166466.25 | 176600.4375 | 86183.98438 | 77869.82813 | 199296.5625 | 150129.9531 | 231667.8281 | 175855.8438 | 217381.7656 | 129098.5938 | 96139.17188 | 147562.0156 | 71219.67969 | 142299.5156 | 181038.1094 | 130535.8906 | 259985.4688 | 213185.6406 | 143804.4531 | 197598.2188 | 204419.9531 | 168568.3281 | 187329.4688 | 143958.7813 | 98016.53906 | 172769.4063 | 222511.5313 |
| P01700 | 2024778 | 1073713.5 | 1551570 | 1141160 | 1677133.375 | 1307136.25 | 837637.25 | 1998648.25 | 1315829.875 | 687839.25 | 856628.9375 | 873028.625 | 656782.375 | 362517.625 | 1278104.125 | 1752201.375 | 2027499.375 | 1519398.875 | 1557054.25 | 2423130 | 1684134.75 | 845627.875 | 1453145.5 | 1652107 | 1102688.25 | 1179452.375 | 1554920.375 | 1101638.125 | 1955475.5 | 2769641.25 | 1808188.375 | 619708.625 | 633657.8125 | 1321585.75 | 924860.375 | 790545.625 | 932348.3125 | 1095576.375 | 891847.125 | 1741526.625 | 1018819.375 | 1647284.25 | 1710570.25 | 1845820.625 | 1489235.75 | 1352108.25 | 1114874.875 | 771152.9375 | 1180069.875 | 2466422.5 | 1193795.375 | 1677344.375 | 1066129.625 | 1347837.125 | 1043747.688 | 991352.5 | 668801.1875 | 2275052.5 | 632741.125 | 1207224.125 | 1101510.125 | 1621221.375 |
| P01701 | 541203.9375 | 521383.5625 | 551546.3125 | 321334.875 | 606608.6875 | 384826 | 309257.6563 | 601307.5 | 738766.3125 | 405483.3125 | 386649.0313 | 277093.1875 | 828605.125 | 316908.2813 | 611160.4375 | 492719.0313 | 203847.9531 | 193716.7656 | 293885.0625 | 439122.125 | 720459.25 | 643945.875 | 406304.5 | 597479.875 | 730790.6875 | 299329.6563 | 50267.66406 | 353445.5 | 778861.1875 | 380667.625 | 241345.9531 | 459020.2813 | 466811.8438 | 422441.0938 | 473463.375 | 317759.5 | 652031 | 309299.2188 | 496989.3125 | 238289.8281 | 439199.0625 | 443440.4063 | 750802.3125 | 635953.625 | 689501.375 | 707698.5625 | 445446.9375 | 340945.8438 | 231142.1094 | 643573.5625 | 552817.25 | 828039.75 | 505021.0625 | 351173.3438 | 413577.9688 | 310115.2188 | 510334.0313 | 619074.9375 | 545588.375 | 435464.25 | 528561.3125 | 1182273.625 |
| P01703 | 139610.9375 | 106092.4219 | 223951.2344 | 96134.3125 | 127406.7344 | 94371.17969 | 129601.8359 | 118887.4922 | 155885.9375 | 185529.1406 | 201330.7344 | 85606.33594 | 52890.125 | 68085.91406 | 93581.70313 | 106672.9609 | 58031.08984 | 127766.0703 | 96962.0625 | 65544.63281 | 170367.2813 | 137689.0313 | 127030.8281 | 118356.3516 | 192857.375 | 127264.375 | 0 | 218070.3438 | 141061.7813 | 152790.0156 | 81489.79688 | 55528.16406 | 95667.64844 | 62765.97656 | 122344.5313 | 152645.3594 | 189527.375 | 116165.8828 | 148252.125 | 99952.40625 | 134411.125 | 124379.2188 | 113178.8203 | 130352.0469 | 172151.3125 | 198031.4219 | 210429.6406 | 68352.97656 | 105089.9141 | 179623.3906 | 163240.3125 | 194462.5938 | 172170.6094 | 117192.9063 | 101093.3203 | 133374.625 | 222830.4219 | 167513.5313 | 175362.3594 | 119149.5 | 112722.7813 | 212321.6406 |
| P01704 | 21120.1543 | 26924.44727 | 37847.78125 | 36369.88672 | 0 | 24539.78516 | 12684.42578 | 86734.89063 | 34097.96875 | 17267.41992 | 32037.4375 | 20424.38477 | 35998.73828 | 25453.95898 | 39976.13281 | 33550.5 | 63954.78906 | 0 | 27317.92969 | 41639.55469 | 15920.08301 | 35566.25 | 29687.10352 | 12987.20117 | 39307.45313 | 14323.13965 | 32837.61719 | 27794.71094 | 78313.85938 | 56841.27344 | 41373.77734 | 35553.41406 | 39656.03125 | 8206.505859 | 42445.28906 | 0 | 39967.07031 | 44978.30859 | 31407.30664 | 22924.04688 | 15065.28418 | 34997.95313 | 64421.6875 | 28792.69141 | 40954.76563 | 31283.21875 | 62517.92969 | 38628.34766 | 26313.32422 | 17543.16211 | 0 | 18836.375 | 55511.33594 | 30853.9375 | 50207.58203 | 11291.43359 | 39112.80859 | 32035.06445 | 89278.17188 | 22507.86719 | 35281.67188 | 62009.01953 |
| P01705 | 113050.6016 | 186185.8125 | 0 | 93064.79688 | 86713.78125 | 112859.4609 | 0 | 267444.9063 | 253911.125 | 61805.24219 | 25982.90625 | 92689.34375 | 177186.5625 | 127056.4219 | 106734.3203 | 74122.71875 | 238752.8438 | 124873.9766 | 0 | 138337.7656 | 147985.7188 | 0 | 70814.77344 | 121068.1094 | 72970.53125 | 102924.2813 | 193214.7969 | 139871.7656 | 65727.50781 | 118175.2344 | 33073.09766 | 83007.96875 | 313204.5 | 95269.39063 | 104177.7734 | 246417.0469 | 90324.94531 | 0 | 67466.75781 | 74000.78906 | 146202.5938 | 55300.28125 | 141097.7969 | 102121.3359 | 22712.14063 | 236150.75 | 89954.1875 | 18918.92383 | 66662.20313 | 19925.91602 | 91687.83594 | 110661.1641 | 0 | 63899.38672 | 0 | 113891.9531 | 83500.00781 | 236826.1719 | 181400.3594 | 124270.5703 | 123726.2344 | 251037.0313 |
| P01706 | 88599.76563 | 55271.78125 | 248828.75 | 139421.4219 | 173082.6719 | 61885.01563 | 136097.3281 | 108808.3281 | 232024.0938 | 115111.4688 | 71643.54688 | 109017.3047 | 112833.9453 | 46424.875 | 64739.22656 | 75848.53906 | 139622.2813 | 137640.2344 | 59682.3125 | 137266.0156 | 274763.3125 | 149690.0625 | 52821.22266 | 175234.6406 | 68050.24219 | 43159.10938 | 271729.6875 | 42719.17188 | 75307.42188 | 45404.66406 | 70753.76563 | 51372.625 | 67500.96094 | 108774.5625 | 163860.0313 | 145541.9531 | 251021.5938 | 68186.40625 | 172992.8906 | 54584.15625 | 163488.0938 | 62145.02734 | 157553.4375 | 177546.7813 | 105307.8594 | 73648.39063 | 164877.125 | 44094.98438 | 56763.40625 | 101051.5703 | 195085.3594 | 384335.3438 | 64150.07031 | 53389.80469 | 44197.03516 | 84193.75 | 45610.72266 | 67197.28906 | 71647.60938 | 125258.9375 | 55296.12891 | 64380.26563 |
| P01709 | 320811.9063 | 71062.42969 | 302150.3438 | 80246.05469 | 128115.1875 | 130597.1563 | 77828.85156 | 156469.5469 | 105177.6328 | 221916.375 | 112718.3672 | 133548.3438 | 89420.85156 | 41776.45313 | 96835.57813 | 141901.9375 | 172905.5156 | 104402.6016 | 109589 | 169266.2344 | 248187.6406 | 167101 | 105591.5859 | 132608.0625 | 136915.3906 | 82527.17188 | 184958.3906 | 47958.23047 | 242131.5625 | 83422.46094 | 146064.3594 | 93700.08594 | 109950.0938 | 62258.56641 | 162608.9688 | 144462.9688 | 93196.03906 | 203155.5313 | 148427.9531 | 133697.1719 | 85654.35156 | 157758.625 | 223212.9219 | 209194.1875 | 182423.0781 | 133287.1563 | 230533.3281 | 90806.61719 | 159187.625 | 103540.9609 | 119881.0078 | 173483.0938 | 162476.2813 | 82158.78906 | 130782.6328 | 76866.11719 | 148172.9375 | 153817.75 | 149753.6563 | 134688.5 | 141127.5625 | 310343.6875 |
| P01715 | 17631.49023 | 12427.72852 | 6824.783203 | 14886.44434 | 9166.55957 | 6792.934082 | 16480.65625 | 13152.99121 | 12467.69922 | 20486.57031 | 21437.81055 | 5065.246582 | 0 | 7585.581055 | 8265.666016 | 11956.08887 | 2696.7771 | 0 | 13118.54004 | 7958.981445 | 13070.00586 | 19791.55469 | 4013.256836 | 0 | 0 | 3560.998535 | 12670.65918 | 14363.41113 | 0 | 9932.789063 | 16808.54492 | 9289.375977 | 15730.72656 | 4118.428223 | 3459.111572 | 20120.96289 | 0 | 5214.598145 | 7507.780273 | 0 | 9110.102539 | 18493.71289 | 0 | 44817.41016 | 12461.12793 | 16280.32227 | 0 | 0 | 5956.861328 | 17743.89648 | 0 | 51417.54297 | 15215.97754 | 4091.55957 | 16178.99316 | 0 | 0 | 0 | 7048.48291 | 0 | 11246.34375 | 7123.342773 |
| P01717 | 7596.211914 | 2123.101563 | 2673.374268 | 6402.170898 | 7821.055176 | 10895.36523 | 3667.392822 | 7778.629883 | 8142.539063 | 0 | 6085.852051 | 5973.211426 | 0 | 2497.657471 | 7626.656738 | 6011.074707 | 5755.550293 | 5207.005859 | 8865.604492 | 6332.658691 | 9426.614258 | 10492.85449 | 0 | 4275.143555 | 0 | 5065.768555 | 4497.263672 | 8313.447266 | 9473.402344 | 0 | 2297.07959 | 0 | 6782.556641 | 3357.395508 | 6289.844727 | 6815.920898 | 0 | 3090.189697 | 7778.54834 | 2851.60376 | 7522.536621 | 10580.74902 | 0 | 9363.71875 | 4840.898926 | 4654.869141 | 8419.355469 | 5111.501953 | 7138.212402 | 14868.07813 | 12995.39648 | 14361.60449 | 9649.21582 | 4704.574219 | 14621.45703 | 0 | 10557.12305 | 0 | 5586.456543 | 1847.678589 | 3050.240967 | 9218.800781 |
| P01718 | 10312.87012 | 0 | 88542.86719 | 6894.376953 | 6484.795898 | 6758.756348 | 4755.318359 | 20345.46094 | 34981.51953 | 25003.86719 | 24829.15625 | 0 | 0 | 6853.299316 | 0 | 21295.8125 | 0 | 16846.66016 | 4543.474121 | 0 | 23432.86523 | 0 | 12031.06934 | 0 | 0 | 0 | 42264.45703 | 13445.18066 | 21644.19336 | 6085.00293 | 0 | 0 | 6279.237305 | 0 | 24021.58789 | 37894.74219 | 7221.88623 | 1009.745117 | 28200.28711 | 36795.32031 | 17669.7793 | 0 | 19182.99219 | 13885.51074 | 13608.63672 | 24804.98633 | 30107.41016 | 25690.34766 | 19190.4375 | 14899.34375 | 26012.52344 | 13370.23438 | 11118.78223 | 0 | 16504.14063 | 37282.80859 | 0 | 63649.73047 | 24217.60352 | 8818.270508 | 37271.88281 | 32550.41797 |
| P01721 | 174353.6563 | 86145.63281 | 64176.88672 | 90811 | 86851.07813 | 76105.94531 | 61932.28906 | 128546.9375 | 94184.05469 | 75438.46875 | 63897.95703 | 54197.17578 | 123643.6797 | 33765.17578 | 84889.42969 | 63805.98438 | 63055.08594 | 117038.5156 | 77307.14063 | 164574.7656 | 98921.74219 | 145902.5938 | 96928.86719 | 148698.2031 | 157691.4063 | 57176.03906 | 71943.54688 | 74159.89844 | 115520.6172 | 75394.02344 | 85292.17188 | 120299.7031 | 90486.94531 | 116079.0938 | 64577.42969 | 75086.73438 | 138312.0313 | 92028.17188 | 68524.45313 | 52065.78516 | 100623.6484 | 111664.9297 | 114150.4375 | 155852.6094 | 97526.63281 | 153215.1563 | 141337.6719 | 42010.47656 | 98986.30469 | 126933.5625 | 101819.8047 | 185443.9688 | 123762.9609 | 122395.7188 | 72857.95313 | 127889.8125 | 153434.8281 | 90251.97656 | 108953.6016 | 55817.07031 | 202129.1719 | 139439.1094 |
| P01742 | 790551.5625 | 672845.375 | 9988223 | 458421.5 | 3589817.25 | 2873660.25 | 426234.1563 | 998954.6875 | 2080063.375 | 3266790 | 2455980 | 312557.3125 | 5543194 | 496532.0938 | 560699.875 | 730401.5 | 747787.25 | 2369099.5 | 2411910 | 62370.80078 | 886570.875 | 621343.8125 | 2097876 | 1788906.25 | 1274099 | 45974.37109 | 670964.625 | 675496.5 | 725406.5 | 1190644.75 | 753822.25 | 2389291 | 1444816.75 | 1024126.625 | 3354600.5 | 120695.0234 | 533923.3125 | 874563.625 | 715938.1875 | 478557.6875 | 782068.6875 | 756211.25 | 1117371.625 | 1202691.875 | 2060171.625 | 1778181.625 | 1072892.375 | 635694.1875 | 389842.3438 | 977197.875 | 669696.0625 | 1147400.375 | 1536816 | 608272.25 | 822907.0625 | 617417.4375 | 1270912.25 | 780982.375 | 7666925 | 551205.625 | 698124.5625 | 1115244.5 |
| P01743 | 14860.52051 | 15586.67383 | 31847.94141 | 16644.38281 | 6775.138672 | 20983.48828 | 20790.0918 | 12603.48047 | 11374.88965 | 17152.06445 | 15211.53223 | 9916.750977 | 20165.0625 | 8246.802734 | 11339.77441 | 15272.99121 | 10527.5752 | 14238.11523 | 12226.72266 | 13125.11328 | 18113.85742 | 14312.53809 | 14151.53906 | 15675.82129 | 20958.66602 | 11797.46094 | 12835.66992 | 9598.650391 | 17242.05859 | 16921.32617 | 24024.3125 | 20938.19727 | 7349.794922 | 8737.705078 | 14615.8623 | 11374.56641 | 8505.06543 | 32362.79688 | 15493.18164 | 9450.050781 | 6881.280762 | 19976.71484 | 38405.19531 | 16092.75879 | 21025.7168 | 14313.34668 | 29547.15234 | 11353.32813 | 12153.01172 | 8634.664063 | 9255.323242 | 17725.18945 | 9165.919922 | 14875.67578 | 11267.77051 | 13986.20898 | 18166.98828 | 10753.70117 | 18754.15625 | 6720.491211 | 32348.01758 | 24906.22266 |
| P01763 | 9844.348633 | 51946.5625 | 29930.65039 | 32311.70898 | 11996.78906 | 18825.99023 | 16156.28711 | 30882.62109 | 35489.64063 | 39385.94531 | 56118.64063 | 24937.49219 | 23314.37109 | 17355.67969 | 40725.40625 | 32966.24609 | 26750.61719 | 47159.69922 | 38109.04688 | 88055.91406 | 13833.0918 | 47176.78125 | 13907.97266 | 31876.00195 | 35605.70703 | 26231 | 28049.58008 | 27056.27734 | 31346.15039 | 27676.81836 | 82325.21094 | 39851.07031 | 46247.88281 | 31430.47266 | 21851.98047 | 42930.44531 | 58526.94531 | 52053.84766 | 15655.99805 | 64850.50391 | 35560.76172 | 44680.03516 | 45628.02734 | 72380.90625 | 15712.04297 | 24315.10156 | 41284.35547 | 35622.0625 | 47638.71484 | 16636.54688 | 44790.88281 | 38975.9375 | 11506.0957 | 31957.44922 | 14115.1582 | 16088.20117 | 52910.82031 | 46382.69922 | 25860.08984 | 3192.761475 | 38713.04297 | 57312.33594 |
| P01764 | 134227.6563 | 37734.93359 | 43242.74219 | 34726.11719 | 26723.52148 | 0 | 20403.49023 | 34292.05469 | 45957.62109 | 58005.14453 | 53239.48828 | 46945.32031 | 25197.16211 | 39932.43359 | 75530.83594 | 28866.26953 | 22330.17969 | 59924.78516 | 0 | 64602.10938 | 37915.03906 | 0 | 21496.35742 | 0 | 82311.35156 | 16029.62793 | 40697.99609 | 53680.61328 | 51701.52734 | 20806.83203 | 63553.69531 | 55737.28906 | 0 | 24914.92773 | 36447.93359 | 22629.00977 | 34458.34766 | 60278.875 | 64707.57813 | 19613.05664 | 0 | 36652.64844 | 86098.33594 | 40470.50781 | 40138.48438 | 34758.13672 | 69219.74219 | 0 | 28433.13281 | 15171.40234 | 23255.17383 | 74555.88281 | 33163.78906 | 36959.53906 | 0 | 0 | 28458.57422 | 45273.95313 | 52209.05859 | 12110.17871 | 62658.30469 | 48861.62891 |
| P01766 | 116307.4453 | 66250.23438 | 108820.0469 | 38092.28125 | 80160.53906 | 72938.61719 | 71244.875 | 116178.4219 | 70118.60938 | 140381.9844 | 85216.47656 | 77848.79688 | 51900.0625 | 48001.02734 | 37061.89844 | 68104.03906 | 199085.75 | 81309.85156 | 113254.3828 | 150140.7344 | 90119.03125 | 61663.52344 | 66892.11719 | 71382.53906 | 98109.9375 | 67260.26563 | 72095.36719 | 84083.42188 | 68745.85938 | 94767.57031 | 206472.875 | 87924.38281 | 60939.14063 | 62882.60547 | 67174.14063 | 22624.42969 | 108467.5391 | 69763.40625 | 63639.01953 | 58272.41016 | 107861.3516 | 70894.3125 | 146599 | 190185.9844 | 100666.5703 | 136375.6406 | 122857.8125 | 71725.59375 | 78080.44531 | 103960.8672 | 70884.69531 | 83594.125 | 58070.95313 | 78487.39844 | 65140.27734 | 60953.35156 | 124729.6875 | 54815.29688 | 71572.38281 | 76184.89063 | 63338.00391 | 99468.0625 |
| P01780 | 2231775.25 | 1423045.25 | 3041677.5 | 1240684.875 | 1537106.5 | 1058214.375 | 1075654.75 | 2644664.5 | 2134568.5 | 2793708.5 | 1126835.75 | 1561656.625 | 2522725.5 | 1781995.5 | 900764.625 | 1629570.125 | 2477197.5 | 1408603.75 | 1133324.375 | 1452551.25 | 3242364 | 1497711.5 | 1473331.75 | 2277235.25 | 1692841.25 | 1068511.75 | 2310832.5 | 1189776.25 | 2247768.5 | 2733781.75 | 2504267.5 | 1903391.25 | 1633087.375 | 1484908.5 | 2426873.5 | 2025487.625 | 1947625 | 841534.125 | 1614045.625 | 1696097.25 | 1831639.5 | 1705529 | 3710788.25 | 2222693.75 | 2109146 | 2486432 | 2550234.75 | 1259414.875 | 1654421 | 1339321.25 | 1951201.25 | 2396947.5 | 1513056 | 1069220.375 | 1536547.75 | 1315382.25 | 1549907.375 | 2023057.5 | 2485072.5 | 2248947 | 2373401 | 3733772.75 |
| P01782 | 275326.4688 | 160901.9375 | 200446.3906 | 161956.0781 | 140829.4375 | 198913.4063 | 172870.2344 | 436635.9688 | 368562.8438 | 429690.8125 | 330791.5 | 44899.75 | 89005.46094 | 149125.3125 | 132487.6406 | 258184.6094 | 260359.5938 | 108837.75 | 169246.1719 | 230829.1094 | 367496.875 | 308779.9375 | 302127.75 | 258366.0938 | 188447.5 | 213994.8125 | 241881.1094 | 177107.2656 | 320218.9688 | 445096.125 | 190368.0313 | 188461.7188 | 134453.4688 | 128828.4531 | 160672.9219 | 241073.1719 | 163799.7969 | 220723.0156 | 98668.46094 | 165149.0156 | 400935.125 | 275401 | 493478.125 | 496331.7813 | 200193.7969 | 189346.6406 | 204506.9531 | 154395.0156 | 179742.3125 | 397428.9375 | 137426.625 | 268100.2813 | 408468.5313 | 205794.8594 | 123820.2344 | 253918.3281 | 233654.9219 | 100570.9297 | 205506.0469 | 141558.3125 | 137381.7188 | 334854.1563 |
| P01814 | 49666.11719 | 27212.91016 | 20096.47656 | 11599.81055 | 43167.89844 | 43078.75781 | 0 | 41951.875 | 36799.57031 | 38861.07031 | 39964.57031 | 21248.56055 | 31692.88477 | 17299.33789 | 28965.14844 | 30594.03516 | 39748.23438 | 23959.04102 | 36273.36328 | 42520.23047 | 36624.82031 | 15960.17871 | 20858.00977 | 50719.39063 | 32307.28516 | 26408.20313 | 34511.03125 | 37954.58203 | 42441.50781 | 25271.18555 | 32297.67188 | 25238.7207 | 28272.28125 | 25696.5332 | 32320.95703 | 13814.51074 | 35621.67969 | 33271.16016 | 24373.20703 | 9497.445313 | 33151.57031 | 47441.39063 | 48390.69531 | 16303.95703 | 18163.53906 | 6264.023926 | 23243.9082 | 10592.38574 | 10423.5332 | 35402.72656 | 39212.10547 | 36146.45703 | 18848.74023 | 41486.58203 | 9411.75 | 28073.15625 | 18154.0918 | 25305.59375 | 39379.75781 | 14211.37109 | 33594.32422 | 36069.44141 |
| P01817 | 52678.9375 | 23407.44531 | 45628.59375 | 43856.53125 | 31726.23438 | 85681.76563 | 64816.28125 | 41359.5 | 63599.95703 | 36461.98438 | 15090.60645 | 21774.05078 | 57962.44141 | 29272.78516 | 20504.18164 | 40413.19531 | 32176.78906 | 23271.97656 | 37499.5 | 18548.35547 | 48970.38672 | 43355.35156 | 21868.30469 | 46317.46484 | 19592.07617 | 21069.9668 | 62443.0625 | 43197.53906 | 42844.04688 | 59852.14844 | 33266.91797 | 57968.28125 | 76322.71094 | 27596.26953 | 62705.04688 | 85582.20313 | 54617.92188 | 39859.76172 | 35494.14063 | 11867.72363 | 35281.14063 | 44956.21875 | 77532.91406 | 40022.625 | 49601.64844 | 50150.65234 | 42681.47656 | 14417.70215 | 24723.69922 | 16612.93555 | 25908.0918 | 59669.00781 | 7847.937012 | 29389.87109 | 33796.16797 | 25977.63281 | 19990.15234 | 25483.89258 | 26399.81641 | 18231.34766 | 31334.01953 | 26441.21289 |
| P01833 | 16881.8457 | 18379.16016 | 14623.60352 | 14735.77832 | 14001.26855 | 7942.68457 | 6914.768555 | 9753.099609 | 9529.338867 | 10891.36523 | 9287.72168 | 7765.927734 | 10943.78711 | 12360.75 | 13465.01953 | 3182.898438 | 13286.16797 | 0 | 15089.89063 | 11751.0918 | 14784.53711 | 9697.289063 | 21039.72461 | 11482.85059 | 14110.5957 | 10938.96289 | 13627.42969 | 12406.15039 | 17797.82031 | 14351.64551 | 15645.75098 | 10643.74121 | 14294.13574 | 13577.95117 | 13412.05176 | 13578.14648 | 7172.835449 | 4031.108154 | 8734.143555 | 0 | 18244.37891 | 9574.613281 | 28652.01172 | 29313.04688 | 19509.85156 | 17308.48242 | 0 | 12378.46289 | 19633.67578 | 10639.34082 | 8649.583984 | 12822.61133 | 16633.82422 | 10021.16016 | 8730.610352 | 6538.486328 | 0 | 0 | 7785.276855 | 12536.13379 | 5754.969727 | 10161.45703 |
| P01834 | 21038844 | 28701352 | 27455010 | 15933832 | 32302470 | 21417180 | 18492096 | 21099128 | 24510340 | 18037704 | 21303100 | 25141564 | 23546348 | 28014676 | 15793361 | 24233696 | 27119276 | 30522044 | 16243662 | 27364832 | 32582992 | 22113122 | 24530340 | 21278864 | 42913584 | 12429133 | 31176878 | 27590032 | 25607408 | 43329136 | 27037016 | 23613604 | 17799408 | 23192588 | 30487880 | 16956638 | 25635104 | 17381544 | 32795984 | 16028705 | 19502384 | 24143428 | 35229216 | 31342440 | 25130532 | 27460890 | 36415576 | 16156646 | 22727652 | 27881388 | 23894908 | 46379788 | 30472820 | 20245092 | 28041922 | 21806620 | 29406272 | 18512450 | 19957812 | 27517152 | 22398648 | 42163376 |
| P01857 | 11281136 | 7181601 | 9179023 | 4975991.5 | 7122705 | 10704572 | 6194182 | 9226559 | 9920346 | 3843006 | 12023236 | 6925487 | 5137304.5 | 5360289 | 5099870.5 | 6535835 | 5483768 | 14208612 | 10338188 | 10630637 | 10660510 | 11647686 | 11744396 | 12123725 | 12989665 | 6404976 | 6995537 | 11193662 | 8929098 | 10130160 | 6509215 | 9758073 | 6718369.5 | 9446676 | 5785807 | 9629361 | 6190682 | 5921213 | 6500617 | 11064244 | 6317818 | 10199159 | 8858436 | 11196776 | 7999746 | 17977556 | 15044667 | 6965152 | 5898106.5 | 12850110 | 16374776 | 16225572 | 13042118 | 5904727 | 13222648 | 4489905 | 10736171 | 12439538 | 8888488 | 5238203.5 | 8747165 | 13599058 |
| P01860 | 6223078 | 1463866.5 | 1058625 | 2417536.5 | 4783286 | 2823555 | 2075423.375 | 2959774.25 | 1785984.75 | 1715437.5 | 2769428 | 1724115.25 | 3952508.75 | 2182315.5 | 3373564.75 | 6125207.5 | 2723039 | 2255615 | 1002224.813 | 3329484.5 | 3893274.75 | 2483498.25 | 2853100.75 | 1437499 | 2535178.75 | 2142720 | 4179779 | 3068185 | 6420921.5 | 1491367.25 | 2664283.5 | 2873546.5 | 2586034 | 2337437 | 5027910.5 | 1327392.125 | 2454559 | 581229.375 | 3550197 | 3129078.75 | 3494109 | 2427226.5 | 3259999 | 3133274.5 | 2833651.5 | 1115726.625 | 5642670 | 4239613.5 | 2334094.5 | 1811315 | 3410184 | 3532761 | 1191254.75 | 2894930 | 3285940.5 | 1705017.75 | 1287578.75 | 978949.75 | 1206256 | 3098057.5 | 6247698 | 3971987.75 |
| P01861 | 292486.5 | 61747.19141 | 801030.5625 | 955693.9375 | 120626.5781 | 83908.4375 | 1073308.25 | 777696.875 | 490170.9688 | 490361.875 | 933465.125 | 50772.84375 | 536971.875 | 132718.125 | 814115.4375 | 143876.4375 | 141734.8281 | 597497.8125 | 1449390.875 | 325704.375 | 55461.22656 | 118238.2734 | 1392649.25 | 127963.7656 | 386918.9375 | 104113.2656 | 221847.2188 | 1222535.5 | 865197 | 276553.8125 | 381437.7188 | 1101925.5 | 113309.5469 | 938777.4375 | 937969.375 | 124790.8359 | 624468.125 | 667185.875 | 91964.27344 | 783390 | 395599.375 | 740021.5625 | 875994.375 | 1068218.125 | 306304.6875 | 677907.875 | 774305.625 | 99246.3125 | 638934.375 | 576470.625 | 79712.13281 | 915190 | 844940.6875 | 790210.0625 | 551641 | 485210.9375 | 691386.5625 | 435564.7188 | 476308.5313 | 516116.625 | 856251.125 | 1202835.125 |
| P01871 | 17474976 | 13188165 | 40325920 | 6572864.5 | 18550352 | 5929914 | 4071815 | 13406414 | 8168282 | 9421033 | 8337344 | 8168525 | 10630136 | 12378790 | 10563003 | 8151509 | 14005734 | 3343160 | 3918377.75 | 9759887 | 20204784 | 12714648 | 5682777 | 7766009 | 5887403 | 2891093.5 | 10232630 | 5036852.5 | 20742500 | 5618846 | 14111490 | 7612773 | 11009637 | 5711264.5 | 5201495 | 15275426 | 9438580 | 6547931 | 13711018 | 5880644 | 18457536 | 8950612 | 41238712 | 11480906 | 14523548 | 10707133 | 12221411 | 9053507 | 14388106 | 5781321 | 13519310 | 18846136 | 4491948.5 | 6436997 | 6871001 | 6059865 | 6351178 | 5531656 | 13132923 | 12536256 | 14195560 | 13816666 |
| P01877 | 398467.5313 | 147341.2188 | 1531543.75 | 258002.8125 | 237936.5938 | 157749.8281 | 128833.2031 | 187036.25 | 167842.5 | 225061.8125 | 349111.0625 | 183970.3281 | 186767.0625 | 133365.8438 | 214445.6563 | 91922.24219 | 210928.0781 | 164072.5313 | 1141624.25 | 218296.875 | 196050.0469 | 89608.21875 | 184385.5938 | 114866.0781 | 280986.5938 | 145756.4688 | 136986.75 | 107713.3281 | 139954.5 | 668102.5 | 316397.4375 | 172922.4531 | 105747.3594 | 124735.8281 | 202353.5156 | 335913.1875 | 158124.6719 | 234677.2656 | 271382.625 | 93115.89844 | 121948.0625 | 214371.4531 | 214988.3281 | 145264.25 | 293400.875 | 154004 | 56620.23438 | 61392.51563 | 256646.625 | 171560.6563 | 619349.6875 | 440599.5 | 209002.5938 | 224091.4375 | 219356.2813 | 65776.97656 | 53268.74219 | 75697.96094 | 367289.2188 | 139235.9531 | 231028.8125 | 300103.25 |
| P02042 | 51266.5625 | 190200.1406 | 29730.54297 | 49600.72266 | 32770.82422 | 70295.09375 | 67156.0625 | 74673.24219 | 68149.9375 | 57133.03125 | 75300.42969 | 39599.89063 | 43187.76172 | 66766.35156 | 65023.15625 | 63812.09766 | 30627.09961 | 51567.82813 | 41162.49219 | 54936.83594 | 43651.27344 | 81879.72656 | 55982.53906 | 60809.36719 | 36445.98438 | 131845.2344 | 112237.5 | 92101.4375 | 102419.1875 | 95238.67188 | 47537.02734 | 41372.98047 | 44497.28516 | 59870.19531 | 34668.17969 | 67886.05469 | 90912.57813 | 115505.6484 | 164099.3438 | 57211.98438 | 86229.9375 | 78556.70313 | 51312.83203 | 50604.82813 | 107018.4844 | 55513.89063 | 46234.35547 | 123949.2031 | 85737.23438 | 47029.78125 | 84929.98438 | 211929.1094 | 56391.46875 | 55860.71875 | 113248.3828 | 167731.2656 | 55007.82813 | 42050.92969 | 42071.55859 | 31449.77344 | 59935.0625 | 41225.20703 |
| P02100 | 15170.18457 | 102339.7734 | 6011.206543 | 9337.990234 | 20022.41016 | 41316.83984 | 23270.23633 | 24533.00391 | 24176.0332 | 16512.75977 | 19740.8418 | 29341.76172 | 11274.89063 | 25746.4082 | 23040.60742 | 9790.874023 | 21722.72266 | 21240.5918 | 25312.16211 | 15083.61328 | 7514.738281 | 21931.5918 | 33116.24219 | 66195.64844 | 24233.10938 | 76675.30469 | 91697.49219 | 59477.16797 | 83063.27344 | 49935.23047 | 16454.6543 | 19140.96875 | 37203.14063 | 33644.75391 | 16000.93945 | 24144.17383 | 61451.44141 | 28454.67383 | 97945.8125 | 37951.99609 | 43379.41406 | 40720.09766 | 28938.75195 | 40830.55859 | 81641.85938 | 30157.20313 | 34414.83203 | 63270.92578 | 59733.02344 | 18641.26367 | 42321.34375 | 37582.68359 | 30661.67773 | 36051.6875 | 37757.0625 | 50074.39453 | 40341.12109 | 27082.80273 | 40723.83203 | 18676.0957 | 14910.13184 | 29681.07227 |
| P02533 | 27389.16211 | 22496.64063 | 43553.03516 | 34151.94141 | 51617.90234 | 37658.90625 | 28134.24805 | 1001.440735 | 28407.80859 | 19284.3457 | 20082.88867 | 22196.96094 | 19088.26367 | 20841.75195 | 26427.2793 | 30933.82031 | 40131.47266 | 34083.28516 | 13492.08105 | 16320.25488 | 31499.83594 | 23231.29297 | 20492.42188 | 10099.55664 | 29367.32227 | 11471.34863 | 16447.17383 | 15228.4375 | 30707.4375 | 29756.14453 | 26030.2832 | 22525.27539 | 20724.15039 | 38193.38672 | 2429099.75 | 26139.96484 | 30145.32422 | 13885.13477 | 19793.71289 | 18845.99219 | 13190.55664 | 11449.3623 | 29869.71289 | 29433.72656 | 19608.55469 | 30595.01953 | 34905.21875 | 21756.39063 | 1162577.875 | 31210.17578 | 22265.80664 | 22707.58789 | 27707.43359 | 20247.56836 | 24259.33008 | 37772.98047 | 23134.49219 | 30632.73828 | 16137.62305 | 15233.50977 | 39748.00391 | 33137.30469 |
| P02538 | 0 | 0 | 14349.78223 | 11473.40234 | 35747.37891 | 8270.667969 | 5948.20166 | 21582.03516 | 0 | 0 | 5360.27832 | 4137.519043 | 0 | 14332.80176 | 5544.336914 | 0 | 5948.762207 | 0 | 0 | 3676.646973 | 26435.5332 | 3856.427002 | 0 | 45493.44141 | 13406.35645 | 7403.465332 | 0 | 0 | 5656.504395 | 6041.071289 | 3757.402832 | 0 | 66030.33594 | 19801.55273 | 3544.236084 | 6085.86377 | 0 | 3113.388428 | 0 | 0 | 5338.576172 | 0 | 8528.96875 | 7261.350098 | 0 | 9289.480469 | 24005.41992 | 4114.952637 | 8593.612305 | 23538.40625 | 0 | 9569.623047 | 2471.241211 | 4305.034668 | 6749.413086 | 23069.96484 | 11983.02539 | 48012.50391 | 2495.108154 | 0 | 3924.068604 | 57155.69922 |
| P02647 | 24837872 | 35310504 | 32475196 | 26267172 | 35343928 | 29575384 | 27053948 | 23015712 | 25203528 | 35260080 | 26554880 | 30189940 | 40566672 | 42834360 | 31740298 | 34523940 | 36047124 | 34870952 | 25551442 | 23197234 | 42654592 | 36229500 | 28481338 | 26984556 | 41263568 | 27474060 | 24331132 | 33435392 | 33615560 | 33769928 | 36773216 | 41107160 | 33869804 | 33834460 | 41066352 | 29330664 | 35165424 | 32686432 | 29175996 | 42921388 | 31265764 | 27304058 | 36151100 | 35869884 | 35927116 | 29961258 | 35833856 | 33236880 | 42805512 | 32090456 | 29351530 | 32180814 | 30792352 | 29519980 | 37310312 | 27107556 | 28212838 | 35650904 | 28226474 | 31718244 | 25638776 | 41106760 |
| P02649 | 1554002 | 1555118.75 | 1336642.75 | 2292547.5 | 1590191 | 966419.5 | 1168561.5 | 2295264 | 1096109.625 | 1491702.5 | 1475827.625 | 1440655.125 | 2800322 | 2381588.75 | 1957400.25 | 1204177.25 | 1032940.875 | 1277195.125 | 1513078 | 1278266.875 | 1535433.25 | 2238366.25 | 1552545 | 1821863.375 | 2505252.75 | 1370146.75 | 1481314.25 | 3122582.25 | 1181959.75 | 1250018.25 | 2017084.5 | 2293072.25 | 2866383.5 | 2717076.5 | 2479722.75 | 2147006.75 | 1975552.25 | 1730144.75 | 1758437.75 | 2681703.25 | 2089503.5 | 1497079.75 | 2089805.25 | 1352747.125 | 1940102.375 | 1983926.5 | 1872467.5 | 1787389 | 4415330 | 1310635.875 | 1372606.25 | 2081931.75 | 2194298 | 1915369 | 1621986.75 | 1651545.875 | 1857038.75 | 2376323 | 2914241 | 3825313.25 | 1905852 | 1080784.875 |
| P02652 | 226728.4375 | 212603.0469 | 511950.9375 | 291078.0938 | 385691.25 | 294206.5625 | 278752.375 | 3219602.25 | 206017.625 | 295684.8125 | 215072 | 302610.9375 | 201903.0938 | 246712.5625 | 234680.625 | 421403.8125 | 368356.375 | 299321.875 | 152126.5938 | 143703.2969 | 489516.25 | 156007.875 | 266927.75 | 459459.8438 | 307662.125 | 404285.4063 | 312655.8125 | 184109.2344 | 402859.5938 | 233188.4375 | 263596.6875 | 316348.3438 | 260516.8125 | 465690.2813 | 167838.0625 | 334455.5625 | 198946.4844 | 244729.9219 | 218731.2188 | 215940.0781 | 489842.625 | 607870.125 | 330182.9375 | 499618.125 | 406772.5313 | 148481.4219 | 303834.6563 | 373869.2188 | 408221.75 | 245510.7969 | 450709.375 | 299635.3125 | 168718.7656 | 529929.0625 | 321905.6875 | 469946.5 | 497480 | 352132.25 | 148668.875 | 269189.375 | 186355.1875 | 329676.7813 |
| P02671 | 7720642 | 6415404 | 7176997 | 6058319 | 8416174 | 8160435 | 8494134 | 5061761.5 | 5801217 | 6745695 | 5371012 | 5923923 | 8517922 | 6682293 | 7604823 | 8596863 | 5996531.5 | 6320120.5 | 9646494 | 5274220.5 | 7595249.5 | 5499621.5 | 4296813 | 6410965 | 4760892 | 5555867.5 | 10528776 | 7185169.5 | 7534844.5 | 4947439.5 | 4779361 | 7193980.5 | 6412638 | 3373987 | 8034037 | 4930748.5 | 7660747.5 | 5760916 | 6430113.5 | 6949566.5 | 7771454 | 4941669.5 | 7266667.5 | 6895025.5 | 8087890.5 | 9373833 | 5162389 | 7338252.5 | 6347075 | 6219740 | 7210814.5 | 3390202.5 | 4822160 | 6119069 | 3941185 | 5466491 | 5833372 | 5306817.5 | 7949201 | 6435292 | 5943512 | 4929861.5 |
| P02675 | 20588008 | 14312182 | 14232715 | 14353304 | 15758395 | 18940834 | 18001008 | 11329018 | 13088386 | 13563420 | 11664508 | 12685016 | 16586262 | 12971902 | 15092872 | 17095426 | 12818350 | 12302861 | 25053860 | 11178910 | 18386280 | 11258140 | 9850054 | 13822650 | 10216885 | 11245528 | 20142428 | 16042046 | 16868112 | 13303656 | 13962698 | 15712191 | 14245372 | 7906865 | 16664646 | 10520632 | 16001968 | 12302017 | 12907545 | 17175504 | 17161492 | 11859490 | 19049128 | 15139056 | 17041128 | 20911952 | 12739301 | 18351940 | 14805350 | 14531496 | 17705510 | 10017188 | 10101805 | 11595556 | 11436372 | 12699235 | 13223866 | 13382898 | 16586258 | 13346288 | 13813510 | 9706292 |
| P02741 | 0 | 0 | 0 | 4565.363281 | 0 | 10440.06152 | 34023.54297 | 0 | 2092.395508 | 0 | 0 | 5824.516602 | 0 | 0 | 0 | 6501.372559 | 0 | 0 | 5249.837402 | 5972.663574 | 0 | 0 | 0 | 0 | 0 | 0 | 9705.833008 | 13739.58789 | 0 | 7684.736328 | 0 | 12474.93555 | 0 | 10532.70117 | 10970.67578 | 0 | 9755.583008 | 0 | 0 | 2606.174805 | 7098.876953 | 0 | 0 | 0 | 6132.756836 | 13709.67969 | 0 | 9936.974609 | 0 | 0 | 0 | 0 | 8505.513672 | 8358.405273 | 0 | 3472.602539 | 9938.806641 | 0 | 26722.24609 | 0 | 0 | 0 |
| P02743 | 517002.1875 | 320111.1563 | 333360.8438 | 339194.875 | 526278.75 | 636412.4375 | 561488.875 | 361882.8125 | 474027.8438 | 462025.125 | 572074.625 | 454856.4375 | 250629.375 | 271476.0625 | 268640.9375 | 327704.0313 | 236396.9375 | 317403 | 575001 | 469413.3125 | 278906.2813 | 287515.75 | 464761.625 | 334890.4688 | 263620.9688 | 395868.7188 | 452830 | 549287.4375 | 271055.1563 | 318548.0313 | 327794.375 | 309140.9688 | 267498.75 | 392096.0313 | 370086.1875 | 217565.5625 | 467889.375 | 474342.5 | 376523.0938 | 394496.625 | 247249.6563 | 381943.6563 | 219289.7969 | 299567.75 | 213886.125 | 414080.1563 | 213697.9375 | 499748.5 | 331807.125 | 407519.9063 | 404120.5 | 363273.125 | 476044.5 | 445519.3125 | 379514.9688 | 488493.7813 | 508953.875 | 389769.0625 | 393974.6875 | 342363.125 | 225943.7188 | 283492.5 |
| P02745 | 37407.87109 | 25160.61133 | 29421.05859 | 23929.32422 | 43901.72266 | 32551.23633 | 35981.25781 | 25874.86133 | 29103.18945 | 26766.10547 | 43549.98047 | 102463.8125 | 26910.22266 | 31289.91602 | 28896.26953 | 40883.85938 | 25583.73438 | 32896.19922 | 32206.05859 | 58932.10547 | 29430.96094 | 39590.84766 | 44373.52344 | 22623.04297 | 27363.91797 | 37180.64453 | 32332.80078 | 26516.8457 | 39092 | 33032.83984 | 30458.67969 | 31928.33594 | 26511.75586 | 28312.90234 | 29168.98047 | 24421.39258 | 26470.58008 | 20657.98828 | 24423.92578 | 23494.23438 | 40949.28125 | 43548.52344 | 31090.19531 | 31703.17773 | 24640.60547 | 17218.89258 | 46675.03125 | 25593.99805 | 30846.34961 | 98355.50781 | 33997.58203 | 25606.59375 | 34811.30859 | 34152.67578 | 27962.15625 | 87922.23438 | 106743.3203 | 36431.34375 | 28360.42188 | 23983.87109 | 31347.93359 | 33715.20313 |
| P02747 | 434735.0313 | 289542.1563 | 306910.875 | 233371.3594 | 553257.8125 | 292580.5938 | 332367.4688 | 406251.0313 | 215790.0938 | 292723.0938 | 488004.8125 | 441910.9063 | 241821.9063 | 264613.9688 | 326322.5313 | 342326.7188 | 426444.2188 | 376221.4063 | 524811.6875 | 764638.375 | 441461.9063 | 566046.75 | 456939.6875 | 377222.1875 | 510679.0313 | 355223.75 | 382191.25 | 576260.8125 | 387216.3438 | 364910.9688 | 293579.9375 | 317453.25 | 223811.5625 | 349096.1563 | 308483.875 | 194328.9063 | 498151.3125 | 267042.7188 | 277022.0938 | 495988.25 | 484695.5625 | 345219.2188 | 466421.2188 | 465615.7813 | 295401.9375 | 429766.125 | 587331.9375 | 526554.5625 | 628252.125 | 565354 | 615500.4375 | 466647.5313 | 683353.1875 | 266328.875 | 656421.0625 | 564228.5 | 519610.0313 | 541319.625 | 436852.2813 | 407619.2188 | 513368.625 | 505100.625 |
| P02748 | 106139.4453 | 132458.125 | 190510.9688 | 145637.7969 | 79627.57813 | 106551.7891 | 234201.1406 | 82927.78125 | 114051.9375 | 86818.46094 | 108073.1875 | 224938.9688 | 130263.7422 | 115821.9375 | 125406.6328 | 166633.5313 | 88582.625 | 108569.2344 | 156185.6563 | 129640.1875 | 137310.0313 | 121286.4219 | 97591.04688 | 146698.25 | 90308.14063 | 89380.39063 | 102853.6563 | 179200.5938 | 120864.7031 | 152579.2969 | 108751.0313 | 193903.8125 | 123403.125 | 124576.8359 | 201405.8125 | 104289.2422 | 171698.625 | 133787.25 | 101481.3594 | 160695.9688 | 130070.9375 | 111905.8984 | 123853.0781 | 138514.2813 | 121805.0391 | 149611.7344 | 111192.8203 | 144806.5938 | 96569.6875 | 118003.5 | 109438.25 | 118291.4219 | 68092.97656 | 134405.0156 | 113460.0469 | 89805.04688 | 103022.4063 | 156777.0625 | 188996.9063 | 116882.2813 | 130333.5156 | 145136.3125 |
| P02749 | 765461.875 | 945763.25 | 549501.75 | 940365 | 908898.0625 | 826687.375 | 1386182.625 | 1063020.875 | 893130.5 | 602080.6875 | 734900.25 | 808747 | 1077011.875 | 579908.875 | 1169064 | 1008838.688 | 550680.3125 | 1186073.5 | 976611.75 | 865985.75 | 557292.3125 | 621568.4375 | 1020863.125 | 895113.4375 | 1239429.875 | 1053737 | 831524.375 | 1355579.5 | 849709.25 | 1121993.75 | 864486.8125 | 1024959.75 | 893615.125 | 1203227.375 | 919850.375 | 717285.5625 | 701462.125 | 612728.5625 | 936709.25 | 1122293.75 | 944998.9375 | 1036761.875 | 966688 | 904428.875 | 939160.625 | 544150.6875 | 1139071.375 | 1046208 | 1093655 | 1128369.625 | 1030788.563 | 1393713 | 1007472.063 | 1059278.625 | 1287130.625 | 1103003.75 | 532169.75 | 540013 | 1402545.875 | 1099072.25 | 1022923.625 | 457621.8125 |
| P02750 | 91579.58594 | 122542.7031 | 188804.8281 | 212488.4531 | 77562.96094 | 146367.2188 | 180812.375 | 99303.08594 | 91758.8125 | 107025.8906 | 48559.84766 | 184414.5625 | 181416.5938 | 112109.1719 | 127874.3516 | 230323.5781 | 138858 | 135235.5781 | 144968.0625 | 124775.0469 | 136126.5 | 110199.1094 | 87871.73438 | 127565.4766 | 132674.3906 | 76180.51563 | 116636.8047 | 143823.4219 | 119556.625 | 185505.9375 | 133882.2656 | 192167.9375 | 110090.5469 | 141344.8438 | 171436.3125 | 116988.5547 | 131554.9375 | 127225.1875 | 79870.04688 | 215590.4531 | 145845.875 | 129845.75 | 142413.5313 | 151313.8906 | 166128.6719 | 160869.9219 | 162758.8125 | 175873.1406 | 124162.5 | 99982.67188 | 159570.3125 | 125822.6563 | 93508.65625 | 152984.5625 | 92296.82813 | 147538 | 163096.9531 | 172699.3125 | 222151.25 | 110642.3672 | 176560.5 | 160081.875 |
| P02751 | 1103761.5 | 758457.125 | 633460.75 | 1239213.75 | 1466219 | 1202736.375 | 777086.1875 | 750298.625 | 799072.625 | 779384 | 641364 | 590715.4375 | 877245.375 | 965567.875 | 784631.625 | 891435.5 | 787262.625 | 809256 | 1258802.125 | 570994.1875 | 937263.25 | 763205.375 | 1064746.25 | 553308.9375 | 877759.3125 | 1232951.125 | 1123859.5 | 851528.0625 | 800133.375 | 406398.5625 | 816707.25 | 774446.75 | 678584.375 | 504430.5625 | 830718.375 | 771950.5625 | 910965.5 | 1039726.688 | 810512.875 | 845277.5 | 740497.875 | 1066449.25 | 703391.25 | 529073.75 | 1301030 | 731655.875 | 431944.4688 | 608315.125 | 699792.625 | 812143.875 | 490444 | 387369.9375 | 408843.125 | 629447 | 187700.7813 | 459085.0313 | 689168.1875 | 241767.2344 | 611981.875 | 741114.625 | 368809.75 | 181248.8125 |
| P02753 | 76016.16406 | 47817.85156 | 24637.77734 | 59113.85938 | 79197 | 66149.10938 | 73458.22656 | 54752.48828 | 70715.14063 | 129511.5469 | 73878.40625 | 68594.53906 | 48191.73828 | 64202.87891 | 66762.76563 | 34052.78125 | 89254.21875 | 80384.98438 | 73767.9375 | 65862.01563 | 45339.58203 | 70182.04688 | 88085.4375 | 65341.38672 | 69249.39844 | 75123.59375 | 58810.88672 | 73533.45313 | 44729.17578 | 94552.85156 | 58821.0625 | 53497.95313 | 91083.35156 | 85266.14844 | 39490.13281 | 68352.41406 | 53356.37891 | 204261.7188 | 54706.60156 | 34355.14453 | 88572.17969 | 72248.28125 | 39706.09766 | 77450.29688 | 47367.60156 | 60208.58203 | 64300.53906 | 48630.96875 | 35641.27734 | 58880.14453 | 59493.33594 | 79238.41406 | 56140.76172 | 76921.29688 | 64456.32813 | 61104.76563 | 45873.67969 | 42897.55469 | 61489.83203 | 55084.27344 | 42217.11719 | 59655.47266 |
| P02760 | 507196.4375 | 395396.9375 | 433317.3125 | 484665.6875 | 582918.125 | 455645.4063 | 620151.5 | 563627.125 | 482137.75 | 498765.6563 | 782982.5 | 352821.75 | 481661.7188 | 342972 | 543397.375 | 315442.0313 | 410487.5625 | 470424.9688 | 536009.0625 | 586452.0625 | 383820.8438 | 423622.375 | 396296.625 | 350264.3125 | 442542 | 423779.2813 | 459525.125 | 454560.625 | 346054.625 | 414055.2188 | 389224.4375 | 343868.625 | 280123.4688 | 361374.4375 | 426058.75 | 299022.2813 | 418818.3125 | 405913.3125 | 497061.9688 | 332802.7188 | 423460.1875 | 391939.0625 | 314725.0938 | 272702.2188 | 405468.125 | 403332.7813 | 344938.0938 | 365577.4063 | 499447.9375 | 343465.1563 | 325857.0625 | 374566.125 | 515232.875 | 573410.3125 | 439633.0313 | 374399.8438 | 405325.3438 | 315588.6875 | 508718.5313 | 474218.875 | 510813.375 | 358100.7813 |
| P02763 | 1133809 | 1209015.125 | 1627957.625 | 1786953.75 | 1206043.625 | 1901639.125 | 2537659 | 1659568.75 | 1281199.5 | 1571986.875 | 1665444.375 | 2103676 | 1829256.5 | 1001136.313 | 1270299.375 | 1478000.125 | 1727100.25 | 1756304.625 | 2779892.5 | 1994041 | 1078003.375 | 1249008.625 | 1605393.375 | 1867478.5 | 1109467.25 | 1414477.75 | 1893551.75 | 3348380.5 | 1526601.5 | 1185793.5 | 1320224 | 1970586 | 1306357.75 | 1704223.125 | 2091111.75 | 1182657 | 2866568.75 | 1570379.375 | 1065504.875 | 2887390 | 1846733.5 | 1152902.875 | 2221391.25 | 1170316.75 | 1064079.375 | 2536930.75 | 1741503.375 | 2868627.5 | 1585941.875 | 1447222.375 | 1655646.25 | 2529679.5 | 1845230.5 | 1806096.875 | 2005799.75 | 1194065.25 | 1749174.25 | 1980612.875 | 3589141.5 | 2025054 | 1589271.75 | 1647317.75 |
| P02766 | 630525.9375 | 824842.6875 | 389674.5625 | 906002.875 | 732544 | 656748.375 | 1126844.5 | 695422 | 546358.125 | 626870.1875 | 661736.125 | 707681.25 | 695889.9375 | 795612.6875 | 885962.25 | 635117 | 715382.375 | 987838.125 | 688682.1875 | 901953 | 629130.4375 | 845777.4375 | 697674.75 | 675438.25 | 708962.875 | 841689 | 698404.125 | 882712.875 | 704213.125 | 638958.625 | 748723.5 | 726660.25 | 639860.875 | 929781.5625 | 723446.5 | 569107.625 | 694210.125 | 668210.875 | 648877.6875 | 925469.875 | 570857.1875 | 964748.1875 | 699236.125 | 725818.75 | 675684.125 | 651431.75 | 691172.125 | 671935.5 | 751492.625 | 476852.3125 | 650660.75 | 1085991.625 | 1168871.75 | 542613.125 | 668086.875 | 700685 | 849549.75 | 648019.375 | 599191.9375 | 713885.5625 | 863846.5 | 591923.9375 |
| P02768 | 459075520 | 497810560 | 509248064 | 437954880 | 424584512 | 399089824 | 449685344 | 513320000 | 459430560 | 453825408 | 401948544 | 440472768 | 490184608 | 432455488 | 424897152 | 494736640 | 481828800 | 529301472 | 464222592 | 532370560 | 530067776 | 570972224 | 480539008 | 473595456 | 550716800 | 422168064 | 451368384 | 528099200 | 465223424 | 569943872 | 562775360 | 452515488 | 302914496 | 491957952 | 461138944 | 375008992 | 526947552 | 382827200 | 408403584 | 475832448 | 362921504 | 506815552 | 498883648 | 442142752 | 423344576 | 481790784 | 490179648 | 448850720 | 484118176 | 431936128 | 505819968 | 536808768 | 480562496 | 404104512 | 491821632 | 456196672 | 462473568 | 498671488 | 467359840 | 475735104 | 587753856 | 482324832 |
| P02775 | 34129.34766 | 61669.53125 | 22657.69531 | 53257.34766 | 31295.80859 | 27298.05469 | 60083.56641 | 17621.16211 | 56394.65625 | 11190.15918 | 21053.31641 | 45236.81641 | 27819.89844 | 92166.40625 | 47703.44922 | 17415.37305 | 18537.92188 | 18797.0625 | 52433.32031 | 53610.37109 | 40186.95703 | 12633.44336 | 115556.1875 | 15275.24219 | 78752.05469 | 37950.95313 | 33740.48438 | 6656.09082 | 19342.05664 | 53365.37891 | 44952.94141 | 10540.00488 | 16390.69141 | 53170.62109 | 10956.54492 | 111796.0234 | 30233.16406 | 27197.75586 | 23995.62109 | 10302.53223 | 17427.60742 | 20061.72266 | 111569.3594 | 41879.85938 | 11373.46484 | 21603.5 | 0 | 84989.67188 | 13731.47461 | 22600.47656 | 14469.84766 | 14115.25879 | 31723.27539 | 12860.11719 | 59867.56641 | 114065.7578 | 33049.11719 | 17324.4043 | 9747.042969 | 20116.40234 | 5409.577148 | 12121.90234 |
| P02776 | 48749.93359 | 72817.73438 | 58168.80469 | 68722.73438 | 89389.54688 | 30843.88867 | 34508.84375 | 29498.62891 | 27698.29688 | 12512.48145 | 34087.17188 | 19226.45313 | 27356.45703 | 44703 | 45047.91406 | 21008.15625 | 20038.98242 | 16978.26172 | 40639.22656 | 30450.07031 | 33394.98438 | 13280.52246 | 65052.29688 | 14814.66406 | 38572.11328 | 50174.23047 | 76873.54688 | 10828.54297 | 30032.11328 | 31812.37305 | 29725.23047 | 7485.069336 | 20266.49219 | 46699.35156 | 23456.06836 | 198775.5625 | 33402.18359 | 19276.32813 | 36973.39844 | 20370.12891 | 37363.77734 | 23845.91211 | 110153.6719 | 42528.94531 | 24184.30469 | 27375.22656 | 0 | 89119.64063 | 20132.48828 | 24345.84961 | 16835.19336 | 13199.29785 | 27883.46484 | 17845.25 | 23872.39844 | 69389.72656 | 46152.75 | 21871.31641 | 15869.40234 | 34293.26563 | 3063.925293 | 15255.31738 |
| P02787 | 13554605 | 18204782 | 25405334 | 14446848 | 14518566 | 12765179 | 22669516 | 15574480 | 15664528 | 11498322 | 12958552 | 16898848 | 14261560 | 20773216 | 19078946 | 20440222 | 21785860 | 18796944 | 18119856 | 21765006 | 21402832 | 18546920 | 13572288 | 15267022 | 18737856 | 13142108 | 21894968 | 25459352 | 20132724 | 17205188 | 18076868 | 15143810 | 15893078 | 22167390 | 23616808 | 18679988 | 21692946 | 13101282 | 16175564 | 19854152 | 14041270 | 18029908 | 31402664 | 23776324 | 15836588 | 17915916 | 21530788 | 21348252 | 16294815 | 20673500 | 22054928 | 21391844 | 24574446 | 14364998 | 26065468 | 18511556 | 22293328 | 21875524 | 20836664 | 23934096 | 17977152 | 20405440 |
| P02790 | 2398039.5 | 2148365.25 | 2983788.5 | 2909264 | 2603224.5 | 2440286 | 4576272 | 2365263 | 2451595.75 | 3065232 | 2749874.5 | 2644153.25 | 3048943.5 | 2381401 | 3346530.25 | 3193724.5 | 3908045 | 3689620 | 2925966.75 | 3022990.5 | 3005706 | 3739671.25 | 2445042 | 3243679.75 | 2739940 | 2449939 | 3898377.5 | 3562039 | 3005083.75 | 2489223.5 | 2905171.25 | 3408918 | 2591091 | 3387833.25 | 3771822 | 2541357.5 | 2982886.5 | 2590070.5 | 2533998.5 | 3245517.25 | 2285927 | 2958775 | 2756519.75 | 3178128 | 2662289.5 | 3450286.5 | 2444906.5 | 2935661 | 3591317.75 | 1874252.25 | 2080591.25 | 2852479 | 2702982.5 | 2518172.75 | 2483691.5 | 2460541.25 | 2833460.25 | 2954871.75 | 3350090.5 | 3206549 | 3066929.5 | 2784997.5 |
| P03950 | 0 | 0 | 0 | 5077.719727 | 0 | 0 | 7323.374512 | 5613.228516 | 0 | 0 | 7637.340332 | 0 | 4927.991699 | 4096.802246 | 3650.058105 | 6434.20752 | 0 | 6256.683105 | 6522.966797 | 8643.305664 | 5123.080078 | 2986.303223 | 7104.82373 | 5949.257324 | 6684.223145 | 0 | 4468.614746 | 8059.280762 | 0 | 3645.844727 | 0 | 0 | 0 | 0 | 0 | 0 | 6733.40625 | 0 | 4996.516113 | 6609.173828 | 0 | 8676.342773 | 0 | 0 | 0 | 8199.542969 | 0 | 0 | 1673.238037 | 6708.792969 | 4031.531982 | 9438.919922 | 9530.564453 | 5835.783203 | 0 | 6287.311523 | 3677.511963 | 0 | 0 | 0 | 4616.969727 | 6467.005859 |
| P03951 | 12419.66797 | 15932.87695 | 10274.21875 | 9590.848633 | 14407.58203 | 14024.81055 | 9064.719727 | 6343.665039 | 14869.02246 | 11780.92773 | 17417.82813 | 16536.46094 | 12981.2832 | 7379.199219 | 13200.24805 | 9891.855469 | 21146.26563 | 11691.21875 | 8991.592773 | 14043.57324 | 16992.14844 | 13025.53613 | 16778.80273 | 7584.24707 | 18046.05469 | 8399.662109 | 18767.13867 | 17912.16406 | 8360.488281 | 15469.92969 | 4385.839355 | 4012.623291 | 11898.37988 | 18837.56836 | 15734.9043 | 7086.980469 | 18170.74609 | 5864.77832 | 14078.36328 | 16020.22168 | 9431.858398 | 10169.03027 | 10661.46582 | 5512.499512 | 6923.146973 | 23419.82422 | 9534.199219 | 8793.71875 | 9286.006836 | 10013.40332 | 11289.44629 | 7982.004395 | 9625.291016 | 6502.460449 | 6101.782227 | 5829.947266 | 14488.8623 | 10178.40625 | 6889.887695 | 14538.43945 | 10692.83008 | 8963.195313 |
| P04003 | 2617916 | 1859974.5 | 3131384.75 | 3574369 | 5401194 | 3208126 | 2157458 | 3401023 | 2050622.25 | 2702329.25 | 2639995.25 | 2264238 | 2868449.5 | 2631262 | 4139618.5 | 2041683.25 | 1812545.375 | 1792826.25 | 4757806.5 | 2633080.75 | 3403818.75 | 1394886 | 2390068 | 2231334.5 | 1503549.75 | 2316872.25 | 3541304.25 | 2498666.5 | 3799387.75 | 1362929.625 | 1617077.75 | 2080163.75 | 2664949.75 | 1480023.25 | 2692114.5 | 2398046 | 2837269.5 | 1538268.5 | 3254728.5 | 2613632 | 4195453 | 2167463.25 | 3565486.5 | 1751781.75 | 2616125.25 | 2378724.25 | 1533793.75 | 3595817.5 | 4369033 | 1392708.25 | 2340186.75 | 1923591.25 | 2933347 | 1820037.75 | 1710618 | 2019564.25 | 1604305.125 | 2384208.5 | 3912618.5 | 3045861.5 | 2956535.5 | 1938560.5 |
| P04004 | 778907 | 761071.875 | 738453.75 | 844009.5 | 825996.6875 | 894454.875 | 1080114.75 | 690980.25 | 759741.625 | 792171.9375 | 945891.125 | 878347.25 | 674434.125 | 762194.5625 | 1066010.75 | 685376.0625 | 863202.375 | 695398.75 | 751638 | 977977.1875 | 705607.25 | 739587.75 | 733446.375 | 881559.625 | 700947.125 | 1275304.25 | 1039199.75 | 1025211 | 724764.25 | 550270.125 | 728662.625 | 962865.375 | 696644.375 | 1285511.75 | 983067 | 667466.5 | 1258478 | 1822280.75 | 730605.6875 | 741354.125 | 755537.75 | 982028.25 | 902361.875 | 978451.9375 | 725329.9375 | 874472.625 | 708164 | 768871.125 | 782398 | 752185.75 | 620438.625 | 723097.375 | 991517.1875 | 878658.75 | 783652.6875 | 879218 | 735711 | 590328.75 | 1069577.125 | 709220.75 | 846726.25 | 644408.5 |
| P04040 | 1928.239014 | 14251.95898 | 0 | 0 | 0 | 5364.431641 | 3482.503174 | 4125.550293 | 0 | 0 | 0 | 3138.376953 | 0 | 0 | 0 | 0 | 3753.932129 | 0 | 3193.070801 | 2958.773193 | 0 | 3983.21167 | 5628.523438 | 2841.179688 | 0 | 7858.764648 | 8256.943359 | 2933.098633 | 5848.894043 | 8405.712891 | 5301.077637 | 0 | 6574.379883 | 5575.652344 | 1584.040771 | 0 | 2509.836914 | 9536.419922 | 8218.014648 | 4928.48584 | 0 | 5810.09668 | 0 | 5314.305664 | 5955.804199 | 7022.474609 | 6436.910156 | 14073.20703 | 6777.359375 | 3103.5625 | 8003.416504 | 9354.623047 | 6378.522461 | 200191.9531 | 5638.295898 | 8448.821289 | 7682.694336 | 6296.583496 | 4886.255371 | 2996.02124 | 6062.012695 | 4350.125977 |
| P04114 | 4976891 | 3125279 | 2626157 | 5542489 | 4077868 | 3175065 | 2909453.75 | 4197087.5 | 4938572.5 | 7607785.5 | 3583994.5 | 4518466.5 | 5507325 | 4525405.5 | 5548182 | 2704452 | 3046772 | 2675411 | 4812079 | 2646232.75 | 3472229.5 | 4255405 | 4227619.5 | 3306881.75 | 4458607 | 4894521 | 3951167 | 1887179 | 3986545 | 2510989.5 | 3810301.5 | 4840789 | 6042129 | 3361629.5 | 3635641.5 | 5012326.5 | 6713065 | 3840901 | 4130617.5 | 4340875 | 4995434 | 4155395.5 | 6116279.5 | 4486420.5 | 3941572.75 | 4028680.5 | 2563928.75 | 5015223.5 | 6218288 | 4168618.5 | 4617100 | 3128475 | 5087366 | 5703133 | 3256907.5 | 3936182.5 | 2588628 | 6079433 | 5010392 | 5756700.5 | 3641573 | 2276854.5 |
| P04180 | 17947.38281 | 20486.64648 | 15939.92871 | 20929.87891 | 19306.75391 | 21231.47461 | 16915.59961 | 19357.69727 | 15858.47363 | 23451.71875 | 20478.26172 | 19488.21484 | 15066.7373 | 19399.78125 | 20058.86719 | 18246.21875 | 17857.61133 | 21343.39453 | 35802.48828 | 18229.80273 | 20181.13086 | 17759.67383 | 14172.93359 | 27163.62695 | 20982.39258 | 21194.82227 | 18486.5 | 23196.4375 | 34740.89063 | 19004.23828 | 23460.26953 | 19611.59766 | 20671.95508 | 24992.03906 | 18673.56445 | 25389.66211 | 14970.58594 | 17004.27734 | 15433.50586 | 17791.29883 | 19101.12891 | 16059.26855 | 25478.84766 | 13058.57422 | 29439.58594 | 13463.77441 | 37743.45313 | 51582.98047 | 28348.62891 | 18307.26367 | 10169.83691 | 8476.177734 | 30538.99219 | 18842.84375 | 22056.50781 | 13954.7168 | 16543.46484 | 27100.5957 | 21048.14453 | 19383.93164 | 15966.04004 | 21398.03906 |
| P04196 | 318623.5 | 711038 | 625062 | 526983.625 | 580159.9375 | 295269.125 | 380608.2188 | 457111.5625 | 607949.5 | 293901.5313 | 678591.3125 | 419919.5 | 841280 | 598537 | 532292 | 624113.625 | 584547.125 | 625411.25 | 636816.125 | 790615.75 | 654015.5625 | 986884.125 | 527593.5625 | 547680.0625 | 954348.375 | 646668.625 | 531433.875 | 940215.25 | 423397.5938 | 554265.8125 | 626182.75 | 517659.5938 | 563181.25 | 561032.9375 | 644236.625 | 478747 | 697942.875 | 751953.625 | 688643.125 | 941749.75 | 513182.3125 | 403990.8125 | 639348 | 750943 | 502364.375 | 659081.375 | 977016.25 | 720488.5 | 419549.3438 | 768361.25 | 602381 | 569561.75 | 549490.5 | 578381.125 | 828055.0625 | 604311.0625 | 339686.875 | 725069.625 | 1021182.313 | 745071.25 | 952499.0625 | 505026.1875 |
| P04211 | 997611.875 | 1014479.938 | 965017.1875 | 414382.6563 | 572630.9375 | 602590.3125 | 632170.0625 | 2250281.5 | 658461.125 | 2546730.75 | 701219.125 | 843402.875 | 932520.8125 | 810569.0625 | 1032355.5 | 2171019.25 | 843455.9375 | 1370707.875 | 594402.125 | 675111.375 | 728307 | 1419999.5 | 786484.4375 | 59109.10938 | 899952.375 | 536719.75 | 939520.0625 | 467747.2188 | 1069143.5 | 658121.9375 | 631704.375 | 792666.25 | 1371297 | 565703.125 | 1376221 | 748538.1875 | 1079004.875 | 737368 | 1184798.5 | 958338.0625 | 1018687.313 | 612609.5 | 1149129.375 | 817351.0625 | 803875.0625 | 1233407.75 | 1033943 | 860178.1875 | 1650772.875 | 458499.9375 | 1637939 | 2082619.875 | 1272367 | 619099.375 | 1087549 | 729857.6875 | 1031613 | 1208717.5 | 367653.7188 | 1031736.875 | 1264309.125 | 1559202 |
| P04217 | 635873.5625 | 904436.25 | 799367.4375 | 972816.5625 | 642203.4375 | 780883.9375 | 1030256.625 | 816839.5 | 592569.4375 | 743875.75 | 704903.625 | 965915 | 919421.3125 | 811122.875 | 927435.25 | 1012704.063 | 807784.1875 | 902735.9375 | 747865.5625 | 837097.75 | 957478.875 | 678553.4375 | 579940.875 | 702343.375 | 788842.625 | 681708.4375 | 777880.5625 | 918129.125 | 744875.5625 | 614294.25 | 755638.875 | 892825.625 | 851147.875 | 876159 | 899110.6875 | 661454.75 | 744098.125 | 643188.125 | 606515.3125 | 942345.8125 | 813809.875 | 689931.3125 | 669140.5 | 840280.1875 | 664685.8125 | 392715.5 | 878964.375 | 812196.5 | 778937.25 | 671519.625 | 703594.5 | 748331.375 | 893317.3125 | 789570.5 | 943809.625 | 741644.875 | 735132.8125 | 409734.0625 | 1043077.25 | 904992.9375 | 570377.0625 | 1026441.938 |
| P04264 | 294773.3125 | 190225.6406 | 239071.2813 | 237651.25 | 324992.3125 | 237403.0313 | 193002.75 | 233599.9688 | 201306.8281 | 261379.9063 | 180237.1563 | 144574.2344 | 186956.375 | 234366.25 | 237134.1563 | 310146.6563 | 345226.625 | 275769.0625 | 147452.375 | 158598.4844 | 277347.75 | 167040.8906 | 197282.5938 | 221045.0313 | 216567.8438 | 172200.9844 | 136378.1563 | 138479.75 | 242610.8125 | 206818.6563 | 233677.5156 | 244831.625 | 195084.7969 | 163496.6563 | 188211.3438 | 211056.0938 | 252317.125 | 141460.0938 | 205999.8438 | 173954.75 | 282499.1875 | 141040.8125 | 247251.6094 | 213659.9375 | 220034.9688 | 421269.125 | 157220.25 | 219704.0156 | 218247.1406 | 235282.9688 | 230036.6094 | 228584.0781 | 201882.5313 | 174500.6875 | 240428.3125 | 374779.0625 | 215758.0625 | 226991.5313 | 181990.5469 | 144262.3594 | 374021.875 | 139883.9219 |
| P04275 | 47623.35938 | 52397.1875 | 49038.09375 | 89885.07813 | 56576.09375 | 38651.03125 | 68020.20313 | 63468.80469 | 56426.47266 | 35807.57422 | 54347.03125 | 109634.4531 | 72191.60938 | 93380.61719 | 52258.19531 | 100279.5547 | 75228.83594 | 170984.5313 | 23879.44727 | 101315.2344 | 54995.97656 | 169686.0313 | 106274.8516 | 11070.62207 | 111795.0313 | 66243.625 | 44997.04688 | 122191.2031 | 43381.86719 | 59636.07031 | 70322.5625 | 83124.3125 | 97768.45313 | 26348.63281 | 65168.70313 | 70804.79688 | 71111.69531 | 79412.10938 | 33766.50391 | 202377.25 | 60866.82422 | 71559.25781 | 18222.03125 | 85421.95313 | 69614.3125 | 165793.8594 | 176670.9063 | 82453.80469 | 18477.5625 | 228641.25 | 94192.42188 | 41464.375 | 36298.375 | 34978.29688 | 26542.25391 | 81817.73438 | 56450.14063 | 66238.64063 | 32605.36719 | 46542.40625 | 31665.99219 | 25080.08203 |
| P04430 | 84490.92188 | 47631.75391 | 100761.2188 | 85394.64063 | 98507.30469 | 70508.29688 | 97272.64063 | 82997.75 | 97039.5625 | 95591.07031 | 126762.5078 | 86040.57031 | 151648.7656 | 126204.1953 | 81709.89844 | 72681.89063 | 97172.46094 | 81340.03125 | 0 | 344092.625 | 181896.7344 | 98977.16406 | 96260.375 | 125622.9219 | 19874.21094 | 22026.89258 | 105390.0859 | 64438.95313 | 91881.82031 | 50190.45703 | 79402.67969 | 61503.14063 | 79416.02344 | 49531.0625 | 90841.46875 | 109866.7813 | 23001.99023 | 47803.51953 | 180741.3125 | 0 | 84372.24219 | 41704.60547 | 22053.27539 | 88901.73438 | 28178.6543 | 114327.1094 | 89107.40625 | 95195.46875 | 13570.96484 | 157621.6719 | 42422.70703 | 37044.70703 | 43461.57031 | 0 | 26886.59961 | 25586.33984 | 42680.99219 | 36206.67188 | 164462.6719 | 0 | 92904.51563 | 120723.0313 |
| P04433 | 101001.6719 | 92921.10156 | 176865.0469 | 68616.57031 | 95464.375 | 118855.8125 | 82390.90625 | 112302.7656 | 151404.625 | 97494.48438 | 71277.49219 | 112719.4922 | 95816.65625 | 97545.80469 | 73756.39844 | 108313.0391 | 107185.9531 | 89407.11719 | 60434.11719 | 66693.42188 | 139598.7031 | 79779 | 86032.85938 | 81871.25 | 128960.7734 | 102945.5156 | 136587.5 | 95222.70313 | 122045.1641 | 114095.4531 | 181234.4688 | 111928.1406 | 96112.54688 | 90850.03125 | 109220.0313 | 119058.7813 | 141481.5 | 86262.21094 | 116833.2578 | 93865.40625 | 104626 | 114021.1953 | 137155.1563 | 133936.1563 | 169814.875 | 107514.7656 | 82423.74219 | 97211.85156 | 62728.375 | 108583.625 | 160864.9531 | 132840.4219 | 84047.57031 | 91509.25 | 73907.16406 | 97478.33594 | 91013.89844 | 93970.875 | 99647.22656 | 80234.9375 | 87042.95313 | 115808.7344 |
| P05109 | 5242.860352 | 37956.96875 | 0 | 12938.97852 | 4193.879395 | 4646.217285 | 15084.84766 | 10547.41602 | 4760.493652 | 0 | 2041.780884 | 6131.286621 | 3145.5271 | 0 | 3191.721924 | 3262.290527 | 5915.380859 | 0 | 3507.871826 | 6750.77002 | 0 | 4861.508789 | 14950.7334 | 0 | 2833.795166 | 6051.537598 | 3049.962158 | 8550.541016 | 4417.658691 | 0 | 0 | 9396.775391 | 4174.199707 | 1588.567383 | 9370.576172 | 0 | 25639.07422 | 7988.943848 | 4568.38916 | 0 | 7204.330566 | 0 | 6413.025391 | 0 | 0 | 9372.896484 | 0 | 0 | 4199.557129 | 3583.808105 | 3402.963867 | 0 | 0 | 4503.883789 | 9129.608398 | 3784.310303 | 14760.77637 | 3309.981201 | 16396.13281 | 5289.669922 | 5856.102539 | 2210.762451 |
| P05154 | 20840.74609 | 14415.75 | 10305.73828 | 12024.4082 | 12089.67578 | 16805.75781 | 8909.771484 | 26174.75781 | 16913.70508 | 19057.78906 | 19819.49609 | 18561.16797 | 22089.47266 | 16611.34375 | 20584.21094 | 14248.12109 | 28673.89844 | 18788.22852 | 21068.70313 | 35654.87109 | 11362.46484 | 19154.76172 | 27417.91016 | 14014.79297 | 18872.8457 | 29594.24609 | 17838.58594 | 18992.66992 | 16774.18945 | 16867.98242 | 13406.65918 | 18413.40625 | 13427.13672 | 18634.45313 | 12870.50195 | 13480.13477 | 19591.86328 | 25068.95508 | 16678.35938 | 20745.46484 | 18054.15234 | 20041.02734 | 19491.16797 | 18400.58789 | 21890.32813 | 22294.31445 | 13018.97266 | 17925.7168 | 18583.36523 | 19525.66797 | 22828.58203 | 30156.30859 | 27166.45508 | 16815.95898 | 26896.55273 | 24248.18945 | 20798.11328 | 20572.57422 | 17634.82617 | 15938.74316 | 19719.75781 | 4535.618164 |
| P05155 | 612744.9375 | 613412.75 | 263443.0313 | 348286.8125 | 255287.0938 | 353905.8125 | 374587.5 | 517197.375 | 266566.5625 | 354684.3438 | 325922.8438 | 303963.4063 | 281147.3125 | 298432.25 | 319230.5625 | 299389.3125 | 312090.6563 | 291767.7188 | 417674.3125 | 294370.8438 | 323088.0625 | 275486.375 | 315464.4063 | 233653 | 252883.9844 | 268372.5625 | 280998.4688 | 340029.125 | 279952.4688 | 515981.1875 | 252392.6719 | 358380.1875 | 305928.2188 | 498309.5625 | 269874.3125 | 253614.3125 | 321312.4375 | 347662.0313 | 242559.8438 | 480317.0938 | 302088.375 | 241508.875 | 345633.6875 | 229926.5469 | 264221.875 | 325825.9375 | 249446.125 | 340667.0625 | 324149.125 | 272269.375 | 283991.1563 | 316932.625 | 471294.7813 | 296425.7813 | 291107.9375 | 322529.3125 | 286214.5625 | 356441.5625 | 402160.0313 | 262073.0625 | 298149.8125 | 263175.9375 |
| P05160 | 31328.26367 | 17121.09961 | 22310.51172 | 33378.17188 | 29840.66016 | 34936.9375 | 30946.68359 | 26384.23828 | 22268.06641 | 23987.44141 | 22770.82617 | 18605.74609 | 22097.96094 | 23686.65625 | 24139.97266 | 22968.36133 | 18527.76172 | 27711.03711 | 28139.86133 | 28156.8125 | 24365.15234 | 18891.97266 | 22931.30469 | 24322.38672 | 32204.05469 | 24256.41406 | 28716.09375 | 25540.03125 | 31187.22656 | 17635.64453 | 23137.14063 | 23578.82227 | 20292.8125 | 20199.5918 | 14404.32031 | 25120.66211 | 21982.34766 | 17713.69336 | 17144.63086 | 18641.07031 | 32551.19531 | 21554.3125 | 31921.75 | 21329.58203 | 29408.5918 | 31238.05469 | 19909.50781 | 24474.12109 | 27654.90234 | 22700.04883 | 27694.91016 | 25782.35547 | 24069.30469 | 23077.66797 | 27695.17773 | 23264.83594 | 23257.98047 | 24399.49219 | 23929.96094 | 26566.91992 | 21828.73633 | 22567.27148 |
| P05543 | 26421.4707 | 31322.33789 | 29261.74805 | 41504.92969 | 16138.89355 | 29069.60156 | 30250.06641 | 28889.21484 | 29524.29297 | 17856.25 | 35526.33594 | 35413.58984 | 19475.83594 | 24189.24805 | 27805.28125 | 34755.76563 | 25347.35938 | 34075.95313 | 28526.95117 | 30104.64648 | 22922.22461 | 32480.75977 | 23423.19922 | 36577.57813 | 30297.86914 | 20431.87305 | 19862.77539 | 28411.48047 | 10651.16797 | 38854.32813 | 28243.81055 | 19281.93359 | 26891.39063 | 44401.67969 | 17409.26367 | 28949.17578 | 25222.96484 | 27341.15625 | 25220.88086 | 46230.86719 | 14924.42578 | 24332.63281 | 26071.28516 | 37815.03125 | 32323.02734 | 30957.36328 | 34543.91797 | 12570.83008 | 26098.17969 | 20698.36719 | 13874.51953 | 18428.77539 | 25350.61328 | 25670.78516 | 9407.666016 | 23940.30469 | 13468.48633 | 28050.89453 | 24657.3457 | 26431.35742 | 34037.81641 | 39753.15625 |
| P05546 | 287014.0625 | 476225.4688 | 456938.9375 | 369782.75 | 644111.375 | 594925.6875 | 722537.75 | 609789.125 | 437803.875 | 615813.375 | 921314 | 481885.1875 | 477390.5625 | 590878.125 | 565878.5 | 713590.5 | 585817.3125 | 496214.9375 | 465130.6875 | 362340.875 | 583730.875 | 425564.9063 | 509046.0938 | 588095.8125 | 457696.6563 | 775905.6875 | 572713.25 | 671338.0625 | 375500.4688 | 389695.1563 | 420178.625 | 483081.0313 | 342925.4063 | 403392 | 728307.375 | 402287.9375 | 530721.5 | 543082.1875 | 422349.375 | 584241.125 | 407960.625 | 281479.4375 | 560817.25 | 475487.75 | 374580.6875 | 509940.75 | 394564.4688 | 676405.375 | 605020.9375 | 390824.4375 | 510354.5625 | 490564.75 | 479625.875 | 530472.25 | 674604.0625 | 440965.1875 | 486971.7813 | 612293.25 | 516470 | 739613.625 | 368377.8438 | 472944 |
| P06312 | 372887.1875 | 354247.1875 | 522618.375 | 239756.875 | 418338 | 379259.0625 | 367979.5 | 515651.6875 | 315985.375 | 293406.5625 | 324923.3438 | 360796.125 | 319908 | 404128.2813 | 295406.7188 | 397426.5 | 610539.9375 | 386147.5 | 309834.6875 | 520368.625 | 560852.4375 | 402156.875 | 315198.9688 | 425406.7188 | 502202.4688 | 385578.0625 | 341281.3438 | 598897.3125 | 349857.9375 | 621543.125 | 554376.6875 | 305082.875 | 258847 | 433629.5625 | 427360.6563 | 413717.6563 | 465213.0938 | 383903.9375 | 457063.6875 | 438532.875 | 311029.4375 | 405951.6875 | 599521.625 | 568941.6875 | 504973.0625 | 335861.1563 | 391103.4063 | 278743.25 | 252267.9688 | 335740.9688 | 660815.0625 | 757368.125 | 836301.375 | 338890.4063 | 473072.625 | 398302.625 | 551612.0625 | 354746.0313 | 387004.9375 | 392914.875 | 473552.0938 | 576323.4375 |
| P06331 | 302977.7813 | 204008.3438 | 213671.9688 | 186483.1563 | 183415.5625 | 207829.5156 | 175498.5156 | 217468.4844 | 196337.6719 | 236792.2969 | 240872.0625 | 1238981.125 | 1626739 | 125594.3047 | 211903.875 | 1981925.875 | 252369.4375 | 2124626.75 | 1728589.25 | 255734.4844 | 311302.0625 | 319197.375 | 239397.6406 | 246860.375 | 428938.5 | 160410.4844 | 203657.0781 | 236470.9375 | 180589.6406 | 309479.6875 | 254685.3438 | 197950.2031 | 250353.5313 | 1397488.25 | 200547.8906 | 309005.125 | 170215.2813 | 2013894.375 | 218806.1406 | 131528.4375 | 2568251.75 | 240076.125 | 348449.2813 | 311344.3438 | 305061.5 | 290782.2813 | 182764.8594 | 144505.2813 | 174446.6094 | 350732.5625 | 298507.5625 | 4132504 | 209210.6719 | 214962.5781 | 235117.5625 | 168789.6563 | 258704.5938 | 252556.5469 | 274608.0313 | 154429.5156 | 284506.4375 | 258106.4531 |
| P06702 | 0 | 17468.15625 | 0 | 0 | 0 | 0 | 0 | 6031.352539 | 0 | 0 | 0 | 7876.259277 | 0 | 0 | 0 | 0 | 0 | 33075.97266 | 0 | 0 | 0 | 0 | 4325.489258 | 0 | 4436.083984 | 0 | 0 | 0 | 0 | 0 | 0 | 0 | 0 | 0 | 0 | 0 | 0 | 0 | 0 | 0 | 0 | 0 | 0 | 0 | 0 | 0 | 0 | 0 | 0 | 2040.431885 | 0 | 0 | 0 | 0 | 0 | 0 | 0 | 0 | 13393.34375 | 0 | 0 | 0 |
| P06727 | 1313856 | 1012128.875 | 867282.0625 | 805126.125 | 806692.625 | 1088827.25 | 1241172.375 | 885929.5 | 1201268.25 | 1072524 | 880797.125 | 628705.25 | 1192526.75 | 1157205.625 | 764339.125 | 899065.625 | 1125675.125 | 897912.25 | 937505.1875 | 990003.625 | 805785.5 | 1052757 | 1356260.125 | 775584.5 | 1184456.375 | 685541 | 547210.125 | 984575.25 | 1183595 | 1046490.563 | 554709.75 | 1385583.75 | 933198.8125 | 925873.25 | 821568.125 | 889307.125 | 818321.125 | 1701645 | 872692.375 | 1010099.063 | 861548.1875 | 1335299.625 | 702279.625 | 1017097 | 868897.875 | 1101830.875 | 866946.5 | 982652.375 | 829509.375 | 1104742.125 | 913441.4375 | 1118687.75 | 750873.75 | 1056522.25 | 1552284.25 | 1277532.125 | 1032234.25 | 1030318.625 | 859539.3125 | 896764.875 | 476698.1875 | 505025.5313 |
| P07357 | 43139.70703 | 56108.11328 | 53343.14063 | 50111.57813 | 48274.36719 | 54602.8125 | 54153.66797 | 46098.10547 | 47948.14063 | 56018.08594 | 50706.625 | 58267.20313 | 56281.94922 | 49096.07031 | 58935.6875 | 64570.28125 | 48545.66016 | 47614.83594 | 65195.27344 | 65251.39453 | 48260.10938 | 24877.97656 | 41161.76953 | 43498.39844 | 53388.54688 | 49661.375 | 56252.16406 | 58297.44531 | 45721.19531 | 48498.14063 | 67278.42188 | 62290.98438 | 62656.48047 | 47737.23438 | 67256.97656 | 49571.80078 | 70226.94531 | 84296.79688 | 42804.72656 | 59191.17188 | 45316.69141 | 47802.18359 | 58122.50781 | 38392.73828 | 60957.24219 | 77286.59375 | 48354.82031 | 55199.10938 | 56095.00781 | 45521.89453 | 46308.58203 | 52634.6875 | 66170.04688 | 50447.89844 | 60445.25 | 53663.69531 | 61426.05469 | 72866.57813 | 75443.82813 | 59199.44531 | 56846.64063 | 55192.91406 |
| P07358 | 27565.03125 | 43820.04688 | 37873.53125 | 32710.15625 | 34136.02734 | 47471.57031 | 45206.66406 | 42217.23438 | 43101.42969 | 55152.39063 | 30375.71094 | 54742.125 | 45514.40625 | 25803.3125 | 31238.88086 | 45417.41797 | 37065.33594 | 35796.73828 | 48508.52734 | 50961.44922 | 33029.45703 | 22385.46484 | 42259.53125 | 34717.16797 | 32288.21094 | 45577.43359 | 42240.47656 | 37405.98828 | 38461.53906 | 32247.64258 | 33158.8125 | 35883.95313 | 48374.74219 | 40400.04688 | 44866.77344 | 40092.76172 | 39002 | 34044.1875 | 33570.83203 | 34933.82813 | 28814.20313 | 43187.60938 | 48337.40625 | 28820.13477 | 32907.60156 | 55608.86719 | 32626.72266 | 35210.16406 | 36290.74219 | 37088.71094 | 30093.40234 | 26742.85742 | 51881.55078 | 41315.92969 | 43389.88281 | 51097.46875 | 42178.84375 | 57044.65625 | 47824.29688 | 31935.32813 | 36176.21094 | 48525.89844 |
| P07360 | 31433.14844 | 40277.33984 | 33506.82813 | 33210.53906 | 38056.44531 | 37614.63281 | 40658.5 | 29251.51953 | 34556.47656 | 33423.63281 | 36581.35938 | 39935.53125 | 38592.02344 | 22648.79688 | 33238.17578 | 38883.48828 | 43295.65625 | 37221.99609 | 51472.01563 | 42935.89063 | 27495.63281 | 17822.19922 | 36008.16406 | 37580.51953 | 36872.02734 | 43723.88281 | 43882.03125 | 48001.53125 | 29156.19922 | 35237.04688 | 31601.67578 | 41184.50781 | 27286.28516 | 18107.48828 | 33098.53516 | 30779.73828 | 46662.79688 | 40092.25391 | 35092.89453 | 46466.23828 | 35423.07813 | 34999.59375 | 45270.3125 | 33032.78125 | 36982.60156 | 60163.42969 | 28136.90234 | 38808.44922 | 37488.00391 | 34086.90234 | 31505.94141 | 47409.51563 | 66347.92188 | 29570.36328 | 59932.09766 | 41237.95313 | 41213.68359 | 75020.39063 | 61073.75 | 35065.39844 | 31385.91016 | 40013.10156 |
| P07437 | 0 | 2120.521729 | 0 | 0 | 0 | 0 | 0 | 0 | 0 | 0 | 0 | 0 | 0 | 0 | 0 | 0 | 0 | 0 | 0 | 0 | 0 | 3651.685791 | 0 | 0 | 0 | 0 | 0 | 0 | 0 | 0 | 0 | 0 | 0 | 0 | 0 | 0 | 0 | 0 | 0 | 0 | 0 | 0 | 0 | 0 | 0 | 0 | 0 | 0 | 0 | 0 | 0 | 0 | 0 | 0 | 0 | 0 | 0 | 0 | 0 | 0 | 0 | 0 |
| P07737 | 6027.759277 | 34457.19922 | 51508.71484 | 18732.55078 | 8449.058594 | 12968.88477 | 11419.09277 | 9725.671875 | 9629.291992 | 9607.986328 | 8450.844727 | 4917.305664 | 19780.23047 | 22500.34961 | 17605.56641 | 13299.66113 | 11745.27344 | 19629.27344 | 9675.84082 | 18694.13477 | 14712.59668 | 13518.74023 | 27921.30859 | 7870.020996 | 16446.81836 | 17348.47852 | 17913.15039 | 2637.579346 | 7441.195313 | 19310.99805 | 14634.14063 | 5839.258789 | 7170.115234 | 24729.33984 | 7677.068359 | 16611.64648 | 15786.7998 | 13456.57617 | 4835.714355 | 4743.964844 | 4400.131348 | 9595.902344 | 15326.33008 | 12028.74609 | 6222.203125 | 7838.399902 | 1891.096924 | 14120.44336 | 1423.34436 | 4566.323242 | 4021.075928 | 14233.25391 | 12393.4707 | 4443.384277 | 15800.2959 | 19837.56836 | 9198.682617 | 7275.291016 | 4088.491211 | 6045.669922 | 2606.457275 | 4168.356445 |
| P07996 | 39601.17969 | 43200.82031 | 29785.83594 | 40953.40625 | 65916.85156 | 19843.30469 | 15648.50391 | 42416.13672 | 21293.71094 | 15359.60156 | 20196.03906 | 8118.855957 | 12336.02148 | 28337.6543 | 21765.94531 | 8300.009766 | 14169.40137 | 4708.720703 | 29574.74219 | 16979.50195 | 26155.07422 | 86838.02344 | 23369.74805 | 15401.48535 | 11019.41895 | 31979.62891 | 30512.58789 | 4958.910645 | 22098.05078 | 16993.69531 | 23200.76953 | 13364.06836 | 15078.2207 | 20202.57031 | 12485.01367 | 154509.9844 | 12511.7002 | 11865.3125 | 35769.125 | 11164.87598 | 45845.61719 | 18587.35742 | 92317.98438 | 63996.19531 | 8702.612305 | 18973.89063 | 16786.64453 | 76006.95313 | 16736.33008 | 21494.96875 | 11162.84863 | 16717.38477 | 12878.47363 | 16792.41016 | 22663.25 | 49069.41406 | 21870.38672 | 7494.642578 | 5011.763672 | 26829.42188 | 13036.30566 | 7002.725586 |
| P08185 | 79127.375 | 95769.46875 | 107862.2969 | 70726.59375 | 94943.45313 | 84185.125 | 120157.3672 | 94734.5 | 103414.2344 | 94350.04688 | 131465.9375 | 101883.4844 | 107601.3672 | 92931.1875 | 99479.82813 | 122395.3906 | 127973.7266 | 129324.8438 | 158261.6406 | 104333.0313 | 100646.9844 | 115447.9063 | 108063.6406 | 123787.125 | 183939.0781 | 84972.05469 | 105569.625 | 153242.5 | 87398.36719 | 143714.0938 | 117892.8047 | 114824.6172 | 48091.54688 | 99669.67188 | 114357.2109 | 96082.60156 | 81578.28125 | 111188.4688 | 85923.09375 | 125782.0156 | 105505.5859 | 89148.32813 | 127009.6016 | 88597.92188 | 80610.99219 | 33011.72656 | 39662.74609 | 34432.03516 | 89097.48438 | 119902.1953 | 109120.9531 | 54978.22266 | 61982.55469 | 71299.11719 | 40251.64453 | 53935.4375 | 74083.20313 | 51475.35938 | 132096.6875 | 122187.5859 | 156207.1563 | 250836.0313 |
| P08514 | 0 | 8042.455566 | 997.6864624 | 4173.163574 | 2074.151855 | 2218.719238 | 0 | 0 | 0 | 0 | 0 | 0 | 1431.664795 | 0 | 263.9187012 | 0 | 0 | 0 | 0 | 0 | 0 | 0 | 1109.03418 | 0 | 0 | 0 | 0 | 0 | 0 | 638.2492065 | 0 | 0 | 0 | 1958.731445 | 0 | 0 | 0 | 0 | 0 | 0 | 0 | 0 | 0 | 0 | 0 | 0 | 0 | 0 | 0 | 0 | 0 | 0 | 0 | 0 | 0 | 1429.825317 | 0 | 0 | 0 | 0 | 0 | 0 |
| P08519 | 26945.80664 | 72448.25781 | 42949.00781 | 111913.1406 | 447595.3438 | 23825.8457 | 48746.55859 | 17231.81836 | 22672.15625 | 198516.6563 | 16435.49219 | 87152.54688 | 58829.58594 | 25098.29297 | 87335.23438 | 84227.58594 | 45763.27344 | 54796.50781 | 60288.80078 | 36875.51953 | 83026.09375 | 76081.86719 | 45300.60547 | 877594.375 | 262283.5313 | 52015.07031 | 25599.8125 | 38886.48828 | 313465.0938 | 52444.94922 | 89678.25 | 99447.28906 | 39993.1875 | 42231.28125 | 51715.48438 | 285231.625 | 43297.53125 | 54474.64844 | 65283.75 | 31560.91602 | 105003.5313 | 33390.78906 | 88794.79688 | 147100.0938 | 81138.64844 | 193315.0781 | 44517.02344 | 155574.9688 | 52832.74219 | 171992.8438 | 1044629.875 | 57665.28906 | 59506.85938 | 389420.5 | 73060.82031 | 91529.09375 | 68796.55469 | 213695.1406 | 103444.5938 | 92634.32813 | 41408.82813 | 344403.4375 |
| P08571 | 9903.008789 | 11572.69922 | 9498.144531 | 12041.97656 | 7932.996094 | 6081.975586 | 16758.60742 | 13604.0293 | 10513.80078 | 13274.37305 | 8154.922852 | 12408.47461 | 11337.15332 | 10859.00586 | 10417.51563 | 8429.994141 | 12902.16797 | 17149.52148 | 13054.83984 | 13664.69824 | 9416.017578 | 14291.17383 | 7927.181641 | 12078.49121 | 11291.76563 | 4954.191406 | 7519.60791 | 17646.57227 | 9390.107422 | 17490.00781 | 12797.69922 | 6482.90918 | 10728.66992 | 11388.47461 | 10920.02734 | 9879.65332 | 18503.03906 | 11272.21875 | 7216.205566 | 9597.87207 | 6912.791016 | 12694.24414 | 13768.87305 | 9097.767578 | 9250.551758 | 10156.49805 | 12117.33398 | 7331.094238 | 7936.040527 | 10335.83594 | 9953.901367 | 11292.46582 | 10209.42188 | 8623.860352 | 13603.74316 | 9307.258789 | 12128.55273 | 12968.48242 | 16293.9707 | 9027.226563 | 15363.86328 | 9293.500977 |
| P08603 | 1094702 | 1374634 | 1589413.75 | 927953.0625 | 1768325.75 | 1433931.5 | 1573599.75 | 1153540.375 | 1261147.5 | 1284156 | 1639076.5 | 1498346.875 | 1249756 | 1058502.625 | 1461638.625 | 1543584 | 1347081.5 | 1198215.875 | 1634359.75 | 1328941.5 | 1159401.25 | 1046675.125 | 1160958.75 | 1265964 | 1261721.625 | 1475838.75 | 1763307.75 | 1505449.25 | 1373034.25 | 990246.875 | 613396.75 | 1346529.75 | 1196941.25 | 1217297.875 | 1361906 | 993707.5625 | 1627686.25 | 1115382 | 1490971.25 | 1263111.875 | 1292282 | 695375.75 | 1074928 | 1213296.25 | 1254896.625 | 1585545.5 | 1088904.5 | 1459167.25 | 1596968.5 | 958140.3125 | 1131510 | 1101113 | 1501240.25 | 1844005 | 1376077.5 | 954657.3125 | 1411964 | 1285713.5 | 1534583.5 | 1444255 | 1275478.375 | 1346190.25 |
| P08697 | 261676.4688 | 212411.9531 | 202323.5938 | 204518.2188 | 239206.4844 | 206775.8125 | 231227.4063 | 256373.8438 | 188093.2031 | 283805.3125 | 270958.3438 | 240074.125 | 185644.1875 | 198201.2813 | 260923.4375 | 229496.1563 | 240867.25 | 221395.5313 | 286586.6875 | 303685.875 | 258308.5625 | 256008.5313 | 215850.9688 | 210380.9688 | 192213.0781 | 216965.75 | 223374.3281 | 228025.0156 | 205843.3438 | 240023.4531 | 239830.5938 | 198444.2188 | 189779.625 | 231235.75 | 218417.8281 | 200591.0313 | 212618.4063 | 225950.5938 | 201389.625 | 191920.0938 | 181298.7188 | 236744.8281 | 233809.3438 | 198089.1563 | 191043.5625 | 166607.6094 | 194922.2969 | 282778.9063 | 211275.7188 | 166915.5781 | 227160.3438 | 132791 | 249026.4844 | 195787.625 | 262211.5313 | 272108 | 284735.7813 | 222966.1563 | 295857.875 | 203116.6406 | 221773.5156 | 238856.8438 |
| P08779 | 22899.38867 | 15795.7373 | 22179.66406 | 105093.7734 | 62239.53125 | 22332.60156 | 16707.75977 | 48625.07813 | 225961.6406 | 120666.7188 | 54135.73828 | 108221.5 | 192222.5313 | 159708.75 | 115949.25 | 18424.79102 | 19505.65625 | 109606.4063 | 109637.9844 | 111742.5078 | 152437.5625 | 17708.92773 | 208976.9688 | 40716.16797 | 29671.92578 | 18880.40234 | 78583.50781 | 262661.8125 | 19140.59375 | 90208.75781 | 19209.46484 | 280568.3438 | 205291.8594 | 24945.1875 | 244436.8438 | 118735.9453 | 252906.7344 | 66619.05469 | 184687.7656 | 99246.375 | 16234.18066 | 178846.7188 | 23656.16406 | 89247.40625 | 208030.0156 | 47928.00781 | 30456.16602 | 12717.16406 | 16840.68359 | 86538.30469 | 173895.8594 | 119874.3359 | 88278.73438 | 100362.5469 | 85882.52344 | 121723.7813 | 81031.1875 | 145621.625 | 175028.1563 | 69641.36719 | 232647.2031 | 14955.49219 |
| P09172 | 0 | 0 | 3660.99292 | 0 | 0 | 0 | 6294.534668 | 2909.706787 | 0 | 11702.60156 | 0 | 3900.102539 | 0 | 6615.715332 | 0 | 1707.149902 | 5455.050293 | 0 | 2952.281006 | 3899.984131 | 3891.115967 | 2133.680664 | 1181.501465 | 3269.453857 | 7630.955078 | 0 | 3105.026367 | 3446.821289 | 5445.437012 | 1316.142944 | 8568.808594 | 1652.244263 | 3629.250977 | 0 | 4907.409668 | 2274.214111 | 0 | 0 | 0 | 0 | 0 | 0 | 6443.171875 | 3154.597656 | 3567.202637 | 0 | 0 | 2954.581299 | 0 | 0 | 3721.644531 | 2391.001221 | 1734.475708 | 0 | 4235.837891 | 1626.008179 | 4834.886719 | 0 | 0 | 2486.333252 | 4250.097168 | 2802.376709 |
| P09486 | 0 | 0 | 0 | 0 | 6530.8125 | 0 | 0 | 0 | 0 | 0 | 0 | 0 | 0 | 0 | 0 | 0 | 0 | 0 | 0 | 0 | 0 | 0 | 2960.182373 | 0 | 0 | 3284.846436 | 0 | 0 | 0 | 1825.438477 | 0 | 0 | 0 | 0 | 0 | 6670.883301 | 0 | 0 | 0 | 0 | 0 | 0 | 0 | 2770.438721 | 0 | 0 | 0 | 0 | 0 | 0 | 0 | 0 | 0 | 0 | 0 | 0 | 0 | 0 | 0 | 0 | 0 | 0 |
| P09871 | 79081.75781 | 96199.42188 | 116362.625 | 94744.21875 | 133440.125 | 72458.92188 | 120270.125 | 76656.96875 | 60514.07422 | 79338.92188 | 74842.55469 | 79463.375 | 138897.8281 | 99253.51563 | 105791.1563 | 83710.10938 | 93783.57813 | 84542.48438 | 84923.44531 | 85109.98438 | 89442.3125 | 100417.2344 | 70620.39063 | 65175.44922 | 73155.46875 | 82351.92188 | 134007.8438 | 110306.1016 | 94304.69531 | 62659.59766 | 99417.21875 | 117288.8672 | 91749.26563 | 121404.6406 | 106180.1094 | 77719.98438 | 137677 | 81551.25 | 113447.7344 | 103234.4609 | 96230.45313 | 101484.9219 | 108621.3438 | 63530.28125 | 130336.2813 | 89396.23438 | 78274.65625 | 101144.0469 | 130748.1484 | 59695.56641 | 94222.16406 | 109246.5313 | 106356.0938 | 92880.0625 | 85427.6875 | 81051.34375 | 81115.86719 | 81518.70313 | 112616.5156 | 100819.6875 | 117444.6016 | 84501.4375 |
| P0C0L4 | 0 | 817.7313843 | 0 | 0 | 61505.79688 | 0 | 506.4406738 | 0 | 0 | 2250.736572 | 0 | 0 | 0 | 0 | 0 | 1348.892822 | 0 | 0 | 0 | 0 | 0 | 0 | 0 | 0 | 0 | 0 | 0 | 84360.41406 | 0 | 0 | 0 | 154144.8906 | 60619.5625 | 1399.863403 | 809.265625 | 0 | 0 | 0 | 0 | 0 | 0 | 0 | 0 | 0 | 0 | 0 | 0 | 0 | 0 | 0 | 0 | 52049.90625 | 0 | 0 | 0 | 0 | 0 | 0 | 0 | 0 | 0 | 51773.05469 |
| P0C0L5 | 1254316.25 | 2669191 | 1849469 | 1758528.25 | 1675305.25 | 2394941 | 1659627.5 | 2026056.25 | 1535968.25 | 3327418.75 | 1609712.375 | 1887403 | 1747204.625 | 1316799.75 | 1618199 | 1275470.5 | 1285472.5 | 1773998.25 | 2710494.25 | 1760729.75 | 1545194.75 | 1702134.5 | 1431337.5 | 1778317 | 1247261 | 1619644.25 | 2814395.25 | 2264964 | 2585608.5 | 965339.125 | 1844001.75 | 1957095.5 | 1853308.375 | 1618505.75 | 2066573.875 | 1158984.25 | 2313837.5 | 1279350.25 | 1713109 | 1337404.875 | 1540905.5 | 1740736.75 | 1379674.25 | 953169.875 | 3350249.5 | 1484869.75 | 1339199.125 | 2603800.5 | 2849455.25 | 958510.75 | 1247603.5 | 2934180.75 | 2181853 | 1418058.5 | 1220048 | 1792065.25 | 2015212.5 | 1882938.25 | 2215282 | 2060735 | 2300739 | 1209167.875 |
| P0CF74 | 0 | 0 | 0 | 10743.89551 | 0 | 0 | 0 | 0 | 7784.590332 | 0 | 0 | 0 | 0 | 0 | 0 | 0 | 0 | 0 | 0 | 0 | 0 | 0 | 0 | 0 | 0 | 0 | 0 | 0 | 0 | 0 | 0 | 0 | 0 | 93455.73438 | 0 | 0 | 0 | 0 | 0 | 0 | 0 | 0 | 0 | 0 | 0 | 0 | 0 | 0 | 0 | 0 | 0 | 0 | 0 | 0 | 0 | 0 | 0 | 0 | 0 | 0 | 0 | 0 |
| P0DJI8 | 27287.30078 | 17702.7832 | 32346.74609 | 22879.39063 | 24313.56445 | 47429.04297 | 26129.92188 | 46040.38672 | 24575.81445 | 46716.23047 | 43212.41406 | 12811.55176 | 22539.05859 | 16194.63867 | 16482.23047 | 52917.60938 | 35718.22656 | 15524.76563 | 43830.58984 | 68064.82813 | 44358.05859 | 32185.92969 | 20312.66797 | 26263.13281 | 20357.66797 | 56436.875 | 15023.51172 | 29175.29297 | 22396.18164 | 64524.77344 | 23504.37305 | 23347.87109 | 15687.48828 | 54264.42578 | 36610.23438 | 41820.21094 | 38396.05078 | 13350.8125 | 22328.98438 | 21158.54688 | 98470.84375 | 28374.33594 | 22672.21094 | 16302.92969 | 28001.35352 | 30539.06641 | 46323.22656 | 25074.94336 | 40591.64453 | 10517.6709 | 41491.86328 | 7666.69873 | 17857.22266 | 38705.04688 | 14328.88086 | 21591.45703 | 30095.67188 | 19473.33594 | 49193.33984 | 38777.40625 | 24503.73828 | 27265.35547 |
| P0DOY3 | 14531187 | 6782938 | 9719655 | 6819884 | 8405028 | 8410421 | 7331037 | 8907542 | 12505010 | 7047725 | 14627642 | 1267717 | 15691012 | 6481702 | 10379275 | 9851255 | 7056685.5 | 8230898 | 8286721.5 | 8880308 | 19944708 | 7464421.5 | 10129291 | 10067210 | 17863860 | 7373194 | 8297560 | 11747028 | 11982276 | 8314625.5 | 11384958 | 9227207 | 2914380.5 | 8752344 | 7191636.5 | 9584559 | 7794611.5 | 7448479 | 11298508 | 7386602 | 9978140 | 11374528 | 16860838 | 8763866 | 10315183 | 11292973 | 10731058 | 2176456.25 | 8507181 | 8892828 | 8319118.5 | 5570330 | 6510908 | 9760281 | 949232.75 | 5636751.5 | 7419694.5 | 7923261.5 | 6927947 | 6876371 | 9188137 | 2922487.5 |
| P0DP01 | 36236.93359 | 28174.25781 | 37339.9375 | 25070.26953 | 28787.30273 | 23529.41992 | 25742.71875 | 56886.57422 | 38213.91797 | 61956.37891 | 30569.55078 | 0 | 13762.67676 | 13012.30762 | 0 | 28212.37695 | 64979.02734 | 0 | 35117.37891 | 51802.24219 | 16040.3125 | 65642.42969 | 15190.25098 | 36468.35156 | 25692.08789 | 0 | 26120.36719 | 17728.95117 | 21090.76367 | 46303.78125 | 0 | 31356.05078 | 48110.40625 | 24112.49609 | 44201.32031 | 24603.70117 | 30291.55078 | 44574.39063 | 25063.04102 | 11608.27344 | 25719.19336 | 26735.62695 | 43381.06641 | 57281.66406 | 19903.97266 | 13099.24023 | 25939.66992 | 16244.92578 | 8682.119141 | 26502.08398 | 16972.20898 | 0 | 0 | 9883.600586 | 42725.59375 | 52023.79688 | 14724.86816 | 24517.83008 | 24037.77344 | 12455.06152 | 0 | 84031.08594 |
| P0DP02 | 289101.8125 | 207417.7188 | 498261.5625 | 270867.125 | 230646.625 | 313204.5 | 246280.3125 | 416548.9063 | 396671.2813 | 265835.9688 | 370230.5313 | 231515.5313 | 146086 | 303515.9688 | 381761.5 | 698163.8125 | 167914.6719 | 610420.5625 | 360377.2188 | 389924.2188 | 468308.7188 | 332256.1875 | 202395 | 442539.8125 | 206853.9063 | 267415.3125 | 280112.1563 | 257409.9219 | 379049.5313 | 227643.9219 | 339775.7813 | 390138.2813 | 205609.2031 | 186003.6406 | 18349.38281 | 298420.9375 | 263317.4063 | 343474.9063 | 52446.28906 | 320479.9063 | 262881.9063 | 680888.8125 | 401881.4375 | 16364.07422 | 358170.5 | 236565.75 | 468722.9063 | 343567.875 | 175103.125 | 25767.55469 | 147602.8281 | 226699.8594 | 224164.2344 | 282369.75 | 201957.9531 | 213145.7344 | 2747855 | 336177.8438 | 159613.2188 | 299992.0313 | 468410.8438 | 280547.6875 |
| P0DPH7 | 0 | 4131.583496 | 0 | 0 | 0 | 0 | 0 | 0 | 0 | 0 | 0 | 0 | 0 | 0 | 0 | 0 | 0 | 0 | 0 | 0 | 0 | 0 | 0 | 0 | 0 | 5331.560059 | 0 | 0 | 0 | 0 | 0 | 0 | 0 | 0 | 0 | 0 | 0 | 0 | 0 | 0 | 0 | 0 | 0 | 0 | 0 | 8952.682617 | 0 | 0 | 0 | 0 | 0 | 0 | 0 | 0 | 0 | 0 | 0 | 0 | 0 | 0 | 0 | 0 |
| P0DTE1 | 12580.01074 | 10238.15234 | 22783.76367 | 0 | 22601.36133 | 13801.12402 | 18729.6875 | 16506.37109 | 15432.64355 | 39135.62109 | 25208.67188 | 27063.23633 | 18456.5625 | 32397.38281 | 0 | 23269.53516 | 12201.12305 | 0 | 10650.30957 | 21441.15234 | 25571.45898 | 12488.5293 | 16207.61523 | 0 | 17710.73242 | 19435.24414 | 17687.28125 | 32058.39453 | 18003.71484 | 14922.07617 | 17191.17383 | 15740.03711 | 12081.75488 | 451273.625 | 13629.74902 | 278473 | 16593.60156 | 29843.54883 | 21169.97266 | 8745.922852 | 13948.07031 | 12775.89453 | 30209.77344 | 13403.79297 | 17216.07227 | 14285.08398 | 25545.61328 | 7599.453613 | 6889.919434 | 16975.76758 | 12224.73828 | 13117.55371 | 29745.94531 | 27822.03906 | 22964.43164 | 2706.554688 | 18530.68359 | 19751.1875 | 18082.79492 | 10884.50977 | 15770.20605 | 17792.66406 |
| P10643 | 91169.28125 | 95739.57813 | 91645.875 | 93734.20313 | 82290.0625 | 82706.42188 | 96924.40625 | 111183.5156 | 91098.6875 | 86685.46875 | 117171.7813 | 81429.51563 | 76826.28906 | 58947.50781 | 92645.64063 | 90356.74219 | 131043.9063 | 128085.1563 | 91619.69531 | 102486.5313 | 78876.78125 | 102468.3203 | 60927.3125 | 91823.01563 | 104194.4531 | 31917.07227 | 81475.51563 | 101316.5313 | 102139.2109 | 132867.5156 | 98792.53125 | 88907.23438 | 107007.6484 | 102985.7656 | 94433.48438 | 67974.1875 | 96656.0625 | 106303.4531 | 103855.1406 | 100388.5078 | 83515.49219 | 78055.53125 | 133560.2188 | 108059.4219 | 63891.07031 | 98425.57813 | 88597.04688 | 54511.96094 | 105972.0313 | 98574.82813 | 102574.7188 | 90144.59375 | 85809.6875 | 89506.45313 | 128931.8594 | 83126.53906 | 103879.1953 | 94703.125 | 102799.5625 | 100497.6797 | 94715.94531 | 122260.2031 |
| P10909 | 762530.625 | 743021.375 | 716286.875 | 1204894.25 | 843864.75 | 779918.75 | 761035.9375 | 1161903.5 | 1117344.75 | 832579.875 | 1055230.625 | 937746.0625 | 911442.4375 | 821448.6875 | 984576.25 | 1027302.063 | 772704.75 | 684147 | 1188316.375 | 807547.75 | 699652.375 | 1014323 | 1073586 | 713954.125 | 1006026.688 | 1024906.25 | 935161.375 | 1040733.5 | 673743 | 894039.375 | 925270.875 | 892073.3125 | 903520.5625 | 901634.5 | 947243.0625 | 945578.5625 | 1493081.75 | 924178 | 1209361.625 | 1024843.063 | 1044010.625 | 1385934.125 | 1071155.75 | 905335.375 | 898288.125 | 1130847.75 | 886163.875 | 1081652 | 1163178.75 | 661112.5625 | 853320.75 | 978471.6875 | 1127726.5 | 821593.125 | 1451142 | 855234.4375 | 747502.125 | 951084.5625 | 1097700.125 | 993216.125 | 1126519.75 | 1100858.5 |
| P11021 | 371066.6875 | 7536.844238 | 466875.375 | 156444.9219 | 108688.1797 | 4037.340332 | 750543.875 | 0 | 0 | 6787.515625 | 0 | 0 | 338.6798401 | 0 | 31405.95117 | 0 | 33511.32422 | 485173.1875 | 5982.214844 | 0 | 871677.875 | 0 | 135514.9219 | 6303.942383 | 1615026.5 | 14624.88184 | 0 | 106451.8984 | 0 | 1149402.75 | 22698.38281 | 774875.5625 | 43351.05859 | 234541.875 | 84407.94531 | 401593.8438 | 10192.74902 | 5318.249512 | 93098.52344 | 23491.45117 | 31036.28711 | 8638.509766 | 0 | 0 | 790435.375 | 168272.7031 | 61811.33203 | 18849.92578 | 880729.125 | 254505.0781 | 35662.26953 | 767146.9375 | 242201.8125 | 0 | 112587.8438 | 0 | 136482.2656 | 475123.6563 | 866954.4375 | 119876.6406 | 108621.875 | 0 |
| P11226 | 252494.7813 | 107684.9688 | 252321.6875 | 112281.1406 | 41132.5625 | 79178.14063 | 112629.9297 | 93794.08594 | 62624.23828 | 20441.25391 | 130864.0703 | 4843.486328 | 116664.2656 | 165973.8594 | 139593.2813 | 108092.3516 | 158303.1875 | 102195.75 | 72820.17969 | 55431.37891 | 0 | 134054.1406 | 73242.32031 | 0 | 119566.5547 | 84272.53906 | 55626.15234 | 0 | 0 | 154944.5313 | 134284.2344 | 89551.03125 | 160908.8281 | 63780.01172 | 0 | 104560.0781 | 185721.0938 | 21044.71875 | 101256.3906 | 0 | 70671.15625 | 111182.0078 | 99065.05469 | 101739.6641 | 130206.8828 | 149281.2188 | 88753.25781 | 98287.91406 | 54070.55078 | 0 | 102798.0391 | 114369.3203 | 91796.48438 | 93009.58594 | 93929.82031 | 125166.2422 | 57755.89844 | 78177.79688 | 104369.5156 | 82149.25 | 0 | 3919.877686 |
| P11597 | 6302.265625 | 2782.868408 | 4628.166992 | 5819.876465 | 6356.601563 | 5147.770508 | 7475.865234 | 6429.916016 | 6587.486816 | 4229.455078 | 10686.13086 | 4525.335449 | 6454.509277 | 3113.739014 | 4915.568359 | 933.3001709 | 15040.86328 | 0 | 7150.126953 | 9480.995117 | 6443.275391 | 7512.743164 | 5030.893555 | 11693.4082 | 1691.729248 | 3361.62207 | 6309.447266 | 7264.134277 | 5676.980469 | 7248.300781 | 3803.201904 | 7743.418457 | 8414.685547 | 6335.88623 | 11267.83105 | 11226.64258 | 4740.15332 | 7047.560547 | 4850.723145 | 13138.78027 | 9076.224609 | 5309.871094 | 11951.23242 | 14676.83594 | 12554.84082 | 3281.92334 | 11471.1543 | 6475.971191 | 5467.637207 | 10400.5332 | 7879.210938 | 9305.598633 | 8587.475586 | 8535.308594 | 7570.150391 | 10306.1543 | 6990.432617 | 4711.851563 | 3748.499023 | 2955.927734 | 6348.554199 | 3077.570313 |
| P13473 | 13501.66309 | 6054.713379 | 11384.50977 | 5222.260254 | 9556.241211 | 4377.346191 | 4543.879395 | 4268.957031 | 7712.848633 | 5202.888672 | 27916.49805 | 7593.469727 | 6892.698242 | 7151.483398 | 7261.09668 | 5577.291504 | 9847.258789 | 9804.515625 | 10541.36328 | 9816.858398 | 7948.895508 | 8192.261719 | 9255.660156 | 8709.489258 | 8475.021484 | 8591.34375 | 12028.89551 | 11696.3916 | 11925.64746 | 7616.052246 | 9931.267578 | 10058.6748 | 6830.674805 | 11849.73926 | 5959.192383 | 5510.51709 | 9807.628906 | 5521.364746 | 7922.652832 | 16978.08984 | 9338.009766 | 9855.65332 | 11478.94531 | 6743.89209 | 10474.48926 | 8624.386719 | 8829.379883 | 13447.01758 | 10958.55859 | 10821.72754 | 7744.002441 | 10419.52734 | 15412.98828 | 8611.97168 | 9381.878906 | 9553.467773 | 6020.224609 | 16900.59375 | 10480.32324 | 7716.884277 | 7443.952637 | 6658.312012 |
| P13645 | 78306.21875 | 34024.3125 | 43102.57031 | 45308.20703 | 52714.13281 | 65866.65625 | 64171.30469 | 49891.82813 | 60160.17188 | 57300.89063 | 34133.42578 | 52852.22266 | 35836.35156 | 37632.85938 | 41943.16016 | 48413.55859 | 70138.10156 | 65923.01563 | 25773.92773 | 35880.38672 | 55842.77344 | 50582.54297 | 51652.09375 | 60068.75781 | 22235.32813 | 48487.67188 | 30394.48438 | 26044.80078 | 87101.83594 | 43207.75 | 60867.55469 | 54249.4375 | 71866.94531 | 63296.39844 | 49986.24609 | 46874.16406 | 66606.65625 | 48892.69531 | 42776.99219 | 29437.76563 | 40556.83594 | 41703.05078 | 77351.17188 | 36195.80859 | 34047.90234 | 79391.91406 | 37758.33984 | 37915.375 | 32748.13477 | 47868.98047 | 49429.60156 | 41554.71484 | 33551.89844 | 44378.60938 | 64340.125 | 70296.85156 | 50299 | 45655.67188 | 38543.95313 | 25210.59766 | 115257.0938 | 37968.64844 |
| P13647 | 28060.05859 | 22499.20313 | 29751.51367 | 26754.7832 | 50165.03125 | 28692.56836 | 30621.36719 | 38139.36719 | 23202.20313 | 24544.40625 | 18540.94922 | 20471.64453 | 26991.73242 | 18725.09766 | 22032.04297 | 22084.23438 | 30505.66016 | 29095.27734 | 12569.60547 | 15666.78711 | 27880.74414 | 20618.62109 | 16009.9375 | 27621.41211 | 28495.68555 | 21456.38867 | 9627.22168 | 18747.98828 | 20754.66016 | 28882.81836 | 28016.27734 | 23904.40234 | 20764.05469 | 22893.53125 | 11936.07129 | 24727.8418 | 26754.86328 | 18280.95117 | 18263.06055 | 24831.49414 | 28005.37695 | 17192.53125 | 35291.8125 | 23212.48828 | 26938.16211 | 42510.80078 | 28065.08008 | 26697.58594 | 23217.3418 | 21421.82813 | 34754.92969 | 37520.98828 | 40259.26563 | 14133.23535 | 25706.71094 | 32145.39063 | 19453.47461 | 23701.82813 | 25077.90039 | 24960.77539 | 31902.72266 | 21962.52344 |
| P13671 | 44473.98438 | 54926.00391 | 51021.03125 | 53710.49219 | 37533.12891 | 53794.85547 | 70712.38281 | 44863.05469 | 44753.375 | 62701.21875 | 55905.16406 | 50691.33203 | 67410.16406 | 47170.51563 | 59003.64063 | 59767.72266 | 56135.55078 | 61400.30859 | 59523.61719 | 49997.26172 | 48116.97656 | 47768.80469 | 40636.28516 | 59721.23828 | 43586.15234 | 42388.66406 | 52015.67188 | 63170.58594 | 46502.32031 | 57847.26953 | 65618.40625 | 60220.17188 | 50011.13281 | 52412.51563 | 62811.16016 | 54175.57031 | 57662.625 | 45013.90625 | 40252.91406 | 79696.65625 | 47099.66406 | 40180.16797 | 61372.66406 | 49143.98438 | 50211 | 62208.03125 | 55675.27344 | 67753.71875 | 48195.93359 | 37437.77344 | 45063.98828 | 61508.61328 | 60803.14844 | 41296.11719 | 62301.65625 | 43595.07813 | 46017.07031 | 61277.79688 | 70611.73438 | 60115.27344 | 69796.73438 | 65000.44531 |
| P13796 | 2008958 | 4835619.5 | 4181.637695 | 2142.611572 | 1431.118896 | 18350.51953 | 11294542 | 5124863 | 1174.486206 | 3760.875977 | 2061.597412 | 2745.283447 | 5202855 | 2658.159424 | 2547.753662 | 9881933 | 5776.644043 | 5078964.5 | 3370.86499 | 8152.134277 | 6598171.5 | 25123.25195 | 5813722 | 2521.827637 | 9207.833984 | 4696.95459 | 4347.28125 | 5124.055664 | 1279.710449 | 4730.298828 | 1634.712036 | 12733.67578 | 3324.283691 | 5042.374023 | 2096.226563 | 4335933.5 | 10784364 | 13260.72754 | 1178.959473 | 6868.103516 | 4147.536133 | 11393.91895 | 8200890.5 | 7209.122559 | 7288.902832 | 11010.02734 | 4394.408691 | 3292.874512 | 7160843 | 2143.013672 | 1855.924805 | 5047309.5 | 2908623.25 | 2552.880371 | 1793.226318 | 8159.897949 | 12746.12988 | 10104.98926 | 5703.550293 | 7331840.5 | 5055.349609 | 32523.46094 |
| P14151 | 33554.09766 | 29281.96289 | 30526.06836 | 28100.55078 | 27451.03125 | 28900.40234 | 27905.46875 | 39933.07031 | 26743.89648 | 29752.92383 | 29486.45703 | 37773.02344 | 21536.36719 | 23892.45703 | 22715.43359 | 27704.16016 | 21941.0293 | 30392.04492 | 31906.29297 | 39497.92578 | 33586.47656 | 32178.0332 | 26079.96875 | 40987.42578 | 24556.42969 | 18437.36133 | 24762.16016 | 33909.96094 | 36075.98438 | 26359.21875 | 39042.70703 | 40034.83594 | 17782.41016 | 32082.02344 | 40606.80859 | 30361.35156 | 36286.10938 | 26446.55469 | 29013.79297 | 37215.94922 | 33674.13281 | 37746.85938 | 34950.44141 | 32588.35156 | 35202.625 | 34570.01563 | 32954.53906 | 30392.92773 | 29021.44922 | 23520.15234 | 35243.46875 | 23509.17188 | 36553.25 | 29461.86523 | 26506.39063 | 32785.52344 | 35813.55859 | 35850.37891 | 28650.27344 | 28166.45508 | 31002.28125 | 25592.91797 |
| P15144 | 0 | 24256.40625 | 0 | 0 | 0 | 0 | 0 | 0 | 0 | 0 | 12223.17871 | 8712.349609 | 0 | 0 | 0 | 0 | 0 | 0 | 4103.476563 | 20175.62695 | 0 | 4269.217773 | 0 | 20612.22266 | 22140.93555 | 1276.217041 | 0 | 0 | 0 | 0 | 0 | 0 | 1967.151001 | 34052.96094 | 3530.86084 | 0 | 0 | 0 | 0 | 0 | 0 | 8634.90625 | 20739.1543 | 0 | 23649.13672 | 22214.00391 | 0 | 0 | 3390.434326 | 0 | 0 | 0 | 13857.19336 | 0 | 0 | 2735.751709 | 31609.81055 | 0 | 28970.52539 | 0 | 0 | 0 |
| P15169 | 30258.03516 | 38622.95313 | 25306.25 | 22808.60938 | 37548.23828 | 30374.03906 | 19591.63672 | 30421.29297 | 20689.22852 | 21562.79688 | 15842.73242 | 34373.32422 | 25258.94922 | 32941.00391 | 24795.08984 | 22418.52734 | 17508.00781 | 26746.27539 | 14942.42578 | 21018.67773 | 17044.26172 | 13794.19922 | 23342.25586 | 38024.03906 | 15472.44238 | 16826.96875 | 19254.85742 | 16452.96875 | 21144.24609 | 25776.51953 | 28202.91406 | 27858.03125 | 26084.01563 | 27672.09766 | 26516.43359 | 26358.07813 | 22498.60742 | 31153.13672 | 30230.98438 | 11617.12598 | 15521.43359 | 22249.19531 | 17002.89844 | 12943.44434 | 21000.75391 | 14765.33594 | 11583.55176 | 19755.09375 | 31718.15234 | 14331.09961 | 16880.65039 | 26828.0625 | 25377.06445 | 24668.5332 | 20122.57227 | 16184.42578 | 20619.85938 | 14805.49414 | 20994.02539 | 16580.26953 | 43607.125 | 24083 |
| P15814 | 15192679 | 11067767 | 11185033 | 8656815 | 9476000 | 9494464 | 836189.9375 | 11909125 | 12921403 | 9027945 | 18957306 | 7652467 | 1362123.25 | 7183049.5 | 12030579 | 9593459 | 9771970 | 11168271 | 9912353 | 12124878 | 12550986 | 9386788 | 11384488 | 11759058 | 12683294 | 7712863.5 | 9771322 | 7808652 | 13372235 | 11169894 | 11292171 | 2455573.5 | 8982157 | 10198284 | 10676020 | 9352268 | 11024011 | 8704210 | 11110067 | 11216232 | 12926434 | 11500146 | 13867903 | 10718940 | 11296639 | 10241794 | 8470982 | 7240984 | 9140532 | 3158242.25 | 12122331 | 17627920 | 13229152 | 10514500 | 5208013.5 | 7792430 | 9440841 | 11720680 | 8201688.5 | 8944484 | 11775292 | 16971446 |
| P16930 | 0 | 0 | 0 | 0 | 0 | 0 | 0 | 0 | 0 | 0 | 0 | 0 | 0 | 0 | 0 | 0 | 0 | 0 | 0 | 2847.031738 | 0 | 0 | 3652.277344 | 0 | 1354.053589 | 0 | 0 | 4631.76416 | 0 | 7077.30127 | 0 | 0 | 0 | 0 | 0 | 0 | 176.0969696 | 0 | 0 | 0 | 0 | 0 | 0 | 0 | 0 | 907.6364746 | 288.2408447 | 0 | 0 | 0 | 0 | 220.2740021 | 6067.381836 | 0 | 0 | 2465.041504 | 0 | 1675.242065 | 0 | 2027.702393 | 0 | 0 |
| P18065 | 0 | 0 | 0 | 0 | 0 | 0 | 0 | 0 | 0 | 0 | 0 | 0 | 16716.58398 | 0 | 0 | 0 | 0 | 0 | 0 | 0 | 0 | 0 | 0 | 0 | 0 | 0 | 0 | 0 | 0 | 0 | 0 | 0 | 9695.979492 | 0 | 0 | 0 | 0 | 0 | 0 | 0 | 0 | 0 | 10249.28711 | 6289.324219 | 4145.376953 | 0 | 0 | 0 | 0 | 7501.508301 | 0 | 0 | 0 | 0 | 0 | 0 | 0 | 0 | 0 | 0 | 0 | 0 |
| P18206 | 0 | 18634.80469 | 10869.46289 | 5714.081543 | 6322.578125 | 2118.39502 | 5492.200684 | 0 | 4873.441895 | 0 | 9877.606445 | 0 | 8386.250977 | 12745.2832 | 9347.870117 | 504.3096313 | 0 | 0 | 10029.98438 | 7128.789063 | 844.4700317 | 2456.098145 | 15431.91797 | 0 | 8595.666016 | 12279.86523 | 15178.7627 | 0 | 9035.918945 | 8772.046875 | 21947.66406 | 7140.210449 | 7092.075684 | 7107.430176 | 5603.123535 | 4308.57666 | 0 | 5423.842285 | 0 | 0 | 0 | 7170.623047 | 24934.40039 | 7203.207031 | 0 | 15546.4873 | 0 | 8742.893555 | 0 | 0 | 0 | 0 | 0 | 8334.503906 | 2068.642578 | 5795.700195 | 592.6098633 | 9967.412109 | 0 | 0 | 0 | 0 |
| P18428 | 9435.550781 | 10129.71289 | 16354.32813 | 23146.63086 | 12130.14453 | 15461.02344 | 26223.3418 | 13950.39258 | 17395.61719 | 13540.77832 | 14468.66699 | 13749.83594 | 7779.152832 | 9132.652344 | 14876.65918 | 17906.67188 | 17324.78125 | 14447.63281 | 26108.89258 | 13688.91211 | 11966.47656 | 8617.6875 | 14738.13477 | 11789.59863 | 18161.78906 | 17198.05664 | 15490.83008 | 20429.62109 | 15022.03125 | 12341.94043 | 17699.75977 | 15280.10059 | 17190.45313 | 19326.66797 | 20120.14648 | 15885.74805 | 20642.5 | 14656.16602 | 16077.07422 | 15770.8916 | 14731.6543 | 22667.48047 | 20078.62695 | 12054.30371 | 10468.11816 | 28574.01758 | 14787.70898 | 13634.41211 | 11176.42383 | 13038.40918 | 11107.23828 | 22143.89063 | 13794.82227 | 17012.55469 | 13379.11523 | 10738.90625 | 14552.65039 | 12410.88086 | 29715.94531 | 15339.33496 | 18385.64844 | 18924.41602 |
| P19320 | 3402.333984 | 3915.572754 | 2758.470215 | 2267.696777 | 2936.41748 | 4727.477051 | 3621.915771 | 0 | 0 | 0 | 5038.572266 | 5023 | 0 | 0 | 1002.979309 | 5186.760742 | 2709.254395 | 5243.172363 | 3151.373291 | 7147.633789 | 5726.400879 | 4380.188965 | 0 | 0 | 0 | 2084.021484 | 4499.413086 | 2987.161621 | 2242.703857 | 0 | 0 | 3292.4729 | 0 | 889.7014771 | 2392.705566 | 1150.572998 | 3435.395996 | 2284.746338 | 4213.957031 | 0 | 5378.692383 | 1791.023315 | 2660.514404 | 2148.42041 | 4386.303223 | 5620.643555 | 4420.729492 | 1588.167236 | 3505.168945 | 4574.682617 | 9115.592773 | 3767.35498 | 0 | 2490.353271 | 3002.58667 | 0 | 4221.656738 | 3417.216797 | 2559.611572 | 35735.14844 | 6501.936035 | 3721.507324 |
| P19652 | 702467.1875 | 667281.125 | 725783.375 | 837615.625 | 752388.625 | 871391.5 | 946117.625 | 906343.5 | 727692.25 | 1122216 | 1021406.875 | 849965.75 | 514086.9375 | 611872.1875 | 783364.4375 | 649929.0625 | 952125.375 | 638780.1875 | 1071947.75 | 998872.125 | 527930.25 | 872298.375 | 763900.125 | 862648.25 | 638665.8125 | 774860.5625 | 845117.6875 | 960103.5 | 678574.25 | 649310.6875 | 839993.5625 | 789017.0625 | 598460.4375 | 922930.125 | 888513.4375 | 745814.0625 | 1040387.875 | 780453.9375 | 872062.125 | 873749.625 | 807026.5 | 755861.0625 | 899532.875 | 238805.2344 | 539806.5625 | 1054243.875 | 966727.4375 | 1298152 | 629541.875 | 729912.9375 | 437585.0625 | 1363799.375 | 1149712 | 769770.5625 | 998932.125 | 1176512.25 | 1075786.625 | 1017161.875 | 1127489.125 | 960469 | 985669.8125 | 592220.75 |
| P19823 | 1540590.125 | 1688012.25 | 1362164.625 | 1451858.125 | 1567207.75 | 1205027 | 1423923.75 | 1480251.75 | 1071226.75 | 1316557.75 | 1606143.75 | 1494352.25 | 1472023.5 | 1273970 | 1687332.75 | 1556715.5 | 1266921.25 | 1185089.625 | 1542250.25 | 1074271 | 1612666.75 | 1453255.75 | 1092156.875 | 1347458.5 | 1391959.25 | 1195800.875 | 1797666.75 | 1549511.375 | 1497160.5 | 1200570.5 | 1288985.625 | 1356737.75 | 1185186.25 | 1349877.75 | 1580782.25 | 1138524.25 | 1227458 | 1119973.5 | 1238839.625 | 1392734 | 1048663.875 | 1312249.75 | 1194590.625 | 1083937 | 1469749.5 | 857221.5625 | 838017.625 | 1067099.75 | 1151430.5 | 1032258.125 | 848273.6875 | 682629.5 | 968390.75 | 1465707 | 900582.375 | 786884.0625 | 1029684.188 | 819104.4375 | 1481304.5 | 1236478 | 1798647 | 1065298.625 |
| P19827 | 365126 | 412595.375 | 323802.0625 | 386029 | 363338.75 | 295164.9688 | 366395.5 | 420150.125 | 282828.5938 | 302083.9063 | 384616 | 357252.9375 | 315185.8125 | 296837.625 | 400115.875 | 382900.4063 | 381175.375 | 327984.25 | 399484.4063 | 356207.5 | 384482.375 | 286004.625 | 287337.2813 | 330001.5625 | 325918.875 | 281385.6563 | 390273 | 408048.75 | 309599.125 | 281781.0938 | 289307.2188 | 274583.1563 | 288247.125 | 304461 | 378657.5625 | 278596.9688 | 302173.5625 | 262068.3125 | 335633.25 | 354282.5625 | 319143.25 | 323012.375 | 340444.0625 | 286685.4063 | 309299.1563 | 270778.125 | 284156.5938 | 383774.75 | 389106.0938 | 225330.4688 | 304855.9063 | 154145.0781 | 266738.375 | 331731.25 | 266652.0625 | 355942.125 | 339182.2188 | 273145.8438 | 408407.375 | 358625.4375 | 397320.1563 | 281068.75 |
| P20742 | 26992.81836 | 0 | 37820.67969 | 28583.10156 | 2374.80127 | 23868.10156 | 0 | 11387.84863 | 0 | 49439.16406 | 12508.58398 | 17449.47461 | 48632.08203 | 25997.1582 | 24807.92188 | 114556.9531 | 19484.99219 | 43753.51563 | 15140.28711 | 9520.318359 | 38728.48438 | 35340.07813 | 0 | 96695.03125 | 0 | 16684.3125 | 166704.2656 | 14438.21191 | 37888.88281 | 0 | 14131.31934 | 24964.35547 | 31350.30859 | 46078.52344 | 168500.1719 | 43033.22656 | 7526.214355 | 0 | 13192.58789 | 62991.64844 | 72610.75 | 8918.032227 | 75120.1875 | 6798.519531 | 74562.64844 | 69399.57813 | 72231.60156 | 23915.29297 | 68633 | 11625.80664 | 0 | 16117.8125 | 0 | 15765.07031 | 0 | 0 | 38076.35156 | 35743.84766 | 65494.45703 | 34442.43359 | 37488.31641 | 329521.6875 |
| P20851 | 51923.73438 | 38750.21484 | 53196.24609 | 47973.87891 | 69962.51563 | 92664.57031 | 56328.64844 | 87328.05469 | 46619.94141 | 68080.69531 | 66375.89063 | 40443.67188 | 37232.64453 | 34742.81641 | 55713.54297 | 56864.71875 | 62621.79688 | 34550.60156 | 84011.8125 | 52613.71094 | 54371.73047 | 37142.51953 | 22757.76563 | 39161.53125 | 51746.74219 | 41163.96484 | 53105.35938 | 42419.74219 | 65144.60156 | 23420.71289 | 33605.01953 | 48677.08984 | 39076.5 | 32749.28906 | 45830.83203 | 57437.46094 | 45047.50391 | 49872.96875 | 77983.46094 | 37457.30469 | 87427.70313 | 56364.08984 | 76638.59375 | 31444.0625 | 38048.04688 | 46027.07031 | 65465.85938 | 58336.92969 | 90187.76563 | 22388.06641 | 46582.42578 | 45984.77734 | 64019.94531 | 31824.20703 | 29278.33789 | 19576.03516 | 45013.21875 | 43334.40234 | 50151.24609 | 58132.21875 | 61384.80469 | 23427.34375 |
| P21333 | 45835.82813 | 14566.26953 | 0 | 4140.039063 | 10043.67871 | 0 | 0 | 0 | 0 | 0 | 0 | 0 | 0 | 0 | 2160.289307 | 37959.3125 | 0 | 35902.87109 | 3684.412842 | 0 | 0 | 0 | 0 | 0 | 0 | 2424.929688 | 0 | 0 | 8063.948242 | 0 | 0 | 2503.288574 | 0 | 5127.784668 | 12501.48926 | 0 | 0 | 7692.157227 | 0 | 0 | 0 | 0 | 0 | 0 | 0 | 0 | 0 | 0 | 0 | 25746.06445 | 25974.87695 | 0 | 0 | 0 | 44189.09375 | 18526.50977 | 12158.58594 | 0 | 0 | 0 | 0 | 29139.45703 |
| P22792 | 102086.9688 | 121825.7109 | 92284.92188 | 64329.78906 | 113778.7891 | 77067.66406 | 79606.50781 | 92052.29688 | 85083.91406 | 112967.8906 | 136019.2344 | 86917.92188 | 119450.8203 | 122887.5547 | 107563.4063 | 97581.71094 | 100096.5391 | 98233.6875 | 91880.32813 | 81146.35938 | 108014.5469 | 96480.55469 | 45658.99219 | 78751.42188 | 114973.7656 | 96263.80469 | 93559.85938 | 56907.98438 | 109135.2188 | 107612.0234 | 76357.26563 | 94778.60938 | 118455.1875 | 111130.9531 | 113941.1953 | 70034.1875 | 102352.6094 | 51981.82813 | 101565.0391 | 116586.8984 | 96531.20313 | 60559.53125 | 130356.6563 | 70635.01563 | 102531.7344 | 59860.61719 | 79101.14063 | 95505.09375 | 140532.5625 | 52976.23828 | 108558.0469 | 119113.7031 | 143782.1875 | 63577.42969 | 112369.875 | 65883.32813 | 63026.125 | 57823.42969 | 87484.125 | 94450.53906 | 55248.79688 | 84397.95313 |
| P22891 | 5389.139648 | 16732.92578 | 8167.505859 | 7893.005859 | 15653.50098 | 14918.53613 | 15567.11426 | 10723.49023 | 12375.85742 | 10506.86133 | 10465.89551 | 13761.62109 | 6062.820801 | 11587.41016 | 11265.54883 | 6006.864746 | 12312.5752 | 14694.31934 | 12663.84766 | 0 | 9527.629883 | 11906.39844 | 9922.793945 | 17361.35938 | 5331.219727 | 11115.50586 | 9418.045898 | 10172.8418 | 4889.026367 | 9429.321289 | 6279.101563 | 13173.99805 | 10691.46191 | 4401.745605 | 11365.9541 | 8067.831543 | 4473.206543 | 2060.274414 | 12743.0498 | 10588.75781 | 7262.534668 | 14356.55078 | 11673.44629 | 9605.676758 | 6072.756836 | 7908.032227 | 6249.237305 | 9600.851563 | 10105.82715 | 5837.579102 | 8977.480469 | 13064.17578 | 28020.98438 | 12397.60352 | 10739.67676 | 11697.13867 | 13794.39648 | 8346.061523 | 5384.864746 | 9838.6875 | 14563.30664 | 8640.87793 |
| P23083 | 770169.125 | 771793.125 | 676234.0625 | 474329.5 | 1359965.875 | 1159377.875 | 415004.0938 | 50205.89844 | 121887.6953 | 684613.0625 | 706034 | 564495.125 | 558177.75 | 484574.5938 | 557806.375 | 769445.25 | 678279.1875 | 829617.125 | 991369.625 | 1404727.125 | 839424.1875 | 606563.125 | 655381.6875 | 1096679 | 114419.0547 | 36607.12891 | 662917.4375 | 1317540.25 | 691326.125 | 1171364.5 | 56033.42578 | 751705.1875 | 726394.9375 | 986510.8125 | 1491050 | 1216449.125 | 1019390.438 | 898158.625 | 50728.12109 | 924621.3125 | 779351.5 | 788912.375 | 1152233.25 | 105717.0469 | 93356.25 | 919068.625 | 1108092.375 | 613533.1875 | 362788.5313 | 934575.0625 | 637559.0625 | 69626.4375 | 771278.1875 | 585702 | 66565.84375 | 593086.1875 | 1248027.375 | 20516.51953 | 57753.66797 | 549391.375 | 1325537.875 | 1063454.125 |
| P23142 | 50469.68359 | 46355.69531 | 40571.6875 | 42658.42578 | 23184.0625 | 30915.33594 | 22601.48438 | 32782.25391 | 35679.46875 | 44870.79688 | 27428.13672 | 34895.40625 | 36588.33594 | 31519.625 | 26348.22266 | 34011.82813 | 52213.16406 | 38507.25781 | 22069.27734 | 31144.45703 | 36400.89844 | 69400.4375 | 37907.20313 | 28777.5625 | 31742.72461 | 24658.03125 | 41986.28906 | 27786.82031 | 33762.48828 | 44209.61719 | 29832.03906 | 35588.55469 | 45943.29688 | 43116.61719 | 24897.60938 | 24046.53125 | 29915.04688 | 28241.88281 | 27944.64648 | 41300.14063 | 32902.08984 | 36392.51563 | 37254.42578 | 46901.91797 | 33019.35547 | 29764.91602 | 42490.67188 | 23439.80273 | 32249.76953 | 41413.71875 | 32235.98438 | 51333.40625 | 22734.79688 | 25636.39844 | 30529.39844 | 31818.83203 | 33656.58984 | 36520.75 | 21228.25391 | 22174.42578 | 22785.33984 | 17206.19922 |
| P25311 | 332518.6875 | 309742.1563 | 243032.1875 | 370122.625 | 304965.7188 | 292090.1875 | 581191 | 295590.9063 | 321804.8125 | 302802.4375 | 392010.4375 | 332940 | 315236.6563 | 292281.7813 | 352624.0313 | 286725 | 355087 | 383701.5938 | 321598.4375 | 452693.7813 | 265956.6563 | 390712.1875 | 304701.875 | 308176.8125 | 337984.8125 | 317427.5625 | 268148.2188 | 434204.625 | 256702.8125 | 522803.75 | 311577.4688 | 327533.8438 | 279954.8125 | 332976.6563 | 324869.8125 | 231998.0156 | 369832.7188 | 286005.625 | 269810.625 | 397712.625 | 295147.125 | 322201.6563 | 275686.5313 | 297027.2188 | 228781.2188 | 335345.625 | 372171.1875 | 314836.4688 | 284706.4375 | 314050.2188 | 364758.875 | 489227.8438 | 316850.9375 | 363924.8125 | 464734.8125 | 332417.0625 | 335119.0625 | 465814.5 | 442040 | 315025.875 | 240831.1094 | 312749.9375 |
| P26038 | 1366.158569 | 9674.637695 | 1401.684204 | 6296.420898 | 1807.668213 | 2456.72583 | 3003.418945 | 0 | 0 | 0 | 1762.236572 | 0 | 3205.396973 | 4635.193848 | 2416.072021 | 0 | 1452.208984 | 0 | 4492.35498 | 2760.442139 | 1925.676392 | 16787.05664 | 7045.561035 | 0 | 4658.374023 | 8542.229492 | 5127.508301 | 11115.27344 | 7458.105957 | 18867.05664 | 2313.72168 | 2313.808105 | 1375.029663 | 0 | 0 | 2968.78833 | 1253.884766 | 4856.71875 | 1444.834595 | 0 | 0 | 2729.86084 | 2497.209229 | 0 | 0 | 1800.659302 | 0 | 4703.617676 | 0 | 2087.888672 | 1333.827637 | 0 | 3565.848389 | 1265.777832 | 3269.942383 | 7374.742188 | 2736.688477 | 2833.669434 | 0 | 0 | 0 | 18124.92383 |
| P27169 | 197472.0156 | 256109 | 179730.5313 | 257733.0781 | 258044.0313 | 225138.5 | 186450.7656 | 197555.6563 | 231617.7969 | 291942.75 | 174635.9063 | 331666.25 | 330546.1563 | 243516.9063 | 216139.8438 | 195078.6875 | 211171.4688 | 251549.1563 | 172924.3438 | 154306.7813 | 208200.9063 | 317456.9375 | 273648.6875 | 229961.2813 | 205923.1875 | 268928.375 | 171666.4375 | 272379.9063 | 227150 | 312836.4375 | 321466.75 | 301276.9688 | 283471.7188 | 233427.6875 | 309529.3125 | 306173.0938 | 234673.1875 | 272747.8438 | 265769.6875 | 248514.0313 | 182377.1563 | 192107.1094 | 268094 | 238145.6094 | 250743.9688 | 197949.5 | 303045.9375 | 274909.1875 | 337645.5 | 196971.2188 | 195487.6563 | 272037.75 | 243440.4688 | 224331.6406 | 287328.5 | 174496.5156 | 242259.8438 | 238804.1563 | 189735.9219 | 300848.8125 | 270548.875 | 169783.5625 |
| P29622 | 56112.64844 | 85203.90625 | 47771.77344 | 88179.64844 | 61554.5625 | 59056.03906 | 88726.5 | 90972.35938 | 69158.59375 | 63838.58203 | 92984.35156 | 50762.48047 | 79373.79688 | 68755.67188 | 97224.75 | 61829.30469 | 56142.8125 | 76432.17969 | 63239.375 | 69491.59375 | 65213.09766 | 66410.6875 | 55566.85938 | 54109.12109 | 101678.4453 | 56855.78906 | 63424.11719 | 70028.3125 | 56331.33984 | 70264.29688 | 67585.875 | 65707.85938 | 60378.07031 | 44589.70703 | 71928.02344 | 70043.82031 | 64714.82813 | 57915.54688 | 60400.83594 | 107190.0156 | 67738.58594 | 57726.66406 | 92517.59375 | 63337.04688 | 73921.17188 | 88871.6875 | 65564.6875 | 52057.11719 | 71639.4375 | 69973.17969 | 86549.6875 | 121643.7578 | 111809.7266 | 62202.40625 | 78888.67969 | 58859.375 | 60285.10938 | 65413.28906 | 84630.69531 | 80056.00781 | 84859.79688 | 96344.33594 |
| P30041 | 0 | 12723.75488 | 0 | 0 | 16462.82422 | 0 | 0 | 0 | 0 | 0 | 0 | 0 | 0 | 0 | 0 | 3596.337402 | 0 | 0 | 0 | 3294.060059 | 0 | 0 | 6078.677246 | 0 | 0 | 6058.323242 | 0 | 0 | 0 | 8410.479492 | 0 | 0 | 0 | 4164.313477 | 0 | 2386.264648 | 0 | 5421.678711 | 0 | 0 | 1902.841431 | 3621.63623 | 1866.774292 | 0 | 2255.40332 | 2002.284912 | 0 | 0 | 0 | 0 | 4658.280762 | 3122.642334 | 3290.899414 | 0 | 5406.828125 | 6520.972656 | 3179.197754 | 0 | 4592.072754 | 0 | 0 | 0 |
| P31151 | 0 | 0 | 0 | 0 | 0 | 0 | 1761.185059 | 0 | 0 | 0 | 0 | 0 | 0 | 0 | 0 | 0 | 0 | 0 | 0 | 0 | 0 | 0 | 0 | 0 | 0 | 0 | 0 | 0 | 0 | 0 | 0 | 0 | 0 | 1927.128784 | 0 | 0 | 0 | 0 | 0 | 0 | 0 | 0 | 0 | 0 | 0 | 0 | 0 | 0 | 0 | 0 | 0 | 0 | 0 | 0 | 0 | 0 | 0 | 0 | 0 | 0 | 0 | 0 |
| P32119 | 6077.759277 | 36530.88672 | 0 | 4556.147461 | 9678.103516 | 9186.264648 | 15587.9707 | 13141.96484 | 6884.430176 | 5527.462891 | 11817.30273 | 8345.269531 | 5273.224121 | 4084.488525 | 8970.917969 | 6577.205078 | 12622.05469 | 9054.484375 | 5884.212402 | 9814.818359 | 4631.80957 | 8514.003906 | 17091.8418 | 3495.384766 | 10751.69141 | 28157.97461 | 13233.60254 | 29060.78516 | 14692.58203 | 37163.40625 | 9772.241211 | 12491.19629 | 14712.70605 | 16117.32227 | 7853.983398 | 10818.01172 | 33662.64063 | 27982.3125 | 28951.10742 | 25471.63672 | 24735.22461 | 24607.26758 | 23591.26367 | 13515.03809 | 14378.56152 | 12819.80078 | 23256.17383 | 26168.75586 | 21071.97266 | 16752.12109 | 23549.1875 | 30701.98047 | 22946.52148 | 18668.82813 | 26473.14063 | 30943.92773 | 29827.85938 | 16830.19531 | 18605.15625 | 16175.0293 | 12197.10938 | 18590.54297 |
| P33908 | 0 | 0 | 0 | 0 | 0 | 0 | 0 | 802.7120361 | 0 | 0 | 0 | 0 | 81496.67969 | 0 | 0 | 5975.54834 | 0 | 0 | 0 | 0 | 0 | 0 | 0 | 0 | 0 | 0 | 0 | 0 | 0 | 0 | 0 | 0 | 0 | 0 | 0 | 46269.66016 | 0 | 0 | 1067.55249 | 0 | 0 | 0 | 0 | 851.2702026 | 0 | 0 | 0 | 0 | 0 | 0 | 0 | 0 | 0 | 0 | 0 | 0 | 0 | 848.7231445 | 2558.335205 | 2013.452393 | 1023.314758 | 0 |
| P35527 | 171917.5938 | 124135.625 | 154362.6563 | 255909.5313 | 177370.0625 | 131927.7969 | 156408.0313 | 126390.9922 | 154227.25 | 100580.3984 | 143285.6094 | 107882.9141 | 142567.0781 | 150098.7813 | 140896.5156 | 193631.5313 | 257366.4688 | 230316.0313 | 77951.03906 | 34824.96094 | 218633.4375 | 88596.61719 | 101309.0156 | 179757.9063 | 128730.1094 | 141359.3438 | 105560.3516 | 63873.82813 | 196420.1875 | 87170.89063 | 234102.0313 | 157367.4688 | 71211.77344 | 169543.0313 | 131782.5156 | 150893.9219 | 220810.7656 | 77541.16406 | 166685.5313 | 90822.64063 | 86523.625 | 131386.9063 | 91203.71094 | 59940.75 | 125206.3828 | 82419.50781 | 62679.4375 | 60447.38281 | 48127.76953 | 178657.2813 | 80572.5 | 73206.95313 | 86648.19531 | 145261.2031 | 53390.20313 | 92354.125 | 80258.20313 | 54111.85938 | 28220.75391 | 47031.82813 | 208099.4063 | 166937.9063 |
| P35579 | 0 | 0 | 0 | 0 | 0 | 0 | 0 | 0 | 0 | 0 | 0 | 0 | 0 | 0 | 0 | 0 | 0 | 0 | 0 | 0 | 0 | 0 | 0 | 0 | 0 | 0 | 0 | 0 | 0 | 0 | 0 | 0 | 0 | 0 | 0 | 0 | 0 | 0 | 0 | 0 | 0 | 0 | 0 | 0 | 0 | 0 | 0 | 0 | 0 | 0 | 0 | 0 | 0 | 0 | 0 | 0 | 0 | 0 | 0 | 0 | 0 | 0 |
| P35858 | 41061.83203 | 42039.18359 | 48664.39844 | 35681.19531 | 59544.52344 | 46564.64453 | 99056.30469 | 63623.30469 | 36224.5 | 85610.07813 | 73506.39063 | 59912.91016 | 32335.39063 | 52310.75781 | 55748.17969 | 38648.38281 | 46710.65234 | 43195.125 | 58039.61719 | 49641.77344 | 44335.39844 | 47109.14063 | 62272.64844 | 66605.75 | 39767.58594 | 41754.88281 | 58155.26172 | 71769.73438 | 56619.61328 | 43694.06641 | 63216.85547 | 42230.21094 | 42811.73828 | 60192.89063 | 72291.71094 | 58939.90625 | 42716.375 | 96291.17188 | 48043.67578 | 46977.47656 | 26212.01172 | 43750.36719 | 45290.71484 | 54260.76172 | 27926.96094 | 24173.39063 | 58841.15625 | 67579.125 | 45547.17578 | 23845.38477 | 44708.33203 | 59493.32031 | 56230.9375 | 60197.32813 | 69636.17969 | 59582.125 | 52423.99219 | 49798.8125 | 57290.69531 | 49343.79297 | 63292.66797 | 36043.17578 |
| P35908 | 118906.6875 | 76620.8125 | 86268.49219 | 97303.15625 | 94235.15625 | 105418.5313 | 101736.8438 | 68369.03906 | 120503.1172 | 93261.59375 | 53436.08594 | 90283.65625 | 119305.375 | 60986.29688 | 91798.46094 | 74511.77344 | 116215.9453 | 117465.0625 | 66008.39844 | 65345.67188 | 113597.7422 | 90527.94531 | 108613.0781 | 102306.0313 | 78422.05469 | 80828.57813 | 105212.2734 | 59842.46875 | 105484.4219 | 70236.60938 | 120841.4141 | 93884.54688 | 30313.67969 | 86664.28906 | 110795.5547 | 99051.34375 | 142239.2813 | 45566.39844 | 86545.76563 | 48128.42188 | 84947.46094 | 82441.52344 | 98816.21875 | 82281.375 | 92458.98438 | 179768.8906 | 74073.0625 | 67369.17188 | 56271.4375 | 87415.53125 | 101295.7656 | 66317.48438 | 91210.32813 | 77722.25 | 85734.34375 | 116978.8438 | 73369.51563 | 91799.51563 | 42179.00391 | 63618.35938 | 202916.1875 | 74054.57031 |
| P36955 | 100131.7813 | 84950.79688 | 68147.28125 | 96583.125 | 102467.9531 | 85729.625 | 155426.5 | 104966.6563 | 110591.5469 | 88927.4375 | 102613.3594 | 77731.71875 | 88707.32031 | 82531.875 | 76078.625 | 78642.75 | 110322.6875 | 118218.375 | 99445.96875 | 113846.5547 | 90512.04688 | 110877.1797 | 105069.4141 | 87016.61719 | 129396.5234 | 92576.71875 | 69220.98438 | 137084.125 | 81909.30469 | 110401.7344 | 90130.79688 | 88919.94531 | 76341.75 | 120570.8281 | 105439.0156 | 54577.94531 | 109705.4766 | 101344.3125 | 99094.29688 | 112692.6172 | 92570.09375 | 130309.7188 | 75050.64844 | 64623.60156 | 83683.51563 | 118100.3906 | 101551.875 | 98961.54688 | 88515.57813 | 101562.4531 | 106147.5781 | 155694.5313 | 141170.5156 | 129659.0625 | 161847.9375 | 143147.0938 | 113914.2031 | 121441.3594 | 123597.3516 | 93916.96094 | 78117.5 | 71183.04688 |
| P37802 | 0 | 8041.862793 | 0 | 5563.827148 | 2346.130127 | 0 | 2300.233887 | 29529.44922 | 0 | 0 | 0 | 0 | 7203.707031 | 9285.613281 | 6975.285156 | 27240.51172 | 0 | 15289.21777 | 10034.36914 | 5454.810059 | 5519.762207 | 3664.078369 | 14347.74023 | 0 | 8642.333984 | 11825.51172 | 7019.790039 | 0 | 2547.342773 | 7808.676758 | 4567.372559 | 0 | 0 | 7564.198242 | 0 | 6700.500977 | 0 | 18390.38672 | 0 | 0 | 0 | 1976.417847 | 0 | 0 | 0 | 0 | 0 | 17131.42188 | 0 | 0 | 0 | 0 | 0 | 0 | 24021.92578 | 3691.801758 | 36753.81641 | 0 | 0 | 4485.979492 | 0 | 0 |
| P41222 | 16751.52148 | 16832.25781 | 0 | 18943.17578 | 16056.4043 | 13197.74512 | 27696.50391 | 0 | 10175.60059 | 16024.70703 | 19167.61133 | 17279.16406 | 15237.76465 | 0 | 20951.54102 | 0 | 13247.00195 | 19398.38867 | 17912.16797 | 19501.71094 | 0 | 27860.91992 | 13508.85742 | 16298.63574 | 18640.23828 | 12950.32227 | 23450.32422 | 13736.81641 | 13811.18457 | 24387.58594 | 24101.56055 | 0 | 15849.17871 | 25490.35938 | 17768.03906 | 14861.20996 | 20599.08008 | 13930.26074 | 0 | 0 | 13626.73438 | 18553.01367 | 0 | 0 | 19656.27539 | 17241.4043 | 17100.71484 | 0 | 20495.82227 | 15902.2666 | 12938.3291 | 17325.33984 | 16243.18262 | 20897.5625 | 22807.30859 | 17314.47266 | 19270.07031 | 21064.34766 | 16857.83008 | 9577.975586 | 11316.20313 | 0 |
| P43121 | 4424.567383 | 7004.693848 | 6513.89209 | 8404.932617 | 5958.804688 | 5121.719727 | 0 | 4289.063477 | 3628.540771 | 0 | 5747.123535 | 0 | 5000.962402 | 3378.621094 | 0 | 4754.677734 | 6365.924316 | 4839.416504 | 5188.029297 | 5653.074707 | 5447.935547 | 8737.582031 | 4983.181152 | 4357.128906 | 4891.196777 | 0 | 0 | 5530.737793 | 0 | 8380.84668 | 0 | 6157.273438 | 3625.654785 | 4853.623535 | 1441.176392 | 0 | 0 | 0 | 2675.453369 | 7515.474609 | 0 | 4558.984863 | 0 | 5434.311523 | 6455.646973 | 8097.577148 | 0 | 4414.532227 | 5397.352051 | 6954.979492 | 0 | 6568.303223 | 0 | 4198.598145 | 7686.09082 | 2626.835449 | 5354.944824 | 7895.474609 | 5061.945801 | 0 | 0 | 1443.527222 |
| P43251 | 28323.55078 | 35726.34766 | 17289.75977 | 29165.4707 | 26979.71875 | 45684.89844 | 36770.73047 | 40352.72266 | 36740.30469 | 34945.14063 | 31685.47656 | 41975.83984 | 28349.27539 | 16711.05469 | 35584.25781 | 32460.42578 | 52193.28125 | 22174.02734 | 36666.39063 | 36676.13281 | 18319.57227 | 27244.72656 | 39987.49219 | 34686.83984 | 39364.375 | 21880.66016 | 28615.66797 | 51127.00781 | 30814.09375 | 18704.72266 | 16730.14453 | 22409.66602 | 22676.64063 | 53611.95313 | 17346.60742 | 32196.64063 | 34069.64453 | 41034.95703 | 33926.45703 | 27319.30469 | 34005.00781 | 24838.12109 | 26081.19922 | 20895.66797 | 35904.91406 | 26910.375 | 19998.48438 | 27393.30078 | 26574.92578 | 27364.92578 | 31081.26953 | 44517.77344 | 63564.71875 | 29175.28125 | 35443.14844 | 27382.31055 | 28491.24219 | 26670.03516 | 36617.26563 | 26114.88867 | 38992.35938 | 40094.5625 |
| P43652 | 203177.0938 | 229563.5469 | 141432 | 112723.6953 | 199572.5938 | 177004.0938 | 222028.4688 | 167534.875 | 167704.6563 | 147223.9844 | 158784.5938 | 109009.7656 | 155479.1875 | 158119.6875 | 180465.5469 | 155913.2969 | 191297.6719 | 120785.4141 | 109542.7344 | 218266.2813 | 139614.9531 | 135782.3125 | 159314 | 157579.25 | 188428.2031 | 153110.9844 | 200251.0156 | 178935.9219 | 139182.125 | 121468.1641 | 148360.5 | 171198.7188 | 71650.1875 | 178437.0313 | 150601.0938 | 127470.9063 | 167123.2344 | 187892.3281 | 171442.875 | 155149.4063 | 101281.1719 | 211981.5625 | 146957.5469 | 135826.2813 | 123923.5078 | 180228.8281 | 108572.2969 | 129874.1641 | 198142.5156 | 112892.1719 | 103958.5391 | 137406.3438 | 206912.2813 | 134248.8281 | 137010.25 | 139774.6563 | 117427.0234 | 125246.7266 | 128517.4219 | 171071.3438 | 162040.6406 | 107839.7344 |
| P48740 | 15560.4541 | 26197.39063 | 22772.61328 | 20942.75391 | 25715.38867 | 26789.85156 | 23430.55469 | 15246.02441 | 17989.39453 | 23584.00391 | 16645.39648 | 11493.16602 | 28185.87891 | 33375.56641 | 26359.58203 | 19467.38086 | 17433.00781 | 14518.17188 | 13800.75781 | 12925.29297 | 21396.75195 | 11087.89063 | 12838.7041 | 13160.01563 | 26991.75586 | 28729.42383 | 16921.9375 | 6917.275879 | 25918.31836 | 16146.48535 | 14994.39063 | 15342.71777 | 17323.00391 | 18285.16211 | 15637.93652 | 16892.31641 | 26050.875 | 20272.44336 | 27763.98828 | 21412.20898 | 20292.14453 | 17339.58594 | 13772.09473 | 13307.50098 | 16005.60547 | 13509.20996 | 19265.94336 | 29615.50977 | 24457.33984 | 14225.69336 | 18449.5 | 30310.23047 | 23501.33008 | 23188.39063 | 33087.32031 | 14334.18164 | 22280.10938 | 22574.4375 | 18396.45703 | 14969.54199 | 15598.46777 | 26065.11133 |
| P51884 | 116293.5313 | 107882.9375 | 70370.98438 | 104359.0156 | 60119.18359 | 85779.74219 | 105334.1875 | 92009.82813 | 101588.3203 | 91445.98438 | 82862.35938 | 67763.53125 | 94569.85156 | 81128.82813 | 60189.65625 | 94918.51563 | 92536.75781 | 84411.70313 | 73012.59375 | 85580.58594 | 92830.54688 | 146410.6094 | 68858.64063 | 59770.92578 | 113113.0781 | 64688.22656 | 58477.52734 | 84598.65625 | 76229.92188 | 125124.8125 | 53712.96094 | 120727.4219 | 105243.0547 | 114797.5313 | 67725.03125 | 72471.10938 | 82168.84375 | 88777.97656 | 63762.75781 | 87188.66406 | 80284.59375 | 108747.875 | 41325.25 | 77997.60156 | 98296.80469 | 120064.0547 | 71174.82031 | 65249.90234 | 75259.00781 | 98021.53125 | 87480.09375 | 125736.1406 | 99961.22656 | 94084.34375 | 86249.70313 | 100604.3438 | 86152.54688 | 104169.2422 | 90627.90625 | 83965.08594 | 64661.88281 | 58298.71094 |
| P55056 | 116351.8047 | 80864.38281 | 88745.97656 | 106920.9609 | 85537.01563 | 143075.0156 | 133444.1406 | 418382.7188 | 73790.57813 | 255031.3594 | 148785.5625 | 77337.1875 | 343998.9688 | 251064.1406 | 118395.3281 | 42993.49609 | 33103.52344 | 78565.125 | 248919.5938 | 131056.0469 | 105435.5313 | 89199.50781 | 164905.0469 | 76555.53125 | 151940.2031 | 190972.4531 | 109246.0859 | 499263.4063 | 43164.17969 | 43532.36328 | 120802.5781 | 82215.51563 | 129231.4531 | 391589.8125 | 140522.9375 | 185751.1563 | 41794.17969 | 359311.4688 | 149354.4844 | 183422.1875 | 80842.92969 | 69518.00781 | 301184.5 | 37154.29688 | 123175.5625 | 151592.0781 | 192817.7656 | 204714.5 | 291860.9688 | 123056.9297 | 120865.1406 | 128010.25 | 387268.9688 | 131962.2188 | 161186.5313 | 186391.125 | 63052 | 305078.4375 | 369182.0625 | 522799 | 110657.3984 | 63189.52734 |
| P55058 | 17746.09961 | 20519.78711 | 34394.47656 | 29713.22266 | 28204.68359 | 24662.64063 | 12535.87695 | 10645.10547 | 16807.16602 | 20080.28906 | 9712.580078 | 9572.412109 | 30067.01172 | 29052.25 | 19040.79102 | 16156.2168 | 16130.50684 | 11592.4043 | 18482.63086 | 10267.35059 | 27707.13281 | 24620.67969 | 14727.53516 | 15410.33691 | 23848.13281 | 14215.67871 | 10522.97949 | 16454.64063 | 16992.09766 | 22201.99414 | 18305.37891 | 30714.55469 | 27021.19141 | 23553.3457 | 18386.34961 | 26045.64063 | 14924.20117 | 25863.61719 | 26477.54102 | 16485.13672 | 14562.49219 | 25727.41406 | 20581.73242 | 14970.81445 | 21903.13281 | 13344.60742 | 11521.64453 | 13725.77734 | 23456.91797 | 12784.77539 | 8995.4375 | 18361.26172 | 12871.99414 | 21206.38281 | 8406.757813 | 15479.39941 | 14966.9209 | 13610.81641 | 15072.80859 | 17229.60352 | 14928.87305 | 10153.03223 |
| P55103 | 0 | 9869.999023 | 4059.434082 | 4586.726563 | 8764.297852 | 10395.03223 | 0 | 11109.33984 | 0 | 0 | 10147.13379 | 0 | 3301.345459 | 7107.21582 | 7979.446777 | 10049.8418 | 5649.500488 | 5012.458496 | 12483.52051 | 9506.484375 | 5898.86084 | 2110.722168 | 8523.359375 | 7033.362305 | 0 | 7270.179199 | 6712.54834 | 7924.054199 | 0 | 0 | 6687.908691 | 0 | 0 | 11395.04688 | 9165.089844 | 0 | 4991.867676 | 8026.072266 | 3272.311523 | 7769.481934 | 8496.700195 | 3201.189209 | 9121.727539 | 5670.54248 | 8819.824219 | 9285.775391 | 5253.145508 | 8453.160156 | 13609.3457 | 8626.868164 | 4365.284668 | 10244.97656 | 10296.6416 | 0 | 9597.662109 | 9426.174805 | 6635.069336 | 3475.002686 | 8907.479492 | 6569.96875 | 6380.378906 | 0 |
| P59665 | 129714.5781 | 1696835.5 | 65316.48047 | 356407.5625 | 104150.5234 | 84688.84375 | 153586.8281 | 97283.53125 | 68814.01563 | 31630.0918 | 161628.7031 | 126366.1016 | 73528.54688 | 58352.41797 | 56846.94531 | 46638.90625 | 94004.50781 | 70696.5 | 40148.08594 | 157244.25 | 145239.0313 | 128756.7969 | 397330.6875 | 144808.75 | 96501.53906 | 110722.6719 | 110496.1172 | 138898.75 | 88577.25781 | 85550.9375 | 132120.3438 | 365712.0313 | 34960.34766 | 66296.65625 | 367263.0313 | 46948.66016 | 208519.6563 | 362348.4375 | 116239 | 101304.5781 | 147128.0313 | 90252.07031 | 65787.33594 | 114045.3594 | 210204.8125 | 131265.6719 | 122756.3984 | 39373.37891 | 76927.95313 | 75383.84375 | 67313.3125 | 46062.46484 | 52229.1875 | 94124.49219 | 113854.7813 | 49929.90625 | 133545.1094 | 86050.51563 | 114276.0313 | 240545.7813 | 79110.77344 | 67907.3125 |
| P60709 | 21069.64648 | 178374.1563 | 14777.93555 | 80629.73438 | 19044.77344 | 31227.57813 | 27458.63281 | 20994.32031 | 15266.02344 | 28764.42578 | 62228.80469 | 8257.186523 | 41411.27344 | 56068.19531 | 59195.92969 | 20569.08594 | 23097.83203 | 27153.41992 | 45395.46875 | 38581.53125 | 37825.17578 | 24881.37891 | 80604.01563 | 17295.36133 | 35779.39844 | 58868.58984 | 78576.8125 | 10300.26855 | 22252.21875 | 48064.01563 | 41632.52344 | 17492.12109 | 21641.45313 | 80773.30469 | 18810.79688 | 42931.88672 | 28451.82813 | 56243.78906 | 15757.47852 | 12642.72852 | 12215.13672 | 32355.94141 | 33959.875 | 20872.5 | 29068.99219 | 21696.32227 | 14417.75684 | 34974.85156 | 8181.422852 | 15674.83594 | 18084.59961 | 30181.71875 | 22988.3418 | 11849.61133 | 38716.57031 | 80405.20313 | 25118.56641 | 23762.07422 | 18053.71875 | 14802.54492 | 13261.91699 | 14317.61719 |
| P60763 | 0 | 17055.20313 | 0 | 5519.250977 | 0 | 0 | 0 | 0 | 0 | 0 | 0 | 0 | 0 | 7743.945801 | 0 | 0 | 0 | 0 | 6299.489746 | 0 | 0 | 1404.532471 | 10873.18848 | 0 | 0 | 10860.28809 | 13850.92773 | 0 | 0 | 0 | 0 | 0 | 0 | 7722.57373 | 0 | 0 | 0 | 5504.160156 | 0 | 0 | 0 | 0 | 0 | 2100.797363 | 0 | 0 | 0 | 0 | 0 | 0 | 0 | 0 | 0 | 0 | 0 | 4776.962891 | 0 | 0 | 0 | 0 | 0 | 0 |
| P61224 | 0 | 16298.43164 | 0 | 6153.405273 | 0 | 4055.948486 | 0 | 0 | 3151.341309 | 0 | 0 | 0 | 2890.917969 | 5677.400391 | 5315.73877 | 0 | 0 | 1731.342163 | 10819.50879 | 3900.418945 | 8731.819336 | 0 | 15762.02539 | 0 | 8880.273438 | 12192.10547 | 9784.544922 | 0 | 0 | 4837.841797 | 3261.446045 | 4129.477051 | 0 | 5156.733398 | 0 | 3451.627686 | 0 | 4603.615234 | 0 | 0 | 0 | 0 | 5832.818848 | 0 | 0 | 0 | 0 | 9992.963867 | 0 | 0 | 2422.04834 | 6079.916016 | 0 | 0 | 7707.22168 | 8745.862305 | 3775.765869 | 3421.34082 | 0 | 0 | 0 | 0 |
| P61626 | 10800.6084 | 9816.854492 | 6658.656738 | 13746.15625 | 4545.459961 | 5059.915527 | 16134.04688 | 6104.453125 | 8076.076172 | 4695.192871 | 9045.654297 | 10405.44238 | 7175.026367 | 8404.993164 | 7855.552246 | 5863.478027 | 12225.19531 | 11612.05078 | 9285.074219 | 26628.82227 | 12730.06055 | 18436.68945 | 12068.15918 | 12731.6582 | 12283.29688 | 7456.62207 | 4376.526367 | 20566.41797 | 13314.23828 | 13949.29297 | 13139.99609 | 11244.375 | 7725.873047 | 12178.43945 | 10395.57031 | 5209.251465 | 5693.399414 | 16532.89063 | 8184.068359 | 7538.981934 | 13701.16211 | 20599.82617 | 7077.881836 | 8096.268555 | 9379.699219 | 19785.21289 | 20377.90234 | 0 | 0 | 10039.69727 | 14432.66797 | 11808.53516 | 10896.83301 | 12554.29883 | 12402.52832 | 10800.60059 | 16652.2793 | 12490.16406 | 8645.110352 | 6272.015137 | 11387.82617 | 9039.242188 |
| P68032 | 0 | 33266.24219 | 0 | 7595.632813 | 1928.384033 | 0 | 16844.70313 | 0 | 0 | 3347.131348 | 248247.7031 | 0 | 0 | 7810.565918 | 3352.887939 | 0 | 0 | 0 | 4578.678711 | 0 | 0 | 0 | 11801.8584 | 0 | 4623.722168 | 0 | 8549.586914 | 0 | 0 | 7337.991699 | 29517.10156 | 0 | 1282.125244 | 10864.81934 | 1399.794434 | 4095.5625 | 0 | 27217.73242 | 0 | 0 | 0 | 0 | 0 | 0 | 0 | 799.3596802 | 0 | 0 | 0 | 1476.795532 | 0 | 1938.314087 | 0 | 0 | 4331.674316 | 11330.68359 | 0 | 0 | 0 | 0 | 0 | 0 |
| P68366 | 0 | 7597.236328 | 0 | 0 | 0 | 0 | 0 | 0 | 0 | 0 | 0 | 0 | 0 | 0 | 0 | 0 | 0 | 0 | 3664.608643 | 0 | 19505.25195 | 0 | 0 | 0 | 0 | 16164.60156 | 0 | 0 | 0 | 0 | 0 | 0 | 0 | 0 | 0 | 0 | 52483.22656 | 0 | 0 | 0 | 0 | 0 | 0 | 25861.99414 | 0 | 0 | 0 | 0 | 41872.71094 | 0 | 0 | 0 | 0 | 0 | 0 | 0 | 0 | 0 | 0 | 44165.10938 | 0 | 0 |
| P68871 | 2501696.5 | 16218720 | 1347734.875 | 2183768.25 | 3973593.5 | 6204500 | 4312140 | 3857932 | 4308432.5 | 2646721.25 | 3780298 | 4990467 | 1783992.625 | 2858932.25 | 4575461 | 2086712.625 | 3320395 | 2802264.5 | 4159128 | 2940429 | 1658053.5 | 3671124.5 | 4961483 | 2441406.5 | 3142711 | 11173694 | 11999062 | 9009039 | 10797341 | 7363099 | 2089495.25 | 3286177.5 | 7374651 | 6546386 | 2867592.25 | 5013615 | 8585462 | 7645326 | 15964588 | 5209345 | 6407725 | 6617615 | 4492047 | 6903386 | 11365306 | 5090979 | 4338175 | 8039786 | 9645416 | 3672987 | 6703064 | 5277256 | 5574119 | 6904533 | 6170384 | 7954038.5 | 6226195 | 3633482.5 | 5813811 | 4058272.5 | 2802163.5 | 4413170.5 |
| P69892 | 0 | 26276.125 | 0 | 0 | 36573.39453 | 0 | 0 | 0 | 0 | 0 | 0 | 15970.88672 | 0 | 0 | 0 | 0 | 0 | 0 | 0 | 0 | 0 | 0 | 0 | 0 | 0 | 0 | 0 | 0 | 0 | 0 | 0 | 0 | 0 | 0 | 41428.30078 | 0 | 0 | 0 | 0 | 0 | 0 | 0 | 0 | 33248.58594 | 0 | 85674.78125 | 0 | 0 | 0 | 0 | 0 | 0 | 0 | 0 | 0 | 0 | 0 | 0 | 0 | 0 | 0 | 0 |
| P69905 | 268610.8438 | 2090172.25 | 130975.6797 | 398575.3438 | 399999.9375 | 609916.3125 | 503545.0625 | 518062.125 | 462684.5 | 316197.25 | 462263.4063 | 534047.3125 | 244838.25 | 324694.6875 | 564088.625 | 227752.4063 | 431627.9375 | 326639.6563 | 561725 | 314159.25 | 209246.4063 | 562211.375 | 610241.5 | 290614.5625 | 438962 | 1250598.125 | 1399247.75 | 1058849.375 | 1049933.75 | 954501.875 | 331152.4375 | 492436.5625 | 954602.875 | 703632.25 | 348058.0625 | 711494.875 | 1075016 | 721429.625 | 1830168.5 | 1107527.125 | 939009.75 | 898803.875 | 849174.75 | 785784.875 | 1297669.25 | 703085 | 639034.125 | 1453596 | 1261732.25 | 387948.375 | 1008262.25 | 966642.9375 | 1192798 | 513655.9375 | 779188.0625 | 793204.75 | 885801.375 | 530036.125 | 765380.75 | 514160.875 | 523486.375 | 587431.4375 |
| P80108 | 78843.20313 | 75109.19531 | 61891.73438 | 54498.76563 | 94477.625 | 70704.67188 | 53816.72656 | 68784.59375 | 61483.07031 | 67824.1875 | 74091.84375 | 70347.03125 | 42543.23828 | 63569.42969 | 59490.89844 | 44309.125 | 56981.07422 | 49524.80859 | 80669.4375 | 57854.58984 | 61940.76953 | 61553.5 | 31563.82813 | 46589.07031 | 101312.7813 | 69114.46094 | 50835.34375 | 88856.625 | 58780.6875 | 71840.09375 | 51911.40234 | 35984.40234 | 69809.5625 | 23510.33789 | 32963.64063 | 50997.98438 | 80226.28125 | 50389.88281 | 66127.04688 | 50132.5625 | 55713.35938 | 40627.49219 | 48037.13281 | 42599.45313 | 39182.0625 | 50942.03906 | 32166.05469 | 68291.60938 | 60835.49219 | 58645.57813 | 84203.47656 | 74795.34375 | 75245.00781 | 45401.8125 | 67956.24219 | 60366.53125 | 59102.14844 | 39757.78906 | 66007.95313 | 61537.65234 | 51111.26563 | 56089.74219 |
| P80748 | 54656.79688 | 427454.8125 | 231439.7813 | 137113.4375 | 379781.5938 | 590623.5 | 185998 | 520158.625 | 531326.8125 | 288040.7188 | 304636.0313 | 239268.8438 | 281981.2813 | 23768.70898 | 55036.78906 | 305461.0313 | 516842.9375 | 896001.4375 | 401832.2813 | 368781.5 | 381406.875 | 393814.7813 | 609459.25 | 666576.625 | 773676.25 | 45236.64844 | 349128.6563 | 466558.375 | 424325.3125 | 397147.5313 | 43300.10547 | 388344.625 | 159484.5 | 22154.71875 | 347135.3438 | 223648.3281 | 307957.7813 | 528806.1875 | 26135.62891 | 543031.0625 | 369775.875 | 176386.1094 | 688322.0625 | 600059.4375 | 347038.625 | 583991.1875 | 552824.375 | 1168439.75 | 329467.5 | 1762150 | 887834.5625 | 821948.0625 | 791053.6875 | 269333.8438 | 404429.9688 | 700803.875 | 782812 | 727227.5 | 626728.375 | 200908.0469 | 434872.3438 | 1053973.375 |
| Q01518 | 0 | 10456.75293 | 0 | 4643.862793 | 0 | 0 | 0 | 0 | 0 | 0 | 0 | 0 | 0 | 0 | 0 | 0 | 0 | 0 | 0 | 0 | 0 | 0 | 0 | 0 | 0 | 3416.04248 | 8660.407227 | 0 | 0 | 0 | 0 | 0 | 0 | 5506.027832 | 0 | 0 | 0 | 0 | 0 | 0 | 0 | 0 | 0 | 0 | 0 | 0 | 0 | 4409.334473 | 0 | 0 | 0 | 0 | 0 | 0 | 0 | 0 | 0 | 0 | 0 | 0 | 0 | 0 |
| Q03591 | 130331.2969 | 132661.6563 | 124293.1641 | 155273.5 | 112852.6094 | 161985.5469 | 246743.75 | 145245.8594 | 178925.4531 | 154018.3594 | 195112.9219 | 151725.0313 | 143740.0625 | 128810.6563 | 96370.25 | 172836.0469 | 148542.2031 | 201351.6875 | 168977.375 | 121197.9766 | 156019.9844 | 137077.4844 | 142336.3594 | 163930.4531 | 150887.5156 | 90703.14844 | 116896.8828 | 173923.2188 | 147604.0625 | 220139.1563 | 148507.3594 | 87890.33594 | 164627.875 | 217279.3438 | 198331.5469 | 145845.875 | 191740.1563 | 133983.5469 | 144836.5938 | 134349.25 | 198455.6719 | 186592.1406 | 152339.1094 | 118252.5625 | 130755.1172 | 182343.4688 | 81601.375 | 155446.4375 | 126995.3438 | 149982.3594 | 194805.2969 | 181241.375 | 74314.67969 | 181952.7656 | 137402.25 | 91186.78125 | 98989.28906 | 156765.125 | 189651.2188 | 151322.7656 | 145350.7969 | 0 |
| Q06033 | 37756.85938 | 27200.42578 | 51466.21484 | 46326.75391 | 22782.14844 | 32202.46094 | 32077.93164 | 27212.41016 | 27434.26953 | 23141.88672 | 33554.32031 | 60430.54688 | 44509.46875 | 33215.65625 | 61484.15625 | 61762.78906 | 33912.71484 | 29607.94336 | 53711.37891 | 49654.39453 | 53121.64453 | 28806.86328 | 14959.40723 | 69906.45313 | 45818.58594 | 9281.833008 | 36114.66797 | 36496.48828 | 40947.90625 | 62533.09766 | 36401.30078 | 28220.23438 | 37555.08984 | 46646.32813 | 58268.92969 | 31044.1582 | 31819.08203 | 15647.87109 | 26861.19141 | 51497.78906 | 50449.77344 | 33464.54688 | 29274.22852 | 46108.01563 | 23213.44141 | 50867.01953 | 51009.74219 | 49085.34375 | 37456.86719 | 34905.24219 | 28532.80469 | 25460.03711 | 48400.13281 | 27733.42773 | 38089.96094 | 28820.90625 | 24182.65234 | 63651.33594 | 49292.41406 | 22446.24609 | 60376.98438 | 39298.32422 |
| Q07954 | 0 | 0 | 0 | 5432.75 | 46693.67188 | 0 | 0 | 0 | 37169.45703 | 0 | 0 | 15309.76758 | 0 | 0 | 32632.81055 | 0 | 12222.34961 | 56493.58594 | 2229.557129 | 0 | 0 | 0 | 0 | 0 | 0 | 14320.80859 | 0 | 15511.9082 | 52698.88281 | 0 | 0 | 0 | 0 | 0 | 556175.6875 | 26308.14063 | 0 | 32938.14844 | 8172.093262 | 65435.26563 | 0 | 0 | 0 | 683.6715698 | 0 | 0 | 0 | 583.7467651 | 0 | 0 | 0 | 0 | 0 | 1848.758179 | 0 | 0 | 0 | 0 | 0 | 0 | 5770.293945 | 35288.61719 |
| Q08380 | 37757.84375 | 24950.88672 | 119547.4219 | 143039.7031 | 114489.3438 | 103978.9688 | 56734.33594 | 69690.77344 | 64794.4375 | 136471.3594 | 66582.78906 | 73433.00781 | 107271.375 | 120389.6875 | 50514.70313 | 52159.28906 | 72193.34375 | 95896.15625 | 50050.75781 | 70618.5625 | 81949.61719 | 53847.89063 | 73451.45313 | 24639.04297 | 44372.83984 | 68146.90625 | 46680.08203 | 90844.97656 | 65104.38281 | 36600.16797 | 94831.71094 | 67198.28125 | 97341.04688 | 123164.7734 | 112387.375 | 115545.9297 | 63162.69531 | 113888.2891 | 47337.17578 | 60974.44531 | 45466.21875 | 131594.625 | 77036.51563 | 70851.46875 | 127516.1563 | 58086.69141 | 47872.65234 | 36810.63672 | 74417.9375 | 89216.00781 | 61635.28125 | 48457.32813 | 55100.07031 | 73605.375 | 39227.00781 | 58202.73438 | 64270.19141 | 72297.4375 | 117198.6953 | 53305.04688 | 59245.59375 | 76615.51563 |
| Q08830 | 0 | 0 | 0 | 0 | 0 | 0 | 0 | 0 | 21925.36914 | 0 | 0 | 0 | 0 | 0 | 0 | 0 | 0 | 0 | 0 | 0 | 0 | 0 | 0 | 0 | 0 | 0 | 0 | 0 | 0 | 11962.09766 | 0 | 0 | 0 | 0 | 0 | 0 | 0 | 0 | 0 | 0 | 0 | 0 | 0 | 0 | 0 | 0 | 0 | 0 | 0 | 0 | 0 | 0 | 0 | 0 | 0 | 0 | 0 | 0 | 0 | 0 | 0 | 0 |
| Q13093 | 8333.527344 | 0 | 6508.196777 | 2649.775635 | 6559.060059 | 3503.082031 | 641.1296997 | 1463.74292 | 5240.904297 | 10647.36914 | 5204.807129 | 3052.534912 | 7848.246094 | 4471.824219 | 4378.551758 | 0 | 0 | 0 | 7531.273926 | 5055.10498 | 10624.47559 | 0 | 6700.308594 | 2693.503418 | 4751.473633 | 3503.025146 | 3160.236572 | 1881.184937 | 5489.921387 | 0 | 9087.524414 | 6321.520508 | 6034.008789 | 5156.827637 | 3876.606934 | 4384.300781 | 6929.226563 | 3979.40332 | 6181.315918 | 13896.46387 | 10172.6582 | 6918.197266 | 13253.24023 | 8041.628906 | 6653.91748 | 8049.297363 | 9752.046875 | 3230.93457 | 12278.21973 | 4166.46582 | 7414.300781 | 8207.042969 | 2442.160645 | 3122.819824 | 4374.4375 | 3615.174561 | 2148.344971 | 8146.743652 | 6225.106445 | 5812.98291 | 5696.197266 | 0 |
| Q13103 | 0 | 0 | 0 | 0 | 1364.629883 | 1225.46582 | 3925.88208 | 2300.560791 | 0 | 0 | 7597.949707 | 4177.822754 | 0 | 8111.400879 | 6230.904785 | 8876.048828 | 0 | 0 | 3916.124756 | 0 | 0 | 2399.237793 | 0 | 0 | 1550.276611 | 5032.185547 | 0 | 7376.618164 | 13052.80176 | 0 | 3297.118164 | 1821.243896 | 0 | 4404.584961 | 0 | 5699.471191 | 0 | 4599.823242 | 1411.141968 | 7032.671387 | 0 | 1021.239014 | 3055.174072 | 5965.166992 | 0 | 2380.548828 | 4441.880371 | 1305.133911 | 5316.206055 | 4203.322266 | 1576.399536 | 0 | 1747.014771 | 0 | 3506.39917 | 5254.872559 | 4827.091309 | 1136.005859 | 2278.111084 | 4074.249023 | 1977.391235 | 6786.121094 |
| Q13201 | 0 | 0 | 49619.9375 | 0 | 0 | 56441.8125 | 47150.86328 | 41557.38281 | 95570.84375 | 0 | 26812.53516 | 60904 | 45791.85156 | 41588.54297 | 43653.63672 | 48773.6875 | 0 | 0 | 0 | 0 | 0 | 31221.11133 | 0 | 0 | 49711.4375 | 39624.55859 | 54391.17578 | 32835.46875 | 54095.08984 | 41836.25781 | 0 | 49106.39453 | 0 | 11426.99219 | 53312.5625 | 6442.662109 | 55576.37109 | 0 | 0 | 18089.03906 | 52492.86719 | 12849.24316 | 77649.09375 | 31144.25 | 45363.97266 | 63917.41406 | 0 | 0 | 120392.6016 | 48598.89063 | 28406.27734 | 16110.41602 | 47820.94922 | 0 | 0 | 20625.97266 | 25741.15234 | 0 | 28255.38672 | 0 | 18441.75977 | 26786.02734 |
| Q13790 | 53880.60547 | 102235.5625 | 112377.7578 | 85502.57813 | 65650.47656 | 59255.01563 | 68108.35156 | 58085.07422 | 43433.8125 | 53690.875 | 42135.17969 | 76361.67188 | 88070.46875 | 55583.96094 | 87562.51563 | 62961.77734 | 75484.27344 | 60906.25 | 57323.64063 | 45801.10156 | 59842.63281 | 85772.94531 | 44537.55469 | 84271.54688 | 71636.60938 | 45631.92578 | 73647.48438 | 62380.53125 | 64270.07031 | 68378.14063 | 87548.5 | 71310.90625 | 56911.04688 | 66767.57813 | 83009.4375 | 78584.07813 | 82259.60938 | 77417.01563 | 69380.17188 | 68465.79688 | 77421.99219 | 77013.60156 | 87421.45313 | 66143.35938 | 92994.95313 | 62781.32813 | 65481.07813 | 62578.60938 | 110795.3672 | 60447.20313 | 88431.375 | 69441.1875 | 103691.6172 | 92823.1875 | 63325.57813 | 49209.00781 | 55608.24219 | 95031.28125 | 81729.03906 | 60027.33594 | 64993.77344 | 63683.32813 |
| Q14520 | 91613.52344 | 105366.0938 | 109811.7188 | 77433.48438 | 101842.6563 | 95730.79688 | 146353.6563 | 82033.8125 | 122494.875 | 50135.29297 | 97782.21875 | 128954.9297 | 94761.09375 | 119198.0938 | 135549.5938 | 123955.2813 | 100522.75 | 117622.75 | 98057.28125 | 117202.9219 | 115813.3359 | 73219.82813 | 109878.625 | 94704.59375 | 91995.28125 | 112797.6641 | 116048.7344 | 146780.8281 | 78131 | 102280 | 78340.17188 | 106822.25 | 88028.34375 | 141883.0781 | 125327.4219 | 115325.9063 | 47365.21094 | 113791.1875 | 96085.75 | 109007.4531 | 76430.42188 | 86431.22656 | 86305.28125 | 84772.375 | 94839.29688 | 85054.48438 | 84867.36719 | 108324.8516 | 87972.07813 | 78124.17188 | 76657.82031 | 104468.9531 | 91732.03125 | 106140.75 | 106514.2813 | 96067.57813 | 97656.58594 | 94128.23438 | 127409.5938 | 101927.25 | 104348.2891 | 109262.125 |
| Q14624 | 0 | 0 | 319774.8125 | 213275.7188 | 181235.0313 | 234617.6406 | 0 | 179280.2188 | 0 | 198735.3906 | 150871.9063 | 0 | 253037.9219 | 0 | 0 | 259837.7813 | 230458.3594 | 280172.0313 | 244864.3594 | 186123.5156 | 271589.6875 | 194862.2813 | 135138.7813 | 242708.6563 | 215442.7344 | 0 | 246471.0625 | 286433.75 | 193835.3125 | 193084.7031 | 0 | 228236.9531 | 205815.4219 | 187342.7969 | 270196 | 250873.375 | 0 | 0 | 116784.1875 | 183283.7969 | 190067.5781 | 143705.8281 | 183926.2813 | 184795.2344 | 0 | 213194 | 167254.6094 | 212301.6406 | 179778.1094 | 136227.2656 | 179239 | 177496.7813 | 178240.5469 | 176127.7656 | 160237.2344 | 132279.6406 | 223747.4531 | 179662.6875 | 340437.7813 | 152579.5938 | 0 | 192896.0313 |
| Q15166 | 19579.7168 | 23377.74023 | 24720.46484 | 22045.60742 | 19955.40234 | 16863.87695 | 30042.99414 | 143764.7344 | 19724.05859 | 8721.351563 | 9398.820313 | 23705.78906 | 17113.23047 | 10066.32617 | 12337.79492 | 16590.41016 | 155006.4688 | 96455.14063 | 4804.891113 | 18927.95313 | 26021.19531 | 17913.61133 | 6529.720215 | 24985.62695 | 16586.61719 | 13196.16016 | 17006.22656 | 110375.6484 | 23454.0625 | 22815.25195 | 109320.2969 | 14078.50488 | 16865.98633 | 15353.58398 | 18252.47852 | 13555.43652 | 92568.05469 | 15225.9541 | 18371.60938 | 18137.16992 | 70112.77344 | 9646.788086 | 29297.95117 | 25355.2207 | 15265.94531 | 7996.3125 | 145008.75 | 11944.06836 | 16191.32129 | 11121.75 | 17722.22266 | 12614.17773 | 99463.61719 | 19167.79102 | 13190.46094 | 73573.3125 | 22307.05273 | 25663.20313 | 11413.02148 | 13530.90918 | 106755.3438 | 14806.97656 |
| Q15485 | 8380.033203 | 0 | 4643.394531 | 7386.457031 | 9665.317383 | 10937.45703 | 0 | 0 | 10793.43652 | 0 | 0 | 0 | 2808.291992 | 0 | 0 | 0 | 0 | 0 | 0 | 0 | 0 | 0 | 0 | 3084.110352 | 0 | 0 | 0 | 0 | 0 | 0 | 0 | 0 | 0 | 0 | 0 | 0 | 0 | 0 | 0 | 0 | 0 | 0 | 0 | 0 | 0 | 0 | 0 | 0 | 0 | 0 | 0 | 0 | 0 | 0 | 0 | 0 | 0 | 0 | 0 | 0 | 0 | 0 |
| Q15582 | 12003.85938 | 16297.49512 | 14830.46094 | 8655.424805 | 15187.08398 | 18694.25195 | 6575.104004 | 9348.216797 | 14627.68555 | 16448.64258 | 15083.93848 | 23751.97656 | 16342.95605 | 21900.14063 | 2433.628418 | 32303.80273 | 12917.08203 | 21977.77539 | 13150.50684 | 18126.57813 | 12169.73438 | 11090.29199 | 12478.2373 | 10417.55273 | 5655.626953 | 21850.49805 | 6192.131348 | 15825.35742 | 12919.67773 | 11981.12305 | 21911.78125 | 4350.435547 | 16595.78125 | 19823.81641 | 17397.48047 | 14573.5 | 22771.38086 | 9541.560547 | 25827.78906 | 4909.418457 | 12460.2207 | 6221.521484 | 17206.94727 | 11068.0752 | 16475.74023 | 4888.193359 | 5271.788574 | 6288.636719 | 13833.49414 | 18554.68555 | 19168.64258 | 6243.548828 | 20611.21484 | 16833.70898 | 7398.583008 | 8508.97168 | 10018.19629 | 5550.75293 | 20717.38672 | 9281.477539 | 9572.078125 | 29502.36719 |
| Q15848 | 10122.46973 | 11556.68359 | 8125.64502 | 14026.59766 | 4758.202637 | 13717.54785 | 4678.169922 | 9706.892578 | 10315.21582 | 11623.21777 | 2914.402832 | 12774.09668 | 34383.45313 | 26763.94727 | 11208.88574 | 15166.24805 | 10298.21191 | 19967.61133 | 10236.57031 | 7215.646484 | 8596.211914 | 10980.01953 | 9749.073242 | 11212.80957 | 13413.61035 | 6455.143555 | 7877.147461 | 8759.435547 | 8956.651367 | 16421.32422 | 26329.91016 | 20974.97852 | 18552.56445 | 11357.24512 | 12123.45313 | 23406.42188 | 4454.687988 | 9821.839844 | 7107.21582 | 16548.19336 | 20745.87305 | 6702.352539 | 7256.48877 | 12260.98633 | 28870.08789 | 23256.99609 | 19249.19727 | 7594.974609 | 13795.25195 | 9597.75 | 13901.34375 | 9404.905273 | 4311.821777 | 12557.06738 | 8358.03418 | 6283.129883 | 6717.59375 | 25957.41992 | 7619.556641 | 21435.92578 | 8235.859375 | 17051.36133 |
| Q16610 | 38087.02344 | 24342.01367 | 40012.75 | 37564.54688 | 42416.67969 | 54168.65234 | 39359.36719 | 33169.72656 | 41985.99219 | 52049.875 | 26462.66016 | 24341.37109 | 41515.68359 | 32114.07422 | 28099.82031 | 43263.39844 | 58137.47266 | 41875.35156 | 41132.26172 | 41034.20313 | 34858.75781 | 46713.03906 | 40056.91406 | 41508.01172 | 40694.59766 | 30426.56445 | 37595.87891 | 34267.72656 | 28558.91406 | 42512.42578 | 35775.90625 | 20315.34375 | 38784.08203 | 33343.67969 | 38725.94922 | 39821.51172 | 34378.23047 | 67975.27344 | 24357.82813 | 42356.95313 | 25441.38281 | 55882.21094 | 34690.83203 | 38800.92578 | 34500.26563 | 42146.0625 | 58952.02344 | 32349.875 | 32626.66016 | 35631.35547 | 38061.42969 | 34814.77344 | 40274.0625 | 19397.49219 | 21219.76172 | 43287.05469 | 47867.0625 | 32946.66406 | 15593.20801 | 39107.98438 | 24976.77734 | 27647.85547 |
| Q16880 | 512559.625 | 830713.8125 | 2176122.5 | 1223162.25 | 1043736.375 | 470924.75 | 372813.1875 | 533376.125 | 534497.875 | 922662.75 | 448960.4688 | 470385.7813 | 1521488.75 | 1009669.375 | 1322609.375 | 818068.25 | 675918.1875 | 346277.6563 | 425260.0313 | 287306.3438 | 1301546.75 | 868977.3125 | 296307.5313 | 748097.9375 | 714144.375 | 280126.375 | 985470.625 | 428165.3438 | 1160386.625 | 478681.625 | 574586 | 701658.75 | 1178959 | 511910.7813 | 759175.8125 | 826107.625 | 667905.0625 | 333604.0625 | 1346838.875 | 774115.9375 | 807243.875 | 350769.1563 | 1116295.75 | 768026.75 | 2224174 | 905916.0625 | 419818.4688 | 784937.5625 | 1561463.375 | 443960.125 | 687565.6875 | 582109.8125 | 407688.8125 | 787137.9375 | 319285.5313 | 247650.7813 | 353448.125 | 782703.25 | 734338.125 | 1019206.563 | 915970.1875 | 726979.875 |
| Q562R1 | 11587.40234 | 72910.38281 | 0 | 32028.70313 | 8321.554688 | 12234.93848 | 10431.80469 | 0 | 6712.319336 | 0 | 0 | 5636.674805 | 14246.6748 | 22279.98242 | 0 | 8277.490234 | 9703.164063 | 0 | 31192.69922 | 17855.68164 | 0 | 0 | 37010.52734 | 0 | 21163.01367 | 27175.95508 | 25970.75977 | 0 | 0 | 27369.66211 | 17773.33594 | 12832.77441 | 0 | 47412.34375 | 10851.66113 | 10743.87305 | 0 | 28510.88281 | 0 | 0 | 0 | 0 | 29010.9707 | 8613.949219 | 0 | 12731.51563 | 0 | 23064.31641 | 0 | 0 | 0 | 0 | 22586.60547 | 0 | 40874.87109 | 31888.36523 | 13735.91406 | 14023.16992 | 0 | 0 | 0 | 0 |
| Q5SRP5 | 0 | 12222.43848 | 17835.95117 | 21750.98438 | 28970.44727 | 13667.74219 | 0 | 10221.01367 | 0 | 0 | 0 | 0 | 0 | 16015.17188 | 28264.8125 | 14612.08105 | 0 | 0 | 15192.72754 | 0 | 27508.48438 | 0 | 0 | 0 | 0 | 0 | 27899.01758 | 14064.81055 | 10387.13379 | 0 | 0 | 27581.43945 | 36076.43359 | 22746.68359 | 0 | 26968.63281 | 27292.90039 | 0 | 0 | 17011.14063 | 10656.18457 | 0 | 23858.94141 | 0 | 0 | 0 | 4746.132813 | 16186.18652 | 30576.0625 | 0 | 0 | 0 | 0 | 0 | 0 | 5744.901855 | 5394.401367 | 0 | 24917.68555 | 18477.14258 | 18063.9668 | 0 |
| Q5T749 | 0 | 0 | 0 | 0 | 0 | 0 | 2050.775879 | 0 | 0 | 0 | 0 | 0 | 0 | 0 | 0 | 0 | 6617.872559 | 0 | 0 | 0 | 0 | 0 | 4461.241699 | 0 | 0 | 0 | 0 | 0 | 0 | 0 | 0 | 0 | 0 | 0 | 0 | 0 | 3649.15918 | 0 | 0 | 0 | 0 | 0 | 0 | 2946.200439 | 0 | 7890.233398 | 0 | 0 | 0 | 0 | 0 | 0 | 0 | 0 | 0 | 0 | 3825.328613 | 0 | 0 | 0 | 20114.43945 | 0 |
| Q6EMK4 | 0 | 11925.63086 | 0 | 8543.171875 | 0 | 5153.776855 | 11431.05957 | 11267.94434 | 8689.782227 | 6747.105957 | 5731.48291 | 9213.582031 | 0 | 2177.708984 | 0 | 0 | 7933.799316 | 0 | 11073.56445 | 10947.84766 | 0 | 13461.69336 | 10783.39746 | 5400.563477 | 0 | 6859.653809 | 13434.27051 | 11871.90039 | 0 | 13497.97656 | 6260.336426 | 12138.61914 | 13107.21191 | 5215.38623 | 11124.32324 | 0 | 8160.69873 | 8775.624023 | 4602.070801 | 12738.20996 | 6251.952148 | 17429.86523 | 2921.35376 | 0 | 3962.831543 | 7653.933105 | 7658.459961 | 5813.174805 | 0 | 10080.77051 | 8741.994141 | 9617.855469 | 7205.355957 | 9494.519531 | 12154.9668 | 6296.505859 | 6610.503418 | 13327.41992 | 7965.242676 | 16034.64844 | 4749.087891 | 3981.707275 |
| Q6UXB8 | 13185.65039 | 20251.74609 | 8387.90625 | 13414.19141 | 11640.42188 | 8799.709961 | 13478.21191 | 8823.821289 | 11183.04297 | 5434.677246 | 3937.883057 | 11777.72363 | 6787.730469 | 9899.123047 | 6791.166992 | 6673.759277 | 8427.492188 | 10774.8623 | 10146.59863 | 18627.62109 | 13026.64648 | 10283.70801 | 7965.155762 | 10329.70801 | 17408.49219 | 12384.1543 | 13118.96191 | 9851.822266 | 13215.64844 | 19202.52539 | 8664.973633 | 9151.597656 | 13478.35449 | 15702.50684 | 11098.0752 | 6978.700195 | 0 | 7263.907227 | 6561.71582 | 10993.94629 | 5200.067871 | 9099.226563 | 5388.502441 | 13482.74805 | 18133.68164 | 15197.32715 | 8147.750488 | 8465.546875 | 9641.114258 | 5403.571777 | 7242.345215 | 18419.19141 | 8723.318359 | 7317.930664 | 15122.21289 | 9659.304688 | 11539.99316 | 8426.949219 | 12055.68945 | 8441.566406 | 6946.433105 | 6161.025391 |
| Q6ZRK6 | 70471.15625 | 0 | 0 | 0 | 0 | 0 | 0 | 0 | 0 | 0 | 0 | 90410.27344 | 0 | 0 | 71079.66406 | 0 | 407290.1563 | 221783.5938 | 0 | 0 | 136445.3281 | 0 | 0 | 180770.4375 | 0 | 0 | 771074.1875 | 1828122.375 | 0 | 0 | 157876.625 | 93994.08594 | 0 | 0 | 50520.83203 | 0 | 0 | 0 | 64906.71875 | 250496.6875 | 417656.0938 | 0 | 0 | 183181.0938 | 829305.375 | 0 | 647432.25 | 0 | 175092.4688 | 163378.875 | 85446.70313 | 0 | 0 | 0 | 0 | 276061.3125 | 0 | 647795.4375 | 0 | 125532.9219 | 0 | 0 |
| Q86UD1 | 4239.133301 | 0 | 0 | 4628.983398 | 0 | 6103.462402 | 12991.85645 | 3413.769775 | 2882.762451 | 0 | 2663.868652 | 4962.292969 | 8515.03125 | 8401.436523 | 7027.141602 | 5058.478027 | 5582.665527 | 0 | 5015.180664 | 7415.441895 | 7253.678223 | 5487.346191 | 0 | 0 | 4076.368652 | 3855.414795 | 0 | 4275.132324 | 5404.624512 | 4233.759277 | 5213.307129 | 0 | 3480.451172 | 6470.158691 | 6766.42041 | 4240.740234 | 3116.572754 | 0 | 5319.036133 | 3210.455811 | 4789.16748 | 6017.881836 | 0 | 6819.880859 | 4104.241211 | 9128.648438 | 5539.602539 | 7159.104004 | 4580.699707 | 0 | 3174.217773 | 4126.492676 | 0 | 0 | 4580.123535 | 5452.723145 | 0 | 8114.112305 | 7568.129395 | 0 | 3214.751465 | 0 |
| Q86UX7 | 2484.451416 | 14639.2168 | 2143.875732 | 5679.069824 | 10154.99023 | 3424.44873 | 17225.75586 | 2736.794189 | 3231.117676 | 3848.575439 | 4254.556641 | 0 | 4128.376465 | 9679.498047 | 7604.092773 | 4292.831055 | 3679.010986 | 15884.43652 | 10290.45117 | 7381.383789 | 27540.84961 | 5076.696289 | 13593.0293 | 1385.740234 | 6586.162109 | 9814.357422 | 9928.916992 | 0 | 3803.94165 | 10386.87402 | 4733.452637 | 0 | 0 | 11746.00488 | 1901.772949 | 19639.81641 | 3595.472412 | 8211.521484 | 2284.286621 | 0 | 0 | 5680.20166 | 4882.454102 | 7841.076172 | 0 | 2400.106445 | 0 | 5390.51123 | 0 | 0 | 0 | 2260.529785 | 2966.306152 | 1579.176758 | 4525.348633 | 7075.57373 | 1931.481934 | 0 | 849.4407349 | 2140.932861 | 0 | 0 |
| Q86YZ3 | 3679.976563 | 0 | 0 | 5006.975098 | 0 | 3693.236328 | 2401.727051 | 0 | 2807.72168 | 0 | 0 | 0 | 2228.007568 | 0 | 0 | 0 | 1840.843994 | 1292.071045 | 0 | 0 | 3584.952393 | 0 | 0 | 0 | 0 | 1824.187012 | 978.0184937 | 0 | 2912.155518 | 0 | 1851.168213 | 1943.812012 | 0 | 1857.161621 | 0 | 1184.859131 | 3252.357666 | 0 | 2290.880859 | 2673.605957 | 1697.136841 | 0 | 0 | 2628.220703 | 3625.379395 | 1910.804443 | 0 | 2396.500732 | 0 | 6146.451172 | 0 | 0 | 0 | 1933.299072 | 0 | 0 | 4615.687012 | 0 | 0 | 0 | 2110.210693 | 2241.100342 |
| Q8IV42 | 33057.22656 | 82928.48438 | 148623.875 | 0 | 106631.1797 | 215486.6875 | 159757.5781 | 117897.6328 | 126323.1172 | 53188.83594 | 187280.9844 | 149859.3594 | 59776.64063 | 144082.4219 | 74050.0625 | 62594.91406 | 46910.89844 | 74244.17188 | 36045.03125 | 0 | 96784 | 72798.63281 | 108871.7422 | 118316.5703 | 43838.35547 | 161671.5 | 143943.375 | 137225.4844 | 72725.95313 | 0 | 0 | 37663.92578 | 0 | 0 | 69427.52344 | 33021.22656 | 144651.25 | 106647.0078 | 126537.8984 | 111266.7031 | 101812.8906 | 38476.78125 | 135948.8281 | 86742.92188 | 43754.57813 | 45391.73438 | 0 | 130412.5078 | 120055.7266 | 63219.14063 | 47104.83203 | 0 | 37185.45313 | 134971.5156 | 96549.95313 | 58149.63672 | 84543.27344 | 53493.72656 | 81057.78125 | 105039.0781 | 0 | 74601.70313 |
| Q8N1N4 | 218397.7969 | 108048.8359 | 142298.6094 | 169051.1719 | 184915.75 | 150830.1563 | 133434.9219 | 124720.9375 | 137050.6719 | 132005.5469 | 183669.3438 | 120481.8438 | 124338.75 | 119038.8672 | 64277.85547 | 155807.7031 | 210915.3594 | 206848.9375 | 81587.39844 | 82981.98438 | 175803.4063 | 104736.5234 | 98464.32813 | 149597.5313 | 140303.6406 | 63317.01563 | 105278.6328 | 80865.9375 | 171685.2188 | 126593.0469 | 183361.5313 | 146604.4844 | 121237.25 | 149304.7031 | 145944.9063 | 85524.55469 | 191616 | 107770.1016 | 64628.25781 | 89430.30469 | 146565.1406 | 112628.8281 | 160289.1563 | 116415.0313 | 115744.625 | 240646.3281 | 136425.0313 | 126069.0781 | 127543.8672 | 149151.8594 | 136025.0469 | 122527.6406 | 138294.8125 | 113257.7578 | 114565.4766 | 104461.1719 | 133510.125 | 175615.7344 | 100317.7969 | 91968.42969 | 256604.2188 | 121356.1641 |
| Q92954 | 35167.92578 | 27549.67969 | 21741.9707 | 52728.3125 | 53452.53125 | 55888.08594 | 42485.14844 | 71613.90625 | 61102.77734 | 83532.51563 | 59191.98438 | 40814.19531 | 41633.71875 | 46286.33594 | 41984.78125 | 36412.17188 | 40228.23828 | 30639.00391 | 65940.73438 | 24667.66797 | 19054.55078 | 36121.01172 | 88373.85938 | 21471.32813 | 27741.46484 | 118340.9063 | 44067.17578 | 84375.71875 | 48736.42188 | 18417.69531 | 28733.56641 | 41525.27344 | 39144.41797 | 38780 | 57017.11719 | 41770.63281 | 68345.5625 | 38384.20313 | 48177.54688 | 32887.07422 | 58049.41406 | 47803.63672 | 28344.05273 | 32330.15625 | 47460.85547 | 31722.38672 | 13626.21484 | 39323.66406 | 72813.64063 | 28146.02734 | 19054.72266 | 27388.36328 | 39903.47656 | 46638.12891 | 25708.57813 | 42622.79688 | 32689.95117 | 33182.40234 | 47619.21875 | 59907.16797 | 24258.90625 | 11634.98633 |
| Q96HR3 | 8188.018555 | 15087.55566 | 16065.63574 | 11423.60254 | 11867.13086 | 8383.597656 | 19241.83594 | 21066.88086 | 15428.58691 | 10733.85254 | 14575.38672 | 9367.714844 | 14037.87207 | 11623.72656 | 16629.46484 | 15672.10742 | 21406.56641 | 17732.03125 | 15468.54785 | 47472.74219 | 20772.42188 | 43338.29297 | 18947.83789 | 18014.6543 | 39241.32813 | 22033.40625 | 23097.2832 | 23285.30078 | 10930.18848 | 12433.10449 | 17056.41797 | 9672.918945 | 10982.48438 | 18743.71289 | 22016.84766 | 15214.44141 | 23873.59375 | 19802.89063 | 17538.42578 | 31593.53516 | 31179.97266 | 11736.43848 | 36720.01172 | 16183.92969 | 17085.06445 | 44005.36328 | 55983.30859 | 45720.90234 | 18060.85352 | 45290.14453 | 42128.41016 | 52601.85938 | 25868.24609 | 18972.82031 | 57277.35938 | 39428.55078 | 12038.64941 | 21074.18555 | 35245.66016 | 39290.48828 | 21369.06055 | 12880.52344 |
| Q96IY4 | 14549.875 | 19075.42578 | 12906.21875 | 13302.47461 | 16102.11914 | 23839.0625 | 27783.06641 | 25625.49609 | 20902.10938 | 29637.89063 | 21458.39844 | 24521.91211 | 23202.83008 | 26156.56641 | 20364.41211 | 23064.10352 | 31257.92188 | 22217.67773 | 26972.85547 | 30179.50586 | 14655.6875 | 15269.43555 | 24871.00391 | 18522.75977 | 28757.11719 | 23744.01953 | 20954.96289 | 24637.15625 | 19370.63672 | 29692.33203 | 12776.89258 | 17093.86719 | 18152.9082 | 24131.51367 | 21714.11328 | 15400.85645 | 23053.15234 | 19802.26172 | 17909.91406 | 32438.5 | 12268.62012 | 13602.80273 | 18430.07617 | 12284.67676 | 15236.90137 | 20265.64648 | 34643.44922 | 21227.19531 | 19930.17969 | 18798.03906 | 29757.73828 | 18849.70508 | 25301.71484 | 19942.73242 | 23415.63672 | 24985.80273 | 25430.98828 | 18402.07813 | 30657.19141 | 28742.36328 | 21212.0625 | 30137.79102 |
| Q96KN2 | 8957.375977 | 6119.029297 | 6692.788086 | 0 | 9503.745117 | 7528.195313 | 7474.724121 | 0 | 5793.750977 | 9249.160156 | 0 | 6025.335449 | 6630.352539 | 7305.088867 | 0 | 4059.356689 | 8207.613281 | 0 | 4192.803711 | 3469.577148 | 6406.030762 | 0 | 0 | 6886.433105 | 0 | 3527.577881 | 9547.46875 | 8888.401367 | 8174.123535 | 0 | 6091.791504 | 5351.207031 | 5628.176758 | 8179.745117 | 7287.905273 | 4265.462402 | 3517.056396 | 6796.085449 | 6631.007813 | 5221.500488 | 0 | 4501.54248 | 0 | 0 | 5872.456055 | 8365.067383 | 4686.470703 | 5810.189941 | 9293.621094 | 3106.655518 | 0 | 6134.241699 | 7233.865723 | 7036.682129 | 0 | 7567.682129 | 9050.266602 | 12123.15527 | 7123.560059 | 8615.206055 | 0 | 0 |
| Q96PD5 | 213206.3594 | 215311.2188 | 207586.6875 | 210379.7813 | 168443.2813 | 147112.625 | 204728.75 | 177197.3125 | 150848.6719 | 151672.7969 | 157070.9063 | 159541.7969 | 230664.7188 | 228350.2188 | 166824.3438 | 219318.4688 | 193845.7656 | 212062.25 | 132604.0938 | 282130.625 | 253896.9531 | 257260.2656 | 161923.1875 | 236857.2969 | 169416.1406 | 179706.4375 | 158235.3125 | 220280.4219 | 174252.5313 | 201104.0938 | 173468.4063 | 204633.4531 | 163872.6563 | 235785 | 239663.9375 | 194043.1406 | 221412.8125 | 229621.7969 | 221808.3594 | 262734.4375 | 195124.2813 | 168142.8594 | 260053.7813 | 275474.4375 | 214113.6094 | 219304.9375 | 170259.2188 | 170899.6875 | 164197.25 | 259794.7344 | 186929.4375 | 133169.8594 | 158166.3594 | 212943.7188 | 180694.4531 | 158000.3438 | 182628.5 | 225698.125 | 193707.25 | 218874.75 | 191243.8594 | 160990.3594 |
| Q9BWP8 | 0 | 0 | 0 | 0 | 0 | 0 | 0 | 0 | 0 | 0 | 0 | 0 | 0 | 0 | 0 | 0 | 0 | 0 | 0 | 0 | 3407.9375 | 4827.848145 | 0 | 0 | 0 | 0 | 1174.426147 | 1951.484619 | 3525.437744 | 0 | 0 | 0 | 0 | 0 | 0 | 0 | 0 | 0 | 0 | 1210.011475 | 0 | 0 | 0 | 59235.95313 | 0 | 0 | 0 | 2478.376465 | 0 | 1704.304321 | 3375.35498 | 1661.640259 | 0 | 0 | 0 | 2484.032715 | 1753.311646 | 0 | 0 | 0 | 0 | 0 |
| Q9H4B7 | 6997.034668 | 3019.646729 | 0 | 8315.939453 | 369.7506714 | 1979.813843 | 0 | 0 | 0 | 10794.1582 | 0 | 0 | 0 | 0 | 2893.597412 | 90910.3125 | 7506.321777 | 166119.4844 | 749.8660889 | 14627.97656 | 0 | 6057.293945 | 4321.954102 | 0 | 113951.6641 | 2832.393066 | 678.7075195 | 0 | 96285.86719 | 760.293335 | 2322.509521 | 0 | 0 | 904.7954712 | 0 | 716.742981 | 0 | 0 | 0 | 0 | 0 | 0 | 70982.21875 | 0 | 0 | 2407.276855 | 0 | 9295.75 | 0 | 69876.24219 | 0 | 132069.625 | 3482.749756 | 102141.5234 | 0 | 0 | 0 | 0 | 0 | 0 | 7381.748047 | 0 |
| Q9HDC9 | 0 | 7510.761719 | 3232.684814 | 2874.02002 | 4064.685547 | 8276.057617 | 4958.578613 | 0 | 5970.727539 | 7317.470215 | 3152.484863 | 0 | 14571.06836 | 1969.265747 | 5005.110352 | 11540.30566 | 3659.06543 | 4366.878906 | 41792.92578 | 0 | 4287.672363 | 2736.625488 | 2826.2146 | 6837.730469 | 8277.660156 | 4583.529785 | 3716.712402 | 0 | 7988.541504 | 7461.210449 | 6030.669434 | 7145.421875 | 7336.133789 | 9382.061523 | 3532.563232 | 6995.310547 | 7231.527344 | 8697.110352 | 6140.115234 | 9374.668945 | 8064.404297 | 0 | 54091.3125 | 1391.457764 | 7670.562012 | 5977.821777 | 5663.542969 | 0 | 6106.638672 | 3759.112549 | 9758.994141 | 5754.016113 | 2725.938721 | 8994.424805 | 5934.995605 | 0 | 4663.322754 | 0 | 6653.295898 | 6773.037109 | 0 | 0 |
| Q9NZP8 | 49155.67969 | 32989.75 | 54625.63281 | 60842.53516 | 46353.73828 | 28485.12109 | 39997.11719 | 19432.91992 | 45653.32813 | 41633.96484 | 7572.281738 | 43305.87891 | 135652.6406 | 55681.41016 | 32640.66992 | 17618.24219 | 33054.37109 | 64416.20313 | 26972.44336 | 39911.88672 | 60614.28125 | 16226.87793 | 106896.0156 | 46233.63672 | 77905.10156 | 47310.75781 | 31160.1875 | 23393.84375 | 42974.86719 | 6419.273438 | 75486.47656 | 39241.30859 | 31122.26172 | 44848.08203 | 36170.91016 | 49848.77344 | 57450.92578 | 24309.74805 | 91383.0625 | 52497.29297 | 41263.44922 | 268758.8438 | 47570.35547 | 4563.978516 | 132710.3438 | 65227.23438 | 43887.23047 | 106334.75 | 78598.92969 | 13565.09766 | 48016.62891 | 6965.226563 | 48890.6875 | 75657.21875 | 55204.62891 | 93113.96094 | 53900.95703 | 8758.587891 | 10380.29492 | 7850.342773 | 25568.25977 | 46545.30078 |
| Q9UGM5 | 21333.80469 | 25728.46484 | 18398.72461 | 25702.82813 | 17965.60156 | 13783.05176 | 25857.12305 | 30331.20508 | 14666.87891 | 15017.2998 | 11234.96289 | 18883.68164 | 13362.49121 | 23484.45117 | 17593.23242 | 19751.61719 | 17703.85938 | 22404.16992 | 24626.85156 | 29384.31055 | 24235.58203 | 19426.25781 | 13687.12207 | 18013.51367 | 28842.07031 | 21513.90234 | 11764.90918 | 41559.64063 | 16196.37109 | 29368.26367 | 21661.85547 | 12151.96289 | 25983.77344 | 19295.33398 | 18330.40234 | 16079.85938 | 28036.46094 | 16412.50391 | 16544.19727 | 29638.73242 | 16960.08594 | 18827.16016 | 43498.57031 | 18232.02539 | 25681.42969 | 23039.38281 | 32946.82813 | 9831.874023 | 22876.06055 | 25309.46094 | 18391.08398 | 33681.82813 | 35974.08594 | 22386.58984 | 31891.98047 | 20102.56445 | 27860.0625 | 31535.79102 | 23995.46875 | 29460.11328 | 19236.28516 | 18916.30469 |
| Q9UHG3 | 15365.26367 | 17203.77539 | 15273.9668 | 31566.90625 | 21455.15625 | 15066.28418 | 13559.33203 | 17740.16992 | 20648.03125 | 28338.42578 | 15733.83398 | 15653.03711 | 29398.58984 | 36578.77344 | 34505.08594 | 17678.35938 | 21093.54688 | 8816.595703 | 17915.21289 | 13132.64063 | 18021.53125 | 27487.09375 | 17438.29297 | 18651.09375 | 18962.19922 | 26331.64844 | 11762.68359 | 14523.60938 | 12532.41016 | 15967.29492 | 18418.01953 | 16699.68164 | 43050.59375 | 20575.14453 | 15119.55273 | 31348.66016 | 21172.76172 | 24394.86328 | 19022.72656 | 20546.69922 | 17324.64063 | 14277.15234 | 26199.28906 | 25037.04492 | 18641.11719 | 11782.26563 | 9929.251953 | 18828.48242 | 23802.97266 | 14366.67969 | 20568.69531 | 21834.12109 | 29220.61133 | 15458.69141 | 15222.0625 | 13786.87695 | 11966.90332 | 15630.49805 | 26768.5 | 22865.83789 | 20277.46094 | 10062.0625 |
| Q9UNN8 | 0 | 0 | 0 | 0 | 0 | 0 | 0 | 0 | 0 | 0 | 0 | 0 | 0 | 0 | 0 | 0 | 0 | 0 | 0 | 0 | 0 | 0 | 0 | 0 | 0 | 2901.078857 | 0 | 0 | 0 | 2985.14502 | 0 | 0 | 0 | 0 | 0 | 0 | 2787.77124 | 0 | 3487.820313 | 0 | 0 | 0 | 0 | 0 | 0 | 0 | 0 | 5019.016113 | 0 | 0 | 0 | 0 | 0 | 0 | 0 | 0 | 0 | 0 | 3999.386719 | 0 | 0 | 0 |
| Q9Y490 | 1638.368896 | 22431.96875 | 1731.870239 | 7155.131348 | 3532.137939 | 3900.649902 | 3147.095459 | 0 | 2427.710449 | 1492.762329 | 27562.97656 | 396853.1875 | 7940.408691 | 13026.46191 | 10061.10742 | 9346.276367 | 29868.38672 | 2852.119141 | 8660.646484 | 7880.400879 | 6782.891113 | 24870.94727 | 10501.51172 | 1938.24646 | 5312.764648 | 9991.990234 | 12795.76465 | 91688.07813 | 952.5836792 | 11939.27734 | 3371.450684 | 0 | 3975.192383 | 8444.96582 | 4494.682617 | 5097.097168 | 159851.6875 | 9772.698242 | 113767.1719 | 8641.40625 | 25088.70703 | 4469.350586 | 2420.939941 | 5464.759766 | 6553.641113 | 4111.088379 | 0 | 7823.401855 | 490.8354187 | 63823.46484 | 0 | 4797.010742 | 2841.863037 | 0 | 8410.4375 | 9373.913086 | 3037.400879 | 2258.338135 | 613.5210571 | 1865.666626 | 354478.9063 | 912905.125 |
| Q9Y5Y7 | 5393.87207 | 5456.712402 | 13673.48047 | 6846.163086 | 8684.839844 | 10122.03613 | 0 | 9288.362305 | 6655.84082 | 10282.625 | 7704.669922 | 7933.291992 | 9919.146484 | 10863.56641 | 9585.042969 | 9570.798828 | 9998.316406 | 10483.61133 | 6801.12793 | 23887.39063 | 17285.87109 | 9521.138672 | 8529.089844 | 8158.274414 | 9916.917969 | 4640.57959 | 10927.23438 | 10870.8457 | 16106.58105 | 13373.7627 | 5637.446289 | 9472.094727 | 8600.19043 | 11140.36719 | 10544.85059 | 8035.660156 | 4564.787109 | 6675.210938 | 11405.13281 | 10493.14941 | 6593.42334 | 15558.29102 | 14727.24902 | 11689.64453 | 15051.26953 | 16868.58398 | 12048.00391 | 17222.11719 | 8512.819336 | 10537.25488 | 12460.94434 | 10917.20313 | 15546.42969 | 5283.056152 | 21391.75 | 11045.875 | 15016.2168 | 23375.75 | 9378.970703 | 10868.3584 | 15895.57422 | 6110.624023 |
| Q9Y6R7 | 29991.48438 | 39333.35547 | 38457.51172 | 75811.71875 | 45543.80859 | 33241.46094 | 31163.68164 | 60012.15234 | 25618.83008 | 48634.32031 | 22549.58789 | 40694.42969 | 57722.72656 | 60342.69141 | 50546.69922 | 44853.44922 | 56628.0625 | 17561.4043 | 20541.3457 | 44219.59375 | 41778.28906 | 50723.15234 | 43457.99219 | 17285.91797 | 25375.04297 | 17809.35938 | 30786.97266 | 33170.96484 | 39353.42188 | 40984.01172 | 40477.63281 | 34549.50391 | 52540.91797 | 33142.3125 | 30046.69531 | 54553.87891 | 43689.34375 | 38336.13281 | 24556.25195 | 30448.50977 | 48364.80469 | 39023.58203 | 30329.41797 | 65679 | 52782.58594 | 39862.26172 | 52800.75391 | 45515.64063 | 41814.03516 | 48661.16406 | 34211.8125 | 68041.70313 | 11470.98145 | 44447.14844 | 28256.47266 | 48005.05859 | 19806.44922 | 61687.27344 | 37539.73047 | 27344.70898 | 56081.19141 | 31882.36914 |

| DC30 | DC31 | DC32 | DC35 | DC37 | DC38 | DC41 | DC42 | DC46 | DC47 | DC48 | DC49 | DC55 | DD1 | DD2 | DD3 | DD4 | DD14 | DP1 | DP2 | DP3 | DP4 | DP5 | DP6 | Description |
| --- | --- | --- | --- | --- | --- | --- | --- | --- | --- | --- | --- | --- | --- | --- | --- | --- | --- | --- | --- | --- | --- | --- | --- | --- |
| 40059.74609 | 92298.64844 | 175300.7813 | 174913.0938 | 169269.4844 | 133241.125 | 95190.22656 | 122992.2656 | 158277.1094 | 193517.5469 | 125206.0938 | 138353.9531 | 197362.7656 | 220329.1875 | 104709.2031 | 65271.32813 | 72009.67188 | 106357.4844 | 188292.5 | 122899.4453 | 41702.83203 | 156641.4063 | 221650.3281 | 133192.3906 | hCG2043206, partial [Homo sapiens] |
| 947965.75 | 653641.25 | 505825.4063 | 0 | 315014.625 | 577636.4375 | 443607.5 | 0 | 1296484.625 | 0 | 455803.8125 | 476620.8125 | 889660.4375 | 718973.6875 | 534100.75 | 509489.4688 | 796332.75 | 822396.625 | 375076.9375 | 824393.9375 | 284929.9688 | 967070.125 | 1198925.25 | 461601.2813 | RecName: Full=Immunoglobulin lambda variable 8-61; Flags: Precursor |
| 82477.28125 | 48841.78125 | 74889.32813 | 187420.9375 | 291523.5 | 81834.67188 | 81522.8125 | 138820.6563 | 113609.5 | 39097.98438 | 76955.69531 | 54467.125 | 93262.1875 | 80881.60938 | 142191.2344 | 86771.75781 | 91029.22656 | 117742.4844 | 74318.46875 | 125641.7031 | 72280.40625 | 142664.0313 | 115945.6094 | 82012.17969 | RecName: Full=Immunoglobulin lambda variable 2-18; Flags: Precursor |
| 4265.483887 | 35931.67578 | 0 | 143624 | 144220.0625 | 5163.5 | 104325.1094 | 0 | 0 | 0 | 0 | 0 | 103791.6875 | 0 | 0 | 0 | 141010.9688 | 0 | 130832.25 | 97406.4375 | 12736.46387 | 0 | 0 | 0 | hCG2041210, partial [Homo sapiens] |
| 25983.94141 | 37867.57422 | 45379.07813 | 73426.80469 | 96322.45313 | 32805.33203 | 27507.75781 | 72719.64063 | 114498.7734 | 80383 | 38103.23438 | 34541.66016 | 173594.9531 | 54390.61328 | 31322.26953 | 117856.75 | 25331.53711 | 32048.27344 | 52555.22656 | 49469.21094 | 179684.8906 | 69005.78906 | 18450.28516 | 46879.41797 | immunoglobulin light chain variable region, partial [Homo sapiens] |
| 14423.53613 | 0 | 0 | 10278.21387 | 18331.80859 | 8353.24707 | 0 | 109005.2813 | 14116.15723 | 20108.30078 | 0 | 0 | 21936.17578 | 15662.64453 | 0 | 73801.00781 | 0 | 0 | 7851.392578 | 9757.530273 | 0 | 14286.46289 | 18593.61133 | 5945.050781 | RecName: Full=Immunoglobulin lambda variable 3-9; Flags: Precursor |
| 56137.8125 | 42089.58594 | 90158.95313 | 74717.15625 | 22079.71484 | 58063.125 | 47303.98438 | 44574.62109 | 145167.125 | 93151.17188 | 0 | 0 | 12583.48633 | 86015.58594 | 82859.71875 | 23197.58398 | 61549.00781 | 75455.88281 | 0 | 56044.14453 | 0 | 62895.99219 | 73711.1875 | 11362.49805 | hCG2036739, partial [Homo sapiens] |
| 49718.14063 | 48607.84375 | 248199.9219 | 199379.125 | 96904.11719 | 66016.07031 | 58575.98828 | 183725.2344 | 126160.0703 | 180713.4531 | 299891.625 | 171478 | 41692.42578 | 136663.1875 | 71125.17969 | 139215.5625 | 268073.375 | 66647.10938 | 44083.23828 | 297001.6875 | 141424.6094 | 136902.875 | 166722.7344 | 407383.9375 | immunoglobulin heavy chain VDJ region, partial [Homo sapiens] |
| 77399.49219 | 183457.625 | 191046.7969 | 273136.7188 | 183673.2188 | 94397.65625 | 88918.875 | 177228.2344 | 211623.2656 | 173595.3438 | 171978.8125 | 126756.6719 | 97014.78125 | 118951.6875 | 132349.5938 | 125441.0391 | 181881.5625 | 255889.0156 | 159414.6719 | 154947.0625 | 126277.7969 | 218960.9063 | 319829.75 | 83687.42969 | RecName: Full=Immunoglobulin kappa variable 2-24; Flags: Precursor |
| 31390.67969 | 33143.44922 | 58535.08984 | 78233.33594 | 112281.5078 | 71523.15625 | 52422.26953 | 69275.71094 | 35660.67188 | 54615.44922 | 37761.57813 | 49687.96484 | 43791.40625 | 36742.89453 | 57382.00391 | 31909.55859 | 45515.5625 | 34557.14453 | 90877.6875 | 50588.30859 | 41341 | 90182.04688 | 115529.9922 | 62557.78125 | monoclonal IgM antibody light chain [Homo sapiens] |
| 0 | 0 | 0 | 4714.898438 | 0 | 0 | 0 | 2579.597656 | 0 | 0 | 1177.921387 | 0 | 710.7200928 | 0 | 639.4700317 | 0 | 0 | 0 | 0 | 2746.349121 | 5540.651367 | 0 | 4455.193359 | 0 | IGKV1D-37 isoform 1, partial [Pan troglodytes] |
| 776368.5 | 441783.3438 | 675821.8125 | 1081728 | 1266818.125 | 931003.5 | 1004075.688 | 612634.25 | 931316.3125 | 791137.5 | 814921.6875 | 692168.375 | 766655.25 | 2955962 | 788508.1875 | 705634.125 | 830972.4375 | 1016376.938 | 1824914.25 | 53231.16797 | 10122.57324 | 2218153.5 | 876939.875 | 479779.2188 | hCG1793095, isoform CRA_a, partial [Homo sapiens] |
| 127398.8047 | 452188.9688 | 458801.5625 | 274036.4375 | 299615.7813 | 238141.1406 | 366330.125 | 271238.8438 | 256047.0469 | 287848.5938 | 444758.75 | 230198.3906 | 69609 | 793471.6875 | 483204.0625 | 616280 | 614054.625 | 541283.25 | 334874.0625 | 397069.5 | 282500.9688 | 157288.5625 | 277440.1875 | 107812.6406 | hCG1728627 [Homo sapiens] |
| 0 | 0 | 0 | 0 | 0 | 0 | 0 | 0 | 0 | 0 | 0 | 0 | 0 | 0 | 0 | 0 | 0 | 0 | 0 | 0 | 0 | 0 | 0 | 0 | hCG2003024, partial [Homo sapiens] |
| 45721.53906 | 62541.13281 | 14922.26855 | 21634.34375 | 48065.10938 | 31842.4707 | 29624.17578 | 12324.92578 | 20465.30078 | 42356.60547 | 18486.70313 | 18115.13281 | 20834.33008 | 43364.49219 | 31761.13281 | 14550.49805 | 9493.301758 | 34575.26172 | 16418.60352 | 13486.16211 | 19615.56836 | 0 | 49471.1875 | 14503.87109 | IGHV3-13 isoform 1, partial [Pan troglodytes] |
| 0 | 0 | 23815.22852 | 18294.91992 | 16755.15625 | 0 | 47440.71484 | 38156.35938 | 23319.23828 | 0 | 17470.1582 | 0 | 17483.04688 | 101120.3984 | 21444.74023 | 0 | 0 | 42385.30078 | 0 | 0 | 20134.80078 | 30958.13477 | 20775.3418 | 22109.4082 | RecName: Full=Immunoglobulin heavy variable 4-30-2; Flags: Precursor |
| 105953.3516 | 209081.5625 | 126127.8125 | 287348.0625 | 343834.5 | 294407.5938 | 404372.75 | 206402.2656 | 115006.7031 | 285104.9063 | 47511.70313 | 267470.5313 | 220994.6875 | 258846.2188 | 109511.1719 | 224450.1563 | 127513.8984 | 204888.9375 | 321764.3438 | 202665.875 | 140497.1406 | 110620.9844 | 148848.5 | 221162.0156 | RecName: Full=Immunoglobulin kappa variable 3D-15; Flags: Precursor |
| 68275.19531 | 193067 | 19049.19727 | 50709.75 | 45584.46875 | 34035.81641 | 38710.1875 | 43073 | 96775.17188 | 39162.10547 | 11691.93164 | 20267.38672 | 33691.80078 | 74666.82031 | 20560.42969 | 132050.5156 | 51931.30859 | 51047.40234 | 36932.63281 | 31945.58594 | 24067.11914 | 65900.07813 | 30687.4668 | 15195.38086 | RecName: Full=Immunoglobulin kappa variable 1D-8; Flags: Precursor |
| 97886.95313 | 57473.57031 | 70344.01563 | 61600.50391 | 48467.62891 | 54955.04297 | 67178.35156 | 59577.74219 | 48601.08203 | 55689.95703 | 74805.70313 | 55802.79688 | 70428.60156 | 52098.43359 | 49667.86328 | 70623.69531 | 64682.52734 | 70026.90625 | 59820.42969 | 81627.05469 | 69485.03906 | 71965.33594 | 56939.57813 | 32794.18359 | ALB protein [Homo sapiens] |
| 0 | 0 | 0 | 0 | 0 | 0 | 0 | 0 | 0 | 0 | 0 | 0 | 0 | 0 | 0 | 0 | 0 | 0 | 0 | 0 | 0 | 0 | 0 | 25692.96094 | TPMsk3, partial [Homo sapiens] |
| 85553.46875 | 0 | 0 | 2737.676514 | 0 | 0 | 3772.150635 | 0 | 0 | 0 | 0 | 1512.176025 | 3128.655762 | 0 | 0 | 0 | 0 | 1329.50415 | 0 | 12006.45801 | 5410.782227 | 4592.258301 | 4861.004883 | 8065.727051 | beta-parvin isoform X3 [Homo sapiens] |
| 89660.125 | 30337.04102 | 155004.6094 | 92175.84375 | 173959.4063 | 75550.77344 | 121096.2578 | 62809.65234 | 133456.8125 | 102648.3516 | 87805.80469 | 110409.4766 | 117761.9844 | 226875.5 | 160612.9688 | 80565.36719 | 134973.3594 | 226468.0938 | 36443.37891 | 24867.17188 | 75693.34375 | 64614.67578 | 96340.28125 | 63412.85547 | RecName: Full=Immunoglobulin kappa variable 2D-40; AltName: Full=Ig kappa chain V-II region Cum; Flags: Precursor |
| 1944.395996 | 0 | 693.447876 | 0 | 1691.896973 | 0 | 0 | 1318.864502 | 0 | 0 | 0 | 0 | 0 | 0 | 0 | 3098.270752 | 0 | 0 | 0 | 0 | 0 | 0 | 0 | 945.7796021 | COL6A1 isoform 2 [Pan troglodytes] |
| 48139.75391 | 43702.69531 | 41483.875 | 54267.8125 | 30694.08398 | 56894.07031 | 55237.17188 | 23791.47461 | 45245.38281 | 76637.94531 | 49255.4375 | 49492.78516 | 41341.78906 | 34002.21875 | 33315.98047 | 42517.26172 | 49925.07813 | 52486.07422 | 56594.58594 | 49782.48828 | 62495.07813 | 66861.32813 | 47798.34766 | 42084.88672 | glutathione peroxidase 3 isoform 1 precursor [Homo sapiens] |
| 4028.823242 | 1131.016235 | 1672.931519 | 1289.710205 | 1661.049438 | 0 | 4150.26709 | 4074.125488 | 3429.391846 | 3609.329346 | 4514.210938 | 2841.254639 | 0 | 2445.561523 | 1875.296265 | 1978.804321 | 2087.90918 | 1462.543579 | 2304.476074 | 2989.926514 | 1270.514648 | 0 | 0 | 0 | ICOS ligand isoform c precursor [Homo sapiens] |
| 881817.125 | 666898.25 | 566700.6875 | 416877.0625 | 560315.1875 | 695399.9375 | 282395.5 | 374059.9688 | 515425.875 | 449680.375 | 491193.625 | 558540.75 | 667148.25 | 390459.3438 | 600503.625 | 520886.125 | 707197.75 | 312969.875 | 344785.3125 | 659600.5625 | 815574.8125 | 750808.3125 | 652582.625 | 610946 | SAA2-SAA4 protein precursor [Homo sapiens] |
| 27206.57422 | 15161.98828 | 16184.05078 | 16894.94727 | 18498.13281 | 9903.927734 | 16613.86523 | 19813.08984 | 28237.95703 | 18683.46094 | 11734.45508 | 19483.63281 | 12719.67676 | 28798.11328 | 24831.95898 | 44363.19141 | 18557.96094 | 18331.81641 | 21833.14844 | 27726.06641 | 20545.78711 | 20458.40234 | 20221.82422 | 24089.60156 | coagulation factor V preproprotein [Homo sapiens] |
| 0 | 0 | 0 | 0 | 3517.772461 | 4450.44873 | 0 | 0 | 0 | 0 | 0 | 0 | 0 | 0 | 0 | 0 | 0 | 0 | 0 | 0 | 0 | 4130.577637 | 0 | 0 | hCG1685763, partial [Homo sapiens] |
| 30621.41016 | 19107.02344 | 0 | 49173.82031 | 90757.55469 | 60450.23438 | 0 | 19227.02539 | 1603.891113 | 12961.09766 | 14016.08008 | 122629.4219 | 19980.92383 | 93965.5625 | 17430.09375 | 53758.89844 | 0 | 48336.28516 | 132487.3594 | 99618.23438 | 74383.21875 | 31444.34375 | 31798.54297 | 0 | immunoglobulin delta-chain, partial [Homo sapiens] |
| 0 | 0 | 0 | 0 | 0 | 0 | 0 | 0 | 0 | 0 | 0 | 0 | 0 | 0 | 0 | 0 | 0 | 0 | 0 | 0 | 0 | 0 | 0 | 0 | hCG1686394, partial [Homo sapiens] |
| 250780.1719 | 137341.625 | 289583.125 | 283487.1875 | 341917.4375 | 184790.4375 | 192153.5625 | 336763.2813 | 328034.3125 | 717248.9375 | 371398.3125 | 324745.2188 | 222939.9219 | 683011.1875 | 272555.125 | 150795.2344 | 152432.5469 | 396913.1563 | 252647.2656 | 233789.4844 | 287403.7188 | 139121.5938 | 324841.4063 | 174490.0781 | RecName: Full=Immunoglobulin heavy variable 3-49; Flags: Precursor |
| 382411.6875 | 224121.5469 | 338690.0313 | 304397.9688 | 184586.9844 | 310744.6875 | 278788.9063 | 259157.7188 | 232005.625 | 243565.0938 | 353655.8438 | 246893.875 | 279474.375 | 247708.8438 | 293190.4688 | 279841.7813 | 280863.6875 | 312375.5 | 298184.9688 | 357917.75 | 228837.3906 | 283463.625 | 210199.6719 | 207783.9375 | gelsolin isoform d [Homo sapiens] |
| 342749.2813 | 77793.54688 | 17440.99219 | 98948.41406 | 323821.625 | 175018.4063 | 133027.8125 | 115525.6875 | 137547.9375 | 144271.5156 | 131653.7344 | 156906.75 | 237358.5313 | 134306.1406 | 69967.78125 | 95751.375 | 71205.30469 | 180905.2344 | 91269.19531 | 152796.0313 | 109914.8594 | 72710.94531 | 93152.78906 | 64266.58203 | RecName: Full=Immunoglobulin kappa variable 6D-21; Flags: Precursor |
| 0 | 2919.806885 | 0 | 0 | 3355.262939 | 0 | 0 | 2273.007813 | 0 | 0 | 0 | 4393.960449 | 0 | 0 | 0 | 0 | 8088.286133 | 0 | 3891.827393 | 7323.257813 | 3582.310059 | 2815.200684 | 9932.4375 | 7398.460938 | integrin-linked protein kinase isoform X1 [Homo sapiens] |
| 45093.84766 | 17631.2168 | 29584.84375 | 32992.37109 | 39214.27344 | 25864.98242 | 33264.94531 | 31948.05859 | 80477.24219 | 53008.55469 | 23262.34375 | 0 | 30620.80859 | 61309.08984 | 43336.51953 | 11028.08105 | 57135.01563 | 52470.25781 | 26661.54492 | 40782.77734 | 24406.5625 | 33103.80859 | 55406.26563 | 28850.01367 | RecName: Full=Immunoglobulin lambda variable 1-36; Flags: Precursor |
| 99311.82813 | 28035.59766 | 23919.50977 | 27004.77539 | 31616.83203 | 25534.23633 | 11980.15039 | 54343.05859 | 18922.40625 | 48110.08984 | 97518.53125 | 94689.42969 | 89370.59375 | 81691.39844 | 11829.75977 | 108681.8203 | 69990.35156 | 32606.89258 | 132104.1875 | 22952.36719 | 67164.14844 | 42194.44531 | 48731.72656 | 91560.61719 | RecName: Full=Immunoglobulin heavy variable 6-1; Flags: Precursor |
| 15939.33691 | 56871.03906 | 42810.32813 | 54550.22656 | 46121.05078 | 34943.35547 | 22746.58203 | 39701.76172 | 39740.04297 | 49921.69141 | 19317.54883 | 21361.38086 | 34231.45313 | 32859.91797 | 43914.08203 | 10319.15625 | 14679.31445 | 35903.75781 | 27867.75195 | 32976.62109 | 11289.80664 | 39869.59766 | 27641.20313 | 23611.5293 | unnamed protein product [Homo sapiens] |
| 0 | 362322.625 | 169412.3906 | 8039.666992 | 153107.4063 | 0 | 0 | 33920.85547 | 78231.07031 | 57244.96875 | 15324.10645 | 0 | 55009.64453 | 116716.2266 | 634091.3125 | 63821.05859 | 30024.96875 | 0 | 197937.9219 | 0 | 0 | 25786.57422 | 78333.91406 | 0 | RecName: Full=Immunoglobulin heavy variable 3-21; Flags: Precursor |
| 120300.3516 | 0 | 47502.69141 | 5984.420898 | 9348.268555 | 8014.646484 | 136318.0781 | 3166.497559 | 10569.58398 | 6579.438477 | 128997.1641 | 8118.852539 | 7752.498535 | 14280.55469 | 6464.922852 | 3530.887207 | 17719.63672 | 12031.34375 | 25680.86719 | 0 | 4141.841309 | 2184.388672 | 7261.76709 | 3391.211426 | RecName: Full=Immunoglobulin heavy variable 2-26; Flags: Precursor |
| 0 | 0 | 95857.10156 | 127607.125 | 97954.92969 | 50305.33203 | 46750.08203 | 35913.44922 | 45624.36328 | 197082.5625 | 45268.26563 | 48561.16016 | 91347.89063 | 160997.7656 | 111206.4141 | 57036.76172 | 11881.88281 | 0 | 103052.4688 | 153524.9688 | 43168.48438 | 29988.18359 | 63246.39063 | 79449 | immunoglobulin heavy chain VH3, partial [Homo sapiens] |
| 128785.6719 | 43618.86719 | 79727.60156 | 165775.6406 | 90831.83594 | 84647.5 | 63058.39844 | 85873.51563 | 151391.7031 | 106137.2031 | 98428.3125 | 135579.8906 | 119441.2422 | 133303.1406 | 93593.13281 | 53737.38281 | 69258.23438 | 133265.5156 | 69318.0625 | 85691.64844 | 50376.10938 | 88304.42188 | 255410.1406 | 46261.35547 | RecName: Full=Immunoglobulin heavy variable 3-43; Flags: Precursor |
| 114153.3984 | 97518.52344 | 133620.7188 | 0 | 170629.125 | 39855.08203 | 72738.61719 | 101308.125 | 142505.2344 | 70767.01563 | 119733.2891 | 41803.01563 | 87083.98438 | 84242.01563 | 105588.7578 | 181482.4688 | 137599.7344 | 136318.3281 | 0 | 70389.9375 | 43517.78906 | 68400.71875 | 149111.3125 | 33005.21094 | Unknown (protein for IMAGE:4575521), partial [Homo sapiens] |
| 6919974 | 8890846 | 14383223 | 9229635 | 29864.23242 | 13886075 | 11929958 | 9979652 | 15738372 | 9176545 | 19000814 | 11237416 | 6299262 | 7447643 | 8160253.5 | 12809598 | 11617129 | 9091742 | 6237145 | 11124149 | 9030593 | 6054720.5 | 8992545 | 14469918 | immunoglobulin lambda-3 surrogate light chain [Homo sapiens] |
| 9023.004883 | 12008.8252 | 14320.0166 | 14344.7041 | 16681.41211 | 11935.83691 | 17736.76563 | 17863.53125 | 14169.95117 | 14279.26172 | 20143.06641 | 8955.933594 | 10879.92969 | 16271.67383 | 8563.142578 | 13543.35156 | 17432.37891 | 25714.07227 | 13592.02344 | 18016.77734 | 15308.18066 | 30092.85742 | 14998.81055 | 8847.288086 | RecName: Full=Immunoglobulin kappa variable 1D-13; Flags: Precursor |
| 12822.58594 | 7612.132813 | 8618.712891 | 11977.18359 | 18135.53906 | 16368.67969 | 11432.17773 | 9647.994141 | 19803.87109 | 7502.34668 | 17586.40234 | 25449.32422 | 16964.5625 | 11296.31348 | 6952.401367 | 15297.28418 | 27873.97266 | 24954.65625 | 10120.89648 | 27550.99805 | 18348.16797 | 20400.94336 | 16019.94434 | 22804.63867 | glycoprotein Ib (platelet), alpha polypeptide [Homo sapiens] |
| 65041.08594 | 19816.17383 | 82835.26563 | 86637.33594 | 42302.07031 | 59575.91016 | 27159.84961 | 56328.81641 | 25550.79102 | 49320.13672 | 41366.57031 | 88026.07813 | 34011.89063 | 67205.21094 | 36203.44531 | 53614.82813 | 35375.87109 | 56796.17969 | 48116.46094 | 40274.85547 | 23074.04297 | 16533.57813 | 28001.91016 | 26388.77539 | complement factor H-related protein 4 [Homo sapiens] |
| 6062.412109 | 11120.86328 | 43560.18359 | 41290.94922 | 92072.89063 | 10980.41211 | 91661.28125 | 27331.71484 | 58906.44531 | 11546.38965 | 22662.78906 | 22633.67578 | 56754.97656 | 85490.34375 | 35086.05469 | 17962.13281 | 93101.70313 | 28339.54492 | 72783.33594 | 52563.86719 | 14086.71484 | 35612.98047 | 26678.86914 | 30546.51367 | RecName: Full=Immunoglobulin kappa variable 6-21; Flags: Precursor |
| 651913.375 | 455105.4688 | 367204.5 | 748100.5 | 775574.9375 | 533642.75 | 573048.5625 | 463835.2188 | 643531.5 | 687640.1875 | 292441.9375 | 654901.8125 | 423136.8438 | 609807.125 | 422759.625 | 306398.9063 | 384162.1563 | 464998.4063 | 380052.4688 | 418556.4375 | 306445.25 | 436868.25 | 837906.125 | 271225.5313 | hCG1686089, partial [Homo sapiens] |
| 59472.98047 | 15201.29102 | 5074.828125 | 37102.76172 | 54724.78906 | 16308.47949 | 47938.70703 | 49468.36719 | 31920.38086 | 27015.38477 | 77431.67188 | 25164.44141 | 38747.71484 | 36860.33203 | 17202.82422 | 34963.92969 | 35318.67578 | 37572.96875 | 26010.1875 | 18336.88477 | 16746.33008 | 24106.07617 | 57683.61328 | 29448.73438 | immunoglobulin heavy chain variable region, partial [Homo sapiens] |
| 31350.20313 | 25863.15625 | 23362.67969 | 23238.12891 | 29395.66992 | 25848.9375 | 24143.72266 | 29104.3125 | 26071.71875 | 20491.35938 | 23444.49609 | 19046.12305 | 29128.71875 | 26561.70313 | 26025.66992 | 30717.49023 | 33792.53516 | 39339.05078 | 22659.75 | 25595.58984 | 24829.01172 | 40681.95703 | 29465.53711 | 19378.5918 | immunoglobulin heavy chain variable region, partial [Homo sapiens] |
| 0 | 64209.91406 | 0 | 30188.03711 | 37788.71484 | 36929.25 | 108125.3516 | 37178.51953 | 57377.125 | 107908.0547 | 62982.66797 | 0 | 29813.60742 | 148386.6563 | 36460.26953 | 89182.09375 | 28020.85352 | 170846.375 | 30005.42188 | 82197.32813 | 97459.21094 | 108735.9922 | 111104.9844 | 33850.56641 | RecName: Full=Immunoglobulin heavy variable 3-20; Flags: Precursor |
| 5324.356934 | 25101.64258 | 2796.508301 | 7235.057617 | 20040 | 7165.12793 | 2413.065918 | 8506.923828 | 4340.528809 | 4648.520996 | 26011.04492 | 1482.472168 | 20050.30273 | 9294.495117 | 3122.996094 | 9830.473633 | 3313.875 | 4379.76123 | 8797.974609 | 8871.62207 | 4556.357422 | 9126.181641 | 16292.78516 | 19258.70313 | immunoglobulin heavy chain variable region, partial [Homo sapiens] |
| 1206763.625 | 724341.875 | 1422820.25 | 1411253.25 | 1361123.625 | 748819.5 | 101712.9844 | 938853.0625 | 362734.3438 | 708331.375 | 1379660.875 | 1050082.125 | 1248761.875 | 923161.4375 | 1194293.25 | 1061516.375 | 146948.2656 | 1302802.875 | 120681.8906 | 1340042.75 | 862918.5 | 1015321.938 | 1640919.625 | 681446.0625 | RecName: Full=Immunoglobulin heavy variable 4-28; Flags: Precursor |
| 62500.22656 | 47063.78906 | 63705.86328 | 59695.37109 | 59354.82031 | 68040.96875 | 63296.29297 | 43898.10156 | 36238.82031 | 55628.32031 | 41265.67969 | 82438.21094 | 54796.4375 | 59997.62109 | 47579.25391 | 44570.03906 | 53110.15234 | 35035.55078 | 59819.90625 | 54520.49219 | 62286.74219 | 69027.90625 | 72874.99219 | 32541.93359 | immunoglobulin heavy chain variable gene IGHV3-38, partial [Homo sapiens] |
| 169627.4063 | 66125.63281 | 42855.74609 | 121143.3516 | 18402.66016 | 40233.63672 | 198699.2188 | 137570.7188 | 174917.6406 | 149994.25 | 113095.8281 | 188902.4375 | 119827.4219 | 207059.375 | 168551.75 | 77113.125 | 310042.1563 | 261144.875 | 62866.03516 | 75146.50781 | 119172.3281 | 199073.5625 | 159006.4531 | 29965.49414 | RecName: Full=Immunoglobulin heavy variable 5-51; Flags: Precursor |
| 3321.731689 | 25807.66797 | 3528.057373 | 64407.48828 | 58193.60156 | 30339.0625 | 38287.45703 | 4894.036133 | 34245.69922 | 29603.42383 | 8636.668945 | 54363.38672 | 50323.89844 | 44233.76953 | 27418.46875 | 36329.59375 | 3029.841553 | 38537.03906 | 7109.566406 | 26110.77734 | 5609.934082 | 53837.13281 | 54909.76953 | 12702.09766 | immunoglobulin heavy chain variable region, partial [Homo sapiens] |
| 73661.02344 | 41907.64063 | 758448.875 | 71074.52344 | 23632.08594 | 646049.9375 | 494786.0313 | 413916.1563 | 628774.5 | 918793.625 | 604060.5 | 404772.6563 | 51913.01563 | 1363770.125 | 553792.0625 | 28477.90039 | 80507.38281 | 876094 | 456959.0625 | 45951.96875 | 652268.0625 | 463024.375 | 764301.625 | 37545.44141 | RecName: Full=Immunoglobulin heavy variable 2-70D; Flags: Precursor |
| 9264044 | 5416517 | 8078554 | 10982505 | 11887404 | 9560545 | 8790751 | 3849596.25 | 7695653.5 | 6739346.5 | 8230571.5 | 5824994 | 10202232 | 8222282 | 7962209 | 8135846.5 | 7056425 | 11143557 | 9571280 | 7742261 | 10087662 | 7164939.5 | 10871252 | 6701130 | RecName: Full=Immunoglobulin kappa variable 3D-7; Flags: Precursor |
| 23101.80469 | 10037.55078 | 43865.15234 | 41284.82813 | 46341.89844 | 33320.16406 | 0 | 38741.89844 | 0 | 42698.875 | 55617 | 4583.979004 | 32415.10938 | 18493.19531 | 24533.14453 | 31229.77539 | 0 | 17690.57031 | 11562.95605 | 24900.42773 | 17640.26758 | 0 | 41574.73438 | 0 | Ig kappa V-region e, partial [Homo sapiens] |
| 106015.7188 | 50328.9375 | 140315.75 | 69520.19531 | 55556.16797 | 176864.5938 | 131830.0781 | 39085.24219 | 69276.29688 | 161048.5625 | 44723.76172 | 97193.79688 | 108077.2344 | 175126.7344 | 94463.01563 | 119389.125 | 90419.47656 | 169052.8594 | 71712.21094 | 59327.80078 | 77888.64063 | 49209.73438 | 126513.4844 | 113418.1328 | immunoglobulin light chain variable region, partial [Homo sapiens] |
| 14555.10156 | 16391.69336 | 0 | 0 | 0 | 0 | 9406.516602 | 0 | 0 | 10852.97168 | 0 | 16628.4043 | 11530.88281 | 0 | 0 | 0 | 5430.648926 | 25738.30469 | 0 | 0 | 16589.04297 | 0 | 0 | 0 | MHC class I antigen, partial [Homo sapiens] |
| 48769.29688 | 33486.72266 | 45729.51172 | 34852.28125 | 33885.17188 | 30654.06641 | 28249.33203 | 35376.75 | 34095.98047 | 31824.12695 | 45160.61328 | 28722.01953 | 45990.28906 | 31287.44922 | 30299.68359 | 25816.8457 | 51445.52734 | 38733.875 | 34019.23438 | 46081.28906 | 42280.21094 | 29060.85547 | 42248.71484 | 33708.39063 | complement C2 isoform 5 [Homo sapiens] |
| 5095241 | 1416293.375 | 1187805.125 | 2820104 | 3066326 | 3766283 | 2884875 | 1437085.5 | 2676315.25 | 1391207 | 2728706 | 2175213.75 | 3291605.5 | 4248754.5 | 1496387.25 | 1763556.125 | 2860444.25 | 4127381 | 1228532.75 | 2796189.5 | 3938516 | 2244934.5 | 3061577 | 3682485.75 | Immunoglobulin heavy chain variant, partial [Homo sapiens] |
| 0 | 61819.34766 | 0 | 0 | 0 | 0 | 0 | 0 | 0 | 0 | 0 | 0 | 0 | 0 | 0 | 0 | 0 | 1350.229614 | 0 | 0 | 0 | 0 | 0 | 0 | RecName: Full=Immunoglobulin heavy variable 1-69-2; Flags: Precursor |
| 492916.8125 | 368160.375 | 442324.375 | 344189.3125 | 367408.4375 | 513360.0625 | 499083.6875 | 165704.8906 | 432169.9375 | 489501 | 320653.4375 | 843569.3125 | 344771.2188 | 352759.1875 | 276740.6563 | 1315896.875 | 604677.9375 | 360696.25 | 529430.75 | 273321.625 | 425570.0938 | 251351.8281 | 388259.7813 | 256476.7188 | complement C4A (Rodgers blood group)-like preproprotein [Homo sapiens] |
| 247468.4063 | 97147.04688 | 188304.7344 | 217476.8438 | 237086.6719 | 342008.6563 | 331716.6875 | 252520.7969 | 224669.5 | 338974.625 | 210824.9531 | 341025.7813 | 254247.9688 | 285187.0625 | 134083.5781 | 237234.4219 | 206076.7969 | 323751.6875 | 358210.9688 | 197629.7969 | 311645.6875 | 226701.3281 | 203709.5156 | 281601.375 | hCG2042707, partial [Homo sapiens] |
| 0 | 28443.73828 | 19566.63086 | 61176.96875 | 9933.757813 | 26536.39453 | 0 | 31603.95508 | 2678.13208 | 57904.95703 | 2834.679199 | 34293.16797 | 48941.13281 | 30563.23047 | 48279.83203 | 64828.32422 | 44078.29688 | 3366.667236 | 0 | 26854.90039 | 20164.65625 | 56882.15625 | 70675.35156 | 22692.73633 | Lambda-V immunoglobulin light chain variable domain precursor, partial [Homo sapiens] |
| 70549.125 | 54428.33984 | 56558.63281 | 54687.875 | 78129.88281 | 0 | 58200.10156 | 0 | 0 | 24335.32617 | 49559.67188 | 0 | 67853.89063 | 0 | 0 | 39219.74609 | 0 | 61210.79297 | 0 | 0 | 0 | 67222.47656 | 70602.23438 | 0 | RecName: Full=Immunoglobulin heavy variable 7-4-1; Flags: Precursor |
| 0 | 0 | 0 | 0 | 0 | 0 | 0 | 0 | 0 | 0 | 0 | 0 | 0 | 0 | 0 | 0 | 0 | 0 | 0 | 0 | 0 | 0 | 14407.34473 | 0 | hypothetical protein EGK_21328, partial [Macaca mulatta] |
| 18244.16797 | 0 | 16159.64355 | 11667.13281 | 37484.12891 | 21580.87695 | 19582.35156 | 23989.97656 | 44568.24219 | 6327.999512 | 15992.21777 | 31203.05273 | 6182.780273 | 15547.77344 | 3422.777588 | 15375.54102 | 13720.72754 | 40018.68359 | 19077.00195 | 72501.24219 | 105393.6875 | 10038.3291 | 70084.10938 | 30753.65234 | RecName: Full=Immunoglobulin heavy variable 3-64D; Flags: Precursor |
| 430558 | 112115.3203 | 314720 | 262340.3438 | 248639.4375 | 404201.4063 | 459647.6563 | 165434.6563 | 547402 | 749860.5 | 405143.1875 | 519830.9063 | 282092.5 | 521241.1563 | 464757.9375 | 172377.5 | 460486 | 175192.7188 | 249276.8125 | 191454 | 350904.5625 | 103118.5859 | 195921.0781 | 117526.0234 | RecName: Full=Immunoglobulin heavy variable 5-10-1; Flags: Precursor |
| 1005064.125 | 18602.00977 | 24938.27734 | 27683.61719 | 30034.125 | 18841.67188 | 29898.20313 | 25556.30469 | 913297.125 | 34459.58594 | 26553.14063 | 35697.67969 | 34319.08594 | 68450.94531 | 55652.27344 | 15714.03223 | 764244.0625 | 60194.44922 | 25869.4668 | 549507.6875 | 605174.8125 | 724066.3125 | 137735.4688 | 16042.4873 | immunoglobulin heavy chain VDJ region, partial [Homo sapiens] |
| 203002.0938 | 206154.3125 | 184574.75 | 198844.125 | 230895.7813 | 133818.4844 | 149046.3125 | 207211.4688 | 259359.9063 | 146812.9063 | 193254.6875 | 173303.1875 | 201755.375 | 224311.2031 | 207565.5 | 394625.125 | 214377 | 175295.1719 | 177764.8125 | 195284.5625 | 278405.7188 | 294528.75 | 248718.4688 | 317844 | vitamin K-dependent protein S isoform 1 precursor [Homo sapiens] |
| 37595.74609 | 28085.50781 | 35320.30469 | 29494.57813 | 23559.80469 | 26319.16797 | 34925.4375 | 25065.63281 | 24250.91406 | 18346.42773 | 32948.58594 | 18692.53906 | 25240.05469 | 35985.85938 | 23779.74805 | 19534.2793 | 27311.40625 | 19573.5957 | 24022.82617 | 23790.22266 | 31067.29102 | 20453.81641 | 23886.8418 | 16295.99316 | EGF-containing fibulin-like extracellular matrix protein 1 isoform X3 [Homo sapiens] |
| 15806.4082 | 12109.61035 | 13129.05078 | 7079.797363 | 7921.185059 | 5821.979492 | 6273.237305 | 7953.083984 | 10526.95313 | 4898.506836 | 5321.135742 | 10867.26953 | 0 | 6287.509277 | 9902.598633 | 6087.253418 | 0 | 0 | 8032.686035 | 10250.49316 | 6958.317383 | 5446.307129 | 5992.23291 | 5786.929688 | tenascin-X isoform 1 precursor [Homo sapiens] |
| 22682.75977 | 17793.34961 | 45772.84766 | 20374.65625 | 17898.63086 | 29941.41016 | 40249.15625 | 23859.54102 | 20308.95313 | 33438.41797 | 45274.51563 | 36907.13672 | 18182.72266 | 47830.13281 | 30594.32422 | 21933.97656 | 36481.88281 | 20531.25781 | 32854.59766 | 26078.29883 | 33637.79297 | 51640.84375 | 21261.97461 | 30152.99219 | Selenoprotein P, plasma, 1 [Homo sapiens] |
| 258492.0156 | 95500.11719 | 153579.5313 | 69066.79688 | 59419.82422 | 59928.5625 | 239869.9375 | 100615.8438 | 168600.8438 | 381322.0938 | 89647.49219 | 165790.9844 | 93465.50781 | 332924.9688 | 83072.24219 | 66289.64063 | 90127.1875 | 191975.7813 | 49499.33594 | 114855.9688 | 73202.13281 | 102098.7578 | 126750.8594 | 79297.03125 | glial fibrillary acidic protein [Mus musculus] |
| 6411095 | 4003640 | 1411621.875 | 3768995 | 4292955 | 4620480.5 | 7490018 | 1883469.5 | 3299595.25 | 6999816.5 | 7265170 | 7897884.5 | 3886913 | 12169378 | 2198148.75 | 2317257.25 | 3994871.25 | 4191763.5 | 3565917.5 | 3827021.75 | 5102112.5 | 6099765 | 8816712 | 4939831 | IGHA1 isoform 1, partial [Pan troglodytes] |
| 313061.7188 | 538985.3125 | 267027.0625 | 771860.75 | 926483.0625 | 510263.5 | 1643046 | 423586.5 | 584537.8125 | 1690302.875 | 734900.8125 | 321388.2188 | 524202.2813 | 726160.5625 | 785305.0625 | 478899.375 | 604150.6875 | 1291563.5 | 872159.5625 | 699354.8125 | 676003.8125 | 537867.25 | 682321.9375 | 460689.1875 | unnamed protein product [Homo sapiens] |
| 242186.9844 | 126110.1016 | 196837 | 164723.5 | 394536.5625 | 490889.1875 | 876276.375 | 234434.3594 | 158294.6094 | 431977.9375 | 207369.6094 | 297000.4688 | 260331.4688 | 505654.4375 | 196378.4063 | 226236.0625 | 649617.6875 | 299158.4375 | 310489.6563 | 118608.875 | 363369.125 | 319602 | 294606.4688 | 211641.125 | IGKV1D-33 isoform 2, partial [Pan troglodytes] |
| 189188.2188 | 196560.9219 | 240905.3125 | 235541.9219 | 247442.875 | 181083.625 | 145079.4375 | 183875.4063 | 157192.2188 | 153501.125 | 204330.5313 | 173337.125 | 227836.6563 | 196785.7188 | 155367.7813 | 265055.625 | 206640.0156 | 164391.9688 | 138280.0781 | 143900.6094 | 159950.7969 | 78893.42188 | 114514.0156 | 137312.3281 | complement factor I isoform X2 [Homo sapiens] |
| 0 | 2920.986572 | 0 | 2178.631348 | 3792.163818 | 0 | 0 | 0 | 0 | 0 | 0 | 0 | 4045.272949 | 0 | 0 | 0 | 0 | 0 | 2168.17749 | 7691.525879 | 4572.293945 | 4482.450684 | 0 | 9762.288086 | tropomyosin alpha-4 chain isoform 4 [Homo sapiens] |
| 0 | 0 | 0 | 0 | 0 | 0 | 0 | 0 | 0 | 0 | 0 | 0 | 0 | 1190.114868 | 0 | 0 | 1693.596436 | 0 | 0 | 0 | 0 | 0 | 0 | 0 | late histone H2B.L4-like [Piliocolobus tephrosceles] |
| 0 | 1755.747314 | 0 | 0 | 0 | 0 | 0 | 0 | 0 | 0 | 0 | 1730.946777 | 0 | 0 | 0 | 0 | 0 | 0 | 0 | 6356.699707 | 3089.51416 | 1530.116455 | 3353.962158 | 4789.153809 | alpha-enolase isoform 3 [Homo sapiens] |
| 27151.08984 | 22401.73047 | 57833.68359 | 27404.97461 | 22179.42773 | 49923.75391 | 54980.86328 | 33440.85156 | 89228.64844 | 55928.94141 | 27753.59375 | 55110.26953 | 18701.57813 | 55661.44922 | 17217.17578 | 15160.60449 | 53201.3125 | 86321.53125 | 48981.42578 | 38547.79688 | 3858.51416 | 13784.41211 | 53982.80469 | 18847.57617 | hemoglobin subunit gamma-2 [Pan troglodytes] |
| 296839.9688 | 366502.8125 | 238528.0625 | 292637.6563 | 388031.0938 | 255254.3125 | 276610.6875 | 372584.8125 | 503727.8125 | 357786.9688 | 256444.875 | 299547.3438 | 195348.625 | 283037.0625 | 259864.25 | 528900.5 | 356181.2813 | 222679.7344 | 286886.5938 | 239503.6563 | 271667.2813 | 305991.0938 | 336486.625 | 317797.7813 | complement C1r subcomponent isoform 1 preproprotein [Homo sapiens] |
| 711315.625 | 143522.875 | 739480.875 | 754812.5625 | 228797.2188 | 604789.5625 | 1229912.125 | 148576.6719 | 1045455.313 | 1142503.5 | 54181.40234 | 560973.5 | 199430.5313 | 817557.3125 | 657561.6875 | 697406.25 | 725731.625 | 1102819.375 | 1274113.875 | 1607372 | 1338569.25 | 116876.6563 | 1743861.75 | 747347.25 | immunoglobulin heavy chain variable region, partial [Homo sapiens] |
| 34560.13672 | 0 | 14120.84961 | 11901.70215 | 26763.45898 | 20141.67383 | 15173.34961 | 6223.390625 | 23861.80859 | 24695.13477 | 0 | 11327.13965 | 0 | 20153.7207 | 11493.78711 | 17121.39063 | 31300.15625 | 24307.44141 | 0 | 0 | 4827.895996 | 0 | 0 | 9450.210938 | RecName: Full=Immunoglobulin lambda constant 7; AltName: Full=Ig lambda-7 chain C region |
| 545410.5625 | 268995.5313 | 470674.625 | 2807030.25 | 704613.1875 | 1880002.5 | 581471.1875 | 467593.2188 | 419815.4688 | 582874.625 | 342668.5313 | 1960193 | 510053.7188 | 672183 | 519889.4688 | 481310.0625 | 1854384.625 | 2578101.25 | 410737.0313 | 447059.0625 | 1582545 | 2369982 | 643645.0625 | 360734.8438 | immunoglobulin kappa chain variable region, partial [Homo sapiens] |
| 113402.4063 | 71568.45313 | 18309.66992 | 72444.55469 | 81200.04688 | 99132.01563 | 90448.82813 | 87972.6875 | 30654.82422 | 121707.7734 | 52805.08984 | 31763.30078 | 108540.5078 | 163106.125 | 61120.22266 | 60780.44922 | 69161.23438 | 12222.50195 | 84472.36719 | 133314.1719 | 136390.0313 | 143153.0938 | 64843.52734 | 137120.8438 | immunoglobulin kappa chain variable region, partial [Homo sapiens] |
| 1297.802124 | 3692.109375 | 0 | 0 | 1783.9646 | 0 | 0 | 3752.822021 | 0 | 0 | 0 | 0 | 0 | 0 | 0 | 2241.529785 | 0 | 0 | 0 | 0 | 1014.933411 | 0 | 4506.060547 | 0 | proprotein convertase subtilisin/kexin type 9 preproprotein [Homo sapiens] |
| 44428.66797 | 38091.17969 | 29979.10742 | 65300.78125 | 43750.29688 | 27264.91406 | 22596.99219 | 24418.07031 | 21067.04297 | 20186.94727 | 41312.21484 | 34229.79688 | 55029.20313 | 21145.40625 | 39536.37891 | 21133.38477 | 27693.43164 | 41296.49219 | 32784.99219 | 25680.91406 | 28243.42773 | 69253.65625 | 20629.03125 | 24345.81641 | insulin-like growth factor binding protein 3 [Homo sapiens] |
| 583888.1875 | 449141.875 | 440358.1875 | 2464564.25 | 2159238.25 | 3754005.75 | 4839585.5 | 1316296.75 | 3192443.25 | 327989.0625 | 6423645 | 4780017.5 | 4335179 | 258758.9688 | 284942.3125 | 434523.1875 | 1419317.625 | 5323746.5 | 541496.4375 | 2237114.25 | 457269.1563 | 2954161 | 1967257.25 | 611222.6875 | apolipoprotein C-III precursor variant 1 [Homo sapiens] |
| 20204.61719 | 10652.81836 | 6660.820313 | 22692.30078 | 15924.91992 | 15665.97168 | 17590.2168 | 16775.99414 | 12175.46191 | 6115.876953 | 18212.73438 | 0 | 24600.69336 | 17787.39063 | 33946.16406 | 18067.88281 | 0 | 0 | 0 | 16757.55273 | 21878.30859 | 21654.88281 | 0 | 22514.24805 | FBLN1 isoform 5 [Pongo abelii] |
| 512365.0938 | 595293.75 | 433384.375 | 726470.75 | 514303.4688 | 618345 | 648924.4375 | 705608 | 457841.5625 | 477753.3125 | 661738.9375 | 486552.9375 | 666738 | 469954.5 | 485965.1563 | 595230.75 | 677027.625 | 459286.8125 | 657500.875 | 627239.5 | 544969.8125 | 555105.625 | 379234.2813 | 347340.6875 | unnamed protein product [Homo sapiens] |
| 614338.9375 | 458361.5 | 522535.3125 | 458265.5625 | 357281.3125 | 508571.5 | 535455.5 | 407650.5625 | 536974 | 631023.875 | 417283.9375 | 561370.75 | 607731.875 | 520558.4063 | 498517.1875 | 528992.75 | 597859.125 | 579869.3125 | 477059.8125 | 569940.5625 | 494249.2813 | 517000.375 | 488855.1563 | 411693.9375 | ITIH4 protein [Homo sapiens] |
| 0 | 0 | 0 | 1009.677734 | 2126.748779 | 0 | 2167.614502 | 1968.413086 | 0 | 0 | 1441.007202 | 3968.427734 | 3396.691895 | 0 | 1555.036987 | 0 | 1891.231934 | 0 | 0 | 5015.477051 | 0 | 0 | 2585.567627 | 2799.739502 | unnamed protein product [Homo sapiens] |
| 4052.620361 | 4544.37207 | 0 | 0 | 0 | 6309.955078 | 0 | 0 | 0 | 0 | 0 | 4213.666016 | 0 | 4575.731445 | 3745.429932 | 3971.450684 | 0 | 0 | 2963.045166 | 0 | 4240.178711 | 2521.699463 | 1783.03833 | 0 | RARRES2 isoform 3, partial [Pan troglodytes] |
| 12467.93945 | 20336.12891 | 16896.22656 | 22599.32422 | 12648.46387 | 15846.64551 | 12015.07715 | 14164.67578 | 7080.715332 | 8119.530762 | 16210.06055 | 11740.31934 | 15840.95801 | 6329.507813 | 17612.16992 | 8272.601563 | 7145.276367 | 12782.5918 | 11732.95703 | 15480.31445 | 16091.16992 | 6145.4375 | 8562.048828 | 11578.38086 | serotransferrin isoform 1 precursor [Homo sapiens] |
| 3948696.25 | 4695059.5 | 4560680.5 | 1915467.625 | 3420839.75 | 5350909 | 3460746.5 | 5735149 | 4946182.5 | 3736858.5 | 3554533.5 | 4858242 | 5653527 | 4393698 | 5321726.5 | 5090099 | 5316520 | 3620402 | 4702538 | 5091218 | 5026679 | 6474119 | 3874174.5 | 7888813 | FGG isoform 6 [Pan troglodytes] |
| 0 | 0 | 0 | 2640.541992 | 1715.181641 | 0 | 0 | 0 | 0 | 0 | 0 | 1762.904785 | 0 | 0 | 0 | 0 | 0 | 0 | 0 | 1566.440063 | 0 | 0 | 1909.333252 | 3984.421143 | PREDICTED: latent-transforming growth factor beta-binding protein 1 isoform X2 [Rhinopithecus roxellana] |
| 1068952.625 | 573198.4375 | 744150.625 | 867429.5 | 596545.125 | 683002.375 | 767815.0625 | 1172845.25 | 946721 | 911456.875 | 826463.4375 | 856180.5 | 747929.875 | 732204.5 | 747762.625 | 1008673.375 | 1027236.375 | 972733.25 | 805386.375 | 778601.3125 | 936477 | 1268095.125 | 913185.875 | 723320.875 | APOD isoform 3, partial [Pan troglodytes] |
| 1732614.625 | 1066797.5 | 1758814.75 | 589882.4375 | 1032982.063 | 1729708.375 | 925396.75 | 1816203.25 | 1709599.5 | 894220.8125 | 1166424 | 1637888.75 | 1403208.25 | 1373049.125 | 2055681.5 | 2869391 | 2203050.75 | 1161989.625 | 2212173.25 | 2523750 | 2140480.75 | 2867153.5 | 2273030 | 3403937 | hypothetical protein, partial [Homo sapiens] |
| 1895017.25 | 2334633.5 | 2117813 | 2721579.5 | 1746230.75 | 1484977 | 1791476.5 | 1784580.75 | 1483847.5 | 1891905.25 | 2265136.75 | 1694473.75 | 2277681.5 | 1497816 | 2570894 | 1776938.75 | 2427911.25 | 2012569.625 | 2924619 | 2899254.5 | 2215754.75 | 2230913 | 1985870.625 | 1554695 | alpha-2-HS-glycoprotein isoform 1 preproprotein [Homo sapiens] |
| 42228.10938 | 106898.4766 | 237995.5313 | 132770.8906 | 147910.6094 | 21054.72461 | 42104.18359 | 14744.74023 | 210910.8125 | 42597.32031 | 127416.2734 | 37652.91406 | 165436.6875 | 30183.20508 | 195663.1406 | 25357 | 45897.49609 | 21829.21094 | 213133.0469 | 16440.92383 | 9696.777344 | 12482.81543 | 184179.0156 | 168910 | transmembrane protein 198 [Macaca mulatta] |
| 91915.92969 | 118318.3281 | 102447.375 | 130832.4688 | 87835.73438 | 109320.0156 | 103585.2344 | 96580.32813 | 83994.15625 | 92216.20313 | 112675.6094 | 89753.03125 | 113619.75 | 94646.85938 | 97936.60156 | 99980.64063 | 82784.77344 | 77288.875 | 136912.8438 | 149767.5625 | 103831.7188 | 105491.2891 | 123918.4375 | 152165.8906 | complement C1q subcomponent subunit B precursor [Homo sapiens] |
| 34800.13672 | 27771.86328 | 40327.16406 | 27116.62891 | 25406.68555 | 17182.01758 | 28195.16797 | 28964.05078 | 24534.87695 | 25301.49023 | 23881.2207 | 42371.14063 | 28528.20117 | 17944.56641 | 23313.07422 | 34966.08984 | 25647.66016 | 27903.13281 | 33399.99219 | 43571.63281 | 28338 | 28540.35938 | 30882.52344 | 29024.41406 | hepatocyte growth factor activator isoform 1 preproprotein [Homo sapiens] |
| 3044214.5 | 1331387.25 | 1913864.625 | 1207144.125 | 4012648 | 2622407 | 707830.75 | 2513733.75 | 3271924.75 | 2153150.75 | 1375371.375 | 825349.9375 | 1634895.25 | 2025363.5 | 2752524.25 | 3202786.5 | 968526.0625 | 2532768 | 1308397.5 | 2892826.75 | 2023906.5 | 3359708.25 | 8085970.5 | 1625166.875 | JCHAIN isoform 4, partial [Pongo abelii] |
| 165497.2188 | 47843.85547 | 296411.9063 | 146489.25 | 90972.96875 | 364478.375 | 117601.9609 | 203472.2188 | 148104.2031 | 305348.0313 | 220571.2188 | 173442.125 | 86823.19531 | 199586.4688 | 191105.6406 | 207603.375 | 121472.3203 | 256537.8281 | 9652.791016 | 8490.455078 | 3319.563965 | 40684.30859 | 55236.37891 | 31877.08008 | PREDICTED: ribosomal RNA processing protein 1 homolog A [Callithrix jacchus] |
| 3392356.25 | 2239155 | 2983891 | 3077532.5 | 2302665 | 3074693.5 | 2112921.5 | 2221703 | 2370161 | 2398934 | 4181838.25 | 2425073.75 | 2466179 | 2321928.5 | 2801700.5 | 2383258 | 3100998 | 3121579.5 | 2975326.5 | 2883654.75 | 2664213.5 | 2723036 | 2476569.25 | 1603936.25 | GC isoform 4 [Pan troglodytes] |
| 21948.10742 | 28396.5957 | 25260.82813 | 24204.95117 | 14090.24219 | 19809.21875 | 15290.05859 | 24382.88281 | 13552.02539 | 20960.21875 | 17756.08594 | 17095.76758 | 21319.57422 | 17558.93164 | 11400.98535 | 18541.85742 | 19868.4668 | 25548.75 | 19410.15234 | 24047.26563 | 23379.67969 | 20206.17578 | 12921.24805 | 14105.04102 | vitamin K-dependent protein C isoform X5 [Homo sapiens] |
| 9001.670898 | 3941.733643 | 0 | 8115.820313 | 7390.317383 | 10376.74121 | 0 | 9735.301758 | 0 | 0 | 0 | 0 | 5991.280273 | 9559.402344 | 0 | 8167.955078 | 0 | 0 | 0 | 0 | 0 | 0 | 0 | 4407.373535 | collagen alpha-3(VI) chain isoform 4 precursor [Homo sapiens] |
| 0 | 38284.29297 | 0 | 0 | 0 | 0 | 0 | 547.6967163 | 0 | 0 | 0 | 0 | 0 | 0 | 0 | 0 | 0 | 0 | 0 | 0 | 0 | 0 | 0 | 0 | lactotransferrin isoform 4 [Homo sapiens] |
| 7425.499023 | 8660.814453 | 7451.050781 | 11546.29297 | 7787.304688 | 7070.233398 | 15457.66992 | 11234.58691 | 6840.601563 | 7207.862305 | 11280.44434 | 19235.47461 | 12766.73633 | 6263.194824 | 6334.995117 | 3779.907227 | 14926.85156 | 7223.417969 | 10133.30957 | 16722.98047 | 5995.751953 | 7170.567871 | 15157.61719 | 12635.48047 | GAPDH isoform 4 [Pan troglodytes] |
| 0 | 0 | 0 | 0 | 0 | 0 | 0 | 0 | 0 | 0 | 0 | 0 | 0 | 0 | 0 | 0 | 0 | 0 | 0 | 0 | 0 | 0 | 27351.43555 | 0 | HYI isoform 11 [Pan troglodytes] |
| 0 | 1788.484497 | 0 | 1203.274902 | 2574.30835 | 1619.631348 | 3529.427734 | 0 | 0 | 0 | 1687.703003 | 4053.92749 | 2588.970947 | 0 | 0 | 0 | 5669.22998 | 0 | 2115.842773 | 5094.812988 | 4554.562012 | 3074.075439 | 3587.941895 | 6428.930176 | YWHAZ isoform 15, partial [Pan troglodytes] |
| 24780.68359 | 24292.45313 | 29494.61914 | 31473.56055 | 19352.01172 | 25254.34375 | 28665.54297 | 24238.82813 | 22861.16992 | 33522.28125 | 29248.32813 | 32454.48047 | 25463.45898 | 29325.69922 | 15044.08789 | 30123.5957 | 42821.04688 | 19388.02734 | 30603.88281 | 29183.24219 | 20700.93359 | 22969.62109 | 25936.42969 | 43537.53516 | properdin precursor [Homo sapiens] |
| 190207.5 | 126065.6563 | 134855.0469 | 143868.25 | 131783.0469 | 140846.0156 | 127208.6406 | 158142.8906 | 115150.3125 | 105423.2031 | 167393 | 134131.4688 | 114453.3359 | 105388.9688 | 122803.125 | 140598.8281 | 174601.6406 | 184679.6406 | 170772.625 | 147971.1719 | 137286.7813 | 132612.2031 | 123818.1484 | 133773.7031 | tetranectin isoform 1precursor [Homo sapiens] |
| 0 | 0 | 0 | 0 | 0 | 0 | 5077.412598 | 0 | 0 | 0 | 0 | 8517.419922 | 3097.204834 | 0 | 0 | 0 | 0 | 0 | 7047.533691 | 11494.12793 | 6121.357422 | 0 | 5681.099609 | 8811.795898 | CFL1 isoform 2, partial [Pan troglodytes] |
| 3030.953125 | 3953.034424 | 7596.537598 | 6962.919434 | 6069.504883 | 8184.916504 | 8279.503906 | 7262.537109 | 4853.192383 | 0 | 9770.791992 | 6175.65625 | 5975.994629 | 9364.097656 | 5360.774902 | 7644.439453 | 8301.981445 | 7980.345215 | 7632.826172 | 8454.242188 | 2260.84082 | 5653.28418 | 8016.700684 | 5738.875488 | coagulation factor VII isoform c precursor [Homo sapiens] |
| 12713.2334 | 4603.249512 | 8787.325195 | 4489.072754 | 0 | 3807.85791 | 9538.103516 | 5350.161621 | 9397.522461 | 15613.87598 | 0 | 10528.02441 | 5170.976563 | 17485.87305 | 0 | 5401.024414 | 5863.946777 | 10361.87109 | 3940.994629 | 8678.038086 | 5224.402344 | 6068.87793 | 10373.52539 | 69638.98438 | KRT74 isoform 2 [Pan troglodytes] |
| 22043.46289 | 27037.83008 | 23917.20703 | 21144.63867 | 21198.02344 | 28647.78906 | 31583.39648 | 29804.4375 | 33540.92969 | 29303.06445 | 30091.35742 | 27147.13672 | 28426.59766 | 28404.85547 | 21489.58984 | 29673.11328 | 44578.03906 | 29270.8125 | 31991.25781 | 32534.20703 | 29768.06641 | 26996.58789 | 28573.66406 | 47092.08594 | unnamed protein product [Homo sapiens] |
| 6786.831055 | 4993.265625 | 5432.422852 | 2251.69165 | 2423.690918 | 2768.42749 | 2595.905029 | 5447.380859 | 4196.333984 | 5314.890137 | 2877.819824 | 3578.00708 | 4590.923828 | 4583.780273 | 4750.28418 | 6090.099121 | 3731.550781 | 4787.220215 | 7999.654297 | 8983.036133 | 4843.567383 | 5634.164063 | 5005.296387 | 5604.422363 | transferrin receptor variant, partial [Homo sapiens] |
| 13144.54199 | 8824.578125 | 12970.96484 | 12499.1543 | 9689.702148 | 8846.271484 | 4682.54248 | 7927.334473 | 4712.515625 | 6807.553223 | 12740.94141 | 5294.262695 | 15941.51563 | 7703.542969 | 12463.33203 | 10919.64063 | 6746.123047 | 6647.792969 | 11711.04883 | 12271.16797 | 14207.16309 | 5658.733887 | 6081.798828 | 11761.375 | protein Z-dependent protease inhibitor isoform X1 [Homo sapiens] |
| 11471.08984 | 24928.46484 | 21608.50391 | 25728.49609 | 16547.24219 | 18619.63086 | 19446.77148 | 16914.99805 | 10229.71777 | 20867.71289 | 22082.83984 | 10832.58398 | 14420.60742 | 10391.94824 | 21315.86914 | 22955.66797 | 14514.52832 | 11663.52148 | 20061.44141 | 17954.11719 | 20398.58008 | 16911.22656 | 13364.17188 | 22057.73828 | hepatocyte growth factor-like protein precursor [Homo sapiens] |
| 10850.25586 | 13530.65234 | 12839.87598 | 13065.66406 | 7308.014648 | 6931.860352 | 0 | 5567.901855 | 9695.936523 | 8106.870117 | 6909.280273 | 7234.197754 | 8823.053711 | 10657.41602 | 9537.633789 | 10548.0791 | 0 | 0 | 0 | 0 | 7817.387207 | 3936.179688 | 6593.80957 | 9300.600586 | unnamed protein product [Homo sapiens] |
| 0 | 6634.89209 | 0 | 0 | 7821.799316 | 0 | 6589.451172 | 6037.584961 | 0 | 0 | 0 | 8636.027344 | 7936.322266 | 0 | 0 | 0 | 0 | 0 | 5991.560059 | 13785.12988 | 10487.35156 | 3271.064697 | 8234.861328 | 6551.17041 | zyxin isoform 2 [Homo sapiens] |
| 17200.14258 | 20312.62109 | 20780.48438 | 17569.64063 | 16121.52344 | 16877.07227 | 16666.85742 | 20819.48828 | 19638.21484 | 15840.84766 | 16044.29785 | 12114.83691 | 14588.81348 | 27281.00195 | 15593.875 | 22404.08984 | 161177.5938 | 18540.16602 | 25637.65625 | 20807.68164 | 19264.94727 | 24588.57422 | 14894.03027 | 24181.82422 | CD44 antigen isoform 8 precursor [Homo sapiens] |
| 8304.46875 | 10677.80762 | 10304.52832 | 5938.064453 | 7486.896484 | 6939.100098 | 7483.880859 | 5549.916016 | 5877.329102 | 4449.59668 | 4528.3125 | 6763.51123 | 3899.9104 | 12592.38672 | 7064.606934 | 9459.109375 | 2680.127197 | 7787.684082 | 4328.022949 | 6746.341309 | 4776.646973 | 2446.007813 | 4625.833008 | 5145.173828 | low affinity immunoglobulin gamma Fc region receptor III-A isoform b [Homo sapiens] |
| 219059.6719 | 184618.9688 | 192763.5625 | 163825.875 | 166396.3438 | 144642.9219 | 140020.6094 | 138917.8438 | 141612.2656 | 157277.2031 | 159839.5 | 195221.25 | 210044.9375 | 140951.1719 | 160570.0938 | 200944.5156 | 260538.4844 | 205286.2188 | 207547.2656 | 156148.9375 | 169731.4688 | 135636.5156 | 139535.0313 | 157866.9531 | KLKB1 isoform 4, partial [Pan troglodytes] |
| 0 | 4328.806152 | 0 | 0 | 4116.959473 | 0 | 0 | 0 | 0 | 77385.42969 | 2832.139648 | 0 | 0 | 0 | 0 | 0 | 0 | 0 | 4193.984375 | 7700.995605 | 5747.351074 | 0 | 0 | 0 | RNH1 isoform 23, partial [Pan troglodytes] |
| 0 | 0 | 0 | 0 | 5722.056641 | 0 | 0 | 0 | 0 | 0 | 0 | 0 | 0 | 0 | 0 | 0 | 0 | 0 | 0 | 11056.03516 | 7556.195313 | 0 | 8517.826172 | 6969.595215 | PREDICTED: rho GDP-dissociation inhibitor 2 isoform X2 [Nomascus leucogenys] |
| 593410.3125 | 996444.25 | 825324.3125 | 731552.8125 | 842770.5 | 607199.1875 | 584544.6875 | 885377.375 | 400106.4375 | 600711.0625 | 623192.8125 | 130446.6172 | 664103.375 | 868035.125 | 569094.625 | 950070.5 | 1315128.5 | 1098246.5 | 763066.0625 | 813734.375 | 961431.25 | 1415350.25 | 683527.625 | 548093.5 | vitronectin, partial [Homo sapiens] |
| 0 | 5359.730957 | 0 | 7271.943359 | 6881.666504 | 0 | 142023.8438 | 0 | 0 | 0 | 0 | 9870.878906 | 9755.512695 | 0 | 748.6489258 | 0 | 5696.921875 | 6050.480957 | 5935.447266 | 10590.05762 | 1196.4823 | 7848.327637 | 2517.631104 | 1221.326172 | pyruvate kinase PKM isoform f [Homo sapiens] |
| 551606.5625 | 219021.1094 | 305156.9375 | 575482.375 | 367210.125 | 295974.4688 | 35148.16797 | 411334.1875 | 618049.5625 | 233217.9375 | 168878.875 | 486717.5 | 65743.99219 | 319728.5938 | 351996.875 | 146157.25 | 346118.75 | 365065.2813 | 279297.5313 | 582402.0625 | 388818.0625 | 260181.5781 | 677499.1875 | 199048.5313 | LOW QUALITY PROTEIN: T0061165 isoform 1, partial [Pan troglodytes] |
| 0 | 5989.367676 | 1704.315674 | 4133.732422 | 6555.041016 | 3449.803955 | 4914.504883 | 3593.105225 | 0 | 38035.91016 | 4974.483398 | 5820.116211 | 6363.765625 | 0 | 2233.52002 | 0 | 6434.056641 | 3894.503906 | 5220.342285 | 10035.67676 | 5139.333008 | 6684.401367 | 4319.440918 | 13963.32617 | alpha-actinin-1 isoform c [Homo sapiens] |
| 16400.92188 | 6457.014648 | 9274.176758 | 3005.622314 | 7953.937012 | 8725.765625 | 9327.797852 | 1122.292847 | 10659.43262 | 6211.819336 | 10825.10254 | 12576.01074 | 5201.461914 | 11248.21875 | 9441.402344 | 8584.947266 | 17377.51367 | 10994.74414 | 25276.77539 | 12647.04395 | 5188.838867 | 5359.520996 | 17578.65625 | 2952.420166 | SHBG protein, partial [Homo sapiens] |
| 0 | 0 | 11717.15918 | 0 | 2820.534668 | 4859.571777 | 0 | 10791.31641 | 0 | 17412.0918 | 0 | 0 | 0 | 10743.09473 | 0 | 0 | 8159.259766 | 0 | 6377.939941 | 4947.132813 | 0 | 5114.758301 | 0 | 7808.951172 | cadherin 5, type 2 preproprotein variant, partial [Homo sapiens] |
| 12944.80664 | 9400.181641 | 18035.32227 | 10931.62793 | 9795.277344 | 14341.25293 | 21310.89453 | 30344.74805 | 15545.35254 | 11042.87988 | 18531.69336 | 27023.38477 | 16675.30859 | 17310.74609 | 14896.47168 | 20222.84766 | 22114.35938 | 25190.85156 | 7890.112793 | 8648.768555 | 15339.05371 | 11373.65137 | 17476.34961 | 21101.7168 | cathelicidin antimicrobial peptide [Homo sapiens] |
| 18925.30664 | 11707.61426 | 14346.43848 | 14258.21875 | 8524.911133 | 6899.961914 | 23605.25195 | 14134.98926 | 13295.33398 | 10049.57129 | 8925.303711 | 15939.14648 | 7537.736328 | 10233.97461 | 11219.60742 | 13707.67578 | 17892.60938 | 11925.9668 | 0 | 0 | 10104.52246 | 7066.230957 | 13163.27148 | 0 | cysteine-rich secretory protein 3 isoform 3 [Homo sapiens] |
| 0 | 4923.725098 | 0 | 0 | 4993.487793 | 0 | 0 | 0 | 0 | 0 | 0 | 3725.859375 | 0 | 0 | 0 | 0 | 0 | 0 | 0 | 8810.698242 | 3439.019775 | 0 | 9662.125 | 11032.9873 | aldolase A, fructose-bisphosphate, isoform CRA_b [Homo sapiens] |
| 34413.08203 | 24063.01172 | 32264.55078 | 30577.86719 | 19620.78906 | 20179.21484 | 22612.97656 | 26161.8125 | 28348.73633 | 10911.86328 | 18969.83203 | 14723.69727 | 28702.55664 | 22972.75195 | 37854.91797 | 14062.23926 | 30234.11133 | 38601.23438 | 29028.63672 | 20901.20898 | 26704.08203 | 24649.02734 | 23555.42969 | 21230.51953 | Carnosine dipeptidase 1 (metallopeptidase M20 family) [Homo sapiens] |
| 432835.0625 | 335288.5 | 308752.9063 | 375184.5 | 260133.9219 | 304569.8125 | 536744.375 | 340810.9063 | 546308.0625 | 286734.0313 | 370396.5313 | 593337.625 | 322286.6563 | 441478.1563 | 243623.4375 | 335994.9375 | 328039.7188 | 280761.2813 | 292040.0938 | 321596.9688 | 518054.2813 | 269999.0938 | 228841.9844 | 189960.1563 | lethal(2) giant larvae protein homolog 2 isoform X4 [Homo sapiens] |
| 52156.47656 | 0 | 0 | 0 | 0 | 0 | 0 | 0 | 85750.47656 | 98079.32031 | 0 | 167992.0625 | 0 | 0 | 0 | 0 | 145957.6094 | 0 | 0 | 0 | 0 | 0 | 0 | 134390.1563 | COL14A1 isoform 7, partial [Pan troglodytes] |
| 720361.0625 | 318473.5625 | 705566.1875 | 343209.375 | 177414.6406 | 275753.0938 | 232976.875 | 371414.0625 | 189961.7344 | 300568 | 480582.9375 | 2088712.75 | 348943.875 | 1366221.5 | 270151.9375 | 244663.625 | 1527538.5 | 978433.375 | 517557.25 | 905819.125 | 216545 | 1058935.875 | 780609.875 | 325809.9063 | apolipoprotein C-II isoform X1 [Mesocricetus auratus] |
| 9242.325195 | 19732.18555 | 8157.501465 | 8169.153809 | 8752.594727 | 14782.4541 | 10087.81543 | 13825.53711 | 27091.31055 | 9286.15625 | 8207.097656 | 11737.16406 | 14922.80273 | 15684.62012 | 14363.61621 | 7521.072266 | 10358.09082 | 16552.01758 | 15321.06445 | 12557.26758 | 10474.50781 | 10022.20313 | 10418.32422 | 12482.49316 | complement factor D isoform 2 precursor [Homo sapiens] |
| 3657264.5 | 2417861 | 3240414.75 | 1808302.125 | 1754050 | 1857499.375 | 1508513.75 | 3095364.25 | 2099432.75 | 1475156.625 | 3525124.75 | 2067123.75 | 2753772.25 | 1438924.875 | 2745955.25 | 3644230.75 | 4952709.5 | 1991166.875 | 2876610.5 | 2596971.75 | 3524857 | 2972365.5 | 2560837 | 3317684.75 | apolipoprotein C-I precursor [Homo sapiens] |
| 7369.316895 | 6739.854004 | 7267.641602 | 8710.527344 | 6023.156738 | 5538.008301 | 0 | 4954.791504 | 3354.773193 | 5327.778809 | 7837.916504 | 8072.021973 | 5700.805176 | 3290.1604 | 5326.814453 | 6202.081055 | 11582.69922 | 2749.817383 | 7213.710449 | 4377.855469 | 7314.885254 | 5394.830078 | 4751.179199 | 5730.674316 | C3 isoform 6, partial [Pan troglodytes] |
| 41027.875 | 37409.53125 | 14351.52246 | 18598.89844 | 32472.81445 | 51058.28125 | 46237.41406 | 48316.65625 | 43282.32422 | 40077.50391 | 51139.64063 | 22340.03711 | 42973.72266 | 40949.48047 | 26646.66602 | 30422.32617 | 38895.28516 | 47098.04688 | 42835.52734 | 19218.09766 | 22837.30469 | 27611.22852 | 41646.66406 | 24649.21484 | mannan-binding lectin serine protease 2 isoform 1 preproprotein [Homo sapiens] |
| 10480.38281 | 8632.038086 | 7858.975586 | 12709.93164 | 5444.433594 | 10363.86914 | 14900.35547 | 13872.01758 | 9458.167969 | 13017.56348 | 19351.81445 | 8776.401367 | 9194.083008 | 10613.31836 | 8619.621094 | 10579.13281 | 12434.48633 | 16318.6582 | 9571.5625 | 21552.89453 | 9396.678711 | 7691.859375 | 9249.033203 | 4583.194336 | sulfhydryl oxidase 1 isoform a precursor [Homo sapiens] |
| 29249.68945 | 64640.35938 | 42722.39844 | 28121.85547 | 59053.94141 | 48312.4375 | 15554.20508 | 58244.82422 | 21685.65625 | 41458.53125 | 22296.19141 | 35711.02344 | 29500.43359 | 34230.40625 | 41227.30078 | 34830.60938 | 55174.77734 | 32565.41211 | 49703.19531 | 24150.68555 | 34890.8125 | 54278.85156 | 65073.49609 | 37734.47656 | apolipoprotein L1 isoform a precursor [Homo sapiens] |
| 210604.2813 | 81787.85938 | 145152.5938 | 69465.14063 | 115326.2969 | 126461.2734 | 84846.88281 | 174283.6875 | 207820.7813 | 211460.8281 | 114447.7656 | 98447.58594 | 121159.8984 | 141812.4844 | 172623.3594 | 212694.375 | 237831.2813 | 199115.8125 | 118614.1172 | 201287.7813 | 114708.4141 | 199095.9531 | 274897.375 | 151734.2188 | CD5 antigen-like isoform 1 precursor [Homo sapiens] |
| 118392.3594 | 108916.1797 | 105845.2969 | 155846.5469 | 91947.01563 | 290403.5938 | 126706.9609 | 87955.14063 | 106297.125 | 158242.1875 | 140979.1563 | 127948.8125 | 116403.9219 | 197149.4063 | 156153.0938 | 138599.3594 | 239636.7344 | 201655.2188 | 60724.03125 | 105302.9766 | 133905.5781 | 162236.8438 | 109390.0469 | 207588.8438 | endoplasmic reticulum to nucleus signalling 1 isoform 1 variant, partial [Homo sapiens] |
| 37637.40625 | 35145.41016 | 23037.17969 | 46292.36328 | 34828.95313 | 17428.35547 | 26602.24219 | 37975.22266 | 51850.99219 | 35486.28125 | 25779.67188 | 29439.04688 | 41116.21094 | 17628.36328 | 35653.48047 | 47537.79297 | 41034.52344 | 32492.62695 | 50575.76172 | 50407.64063 | 105836.0313 | 91452.73438 | 67083.85938 | 84062.5625 | ficolin-3 isoform 1 precursor [Homo sapiens] |
| 47729.24609 | 54257.25391 | 36589.41406 | 44092.58984 | 46506.39063 | 40875.94531 | 24748.13672 | 69283.125 | 31547.67188 | 26681.68359 | 65520.59375 | 34116.71484 | 50103.53125 | 32477.69531 | 42891.66406 | 32377.71484 | 53047.87891 | 35737.46875 | 46430.74219 | 51397.40234 | 58922.07813 | 55911.46094 | 39553.22266 | 66329.21094 | attractin isoform 1 preproprotein [Homo sapiens] |
| 59612.15234 | 56693.29297 | 48462.29297 | 43301.44141 | 70150.375 | 61284.46875 | 44367.80859 | 51209.20313 | 43299.83594 | 71117.58594 | 77055.30469 | 49233.50391 | 58117.63672 | 47098.16016 | 68381.23438 | 62962.16406 | 76914.22656 | 35683.38672 | 84262.71094 | 71552.73438 | 79850.70313 | 83653.45313 | 80977.1875 | 62909.98828 | apolipoprotein M isoform 1 [Homo sapiens] |
| 14551.2373 | 18621.66992 | 4783.652832 | 5471.065918 | 7764.680664 | 0 | 0 | 0 | 0 | 3446.869629 | 0 | 0 | 0 | 0 | 0 | 28360.32617 | 0 | 0 | 34257.07813 | 0 | 0 | 0 | 0 | 0 | pantetheinase precursor [Homo sapiens] |
| 754271.3125 | 492424.7813 | 323720.4063 | 815568 | 602529.625 | 730381.125 | 570987.4375 | 491621.8125 | 646631.625 | 554788.5 | 600380.75 | 629775.5 | 730733.25 | 599236.25 | 887818.1875 | 801453.5 | 681441.375 | 639052.375 | 603539.25 | 560620.625 | 605499.625 | 677397.25 | 548469 | 486518.625 | RecName: Full=Ceruloplasmin; AltName: Full=Ferroxidase; Flags: Precursor |
| 18615.95313 | 22063.34375 | 25872.32031 | 14036.73633 | 27695.31641 | 37225.37109 | 19702.58398 | 16228.48828 | 25707.5293 | 17409.125 | 30822.75195 | 22031.41016 | 10025.01855 | 31947.57813 | 12613.85352 | 21092.85742 | 34177.66797 | 27929.51563 | 12736.69922 | 20985.20313 | 24175.875 | 26818.67188 | 20462.63867 | 41311.66797 | RecName: Full=Coagulation factor XIII A chain; Short=Coagulation factor XIIIa; AltName: Full=Protein-glutamine gamma-glutamyltransferase A chain; AltName: Full=Transglutaminase A chain; Flags: Precursor |
| 573768.125 | 580798.625 | 766135.1875 | 622930.25 | 450243.25 | 564804.6875 | 501829.375 | 511166.5 | 425489.625 | 492530.8125 | 760534.0625 | 568524.875 | 716726.25 | 422646.3438 | 612446.125 | 568349.75 | 680980.25 | 554801.6875 | 635769.625 | 612512.375 | 622708.4375 | 666162.625 | 544197 | 487774.3438 | prothrombin isoform 1 preproprotein [Homo sapiens] |
| 6926112 | 6377182 | 12124306 | 11010740 | 8071730.5 | 19214576 | 6877861 | 9731100 | 8243423 | 6236414.5 | 14786577 | 9447688 | 11891430 | 11099483 | 8181156.5 | 13566641 | 13047832 | 14724672 | 6423194 | 11678401 | 11150663 | 15214645 | 11662402 | 19719446 | haptoglobin isoform 1 preproprotein [Homo sapiens] |
| 55317.44531 | 84431.82813 | 23882.05273 | 61889.05469 | 69976.19531 | 27459.79688 | 15481.53125 | 41763.59375 | 53063.5625 | 106535.2813 | 29475.4707 | 84979.44531 | 45463.55469 | 34480.95313 | 71188.61719 | 69819.22656 | 41640.44531 | 34057.64844 | 52745.00781 | 39602.21484 | 28514.50586 | 23270.26172 | 82121.32031 | 27837.34375 | haptoglobin-related protein precursor [Homo sapiens] |
| 33885.42969 | 45600.39063 | 38225.05859 | 36818.82813 | 37915.35156 | 38014.88281 | 28091.60938 | 42212.16797 | 32432.10742 | 27026.81641 | 43597.25391 | 35723.84766 | 37443.21094 | 26875.06836 | 27282.1875 | 40492.58984 | 46512.08203 | 46106.06641 | 37145.72656 | 32644.11133 | 34709.23828 | 34721.85156 | 23280.68945 | 30202.04297 | coagulation factor IX isoform 1 preproprotein [Homo sapiens] |
| 67468.35156 | 98872.39844 | 82004.76563 | 86930.28125 | 76675.15625 | 75130.63281 | 63519.21094 | 106255.7188 | 90975.42188 | 55030.53125 | 79284.625 | 61622.97266 | 99371.03125 | 82596.40625 | 63593.92969 | 110308.1563 | 98272.03125 | 96522.14844 | 82341.1875 | 79668.79688 | 127323.0469 | 72201.28125 | 88150.875 | 61644.32031 | coagulation factor X isoform 1 preproprotein [Homo sapiens] |
| 1350411.75 | 1031375.75 | 1344783.125 | 1346812.625 | 891554.75 | 1174092.25 | 858014.875 | 1109560.25 | 781329.25 | 724967.25 | 1434046 | 988301.8125 | 1334111.5 | 726427.25 | 1008980.563 | 1221007.875 | 1082933.25 | 1132853.875 | 1278061 | 1371017 | 1402249.25 | 1277683.75 | 967057.3125 | 1038558.875 | plasminogen isoform 1 precursor [Homo sapiens] |
| 75727.90625 | 107727.2969 | 63210.26563 | 97039.63281 | 46734.19531 | 50482.92188 | 39204.20313 | 50422.72656 | 79059.86719 | 73489.21094 | 80824.35938 | 65841.48438 | 54666.02344 | 55446.64844 | 64433.57813 | 34687.38281 | 65459.26563 | 51144.83594 | 74536.39063 | 33688.51953 | 78677.60156 | 30824.36914 | 50106.80078 | 49601.75781 | coagulation factor XII preproprotein [Homo sapiens] |
| 23950.53906 | 29633.96484 | 8503.864258 | 14655.12109 | 20652.60742 | 20158.86133 | 40366.22656 | 29835.86719 | 43265.08203 | 35625.32813 | 22487.17188 | 36775.875 | 17188.41602 | 28047.69531 | 3853.734863 | 21643.21875 | 14929.38867 | 18466.57813 | 15009.05469 | 22173.03125 | 5568.240234 | 5351.169434 | 16524.42773 | 12208.40234 | carbonic anhydrase 1 isoform a [Homo sapiens] |
| 11314.27539 | 12121.68359 | 8669.06543 | 5965.459961 | 10236.89453 | 8080.067383 | 18463.83789 | 10246.99512 | 9249.496094 | 11342.22363 | 14162.94141 | 13683.0459 | 4597.040039 | 0 | 0 | 0 | 9840.969727 | 11008.76855 | 6831.092773 | 12884.19727 | 5444.330566 | 0 | 9249.25 | 6100.741211 | carbonic anhydrase 2 isoform 1 [Homo sapiens] |
| 640534.1875 | 597322.875 | 675603.375 | 582383.25 | 484542.9063 | 523129.125 | 494204.25 | 495693.125 | 438718.125 | 473754.0625 | 511857 | 486699.3125 | 567100.625 | 387322.5 | 427846.0625 | 533661.5 | 691503.6875 | 554084.125 | 586577.25 | 563019.5 | 449605.4688 | 441729.5 | 374363.3125 | 340775.375 | antithrombin-III isoform 1 precursor [Homo sapiens] |
| 8472360 | 3997765.5 | 5044118 | 5343623.5 | 3709637.25 | 6969993.5 | 5208994.5 | 4310263 | 5472203 | 5271302 | 5724330 | 6552513.5 | 5374454 | 4631344 | 5443254.5 | 5710290.5 | 7885265 | 8055398 | 6151433 | 7247723.5 | 3528766.75 | 4903759.5 | 4944420.5 | 3923876.5 | alpha-1-antitrypsin precursor [Homo sapiens] |
| 808301.5 | 713722.5 | 881769.625 | 779267.875 | 518016.2813 | 894321 | 609343.625 | 619607.625 | 692391.75 | 574335.125 | 797942 | 703316.875 | 827768.375 | 682734.75 | 662100.0625 | 838250.25 | 884014.4375 | 648906.125 | 790606.25 | 795576.9375 | 659105.875 | 829696 | 693152.625 | 434522.75 | serpin peptidase inhibitor, clade A (alpha-1 antiproteinase, antitrypsin), member 3, isoform CRA_b [Homo sapiens] |
| 287318.3438 | 235471.8438 | 243673.5938 | 158400.1875 | 159355.2031 | 222839.7656 | 214493.375 | 180040.2969 | 189539.2813 | 284265.25 | 188545.9063 | 223780.7969 | 185234.3125 | 238231.0625 | 221611.625 | 189259.5313 | 239512.7188 | 329795.375 | 190471.125 | 152781.3906 | 205221.4844 | 142357.4688 | 109654.2656 | 140345.4375 | angiotensinogen preproprotein [Homo sapiens] |
| 8796190 | 5906484 | 8827946 | 5851421 | 6910634 | 3705678.25 | 5406454 | 6650643 | 11909838 | 5407756 | 6426515 | 6364938 | 4429940 | 6335962 | 7196993 | 10738009 | 5297509 | 6615054 | 6419452 | 11864440 | 6652189 | 15978734 | 12602853 | 12369801 | alpha-2-macroglobulin isoform a precursor [Homo sapiens] |
| 7385265.5 | 7890156 | 8346101.5 | 8465122 | 6643374 | 6537231 | 6672614 | 6630969 | 5372284.5 | 5164078 | 9850834 | 6937408 | 7910508 | 4737418 | 6621838.5 | 7537769 | 9999250 | 5972414 | 8106738 | 7759946 | 8609635 | 6433565.5 | 6329309 | 6718336 | complement C3 preproprotein [Homo sapiens] |
| 348415.875 | 181464.1719 | 277460.1875 | 241405.375 | 174892.8906 | 266825.6875 | 74865.77344 | 180000.9688 | 194733.8125 | 168218.625 | 261096.0156 | 222011.9375 | 234190.4688 | 173132.3281 | 163540.4375 | 249800.4688 | 256416.4063 | 177643.9063 | 204843.1094 | 236954.375 | 220690.9531 | 239211.3906 | 184576.9063 | 158917.5313 | complement C5 isoform 1 preproprotein [Homo sapiens] |
| 13474.42285 | 12593.94629 | 13285.19824 | 11838.16016 | 12284.31836 | 12046.59375 | 14565.0918 | 11810.53125 | 8797.308594 | 11397.34473 | 15725.51172 | 15939.69043 | 10618.59766 | 21377.3418 | 10682.68359 | 10311.21777 | 10413.55078 | 14138.05078 | 15163.10449 | 11750.23926 | 11375.6377 | 12029.90137 | 7890.916992 | 6036.597656 | cystatin-C precursor [Homo sapiens] |
| 1506865.375 | 1359761.875 | 1387883.75 | 1334985.25 | 1229990.375 | 1130014.75 | 1137834.875 | 1264622.75 | 929676.875 | 1011588.813 | 1334372.5 | 1082366 | 1285545.25 | 1088596.75 | 1012651.875 | 1422455 | 1664724.625 | 1117036.375 | 1359237.625 | 1178417.25 | 1136235.125 | 1167379.375 | 1141638.75 | 1112455.875 | kininogen-1 isoform 1 precursor [Homo sapiens] |
| 69586.57813 | 60721.71875 | 60683.8125 | 57728.25 | 56617.22656 | 47652.70703 | 27956 | 54527.28516 | 19023.33594 | 39552.07813 | 51540.18359 | 54823.03516 | 75758.54688 | 32505.52344 | 55373.51953 | 62320.89063 | 64706.20313 | 57605.71484 | 55109.04297 | 21602.16016 | 45545.48438 | 44637.21875 | 37202.55078 | 40710 | insulin-like growth factor II isoform 2 [Homo sapiens] |
| 23198.40234 | 29641.41797 | 59566.58594 | 48721.86719 | 56135.10156 | 56541.17969 | 29316.02539 | 54500.43359 | 41665.71875 | 38997.57422 | 55579.76172 | 18928.79297 | 48758.01172 | 38740.70313 | 37779.21875 | 47862.90625 | 29558.29688 | 55444.35938 | 41452.375 | 39033.44531 | 26134.40234 | 44237.19531 | 45358.21875 | 29767.87109 | immunoglobulin kappa light chain VC region, partial [Homo sapiens] |
| 129248.6094 | 114891.4219 | 223509.6875 | 172561.9219 | 132406.0625 | 64066.95313 | 176110.3438 | 114018.375 | 193986.3125 | 67220.82813 | 161131.375 | 136807.6563 | 69944.60938 | 127203.5313 | 142480.375 | 129956.6328 | 109608.5391 | 162853.2344 | 172532.75 | 114378.0313 | 53436.80469 | 134186.1563 | 200384.8281 | 85380.05469 | RecName: Full=Immunoglobulin kappa variable 1-17; AltName: Full=Ig kappa chain V-I region Gal; AltName: Full=Ig kappa chain V-I region WEA; Flags: Precursor |
| 102975.7734 | 61339.47656 | 82136.3125 | 105513.0781 | 52358.45313 | 81549.01563 | 90984.82031 | 76941.91406 | 103308.9297 | 90561.78125 | 91977.9375 | 92545.42188 | 95202.96875 | 101121.0156 | 82786.32813 | 80550.0625 | 74452.07031 | 118586.0938 | 253125.8594 | 62879.43359 | 71687.8125 | 77657.35938 | 122613.2813 | 71151.91406 | RecName: Full=Immunoglobulin kappa variable 1D-16; AltName: Full=Ig kappa chain V-I region HK146; AltName: Full=Ig kappa chain V-I region HK189; Flags: Precursor |
| 383838.875 | 52980.47656 | 45275.96094 | 117953.5078 | 84031.91406 | 59805.17969 | 293199.0625 | 116820.0469 | 65298.30469 | 231781.3594 | 611161.5625 | 644349.25 | 105510.1094 | 117506.2656 | 73341.30469 | 41982.64453 | 78395.44531 | 130122.4297 | 77885.70313 | 87027.26563 | 672710 | 104624.7031 | 1106028.375 | 485666.875 | hCG2043208, partial [Homo sapiens] |
| 3653316.5 | 2176458.5 | 1804883.25 | 4103098.5 | 1630697.375 | 3380488 | 4545885 | 2528326.75 | 3274807 | 3375831 | 2600237.25 | 4735219 | 2497066.75 | 4064313.25 | 2634767.5 | 2953434.75 | 3085731 | 3185482.25 | 3192478.75 | 2936394.75 | 3422067 | 3339432.25 | 4479109 | 2341136.75 | immunoglobulin light chain variable region, partial [Homo sapiens] |
| 121592.4375 | 124882.6484 | 193281.6563 | 185149.9063 | 164179.3438 | 193776.4844 | 176812.2656 | 139998.75 | 180020.8125 | 180395.6094 | 129714.3125 | 113322.7188 | 310496.8438 | 157614.9844 | 241844.5625 | 173606.5938 | 35430.02734 | 173239.1094 | 94578.21875 | 187874.8281 | 129483.3984 | 174150.0781 | 248924.9844 | 127842.3594 | hCG2043214, partial [Homo sapiens] |
| 1351512.875 | 1453304 | 1052031.25 | 2609466.25 | 1398332.625 | 1745433 | 2063389.875 | 1556031.25 | 972430.1875 | 3108454.25 | 1216370.875 | 1426471.875 | 1167364.375 | 1915136.625 | 1226683.375 | 995468.875 | 902436.5625 | 2021230.625 | 614348.5 | 1626959.5 | 1631696.125 | 1448689.125 | 1715054.625 | 1030089.938 | RecName: Full=Immunoglobulin lambda variable 1-47; AltName: Full=Ig lambda chain V-I region HA; AltName: Full=Ig lambda chain V-I region WAH; Flags: Precursor |
| 431399.2188 | 256358.5625 | 816878.9375 | 536672.1875 | 102542.4375 | 462436.0625 | 289955.0625 | 362095.0625 | 524752.25 | 442928.0313 | 452599.3438 | 356387.5 | 413165.8125 | 533338.125 | 563578.3125 | 314958.3125 | 44094.09375 | 525192.125 | 287128.9688 | 407215.7188 | 482001.8125 | 502381 | 937142.75 | 331004.0313 | RecName: Full=Immunoglobulin lambda variable 1-51; AltName: Full=Ig lambda chain V-I region BL2; AltName: Full=Ig lambda chain V-I region EPS; AltName: Full=Ig lambda chain V-I region NEW; AltName: Full=Ig lambda chain V-I region NIG-64; Flags: Precursor |
| 104173.8594 | 158057.0313 | 330988.3438 | 171598.5781 | 175600.5625 | 198336.6875 | 39470.18359 | 133949.2813 | 167839.9219 | 135234.8438 | 195462.3906 | 69201.35156 | 158659.2813 | 199442.7344 | 144467.875 | 174060.2813 | 0 | 108107.2734 | 114779.0156 | 146851.2969 | 86504.40625 | 96978.90625 | 178347.375 | 125520.7188 | Unknown (protein for MGC:31936) [Homo sapiens] |
| 40842.83594 | 8470.942383 | 43287.36328 | 25300.36719 | 27845.64063 | 38763.05078 | 51710.08984 | 25668.22461 | 24789.87891 | 41112.0625 | 83326.16406 | 17163.17969 | 36336.22266 | 18776.32031 | 26007.77539 | 28884.54688 | 22282.63281 | 20205.30664 | 30539.33008 | 25596.73633 | 28647.66406 | 49613.95313 | 46917.04297 | 35898.25 | RecName: Full=Immunoglobulin lambda variable 2-14; AltName: Full=Ig lambda chain V-II region NIG-84; AltName: Full=Ig lambda chain V-II region TOG; AltName: Full=Ig lambda chain V-II region VIL; Flags: Precursor |
| 35034.41406 | 32146.68945 | 0 | 0 | 98456.48438 | 63490.05469 | 36646.78516 | 0 | 17817.70508 | 98189.89844 | 78811.16406 | 33828.45313 | 113703.9531 | 93495.89063 | 66257.67969 | 0 | 63361.62109 | 145685.8906 | 88293.10156 | 68069.51563 | 0 | 166790.75 | 77105.17969 | 147779.4219 | immunoglobulin lambda-chain, partial [Homo sapiens] |
| 133258.1875 | 160488.6406 | 74881.14063 | 159007.4063 | 194977.7344 | 72312.59375 | 80812.29688 | 175085.0469 | 210326.0625 | 118691.6484 | 107600.6953 | 41410.10156 | 111849.9063 | 93860.25781 | 109830.5156 | 130537.0469 | 52978.19531 | 115891.6875 | 40481.67578 | 168648.5469 | 38375.41406 | 66352.90625 | 287631 | 56526.95313 | hCG2043237, partial [Homo sapiens] |
| 121702.7109 | 97568.71875 | 241047.5156 | 226231.0469 | 147975.5313 | 115077.4609 | 89195.94531 | 168616.125 | 248811.9531 | 228019.8594 | 79445.92188 | 125279.3125 | 150989.8438 | 153831.3125 | 116671.2969 | 278259.5938 | 82064.42969 | 85624.46875 | 115036.6094 | 241866.7969 | 61954 | 289710.0313 | 203737.4531 | 132281.0469 | hCG2043240, partial [Homo sapiens] |
| 0 | 26723.14258 | 11785.65137 | 0 | 28094.5625 | 11795.66797 | 9155.676758 | 48859.03125 | 6948.422852 | 12903.4209 | 37099.80078 | 8570.313477 | 69477 | 11670.58887 | 0 | 7364.438477 | 0 | 0 | 0 | 4315.276855 | 0 | 0 | 8398.09082 | 7953.170898 | hCG2040023, partial [Homo sapiens] |
| 3905.838623 | 6800.261719 | 6213.526367 | 9695.310547 | 13767.50098 | 11596.27051 | 4573.105957 | 10511.68164 | 8544.397461 | 7590.017578 | 16729.53125 | 0 | 20347.92578 | 12263.6543 | 4034.869873 | 5049.127441 | 0 | 4820.050781 | 5913.166992 | 6999.955078 | 0 | 5392.924805 | 2345.80835 | 10676.53613 | immunoglobulin lambda light chain variable region, partial [Homo sapiens] |
| 7582.428223 | 203032.125 | 27256.07617 | 10268.18262 | 11182.75586 | 17484.68945 | 0 | 9412.041016 | 41134.0625 | 19387.37109 | 25221.67578 | 27976.70313 | 55373.20313 | 35619.07813 | 26536.89063 | 70900.07813 | 8799.810547 | 36278.45313 | 0 | 3932.32666 | 10874.91309 | 5922.651855 | 56086 | 24186.51367 | immunoglobulin light chain variable region, partial [Homo sapiens] |
| 76310.79688 | 93036.80469 | 80557.17188 | 125464.125 | 83708.97656 | 114735.9766 | 98312.17188 | 55121.97266 | 83992.91406 | 148767.625 | 113109.4844 | 82863.32031 | 100206.875 | 287243.4688 | 143786.2656 | 108522.2188 | 79169.41406 | 102179.2344 | 113707.9531 | 90626.25 | 124819.8906 | 126726.3203 | 122314.8516 | 67966.07031 | RecName: Full=Immunoglobulin lambda variable 6-57; AltName: Full=Ig lambda chain V-VI region AR; AltName: Full=Ig lambda chain V-VI region EB4; AltName: Full=Ig lambda chain V-VI region NIG-48; AltName: Full=Ig lambda chain V-VI region SUT; AltName: Full=Ig lambda chain V-VI region WLT; Flags: Precursor |
| 533762.1875 | 793865.4375 | 704985.5625 | 736388.5 | 961471.75 | 799766.25 | 770058.4375 | 2789705.25 | 814038.125 | 2367869.75 | 833064.4375 | 523730.375 | 1097756.5 | 896198.25 | 726519.6875 | 3873830.75 | 1817165.25 | 629662 | 670020.5 | 976211.125 | 777248.25 | 1040946.125 | 559248.1875 | 4356871 | IgM heavy chain VH1 region precursor, partial [Homo sapiens] |
| 30536.53711 | 7586.431641 | 8817.163086 | 22156.26172 | 18466.82813 | 23414.57617 | 30930.36133 | 12838.9043 | 14971.02637 | 13112.67773 | 13756.42969 | 20535.875 | 16428.19336 | 21171.37109 | 8980.75 | 11278.18945 | 15033.09277 | 29151.22461 | 8631.138672 | 12761.15332 | 11028.89746 | 27327.97852 | 15976.23242 | 20417.21875 | IgM heavy chain VH1 region precursor, partial [Homo sapiens] |
| 0 | 33636.33203 | 24258.43945 | 31875.12109 | 35886.76563 | 24612.94727 | 47001.50781 | 30857.55078 | 61258.67578 | 28389.3457 | 28573.07031 | 26109.8125 | 33693.02344 | 144525.8438 | 62888.15625 | 49568.97656 | 9098.932617 | 33042.44922 | 62538.61719 | 39481.92969 | 19964.73438 | 12305.67871 | 22790.17969 | 14646.2666 | immunoglobulin heavy chain variable region, partial [Homo sapiens] |
| 0 | 16900.25391 | 33615.10547 | 0 | 42220.99219 | 26124.0918 | 30608.00391 | 0 | 0 | 0 | 43444.62891 | 0 | 38960.22656 | 81617.85156 | 0 | 34125.17578 | 0 | 19202.0957 | 24417.90625 | 59125.19922 | 36626.26563 | 0 | 80292.875 | 27381.73633 | RecName: Full=Immunoglobulin heavy variable 3-23; AltName: Full=Ig heavy chain V-III region LAY; AltName: Full=Ig heavy chain V-III region POM; AltName: Full=Ig heavy chain V-III region TEI; AltName: Full=Ig heavy chain V-III region TIL; AltName: Full=Ig heavy chain V-III region TUR; AltName: Full=Ig heavy chain V-III region VH26; AltName: Full=Ig heavy chain V-III region WAS; AltName: Full=Ig heavy chain V-III region ZAP; Flags: Precursor |
| 73243.63281 | 73376.88281 | 66277.20313 | 58655.05859 | 104859.8594 | 85044.17969 | 78414.85156 | 63831.88672 | 63348.47656 | 75716.38281 | 100625.6797 | 49814.96094 | 104912.5313 | 82531.0625 | 118439.4453 | 59922.21094 | 32148.0918 | 69938.84375 | 59572.53125 | 215247.8281 | 65260.33203 | 63937.99609 | 51145.96875 | 86350.22656 | RecName: Full=Immunoglobulin heavy variable 3-13; AltName: Full=Ig heavy chain V-III region BRO; Flags: Precursor |
| 2190081 | 1495303.25 | 1668305.875 | 2497426.5 | 2048628.25 | 1629659.375 | 1292962.25 | 1107091.375 | 2608483.5 | 1721617.5 | 1406541.5 | 1482432.375 | 1991674.625 | 1620768.75 | 1534550.875 | 1839337.25 | 1534814.25 | 1839872.625 | 1310849 | 1664221.25 | 1181163.875 | 2604972.75 | 2855145.75 | 1185831.75 | immunoglobulin heavy chain variable region precursor, partial [Homo sapiens] |
| 178145.75 | 154158.0469 | 168705.3438 | 373536.6875 | 357450.2188 | 172800.5156 | 220695.6563 | 160200.7656 | 195982.6875 | 529186.4375 | 279616.5 | 132646.6719 | 414613.3125 | 304347 | 181728.9844 | 214646.8281 | 181141.8438 | 196215.2656 | 178129.0156 | 226952.5781 | 150522.4375 | 438102.5625 | 366088.2813 | 190873.6875 | hCG2038940, partial [Homo sapiens] |
| 19852.78711 | 12024.86523 | 19811.05859 | 17598.375 | 24864.01758 | 26242.09375 | 23704.50195 | 38956.52734 | 26882.67969 | 31964.125 | 33838.78906 | 24462.86523 | 19180.49219 | 42834.34766 | 33482.65625 | 16103.51758 | 8907.186523 | 23479.45117 | 22108.45703 | 16916.91211 | 27585.38672 | 51396.39453 | 51899.07813 | 17231.68359 | RecName: Full=Immunoglobulin heavy variable 2-70; AltName: Full=Ig heavy chain V-II region COR; AltName: Full=Ig heavy chain V-II region DAW; AltName: Full=Ig heavy chain V-II region OU; AltName: Full=Ig heavy chain V-II region SESS; Flags: Precursor |
| 41046.95313 | 26598.1875 | 37597.59375 | 31141.77734 | 35277.60156 | 49631.14453 | 24950.07031 | 23232.77148 | 23393.18164 | 8493.320313 | 41480.47656 | 34497.46484 | 35777.28516 | 14811.61328 | 29497.74219 | 18526.01563 | 7244.512207 | 11973.14258 | 24107.89648 | 79064.25 | 19500.73828 | 34666.10938 | 81843.97656 | 44777.90625 | RecName: Full=Immunoglobulin heavy variable 2-5; AltName: Full=Ig heavy chain V-II region HE; AltName: Full=Ig heavy chain V-II region MCE; Flags: Precursor |
| 11190.63281 | 22031.26563 | 15553.4834 | 11654.78809 | 22287.83203 | 15507.74121 | 89612.35938 | 12781.54492 | 45536.46094 | 9641.932617 | 9783.408203 | 13145.87598 | 0 | 11793.00684 | 10654.01367 | 10007.7832 | 23267.83203 | 10944.39063 | 0 | 8902.412109 | 10070.49219 | 0 | 14983.31348 | 10807.2998 | polymeric immunoglobulin receptor precursor [Homo sapiens] |
| 26325362 | 14488814 | 21690970 | 28094976 | 35823628 | 27633224 | 36814280 | 16498306 | 27055968 | 24172138 | 18317258 | 28055108 | 27011112 | 26797068 | 25438506 | 21970688 | 25830484 | 28200804 | 32968540 | 22433820 | 30975810 | 25681080 | 23613124 | 20814856 | light chain kappa Sci, k Sci=Bence Jones protein [human, Peptide, 214 aa] |
| 8853220 | 5203211 | 9876134 | 11555482 | 12278912 | 9539064 | 11991841 | 6649127 | 5189348 | 7840486 | 10189019 | 9913473 | 10093550 | 12103574 | 7647608 | 6769732 | 8554568 | 5552320.5 | 10675140 | 9173236 | 8050763.5 | 6613459 | 6864265 | 3570769.25 | IGH@ protein [Homo sapiens] |
| 4204414 | 1055786.875 | 3858803 | 3050065 | 3272569 | 3471432.5 | 2088598.5 | 1470449.25 | 2950524.5 | 5095791 | 2686286 | 2730105.5 | 1856611.75 | 2827907 | 3664245.5 | 2574058.75 | 2154965.5 | 1236161.75 | 2485974.5 | 3531252.75 | 2192135 | 1245416.25 | 2345676.75 | 1851016.875 | Unknown (protein for MGC:105008) [Homo sapiens] |
| 1380028.25 | 279750.0625 | 267862.4688 | 701379 | 435935.9375 | 1290627.25 | 98420.89063 | 118390.3594 | 1199921.125 | 624351.8125 | 1101748.625 | 1286775.875 | 236088.6875 | 76427.1875 | 1281345.25 | 649214.8125 | 924850.375 | 670104.375 | 262762.1875 | 921335 | 92432.73438 | 957234.9375 | 583050.75 | 94337.1875 | RecName: Full=Immunoglobulin heavy constant gamma 4; AltName: Full=Ig gamma-4 chain C region |
| 13453227 | 7735052.5 | 11062737 | 3314392 | 12259434 | 10670038 | 3438140 | 16157423 | 17745132 | 16930056 | 6424574 | 5271028 | 7951520 | 10910546 | 14775674 | 18006420 | 4275622 | 14433919 | 5957005.5 | 12802314 | 6951333 | 13157916 | 32870560 | 8911312 | immunoglobulin heavy chain [Homo sapiens] |
| 273552.375 | 91899.91406 | 55472.77344 | 298445.5625 | 227839.9375 | 217250.5625 | 103478.8594 | 73448.57813 | 187019.5781 | 102533.5 | 198379.8125 | 129233.5 | 100335.1016 | 196996.7031 | 68021.17969 | 103982.4531 | 177687.1719 | 1751682.75 | 103649.0234 | 201456.375 | 418021.0938 | 80359.82031 | 230943.8281 | 253220.7813 | RecName: Full=Immunoglobulin heavy constant alpha 2; AltName: Full=Ig alpha-2 chain C region; AltName: Full=Ig alpha-2 chain C region BUT; AltName: Full=Ig alpha-2 chain C region LAN |
| 66177.65625 | 77458.94531 | 54969.28125 | 58469.09375 | 84380 | 102930.7969 | 131273.875 | 122581.1563 | 160940.6563 | 122019.9375 | 52076.42578 | 131053.0547 | 34635.98438 | 112155.6328 | 25097.00391 | 77843.03906 | 50842.23438 | 158726 | 60511.85938 | 61431.03906 | 40245.27344 | 54624.95313 | 103037.4219 | 92916.09375 | hemoglobin subunit delta [Homo sapiens] |
| 29132.1875 | 45663.46484 | 30649.82227 | 20955.77734 | 52437.34766 | 34678.38281 | 52232.17188 | 70135.27344 | 88137.34375 | 53537.94922 | 36246.73828 | 62105.84375 | 17855.81836 | 26067.93164 | 4716.468262 | 34882.78125 | 32819.99609 | 68332.0625 | 31589.8125 | 41995.72266 | 7441.751953 | 17296.8125 | 36199.58203 | 69373.84375 | hemoglobin subunit epsilon [Homo sapiens] |
| 27824.21484 | 28984.94922 | 22533.86523 | 25770.71094 | 14897.9834 | 14028.08887 | 39618.09766 | 17300.83398 | 23282.55859 | 11147.62305 | 22959.9082 | 27012.12109 | 26081.03711 | 29597.41406 | 25753.01758 | 15870.92676 | 46731.78125 | 43673.42188 | 16999.10938 | 41292.91016 | 26799.51172 | 32789.28516 | 39155.29688 | 22560.68555 | keratin, type I cytoskeletal 14 [Homo sapiens] |
| 8848.458008 | 12221.76563 | 29957.59375 | 5119.152344 | 0 | 0 | 5108.23584 | 9099.850586 | 9880.887695 | 51317.86719 | 3773.601318 | 0 | 22386.13281 | 14949.26563 | 13111.75195 | 3333.041992 | 3345.014404 | 15859.8125 | 1443.935913 | 15763.46875 | 4156.996094 | 0 | 9468.563477 | 31658.08008 | keratin, type II cytoskeletal 6A [Homo sapiens] |
| 50476584 | 27319284 | 38969536 | 25911082 | 23536992 | 20397708 | 25700032 | 33226348 | 22905720 | 26413104 | 36737616 | 27473886 | 30532908 | 23162982 | 27567924 | 35763564 | 33334864 | 38001852 | 38111172 | 42101180 | 41367224 | 47634496 | 33996200 | 28875552 | apolipoprotein A-I isoform 1 preproprotein [Homo sapiens] |
| 3478304.75 | 3341721 | 2671816.75 | 1577346.875 | 1555037.25 | 1280993.25 | 1929443.125 | 2561106.5 | 2566037.5 | 1245928.5 | 3243135.75 | 2595055 | 1717081.25 | 1681281.25 | 1753992 | 4731176 | 5446511 | 2167278.5 | 2360253.5 | 1870946.75 | 2893788 | 2221524 | 2059219.25 | 2966277 | apolipoprotein E [Homo sapiens] |
| 419728.625 | 498360.6875 | 477636.5625 | 176994.7031 | 231648.25 | 392446.875 | 534020.0625 | 322389.8438 | 204871.9219 | 563762.6875 | 465383.3125 | 189688.5156 | 206376.7656 | 313926.3438 | 400918.1563 | 201694.1875 | 4466665 | 285540.8125 | 492423.4375 | 245320.5469 | 368377.5 | 467814.0625 | 579812.3125 | 361648.2188 | apolipoprotein A-II preproprotein [Homo sapiens] |
| 5928893 | 5906608 | 6211124.5 | 2276590 | 4050530.5 | 6443586.5 | 4905177 | 7131942 | 7147239 | 4375697 | 4004509 | 6207002 | 7054492.5 | 5658427.5 | 6576353 | 7854298 | 7300485.5 | 5442629.5 | 5884392.5 | 6884957 | 6450799 | 8972720 | 5169572.5 | 11243878 | fibrinogen alpha chain isoform alpha-E preproprotein [Homo sapiens] |
| 12855408 | 13654373 | 14286071 | 5512079 | 9006668 | 15797707 | 11105228 | 14868691 | 15530452 | 10882096 | 8379343 | 14051386 | 15150206 | 13275018 | 15866516 | 18946188 | 17254100 | 11260012 | 14259581 | 16275540 | 13356637 | 18770902 | 12507140 | 22439148 | fibrinogen beta chain isoform 1 preproprotein [Homo sapiens] |
| 0 | 0 | 0 | 3812.092041 | 0 | 18470.58203 | 0 | 0 | 0 | 0 | 6812.767578 | 5121.984863 | 4635.867676 | 0 | 0 | 11723.48047 | 18123.10938 | 0 | 0 | 0 | 0 | 0 | 0 | 0 | C-reactive protein isoform 1 precursor [Homo sapiens] |
| 239878.7969 | 511030.125 | 386922.8438 | 571817.875 | 451546.3438 | 683232 | 520324.75 | 474062.4063 | 335357.9375 | 496202.5625 | 550337.625 | 395316.1563 | 565924 | 396586.6875 | 321740.1563 | 469627.25 | 727437.75 | 491407.625 | 442924.5938 | 294577.375 | 446121.5625 | 505221.9375 | 298575.625 | 444433.75 | serum amyloid P-component precursor [Homo sapiens] |
| 183013.7969 | 43218.78125 | 30863.38867 | 32460.16211 | 29879.0957 | 24529.14453 | 56000.41797 | 29219.34375 | 24004.57031 | 56971.29297 | 46004.60547 | 43173.14453 | 40284.81641 | 40984.89063 | 46820.23828 | 35647.62109 | 37639.84375 | 140789.125 | 30485.02734 | 35116.56641 | 35663.91797 | 25196.04492 | 33338.80078 | 33427.50781 | complement C1q subcomponent subunit A precursor [Homo sapiens] |
| 384296.375 | 677337.375 | 575277.875 | 636497.5 | 400985.0938 | 487929.9063 | 358668.2813 | 634743.375 | 347882.9688 | 357401.5625 | 510344.2188 | 357702.6875 | 483674.375 | 390317.9688 | 423137.9375 | 504413.375 | 497291.0625 | 368566.8125 | 501852.0938 | 547271.125 | 388261.6563 | 372778.0938 | 338183.9375 | 302673.75 | complement C1q subcomponent subunit C isoform X1 [Pongo abelii] |
| 101468.0938 | 74807.01563 | 122683.4688 | 154321.875 | 71775.01563 | 100261.9609 | 94726.69531 | 92103.125 | 120191.875 | 78970.76563 | 161088.1875 | 123213.6563 | 99356.03906 | 142144.0625 | 112288.4531 | 212009.8438 | 101283.6641 | 113204.3906 | 134201.875 | 137606.0781 | 87315.375 | 109038.7656 | 89941.05469 | 65555.02344 | complement component C9 preproprotein [Homo sapiens] |
| 1263584.75 | 732439.625 | 683439.125 | 1211478.75 | 998937.5625 | 1139690.875 | 722707.75 | 979822.75 | 926282 | 1225226.75 | 1339118.5 | 868608.75 | 1277854.25 | 1252927.5 | 994481.25 | 1047196.313 | 1218578.75 | 1257914 | 1293446 | 1087306.25 | 1156306.25 | 743065.3125 | 728540.875 | 752742.375 | beta-2-glycoprotein 1 precursor [Homo sapiens] |
| 178047.5313 | 97390.46875 | 141122.5938 | 99862.71875 | 92314.625 | 164307.2813 | 82511.82813 | 86606.5625 | 114105.5781 | 85948.21875 | 146816.2344 | 118421.8672 | 111901.2188 | 144196.5313 | 155410.4844 | 146709.9688 | 180907.0938 | 231022.7188 | 134407.25 | 131323.8125 | 84474.66406 | 138122.4844 | 152790.4375 | 80293.42188 | leucine-rich alpha-2-glycoprotein precursor [Homo sapiens] |
| 159014.125 | 578516.5 | 383582.25 | 204167.3438 | 438051.375 | 913951.5625 | 662064 | 1341123.625 | 629513.9375 | 386190.625 | 126019.1172 | 695912.25 | 603546.5625 | 608150.0625 | 842838.5 | 716160 | 1264753.5 | 712232 | 811685.9375 | 840640.375 | 999780 | 918483.5 | 337578.125 | 1589404 | fibronectin isoform 1 precursor [Homo sapiens] |
| 55211.97656 | 104496.8125 | 66551.32031 | 48946.99609 | 80756.29688 | 59201.98438 | 62308.58203 | 78410.88281 | 56248.19922 | 60942.39063 | 89117.85156 | 53626.47656 | 60909.30469 | 62778.57813 | 53300.89453 | 81842.0625 | 43678.00781 | 49608.51563 | 96947.28125 | 49035.28125 | 84396.40625 | 77259.63281 | 36498.11719 | 55422.05859 | retinol-binding protein 4 isoform a precursor [Homo sapiens] |
| 503325.625 | 414678.5625 | 397151.75 | 386826.6875 | 365267.5 | 424534.625 | 404061.5938 | 421949.5 | 348226.6875 | 352166.25 | 431630.125 | 439255.0313 | 415062.8125 | 531155.125 | 368980.5313 | 411519.25 | 326078.3125 | 405705.875 | 385639.375 | 381334.5 | 575633.625 | 427716.4063 | 368066.7188 | 538938.125 | protein AMBP preproprotein [Homo sapiens] |
| 1946483 | 1952170.25 | 3417004.5 | 2403905 | 1666091.5 | 2296641 | 1769097.25 | 1836885.25 | 1789929 | 1588107.25 | 2784324.75 | 2390674.75 | 2204300 | 2122631.25 | 1492505.625 | 2892199.5 | 3748525.75 | 2733213.5 | 1600170.125 | 2092053.625 | 1496146.25 | 1396138.625 | 1337536.25 | 1219226 | RecName: Full=Alpha-1-acid glycoprotein 1; Short=AGP 1; AltName: Full=Orosomucoid-1; Short=OMD 1; Flags: Precursor |
| 1129980 | 695272.5 | 832900.625 | 809292.4375 | 734143.75 | 832468.8125 | 871740.4375 | 787071.75 | 1043437.75 | 1029923.25 | 887684.75 | 1241165.75 | 938640.3125 | 926642.875 | 750048.625 | 1089332.5 | 1297783.5 | 1087917.875 | 809252.0625 | 621531.9375 | 748690 | 673453.75 | 620907.25 | 646039.9375 | transthyretin precursor [Homo sapiens] |
| 535587872 | 454998848 | 475412800 | 480977024 | 413830240 | 486421632 | 467814720 | 463233600 | 407651904 | 390904096 | 558591040 | 460347200 | 493708992 | 363529856 | 397448384 | 466694432 | 554724800 | 526727936 | 543688896 | 653722560 | 497794304 | 553186880 | 487580224 | 347120384 | serum albumin preproprotein [Homo sapiens] |
| 39434.20703 | 21303.61328 | 18984.09375 | 232010.7188 | 59116.61719 | 30922.40234 | 56902.38672 | 13296.3125 | 16946.53125 | 0 | 31430.61328 | 89828.46875 | 45503.22656 | 0 | 11281.43652 | 16329.14551 | 67985.3125 | 65089.84375 | 65882.91406 | 96332.27344 | 97933 | 58713.96484 | 55573.54688 | 50236.96094 | platelet basic protein preproprotein [Homo sapiens] |
| 20585.10742 | 37381.92578 | 17626.95898 | 151373.9375 | 51930.24219 | 29100.86719 | 38554.28516 | 25423.34961 | 24448.10352 | 4746.481445 | 21973.54883 | 66603.78906 | 28938.50977 | 7121.949219 | 18740.11328 | 29177.44531 | 74730.92188 | 25533.78516 | 59568.17969 | 117870.0938 | 46080.71094 | 33256.90625 | 61754.80469 | 104020.1719 | platelet factor 4 isoform 1 precursor [Homo sapiens] |
| 26115824 | 21483380 | 23848896 | 29173996 | 16176014 | 19427140 | 15966626 | 17563200 | 14148647 | 15037300 | 21821292 | 16155692 | 18838960 | 12578976 | 25925382 | 19799500 | 22378590 | 23109102 | 18860516 | 25924864 | 20696370 | 16605656 | 16485816 | 14759710 | serotransferrin isoform 1 precursor [Homo sapiens] |
| 3248052.5 | 2447079.5 | 4088099 | 2807119 | 2127160.25 | 2574785.75 | 2728899 | 2782513.5 | 2512498 | 2759691.5 | 3619198.5 | 4011305.5 | 2851063 | 3299176 | 2716117.5 | 3663873 | 3889304.5 | 3381110.5 | 2897278 | 3696894.5 | 2729940 | 2850043 | 2292639 | 2138477 | hemopexin precursor [Homo sapiens] |
| 0 | 3884.67041 | 6897.8125 | 0 | 0 | 8793.758789 | 0 | 11228.67383 | 0 | 0 | 0 | 4150.135254 | 0 | 6071.359863 | 0 | 0 | 0 | 0 | 5846.989746 | 0 | 8940.678711 | 0 | 3263.383301 | 5042.208984 | angiogenin precursor [Homo sapiens] |
| 10284.01563 | 8506.447266 | 5302.126953 | 6438.81543 | 18294.99219 | 10170.9668 | 5195.310547 | 6689.550293 | 7630.100098 | 5877.462891 | 5961.876465 | 8085.40332 | 11656.53223 | 5907.23584 | 5075.334473 | 10523.0918 | 15232.77637 | 8775.779297 | 22623.41211 | 15134.19629 | 9048.992188 | 14360.57715 | 12808.17383 | 11752.44043 | coagulation factor XI isoform 1 preproprotein [Homo sapiens] |
| 2928946 | 3035332 | 2884251.75 | 3186530.5 | 3556162.5 | 1667997 | 1563169 | 3011240.5 | 4117855.5 | 1711722.5 | 2190961 | 2239056.25 | 2353603.25 | 3471967.5 | 3140864.25 | 6950511 | 3040141.5 | 2182300.75 | 2707176.25 | 2690908.5 | 3238714.5 | 3491521.5 | 3352694.5 | 4218401.5 | C4b-binding protein alpha chain precursor [Homo sapiens] |
| 662756.25 | 933669.125 | 933751.6875 | 739608.875 | 816548.375 | 787296.125 | 868617.4375 | 971323.875 | 464010.625 | 836658.6875 | 709734.5625 | 737036.5625 | 801966.125 | 632402.8125 | 690562.25 | 967754.625 | 458537.3125 | 707736.375 | 769859.875 | 874362.75 | 877991.125 | 661663.5 | 666790.8125 | 658014.8125 | vitronectin precursor [Homo sapiens] |
| 434929.2188 | 9313.248047 | 7112.369141 | 6614.808105 | 7302.779297 | 5018.083984 | 10756.01563 | 7064.944824 | 10761.6875 | 8834.946289 | 8585.177734 | 11290.11719 | 0 | 8269.529297 | 23712.6582 | 0 | 6355.354492 | 8082.695801 | 0 | 4183.364746 | 0 | 0 | 6541.522461 | 5801.438965 | catalase [Homo sapiens] |
| 3649240.5 | 4576699 | 3383965.5 | 4173759.5 | 6040662.5 | 2443901.5 | 2845106.25 | 3523747 | 6163791.5 | 4137316.25 | 3628270.5 | 3567130.5 | 3194275.5 | 3910097.25 | 4018624 | 6873276 | 5115857.5 | 3278514.5 | 3880109.5 | 2078301.5 | 5224927.5 | 4150574.25 | 5380167 | 5024207 | RecName: Full=Apolipoprotein B-100; Short=Apo B-100; Contains: RecName: Full=Apolipoprotein B-48; Short=Apo B-48; Flags: Precursor |
| 19833.23047 | 16463.20898 | 15957.83008 | 12750.32617 | 11937.79297 | 14341.81152 | 18012.6543 | 19417.79102 | 20295.03516 | 11137.96191 | 13464.25684 | 15562.09375 | 26170.04102 | 17902.63086 | 15419.90137 | 15361.27637 | 18034.45117 | 13764.41406 | 20194.80078 | 28080.57422 | 23032.23047 | 18845.17773 | 20719.65234 | 21647.04297 | phosphatidylcholine-sterol acyltransferase precursor [Homo sapiens] |
| 1352722.75 | 674195.375 | 683404.5625 | 771401.75 | 435356.4375 | 790951.4375 | 549873.875 | 721384.6875 | 714938.625 | 436180.375 | 454299.625 | 625877.25 | 903827.125 | 320322.0625 | 604755.75 | 556729.75 | 596389.4375 | 821523.75 | 657468.375 | 1022681.563 | 690325.25 | 452923.25 | 685440.6875 | 550162.9375 | histidine-rich glycoprotein precursor [Homo sapiens] |
| 1473740.5 | 613190 | 659920.25 | 848183.0625 | 930334 | 772283.1875 | 1356456.125 | 505153.7188 | 1450881.25 | 964303.25 | 2128863.25 | 1162233.875 | 652541.25 | 2081426 | 937924.625 | 732089.5 | 1674170.5 | 2335641.5 | 751045.0625 | 1160167.75 | 763140.1875 | 831153.9375 | 1060046.125 | 789522 | hCG1731877, partial [Homo sapiens] |
| 1167174.25 | 750470.5 | 894190.1875 | 622876.9375 | 634652.125 | 915151.75 | 785923.625 | 792046 | 775173.625 | 734544.0625 | 953042.125 | 909052.5625 | 731420.875 | 689888.25 | 748605 | 911353.6875 | 861936.8125 | 692049.8125 | 714985.75 | 910157.25 | 886004.625 | 714054.125 | 662913.125 | 618162.9375 | alpha-1B-glycoprotein precursor [Homo sapiens] |
| 589688.125 | 288897.6875 | 376664.9375 | 185878.6563 | 136172.1406 | 135779.5313 | 431128.4375 | 302623.1563 | 422634.7188 | 680320.25 | 191804.6875 | 347082.3125 | 213656.5469 | 666115.875 | 206951.0625 | 162059.9688 | 534232.875 | 466734.7188 | 123260.25 | 317530.625 | 232367.2656 | 239757.8438 | 341581.375 | 170735.7969 | keratin 1 [Homo sapiens] |
| 15131.85352 | 114039.7344 | 17130.17969 | 19442.85938 | 19855.71094 | 29375.46875 | 139872.7188 | 190536.8125 | 34236.01563 | 28461.61133 | 5543.116211 | 76554.29688 | 41972.97656 | 117287.9844 | 81479.51563 | 93413.35938 | 69298.10156 | 45384.64844 | 56179.61719 | 40323.33594 | 68471.50781 | 60736.84375 | 10871.12793 | 62625.36719 | RecName: Full=von Willebrand factor; Short=vWF; Contains: RecName: Full=von Willebrand antigen 2; AltName: Full=von Willebrand antigen II; Flags: Precursor |
| 0 | 22339.59766 | 0 | 46215.25 | 168785.2656 | 84270.70313 | 31692.93359 | 88387.05469 | 26449.91016 | 74618.83594 | 36092.63281 | 21359.71094 | 162690 | 32961.90234 | 38107.35938 | 36959.20313 | 0 | 0 | 122863.0391 | 58458.54688 | 64124.55469 | 116383.0156 | 170540.4531 | 91546.98438 | RecName: Full=Immunoglobulin kappa variable 1-16; AltName: Full=Ig kappa chain V-I region BAN; Flags: Precursor |
| 86954.51563 | 61396.41016 | 86314.46875 | 106754.7109 | 102918.3438 | 74949.19531 | 116984.8672 | 113212.2109 | 98336.94531 | 108290.9141 | 84330.76563 | 77325.19531 | 91326.54688 | 111370.9375 | 103796.6094 | 79710.84375 | 65547.14063 | 71034.53906 | 63838.01172 | 75168.5 | 88245.21875 | 95232.32813 | 141007.4844 | 73081.33594 | rheumatoid factor D1 IgG light chain VK3 region, partial [Homo sapiens] |
| 20402.92969 | 8187.952637 | 5776.078613 | 12424.61035 | 7179.066406 | 5925.213379 | 6685.615723 | 7892.078613 | 0 | 6363.488281 | 26848.74609 | 14000.00781 | 8505.188477 | 5292.839844 | 22861.02539 | 2991.272949 | 10092.3457 | 7143.131348 | 5166.192383 | 0 | 0 | 0 | 4191.349121 | 7974.687012 | protein S100-A8 isoform d [Homo sapiens] |
| 19399.50781 | 10147.31152 | 21107.70313 | 16758.55664 | 29551.35156 | 18209.73047 | 20729.82031 | 28795.20313 | 12421.91602 | 26072.96094 | 25699.5293 | 28510.92383 | 23391.48828 | 22196.03516 | 18832.90039 | 17681.26172 | 18178.12109 | 28821.93555 | 20174.95703 | 13058.08398 | 19386.41016 | 15976.97852 | 13294.82227 | 22464.69922 | plasma serine protease inhibitor preproprotein [Homo sapiens] |
| 332280.9063 | 300231.1875 | 358749.3125 | 292074.25 | 229720.125 | 343852.125 | 312251.375 | 314977.8438 | 340008.625 | 346798.7188 | 362376.5 | 340823.5 | 376645.4375 | 300247.125 | 316712.25 | 412864.8438 | 455191.25 | 365038.625 | 312215.6875 | 558466.625 | 278928.4375 | 373344.0625 | 244220.2188 | 335775.5313 | unnamed protein product [Homo sapiens] |
| 35155.51953 | 25549.56641 | 26696.71094 | 19687.53125 | 21000.70313 | 23027.25 | 24478.39063 | 26627.10156 | 24696.64453 | 24890.94922 | 22568.86914 | 31036.61719 | 27060.93164 | 29515.34375 | 22446.77539 | 29481.00977 | 45750.22656 | 27636.67188 | 36336.28125 | 32834.32422 | 36271.75 | 26336.75 | 26230.58203 | 44819.51563 | coagulation factor XIII B chain precursor [Homo sapiens] |
| 25762.12109 | 11803.08105 | 38449.11719 | 24829.83594 | 20113.33594 | 32978.83203 | 29447.20117 | 21690.78711 | 17142.77539 | 33390.50781 | 20375.39844 | 19260.92773 | 22727.46094 | 32060.03516 | 28573.06836 | 22974.9043 | 38277.66406 | 25243.98633 | 28440.17578 | 25992.87109 | 24184.9375 | 20928.32422 | 12076.99023 | 18099.28711 | thyroxine-binding globulin precursor [Homo sapiens] |
| 573716.125 | 642476.6875 | 681422.625 | 705247.0625 | 453415.125 | 682880.9375 | 381318.8438 | 504427.625 | 346351.4063 | 442410.375 | 774963.375 | 540970.5 | 737596.25 | 395259.1875 | 498847.8125 | 450196.875 | 715134.875 | 535797.9375 | 654128.25 | 691411.75 | 645404.5 | 609509.0625 | 450568.7188 | 578983.1875 | heparin cofactor 2 precursor [Homo sapiens] |
| 200714.2656 | 136155.6719 | 215764.4063 | 321305.875 | 410108.5625 | 435336.125 | 346494.2188 | 413252.0625 | 427510.0938 | 518412.5313 | 455680.125 | 386092.25 | 289839.6875 | 423087.5 | 511549.75 | 257092.4844 | 407556.75 | 426645.9375 | 354662.0625 | 405009 | 298762.0625 | 568344.8125 | 501625.9375 | 305378.5 | immunoglobulin kappa chain, partial [Homo sapiens] |
| 258728.0156 | 1271168.25 | 228253.2188 | 243979.1094 | 226603.4063 | 178281.5938 | 228717.2188 | 1580711.25 | 236321.9063 | 281413.4688 | 277228.5625 | 233760.6406 | 253913.0938 | 1577428.125 | 243498.125 | 130197.8359 | 273529.5 | 2180320.25 | 176118.25 | 289960.75 | 178020.0625 | 1719007.875 | 282586.2813 | 142569.1563 | hCG1793614, partial [Homo sapiens] |
| 0 | 7346.785156 | 0 | 3855.356934 | 0 | 0 | 3201.015869 | 0 | 0 | 0 | 20493.97852 | 0 | 0 | 0 | 5821.447754 | 0 | 0 | 0 | 0 | 0 | 0 | 0 | 0 | 0 | protein S100-A9 [Homo sapiens] |
| 1149536 | 1166258.125 | 1122162 | 773225.5625 | 608303.125 | 734746.3125 | 808980 | 693519.1875 | 893104.625 | 746947.625 | 1297721.125 | 712446.875 | 936791.5625 | 1733737.75 | 801238.375 | 888856.625 | 1235710.875 | 1742867.125 | 1175793.75 | 956606.125 | 1088623.625 | 1093288.375 | 708270.5 | 685845.75 | RecName: Full=Apolipoprotein A-IV; Short=Apo-AIV; Short=ApoA-IV; AltName: Full=Apolipoprotein A4; Flags: Precursor |
| 46370.34766 | 56540.38672 | 57774.75781 | 66814 | 64499.54688 | 52371.13281 | 55999.20703 | 58353.26563 | 38301.64063 | 46900.8125 | 68530.98438 | 60335.50781 | 63675.55469 | 50260.46094 | 60392.875 | 64769.17188 | 74973.39063 | 51350.70703 | 64451.61328 | 49792.00781 | 44356.57813 | 57152.92578 | 53519.79688 | 37101.36719 | complement component C8 alpha chain preproprotein [Homo sapiens] |
| 38181.46094 | 46099.75391 | 38299.85156 | 50760.25 | 37484.42188 | 42146.38672 | 42364.77344 | 42804.45313 | 40169.87891 | 44503.33594 | 41236.69531 | 54971.20313 | 44500.74219 | 54802.33203 | 48071.36719 | 39576.15234 | 36198.15234 | 59464.39453 | 48555.14844 | 25874.67188 | 34307.36719 | 34708.59375 | 35563.29297 | 35224.07031 | RecName: Full=Complement component C8 beta chain; AltName: Full=Complement component 8 subunit beta; Flags: Precursor |
| 28738.78906 | 42754.00781 | 36343.64063 | 54288.40625 | 46837.42188 | 43880.46875 | 41720.25781 | 53639.74219 | 30310.97266 | 45811.32031 | 49712.875 | 39837.45313 | 50161.32813 | 36453.42188 | 43201.08594 | 39619.19531 | 61106.59766 | 41832.97656 | 47120.24219 | 34731.73828 | 34127.15625 | 28955.39063 | 32099.81641 | 27201.23828 | complement component C8 gamma chain precursor [Homo sapiens] |
| 0 | 0 | 0 | 0 | 0 | 0 | 0 | 0 | 0 | 1236.629639 | 0 | 0 | 0 | 0 | 0 | 0 | 0 | 0 | 6539.580078 | 10170.34473 | 7699.487793 | 5170.303223 | 7106.15625 | 12883.33203 | tubulin, beta 5, partial [Mus musculus] |
| 7252.268555 | 15708.36816 | 8505.632813 | 14362.88672 | 22140.75391 | 12327.65625 | 24901.41406 | 9162.80957 | 6377.092773 | 2190.99585 | 15252.75488 | 33582.89453 | 14578.91016 | 5020.012207 | 8752.191406 | 6973.483398 | 17334.94727 | 10983.94336 | 19252.30469 | 42985.27344 | 23181.44922 | 16389.83594 | 22586.07031 | 20619.9707 | PREDICTED: profilin-1 isoform X2 [Nomascus leucogenys] |
| 16386.70703 | 28968.01563 | 7974.1875 | 92818.8125 | 34337.69141 | 9009.322266 | 26572.84375 | 14650.46484 | 27112.90234 | 18894.52344 | 6421.652344 | 36610.13281 | 16339.0957 | 6309.328125 | 9709.178711 | 22355.41016 | 19928.75586 | 16902.93555 | 25034.625 | 57262.05859 | 32875.40625 | 22916.88672 | 43697.96875 | 92691.89063 | thrombospondin-1 precursor [Homo sapiens] |
| 45417.44531 | 40142.39063 | 46221.50781 | 59349.60156 | 112061.9453 | 115168.6719 | 33919.93359 | 107326.6172 | 26859.57617 | 36060.36719 | 143903.9375 | 31587.1543 | 113872.9219 | 44432.02344 | 75999.21875 | 32442.78516 | 41716.82422 | 51259.17578 | 154884.6563 | 127512.6563 | 129253.8906 | 133857.9219 | 100473.5625 | 56091.55469 | corticosteroid-binding globulin precursor [Homo sapiens] |
| 0 | 652.4032593 | 0 | 0 | 0 | 0 | 0 | 0 | 0 | 0 | 0 | 0 | 0 | 0 | 0 | 0 | 923.8422852 | 0 | 2558.61792 | 11014.29199 | 3902.144287 | 0 | 3844.881836 | 7463.073242 | integrin alpha-IIb preproprotein [Homo sapiens] |
| 58467.60156 | 35306.74609 | 121683.6172 | 67134.45313 | 56356.86719 | 142735.9375 | 47652.86719 | 31540.65234 | 639287.375 | 133440.3438 | 115876.5781 | 56053.79688 | 33796.25781 | 49414.39844 | 17732.17773 | 60765.27734 | 43356.80469 | 23806.51172 | 26731.98633 | 36990.92969 | 52313.29688 | 32675.5293 | 800727.375 | 64406.24219 | RecName: Full=Apolipoprotein(a); Short=Apo(a); Short=Lp(a); Flags: Precursor |
| 12573.42383 | 10244.57715 | 5834.999512 | 12859.94238 | 8749.010742 | 10378.77637 | 9206.229492 | 10618.9043 | 8634.548828 | 15168.87988 | 12218.81641 | 8572.041016 | 9426.730469 | 9437.429688 | 12257.83301 | 7982.820313 | 10841.77246 | 12494.89453 | 14535.58984 | 12111.31934 | 12069.41797 | 10292.55273 | 12739.32422 | 4244.029297 | monocyte differentiation antigen CD14 precursor [Homo sapiens] |
| 1355048.25 | 1340270 | 1505608 | 1655420.75 | 1256665.75 | 1232410.25 | 1040862.25 | 1462139.375 | 1110006.25 | 805995.6875 | 1322564.5 | 1141433 | 1305169.5 | 1009239.813 | 924360 | 1572166.75 | 1448908.25 | 1028537.125 | 1253879 | 1346991 | 1353366.875 | 1069329.25 | 1100312.375 | 1696349 | RecName: Full=Complement factor H; AltName: Full=H factor 1; Flags: Precursor |
| 249402.4375 | 241939.6719 | 257467.4844 | 226630.5 | 188645.2813 | 278004.0313 | 313918.75 | 195963.9688 | 222626.1875 | 307670.2188 | 210715.5781 | 225854.6719 | 240504.7656 | 258364.25 | 221446.375 | 265355.6563 | 318532.4063 | 260214.4688 | 300300.2813 | 343397.625 | 240425.125 | 214831.875 | 238013.8438 | 180960.8125 | alpha-2-antiplasmin isoform X1 [Homo sapiens] |
| 34417.67578 | 100847.5625 | 30410.01172 | 83037.4375 | 100869.8438 | 84523.71094 | 44584.21484 | 20504.60156 | 35654.57422 | 104951.3594 | 141773.5781 | 23191.60156 | 54991.66016 | 37089.82031 | 59024.46875 | 116440.7422 | 34192.95313 | 31682.55859 | 79924.85938 | 268450.125 | 16545.33984 | 482869.4063 | 25826.45313 | 18681.72461 | keratin, type I cytoskeletal 16 [Homo sapiens] |
| 2897.793945 | 4323.808105 | 0 | 2360.475342 | 0 | 0 | 4230.950195 | 8610.535156 | 0 | 3275.859619 | 1630.779053 | 0 | 1585.327148 | 2703.059326 | 0 | 5263.240234 | 2557.312744 | 7197.940918 | 0 | 0 | 4007.282959 | 5404.182617 | 1653.563354 | 3229.155029 | dopamine beta-hydroxylase precursor [Homo sapiens] |
| 0 | 0 | 0 | 10544.52344 | 0 | 0 | 0 | 0 | 0 | 0 | 0 | 1922.342163 | 0 | 0 | 0 | 0 | 0 | 0 | 0 | 3713.807617 | 0 | 0 | 0 | 5883.597656 | SPARC isoform 1 precursor [Homo sapiens] |
| 112087.8906 | 87677.70313 | 89165.89844 | 91870.54688 | 75945.09375 | 81532.44531 | 81476.07031 | 90503.89844 | 100293.8281 | 81084.91406 | 88529.51563 | 104625.3906 | 75544.4375 | 88744.625 | 75570.25781 | 141217.7188 | 97383.73438 | 83735.03125 | 95449 | 108503.8594 | 107806.8438 | 88059.11719 | 85164.05469 | 84332.4375 | complement C1s subcomponent isoform 1 preproprotein [Homo sapiens] |
| 0 | 0 | 0 | 26855.66406 | 0 | 0 | 0 | 0 | 0 | 26308.82813 | 0 | 0 | 0 | 0 | 0 | 2086.176758 | 4151.487793 | 21985.54297 | 0 | 0 | 885.4891968 | 59314.16016 | 0 | 0 | complement C4-A isoform 1 preproprotein [Homo sapiens] |
| 1911321.75 | 1569389.75 | 1845794.75 | 1159715.125 | 1592193.5 | 2278331.75 | 1560900.875 | 2968363.25 | 1232793.375 | 1065250 | 1600056 | 1968661.875 | 1809786.75 | 1460675 | 1673813.625 | 4921905 | 848844.375 | 1123670 | 1561813.25 | 1727734.875 | 1584298 | 2005207.25 | 1976303.375 | 1810395.25 | complement C4-B preproprotein [Homo sapiens] |
| 0 | 0 | 0 | 0 | 0 | 0 | 0 | 0 | 5646.003906 | 0 | 0 | 0 | 0 | 0 | 0 | 0 | 0 | 0 | 0 | 0 | 0 | 0 | 0 | 0 | RecName: Full=Immunoglobulin lambda constant 6; AltName: Full=Ig lambda-6 chain C region |
| 25699.45313 | 26961.45117 | 10895.09082 | 22812.36133 | 27522.76563 | 12891.04492 | 14455.53418 | 85544.15625 | 11580.62207 | 28078.68945 | 20839.63672 | 47744.73438 | 43878.94922 | 30521.42773 | 21045.00977 | 32572.13086 | 36507.83984 | 22619.00195 | 41967.51172 | 34542.31641 | 53193.93359 | 63430.83984 | 15779.63086 | 19598.73828 | RecName: Full=Serum amyloid A-1 protein; Short=SAA; Contains: RecName: Full=Amyloid protein A; AltName: Full=Amyloid fibril protein AA; Contains: RecName: Full=Serum amyloid protein A(2-104); Contains: RecName: Full=Serum amyloid protein A(3-104); Contains: RecName: Full=Serum amyloid protein A(2-103); Contains: RecName: Full=Serum amyloid protein A(2-102); Contains: RecName: Full=Serum amyloid protein A(4-101); Flags: Precursor |
| 9614351 | 4766568 | 8002974 | 5722056 | 11553848 | 9502853 | 9033781 | 1193892.75 | 9098345 | 9329487 | 10372131 | 8323205 | 8372825.5 | 12415910 | 10061390 | 8301053 | 19815726 | 9955400 | 6737246 | 10553771 | 10192443 | 7649513 | 10110834 | 8569576 | RecName: Full=Immunoglobulin lambda constant 3; AltName: Full=Ig lambda chain C region DOT; AltName: Full=Ig lambda chain C region NEWM; AltName: Full=Ig lambda-3 chain C regions |
| 20026.05273 | 37740.03125 | 42193.87109 | 35346.91016 | 69761.72656 | 29338.02148 | 31336.21289 | 0 | 13780.00586 | 28309.05273 | 36147.58984 | 9803.410156 | 63801.01953 | 17405.61133 | 37186.30078 | 24282.14844 | 25255.12305 | 0 | 30006.7168 | 29408.37109 | 15348.90332 | 26316.78711 | 145008.3906 | 26730.60156 | immunoglobulin heavy chain variable region, partial [Homo sapiens] |
[truncated: 38,074 more chars]
