# Supplementary material for: Proteomics of extracellular vesicles in plasma reveals the characteristics and residual traces of COVID-19 patients without underlying diseases after 3 months of recovery
Source: Cell Death Dis. 2021 May 25;12(6):541. doi: 10.1038/s41419-021-03816-3 (PMC8146187; doi:10.1038/s41419-021-03816-3)
Supplement: Supplementary file 15 — Table S2 [file 41419_2021_3816_MOESM15_ESM.docx]

| Table S2-1 Proteins identified in A vs C group. | | | | | | | | | | | |
| --- | --- | --- | --- | --- | --- | --- | --- | --- | --- | --- | --- |
| Accession | C | A | FC(C/A) | log2FC(C/A) | Pvalue(C/A) | significant | regulate | GO | KEGG | COG | Description |
| Q15166 | 33504.54737 | 27697.90895 | 0.826691035 | -0.274579853 | 0.67629952 | no | down | GO:0016787;GO:0004063;GO:0070062;GO:0005615;GO:0046395;GO:0019372;GO:0009636;GO:0016311;GO:0043231;GO:0019439;GO:0046226;GO:0005576;GO:0010124;GO:0032929;GO:0102007;GO:0046872;GO:0004064;GO:0018733;GO:0042803 | hsa:5446 |  | serum paraoxonase/lactonase 3 [Homo sapiens] |
| A0A0C4DH32 | 74133.90813 | 61163.57889 | 0.825041879 | -0.277460742 | 0.281559762 | no | down | GO:0009897;GO:0050853;GO:0045087;GO:0016020;GO:0006910;GO:0006911;GO:0002376;GO:0005576;GO:0002250;GO:0003823;GO:0005886;GO:0072562;GO:0042571;GO:0006958;GO:0050871;GO:0034987;GO:0042742 | hsa:102723407 | | RecName: Full=Immunoglobulin heavy variable 3-20; Flags: Precursor |
| P05543 | 28069.67421 | 23109.91774 | 0.823305521 | -0.280500195 | 0.076825336 | no | down | GO:0005615;GO:0070327;GO:0005576;GO:0004867;GO:0010951;GO:0070062 | hsa:6906 | COG4826 | thyroxine-binding globulin precursor [Homo sapiens] |
| P60709 | 33189.38895 | 27249.58842 | 0.821033146 | -0.284487629 | 0.34372667 | no | down | GO:0043044;GO:0019894;GO:0015629;GO:0072749;GO:0061024;GO:0005200;GO:0021762;GO:0036464;GO:0005925;GO:0048488;GO:0030957;GO:0032091;GO:0005615;GO:0043209;GO:0005634;GO:0016020;GO:0097433;GO:0070062;GO:0098871;GO:0005654;GO:0045815;GO:0016579;GO:0005524;GO:0000980;GO:0005856;GO:0070527;GO:0000166;GO:1903076;GO:0019901;GO:0000790;GO:0005886;GO:0048013;GO:0001895;GO:0098685;GO:0034329;GO:0031982;GO:0038096;GO:0050998;GO:0044305;GO:0032991;GO:0051621;GO:0048870;GO:0030863;GO:0022898;GO:0005829;GO:0051623;GO:1990904;GO:0005737;GO:0098973;GO:0031492;GO:0098793;GO:0042802;GO:0005515;GO:0098974;GO:0072562;GO:0000978;GO:0098978;GO:0000079;GO:0035267 | hsa:71;hsa:60 | COG5277 | cytoskeletal beta actin, partial [Sus scrofa] |
| Q08380 | 86284.11158 | 70615.65263 | 0.818408527 | -0.289106919 | 0.352715384 | no | down | GO:0006968;GO:0070062;GO:0006898;GO:0005044;GO:0002576;GO:0016020;GO:0072562;GO:0031089;GO:0062023;GO:0005615;GO:0007165;GO:0005515;GO:0007155;GO:0005576 | hsa:3959 |  | galectin-3-binding protein precursor [Homo sapiens] |
| P61626 | 14455.72159 | 11785.74744 | 0.815299836 | -0.29459737 | 0.13955286 | no | down | GO:0006954;GO:0019730;GO:0016798;GO:0003796;GO:0035578;GO:0042742;GO:0050829;GO:0019835;GO:0044267;GO:0070062;GO:0016998;GO:0035580;GO:0042802;GO:0016787;GO:1904724;GO:0003824;GO:0050830;GO:0008152;GO:0001895;GO:0005615;GO:0031640;GO:0043312;GO:0005576 | hsa:4069 |  | lysozyme C precursor [Homo sapiens] |
| A0A0C4DH21 | 50413.95316 | 40816.30053 | 0.80962309 | -0.30467766 | 0.122375148 | no | down |  | hsa:10877 |  | complement factor H-related protein 4 [Homo sapiens] |
| P0DOY3 | 9264277.474 | 7486774.005 | 0.808133611 | -0.307334258 | 0.130565776 | no | down | GO:0005615;GO:0016020;GO:0072562;GO:0002376;GO:0005576;GO:0002250;GO:0003823;GO:0005886;GO:0070062 | hsa:100423062 | | RecName: Full=Immunoglobulin lambda constant 3; AltName: Full=Ig lambda chain C region DOT; AltName: Full=Ig lambda chain C region NEWM; AltName: Full=Ig lambda-3 chain C regions |
| A0A4W8ZXM2 | 817147.1153 | 659986.1158 | 0.807671108 | -0.308160163 | 0.276925967 | no | down |  | hsa:102723407 | | immunoglobulin heavy chain variable region, partial [Homo sapiens] |
| P55056 | 293654.3416 | 234977.8321 | 0.800185112 | -0.321594309 | 0.382851077 | no | down | GO:0005319;GO:0034361;GO:0070328;GO:0006629;GO:0034447;GO:0006869;GO:0034379;GO:0010890;GO:0005576;GO:0034364 | hsa:346 |  | apolipoprotein C-IV precursor [Homo sapiens] |
| P02042 | 91669.92789 | 73247.19789 | 0.799031913 | -0.323674969 | 0.20375821 | no | down | GO:0005344;GO:0019825;GO:0020037;GO:0043177;GO:0031721;GO:0007596;GO:0072562;GO:0005833;GO:0098869;GO:0042744;GO:0005515;GO:0031838;GO:0046872;GO:0005829;GO:0015671 | hsa:3045 | COG1018 | hemoglobin subunit delta [Homo sapiens] |
| A0A0A0MS15 | 304227.7211 | 242347.3158 | 0.7965984 | -0.328075513 | 0.291568468 | no | down | GO:0009897;GO:0050853;GO:0045087;GO:0016020;GO:0006910;GO:0006911;GO:0002376;GO:0005576;GO:0002250;GO:0003823;GO:0005886;GO:0072562;GO:0042571;GO:0006958;GO:0050871;GO:0034987;GO:0042742 | hsa:102723407 | | RecName: Full=Immunoglobulin heavy variable 3-49; Flags: Precursor |
| P11021 | 292663.4498 | 232966.6897 | 0.796022496 | -0.329118893 | 0.618368353 | no | down | GO:1990090;GO:0051087;GO:0005783;GO:0071480;GO:1990440;GO:0035437;GO:0009314;GO:0030433;GO:0019899;GO:0036500;GO:0005789;GO:0042149;GO:0035690;GO:0021762;GO:0045296;GO:0016887;GO:0031204;GO:0034663;GO:0005925;GO:1901998;GO:0019904;GO:0008180;GO:0005737;GO:0005793;GO:0006983;GO:0042623;GO:0043209;GO:0005634;GO:0016020;GO:0071353;GO:0005739;GO:0000166;GO:0031625;GO:0043066;GO:0005509;GO:0031398;GO:0043022;GO:0005524;GO:0016787;GO:0030335;GO:0071236;GO:0030176;GO:0005790;GO:0030182;GO:0042470;GO:0097501;GO:0009986;GO:0071277;GO:0036498;GO:0036499;GO:0051402;GO:0051082;GO:0005788;GO:0005886;GO:0042220;GO:0030512;GO:0001554;GO:0051603;GO:1904313;GO:0032991;GO:0043231;GO:0030968;GO:0071287;GO:0070062;GO:0005829;GO:0071320;GO:0030496;GO:0060904;GO:0090074;GO:0021589;GO:1903897;GO:1903894;GO:1903895;GO:0051787;GO:0005515;GO:1903891;GO:0034976;GO:0034975;GO:0021680;GO:0010976 | hsa:3309 | COG0443 | 78 kDa glucose-regulated protein [Otolemur garnettii] |
| H0Y5E4 | 25163.22316 | 19693.86579 | 0.782644801 | -0.353570397 | 0.478966556 | no | down | GO:0005540;GO:0007155;GO:0016021;GO:0016020 | hsa:960 |  | CD44 antigen isoform 8 precursor [Homo sapiens] |
| A0A0G2JPR0 | 503231.4947 | 392835.4947 | 0.780625813 | -0.357296926 | 0.101078931 | no | down | GO:0004866;GO:0005576;GO:0006956;GO:0006954;GO:0010951;GO:0005615 | hsa:100293534;hsa:110384692;hsa:720;hsa:721 | | complement C4A (Rodgers blood group)-like preproprotein [Homo sapiens] |
| P01763 | 44011.60167 | 34043.84868 | 0.773519876 | -0.370489731 | 0.213272822 | no | down | GO:0038096;GO:0004252;GO:0016020;GO:0030449;GO:0050776;GO:0006898;GO:0038095;GO:0002376;GO:0050900;GO:0005576;GO:0006956;GO:0002250;GO:0003823;GO:0005886;GO:0006508;GO:0006958;GO:0006955 | hsa:102723407 | | immunoglobulin heavy chain variable region, partial [Homo sapiens] |
| A0A087WSY4 | 35933.089 | 27725.29313 | 0.771581122 | -0.374110249 | 0.272141672 | no | down | GO:0009897;GO:0050853;GO:0045087;GO:0016020;GO:0006910;GO:0006911;GO:0002376;GO:0005576;GO:0002250;GO:0003823;GO:0005886;GO:0072562;GO:0042571;GO:0006958;GO:0050871;GO:0034987;GO:0042742 | hsa:102723407 | | RecName: Full=Immunoglobulin heavy variable 4-30-2; Flags: Precursor |
| Q15848 | 13433.81232 | 10299.76947 | 0.766704881 | -0.38325673 | 0.156310007 | no | down | GO:0034115;GO:0005783;GO:0010804;GO:0050728;GO:0009967;GO:0009617;GO:0030853;GO:0043124;GO:0033034;GO:0032720;GO:0043123;GO:2000481;GO:0014823;GO:0072659;GO:0010875;GO:0045650;GO:0071320;GO:0031667;GO:0050731;GO:1904753;GO:0045715;GO:0005125;GO:0033691;GO:0031953;GO:0010906;GO:0046326;GO:0070994;GO:0045721;GO:0010739;GO:0007623;GO:2000467;GO:2000279;GO:0070208;GO:0071872;GO:0019395;GO:0042304;GO:0006006;GO:0034383;GO:0006635;GO:2000478;GO:0050805;GO:0043407;GO:0001666;GO:0010642;GO:0050765;GO:0005581;GO:0032270;GO:0042802;GO:0042803;GO:0009986;GO:0050873;GO:0045892;GO:0046888;GO:0045599;GO:0007584;GO:0120162;GO:0120163;GO:0070543;GO:0042493;GO:1900121;GO:0005515;GO:0005102;GO:0045860;GO:0070373;GO:0045923;GO:0051384;GO:0042593;GO:0035690;GO:0032869;GO:0006091;GO:0005615;GO:0051260;GO:2000590;GO:0010745;GO:0010469;GO:1904706;GO:0005179;GO:0030336;GO:2000534;GO:0034612;GO:0090317;GO:0045471;GO:0009744;GO:0032991;GO:0045776;GO:0009749;GO:0071639;GO:0005576;GO:2000584;GO:0032757;GO:0001934 | hsa:9370 |  | TPA: adiponectin D [Homo sapiens] |
| P11226 | 108637.3736 | 82655.61287 | 0.760839572 | -0.394335811 | 0.060646387 | no | down | GO:0004252;GO:0048306;GO:0030246;GO:0042742;GO:0044130;GO:0050766;GO:0005581;GO:0001867;GO:0005509;GO:0051873;GO:0006508;GO:0009986;GO:0045087;GO:0006953;GO:0008228;GO:0006956;GO:0006958;GO:0006979;GO:0005615;GO:0050830;GO:0002376;GO:0005576;GO:0005515;GO:0048525;GO:0005102;GO:0005537 | hsa:4153 |  | mannose-binding lectin [Homo sapiens] |
| A0A0A0MS09 | 66174.83007 | 49809.89976 | 0.752701589 | -0.409850077 | 0.365805822 | no | down | GO:0016021;GO:0016020 | | | immunoglobulin delta-chain, partial [Homo sapiens] |
| P01833 | 20205.39627 | 14894.20894 | 0.737140155 | -0.439989144 | 0.326716921 | no | down | GO:0043235;GO:0001895;GO:0005615;GO:0002415;GO:0001580;GO:0070062;GO:0016021;GO:0016020;GO:0043312;GO:0005576;GO:0007173;GO:0005887;GO:0005886;GO:0043113;GO:0001792;GO:0035577;GO:0038093 | hsa:5284 |  | polymeric immunoglobulin receptor precursor [Homo sapiens] |
| Q86UD1 | 7321.776929 | 5391.175643 | 0.736320663 | -0.441593907 | 0.096522283 | no | down |  | hsa:220323 |  | out at first protein homolog precursor [Homo sapiens] |
| P61224 | 10046.39377 | 7364.3994 | 0.733039095 | -0.448037951 | 0.177175944 | no | down | GO:0005811;GO:0030033;GO:0044877;GO:0007165;GO:0061028;GO:0030054;GO:0005737;GO:0070062;GO:2000114;GO:0016020;GO:0000166;GO:0070382;GO:0045955;GO:0035722;GO:0005525;GO:0008283;GO:0003924;GO:0035577;GO:0032486;GO:0005911;GO:0005886;GO:0045121;GO:0007264;GO:0005829;GO:0071320;GO:0043312;GO:0005622;GO:0019003;GO:0005515;GO:1901888;GO:0070374;GO:2000301 | hsa:5908 | COG1100 | Ras-related protein Rap-1b, partial [Bos mutus] |
| A0A0J9YXX1 | 357816.2368 | 260636.5474 | 0.728408944 | -0.457179457 | 0.066747883 | no | down | GO:0016020;GO:0002376;GO:0005576;GO:0002250;GO:0003823;GO:0005886 | hsa:102724971 | | RecName: Full=Immunoglobulin heavy variable 5-10-1; Flags: Precursor |
| P04275 | 83127.79421 | 59863.79484 | 0.720141746 | -0.473647194 | 0.22117883 | no | down | GO:0005201;GO:0007599;GO:0005783;GO:0007597;GO:0007596;GO:0031012;GO:0031091;GO:0070062;GO:0031093;GO:0002576;GO:0051087;GO:0051260;GO:0009611;GO:0005576;GO:0002020;GO:0042802;GO:0042803;GO:0005178;GO:0031589;GO:0033093;GO:0062023;GO:0007155;GO:0030168;GO:0047485;GO:0019865;GO:0030198;GO:0005515;GO:0005518 | hsa:7450 |  | RecName: Full=von Willebrand factor; Short=vWF; Contains: RecName: Full=von Willebrand antigen 2; AltName: Full=von Willebrand antigen II; Flags: Precursor |
| Q9Y5Y7 | 16941.61205 | 11788.65695 | 0.695840332 | -0.523171794 | 0.05204988 | no | down | GO:0038023;GO:0070062;GO:0005540;GO:0016021;GO:0016020;GO:0009611;GO:0007160;GO:0004888;GO:0071944;GO:0005515;GO:0005887;GO:0005886;GO:0007155;GO:0030214;GO:0009653;GO:0006027 | hsa:10894 |  | lymphatic vessel endothelial hyaluronic acid receptor 1 precursor [Homo sapiens] |
| P02538 | 17261.70475 | 11764.16056 | 0.681517888 | -0.553176571 | 0.282959137 | no | down | GO:0008284;GO:0002009;GO:0005200;GO:0007010;GO:0005829;GO:0031424;GO:0070062;GO:2000536;GO:0030154;GO:0045095;GO:0070268;GO:0042060;GO:0005198;GO:0050830;GO:0005882;GO:0005515;GO:0001899;GO:0016020;GO:0005634;GO:0051801;GO:0061844 | hsa:3853 |  | keratin, type II cytoskeletal 6A [Homo sapiens] |
| K7ER74 | 581391.7695 | 395514.3247 | 0.680288827 | -0.555780701 | 0.183344495 | no | down | GO:0034375;GO:0034372;GO:0034371;GO:0034370;GO:0042627;GO:0034378;GO:0051006;GO:0010902;GO:0008047;GO:0048261;GO:0001523;GO:0043085;GO:0055102;GO:0005615;GO:0032375;GO:0005576;GO:0016042;GO:0045833;GO:0016004;GO:0010898;GO:0033700;GO:0034361;GO:0034362;GO:0034363;GO:0070328;GO:0006629;GO:0034366;GO:0060230;GO:0008289;GO:0006869;GO:0042803;GO:0010916;GO:0043274;GO:0042632;GO:0043691;GO:0034382;GO:0042493;GO:0034384;GO:0010518;GO:0033344;GO:0042953;GO:0060697;GO:0045723;GO:0005769 | hsa:344 |  | apolipoprotein C-II isoform X1 [Mesocricetus auratus] |
| P02745 | 56027.18421 | 36515.59947 | 0.651747897 | -0.617614073 | 0.077395877 | no | down | GO:0004252;GO:0030449;GO:0005581;GO:0045087;GO:0002376;GO:0005576;GO:0006956;GO:0005515;GO:0010039;GO:0007267;GO:0006508;GO:0006958;GO:0005602 | hsa:712 |  | complement C1q subcomponent subunit A precursor [Homo sapiens] |
| A0A0B4J1V2 | 27879.93279 | 17139.59753 | 0.614764665 | -0.701893851 | 0.38276734 | no | down | GO:0009897;GO:0050853;GO:0045087;GO:0016020;GO:0006910;GO:0006911;GO:0002376;GO:0005576;GO:0002250;GO:0003823;GO:0005886;GO:0072562;GO:0042571;GO:0006958;GO:0050871;GO:0034987;GO:0042742 | hsa:102723407 | | RecName: Full=Immunoglobulin heavy variable 2-26; Flags: Precursor |
| P02652 | 569083.3632 | 318388.9053 | 0.559476741 | -0.83784994 | 0.262263922 | no | down | GO:0060192;GO:0034384;GO:0034375;GO:0005543;GO:0034374;GO:0034371;GO:0006869;GO:0031072;GO:0031647;GO:0017127;GO:0030301;GO:0006656;GO:0010903;GO:0010873;GO:0002740;GO:0060228;GO:0001523;GO:0043085;GO:0034190;GO:0009395;GO:0044267;GO:0042627;GO:0005615;GO:0032375;GO:0031100;GO:0055102;GO:0072562;GO:0033344;GO:0034361;GO:0070062;GO:0033700;GO:0005319;GO:0018158;GO:0008035;GO:0034364;GO:0016032;GO:0034366;GO:0006641;GO:0060621;GO:0043687;GO:0043691;GO:0008289;GO:0031210;GO:0005788;GO:0042632;GO:0050996;GO:0043627;GO:0002526;GO:0051384;GO:0034378;GO:0008203;GO:0030300;GO:0046982;GO:0045416;GO:0015485;GO:0019216;GO:0050995;GO:0046340;GO:0005829;GO:0018206;GO:0042493;GO:0034380;GO:0009749;GO:0005576;GO:0005515;GO:0070653;GO:0060695;GO:0042803;GO:0034370;GO:0005769;GO:0042157 | hsa:336 |  | apolipoprotein A-II preproprotein [Homo sapiens] |
| A0A0B4J1V1 | 123262.3727 | 61207.74753 | 0.496564736 | -1.009946285 | 0.175615375 | no | down | GO:0009897;GO:0050853;GO:0045087;GO:0016020;GO:0006910;GO:0006911;GO:0002376;GO:0005576;GO:0002250;GO:0003823;GO:0005886;GO:0072562;GO:0042571;GO:0006958;GO:0050871;GO:0034987;GO:0042742 | hsa:102723407 | | RecName: Full=Immunoglobulin heavy variable 3-21; Flags: Precursor |
| P0DP02 | 532154.5174 | 236468.2979 | 0.444360219 | -1.170198428 | 0.07723 | no | down | GO:0016020;GO:0002376;GO:0005576;GO:0002250;GO:0003823;GO:0005886 | hsa:102723407 | | immunoglobulin heavy chain [Homo sapiens] |
| E7ENL6 | 19482.6817 | 7959.001818 | 0.40851675 | -1.29153286 | 0.303502983 | no | down | GO:0010951;GO:0004867 | hsa:1293 |  | collagen alpha-3(VI) chain isoform 4 precursor [Homo sapiens] |
| P04040 | 32778.43622 | 6359.091125 | 0.194002273 | -2.365854541 | 0.301595024 | no | down | GO:0016491;GO:0005829;GO:0016020;GO:0005782;GO:0020027;GO:0033189;GO:0051289;GO:0009314;GO:0005886;GO:0019899;GO:0000302;GO:1904813;GO:0009060;GO:0005739;GO:0004601;GO:0009650;GO:0032868;GO:0001666;GO:0055093;GO:0001649;GO:0005783;GO:0050661;GO:0005778;GO:0009636;GO:0070062;GO:0005615;GO:0033591;GO:0005777;GO:0001822;GO:0051262;GO:0006979;GO:0098869;GO:0043066;GO:0046686;GO:0005758;GO:0034774;GO:0046872;GO:0014823;GO:0042802;GO:0042803;GO:0004046;GO:0005794;GO:0005925;GO:0010288;GO:0020037;GO:0006641;GO:0009411;GO:0006625;GO:0016209;GO:0032355;GO:0045471;GO:0014068;GO:0001657;GO:0071363;GO:0034599;GO:0009642;GO:0055114;GO:0008203;GO:0051092;GO:0016684;GO:0007568;GO:0043231;GO:0042542;GO:0070542;GO:0042493;GO:0043312;GO:0032088;GO:0005764;GO:0051781;GO:0042744;GO:0033197;GO:0010193;GO:0004096;GO:0005576;GO:0005102;GO:0014854;GO:0080184 | hsa:847 | COG0753 | catalase [Homo sapiens] |
| A0A0A0MT36 | 116382.66 | 139614.64 | 1.199617194 | 0.262574105 | 0.435705262 | no | no change | GO:0016020;GO:0005615;GO:0006955;GO:0002376;GO:0002377;GO:0005576;GO:0002250;GO:0003823;GO:0005886 | hsa:7441 |  | RecName: Full=Immunoglobulin kappa variable 6D-21; Flags: Precursor |
| P26038 | 3912.521765 | 4686.3125 | 1.19777289 | 0.260354384 | 0.539468235 | no | no change | GO:0005200;GO:0019899;GO:0030175;GO:0050900;GO:0010628;GO:0005925;GO:0061028;GO:0005737;GO:0071803;GO:0045177;GO:0042995;GO:0070062;GO:1902115;GO:0043209;GO:0005634;GO:0042098;GO:0072562;GO:0003779;GO:2000401;GO:0035722;GO:0022612;GO:0048471;GO:2000643;GO:0022614;GO:1903364;GO:0005856;GO:0005515;GO:0016324;GO:0009986;GO:0071944;GO:0031982;GO:0071394;GO:0016323;GO:0019901;GO:0008361;GO:0008360;GO:0045198;GO:0001771;GO:0005886;GO:0016020;GO:0050839;GO:0072678;GO:0005615;GO:0031528;GO:0007010;GO:0071437;GO:0005829;GO:0008092;GO:0005902;GO:0003725;GO:0016032;GO:0001931;GO:0007159;GO:0005102;GO:0070489;GO:0031143;GO:1902966 | hsa:4478 |  | moesin [Homo sapiens] |
| P01704 | 32190.41579 | 38484.2075 | 1.195517565 | 0.257635327 | 0.317617132 | no | no change | GO:0038096;GO:0004252;GO:0016020;GO:0030449;GO:0050776;GO:0006898;GO:0038095;GO:0002376;GO:0050900;GO:0005576;GO:0006956;GO:0002250;GO:0003823;GO:0005886;GO:0006508;GO:0006958;GO:0006955;GO:0070062 | hsa:7441 |  | RecName: Full=Immunoglobulin lambda variable 2-14; AltName: Full=Ig lambda chain V-II region NIG-84; AltName: Full=Ig lambda chain V-II region TOG; AltName: Full=Ig lambda chain V-II region VIL; Flags: Precursor |
| Q92954 | 34921.39316 | 41636.00263 | 1.192277824 | 0.253720451 | 0.236010184 | no | no change | GO:0008283;GO:0005044;GO:0006898;GO:0030247;GO:0030021;GO:0005576;GO:0006955;GO:0062023 | hsa:10216 |  | unnamed protein product [Homo sapiens] |
| P06312 | 384481.9842 | 455326.1211 | 1.184258664 | 0.243984227 | 0.134250997 | no | no change | GO:0038096;GO:0004252;GO:0016020;GO:0030449;GO:0050776;GO:0006898;GO:0038095;GO:0072562;GO:0002376;GO:0002377;GO:0050900;GO:0005576;GO:0006956;GO:0002250;GO:0003823;GO:0005886;GO:0006508;GO:0006958;GO:0006955 | hsa:7441 |  | immunoglobulin kappa chain, partial [Homo sapiens] |
| A0A0G2JMB2 | 2454396.105 | 2906175.368 | 1.184069418 | 0.243753664 | 0.205675741 | no | no change | GO:0009897;GO:0050853;GO:0045087;GO:0006910;GO:0006911;GO:0042742;GO:0003823;GO:0034987;GO:0042571;GO:0006958;GO:0050871;GO:0072562 | hsa:55423 |  | Immunoglobulin heavy chain variant, partial [Homo sapiens] |
| A0A0C4DH39 | 29197.24784 | 34557.78826 | 1.183597456 | 0.243178501 | 0.514943651 | no | no change | GO:0009897;GO:0050853;GO:0045087;GO:0016020;GO:0006910;GO:0006911;GO:0002376;GO:0005576;GO:0002250;GO:0003823;GO:0005886;GO:0072562;GO:0042571;GO:0006958;GO:0050871;GO:0034987;GO:0042742 | hsa:102723407 | | immunoglobulin heavy chain variable region, partial [Homo sapiens] |
| P00739 | 46650.73105 | 54761.93632 | 1.173870914 | 0.23127377 | 0.334667166 | no | no change | GO:0004252;GO:0070062;GO:0034366;GO:0030492;GO:0006898;GO:0072562;GO:0005576;GO:0010942;GO:0002526;GO:0010033;GO:0005615 | hsa:3250 | COG5640 | haptoglobin-related protein precursor [Homo sapiens] |
| O75636 | 31221.79526 | 36592.94316 | 1.172032 | 0.229011961 | 0.149808171 | no | no change | GO:0004252;GO:0051607;GO:0006956;GO:0003823;GO:1902679;GO:0045087;GO:0030246;GO:0043654;GO:0002376;GO:0046597;GO:0005576;GO:0001867;GO:0072562;GO:0005515;GO:0006508;GO:0046872;GO:0005581 | hsa:8547 |  | ficolin-3 isoform 1 precursor [Homo sapiens] |
| P01780 | 1781232.211 | 2081288.158 | 1.168454144 | 0.224601117 | 0.121077817 | no | no change | GO:0038096;GO:0004252;GO:0016020;GO:0030449;GO:0050776;GO:0006898;GO:0038095;GO:0072562;GO:0002376;GO:0050900;GO:0005576;GO:0006956;GO:0002250;GO:0003823;GO:0005886;GO:0006508;GO:0006958;GO:0006955;GO:0070062 | hsa:102723407 | | immunoglobulin heavy chain variable region precursor, partial [Homo sapiens] |
| P02753 | 55930.01158 | 65298.08789 | 1.167496413 | 0.223418117 | 0.061825179 | no | no change | GO:0060347;GO:0032024;GO:0048562;GO:0030324;GO:0060059;GO:0060044;GO:0042593;GO:0051024;GO:0060065;GO:0042572;GO:0001523;GO:0016918;GO:0005615;GO:0048807;GO:0006094;GO:0005501;GO:0032526;GO:0048706;GO:0007601;GO:0045471;GO:0001654;GO:0034633;GO:0070062;GO:0007507;GO:0032991;GO:0060157;GO:0005829;GO:0019841;GO:0048738;GO:0005576;GO:0005515;GO:0060068;GO:0034632;GO:0046982;GO:0050896;GO:0030277 | hsa:5950 |  | retinol-binding protein 4 isoform a precursor [Homo sapiens] |
| G3V2W1 | 8948.705105 | 10439.47474 | 1.166590542 | 0.222298283 | 0.26836013 | no | no change | GO:0010951;GO:0007596;GO:0005615;GO:0004867 | hsa:51156 | COG4826 | protein Z-dependent protease inhibitor isoform X1 [Homo sapiens] |
| A6XND0 | 28409.03947 | 33136.64211 | 1.166411914 | 0.222077361 | 0.171472383 | no | no change | GO:0005520;GO:0001558;GO:0005576 | hsa:3486 |  | insulin-like growth factor binding protein 3 [Homo sapiens] |
| H0YJW9 | 684252.6316 | 796192.4947 | 1.163594348 | 0.218588194 | 0.150001284 | no | no change |  | hsa:7448 |  | vitronectin, partial [Homo sapiens] |
| K7ERI9 | 2356485.368 | 2741538.053 | 1.163401262 | 0.218348775 | 0.224256983 | no | no change | GO:0005576;GO:0042157 | hsa:341 |  | apolipoprotein C-I precursor [Homo sapiens] |
| O95445 | 55209.75105 | 64039.99368 | 1.15993991 | 0.21405007 | 0.052446549 | no | no change | GO:0034375;GO:0034445;GO:0006869;GO:0043691;GO:0042157;GO:0001523;GO:0005615;GO:0005543;GO:0098869;GO:0005576;GO:0005319;GO:0034361;GO:0034362;GO:0034364;GO:0034365;GO:0034366;GO:0016209;GO:0042632;GO:0034380;GO:0034384;GO:0009749;GO:0033344 | hsa:55937 |  | apolipoprotein M isoform 1 [Homo sapiens] |
| P01766 | 78135.21684 | 90589.62789 | 1.159395616 | 0.213372936 | 0.219681757 | no | no change | GO:0038096;GO:0004252;GO:0016020;GO:0030449;GO:0005615;GO:0006898;GO:0038095;GO:0050900;GO:0072562;GO:0002376;GO:0050776;GO:0005576;GO:0006956;GO:0002250;GO:0003823;GO:0005886;GO:0006508;GO:0006958;GO:0006955 | hsa:102723407 | | RecName: Full=Immunoglobulin heavy variable 3-13; AltName: Full=Ig heavy chain V-III region BRO; Flags: Precursor |
| Q16880 | 566498.0211 | 655931.7579 | 1.157871226 | 0.211474811 | 0.34846975 | no | no change | GO:0008489;GO:0007417;GO:0030913;GO:0016021;GO:0016020;GO:0006682;GO:0016740;GO:0006687;GO:0015020;GO:0006665;GO:0006629;GO:0002175;GO:0008152;GO:0005886;GO:0008194;GO:0007010;GO:0043231;GO:0007422;GO:0048812;GO:0016757;GO:0047263;GO:0016758 | hsa:7368 | COG1819 | 2-hydroxyacylsphingosine 1-beta-galactosyltransferase precursor [Homo sapiens] |
| P01701 | 446200.1774 | 516602.5105 | 1.157781948 | 0.211363567 | 0.305535611 | no | no change | GO:0038096;GO:0004252;GO:0016020;GO:0030449;GO:0050776;GO:0006898;GO:0038095;GO:0050900;GO:0005576;GO:0006956;GO:0003823;GO:0005886;GO:0006508;GO:0006958;GO:0006955;GO:0070062 | hsa:7441 |  | RecName: Full=Immunoglobulin lambda variable 1-51; AltName: Full=Ig lambda chain V-I region BL2; AltName: Full=Ig lambda chain V-I region EPS; AltName: Full=Ig lambda chain V-I region NEW; AltName: Full=Ig lambda chain V-I region NIG-64; Flags: Precursor |
| A0A075B7F0 | 23855.47089 | 27609.92778 | 1.157383474 | 0.210866949 | 0.401936689 | no | no change | GO:0009897;GO:0050853;GO:0045087;GO:0006910;GO:0006911;GO:0042742;GO:0003823;GO:0034987;GO:0042571;GO:0006958;GO:0050871;GO:0072562 | hsa:102723407 | | IGHV3-13 isoform 1, partial [Pan troglodytes] |
| P01619 | 3394970 | 3928846.211 | 1.15725506 | 0.210706871 | 0.418727069 | no | no change | GO:0004252;GO:0030449;GO:0019731;GO:0006955;GO:0050900;GO:0005615;GO:0016020;GO:0003094;GO:0072562;GO:0071748;GO:0006508;GO:0050776;GO:0006956;GO:0003823;GO:0005886;GO:0006958;GO:0070062;GO:0038096;GO:0038095;GO:0006898;GO:0002376;GO:0005576;GO:0002250;GO:0071751;GO:0071756 | hsa:29802 |  | immunoglobulin light chain variable region, partial [Homo sapiens] |
| A0A0B4J1V0 | 31502.57684 | 36433.38526 | 1.156520797 | 0.209791209 | 0.23337577 | no | no change | GO:0009897;GO:0050853;GO:0045087;GO:0016020;GO:0006910;GO:0006911;GO:0002376;GO:0005576;GO:0002250;GO:0003823;GO:0005886;GO:0072562;GO:0042571;GO:0006958;GO:0050871;GO:0034987;GO:0042742 | hsa:102724971 | | unnamed protein product [Homo sapiens] |
| C9JXI5 | 131802.1526 | 152351.3726 | 1.155909593 | 0.209028565 | 0.501856813 | no | no change | GO:0090263;GO:0031410;GO:0016021;GO:0016020;GO:0005886 | hsa:130612 |  | transmembrane protein 198 [Macaca mulatta] |
| P15169 | 17697.84421 | 20256.22737 | 1.144559028 | 0.194791869 | 0.242662914 | no | no change | GO:0097060;GO:0005794;GO:0051384;GO:0030449;GO:0005615;GO:0016787;GO:0030141;GO:0004181;GO:0004180;GO:0010815;GO:0004185;GO:0005576;GO:0043025;GO:0008270;GO:0016485;GO:0006508;GO:0006518;GO:0046872;GO:0030070;GO:0008233;GO:0008237 | hsa:1369 |  | carboxypeptidase N catalytic chain precursor [Homo sapiens] |
| A0A286YEY1 | 4509518.632 | 5144417.211 | 1.140790765 | 0.190034208 | 0.43923486 | no | no change |  |  |  | IGHA1 isoform 1, partial [Pan troglodytes] |
| B0YIW2 | 1944324.595 | 2192579.847 | 1.127682 | 0.173360293 | 0.672704834 | no | no change | GO:0034375;GO:0042627;GO:0034371;GO:0006869;GO:0034379;GO:0034378;GO:0051005;GO:0042157;GO:0005576;GO:0048261;GO:0001523;GO:0055102;GO:0070062;GO:0005543;GO:0045833;GO:0010897;GO:0007186;GO:0062023;GO:0033700;GO:0034361;GO:0034363;GO:0070328;GO:0034366;GO:0006641;GO:0060621;GO:0030234;GO:0070653;GO:0008289;GO:0010916;GO:0042632;GO:0032489;GO:0043691;GO:0005615;GO:0015485;GO:0034382;GO:0050995;GO:0019433;GO:0033344;GO:0010989;GO:0010987;GO:0005769;GO:0045717;GO:0010903 | hsa:345 |  | apolipoprotein C-III precursor variant 1 [Homo sapiens] |
| P01877 | 206673.0384 | 232985.3742 | 1.127313829 | 0.172889198 | 0.777107059 | no | no change | GO:0009897;GO:0019731;GO:0006955;GO:0050900;GO:0034987;GO:0001895;GO:0005615;GO:0016020;GO:0003094;GO:0072562;GO:0071748;GO:0050853;GO:0045087;GO:0006910;GO:0006911;GO:0003823;GO:0005886;GO:0006958;GO:0050871;GO:0060267;GO:0070062;GO:0006898;GO:0002376;GO:0005576;GO:0002250;GO:0071752;GO:0071751 | hsa:55423 |  | RecName: Full=Immunoglobulin heavy constant alpha 2; AltName: Full=Ig alpha-2 chain C region; AltName: Full=Ig alpha-2 chain C region BUT; AltName: Full=Ig alpha-2 chain C region LAN |
| A0A0B4J2D9 | 15440.17733 | 17281.17761 | 1.1192344 | 0.16251221 | 0.528486124 | no | no change | GO:0016020;GO:0005615;GO:0006955;GO:0002376;GO:0002377;GO:0005576;GO:0002250;GO:0003823;GO:0005886 | hsa:7441 |  | RecName: Full=Immunoglobulin kappa variable 1D-13; Flags: Precursor |
| P01024 | 6968412.895 | 7798733.316 | 1.119154883 | 0.162409708 | 0.066935314 | no | no change | GO:0045087;GO:0004252;GO:0006631;GO:0030449;GO:0004866;GO:0005886;GO:0005788;GO:0035578;GO:0007165;GO:0031715;GO:0048260;GO:0010828;GO:0010866;GO:0050766;GO:0044267;GO:0070062;GO:0009617;GO:0045766;GO:1905114;GO:0045745;GO:0097242;GO:2000427;GO:0034774;GO:0007186;GO:0009986;GO:0005576;GO:0006629;GO:0150064;GO:0043687;GO:0016322;GO:0006911;GO:0150062;GO:0006956;GO:0006957;GO:0006954;GO:0006955;GO:0010951;GO:0060100;GO:0006958;GO:0006508;GO:0005615;GO:0050776;GO:0032991;GO:0010575;GO:0043312;GO:0002376;GO:0001798;GO:0010884;GO:0001970;GO:0005515;GO:0097278;GO:0005102;GO:0001934;GO:0072562 | hsa:718 |  | complement C3 preproprotein [Homo sapiens] |
| P22792 | 89832.95895 | 100444.6111 | 1.11812649 | 0.161083404 | 0.236809529 | no | no change | GO:0030449;GO:0070062;GO:0004181;GO:0050790;GO:0072562;GO:0050821;GO:0005576;GO:0006508;GO:0030234 | hsa:1370 | COG4886 | carboxypeptidase N subunit 2 precursor [Homo sapiens] |
| Q8IV42 | 84821.05625 | 94837.45412 | 1.118088577 | 0.161034486 | 0.420303487 | no | no change | GO:0000049;GO:0016310;GO:0016301;GO:0097056;GO:0016740;GO:0001514;GO:0000166;GO:0006412;GO:0005524 | hsa:118672 |  | L-seryl-tRNA(Sec) kinase isoform 2 [Homo sapiens] |
| P29622 | 76645.57316 | 85693.02526 | 1.118042722 | 0.160975317 | 0.124911729 | no | no change | GO:0005615;GO:0070062;GO:0002576;GO:0031089;GO:0010466;GO:0030414;GO:0004867;GO:0010951;GO:0005576 | hsa:5267 | COG4826 | kallistatin isoform 1 [Homo sapiens] |
| A0A075B6I0 | 624553.5278 | 696118.8067 | 1.114586302 | 0.156508328 | 0.560814252 | no | no change | GO:0016020;GO:0005615;GO:0006955;GO:0002376;GO:0002377;GO:0005576;GO:0002250;GO:0003823;GO:0005886 | hsa:7441 |  | RecName: Full=Immunoglobulin lambda variable 8-61; Flags: Precursor |
| P01699 | 158709.0758 | 176825.7089 | 1.114149951 | 0.155943414 | 0.2821357 | no | no change | GO:0038096;GO:0004252;GO:0016020;GO:0030449;GO:0050776;GO:0006898;GO:0038095;GO:0002376;GO:0050900;GO:0005576;GO:0006956;GO:0002250;GO:0003823;GO:0005886;GO:0006508;GO:0006958;GO:0006955 | hsa:7441 |  | hCG2043214, partial [Homo sapiens] |
| A0A2Q2TTZ9 | 318978.3258 | 355325.4426 | 1.113948547 | 0.155682597 | 0.681468601 | no | no change |  | hsa:7441 |  | IGKV1D-33 isoform 2, partial [Pan troglodytes] |
| P04433 | 91794.47789 | 102176.9611 | 1.113105749 | 0.15459066 | 0.140006993 | no | no change | GO:0038096;GO:0004252;GO:0016020;GO:0030449;GO:0050776;GO:0006898;GO:0038095;GO:0072562;GO:0002376;GO:0050900;GO:0005576;GO:0006956;GO:0002250;GO:0003823;GO:0005886;GO:0006508;GO:0006958;GO:0006955;GO:0070062 | hsa:7441 |  | rheumatoid factor D1 IgG light chain VK3 region, partial [Homo sapiens] |
| P04004 | 739223.1684 | 817496.0474 | 1.105885316 | 0.145201781 | 0.078401536 | no | no change | GO:0005201;GO:0030449;GO:0005783;GO:0061302;GO:0050840;GO:0014911;GO:0030949;GO:0030247;GO:0031012;GO:0007160;GO:0007155;GO:0090303;GO:0048260;GO:0005737;GO:0032092;GO:0070062;GO:0072562;GO:0005576;GO:0016477;GO:0042802;GO:0033627;GO:0005178;GO:0005796;GO:0008283;GO:0010811;GO:0048709;GO:0048237;GO:0062023;GO:0006955;GO:0010951;GO:0035987;GO:0008201;GO:0005615;GO:0051258;GO:0005604;GO:0043231;GO:0050731;GO:0005044;GO:0006898;GO:0030198;GO:0005515;GO:0097421;GO:0071062;GO:0005518;GO:0030195 | hsa:7448 |  | vitronectin precursor [Homo sapiens] |
| M0R0Q9 | 5830.902444 | 6398.391789 | 1.097324445 | 0.133990149 | 0.303284958 | no | no change | GO:0006631;GO:0030449;GO:0004866;GO:0005886;GO:0005788;GO:0035578;GO:0007165;GO:0031715;GO:0048260;GO:0010828;GO:0010866;GO:0044267;GO:0070062;GO:0009617;GO:0045766;GO:1905114;GO:0045745;GO:0097242;GO:2000427;GO:0034774;GO:0007186;GO:0009986;GO:0005576;GO:0150064;GO:0043687;GO:0016322;GO:0150062;GO:0006956;GO:0006957;GO:0006954;GO:0006955;GO:0060100;GO:0006958;GO:0005615;GO:0050776;GO:0032991;GO:0010575;GO:0043312;GO:0001798;GO:0010884;GO:0001970;GO:0097278;GO:0005102;GO:0001934;GO:0072562 | hsa:718 |  | C3 isoform 6, partial [Pan troglodytes] |
| H9KV75 | 6787.488923 | 7426.674333 | 1.094171117 | 0.129838378 | 0.856774508 | no | no change | GO:0019894;GO:0032029;GO:0032391;GO:0030036;GO:0017166;GO:0007041;GO:0042383;GO:0005925;GO:0005923;GO:0030507;GO:0005737;GO:0001725;GO:0097433;GO:0045505;GO:0005815;GO:0003779;GO:0030027;GO:0005509;GO:0042803;GO:0034452;GO:0031941;GO:0051393;GO:0016328;GO:0030486;GO:0048741;GO:1990357;GO:0051017;GO:0031252;GO:0051015;GO:0005915;GO:0045214;GO:0051764;GO:0097381;GO:0007030;GO:0090636;GO:0090637 | hsa:87 | COG5069 | alpha-actinin-1 isoform c [Homo sapiens] |
| P00747 | 1059033.553 | 1155272.695 | 1.090874498 | 0.125485133 | 0.115074487 | no | no change | GO:0004252;GO:0004175;GO:0007599;GO:0016787;GO:1904854;GO:0048771;GO:0019899;GO:0008233;GO:0008236;GO:0044267;GO:0070062;GO:0043536;GO:0052182;GO:0051087;GO:0072562;GO:1990405;GO:0006508;GO:0022617;GO:0009986;GO:0051918;GO:0051919;GO:0008285;GO:0007596;GO:0010812;GO:0019900;GO:0062023;GO:0019904;GO:0005886;GO:0051702;GO:0031232;GO:0034185;GO:0005615;GO:0042730;GO:0031093;GO:0052213;GO:2000048;GO:0044218;GO:0005576;GO:0005515;GO:0005102;GO:0002576 | hsa:5340 | COG5640 | plasminogen isoform 1 precursor [Homo sapiens] |
| P02749 | 980397.2895 | 1067318.932 | 1.088659611 | 0.12255294 | 0.28181431 | no | no change | GO:0034392;GO:0007597;GO:0051006;GO:0034197;GO:0042627;GO:0070062;GO:0002576;GO:0005543;GO:0031639;GO:0051917;GO:0042802;GO:0051918;GO:0034361;GO:0034364;GO:0006641;GO:0009986;GO:0060230;GO:0008289;GO:0062023;GO:0033033;GO:0008201;GO:0005615;GO:0010596;GO:0031089;GO:0005576;GO:0030195;GO:0005515;GO:0030193;GO:0016525;GO:0001937;GO:0030194 | hsa:350 |  | beta-2-glycoprotein 1 precursor [Homo sapiens] |
| A0A0G2JRQ6 | 270236.3158 | 294034.5047 | 1.088064363 | 0.1217639 | 0.431474515 | no | no change | GO:0002377;GO:0005615;GO:0006955 | hsa:7441 |  | hCG2042707, partial [Homo sapiens] |
| A0A0B4J1Y8 | 106914.9979 | 116112.7792 | 1.086028916 | 0.119062516 | 0.617412266 | no | no change | GO:0016020;GO:0005615;GO:0006955;GO:0002376;GO:0002377;GO:0005576;GO:0002250;GO:0003823;GO:0005886 | hsa:7441 |  | Unknown (protein for IMAGE:4575521), partial [Homo sapiens] |
| P00740 | 34031.94947 | 36940.94105 | 1.085478253 | 0.118330822 | 0.115349986 | no | no change | GO:0004252;GO:0004175;GO:0006888;GO:0005615;GO:0016787;GO:0070062;GO:0007597;GO:0007596;GO:0031638;GO:0005788;GO:0005796;GO:0005576;GO:0005509;GO:0005515;GO:0005886;GO:0006508;GO:0008233;GO:0046872;GO:0007599;GO:0008236 | hsa:2158 | COG5640 | coagulation factor IX isoform 1 preproprotein [Homo sapiens] |
| P10909 | 1002494.932 | 1087859.132 | 1.085151752 | 0.117896809 | 0.137054376 | no | no change | GO:0032436;GO:0005783;GO:0019730;GO:1903573;GO:0009615;GO:0016020;GO:1902949;GO:0042127;GO:0005794;GO:0034366;GO:0060548;GO:0031966;GO:0043691;GO:0043231;GO:0005829;GO:0002376;GO:0051787;GO:1902004;GO:0051788;GO:0030449;GO:1902230;GO:0050821;GO:0010628;GO:0044877;GO:0099020;GO:0048260;GO:0005856;GO:0000902;GO:0002434;GO:0072562;GO:0048471;GO:0097418;GO:0051131;GO:0032760;GO:0006629;GO:1905895;GO:0048156;GO:0001774;GO:0006956;GO:1905892;GO:1901216;GO:0006958;GO:0061077;GO:2000060;GO:1902430;GO:1902847;GO:0090201;GO:0031012;GO:0045429;GO:0051082;GO:1902998;GO:1901214;GO:0005737;GO:0001540;GO:0031093;GO:0031410;GO:0005634;GO:0051087;GO:0005739;GO:1900221;GO:0009986;GO:0017038;GO:0061740;GO:0061741;GO:0051092;GO:0031625;GO:0070062;GO:0005622;GO:0005743;GO:0005515;GO:0097440;GO:0032286;GO:0016887;GO:0071944;GO:0045202;GO:0061518;GO:0005615;GO:0002576;GO:0043065;GO:0032464;GO:0032463;GO:0045087;GO:0006915;GO:0042583;GO:0062023;GO:0050750;GO:0032991;GO:0001836;GO:0005576;GO:1905907;GO:1905908 | hsa:1191 |  | clusterin preproprotein [Homo sapiens] |
| P08603 | 1222296.805 | 1325699.621 | 1.084597141 | 0.117159273 | 0.137901588 | no | no change | GO:1903659;GO:0030449;GO:0070062;GO:0005515;GO:0045087;GO:0072562;GO:0002376;GO:0043395;GO:0005576;GO:0006956;GO:0006957;GO:0016032;GO:0008201;GO:0005615 | hsa:3075 |  | RecName: Full=Complement factor H; AltName: Full=H factor 1; Flags: Precursor |
| P22891 | 9700.652421 | 10520.92905 | 1.084558914 | 0.117108423 | 0.55703065 | no | no change | GO:0004252;GO:0005796;GO:0006888;GO:0005615;GO:0007596;GO:0005788;GO:0005576;GO:0005509;GO:0006508;GO:0030195;GO:0007599;GO:0070062 | hsa:8858 | COG5640 | vitamin K-dependent protein Z isoform 2 precursor [Homo sapiens] |
| I3L145 | 11796.79926 | 12792.186 | 1.084377696 | 0.116867344 | 0.76502926 | no | no change | GO:0005496 | hsa:6462 |  | SHBG protein, partial [Homo sapiens] |
| P00742 | 77559.30526 | 84047.87211 | 1.083659424 | 0.115911414 | 0.235221123 | no | no change | GO:0004252;GO:0030335;GO:0005543;GO:0006888;GO:0005615;GO:0016787;GO:0007596;GO:0005788;GO:0005796;GO:0005576;GO:0005509;GO:0005515;GO:0005886;GO:0006508;GO:0008233;GO:0008236;GO:0051897;GO:0031233;GO:0007599;GO:0007598 | hsa:2159 | COG5640 | coagulation factor X isoform 1 preproprotein [Homo sapiens] |
| A0A0C4DH55 | 8471991.474 | 9163152.211 | 1.08158185 | 0.113142847 | 0.465238096 | no | no change | GO:0016020;GO:0005615;GO:0006955;GO:0002376;GO:0002377;GO:0005576;GO:0002250;GO:0003823;GO:0005886 | hsa:29802 |  | RecName: Full=Immunoglobulin kappa variable 3D-7; Flags: Precursor |
| P02787 | 19885325.84 | 21499234.37 | 1.081160779 | 0.112581081 | 0.236417201 | no | no change | GO:0009617;GO:0016020;GO:0055037;GO:1990459;GO:1900390;GO:0005788;GO:0030139;GO:0061024;GO:0055072;GO:0006826;GO:0005905;GO:0034986;GO:1990712;GO:0048260;GO:0010008;GO:0030665;GO:0007257;GO:0045780;GO:0031232;GO:0005615;GO:0031410;GO:0002576;GO:0005770;GO:0045178;GO:0070062;GO:2000147;GO:0009925;GO:0044267;GO:0048471;GO:0046872;GO:0043687;GO:0009986;GO:0006879;GO:0033572;GO:0007015;GO:0016324;GO:0006811;GO:0030316;GO:0031982;GO:0008198;GO:0008199;GO:0045893;GO:0034774;GO:0015091;GO:0001895;GO:0034756;GO:0071281;GO:0042327;GO:0060395;GO:0031647;GO:0005623;GO:0005576;GO:0005515;GO:0005768;GO:0005769;GO:0070371;GO:0072562 | hsa:7018 |  | serotransferrin isoform 1 precursor [Homo sapiens] |
| A0A0C4DH25 | 534346.1842 | 577141.8526 | 1.08008978 | 0.111151238 | 0.350540045 | no | no change | GO:0038096;GO:0004252;GO:0016020;GO:0030449;GO:0005615;GO:0006898;GO:0038095;GO:0050900;GO:0002376;GO:0002377;GO:0050776;GO:0005576;GO:0006956;GO:0002250;GO:0003823;GO:0005886;GO:0006508;GO:0006958;GO:0006955 | hsa:29802 |  | hCG1686089, partial [Homo sapiens] |
| A0A0C4DH43 | 454010.2942 | 488918.7659 | 1.076889163 | 0.106869771 | 0.81853659 | no | no change | GO:0009897;GO:0050853;GO:0045087;GO:0016020;GO:0006910;GO:0006911;GO:0002376;GO:0005576;GO:0002250;GO:0003823;GO:0005886;GO:0072562;GO:0042571;GO:0006958;GO:0050871;GO:0034987;GO:0042742 | hsa:102723407 | | RecName: Full=Immunoglobulin heavy variable 2-70D; Flags: Precursor |
| P04114 | 4102093.263 | 4415674.842 | 1.076444283 | 0.106273647 | 0.424351149 | no | no change | GO:0034360;GO:0034374;GO:0034359;GO:0005783;GO:0034371;GO:0006869;GO:0050750;GO:0005788;GO:0005789;GO:0009791;GO:0061024;GO:0050900;GO:0010628;GO:0042157;GO:0034378;GO:0032496;GO:0016042;GO:0001523;GO:0006629;GO:0005737;GO:0043202;GO:0032355;GO:0001701;GO:0031983;GO:0005615;GO:0071682;GO:0071356;GO:0009615;GO:0048844;GO:0005543;GO:0030669;GO:0071379;GO:0070062;GO:0010033;GO:0042953;GO:0010884;GO:0042158;GO:0002224;GO:0010008;GO:0043025;GO:0043687;GO:0070971;GO:0005319;GO:0034361;GO:0034362;GO:0034363;GO:0006642;GO:0005790;GO:0044267;GO:0030317;GO:0008289;GO:0034379;GO:0010744;GO:0007283;GO:0042627;GO:0042159;GO:0017127;GO:0012506;GO:0005886;GO:0042632;GO:0030301;GO:0008201;GO:0008202;GO:0008203;GO:0035473;GO:0031904;GO:0009743;GO:0007399;GO:0034382;GO:0034383;GO:0005829;GO:0006898;GO:0009566;GO:0043231;GO:0010886;GO:0019433;GO:0005576;GO:0045540;GO:0005515;GO:0034447;GO:0010269;GO:0005769;GO:0033344 | hsa:338 |  | RecName: Full=Apolipoprotein B-100; Short=Apo B-100; Contains: RecName: Full=Apolipoprotein B-48; Short=Apo B-48; Flags: Precursor |
| P01857 | 9607821.105 | 10338346.11 | 1.076034409 | 0.105724213 | 0.517592974 | no | no change | GO:0004252;GO:0030449;GO:0019221;GO:0009897;GO:0042742;GO:0034987;GO:0042571;GO:0005615;GO:0016020;GO:0072562;GO:0006508;GO:0050853;GO:0045087;GO:0006910;GO:0006911;GO:0006956;GO:0003823;GO:0005886;GO:0006958;GO:0050871;GO:0070062;GO:0038096;GO:0002376;GO:0005576;GO:0002250;GO:0005515 | hsa:100423062 | | IGH@ protein [Homo sapiens] |
| Q9UHG3 | 19234.01847 | 20695.87421 | 1.076003657 | 0.105682981 | 0.467962619 | no | no change | GO:0034361;GO:1902476;GO:0070062;GO:0030327;GO:0030328;GO:0030329;GO:0099133;GO:0005774;GO:0005764;GO:0016670;GO:0006821;GO:0055114;GO:0005886;GO:0008555;GO:0001735;GO:0016491 | hsa:51449 |  | prenylcysteine oxidase 1 precursor [Homo sapiens] |
| P01764 | 38419.61727 | 41234.23267 | 1.07325985 | 0.101999413 | 0.736299964 | no | no change | GO:0004252;GO:0030449;GO:0009897;GO:0006955;GO:0050900;GO:0042742;GO:0034987;GO:0042571;GO:0005615;GO:0016020;GO:0072562;GO:0006508;GO:0050853;GO:0045087;GO:0006910;GO:0006911;GO:0050776;GO:0006956;GO:0003823;GO:0005886;GO:0006958;GO:0050871;GO:0070062;GO:0038096;GO:0038095;GO:0006898;GO:0002376;GO:0005576;GO:0002250 | hsa:102723407 | | RecName: Full=Immunoglobulin heavy variable 3-23; AltName: Full=Ig heavy chain V-III region LAY; AltName: Full=Ig heavy chain V-III region POM; AltName: Full=Ig heavy chain V-III region TEI; AltName: Full=Ig heavy chain V-III region TIL; AltName: Full=Ig heavy chain V-III region TUR; AltName: Full=Ig heavy chain V-III region VH26; AltName: Full=Ig heavy chain V-III region WAS; AltName: Full=Ig heavy chain V-III region ZAP; Flags: Precursor |
| P01023 | 7676298 | 8232157.474 | 1.072412441 | 0.100859861 | 0.526074254 | no | no change | GO:0051056;GO:0030414;GO:0019959;GO:0007597;GO:0019899;GO:0072562;GO:0048863;GO:0005615;GO:0031093;GO:0002576;GO:0048306;GO:0019838;GO:0070062;GO:0010466;GO:0002020;GO:0001869;GO:0022617;GO:0019966;GO:0005096;GO:0004866;GO:0004867;GO:0010951;GO:0043547;GO:0043120;GO:0005829;GO:0005576;GO:0005515;GO:0005102 | hsa:2 | COG2373 | alpha-2-macroglobulin isoform a precursor [Homo sapiens] |
| A0A096LPE2 | 482254.3211 | 517015.4895 | 1.072080574 | 0.100413338 | 0.477763848 | no | no change | GO:0042056;GO:0034364;GO:0005615;GO:0006953;GO:0005576;GO:0060326;GO:0050918;GO:0070062 | hsa:6291;hsa:100528017 | | SAA2-SAA4 protein precursor [Homo sapiens] |
| A0A0C4DH29 | 32525.15347 | 34843.57474 | 1.071280871 | 0.099336779 | 0.647998244 | no | no change | GO:0009897;GO:0050853;GO:0045087;GO:0016020;GO:0006910;GO:0006911;GO:0002376;GO:0005576;GO:0002250;GO:0003823;GO:0005886;GO:0072562;GO:0042571;GO:0006958;GO:0050871;GO:0034987;GO:0042742 | hsa:102723407 | | immunoglobulin heavy chain variable region, partial [Homo sapiens] |
| A0A0B4J1X8 | 99827.09421 | 106788.1384 | 1.069731011 | 0.09724807 | 0.575108731 | no | no change | GO:0009897;GO:0050853;GO:0045087;GO:0016020;GO:0006910;GO:0006911;GO:0002376;GO:0005576;GO:0002250;GO:0003823;GO:0005886;GO:0072562;GO:0042571;GO:0006958;GO:0050871;GO:0034987;GO:0042742 | hsa:102723407 | | RecName: Full=Immunoglobulin heavy variable 3-43; Flags: Precursor |
| O14791 | 35749.16842 | 38175.58632 | 1.067873408 | 0.094740632 | 0.536398189 | no | no change | GO:0006869;GO:0005788;GO:0042157;GO:0031224;GO:0019835;GO:0044267;GO:0005615;GO:0072562;GO:0005254;GO:0045087;GO:0034361;GO:0034364;GO:0006629;GO:1902476;GO:0043687;GO:0008289;GO:0008202;GO:0008203;GO:0006898;GO:0031640;GO:0005576;GO:0005515 | hsa:8542 |  | apolipoprotein L1 isoform a precursor [Homo sapiens] |
| O00391 | 10480.24147 | 11190.81111 | 1.067800884 | 0.094642648 | 0.562904732 | no | no change | GO:0005788;GO:0030173;GO:0071949;GO:0016491;GO:0016971;GO:0044267;GO:0016972;GO:0045171;GO:0070062;GO:0031093;GO:0016021;GO:0016020;GO:0003756;GO:0035580;GO:0016242;GO:0005794;GO:1904724;GO:0043687;GO:0045454;GO:0055114;GO:0005615;GO:0043231;GO:0043312;GO:0000139;GO:0005576;GO:0085029;GO:0002576 | hsa:5768 |  | sulfhydryl oxidase 1 isoform a precursor [Homo sapiens] |
| P01834 | 25566934.68 | 27249663.16 | 1.065816591 | 0.091959196 | 0.472720315 | no | no change | GO:0004252;GO:0030449;GO:0009897;GO:0006955;GO:0050871;GO:0042742;GO:0034987;GO:0042571;GO:0005615;GO:0016020;GO:0072562;GO:0006508;GO:0050853;GO:0045087;GO:0006910;GO:0006911;GO:0050776;GO:0006956;GO:0003823;GO:0005886;GO:0006958;GO:0001895;GO:0070062;GO:0038096;GO:0038095;GO:0006898;GO:0050900;GO:0002376;GO:0005576;GO:0002250 | hsa:100423062 | | light chain kappa Sci, k Sci=Bence Jones protein [human, Peptide, 214 aa] |
| P11597 | 7393.141222 | 7833.990842 | 1.059629541 | 0.08355997 | 0.650805181 | no | no change | GO:0017129;GO:0034375;GO:0034374;GO:0034372;GO:0006869;GO:0015914;GO:0017127;GO:0030301;GO:0034197;GO:0034364;GO:0055091;GO:0005548;GO:0070062;GO:0005615;GO:0008202;GO:0010745;GO:0031982;GO:0005319;GO:0070328;GO:0006629;GO:0006641;GO:0008289;GO:0055088;GO:0031210;GO:0042632;GO:0043691;GO:0010874;GO:0008203;GO:0015485;GO:0005576;GO:0046470 | hsa:1071 |  | cholesteryl ester transfer protein isoform 1 precursor [Homo sapiens] |
| A0A0B4J231 | 11148517 | 11789687.64 | 1.057511743 | 0.080673684 | 0.661409284 | no | no change | GO:0009897;GO:0050853;GO:0070062;GO:0045087;GO:0006910;GO:0006911;GO:0042742;GO:0003823;GO:0034987;GO:0042571;GO:0006958;GO:0050871 | hsa:100423062 | | immunoglobulin lambda-3 surrogate light chain [Homo sapiens] |
| A0A0G2JL69 | 36053.48895 | 38095.56632 | 1.056640215 | 0.079484224 | 0.459369432 | no | no change | GO:0004252;GO:0016787;GO:0045087;GO:0005576;GO:0006956;GO:0006508;GO:0006958;GO:0046872;GO:0008233;GO:0008236 | hsa:717 | COG5640 | complement C2 isoform 5 [Homo sapiens] |
| P05546 | 523667.8632 | 553199.5684 | 1.056393961 | 0.07914796 | 0.452039018 | no | no change | GO:0044267;GO:0007599;GO:0006935;GO:0005615;GO:0043687;GO:0007596;GO:0005788;GO:0005576;GO:0030414;GO:0004866;GO:0004867;GO:0010951;GO:0010466;GO:0008201;GO:0070062 | hsa:3053 | COG4826 | heparin cofactor 2 precursor [Homo sapiens] |
| E9PHK0 | 142852.4105 | 150859.3505 | 1.056050437 | 0.078678739 | 0.416856072 | no | no change | GO:0005737;GO:0036143;GO:0070062;GO:0002576;GO:0001652;GO:0030246;GO:0008201;GO:0071560;GO:0071310;GO:0001503;GO:0005615;GO:0005509;GO:0005576;GO:0031089;GO:0030282;GO:0062023;GO:0010756 | hsa:7123 |  | tetranectin isoform 1precursor [Homo sapiens] |
| A0A087WWT3 | 62502.87474 | 65991.80895 | 1.05582038 | 0.078364419 | 0.359622843 | no | no change | GO:0005794;GO:0005615;GO:0005783 | hsa:213 |  | ALB protein [Homo sapiens] |
| H3BUA5 | 380376.8626 | 401534.5821 | 1.055623045 | 0.078094752 | 0.737235818 | no | no change |  | hsa:10326 |  | LOW QUALITY PROTEIN: T0061165 isoform 1, partial [Pan troglodytes] |
| Q9UGM5 | 24205.02789 | 25507.93 | 1.053827746 | 0.07563907 | 0.651059453 | no | no change | GO:0005615;GO:0007339;GO:0008150;GO:0005576;GO:0003674;GO:0007338;GO:0030414;GO:0008191;GO:0010951;GO:0004869;GO:0004857;GO:0010466;GO:0070062 | hsa:26998 |  | fetuin-B isoform 1 precursor [Homo sapiens] |
| P02776 | 35069.36644 | 36937.81637 | 1.053278691 | 0.074887214 | 0.854011348 | no | no change | GO:0020005;GO:0070098;GO:0005125;GO:0071222;GO:0019221;GO:0008009;GO:0010469;GO:0032760;GO:0010628;GO:0048248;GO:0032496;GO:0045918;GO:0061844;GO:0005737;GO:0045347;GO:0031093;GO:0002576;GO:0007189;GO:0051873;GO:0010744;GO:0007186;GO:2001240;GO:0042127;GO:0030595;GO:0030593;GO:0006935;GO:0043950;GO:0006952;GO:0062023;GO:0006954;GO:0006955;GO:0008201;GO:0030168;GO:0005615;GO:0090023;GO:0045651;GO:0045652;GO:0045653;GO:0031640;GO:0005576;GO:0045944;GO:0005515;GO:0097679;GO:0042832;GO:0016525 | hsa:5196 |  | platelet factor 4 isoform 1 precursor [Homo sapiens] |
| P08571 | 10367.93374 | 10905.65174 | 1.051863564 | 0.072947587 | 0.495065894 | no | no change | GO:0006954;GO:0009897;GO:0045121;GO:0071222;GO:0071223;GO:0097190;GO:0031362;GO:1901224;GO:0007166;GO:0006909;GO:0032496;GO:0045471;GO:0010008;GO:0034612;GO:0016020;GO:0030667;GO:0005615;GO:0071727;GO:0071726;GO:0009617;GO:0071723;GO:0071219;GO:0032729;GO:0002224;GO:0031225;GO:0009986;GO:0001847;GO:0005794;GO:0032760;GO:0070266;GO:0034128;GO:0038124;GO:0045087;GO:0006915;GO:2000484;GO:0035666;GO:0032481;GO:0070891;GO:0034142;GO:0005886;GO:0009408;GO:0001530;GO:0032026;GO:0070062;GO:0051602;GO:0002755;GO:0002756;GO:0016019;GO:0050715;GO:0038123;GO:0031663;GO:0006898;GO:0007249;GO:0043312;GO:0002376;GO:0005576;GO:0002237;GO:0005515;GO:0045807;GO:0046696 | hsa:929 |  | monocyte differentiation antigen CD14 precursor [Homo sapiens] |
| A0A0S2Z4L3 | 194502.2211 | 203988.0474 | 1.048769758 | 0.068697991 | 0.656604439 | no | no change | GO:0005576;GO:0005509;GO:0030195 | hsa:5627 |  | vitamin K-dependent protein S isoform 1 precursor [Homo sapiens] |
| A0A075B7D0 | 336157.6105 | 351833.2974 | 1.046631956 | 0.065754213 | 0.831737141 | no | no change | GO:0009897;GO:0050853;GO:0045087;GO:0006910;GO:0006911;GO:0042742;GO:0003823;GO:0034987;GO:0042571;GO:0006958;GO:0050871;GO:0072562 | hsa:102723407 | | hCG1728627 [Homo sapiens] |
| P02760 | 392322.3842 | 410334.5526 | 1.045911651 | 0.064760991 | 0.391956712 | no | no change | GO:0005886;GO:0010951;GO:0046329;GO:0070062;GO:0019855;GO:0072562;GO:0010466;GO:0042803;GO:0005515;GO:0020037;GO:0009986;GO:0042167;GO:0018298;GO:0062023;GO:0030163;GO:0030414;GO:0004867;GO:0007155;GO:0005615;GO:0050777;GO:0046904;GO:0019862;GO:0007565;GO:0006898;GO:0043231;GO:0005576;GO:0016032 | hsa:259 |  | protein AMBP preproprotein [Homo sapiens] |
| P07360 | 43152.80158 | 45022.55263 | 1.043328613 | 0.06119363 | 0.607446151 | no | no change | GO:0019835;GO:0036094;GO:0030449;GO:0070062;GO:0045087;GO:0019841;GO:0072562;GO:0002376;GO:0001848;GO:0005576;GO:0044877;GO:0006957;GO:0006958;GO:0005579;GO:0005615 | hsa:733 |  | complement component C8 gamma chain precursor [Homo sapiens] |
| P01709 | 150145.6289 | 156534.4021 | 1.04255051 | 0.060117282 | 0.744132464 | no | no change | GO:0038096;GO:0004252;GO:0016020;GO:0030449;GO:0050776;GO:0006898;GO:0038095;GO:0002376;GO:0050900;GO:0005576;GO:0006956;GO:0002250;GO:0003823;GO:0005886;GO:0006508;GO:0006958;GO:0006955 | hsa:7441 |  | hCG2043240, partial [Homo sapiens] |
| P01597 | 42072.93842 | 43764.09368 | 1.040195796 | 0.056855113 | 0.686808005 | no | no change | GO:0038096;GO:0004252;GO:0016020;GO:0030449;GO:0050776;GO:0006898;GO:0038095;GO:0072562;GO:0002376;GO:0050900;GO:0005576;GO:0006956;GO:0002250;GO:0003823;GO:0005886;GO:0006508;GO:0006958;GO:0006955;GO:0070062 | hsa:7441 |  | immunoglobulin kappa light chain VC region, partial [Homo sapiens] |
| Q14520 | 92805.35211 | 96249.33684 | 1.037109764 | 0.052568592 | 0.493938739 | no | no change | GO:0004252;GO:0005615;GO:0016787;GO:0005539;GO:0005576;GO:0005509;GO:0007155;GO:0006508;GO:0008233;GO:0008236 | hsa:3026 | COG5640 | hyaluronan-binding protein 2 isoform 1 preproprotein [Homo sapiens] |
| B1AHL2 | 16086.271 | 16650.34933 | 1.035065823 | 0.049722516 | 0.819816122 | no | no change | GO:0016504;GO:0005576;GO:0005509;GO:0010952;GO:0030198 | hsa:2192 |  | FBLN1 isoform 5 [Pongo abelii] |
| A0A0C4DH31 | 26625.47789 | 27515.99632 | 1.033446101 | 0.047463147 | 0.719257663 | no | no change | GO:0009897;GO:0050853;GO:0045087;GO:0016020;GO:0006910;GO:0006911;GO:0002376;GO:0005576;GO:0002250;GO:0003823;GO:0005886;GO:0072562;GO:0042571;GO:0006958;GO:0050871;GO:0034987;GO:0042742 | hsa:102723407 | | immunoglobulin heavy chain variable region, partial [Homo sapiens] |
| P01042 | 1234049.968 | 1274189.979 | 1.032527054 | 0.046179584 | 0.566888934 | no | no change | GO:0007599;GO:0030414;GO:0007597;GO:0007596;GO:0045861;GO:0005788;GO:0007162;GO:0008270;GO:0042311;GO:0044267;GO:0050880;GO:0005615;GO:0031093;GO:0002576;GO:0072562;GO:0043065;GO:0010466;GO:0007186;GO:0043687;GO:0062023;GO:0006954;GO:0005886;GO:0010951;GO:0004869;GO:0008201;GO:0070062;GO:0007204;GO:0005576;GO:0005515;GO:0005102;GO:0030195 | hsa:3827 |  | kininogen-1 isoform 1 precursor [Homo sapiens] |
| P02768 | 465645313.7 | 479659392 | 1.030096036 | 0.042778847 | 0.39045119 | no | no change | GO:0034375;GO:0015643;GO:0005788;GO:0008144;GO:0051659;GO:0030170;GO:0003677;GO:0005783;GO:0005737;GO:0044267;GO:0019836;GO:0070062;GO:0031093;GO:0043209;GO:0002576;GO:0051087;GO:0072562;GO:0098869;GO:0043066;GO:0005504;GO:0005507;GO:0043687;GO:0032460;GO:0042802;GO:0005794;GO:0140272;GO:0016209;GO:0008289;GO:0019825;GO:0009267;GO:0001895;GO:0005615;GO:0046872;GO:0032991;GO:0043069;GO:0006898;GO:1903981;GO:0005576;GO:0005515;GO:0005634 | hsa:213 |  | serum albumin preproprotein [Homo sapiens] |
| Q13790 | 68167.64368 | 70172.31053 | 1.029407894 | 0.041814751 | 0.689299214 | no | no change | GO:0005319;GO:0034362;GO:0034364;GO:0006629;GO:0006869;GO:0005615;GO:0005576;GO:0005102;GO:0008203;GO:0008202;GO:0015485 | hsa:319 |  | apolipoprotein F preproprotein [Homo sapiens] |
| Q96HR3 | 35719.59263 | 36736.91842 | 1.02848089 | 0.040514988 | 0.841567558 | no | no change | GO:0019827;GO:0030521;GO:0038023;GO:0005515;GO:0006355;GO:0005634;GO:0030518;GO:0003712;GO:0000151;GO:0005654;GO:0046966;GO:0045893;GO:0006367;GO:0016567;GO:0016592;GO:0030374;GO:0061630;GO:0042809;GO:0006351 | hsa:90390 |  | mediator of RNA polymerase II transcription subunit 30 isoform 1 [Homo sapiens] |
| E7END6 | 19835.25474 | 20400.11526 | 1.028477604 | 0.040510378 | 0.702683187 | no | no change | GO:0004252;GO:0016787;GO:0007596;GO:0005576;GO:0005509;GO:0006508;GO:0008233;GO:0008236 | hsa:5624 | COG5640 | vitamin K-dependent protein C isoform X5 [Homo sapiens] |
| P01601 | 100026.1226 | 102837.5474 | 1.028106905 | 0.039990287 | 0.703539793 | no | no change | GO:0038096;GO:0004252;GO:0016020;GO:0030449;GO:0005615;GO:0006898;GO:0038095;GO:0050900;GO:0002376;GO:0002377;GO:0050776;GO:0005576;GO:0006956;GO:0002250;GO:0003823;GO:0005886;GO:0006508;GO:0006958;GO:0006955 | hsa:7441 |  | RecName: Full=Immunoglobulin kappa variable 1D-16; AltName: Full=Ig kappa chain V-I region HK146; AltName: Full=Ig kappa chain V-I region HK189; Flags: Precursor |
| P01344 | 49122.98947 | 50416.95737 | 1.026341391 | 0.037510694 | 0.763099036 | no | no change | GO:0046628;GO:0045840;GO:0031017;GO:0051146;GO:0008286;GO:0038028;GO:0008284;GO:0001501;GO:0001503;GO:0051147;GO:0031056;GO:0071902;GO:0051897;GO:0007275;GO:0001649;GO:0044267;GO:0006349;GO:0001701;GO:0008083;GO:0031093;GO:0000122;GO:0002576;GO:0043085;GO:0045725;GO:0010469;GO:0005179;GO:0005178;GO:0009887;GO:2000467;GO:0042104;GO:0005159;GO:0005158;GO:0043410;GO:0048018;GO:0060669;GO:0001892;GO:0043539;GO:0005615;GO:0005975;GO:0050731;GO:0040018;GO:0006355;GO:0051781;GO:0005576;GO:0045944;GO:0005515;GO:0006006;GO:0001934 | hsa:3481 |  | insulin-like growth factor II isoform 2 [Homo sapiens] |
| P04003 | 2738359.421 | 2808926.789 | 1.025769944 | 0.036707204 | 0.842658864 | no | no change | GO:0030449;GO:0005615;GO:0045087;GO:0072562;GO:0002376;GO:0045732;GO:0005576;GO:0003723;GO:0005515;GO:0005886;GO:1903027;GO:0006958;GO:0045959;GO:0044216 | hsa:722 |  | C4b-binding protein alpha chain precursor [Homo sapiens] |
| P69905 | 816929.0632 | 836201.7737 | 1.023591657 | 0.033640294 | 0.867677793 | no | no change | GO:0005344;GO:0022627;GO:0015701;GO:0042744;GO:0010942;GO:0005615;GO:0071682;GO:0016020;GO:0072562;GO:0005833;GO:0098869;GO:0005506;GO:0046872;GO:0015671;GO:0020037;GO:0031720;GO:0051291;GO:0070062;GO:0019825;GO:0042542;GO:0005829;GO:0006898;GO:0005576;GO:0005515;GO:0031838;GO:0004601 | hsa:3040;hsa:3039 | COG1018 | TPA: globin C1 [Homo sapiens] |
| A0A5H1ZRS2 | 1037270.826 | 1060716.079 | 1.022602827 | 0.032245919 | 0.938067268 | no | no change |  |  |  | immunoglobulin kappa chain variable region, partial [Homo sapiens] |
| P43652 | 145866.8226 | 149067.1842 | 1.021940298 | 0.031310916 | 0.773924607 | no | no change | GO:0008431;GO:0051180;GO:0005615;GO:0072562;GO:0050821;GO:0015031;GO:0005576;GO:0005515;GO:0071693;GO:0046872;GO:0070062 | hsa:173 |  | afamin precursor [Homo sapiens] |
| G3V0E5 | 5212.642421 | 5320.957789 | 1.020779359 | 0.029671062 | 0.891885832 | no | no change | GO:0009897;GO:0055037;GO:0035690;GO:0005905;GO:1990712;GO:0010008;GO:0045780;GO:1990830;GO:0070062;GO:0016021;GO:0045830;GO:0004998;GO:0048471;GO:0042803;GO:0006879;GO:0030890;GO:0030316;GO:0042102;GO:0005887;GO:0005886;GO:0016323;GO:0042470;GO:0031623;GO:0006898;GO:0003725;GO:0033570;GO:0033572;GO:0005769 | hsa:7037 | COG2234 | transferrin receptor variant, partial [Homo sapiens] |
| P19827 | 314246.0632 | 319962 | 1.018189367 | 0.026005904 | 0.81165285 | no | no change | GO:0030212;GO:0070062;GO:0072562;GO:0010466;GO:0005509;GO:0030414;GO:0004867;GO:0010951;GO:0005576 | hsa:3697 | COG2304 | inter-alpha-trypsin inhibitor heavy chain H1 isoform a preproprotein [Homo sapiens] |
| P09871 | 92244.90053 | 93659.56368 | 1.01533595 | 0.021957159 | 0.799453628 | no | no change | GO:0004252;GO:0006956;GO:0016787;GO:0045087;GO:0072562;GO:0002376;GO:0030449;GO:0005576;GO:0001867;GO:0005509;GO:0005515;GO:0006508;GO:0006958;GO:0046872;GO:0008233;GO:0042802;GO:0008236 | hsa:716 | COG5640 | complement C1s subcomponent isoform 1 preproprotein [Homo sapiens] |
| P02647 | 31741707.16 | 32198537.26 | 1.014392109 | 0.020615428 | 0.829574913 | no | no change | GO:0034115;GO:0010804;GO:0005788;GO:0019915;GO:0034191;GO:0034190;GO:0005548;GO:0050728;GO:0071682;GO:0051496;GO:0005543;GO:0010898;GO:0005319;GO:0034361;GO:0034362;GO:0034363;GO:0034364;GO:0031102;GO:0034366;GO:0031100;GO:0043534;GO:0030300;GO:0034774;GO:0010873;GO:0043691;GO:0007179;GO:0015485;GO:0005829;GO:0018206;GO:0019433;GO:0045499;GO:0070508;GO:0045723;GO:0034375;GO:0034371;GO:0031072;GO:0015914;GO:0034378;GO:0050821;GO:0010903;GO:0042158;GO:0055102;GO:0044267;GO:0042627;GO:0051180;GO:0014012;GO:0072562;GO:0050919;GO:0007186;GO:0033700;GO:0018158;GO:0006629;GO:0060761;GO:0042632;GO:0032489;GO:0050713;GO:0007229;GO:0006898;GO:0034384;GO:0062023;GO:0006695;GO:0060192;GO:0006869;GO:0019899;GO:0030301;GO:0035025;GO:0051006;GO:0002740;GO:0001540;GO:1900026;GO:0031410;GO:0005634;GO:0042802;GO:0008035;GO:0060354;GO:0009986;GO:0008289;GO:0008202;GO:0008203;GO:0071813;GO:0070062;GO:0042493;GO:0005515;GO:0034380;GO:0070371;GO:0042157;GO:0006656;GO:0030325;GO:0006644;GO:0017127;GO:0030139;GO:0008211;GO:0060228;GO:0001523;GO:0055091;GO:0034365;GO:0005615;GO:0002576;GO:0005576;GO:0043687;GO:0070328;GO:0019216;GO:0007584;GO:0031210;GO:0005886;GO:0043627;GO:1903561;GO:0051346;GO:0051345;GO:0033344;GO:0070653;GO:0001932;GO:0001935;GO:0005769 | hsa:335 |  | apolipoprotein A-I isoform 1 preproprotein [Homo sapiens] |
| P01602 | 182768.7774 | 185182.1458 | 1.01320449 | 0.018925375 | 0.969225495 | no | no change | GO:0038096;GO:0004252;GO:0016020;GO:0030449;GO:0050776;GO:0006898;GO:0038095;GO:0072562;GO:0002376;GO:0002377;GO:0050900;GO:0005576;GO:0006956;GO:0002250;GO:0003823;GO:0005886;GO:0006508;GO:0006958;GO:0006955;GO:0070062 | hsa:7441 |  | hCG2043208, partial [Homo sapiens] |
| P27169 | 245743.4632 | 248603.3211 | 1.011637575 | 0.016692528 | 0.844724941 | no | no change | GO:0034445;GO:0032411;GO:0016311;GO:0046872;GO:0004064;GO:0046434;GO:0070062;GO:0005615;GO:0005543;GO:0009636;GO:0072562;GO:0005509;GO:0102007;GO:0010875;GO:0019372;GO:0042803;GO:0016787;GO:0034364;GO:0006629;GO:0034366;GO:1902617;GO:0008203;GO:0004063;GO:0043231;GO:0046395;GO:0051099;GO:0031667;GO:0046470;GO:0005576;GO:0019439;GO:0070542 | hsa:5444 |  | serum paraoxonase/arylesterase 1 precursor [Homo sapiens] |
| G3XAK1 | 17282.07211 | 17478.04947 | 1.011339923 | 0.016267985 | 0.89961913 | no | no change | GO:0004252;GO:2000479;GO:0019899;GO:0010628;GO:0005737;GO:0005615;GO:0005773;GO:0033601;GO:0030971;GO:1904036;GO:0006508;GO:0045721;GO:0046425;GO:0030317;GO:0071456;GO:0007283;GO:0062023;GO:0060763;GO:0048012;GO:0007566;GO:0030879;GO:0005576;GO:0010758 | hsa:4485 | COG5640 | hepatocyte growth factor-like protein precursor [Homo sapiens] |
| P32119 | 22190.79347 | 22429.17211 | 1.010742231 | 0.015415115 | 0.929326271 | no | no change | GO:0000187;GO:0045581;GO:0042744;GO:0008379;GO:0032496;GO:0032088;GO:0042981;GO:0034599;GO:0002536;GO:0016491;GO:0005737;GO:0010310;GO:0070062;GO:0042098;GO:0043066;GO:0048538;GO:2001240;GO:0016209;GO:0045454;GO:0006979;GO:0055114;GO:0048872;GO:0045321;GO:0005829;GO:0005623;GO:0031665;GO:0019430;GO:0005515;GO:0051920;GO:0004601;GO:0030194 | hsa:7001 | COG0450 | peroxiredoxin-2 [Homo sapiens] |
| P68871 | 5812417.526 | 5874401.842 | 1.01066412 | 0.015303617 | 0.935709184 | no | no change | GO:0005344;GO:0007596;GO:0015701;GO:1904813;GO:0045429;GO:0042744;GO:0008217;GO:0010942;GO:0050880;GO:0005615;GO:0071682;GO:0072562;GO:0005833;GO:0098869;GO:0046872;GO:0015671;GO:0070293;GO:0030185;GO:1904724;GO:0020037;GO:0031721;GO:0031720;GO:0051291;GO:0070062;GO:0019825;GO:0042542;GO:0030492;GO:0005829;GO:0006898;GO:0043312;GO:0070527;GO:0043177;GO:0005576;GO:0005515;GO:0031838;GO:0004601 | hsa:3043 | COG1018 | PREDICTED: hemoglobin subunit beta [Gorilla gorilla gorilla] |
| P10643 | 100947.3453 | 101851.2711 | 1.008954429 | 0.012861014 | 0.882425566 | no | no change | GO:0019835;GO:0030449;GO:0070062;GO:0045087;GO:0006883;GO:0002376;GO:0005576;GO:0006956;GO:0006957;GO:0006955;GO:0006958;GO:0005579 | hsa:730 |  | complement component C7 precursor [Homo sapiens] |
| A0A0U1RQV3 | 27699.22579 | 27943.68526 | 1.008825498 | 0.012676646 | 0.901745295 | no | no change | GO:0062023;GO:0007173;GO:0005509;GO:0005006 | hsa:2202 |  | EGF-containing fibulin-like extracellular matrix protein 1 isoform X3 [Homo sapiens] |
| A0A075B6Q5 | 61209.78353 | 61693.16944 | 1.0078972 | 0.0113485 | 0.972816656 | no | no change | GO:0009897;GO:0050853;GO:0045087;GO:0016020;GO:0006910;GO:0006911;GO:0002376;GO:0005576;GO:0002250;GO:0003823;GO:0005886;GO:0072562;GO:0042571;GO:0006958;GO:0050871;GO:0034987;GO:0042742 | hsa:102723407 | | hCG2036739, partial [Homo sapiens] |
| A0A0C4DH36 | 52856.89 | 53260.81474 | 1.007641856 | 0.010982956 | 0.923878071 | no | no change | GO:0009897;GO:0050853;GO:0045087;GO:0006910;GO:0006911;GO:0042742;GO:0003823;GO:0034987;GO:0042571;GO:0006958;GO:0050871;GO:0072562 | hsa:102723407 | | immunoglobulin heavy chain variable gene IGHV3-38, partial [Homo sapiens] |
| P0DTE1 | 16825.00479 | 16931.36632 | 1.006321634 | 0.009091484 | 0.961306042 | no | no change |  | hsa:102723407 | | immunoglobulin heavy chain variable region, partial [Homo sapiens] |
| P04217 | 789356.7158 | 794323.0421 | 1.006291612 | 0.009048443 | 0.923707625 | no | no change | GO:0070062;GO:0031093;GO:0002576;GO:0043312;GO:0072562;GO:1904813;GO:0005576;GO:0003674;GO:0034774;GO:0008150;GO:0062023;GO:0005615 | hsa:1 |  | alpha-1B-glycoprotein precursor [Homo sapiens] |
| P07357 | 57252.00211 | 57584.42 | 1.005806223 | 0.008352385 | 0.916207897 | no | no change | GO:0019835;GO:0030449;GO:0070062;GO:0016021;GO:0016020;GO:0072562;GO:0002376;GO:0001848;GO:0005576;GO:0006956;GO:0006957;GO:0005886;GO:0006955;GO:0044877;GO:0006958;GO:0005579;GO:0045087;GO:0005615 | hsa:731 |  | complement component C8 alpha chain preproprotein [Homo sapiens] |
| P05154 | 20619.27105 | 20698.69611 | 1.003851982 | 0.005546559 | 0.965334677 | no | no change | GO:0009897;GO:0006869;GO:0007596;GO:0036024;GO:0002080;GO:0061107;GO:0036029;GO:0031094;GO:0031091;GO:0051346;GO:0007342;GO:0016020;GO:0036025;GO:0032190;GO:0036027;GO:0036026;GO:0010466;GO:0002020;GO:0097183;GO:0097182;GO:0097181;GO:0036028;GO:0007283;GO:0031210;GO:0007338;GO:0030414;GO:0004867;GO:0010951;GO:0008201;GO:0005615;GO:0032991;GO:0070062;GO:0036030;GO:0005539;GO:0005576;GO:0005515;GO:0001972;GO:0045861 | hsa:5104 | COG4826 | plasma serine protease inhibitor preproprotein [Homo sapiens] |
| P30041 | 4211.5429 | 4216.224692 | 1.001111657 | 0.001602892 | 0.995178392 | no | no change | GO:0035578;GO:0042744;GO:0005623;GO:0048026;GO:0045454;GO:0016491;GO:0005737;GO:0047499;GO:0005615;GO:0005634;GO:0016020;GO:0016042;GO:0098869;GO:0048471;GO:0042803;GO:0016787;GO:0006629;GO:0016209;GO:0102568;GO:0008152;GO:0034599;GO:0006979;GO:0055114;GO:0070062;GO:0031625;GO:0003824;GO:0005829;GO:0043312;GO:0045296;GO:0051920;GO:0005764;GO:0005576;GO:0005515;GO:0102567;GO:0046475;GO:0004623;GO:0004601;GO:0004602 | hsa:9588 | COG0450 | peroxiredoxin-6 [Homo sapiens] |
| P00450 | 660068.4105 | 659652.3579 | 0.999369683 | -0.000909643 | 0.993441501 | no | no change | GO:0016491;GO:0044267;GO:0046872;GO:0006879;GO:0005615;GO:0006825;GO:0004322;GO:0006811;GO:0051087;GO:0070062;GO:0005623;GO:0005765;GO:0055072;GO:0006826;GO:0005788;GO:0005886;GO:0072562;GO:0005507;GO:0043687;GO:0055114;GO:0005576 | hsa:1356 | COG2132 | RecName: Full=Ceruloplasmin; AltName: Full=Ferroxidase; Flags: Precursor |
| A0A0C4DGZ8 | 15936.75382 | 15921.57261 | 0.999047409 | -0.001374954 | 0.99511305 | no | no change | GO:0016021;GO:0016020 | hsa:2811 | COG4886 | glycoprotein Ib (platelet), alpha polypeptide [Homo sapiens] |
| C9JC84 | 4693296.895 | 4677622.579 | 0.996660276 | -0.004826267 | 0.960372252 | no | no change | GO:0051258;GO:0005102;GO:0007596;GO:0005577;GO:0030168 | hsa:2266 |  | FGG isoform 6 [Pan troglodytes] |
| J3KNB4 | 16019.31779 | 15955.17689 | 0.995996028 | -0.005788106 | 0.98065433 | no | no change | GO:0071224;GO:0071222;GO:0042742;GO:0050829;GO:0044130;GO:0061844;GO:0005737;GO:0042995;GO:0005615;GO:0071354;GO:0071356;GO:0016021;GO:0045766;GO:0051873;GO:0008284;GO:0045087;GO:0006952;GO:0050830;GO:0044140;GO:0001530;GO:0042581;GO:0071347;GO:0005576;GO:0001934 | hsa:820 |  | cathelicidin antimicrobial peptide [Homo sapiens] |
| P01721 | 114034.0153 | 113548.3379 | 0.995740943 | -0.006157643 | 0.974537554 | no | no change | GO:0038096;GO:0004252;GO:0016020;GO:0030449;GO:0050776;GO:0006898;GO:0038095;GO:0002376;GO:0050900;GO:0005576;GO:0006956;GO:0002250;GO:0003823;GO:0005886;GO:0006508;GO:0006958;GO:0006955 | hsa:7441 |  | RecName: Full=Immunoglobulin lambda variable 6-57; AltName: Full=Ig lambda chain V-VI region AR; AltName: Full=Ig lambda chain V-VI region EB4; AltName: Full=Ig lambda chain V-VI region NIG-48; AltName: Full=Ig lambda chain V-VI region SUT; AltName: Full=Ig lambda chain V-VI region WLT; Flags: Precursor |
| P55103 | 8508.933474 | 8468.860111 | 0.995290436 | -0.006810514 | 0.959673735 | no | no change | GO:0005179;GO:0005125;GO:0008083;GO:0060395;GO:0005160;GO:0010469;GO:0048468;GO:0005576;GO:0005615;GO:0010862;GO:0042981;GO:0043408 | hsa:3626 |  | inhibin beta C chain preproprotein [Homo sapiens] |
| P01703 | 153989.3533 | 152973.9737 | 0.99340617 | -0.009544388 | 0.954771714 | no | no change | GO:0038096;GO:0004252;GO:0016020;GO:0030449;GO:0050776;GO:0006898;GO:0038095;GO:0002376;GO:0050900;GO:0005576;GO:0006956;GO:0002250;GO:0003823;GO:0005886;GO:0006508;GO:0006958;GO:0006955 | hsa:7441 |  | Unknown (protein for MGC:31936) [Homo sapiens] |
| Q14624 | 181299.5368 | 179441.1722 | 0.989749755 | -0.014864289 | 0.897710541 | no | no change | GO:0005737;GO:0030212;GO:0034097;GO:0002576;GO:0004867;GO:0072562;GO:0031089;GO:0006953;GO:0005576;GO:0030414;GO:0005515;GO:0004866;GO:0005886;GO:0010951;GO:0010466;GO:0070062 | hsa:3700 | COG2304 | inter-alpha-trypsin inhibitor heavy chain H4 isoform 1 precursor [Homo sapiens] |
| O43866 | 151022.4368 | 149405.1953 | 0.989291382 | -0.015532586 | 0.943075481 | no | no change | GO:0005737;GO:0006898;GO:0005615;GO:0005044;GO:0009986;GO:0016020;GO:0072562;GO:0002376;GO:0005576;GO:0006954;GO:0006968;GO:0006915 | hsa:922 |  | CD5 antigen-like isoform 1 precursor [Homo sapiens] |
| A0A3B3ISR2 | 308661.1105 | 305276.5737 | 0.98903478 | -0.015906839 | 0.899239284 | no | no change | GO:0004252;GO:0005615;GO:0045087;GO:0031638;GO:0005509;GO:0006958 | hsa:715 | COG5640 | complement C1r subcomponent isoform 1 preproprotein [Homo sapiens] |
| F8WF14 | 25632.02316 | 25332.67737 | 0.988321414 | -0.016947795 | 0.897093071 | no | no change | GO:0019899;GO:0051384;GO:0005783;GO:0016787;GO:0007612;GO:0050805;GO:0050783;GO:0014016;GO:0001540;GO:0016021;GO:0016020;GO:0072562;GO:0042802;GO:0008285;GO:0004104;GO:0003824;GO:0005788;GO:0043279;GO:0051593;GO:0019695;GO:0005641;GO:0005576;GO:0033265;GO:0003990 | hsa:590 | COG2272 | unnamed protein product [Homo sapiens] |
| K7ERG9 | 12504.43326 | 12339.92889 | 0.986844316 | -0.019105591 | 0.905537921 | no | no change | GO:0004252;GO:0007219;GO:0005615;GO:0016787;GO:0009617;GO:0006957;GO:0006508;GO:0008233;GO:0008236 | hsa:1675 | COG5640 | complement factor D isoform 2 precursor [Homo sapiens] |
| D6RF35 | 2747335.053 | 2707468.579 | 0.985489038 | -0.021088271 | 0.786414925 | no | no change | GO:0051180;GO:0090482;GO:0035461;GO:0005615;GO:0005499 | hsa:2638 |  | GC isoform 4 [Pan troglodytes] |
| P04196 | 685319.6474 | 675006.1053 | 0.984950757 | -0.021876497 | 0.879782042 | no | no change | GO:0002839;GO:0030308;GO:0032956;GO:2000504;GO:0007162;GO:0008270;GO:0051894;GO:0007599;GO:0001525;GO:0061844;GO:0051715;GO:0070062;GO:0031093;GO:0002576;GO:0072562;GO:0043065;GO:0033629;GO:0030168;GO:0005886;GO:0046872;GO:0010468;GO:0051918;GO:0010543;GO:0008285;GO:0007596;GO:0006935;GO:0020037;GO:0009986;GO:0010593;GO:0043537;GO:0043395;GO:0062023;GO:0004867;GO:0010951;GO:0004869;GO:0043254;GO:0008201;GO:2001027;GO:0050832;GO:0042730;GO:0036019;GO:0050730;GO:0015886;GO:0019865;GO:0005576;GO:1900747;GO:0005515;GO:0030193;GO:0005102;GO:0016525 | hsa:3273 |  | histidine-rich glycoprotein precursor [Homo sapiens] |
| P80748 | 678144.7579 | 667222.8947 | 0.983894496 | -0.023424473 | 0.902830573 | no | no change | GO:0038096;GO:0004252;GO:0016020;GO:0030449;GO:0050776;GO:0006898;GO:0038095;GO:0072562;GO:0002376;GO:0050900;GO:0005576;GO:0006956;GO:0002250;GO:0003823;GO:0005886;GO:0006508;GO:0006958;GO:0006955;GO:0070062 | hsa:7441 |  | hCG2040021, partial [Homo sapiens] |
| P0C0L5 | 1807760.674 | 1776359.432 | 0.982629757 | -0.025280166 | 0.900427863 | no | no change | GO:0004252;GO:0030449;GO:0006954;GO:0030246;GO:0030425;GO:0030424;GO:0045202;GO:0032490;GO:0030054;GO:0005615;GO:0072562;GO:0001848;GO:0006508;GO:0045087;GO:0008228;GO:0006956;GO:2000427;GO:0004866;GO:0005886;GO:0010951;GO:0006958;GO:0042995;GO:0070062;GO:0044216;GO:0002376;GO:0005576 | hsa:100293534;hsa:110384692;hsa:720;hsa:721 | | complement C4-B preproprotein [Homo sapiens] |
| P01700 | 1457660.432 | 1429804.511 | 0.980889979 | -0.027836768 | 0.878451845 | no | no change | GO:0038096;GO:0004252;GO:0016020;GO:0030449;GO:0050776;GO:0006898;GO:0038095;GO:0072562;GO:0002376;GO:0050900;GO:0005576;GO:0006956;GO:0002250;GO:0003823;GO:0005886;GO:0006508;GO:0006958;GO:0006955 | hsa:7441 |  | RecName: Full=Immunoglobulin lambda variable 1-47; AltName: Full=Ig lambda chain V-I region HA; AltName: Full=Ig lambda chain V-I region WAH; Flags: Precursor |
| A0A0J9YX35 | 22457.00516 | 22011.14278 | 0.980145955 | -0.028931495 | 0.918379214 | no | no change | GO:0016020;GO:0002376;GO:0005576;GO:0002250;GO:0003823;GO:0005886 | hsa:102723407 | | RecName: Full=Immunoglobulin heavy variable 3-64D; Flags: Precursor |
| E9PAQ1 | 27865.30579 | 27298.39105 | 0.979655176 | -0.029654063 | 0.770780653 | no | no change |  | hsa:5199 |  | properdin precursor [Homo sapiens] |
| C9JF17 | 842503.8 | 825317.6895 | 0.979601148 | -0.029733631 | 0.815529555 | no | no change | GO:0022626;GO:0005783;GO:0006869;GO:0042493;GO:0048678;GO:0030425;GO:0000302;GO:0001525;GO:0051895;GO:0010642;GO:0005737;GO:0070062;GO:0014012;GO:0005615;GO:2000405;GO:0048471;GO:0043025;GO:0005319;GO:0048662;GO:0006629;GO:2000098;GO:0008289;GO:0042308;GO:0060588;GO:0007420;GO:1900016;GO:0007568;GO:0015485;GO:0042246;GO:0071638;GO:0005576;GO:0006006 | hsa:347 | COG3040 | APOD isoform 3, partial [Pan troglodytes] |
| B4E1Z4 | 548596.7158 | 536278.9316 | 0.977546741 | -0.032762409 | 0.711068253 | no | no change | GO:0004252;GO:0030449;GO:0070062;GO:0016787;GO:0072562;GO:0001848;GO:0005576;GO:0006956;GO:0006957;GO:0005886;GO:0006508;GO:0008233;GO:0008236;GO:0005615 | hsa:629 | COG5640 | unnamed protein product [Homo sapiens] |
| A0A0A0MRJ7 | 20750.63968 | 20252.18995 | 0.975979066 | -0.035077891 | 0.839479443 | no | no change | GO:0048208;GO:0044267;GO:1903561;GO:0006888;GO:0005615;GO:0031093;GO:0008015;GO:0002576;GO:0007596;GO:0033116;GO:0005788;GO:0030134;GO:0046872;GO:0005576;GO:0005886;GO:0016020;GO:0005507;GO:0043687;GO:0000139 | hsa:2153 |  | coagulation factor V preproprotein [Homo sapiens] |
| J3QRV5 | 399653.4421 | 389926.1947 | 0.975660794 | -0.035548438 | 0.846472201 | no | no change | GO:0005737;GO:0043231;GO:0005829;GO:0006887;GO:0007049;GO:0051301 | hsa:3993 |  | lethal(2) giant larvae protein homolog 2 isoform X4 [Homo sapiens] |
| P00488 | 24389.30105 | 23771.56053 | 0.974671659 | -0.037011799 | 0.83023297 | no | no change | GO:0005737;GO:0018149;GO:0007599;GO:0019221;GO:0031093;GO:0003810;GO:0072378;GO:0002576;GO:0007596;GO:0072562;GO:0016740;GO:0062023;GO:0016746;GO:0046872;GO:0005576 | hsa:2162 |  | RecName: Full=Coagulation factor XIII A chain; Short=Coagulation factor XIIIa; AltName: Full=Protein-glutamine gamma-glutamyltransferase A chain; AltName: Full=Transglutaminase A chain; Flags: Precursor |
| A0A2R8Y3M9 | 189805.2842 | 184972.8526 | 0.974540058 | -0.037206607 | 0.674272986 | no | no change | GO:0004252;GO:0006898;GO:0005044;GO:0016020;GO:0016042;GO:0005576;GO:0005509;GO:0006508;GO:0004623 | hsa:81579;hsa:3426 | COG5640 | complement factor I isoform X2 [Homo sapiens] |
| P01008 | 531044.1632 | 517418.1526 | 0.974341097 | -0.037501176 | 0.661939175 | no | no change | GO:0007599;GO:0007595;GO:2000266;GO:0007596;GO:0005788;GO:0002438;GO:0070062;GO:0072562;GO:0010466;GO:0002020;GO:0043687;GO:0042802;GO:0044267;GO:0007584;GO:0062023;GO:0030414;GO:0004867;GO:0010951;GO:0005886;GO:0008201;GO:0005615;GO:0005576;GO:0005515;GO:0030193 | hsa:462 | COG4826 | antithrombin-III isoform 1 precursor [Homo sapiens] |
| P00734 | 573057.1632 | 557441.9474 | 0.972751033 | -0.039857488 | 0.605022477 | no | no change | GO:0004252;GO:0048712;GO:0009897;GO:0030307;GO:0007597;GO:0007596;GO:0005788;GO:0051281;GO:0030449;GO:0032967;GO:0008047;GO:0007166;GO:0007599;GO:0008233;GO:0007275;GO:0061844;GO:0044267;GO:0006888;GO:0005615;GO:0005102;GO:0009611;GO:0070062;GO:0001530;GO:0051838;GO:0010544;GO:0005509;GO:0006508;GO:0008236;GO:0007186;GO:0010468;GO:1900738;GO:0008284;GO:0016787;GO:2000379;GO:0046427;GO:0090218;GO:0070945;GO:0006953;GO:0005796;GO:0008360;GO:0014068;GO:0005886;GO:0008201;GO:0030168;GO:0008083;GO:1900016;GO:0042730;GO:1900182;GO:0051480;GO:0010469;GO:0070053;GO:0005576;GO:0051918;GO:0005515;GO:0072378;GO:0030193;GO:0045861;GO:0072562;GO:0001934;GO:0030194 | hsa:2147 | COG5640 | prothrombin isoform 1 preproprotein [Homo sapiens] |
| P01860 | 2892402.258 | 2808878.895 | 0.971123186 | -0.042273784 | 0.843535306 | no | no change | GO:0004252;GO:0030449;GO:0009897;GO:0050871;GO:0042742;GO:0034987;GO:0042571;GO:0005615;GO:0016020;GO:0072562;GO:0006508;GO:0050853;GO:0045087;GO:0006910;GO:0006911;GO:0006956;GO:0003823;GO:0005886;GO:0006958;GO:0001895;GO:0070062;GO:0038096;GO:0002376;GO:0005576;GO:0002250 | hsa:100423062 | | Unknown (protein for MGC:105008) [Homo sapiens] |
| P13671 | 57976.29579 | 56036.18947 | 0.966536215 | -0.049104306 | 0.531808919 | no | no change | GO:0019835;GO:0001701;GO:0030449;GO:0070062;GO:0045917;GO:0045087;GO:0045766;GO:0002376;GO:0005576;GO:0006956;GO:0001970;GO:0005515;GO:0006955;GO:0006958;GO:0005579 | hsa:729 |  | complement component C6 precursor [Homo sapiens] |
| P51884 | 86643.76368 | 83551.94263 | 0.964315712 | -0.052422541 | 0.583152851 | no | no change | GO:0005201;GO:0018146;GO:0031012;GO:0014070;GO:0043202;GO:0070062;GO:0005583;GO:0070848;GO:0005576;GO:0005796;GO:0032914;GO:0007601;GO:0030021;GO:0062023;GO:0007409;GO:0005615;GO:0051216;GO:0030199;GO:0030198;GO:0045944;GO:0005515;GO:0005518;GO:0042340 | hsa:4060 | COG4886 | lumican precursor [Homo sapiens] |
| P02748 | 123071.5032 | 118478.6568 | 0.96268148 | -0.054869558 | 0.656421233 | no | no change | GO:0030449;GO:0006955;GO:0019835;GO:0019836;GO:0070062;GO:0016021;GO:0016020;GO:0051260;GO:0072562;GO:0001906;GO:0045087;GO:0006957;GO:0005887;GO:0005886;GO:0006958;GO:0005615;GO:0005829;GO:0044218;GO:0044279;GO:0002376;GO:0005576;GO:0005579 | hsa:735 |  | complement component C9 preproprotein [Homo sapiens] |
| B7ZKJ8 | 544967.0421 | 523845.2211 | 0.961242021 | -0.057028377 | 0.376868734 | no | no change | GO:0005737;GO:0030212;GO:0034097;GO:0004867;GO:0006953;GO:0005886;GO:0010951 | hsa:3700 | COG2304 | ITIH4 protein [Homo sapiens] |
| P20851 | 56854.73632 | 54623.62684 | 0.96075772 | -0.057755431 | 0.714681795 | no | no change | GO:0030449;GO:0005615;GO:0045087;GO:0007596;GO:0002376;GO:0045732;GO:0005576;GO:0005515;GO:0005886;GO:1903027;GO:0006958;GO:0045959;GO:0044216 | hsa:725 |  | C4b-binding protein beta chain isoform 1 precursor [Homo sapiens] |
| P03951 | 9806.567526 | 9396.648474 | 0.958199538 | -0.061601977 | 0.754276747 | no | no change | GO:0004252;GO:0016020;GO:0007599;GO:0070062;GO:0016787;GO:0005615;GO:0007597;GO:0007596;GO:0031639;GO:0030193;GO:0005576;GO:0005515;GO:0005886;GO:0070009;GO:0006508;GO:0008233;GO:0008236;GO:0008201;GO:0042802;GO:0051919 | hsa:2160 | COG5640 | coagulation factor XI isoform 1 preproprotein [Homo sapiens] |
| O00187 | 37616.25611 | 36016.13684 | 0.957462027 | -0.062712823 | 0.68747669 | no | no change | GO:0045087;GO:0004252;GO:0046872;GO:0006956;GO:0005615;GO:0048306;GO:0070062;GO:0016787;GO:0002376;GO:0005576;GO:0001867;GO:0005509;GO:0005515;GO:0006508;GO:0006958;GO:0008236;GO:0008233;GO:0001855 | hsa:10747 | COG5640 | mannan-binding lectin serine protease 2 isoform 1 preproprotein [Homo sapiens] |
| Q6UXB8 | 10108.85542 | 9626.388737 | 0.952272867 | -0.070553068 | 0.632764423 | no | no change | GO:0010466;GO:0005576;GO:0030414;GO:0005615;GO:0061052 | hsa:221476 | COG2340 | peptidase inhibitor 16 precursor [Homo sapiens] |
| A0A087WSZ0 | 53286.55 | 50574.93474 | 0.949112576 | -0.075348877 | 0.840984387 | no | no change | GO:0016020;GO:0005615;GO:0006955;GO:0002376;GO:0002377;GO:0005576;GO:0002250;GO:0003823;GO:0005886 | | | RecName: Full=Immunoglobulin kappa variable 1D-8; Flags: Precursor |
| P01718 | 31605.07312 | 29956.94232 | 0.947852334 | -0.077265776 | 0.884029442 | no | no change | GO:0038096;GO:0004252;GO:0016020;GO:0030449;GO:0050776;GO:0006898;GO:0038095;GO:0002376;GO:0050900;GO:0005576;GO:0006956;GO:0002250;GO:0003823;GO:0005886;GO:0006508;GO:0006958;GO:0006955 | hsa:29802 |  | immunoglobulin light chain variable region, partial [Homo sapiens] |
| Q13093 | 7030.992333 | 6661.107222 | 0.94739219 | -0.077966317 | 0.753972366 | no | no change | GO:0034441;GO:0034440;GO:0034362;GO:0034374;GO:0047499;GO:0016787;GO:0006629;GO:0005615;GO:0005543;GO:0005737;GO:0016788;GO:0016042;GO:0050729;GO:0090026;GO:0003847;GO:0046469;GO:0005576 | hsa:7941 |  | platelet-activating factor acetylhydrolase precursor [Homo sapiens] |
| P09172 | 3754.1633 | 3552.175786 | 0.946196396 | -0.079788429 | 0.794354942 | no | no change | GO:0016491;GO:0042127;GO:2001236;GO:0045907;GO:0005783;GO:0048149;GO:0034774;GO:0007613;GO:0042593;GO:0050900;GO:0042596;GO:0005815;GO:0042423;GO:0042420;GO:0042421;GO:0005737;GO:0031418;GO:0030667;GO:0005615;GO:0004500;GO:0031410;GO:0016021;GO:0016020;GO:0008542;GO:0005507;GO:0006589;GO:0007626;GO:0004497;GO:0030658;GO:0003824;GO:0008306;GO:0042584;GO:0042309;GO:0007268;GO:0002443;GO:0055114;GO:0043231;GO:0048265;GO:0042711;GO:0120162;GO:0046872;GO:0016715;GO:0001975;GO:0001816;GO:0001974;GO:0005576;GO:0034466 | hsa:1621 |  | dopamine beta-hydroxylase precursor [Homo sapiens] |
| P04180 | 19673.87158 | 18607.13326 | 0.945778933 | -0.080425089 | 0.688789878 | no | no change | GO:0034375;GO:0006656;GO:0034372;GO:0043691;GO:0008374;GO:0042158;GO:0090107;GO:0070062;GO:0016740;GO:0016746;GO:0034435;GO:0008203;GO:0006644;GO:0034364;GO:0006629;GO:0042632;GO:0030301;GO:0008202;GO:0005615;GO:0005576;GO:0005515;GO:0034186;GO:0004607;GO:0046470 | hsa:3931 |  | phosphatidylcholine-sterol acyltransferase precursor [Homo sapiens] |
| H0Y755 | 7768.903474 | 7334.283579 | 0.944056469 | -0.083054938 | 0.591646031 | no | no change | GO:0016021;GO:0016020 | hsa:2214 |  | low affinity immunoglobulin gamma Fc region receptor III-A isoform b [Homo sapiens] |
| P02775 | 43439.92563 | 40917.93421 | 0.941943008 | -0.086288322 | 0.868823199 | no | no change | GO:0042127;GO:0070098;GO:0005125;GO:0071222;GO:0008009;GO:0042742;GO:0060326;GO:0032496;GO:0061844;GO:0031091;GO:0008083;GO:0031093;GO:0002576;GO:0007186;GO:0005355;GO:0030595;GO:0030593;GO:1904724;GO:0006952;GO:0006954;GO:0006955;GO:0045236;GO:0005615;GO:0090023;GO:0010469;GO:0031640;GO:0043312;GO:0006935;GO:0051781;GO:0005576;GO:1904659;GO:0005515 | hsa:5473 |  | platelet basic protein preproprotein [Homo sapiens] |
| P15814 | 11435450.37 | 10754431.68 | 0.940446711 | -0.088581897 | 0.503725643 | no | no change | GO:0009897;GO:0050853;GO:0045087;GO:0016020;GO:0006910;GO:0006911;GO:0050900;GO:0005576;GO:0042742;GO:0003823;GO:0006955;GO:0042571;GO:0006958;GO:0050871;GO:0034987;GO:0072562 | hsa:3543 |  | immunoglobulin lambda-like polypeptide 1 isoform a precursor [Homo sapiens] |
| A0A0C4DH67 | 32356.6928 | 30398.61047 | 0.939484473 | -0.090058777 | 0.703647427 | no | no change | GO:0016020;GO:0002376;GO:0005576;GO:0002250;GO:0003823;GO:0005886 | | | Ig kappa V-region e, partial [Homo sapiens] |
| A0A0A0MS51 | 291283.5526 | 273651.6684 | 0.939468315 | -0.090083589 | 0.300969903 | no | no change | GO:0051127;GO:0015629;GO:1902174;GO:0030155;GO:0030041;GO:0042989;GO:0045159;GO:1990000;GO:0005925;GO:1903923;GO:0001726;GO:0071801;GO:0005615;GO:0045010;GO:1903903;GO:0043209;GO:0005634;GO:1903909;GO:0030478;GO:0005509;GO:0048471;GO:1903906;GO:0097284;GO:2001269;GO:0060271;GO:0030027;GO:0006915;GO:0051016;GO:0051015;GO:0051014;GO:0045471;GO:0045335;GO:0002102;GO:0005886;GO:0014003;GO:0032991;GO:0006911;GO:0048015;GO:0051693;GO:0046597;GO:0090527;GO:0007568;GO:0051593;GO:0005829;GO:0071346;GO:0042246;GO:0031648;GO:0016528;GO:0014891;GO:0097017;GO:0071276 | hsa:2934 |  | gelsolin isoform d [Homo sapiens] |
| P05160 | 26841.95632 | 25199.30632 | 0.938802896 | -0.091105803 | 0.318435373 | no | no change | GO:0072378;GO:0005576;GO:0007596;GO:0007599;GO:1903363 | hsa:2165 |  | coagulation factor XIII B chain precursor [Homo sapiens] |
| Q03591 | 171000.5442 | 159912.5561 | 0.935158171 | -0.096717695 | 0.507457916 | no | no change | GO:0032091;GO:0030449;GO:0005615;GO:0072562;GO:0045919;GO:0005576;GO:0006956;GO:0005515;GO:0032991;GO:0046982;GO:0042803 | hsa:3078 |  | complement factor H-related protein 1 precursor [Homo sapiens] |
| P01011 | 768373.9316 | 718008.5 | 0.934451926 | -0.097807651 | 0.201738203 | no | no change | GO:0006954;GO:0034774;GO:0035578;GO:0003677;GO:0070062;GO:0031093;GO:0002576;GO:0072562;GO:0010466;GO:0019216;GO:0006953;GO:0062023;GO:0030277;GO:0030414;GO:0004867;GO:0010951;GO:0005615;GO:0043312;GO:0005622;GO:0005576;GO:0005515;GO:0005634 | hsa:12 | COG4826 | serpin peptidase inhibitor, clade A (alpha-1 antiproteinase, antitrypsin), member 3, isoform CRA_b [Homo sapiens] |
| P23142 | 34465.29789 | 32198.87526 | 0.934240446 | -0.098134191 | 0.485188368 | no | no change | GO:0005201;GO:0008022;GO:0031012;GO:0007162;GO:0044877;GO:0005615;GO:0072378;GO:1900025;GO:0005576;GO:0005509;GO:2000647;GO:0042802;GO:0062023;GO:0071953;GO:0010952;GO:0070062;GO:0005604;GO:0007566;GO:0070051;GO:0005577;GO:0030198;GO:0001968;GO:0016032;GO:2000146;GO:0016504;GO:0001933;GO:0070373;GO:0007229 | hsa:2192 |  | fibulin-1 isoform D precursor [Homo sapiens] |
| A0A0G2JSC0 | 30103.95338 | 28074.66676 | 0.932590694 | -0.100684062 | 0.778944865 | no | no change |  | hsa:7441 |  | Lambda-V immunoglobulin light chain variable domain precursor, partial [Homo sapiens] |
| P04211 | 1192480.3 | 1109267.495 | 0.930218717 | -0.104358126 | 0.601528033 | no | no change | GO:0038096;GO:0004252;GO:0016020;GO:0030449;GO:0050776;GO:0006898;GO:0038095;GO:0002376;GO:0050900;GO:0005576;GO:0006956;GO:0002250;GO:0003823;GO:0005886;GO:0006508;GO:0006958;GO:0006955 | hsa:7441 |  | hCG1731877, partial [Homo sapiens] |
| P01034 | 13011.39337 | 12066.96882 | 0.927415572 | -0.108712144 | 0.334981843 | no | no change | GO:0005783;GO:0060009;GO:0048678;GO:0005788;GO:0008584;GO:0042747;GO:0030414;GO:0070301;GO:0001666;GO:0014070;GO:1904724;GO:0005737;GO:0044267;GO:0032355;GO:0042995;GO:0001540;GO:0005615;GO:0007431;GO:2000117;GO:0005771;GO:0009636;GO:0070062;GO:0043067;GO:0010466;GO:0002020;GO:0030424;GO:0048471;GO:0043025;GO:0042802;GO:0008284;GO:0034103;GO:0045740;GO:0060548;GO:0043687;GO:0006915;GO:0031965;GO:0031982;GO:0006952;GO:0001775;GO:0001654;GO:0060311;GO:0004866;GO:0060313;GO:0004869;GO:0034599;GO:0010035;GO:0006979;GO:0009743;GO:1904813;GO:0005604;GO:0007420;GO:0010716;GO:0007566;GO:0042493;GO:0031667;GO:0010711;GO:0005764;GO:0005576;GO:0005515;GO:0043312;GO:0043292;GO:0045861;GO:0097435 | hsa:1471 |  | cystatin-C precursor [Homo sapiens] |
| P25311 | 376893.9737 | 349100.7526 | 0.926257189 | -0.11051526 | 0.200600377 | no | no change | GO:0008285;GO:0090501;GO:0071806;GO:0070062;GO:0009897;GO:0001580;GO:0005615;GO:0005634;GO:0004540;GO:0008320;GO:0062023;GO:0005515;GO:0005886;GO:0006955;GO:0007155;GO:0001895;GO:0055085;GO:0005576 | hsa:563 |  | zinc-alpha-2-glycoprotein precursor [Homo sapiens] |
| C9JPQ9 | 1733016.668 | 1603108.447 | 0.925039255 | -0.112413506 | 0.510573131 | no | no change | GO:0051258;GO:0005102;GO:0007596;GO:0005577;GO:0030168 | hsa:2266 |  | hypothetical protein, partial [Homo sapiens] |
| P02649 | 2408326.895 | 2223708.632 | 0.923341693 | -0.115063463 | 0.578508737 | no | no change | GO:0051044;GO:0005783;GO:0034447;GO:0007616;GO:0005788;GO:0072358;GO:0043524;GO:0010873;GO:0051651;GO:0050728;GO:1905855;GO:0071682;GO:0016020;GO:0008201;GO:0098869;GO:0060999;GO:0045541;GO:0044794;GO:0005319;GO:0034361;GO:0034362;GO:0034363;GO:0034364;GO:0031102;GO:0016209;GO:0071813;GO:0043537;GO:0010977;GO:0030425;GO:0030516;GO:0043691;GO:0010877;GO:0010875;GO:0061771;GO:0015485;GO:0043025;GO:0019433;GO:1990777;GO:0030195;GO:0034375;GO:0034374;GO:0034372;GO:0034371;GO:1903002;GO:0000302;GO:0019934;GO:0010629;GO:0044877;GO:0042157;GO:0042311;GO:0042158;GO:0042159;GO:0048168;GO:0042982;GO:0044267;GO:0042627;GO:0072562;GO:0035641;GO:0010544;GO:0097006;GO:0007186;GO:0062023;GO:0033700;GO:0015909;GO:0006629;GO:1905890;GO:0048156;GO:1902430;GO:0042632;GO:0032489;GO:1901215;GO:0046907;GO:0034382;GO:0034380;GO:0006898;GO:0034384;GO:0006357;GO:0046983;GO:0032805;GO:0005543;GO:0090209;GO:0006869;GO:0051246;GO:0019068;GO:0050807;GO:0031012;GO:0043407;GO:0051000;GO:1902995;GO:0043083;GO:0005737;GO:0001540;GO:0005634;GO:0030669;GO:1900221;GO:0002021;GO:0090181;GO:0042802;GO:0042803;GO:0006641;GO:0006874;GO:0043687;GO:0017038;GO:0008289;GO:0055088;GO:0055089;GO:0046889;GO:0034378;GO:0006979;GO:0008202;GO:0008203;GO:0032269;GO:0070062;GO:1900272;GO:1902952;GO:0005515;GO:1901630;GO:0005794;GO:0017127;GO:0007271;GO:0060228;GO:0001523;GO:0034365;GO:0005615;GO:0048844;GO:0005198;GO:0033344;GO:1901628;GO:0010468;GO:0032462;GO:0090090;GO:0006707;GO:0070326;GO:0046911;GO:0005886;GO:2000822;GO:1903561;GO:0007263;GO:1905908;GO:0050750;GO:0007010;GO:1905860;GO:0097114;GO:0005576;GO:1905906;GO:0097113;GO:0045807;GO:0098978;GO:0070328;GO:0005769;GO:0001937;GO:0010976 | hsa:348 |  | apolipoprotein E [Homo sapiens] |
| P01031 | 225098.3579 | 207439.2947 | 0.921549569 | -0.117866326 | 0.307894747 | no | no change | GO:0000187;GO:0030449;GO:0006954;GO:0008009;GO:0007166;GO:0060326;GO:0019835;GO:0001701;GO:0005615;GO:0045766;GO:0010760;GO:0007186;GO:0045087;GO:0006935;GO:0090197;GO:0006956;GO:0006957;GO:0004866;GO:0010951;GO:0006958;GO:0070062;GO:0010575;GO:0002376;GO:0005576;GO:0005515;GO:0005102;GO:0005579 | hsa:727 |  | complement C5 isoform 1 preproprotein [Homo sapiens] |
| P07737 | 11005.68311 | 10121.49626 | 0.919660885 | -0.120826114 | 0.702648286 | no | no change | GO:0017048;GO:0050821;GO:0060074;GO:0045202;GO:0050434;GO:0005925;GO:0005737;GO:0032232;GO:0032233;GO:0070062;GO:0030837;GO:0030838;GO:0051496;GO:0005634;GO:0016020;GO:0005546;GO:0003779;GO:0098794;GO:0043005;GO:0098793;GO:0001843;GO:0005856;GO:0051054;GO:0098685;GO:0005515;GO:0032781;GO:0060071;GO:0098688;GO:0070064;GO:0005938;GO:0000774;GO:0010033;GO:0010634;GO:0071363;GO:0005829;GO:1900029;GO:0098885;GO:0045296;GO:0051497;GO:0006357;GO:0045944;GO:0003723;GO:0030036;GO:0072562;GO:0005102;GO:0098978;GO:0003785 | hsa:5216 |  | PREDICTED: profilin-1 isoform X2 [Nomascus leucogenys] |
| J3KRP0 | 29145.70421 | 26790.15789 | 0.919180326 | -0.121580176 | 0.534368062 | no | no change | GO:0016787;GO:0032268;GO:0005829;GO:0004180;GO:0016805;GO:0005576;GO:0008152;GO:0006508;GO:0046872;GO:0008237 | hsa:84735 | COG0624 | Carnosine dipeptidase 1 (metallopeptidase M20 family) [Homo sapiens] |
| P00915 | 22432.51958 | 20567.45463 | 0.916858874 | -0.125228409 | 0.538246102 | no | no change | GO:0005737;GO:0015701;GO:0046872;GO:0070062;GO:0006730;GO:0016829;GO:0008270;GO:0005515;GO:0016836;GO:0004089;GO:0004064;GO:0005829;GO:0035722 | hsa:759 | COG3338 | carbonic anhydrase 1 isoform a [Homo sapiens] |
| P02671 | 6288507.053 | 5765624.316 | 0.916851053 | -0.125240714 | 0.265464312 | no | no change | GO:0045087;GO:0009897;GO:0045907;GO:0007599;GO:0034116;GO:0007596;GO:0005788;GO:0007160;GO:0045921;GO:0045202;GO:0034622;GO:1902042;GO:0044267;GO:0050839;GO:0031091;GO:0005615;GO:0031093;GO:1900026;GO:0072378;GO:0002576;GO:0031639;GO:0072562;GO:0005198;GO:0065003;GO:0030198;GO:0002224;GO:0046872;GO:0043687;GO:0009986;GO:2000352;GO:0051592;GO:0090277;GO:0005938;GO:0005886;GO:1903561;GO:0030168;GO:0070062;GO:0051258;GO:0042730;GO:0072377;GO:0050714;GO:0002376;GO:0005577;GO:0005576;GO:0002250;GO:0005515;GO:0043152;GO:0005102;GO:0070374;GO:0070527 | hsa:2243 |  | fibrinogen alpha chain isoform alpha-E preproprotein [Homo sapiens] |
| Q86UX7 | 5055.186455 | 4622.435408 | 0.914394642 | -0.129111145 | 0.699648695 | no | no change | GO:0005178;GO:0030335;GO:0002102;GO:0070062;GO:0031093;GO:0034446;GO:0007229;GO:0016020;GO:0033632;GO:0005576;GO:0007159;GO:0007155;GO:0033622;GO:0042995;GO:0070527;GO:0030054;GO:0002576 | hsa:83706 |  | fermitin family homolog 3 long isoform [Homo sapiens] |
| Q13103 | 4606.068429 | 4202.4675 | 0.912376263 | -0.132299182 | 0.56983099 | no | no change | GO:0044267;GO:0002576;GO:0005788;GO:0031089;GO:0001501;GO:0005576;GO:0046849;GO:0004866;GO:0010951;GO:0043687;GO:0062023 | hsa:6694 |  | secreted phosphoprotein 24 precursor [Homo sapiens] |
| Q96PD5 | 214713.8632 | 195693.1421 | 0.911413633 | -0.133822144 | 0.182943277 | no | no change | GO:0019730;GO:0008745;GO:0032827;GO:0009253;GO:0008270;GO:0044117;GO:0005615;GO:0016045;GO:0016020;GO:0050727;GO:0046872;GO:0002221;GO:0016787;GO:0045087;GO:0050830;GO:0001519;GO:0070062;GO:0005622;GO:0002376;GO:0005576;GO:0032689;GO:0016019;GO:0042834 | hsa:114770 |  | N-acetylmuramoyl-L-alanine amidase isoform 1 precursor [Homo sapiens] |
| P05109 | 9250.794133 | 8420.225188 | 0.910216471 | -0.135718402 | 0.706927176 | no | no change | GO:0045087;GO:0019730;GO:0030307;GO:0032602;GO:0043312;GO:0050786;GO:0008270;GO:0014002;GO:0010043;GO:0032496;GO:0005737;GO:0001816;GO:0045111;GO:0050729;GO:0005615;GO:0005634;GO:0016020;GO:0051493;GO:0050727;GO:0005509;GO:0002224;GO:2001244;GO:0034774;GO:0046872;GO:0035662;GO:0008017;GO:0005856;GO:0030593;GO:0006935;GO:0006919;GO:0032119;GO:0006914;GO:0006915;GO:0018119;GO:0042060;GO:0050832;GO:0045471;GO:0070488;GO:0006954;GO:0005886;GO:0002526;GO:0002523;GO:0070062;GO:0051092;GO:0050544;GO:0005829;GO:0002793;GO:0002544;GO:0002376;GO:0005576;GO:0005515;GO:0042742 | hsa:6279 |  | protein S100-A8 isoform d [Homo sapiens] |
| P36955 | 119127.5616 | 107954.0368 | 0.906205377 | -0.142090044 | 0.186922338 | no | no change | GO:0007614;GO:0030424;GO:0010629;GO:0060041;GO:0060770;GO:0007275;GO:0043203;GO:0005615;GO:0071300;GO:0010447;GO:0001822;GO:0050769;GO:0071333;GO:0048471;GO:0046685;GO:0043025;GO:0071279;GO:0008283;GO:0050728;GO:0062023;GO:0010976;GO:0004867;GO:0010951;GO:1901215;GO:0070062;GO:0042470;GO:0071549;GO:0005604;GO:0007568;GO:0010596;GO:0042698;GO:0005576;GO:0005515;GO:0016525 | hsa:5176 | COG4826 | pigment epithelium-derived factor isoform 1 precursor [Homo sapiens] |
| P35858 | 58197.45158 | 52673.09368 | 0.905075605 | -0.143889783 | 0.241379322 | no | no change | GO:0044267;GO:0005615;GO:0031012;GO:0005654;GO:0007155;GO:0005576;GO:0007165;GO:0042567;GO:0005520;GO:0070062 | hsa:3483 | COG4886 | Insulin-like growth factor binding protein, acid labile subunit [Homo sapiens] |
| P01743 | 17658.08526 | 15975.95005 | 0.904738527 | -0.144427187 | 0.531135481 | no | no change | GO:0038096;GO:0004252;GO:0016020;GO:0030449;GO:0050776;GO:0006898;GO:0038095;GO:0002376;GO:0050900;GO:0005576;GO:0006956;GO:0002250;GO:0003823;GO:0005886;GO:0006508;GO:0006958;GO:0006955 | hsa:102723407 | | IgM heavy chain VH1 region precursor, partial [Homo sapiens] |
| Q06033 | 41468.19842 | 37503.02105 | 0.904380284 | -0.144998554 | 0.299509374 | no | no change | GO:0030212;GO:0070062;GO:0002576;GO:0031089;GO:0005576;GO:0030414;GO:0004866;GO:0004867;GO:0010951;GO:0010466 | hsa:3699 | COG2304 | inter-alpha-trypsin inhibitor heavy chain H3 preproprotein [Homo sapiens] |
| G3XAP6 | 10099.62869 | 9133.701 | 0.904360079 | -0.145030786 | 0.372174077 | no | no change | GO:0005201;GO:0060173;GO:0062023;GO:0030509;GO:0035264;GO:0050905;GO:0030500;GO:0030282;GO:0050881;GO:0010259;GO:1900047;GO:0005615;GO:0048844;GO:0036122;GO:0030198;GO:0005509;GO:0002020;GO:0002063;GO:0003417;GO:0043588;GO:0005576;GO:0014829;GO:0005178;GO:0009887;GO:1902732;GO:0009306;GO:0006915;GO:0043066;GO:0048747;GO:0006986;GO:0043395;GO:0035988;GO:0007155;GO:0097084;GO:0016485;GO:0031012;GO:0008201;GO:0070062;GO:0010260;GO:0032991;GO:0001501;GO:0035989;GO:0030199;GO:0043394;GO:0005518;GO:0070527 | hsa:1311 |  | unnamed protein product [Homo sapiens] |
| A0A0B4J1U7 | 59076.67632 | 53333.70684 | 0.902787871 | -0.147541059 | 0.621303361 | no | no change | GO:0009897;GO:0050853;GO:0045087;GO:0016020;GO:0006910;GO:0006911;GO:0002376;GO:0005576;GO:0002250;GO:0003823;GO:0005886;GO:0072562;GO:0042571;GO:0006958;GO:0050871;GO:0034987;GO:0042742 | hsa:102723407 | | RecName: Full=Immunoglobulin heavy variable 6-1; Flags: Precursor |
| P05155 | 346645.2684 | 312577.7684 | 0.901722299 | -0.149244896 | 0.073536084 | no | no change | GO:0030449;GO:0007597;GO:0007596;GO:0007599;GO:0005615;GO:0031093;GO:0002576;GO:0072562;GO:0010466;GO:0001869;GO:0045916;GO:0008015;GO:0045087;GO:0030414;GO:0004867;GO:0010951;GO:0006958;GO:0070062;GO:0042730;GO:0007568;GO:0002376;GO:0005576;GO:0005515;GO:0030193 | hsa:710 | COG4826 | unnamed protein product [Homo sapiens] |
| P35527 | 98069.04789 | 88404.13842 | 0.901447912 | -0.149683964 | 0.516608195 | no | no change | GO:0005200;GO:0045109;GO:0031424;GO:0005615;GO:0005829;GO:0016020;GO:0070268;GO:0005198;GO:0007283;GO:0005882;GO:0008544;GO:0005634;GO:0043588;GO:0070062 | hsa:3857 |  | keratin, type I cytoskeletal 9 [Homo sapiens] |
| Q96KN2 | 6516.0505 | 5848.279083 | 0.897518993 | -0.155985626 | 0.446816747 | no | no change | GO:0016787;GO:0032268;GO:0005829;GO:0004180;GO:0016805;GO:0005576;GO:0008152;GO:0006508;GO:0008233;GO:0046872;GO:0008237 | hsa:84735 | COG0624 | RecName: Full=Beta-Ala-His dipeptidase; AltName: Full=CNDP dipeptidase 1; AltName: Full=Carnosine dipeptidase 1; AltName: Full=Glutamate carboxypeptidase-like protein 2; AltName: Full=Serum carnosinase; Flags: Precursor |
| P01009 | 5993319.158 | 5372299.105 | 0.896381281 | -0.157815573 | 0.099700955 | no | no change | GO:0048208;GO:0007599;GO:0005783;GO:0007596;GO:0033116;GO:0005788;GO:1904813;GO:0030134;GO:0044267;GO:0006888;GO:0005615;GO:0031093;GO:0002576;GO:0010466;GO:0002020;GO:0042802;GO:0005794;GO:0043687;GO:0006953;GO:0030414;GO:0004867;GO:0010951;GO:0070062;GO:0043231;GO:0043312;GO:0000139;GO:0005576;GO:0005515 | hsa:5265 | COG4826 | alpha-1-antitrypsin precursor [Homo sapiens] |
| P02743 | 440698.6632 | 394677.8789 | 0.895573125 | -0.159116859 | 0.245161746 | no | no change | GO:0006457;GO:0030246;GO:0046597;GO:0044871;GO:0072562;GO:0061045;GO:0044267;GO:0070062;GO:0005615;GO:0005634;GO:0051082;GO:0001849;GO:0005509;GO:0030169;GO:0042802;GO:1903016;GO:0051131;GO:0045087;GO:1903019;GO:0006953;GO:0062023;GO:0044869;GO:0006958;GO:0002674;GO:0046872;GO:0045656;GO:0046790;GO:0005576;GO:0048525 | hsa:325 |  | serum amyloid P-component precursor [Homo sapiens] |
| P07358 | 44602.62263 | 39937.20684 | 0.895400416 | -0.159395107 | 0.091239243 | no | no change | GO:0019835;GO:0030449;GO:0005615;GO:0045087;GO:0016020;GO:0002376;GO:0005576;GO:0006956;GO:0006957;GO:0006955;GO:0044877;GO:0006958;GO:1903561;GO:0005579;GO:0070062 | hsa:732 |  | RecName: Full=Complement component C8 beta chain; AltName: Full=Complement component 8 subunit beta; Flags: Precursor |
| P02675 | 14715519.89 | 13156373.95 | 0.894047512 | -0.161576593 | 0.145146644 | no | no change | GO:1902042;GO:0034116;GO:0045921;GO:0007599;GO:0005783;GO:0009897;GO:0007596;GO:0007160;GO:0045907;GO:0045202;GO:0034622;GO:0044320;GO:0005737;GO:1903561;GO:0031091;GO:0005615;GO:0031093;GO:1900026;GO:0072378;GO:0002576;GO:0051087;GO:0031639;GO:0072562;GO:0005198;GO:0030198;GO:0002224;GO:0045087;GO:0009986;GO:2000352;GO:0051592;GO:0090277;GO:0005938;GO:0005886;GO:0050839;GO:0030168;GO:0070062;GO:0051258;GO:0042730;GO:0050714;GO:0071347;GO:0002376;GO:0005577;GO:0005576;GO:0002250;GO:0005515;GO:0043152;GO:0005102;GO:0070374;GO:0070527 | hsa:2244 |  | fibrinogen beta chain isoform 1 preproprotein [Homo sapiens] |
| P02790 | 3115692.421 | 2781504.737 | 0.892740477 | -0.163687256 | 0.050314502 | no | no change | GO:0020027;GO:0002925;GO:0015232;GO:0071682;GO:0005615;GO:0051246;GO:0042531;GO:0072562;GO:0046872;GO:0042168;GO:0006879;GO:0016032;GO:0060332;GO:0062023;GO:0060335;GO:0002639;GO:0070062;GO:0015886;GO:0006898;GO:0005623;GO:0005576;GO:0005515 | hsa:3263 |  | hemopexin precursor [Homo sapiens] |
| P48740 | 21827.52947 | 19459.41895 | 0.891508083 | -0.165680217 | 0.201274256 | no | no change | GO:0004252;GO:0006898;GO:0006956;GO:0005615;GO:0016787;GO:0048306;GO:0005829;GO:0005654;GO:0002376;GO:0046872;GO:0005576;GO:0001867;GO:0005509;GO:0005515;GO:0006508;GO:0008233;GO:0042803;GO:0045087;GO:0008236 | hsa:5648 | COG5640 | mannan-binding lectin serine protease 1 isoform 1 precursor [Homo sapiens] |
| P00918 | 10796.67714 | 9617.187471 | 0.890754381 | -0.16690042 | 0.381197945 | no | no change | 3P3J:A;1IF6:A;1H9N:A;3RYJ:B;5FLQ:A;3P5A:A;1AVN:A;GO:0010043;6BC9:A;3V3H:B;4Q78:A;5JGS:B;5M78:A;1CAM:A;GO:0043209;5TY9:A;3RZ1:B;5EOI:A;5JN3:A;1CNI:A;4ZWY:A;3PJJ:A;3TMJ:A;5LL4:A;4CAC:A;1CNK:A;3T5U:A;6EQU:A;5EH8:A;1G52:A;5JEG:B;1AM6:A;3SBH:A;1CVD:A;4BF1:A;1BNU:A;1I91:A;4PYY:A;3MHL:A;4HEY:A;5FLO:A;5NXP:A;3S78:B;4Q08:A;4K1Q:A;2O4Z:A;2Q38:A;3DD8:A;5CAC:A;4YYT:A;2VVB:X;1CNC:A;2FNN:A;5G0C:A;1CVB:A;1FR7:B;2FOQ:A;3RZ7:A;4KNI:A;6H2Z:A;4QTL:A;4QEF:A;4FVO:A;6BCC:A;3M1Q:A;5LLG:A;6E92:A;3T83:A;5WEX:A;3D8W:A;4YXI:A;5WLV:A;4Q6D:A;5JQT:A;4YXO:A;5TFX:A;2HKK:A;2POU:A;3M1W:A;6MBY:A;5DOH:B;5OGO:A;4QSI:A;1BNV:A;3DBU:A;1IF9:A;3PYK:A;1CAK:A;1G46:A;6D1L:A;5TY8:A;5DRS:A;5E28:A;4Q9Y:A;5LLC:A;6FJI:A;3MNH:A;1I9P:A;1CIL:A;4FIK:A;6BBS:A;3OIL:A;1LG6:A;4BF6:A;6IC2:A;6C7W:A;1CIN:A;1G4O:A;1BNM:A;4YGL:A;4HF3:A;2CBE:A;3KOK:A;GO:0016829;4MDG:A;3HS4:A;5DSM:A;4K0Z:A;2F14:A;5FNL:A;4JSW:A;GO:0045177;5BRU:A;5DSO:A;3DVC:A;2WD2:A;3RG3:A;4KUV:A;4HEZ:A;5LL4:B;1I9M:A;1RZD:A;5DSL:A;3B4F:A;1TE3:X;5BNL:A;4YGK:A;GO:0016323;5U0G:A;5LMD:A;5CA2:A;4DZ9:A;5SZ1:A;1ZSB:A;1YDB:A;1YO0:A;4E49:A;4YVY:A;4KV0:A;2X7S:A;5THN:A;4KUW:A;1YDD:A;1YO2:A;1I8Z:A;2X7U:A;2CBC:A;5JQ0:A;4QSA:A;3S74:B;3P58:A;3M67:A;3IGP:A;3S76:A;3EFT:A;5TYA:A;3IEO:A;4JSZ:A;3V7X:A;5N24:A;3HKU:A;3KKX:A;5ZXW:A;1BN3:A;2EZ7:A;5JDV:B;5FLT:A;4E4A:A;GO:0030424;1CVH:A;GO:0004064;1XEG:A;5G03:A;2AW1:A;3F8E:A;1RAZ:A;5W8B:A;5G01:A;5N1R:A;5L70:B;2ABE:A;2EU3:A;5L70:A;3IBU:A;GO:0005615;6EDA:A;2GEH:A;5N1S:A;4Q7W:A;1CAY:A;5EKM:A;3D93:A;3M2Z:A;5MJN:A;1ZFQ:A;2NNO:A;GO:0038166;3R17:B;3S9T:A;1BV3:A;5VGY:A;4IWZ:A;1G3Z:A;5DSP:A;4Z0Q:A;GO:0045780;1XPZ:A;3RZ5:A;1UGD:A;GO:0016020;5AMD:A;1RAY:A;4M2U:A;1UGF:A;3DV7:A;3S75:B;1RZE:A;4PXX:A;1BNT:A;5DOG:A;3P4V:A;2WEG:A;6EBE:A;3KS3:A;5WLR:A;5SZ2:A;1KWQ:A;1CIM:A;1CNG:A;5AML:A;2NNV:A;GO:0005829;1CCS:A;5OGP:A;GO:0043627;1CVF:A;1TTM:A;4FVN:A;4JSA:A;1TBT:X;1FSQ:A;5SZ3:A;4RFC:A;1FSQ:B;3MNJ:A;2NWP:A;5FNJ:A;1OKM:A;5LLE:A;4ITP:A;2WD3:A;3K7K:A;1G4J:A;2H4N:A;4HBA:A;1ZH9:A;4R5B:A;5T71:A;6ECZ:A;5L6T:A;5L6T:B;2AX2:A;3P44:A;5TI0:A;GO:2001225;2QOA:A;6CA2:A;4G0C:A;1CA2:A;1CAO:A;3V3F:A;4Z1K:A;1FR7:A;4QK3:A;1CAI:A;5N0E:A;4PQ7:A;3HKN:A;1RZC:A;1ZGF:A;4QK1:A;5GMN:A;4E3D:A;1CNW:A;5ULN:A;GO:0048545;2FOV:A;4FRC:A;2FNK:A;2HD6:A;2WEO:A;1TG9:A;3M40:A;5BRV:A;4Q7P:A;4Q8Z:A;2QO8:A;5DSK:A;4RIV:A;3KOI:A;5Y2S:A;4Q8X:A;3HLJ:A;1LZV:A;1G54:A;5C8I:A;3L14:A;3N2P:A;3OYQ:A;3CAJ:A;2POV:A;6EEO:A;4WL4:A;6B4D:A;5YUJ:A;4FPT:A;3GZ0:A;5NXV:A;1OQ5:A;6GOT:A;5J8Z:A;3ZP9:A;4RUY:A;6B5A:A;2NWZ:A;1I9O:A;1THK:A;2EU2:A;5SZ7:A;4L5W:A;4M2W:A;6H6S:A;5JMZ:A;4Q7V:A;2Q1Q:A;4HT0:A;4QSB:A;4QJM:A;1BNN:A;2NXS:A;GO:0005515;3M1K:A;4Q99:A;3M2X:A;1H9Q:A;6G6T:A;4MDM:A;1FQM:A;5FDC:A;1H4N:A;5SZ5:A;4Q6E:A;3SAP:A;1EOU:A;1TEQ:X;5FLP:A;5T75:A;5NXI:A;3K34:A;2HOC:A;6CJV:A;6FJJ:A;1CNJ:A;3P5L:A;1G53:A;3V5G:A;4MDL:A;1ZSC:A;6HX5:A;4XE1:A;1ZSA:A;1TG3:A;6GXE:A;5JEH:B;4MO8:A;2X7T:A;4CA2:A;1IF7:A;6CEH:A;4QY3:A;GO:0046903;1LGD:A;4Q09:A;2CBD:A;1IF5:A;1CNB:A;3FFP:X;3DCW:A;5NXO:A;5LL8:A;3RYY:A;4Z1E:A;3R16:A;3BET:A;GO:0032849;5JEP:B;5G0B:A;4ZAO:A;3ML2:A;1BCD:A;3TVN:X;4Y0J:A;5NXW:A;6EEA:A;1BN4:A;1UGB:A;4K13:A;5DOH:A;3C7P:A;4RFD:A;GO:0001822;1TB0:X;5SZ4:A;5DSQ:A;GO:0046872;5L6K:B;3N3J:A;4Q07:A;3T84:A;1IF8:A;12CA:A;1DCA:A;4QK2:A;5E2K:A;4Q83:A;3SAX:A;5LVS:B;5T74:A;1CAZ:A;6D1M:A;6GXB:A;3RYZ:A;5LVS:A;2FMZ:A;3IBI:A;2QP6:A;1I9Q:A;4E5Q:A;3OIM:A;5NEA:A;6B59:A;6H29:A;2FOU:A;3M5T:A;5THI:A;4GL1:X;4Q87:A;2WEJ:A;1G0F:A;GO:2001150;6H34:A;6G3Q:A;3CYU:A;5O07:A;6QEB:A;3RGE:A;5E2R:A;5YUK:A;5EH5:A;4MLT:A;5EIJ:A;3RZ0:B;5FLS:A;3NB5:A;5EH7:A;1MOO:A;3U3A:X;4Z1J:A;3SBI:A;4Q90:A;2VVA:X;1I90:A;1XQ0:A;5NXG:A;1ZFK:A;3MHO:A;5FNI:A;6H6S:B;2OSM:A;3VBD:A;2FNM:A;3MHM:A;4K0S:A;3MNU:A;5EHV:A;5FNG:A;4E3G:A;3RZ8:A;4YWP:A;5THJ:A;1CRA:A;1RZA:A;2NNS:A;1FR4:A;3T5Z:A;1F2W:A;1CVC:A;3RG4:A;5TH4:A;3PO6:A;5WLU:A;5JN7:A;6E91:A;4ZX0:A;1Z9Y:A;5JN1:A;1HVA:A;GO:0009268;3NI5:A;5A6H:A;3S77:B;GO:0005737;3S71:B;4Q49:A;4ILX:A;3HKT:A;5U0F:A;1BIC:A;3OYS:A;3M96:A;2FMG:A;3KIG:A;5FLR:A;5OGN:B;6GCY:A;2NXT:A;1CAH:A;5SZ0:A;4E3H:A;4ITO:A;4QIY:C;4QIY:B;4QIY:A;4R5A:A;3DCC:A;1CNX:A;1CAJ:A;4MLX:A;2Q1B:A;3M04:A;4L5U:A;1ZGE:A;1LG5:A;5FDI:A;GO:0070062;3D92:A;2H15:A;3MZC:A;3F4X:A;1FQL:A;8CA2:A;5JG3:B;1UGA:A;5E2S:A;4LHI:A;5NY1:A;3M14:A;5LJT:A;3IBN:A;3P55:A;3V3G:B;1CVA:A;3D9Z:A;1HCA:A;5NXM:A;1BNQ:A;GO:0044070;3DVB:A;5JGT:B;4YGN:A;1UGG:A;3CA2:A;1BNW:A;3V2J:A;GO:0010033;1YDA:A;1I9L:A;4ZWZ:A;3RJ7:A;1YDC:A;3DAZ:A;6EEH:A;5WLT:A;6E8P:A;4M2R:A;5L6K:A;1TEU:X;3DCS:A;2CBB:A;3BL0:A;1FSR:B;1FSR:A;1LUG:A;2OSF:A;2ILI:A;1MUA:A;4N16:A;2GD8:A;3MMF:A;5U0D:A;4E3F:A;2HL4:A;3OKV:A;1ZE8:A;5TY1:A;GO:0008270;5JE7:B;3DC9:A;3V3J:A;3S73:B;GO:0032230;3M5S:A;1T9N:A;4Q06:A;1CCU:A;5AMG:A;3MWO:B;7CA2:A;3MWO:A;5T72:A;GO:0004089;5BYI:A;3DC3:A;1CNH:A;2NNG:A;1CA3:A;5EKH:A;5WG7:A;3OY0:A;5NY3:A;1FQR:A;1HEC:A;9CA2:A;2FOS:A;5EKJ:A;3V2M:A;3N4B:A;1HEA:A;2NWO:A;5NEE:A;3K2F:A;6E8X:A;3M2Y:A;3P3H:A;3U7C:A;4FL7:A;4Q81:A;4WW6:A;GO:0015701;3RYV:B;3S8X:A;5U0E:A;GO:0051453;3EFI:A;4RH2:A;5EHW:A;2WEH:A;4Q8Y:A;2POW:A;4Z1N:A;1HEB:A;4RIU:A;1CAL:A;5FNM:A;1I9N:A;4ZWX:A;1G1D:A;1KWR:A;5TXY:A;3MHI:A;5LLH:A;1CCT:A;4RN4:A;3KWA:A;4CQ0:A;4YXU:A;1CVE:A;5SZ6:A;4KAP:A;3QYK:A;4PYX:A;4YGJ:A;2NWY:A;1FSN:B;1FSN:A;4HEW:A;3V3I:B;4N0X:B;1YO1:A;4KNJ:A;3MNK:A;3HFP:A;4FU5:A;5BRW:A;GO:0005886;1G0E:A;1OKN:A;5N25:A;5CLU:A;4JS6:A;GO:0005902;4PZH:A;1OKL:A;5L9E:C;3M2N:A;5LJQ:A;3T82:A;1FQN:A;6MBV:A;4RUZ:A;4DZ7:A;5DSR:A;1A42:A;4RUX:A;3MNA:A;5L3O:B;1CNY:A;4ZWI:A;1CAN:A;3U45:X;3OIK:A;4KUY:A;5NY6:A;3U47:A;5YUI:A;2CBA:A;5N0D:A;5FNH:A;6H33:A;4K0T:A;3N0N:A;3RLD:A;3BL1:A;4ZX1:A;5UMC:A;1RZB:A;3M5E:A;5OGN:A;4LP6:B;4LP6:A;1G45:A;3MNI:A;6GDC:A;3RYX:B;GO:0015670;4YX4:A;1IF4:A;5JG5:B;4JSS:A;3DVD:A;4BCW:A;5WGP:A;5Y2R:A;3TVO:X;3IBL:A;6C7X:A;GO:0002009;3DD0:A;3S72:B;3M3X:A;1UGC:A;4R59:A;3HKQ:A;1UGE:A;3T85:A;4QIY:D;3MYQ:A;4M2V:A;1G48:A;GO:0071498;1XEV:B;2HNC:A;5JES:B;4L5V:A;3NJ9:A;1TH9:A;5L9E:A;1DCB:A;3M98:A;5L9E:B;5L3O:A;5L9E:D;5FNK:A;2CA2:A;GO:0042475;4IDR:X;3KNE:A;5NYA:A;3M1J:A;1HED:A;3MHC:A;2NXR:A;GO:0045672;4Q7S:A;3OKU:A;3KON:A;1XEV:C;5EHE:A;1XEV:A;4MTY:A;1XEV:D;1BN1:A | hsa:760 | COG3338 | carbonic anhydrase 2 isoform 1 [Homo sapiens] |
| P00748 | 68588.26368 | 60677.42474 | 0.884661916 | -0.176801878 | 0.336813211 | no | no change | GO:0004252;GO:0002542;GO:0007599;GO:0007597;GO:0007596;GO:0008233;GO:0008236;GO:0070062;GO:0031638;GO:0005509;GO:0006508;GO:0051919;GO:0016787;GO:0005791;GO:0045087;GO:0062023;GO:0005886;GO:0016485;GO:0005615;GO:0042730;GO:0016540;GO:0002353;GO:0005576;GO:0051787;GO:0010756;GO:0005515;GO:0030193;GO:0051788;GO:0030194 | hsa:2161 | COG5640 | coagulation factor XII preproprotein [Homo sapiens] |
| P18428 | 17813.44737 | 15726.31684 | 0.882833991 | -0.179785917 | 0.299130645 | no | no change | GO:0006968;GO:0071222;GO:0071223;GO:0006869;GO:0042742;GO:0050829;GO:0032496;GO:0032490;GO:0044130;GO:0019221;GO:0060265;GO:0002281;GO:0070062;GO:0090023;GO:0042535;GO:0016020;GO:0071723;GO:0045919;GO:0002224;GO:0033036;GO:0032722;GO:0009986;GO:0032720;GO:0032760;GO:0015920;GO:0045087;GO:0008289;GO:0006953;GO:0008228;GO:0070891;GO:0034142;GO:0001530;GO:0005615;GO:0034145;GO:0031663;GO:0050830;GO:0002376;GO:0002232;GO:0005576;GO:0005515;GO:0043032;GO:0005102;GO:0032757;GO:0032755 | hsa:3929 |  | lipopolysaccharide-binding protein precursor [Homo sapiens] |
| A0A087X0Q4 | 130877.2211 | 115433.0263 | 0.881994784 | -0.181157971 | 0.441183281 | no | no change |  |  |  | RecName: Full=Immunoglobulin kappa variable 2D-40; AltName: Full=Ig kappa chain V-II region Cum; Flags: Precursor |
| P02763 | 2270806.158 | 2000338.158 | 0.8808934 | -0.18296065 | 0.187183599 | no | no change | GO:1904469;GO:0050716;GO:0031093;GO:0005615;GO:0002576;GO:0050718;GO:0043312;GO:0072562;GO:0006953;GO:0032715;GO:0062023;GO:0002682;GO:0005515;GO:0006954;GO:0035580;GO:0005576;GO:1904724;GO:0032720;GO:0070062 | hsa:5004 |  | RecName: Full=Alpha-1-acid glycoprotein 1; Short=AGP 1; AltName: Full=Orosomucoid-1; Short=OMD 1; Flags: Precursor |
| A0A286YEY4 | 733018.9684 | 644354.6316 | 0.879042234 | -0.185995613 | 0.412607229 | no | no change | GO:0016021;GO:0016020 | hsa:100423062 | | unnamed protein product [Homo sapiens] |
| F5H8B0 | 6778.0535 | 5957.909684 | 0.879000097 | -0.18606477 | 0.144759918 | no | no change | GO:0004252;GO:0016787;GO:0007596;GO:0005576;GO:0005509;GO:0006508;GO:0008233;GO:0008236 | hsa:2155 | COG5640 | coagulation factor VII isoform c precursor [Homo sapiens] |
| P23083 | 681387.4784 | 598812.4874 | 0.87881346 | -0.186371129 | 0.603866397 | no | no change | GO:0038096;GO:0004252;GO:0016020;GO:0030449;GO:0050776;GO:0006898;GO:0038095;GO:0002376;GO:0050900;GO:0005576;GO:0006956;GO:0002250;GO:0003823;GO:0005886;GO:0006508;GO:0006958;GO:0006955 | hsa:102723407 | | IgM heavy chain VH1 region precursor, partial [Homo sapiens] |
| P55058 | 15843.73116 | 13904.45947 | 0.877600064 | -0.188364464 | 0.093317661 | no | no change | GO:0034375;GO:0006869;GO:0015914;GO:1990050;GO:0035627;GO:0005548;GO:0005615;GO:0019992;GO:0008525;GO:0097001;GO:0010875;GO:0030317;GO:0010189;GO:0005319;GO:0035620;GO:0034364;GO:0006629;GO:0070300;GO:0008289;GO:0031210;GO:0008429;GO:1904121;GO:0005576;GO:1901611 | hsa:5360 |  | phospholipid transfer protein, isoform CRA_c [Homo sapiens] |
| Q6EMK4 | 9798.095632 | 8586.009118 | 0.876293664 | -0.190513668 | 0.285568716 | no | no change | GO:0016020;GO:0016021;GO:0070062;GO:0010719;GO:0005615;GO:0009986;GO:0005886;GO:0045296;GO:0031012;GO:0005765;GO:0005739;GO:0071461;GO:0005515;GO:0071456;GO:0005576;GO:0030512;GO:0050431 | hsa:114990 | COG4886 | vasorin precursor [Homo sapiens] |
| H0YAC1 | 177889.1368 | 155518.0774 | 0.874241565 | -0.193896124 | 0.057852508 | no | no change | GO:0004252;GO:0004497;GO:0007597;GO:0008236;GO:0008233;GO:0016491;GO:0070062;GO:0016705;GO:0031639;GO:0031638;GO:0006508;GO:0005506;GO:0046872;GO:0022617;GO:0051919;GO:0016787;GO:0020037;GO:0005886;GO:0055114;GO:0005615;GO:0042730;GO:0002542;GO:0005576 | hsa:3818 | COG5640 | KLKB1 isoform 4, partial [Pan troglodytes] |
| A0A0B4J1X5 | 77520.55882 | 67415.87611 | 0.869651575 | -0.201490592 | 0.483771449 | no | no change | GO:0009897;GO:0050853;GO:0045087;GO:0016020;GO:0006910;GO:0006911;GO:0002376;GO:0005576;GO:0002250;GO:0003823;GO:0005886;GO:0072562;GO:0042571;GO:0006958;GO:0050871;GO:0034987;GO:0042742 | hsa:102723407 | | immunoglobulin heavy chain VH3, partial [Homo sapiens] |
| Q9Y6R7 | 47058.71 | 40889.26737 | 0.868899028 | -0.202739559 | 0.35340666 | no | no change | GO:0005576;GO:0070062;GO:0005515 | hsa:8857 |  | IgGFc-binding protein precursor [Homo sapiens] |
| P01861 | 720891.6689 | 624816.04 | 0.866726676 | -0.206350986 | 0.440527777 | no | no change | GO:0004252;GO:0030449;GO:0019221;GO:0009897;GO:0042742;GO:0034987;GO:0042571;GO:0005615;GO:0016020;GO:0072562;GO:0006508;GO:0050853;GO:0045087;GO:0006910;GO:0006911;GO:0006956;GO:0003823;GO:0005886;GO:0006958;GO:0050871;GO:0070062;GO:0038096;GO:0002376;GO:0005576;GO:0002250 | hsa:100423062 | | RecName: Full=Immunoglobulin heavy constant gamma 4; AltName: Full=Ig gamma-4 chain C region |
| E7EUT5 | 9026.696316 | 7822.728632 | 0.866621448 | -0.206526153 | 0.307450846 | no | no change | GO:0051287;GO:0050821;GO:0000226;GO:0097718;GO:0006096;GO:0061844;GO:0005737;GO:0004365;GO:0005634;GO:0005811;GO:0016620;GO:0051873;GO:0097452;GO:0042802;GO:0035605;GO:0035606;GO:0015630;GO:0008017;GO:0031965;GO:0050661;GO:0050832;GO:0051402;GO:0052501;GO:0005886;GO:0055114;GO:0043231;GO:0050715;GO:0005829;GO:0071346;GO:1990904;GO:0019828;GO:0017148;GO:0006417;GO:0006006 | hsa:2597 | COG0057 | GAPDH isoform 4 [Pan troglodytes] |
| Q96IY4 | 27470.42211 | 23595.77158 | 0.858951912 | -0.219350731 | 0.054305372 | no | no change | GO:0007599;GO:2000346;GO:0007596;GO:0008270;GO:0030449;GO:0008233;GO:0008237;GO:0005615;GO:0004181;GO:0004180;GO:0071333;GO:0006508;GO:0046872;GO:0051918;GO:0016787;GO:0009408;GO:0070062;GO:0042730;GO:0003331;GO:0097421;GO:0042493;GO:0005623;GO:0005576;GO:0010757 | hsa:1361 | COG2866 | carboxypeptidase B2 isoform 1 preproprotein [Homo sapiens] |
| P06727 | 1045892 | 898121.5842 | 0.858713504 | -0.219751215 | 0.115778531 | no | no change | GO:0034375;GO:0034445;GO:0034372;GO:0034371;GO:0006869;GO:0034380;GO:0045723;GO:0005788;GO:0034378;GO:0051006;GO:0042744;GO:0042157;GO:0042632;GO:0070328;GO:0060228;GO:0001523;GO:0035634;GO:0044267;GO:0042627;GO:0005615;GO:0006982;GO:0032374;GO:0072562;GO:0016042;GO:0033344;GO:0065005;GO:0010898;GO:0002227;GO:0009986;GO:0042802;GO:0033700;GO:0005319;GO:0034361;GO:0034364;GO:0031102;GO:0062023;GO:0016209;GO:0008289;GO:0055088;GO:0031210;GO:0007159;GO:0017127;GO:0010873;GO:0043691;GO:0008203;GO:0030300;GO:0005507;GO:0015485;GO:0070062;GO:0005829;GO:0046470;GO:0019430;GO:0005576;GO:0006695;GO:0005515;GO:0042803;GO:0005769 | hsa:337 |  | RecName: Full=Apolipoprotein A-IV; Short=Apo-AIV; Short=ApoA-IV; AltName: Full=Apolipoprotein A4; Flags: Precursor |
| P00738 | 10340270.74 | 8878679.147 | 0.858650549 | -0.219856987 | 0.232366487 | no | no change | GO:0042742;GO:0010942;GO:0005615;GO:0071682;GO:2000296;GO:0072562;GO:0098869;GO:0035580;GO:1904724;GO:0016209;GO:0006952;GO:0006953;GO:0070062;GO:0042542;GO:0051354;GO:0006898;GO:0043312;GO:0002376;GO:0030492;GO:0005576;GO:0005515;GO:0031838 | hsa:3240 | COG5640 | haptoglobin isoform 1 preproprotein [Homo sapiens] |
| P02100 | 42136.11668 | 36164.80579 | 0.858285211 | -0.220470956 | 0.288459425 | no | no change | GO:0005344;GO:0019825;GO:0020037;GO:0043177;GO:0031721;GO:0007596;GO:0051291;GO:0072562;GO:0005833;GO:0098869;GO:0042744;GO:0015671;GO:0005515;GO:0031838;GO:0046872;GO:0005829;GO:0014070 | hsa:3046 | COG1018 | hemoglobin subunit epsilon [Homo sapiens] |
| A0A0B4J1U3 | 40123.38389 | 34123.82421 | 0.850472241 | -0.233663947 | 0.270382567 | no | no change | GO:0016020;GO:0005615;GO:0006955;GO:0002376;GO:0002377;GO:0005576;GO:0002250;GO:0003823;GO:0005886 | hsa:7441 |  | RecName: Full=Immunoglobulin lambda variable 1-36; Flags: Precursor |
| P02751 | 630609.2684 | 534412.4632 | 0.847454184 | -0.238792721 | 0.281665066 | no | no change | GO:0008022;GO:0018149;GO:0019221;GO:0034446;GO:0048146;GO:0005518;GO:0005201;GO:0005788;GO:0062023;GO:0007160;GO:0007161;GO:0050900;GO:0010628;GO:0097718;GO:0001525;GO:0044267;GO:0070062;GO:0031093;GO:0002576;GO:0051087;GO:0009611;GO:0072562;GO:0008201;GO:0005576;GO:0002020;GO:0030198;GO:1904237;GO:0033622;GO:0042802;GO:0008284;GO:0005178;GO:2001202;GO:0008360;GO:0005793;GO:0016324;GO:0043687;GO:0042060;GO:0006953;GO:0043394;GO:0019899;GO:0007155;GO:0051702;GO:0010952;GO:0035987;GO:1901166;GO:0005615;GO:0052047;GO:0005604;GO:0045773;GO:0031012;GO:0005577;GO:0007044;GO:0005515;GO:0016504;GO:0005102;GO:0001932;GO:0070372 | hsa:2335 |  | fibronectin isoform 1 precursor [Homo sapiens] |
| A0A087X1J7 | 47417.00632 | 40054.36895 | 0.844725807 | -0.243444967 | 0.06963622 | no | no change | GO:0008430;GO:0005615;GO:0051289;GO:0006979;GO:0098869;GO:0055114;GO:0004601;GO:0004602;GO:0016491 | hsa:2878 | COG0386 | glutathione peroxidase 3 isoform 1 precursor [Homo sapiens] |
| A0A0J9YY99 | 222303.1363 | 187520.5705 | 0.843535425 | -0.245479438 | 0.789495904 | no | no change |  | hsa:102723407 | | immunoglobulin heavy chain VDJ region, partial [Homo sapiens] |
| A0A075B7B8 | 1150906.189 | 969976.1632 | 0.84279342 | -0.246749045 | 0.341605712 | no | no change | GO:0009897;GO:0050853;GO:0045087;GO:0006910;GO:0006911;GO:0042742;GO:0003823;GO:0034987;GO:0042571;GO:0006958;GO:0050871;GO:0072562 | hsa:102723407 | | hCG1793095, isoform CRA_a, partial [Homo sapiens] |
| A0A140T8Y3 | 10061.29975 | 8465.4935 | 0.84139164 | -0.249150611 | 0.31989776 | no | no change | GO:0005201;GO:0062023;GO:0030199;GO:0030198;GO:0031012 | hsa:7148 |  | tenascin-X isoform 1 precursor [Homo sapiens] |
| Q9HDC9 | 9404.359133 | 7890.958176 | 0.839074525 | -0.25312914 | 0.684584631 | no | no change | GO:0016844;GO:0005783;GO:0009986;GO:0016020;GO:0009058;GO:0008150;GO:0004064;GO:0016021 | hsa:57136 | COG3386 | adipocyte plasma membrane-associated protein [Homo sapiens] |
| A0A087X1L8 | 2540.625867 | 2110.5175 | 0.830707712 | -0.267587147 | 0.274171931 | no | no change | GO:0016021;GO:0016020 | hsa:102723996;hsa:23308 | | ICOS ligand isoform c precursor [Homo sapiens] |
| J3KPA1 | 13651.48432 | 11335.57081 | 0.830354455 | -0.268200782 | 0.058150906 | no | no change | GO:0016020;GO:0016021;GO:0005576 | hsa:10321 | COG2340 | cysteine-rich secretory protein 3 isoform 3 [Homo sapiens] |
| Q9Y490 | 10605.37706 | 79157.93965 | 7.463943923 | 2.899938147 | 0.216274155 | no | up | GO:0005200;GO:0017166;GO:0007043;GO:0005925;GO:0030054;GO:0005856;GO:0001726;GO:0070062;GO:0002576;GO:0016020;GO:0005737;GO:0003779;GO:0005576;GO:0033622;GO:0001786;GO:0005178;GO:0005515;GO:0006936;GO:0009986;GO:0051015;GO:0036498;GO:0030274;GO:0005886;GO:0007155;GO:0042995;GO:0032587;GO:0007016;GO:0035091;GO:0005829;GO:0030866;GO:0045296;GO:0070527;GO:0007044;GO:0016032;GO:0044877;GO:0007229 | hsa:7094 |  | talin-1 [Homo sapiens] |
| P02533 | 27616.62947 | 84393.37211 | 3.055889647 | 1.611592447 | 0.35001738 | no | up | GO:0005200;GO:0045095;GO:0071944;GO:0010043;GO:0008544;GO:0005737;GO:0045110;GO:0010212;GO:0070062;GO:0005634;GO:0045178;GO:0005198;GO:0030855;GO:1990254;GO:0031581;GO:0070268;GO:0005882;GO:0042633;GO:0007568;GO:0031424;GO:0005829;GO:0005622;GO:0005515 | hsa:3861 |  | keratin, type I cytoskeletal 14 [Homo sapiens] |
| P19320 | 2930.442067 | 5751.492059 | 1.962670453 | 0.972817954 | 0.185612607 | no | up | GO:0009897;GO:0034113;GO:0005783;GO:0005794;GO:0035094;GO:0030175;GO:0060945;GO:0005902;GO:0050901;GO:0060326;GO:0010043;GO:0032496;GO:0001666;GO:0019221;GO:0045177;GO:0002102;GO:0010212;GO:0005615;GO:0060384;GO:0071356;GO:0016021;GO:0016020;GO:0050839;GO:0035584;GO:1904646;GO:0140039;GO:0022614;GO:0005178;GO:0042383;GO:0009308;GO:0030183;GO:0098609;GO:0007584;GO:0008131;GO:0045471;GO:0007159;GO:0005887;GO:0005886;GO:0007155;GO:0002526;GO:0007157;GO:0042102;GO:0009986;GO:0055114;GO:0070062;GO:0007160;GO:0050776;GO:0007568;GO:0060333;GO:0002544;GO:0005769;GO:0030198;GO:0035924;GO:0071065 | hsa:7412 |  | vascular cell adhesion protein 1 isoform a precursor [Homo sapiens] |
| P06331 | 393688.4947 | 708095.7105 | 1.798619264 | 0.846889826 | 0.247612362 | no | up | GO:0038096;GO:0004252;GO:0016020;GO:0030449;GO:0050776;GO:0006898;GO:0038095;GO:0002376;GO:0050900;GO:0005576;GO:0006956;GO:0002250;GO:0003823;GO:0005886;GO:0006508;GO:0006958;GO:0006955 | hsa:102724971 | | hCG1793614, partial [Homo sapiens] |
| P59665 | 93970.19158 | 138895.9084 | 1.478084764 | 0.563729006 | 0.124584624 | no | up | GO:0071222;GO:0019730;GO:0019731;GO:0035578;GO:0051673;GO:0042742;GO:0050829;GO:0061844;GO:0005615;GO:0030520;GO:0051852;GO:0044657;GO:0002227;GO:0042803;GO:0010818;GO:0005796;GO:0006935;GO:0006952;GO:0050830;GO:0062023;GO:0006955;GO:0070062;GO:0050832;GO:0051607;GO:0031640;GO:0043312;GO:0005576;GO:0042832 | hsa:1667;hsa:728358;hsa:1668 | | neutrophil defensin 1 preproprotein [Homo sapiens] |
| P04430 | 53537.46714 | 78829.74944 | 1.472422093 | 0.558191302 | 0.134539479 | no | up | GO:0038096;GO:0004252;GO:0016020;GO:0030449;GO:0050776;GO:0006898;GO:0038095;GO:0050900;GO:0005576;GO:0006956;GO:0003823;GO:0005886;GO:0006508;GO:0006958;GO:0006955 | hsa:7441 |  | RecName: Full=Immunoglobulin kappa variable 1-16; AltName: Full=Ig kappa chain V-I region BAN; Flags: Precursor |
| P20742 | 35256.6036 | 51671.45238 | 1.465582248 | 0.551473934 | 0.438637974 | no | up | GO:0004866;GO:0007565;GO:0070062;GO:0005576;GO:0030414;GO:0072562;GO:0002020;GO:0004867;GO:0010951;GO:0010466;GO:0005615 | hsa:5858 | COG2373 | pregnancy zone protein precursor [Homo sapiens] |
| P01706 | 93105.06105 | 135250.71 | 1.452667647 | 0.53870467 | 0.050550522 | no | up | GO:0038096;GO:0004252;GO:0016020;GO:0030449;GO:0050776;GO:0006898;GO:0038095;GO:0002376;GO:0050900;GO:0005576;GO:0006956;GO:0002250;GO:0003823;GO:0005886;GO:0006508;GO:0006958;GO:0006955 | hsa:7441 |  | hCG2043237, partial [Homo sapiens] |
| P08779 | 61836.21 | 89197.10947 | 1.442473746 | 0.528545061 | 0.106883257 | no | up | GO:0005200;GO:0061436;GO:0008544;GO:0005856;GO:0002009;GO:0051546;GO:0070062;GO:0005634;GO:0005198;GO:0030336;GO:0030216;GO:0008283;GO:0045087;GO:0070268;GO:0005882;GO:0006954;GO:0042633;GO:0007568;GO:0007010;GO:0045104;GO:0005829;GO:0031424;GO:0005515 | hsa:3868 |  | keratin, type I cytoskeletal 16 [Homo sapiens] |
| A0A075B6K5 | 21206.54655 | 29325.31315 | 1.382842468 | 0.467636815 | 0.447391453 | no | up | GO:0016020;GO:0005615;GO:0006955;GO:0002376;GO:0002377;GO:0005576;GO:0002250;GO:0003823;GO:0005886 | hsa:7441 |  | RecName: Full=Immunoglobulin lambda variable 3-9; Flags: Precursor |
| D6RD17 | 1986535.632 | 2656375.211 | 1.337189813 | 0.41920427 | 0.091198461 | no | up |  | hsa:3512 |  | JCHAIN isoform 4, partial [Pongo abelii] |
| A0A075B6R9 | 176609.7653 | 233860.0358 | 1.324162542 | 0.405080225 | 0.193536562 | no | up | GO:0002377;GO:0005615;GO:0006955 | hsa:7441 |  | RecName: Full=Immunoglobulin kappa variable 2-24; Flags: Precursor |
| P07996 | 22059.63353 | 28906.78963 | 1.310393013 | 0.389999569 | 0.350202081 | no | up | GO:0032026;GO:0005783;GO:0050840;GO:0034605;GO:0005788;GO:2001237;GO:0050921;GO:1903671;GO:0048266;GO:0051895;GO:0006986;GO:0043652;GO:0042535;GO:0009612;GO:0030511;GO:0002581;GO:0042327;GO:0051592;GO:0043536;GO:0040037;GO:2001027;GO:0030169;GO:0043236;GO:0045727;GO:0045652;GO:0030198;GO:0043032;GO:0030194;GO:0009897;GO:0018149;GO:0042493;GO:0002605;GO:0071356;GO:0051897;GO:0016477;GO:0002040;GO:0001968;GO:2000379;GO:0030141;GO:0007050;GO:0043394;GO:0006954;GO:0006955;GO:0070052;GO:0090051;GO:0070051;GO:0033574;GO:0043154;GO:0034976;GO:1902043;GO:0031012;GO:0001666;GO:0050431;GO:0005737;GO:0031091;GO:0031093;GO:0005509;GO:0010763;GO:0001786;GO:0051918;GO:0008284;GO:0009986;GO:0017134;GO:2000353;GO:0008201;GO:0071363;GO:0001953;GO:0002544;GO:0016529;GO:0042802;GO:0005515;GO:0032570;GO:0016525;GO:0000187;GO:0005201;GO:0005615;GO:0002576;GO:0010751;GO:0045766;GO:0043066;GO:0010748;GO:0032695;GO:1903588;GO:0005178;GO:0030335;GO:0048661;GO:0032914;GO:0062023;GO:0007155;GO:0070062;GO:0043537;GO:0010595;GO:0010596;GO:0009749;GO:0005577;GO:0005576;GO:0010757;GO:0010754;GO:0010759;GO:0001937;GO:0071636 | hsa:7057 |  | thrombospondin-1 precursor [Homo sapiens] |
| P0DJI8 | 25729.83263 | 33536.79611 | 1.303420686 | 0.382302797 | 0.197350162 | no | up | GO:0000187;GO:0042056;GO:0019221;GO:0001664;GO:0034364;GO:0048246;GO:0048247;GO:0044267;GO:0050728;GO:0050708;GO:0005615;GO:0071682;GO:0045785;GO:0007186;GO:0030593;GO:0045087;GO:0005881;GO:0006953;GO:0050918;GO:0008201;GO:0030168;GO:0070062;GO:0050716;GO:0050715;GO:0006898;GO:0007204;GO:0005576 | hsa:6288 |  | RecName: Full=Serum amyloid A-1 protein; Short=SAA; Contains: RecName: Full=Amyloid protein A; AltName: Full=Amyloid fibril protein AA; Contains: RecName: Full=Serum amyloid protein A(2-104); Contains: RecName: Full=Serum amyloid protein A(3-104); Contains: RecName: Full=Serum amyloid protein A(2-103); Contains: RecName: Full=Serum amyloid protein A(2-102); Contains: RecName: Full=Serum amyloid protein A(4-101); Flags: Precursor |
| A0A075B6K4 | 57859.10211 | 74879.95526 | 1.294177624 | 0.372035638 | 0.133472004 | no | up | GO:0016020;GO:0005615;GO:0006955;GO:0002376;GO:0002377;GO:0005576;GO:0002250;GO:0003823;GO:0005886 | hsa:7441 |  | immunoglobulin light chain variable region, partial [Homo sapiens] |
| A0A075B6H7 | 138596.2984 | 179338.0268 | 1.293959715 | 0.371792703 | 0.070747843 | no | up | GO:0002377;GO:0005615;GO:0006955 | hsa:7441 |  | hCG2043206, partial [Homo sapiens] |
| Q13201 | 36145.25167 | 46496.28313 | 1.286373202 | 0.363309257 | 0.317549492 | no | up | GO:0005201;GO:0031093;GO:0010811;GO:0002576;GO:0007596;GO:0031012;GO:0005576;GO:0005509;GO:0005515;GO:0007155;GO:0062023 | hsa:22915 |  | multimerin-1 precursor [Homo sapiens] |
| A0A5H1ZRS9 | 66724.68526 | 84803.10211 | 1.270940459 | 0.345896444 | 0.160041661 | no | up |  | hsa:7441 |  | immunoglobulin kappa chain variable region, partial [Homo sapiens] |
| P01705 | 88650.06882 | 112127.4213 | 1.264831746 | 0.338945483 | 0.289563883 | no | up | GO:0038096;GO:0004252;GO:0016020;GO:0030449;GO:0050776;GO:0006898;GO:0038095;GO:0002376;GO:0050900;GO:0005576;GO:0006956;GO:0002250;GO:0003823;GO:0005886;GO:0006508;GO:0006958;GO:0006955 | hsa:7441 |  | immunoglobulin lambda-chain, partial [Homo sapiens] |
| P01817 | 25906.94674 | 32489.94826 | 1.25410179 | 0.32665445 | 0.170468096 | no | up | GO:0038096;GO:0004252;GO:0016020;GO:0030449;GO:0050776;GO:0006898;GO:0038095;GO:0002376;GO:0050900;GO:0005576;GO:0006956;GO:0002250;GO:0003823;GO:0005886;GO:0006508;GO:0006958;GO:0006955 | hsa:102723407 | | RecName: Full=Immunoglobulin heavy variable 2-5; AltName: Full=Ig heavy chain V-II region HE; AltName: Full=Ig heavy chain V-II region MCE; Flags: Precursor |
| P08519 | 115441.3416 | 143288.9263 | 1.241227141 | 0.311767148 | 0.654198366 | no | up | GO:0004252;GO:0034374;GO:0005515;GO:0006869;GO:0008015;GO:0016787;GO:0034185;GO:0005576;GO:0004866;GO:0010951;GO:0006508;GO:0008233;GO:0008236;GO:0034358;GO:0008201;GO:0001968;GO:0006629 | hsa:4018 | COG5640 | RecName: Full=Apolipoprotein(a); Short=Apo(a); Short=Lp(a); Flags: Precursor |
| A0A0C4DH24 | 40461.87842 | 50128.34316 | 1.238903015 | 0.309063253 | 0.330982797 | no | up | GO:0016020;GO:0005615;GO:0006955;GO:0002376;GO:0002377;GO:0005576;GO:0002250;GO:0003823;GO:0005886 | hsa:7441 |  | RecName: Full=Immunoglobulin kappa variable 6-21; Flags: Precursor |
| P01814 | 22045.15142 | 27211.02737 | 1.234331616 | 0.303730041 | 0.144677265 | no | up | GO:0038096;GO:0004252;GO:0016020;GO:0030449;GO:0050776;GO:0006898;GO:0038095;GO:0002376;GO:0050900;GO:0005576;GO:0006956;GO:0002250;GO:0003823;GO:0005886;GO:0006508;GO:0006958;GO:0006955 | hsa:102723407 | | RecName: Full=Immunoglobulin heavy variable 2-70; AltName: Full=Ig heavy chain V-II region COR; AltName: Full=Ig heavy chain V-II region DAW; AltName: Full=Ig heavy chain V-II region OU; AltName: Full=Ig heavy chain V-II region SESS; Flags: Precursor |
| P01871 | 10356733.32 | 12768356.63 | 1.232855597 | 0.302003828 | 0.276174848 | no | up | GO:0009897;GO:0042834;GO:0019731;GO:0050900;GO:0034987;GO:0050829;GO:0005615;GO:0016021;GO:0016020;GO:0072562;GO:0009986;GO:0050853;GO:0045087;GO:0006910;GO:0006911;GO:0031210;GO:0003823;GO:0005886;GO:0006958;GO:0050871;GO:0070062;GO:0003697;GO:0002376;GO:0005576;GO:0002250;GO:0005515;GO:0071756;GO:0071757 | hsa:3543 |  | immunoglobulin heavy chain [Homo sapiens] |
| P01742 | 1110117.079 | 1362618.079 | 1.227454387 | 0.295669414 | 0.547952724 | no | up | GO:0038096;GO:0004252;GO:0016020;GO:0030449;GO:0050776;GO:0006898;GO:0038095;GO:0002376;GO:0050900;GO:0005576;GO:0006956;GO:0002250;GO:0003823;GO:0005886;GO:0006508;GO:0006958;GO:0006955 | hsa:102724971 | | IgM heavy chain VH1 region precursor, partial [Homo sapiens] |
| C9JB55 | 12257.70416 | 14991.58368 | 1.223033571 | 0.290464004 | 0.057632146 | no | up | GO:0055037;GO:1990459;GO:0030139;GO:0005905;GO:0034986;GO:1990712;GO:0004857;GO:0048260;GO:0005615;GO:0005770;GO:0043086;GO:0009925;GO:0048471;GO:0006879;GO:0016324;GO:0008198;GO:0008199;GO:0015091;GO:0031232;GO:0034756;GO:0071281;GO:0005623;GO:0005769 | hsa:7018 |  | serotransferrin isoform 1 precursor [Homo sapiens] |
| Q562R1 | 19868.54273 | 24097.0568 | 1.212824571 | 0.278370886 | 0.329306467 | no | up | GO:0005737;GO:0005856;GO:0005615;GO:0070062;GO:0000166;GO:0003674;GO:0045202;GO:0005515;GO:0008150;GO:0098978;GO:0005524;GO:0015629 | hsa:345651 | COG5277 | beta-actin-like protein 2 [Homo sapiens] |
| P01599 | 138053.15 | 167274.5911 | 1.21166805 | 0.27699451 | 0.142253998 | no | up | GO:0038096;GO:0004252;GO:0016020;GO:0030449;GO:0050776;GO:0006898;GO:0038095;GO:0072562;GO:0002376;GO:0050900;GO:0005576;GO:0006956;GO:0002250;GO:0003823;GO:0005886;GO:0006508;GO:0006958;GO:0006955;GO:0070062 | hsa:7441 |  | RecName: Full=Immunoglobulin kappa variable 1-17; AltName: Full=Ig kappa chain V-I region Gal; AltName: Full=Ig kappa chain V-I region WEA; Flags: Precursor |
| P02750 | 154796.7511 | 128381.3026 | 0.829353987 | -0.269940087 | 0.032648526 | yes | down | GO:0043231;GO:0016020;GO:0070062;GO:0005160;GO:0009617;GO:0050873;GO:0045766;GO:0043312;GO:1904813;GO:0001938;GO:1904724;GO:0005576;GO:0003674;GO:0005515;GO:0035580;GO:0008150;GO:0030511;GO:0005615 | hsa:116844 | COG4886 | leucine-rich alpha-2-glycoprotein precursor [Homo sapiens] |
| P19652 | 1147756.379 | 944377.3947 | 0.822803003 | -0.281381036 | 0.041824951 | yes | down | GO:1904469;GO:0050716;GO:0031093;GO:0005615;GO:0002576;GO:0050718;GO:0043312;GO:0072562;GO:0006953;GO:0035578;GO:0062023;GO:0002682;GO:0035580;GO:0005576;GO:0070062 | hsa:5005 |  | alpha-1-acid glycoprotein 2 precursor [Homo sapiens] |
| P41222 | 20407.13071 | 16649.46113 | 0.815864874 | -0.293597867 | 0.006687874 | yes | down | GO:0006633;GO:0006631;GO:0005783;GO:0005789;GO:0005737;GO:0070062;GO:0005634;GO:0016020;GO:0005504;GO:0048471;GO:0005501;GO:0019371;GO:0005794;GO:0005791;GO:0006629;GO:0031965;GO:2000255;GO:0036094;GO:0001516;GO:0045187;GO:0005615;GO:0016853;GO:0004667;GO:0006693;GO:0005576;GO:0005515 | hsa:5730 |  | prostaglandin-H2 D-isomerase precursor [Homo sapiens] |
| D6RAR4 | 31434.37947 | 25599.32632 | 0.814373522 | -0.29623744 | 0.018577374 | yes | down | GO:0005737;GO:0004252;GO:0005791;GO:0005615;GO:0016787;GO:0005576;GO:0006508;GO:0008233;GO:0008236 | hsa:3083 | COG5640 | hepatocyte growth factor activator isoform 1 preproprotein [Homo sapiens] |
| P01019 | 227177.4053 | 181866.2316 | 0.800547182 | -0.320941661 | 0.000296383 | yes | down | GO:1903779;GO:0038166;GO:2001238;GO:0035106;GO:0003014;GO:0007166;GO:0050729;GO:0001822;GO:0042127;GO:0032930;GO:0006606;GO:0014824;GO:0019229;GO:0010976;GO:0010873;GO:0050731;GO:0035815;GO:0005829;GO:0007202;GO:0007204;GO:0003081;GO:1904754;GO:0010536;GO:0007565;GO:0005179;GO:0034374;GO:0007200;GO:0071260;GO:0042310;GO:0042311;GO:0048169;GO:0008083;GO:1901201;GO:0072562;GO:0006883;GO:0061098;GO:0007186;GO:2000379;GO:0090190;GO:0048018;GO:0008306;GO:0003331;GO:0033864;GO:0007199;GO:0001974;GO:0062023;GO:0048659;GO:0051969;GO:0016525;GO:0014873;GO:0030308;GO:0048146;GO:0048144;GO:0007267;GO:0045429;GO:1905010;GO:0043085;GO:0005737;GO:0050880;GO:0003051;GO:0002027;GO:0045742;GO:0032270;GO:0070471;GO:0008284;GO:0031701;GO:0031703;GO:0001558;GO:0014068;GO:0004867;GO:0014061;GO:0051092;GO:1904385;GO:0007568;GO:0070062;GO:0002034;GO:2001275;GO:0005515;GO:0070371;GO:0001819;GO:0051387;GO:0010469;GO:0010666;GO:0008217;GO:0061049;GO:0050663;GO:0032355;GO:0005615;GO:0097755;GO:0042981;GO:0010744;GO:0035813;GO:1904707;GO:0046628;GO:0034104;GO:0019216;GO:0051403;GO:0010951;GO:0007263;GO:1905589;GO:0010613;GO:0010595;GO:0002019;GO:0002018;GO:0045777;GO:0002016;GO:0031702;GO:0051924;GO:0005576;GO:1903598;GO:0045893 | hsa:183 | COG4826 | angiotensinogen preproprotein [Homo sapiens] |
| Q8N1N4 | 172097.1737 | 133954.8405 | 0.778367463 | -0.36147669 | 0.027558663 | yes | down | GO:0005615;GO:0031424;GO:0005829;GO:0045095;GO:0070268;GO:0005198;GO:0005882;GO:0070062 | hsa:196374 |  | keratin, type II cytoskeletal 78 isoform 1 [Homo sapiens] |
| P13473 | 12021.42644 | 9220.985842 | 0.767045898 | -0.382615188 | 0.008149094 | yes | down | GO:0005770;GO:0061684;GO:0101003;GO:0019899;GO:0050821;GO:0044754;GO:0035577;GO:0010008;GO:1990836;GO:0043202;GO:0005615;GO:0097637;GO:0031410;GO:0016021;GO:0016020;GO:0097352;GO:0031902;GO:0046716;GO:0045121;GO:0006914;GO:0017038;GO:0006605;GO:1905146;GO:0061740;GO:0019904;GO:0009267;GO:0005886;GO:0070062;GO:0000421;GO:0030670;GO:0031647;GO:0043312;GO:0031088;GO:0005764;GO:0005765;GO:0005515;GO:0098857;GO:0005768;GO:0002576 | hsa:3920 |  | lysosome-associated membrane glycoprotein 2 isoform A precursor [Homo sapiens] |
| A0A182DWH7 | 33602.84316 | 24868.99737 | 0.740086107 | -0.434234961 | 0.015369372 | yes | down | GO:0008430 | hsa:6414 |  | Selenoprotein P, plasma, 1 [Homo sapiens] |
| P35908 | 117316.6737 | 86320.11789 | 0.735787294 | -0.442639331 | 0.031002622 | yes | down | GO:0005200;GO:0045095;GO:0018149;GO:0008544;GO:0045684;GO:0043616;GO:0051546;GO:0070062;GO:0032980;GO:0005634;GO:0016020;GO:0005198;GO:0030280;GO:0070268;GO:0005882;GO:0001533;GO:0005615;GO:0045109;GO:0008092;GO:0031424;GO:0005829;GO:0003334;GO:0005515 | hsa:3849 |  | keratin, type II cytoskeletal 2 epidermal [Homo sapiens] |
| P13647 | 37475.64211 | 27473.64947 | 0.733106838 | -0.447904633 | 0.013049212 | yes | down | GO:0005737;GO:0016020;GO:0005200;GO:0007010;GO:0070062;GO:0031424;GO:0031581;GO:0045095;GO:0070268;GO:0005198;GO:0005634;GO:0005882;GO:0097110;GO:0005515;GO:0005886;GO:0008544;GO:0005829 | hsa:3852 |  | Keratin 5 [Homo sapiens] |
| A0A0C4DH38 | 152379.5311 | 109825.7963 | 0.720738511 | -0.47245216 | 0.04335322 | yes | down | GO:0009897;GO:0050853;GO:0045087;GO:0016020;GO:0006910;GO:0006911;GO:0002376;GO:0005576;GO:0002250;GO:0003823;GO:0005886;GO:0072562;GO:0042571;GO:0006958;GO:0050871;GO:0034987;GO:0042742 | hsa:102723407 | | RecName: Full=Immunoglobulin heavy variable 5-51; Flags: Precursor |
| O75460 | 151139.3916 | 97874.42944 | 0.647577236 | -0.626875823 | 0.00177573 | yes | down | GO:0005739;GO:0004540;GO:0005783;GO:0016787;GO:0016310;GO:0005161;GO:0019899;GO:0005789;GO:0008152;GO:1990604;GO:0030176;GO:1990597;GO:0034620;GO:1990630;GO:0046777;GO:0036289;GO:0007257;GO:0006986;GO:0098787;GO:0016241;GO:0030544;GO:0005637;GO:0016021;GO:0000287;GO:0004521;GO:0051082;GO:0000166;GO:0004674;GO:0016740;GO:0071333;GO:1901142;GO:0006402;GO:0046872;GO:0051879;GO:0042802;GO:0042803;GO:0005737;GO:0006468;GO:0033120;GO:0006397;GO:1990332;GO:0004672;GO:0007050;GO:0003824;GO:0043531;GO:0016020;GO:0090502;GO:0036498;GO:0006351;GO:0030968;GO:0070059;GO:1904707;GO:0070054;GO:0016301;GO:0006355;GO:1990579;GO:0005524;GO:0005515;GO:0004519;GO:1900103;GO:0006379;GO:0001935;GO:0035924;GO:0006915;GO:0034976 | hsa:2081 | COG0515 | endoplasmic reticulum to nucleus signalling 1 isoform 1 variant, partial [Homo sapiens] |
| A0A5H1ZRQ7 | 20376.10105 | 13185.15492 | 0.647089199 | -0.627963498 | 0.018472777 | yes | down |  | hsa:100423062 | | RecName: Full=Immunoglobulin lambda constant 7; AltName: Full=Ig lambda-7 chain C region |
| F8W1S1 | 8692.160882 | 5466.170615 | 0.628862108 | -0.669184386 | 0.016775626 | yes | down | GO:0005882;GO:0045095;GO:0005198 | hsa:121391 |  | KRT74 isoform 2 [Pan troglodytes] |
| D6RE82 | 209939.3632 | 131886.3826 | 0.628211787 | -0.670677082 | 0.002640396 | yes | down | GO:0030688;GO:0005730;GO:0005634;GO:0030687;GO:0006364;GO:0003723 | hsa:8568 |  | PREDICTED: ribosomal RNA processing protein 1 homolog A [Callithrix jacchus] |
| P13645 | 75944.19947 | 47574.99368 | 0.626446707 | -0.674736313 | 0.012382928 | yes | down | GO:0005737;GO:0018149;GO:0045684;GO:0070062;GO:0030216;GO:0031424;GO:0005615;GO:0009986;GO:0016020;GO:0070268;GO:0051290;GO:0005634;GO:0005882;GO:0005576;GO:0046982;GO:0005198;GO:0005829;GO:0001533;GO:0030280 | hsa:3858 |  | Keratin 10 [Homo sapiens] |
| P04264 | 358372.0789 | 224055.9947 | 0.625204942 | -0.677598913 | 0.003117062 | yes | down | GO:0018149;GO:0038023;GO:0045095;GO:0030246;GO:0031012;GO:1904813;GO:0061436;GO:0030280;GO:0005856;GO:0050728;GO:0070062;GO:0005634;GO:0016020;GO:0045765;GO:0072562;GO:0005198;GO:0001867;GO:0005882;GO:0051290;GO:0070268;GO:0062023;GO:0005886;GO:0001895;GO:0006979;GO:0001533;GO:0005615;GO:0042730;GO:0031424;GO:0005829;GO:0043312;GO:0005576;GO:0005515;GO:0046982 | hsa:3848 |  | keratin 1 [Homo sapiens] |
| A0A2R8Y7X9 | 47762.113 | 29304.87822 | 0.613559082 | -0.704725821 | 0.008090901 | yes | down | GO:0005344;GO:0019825;GO:0020037;GO:0005833;GO:0046872;GO:0015671 | hsa:3048 | COG1018 | hemoglobin subunit gamma-2 [Pan troglodytes] |
| A0A1W2PQU7 | 150467.4732 | 86400.39789 | 0.574213124 | -0.800341789 | 0.007169534 | yes | down | GO:1904714;GO:0005737;GO:0045111;GO:0061564;GO:0045109;GO:0060291;GO:0016020;GO:0044297;GO:0060020;GO:0005198;GO:0051580;GO:0005883;GO:0005882;GO:0010977;GO:0030198;GO:0014002;GO:0097450;GO:0010625;GO:0031102 | hsa:2670 |  | glial fibrillary acidic protein [Mus musculus] |
| A0A0C4DH73 | 127967.1195 | 64750.49632 | 0.505993232 | -0.982810008 | 2.82E-08 | yes | down | GO:0038096;GO:0004252;GO:0016020;GO:0030449;GO:0005615;GO:0006898;GO:0038095;GO:0050900;GO:0002376;GO:0002377;GO:0050776;GO:0005576;GO:0006956;GO:0002250;GO:0003823;GO:0005886;GO:0006508;GO:0006958;GO:0006955 | hsa:7441 |  | immunoglobulin light chain variable region, partial [Homo sapiens] |
| Q9NZP8 | 74769.25305 | 30579.27511 | 0.408981953 | -1.289890913 | 9.95E-06 | yes | down | GO:0004252;GO:0005615;GO:0016787;GO:0070062;GO:0045087;GO:0031638;GO:0002376;GO:0005576;GO:0006508;GO:0006958;GO:0008233;GO:0008236 | hsa:51279 | COG5640 | complement C1r subcomponent-like protein isoform 1 precursor [Homo sapiens] |
| P19823 | 928446.2053 | 1107498.8 | 1.192851878 | 0.254414909 | 0.029074177 | yes | no change | GO:0044267;GO:0030212;GO:0070062;GO:0043687;GO:0072562;GO:0062023;GO:0030414;GO:0005788;GO:0004866;GO:0004867;GO:0010951;GO:0010466;GO:0005576 | hsa:3698 | COG2304 | inter-alpha-trypsin inhibitor heavy chain H2 precursor [Homo sapiens] |
| P02747 | 458825.5737 | 538883.3474 | 1.174484114 | 0.2320272 | 0.008591019 | yes | no change | GO:0004252;GO:0030449;GO:0005581;GO:0045650;GO:0045087;GO:0072562;GO:0002376;GO:0030853;GO:0005576;GO:0006956;GO:0005515;GO:0006955;GO:0006508;GO:0006958;GO:0005615 | hsa:714 |  | complement C1q subcomponent subunit C isoform X1 [Pongo abelii] |
| C9JV77 | 1898157.316 | 2167220.737 | 1.141749801 | 0.191246538 | 0.023034148 | yes | no change | GO:0019210;GO:0006907;GO:0005788;GO:0001501;GO:0030500;GO:0030502;GO:0050766;GO:0044267;GO:0070062;GO:0031093;GO:0002576;GO:0050727;GO:0072562;GO:0005794;GO:0043687;GO:0046627;GO:0006953;GO:0062023;GO:0034774;GO:0010951;GO:0004869;GO:0005615;GO:0043312;GO:0005576 | hsa:197 |  | alpha-2-HS-glycoprotein isoform 1 preproprotein [Homo sapiens] |
| D6R934 | 95974.26 | 107963.7168 | 1.124923671 | 0.169827114 | 0.015139953 | yes | no change | GO:0006958;GO:0005576;GO:0005581 | hsa:713 |  | complement C1q subcomponent subunit B precursor [Homo sapiens] |
| P08697 | 252362.9421 | 217188.5526 | 0.860619831 | -0.21655201 | 0.009548879 | yes | no change | GO:0030414;GO:0032967;GO:0005615;GO:0031093;GO:0051496;GO:0002576;GO:0030199;GO:0072562;GO:0010466;GO:0048514;GO:0002020;GO:0010757;GO:0051918;GO:0042803;GO:0048661;GO:0009986;GO:0045597;GO:0006953;GO:0046330;GO:0004866;GO:0004867;GO:0010951;GO:0010033;GO:0070062;GO:0042730;GO:2000049;GO:0002034;GO:0005577;GO:0005576;GO:0045944;GO:0005515;GO:0070374;GO:0071636 | hsa:5345 | COG4826 | alpha-2-antiplasmin isoform X1 [Homo sapiens] |
| P14151 | 34739.74474 | 29660.54053 | 0.853792702 | -0.228042264 | 0.005786808 | yes | no change | GO:0009897;GO:0016339;GO:0030246;GO:0050900;GO:0050901;GO:0070492;GO:0030667;GO:0043208;GO:0016021;GO:0016020;GO:0005509;GO:0002020;GO:0046872;GO:0009986;GO:0005887;GO:0005886;GO:0007155;GO:0050839;GO:0008201;GO:0050776;GO:0043312;GO:0005515;GO:0033198 | hsa:6402 |  | L-selectin [Homo sapiens] |
| P02766 | 909031.8684 | 758885.3474 | 0.8348281 | -0.260448933 | 0.018627945 | yes | no change | GO:0005179;GO:0044267;GO:0032991;GO:0070062;GO:0042562;GO:0070324;GO:0005615;GO:0070327;GO:0005737;GO:0043312;GO:0001523;GO:0035578;GO:0030198;GO:0005515;GO:0042572;GO:0046982;GO:0010469;GO:0006144;GO:0042802;GO:0005576 | hsa:7276 | COG2351 | transthyretin precursor [Homo sapiens] |
| Q16610 | 39667.41 | 32948.33211 | 0.830614656 | -0.267748767 | 0.016366841 | yes | no change | GO:0008022;GO:0005201;GO:0019899;GO:0031012;GO:0001503;GO:0007165;GO:0030500;GO:0030502;GO:0001525;GO:0005615;GO:0002576;GO:0045766;GO:0010466;GO:2000404;GO:0001960;GO:0002063;GO:0003416;GO:0005576;GO:0005134;GO:0002020;GO:0002828;GO:0062023;GO:0006954;GO:0031214;GO:0070062;GO:0043236;GO:0031089;GO:0001938;GO:0006357;GO:0005515;GO:0043123 | hsa:1893 |  | extracellular matrix protein 1 isoform 1 precursor [Homo sapiens] |
| P13796 | 685041.9409 | 2568131.265 | 3.748867202 | 1.906454721 | 0.049265982 | yes | up | GO:0032432;GO:0033157;GO:0015629;GO:0030175;GO:0051020;GO:0005925;GO:0030054;GO:0005737;GO:0071803;GO:0001726;GO:0002102;GO:0002286;GO:0005615;GO:0016020;GO:0003779;GO:0005509;GO:0048471;GO:0016477;GO:0022617;GO:0042802;GO:0035722;GO:0005178;GO:0031100;GO:0051017;GO:0051015;GO:0005884;GO:0005886;GO:0051764;GO:0042995;GO:0032587;GO:0001891;GO:0046872;GO:0070062;GO:0005829;GO:0005856;GO:0051639;GO:0044319;GO:0001725;GO:0010737 | hsa:3936 | COG5069 | plastin-2 [Homo sapiens] |
| P08185 | 52287.74895 | 107075.3179 | 2.047808905 | 1.034081094 | 0.000133636 | yes | up | GO:0005615;GO:0008289;GO:0010951;GO:0008211;GO:0005576;GO:0004867;GO:0005496;GO:0070062 | hsa:866 | COG4826 | corticosteroid-binding globulin precursor [Homo sapiens] |
| A0A0C4DH33 | 6490.322412 | 12642.38142 | 1.94788188 | 0.961906195 | 0.002744043 | yes | up | GO:0009897;GO:0050853;GO:0045087;GO:0016020;GO:0006910;GO:0006911;GO:0002376;GO:0005576;GO:0002250;GO:0003823;GO:0005886;GO:0072562;GO:0042571;GO:0006958;GO:0050871;GO:0034987;GO:0042742 | hsa:102723407 | | immunoglobulin heavy chain variable region, partial [Homo sapiens] |
| Q15582 | 7728.168526 | 14484.47174 | 1.874243773 | 0.906308609 | 2.21E-05 | yes | up | GO:0005201;GO:0050840;GO:0031012;GO:0007162;GO:0001525;GO:0044267;GO:0070062;GO:0005576;GO:0002062;GO:0005178;GO:0008283;GO:0007601;GO:0062023;GO:0005886;GO:0007155;GO:0050839;GO:0005615;GO:0005604;GO:0050896;GO:0005802;GO:0030198;GO:0005515;GO:0005518 | hsa:7045 | COG2335 | transforming growth factor-beta-induced protein ig-h3 precursor [Homo sapiens] |
| P0DP01 | 24281.91167 | 38972.34187 | 1.604994796 | 0.68256862 | 0.018318338 | yes | up | GO:0016020;GO:0002376;GO:0005576;GO:0002250;GO:0003823;GO:0005886 | hsa:102723407 | | immunoglobulin heavy chain variable region, partial [Homo sapiens] |
| A0A075B6R2 | 129021.1653 | 205343.2858 | 1.591547289 | 0.670430024 | 0.01840245 | yes | up | GO:0009897;GO:0050853;GO:0045087;GO:0016020;GO:0006910;GO:0006911;GO:0002376;GO:0005576;GO:0002250;GO:0003823;GO:0005886;GO:0072562;GO:0042571;GO:0006958;GO:0050871;GO:0034987;GO:0042742 | hsa:102724971 | | immunoglobulin heavy chain VDJ region, partial [Homo sapiens] |
| P01717 | 6679.0208 | 10448.645 | 1.564397733 | 0.645607351 | 0.013143718 | yes | up | GO:0038096;GO:0004252;GO:0016020;GO:0030449;GO:0050776;GO:0006898;GO:0038095;GO:0072562;GO:0002376;GO:0050900;GO:0005576;GO:0006956;GO:0002250;GO:0003823;GO:0005886;GO:0006508;GO:0006958;GO:0006955 | hsa:7441 |  | immunoglobulin lambda light chain variable region, partial [Homo sapiens] |
| A0A0C4DH34 | 941572.7789 | 1442302.121 | 1.531800996 | 0.615228882 | 0.00131395 | yes | up | GO:0009897;GO:0050853;GO:0045087;GO:0016020;GO:0006910;GO:0006911;GO:0002376;GO:0005576;GO:0002250;GO:0003823;GO:0005886;GO:0072562;GO:0042571;GO:0006958;GO:0050871;GO:0034987;GO:0042742 | hsa:102723407 | | RecName: Full=Immunoglobulin heavy variable 4-28; Flags: Precursor |
| A0A075B6J9 | 86557.38105 | 130416.4753 | 1.506705421 | 0.59139738 | 0.01257421 | yes | up | GO:0016020;GO:0005615;GO:0006955;GO:0002376;GO:0002377;GO:0005576;GO:0002250;GO:0003823;GO:0005886 | hsa:7441 |  | RecName: Full=Immunoglobulin lambda variable 2-18; Flags: Precursor |
| A0A075B6S5 | 48086.14368 | 71694.97316 | 1.490969491 | 0.576250736 | 0.003344198 | yes | up | GO:0016020;GO:0005615;GO:0006955;GO:0002376;GO:0002377;GO:0005576;GO:0002250;GO:0003823;GO:0005886 | hsa:7441 |  | monoclonal IgM antibody light chain [Homo sapiens] |
| P01782 | 209357.0895 | 287610.9211 | 1.373781618 | 0.458152685 | 0.036786164 | yes | up | GO:0038096;GO:0004252;GO:0016020;GO:0030449;GO:0050776;GO:0006898;GO:0038095;GO:0002376;GO:0050900;GO:0005576;GO:0006956;GO:0002250;GO:0003823;GO:0005886;GO:0006508;GO:0006958;GO:0006955;GO:0070062 | hsa:102723407 | | hCG2038940, partial [Homo sapiens] |
| A0A087WSY6 | 205787.9 | 277516.2737 | 1.348554865 | 0.431414218 | 0.027990243 | yes | up | GO:0016020;GO:0005615;GO:0006955;GO:0002376;GO:0002377;GO:0005576;GO:0002250;GO:0003823;GO:0005886 | hsa:29802 |  | RecName: Full=Immunoglobulin kappa variable 3D-15; Flags: Precursor |
| A0A0J9YVY3 | 48104.57 | 60778.144 | 1.263458836 | 0.337378661 | 0.021406404 | yes | up | GO:0016020;GO:0002376;GO:0005576;GO:0002250;GO:0003823;GO:0005886 | hsa:102723407 | | RecName: Full=Immunoglobulin heavy variable 7-4-1; Flags: Precursor |
| P80108 | 54402.06105 | 67531.23053 | 1.241335884 | 0.311893536 | 0.009504302 | yes | up | GO:0035774;GO:0051044;GO:0051047;GO:0035690;GO:0031012;GO:0010907;GO:0001503;GO:0032869;GO:0009749;GO:0010867;GO:0005737;GO:0043065;GO:0070062;GO:0002430;GO:0006501;GO:0045919;GO:0005576;GO:0097241;GO:0006507;GO:0010897;GO:0002062;GO:0004630;GO:0035701;GO:0002042;GO:0008285;GO:0008286;GO:0010983;GO:0071397;GO:0071277;GO:0005615;GO:0010595;GO:0043231;GO:0016787;GO:0070633;GO:0071401;GO:0005765;GO:0017080;GO:0071467;GO:1900076;GO:0004621;GO:0046470;GO:0010694;GO:0005622 | hsa:2822 |  | phosphatidylinositol-glycan-specific phospholipase D precursor [Homo sapiens] |
| P43251 | 31395.26105 | 38404.83263 | 1.22326846 | 0.290741054 | 0.038349735 | yes | up | GO:0016787;GO:0005615;GO:0047708;GO:0070062;GO:0016810;GO:0016811;GO:0006768;GO:0006807;GO:0005759;GO:0007417;GO:0005576 | hsa:686 | COG0388 | RecName: Full=Biotinidase; Short=Biotinase; Flags: Precursor |
| O75882 | 38134.91316 | 46564.93 | 1.221057717 | 0.288131395 | 0.018777594 | yes | up | GO:0005737;GO:0043473;GO:0042552;GO:0005615;GO:0070062;GO:0016021;GO:0016020;GO:0030246;GO:0006954;GO:0005576;GO:0005887;GO:0005886;GO:0038023;GO:0021549;GO:0006979;GO:0040014 | hsa:8455 |  | attractin isoform 1 preproprotein [Homo sapiens] |

| Table S2-2 Proteins identified in M vs C group. | | | | | | | | | | | |
| --- | --- | --- | --- | --- | --- | --- | --- | --- | --- | --- | --- |
| Accession | C | M | FC(M/C) | log2FC(M/C) | Pvalue(M/C) | significant | regulate | GO | KEGG | COG | Description |
| A0A0J9YX35 | 22457.00516 | 55634.76146 | 2.477390065 | 1.308821039 | 0.000548222 | yes | up | GO:0016020;GO:0002376;GO:0005576;GO:0002250;GO:0003823;GO:0005886 | hsa:102723407 | | RecName: Full=Immunoglobulin heavy variable 3-64D; Flags: Precursor |
| P08185 | 52287.74895 | 120878.3283 | 2.311790635 | 1.209010747 | 3.61E-10 | yes | up | GO:0005615;GO:0008289;GO:0010951;GO:0008211;GO:0005576;GO:0004867;GO:0005496;GO:0070062 | hsa:866 | COG4826 | corticosteroid-binding globulin precursor [Homo sapiens] |
| Q15582 | 7728.168526 | 15606.87442 | 2.019479048 | 1.013983178 | 0.000202411 | yes | up | GO:0005201;GO:0050840;GO:0031012;GO:0007162;GO:0001525;GO:0044267;GO:0070062;GO:0005576;GO:0002062;GO:0005178;GO:0008283;GO:0007601;GO:0062023;GO:0005886;GO:0007155;GO:0050839;GO:0005615;GO:0005604;GO:0050896;GO:0005802;GO:0030198;GO:0005515;GO:0005518 | hsa:7045 | COG2335 | transforming growth factor-beta-induced protein ig-h3 precursor [Homo sapiens] |
| P04430 | 53537.46714 | 106518.657 | 1.98960957 | 0.992485352 | 0.005972462 | yes | up | GO:0038096;GO:0004252;GO:0016020;GO:0030449;GO:0050776;GO:0006898;GO:0038095;GO:0050900;GO:0005576;GO:0006956;GO:0003823;GO:0005886;GO:0006508;GO:0006958;GO:0006955 | hsa:7441 |  | RecName: Full=Immunoglobulin kappa variable 1-16; AltName: Full=Ig kappa chain V-I region BAN; Flags: Precursor |
| P26038 | 3912.521765 | 7080.574278 | 1.809721378 | 0.855767599 | 0.019531633 | yes | up | GO:0005200;GO:0019899;GO:0030175;GO:0050900;GO:0010628;GO:0005925;GO:0061028;GO:0005737;GO:0071803;GO:0045177;GO:0042995;GO:0070062;GO:1902115;GO:0043209;GO:0005634;GO:0042098;GO:0072562;GO:0003779;GO:2000401;GO:0035722;GO:0022612;GO:0048471;GO:2000643;GO:0022614;GO:1903364;GO:0005856;GO:0005515;GO:0016324;GO:0009986;GO:0071944;GO:0031982;GO:0071394;GO:0016323;GO:0019901;GO:0008361;GO:0008360;GO:0045198;GO:0001771;GO:0005886;GO:0016020;GO:0050839;GO:0072678;GO:0005615;GO:0031528;GO:0007010;GO:0071437;GO:0005829;GO:0008092;GO:0005902;GO:0003725;GO:0016032;GO:0001931;GO:0007159;GO:0005102;GO:0070489;GO:0031143;GO:1902966 | hsa:4478 |  | moesin [Homo sapiens] |
| A0A5H1ZRS9 | 66724.68526 | 117885.5538 | 1.766745745 | 0.821094434 | 0.000128418 | yes | up |  | hsa:7441 |  | immunoglobulin kappa chain variable region, partial [Homo sapiens] |
| O75636 | 31221.79526 | 54872.66833 | 1.757511632 | 0.813534237 | 0.000152589 | yes | up | GO:0004252;GO:0051607;GO:0006956;GO:0003823;GO:1902679;GO:0045087;GO:0030246;GO:0043654;GO:0002376;GO:0046597;GO:0005576;GO:0001867;GO:0072562;GO:0005515;GO:0006508;GO:0046872;GO:0005581 | hsa:8547 |  | ficolin-3 isoform 1 precursor [Homo sapiens] |
| A0A0C4DH33 | 6490.322412 | 10923.81908 | 1.683093441 | 0.751115274 | 0.016167913 | yes | up | GO:0009897;GO:0050853;GO:0045087;GO:0016020;GO:0006910;GO:0006911;GO:0002376;GO:0005576;GO:0002250;GO:0003823;GO:0005886;GO:0072562;GO:0042571;GO:0006958;GO:0050871;GO:0034987;GO:0042742 | hsa:102723407 | | immunoglobulin heavy chain variable region, partial [Homo sapiens] |
| P0DP01 | 24281.91167 | 39861.82217 | 1.641626192 | 0.715125654 | 0.032061739 | yes | up | GO:0016020;GO:0002376;GO:0005576;GO:0002250;GO:0003823;GO:0005886 | hsa:102723407 | | immunoglobulin heavy chain variable region, partial [Homo sapiens] |
| P01817 | 25906.94674 | 40830.34333 | 1.576038417 | 0.656302702 | 0.005026666 | yes | up | GO:0038096;GO:0004252;GO:0016020;GO:0030449;GO:0050776;GO:0006898;GO:0038095;GO:0002376;GO:0050900;GO:0005576;GO:0006956;GO:0002250;GO:0003823;GO:0005886;GO:0006508;GO:0006958;GO:0006955 | hsa:102723407 | | RecName: Full=Immunoglobulin heavy variable 2-5; AltName: Full=Ig heavy chain V-II region HE; AltName: Full=Ig heavy chain V-II region MCE; Flags: Precursor |
| Q5SRP5 | 12873.06981 | 20006.02615 | 1.554099096 | 0.636078499 | 0.01204523 | yes | up | GO:0005319;GO:0034361;GO:0034375;GO:0034445;GO:0034365;GO:0034366;GO:0016209;GO:0005543;GO:0034362;GO:0034384;GO:0043691;GO:0033344;GO:0042157;GO:0034364;GO:0034380 | hsa:55937 |  | APOM isoform 1 [Pan troglodytes] |
| P19823 | 928446.2053 | 1441078.625 | 1.552140142 | 0.634258824 | 6.39E-11 | yes | up | GO:0044267;GO:0030212;GO:0070062;GO:0043687;GO:0072562;GO:0062023;GO:0030414;GO:0005788;GO:0004866;GO:0004867;GO:0010951;GO:0010466;GO:0005576 | hsa:3698 | COG2304 | inter-alpha-trypsin inhibitor heavy chain H2 precursor [Homo sapiens] |
| P35527 | 98069.04789 | 150298.3288 | 1.532576608 | 0.615959191 | 0.005096177 | yes | up | GO:0005200;GO:0045109;GO:0031424;GO:0005615;GO:0005829;GO:0016020;GO:0070268;GO:0005198;GO:0007283;GO:0005882;GO:0008544;GO:0005634;GO:0043588;GO:0070062 | hsa:3857 |  | keratin, type I cytoskeletal 9 [Homo sapiens] |
| P01814 | 22045.15142 | 33749.13708 | 1.530909742 | 0.614389229 | 0.000527967 | yes | up | GO:0038096;GO:0004252;GO:0016020;GO:0030449;GO:0050776;GO:0006898;GO:0038095;GO:0002376;GO:0050900;GO:0005576;GO:0006956;GO:0002250;GO:0003823;GO:0005886;GO:0006508;GO:0006958;GO:0006955 | hsa:102723407 | | RecName: Full=Immunoglobulin heavy variable 2-70; AltName: Full=Ig heavy chain V-II region COR; AltName: Full=Ig heavy chain V-II region DAW; AltName: Full=Ig heavy chain V-II region OU; AltName: Full=Ig heavy chain V-II region SESS; Flags: Precursor |
| P0DJI8 | 25729.83263 | 37156.06125 | 1.44408484 | 0.530155503 | 0.010599329 | yes | up | GO:0000187;GO:0042056;GO:0019221;GO:0001664;GO:0034364;GO:0048246;GO:0048247;GO:0044267;GO:0050728;GO:0050708;GO:0005615;GO:0071682;GO:0045785;GO:0007186;GO:0030593;GO:0045087;GO:0005881;GO:0006953;GO:0050918;GO:0008201;GO:0030168;GO:0070062;GO:0050716;GO:0050715;GO:0006898;GO:0007204;GO:0005576 | hsa:6288 |  | RecName: Full=Serum amyloid A-1 protein; Short=SAA; Contains: RecName: Full=Amyloid protein A; AltName: Full=Amyloid fibril protein AA; Contains: RecName: Full=Serum amyloid protein A(2-104); Contains: RecName: Full=Serum amyloid protein A(3-104); Contains: RecName: Full=Serum amyloid protein A(2-103); Contains: RecName: Full=Serum amyloid protein A(2-102); Contains: RecName: Full=Serum amyloid protein A(4-101); Flags: Precursor |
| P03951 | 9806.567526 | 13987.7555 | 1.426366102 | 0.512344323 | 0.004204054 | yes | up | GO:0004252;GO:0016020;GO:0007599;GO:0070062;GO:0016787;GO:0005615;GO:0007597;GO:0007596;GO:0031639;GO:0030193;GO:0005576;GO:0005515;GO:0005886;GO:0070009;GO:0006508;GO:0008233;GO:0008236;GO:0008201;GO:0042802;GO:0051919 | hsa:2160 | COG5640 | coagulation factor XI isoform 1 preproprotein [Homo sapiens] |
| A0A0J9YVY3 | 48104.57 | 68573.47571 | 1.425508548 | 0.51147669 | 0.012173375 | yes | up | GO:0016020;GO:0002376;GO:0005576;GO:0002250;GO:0003823;GO:0005886 | hsa:102723407 | | RecName: Full=Immunoglobulin heavy variable 7-4-1; Flags: Precursor |
| Q92954 | 34921.39316 | 47163.22458 | 1.350553925 | 0.433551245 | 0.03777674 | yes | up | GO:0008283;GO:0005044;GO:0006898;GO:0030247;GO:0030021;GO:0005576;GO:0006955;GO:0062023 | hsa:10216 |  | unnamed protein product [Homo sapiens] |
| Q16880 | 566498.0211 | 760697.2958 | 1.342806625 | 0.42525156 | 0.041491385 | yes | up | GO:0008489;GO:0007417;GO:0030913;GO:0016021;GO:0016020;GO:0006682;GO:0016740;GO:0006687;GO:0015020;GO:0006665;GO:0006629;GO:0002175;GO:0008152;GO:0005886;GO:0008194;GO:0007010;GO:0043231;GO:0007422;GO:0048812;GO:0016757;GO:0047263;GO:0016758 | hsa:7368 | COG1819 | 2-hydroxyacylsphingosine 1-beta-galactosyltransferase precursor [Homo sapiens] |
| P02751 | 630609.2684 | 841470.4125 | 1.334376855 | 0.416166171 | 0.011238715 | yes | up | GO:0008022;GO:0018149;GO:0019221;GO:0034446;GO:0048146;GO:0005518;GO:0005201;GO:0005788;GO:0062023;GO:0007160;GO:0007161;GO:0050900;GO:0010628;GO:0097718;GO:0001525;GO:0044267;GO:0070062;GO:0031093;GO:0002576;GO:0051087;GO:0009611;GO:0072562;GO:0008201;GO:0005576;GO:0002020;GO:0030198;GO:1904237;GO:0033622;GO:0042802;GO:0008284;GO:0005178;GO:2001202;GO:0008360;GO:0005793;GO:0016324;GO:0043687;GO:0042060;GO:0006953;GO:0043394;GO:0019899;GO:0007155;GO:0051702;GO:0010952;GO:0035987;GO:1901166;GO:0005615;GO:0052047;GO:0005604;GO:0045773;GO:0031012;GO:0005577;GO:0007044;GO:0005515;GO:0016504;GO:0005102;GO:0001932;GO:0070372 | hsa:2335 |  | fibronectin isoform 1 precursor [Homo sapiens] |
| A0A0C4DH36 | 52856.89 | 69261.3875 | 1.310356843 | 0.389959747 | 0.003920642 | yes | up | GO:0009897;GO:0050853;GO:0045087;GO:0006910;GO:0006911;GO:0042742;GO:0003823;GO:0034987;GO:0042571;GO:0006958;GO:0050871;GO:0072562 | hsa:102723407 | | immunoglobulin heavy chain variable gene IGHV3-38, partial [Homo sapiens] |
| P01782 | 209357.0895 | 272726.6167 | 1.302686321 | 0.381489733 | 0.037912195 | yes | up | GO:0038096;GO:0004252;GO:0016020;GO:0030449;GO:0050776;GO:0006898;GO:0038095;GO:0002376;GO:0050900;GO:0005576;GO:0006956;GO:0002250;GO:0003823;GO:0005886;GO:0006508;GO:0006958;GO:0006955;GO:0070062 | hsa:102723407 | | hCG2038940, partial [Homo sapiens] |
| O75882 | 38134.91316 | 49620.2125 | 1.301175442 | 0.379815499 | 0.000159275 | yes | up | GO:0005737;GO:0043473;GO:0042552;GO:0005615;GO:0070062;GO:0016021;GO:0016020;GO:0030246;GO:0006954;GO:0005576;GO:0005887;GO:0005886;GO:0038023;GO:0021549;GO:0006979;GO:0040014 | hsa:8455 |  | attractin isoform 1 preproprotein [Homo sapiens] |
| A0A075B6S5 | 48086.14368 | 62380.625 | 1.297268199 | 0.375476775 | 0.026581515 | yes | up | GO:0016020;GO:0005615;GO:0006955;GO:0002376;GO:0002377;GO:0005576;GO:0002250;GO:0003823;GO:0005886 | hsa:7441 |  | monoclonal IgM antibody light chain [Homo sapiens] |
| A0A096LPE2 | 482254.3211 | 623219.7375 | 1.292305139 | 0.36994676 | 0.016161466 | yes | up | GO:0042056;GO:0034364;GO:0005615;GO:0006953;GO:0005576;GO:0060326;GO:0050918;GO:0070062 | hsa:6291;hsa:100528017 | | SAA2-SAA4 protein precursor [Homo sapiens] |
| D6R934 | 95974.26 | 119813.6633 | 1.248393719 | 0.320073003 | 5.98E-05 | yes | up | GO:0006958;GO:0005576;GO:0005581 | hsa:713 |  | complement C1q subcomponent subunit B precursor [Homo sapiens] |
| P02753 | 55930.01158 | 68764.06375 | 1.229466288 | 0.298032177 | 0.02692932 | yes | up | GO:0060347;GO:0032024;GO:0048562;GO:0030324;GO:0060059;GO:0060044;GO:0042593;GO:0051024;GO:0060065;GO:0042572;GO:0001523;GO:0016918;GO:0005615;GO:0048807;GO:0006094;GO:0005501;GO:0032526;GO:0048706;GO:0007601;GO:0045471;GO:0001654;GO:0034633;GO:0070062;GO:0007507;GO:0032991;GO:0060157;GO:0005829;GO:0019841;GO:0048738;GO:0005576;GO:0005515;GO:0060068;GO:0034632;GO:0046982;GO:0050896;GO:0030277 | hsa:5950 |  | retinol-binding protein 4 isoform a precursor [Homo sapiens] |
| P15169 | 17697.84421 | 21634.38792 | 1.22243069 | 0.289752669 | 0.027464102 | yes | up | GO:0097060;GO:0005794;GO:0051384;GO:0030449;GO:0005615;GO:0016787;GO:0030141;GO:0004181;GO:0004180;GO:0010815;GO:0004185;GO:0005576;GO:0043025;GO:0008270;GO:0016485;GO:0006508;GO:0006518;GO:0046872;GO:0030070;GO:0008233;GO:0008237 | hsa:1369 |  | carboxypeptidase N catalytic chain precursor [Homo sapiens] |
| C9JV77 | 1898157.316 | 2296220.083 | 1.209710104 | 0.27466136 | 0.001594018 | yes | up | GO:0019210;GO:0006907;GO:0005788;GO:0001501;GO:0030500;GO:0030502;GO:0050766;GO:0044267;GO:0070062;GO:0031093;GO:0002576;GO:0050727;GO:0072562;GO:0005794;GO:0043687;GO:0046627;GO:0006953;GO:0062023;GO:0034774;GO:0010951;GO:0004869;GO:0005615;GO:0043312;GO:0005576 | hsa:197 |  | alpha-2-HS-glycoprotein isoform 1 preproprotein [Homo sapiens] |
| Q14624 | 181299.5368 | 215482.1778 | 1.188542351 | 0.249193312 | 0.019938276 | yes | no change | GO:0005737;GO:0030212;GO:0034097;GO:0002576;GO:0004867;GO:0072562;GO:0031089;GO:0006953;GO:0005576;GO:0030414;GO:0005515;GO:0004866;GO:0005886;GO:0010951;GO:0010466;GO:0070062 | hsa:3700 | COG2304 | inter-alpha-trypsin inhibitor heavy chain H4 isoform 1 precursor [Homo sapiens] |
| P02760 | 392322.3842 | 455763.3417 | 1.161706189 | 0.216245238 | 0.020078849 | yes | no change | GO:0005886;GO:0010951;GO:0046329;GO:0070062;GO:0019855;GO:0072562;GO:0010466;GO:0042803;GO:0005515;GO:0020037;GO:0009986;GO:0042167;GO:0018298;GO:0062023;GO:0030163;GO:0030414;GO:0004867;GO:0007155;GO:0005615;GO:0050777;GO:0046904;GO:0019862;GO:0007565;GO:0006898;GO:0043231;GO:0005576;GO:0016032 | hsa:259 |  | protein AMBP preproprotein [Homo sapiens] |
| P01024 | 6968412.895 | 7993185.208 | 1.147059643 | 0.197940408 | 0.016598274 | yes | no change | GO:0045087;GO:0004252;GO:0006631;GO:0030449;GO:0004866;GO:0005886;GO:0005788;GO:0035578;GO:0007165;GO:0031715;GO:0048260;GO:0010828;GO:0010866;GO:0050766;GO:0044267;GO:0070062;GO:0009617;GO:0045766;GO:1905114;GO:0045745;GO:0097242;GO:2000427;GO:0034774;GO:0007186;GO:0009986;GO:0005576;GO:0006629;GO:0150064;GO:0043687;GO:0016322;GO:0006911;GO:0150062;GO:0006956;GO:0006957;GO:0006954;GO:0006955;GO:0010951;GO:0060100;GO:0006958;GO:0006508;GO:0005615;GO:0050776;GO:0032991;GO:0010575;GO:0043312;GO:0002376;GO:0001798;GO:0010884;GO:0001970;GO:0005515;GO:0097278;GO:0005102;GO:0001934;GO:0072562 | hsa:718 |  | complement C3 preproprotein [Homo sapiens] |
| C9JC84 | 4693296.895 | 5372966.625 | 1.144817118 | 0.195117149 | 0.040201339 | yes | no change | GO:0051258;GO:0005102;GO:0007596;GO:0005577;GO:0030168 | hsa:2266 |  | FGG isoform 6 [Pan troglodytes] |
| P19827 | 314246.0632 | 355189.1833 | 1.130290002 | 0.176692977 | 0.040590813 | yes | no change | GO:0030212;GO:0070062;GO:0072562;GO:0010466;GO:0005509;GO:0030414;GO:0004867;GO:0010951;GO:0005576 | hsa:3697 | COG2304 | inter-alpha-trypsin inhibitor heavy chain H1 isoform a preproprotein [Homo sapiens] |
| P02768 | 465645313.7 | 501513014.7 | 1.077027944 | 0.107055681 | 0.045347667 | yes | no change | GO:0034375;GO:0015643;GO:0005788;GO:0008144;GO:0051659;GO:0030170;GO:0003677;GO:0005783;GO:0005737;GO:0044267;GO:0019836;GO:0070062;GO:0031093;GO:0043209;GO:0002576;GO:0051087;GO:0072562;GO:0098869;GO:0043066;GO:0005504;GO:0005507;GO:0043687;GO:0032460;GO:0042802;GO:0005794;GO:0140272;GO:0016209;GO:0008289;GO:0019825;GO:0009267;GO:0001895;GO:0005615;GO:0046872;GO:0032991;GO:0043069;GO:0006898;GO:1903981;GO:0005576;GO:0005515;GO:0005634 | hsa:213 |  | serum albumin preproprotein [Homo sapiens] |
| B7ZKJ8 | 544967.0421 | 489822.0417 | 0.898810394 | -0.153911287 | 0.004399043 | yes | no change | GO:0005737;GO:0030212;GO:0034097;GO:0004867;GO:0006953;GO:0005886;GO:0010951 | hsa:3700 | COG2304 | ITIH4 protein [Homo sapiens] |
| P25311 | 376893.9737 | 333564.1083 | 0.885034338 | -0.176194665 | 0.036813591 | yes | no change | GO:0008285;GO:0090501;GO:0071806;GO:0070062;GO:0009897;GO:0001580;GO:0005615;GO:0005634;GO:0004540;GO:0008320;GO:0062023;GO:0005515;GO:0005886;GO:0006955;GO:0007155;GO:0001895;GO:0055085;GO:0005576 | hsa:563 |  | zinc-alpha-2-glycoprotein precursor [Homo sapiens] |
| P14151 | 34739.74474 | 30580.76667 | 0.880281847 | -0.183962578 | 0.02542557 | yes | no change | GO:0009897;GO:0016339;GO:0030246;GO:0050900;GO:0050901;GO:0070492;GO:0030667;GO:0043208;GO:0016021;GO:0016020;GO:0005509;GO:0002020;GO:0046872;GO:0009986;GO:0005887;GO:0005886;GO:0007155;GO:0050839;GO:0008201;GO:0050776;GO:0043312;GO:0005515;GO:0033198 | hsa:6402 |  | L-selectin [Homo sapiens] |
| P01008 | 531044.1632 | 459821.625 | 0.865882081 | -0.207757528 | 0.012524322 | yes | no change | GO:0007599;GO:0007595;GO:2000266;GO:0007596;GO:0005788;GO:0002438;GO:0070062;GO:0072562;GO:0010466;GO:0002020;GO:0043687;GO:0042802;GO:0044267;GO:0007584;GO:0062023;GO:0030414;GO:0004867;GO:0010951;GO:0005886;GO:0008201;GO:0005615;GO:0005576;GO:0005515;GO:0030193 | hsa:462 | COG4826 | antithrombin-III isoform 1 precursor [Homo sapiens] |
| P07358 | 44602.62263 | 38317.925 | 0.859095783 | -0.219109105 | 0.01625141 | yes | no change | GO:0019835;GO:0030449;GO:0005615;GO:0045087;GO:0016020;GO:0002376;GO:0005576;GO:0006956;GO:0006957;GO:0006955;GO:0044877;GO:0006958;GO:1903561;GO:0005579;GO:0070062 | hsa:732 |  | RecName: Full=Complement component C8 beta chain; AltName: Full=Complement component 8 subunit beta; Flags: Precursor |
| H0YAC1 | 177889.1368 | 151327.6013 | 0.850684892 | -0.233303262 | 0.012859706 | yes | no change | GO:0004252;GO:0004497;GO:0007597;GO:0008236;GO:0008233;GO:0016491;GO:0070062;GO:0016705;GO:0031639;GO:0031638;GO:0006508;GO:0005506;GO:0046872;GO:0022617;GO:0051919;GO:0016787;GO:0020037;GO:0005886;GO:0055114;GO:0005615;GO:0042730;GO:0002542;GO:0005576 | hsa:3818 | COG5640 | KLKB1 isoform 4, partial [Pan troglodytes] |
| Q96IY4 | 27470.42211 | 23241.97958 | 0.846072896 | -0.241146126 | 0.023298642 | yes | no change | GO:0007599;GO:2000346;GO:0007596;GO:0008270;GO:0030449;GO:0008233;GO:0008237;GO:0005615;GO:0004181;GO:0004180;GO:0071333;GO:0006508;GO:0046872;GO:0051918;GO:0016787;GO:0009408;GO:0070062;GO:0042730;GO:0003331;GO:0097421;GO:0042493;GO:0005623;GO:0005576;GO:0010757 | hsa:1361 | COG2866 | carboxypeptidase B2 isoform 1 preproprotein [Homo sapiens] |
| P36955 | 119127.5616 | 100758.2771 | 0.845801557 | -0.241608878 | 0.004275651 | yes | no change | GO:0007614;GO:0030424;GO:0010629;GO:0060041;GO:0060770;GO:0007275;GO:0043203;GO:0005615;GO:0071300;GO:0010447;GO:0001822;GO:0050769;GO:0071333;GO:0048471;GO:0046685;GO:0043025;GO:0071279;GO:0008283;GO:0050728;GO:0062023;GO:0010976;GO:0004867;GO:0010951;GO:1901215;GO:0070062;GO:0042470;GO:0071549;GO:0005604;GO:0007568;GO:0010596;GO:0042698;GO:0005576;GO:0005515;GO:0016525 | hsa:5176 | COG4826 | pigment epithelium-derived factor isoform 1 precursor [Homo sapiens] |
| P07360 | 43152.80158 | 35935.62708 | 0.832753049 | -0.264039364 | 0.01803123 | yes | no change | GO:0019835;GO:0036094;GO:0030449;GO:0070062;GO:0045087;GO:0019841;GO:0072562;GO:0002376;GO:0001848;GO:0005576;GO:0044877;GO:0006957;GO:0006958;GO:0005579;GO:0005615 | hsa:733 |  | complement component C8 gamma chain precursor [Homo sapiens] |
| P01009 | 5993319.158 | 4974463.417 | 0.830001421 | -0.268814289 | 0.003609873 | yes | no change | GO:0048208;GO:0007599;GO:0005783;GO:0007596;GO:0033116;GO:0005788;GO:1904813;GO:0030134;GO:0044267;GO:0006888;GO:0005615;GO:0031093;GO:0002576;GO:0010466;GO:0002020;GO:0042802;GO:0005794;GO:0043687;GO:0006953;GO:0030414;GO:0004867;GO:0010951;GO:0070062;GO:0043231;GO:0043312;GO:0000139;GO:0005576;GO:0005515 | hsa:5265 | COG4826 | alpha-1-antitrypsin precursor [Homo sapiens] |
| P02750 | 154796.7511 | 128410.7275 | 0.829544074 | -0.269609461 | 0.025374935 | yes | down | GO:0043231;GO:0016020;GO:0070062;GO:0005160;GO:0009617;GO:0050873;GO:0045766;GO:0043312;GO:1904813;GO:0001938;GO:1904724;GO:0005576;GO:0003674;GO:0005515;GO:0035580;GO:0008150;GO:0030511;GO:0005615 | hsa:116844 | COG4886 | leucine-rich alpha-2-glycoprotein precursor [Homo sapiens] |
| Q8N1N4 | 172097.1737 | 137721.1304 | 0.800252134 | -0.321473477 | 0.037014957 | yes | down | GO:0005615;GO:0031424;GO:0005829;GO:0045095;GO:0070268;GO:0005198;GO:0005882;GO:0070062 | hsa:196374 |  | keratin, type II cytoskeletal 78 isoform 1 [Homo sapiens] |
| A0A2R8Y3M9 | 189805.2842 | 151192.2096 | 0.796564807 | -0.328136353 | 0.000474955 | yes | down | GO:0004252;GO:0006898;GO:0005044;GO:0016020;GO:0016042;GO:0005576;GO:0005509;GO:0006508;GO:0004623 | hsa:81579;hsa:3426 | COG5640 | complement factor I isoform X2 [Homo sapiens] |
| P02766 | 909031.8684 | 718233.1167 | 0.790107742 | -0.339878698 | 0.000206185 | yes | down | GO:0005179;GO:0044267;GO:0032991;GO:0070062;GO:0042562;GO:0070324;GO:0005615;GO:0070327;GO:0005737;GO:0043312;GO:0001523;GO:0035578;GO:0030198;GO:0005515;GO:0042572;GO:0046982;GO:0010469;GO:0006144;GO:0042802;GO:0005576 | hsa:7276 | COG2351 | transthyretin precursor [Homo sapiens] |
| P13473 | 12021.42644 | 9403.000792 | 0.782186776 | -0.35441495 | 0.049512521 | yes | down | GO:0005770;GO:0061684;GO:0101003;GO:0019899;GO:0050821;GO:0044754;GO:0035577;GO:0010008;GO:1990836;GO:0043202;GO:0005615;GO:0097637;GO:0031410;GO:0016021;GO:0016020;GO:0097352;GO:0031902;GO:0046716;GO:0045121;GO:0006914;GO:0017038;GO:0006605;GO:1905146;GO:0061740;GO:0019904;GO:0009267;GO:0005886;GO:0070062;GO:0000421;GO:0030670;GO:0031647;GO:0043312;GO:0031088;GO:0005764;GO:0005765;GO:0005515;GO:0098857;GO:0005768;GO:0002576 | hsa:3920 |  | lysosome-associated membrane glycoprotein 2 isoform A precursor [Homo sapiens] |
| P35908 | 117316.6737 | 90491.12417 | 0.771340691 | -0.374559874 | 0.024650983 | yes | down | GO:0005200;GO:0045095;GO:0018149;GO:0008544;GO:0045684;GO:0043616;GO:0051546;GO:0070062;GO:0032980;GO:0005634;GO:0016020;GO:0005198;GO:0030280;GO:0070268;GO:0005882;GO:0001533;GO:0005615;GO:0045109;GO:0008092;GO:0031424;GO:0005829;GO:0003334;GO:0005515 | hsa:3849 |  | keratin, type II cytoskeletal 2 epidermal [Homo sapiens] |
| A0A140T8Y3 | 10061.29975 | 7673.41365 | 0.76266624 | -0.390876256 | 0.041255888 | yes | down | GO:0005201;GO:0062023;GO:0030199;GO:0030198;GO:0031012 | hsa:7148 |  | tenascin-X isoform 1 precursor [Homo sapiens] |
| P02763 | 2270806.158 | 1697016.625 | 0.747319017 | -0.420203861 | 0.003169774 | yes | down | GO:1904469;GO:0050716;GO:0031093;GO:0005615;GO:0002576;GO:0050718;GO:0043312;GO:0072562;GO:0006953;GO:0032715;GO:0062023;GO:0002682;GO:0005515;GO:0006954;GO:0035580;GO:0005576;GO:1904724;GO:0032720;GO:0070062 | hsa:5004 |  | RecName: Full=Alpha-1-acid glycoprotein 1; Short=AGP 1; AltName: Full=Orosomucoid-1; Short=OMD 1; Flags: Precursor |
| A0A0B4J1Y8 | 106914.9979 | 78605.33 | 0.735213315 | -0.443765201 | 0.029430541 | yes | down | GO:0016020;GO:0005615;GO:0006955;GO:0002376;GO:0002377;GO:0005576;GO:0002250;GO:0003823;GO:0005886 | hsa:7441 |  | Unknown (protein for IMAGE:4575521), partial [Homo sapiens] |
| K7ERG9 | 12504.43326 | 9029.719 | 0.722121412 | -0.469686674 | 0.006770387 | yes | down | GO:0004252;GO:0007219;GO:0005615;GO:0016787;GO:0009617;GO:0006957;GO:0006508;GO:0008233;GO:0008236 | hsa:1675 | COG5640 | complement factor D isoform 2 precursor [Homo sapiens] |
| P19652 | 1147756.379 | 828048.6208 | 0.721449809 | -0.471029064 | 4.22E-05 | yes | down | GO:1904469;GO:0050716;GO:0031093;GO:0005615;GO:0002576;GO:0050718;GO:0043312;GO:0072562;GO:0006953;GO:0035578;GO:0062023;GO:0002682;GO:0035580;GO:0005576;GO:0070062 | hsa:5005 |  | alpha-1-acid glycoprotein 2 precursor [Homo sapiens] |
| P02042 | 91669.92789 | 65725.565 | 0.716980656 | -0.4799939 | 0.020629144 | yes | down | GO:0005344;GO:0019825;GO:0020037;GO:0043177;GO:0031721;GO:0007596;GO:0072562;GO:0005833;GO:0098869;GO:0042744;GO:0005515;GO:0031838;GO:0046872;GO:0005829;GO:0015671 | hsa:3045 | COG1018 | hemoglobin subunit delta [Homo sapiens] |
| P80748 | 678144.7579 | 482747.5292 | 0.711865016 | -0.490324391 | 0.001643171 | yes | down | GO:0038096;GO:0004252;GO:0016020;GO:0030449;GO:0050776;GO:0006898;GO:0038095;GO:0072562;GO:0002376;GO:0050900;GO:0005576;GO:0006956;GO:0002250;GO:0003823;GO:0005886;GO:0006508;GO:0006958;GO:0006955;GO:0070062 | hsa:7441 |  | hCG2040021, partial [Homo sapiens] |
| P69905 | 816929.0632 | 554854.3042 | 0.679195207 | -0.558101817 | 0.021820518 | yes | down | GO:0005344;GO:0022627;GO:0015701;GO:0042744;GO:0010942;GO:0005615;GO:0071682;GO:0016020;GO:0072562;GO:0005833;GO:0098869;GO:0005506;GO:0046872;GO:0015671;GO:0020037;GO:0031720;GO:0051291;GO:0070062;GO:0019825;GO:0042542;GO:0005829;GO:0006898;GO:0005576;GO:0005515;GO:0031838;GO:0004601 | hsa:3040;hsa:3039 | COG1018 | TPA: globin C1 [Homo sapiens] |
| A0A0A0MS15 | 304227.7211 | 206527.4629 | 0.678858134 | -0.55881798 | 0.021919445 | yes | down | GO:0009897;GO:0050853;GO:0045087;GO:0016020;GO:0006910;GO:0006911;GO:0002376;GO:0005576;GO:0002250;GO:0003823;GO:0005886;GO:0072562;GO:0042571;GO:0006958;GO:0050871;GO:0034987;GO:0042742 | hsa:102723407 | | RecName: Full=Immunoglobulin heavy variable 3-49; Flags: Precursor |
| P01019 | 227177.4053 | 153225.3458 | 0.674474408 | -0.568164393 | 5.70E-09 | yes | down | GO:1903779;GO:0038166;GO:2001238;GO:0035106;GO:0003014;GO:0007166;GO:0050729;GO:0001822;GO:0042127;GO:0032930;GO:0006606;GO:0014824;GO:0019229;GO:0010976;GO:0010873;GO:0050731;GO:0035815;GO:0005829;GO:0007202;GO:0007204;GO:0003081;GO:1904754;GO:0010536;GO:0007565;GO:0005179;GO:0034374;GO:0007200;GO:0071260;GO:0042310;GO:0042311;GO:0048169;GO:0008083;GO:1901201;GO:0072562;GO:0006883;GO:0061098;GO:0007186;GO:2000379;GO:0090190;GO:0048018;GO:0008306;GO:0003331;GO:0033864;GO:0007199;GO:0001974;GO:0062023;GO:0048659;GO:0051969;GO:0016525;GO:0014873;GO:0030308;GO:0048146;GO:0048144;GO:0007267;GO:0045429;GO:1905010;GO:0043085;GO:0005737;GO:0050880;GO:0003051;GO:0002027;GO:0045742;GO:0032270;GO:0070471;GO:0008284;GO:0031701;GO:0031703;GO:0001558;GO:0014068;GO:0004867;GO:0014061;GO:0051092;GO:1904385;GO:0007568;GO:0070062;GO:0002034;GO:2001275;GO:0005515;GO:0070371;GO:0001819;GO:0051387;GO:0010469;GO:0010666;GO:0008217;GO:0061049;GO:0050663;GO:0032355;GO:0005615;GO:0097755;GO:0042981;GO:0010744;GO:0035813;GO:1904707;GO:0046628;GO:0034104;GO:0019216;GO:0051403;GO:0010951;GO:0007263;GO:1905589;GO:0010613;GO:0010595;GO:0002019;GO:0002018;GO:0045777;GO:0002016;GO:0031702;GO:0051924;GO:0005576;GO:1903598;GO:0045893 | hsa:183 | COG4826 | angiotensinogen preproprotein [Homo sapiens] |
| Q9Y5Y7 | 16941.61205 | 11422.03071 | 0.674199756 | -0.56875199 | 0.021285394 | yes | down | GO:0038023;GO:0070062;GO:0005540;GO:0016021;GO:0016020;GO:0009611;GO:0007160;GO:0004888;GO:0071944;GO:0005515;GO:0005887;GO:0005886;GO:0007155;GO:0030214;GO:0009653;GO:0006027 | hsa:10894 |  | lymphatic vessel endothelial hyaluronic acid receptor 1 precursor [Homo sapiens] |
| A0A0C4DH73 | 127967.1195 | 86163.89625 | 0.673328403 | -0.570617771 | 0.000313951 | yes | down | GO:0038096;GO:0004252;GO:0016020;GO:0030449;GO:0005615;GO:0006898;GO:0038095;GO:0050900;GO:0002376;GO:0002377;GO:0050776;GO:0005576;GO:0006956;GO:0002250;GO:0003823;GO:0005886;GO:0006508;GO:0006958;GO:0006955 | hsa:7441 |  | immunoglobulin light chain variable region, partial [Homo sapiens] |
| P00918 | 10796.67714 | 7127.791125 | 0.66018378 | -0.599060402 | 0.006739905 | yes | down | 3P3J:A;1IF6:A;1H9N:A;3RYJ:B;5FLQ:A;3P5A:A;1AVN:A;GO:0010043;6BC9:A;3V3H:B;4Q78:A;5JGS:B;5M78:A;1CAM:A;GO:0043209;5TY9:A;3RZ1:B;5EOI:A;5JN3:A;1CNI:A;4ZWY:A;3PJJ:A;3TMJ:A;5LL4:A;4CAC:A;1CNK:A;3T5U:A;6EQU:A;5EH8:A;1G52:A;5JEG:B;1AM6:A;3SBH:A;1CVD:A;4BF1:A;1BNU:A;1I91:A;4PYY:A;3MHL:A;4HEY:A;5FLO:A;5NXP:A;3S78:B;4Q08:A;4K1Q:A;2O4Z:A;2Q38:A;3DD8:A;5CAC:A;4YYT:A;2VVB:X;1CNC:A;2FNN:A;5G0C:A;1CVB:A;1FR7:B;2FOQ:A;3RZ7:A;4KNI:A;6H2Z:A;4QTL:A;4QEF:A;4FVO:A;6BCC:A;3M1Q:A;5LLG:A;6E92:A;3T83:A;5WEX:A;3D8W:A;4YXI:A;5WLV:A;4Q6D:A;5JQT:A;4YXO:A;5TFX:A;2HKK:A;2POU:A;3M1W:A;6MBY:A;5DOH:B;5OGO:A;4QSI:A;1BNV:A;3DBU:A;1IF9:A;3PYK:A;1CAK:A;1G46:A;6D1L:A;5TY8:A;5DRS:A;5E28:A;4Q9Y:A;5LLC:A;6FJI:A;3MNH:A;1I9P:A;1CIL:A;4FIK:A;6BBS:A;3OIL:A;1LG6:A;4BF6:A;6IC2:A;6C7W:A;1CIN:A;1G4O:A;1BNM:A;4YGL:A;4HF3:A;2CBE:A;3KOK:A;GO:0016829;4MDG:A;3HS4:A;5DSM:A;4K0Z:A;2F14:A;5FNL:A;4JSW:A;GO:0045177;5BRU:A;5DSO:A;3DVC:A;2WD2:A;3RG3:A;4KUV:A;4HEZ:A;5LL4:B;1I9M:A;1RZD:A;5DSL:A;3B4F:A;1TE3:X;5BNL:A;4YGK:A;GO:0016323;5U0G:A;5LMD:A;5CA2:A;4DZ9:A;5SZ1:A;1ZSB:A;1YDB:A;1YO0:A;4E49:A;4YVY:A;4KV0:A;2X7S:A;5THN:A;4KUW:A;1YDD:A;1YO2:A;1I8Z:A;2X7U:A;2CBC:A;5JQ0:A;4QSA:A;3S74:B;3P58:A;3M67:A;3IGP:A;3S76:A;3EFT:A;5TYA:A;3IEO:A;4JSZ:A;3V7X:A;5N24:A;3HKU:A;3KKX:A;5ZXW:A;1BN3:A;2EZ7:A;5JDV:B;5FLT:A;4E4A:A;GO:0030424;1CVH:A;GO:0004064;1XEG:A;5G03:A;2AW1:A;3F8E:A;1RAZ:A;5W8B:A;5G01:A;5N1R:A;5L70:B;2ABE:A;2EU3:A;5L70:A;3IBU:A;GO:0005615;6EDA:A;2GEH:A;5N1S:A;4Q7W:A;1CAY:A;5EKM:A;3D93:A;3M2Z:A;5MJN:A;1ZFQ:A;2NNO:A;GO:0038166;3R17:B;3S9T:A;1BV3:A;5VGY:A;4IWZ:A;1G3Z:A;5DSP:A;4Z0Q:A;GO:0045780;1XPZ:A;3RZ5:A;1UGD:A;GO:0016020;5AMD:A;1RAY:A;4M2U:A;1UGF:A;3DV7:A;3S75:B;1RZE:A;4PXX:A;1BNT:A;5DOG:A;3P4V:A;2WEG:A;6EBE:A;3KS3:A;5WLR:A;5SZ2:A;1KWQ:A;1CIM:A;1CNG:A;5AML:A;2NNV:A;GO:0005829;1CCS:A;5OGP:A;GO:0043627;1CVF:A;1TTM:A;4FVN:A;4JSA:A;1TBT:X;1FSQ:A;5SZ3:A;4RFC:A;1FSQ:B;3MNJ:A;2NWP:A;5FNJ:A;1OKM:A;5LLE:A;4ITP:A;2WD3:A;3K7K:A;1G4J:A;2H4N:A;4HBA:A;1ZH9:A;4R5B:A;5T71:A;6ECZ:A;5L6T:A;5L6T:B;2AX2:A;3P44:A;5TI0:A;GO:2001225;2QOA:A;6CA2:A;4G0C:A;1CA2:A;1CAO:A;3V3F:A;4Z1K:A;1FR7:A;4QK3:A;1CAI:A;5N0E:A;4PQ7:A;3HKN:A;1RZC:A;1ZGF:A;4QK1:A;5GMN:A;4E3D:A;1CNW:A;5ULN:A;GO:0048545;2FOV:A;4FRC:A;2FNK:A;2HD6:A;2WEO:A;1TG9:A;3M40:A;5BRV:A;4Q7P:A;4Q8Z:A;2QO8:A;5DSK:A;4RIV:A;3KOI:A;5Y2S:A;4Q8X:A;3HLJ:A;1LZV:A;1G54:A;5C8I:A;3L14:A;3N2P:A;3OYQ:A;3CAJ:A;2POV:A;6EEO:A;4WL4:A;6B4D:A;5YUJ:A;4FPT:A;3GZ0:A;5NXV:A;1OQ5:A;6GOT:A;5J8Z:A;3ZP9:A;4RUY:A;6B5A:A;2NWZ:A;1I9O:A;1THK:A;2EU2:A;5SZ7:A;4L5W:A;4M2W:A;6H6S:A;5JMZ:A;4Q7V:A;2Q1Q:A;4HT0:A;4QSB:A;4QJM:A;1BNN:A;2NXS:A;GO:0005515;3M1K:A;4Q99:A;3M2X:A;1H9Q:A;6G6T:A;4MDM:A;1FQM:A;5FDC:A;1H4N:A;5SZ5:A;4Q6E:A;3SAP:A;1EOU:A;1TEQ:X;5FLP:A;5T75:A;5NXI:A;3K34:A;2HOC:A;6CJV:A;6FJJ:A;1CNJ:A;3P5L:A;1G53:A;3V5G:A;4MDL:A;1ZSC:A;6HX5:A;4XE1:A;1ZSA:A;1TG3:A;6GXE:A;5JEH:B;4MO8:A;2X7T:A;4CA2:A;1IF7:A;6CEH:A;4QY3:A;GO:0046903;1LGD:A;4Q09:A;2CBD:A;1IF5:A;1CNB:A;3FFP:X;3DCW:A;5NXO:A;5LL8:A;3RYY:A;4Z1E:A;3R16:A;3BET:A;GO:0032849;5JEP:B;5G0B:A;4ZAO:A;3ML2:A;1BCD:A;3TVN:X;4Y0J:A;5NXW:A;6EEA:A;1BN4:A;1UGB:A;4K13:A;5DOH:A;3C7P:A;4RFD:A;GO:0001822;1TB0:X;5SZ4:A;5DSQ:A;GO:0046872;5L6K:B;3N3J:A;4Q07:A;3T84:A;1IF8:A;12CA:A;1DCA:A;4QK2:A;5E2K:A;4Q83:A;3SAX:A;5LVS:B;5T74:A;1CAZ:A;6D1M:A;6GXB:A;3RYZ:A;5LVS:A;2FMZ:A;3IBI:A;2QP6:A;1I9Q:A;4E5Q:A;3OIM:A;5NEA:A;6B59:A;6H29:A;2FOU:A;3M5T:A;5THI:A;4GL1:X;4Q87:A;2WEJ:A;1G0F:A;GO:2001150;6H34:A;6G3Q:A;3CYU:A;5O07:A;6QEB:A;3RGE:A;5E2R:A;5YUK:A;5EH5:A;4MLT:A;5EIJ:A;3RZ0:B;5FLS:A;3NB5:A;5EH7:A;1MOO:A;3U3A:X;4Z1J:A;3SBI:A;4Q90:A;2VVA:X;1I90:A;1XQ0:A;5NXG:A;1ZFK:A;3MHO:A;5FNI:A;6H6S:B;2OSM:A;3VBD:A;2FNM:A;3MHM:A;4K0S:A;3MNU:A;5EHV:A;5FNG:A;4E3G:A;3RZ8:A;4YWP:A;5THJ:A;1CRA:A;1RZA:A;2NNS:A;1FR4:A;3T5Z:A;1F2W:A;1CVC:A;3RG4:A;5TH4:A;3PO6:A;5WLU:A;5JN7:A;6E91:A;4ZX0:A;1Z9Y:A;5JN1:A;1HVA:A;GO:0009268;3NI5:A;5A6H:A;3S77:B;GO:0005737;3S71:B;4Q49:A;4ILX:A;3HKT:A;5U0F:A;1BIC:A;3OYS:A;3M96:A;2FMG:A;3KIG:A;5FLR:A;5OGN:B;6GCY:A;2NXT:A;1CAH:A;5SZ0:A;4E3H:A;4ITO:A;4QIY:C;4QIY:B;4QIY:A;4R5A:A;3DCC:A;1CNX:A;1CAJ:A;4MLX:A;2Q1B:A;3M04:A;4L5U:A;1ZGE:A;1LG5:A;5FDI:A;GO:0070062;3D92:A;2H15:A;3MZC:A;3F4X:A;1FQL:A;8CA2:A;5JG3:B;1UGA:A;5E2S:A;4LHI:A;5NY1:A;3M14:A;5LJT:A;3IBN:A;3P55:A;3V3G:B;1CVA:A;3D9Z:A;1HCA:A;5NXM:A;1BNQ:A;GO:0044070;3DVB:A;5JGT:B;4YGN:A;1UGG:A;3CA2:A;1BNW:A;3V2J:A;GO:0010033;1YDA:A;1I9L:A;4ZWZ:A;3RJ7:A;1YDC:A;3DAZ:A;6EEH:A;5WLT:A;6E8P:A;4M2R:A;5L6K:A;1TEU:X;3DCS:A;2CBB:A;3BL0:A;1FSR:B;1FSR:A;1LUG:A;2OSF:A;2ILI:A;1MUA:A;4N16:A;2GD8:A;3MMF:A;5U0D:A;4E3F:A;2HL4:A;3OKV:A;1ZE8:A;5TY1:A;GO:0008270;5JE7:B;3DC9:A;3V3J:A;3S73:B;GO:0032230;3M5S:A;1T9N:A;4Q06:A;1CCU:A;5AMG:A;3MWO:B;7CA2:A;3MWO:A;5T72:A;GO:0004089;5BYI:A;3DC3:A;1CNH:A;2NNG:A;1CA3:A;5EKH:A;5WG7:A;3OY0:A;5NY3:A;1FQR:A;1HEC:A;9CA2:A;2FOS:A;5EKJ:A;3V2M:A;3N4B:A;1HEA:A;2NWO:A;5NEE:A;3K2F:A;6E8X:A;3M2Y:A;3P3H:A;3U7C:A;4FL7:A;4Q81:A;4WW6:A;GO:0015701;3RYV:B;3S8X:A;5U0E:A;GO:0051453;3EFI:A;4RH2:A;5EHW:A;2WEH:A;4Q8Y:A;2POW:A;4Z1N:A;1HEB:A;4RIU:A;1CAL:A;5FNM:A;1I9N:A;4ZWX:A;1G1D:A;1KWR:A;5TXY:A;3MHI:A;5LLH:A;1CCT:A;4RN4:A;3KWA:A;4CQ0:A;4YXU:A;1CVE:A;5SZ6:A;4KAP:A;3QYK:A;4PYX:A;4YGJ:A;2NWY:A;1FSN:B;1FSN:A;4HEW:A;3V3I:B;4N0X:B;1YO1:A;4KNJ:A;3MNK:A;3HFP:A;4FU5:A;5BRW:A;GO:0005886;1G0E:A;1OKN:A;5N25:A;5CLU:A;4JS6:A;GO:0005902;4PZH:A;1OKL:A;5L9E:C;3M2N:A;5LJQ:A;3T82:A;1FQN:A;6MBV:A;4RUZ:A;4DZ7:A;5DSR:A;1A42:A;4RUX:A;3MNA:A;5L3O:B;1CNY:A;4ZWI:A;1CAN:A;3U45:X;3OIK:A;4KUY:A;5NY6:A;3U47:A;5YUI:A;2CBA:A;5N0D:A;5FNH:A;6H33:A;4K0T:A;3N0N:A;3RLD:A;3BL1:A;4ZX1:A;5UMC:A;1RZB:A;3M5E:A;5OGN:A;4LP6:B;4LP6:A;1G45:A;3MNI:A;6GDC:A;3RYX:B;GO:0015670;4YX4:A;1IF4:A;5JG5:B;4JSS:A;3DVD:A;4BCW:A;5WGP:A;5Y2R:A;3TVO:X;3IBL:A;6C7X:A;GO:0002009;3DD0:A;3S72:B;3M3X:A;1UGC:A;4R59:A;3HKQ:A;1UGE:A;3T85:A;4QIY:D;3MYQ:A;4M2V:A;1G48:A;GO:0071498;1XEV:B;2HNC:A;5JES:B;4L5V:A;3NJ9:A;1TH9:A;5L9E:A;1DCB:A;3M98:A;5L9E:B;5L3O:A;5L9E:D;5FNK:A;2CA2:A;GO:0042475;4IDR:X;3KNE:A;5NYA:A;3M1J:A;1HED:A;3MHC:A;2NXR:A;GO:0045672;4Q7S:A;3OKU:A;3KON:A;1XEV:C;5EHE:A;1XEV:A;4MTY:A;1XEV:D;1BN1:A | hsa:760 | COG3338 | carbonic anhydrase 2 isoform 1 [Homo sapiens] |
| Q86UD1 | 7321.776929 | 4825.3435 | 0.65903995 | -0.601562173 | 0.013474237 | yes | down |  | hsa:220323 |  | out at first protein homolog precursor [Homo sapiens] |
| A0A087X0Q4 | 130877.2211 | 85608.28125 | 0.654111392 | -0.612391754 | 0.006579228 | yes | down |  |  |  | RecName: Full=Immunoglobulin kappa variable 2D-40; AltName: Full=Ig kappa chain V-II region Cum; Flags: Precursor |
| P00915 | 22432.51958 | 14078.96017 | 0.627613859 | -0.672050885 | 0.004762743 | yes | down | GO:0005737;GO:0015701;GO:0046872;GO:0070062;GO:0006730;GO:0016829;GO:0008270;GO:0005515;GO:0016836;GO:0004089;GO:0004064;GO:0005829;GO:0035722 | hsa:759 | COG3338 | carbonic anhydrase 1 isoform a [Homo sapiens] |
| A0A2R8Y7X9 | 47762.113 | 29958.3093 | 0.627240032 | -0.672910455 | 0.012223852 | yes | down | GO:0005344;GO:0019825;GO:0020037;GO:0005833;GO:0046872;GO:0015671 | hsa:3048 | COG1018 | hemoglobin subunit gamma-2 [Pan troglodytes] |
| P01718 | 31605.07312 | 19821.73289 | 0.627169341 | -0.673073059 | 0.025613852 | yes | down | GO:0038096;GO:0004252;GO:0016020;GO:0030449;GO:0050776;GO:0006898;GO:0038095;GO:0002376;GO:0050900;GO:0005576;GO:0006956;GO:0002250;GO:0003823;GO:0005886;GO:0006508;GO:0006958;GO:0006955 | hsa:29802 |  | immunoglobulin light chain variable region, partial [Homo sapiens] |
| A0A0J9YXX1 | 357816.2368 | 222593.1875 | 0.622088001 | -0.684809415 | 0.00283349 | yes | down | GO:0016020;GO:0002376;GO:0005576;GO:0002250;GO:0003823;GO:0005886 | hsa:102724971 | | RecName: Full=Immunoglobulin heavy variable 5-10-1; Flags: Precursor |
| Q96HR3 | 35719.59263 | 22188.84 | 0.621195214 | -0.68688138 | 0.00236229 | yes | down | GO:0019827;GO:0030521;GO:0038023;GO:0005515;GO:0006355;GO:0005634;GO:0030518;GO:0003712;GO:0000151;GO:0005654;GO:0046966;GO:0045893;GO:0006367;GO:0016567;GO:0016592;GO:0030374;GO:0061630;GO:0042809;GO:0006351 | hsa:90390 |  | mediator of RNA polymerase II transcription subunit 30 isoform 1 [Homo sapiens] |
| P04264 | 358372.0789 | 222059.4333 | 0.619633745 | -0.69051238 | 0.001046219 | yes | down | GO:0018149;GO:0038023;GO:0045095;GO:0030246;GO:0031012;GO:1904813;GO:0061436;GO:0030280;GO:0005856;GO:0050728;GO:0070062;GO:0005634;GO:0016020;GO:0045765;GO:0072562;GO:0005198;GO:0001867;GO:0005882;GO:0051290;GO:0070268;GO:0062023;GO:0005886;GO:0001895;GO:0006979;GO:0001533;GO:0005615;GO:0042730;GO:0031424;GO:0005829;GO:0043312;GO:0005576;GO:0005515;GO:0046982 | hsa:3848 |  | keratin 1 [Homo sapiens] |
| P13645 | 75944.19947 | 46754.77875 | 0.615646476 | -0.699825949 | 0.003189359 | yes | down | GO:0005737;GO:0018149;GO:0045684;GO:0070062;GO:0030216;GO:0031424;GO:0005615;GO:0009986;GO:0016020;GO:0070268;GO:0051290;GO:0005634;GO:0005882;GO:0005576;GO:0046982;GO:0005198;GO:0005829;GO:0001533;GO:0030280 | hsa:3858 |  | Keratin 10 [Homo sapiens] |
| P02745 | 56027.18421 | 33476.93917 | 0.597512433 | -0.742959361 | 0.01774393 | yes | down | GO:0004252;GO:0030449;GO:0005581;GO:0045087;GO:0002376;GO:0005576;GO:0006956;GO:0005515;GO:0010039;GO:0007267;GO:0006508;GO:0006958;GO:0005602 | hsa:712 |  | complement C1q subcomponent subunit A precursor [Homo sapiens] |
| P13647 | 37475.64211 | 22368.1385 | 0.596871387 | -0.744507999 | 0.000109094 | yes | down | GO:0005737;GO:0016020;GO:0005200;GO:0007010;GO:0070062;GO:0031424;GO:0031581;GO:0045095;GO:0070268;GO:0005198;GO:0005634;GO:0005882;GO:0097110;GO:0005515;GO:0005886;GO:0008544;GO:0005829 | hsa:3852 |  | Keratin 5 [Homo sapiens] |
| P55056 | 293654.3416 | 171445.4054 | 0.583834056 | -0.776369726 | 0.044745529 | yes | down | GO:0005319;GO:0034361;GO:0070328;GO:0006629;GO:0034447;GO:0006869;GO:0034379;GO:0010890;GO:0005576;GO:0034364 | hsa:346 |  | apolipoprotein C-IV precursor [Homo sapiens] |
| P32119 | 22190.79347 | 12268.95304 | 0.552884828 | -0.854949111 | 0.000355494 | yes | down | GO:0000187;GO:0045581;GO:0042744;GO:0008379;GO:0032496;GO:0032088;GO:0042981;GO:0034599;GO:0002536;GO:0016491;GO:0005737;GO:0010310;GO:0070062;GO:0042098;GO:0043066;GO:0048538;GO:2001240;GO:0016209;GO:0045454;GO:0006979;GO:0055114;GO:0048872;GO:0045321;GO:0005829;GO:0005623;GO:0031665;GO:0019430;GO:0005515;GO:0051920;GO:0004601;GO:0030194 | hsa:7001 | COG0450 | peroxiredoxin-2 [Homo sapiens] |
| A0A1W2PQU7 | 150467.4732 | 81805.52458 | 0.543675805 | -0.879181469 | 0.001515363 | yes | down | GO:1904714;GO:0005737;GO:0045111;GO:0061564;GO:0045109;GO:0060291;GO:0016020;GO:0044297;GO:0060020;GO:0005198;GO:0051580;GO:0005883;GO:0005882;GO:0010977;GO:0030198;GO:0014002;GO:0097450;GO:0010625;GO:0031102 | hsa:2670 |  | glial fibrillary acidic protein [Mus musculus] |
| Q9NZP8 | 74769.25305 | 38089.40583 | 0.509426058 | -0.973055337 | 3.20E-05 | yes | down | GO:0004252;GO:0005615;GO:0016787;GO:0070062;GO:0045087;GO:0031638;GO:0002376;GO:0005576;GO:0006508;GO:0006958;GO:0008233;GO:0008236 | hsa:51279 | COG5640 | complement C1r subcomponent-like protein isoform 1 precursor [Homo sapiens] |
| D6RE82 | 209939.3632 | 73169.90329 | 0.348528747 | -1.520650437 | 5.30E-06 | yes | down | GO:0030688;GO:0005730;GO:0005634;GO:0030687;GO:0006364;GO:0003723 | hsa:8568 |  | PREDICTED: ribosomal RNA processing protein 1 homolog A [Callithrix jacchus] |
| A0A0B4J1V2 | 27879.93279 | 6906.333455 | 0.247717005 | -2.013235185 | 0.017725368 | yes | down | GO:0009897;GO:0050853;GO:0045087;GO:0016020;GO:0006910;GO:0006911;GO:0002376;GO:0005576;GO:0002250;GO:0003823;GO:0005886;GO:0072562;GO:0042571;GO:0006958;GO:0050871;GO:0034987;GO:0042742 | hsa:102723407 | | RecName: Full=Immunoglobulin heavy variable 2-26; Flags: Precursor |
| P02533 | 27616.62947 | 124618.9902 | 4.512461967 | 2.173914773 | 0.395381814 | no | up | GO:0005200;GO:0045095;GO:0071944;GO:0010043;GO:0008544;GO:0005737;GO:0045110;GO:0010212;GO:0070062;GO:0005634;GO:0045178;GO:0005198;GO:0030855;GO:1990254;GO:0031581;GO:0070268;GO:0005882;GO:0042633;GO:0007568;GO:0031424;GO:0005829;GO:0005622;GO:0005515 | hsa:3861 |  | keratin, type I cytoskeletal 14 [Homo sapiens] |
| P68032 | 9576.84905 | 27224.60462 | 2.842751773 | 1.507288129 | 0.41560868 | no | up | GO:0055008;GO:0009991;GO:0005200;GO:0015629;GO:0030240;GO:0017022;GO:0030175;GO:0030017;GO:0010628;GO:0009612;GO:0016887;GO:0005925;GO:0060048;GO:0043503;GO:0031032;GO:0005737;GO:0001725;GO:0070062;GO:0071417;GO:0016020;GO:0031674;GO:0072562;GO:0000166;GO:0030027;GO:0033275;GO:0043066;GO:0005524;GO:0005856;GO:0055003;GO:0006936;GO:0048741;GO:0044297;GO:0030049;GO:0030048;GO:0005884;GO:0043531;GO:0010226;GO:0005865;GO:0090131;GO:0005615;GO:0045471;GO:0005829;GO:0042493;GO:0060047;GO:0042643;GO:0070252;GO:0098978;GO:0048545;GO:0045202 | hsa:58;hsa:70;hsa:59;hsa:72 | COG5277 | ACTC1, partial [Cricetulus griseus] |
| P13796 | 685041.9409 | 1360041.786 | 1.985340904 | 0.989386755 | 0.358985301 | no | up | GO:0032432;GO:0033157;GO:0015629;GO:0030175;GO:0051020;GO:0005925;GO:0030054;GO:0005737;GO:0071803;GO:0001726;GO:0002102;GO:0002286;GO:0005615;GO:0016020;GO:0003779;GO:0005509;GO:0048471;GO:0016477;GO:0022617;GO:0042802;GO:0035722;GO:0005178;GO:0031100;GO:0051017;GO:0051015;GO:0005884;GO:0005886;GO:0051764;GO:0042995;GO:0032587;GO:0001891;GO:0046872;GO:0070062;GO:0005829;GO:0005856;GO:0051639;GO:0044319;GO:0001725;GO:0010737 | hsa:3936 | COG5069 | plastin-2 [Homo sapiens] |
| P08779 | 61836.21 | 109549.4108 | 1.771606165 | 0.825057922 | 0.08749544 | no | up | GO:0005200;GO:0061436;GO:0008544;GO:0005856;GO:0002009;GO:0051546;GO:0070062;GO:0005634;GO:0005198;GO:0030336;GO:0030216;GO:0008283;GO:0045087;GO:0070268;GO:0005882;GO:0006954;GO:0042633;GO:0007568;GO:0007010;GO:0045104;GO:0005829;GO:0031424;GO:0005515 | hsa:3868 |  | keratin, type I cytoskeletal 16 [Homo sapiens] |
| Q86UX7 | 5055.186455 | 8737.222565 | 1.728368012 | 0.789410436 | 0.089286625 | no | up | GO:0005178;GO:0030335;GO:0002102;GO:0070062;GO:0031093;GO:0034446;GO:0007229;GO:0016020;GO:0033632;GO:0005576;GO:0007159;GO:0007155;GO:0033622;GO:0042995;GO:0070527;GO:0030054;GO:0002576 | hsa:83706 |  | fermitin family homolog 3 long isoform [Homo sapiens] |
| P01602 | 182768.7774 | 293847.575 | 1.607755872 | 0.685048358 | 0.17829289 | no | up | GO:0038096;GO:0004252;GO:0016020;GO:0030449;GO:0050776;GO:0006898;GO:0038095;GO:0072562;GO:0002376;GO:0002377;GO:0050900;GO:0005576;GO:0006956;GO:0002250;GO:0003823;GO:0005886;GO:0006508;GO:0006958;GO:0006955;GO:0070062 | hsa:7441 |  | hCG2043208, partial [Homo sapiens] |
| P19320 | 2930.442067 | 4600.265593 | 1.569819668 | 0.65059884 | 0.068172216 | no | up | GO:0009897;GO:0034113;GO:0005783;GO:0005794;GO:0035094;GO:0030175;GO:0060945;GO:0005902;GO:0050901;GO:0060326;GO:0010043;GO:0032496;GO:0001666;GO:0019221;GO:0045177;GO:0002102;GO:0010212;GO:0005615;GO:0060384;GO:0071356;GO:0016021;GO:0016020;GO:0050839;GO:0035584;GO:1904646;GO:0140039;GO:0022614;GO:0005178;GO:0042383;GO:0009308;GO:0030183;GO:0098609;GO:0007584;GO:0008131;GO:0045471;GO:0007159;GO:0005887;GO:0005886;GO:0007155;GO:0002526;GO:0007157;GO:0042102;GO:0009986;GO:0055114;GO:0070062;GO:0007160;GO:0050776;GO:0007568;GO:0060333;GO:0002544;GO:0005769;GO:0030198;GO:0035924;GO:0071065 | hsa:7412 |  | vascular cell adhesion protein 1 isoform a precursor [Homo sapiens] |
| A0A0C4DH34 | 941572.7789 | 1456116.231 | 1.54647231 | 0.628981002 | 0.157980734 | no | up | GO:0009897;GO:0050853;GO:0045087;GO:0016020;GO:0006910;GO:0006911;GO:0002376;GO:0005576;GO:0002250;GO:0003823;GO:0005886;GO:0072562;GO:0042571;GO:0006958;GO:0050871;GO:0034987;GO:0042742 | hsa:102723407 | | RecName: Full=Immunoglobulin heavy variable 4-28; Flags: Precursor |
| Q9Y490 | 10605.37706 | 15657.33965 | 1.476358602 | 0.56204319 | 0.322836194 | no | up | GO:0005200;GO:0017166;GO:0007043;GO:0005925;GO:0030054;GO:0005856;GO:0001726;GO:0070062;GO:0002576;GO:0016020;GO:0005737;GO:0003779;GO:0005576;GO:0033622;GO:0001786;GO:0005178;GO:0005515;GO:0006936;GO:0009986;GO:0051015;GO:0036498;GO:0030274;GO:0005886;GO:0007155;GO:0042995;GO:0032587;GO:0007016;GO:0035091;GO:0005829;GO:0030866;GO:0045296;GO:0070527;GO:0007044;GO:0016032;GO:0044877;GO:0007229 | hsa:7094 |  | talin-1 [Homo sapiens] |
| A0A075B6R2 | 129021.1653 | 189176.7156 | 1.466245598 | 0.552126777 | 0.070766791 | no | up | GO:0009897;GO:0050853;GO:0045087;GO:0016020;GO:0006910;GO:0006911;GO:0002376;GO:0005576;GO:0002250;GO:0003823;GO:0005886;GO:0072562;GO:0042571;GO:0006958;GO:0050871;GO:0034987;GO:0042742 | hsa:102724971 | | immunoglobulin heavy chain VDJ region, partial [Homo sapiens] |
| P07737 | 11005.68311 | 15887.21104 | 1.443546111 | 0.529617192 | 0.060848715 | no | up | GO:0017048;GO:0050821;GO:0060074;GO:0045202;GO:0050434;GO:0005925;GO:0005737;GO:0032232;GO:0032233;GO:0070062;GO:0030837;GO:0030838;GO:0051496;GO:0005634;GO:0016020;GO:0005546;GO:0003779;GO:0098794;GO:0043005;GO:0098793;GO:0001843;GO:0005856;GO:0051054;GO:0098685;GO:0005515;GO:0032781;GO:0060071;GO:0098688;GO:0070064;GO:0005938;GO:0000774;GO:0010033;GO:0010634;GO:0071363;GO:0005829;GO:1900029;GO:0098885;GO:0045296;GO:0051497;GO:0006357;GO:0045944;GO:0003723;GO:0030036;GO:0072562;GO:0005102;GO:0098978;GO:0003785 | hsa:5216 |  | PREDICTED: profilin-1 isoform X2 [Nomascus leucogenys] |
| P60709 | 33189.38895 | 47389.20958 | 1.427842183 | 0.51383653 | 0.105601461 | no | up | GO:0043044;GO:0019894;GO:0015629;GO:0072749;GO:0061024;GO:0005200;GO:0021762;GO:0036464;GO:0005925;GO:0048488;GO:0030957;GO:0032091;GO:0005615;GO:0043209;GO:0005634;GO:0016020;GO:0097433;GO:0070062;GO:0098871;GO:0005654;GO:0045815;GO:0016579;GO:0005524;GO:0000980;GO:0005856;GO:0070527;GO:0000166;GO:1903076;GO:0019901;GO:0000790;GO:0005886;GO:0048013;GO:0001895;GO:0098685;GO:0034329;GO:0031982;GO:0038096;GO:0050998;GO:0044305;GO:0032991;GO:0051621;GO:0048870;GO:0030863;GO:0022898;GO:0005829;GO:0051623;GO:1990904;GO:0005737;GO:0098973;GO:0031492;GO:0098793;GO:0042802;GO:0005515;GO:0098974;GO:0072562;GO:0000978;GO:0098978;GO:0000079;GO:0035267 | hsa:71;hsa:60 | COG5277 | cytoskeletal beta actin, partial [Sus scrofa] |
| A0A0G2JSC0 | 30103.95338 | 41204.08064 | 1.368726563 | 0.452834261 | 0.21586754 | no | up |  | hsa:7441 |  | Lambda-V immunoglobulin light chain variable domain precursor, partial [Homo sapiens] |
| P01705 | 88650.06882 | 119684.0262 | 1.350072569 | 0.433036957 | 0.138078357 | no | up | GO:0038096;GO:0004252;GO:0016020;GO:0030449;GO:0050776;GO:0006898;GO:0038095;GO:0002376;GO:0050900;GO:0005576;GO:0006956;GO:0002250;GO:0003823;GO:0005886;GO:0006508;GO:0006958;GO:0006955 | hsa:7441 |  | immunoglobulin lambda-chain, partial [Homo sapiens] |
| A0A286YEY1 | 4509518.632 | 5983712.083 | 1.326907054 | 0.408067318 | 0.100803962 | no | up |  |  |  | IGHA1 isoform 1, partial [Pan troglodytes] |
| P06331 | 393688.4947 | 520961.7667 | 1.323284205 | 0.404122946 | 0.487262984 | no | up | GO:0038096;GO:0004252;GO:0016020;GO:0030449;GO:0050776;GO:0006898;GO:0038095;GO:0002376;GO:0050900;GO:0005576;GO:0006956;GO:0002250;GO:0003823;GO:0005886;GO:0006508;GO:0006958;GO:0006955 | hsa:102724971 | | hCG1793614, partial [Homo sapiens] |
| P20742 | 35256.6036 | 46596.61316 | 1.321642144 | 0.402331596 | 0.43429776 | no | up | GO:0004866;GO:0007565;GO:0070062;GO:0005576;GO:0030414;GO:0072562;GO:0002020;GO:0004867;GO:0010951;GO:0010466;GO:0005615 | hsa:5858 | COG2373 | pregnancy zone protein precursor [Homo sapiens] |
| A0A075B6K4 | 57859.10211 | 76207.42708 | 1.317120804 | 0.397387673 | 0.16211377 | no | up | GO:0016020;GO:0005615;GO:0006955;GO:0002376;GO:0002377;GO:0005576;GO:0002250;GO:0003823;GO:0005886 | hsa:7441 |  | immunoglobulin light chain variable region, partial [Homo sapiens] |
| P01742 | 1110117.079 | 1446239.483 | 1.302781041 | 0.381594629 | 0.272149129 | no | up | GO:0038096;GO:0004252;GO:0016020;GO:0030449;GO:0050776;GO:0006898;GO:0038095;GO:0002376;GO:0050900;GO:0005576;GO:0006956;GO:0002250;GO:0003823;GO:0005886;GO:0006508;GO:0006958;GO:0006955 | hsa:102724971 | | IgM heavy chain VH1 region precursor, partial [Homo sapiens] |
| D6RD17 | 1986535.632 | 2585204.783 | 1.30136341 | 0.380023895 | 0.129349387 | no | up |  | hsa:3512 |  | JCHAIN isoform 4, partial [Pongo abelii] |
| P11021 | 292663.4498 | 378243.2118 | 1.292416979 | 0.37007161 | 0.579250982 | no | up | GO:1990090;GO:0051087;GO:0005783;GO:0071480;GO:1990440;GO:0035437;GO:0009314;GO:0030433;GO:0019899;GO:0036500;GO:0005789;GO:0042149;GO:0035690;GO:0021762;GO:0045296;GO:0016887;GO:0031204;GO:0034663;GO:0005925;GO:1901998;GO:0019904;GO:0008180;GO:0005737;GO:0005793;GO:0006983;GO:0042623;GO:0043209;GO:0005634;GO:0016020;GO:0071353;GO:0005739;GO:0000166;GO:0031625;GO:0043066;GO:0005509;GO:0031398;GO:0043022;GO:0005524;GO:0016787;GO:0030335;GO:0071236;GO:0030176;GO:0005790;GO:0030182;GO:0042470;GO:0097501;GO:0009986;GO:0071277;GO:0036498;GO:0036499;GO:0051402;GO:0051082;GO:0005788;GO:0005886;GO:0042220;GO:0030512;GO:0001554;GO:0051603;GO:1904313;GO:0032991;GO:0043231;GO:0030968;GO:0071287;GO:0070062;GO:0005829;GO:0071320;GO:0030496;GO:0060904;GO:0090074;GO:0021589;GO:1903897;GO:1903894;GO:1903895;GO:0051787;GO:0005515;GO:1903891;GO:0034976;GO:0034975;GO:0021680;GO:0010976 | hsa:3309 | COG0443 | 78 kDa glucose-regulated protein [Otolemur garnettii] |
| A0A0A0MT36 | 116382.66 | 149523.145 | 1.28475449 | 0.361492694 | 0.363411699 | no | up | GO:0016020;GO:0005615;GO:0006955;GO:0002376;GO:0002377;GO:0005576;GO:0002250;GO:0003823;GO:0005886 | hsa:7441 |  | RecName: Full=Immunoglobulin kappa variable 6D-21; Flags: Precursor |
| P07996 | 22059.63353 | 28167.29163 | 1.276870334 | 0.352612027 | 0.334355413 | no | up | GO:0032026;GO:0005783;GO:0050840;GO:0034605;GO:0005788;GO:2001237;GO:0050921;GO:1903671;GO:0048266;GO:0051895;GO:0006986;GO:0043652;GO:0042535;GO:0009612;GO:0030511;GO:0002581;GO:0042327;GO:0051592;GO:0043536;GO:0040037;GO:2001027;GO:0030169;GO:0043236;GO:0045727;GO:0045652;GO:0030198;GO:0043032;GO:0030194;GO:0009897;GO:0018149;GO:0042493;GO:0002605;GO:0071356;GO:0051897;GO:0016477;GO:0002040;GO:0001968;GO:2000379;GO:0030141;GO:0007050;GO:0043394;GO:0006954;GO:0006955;GO:0070052;GO:0090051;GO:0070051;GO:0033574;GO:0043154;GO:0034976;GO:1902043;GO:0031012;GO:0001666;GO:0050431;GO:0005737;GO:0031091;GO:0031093;GO:0005509;GO:0010763;GO:0001786;GO:0051918;GO:0008284;GO:0009986;GO:0017134;GO:2000353;GO:0008201;GO:0071363;GO:0001953;GO:0002544;GO:0016529;GO:0042802;GO:0005515;GO:0032570;GO:0016525;GO:0000187;GO:0005201;GO:0005615;GO:0002576;GO:0010751;GO:0045766;GO:0043066;GO:0010748;GO:0032695;GO:1903588;GO:0005178;GO:0030335;GO:0048661;GO:0032914;GO:0062023;GO:0007155;GO:0070062;GO:0043537;GO:0010595;GO:0010596;GO:0009749;GO:0005577;GO:0005576;GO:0010757;GO:0010754;GO:0010759;GO:0001937;GO:0071636 | hsa:7057 |  | thrombospondin-1 precursor [Homo sapiens] |
| A0A0G2JMB2 | 2454396.105 | 3112496.792 | 1.268131409 | 0.342704251 | 0.084736797 | no | up | GO:0009897;GO:0050853;GO:0045087;GO:0006910;GO:0006911;GO:0042742;GO:0003823;GO:0034987;GO:0042571;GO:0006958;GO:0050871;GO:0072562 | hsa:55423 |  | Immunoglobulin heavy chain variant, partial [Homo sapiens] |
| C9JPQ9 | 1733016.668 | 2195261.946 | 1.266728697 | 0.341107566 | 0.05820135 | no | up | GO:0051258;GO:0005102;GO:0007596;GO:0005577;GO:0030168 | hsa:2266 |  | hypothetical protein, partial [Homo sapiens] |
| P01706 | 93105.06105 | 117634.1529 | 1.263456053 | 0.337375483 | 0.220100293 | no | up | GO:0038096;GO:0004252;GO:0016020;GO:0030449;GO:0050776;GO:0006898;GO:0038095;GO:0002376;GO:0050900;GO:0005576;GO:0006956;GO:0002250;GO:0003823;GO:0005886;GO:0006508;GO:0006958;GO:0006955 | hsa:7441 |  | hCG2043237, partial [Homo sapiens] |
| Q562R1 | 19868.54273 | 24894.69554 | 1.25297038 | 0.32535231 | 0.297849351 | no | up | GO:0005737;GO:0005856;GO:0005615;GO:0070062;GO:0000166;GO:0003674;GO:0045202;GO:0005515;GO:0008150;GO:0098978;GO:0005524;GO:0015629 | hsa:345651 | COG5277 | beta-actin-like protein 2 [Homo sapiens] |
| P59665 | 93970.19158 | 117345.0063 | 1.248747122 | 0.320481353 | 0.331306542 | no | up | GO:0071222;GO:0019730;GO:0019731;GO:0035578;GO:0051673;GO:0042742;GO:0050829;GO:0061844;GO:0005615;GO:0030520;GO:0051852;GO:0044657;GO:0002227;GO:0042803;GO:0010818;GO:0005796;GO:0006935;GO:0006952;GO:0050830;GO:0062023;GO:0006955;GO:0070062;GO:0050832;GO:0051607;GO:0031640;GO:0043312;GO:0005576;GO:0042832 | hsa:1667;hsa:728358;hsa:1668 | | neutrophil defensin 1 preproprotein [Homo sapiens] |
| Q13103 | 4606.068429 | 5740.203923 | 1.246226367 | 0.317566146 | 0.259865819 | no | up | GO:0044267;GO:0002576;GO:0005788;GO:0031089;GO:0001501;GO:0005576;GO:0046849;GO:0004866;GO:0010951;GO:0043687;GO:0062023 | hsa:6694 |  | secreted phosphoprotein 24 precursor [Homo sapiens] |
| A0A087WSY6 | 205787.9 | 253644.4483 | 1.23255278 | 0.301649427 | 0.137610785 | no | up | GO:0016020;GO:0005615;GO:0006955;GO:0002376;GO:0002377;GO:0005576;GO:0002250;GO:0003823;GO:0005886 | hsa:29802 |  | RecName: Full=Immunoglobulin kappa variable 3D-15; Flags: Precursor |
| A0A0B4J1U7 | 59076.67632 | 72494.305 | 1.227122268 | 0.295279003 | 0.276237567 | no | up | GO:0009897;GO:0050853;GO:0045087;GO:0016020;GO:0006910;GO:0006911;GO:0002376;GO:0005576;GO:0002250;GO:0003823;GO:0005886;GO:0072562;GO:0042571;GO:0006958;GO:0050871;GO:0034987;GO:0042742 | hsa:102723407 | | RecName: Full=Immunoglobulin heavy variable 6-1; Flags: Precursor |
| P08519 | 115441.3416 | 139765.8054 | 1.21070843 | 0.275851468 | 0.685702613 | no | up | GO:0004252;GO:0034374;GO:0005515;GO:0006869;GO:0008015;GO:0016787;GO:0034185;GO:0005576;GO:0004866;GO:0010951;GO:0006508;GO:0008233;GO:0008236;GO:0034358;GO:0008201;GO:0001968;GO:0006629 | hsa:4018 | COG5640 | RecName: Full=Apolipoprotein(a); Short=Apo(a); Short=Lp(a); Flags: Precursor |
| P01766 | 78135.21684 | 94592.50125 | 1.210625696 | 0.275752878 | 0.155042358 | no | up | GO:0038096;GO:0004252;GO:0016020;GO:0030449;GO:0005615;GO:0006898;GO:0038095;GO:0050900;GO:0072562;GO:0002376;GO:0050776;GO:0005576;GO:0006956;GO:0002250;GO:0003823;GO:0005886;GO:0006508;GO:0006958;GO:0006955 | hsa:102723407 | | RecName: Full=Immunoglobulin heavy variable 3-13; AltName: Full=Ig heavy chain V-III region BRO; Flags: Precursor |
| A0A075B6H7 | 138596.2984 | 167637.5396 | 1.209538361 | 0.274456525 | 0.127523679 | no | up | GO:0002377;GO:0005615;GO:0006955 | hsa:7441 |  | hCG2043206, partial [Homo sapiens] |
| P01877 | 206673.0384 | 249862.9646 | 1.208977071 | 0.273786883 | 0.646218066 | no | up | GO:0009897;GO:0019731;GO:0006955;GO:0050900;GO:0034987;GO:0001895;GO:0005615;GO:0016020;GO:0003094;GO:0072562;GO:0071748;GO:0050853;GO:0045087;GO:0006910;GO:0006911;GO:0003823;GO:0005886;GO:0006958;GO:0050871;GO:0060267;GO:0070062;GO:0006898;GO:0002376;GO:0005576;GO:0002250;GO:0071752;GO:0071751 | hsa:55423 |  | RecName: Full=Immunoglobulin heavy constant alpha 2; AltName: Full=Ig alpha-2 chain C region; AltName: Full=Ig alpha-2 chain C region BUT; AltName: Full=Ig alpha-2 chain C region LAN |
| A0A075B7F0 | 23855.47089 | 28762.55174 | 1.205700439 | 0.269871509 | 0.332720984 | no | up | GO:0009897;GO:0050853;GO:0045087;GO:0006910;GO:0006911;GO:0042742;GO:0003823;GO:0034987;GO:0042571;GO:0006958;GO:0050871;GO:0072562 | hsa:102723407 | | IGHV3-13 isoform 1, partial [Pan troglodytes] |
| P01704 | 32190.41579 | 38757.2313 | 1.20399909 | 0.267834301 | 0.197925311 | no | up | GO:0038096;GO:0004252;GO:0016020;GO:0030449;GO:0050776;GO:0006898;GO:0038095;GO:0002376;GO:0050900;GO:0005576;GO:0006956;GO:0002250;GO:0003823;GO:0005886;GO:0006508;GO:0006958;GO:0006955;GO:0070062 | hsa:7441 |  | RecName: Full=Immunoglobulin lambda variable 2-14; AltName: Full=Ig lambda chain V-II region NIG-84; AltName: Full=Ig lambda chain V-II region TOG; AltName: Full=Ig lambda chain V-II region VIL; Flags: Precursor |
| Q6EMK4 | 9798.095632 | 11516.98463 | 1.175430926 | 0.233189762 | 0.239345232 | no | no change | GO:0016020;GO:0016021;GO:0070062;GO:0010719;GO:0005615;GO:0009986;GO:0005886;GO:0045296;GO:0031012;GO:0005765;GO:0005739;GO:0071461;GO:0005515;GO:0071456;GO:0005576;GO:0030512;GO:0050431 | hsa:114990 | COG4886 | vasorin precursor [Homo sapiens] |
| A0A0C4DH31 | 26625.47789 | 31057.25 | 1.166448547 | 0.22212267 | 0.072922973 | no | no change | GO:0009897;GO:0050853;GO:0045087;GO:0016020;GO:0006910;GO:0006911;GO:0002376;GO:0005576;GO:0002250;GO:0003823;GO:0005886;GO:0072562;GO:0042571;GO:0006958;GO:0050871;GO:0034987;GO:0042742 | hsa:102723407 | | immunoglobulin heavy chain variable region, partial [Homo sapiens] |
| P01764 | 38419.61727 | 44708.084 | 1.163678536 | 0.218692572 | 0.390812783 | no | no change | GO:0004252;GO:0030449;GO:0009897;GO:0006955;GO:0050900;GO:0042742;GO:0034987;GO:0042571;GO:0005615;GO:0016020;GO:0072562;GO:0006508;GO:0050853;GO:0045087;GO:0006910;GO:0006911;GO:0050776;GO:0006956;GO:0003823;GO:0005886;GO:0006958;GO:0050871;GO:0070062;GO:0038096;GO:0038095;GO:0006898;GO:0002376;GO:0005576;GO:0002250 | hsa:102723407 | | RecName: Full=Immunoglobulin heavy variable 3-23; AltName: Full=Ig heavy chain V-III region LAY; AltName: Full=Ig heavy chain V-III region POM; AltName: Full=Ig heavy chain V-III region TEI; AltName: Full=Ig heavy chain V-III region TIL; AltName: Full=Ig heavy chain V-III region TUR; AltName: Full=Ig heavy chain V-III region VH26; AltName: Full=Ig heavy chain V-III region WAS; AltName: Full=Ig heavy chain V-III region ZAP; Flags: Precursor |
| A0A0C4DH43 | 454010.2942 | 524811.8396 | 1.155947004 | 0.209075258 | 0.520617091 | no | no change | GO:0009897;GO:0050853;GO:0045087;GO:0016020;GO:0006910;GO:0006911;GO:0002376;GO:0005576;GO:0002250;GO:0003823;GO:0005886;GO:0072562;GO:0042571;GO:0006958;GO:0050871;GO:0034987;GO:0042742 | hsa:102723407 | | RecName: Full=Immunoglobulin heavy variable 2-70D; Flags: Precursor |
| G3V2W1 | 8948.705105 | 10282.13542 | 1.149008186 | 0.200389076 | 0.174767307 | no | no change | GO:0010951;GO:0007596;GO:0005615;GO:0004867 | hsa:51156 | COG4826 | protein Z-dependent protease inhibitor isoform X1 [Homo sapiens] |
| C9JB55 | 12257.70416 | 14073.08717 | 1.148101389 | 0.199250052 | 0.162201411 | no | no change | GO:0055037;GO:1990459;GO:0030139;GO:0005905;GO:0034986;GO:1990712;GO:0004857;GO:0048260;GO:0005615;GO:0005770;GO:0043086;GO:0009925;GO:0048471;GO:0006879;GO:0016324;GO:0008198;GO:0008199;GO:0015091;GO:0031232;GO:0034756;GO:0071281;GO:0005623;GO:0005769 | hsa:7018 |  | serotransferrin isoform 1 precursor [Homo sapiens] |
| P02776 | 35069.36644 | 40245.04375 | 1.147583998 | 0.198599756 | 0.534405074 | no | no change | GO:0020005;GO:0070098;GO:0005125;GO:0071222;GO:0019221;GO:0008009;GO:0010469;GO:0032760;GO:0010628;GO:0048248;GO:0032496;GO:0045918;GO:0061844;GO:0005737;GO:0045347;GO:0031093;GO:0002576;GO:0007189;GO:0051873;GO:0010744;GO:0007186;GO:2001240;GO:0042127;GO:0030595;GO:0030593;GO:0006935;GO:0043950;GO:0006952;GO:0062023;GO:0006954;GO:0006955;GO:0008201;GO:0030168;GO:0005615;GO:0090023;GO:0045651;GO:0045652;GO:0045653;GO:0031640;GO:0005576;GO:0045944;GO:0005515;GO:0097679;GO:0042832;GO:0016525 | hsa:5196 |  | platelet factor 4 isoform 1 precursor [Homo sapiens] |
| P80108 | 54402.06105 | 62317.795 | 1.145504302 | 0.195982877 | 0.127511504 | no | no change | GO:0035774;GO:0051044;GO:0051047;GO:0035690;GO:0031012;GO:0010907;GO:0001503;GO:0032869;GO:0009749;GO:0010867;GO:0005737;GO:0043065;GO:0070062;GO:0002430;GO:0006501;GO:0045919;GO:0005576;GO:0097241;GO:0006507;GO:0010897;GO:0002062;GO:0004630;GO:0035701;GO:0002042;GO:0008285;GO:0008286;GO:0010983;GO:0071397;GO:0071277;GO:0005615;GO:0010595;GO:0043231;GO:0016787;GO:0070633;GO:0071401;GO:0005765;GO:0017080;GO:0071467;GO:1900076;GO:0004621;GO:0046470;GO:0010694;GO:0005622 | hsa:2822 |  | phosphatidylinositol-glycan-specific phospholipase D precursor [Homo sapiens] |
| A6XND0 | 28409.03947 | 32510.07375 | 1.144356668 | 0.194536775 | 0.208212498 | no | no change | GO:0005520;GO:0001558;GO:0005576 | hsa:3486 |  | insulin-like growth factor binding protein 3 [Homo sapiens] |
| H0YJW9 | 684252.6316 | 782399.1917 | 1.143436146 | 0.193375802 | 0.1845183 | no | no change |  | hsa:7448 |  | vitronectin, partial [Homo sapiens] |
| P08571 | 10367.93374 | 11841.57217 | 1.142134245 | 0.191732233 | 0.106345804 | no | no change | GO:0006954;GO:0009897;GO:0045121;GO:0071222;GO:0071223;GO:0097190;GO:0031362;GO:1901224;GO:0007166;GO:0006909;GO:0032496;GO:0045471;GO:0010008;GO:0034612;GO:0016020;GO:0030667;GO:0005615;GO:0071727;GO:0071726;GO:0009617;GO:0071723;GO:0071219;GO:0032729;GO:0002224;GO:0031225;GO:0009986;GO:0001847;GO:0005794;GO:0032760;GO:0070266;GO:0034128;GO:0038124;GO:0045087;GO:0006915;GO:2000484;GO:0035666;GO:0032481;GO:0070891;GO:0034142;GO:0005886;GO:0009408;GO:0001530;GO:0032026;GO:0070062;GO:0051602;GO:0002755;GO:0002756;GO:0016019;GO:0050715;GO:0038123;GO:0031663;GO:0006898;GO:0007249;GO:0043312;GO:0002376;GO:0005576;GO:0002237;GO:0005515;GO:0045807;GO:0046696 | hsa:929 |  | monocyte differentiation antigen CD14 precursor [Homo sapiens] |
| P23083 | 681387.4784 | 772577.5967 | 1.133830047 | 0.181204407 | 0.493248377 | no | no change | GO:0038096;GO:0004252;GO:0016020;GO:0030449;GO:0050776;GO:0006898;GO:0038095;GO:0002376;GO:0050900;GO:0005576;GO:0006956;GO:0002250;GO:0003823;GO:0005886;GO:0006508;GO:0006958;GO:0006955 | hsa:102723407 | | IgM heavy chain VH1 region precursor, partial [Homo sapiens] |
| G3V0E5 | 5212.642421 | 5906.334542 | 1.133078785 | 0.180248178 | 0.188846529 | no | no change | GO:0009897;GO:0055037;GO:0035690;GO:0005905;GO:1990712;GO:0010008;GO:0045780;GO:1990830;GO:0070062;GO:0016021;GO:0045830;GO:0004998;GO:0048471;GO:0042803;GO:0006879;GO:0030890;GO:0030316;GO:0042102;GO:0005887;GO:0005886;GO:0016323;GO:0042470;GO:0031623;GO:0006898;GO:0003725;GO:0033570;GO:0033572;GO:0005769 | hsa:7037 | COG2234 | transferrin receptor variant, partial [Homo sapiens] |
| P01023 | 7676298 | 8692043.75 | 1.132322345 | 0.179284717 | 0.265216593 | no | no change | GO:0051056;GO:0030414;GO:0019959;GO:0007597;GO:0019899;GO:0072562;GO:0048863;GO:0005615;GO:0031093;GO:0002576;GO:0048306;GO:0019838;GO:0070062;GO:0010466;GO:0002020;GO:0001869;GO:0022617;GO:0019966;GO:0005096;GO:0004866;GO:0004867;GO:0010951;GO:0043547;GO:0043120;GO:0005829;GO:0005576;GO:0005515;GO:0005102 | hsa:2 | COG2373 | alpha-2-macroglobulin isoform a precursor [Homo sapiens] |
| A0A0C4DH29 | 32525.15347 | 36525.98045 | 1.123007167 | 0.167367135 | 0.410840946 | no | no change | GO:0009897;GO:0050853;GO:0045087;GO:0016020;GO:0006910;GO:0006911;GO:0002376;GO:0005576;GO:0002250;GO:0003823;GO:0005886;GO:0072562;GO:0042571;GO:0006958;GO:0050871;GO:0034987;GO:0042742 | hsa:102723407 | | immunoglobulin heavy chain variable region, partial [Homo sapiens] |
| P0DTE1 | 16825.00479 | 18878.19 | 1.122031776 | 0.166113533 | 0.377807507 | no | no change |  | hsa:102723407 | | immunoglobulin heavy chain variable region, partial [Homo sapiens] |
| H3BUA5 | 380376.8626 | 426551.9625 | 1.12139303 | 0.165292008 | 0.395124708 | no | no change |  | hsa:10326 |  | LOW QUALITY PROTEIN: T0061165 isoform 1, partial [Pan troglodytes] |
| P06312 | 384481.9842 | 430701.625 | 1.120212761 | 0.163772768 | 0.154819289 | no | no change | GO:0038096;GO:0004252;GO:0016020;GO:0030449;GO:0050776;GO:0006898;GO:0038095;GO:0072562;GO:0002376;GO:0002377;GO:0050900;GO:0005576;GO:0006956;GO:0002250;GO:0003823;GO:0005886;GO:0006508;GO:0006958;GO:0006955 | hsa:7441 |  | immunoglobulin kappa chain, partial [Homo sapiens] |
| A0A0C4DGZ8 | 15936.75382 | 17803.04204 | 1.11710592 | 0.159765983 | 0.349666334 | no | no change | GO:0016021;GO:0016020 | hsa:2811 | COG4886 | glycoprotein Ib (platelet), alpha polypeptide [Homo sapiens] |
| P00738 | 10340270.74 | 11543587 | 1.116371833 | 0.15881763 | 0.437253284 | no | no change | GO:0042742;GO:0010942;GO:0005615;GO:0071682;GO:2000296;GO:0072562;GO:0098869;GO:0035580;GO:1904724;GO:0016209;GO:0006952;GO:0006953;GO:0070062;GO:0042542;GO:0051354;GO:0006898;GO:0043312;GO:0002376;GO:0030492;GO:0005576;GO:0005515;GO:0031838 | hsa:3240 | COG5640 | haptoglobin isoform 1 preproprotein [Homo sapiens] |
| P55058 | 15843.73116 | 17563.92375 | 1.108572443 | 0.14870305 | 0.191470497 | no | no change | GO:0034375;GO:0006869;GO:0015914;GO:1990050;GO:0035627;GO:0005548;GO:0005615;GO:0019992;GO:0008525;GO:0097001;GO:0010875;GO:0030317;GO:0010189;GO:0005319;GO:0035620;GO:0034364;GO:0006629;GO:0070300;GO:0008289;GO:0031210;GO:0008429;GO:1904121;GO:0005576;GO:1901611 | hsa:5360 |  | phospholipid transfer protein, isoform CRA_c [Homo sapiens] |
| P04180 | 19673.87158 | 21735.335 | 1.104781787 | 0.143761441 | 0.375597574 | no | no change | GO:0034375;GO:0006656;GO:0034372;GO:0043691;GO:0008374;GO:0042158;GO:0090107;GO:0070062;GO:0016740;GO:0016746;GO:0034435;GO:0008203;GO:0006644;GO:0034364;GO:0006629;GO:0042632;GO:0030301;GO:0008202;GO:0005615;GO:0005576;GO:0005515;GO:0034186;GO:0004607;GO:0046470 | hsa:3931 |  | phosphatidylcholine-sterol acyltransferase precursor [Homo sapiens] |
| Q8IV42 | 84821.05625 | 93699.78955 | 1.104676052 | 0.143623359 | 0.488100828 | no | no change | GO:0000049;GO:0016310;GO:0016301;GO:0097056;GO:0016740;GO:0001514;GO:0000166;GO:0006412;GO:0005524 | hsa:118672 |  | L-seryl-tRNA(Sec) kinase isoform 2 [Homo sapiens] |
| A0A0B4J2D9 | 15440.17733 | 16980.78446 | 1.099779108 | 0.137213786 | 0.497797379 | no | no change | GO:0016020;GO:0005615;GO:0006955;GO:0002376;GO:0002377;GO:0005576;GO:0002250;GO:0003823;GO:0005886 | hsa:7441 |  | RecName: Full=Immunoglobulin kappa variable 1D-13; Flags: Precursor |
| A0A0S2Z4L3 | 194502.2211 | 213795.2708 | 1.099191925 | 0.136443311 | 0.348434709 | no | no change | GO:0005576;GO:0005509;GO:0030195 | hsa:5627 |  | vitamin K-dependent protein S isoform 1 precursor [Homo sapiens] |
| P00742 | 77559.30526 | 85201.01083 | 1.098527257 | 0.135570666 | 0.174248234 | no | no change | GO:0004252;GO:0030335;GO:0005543;GO:0006888;GO:0005615;GO:0016787;GO:0007596;GO:0005788;GO:0005796;GO:0005576;GO:0005509;GO:0005515;GO:0005886;GO:0006508;GO:0008233;GO:0008236;GO:0051897;GO:0031233;GO:0007599;GO:0007598 | hsa:2159 | COG5640 | coagulation factor X isoform 1 preproprotein [Homo sapiens] |
| Q13201 | 36145.25167 | 39688.43057 | 1.098026124 | 0.134912378 | 0.675488855 | no | no change | GO:0005201;GO:0031093;GO:0010811;GO:0002576;GO:0007596;GO:0031012;GO:0005576;GO:0005509;GO:0005515;GO:0007155;GO:0062023 | hsa:22915 |  | multimerin-1 precursor [Homo sapiens] |
| A0A0G2JRQ6 | 270236.3158 | 296726.0333 | 1.09802427 | 0.134909943 | 0.299393892 | no | no change | GO:0002377;GO:0005615;GO:0006955 | hsa:7441 |  | hCG2042707, partial [Homo sapiens] |
| F8W1S1 | 8692.160882 | 9501.73105 | 1.093137964 | 0.128475494 | 0.823266363 | no | no change | GO:0005882;GO:0045095;GO:0005198 | hsa:121391 |  | KRT74 isoform 2 [Pan troglodytes] |
| Q6UXB8 | 10108.85542 | 11033.6725 | 1.091485835 | 0.126293408 | 0.359317488 | no | no change | GO:0010466;GO:0005576;GO:0030414;GO:0005615;GO:0061052 | hsa:221476 | COG2340 | peptidase inhibitor 16 precursor [Homo sapiens] |
| P05546 | 523667.8632 | 570904.3792 | 1.090203198 | 0.124597058 | 0.219828462 | no | no change | GO:0044267;GO:0007599;GO:0006935;GO:0005615;GO:0043687;GO:0007596;GO:0005788;GO:0005576;GO:0030414;GO:0004866;GO:0004867;GO:0010951;GO:0010466;GO:0008201;GO:0070062 | hsa:3053 | COG4826 | heparin cofactor 2 precursor [Homo sapiens] |
| P00747 | 1059033.553 | 1153996.371 | 1.08966932 | 0.123890389 | 0.103754054 | no | no change | GO:0004252;GO:0004175;GO:0007599;GO:0016787;GO:1904854;GO:0048771;GO:0019899;GO:0008233;GO:0008236;GO:0044267;GO:0070062;GO:0043536;GO:0052182;GO:0051087;GO:0072562;GO:1990405;GO:0006508;GO:0022617;GO:0009986;GO:0051918;GO:0051919;GO:0008285;GO:0007596;GO:0010812;GO:0019900;GO:0062023;GO:0019904;GO:0005886;GO:0051702;GO:0031232;GO:0034185;GO:0005615;GO:0042730;GO:0031093;GO:0052213;GO:2000048;GO:0044218;GO:0005576;GO:0005515;GO:0005102;GO:0002576 | hsa:5340 | COG5640 | plasminogen isoform 1 precursor [Homo sapiens] |
| A0A0C4DH55 | 8471991.474 | 9231465.583 | 1.089645287 | 0.12385857 | 0.362543124 | no | no change | GO:0016020;GO:0005615;GO:0006955;GO:0002376;GO:0002377;GO:0005576;GO:0002250;GO:0003823;GO:0005886 | hsa:29802 |  | RecName: Full=Immunoglobulin kappa variable 3D-7; Flags: Precursor |
| P02671 | 6288507.053 | 6850534.375 | 1.089373729 | 0.123498981 | 0.260284587 | no | no change | GO:0045087;GO:0009897;GO:0045907;GO:0007599;GO:0034116;GO:0007596;GO:0005788;GO:0007160;GO:0045921;GO:0045202;GO:0034622;GO:1902042;GO:0044267;GO:0050839;GO:0031091;GO:0005615;GO:0031093;GO:1900026;GO:0072378;GO:0002576;GO:0031639;GO:0072562;GO:0005198;GO:0065003;GO:0030198;GO:0002224;GO:0046872;GO:0043687;GO:0009986;GO:2000352;GO:0051592;GO:0090277;GO:0005938;GO:0005886;GO:1903561;GO:0030168;GO:0070062;GO:0051258;GO:0042730;GO:0072377;GO:0050714;GO:0002376;GO:0005577;GO:0005576;GO:0002250;GO:0005515;GO:0043152;GO:0005102;GO:0070374;GO:0070527 | hsa:2243 |  | fibrinogen alpha chain isoform alpha-E preproprotein [Homo sapiens] |
| P01780 | 1781232.211 | 1940048.167 | 1.089160725 | 0.123216866 | 0.370135042 | no | no change | GO:0038096;GO:0004252;GO:0016020;GO:0030449;GO:0050776;GO:0006898;GO:0038095;GO:0072562;GO:0002376;GO:0050900;GO:0005576;GO:0006956;GO:0002250;GO:0003823;GO:0005886;GO:0006508;GO:0006958;GO:0006955;GO:0070062 | hsa:102723407 | | immunoglobulin heavy chain variable region precursor, partial [Homo sapiens] |
| A0A075B6J9 | 86557.38105 | 93959.55042 | 1.085517483 | 0.118382962 | 0.489933099 | no | no change | GO:0016020;GO:0005615;GO:0006955;GO:0002376;GO:0002377;GO:0005576;GO:0002250;GO:0003823;GO:0005886 | hsa:7441 |  | RecName: Full=Immunoglobulin lambda variable 2-18; Flags: Precursor |
| P08603 | 1222296.805 | 1326576.996 | 1.08531495 | 0.118113762 | 0.133481674 | no | no change | GO:1903659;GO:0030449;GO:0070062;GO:0005515;GO:0045087;GO:0072562;GO:0002376;GO:0043395;GO:0005576;GO:0006956;GO:0006957;GO:0016032;GO:0008201;GO:0005615 | hsa:3075 |  | RecName: Full=Complement factor H; AltName: Full=H factor 1; Flags: Precursor |
| O95445 | 55209.75105 | 59763.54667 | 1.082481727 | 0.114342672 | 0.27934882 | no | no change | GO:0034375;GO:0034445;GO:0006869;GO:0043691;GO:0042157;GO:0001523;GO:0005615;GO:0005543;GO:0098869;GO:0005576;GO:0005319;GO:0034361;GO:0034362;GO:0034364;GO:0034365;GO:0034366;GO:0016209;GO:0042632;GO:0034380;GO:0034384;GO:0009749;GO:0033344 | hsa:55937 |  | apolipoprotein M isoform 1 [Homo sapiens] |
| P22891 | 9700.652421 | 10479.47265 | 1.080285345 | 0.111412435 | 0.460563842 | no | no change | GO:0004252;GO:0005796;GO:0006888;GO:0005615;GO:0007596;GO:0005788;GO:0005576;GO:0005509;GO:0006508;GO:0030195;GO:0007599;GO:0070062 | hsa:8858 | COG5640 | vitamin K-dependent protein Z isoform 2 precursor [Homo sapiens] |
| P22792 | 89832.95895 | 97010.12417 | 1.079894565 | 0.110890463 | 0.343395776 | no | no change | GO:0030449;GO:0070062;GO:0004181;GO:0050790;GO:0072562;GO:0050821;GO:0005576;GO:0006508;GO:0030234 | hsa:1370 | COG4886 | carboxypeptidase N subunit 2 precursor [Homo sapiens] |
| Q14520 | 92805.35211 | 100176.1446 | 1.079422063 | 0.11025908 | 0.233204846 | no | no change | GO:0004252;GO:0005615;GO:0016787;GO:0005539;GO:0005576;GO:0005509;GO:0007155;GO:0006508;GO:0008233;GO:0008236 | hsa:3026 | COG5640 | hyaluronan-binding protein 2 isoform 1 preproprotein [Homo sapiens] |
| P43251 | 31395.26105 | 33854.93875 | 1.078345509 | 0.108819501 | 0.361309296 | no | no change | GO:0016787;GO:0005615;GO:0047708;GO:0070062;GO:0016810;GO:0016811;GO:0006768;GO:0006807;GO:0005759;GO:0007417;GO:0005576 | hsa:686 | COG0388 | RecName: Full=Biotinidase; Short=Biotinase; Flags: Precursor |
| A0A087X1J7 | 47417.00632 | 51128.205 | 1.07826725 | 0.108714796 | 0.309939827 | no | no change | GO:0008430;GO:0005615;GO:0051289;GO:0006979;GO:0098869;GO:0055114;GO:0004601;GO:0004602;GO:0016491 | hsa:2878 | COG0386 | glutathione peroxidase 3 isoform 1 precursor [Homo sapiens] |
| P0DOY3 | 9264277.474 | 9987699.083 | 1.078087213 | 0.108473891 | 0.484947879 | no | no change | GO:0005615;GO:0016020;GO:0072562;GO:0002376;GO:0005576;GO:0002250;GO:0003823;GO:0005886;GO:0070062 | hsa:100423062 | | RecName: Full=Immunoglobulin lambda constant 3; AltName: Full=Ig lambda chain C region DOT; AltName: Full=Ig lambda chain C region NEWM; AltName: Full=Ig lambda-3 chain C regions |
| P43652 | 145866.8226 | 157090.7458 | 1.076946375 | 0.106946414 | 0.25167757 | no | no change | GO:0008431;GO:0051180;GO:0005615;GO:0072562;GO:0050821;GO:0015031;GO:0005576;GO:0005515;GO:0071693;GO:0046872;GO:0070062 | hsa:173 |  | afamin precursor [Homo sapiens] |
| P04004 | 739223.1684 | 791457.45 | 1.070661045 | 0.098501817 | 0.219135931 | no | no change | GO:0005201;GO:0030449;GO:0005783;GO:0061302;GO:0050840;GO:0014911;GO:0030949;GO:0030247;GO:0031012;GO:0007160;GO:0007155;GO:0090303;GO:0048260;GO:0005737;GO:0032092;GO:0070062;GO:0072562;GO:0005576;GO:0016477;GO:0042802;GO:0033627;GO:0005178;GO:0005796;GO:0008283;GO:0010811;GO:0048709;GO:0048237;GO:0062023;GO:0006955;GO:0010951;GO:0035987;GO:0008201;GO:0005615;GO:0051258;GO:0005604;GO:0043231;GO:0050731;GO:0005044;GO:0006898;GO:0030198;GO:0005515;GO:0097421;GO:0071062;GO:0005518;GO:0030195 | hsa:7448 |  | vitronectin precursor [Homo sapiens] |
| A0A4W8ZXM2 | 817147.1153 | 872953.3875 | 1.068294033 | 0.095308784 | 0.667374487 | no | no change |  | hsa:102723407 | | immunoglobulin heavy chain variable region, partial [Homo sapiens] |
| P02647 | 31741707.16 | 33872462.75 | 1.067127946 | 0.093733162 | 0.33956822 | no | no change | GO:0034115;GO:0010804;GO:0005788;GO:0019915;GO:0034191;GO:0034190;GO:0005548;GO:0050728;GO:0071682;GO:0051496;GO:0005543;GO:0010898;GO:0005319;GO:0034361;GO:0034362;GO:0034363;GO:0034364;GO:0031102;GO:0034366;GO:0031100;GO:0043534;GO:0030300;GO:0034774;GO:0010873;GO:0043691;GO:0007179;GO:0015485;GO:0005829;GO:0018206;GO:0019433;GO:0045499;GO:0070508;GO:0045723;GO:0034375;GO:0034371;GO:0031072;GO:0015914;GO:0034378;GO:0050821;GO:0010903;GO:0042158;GO:0055102;GO:0044267;GO:0042627;GO:0051180;GO:0014012;GO:0072562;GO:0050919;GO:0007186;GO:0033700;GO:0018158;GO:0006629;GO:0060761;GO:0042632;GO:0032489;GO:0050713;GO:0007229;GO:0006898;GO:0034384;GO:0062023;GO:0006695;GO:0060192;GO:0006869;GO:0019899;GO:0030301;GO:0035025;GO:0051006;GO:0002740;GO:0001540;GO:1900026;GO:0031410;GO:0005634;GO:0042802;GO:0008035;GO:0060354;GO:0009986;GO:0008289;GO:0008202;GO:0008203;GO:0071813;GO:0070062;GO:0042493;GO:0005515;GO:0034380;GO:0070371;GO:0042157;GO:0006656;GO:0030325;GO:0006644;GO:0017127;GO:0030139;GO:0008211;GO:0060228;GO:0001523;GO:0055091;GO:0034365;GO:0005615;GO:0002576;GO:0005576;GO:0043687;GO:0070328;GO:0019216;GO:0007584;GO:0031210;GO:0005886;GO:0043627;GO:1903561;GO:0051346;GO:0051345;GO:0033344;GO:0070653;GO:0001932;GO:0001935;GO:0005769 | hsa:335 |  | apolipoprotein A-I isoform 1 preproprotein [Homo sapiens] |
| P04433 | 91794.47789 | 97628.94542 | 1.063560114 | 0.088901579 | 0.36195017 | no | no change | GO:0038096;GO:0004252;GO:0016020;GO:0030449;GO:0050776;GO:0006898;GO:0038095;GO:0072562;GO:0002376;GO:0050900;GO:0005576;GO:0006956;GO:0002250;GO:0003823;GO:0005886;GO:0006508;GO:0006958;GO:0006955;GO:0070062 | hsa:7441 |  | rheumatoid factor D1 IgG light chain VK3 region, partial [Homo sapiens] |
| K7ERI9 | 2356485.368 | 2494084.083 | 1.0583915 | 0.08187338 | 0.575663514 | no | no change | GO:0005576;GO:0042157 | hsa:341 |  | apolipoprotein C-I precursor [Homo sapiens] |
| Q13790 | 68167.64368 | 72003.63667 | 1.056272929 | 0.078982659 | 0.500589685 | no | no change | GO:0005319;GO:0034362;GO:0034364;GO:0006629;GO:0006869;GO:0005615;GO:0005576;GO:0005102;GO:0008203;GO:0008202;GO:0015485 | hsa:319 |  | apolipoprotein F preproprotein [Homo sapiens] |
| A0A087WWT3 | 62502.87474 | 66017.89583 | 1.056237751 | 0.078934611 | 0.488032114 | no | no change | GO:0005794;GO:0005615;GO:0005783 | hsa:213 |  | ALB protein [Homo sapiens] |
| Q15166 | 33504.54737 | 35382.2105 | 1.056042038 | 0.078667266 | 0.897923562 | no | no change | GO:0016787;GO:0004063;GO:0070062;GO:0005615;GO:0046395;GO:0019372;GO:0009636;GO:0016311;GO:0043231;GO:0019439;GO:0046226;GO:0005576;GO:0010124;GO:0032929;GO:0102007;GO:0046872;GO:0004064;GO:0018733;GO:0042803 | hsa:5446 |  | serum paraoxonase/lactonase 3 [Homo sapiens] |
| P09172 | 3754.1633 | 3962.4251 | 1.055474891 | 0.077892258 | 0.81012397 | no | no change | GO:0016491;GO:0042127;GO:2001236;GO:0045907;GO:0005783;GO:0048149;GO:0034774;GO:0007613;GO:0042593;GO:0050900;GO:0042596;GO:0005815;GO:0042423;GO:0042420;GO:0042421;GO:0005737;GO:0031418;GO:0030667;GO:0005615;GO:0004500;GO:0031410;GO:0016021;GO:0016020;GO:0008542;GO:0005507;GO:0006589;GO:0007626;GO:0004497;GO:0030658;GO:0003824;GO:0008306;GO:0042584;GO:0042309;GO:0007268;GO:0002443;GO:0055114;GO:0043231;GO:0048265;GO:0042711;GO:0120162;GO:0046872;GO:0016715;GO:0001975;GO:0001816;GO:0001974;GO:0005576;GO:0034466 | hsa:1621 |  | dopamine beta-hydroxylase precursor [Homo sapiens] |
| F8WF14 | 25632.02316 | 26920.89083 | 1.050283494 | 0.070778794 | 0.568825826 | no | no change | GO:0019899;GO:0051384;GO:0005783;GO:0016787;GO:0007612;GO:0050805;GO:0050783;GO:0014016;GO:0001540;GO:0016021;GO:0016020;GO:0072562;GO:0042802;GO:0008285;GO:0004104;GO:0003824;GO:0005788;GO:0043279;GO:0051593;GO:0019695;GO:0005641;GO:0005576;GO:0033265;GO:0003990 | hsa:590 | COG2272 | unnamed protein product [Homo sapiens] |
| G3XAK1 | 17282.07211 | 18147.16042 | 1.050056978 | 0.070467614 | 0.541454818 | no | no change | GO:0004252;GO:2000479;GO:0019899;GO:0010628;GO:0005737;GO:0005615;GO:0005773;GO:0033601;GO:0030971;GO:1904036;GO:0006508;GO:0045721;GO:0046425;GO:0030317;GO:0071456;GO:0007283;GO:0062023;GO:0060763;GO:0048012;GO:0007566;GO:0030879;GO:0005576;GO:0010758 | hsa:4485 | COG5640 | hepatocyte growth factor-like protein precursor [Homo sapiens] |
| P01834 | 25566934.68 | 26834204.5 | 1.049566748 | 0.069793919 | 0.51384641 | no | no change | GO:0004252;GO:0030449;GO:0009897;GO:0006955;GO:0050871;GO:0042742;GO:0034987;GO:0042571;GO:0005615;GO:0016020;GO:0072562;GO:0006508;GO:0050853;GO:0045087;GO:0006910;GO:0006911;GO:0050776;GO:0006956;GO:0003823;GO:0005886;GO:0006958;GO:0001895;GO:0070062;GO:0038096;GO:0038095;GO:0006898;GO:0050900;GO:0002376;GO:0005576;GO:0002250 | hsa:100423062 | | light chain kappa Sci, k Sci=Bence Jones protein [human, Peptide, 214 aa] |
| P01709 | 150145.6289 | 156051.6175 | 1.039335068 | 0.055660835 | 0.759977752 | no | no change | GO:0038096;GO:0004252;GO:0016020;GO:0030449;GO:0050776;GO:0006898;GO:0038095;GO:0002376;GO:0050900;GO:0005576;GO:0006956;GO:0002250;GO:0003823;GO:0005886;GO:0006508;GO:0006958;GO:0006955 | hsa:7441 |  | hCG2043240, partial [Homo sapiens] |
| B1AHL2 | 16086.271 | 16703.32688 | 1.038359162 | 0.054305549 | 0.785043165 | no | no change | GO:0016504;GO:0005576;GO:0005509;GO:0010952;GO:0030198 | hsa:2192 |  | FBLN1 isoform 5 [Pongo abelii] |
| A0A0U1RQV3 | 27699.22579 | 28724.69542 | 1.037021599 | 0.052445943 | 0.60410828 | no | no change | GO:0062023;GO:0007173;GO:0005509;GO:0005006 | hsa:2202 |  | EGF-containing fibulin-like extracellular matrix protein 1 isoform X3 [Homo sapiens] |
| P01701 | 446200.1774 | 462470.4417 | 1.036464047 | 0.051670073 | 0.784250074 | no | no change | GO:0038096;GO:0004252;GO:0016020;GO:0030449;GO:0050776;GO:0006898;GO:0038095;GO:0050900;GO:0005576;GO:0006956;GO:0003823;GO:0005886;GO:0006508;GO:0006958;GO:0006955;GO:0070062 | hsa:7441 |  | RecName: Full=Immunoglobulin lambda variable 1-51; AltName: Full=Ig lambda chain V-I region BL2; AltName: Full=Ig lambda chain V-I region EPS; AltName: Full=Ig lambda chain V-I region NEW; AltName: Full=Ig lambda chain V-I region NIG-64; Flags: Precursor |
| E7EUT5 | 9026.696316 | 9330.010583 | 1.033601913 | 0.047680646 | 0.806657921 | no | no change | GO:0051287;GO:0050821;GO:0000226;GO:0097718;GO:0006096;GO:0061844;GO:0005737;GO:0004365;GO:0005634;GO:0005811;GO:0016620;GO:0051873;GO:0097452;GO:0042802;GO:0035605;GO:0035606;GO:0015630;GO:0008017;GO:0031965;GO:0050661;GO:0050832;GO:0051402;GO:0052501;GO:0005886;GO:0055114;GO:0043231;GO:0050715;GO:0005829;GO:0071346;GO:1990904;GO:0019828;GO:0017148;GO:0006417;GO:0006006 | hsa:2597 | COG0057 | GAPDH isoform 4 [Pan troglodytes] |
| P0C0L5 | 1807760.674 | 1868176.25 | 1.033420119 | 0.047426876 | 0.783063095 | no | no change | GO:0004252;GO:0030449;GO:0006954;GO:0030246;GO:0030425;GO:0030424;GO:0045202;GO:0032490;GO:0030054;GO:0005615;GO:0072562;GO:0001848;GO:0006508;GO:0045087;GO:0008228;GO:0006956;GO:2000427;GO:0004866;GO:0005886;GO:0010951;GO:0006958;GO:0042995;GO:0070062;GO:0044216;GO:0002376;GO:0005576 | hsa:100293534;hsa:110384692;hsa:720;hsa:721 | | complement C4-B preproprotein [Homo sapiens] |
| M0R0Q9 | 5830.902444 | 6022.777783 | 1.032906628 | 0.046709844 | 0.709996344 | no | no change | GO:0006631;GO:0030449;GO:0004866;GO:0005886;GO:0005788;GO:0035578;GO:0007165;GO:0031715;GO:0048260;GO:0010828;GO:0010866;GO:0044267;GO:0070062;GO:0009617;GO:0045766;GO:1905114;GO:0045745;GO:0097242;GO:2000427;GO:0034774;GO:0007186;GO:0009986;GO:0005576;GO:0150064;GO:0043687;GO:0016322;GO:0150062;GO:0006956;GO:0006957;GO:0006954;GO:0006955;GO:0060100;GO:0006958;GO:0005615;GO:0050776;GO:0032991;GO:0010575;GO:0043312;GO:0001798;GO:0010884;GO:0001970;GO:0097278;GO:0005102;GO:0001934;GO:0072562 | hsa:718 |  | C3 isoform 6, partial [Pan troglodytes] |
| P01700 | 1457660.432 | 1497228.204 | 1.027144712 | 0.038639454 | 0.823070206 | no | no change | GO:0038096;GO:0004252;GO:0016020;GO:0030449;GO:0050776;GO:0006898;GO:0038095;GO:0072562;GO:0002376;GO:0050900;GO:0005576;GO:0006956;GO:0002250;GO:0003823;GO:0005886;GO:0006508;GO:0006958;GO:0006955 | hsa:7441 |  | RecName: Full=Immunoglobulin lambda variable 1-47; AltName: Full=Ig lambda chain V-I region HA; AltName: Full=Ig lambda chain V-I region WAH; Flags: Precursor |
| P01717 | 6679.0208 | 6849.123 | 1.025468134 | 0.036282662 | 0.850214126 | no | no change | GO:0038096;GO:0004252;GO:0016020;GO:0030449;GO:0050776;GO:0006898;GO:0038095;GO:0072562;GO:0002376;GO:0050900;GO:0005576;GO:0006956;GO:0002250;GO:0003823;GO:0005886;GO:0006508;GO:0006958;GO:0006955 | hsa:7441 |  | immunoglobulin lambda light chain variable region, partial [Homo sapiens] |
| A0A0C4DH25 | 534346.1842 | 544631 | 1.019247477 | 0.027504385 | 0.866454351 | no | no change | GO:0038096;GO:0004252;GO:0016020;GO:0030449;GO:0005615;GO:0006898;GO:0038095;GO:0050900;GO:0002376;GO:0002377;GO:0050776;GO:0005576;GO:0006956;GO:0002250;GO:0003823;GO:0005886;GO:0006508;GO:0006958;GO:0006955 | hsa:29802 |  | hCG1686089, partial [Homo sapiens] |
| P01619 | 3394970 | 3456730.083 | 1.018191643 | 0.02600913 | 0.796711647 | no | no change | GO:0004252;GO:0030449;GO:0019731;GO:0006955;GO:0050900;GO:0005615;GO:0016020;GO:0003094;GO:0072562;GO:0071748;GO:0006508;GO:0050776;GO:0006956;GO:0003823;GO:0005886;GO:0006958;GO:0070062;GO:0038096;GO:0038095;GO:0006898;GO:0002376;GO:0005576;GO:0002250;GO:0071751;GO:0071756 | hsa:29802 |  | immunoglobulin light chain variable region, partial [Homo sapiens] |
| P02675 | 14715519.89 | 14965424.92 | 1.016982412 | 0.024294729 | 0.816038063 | no | no change | GO:1902042;GO:0034116;GO:0045921;GO:0007599;GO:0005783;GO:0009897;GO:0007596;GO:0007160;GO:0045907;GO:0045202;GO:0034622;GO:0044320;GO:0005737;GO:1903561;GO:0031091;GO:0005615;GO:0031093;GO:1900026;GO:0072378;GO:0002576;GO:0051087;GO:0031639;GO:0072562;GO:0005198;GO:0030198;GO:0002224;GO:0045087;GO:0009986;GO:2000352;GO:0051592;GO:0090277;GO:0005938;GO:0005886;GO:0050839;GO:0030168;GO:0070062;GO:0051258;GO:0042730;GO:0050714;GO:0071347;GO:0002376;GO:0005577;GO:0005576;GO:0002250;GO:0005515;GO:0043152;GO:0005102;GO:0070374;GO:0070527 | hsa:2244 |  | fibrinogen beta chain isoform 1 preproprotein [Homo sapiens] |
| P00488 | 24389.30105 | 24800.94633 | 1.016878109 | 0.024146757 | 0.896018587 | no | no change | GO:0005737;GO:0018149;GO:0007599;GO:0019221;GO:0031093;GO:0003810;GO:0072378;GO:0002576;GO:0007596;GO:0072562;GO:0016740;GO:0062023;GO:0016746;GO:0046872;GO:0005576 | hsa:2162 |  | RecName: Full=Coagulation factor XIII A chain; Short=Coagulation factor XIIIa; AltName: Full=Protein-glutamine gamma-glutamyltransferase A chain; AltName: Full=Transglutaminase A chain; Flags: Precursor |
| O14791 | 35749.16842 | 36310.42333 | 1.015699803 | 0.022474067 | 0.889761726 | no | no change | GO:0006869;GO:0005788;GO:0042157;GO:0031224;GO:0019835;GO:0044267;GO:0005615;GO:0072562;GO:0005254;GO:0045087;GO:0034361;GO:0034364;GO:0006629;GO:1902476;GO:0043687;GO:0008289;GO:0008202;GO:0008203;GO:0006898;GO:0031640;GO:0005576;GO:0005515 | hsa:8542 |  | apolipoprotein L1 isoform a precursor [Homo sapiens] |
| P00734 | 573057.1632 | 579945.2667 | 1.012019924 | 0.017237693 | 0.770625717 | no | no change | GO:0004252;GO:0048712;GO:0009897;GO:0030307;GO:0007597;GO:0007596;GO:0005788;GO:0051281;GO:0030449;GO:0032967;GO:0008047;GO:0007166;GO:0007599;GO:0008233;GO:0007275;GO:0061844;GO:0044267;GO:0006888;GO:0005615;GO:0005102;GO:0009611;GO:0070062;GO:0001530;GO:0051838;GO:0010544;GO:0005509;GO:0006508;GO:0008236;GO:0007186;GO:0010468;GO:1900738;GO:0008284;GO:0016787;GO:2000379;GO:0046427;GO:0090218;GO:0070945;GO:0006953;GO:0005796;GO:0008360;GO:0014068;GO:0005886;GO:0008201;GO:0030168;GO:0008083;GO:1900016;GO:0042730;GO:1900182;GO:0051480;GO:0010469;GO:0070053;GO:0005576;GO:0051918;GO:0005515;GO:0072378;GO:0030193;GO:0045861;GO:0072562;GO:0001934;GO:0030194 | hsa:2147 | COG5640 | prothrombin isoform 1 preproprotein [Homo sapiens] |
| A0A0G2JL69 | 36053.48895 | 36451.96917 | 1.011052473 | 0.015857874 | 0.871071904 | no | no change | GO:0004252;GO:0016787;GO:0045087;GO:0005576;GO:0006956;GO:0006508;GO:0006958;GO:0046872;GO:0008233;GO:0008236 | hsa:717 | COG5640 | complement C2 isoform 5 [Homo sapiens] |
| O00391 | 10480.24147 | 10590.16629 | 1.010488768 | 0.015053285 | 0.920787138 | no | no change | GO:0005788;GO:0030173;GO:0071949;GO:0016491;GO:0016971;GO:0044267;GO:0016972;GO:0045171;GO:0070062;GO:0031093;GO:0016021;GO:0016020;GO:0003756;GO:0035580;GO:0016242;GO:0005794;GO:1904724;GO:0043687;GO:0045454;GO:0055114;GO:0005615;GO:0043231;GO:0043312;GO:0000139;GO:0005576;GO:0085029;GO:0002576 | hsa:5768 |  | sulfhydryl oxidase 1 isoform a precursor [Homo sapiens] |
| P23142 | 34465.29789 | 34770.50708 | 1.008855551 | 0.012719622 | 0.922209472 | no | no change | GO:0005201;GO:0008022;GO:0031012;GO:0007162;GO:0044877;GO:0005615;GO:0072378;GO:1900025;GO:0005576;GO:0005509;GO:2000647;GO:0042802;GO:0062023;GO:0071953;GO:0010952;GO:0070062;GO:0005604;GO:0007566;GO:0070051;GO:0005577;GO:0030198;GO:0001968;GO:0016032;GO:2000146;GO:0016504;GO:0001933;GO:0070373;GO:0007229 | hsa:2192 |  | fibulin-1 isoform D precursor [Homo sapiens] |
| P04003 | 2738359.421 | 2762448.458 | 1.008796887 | 0.012635728 | 0.94189786 | no | no change | GO:0030449;GO:0005615;GO:0045087;GO:0072562;GO:0002376;GO:0045732;GO:0005576;GO:0003723;GO:0005515;GO:0005886;GO:1903027;GO:0006958;GO:0045959;GO:0044216 | hsa:722 |  | C4b-binding protein alpha chain precursor [Homo sapiens] |
| P11597 | 7393.141222 | 7446.994248 | 1.007284187 | 0.010470772 | 0.955603557 | no | no change | GO:0017129;GO:0034375;GO:0034374;GO:0034372;GO:0006869;GO:0015914;GO:0017127;GO:0030301;GO:0034197;GO:0034364;GO:0055091;GO:0005548;GO:0070062;GO:0005615;GO:0008202;GO:0010745;GO:0031982;GO:0005319;GO:0070328;GO:0006629;GO:0006641;GO:0008289;GO:0055088;GO:0031210;GO:0042632;GO:0043691;GO:0010874;GO:0008203;GO:0015485;GO:0005576;GO:0046470 | hsa:1071 |  | cholesteryl ester transfer protein isoform 1 precursor [Homo sapiens] |
| P01860 | 2892402.258 | 2913433.25 | 1.007271116 | 0.01045205 | 0.960646302 | no | no change | GO:0004252;GO:0030449;GO:0009897;GO:0050871;GO:0042742;GO:0034987;GO:0042571;GO:0005615;GO:0016020;GO:0072562;GO:0006508;GO:0050853;GO:0045087;GO:0006910;GO:0006911;GO:0006956;GO:0003823;GO:0005886;GO:0006958;GO:0001895;GO:0070062;GO:0038096;GO:0002376;GO:0005576;GO:0002250 | hsa:100423062 | | Unknown (protein for MGC:105008) [Homo sapiens] |
| P01871 | 10356733.32 | 10420076.83 | 1.006116168 | 0.00879689 | 0.972112467 | no | no change | GO:0009897;GO:0042834;GO:0019731;GO:0050900;GO:0034987;GO:0050829;GO:0005615;GO:0016021;GO:0016020;GO:0072562;GO:0009986;GO:0050853;GO:0045087;GO:0006910;GO:0006911;GO:0031210;GO:0003823;GO:0005886;GO:0006958;GO:0050871;GO:0070062;GO:0003697;GO:0002376;GO:0005576;GO:0002250;GO:0005515;GO:0071756;GO:0071757 | hsa:3543 |  | immunoglobulin heavy chain [Homo sapiens] |
| P00740 | 34031.94947 | 34206.12833 | 1.005118098 | 0.007365023 | 0.918146603 | no | no change | GO:0004252;GO:0004175;GO:0006888;GO:0005615;GO:0016787;GO:0070062;GO:0007597;GO:0007596;GO:0031638;GO:0005788;GO:0005796;GO:0005576;GO:0005509;GO:0005515;GO:0005886;GO:0006508;GO:0008233;GO:0046872;GO:0007599;GO:0008236 | hsa:2158 | COG5640 | coagulation factor IX isoform 1 preproprotein [Homo sapiens] |
| P51884 | 86643.76368 | 86967.095 | 1.003731732 | 0.005373731 | 0.954735505 | no | no change | GO:0005201;GO:0018146;GO:0031012;GO:0014070;GO:0043202;GO:0070062;GO:0005583;GO:0070848;GO:0005576;GO:0005796;GO:0032914;GO:0007601;GO:0030021;GO:0062023;GO:0007409;GO:0005615;GO:0051216;GO:0030199;GO:0030198;GO:0045944;GO:0005515;GO:0005518;GO:0042340 | hsa:4060 | COG4886 | lumican precursor [Homo sapiens] |
| A0A0J9YY99 | 222303.1363 | 222880.8396 | 1.002598718 | 0.003744295 | 0.9954339 | no | no change |  | hsa:102723407 | | immunoglobulin heavy chain VDJ region, partial [Homo sapiens] |
| P01597 | 42072.93842 | 42113.01583 | 1.00095257 | 0.001373614 | 0.990552832 | no | no change | GO:0038096;GO:0004252;GO:0016020;GO:0030449;GO:0050776;GO:0006898;GO:0038095;GO:0072562;GO:0002376;GO:0050900;GO:0005576;GO:0006956;GO:0002250;GO:0003823;GO:0005886;GO:0006508;GO:0006958;GO:0006955;GO:0070062 | hsa:7441 |  | immunoglobulin kappa light chain VC region, partial [Homo sapiens] |
| Q16610 | 39667.41 | 39692.97458 | 1.000644473 | 0.000929479 | 0.991367212 | no | no change | GO:0008022;GO:0005201;GO:0019899;GO:0031012;GO:0001503;GO:0007165;GO:0030500;GO:0030502;GO:0001525;GO:0005615;GO:0002576;GO:0045766;GO:0010466;GO:2000404;GO:0001960;GO:0002063;GO:0003416;GO:0005576;GO:0005134;GO:0002020;GO:0002828;GO:0062023;GO:0006954;GO:0031214;GO:0070062;GO:0043236;GO:0031089;GO:0001938;GO:0006357;GO:0005515;GO:0043123 | hsa:1893 |  | extracellular matrix protein 1 isoform 1 precursor [Homo sapiens] |
| O43866 | 151022.4368 | 150706.8717 | 0.997910475 | -0.003017701 | 0.984702231 | no | no change | GO:0005737;GO:0006898;GO:0005615;GO:0005044;GO:0009986;GO:0016020;GO:0072562;GO:0002376;GO:0005576;GO:0006954;GO:0006968;GO:0006915 | hsa:922 |  | CD5 antigen-like isoform 1 precursor [Homo sapiens] |
| E9PAQ1 | 27865.30579 | 27762.39167 | 0.996306729 | -0.005338127 | 0.959041815 | no | no change |  | hsa:5199 |  | properdin precursor [Homo sapiens] |
| P05160 | 26841.95632 | 26736.40458 | 0.996067659 | -0.005684353 | 0.957857971 | no | no change | GO:0072378;GO:0005576;GO:0007596;GO:0007599;GO:1903363 | hsa:2165 |  | coagulation factor XIII B chain precursor [Homo sapiens] |
| A0A0A0MRJ7 | 20750.63968 | 20650.47167 | 0.995172774 | -0.006981078 | 0.961046032 | no | no change | GO:0048208;GO:0044267;GO:1903561;GO:0006888;GO:0005615;GO:0031093;GO:0008015;GO:0002576;GO:0007596;GO:0033116;GO:0005788;GO:0030134;GO:0046872;GO:0005576;GO:0005886;GO:0016020;GO:0005507;GO:0043687;GO:0000139 | hsa:2153 |  | coagulation factor V preproprotein [Homo sapiens] |
| E9PHK0 | 142852.4105 | 142086.1017 | 0.99463566 | -0.007759938 | 0.910964449 | no | no change | GO:0005737;GO:0036143;GO:0070062;GO:0002576;GO:0001652;GO:0030246;GO:0008201;GO:0071560;GO:0071310;GO:0001503;GO:0005615;GO:0005509;GO:0005576;GO:0031089;GO:0030282;GO:0062023;GO:0010756 | hsa:7123 |  | tetranectin isoform 1precursor [Homo sapiens] |
| P11226 | 108637.3736 | 107708.6689 | 0.991451333 | -0.012386138 | 0.951842786 | no | no change | GO:0004252;GO:0048306;GO:0030246;GO:0042742;GO:0044130;GO:0050766;GO:0005581;GO:0001867;GO:0005509;GO:0051873;GO:0006508;GO:0009986;GO:0045087;GO:0006953;GO:0008228;GO:0006956;GO:0006958;GO:0006979;GO:0005615;GO:0050830;GO:0002376;GO:0005576;GO:0005515;GO:0048525;GO:0005102;GO:0005537 | hsa:4153 |  | mannose-binding lectin [Homo sapiens] |
| A0A075B6I0 | 624553.5278 | 618177.3828 | 0.989790875 | -0.014804353 | 0.949157432 | no | no change | GO:0016020;GO:0005615;GO:0006955;GO:0002376;GO:0002377;GO:0005576;GO:0002250;GO:0003823;GO:0005886 | hsa:7441 |  | RecName: Full=Immunoglobulin lambda variable 8-61; Flags: Precursor |
| P04217 | 789356.7158 | 780897.2333 | 0.989283068 | -0.01554471 | 0.851440953 | no | no change | GO:0070062;GO:0031093;GO:0002576;GO:0043312;GO:0072562;GO:1904813;GO:0005576;GO:0003674;GO:0034774;GO:0008150;GO:0062023;GO:0005615 | hsa:1 |  | alpha-1B-glycoprotein precursor [Homo sapiens] |
| P18206 | 8854.195455 | 8736.734328 | 0.986733845 | -0.0192671 | 0.949229174 | no | no change | GO:0048675;GO:0015629;GO:0030336;GO:0034333;GO:0030032;GO:0034774;GO:0007160;GO:0002162;GO:0005925;GO:0030054;GO:0030055;GO:0005737;GO:0002009;GO:0002102;GO:0070062;GO:0002576;GO:0016020;GO:0034394;GO:0005198;GO:0003779;GO:0035580;GO:0005856;GO:0030334;GO:0042383;GO:0006936;GO:0051015;GO:0005916;GO:0005913;GO:0005912;GO:0005911;GO:0005886;GO:0007155;GO:0090136;GO:1903561;GO:1904813;GO:0031625;GO:0032991;GO:0008013;GO:0005829;GO:0045294;GO:0043312;GO:0045296;GO:0070527;GO:0005576;GO:0005515;GO:0043297;GO:0043034 | hsa:7414 |  | vinculin isoform meta-VCL [Homo sapiens] |
| P01601 | 100026.1226 | 98663.38208 | 0.986376153 | -0.019790173 | 0.895998512 | no | no change | GO:0038096;GO:0004252;GO:0016020;GO:0030449;GO:0005615;GO:0006898;GO:0038095;GO:0050900;GO:0002376;GO:0002377;GO:0050776;GO:0005576;GO:0006956;GO:0002250;GO:0003823;GO:0005886;GO:0006508;GO:0006958;GO:0006955 | hsa:7441 |  | RecName: Full=Immunoglobulin kappa variable 1D-16; AltName: Full=Ig kappa chain V-I region HK146; AltName: Full=Ig kappa chain V-I region HK189; Flags: Precursor |
| H0Y755 | 7768.903474 | 7651.50013 | 0.984888042 | -0.021968361 | 0.902360963 | no | no change | GO:0016021;GO:0016020 | hsa:2214 |  | low affinity immunoglobulin gamma Fc region receptor III-A isoform b [Homo sapiens] |
| P02748 | 123071.5032 | 120882.7829 | 0.982215865 | -0.02588797 | 0.830410351 | no | no change | GO:0030449;GO:0006955;GO:0019835;GO:0019836;GO:0070062;GO:0016021;GO:0016020;GO:0051260;GO:0072562;GO:0001906;GO:0045087;GO:0006957;GO:0005887;GO:0005886;GO:0006958;GO:0005615;GO:0005829;GO:0044218;GO:0044279;GO:0002376;GO:0005576;GO:0005579 | hsa:735 |  | complement component C9 preproprotein [Homo sapiens] |
| P02790 | 3115692.421 | 3059440.5 | 0.981945612 | -0.026284977 | 0.731548624 | no | no change | GO:0020027;GO:0002925;GO:0015232;GO:0071682;GO:0005615;GO:0051246;GO:0042531;GO:0072562;GO:0046872;GO:0042168;GO:0006879;GO:0016032;GO:0060332;GO:0062023;GO:0060335;GO:0002639;GO:0070062;GO:0015886;GO:0006898;GO:0005623;GO:0005576;GO:0005515 | hsa:3263 |  | hemopexin precursor [Homo sapiens] |
| P02775 | 43439.92563 | 42574.82442 | 0.980085113 | -0.029021053 | 0.932969211 | no | no change | GO:0042127;GO:0070098;GO:0005125;GO:0071222;GO:0008009;GO:0042742;GO:0060326;GO:0032496;GO:0061844;GO:0031091;GO:0008083;GO:0031093;GO:0002576;GO:0007186;GO:0005355;GO:0030595;GO:0030593;GO:1904724;GO:0006952;GO:0006954;GO:0006955;GO:0045236;GO:0005615;GO:0090023;GO:0010469;GO:0031640;GO:0043312;GO:0006935;GO:0051781;GO:0005576;GO:1904659;GO:0005515 | hsa:5473 |  | platelet basic protein preproprotein [Homo sapiens] |
| P01599 | 138053.15 | 135019.8892 | 0.978028311 | -0.032051867 | 0.85856715 | no | no change | GO:0038096;GO:0004252;GO:0016020;GO:0030449;GO:0050776;GO:0006898;GO:0038095;GO:0072562;GO:0002376;GO:0050900;GO:0005576;GO:0006956;GO:0002250;GO:0003823;GO:0005886;GO:0006508;GO:0006958;GO:0006955;GO:0070062 | hsa:7441 |  | RecName: Full=Immunoglobulin kappa variable 1-17; AltName: Full=Ig kappa chain V-I region Gal; AltName: Full=Ig kappa chain V-I region WEA; Flags: Precursor |
| Q06033 | 41468.19842 | 40549.3975 | 0.97784324 | -0.032324893 | 0.824833293 | no | no change | GO:0030212;GO:0070062;GO:0002576;GO:0031089;GO:0005576;GO:0030414;GO:0004866;GO:0004867;GO:0010951;GO:0010466 | hsa:3699 | COG2304 | inter-alpha-trypsin inhibitor heavy chain H3 preproprotein [Homo sapiens] |
| C9JF17 | 842503.8 | 820149.85 | 0.973467241 | -0.038795664 | 0.706158246 | no | no change | GO:0022626;GO:0005783;GO:0006869;GO:0042493;GO:0048678;GO:0030425;GO:0000302;GO:0001525;GO:0051895;GO:0010642;GO:0005737;GO:0070062;GO:0014012;GO:0005615;GO:2000405;GO:0048471;GO:0043025;GO:0005319;GO:0048662;GO:0006629;GO:2000098;GO:0008289;GO:0042308;GO:0060588;GO:0007420;GO:1900016;GO:0007568;GO:0015485;GO:0042246;GO:0071638;GO:0005576;GO:0006006 | hsa:347 | COG3040 | APOD isoform 3, partial [Pan troglodytes] |
| Q9UHG3 | 19234.01847 | 18711.54929 | 0.972836192 | -0.039731193 | 0.788208286 | no | no change | GO:0034361;GO:1902476;GO:0070062;GO:0030327;GO:0030328;GO:0030329;GO:0099133;GO:0005774;GO:0005764;GO:0016670;GO:0006821;GO:0055114;GO:0005886;GO:0008555;GO:0001735;GO:0016491 | hsa:51449 |  | prenylcysteine oxidase 1 precursor [Homo sapiens] |
| P27169 | 245743.4632 | 238200.1833 | 0.96930425 | -0.044978517 | 0.600674962 | no | no change | GO:0034445;GO:0032411;GO:0016311;GO:0046872;GO:0004064;GO:0046434;GO:0070062;GO:0005615;GO:0005543;GO:0009636;GO:0072562;GO:0005509;GO:0102007;GO:0010875;GO:0019372;GO:0042803;GO:0016787;GO:0034364;GO:0006629;GO:0034366;GO:1902617;GO:0008203;GO:0004063;GO:0043231;GO:0046395;GO:0051099;GO:0031667;GO:0046470;GO:0005576;GO:0019439;GO:0070542 | hsa:5444 |  | serum paraoxonase/arylesterase 1 precursor [Homo sapiens] |
| P15814 | 11435450.37 | 11078226.17 | 0.968761685 | -0.045786288 | 0.623636758 | no | no change | GO:0009897;GO:0050853;GO:0045087;GO:0016020;GO:0006910;GO:0006911;GO:0050900;GO:0005576;GO:0042742;GO:0003823;GO:0006955;GO:0042571;GO:0006958;GO:0050871;GO:0034987;GO:0072562 | hsa:3543 |  | immunoglobulin lambda-like polypeptide 1 isoform a precursor [Homo sapiens] |
| P01031 | 225098.3579 | 217947.0667 | 0.968230371 | -0.046577746 | 0.634438748 | no | no change | GO:0000187;GO:0030449;GO:0006954;GO:0008009;GO:0007166;GO:0060326;GO:0019835;GO:0001701;GO:0005615;GO:0045766;GO:0010760;GO:0007186;GO:0045087;GO:0006935;GO:0090197;GO:0006956;GO:0006957;GO:0004866;GO:0010951;GO:0006958;GO:0070062;GO:0010575;GO:0002376;GO:0005576;GO:0005515;GO:0005102;GO:0005579 | hsa:727 |  | complement C5 isoform 1 preproprotein [Homo sapiens] |
| B4E1Z4 | 548596.7158 | 531061.825 | 0.968036829 | -0.046866159 | 0.579032115 | no | no change | GO:0004252;GO:0030449;GO:0070062;GO:0016787;GO:0072562;GO:0001848;GO:0005576;GO:0006956;GO:0006957;GO:0005886;GO:0006508;GO:0008233;GO:0008236;GO:0005615 | hsa:629 | COG5640 | unnamed protein product [Homo sapiens] |
| Q96KN2 | 6516.0505 | 6305.209933 | 0.967642889 | -0.047453379 | 0.802974293 | no | no change | GO:0016787;GO:0032268;GO:0005829;GO:0004180;GO:0016805;GO:0005576;GO:0008152;GO:0006508;GO:0008233;GO:0046872;GO:0008237 | hsa:84735 | COG0624 | RecName: Full=Beta-Ala-His dipeptidase; AltName: Full=CNDP dipeptidase 1; AltName: Full=Carnosine dipeptidase 1; AltName: Full=Glutamate carboxypeptidase-like protein 2; AltName: Full=Serum carnosinase; Flags: Precursor |
| P08697 | 252362.9421 | 244147.6875 | 0.967446668 | -0.047745962 | 0.510281492 | no | no change | GO:0030414;GO:0032967;GO:0005615;GO:0031093;GO:0051496;GO:0002576;GO:0030199;GO:0072562;GO:0010466;GO:0048514;GO:0002020;GO:0010757;GO:0051918;GO:0042803;GO:0048661;GO:0009986;GO:0045597;GO:0006953;GO:0046330;GO:0004866;GO:0004867;GO:0010951;GO:0010033;GO:0070062;GO:0042730;GO:2000049;GO:0002034;GO:0005577;GO:0005576;GO:0045944;GO:0005515;GO:0070374;GO:0071636 | hsa:5345 | COG4826 | alpha-2-antiplasmin isoform X1 [Homo sapiens] |
| P09871 | 92244.90053 | 89103.85625 | 0.965948857 | -0.049981288 | 0.529690064 | no | no change | GO:0004252;GO:0006956;GO:0016787;GO:0045087;GO:0072562;GO:0002376;GO:0030449;GO:0005576;GO:0001867;GO:0005509;GO:0005515;GO:0006508;GO:0006958;GO:0046872;GO:0008233;GO:0042802;GO:0008236 | hsa:716 | COG5640 | complement C1s subcomponent isoform 1 preproprotein [Homo sapiens] |
| P61224 | 10046.39377 | 9703.622615 | 0.965881175 | -0.050082379 | 0.870982968 | no | no change | GO:0005811;GO:0030033;GO:0044877;GO:0007165;GO:0061028;GO:0030054;GO:0005737;GO:0070062;GO:2000114;GO:0016020;GO:0000166;GO:0070382;GO:0045955;GO:0035722;GO:0005525;GO:0008283;GO:0003924;GO:0035577;GO:0032486;GO:0005911;GO:0005886;GO:0045121;GO:0007264;GO:0005829;GO:0071320;GO:0043312;GO:0005622;GO:0019003;GO:0005515;GO:1901888;GO:0070374;GO:2000301 | hsa:5908 | COG1100 | Ras-related protein Rap-1b, partial [Bos mutus] |
| P35858 | 58197.45158 | 56035.23375 | 0.962846864 | -0.054621732 | 0.608412216 | no | no change | GO:0044267;GO:0005615;GO:0031012;GO:0005654;GO:0007155;GO:0005576;GO:0007165;GO:0042567;GO:0005520;GO:0070062 | hsa:3483 | COG4886 | Insulin-like growth factor binding protein, acid labile subunit [Homo sapiens] |
| P05155 | 346645.2684 | 333477.4708 | 0.962013624 | -0.05587077 | 0.567065786 | no | no change | GO:0030449;GO:0007597;GO:0007596;GO:0007599;GO:0005615;GO:0031093;GO:0002576;GO:0072562;GO:0010466;GO:0001869;GO:0045916;GO:0008015;GO:0045087;GO:0030414;GO:0004867;GO:0010951;GO:0006958;GO:0070062;GO:0042730;GO:0007568;GO:0002376;GO:0005576;GO:0005515;GO:0030193 | hsa:710 | COG4826 | unnamed protein product [Homo sapiens] |
| J3QRV5 | 399653.4421 | 384036.1792 | 0.960922987 | -0.057507284 | 0.734947349 | no | no change | GO:0005737;GO:0043231;GO:0005829;GO:0006887;GO:0007049;GO:0051301 | hsa:3993 |  | lethal(2) giant larvae protein homolog 2 isoform X4 [Homo sapiens] |
| P01699 | 158709.0758 | 152317.1246 | 0.959725358 | -0.059306482 | 0.650892629 | no | no change | GO:0038096;GO:0004252;GO:0016020;GO:0030449;GO:0050776;GO:0006898;GO:0038095;GO:0002376;GO:0050900;GO:0005576;GO:0006956;GO:0002250;GO:0003823;GO:0005886;GO:0006508;GO:0006958;GO:0006955 | hsa:7441 |  | hCG2043214, partial [Homo sapiens] |
| P20851 | 56854.73632 | 54330.14875 | 0.955595827 | -0.065527542 | 0.653615737 | no | no change | GO:0030449;GO:0005615;GO:0045087;GO:0007596;GO:0002376;GO:0045732;GO:0005576;GO:0005515;GO:0005886;GO:1903027;GO:0006958;GO:0045959;GO:0044216 | hsa:725 |  | C4b-binding protein beta chain isoform 1 precursor [Homo sapiens] |
| P01042 | 1234049.968 | 1178250.821 | 0.954783721 | -0.066754126 | 0.289736413 | no | no change | GO:0007599;GO:0030414;GO:0007597;GO:0007596;GO:0045861;GO:0005788;GO:0007162;GO:0008270;GO:0042311;GO:0044267;GO:0050880;GO:0005615;GO:0031093;GO:0002576;GO:0072562;GO:0043065;GO:0010466;GO:0007186;GO:0043687;GO:0062023;GO:0006954;GO:0005886;GO:0010951;GO:0004869;GO:0008201;GO:0070062;GO:0007204;GO:0005576;GO:0005515;GO:0005102;GO:0030195 | hsa:3827 |  | kininogen-1 isoform 1 precursor [Homo sapiens] |
| A0A0B4J231 | 11148517 | 10631262.05 | 0.953603251 | -0.068538941 | 0.723085239 | no | no change | GO:0009897;GO:0050853;GO:0070062;GO:0045087;GO:0006910;GO:0006911;GO:0042742;GO:0003823;GO:0034987;GO:0042571;GO:0006958;GO:0050871 | hsa:100423062 | | immunoglobulin lambda-3 surrogate light chain [Homo sapiens] |
| P01857 | 9607821.105 | 9143238.333 | 0.951645356 | -0.071504062 | 0.631193069 | no | no change | GO:0004252;GO:0030449;GO:0019221;GO:0009897;GO:0042742;GO:0034987;GO:0042571;GO:0005615;GO:0016020;GO:0072562;GO:0006508;GO:0050853;GO:0045087;GO:0006910;GO:0006911;GO:0006956;GO:0003823;GO:0005886;GO:0006958;GO:0050871;GO:0070062;GO:0038096;GO:0002376;GO:0005576;GO:0002250;GO:0005515 | hsa:100423062 | | IGH@ protein [Homo sapiens] |
| P01011 | 768373.9316 | 729900.2542 | 0.949928445 | -0.074109251 | 0.360074181 | no | no change | GO:0006954;GO:0034774;GO:0035578;GO:0003677;GO:0070062;GO:0031093;GO:0002576;GO:0072562;GO:0010466;GO:0019216;GO:0006953;GO:0062023;GO:0030277;GO:0030414;GO:0004867;GO:0010951;GO:0005615;GO:0043312;GO:0005622;GO:0005576;GO:0005515;GO:0005634 | hsa:12 | COG4826 | serpin peptidase inhibitor, clade A (alpha-1 antiproteinase, antitrypsin), member 3, isoform CRA_b [Homo sapiens] |
| A0A0B4J1V0 | 31502.57684 | 29888.46875 | 0.948762665 | -0.075880856 | 0.654404949 | no | no change | GO:0009897;GO:0050853;GO:0045087;GO:0016020;GO:0006910;GO:0006911;GO:0002376;GO:0005576;GO:0002250;GO:0003823;GO:0005886;GO:0072562;GO:0042571;GO:0006958;GO:0050871;GO:0034987;GO:0042742 | hsa:102724971 | | unnamed protein product [Homo sapiens] |
| P02747 | 458825.5737 | 435160.6167 | 0.948422759 | -0.076397811 | 0.440234942 | no | no change | GO:0004252;GO:0030449;GO:0005581;GO:0045650;GO:0045087;GO:0072562;GO:0002376;GO:0030853;GO:0005576;GO:0006956;GO:0005515;GO:0006955;GO:0006508;GO:0006958;GO:0005615 | hsa:714 |  | complement C1q subcomponent subunit C isoform X1 [Pongo abelii] |
| P00450 | 660068.4105 | 625856.0042 | 0.948168393 | -0.076784793 | 0.375623064 | no | no change | GO:0016491;GO:0044267;GO:0046872;GO:0006879;GO:0005615;GO:0006825;GO:0004322;GO:0006811;GO:0051087;GO:0070062;GO:0005623;GO:0005765;GO:0055072;GO:0006826;GO:0005788;GO:0005886;GO:0072562;GO:0005507;GO:0043687;GO:0055114;GO:0005576 | hsa:1356 | COG2132 | RecName: Full=Ceruloplasmin; AltName: Full=Ferroxidase; Flags: Precursor |
| P04196 | 685319.6474 | 646424.2125 | 0.943244827 | -0.084295811 | 0.546113052 | no | no change | GO:0002839;GO:0030308;GO:0032956;GO:2000504;GO:0007162;GO:0008270;GO:0051894;GO:0007599;GO:0001525;GO:0061844;GO:0051715;GO:0070062;GO:0031093;GO:0002576;GO:0072562;GO:0043065;GO:0033629;GO:0030168;GO:0005886;GO:0046872;GO:0010468;GO:0051918;GO:0010543;GO:0008285;GO:0007596;GO:0006935;GO:0020037;GO:0009986;GO:0010593;GO:0043537;GO:0043395;GO:0062023;GO:0004867;GO:0010951;GO:0004869;GO:0043254;GO:0008201;GO:2001027;GO:0050832;GO:0042730;GO:0036019;GO:0050730;GO:0015886;GO:0019865;GO:0005576;GO:1900747;GO:0005515;GO:0030193;GO:0005102;GO:0016525 | hsa:3273 |  | histidine-rich glycoprotein precursor [Homo sapiens] |
| P02787 | 19885325.84 | 18754505.5 | 0.943132924 | -0.084466978 | 0.339333141 | no | no change | GO:0009617;GO:0016020;GO:0055037;GO:1990459;GO:1900390;GO:0005788;GO:0030139;GO:0061024;GO:0055072;GO:0006826;GO:0005905;GO:0034986;GO:1990712;GO:0048260;GO:0010008;GO:0030665;GO:0007257;GO:0045780;GO:0031232;GO:0005615;GO:0031410;GO:0002576;GO:0005770;GO:0045178;GO:0070062;GO:2000147;GO:0009925;GO:0044267;GO:0048471;GO:0046872;GO:0043687;GO:0009986;GO:0006879;GO:0033572;GO:0007015;GO:0016324;GO:0006811;GO:0030316;GO:0031982;GO:0008198;GO:0008199;GO:0045893;GO:0034774;GO:0015091;GO:0001895;GO:0034756;GO:0071281;GO:0042327;GO:0060395;GO:0031647;GO:0005623;GO:0005576;GO:0005515;GO:0005768;GO:0005769;GO:0070371;GO:0072562 | hsa:7018 |  | serotransferrin isoform 1 precursor [Homo sapiens] |
| A0A075B7D0 | 336157.6105 | 316636.0583 | 0.941927383 | -0.086312254 | 0.761712021 | no | no change | GO:0009897;GO:0050853;GO:0045087;GO:0006910;GO:0006911;GO:0042742;GO:0003823;GO:0034987;GO:0042571;GO:0006958;GO:0050871;GO:0072562 | hsa:102723407 | | hCG1728627 [Homo sapiens] |
| P02749 | 980397.2895 | 923438.4708 | 0.941902309 | -0.086350659 | 0.45421165 | no | no change | GO:0034392;GO:0007597;GO:0051006;GO:0034197;GO:0042627;GO:0070062;GO:0002576;GO:0005543;GO:0031639;GO:0051917;GO:0042802;GO:0051918;GO:0034361;GO:0034364;GO:0006641;GO:0009986;GO:0060230;GO:0008289;GO:0062023;GO:0033033;GO:0008201;GO:0005615;GO:0010596;GO:0031089;GO:0005576;GO:0030195;GO:0005515;GO:0030193;GO:0016525;GO:0001937;GO:0030194 | hsa:350 |  | beta-2-glycoprotein 1 precursor [Homo sapiens] |
| P04114 | 4102093.263 | 3862682.083 | 0.941636827 | -0.086757351 | 0.536720937 | no | no change | GO:0034360;GO:0034374;GO:0034359;GO:0005783;GO:0034371;GO:0006869;GO:0050750;GO:0005788;GO:0005789;GO:0009791;GO:0061024;GO:0050900;GO:0010628;GO:0042157;GO:0034378;GO:0032496;GO:0016042;GO:0001523;GO:0006629;GO:0005737;GO:0043202;GO:0032355;GO:0001701;GO:0031983;GO:0005615;GO:0071682;GO:0071356;GO:0009615;GO:0048844;GO:0005543;GO:0030669;GO:0071379;GO:0070062;GO:0010033;GO:0042953;GO:0010884;GO:0042158;GO:0002224;GO:0010008;GO:0043025;GO:0043687;GO:0070971;GO:0005319;GO:0034361;GO:0034362;GO:0034363;GO:0006642;GO:0005790;GO:0044267;GO:0030317;GO:0008289;GO:0034379;GO:0010744;GO:0007283;GO:0042627;GO:0042159;GO:0017127;GO:0012506;GO:0005886;GO:0042632;GO:0030301;GO:0008201;GO:0008202;GO:0008203;GO:0035473;GO:0031904;GO:0009743;GO:0007399;GO:0034382;GO:0034383;GO:0005829;GO:0006898;GO:0009566;GO:0043231;GO:0010886;GO:0019433;GO:0005576;GO:0045540;GO:0005515;GO:0034447;GO:0010269;GO:0005769;GO:0033344 | hsa:338 |  | RecName: Full=Apolipoprotein B-100; Short=Apo B-100; Contains: RecName: Full=Apolipoprotein B-48; Short=Apo B-48; Flags: Precursor |
| P10643 | 100947.3453 | 94850.89417 | 0.939607613 | -0.089869692 | 0.357189371 | no | no change | GO:0019835;GO:0030449;GO:0070062;GO:0045087;GO:0006883;GO:0002376;GO:0005576;GO:0006956;GO:0006957;GO:0006955;GO:0006958;GO:0005579 | hsa:730 |  | complement component C7 precursor [Homo sapiens] |
| D6RF35 | 2747335.053 | 2574258.708 | 0.937002098 | -0.093875817 | 0.167641578 | no | no change | GO:0051180;GO:0090482;GO:0035461;GO:0005615;GO:0005499 | hsa:2638 |  | GC isoform 4 [Pan troglodytes] |
| Q03591 | 171000.5442 | 160150.8988 | 0.936551983 | -0.094569022 | 0.473814721 | no | no change | GO:0032091;GO:0030449;GO:0005615;GO:0072562;GO:0045919;GO:0005576;GO:0006956;GO:0005515;GO:0032991;GO:0046982;GO:0042803 | hsa:3078 |  | complement factor H-related protein 1 precursor [Homo sapiens] |
| P05154 | 20619.27105 | 19250.355 | 0.933609872 | -0.099108279 | 0.38946594 | no | no change | GO:0009897;GO:0006869;GO:0007596;GO:0036024;GO:0002080;GO:0061107;GO:0036029;GO:0031094;GO:0031091;GO:0051346;GO:0007342;GO:0016020;GO:0036025;GO:0032190;GO:0036027;GO:0036026;GO:0010466;GO:0002020;GO:0097183;GO:0097182;GO:0097181;GO:0036028;GO:0007283;GO:0031210;GO:0007338;GO:0030414;GO:0004867;GO:0010951;GO:0008201;GO:0005615;GO:0032991;GO:0070062;GO:0036030;GO:0005539;GO:0005576;GO:0005515;GO:0001972;GO:0045861 | hsa:5104 | COG4826 | plasma serine protease inhibitor preproprotein [Homo sapiens] |
| P13671 | 57976.29579 | 54120.68 | 0.933496686 | -0.099283194 | 0.177801084 | no | no change | GO:0019835;GO:0001701;GO:0030449;GO:0070062;GO:0045917;GO:0045087;GO:0045766;GO:0002376;GO:0005576;GO:0006956;GO:0001970;GO:0005515;GO:0006955;GO:0006958;GO:0005579 | hsa:729 |  | complement component C6 precursor [Homo sapiens] |
| I3L145 | 11796.79926 | 10979.04358 | 0.930679868 | -0.103643095 | 0.682489951 | no | no change | GO:0005496 | hsa:6462 |  | SHBG protein, partial [Homo sapiens] |
| P05543 | 28069.67421 | 26070.655 | 0.928783669 | -0.10658549 | 0.42689045 | no | no change | GO:0005615;GO:0070327;GO:0005576;GO:0004867;GO:0010951;GO:0070062 | hsa:6906 | COG4826 | thyroxine-binding globulin precursor [Homo sapiens] |
| P55103 | 8508.933474 | 7873.938294 | 0.925373117 | -0.111892906 | 0.469757168 | no | no change | GO:0005179;GO:0005125;GO:0008083;GO:0060395;GO:0005160;GO:0010469;GO:0048468;GO:0005576;GO:0005615;GO:0010862;GO:0042981;GO:0043408 | hsa:3626 |  | inhibin beta C chain preproprotein [Homo sapiens] |
| Q96PD5 | 214713.8632 | 198554.5458 | 0.924740224 | -0.112879952 | 0.244284377 | no | no change | GO:0019730;GO:0008745;GO:0032827;GO:0009253;GO:0008270;GO:0044117;GO:0005615;GO:0016045;GO:0016020;GO:0050727;GO:0046872;GO:0002221;GO:0016787;GO:0045087;GO:0050830;GO:0001519;GO:0070062;GO:0005622;GO:0002376;GO:0005576;GO:0032689;GO:0016019;GO:0042834 | hsa:114770 |  | N-acetylmuramoyl-L-alanine amidase isoform 1 precursor [Homo sapiens] |
| P06727 | 1045892 | 964838.4583 | 0.922502953 | -0.116374566 | 0.278531315 | no | no change | GO:0034375;GO:0034445;GO:0034372;GO:0034371;GO:0006869;GO:0034380;GO:0045723;GO:0005788;GO:0034378;GO:0051006;GO:0042744;GO:0042157;GO:0042632;GO:0070328;GO:0060228;GO:0001523;GO:0035634;GO:0044267;GO:0042627;GO:0005615;GO:0006982;GO:0032374;GO:0072562;GO:0016042;GO:0033344;GO:0065005;GO:0010898;GO:0002227;GO:0009986;GO:0042802;GO:0033700;GO:0005319;GO:0034361;GO:0034364;GO:0031102;GO:0062023;GO:0016209;GO:0008289;GO:0055088;GO:0031210;GO:0007159;GO:0017127;GO:0010873;GO:0043691;GO:0008203;GO:0030300;GO:0005507;GO:0015485;GO:0070062;GO:0005829;GO:0046470;GO:0019430;GO:0005576;GO:0006695;GO:0005515;GO:0042803;GO:0005769 | hsa:337 |  | RecName: Full=Apolipoprotein A-IV; Short=Apo-AIV; Short=ApoA-IV; AltName: Full=Apolipoprotein A4; Flags: Precursor |
| E7END6 | 19835.25474 | 18291.93458 | 0.922193076 | -0.116859261 | 0.222103206 | no | no change | GO:0004252;GO:0016787;GO:0007596;GO:0005576;GO:0005509;GO:0006508;GO:0008233;GO:0008236 | hsa:5624 | COG5640 | vitamin K-dependent protein C isoform X5 [Homo sapiens] |
| P41222 | 20407.13071 | 18677.39048 | 0.91523844 | -0.127780449 | 0.227531625 | no | no change | GO:0006633;GO:0006631;GO:0005783;GO:0005789;GO:0005737;GO:0070062;GO:0005634;GO:0016020;GO:0005504;GO:0048471;GO:0005501;GO:0019371;GO:0005794;GO:0005791;GO:0006629;GO:0031965;GO:2000255;GO:0036094;GO:0001516;GO:0045187;GO:0005615;GO:0016853;GO:0004667;GO:0006693;GO:0005576;GO:0005515 | hsa:5730 |  | prostaglandin-H2 D-isomerase precursor [Homo sapiens] |
| P29622 | 76645.57316 | 69966.39042 | 0.912856249 | -0.131540404 | 0.21590079 | no | no change | GO:0005615;GO:0070062;GO:0002576;GO:0031089;GO:0010466;GO:0030414;GO:0004867;GO:0010951;GO:0005576 | hsa:5267 | COG4826 | kallistatin isoform 1 [Homo sapiens] |
| F5H8B0 | 6778.0535 | 6187.351708 | 0.912850822 | -0.131548981 | 0.332855168 | no | no change | GO:0004252;GO:0016787;GO:0007596;GO:0005576;GO:0005509;GO:0006508;GO:0008233;GO:0008236 | hsa:2155 | COG5640 | coagulation factor VII isoform c precursor [Homo sapiens] |
| P10909 | 1002494.932 | 913876.8833 | 0.911602497 | -0.133523218 | 0.084421562 | no | no change | GO:0032436;GO:0005783;GO:0019730;GO:1903573;GO:0009615;GO:0016020;GO:1902949;GO:0042127;GO:0005794;GO:0034366;GO:0060548;GO:0031966;GO:0043691;GO:0043231;GO:0005829;GO:0002376;GO:0051787;GO:1902004;GO:0051788;GO:0030449;GO:1902230;GO:0050821;GO:0010628;GO:0044877;GO:0099020;GO:0048260;GO:0005856;GO:0000902;GO:0002434;GO:0072562;GO:0048471;GO:0097418;GO:0051131;GO:0032760;GO:0006629;GO:1905895;GO:0048156;GO:0001774;GO:0006956;GO:1905892;GO:1901216;GO:0006958;GO:0061077;GO:2000060;GO:1902430;GO:1902847;GO:0090201;GO:0031012;GO:0045429;GO:0051082;GO:1902998;GO:1901214;GO:0005737;GO:0001540;GO:0031093;GO:0031410;GO:0005634;GO:0051087;GO:0005739;GO:1900221;GO:0009986;GO:0017038;GO:0061740;GO:0061741;GO:0051092;GO:0031625;GO:0070062;GO:0005622;GO:0005743;GO:0005515;GO:0097440;GO:0032286;GO:0016887;GO:0071944;GO:0045202;GO:0061518;GO:0005615;GO:0002576;GO:0043065;GO:0032464;GO:0032463;GO:0045087;GO:0006915;GO:0042583;GO:0062023;GO:0050750;GO:0032991;GO:0001836;GO:0005576;GO:1905907;GO:1905908 | hsa:1191 |  | clusterin preproprotein [Homo sapiens] |
| A0A182DWH7 | 33602.84316 | 30627.1725 | 0.911445867 | -0.133771122 | 0.310303936 | no | no change | GO:0008430 | hsa:6414 |  | Selenoprotein P, plasma, 1 [Homo sapiens] |
| A0A075B7B8 | 1150906.189 | 1048197.338 | 0.910758277 | -0.134859894 | 0.621565418 | no | no change | GO:0009897;GO:0050853;GO:0045087;GO:0006910;GO:0006911;GO:0042742;GO:0003823;GO:0034987;GO:0042571;GO:0006958;GO:0050871;GO:0072562 | hsa:102723407 | | hCG1793095, isoform CRA_a, partial [Homo sapiens] |
| A0A0A0MS09 | 66174.83007 | 59805.41636 | 0.903748696 | -0.146006435 | 0.747774809 | no | no change | GO:0016021;GO:0016020 | | | immunoglobulin delta-chain, partial [Homo sapiens] |
| P07357 | 57252.00211 | 51566.1375 | 0.900687061 | -0.150902159 | 0.074159772 | no | no change | GO:0019835;GO:0030449;GO:0070062;GO:0016021;GO:0016020;GO:0072562;GO:0002376;GO:0001848;GO:0005576;GO:0006956;GO:0006957;GO:0005886;GO:0006955;GO:0044877;GO:0006958;GO:0005579;GO:0045087;GO:0005615 | hsa:731 |  | complement component C8 alpha chain preproprotein [Homo sapiens] |
| G3XAP6 | 10099.62869 | 9080.6428 | 0.899106599 | -0.153435922 | 0.310167074 | no | no change | GO:0005201;GO:0060173;GO:0062023;GO:0030509;GO:0035264;GO:0050905;GO:0030500;GO:0030282;GO:0050881;GO:0010259;GO:1900047;GO:0005615;GO:0048844;GO:0036122;GO:0030198;GO:0005509;GO:0002020;GO:0002063;GO:0003417;GO:0043588;GO:0005576;GO:0014829;GO:0005178;GO:0009887;GO:1902732;GO:0009306;GO:0006915;GO:0043066;GO:0048747;GO:0006986;GO:0043395;GO:0035988;GO:0007155;GO:0097084;GO:0016485;GO:0031012;GO:0008201;GO:0070062;GO:0010260;GO:0032991;GO:0001501;GO:0035989;GO:0030199;GO:0043394;GO:0005518;GO:0070527 | hsa:1311 |  | unnamed protein product [Homo sapiens] |
| J3KRP0 | 29145.70421 | 26132.74042 | 0.896624087 | -0.157424839 | 0.350092613 | no | no change | GO:0016787;GO:0032268;GO:0005829;GO:0004180;GO:0016805;GO:0005576;GO:0008152;GO:0006508;GO:0046872;GO:0008237 | hsa:84735 | COG0624 | Carnosine dipeptidase 1 (metallopeptidase M20 family) [Homo sapiens] |
| P01721 | 114034.0153 | 102065.1888 | 0.895041611 | -0.159973339 | 0.372268918 | no | no change | GO:0038096;GO:0004252;GO:0016020;GO:0030449;GO:0050776;GO:0006898;GO:0038095;GO:0002376;GO:0050900;GO:0005576;GO:0006956;GO:0002250;GO:0003823;GO:0005886;GO:0006508;GO:0006958;GO:0006955 | hsa:7441 |  | RecName: Full=Immunoglobulin lambda variable 6-57; AltName: Full=Ig lambda chain V-VI region AR; AltName: Full=Ig lambda chain V-VI region EB4; AltName: Full=Ig lambda chain V-VI region NIG-48; AltName: Full=Ig lambda chain V-VI region SUT; AltName: Full=Ig lambda chain V-VI region WLT; Flags: Precursor |
| A0A0B4J1X8 | 99827.09421 | 89254.54208 | 0.894091357 | -0.161505844 | 0.414649632 | no | no change | GO:0009897;GO:0050853;GO:0045087;GO:0016020;GO:0006910;GO:0006911;GO:0002376;GO:0005576;GO:0002250;GO:0003823;GO:0005886;GO:0072562;GO:0042571;GO:0006958;GO:0050871;GO:0034987;GO:0042742 | hsa:102723407 | | RecName: Full=Immunoglobulin heavy variable 3-43; Flags: Precursor |
| A0A0C4DH39 | 29197.24784 | 26098.06167 | 0.893853483 | -0.161889726 | 0.618776371 | no | no change | GO:0009897;GO:0050853;GO:0045087;GO:0016020;GO:0006910;GO:0006911;GO:0002376;GO:0005576;GO:0002250;GO:0003823;GO:0005886;GO:0072562;GO:0042571;GO:0006958;GO:0050871;GO:0034987;GO:0042742 | hsa:102723407 | | immunoglobulin heavy chain variable region, partial [Homo sapiens] |
| O00187 | 37616.25611 | 33615.62133 | 0.89364612 | -0.162224451 | 0.340889354 | no | no change | GO:0045087;GO:0004252;GO:0046872;GO:0006956;GO:0005615;GO:0048306;GO:0070062;GO:0016787;GO:0002376;GO:0005576;GO:0001867;GO:0005509;GO:0005515;GO:0006508;GO:0006958;GO:0008236;GO:0008233;GO:0001855 | hsa:10747 | COG5640 | mannan-binding lectin serine protease 2 isoform 1 preproprotein [Homo sapiens] |
| A0A0A0MS51 | 291283.5526 | 259748.9917 | 0.891739301 | -0.165306093 | 0.093560538 | no | no change | GO:0051127;GO:0015629;GO:1902174;GO:0030155;GO:0030041;GO:0042989;GO:0045159;GO:1990000;GO:0005925;GO:1903923;GO:0001726;GO:0071801;GO:0005615;GO:0045010;GO:1903903;GO:0043209;GO:0005634;GO:1903909;GO:0030478;GO:0005509;GO:0048471;GO:1903906;GO:0097284;GO:2001269;GO:0060271;GO:0030027;GO:0006915;GO:0051016;GO:0051015;GO:0051014;GO:0045471;GO:0045335;GO:0002102;GO:0005886;GO:0014003;GO:0032991;GO:0006911;GO:0048015;GO:0051693;GO:0046597;GO:0090527;GO:0007568;GO:0051593;GO:0005829;GO:0071346;GO:0042246;GO:0031648;GO:0016528;GO:0014891;GO:0097017;GO:0071276 | hsa:2934 |  | gelsolin isoform d [Homo sapiens] |
| H9KV75 | 6787.488923 | 6051.9458 | 0.891632512 | -0.165478871 | 0.779566865 | no | no change | GO:0019894;GO:0032029;GO:0032391;GO:0030036;GO:0017166;GO:0007041;GO:0042383;GO:0005925;GO:0005923;GO:0030507;GO:0005737;GO:0001725;GO:0097433;GO:0045505;GO:0005815;GO:0003779;GO:0030027;GO:0005509;GO:0042803;GO:0034452;GO:0031941;GO:0051393;GO:0016328;GO:0030486;GO:0048741;GO:1990357;GO:0051017;GO:0031252;GO:0051015;GO:0005915;GO:0045214;GO:0051764;GO:0097381;GO:0007030;GO:0090636;GO:0090637 | hsa:87 | COG5069 | alpha-actinin-1 isoform c [Homo sapiens] |
| P01344 | 49122.98947 | 43611.33875 | 0.887798956 | -0.171695083 | 0.144453081 | no | no change | GO:0046628;GO:0045840;GO:0031017;GO:0051146;GO:0008286;GO:0038028;GO:0008284;GO:0001501;GO:0001503;GO:0051147;GO:0031056;GO:0071902;GO:0051897;GO:0007275;GO:0001649;GO:0044267;GO:0006349;GO:0001701;GO:0008083;GO:0031093;GO:0000122;GO:0002576;GO:0043085;GO:0045725;GO:0010469;GO:0005179;GO:0005178;GO:0009887;GO:2000467;GO:0042104;GO:0005159;GO:0005158;GO:0043410;GO:0048018;GO:0060669;GO:0001892;GO:0043539;GO:0005615;GO:0005975;GO:0050731;GO:0040018;GO:0006355;GO:0051781;GO:0005576;GO:0045944;GO:0005515;GO:0006006;GO:0001934 | hsa:3481 |  | insulin-like growth factor II isoform 2 [Homo sapiens] |
| P48740 | 21827.52947 | 19313.20979 | 0.884809699 | -0.176560894 | 0.194150967 | no | no change | GO:0004252;GO:0006898;GO:0006956;GO:0005615;GO:0016787;GO:0048306;GO:0005829;GO:0005654;GO:0002376;GO:0046872;GO:0005576;GO:0001867;GO:0005509;GO:0005515;GO:0006508;GO:0008233;GO:0042803;GO:0045087;GO:0008236 | hsa:5648 | COG5640 | mannan-binding lectin serine protease 1 isoform 1 precursor [Homo sapiens] |
| A0A075B6Q5 | 61209.78353 | 54111.40905 | 0.884032028 | -0.177829457 | 0.510567519 | no | no change | GO:0009897;GO:0050853;GO:0045087;GO:0016020;GO:0006910;GO:0006911;GO:0002376;GO:0005576;GO:0002250;GO:0003823;GO:0005886;GO:0072562;GO:0042571;GO:0006958;GO:0050871;GO:0034987;GO:0042742 | hsa:102723407 | | hCG2036739, partial [Homo sapiens] |
| P02743 | 440698.6632 | 389385.7333 | 0.883564589 | -0.178592494 | 0.153816928 | no | no change | GO:0006457;GO:0030246;GO:0046597;GO:0044871;GO:0072562;GO:0061045;GO:0044267;GO:0070062;GO:0005615;GO:0005634;GO:0051082;GO:0001849;GO:0005509;GO:0030169;GO:0042802;GO:1903016;GO:0051131;GO:0045087;GO:1903019;GO:0006953;GO:0062023;GO:0044869;GO:0006958;GO:0002674;GO:0046872;GO:0045656;GO:0046790;GO:0005576;GO:0048525 | hsa:325 |  | serum amyloid P-component precursor [Homo sapiens] |
| D6RAR4 | 31434.37947 | 27468.56833 | 0.873838415 | -0.194561565 | 0.131873604 | no | no change | GO:0005737;GO:0004252;GO:0005791;GO:0005615;GO:0016787;GO:0005576;GO:0006508;GO:0008233;GO:0008236 | hsa:3083 | COG5640 | hepatocyte growth factor activator isoform 1 preproprotein [Homo sapiens] |
| P01703 | 153989.3533 | 134359.7152 | 0.872526005 | -0.196729965 | 0.269516372 | no | no change | GO:0038096;GO:0004252;GO:0016020;GO:0030449;GO:0050776;GO:0006898;GO:0038095;GO:0002376;GO:0050900;GO:0005576;GO:0006956;GO:0002250;GO:0003823;GO:0005886;GO:0006508;GO:0006958;GO:0006955 | hsa:7441 |  | Unknown (protein for MGC:31936) [Homo sapiens] |
| P18428 | 17813.44737 | 15522.83508 | 0.871411062 | -0.198574668 | 0.198935725 | no | no change | GO:0006968;GO:0071222;GO:0071223;GO:0006869;GO:0042742;GO:0050829;GO:0032496;GO:0032490;GO:0044130;GO:0019221;GO:0060265;GO:0002281;GO:0070062;GO:0090023;GO:0042535;GO:0016020;GO:0071723;GO:0045919;GO:0002224;GO:0033036;GO:0032722;GO:0009986;GO:0032720;GO:0032760;GO:0015920;GO:0045087;GO:0008289;GO:0006953;GO:0008228;GO:0070891;GO:0034142;GO:0001530;GO:0005615;GO:0034145;GO:0031663;GO:0050830;GO:0002376;GO:0002232;GO:0005576;GO:0005515;GO:0043032;GO:0005102;GO:0032757;GO:0032755 | hsa:3929 |  | lipopolysaccharide-binding protein precursor [Homo sapiens] |
| P04211 | 1192480.3 | 1038025.381 | 0.870475916 | -0.200123713 | 0.347797457 | no | no change | GO:0038096;GO:0004252;GO:0016020;GO:0030449;GO:0050776;GO:0006898;GO:0038095;GO:0002376;GO:0050900;GO:0005576;GO:0006956;GO:0002250;GO:0003823;GO:0005886;GO:0006508;GO:0006958;GO:0006955 | hsa:7441 |  | hCG1731877, partial [Homo sapiens] |
| A0A075B6K2 | 58179.6602 | 50602.50513 | 0.869762817 | -0.201306061 | 0.728752022 | no | no change | GO:0016020;GO:0005615;GO:0006955;GO:0002376;GO:0002377;GO:0005576;GO:0002250;GO:0003823;GO:0005886 | hsa:7441 |  | hCG2041210, partial [Homo sapiens] |
| A0A0C4DH32 | 74133.90813 | 64458.94955 | 0.869493477 | -0.20175289 | 0.414628135 | no | no change | GO:0009897;GO:0050853;GO:0045087;GO:0016020;GO:0006910;GO:0006911;GO:0002376;GO:0005576;GO:0002250;GO:0003823;GO:0005886;GO:0072562;GO:0042571;GO:0006958;GO:0050871;GO:0034987;GO:0042742 | hsa:102723407 | | RecName: Full=Immunoglobulin heavy variable 3-20; Flags: Precursor |
| P01034 | 13011.39337 | 11225.5915 | 0.862750912 | -0.212984001 | 0.098285162 | no | no change | GO:0005783;GO:0060009;GO:0048678;GO:0005788;GO:0008584;GO:0042747;GO:0030414;GO:0070301;GO:0001666;GO:0014070;GO:1904724;GO:0005737;GO:0044267;GO:0032355;GO:0042995;GO:0001540;GO:0005615;GO:0007431;GO:2000117;GO:0005771;GO:0009636;GO:0070062;GO:0043067;GO:0010466;GO:0002020;GO:0030424;GO:0048471;GO:0043025;GO:0042802;GO:0008284;GO:0034103;GO:0045740;GO:0060548;GO:0043687;GO:0006915;GO:0031965;GO:0031982;GO:0006952;GO:0001775;GO:0001654;GO:0060311;GO:0004866;GO:0060313;GO:0004869;GO:0034599;GO:0010035;GO:0006979;GO:0009743;GO:1904813;GO:0005604;GO:0007420;GO:0010716;GO:0007566;GO:0042493;GO:0031667;GO:0010711;GO:0005764;GO:0005576;GO:0005515;GO:0043312;GO:0043292;GO:0045861;GO:0097435 | hsa:1471 |  | cystatin-C precursor [Homo sapiens] |
| P04275 | 83127.79421 | 71404.84208 | 0.858976745 | -0.219309021 | 0.43340406 | no | no change | GO:0005201;GO:0007599;GO:0005783;GO:0007597;GO:0007596;GO:0031012;GO:0031091;GO:0070062;GO:0031093;GO:0002576;GO:0051087;GO:0051260;GO:0009611;GO:0005576;GO:0002020;GO:0042802;GO:0042803;GO:0005178;GO:0031589;GO:0033093;GO:0062023;GO:0007155;GO:0030168;GO:0047485;GO:0019865;GO:0030198;GO:0005515;GO:0005518 | hsa:7450 |  | RecName: Full=von Willebrand factor; Short=vWF; Contains: RecName: Full=von Willebrand antigen 2; AltName: Full=von Willebrand antigen II; Flags: Precursor |
| A0A3B3ISR2 | 308661.1105 | 264784.65 | 0.857849081 | -0.221204234 | 0.056636253 | no | no change | GO:0004252;GO:0005615;GO:0045087;GO:0031638;GO:0005509;GO:0006958 | hsa:715 | COG5640 | complement C1r subcomponent isoform 1 preproprotein [Homo sapiens] |
| B0YIW2 | 1944324.595 | 1664405.242 | 0.856032602 | -0.224262352 | 0.541857111 | no | no change | GO:0034375;GO:0042627;GO:0034371;GO:0006869;GO:0034379;GO:0034378;GO:0051005;GO:0042157;GO:0005576;GO:0048261;GO:0001523;GO:0055102;GO:0070062;GO:0005543;GO:0045833;GO:0010897;GO:0007186;GO:0062023;GO:0033700;GO:0034361;GO:0034363;GO:0070328;GO:0034366;GO:0006641;GO:0060621;GO:0030234;GO:0070653;GO:0008289;GO:0010916;GO:0042632;GO:0032489;GO:0043691;GO:0005615;GO:0015485;GO:0034382;GO:0050995;GO:0019433;GO:0033344;GO:0010989;GO:0010987;GO:0005769;GO:0045717;GO:0010903 | hsa:345 |  | apolipoprotein C-III precursor variant 1 [Homo sapiens] |
| A0A0B4J1U3 | 40123.38389 | 34324.28318 | 0.855468304 | -0.225213692 | 0.269029297 | no | no change | GO:0016020;GO:0005615;GO:0006955;GO:0002376;GO:0002377;GO:0005576;GO:0002250;GO:0003823;GO:0005886 | hsa:7441 |  | RecName: Full=Immunoglobulin lambda variable 1-36; Flags: Precursor |
| A0A0B4J1X5 | 77520.55882 | 66294.06174 | 0.85518039 | -0.225699323 | 0.400620052 | no | no change | GO:0009897;GO:0050853;GO:0045087;GO:0016020;GO:0006910;GO:0006911;GO:0002376;GO:0005576;GO:0002250;GO:0003823;GO:0005886;GO:0072562;GO:0042571;GO:0006958;GO:0050871;GO:0034987;GO:0042742 | hsa:102723407 | | immunoglobulin heavy chain VH3, partial [Homo sapiens] |
| J3KNB4 | 16019.31779 | 13648.52625 | 0.852004213 | -0.23106753 | 0.212496654 | no | no change | GO:0071224;GO:0071222;GO:0042742;GO:0050829;GO:0044130;GO:0061844;GO:0005737;GO:0042995;GO:0005615;GO:0071354;GO:0071356;GO:0016021;GO:0045766;GO:0051873;GO:0008284;GO:0045087;GO:0006952;GO:0050830;GO:0044140;GO:0001530;GO:0042581;GO:0071347;GO:0005576;GO:0001934 | hsa:820 |  | cathelicidin antimicrobial peptide [Homo sapiens] |
| P01743 | 17658.08526 | 15038.59167 | 0.85165472 | -0.231659446 | 0.159503435 | no | no change | GO:0038096;GO:0004252;GO:0016020;GO:0030449;GO:0050776;GO:0006898;GO:0038095;GO:0002376;GO:0050900;GO:0005576;GO:0006956;GO:0002250;GO:0003823;GO:0005886;GO:0006508;GO:0006958;GO:0006955 | hsa:102723407 | | IgM heavy chain VH1 region precursor, partial [Homo sapiens] |
| A0A075B6R9 | 176609.7653 | 150302.1738 | 0.851041127 | -0.232699242 | 0.287217602 | no | no change | GO:0002377;GO:0005615;GO:0006955 | hsa:7441 |  | RecName: Full=Immunoglobulin kappa variable 2-24; Flags: Precursor |
| A0A0C4DH38 | 152379.5311 | 129219.8125 | 0.848012929 | -0.237841834 | 0.248567916 | no | no change | GO:0009897;GO:0050853;GO:0045087;GO:0016020;GO:0006910;GO:0006911;GO:0002376;GO:0005576;GO:0002250;GO:0003823;GO:0005886;GO:0072562;GO:0042571;GO:0006958;GO:0050871;GO:0034987;GO:0042742 | hsa:102723407 | | RecName: Full=Immunoglobulin heavy variable 5-51; Flags: Precursor |
| A0A0C4DH24 | 40461.87842 | 34211.68261 | 0.845528778 | -0.242074237 | 0.371623647 | no | no change | GO:0016020;GO:0005615;GO:0006955;GO:0002376;GO:0002377;GO:0005576;GO:0002250;GO:0003823;GO:0005886 | hsa:7441 |  | RecName: Full=Immunoglobulin kappa variable 6-21; Flags: Precursor |
| A0A2Q2TTZ9 | 318978.3258 | 269566.5879 | 0.845093745 | -0.242816709 | 0.352364178 | no | no change |  | hsa:7441 |  | IGKV1D-33 isoform 2, partial [Pan troglodytes] |
| Q9UGM5 | 24205.02789 | 20334.905 | 0.840110786 | -0.251348506 | 0.145723156 | no | no change | GO:0005615;GO:0007339;GO:0008150;GO:0005576;GO:0003674;GO:0007338;GO:0030414;GO:0008191;GO:0010951;GO:0004869;GO:0004857;GO:0010466;GO:0070062 | hsa:26998 |  | fetuin-B isoform 1 precursor [Homo sapiens] |
| Q15848 | 13433.81232 | 11252.50825 | 0.837625834 | -0.255622156 | 0.247118181 | no | no change | GO:0034115;GO:0005783;GO:0010804;GO:0050728;GO:0009967;GO:0009617;GO:0030853;GO:0043124;GO:0033034;GO:0032720;GO:0043123;GO:2000481;GO:0014823;GO:0072659;GO:0010875;GO:0045650;GO:0071320;GO:0031667;GO:0050731;GO:1904753;GO:0045715;GO:0005125;GO:0033691;GO:0031953;GO:0010906;GO:0046326;GO:0070994;GO:0045721;GO:0010739;GO:0007623;GO:2000467;GO:2000279;GO:0070208;GO:0071872;GO:0019395;GO:0042304;GO:0006006;GO:0034383;GO:0006635;GO:2000478;GO:0050805;GO:0043407;GO:0001666;GO:0010642;GO:0050765;GO:0005581;GO:0032270;GO:0042802;GO:0042803;GO:0009986;GO:0050873;GO:0045892;GO:0046888;GO:0045599;GO:0007584;GO:0120162;GO:0120163;GO:0070543;GO:0042493;GO:1900121;GO:0005515;GO:0005102;GO:0045860;GO:0070373;GO:0045923;GO:0051384;GO:0042593;GO:0035690;GO:0032869;GO:0006091;GO:0005615;GO:0051260;GO:2000590;GO:0010745;GO:0010469;GO:1904706;GO:0005179;GO:0030336;GO:2000534;GO:0034612;GO:0090317;GO:0045471;GO:0009744;GO:0032991;GO:0045776;GO:0009749;GO:0071639;GO:0005576;GO:2000584;GO:0032757;GO:0001934 | hsa:9370 |  | TPA: adiponectin D [Homo sapiens] |
| J3KPA1 | 13651.48432 | 11406.30075 | 0.835535572 | -0.259226845 | 0.064313477 | no | no change | GO:0016020;GO:0016021;GO:0005576 | hsa:10321 | COG2340 | cysteine-rich secretory protein 3 isoform 3 [Homo sapiens] |
| P00739 | 46650.73105 | 38869.02583 | 0.833192213 | -0.263278739 | 0.2617676 | no | no change | GO:0004252;GO:0070062;GO:0034366;GO:0030492;GO:0006898;GO:0072562;GO:0005576;GO:0010942;GO:0002526;GO:0010033;GO:0005615 | hsa:3250 | COG5640 | haptoglobin-related protein precursor [Homo sapiens] |
| A0A0G2JPR0 | 503231.4947 | 414208.8667 | 0.823098059 | -0.280863779 | 0.128756202 | no | down | GO:0004866;GO:0005576;GO:0006956;GO:0006954;GO:0010951;GO:0005615 | hsa:100293534;hsa:110384692;hsa:720;hsa:721 | | complement C4A (Rodgers blood group)-like preproprotein [Homo sapiens] |
| A0A087WSZ0 | 53286.55 | 43621.46442 | 0.818620542 | -0.288733226 | 0.467062196 | no | down | GO:0016020;GO:0005615;GO:0006955;GO:0002376;GO:0002377;GO:0005576;GO:0002250;GO:0003823;GO:0005886 | | | RecName: Full=Immunoglobulin kappa variable 1D-8; Flags: Precursor |
| P68871 | 5812417.526 | 4757867.333 | 0.818569435 | -0.288823296 | 0.232227685 | no | down | GO:0005344;GO:0007596;GO:0015701;GO:1904813;GO:0045429;GO:0042744;GO:0008217;GO:0010942;GO:0050880;GO:0005615;GO:0071682;GO:0072562;GO:0005833;GO:0098869;GO:0046872;GO:0015671;GO:0070293;GO:0030185;GO:1904724;GO:0020037;GO:0031721;GO:0031720;GO:0051291;GO:0070062;GO:0019825;GO:0042542;GO:0030492;GO:0005829;GO:0006898;GO:0043312;GO:0070527;GO:0043177;GO:0005576;GO:0005515;GO:0031838;GO:0004601 | hsa:3043 | COG1018 | PREDICTED: hemoglobin subunit beta [Gorilla gorilla gorilla] |
| A0A075B6K5 | 21206.54655 | 17224.64667 | 0.812232516 | -0.30003531 | 0.638678894 | no | down | GO:0016020;GO:0005615;GO:0006955;GO:0002376;GO:0002377;GO:0005576;GO:0002250;GO:0003823;GO:0005886 | hsa:7441 |  | RecName: Full=Immunoglobulin lambda variable 3-9; Flags: Precursor |
| A0A087X1L8 | 2540.625867 | 2061.326993 | 0.811346141 | -0.301610558 | 0.186765801 | no | down | GO:0016021;GO:0016020 | hsa:102723996;hsa:23308 | | ICOS ligand isoform c precursor [Homo sapiens] |
| P61626 | 14455.72159 | 11668.72709 | 0.807204747 | -0.308993436 | 0.142579336 | no | down | GO:0006954;GO:0019730;GO:0016798;GO:0003796;GO:0035578;GO:0042742;GO:0050829;GO:0019835;GO:0044267;GO:0070062;GO:0016998;GO:0035580;GO:0042802;GO:0016787;GO:1904724;GO:0003824;GO:0050830;GO:0008152;GO:0001895;GO:0005615;GO:0031640;GO:0043312;GO:0005576 | hsa:4069 |  | lysozyme C precursor [Homo sapiens] |
| A0A5H1ZRQ7 | 20376.10105 | 16360.20347 | 0.802911383 | -0.316687329 | 0.127439214 | no | down |  | hsa:100423062 | | RecName: Full=Immunoglobulin lambda constant 7; AltName: Full=Ig lambda-7 chain C region |
| P00748 | 68588.26368 | 55029.87542 | 0.802322037 | -0.317746672 | 0.060595233 | no | down | GO:0004252;GO:0002542;GO:0007599;GO:0007597;GO:0007596;GO:0008233;GO:0008236;GO:0070062;GO:0031638;GO:0005509;GO:0006508;GO:0051919;GO:0016787;GO:0005791;GO:0045087;GO:0062023;GO:0005886;GO:0016485;GO:0005615;GO:0042730;GO:0016540;GO:0002353;GO:0005576;GO:0051787;GO:0010756;GO:0005515;GO:0030193;GO:0051788;GO:0030194 | hsa:2161 | COG5640 | coagulation factor XII preproprotein [Homo sapiens] |
| P02100 | 42136.11668 | 33791.64692 | 0.801963958 | -0.318390694 | 0.222510229 | no | down | GO:0005344;GO:0019825;GO:0020037;GO:0043177;GO:0031721;GO:0007596;GO:0051291;GO:0072562;GO:0005833;GO:0098869;GO:0042744;GO:0015671;GO:0005515;GO:0031838;GO:0046872;GO:0005829;GO:0014070 | hsa:3046 | COG1018 | hemoglobin subunit epsilon [Homo sapiens] |
| H0Y5E4 | 25163.22316 | 20060.71042 | 0.797223404 | -0.326944031 | 0.45666253 | no | down | GO:0005540;GO:0007155;GO:0016021;GO:0016020 | hsa:960 |  | CD44 antigen isoform 8 precursor [Homo sapiens] |
| O75460 | 151139.3916 | 120191.0938 | 0.79523341 | -0.330549725 | 0.11711669 | no | down | GO:0005739;GO:0004540;GO:0005783;GO:0016787;GO:0016310;GO:0005161;GO:0019899;GO:0005789;GO:0008152;GO:1990604;GO:0030176;GO:1990597;GO:0034620;GO:1990630;GO:0046777;GO:0036289;GO:0007257;GO:0006986;GO:0098787;GO:0016241;GO:0030544;GO:0005637;GO:0016021;GO:0000287;GO:0004521;GO:0051082;GO:0000166;GO:0004674;GO:0016740;GO:0071333;GO:1901142;GO:0006402;GO:0046872;GO:0051879;GO:0042802;GO:0042803;GO:0005737;GO:0006468;GO:0033120;GO:0006397;GO:1990332;GO:0004672;GO:0007050;GO:0003824;GO:0043531;GO:0016020;GO:0090502;GO:0036498;GO:0006351;GO:0030968;GO:0070059;GO:1904707;GO:0070054;GO:0016301;GO:0006355;GO:1990579;GO:0005524;GO:0005515;GO:0004519;GO:1900103;GO:0006379;GO:0001935;GO:0035924;GO:0006915;GO:0034976 | hsa:2081 | COG0515 | endoplasmic reticulum to nucleus signalling 1 isoform 1 variant, partial [Homo sapiens] |
| A0A0C4DH21 | 50413.95316 | 40072.155 | 0.794862384 | -0.331222989 | 0.100285954 | no | down |  | hsa:10877 |  | complement factor H-related protein 4 [Homo sapiens] |
| A0A286YEY4 | 733018.9684 | 577230.3292 | 0.787469839 | -0.344703427 | 0.108455951 | no | down | GO:0016021;GO:0016020 | hsa:100423062 | | unnamed protein product [Homo sapiens] |
| P02649 | 2408326.895 | 1879563.25 | 0.780443574 | -0.357633765 | 0.052096714 | no | down | GO:0051044;GO:0005783;GO:0034447;GO:0007616;GO:0005788;GO:0072358;GO:0043524;GO:0010873;GO:0051651;GO:0050728;GO:1905855;GO:0071682;GO:0016020;GO:0008201;GO:0098869;GO:0060999;GO:0045541;GO:0044794;GO:0005319;GO:0034361;GO:0034362;GO:0034363;GO:0034364;GO:0031102;GO:0016209;GO:0071813;GO:0043537;GO:0010977;GO:0030425;GO:0030516;GO:0043691;GO:0010877;GO:0010875;GO:0061771;GO:0015485;GO:0043025;GO:0019433;GO:1990777;GO:0030195;GO:0034375;GO:0034374;GO:0034372;GO:0034371;GO:1903002;GO:0000302;GO:0019934;GO:0010629;GO:0044877;GO:0042157;GO:0042311;GO:0042158;GO:0042159;GO:0048168;GO:0042982;GO:0044267;GO:0042627;GO:0072562;GO:0035641;GO:0010544;GO:0097006;GO:0007186;GO:0062023;GO:0033700;GO:0015909;GO:0006629;GO:1905890;GO:0048156;GO:1902430;GO:0042632;GO:0032489;GO:1901215;GO:0046907;GO:0034382;GO:0034380;GO:0006898;GO:0034384;GO:0006357;GO:0046983;GO:0032805;GO:0005543;GO:0090209;GO:0006869;GO:0051246;GO:0019068;GO:0050807;GO:0031012;GO:0043407;GO:0051000;GO:1902995;GO:0043083;GO:0005737;GO:0001540;GO:0005634;GO:0030669;GO:1900221;GO:0002021;GO:0090181;GO:0042802;GO:0042803;GO:0006641;GO:0006874;GO:0043687;GO:0017038;GO:0008289;GO:0055088;GO:0055089;GO:0046889;GO:0034378;GO:0006979;GO:0008202;GO:0008203;GO:0032269;GO:0070062;GO:1900272;GO:1902952;GO:0005515;GO:1901630;GO:0005794;GO:0017127;GO:0007271;GO:0060228;GO:0001523;GO:0034365;GO:0005615;GO:0048844;GO:0005198;GO:0033344;GO:1901628;GO:0010468;GO:0032462;GO:0090090;GO:0006707;GO:0070326;GO:0046911;GO:0005886;GO:2000822;GO:1903561;GO:0007263;GO:1905908;GO:0050750;GO:0007010;GO:1905860;GO:0097114;GO:0005576;GO:1905906;GO:0097113;GO:0045807;GO:0098978;GO:0070328;GO:0005769;GO:0001937;GO:0010976 | hsa:348 |  | apolipoprotein E [Homo sapiens] |
| P02652 | 569083.3632 | 442120.4958 | 0.776899352 | -0.364200387 | 0.597189974 | no | down | GO:0060192;GO:0034384;GO:0034375;GO:0005543;GO:0034374;GO:0034371;GO:0006869;GO:0031072;GO:0031647;GO:0017127;GO:0030301;GO:0006656;GO:0010903;GO:0010873;GO:0002740;GO:0060228;GO:0001523;GO:0043085;GO:0034190;GO:0009395;GO:0044267;GO:0042627;GO:0005615;GO:0032375;GO:0031100;GO:0055102;GO:0072562;GO:0033344;GO:0034361;GO:0070062;GO:0033700;GO:0005319;GO:0018158;GO:0008035;GO:0034364;GO:0016032;GO:0034366;GO:0006641;GO:0060621;GO:0043687;GO:0043691;GO:0008289;GO:0031210;GO:0005788;GO:0042632;GO:0050996;GO:0043627;GO:0002526;GO:0051384;GO:0034378;GO:0008203;GO:0030300;GO:0046982;GO:0045416;GO:0015485;GO:0019216;GO:0050995;GO:0046340;GO:0005829;GO:0018206;GO:0042493;GO:0034380;GO:0009749;GO:0005576;GO:0005515;GO:0070653;GO:0060695;GO:0042803;GO:0034370;GO:0005769;GO:0042157 | hsa:336 |  | apolipoprotein A-II preproprotein [Homo sapiens] |
| Q9Y6R7 | 47058.71 | 36556.8925 | 0.77683584 | -0.364318333 | 0.080800808 | no | down | GO:0005576;GO:0070062;GO:0005515 | hsa:8857 |  | IgGFc-binding protein precursor [Homo sapiens] |
| P01861 | 720891.6689 | 557342.3079 | 0.773129073 | -0.371218804 | 0.224644384 | no | down | GO:0004252;GO:0030449;GO:0019221;GO:0009897;GO:0042742;GO:0034987;GO:0042571;GO:0005615;GO:0016020;GO:0072562;GO:0006508;GO:0050853;GO:0045087;GO:0006910;GO:0006911;GO:0006956;GO:0003823;GO:0005886;GO:0006958;GO:0050871;GO:0070062;GO:0038096;GO:0002376;GO:0005576;GO:0002250 | hsa:100423062 | | RecName: Full=Immunoglobulin heavy constant gamma 4; AltName: Full=Ig gamma-4 chain C region |
| Q9HDC9 | 9404.359133 | 7222.64681 | 0.768010527 | -0.38080201 | 0.436123707 | no | down | GO:0016844;GO:0005783;GO:0009986;GO:0016020;GO:0009058;GO:0008150;GO:0004064;GO:0016021 | hsa:57136 | COG3386 | adipocyte plasma membrane-associated protein [Homo sapiens] |
| P01763 | 44011.60167 | 33730.68792 | 0.766404462 | -0.383822134 | 0.169391045 | no | down | GO:0038096;GO:0004252;GO:0016020;GO:0030449;GO:0050776;GO:0006898;GO:0038095;GO:0002376;GO:0050900;GO:0005576;GO:0006956;GO:0002250;GO:0003823;GO:0005886;GO:0006508;GO:0006958;GO:0006955 | hsa:102723407 | | immunoglobulin heavy chain variable region, partial [Homo sapiens] |
| Q13093 | 7030.992333 | 5369.202353 | 0.763647875 | -0.389020544 | 0.149901938 | no | down | GO:0034441;GO:0034440;GO:0034362;GO:0034374;GO:0047499;GO:0016787;GO:0006629;GO:0005615;GO:0005543;GO:0005737;GO:0016788;GO:0016042;GO:0050729;GO:0090026;GO:0003847;GO:0046469;GO:0005576 | hsa:7941 |  | platelet-activating factor acetylhydrolase precursor [Homo sapiens] |
| A0A5H1ZRS2 | 1037270.826 | 792074.7708 | 0.763614237 | -0.389084094 | 0.273957591 | no | down |  |  |  | immunoglobulin kappa chain variable region, partial [Homo sapiens] |
| Q08380 | 86284.11158 | 65083.83583 | 0.754296876 | -0.406795644 | 0.172159055 | no | down | GO:0006968;GO:0070062;GO:0006898;GO:0005044;GO:0002576;GO:0016020;GO:0072562;GO:0031089;GO:0062023;GO:0005615;GO:0007165;GO:0005515;GO:0007155;GO:0005576 | hsa:3959 |  | galectin-3-binding protein precursor [Homo sapiens] |
| A0A0C4DH67 | 32356.6928 | 24215.4464 | 0.748390651 | -0.418136558 | 0.096953919 | no | down | GO:0016020;GO:0002376;GO:0005576;GO:0002250;GO:0003823;GO:0005886 | | | Ig kappa V-region e, partial [Homo sapiens] |
| K7ER74 | 581391.7695 | 428761.3892 | 0.73747413 | -0.439335654 | 0.290698252 | no | down | GO:0034375;GO:0034372;GO:0034371;GO:0034370;GO:0042627;GO:0034378;GO:0051006;GO:0010902;GO:0008047;GO:0048261;GO:0001523;GO:0043085;GO:0055102;GO:0005615;GO:0032375;GO:0005576;GO:0016042;GO:0045833;GO:0016004;GO:0010898;GO:0033700;GO:0034361;GO:0034362;GO:0034363;GO:0070328;GO:0006629;GO:0034366;GO:0060230;GO:0008289;GO:0006869;GO:0042803;GO:0010916;GO:0043274;GO:0042632;GO:0043691;GO:0034382;GO:0042493;GO:0034384;GO:0010518;GO:0033344;GO:0042953;GO:0060697;GO:0045723;GO:0005769 | hsa:344 |  | apolipoprotein C-II isoform X1 [Mesocricetus auratus] |
| P02538 | 17261.70475 | 12718.27088 | 0.736791126 | -0.44067241 | 0.356729277 | no | down | GO:0008284;GO:0002009;GO:0005200;GO:0007010;GO:0005829;GO:0031424;GO:0070062;GO:2000536;GO:0030154;GO:0045095;GO:0070268;GO:0042060;GO:0005198;GO:0050830;GO:0005882;GO:0005515;GO:0001899;GO:0016020;GO:0005634;GO:0051801;GO:0061844 | hsa:3853 |  | keratin, type II cytoskeletal 6A [Homo sapiens] |
| A0A087WSY4 | 35933.089 | 26257.91982 | 0.730744853 | -0.452560333 | 0.213437997 | no | down | GO:0009897;GO:0050853;GO:0045087;GO:0016020;GO:0006910;GO:0006911;GO:0002376;GO:0005576;GO:0002250;GO:0003823;GO:0005886;GO:0072562;GO:0042571;GO:0006958;GO:0050871;GO:0034987;GO:0042742 | hsa:102723407 | | RecName: Full=Immunoglobulin heavy variable 4-30-2; Flags: Precursor |
| P05109 | 9250.794133 | 6087.032125 | 0.658001036 | -0.603838239 | 0.08458577 | no | down | GO:0045087;GO:0019730;GO:0030307;GO:0032602;GO:0043312;GO:0050786;GO:0008270;GO:0014002;GO:0010043;GO:0032496;GO:0005737;GO:0001816;GO:0045111;GO:0050729;GO:0005615;GO:0005634;GO:0016020;GO:0051493;GO:0050727;GO:0005509;GO:0002224;GO:2001244;GO:0034774;GO:0046872;GO:0035662;GO:0008017;GO:0005856;GO:0030593;GO:0006935;GO:0006919;GO:0032119;GO:0006914;GO:0006915;GO:0018119;GO:0042060;GO:0050832;GO:0045471;GO:0070488;GO:0006954;GO:0005886;GO:0002526;GO:0002523;GO:0070062;GO:0051092;GO:0050544;GO:0005829;GO:0002793;GO:0002544;GO:0002376;GO:0005576;GO:0005515;GO:0042742 | hsa:6279 |  | protein S100-A8 isoform d [Homo sapiens] |
| A0A0B4J1V1 | 123262.3727 | 79740.12053 | 0.646913724 | -0.628354776 | 0.328814739 | no | down | GO:0009897;GO:0050853;GO:0045087;GO:0016020;GO:0006910;GO:0006911;GO:0002376;GO:0005576;GO:0002250;GO:0003823;GO:0005886;GO:0072562;GO:0042571;GO:0006958;GO:0050871;GO:0034987;GO:0042742 | hsa:102723407 | | RecName: Full=Immunoglobulin heavy variable 3-21; Flags: Precursor |
| P01833 | 20205.39627 | 12415.04043 | 0.614441819 | -0.702651686 | 0.108081813 | no | down | GO:0043235;GO:0001895;GO:0005615;GO:0002415;GO:0001580;GO:0070062;GO:0016021;GO:0016020;GO:0043312;GO:0005576;GO:0007173;GO:0005887;GO:0005886;GO:0043113;GO:0001792;GO:0035577;GO:0038093 | hsa:5284 |  | polymeric immunoglobulin receptor precursor [Homo sapiens] |
| P0DP02 | 532154.5174 | 315646.3906 | 0.593148006 | -0.753535956 | 0.148185078 | no | down | GO:0016020;GO:0002376;GO:0005576;GO:0002250;GO:0003823;GO:0005886 | hsa:102723407 | | immunoglobulin heavy chain [Homo sapiens] |
| C9JXI5 | 131802.1526 | 76099.971 | 0.577380335 | -0.792406124 | 0.084751517 | no | down | GO:0090263;GO:0031410;GO:0016021;GO:0016020;GO:0005886 | hsa:130612 |  | transmembrane protein 198 [Macaca mulatta] |
| P04040 | 32778.43622 | 4669.2838 | 0.142449865 | -2.811473845 | 0.288195611 | no | down | GO:0016491;GO:0005829;GO:0016020;GO:0005782;GO:0020027;GO:0033189;GO:0051289;GO:0009314;GO:0005886;GO:0019899;GO:0000302;GO:1904813;GO:0009060;GO:0005739;GO:0004601;GO:0009650;GO:0032868;GO:0001666;GO:0055093;GO:0001649;GO:0005783;GO:0050661;GO:0005778;GO:0009636;GO:0070062;GO:0005615;GO:0033591;GO:0005777;GO:0001822;GO:0051262;GO:0006979;GO:0098869;GO:0043066;GO:0046686;GO:0005758;GO:0034774;GO:0046872;GO:0014823;GO:0042802;GO:0042803;GO:0004046;GO:0005794;GO:0005925;GO:0010288;GO:0020037;GO:0006641;GO:0009411;GO:0006625;GO:0016209;GO:0032355;GO:0045471;GO:0014068;GO:0001657;GO:0071363;GO:0034599;GO:0009642;GO:0055114;GO:0008203;GO:0051092;GO:0016684;GO:0007568;GO:0043231;GO:0042542;GO:0070542;GO:0042493;GO:0043312;GO:0032088;GO:0005764;GO:0051781;GO:0042744;GO:0033197;GO:0010193;GO:0004096;GO:0005576;GO:0005102;GO:0014854;GO:0080184 | hsa:847 | COG0753 | catalase [Homo sapiens] |

| Table S2-3 Proteins identified in S vs C group. | | | | | | | | | | | |
| --- | --- | --- | --- | --- | --- | --- | --- | --- | --- | --- | --- |
| Accession | C | S | FC(S/C) | log2FC(S/C) | Pvalue(S/C) | significant | regulate | GO | KEGG | COG | Description |
| A0A075B6R2 | 129021.1653 | 185933.5563 | 1.441108952 | 0.527179411 | 0.027809374 | yes | up | GO:0009897;GO:0050853;GO:0045087;GO:0016020;GO:0006910;GO:0006911;GO:0002376;GO:0005576;GO:0002250;GO:0003823;GO:0005886;GO:0072562;GO:0042571;GO:0006958;GO:0050871;GO:0034987;GO:0042742 | hsa:102724971 | | immunoglobulin heavy chain VDJ region, partial [Homo sapiens] |
| A0A096LPE2 | 482254.3211 | 600397.2708 | 1.244980593 | 0.316123254 | 0.023239338 | yes | up | GO:0042056;GO:0034364;GO:0005615;GO:0006953;GO:0005576;GO:0060326;GO:0050918;GO:0070062 | hsa:6291;hsa:100528017 | | SAA2-SAA4 protein precursor [Homo sapiens] |
| A0A0C4DH33 | 6490.322412 | 10801.41761 | 1.664234367 | 0.734858616 | 0.034820244 | yes | up | GO:0009897;GO:0050853;GO:0045087;GO:0016020;GO:0006910;GO:0006911;GO:0002376;GO:0005576;GO:0002250;GO:0003823;GO:0005886;GO:0072562;GO:0042571;GO:0006958;GO:0050871;GO:0034987;GO:0042742 | hsa:102723407 | | immunoglobulin heavy chain variable region, partial [Homo sapiens] |
| A0A0J9YX35 | 22457.00516 | 62925.73136 | 2.802053565 | 1.486484535 | 1.20E-06 | yes | up | GO:0016020;GO:0002376;GO:0005576;GO:0002250;GO:0003823;GO:0005886 | hsa:102723407 | | RecName: Full=Immunoglobulin heavy variable 3-64D; Flags: Precursor |
| A0A5H1ZRS9 | 66724.68526 | 95647.84275 | 1.433470122 | 0.519511835 | 0.036385688 | yes | up |  | hsa:7441 |  | immunoglobulin kappa chain variable region, partial [Homo sapiens] |
| C9JPQ9 | 1733016.668 | 2232012.625 | 1.287934886 | 0.365059657 | 0.023813269 | yes | up | GO:0051258;GO:0005102;GO:0007596;GO:0005577;GO:0030168 | hsa:2266 |  | hypothetical protein, partial [Homo sapiens] |
| D6RD17 | 1986535.632 | 2865348 | 1.442384397 | 0.528455696 | 0.039601783 | yes | up |  | hsa:3512 |  | JCHAIN isoform 4, partial [Pongo abelii] |
| H0YJW9 | 684252.6316 | 939131.4021 | 1.372492204 | 0.456797954 | 0.024601914 | yes | up |  | hsa:7448 |  | vitronectin, partial [Homo sapiens] |
| O14791 | 35749.16842 | 43243.82 | 1.20964548 | 0.274584288 | 0.049602704 | yes | up | GO:0006869;GO:0005788;GO:0042157;GO:0031224;GO:0019835;GO:0044267;GO:0005615;GO:0072562;GO:0005254;GO:0045087;GO:0034361;GO:0034364;GO:0006629;GO:1902476;GO:0043687;GO:0008289;GO:0008202;GO:0008203;GO:0006898;GO:0031640;GO:0005576;GO:0005515 | hsa:8542 |  | apolipoprotein L1 isoform a precursor [Homo sapiens] |
| O75636 | 31221.79526 | 62773.78417 | 2.010575742 | 1.007608686 | 3.22E-05 | yes | up | GO:0004252;GO:0051607;GO:0006956;GO:0003823;GO:1902679;GO:0045087;GO:0030246;GO:0043654;GO:0002376;GO:0046597;GO:0005576;GO:0001867;GO:0072562;GO:0005515;GO:0006508;GO:0046872;GO:0005581 | hsa:8547 |  | ficolin-3 isoform 1 precursor [Homo sapiens] |
| P01814 | 22045.15142 | 29691.86739 | 1.346866112 | 0.429606444 | 0.017596336 | yes | up | GO:0038096;GO:0004252;GO:0016020;GO:0030449;GO:0050776;GO:0006898;GO:0038095;GO:0002376;GO:0050900;GO:0005576;GO:0006956;GO:0002250;GO:0003823;GO:0005886;GO:0006508;GO:0006958;GO:0006955 | hsa:102723407 | | RecName: Full=Immunoglobulin heavy variable 2-70; AltName: Full=Ig heavy chain V-II region COR; AltName: Full=Ig heavy chain V-II region DAW; AltName: Full=Ig heavy chain V-II region OU; AltName: Full=Ig heavy chain V-II region SESS; Flags: Precursor |
| P01817 | 25906.94674 | 45693.13 | 1.763740454 | 0.818638274 | 0.000572258 | yes | up | GO:0038096;GO:0004252;GO:0016020;GO:0030449;GO:0050776;GO:0006898;GO:0038095;GO:0002376;GO:0050900;GO:0005576;GO:0006956;GO:0002250;GO:0003823;GO:0005886;GO:0006508;GO:0006958;GO:0006955 | hsa:102723407 | | RecName: Full=Immunoglobulin heavy variable 2-5; AltName: Full=Ig heavy chain V-II region HE; AltName: Full=Ig heavy chain V-II region MCE; Flags: Precursor |
| P02751 | 630609.2684 | 905640.4042 | 1.436135575 | 0.52219195 | 0.000991178 | yes | up | GO:0008022;GO:0018149;GO:0019221;GO:0034446;GO:0048146;GO:0005518;GO:0005201;GO:0005788;GO:0062023;GO:0007160;GO:0007161;GO:0050900;GO:0010628;GO:0097718;GO:0001525;GO:0044267;GO:0070062;GO:0031093;GO:0002576;GO:0051087;GO:0009611;GO:0072562;GO:0008201;GO:0005576;GO:0002020;GO:0030198;GO:1904237;GO:0033622;GO:0042802;GO:0008284;GO:0005178;GO:2001202;GO:0008360;GO:0005793;GO:0016324;GO:0043687;GO:0042060;GO:0006953;GO:0043394;GO:0019899;GO:0007155;GO:0051702;GO:0010952;GO:0035987;GO:1901166;GO:0005615;GO:0052047;GO:0005604;GO:0045773;GO:0031012;GO:0005577;GO:0007044;GO:0005515;GO:0016504;GO:0005102;GO:0001932;GO:0070372 | hsa:2335 |  | fibronectin isoform 1 precursor [Homo sapiens] |
| P04004 | 739223.1684 | 919898.8833 | 1.244412949 | 0.315465313 | 0.011524643 | yes | up | GO:0005201;GO:0030449;GO:0005783;GO:0061302;GO:0050840;GO:0014911;GO:0030949;GO:0030247;GO:0031012;GO:0007160;GO:0007155;GO:0090303;GO:0048260;GO:0005737;GO:0032092;GO:0070062;GO:0072562;GO:0005576;GO:0016477;GO:0042802;GO:0033627;GO:0005178;GO:0005796;GO:0008283;GO:0010811;GO:0048709;GO:0048237;GO:0062023;GO:0006955;GO:0010951;GO:0035987;GO:0008201;GO:0005615;GO:0051258;GO:0005604;GO:0043231;GO:0050731;GO:0005044;GO:0006898;GO:0030198;GO:0005515;GO:0097421;GO:0071062;GO:0005518;GO:0030195 | hsa:7448 |  | vitronectin precursor [Homo sapiens] |
| P04430 | 53537.46714 | 80451.58348 | 1.502715533 | 0.58757193 | 0.036076239 | yes | up | GO:0038096;GO:0004252;GO:0016020;GO:0030449;GO:0050776;GO:0006898;GO:0038095;GO:0050900;GO:0005576;GO:0006956;GO:0003823;GO:0005886;GO:0006508;GO:0006958;GO:0006955 | hsa:7441 |  | RecName: Full=Immunoglobulin kappa variable 1-16; AltName: Full=Ig kappa chain V-I region BAN; Flags: Precursor |
| P04433 | 91794.47789 | 112058.7604 | 1.220757098 | 0.287776166 | 0.01210448 | yes | up | GO:0038096;GO:0004252;GO:0016020;GO:0030449;GO:0050776;GO:0006898;GO:0038095;GO:0072562;GO:0002376;GO:0050900;GO:0005576;GO:0006956;GO:0002250;GO:0003823;GO:0005886;GO:0006508;GO:0006958;GO:0006955;GO:0070062 | hsa:7441 |  | rheumatoid factor D1 IgG light chain VK3 region, partial [Homo sapiens] |
| P08185 | 52287.74895 | 93306.80625 | 1.784486962 | 0.83550936 | 3.95E-07 | yes | up | GO:0005615;GO:0008289;GO:0010951;GO:0008211;GO:0005576;GO:0004867;GO:0005496;GO:0070062 | hsa:866 | COG4826 | corticosteroid-binding globulin precursor [Homo sapiens] |
| P08779 | 61836.21 | 113683.1733 | 1.838456356 | 0.878494928 | 0.019423168 | yes | up | GO:0005200;GO:0061436;GO:0008544;GO:0005856;GO:0002009;GO:0051546;GO:0070062;GO:0005634;GO:0005198;GO:0030336;GO:0030216;GO:0008283;GO:0045087;GO:0070268;GO:0005882;GO:0006954;GO:0042633;GO:0007568;GO:0007010;GO:0045104;GO:0005829;GO:0031424;GO:0005515 | hsa:3868 |  | keratin, type I cytoskeletal 16 [Homo sapiens] |
| P15169 | 17697.84421 | 26973.77833 | 1.524127911 | 0.607983985 | 1.84E-06 | yes | up | GO:0097060;GO:0005794;GO:0051384;GO:0030449;GO:0005615;GO:0016787;GO:0030141;GO:0004181;GO:0004180;GO:0010815;GO:0004185;GO:0005576;GO:0043025;GO:0008270;GO:0016485;GO:0006508;GO:0006518;GO:0046872;GO:0030070;GO:0008233;GO:0008237 | hsa:1369 |  | carboxypeptidase N catalytic chain precursor [Homo sapiens] |
| P19823 | 928446.2053 | 1357782.583 | 1.462424614 | 0.548362258 | 2.88E-09 | yes | up | GO:0044267;GO:0030212;GO:0070062;GO:0043687;GO:0072562;GO:0062023;GO:0030414;GO:0005788;GO:0004866;GO:0004867;GO:0010951;GO:0010466;GO:0005576 | hsa:3698 | COG2304 | inter-alpha-trypsin inhibitor heavy chain H2 precursor [Homo sapiens] |
| P35527 | 98069.04789 | 152461.3892 | 1.554633112 | 0.63657415 | 0.000175177 | yes | up | GO:0005200;GO:0045109;GO:0031424;GO:0005615;GO:0005829;GO:0016020;GO:0070268;GO:0005198;GO:0007283;GO:0005882;GO:0008544;GO:0005634;GO:0043588;GO:0070062 | hsa:3857 |  | keratin, type I cytoskeletal 9 [Homo sapiens] |
| P55058 | 15843.73116 | 22844.77083 | 1.441880742 | 0.527951844 | 7.40E-05 | yes | up | GO:0034375;GO:0006869;GO:0015914;GO:1990050;GO:0035627;GO:0005548;GO:0005615;GO:0019992;GO:0008525;GO:0097001;GO:0010875;GO:0030317;GO:0010189;GO:0005319;GO:0035620;GO:0034364;GO:0006629;GO:0070300;GO:0008289;GO:0031210;GO:0008429;GO:1904121;GO:0005576;GO:1901611 | hsa:5360 |  | phospholipid transfer protein, isoform CRA_c [Homo sapiens] |
| Q15582 | 7728.168526 | 15336.56588 | 1.984501997 | 0.988777014 | 1.34E-05 | yes | up | GO:0005201;GO:0050840;GO:0031012;GO:0007162;GO:0001525;GO:0044267;GO:0070062;GO:0005576;GO:0002062;GO:0005178;GO:0008283;GO:0007601;GO:0062023;GO:0005886;GO:0007155;GO:0050839;GO:0005615;GO:0005604;GO:0050896;GO:0005802;GO:0030198;GO:0005515;GO:0005518 | hsa:7045 | COG2335 | transforming growth factor-beta-induced protein ig-h3 precursor [Homo sapiens] |
| Q16880 | 566498.0211 | 886352.5667 | 1.564617234 | 0.645809761 | 0.021365194 | yes | up | GO:0008489;GO:0007417;GO:0030913;GO:0016021;GO:0016020;GO:0006682;GO:0016740;GO:0006687;GO:0015020;GO:0006665;GO:0006629;GO:0002175;GO:0008152;GO:0005886;GO:0008194;GO:0007010;GO:0043231;GO:0007422;GO:0048812;GO:0016757;GO:0047263;GO:0016758 | hsa:7368 | COG1819 | 2-hydroxyacylsphingosine 1-beta-galactosyltransferase precursor [Homo sapiens] |
| Q5SRP5 | 12873.06981 | 23282.80167 | 1.808644092 | 0.85490854 | 0.000954558 | yes | up | GO:0005319;GO:0034361;GO:0034375;GO:0034445;GO:0034365;GO:0034366;GO:0016209;GO:0005543;GO:0034362;GO:0034384;GO:0043691;GO:0033344;GO:0042157;GO:0034364;GO:0034380 | hsa:55937 |  | APOM isoform 1 [Pan troglodytes] |
| Q92954 | 34921.39316 | 46914.17583 | 1.343422229 | 0.425912806 | 0.023154068 | yes | up | GO:0008283;GO:0005044;GO:0006898;GO:0030247;GO:0030021;GO:0005576;GO:0006955;GO:0062023 | hsa:10216 |  | unnamed protein product [Homo sapiens] |
| A0A087WWT3 | 62502.87474 | 72358.50375 | 1.157682811 | 0.21124003 | 0.03560828 | yes | no change | GO:0005794;GO:0005615;GO:0005783 | hsa:213 |  | ALB protein [Homo sapiens] |
| A0A0A0MS51 | 291283.5526 | 246227.2667 | 0.845318125 | -0.24243371 | 0.000965915 | yes | no change | GO:0051127;GO:0015629;GO:1902174;GO:0030155;GO:0030041;GO:0042989;GO:0045159;GO:1990000;GO:0005925;GO:1903923;GO:0001726;GO:0071801;GO:0005615;GO:0045010;GO:1903903;GO:0043209;GO:0005634;GO:1903909;GO:0030478;GO:0005509;GO:0048471;GO:1903906;GO:0097284;GO:2001269;GO:0060271;GO:0030027;GO:0006915;GO:0051016;GO:0051015;GO:0051014;GO:0045471;GO:0045335;GO:0002102;GO:0005886;GO:0014003;GO:0032991;GO:0006911;GO:0048015;GO:0051693;GO:0046597;GO:0090527;GO:0007568;GO:0051593;GO:0005829;GO:0071346;GO:0042246;GO:0031648;GO:0016528;GO:0014891;GO:0097017;GO:0071276 | hsa:2934 |  | gelsolin isoform d [Homo sapiens] |
| A0A0C4DH36 | 52856.89 | 62414.52333 | 1.180820955 | 0.239790229 | 0.034622696 | yes | no change | GO:0009897;GO:0050853;GO:0045087;GO:0006910;GO:0006911;GO:0042742;GO:0003823;GO:0034987;GO:0042571;GO:0006958;GO:0050871;GO:0072562 | hsa:102723407 | | immunoglobulin heavy chain variable gene IGHV3-38, partial [Homo sapiens] |
| A0A0G2JL69 | 36053.48895 | 31058.21125 | 0.861448147 | -0.215164136 | 0.045089068 | yes | no change | GO:0004252;GO:0016787;GO:0045087;GO:0005576;GO:0006956;GO:0006508;GO:0006958;GO:0046872;GO:0008233;GO:0008236 | hsa:717 | COG5640 | complement C2 isoform 5 [Homo sapiens] |
| A0A0U1RQV3 | 27699.22579 | 23377.98833 | 0.843994287 | -0.244694861 | 0.012055416 | yes | no change | GO:0062023;GO:0007173;GO:0005509;GO:0005006 | hsa:2202 |  | EGF-containing fibulin-like extracellular matrix protein 1 isoform X3 [Homo sapiens] |
| B7ZKJ8 | 544967.0421 | 482377.3375 | 0.88514956 | -0.176006853 | 0.008918391 | yes | no change | GO:0005737;GO:0030212;GO:0034097;GO:0004867;GO:0006953;GO:0005886;GO:0010951 | hsa:3700 | COG2304 | ITIH4 protein [Homo sapiens] |
| D6RF35 | 2747335.053 | 2487710.75 | 0.905499585 | -0.143214115 | 0.036056817 | yes | no change | GO:0051180;GO:0090482;GO:0035461;GO:0005615;GO:0005499 | hsa:2638 |  | GC isoform 4 [Pan troglodytes] |
| E7END6 | 19835.25474 | 16707.21046 | 0.842298759 | -0.247596054 | 0.009104736 | yes | no change | GO:0004252;GO:0016787;GO:0007596;GO:0005576;GO:0005509;GO:0006508;GO:0008233;GO:0008236 | hsa:5624 | COG5640 | vitamin K-dependent protein C isoform X5 [Homo sapiens] |
| O75882 | 38134.91316 | 44478.78583 | 1.166353406 | 0.222004992 | 0.039309238 | yes | no change | GO:0005737;GO:0043473;GO:0042552;GO:0005615;GO:0070062;GO:0016021;GO:0016020;GO:0030246;GO:0006954;GO:0005576;GO:0005887;GO:0005886;GO:0038023;GO:0021549;GO:0006979;GO:0040014 | hsa:8455 |  | attractin isoform 1 preproprotein [Homo sapiens] |
| P01344 | 49122.98947 | 41745.06167 | 0.849807028 | -0.234792821 | 0.028961014 | yes | no change | GO:0046628;GO:0045840;GO:0031017;GO:0051146;GO:0008286;GO:0038028;GO:0008284;GO:0001501;GO:0001503;GO:0051147;GO:0031056;GO:0071902;GO:0051897;GO:0007275;GO:0001649;GO:0044267;GO:0006349;GO:0001701;GO:0008083;GO:0031093;GO:0000122;GO:0002576;GO:0043085;GO:0045725;GO:0010469;GO:0005179;GO:0005178;GO:0009887;GO:2000467;GO:0042104;GO:0005159;GO:0005158;GO:0043410;GO:0048018;GO:0060669;GO:0001892;GO:0043539;GO:0005615;GO:0005975;GO:0050731;GO:0040018;GO:0006355;GO:0051781;GO:0005576;GO:0045944;GO:0005515;GO:0006006;GO:0001934 | hsa:3481 |  | insulin-like growth factor II isoform 2 [Homo sapiens] |
| P01619 | 3394970 | 2890104.333 | 0.851290095 | -0.232277251 | 0.024100455 | yes | no change | GO:0004252;GO:0030449;GO:0019731;GO:0006955;GO:0050900;GO:0005615;GO:0016020;GO:0003094;GO:0072562;GO:0071748;GO:0006508;GO:0050776;GO:0006956;GO:0003823;GO:0005886;GO:0006958;GO:0070062;GO:0038096;GO:0038095;GO:0006898;GO:0002376;GO:0005576;GO:0002250;GO:0071751;GO:0071756 | hsa:29802 |  | immunoglobulin light chain variable region, partial [Homo sapiens] |
| P02787 | 19885325.84 | 17106102.33 | 0.860237467 | -0.217193127 | 0.016676331 | yes | no change | GO:0009617;GO:0016020;GO:0055037;GO:1990459;GO:1900390;GO:0005788;GO:0030139;GO:0061024;GO:0055072;GO:0006826;GO:0005905;GO:0034986;GO:1990712;GO:0048260;GO:0010008;GO:0030665;GO:0007257;GO:0045780;GO:0031232;GO:0005615;GO:0031410;GO:0002576;GO:0005770;GO:0045178;GO:0070062;GO:2000147;GO:0009925;GO:0044267;GO:0048471;GO:0046872;GO:0043687;GO:0009986;GO:0006879;GO:0033572;GO:0007015;GO:0016324;GO:0006811;GO:0030316;GO:0031982;GO:0008198;GO:0008199;GO:0045893;GO:0034774;GO:0015091;GO:0001895;GO:0034756;GO:0071281;GO:0042327;GO:0060395;GO:0031647;GO:0005623;GO:0005576;GO:0005515;GO:0005768;GO:0005769;GO:0070371;GO:0072562 | hsa:7018 |  | serotransferrin isoform 1 precursor [Homo sapiens] |
| P05154 | 20619.27105 | 17383.09479 | 0.843050889 | -0.246308376 | 0.026569211 | yes | no change | GO:0009897;GO:0006869;GO:0007596;GO:0036024;GO:0002080;GO:0061107;GO:0036029;GO:0031094;GO:0031091;GO:0051346;GO:0007342;GO:0016020;GO:0036025;GO:0032190;GO:0036027;GO:0036026;GO:0010466;GO:0002020;GO:0097183;GO:0097182;GO:0097181;GO:0036028;GO:0007283;GO:0031210;GO:0007338;GO:0030414;GO:0004867;GO:0010951;GO:0008201;GO:0005615;GO:0032991;GO:0070062;GO:0036030;GO:0005539;GO:0005576;GO:0005515;GO:0001972;GO:0045861 | hsa:5104 | COG4826 | plasma serine protease inhibitor preproprotein [Homo sapiens] |
| P07358 | 44602.62263 | 39029.12833 | 0.875041108 | -0.192577301 | 0.024148352 | yes | no change | GO:0019835;GO:0030449;GO:0005615;GO:0045087;GO:0016020;GO:0002376;GO:0005576;GO:0006956;GO:0006957;GO:0006955;GO:0044877;GO:0006958;GO:1903561;GO:0005579;GO:0070062 | hsa:732 |  | RecName: Full=Complement component C8 beta chain; AltName: Full=Complement component 8 subunit beta; Flags: Precursor |
| P08697 | 252362.9421 | 215477.4083 | 0.853839342 | -0.227963457 | 0.000696472 | yes | no change | GO:0030414;GO:0032967;GO:0005615;GO:0031093;GO:0051496;GO:0002576;GO:0030199;GO:0072562;GO:0010466;GO:0048514;GO:0002020;GO:0010757;GO:0051918;GO:0042803;GO:0048661;GO:0009986;GO:0045597;GO:0006953;GO:0046330;GO:0004866;GO:0004867;GO:0010951;GO:0010033;GO:0070062;GO:0042730;GO:2000049;GO:0002034;GO:0005577;GO:0005576;GO:0045944;GO:0005515;GO:0070374;GO:0071636 | hsa:5345 | COG4826 | alpha-2-antiplasmin isoform X1 [Homo sapiens] |
| P10643 | 100947.3453 | 86292.0675 | 0.854822554 | -0.226303122 | 0.018528826 | yes | no change | GO:0019835;GO:0030449;GO:0070062;GO:0045087;GO:0006883;GO:0002376;GO:0005576;GO:0006956;GO:0006957;GO:0006955;GO:0006958;GO:0005579 | hsa:730 |  | complement component C7 precursor [Homo sapiens] |
| P13671 | 57976.29579 | 51443.50375 | 0.887319603 | -0.172474254 | 0.024852519 | yes | no change | GO:0019835;GO:0001701;GO:0030449;GO:0070062;GO:0045917;GO:0045087;GO:0045766;GO:0002376;GO:0005576;GO:0006956;GO:0001970;GO:0005515;GO:0006955;GO:0006958;GO:0005579 | hsa:729 |  | complement component C6 precursor [Homo sapiens] |
| P14151 | 34739.74474 | 29594.96875 | 0.851905187 | -0.23123522 | 0.008376921 | yes | no change | GO:0009897;GO:0016339;GO:0030246;GO:0050900;GO:0050901;GO:0070492;GO:0030667;GO:0043208;GO:0016021;GO:0016020;GO:0005509;GO:0002020;GO:0046872;GO:0009986;GO:0005887;GO:0005886;GO:0007155;GO:0050839;GO:0008201;GO:0050776;GO:0043312;GO:0005515;GO:0033198 | hsa:6402 |  | L-selectin [Homo sapiens] |
| P25311 | 376893.9737 | 320440.6208 | 0.850214233 | -0.234101684 | 0.006410335 | yes | no change | GO:0008285;GO:0090501;GO:0071806;GO:0070062;GO:0009897;GO:0001580;GO:0005615;GO:0005634;GO:0004540;GO:0008320;GO:0062023;GO:0005515;GO:0005886;GO:0006955;GO:0007155;GO:0001895;GO:0055085;GO:0005576 | hsa:563 |  | zinc-alpha-2-glycoprotein precursor [Homo sapiens] |
| A0A0A0MS15 | 304227.7211 | 203933.7596 | 0.670332601 | -0.577050995 | 0.017946032 | yes | down | GO:0009897;GO:0050853;GO:0045087;GO:0016020;GO:0006910;GO:0006911;GO:0002376;GO:0005576;GO:0002250;GO:0003823;GO:0005886;GO:0072562;GO:0042571;GO:0006958;GO:0050871;GO:0034987;GO:0042742 | hsa:102723407 | | RecName: Full=Immunoglobulin heavy variable 3-49; Flags: Precursor |
| A0A0B4J1U3 | 40123.38389 | 27657.68086 | 0.68931576 | -0.536763094 | 0.043440287 | yes | down | GO:0016020;GO:0005615;GO:0006955;GO:0002376;GO:0002377;GO:0005576;GO:0002250;GO:0003823;GO:0005886 | hsa:7441 |  | RecName: Full=Immunoglobulin lambda variable 1-36; Flags: Precursor |
| A0A0B4J1V2 | 27879.93279 | 8304.0655 | 0.297850987 | -1.747337355 | 0.030364267 | yes | down | GO:0009897;GO:0050853;GO:0045087;GO:0016020;GO:0006910;GO:0006911;GO:0002376;GO:0005576;GO:0002250;GO:0003823;GO:0005886;GO:0072562;GO:0042571;GO:0006958;GO:0050871;GO:0034987;GO:0042742 | hsa:102723407 | | RecName: Full=Immunoglobulin heavy variable 2-26; Flags: Precursor |
| A0A0C4DH21 | 50413.95316 | 37914.02208 | 0.752054138 | -0.411091574 | 0.017377711 | yes | down |  | hsa:10877 |  | complement factor H-related protein 4 [Homo sapiens] |
| A0A0C4DH25 | 534346.1842 | 396296.725 | 0.741647899 | -0.431193672 | 0.002404695 | yes | down | GO:0038096;GO:0004252;GO:0016020;GO:0030449;GO:0005615;GO:0006898;GO:0038095;GO:0050900;GO:0002376;GO:0002377;GO:0050776;GO:0005576;GO:0006956;GO:0002250;GO:0003823;GO:0005886;GO:0006508;GO:0006958;GO:0006955 | hsa:29802 |  | hCG1686089, partial [Homo sapiens] |
| A0A0C4DH38 | 152379.5311 | 101988.5363 | 0.669306012 | -0.579262121 | 0.018891176 | yes | down | GO:0009897;GO:0050853;GO:0045087;GO:0016020;GO:0006910;GO:0006911;GO:0002376;GO:0005576;GO:0002250;GO:0003823;GO:0005886;GO:0072562;GO:0042571;GO:0006958;GO:0050871;GO:0034987;GO:0042742 | hsa:102723407 | | RecName: Full=Immunoglobulin heavy variable 5-51; Flags: Precursor |
| A0A0C4DH67 | 32356.6928 | 17966.08429 | 0.555250946 | -0.848788149 | 0.005321411 | yes | down | GO:0016020;GO:0002376;GO:0005576;GO:0002250;GO:0003823;GO:0005886 | | | Ig kappa V-region e, partial [Homo sapiens] |
| A0A0C4DH73 | 127967.1195 | 84374.96458 | 0.659348784 | -0.600886268 | 0.000144769 | yes | down | GO:0038096;GO:0004252;GO:0016020;GO:0030449;GO:0005615;GO:0006898;GO:0038095;GO:0050900;GO:0002376;GO:0002377;GO:0050776;GO:0005576;GO:0006956;GO:0002250;GO:0003823;GO:0005886;GO:0006508;GO:0006958;GO:0006955 | hsa:7441 |  | immunoglobulin light chain variable region, partial [Homo sapiens] |
| A0A0G2JRQ6 | 270236.3158 | 220779.3208 | 0.816986126 | -0.291616515 | 0.029501207 | yes | down | GO:0002377;GO:0005615;GO:0006955 | hsa:7441 |  | hCG2042707, partial [Homo sapiens] |
| A0A0J9YXX1 | 357816.2368 | 197626.7463 | 0.552313523 | -0.856440644 | 0.000245181 | yes | down | GO:0016020;GO:0002376;GO:0005576;GO:0002250;GO:0003823;GO:0005886 | hsa:102724971 | | RecName: Full=Immunoglobulin heavy variable 5-10-1; Flags: Precursor |
| A0A140T8Y3 | 10061.29975 | 6675.028227 | 0.663435977 | -0.591970846 | 0.004719433 | yes | down | GO:0005201;GO:0062023;GO:0030199;GO:0030198;GO:0031012 | hsa:7148 |  | tenascin-X isoform 1 precursor [Homo sapiens] |
| A0A1W2PQU7 | 150467.4732 | 87935.20958 | 0.584413413 | -0.774938804 | 0.002797277 | yes | down | GO:1904714;GO:0005737;GO:0045111;GO:0061564;GO:0045109;GO:0060291;GO:0016020;GO:0044297;GO:0060020;GO:0005198;GO:0051580;GO:0005883;GO:0005882;GO:0010977;GO:0030198;GO:0014002;GO:0097450;GO:0010625;GO:0031102 | hsa:2670 |  | glial fibrillary acidic protein [Mus musculus] |
| A0A286YEY4 | 733018.9684 | 536669.5125 | 0.732135914 | -0.449816599 | 0.043854697 | yes | down | GO:0016021;GO:0016020 | hsa:100423062 | | unnamed protein product [Homo sapiens] |
| A0A2R8Y3M9 | 189805.2842 | 144395.1796 | 0.760754266 | -0.394497577 | 3.32E-05 | yes | down | GO:0004252;GO:0006898;GO:0005044;GO:0016020;GO:0016042;GO:0005576;GO:0005509;GO:0006508;GO:0004623 | hsa:81579;hsa:3426 | COG5640 | complement factor I isoform X2 [Homo sapiens] |
| C9JB55 | 12257.70416 | 9811.448136 | 0.800431142 | -0.321150796 | 0.044196664 | yes | down | GO:0055037;GO:1990459;GO:0030139;GO:0005905;GO:0034986;GO:1990712;GO:0004857;GO:0048260;GO:0005615;GO:0005770;GO:0043086;GO:0009925;GO:0048471;GO:0006879;GO:0016324;GO:0008198;GO:0008199;GO:0015091;GO:0031232;GO:0034756;GO:0071281;GO:0005623;GO:0005769 | hsa:7018 |  | serotransferrin isoform 1 precursor [Homo sapiens] |
| D6RAR4 | 31434.37947 | 25873.07667 | 0.823082151 | -0.280891663 | 0.031081573 | yes | down | GO:0005737;GO:0004252;GO:0005791;GO:0005615;GO:0016787;GO:0005576;GO:0006508;GO:0008233;GO:0008236 | hsa:3083 | COG5640 | hepatocyte growth factor activator isoform 1 preproprotein [Homo sapiens] |
| F8W1S1 | 8692.160882 | 5528.127 | 0.635989954 | -0.652924118 | 0.001542644 | yes | down | GO:0005882;GO:0045095;GO:0005198 | hsa:121391 | | KRT74 isoform 2 [Pan troglodytes] |
| H0YAC1 | 177889.1368 | 133550.6958 | 0.750752397 | -0.413590919 | 6.61E-06 | yes | down | GO:0004252;GO:0004497;GO:0007597;GO:0008236;GO:0008233;GO:0016491;GO:0070062;GO:0016705;GO:0031639;GO:0031638;GO:0006508;GO:0005506;GO:0046872;GO:0022617;GO:0051919;GO:0016787;GO:0020037;GO:0005886;GO:0055114;GO:0005615;GO:0042730;GO:0002542;GO:0005576 | hsa:3818 | COG5640 | KLKB1 isoform 4, partial [Pan troglodytes] |
| J3KPA1 | 13651.48432 | 10629.90188 | 0.778662717 | -0.360929543 | 0.045066027 | yes | down | GO:0016020;GO:0016021;GO:0005576 | hsa:10321 | COG2340 | cysteine-rich secretory protein 3 isoform 3 [Homo sapiens] |
| J3QRV5 | 399653.4421 | 278678.4542 | 0.697300273 | -0.520148049 | 0.001938507 | yes | down | GO:0005737;GO:0043231;GO:0005829;GO:0006887;GO:0007049;GO:0051301 | hsa:3993 |  | lethal(2) giant larvae protein homolog 2 isoform X4 [Homo sapiens] |
| K7ERG9 | 12504.43326 | 9928.051708 | 0.793962549 | -0.332857137 | 0.035686544 | yes | down | GO:0004252;GO:0007219;GO:0005615;GO:0016787;GO:0009617;GO:0006957;GO:0006508;GO:0008233;GO:0008236 | hsa:1675 | COG5640 | complement factor D isoform 2 precursor [Homo sapiens] |
| O00187 | 37616.25611 | 24618.28871 | 0.65445877 | -0.611625788 | 0.002316392 | yes | down | GO:0045087;GO:0004252;GO:0046872;GO:0006956;GO:0005615;GO:0048306;GO:0070062;GO:0016787;GO:0002376;GO:0005576;GO:0001867;GO:0005509;GO:0005515;GO:0006508;GO:0006958;GO:0008236;GO:0008233;GO:0001855 | hsa:10747 | COG5640 | mannan-binding lectin serine protease 2 isoform 1 preproprotein [Homo sapiens] |
| P00748 | 68588.26368 | 40103.85458 | 0.584704327 | -0.774220826 | 4.51E-05 | yes | down | GO:0004252;GO:0002542;GO:0007599;GO:0007597;GO:0007596;GO:0008233;GO:0008236;GO:0070062;GO:0031638;GO:0005509;GO:0006508;GO:0051919;GO:0016787;GO:0005791;GO:0045087;GO:0062023;GO:0005886;GO:0016485;GO:0005615;GO:0042730;GO:0016540;GO:0002353;GO:0005576;GO:0051787;GO:0010756;GO:0005515;GO:0030193;GO:0051788;GO:0030194 | hsa:2161 | COG5640 | coagulation factor XII preproprotein [Homo sapiens] |
| P00915 | 22432.51958 | 14246.79221 | 0.635095499 | -0.65495455 | 0.009893109 | yes | down | GO:0005737;GO:0015701;GO:0046872;GO:0070062;GO:0006730;GO:0016829;GO:0008270;GO:0005515;GO:0016836;GO:0004089;GO:0004064;GO:0005829;GO:0035722 | hsa:759 | COG3338 | carbonic anhydrase 1 isoform a [Homo sapiens] |
| P00918 | 10796.67714 | 6637.175053 | 0.614742385 | -0.701946137 | 0.000293531 | yes | down | 3P3J:A;1IF6:A;1H9N:A;3RYJ:B;5FLQ:A;3P5A:A;1AVN:A;GO:0010043;6BC9:A;3V3H:B;4Q78:A;5JGS:B;5M78:A;1CAM:A;GO:0043209;5TY9:A;3RZ1:B;5EOI:A;5JN3:A;1CNI:A;4ZWY:A;3PJJ:A;3TMJ:A;5LL4:A;4CAC:A;1CNK:A;3T5U:A;6EQU:A;5EH8:A;1G52:A;5JEG:B;1AM6:A;3SBH:A;1CVD:A;4BF1:A;1BNU:A;1I91:A;4PYY:A;3MHL:A;4HEY:A;5FLO:A;5NXP:A;3S78:B;4Q08:A;4K1Q:A;2O4Z:A;2Q38:A;3DD8:A;5CAC:A;4YYT:A;2VVB:X;1CNC:A;2FNN:A;5G0C:A;1CVB:A;1FR7:B;2FOQ:A;3RZ7:A;4KNI:A;6H2Z:A;4QTL:A;4QEF:A;4FVO:A;6BCC:A;3M1Q:A;5LLG:A;6E92:A;3T83:A;5WEX:A;3D8W:A;4YXI:A;5WLV:A;4Q6D:A;5JQT:A;4YXO:A;5TFX:A;2HKK:A;2POU:A;3M1W:A;6MBY:A;5DOH:B;5OGO:A;4QSI:A;1BNV:A;3DBU:A;1IF9:A;3PYK:A;1CAK:A;1G46:A;6D1L:A;5TY8:A;5DRS:A;5E28:A;4Q9Y:A;5LLC:A;6FJI:A;3MNH:A;1I9P:A;1CIL:A;4FIK:A;6BBS:A;3OIL:A;1LG6:A;4BF6:A;6IC2:A;6C7W:A;1CIN:A;1G4O:A;1BNM:A;4YGL:A;4HF3:A;2CBE:A;3KOK:A;GO:0016829;4MDG:A;3HS4:A;5DSM:A;4K0Z:A;2F14:A;5FNL:A;4JSW:A;GO:0045177;5BRU:A;5DSO:A;3DVC:A;2WD2:A;3RG3:A;4KUV:A;4HEZ:A;5LL4:B;1I9M:A;1RZD:A;5DSL:A;3B4F:A;1TE3:X;5BNL:A;4YGK:A;GO:0016323;5U0G:A;5LMD:A;5CA2:A;4DZ9:A;5SZ1:A;1ZSB:A;1YDB:A;1YO0:A;4E49:A;4YVY:A;4KV0:A;2X7S:A;5THN:A;4KUW:A;1YDD:A;1YO2:A;1I8Z:A;2X7U:A;2CBC:A;5JQ0:A;4QSA:A;3S74:B;3P58:A;3M67:A;3IGP:A;3S76:A;3EFT:A;5TYA:A;3IEO:A;4JSZ:A;3V7X:A;5N24:A;3HKU:A;3KKX:A;5ZXW:A;1BN3:A;2EZ7:A;5JDV:B;5FLT:A;4E4A:A;GO:0030424;1CVH:A;GO:0004064;1XEG:A;5G03:A;2AW1:A;3F8E:A;1RAZ:A;5W8B:A;5G01:A;5N1R:A;5L70:B;2ABE:A;2EU3:A;5L70:A;3IBU:A;GO:0005615;6EDA:A;2GEH:A;5N1S:A;4Q7W:A;1CAY:A;5EKM:A;3D93:A;3M2Z:A;5MJN:A;1ZFQ:A;2NNO:A;GO:0038166;3R17:B;3S9T:A;1BV3:A;5VGY:A;4IWZ:A;1G3Z:A;5DSP:A;4Z0Q:A;GO:0045780;1XPZ:A;3RZ5:A;1UGD:A;GO:0016020;5AMD:A;1RAY:A;4M2U:A;1UGF:A;3DV7:A;3S75:B;1RZE:A;4PXX:A;1BNT:A;5DOG:A;3P4V:A;2WEG:A;6EBE:A;3KS3:A;5WLR:A;5SZ2:A;1KWQ:A;1CIM:A;1CNG:A;5AML:A;2NNV:A;GO:0005829;1CCS:A;5OGP:A;GO:0043627;1CVF:A;1TTM:A;4FVN:A;4JSA:A;1TBT:X;1FSQ:A;5SZ3:A;4RFC:A;1FSQ:B;3MNJ:A;2NWP:A;5FNJ:A;1OKM:A;5LLE:A;4ITP:A;2WD3:A;3K7K:A;1G4J:A;2H4N:A;4HBA:A;1ZH9:A;4R5B:A;5T71:A;6ECZ:A;5L6T:A;5L6T:B;2AX2:A;3P44:A;5TI0:A;GO:2001225;2QOA:A;6CA2:A;4G0C:A;1CA2:A;1CAO:A;3V3F:A;4Z1K:A;1FR7:A;4QK3:A;1CAI:A;5N0E:A;4PQ7:A;3HKN:A;1RZC:A;1ZGF:A;4QK1:A;5GMN:A;4E3D:A;1CNW:A;5ULN:A;GO:0048545;2FOV:A;4FRC:A;2FNK:A;2HD6:A;2WEO:A;1TG9:A;3M40:A;5BRV:A;4Q7P:A;4Q8Z:A;2QO8:A;5DSK:A;4RIV:A;3KOI:A;5Y2S:A;4Q8X:A;3HLJ:A;1LZV:A;1G54:A;5C8I:A;3L14:A;3N2P:A;3OYQ:A;3CAJ:A;2POV:A;6EEO:A;4WL4:A;6B4D:A;5YUJ:A;4FPT:A;3GZ0:A;5NXV:A;1OQ5:A;6GOT:A;5J8Z:A;3ZP9:A;4RUY:A;6B5A:A;2NWZ:A;1I9O:A;1THK:A;2EU2:A;5SZ7:A;4L5W:A;4M2W:A;6H6S:A;5JMZ:A;4Q7V:A;2Q1Q:A;4HT0:A;4QSB:A;4QJM:A;1BNN:A;2NXS:A;GO:0005515;3M1K:A;4Q99:A;3M2X:A;1H9Q:A;6G6T:A;4MDM:A;1FQM:A;5FDC:A;1H4N:A;5SZ5:A;4Q6E:A;3SAP:A;1EOU:A;1TEQ:X;5FLP:A;5T75:A;5NXI:A;3K34:A;2HOC:A;6CJV:A;6FJJ:A;1CNJ:A;3P5L:A;1G53:A;3V5G:A;4MDL:A;1ZSC:A;6HX5:A;4XE1:A;1ZSA:A;1TG3:A;6GXE:A;5JEH:B;4MO8:A;2X7T:A;4CA2:A;1IF7:A;6CEH:A;4QY3:A;GO:0046903;1LGD:A;4Q09:A;2CBD:A;1IF5:A;1CNB:A;3FFP:X;3DCW:A;5NXO:A;5LL8:A;3RYY:A;4Z1E:A;3R16:A;3BET:A;GO:0032849;5JEP:B;5G0B:A;4ZAO:A;3ML2:A;1BCD:A;3TVN:X;4Y0J:A;5NXW:A;6EEA:A;1BN4:A;1UGB:A;4K13:A;5DOH:A;3C7P:A;4RFD:A;GO:0001822;1TB0:X;5SZ4:A;5DSQ:A;GO:0046872;5L6K:B;3N3J:A;4Q07:A;3T84:A;1IF8:A;12CA:A;1DCA:A;4QK2:A;5E2K:A;4Q83:A;3SAX:A;5LVS:B;5T74:A;1CAZ:A;6D1M:A;6GXB:A;3RYZ:A;5LVS:A;2FMZ:A;3IBI:A;2QP6:A;1I9Q:A;4E5Q:A;3OIM:A;5NEA:A;6B59:A;6H29:A;2FOU:A;3M5T:A;5THI:A;4GL1:X;4Q87:A;2WEJ:A;1G0F:A;GO:2001150;6H34:A;6G3Q:A;3CYU:A;5O07:A;6QEB:A;3RGE:A;5E2R:A;5YUK:A;5EH5:A;4MLT:A;5EIJ:A;3RZ0:B;5FLS:A;3NB5:A;5EH7:A;1MOO:A;3U3A:X;4Z1J:A;3SBI:A;4Q90:A;2VVA:X;1I90:A;1XQ0:A;5NXG:A;1ZFK:A;3MHO:A;5FNI:A;6H6S:B;2OSM:A;3VBD:A;2FNM:A;3MHM:A;4K0S:A;3MNU:A;5EHV:A;5FNG:A;4E3G:A;3RZ8:A;4YWP:A;5THJ:A;1CRA:A;1RZA:A;2NNS:A;1FR4:A;3T5Z:A;1F2W:A;1CVC:A;3RG4:A;5TH4:A;3PO6:A;5WLU:A;5JN7:A;6E91:A;4ZX0:A;1Z9Y:A;5JN1:A;1HVA:A;GO:0009268;3NI5:A;5A6H:A;3S77:B;GO:0005737;3S71:B;4Q49:A;4ILX:A;3HKT:A;5U0F:A;1BIC:A;3OYS:A;3M96:A;2FMG:A;3KIG:A;5FLR:A;5OGN:B;6GCY:A;2NXT:A;1CAH:A;5SZ0:A;4E3H:A;4ITO:A;4QIY:C;4QIY:B;4QIY:A;4R5A:A;3DCC:A;1CNX:A;1CAJ:A;4MLX:A;2Q1B:A;3M04:A;4L5U:A;1ZGE:A;1LG5:A;5FDI:A;GO:0070062;3D92:A;2H15:A;3MZC:A;3F4X:A;1FQL:A;8CA2:A;5JG3:B;1UGA:A;5E2S:A;4LHI:A;5NY1:A;3M14:A;5LJT:A;3IBN:A;3P55:A;3V3G:B;1CVA:A;3D9Z:A;1HCA:A;5NXM:A;1BNQ:A;GO:0044070;3DVB:A;5JGT:B;4YGN:A;1UGG:A;3CA2:A;1BNW:A;3V2J:A;GO:0010033;1YDA:A;1I9L:A;4ZWZ:A;3RJ7:A;1YDC:A;3DAZ:A;6EEH:A;5WLT:A;6E8P:A;4M2R:A;5L6K:A;1TEU:X;3DCS:A;2CBB:A;3BL0:A;1FSR:B;1FSR:A;1LUG:A;2OSF:A;2ILI:A;1MUA:A;4N16:A;2GD8:A;3MMF:A;5U0D:A;4E3F:A;2HL4:A;3OKV:A;1ZE8:A;5TY1:A;GO:0008270;5JE7:B;3DC9:A;3V3J:A;3S73:B;GO:0032230;3M5S:A;1T9N:A;4Q06:A;1CCU:A;5AMG:A;3MWO:B;7CA2:A;3MWO:A;5T72:A;GO:0004089;5BYI:A;3DC3:A;1CNH:A;2NNG:A;1CA3:A;5EKH:A;5WG7:A;3OY0:A;5NY3:A;1FQR:A;1HEC:A;9CA2:A;2FOS:A;5EKJ:A;3V2M:A;3N4B:A;1HEA:A;2NWO:A;5NEE:A;3K2F:A;6E8X:A;3M2Y:A;3P3H:A;3U7C:A;4FL7:A;4Q81:A;4WW6:A;GO:0015701;3RYV:B;3S8X:A;5U0E:A;GO:0051453;3EFI:A;4RH2:A;5EHW:A;2WEH:A;4Q8Y:A;2POW:A;4Z1N:A;1HEB:A;4RIU:A;1CAL:A;5FNM:A;1I9N:A;4ZWX:A;1G1D:A;1KWR:A;5TXY:A;3MHI:A;5LLH:A;1CCT:A;4RN4:A;3KWA:A;4CQ0:A;4YXU:A;1CVE:A;5SZ6:A;4KAP:A;3QYK:A;4PYX:A;4YGJ:A;2NWY:A;1FSN:B;1FSN:A;4HEW:A;3V3I:B;4N0X:B;1YO1:A;4KNJ:A;3MNK:A;3HFP:A;4FU5:A;5BRW:A;GO:0005886;1G0E:A;1OKN:A;5N25:A;5CLU:A;4JS6:A;GO:0005902;4PZH:A;1OKL:A;5L9E:C;3M2N:A;5LJQ:A;3T82:A;1FQN:A;6MBV:A;4RUZ:A;4DZ7:A;5DSR:A;1A42:A;4RUX:A;3MNA:A;5L3O:B;1CNY:A;4ZWI:A;1CAN:A;3U45:X;3OIK:A;4KUY:A;5NY6:A;3U47:A;5YUI:A;2CBA:A;5N0D:A;5FNH:A;6H33:A;4K0T:A;3N0N:A;3RLD:A;3BL1:A;4ZX1:A;5UMC:A;1RZB:A;3M5E:A;5OGN:A;4LP6:B;4LP6:A;1G45:A;3MNI:A;6GDC:A;3RYX:B;GO:0015670;4YX4:A;1IF4:A;5JG5:B;4JSS:A;3DVD:A;4BCW:A;5WGP:A;5Y2R:A;3TVO:X;3IBL:A;6C7X:A;GO:0002009;3DD0:A;3S72:B;3M3X:A;1UGC:A;4R59:A;3HKQ:A;1UGE:A;3T85:A;4QIY:D;3MYQ:A;4M2V:A;1G48:A;GO:0071498;1XEV:B;2HNC:A;5JES:B;4L5V:A;3NJ9:A;1TH9:A;5L9E:A;1DCB:A;3M98:A;5L9E:B;5L3O:A;5L9E:D;5FNK:A;2CA2:A;GO:0042475;4IDR:X;3KNE:A;5NYA:A;3M1J:A;1HED:A;3MHC:A;2NXR:A;GO:0045672;4Q7S:A;3OKU:A;3KON:A;1XEV:C;5EHE:A;1XEV:A;4MTY:A;1XEV:D;1BN1:A | hsa:760 | COG3338 | carbonic anhydrase 2 isoform 1 [Homo sapiens] |
| P01008 | 531044.1632 | 406228.2042 | 0.764961245 | -0.386541436 | 3.63E-05 | yes | down | GO:0007599;GO:0007595;GO:2000266;GO:0007596;GO:0005788;GO:0002438;GO:0070062;GO:0072562;GO:0010466;GO:0002020;GO:0043687;GO:0042802;GO:0044267;GO:0007584;GO:0062023;GO:0030414;GO:0004867;GO:0010951;GO:0005886;GO:0008201;GO:0005615;GO:0005576;GO:0005515;GO:0030193 | hsa:462 | COG4826 | antithrombin-III isoform 1 precursor [Homo sapiens] |
| P01009 | 5993319.158 | 4427646.083 | 0.738763608 | -0.436815295 | 2.03E-05 | yes | down | GO:0048208;GO:0007599;GO:0005783;GO:0007596;GO:0033116;GO:0005788;GO:1904813;GO:0030134;GO:0044267;GO:0006888;GO:0005615;GO:0031093;GO:0002576;GO:0010466;GO:0002020;GO:0042802;GO:0005794;GO:0043687;GO:0006953;GO:0030414;GO:0004867;GO:0010951;GO:0070062;GO:0043231;GO:0043312;GO:0000139;GO:0005576;GO:0005515 | hsa:5265 | COG4826 | alpha-1-antitrypsin precursor [Homo sapiens] |
| P01019 | 227177.4053 | 187123.3125 | 0.823688044 | -0.279830048 | 0.001524574 | yes | down | GO:1903779;GO:0038166;GO:2001238;GO:0035106;GO:0003014;GO:0007166;GO:0050729;GO:0001822;GO:0042127;GO:0032930;GO:0006606;GO:0014824;GO:0019229;GO:0010976;GO:0010873;GO:0050731;GO:0035815;GO:0005829;GO:0007202;GO:0007204;GO:0003081;GO:1904754;GO:0010536;GO:0007565;GO:0005179;GO:0034374;GO:0007200;GO:0071260;GO:0042310;GO:0042311;GO:0048169;GO:0008083;GO:1901201;GO:0072562;GO:0006883;GO:0061098;GO:0007186;GO:2000379;GO:0090190;GO:0048018;GO:0008306;GO:0003331;GO:0033864;GO:0007199;GO:0001974;GO:0062023;GO:0048659;GO:0051969;GO:0016525;GO:0014873;GO:0030308;GO:0048146;GO:0048144;GO:0007267;GO:0045429;GO:1905010;GO:0043085;GO:0005737;GO:0050880;GO:0003051;GO:0002027;GO:0045742;GO:0032270;GO:0070471;GO:0008284;GO:0031701;GO:0031703;GO:0001558;GO:0014068;GO:0004867;GO:0014061;GO:0051092;GO:1904385;GO:0007568;GO:0070062;GO:0002034;GO:2001275;GO:0005515;GO:0070371;GO:0001819;GO:0051387;GO:0010469;GO:0010666;GO:0008217;GO:0061049;GO:0050663;GO:0032355;GO:0005615;GO:0097755;GO:0042981;GO:0010744;GO:0035813;GO:1904707;GO:0046628;GO:0034104;GO:0019216;GO:0051403;GO:0010951;GO:0007263;GO:1905589;GO:0010613;GO:0010595;GO:0002019;GO:0002018;GO:0045777;GO:0002016;GO:0031702;GO:0051924;GO:0005576;GO:1903598;GO:0045893 | hsa:183 | COG4826 | angiotensinogen preproprotein [Homo sapiens] |
| P01601 | 100026.1226 | 65756.58375 | 0.657394109 | -0.605169567 | 5.60E-06 | yes | down | GO:0038096;GO:0004252;GO:0016020;GO:0030449;GO:0005615;GO:0006898;GO:0038095;GO:0050900;GO:0002376;GO:0002377;GO:0050776;GO:0005576;GO:0006956;GO:0002250;GO:0003823;GO:0005886;GO:0006508;GO:0006958;GO:0006955 | hsa:7441 |  | RecName: Full=Immunoglobulin kappa variable 1D-16; AltName: Full=Ig kappa chain V-I region HK146; AltName: Full=Ig kappa chain V-I region HK189; Flags: Precursor |
| P01703 | 153989.3533 | 117343.7004 | 0.762024763 | -0.392090215 | 0.041075775 | yes | down | GO:0038096;GO:0004252;GO:0016020;GO:0030449;GO:0050776;GO:0006898;GO:0038095;GO:0002376;GO:0050900;GO:0005576;GO:0006956;GO:0002250;GO:0003823;GO:0005886;GO:0006508;GO:0006958;GO:0006955 | hsa:7441 |  | Unknown (protein for MGC:31936) [Homo sapiens] |
| P01857 | 9607821.105 | 7511055.083 | 0.781764669 | -0.35519371 | 0.015284332 | yes | down | GO:0004252;GO:0030449;GO:0019221;GO:0009897;GO:0042742;GO:0034987;GO:0042571;GO:0005615;GO:0016020;GO:0072562;GO:0006508;GO:0050853;GO:0045087;GO:0006910;GO:0006911;GO:0006956;GO:0003823;GO:0005886;GO:0006958;GO:0050871;GO:0070062;GO:0038096;GO:0002376;GO:0005576;GO:0002250;GO:0005515 | hsa:100423062 | | IGH@ protein [Homo sapiens] |
| P01861 | 720891.6689 | 474919.1504 | 0.658794061 | -0.602100545 | 0.046276916 | yes | down | GO:0004252;GO:0030449;GO:0019221;GO:0009897;GO:0042742;GO:0034987;GO:0042571;GO:0005615;GO:0016020;GO:0072562;GO:0006508;GO:0050853;GO:0045087;GO:0006910;GO:0006911;GO:0006956;GO:0003823;GO:0005886;GO:0006958;GO:0050871;GO:0070062;GO:0038096;GO:0002376;GO:0005576;GO:0002250 | hsa:100423062 | | RecName: Full=Immunoglobulin heavy constant gamma 4; AltName: Full=Ig gamma-4 chain C region |
| P02649 | 2408326.895 | 1848625.629 | 0.767597469 | -0.38157814 | 0.032943933 | yes | down | GO:0051044;GO:0005783;GO:0034447;GO:0007616;GO:0005788;GO:0072358;GO:0043524;GO:0010873;GO:0051651;GO:0050728;GO:1905855;GO:0071682;GO:0016020;GO:0008201;GO:0098869;GO:0060999;GO:0045541;GO:0044794;GO:0005319;GO:0034361;GO:0034362;GO:0034363;GO:0034364;GO:0031102;GO:0016209;GO:0071813;GO:0043537;GO:0010977;GO:0030425;GO:0030516;GO:0043691;GO:0010877;GO:0010875;GO:0061771;GO:0015485;GO:0043025;GO:0019433;GO:1990777;GO:0030195;GO:0034375;GO:0034374;GO:0034372;GO:0034371;GO:1903002;GO:0000302;GO:0019934;GO:0010629;GO:0044877;GO:0042157;GO:0042311;GO:0042158;GO:0042159;GO:0048168;GO:0042982;GO:0044267;GO:0042627;GO:0072562;GO:0035641;GO:0010544;GO:0097006;GO:0007186;GO:0062023;GO:0033700;GO:0015909;GO:0006629;GO:1905890;GO:0048156;GO:1902430;GO:0042632;GO:0032489;GO:1901215;GO:0046907;GO:0034382;GO:0034380;GO:0006898;GO:0034384;GO:0006357;GO:0046983;GO:0032805;GO:0005543;GO:0090209;GO:0006869;GO:0051246;GO:0019068;GO:0050807;GO:0031012;GO:0043407;GO:0051000;GO:1902995;GO:0043083;GO:0005737;GO:0001540;GO:0005634;GO:0030669;GO:1900221;GO:0002021;GO:0090181;GO:0042802;GO:0042803;GO:0006641;GO:0006874;GO:0043687;GO:0017038;GO:0008289;GO:0055088;GO:0055089;GO:0046889;GO:0034378;GO:0006979;GO:0008202;GO:0008203;GO:0032269;GO:0070062;GO:1900272;GO:1902952;GO:0005515;GO:1901630;GO:0005794;GO:0017127;GO:0007271;GO:0060228;GO:0001523;GO:0034365;GO:0005615;GO:0048844;GO:0005198;GO:0033344;GO:1901628;GO:0010468;GO:0032462;GO:0090090;GO:0006707;GO:0070326;GO:0046911;GO:0005886;GO:2000822;GO:1903561;GO:0007263;GO:1905908;GO:0050750;GO:0007010;GO:1905860;GO:0097114;GO:0005576;GO:1905906;GO:0097113;GO:0045807;GO:0098978;GO:0070328;GO:0005769;GO:0001937;GO:0010976 | hsa:348 |  | apolipoprotein E [Homo sapiens] |
| P02745 | 56027.18421 | 33321.85458 | 0.594744409 | -0.74965829 | 0.023615143 | yes | down | GO:0004252;GO:0030449;GO:0005581;GO:0045087;GO:0002376;GO:0005576;GO:0006956;GO:0005515;GO:0010039;GO:0007267;GO:0006508;GO:0006958;GO:0005602 | hsa:712 |  | complement C1q subcomponent subunit A precursor [Homo sapiens] |
| P02747 | 458825.5737 | 317328.5208 | 0.691610361 | -0.531968612 | 3.77E-06 | yes | down | GO:0004252;GO:0030449;GO:0005581;GO:0045650;GO:0045087;GO:0072562;GO:0002376;GO:0030853;GO:0005576;GO:0006956;GO:0005515;GO:0006955;GO:0006508;GO:0006958;GO:0005615 | hsa:714 |  | complement C1q subcomponent subunit C isoform X1 [Pongo abelii] |
| P02763 | 2270806.158 | 1554695.958 | 0.684644946 | -0.546572089 | 0.000232716 | yes | down | GO:1904469;GO:0050716;GO:0031093;GO:0005615;GO:0002576;GO:0050718;GO:0043312;GO:0072562;GO:0006953;GO:0032715;GO:0062023;GO:0002682;GO:0005515;GO:0006954;GO:0035580;GO:0005576;GO:1904724;GO:0032720;GO:0070062 | hsa:5004 |  | RecName: Full=Alpha-1-acid glycoprotein 1; Short=AGP 1; AltName: Full=Orosomucoid-1; Short=OMD 1; Flags: Precursor |
| P02766 | 909031.8684 | 731202.25 | 0.804374715 | -0.314060363 | 0.002421706 | yes | down | GO:0005179;GO:0044267;GO:0032991;GO:0070062;GO:0042562;GO:0070324;GO:0005615;GO:0070327;GO:0005737;GO:0043312;GO:0001523;GO:0035578;GO:0030198;GO:0005515;GO:0042572;GO:0046982;GO:0010469;GO:0006144;GO:0042802;GO:0005576 | hsa:7276 | COG2351 | transthyretin precursor [Homo sapiens] |
| P04196 | 685319.6474 | 560602.9292 | 0.818016719 | -0.289797766 | 0.034722036 | yes | down | GO:0002839;GO:0030308;GO:0032956;GO:2000504;GO:0007162;GO:0008270;GO:0051894;GO:0007599;GO:0001525;GO:0061844;GO:0051715;GO:0070062;GO:0031093;GO:0002576;GO:0072562;GO:0043065;GO:0033629;GO:0030168;GO:0005886;GO:0046872;GO:0010468;GO:0051918;GO:0010543;GO:0008285;GO:0007596;GO:0006935;GO:0020037;GO:0009986;GO:0010593;GO:0043537;GO:0043395;GO:0062023;GO:0004867;GO:0010951;GO:0004869;GO:0043254;GO:0008201;GO:2001027;GO:0050832;GO:0042730;GO:0036019;GO:0050730;GO:0015886;GO:0019865;GO:0005576;GO:1900747;GO:0005515;GO:0030193;GO:0005102;GO:0016525 | hsa:3273 |  | histidine-rich glycoprotein precursor [Homo sapiens] |
| P04211 | 1192480.3 | 798315.6875 | 0.669458177 | -0.578934166 | 0.000664304 | yes | down | GO:0038096;GO:0004252;GO:0016020;GO:0030449;GO:0050776;GO:0006898;GO:0038095;GO:0002376;GO:0050900;GO:0005576;GO:0006956;GO:0002250;GO:0003823;GO:0005886;GO:0006508;GO:0006958;GO:0006955 | hsa:7441 |  | hCG1731877, partial [Homo sapiens] |
| P04264 | 358372.0789 | 211548.2708 | 0.59030344 | -0.760471347 | 0.000294948 | yes | down | GO:0018149;GO:0038023;GO:0045095;GO:0030246;GO:0031012;GO:1904813;GO:0061436;GO:0030280;GO:0005856;GO:0050728;GO:0070062;GO:0005634;GO:0016020;GO:0045765;GO:0072562;GO:0005198;GO:0001867;GO:0005882;GO:0051290;GO:0070268;GO:0062023;GO:0005886;GO:0001895;GO:0006979;GO:0001533;GO:0005615;GO:0042730;GO:0031424;GO:0005829;GO:0043312;GO:0005576;GO:0005515;GO:0046982 | hsa:3848 |  | keratin 1 [Homo sapiens] |
| P07360 | 43152.80158 | 34992.18875 | 0.810890312 | -0.302421318 | 0.005436117 | yes | down | GO:0019835;GO:0036094;GO:0030449;GO:0070062;GO:0045087;GO:0019841;GO:0072562;GO:0002376;GO:0001848;GO:0005576;GO:0044877;GO:0006957;GO:0006958;GO:0005579;GO:0005615 | hsa:733 |  | complement component C8 gamma chain precursor [Homo sapiens] |
| P13473 | 12021.42644 | 8175.757833 | 0.680098811 | -0.556183725 | 0.0001766 | yes | down | GO:0005770;GO:0061684;GO:0101003;GO:0019899;GO:0050821;GO:0044754;GO:0035577;GO:0010008;GO:1990836;GO:0043202;GO:0005615;GO:0097637;GO:0031410;GO:0016021;GO:0016020;GO:0097352;GO:0031902;GO:0046716;GO:0045121;GO:0006914;GO:0017038;GO:0006605;GO:1905146;GO:0061740;GO:0019904;GO:0009267;GO:0005886;GO:0070062;GO:0000421;GO:0030670;GO:0031647;GO:0043312;GO:0031088;GO:0005764;GO:0005765;GO:0005515;GO:0098857;GO:0005768;GO:0002576 | hsa:3920 |  | lysosome-associated membrane glycoprotein 2 isoform A precursor [Homo sapiens] |
| P13645 | 75944.19947 | 51498.59333 | 0.678110951 | -0.560406752 | 0.009936738 | yes | down | GO:0005737;GO:0018149;GO:0045684;GO:0070062;GO:0030216;GO:0031424;GO:0005615;GO:0009986;GO:0016020;GO:0070268;GO:0051290;GO:0005634;GO:0005882;GO:0005576;GO:0046982;GO:0005198;GO:0005829;GO:0001533;GO:0030280 | hsa:3858 |  | Keratin 10 [Homo sapiens] |
| P13647 | 37475.64211 | 24637.18833 | 0.657418711 | -0.605115577 | 0.000813923 | yes | down | GO:0005737;GO:0016020;GO:0005200;GO:0007010;GO:0070062;GO:0031424;GO:0031581;GO:0045095;GO:0070268;GO:0005198;GO:0005634;GO:0005882;GO:0097110;GO:0005515;GO:0005886;GO:0008544;GO:0005829 | hsa:3852 |  | Keratin 5 [Homo sapiens] |
| P15814 | 11435450.37 | 9216727.483 | 0.80597853 | -0.311186686 | 0.022347037 | yes | down | GO:0009897;GO:0050853;GO:0045087;GO:0016020;GO:0006910;GO:0006911;GO:0050900;GO:0005576;GO:0042742;GO:0003823;GO:0006955;GO:0042571;GO:0006958;GO:0050871;GO:0034987;GO:0072562 | hsa:3543 |  | immunoglobulin lambda-like polypeptide 1 isoform a precursor [Homo sapiens] |
| P19652 | 1147756.379 | 767476.8583 | 0.668675751 | -0.580621295 | 9.83E-07 | yes | down | GO:1904469;GO:0050716;GO:0031093;GO:0005615;GO:0002576;GO:0050718;GO:0043312;GO:0072562;GO:0006953;GO:0035578;GO:0062023;GO:0002682;GO:0035580;GO:0005576;GO:0070062 | hsa:5005 |  | alpha-1-acid glycoprotein 2 precursor [Homo sapiens] |
| P32119 | 22190.79347 | 15456.33191 | 0.69652002 | -0.521763274 | 0.023868191 | yes | down | GO:0000187;GO:0045581;GO:0042744;GO:0008379;GO:0032496;GO:0032088;GO:0042981;GO:0034599;GO:0002536;GO:0016491;GO:0005737;GO:0010310;GO:0070062;GO:0042098;GO:0043066;GO:0048538;GO:2001240;GO:0016209;GO:0045454;GO:0006979;GO:0055114;GO:0048872;GO:0045321;GO:0005829;GO:0005623;GO:0031665;GO:0019430;GO:0005515;GO:0051920;GO:0004601;GO:0030194 | hsa:7001 | COG0450 | peroxiredoxin-2 [Homo sapiens] |
| P35908 | 117316.6737 | 91746.86042 | 0.782044509 | -0.354677377 | 0.033927539 | yes | down | GO:0005200;GO:0045095;GO:0018149;GO:0008544;GO:0045684;GO:0043616;GO:0051546;GO:0070062;GO:0032980;GO:0005634;GO:0016020;GO:0005198;GO:0030280;GO:0070268;GO:0005882;GO:0001533;GO:0005615;GO:0045109;GO:0008092;GO:0031424;GO:0005829;GO:0003334;GO:0005515 | hsa:3849 |  | keratin, type II cytoskeletal 2 epidermal [Homo sapiens] |
| P36955 | 119127.5616 | 96083.02208 | 0.806555769 | -0.310153803 | 0.001404985 | yes | down | GO:0007614;GO:0030424;GO:0010629;GO:0060041;GO:0060770;GO:0007275;GO:0043203;GO:0005615;GO:0071300;GO:0010447;GO:0001822;GO:0050769;GO:0071333;GO:0048471;GO:0046685;GO:0043025;GO:0071279;GO:0008283;GO:0050728;GO:0062023;GO:0010976;GO:0004867;GO:0010951;GO:1901215;GO:0070062;GO:0042470;GO:0071549;GO:0005604;GO:0007568;GO:0010596;GO:0042698;GO:0005576;GO:0005515;GO:0016525 | hsa:5176 | COG4826 | pigment epithelium-derived factor isoform 1 precursor [Homo sapiens] |
| P55056 | 293654.3416 | 149800.1833 | 0.510124191 | -0.971079578 | 0.013574306 | yes | down | GO:0005319;GO:0034361;GO:0070328;GO:0006629;GO:0034447;GO:0006869;GO:0034379;GO:0010890;GO:0005576;GO:0034364 | hsa:346 |  | apolipoprotein C-IV precursor [Homo sapiens] |
| P61224 | 10046.39377 | 5872.168231 | 0.584505084 | -0.774712522 | 0.035843774 | yes | down | GO:0005811;GO:0030033;GO:0044877;GO:0007165;GO:0061028;GO:0030054;GO:0005737;GO:0070062;GO:2000114;GO:0016020;GO:0000166;GO:0070382;GO:0045955;GO:0035722;GO:0005525;GO:0008283;GO:0003924;GO:0035577;GO:0032486;GO:0005911;GO:0005886;GO:0045121;GO:0007264;GO:0005829;GO:0071320;GO:0043312;GO:0005622;GO:0019003;GO:0005515;GO:1901888;GO:0070374;GO:2000301 | hsa:5908 | COG1100 | Ras-related protein Rap-1b, partial [Bos mutus] |
| P61626 | 14455.72159 | 9940.730208 | 0.68766752 | -0.54021689 | 0.010174012 | yes | down | GO:0006954;GO:0019730;GO:0016798;GO:0003796;GO:0035578;GO:0042742;GO:0050829;GO:0019835;GO:0044267;GO:0070062;GO:0016998;GO:0035580;GO:0042802;GO:0016787;GO:1904724;GO:0003824;GO:0050830;GO:0008152;GO:0001895;GO:0005615;GO:0031640;GO:0043312;GO:0005576 | hsa:4069 |  | lysozyme C precursor [Homo sapiens] |
| P80748 | 678144.7579 | 236511.5604 | 0.348762646 | -1.519682564 | 1.84E-10 | yes | down | GO:0038096;GO:0004252;GO:0016020;GO:0030449;GO:0050776;GO:0006898;GO:0038095;GO:0072562;GO:0002376;GO:0050900;GO:0005576;GO:0006956;GO:0002250;GO:0003823;GO:0005886;GO:0006508;GO:0006958;GO:0006955;GO:0070062 | hsa:7441 |  | hCG2040021, partial [Homo sapiens] |
| Q86YZ3 | 5980.4445 | 2638.716667 | 0.441224171 | -1.180416269 | 0.004894938 | yes | down | GO:0005737;GO:0070062;GO:0046914;GO:0031424;GO:0005634;GO:0043163;GO:0043312;GO:0007275;GO:0035578;GO:0062023;GO:0061436;GO:0005509;GO:0005576;GO:0048471;GO:0046872;GO:0036457;GO:0001533;GO:0030280 | hsa:388697 | | TPA: Hornerin [Homo sapiens] |
| Q8N1N4 | 172097.1737 | 130298.3475 | 0.757120787 | -0.401404617 | 0.011501879 | yes | down | GO:0005615;GO:0031424;GO:0005829;GO:0045095;GO:0070268;GO:0005198;GO:0005882;GO:0070062 | hsa:196374 | | keratin, type II cytoskeletal 78 isoform 1 [Homo sapiens] |
| Q96HR3 | 35719.59263 | 15002.38996 | 0.420004509 | -1.251523279 | 6.72E-07 | yes | down | GO:0019827;GO:0030521;GO:0038023;GO:0005515;GO:0006355;GO:0005634;GO:0030518;GO:0003712;GO:0000151;GO:0005654;GO:0046966;GO:0045893;GO:0006367;GO:0016567;GO:0016592;GO:0030374;GO:0061630;GO:0042809;GO:0006351 | hsa:90390 |  | mediator of RNA polymerase II transcription subunit 30 isoform 1 [Homo sapiens] |
| Q96IY4 | 27470.42211 | 19314.74542 | 0.703110616 | -0.508176417 | 9.11E-06 | yes | down | GO:0007599;GO:2000346;GO:0007596;GO:0008270;GO:0030449;GO:0008233;GO:0008237;GO:0005615;GO:0004181;GO:0004180;GO:0071333;GO:0006508;GO:0046872;GO:0051918;GO:0016787;GO:0009408;GO:0070062;GO:0042730;GO:0003331;GO:0097421;GO:0042493;GO:0005623;GO:0005576;GO:0010757 | hsa:1361 | COG2866 | carboxypeptidase B2 isoform 1 preproprotein [Homo sapiens] |
| Q9Y5Y7 | 16941.61205 | 8747.742478 | 0.516346523 | -0.953588503 | 0.000641998 | yes | down | GO:0038023;GO:0070062;GO:0005540;GO:0016021;GO:0016020;GO:0009611;GO:0007160;GO:0004888;GO:0071944;GO:0005515;GO:0005887;GO:0005886;GO:0007155;GO:0030214;GO:0009653;GO:0006027 | hsa:10894 |  | lymphatic vessel endothelial hyaluronic acid receptor 1 precursor [Homo sapiens] |
| A0A0G2JSC0 | 30103.95338 | 49210.14461 | 1.634673825 | 0.709002796 | 0.066478472 | no | up |  | hsa:7441 |  | Lambda-V immunoglobulin light chain variable domain precursor, partial [Homo sapiens] |
| D6RE82 | 209939.3632 | 2667021.277 | 12.70376949 | 3.667184736 | 0.338541164 | no | up | GO:0030688;GO:0005730;GO:0005634;GO:0030687;GO:0006364;GO:0003723 | hsa:8568 |  | PREDICTED: ribosomal RNA processing protein 1 homolog A [Callithrix jacchus] |
| P01602 | 182768.7774 | 313495.2904 | 1.715256265 | 0.778424136 | 0.140115452 | no | up | GO:0038096;GO:0004252;GO:0016020;GO:0030449;GO:0050776;GO:0006898;GO:0038095;GO:0072562;GO:0002376;GO:0002377;GO:0050900;GO:0005576;GO:0006956;GO:0002250;GO:0003823;GO:0005886;GO:0006508;GO:0006958;GO:0006955;GO:0070062 | hsa:7441 |  | hCG2043208, partial [Homo sapiens] |
| P01705 | 88650.06882 | 120145.3343 | 1.355276266 | 0.438586968 | 0.180963239 | no | up | GO:0038096;GO:0004252;GO:0016020;GO:0030449;GO:0050776;GO:0006898;GO:0038095;GO:0002376;GO:0050900;GO:0005576;GO:0006956;GO:0002250;GO:0003823;GO:0005886;GO:0006508;GO:0006958;GO:0006955 | hsa:7441 |  | immunoglobulin lambda-chain, partial [Homo sapiens] |
| P01742 | 1110117.079 | 1630025.25 | 1.468336342 | 0.554182474 | 0.334511795 | no | up | GO:0038096;GO:0004252;GO:0016020;GO:0030449;GO:0050776;GO:0006898;GO:0038095;GO:0002376;GO:0050900;GO:0005576;GO:0006956;GO:0002250;GO:0003823;GO:0005886;GO:0006508;GO:0006958;GO:0006955 | hsa:102724971 | | IgM heavy chain VH1 region precursor, partial [Homo sapiens] |
| P01877 | 206673.0384 | 264324.4792 | 1.278949984 | 0.354959846 | 0.569466043 | no | up | GO:0009897;GO:0019731;GO:0006955;GO:0050900;GO:0034987;GO:0001895;GO:0005615;GO:0016020;GO:0003094;GO:0072562;GO:0071748;GO:0050853;GO:0045087;GO:0006910;GO:0006911;GO:0003823;GO:0005886;GO:0006958;GO:0050871;GO:0060267;GO:0070062;GO:0006898;GO:0002376;GO:0005576;GO:0002250;GO:0071752;GO:0071751 | hsa:55423 |  | RecName: Full=Immunoglobulin heavy constant alpha 2; AltName: Full=Ig alpha-2 chain C region; AltName: Full=Ig alpha-2 chain C region BUT; AltName: Full=Ig alpha-2 chain C region LAN |
| P02753 | 55930.01158 | 70244.31042 | 1.255932342 | 0.328758747 | 0.069062504 | no | up | GO:0060347;GO:0032024;GO:0048562;GO:0030324;GO:0060059;GO:0060044;GO:0042593;GO:0051024;GO:0060065;GO:0042572;GO:0001523;GO:0016918;GO:0005615;GO:0048807;GO:0006094;GO:0005501;GO:0032526;GO:0048706;GO:0007601;GO:0045471;GO:0001654;GO:0034633;GO:0070062;GO:0007507;GO:0032991;GO:0060157;GO:0005829;GO:0019841;GO:0048738;GO:0005576;GO:0005515;GO:0060068;GO:0034632;GO:0046982;GO:0050896;GO:0030277 | hsa:5950 |  | retinol-binding protein 4 isoform a precursor [Homo sapiens] |
| P02776 | 35069.36644 | 44828.62167 | 1.278284332 | 0.354208775 | 0.348226062 | no | up | GO:0020005;GO:0070098;GO:0005125;GO:0071222;GO:0019221;GO:0008009;GO:0010469;GO:0032760;GO:0010628;GO:0048248;GO:0032496;GO:0045918;GO:0061844;GO:0005737;GO:0045347;GO:0031093;GO:0002576;GO:0007189;GO:0051873;GO:0010744;GO:0007186;GO:2001240;GO:0042127;GO:0030595;GO:0030593;GO:0006935;GO:0043950;GO:0006952;GO:0062023;GO:0006954;GO:0006955;GO:0008201;GO:0030168;GO:0005615;GO:0090023;GO:0045651;GO:0045652;GO:0045653;GO:0031640;GO:0005576;GO:0045944;GO:0005515;GO:0097679;GO:0042832;GO:0016525 | hsa:5196 |  | platelet factor 4 isoform 1 precursor [Homo sapiens] |
| P07737 | 11005.68311 | 14944.56517 | 1.357895282 | 0.441372227 | 0.190824339 | no | up | GO:0017048;GO:0050821;GO:0060074;GO:0045202;GO:0050434;GO:0005925;GO:0005737;GO:0032232;GO:0032233;GO:0070062;GO:0030837;GO:0030838;GO:0051496;GO:0005634;GO:0016020;GO:0005546;GO:0003779;GO:0098794;GO:0043005;GO:0098793;GO:0001843;GO:0005856;GO:0051054;GO:0098685;GO:0005515;GO:0032781;GO:0060071;GO:0098688;GO:0070064;GO:0005938;GO:0000774;GO:0010033;GO:0010634;GO:0071363;GO:0005829;GO:1900029;GO:0098885;GO:0045296;GO:0051497;GO:0006357;GO:0045944;GO:0003723;GO:0030036;GO:0072562;GO:0005102;GO:0098978;GO:0003785 | hsa:5216 |  | PREDICTED: profilin-1 isoform X2 [Nomascus leucogenys] |
| P07996 | 22059.63353 | 29556.91033 | 1.339864069 | 0.422086645 | 0.334276208 | no | up | GO:0032026;GO:0005783;GO:0050840;GO:0034605;GO:0005788;GO:2001237;GO:0050921;GO:1903671;GO:0048266;GO:0051895;GO:0006986;GO:0043652;GO:0042535;GO:0009612;GO:0030511;GO:0002581;GO:0042327;GO:0051592;GO:0043536;GO:0040037;GO:2001027;GO:0030169;GO:0043236;GO:0045727;GO:0045652;GO:0030198;GO:0043032;GO:0030194;GO:0009897;GO:0018149;GO:0042493;GO:0002605;GO:0071356;GO:0051897;GO:0016477;GO:0002040;GO:0001968;GO:2000379;GO:0030141;GO:0007050;GO:0043394;GO:0006954;GO:0006955;GO:0070052;GO:0090051;GO:0070051;GO:0033574;GO:0043154;GO:0034976;GO:1902043;GO:0031012;GO:0001666;GO:0050431;GO:0005737;GO:0031091;GO:0031093;GO:0005509;GO:0010763;GO:0001786;GO:0051918;GO:0008284;GO:0009986;GO:0017134;GO:2000353;GO:0008201;GO:0071363;GO:0001953;GO:0002544;GO:0016529;GO:0042802;GO:0005515;GO:0032570;GO:0016525;GO:0000187;GO:0005201;GO:0005615;GO:0002576;GO:0010751;GO:0045766;GO:0043066;GO:0010748;GO:0032695;GO:1903588;GO:0005178;GO:0030335;GO:0048661;GO:0032914;GO:0062023;GO:0007155;GO:0070062;GO:0043537;GO:0010595;GO:0010596;GO:0009749;GO:0005577;GO:0005576;GO:0010757;GO:0010754;GO:0010759;GO:0001937;GO:0071636 | hsa:7057 |  | thrombospondin-1 precursor [Homo sapiens] |
| P0DTE1 | 16825.00479 | 50622.72045 | 3.008778963 | 1.589178124 | 0.171369051 | no | up |  | hsa:102723407 | | immunoglobulin heavy chain variable region, partial [Homo sapiens] |
| P13796 | 685041.9409 | 1606692.123 | 2.345392344 | 1.229829281 | 0.27579646 | no | up | GO:0032432;GO:0033157;GO:0015629;GO:0030175;GO:0051020;GO:0005925;GO:0030054;GO:0005737;GO:0071803;GO:0001726;GO:0002102;GO:0002286;GO:0005615;GO:0016020;GO:0003779;GO:0005509;GO:0048471;GO:0016477;GO:0022617;GO:0042802;GO:0035722;GO:0005178;GO:0031100;GO:0051017;GO:0051015;GO:0005884;GO:0005886;GO:0051764;GO:0042995;GO:0032587;GO:0001891;GO:0046872;GO:0070062;GO:0005829;GO:0005856;GO:0051639;GO:0044319;GO:0001725;GO:0010737 | hsa:3936 | COG5069 | plastin-2 [Homo sapiens] |
| P59665 | 93970.19158 | 200544.0638 | 2.134124241 | 1.093644167 | 0.176967572 | no | up | GO:0071222;GO:0019730;GO:0019731;GO:0035578;GO:0051673;GO:0042742;GO:0050829;GO:0061844;GO:0005615;GO:0030520;GO:0051852;GO:0044657;GO:0002227;GO:0042803;GO:0010818;GO:0005796;GO:0006935;GO:0006952;GO:0050830;GO:0062023;GO:0006955;GO:0070062;GO:0050832;GO:0051607;GO:0031640;GO:0043312;GO:0005576;GO:0042832 | hsa:1667;hsa:728358;hsa:1668 | | neutrophil defensin 1 preproprotein [Homo sapiens] |
| P60709 | 33189.38895 | 41243.69096 | 1.24267702 | 0.313451379 | 0.397054574 | no | up | GO:0043044;GO:0019894;GO:0015629;GO:0072749;GO:0061024;GO:0005200;GO:0021762;GO:0036464;GO:0005925;GO:0048488;GO:0030957;GO:0032091;GO:0005615;GO:0043209;GO:0005634;GO:0016020;GO:0097433;GO:0070062;GO:0098871;GO:0005654;GO:0045815;GO:0016579;GO:0005524;GO:0000980;GO:0005856;GO:0070527;GO:0000166;GO:1903076;GO:0019901;GO:0000790;GO:0005886;GO:0048013;GO:0001895;GO:0098685;GO:0034329;GO:0031982;GO:0038096;GO:0050998;GO:0044305;GO:0032991;GO:0051621;GO:0048870;GO:0030863;GO:0022898;GO:0005829;GO:0051623;GO:1990904;GO:0005737;GO:0098973;GO:0031492;GO:0098793;GO:0042802;GO:0005515;GO:0098974;GO:0072562;GO:0000978;GO:0098978;GO:0000079;GO:0035267 | hsa:71;hsa:60 | COG5277 | cytoskeletal beta actin, partial [Sus scrofa] |
| Q13201 | 36145.25167 | 44074.11227 | 1.219361057 | 0.286125376 | 0.378597744 | no | up | GO:0005201;GO:0031093;GO:0010811;GO:0002576;GO:0007596;GO:0031012;GO:0005576;GO:0005509;GO:0005515;GO:0007155;GO:0062023 | hsa:22915 |  | multimerin-1 precursor [Homo sapiens] |
| Q86UX7 | 5055.186455 | 7383.9592 | 1.46067 | 0.546630276 | 0.186603621 | no | up | GO:0005178;GO:0030335;GO:0002102;GO:0070062;GO:0031093;GO:0034446;GO:0007229;GO:0016020;GO:0033632;GO:0005576;GO:0007159;GO:0007155;GO:0033622;GO:0042995;GO:0070527;GO:0030054;GO:0002576 | hsa:83706 |  | fermitin family homolog 3 long isoform [Homo sapiens] |
| Q8IV42 | 84821.05625 | 106398.617 | 1.254389201 | 0.326985045 | 0.179128575 | no | up | GO:0000049;GO:0016310;GO:0016301;GO:0097056;GO:0016740;GO:0001514;GO:0000166;GO:0006412;GO:0005524 | hsa:118672 | | L-seryl-tRNA(Sec) kinase isoform 2 [Homo sapiens] |
| Q9Y490 | 10605.37706 | 36324.60655 | 3.425112218 | 1.776151256 | 0.247522621 | no | up | GO:0005200;GO:0017166;GO:0007043;GO:0005925;GO:0030054;GO:0005856;GO:0001726;GO:0070062;GO:0002576;GO:0016020;GO:0005737;GO:0003779;GO:0005576;GO:0033622;GO:0001786;GO:0005178;GO:0005515;GO:0006936;GO:0009986;GO:0051015;GO:0036498;GO:0030274;GO:0005886;GO:0007155;GO:0042995;GO:0032587;GO:0007016;GO:0035091;GO:0005829;GO:0030866;GO:0045296;GO:0070527;GO:0007044;GO:0016032;GO:0044877;GO:0007229 | hsa:7094 |  | talin-1 [Homo sapiens] |
| A0A075B6H7 | 138596.2984 | 138915.8046 | 1.002305301 | 0.003322018 | 0.986309573 | no | no change | GO:0002377;GO:0005615;GO:0006955 | hsa:7441 |  | hCG2043206, partial [Homo sapiens] |
| A0A075B6J9 | 86557.38105 | 76612.62958 | 0.88510799 | -0.176074609 | 0.33085704 | no | no change | GO:0016020;GO:0005615;GO:0006955;GO:0002376;GO:0002377;GO:0005576;GO:0002250;GO:0003823;GO:0005886 | hsa:7441 |  | RecName: Full=Immunoglobulin lambda variable 2-18; Flags: Precursor |
| A0A075B6K2 | 58179.6602 | 60776.27556 | 1.044630982 | 0.062993397 | 0.917033626 | no | no change | GO:0016020;GO:0005615;GO:0006955;GO:0002376;GO:0002377;GO:0005576;GO:0002250;GO:0003823;GO:0005886 | hsa:7441 |  | hCG2041210, partial [Homo sapiens] |
| A0A075B6K4 | 57859.10211 | 49739.26917 | 0.859661961 | -0.218158625 | 0.339957256 | no | no change | GO:0016020;GO:0005615;GO:0006955;GO:0002376;GO:0002377;GO:0005576;GO:0002250;GO:0003823;GO:0005886 | hsa:7441 |  | immunoglobulin light chain variable region, partial [Homo sapiens] |
| A0A075B6K5 | 21206.54655 | 21335.71587 | 1.006091012 | 0.008760818 | 0.989618982 | no | no change | GO:0016020;GO:0005615;GO:0006955;GO:0002376;GO:0002377;GO:0005576;GO:0002250;GO:0003823;GO:0005886 | hsa:7441 |  | RecName: Full=Immunoglobulin lambda variable 3-9; Flags: Precursor |
| A0A075B6S5 | 48086.14368 | 55566.12583 | 1.155553795 | 0.208584424 | 0.23631134 | no | no change | GO:0016020;GO:0005615;GO:0006955;GO:0002376;GO:0002377;GO:0005576;GO:0002250;GO:0003823;GO:0005886 | hsa:7441 |  | monoclonal IgM antibody light chain [Homo sapiens] |
| A0A075B7D0 | 336157.6105 | 280345.8871 | 0.833971561 | -0.261929908 | 0.32190521 | no | no change | GO:0009897;GO:0050853;GO:0045087;GO:0006910;GO:0006911;GO:0042742;GO:0003823;GO:0034987;GO:0042571;GO:0006958;GO:0050871;GO:0072562 | hsa:102723407 | | hCG1728627 [Homo sapiens] |
| A0A075B7F0 | 23855.47089 | 26816.23604 | 1.124112626 | 0.168786588 | 0.575556468 | no | no change | GO:0009897;GO:0050853;GO:0045087;GO:0006910;GO:0006911;GO:0042742;GO:0003823;GO:0034987;GO:0042571;GO:0006958;GO:0050871;GO:0072562 | hsa:102723407 | | IGHV3-13 isoform 1, partial [Pan troglodytes] |
| A0A087WSY6 | 205787.9 | 191836.3392 | 0.932204173 | -0.101282124 | 0.682774897 | no | no change | GO:0016020;GO:0005615;GO:0006955;GO:0002376;GO:0002377;GO:0005576;GO:0002250;GO:0003823;GO:0005886 | hsa:29802 |  | RecName: Full=Immunoglobulin kappa variable 3D-15; Flags: Precursor |
| A0A087WSZ0 | 53286.55 | 47885.78267 | 0.898646707 | -0.154174048 | 0.698517645 | no | no change | GO:0016020;GO:0005615;GO:0006955;GO:0002376;GO:0002377;GO:0005576;GO:0002250;GO:0003823;GO:0005886 | | | RecName: Full=Immunoglobulin kappa variable 1D-8; Flags: Precursor |
| A0A087X0Q4 | 130877.2211 | 126820.3292 | 0.969002307 | -0.045427994 | 0.801673853 | no | no change |  |  |  | RecName: Full=Immunoglobulin kappa variable 2D-40; AltName: Full=Ig kappa chain V-II region Cum; Flags: Precursor |
| A0A087X1J7 | 47417.00632 | 43706.02333 | 0.921737299 | -0.117572463 | 0.408328528 | no | no change | GO:0008430;GO:0005615;GO:0051289;GO:0006979;GO:0098869;GO:0055114;GO:0004601;GO:0004602;GO:0016491 | hsa:2878 | COG0386 | glutathione peroxidase 3 isoform 1 precursor [Homo sapiens] |
| A0A087X1L8 | 2540.625867 | 2639.587619 | 1.038951722 | 0.055128617 | 0.7517361 | no | no change | GO:0016021;GO:0016020 | hsa:102723996;hsa:23308 | | ICOS ligand isoform c precursor [Homo sapiens] |
| A0A0A0MRJ7 | 20750.63968 | 23400.44375 | 1.127697464 | 0.173380077 | 0.242656427 | no | no change | GO:0048208;GO:0044267;GO:1903561;GO:0006888;GO:0005615;GO:0031093;GO:0008015;GO:0002576;GO:0007596;GO:0033116;GO:0005788;GO:0030134;GO:0046872;GO:0005576;GO:0005886;GO:0016020;GO:0005507;GO:0043687;GO:0000139 | hsa:2153 |  | coagulation factor V preproprotein [Homo sapiens] |
| A0A0A0MT36 | 116382.66 | 129700.0317 | 1.114427456 | 0.156302707 | 0.625108193 | no | no change | GO:0016020;GO:0005615;GO:0006955;GO:0002376;GO:0002377;GO:0005576;GO:0002250;GO:0003823;GO:0005886 | hsa:7441 |  | RecName: Full=Immunoglobulin kappa variable 6D-21; Flags: Precursor |
| A0A0B4J1U7 | 59076.67632 | 57424.72708 | 0.972037201 | -0.040916567 | 0.898972022 | no | no change | GO:0009897;GO:0050853;GO:0045087;GO:0016020;GO:0006910;GO:0006911;GO:0002376;GO:0005576;GO:0002250;GO:0003823;GO:0005886;GO:0072562;GO:0042571;GO:0006958;GO:0050871;GO:0034987;GO:0042742 | hsa:102723407 | | RecName: Full=Immunoglobulin heavy variable 6-1; Flags: Precursor |
| A0A0B4J1X8 | 99827.09421 | 94104.9375 | 0.942679322 | -0.085161012 | 0.687619645 | no | no change | GO:0009897;GO:0050853;GO:0045087;GO:0016020;GO:0006910;GO:0006911;GO:0002376;GO:0005576;GO:0002250;GO:0003823;GO:0005886;GO:0072562;GO:0042571;GO:0006958;GO:0050871;GO:0034987;GO:0042742 | hsa:102723407 | | RecName: Full=Immunoglobulin heavy variable 3-43; Flags: Precursor |
| A0A0B4J1Y8 | 106914.9979 | 124365.8168 | 1.163221431 | 0.218125754 | 0.43155464 | no | no change | GO:0016020;GO:0005615;GO:0006955;GO:0002376;GO:0002377;GO:0005576;GO:0002250;GO:0003823;GO:0005886 | hsa:7441 |  | Unknown (protein for IMAGE:4575521), partial [Homo sapiens] |
| A0A0B4J231 | 11148517 | 13135041 | 1.178187287 | 0.236568891 | 0.129463346 | no | no change | GO:0009897;GO:0050853;GO:0070062;GO:0045087;GO:0006910;GO:0006911;GO:0042742;GO:0003823;GO:0034987;GO:0042571;GO:0006958;GO:0050871 | hsa:100423062 | | immunoglobulin lambda-3 surrogate light chain [Homo sapiens] |
| A0A0B4J2D9 | 15440.17733 | 13928.09432 | 0.902068287 | -0.148691445 | 0.493583406 | no | no change | GO:0016020;GO:0005615;GO:0006955;GO:0002376;GO:0002377;GO:0005576;GO:0002250;GO:0003823;GO:0005886 | hsa:7441 |  | RecName: Full=Immunoglobulin kappa variable 1D-13; Flags: Precursor |
| A0A0C4DGZ8 | 15936.75382 | 15971.72671 | 1.00219448 | 0.003162496 | 0.990955643 | no | no change | GO:0016021;GO:0016020 | hsa:2811 | COG4886 | glycoprotein Ib (platelet), alpha polypeptide [Homo sapiens] |
| A0A0C4DH24 | 40461.87842 | 34415.31354 | 0.850561439 | -0.233512644 | 0.462763152 | no | no change | GO:0016020;GO:0005615;GO:0006955;GO:0002376;GO:0002377;GO:0005576;GO:0002250;GO:0003823;GO:0005886 | hsa:7441 |  | RecName: Full=Immunoglobulin kappa variable 6-21; Flags: Precursor |
| A0A0C4DH29 | 32525.15347 | 32287.37333 | 0.992689346 | -0.010585786 | 0.956991559 | no | no change | GO:0009897;GO:0050853;GO:0045087;GO:0016020;GO:0006910;GO:0006911;GO:0002376;GO:0005576;GO:0002250;GO:0003823;GO:0005886;GO:0072562;GO:0042571;GO:0006958;GO:0050871;GO:0034987;GO:0042742 | hsa:102723407 | | immunoglobulin heavy chain variable region, partial [Homo sapiens] |
| A0A0C4DH31 | 26625.47789 | 31109.19917 | 1.168399654 | 0.224533836 | 0.068220699 | no | no change | GO:0009897;GO:0050853;GO:0045087;GO:0016020;GO:0006910;GO:0006911;GO:0002376;GO:0005576;GO:0002250;GO:0003823;GO:0005886;GO:0072562;GO:0042571;GO:0006958;GO:0050871;GO:0034987;GO:0042742 | hsa:102723407 | | immunoglobulin heavy chain variable region, partial [Homo sapiens] |
| A0A0C4DH32 | 74133.90813 | 79619.69304 | 1.073998324 | 0.102991741 | 0.643457371 | no | no change | GO:0009897;GO:0050853;GO:0045087;GO:0016020;GO:0006910;GO:0006911;GO:0002376;GO:0005576;GO:0002250;GO:0003823;GO:0005886;GO:0072562;GO:0042571;GO:0006958;GO:0050871;GO:0034987;GO:0042742 | hsa:102723407 | | RecName: Full=Immunoglobulin heavy variable 3-20; Flags: Precursor |
| A0A0C4DH34 | 941572.7789 | 901875.0667 | 0.957838934 | -0.062145016 | 0.778174402 | no | no change | GO:0009897;GO:0050853;GO:0045087;GO:0016020;GO:0006910;GO:0006911;GO:0002376;GO:0005576;GO:0002250;GO:0003823;GO:0005886;GO:0072562;GO:0042571;GO:0006958;GO:0050871;GO:0034987;GO:0042742 | hsa:102723407 | | RecName: Full=Immunoglobulin heavy variable 4-28; Flags: Precursor |
| A0A0C4DH39 | 29197.24784 | 28223.76042 | 0.966658247 | -0.048922166 | 0.88084134 | no | no change | GO:0009897;GO:0050853;GO:0045087;GO:0016020;GO:0006910;GO:0006911;GO:0002376;GO:0005576;GO:0002250;GO:0003823;GO:0005886;GO:0072562;GO:0042571;GO:0006958;GO:0050871;GO:0034987;GO:0042742 | hsa:102723407 | | immunoglobulin heavy chain variable region, partial [Homo sapiens] |
| A0A0C4DH55 | 8471991.474 | 8138870.833 | 0.960679772 | -0.057872485 | 0.615186037 | no | no change | GO:0016020;GO:0005615;GO:0006955;GO:0002376;GO:0002377;GO:0005576;GO:0002250;GO:0003823;GO:0005886 | hsa:29802 |  | RecName: Full=Immunoglobulin kappa variable 3D-7; Flags: Precursor |
| A0A0G2JMB2 | 2454396.105 | 2504752.125 | 1.020516664 | 0.02929974 | 0.87543325 | no | no change | GO:0009897;GO:0050853;GO:0045087;GO:0006910;GO:0006911;GO:0042742;GO:0003823;GO:0034987;GO:0042571;GO:0006958;GO:0050871;GO:0072562 | hsa:55423 |  | Immunoglobulin heavy chain variant, partial [Homo sapiens] |
| A0A0G2JPR0 | 503231.4947 | 441040.4542 | 0.876416637 | -0.190311224 | 0.358599649 | no | no change | GO:0004866;GO:0005576;GO:0006956;GO:0006954;GO:0010951;GO:0005615 | hsa:100293534;hsa:110384692;hsa:720;hsa:721 | | complement C4A (Rodgers blood group)-like preproprotein [Homo sapiens] |
| A0A0J9YVY3 | 48104.57 | 52176.18824 | 1.084640986 | 0.117217592 | 0.590232676 | no | no change | GO:0016020;GO:0002376;GO:0005576;GO:0002250;GO:0003823;GO:0005886 | hsa:102723407 | | RecName: Full=Immunoglobulin heavy variable 7-4-1; Flags: Precursor |
| A0A0J9YY99 | 222303.1363 | 212404.6646 | 0.95547309 | -0.065712853 | 0.929438348 | no | no change |  | hsa:102723407 | | immunoglobulin heavy chain VDJ region, partial [Homo sapiens] |
| A0A0S2Z4L3 | 194502.2211 | 199108.0708 | 1.023680191 | 0.033765072 | 0.769985165 | no | no change | GO:0005576;GO:0005509;GO:0030195 | hsa:5627 |  | vitamin K-dependent protein S isoform 1 precursor [Homo sapiens] |
| A0A182DWH7 | 33602.84316 | 32971.73625 | 0.981218646 | -0.027353446 | 0.823893989 | no | no change | GO:0008430 | hsa:6414 |  | Selenoprotein P, plasma, 1 [Homo sapiens] |
| A0A286YEY1 | 4509518.632 | 4794880.125 | 1.063279812 | 0.088521306 | 0.732588895 | no | no change |  |  |  | IGHA1 isoform 1, partial [Pan troglodytes] |
| A0A2R8Y7X9 | 47762.113 | 44102.83375 | 0.923385315 | -0.114995306 | 0.665550326 | no | no change | GO:0005344;GO:0019825;GO:0020037;GO:0005833;GO:0046872;GO:0015671 | hsa:3048 | COG1018 | hemoglobin subunit gamma-2 [Pan troglodytes] |
| A0A3B3ISR2 | 308661.1105 | 277518.2125 | 0.899103266 | -0.15344127 | 0.232447782 | no | no change | GO:0004252;GO:0005615;GO:0045087;GO:0031638;GO:0005509;GO:0006958 | hsa:715 | COG5640 | complement C1r subcomponent isoform 1 preproprotein [Homo sapiens] |
| A0A4W8ZXM2 | 817147.1153 | 809666.7421 | 0.990845745 | -0.013267619 | 0.946417701 | no | no change |  | hsa:102723407 | | immunoglobulin heavy chain variable region, partial [Homo sapiens] |
| A0A5H1ZRQ7 | 20376.10105 | 19808.58905 | 0.972148155 | -0.040751898 | 0.825429645 | no | no change |  | hsa:100423062 | | RecName: Full=Immunoglobulin lambda constant 7; AltName: Full=Ig lambda-7 chain C region |
| A6XND0 | 28409.03947 | 29247.19583 | 1.029503157 | 0.041948254 | 0.774311047 | no | no change | GO:0005520;GO:0001558;GO:0005576 | hsa:3486 |  | insulin-like growth factor binding protein 3 [Homo sapiens] |
| B0YIW2 | 1944324.595 | 1884424.854 | 0.96919252 | -0.045144824 | 0.891874022 | no | no change | GO:0034375;GO:0042627;GO:0034371;GO:0006869;GO:0034379;GO:0034378;GO:0051005;GO:0042157;GO:0005576;GO:0048261;GO:0001523;GO:0055102;GO:0070062;GO:0005543;GO:0045833;GO:0010897;GO:0007186;GO:0062023;GO:0033700;GO:0034361;GO:0034363;GO:0070328;GO:0034366;GO:0006641;GO:0060621;GO:0030234;GO:0070653;GO:0008289;GO:0010916;GO:0042632;GO:0032489;GO:0043691;GO:0005615;GO:0015485;GO:0034382;GO:0050995;GO:0019433;GO:0033344;GO:0010989;GO:0010987;GO:0005769;GO:0045717;GO:0010903 | hsa:345 |  | apolipoprotein C-III precursor variant 1 [Homo sapiens] |
| B1AHL2 | 16086.271 | 14313.19365 | 0.889776981 | -0.168484319 | 0.346753082 | no | no change | GO:0016504;GO:0005576;GO:0005509;GO:0010952;GO:0030198 | hsa:2192 |  | FBLN1 isoform 5 [Pongo abelii] |
| B4E1Z4 | 548596.7158 | 496005.725 | 0.904135425 | -0.145389213 | 0.08102549 | no | no change | GO:0004252;GO:0030449;GO:0070062;GO:0016787;GO:0072562;GO:0001848;GO:0005576;GO:0006956;GO:0006957;GO:0005886;GO:0006508;GO:0008233;GO:0008236;GO:0005615 | hsa:629 | COG5640 | unnamed protein product [Homo sapiens] |
| C9JC84 | 4693296.895 | 5148328.417 | 1.096953492 | 0.13350236 | 0.111956134 | no | no change | GO:0051258;GO:0005102;GO:0007596;GO:0005577;GO:0030168 | hsa:2266 |  | FGG isoform 6 [Pan troglodytes] |
| C9JF17 | 842503.8 | 919032.8292 | 1.090835233 | 0.125433204 | 0.327088524 | no | no change | GO:0022626;GO:0005783;GO:0006869;GO:0042493;GO:0048678;GO:0030425;GO:0000302;GO:0001525;GO:0051895;GO:0010642;GO:0005737;GO:0070062;GO:0014012;GO:0005615;GO:2000405;GO:0048471;GO:0043025;GO:0005319;GO:0048662;GO:0006629;GO:2000098;GO:0008289;GO:0042308;GO:0060588;GO:0007420;GO:1900016;GO:0007568;GO:0015485;GO:0042246;GO:0071638;GO:0005576;GO:0006006 | hsa:347 | COG3040 | APOD isoform 3, partial [Pan troglodytes] |
| C9JV77 | 1898157.316 | 2053469 | 1.081822346 | 0.113463603 | 0.14752989 | no | no change | GO:0019210;GO:0006907;GO:0005788;GO:0001501;GO:0030500;GO:0030502;GO:0050766;GO:0044267;GO:0070062;GO:0031093;GO:0002576;GO:0050727;GO:0072562;GO:0005794;GO:0043687;GO:0046627;GO:0006953;GO:0062023;GO:0034774;GO:0010951;GO:0004869;GO:0005615;GO:0043312;GO:0005576 | hsa:197 |  | alpha-2-HS-glycoprotein isoform 1 preproprotein [Homo sapiens] |
| D6R934 | 95974.26 | 96657.57542 | 1.007119778 | 0.010235275 | 0.894534206 | no | no change | GO:0006958;GO:0005576;GO:0005581 | hsa:713 |  | complement C1q subcomponent subunit B precursor [Homo sapiens] |
| E7EUT5 | 9026.696316 | 10620.43657 | 1.176558532 | 0.234573094 | 0.238035859 | no | no change | GO:0051287;GO:0050821;GO:0000226;GO:0097718;GO:0006096;GO:0061844;GO:0005737;GO:0004365;GO:0005634;GO:0005811;GO:0016620;GO:0051873;GO:0097452;GO:0042802;GO:0035605;GO:0035606;GO:0015630;GO:0008017;GO:0031965;GO:0050661;GO:0050832;GO:0051402;GO:0052501;GO:0005886;GO:0055114;GO:0043231;GO:0050715;GO:0005829;GO:0071346;GO:1990904;GO:0019828;GO:0017148;GO:0006417;GO:0006006 | hsa:2597 | COG0057 | GAPDH isoform 4 [Pan troglodytes] |
| E9PAQ1 | 27865.30579 | 27897.92625 | 1.001170648 | 0.0016879 | 0.98520898 | no | no change |  | hsa:5199 |  | properdin precursor [Homo sapiens] |
| E9PHK0 | 142852.4105 | 149272.3833 | 1.044941298 | 0.063421898 | 0.391381067 | no | no change | GO:0005737;GO:0036143;GO:0070062;GO:0002576;GO:0001652;GO:0030246;GO:0008201;GO:0071560;GO:0071310;GO:0001503;GO:0005615;GO:0005509;GO:0005576;GO:0031089;GO:0030282;GO:0062023;GO:0010756 | hsa:7123 |  | tetranectin isoform 1precursor [Homo sapiens] |
| F5H8B0 | 6778.0535 | 6175.647174 | 0.911123994 | -0.134280693 | 0.265790627 | no | no change | GO:0004252;GO:0016787;GO:0007596;GO:0005576;GO:0005509;GO:0006508;GO:0008233;GO:0008236 | hsa:2155 | COG5640 | coagulation factor VII isoform c precursor [Homo sapiens] |
| F8WF14 | 25632.02316 | 26077.91 | 1.017395694 | 0.024880894 | 0.843585372 | no | no change | GO:0019899;GO:0051384;GO:0005783;GO:0016787;GO:0007612;GO:0050805;GO:0050783;GO:0014016;GO:0001540;GO:0016021;GO:0016020;GO:0072562;GO:0042802;GO:0008285;GO:0004104;GO:0003824;GO:0005788;GO:0043279;GO:0051593;GO:0019695;GO:0005641;GO:0005576;GO:0033265;GO:0003990 | hsa:590 | COG2272 | unnamed protein product [Homo sapiens] |
| G3V0E5 | 5212.642421 | 5941.735708 | 1.13987019 | 0.188869538 | 0.24086789 | no | no change | GO:0009897;GO:0055037;GO:0035690;GO:0005905;GO:1990712;GO:0010008;GO:0045780;GO:1990830;GO:0070062;GO:0016021;GO:0045830;GO:0004998;GO:0048471;GO:0042803;GO:0006879;GO:0030890;GO:0030316;GO:0042102;GO:0005887;GO:0005886;GO:0016323;GO:0042470;GO:0031623;GO:0006898;GO:0003725;GO:0033570;GO:0033572;GO:0005769 | hsa:7037 | COG2234 | transferrin receptor variant, partial [Homo sapiens] |
| G3V2W1 | 8948.705105 | 9160.647292 | 1.023684118 | 0.033770606 | 0.808587275 | no | no change | GO:0010951;GO:0007596;GO:0005615;GO:0004867 | hsa:51156 | COG4826 | protein Z-dependent protease inhibitor isoform X1 [Homo sapiens] |
| G3XAK1 | 17282.07211 | 15567.44625 | 0.900785864 | -0.150743908 | 0.189594975 | no | no change | GO:0004252;GO:2000479;GO:0019899;GO:0010628;GO:0005737;GO:0005615;GO:0005773;GO:0033601;GO:0030971;GO:1904036;GO:0006508;GO:0045721;GO:0046425;GO:0030317;GO:0071456;GO:0007283;GO:0062023;GO:0060763;GO:0048012;GO:0007566;GO:0030879;GO:0005576;GO:0010758 | hsa:4485 | COG5640 | hepatocyte growth factor-like protein precursor [Homo sapiens] |
| G3XAP6 | 10099.62869 | 9223.943667 | 0.913295325 | -0.130846647 | 0.366055124 | no | no change | GO:0005201;GO:0060173;GO:0062023;GO:0030509;GO:0035264;GO:0050905;GO:0030500;GO:0030282;GO:0050881;GO:0010259;GO:1900047;GO:0005615;GO:0048844;GO:0036122;GO:0030198;GO:0005509;GO:0002020;GO:0002063;GO:0003417;GO:0043588;GO:0005576;GO:0014829;GO:0005178;GO:0009887;GO:1902732;GO:0009306;GO:0006915;GO:0043066;GO:0048747;GO:0006986;GO:0043395;GO:0035988;GO:0007155;GO:0097084;GO:0016485;GO:0031012;GO:0008201;GO:0070062;GO:0010260;GO:0032991;GO:0001501;GO:0035989;GO:0030199;GO:0043394;GO:0005518;GO:0070527 | hsa:1311 |  | unnamed protein product [Homo sapiens] |
| H0Y755 | 7768.903474 | 8253.877304 | 1.062425004 | 0.087361006 | 0.554198901 | no | no change | GO:0016021;GO:0016020 | hsa:2214 |  | low affinity immunoglobulin gamma Fc region receptor III-A isoform b [Homo sapiens] |
| H3BUA5 | 380376.8626 | 405591.1083 | 1.066287538 | 0.092596532 | 0.572648217 | no | no change |  | hsa:10326 |  | LOW QUALITY PROTEIN: T0061165 isoform 1, partial [Pan troglodytes] |
| I3L145 | 11796.79926 | 12641.29492 | 1.071586846 | 0.099748777 | 0.64385219 | no | no change | GO:0005496 | hsa:6462 |  | SHBG protein, partial [Homo sapiens] |
| J3KNB4 | 16019.31779 | 14444.01538 | 0.901662328 | -0.149340848 | 0.305215295 | no | no change | GO:0071224;GO:0071222;GO:0042742;GO:0050829;GO:0044130;GO:0061844;GO:0005737;GO:0042995;GO:0005615;GO:0071354;GO:0071356;GO:0016021;GO:0045766;GO:0051873;GO:0008284;GO:0045087;GO:0006952;GO:0050830;GO:0044140;GO:0001530;GO:0042581;GO:0071347;GO:0005576;GO:0001934 | hsa:820 |  | cathelicidin antimicrobial peptide [Homo sapiens] |
| J3KRP0 | 29145.70421 | 29721.22042 | 1.019746176 | 0.028210097 | 0.87204573 | no | no change | GO:0016787;GO:0032268;GO:0005829;GO:0004180;GO:0016805;GO:0005576;GO:0008152;GO:0006508;GO:0046872;GO:0008237 | hsa:84735 | COG0624 | Carnosine dipeptidase 1 (metallopeptidase M20 family) [Homo sapiens] |
| K7ER74 | 581391.7695 | 692444.0838 | 1.191011157 | 0.252186927 | 0.489090191 | no | no change | GO:0034375;GO:0034372;GO:0034371;GO:0034370;GO:0042627;GO:0034378;GO:0051006;GO:0010902;GO:0008047;GO:0048261;GO:0001523;GO:0043085;GO:0055102;GO:0005615;GO:0032375;GO:0005576;GO:0016042;GO:0045833;GO:0016004;GO:0010898;GO:0033700;GO:0034361;GO:0034362;GO:0034363;GO:0070328;GO:0006629;GO:0034366;GO:0060230;GO:0008289;GO:0006869;GO:0042803;GO:0010916;GO:0043274;GO:0042632;GO:0043691;GO:0034382;GO:0042493;GO:0034384;GO:0010518;GO:0033344;GO:0042953;GO:0060697;GO:0045723;GO:0005769 | hsa:344 |  | apolipoprotein C-II isoform X1 [Mesocricetus auratus] |
| K7ERI9 | 2356485.368 | 2179113.75 | 0.924730439 | -0.112895216 | 0.462145182 | no | no change | GO:0005576;GO:0042157 | hsa:341 |  | apolipoprotein C-I precursor [Homo sapiens] |
| M0R0Q9 | 5830.902444 | 5207.888087 | 0.893153013 | -0.163020739 | 0.223305033 | no | no change | GO:0006631;GO:0030449;GO:0004866;GO:0005886;GO:0005788;GO:0035578;GO:0007165;GO:0031715;GO:0048260;GO:0010828;GO:0010866;GO:0044267;GO:0070062;GO:0009617;GO:0045766;GO:1905114;GO:0045745;GO:0097242;GO:2000427;GO:0034774;GO:0007186;GO:0009986;GO:0005576;GO:0150064;GO:0043687;GO:0016322;GO:0150062;GO:0006956;GO:0006957;GO:0006954;GO:0006955;GO:0060100;GO:0006958;GO:0005615;GO:0050776;GO:0032991;GO:0010575;GO:0043312;GO:0001798;GO:0010884;GO:0001970;GO:0097278;GO:0005102;GO:0001934;GO:0072562 | hsa:718 |  | C3 isoform 6, partial [Pan troglodytes] |
| O00391 | 10480.24147 | 9566.577958 | 0.912820376 | -0.1315971 | 0.207740942 | no | no change | GO:0005788;GO:0030173;GO:0071949;GO:0016491;GO:0016971;GO:0044267;GO:0016972;GO:0045171;GO:0070062;GO:0031093;GO:0016021;GO:0016020;GO:0003756;GO:0035580;GO:0016242;GO:0005794;GO:1904724;GO:0043687;GO:0045454;GO:0055114;GO:0005615;GO:0043231;GO:0043312;GO:0000139;GO:0005576;GO:0085029;GO:0002576 | hsa:5768 |  | sulfhydryl oxidase 1 isoform a precursor [Homo sapiens] |
| O43866 | 151022.4368 | 171118.175 | 1.133064587 | 0.1802301 | 0.292053446 | no | no change | GO:0005737;GO:0006898;GO:0005615;GO:0005044;GO:0009986;GO:0016020;GO:0072562;GO:0002376;GO:0005576;GO:0006954;GO:0006968;GO:0006915 | hsa:922 |  | CD5 antigen-like isoform 1 precursor [Homo sapiens] |
| O95445 | 55209.75105 | 57454.5025 | 1.040658605 | 0.05749686 | 0.538483167 | no | no change | GO:0034375;GO:0034445;GO:0006869;GO:0043691;GO:0042157;GO:0001523;GO:0005615;GO:0005543;GO:0098869;GO:0005576;GO:0005319;GO:0034361;GO:0034362;GO:0034364;GO:0034365;GO:0034366;GO:0016209;GO:0042632;GO:0034380;GO:0034384;GO:0009749;GO:0033344 | hsa:55937 |  | apolipoprotein M isoform 1 [Homo sapiens] |
| P00450 | 660068.4105 | 629645.5833 | 0.953909585 | -0.068075567 | 0.451186855 | no | no change | GO:0016491;GO:0044267;GO:0046872;GO:0006879;GO:0005615;GO:0006825;GO:0004322;GO:0006811;GO:0051087;GO:0070062;GO:0005623;GO:0005765;GO:0055072;GO:0006826;GO:0005788;GO:0005886;GO:0072562;GO:0005507;GO:0043687;GO:0055114;GO:0005576 | hsa:1356 | COG2132 | RecName: Full=Ceruloplasmin; AltName: Full=Ferroxidase; Flags: Precursor |
| P00488 | 24389.30105 | 22051.47217 | 0.90414531 | -0.14537344 | 0.443405515 | no | no change | GO:0005737;GO:0018149;GO:0007599;GO:0019221;GO:0031093;GO:0003810;GO:0072378;GO:0002576;GO:0007596;GO:0072562;GO:0016740;GO:0062023;GO:0016746;GO:0046872;GO:0005576 | hsa:2162 |  | RecName: Full=Coagulation factor XIII A chain; Short=Coagulation factor XIIIa; AltName: Full=Protein-glutamine gamma-glutamyltransferase A chain; AltName: Full=Transglutaminase A chain; Flags: Precursor |
| P00734 | 573057.1632 | 556802.7667 | 0.971635646 | -0.041512677 | 0.467029862 | no | no change | GO:0004252;GO:0048712;GO:0009897;GO:0030307;GO:0007597;GO:0007596;GO:0005788;GO:0051281;GO:0030449;GO:0032967;GO:0008047;GO:0007166;GO:0007599;GO:0008233;GO:0007275;GO:0061844;GO:0044267;GO:0006888;GO:0005615;GO:0005102;GO:0009611;GO:0070062;GO:0001530;GO:0051838;GO:0010544;GO:0005509;GO:0006508;GO:0008236;GO:0007186;GO:0010468;GO:1900738;GO:0008284;GO:0016787;GO:2000379;GO:0046427;GO:0090218;GO:0070945;GO:0006953;GO:0005796;GO:0008360;GO:0014068;GO:0005886;GO:0008201;GO:0030168;GO:0008083;GO:1900016;GO:0042730;GO:1900182;GO:0051480;GO:0010469;GO:0070053;GO:0005576;GO:0051918;GO:0005515;GO:0072378;GO:0030193;GO:0045861;GO:0072562;GO:0001934;GO:0030194 | hsa:2147 | COG5640 | prothrombin isoform 1 preproprotein [Homo sapiens] |
| P00738 | 10340270.74 | 11548709.83 | 1.116867259 | 0.15945773 | 0.400501511 | no | no change | GO:0042742;GO:0010942;GO:0005615;GO:0071682;GO:2000296;GO:0072562;GO:0098869;GO:0035580;GO:1904724;GO:0016209;GO:0006952;GO:0006953;GO:0070062;GO:0042542;GO:0051354;GO:0006898;GO:0043312;GO:0002376;GO:0030492;GO:0005576;GO:0005515;GO:0031838 | hsa:3240 | COG5640 | haptoglobin isoform 1 preproprotein [Homo sapiens] |
| P00739 | 46650.73105 | 42310.0025 | 0.906952615 | -0.140900918 | 0.570207504 | no | no change | GO:0004252;GO:0070062;GO:0034366;GO:0030492;GO:0006898;GO:0072562;GO:0005576;GO:0010942;GO:0002526;GO:0010033;GO:0005615 | hsa:3250 | COG5640 | haptoglobin-related protein precursor [Homo sapiens] |
| P00740 | 34031.94947 | 31076.98208 | 0.913170787 | -0.131043387 | 0.084356385 | no | no change | GO:0004252;GO:0004175;GO:0006888;GO:0005615;GO:0016787;GO:0070062;GO:0007597;GO:0007596;GO:0031638;GO:0005788;GO:0005796;GO:0005576;GO:0005509;GO:0005515;GO:0005886;GO:0006508;GO:0008233;GO:0046872;GO:0007599;GO:0008236 | hsa:2158 | COG5640 | coagulation factor IX isoform 1 preproprotein [Homo sapiens] |
| P00742 | 77559.30526 | 77988.13 | 1.005528991 | 0.007954678 | 0.935122359 | no | no change | GO:0004252;GO:0030335;GO:0005543;GO:0006888;GO:0005615;GO:0016787;GO:0007596;GO:0005788;GO:0005796;GO:0005576;GO:0005509;GO:0005515;GO:0005886;GO:0006508;GO:0008233;GO:0008236;GO:0051897;GO:0031233;GO:0007599;GO:0007598 | hsa:2159 | COG5640 | coagulation factor X isoform 1 preproprotein [Homo sapiens] |
| P00747 | 1059033.553 | 1161562.133 | 1.096813345 | 0.13331803 | 0.053554332 | no | no change | GO:0004252;GO:0004175;GO:0007599;GO:0016787;GO:1904854;GO:0048771;GO:0019899;GO:0008233;GO:0008236;GO:0044267;GO:0070062;GO:0043536;GO:0052182;GO:0051087;GO:0072562;GO:1990405;GO:0006508;GO:0022617;GO:0009986;GO:0051918;GO:0051919;GO:0008285;GO:0007596;GO:0010812;GO:0019900;GO:0062023;GO:0019904;GO:0005886;GO:0051702;GO:0031232;GO:0034185;GO:0005615;GO:0042730;GO:0031093;GO:0052213;GO:2000048;GO:0044218;GO:0005576;GO:0005515;GO:0005102;GO:0002576 | hsa:5340 | COG5640 | plasminogen isoform 1 precursor [Homo sapiens] |
| P01011 | 768373.9316 | 747812.3792 | 0.973240174 | -0.03913222 | 0.585406937 | no | no change | GO:0006954;GO:0034774;GO:0035578;GO:0003677;GO:0070062;GO:0031093;GO:0002576;GO:0072562;GO:0010466;GO:0019216;GO:0006953;GO:0062023;GO:0030277;GO:0030414;GO:0004867;GO:0010951;GO:0005615;GO:0043312;GO:0005622;GO:0005576;GO:0005515;GO:0005634 | hsa:12 | COG4826 | serpin peptidase inhibitor, clade A (alpha-1 antiproteinase, antitrypsin), member 3, isoform CRA_b [Homo sapiens] |
| P01023 | 7676298 | 7746371.708 | 1.009128581 | 0.013110011 | 0.942685298 | no | no change | GO:0051056;GO:0030414;GO:0019959;GO:0007597;GO:0019899;GO:0072562;GO:0048863;GO:0005615;GO:0031093;GO:0002576;GO:0048306;GO:0019838;GO:0070062;GO:0010466;GO:0002020;GO:0001869;GO:0022617;GO:0019966;GO:0005096;GO:0004866;GO:0004867;GO:0010951;GO:0043547;GO:0043120;GO:0005829;GO:0005576;GO:0005515;GO:0005102 | hsa:2 | COG2373 | alpha-2-macroglobulin isoform a precursor [Homo sapiens] |
| P01024 | 6968412.895 | 7683627.625 | 1.102636675 | 0.140957493 | 0.089841128 | no | no change | GO:0045087;GO:0004252;GO:0006631;GO:0030449;GO:0004866;GO:0005886;GO:0005788;GO:0035578;GO:0007165;GO:0031715;GO:0048260;GO:0010828;GO:0010866;GO:0050766;GO:0044267;GO:0070062;GO:0009617;GO:0045766;GO:1905114;GO:0045745;GO:0097242;GO:2000427;GO:0034774;GO:0007186;GO:0009986;GO:0005576;GO:0006629;GO:0150064;GO:0043687;GO:0016322;GO:0006911;GO:0150062;GO:0006956;GO:0006957;GO:0006954;GO:0006955;GO:0010951;GO:0060100;GO:0006958;GO:0006508;GO:0005615;GO:0050776;GO:0032991;GO:0010575;GO:0043312;GO:0002376;GO:0001798;GO:0010884;GO:0001970;GO:0005515;GO:0097278;GO:0005102;GO:0001934;GO:0072562 | hsa:718 |  | complement C3 preproprotein [Homo sapiens] |
| P01031 | 225098.3579 | 216864.45 | 0.963420844 | -0.053761957 | 0.597300733 | no | no change | GO:0000187;GO:0030449;GO:0006954;GO:0008009;GO:0007166;GO:0060326;GO:0019835;GO:0001701;GO:0005615;GO:0045766;GO:0010760;GO:0007186;GO:0045087;GO:0006935;GO:0090197;GO:0006956;GO:0006957;GO:0004866;GO:0010951;GO:0006958;GO:0070062;GO:0010575;GO:0002376;GO:0005576;GO:0005515;GO:0005102;GO:0005579 | hsa:727 |  | complement C5 isoform 1 preproprotein [Homo sapiens] |
| P01034 | 13011.39337 | 11943.61057 | 0.917934785 | -0.123536435 | 0.300879149 | no | no change | GO:0005783;GO:0060009;GO:0048678;GO:0005788;GO:0008584;GO:0042747;GO:0030414;GO:0070301;GO:0001666;GO:0014070;GO:1904724;GO:0005737;GO:0044267;GO:0032355;GO:0042995;GO:0001540;GO:0005615;GO:0007431;GO:2000117;GO:0005771;GO:0009636;GO:0070062;GO:0043067;GO:0010466;GO:0002020;GO:0030424;GO:0048471;GO:0043025;GO:0042802;GO:0008284;GO:0034103;GO:0045740;GO:0060548;GO:0043687;GO:0006915;GO:0031965;GO:0031982;GO:0006952;GO:0001775;GO:0001654;GO:0060311;GO:0004866;GO:0060313;GO:0004869;GO:0034599;GO:0010035;GO:0006979;GO:0009743;GO:1904813;GO:0005604;GO:0007420;GO:0010716;GO:0007566;GO:0042493;GO:0031667;GO:0010711;GO:0005764;GO:0005576;GO:0005515;GO:0043312;GO:0043292;GO:0045861;GO:0097435 | hsa:1471 |  | cystatin-C precursor [Homo sapiens] |
| P01042 | 1234049.968 | 1153652.767 | 0.934850935 | -0.097191754 | 0.176087346 | no | no change | GO:0007599;GO:0030414;GO:0007597;GO:0007596;GO:0045861;GO:0005788;GO:0007162;GO:0008270;GO:0042311;GO:0044267;GO:0050880;GO:0005615;GO:0031093;GO:0002576;GO:0072562;GO:0043065;GO:0010466;GO:0007186;GO:0043687;GO:0062023;GO:0006954;GO:0005886;GO:0010951;GO:0004869;GO:0008201;GO:0070062;GO:0007204;GO:0005576;GO:0005515;GO:0005102;GO:0030195 | hsa:3827 |  | kininogen-1 isoform 1 precursor [Homo sapiens] |
| P01597 | 42072.93842 | 41109.17542 | 0.977093043 | -0.033432147 | 0.771852663 | no | no change | GO:0038096;GO:0004252;GO:0016020;GO:0030449;GO:0050776;GO:0006898;GO:0038095;GO:0072562;GO:0002376;GO:0050900;GO:0005576;GO:0006956;GO:0002250;GO:0003823;GO:0005886;GO:0006508;GO:0006958;GO:0006955;GO:0070062 | hsa:7441 |  | immunoglobulin kappa light chain VC region, partial [Homo sapiens] |
| P01701 | 446200.1774 | 464909.8917 | 1.041931212 | 0.059260035 | 0.718691015 | no | no change | GO:0038096;GO:0004252;GO:0016020;GO:0030449;GO:0050776;GO:0006898;GO:0038095;GO:0050900;GO:0005576;GO:0006956;GO:0003823;GO:0005886;GO:0006508;GO:0006958;GO:0006955;GO:0070062 | hsa:7441 |  | RecName: Full=Immunoglobulin lambda variable 1-51; AltName: Full=Ig lambda chain V-I region BL2; AltName: Full=Ig lambda chain V-I region EPS; AltName: Full=Ig lambda chain V-I region NEW; AltName: Full=Ig lambda chain V-I region NIG-64; Flags: Precursor |
| P01704 | 32190.41579 | 30804.99432 | 0.956961678 | -0.063466942 | 0.703781744 | no | no change | GO:0038096;GO:0004252;GO:0016020;GO:0030449;GO:0050776;GO:0006898;GO:0038095;GO:0002376;GO:0050900;GO:0005576;GO:0006956;GO:0002250;GO:0003823;GO:0005886;GO:0006508;GO:0006958;GO:0006955;GO:0070062 | hsa:7441 |  | RecName: Full=Immunoglobulin lambda variable 2-14; AltName: Full=Ig lambda chain V-II region NIG-84; AltName: Full=Ig lambda chain V-II region TOG; AltName: Full=Ig lambda chain V-II region VIL; Flags: Precursor |
| P01706 | 93105.06105 | 111182.1975 | 1.194158473 | 0.255994304 | 0.303752648 | no | no change | GO:0038096;GO:0004252;GO:0016020;GO:0030449;GO:0050776;GO:0006898;GO:0038095;GO:0002376;GO:0050900;GO:0005576;GO:0006956;GO:0002250;GO:0003823;GO:0005886;GO:0006508;GO:0006958;GO:0006955 | hsa:7441 |  | hCG2043237, partial [Homo sapiens] |
| P01709 | 150145.6289 | 128485.585 | 0.855739763 | -0.224755965 | 0.293855976 | no | no change | GO:0038096;GO:0004252;GO:0016020;GO:0030449;GO:0050776;GO:0006898;GO:0038095;GO:0002376;GO:0050900;GO:0005576;GO:0006956;GO:0002250;GO:0003823;GO:0005886;GO:0006508;GO:0006958;GO:0006955 | hsa:7441 |  | hCG2043240, partial [Homo sapiens] |
| P01717 | 6679.0208 | 5749.16281 | 0.860779294 | -0.21628472 | 0.329289832 | no | no change | GO:0038096;GO:0004252;GO:0016020;GO:0030449;GO:0050776;GO:0006898;GO:0038095;GO:0072562;GO:0002376;GO:0050900;GO:0005576;GO:0006956;GO:0002250;GO:0003823;GO:0005886;GO:0006508;GO:0006958;GO:0006955 | hsa:7441 |  | immunoglobulin lambda light chain variable region, partial [Homo sapiens] |
| P01743 | 17658.08526 | 16041.36275 | 0.908442932 | -0.138532207 | 0.480638413 | no | no change | GO:0038096;GO:0004252;GO:0016020;GO:0030449;GO:0050776;GO:0006898;GO:0038095;GO:0002376;GO:0050900;GO:0005576;GO:0006956;GO:0002250;GO:0003823;GO:0005886;GO:0006508;GO:0006958;GO:0006955 | hsa:102723407 | | IgM heavy chain VH1 region precursor, partial [Homo sapiens] |
| P01764 | 38419.61727 | 44849.17591 | 1.167350929 | 0.223238329 | 0.468683738 | no | no change | GO:0004252;GO:0030449;GO:0009897;GO:0006955;GO:0050900;GO:0042742;GO:0034987;GO:0042571;GO:0005615;GO:0016020;GO:0072562;GO:0006508;GO:0050853;GO:0045087;GO:0006910;GO:0006911;GO:0050776;GO:0006956;GO:0003823;GO:0005886;GO:0006958;GO:0050871;GO:0070062;GO:0038096;GO:0038095;GO:0006898;GO:0002376;GO:0005576;GO:0002250 | hsa:102723407 | | RecName: Full=Immunoglobulin heavy variable 3-23; AltName: Full=Ig heavy chain V-III region LAY; AltName: Full=Ig heavy chain V-III region POM; AltName: Full=Ig heavy chain V-III region TEI; AltName: Full=Ig heavy chain V-III region TIL; AltName: Full=Ig heavy chain V-III region TUR; AltName: Full=Ig heavy chain V-III region VH26; AltName: Full=Ig heavy chain V-III region WAS; AltName: Full=Ig heavy chain V-III region ZAP; Flags: Precursor |
| P01766 | 78135.21684 | 76615.26708 | 0.980547187 | -0.028341036 | 0.880378035 | no | no change | GO:0038096;GO:0004252;GO:0016020;GO:0030449;GO:0005615;GO:0006898;GO:0038095;GO:0050900;GO:0072562;GO:0002376;GO:0050776;GO:0005576;GO:0006956;GO:0002250;GO:0003823;GO:0005886;GO:0006508;GO:0006958;GO:0006955 | hsa:102723407 | | RecName: Full=Immunoglobulin heavy variable 3-13; AltName: Full=Ig heavy chain V-III region BRO; Flags: Precursor |
| P01780 | 1781232.211 | 1684025.971 | 0.945427531 | -0.080961218 | 0.53329802 | no | no change | GO:0038096;GO:0004252;GO:0016020;GO:0030449;GO:0050776;GO:0006898;GO:0038095;GO:0072562;GO:0002376;GO:0050900;GO:0005576;GO:0006956;GO:0002250;GO:0003823;GO:0005886;GO:0006508;GO:0006958;GO:0006955;GO:0070062 | hsa:102723407 | | immunoglobulin heavy chain variable region precursor, partial [Homo sapiens] |
| P01782 | 209357.0895 | 181545.2313 | 0.86715588 | -0.205636738 | 0.251779563 | no | no change | GO:0038096;GO:0004252;GO:0016020;GO:0030449;GO:0050776;GO:0006898;GO:0038095;GO:0002376;GO:0050900;GO:0005576;GO:0006956;GO:0002250;GO:0003823;GO:0005886;GO:0006508;GO:0006958;GO:0006955;GO:0070062 | hsa:102723407 | | hCG2038940, partial [Homo sapiens] |
| P01834 | 25566934.68 | 22862797.67 | 0.894233038 | -0.161277247 | 0.110795219 | no | no change | GO:0004252;GO:0030449;GO:0009897;GO:0006955;GO:0050871;GO:0042742;GO:0034987;GO:0042571;GO:0005615;GO:0016020;GO:0072562;GO:0006508;GO:0050853;GO:0045087;GO:0006910;GO:0006911;GO:0050776;GO:0006956;GO:0003823;GO:0005886;GO:0006958;GO:0001895;GO:0070062;GO:0038096;GO:0038095;GO:0006898;GO:0050900;GO:0002376;GO:0005576;GO:0002250 | hsa:100423062 | | light chain kappa Sci, k Sci=Bence Jones protein [human, Peptide, 214 aa] |
| P01860 | 2892402.258 | 2605711.083 | 0.900881292 | -0.150591078 | 0.465497118 | no | no change | GO:0004252;GO:0030449;GO:0009897;GO:0050871;GO:0042742;GO:0034987;GO:0042571;GO:0005615;GO:0016020;GO:0072562;GO:0006508;GO:0050853;GO:0045087;GO:0006910;GO:0006911;GO:0006956;GO:0003823;GO:0005886;GO:0006958;GO:0001895;GO:0070062;GO:0038096;GO:0002376;GO:0005576;GO:0002250 | hsa:100423062 | | Unknown (protein for MGC:105008) [Homo sapiens] |
| P01871 | 10356733.32 | 11343463 | 1.09527422 | 0.131292117 | 0.617266737 | no | no change | GO:0009897;GO:0042834;GO:0019731;GO:0050900;GO:0034987;GO:0050829;GO:0005615;GO:0016021;GO:0016020;GO:0072562;GO:0009986;GO:0050853;GO:0045087;GO:0006910;GO:0006911;GO:0031210;GO:0003823;GO:0005886;GO:0006958;GO:0050871;GO:0070062;GO:0003697;GO:0002376;GO:0005576;GO:0002250;GO:0005515;GO:0071756;GO:0071757 | hsa:3543 |  | immunoglobulin heavy chain [Homo sapiens] |
| P02100 | 42136.11668 | 36857.37129 | 0.874721597 | -0.19310418 | 0.477791685 | no | no change | GO:0005344;GO:0019825;GO:0020037;GO:0043177;GO:0031721;GO:0007596;GO:0051291;GO:0072562;GO:0005833;GO:0098869;GO:0042744;GO:0015671;GO:0005515;GO:0031838;GO:0046872;GO:0005829;GO:0014070 | hsa:3046 | COG1018 | hemoglobin subunit epsilon [Homo sapiens] |
| P02533 | 27616.62947 | 25924.08125 | 0.938712716 | -0.091244393 | 0.574435326 | no | no change | GO:0005200;GO:0045095;GO:0071944;GO:0010043;GO:0008544;GO:0005737;GO:0045110;GO:0010212;GO:0070062;GO:0005634;GO:0045178;GO:0005198;GO:0030855;GO:1990254;GO:0031581;GO:0070268;GO:0005882;GO:0042633;GO:0007568;GO:0031424;GO:0005829;GO:0005622;GO:0005515 | hsa:3861 |  | keratin, type I cytoskeletal 14 [Homo sapiens] |
| P02647 | 31741707.16 | 32231965.5 | 1.015445242 | 0.022112444 | 0.800568645 | no | no change | GO:0034115;GO:0010804;GO:0005788;GO:0019915;GO:0034191;GO:0034190;GO:0005548;GO:0050728;GO:0071682;GO:0051496;GO:0005543;GO:0010898;GO:0005319;GO:0034361;GO:0034362;GO:0034363;GO:0034364;GO:0031102;GO:0034366;GO:0031100;GO:0043534;GO:0030300;GO:0034774;GO:0010873;GO:0043691;GO:0007179;GO:0015485;GO:0005829;GO:0018206;GO:0019433;GO:0045499;GO:0070508;GO:0045723;GO:0034375;GO:0034371;GO:0031072;GO:0015914;GO:0034378;GO:0050821;GO:0010903;GO:0042158;GO:0055102;GO:0044267;GO:0042627;GO:0051180;GO:0014012;GO:0072562;GO:0050919;GO:0007186;GO:0033700;GO:0018158;GO:0006629;GO:0060761;GO:0042632;GO:0032489;GO:0050713;GO:0007229;GO:0006898;GO:0034384;GO:0062023;GO:0006695;GO:0060192;GO:0006869;GO:0019899;GO:0030301;GO:0035025;GO:0051006;GO:0002740;GO:0001540;GO:1900026;GO:0031410;GO:0005634;GO:0042802;GO:0008035;GO:0060354;GO:0009986;GO:0008289;GO:0008202;GO:0008203;GO:0071813;GO:0070062;GO:0042493;GO:0005515;GO:0034380;GO:0070371;GO:0042157;GO:0006656;GO:0030325;GO:0006644;GO:0017127;GO:0030139;GO:0008211;GO:0060228;GO:0001523;GO:0055091;GO:0034365;GO:0005615;GO:0002576;GO:0005576;GO:0043687;GO:0070328;GO:0019216;GO:0007584;GO:0031210;GO:0005886;GO:0043627;GO:1903561;GO:0051346;GO:0051345;GO:0033344;GO:0070653;GO:0001932;GO:0001935;GO:0005769 | hsa:335 |  | apolipoprotein A-I isoform 1 preproprotein [Homo sapiens] |
| P02671 | 6288507.053 | 6592469.667 | 1.048336213 | 0.068101479 | 0.44448021 | no | no change | GO:0045087;GO:0009897;GO:0045907;GO:0007599;GO:0034116;GO:0007596;GO:0005788;GO:0007160;GO:0045921;GO:0045202;GO:0034622;GO:1902042;GO:0044267;GO:0050839;GO:0031091;GO:0005615;GO:0031093;GO:1900026;GO:0072378;GO:0002576;GO:0031639;GO:0072562;GO:0005198;GO:0065003;GO:0030198;GO:0002224;GO:0046872;GO:0043687;GO:0009986;GO:2000352;GO:0051592;GO:0090277;GO:0005938;GO:0005886;GO:1903561;GO:0030168;GO:0070062;GO:0051258;GO:0042730;GO:0072377;GO:0050714;GO:0002376;GO:0005577;GO:0005576;GO:0002250;GO:0005515;GO:0043152;GO:0005102;GO:0070374;GO:0070527 | hsa:2243 |  | fibrinogen alpha chain isoform alpha-E preproprotein [Homo sapiens] |
| P02675 | 14715519.89 | 14246328.08 | 0.968115852 | -0.046748393 | 0.591824132 | no | no change | GO:1902042;GO:0034116;GO:0045921;GO:0007599;GO:0005783;GO:0009897;GO:0007596;GO:0007160;GO:0045907;GO:0045202;GO:0034622;GO:0044320;GO:0005737;GO:1903561;GO:0031091;GO:0005615;GO:0031093;GO:1900026;GO:0072378;GO:0002576;GO:0051087;GO:0031639;GO:0072562;GO:0005198;GO:0030198;GO:0002224;GO:0045087;GO:0009986;GO:2000352;GO:0051592;GO:0090277;GO:0005938;GO:0005886;GO:0050839;GO:0030168;GO:0070062;GO:0051258;GO:0042730;GO:0050714;GO:0071347;GO:0002376;GO:0005577;GO:0005576;GO:0002250;GO:0005515;GO:0043152;GO:0005102;GO:0070374;GO:0070527 | hsa:2244 |  | fibrinogen beta chain isoform 1 preproprotein [Homo sapiens] |
| P02743 | 440698.6632 | 384314.5167 | 0.872057369 | -0.197505048 | 0.13374728 | no | no change | GO:0006457;GO:0030246;GO:0046597;GO:0044871;GO:0072562;GO:0061045;GO:0044267;GO:0070062;GO:0005615;GO:0005634;GO:0051082;GO:0001849;GO:0005509;GO:0030169;GO:0042802;GO:1903016;GO:0051131;GO:0045087;GO:1903019;GO:0006953;GO:0062023;GO:0044869;GO:0006958;GO:0002674;GO:0046872;GO:0045656;GO:0046790;GO:0005576;GO:0048525 | hsa:325 |  | serum amyloid P-component precursor [Homo sapiens] |
| P02748 | 123071.5032 | 134374.9038 | 1.091844174 | 0.126766973 | 0.322697542 | no | no change | GO:0030449;GO:0006955;GO:0019835;GO:0019836;GO:0070062;GO:0016021;GO:0016020;GO:0051260;GO:0072562;GO:0001906;GO:0045087;GO:0006957;GO:0005887;GO:0005886;GO:0006958;GO:0005615;GO:0005829;GO:0044218;GO:0044279;GO:0002376;GO:0005576;GO:0005579 | hsa:735 |  | complement component C9 preproprotein [Homo sapiens] |
| P02749 | 980397.2895 | 912255.625 | 0.930495866 | -0.103928354 | 0.336014493 | no | no change | GO:0034392;GO:0007597;GO:0051006;GO:0034197;GO:0042627;GO:0070062;GO:0002576;GO:0005543;GO:0031639;GO:0051917;GO:0042802;GO:0051918;GO:0034361;GO:0034364;GO:0006641;GO:0009986;GO:0060230;GO:0008289;GO:0062023;GO:0033033;GO:0008201;GO:0005615;GO:0010596;GO:0031089;GO:0005576;GO:0030195;GO:0005515;GO:0030193;GO:0016525;GO:0001937;GO:0030194 | hsa:350 |  | beta-2-glycoprotein 1 precursor [Homo sapiens] |
| P02750 | 154796.7511 | 136499.81 | 0.881800226 | -0.181476249 | 0.129217883 | no | no change | GO:0043231;GO:0016020;GO:0070062;GO:0005160;GO:0009617;GO:0050873;GO:0045766;GO:0043312;GO:1904813;GO:0001938;GO:1904724;GO:0005576;GO:0003674;GO:0005515;GO:0035580;GO:0008150;GO:0030511;GO:0005615 | hsa:116844 | COG4886 | leucine-rich alpha-2-glycoprotein precursor [Homo sapiens] |
| P02760 | 392322.3842 | 436345.2333 | 1.112210903 | 0.153430385 | 0.067811763 | no | no change | GO:0005886;GO:0010951;GO:0046329;GO:0070062;GO:0019855;GO:0072562;GO:0010466;GO:0042803;GO:0005515;GO:0020037;GO:0009986;GO:0042167;GO:0018298;GO:0062023;GO:0030163;GO:0030414;GO:0004867;GO:0007155;GO:0005615;GO:0050777;GO:0046904;GO:0019862;GO:0007565;GO:0006898;GO:0043231;GO:0005576;GO:0016032 | hsa:259 |  | protein AMBP preproprotein [Homo sapiens] |
| P02768 | 465645313.7 | 445194692 | 0.956081118 | -0.064795067 | 0.2171667 | no | no change | GO:0034375;GO:0015643;GO:0005788;GO:0008144;GO:0051659;GO:0030170;GO:0003677;GO:0005783;GO:0005737;GO:0044267;GO:0019836;GO:0070062;GO:0031093;GO:0043209;GO:0002576;GO:0051087;GO:0072562;GO:0098869;GO:0043066;GO:0005504;GO:0005507;GO:0043687;GO:0032460;GO:0042802;GO:0005794;GO:0140272;GO:0016209;GO:0008289;GO:0019825;GO:0009267;GO:0001895;GO:0005615;GO:0046872;GO:0032991;GO:0043069;GO:0006898;GO:1903981;GO:0005576;GO:0005515;GO:0005634 | hsa:213 |  | serum albumin preproprotein [Homo sapiens] |
| P02775 | 43439.92563 | 40009.81917 | 0.92103793 | -0.118667525 | 0.706526883 | no | no change | GO:0042127;GO:0070098;GO:0005125;GO:0071222;GO:0008009;GO:0042742;GO:0060326;GO:0032496;GO:0061844;GO:0031091;GO:0008083;GO:0031093;GO:0002576;GO:0007186;GO:0005355;GO:0030595;GO:0030593;GO:1904724;GO:0006952;GO:0006954;GO:0006955;GO:0045236;GO:0005615;GO:0090023;GO:0010469;GO:0031640;GO:0043312;GO:0006935;GO:0051781;GO:0005576;GO:1904659;GO:0005515 | hsa:5473 |  | platelet basic protein preproprotein [Homo sapiens] |
| P02790 | 3115692.421 | 2810931.875 | 0.902185291 | -0.148504329 | 0.060903367 | no | no change | GO:0020027;GO:0002925;GO:0015232;GO:0071682;GO:0005615;GO:0051246;GO:0042531;GO:0072562;GO:0046872;GO:0042168;GO:0006879;GO:0016032;GO:0060332;GO:0062023;GO:0060335;GO:0002639;GO:0070062;GO:0015886;GO:0006898;GO:0005623;GO:0005576;GO:0005515 | hsa:3263 |  | hemopexin precursor [Homo sapiens] |
| P03951 | 9806.567526 | 11125.43813 | 1.134488504 | 0.18204199 | 0.34213502 | no | no change | GO:0004252;GO:0016020;GO:0007599;GO:0070062;GO:0016787;GO:0005615;GO:0007597;GO:0007596;GO:0031639;GO:0030193;GO:0005576;GO:0005515;GO:0005886;GO:0070009;GO:0006508;GO:0008233;GO:0008236;GO:0008201;GO:0042802;GO:0051919 | hsa:2160 | COG5640 | coagulation factor XI isoform 1 preproprotein [Homo sapiens] |
| P04003 | 2738359.421 | 2612318.208 | 0.953971998 | -0.067981175 | 0.703324051 | no | no change | GO:0030449;GO:0005615;GO:0045087;GO:0072562;GO:0002376;GO:0045732;GO:0005576;GO:0003723;GO:0005515;GO:0005886;GO:1903027;GO:0006958;GO:0045959;GO:0044216 | hsa:722 |  | C4b-binding protein alpha chain precursor [Homo sapiens] |
| P04114 | 4102093.263 | 4496564.042 | 1.096163289 | 0.132462724 | 0.269618043 | no | no change | GO:0034360;GO:0034374;GO:0034359;GO:0005783;GO:0034371;GO:0006869;GO:0050750;GO:0005788;GO:0005789;GO:0009791;GO:0061024;GO:0050900;GO:0010628;GO:0042157;GO:0034378;GO:0032496;GO:0016042;GO:0001523;GO:0006629;GO:0005737;GO:0043202;GO:0032355;GO:0001701;GO:0031983;GO:0005615;GO:0071682;GO:0071356;GO:0009615;GO:0048844;GO:0005543;GO:0030669;GO:0071379;GO:0070062;GO:0010033;GO:0042953;GO:0010884;GO:0042158;GO:0002224;GO:0010008;GO:0043025;GO:0043687;GO:0070971;GO:0005319;GO:0034361;GO:0034362;GO:0034363;GO:0006642;GO:0005790;GO:0044267;GO:0030317;GO:0008289;GO:0034379;GO:0010744;GO:0007283;GO:0042627;GO:0042159;GO:0017127;GO:0012506;GO:0005886;GO:0042632;GO:0030301;GO:0008201;GO:0008202;GO:0008203;GO:0035473;GO:0031904;GO:0009743;GO:0007399;GO:0034382;GO:0034383;GO:0005829;GO:0006898;GO:0009566;GO:0043231;GO:0010886;GO:0019433;GO:0005576;GO:0045540;GO:0005515;GO:0034447;GO:0010269;GO:0005769;GO:0033344 | hsa:338 |  | RecName: Full=Apolipoprotein B-100; Short=Apo B-100; Contains: RecName: Full=Apolipoprotein B-48; Short=Apo B-48; Flags: Precursor |
| P04180 | 19673.87158 | 19570.84 | 0.994763025 | -0.007575211 | 0.961891313 | no | no change | GO:0034375;GO:0006656;GO:0034372;GO:0043691;GO:0008374;GO:0042158;GO:0090107;GO:0070062;GO:0016740;GO:0016746;GO:0034435;GO:0008203;GO:0006644;GO:0034364;GO:0006629;GO:0042632;GO:0030301;GO:0008202;GO:0005615;GO:0005576;GO:0005515;GO:0034186;GO:0004607;GO:0046470 | hsa:3931 |  | phosphatidylcholine-sterol acyltransferase precursor [Homo sapiens] |
| P04217 | 789356.7158 | 784967.7167 | 0.994439777 | -0.00804409 | 0.925902569 | no | no change | GO:0070062;GO:0031093;GO:0002576;GO:0043312;GO:0072562;GO:1904813;GO:0005576;GO:0003674;GO:0034774;GO:0008150;GO:0062023;GO:0005615 | hsa:1 |  | alpha-1B-glycoprotein precursor [Homo sapiens] |
| P05109 | 9250.794133 | 8955.786278 | 0.968109997 | -0.046757119 | 0.916421838 | no | no change | GO:0045087;GO:0019730;GO:0030307;GO:0032602;GO:0043312;GO:0050786;GO:0008270;GO:0014002;GO:0010043;GO:0032496;GO:0005737;GO:0001816;GO:0045111;GO:0050729;GO:0005615;GO:0005634;GO:0016020;GO:0051493;GO:0050727;GO:0005509;GO:0002224;GO:2001244;GO:0034774;GO:0046872;GO:0035662;GO:0008017;GO:0005856;GO:0030593;GO:0006935;GO:0006919;GO:0032119;GO:0006914;GO:0006915;GO:0018119;GO:0042060;GO:0050832;GO:0045471;GO:0070488;GO:0006954;GO:0005886;GO:0002526;GO:0002523;GO:0070062;GO:0051092;GO:0050544;GO:0005829;GO:0002793;GO:0002544;GO:0002376;GO:0005576;GO:0005515;GO:0042742 | hsa:6279 |  | protein S100-A8 isoform d [Homo sapiens] |
| P05155 | 346645.2684 | 330904.0167 | 0.95458974 | -0.067047265 | 0.553169008 | no | no change | GO:0030449;GO:0007597;GO:0007596;GO:0007599;GO:0005615;GO:0031093;GO:0002576;GO:0072562;GO:0010466;GO:0001869;GO:0045916;GO:0008015;GO:0045087;GO:0030414;GO:0004867;GO:0010951;GO:0006958;GO:0070062;GO:0042730;GO:0007568;GO:0002376;GO:0005576;GO:0005515;GO:0030193 | hsa:710 | COG4826 | unnamed protein product [Homo sapiens] |
| P05160 | 26841.95632 | 24088.64167 | 0.897424963 | -0.156136779 | 0.114198199 | no | no change | GO:0072378;GO:0005576;GO:0007596;GO:0007599;GO:1903363 | hsa:2165 |  | coagulation factor XIII B chain precursor [Homo sapiens] |
| P05543 | 28069.67421 | 27862.02542 | 0.99260238 | -0.010712182 | 0.928742406 | no | no change | GO:0005615;GO:0070327;GO:0005576;GO:0004867;GO:0010951;GO:0070062 | hsa:6906 | COG4826 | thyroxine-binding globulin precursor [Homo sapiens] |
| P05546 | 523667.8632 | 483992.8542 | 0.924236311 | -0.113666324 | 0.285868649 | no | no change | GO:0044267;GO:0007599;GO:0006935;GO:0005615;GO:0043687;GO:0007596;GO:0005788;GO:0005576;GO:0030414;GO:0004866;GO:0004867;GO:0010951;GO:0010466;GO:0008201;GO:0070062 | hsa:3053 | COG4826 | heparin cofactor 2 precursor [Homo sapiens] |
| P06312 | 384481.9842 | 385772.4458 | 1.003356364 | 0.004834102 | 0.961753152 | no | no change | GO:0038096;GO:0004252;GO:0016020;GO:0030449;GO:0050776;GO:0006898;GO:0038095;GO:0072562;GO:0002376;GO:0002377;GO:0050900;GO:0005576;GO:0006956;GO:0002250;GO:0003823;GO:0005886;GO:0006508;GO:0006958;GO:0006955 | hsa:7441 |  | immunoglobulin kappa chain, partial [Homo sapiens] |
| P06331 | 393688.4947 | 441931.2375 | 1.122540393 | 0.166767359 | 0.769615551 | no | no change | GO:0038096;GO:0004252;GO:0016020;GO:0030449;GO:0050776;GO:0006898;GO:0038095;GO:0002376;GO:0050900;GO:0005576;GO:0006956;GO:0002250;GO:0003823;GO:0005886;GO:0006508;GO:0006958;GO:0006955 | hsa:102724971 | | hCG1793614, partial [Homo sapiens] |
| P06727 | 1045892 | 1004446.754 | 0.960373303 | -0.058332796 | 0.634875489 | no | no change | GO:0034375;GO:0034445;GO:0034372;GO:0034371;GO:0006869;GO:0034380;GO:0045723;GO:0005788;GO:0034378;GO:0051006;GO:0042744;GO:0042157;GO:0042632;GO:0070328;GO:0060228;GO:0001523;GO:0035634;GO:0044267;GO:0042627;GO:0005615;GO:0006982;GO:0032374;GO:0072562;GO:0016042;GO:0033344;GO:0065005;GO:0010898;GO:0002227;GO:0009986;GO:0042802;GO:0033700;GO:0005319;GO:0034361;GO:0034364;GO:0031102;GO:0062023;GO:0016209;GO:0008289;GO:0055088;GO:0031210;GO:0007159;GO:0017127;GO:0010873;GO:0043691;GO:0008203;GO:0030300;GO:0005507;GO:0015485;GO:0070062;GO:0005829;GO:0046470;GO:0019430;GO:0005576;GO:0006695;GO:0005515;GO:0042803;GO:0005769 | hsa:337 |  | RecName: Full=Apolipoprotein A-IV; Short=Apo-AIV; Short=ApoA-IV; AltName: Full=Apolipoprotein A4; Flags: Precursor |
| P07357 | 57252.00211 | 55249.77208 | 0.965027773 | -0.051357632 | 0.5071242 | no | no change | GO:0019835;GO:0030449;GO:0070062;GO:0016021;GO:0016020;GO:0072562;GO:0002376;GO:0001848;GO:0005576;GO:0006956;GO:0006957;GO:0005886;GO:0006955;GO:0044877;GO:0006958;GO:0005579;GO:0045087;GO:0005615 | hsa:731 |  | complement component C8 alpha chain preproprotein [Homo sapiens] |
| P08519 | 115441.3416 | 97129.765 | 0.841377653 | -0.249174595 | 0.635797202 | no | no change | GO:0004252;GO:0034374;GO:0005515;GO:0006869;GO:0008015;GO:0016787;GO:0034185;GO:0005576;GO:0004866;GO:0010951;GO:0006508;GO:0008233;GO:0008236;GO:0034358;GO:0008201;GO:0001968;GO:0006629 | hsa:4018 | COG5640 | RecName: Full=Apolipoprotein(a); Short=Apo(a); Short=Lp(a); Flags: Precursor |
| P08571 | 10367.93374 | 10546.54488 | 1.017227265 | 0.024642036 | 0.833029308 | no | no change | GO:0006954;GO:0009897;GO:0045121;GO:0071222;GO:0071223;GO:0097190;GO:0031362;GO:1901224;GO:0007166;GO:0006909;GO:0032496;GO:0045471;GO:0010008;GO:0034612;GO:0016020;GO:0030667;GO:0005615;GO:0071727;GO:0071726;GO:0009617;GO:0071723;GO:0071219;GO:0032729;GO:0002224;GO:0031225;GO:0009986;GO:0001847;GO:0005794;GO:0032760;GO:0070266;GO:0034128;GO:0038124;GO:0045087;GO:0006915;GO:2000484;GO:0035666;GO:0032481;GO:0070891;GO:0034142;GO:0005886;GO:0009408;GO:0001530;GO:0032026;GO:0070062;GO:0051602;GO:0002755;GO:0002756;GO:0016019;GO:0050715;GO:0038123;GO:0031663;GO:0006898;GO:0007249;GO:0043312;GO:0002376;GO:0005576;GO:0002237;GO:0005515;GO:0045807;GO:0046696 | hsa:929 |  | monocyte differentiation antigen CD14 precursor [Homo sapiens] |
| P08603 | 1222296.805 | 1298499.188 | 1.062343599 | 0.08725046 | 0.376246191 | no | no change | GO:1903659;GO:0030449;GO:0070062;GO:0005515;GO:0045087;GO:0072562;GO:0002376;GO:0043395;GO:0005576;GO:0006956;GO:0006957;GO:0016032;GO:0008201;GO:0005615 | hsa:3075 |  | RecName: Full=Complement factor H; AltName: Full=H factor 1; Flags: Precursor |
| P09871 | 92244.90053 | 101824.4283 | 1.103848861 | 0.142542651 | 0.117004507 | no | no change | GO:0004252;GO:0006956;GO:0016787;GO:0045087;GO:0072562;GO:0002376;GO:0030449;GO:0005576;GO:0001867;GO:0005509;GO:0005515;GO:0006508;GO:0006958;GO:0046872;GO:0008233;GO:0042802;GO:0008236 | hsa:716 | COG5640 | complement C1s subcomponent isoform 1 preproprotein [Homo sapiens] |
| P0C0L5 | 1807760.674 | 1801409.792 | 0.996486879 | -0.005077286 | 0.975941372 | no | no change | GO:0004252;GO:0030449;GO:0006954;GO:0030246;GO:0030425;GO:0030424;GO:0045202;GO:0032490;GO:0030054;GO:0005615;GO:0072562;GO:0001848;GO:0006508;GO:0045087;GO:0008228;GO:0006956;GO:2000427;GO:0004866;GO:0005886;GO:0010951;GO:0006958;GO:0042995;GO:0070062;GO:0044216;GO:0002376;GO:0005576 | hsa:100293534;hsa:110384692;hsa:720;hsa:721 | | complement C4-B preproprotein [Homo sapiens] |
| P0DJI8 | 25729.83263 | 28121.24417 | 1.092943144 | 0.128218352 | 0.501474601 | no | no change | GO:0000187;GO:0042056;GO:0019221;GO:0001664;GO:0034364;GO:0048246;GO:0048247;GO:0044267;GO:0050728;GO:0050708;GO:0005615;GO:0071682;GO:0045785;GO:0007186;GO:0030593;GO:0045087;GO:0005881;GO:0006953;GO:0050918;GO:0008201;GO:0030168;GO:0070062;GO:0050716;GO:0050715;GO:0006898;GO:0007204;GO:0005576 | hsa:6288 |  | RecName: Full=Serum amyloid A-1 protein; Short=SAA; Contains: RecName: Full=Amyloid protein A; AltName: Full=Amyloid fibril protein AA; Contains: RecName: Full=Serum amyloid protein A(2-104); Contains: RecName: Full=Serum amyloid protein A(3-104); Contains: RecName: Full=Serum amyloid protein A(2-103); Contains: RecName: Full=Serum amyloid protein A(2-102); Contains: RecName: Full=Serum amyloid protein A(4-101); Flags: Precursor |
| P0DOY3 | 9264277.474 | 8981379.125 | 0.969463528 | -0.044741471 | 0.779148707 | no | no change | GO:0005615;GO:0016020;GO:0072562;GO:0002376;GO:0005576;GO:0002250;GO:0003823;GO:0005886;GO:0070062 | hsa:100423062 | | RecName: Full=Immunoglobulin lambda constant 3; AltName: Full=Ig lambda chain C region DOT; AltName: Full=Ig lambda chain C region NEWM; AltName: Full=Ig lambda-3 chain C regions |
| P0DP01 | 24281.91167 | 27725.22915 | 1.141805865 | 0.191317378 | 0.32288093 | no | no change | GO:0016020;GO:0002376;GO:0005576;GO:0002250;GO:0003823;GO:0005886 | hsa:102723407 | | immunoglobulin heavy chain variable region, partial [Homo sapiens] |
| P10909 | 1002494.932 | 954563.8167 | 0.952188172 | -0.070681386 | 0.4044684 | no | no change | GO:0032436;GO:0005783;GO:0019730;GO:1903573;GO:0009615;GO:0016020;GO:1902949;GO:0042127;GO:0005794;GO:0034366;GO:0060548;GO:0031966;GO:0043691;GO:0043231;GO:0005829;GO:0002376;GO:0051787;GO:1902004;GO:0051788;GO:0030449;GO:1902230;GO:0050821;GO:0010628;GO:0044877;GO:0099020;GO:0048260;GO:0005856;GO:0000902;GO:0002434;GO:0072562;GO:0048471;GO:0097418;GO:0051131;GO:0032760;GO:0006629;GO:1905895;GO:0048156;GO:0001774;GO:0006956;GO:1905892;GO:1901216;GO:0006958;GO:0061077;GO:2000060;GO:1902430;GO:1902847;GO:0090201;GO:0031012;GO:0045429;GO:0051082;GO:1902998;GO:1901214;GO:0005737;GO:0001540;GO:0031093;GO:0031410;GO:0005634;GO:0051087;GO:0005739;GO:1900221;GO:0009986;GO:0017038;GO:0061740;GO:0061741;GO:0051092;GO:0031625;GO:0070062;GO:0005622;GO:0005743;GO:0005515;GO:0097440;GO:0032286;GO:0016887;GO:0071944;GO:0045202;GO:0061518;GO:0005615;GO:0002576;GO:0043065;GO:0032464;GO:0032463;GO:0045087;GO:0006915;GO:0042583;GO:0062023;GO:0050750;GO:0032991;GO:0001836;GO:0005576;GO:1905907;GO:1905908 | hsa:1191 |  | clusterin preproprotein [Homo sapiens] |
| P11226 | 108637.3736 | 113633.3227 | 1.045987388 | 0.064865457 | 0.774758532 | no | no change | GO:0004252;GO:0048306;GO:0030246;GO:0042742;GO:0044130;GO:0050766;GO:0005581;GO:0001867;GO:0005509;GO:0051873;GO:0006508;GO:0009986;GO:0045087;GO:0006953;GO:0008228;GO:0006956;GO:0006958;GO:0006979;GO:0005615;GO:0050830;GO:0002376;GO:0005576;GO:0005515;GO:0048525;GO:0005102;GO:0005537 | hsa:4153 |  | mannose-binding lectin [Homo sapiens] |
| P11597 | 7393.141222 | 6168.082 | 0.834297873 | -0.261365527 | 0.124320742 | no | no change | GO:0017129;GO:0034375;GO:0034374;GO:0034372;GO:0006869;GO:0015914;GO:0017127;GO:0030301;GO:0034197;GO:0034364;GO:0055091;GO:0005548;GO:0070062;GO:0005615;GO:0008202;GO:0010745;GO:0031982;GO:0005319;GO:0070328;GO:0006629;GO:0006641;GO:0008289;GO:0055088;GO:0031210;GO:0042632;GO:0043691;GO:0010874;GO:0008203;GO:0015485;GO:0005576;GO:0046470 | hsa:1071 |  | cholesteryl ester transfer protein isoform 1 precursor [Homo sapiens] |
| P18206 | 8854.195455 | 8700.482158 | 0.982639496 | -0.025265867 | 0.933872033 | no | no change | GO:0048675;GO:0015629;GO:0030336;GO:0034333;GO:0030032;GO:0034774;GO:0007160;GO:0002162;GO:0005925;GO:0030054;GO:0030055;GO:0005737;GO:0002009;GO:0002102;GO:0070062;GO:0002576;GO:0016020;GO:0034394;GO:0005198;GO:0003779;GO:0035580;GO:0005856;GO:0030334;GO:0042383;GO:0006936;GO:0051015;GO:0005916;GO:0005913;GO:0005912;GO:0005911;GO:0005886;GO:0007155;GO:0090136;GO:1903561;GO:1904813;GO:0031625;GO:0032991;GO:0008013;GO:0005829;GO:0045294;GO:0043312;GO:0045296;GO:0070527;GO:0005576;GO:0005515;GO:0043297;GO:0043034 | hsa:7414 |  | vinculin isoform meta-VCL [Homo sapiens] |
| P18428 | 17813.44737 | 15829.97208 | 0.888652924 | -0.170308031 | 0.284312928 | no | no change | GO:0006968;GO:0071222;GO:0071223;GO:0006869;GO:0042742;GO:0050829;GO:0032496;GO:0032490;GO:0044130;GO:0019221;GO:0060265;GO:0002281;GO:0070062;GO:0090023;GO:0042535;GO:0016020;GO:0071723;GO:0045919;GO:0002224;GO:0033036;GO:0032722;GO:0009986;GO:0032720;GO:0032760;GO:0015920;GO:0045087;GO:0008289;GO:0006953;GO:0008228;GO:0070891;GO:0034142;GO:0001530;GO:0005615;GO:0034145;GO:0031663;GO:0050830;GO:0002376;GO:0002232;GO:0005576;GO:0005515;GO:0043032;GO:0005102;GO:0032757;GO:0032755 | hsa:3929 |  | lipopolysaccharide-binding protein precursor [Homo sapiens] |
| P19320 | 2930.442067 | 2930.232184 | 0.999928379 | -0.000103332 | 0.999635104 | no | no change | GO:0009897;GO:0034113;GO:0005783;GO:0005794;GO:0035094;GO:0030175;GO:0060945;GO:0005902;GO:0050901;GO:0060326;GO:0010043;GO:0032496;GO:0001666;GO:0019221;GO:0045177;GO:0002102;GO:0010212;GO:0005615;GO:0060384;GO:0071356;GO:0016021;GO:0016020;GO:0050839;GO:0035584;GO:1904646;GO:0140039;GO:0022614;GO:0005178;GO:0042383;GO:0009308;GO:0030183;GO:0098609;GO:0007584;GO:0008131;GO:0045471;GO:0007159;GO:0005887;GO:0005886;GO:0007155;GO:0002526;GO:0007157;GO:0042102;GO:0009986;GO:0055114;GO:0070062;GO:0007160;GO:0050776;GO:0007568;GO:0060333;GO:0002544;GO:0005769;GO:0030198;GO:0035924;GO:0071065 | hsa:7412 |  | vascular cell adhesion protein 1 isoform a precursor [Homo sapiens] |
| P19827 | 314246.0632 | 322715.4875 | 1.026951569 | 0.038368145 | 0.650026526 | no | no change | GO:0030212;GO:0070062;GO:0072562;GO:0010466;GO:0005509;GO:0030414;GO:0004867;GO:0010951;GO:0005576 | hsa:3697 | COG2304 | inter-alpha-trypsin inhibitor heavy chain H1 isoform a preproprotein [Homo sapiens] |
| P20851 | 56854.73632 | 49058.4175 | 0.862873011 | -0.212779842 | 0.123690294 | no | no change | GO:0030449;GO:0005615;GO:0045087;GO:0007596;GO:0002376;GO:0045732;GO:0005576;GO:0005515;GO:0005886;GO:1903027;GO:0006958;GO:0045959;GO:0044216 | hsa:725 |  | C4b-binding protein beta chain isoform 1 precursor [Homo sapiens] |
| P22792 | 89832.95895 | 92603.00583 | 1.03083553 | 0.043814169 | 0.713069723 | no | no change | GO:0030449;GO:0070062;GO:0004181;GO:0050790;GO:0072562;GO:0050821;GO:0005576;GO:0006508;GO:0030234 | hsa:1370 | COG4886 | carboxypeptidase N subunit 2 precursor [Homo sapiens] |
| P22891 | 9700.652421 | 10217.00317 | 1.053228455 | 0.074818405 | 0.662757128 | no | no change | GO:0004252;GO:0005796;GO:0006888;GO:0005615;GO:0007596;GO:0005788;GO:0005576;GO:0005509;GO:0006508;GO:0030195;GO:0007599;GO:0070062 | hsa:8858 | COG5640 | vitamin K-dependent protein Z isoform 2 precursor [Homo sapiens] |
| P23083 | 681387.4784 | 630156.8175 | 0.924814203 | -0.112764541 | 0.685508308 | no | no change | GO:0038096;GO:0004252;GO:0016020;GO:0030449;GO:0050776;GO:0006898;GO:0038095;GO:0002376;GO:0050900;GO:0005576;GO:0006956;GO:0002250;GO:0003823;GO:0005886;GO:0006508;GO:0006958;GO:0006955 | hsa:102723407 | | IgM heavy chain VH1 region precursor, partial [Homo sapiens] |
| P23142 | 34465.29789 | 33588.43208 | 0.974558009 | -0.037180033 | 0.748898772 | no | no change | GO:0005201;GO:0008022;GO:0031012;GO:0007162;GO:0044877;GO:0005615;GO:0072378;GO:1900025;GO:0005576;GO:0005509;GO:2000647;GO:0042802;GO:0062023;GO:0071953;GO:0010952;GO:0070062;GO:0005604;GO:0007566;GO:0070051;GO:0005577;GO:0030198;GO:0001968;GO:0016032;GO:2000146;GO:0016504;GO:0001933;GO:0070373;GO:0007229 | hsa:2192 |  | fibulin-1 isoform D precursor [Homo sapiens] |
| P26038 | 3912.521765 | 3266.40165 | 0.834858397 | -0.260396576 | 0.409099712 | no | no change | GO:0005200;GO:0019899;GO:0030175;GO:0050900;GO:0010628;GO:0005925;GO:0061028;GO:0005737;GO:0071803;GO:0045177;GO:0042995;GO:0070062;GO:1902115;GO:0043209;GO:0005634;GO:0042098;GO:0072562;GO:0003779;GO:2000401;GO:0035722;GO:0022612;GO:0048471;GO:2000643;GO:0022614;GO:1903364;GO:0005856;GO:0005515;GO:0016324;GO:0009986;GO:0071944;GO:0031982;GO:0071394;GO:0016323;GO:0019901;GO:0008361;GO:0008360;GO:0045198;GO:0001771;GO:0005886;GO:0016020;GO:0050839;GO:0072678;GO:0005615;GO:0031528;GO:0007010;GO:0071437;GO:0005829;GO:0008092;GO:0005902;GO:0003725;GO:0016032;GO:0001931;GO:0007159;GO:0005102;GO:0070489;GO:0031143;GO:1902966 | hsa:4478 |  | moesin [Homo sapiens] |
| P27169 | 245743.4632 | 252886.7875 | 1.029068217 | 0.041338622 | 0.592271753 | no | no change | GO:0034445;GO:0032411;GO:0016311;GO:0046872;GO:0004064;GO:0046434;GO:0070062;GO:0005615;GO:0005543;GO:0009636;GO:0072562;GO:0005509;GO:0102007;GO:0010875;GO:0019372;GO:0042803;GO:0016787;GO:0034364;GO:0006629;GO:0034366;GO:1902617;GO:0008203;GO:0004063;GO:0043231;GO:0046395;GO:0051099;GO:0031667;GO:0046470;GO:0005576;GO:0019439;GO:0070542 | hsa:5444 |  | serum paraoxonase/arylesterase 1 precursor [Homo sapiens] |
| P29622 | 76645.57316 | 66413.45667 | 0.866500881 | -0.206726879 | 0.054857633 | no | no change | GO:0005615;GO:0070062;GO:0002576;GO:0031089;GO:0010466;GO:0030414;GO:0004867;GO:0010951;GO:0005576 | hsa:5267 | COG4826 | kallistatin isoform 1 [Homo sapiens] |
| P35858 | 58197.45158 | 51550.67458 | 0.885789209 | -0.174964673 | 0.191908111 | no | no change | GO:0044267;GO:0005615;GO:0031012;GO:0005654;GO:0007155;GO:0005576;GO:0007165;GO:0042567;GO:0005520;GO:0070062 | hsa:3483 | COG4886 | Insulin-like growth factor binding protein, acid labile subunit [Homo sapiens] |
[truncated: 25,097 more chars]
