# Supplementary material for: Proteomics of extracellular vesicles in plasma reveals the characteristics and residual traces of COVID-19 patients without underlying diseases after 3 months of recovery
Source: Cell Death Dis. 2021 May 25;12(6):541. doi: 10.1038/s41419-021-03816-3 (PMC8146187; doi:10.1038/s41419-021-03816-3)
Supplement: Supplementary file 16 — Table S3 [file 41419_2021_3816_MOESM16_ESM.docx]

| Table S3-1 KEGG pathway enrichment of DEPs in A vs C group. | | | | | | | | | |
| --- | --- | --- | --- | --- | --- | --- | --- | --- | --- |
| pathway | pathway_name | class | all_number_of_accs | all_KO2acc | diff_number_of_accs | diff_KO2acc | p_value | FDR | url |
| hsa04145 | Phagosome | Cellular Processes | 56\|228 | hsa:203068(P07437);hsa:3105(A0A0G2JI36);hsa:7278(P0DPH7);hsa:81027(Q9H4B7);hsa:102723407(A0A0C4DH29;A0A0C4DH32;A0A0C4DH33;A0A0J9YX35;A0A0C4DH31;A0A0C4DH36;A0A0C4DH34;A0A0C4DH38;A0A0C4DH39;A0A0J9YY99;A0A0G2JMI3;A0A0B4J1U7;P01763;P01764;P01743;P01766;A0A0B4J1X5;A0A0C4DH43;P01782;P01780;A0A087WSY4;P01814;A0A0B4J1X8;A0A4W8ZXM2;A0A075B7D0;P0DP02;P0DP01;A0A075B7F0;A0A075B7D8;P01817;A0A0A0MS14;A0A0A0MS15;A0A0B4J1V1;A0A075B7B8;A0A0B4J1V2;P0DTE1;A0A075B6Q5;A0A0J9YVY3;P23083);hsa:1311(G3XAP6);hsa:71(P60709);hsa:7277(P68366);hsa:113457(P0DPH7);hsa:3920(P13473);hsa:718(P01024;M0R0Q9);hsa:7057(P07996);hsa:112714(P0DPH7);hsa:2214(H0Y755);hsa:4153(P11226);hsa:78989(Q9BWP8);hsa:7037(G3V0E5);hsa:929(P08571);hsa:715(A0A3B3ISR2);hsa:60(P60709) | 8\|17 | hsa:3920(P13473);hsa:102723407(A0A0C4DH33;A0A0C4DH34;A0A0C4DH38;A0A0J9YVY3;P01782;P0DP01);hsa:3105(A0A0G2JI36) | 0.02247566 | 0.185424199 | http://www.kegg.jp/kegg-bin/show_pathway?hsa04145+hsa:3920%09green+hsa:102723407%09salmon+hsa:102723407%09salmon+hsa:102723407%09green+hsa:102723407%09salmon+hsa:102723407%09salmon+hsa:102723407%09salmon+hsa:3105%09green |
| hsa05150 | Staphylococcus aureus infection | Human Diseases | 63\|228 | hsa:102723407(A0A0C4DH29;A0A0C4DH32;A0A0C4DH33;A0A0J9YX35;A0A0C4DH31;A0A0C4DH36;A0A0C4DH34;A0A0C4DH38;A0A0C4DH39;A0A0J9YY99;A0A0G2JMI3;A0A0B4J1U7;P01763;P01764;P01743;P01766;A0A0B4J1X5;A0A0C4DH43;P01782;P01780;A0A087WSY4;P01814;A0A0B4J1X8;A0A4W8ZXM2;A0A075B7D0;P0DP02;P0DP01;A0A075B7F0;A0A075B7D8;P01817;A0A0A0MS14;A0A0A0MS15;A0A0B4J1V1;A0A075B7B8;A0A0B4J1V2;P0DTE1;A0A075B6Q5;A0A0J9YVY3;P23083);hsa:5340(P00747);hsa:629(B4E1Z4);hsa:3858(P13645);hsa:727(P01031);hsa:720(A0A0G2JPR0;P0C0L5;P0C0L4);hsa:721(A0A0G2JPR0;P0C0L5;P0C0L4);hsa:1675(K7ERG9);hsa:5648(P48740);hsa:2214(H0Y755);hsa:718(P01024;M0R0Q9);hsa:10747(O00187);hsa:713(D6R934);hsa:712(P02745);hsa:715(A0A3B3ISR2);hsa:714(P02747);hsa:717(A0A0G2JL69);hsa:716(P09871);hsa:4153(P11226);hsa:3426(A0A2R8Y3M9);hsa:2266(C9JPQ9;C9JC84);hsa:3075(P08603) | 7\|17 | hsa:3858(P13645);hsa:102723407(A0A0C4DH33;A0A0C4DH34;A0A0C4DH38;A0A0J9YVY3;P01782;P0DP01) | 0.093098696 | 0.175557542 | http://www.kegg.jp/kegg-bin/show_pathway?hsa05150+hsa:3858%09green+hsa:102723407%09salmon+hsa:102723407%09salmon+hsa:102723407%09green+hsa:102723407%09salmon+hsa:102723407%09salmon+hsa:102723407%09salmon |
| hsa05146 | Amoebiasis | Human Diseases | 47\|228 | hsa:102723407(A0A0C4DH29;A0A0C4DH32;A0A0C4DH33;A0A0J9YX35;A0A0C4DH31;A0A0C4DH36;A0A0C4DH34;A0A0C4DH38;A0A0C4DH39;A0A0J9YY99;A0A0G2JMI3;A0A0B4J1U7;P01763;P01764;P01743;P01766;A0A0B4J1X5;A0A0C4DH43;P01782;P01780;A0A087WSY4;P01814;A0A0B4J1X8;A0A4W8ZXM2;A0A075B7D0;P0DP02;P0DP01;A0A075B7F0;A0A075B7D8;P01817;A0A0A0MS14;A0A0A0MS15;A0A0B4J1V1;A0A075B7B8;A0A0B4J1V2;P0DTE1;A0A075B6Q5;A0A0J9YVY3;P23083);hsa:2335(P02751);hsa:87(H9KV75);hsa:7414(P18206);hsa:735(P02748);hsa:929(P08571);hsa:733(P07360);hsa:732(P07358);hsa:731(P07357) | 7\|17 | hsa:7414(P18206);hsa:102723407(A0A0C4DH33;A0A0C4DH34;A0A0C4DH38;A0A0J9YVY3;P01782;P0DP01) | 0.027378841 | 0.180700351 | http://www.kegg.jp/kegg-bin/show_pathway?hsa05146+hsa:7414%09green+hsa:102723407%09salmon+hsa:102723407%09salmon+hsa:102723407%09green+hsa:102723407%09salmon+hsa:102723407%09salmon+hsa:102723407%09salmon |
| hsa05152 | Tuberculosis | Human Diseases | 46\|228 | hsa:102723407(A0A0C4DH29;A0A0C4DH32;A0A0C4DH33;A0A0J9YX35;A0A0C4DH31;A0A0C4DH36;A0A0C4DH34;A0A0C4DH38;A0A0C4DH39;A0A0J9YY99;A0A0G2JMI3;A0A0B4J1U7;P01763;P01764;P01743;P01766;A0A0B4J1X5;A0A0C4DH43;P01782;P01780;A0A087WSY4;P01814;A0A0B4J1X8;A0A4W8ZXM2;A0A075B7D0;P0DP02;P0DP01;A0A075B7F0;A0A075B7D8;P01817;A0A0A0MS14;A0A0A0MS15;A0A0B4J1V1;A0A075B7B8;A0A0B4J1V2;P0DTE1;A0A075B6Q5;A0A0J9YVY3;P23083);hsa:820(J3KNB4);hsa:3920(P13473);hsa:718(P01024;M0R0Q9);hsa:2214(H0Y755);hsa:3929(P18428);hsa:929(P08571) | 7\|17 | hsa:3920(P13473);hsa:102723407(A0A0C4DH33;A0A0C4DH34;A0A0C4DH38;A0A0J9YVY3;P01782;P0DP01) | 0.024655859 | 0.180809631 | http://www.kegg.jp/kegg-bin/show_pathway?hsa05152+hsa:3920%09green+hsa:102723407%09salmon+hsa:102723407%09salmon+hsa:102723407%09green+hsa:102723407%09salmon+hsa:102723407%09salmon+hsa:102723407%09salmon |
| hsa05414 | Dilated cardiomyopathy (DCM) | Human Diseases | 44\|228 | hsa:3674(P08514);hsa:102723407(A0A0C4DH29;A0A0C4DH32;A0A0C4DH33;A0A0J9YX35;A0A0C4DH31;A0A0C4DH36;A0A0C4DH34;A0A0C4DH38;A0A0C4DH39;A0A0J9YY99;A0A0G2JMI3;A0A0B4J1U7;P01763;P01764;P01743;P01766;A0A0B4J1X5;A0A0C4DH43;P01782;P01780;A0A087WSY4;P01814;A0A0B4J1X8;A0A4W8ZXM2;A0A075B7D0;P0DP02;P0DP01;A0A075B7F0;A0A075B7D8;P01817;A0A0A0MS14;A0A0A0MS15;A0A0B4J1V1;A0A075B7B8;A0A0B4J1V2;P0DTE1;A0A075B6Q5;A0A0J9YVY3;P23083);hsa:7171(A0A087WWU8;A0A2R8Y5V9);hsa:71(P60709);hsa:70(P68032);hsa:60(P60709) | 7\|17 | hsa:102723407(A0A0C4DH33;A0A0C4DH34;A0A0C4DH38;A0A0J9YVY3;P01782;P0DP01);hsa:70(P68032) | 0.019745655 | 0.186173323 | http://www.kegg.jp/kegg-bin/show_pathway?hsa05414+hsa:102723407%09salmon+hsa:102723407%09salmon+hsa:102723407%09green+hsa:102723407%09salmon+hsa:102723407%09salmon+hsa:102723407%09salmon+hsa:70%09green |
| hsa05416 | Viral myocarditis | Human Diseases | 42\|228 | hsa:5881(P60763);hsa:102723407(A0A0C4DH29;A0A0C4DH32;A0A0C4DH33;A0A0J9YX35;A0A0C4DH31;A0A0C4DH36;A0A0C4DH34;A0A0C4DH38;A0A0C4DH39;A0A0J9YY99;A0A0G2JMI3;A0A0B4J1U7;P01763;P01764;P01743;P01766;A0A0B4J1X5;A0A0C4DH43;P01782;P01780;A0A087WSY4;P01814;A0A0B4J1X8;A0A4W8ZXM2;A0A075B7D0;P0DP02;P0DP01;A0A075B7F0;A0A075B7D8;P01817;A0A0A0MS14;A0A0A0MS15;A0A0B4J1V1;A0A075B7B8;A0A0B4J1V2;P0DTE1;A0A075B6Q5;A0A0J9YVY3;P23083);hsa:3105(A0A0G2JI36);hsa:71(P60709);hsa:60(P60709) | 7\|17 | hsa:102723407(A0A0C4DH33;A0A0C4DH34;A0A0C4DH38;A0A0J9YVY3;P01782;P0DP01);hsa:3105(A0A0G2JI36) | 0.015530613 | 0.205004094 | http://www.kegg.jp/kegg-bin/show_pathway?hsa05416+hsa:102723407%09salmon+hsa:102723407%09salmon+hsa:102723407%09green+hsa:102723407%09salmon+hsa:102723407%09salmon+hsa:102723407%09salmon+hsa:3105%09green |
| hsa04650 | Natural killer cell mediated cytotoxicity | Organismal Systems | 42\|228 | hsa:5881(P60763);hsa:102723407(A0A0C4DH29;A0A0C4DH32;A0A0C4DH33;A0A0J9YX35;A0A0C4DH31;A0A0C4DH36;A0A0C4DH34;A0A0C4DH38;A0A0C4DH39;A0A0J9YY99;A0A0G2JMI3;A0A0B4J1U7;P01763;P01764;P01743;P01766;A0A0B4J1X5;A0A0C4DH43;P01782;P01780;A0A087WSY4;P01814;A0A0B4J1X8;A0A4W8ZXM2;A0A075B7D0;P0DP02;P0DP01;A0A075B7F0;A0A075B7D8;P01817;A0A0A0MS14;A0A0A0MS15;A0A0B4J1V1;A0A075B7B8;A0A0B4J1V2;P0DTE1;A0A075B6Q5;A0A0J9YVY3;P23083);hsa:3105(A0A0G2JI36);hsa:2214(H0Y755) | 7\|17 | hsa:102723407(A0A0C4DH33;A0A0C4DH34;A0A0C4DH38;A0A0J9YVY3;P01782;P0DP01);hsa:3105(A0A0G2JI36) | 0.015530613 | 0.205004094 | http://www.kegg.jp/kegg-bin/show_pathway?hsa04650+hsa:102723407%09salmon+hsa:102723407%09salmon+hsa:102723407%09green+hsa:102723407%09salmon+hsa:102723407%09salmon+hsa:102723407%09salmon+hsa:3105%09green |
| hsa05169 | Epstein-Barr virus infection | Human Diseases | 41\|228 | hsa:102723407(A0A0C4DH29;A0A0C4DH32;A0A0C4DH33;A0A0J9YX35;A0A0C4DH31;A0A0C4DH36;A0A0C4DH34;A0A0C4DH38;A0A0C4DH39;A0A0J9YY99;A0A0G2JMI3;A0A0B4J1U7;P01763;P01764;P01743;P01766;A0A0B4J1X5;A0A0C4DH43;P01782;P01780;A0A087WSY4;P01814;A0A0B4J1X8;A0A4W8ZXM2;A0A075B7D0;P0DP02;P0DP01;A0A075B7F0;A0A075B7D8;P01817;A0A0A0MS14;A0A0A0MS15;A0A0B4J1V1;A0A075B7B8;A0A0B4J1V2;P0DTE1;A0A075B6Q5;A0A0J9YVY3;P23083);hsa:3105(A0A0G2JI36);hsa:960(H0Y5E4) | 7\|17 | hsa:102723407(A0A0C4DH33;A0A0C4DH34;A0A0C4DH38;A0A0J9YVY3;P01782;P0DP01);hsa:3105(A0A0G2JI36) | 0.013673374 | 0.225610673 | http://www.kegg.jp/kegg-bin/show_pathway?hsa05169+hsa:102723407%09salmon+hsa:102723407%09salmon+hsa:102723407%09green+hsa:102723407%09salmon+hsa:102723407%09salmon+hsa:102723407%09salmon+hsa:3105%09green |
| hsa05320 | Autoimmune thyroid disease | Human Diseases | 40\|228 | hsa:102723407(A0A0C4DH29;A0A0C4DH32;A0A0C4DH33;A0A0J9YX35;A0A0C4DH31;A0A0C4DH36;A0A0C4DH34;A0A0C4DH38;A0A0C4DH39;A0A0J9YY99;A0A0G2JMI3;A0A0B4J1U7;P01763;P01764;P01743;P01766;A0A0B4J1X5;A0A0C4DH43;P01782;P01780;A0A087WSY4;P01814;A0A0B4J1X8;A0A4W8ZXM2;A0A075B7D0;P0DP02;P0DP01;A0A075B7F0;A0A075B7D8;P01817;A0A0A0MS14;A0A0A0MS15;A0A0B4J1V1;A0A075B7B8;A0A0B4J1V2;P0DTE1;A0A075B6Q5;A0A0J9YVY3;P23083);hsa:3105(A0A0G2JI36) | 7\|17 | hsa:102723407(A0A0C4DH33;A0A0C4DH34;A0A0C4DH38;A0A0J9YVY3;P01782;P0DP01);hsa:3105(A0A0G2JI36) | 0.011975912 | 0.395205103 | http://www.kegg.jp/kegg-bin/show_pathway?hsa05320+hsa:102723407%09salmon+hsa:102723407%09salmon+hsa:102723407%09green+hsa:102723407%09salmon+hsa:102723407%09salmon+hsa:102723407%09salmon+hsa:3105%09green |
| hsa05330 | Allograft rejection | Human Diseases | 40\|228 | hsa:102723407(A0A0C4DH29;A0A0C4DH32;A0A0C4DH33;A0A0J9YX35;A0A0C4DH31;A0A0C4DH36;A0A0C4DH34;A0A0C4DH38;A0A0C4DH39;A0A0J9YY99;A0A0G2JMI3;A0A0B4J1U7;P01763;P01764;P01743;P01766;A0A0B4J1X5;A0A0C4DH43;P01782;P01780;A0A087WSY4;P01814;A0A0B4J1X8;A0A4W8ZXM2;A0A075B7D0;P0DP02;P0DP01;A0A075B7F0;A0A075B7D8;P01817;A0A0A0MS14;A0A0A0MS15;A0A0B4J1V1;A0A075B7B8;A0A0B4J1V2;P0DTE1;A0A075B6Q5;A0A0J9YVY3;P23083);hsa:3105(A0A0G2JI36) | 7\|17 | hsa:102723407(A0A0C4DH33;A0A0C4DH34;A0A0C4DH38;A0A0J9YVY3;P01782;P0DP01);hsa:3105(A0A0G2JI36) | 0.011975912 | 0.395205103 | http://www.kegg.jp/kegg-bin/show_pathway?hsa05330+hsa:102723407%09salmon+hsa:102723407%09salmon+hsa:102723407%09green+hsa:102723407%09salmon+hsa:102723407%09salmon+hsa:102723407%09salmon+hsa:3105%09green |
| hsa05322 | Systemic lupus erythematosus | Human Diseases | 60\|228 | hsa:735(P02748);hsa:102723407(A0A0C4DH29;A0A0C4DH32;A0A0C4DH33;A0A0J9YX35;A0A0C4DH31;A0A0C4DH36;A0A0C4DH34;A0A0C4DH38;A0A0C4DH39;A0A0J9YY99;A0A0G2JMI3;A0A0B4J1U7;P01763;P01764;P01743;P01766;A0A0B4J1X5;A0A0C4DH43;P01782;P01780;A0A087WSY4;P01814;A0A0B4J1X8;A0A4W8ZXM2;A0A075B7D0;P0DP02;P0DP01;A0A075B7F0;A0A075B7D8;P01817;A0A0A0MS14;A0A0A0MS15;A0A0B4J1V1;A0A075B7B8;A0A0B4J1V2;P0DTE1;A0A075B6Q5;A0A0J9YVY3;P23083);hsa:712(P02745);hsa:733(P07360);hsa:128312(A0A2R8Y619);hsa:715(A0A3B3ISR2);hsa:721(A0A0G2JPR0;P0C0L5;P0C0L4);hsa:727(P01031);hsa:718(P01024;M0R0Q9);hsa:732(P07358);hsa:87(H9KV75);hsa:2214(H0Y755);hsa:730(P10643);hsa:731(P07357);hsa:713(D6R934);hsa:716(P09871);hsa:720(A0A0G2JPR0;P0C0L5;P0C0L4);hsa:714(P02747);hsa:717(A0A0G2JL69);hsa:729(P13671) | 6\|17 | hsa:102723407(A0A0C4DH33;A0A0C4DH34;A0A0C4DH38;A0A0J9YVY3;P01782;P0DP01) | 0.145628772 | 0.213588866 | http://www.kegg.jp/kegg-bin/show_pathway?hsa05322+hsa:102723407%09salmon+hsa:102723407%09salmon+hsa:102723407%09green+hsa:102723407%09salmon+hsa:102723407%09salmon+hsa:102723407%09salmon |
| hsa04151 | PI3K-Akt signaling pathway | Environmental Information Processing | 51\|228 | hsa:7450(P04275);hsa:102723407(A0A0C4DH29;A0A0C4DH32;A0A0C4DH33;A0A0J9YX35;A0A0C4DH31;A0A0C4DH36;A0A0C4DH34;A0A0C4DH38;A0A0C4DH39;A0A0J9YY99;A0A0G2JMI3;A0A0B4J1U7;P01763;P01764;P01743;P01766;A0A0B4J1X5;A0A0C4DH43;P01782;P01780;A0A087WSY4;P01814;A0A0B4J1X8;A0A4W8ZXM2;A0A075B7D0;P0DP02;P0DP01;A0A075B7F0;A0A075B7D8;P01817;A0A0A0MS14;A0A0A0MS15;A0A0B4J1V1;A0A075B7B8;A0A0B4J1V2;P0DTE1;A0A075B6Q5;A0A0J9YVY3;P23083);hsa:1291(A0A087X0S5);hsa:7534(E7EX29);hsa:1311(G3XAP6);hsa:3481(P01344);hsa:7448(H0YJW9;P04004);hsa:2335(P02751);hsa:3674(P08514);hsa:7057(P07996);hsa:1293(E7ENL6);hsa:7148(A0A140T8Y3) | 6\|17 | hsa:102723407(A0A0C4DH33;A0A0C4DH34;A0A0C4DH38;A0A0J9YVY3;P01782;P0DP01) | 0.094637769 | 0.173502577 | http://www.kegg.jp/kegg-bin/show_pathway?hsa04151+hsa:102723407%09salmon+hsa:102723407%09salmon+hsa:102723407%09green+hsa:102723407%09salmon+hsa:102723407%09salmon+hsa:102723407%09salmon |
| hsa05143 | African trypanosomiasis | Human Diseases | 45\|228 | hsa:102723407(A0A0C4DH29;A0A0C4DH32;A0A0C4DH33;A0A0J9YX35;A0A0C4DH31;A0A0C4DH36;A0A0C4DH34;A0A0C4DH38;A0A0C4DH39;A0A0J9YY99;A0A0G2JMI3;A0A0B4J1U7;P01763;P01764;P01743;P01766;A0A0B4J1X5;A0A0C4DH43;P01782;P01780;A0A087WSY4;P01814;A0A0B4J1X8;A0A4W8ZXM2;A0A075B7D0;P0DP02;P0DP01;A0A075B7F0;A0A075B7D8;P01817;A0A0A0MS14;A0A0A0MS15;A0A0B4J1V1;A0A075B7B8;A0A0B4J1V2;P0DTE1;A0A075B6Q5;A0A0J9YVY3;P23083);hsa:3039(P69905);hsa:8542(O14791);hsa:335(P02647);hsa:3250(P00739);hsa:3043(P68871);hsa:3040(P69905);hsa:7412(P19320) | 6\|17 | hsa:102723407(A0A0C4DH33;A0A0C4DH34;A0A0C4DH38;A0A0J9YVY3;P01782;P0DP01) | 0.062419279 | 0.171653017 | http://www.kegg.jp/kegg-bin/show_pathway?hsa05143+hsa:102723407%09salmon+hsa:102723407%09salmon+hsa:102723407%09green+hsa:102723407%09salmon+hsa:102723407%09salmon+hsa:102723407%09salmon |
| hsa04640 | Hematopoietic cell lineage | Organismal Systems | 45\|228 | hsa:3674(P08514);hsa:102723407(A0A0C4DH29;A0A0C4DH32;A0A0C4DH33;A0A0J9YX35;A0A0C4DH31;A0A0C4DH36;A0A0C4DH34;A0A0C4DH38;A0A0C4DH39;A0A0J9YY99;A0A0G2JMI3;A0A0B4J1U7;P01763;P01764;P01743;P01766;A0A0B4J1X5;A0A0C4DH43;P01782;P01780;A0A087WSY4;P01814;A0A0B4J1X8;A0A4W8ZXM2;A0A075B7D0;P0DP02;P0DP01;A0A075B7F0;A0A075B7D8;P01817;A0A0A0MS14;A0A0A0MS15;A0A0B4J1V1;A0A075B7B8;A0A0B4J1V2;P0DTE1;A0A075B6Q5;A0A0J9YVY3;P23083);hsa:2811(A0A0C4DGZ8);hsa:960(H0Y5E4);hsa:290(P15144);hsa:7037(G3V0E5);hsa:929(P08571) | 6\|17 | hsa:102723407(A0A0C4DH33;A0A0C4DH34;A0A0C4DH38;A0A0J9YVY3;P01782;P0DP01) | 0.062419279 | 0.171653017 | http://www.kegg.jp/kegg-bin/show_pathway?hsa04640+hsa:102723407%09salmon+hsa:102723407%09salmon+hsa:102723407%09green+hsa:102723407%09salmon+hsa:102723407%09salmon+hsa:102723407%09salmon |
| hsa04064 | NF-kappa B signaling pathway | Environmental Information Processing | 42\|228 | hsa:102723407(A0A0C4DH29;A0A0C4DH32;A0A0C4DH33;A0A0J9YX35;A0A0C4DH31;A0A0C4DH36;A0A0C4DH34;A0A0C4DH38;A0A0C4DH39;A0A0J9YY99;A0A0G2JMI3;A0A0B4J1U7;P01763;P01764;P01743;P01766;A0A0B4J1X5;A0A0C4DH43;P01782;P01780;A0A087WSY4;P01814;A0A0B4J1X8;A0A4W8ZXM2;A0A075B7D0;P0DP02;P0DP01;A0A075B7F0;A0A075B7D8;P01817;A0A0A0MS14;A0A0A0MS15;A0A0B4J1V1;A0A075B7B8;A0A0B4J1V2;P0DTE1;A0A075B6Q5;A0A0J9YVY3;P23083);hsa:929(P08571);hsa:7412(P19320);hsa:3929(P18428) | 6\|17 | hsa:102723407(A0A0C4DH33;A0A0C4DH34;A0A0C4DH38;A0A0J9YVY3;P01782;P0DP01) | 0.048317463 | 0.151854884 | http://www.kegg.jp/kegg-bin/show_pathway?hsa04064+hsa:102723407%09salmon+hsa:102723407%09salmon+hsa:102723407%09green+hsa:102723407%09salmon+hsa:102723407%09salmon+hsa:102723407%09salmon |
| hsa05202 | Transcriptional misregulation in cancer | Human Diseases | 42\|228 | hsa:102723407(A0A0C4DH29;A0A0C4DH32;A0A0C4DH33;A0A0J9YX35;A0A0C4DH31;A0A0C4DH36;A0A0C4DH34;A0A0C4DH38;A0A0C4DH39;A0A0J9YY99;A0A0G2JMI3;A0A0B4J1U7;P01763;P01764;P01743;P01766;A0A0B4J1X5;A0A0C4DH43;P01782;P01780;A0A087WSY4;P01814;A0A0B4J1X8;A0A4W8ZXM2;A0A075B7D0;P0DP02;P0DP01;A0A075B7F0;A0A075B7D8;P01817;A0A0A0MS14;A0A0A0MS15;A0A0B4J1V1;A0A075B7B8;A0A0B4J1V2;P0DTE1;A0A075B6Q5;A0A0J9YVY3;P23083);hsa:929(P08571);hsa:3486(A6XND0);hsa:1668(P59665) | 6\|17 | hsa:102723407(A0A0C4DH33;A0A0C4DH34;A0A0C4DH38;A0A0J9YVY3;P01782;P0DP01) | 0.048317463 | 0.151854884 | http://www.kegg.jp/kegg-bin/show_pathway?hsa05202+hsa:102723407%09salmon+hsa:102723407%09salmon+hsa:102723407%09green+hsa:102723407%09salmon+hsa:102723407%09salmon+hsa:102723407%09salmon |
| hsa05140 | Leishmaniasis | Human Diseases | 42\|228 | hsa:718(P01024;M0R0Q9);hsa:2214(H0Y755);hsa:102723407(A0A0C4DH29;A0A0C4DH32;A0A0C4DH33;A0A0J9YX35;A0A0C4DH31;A0A0C4DH36;A0A0C4DH34;A0A0C4DH38;A0A0C4DH39;A0A0J9YY99;A0A0G2JMI3;A0A0B4J1U7;P01763;P01764;P01743;P01766;A0A0B4J1X5;A0A0C4DH43;P01782;P01780;A0A087WSY4;P01814;A0A0B4J1X8;A0A4W8ZXM2;A0A075B7D0;P0DP02;P0DP01;A0A075B7F0;A0A075B7D8;P01817;A0A0A0MS14;A0A0A0MS15;A0A0B4J1V1;A0A075B7B8;A0A0B4J1V2;P0DTE1;A0A075B6Q5;A0A0J9YVY3;P23083) | 6\|17 | hsa:102723407(A0A0C4DH33;A0A0C4DH34;A0A0C4DH38;A0A0J9YVY3;P01782;P0DP01) | 0.048317463 | 0.151854884 | http://www.kegg.jp/kegg-bin/show_pathway?hsa05140+hsa:102723407%09salmon+hsa:102723407%09salmon+hsa:102723407%09green+hsa:102723407%09salmon+hsa:102723407%09salmon+hsa:102723407%09salmon |
| hsa04666 | Fc gamma R-mediated phagocytosis | Organismal Systems | 42\|228 | hsa:2934(A0A0A0MS51);hsa:102723407(A0A0C4DH29;A0A0C4DH32;A0A0C4DH33;A0A0J9YX35;A0A0C4DH31;A0A0C4DH36;A0A0C4DH34;A0A0C4DH38;A0A0C4DH39;A0A0J9YY99;A0A0G2JMI3;A0A0B4J1U7;P01763;P01764;P01743;P01766;A0A0B4J1X5;A0A0C4DH43;P01782;P01780;A0A087WSY4;P01814;A0A0B4J1X8;A0A4W8ZXM2;A0A075B7D0;P0DP02;P0DP01;A0A075B7F0;A0A075B7D8;P01817;A0A0A0MS14;A0A0A0MS15;A0A0B4J1V1;A0A075B7B8;A0A0B4J1V2;P0DTE1;A0A075B6Q5;A0A0J9YVY3;P23083);hsa:1072(E9PK25);hsa:2214(H0Y755) | 6\|17 | hsa:102723407(A0A0C4DH33;A0A0C4DH34;A0A0C4DH38;A0A0J9YVY3;P01782;P0DP01) | 0.048317463 | 0.151854884 | http://www.kegg.jp/kegg-bin/show_pathway?hsa04666+hsa:102723407%09salmon+hsa:102723407%09salmon+hsa:102723407%09green+hsa:102723407%09salmon+hsa:102723407%09salmon+hsa:102723407%09salmon |
| hsa04672 | Intestinal immune network for IgA production | Organismal Systems | 41\|228 | hsa:102723407(A0A0C4DH29;A0A0C4DH32;A0A0C4DH33;A0A0J9YX35;A0A0C4DH31;A0A0C4DH36;A0A0C4DH34;A0A0C4DH38;A0A0C4DH39;A0A0J9YY99;A0A0G2JMI3;A0A0B4J1U7;P01763;P01764;P01743;P01766;A0A0B4J1X5;A0A0C4DH43;P01782;P01780;A0A087WSY4;P01814;A0A0B4J1X8;A0A4W8ZXM2;A0A075B7D0;P0DP02;P0DP01;A0A075B7F0;A0A075B7D8;P01817;A0A0A0MS14;A0A0A0MS15;A0A0B4J1V1;A0A075B7B8;A0A0B4J1V2;P0DTE1;A0A075B6Q5;A0A0J9YVY3;P23083);hsa:102723996(A0A087X1L8);hsa:5284(P01833);hsa:23308(A0A087X1L8) | 6\|17 | hsa:102723407(A0A0C4DH33;A0A0C4DH34;A0A0C4DH38;A0A0J9YVY3;P01782;P0DP01) | 0.044003404 | 0.161345815 | http://www.kegg.jp/kegg-bin/show_pathway?hsa04672+hsa:102723407%09salmon+hsa:102723407%09salmon+hsa:102723407%09green+hsa:102723407%09salmon+hsa:102723407%09salmon+hsa:102723407%09salmon |
| hsa05340 | Primary immunodeficiency | Human Diseases | 41\|228 | hsa:102723407(A0A0C4DH29;A0A0C4DH32;A0A0C4DH33;A0A0J9YX35;A0A0C4DH31;A0A0C4DH36;A0A0C4DH34;A0A0C4DH38;A0A0C4DH39;A0A0J9YY99;A0A0G2JMI3;A0A0B4J1U7;P01763;P01764;P01743;P01766;A0A0B4J1X5;A0A0C4DH43;P01782;P01780;A0A087WSY4;P01814;A0A0B4J1X8;A0A4W8ZXM2;A0A075B7D0;P0DP02;P0DP01;A0A075B7F0;A0A075B7D8;P01817;A0A0A0MS14;A0A0A0MS15;A0A0B4J1V1;A0A075B7B8;A0A0B4J1V2;P0DTE1;A0A075B6Q5;A0A0J9YVY3;P23083);hsa:3543(P01871;P15814) | 6\|17 | hsa:102723407(A0A0C4DH33;A0A0C4DH34;A0A0C4DH38;A0A0J9YVY3;P01782;P0DP01) | 0.044003404 | 0.161345815 | http://www.kegg.jp/kegg-bin/show_pathway?hsa05340+hsa:102723407%09salmon+hsa:102723407%09salmon+hsa:102723407%09green+hsa:102723407%09salmon+hsa:102723407%09salmon+hsa:102723407%09salmon |
| hsa04662 | B cell receptor signaling pathway | Organismal Systems | 40\|228 | hsa:5881(P60763);hsa:102723407(A0A0C4DH29;A0A0C4DH32;A0A0C4DH33;A0A0J9YX35;A0A0C4DH31;A0A0C4DH36;A0A0C4DH34;A0A0C4DH38;A0A0C4DH39;A0A0J9YY99;A0A0G2JMI3;A0A0B4J1U7;P01763;P01764;P01743;P01766;A0A0B4J1X5;A0A0C4DH43;P01782;P01780;A0A087WSY4;P01814;A0A0B4J1X8;A0A4W8ZXM2;A0A075B7D0;P0DP02;P0DP01;A0A075B7F0;A0A075B7D8;P01817;A0A0A0MS14;A0A0A0MS15;A0A0B4J1V1;A0A075B7B8;A0A0B4J1V2;P0DTE1;A0A075B6Q5;A0A0J9YVY3;P23083) | 6\|17 | hsa:102723407(A0A0C4DH33;A0A0C4DH34;A0A0C4DH38;A0A0J9YVY3;P01782;P0DP01) | 0.03989836 | 0.164580735 | http://www.kegg.jp/kegg-bin/show_pathway?hsa04662+hsa:102723407%09salmon+hsa:102723407%09salmon+hsa:102723407%09green+hsa:102723407%09salmon+hsa:102723407%09salmon+hsa:102723407%09salmon |
| hsa05162 | Measles | Human Diseases | 40\|228 | hsa:102723407(A0A0C4DH29;A0A0C4DH32;A0A0C4DH33;A0A0J9YX35;A0A0C4DH31;A0A0C4DH36;A0A0C4DH34;A0A0C4DH38;A0A0C4DH39;A0A0J9YY99;A0A0G2JMI3;A0A0B4J1U7;P01763;P01764;P01743;P01766;A0A0B4J1X5;A0A0C4DH43;P01782;P01780;A0A087WSY4;P01814;A0A0B4J1X8;A0A4W8ZXM2;A0A075B7D0;P0DP02;P0DP01;A0A075B7F0;A0A075B7D8;P01817;A0A0A0MS14;A0A0A0MS15;A0A0B4J1V1;A0A075B7B8;A0A0B4J1V2;P0DTE1;A0A075B6Q5;A0A0J9YVY3;P23083);hsa:4478(P26038) | 6\|17 | hsa:102723407(A0A0C4DH33;A0A0C4DH34;A0A0C4DH38;A0A0J9YVY3;P01782;P0DP01) | 0.03989836 | 0.164580735 | http://www.kegg.jp/kegg-bin/show_pathway?hsa05162+hsa:102723407%09salmon+hsa:102723407%09salmon+hsa:102723407%09green+hsa:102723407%09salmon+hsa:102723407%09salmon+hsa:102723407%09salmon |
| hsa04664 | Fc epsilon RI signaling pathway | Organismal Systems | 40\|228 | hsa:5881(P60763);hsa:102723407(A0A0C4DH29;A0A0C4DH32;A0A0C4DH33;A0A0J9YX35;A0A0C4DH31;A0A0C4DH36;A0A0C4DH34;A0A0C4DH38;A0A0C4DH39;A0A0J9YY99;A0A0G2JMI3;A0A0B4J1U7;P01763;P01764;P01743;P01766;A0A0B4J1X5;A0A0C4DH43;P01782;P01780;A0A087WSY4;P01814;A0A0B4J1X8;A0A4W8ZXM2;A0A075B7D0;P0DP02;P0DP01;A0A075B7F0;A0A075B7D8;P01817;A0A0A0MS14;A0A0A0MS15;A0A0B4J1V1;A0A075B7B8;A0A0B4J1V2;P0DTE1;A0A075B6Q5;A0A0J9YVY3;P23083) | 6\|17 | hsa:102723407(A0A0C4DH33;A0A0C4DH34;A0A0C4DH38;A0A0J9YVY3;P01782;P0DP01) | 0.03989836 | 0.164580735 | http://www.kegg.jp/kegg-bin/show_pathway?hsa04664+hsa:102723407%09salmon+hsa:102723407%09salmon+hsa:102723407%09green+hsa:102723407%09salmon+hsa:102723407%09salmon+hsa:102723407%09salmon |
| hsa05323 | Rheumatoid arthritis | Human Diseases | 39\|228 | hsa:102723407(A0A0C4DH29;A0A0C4DH32;A0A0C4DH33;A0A0J9YX35;A0A0C4DH31;A0A0C4DH36;A0A0C4DH34;A0A0C4DH38;A0A0C4DH39;A0A0J9YY99;A0A0G2JMI3;A0A0B4J1U7;P01763;P01764;P01743;P01766;A0A0B4J1X5;A0A0C4DH43;P01782;P01780;A0A087WSY4;P01814;A0A0B4J1X8;A0A4W8ZXM2;A0A075B7D0;P0DP02;P0DP01;A0A075B7F0;A0A075B7D8;P01817;A0A0A0MS14;A0A0A0MS15;A0A0B4J1V1;A0A075B7B8;A0A0B4J1V2;P0DTE1;A0A075B6Q5;A0A0J9YVY3;P23083) | 6\|17 | hsa:102723407(A0A0C4DH33;A0A0C4DH34;A0A0C4DH38;A0A0J9YVY3;P01782;P0DP01) | 0.036009391 | 0.198051648 | http://www.kegg.jp/kegg-bin/show_pathway?hsa05323+hsa:102723407%09salmon+hsa:102723407%09salmon+hsa:102723407%09green+hsa:102723407%09salmon+hsa:102723407%09salmon+hsa:102723407%09salmon |
| hsa04072 | Phospholipase D signaling pathway | Environmental Information Processing | 39\|228 | hsa:102723407(A0A0C4DH29;A0A0C4DH32;A0A0C4DH33;A0A0J9YX35;A0A0C4DH31;A0A0C4DH36;A0A0C4DH34;A0A0C4DH38;A0A0C4DH39;A0A0J9YY99;A0A0G2JMI3;A0A0B4J1U7;P01763;P01764;P01743;P01766;A0A0B4J1X5;A0A0C4DH43;P01782;P01780;A0A087WSY4;P01814;A0A0B4J1X8;A0A4W8ZXM2;A0A075B7D0;P0DP02;P0DP01;A0A075B7F0;A0A075B7D8;P01817;A0A0A0MS14;A0A0A0MS15;A0A0B4J1V1;A0A075B7B8;A0A0B4J1V2;P0DTE1;A0A075B6Q5;A0A0J9YVY3;P23083) | 6\|17 | hsa:102723407(A0A0C4DH33;A0A0C4DH34;A0A0C4DH38;A0A0J9YVY3;P01782;P0DP01) | 0.036009391 | 0.198051648 | http://www.kegg.jp/kegg-bin/show_pathway?hsa04072+hsa:102723407%09salmon+hsa:102723407%09salmon+hsa:102723407%09green+hsa:102723407%09salmon+hsa:102723407%09salmon+hsa:102723407%09salmon |
| hsa04020 | Calcium signaling pathway | Environmental Information Processing | 39\|228 | hsa:102723407(A0A0C4DH29;A0A0C4DH32;A0A0C4DH33;A0A0J9YX35;A0A0C4DH31;A0A0C4DH36;A0A0C4DH34;A0A0C4DH38;A0A0C4DH39;A0A0J9YY99;A0A0G2JMI3;A0A0B4J1U7;P01763;P01764;P01743;P01766;A0A0B4J1X5;A0A0C4DH43;P01782;P01780;A0A087WSY4;P01814;A0A0B4J1X8;A0A4W8ZXM2;A0A075B7D0;P0DP02;P0DP01;A0A075B7F0;A0A075B7D8;P01817;A0A0A0MS14;A0A0A0MS15;A0A0B4J1V1;A0A075B7B8;A0A0B4J1V2;P0DTE1;A0A075B6Q5;A0A0J9YVY3;P23083) | 6\|17 | hsa:102723407(A0A0C4DH33;A0A0C4DH34;A0A0C4DH38;A0A0J9YVY3;P01782;P0DP01) | 0.036009391 | 0.198051648 | http://www.kegg.jp/kegg-bin/show_pathway?hsa04020+hsa:102723407%09salmon+hsa:102723407%09salmon+hsa:102723407%09green+hsa:102723407%09salmon+hsa:102723407%09salmon+hsa:102723407%09salmon |
| hsa05310 | Asthma | Human Diseases | 39\|228 | hsa:102723407(A0A0C4DH29;A0A0C4DH32;A0A0C4DH33;A0A0J9YX35;A0A0C4DH31;A0A0C4DH36;A0A0C4DH34;A0A0C4DH38;A0A0C4DH39;A0A0J9YY99;A0A0G2JMI3;A0A0B4J1U7;P01763;P01764;P01743;P01766;A0A0B4J1X5;A0A0C4DH43;P01782;P01780;A0A087WSY4;P01814;A0A0B4J1X8;A0A4W8ZXM2;A0A075B7D0;P0DP02;P0DP01;A0A075B7F0;A0A075B7D8;P01817;A0A0A0MS14;A0A0A0MS15;A0A0B4J1V1;A0A075B7B8;A0A0B4J1V2;P0DTE1;A0A075B6Q5;A0A0J9YVY3;P23083) | 6\|17 | hsa:102723407(A0A0C4DH33;A0A0C4DH34;A0A0C4DH38;A0A0J9YVY3;P01782;P0DP01) | 0.036009391 | 0.198051648 | http://www.kegg.jp/kegg-bin/show_pathway?hsa05310+hsa:102723407%09salmon+hsa:102723407%09salmon+hsa:102723407%09green+hsa:102723407%09salmon+hsa:102723407%09salmon+hsa:102723407%09salmon |
| hsa04140 | Autophagy - animal | Cellular Processes | 2\|228 | hsa:3920(P13473);hsa:2081(O75460) | 2\|17 | hsa:3920(P13473);hsa:2081(O75460) | 0.005255429 | 0.346858335 | http://www.kegg.jp/kegg-bin/show_pathway?hsa04140+hsa:3920%09green+hsa:2081%09green |
| hsa05203 | Viral carcinogenesis | Human Diseases | 8\|228 | hsa:2934(A0A0A0MS51);hsa:128312(A0A2R8Y619);hsa:7534(E7EX29);hsa:718(P01024;M0R0Q9);hsa:87(H9KV75);hsa:5315(H3BTN5);hsa:3105(A0A0G2JI36) | 1\|17 | hsa:3105(A0A0G2JI36) | 0.355051961 | 0.366147335 | http://www.kegg.jp/kegg-bin/show_pathway?hsa05203+hsa:3105%09green |
| hsa04670 | Leukocyte transendothelial migration | Organismal Systems | 7\|228 | hsa:5908(P61224);hsa:87(H9KV75);hsa:7412(P19320);hsa:60(P60709);hsa:7414(P18206);hsa:4478(P26038);hsa:71(P60709);hsa:1003(I3L1J2) | 1\|17 | hsa:7414(P18206) | 0.334917917 | 0.356525524 | http://www.kegg.jp/kegg-bin/show_pathway?hsa04670+hsa:7414%09green |
| hsa04514 | Cell adhesion molecules (CAMs) | Environmental Information Processing | 5\|228 | hsa:102723996(A0A087X1L8);hsa:7412(P19320);hsa:6402(P14151);hsa:23308(A0A087X1L8);hsa:3105(A0A0G2JI36);hsa:1003(I3L1J2) | 1\|17 | hsa:3105(A0A0G2JI36) | 0.277735003 | 0.305508503 | http://www.kegg.jp/kegg-bin/show_pathway?hsa04514+hsa:3105%09green |
| hsa05410 | Hypertrophic cardiomyopathy (HCM) | Human Diseases | 5\|228 | hsa:3674(P08514);hsa:7171(A0A087WWU8;A0A2R8Y5V9);hsa:71(P60709);hsa:70(P68032);hsa:60(P60709) | 1\|17 | hsa:70(P68032) | 0.277735003 | 0.305508503 | http://www.kegg.jp/kegg-bin/show_pathway?hsa05410+hsa:70%09green |
| hsa05168 | Herpes simplex infection | Human Diseases | 5\|228 | hsa:718(P01024;M0R0Q9);hsa:5199(E9PAQ1);hsa:727(P01031);hsa:3105(A0A0G2JI36) | 1\|17 | hsa:3105(A0A0G2JI36) | 0.277735003 | 0.305508503 | http://www.kegg.jp/kegg-bin/show_pathway?hsa05168+hsa:3105%09green |
| hsa05100 | Bacterial invasion of epithelial cells | Human Diseases | 4\|228 | hsa:7414(P18206);hsa:3611(A0A0A0MTH3);hsa:71(P60709);hsa:60(P60709);hsa:2335(P02751) | 1\|17 | hsa:7414(P18206) | 0.239279387 | 0.287135264 | http://www.kegg.jp/kegg-bin/show_pathway?hsa05100+hsa:7414%09green |
| hsa05131 | Shigellosis | Human Diseases | 4\|228 | hsa:7414(P18206);hsa:71(P60709);hsa:960(H0Y5E4);hsa:5216(P07737);hsa:60(P60709) | 1\|17 | hsa:7414(P18206) | 0.239279387 | 0.287135264 | http://www.kegg.jp/kegg-bin/show_pathway?hsa05131+hsa:7414%09green |
| hsa04915 | Estrogen signaling pathway | Organismal Systems | 4\|228 | hsa:3858(P13645);hsa:3868(P08779);hsa:3861(P02533);hsa:3857(P35527) | 1\|17 | hsa:3858(P13645) | 0.239279387 | 0.287135264 | http://www.kegg.jp/kegg-bin/show_pathway?hsa04915+hsa:3858%09green |
| hsa04520 | Adherens junction | Cellular Processes | 4\|228 | hsa:5881(P60763);hsa:7414(P18206);hsa:87(H9KV75);hsa:71(P60709);hsa:60(P60709) | 1\|17 | hsa:7414(P18206) | 0.239279387 | 0.287135264 | http://www.kegg.jp/kegg-bin/show_pathway?hsa04520+hsa:7414%09green |
| hsa04977 | Vitamin digestion and absorption | Organismal Systems | 4\|228 | hsa:686(P43251);hsa:338(P04114);hsa:337(P06727);hsa:335(P02647) | 1\|17 | hsa:686(P43251) | 0.239279387 | 0.287135264 | http://www.kegg.jp/kegg-bin/show_pathway?hsa04977+hsa:686%09salmon |
| hsa04210 | Apoptosis | Cellular Processes | 4\|228 | hsa:7278(P0DPH7);hsa:2081(O75460);hsa:7277(P68366);hsa:113457(P0DPH7);hsa:112714(P0DPH7);hsa:71(P60709);hsa:60(P60709) | 1\|17 | hsa:2081(O75460) | 0.239279387 | 0.287135264 | http://www.kegg.jp/kegg-bin/show_pathway?hsa04210+hsa:2081%09green |
| hsa05010 | Alzheimer disease | Human Diseases | 4\|228 | hsa:2597(E7EUT5);hsa:4035(Q07954);hsa:348(P02649);hsa:2081(O75460) | 1\|17 | hsa:2081(O75460) | 0.239279387 | 0.287135264 | http://www.kegg.jp/kegg-bin/show_pathway?hsa05010+hsa:2081%09green |
| hsa00590 | Arachidonic acid metabolism | Metabolism | 3\|228 | hsa:2878(A0A087X1J7);hsa:5730(P41222);hsa:81579(A0A2R8Y3M9) | 1\|17 | hsa:5730(P41222) | 0.19319807 | 0.265647346 | http://www.kegg.jp/kegg-bin/show_pathway?hsa00590+hsa:5730%09green |
| hsa04141 | Protein processing in endoplasmic reticulum | Genetic Information Processing | 3\|228 | hsa:4121(P33908);hsa:2081(O75460);hsa:3309(P11021) | 1\|17 | hsa:2081(O75460) | 0.19319807 | 0.265647346 | http://www.kegg.jp/kegg-bin/show_pathway?hsa04141+hsa:2081%09green |
| hsa04261 | Adrenergic signaling in cardiomyocytes | Organismal Systems | 3\|228 | hsa:7171(A0A087WWU8;A0A2R8Y5V9);hsa:70(P68032) | 1\|17 | hsa:70(P68032) | 0.19319807 | 0.265647346 | http://www.kegg.jp/kegg-bin/show_pathway?hsa04261+hsa:70%09green |
| hsa05170 | Human immunodeficiency virus 1 infection | Human Diseases | 3\|228 | hsa:5881(P60763);hsa:1072(E9PK25);hsa:3105(A0A0G2JI36) | 1\|17 | hsa:3105(A0A0G2JI36) | 0.19319807 | 0.265647346 | http://www.kegg.jp/kegg-bin/show_pathway?hsa05170+hsa:3105%09green |
| hsa04260 | Cardiac muscle contraction | Organismal Systems | 3\|228 | hsa:7171(A0A087WWU8;A0A2R8Y5V9);hsa:70(P68032) | 1\|17 | hsa:70(P68032) | 0.19319807 | 0.265647346 | http://www.kegg.jp/kegg-bin/show_pathway?hsa04260+hsa:70%09green |
| hsa05167 | Kaposi sarcoma-associated herpesvirus infection | Human Diseases | 3\|228 | hsa:718(P01024;M0R0Q9);hsa:3105(A0A0G2JI36) | 1\|17 | hsa:3105(A0A0G2JI36) | 0.19319807 | 0.265647346 | http://www.kegg.jp/kegg-bin/show_pathway?hsa05167+hsa:3105%09green |
| hsa04510 | Focal adhesion | Cellular Processes | 20\|228 | hsa:7450(P04275);hsa:29780(A0A087WZB5);hsa:7094(Q9Y490);hsa:1291(A0A087X0S5);hsa:1293(E7ENL6);hsa:5908(P61224);hsa:87(H9KV75);hsa:3674(P08514);hsa:7448(H0YJW9;P04004);hsa:3611(A0A0A0MTH3);hsa:5881(P60763);hsa:1311(G3XAP6);hsa:2316(P21333);hsa:7057(P07996);hsa:7791(H0Y2Y8);hsa:60(P60709);hsa:7414(P18206);hsa:2335(P02751);hsa:71(P60709);hsa:7148(A0A140T8Y3) | 1\|17 | hsa:7414(P18206) | 0.350039664 | 0.366708219 | http://www.kegg.jp/kegg-bin/show_pathway?hsa04510+hsa:7414%09green |
| hsa04218 | Cellular senescence | Cellular Processes | 2\|228 | hsa:3105(A0A0G2JI36);hsa:3486(A6XND0) | 1\|17 | hsa:3105(A0A0G2JI36) | 0.138611948 | 0.228709715 | http://www.kegg.jp/kegg-bin/show_pathway?hsa04218+hsa:3105%09green |
| hsa04144 | Endocytosis | Cellular Processes | 2\|228 | hsa:7037(G3V0E5);hsa:3105(A0A0G2JI36) | 1\|17 | hsa:3105(A0A0G2JI36) | 0.138611948 | 0.228709715 | http://www.kegg.jp/kegg-bin/show_pathway?hsa04144+hsa:3105%09green |
| hsa05163 | Human cytomegalovirus infection | Human Diseases | 2\|228 | hsa:5881(P60763);hsa:3105(A0A0G2JI36) | 1\|17 | hsa:3105(A0A0G2JI36) | 0.138611948 | 0.228709715 | http://www.kegg.jp/kegg-bin/show_pathway?hsa05163+hsa:3105%09green |
| hsa05166 | Human T-cell leukemia virus 1 infection | Human Diseases | 2\|228 | hsa:3105(A0A0G2JI36);hsa:7094(Q9Y490) | 1\|17 | hsa:3105(A0A0G2JI36) | 0.138611948 | 0.228709715 | http://www.kegg.jp/kegg-bin/show_pathway?hsa05166+hsa:3105%09green |
| hsa04270 | Vascular smooth muscle contraction | Organismal Systems | 2\|228 | hsa:81579(A0A2R8Y3M9);hsa:59(P68032);hsa:72(P68032) | 1\|17 | hsa:59(P68032);hsa:72(P68032) | 0.138611948 | 0.228709715 | http://www.kegg.jp/kegg-bin/show_pathway?hsa04270+hsa:59%09green+hsa:72%09green |
| hsa04614 | Renin-angiotensin system | Organismal Systems | 2\|228 | hsa:290(P15144);hsa:183(P01019) | 1\|17 | hsa:183(P01019) | 0.138611948 | 0.228709715 | http://www.kegg.jp/kegg-bin/show_pathway?hsa04614+hsa:183%09green |
| hsa04612 | Antigen processing and presentation | Organismal Systems | 2\|228 | hsa:3105(A0A0G2JI36);hsa:3309(P11021) | 1\|17 | hsa:3105(A0A0G2JI36) | 0.138611948 | 0.228709715 | http://www.kegg.jp/kegg-bin/show_pathway?hsa04612+hsa:3105%09green |
| hsa04932 | Non-alcoholic fatty liver disease (NAFLD) | Human Diseases | 2\|228 | hsa:2081(O75460);hsa:9370(Q15848) | 1\|17 | hsa:2081(O75460) | 0.138611948 | 0.228709715 | http://www.kegg.jp/kegg-bin/show_pathway?hsa04932+hsa:2081%09green |
| hsa05165 | Human papillomavirus infection | Human Diseases | 13\|228 | hsa:7450(P04275);hsa:3674(P08514);hsa:3993(J3QRV5);hsa:1291(A0A087X0S5);hsa:1293(E7ENL6);hsa:1311(G3XAP6);hsa:7448(H0YJW9;P04004);hsa:2335(P02751);hsa:7057(P07996);hsa:5315(H3BTN5);hsa:3105(A0A0G2JI36);hsa:7148(A0A140T8Y3) | 1\|17 | hsa:3105(A0A0G2JI36) | 0.394105172 | 0.394105172 | http://www.kegg.jp/kegg-bin/show_pathway?hsa05165+hsa:3105%09green |
| hsa04810 | Regulation of actin cytoskeleton | Cellular Processes | 12\|228 | hsa:3674(P08514);hsa:2934(A0A0A0MS51);hsa:1072(E9PK25);hsa:2147(P00734);hsa:2335(P02751);hsa:5881(P60763);hsa:4627(P35579);hsa:87(H9KV75);hsa:5216(P07737);hsa:7414(P18206);hsa:4478(P26038);hsa:71(P60709);hsa:60(P60709) | 1\|17 | hsa:7414(P18206) | 0.392892541 | 0.398937041 | http://www.kegg.jp/kegg-bin/show_pathway?hsa04810+hsa:7414%09green |
| hsa04940 | Type I diabetes mellitus | Human Diseases | 1\|228 | hsa:3105(A0A0G2JI36) | 1\|17 | hsa:3105(A0A0G2JI36) | 0.074561404 | 0.164035088 | http://www.kegg.jp/kegg-bin/show_pathway?hsa04940+hsa:3105%09green |
| hsa05332 | Graft-versus-host disease | Human Diseases | 1\|228 | hsa:3105(A0A0G2JI36) | 1\|17 | hsa:3105(A0A0G2JI36) | 0.074561404 | 0.164035088 | http://www.kegg.jp/kegg-bin/show_pathway?hsa05332+hsa:3105%09green |
| hsa00780 | Biotin metabolism | Metabolism | 1\|228 | hsa:686(P43251) | 1\|17 | hsa:686(P43251) | 0.074561404 | 0.164035088 | http://www.kegg.jp/kegg-bin/show_pathway?hsa00780+hsa:686%09salmon |
| hsa04142 | Lysosome | Cellular Processes | 1\|228 | hsa:3920(P13473) | 1\|17 | hsa:3920(P13473) | 0.074561404 | 0.164035088 | http://www.kegg.jp/kegg-bin/show_pathway?hsa04142+hsa:3920%09green |
| hsa04926 | Relaxin signaling pathway | Organismal Systems | 1\|228 | hsa:59(P68032) | 1\|17 | hsa:59(P68032) | 0.074561404 | 0.164035088 | http://www.kegg.jp/kegg-bin/show_pathway?hsa04926+hsa:59%09green |
| hsa04371 | Apelin signaling pathway | Environmental Information Processing | 1\|228 | hsa:59(P68032) | 1\|17 | hsa:59(P68032) | 0.074561404 | 0.164035088 | http://www.kegg.jp/kegg-bin/show_pathway?hsa04371+hsa:59%09green |
| hsa00563 | Glycosylphosphatidylinositol (GPI)-anchor biosynthesis | Metabolism | 1\|228 | hsa:2822(P80108) | 1\|17 | hsa:2822(P80108) | 0.074561404 | 0.164035088 | http://www.kegg.jp/kegg-bin/show_pathway?hsa00563+hsa:2822%09salmon |
| hsa04630 | JAK-STAT signaling pathway | Environmental Information Processing | 1\|228 | hsa:2670(A0A1W2PQU7) | 1\|17 | hsa:2670(A0A1W2PQU7) | 0.074561404 | 0.164035088 | http://www.kegg.jp/kegg-bin/show_pathway?hsa04630+hsa:2670%09green |
| hsa04924 | Renin secretion | Organismal Systems | 1\|228 | hsa:183(P01019) | 1\|17 | hsa:183(P01019) | 0.074561404 | 0.164035088 | http://www.kegg.jp/kegg-bin/show_pathway?hsa04924+hsa:183%09green |

| Table S3-2 KEGG pathway enrichment of DEPs in M vs C group. | | | | | | | | | |
| --- | --- | --- | --- | --- | --- | --- | --- | --- | --- |
| pathway | pathway_name | class | all_number_of_accs | all_KO2acc | diff_number_of_accs | diff_KO2acc | p_value | FDR | url |
| hsa04218 | Cellular senescence | Cellular Processes | 2\|228 | hsa:3105(A0A0G2JI36);hsa:3486(A6XND0) | 1\|36 | hsa:3105(A0A0G2JI36) | 0.2670995 | 0.3827097 | http://www.kegg.jp/kegg-bin/show_pathway?hsa04218+hsa:3105%09green |
| hsa05323 | Rheumatoid arthritis | Human Diseases | 39\|228 | hsa:102723407(A0A0C4DH29;A0A0C4DH32;A0A0C4DH33;A0A0J9YX35;A0A0C4DH31;A0A0C4DH36;A0A0C4DH34;A0A0C4DH38;A0A0C4DH39;A0A0J9YY99;A0A0G2JMI3;A0A0B4J1U7;P01763;P01764;P01743;P01766;A0A0B4J1X5;A0A0C4DH43;P01782;P01780;A0A087WSY4;P01814;A0A0B4J1X8;A0A4W8ZXM2;A0A075B7D0;P0DP02;P0DP01;A0A075B7F0;A0A075B7D8;P01817;A0A0A0MS14;A0A0A0MS15;A0A0B4J1V1;A0A075B7B8;A0A0B4J1V2;P0DTE1;A0A075B6Q5;A0A0J9YVY3;P23083) | 10\|36 | hsa:102723407(A0A0A0MS15;A0A0B4J1V2;A0A0C4DH33;A0A0C4DH36;A0A0J9YVY3;A0A0J9YX35;P01782;P01814;P01817;P0DP01) | 0.0359216 | 0.287373 | http://www.kegg.jp/kegg-bin/show_pathway?hsa05323+hsa:102723407%09green+hsa:102723407%09green+hsa:102723407%09salmon+hsa:102723407%09salmon+hsa:102723407%09salmon+hsa:102723407%09salmon+hsa:102723407%09salmon+hsa:102723407%09salmon+hsa:102723407%09salmon+hsa:102723407%09salmon |
| hsa05100 | Bacterial invasion of epithelial cells | Human Diseases | 4\|228 | hsa:7414(P18206);hsa:3611(A0A0A0MTH3);hsa:71(P60709);hsa:60(P60709);hsa:2335(P02751) | 1\|36 | hsa:2335(P02751) | 0.3812405 | 0.4255708 | http://www.kegg.jp/kegg-bin/show_pathway?hsa05100+hsa:2335%09salmon |
| hsa04144 | Endocytosis | Cellular Processes | 2\|228 | hsa:7037(G3V0E5);hsa:3105(A0A0G2JI36) | 1\|36 | hsa:3105(A0A0G2JI36) | 0.2670995 | 0.3827097 | http://www.kegg.jp/kegg-bin/show_pathway?hsa04144+hsa:3105%09green |
| hsa04140 | Autophagy - animal | Cellular Processes | 2\|228 | hsa:3920(P13473);hsa:2081(O75460) | 1\|36 | hsa:3920(P13473) | 0.2670995 | 0.3827097 | http://www.kegg.jp/kegg-bin/show_pathway?hsa04140+hsa:3920%09green |
| hsa05142 | Chagas disease (American trypanosomiasis) | Human Diseases | 5\|228 | hsa:718(P01024;M0R0Q9);hsa:713(D6R934);hsa:712(P02745);hsa:714(P02747) | 2\|36 | hsa:713(D6R934);hsa:712(P02745) | 0.1489221 | 0.408472 | http://www.kegg.jp/kegg-bin/show_pathway?hsa05142+hsa:713%09salmon+hsa:712%09green |
| hsa04971 | Gastric acid secretion | Organismal Systems | 2\|228 | hsa:760(P00918);hsa:60(P60709) | 1\|36 | hsa:760(P00918) | 0.2670995 | 0.3827097 | http://www.kegg.jp/kegg-bin/show_pathway?hsa04971+hsa:760%09green |
| hsa05169 | Epstein-Barr virus infection | Human Diseases | 41\|228 | hsa:102723407(A0A0C4DH29;A0A0C4DH32;A0A0C4DH33;A0A0J9YX35;A0A0C4DH31;A0A0C4DH36;A0A0C4DH34;A0A0C4DH38;A0A0C4DH39;A0A0J9YY99;A0A0G2JMI3;A0A0B4J1U7;P01763;P01764;P01743;P01766;A0A0B4J1X5;A0A0C4DH43;P01782;P01780;A0A087WSY4;P01814;A0A0B4J1X8;A0A4W8ZXM2;A0A075B7D0;P0DP02;P0DP01;A0A075B7F0;A0A075B7D8;P01817;A0A0A0MS14;A0A0A0MS15;A0A0B4J1V1;A0A075B7B8;A0A0B4J1V2;P0DTE1;A0A075B6Q5;A0A0J9YVY3;P23083);hsa:3105(A0A0G2JI36);hsa:960(H0Y5E4) | 11\|36 | hsa:102723407(A0A0A0MS15;A0A0B4J1V2;A0A0C4DH33;A0A0C4DH36;A0A0J9YVY3;A0A0J9YX35;P01782;P01814;P01817;P0DP01);hsa:3105(A0A0G2JI36) | 0.0212925 | 0.3406804 | http://www.kegg.jp/kegg-bin/show_pathway?hsa05169+hsa:102723407%09green+hsa:102723407%09green+hsa:102723407%09salmon+hsa:102723407%09salmon+hsa:102723407%09salmon+hsa:102723407%09salmon+hsa:102723407%09salmon+hsa:102723407%09salmon+hsa:102723407%09salmon+hsa:102723407%09salmon+hsa:3105%09green |
| hsa05130 | Pathogenic Escherichia coli infection | Human Diseases | 7\|228 | hsa:203068(P07437);hsa:7278(P0DPH7);hsa:81027(Q9H4B7);hsa:7534(E7EX29);hsa:7277(P68366);hsa:113457(P0DPH7);hsa:112714(P0DPH7);hsa:929(P08571);hsa:71(P60709);hsa:60(P60709) | 2\|36 | hsa:81027(Q9H4B7);hsa:7534(E7EX29) | 0.2244606 | 0.378039 | http://www.kegg.jp/kegg-bin/show_pathway?hsa05130+hsa:81027%09salmon+hsa:7534%09salmon |
| hsa04110 | Cell cycle | Cellular Processes | 1\|228 | hsa:7534(E7EX29) | 1\|36 | hsa:7534(E7EX29) | 0.1578947 | 0.3295195 | http://www.kegg.jp/kegg-bin/show_pathway?hsa04110+hsa:7534%09salmon |
| hsa05416 | Viral myocarditis | Human Diseases | 42\|228 | hsa:5881(P60763);hsa:102723407(A0A0C4DH29;A0A0C4DH32;A0A0C4DH33;A0A0J9YX35;A0A0C4DH31;A0A0C4DH36;A0A0C4DH34;A0A0C4DH38;A0A0C4DH39;A0A0J9YY99;A0A0G2JMI3;A0A0B4J1U7;P01763;P01764;P01743;P01766;A0A0B4J1X5;A0A0C4DH43;P01782;P01780;A0A087WSY4;P01814;A0A0B4J1X8;A0A4W8ZXM2;A0A075B7D0;P0DP02;P0DP01;A0A075B7F0;A0A075B7D8;P01817;A0A0A0MS14;A0A0A0MS15;A0A0B4J1V1;A0A075B7B8;A0A0B4J1V2;P0DTE1;A0A075B6Q5;A0A0J9YVY3;P23083);hsa:3105(A0A0G2JI36);hsa:71(P60709);hsa:60(P60709) | 11\|36 | hsa:102723407(A0A0A0MS15;A0A0B4J1V2;A0A0C4DH33;A0A0C4DH36;A0A0J9YVY3;A0A0J9YX35;P01782;P01814;P01817;P0DP01);hsa:3105(A0A0G2JI36) | 0.0249913 | 0.2998951 | http://www.kegg.jp/kegg-bin/show_pathway?hsa05416+hsa:102723407%09green+hsa:102723407%09green+hsa:102723407%09salmon+hsa:102723407%09salmon+hsa:102723407%09salmon+hsa:102723407%09salmon+hsa:102723407%09salmon+hsa:102723407%09salmon+hsa:102723407%09salmon+hsa:102723407%09salmon+hsa:3105%09green |
| hsa00590 | Arachidonic acid metabolism | Metabolism | 3\|228 | hsa:2878(A0A087X1J7);hsa:5730(P41222);hsa:81579(A0A2R8Y3M9) | 1\|36 | hsa:81579(A0A2R8Y3M9) | 0.3386018 | 0.4063221 | http://www.kegg.jp/kegg-bin/show_pathway?hsa00590+hsa:81579%09green |
| hsa05163 | Human cytomegalovirus infection | Human Diseases | 2\|228 | hsa:5881(P60763);hsa:3105(A0A0G2JI36) | 1\|36 | hsa:3105(A0A0G2JI36) | 0.2670995 | 0.3827097 | http://www.kegg.jp/kegg-bin/show_pathway?hsa05163+hsa:3105%09green |
| hsa05150 | Staphylococcus aureus infection | Human Diseases | 63\|228 | hsa:102723407(A0A0C4DH29;A0A0C4DH32;A0A0C4DH33;A0A0J9YX35;A0A0C4DH31;A0A0C4DH36;A0A0C4DH34;A0A0C4DH38;A0A0C4DH39;A0A0J9YY99;A0A0G2JMI3;A0A0B4J1U7;P01763;P01764;P01743;P01766;A0A0B4J1X5;A0A0C4DH43;P01782;P01780;A0A087WSY4;P01814;A0A0B4J1X8;A0A4W8ZXM2;A0A075B7D0;P0DP02;P0DP01;A0A075B7F0;A0A075B7D8;P01817;A0A0A0MS14;A0A0A0MS15;A0A0B4J1V1;A0A075B7B8;A0A0B4J1V2;P0DTE1;A0A075B6Q5;A0A0J9YVY3;P23083);hsa:5340(P00747);hsa:629(B4E1Z4);hsa:3858(P13645);hsa:727(P01031);hsa:720(A0A0G2JPR0;P0C0L5;P0C0L4);hsa:721(A0A0G2JPR0;P0C0L5;P0C0L4);hsa:1675(K7ERG9);hsa:5648(P48740);hsa:2214(H0Y755);hsa:718(P01024;M0R0Q9);hsa:10747(O00187);hsa:713(D6R934);hsa:712(P02745);hsa:715(A0A3B3ISR2);hsa:714(P02747);hsa:717(A0A0G2JL69);hsa:716(P09871);hsa:4153(P11226);hsa:3426(A0A2R8Y3M9);hsa:2266(C9JPQ9;C9JC84);hsa:3075(P08603) | 15\|36 | hsa:102723407(A0A0A0MS15;A0A0B4J1V2;A0A0C4DH33;A0A0C4DH36;A0A0J9YVY3;A0A0J9YX35;P01782;P01814;P01817;P0DP01);hsa:3426(A0A2R8Y3M9);hsa:3858(P13645);hsa:713(D6R934);hsa:712(P02745);hsa:1675(K7ERG9) | 0.0209422 | 0.4020894 | http://www.kegg.jp/kegg-bin/show_pathway?hsa05150+hsa:102723407%09green+hsa:102723407%09green+hsa:102723407%09salmon+hsa:102723407%09salmon+hsa:102723407%09salmon+hsa:102723407%09salmon+hsa:102723407%09salmon+hsa:102723407%09salmon+hsa:102723407%09salmon+hsa:102723407%09salmon+hsa:3426%09green+hsa:3858%09green+hsa:713%09salmon+hsa:712%09green+hsa:1675%09green |
| hsa04810 | Regulation of actin cytoskeleton | Cellular Processes | 12\|228 | hsa:3674(P08514);hsa:2934(A0A0A0MS51);hsa:1072(E9PK25);hsa:2147(P00734);hsa:2335(P02751);hsa:5881(P60763);hsa:4627(P35579);hsa:87(H9KV75);hsa:5216(P07737);hsa:7414(P18206);hsa:4478(P26038);hsa:71(P60709);hsa:60(P60709) | 2\|36 | hsa:4478(P26038);hsa:2335(P02751) | 0.3034483 | 0.3783252 | http://www.kegg.jp/kegg-bin/show_pathway?hsa04810+hsa:4478%09salmon+hsa:2335%09salmon |
| hsa05143 | African trypanosomiasis | Human Diseases | 45\|228 | hsa:102723407(A0A0C4DH29;A0A0C4DH32;A0A0C4DH33;A0A0J9YX35;A0A0C4DH31;A0A0C4DH36;A0A0C4DH34;A0A0C4DH38;A0A0C4DH39;A0A0J9YY99;A0A0G2JMI3;A0A0B4J1U7;P01763;P01764;P01743;P01766;A0A0B4J1X5;A0A0C4DH43;P01782;P01780;A0A087WSY4;P01814;A0A0B4J1X8;A0A4W8ZXM2;A0A075B7D0;P0DP02;P0DP01;A0A075B7F0;A0A075B7D8;P01817;A0A0A0MS14;A0A0A0MS15;A0A0B4J1V1;A0A075B7B8;A0A0B4J1V2;P0DTE1;A0A075B6Q5;A0A0J9YVY3;P23083);hsa:3039(P69905);hsa:8542(O14791);hsa:335(P02647);hsa:3250(P00739);hsa:3043(P68871);hsa:3040(P69905);hsa:7412(P19320) | 11\|36 | hsa:102723407(A0A0A0MS15;A0A0B4J1V2;A0A0C4DH33;A0A0C4DH36;A0A0J9YVY3;A0A0J9YX35;P01782;P01814;P01817;P0DP01);hsa:3039(P69905);hsa:3040(P69905) | 0.0383371 | 0.216492 | http://www.kegg.jp/kegg-bin/show_pathway?hsa05143+hsa:102723407%09green+hsa:102723407%09green+hsa:102723407%09salmon+hsa:102723407%09salmon+hsa:102723407%09salmon+hsa:102723407%09salmon+hsa:102723407%09salmon+hsa:102723407%09salmon+hsa:102723407%09salmon+hsa:102723407%09salmon+hsa:3039%09green+hsa:3040%09green |
| hsa05200 | Pathways in cancer | Human Diseases | 4\|228 | hsa:5881(P60763);hsa:3674(P08514);hsa:3481(P01344);hsa:2335(P02751) | 1\|36 | hsa:2335(P02751) | 0.3812405 | 0.4255708 | http://www.kegg.jp/kegg-bin/show_pathway?hsa05200+hsa:2335%09salmon |
| hsa00564 | Glycerophospholipid metabolism | Metabolism | 2\|228 | hsa:81579(A0A2R8Y3M9);hsa:3931(P04180) | 1\|36 | hsa:81579(A0A2R8Y3M9) | 0.2670995 | 0.3827097 | http://www.kegg.jp/kegg-bin/show_pathway?hsa00564+hsa:81579%09green |
| hsa04072 | Phospholipase D signaling pathway | Environmental Information Processing | 39\|228 | hsa:102723407(A0A0C4DH29;A0A0C4DH32;A0A0C4DH33;A0A0J9YX35;A0A0C4DH31;A0A0C4DH36;A0A0C4DH34;A0A0C4DH38;A0A0C4DH39;A0A0J9YY99;A0A0G2JMI3;A0A0B4J1U7;P01763;P01764;P01743;P01766;A0A0B4J1X5;A0A0C4DH43;P01782;P01780;A0A087WSY4;P01814;A0A0B4J1X8;A0A4W8ZXM2;A0A075B7D0;P0DP02;P0DP01;A0A075B7F0;A0A075B7D8;P01817;A0A0A0MS14;A0A0A0MS15;A0A0B4J1V1;A0A075B7B8;A0A0B4J1V2;P0DTE1;A0A075B6Q5;A0A0J9YVY3;P23083) | 10\|36 | hsa:102723407(A0A0A0MS15;A0A0B4J1V2;A0A0C4DH33;A0A0C4DH36;A0A0J9YVY3;A0A0J9YX35;P01782;P01814;P01817;P0DP01) | 0.0359216 | 0.287373 | http://www.kegg.jp/kegg-bin/show_pathway?hsa04072+hsa:102723407%09green+hsa:102723407%09green+hsa:102723407%09salmon+hsa:102723407%09salmon+hsa:102723407%09salmon+hsa:102723407%09salmon+hsa:102723407%09salmon+hsa:102723407%09salmon+hsa:102723407%09salmon+hsa:102723407%09salmon |
| hsa05203 | Viral carcinogenesis | Human Diseases | 8\|228 | hsa:2934(A0A0A0MS51);hsa:128312(A0A2R8Y619);hsa:7534(E7EX29);hsa:718(P01024;M0R0Q9);hsa:87(H9KV75);hsa:5315(H3BTN5);hsa:3105(A0A0G2JI36) | 3\|36 | hsa:5315(H3BTN5);hsa:3105(A0A0G2JI36);hsa:7534(E7EX29) | 0.0920864 | 0.2762592 | http://www.kegg.jp/kegg-bin/show_pathway?hsa05203+hsa:5315%09salmon+hsa:3105%09green+hsa:7534%09salmon |
| hsa04915 | Estrogen signaling pathway | Organismal Systems | 4\|228 | hsa:3858(P13645);hsa:3868(P08779);hsa:3861(P02533);hsa:3857(P35527) | 2\|36 | hsa:3858(P13645);hsa:3857(P35527) | 0.1053428 | 0.3064517 | http://www.kegg.jp/kegg-bin/show_pathway?hsa04915+hsa:3858%09green+hsa:3857%09salmon |
| hsa05206 | MicroRNAs in cancer | Human Diseases | 3\|228 | hsa:7057(P07996);hsa:960(H0Y5E4);hsa:7148(A0A140T8Y3) | 1\|36 | hsa:7148(A0A140T8Y3) | 0.3386018 | 0.4063221 | http://www.kegg.jp/kegg-bin/show_pathway?hsa05206+hsa:7148%09green |
| hsa05418 | Fluid shear stress and atherosclerosis | Human Diseases | 5\|228 | hsa:3674(P08514);hsa:5881(P60763);hsa:7412(P19320);hsa:60(P60709);hsa:71(P60709);hsa:1003(I3L1J2) | 1\|36 | hsa:1003(I3L1J2) | 0.4020896 | 0.4150602 | http://www.kegg.jp/kegg-bin/show_pathway?hsa05418+hsa:1003%09salmon |
| hsa00565 | Ether lipid metabolism | Metabolism | 3\|228 | hsa:81579(A0A2R8Y3M9);hsa:7368(Q16880);hsa:7941(Q13093) | 2\|36 | hsa:81579(A0A2R8Y3M9);hsa:7368(Q16880) | 0.0620474 | 0.2127341 | http://www.kegg.jp/kegg-bin/show_pathway?hsa00565+hsa:81579%09green+hsa:7368%09salmon |
| hsa04940 | Type I diabetes mellitus | Human Diseases | 1\|228 | hsa:3105(A0A0G2JI36) | 1\|36 | hsa:3105(A0A0G2JI36) | 0.1578947 | 0.3295195 | http://www.kegg.jp/kegg-bin/show_pathway?hsa04940+hsa:3105%09green |
| hsa05152 | Tuberculosis | Human Diseases | 46\|228 | hsa:102723407(A0A0C4DH29;A0A0C4DH32;A0A0C4DH33;A0A0J9YX35;A0A0C4DH31;A0A0C4DH36;A0A0C4DH34;A0A0C4DH38;A0A0C4DH39;A0A0J9YY99;A0A0G2JMI3;A0A0B4J1U7;P01763;P01764;P01743;P01766;A0A0B4J1X5;A0A0C4DH43;P01782;P01780;A0A087WSY4;P01814;A0A0B4J1X8;A0A4W8ZXM2;A0A075B7D0;P0DP02;P0DP01;A0A075B7F0;A0A075B7D8;P01817;A0A0A0MS14;A0A0A0MS15;A0A0B4J1V1;A0A075B7B8;A0A0B4J1V2;P0DTE1;A0A075B6Q5;A0A0J9YVY3;P23083);hsa:820(J3KNB4);hsa:3920(P13473);hsa:718(P01024;M0R0Q9);hsa:2214(H0Y755);hsa:3929(P18428);hsa:929(P08571) | 11\|36 | hsa:3920(P13473);hsa:102723407(A0A0A0MS15;A0A0B4J1V2;A0A0C4DH33;A0A0C4DH36;A0A0J9YVY3;A0A0J9YX35;P01782;P01814;P01817;P0DP01) | 0.0435026 | 0.2088125 | http://www.kegg.jp/kegg-bin/show_pathway?hsa05152+hsa:3920%09green+hsa:102723407%09green+hsa:102723407%09green+hsa:102723407%09salmon+hsa:102723407%09salmon+hsa:102723407%09salmon+hsa:102723407%09salmon+hsa:102723407%09salmon+hsa:102723407%09salmon+hsa:102723407%09salmon+hsa:102723407%09salmon |
| hsa00591 | Linoleic acid metabolism | Metabolism | 1\|228 | hsa:81579(A0A2R8Y3M9) | 1\|36 | hsa:81579(A0A2R8Y3M9) | 0.1578947 | 0.3295195 | http://www.kegg.jp/kegg-bin/show_pathway?hsa00591+hsa:81579%09green |
| hsa05332 | Graft-versus-host disease | Human Diseases | 1\|228 | hsa:3105(A0A0G2JI36) | 1\|36 | hsa:3105(A0A0G2JI36) | 0.1578947 | 0.3295195 | http://www.kegg.jp/kegg-bin/show_pathway?hsa05332+hsa:3105%09green |
| hsa05230 | Central carbon metabolism in cancer | Human Diseases | 1\|228 | hsa:5315(H3BTN5) | 1\|36 | hsa:5315(H3BTN5) | 0.1578947 | 0.3295195 | http://www.kegg.jp/kegg-bin/show_pathway?hsa05230+hsa:5315%09salmon |
| hsa05166 | Human T-cell leukemia virus 1 infection | Human Diseases | 2\|228 | hsa:3105(A0A0G2JI36);hsa:7094(Q9Y490) | 1\|36 | hsa:3105(A0A0G2JI36) | 0.2670995 | 0.3827097 | http://www.kegg.jp/kegg-bin/show_pathway?hsa05166+hsa:3105%09green |
| hsa04672 | Intestinal immune network for IgA production | Organismal Systems | 41\|228 | hsa:102723407(A0A0C4DH29;A0A0C4DH32;A0A0C4DH33;A0A0J9YX35;A0A0C4DH31;A0A0C4DH36;A0A0C4DH34;A0A0C4DH38;A0A0C4DH39;A0A0J9YY99;A0A0G2JMI3;A0A0B4J1U7;P01763;P01764;P01743;P01766;A0A0B4J1X5;A0A0C4DH43;P01782;P01780;A0A087WSY4;P01814;A0A0B4J1X8;A0A4W8ZXM2;A0A075B7D0;P0DP02;P0DP01;A0A075B7F0;A0A075B7D8;P01817;A0A0A0MS14;A0A0A0MS15;A0A0B4J1V1;A0A075B7B8;A0A0B4J1V2;P0DTE1;A0A075B6Q5;A0A0J9YVY3;P23083);hsa:102723996(A0A087X1L8);hsa:5284(P01833);hsa:23308(A0A087X1L8) | 10\|36 | hsa:102723407(A0A0A0MS15;A0A0B4J1V2;A0A0C4DH33;A0A0C4DH36;A0A0J9YVY3;A0A0J9YX35;P01782;P01814;P01817;P0DP01) | 0.047076 | 0.2152047 | http://www.kegg.jp/kegg-bin/show_pathway?hsa04672+hsa:102723407%09green+hsa:102723407%09green+hsa:102723407%09salmon+hsa:102723407%09salmon+hsa:102723407%09salmon+hsa:102723407%09salmon+hsa:102723407%09salmon+hsa:102723407%09salmon+hsa:102723407%09salmon+hsa:102723407%09salmon |
| hsa04933 | AGE-RAGE signaling pathway in diabetic complications | Human Diseases | 2\|228 | hsa:7412(P19320);hsa:2335(P02751) | 1\|36 | hsa:2335(P02751) | 0.2670995 | 0.3827097 | http://www.kegg.jp/kegg-bin/show_pathway?hsa04933+hsa:2335%09salmon |
| hsa04975 | Fat digestion and absorption | Organismal Systems | 4\|228 | hsa:81579(A0A2R8Y3M9);hsa:338(P04114);hsa:337(P06727);hsa:335(P02647) | 1\|36 | hsa:81579(A0A2R8Y3M9) | 0.3812405 | 0.4255708 | http://www.kegg.jp/kegg-bin/show_pathway?hsa04975+hsa:81579%09green |
| hsa04064 | NF-kappa B signaling pathway | Environmental Information Processing | 42\|228 | hsa:102723407(A0A0C4DH29;A0A0C4DH32;A0A0C4DH33;A0A0J9YX35;A0A0C4DH31;A0A0C4DH36;A0A0C4DH34;A0A0C4DH38;A0A0C4DH39;A0A0J9YY99;A0A0G2JMI3;A0A0B4J1U7;P01763;P01764;P01743;P01766;A0A0B4J1X5;A0A0C4DH43;P01782;P01780;A0A087WSY4;P01814;A0A0B4J1X8;A0A4W8ZXM2;A0A075B7D0;P0DP02;P0DP01;A0A075B7F0;A0A075B7D8;P01817;A0A0A0MS14;A0A0A0MS15;A0A0B4J1V1;A0A075B7B8;A0A0B4J1V2;P0DTE1;A0A075B6Q5;A0A0J9YVY3;P23083);hsa:929(P08571);hsa:7412(P19320);hsa:3929(P18428) | 10\|36 | hsa:102723407(A0A0A0MS15;A0A0B4J1V2;A0A0C4DH33;A0A0C4DH36;A0A0J9YVY3;A0A0J9YX35;P01782;P01814;P01817;P0DP01) | 0.0531965 | 0.2042747 | http://www.kegg.jp/kegg-bin/show_pathway?hsa04064+hsa:102723407%09green+hsa:102723407%09green+hsa:102723407%09salmon+hsa:102723407%09salmon+hsa:102723407%09salmon+hsa:102723407%09salmon+hsa:102723407%09salmon+hsa:102723407%09salmon+hsa:102723407%09salmon+hsa:102723407%09salmon |
| hsa01230 | Biosynthesis of amino acids | Metabolism | 4\|228 | hsa:5315(H3BTN5);hsa:2023(A0A2R8Y6G6);hsa:226(J3KPS3);hsa:2597(E7EUT5) | 1\|36 | hsa:5315(H3BTN5) | 0.3812405 | 0.4255708 | http://www.kegg.jp/kegg-bin/show_pathway?hsa01230+hsa:5315%09salmon |
| hsa05165 | Human papillomavirus infection | Human Diseases | 13\|228 | hsa:7450(P04275);hsa:3674(P08514);hsa:3993(J3QRV5);hsa:1291(A0A087X0S5);hsa:1293(E7ENL6);hsa:1311(G3XAP6);hsa:7448(H0YJW9;P04004);hsa:2335(P02751);hsa:7057(P07996);hsa:5315(H3BTN5);hsa:3105(A0A0G2JI36);hsa:7148(A0A140T8Y3) | 5\|36 | hsa:5315(H3BTN5);hsa:3105(A0A0G2JI36);hsa:1293(E7ENL6);hsa:7148(A0A140T8Y3);hsa:2335(P02751) | 0.0292105 | 0.2804211 | http://www.kegg.jp/kegg-bin/show_pathway?hsa05165+hsa:5315%09salmon+hsa:3105%09green+hsa:1293%09green+hsa:7148%09green+hsa:2335%09salmon |
| hsa05320 | Autoimmune thyroid disease | Human Diseases | 40\|228 | hsa:102723407(A0A0C4DH29;A0A0C4DH32;A0A0C4DH33;A0A0J9YX35;A0A0C4DH31;A0A0C4DH36;A0A0C4DH34;A0A0C4DH38;A0A0C4DH39;A0A0J9YY99;A0A0G2JMI3;A0A0B4J1U7;P01763;P01764;P01743;P01766;A0A0B4J1X5;A0A0C4DH43;P01782;P01780;A0A087WSY4;P01814;A0A0B4J1X8;A0A4W8ZXM2;A0A075B7D0;P0DP02;P0DP01;A0A075B7F0;A0A075B7D8;P01817;A0A0A0MS14;A0A0A0MS15;A0A0B4J1V1;A0A075B7B8;A0A0B4J1V2;P0DTE1;A0A075B6Q5;A0A0J9YVY3;P23083);hsa:3105(A0A0G2JI36) | 11\|36 | hsa:102723407(A0A0A0MS15;A0A0B4J1V2;A0A0C4DH33;A0A0C4DH36;A0A0J9YVY3;A0A0J9YX35;P01782;P01814;P01817;P0DP01);hsa:3105(A0A0G2JI36) | 0.0179695 | 0.5750224 | http://www.kegg.jp/kegg-bin/show_pathway?hsa05320+hsa:102723407%09green+hsa:102723407%09green+hsa:102723407%09salmon+hsa:102723407%09salmon+hsa:102723407%09salmon+hsa:102723407%09salmon+hsa:102723407%09salmon+hsa:102723407%09salmon+hsa:102723407%09salmon+hsa:102723407%09salmon+hsa:3105%09green |
| hsa05202 | Transcriptional misregulation in cancer | Human Diseases | 42\|228 | hsa:102723407(A0A0C4DH29;A0A0C4DH32;A0A0C4DH33;A0A0J9YX35;A0A0C4DH31;A0A0C4DH36;A0A0C4DH34;A0A0C4DH38;A0A0C4DH39;A0A0J9YY99;A0A0G2JMI3;A0A0B4J1U7;P01763;P01764;P01743;P01766;A0A0B4J1X5;A0A0C4DH43;P01782;P01780;A0A087WSY4;P01814;A0A0B4J1X8;A0A4W8ZXM2;A0A075B7D0;P0DP02;P0DP01;A0A075B7F0;A0A075B7D8;P01817;A0A0A0MS14;A0A0A0MS15;A0A0B4J1V1;A0A075B7B8;A0A0B4J1V2;P0DTE1;A0A075B6Q5;A0A0J9YVY3;P23083);hsa:929(P08571);hsa:3486(A6XND0);hsa:1668(P59665) | 10\|36 | hsa:102723407(A0A0A0MS15;A0A0B4J1V2;A0A0C4DH33;A0A0C4DH36;A0A0J9YVY3;A0A0J9YX35;P01782;P01814;P01817;P0DP01) | 0.0531965 | 0.2042747 | http://www.kegg.jp/kegg-bin/show_pathway?hsa05202+hsa:102723407%09green+hsa:102723407%09green+hsa:102723407%09salmon+hsa:102723407%09salmon+hsa:102723407%09salmon+hsa:102723407%09salmon+hsa:102723407%09salmon+hsa:102723407%09salmon+hsa:102723407%09salmon+hsa:102723407%09salmon |
| hsa04145 | Phagosome | Cellular Processes | 56\|228 | hsa:203068(P07437);hsa:3105(A0A0G2JI36);hsa:7278(P0DPH7);hsa:81027(Q9H4B7);hsa:102723407(A0A0C4DH29;A0A0C4DH32;A0A0C4DH33;A0A0J9YX35;A0A0C4DH31;A0A0C4DH36;A0A0C4DH34;A0A0C4DH38;A0A0C4DH39;A0A0J9YY99;A0A0G2JMI3;A0A0B4J1U7;P01763;P01764;P01743;P01766;A0A0B4J1X5;A0A0C4DH43;P01782;P01780;A0A087WSY4;P01814;A0A0B4J1X8;A0A4W8ZXM2;A0A075B7D0;P0DP02;P0DP01;A0A075B7F0;A0A075B7D8;P01817;A0A0A0MS14;A0A0A0MS15;A0A0B4J1V1;A0A075B7B8;A0A0B4J1V2;P0DTE1;A0A075B6Q5;A0A0J9YVY3;P23083);hsa:1311(G3XAP6);hsa:71(P60709);hsa:7277(P68366);hsa:113457(P0DPH7);hsa:3920(P13473);hsa:718(P01024;M0R0Q9);hsa:7057(P07996);hsa:112714(P0DPH7);hsa:2214(H0Y755);hsa:4153(P11226);hsa:78989(Q9BWP8);hsa:7037(G3V0E5);hsa:929(P08571);hsa:715(A0A3B3ISR2);hsa:60(P60709) | 13\|36 | hsa:3920(P13473);hsa:102723407(A0A0A0MS15;A0A0B4J1V2;A0A0C4DH33;A0A0C4DH36;A0A0J9YVY3;A0A0J9YX35;P01782;P01814;P01817;P0DP01);hsa:3105(A0A0G2JI36);hsa:81027(Q9H4B7) | 0.0367276 | 0.2350563 | http://www.kegg.jp/kegg-bin/show_pathway?hsa04145+hsa:3920%09green+hsa:102723407%09green+hsa:102723407%09green+hsa:102723407%09salmon+hsa:102723407%09salmon+hsa:102723407%09salmon+hsa:102723407%09salmon+hsa:102723407%09salmon+hsa:102723407%09salmon+hsa:102723407%09salmon+hsa:102723407%09salmon+hsa:3105%09green+hsa:81027%09salmon |
| hsa04142 | Lysosome | Cellular Processes | 1\|228 | hsa:3920(P13473) | 1\|36 | hsa:3920(P13473) | 0.1578947 | 0.3295195 | http://www.kegg.jp/kegg-bin/show_pathway?hsa04142+hsa:3920%09green |
| hsa05146 | Amoebiasis | Human Diseases | 47\|228 | hsa:102723407(A0A0C4DH29;A0A0C4DH32;A0A0C4DH33;A0A0J9YX35;A0A0C4DH31;A0A0C4DH36;A0A0C4DH34;A0A0C4DH38;A0A0C4DH39;A0A0J9YY99;A0A0G2JMI3;A0A0B4J1U7;P01763;P01764;P01743;P01766;A0A0B4J1X5;A0A0C4DH43;P01782;P01780;A0A087WSY4;P01814;A0A0B4J1X8;A0A4W8ZXM2;A0A075B7D0;P0DP02;P0DP01;A0A075B7F0;A0A075B7D8;P01817;A0A0A0MS14;A0A0A0MS15;A0A0B4J1V1;A0A075B7B8;A0A0B4J1V2;P0DTE1;A0A075B6Q5;A0A0J9YVY3;P23083);hsa:2335(P02751);hsa:87(H9KV75);hsa:7414(P18206);hsa:735(P02748);hsa:929(P08571);hsa:733(P07360);hsa:732(P07358);hsa:731(P07357) | 11\|36 | hsa:102723407(A0A0A0MS15;A0A0B4J1V2;A0A0C4DH33;A0A0C4DH36;A0A0J9YVY3;A0A0J9YX35;P01782;P01814;P01817;P0DP01);hsa:2335(P02751) | 0.0489935 | 0.2044948 | http://www.kegg.jp/kegg-bin/show_pathway?hsa05146+hsa:102723407%09green+hsa:102723407%09green+hsa:102723407%09salmon+hsa:102723407%09salmon+hsa:102723407%09salmon+hsa:102723407%09salmon+hsa:102723407%09salmon+hsa:102723407%09salmon+hsa:102723407%09salmon+hsa:102723407%09salmon+hsa:2335%09salmon |
| hsa00010 | Glycolysis / Gluconeogenesis | Metabolism | 4\|228 | hsa:5315(H3BTN5);hsa:2023(A0A2R8Y6G6);hsa:226(J3KPS3);hsa:2597(E7EUT5) | 1\|36 | hsa:5315(H3BTN5) | 0.3812405 | 0.4255708 | http://www.kegg.jp/kegg-bin/show_pathway?hsa00010+hsa:5315%09salmon |
| hsa05205 | Proteoglycans in cancer | Human Diseases | 10\|228 | hsa:71(P60709);hsa:3481(P01344);hsa:7448(H0YJW9;P04004);hsa:960(H0Y5E4);hsa:2335(P02751);hsa:2316(P21333);hsa:7057(P07996);hsa:4478(P26038);hsa:4060(P51884);hsa:60(P60709) | 2\|36 | hsa:4478(P26038);hsa:2335(P02751) | 0.2906703 | 0.3720579 | http://www.kegg.jp/kegg-bin/show_pathway?hsa05205+hsa:4478%09salmon+hsa:2335%09salmon |
| hsa04514 | Cell adhesion molecules (CAMs) | Environmental Information Processing | 5\|228 | hsa:102723996(A0A087X1L8);hsa:7412(P19320);hsa:6402(P14151);hsa:23308(A0A087X1L8);hsa:3105(A0A0G2JI36);hsa:1003(I3L1J2) | 2\|36 | hsa:3105(A0A0G2JI36);hsa:1003(I3L1J2) | 0.1489221 | 0.408472 | http://www.kegg.jp/kegg-bin/show_pathway?hsa04514+hsa:3105%09green+hsa:1003%09salmon |
| hsa00592 | alpha-Linolenic acid metabolism | Metabolism | 1\|228 | hsa:81579(A0A2R8Y3M9) | 1\|36 | hsa:81579(A0A2R8Y3M9) | 0.1578947 | 0.3295195 | http://www.kegg.jp/kegg-bin/show_pathway?hsa00592+hsa:81579%09green |
| hsa04270 | Vascular smooth muscle contraction | Organismal Systems | 2\|228 | hsa:81579(A0A2R8Y3M9);hsa:59(P68032);hsa:72(P68032) | 1\|36 | hsa:81579(A0A2R8Y3M9) | 0.2670995 | 0.3827097 | http://www.kegg.jp/kegg-bin/show_pathway?hsa04270+hsa:81579%09green |
| hsa00230 | Purine metabolism | Metabolism | 1\|228 | hsa:5315(H3BTN5) | 1\|36 | hsa:5315(H3BTN5) | 0.1578947 | 0.3295195 | http://www.kegg.jp/kegg-bin/show_pathway?hsa00230+hsa:5315%09salmon |
| hsa04662 | B cell receptor signaling pathway | Organismal Systems | 40\|228 | hsa:5881(P60763);hsa:102723407(A0A0C4DH29;A0A0C4DH32;A0A0C4DH33;A0A0J9YX35;A0A0C4DH31;A0A0C4DH36;A0A0C4DH34;A0A0C4DH38;A0A0C4DH39;A0A0J9YY99;A0A0G2JMI3;A0A0B4J1U7;P01763;P01764;P01743;P01766;A0A0B4J1X5;A0A0C4DH43;P01782;P01780;A0A087WSY4;P01814;A0A0B4J1X8;A0A4W8ZXM2;A0A075B7D0;P0DP02;P0DP01;A0A075B7F0;A0A075B7D8;P01817;A0A0A0MS14;A0A0A0MS15;A0A0B4J1V1;A0A075B7B8;A0A0B4J1V2;P0DTE1;A0A075B6Q5;A0A0J9YVY3;P23083) | 10\|36 | hsa:102723407(A0A0A0MS15;A0A0B4J1V2;A0A0C4DH33;A0A0C4DH36;A0A0J9YVY3;A0A0J9YX35;P01782;P01814;P01817;P0DP01) | 0.0413067 | 0.2203024 | http://www.kegg.jp/kegg-bin/show_pathway?hsa04662+hsa:102723407%09green+hsa:102723407%09green+hsa:102723407%09salmon+hsa:102723407%09salmon+hsa:102723407%09salmon+hsa:102723407%09salmon+hsa:102723407%09salmon+hsa:102723407%09salmon+hsa:102723407%09salmon+hsa:102723407%09salmon |
| hsa04614 | Renin-angiotensin system | Organismal Systems | 2\|228 | hsa:290(P15144);hsa:183(P01019) | 1\|36 | hsa:183(P01019) | 0.2670995 | 0.3827097 | http://www.kegg.jp/kegg-bin/show_pathway?hsa04614+hsa:183%09green |
| hsa05170 | Human immunodeficiency virus 1 infection | Human Diseases | 3\|228 | hsa:5881(P60763);hsa:1072(E9PK25);hsa:3105(A0A0G2JI36) | 1\|36 | hsa:3105(A0A0G2JI36) | 0.3386018 | 0.4063221 | http://www.kegg.jp/kegg-bin/show_pathway?hsa05170+hsa:3105%09green |
| hsa05144 | Malaria | Human Diseases | 6\|228 | hsa:3039(P69905);hsa:4035(Q07954);hsa:1311(G3XAP6);hsa:7057(P07996);hsa:3043(P68871);hsa:3040(P69905);hsa:7412(P19320) | 1\|36 | hsa:3040(P69905);hsa:3039(P69905) | 0.4067776 | 0.4110595 | http://www.kegg.jp/kegg-bin/show_pathway?hsa05144+hsa:3040%09green+hsa:3039%09green |
| hsa04512 | ECM-receptor interaction | Environmental Information Processing | 12\|228 | hsa:7450(P04275);hsa:3674(P08514);hsa:1291(A0A087X0S5);hsa:2811(A0A0C4DGZ8);hsa:1293(E7ENL6);hsa:1311(G3XAP6);hsa:7448(H0YJW9;P04004);hsa:960(H0Y5E4);hsa:2335(P02751);hsa:7057(P07996);hsa:7148(A0A140T8Y3) | 3\|36 | hsa:1293(E7ENL6);hsa:7148(A0A140T8Y3);hsa:2335(P02751) | 0.1879279 | 0.3221621 | http://www.kegg.jp/kegg-bin/show_pathway?hsa04512+hsa:1293%09green+hsa:7148%09green+hsa:2335%09salmon |
| hsa04612 | Antigen processing and presentation | Organismal Systems | 2\|228 | hsa:3105(A0A0G2JI36);hsa:3309(P11021) | 1\|36 | hsa:3105(A0A0G2JI36) | 0.2670995 | 0.3827097 | http://www.kegg.jp/kegg-bin/show_pathway?hsa04612+hsa:3105%09green |
| hsa00600 | Sphingolipid metabolism | Metabolism | 1\|228 | hsa:7368(Q16880) | 1\|36 | hsa:7368(Q16880) | 0.1578947 | 0.3295195 | http://www.kegg.jp/kegg-bin/show_pathway?hsa00600+hsa:7368%09salmon |
| hsa04650 | Natural killer cell mediated cytotoxicity | Organismal Systems | 42\|228 | hsa:5881(P60763);hsa:102723407(A0A0C4DH29;A0A0C4DH32;A0A0C4DH33;A0A0J9YX35;A0A0C4DH31;A0A0C4DH36;A0A0C4DH34;A0A0C4DH38;A0A0C4DH39;A0A0J9YY99;A0A0G2JMI3;A0A0B4J1U7;P01763;P01764;P01743;P01766;A0A0B4J1X5;A0A0C4DH43;P01782;P01780;A0A087WSY4;P01814;A0A0B4J1X8;A0A4W8ZXM2;A0A075B7D0;P0DP02;P0DP01;A0A075B7F0;A0A075B7D8;P01817;A0A0A0MS14;A0A0A0MS15;A0A0B4J1V1;A0A075B7B8;A0A0B4J1V2;P0DTE1;A0A075B6Q5;A0A0J9YVY3;P23083);hsa:3105(A0A0G2JI36);hsa:2214(H0Y755) | 11\|36 | hsa:102723407(A0A0A0MS15;A0A0B4J1V2;A0A0C4DH33;A0A0C4DH36;A0A0J9YVY3;A0A0J9YX35;P01782;P01814;P01817;P0DP01);hsa:3105(A0A0G2JI36) | 0.0249913 | 0.2998951 | http://www.kegg.jp/kegg-bin/show_pathway?hsa04650+hsa:102723407%09green+hsa:102723407%09green+hsa:102723407%09salmon+hsa:102723407%09salmon+hsa:102723407%09salmon+hsa:102723407%09salmon+hsa:102723407%09salmon+hsa:102723407%09salmon+hsa:102723407%09salmon+hsa:102723407%09salmon+hsa:3105%09green |
| hsa00910 | Nitrogen metabolism | Metabolism | 2\|228 | hsa:760(P00918);hsa:759(P00915) | 2\|36 | hsa:760(P00918);hsa:759(P00915) | 0.024345 | 0.3338743 | http://www.kegg.jp/kegg-bin/show_pathway?hsa00910+hsa:760%09green+hsa:759%09green |
| hsa05140 | Leishmaniasis | Human Diseases | 42\|228 | hsa:718(P01024;M0R0Q9);hsa:2214(H0Y755);hsa:102723407(A0A0C4DH29;A0A0C4DH32;A0A0C4DH33;A0A0J9YX35;A0A0C4DH31;A0A0C4DH36;A0A0C4DH34;A0A0C4DH38;A0A0C4DH39;A0A0J9YY99;A0A0G2JMI3;A0A0B4J1U7;P01763;P01764;P01743;P01766;A0A0B4J1X5;A0A0C4DH43;P01782;P01780;A0A087WSY4;P01814;A0A0B4J1X8;A0A4W8ZXM2;A0A075B7D0;P0DP02;P0DP01;A0A075B7F0;A0A075B7D8;P01817;A0A0A0MS14;A0A0A0MS15;A0A0B4J1V1;A0A075B7B8;A0A0B4J1V2;P0DTE1;A0A075B6Q5;A0A0J9YVY3;P23083) | 10\|36 | hsa:102723407(A0A0A0MS15;A0A0B4J1V2;A0A0C4DH33;A0A0C4DH36;A0A0J9YVY3;A0A0J9YX35;P01782;P01814;P01817;P0DP01) | 0.0531965 | 0.2042747 | http://www.kegg.jp/kegg-bin/show_pathway?hsa05140+hsa:102723407%09green+hsa:102723407%09green+hsa:102723407%09salmon+hsa:102723407%09salmon+hsa:102723407%09salmon+hsa:102723407%09salmon+hsa:102723407%09salmon+hsa:102723407%09salmon+hsa:102723407%09salmon+hsa:102723407%09salmon |
| hsa04510 | Focal adhesion | Cellular Processes | 20\|228 | hsa:7450(P04275);hsa:29780(A0A087WZB5);hsa:7094(Q9Y490);hsa:1291(A0A087X0S5);hsa:1293(E7ENL6);hsa:5908(P61224);hsa:87(H9KV75);hsa:3674(P08514);hsa:7448(H0YJW9;P04004);hsa:3611(A0A0A0MTH3);hsa:5881(P60763);hsa:1311(G3XAP6);hsa:2316(P21333);hsa:7057(P07996);hsa:7791(H0Y2Y8);hsa:60(P60709);hsa:7414(P18206);hsa:2335(P02751);hsa:71(P60709);hsa:7148(A0A140T8Y3) | 3\|36 | hsa:1293(E7ENL6);hsa:7148(A0A140T8Y3);hsa:2335(P02751) | 0.2523693 | 0.4106348 | http://www.kegg.jp/kegg-bin/show_pathway?hsa04510+hsa:1293%09green+hsa:7148%09green+hsa:2335%09salmon |
| hsa05133 | Pertussis | Human Diseases | 17\|228 | hsa:712(P02745);hsa:715(A0A3B3ISR2);hsa:1072(E9PK25);hsa:721(A0A0G2JPR0;P0C0L5;P0C0L4);hsa:727(P01031);hsa:718(P01024;M0R0Q9);hsa:717(A0A0G2JL69);hsa:710(P05155);hsa:725(P20851);hsa:713(D6R934);hsa:929(P08571);hsa:720(A0A0G2JPR0;P0C0L5;P0C0L4);hsa:714(P02747);hsa:722(P04003);hsa:716(P09871) | 2\|36 | hsa:713(D6R934);hsa:712(P02745) | 0.2632411 | 0.4211858 | http://www.kegg.jp/kegg-bin/show_pathway?hsa05133+hsa:713%09salmon+hsa:712%09green |
| hsa04114 | Oocyte meiosis | Cellular Processes | 1\|228 | hsa:7534(E7EX29) | 1\|36 | hsa:7534(E7EX29) | 0.1578947 | 0.3295195 | http://www.kegg.jp/kegg-bin/show_pathway?hsa04114+hsa:7534%09salmon |
| hsa04020 | Calcium signaling pathway | Environmental Information Processing | 39\|228 | hsa:102723407(A0A0C4DH29;A0A0C4DH32;A0A0C4DH33;A0A0J9YX35;A0A0C4DH31;A0A0C4DH36;A0A0C4DH34;A0A0C4DH38;A0A0C4DH39;A0A0J9YY99;A0A0G2JMI3;A0A0B4J1U7;P01763;P01764;P01743;P01766;A0A0B4J1X5;A0A0C4DH43;P01782;P01780;A0A087WSY4;P01814;A0A0B4J1X8;A0A4W8ZXM2;A0A075B7D0;P0DP02;P0DP01;A0A075B7F0;A0A075B7D8;P01817;A0A0A0MS14;A0A0A0MS15;A0A0B4J1V1;A0A075B7B8;A0A0B4J1V2;P0DTE1;A0A075B6Q5;A0A0J9YVY3;P23083) | 10\|36 | hsa:102723407(A0A0A0MS15;A0A0B4J1V2;A0A0C4DH33;A0A0C4DH36;A0A0J9YVY3;A0A0J9YX35;P01782;P01814;P01817;P0DP01) | 0.0359216 | 0.287373 | http://www.kegg.jp/kegg-bin/show_pathway?hsa04020+hsa:102723407%09green+hsa:102723407%09green+hsa:102723407%09salmon+hsa:102723407%09salmon+hsa:102723407%09salmon+hsa:102723407%09salmon+hsa:102723407%09salmon+hsa:102723407%09salmon+hsa:102723407%09salmon+hsa:102723407%09salmon |
| hsa05340 | Primary immunodeficiency | Human Diseases | 41\|228 | hsa:102723407(A0A0C4DH29;A0A0C4DH32;A0A0C4DH33;A0A0J9YX35;A0A0C4DH31;A0A0C4DH36;A0A0C4DH34;A0A0C4DH38;A0A0C4DH39;A0A0J9YY99;A0A0G2JMI3;A0A0B4J1U7;P01763;P01764;P01743;P01766;A0A0B4J1X5;A0A0C4DH43;P01782;P01780;A0A087WSY4;P01814;A0A0B4J1X8;A0A4W8ZXM2;A0A075B7D0;P0DP02;P0DP01;A0A075B7F0;A0A075B7D8;P01817;A0A0A0MS14;A0A0A0MS15;A0A0B4J1V1;A0A075B7B8;A0A0B4J1V2;P0DTE1;A0A075B6Q5;A0A0J9YVY3;P23083);hsa:3543(P01871;P15814) | 10\|36 | hsa:102723407(A0A0A0MS15;A0A0B4J1V2;A0A0C4DH33;A0A0C4DH36;A0A0J9YVY3;A0A0J9YX35;P01782;P01814;P01817;P0DP01) | 0.047076 | 0.2152047 | http://www.kegg.jp/kegg-bin/show_pathway?hsa05340+hsa:102723407%09green+hsa:102723407%09green+hsa:102723407%09salmon+hsa:102723407%09salmon+hsa:102723407%09salmon+hsa:102723407%09salmon+hsa:102723407%09salmon+hsa:102723407%09salmon+hsa:102723407%09salmon+hsa:102723407%09salmon |
| hsa05414 | Dilated cardiomyopathy (DCM) | Human Diseases | 44\|228 | hsa:3674(P08514);hsa:102723407(A0A0C4DH29;A0A0C4DH32;A0A0C4DH33;A0A0J9YX35;A0A0C4DH31;A0A0C4DH36;A0A0C4DH34;A0A0C4DH38;A0A0C4DH39;A0A0J9YY99;A0A0G2JMI3;A0A0B4J1U7;P01763;P01764;P01743;P01766;A0A0B4J1X5;A0A0C4DH43;P01782;P01780;A0A087WSY4;P01814;A0A0B4J1X8;A0A4W8ZXM2;A0A075B7D0;P0DP02;P0DP01;A0A075B7F0;A0A075B7D8;P01817;A0A0A0MS14;A0A0A0MS15;A0A0B4J1V1;A0A075B7B8;A0A0B4J1V2;P0DTE1;A0A075B6Q5;A0A0J9YVY3;P23083);hsa:7171(A0A087WWU8;A0A2R8Y5V9);hsa:71(P60709);hsa:70(P68032);hsa:60(P60709) | 10\|36 | hsa:102723407(A0A0A0MS15;A0A0B4J1V2;A0A0C4DH33;A0A0C4DH36;A0A0J9YVY3;A0A0J9YX35;P01782;P01814;P01817;P0DP01) | 0.0663199 | 0.2195419 | http://www.kegg.jp/kegg-bin/show_pathway?hsa05414+hsa:102723407%09green+hsa:102723407%09green+hsa:102723407%09salmon+hsa:102723407%09salmon+hsa:102723407%09salmon+hsa:102723407%09salmon+hsa:102723407%09salmon+hsa:102723407%09salmon+hsa:102723407%09salmon+hsa:102723407%09salmon |
| hsa05020 | Prion diseases | Human Diseases | 11\|228 | hsa:735(P02748);hsa:712(P02745);hsa:3309(P11021);hsa:714(P02747);hsa:729(P13671);hsa:713(D6R934);hsa:727(P01031);hsa:733(P07360);hsa:732(P07358);hsa:731(P07357);hsa:730(P10643) | 2\|36 | hsa:713(D6R934);hsa:712(P02745) | 0.2998556 | 0.3787649 | http://www.kegg.jp/kegg-bin/show_pathway?hsa05020+hsa:713%09salmon+hsa:712%09green |
| hsa05160 | Hepatitis C | Human Diseases | 1\|228 | hsa:7534(E7EX29) | 1\|36 | hsa:7534(E7EX29) | 0.1578947 | 0.3295195 | http://www.kegg.jp/kegg-bin/show_pathway?hsa05160+hsa:7534%09salmon |
| hsa00620 | Pyruvate metabolism | Metabolism | 1\|228 | hsa:5315(H3BTN5) | 1\|36 | hsa:5315(H3BTN5) | 0.1578947 | 0.3295195 | http://www.kegg.jp/kegg-bin/show_pathway?hsa00620+hsa:5315%09salmon |
| hsa04151 | PI3K-Akt signaling pathway | Environmental Information Processing | 51\|228 | hsa:7450(P04275);hsa:102723407(A0A0C4DH29;A0A0C4DH32;A0A0C4DH33;A0A0J9YX35;A0A0C4DH31;A0A0C4DH36;A0A0C4DH34;A0A0C4DH38;A0A0C4DH39;A0A0J9YY99;A0A0G2JMI3;A0A0B4J1U7;P01763;P01764;P01743;P01766;A0A0B4J1X5;A0A0C4DH43;P01782;P01780;A0A087WSY4;P01814;A0A0B4J1X8;A0A4W8ZXM2;A0A075B7D0;P0DP02;P0DP01;A0A075B7F0;A0A075B7D8;P01817;A0A0A0MS14;A0A0A0MS15;A0A0B4J1V1;A0A075B7B8;A0A0B4J1V2;P0DTE1;A0A075B6Q5;A0A0J9YVY3;P23083);hsa:1291(A0A087X0S5);hsa:7534(E7EX29);hsa:1311(G3XAP6);hsa:3481(P01344);hsa:7448(H0YJW9;P04004);hsa:2335(P02751);hsa:3674(P08514);hsa:7057(P07996);hsa:1293(E7ENL6);hsa:7148(A0A140T8Y3) | 14\|36 | hsa:102723407(A0A0A0MS15;A0A0B4J1V2;A0A0C4DH33;A0A0C4DH36;A0A0J9YVY3;A0A0J9YX35;P01782;P01814;P01817;P0DP01);hsa:2335(P02751);hsa:1293(E7ENL6);hsa:7148(A0A140T8Y3);hsa:7534(E7EX29) | 0.0075412 | 0.723957 | http://www.kegg.jp/kegg-bin/show_pathway?hsa04151+hsa:102723407%09green+hsa:102723407%09green+hsa:102723407%09salmon+hsa:102723407%09salmon+hsa:102723407%09salmon+hsa:102723407%09salmon+hsa:102723407%09salmon+hsa:102723407%09salmon+hsa:102723407%09salmon+hsa:102723407%09salmon+hsa:2335%09salmon+hsa:1293%09green+hsa:7148%09green+hsa:7534%09salmon |
| hsa04390 | Hippo signaling pathway | Environmental Information Processing | 3\|228 | hsa:3993(J3QRV5);hsa:71(P60709);hsa:60(P60709);hsa:7534(E7EX29) | 1\|36 | hsa:7534(E7EX29) | 0.3386018 | 0.4063221 | http://www.kegg.jp/kegg-bin/show_pathway?hsa04390+hsa:7534%09salmon |
| hsa04919 | Thyroid hormone signaling pathway | Organismal Systems | 2\|228 | hsa:71(P60709);hsa:60(P60709);hsa:90390(Q96HR3) | 1\|36 | hsa:90390(Q96HR3) | 0.2670995 | 0.3827097 | http://www.kegg.jp/kegg-bin/show_pathway?hsa04919+hsa:90390%09green |
| hsa04014 | Ras signaling pathway | Environmental Information Processing | 4\|228 | hsa:5881(P60763);hsa:81579(A0A2R8Y3M9);hsa:3481(P01344);hsa:5908(P61224) | 1\|36 | hsa:81579(A0A2R8Y3M9) | 0.3812405 | 0.4255708 | http://www.kegg.jp/kegg-bin/show_pathway?hsa04014+hsa:81579%09green |
| hsa04974 | Protein digestion and absorption | Organismal Systems | 4\|228 | hsa:1293(E7ENL6);hsa:1361(Q96IY4);hsa:7373(J3QT83);hsa:1291(A0A087X0S5) | 1\|36 | hsa:1293(E7ENL6) | 0.3812405 | 0.4255708 | http://www.kegg.jp/kegg-bin/show_pathway?hsa04974+hsa:1293%09green |
| hsa01200 | Carbon metabolism | Metabolism | 5\|228 | hsa:5315(H3BTN5);hsa:2023(A0A2R8Y6G6);hsa:847(P04040);hsa:226(J3KPS3);hsa:2597(E7EUT5) | 1\|36 | hsa:5315(H3BTN5) | 0.4020896 | 0.4150602 | http://www.kegg.jp/kegg-bin/show_pathway?hsa01200+hsa:5315%09salmon |
| hsa04918 | Thyroid hormone synthesis | Organismal Systems | 6\|228 | hsa:2878(A0A087X1J7);hsa:7276(P02766);hsa:213(P02768;A0A087WWT3);hsa:3309(P11021);hsa:6906(P05543) | 1\|36 | hsa:7276(P02766) | 0.4067776 | 0.4110595 | http://www.kegg.jp/kegg-bin/show_pathway?hsa04918+hsa:7276%09green |
| hsa04976 | Bile secretion | Organismal Systems | 1\|228 | hsa:760(P00918) | 1\|36 | hsa:760(P00918) | 0.1578947 | 0.3295195 | http://www.kegg.jp/kegg-bin/show_pathway?hsa04976+hsa:760%09green |
| hsa05168 | Herpes simplex infection | Human Diseases | 5\|228 | hsa:718(P01024;M0R0Q9);hsa:5199(E9PAQ1);hsa:727(P01031);hsa:3105(A0A0G2JI36) | 1\|36 | hsa:3105(A0A0G2JI36) | 0.4020896 | 0.4150602 | http://www.kegg.jp/kegg-bin/show_pathway?hsa05168+hsa:3105%09green |
| hsa04972 | Pancreatic secretion | Organismal Systems | 4\|228 | hsa:760(P00918);hsa:81579(A0A2R8Y3M9);hsa:1361(Q96IY4);hsa:5908(P61224) | 2\|36 | hsa:760(P00918);hsa:81579(A0A2R8Y3M9) | 0.1053428 | 0.3064517 | http://www.kegg.jp/kegg-bin/show_pathway?hsa04972+hsa:760%09green+hsa:81579%09green |
| hsa04610 | Complement and coagulation cascades | Organismal Systems | 58\|228 | hsa:3818(H0YAC1);hsa:5345(P08697);hsa:5104(P05154);hsa:5340(P00747);hsa:629(B4E1Z4);hsa:729(P13671);hsa:2(P01023);hsa:725(P20851);hsa:732(P07358);hsa:727(P01031);hsa:720(A0A0G2JPR0;P0C0L5;P0C0L4);hsa:462(P01008);hsa:722(P04003);hsa:1675(K7ERG9);hsa:7450(P04275);hsa:10544(Q9UNN8);hsa:5624(E7END6);hsa:5627(A0A0S2Z4L3);hsa:2147(P00734);hsa:721(A0A0G2JPR0;P0C0L5;P0C0L4);hsa:2162(P00488);hsa:2161(P00748);hsa:2160(P03951);hsa:731(P07357);hsa:2165(P05160);hsa:730(P10643);hsa:735(P02748);hsa:3827(P01042);hsa:5648(P48740);hsa:1361(Q96IY4);hsa:718(P01024;M0R0Q9);hsa:10747(O00187);hsa:4153(P11226);hsa:1191(P10909);hsa:710(P05155);hsa:713(D6R934);hsa:712(P02745);hsa:715(A0A3B3ISR2);hsa:714(P02747);hsa:717(A0A0G2JL69);hsa:716(P09871);hsa:5265(P01009);hsa:3075(P08603);hsa:2243(P02671);hsa:7448(H0YJW9;P04004);hsa:2244(P02675);hsa:3426(A0A2R8Y3M9);hsa:2266(C9JPQ9;C9JC84);hsa:2155(F5H8B0);hsa:2153(A0A0A0MRJ7);hsa:3053(P05546);hsa:733(P07360);hsa:2158(P00740);hsa:2159(P00742) | 5\|36 | hsa:713(D6R934);hsa:2160(P03951);hsa:3426(A0A2R8Y3M9);hsa:712(P02745);hsa:1675(K7ERG9) | 0.0377038 | 0.2262225 | http://www.kegg.jp/kegg-bin/show_pathway?hsa04610+hsa:713%09salmon+hsa:2160%09salmon+hsa:3426%09green+hsa:712%09green+hsa:1675%09green |
| hsa04922 | Glucagon signaling pathway | Organismal Systems | 1\|228 | hsa:5315(H3BTN5) | 1\|36 | hsa:5315(H3BTN5) | 0.1578947 | 0.3295195 | http://www.kegg.jp/kegg-bin/show_pathway?hsa04922+hsa:5315%09salmon |
| hsa05162 | Measles | Human Diseases | 40\|228 | hsa:102723407(A0A0C4DH29;A0A0C4DH32;A0A0C4DH33;A0A0J9YX35;A0A0C4DH31;A0A0C4DH36;A0A0C4DH34;A0A0C4DH38;A0A0C4DH39;A0A0J9YY99;A0A0G2JMI3;A0A0B4J1U7;P01763;P01764;P01743;P01766;A0A0B4J1X5;A0A0C4DH43;P01782;P01780;A0A087WSY4;P01814;A0A0B4J1X8;A0A4W8ZXM2;A0A075B7D0;P0DP02;P0DP01;A0A075B7F0;A0A075B7D8;P01817;A0A0A0MS14;A0A0A0MS15;A0A0B4J1V1;A0A075B7B8;A0A0B4J1V2;P0DTE1;A0A075B6Q5;A0A0J9YVY3;P23083);hsa:4478(P26038) | 11\|36 | hsa:102723407(A0A0A0MS15;A0A0B4J1V2;A0A0C4DH33;A0A0C4DH36;A0A0J9YVY3;A0A0J9YX35;P01782;P01814;P01817;P0DP01);hsa:4478(P26038) | 0.0179695 | 0.5750224 | http://www.kegg.jp/kegg-bin/show_pathway?hsa05162+hsa:102723407%09green+hsa:102723407%09green+hsa:102723407%09salmon+hsa:102723407%09salmon+hsa:102723407%09salmon+hsa:102723407%09salmon+hsa:102723407%09salmon+hsa:102723407%09salmon+hsa:102723407%09salmon+hsa:102723407%09salmon+hsa:4478%09salmon |
| hsa04670 | Leukocyte transendothelial migration | Organismal Systems | 7\|228 | hsa:5908(P61224);hsa:87(H9KV75);hsa:7412(P19320);hsa:60(P60709);hsa:7414(P18206);hsa:4478(P26038);hsa:71(P60709);hsa:1003(I3L1J2) | 2\|36 | hsa:4478(P26038);hsa:1003(I3L1J2) | 0.2244606 | 0.378039 | http://www.kegg.jp/kegg-bin/show_pathway?hsa04670+hsa:4478%09salmon+hsa:1003%09salmon |
| hsa05330 | Allograft rejection | Human Diseases | 40\|228 | hsa:102723407(A0A0C4DH29;A0A0C4DH32;A0A0C4DH33;A0A0J9YX35;A0A0C4DH31;A0A0C4DH36;A0A0C4DH34;A0A0C4DH38;A0A0C4DH39;A0A0J9YY99;A0A0G2JMI3;A0A0B4J1U7;P01763;P01764;P01743;P01766;A0A0B4J1X5;A0A0C4DH43;P01782;P01780;A0A087WSY4;P01814;A0A0B4J1X8;A0A4W8ZXM2;A0A075B7D0;P0DP02;P0DP01;A0A075B7F0;A0A075B7D8;P01817;A0A0A0MS14;A0A0A0MS15;A0A0B4J1V1;A0A075B7B8;A0A0B4J1V2;P0DTE1;A0A075B6Q5;A0A0J9YVY3;P23083);hsa:3105(A0A0G2JI36) | 11\|36 | hsa:102723407(A0A0A0MS15;A0A0B4J1V2;A0A0C4DH33;A0A0C4DH36;A0A0J9YVY3;A0A0J9YX35;P01782;P01814;P01817;P0DP01);hsa:3105(A0A0G2JI36) | 0.0179695 | 0.5750224 | http://www.kegg.jp/kegg-bin/show_pathway?hsa05330+hsa:102723407%09green+hsa:102723407%09green+hsa:102723407%09salmon+hsa:102723407%09salmon+hsa:102723407%09salmon+hsa:102723407%09salmon+hsa:102723407%09salmon+hsa:102723407%09salmon+hsa:102723407%09salmon+hsa:102723407%09salmon+hsa:3105%09green |
| hsa04640 | Hematopoietic cell lineage | Organismal Systems | 45\|228 | hsa:3674(P08514);hsa:102723407(A0A0C4DH29;A0A0C4DH32;A0A0C4DH33;A0A0J9YX35;A0A0C4DH31;A0A0C4DH36;A0A0C4DH34;A0A0C4DH38;A0A0C4DH39;A0A0J9YY99;A0A0G2JMI3;A0A0B4J1U7;P01763;P01764;P01743;P01766;A0A0B4J1X5;A0A0C4DH43;P01782;P01780;A0A087WSY4;P01814;A0A0B4J1X8;A0A4W8ZXM2;A0A075B7D0;P0DP02;P0DP01;A0A075B7F0;A0A075B7D8;P01817;A0A0A0MS14;A0A0A0MS15;A0A0B4J1V1;A0A075B7B8;A0A0B4J1V2;P0DTE1;A0A075B6Q5;A0A0J9YVY3;P23083);hsa:2811(A0A0C4DGZ8);hsa:960(H0Y5E4);hsa:290(P15144);hsa:7037(G3V0E5);hsa:929(P08571) | 10\|36 | hsa:102723407(A0A0A0MS15;A0A0B4J1V2;A0A0C4DH33;A0A0C4DH36;A0A0J9YVY3;A0A0J9YX35;P01782;P01814;P01817;P0DP01) | 0.0732197 | 0.234303 | http://www.kegg.jp/kegg-bin/show_pathway?hsa04640+hsa:102723407%09green+hsa:102723407%09green+hsa:102723407%09salmon+hsa:102723407%09salmon+hsa:102723407%09salmon+hsa:102723407%09salmon+hsa:102723407%09salmon+hsa:102723407%09salmon+hsa:102723407%09salmon+hsa:102723407%09salmon |
| hsa04964 | Proximal tubule bicarbonate reclamation | Organismal Systems | 1\|228 | hsa:760(P00918) | 1\|36 | hsa:760(P00918) | 0.1578947 | 0.3295195 | http://www.kegg.jp/kegg-bin/show_pathway?hsa04964+hsa:760%09green |
| hsa04630 | JAK-STAT signaling pathway | Environmental Information Processing | 1\|228 | hsa:2670(A0A1W2PQU7) | 1\|36 | hsa:2670(A0A1W2PQU7) | 0.1578947 | 0.3295195 | http://www.kegg.jp/kegg-bin/show_pathway?hsa04630+hsa:2670%09green |
| hsa04966 | Collecting duct acid secretion | Organismal Systems | 1\|228 | hsa:760(P00918) | 1\|36 | hsa:760(P00918) | 0.1578947 | 0.3295195 | http://www.kegg.jp/kegg-bin/show_pathway?hsa04966+hsa:760%09green |
| hsa05161 | Hepatitis B | Human Diseases | 1\|228 | hsa:7534(E7EX29) | 1\|36 | hsa:7534(E7EX29) | 0.1578947 | 0.3295195 | http://www.kegg.jp/kegg-bin/show_pathway?hsa05161+hsa:7534%09salmon |
| hsa04540 | Gap junction | Cellular Processes | 4\|228 | hsa:203068(P07437);hsa:7278(P0DPH7);hsa:81027(Q9H4B7);hsa:7277(P68366);hsa:113457(P0DPH7);hsa:112714(P0DPH7) | 1\|36 | hsa:81027(Q9H4B7) | 0.3812405 | 0.4255708 | http://www.kegg.jp/kegg-bin/show_pathway?hsa04540+hsa:81027%09salmon |
| hsa05322 | Systemic lupus erythematosus | Human Diseases | 60\|228 | hsa:735(P02748);hsa:102723407(A0A0C4DH29;A0A0C4DH32;A0A0C4DH33;A0A0J9YX35;A0A0C4DH31;A0A0C4DH36;A0A0C4DH34;A0A0C4DH38;A0A0C4DH39;A0A0J9YY99;A0A0G2JMI3;A0A0B4J1U7;P01763;P01764;P01743;P01766;A0A0B4J1X5;A0A0C4DH43;P01782;P01780;A0A087WSY4;P01814;A0A0B4J1X8;A0A4W8ZXM2;A0A075B7D0;P0DP02;P0DP01;A0A075B7F0;A0A075B7D8;P01817;A0A0A0MS14;A0A0A0MS15;A0A0B4J1V1;A0A075B7B8;A0A0B4J1V2;P0DTE1;A0A075B6Q5;A0A0J9YVY3;P23083);hsa:712(P02745);hsa:733(P07360);hsa:128312(A0A2R8Y619);hsa:715(A0A3B3ISR2);hsa:721(A0A0G2JPR0;P0C0L5;P0C0L4);hsa:727(P01031);hsa:718(P01024;M0R0Q9);hsa:732(P07358);hsa:87(H9KV75);hsa:2214(H0Y755);hsa:730(P10643);hsa:731(P07357);hsa:713(D6R934);hsa:716(P09871);hsa:720(A0A0G2JPR0;P0C0L5;P0C0L4);hsa:714(P02747);hsa:717(A0A0G2JL69);hsa:729(P13671) | 12\|36 | hsa:102723407(A0A0A0MS15;A0A0B4J1V2;A0A0C4DH33;A0A0C4DH36;A0A0J9YVY3;A0A0J9YX35;P01782;P01814;P01817;P0DP01);hsa:713(D6R934);hsa:712(P02745) | 0.0920225 | 0.2849729 | http://www.kegg.jp/kegg-bin/show_pathway?hsa05322+hsa:102723407%09green+hsa:102723407%09green+hsa:102723407%09salmon+hsa:102723407%09salmon+hsa:102723407%09salmon+hsa:102723407%09salmon+hsa:102723407%09salmon+hsa:102723407%09salmon+hsa:102723407%09salmon+hsa:102723407%09salmon+hsa:713%09salmon+hsa:712%09green |
| hsa04666 | Fc gamma R-mediated phagocytosis | Organismal Systems | 42\|228 | hsa:2934(A0A0A0MS51);hsa:102723407(A0A0C4DH29;A0A0C4DH32;A0A0C4DH33;A0A0J9YX35;A0A0C4DH31;A0A0C4DH36;A0A0C4DH34;A0A0C4DH38;A0A0C4DH39;A0A0J9YY99;A0A0G2JMI3;A0A0B4J1U7;P01763;P01764;P01743;P01766;A0A0B4J1X5;A0A0C4DH43;P01782;P01780;A0A087WSY4;P01814;A0A0B4J1X8;A0A4W8ZXM2;A0A075B7D0;P0DP02;P0DP01;A0A075B7F0;A0A075B7D8;P01817;A0A0A0MS14;A0A0A0MS15;A0A0B4J1V1;A0A075B7B8;A0A0B4J1V2;P0DTE1;A0A075B6Q5;A0A0J9YVY3;P23083);hsa:1072(E9PK25);hsa:2214(H0Y755) | 10\|36 | hsa:102723407(A0A0A0MS15;A0A0B4J1V2;A0A0C4DH33;A0A0C4DH36;A0A0J9YVY3;A0A0J9YX35;P01782;P01814;P01817;P0DP01) | 0.0531965 | 0.2042747 | http://www.kegg.jp/kegg-bin/show_pathway?hsa04666+hsa:102723407%09green+hsa:102723407%09green+hsa:102723407%09salmon+hsa:102723407%09salmon+hsa:102723407%09salmon+hsa:102723407%09salmon+hsa:102723407%09salmon+hsa:102723407%09salmon+hsa:102723407%09salmon+hsa:102723407%09salmon |
| hsa05222 | Small cell lung cancer | Human Diseases | 2\|228 | hsa:3674(P08514);hsa:2335(P02751) | 1\|36 | hsa:2335(P02751) | 0.2670995 | 0.3827097 | http://www.kegg.jp/kegg-bin/show_pathway?hsa05222+hsa:2335%09salmon |
| hsa04930 | Type II diabetes mellitus | Human Diseases | 2\|228 | hsa:5315(H3BTN5);hsa:9370(Q15848) | 1\|36 | hsa:5315(H3BTN5) | 0.2670995 | 0.3827097 | http://www.kegg.jp/kegg-bin/show_pathway?hsa04930+hsa:5315%09salmon |
| hsa05167 | Kaposi sarcoma-associated herpesvirus infection | Human Diseases | 3\|228 | hsa:718(P01024;M0R0Q9);hsa:3105(A0A0G2JI36) | 1\|36 | hsa:3105(A0A0G2JI36) | 0.3386018 | 0.4063221 | http://www.kegg.jp/kegg-bin/show_pathway?hsa05167+hsa:3105%09green |
| hsa04924 | Renin secretion | Organismal Systems | 1\|228 | hsa:183(P01019) | 1\|36 | hsa:183(P01019) | 0.1578947 | 0.3295195 | http://www.kegg.jp/kegg-bin/show_pathway?hsa04924+hsa:183%09green |
| hsa04530 | Tight junction | Cellular Processes | 7\|228 | hsa:3993(J3QRV5);hsa:7278(P0DPH7);hsa:7277(P68366);hsa:113457(P0DPH7);hsa:4627(P35579);hsa:87(H9KV75);hsa:112714(P0DPH7);hsa:4478(P26038);hsa:71(P60709);hsa:60(P60709) | 1\|36 | hsa:4478(P26038) | 0.3997537 | 0.4217182 | http://www.kegg.jp/kegg-bin/show_pathway?hsa04530+hsa:4478%09salmon |
| hsa05310 | Asthma | Human Diseases | 39\|228 | hsa:102723407(A0A0C4DH29;A0A0C4DH32;A0A0C4DH33;A0A0J9YX35;A0A0C4DH31;A0A0C4DH36;A0A0C4DH34;A0A0C4DH38;A0A0C4DH39;A0A0J9YY99;A0A0G2JMI3;A0A0B4J1U7;P01763;P01764;P01743;P01766;A0A0B4J1X5;A0A0C4DH43;P01782;P01780;A0A087WSY4;P01814;A0A0B4J1X8;A0A4W8ZXM2;A0A075B7D0;P0DP02;P0DP01;A0A075B7F0;A0A075B7D8;P01817;A0A0A0MS14;A0A0A0MS15;A0A0B4J1V1;A0A075B7B8;A0A0B4J1V2;P0DTE1;A0A075B6Q5;A0A0J9YVY3;P23083) | 10\|36 | hsa:102723407(A0A0A0MS15;A0A0B4J1V2;A0A0C4DH33;A0A0C4DH36;A0A0J9YVY3;A0A0J9YX35;P01782;P01814;P01817;P0DP01) | 0.0359216 | 0.287373 | http://www.kegg.jp/kegg-bin/show_pathway?hsa05310+hsa:102723407%09green+hsa:102723407%09green+hsa:102723407%09salmon+hsa:102723407%09salmon+hsa:102723407%09salmon+hsa:102723407%09salmon+hsa:102723407%09salmon+hsa:102723407%09salmon+hsa:102723407%09salmon+hsa:102723407%09salmon |
| hsa04664 | Fc epsilon RI signaling pathway | Organismal Systems | 40\|228 | hsa:5881(P60763);hsa:102723407(A0A0C4DH29;A0A0C4DH32;A0A0C4DH33;A0A0J9YX35;A0A0C4DH31;A0A0C4DH36;A0A0C4DH34;A0A0C4DH38;A0A0C4DH39;A0A0J9YY99;A0A0G2JMI3;A0A0B4J1U7;P01763;P01764;P01743;P01766;A0A0B4J1X5;A0A0C4DH43;P01782;P01780;A0A087WSY4;P01814;A0A0B4J1X8;A0A4W8ZXM2;A0A075B7D0;P0DP02;P0DP01;A0A075B7F0;A0A075B7D8;P01817;A0A0A0MS14;A0A0A0MS15;A0A0B4J1V1;A0A075B7B8;A0A0B4J1V2;P0DTE1;A0A075B6Q5;A0A0J9YVY3;P23083) | 10\|36 | hsa:102723407(A0A0A0MS15;A0A0B4J1V2;A0A0C4DH33;A0A0C4DH36;A0A0J9YVY3;A0A0J9YX35;P01782;P01814;P01817;P0DP01) | 0.0413067 | 0.2203024 | http://www.kegg.jp/kegg-bin/show_pathway?hsa04664+hsa:102723407%09green+hsa:102723407%09green+hsa:102723407%09salmon+hsa:102723407%09salmon+hsa:102723407%09salmon+hsa:102723407%09salmon+hsa:102723407%09salmon+hsa:102723407%09salmon+hsa:102723407%09salmon+hsa:102723407%09salmon |

| Table S3-3 KEGG pathway enrichment of DEPs in S vs C group. | | | | | | | | | |
| --- | --- | --- | --- | --- | --- | --- | --- | --- | --- |
| pathway | pathway_name | class | all_number_of_accs | all_KO2acc | diff_number_of_accs | diff_KO2acc | p_value | FDR | url |
| hsa04145 | Phagosome | Cellular Processes | 56\|228 | hsa:203068(P07437);hsa:3105(A0A0G2JI36);hsa:7278(P0DPH7);hsa:81027(Q9H4B7);hsa:102723407(A0A0C4DH29;A0A0C4DH32;A0A0C4DH33;A0A0J9YX35;A0A0C4DH31;A0A0C4DH36;A0A0C4DH34;A0A0C4DH38;A0A0C4DH39;A0A0J9YY99;A0A0G2JMI3;A0A0B4J1U7;P01763;P01764;P01743;P01766;A0A0B4J1X5;A0A0C4DH43;P01782;P01780;A0A087WSY4;P01814;A0A0B4J1X8;A0A4W8ZXM2;A0A075B7D0;P0DP02;P0DP01;A0A075B7F0;A0A075B7D8;P01817;A0A0A0MS14;A0A0A0MS15;A0A0B4J1V1;A0A075B7B8;A0A0B4J1V2;P0DTE1;A0A075B6Q5;A0A0J9YVY3;P23083);hsa:1311(G3XAP6);hsa:71(P60709);hsa:7277(P68366);hsa:113457(P0DPH7);hsa:3920(P13473);hsa:718(P01024;M0R0Q9);hsa:7057(P07996);hsa:112714(P0DPH7);hsa:2214(H0Y755);hsa:4153(P11226);hsa:78989(Q9BWP8);hsa:7037(G3V0E5);hsa:929(P08571);hsa:715(A0A3B3ISR2);hsa:60(P60709) | 9\|53 | hsa:3920(P13473);hsa:102723407(A0A0A0MS15;A0A0B4J1V2;A0A0C4DH33;A0A0C4DH38;A0A0J9YX35;P01814;P01817);hsa:3105(A0A0G2JI36) | 0.051479005 | 1 | http://www.kegg.jp/kegg-bin/show_pathway?hsa04145+hsa:3920%09green+hsa:102723407%09green+hsa:102723407%09green+hsa:102723407%09salmon+hsa:102723407%09green+hsa:102723407%09salmon+hsa:102723407%09salmon+hsa:102723407%09salmon+hsa:3105%09green |
| hsa05146 | Amoebiasis | Human Diseases | 47\|228 | hsa:102723407(A0A0C4DH29;A0A0C4DH32;A0A0C4DH33;A0A0J9YX35;A0A0C4DH31;A0A0C4DH36;A0A0C4DH34;A0A0C4DH38;A0A0C4DH39;A0A0J9YY99;A0A0G2JMI3;A0A0B4J1U7;P01763;P01764;P01743;P01766;A0A0B4J1X5;A0A0C4DH43;P01782;P01780;A0A087WSY4;P01814;A0A0B4J1X8;A0A4W8ZXM2;A0A075B7D0;P0DP02;P0DP01;A0A075B7F0;A0A075B7D8;P01817;A0A0A0MS14;A0A0A0MS15;A0A0B4J1V1;A0A075B7B8;A0A0B4J1V2;P0DTE1;A0A075B6Q5;A0A0J9YVY3;P23083);hsa:2335(P02751);hsa:87(H9KV75);hsa:7414(P18206);hsa:735(P02748);hsa:929(P08571);hsa:733(P07360);hsa:732(P07358);hsa:731(P07357) | 9\|53 | hsa:102723407(A0A0A0MS15;A0A0B4J1V2;A0A0C4DH33;A0A0C4DH38;A0A0J9YX35;P01814;P01817);hsa:733(P07360);hsa:2335(P02751) | 0.121336514 | 0.556125691 | http://www.kegg.jp/kegg-bin/show_pathway?hsa05146+hsa:102723407%09green+hsa:102723407%09green+hsa:102723407%09salmon+hsa:102723407%09green+hsa:102723407%09salmon+hsa:102723407%09salmon+hsa:102723407%09salmon+hsa:733%09green+hsa:2335%09salmon |
| hsa05169 | Epstein-Barr virus infection | Human Diseases | 41\|228 | hsa:102723407(A0A0C4DH29;A0A0C4DH32;A0A0C4DH33;A0A0J9YX35;A0A0C4DH31;A0A0C4DH36;A0A0C4DH34;A0A0C4DH38;A0A0C4DH39;A0A0J9YY99;A0A0G2JMI3;A0A0B4J1U7;P01763;P01764;P01743;P01766;A0A0B4J1X5;A0A0C4DH43;P01782;P01780;A0A087WSY4;P01814;A0A0B4J1X8;A0A4W8ZXM2;A0A075B7D0;P0DP02;P0DP01;A0A075B7F0;A0A075B7D8;P01817;A0A0A0MS14;A0A0A0MS15;A0A0B4J1V1;A0A075B7B8;A0A0B4J1V2;P0DTE1;A0A075B6Q5;A0A0J9YVY3;P23083);hsa:3105(A0A0G2JI36);hsa:960(H0Y5E4) | 8\|53 | hsa:102723407(A0A0A0MS15;A0A0B4J1V2;A0A0C4DH33;A0A0C4DH38;A0A0J9YX35;P01814;P01817);hsa:3105(A0A0G2JI36) | 0.138661172 | 0.492023515 | http://www.kegg.jp/kegg-bin/show_pathway?hsa05169+hsa:102723407%09green+hsa:102723407%09green+hsa:102723407%09salmon+hsa:102723407%09green+hsa:102723407%09salmon+hsa:102723407%09salmon+hsa:102723407%09salmon+hsa:3105%09green |
| hsa05416 | Viral myocarditis | Human Diseases | 42\|228 | hsa:5881(P60763);hsa:102723407(A0A0C4DH29;A0A0C4DH32;A0A0C4DH33;A0A0J9YX35;A0A0C4DH31;A0A0C4DH36;A0A0C4DH34;A0A0C4DH38;A0A0C4DH39;A0A0J9YY99;A0A0G2JMI3;A0A0B4J1U7;P01763;P01764;P01743;P01766;A0A0B4J1X5;A0A0C4DH43;P01782;P01780;A0A087WSY4;P01814;A0A0B4J1X8;A0A4W8ZXM2;A0A075B7D0;P0DP02;P0DP01;A0A075B7F0;A0A075B7D8;P01817;A0A0A0MS14;A0A0A0MS15;A0A0B4J1V1;A0A075B7B8;A0A0B4J1V2;P0DTE1;A0A075B6Q5;A0A0J9YVY3;P23083);hsa:3105(A0A0G2JI36);hsa:71(P60709);hsa:60(P60709) | 8\|53 | hsa:102723407(A0A0A0MS15;A0A0B4J1V2;A0A0C4DH33;A0A0C4DH38;A0A0J9YX35;P01814;P01817);hsa:3105(A0A0G2JI36) | 0.130068454 | 0.529908518 | http://www.kegg.jp/kegg-bin/show_pathway?hsa05416+hsa:102723407%09green+hsa:102723407%09green+hsa:102723407%09salmon+hsa:102723407%09green+hsa:102723407%09salmon+hsa:102723407%09salmon+hsa:102723407%09salmon+hsa:3105%09green |
| hsa05143 | African trypanosomiasis | Human Diseases | 45\|228 | hsa:102723407(A0A0C4DH29;A0A0C4DH32;A0A0C4DH33;A0A0J9YX35;A0A0C4DH31;A0A0C4DH36;A0A0C4DH34;A0A0C4DH38;A0A0C4DH39;A0A0J9YY99;A0A0G2JMI3;A0A0B4J1U7;P01763;P01764;P01743;P01766;A0A0B4J1X5;A0A0C4DH43;P01782;P01780;A0A087WSY4;P01814;A0A0B4J1X8;A0A4W8ZXM2;A0A075B7D0;P0DP02;P0DP01;A0A075B7F0;A0A075B7D8;P01817;A0A0A0MS14;A0A0A0MS15;A0A0B4J1V1;A0A075B7B8;A0A0B4J1V2;P0DTE1;A0A075B6Q5;A0A0J9YVY3;P23083);hsa:3039(P69905);hsa:8542(O14791);hsa:335(P02647);hsa:3250(P00739);hsa:3043(P68871);hsa:3040(P69905);hsa:7412(P19320) | 8\|53 | hsa:102723407(A0A0A0MS15;A0A0B4J1V2;A0A0C4DH33;A0A0C4DH38;A0A0J9YX35;P01814;P01817);hsa:8542(O14791) | 0.102942049 | 0.75490836 | http://www.kegg.jp/kegg-bin/show_pathway?hsa05143+hsa:102723407%09green+hsa:102723407%09green+hsa:102723407%09salmon+hsa:102723407%09green+hsa:102723407%09salmon+hsa:102723407%09salmon+hsa:102723407%09salmon+hsa:8542%09salmon |
| hsa05152 | Tuberculosis | Human Diseases | 46\|228 | hsa:102723407(A0A0C4DH29;A0A0C4DH32;A0A0C4DH33;A0A0J9YX35;A0A0C4DH31;A0A0C4DH36;A0A0C4DH34;A0A0C4DH38;A0A0C4DH39;A0A0J9YY99;A0A0G2JMI3;A0A0B4J1U7;P01763;P01764;P01743;P01766;A0A0B4J1X5;A0A0C4DH43;P01782;P01780;A0A087WSY4;P01814;A0A0B4J1X8;A0A4W8ZXM2;A0A075B7D0;P0DP02;P0DP01;A0A075B7F0;A0A075B7D8;P01817;A0A0A0MS14;A0A0A0MS15;A0A0B4J1V1;A0A075B7B8;A0A0B4J1V2;P0DTE1;A0A075B6Q5;A0A0J9YVY3;P23083);hsa:820(J3KNB4);hsa:3920(P13473);hsa:718(P01024;M0R0Q9);hsa:2214(H0Y755);hsa:3929(P18428);hsa:929(P08571) | 8\|53 | hsa:3920(P13473);hsa:102723407(A0A0A0MS15;A0A0B4J1V2;A0A0C4DH33;A0A0C4DH38;A0A0J9YX35;P01814;P01817) | 0.093971258 | 0.795141414 | http://www.kegg.jp/kegg-bin/show_pathway?hsa05152+hsa:3920%09green+hsa:102723407%09green+hsa:102723407%09green+hsa:102723407%09salmon+hsa:102723407%09green+hsa:102723407%09salmon+hsa:102723407%09salmon+hsa:102723407%09salmon |
| hsa05320 | Autoimmune thyroid disease | Human Diseases | 40\|228 | hsa:102723407(A0A0C4DH29;A0A0C4DH32;A0A0C4DH33;A0A0J9YX35;A0A0C4DH31;A0A0C4DH36;A0A0C4DH34;A0A0C4DH38;A0A0C4DH39;A0A0J9YY99;A0A0G2JMI3;A0A0B4J1U7;P01763;P01764;P01743;P01766;A0A0B4J1X5;A0A0C4DH43;P01782;P01780;A0A087WSY4;P01814;A0A0B4J1X8;A0A4W8ZXM2;A0A075B7D0;P0DP02;P0DP01;A0A075B7F0;A0A075B7D8;P01817;A0A0A0MS14;A0A0A0MS15;A0A0B4J1V1;A0A075B7B8;A0A0B4J1V2;P0DTE1;A0A075B6Q5;A0A0J9YVY3;P23083);hsa:3105(A0A0G2JI36) | 8\|53 | hsa:102723407(A0A0A0MS15;A0A0B4J1V2;A0A0C4DH33;A0A0C4DH38;A0A0J9YX35;P01814;P01817);hsa:3105(A0A0G2JI36) | 0.146725894 | 0.47470142 | http://www.kegg.jp/kegg-bin/show_pathway?hsa05320+hsa:102723407%09green+hsa:102723407%09green+hsa:102723407%09salmon+hsa:102723407%09green+hsa:102723407%09salmon+hsa:102723407%09salmon+hsa:102723407%09salmon+hsa:3105%09green |
| hsa04650 | Natural killer cell mediated cytotoxicity | Organismal Systems | 42\|228 | hsa:5881(P60763);hsa:102723407(A0A0C4DH29;A0A0C4DH32;A0A0C4DH33;A0A0J9YX35;A0A0C4DH31;A0A0C4DH36;A0A0C4DH34;A0A0C4DH38;A0A0C4DH39;A0A0J9YY99;A0A0G2JMI3;A0A0B4J1U7;P01763;P01764;P01743;P01766;A0A0B4J1X5;A0A0C4DH43;P01782;P01780;A0A087WSY4;P01814;A0A0B4J1X8;A0A4W8ZXM2;A0A075B7D0;P0DP02;P0DP01;A0A075B7F0;A0A075B7D8;P01817;A0A0A0MS14;A0A0A0MS15;A0A0B4J1V1;A0A075B7B8;A0A0B4J1V2;P0DTE1;A0A075B6Q5;A0A0J9YVY3;P23083);hsa:3105(A0A0G2JI36);hsa:2214(H0Y755) | 8\|53 | hsa:102723407(A0A0A0MS15;A0A0B4J1V2;A0A0C4DH33;A0A0C4DH38;A0A0J9YX35;P01814;P01817);hsa:3105(A0A0G2JI36) | 0.130068454 | 0.529908518 | http://www.kegg.jp/kegg-bin/show_pathway?hsa04650+hsa:102723407%09green+hsa:102723407%09green+hsa:102723407%09salmon+hsa:102723407%09green+hsa:102723407%09salmon+hsa:102723407%09salmon+hsa:102723407%09salmon+hsa:3105%09green |
| hsa05340 | Primary immunodeficiency | Human Diseases | 41\|228 | hsa:102723407(A0A0C4DH29;A0A0C4DH32;A0A0C4DH33;A0A0J9YX35;A0A0C4DH31;A0A0C4DH36;A0A0C4DH34;A0A0C4DH38;A0A0C4DH39;A0A0J9YY99;A0A0G2JMI3;A0A0B4J1U7;P01763;P01764;P01743;P01766;A0A0B4J1X5;A0A0C4DH43;P01782;P01780;A0A087WSY4;P01814;A0A0B4J1X8;A0A4W8ZXM2;A0A075B7D0;P0DP02;P0DP01;A0A075B7F0;A0A075B7D8;P01817;A0A0A0MS14;A0A0A0MS15;A0A0B4J1V1;A0A075B7B8;A0A0B4J1V2;P0DTE1;A0A075B6Q5;A0A0J9YVY3;P23083);hsa:3543(P01871;P15814) | 8\|53 | hsa:102723407(A0A0A0MS15;A0A0B4J1V2;A0A0C4DH33;A0A0C4DH38;A0A0J9YX35;P01814;P01817);hsa:3543(P15814) | 0.138661172 | 0.492023515 | http://www.kegg.jp/kegg-bin/show_pathway?hsa05340+hsa:102723407%09green+hsa:102723407%09green+hsa:102723407%09salmon+hsa:102723407%09green+hsa:102723407%09salmon+hsa:102723407%09salmon+hsa:102723407%09salmon+hsa:3543%09green |
| hsa05414 | Dilated cardiomyopathy (DCM) | Human Diseases | 44\|228 | hsa:3674(P08514);hsa:102723407(A0A0C4DH29;A0A0C4DH32;A0A0C4DH33;A0A0J9YX35;A0A0C4DH31;A0A0C4DH36;A0A0C4DH34;A0A0C4DH38;A0A0C4DH39;A0A0J9YY99;A0A0G2JMI3;A0A0B4J1U7;P01763;P01764;P01743;P01766;A0A0B4J1X5;A0A0C4DH43;P01782;P01780;A0A087WSY4;P01814;A0A0B4J1X8;A0A4W8ZXM2;A0A075B7D0;P0DP02;P0DP01;A0A075B7F0;A0A075B7D8;P01817;A0A0A0MS14;A0A0A0MS15;A0A0B4J1V1;A0A075B7B8;A0A0B4J1V2;P0DTE1;A0A075B6Q5;A0A0J9YVY3;P23083);hsa:7171(A0A087WWU8;A0A2R8Y5V9);hsa:71(P60709);hsa:70(P68032);hsa:60(P60709) | 8\|53 | hsa:102723407(A0A0A0MS15;A0A0B4J1V2;A0A0C4DH33;A0A0C4DH38;A0A0J9YX35;P01814;P01817);hsa:70(P68032) | 0.112043081 | 0.648670468 | http://www.kegg.jp/kegg-bin/show_pathway?hsa05414+hsa:102723407%09green+hsa:102723407%09green+hsa:102723407%09salmon+hsa:102723407%09green+hsa:102723407%09salmon+hsa:102723407%09salmon+hsa:102723407%09salmon+hsa:70%09green |
| hsa05330 | Allograft rejection | Human Diseases | 40\|228 | hsa:102723407(A0A0C4DH29;A0A0C4DH32;A0A0C4DH33;A0A0J9YX35;A0A0C4DH31;A0A0C4DH36;A0A0C4DH34;A0A0C4DH38;A0A0C4DH39;A0A0J9YY99;A0A0G2JMI3;A0A0B4J1U7;P01763;P01764;P01743;P01766;A0A0B4J1X5;A0A0C4DH43;P01782;P01780;A0A087WSY4;P01814;A0A0B4J1X8;A0A4W8ZXM2;A0A075B7D0;P0DP02;P0DP01;A0A075B7F0;A0A075B7D8;P01817;A0A0A0MS14;A0A0A0MS15;A0A0B4J1V1;A0A075B7B8;A0A0B4J1V2;P0DTE1;A0A075B6Q5;A0A0J9YVY3;P23083);hsa:3105(A0A0G2JI36) | 8\|53 | hsa:102723407(A0A0A0MS15;A0A0B4J1V2;A0A0C4DH33;A0A0C4DH38;A0A0J9YX35;P01814;P01817);hsa:3105(A0A0G2JI36) | 0.146725894 | 0.47470142 | http://www.kegg.jp/kegg-bin/show_pathway?hsa05330+hsa:102723407%09green+hsa:102723407%09green+hsa:102723407%09salmon+hsa:102723407%09green+hsa:102723407%09salmon+hsa:102723407%09salmon+hsa:102723407%09salmon+hsa:3105%09green |
| hsa05323 | Rheumatoid arthritis | Human Diseases | 39\|228 | hsa:102723407(A0A0C4DH29;A0A0C4DH32;A0A0C4DH33;A0A0J9YX35;A0A0C4DH31;A0A0C4DH36;A0A0C4DH34;A0A0C4DH38;A0A0C4DH39;A0A0J9YY99;A0A0G2JMI3;A0A0B4J1U7;P01763;P01764;P01743;P01766;A0A0B4J1X5;A0A0C4DH43;P01782;P01780;A0A087WSY4;P01814;A0A0B4J1X8;A0A4W8ZXM2;A0A075B7D0;P0DP02;P0DP01;A0A075B7F0;A0A075B7D8;P01817;A0A0A0MS14;A0A0A0MS15;A0A0B4J1V1;A0A075B7B8;A0A0B4J1V2;P0DTE1;A0A075B6Q5;A0A0J9YVY3;P23083) | 7\|53 | hsa:102723407(A0A0A0MS15;A0A0B4J1V2;A0A0C4DH33;A0A0C4DH38;A0A0J9YX35;P01814;P01817) | 0.120570408 | 0.631559281 | http://www.kegg.jp/kegg-bin/show_pathway?hsa05323+hsa:102723407%09green+hsa:102723407%09green+hsa:102723407%09salmon+hsa:102723407%09green+hsa:102723407%09salmon+hsa:102723407%09salmon+hsa:102723407%09salmon |
| hsa04072 | Phospholipase D signaling pathway | Environmental Information Processing | 39\|228 | hsa:102723407(A0A0C4DH29;A0A0C4DH32;A0A0C4DH33;A0A0J9YX35;A0A0C4DH31;A0A0C4DH36;A0A0C4DH34;A0A0C4DH38;A0A0C4DH39;A0A0J9YY99;A0A0G2JMI3;A0A0B4J1U7;P01763;P01764;P01743;P01766;A0A0B4J1X5;A0A0C4DH43;P01782;P01780;A0A087WSY4;P01814;A0A0B4J1X8;A0A4W8ZXM2;A0A075B7D0;P0DP02;P0DP01;A0A075B7F0;A0A075B7D8;P01817;A0A0A0MS14;A0A0A0MS15;A0A0B4J1V1;A0A075B7B8;A0A0B4J1V2;P0DTE1;A0A075B6Q5;A0A0J9YVY3;P23083) | 7\|53 | hsa:102723407(A0A0A0MS15;A0A0B4J1V2;A0A0C4DH33;A0A0C4DH38;A0A0J9YX35;P01814;P01817) | 0.120570408 | 0.631559281 | http://www.kegg.jp/kegg-bin/show_pathway?hsa04072+hsa:102723407%09green+hsa:102723407%09green+hsa:102723407%09salmon+hsa:102723407%09green+hsa:102723407%09salmon+hsa:102723407%09salmon+hsa:102723407%09salmon |
| hsa04672 | Intestinal immune network for IgA production | Organismal Systems | 41\|228 | hsa:102723407(A0A0C4DH29;A0A0C4DH32;A0A0C4DH33;A0A0J9YX35;A0A0C4DH31;A0A0C4DH36;A0A0C4DH34;A0A0C4DH38;A0A0C4DH39;A0A0J9YY99;A0A0G2JMI3;A0A0B4J1U7;P01763;P01764;P01743;P01766;A0A0B4J1X5;A0A0C4DH43;P01782;P01780;A0A087WSY4;P01814;A0A0B4J1X8;A0A4W8ZXM2;A0A075B7D0;P0DP02;P0DP01;A0A075B7F0;A0A075B7D8;P01817;A0A0A0MS14;A0A0A0MS15;A0A0B4J1V1;A0A075B7B8;A0A0B4J1V2;P0DTE1;A0A075B6Q5;A0A0J9YVY3;P23083);hsa:102723996(A0A087X1L8);hsa:5284(P01833);hsa:23308(A0A087X1L8) | 7\|53 | hsa:102723407(A0A0A0MS15;A0A0B4J1V2;A0A0C4DH33;A0A0C4DH38;A0A0J9YX35;P01814;P01817) | 0.100715532 | 0.791336322 | http://www.kegg.jp/kegg-bin/show_pathway?hsa04672+hsa:102723407%09green+hsa:102723407%09green+hsa:102723407%09salmon+hsa:102723407%09green+hsa:102723407%09salmon+hsa:102723407%09salmon+hsa:102723407%09salmon |
| hsa04064 | NF-kappa B signaling pathway | Environmental Information Processing | 42\|228 | hsa:102723407(A0A0C4DH29;A0A0C4DH32;A0A0C4DH33;A0A0J9YX35;A0A0C4DH31;A0A0C4DH36;A0A0C4DH34;A0A0C4DH38;A0A0C4DH39;A0A0J9YY99;A0A0G2JMI3;A0A0B4J1U7;P01763;P01764;P01743;P01766;A0A0B4J1X5;A0A0C4DH43;P01782;P01780;A0A087WSY4;P01814;A0A0B4J1X8;A0A4W8ZXM2;A0A075B7D0;P0DP02;P0DP01;A0A075B7F0;A0A075B7D8;P01817;A0A0A0MS14;A0A0A0MS15;A0A0B4J1V1;A0A075B7B8;A0A0B4J1V2;P0DTE1;A0A075B6Q5;A0A0J9YVY3;P23083);hsa:929(P08571);hsa:7412(P19320);hsa:3929(P18428) | 7\|53 | hsa:102723407(A0A0A0MS15;A0A0B4J1V2;A0A0C4DH33;A0A0C4DH38;A0A0J9YX35;P01814;P01817) | 0.091128706 | 1 | http://www.kegg.jp/kegg-bin/show_pathway?hsa04064+hsa:102723407%09green+hsa:102723407%09green+hsa:102723407%09salmon+hsa:102723407%09green+hsa:102723407%09salmon+hsa:102723407%09salmon+hsa:102723407%09salmon |
| hsa05202 | Transcriptional misregulation in cancer | Human Diseases | 42\|228 | hsa:102723407(A0A0C4DH29;A0A0C4DH32;A0A0C4DH33;A0A0J9YX35;A0A0C4DH31;A0A0C4DH36;A0A0C4DH34;A0A0C4DH38;A0A0C4DH39;A0A0J9YY99;A0A0G2JMI3;A0A0B4J1U7;P01763;P01764;P01743;P01766;A0A0B4J1X5;A0A0C4DH43;P01782;P01780;A0A087WSY4;P01814;A0A0B4J1X8;A0A4W8ZXM2;A0A075B7D0;P0DP02;P0DP01;A0A075B7F0;A0A075B7D8;P01817;A0A0A0MS14;A0A0A0MS15;A0A0B4J1V1;A0A075B7B8;A0A0B4J1V2;P0DTE1;A0A075B6Q5;A0A0J9YVY3;P23083);hsa:929(P08571);hsa:3486(A6XND0);hsa:1668(P59665) | 7\|53 | hsa:102723407(A0A0A0MS15;A0A0B4J1V2;A0A0C4DH33;A0A0C4DH38;A0A0J9YX35;P01814;P01817) | 0.091128706 | 1 | http://www.kegg.jp/kegg-bin/show_pathway?hsa05202+hsa:102723407%09green+hsa:102723407%09green+hsa:102723407%09salmon+hsa:102723407%09green+hsa:102723407%09salmon+hsa:102723407%09salmon+hsa:102723407%09salmon |
| hsa04662 | B cell receptor signaling pathway | Organismal Systems | 40\|228 | hsa:5881(P60763);hsa:102723407(A0A0C4DH29;A0A0C4DH32;A0A0C4DH33;A0A0J9YX35;A0A0C4DH31;A0A0C4DH36;A0A0C4DH34;A0A0C4DH38;A0A0C4DH39;A0A0J9YY99;A0A0G2JMI3;A0A0B4J1U7;P01763;P01764;P01743;P01766;A0A0B4J1X5;A0A0C4DH43;P01782;P01780;A0A087WSY4;P01814;A0A0B4J1X8;A0A4W8ZXM2;A0A075B7D0;P0DP02;P0DP01;A0A075B7F0;A0A075B7D8;P01817;A0A0A0MS14;A0A0A0MS15;A0A0B4J1V1;A0A075B7B8;A0A0B4J1V2;P0DTE1;A0A075B6Q5;A0A0J9YVY3;P23083) | 7\|53 | hsa:102723407(A0A0A0MS15;A0A0B4J1V2;A0A0C4DH33;A0A0C4DH38;A0A0J9YX35;P01814;P01817) | 0.110576036 | 0.715491996 | http://www.kegg.jp/kegg-bin/show_pathway?hsa04662+hsa:102723407%09green+hsa:102723407%09green+hsa:102723407%09salmon+hsa:102723407%09green+hsa:102723407%09salmon+hsa:102723407%09salmon+hsa:102723407%09salmon |
| hsa05140 | Leishmaniasis | Human Diseases | 42\|228 | hsa:718(P01024;M0R0Q9);hsa:2214(H0Y755);hsa:102723407(A0A0C4DH29;A0A0C4DH32;A0A0C4DH33;A0A0J9YX35;A0A0C4DH31;A0A0C4DH36;A0A0C4DH34;A0A0C4DH38;A0A0C4DH39;A0A0J9YY99;A0A0G2JMI3;A0A0B4J1U7;P01763;P01764;P01743;P01766;A0A0B4J1X5;A0A0C4DH43;P01782;P01780;A0A087WSY4;P01814;A0A0B4J1X8;A0A4W8ZXM2;A0A075B7D0;P0DP02;P0DP01;A0A075B7F0;A0A075B7D8;P01817;A0A0A0MS14;A0A0A0MS15;A0A0B4J1V1;A0A075B7B8;A0A0B4J1V2;P0DTE1;A0A075B6Q5;A0A0J9YVY3;P23083) | 7\|53 | hsa:102723407(A0A0A0MS15;A0A0B4J1V2;A0A0C4DH33;A0A0C4DH38;A0A0J9YX35;P01814;P01817) | 0.091128706 | 1 | http://www.kegg.jp/kegg-bin/show_pathway?hsa05140+hsa:102723407%09green+hsa:102723407%09green+hsa:102723407%09salmon+hsa:102723407%09green+hsa:102723407%09salmon+hsa:102723407%09salmon+hsa:102723407%09salmon |
| hsa04020 | Calcium signaling pathway | Environmental Information Processing | 39\|228 | hsa:102723407(A0A0C4DH29;A0A0C4DH32;A0A0C4DH33;A0A0J9YX35;A0A0C4DH31;A0A0C4DH36;A0A0C4DH34;A0A0C4DH38;A0A0C4DH39;A0A0J9YY99;A0A0G2JMI3;A0A0B4J1U7;P01763;P01764;P01743;P01766;A0A0B4J1X5;A0A0C4DH43;P01782;P01780;A0A087WSY4;P01814;A0A0B4J1X8;A0A4W8ZXM2;A0A075B7D0;P0DP02;P0DP01;A0A075B7F0;A0A075B7D8;P01817;A0A0A0MS14;A0A0A0MS15;A0A0B4J1V1;A0A075B7B8;A0A0B4J1V2;P0DTE1;A0A075B6Q5;A0A0J9YVY3;P23083) | 7\|53 | hsa:102723407(A0A0A0MS15;A0A0B4J1V2;A0A0C4DH33;A0A0C4DH38;A0A0J9YX35;P01814;P01817) | 0.120570408 | 0.631559281 | http://www.kegg.jp/kegg-bin/show_pathway?hsa04020+hsa:102723407%09green+hsa:102723407%09green+hsa:102723407%09salmon+hsa:102723407%09green+hsa:102723407%09salmon+hsa:102723407%09salmon+hsa:102723407%09salmon |
| hsa05162 | Measles | Human Diseases | 40\|228 | hsa:102723407(A0A0C4DH29;A0A0C4DH32;A0A0C4DH33;A0A0J9YX35;A0A0C4DH31;A0A0C4DH36;A0A0C4DH34;A0A0C4DH38;A0A0C4DH39;A0A0J9YY99;A0A0G2JMI3;A0A0B4J1U7;P01763;P01764;P01743;P01766;A0A0B4J1X5;A0A0C4DH43;P01782;P01780;A0A087WSY4;P01814;A0A0B4J1X8;A0A4W8ZXM2;A0A075B7D0;P0DP02;P0DP01;A0A075B7F0;A0A075B7D8;P01817;A0A0A0MS14;A0A0A0MS15;A0A0B4J1V1;A0A075B7B8;A0A0B4J1V2;P0DTE1;A0A075B6Q5;A0A0J9YVY3;P23083);hsa:4478(P26038) | 7\|53 | hsa:102723407(A0A0A0MS15;A0A0B4J1V2;A0A0C4DH33;A0A0C4DH38;A0A0J9YX35;P01814;P01817) | 0.110576036 | 0.715491996 | http://www.kegg.jp/kegg-bin/show_pathway?hsa05162+hsa:102723407%09green+hsa:102723407%09green+hsa:102723407%09salmon+hsa:102723407%09green+hsa:102723407%09salmon+hsa:102723407%09salmon+hsa:102723407%09salmon |
| hsa04640 | Hematopoietic cell lineage | Organismal Systems | 45\|228 | hsa:3674(P08514);hsa:102723407(A0A0C4DH29;A0A0C4DH32;A0A0C4DH33;A0A0J9YX35;A0A0C4DH31;A0A0C4DH36;A0A0C4DH34;A0A0C4DH38;A0A0C4DH39;A0A0J9YY99;A0A0G2JMI3;A0A0B4J1U7;P01763;P01764;P01743;P01766;A0A0B4J1X5;A0A0C4DH43;P01782;P01780;A0A087WSY4;P01814;A0A0B4J1X8;A0A4W8ZXM2;A0A075B7D0;P0DP02;P0DP01;A0A075B7F0;A0A075B7D8;P01817;A0A0A0MS14;A0A0A0MS15;A0A0B4J1V1;A0A075B7B8;A0A0B4J1V2;P0DTE1;A0A075B6Q5;A0A0J9YVY3;P23083);hsa:2811(A0A0C4DGZ8);hsa:960(H0Y5E4);hsa:290(P15144);hsa:7037(G3V0E5);hsa:929(P08571) | 7\|53 | hsa:102723407(A0A0A0MS15;A0A0B4J1V2;A0A0C4DH33;A0A0C4DH38;A0A0J9YX35;P01814;P01817) | 0.065016031 | 0.893970427 | http://www.kegg.jp/kegg-bin/show_pathway?hsa04640+hsa:102723407%09green+hsa:102723407%09green+hsa:102723407%09salmon+hsa:102723407%09green+hsa:102723407%09salmon+hsa:102723407%09salmon+hsa:102723407%09salmon |
| hsa04666 | Fc gamma R-mediated phagocytosis | Organismal Systems | 42\|228 | hsa:2934(A0A0A0MS51);hsa:102723407(A0A0C4DH29;A0A0C4DH32;A0A0C4DH33;A0A0J9YX35;A0A0C4DH31;A0A0C4DH36;A0A0C4DH34;A0A0C4DH38;A0A0C4DH39;A0A0J9YY99;A0A0G2JMI3;A0A0B4J1U7;P01763;P01764;P01743;P01766;A0A0B4J1X5;A0A0C4DH43;P01782;P01780;A0A087WSY4;P01814;A0A0B4J1X8;A0A4W8ZXM2;A0A075B7D0;P0DP02;P0DP01;A0A075B7F0;A0A075B7D8;P01817;A0A0A0MS14;A0A0A0MS15;A0A0B4J1V1;A0A075B7B8;A0A0B4J1V2;P0DTE1;A0A075B6Q5;A0A0J9YVY3;P23083);hsa:1072(E9PK25);hsa:2214(H0Y755) | 7\|53 | hsa:102723407(A0A0A0MS15;A0A0B4J1V2;A0A0C4DH33;A0A0C4DH38;A0A0J9YX35;P01814;P01817) | 0.091128706 | 1 | http://www.kegg.jp/kegg-bin/show_pathway?hsa04666+hsa:102723407%09green+hsa:102723407%09green+hsa:102723407%09salmon+hsa:102723407%09green+hsa:102723407%09salmon+hsa:102723407%09salmon+hsa:102723407%09salmon |
| hsa05310 | Asthma | Human Diseases | 39\|228 | hsa:102723407(A0A0C4DH29;A0A0C4DH32;A0A0C4DH33;A0A0J9YX35;A0A0C4DH31;A0A0C4DH36;A0A0C4DH34;A0A0C4DH38;A0A0C4DH39;A0A0J9YY99;A0A0G2JMI3;A0A0B4J1U7;P01763;P01764;P01743;P01766;A0A0B4J1X5;A0A0C4DH43;P01782;P01780;A0A087WSY4;P01814;A0A0B4J1X8;A0A4W8ZXM2;A0A075B7D0;P0DP02;P0DP01;A0A075B7F0;A0A075B7D8;P01817;A0A0A0MS14;A0A0A0MS15;A0A0B4J1V1;A0A075B7B8;A0A0B4J1V2;P0DTE1;A0A075B6Q5;A0A0J9YVY3;P23083) | 7\|53 | hsa:102723407(A0A0A0MS15;A0A0B4J1V2;A0A0C4DH33;A0A0C4DH38;A0A0J9YX35;P01814;P01817) | 0.120570408 | 0.631559281 | http://www.kegg.jp/kegg-bin/show_pathway?hsa05310+hsa:102723407%09green+hsa:102723407%09green+hsa:102723407%09salmon+hsa:102723407%09green+hsa:102723407%09salmon+hsa:102723407%09salmon+hsa:102723407%09salmon |
| hsa04664 | Fc epsilon RI signaling pathway | Organismal Systems | 40\|228 | hsa:5881(P60763);hsa:102723407(A0A0C4DH29;A0A0C4DH32;A0A0C4DH33;A0A0J9YX35;A0A0C4DH31;A0A0C4DH36;A0A0C4DH34;A0A0C4DH38;A0A0C4DH39;A0A0J9YY99;A0A0G2JMI3;A0A0B4J1U7;P01763;P01764;P01743;P01766;A0A0B4J1X5;A0A0C4DH43;P01782;P01780;A0A087WSY4;P01814;A0A0B4J1X8;A0A4W8ZXM2;A0A075B7D0;P0DP02;P0DP01;A0A075B7F0;A0A075B7D8;P01817;A0A0A0MS14;A0A0A0MS15;A0A0B4J1V1;A0A075B7B8;A0A0B4J1V2;P0DTE1;A0A075B6Q5;A0A0J9YVY3;P23083) | 7\|53 | hsa:102723407(A0A0A0MS15;A0A0B4J1V2;A0A0C4DH33;A0A0C4DH38;A0A0J9YX35;P01814;P01817) | 0.110576036 | 0.715491996 | http://www.kegg.jp/kegg-bin/show_pathway?hsa04664+hsa:102723407%09green+hsa:102723407%09green+hsa:102723407%09salmon+hsa:102723407%09green+hsa:102723407%09salmon+hsa:102723407%09salmon+hsa:102723407%09salmon |
| hsa05165 | Human papillomavirus infection | Human Diseases | 13\|228 | hsa:7450(P04275);hsa:3674(P08514);hsa:3993(J3QRV5);hsa:1291(A0A087X0S5);hsa:1293(E7ENL6);hsa:1311(G3XAP6);hsa:7448(H0YJW9;P04004);hsa:2335(P02751);hsa:7057(P07996);hsa:5315(H3BTN5);hsa:3105(A0A0G2JI36);hsa:7148(A0A140T8Y3) | 6\|53 | hsa:7448(H0YJW9;P04004);hsa:3993(J3QRV5);hsa:3105(A0A0G2JI36);hsa:7148(A0A140T8Y3);hsa:2335(P02751) | 0.039763171 | 1 | http://www.kegg.jp/kegg-bin/show_pathway?hsa05165+hsa:7448%09salmon+hsa:7448%09salmon+hsa:3993%09green+hsa:3105%09green+hsa:7148%09green+hsa:2335%09salmon |
| hsa04510 | Focal adhesion | Cellular Processes | 20\|228 | hsa:7450(P04275);hsa:29780(A0A087WZB5);hsa:7094(Q9Y490);hsa:1291(A0A087X0S5);hsa:1293(E7ENL6);hsa:5908(P61224);hsa:87(H9KV75);hsa:3674(P08514);hsa:7448(H0YJW9;P04004);hsa:3611(A0A0A0MTH3);hsa:5881(P60763);hsa:1311(G3XAP6);hsa:2316(P21333);hsa:7057(P07996);hsa:7791(H0Y2Y8);hsa:60(P60709);hsa:7414(P18206);hsa:2335(P02751);hsa:71(P60709);hsa:7148(A0A140T8Y3) | 6\|53 | hsa:5908(P61224);hsa:29780(A0A087WZB5);hsa:7448(H0YJW9;P04004);hsa:7148(A0A140T8Y3);hsa:2335(P02751) | 0.155344548 | 0.449681586 | http://www.kegg.jp/kegg-bin/show_pathway?hsa04510+hsa:5908%09green+hsa:29780%09salmon+hsa:7448%09salmon+hsa:7448%09salmon+hsa:7148%09green+hsa:2335%09salmon |
| hsa04512 | ECM-receptor interaction | Environmental Information Processing | 12\|228 | hsa:7450(P04275);hsa:3674(P08514);hsa:1291(A0A087X0S5);hsa:2811(A0A0C4DGZ8);hsa:1293(E7ENL6);hsa:1311(G3XAP6);hsa:7448(H0YJW9;P04004);hsa:960(H0Y5E4);hsa:2335(P02751);hsa:7057(P07996);hsa:7148(A0A140T8Y3) | 4\|53 | hsa:7448(H0YJW9;P04004);hsa:7148(A0A140T8Y3);hsa:2335(P02751) | 0.17696796 | 0.499140399 | http://www.kegg.jp/kegg-bin/show_pathway?hsa04512+hsa:7448%09salmon+hsa:7448%09salmon+hsa:7148%09green+hsa:2335%09salmon |
| hsa04972 | Pancreatic secretion | Organismal Systems | 4\|228 | hsa:760(P00918);hsa:81579(A0A2R8Y3M9);hsa:1361(Q96IY4);hsa:5908(P61224) | 4\|53 | hsa:760(P00918);hsa:5908(P61224);hsa:1361(Q96IY4);hsa:81579(A0A2R8Y3M9) | 0.002670347 | 0.293738192 | http://www.kegg.jp/kegg-bin/show_pathway?hsa04972+hsa:760%09green+hsa:5908%09green+hsa:1361%09green+hsa:81579%09green |
| hsa04915 | Estrogen signaling pathway | Organismal Systems | 4\|228 | hsa:3858(P13645);hsa:3868(P08779);hsa:3861(P02533);hsa:3857(P35527) | 3\|53 | hsa:3858(P13645);hsa:3868(P08779);hsa:3857(P35527) | 0.037384861 | 1 | http://www.kegg.jp/kegg-bin/show_pathway?hsa04915+hsa:3858%09green+hsa:3868%09salmon+hsa:3857%09salmon |
| hsa04979 | Cholesterol metabolism | Organismal Systems | 15\|228 | hsa:255738(A0A669KAY4);hsa:4035(Q07954);hsa:350(P02749);hsa:5360(P55058);hsa:1071(P11597);hsa:4018(P08519);hsa:3931(P04180);hsa:338(P04114);hsa:348(P02649);hsa:344(K7ER74);hsa:345(B0YIW2);hsa:337(P06727);hsa:336(P02652);hsa:335(P02647);hsa:341(K7ERI9) | 3\|53 | hsa:5360(P55058);hsa:348(P02649);hsa:255738(A0A669KAY4) | 0.245571071 | 0.428774886 | http://www.kegg.jp/kegg-bin/show_pathway?hsa04979+hsa:5360%09salmon+hsa:348%09green+hsa:255738%09salmon |
| hsa05205 | Proteoglycans in cancer | Human Diseases | 10\|228 | hsa:71(P60709);hsa:3481(P01344);hsa:7448(H0YJW9;P04004);hsa:960(H0Y5E4);hsa:2335(P02751);hsa:2316(P21333);hsa:7057(P07996);hsa:4478(P26038);hsa:4060(P51884);hsa:60(P60709) | 3\|53 | hsa:7448(H0YJW9;P04004);hsa:2335(P02751) | 0.241605954 | 0.428655724 | http://www.kegg.jp/kegg-bin/show_pathway?hsa05205+hsa:7448%09salmon+hsa:7448%09salmon+hsa:2335%09salmon |
| hsa05020 | Prion diseases | Human Diseases | 11\|228 | hsa:735(P02748);hsa:712(P02745);hsa:3309(P11021);hsa:714(P02747);hsa:729(P13671);hsa:713(D6R934);hsa:727(P01031);hsa:733(P07360);hsa:732(P07358);hsa:731(P07357);hsa:730(P10643) | 3\|53 | hsa:712(P02745);hsa:733(P07360);hsa:714(P02747) | 0.256013648 | 0.426689414 | http://www.kegg.jp/kegg-bin/show_pathway?hsa05020+hsa:712%09green+hsa:733%09green+hsa:714%09green |
| hsa05142 | Chagas disease (American trypanosomiasis) | Human Diseases | 5\|228 | hsa:718(P01024;M0R0Q9);hsa:713(D6R934);hsa:712(P02745);hsa:714(P02747) | 2\|53 | hsa:712(P02745);hsa:714(P02747) | 0.246270807 | 0.42327795 | http://www.kegg.jp/kegg-bin/show_pathway?hsa05142+hsa:712%09green+hsa:714%09green |
| hsa05203 | Viral carcinogenesis | Human Diseases | 8\|228 | hsa:2934(A0A0A0MS51);hsa:128312(A0A2R8Y619);hsa:7534(E7EX29);hsa:718(P01024;M0R0Q9);hsa:87(H9KV75);hsa:5315(H3BTN5);hsa:3105(A0A0G2JI36) | 2\|53 | hsa:3105(A0A0G2JI36);hsa:7534(E7EX29) | 0.315133325 | 0.502386461 | http://www.kegg.jp/kegg-bin/show_pathway?hsa05203+hsa:3105%09green+hsa:7534%09salmon |
| hsa00565 | Ether lipid metabolism | Metabolism | 3\|228 | hsa:81579(A0A2R8Y3M9);hsa:7368(Q16880);hsa:7941(Q13093) | 2\|53 | hsa:81579(A0A2R8Y3M9);hsa:7368(Q16880) | 0.123699907 | 0.544279591 | http://www.kegg.jp/kegg-bin/show_pathway?hsa00565+hsa:81579%09green+hsa:7368%09salmon |
| hsa04514 | Cell adhesion molecules (CAMs) | Environmental Information Processing | 5\|228 | hsa:102723996(A0A087X1L8);hsa:7412(P19320);hsa:6402(P14151);hsa:23308(A0A087X1L8);hsa:3105(A0A0G2JI36);hsa:1003(I3L1J2) | 2\|53 | hsa:3105(A0A0G2JI36);hsa:1003(I3L1J2) | 0.246270807 | 0.42327795 | http://www.kegg.jp/kegg-bin/show_pathway?hsa04514+hsa:3105%09green+hsa:1003%09salmon |
| hsa04270 | Vascular smooth muscle contraction | Organismal Systems | 2\|228 | hsa:81579(A0A2R8Y3M9);hsa:59(P68032);hsa:72(P68032) | 2\|53 | hsa:81579(A0A2R8Y3M9);hsa:59(P68032);hsa:72(P68032) | 0.053249865 | 1 | http://www.kegg.jp/kegg-bin/show_pathway?hsa04270+hsa:81579%09green+hsa:59%09green+hsa:72%09green |
| hsa00910 | Nitrogen metabolism | Metabolism | 2\|228 | hsa:760(P00918);hsa:759(P00915) | 2\|53 | hsa:760(P00918);hsa:759(P00915) | 0.053249865 | 1 | http://www.kegg.jp/kegg-bin/show_pathway?hsa00910+hsa:760%09green+hsa:759%09green |
| hsa05133 | Pertussis | Human Diseases | 17\|228 | hsa:712(P02745);hsa:715(A0A3B3ISR2);hsa:1072(E9PK25);hsa:721(A0A0G2JPR0;P0C0L5;P0C0L4);hsa:727(P01031);hsa:718(P01024;M0R0Q9);hsa:717(A0A0G2JL69);hsa:710(P05155);hsa:725(P20851);hsa:713(D6R934);hsa:929(P08571);hsa:720(A0A0G2JPR0;P0C0L5;P0C0L4);hsa:714(P02747);hsa:722(P04003);hsa:716(P09871) | 2\|53 | hsa:712(P02745);hsa:714(P02747) | 0.135502582 | 0.51397531 | http://www.kegg.jp/kegg-bin/show_pathway?hsa05133+hsa:712%09green+hsa:714%09green |
| hsa04390 | Hippo signaling pathway | Environmental Information Processing | 3\|228 | hsa:3993(J3QRV5);hsa:71(P60709);hsa:60(P60709);hsa:7534(E7EX29) | 2\|53 | hsa:3993(J3QRV5);hsa:7534(E7EX29) | 0.123699907 | 0.544279591 | http://www.kegg.jp/kegg-bin/show_pathway?hsa04390+hsa:3993%09green+hsa:7534%09salmon |
| hsa04014 | Ras signaling pathway | Environmental Information Processing | 4\|228 | hsa:5881(P60763);hsa:81579(A0A2R8Y3M9);hsa:3481(P01344);hsa:5908(P61224) | 2\|53 | hsa:5908(P61224);hsa:81579(A0A2R8Y3M9) | 0.191322523 | 0.526136938 | http://www.kegg.jp/kegg-bin/show_pathway?hsa04014+hsa:5908%09green+hsa:81579%09green |
| hsa04974 | Protein digestion and absorption | Organismal Systems | 4\|228 | hsa:1293(E7ENL6);hsa:1361(Q96IY4);hsa:7373(J3QT83);hsa:1291(A0A087X0S5) | 2\|53 | hsa:1361(Q96IY4);hsa:7373(J3QT83) | 0.191322523 | 0.526136938 | http://www.kegg.jp/kegg-bin/show_pathway?hsa04974+hsa:1361%09green+hsa:7373%09salmon |
| hsa04670 | Leukocyte transendothelial migration | Organismal Systems | 7\|228 | hsa:5908(P61224);hsa:87(H9KV75);hsa:7412(P19320);hsa:60(P60709);hsa:7414(P18206);hsa:4478(P26038);hsa:71(P60709);hsa:1003(I3L1J2) | 2\|53 | hsa:5908(P61224);hsa:1003(I3L1J2) | 0.307254992 | 0.497030135 | http://www.kegg.jp/kegg-bin/show_pathway?hsa04670+hsa:5908%09green+hsa:1003%09salmon |
| hsa04611 | Platelet activation | Organismal Systems | 11\|228 | hsa:7450(P04275);hsa:3674(P08514);hsa:7094(Q9Y490);hsa:2811(A0A0C4DGZ8);hsa:5908(P61224);hsa:2243(P02671);hsa:83706(Q86UX7);hsa:2244(P02675);hsa:2266(C9JPQ9;C9JC84);hsa:71(P60709);hsa:60(P60709) | 2\|53 | hsa:5908(P61224);hsa:2266(C9JPQ9) | 0.279439734 | 0.458781652 | http://www.kegg.jp/kegg-bin/show_pathway?hsa04611+hsa:5908%09green+hsa:2266%09salmon |
| hsa05150 | Staphylococcus aureus infection | Human Diseases | 63\|228 | hsa:102723407(A0A0C4DH29;A0A0C4DH32;A0A0C4DH33;A0A0J9YX35;A0A0C4DH31;A0A0C4DH36;A0A0C4DH34;A0A0C4DH38;A0A0C4DH39;A0A0J9YY99;A0A0G2JMI3;A0A0B4J1U7;P01763;P01764;P01743;P01766;A0A0B4J1X5;A0A0C4DH43;P01782;P01780;A0A087WSY4;P01814;A0A0B4J1X8;A0A4W8ZXM2;A0A075B7D0;P0DP02;P0DP01;A0A075B7F0;A0A075B7D8;P01817;A0A0A0MS14;A0A0A0MS15;A0A0B4J1V1;A0A075B7B8;A0A0B4J1V2;P0DTE1;A0A075B6Q5;A0A0J9YVY3;P23083);hsa:5340(P00747);hsa:629(B4E1Z4);hsa:3858(P13645);hsa:727(P01031);hsa:720(A0A0G2JPR0;P0C0L5;P0C0L4);hsa:721(A0A0G2JPR0;P0C0L5;P0C0L4);hsa:1675(K7ERG9);hsa:5648(P48740);hsa:2214(H0Y755);hsa:718(P01024;M0R0Q9);hsa:10747(O00187);hsa:713(D6R934);hsa:712(P02745);hsa:715(A0A3B3ISR2);hsa:714(P02747);hsa:717(A0A0G2JL69);hsa:716(P09871);hsa:4153(P11226);hsa:3426(A0A2R8Y3M9);hsa:2266(C9JPQ9;C9JC84);hsa:3075(P08603) | 14\|53 | hsa:102723407(A0A0A0MS15;A0A0B4J1V2;A0A0C4DH33;A0A0C4DH38;A0A0J9YX35;P01814;P01817);hsa:2266(C9JPQ9);hsa:3426(A0A2R8Y3M9);hsa:10747(O00187);hsa:3858(P13645);hsa:712(P02745);hsa:714(P02747);hsa:1675(K7ERG9) | 0.136757021 | 0.501442411 | http://www.kegg.jp/kegg-bin/show_pathway?hsa05150+hsa:102723407%09green+hsa:102723407%09green+hsa:102723407%09salmon+hsa:102723407%09green+hsa:102723407%09salmon+hsa:102723407%09salmon+hsa:102723407%09salmon+hsa:2266%09salmon+hsa:3426%09green+hsa:10747%09green+hsa:3858%09green+hsa:712%09green+hsa:714%09green+hsa:1675%09green |
| hsa04610 | Complement and coagulation cascades | Organismal Systems | 58\|228 | hsa:3818(H0YAC1);hsa:5345(P08697);hsa:5104(P05154);hsa:5340(P00747);hsa:629(B4E1Z4);hsa:729(P13671);hsa:2(P01023);hsa:725(P20851);hsa:732(P07358);hsa:727(P01031);hsa:720(A0A0G2JPR0;P0C0L5;P0C0L4);hsa:462(P01008);hsa:722(P04003);hsa:1675(K7ERG9);hsa:7450(P04275);hsa:10544(Q9UNN8);hsa:5624(E7END6);hsa:5627(A0A0S2Z4L3);hsa:2147(P00734);hsa:721(A0A0G2JPR0;P0C0L5;P0C0L4);hsa:2162(P00488);hsa:2161(P00748);hsa:2160(P03951);hsa:731(P07357);hsa:2165(P05160);hsa:730(P10643);hsa:735(P02748);hsa:3827(P01042);hsa:5648(P48740);hsa:1361(Q96IY4);hsa:718(P01024;M0R0Q9);hsa:10747(O00187);hsa:4153(P11226);hsa:1191(P10909);hsa:710(P05155);hsa:713(D6R934);hsa:712(P02745);hsa:715(A0A3B3ISR2);hsa:714(P02747);hsa:717(A0A0G2JL69);hsa:716(P09871);hsa:5265(P01009);hsa:3075(P08603);hsa:2243(P02671);hsa:7448(H0YJW9;P04004);hsa:2244(P02675);hsa:3426(A0A2R8Y3M9);hsa:2266(C9JPQ9;C9JC84);hsa:2155(F5H8B0);hsa:2153(A0A0A0MRJ7);hsa:3053(P05546);hsa:733(P07360);hsa:2158(P00740);hsa:2159(P00742) | 14\|53 | hsa:5265(P01009);hsa:10747(O00187);hsa:3818(H0YAC1);hsa:733(P07360);hsa:2266(C9JPQ9);hsa:7448(H0YJW9;P04004);hsa:1361(Q96IY4);hsa:714(P02747);hsa:2161(P00748);hsa:712(P02745);hsa:3426(A0A2R8Y3M9);hsa:462(P01008);hsa:1675(K7ERG9) | 0.139008461 | 0.463361537 | http://www.kegg.jp/kegg-bin/show_pathway?hsa04610+hsa:5265%09green+hsa:10747%09green+hsa:3818%09green+hsa:733%09green+hsa:2266%09salmon+hsa:7448%09salmon+hsa:7448%09salmon+hsa:1361%09green+hsa:714%09green+hsa:2161%09green+hsa:712%09green+hsa:3426%09green+hsa:462%09green+hsa:1675%09green |
| hsa04151 | PI3K-Akt signaling pathway | Environmental Information Processing | 51\|228 | hsa:7450(P04275);hsa:102723407(A0A0C4DH29;A0A0C4DH32;A0A0C4DH33;A0A0J9YX35;A0A0C4DH31;A0A0C4DH36;A0A0C4DH34;A0A0C4DH38;A0A0C4DH39;A0A0J9YY99;A0A0G2JMI3;A0A0B4J1U7;P01763;P01764;P01743;P01766;A0A0B4J1X5;A0A0C4DH43;P01782;P01780;A0A087WSY4;P01814;A0A0B4J1X8;A0A4W8ZXM2;A0A075B7D0;P0DP02;P0DP01;A0A075B7F0;A0A075B7D8;P01817;A0A0A0MS14;A0A0A0MS15;A0A0B4J1V1;A0A075B7B8;A0A0B4J1V2;P0DTE1;A0A075B6Q5;A0A0J9YVY3;P23083);hsa:1291(A0A087X0S5);hsa:7534(E7EX29);hsa:1311(G3XAP6);hsa:3481(P01344);hsa:7448(H0YJW9;P04004);hsa:2335(P02751);hsa:3674(P08514);hsa:7057(P07996);hsa:1293(E7ENL6);hsa:7148(A0A140T8Y3) | 12\|53 | hsa:7448(H0YJW9;P04004);hsa:102723407(A0A0A0MS15;A0A0B4J1V2;A0A0C4DH33;A0A0C4DH38;A0A0J9YX35;P01814;P01817);hsa:2335(P02751);hsa:7148(A0A140T8Y3);hsa:7534(E7EX29) | 0.148250904 | 0.440745931 | http://www.kegg.jp/kegg-bin/show_pathway?hsa04151+hsa:7448%09salmon+hsa:7448%09salmon+hsa:102723407%09green+hsa:102723407%09green+hsa:102723407%09salmon+hsa:102723407%09green+hsa:102723407%09salmon+hsa:102723407%09salmon+hsa:102723407%09salmon+hsa:2335%09salmon+hsa:7148%09green+hsa:7534%09salmon |
| hsa05322 | Systemic lupus erythematosus | Human Diseases | 60\|228 | hsa:735(P02748);hsa:102723407(A0A0C4DH29;A0A0C4DH32;A0A0C4DH33;A0A0J9YX35;A0A0C4DH31;A0A0C4DH36;A0A0C4DH34;A0A0C4DH38;A0A0C4DH39;A0A0J9YY99;A0A0G2JMI3;A0A0B4J1U7;P01763;P01764;P01743;P01766;A0A0B4J1X5;A0A0C4DH43;P01782;P01780;A0A087WSY4;P01814;A0A0B4J1X8;A0A4W8ZXM2;A0A075B7D0;P0DP02;P0DP01;A0A075B7F0;A0A075B7D8;P01817;A0A0A0MS14;A0A0A0MS15;A0A0B4J1V1;A0A075B7B8;A0A0B4J1V2;P0DTE1;A0A075B6Q5;A0A0J9YVY3;P23083);hsa:712(P02745);hsa:733(P07360);hsa:128312(A0A2R8Y619);hsa:715(A0A3B3ISR2);hsa:721(A0A0G2JPR0;P0C0L5;P0C0L4);hsa:727(P01031);hsa:718(P01024;M0R0Q9);hsa:732(P07358);hsa:87(H9KV75);hsa:2214(H0Y755);hsa:730(P10643);hsa:731(P07357);hsa:713(D6R934);hsa:716(P09871);hsa:720(A0A0G2JPR0;P0C0L5;P0C0L4);hsa:714(P02747);hsa:717(A0A0G2JL69);hsa:729(P13671) | 10\|53 | hsa:102723407(A0A0A0MS15;A0A0B4J1V2;A0A0C4DH33;A0A0C4DH38;A0A0J9YX35;P01814;P01817);hsa:712(P02745);hsa:733(P07360);hsa:714(P02747) | 0.054639368 | 0.858618636 | http://www.kegg.jp/kegg-bin/show_pathway?hsa05322+hsa:102723407%09green+hsa:102723407%09green+hsa:102723407%09salmon+hsa:102723407%09green+hsa:102723407%09salmon+hsa:102723407%09salmon+hsa:102723407%09salmon+hsa:712%09green+hsa:733%09green+hsa:714%09green |
| hsa04218 | Cellular senescence | Cellular Processes | 2\|228 | hsa:3105(A0A0G2JI36);hsa:3486(A6XND0) | 1\|53 | hsa:3105(A0A0G2JI36) | 0.358412551 | 0.486733094 | http://www.kegg.jp/kegg-bin/show_pathway?hsa04218+hsa:3105%09green |
| hsa05100 | Bacterial invasion of epithelial cells | Human Diseases | 4\|228 | hsa:7414(P18206);hsa:3611(A0A0A0MTH3);hsa:71(P60709);hsa:60(P60709);hsa:2335(P02751) | 1\|53 | hsa:2335(P02751) | 0.424343544 | 0.436241027 | http://www.kegg.jp/kegg-bin/show_pathway?hsa05100+hsa:2335%09salmon |
| hsa04144 | Endocytosis | Cellular Processes | 2\|228 | hsa:7037(G3V0E5);hsa:3105(A0A0G2JI36) | 1\|53 | hsa:3105(A0A0G2JI36) | 0.358412551 | 0.486733094 | http://www.kegg.jp/kegg-bin/show_pathway?hsa04144+hsa:3105%09green |
| hsa04140 | Autophagy - animal | Cellular Processes | 2\|228 | hsa:3920(P13473);hsa:2081(O75460) | 1\|53 | hsa:3920(P13473) | 0.358412551 | 0.486733094 | http://www.kegg.jp/kegg-bin/show_pathway?hsa04140+hsa:3920%09green |
| hsa04971 | Gastric acid secretion | Organismal Systems | 2\|228 | hsa:760(P00918);hsa:60(P60709) | 1\|53 | hsa:760(P00918) | 0.358412551 | 0.486733094 | http://www.kegg.jp/kegg-bin/show_pathway?hsa04971+hsa:760%09green |
| hsa05130 | Pathogenic Escherichia coli infection | Human Diseases | 7\|228 | hsa:203068(P07437);hsa:7278(P0DPH7);hsa:81027(Q9H4B7);hsa:7534(E7EX29);hsa:7277(P68366);hsa:113457(P0DPH7);hsa:112714(P0DPH7);hsa:929(P08571);hsa:71(P60709);hsa:60(P60709) | 1\|53 | hsa:7534(E7EX29) | 0.334829158 | 0.5187494 | http://www.kegg.jp/kegg-bin/show_pathway?hsa05130+hsa:7534%09salmon |
| hsa04110 | Cell cycle | Cellular Processes | 1\|228 | hsa:7534(E7EX29) | 1\|53 | hsa:7534(E7EX29) | 0.23245614 | 0.501375989 | http://www.kegg.jp/kegg-bin/show_pathway?hsa04110+hsa:7534%09salmon |
| hsa00590 | Arachidonic acid metabolism | Metabolism | 3\|228 | hsa:2878(A0A087X1J7);hsa:5730(P41222);hsa:81579(A0A2R8Y3M9) | 1\|53 | hsa:81579(A0A2R8Y3M9) | 0.41391892 | 0.450802784 | http://www.kegg.jp/kegg-bin/show_pathway?hsa00590+hsa:81579%09green |
| hsa05163 | Human cytomegalovirus infection | Human Diseases | 2\|228 | hsa:5881(P60763);hsa:3105(A0A0G2JI36) | 1\|53 | hsa:3105(A0A0G2JI36) | 0.358412551 | 0.486733094 | http://www.kegg.jp/kegg-bin/show_pathway?hsa05163+hsa:3105%09green |
| hsa04310 | Wnt signaling pathway | Environmental Information Processing | 2\|228 | hsa:5881(P60763);hsa:5176(P36955) | 1\|53 | hsa:5176(P36955) | 0.358412551 | 0.486733094 | http://www.kegg.jp/kegg-bin/show_pathway?hsa04310+hsa:5176%09green |
| hsa04810 | Regulation of actin cytoskeleton | Cellular Processes | 12\|228 | hsa:3674(P08514);hsa:2934(A0A0A0MS51);hsa:1072(E9PK25);hsa:2147(P00734);hsa:2335(P02751);hsa:5881(P60763);hsa:4627(P35579);hsa:87(H9KV75);hsa:5216(P07737);hsa:7414(P18206);hsa:4478(P26038);hsa:71(P60709);hsa:60(P60709) | 1\|53 | hsa:2335(P02751) | 0.147991124 | 0.452195103 | http://www.kegg.jp/kegg-bin/show_pathway?hsa04810+hsa:2335%09salmon |
| hsa05200 | Pathways in cancer | Human Diseases | 4\|228 | hsa:5881(P60763);hsa:3674(P08514);hsa:3481(P01344);hsa:2335(P02751) | 1\|53 | hsa:2335(P02751) | 0.424343544 | 0.436241027 | http://www.kegg.jp/kegg-bin/show_pathway?hsa05200+hsa:2335%09salmon |
| hsa00564 | Glycerophospholipid metabolism | Metabolism | 2\|228 | hsa:81579(A0A2R8Y3M9);hsa:3931(P04180) | 1\|53 | hsa:81579(A0A2R8Y3M9) | 0.358412551 | 0.486733094 | http://www.kegg.jp/kegg-bin/show_pathway?hsa00564+hsa:81579%09green |
| hsa05211 | Renal cell carcinoma | Human Diseases | 1\|228 | hsa:5908(P61224) | 1\|53 | hsa:5908(P61224) | 0.23245614 | 0.501375989 | http://www.kegg.jp/kegg-bin/show_pathway?hsa05211+hsa:5908%09green |
| hsa05206 | MicroRNAs in cancer | Human Diseases | 3\|228 | hsa:7057(P07996);hsa:960(H0Y5E4);hsa:7148(A0A140T8Y3) | 1\|53 | hsa:7148(A0A140T8Y3) | 0.41391892 | 0.450802784 | http://www.kegg.jp/kegg-bin/show_pathway?hsa05206+hsa:7148%09green |
| hsa00350 | Tyrosine metabolism | Metabolism | 2\|228 | hsa:1621(P09172);hsa:2184(P16930) | 1\|53 | hsa:1621(P09172) | 0.358412551 | 0.486733094 | http://www.kegg.jp/kegg-bin/show_pathway?hsa00350+hsa:1621%09green |
| hsa05418 | Fluid shear stress and atherosclerosis | Human Diseases | 5\|228 | hsa:3674(P08514);hsa:5881(P60763);hsa:7412(P19320);hsa:60(P60709);hsa:71(P60709);hsa:1003(I3L1J2) | 1\|53 | hsa:1003(I3L1J2) | 0.407294027 | 0.47160361 | http://www.kegg.jp/kegg-bin/show_pathway?hsa05418+hsa:1003%09salmon |
| hsa04010 | MAPK signaling pathway | Environmental Information Processing | 5\|228 | hsa:5881(P60763);hsa:5908(P61224);hsa:3481(P01344);hsa:929(P08571);hsa:2316(P21333) | 1\|53 | hsa:5908(P61224) | 0.407294027 | 0.47160361 | http://www.kegg.jp/kegg-bin/show_pathway?hsa04010+hsa:5908%09green |
| hsa04940 | Type I diabetes mellitus | Human Diseases | 1\|228 | hsa:3105(A0A0G2JI36) | 1\|53 | hsa:3105(A0A0G2JI36) | 0.23245614 | 0.501375989 | http://www.kegg.jp/kegg-bin/show_pathway?hsa04940+hsa:3105%09green |
| hsa00591 | Linoleic acid metabolism | Metabolism | 1\|228 | hsa:81579(A0A2R8Y3M9) | 1\|53 | hsa:81579(A0A2R8Y3M9) | 0.23245614 | 0.501375989 | http://www.kegg.jp/kegg-bin/show_pathway?hsa00591+hsa:81579%09green |
| hsa05332 | Graft-versus-host disease | Human Diseases | 1\|228 | hsa:3105(A0A0G2JI36) | 1\|53 | hsa:3105(A0A0G2JI36) | 0.23245614 | 0.501375989 | http://www.kegg.jp/kegg-bin/show_pathway?hsa05332+hsa:3105%09green |
| hsa04970 | Salivary secretion | Organismal Systems | 3\|228 | hsa:1471(P01034);hsa:4069(P61626);hsa:820(J3KNB4) | 1\|53 | hsa:4069(P61626) | 0.41391892 | 0.450802784 | http://www.kegg.jp/kegg-bin/show_pathway?hsa04970+hsa:4069%09green |
| hsa04720 | Long-term potentiation | Organismal Systems | 1\|228 | hsa:5908(P61224) | 1\|53 | hsa:5908(P61224) | 0.23245614 | 0.501375989 | http://www.kegg.jp/kegg-bin/show_pathway?hsa04720+hsa:5908%09green |
| hsa04722 | Neurotrophin signaling pathway | Organismal Systems | 2\|228 | hsa:5908(P61224);hsa:397(H0YGX7) | 1\|53 | hsa:5908(P61224) | 0.358412551 | 0.486733094 | http://www.kegg.jp/kegg-bin/show_pathway?hsa04722+hsa:5908%09green |
| hsa05166 | Human T-cell leukemia virus 1 infection | Human Diseases | 2\|228 | hsa:3105(A0A0G2JI36);hsa:7094(Q9Y490) | 1\|53 | hsa:3105(A0A0G2JI36) | 0.358412551 | 0.486733094 | http://www.kegg.jp/kegg-bin/show_pathway?hsa05166+hsa:3105%09green |
| hsa04933 | AGE-RAGE signaling pathway in diabetic complications | Human Diseases | 2\|228 | hsa:7412(P19320);hsa:2335(P02751) | 1\|53 | hsa:2335(P02751) | 0.358412551 | 0.486733094 | http://www.kegg.jp/kegg-bin/show_pathway?hsa04933+hsa:2335%09salmon |
| hsa04975 | Fat digestion and absorption | Organismal Systems | 4\|228 | hsa:81579(A0A2R8Y3M9);hsa:338(P04114);hsa:337(P06727);hsa:335(P02647) | 1\|53 | hsa:81579(A0A2R8Y3M9) | 0.424343544 | 0.436241027 | http://www.kegg.jp/kegg-bin/show_pathway?hsa04975+hsa:81579%09green |
| hsa04142 | Lysosome | Cellular Processes | 1\|228 | hsa:3920(P13473) | 1\|53 | hsa:3920(P13473) | 0.23245614 | 0.501375989 | http://www.kegg.jp/kegg-bin/show_pathway?hsa04142+hsa:3920%09green |
| hsa00592 | alpha-Linolenic acid metabolism | Metabolism | 1\|228 | hsa:81579(A0A2R8Y3M9) | 1\|53 | hsa:81579(A0A2R8Y3M9) | 0.23245614 | 0.501375989 | http://www.kegg.jp/kegg-bin/show_pathway?hsa00592+hsa:81579%09green |
| hsa04062 | Chemokine signaling pathway | Organismal Systems | 4\|228 | hsa:5881(P60763);hsa:5908(P61224);hsa:5473(P02775);hsa:5196(P02776) | 1\|53 | hsa:5908(P61224) | 0.424343544 | 0.436241027 | http://www.kegg.jp/kegg-bin/show_pathway?hsa04062+hsa:5908%09green |
| hsa04261 | Adrenergic signaling in cardiomyocytes | Organismal Systems | 3\|228 | hsa:7171(A0A087WWU8;A0A2R8Y5V9);hsa:70(P68032) | 1\|53 | hsa:70(P68032) | 0.41391892 | 0.450802784 | http://www.kegg.jp/kegg-bin/show_pathway?hsa04261+hsa:70%09green |
| hsa04614 | Renin-angiotensin system | Organismal Systems | 2\|228 | hsa:290(P15144);hsa:183(P01019) | 1\|53 | hsa:183(P01019) | 0.358412551 | 0.486733094 | http://www.kegg.jp/kegg-bin/show_pathway?hsa04614+hsa:183%09green |
| hsa05170 | Human immunodeficiency virus 1 infection | Human Diseases | 3\|228 | hsa:5881(P60763);hsa:1072(E9PK25);hsa:3105(A0A0G2JI36) | 1\|53 | hsa:3105(A0A0G2JI36) | 0.41391892 | 0.450802784 | http://www.kegg.jp/kegg-bin/show_pathway?hsa05170+hsa:3105%09green |
| hsa04612 | Antigen processing and presentation | Organismal Systems | 2\|228 | hsa:3105(A0A0G2JI36);hsa:3309(P11021) | 1\|53 | hsa:3105(A0A0G2JI36) | 0.358412551 | 0.486733094 | http://www.kegg.jp/kegg-bin/show_pathway?hsa04612+hsa:3105%09green |
| hsa00600 | Sphingolipid metabolism | Metabolism | 1\|228 | hsa:7368(Q16880) | 1\|53 | hsa:7368(Q16880) | 0.23245614 | 0.501375989 | http://www.kegg.jp/kegg-bin/show_pathway?hsa00600+hsa:7368%09salmon |
| hsa00630 | Glyoxylate and dicarboxylate metabolism | Metabolism | 2\|228 | hsa:81888(E7EWH8);hsa:847(P04040) | 1\|53 | hsa:81888(E7EWH8) | 0.358412551 | 0.486733094 | http://www.kegg.jp/kegg-bin/show_pathway?hsa00630+hsa:81888%09salmon |
| hsa04114 | Oocyte meiosis | Cellular Processes | 1\|228 | hsa:7534(E7EX29) | 1\|53 | hsa:7534(E7EX29) | 0.23245614 | 0.501375989 | http://www.kegg.jp/kegg-bin/show_pathway?hsa04114+hsa:7534%09salmon |
| hsa04978 | Mineral absorption | Organismal Systems | 2\|228 | hsa:7018(C9JB55;P02787) | 1\|53 | hsa:7018(C9JB55) | 0.358412551 | 0.486733094 | http://www.kegg.jp/kegg-bin/show_pathway?hsa04978+hsa:7018%09green |
| hsa05160 | Hepatitis C | Human Diseases | 1\|228 | hsa:7534(E7EX29) | 1\|53 | hsa:7534(E7EX29) | 0.23245614 | 0.501375989 | http://www.kegg.jp/kegg-bin/show_pathway?hsa05160+hsa:7534%09salmon |
| hsa03320 | PPAR signaling pathway | Organismal Systems | 6\|228 | hsa:9370(Q15848);hsa:5360(P55058);hsa:3611(A0A0A0MTH3);hsa:345(B0YIW2);hsa:336(P02652);hsa:335(P02647) | 1\|53 | hsa:5360(P55058) | 0.374783562 | 0.448110781 | http://www.kegg.jp/kegg-bin/show_pathway?hsa03320+hsa:5360%09salmon |
| hsa04934 | Cushing syndrome | Human Diseases | 1\|228 | hsa:5908(P61224) | 1\|53 | hsa:5908(P61224) | 0.23245614 | 0.501375989 | http://www.kegg.jp/kegg-bin/show_pathway?hsa04934+hsa:5908%09green |
| hsa04926 | Relaxin signaling pathway | Organismal Systems | 1\|228 | hsa:59(P68032) | 1\|53 | hsa:59(P68032) | 0.23245614 | 0.501375989 | http://www.kegg.jp/kegg-bin/show_pathway?hsa04926+hsa:59%09green |
| hsa04919 | Thyroid hormone signaling pathway | Organismal Systems | 2\|228 | hsa:71(P60709);hsa:60(P60709);hsa:90390(Q96HR3) | 1\|53 | hsa:90390(Q96HR3) | 0.358412551 | 0.486733094 | http://www.kegg.jp/kegg-bin/show_pathway?hsa04919+hsa:90390%09green |
| hsa04015 | Rap1 signaling pathway | Environmental Information Processing | 7\|228 | hsa:3674(P08514);hsa:7094(Q9Y490);hsa:5908(P61224);hsa:5881(P60763);hsa:7057(P07996);hsa:5216(P07737);hsa:71(P60709);hsa:60(P60709) | 1\|53 | hsa:5908(P61224) | 0.334829158 | 0.5187494 | http://www.kegg.jp/kegg-bin/show_pathway?hsa04015+hsa:5908%09green |
| hsa04216 | Ferroptosis | Cellular Processes | 4\|228 | hsa:7018(C9JB55;P02787);hsa:7037(G3V0E5);hsa:1356(P00450) | 1\|53 | hsa:7018(C9JB55) | 0.424343544 | 0.436241027 | http://www.kegg.jp/kegg-bin/show_pathway?hsa04216+hsa:7018%09green |
| hsa04918 | Thyroid hormone synthesis | Organismal Systems | 6\|228 | hsa:2878(A0A087X1J7);hsa:7276(P02766);hsa:213(P02768;A0A087WWT3);hsa:3309(P11021);hsa:6906(P05543) | 1\|53 | hsa:7276(P02766) | 0.374783562 | 0.448110781 | http://www.kegg.jp/kegg-bin/show_pathway?hsa04918+hsa:7276%09green |
| hsa04066 | HIF-1 signaling pathway | Environmental Information Processing | 6\|228 | hsa:7018(C9JB55;P02787);hsa:2023(A0A2R8Y6G6);hsa:226(J3KPS3);hsa:2597(E7EUT5);hsa:7037(G3V0E5) | 1\|53 | hsa:7018(C9JB55) | 0.374783562 | 0.448110781 | http://www.kegg.jp/kegg-bin/show_pathway?hsa04066+hsa:7018%09green |
| hsa05010 | Alzheimer disease | Human Diseases | 4\|228 | hsa:2597(E7EUT5);hsa:4035(Q07954);hsa:348(P02649);hsa:2081(O75460) | 1\|53 | hsa:348(P02649) | 0.424343544 | 0.436241027 | http://www.kegg.jp/kegg-bin/show_pathway?hsa05010+hsa:348%09green |
| hsa04976 | Bile secretion | Organismal Systems | 1\|228 | hsa:760(P00918) | 1\|53 | hsa:760(P00918) | 0.23245614 | 0.501375989 | http://www.kegg.jp/kegg-bin/show_pathway?hsa04976+hsa:760%09green |
| hsa05410 | Hypertrophic cardiomyopathy (HCM) | Human Diseases | 5\|228 | hsa:3674(P08514);hsa:7171(A0A087WWU8;A0A2R8Y5V9);hsa:71(P60709);hsa:70(P68032);hsa:60(P60709) | 1\|53 | hsa:70(P68032) | 0.407294027 | 0.47160361 | http://www.kegg.jp/kegg-bin/show_pathway?hsa05410+hsa:70%09green |
| hsa05168 | Herpes simplex infection | Human Diseases | 5\|228 | hsa:718(P01024;M0R0Q9);hsa:5199(E9PAQ1);hsa:727(P01031);hsa:3105(A0A0G2JI36) | 1\|53 | hsa:3105(A0A0G2JI36) | 0.407294027 | 0.47160361 | http://www.kegg.jp/kegg-bin/show_pathway?hsa05168+hsa:3105%09green |
| hsa04371 | Apelin signaling pathway | Environmental Information Processing | 1\|228 | hsa:59(P68032) | 1\|53 | hsa:59(P68032) | 0.23245614 | 0.501375989 | http://www.kegg.jp/kegg-bin/show_pathway?hsa04371+hsa:59%09green |
| hsa04260 | Cardiac muscle contraction | Organismal Systems | 3\|228 | hsa:7171(A0A087WWU8;A0A2R8Y5V9);hsa:70(P68032) | 1\|53 | hsa:70(P68032) | 0.41391892 | 0.450802784 | http://www.kegg.jp/kegg-bin/show_pathway?hsa04260+hsa:70%09green |
| hsa04964 | Proximal tubule bicarbonate reclamation | Organismal Systems | 1\|228 | hsa:760(P00918) | 1\|53 | hsa:760(P00918) | 0.23245614 | 0.501375989 | http://www.kegg.jp/kegg-bin/show_pathway?hsa04964+hsa:760%09green |
| hsa04630 | JAK-STAT signaling pathway | Environmental Information Processing | 1\|228 | hsa:2670(A0A1W2PQU7) | 1\|53 | hsa:2670(A0A1W2PQU7) | 0.23245614 | 0.501375989 | http://www.kegg.jp/kegg-bin/show_pathway?hsa04630+hsa:2670%09green |
| hsa04966 | Collecting duct acid secretion | Organismal Systems | 1\|228 | hsa:760(P00918) | 1\|53 | hsa:760(P00918) | 0.23245614 | 0.501375989 | http://www.kegg.jp/kegg-bin/show_pathway?hsa04966+hsa:760%09green |
| hsa05161 | Hepatitis B | Human Diseases | 1\|228 | hsa:7534(E7EX29) | 1\|53 | hsa:7534(E7EX29) | 0.23245614 | 0.501375989 | http://www.kegg.jp/kegg-bin/show_pathway?hsa05161+hsa:7534%09salmon |
| hsa05222 | Small cell lung cancer | Human Diseases | 2\|228 | hsa:3674(P08514);hsa:2335(P02751) | 1\|53 | hsa:2335(P02751) | 0.358412551 | 0.486733094 | http://www.kegg.jp/kegg-bin/show_pathway?hsa05222+hsa:2335%09salmon |
| hsa05167 | Kaposi sarcoma-associated herpesvirus infection | Human Diseases | 3\|228 | hsa:718(P01024;M0R0Q9);hsa:3105(A0A0G2JI36) | 1\|53 | hsa:3105(A0A0G2JI36) | 0.41391892 | 0.450802784 | http://www.kegg.jp/kegg-bin/show_pathway?hsa05167+hsa:3105%09green |
| hsa04924 | Renin secretion | Organismal Systems | 1\|228 | hsa:183(P01019) | 1\|53 | hsa:183(P01019) | 0.23245614 | 0.501375989 | http://www.kegg.jp/kegg-bin/show_pathway?hsa04924+hsa:183%09green |
| hsa04024 | cAMP signaling pathway | Environmental Information Processing | 2\|228 | hsa:5881(P60763);hsa:5908(P61224) | 1\|53 | hsa:5908(P61224) | 0.358412551 | 0.486733094 | http://www.kegg.jp/kegg-bin/show_pathway?hsa04024+hsa:5908%09green |
| hsa04530 | Tight junction | Cellular Processes | 7\|228 | hsa:3993(J3QRV5);hsa:7278(P0DPH7);hsa:7277(P68366);hsa:113457(P0DPH7);hsa:4627(P35579);hsa:87(H9KV75);hsa:112714(P0DPH7);hsa:4478(P26038);hsa:71(P60709);hsa:60(P60709) | 1\|53 | hsa:3993(J3QRV5) | 0.334829158 | 0.5187494 | http://www.kegg.jp/kegg-bin/show_pathway?hsa04530+hsa:3993%09green |
