# Supplementary material for: Proteomics of extracellular vesicles in plasma reveals the characteristics and residual traces of COVID-19 patients without underlying diseases after 3 months of recovery
Source: Cell Death Dis. 2021 May 25;12(6):541. doi: 10.1038/s41419-021-03816-3 (PMC8146187; doi:10.1038/s41419-021-03816-3)
Supplement: Supplementary file 17 — Table S4 [file 41419_2021_3816_MOESM17_ESM.docx]

| Table S4-1 Proteins identified in M vs A group. | | | | | | | | | | | |
| --- | --- | --- | --- | --- | --- | --- | --- | --- | --- | --- | --- |
| Accession | A | M | FC(M/A) | log2FC(M/A) | Pvalue(M/A) | significant | regulate | GO | KEGG | COG | Description |
| A0A087X1J7 | 40054.36895 | 51128.205 | 1.276470117 | 0.352159763 | 0.004739324 | yes | up | GO:0008430;GO:0005615;GO:0051289;GO:0006979;GO:0098869;GO:0055114;GO:0004601;GO:0004602;GO:0016491 | hsa:2878 | COG0386 | glutathione peroxidase 3 isoform 1 precursor [Homo sapiens] |
| A0A0C4DH36 | 53260.81474 | 69261.3875 | 1.300419226 | 0.378976791 | 0.005598824 | yes | up | GO:0009897;GO:0050853;GO:0045087;GO:0006910;GO:0006911;GO:0042742;GO:0003823;GO:0034987;GO:0042571;GO:0006958;GO:0050871;GO:0072562 | hsa:102723407 | | immunoglobulin heavy chain variable gene IGHV3-38, partial [Homo sapiens] |
| A0A0C4DH73 | 64750.49632 | 86163.89625 | 1.330706344 | 0.412192237 | 0.033617327 | yes | up | GO:0038096;GO:0004252;GO:0016020;GO:0030449;GO:0005615;GO:0006898;GO:0038095;GO:0050900;GO:0002376;GO:0002377;GO:0050776;GO:0005576;GO:0006956;GO:0002250;GO:0003823;GO:0005886;GO:0006508;GO:0006958;GO:0006955 | hsa:7441 |  | immunoglobulin light chain variable region, partial [Homo sapiens] |
| A0A0J9YX35 | 22011.14278 | 55634.76146 | 2.527572604 | 1.337752534 | 0.000573973 | yes | up | GO:0016020;GO:0002376;GO:0005576;GO:0002250;GO:0003823;GO:0005886 | hsa:102723407 | | RecName: Full=Immunoglobulin heavy variable 3-64D; Flags: Precursor |
| A0A182DWH7 | 24868.99737 | 30627.1725 | 1.231540301 | 0.300463839 | 0.029653576 | yes | up | GO:0008430 | hsa:6414 |  | Selenoprotein P, plasma, 1 [Homo sapiens] |
| A0A5H1ZRS9 | 84803.10211 | 117885.5538 | 1.39010898 | 0.47519799 | 0.004734909 | yes | up |  | hsa:7441 |  | immunoglobulin kappa chain variable region, partial [Homo sapiens] |
| C9JPQ9 | 1603108.447 | 2195261.946 | 1.369378316 | 0.453521073 | 0.012680625 | yes | up | GO:0051258;GO:0005102;GO:0007596;GO:0005577;GO:0030168 | hsa:2266 |  | hypothetical protein, partial [Homo sapiens] |
| O75636 | 36592.94316 | 54872.66833 | 1.49954236 | 0.584522277 | 0.002771049 | yes | up | GO:0004252;GO:0051607;GO:0006956;GO:0003823;GO:1902679;GO:0045087;GO:0030246;GO:0043654;GO:0002376;GO:0046597;GO:0005576;GO:0001867;GO:0072562;GO:0005515;GO:0006508;GO:0046872;GO:0005581 | hsa:8547 |  | ficolin-3 isoform 1 precursor [Homo sapiens] |
| P02751 | 534412.4632 | 841470.4125 | 1.574571086 | 0.654958891 | 0.000700959 | yes | up | GO:0008022;GO:0018149;GO:0019221;GO:0034446;GO:0048146;GO:0005518;GO:0005201;GO:0005788;GO:0062023;GO:0007160;GO:0007161;GO:0050900;GO:0010628;GO:0097718;GO:0001525;GO:0044267;GO:0070062;GO:0031093;GO:0002576;GO:0051087;GO:0009611;GO:0072562;GO:0008201;GO:0005576;GO:0002020;GO:0030198;GO:1904237;GO:0033622;GO:0042802;GO:0008284;GO:0005178;GO:2001202;GO:0008360;GO:0005793;GO:0016324;GO:0043687;GO:0042060;GO:0006953;GO:0043394;GO:0019899;GO:0007155;GO:0051702;GO:0010952;GO:0035987;GO:1901166;GO:0005615;GO:0052047;GO:0005604;GO:0045773;GO:0031012;GO:0005577;GO:0007044;GO:0005515;GO:0016504;GO:0005102;GO:0001932;GO:0070372 | hsa:2335 |  | fibronectin isoform 1 precursor [Homo sapiens] |
| P03951 | 9396.648474 | 13987.7555 | 1.488589846 | 0.5739463 | 0.000381538 | yes | up | GO:0004252;GO:0016020;GO:0007599;GO:0070062;GO:0016787;GO:0005615;GO:0007597;GO:0007596;GO:0031639;GO:0030193;GO:0005576;GO:0005515;GO:0005886;GO:0070009;GO:0006508;GO:0008233;GO:0008236;GO:0008201;GO:0042802;GO:0051919 | hsa:2160 | COG5640 | coagulation factor XI isoform 1 preproprotein [Homo sapiens] |
| P07737 | 10121.49626 | 15887.21104 | 1.569650438 | 0.650443306 | 0.015746501 | yes | up | GO:0017048;GO:0050821;GO:0060074;GO:0045202;GO:0050434;GO:0005925;GO:0005737;GO:0032232;GO:0032233;GO:0070062;GO:0030837;GO:0030838;GO:0051496;GO:0005634;GO:0016020;GO:0005546;GO:0003779;GO:0098794;GO:0043005;GO:0098793;GO:0001843;GO:0005856;GO:0051054;GO:0098685;GO:0005515;GO:0032781;GO:0060071;GO:0098688;GO:0070064;GO:0005938;GO:0000774;GO:0010033;GO:0010634;GO:0071363;GO:0005829;GO:1900029;GO:0098885;GO:0045296;GO:0051497;GO:0006357;GO:0045944;GO:0003723;GO:0030036;GO:0072562;GO:0005102;GO:0098978;GO:0003785 | hsa:5216 |  | PREDICTED: profilin-1 isoform X2 [Nomascus leucogenys] |
| P0DOY3 | 7486774.005 | 9987699.083 | 1.334045755 | 0.415808149 | 0.024772618 | yes | up | GO:0005615;GO:0016020;GO:0072562;GO:0002376;GO:0005576;GO:0002250;GO:0003823;GO:0005886;GO:0070062 | hsa:100423062 | | RecName: Full=Immunoglobulin lambda constant 3; AltName: Full=Ig lambda chain C region DOT; AltName: Full=Ig lambda chain C region NEWM; AltName: Full=Ig lambda-3 chain C regions |
| P19823 | 1107498.8 | 1441078.625 | 1.301201071 | 0.379843915 | 2.51E-05 | yes | up | GO:0044267;GO:0030212;GO:0070062;GO:0043687;GO:0072562;GO:0062023;GO:0030414;GO:0005788;GO:0004866;GO:0004867;GO:0010951;GO:0010466;GO:0005576 | hsa:3698 | COG2304 | inter-alpha-trypsin inhibitor heavy chain H2 precursor [Homo sapiens] |
| P35527 | 88404.13842 | 150298.3288 | 1.700127748 | 0.765643155 | 0.001316609 | yes | up | GO:0005200;GO:0045109;GO:0031424;GO:0005615;GO:0005829;GO:0016020;GO:0070268;GO:0005198;GO:0007283;GO:0005882;GO:0008544;GO:0005634;GO:0043588;GO:0070062 | hsa:3857 |  | keratin, type I cytoskeletal 9 [Homo sapiens] |
| P55058 | 13904.45947 | 17563.92375 | 1.263186374 | 0.337067514 | 0.013172587 | yes | up | GO:0034375;GO:0006869;GO:0015914;GO:1990050;GO:0035627;GO:0005548;GO:0005615;GO:0019992;GO:0008525;GO:0097001;GO:0010875;GO:0030317;GO:0010189;GO:0005319;GO:0035620;GO:0034364;GO:0006629;GO:0070300;GO:0008289;GO:0031210;GO:0008429;GO:1904121;GO:0005576;GO:1901611 | hsa:5360 |  | phospholipid transfer protein, isoform CRA_c [Homo sapiens] |
| P60709 | 27249.58842 | 47389.20958 | 1.739079829 | 0.798324159 | 0.014604211 | yes | up | GO:0043044;GO:0019894;GO:0015629;GO:0072749;GO:0061024;GO:0005200;GO:0021762;GO:0036464;GO:0005925;GO:0048488;GO:0030957;GO:0032091;GO:0005615;GO:0043209;GO:0005634;GO:0016020;GO:0097433;GO:0070062;GO:0098871;GO:0005654;GO:0045815;GO:0016579;GO:0005524;GO:0000980;GO:0005856;GO:0070527;GO:0000166;GO:1903076;GO:0019901;GO:0000790;GO:0005886;GO:0048013;GO:0001895;GO:0098685;GO:0034329;GO:0031982;GO:0038096;GO:0050998;GO:0044305;GO:0032991;GO:0051621;GO:0048870;GO:0030863;GO:0022898;GO:0005829;GO:0051623;GO:1990904;GO:0005737;GO:0098973;GO:0031492;GO:0098793;GO:0042802;GO:0005515;GO:0098974;GO:0072562;GO:0000978;GO:0098978;GO:0000079;GO:0035267 | hsa:71;hsa:60 | COG5277 | cytoskeletal beta actin, partial [Sus scrofa] |
| Q14624 | 179441.1722 | 215482.1778 | 1.200851372 | 0.264057601 | 0.026371973 | yes | up | GO:0005737;GO:0030212;GO:0034097;GO:0002576;GO:0004867;GO:0072562;GO:0031089;GO:0006953;GO:0005576;GO:0030414;GO:0005515;GO:0004866;GO:0005886;GO:0010951;GO:0010466;GO:0070062 | hsa:3700 | COG2304 | inter-alpha-trypsin inhibitor heavy chain H4 isoform 1 precursor [Homo sapiens] |
| Q16610 | 32948.33211 | 39692.97458 | 1.204703609 | 0.268678246 | 0.012896172 | yes | up | GO:0008022;GO:0005201;GO:0019899;GO:0031012;GO:0001503;GO:0007165;GO:0030500;GO:0030502;GO:0001525;GO:0005615;GO:0002576;GO:0045766;GO:0010466;GO:2000404;GO:0001960;GO:0002063;GO:0003416;GO:0005576;GO:0005134;GO:0002020;GO:0002828;GO:0062023;GO:0006954;GO:0031214;GO:0070062;GO:0043236;GO:0031089;GO:0001938;GO:0006357;GO:0005515;GO:0043123 | hsa:1893 |  | extracellular matrix protein 1 isoform 1 precursor [Homo sapiens] |
| Q86UX7 | 4622.435408 | 8737.222565 | 1.890177319 | 0.918521581 | 0.038582981 | yes | up | GO:0005178;GO:0030335;GO:0002102;GO:0070062;GO:0031093;GO:0034446;GO:0007229;GO:0016020;GO:0033632;GO:0005576;GO:0007159;GO:0007155;GO:0033622;GO:0042995;GO:0070527;GO:0030054;GO:0002576 | hsa:83706 |  | fermitin family homolog 3 long isoform [Homo sapiens] |
| P01008 | 517418.1526 | 459821.625 | 0.888684757 | -0.170256353 | 0.0275401 | yes | no change | GO:0007599;GO:0007595;GO:2000266;GO:0007596;GO:0005788;GO:0002438;GO:0070062;GO:0072562;GO:0010466;GO:0002020;GO:0043687;GO:0042802;GO:0044267;GO:0007584;GO:0062023;GO:0030414;GO:0004867;GO:0010951;GO:0005886;GO:0008201;GO:0005615;GO:0005576;GO:0005515;GO:0030193 | hsa:462 | COG4826 | antithrombin-III isoform 1 precursor [Homo sapiens] |
| P01019 | 181866.2316 | 153225.3458 | 0.842516747 | -0.247222731 | 0.002333149 | yes | no change | GO:1903779;GO:0038166;GO:2001238;GO:0035106;GO:0003014;GO:0007166;GO:0050729;GO:0001822;GO:0042127;GO:0032930;GO:0006606;GO:0014824;GO:0019229;GO:0010976;GO:0010873;GO:0050731;GO:0035815;GO:0005829;GO:0007202;GO:0007204;GO:0003081;GO:1904754;GO:0010536;GO:0007565;GO:0005179;GO:0034374;GO:0007200;GO:0071260;GO:0042310;GO:0042311;GO:0048169;GO:0008083;GO:1901201;GO:0072562;GO:0006883;GO:0061098;GO:0007186;GO:2000379;GO:0090190;GO:0048018;GO:0008306;GO:0003331;GO:0033864;GO:0007199;GO:0001974;GO:0062023;GO:0048659;GO:0051969;GO:0016525;GO:0014873;GO:0030308;GO:0048146;GO:0048144;GO:0007267;GO:0045429;GO:1905010;GO:0043085;GO:0005737;GO:0050880;GO:0003051;GO:0002027;GO:0045742;GO:0032270;GO:0070471;GO:0008284;GO:0031701;GO:0031703;GO:0001558;GO:0014068;GO:0004867;GO:0014061;GO:0051092;GO:1904385;GO:0007568;GO:0070062;GO:0002034;GO:2001275;GO:0005515;GO:0070371;GO:0001819;GO:0051387;GO:0010469;GO:0010666;GO:0008217;GO:0061049;GO:0050663;GO:0032355;GO:0005615;GO:0097755;GO:0042981;GO:0010744;GO:0035813;GO:1904707;GO:0046628;GO:0034104;GO:0019216;GO:0051403;GO:0010951;GO:0007263;GO:1905589;GO:0010613;GO:0010595;GO:0002019;GO:0002018;GO:0045777;GO:0002016;GO:0031702;GO:0051924;GO:0005576;GO:1903598;GO:0045893 | hsa:183 | COG4826 | angiotensinogen preproprotein [Homo sapiens] |
| P02787 | 21499234.37 | 18754505.5 | 0.872333646 | -0.197048059 | 0.033253952 | yes | no change | GO:0009617;GO:0016020;GO:0055037;GO:1990459;GO:1900390;GO:0005788;GO:0030139;GO:0061024;GO:0055072;GO:0006826;GO:0005905;GO:0034986;GO:1990712;GO:0048260;GO:0010008;GO:0030665;GO:0007257;GO:0045780;GO:0031232;GO:0005615;GO:0031410;GO:0002576;GO:0005770;GO:0045178;GO:0070062;GO:2000147;GO:0009925;GO:0044267;GO:0048471;GO:0046872;GO:0043687;GO:0009986;GO:0006879;GO:0033572;GO:0007015;GO:0016324;GO:0006811;GO:0030316;GO:0031982;GO:0008198;GO:0008199;GO:0045893;GO:0034774;GO:0015091;GO:0001895;GO:0034756;GO:0071281;GO:0042327;GO:0060395;GO:0031647;GO:0005623;GO:0005576;GO:0005515;GO:0005768;GO:0005769;GO:0070371;GO:0072562 | hsa:7018 |  | serotransferrin isoform 1 precursor [Homo sapiens] |
| P07357 | 57584.42 | 51566.1375 | 0.89548766 | -0.159254544 | 0.049951686 | yes | no change | GO:0019835;GO:0030449;GO:0070062;GO:0016021;GO:0016020;GO:0072562;GO:0002376;GO:0001848;GO:0005576;GO:0006956;GO:0006957;GO:0005886;GO:0006955;GO:0044877;GO:0006958;GO:0005579;GO:0045087;GO:0005615 | hsa:731 |  | complement component C8 alpha chain preproprotein [Homo sapiens] |
| P08697 | 217188.5526 | 244147.6875 | 1.124127789 | 0.168806048 | 0.025078204 | yes | no change | GO:0030414;GO:0032967;GO:0005615;GO:0031093;GO:0051496;GO:0002576;GO:0030199;GO:0072562;GO:0010466;GO:0048514;GO:0002020;GO:0010757;GO:0051918;GO:0042803;GO:0048661;GO:0009986;GO:0045597;GO:0006953;GO:0046330;GO:0004866;GO:0004867;GO:0010951;GO:0010033;GO:0070062;GO:0042730;GO:2000049;GO:0002034;GO:0005577;GO:0005576;GO:0045944;GO:0005515;GO:0070374;GO:0071636 | hsa:5345 | COG4826 | alpha-2-antiplasmin isoform X1 [Homo sapiens] |
| P10909 | 1087859.132 | 913876.8833 | 0.840069138 | -0.251420027 | 0.00170311 | yes | no change | GO:0032436;GO:0005783;GO:0019730;GO:1903573;GO:0009615;GO:0016020;GO:1902949;GO:0042127;GO:0005794;GO:0034366;GO:0060548;GO:0031966;GO:0043691;GO:0043231;GO:0005829;GO:0002376;GO:0051787;GO:1902004;GO:0051788;GO:0030449;GO:1902230;GO:0050821;GO:0010628;GO:0044877;GO:0099020;GO:0048260;GO:0005856;GO:0000902;GO:0002434;GO:0072562;GO:0048471;GO:0097418;GO:0051131;GO:0032760;GO:0006629;GO:1905895;GO:0048156;GO:0001774;GO:0006956;GO:1905892;GO:1901216;GO:0006958;GO:0061077;GO:2000060;GO:1902430;GO:1902847;GO:0090201;GO:0031012;GO:0045429;GO:0051082;GO:1902998;GO:1901214;GO:0005737;GO:0001540;GO:0031093;GO:0031410;GO:0005634;GO:0051087;GO:0005739;GO:1900221;GO:0009986;GO:0017038;GO:0061740;GO:0061741;GO:0051092;GO:0031625;GO:0070062;GO:0005622;GO:0005743;GO:0005515;GO:0097440;GO:0032286;GO:0016887;GO:0071944;GO:0045202;GO:0061518;GO:0005615;GO:0002576;GO:0043065;GO:0032464;GO:0032463;GO:0045087;GO:0006915;GO:0042583;GO:0062023;GO:0050750;GO:0032991;GO:0001836;GO:0005576;GO:1905907;GO:1905908 | hsa:1191 |  | clusterin preproprotein [Homo sapiens] |
| A0A075B6J9 | 130416.4753 | 93959.55042 | 0.720457674 | -0.473014419 | 0.025992844 | yes | down | GO:0016020;GO:0005615;GO:0006955;GO:0002376;GO:0002377;GO:0005576;GO:0002250;GO:0003823;GO:0005886 | hsa:7441 |  | RecName: Full=Immunoglobulin lambda variable 2-18; Flags: Precursor |
| A0A075B6R9 | 233860.0358 | 150302.1738 | 0.642701406 | -0.637779467 | 0.026693688 | yes | down | GO:0002377;GO:0005615;GO:0006955 | hsa:7441 |  | RecName: Full=Immunoglobulin kappa variable 2-24; Flags: Precursor |
| A0A0B4J1Y8 | 116112.7792 | 78605.33 | 0.676973978 | -0.562827716 | 0.02765559 | yes | down | GO:0016020;GO:0005615;GO:0006955;GO:0002376;GO:0002377;GO:0005576;GO:0002250;GO:0003823;GO:0005886 | hsa:7441 |  | Unknown (protein for IMAGE:4575521), partial [Homo sapiens] |
| A0A2R8Y3M9 | 184972.8526 | 151192.2096 | 0.817375131 | -0.290929746 | 0.001701729 | yes | down | GO:0004252;GO:0006898;GO:0005044;GO:0016020;GO:0016042;GO:0005576;GO:0005509;GO:0006508;GO:0004623 | hsa:81579;hsa:3426 | COG5640 | complement factor I isoform X2 [Homo sapiens] |
| C9JXI5 | 152351.3726 | 76099.971 | 0.499503022 | -1.001434689 | 0.006855077 | yes | down | GO:0090263;GO:0031410;GO:0016021;GO:0016020;GO:0005886 | hsa:130612 | | transmembrane protein 198 [Macaca mulatta] |
| D6RE82 | 131886.3826 | 73169.90329 | 0.554794982 | -0.849973355 | 0.02719182 | yes | down | GO:0030688;GO:0005730;GO:0005634;GO:0030687;GO:0006364;GO:0003723 | hsa:8568 |  | PREDICTED: ribosomal RNA processing protein 1 homolog A [Callithrix jacchus] |
| K7ERG9 | 12339.92889 | 9029.719 | 0.731748058 | -0.450581084 | 0.001431633 | yes | down | GO:0004252;GO:0007219;GO:0005615;GO:0016787;GO:0009617;GO:0006957;GO:0006508;GO:0008233;GO:0008236 | hsa:1675 | COG5640 | complement factor D isoform 2 precursor [Homo sapiens] |
| P00739 | 54761.93632 | 38869.02583 | 0.709781802 | -0.494552509 | 0.031196936 | yes | down | GO:0004252;GO:0070062;GO:0034366;GO:0030492;GO:0006898;GO:0072562;GO:0005576;GO:0010942;GO:0002526;GO:0010033;GO:0005615 | hsa:3250 | COG5640 | haptoglobin-related protein precursor [Homo sapiens] |
| P00915 | 20567.45463 | 14078.96017 | 0.684526132 | -0.546822476 | 0.004652707 | yes | down | GO:0005737;GO:0015701;GO:0046872;GO:0070062;GO:0006730;GO:0016829;GO:0008270;GO:0005515;GO:0016836;GO:0004089;GO:0004064;GO:0005829;GO:0035722 | hsa:759 | COG3338 | carbonic anhydrase 1 isoform a [Homo sapiens] |
| P01715 | 26407.4994 | 11104.41688 | 0.4205024 | -1.249814059 | 0.004664917 | yes | down | GO:0038096;GO:0004252;GO:0016020;GO:0030449;GO:0050776;GO:0006898;GO:0038095;GO:0002376;GO:0050900;GO:0005576;GO:0006956;GO:0002250;GO:0003823;GO:0005886;GO:0006508;GO:0006958;GO:0006955 | hsa:29802 |  | hCG2040023, partial [Homo sapiens] |
| P01717 | 10448.645 | 6849.123 | 0.655503465 | -0.609324689 | 0.005830596 | yes | down | GO:0038096;GO:0004252;GO:0016020;GO:0030449;GO:0050776;GO:0006898;GO:0038095;GO:0072562;GO:0002376;GO:0050900;GO:0005576;GO:0006956;GO:0002250;GO:0003823;GO:0005886;GO:0006508;GO:0006958;GO:0006955 | hsa:7441 |  | immunoglobulin lambda light chain variable region, partial [Homo sapiens] |
| P02747 | 538883.3474 | 435160.6167 | 0.80752285 | -0.308425011 | 0.002097669 | yes | down | GO:0004252;GO:0030449;GO:0005581;GO:0045650;GO:0045087;GO:0072562;GO:0002376;GO:0030853;GO:0005576;GO:0006956;GO:0005515;GO:0006955;GO:0006508;GO:0006958;GO:0005615 | hsa:714 |  | complement C1q subcomponent subunit C isoform X1 [Pongo abelii] |
| P04040 | 6359.091125 | 4669.2838 | 0.73426905 | -0.445619304 | 0.024320235 | yes | down | GO:0016491;GO:0005829;GO:0016020;GO:0005782;GO:0020027;GO:0033189;GO:0051289;GO:0009314;GO:0005886;GO:0019899;GO:0000302;GO:1904813;GO:0009060;GO:0005739;GO:0004601;GO:0009650;GO:0032868;GO:0001666;GO:0055093;GO:0001649;GO:0005783;GO:0050661;GO:0005778;GO:0009636;GO:0070062;GO:0005615;GO:0033591;GO:0005777;GO:0001822;GO:0051262;GO:0006979;GO:0098869;GO:0043066;GO:0046686;GO:0005758;GO:0034774;GO:0046872;GO:0014823;GO:0042802;GO:0042803;GO:0004046;GO:0005794;GO:0005925;GO:0010288;GO:0020037;GO:0006641;GO:0009411;GO:0006625;GO:0016209;GO:0032355;GO:0045471;GO:0014068;GO:0001657;GO:0071363;GO:0034599;GO:0009642;GO:0055114;GO:0008203;GO:0051092;GO:0016684;GO:0007568;GO:0043231;GO:0042542;GO:0070542;GO:0042493;GO:0043312;GO:0032088;GO:0005764;GO:0051781;GO:0042744;GO:0033197;GO:0010193;GO:0004096;GO:0005576;GO:0005102;GO:0014854;GO:0080184 | hsa:847 | COG0753 | catalase [Homo sapiens] |
| P07360 | 45022.55263 | 35935.62708 | 0.798169472 | -0.325232994 | 0.002325887 | yes | down | GO:0019835;GO:0036094;GO:0030449;GO:0070062;GO:0045087;GO:0019841;GO:0072562;GO:0002376;GO:0001848;GO:0005576;GO:0044877;GO:0006957;GO:0006958;GO:0005579;GO:0005615 | hsa:733 |  | complement component C8 gamma chain precursor [Homo sapiens] |
| P13647 | 27473.64947 | 22368.1385 | 0.81416699 | -0.296603365 | 0.013235149 | yes | down | GO:0005737;GO:0016020;GO:0005200;GO:0007010;GO:0070062;GO:0031424;GO:0031581;GO:0045095;GO:0070268;GO:0005198;GO:0005634;GO:0005882;GO:0097110;GO:0005515;GO:0005886;GO:0008544;GO:0005829 | hsa:3852 |  | Keratin 5 [Homo sapiens] |
| P29622 | 85693.02526 | 69966.39042 | 0.816477073 | -0.292515721 | 0.000985088 | yes | down | GO:0005615;GO:0070062;GO:0002576;GO:0031089;GO:0010466;GO:0030414;GO:0004867;GO:0010951;GO:0005576 | hsa:5267 | COG4826 | kallistatin isoform 1 [Homo sapiens] |
| P32119 | 22429.17211 | 12268.95304 | 0.547008734 | -0.870364226 | 0.000174905 | yes | down | GO:0000187;GO:0045581;GO:0042744;GO:0008379;GO:0032496;GO:0032088;GO:0042981;GO:0034599;GO:0002536;GO:0016491;GO:0005737;GO:0010310;GO:0070062;GO:0042098;GO:0043066;GO:0048538;GO:2001240;GO:0016209;GO:0045454;GO:0006979;GO:0055114;GO:0048872;GO:0045321;GO:0005829;GO:0005623;GO:0031665;GO:0019430;GO:0005515;GO:0051920;GO:0004601;GO:0030194 | hsa:7001 | COG0450 | peroxiredoxin-2 [Homo sapiens] |
| P69905 | 836201.7737 | 554854.3042 | 0.663541171 | -0.591742111 | 0.004628895 | yes | down | GO:0005344;GO:0022627;GO:0015701;GO:0042744;GO:0010942;GO:0005615;GO:0071682;GO:0016020;GO:0072562;GO:0005833;GO:0098869;GO:0005506;GO:0046872;GO:0015671;GO:0020037;GO:0031720;GO:0051291;GO:0070062;GO:0019825;GO:0042542;GO:0005829;GO:0006898;GO:0005576;GO:0005515;GO:0031838;GO:0004601 | hsa:3040;hsa:3039 | COG1018 | TPA: globin C1 [Homo sapiens] |
| P80748 | 667222.8947 | 482747.5292 | 0.723517633 | -0.466899918 | 0.036119355 | yes | down | GO:0038096;GO:0004252;GO:0016020;GO:0030449;GO:0050776;GO:0006898;GO:0038095;GO:0072562;GO:0002376;GO:0050900;GO:0005576;GO:0006956;GO:0002250;GO:0003823;GO:0005886;GO:0006508;GO:0006958;GO:0006955;GO:0070062 | hsa:7441 |  | hCG2040021, partial [Homo sapiens] |
| Q96HR3 | 36736.91842 | 22188.84 | 0.603992957 | -0.727396367 | 0.000449145 | yes | down | GO:0019827;GO:0030521;GO:0038023;GO:0005515;GO:0006355;GO:0005634;GO:0030518;GO:0003712;GO:0000151;GO:0005654;GO:0046966;GO:0045893;GO:0006367;GO:0016567;GO:0016592;GO:0030374;GO:0061630;GO:0042809;GO:0006351 | hsa:90390 |  | mediator of RNA polymerase II transcription subunit 30 isoform 1 [Homo sapiens] |
| Q9UGM5 | 25507.93 | 20334.905 | 0.797199342 | -0.326987576 | 0.030612361 | yes | down | GO:0005615;GO:0007339;GO:0008150;GO:0005576;GO:0003674;GO:0007338;GO:0030414;GO:0008191;GO:0010951;GO:0004869;GO:0004857;GO:0010466;GO:0070062 | hsa:26998 |  | fetuin-B isoform 1 precursor [Homo sapiens] |
| A0A096LPE2 | 517015.4895 | 623219.7375 | 1.205417923 | 0.269533421 | 0.055906592 | no | up | GO:0042056;GO:0034364;GO:0005615;GO:0006953;GO:0005576;GO:0060326;GO:0050918;GO:0070062 | hsa:6291;hsa:100528017 | | SAA2-SAA4 protein precursor [Homo sapiens] |
| A0A0A0MS09 | 49809.89976 | 59805.41636 | 1.200673293 | 0.263843642 | 0.555714268 | no | up | GO:0016021;GO:0016020 | | | immunoglobulin delta-chain, partial [Homo sapiens] |
| A0A0B4J1U7 | 53333.70684 | 72494.305 | 1.359258699 | 0.442820062 | 0.102583274 | no | up | GO:0009897;GO:0050853;GO:0045087;GO:0016020;GO:0006910;GO:0006911;GO:0002376;GO:0005576;GO:0002250;GO:0003823;GO:0005886;GO:0072562;GO:0042571;GO:0006958;GO:0050871;GO:0034987;GO:0042742 | hsa:102723407 | | RecName: Full=Immunoglobulin heavy variable 6-1; Flags: Precursor |
| A0A0B4J1V1 | 61207.74753 | 79740.12053 | 1.302778223 | 0.381591509 | 0.46399297 | no | up | GO:0009897;GO:0050853;GO:0045087;GO:0016020;GO:0006910;GO:0006911;GO:0002376;GO:0005576;GO:0002250;GO:0003823;GO:0005886;GO:0072562;GO:0042571;GO:0006958;GO:0050871;GO:0034987;GO:0042742 | hsa:102723407 | | RecName: Full=Immunoglobulin heavy variable 3-21; Flags: Precursor |
| A0A0G2JSC0 | 28074.66676 | 41204.08064 | 1.467660542 | 0.553518323 | 0.110116198 | no | up |  | hsa:7441 |  | Lambda-V immunoglobulin light chain variable domain precursor, partial [Homo sapiens] |
| A0A4W8ZXM2 | 659986.1158 | 872953.3875 | 1.322684473 | 0.403468947 | 0.148178201 | no | up |  | hsa:102723407 | | immunoglobulin heavy chain variable region, partial [Homo sapiens] |
| A0A5H1ZRQ7 | 13185.15492 | 16360.20347 | 1.240804797 | 0.311276169 | 0.288193793 | no | up |  | hsa:100423062 | | RecName: Full=Immunoglobulin lambda constant 7; AltName: Full=Ig lambda-7 chain C region |
| F8W1S1 | 5466.170615 | 9501.73105 | 1.738279267 | 0.79765988 | 0.323315783 | no | up | GO:0005882;GO:0045095;GO:0005198 | hsa:121391 | | KRT74 isoform 2 [Pan troglodytes] |
| O75460 | 97874.42944 | 120191.0938 | 1.228013225 | 0.296326098 | 0.182058991 | no | up | GO:0005739;GO:0004540;GO:0005783;GO:0016787;GO:0016310;GO:0005161;GO:0019899;GO:0005789;GO:0008152;GO:1990604;GO:0030176;GO:1990597;GO:0034620;GO:1990630;GO:0046777;GO:0036289;GO:0007257;GO:0006986;GO:0098787;GO:0016241;GO:0030544;GO:0005637;GO:0016021;GO:0000287;GO:0004521;GO:0051082;GO:0000166;GO:0004674;GO:0016740;GO:0071333;GO:1901142;GO:0006402;GO:0046872;GO:0051879;GO:0042802;GO:0042803;GO:0005737;GO:0006468;GO:0033120;GO:0006397;GO:1990332;GO:0004672;GO:0007050;GO:0003824;GO:0043531;GO:0016020;GO:0090502;GO:0036498;GO:0006351;GO:0030968;GO:0070059;GO:1904707;GO:0070054;GO:0016301;GO:0006355;GO:1990579;GO:0005524;GO:0005515;GO:0004519;GO:1900103;GO:0006379;GO:0001935;GO:0035924;GO:0006915;GO:0034976 | hsa:2081 | COG0515 | endoplasmic reticulum to nucleus signalling 1 isoform 1 variant, partial [Homo sapiens] |
| P00738 | 8878679.147 | 11543587 | 1.300146881 | 0.378674617 | 0.074638396 | no | up | GO:0042742;GO:0010942;GO:0005615;GO:0071682;GO:2000296;GO:0072562;GO:0098869;GO:0035580;GO:1904724;GO:0016209;GO:0006952;GO:0006953;GO:0070062;GO:0042542;GO:0051354;GO:0006898;GO:0043312;GO:0002376;GO:0030492;GO:0005576;GO:0005515;GO:0031838 | hsa:3240 | COG5640 | haptoglobin isoform 1 preproprotein [Homo sapiens] |
| P01602 | 185182.1458 | 293847.575 | 1.586802949 | 0.666122984 | 0.189355228 | no | up | GO:0038096;GO:0004252;GO:0016020;GO:0030449;GO:0050776;GO:0006898;GO:0038095;GO:0072562;GO:0002376;GO:0002377;GO:0050900;GO:0005576;GO:0006956;GO:0002250;GO:0003823;GO:0005886;GO:0006508;GO:0006958;GO:0006955;GO:0070062 | hsa:7441 |  | hCG2043208, partial [Homo sapiens] |
| P01814 | 27211.02737 | 33749.13708 | 1.240274269 | 0.310659188 | 0.067009403 | no | up | GO:0038096;GO:0004252;GO:0016020;GO:0030449;GO:0050776;GO:0006898;GO:0038095;GO:0002376;GO:0050900;GO:0005576;GO:0006956;GO:0002250;GO:0003823;GO:0005886;GO:0006508;GO:0006958;GO:0006955 | hsa:102723407 | | RecName: Full=Immunoglobulin heavy variable 2-70; AltName: Full=Ig heavy chain V-II region COR; AltName: Full=Ig heavy chain V-II region DAW; AltName: Full=Ig heavy chain V-II region OU; AltName: Full=Ig heavy chain V-II region SESS; Flags: Precursor |
| P01817 | 32489.94826 | 40830.34333 | 1.256706936 | 0.329648253 | 0.12054538 | no | up | GO:0038096;GO:0004252;GO:0016020;GO:0030449;GO:0050776;GO:0006898;GO:0038095;GO:0002376;GO:0050900;GO:0005576;GO:0006956;GO:0002250;GO:0003823;GO:0005886;GO:0006508;GO:0006958;GO:0006955 | hsa:102723407 | | RecName: Full=Immunoglobulin heavy variable 2-5; AltName: Full=Ig heavy chain V-II region HE; AltName: Full=Ig heavy chain V-II region MCE; Flags: Precursor |
| P02533 | 84393.37211 | 124618.9902 | 1.47664428 | 0.562322326 | 0.748845821 | no | up | GO:0005200;GO:0045095;GO:0071944;GO:0010043;GO:0008544;GO:0005737;GO:0045110;GO:0010212;GO:0070062;GO:0005634;GO:0045178;GO:0005198;GO:0030855;GO:1990254;GO:0031581;GO:0070268;GO:0005882;GO:0042633;GO:0007568;GO:0031424;GO:0005829;GO:0005622;GO:0005515 | hsa:3861 |  | keratin, type I cytoskeletal 14 [Homo sapiens] |
| P02652 | 318388.9053 | 442120.4958 | 1.388617783 | 0.473649552 | 0.385621882 | no | up | GO:0060192;GO:0034384;GO:0034375;GO:0005543;GO:0034374;GO:0034371;GO:0006869;GO:0031072;GO:0031647;GO:0017127;GO:0030301;GO:0006656;GO:0010903;GO:0010873;GO:0002740;GO:0060228;GO:0001523;GO:0043085;GO:0034190;GO:0009395;GO:0044267;GO:0042627;GO:0005615;GO:0032375;GO:0031100;GO:0055102;GO:0072562;GO:0033344;GO:0034361;GO:0070062;GO:0033700;GO:0005319;GO:0018158;GO:0008035;GO:0034364;GO:0016032;GO:0034366;GO:0006641;GO:0060621;GO:0043687;GO:0043691;GO:0008289;GO:0031210;GO:0005788;GO:0042632;GO:0050996;GO:0043627;GO:0002526;GO:0051384;GO:0034378;GO:0008203;GO:0030300;GO:0046982;GO:0045416;GO:0015485;GO:0019216;GO:0050995;GO:0046340;GO:0005829;GO:0018206;GO:0042493;GO:0034380;GO:0009749;GO:0005576;GO:0005515;GO:0070653;GO:0060695;GO:0042803;GO:0034370;GO:0005769;GO:0042157 | hsa:336 |  | apolipoprotein A-II preproprotein [Homo sapiens] |
| P04430 | 78829.74944 | 106518.657 | 1.351249467 | 0.434294049 | 0.148180351 | no | up | GO:0038096;GO:0004252;GO:0016020;GO:0030449;GO:0050776;GO:0006898;GO:0038095;GO:0050900;GO:0005576;GO:0006956;GO:0003823;GO:0005886;GO:0006508;GO:0006958;GO:0006955 | hsa:7441 |  | RecName: Full=Immunoglobulin kappa variable 1-16; AltName: Full=Ig kappa chain V-I region BAN; Flags: Precursor |
| P08779 | 89197.10947 | 109549.4108 | 1.228172207 | 0.296512861 | 0.483953513 | no | up | GO:0005200;GO:0061436;GO:0008544;GO:0005856;GO:0002009;GO:0051546;GO:0070062;GO:0005634;GO:0005198;GO:0030336;GO:0030216;GO:0008283;GO:0045087;GO:0070268;GO:0005882;GO:0006954;GO:0042633;GO:0007568;GO:0007010;GO:0045104;GO:0005829;GO:0031424;GO:0005515 | hsa:3868 |  | keratin, type I cytoskeletal 16 [Homo sapiens] |
| P0DP02 | 236468.2979 | 315646.3906 | 1.334835974 | 0.416662472 | 0.101769276 | no | up | GO:0016020;GO:0002376;GO:0005576;GO:0002250;GO:0003823;GO:0005886 | hsa:102723407 | | immunoglobulin heavy chain [Homo sapiens] |
| P11021 | 232966.6897 | 378243.2118 | 1.623593537 | 0.699190503 | 0.33372392 | no | up | GO:1990090;GO:0051087;GO:0005783;GO:0071480;GO:1990440;GO:0035437;GO:0009314;GO:0030433;GO:0019899;GO:0036500;GO:0005789;GO:0042149;GO:0035690;GO:0021762;GO:0045296;GO:0016887;GO:0031204;GO:0034663;GO:0005925;GO:1901998;GO:0019904;GO:0008180;GO:0005737;GO:0005793;GO:0006983;GO:0042623;GO:0043209;GO:0005634;GO:0016020;GO:0071353;GO:0005739;GO:0000166;GO:0031625;GO:0043066;GO:0005509;GO:0031398;GO:0043022;GO:0005524;GO:0016787;GO:0030335;GO:0071236;GO:0030176;GO:0005790;GO:0030182;GO:0042470;GO:0097501;GO:0009986;GO:0071277;GO:0036498;GO:0036499;GO:0051402;GO:0051082;GO:0005788;GO:0005886;GO:0042220;GO:0030512;GO:0001554;GO:0051603;GO:1904313;GO:0032991;GO:0043231;GO:0030968;GO:0071287;GO:0070062;GO:0005829;GO:0071320;GO:0030496;GO:0060904;GO:0090074;GO:0021589;GO:1903897;GO:1903894;GO:1903895;GO:0051787;GO:0005515;GO:1903891;GO:0034976;GO:0034975;GO:0021680;GO:0010976 | hsa:3309 | COG0443 | 78 kDa glucose-regulated protein [Otolemur garnettii] |
| P11226 | 82655.61287 | 107708.6689 | 1.303101691 | 0.381949673 | 0.089908716 | no | up | GO:0004252;GO:0048306;GO:0030246;GO:0042742;GO:0044130;GO:0050766;GO:0005581;GO:0001867;GO:0005509;GO:0051873;GO:0006508;GO:0009986;GO:0045087;GO:0006953;GO:0008228;GO:0006956;GO:0006958;GO:0006979;GO:0005615;GO:0050830;GO:0002376;GO:0005576;GO:0005515;GO:0048525;GO:0005102;GO:0005537 | hsa:4153 |  | mannose-binding lectin [Homo sapiens] |
| P23083 | 598812.4874 | 772577.5967 | 1.290182842 | 0.367575536 | 0.231380157 | no | up | GO:0038096;GO:0004252;GO:0016020;GO:0030449;GO:0050776;GO:0006898;GO:0038095;GO:0002376;GO:0050900;GO:0005576;GO:0006956;GO:0002250;GO:0003823;GO:0005886;GO:0006508;GO:0006958;GO:0006955 | hsa:102723407 | | IgM heavy chain VH1 region precursor, partial [Homo sapiens] |
| P26038 | 4686.3125 | 7080.574278 | 1.510905275 | 0.595413215 | 0.179866189 | no | up | GO:0005200;GO:0019899;GO:0030175;GO:0050900;GO:0010628;GO:0005925;GO:0061028;GO:0005737;GO:0071803;GO:0045177;GO:0042995;GO:0070062;GO:1902115;GO:0043209;GO:0005634;GO:0042098;GO:0072562;GO:0003779;GO:2000401;GO:0035722;GO:0022612;GO:0048471;GO:2000643;GO:0022614;GO:1903364;GO:0005856;GO:0005515;GO:0016324;GO:0009986;GO:0071944;GO:0031982;GO:0071394;GO:0016323;GO:0019901;GO:0008361;GO:0008360;GO:0045198;GO:0001771;GO:0005886;GO:0016020;GO:0050839;GO:0072678;GO:0005615;GO:0031528;GO:0007010;GO:0071437;GO:0005829;GO:0008092;GO:0005902;GO:0003725;GO:0016032;GO:0001931;GO:0007159;GO:0005102;GO:0070489;GO:0031143;GO:1902966 | hsa:4478 |  | moesin [Homo sapiens] |
| P61224 | 7364.3994 | 9703.622615 | 1.317639374 | 0.397955572 | 0.211758977 | no | up | GO:0005811;GO:0030033;GO:0044877;GO:0007165;GO:0061028;GO:0030054;GO:0005737;GO:0070062;GO:2000114;GO:0016020;GO:0000166;GO:0070382;GO:0045955;GO:0035722;GO:0005525;GO:0008283;GO:0003924;GO:0035577;GO:0032486;GO:0005911;GO:0005886;GO:0045121;GO:0007264;GO:0005829;GO:0071320;GO:0043312;GO:0005622;GO:0019003;GO:0005515;GO:1901888;GO:0070374;GO:2000301 | hsa:5908 | COG1100 | Ras-related protein Rap-1b, partial [Bos mutus] |
| Q13103 | 4202.4675 | 5740.203923 | 1.365912746 | 0.449865328 | 0.13211723 | no | up | GO:0044267;GO:0002576;GO:0005788;GO:0031089;GO:0001501;GO:0005576;GO:0046849;GO:0004866;GO:0010951;GO:0043687;GO:0062023 | hsa:6694 |  | secreted phosphoprotein 24 precursor [Homo sapiens] |
| Q15166 | 27697.90895 | 35382.2105 | 1.277432552 | 0.353247119 | 0.512293208 | no | up | GO:0016787;GO:0004063;GO:0070062;GO:0005615;GO:0046395;GO:0019372;GO:0009636;GO:0016311;GO:0043231;GO:0019439;GO:0046226;GO:0005576;GO:0010124;GO:0032929;GO:0102007;GO:0046872;GO:0004064;GO:0018733;GO:0042803 | hsa:5446 |  | serum paraoxonase/lactonase 3 [Homo sapiens] |
| Q6EMK4 | 8586.009118 | 11516.98463 | 1.341366456 | 0.42370343 | 0.068496617 | no | up | GO:0016020;GO:0016021;GO:0070062;GO:0010719;GO:0005615;GO:0009986;GO:0005886;GO:0045296;GO:0031012;GO:0005765;GO:0005739;GO:0071461;GO:0005515;GO:0071456;GO:0005576;GO:0030512;GO:0050431 | hsa:114990 | COG4886 | vasorin precursor [Homo sapiens] |
| Q9NZP8 | 30579.27511 | 38089.40583 | 1.245595447 | 0.316835576 | 0.306472614 | no | up | GO:0004252;GO:0005615;GO:0016787;GO:0070062;GO:0045087;GO:0031638;GO:0002376;GO:0005576;GO:0006508;GO:0006958;GO:0008233;GO:0008236 | hsa:51279 | COG5640 | complement C1r subcomponent-like protein isoform 1 precursor [Homo sapiens] |
| A0A075B6H7 | 179338.0268 | 167637.5396 | 0.934757355 | -0.097336177 | 0.543844883 | no | no change | GO:0002377;GO:0005615;GO:0006955 | hsa:7441 |  | hCG2043206, partial [Homo sapiens] |
| A0A075B6I0 | 696118.8067 | 618177.3828 | 0.888034308 | -0.171312681 | 0.542652761 | no | no change | GO:0016020;GO:0005615;GO:0006955;GO:0002376;GO:0002377;GO:0005576;GO:0002250;GO:0003823;GO:0005886 | hsa:7441 |  | RecName: Full=Immunoglobulin lambda variable 8-61; Flags: Precursor |
| A0A075B6K4 | 74879.95526 | 76207.42708 | 1.017728 | 0.025352035 | 0.921472392 | no | no change | GO:0016020;GO:0005615;GO:0006955;GO:0002376;GO:0002377;GO:0005576;GO:0002250;GO:0003823;GO:0005886 | hsa:7441 |  | immunoglobulin light chain variable region, partial [Homo sapiens] |
| A0A075B6Q5 | 61693.16944 | 54111.40905 | 0.877105351 | -0.189177956 | 0.569019714 | no | no change | GO:0009897;GO:0050853;GO:0045087;GO:0016020;GO:0006910;GO:0006911;GO:0002376;GO:0005576;GO:0002250;GO:0003823;GO:0005886;GO:0072562;GO:0042571;GO:0006958;GO:0050871;GO:0034987;GO:0042742 | hsa:102723407 | | hCG2036739, partial [Homo sapiens] |
| A0A075B6R2 | 205343.2858 | 189176.7156 | 0.92127052 | -0.118303247 | 0.656397071 | no | no change | GO:0009897;GO:0050853;GO:0045087;GO:0016020;GO:0006910;GO:0006911;GO:0002376;GO:0005576;GO:0002250;GO:0003823;GO:0005886;GO:0072562;GO:0042571;GO:0006958;GO:0050871;GO:0034987;GO:0042742 | hsa:102724971 | | immunoglobulin heavy chain VDJ region, partial [Homo sapiens] |
| A0A075B6S5 | 71694.97316 | 62380.625 | 0.870083665 | -0.200773962 | 0.222636779 | no | no change | GO:0016020;GO:0005615;GO:0006955;GO:0002376;GO:0002377;GO:0005576;GO:0002250;GO:0003823;GO:0005886 | hsa:7441 |  | monoclonal IgM antibody light chain [Homo sapiens] |
| A0A075B7B8 | 969976.1632 | 1048197.338 | 1.080642368 | 0.111889151 | 0.594344537 | no | no change | GO:0009897;GO:0050853;GO:0045087;GO:0006910;GO:0006911;GO:0042742;GO:0003823;GO:0034987;GO:0042571;GO:0006958;GO:0050871;GO:0072562 | hsa:102723407 | | hCG1793095, isoform CRA_a, partial [Homo sapiens] |
| A0A075B7D0 | 351833.2974 | 316636.0583 | 0.899960466 | -0.152066467 | 0.62302464 | no | no change | GO:0009897;GO:0050853;GO:0045087;GO:0006910;GO:0006911;GO:0042742;GO:0003823;GO:0034987;GO:0042571;GO:0006958;GO:0050871;GO:0072562 | hsa:102723407 | | hCG1728627 [Homo sapiens] |
| A0A075B7F0 | 27609.92778 | 28762.55174 | 1.041746721 | 0.05900456 | 0.836662774 | no | no change | GO:0009897;GO:0050853;GO:0045087;GO:0006910;GO:0006911;GO:0042742;GO:0003823;GO:0034987;GO:0042571;GO:0006958;GO:0050871;GO:0072562 | hsa:102723407 | | IGHV3-13 isoform 1, partial [Pan troglodytes] |
| A0A087WSY4 | 27725.29313 | 26257.91982 | 0.947074561 | -0.078450084 | 0.711384754 | no | no change | GO:0009897;GO:0050853;GO:0045087;GO:0016020;GO:0006910;GO:0006911;GO:0002376;GO:0005576;GO:0002250;GO:0003823;GO:0005886;GO:0072562;GO:0042571;GO:0006958;GO:0050871;GO:0034987;GO:0042742 | hsa:102723407 | | RecName: Full=Immunoglobulin heavy variable 4-30-2; Flags: Precursor |
| A0A087WSY6 | 277516.2737 | 253644.4483 | 0.913980449 | -0.129764791 | 0.499853544 | no | no change | GO:0016020;GO:0005615;GO:0006955;GO:0002376;GO:0002377;GO:0005576;GO:0002250;GO:0003823;GO:0005886 | hsa:29802 |  | RecName: Full=Immunoglobulin kappa variable 3D-15; Flags: Precursor |
| A0A087WSZ0 | 50574.93474 | 43621.46442 | 0.862511532 | -0.21338435 | 0.634515244 | no | no change | GO:0016020;GO:0005615;GO:0006955;GO:0002376;GO:0002377;GO:0005576;GO:0002250;GO:0003823;GO:0005886 | | | RecName: Full=Immunoglobulin kappa variable 1D-8; Flags: Precursor |
| A0A087WWT3 | 65991.80895 | 66017.89583 | 1.000395305 | 0.000570192 | 0.995956246 | no | no change | GO:0005794;GO:0005615;GO:0005783 | hsa:213 |  | ALB protein [Homo sapiens] |
| A0A087X1L8 | 2110.5175 | 2061.326993 | 0.97669268 | -0.034023411 | 0.900072094 | no | no change | GO:0016021;GO:0016020 | hsa:102723996;hsa:23308 | | ICOS ligand isoform c precursor [Homo sapiens] |
| A0A0A0MRJ7 | 20252.18995 | 20650.47167 | 1.019666106 | 0.028096813 | 0.836163509 | no | no change | GO:0048208;GO:0044267;GO:1903561;GO:0006888;GO:0005615;GO:0031093;GO:0008015;GO:0002576;GO:0007596;GO:0033116;GO:0005788;GO:0030134;GO:0046872;GO:0005576;GO:0005886;GO:0016020;GO:0005507;GO:0043687;GO:0000139 | hsa:2153 |  | coagulation factor V preproprotein [Homo sapiens] |
| A0A0A0MS15 | 242347.3158 | 206527.4629 | 0.852196205 | -0.230742467 | 0.448945983 | no | no change | GO:0009897;GO:0050853;GO:0045087;GO:0016020;GO:0006910;GO:0006911;GO:0002376;GO:0005576;GO:0002250;GO:0003823;GO:0005886;GO:0072562;GO:0042571;GO:0006958;GO:0050871;GO:0034987;GO:0042742 | hsa:102723407 | | RecName: Full=Immunoglobulin heavy variable 3-49; Flags: Precursor |
| A0A0A0MS51 | 273651.6684 | 259748.9917 | 0.949195717 | -0.075222504 | 0.492059079 | no | no change | GO:0051127;GO:0015629;GO:1902174;GO:0030155;GO:0030041;GO:0042989;GO:0045159;GO:1990000;GO:0005925;GO:1903923;GO:0001726;GO:0071801;GO:0005615;GO:0045010;GO:1903903;GO:0043209;GO:0005634;GO:1903909;GO:0030478;GO:0005509;GO:0048471;GO:1903906;GO:0097284;GO:2001269;GO:0060271;GO:0030027;GO:0006915;GO:0051016;GO:0051015;GO:0051014;GO:0045471;GO:0045335;GO:0002102;GO:0005886;GO:0014003;GO:0032991;GO:0006911;GO:0048015;GO:0051693;GO:0046597;GO:0090527;GO:0007568;GO:0051593;GO:0005829;GO:0071346;GO:0042246;GO:0031648;GO:0016528;GO:0014891;GO:0097017;GO:0071276 | hsa:2934 |  | gelsolin isoform d [Homo sapiens] |
| A0A0A0MT36 | 139614.64 | 149523.145 | 1.070970387 | 0.098918589 | 0.800940259 | no | no change | GO:0016020;GO:0005615;GO:0006955;GO:0002376;GO:0002377;GO:0005576;GO:0002250;GO:0003823;GO:0005886 | hsa:7441 |  | RecName: Full=Immunoglobulin kappa variable 6D-21; Flags: Precursor |
| A0A0B4J1U3 | 34123.82421 | 34324.28318 | 1.005874458 | 0.008450255 | 0.96768326 | no | no change | GO:0016020;GO:0005615;GO:0006955;GO:0002376;GO:0002377;GO:0005576;GO:0002250;GO:0003823;GO:0005886 | hsa:7441 |  | RecName: Full=Immunoglobulin lambda variable 1-36; Flags: Precursor |
| A0A0B4J1X5 | 67415.87611 | 66294.06174 | 0.983359789 | -0.024208732 | 0.920344129 | no | no change | GO:0009897;GO:0050853;GO:0045087;GO:0016020;GO:0006910;GO:0006911;GO:0002376;GO:0005576;GO:0002250;GO:0003823;GO:0005886;GO:0072562;GO:0042571;GO:0006958;GO:0050871;GO:0034987;GO:0042742 | hsa:102723407 | | immunoglobulin heavy chain VH3, partial [Homo sapiens] |
| A0A0B4J1X8 | 106788.1384 | 89254.54208 | 0.835809514 | -0.258753914 | 0.157626286 | no | no change | GO:0009897;GO:0050853;GO:0045087;GO:0016020;GO:0006910;GO:0006911;GO:0002376;GO:0005576;GO:0002250;GO:0003823;GO:0005886;GO:0072562;GO:0042571;GO:0006958;GO:0050871;GO:0034987;GO:0042742 | hsa:102723407 | | RecName: Full=Immunoglobulin heavy variable 3-43; Flags: Precursor |
| A0A0B4J231 | 11789687.64 | 10631262.05 | 0.90174247 | -0.149212625 | 0.486250525 | no | no change | GO:0009897;GO:0050853;GO:0070062;GO:0045087;GO:0006910;GO:0006911;GO:0042742;GO:0003823;GO:0034987;GO:0042571;GO:0006958;GO:0050871 | hsa:100423062 | | immunoglobulin lambda-3 surrogate light chain [Homo sapiens] |
| A0A0B4J2D9 | 17281.17761 | 16980.78446 | 0.982617322 | -0.025298424 | 0.894169052 | no | no change | GO:0016020;GO:0005615;GO:0006955;GO:0002376;GO:0002377;GO:0005576;GO:0002250;GO:0003823;GO:0005886 | hsa:7441 |  | RecName: Full=Immunoglobulin kappa variable 1D-13; Flags: Precursor |
| A0A0C4DGZ8 | 15921.57261 | 17803.04204 | 1.11817108 | 0.161140937 | 0.386668852 | no | no change | GO:0016021;GO:0016020 | hsa:2811 | COG4886 | glycoprotein Ib (platelet), alpha polypeptide [Homo sapiens] |
| A0A0C4DH21 | 40816.30053 | 40072.155 | 0.981768423 | -0.026545329 | 0.90515815 | no | no change |  | hsa:10877 |  | complement factor H-related protein 4 [Homo sapiens] |
| A0A0C4DH25 | 577141.8526 | 544631 | 0.943669217 | -0.083646853 | 0.59224915 | no | no change | GO:0038096;GO:0004252;GO:0016020;GO:0030449;GO:0005615;GO:0006898;GO:0038095;GO:0050900;GO:0002376;GO:0002377;GO:0050776;GO:0005576;GO:0006956;GO:0002250;GO:0003823;GO:0005886;GO:0006508;GO:0006958;GO:0006955 | hsa:29802 |  | hCG1686089, partial [Homo sapiens] |
| A0A0C4DH29 | 34843.57474 | 36525.98045 | 1.048284533 | 0.068030356 | 0.725604377 | no | no change | GO:0009897;GO:0050853;GO:0045087;GO:0016020;GO:0006910;GO:0006911;GO:0002376;GO:0005576;GO:0002250;GO:0003823;GO:0005886;GO:0072562;GO:0042571;GO:0006958;GO:0050871;GO:0034987;GO:0042742 | hsa:102723407 | | immunoglobulin heavy chain variable region, partial [Homo sapiens] |
| A0A0C4DH31 | 27515.99632 | 31057.25 | 1.128698 | 0.174659523 | 0.182685131 | no | no change | GO:0009897;GO:0050853;GO:0045087;GO:0016020;GO:0006910;GO:0006911;GO:0002376;GO:0005576;GO:0002250;GO:0003823;GO:0005886;GO:0072562;GO:0042571;GO:0006958;GO:0050871;GO:0034987;GO:0042742 | hsa:102723407 | | immunoglobulin heavy chain variable region, partial [Homo sapiens] |
| A0A0C4DH32 | 61163.57889 | 64458.94955 | 1.053877989 | 0.075707852 | 0.704964056 | no | no change | GO:0009897;GO:0050853;GO:0045087;GO:0016020;GO:0006910;GO:0006911;GO:0002376;GO:0005576;GO:0002250;GO:0003823;GO:0005886;GO:0072562;GO:0042571;GO:0006958;GO:0050871;GO:0034987;GO:0042742 | hsa:102723407 | | RecName: Full=Immunoglobulin heavy variable 3-20; Flags: Precursor |
| A0A0C4DH33 | 12642.38142 | 10923.81908 | 0.864063401 | -0.210790921 | 0.406833211 | no | no change | GO:0009897;GO:0050853;GO:0045087;GO:0016020;GO:0006910;GO:0006911;GO:0002376;GO:0005576;GO:0002250;GO:0003823;GO:0005886;GO:0072562;GO:0042571;GO:0006958;GO:0050871;GO:0034987;GO:0042742 | hsa:102723407 | | immunoglobulin heavy chain variable region, partial [Homo sapiens] |
| A0A0C4DH34 | 1442302.121 | 1456116.231 | 1.00957782 | 0.01375212 | 0.968869581 | no | no change | GO:0009897;GO:0050853;GO:0045087;GO:0016020;GO:0006910;GO:0006911;GO:0002376;GO:0005576;GO:0002250;GO:0003823;GO:0005886;GO:0072562;GO:0042571;GO:0006958;GO:0050871;GO:0034987;GO:0042742 | hsa:102723407 | | RecName: Full=Immunoglobulin heavy variable 4-28; Flags: Precursor |
| A0A0C4DH38 | 109825.7963 | 129219.8125 | 1.176588897 | 0.234610327 | 0.196940054 | no | no change | GO:0009897;GO:0050853;GO:0045087;GO:0016020;GO:0006910;GO:0006911;GO:0002376;GO:0005576;GO:0002250;GO:0003823;GO:0005886;GO:0072562;GO:0042571;GO:0006958;GO:0050871;GO:0034987;GO:0042742 | hsa:102723407 | | RecName: Full=Immunoglobulin heavy variable 5-51; Flags: Precursor |
| A0A0C4DH43 | 488918.7659 | 524811.8396 | 1.073413164 | 0.102205487 | 0.793090343 | no | no change | GO:0009897;GO:0050853;GO:0045087;GO:0016020;GO:0006910;GO:0006911;GO:0002376;GO:0005576;GO:0002250;GO:0003823;GO:0005886;GO:0072562;GO:0042571;GO:0006958;GO:0050871;GO:0034987;GO:0042742 | hsa:102723407 | | RecName: Full=Immunoglobulin heavy variable 2-70D; Flags: Precursor |
| A0A0C4DH55 | 9163152.211 | 9231465.583 | 1.007455226 | 0.010715723 | 0.946292003 | no | no change | GO:0016020;GO:0005615;GO:0006955;GO:0002376;GO:0002377;GO:0005576;GO:0002250;GO:0003823;GO:0005886 | hsa:29802 |  | RecName: Full=Immunoglobulin kappa variable 3D-7; Flags: Precursor |
| A0A0G2JL69 | 38095.56632 | 36451.96917 | 0.956855947 | -0.06362635 | 0.411828806 | no | no change | GO:0004252;GO:0016787;GO:0045087;GO:0005576;GO:0006956;GO:0006508;GO:0006958;GO:0046872;GO:0008233;GO:0008236 | hsa:717 | COG5640 | complement C2 isoform 5 [Homo sapiens] |
| A0A0G2JMB2 | 2906175.368 | 3112496.792 | 1.070994141 | 0.098950587 | 0.567341872 | no | no change | GO:0009897;GO:0050853;GO:0045087;GO:0006910;GO:0006911;GO:0042742;GO:0003823;GO:0034987;GO:0042571;GO:0006958;GO:0050871;GO:0072562 | hsa:55423 |  | Immunoglobulin heavy chain variant, partial [Homo sapiens] |
| A0A0G2JPR0 | 392835.4947 | 414208.8667 | 1.054407945 | 0.076433146 | 0.646706959 | no | no change | GO:0004866;GO:0005576;GO:0006956;GO:0006954;GO:0010951;GO:0005615 | hsa:100293534;hsa:110384692;hsa:720;hsa:721 | | complement C4A (Rodgers blood group)-like preproprotein [Homo sapiens] |
| A0A0G2JRQ6 | 294034.5047 | 296726.0333 | 1.009153785 | 0.013146043 | 0.928888818 | no | no change | GO:0002377;GO:0005615;GO:0006955 | hsa:7441 |  | hCG2042707, partial [Homo sapiens] |
| A0A0J9YVY3 | 60778.144 | 68573.47571 | 1.128258798 | 0.174098029 | 0.313737467 | no | no change | GO:0016020;GO:0002376;GO:0005576;GO:0002250;GO:0003823;GO:0005886 | hsa:102723407 | | RecName: Full=Immunoglobulin heavy variable 7-4-1; Flags: Precursor |
| A0A0J9YXX1 | 260636.5474 | 222593.1875 | 0.854036741 | -0.227629958 | 0.327335042 | no | no change | GO:0016020;GO:0002376;GO:0005576;GO:0002250;GO:0003823;GO:0005886 | hsa:102724971 | | RecName: Full=Immunoglobulin heavy variable 5-10-1; Flags: Precursor |
| A0A0J9YY99 | 187520.5705 | 222880.8396 | 1.188567414 | 0.249223733 | 0.76168765 | no | no change |  | hsa:102723407 | | immunoglobulin heavy chain VDJ region, partial [Homo sapiens] |
| A0A0S2Z4L3 | 203988.0474 | 213795.2708 | 1.048077442 | 0.067745321 | 0.645156399 | no | no change | GO:0005576;GO:0005509;GO:0030195 | hsa:5627 |  | vitamin K-dependent protein S isoform 1 precursor [Homo sapiens] |
| A0A0U1RQV3 | 27943.68526 | 28724.69542 | 1.027949433 | 0.039769297 | 0.673957225 | no | no change | GO:0062023;GO:0007173;GO:0005509;GO:0005006 | hsa:2202 |  | EGF-containing fibulin-like extracellular matrix protein 1 isoform X3 [Homo sapiens] |
| A0A140T8Y3 | 8465.4935 | 7673.41365 | 0.906434297 | -0.141725645 | 0.487570116 | no | no change | GO:0005201;GO:0062023;GO:0030199;GO:0030198;GO:0031012 | hsa:7148 |  | tenascin-X isoform 1 precursor [Homo sapiens] |
| A0A1W2PQU7 | 86400.39789 | 81805.52458 | 0.946818841 | -0.07883968 | 0.590829469 | no | no change | GO:1904714;GO:0005737;GO:0045111;GO:0061564;GO:0045109;GO:0060291;GO:0016020;GO:0044297;GO:0060020;GO:0005198;GO:0051580;GO:0005883;GO:0005882;GO:0010977;GO:0030198;GO:0014002;GO:0097450;GO:0010625;GO:0031102 | hsa:2670 |  | glial fibrillary acidic protein [Mus musculus] |
| A0A286YEY1 | 5144417.211 | 5983712.083 | 1.163146735 | 0.21803311 | 0.318088067 | no | no change |  |  |  | IGHA1 isoform 1, partial [Pan troglodytes] |
| A0A286YEY4 | 644354.6316 | 577230.3292 | 0.895827082 | -0.158707814 | 0.284027824 | no | no change | GO:0016021;GO:0016020 | hsa:100423062 | | unnamed protein product [Homo sapiens] |
| A0A2R8Y7X9 | 29304.87822 | 29958.3093 | 1.02229769 | 0.031815366 | 0.912206939 | no | no change | GO:0005344;GO:0019825;GO:0020037;GO:0005833;GO:0046872;GO:0015671 | hsa:3048 | COG1018 | hemoglobin subunit gamma-2 [Pan troglodytes] |
| A0A3B3ISR2 | 305276.5737 | 264784.65 | 0.867359872 | -0.205297395 | 0.064029683 | no | no change | GO:0004252;GO:0005615;GO:0045087;GO:0031638;GO:0005509;GO:0006958 | hsa:715 | COG5640 | complement C1r subcomponent isoform 1 preproprotein [Homo sapiens] |
| A6XND0 | 33136.64211 | 32510.07375 | 0.981091375 | -0.027540586 | 0.86829965 | no | no change | GO:0005520;GO:0001558;GO:0005576 | hsa:3486 |  | insulin-like growth factor binding protein 3 [Homo sapiens] |
| B1AHL2 | 16650.34933 | 16703.32688 | 1.003181768 | 0.004583033 | 0.977855876 | no | no change | GO:0016504;GO:0005576;GO:0005509;GO:0010952;GO:0030198 | hsa:2192 |  | FBLN1 isoform 5 [Pongo abelii] |
| B4E1Z4 | 536278.9316 | 531061.825 | 0.990271655 | -0.01410375 | 0.883637262 | no | no change | GO:0004252;GO:0030449;GO:0070062;GO:0016787;GO:0072562;GO:0001848;GO:0005576;GO:0006956;GO:0006957;GO:0005886;GO:0006508;GO:0008233;GO:0008236;GO:0005615 | hsa:629 | COG5640 | unnamed protein product [Homo sapiens] |
| B7ZKJ8 | 523845.2211 | 489822.0417 | 0.935051084 | -0.09688291 | 0.164322615 | no | no change | GO:0005737;GO:0030212;GO:0034097;GO:0004867;GO:0006953;GO:0005886;GO:0010951 | hsa:3700 | COG2304 | ITIH4 protein [Homo sapiens] |
| C9JB55 | 14991.58368 | 14073.08717 | 0.938732522 | -0.091213953 | 0.466823902 | no | no change | GO:0055037;GO:1990459;GO:0030139;GO:0005905;GO:0034986;GO:1990712;GO:0004857;GO:0048260;GO:0005615;GO:0005770;GO:0043086;GO:0009925;GO:0048471;GO:0006879;GO:0016324;GO:0008198;GO:0008199;GO:0015091;GO:0031232;GO:0034756;GO:0071281;GO:0005623;GO:0005769 | hsa:7018 |  | serotransferrin isoform 1 precursor [Homo sapiens] |
| C9JC84 | 4677622.579 | 5372966.625 | 1.148653303 | 0.199943416 | 0.062601531 | no | no change | GO:0051258;GO:0005102;GO:0007596;GO:0005577;GO:0030168 | hsa:2266 |  | FGG isoform 6 [Pan troglodytes] |
| C9JF17 | 825317.6895 | 820149.85 | 0.993738363 | -0.009062033 | 0.945509777 | no | no change | GO:0022626;GO:0005783;GO:0006869;GO:0042493;GO:0048678;GO:0030425;GO:0000302;GO:0001525;GO:0051895;GO:0010642;GO:0005737;GO:0070062;GO:0014012;GO:0005615;GO:2000405;GO:0048471;GO:0043025;GO:0005319;GO:0048662;GO:0006629;GO:2000098;GO:0008289;GO:0042308;GO:0060588;GO:0007420;GO:1900016;GO:0007568;GO:0015485;GO:0042246;GO:0071638;GO:0005576;GO:0006006 | hsa:347 | COG3040 | APOD isoform 3, partial [Pan troglodytes] |
| C9JV77 | 2167220.737 | 2296220.083 | 1.059522938 | 0.083414822 | 0.309432384 | no | no change | GO:0019210;GO:0006907;GO:0005788;GO:0001501;GO:0030500;GO:0030502;GO:0050766;GO:0044267;GO:0070062;GO:0031093;GO:0002576;GO:0050727;GO:0072562;GO:0005794;GO:0043687;GO:0046627;GO:0006953;GO:0062023;GO:0034774;GO:0010951;GO:0004869;GO:0005615;GO:0043312;GO:0005576 | hsa:197 |  | alpha-2-HS-glycoprotein isoform 1 preproprotein [Homo sapiens] |
| D6R934 | 107963.7168 | 119813.6633 | 1.109758601 | 0.15024589 | 0.058571376 | no | no change | GO:0006958;GO:0005576;GO:0005581 | hsa:713 |  | complement C1q subcomponent subunit B precursor [Homo sapiens] |
| D6RAR4 | 25599.32632 | 27468.56833 | 1.073019188 | 0.101675875 | 0.361339755 | no | no change | GO:0005737;GO:0004252;GO:0005791;GO:0005615;GO:0016787;GO:0005576;GO:0006508;GO:0008233;GO:0008236 | hsa:3083 | COG5640 | hepatocyte growth factor activator isoform 1 preproprotein [Homo sapiens] |
| D6RD17 | 2656375.211 | 2585204.783 | 0.97320769 | -0.039180374 | 0.876861605 | no | no change |  | hsa:3512 |  | JCHAIN isoform 4, partial [Pongo abelii] |
| D6RF35 | 2707468.579 | 2574258.708 | 0.950799107 | -0.072787546 | 0.352752436 | no | no change | GO:0051180;GO:0090482;GO:0035461;GO:0005615;GO:0005499 | hsa:2638 |  | GC isoform 4 [Pan troglodytes] |
| E7END6 | 20400.11526 | 18291.93458 | 0.896658394 | -0.157369639 | 0.091483469 | no | no change | GO:0004252;GO:0016787;GO:0007596;GO:0005576;GO:0005509;GO:0006508;GO:0008233;GO:0008236 | hsa:5624 | COG5640 | vitamin K-dependent protein C isoform X5 [Homo sapiens] |
| E7EUT5 | 7822.728632 | 9330.010583 | 1.192679821 | 0.254206798 | 0.173007723 | no | no change | GO:0051287;GO:0050821;GO:0000226;GO:0097718;GO:0006096;GO:0061844;GO:0005737;GO:0004365;GO:0005634;GO:0005811;GO:0016620;GO:0051873;GO:0097452;GO:0042802;GO:0035605;GO:0035606;GO:0015630;GO:0008017;GO:0031965;GO:0050661;GO:0050832;GO:0051402;GO:0052501;GO:0005886;GO:0055114;GO:0043231;GO:0050715;GO:0005829;GO:0071346;GO:1990904;GO:0019828;GO:0017148;GO:0006417;GO:0006006 | hsa:2597 | COG0057 | GAPDH isoform 4 [Pan troglodytes] |
| E9PAQ1 | 27298.39105 | 27762.39167 | 1.016997361 | 0.024315936 | 0.80419617 | no | no change |  | hsa:5199 |  | properdin precursor [Homo sapiens] |
| E9PHK0 | 150859.3505 | 142086.1017 | 0.941844845 | -0.086438678 | 0.288202905 | no | no change | GO:0005737;GO:0036143;GO:0070062;GO:0002576;GO:0001652;GO:0030246;GO:0008201;GO:0071560;GO:0071310;GO:0001503;GO:0005615;GO:0005509;GO:0005576;GO:0031089;GO:0030282;GO:0062023;GO:0010756 | hsa:7123 |  | tetranectin isoform 1precursor [Homo sapiens] |
| F5H8B0 | 5957.909684 | 6187.351708 | 1.03851049 | 0.05451579 | 0.699136087 | no | no change | GO:0004252;GO:0016787;GO:0007596;GO:0005576;GO:0005509;GO:0006508;GO:0008233;GO:0008236 | hsa:2155 | COG5640 | coagulation factor VII isoform c precursor [Homo sapiens] |
| F8WF14 | 25332.67737 | 26920.89083 | 1.06269426 | 0.08772659 | 0.478182782 | no | no change | GO:0019899;GO:0051384;GO:0005783;GO:0016787;GO:0007612;GO:0050805;GO:0050783;GO:0014016;GO:0001540;GO:0016021;GO:0016020;GO:0072562;GO:0042802;GO:0008285;GO:0004104;GO:0003824;GO:0005788;GO:0043279;GO:0051593;GO:0019695;GO:0005641;GO:0005576;GO:0033265;GO:0003990 | hsa:590 | COG2272 | unnamed protein product [Homo sapiens] |
| G3V0E5 | 5320.957789 | 5906.334542 | 1.110013418 | 0.150577116 | 0.395544582 | no | no change | GO:0009897;GO:0055037;GO:0035690;GO:0005905;GO:1990712;GO:0010008;GO:0045780;GO:1990830;GO:0070062;GO:0016021;GO:0045830;GO:0004998;GO:0048471;GO:0042803;GO:0006879;GO:0030890;GO:0030316;GO:0042102;GO:0005887;GO:0005886;GO:0016323;GO:0042470;GO:0031623;GO:0006898;GO:0003725;GO:0033570;GO:0033572;GO:0005769 | hsa:7037 | COG2234 | transferrin receptor variant, partial [Homo sapiens] |
| G3V2W1 | 10439.47474 | 10282.13542 | 0.984928426 | -0.021909207 | 0.903128103 | no | no change | GO:0010951;GO:0007596;GO:0005615;GO:0004867 | hsa:51156 | COG4826 | protein Z-dependent protease inhibitor isoform X1 [Homo sapiens] |
| G3XAK1 | 17478.04947 | 18147.16042 | 1.03828293 | 0.054199629 | 0.586178933 | no | no change | GO:0004252;GO:2000479;GO:0019899;GO:0010628;GO:0005737;GO:0005615;GO:0005773;GO:0033601;GO:0030971;GO:1904036;GO:0006508;GO:0045721;GO:0046425;GO:0030317;GO:0071456;GO:0007283;GO:0062023;GO:0060763;GO:0048012;GO:0007566;GO:0030879;GO:0005576;GO:0010758 | hsa:4485 | COG5640 | hepatocyte growth factor-like protein precursor [Homo sapiens] |
| G3XAP6 | 9133.701 | 9080.6428 | 0.994190942 | -0.008405136 | 0.959056456 | no | no change | GO:0005201;GO:0060173;GO:0062023;GO:0030509;GO:0035264;GO:0050905;GO:0030500;GO:0030282;GO:0050881;GO:0010259;GO:1900047;GO:0005615;GO:0048844;GO:0036122;GO:0030198;GO:0005509;GO:0002020;GO:0002063;GO:0003417;GO:0043588;GO:0005576;GO:0014829;GO:0005178;GO:0009887;GO:1902732;GO:0009306;GO:0006915;GO:0043066;GO:0048747;GO:0006986;GO:0043395;GO:0035988;GO:0007155;GO:0097084;GO:0016485;GO:0031012;GO:0008201;GO:0070062;GO:0010260;GO:0032991;GO:0001501;GO:0035989;GO:0030199;GO:0043394;GO:0005518;GO:0070527 | hsa:1311 |  | unnamed protein product [Homo sapiens] |
| H0Y5E4 | 19693.86579 | 20060.71042 | 1.018627355 | 0.026626366 | 0.738521627 | no | no change | GO:0005540;GO:0007155;GO:0016021;GO:0016020 | hsa:960 |  | CD44 antigen isoform 8 precursor [Homo sapiens] |
| H0Y755 | 7334.283579 | 7651.50013 | 1.043251198 | 0.061086577 | 0.739652865 | no | no change | GO:0016021;GO:0016020 | hsa:2214 |  | low affinity immunoglobulin gamma Fc region receptor III-A isoform b [Homo sapiens] |
| H0YAC1 | 155518.0774 | 151327.6013 | 0.973054733 | -0.039407138 | 0.688362843 | no | no change | GO:0004252;GO:0004497;GO:0007597;GO:0008236;GO:0008233;GO:0016491;GO:0070062;GO:0016705;GO:0031639;GO:0031638;GO:0006508;GO:0005506;GO:0046872;GO:0022617;GO:0051919;GO:0016787;GO:0020037;GO:0005886;GO:0055114;GO:0005615;GO:0042730;GO:0002542;GO:0005576 | hsa:3818 | COG5640 | KLKB1 isoform 4, partial [Pan troglodytes] |
| H0YJW9 | 796192.4947 | 782399.1917 | 0.982675919 | -0.025212392 | 0.834836496 | no | no change |  | hsa:7448 |  | vitronectin, partial [Homo sapiens] |
| H3BUA5 | 401534.5821 | 426551.9625 | 1.062304423 | 0.087197256 | 0.647958971 | no | no change |  | hsa:10326 |  | LOW QUALITY PROTEIN: T0061165 isoform 1, partial [Pan troglodytes] |
| I3L145 | 12792.186 | 10979.04358 | 0.858261722 | -0.220510439 | 0.587502341 | no | no change | GO:0005496 | hsa:6462 |  | SHBG protein, partial [Homo sapiens] |
| J3KNB4 | 15955.17689 | 13648.52625 | 0.855429328 | -0.225279425 | 0.372485734 | no | no change | GO:0071224;GO:0071222;GO:0042742;GO:0050829;GO:0044130;GO:0061844;GO:0005737;GO:0042995;GO:0005615;GO:0071354;GO:0071356;GO:0016021;GO:0045766;GO:0051873;GO:0008284;GO:0045087;GO:0006952;GO:0050830;GO:0044140;GO:0001530;GO:0042581;GO:0071347;GO:0005576;GO:0001934 | hsa:820 |  | cathelicidin antimicrobial peptide [Homo sapiens] |
| J3KPA1 | 11335.57081 | 11406.30075 | 1.006239645 | 0.008973937 | 0.940710547 | no | no change | GO:0016020;GO:0016021;GO:0005576 | hsa:10321 | COG2340 | cysteine-rich secretory protein 3 isoform 3 [Homo sapiens] |
| J3KRP0 | 26790.15789 | 26132.74042 | 0.975460485 | -0.035844663 | 0.83180753 | no | no change | GO:0016787;GO:0032268;GO:0005829;GO:0004180;GO:0016805;GO:0005576;GO:0008152;GO:0006508;GO:0046872;GO:0008237 | hsa:84735 | COG0624 | Carnosine dipeptidase 1 (metallopeptidase M20 family) [Homo sapiens] |
| J3QRV5 | 389926.1947 | 384036.1792 | 0.984894537 | -0.021958846 | 0.896331476 | no | no change | GO:0005737;GO:0043231;GO:0005829;GO:0006887;GO:0007049;GO:0051301 | hsa:3993 |  | lethal(2) giant larvae protein homolog 2 isoform X4 [Homo sapiens] |
| K7ER74 | 395514.3247 | 428761.3892 | 1.084060329 | 0.116445047 | 0.751729146 | no | no change | GO:0034375;GO:0034372;GO:0034371;GO:0034370;GO:0042627;GO:0034378;GO:0051006;GO:0010902;GO:0008047;GO:0048261;GO:0001523;GO:0043085;GO:0055102;GO:0005615;GO:0032375;GO:0005576;GO:0016042;GO:0045833;GO:0016004;GO:0010898;GO:0033700;GO:0034361;GO:0034362;GO:0034363;GO:0070328;GO:0006629;GO:0034366;GO:0060230;GO:0008289;GO:0006869;GO:0042803;GO:0010916;GO:0043274;GO:0042632;GO:0043691;GO:0034382;GO:0042493;GO:0034384;GO:0010518;GO:0033344;GO:0042953;GO:0060697;GO:0045723;GO:0005769 | hsa:344 |  | apolipoprotein C-II isoform X1 [Mesocricetus auratus] |
| K7ERI9 | 2741538.053 | 2494084.083 | 0.909738999 | -0.136475395 | 0.317930261 | no | no change | GO:0005576;GO:0042157 | hsa:341 |  | apolipoprotein C-I precursor [Homo sapiens] |
| M0R0Q9 | 6398.391789 | 6022.777783 | 0.94129556 | -0.087280305 | 0.323868917 | no | no change | GO:0006631;GO:0030449;GO:0004866;GO:0005886;GO:0005788;GO:0035578;GO:0007165;GO:0031715;GO:0048260;GO:0010828;GO:0010866;GO:0044267;GO:0070062;GO:0009617;GO:0045766;GO:1905114;GO:0045745;GO:0097242;GO:2000427;GO:0034774;GO:0007186;GO:0009986;GO:0005576;GO:0150064;GO:0043687;GO:0016322;GO:0150062;GO:0006956;GO:0006957;GO:0006954;GO:0006955;GO:0060100;GO:0006958;GO:0005615;GO:0050776;GO:0032991;GO:0010575;GO:0043312;GO:0001798;GO:0010884;GO:0001970;GO:0097278;GO:0005102;GO:0001934;GO:0072562 | hsa:718 |  | C3 isoform 6, partial [Pan troglodytes] |
| O00187 | 36016.13684 | 33615.62133 | 0.93334889 | -0.099511628 | 0.582492312 | no | no change | GO:0045087;GO:0004252;GO:0046872;GO:0006956;GO:0005615;GO:0048306;GO:0070062;GO:0016787;GO:0002376;GO:0005576;GO:0001867;GO:0005509;GO:0005515;GO:0006508;GO:0006958;GO:0008236;GO:0008233;GO:0001855 | hsa:10747 | COG5640 | mannan-binding lectin serine protease 2 isoform 1 preproprotein [Homo sapiens] |
| O00391 | 11190.81111 | 10590.16629 | 0.946326963 | -0.079589363 | 0.643376257 | no | no change | GO:0005788;GO:0030173;GO:0071949;GO:0016491;GO:0016971;GO:0044267;GO:0016972;GO:0045171;GO:0070062;GO:0031093;GO:0016021;GO:0016020;GO:0003756;GO:0035580;GO:0016242;GO:0005794;GO:1904724;GO:0043687;GO:0045454;GO:0055114;GO:0005615;GO:0043231;GO:0043312;GO:0000139;GO:0005576;GO:0085029;GO:0002576 | hsa:5768 |  | sulfhydryl oxidase 1 isoform a precursor [Homo sapiens] |
| O14791 | 38175.58632 | 36310.42333 | 0.951142519 | -0.072266564 | 0.65158566 | no | no change | GO:0006869;GO:0005788;GO:0042157;GO:0031224;GO:0019835;GO:0044267;GO:0005615;GO:0072562;GO:0005254;GO:0045087;GO:0034361;GO:0034364;GO:0006629;GO:1902476;GO:0043687;GO:0008289;GO:0008202;GO:0008203;GO:0006898;GO:0031640;GO:0005576;GO:0005515 | hsa:8542 |  | apolipoprotein L1 isoform a precursor [Homo sapiens] |
| O43866 | 149405.1953 | 150706.8717 | 1.00871239 | 0.012514884 | 0.951376206 | no | no change | GO:0005737;GO:0006898;GO:0005615;GO:0005044;GO:0009986;GO:0016020;GO:0072562;GO:0002376;GO:0005576;GO:0006954;GO:0006968;GO:0006915 | hsa:922 |  | CD5 antigen-like isoform 1 precursor [Homo sapiens] |
| O75882 | 46564.93 | 49620.2125 | 1.065613381 | 0.091684104 | 0.366681788 | no | no change | GO:0005737;GO:0043473;GO:0042552;GO:0005615;GO:0070062;GO:0016021;GO:0016020;GO:0030246;GO:0006954;GO:0005576;GO:0005887;GO:0005886;GO:0038023;GO:0021549;GO:0006979;GO:0040014 | hsa:8455 |  | attractin isoform 1 preproprotein [Homo sapiens] |
| O95445 | 64039.99368 | 59763.54667 | 0.933222245 | -0.099707398 | 0.329882394 | no | no change | GO:0034375;GO:0034445;GO:0006869;GO:0043691;GO:0042157;GO:0001523;GO:0005615;GO:0005543;GO:0098869;GO:0005576;GO:0005319;GO:0034361;GO:0034362;GO:0034364;GO:0034365;GO:0034366;GO:0016209;GO:0042632;GO:0034380;GO:0034384;GO:0009749;GO:0033344 | hsa:55937 |  | apolipoprotein M isoform 1 [Homo sapiens] |
| P00450 | 659652.3579 | 625856.0042 | 0.948766417 | -0.07587515 | 0.456451847 | no | no change | GO:0016491;GO:0044267;GO:0046872;GO:0006879;GO:0005615;GO:0006825;GO:0004322;GO:0006811;GO:0051087;GO:0070062;GO:0005623;GO:0005765;GO:0055072;GO:0006826;GO:0005788;GO:0005886;GO:0072562;GO:0005507;GO:0043687;GO:0055114;GO:0005576 | hsa:1356 | COG2132 | RecName: Full=Ceruloplasmin; AltName: Full=Ferroxidase; Flags: Precursor |
| P00488 | 23771.56053 | 24800.94633 | 1.043303249 | 0.061158556 | 0.776024004 | no | no change | GO:0005737;GO:0018149;GO:0007599;GO:0019221;GO:0031093;GO:0003810;GO:0072378;GO:0002576;GO:0007596;GO:0072562;GO:0016740;GO:0062023;GO:0016746;GO:0046872;GO:0005576 | hsa:2162 |  | RecName: Full=Coagulation factor XIII A chain; Short=Coagulation factor XIIIa; AltName: Full=Protein-glutamine gamma-glutamyltransferase A chain; AltName: Full=Transglutaminase A chain; Flags: Precursor |
| P00734 | 557441.9474 | 579945.2667 | 1.040368902 | 0.057095181 | 0.369891554 | no | no change | GO:0004252;GO:0048712;GO:0009897;GO:0030307;GO:0007597;GO:0007596;GO:0005788;GO:0051281;GO:0030449;GO:0032967;GO:0008047;GO:0007166;GO:0007599;GO:0008233;GO:0007275;GO:0061844;GO:0044267;GO:0006888;GO:0005615;GO:0005102;GO:0009611;GO:0070062;GO:0001530;GO:0051838;GO:0010544;GO:0005509;GO:0006508;GO:0008236;GO:0007186;GO:0010468;GO:1900738;GO:0008284;GO:0016787;GO:2000379;GO:0046427;GO:0090218;GO:0070945;GO:0006953;GO:0005796;GO:0008360;GO:0014068;GO:0005886;GO:0008201;GO:0030168;GO:0008083;GO:1900016;GO:0042730;GO:1900182;GO:0051480;GO:0010469;GO:0070053;GO:0005576;GO:0051918;GO:0005515;GO:0072378;GO:0030193;GO:0045861;GO:0072562;GO:0001934;GO:0030194 | hsa:2147 | COG5640 | prothrombin isoform 1 preproprotein [Homo sapiens] |
| P00740 | 36940.94105 | 34206.12833 | 0.925967974 | -0.110965799 | 0.05712521 | no | no change | GO:0004252;GO:0004175;GO:0006888;GO:0005615;GO:0016787;GO:0070062;GO:0007597;GO:0007596;GO:0031638;GO:0005788;GO:0005796;GO:0005576;GO:0005509;GO:0005515;GO:0005886;GO:0006508;GO:0008233;GO:0046872;GO:0007599;GO:0008236 | hsa:2158 | COG5640 | coagulation factor IX isoform 1 preproprotein [Homo sapiens] |
| P00742 | 84047.87211 | 85201.01083 | 1.013720023 | 0.019659253 | 0.843502707 | no | no change | GO:0004252;GO:0030335;GO:0005543;GO:0006888;GO:0005615;GO:0016787;GO:0007596;GO:0005788;GO:0005796;GO:0005576;GO:0005509;GO:0005515;GO:0005886;GO:0006508;GO:0008233;GO:0008236;GO:0051897;GO:0031233;GO:0007599;GO:0007598 | hsa:2159 | COG5640 | coagulation factor X isoform 1 preproprotein [Homo sapiens] |
| P00747 | 1155272.695 | 1153996.371 | 0.998895218 | -0.001594744 | 0.981725311 | no | no change | GO:0004252;GO:0004175;GO:0007599;GO:0016787;GO:1904854;GO:0048771;GO:0019899;GO:0008233;GO:0008236;GO:0044267;GO:0070062;GO:0043536;GO:0052182;GO:0051087;GO:0072562;GO:1990405;GO:0006508;GO:0022617;GO:0009986;GO:0051918;GO:0051919;GO:0008285;GO:0007596;GO:0010812;GO:0019900;GO:0062023;GO:0019904;GO:0005886;GO:0051702;GO:0031232;GO:0034185;GO:0005615;GO:0042730;GO:0031093;GO:0052213;GO:2000048;GO:0044218;GO:0005576;GO:0005515;GO:0005102;GO:0002576 | hsa:5340 | COG5640 | plasminogen isoform 1 precursor [Homo sapiens] |
| P00748 | 60677.42474 | 55029.87542 | 0.906925033 | -0.140944794 | 0.42675655 | no | no change | GO:0004252;GO:0002542;GO:0007599;GO:0007597;GO:0007596;GO:0008233;GO:0008236;GO:0070062;GO:0031638;GO:0005509;GO:0006508;GO:0051919;GO:0016787;GO:0005791;GO:0045087;GO:0062023;GO:0005886;GO:0016485;GO:0005615;GO:0042730;GO:0016540;GO:0002353;GO:0005576;GO:0051787;GO:0010756;GO:0005515;GO:0030193;GO:0051788;GO:0030194 | hsa:2161 | COG5640 | coagulation factor XII preproprotein [Homo sapiens] |
| P01009 | 5372299.105 | 4974463.417 | 0.925946847 | -0.110998716 | 0.200123045 | no | no change | GO:0048208;GO:0007599;GO:0005783;GO:0007596;GO:0033116;GO:0005788;GO:1904813;GO:0030134;GO:0044267;GO:0006888;GO:0005615;GO:0031093;GO:0002576;GO:0010466;GO:0002020;GO:0042802;GO:0005794;GO:0043687;GO:0006953;GO:0030414;GO:0004867;GO:0010951;GO:0070062;GO:0043231;GO:0043312;GO:0000139;GO:0005576;GO:0005515 | hsa:5265 | COG4826 | alpha-1-antitrypsin precursor [Homo sapiens] |
| P01011 | 718008.5 | 729900.2542 | 1.016562136 | 0.0236984 | 0.770315548 | no | no change | GO:0006954;GO:0034774;GO:0035578;GO:0003677;GO:0070062;GO:0031093;GO:0002576;GO:0072562;GO:0010466;GO:0019216;GO:0006953;GO:0062023;GO:0030277;GO:0030414;GO:0004867;GO:0010951;GO:0005615;GO:0043312;GO:0005622;GO:0005576;GO:0005515;GO:0005634 | hsa:12 | COG4826 | serpin peptidase inhibitor, clade A (alpha-1 antiproteinase, antitrypsin), member 3, isoform CRA_b [Homo sapiens] |
| P01023 | 8232157.474 | 8692043.75 | 1.055864611 | 0.078424856 | 0.606943635 | no | no change | GO:0051056;GO:0030414;GO:0019959;GO:0007597;GO:0019899;GO:0072562;GO:0048863;GO:0005615;GO:0031093;GO:0002576;GO:0048306;GO:0019838;GO:0070062;GO:0010466;GO:0002020;GO:0001869;GO:0022617;GO:0019966;GO:0005096;GO:0004866;GO:0004867;GO:0010951;GO:0043547;GO:0043120;GO:0005829;GO:0005576;GO:0005515;GO:0005102 | hsa:2 | COG2373 | alpha-2-macroglobulin isoform a precursor [Homo sapiens] |
| P01024 | 7798733.316 | 7993185.208 | 1.024933779 | 0.0355307 | 0.641484401 | no | no change | GO:0045087;GO:0004252;GO:0006631;GO:0030449;GO:0004866;GO:0005886;GO:0005788;GO:0035578;GO:0007165;GO:0031715;GO:0048260;GO:0010828;GO:0010866;GO:0050766;GO:0044267;GO:0070062;GO:0009617;GO:0045766;GO:1905114;GO:0045745;GO:0097242;GO:2000427;GO:0034774;GO:0007186;GO:0009986;GO:0005576;GO:0006629;GO:0150064;GO:0043687;GO:0016322;GO:0006911;GO:0150062;GO:0006956;GO:0006957;GO:0006954;GO:0006955;GO:0010951;GO:0060100;GO:0006958;GO:0006508;GO:0005615;GO:0050776;GO:0032991;GO:0010575;GO:0043312;GO:0002376;GO:0001798;GO:0010884;GO:0001970;GO:0005515;GO:0097278;GO:0005102;GO:0001934;GO:0072562 | hsa:718 |  | complement C3 preproprotein [Homo sapiens] |
| P01031 | 207439.2947 | 217947.0667 | 1.050654684 | 0.07128858 | 0.352392828 | no | no change | GO:0000187;GO:0030449;GO:0006954;GO:0008009;GO:0007166;GO:0060326;GO:0019835;GO:0001701;GO:0005615;GO:0045766;GO:0010760;GO:0007186;GO:0045087;GO:0006935;GO:0090197;GO:0006956;GO:0006957;GO:0004866;GO:0010951;GO:0006958;GO:0070062;GO:0010575;GO:0002376;GO:0005576;GO:0005515;GO:0005102;GO:0005579 | hsa:727 |  | complement C5 isoform 1 preproprotein [Homo sapiens] |
| P01034 | 12066.96882 | 11225.5915 | 0.930274343 | -0.104271857 | 0.394756469 | no | no change | GO:0005783;GO:0060009;GO:0048678;GO:0005788;GO:0008584;GO:0042747;GO:0030414;GO:0070301;GO:0001666;GO:0014070;GO:1904724;GO:0005737;GO:0044267;GO:0032355;GO:0042995;GO:0001540;GO:0005615;GO:0007431;GO:2000117;GO:0005771;GO:0009636;GO:0070062;GO:0043067;GO:0010466;GO:0002020;GO:0030424;GO:0048471;GO:0043025;GO:0042802;GO:0008284;GO:0034103;GO:0045740;GO:0060548;GO:0043687;GO:0006915;GO:0031965;GO:0031982;GO:0006952;GO:0001775;GO:0001654;GO:0060311;GO:0004866;GO:0060313;GO:0004869;GO:0034599;GO:0010035;GO:0006979;GO:0009743;GO:1904813;GO:0005604;GO:0007420;GO:0010716;GO:0007566;GO:0042493;GO:0031667;GO:0010711;GO:0005764;GO:0005576;GO:0005515;GO:0043312;GO:0043292;GO:0045861;GO:0097435 | hsa:1471 |  | cystatin-C precursor [Homo sapiens] |
| P01042 | 1274189.979 | 1178250.821 | 0.924705766 | -0.11293371 | 0.080781225 | no | no change | GO:0007599;GO:0030414;GO:0007597;GO:0007596;GO:0045861;GO:0005788;GO:0007162;GO:0008270;GO:0042311;GO:0044267;GO:0050880;GO:0005615;GO:0031093;GO:0002576;GO:0072562;GO:0043065;GO:0010466;GO:0007186;GO:0043687;GO:0062023;GO:0006954;GO:0005886;GO:0010951;GO:0004869;GO:0008201;GO:0070062;GO:0007204;GO:0005576;GO:0005515;GO:0005102;GO:0030195 | hsa:3827 |  | kininogen-1 isoform 1 precursor [Homo sapiens] |
| P01344 | 50416.95737 | 43611.33875 | 0.865013302 | -0.209205777 | 0.066247477 | no | no change | GO:0046628;GO:0045840;GO:0031017;GO:0051146;GO:0008286;GO:0038028;GO:0008284;GO:0001501;GO:0001503;GO:0051147;GO:0031056;GO:0071902;GO:0051897;GO:0007275;GO:0001649;GO:0044267;GO:0006349;GO:0001701;GO:0008083;GO:0031093;GO:0000122;GO:0002576;GO:0043085;GO:0045725;GO:0010469;GO:0005179;GO:0005178;GO:0009887;GO:2000467;GO:0042104;GO:0005159;GO:0005158;GO:0043410;GO:0048018;GO:0060669;GO:0001892;GO:0043539;GO:0005615;GO:0005975;GO:0050731;GO:0040018;GO:0006355;GO:0051781;GO:0005576;GO:0045944;GO:0005515;GO:0006006;GO:0001934 | hsa:3481 |  | insulin-like growth factor II isoform 2 [Homo sapiens] |
| P01597 | 43764.09368 | 42113.01583 | 0.962273231 | -0.055481499 | 0.648299772 | no | no change | GO:0038096;GO:0004252;GO:0016020;GO:0030449;GO:0050776;GO:0006898;GO:0038095;GO:0072562;GO:0002376;GO:0050900;GO:0005576;GO:0006956;GO:0002250;GO:0003823;GO:0005886;GO:0006508;GO:0006958;GO:0006955;GO:0070062 | hsa:7441 |  | immunoglobulin kappa light chain VC region, partial [Homo sapiens] |
| P01601 | 102837.5474 | 98663.38208 | 0.959410105 | -0.059780461 | 0.709977104 | no | no change | GO:0038096;GO:0004252;GO:0016020;GO:0030449;GO:0005615;GO:0006898;GO:0038095;GO:0050900;GO:0002376;GO:0002377;GO:0050776;GO:0005576;GO:0006956;GO:0002250;GO:0003823;GO:0005886;GO:0006508;GO:0006958;GO:0006955 | hsa:7441 |  | RecName: Full=Immunoglobulin kappa variable 1D-16; AltName: Full=Ig kappa chain V-I region HK146; AltName: Full=Ig kappa chain V-I region HK189; Flags: Precursor |
| P01619 | 3928846.211 | 3456730.083 | 0.879833391 | -0.18469774 | 0.426086524 | no | no change | GO:0004252;GO:0030449;GO:0019731;GO:0006955;GO:0050900;GO:0005615;GO:0016020;GO:0003094;GO:0072562;GO:0071748;GO:0006508;GO:0050776;GO:0006956;GO:0003823;GO:0005886;GO:0006958;GO:0070062;GO:0038096;GO:0038095;GO:0006898;GO:0002376;GO:0005576;GO:0002250;GO:0071751;GO:0071756 | hsa:29802 |  | immunoglobulin light chain variable region, partial [Homo sapiens] |
| P01699 | 176825.7089 | 152317.1246 | 0.86139694 | -0.215249896 | 0.091798623 | no | no change | GO:0038096;GO:0004252;GO:0016020;GO:0030449;GO:0050776;GO:0006898;GO:0038095;GO:0002376;GO:0050900;GO:0005576;GO:0006956;GO:0002250;GO:0003823;GO:0005886;GO:0006508;GO:0006958;GO:0006955 | hsa:7441 |  | hCG2043214, partial [Homo sapiens] |
| P01700 | 1429804.511 | 1497228.204 | 1.047155883 | 0.066476222 | 0.672078123 | no | no change | GO:0038096;GO:0004252;GO:0016020;GO:0030449;GO:0050776;GO:0006898;GO:0038095;GO:0072562;GO:0002376;GO:0050900;GO:0005576;GO:0006956;GO:0002250;GO:0003823;GO:0005886;GO:0006508;GO:0006958;GO:0006955 | hsa:7441 |  | RecName: Full=Immunoglobulin lambda variable 1-47; AltName: Full=Ig lambda chain V-I region HA; AltName: Full=Ig lambda chain V-I region WAH; Flags: Precursor |
| P01701 | 516602.5105 | 462470.4417 | 0.895215242 | -0.159693494 | 0.425677205 | no | no change | GO:0038096;GO:0004252;GO:0016020;GO:0030449;GO:0050776;GO:0006898;GO:0038095;GO:0050900;GO:0005576;GO:0006956;GO:0003823;GO:0005886;GO:0006508;GO:0006958;GO:0006955;GO:0070062 | hsa:7441 |  | RecName: Full=Immunoglobulin lambda variable 1-51; AltName: Full=Ig lambda chain V-I region BL2; AltName: Full=Ig lambda chain V-I region EPS; AltName: Full=Ig lambda chain V-I region NEW; AltName: Full=Ig lambda chain V-I region NIG-64; Flags: Precursor |
| P01703 | 152973.9737 | 134359.7152 | 0.878317481 | -0.187185577 | 0.126246522 | no | no change | GO:0038096;GO:0004252;GO:0016020;GO:0030449;GO:0050776;GO:0006898;GO:0038095;GO:0002376;GO:0050900;GO:0005576;GO:0006956;GO:0002250;GO:0003823;GO:0005886;GO:0006508;GO:0006958;GO:0006955 | hsa:7441 |  | Unknown (protein for MGC:31936) [Homo sapiens] |
| P01704 | 38484.2075 | 38757.2313 | 1.007094437 | 0.010198974 | 0.96703312 | no | no change | GO:0038096;GO:0004252;GO:0016020;GO:0030449;GO:0050776;GO:0006898;GO:0038095;GO:0002376;GO:0050900;GO:0005576;GO:0006956;GO:0002250;GO:0003823;GO:0005886;GO:0006508;GO:0006958;GO:0006955;GO:0070062 | hsa:7441 |  | RecName: Full=Immunoglobulin lambda variable 2-14; AltName: Full=Ig lambda chain V-II region NIG-84; AltName: Full=Ig lambda chain V-II region TOG; AltName: Full=Ig lambda chain V-II region VIL; Flags: Precursor |
| P01705 | 112127.4213 | 119684.0262 | 1.067393014 | 0.094091474 | 0.707395869 | no | no change | GO:0038096;GO:0004252;GO:0016020;GO:0030449;GO:0050776;GO:0006898;GO:0038095;GO:0002376;GO:0050900;GO:0005576;GO:0006956;GO:0002250;GO:0003823;GO:0005886;GO:0006508;GO:0006958;GO:0006955 | hsa:7441 |  | immunoglobulin lambda-chain, partial [Homo sapiens] |
| P01706 | 135250.71 | 117634.1529 | 0.869748875 | -0.201329187 | 0.462677209 | no | no change | GO:0038096;GO:0004252;GO:0016020;GO:0030449;GO:0050776;GO:0006898;GO:0038095;GO:0002376;GO:0050900;GO:0005576;GO:0006956;GO:0002250;GO:0003823;GO:0005886;GO:0006508;GO:0006958;GO:0006955 | hsa:7441 |  | hCG2043237, partial [Homo sapiens] |
| P01709 | 156534.4021 | 156051.6175 | 0.996915792 | -0.004456447 | 0.978961541 | no | no change | GO:0038096;GO:0004252;GO:0016020;GO:0030449;GO:0050776;GO:0006898;GO:0038095;GO:0002376;GO:0050900;GO:0005576;GO:0006956;GO:0002250;GO:0003823;GO:0005886;GO:0006508;GO:0006958;GO:0006955 | hsa:7441 |  | hCG2043240, partial [Homo sapiens] |
| P01721 | 113548.3379 | 102065.1888 | 0.89886995 | -0.153815696 | 0.295712253 | no | no change | GO:0038096;GO:0004252;GO:0016020;GO:0030449;GO:0050776;GO:0006898;GO:0038095;GO:0002376;GO:0050900;GO:0005576;GO:0006956;GO:0002250;GO:0003823;GO:0005886;GO:0006508;GO:0006958;GO:0006955 | hsa:7441 |  | RecName: Full=Immunoglobulin lambda variable 6-57; AltName: Full=Ig lambda chain V-VI region AR; AltName: Full=Ig lambda chain V-VI region EB4; AltName: Full=Ig lambda chain V-VI region NIG-48; AltName: Full=Ig lambda chain V-VI region SUT; AltName: Full=Ig lambda chain V-VI region WLT; Flags: Precursor |
| P01742 | 1362618.079 | 1446239.483 | 1.061368189 | 0.085925215 | 0.839787674 | no | no change | GO:0038096;GO:0004252;GO:0016020;GO:0030449;GO:0050776;GO:0006898;GO:0038095;GO:0002376;GO:0050900;GO:0005576;GO:0006956;GO:0002250;GO:0003823;GO:0005886;GO:0006508;GO:0006958;GO:0006955 | hsa:102724971 | | IgM heavy chain VH1 region precursor, partial [Homo sapiens] |
| P01743 | 15975.95005 | 15038.59167 | 0.941326908 | -0.08723226 | 0.639690014 | no | no change | GO:0038096;GO:0004252;GO:0016020;GO:0030449;GO:0050776;GO:0006898;GO:0038095;GO:0002376;GO:0050900;GO:0005576;GO:0006956;GO:0002250;GO:0003823;GO:0005886;GO:0006508;GO:0006958;GO:0006955 | hsa:102723407 | | IgM heavy chain VH1 region precursor, partial [Homo sapiens] |
| P01763 | 34043.84868 | 33730.68792 | 0.990801253 | -0.013332403 | 0.952242745 | no | no change | GO:0038096;GO:0004252;GO:0016020;GO:0030449;GO:0050776;GO:0006898;GO:0038095;GO:0002376;GO:0050900;GO:0005576;GO:0006956;GO:0002250;GO:0003823;GO:0005886;GO:0006508;GO:0006958;GO:0006955 | hsa:102723407 | | immunoglobulin heavy chain variable region, partial [Homo sapiens] |
| P01764 | 41234.23267 | 44708.084 | 1.08424678 | 0.116693159 | 0.614693881 | no | no change | GO:0004252;GO:0030449;GO:0009897;GO:0006955;GO:0050900;GO:0042742;GO:0034987;GO:0042571;GO:0005615;GO:0016020;GO:0072562;GO:0006508;GO:0050853;GO:0045087;GO:0006910;GO:0006911;GO:0050776;GO:0006956;GO:0003823;GO:0005886;GO:0006958;GO:0050871;GO:0070062;GO:0038096;GO:0038095;GO:0006898;GO:0002376;GO:0005576;GO:0002250 | hsa:102723407 | | RecName: Full=Immunoglobulin heavy variable 3-23; AltName: Full=Ig heavy chain V-III region LAY; AltName: Full=Ig heavy chain V-III region POM; AltName: Full=Ig heavy chain V-III region TEI; AltName: Full=Ig heavy chain V-III region TIL; AltName: Full=Ig heavy chain V-III region TUR; AltName: Full=Ig heavy chain V-III region VH26; AltName: Full=Ig heavy chain V-III region WAS; AltName: Full=Ig heavy chain V-III region ZAP; Flags: Precursor |
| P01766 | 90589.62789 | 94592.50125 | 1.044186884 | 0.062379942 | 0.738454331 | no | no change | GO:0038096;GO:0004252;GO:0016020;GO:0030449;GO:0005615;GO:0006898;GO:0038095;GO:0050900;GO:0072562;GO:0002376;GO:0050776;GO:0005576;GO:0006956;GO:0002250;GO:0003823;GO:0005886;GO:0006508;GO:0006958;GO:0006955 | hsa:102723407 | | RecName: Full=Immunoglobulin heavy variable 3-13; AltName: Full=Ig heavy chain V-III region BRO; Flags: Precursor |
| P01780 | 2081288.158 | 1940048.167 | 0.932138185 | -0.101384251 | 0.507780095 | no | no change | GO:0038096;GO:0004252;GO:0016020;GO:0030449;GO:0050776;GO:0006898;GO:0038095;GO:0072562;GO:0002376;GO:0050900;GO:0005576;GO:0006956;GO:0002250;GO:0003823;GO:0005886;GO:0006508;GO:0006958;GO:0006955;GO:0070062 | hsa:102723407 | | immunoglobulin heavy chain variable region precursor, partial [Homo sapiens] |
| P01782 | 287610.9211 | 272726.6167 | 0.948248473 | -0.076662952 | 0.674580552 | no | no change | GO:0038096;GO:0004252;GO:0016020;GO:0030449;GO:0050776;GO:0006898;GO:0038095;GO:0002376;GO:0050900;GO:0005576;GO:0006956;GO:0002250;GO:0003823;GO:0005886;GO:0006508;GO:0006958;GO:0006955;GO:0070062 | hsa:102723407 | | hCG2038940, partial [Homo sapiens] |
| P01833 | 14894.20894 | 12415.04043 | 0.833548158 | -0.262662542 | 0.166781769 | no | no change | GO:0043235;GO:0001895;GO:0005615;GO:0002415;GO:0001580;GO:0070062;GO:0016021;GO:0016020;GO:0043312;GO:0005576;GO:0007173;GO:0005887;GO:0005886;GO:0043113;GO:0001792;GO:0035577;GO:0038093 | hsa:5284 |  | polymeric immunoglobulin receptor precursor [Homo sapiens] |
| P01834 | 27249663.16 | 26834204.5 | 0.984753622 | -0.022165276 | 0.858557553 | no | no change | GO:0004252;GO:0030449;GO:0009897;GO:0006955;GO:0050871;GO:0042742;GO:0034987;GO:0042571;GO:0005615;GO:0016020;GO:0072562;GO:0006508;GO:0050853;GO:0045087;GO:0006910;GO:0006911;GO:0050776;GO:0006956;GO:0003823;GO:0005886;GO:0006958;GO:0001895;GO:0070062;GO:0038096;GO:0038095;GO:0006898;GO:0050900;GO:0002376;GO:0005576;GO:0002250 | hsa:100423062 | | light chain kappa Sci, k Sci=Bence Jones protein [human, Peptide, 214 aa] |
| P01857 | 10338346.11 | 9143238.333 | 0.884400487 | -0.177228275 | 0.225794593 | no | no change | GO:0004252;GO:0030449;GO:0019221;GO:0009897;GO:0042742;GO:0034987;GO:0042571;GO:0005615;GO:0016020;GO:0072562;GO:0006508;GO:0050853;GO:0045087;GO:0006910;GO:0006911;GO:0006956;GO:0003823;GO:0005886;GO:0006958;GO:0050871;GO:0070062;GO:0038096;GO:0002376;GO:0005576;GO:0002250;GO:0005515 | hsa:100423062 | | IGH@ protein [Homo sapiens] |
| P01860 | 2808878.895 | 2913433.25 | 1.037222806 | 0.052725834 | 0.799735352 | no | no change | GO:0004252;GO:0030449;GO:0009897;GO:0050871;GO:0042742;GO:0034987;GO:0042571;GO:0005615;GO:0016020;GO:0072562;GO:0006508;GO:0050853;GO:0045087;GO:0006910;GO:0006911;GO:0006956;GO:0003823;GO:0005886;GO:0006958;GO:0001895;GO:0070062;GO:0038096;GO:0002376;GO:0005576;GO:0002250 | hsa:100423062 | | Unknown (protein for MGC:105008) [Homo sapiens] |
| P01861 | 624816.04 | 557342.3079 | 0.892010243 | -0.164867817 | 0.581024553 | no | no change | GO:0004252;GO:0030449;GO:0019221;GO:0009897;GO:0042742;GO:0034987;GO:0042571;GO:0005615;GO:0016020;GO:0072562;GO:0006508;GO:0050853;GO:0045087;GO:0006910;GO:0006911;GO:0006956;GO:0003823;GO:0005886;GO:0006958;GO:0050871;GO:0070062;GO:0038096;GO:0002376;GO:0005576;GO:0002250 | hsa:100423062 | | RecName: Full=Immunoglobulin heavy constant gamma 4; AltName: Full=Ig gamma-4 chain C region |
| P01877 | 232985.3742 | 249862.9646 | 1.072440557 | 0.100897685 | 0.776555162 | no | no change | GO:0009897;GO:0019731;GO:0006955;GO:0050900;GO:0034987;GO:0001895;GO:0005615;GO:0016020;GO:0003094;GO:0072562;GO:0071748;GO:0050853;GO:0045087;GO:0006910;GO:0006911;GO:0003823;GO:0005886;GO:0006958;GO:0050871;GO:0060267;GO:0070062;GO:0006898;GO:0002376;GO:0005576;GO:0002250;GO:0071752;GO:0071751 | hsa:55423 |  | RecName: Full=Immunoglobulin heavy constant alpha 2; AltName: Full=Ig alpha-2 chain C region; AltName: Full=Ig alpha-2 chain C region BUT; AltName: Full=Ig alpha-2 chain C region LAN |
| P02042 | 73247.19789 | 65725.565 | 0.897311664 | -0.156318931 | 0.464059609 | no | no change | GO:0005344;GO:0019825;GO:0020037;GO:0043177;GO:0031721;GO:0007596;GO:0072562;GO:0005833;GO:0098869;GO:0042744;GO:0005515;GO:0031838;GO:0046872;GO:0005829;GO:0015671 | hsa:3045 | COG1018 | hemoglobin subunit delta [Homo sapiens] |
| P02100 | 36164.80579 | 33791.64692 | 0.934379328 | -0.097919739 | 0.707409723 | no | no change | GO:0005344;GO:0019825;GO:0020037;GO:0043177;GO:0031721;GO:0007596;GO:0051291;GO:0072562;GO:0005833;GO:0098869;GO:0042744;GO:0015671;GO:0005515;GO:0031838;GO:0046872;GO:0005829;GO:0014070 | hsa:3046 | COG1018 | hemoglobin subunit epsilon [Homo sapiens] |
| P02538 | 11764.16056 | 12718.27088 | 1.081103136 | 0.112504161 | 0.837930545 | no | no change | GO:0008284;GO:0002009;GO:0005200;GO:0007010;GO:0005829;GO:0031424;GO:0070062;GO:2000536;GO:0030154;GO:0045095;GO:0070268;GO:0042060;GO:0005198;GO:0050830;GO:0005882;GO:0005515;GO:0001899;GO:0016020;GO:0005634;GO:0051801;GO:0061844 | hsa:3853 |  | keratin, type II cytoskeletal 6A [Homo sapiens] |
| P02647 | 32198537.26 | 33872462.75 | 1.051987625 | 0.073117734 | 0.381348406 | no | no change | GO:0034115;GO:0010804;GO:0005788;GO:0019915;GO:0034191;GO:0034190;GO:0005548;GO:0050728;GO:0071682;GO:0051496;GO:0005543;GO:0010898;GO:0005319;GO:0034361;GO:0034362;GO:0034363;GO:0034364;GO:0031102;GO:0034366;GO:0031100;GO:0043534;GO:0030300;GO:0034774;GO:0010873;GO:0043691;GO:0007179;GO:0015485;GO:0005829;GO:0018206;GO:0019433;GO:0045499;GO:0070508;GO:0045723;GO:0034375;GO:0034371;GO:0031072;GO:0015914;GO:0034378;GO:0050821;GO:0010903;GO:0042158;GO:0055102;GO:0044267;GO:0042627;GO:0051180;GO:0014012;GO:0072562;GO:0050919;GO:0007186;GO:0033700;GO:0018158;GO:0006629;GO:0060761;GO:0042632;GO:0032489;GO:0050713;GO:0007229;GO:0006898;GO:0034384;GO:0062023;GO:0006695;GO:0060192;GO:0006869;GO:0019899;GO:0030301;GO:0035025;GO:0051006;GO:0002740;GO:0001540;GO:1900026;GO:0031410;GO:0005634;GO:0042802;GO:0008035;GO:0060354;GO:0009986;GO:0008289;GO:0008202;GO:0008203;GO:0071813;GO:0070062;GO:0042493;GO:0005515;GO:0034380;GO:0070371;GO:0042157;GO:0006656;GO:0030325;GO:0006644;GO:0017127;GO:0030139;GO:0008211;GO:0060228;GO:0001523;GO:0055091;GO:0034365;GO:0005615;GO:0002576;GO:0005576;GO:0043687;GO:0070328;GO:0019216;GO:0007584;GO:0031210;GO:0005886;GO:0043627;GO:1903561;GO:0051346;GO:0051345;GO:0033344;GO:0070653;GO:0001932;GO:0001935;GO:0005769 | hsa:335 |  | apolipoprotein A-I isoform 1 preproprotein [Homo sapiens] |
| P02649 | 2223708.632 | 1879563.25 | 0.845238096 | -0.242570302 | 0.153218652 | no | no change | GO:0051044;GO:0005783;GO:0034447;GO:0007616;GO:0005788;GO:0072358;GO:0043524;GO:0010873;GO:0051651;GO:0050728;GO:1905855;GO:0071682;GO:0016020;GO:0008201;GO:0098869;GO:0060999;GO:0045541;GO:0044794;GO:0005319;GO:0034361;GO:0034362;GO:0034363;GO:0034364;GO:0031102;GO:0016209;GO:0071813;GO:0043537;GO:0010977;GO:0030425;GO:0030516;GO:0043691;GO:0010877;GO:0010875;GO:0061771;GO:0015485;GO:0043025;GO:0019433;GO:1990777;GO:0030195;GO:0034375;GO:0034374;GO:0034372;GO:0034371;GO:1903002;GO:0000302;GO:0019934;GO:0010629;GO:0044877;GO:0042157;GO:0042311;GO:0042158;GO:0042159;GO:0048168;GO:0042982;GO:0044267;GO:0042627;GO:0072562;GO:0035641;GO:0010544;GO:0097006;GO:0007186;GO:0062023;GO:0033700;GO:0015909;GO:0006629;GO:1905890;GO:0048156;GO:1902430;GO:0042632;GO:0032489;GO:1901215;GO:0046907;GO:0034382;GO:0034380;GO:0006898;GO:0034384;GO:0006357;GO:0046983;GO:0032805;GO:0005543;GO:0090209;GO:0006869;GO:0051246;GO:0019068;GO:0050807;GO:0031012;GO:0043407;GO:0051000;GO:1902995;GO:0043083;GO:0005737;GO:0001540;GO:0005634;GO:0030669;GO:1900221;GO:0002021;GO:0090181;GO:0042802;GO:0042803;GO:0006641;GO:0006874;GO:0043687;GO:0017038;GO:0008289;GO:0055088;GO:0055089;GO:0046889;GO:0034378;GO:0006979;GO:0008202;GO:0008203;GO:0032269;GO:0070062;GO:1900272;GO:1902952;GO:0005515;GO:1901630;GO:0005794;GO:0017127;GO:0007271;GO:0060228;GO:0001523;GO:0034365;GO:0005615;GO:0048844;GO:0005198;GO:0033344;GO:1901628;GO:0010468;GO:0032462;GO:0090090;GO:0006707;GO:0070326;GO:0046911;GO:0005886;GO:2000822;GO:1903561;GO:0007263;GO:1905908;GO:0050750;GO:0007010;GO:1905860;GO:0097114;GO:0005576;GO:1905906;GO:0097113;GO:0045807;GO:0098978;GO:0070328;GO:0005769;GO:0001937;GO:0010976 | hsa:348 |  | apolipoprotein E [Homo sapiens] |
| P02671 | 5765624.316 | 6850534.375 | 1.188168705 | 0.248739695 | 0.052064126 | no | no change | GO:0045087;GO:0009897;GO:0045907;GO:0007599;GO:0034116;GO:0007596;GO:0005788;GO:0007160;GO:0045921;GO:0045202;GO:0034622;GO:1902042;GO:0044267;GO:0050839;GO:0031091;GO:0005615;GO:0031093;GO:1900026;GO:0072378;GO:0002576;GO:0031639;GO:0072562;GO:0005198;GO:0065003;GO:0030198;GO:0002224;GO:0046872;GO:0043687;GO:0009986;GO:2000352;GO:0051592;GO:0090277;GO:0005938;GO:0005886;GO:1903561;GO:0030168;GO:0070062;GO:0051258;GO:0042730;GO:0072377;GO:0050714;GO:0002376;GO:0005577;GO:0005576;GO:0002250;GO:0005515;GO:0043152;GO:0005102;GO:0070374;GO:0070527 | hsa:2243 |  | fibrinogen alpha chain isoform alpha-E preproprotein [Homo sapiens] |
| P02675 | 13156373.95 | 14965424.92 | 1.137503766 | 0.185871322 | 0.12629761 | no | no change | GO:1902042;GO:0034116;GO:0045921;GO:0007599;GO:0005783;GO:0009897;GO:0007596;GO:0007160;GO:0045907;GO:0045202;GO:0034622;GO:0044320;GO:0005737;GO:1903561;GO:0031091;GO:0005615;GO:0031093;GO:1900026;GO:0072378;GO:0002576;GO:0051087;GO:0031639;GO:0072562;GO:0005198;GO:0030198;GO:0002224;GO:0045087;GO:0009986;GO:2000352;GO:0051592;GO:0090277;GO:0005938;GO:0005886;GO:0050839;GO:0030168;GO:0070062;GO:0051258;GO:0042730;GO:0050714;GO:0071347;GO:0002376;GO:0005577;GO:0005576;GO:0002250;GO:0005515;GO:0043152;GO:0005102;GO:0070374;GO:0070527 | hsa:2244 |  | fibrinogen beta chain isoform 1 preproprotein [Homo sapiens] |
| P02743 | 394677.8789 | 389385.7333 | 0.986591228 | -0.019475635 | 0.873397273 | no | no change | GO:0006457;GO:0030246;GO:0046597;GO:0044871;GO:0072562;GO:0061045;GO:0044267;GO:0070062;GO:0005615;GO:0005634;GO:0051082;GO:0001849;GO:0005509;GO:0030169;GO:0042802;GO:1903016;GO:0051131;GO:0045087;GO:1903019;GO:0006953;GO:0062023;GO:0044869;GO:0006958;GO:0002674;GO:0046872;GO:0045656;GO:0046790;GO:0005576;GO:0048525 | hsa:325 |  | serum amyloid P-component precursor [Homo sapiens] |
| P02745 | 36515.59947 | 33476.93917 | 0.916784598 | -0.125345289 | 0.42322347 | no | no change | GO:0004252;GO:0030449;GO:0005581;GO:0045087;GO:0002376;GO:0005576;GO:0006956;GO:0005515;GO:0010039;GO:0007267;GO:0006508;GO:0006958;GO:0005602 | hsa:712 |  | complement C1q subcomponent subunit A precursor [Homo sapiens] |
| P02748 | 118478.6568 | 120882.7829 | 1.020291638 | 0.028981589 | 0.813374403 | no | no change | GO:0030449;GO:0006955;GO:0019835;GO:0019836;GO:0070062;GO:0016021;GO:0016020;GO:0051260;GO:0072562;GO:0001906;GO:0045087;GO:0006957;GO:0005887;GO:0005886;GO:0006958;GO:0005615;GO:0005829;GO:0044218;GO:0044279;GO:0002376;GO:0005576;GO:0005579 | hsa:735 |  | complement component C9 preproprotein [Homo sapiens] |
| P02749 | 1067318.932 | 923438.4708 | 0.865194501 | -0.208903598 | 0.051597534 | no | no change | GO:0034392;GO:0007597;GO:0051006;GO:0034197;GO:0042627;GO:0070062;GO:0002576;GO:0005543;GO:0031639;GO:0051917;GO:0042802;GO:0051918;GO:0034361;GO:0034364;GO:0006641;GO:0009986;GO:0060230;GO:0008289;GO:0062023;GO:0033033;GO:0008201;GO:0005615;GO:0010596;GO:0031089;GO:0005576;GO:0030195;GO:0005515;GO:0030193;GO:0016525;GO:0001937;GO:0030194 | hsa:350 |  | beta-2-glycoprotein 1 precursor [Homo sapiens] |
| P02750 | 128381.3026 | 128410.7275 | 1.000229199 | 0.000330626 | 0.997912352 | no | no change | GO:0043231;GO:0016020;GO:0070062;GO:0005160;GO:0009617;GO:0050873;GO:0045766;GO:0043312;GO:1904813;GO:0001938;GO:1904724;GO:0005576;GO:0003674;GO:0005515;GO:0035580;GO:0008150;GO:0030511;GO:0005615 | hsa:116844 | COG4886 | leucine-rich alpha-2-glycoprotein precursor [Homo sapiens] |
| P02753 | 65298.08789 | 68764.06375 | 1.053079286 | 0.07461406 | 0.589340823 | no | no change | GO:0060347;GO:0032024;GO:0048562;GO:0030324;GO:0060059;GO:0060044;GO:0042593;GO:0051024;GO:0060065;GO:0042572;GO:0001523;GO:0016918;GO:0005615;GO:0048807;GO:0006094;GO:0005501;GO:0032526;GO:0048706;GO:0007601;GO:0045471;GO:0001654;GO:0034633;GO:0070062;GO:0007507;GO:0032991;GO:0060157;GO:0005829;GO:0019841;GO:0048738;GO:0005576;GO:0005515;GO:0060068;GO:0034632;GO:0046982;GO:0050896;GO:0030277 | hsa:5950 |  | retinol-binding protein 4 isoform a precursor [Homo sapiens] |
| P02760 | 410334.5526 | 455763.3417 | 1.110711586 | 0.151484246 | 0.108193146 | no | no change | GO:0005886;GO:0010951;GO:0046329;GO:0070062;GO:0019855;GO:0072562;GO:0010466;GO:0042803;GO:0005515;GO:0020037;GO:0009986;GO:0042167;GO:0018298;GO:0062023;GO:0030163;GO:0030414;GO:0004867;GO:0007155;GO:0005615;GO:0050777;GO:0046904;GO:0019862;GO:0007565;GO:0006898;GO:0043231;GO:0005576;GO:0016032 | hsa:259 |  | protein AMBP preproprotein [Homo sapiens] |
| P02763 | 2000338.158 | 1697016.625 | 0.848364872 | -0.23724321 | 0.070544306 | no | no change | GO:1904469;GO:0050716;GO:0031093;GO:0005615;GO:0002576;GO:0050718;GO:0043312;GO:0072562;GO:0006953;GO:0032715;GO:0062023;GO:0002682;GO:0005515;GO:0006954;GO:0035580;GO:0005576;GO:1904724;GO:0032720;GO:0070062 | hsa:5004 |  | RecName: Full=Alpha-1-acid glycoprotein 1; Short=AGP 1; AltName: Full=Orosomucoid-1; Short=OMD 1; Flags: Precursor |
| P02766 | 758885.3474 | 718233.1167 | 0.946431657 | -0.079429764 | 0.336585689 | no | no change | GO:0005179;GO:0044267;GO:0032991;GO:0070062;GO:0042562;GO:0070324;GO:0005615;GO:0070327;GO:0005737;GO:0043312;GO:0001523;GO:0035578;GO:0030198;GO:0005515;GO:0042572;GO:0046982;GO:0010469;GO:0006144;GO:0042802;GO:0005576 | hsa:7276 | COG2351 | transthyretin precursor [Homo sapiens] |
| P02768 | 479659392 | 501513014.7 | 1.04556071 | 0.064276834 | 0.218513152 | no | no change | GO:0034375;GO:0015643;GO:0005788;GO:0008144;GO:0051659;GO:0030170;GO:0003677;GO:0005783;GO:0005737;GO:0044267;GO:0019836;GO:0070062;GO:0031093;GO:0043209;GO:0002576;GO:0051087;GO:0072562;GO:0098869;GO:0043066;GO:0005504;GO:0005507;GO:0043687;GO:0032460;GO:0042802;GO:0005794;GO:0140272;GO:0016209;GO:0008289;GO:0019825;GO:0009267;GO:0001895;GO:0005615;GO:0046872;GO:0032991;GO:0043069;GO:0006898;GO:1903981;GO:0005576;GO:0005515;GO:0005634 | hsa:213 |  | serum albumin preproprotein [Homo sapiens] |
| P02775 | 40917.93421 | 42574.82442 | 1.040493007 | 0.057267269 | 0.898487952 | no | no change | GO:0042127;GO:0070098;GO:0005125;GO:0071222;GO:0008009;GO:0042742;GO:0060326;GO:0032496;GO:0061844;GO:0031091;GO:0008083;GO:0031093;GO:0002576;GO:0007186;GO:0005355;GO:0030595;GO:0030593;GO:1904724;GO:0006952;GO:0006954;GO:0006955;GO:0045236;GO:0005615;GO:0090023;GO:0010469;GO:0031640;GO:0043312;GO:0006935;GO:0051781;GO:0005576;GO:1904659;GO:0005515 | hsa:5473 |  | platelet basic protein preproprotein [Homo sapiens] |
| P02776 | 36937.81637 | 40245.04375 | 1.089535 | 0.123712542 | 0.734902179 | no | no change | GO:0020005;GO:0070098;GO:0005125;GO:0071222;GO:0019221;GO:0008009;GO:0010469;GO:0032760;GO:0010628;GO:0048248;GO:0032496;GO:0045918;GO:0061844;GO:0005737;GO:0045347;GO:0031093;GO:0002576;GO:0007189;GO:0051873;GO:0010744;GO:0007186;GO:2001240;GO:0042127;GO:0030595;GO:0030593;GO:0006935;GO:0043950;GO:0006952;GO:0062023;GO:0006954;GO:0006955;GO:0008201;GO:0030168;GO:0005615;GO:0090023;GO:0045651;GO:0045652;GO:0045653;GO:0031640;GO:0005576;GO:0045944;GO:0005515;GO:0097679;GO:0042832;GO:0016525 | hsa:5196 |  | platelet factor 4 isoform 1 precursor [Homo sapiens] |
| P02790 | 2781504.737 | 3059440.5 | 1.099922808 | 0.137402279 | 0.085699066 | no | no change | GO:0020027;GO:0002925;GO:0015232;GO:0071682;GO:0005615;GO:0051246;GO:0042531;GO:0072562;GO:0046872;GO:0042168;GO:0006879;GO:0016032;GO:0060332;GO:0062023;GO:0060335;GO:0002639;GO:0070062;GO:0015886;GO:0006898;GO:0005623;GO:0005576;GO:0005515 | hsa:3263 |  | hemopexin precursor [Homo sapiens] |
| P04003 | 2808926.789 | 2762448.458 | 0.983453349 | -0.024071476 | 0.864258634 | no | no change | GO:0030449;GO:0005615;GO:0045087;GO:0072562;GO:0002376;GO:0045732;GO:0005576;GO:0003723;GO:0005515;GO:0005886;GO:1903027;GO:0006958;GO:0045959;GO:0044216 | hsa:722 |  | C4b-binding protein alpha chain precursor [Homo sapiens] |
| P04004 | 817496.0474 | 791457.45 | 0.968148351 | -0.046699964 | 0.51664689 | no | no change | GO:0005201;GO:0030449;GO:0005783;GO:0061302;GO:0050840;GO:0014911;GO:0030949;GO:0030247;GO:0031012;GO:0007160;GO:0007155;GO:0090303;GO:0048260;GO:0005737;GO:0032092;GO:0070062;GO:0072562;GO:0005576;GO:0016477;GO:0042802;GO:0033627;GO:0005178;GO:0005796;GO:0008283;GO:0010811;GO:0048709;GO:0048237;GO:0062023;GO:0006955;GO:0010951;GO:0035987;GO:0008201;GO:0005615;GO:0051258;GO:0005604;GO:0043231;GO:0050731;GO:0005044;GO:0006898;GO:0030198;GO:0005515;GO:0097421;GO:0071062;GO:0005518;GO:0030195 | hsa:7448 |  | vitronectin precursor [Homo sapiens] |
| P04114 | 4415674.842 | 3862682.083 | 0.874765969 | -0.193030998 | 0.139951765 | no | no change | GO:0034360;GO:0034374;GO:0034359;GO:0005783;GO:0034371;GO:0006869;GO:0050750;GO:0005788;GO:0005789;GO:0009791;GO:0061024;GO:0050900;GO:0010628;GO:0042157;GO:0034378;GO:0032496;GO:0016042;GO:0001523;GO:0006629;GO:0005737;GO:0043202;GO:0032355;GO:0001701;GO:0031983;GO:0005615;GO:0071682;GO:0071356;GO:0009615;GO:0048844;GO:0005543;GO:0030669;GO:0071379;GO:0070062;GO:0010033;GO:0042953;GO:0010884;GO:0042158;GO:0002224;GO:0010008;GO:0043025;GO:0043687;GO:0070971;GO:0005319;GO:0034361;GO:0034362;GO:0034363;GO:0006642;GO:0005790;GO:0044267;GO:0030317;GO:0008289;GO:0034379;GO:0010744;GO:0007283;GO:0042627;GO:0042159;GO:0017127;GO:0012506;GO:0005886;GO:0042632;GO:0030301;GO:0008201;GO:0008202;GO:0008203;GO:0035473;GO:0031904;GO:0009743;GO:0007399;GO:0034382;GO:0034383;GO:0005829;GO:0006898;GO:0009566;GO:0043231;GO:0010886;GO:0019433;GO:0005576;GO:0045540;GO:0005515;GO:0034447;GO:0010269;GO:0005769;GO:0033344 | hsa:338 |  | RecName: Full=Apolipoprotein B-100; Short=Apo B-100; Contains: RecName: Full=Apolipoprotein B-48; Short=Apo B-48; Flags: Precursor |
| P04180 | 18607.13326 | 21735.335 | 1.168118414 | 0.22418653 | 0.077318184 | no | no change | GO:0034375;GO:0006656;GO:0034372;GO:0043691;GO:0008374;GO:0042158;GO:0090107;GO:0070062;GO:0016740;GO:0016746;GO:0034435;GO:0008203;GO:0006644;GO:0034364;GO:0006629;GO:0042632;GO:0030301;GO:0008202;GO:0005615;GO:0005576;GO:0005515;GO:0034186;GO:0004607;GO:0046470 | hsa:3931 |  | phosphatidylcholine-sterol acyltransferase precursor [Homo sapiens] |
| P04196 | 675006.1053 | 646424.2125 | 0.957656838 | -0.062419314 | 0.608682132 | no | no change | GO:0002839;GO:0030308;GO:0032956;GO:2000504;GO:0007162;GO:0008270;GO:0051894;GO:0007599;GO:0001525;GO:0061844;GO:0051715;GO:0070062;GO:0031093;GO:0002576;GO:0072562;GO:0043065;GO:0033629;GO:0030168;GO:0005886;GO:0046872;GO:0010468;GO:0051918;GO:0010543;GO:0008285;GO:0007596;GO:0006935;GO:0020037;GO:0009986;GO:0010593;GO:0043537;GO:0043395;GO:0062023;GO:0004867;GO:0010951;GO:0004869;GO:0043254;GO:0008201;GO:2001027;GO:0050832;GO:0042730;GO:0036019;GO:0050730;GO:0015886;GO:0019865;GO:0005576;GO:1900747;GO:0005515;GO:0030193;GO:0005102;GO:0016525 | hsa:3273 |  | histidine-rich glycoprotein precursor [Homo sapiens] |
| P04211 | 1109267.495 | 1038025.381 | 0.935775533 | -0.095765587 | 0.677556529 | no | no change | GO:0038096;GO:0004252;GO:0016020;GO:0030449;GO:0050776;GO:0006898;GO:0038095;GO:0002376;GO:0050900;GO:0005576;GO:0006956;GO:0002250;GO:0003823;GO:0005886;GO:0006508;GO:0006958;GO:0006955 | hsa:7441 |  | hCG1731877, partial [Homo sapiens] |
| P04217 | 794323.0421 | 780897.2333 | 0.983097798 | -0.024593153 | 0.730030862 | no | no change | GO:0070062;GO:0031093;GO:0002576;GO:0043312;GO:0072562;GO:1904813;GO:0005576;GO:0003674;GO:0034774;GO:0008150;GO:0062023;GO:0005615 | hsa:1 |  | alpha-1B-glycoprotein precursor [Homo sapiens] |
| P04264 | 224055.9947 | 222059.4333 | 0.991089007 | -0.012913467 | 0.917961519 | no | no change | GO:0018149;GO:0038023;GO:0045095;GO:0030246;GO:0031012;GO:1904813;GO:0061436;GO:0030280;GO:0005856;GO:0050728;GO:0070062;GO:0005634;GO:0016020;GO:0045765;GO:0072562;GO:0005198;GO:0001867;GO:0005882;GO:0051290;GO:0070268;GO:0062023;GO:0005886;GO:0001895;GO:0006979;GO:0001533;GO:0005615;GO:0042730;GO:0031424;GO:0005829;GO:0043312;GO:0005576;GO:0005515;GO:0046982 | hsa:3848 |  | keratin 1 [Homo sapiens] |
| P04275 | 59863.79484 | 71404.84208 | 1.192788434 | 0.254338173 | 0.465001588 | no | no change | GO:0005201;GO:0007599;GO:0005783;GO:0007597;GO:0007596;GO:0031012;GO:0031091;GO:0070062;GO:0031093;GO:0002576;GO:0051087;GO:0051260;GO:0009611;GO:0005576;GO:0002020;GO:0042802;GO:0042803;GO:0005178;GO:0031589;GO:0033093;GO:0062023;GO:0007155;GO:0030168;GO:0047485;GO:0019865;GO:0030198;GO:0005515;GO:0005518 | hsa:7450 |  | RecName: Full=von Willebrand factor; Short=vWF; Contains: RecName: Full=von Willebrand antigen 2; AltName: Full=von Willebrand antigen II; Flags: Precursor |
| P04433 | 102176.9611 | 97628.94542 | 0.955488834 | -0.065689081 | 0.561242748 | no | no change | GO:0038096;GO:0004252;GO:0016020;GO:0030449;GO:0050776;GO:0006898;GO:0038095;GO:0072562;GO:0002376;GO:0050900;GO:0005576;GO:0006956;GO:0002250;GO:0003823;GO:0005886;GO:0006508;GO:0006958;GO:0006955;GO:0070062 | hsa:7441 |  | rheumatoid factor D1 IgG light chain VK3 region, partial [Homo sapiens] |
| P05154 | 20698.69611 | 19250.355 | 0.930027423 | -0.104654838 | 0.44488341 | no | no change | GO:0009897;GO:0006869;GO:0007596;GO:0036024;GO:0002080;GO:0061107;GO:0036029;GO:0031094;GO:0031091;GO:0051346;GO:0007342;GO:0016020;GO:0036025;GO:0032190;GO:0036027;GO:0036026;GO:0010466;GO:0002020;GO:0097183;GO:0097182;GO:0097181;GO:0036028;GO:0007283;GO:0031210;GO:0007338;GO:0030414;GO:0004867;GO:0010951;GO:0008201;GO:0005615;GO:0032991;GO:0070062;GO:0036030;GO:0005539;GO:0005576;GO:0005515;GO:0001972;GO:0045861 | hsa:5104 | COG4826 | plasma serine protease inhibitor preproprotein [Homo sapiens] |
| P05155 | 312577.7684 | 333477.4708 | 1.066862408 | 0.093374126 | 0.375572607 | no | no change | GO:0030449;GO:0007597;GO:0007596;GO:0007599;GO:0005615;GO:0031093;GO:0002576;GO:0072562;GO:0010466;GO:0001869;GO:0045916;GO:0008015;GO:0045087;GO:0030414;GO:0004867;GO:0010951;GO:0006958;GO:0070062;GO:0042730;GO:0007568;GO:0002376;GO:0005576;GO:0005515;GO:0030193 | hsa:710 | COG4826 | unnamed protein product [Homo sapiens] |
| P05160 | 25199.30632 | 26736.40458 | 1.060997642 | 0.08542145 | 0.371443362 | no | no change | GO:0072378;GO:0005576;GO:0007596;GO:0007599;GO:1903363 | hsa:2165 |  | coagulation factor XIII B chain precursor [Homo sapiens] |
| P05543 | 23109.91774 | 26070.655 | 1.128115439 | 0.173914705 | 0.223476319 | no | no change | GO:0005615;GO:0070327;GO:0005576;GO:0004867;GO:0010951;GO:0070062 | hsa:6906 | COG4826 | thyroxine-binding globulin precursor [Homo sapiens] |
| P05546 | 553199.5684 | 570904.3792 | 1.032004383 | 0.045449098 | 0.656679099 | no | no change | GO:0044267;GO:0007599;GO:0006935;GO:0005615;GO:0043687;GO:0007596;GO:0005788;GO:0005576;GO:0030414;GO:0004866;GO:0004867;GO:0010951;GO:0010466;GO:0008201;GO:0070062 | hsa:3053 | COG4826 | heparin cofactor 2 precursor [Homo sapiens] |
| P06312 | 455326.1211 | 430701.625 | 0.945918991 | -0.080211459 | 0.577894493 | no | no change | GO:0038096;GO:0004252;GO:0016020;GO:0030449;GO:0050776;GO:0006898;GO:0038095;GO:0072562;GO:0002376;GO:0002377;GO:0050900;GO:0005576;GO:0006956;GO:0002250;GO:0003823;GO:0005886;GO:0006508;GO:0006958;GO:0006955 | hsa:7441 |  | immunoglobulin kappa chain, partial [Homo sapiens] |
| P06727 | 898121.5842 | 964838.4583 | 1.074284902 | 0.103376649 | 0.343710724 | no | no change | GO:0034375;GO:0034445;GO:0034372;GO:0034371;GO:0006869;GO:0034380;GO:0045723;GO:0005788;GO:0034378;GO:0051006;GO:0042744;GO:0042157;GO:0042632;GO:0070328;GO:0060228;GO:0001523;GO:0035634;GO:0044267;GO:0042627;GO:0005615;GO:0006982;GO:0032374;GO:0072562;GO:0016042;GO:0033344;GO:0065005;GO:0010898;GO:0002227;GO:0009986;GO:0042802;GO:0033700;GO:0005319;GO:0034361;GO:0034364;GO:0031102;GO:0062023;GO:0016209;GO:0008289;GO:0055088;GO:0031210;GO:0007159;GO:0017127;GO:0010873;GO:0043691;GO:0008203;GO:0030300;GO:0005507;GO:0015485;GO:0070062;GO:0005829;GO:0046470;GO:0019430;GO:0005576;GO:0006695;GO:0005515;GO:0042803;GO:0005769 | hsa:337 |  | RecName: Full=Apolipoprotein A-IV; Short=Apo-AIV; Short=ApoA-IV; AltName: Full=Apolipoprotein A4; Flags: Precursor |
| P07358 | 39937.20684 | 38317.925 | 0.959454304 | -0.059713998 | 0.511815236 | no | no change | GO:0019835;GO:0030449;GO:0005615;GO:0045087;GO:0016020;GO:0002376;GO:0005576;GO:0006956;GO:0006957;GO:0006955;GO:0044877;GO:0006958;GO:1903561;GO:0005579;GO:0070062 | hsa:732 |  | RecName: Full=Complement component C8 beta chain; AltName: Full=Complement component 8 subunit beta; Flags: Precursor |
| P07996 | 28906.78963 | 28167.29163 | 0.974417844 | -0.037387542 | 0.922284183 | no | no change | GO:0032026;GO:0005783;GO:0050840;GO:0034605;GO:0005788;GO:2001237;GO:0050921;GO:1903671;GO:0048266;GO:0051895;GO:0006986;GO:0043652;GO:0042535;GO:0009612;GO:0030511;GO:0002581;GO:0042327;GO:0051592;GO:0043536;GO:0040037;GO:2001027;GO:0030169;GO:0043236;GO:0045727;GO:0045652;GO:0030198;GO:0043032;GO:0030194;GO:0009897;GO:0018149;GO:0042493;GO:0002605;GO:0071356;GO:0051897;GO:0016477;GO:0002040;GO:0001968;GO:2000379;GO:0030141;GO:0007050;GO:0043394;GO:0006954;GO:0006955;GO:0070052;GO:0090051;GO:0070051;GO:0033574;GO:0043154;GO:0034976;GO:1902043;GO:0031012;GO:0001666;GO:0050431;GO:0005737;GO:0031091;GO:0031093;GO:0005509;GO:0010763;GO:0001786;GO:0051918;GO:0008284;GO:0009986;GO:0017134;GO:2000353;GO:0008201;GO:0071363;GO:0001953;GO:0002544;GO:0016529;GO:0042802;GO:0005515;GO:0032570;GO:0016525;GO:0000187;GO:0005201;GO:0005615;GO:0002576;GO:0010751;GO:0045766;GO:0043066;GO:0010748;GO:0032695;GO:1903588;GO:0005178;GO:0030335;GO:0048661;GO:0032914;GO:0062023;GO:0007155;GO:0070062;GO:0043537;GO:0010595;GO:0010596;GO:0009749;GO:0005577;GO:0005576;GO:0010757;GO:0010754;GO:0010759;GO:0001937;GO:0071636 | hsa:7057 |  | thrombospondin-1 precursor [Homo sapiens] |
| P08185 | 107075.3179 | 120878.3283 | 1.128909358 | 0.174929654 | 0.244708939 | no | no change | GO:0005615;GO:0008289;GO:0010951;GO:0008211;GO:0005576;GO:0004867;GO:0005496;GO:0070062 | hsa:866 | COG4826 | corticosteroid-binding globulin precursor [Homo sapiens] |
| P08519 | 143288.9263 | 139765.8054 | 0.975412469 | -0.03591568 | 0.960280059 | no | no change | GO:0004252;GO:0034374;GO:0005515;GO:0006869;GO:0008015;GO:0016787;GO:0034185;GO:0005576;GO:0004866;GO:0010951;GO:0006508;GO:0008233;GO:0008236;GO:0034358;GO:0008201;GO:0001968;GO:0006629 | hsa:4018 | COG5640 | RecName: Full=Apolipoprotein(a); Short=Apo(a); Short=Lp(a); Flags: Precursor |
| P08571 | 10905.65174 | 11841.57217 | 1.085819761 | 0.118784646 | 0.31289046 | no | no change | GO:0006954;GO:0009897;GO:0045121;GO:0071222;GO:0071223;GO:0097190;GO:0031362;GO:1901224;GO:0007166;GO:0006909;GO:0032496;GO:0045471;GO:0010008;GO:0034612;GO:0016020;GO:0030667;GO:0005615;GO:0071727;GO:0071726;GO:0009617;GO:0071723;GO:0071219;GO:0032729;GO:0002224;GO:0031225;GO:0009986;GO:0001847;GO:0005794;GO:0032760;GO:0070266;GO:0034128;GO:0038124;GO:0045087;GO:0006915;GO:2000484;GO:0035666;GO:0032481;GO:0070891;GO:0034142;GO:0005886;GO:0009408;GO:0001530;GO:0032026;GO:0070062;GO:0051602;GO:0002755;GO:0002756;GO:0016019;GO:0050715;GO:0038123;GO:0031663;GO:0006898;GO:0007249;GO:0043312;GO:0002376;GO:0005576;GO:0002237;GO:0005515;GO:0045807;GO:0046696 | hsa:929 |  | monocyte differentiation antigen CD14 precursor [Homo sapiens] |
| P08603 | 1325699.621 | 1326576.996 | 1.00066182 | 0.000954489 | 0.988635541 | no | no change | GO:1903659;GO:0030449;GO:0070062;GO:0005515;GO:0045087;GO:0072562;GO:0002376;GO:0043395;GO:0005576;GO:0006956;GO:0006957;GO:0016032;GO:0008201;GO:0005615 | hsa:3075 |  | RecName: Full=Complement factor H; AltName: Full=H factor 1; Flags: Precursor |
| P09172 | 3552.175786 | 3962.4251 | 1.115492402 | 0.157680687 | 0.607087536 | no | no change | GO:0016491;GO:0042127;GO:2001236;GO:0045907;GO:0005783;GO:0048149;GO:0034774;GO:0007613;GO:0042593;GO:0050900;GO:0042596;GO:0005815;GO:0042423;GO:0042420;GO:0042421;GO:0005737;GO:0031418;GO:0030667;GO:0005615;GO:0004500;GO:0031410;GO:0016021;GO:0016020;GO:0008542;GO:0005507;GO:0006589;GO:0007626;GO:0004497;GO:0030658;GO:0003824;GO:0008306;GO:0042584;GO:0042309;GO:0007268;GO:0002443;GO:0055114;GO:0043231;GO:0048265;GO:0042711;GO:0120162;GO:0046872;GO:0016715;GO:0001975;GO:0001816;GO:0001974;GO:0005576;GO:0034466 | hsa:1621 |  | dopamine beta-hydroxylase precursor [Homo sapiens] |
| P09871 | 93659.56368 | 89103.85625 | 0.951358866 | -0.071938447 | 0.392108931 | no | no change | GO:0004252;GO:0006956;GO:0016787;GO:0045087;GO:0072562;GO:0002376;GO:0030449;GO:0005576;GO:0001867;GO:0005509;GO:0005515;GO:0006508;GO:0006958;GO:0046872;GO:0008233;GO:0042802;GO:0008236 | hsa:716 | COG5640 | complement C1s subcomponent isoform 1 preproprotein [Homo sapiens] |
| P0C0L5 | 1776359.432 | 1868176.25 | 1.051688198 | 0.072707042 | 0.618931387 | no | no change | GO:0004252;GO:0030449;GO:0006954;GO:0030246;GO:0030425;GO:0030424;GO:0045202;GO:0032490;GO:0030054;GO:0005615;GO:0072562;GO:0001848;GO:0006508;GO:0045087;GO:0008228;GO:0006956;GO:2000427;GO:0004866;GO:0005886;GO:0010951;GO:0006958;GO:0042995;GO:0070062;GO:0044216;GO:0002376;GO:0005576 | hsa:100293534;hsa:110384692;hsa:720;hsa:721 | | complement C4-B preproprotein [Homo sapiens] |
| P0DJI8 | 33536.79611 | 37156.06125 | 1.107919228 | 0.147852707 | 0.554890832 | no | no change | GO:0000187;GO:0042056;GO:0019221;GO:0001664;GO:0034364;GO:0048246;GO:0048247;GO:0044267;GO:0050728;GO:0050708;GO:0005615;GO:0071682;GO:0045785;GO:0007186;GO:0030593;GO:0045087;GO:0005881;GO:0006953;GO:0050918;GO:0008201;GO:0030168;GO:0070062;GO:0050716;GO:0050715;GO:0006898;GO:0007204;GO:0005576 | hsa:6288 |  | RecName: Full=Serum amyloid A-1 protein; Short=SAA; Contains: RecName: Full=Amyloid protein A; AltName: Full=Amyloid fibril protein AA; Contains: RecName: Full=Serum amyloid protein A(2-104); Contains: RecName: Full=Serum amyloid protein A(3-104); Contains: RecName: Full=Serum amyloid protein A(2-103); Contains: RecName: Full=Serum amyloid protein A(2-102); Contains: RecName: Full=Serum amyloid protein A(4-101); Flags: Precursor |
| P0DP01 | 38972.34187 | 39861.82217 | 1.022823373 | 0.032557034 | 0.917568507 | no | no change | GO:0016020;GO:0002376;GO:0005576;GO:0002250;GO:0003823;GO:0005886 | hsa:102723407 | | immunoglobulin heavy chain variable region, partial [Homo sapiens] |
| P0DTE1 | 16931.36632 | 18878.19 | 1.114983259 | 0.157022049 | 0.36220918 | no | no change |  | hsa:102723407 | | immunoglobulin heavy chain variable region, partial [Homo sapiens] |
| P10643 | 101851.2711 | 94850.89417 | 0.931268635 | -0.102730705 | 0.221515624 | no | no change | GO:0019835;GO:0030449;GO:0070062;GO:0045087;GO:0006883;GO:0002376;GO:0005576;GO:0006956;GO:0006957;GO:0006955;GO:0006958;GO:0005579 | hsa:730 |  | complement component C7 precursor [Homo sapiens] |
| P11597 | 7833.990842 | 7446.994248 | 0.950600326 | -0.073089199 | 0.702824486 | no | no change | GO:0017129;GO:0034375;GO:0034374;GO:0034372;GO:0006869;GO:0015914;GO:0017127;GO:0030301;GO:0034197;GO:0034364;GO:0055091;GO:0005548;GO:0070062;GO:0005615;GO:0008202;GO:0010745;GO:0031982;GO:0005319;GO:0070328;GO:0006629;GO:0006641;GO:0008289;GO:0055088;GO:0031210;GO:0042632;GO:0043691;GO:0010874;GO:0008203;GO:0015485;GO:0005576;GO:0046470 | hsa:1071 |  | cholesteryl ester transfer protein isoform 1 precursor [Homo sapiens] |
| P13473 | 9220.985842 | 9403.000792 | 1.019739207 | 0.028200238 | 0.873781522 | no | no change | GO:0005770;GO:0061684;GO:0101003;GO:0019899;GO:0050821;GO:0044754;GO:0035577;GO:0010008;GO:1990836;GO:0043202;GO:0005615;GO:0097637;GO:0031410;GO:0016021;GO:0016020;GO:0097352;GO:0031902;GO:0046716;GO:0045121;GO:0006914;GO:0017038;GO:0006605;GO:1905146;GO:0061740;GO:0019904;GO:0009267;GO:0005886;GO:0070062;GO:0000421;GO:0030670;GO:0031647;GO:0043312;GO:0031088;GO:0005764;GO:0005765;GO:0005515;GO:0098857;GO:0005768;GO:0002576 | hsa:3920 |  | lysosome-associated membrane glycoprotein 2 isoform A precursor [Homo sapiens] |
| P13645 | 47574.99368 | 46754.77875 | 0.982759537 | -0.025089636 | 0.883445064 | no | no change | GO:0005737;GO:0018149;GO:0045684;GO:0070062;GO:0030216;GO:0031424;GO:0005615;GO:0009986;GO:0016020;GO:0070268;GO:0051290;GO:0005634;GO:0005882;GO:0005576;GO:0046982;GO:0005198;GO:0005829;GO:0001533;GO:0030280 | hsa:3858 |  | Keratin 10 [Homo sapiens] |
| P13671 | 56036.18947 | 54120.68 | 0.965816564 | -0.050178888 | 0.501274263 | no | no change | GO:0019835;GO:0001701;GO:0030449;GO:0070062;GO:0045917;GO:0045087;GO:0045766;GO:0002376;GO:0005576;GO:0006956;GO:0001970;GO:0005515;GO:0006955;GO:0006958;GO:0005579 | hsa:729 |  | complement component C6 precursor [Homo sapiens] |
| P14151 | 29660.54053 | 30580.76667 | 1.031025265 | 0.044079686 | 0.576356154 | no | no change | GO:0009897;GO:0016339;GO:0030246;GO:0050900;GO:0050901;GO:0070492;GO:0030667;GO:0043208;GO:0016021;GO:0016020;GO:0005509;GO:0002020;GO:0046872;GO:0009986;GO:0005887;GO:0005886;GO:0007155;GO:0050839;GO:0008201;GO:0050776;GO:0043312;GO:0005515;GO:0033198 | hsa:6402 |  | L-selectin [Homo sapiens] |
| P15169 | 20256.22737 | 21634.38792 | 1.068036388 | 0.094960801 | 0.508173178 | no | no change | GO:0097060;GO:0005794;GO:0051384;GO:0030449;GO:0005615;GO:0016787;GO:0030141;GO:0004181;GO:0004180;GO:0010815;GO:0004185;GO:0005576;GO:0043025;GO:0008270;GO:0016485;GO:0006508;GO:0006518;GO:0046872;GO:0030070;GO:0008233;GO:0008237 | hsa:1369 |  | carboxypeptidase N catalytic chain precursor [Homo sapiens] |
| P15814 | 10754431.68 | 11078226.17 | 1.030108005 | 0.042795609 | 0.725904582 | no | no change | GO:0009897;GO:0050853;GO:0045087;GO:0016020;GO:0006910;GO:0006911;GO:0050900;GO:0005576;GO:0042742;GO:0003823;GO:0006955;GO:0042571;GO:0006958;GO:0050871;GO:0034987;GO:0072562 | hsa:3543 |  | immunoglobulin lambda-like polypeptide 1 isoform a precursor [Homo sapiens] |
| P18428 | 15726.31684 | 15522.83508 | 0.987061067 | -0.018788751 | 0.877558886 | no | no change | GO:0006968;GO:0071222;GO:0071223;GO:0006869;GO:0042742;GO:0050829;GO:0032496;GO:0032490;GO:0044130;GO:0019221;GO:0060265;GO:0002281;GO:0070062;GO:0090023;GO:0042535;GO:0016020;GO:0071723;GO:0045919;GO:0002224;GO:0033036;GO:0032722;GO:0009986;GO:0032720;GO:0032760;GO:0015920;GO:0045087;GO:0008289;GO:0006953;GO:0008228;GO:0070891;GO:0034142;GO:0001530;GO:0005615;GO:0034145;GO:0031663;GO:0050830;GO:0002376;GO:0002232;GO:0005576;GO:0005515;GO:0043032;GO:0005102;GO:0032757;GO:0032755 | hsa:3929 |  | lipopolysaccharide-binding protein precursor [Homo sapiens] |
| P19652 | 944377.3947 | 828048.6208 | 0.876819612 | -0.189648027 | 0.109656199 | no | no change | GO:1904469;GO:0050716;GO:0031093;GO:0005615;GO:0002576;GO:0050718;GO:0043312;GO:0072562;GO:0006953;GO:0035578;GO:0062023;GO:0002682;GO:0035580;GO:0005576;GO:0070062 | hsa:5005 |  | alpha-1-acid glycoprotein 2 precursor [Homo sapiens] |
| P19827 | 319962 | 355189.1833 | 1.110098022 | 0.150687073 | 0.056464196 | no | no change | GO:0030212;GO:0070062;GO:0072562;GO:0010466;GO:0005509;GO:0030414;GO:0004867;GO:0010951;GO:0005576 | hsa:3697 | COG2304 | inter-alpha-trypsin inhibitor heavy chain H1 isoform a preproprotein [Homo sapiens] |
| P20742 | 51671.45238 | 46596.61316 | 0.901786403 | -0.149142338 | 0.819265825 | no | no change | GO:0004866;GO:0007565;GO:0070062;GO:0005576;GO:0030414;GO:0072562;GO:0002020;GO:0004867;GO:0010951;GO:0010466;GO:0005615 | hsa:5858 | COG2373 | pregnancy zone protein precursor [Homo sapiens] |
| P20851 | 54623.62684 | 54330.14875 | 0.994627268 | -0.007772111 | 0.960147521 | no | no change | GO:0030449;GO:0005615;GO:0045087;GO:0007596;GO:0002376;GO:0045732;GO:0005576;GO:0005515;GO:0005886;GO:1903027;GO:0006958;GO:0045959;GO:0044216 | hsa:725 |  | C4b-binding protein beta chain isoform 1 precursor [Homo sapiens] |
| P22792 | 100444.6111 | 97010.12417 | 0.965807156 | -0.050192941 | 0.636481884 | no | no change | GO:0030449;GO:0070062;GO:0004181;GO:0050790;GO:0072562;GO:0050821;GO:0005576;GO:0006508;GO:0030234 | hsa:1370 | COG4886 | carboxypeptidase N subunit 2 precursor [Homo sapiens] |
| P22891 | 10520.92905 | 10479.47265 | 0.996059626 | -0.005695988 | 0.974868133 | no | no change | GO:0004252;GO:0005796;GO:0006888;GO:0005615;GO:0007596;GO:0005788;GO:0005576;GO:0005509;GO:0006508;GO:0030195;GO:0007599;GO:0070062 | hsa:8858 | COG5640 | vitamin K-dependent protein Z isoform 2 precursor [Homo sapiens] |
| P23142 | 32198.87526 | 34770.50708 | 1.079867132 | 0.110853813 | 0.413784918 | no | no change | GO:0005201;GO:0008022;GO:0031012;GO:0007162;GO:0044877;GO:0005615;GO:0072378;GO:1900025;GO:0005576;GO:0005509;GO:2000647;GO:0042802;GO:0062023;GO:0071953;GO:0010952;GO:0070062;GO:0005604;GO:0007566;GO:0070051;GO:0005577;GO:0030198;GO:0001968;GO:0016032;GO:2000146;GO:0016504;GO:0001933;GO:0070373;GO:0007229 | hsa:2192 |  | fibulin-1 isoform D precursor [Homo sapiens] |
| P25311 | 349100.7526 | 333564.1083 | 0.955495243 | -0.065679405 | 0.480432085 | no | no change | GO:0008285;GO:0090501;GO:0071806;GO:0070062;GO:0009897;GO:0001580;GO:0005615;GO:0005634;GO:0004540;GO:0008320;GO:0062023;GO:0005515;GO:0005886;GO:0006955;GO:0007155;GO:0001895;GO:0055085;GO:0005576 | hsa:563 |  | zinc-alpha-2-glycoprotein precursor [Homo sapiens] |
| P27169 | 248603.3211 | 238200.1833 | 0.958153666 | -0.061671046 | 0.49181589 | no | no change | GO:0034445;GO:0032411;GO:0016311;GO:0046872;GO:0004064;GO:0046434;GO:0070062;GO:0005615;GO:0005543;GO:0009636;GO:0072562;GO:0005509;GO:0102007;GO:0010875;GO:0019372;GO:0042803;GO:0016787;GO:0034364;GO:0006629;GO:0034366;GO:1902617;GO:0008203;GO:0004063;GO:0043231;GO:0046395;GO:0051099;GO:0031667;GO:0046470;GO:0005576;GO:0019439;GO:0070542 | hsa:5444 |  | serum paraoxonase/arylesterase 1 precursor [Homo sapiens] |
| P35858 | 52673.09368 | 56035.23375 | 1.063830313 | 0.089268051 | 0.399361181 | no | no change | GO:0044267;GO:0005615;GO:0031012;GO:0005654;GO:0007155;GO:0005576;GO:0007165;GO:0042567;GO:0005520;GO:0070062 | hsa:3483 | COG4886 | Insulin-like growth factor binding protein, acid labile subunit [Homo sapiens] |
| P35908 | 86320.11789 | 90491.12417 | 1.048320211 | 0.068079457 | 0.629252006 | no | no change | GO:0005200;GO:0045095;GO:0018149;GO:0008544;GO:0045684;GO:0043616;GO:0051546;GO:0070062;GO:0032980;GO:0005634;GO:0016020;GO:0005198;GO:0030280;GO:0070268;GO:0005882;GO:0001533;GO:0005615;GO:0045109;GO:0008092;GO:0031424;GO:0005829;GO:0003334;GO:0005515 | hsa:3849 |  | keratin, type II cytoskeletal 2 epidermal [Homo sapiens] |
| P36955 | 107954.0368 | 100758.2771 | 0.933344227 | -0.099518835 | 0.326199181 | no | no change | GO:0007614;GO:0030424;GO:0010629;GO:0060041;GO:0060770;GO:0007275;GO:0043203;GO:0005615;GO:0071300;GO:0010447;GO:0001822;GO:0050769;GO:0071333;GO:0048471;GO:0046685;GO:0043025;GO:0071279;GO:0008283;GO:0050728;GO:0062023;GO:0010976;GO:0004867;GO:0010951;GO:1901215;GO:0070062;GO:0042470;GO:0071549;GO:0005604;GO:0007568;GO:0010596;GO:0042698;GO:0005576;GO:0005515;GO:0016525 | hsa:5176 | COG4826 | pigment epithelium-derived factor isoform 1 precursor [Homo sapiens] |
| P41222 | 16649.46113 | 18677.39048 | 1.121801501 | 0.165817418 | 0.155932478 | no | no change | GO:0006633;GO:0006631;GO:0005783;GO:0005789;GO:0005737;GO:0070062;GO:0005634;GO:0016020;GO:0005504;GO:0048471;GO:0005501;GO:0019371;GO:0005794;GO:0005791;GO:0006629;GO:0031965;GO:2000255;GO:0036094;GO:0001516;GO:0045187;GO:0005615;GO:0016853;GO:0004667;GO:0006693;GO:0005576;GO:0005515 | hsa:5730 |  | prostaglandin-H2 D-isomerase precursor [Homo sapiens] |
| P43121 | 5617.6761 | 5235.567056 | 0.931980941 | -0.101627643 | 0.594885982 | no | no change | GO:0009897;GO:0030335;GO:0001525;GO:0005925;GO:0005615;GO:0016021;GO:0016020;GO:0003094;GO:0061042;GO:0005576;GO:0005886;GO:0007155;GO:0009653;GO:0005634 | hsa:4162 |  | cell surface glycoprotein MUC18 precursor [Homo sapiens] |
| P43251 | 38404.83263 | 33854.93875 | 0.88152809 | -0.181921553 | 0.169382487 | no | no change | GO:0016787;GO:0005615;GO:0047708;GO:0070062;GO:0016810;GO:0016811;GO:0006768;GO:0006807;GO:0005759;GO:0007417;GO:0005576 | hsa:686 | COG0388 | RecName: Full=Biotinidase; Short=Biotinase; Flags: Precursor |
| P43652 | 149067.1842 | 157090.7458 | 1.053825137 | 0.075635498 | 0.377289057 | no | no change | GO:0008431;GO:0051180;GO:0005615;GO:0072562;GO:0050821;GO:0015031;GO:0005576;GO:0005515;GO:0071693;GO:0046872;GO:0070062 | hsa:173 |  | afamin precursor [Homo sapiens] |
| P48740 | 19459.41895 | 19313.20979 | 0.992486458 | -0.010880677 | 0.943456463 | no | no change | GO:0004252;GO:0006898;GO:0006956;GO:0005615;GO:0016787;GO:0048306;GO:0005829;GO:0005654;GO:0002376;GO:0046872;GO:0005576;GO:0001867;GO:0005509;GO:0005515;GO:0006508;GO:0008233;GO:0042803;GO:0045087;GO:0008236 | hsa:5648 | COG5640 | mannan-binding lectin serine protease 1 isoform 1 precursor [Homo sapiens] |
| P51884 | 83551.94263 | 86967.095 | 1.040874602 | 0.057796272 | 0.578286591 | no | no change | GO:0005201;GO:0018146;GO:0031012;GO:0014070;GO:0043202;GO:0070062;GO:0005583;GO:0070848;GO:0005576;GO:0005796;GO:0032914;GO:0007601;GO:0030021;GO:0062023;GO:0007409;GO:0005615;GO:0051216;GO:0030199;GO:0030198;GO:0045944;GO:0005515;GO:0005518;GO:0042340 | hsa:4060 | COG4886 | lumican precursor [Homo sapiens] |
| P55103 | 8468.860111 | 7873.938294 | 0.929751843 | -0.105082393 | 0.456963312 | no | no change | GO:0005179;GO:0005125;GO:0008083;GO:0060395;GO:0005160;GO:0010469;GO:0048468;GO:0005576;GO:0005615;GO:0010862;GO:0042981;GO:0043408 | hsa:3626 |  | inhibin beta C chain preproprotein [Homo sapiens] |
| P59665 | 138895.9084 | 117345.0063 | 0.844841346 | -0.243247654 | 0.484806057 | no | no change | GO:0071222;GO:0019730;GO:0019731;GO:0035578;GO:0051673;GO:0042742;GO:0050829;GO:0061844;GO:0005615;GO:0030520;GO:0051852;GO:0044657;GO:0002227;GO:0042803;GO:0010818;GO:0005796;GO:0006935;GO:0006952;GO:0050830;GO:0062023;GO:0006955;GO:0070062;GO:0050832;GO:0051607;GO:0031640;GO:0043312;GO:0005576;GO:0042832 | hsa:1667;hsa:728358;hsa:1668 | | neutrophil defensin 1 preproprotein [Homo sapiens] |
| P61626 | 11785.74744 | 11668.72709 | 0.990071028 | -0.014396067 | 0.933199596 | no | no change | GO:0006954;GO:0019730;GO:0016798;GO:0003796;GO:0035578;GO:0042742;GO:0050829;GO:0019835;GO:0044267;GO:0070062;GO:0016998;GO:0035580;GO:0042802;GO:0016787;GO:1904724;GO:0003824;GO:0050830;GO:0008152;GO:0001895;GO:0005615;GO:0031640;GO:0043312;GO:0005576 | hsa:4069 |  | lysozyme C precursor [Homo sapiens] |
| P80108 | 67531.23053 | 62317.795 | 0.922799637 | -0.115910659 | 0.322004251 | no | no change | GO:0035774;GO:0051044;GO:0051047;GO:0035690;GO:0031012;GO:0010907;GO:0001503;GO:0032869;GO:0009749;GO:0010867;GO:0005737;GO:0043065;GO:0070062;GO:0002430;GO:0006501;GO:0045919;GO:0005576;GO:0097241;GO:0006507;GO:0010897;GO:0002062;GO:0004630;GO:0035701;GO:0002042;GO:0008285;GO:0008286;GO:0010983;GO:0071397;GO:0071277;GO:0005615;GO:0010595;GO:0043231;GO:0016787;GO:0070633;GO:0071401;GO:0005765;GO:0017080;GO:0071467;GO:1900076;GO:0004621;GO:0046470;GO:0010694;GO:0005622 | hsa:2822 |  | phosphatidylinositol-glycan-specific phospholipase D precursor [Homo sapiens] |
| Q03591 | 159912.5561 | 160150.8988 | 1.001490456 | 0.002148673 | 0.982454263 | no | no change | GO:0032091;GO:0030449;GO:0005615;GO:0072562;GO:0045919;GO:0005576;GO:0006956;GO:0005515;GO:0032991;GO:0046982;GO:0042803 | hsa:3078 |  | complement factor H-related protein 1 precursor [Homo sapiens] |
| Q06033 | 37503.02105 | 40549.3975 | 1.081230161 | 0.112673662 | 0.428576488 | no | no change | GO:0030212;GO:0070062;GO:0002576;GO:0031089;GO:0005576;GO:0030414;GO:0004866;GO:0004867;GO:0010951;GO:0010466 | hsa:3699 | COG2304 | inter-alpha-trypsin inhibitor heavy chain H3 preproprotein [Homo sapiens] |
| Q08380 | 70615.65263 | 65083.83583 | 0.921663022 | -0.117688725 | 0.476698871 | no | no change | GO:0006968;GO:0070062;GO:0006898;GO:0005044;GO:0002576;GO:0016020;GO:0072562;GO:0031089;GO:0062023;GO:0005615;GO:0007165;GO:0005515;GO:0007155;GO:0005576 | hsa:3959 |  | galectin-3-binding protein precursor [Homo sapiens] |
| Q13201 | 46496.28313 | 39688.43057 | 0.853582865 | -0.228396879 | 0.452599232 | no | no change | GO:0005201;GO:0031093;GO:0010811;GO:0002576;GO:0007596;GO:0031012;GO:0005576;GO:0005509;GO:0005515;GO:0007155;GO:0062023 | hsa:22915 |  | multimerin-1 precursor [Homo sapiens] |
| Q13790 | 70172.31053 | 72003.63667 | 1.026097561 | 0.037167908 | 0.773186656 | no | no change | GO:0005319;GO:0034362;GO:0034364;GO:0006629;GO:0006869;GO:0005615;GO:0005576;GO:0005102;GO:0008203;GO:0008202;GO:0015485 | hsa:319 |  | apolipoprotein F preproprotein [Homo sapiens] |
| Q14520 | 96249.33684 | 100176.1446 | 1.040798284 | 0.057690488 | 0.502627783 | no | no change | GO:0004252;GO:0005615;GO:0016787;GO:0005539;GO:0005576;GO:0005509;GO:0007155;GO:0006508;GO:0008233;GO:0008236 | hsa:3026 | COG5640 | hyaluronan-binding protein 2 isoform 1 preproprotein [Homo sapiens] |
| Q15582 | 14484.47174 | 15606.87442 | 1.077490067 | 0.107674569 | 0.612494075 | no | no change | GO:0005201;GO:0050840;GO:0031012;GO:0007162;GO:0001525;GO:0044267;GO:0070062;GO:0005576;GO:0002062;GO:0005178;GO:0008283;GO:0007601;GO:0062023;GO:0005886;GO:0007155;GO:0050839;GO:0005615;GO:0005604;GO:0050896;GO:0005802;GO:0030198;GO:0005515;GO:0005518 | hsa:7045 | COG2335 | transforming growth factor-beta-induced protein ig-h3 precursor [Homo sapiens] |
| Q15848 | 10299.76947 | 11252.50825 | 1.092500981 | 0.127634575 | 0.511627403 | no | no change | GO:0034115;GO:0005783;GO:0010804;GO:0050728;GO:0009967;GO:0009617;GO:0030853;GO:0043124;GO:0033034;GO:0032720;GO:0043123;GO:2000481;GO:0014823;GO:0072659;GO:0010875;GO:0045650;GO:0071320;GO:0031667;GO:0050731;GO:1904753;GO:0045715;GO:0005125;GO:0033691;GO:0031953;GO:0010906;GO:0046326;GO:0070994;GO:0045721;GO:0010739;GO:0007623;GO:2000467;GO:2000279;GO:0070208;GO:0071872;GO:0019395;GO:0042304;GO:0006006;GO:0034383;GO:0006635;GO:2000478;GO:0050805;GO:0043407;GO:0001666;GO:0010642;GO:0050765;GO:0005581;GO:0032270;GO:0042802;GO:0042803;GO:0009986;GO:0050873;GO:0045892;GO:0046888;GO:0045599;GO:0007584;GO:0120162;GO:0120163;GO:0070543;GO:0042493;GO:1900121;GO:0005515;GO:0005102;GO:0045860;GO:0070373;GO:0045923;GO:0051384;GO:0042593;GO:0035690;GO:0032869;GO:0006091;GO:0005615;GO:0051260;GO:2000590;GO:0010745;GO:0010469;GO:1904706;GO:0005179;GO:0030336;GO:2000534;GO:0034612;GO:0090317;GO:0045471;GO:0009744;GO:0032991;GO:0045776;GO:0009749;GO:0071639;GO:0005576;GO:2000584;GO:0032757;GO:0001934 | hsa:9370 |  | TPA: adiponectin D [Homo sapiens] |
| Q16880 | 655931.7579 | 760697.2958 | 1.159720179 | 0.21377675 | 0.310878157 | no | no change | GO:0008489;GO:0007417;GO:0030913;GO:0016021;GO:0016020;GO:0006682;GO:0016740;GO:0006687;GO:0015020;GO:0006665;GO:0006629;GO:0002175;GO:0008152;GO:0005886;GO:0008194;GO:0007010;GO:0043231;GO:0007422;GO:0048812;GO:0016757;GO:0047263;GO:0016758 | hsa:7368 | COG1819 | 2-hydroxyacylsphingosine 1-beta-galactosyltransferase precursor [Homo sapiens] |
| Q562R1 | 24097.0568 | 24894.69554 | 1.033101086 | 0.046981424 | 0.869526854 | no | no change | GO:0005737;GO:0005856;GO:0005615;GO:0070062;GO:0000166;GO:0003674;GO:0045202;GO:0005515;GO:0008150;GO:0098978;GO:0005524;GO:0015629 | hsa:345651 | COG5277 | beta-actin-like protein 2 [Homo sapiens] |
| Q6UXB8 | 9626.388737 | 11033.6725 | 1.146190207 | 0.196846475 | 0.235048175 | no | no change | GO:0010466;GO:0005576;GO:0030414;GO:0005615;GO:0061052 | hsa:221476 | COG2340 | peptidase inhibitor 16 precursor [Homo sapiens] |
| Q86UD1 | 5391.175643 | 4825.3435 | 0.895044758 | -0.159968266 | 0.323523775 | no | no change |  | hsa:220323 | | out at first protein homolog precursor [Homo sapiens] |
| Q8IV42 | 94837.45412 | 93699.78955 | 0.988004058 | -0.017411127 | 0.9241547 | no | no change | GO:0000049;GO:0016310;GO:0016301;GO:0097056;GO:0016740;GO:0001514;GO:0000166;GO:0006412;GO:0005524 | hsa:118672 | | L-seryl-tRNA(Sec) kinase isoform 2 [Homo sapiens] |
| Q8N1N4 | 133954.8405 | 137721.1304 | 1.028116116 | 0.040003213 | 0.755266491 | no | no change | GO:0005615;GO:0031424;GO:0005829;GO:0045095;GO:0070268;GO:0005198;GO:0005882;GO:0070062 | hsa:196374 | | keratin, type II cytoskeletal 78 isoform 1 [Homo sapiens] |
| Q92954 | 41636.00263 | 47163.22458 | 1.132751023 | 0.179830794 | 0.392872297 | no | no change | GO:0008283;GO:0005044;GO:0006898;GO:0030247;GO:0030021;GO:0005576;GO:0006955;GO:0062023 | hsa:10216 |  | unnamed protein product [Homo sapiens] |
| Q96IY4 | 23595.77158 | 23241.97958 | 0.985006127 | -0.021795396 | 0.843485297 | no | no change | GO:0007599;GO:2000346;GO:0007596;GO:0008270;GO:0030449;GO:0008233;GO:0008237;GO:0005615;GO:0004181;GO:0004180;GO:0071333;GO:0006508;GO:0046872;GO:0051918;GO:0016787;GO:0009408;GO:0070062;GO:0042730;GO:0003331;GO:0097421;GO:0042493;GO:0005623;GO:0005576;GO:0010757 | hsa:1361 | COG2866 | carboxypeptidase B2 isoform 1 preproprotein [Homo sapiens] |
| Q96KN2 | 5848.279083 | 6305.209933 | 1.078130822 | 0.108532247 | 0.576623827 | no | no change | GO:0016787;GO:0032268;GO:0005829;GO:0004180;GO:0016805;GO:0005576;GO:0008152;GO:0006508;GO:0008233;GO:0046872;GO:0008237 | hsa:84735 | COG0624 | RecName: Full=Beta-Ala-His dipeptidase; AltName: Full=CNDP dipeptidase 1; AltName: Full=Carnosine dipeptidase 1; AltName: Full=Glutamate carboxypeptidase-like protein 2; AltName: Full=Serum carnosinase; Flags: Precursor |
| Q96PD5 | 195693.1421 | 198554.5458 | 1.014621891 | 0.020942192 | 0.824814069 | no | no change | GO:0019730;GO:0008745;GO:0032827;GO:0009253;GO:0008270;GO:0044117;GO:0005615;GO:0016045;GO:0016020;GO:0050727;GO:0046872;GO:0002221;GO:0016787;GO:0045087;GO:0050830;GO:0001519;GO:0070062;GO:0005622;GO:0002376;GO:0005576;GO:0032689;GO:0016019;GO:0042834 | hsa:114770 | | N-acetylmuramoyl-L-alanine amidase isoform 1 precursor [Homo sapiens] |
| Q9HDC9 | 7890.958176 | 7222.64681 | 0.91530669 | -0.127672869 | 0.841538568 | no | no change | GO:0016844;GO:0005783;GO:0009986;GO:0016020;GO:0009058;GO:0008150;GO:0004064;GO:0016021 | hsa:57136 | COG3386 | adipocyte plasma membrane-associated protein [Homo sapiens] |
| Q9UHG3 | 20695.87421 | 18711.54929 | 0.904119783 | -0.145414174 | 0.26217332 | no | no change | GO:0034361;GO:1902476;GO:0070062;GO:0030327;GO:0030328;GO:0030329;GO:0099133;GO:0005774;GO:0005764;GO:0016670;GO:0006821;GO:0055114;GO:0005886;GO:0008555;GO:0001735;GO:0016491 | hsa:51449 |  | prenylcysteine oxidase 1 precursor [Homo sapiens] |
| Q9Y5Y7 | 11788.65695 | 11422.03071 | 0.968900084 | -0.045580197 | 0.80351903 | no | no change | GO:0038023;GO:0070062;GO:0005540;GO:0016021;GO:0016020;GO:0009611;GO:0007160;GO:0004888;GO:0071944;GO:0005515;GO:0005887;GO:0005886;GO:0007155;GO:0030214;GO:0009653;GO:0006027 | hsa:10894 |  | lymphatic vessel endothelial hyaluronic acid receptor 1 precursor [Homo sapiens] |
| Q9Y6R7 | 40889.26737 | 36556.8925 | 0.894046161 | -0.161578774 | 0.382323616 | no | no change | GO:0005576;GO:0070062;GO:0005515 | hsa:8857 |  | IgGFc-binding protein precursor [Homo sapiens] |
| A0A075B6K5 | 29325.31315 | 17224.64667 | 0.587364458 | -0.767672125 | 0.206768116 | no | down | GO:0016020;GO:0005615;GO:0006955;GO:0002376;GO:0002377;GO:0005576;GO:0002250;GO:0003823;GO:0005886 | hsa:7441 |  | RecName: Full=Immunoglobulin lambda variable 3-9; Flags: Precursor |
| A0A075B6S9 | 6947.50055 | 4383.2855 | 0.630915459 | -0.664481394 | 0.361934823 | no | down | GO:0002377;GO:0005615;GO:0006955 | hsa:7441 |  | IGKV1D-37 isoform 1, partial [Pan troglodytes] |
| A0A087X0Q4 | 115433.0263 | 85608.28125 | 0.741627279 | -0.431233783 | 0.073597388 | no | down |  |  |  | RecName: Full=Immunoglobulin kappa variable 2D-40; AltName: Full=Ig kappa chain V-II region Cum; Flags: Precursor |
| A0A0B4J1V0 | 36433.38526 | 29888.46875 | 0.820359364 | -0.285672065 | 0.06122355 | no | down | GO:0009897;GO:0050853;GO:0045087;GO:0016020;GO:0006910;GO:0006911;GO:0002376;GO:0005576;GO:0002250;GO:0003823;GO:0005886;GO:0072562;GO:0042571;GO:0006958;GO:0050871;GO:0034987;GO:0042742 | hsa:102724971 | | unnamed protein product [Homo sapiens] |
| A0A0B4J1V2 | 17139.59753 | 6906.333455 | 0.402946069 | -1.311341335 | 0.158795194 | no | down | GO:0009897;GO:0050853;GO:0045087;GO:0016020;GO:0006910;GO:0006911;GO:0002376;GO:0005576;GO:0002250;GO:0003823;GO:0005886;GO:0072562;GO:0042571;GO:0006958;GO:0050871;GO:0034987;GO:0042742 | hsa:102723407 | | RecName: Full=Immunoglobulin heavy variable 2-26; Flags: Precursor |
| A0A0C4DH24 | 50128.34316 | 34211.68261 | 0.682481815 | -0.55113749 | 0.054633578 | no | down | GO:0016020;GO:0005615;GO:0006955;GO:0002376;GO:0002377;GO:0005576;GO:0002250;GO:0003823;GO:0005886 | hsa:7441 |  | RecName: Full=Immunoglobulin kappa variable 6-21; Flags: Precursor |
| A0A0C4DH39 | 34557.78826 | 26098.06167 | 0.755200578 | -0.405068226 | 0.272361063 | no | down | GO:0009897;GO:0050853;GO:0045087;GO:0016020;GO:0006910;GO:0006911;GO:0002376;GO:0005576;GO:0002250;GO:0003823;GO:0005886;GO:0072562;GO:0042571;GO:0006958;GO:0050871;GO:0034987;GO:0042742 | hsa:102723407 | | immunoglobulin heavy chain variable region, partial [Homo sapiens] |
| A0A0C4DH67 | 30398.61047 | 24215.4464 | 0.796597148 | -0.328077781 | 0.137711386 | no | down | GO:0016020;GO:0002376;GO:0005576;GO:0002250;GO:0003823;GO:0005886 | | | Ig kappa V-region e, partial [Homo sapiens] |
| A0A2Q2TTZ9 | 355325.4426 | 269566.5879 | 0.758647019 | -0.398499305 | 0.239790072 | no | down |  | hsa:7441 |  | IGKV1D-33 isoform 2, partial [Pan troglodytes] |
| A0A5H1ZRS2 | 1060716.079 | 792074.7708 | 0.746735895 | -0.421330012 | 0.321234305 | no | down |  |  |  | immunoglobulin kappa chain variable region, partial [Homo sapiens] |
| B0YIW2 | 2192579.847 | 1664405.242 | 0.759108155 | -0.397622645 | 0.247025861 | no | down | GO:0034375;GO:0042627;GO:0034371;GO:0006869;GO:0034379;GO:0034378;GO:0051005;GO:0042157;GO:0005576;GO:0048261;GO:0001523;GO:0055102;GO:0070062;GO:0005543;GO:0045833;GO:0010897;GO:0007186;GO:0062023;GO:0033700;GO:0034361;GO:0034363;GO:0070328;GO:0034366;GO:0006641;GO:0060621;GO:0030234;GO:0070653;GO:0008289;GO:0010916;GO:0042632;GO:0032489;GO:0043691;GO:0005615;GO:0015485;GO:0034382;GO:0050995;GO:0019433;GO:0033344;GO:0010989;GO:0010987;GO:0005769;GO:0045717;GO:0010903 | hsa:345 |  | apolipoprotein C-III precursor variant 1 [Homo sapiens] |
| H9KV75 | 7426.674333 | 6051.9458 | 0.814893117 | -0.295317249 | 0.537327228 | no | down | GO:0019894;GO:0032029;GO:0032391;GO:0030036;GO:0017166;GO:0007041;GO:0042383;GO:0005925;GO:0005923;GO:0030507;GO:0005737;GO:0001725;GO:0097433;GO:0045505;GO:0005815;GO:0003779;GO:0030027;GO:0005509;GO:0042803;GO:0034452;GO:0031941;GO:0051393;GO:0016328;GO:0030486;GO:0048741;GO:1990357;GO:0051017;GO:0031252;GO:0051015;GO:0005915;GO:0045214;GO:0051764;GO:0097381;GO:0007030;GO:0090636;GO:0090637 | hsa:87 | COG5069 | alpha-actinin-1 isoform c [Homo sapiens] |
| P00918 | 9617.187471 | 7127.791125 | 0.741151313 | -0.432159982 | 0.086743044 | no | down | 3P3J:A;1IF6:A;1H9N:A;3RYJ:B;5FLQ:A;3P5A:A;1AVN:A;GO:0010043;6BC9:A;3V3H:B;4Q78:A;5JGS:B;5M78:A;1CAM:A;GO:0043209;5TY9:A;3RZ1:B;5EOI:A;5JN3:A;1CNI:A;4ZWY:A;3PJJ:A;3TMJ:A;5LL4:A;4CAC:A;1CNK:A;3T5U:A;6EQU:A;5EH8:A;1G52:A;5JEG:B;1AM6:A;3SBH:A;1CVD:A;4BF1:A;1BNU:A;1I91:A;4PYY:A;3MHL:A;4HEY:A;5FLO:A;5NXP:A;3S78:B;4Q08:A;4K1Q:A;2O4Z:A;2Q38:A;3DD8:A;5CAC:A;4YYT:A;2VVB:X;1CNC:A;2FNN:A;5G0C:A;1CVB:A;1FR7:B;2FOQ:A;3RZ7:A;4KNI:A;6H2Z:A;4QTL:A;4QEF:A;4FVO:A;6BCC:A;3M1Q:A;5LLG:A;6E92:A;3T83:A;5WEX:A;3D8W:A;4YXI:A;5WLV:A;4Q6D:A;5JQT:A;4YXO:A;5TFX:A;2HKK:A;2POU:A;3M1W:A;6MBY:A;5DOH:B;5OGO:A;4QSI:A;1BNV:A;3DBU:A;1IF9:A;3PYK:A;1CAK:A;1G46:A;6D1L:A;5TY8:A;5DRS:A;5E28:A;4Q9Y:A;5LLC:A;6FJI:A;3MNH:A;1I9P:A;1CIL:A;4FIK:A;6BBS:A;3OIL:A;1LG6:A;4BF6:A;6IC2:A;6C7W:A;1CIN:A;1G4O:A;1BNM:A;4YGL:A;4HF3:A;2CBE:A;3KOK:A;GO:0016829;4MDG:A;3HS4:A;5DSM:A;4K0Z:A;2F14:A;5FNL:A;4JSW:A;GO:0045177;5BRU:A;5DSO:A;3DVC:A;2WD2:A;3RG3:A;4KUV:A;4HEZ:A;5LL4:B;1I9M:A;1RZD:A;5DSL:A;3B4F:A;1TE3:X;5BNL:A;4YGK:A;GO:0016323;5U0G:A;5LMD:A;5CA2:A;4DZ9:A;5SZ1:A;1ZSB:A;1YDB:A;1YO0:A;4E49:A;4YVY:A;4KV0:A;2X7S:A;5THN:A;4KUW:A;1YDD:A;1YO2:A;1I8Z:A;2X7U:A;2CBC:A;5JQ0:A;4QSA:A;3S74:B;3P58:A;3M67:A;3IGP:A;3S76:A;3EFT:A;5TYA:A;3IEO:A;4JSZ:A;3V7X:A;5N24:A;3HKU:A;3KKX:A;5ZXW:A;1BN3:A;2EZ7:A;5JDV:B;5FLT:A;4E4A:A;GO:0030424;1CVH:A;GO:0004064;1XEG:A;5G03:A;2AW1:A;3F8E:A;1RAZ:A;5W8B:A;5G01:A;5N1R:A;5L70:B;2ABE:A;2EU3:A;5L70:A;3IBU:A;GO:0005615;6EDA:A;2GEH:A;5N1S:A;4Q7W:A;1CAY:A;5EKM:A;3D93:A;3M2Z:A;5MJN:A;1ZFQ:A;2NNO:A;GO:0038166;3R17:B;3S9T:A;1BV3:A;5VGY:A;4IWZ:A;1G3Z:A;5DSP:A;4Z0Q:A;GO:0045780;1XPZ:A;3RZ5:A;1UGD:A;GO:0016020;5AMD:A;1RAY:A;4M2U:A;1UGF:A;3DV7:A;3S75:B;1RZE:A;4PXX:A;1BNT:A;5DOG:A;3P4V:A;2WEG:A;6EBE:A;3KS3:A;5WLR:A;5SZ2:A;1KWQ:A;1CIM:A;1CNG:A;5AML:A;2NNV:A;GO:0005829;1CCS:A;5OGP:A;GO:0043627;1CVF:A;1TTM:A;4FVN:A;4JSA:A;1TBT:X;1FSQ:A;5SZ3:A;4RFC:A;1FSQ:B;3MNJ:A;2NWP:A;5FNJ:A;1OKM:A;5LLE:A;4ITP:A;2WD3:A;3K7K:A;1G4J:A;2H4N:A;4HBA:A;1ZH9:A;4R5B:A;5T71:A;6ECZ:A;5L6T:A;5L6T:B;2AX2:A;3P44:A;5TI0:A;GO:2001225;2QOA:A;6CA2:A;4G0C:A;1CA2:A;1CAO:A;3V3F:A;4Z1K:A;1FR7:A;4QK3:A;1CAI:A;5N0E:A;4PQ7:A;3HKN:A;1RZC:A;1ZGF:A;4QK1:A;5GMN:A;4E3D:A;1CNW:A;5ULN:A;GO:0048545;2FOV:A;4FRC:A;2FNK:A;2HD6:A;2WEO:A;1TG9:A;3M40:A;5BRV:A;4Q7P:A;4Q8Z:A;2QO8:A;5DSK:A;4RIV:A;3KOI:A;5Y2S:A;4Q8X:A;3HLJ:A;1LZV:A;1G54:A;5C8I:A;3L14:A;3N2P:A;3OYQ:A;3CAJ:A;2POV:A;6EEO:A;4WL4:A;6B4D:A;5YUJ:A;4FPT:A;3GZ0:A;5NXV:A;1OQ5:A;6GOT:A;5J8Z:A;3ZP9:A;4RUY:A;6B5A:A;2NWZ:A;1I9O:A;1THK:A;2EU2:A;5SZ7:A;4L5W:A;4M2W:A;6H6S:A;5JMZ:A;4Q7V:A;2Q1Q:A;4HT0:A;4QSB:A;4QJM:A;1BNN:A;2NXS:A;GO:0005515;3M1K:A;4Q99:A;3M2X:A;1H9Q:A;6G6T:A;4MDM:A;1FQM:A;5FDC:A;1H4N:A;5SZ5:A;4Q6E:A;3SAP:A;1EOU:A;1TEQ:X;5FLP:A;5T75:A;5NXI:A;3K34:A;2HOC:A;6CJV:A;6FJJ:A;1CNJ:A;3P5L:A;1G53:A;3V5G:A;4MDL:A;1ZSC:A;6HX5:A;4XE1:A;1ZSA:A;1TG3:A;6GXE:A;5JEH:B;4MO8:A;2X7T:A;4CA2:A;1IF7:A;6CEH:A;4QY3:A;GO:0046903;1LGD:A;4Q09:A;2CBD:A;1IF5:A;1CNB:A;3FFP:X;3DCW:A;5NXO:A;5LL8:A;3RYY:A;4Z1E:A;3R16:A;3BET:A;GO:0032849;5JEP:B;5G0B:A;4ZAO:A;3ML2:A;1BCD:A;3TVN:X;4Y0J:A;5NXW:A;6EEA:A;1BN4:A;1UGB:A;4K13:A;5DOH:A;3C7P:A;4RFD:A;GO:0001822;1TB0:X;5SZ4:A;5DSQ:A;GO:0046872;5L6K:B;3N3J:A;4Q07:A;3T84:A;1IF8:A;12CA:A;1DCA:A;4QK2:A;5E2K:A;4Q83:A;3SAX:A;5LVS:B;5T74:A;1CAZ:A;6D1M:A;6GXB:A;3RYZ:A;5LVS:A;2FMZ:A;3IBI:A;2QP6:A;1I9Q:A;4E5Q:A;3OIM:A;5NEA:A;6B59:A;6H29:A;2FOU:A;3M5T:A;5THI:A;4GL1:X;4Q87:A;2WEJ:A;1G0F:A;GO:2001150;6H34:A;6G3Q:A;3CYU:A;5O07:A;6QEB:A;3RGE:A;5E2R:A;5YUK:A;5EH5:A;4MLT:A;5EIJ:A;3RZ0:B;5FLS:A;3NB5:A;5EH7:A;1MOO:A;3U3A:X;4Z1J:A;3SBI:A;4Q90:A;2VVA:X;1I90:A;1XQ0:A;5NXG:A;1ZFK:A;3MHO:A;5FNI:A;6H6S:B;2OSM:A;3VBD:A;2FNM:A;3MHM:A;4K0S:A;3MNU:A;5EHV:A;5FNG:A;4E3G:A;3RZ8:A;4YWP:A;5THJ:A;1CRA:A;1RZA:A;2NNS:A;1FR4:A;3T5Z:A;1F2W:A;1CVC:A;3RG4:A;5TH4:A;3PO6:A;5WLU:A;5JN7:A;6E91:A;4ZX0:A;1Z9Y:A;5JN1:A;1HVA:A;GO:0009268;3NI5:A;5A6H:A;3S77:B;GO:0005737;3S71:B;4Q49:A;4ILX:A;3HKT:A;5U0F:A;1BIC:A;3OYS:A;3M96:A;2FMG:A;3KIG:A;5FLR:A;5OGN:B;6GCY:A;2NXT:A;1CAH:A;5SZ0:A;4E3H:A;4ITO:A;4QIY:C;4QIY:B;4QIY:A;4R5A:A;3DCC:A;1CNX:A;1CAJ:A;4MLX:A;2Q1B:A;3M04:A;4L5U:A;1ZGE:A;1LG5:A;5FDI:A;GO:0070062;3D92:A;2H15:A;3MZC:A;3F4X:A;1FQL:A;8CA2:A;5JG3:B;1UGA:A;5E2S:A;4LHI:A;5NY1:A;3M14:A;5LJT:A;3IBN:A;3P55:A;3V3G:B;1CVA:A;3D9Z:A;1HCA:A;5NXM:A;1BNQ:A;GO:0044070;3DVB:A;5JGT:B;4YGN:A;1UGG:A;3CA2:A;1BNW:A;3V2J:A;GO:0010033;1YDA:A;1I9L:A;4ZWZ:A;3RJ7:A;1YDC:A;3DAZ:A;6EEH:A;5WLT:A;6E8P:A;4M2R:A;5L6K:A;1TEU:X;3DCS:A;2CBB:A;3BL0:A;1FSR:B;1FSR:A;1LUG:A;2OSF:A;2ILI:A;1MUA:A;4N16:A;2GD8:A;3MMF:A;5U0D:A;4E3F:A;2HL4:A;3OKV:A;1ZE8:A;5TY1:A;GO:0008270;5JE7:B;3DC9:A;3V3J:A;3S73:B;GO:0032230;3M5S:A;1T9N:A;4Q06:A;1CCU:A;5AMG:A;3MWO:B;7CA2:A;3MWO:A;5T72:A;GO:0004089;5BYI:A;3DC3:A;1CNH:A;2NNG:A;1CA3:A;5EKH:A;5WG7:A;3OY0:A;5NY3:A;1FQR:A;1HEC:A;9CA2:A;2FOS:A;5EKJ:A;3V2M:A;3N4B:A;1HEA:A;2NWO:A;5NEE:A;3K2F:A;6E8X:A;3M2Y:A;3P3H:A;3U7C:A;4FL7:A;4Q81:A;4WW6:A;GO:0015701;3RYV:B;3S8X:A;5U0E:A;GO:0051453;3EFI:A;4RH2:A;5EHW:A;2WEH:A;4Q8Y:A;2POW:A;4Z1N:A;1HEB:A;4RIU:A;1CAL:A;5FNM:A;1I9N:A;4ZWX:A;1G1D:A;1KWR:A;5TXY:A;3MHI:A;5LLH:A;1CCT:A;4RN4:A;3KWA:A;4CQ0:A;4YXU:A;1CVE:A;5SZ6:A;4KAP:A;3QYK:A;4PYX:A;4YGJ:A;2NWY:A;1FSN:B;1FSN:A;4HEW:A;3V3I:B;4N0X:B;1YO1:A;4KNJ:A;3MNK:A;3HFP:A;4FU5:A;5BRW:A;GO:0005886;1G0E:A;1OKN:A;5N25:A;5CLU:A;4JS6:A;GO:0005902;4PZH:A;1OKL:A;5L9E:C;3M2N:A;5LJQ:A;3T82:A;1FQN:A;6MBV:A;4RUZ:A;4DZ7:A;5DSR:A;1A42:A;4RUX:A;3MNA:A;5L3O:B;1CNY:A;4ZWI:A;1CAN:A;3U45:X;3OIK:A;4KUY:A;5NY6:A;3U47:A;5YUI:A;2CBA:A;5N0D:A;5FNH:A;6H33:A;4K0T:A;3N0N:A;3RLD:A;3BL1:A;4ZX1:A;5UMC:A;1RZB:A;3M5E:A;5OGN:A;4LP6:B;4LP6:A;1G45:A;3MNI:A;6GDC:A;3RYX:B;GO:0015670;4YX4:A;1IF4:A;5JG5:B;4JSS:A;3DVD:A;4BCW:A;5WGP:A;5Y2R:A;3TVO:X;3IBL:A;6C7X:A;GO:0002009;3DD0:A;3S72:B;3M3X:A;1UGC:A;4R59:A;3HKQ:A;1UGE:A;3T85:A;4QIY:D;3MYQ:A;4M2V:A;1G48:A;GO:0071498;1XEV:B;2HNC:A;5JES:B;4L5V:A;3NJ9:A;1TH9:A;5L9E:A;1DCB:A;3M98:A;5L9E:B;5L3O:A;5L9E:D;5FNK:A;2CA2:A;GO:0042475;4IDR:X;3KNE:A;5NYA:A;3M1J:A;1HED:A;3MHC:A;2NXR:A;GO:0045672;4Q7S:A;3OKU:A;3KON:A;1XEV:C;5EHE:A;1XEV:A;4MTY:A;1XEV:D;1BN1:A | hsa:760 | COG3338 | carbonic anhydrase 2 isoform 1 [Homo sapiens] |
| P01599 | 167274.5911 | 135019.8892 | 0.807175126 | -0.309046377 | 0.11703361 | no | down | GO:0038096;GO:0004252;GO:0016020;GO:0030449;GO:0050776;GO:0006898;GO:0038095;GO:0072562;GO:0002376;GO:0050900;GO:0005576;GO:0006956;GO:0002250;GO:0003823;GO:0005886;GO:0006508;GO:0006958;GO:0006955;GO:0070062 | hsa:7441 |  | RecName: Full=Immunoglobulin kappa variable 1-17; AltName: Full=Ig kappa chain V-I region Gal; AltName: Full=Ig kappa chain V-I region WEA; Flags: Precursor |
| P01718 | 29956.94232 | 19821.73289 | 0.661674101 | -0.595807283 | 0.349800508 | no | down | GO:0038096;GO:0004252;GO:0016020;GO:0030449;GO:0050776;GO:0006898;GO:0038095;GO:0002376;GO:0050900;GO:0005576;GO:0006956;GO:0002250;GO:0003823;GO:0005886;GO:0006508;GO:0006958;GO:0006955 | hsa:29802 |  | immunoglobulin light chain variable region, partial [Homo sapiens] |
| P01871 | 12768356.63 | 10420076.83 | 0.81608598 | -0.293206938 | 0.307288394 | no | down | GO:0009897;GO:0042834;GO:0019731;GO:0050900;GO:0034987;GO:0050829;GO:0005615;GO:0016021;GO:0016020;GO:0072562;GO:0009986;GO:0050853;GO:0045087;GO:0006910;GO:0006911;GO:0031210;GO:0003823;GO:0005886;GO:0006958;GO:0050871;GO:0070062;GO:0003697;GO:0002376;GO:0005576;GO:0002250;GO:0005515;GO:0071756;GO:0071757 | hsa:3543 |  | immunoglobulin heavy chain [Homo sapiens] |
| P05109 | 8420.225188 | 6087.032125 | 0.722906097 | -0.468119837 | 0.190224859 | no | down | GO:0045087;GO:0019730;GO:0030307;GO:0032602;GO:0043312;GO:0050786;GO:0008270;GO:0014002;GO:0010043;GO:0032496;GO:0005737;GO:0001816;GO:0045111;GO:0050729;GO:0005615;GO:0005634;GO:0016020;GO:0051493;GO:0050727;GO:0005509;GO:0002224;GO:2001244;GO:0034774;GO:0046872;GO:0035662;GO:0008017;GO:0005856;GO:0030593;GO:0006935;GO:0006919;GO:0032119;GO:0006914;GO:0006915;GO:0018119;GO:0042060;GO:0050832;GO:0045471;GO:0070488;GO:0006954;GO:0005886;GO:0002526;GO:0002523;GO:0070062;GO:0051092;GO:0050544;GO:0005829;GO:0002793;GO:0002544;GO:0002376;GO:0005576;GO:0005515;GO:0042742 | hsa:6279 |  | protein S100-A8 isoform d [Homo sapiens] |
| P06331 | 708095.7105 | 520961.7667 | 0.735722246 | -0.44276688 | 0.468955913 | no | down | GO:0038096;GO:0004252;GO:0016020;GO:0030449;GO:0050776;GO:0006898;GO:0038095;GO:0002376;GO:0050900;GO:0005576;GO:0006956;GO:0002250;GO:0003823;GO:0005886;GO:0006508;GO:0006958;GO:0006955 | hsa:102724971 | | hCG1793614, partial [Homo sapiens] |
| P13796 | 2568131.265 | 1360041.786 | 0.529584218 | -0.917067967 | 0.230317143 | no | down | GO:0032432;GO:0033157;GO:0015629;GO:0030175;GO:0051020;GO:0005925;GO:0030054;GO:0005737;GO:0071803;GO:0001726;GO:0002102;GO:0002286;GO:0005615;GO:0016020;GO:0003779;GO:0005509;GO:0048471;GO:0016477;GO:0022617;GO:0042802;GO:0035722;GO:0005178;GO:0031100;GO:0051017;GO:0051015;GO:0005884;GO:0005886;GO:0051764;GO:0042995;GO:0032587;GO:0001891;GO:0046872;GO:0070062;GO:0005829;GO:0005856;GO:0051639;GO:0044319;GO:0001725;GO:0010737 | hsa:3936 | COG5069 | plastin-2 [Homo sapiens] |
| P19320 | 5751.492059 | 4600.265593 | 0.799838641 | -0.322219114 | 0.602450353 | no | down | GO:0009897;GO:0034113;GO:0005783;GO:0005794;GO:0035094;GO:0030175;GO:0060945;GO:0005902;GO:0050901;GO:0060326;GO:0010043;GO:0032496;GO:0001666;GO:0019221;GO:0045177;GO:0002102;GO:0010212;GO:0005615;GO:0060384;GO:0071356;GO:0016021;GO:0016020;GO:0050839;GO:0035584;GO:1904646;GO:0140039;GO:0022614;GO:0005178;GO:0042383;GO:0009308;GO:0030183;GO:0098609;GO:0007584;GO:0008131;GO:0045471;GO:0007159;GO:0005887;GO:0005886;GO:0007155;GO:0002526;GO:0007157;GO:0042102;GO:0009986;GO:0055114;GO:0070062;GO:0007160;GO:0050776;GO:0007568;GO:0060333;GO:0002544;GO:0005769;GO:0030198;GO:0035924;GO:0071065 | hsa:7412 |  | vascular cell adhesion protein 1 isoform a precursor [Homo sapiens] |
| P55056 | 234977.8321 | 171445.4054 | 0.729623743 | -0.454775417 | 0.129086802 | no | down | GO:0005319;GO:0034361;GO:0070328;GO:0006629;GO:0034447;GO:0006869;GO:0034379;GO:0010890;GO:0005576;GO:0034364 | hsa:346 |  | apolipoprotein C-IV precursor [Homo sapiens] |
| P68871 | 5874401.842 | 4757867.333 | 0.809932221 | -0.304126913 | 0.181582133 | no | down | GO:0005344;GO:0007596;GO:0015701;GO:1904813;GO:0045429;GO:0042744;GO:0008217;GO:0010942;GO:0050880;GO:0005615;GO:0071682;GO:0072562;GO:0005833;GO:0098869;GO:0046872;GO:0015671;GO:0070293;GO:0030185;GO:1904724;GO:0020037;GO:0031721;GO:0031720;GO:0051291;GO:0070062;GO:0019825;GO:0042542;GO:0030492;GO:0005829;GO:0006898;GO:0043312;GO:0070527;GO:0043177;GO:0005576;GO:0005515;GO:0031838;GO:0004601 | hsa:3043 | COG1018 | PREDICTED: hemoglobin subunit beta [Gorilla gorilla gorilla] |
| Q13093 | 6661.107222 | 5369.202353 | 0.806052534 | -0.311054227 | 0.208323912 | no | down | GO:0034441;GO:0034440;GO:0034362;GO:0034374;GO:0047499;GO:0016787;GO:0006629;GO:0005615;GO:0005543;GO:0005737;GO:0016788;GO:0016042;GO:0050729;GO:0090026;GO:0003847;GO:0046469;GO:0005576 | hsa:7941 |  | platelet-activating factor acetylhydrolase precursor [Homo sapiens] |
| Q9Y490 | 79157.93965 | 15657.33965 | 0.197798726 | -2.337894958 | 0.181785436 | no | down | GO:0005200;GO:0017166;GO:0007043;GO:0005925;GO:0030054;GO:0005856;GO:0001726;GO:0070062;GO:0002576;GO:0016020;GO:0005737;GO:0003779;GO:0005576;GO:0033622;GO:0001786;GO:0005178;GO:0005515;GO:0006936;GO:0009986;GO:0051015;GO:0036498;GO:0030274;GO:0005886;GO:0007155;GO:0042995;GO:0032587;GO:0007016;GO:0035091;GO:0005829;GO:0030866;GO:0045296;GO:0070527;GO:0007044;GO:0016032;GO:0044877;GO:0007229 | hsa:7094 |  | talin-1 [Homo sapiens] |

| Table S4-2 Proteins identified in S vs A group. | | | | | | | | | | | |
| --- | --- | --- | --- | --- | --- | --- | --- | --- | --- | --- | --- |
| Accession | A | S | FC(S/A) | log2FC(S/A) | Pvalue(S/A) | significant | regulate | GO | KEGG | COG | Description |
| A0A0C4DH32 | 61163.57889 | 79619.69304 | 1.301750069 | 0.380452483 | 0.042460935 | yes | up | GO:0009897;GO:0050853;GO:0045087;GO:0016020;GO:0006910;GO:0006911;GO:0002376;GO:0005576;GO:0002250;GO:0003823;GO:0005886;GO:0072562;GO:0042571;GO:0006958;GO:0050871;GO:0034987;GO:0042742 | hsa:102723407 | | RecName: Full=Immunoglobulin heavy variable 3-20; Flags: Precursor |
| A0A0C4DH73 | 64750.49632 | 84374.96458 | 1.303078268 | 0.38192374 | 0.045286937 | yes | up | GO:0038096;GO:0004252;GO:0016020;GO:0030449;GO:0005615;GO:0006898;GO:0038095;GO:0050900;GO:0002376;GO:0002377;GO:0050776;GO:0005576;GO:0006956;GO:0002250;GO:0003823;GO:0005886;GO:0006508;GO:0006958;GO:0006955 | hsa:7441 |  | immunoglobulin light chain variable region, partial [Homo sapiens] |
| A0A0G2JSC0 | 28074.66676 | 49210.14461 | 1.752830943 | 0.809686857 | 0.029846821 | yes | up |  | hsa:7441 |  | Lambda-V immunoglobulin light chain variable domain precursor, partial [Homo sapiens] |
| A0A0J9YX35 | 22011.14278 | 62925.73136 | 2.858812557 | 1.51541603 | 1.20E-06 | yes | up | GO:0016020;GO:0002376;GO:0005576;GO:0002250;GO:0003823;GO:0005886 | hsa:102723407 | | RecName: Full=Immunoglobulin heavy variable 3-64D; Flags: Precursor |
| A0A182DWH7 | 24868.99737 | 32971.73625 | 1.32581687 | 0.406881515 | 0.002087313 | yes | up | GO:0008430 | hsa:6414 |  | Selenoprotein P, plasma, 1 [Homo sapiens] |
| A0A5H1ZRQ7 | 13185.15492 | 19808.58905 | 1.502340259 | 0.5872116 | 0.033223998 | yes | up |  | hsa:100423062 | | RecName: Full=Immunoglobulin lambda constant 7; AltName: Full=Ig lambda-7 chain C region |
| C9JPQ9 | 1603108.447 | 2232012.625 | 1.392302953 | 0.477473163 | 0.00326496 | yes | up | GO:0051258;GO:0005102;GO:0007596;GO:0005577;GO:0030168 | hsa:2266 |  | hypothetical protein, partial [Homo sapiens] |
| E7EUT5 | 7822.728632 | 10620.43657 | 1.357638372 | 0.441099247 | 0.023942148 | yes | up | GO:0051287;GO:0050821;GO:0000226;GO:0097718;GO:0006096;GO:0061844;GO:0005737;GO:0004365;GO:0005634;GO:0005811;GO:0016620;GO:0051873;GO:0097452;GO:0042802;GO:0035605;GO:0035606;GO:0015630;GO:0008017;GO:0031965;GO:0050661;GO:0050832;GO:0051402;GO:0052501;GO:0005886;GO:0055114;GO:0043231;GO:0050715;GO:0005829;GO:0071346;GO:1990904;GO:0019828;GO:0017148;GO:0006417;GO:0006006 | hsa:2597 | COG0057 | GAPDH isoform 4 [Pan troglodytes] |
| K7ER74 | 395514.3247 | 692444.0838 | 1.750743375 | 0.807967628 | 0.023297767 | yes | up | GO:0034375;GO:0034372;GO:0034371;GO:0034370;GO:0042627;GO:0034378;GO:0051006;GO:0010902;GO:0008047;GO:0048261;GO:0001523;GO:0043085;GO:0055102;GO:0005615;GO:0032375;GO:0005576;GO:0016042;GO:0045833;GO:0016004;GO:0010898;GO:0033700;GO:0034361;GO:0034362;GO:0034363;GO:0070328;GO:0006629;GO:0034366;GO:0060230;GO:0008289;GO:0006869;GO:0042803;GO:0010916;GO:0043274;GO:0042632;GO:0043691;GO:0034382;GO:0042493;GO:0034384;GO:0010518;GO:0033344;GO:0042953;GO:0060697;GO:0045723;GO:0005769 | hsa:344 |  | apolipoprotein C-II isoform X1 [Mesocricetus auratus] |
| O75636 | 36592.94316 | 62773.78417 | 1.715461473 | 0.778596725 | 0.000417904 | yes | up | GO:0004252;GO:0051607;GO:0006956;GO:0003823;GO:1902679;GO:0045087;GO:0030246;GO:0043654;GO:0002376;GO:0046597;GO:0005576;GO:0001867;GO:0072562;GO:0005515;GO:0006508;GO:0046872;GO:0005581 | hsa:8547 |  | ficolin-3 isoform 1 precursor [Homo sapiens] |
| P01817 | 32489.94826 | 45693.13 | 1.406377432 | 0.491983825 | 0.021302828 | yes | up | GO:0038096;GO:0004252;GO:0016020;GO:0030449;GO:0050776;GO:0006898;GO:0038095;GO:0002376;GO:0050900;GO:0005576;GO:0006956;GO:0002250;GO:0003823;GO:0005886;GO:0006508;GO:0006958;GO:0006955 | hsa:102723407 | | RecName: Full=Immunoglobulin heavy variable 2-5; AltName: Full=Ig heavy chain V-II region HE; AltName: Full=Ig heavy chain V-II region MCE; Flags: Precursor |
| P02751 | 534412.4632 | 905640.4042 | 1.694646863 | 0.76098467 | 5.03E-05 | yes | up | GO:0008022;GO:0018149;GO:0019221;GO:0034446;GO:0048146;GO:0005518;GO:0005201;GO:0005788;GO:0062023;GO:0007160;GO:0007161;GO:0050900;GO:0010628;GO:0097718;GO:0001525;GO:0044267;GO:0070062;GO:0031093;GO:0002576;GO:0051087;GO:0009611;GO:0072562;GO:0008201;GO:0005576;GO:0002020;GO:0030198;GO:1904237;GO:0033622;GO:0042802;GO:0008284;GO:0005178;GO:2001202;GO:0008360;GO:0005793;GO:0016324;GO:0043687;GO:0042060;GO:0006953;GO:0043394;GO:0019899;GO:0007155;GO:0051702;GO:0010952;GO:0035987;GO:1901166;GO:0005615;GO:0052047;GO:0005604;GO:0045773;GO:0031012;GO:0005577;GO:0007044;GO:0005515;GO:0016504;GO:0005102;GO:0001932;GO:0070372 | hsa:2335 |  | fibronectin isoform 1 precursor [Homo sapiens] |
| P05543 | 23109.91774 | 27862.02542 | 1.205630662 | 0.269788013 | 0.037004927 | yes | up | GO:0005615;GO:0070327;GO:0005576;GO:0004867;GO:0010951;GO:0070062 | hsa:6906 | COG4826 | thyroxine-binding globulin precursor [Homo sapiens] |
| P15169 | 20256.22737 | 26973.77833 | 1.331628928 | 0.413192117 | 0.001876458 | yes | up | GO:0097060;GO:0005794;GO:0051384;GO:0030449;GO:0005615;GO:0016787;GO:0030141;GO:0004181;GO:0004180;GO:0010815;GO:0004185;GO:0005576;GO:0043025;GO:0008270;GO:0016485;GO:0006508;GO:0006518;GO:0046872;GO:0030070;GO:0008233;GO:0008237 | hsa:1369 |  | carboxypeptidase N catalytic chain precursor [Homo sapiens] |
| P19823 | 1107498.8 | 1357782.583 | 1.225990117 | 0.293947349 | 0.000769918 | yes | up | GO:0044267;GO:0030212;GO:0070062;GO:0043687;GO:0072562;GO:0062023;GO:0030414;GO:0005788;GO:0004866;GO:0004867;GO:0010951;GO:0010466;GO:0005576 | hsa:3698 | COG2304 | inter-alpha-trypsin inhibitor heavy chain H2 precursor [Homo sapiens] |
| P35527 | 88404.13842 | 152461.3892 | 1.724595612 | 0.786258114 | 2.77E-05 | yes | up | GO:0005200;GO:0045109;GO:0031424;GO:0005615;GO:0005829;GO:0016020;GO:0070268;GO:0005198;GO:0007283;GO:0005882;GO:0008544;GO:0005634;GO:0043588;GO:0070062 | hsa:3857 |  | keratin, type I cytoskeletal 9 [Homo sapiens] |
| P55058 | 13904.45947 | 22844.77083 | 1.642981583 | 0.716316308 | 4.20E-06 | yes | up | GO:0034375;GO:0006869;GO:0015914;GO:1990050;GO:0035627;GO:0005548;GO:0005615;GO:0019992;GO:0008525;GO:0097001;GO:0010875;GO:0030317;GO:0010189;GO:0005319;GO:0035620;GO:0034364;GO:0006629;GO:0070300;GO:0008289;GO:0031210;GO:0008429;GO:1904121;GO:0005576;GO:1901611 | hsa:5360 |  | phospholipid transfer protein, isoform CRA_c [Homo sapiens] |
| Q08380 | 70615.65263 | 89176.44583 | 1.262842479 | 0.336674696 | 0.045558323 | yes | up | GO:0006968;GO:0070062;GO:0006898;GO:0005044;GO:0002576;GO:0016020;GO:0072562;GO:0031089;GO:0062023;GO:0005615;GO:0007165;GO:0005515;GO:0007155;GO:0005576 | hsa:3959 |  | galectin-3-binding protein precursor [Homo sapiens] |
| Q9NZP8 | 30579.27511 | 65146.30458 | 2.130407093 | 1.091129137 | 0.010821113 | yes | up | GO:0004252;GO:0005615;GO:0016787;GO:0070062;GO:0045087;GO:0031638;GO:0002376;GO:0005576;GO:0006508;GO:0006958;GO:0008233;GO:0008236 | hsa:51279 | COG5640 | complement C1r subcomponent-like protein isoform 1 precursor [Homo sapiens] |
| A0A0C4DH36 | 53260.81474 | 62414.52333 | 1.171865726 | 0.228807273 | 0.048555737 | yes | no change | GO:0009897;GO:0050853;GO:0045087;GO:0006910;GO:0006911;GO:0042742;GO:0003823;GO:0034987;GO:0042571;GO:0006958;GO:0050871;GO:0072562 | hsa:102723407 | | immunoglobulin heavy chain variable gene IGHV3-38, partial [Homo sapiens] |
| A0A0U1RQV3 | 27943.68526 | 23377.98833 | 0.83661078 | -0.257371507 | 0.004074767 | yes | no change | GO:0062023;GO:0007173;GO:0005509;GO:0005006 | hsa:2202 |  | EGF-containing fibulin-like extracellular matrix protein 1 isoform X3 [Homo sapiens] |
| H0YAC1 | 155518.0774 | 133550.6958 | 0.858747086 | -0.219694796 | 0.016436019 | yes | no change | GO:0004252;GO:0004497;GO:0007597;GO:0008236;GO:0008233;GO:0016491;GO:0070062;GO:0016705;GO:0031639;GO:0031638;GO:0006508;GO:0005506;GO:0046872;GO:0022617;GO:0051919;GO:0016787;GO:0020037;GO:0005886;GO:0055114;GO:0005615;GO:0042730;GO:0002542;GO:0005576 | hsa:3818 | COG5640 | KLKB1 isoform 4, partial [Pan troglodytes] |
| P00740 | 36940.94105 | 31076.98208 | 0.841261246 | -0.24937421 | 0.000120828 | yes | no change | GO:0004252;GO:0004175;GO:0006888;GO:0005615;GO:0016787;GO:0070062;GO:0007597;GO:0007596;GO:0031638;GO:0005788;GO:0005796;GO:0005576;GO:0005509;GO:0005515;GO:0005886;GO:0006508;GO:0008233;GO:0046872;GO:0007599;GO:0008236 | hsa:2158 | COG5640 | coagulation factor IX isoform 1 preproprotein [Homo sapiens] |
| P01834 | 27249663.16 | 22862797.67 | 0.83901212 | -0.253236443 | 0.043113819 | yes | no change | GO:0004252;GO:0030449;GO:0009897;GO:0006955;GO:0050871;GO:0042742;GO:0034987;GO:0042571;GO:0005615;GO:0016020;GO:0072562;GO:0006508;GO:0050853;GO:0045087;GO:0006910;GO:0006911;GO:0050776;GO:0006956;GO:0003823;GO:0005886;GO:0006958;GO:0001895;GO:0070062;GO:0038096;GO:0038095;GO:0006898;GO:0050900;GO:0002376;GO:0005576;GO:0002250 | hsa:100423062 | | light chain kappa Sci, k Sci=Bence Jones protein [human, Peptide, 214 aa] |
| P02749 | 1067318.932 | 912255.625 | 0.85471699 | -0.226481294 | 0.023860219 | yes | no change | GO:0034392;GO:0007597;GO:0051006;GO:0034197;GO:0042627;GO:0070062;GO:0002576;GO:0005543;GO:0031639;GO:0051917;GO:0042802;GO:0051918;GO:0034361;GO:0034364;GO:0006641;GO:0009986;GO:0060230;GO:0008289;GO:0062023;GO:0033033;GO:0008201;GO:0005615;GO:0010596;GO:0031089;GO:0005576;GO:0030195;GO:0005515;GO:0030193;GO:0016525;GO:0001937;GO:0030194 | hsa:350 |  | beta-2-glycoprotein 1 precursor [Homo sapiens] |
| P02768 | 479659392 | 445194692 | 0.928147555 | -0.107573914 | 0.042221842 | yes | no change | GO:0034375;GO:0015643;GO:0005788;GO:0008144;GO:0051659;GO:0030170;GO:0003677;GO:0005783;GO:0005737;GO:0044267;GO:0019836;GO:0070062;GO:0031093;GO:0043209;GO:0002576;GO:0051087;GO:0072562;GO:0098869;GO:0043066;GO:0005504;GO:0005507;GO:0043687;GO:0032460;GO:0042802;GO:0005794;GO:0140272;GO:0016209;GO:0008289;GO:0019825;GO:0009267;GO:0001895;GO:0005615;GO:0046872;GO:0032991;GO:0043069;GO:0006898;GO:1903981;GO:0005576;GO:0005515;GO:0005634 | hsa:213 |  | serum albumin preproprotein [Homo sapiens] |
| P04196 | 675006.1053 | 560602.9292 | 0.830515346 | -0.267921269 | 0.020323181 | yes | no change | GO:0002839;GO:0030308;GO:0032956;GO:2000504;GO:0007162;GO:0008270;GO:0051894;GO:0007599;GO:0001525;GO:0061844;GO:0051715;GO:0070062;GO:0031093;GO:0002576;GO:0072562;GO:0043065;GO:0033629;GO:0030168;GO:0005886;GO:0046872;GO:0010468;GO:0051918;GO:0010543;GO:0008285;GO:0007596;GO:0006935;GO:0020037;GO:0009986;GO:0010593;GO:0043537;GO:0043395;GO:0062023;GO:0004867;GO:0010951;GO:0004869;GO:0043254;GO:0008201;GO:2001027;GO:0050832;GO:0042730;GO:0036019;GO:0050730;GO:0015886;GO:0019865;GO:0005576;GO:1900747;GO:0005515;GO:0030193;GO:0005102;GO:0016525 | hsa:3273 |  | histidine-rich glycoprotein precursor [Homo sapiens] |
| P10643 | 101851.2711 | 86292.0675 | 0.847236039 | -0.239164135 | 0.003181387 | yes | no change | GO:0019835;GO:0030449;GO:0070062;GO:0045087;GO:0006883;GO:0002376;GO:0005576;GO:0006956;GO:0006957;GO:0006955;GO:0006958;GO:0005579 | hsa:730 |  | complement component C7 precursor [Homo sapiens] |
| P10909 | 1087859.132 | 954563.8167 | 0.877470059 | -0.188578196 | 0.027721682 | yes | no change | GO:0032436;GO:0005783;GO:0019730;GO:1903573;GO:0009615;GO:0016020;GO:1902949;GO:0042127;GO:0005794;GO:0034366;GO:0060548;GO:0031966;GO:0043691;GO:0043231;GO:0005829;GO:0002376;GO:0051787;GO:1902004;GO:0051788;GO:0030449;GO:1902230;GO:0050821;GO:0010628;GO:0044877;GO:0099020;GO:0048260;GO:0005856;GO:0000902;GO:0002434;GO:0072562;GO:0048471;GO:0097418;GO:0051131;GO:0032760;GO:0006629;GO:1905895;GO:0048156;GO:0001774;GO:0006956;GO:1905892;GO:1901216;GO:0006958;GO:0061077;GO:2000060;GO:1902430;GO:1902847;GO:0090201;GO:0031012;GO:0045429;GO:0051082;GO:1902998;GO:1901214;GO:0005737;GO:0001540;GO:0031093;GO:0031410;GO:0005634;GO:0051087;GO:0005739;GO:1900221;GO:0009986;GO:0017038;GO:0061740;GO:0061741;GO:0051092;GO:0031625;GO:0070062;GO:0005622;GO:0005743;GO:0005515;GO:0097440;GO:0032286;GO:0016887;GO:0071944;GO:0045202;GO:0061518;GO:0005615;GO:0002576;GO:0043065;GO:0032464;GO:0032463;GO:0045087;GO:0006915;GO:0042583;GO:0062023;GO:0050750;GO:0032991;GO:0001836;GO:0005576;GO:1905907;GO:1905908 | hsa:1191 |  | clusterin preproprotein [Homo sapiens] |
| A0A075B6H7 | 179338.0268 | 138915.8046 | 0.774603173 | -0.368470684 | 0.039000262 | yes | down | GO:0002377;GO:0005615;GO:0006955 | hsa:7441 |  | hCG2043206, partial [Homo sapiens] |
| A0A075B6J9 | 130416.4753 | 76612.62958 | 0.587445945 | -0.767471989 | 0.001184943 | yes | down | GO:0016020;GO:0005615;GO:0006955;GO:0002376;GO:0002377;GO:0005576;GO:0002250;GO:0003823;GO:0005886 | hsa:7441 |  | RecName: Full=Immunoglobulin lambda variable 2-18; Flags: Precursor |
| A0A075B6K4 | 74879.95526 | 49739.26917 | 0.664253457 | -0.590194263 | 0.008886507 | yes | down | GO:0016020;GO:0005615;GO:0006955;GO:0002376;GO:0002377;GO:0005576;GO:0002250;GO:0003823;GO:0005886 | hsa:7441 |  | immunoglobulin light chain variable region, partial [Homo sapiens] |
| A0A075B6R9 | 233860.0358 | 142817.3904 | 0.610696009 | -0.711473679 | 0.019912319 | yes | down | GO:0002377;GO:0005615;GO:0006955 | hsa:7441 |  | RecName: Full=Immunoglobulin kappa variable 2-24; Flags: Precursor |
| A0A075B6S5 | 71694.97316 | 55566.12583 | 0.775035172 | -0.367666312 | 0.038185319 | yes | down | GO:0016020;GO:0005615;GO:0006955;GO:0002376;GO:0002377;GO:0005576;GO:0002250;GO:0003823;GO:0005886 | hsa:7441 |  | monoclonal IgM antibody light chain [Homo sapiens] |
| A0A087WSY6 | 277516.2737 | 191836.3392 | 0.691261585 | -0.532696341 | 0.026210666 | yes | down | GO:0016020;GO:0005615;GO:0006955;GO:0002376;GO:0002377;GO:0005576;GO:0002250;GO:0003823;GO:0005886 | hsa:29802 |  | RecName: Full=Immunoglobulin kappa variable 3D-15; Flags: Precursor |
| A0A0B4J1V0 | 36433.38526 | 24214.50667 | 0.664624121 | -0.589389441 | 0.001841382 | yes | down | GO:0009897;GO:0050853;GO:0045087;GO:0016020;GO:0006910;GO:0006911;GO:0002376;GO:0005576;GO:0002250;GO:0003823;GO:0005886;GO:0072562;GO:0042571;GO:0006958;GO:0050871;GO:0034987;GO:0042742 | hsa:102724971 | | unnamed protein product [Homo sapiens] |
| A0A0C4DH25 | 577141.8526 | 396296.725 | 0.686653936 | -0.54234491 | 9.73E-05 | yes | down | GO:0038096;GO:0004252;GO:0016020;GO:0030449;GO:0005615;GO:0006898;GO:0038095;GO:0050900;GO:0002376;GO:0002377;GO:0050776;GO:0005576;GO:0006956;GO:0002250;GO:0003823;GO:0005886;GO:0006508;GO:0006958;GO:0006955 | hsa:29802 |  | hCG1686089, partial [Homo sapiens] |
| A0A0C4DH34 | 1442302.121 | 901875.0667 | 0.625302462 | -0.677373898 | 8.55E-05 | yes | down | GO:0009897;GO:0050853;GO:0045087;GO:0016020;GO:0006910;GO:0006911;GO:0002376;GO:0005576;GO:0002250;GO:0003823;GO:0005886;GO:0072562;GO:0042571;GO:0006958;GO:0050871;GO:0034987;GO:0042742 | hsa:102723407 | | RecName: Full=Immunoglobulin heavy variable 4-28; Flags: Precursor |
| A0A0C4DH67 | 30398.61047 | 17966.08429 | 0.591016629 | -0.758729372 | 0.004751946 | yes | down | GO:0016020;GO:0002376;GO:0005576;GO:0002250;GO:0003823;GO:0005886 | | | Ig kappa V-region e, partial [Homo sapiens] |
| A0A0G2JL69 | 38095.56632 | 31058.21125 | 0.815271021 | -0.29464836 | 0.000844887 | yes | down | GO:0004252;GO:0016787;GO:0045087;GO:0005576;GO:0006956;GO:0006508;GO:0006958;GO:0046872;GO:0008233;GO:0008236 | hsa:717 | COG5640 | complement C2 isoform 5 [Homo sapiens] |
| A0A0G2JRQ6 | 294034.5047 | 220779.3208 | 0.750861947 | -0.413380415 | 0.010405703 | yes | down | GO:0002377;GO:0005615;GO:0006955 | hsa:7441 |  | hCG2042707, partial [Homo sapiens] |
| A0A2R8Y3M9 | 184972.8526 | 144395.1796 | 0.780629036 | -0.35729097 | 0.000134429 | yes | down | GO:0004252;GO:0006898;GO:0005044;GO:0016020;GO:0016042;GO:0005576;GO:0005509;GO:0006508;GO:0004623 | hsa:81579;hsa:3426 | COG5640 | complement factor I isoform X2 [Homo sapiens] |
| C9JB55 | 14991.58368 | 9811.448136 | 0.654463754 | -0.611614801 | 5.67E-05 | yes | down | GO:0055037;GO:1990459;GO:0030139;GO:0005905;GO:0034986;GO:1990712;GO:0004857;GO:0048260;GO:0005615;GO:0005770;GO:0043086;GO:0009925;GO:0048471;GO:0006879;GO:0016324;GO:0008198;GO:0008199;GO:0015091;GO:0031232;GO:0034756;GO:0071281;GO:0005623;GO:0005769 | hsa:7018 |  | serotransferrin isoform 1 precursor [Homo sapiens] |
| E7END6 | 20400.11526 | 16707.21046 | 0.818976277 | -0.288106432 | 0.00194488 | yes | down | GO:0004252;GO:0016787;GO:0007596;GO:0005576;GO:0005509;GO:0006508;GO:0008233;GO:0008236 | hsa:5624 | COG5640 | vitamin K-dependent protein C isoform X5 [Homo sapiens] |
| J3QRV5 | 389926.1947 | 278678.4542 | 0.714695391 | -0.484599611 | 0.003105236 | yes | down | GO:0005737;GO:0043231;GO:0005829;GO:0006887;GO:0007049;GO:0051301 | hsa:3993 |  | lethal(2) giant larvae protein homolog 2 isoform X4 [Homo sapiens] |
| K7ERG9 | 12339.92889 | 9928.051708 | 0.804546914 | -0.313751547 | 0.012879054 | yes | down | GO:0004252;GO:0007219;GO:0005615;GO:0016787;GO:0009617;GO:0006957;GO:0006508;GO:0008233;GO:0008236 | hsa:1675 | COG5640 | complement factor D isoform 2 precursor [Homo sapiens] |
| K7ERI9 | 2741538.053 | 2179113.75 | 0.794850813 | -0.331243991 | 0.023959727 | yes | down | GO:0005576;GO:0042157 | hsa:341 |  | apolipoprotein C-I precursor [Homo sapiens] |
| M0R0Q9 | 6398.391789 | 5207.888087 | 0.813937042 | -0.297010888 | 0.002249161 | yes | down | GO:0006631;GO:0030449;GO:0004866;GO:0005886;GO:0005788;GO:0035578;GO:0007165;GO:0031715;GO:0048260;GO:0010828;GO:0010866;GO:0044267;GO:0070062;GO:0009617;GO:0045766;GO:1905114;GO:0045745;GO:0097242;GO:2000427;GO:0034774;GO:0007186;GO:0009986;GO:0005576;GO:0150064;GO:0043687;GO:0016322;GO:0150062;GO:0006956;GO:0006957;GO:0006954;GO:0006955;GO:0060100;GO:0006958;GO:0005615;GO:0050776;GO:0032991;GO:0010575;GO:0043312;GO:0001798;GO:0010884;GO:0001970;GO:0097278;GO:0005102;GO:0001934;GO:0072562 | hsa:718 |  | C3 isoform 6, partial [Pan troglodytes] |
| O00187 | 36016.13684 | 24618.28871 | 0.683534961 | -0.548912964 | 0.009536382 | yes | down | GO:0045087;GO:0004252;GO:0046872;GO:0006956;GO:0005615;GO:0048306;GO:0070062;GO:0016787;GO:0002376;GO:0005576;GO:0001867;GO:0005509;GO:0005515;GO:0006508;GO:0006958;GO:0008236;GO:0008233;GO:0001855 | hsa:10747 | COG5640 | mannan-binding lectin serine protease 2 isoform 1 preproprotein [Homo sapiens] |
| P00748 | 60677.42474 | 40103.85458 | 0.660935344 | -0.597418948 | 0.002058938 | yes | down | GO:0004252;GO:0002542;GO:0007599;GO:0007597;GO:0007596;GO:0008233;GO:0008236;GO:0070062;GO:0031638;GO:0005509;GO:0006508;GO:0051919;GO:0016787;GO:0005791;GO:0045087;GO:0062023;GO:0005886;GO:0016485;GO:0005615;GO:0042730;GO:0016540;GO:0002353;GO:0005576;GO:0051787;GO:0010756;GO:0005515;GO:0030193;GO:0051788;GO:0030194 | hsa:2161 | COG5640 | coagulation factor XII preproprotein [Homo sapiens] |
| P00915 | 20567.45463 | 14246.79221 | 0.69268621 | -0.529726141 | 0.014753102 | yes | down | GO:0005737;GO:0015701;GO:0046872;GO:0070062;GO:0006730;GO:0016829;GO:0008270;GO:0005515;GO:0016836;GO:0004089;GO:0004064;GO:0005829;GO:0035722 | hsa:759 | COG3338 | carbonic anhydrase 1 isoform a [Homo sapiens] |
| P00918 | 9617.187471 | 6637.175053 | 0.690136807 | -0.535045717 | 0.017860176 | yes | down | 3P3J:A;1IF6:A;1H9N:A;3RYJ:B;5FLQ:A;3P5A:A;1AVN:A;GO:0010043;6BC9:A;3V3H:B;4Q78:A;5JGS:B;5M78:A;1CAM:A;GO:0043209;5TY9:A;3RZ1:B;5EOI:A;5JN3:A;1CNI:A;4ZWY:A;3PJJ:A;3TMJ:A;5LL4:A;4CAC:A;1CNK:A;3T5U:A;6EQU:A;5EH8:A;1G52:A;5JEG:B;1AM6:A;3SBH:A;1CVD:A;4BF1:A;1BNU:A;1I91:A;4PYY:A;3MHL:A;4HEY:A;5FLO:A;5NXP:A;3S78:B;4Q08:A;4K1Q:A;2O4Z:A;2Q38:A;3DD8:A;5CAC:A;4YYT:A;2VVB:X;1CNC:A;2FNN:A;5G0C:A;1CVB:A;1FR7:B;2FOQ:A;3RZ7:A;4KNI:A;6H2Z:A;4QTL:A;4QEF:A;4FVO:A;6BCC:A;3M1Q:A;5LLG:A;6E92:A;3T83:A;5WEX:A;3D8W:A;4YXI:A;5WLV:A;4Q6D:A;5JQT:A;4YXO:A;5TFX:A;2HKK:A;2POU:A;3M1W:A;6MBY:A;5DOH:B;5OGO:A;4QSI:A;1BNV:A;3DBU:A;1IF9:A;3PYK:A;1CAK:A;1G46:A;6D1L:A;5TY8:A;5DRS:A;5E28:A;4Q9Y:A;5LLC:A;6FJI:A;3MNH:A;1I9P:A;1CIL:A;4FIK:A;6BBS:A;3OIL:A;1LG6:A;4BF6:A;6IC2:A;6C7W:A;1CIN:A;1G4O:A;1BNM:A;4YGL:A;4HF3:A;2CBE:A;3KOK:A;GO:0016829;4MDG:A;3HS4:A;5DSM:A;4K0Z:A;2F14:A;5FNL:A;4JSW:A;GO:0045177;5BRU:A;5DSO:A;3DVC:A;2WD2:A;3RG3:A;4KUV:A;4HEZ:A;5LL4:B;1I9M:A;1RZD:A;5DSL:A;3B4F:A;1TE3:X;5BNL:A;4YGK:A;GO:0016323;5U0G:A;5LMD:A;5CA2:A;4DZ9:A;5SZ1:A;1ZSB:A;1YDB:A;1YO0:A;4E49:A;4YVY:A;4KV0:A;2X7S:A;5THN:A;4KUW:A;1YDD:A;1YO2:A;1I8Z:A;2X7U:A;2CBC:A;5JQ0:A;4QSA:A;3S74:B;3P58:A;3M67:A;3IGP:A;3S76:A;3EFT:A;5TYA:A;3IEO:A;4JSZ:A;3V7X:A;5N24:A;3HKU:A;3KKX:A;5ZXW:A;1BN3:A;2EZ7:A;5JDV:B;5FLT:A;4E4A:A;GO:0030424;1CVH:A;GO:0004064;1XEG:A;5G03:A;2AW1:A;3F8E:A;1RAZ:A;5W8B:A;5G01:A;5N1R:A;5L70:B;2ABE:A;2EU3:A;5L70:A;3IBU:A;GO:0005615;6EDA:A;2GEH:A;5N1S:A;4Q7W:A;1CAY:A;5EKM:A;3D93:A;3M2Z:A;5MJN:A;1ZFQ:A;2NNO:A;GO:0038166;3R17:B;3S9T:A;1BV3:A;5VGY:A;4IWZ:A;1G3Z:A;5DSP:A;4Z0Q:A;GO:0045780;1XPZ:A;3RZ5:A;1UGD:A;GO:0016020;5AMD:A;1RAY:A;4M2U:A;1UGF:A;3DV7:A;3S75:B;1RZE:A;4PXX:A;1BNT:A;5DOG:A;3P4V:A;2WEG:A;6EBE:A;3KS3:A;5WLR:A;5SZ2:A;1KWQ:A;1CIM:A;1CNG:A;5AML:A;2NNV:A;GO:0005829;1CCS:A;5OGP:A;GO:0043627;1CVF:A;1TTM:A;4FVN:A;4JSA:A;1TBT:X;1FSQ:A;5SZ3:A;4RFC:A;1FSQ:B;3MNJ:A;2NWP:A;5FNJ:A;1OKM:A;5LLE:A;4ITP:A;2WD3:A;3K7K:A;1G4J:A;2H4N:A;4HBA:A;1ZH9:A;4R5B:A;5T71:A;6ECZ:A;5L6T:A;5L6T:B;2AX2:A;3P44:A;5TI0:A;GO:2001225;2QOA:A;6CA2:A;4G0C:A;1CA2:A;1CAO:A;3V3F:A;4Z1K:A;1FR7:A;4QK3:A;1CAI:A;5N0E:A;4PQ7:A;3HKN:A;1RZC:A;1ZGF:A;4QK1:A;5GMN:A;4E3D:A;1CNW:A;5ULN:A;GO:0048545;2FOV:A;4FRC:A;2FNK:A;2HD6:A;2WEO:A;1TG9:A;3M40:A;5BRV:A;4Q7P:A;4Q8Z:A;2QO8:A;5DSK:A;4RIV:A;3KOI:A;5Y2S:A;4Q8X:A;3HLJ:A;1LZV:A;1G54:A;5C8I:A;3L14:A;3N2P:A;3OYQ:A;3CAJ:A;2POV:A;6EEO:A;4WL4:A;6B4D:A;5YUJ:A;4FPT:A;3GZ0:A;5NXV:A;1OQ5:A;6GOT:A;5J8Z:A;3ZP9:A;4RUY:A;6B5A:A;2NWZ:A;1I9O:A;1THK:A;2EU2:A;5SZ7:A;4L5W:A;4M2W:A;6H6S:A;5JMZ:A;4Q7V:A;2Q1Q:A;4HT0:A;4QSB:A;4QJM:A;1BNN:A;2NXS:A;GO:0005515;3M1K:A;4Q99:A;3M2X:A;1H9Q:A;6G6T:A;4MDM:A;1FQM:A;5FDC:A;1H4N:A;5SZ5:A;4Q6E:A;3SAP:A;1EOU:A;1TEQ:X;5FLP:A;5T75:A;5NXI:A;3K34:A;2HOC:A;6CJV:A;6FJJ:A;1CNJ:A;3P5L:A;1G53:A;3V5G:A;4MDL:A;1ZSC:A;6HX5:A;4XE1:A;1ZSA:A;1TG3:A;6GXE:A;5JEH:B;4MO8:A;2X7T:A;4CA2:A;1IF7:A;6CEH:A;4QY3:A;GO:0046903;1LGD:A;4Q09:A;2CBD:A;1IF5:A;1CNB:A;3FFP:X;3DCW:A;5NXO:A;5LL8:A;3RYY:A;4Z1E:A;3R16:A;3BET:A;GO:0032849;5JEP:B;5G0B:A;4ZAO:A;3ML2:A;1BCD:A;3TVN:X;4Y0J:A;5NXW:A;6EEA:A;1BN4:A;1UGB:A;4K13:A;5DOH:A;3C7P:A;4RFD:A;GO:0001822;1TB0:X;5SZ4:A;5DSQ:A;GO:0046872;5L6K:B;3N3J:A;4Q07:A;3T84:A;1IF8:A;12CA:A;1DCA:A;4QK2:A;5E2K:A;4Q83:A;3SAX:A;5LVS:B;5T74:A;1CAZ:A;6D1M:A;6GXB:A;3RYZ:A;5LVS:A;2FMZ:A;3IBI:A;2QP6:A;1I9Q:A;4E5Q:A;3OIM:A;5NEA:A;6B59:A;6H29:A;2FOU:A;3M5T:A;5THI:A;4GL1:X;4Q87:A;2WEJ:A;1G0F:A;GO:2001150;6H34:A;6G3Q:A;3CYU:A;5O07:A;6QEB:A;3RGE:A;5E2R:A;5YUK:A;5EH5:A;4MLT:A;5EIJ:A;3RZ0:B;5FLS:A;3NB5:A;5EH7:A;1MOO:A;3U3A:X;4Z1J:A;3SBI:A;4Q90:A;2VVA:X;1I90:A;1XQ0:A;5NXG:A;1ZFK:A;3MHO:A;5FNI:A;6H6S:B;2OSM:A;3VBD:A;2FNM:A;3MHM:A;4K0S:A;3MNU:A;5EHV:A;5FNG:A;4E3G:A;3RZ8:A;4YWP:A;5THJ:A;1CRA:A;1RZA:A;2NNS:A;1FR4:A;3T5Z:A;1F2W:A;1CVC:A;3RG4:A;5TH4:A;3PO6:A;5WLU:A;5JN7:A;6E91:A;4ZX0:A;1Z9Y:A;5JN1:A;1HVA:A;GO:0009268;3NI5:A;5A6H:A;3S77:B;GO:0005737;3S71:B;4Q49:A;4ILX:A;3HKT:A;5U0F:A;1BIC:A;3OYS:A;3M96:A;2FMG:A;3KIG:A;5FLR:A;5OGN:B;6GCY:A;2NXT:A;1CAH:A;5SZ0:A;4E3H:A;4ITO:A;4QIY:C;4QIY:B;4QIY:A;4R5A:A;3DCC:A;1CNX:A;1CAJ:A;4MLX:A;2Q1B:A;3M04:A;4L5U:A;1ZGE:A;1LG5:A;5FDI:A;GO:0070062;3D92:A;2H15:A;3MZC:A;3F4X:A;1FQL:A;8CA2:A;5JG3:B;1UGA:A;5E2S:A;4LHI:A;5NY1:A;3M14:A;5LJT:A;3IBN:A;3P55:A;3V3G:B;1CVA:A;3D9Z:A;1HCA:A;5NXM:A;1BNQ:A;GO:0044070;3DVB:A;5JGT:B;4YGN:A;1UGG:A;3CA2:A;1BNW:A;3V2J:A;GO:0010033;1YDA:A;1I9L:A;4ZWZ:A;3RJ7:A;1YDC:A;3DAZ:A;6EEH:A;5WLT:A;6E8P:A;4M2R:A;5L6K:A;1TEU:X;3DCS:A;2CBB:A;3BL0:A;1FSR:B;1FSR:A;1LUG:A;2OSF:A;2ILI:A;1MUA:A;4N16:A;2GD8:A;3MMF:A;5U0D:A;4E3F:A;2HL4:A;3OKV:A;1ZE8:A;5TY1:A;GO:0008270;5JE7:B;3DC9:A;3V3J:A;3S73:B;GO:0032230;3M5S:A;1T9N:A;4Q06:A;1CCU:A;5AMG:A;3MWO:B;7CA2:A;3MWO:A;5T72:A;GO:0004089;5BYI:A;3DC3:A;1CNH:A;2NNG:A;1CA3:A;5EKH:A;5WG7:A;3OY0:A;5NY3:A;1FQR:A;1HEC:A;9CA2:A;2FOS:A;5EKJ:A;3V2M:A;3N4B:A;1HEA:A;2NWO:A;5NEE:A;3K2F:A;6E8X:A;3M2Y:A;3P3H:A;3U7C:A;4FL7:A;4Q81:A;4WW6:A;GO:0015701;3RYV:B;3S8X:A;5U0E:A;GO:0051453;3EFI:A;4RH2:A;5EHW:A;2WEH:A;4Q8Y:A;2POW:A;4Z1N:A;1HEB:A;4RIU:A;1CAL:A;5FNM:A;1I9N:A;4ZWX:A;1G1D:A;1KWR:A;5TXY:A;3MHI:A;5LLH:A;1CCT:A;4RN4:A;3KWA:A;4CQ0:A;4YXU:A;1CVE:A;5SZ6:A;4KAP:A;3QYK:A;4PYX:A;4YGJ:A;2NWY:A;1FSN:B;1FSN:A;4HEW:A;3V3I:B;4N0X:B;1YO1:A;4KNJ:A;3MNK:A;3HFP:A;4FU5:A;5BRW:A;GO:0005886;1G0E:A;1OKN:A;5N25:A;5CLU:A;4JS6:A;GO:0005902;4PZH:A;1OKL:A;5L9E:C;3M2N:A;5LJQ:A;3T82:A;1FQN:A;6MBV:A;4RUZ:A;4DZ7:A;5DSR:A;1A42:A;4RUX:A;3MNA:A;5L3O:B;1CNY:A;4ZWI:A;1CAN:A;3U45:X;3OIK:A;4KUY:A;5NY6:A;3U47:A;5YUI:A;2CBA:A;5N0D:A;5FNH:A;6H33:A;4K0T:A;3N0N:A;3RLD:A;3BL1:A;4ZX1:A;5UMC:A;1RZB:A;3M5E:A;5OGN:A;4LP6:B;4LP6:A;1G45:A;3MNI:A;6GDC:A;3RYX:B;GO:0015670;4YX4:A;1IF4:A;5JG5:B;4JSS:A;3DVD:A;4BCW:A;5WGP:A;5Y2R:A;3TVO:X;3IBL:A;6C7X:A;GO:0002009;3DD0:A;3S72:B;3M3X:A;1UGC:A;4R59:A;3HKQ:A;1UGE:A;3T85:A;4QIY:D;3MYQ:A;4M2V:A;1G48:A;GO:0071498;1XEV:B;2HNC:A;5JES:B;4L5V:A;3NJ9:A;1TH9:A;5L9E:A;1DCB:A;3M98:A;5L9E:B;5L3O:A;5L9E:D;5FNK:A;2CA2:A;GO:0042475;4IDR:X;3KNE:A;5NYA:A;3M1J:A;1HED:A;3MHC:A;2NXR:A;GO:0045672;4Q7S:A;3OKU:A;3KON:A;1XEV:C;5EHE:A;1XEV:A;4MTY:A;1XEV:D;1BN1:A | hsa:760 | COG3338 | carbonic anhydrase 2 isoform 1 [Homo sapiens] |
| P01008 | 517418.1526 | 406228.2042 | 0.785106209 | -0.349040261 | 5.98E-05 | yes | down | GO:0007599;GO:0007595;GO:2000266;GO:0007596;GO:0005788;GO:0002438;GO:0070062;GO:0072562;GO:0010466;GO:0002020;GO:0043687;GO:0042802;GO:0044267;GO:0007584;GO:0062023;GO:0030414;GO:0004867;GO:0010951;GO:0005886;GO:0008201;GO:0005615;GO:0005576;GO:0005515;GO:0030193 | hsa:462 | COG4826 | antithrombin-III isoform 1 precursor [Homo sapiens] |
| P01009 | 5372299.105 | 4427646.083 | 0.824162243 | -0.278999722 | 0.00307911 | yes | down | GO:0048208;GO:0007599;GO:0005783;GO:0007596;GO:0033116;GO:0005788;GO:1904813;GO:0030134;GO:0044267;GO:0006888;GO:0005615;GO:0031093;GO:0002576;GO:0010466;GO:0002020;GO:0042802;GO:0005794;GO:0043687;GO:0006953;GO:0030414;GO:0004867;GO:0010951;GO:0070062;GO:0043231;GO:0043312;GO:0000139;GO:0005576;GO:0005515 | hsa:5265 | COG4826 | alpha-1-antitrypsin precursor [Homo sapiens] |
| P01344 | 50416.95737 | 41745.06167 | 0.827996449 | -0.272303515 | 0.008711672 | yes | down | GO:0046628;GO:0045840;GO:0031017;GO:0051146;GO:0008286;GO:0038028;GO:0008284;GO:0001501;GO:0001503;GO:0051147;GO:0031056;GO:0071902;GO:0051897;GO:0007275;GO:0001649;GO:0044267;GO:0006349;GO:0001701;GO:0008083;GO:0031093;GO:0000122;GO:0002576;GO:0043085;GO:0045725;GO:0010469;GO:0005179;GO:0005178;GO:0009887;GO:2000467;GO:0042104;GO:0005159;GO:0005158;GO:0043410;GO:0048018;GO:0060669;GO:0001892;GO:0043539;GO:0005615;GO:0005975;GO:0050731;GO:0040018;GO:0006355;GO:0051781;GO:0005576;GO:0045944;GO:0005515;GO:0006006;GO:0001934 | hsa:3481 |  | insulin-like growth factor II isoform 2 [Homo sapiens] |
| P01599 | 167274.5911 | 110797.8304 | 0.662370954 | -0.594288686 | 0.002832631 | yes | down | GO:0038096;GO:0004252;GO:0016020;GO:0030449;GO:0050776;GO:0006898;GO:0038095;GO:0072562;GO:0002376;GO:0050900;GO:0005576;GO:0006956;GO:0002250;GO:0003823;GO:0005886;GO:0006508;GO:0006958;GO:0006955;GO:0070062 | hsa:7441 |  | RecName: Full=Immunoglobulin kappa variable 1-17; AltName: Full=Ig kappa chain V-I region Gal; AltName: Full=Ig kappa chain V-I region WEA; Flags: Precursor |
| P01601 | 102837.5474 | 65756.58375 | 0.639421937 | -0.645159854 | 2.24E-05 | yes | down | GO:0038096;GO:0004252;GO:0016020;GO:0030449;GO:0005615;GO:0006898;GO:0038095;GO:0050900;GO:0002376;GO:0002377;GO:0050776;GO:0005576;GO:0006956;GO:0002250;GO:0003823;GO:0005886;GO:0006508;GO:0006958;GO:0006955 | hsa:7441 |  | RecName: Full=Immunoglobulin kappa variable 1D-16; AltName: Full=Ig kappa chain V-I region HK146; AltName: Full=Ig kappa chain V-I region HK189; Flags: Precursor |
| P01699 | 176825.7089 | 130471.1858 | 0.737851903 | -0.438596818 | 0.002981848 | yes | down | GO:0038096;GO:0004252;GO:0016020;GO:0030449;GO:0050776;GO:0006898;GO:0038095;GO:0002376;GO:0050900;GO:0005576;GO:0006956;GO:0002250;GO:0003823;GO:0005886;GO:0006508;GO:0006958;GO:0006955 | hsa:7441 |  | hCG2043214, partial [Homo sapiens] |
| P01703 | 152973.9737 | 117343.7004 | 0.767082776 | -0.382545827 | 0.004835106 | yes | down | GO:0038096;GO:0004252;GO:0016020;GO:0030449;GO:0050776;GO:0006898;GO:0038095;GO:0002376;GO:0050900;GO:0005576;GO:0006956;GO:0002250;GO:0003823;GO:0005886;GO:0006508;GO:0006958;GO:0006955 | hsa:7441 |  | Unknown (protein for MGC:31936) [Homo sapiens] |
| P01715 | 26407.4994 | 10681.48209 | 0.404486693 | -1.305835856 | 0.00102876 | yes | down | GO:0038096;GO:0004252;GO:0016020;GO:0030449;GO:0050776;GO:0006898;GO:0038095;GO:0002376;GO:0050900;GO:0005576;GO:0006956;GO:0002250;GO:0003823;GO:0005886;GO:0006508;GO:0006958;GO:0006955 | hsa:29802 |  | hCG2040023, partial [Homo sapiens] |
| P01717 | 10448.645 | 5749.16281 | 0.550230466 | -0.861892071 | 0.000452956 | yes | down | GO:0038096;GO:0004252;GO:0016020;GO:0030449;GO:0050776;GO:0006898;GO:0038095;GO:0072562;GO:0002376;GO:0050900;GO:0005576;GO:0006956;GO:0002250;GO:0003823;GO:0005886;GO:0006508;GO:0006958;GO:0006955 | hsa:7441 |  | immunoglobulin lambda light chain variable region, partial [Homo sapiens] |
| P01721 | 113548.3379 | 91913.69292 | 0.809467533 | -0.304954879 | 0.045762991 | yes | down | GO:0038096;GO:0004252;GO:0016020;GO:0030449;GO:0050776;GO:0006898;GO:0038095;GO:0002376;GO:0050900;GO:0005576;GO:0006956;GO:0002250;GO:0003823;GO:0005886;GO:0006508;GO:0006958;GO:0006955 | hsa:7441 |  | RecName: Full=Immunoglobulin lambda variable 6-57; AltName: Full=Ig lambda chain V-VI region AR; AltName: Full=Ig lambda chain V-VI region EB4; AltName: Full=Ig lambda chain V-VI region NIG-48; AltName: Full=Ig lambda chain V-VI region SUT; AltName: Full=Ig lambda chain V-VI region WLT; Flags: Precursor |
| P01780 | 2081288.158 | 1684025.971 | 0.809126773 | -0.305562335 | 0.047783513 | yes | down | GO:0038096;GO:0004252;GO:0016020;GO:0030449;GO:0050776;GO:0006898;GO:0038095;GO:0072562;GO:0002376;GO:0050900;GO:0005576;GO:0006956;GO:0002250;GO:0003823;GO:0005886;GO:0006508;GO:0006958;GO:0006955;GO:0070062 | hsa:102723407 | | immunoglobulin heavy chain variable region precursor, partial [Homo sapiens] |
| P01782 | 287610.9211 | 181545.2313 | 0.631218142 | -0.663789424 | 0.001266731 | yes | down | GO:0038096;GO:0004252;GO:0016020;GO:0030449;GO:0050776;GO:0006898;GO:0038095;GO:0002376;GO:0050900;GO:0005576;GO:0006956;GO:0002250;GO:0003823;GO:0005886;GO:0006508;GO:0006958;GO:0006955;GO:0070062 | hsa:102723407 | | hCG2038940, partial [Homo sapiens] |
| P01857 | 10338346.11 | 7511055.083 | 0.726523857 | -0.460917923 | 0.001691891 | yes | down | GO:0004252;GO:0030449;GO:0019221;GO:0009897;GO:0042742;GO:0034987;GO:0042571;GO:0005615;GO:0016020;GO:0072562;GO:0006508;GO:0050853;GO:0045087;GO:0006910;GO:0006911;GO:0006956;GO:0003823;GO:0005886;GO:0006958;GO:0050871;GO:0070062;GO:0038096;GO:0002376;GO:0005576;GO:0002250;GO:0005515 | hsa:100423062 | | IGH@ protein [Homo sapiens] |
| P02747 | 538883.3474 | 317328.5208 | 0.588863104 | -0.763995812 | 7.99E-10 | yes | down | GO:0004252;GO:0030449;GO:0005581;GO:0045650;GO:0045087;GO:0072562;GO:0002376;GO:0030853;GO:0005576;GO:0006956;GO:0005515;GO:0006955;GO:0006508;GO:0006958;GO:0005615 | hsa:714 |  | complement C1q subcomponent subunit C isoform X1 [Pongo abelii] |
| P02763 | 2000338.158 | 1554695.958 | 0.777216568 | -0.363611439 | 0.007073481 | yes | down | GO:1904469;GO:0050716;GO:0031093;GO:0005615;GO:0002576;GO:0050718;GO:0043312;GO:0072562;GO:0006953;GO:0032715;GO:0062023;GO:0002682;GO:0005515;GO:0006954;GO:0035580;GO:0005576;GO:1904724;GO:0032720;GO:0070062 | hsa:5004 |  | RecName: Full=Alpha-1-acid glycoprotein 1; Short=AGP 1; AltName: Full=Orosomucoid-1; Short=OMD 1; Flags: Precursor |
| P02787 | 21499234.37 | 17106102.33 | 0.795661001 | -0.329774208 | 0.000668795 | yes | down | GO:0009617;GO:0016020;GO:0055037;GO:1990459;GO:1900390;GO:0005788;GO:0030139;GO:0061024;GO:0055072;GO:0006826;GO:0005905;GO:0034986;GO:1990712;GO:0048260;GO:0010008;GO:0030665;GO:0007257;GO:0045780;GO:0031232;GO:0005615;GO:0031410;GO:0002576;GO:0005770;GO:0045178;GO:0070062;GO:2000147;GO:0009925;GO:0044267;GO:0048471;GO:0046872;GO:0043687;GO:0009986;GO:0006879;GO:0033572;GO:0007015;GO:0016324;GO:0006811;GO:0030316;GO:0031982;GO:0008198;GO:0008199;GO:0045893;GO:0034774;GO:0015091;GO:0001895;GO:0034756;GO:0071281;GO:0042327;GO:0060395;GO:0031647;GO:0005623;GO:0005576;GO:0005515;GO:0005768;GO:0005769;GO:0070371;GO:0072562 | hsa:7018 |  | serotransferrin isoform 1 precursor [Homo sapiens] |
| P04211 | 1109267.495 | 798315.6875 | 0.719678248 | -0.47457604 | 0.011855527 | yes | down | GO:0038096;GO:0004252;GO:0016020;GO:0030449;GO:0050776;GO:0006898;GO:0038095;GO:0002376;GO:0050900;GO:0005576;GO:0006956;GO:0002250;GO:0003823;GO:0005886;GO:0006508;GO:0006958;GO:0006955 | hsa:7441 |  | hCG1731877, partial [Homo sapiens] |
| P07360 | 45022.55263 | 34992.18875 | 0.777214678 | -0.363614948 | 0.000475582 | yes | down | GO:0019835;GO:0036094;GO:0030449;GO:0070062;GO:0045087;GO:0019841;GO:0072562;GO:0002376;GO:0001848;GO:0005576;GO:0044877;GO:0006957;GO:0006958;GO:0005579;GO:0005615 | hsa:733 |  | complement component C8 gamma chain precursor [Homo sapiens] |
| P0DP01 | 38972.34187 | 27725.22915 | 0.71140783 | -0.491251242 | 0.048088938 | yes | down | GO:0016020;GO:0002376;GO:0005576;GO:0002250;GO:0003823;GO:0005886 | hsa:102723407 | | immunoglobulin heavy chain variable region, partial [Homo sapiens] |
| P19652 | 944377.3947 | 767476.8583 | 0.812680251 | -0.299240259 | 0.012402288 | yes | down | GO:1904469;GO:0050716;GO:0031093;GO:0005615;GO:0002576;GO:0050718;GO:0043312;GO:0072562;GO:0006953;GO:0035578;GO:0062023;GO:0002682;GO:0035580;GO:0005576;GO:0070062 | hsa:5005 |  | alpha-1-acid glycoprotein 2 precursor [Homo sapiens] |
| P29622 | 85693.02526 | 66413.45667 | 0.775015895 | -0.367702196 | 5.18E-05 | yes | down | GO:0005615;GO:0070062;GO:0002576;GO:0031089;GO:0010466;GO:0030414;GO:0004867;GO:0010951;GO:0005576 | hsa:5267 | COG4826 | kallistatin isoform 1 [Homo sapiens] |
| P32119 | 22429.17211 | 15456.33191 | 0.689117362 | -0.537178389 | 0.016675595 | yes | down | GO:0000187;GO:0045581;GO:0042744;GO:0008379;GO:0032496;GO:0032088;GO:0042981;GO:0034599;GO:0002536;GO:0016491;GO:0005737;GO:0010310;GO:0070062;GO:0042098;GO:0043066;GO:0048538;GO:2001240;GO:0016209;GO:0045454;GO:0006979;GO:0055114;GO:0048872;GO:0045321;GO:0005829;GO:0005623;GO:0031665;GO:0019430;GO:0005515;GO:0051920;GO:0004601;GO:0030194 | hsa:7001 | COG0450 | peroxiredoxin-2 [Homo sapiens] |
| P43251 | 38404.83263 | 31156.51167 | 0.811265394 | -0.301754146 | 0.030910851 | yes | down | GO:0016787;GO:0005615;GO:0047708;GO:0070062;GO:0016810;GO:0016811;GO:0006768;GO:0006807;GO:0005759;GO:0007417;GO:0005576 | hsa:686 | COG0388 | RecName: Full=Biotinidase; Short=Biotinase; Flags: Precursor |
| P55056 | 234977.8321 | 149800.1833 | 0.637507726 | -0.649485269 | 0.023397656 | yes | down | GO:0005319;GO:0034361;GO:0070328;GO:0006629;GO:0034447;GO:0006869;GO:0034379;GO:0010890;GO:0005576;GO:0034364 | hsa:346 |  | apolipoprotein C-IV precursor [Homo sapiens] |
| P80748 | 667222.8947 | 236511.5604 | 0.35447159 | -1.496258091 | 4.50E-06 | yes | down | GO:0038096;GO:0004252;GO:0016020;GO:0030449;GO:0050776;GO:0006898;GO:0038095;GO:0072562;GO:0002376;GO:0050900;GO:0005576;GO:0006956;GO:0002250;GO:0003823;GO:0005886;GO:0006508;GO:0006958;GO:0006955;GO:0070062 | hsa:7441 |  | hCG2040021, partial [Homo sapiens] |
| Q96HR3 | 36736.91842 | 15002.38996 | 0.408373663 | -1.292038267 | 1.54E-08 | yes | down | GO:0019827;GO:0030521;GO:0038023;GO:0005515;GO:0006355;GO:0005634;GO:0030518;GO:0003712;GO:0000151;GO:0005654;GO:0046966;GO:0045893;GO:0006367;GO:0016567;GO:0016592;GO:0030374;GO:0061630;GO:0042809;GO:0006351 | hsa:90390 |  | mediator of RNA polymerase II transcription subunit 30 isoform 1 [Homo sapiens] |
| Q96IY4 | 23595.77158 | 19314.74542 | 0.818568079 | -0.288825686 | 0.010449485 | yes | down | GO:0007599;GO:2000346;GO:0007596;GO:0008270;GO:0030449;GO:0008233;GO:0008237;GO:0005615;GO:0004181;GO:0004180;GO:0071333;GO:0006508;GO:0046872;GO:0051918;GO:0016787;GO:0009408;GO:0070062;GO:0042730;GO:0003331;GO:0097421;GO:0042493;GO:0005623;GO:0005576;GO:0010757 | hsa:1361 | COG2866 | carboxypeptidase B2 isoform 1 preproprotein [Homo sapiens] |
| Q9UGM5 | 25507.93 | 20055.63875 | 0.786251129 | -0.346937912 | 0.006326331 | yes | down | GO:0005615;GO:0007339;GO:0008150;GO:0005576;GO:0003674;GO:0007338;GO:0030414;GO:0008191;GO:0010951;GO:0004869;GO:0004857;GO:0010466;GO:0070062 | hsa:26998 |  | fetuin-B isoform 1 precursor [Homo sapiens] |
| Q9Y5Y7 | 11788.65695 | 8747.742478 | 0.74204742 | -0.43041671 | 0.020365618 | yes | down | GO:0038023;GO:0070062;GO:0005540;GO:0016021;GO:0016020;GO:0009611;GO:0007160;GO:0004888;GO:0071944;GO:0005515;GO:0005887;GO:0005886;GO:0007155;GO:0030214;GO:0009653;GO:0006027 | hsa:10894 |  | lymphatic vessel endothelial hyaluronic acid receptor 1 precursor [Homo sapiens] |
| A0A087X1L8 | 2110.5175 | 2639.587619 | 1.25068265 | 0.322715764 | 0.127527879 | no | up | GO:0016021;GO:0016020 | hsa:102723996;hsa:23308 | | ICOS ligand isoform c precursor [Homo sapiens] |
| A0A0B4J1V1 | 61207.74753 | 78668.15895 | 1.285264728 | 0.362065544 | 0.450113168 | no | up | GO:0009897;GO:0050853;GO:0045087;GO:0016020;GO:0006910;GO:0006911;GO:0002376;GO:0005576;GO:0002250;GO:0003823;GO:0005886;GO:0072562;GO:0042571;GO:0006958;GO:0050871;GO:0034987;GO:0042742 | hsa:102723407 | | RecName: Full=Immunoglobulin heavy variable 3-21; Flags: Precursor |
| A0A2R8Y7X9 | 29304.87822 | 44102.83375 | 1.504965604 | 0.589730514 | 0.065977082 | no | up | GO:0005344;GO:0019825;GO:0020037;GO:0005833;GO:0046872;GO:0015671 | hsa:3048 | COG1018 | hemoglobin subunit gamma-2 [Pan troglodytes] |
| A0A4W8ZXM2 | 659986.1158 | 809666.7421 | 1.226793599 | 0.294892544 | 0.250634499 | no | up |  | hsa:102723407 | | immunoglobulin heavy chain variable region, partial [Homo sapiens] |
| D6RE82 | 131886.3826 | 2667021.277 | 20.22211259 | 4.337861817 | 0.323593955 | no | up | GO:0030688;GO:0005730;GO:0005634;GO:0030687;GO:0006364;GO:0003723 | hsa:8568 |  | PREDICTED: ribosomal RNA processing protein 1 homolog A [Callithrix jacchus] |
| O75460 | 97874.42944 | 120563.645 | 1.231819646 | 0.300791042 | 0.144172792 | no | up | GO:0005739;GO:0004540;GO:0005783;GO:0016787;GO:0016310;GO:0005161;GO:0019899;GO:0005789;GO:0008152;GO:1990604;GO:0030176;GO:1990597;GO:0034620;GO:1990630;GO:0046777;GO:0036289;GO:0007257;GO:0006986;GO:0098787;GO:0016241;GO:0030544;GO:0005637;GO:0016021;GO:0000287;GO:0004521;GO:0051082;GO:0000166;GO:0004674;GO:0016740;GO:0071333;GO:1901142;GO:0006402;GO:0046872;GO:0051879;GO:0042802;GO:0042803;GO:0005737;GO:0006468;GO:0033120;GO:0006397;GO:1990332;GO:0004672;GO:0007050;GO:0003824;GO:0043531;GO:0016020;GO:0090502;GO:0036498;GO:0006351;GO:0030968;GO:0070059;GO:1904707;GO:0070054;GO:0016301;GO:0006355;GO:1990579;GO:0005524;GO:0005515;GO:0004519;GO:1900103;GO:0006379;GO:0001935;GO:0035924;GO:0006915;GO:0034976 | hsa:2081 | COG0515 | endoplasmic reticulum to nucleus signalling 1 isoform 1 variant, partial [Homo sapiens] |
| P00738 | 8878679.147 | 11548709.83 | 1.300723862 | 0.379314717 | 0.052824444 | no | up | GO:0042742;GO:0010942;GO:0005615;GO:0071682;GO:2000296;GO:0072562;GO:0098869;GO:0035580;GO:1904724;GO:0016209;GO:0006952;GO:0006953;GO:0070062;GO:0042542;GO:0051354;GO:0006898;GO:0043312;GO:0002376;GO:0030492;GO:0005576;GO:0005515;GO:0031838 | hsa:3240 | COG5640 | haptoglobin isoform 1 preproprotein [Homo sapiens] |
| P01602 | 185182.1458 | 313495.2904 | 1.692902353 | 0.759498761 | 0.148703466 | no | up | GO:0038096;GO:0004252;GO:0016020;GO:0030449;GO:0050776;GO:0006898;GO:0038095;GO:0072562;GO:0002376;GO:0002377;GO:0050900;GO:0005576;GO:0006956;GO:0002250;GO:0003823;GO:0005886;GO:0006508;GO:0006958;GO:0006955;GO:0070062 | hsa:7441 |  | hCG2043208, partial [Homo sapiens] |
| P02776 | 36937.81637 | 44828.62167 | 1.213624033 | 0.27932156 | 0.492135926 | no | up | GO:0020005;GO:0070098;GO:0005125;GO:0071222;GO:0019221;GO:0008009;GO:0010469;GO:0032760;GO:0010628;GO:0048248;GO:0032496;GO:0045918;GO:0061844;GO:0005737;GO:0045347;GO:0031093;GO:0002576;GO:0007189;GO:0051873;GO:0010744;GO:0007186;GO:2001240;GO:0042127;GO:0030595;GO:0030593;GO:0006935;GO:0043950;GO:0006952;GO:0062023;GO:0006954;GO:0006955;GO:0008201;GO:0030168;GO:0005615;GO:0090023;GO:0045651;GO:0045652;GO:0045653;GO:0031640;GO:0005576;GO:0045944;GO:0005515;GO:0097679;GO:0042832;GO:0016525 | hsa:5196 |  | platelet factor 4 isoform 1 precursor [Homo sapiens] |
| P04040 | 6359.091125 | 19046.50073 | 2.995160843 | 1.582633479 | 0.320307719 | no | up | GO:0016491;GO:0005829;GO:0016020;GO:0005782;GO:0020027;GO:0033189;GO:0051289;GO:0009314;GO:0005886;GO:0019899;GO:0000302;GO:1904813;GO:0009060;GO:0005739;GO:0004601;GO:0009650;GO:0032868;GO:0001666;GO:0055093;GO:0001649;GO:0005783;GO:0050661;GO:0005778;GO:0009636;GO:0070062;GO:0005615;GO:0033591;GO:0005777;GO:0001822;GO:0051262;GO:0006979;GO:0098869;GO:0043066;GO:0046686;GO:0005758;GO:0034774;GO:0046872;GO:0014823;GO:0042802;GO:0042803;GO:0004046;GO:0005794;GO:0005925;GO:0010288;GO:0020037;GO:0006641;GO:0009411;GO:0006625;GO:0016209;GO:0032355;GO:0045471;GO:0014068;GO:0001657;GO:0071363;GO:0034599;GO:0009642;GO:0055114;GO:0008203;GO:0051092;GO:0016684;GO:0007568;GO:0043231;GO:0042542;GO:0070542;GO:0042493;GO:0043312;GO:0032088;GO:0005764;GO:0051781;GO:0042744;GO:0033197;GO:0010193;GO:0004096;GO:0005576;GO:0005102;GO:0014854;GO:0080184 | hsa:847 | COG0753 | catalase [Homo sapiens] |
| P07737 | 10121.49626 | 14944.56517 | 1.476517382 | 0.562198341 | 0.087548454 | no | up | GO:0017048;GO:0050821;GO:0060074;GO:0045202;GO:0050434;GO:0005925;GO:0005737;GO:0032232;GO:0032233;GO:0070062;GO:0030837;GO:0030838;GO:0051496;GO:0005634;GO:0016020;GO:0005546;GO:0003779;GO:0098794;GO:0043005;GO:0098793;GO:0001843;GO:0005856;GO:0051054;GO:0098685;GO:0005515;GO:0032781;GO:0060071;GO:0098688;GO:0070064;GO:0005938;GO:0000774;GO:0010033;GO:0010634;GO:0071363;GO:0005829;GO:1900029;GO:0098885;GO:0045296;GO:0051497;GO:0006357;GO:0045944;GO:0003723;GO:0030036;GO:0072562;GO:0005102;GO:0098978;GO:0003785 | hsa:5216 |  | PREDICTED: profilin-1 isoform X2 [Nomascus leucogenys] |
| P08779 | 89197.10947 | 113683.1733 | 1.274516338 | 0.349949867 | 0.298811955 | no | up | GO:0005200;GO:0061436;GO:0008544;GO:0005856;GO:0002009;GO:0051546;GO:0070062;GO:0005634;GO:0005198;GO:0030336;GO:0030216;GO:0008283;GO:0045087;GO:0070268;GO:0005882;GO:0006954;GO:0042633;GO:0007568;GO:0007010;GO:0045104;GO:0005829;GO:0031424;GO:0005515 | hsa:3868 |  | keratin, type I cytoskeletal 16 [Homo sapiens] |
| P0DP02 | 236468.2979 | 299306.6975 | 1.265737099 | 0.33997778 | 0.114411912 | no | up | GO:0016020;GO:0002376;GO:0005576;GO:0002250;GO:0003823;GO:0005886 | hsa:102723407 | | immunoglobulin heavy chain [Homo sapiens] |
| P0DTE1 | 16931.36632 | 50622.72045 | 2.989878047 | 1.58008664 | 0.172389786 | no | up |  | hsa:102723407 | | immunoglobulin heavy chain variable region, partial [Homo sapiens] |
| P11226 | 82655.61287 | 113633.3227 | 1.374780475 | 0.459201267 | 0.076174399 | no | up | GO:0004252;GO:0048306;GO:0030246;GO:0042742;GO:0044130;GO:0050766;GO:0005581;GO:0001867;GO:0005509;GO:0051873;GO:0006508;GO:0009986;GO:0045087;GO:0006953;GO:0008228;GO:0006956;GO:0006958;GO:0006979;GO:0005615;GO:0050830;GO:0002376;GO:0005576;GO:0005515;GO:0048525;GO:0005102;GO:0005537 | hsa:4153 |  | mannose-binding lectin [Homo sapiens] |
| P59665 | 138895.9084 | 200544.0638 | 1.443844286 | 0.529915161 | 0.444908391 | no | up | GO:0071222;GO:0019730;GO:0019731;GO:0035578;GO:0051673;GO:0042742;GO:0050829;GO:0061844;GO:0005615;GO:0030520;GO:0051852;GO:0044657;GO:0002227;GO:0042803;GO:0010818;GO:0005796;GO:0006935;GO:0006952;GO:0050830;GO:0062023;GO:0006955;GO:0070062;GO:0050832;GO:0051607;GO:0031640;GO:0043312;GO:0005576;GO:0042832 | hsa:1667;hsa:728358;hsa:1668 | | neutrophil defensin 1 preproprotein [Homo sapiens] |
| P60709 | 27249.58842 | 41243.69096 | 1.513552804 | 0.597939008 | 0.119151126 | no | up | GO:0043044;GO:0019894;GO:0015629;GO:0072749;GO:0061024;GO:0005200;GO:0021762;GO:0036464;GO:0005925;GO:0048488;GO:0030957;GO:0032091;GO:0005615;GO:0043209;GO:0005634;GO:0016020;GO:0097433;GO:0070062;GO:0098871;GO:0005654;GO:0045815;GO:0016579;GO:0005524;GO:0000980;GO:0005856;GO:0070527;GO:0000166;GO:1903076;GO:0019901;GO:0000790;GO:0005886;GO:0048013;GO:0001895;GO:0098685;GO:0034329;GO:0031982;GO:0038096;GO:0050998;GO:0044305;GO:0032991;GO:0051621;GO:0048870;GO:0030863;GO:0022898;GO:0005829;GO:0051623;GO:1990904;GO:0005737;GO:0098973;GO:0031492;GO:0098793;GO:0042802;GO:0005515;GO:0098974;GO:0072562;GO:0000978;GO:0098978;GO:0000079;GO:0035267 | hsa:71;hsa:60 | COG5277 | cytoskeletal beta actin, partial [Sus scrofa] |
| Q15848 | 10299.76947 | 14125.85138 | 1.371472576 | 0.455725775 | 0.091853646 | no | up | GO:0034115;GO:0005783;GO:0010804;GO:0050728;GO:0009967;GO:0009617;GO:0030853;GO:0043124;GO:0033034;GO:0032720;GO:0043123;GO:2000481;GO:0014823;GO:0072659;GO:0010875;GO:0045650;GO:0071320;GO:0031667;GO:0050731;GO:1904753;GO:0045715;GO:0005125;GO:0033691;GO:0031953;GO:0010906;GO:0046326;GO:0070994;GO:0045721;GO:0010739;GO:0007623;GO:2000467;GO:2000279;GO:0070208;GO:0071872;GO:0019395;GO:0042304;GO:0006006;GO:0034383;GO:0006635;GO:2000478;GO:0050805;GO:0043407;GO:0001666;GO:0010642;GO:0050765;GO:0005581;GO:0032270;GO:0042802;GO:0042803;GO:0009986;GO:0050873;GO:0045892;GO:0046888;GO:0045599;GO:0007584;GO:0120162;GO:0120163;GO:0070543;GO:0042493;GO:1900121;GO:0005515;GO:0005102;GO:0045860;GO:0070373;GO:0045923;GO:0051384;GO:0042593;GO:0035690;GO:0032869;GO:0006091;GO:0005615;GO:0051260;GO:2000590;GO:0010745;GO:0010469;GO:1904706;GO:0005179;GO:0030336;GO:2000534;GO:0034612;GO:0090317;GO:0045471;GO:0009744;GO:0032991;GO:0045776;GO:0009749;GO:0071639;GO:0005576;GO:2000584;GO:0032757;GO:0001934 | hsa:9370 |  | TPA: adiponectin D [Homo sapiens] |
| Q16880 | 655931.7579 | 886352.5667 | 1.351287777 | 0.43433495 | 0.108953621 | no | up | GO:0008489;GO:0007417;GO:0030913;GO:0016021;GO:0016020;GO:0006682;GO:0016740;GO:0006687;GO:0015020;GO:0006665;GO:0006629;GO:0002175;GO:0008152;GO:0005886;GO:0008194;GO:0007010;GO:0043231;GO:0007422;GO:0048812;GO:0016757;GO:0047263;GO:0016758 | hsa:7368 | COG1819 | 2-hydroxyacylsphingosine 1-beta-galactosyltransferase precursor [Homo sapiens] |
| Q86UX7 | 4622.435408 | 7383.9592 | 1.597417497 | 0.675741421 | 0.085697643 | no | up | GO:0005178;GO:0030335;GO:0002102;GO:0070062;GO:0031093;GO:0034446;GO:0007229;GO:0016020;GO:0033632;GO:0005576;GO:0007159;GO:0007155;GO:0033622;GO:0042995;GO:0070527;GO:0030054;GO:0002576 | hsa:83706 |  | fermitin family homolog 3 long isoform [Homo sapiens] |
| A0A075B6R2 | 205343.2858 | 185933.5563 | 0.905476678 | -0.143250612 | 0.514752996 | no | no change | GO:0009897;GO:0050853;GO:0045087;GO:0016020;GO:0006910;GO:0006911;GO:0002376;GO:0005576;GO:0002250;GO:0003823;GO:0005886;GO:0072562;GO:0042571;GO:0006958;GO:0050871;GO:0034987;GO:0042742 | hsa:102724971 | | immunoglobulin heavy chain VDJ region, partial [Homo sapiens] |
| A0A075B7B8 | 969976.1632 | 817348.4688 | 0.842647995 | -0.246998005 | 0.157103871 | no | no change | GO:0009897;GO:0050853;GO:0045087;GO:0006910;GO:0006911;GO:0042742;GO:0003823;GO:0034987;GO:0042571;GO:0006958;GO:0050871;GO:0072562 | hsa:102723407 | | hCG1793095, isoform CRA_a, partial [Homo sapiens] |
| A0A075B7F0 | 27609.92778 | 26816.23604 | 0.971253393 | -0.042080361 | 0.891240523 | no | no change | GO:0009897;GO:0050853;GO:0045087;GO:0006910;GO:0006911;GO:0042742;GO:0003823;GO:0034987;GO:0042571;GO:0006958;GO:0050871;GO:0072562 | hsa:102723407 | | IGHV3-13 isoform 1, partial [Pan troglodytes] |
| A0A087WSZ0 | 50574.93474 | 47885.78267 | 0.946828363 | -0.078825171 | 0.859876994 | no | no change | GO:0016020;GO:0005615;GO:0006955;GO:0002376;GO:0002377;GO:0005576;GO:0002250;GO:0003823;GO:0005886 | | | RecName: Full=Immunoglobulin kappa variable 1D-8; Flags: Precursor |
| A0A087WWT3 | 65991.80895 | 72358.50375 | 1.096477046 | 0.132875611 | 0.177291665 | no | no change | GO:0005794;GO:0005615;GO:0005783 | hsa:213 |  | ALB protein [Homo sapiens] |
| A0A087X0Q4 | 115433.0263 | 126820.3292 | 1.098648569 | 0.135729977 | 0.493302984 | no | no change |  |  |  | RecName: Full=Immunoglobulin kappa variable 2D-40; AltName: Full=Ig kappa chain V-II region Cum; Flags: Precursor |
| A0A087X1J7 | 40054.36895 | 43706.02333 | 1.091167443 | 0.125872504 | 0.423992227 | no | no change | GO:0008430;GO:0005615;GO:0051289;GO:0006979;GO:0098869;GO:0055114;GO:0004601;GO:0004602;GO:0016491 | hsa:2878 | COG0386 | glutathione peroxidase 3 isoform 1 precursor [Homo sapiens] |
| A0A096LPE2 | 517015.4895 | 600397.2708 | 1.161275209 | 0.215709916 | 0.087401406 | no | no change | GO:0042056;GO:0034364;GO:0005615;GO:0006953;GO:0005576;GO:0060326;GO:0050918;GO:0070062 | hsa:6291;hsa:100528017 | | SAA2-SAA4 protein precursor [Homo sapiens] |
| A0A0A0MRJ7 | 20252.18995 | 23400.44375 | 1.155452512 | 0.208457968 | 0.145576254 | no | no change | GO:0048208;GO:0044267;GO:1903561;GO:0006888;GO:0005615;GO:0031093;GO:0008015;GO:0002576;GO:0007596;GO:0033116;GO:0005788;GO:0030134;GO:0046872;GO:0005576;GO:0005886;GO:0016020;GO:0005507;GO:0043687;GO:0000139 | hsa:2153 |  | coagulation factor V preproprotein [Homo sapiens] |
| A0A0A0MS15 | 242347.3158 | 203933.7596 | 0.841493783 | -0.248975482 | 0.414141969 | no | no change | GO:0009897;GO:0050853;GO:0045087;GO:0016020;GO:0006910;GO:0006911;GO:0002376;GO:0005576;GO:0002250;GO:0003823;GO:0005886;GO:0072562;GO:0042571;GO:0006958;GO:0050871;GO:0034987;GO:0042742 | hsa:102723407 | | RecName: Full=Immunoglobulin heavy variable 3-49; Flags: Precursor |
| A0A0A0MS51 | 273651.6684 | 246227.2667 | 0.899783539 | -0.152350121 | 0.07498235 | no | no change | GO:0051127;GO:0015629;GO:1902174;GO:0030155;GO:0030041;GO:0042989;GO:0045159;GO:1990000;GO:0005925;GO:1903923;GO:0001726;GO:0071801;GO:0005615;GO:0045010;GO:1903903;GO:0043209;GO:0005634;GO:1903909;GO:0030478;GO:0005509;GO:0048471;GO:1903906;GO:0097284;GO:2001269;GO:0060271;GO:0030027;GO:0006915;GO:0051016;GO:0051015;GO:0051014;GO:0045471;GO:0045335;GO:0002102;GO:0005886;GO:0014003;GO:0032991;GO:0006911;GO:0048015;GO:0051693;GO:0046597;GO:0090527;GO:0007568;GO:0051593;GO:0005829;GO:0071346;GO:0042246;GO:0031648;GO:0016528;GO:0014891;GO:0097017;GO:0071276 | hsa:2934 |  | gelsolin isoform d [Homo sapiens] |
| A0A0A0MT36 | 139614.64 | 129700.0317 | 0.928985898 | -0.106271399 | 0.750104807 | no | no change | GO:0016020;GO:0005615;GO:0006955;GO:0002376;GO:0002377;GO:0005576;GO:0002250;GO:0003823;GO:0005886 | hsa:7441 |  | RecName: Full=Immunoglobulin kappa variable 6D-21; Flags: Precursor |
| A0A0B4J1U7 | 53333.70684 | 57424.72708 | 1.076706092 | 0.106624492 | 0.740930855 | no | no change | GO:0009897;GO:0050853;GO:0045087;GO:0016020;GO:0006910;GO:0006911;GO:0002376;GO:0005576;GO:0002250;GO:0003823;GO:0005886;GO:0072562;GO:0042571;GO:0006958;GO:0050871;GO:0034987;GO:0042742 | hsa:102723407 | | RecName: Full=Immunoglobulin heavy variable 6-1; Flags: Precursor |
| A0A0B4J1X5 | 67415.87611 | 59118.22 | 0.876918367 | -0.189485547 | 0.413314531 | no | no change | GO:0009897;GO:0050853;GO:0045087;GO:0016020;GO:0006910;GO:0006911;GO:0002376;GO:0005576;GO:0002250;GO:0003823;GO:0005886;GO:0072562;GO:0042571;GO:0006958;GO:0050871;GO:0034987;GO:0042742 | hsa:102723407 | | immunoglobulin heavy chain VH3, partial [Homo sapiens] |
| A0A0B4J1X8 | 106788.1384 | 94104.9375 | 0.881230246 | -0.182409082 | 0.354559085 | no | no change | GO:0009897;GO:0050853;GO:0045087;GO:0016020;GO:0006910;GO:0006911;GO:0002376;GO:0005576;GO:0002250;GO:0003823;GO:0005886;GO:0072562;GO:0042571;GO:0006958;GO:0050871;GO:0034987;GO:0042742 | hsa:102723407 | | RecName: Full=Immunoglobulin heavy variable 3-43; Flags: Precursor |
| A0A0B4J1Y8 | 116112.7792 | 124365.8168 | 1.071077771 | 0.099063239 | 0.764217776 | no | no change | GO:0016020;GO:0005615;GO:0006955;GO:0002376;GO:0002377;GO:0005576;GO:0002250;GO:0003823;GO:0005886 | hsa:7441 |  | Unknown (protein for IMAGE:4575521), partial [Homo sapiens] |
| A0A0B4J231 | 11789687.64 | 13135041 | 1.114112723 | 0.155895208 | 0.376522215 | no | no change | GO:0009897;GO:0050853;GO:0070062;GO:0045087;GO:0006910;GO:0006911;GO:0042742;GO:0003823;GO:0034987;GO:0042571;GO:0006958;GO:0050871 | hsa:100423062 | | immunoglobulin lambda-3 surrogate light chain [Homo sapiens] |
| A0A0C4DGZ8 | 15921.57261 | 15971.72671 | 1.003150072 | 0.00453745 | 0.987309352 | no | no change | GO:0016021;GO:0016020 | hsa:2811 | COG4886 | glycoprotein Ib (platelet), alpha polypeptide [Homo sapiens] |
| A0A0C4DH21 | 40816.30053 | 37914.02208 | 0.928894133 | -0.106413915 | 0.573226915 | no | no change |  | hsa:10877 |  | complement factor H-related protein 4 [Homo sapiens] |
| A0A0C4DH29 | 34843.57474 | 32287.37333 | 0.926637797 | -0.109922565 | 0.558065743 | no | no change | GO:0009897;GO:0050853;GO:0045087;GO:0016020;GO:0006910;GO:0006911;GO:0002376;GO:0005576;GO:0002250;GO:0003823;GO:0005886;GO:0072562;GO:0042571;GO:0006958;GO:0050871;GO:0034987;GO:0042742 | hsa:102723407 | | immunoglobulin heavy chain variable region, partial [Homo sapiens] |
| A0A0C4DH31 | 27515.99632 | 31109.19917 | 1.130585962 | 0.177070689 | 0.174428312 | no | no change | GO:0009897;GO:0050853;GO:0045087;GO:0016020;GO:0006910;GO:0006911;GO:0002376;GO:0005576;GO:0002250;GO:0003823;GO:0005886;GO:0072562;GO:0042571;GO:0006958;GO:0050871;GO:0034987;GO:0042742 | hsa:102723407 | | immunoglobulin heavy chain variable region, partial [Homo sapiens] |
| A0A0C4DH33 | 12642.38142 | 10801.41761 | 0.854381564 | -0.227047579 | 0.413126513 | no | no change | GO:0009897;GO:0050853;GO:0045087;GO:0016020;GO:0006910;GO:0006911;GO:0002376;GO:0005576;GO:0002250;GO:0003823;GO:0005886;GO:0072562;GO:0042571;GO:0006958;GO:0050871;GO:0034987;GO:0042742 | hsa:102723407 | | immunoglobulin heavy chain variable region, partial [Homo sapiens] |
| A0A0C4DH38 | 109825.7963 | 101988.5363 | 0.928639169 | -0.106809961 | 0.624185917 | no | no change | GO:0009897;GO:0050853;GO:0045087;GO:0016020;GO:0006910;GO:0006911;GO:0002376;GO:0005576;GO:0002250;GO:0003823;GO:0005886;GO:0072562;GO:0042571;GO:0006958;GO:0050871;GO:0034987;GO:0042742 | hsa:102723407 | | RecName: Full=Immunoglobulin heavy variable 5-51; Flags: Precursor |
| A0A0C4DH55 | 9163152.211 | 8138870.833 | 0.888217356 | -0.171015332 | 0.249251241 | no | no change | GO:0016020;GO:0005615;GO:0006955;GO:0002376;GO:0002377;GO:0005576;GO:0002250;GO:0003823;GO:0005886 | hsa:29802 |  | RecName: Full=Immunoglobulin kappa variable 3D-7; Flags: Precursor |
| A0A0G2JMB2 | 2906175.368 | 2504752.125 | 0.861872326 | -0.214453924 | 0.191019655 | no | no change | GO:0009897;GO:0050853;GO:0045087;GO:0006910;GO:0006911;GO:0042742;GO:0003823;GO:0034987;GO:0042571;GO:0006958;GO:0050871;GO:0072562 | hsa:55423 |  | Immunoglobulin heavy chain variant, partial [Homo sapiens] |
| A0A0G2JPR0 | 392835.4947 | 441040.4542 | 1.122710295 | 0.166985702 | 0.408492642 | no | no change | GO:0004866;GO:0005576;GO:0006956;GO:0006954;GO:0010951;GO:0005615 | hsa:100293534;hsa:110384692;hsa:720;hsa:721 | | complement C4A (Rodgers blood group)-like preproprotein [Homo sapiens] |
| A0A0J9YVY3 | 60778.144 | 52176.18824 | 0.858469588 | -0.220161069 | 0.266177185 | no | no change | GO:0016020;GO:0002376;GO:0005576;GO:0002250;GO:0003823;GO:0005886 | hsa:102723407 | | RecName: Full=Immunoglobulin heavy variable 7-4-1; Flags: Precursor |
| A0A0J9YY99 | 187520.5705 | 212404.6646 | 1.13270061 | 0.179766585 | 0.843514813 | no | no change |  | hsa:102723407 | | immunoglobulin heavy chain VDJ region, partial [Homo sapiens] |
| A0A0S2Z4L3 | 203988.0474 | 199108.0708 | 0.976077145 | -0.034932918 | 0.771246261 | no | no change | GO:0005576;GO:0005509;GO:0030195 | hsa:5627 |  | vitamin K-dependent protein S isoform 1 precursor [Homo sapiens] |
| A0A1W2PQU7 | 86400.39789 | 87935.20958 | 1.017763942 | 0.025402985 | 0.829948 | no | no change | GO:1904714;GO:0005737;GO:0045111;GO:0061564;GO:0045109;GO:0060291;GO:0016020;GO:0044297;GO:0060020;GO:0005198;GO:0051580;GO:0005883;GO:0005882;GO:0010977;GO:0030198;GO:0014002;GO:0097450;GO:0010625;GO:0031102 | hsa:2670 |  | glial fibrillary acidic protein [Mus musculus] |
| A0A286YEY1 | 5144417.211 | 4794880.125 | 0.932055066 | -0.101512902 | 0.655921866 | no | no change |  |  |  | IGHA1 isoform 1, partial [Pan troglodytes] |
| A0A286YEY4 | 644354.6316 | 536669.5125 | 0.832879111 | -0.263820986 | 0.085115441 | no | no change | GO:0016021;GO:0016020 | hsa:100423062 | | unnamed protein product [Homo sapiens] |
| A0A3B3ISR2 | 305276.5737 | 277518.2125 | 0.909071434 | -0.137534431 | 0.268539102 | no | no change | GO:0004252;GO:0005615;GO:0045087;GO:0031638;GO:0005509;GO:0006958 | hsa:715 | COG5640 | complement C1r subcomponent isoform 1 preproprotein [Homo sapiens] |
| A0A5H1ZRS9 | 84803.10211 | 95647.84275 | 1.127881414 | 0.173615391 | 0.388755365 | no | no change |  | hsa:7441 |  | immunoglobulin kappa chain variable region, partial [Homo sapiens] |
| A6XND0 | 33136.64211 | 29247.19583 | 0.882624007 | -0.180129106 | 0.272838186 | no | no change | GO:0005520;GO:0001558;GO:0005576 | hsa:3486 |  | insulin-like growth factor binding protein 3 [Homo sapiens] |
| B0YIW2 | 2192579.847 | 1884424.854 | 0.859455521 | -0.218505117 | 0.480119031 | no | no change | GO:0034375;GO:0042627;GO:0034371;GO:0006869;GO:0034379;GO:0034378;GO:0051005;GO:0042157;GO:0005576;GO:0048261;GO:0001523;GO:0055102;GO:0070062;GO:0005543;GO:0045833;GO:0010897;GO:0007186;GO:0062023;GO:0033700;GO:0034361;GO:0034363;GO:0070328;GO:0034366;GO:0006641;GO:0060621;GO:0030234;GO:0070653;GO:0008289;GO:0010916;GO:0042632;GO:0032489;GO:0043691;GO:0005615;GO:0015485;GO:0034382;GO:0050995;GO:0019433;GO:0033344;GO:0010989;GO:0010987;GO:0005769;GO:0045717;GO:0010903 | hsa:345 |  | apolipoprotein C-III precursor variant 1 [Homo sapiens] |
| B1AHL2 | 16650.34933 | 14313.19365 | 0.859633234 | -0.218206835 | 0.145466183 | no | no change | GO:0016504;GO:0005576;GO:0005509;GO:0010952;GO:0030198 | hsa:2192 |  | FBLN1 isoform 5 [Pongo abelii] |
| B4E1Z4 | 536278.9316 | 496005.725 | 0.924902501 | -0.112626804 | 0.239017933 | no | no change | GO:0004252;GO:0030449;GO:0070062;GO:0016787;GO:0072562;GO:0001848;GO:0005576;GO:0006956;GO:0006957;GO:0005886;GO:0006508;GO:0008233;GO:0008236;GO:0005615 | hsa:629 | COG5640 | unnamed protein product [Homo sapiens] |
| B7ZKJ8 | 523845.2211 | 482377.3375 | 0.920839435 | -0.118978476 | 0.140767614 | no | no change | GO:0005737;GO:0030212;GO:0034097;GO:0004867;GO:0006953;GO:0005886;GO:0010951 | hsa:3700 | COG2304 | ITIH4 protein [Homo sapiens] |
| C9JC84 | 4677622.579 | 5148328.417 | 1.10062929 | 0.138328627 | 0.158776544 | no | no change | GO:0051258;GO:0005102;GO:0007596;GO:0005577;GO:0030168 | hsa:2266 |  | FGG isoform 6 [Pan troglodytes] |
| C9JF17 | 825317.6895 | 919032.8292 | 1.113550383 | 0.155166835 | 0.304852984 | no | no change | GO:0022626;GO:0005783;GO:0006869;GO:0042493;GO:0048678;GO:0030425;GO:0000302;GO:0001525;GO:0051895;GO:0010642;GO:0005737;GO:0070062;GO:0014012;GO:0005615;GO:2000405;GO:0048471;GO:0043025;GO:0005319;GO:0048662;GO:0006629;GO:2000098;GO:0008289;GO:0042308;GO:0060588;GO:0007420;GO:1900016;GO:0007568;GO:0015485;GO:0042246;GO:0071638;GO:0005576;GO:0006006 | hsa:347 | COG3040 | APOD isoform 3, partial [Pan troglodytes] |
| C9JV77 | 2167220.737 | 2053469 | 0.947512621 | -0.077782935 | 0.322882578 | no | no change | GO:0019210;GO:0006907;GO:0005788;GO:0001501;GO:0030500;GO:0030502;GO:0050766;GO:0044267;GO:0070062;GO:0031093;GO:0002576;GO:0050727;GO:0072562;GO:0005794;GO:0043687;GO:0046627;GO:0006953;GO:0062023;GO:0034774;GO:0010951;GO:0004869;GO:0005615;GO:0043312;GO:0005576 | hsa:197 |  | alpha-2-HS-glycoprotein isoform 1 preproprotein [Homo sapiens] |
| D6R934 | 107963.7168 | 96657.57542 | 0.895278324 | -0.159591839 | 0.062783202 | no | no change | GO:0006958;GO:0005576;GO:0005581 | hsa:713 |  | complement C1q subcomponent subunit B precursor [Homo sapiens] |
| D6RAR4 | 25599.32632 | 25873.07667 | 1.010693654 | 0.015345777 | 0.886762298 | no | no change | GO:0005737;GO:0004252;GO:0005791;GO:0005615;GO:0016787;GO:0005576;GO:0006508;GO:0008233;GO:0008236 | hsa:3083 | COG5640 | hepatocyte growth factor activator isoform 1 preproprotein [Homo sapiens] |
| D6RD17 | 2656375.211 | 2865348 | 1.078668401 | 0.109251426 | 0.665091934 | no | no change |  | hsa:3512 |  | JCHAIN isoform 4, partial [Pongo abelii] |
| D6RF35 | 2707468.579 | 2487710.75 | 0.918832732 | -0.122125844 | 0.120909542 | no | no change | GO:0051180;GO:0090482;GO:0035461;GO:0005615;GO:0005499 | hsa:2638 |  | GC isoform 4 [Pan troglodytes] |
| E9PAQ1 | 27298.39105 | 27897.92625 | 1.021962291 | 0.031341963 | 0.709228934 | no | no change |  | hsa:5199 |  | properdin precursor [Homo sapiens] |
| E9PHK0 | 150859.3505 | 149272.3833 | 0.989480485 | -0.015256841 | 0.855520019 | no | no change | GO:0005737;GO:0036143;GO:0070062;GO:0002576;GO:0001652;GO:0030246;GO:0008201;GO:0071560;GO:0071310;GO:0001503;GO:0005615;GO:0005509;GO:0005576;GO:0031089;GO:0030282;GO:0062023;GO:0010756 | hsa:7123 |  | tetranectin isoform 1precursor [Homo sapiens] |
| F5H8B0 | 5957.909684 | 6175.647174 | 1.036545953 | 0.051784077 | 0.67936039 | no | no change | GO:0004252;GO:0016787;GO:0007596;GO:0005576;GO:0005509;GO:0006508;GO:0008233;GO:0008236 | hsa:2155 | COG5640 | coagulation factor VII isoform c precursor [Homo sapiens] |
| F8W1S1 | 5466.170615 | 5528.127 | 1.011334513 | 0.016260268 | 0.919806547 | no | no change | GO:0005882;GO:0045095;GO:0005198 | hsa:121391 | | KRT74 isoform 2 [Pan troglodytes] |
| F8WF14 | 25332.67737 | 26077.91 | 1.029417839 | 0.041828689 | 0.738888148 | no | no change | GO:0019899;GO:0051384;GO:0005783;GO:0016787;GO:0007612;GO:0050805;GO:0050783;GO:0014016;GO:0001540;GO:0016021;GO:0016020;GO:0072562;GO:0042802;GO:0008285;GO:0004104;GO:0003824;GO:0005788;GO:0043279;GO:0051593;GO:0019695;GO:0005641;GO:0005576;GO:0033265;GO:0003990 | hsa:590 | COG2272 | unnamed protein product [Homo sapiens] |
| G3V0E5 | 5320.957789 | 5941.735708 | 1.116666575 | 0.159198476 | 0.415955258 | no | no change | GO:0009897;GO:0055037;GO:0035690;GO:0005905;GO:1990712;GO:0010008;GO:0045780;GO:1990830;GO:0070062;GO:0016021;GO:0045830;GO:0004998;GO:0048471;GO:0042803;GO:0006879;GO:0030890;GO:0030316;GO:0042102;GO:0005887;GO:0005886;GO:0016323;GO:0042470;GO:0031623;GO:0006898;GO:0003725;GO:0033570;GO:0033572;GO:0005769 | hsa:7037 | COG2234 | transferrin receptor variant, partial [Homo sapiens] |
| G3V2W1 | 10439.47474 | 9160.647292 | 0.877500786 | -0.188527676 | 0.298341697 | no | no change | GO:0010951;GO:0007596;GO:0005615;GO:0004867 | hsa:51156 | COG4826 | protein Z-dependent protease inhibitor isoform X1 [Homo sapiens] |
| G3XAK1 | 17478.04947 | 15567.44625 | 0.890685558 | -0.167011893 | 0.084204771 | no | no change | GO:0004252;GO:2000479;GO:0019899;GO:0010628;GO:0005737;GO:0005615;GO:0005773;GO:0033601;GO:0030971;GO:1904036;GO:0006508;GO:0045721;GO:0046425;GO:0030317;GO:0071456;GO:0007283;GO:0062023;GO:0060763;GO:0048012;GO:0007566;GO:0030879;GO:0005576;GO:0010758 | hsa:4485 | COG5640 | hepatocyte growth factor-like protein precursor [Homo sapiens] |
| G3XAP6 | 9133.701 | 9223.943667 | 1.009880186 | 0.014184139 | 0.926696997 | no | no change | GO:0005201;GO:0060173;GO:0062023;GO:0030509;GO:0035264;GO:0050905;GO:0030500;GO:0030282;GO:0050881;GO:0010259;GO:1900047;GO:0005615;GO:0048844;GO:0036122;GO:0030198;GO:0005509;GO:0002020;GO:0002063;GO:0003417;GO:0043588;GO:0005576;GO:0014829;GO:0005178;GO:0009887;GO:1902732;GO:0009306;GO:0006915;GO:0043066;GO:0048747;GO:0006986;GO:0043395;GO:0035988;GO:0007155;GO:0097084;GO:0016485;GO:0031012;GO:0008201;GO:0070062;GO:0010260;GO:0032991;GO:0001501;GO:0035989;GO:0030199;GO:0043394;GO:0005518;GO:0070527 | hsa:1311 |  | unnamed protein product [Homo sapiens] |
| H0Y5E4 | 19693.86579 | 18186.77875 | 0.923474291 | -0.114856297 | 0.220967605 | no | no change | GO:0005540;GO:0007155;GO:0016021;GO:0016020 | hsa:960 |  | CD44 antigen isoform 8 precursor [Homo sapiens] |
| H0Y755 | 7334.283579 | 8253.877304 | 1.125382897 | 0.170415944 | 0.262739793 | no | no change | GO:0016021;GO:0016020 | hsa:2214 |  | low affinity immunoglobulin gamma Fc region receptor III-A isoform b [Homo sapiens] |
| H0YJW9 | 796192.4947 | 939131.4021 | 1.179528077 | 0.23820976 | 0.179642644 | no | no change |  | hsa:7448 |  | vitronectin, partial [Homo sapiens] |
| H3BUA5 | 401534.5821 | 405591.1083 | 1.010102558 | 0.01450178 | 0.928815128 | no | no change |  | hsa:10326 |  | LOW QUALITY PROTEIN: T0061165 isoform 1, partial [Pan troglodytes] |
| I3L145 | 12792.186 | 12641.29492 | 0.988204433 | -0.017118567 | 0.962808874 | no | no change | GO:0005496 | hsa:6462 |  | SHBG protein, partial [Homo sapiens] |
| J3KNB4 | 15955.17689 | 14444.01538 | 0.905287072 | -0.143552743 | 0.516860976 | no | no change | GO:0071224;GO:0071222;GO:0042742;GO:0050829;GO:0044130;GO:0061844;GO:0005737;GO:0042995;GO:0005615;GO:0071354;GO:0071356;GO:0016021;GO:0045766;GO:0051873;GO:0008284;GO:0045087;GO:0006952;GO:0050830;GO:0044140;GO:0001530;GO:0042581;GO:0071347;GO:0005576;GO:0001934 | hsa:820 |  | cathelicidin antimicrobial peptide [Homo sapiens] |
| J3KPA1 | 11335.57081 | 10629.90188 | 0.937747384 | -0.092728762 | 0.598586153 | no | no change | GO:0016020;GO:0016021;GO:0005576 | hsa:10321 | COG2340 | cysteine-rich secretory protein 3 isoform 3 [Homo sapiens] |
| J3KRP0 | 26790.15789 | 29721.22042 | 1.109408184 | 0.149790273 | 0.400614936 | no | no change | GO:0016787;GO:0032268;GO:0005829;GO:0004180;GO:0016805;GO:0005576;GO:0008152;GO:0006508;GO:0046872;GO:0008237 | hsa:84735 | COG0624 | Carnosine dipeptidase 1 (metallopeptidase M20 family) [Homo sapiens] |
| O00391 | 11190.81111 | 9566.577958 | 0.854860105 | -0.226239748 | 0.105150209 | no | no change | GO:0005788;GO:0030173;GO:0071949;GO:0016491;GO:0016971;GO:0044267;GO:0016972;GO:0045171;GO:0070062;GO:0031093;GO:0016021;GO:0016020;GO:0003756;GO:0035580;GO:0016242;GO:0005794;GO:1904724;GO:0043687;GO:0045454;GO:0055114;GO:0005615;GO:0043231;GO:0043312;GO:0000139;GO:0005576;GO:0085029;GO:0002576 | hsa:5768 |  | sulfhydryl oxidase 1 isoform a precursor [Homo sapiens] |
| O14791 | 38175.58632 | 43243.82 | 1.132761122 | 0.179843656 | 0.188138113 | no | no change | GO:0006869;GO:0005788;GO:0042157;GO:0031224;GO:0019835;GO:0044267;GO:0005615;GO:0072562;GO:0005254;GO:0045087;GO:0034361;GO:0034364;GO:0006629;GO:1902476;GO:0043687;GO:0008289;GO:0008202;GO:0008203;GO:0006898;GO:0031640;GO:0005576;GO:0005515 | hsa:8542 |  | apolipoprotein L1 isoform a precursor [Homo sapiens] |
| O43866 | 149405.1953 | 171118.175 | 1.145329483 | 0.195762685 | 0.354263533 | no | no change | GO:0005737;GO:0006898;GO:0005615;GO:0005044;GO:0009986;GO:0016020;GO:0072562;GO:0002376;GO:0005576;GO:0006954;GO:0006968;GO:0006915 | hsa:922 |  | CD5 antigen-like isoform 1 precursor [Homo sapiens] |
| O75882 | 46564.93 | 44478.78583 | 0.955199242 | -0.066126403 | 0.557682788 | no | no change | GO:0005737;GO:0043473;GO:0042552;GO:0005615;GO:0070062;GO:0016021;GO:0016020;GO:0030246;GO:0006954;GO:0005576;GO:0005887;GO:0005886;GO:0038023;GO:0021549;GO:0006979;GO:0040014 | hsa:8455 |  | attractin isoform 1 preproprotein [Homo sapiens] |
| O95445 | 64039.99368 | 57454.5025 | 0.897165961 | -0.15655321 | 0.092863995 | no | no change | GO:0034375;GO:0034445;GO:0006869;GO:0043691;GO:0042157;GO:0001523;GO:0005615;GO:0005543;GO:0098869;GO:0005576;GO:0005319;GO:0034361;GO:0034362;GO:0034364;GO:0034365;GO:0034366;GO:0016209;GO:0042632;GO:0034380;GO:0034384;GO:0009749;GO:0033344 | hsa:55937 |  | apolipoprotein M isoform 1 [Homo sapiens] |
| P00450 | 659652.3579 | 629645.5833 | 0.95451123 | -0.067165924 | 0.522235251 | no | no change | GO:0016491;GO:0044267;GO:0046872;GO:0006879;GO:0005615;GO:0006825;GO:0004322;GO:0006811;GO:0051087;GO:0070062;GO:0005623;GO:0005765;GO:0055072;GO:0006826;GO:0005788;GO:0005886;GO:0072562;GO:0005507;GO:0043687;GO:0055114;GO:0005576 | hsa:1356 | COG2132 | RecName: Full=Ceruloplasmin; AltName: Full=Ferroxidase; Flags: Precursor |
| P00488 | 23771.56053 | 22051.47217 | 0.927640915 | -0.108361641 | 0.625665398 | no | no change | GO:0005737;GO:0018149;GO:0007599;GO:0019221;GO:0031093;GO:0003810;GO:0072378;GO:0002576;GO:0007596;GO:0072562;GO:0016740;GO:0062023;GO:0016746;GO:0046872;GO:0005576 | hsa:2162 |  | RecName: Full=Coagulation factor XIII A chain; Short=Coagulation factor XIIIa; AltName: Full=Protein-glutamine gamma-glutamyltransferase A chain; AltName: Full=Transglutaminase A chain; Flags: Precursor |
| P00734 | 557441.9474 | 556802.7667 | 0.998853368 | -0.001655189 | 0.978494575 | no | no change | GO:0004252;GO:0048712;GO:0009897;GO:0030307;GO:0007597;GO:0007596;GO:0005788;GO:0051281;GO:0030449;GO:0032967;GO:0008047;GO:0007166;GO:0007599;GO:0008233;GO:0007275;GO:0061844;GO:0044267;GO:0006888;GO:0005615;GO:0005102;GO:0009611;GO:0070062;GO:0001530;GO:0051838;GO:0010544;GO:0005509;GO:0006508;GO:0008236;GO:0007186;GO:0010468;GO:1900738;GO:0008284;GO:0016787;GO:2000379;GO:0046427;GO:0090218;GO:0070945;GO:0006953;GO:0005796;GO:0008360;GO:0014068;GO:0005886;GO:0008201;GO:0030168;GO:0008083;GO:1900016;GO:0042730;GO:1900182;GO:0051480;GO:0010469;GO:0070053;GO:0005576;GO:0051918;GO:0005515;GO:0072378;GO:0030193;GO:0045861;GO:0072562;GO:0001934;GO:0030194 | hsa:2147 | COG5640 | prothrombin isoform 1 preproprotein [Homo sapiens] |
| P00742 | 84047.87211 | 77988.13 | 0.927901303 | -0.107956736 | 0.279387782 | no | no change | GO:0004252;GO:0030335;GO:0005543;GO:0006888;GO:0005615;GO:0016787;GO:0007596;GO:0005788;GO:0005796;GO:0005576;GO:0005509;GO:0005515;GO:0005886;GO:0006508;GO:0008233;GO:0008236;GO:0051897;GO:0031233;GO:0007599;GO:0007598 | hsa:2159 | COG5640 | coagulation factor X isoform 1 preproprotein [Homo sapiens] |
| P00747 | 1155272.695 | 1161562.133 | 1.005444116 | 0.007832897 | 0.899927827 | no | no change | GO:0004252;GO:0004175;GO:0007599;GO:0016787;GO:1904854;GO:0048771;GO:0019899;GO:0008233;GO:0008236;GO:0044267;GO:0070062;GO:0043536;GO:0052182;GO:0051087;GO:0072562;GO:1990405;GO:0006508;GO:0022617;GO:0009986;GO:0051918;GO:0051919;GO:0008285;GO:0007596;GO:0010812;GO:0019900;GO:0062023;GO:0019904;GO:0005886;GO:0051702;GO:0031232;GO:0034185;GO:0005615;GO:0042730;GO:0031093;GO:0052213;GO:2000048;GO:0044218;GO:0005576;GO:0005515;GO:0005102;GO:0002576 | hsa:5340 | COG5640 | plasminogen isoform 1 precursor [Homo sapiens] |
| P01011 | 718008.5 | 747812.3792 | 1.04150909 | 0.05867543 | 0.414619064 | no | no change | GO:0006954;GO:0034774;GO:0035578;GO:0003677;GO:0070062;GO:0031093;GO:0002576;GO:0072562;GO:0010466;GO:0019216;GO:0006953;GO:0062023;GO:0030277;GO:0030414;GO:0004867;GO:0010951;GO:0005615;GO:0043312;GO:0005622;GO:0005576;GO:0005515;GO:0005634 | hsa:12 | COG4826 | serpin peptidase inhibitor, clade A (alpha-1 antiproteinase, antitrypsin), member 3, isoform CRA_b [Homo sapiens] |
| P01019 | 181866.2316 | 187123.3125 | 1.028906306 | 0.041111614 | 0.62657888 | no | no change | GO:1903779;GO:0038166;GO:2001238;GO:0035106;GO:0003014;GO:0007166;GO:0050729;GO:0001822;GO:0042127;GO:0032930;GO:0006606;GO:0014824;GO:0019229;GO:0010976;GO:0010873;GO:0050731;GO:0035815;GO:0005829;GO:0007202;GO:0007204;GO:0003081;GO:1904754;GO:0010536;GO:0007565;GO:0005179;GO:0034374;GO:0007200;GO:0071260;GO:0042310;GO:0042311;GO:0048169;GO:0008083;GO:1901201;GO:0072562;GO:0006883;GO:0061098;GO:0007186;GO:2000379;GO:0090190;GO:0048018;GO:0008306;GO:0003331;GO:0033864;GO:0007199;GO:0001974;GO:0062023;GO:0048659;GO:0051969;GO:0016525;GO:0014873;GO:0030308;GO:0048146;GO:0048144;GO:0007267;GO:0045429;GO:1905010;GO:0043085;GO:0005737;GO:0050880;GO:0003051;GO:0002027;GO:0045742;GO:0032270;GO:0070471;GO:0008284;GO:0031701;GO:0031703;GO:0001558;GO:0014068;GO:0004867;GO:0014061;GO:0051092;GO:1904385;GO:0007568;GO:0070062;GO:0002034;GO:2001275;GO:0005515;GO:0070371;GO:0001819;GO:0051387;GO:0010469;GO:0010666;GO:0008217;GO:0061049;GO:0050663;GO:0032355;GO:0005615;GO:0097755;GO:0042981;GO:0010744;GO:0035813;GO:1904707;GO:0046628;GO:0034104;GO:0019216;GO:0051403;GO:0010951;GO:0007263;GO:1905589;GO:0010613;GO:0010595;GO:0002019;GO:0002018;GO:0045777;GO:0002016;GO:0031702;GO:0051924;GO:0005576;GO:1903598;GO:0045893 | hsa:183 | COG4826 | angiotensinogen preproprotein [Homo sapiens] |
| P01023 | 8232157.474 | 7746371.708 | 0.940989253 | -0.087749849 | 0.614607777 | no | no change | GO:0051056;GO:0030414;GO:0019959;GO:0007597;GO:0019899;GO:0072562;GO:0048863;GO:0005615;GO:0031093;GO:0002576;GO:0048306;GO:0019838;GO:0070062;GO:0010466;GO:0002020;GO:0001869;GO:0022617;GO:0019966;GO:0005096;GO:0004866;GO:0004867;GO:0010951;GO:0043547;GO:0043120;GO:0005829;GO:0005576;GO:0005515;GO:0005102 | hsa:2 | COG2373 | alpha-2-macroglobulin isoform a precursor [Homo sapiens] |
| P01024 | 7798733.316 | 7683627.625 | 0.985240463 | -0.021452215 | 0.783368966 | no | no change | GO:0045087;GO:0004252;GO:0006631;GO:0030449;GO:0004866;GO:0005886;GO:0005788;GO:0035578;GO:0007165;GO:0031715;GO:0048260;GO:0010828;GO:0010866;GO:0050766;GO:0044267;GO:0070062;GO:0009617;GO:0045766;GO:1905114;GO:0045745;GO:0097242;GO:2000427;GO:0034774;GO:0007186;GO:0009986;GO:0005576;GO:0006629;GO:0150064;GO:0043687;GO:0016322;GO:0006911;GO:0150062;GO:0006956;GO:0006957;GO:0006954;GO:0006955;GO:0010951;GO:0060100;GO:0006958;GO:0006508;GO:0005615;GO:0050776;GO:0032991;GO:0010575;GO:0043312;GO:0002376;GO:0001798;GO:0010884;GO:0001970;GO:0005515;GO:0097278;GO:0005102;GO:0001934;GO:0072562 | hsa:718 |  | complement C3 preproprotein [Homo sapiens] |
| P01031 | 207439.2947 | 216864.45 | 1.045435727 | 0.064104369 | 0.432091186 | no | no change | GO:0000187;GO:0030449;GO:0006954;GO:0008009;GO:0007166;GO:0060326;GO:0019835;GO:0001701;GO:0005615;GO:0045766;GO:0010760;GO:0007186;GO:0045087;GO:0006935;GO:0090197;GO:0006956;GO:0006957;GO:0004866;GO:0010951;GO:0006958;GO:0070062;GO:0010575;GO:0002376;GO:0005576;GO:0005515;GO:0005102;GO:0005579 | hsa:727 |  | complement C5 isoform 1 preproprotein [Homo sapiens] |
| P01034 | 12066.96882 | 11943.61057 | 0.989777196 | -0.014824291 | 0.892796656 | no | no change | GO:0005783;GO:0060009;GO:0048678;GO:0005788;GO:0008584;GO:0042747;GO:0030414;GO:0070301;GO:0001666;GO:0014070;GO:1904724;GO:0005737;GO:0044267;GO:0032355;GO:0042995;GO:0001540;GO:0005615;GO:0007431;GO:2000117;GO:0005771;GO:0009636;GO:0070062;GO:0043067;GO:0010466;GO:0002020;GO:0030424;GO:0048471;GO:0043025;GO:0042802;GO:0008284;GO:0034103;GO:0045740;GO:0060548;GO:0043687;GO:0006915;GO:0031965;GO:0031982;GO:0006952;GO:0001775;GO:0001654;GO:0060311;GO:0004866;GO:0060313;GO:0004869;GO:0034599;GO:0010035;GO:0006979;GO:0009743;GO:1904813;GO:0005604;GO:0007420;GO:0010716;GO:0007566;GO:0042493;GO:0031667;GO:0010711;GO:0005764;GO:0005576;GO:0005515;GO:0043312;GO:0043292;GO:0045861;GO:0097435 | hsa:1471 |  | cystatin-C precursor [Homo sapiens] |
| P01042 | 1274189.979 | 1153652.767 | 0.90540091 | -0.143371338 | 0.050406077 | no | no change | GO:0007599;GO:0030414;GO:0007597;GO:0007596;GO:0045861;GO:0005788;GO:0007162;GO:0008270;GO:0042311;GO:0044267;GO:0050880;GO:0005615;GO:0031093;GO:0002576;GO:0072562;GO:0043065;GO:0010466;GO:0007186;GO:0043687;GO:0062023;GO:0006954;GO:0005886;GO:0010951;GO:0004869;GO:0008201;GO:0070062;GO:0007204;GO:0005576;GO:0005515;GO:0005102;GO:0030195 | hsa:3827 |  | kininogen-1 isoform 1 precursor [Homo sapiens] |
| P01597 | 43764.09368 | 41109.17542 | 0.939335696 | -0.09028726 | 0.456916488 | no | no change | GO:0038096;GO:0004252;GO:0016020;GO:0030449;GO:0050776;GO:0006898;GO:0038095;GO:0072562;GO:0002376;GO:0050900;GO:0005576;GO:0006956;GO:0002250;GO:0003823;GO:0005886;GO:0006508;GO:0006958;GO:0006955;GO:0070062 | hsa:7441 |  | immunoglobulin kappa light chain VC region, partial [Homo sapiens] |
| P01701 | 516602.5105 | 464909.8917 | 0.899937345 | -0.152103533 | 0.400451035 | no | no change | GO:0038096;GO:0004252;GO:0016020;GO:0030449;GO:0050776;GO:0006898;GO:0038095;GO:0050900;GO:0005576;GO:0006956;GO:0003823;GO:0005886;GO:0006508;GO:0006958;GO:0006955;GO:0070062 | hsa:7441 |  | RecName: Full=Immunoglobulin lambda variable 1-51; AltName: Full=Ig lambda chain V-I region BL2; AltName: Full=Ig lambda chain V-I region EPS; AltName: Full=Ig lambda chain V-I region NEW; AltName: Full=Ig lambda chain V-I region NIG-64; Flags: Precursor |
| P01705 | 112127.4213 | 120145.3343 | 1.071507155 | 0.099641484 | 0.729409091 | no | no change | GO:0038096;GO:0004252;GO:0016020;GO:0030449;GO:0050776;GO:0006898;GO:0038095;GO:0002376;GO:0050900;GO:0005576;GO:0006956;GO:0002250;GO:0003823;GO:0005886;GO:0006508;GO:0006958;GO:0006955 | hsa:7441 |  | immunoglobulin lambda-chain, partial [Homo sapiens] |
| P01742 | 1362618.079 | 1630025.25 | 1.196245137 | 0.25851306 | 0.658695455 | no | no change | GO:0038096;GO:0004252;GO:0016020;GO:0030449;GO:0050776;GO:0006898;GO:0038095;GO:0002376;GO:0050900;GO:0005576;GO:0006956;GO:0002250;GO:0003823;GO:0005886;GO:0006508;GO:0006958;GO:0006955 | hsa:102724971 | | IgM heavy chain VH1 region precursor, partial [Homo sapiens] |
| P01743 | 15975.95005 | 16041.36275 | 1.004094448 | 0.00589498 | 0.978401177 | no | no change | GO:0038096;GO:0004252;GO:0016020;GO:0030449;GO:0050776;GO:0006898;GO:0038095;GO:0002376;GO:0050900;GO:0005576;GO:0006956;GO:0002250;GO:0003823;GO:0005886;GO:0006508;GO:0006958;GO:0006955 | hsa:102723407 | | IgM heavy chain VH1 region precursor, partial [Homo sapiens] |
| P01763 | 34043.84868 | 33351.55525 | 0.979664654 | -0.029640105 | 0.894129556 | no | no change | GO:0038096;GO:0004252;GO:0016020;GO:0030449;GO:0050776;GO:0006898;GO:0038095;GO:0002376;GO:0050900;GO:0005576;GO:0006956;GO:0002250;GO:0003823;GO:0005886;GO:0006508;GO:0006958;GO:0006955 | hsa:102723407 | | immunoglobulin heavy chain variable region, partial [Homo sapiens] |
| P01764 | 41234.23267 | 44849.17591 | 1.087668498 | 0.121238916 | 0.653955289 | no | no change | GO:0004252;GO:0030449;GO:0009897;GO:0006955;GO:0050900;GO:0042742;GO:0034987;GO:0042571;GO:0005615;GO:0016020;GO:0072562;GO:0006508;GO:0050853;GO:0045087;GO:0006910;GO:0006911;GO:0050776;GO:0006956;GO:0003823;GO:0005886;GO:0006958;GO:0050871;GO:0070062;GO:0038096;GO:0038095;GO:0006898;GO:0002376;GO:0005576;GO:0002250 | hsa:102723407 | | RecName: Full=Immunoglobulin heavy variable 3-23; AltName: Full=Ig heavy chain V-III region LAY; AltName: Full=Ig heavy chain V-III region POM; AltName: Full=Ig heavy chain V-III region TEI; AltName: Full=Ig heavy chain V-III region TIL; AltName: Full=Ig heavy chain V-III region TUR; AltName: Full=Ig heavy chain V-III region VH26; AltName: Full=Ig heavy chain V-III region WAS; AltName: Full=Ig heavy chain V-III region ZAP; Flags: Precursor |
| P01766 | 90589.62789 | 76615.26708 | 0.845739947 | -0.241713972 | 0.196853128 | no | no change | GO:0038096;GO:0004252;GO:0016020;GO:0030449;GO:0005615;GO:0006898;GO:0038095;GO:0050900;GO:0072562;GO:0002376;GO:0050776;GO:0005576;GO:0006956;GO:0002250;GO:0003823;GO:0005886;GO:0006508;GO:0006958;GO:0006955 | hsa:102723407 | | RecName: Full=Immunoglobulin heavy variable 3-13; AltName: Full=Ig heavy chain V-III region BRO; Flags: Precursor |
| P01814 | 27211.02737 | 29691.86739 | 1.091170392 | 0.125876403 | 0.47872388 | no | no change | GO:0038096;GO:0004252;GO:0016020;GO:0030449;GO:0050776;GO:0006898;GO:0038095;GO:0002376;GO:0050900;GO:0005576;GO:0006956;GO:0002250;GO:0003823;GO:0005886;GO:0006508;GO:0006958;GO:0006955 | hsa:102723407 | | RecName: Full=Immunoglobulin heavy variable 2-70; AltName: Full=Ig heavy chain V-II region COR; AltName: Full=Ig heavy chain V-II region DAW; AltName: Full=Ig heavy chain V-II region OU; AltName: Full=Ig heavy chain V-II region SESS; Flags: Precursor |
| P01860 | 2808878.895 | 2605711.083 | 0.92766943 | -0.108317294 | 0.589606102 | no | no change | GO:0004252;GO:0030449;GO:0009897;GO:0050871;GO:0042742;GO:0034987;GO:0042571;GO:0005615;GO:0016020;GO:0072562;GO:0006508;GO:0050853;GO:0045087;GO:0006910;GO:0006911;GO:0006956;GO:0003823;GO:0005886;GO:0006958;GO:0001895;GO:0070062;GO:0038096;GO:0002376;GO:0005576;GO:0002250 | hsa:100423062 | | Unknown (protein for MGC:105008) [Homo sapiens] |
| P01871 | 12768356.63 | 11343463 | 0.888404305 | -0.170711711 | 0.555837979 | no | no change | GO:0009897;GO:0042834;GO:0019731;GO:0050900;GO:0034987;GO:0050829;GO:0005615;GO:0016021;GO:0016020;GO:0072562;GO:0009986;GO:0050853;GO:0045087;GO:0006910;GO:0006911;GO:0031210;GO:0003823;GO:0005886;GO:0006958;GO:0050871;GO:0070062;GO:0003697;GO:0002376;GO:0005576;GO:0002250;GO:0005515;GO:0071756;GO:0071757 | hsa:3543 |  | immunoglobulin heavy chain [Homo sapiens] |
| P01877 | 232985.3742 | 264324.4792 | 1.13451104 | 0.182070648 | 0.655863792 | no | no change | GO:0009897;GO:0019731;GO:0006955;GO:0050900;GO:0034987;GO:0001895;GO:0005615;GO:0016020;GO:0003094;GO:0072562;GO:0071748;GO:0050853;GO:0045087;GO:0006910;GO:0006911;GO:0003823;GO:0005886;GO:0006958;GO:0050871;GO:0060267;GO:0070062;GO:0006898;GO:0002376;GO:0005576;GO:0002250;GO:0071752;GO:0071751 | hsa:55423 |  | RecName: Full=Immunoglobulin heavy constant alpha 2; AltName: Full=Ig alpha-2 chain C region; AltName: Full=Ig alpha-2 chain C region BUT; AltName: Full=Ig alpha-2 chain C region LAN |
| P02042 | 73247.19789 | 74112.88667 | 1.011818729 | 0.016950849 | 0.946064926 | no | no change | GO:0005344;GO:0019825;GO:0020037;GO:0043177;GO:0031721;GO:0007596;GO:0072562;GO:0005833;GO:0098869;GO:0042744;GO:0005515;GO:0031838;GO:0046872;GO:0005829;GO:0015671 | hsa:3045 | COG1018 | hemoglobin subunit delta [Homo sapiens] |
| P02100 | 36164.80579 | 36857.37129 | 1.019150262 | 0.027366776 | 0.921132596 | no | no change | GO:0005344;GO:0019825;GO:0020037;GO:0043177;GO:0031721;GO:0007596;GO:0051291;GO:0072562;GO:0005833;GO:0098869;GO:0042744;GO:0015671;GO:0005515;GO:0031838;GO:0046872;GO:0005829;GO:0014070 | hsa:3046 | COG1018 | hemoglobin subunit epsilon [Homo sapiens] |
| P02538 | 11764.16056 | 14020.07567 | 1.191761672 | 0.253095756 | 0.682299497 | no | no change | GO:0008284;GO:0002009;GO:0005200;GO:0007010;GO:0005829;GO:0031424;GO:0070062;GO:2000536;GO:0030154;GO:0045095;GO:0070268;GO:0042060;GO:0005198;GO:0050830;GO:0005882;GO:0005515;GO:0001899;GO:0016020;GO:0005634;GO:0051801;GO:0061844 | hsa:3853 |  | keratin, type II cytoskeletal 6A [Homo sapiens] |
| P02647 | 32198537.26 | 32231965.5 | 1.001038191 | 0.001497016 | 0.983007582 | no | no change | GO:0034115;GO:0010804;GO:0005788;GO:0019915;GO:0034191;GO:0034190;GO:0005548;GO:0050728;GO:0071682;GO:0051496;GO:0005543;GO:0010898;GO:0005319;GO:0034361;GO:0034362;GO:0034363;GO:0034364;GO:0031102;GO:0034366;GO:0031100;GO:0043534;GO:0030300;GO:0034774;GO:0010873;GO:0043691;GO:0007179;GO:0015485;GO:0005829;GO:0018206;GO:0019433;GO:0045499;GO:0070508;GO:0045723;GO:0034375;GO:0034371;GO:0031072;GO:0015914;GO:0034378;GO:0050821;GO:0010903;GO:0042158;GO:0055102;GO:0044267;GO:0042627;GO:0051180;GO:0014012;GO:0072562;GO:0050919;GO:0007186;GO:0033700;GO:0018158;GO:0006629;GO:0060761;GO:0042632;GO:0032489;GO:0050713;GO:0007229;GO:0006898;GO:0034384;GO:0062023;GO:0006695;GO:0060192;GO:0006869;GO:0019899;GO:0030301;GO:0035025;GO:0051006;GO:0002740;GO:0001540;GO:1900026;GO:0031410;GO:0005634;GO:0042802;GO:0008035;GO:0060354;GO:0009986;GO:0008289;GO:0008202;GO:0008203;GO:0071813;GO:0070062;GO:0042493;GO:0005515;GO:0034380;GO:0070371;GO:0042157;GO:0006656;GO:0030325;GO:0006644;GO:0017127;GO:0030139;GO:0008211;GO:0060228;GO:0001523;GO:0055091;GO:0034365;GO:0005615;GO:0002576;GO:0005576;GO:0043687;GO:0070328;GO:0019216;GO:0007584;GO:0031210;GO:0005886;GO:0043627;GO:1903561;GO:0051346;GO:0051345;GO:0033344;GO:0070653;GO:0001932;GO:0001935;GO:0005769 | hsa:335 |  | apolipoprotein A-I isoform 1 preproprotein [Homo sapiens] |
| P02649 | 2223708.632 | 1848625.629 | 0.831325473 | -0.266514677 | 0.10225094 | no | no change | GO:0051044;GO:0005783;GO:0034447;GO:0007616;GO:0005788;GO:0072358;GO:0043524;GO:0010873;GO:0051651;GO:0050728;GO:1905855;GO:0071682;GO:0016020;GO:0008201;GO:0098869;GO:0060999;GO:0045541;GO:0044794;GO:0005319;GO:0034361;GO:0034362;GO:0034363;GO:0034364;GO:0031102;GO:0016209;GO:0071813;GO:0043537;GO:0010977;GO:0030425;GO:0030516;GO:0043691;GO:0010877;GO:0010875;GO:0061771;GO:0015485;GO:0043025;GO:0019433;GO:1990777;GO:0030195;GO:0034375;GO:0034374;GO:0034372;GO:0034371;GO:1903002;GO:0000302;GO:0019934;GO:0010629;GO:0044877;GO:0042157;GO:0042311;GO:0042158;GO:0042159;GO:0048168;GO:0042982;GO:0044267;GO:0042627;GO:0072562;GO:0035641;GO:0010544;GO:0097006;GO:0007186;GO:0062023;GO:0033700;GO:0015909;GO:0006629;GO:1905890;GO:0048156;GO:1902430;GO:0042632;GO:0032489;GO:1901215;GO:0046907;GO:0034382;GO:0034380;GO:0006898;GO:0034384;GO:0006357;GO:0046983;GO:0032805;GO:0005543;GO:0090209;GO:0006869;GO:0051246;GO:0019068;GO:0050807;GO:0031012;GO:0043407;GO:0051000;GO:1902995;GO:0043083;GO:0005737;GO:0001540;GO:0005634;GO:0030669;GO:1900221;GO:0002021;GO:0090181;GO:0042802;GO:0042803;GO:0006641;GO:0006874;GO:0043687;GO:0017038;GO:0008289;GO:0055088;GO:0055089;GO:0046889;GO:0034378;GO:0006979;GO:0008202;GO:0008203;GO:0032269;GO:0070062;GO:1900272;GO:1902952;GO:0005515;GO:1901630;GO:0005794;GO:0017127;GO:0007271;GO:0060228;GO:0001523;GO:0034365;GO:0005615;GO:0048844;GO:0005198;GO:0033344;GO:1901628;GO:0010468;GO:0032462;GO:0090090;GO:0006707;GO:0070326;GO:0046911;GO:0005886;GO:2000822;GO:1903561;GO:0007263;GO:1905908;GO:0050750;GO:0007010;GO:1905860;GO:0097114;GO:0005576;GO:1905906;GO:0097113;GO:0045807;GO:0098978;GO:0070328;GO:0005769;GO:0001937;GO:0010976 | hsa:348 |  | apolipoprotein E [Homo sapiens] |
| P02652 | 318388.9053 | 318533.6583 | 1.000454642 | 0.000655761 | 0.996811735 | no | no change | GO:0060192;GO:0034384;GO:0034375;GO:0005543;GO:0034374;GO:0034371;GO:0006869;GO:0031072;GO:0031647;GO:0017127;GO:0030301;GO:0006656;GO:0010903;GO:0010873;GO:0002740;GO:0060228;GO:0001523;GO:0043085;GO:0034190;GO:0009395;GO:0044267;GO:0042627;GO:0005615;GO:0032375;GO:0031100;GO:0055102;GO:0072562;GO:0033344;GO:0034361;GO:0070062;GO:0033700;GO:0005319;GO:0018158;GO:0008035;GO:0034364;GO:0016032;GO:0034366;GO:0006641;GO:0060621;GO:0043687;GO:0043691;GO:0008289;GO:0031210;GO:0005788;GO:0042632;GO:0050996;GO:0043627;GO:0002526;GO:0051384;GO:0034378;GO:0008203;GO:0030300;GO:0046982;GO:0045416;GO:0015485;GO:0019216;GO:0050995;GO:0046340;GO:0005829;GO:0018206;GO:0042493;GO:0034380;GO:0009749;GO:0005576;GO:0005515;GO:0070653;GO:0060695;GO:0042803;GO:0034370;GO:0005769;GO:0042157 | hsa:336 |  | apolipoprotein A-II preproprotein [Homo sapiens] |
| P02671 | 5765624.316 | 6592469.667 | 1.143409509 | 0.193342193 | 0.076195495 | no | no change | GO:0045087;GO:0009897;GO:0045907;GO:0007599;GO:0034116;GO:0007596;GO:0005788;GO:0007160;GO:0045921;GO:0045202;GO:0034622;GO:1902042;GO:0044267;GO:0050839;GO:0031091;GO:0005615;GO:0031093;GO:1900026;GO:0072378;GO:0002576;GO:0031639;GO:0072562;GO:0005198;GO:0065003;GO:0030198;GO:0002224;GO:0046872;GO:0043687;GO:0009986;GO:2000352;GO:0051592;GO:0090277;GO:0005938;GO:0005886;GO:1903561;GO:0030168;GO:0070062;GO:0051258;GO:0042730;GO:0072377;GO:0050714;GO:0002376;GO:0005577;GO:0005576;GO:0002250;GO:0005515;GO:0043152;GO:0005102;GO:0070374;GO:0070527 | hsa:2243 |  | fibrinogen alpha chain isoform alpha-E preproprotein [Homo sapiens] |
| P02675 | 13156373.95 | 14246328.08 | 1.08284609 | 0.1148282 | 0.272011202 | no | no change | GO:1902042;GO:0034116;GO:0045921;GO:0007599;GO:0005783;GO:0009897;GO:0007596;GO:0007160;GO:0045907;GO:0045202;GO:0034622;GO:0044320;GO:0005737;GO:1903561;GO:0031091;GO:0005615;GO:0031093;GO:1900026;GO:0072378;GO:0002576;GO:0051087;GO:0031639;GO:0072562;GO:0005198;GO:0030198;GO:0002224;GO:0045087;GO:0009986;GO:2000352;GO:0051592;GO:0090277;GO:0005938;GO:0005886;GO:0050839;GO:0030168;GO:0070062;GO:0051258;GO:0042730;GO:0050714;GO:0071347;GO:0002376;GO:0005577;GO:0005576;GO:0002250;GO:0005515;GO:0043152;GO:0005102;GO:0070374;GO:0070527 | hsa:2244 |  | fibrinogen beta chain isoform 1 preproprotein [Homo sapiens] |
| P02743 | 394677.8789 | 384314.5167 | 0.973742227 | -0.038388189 | 0.766395485 | no | no change | GO:0006457;GO:0030246;GO:0046597;GO:0044871;GO:0072562;GO:0061045;GO:0044267;GO:0070062;GO:0005615;GO:0005634;GO:0051082;GO:0001849;GO:0005509;GO:0030169;GO:0042802;GO:1903016;GO:0051131;GO:0045087;GO:1903019;GO:0006953;GO:0062023;GO:0044869;GO:0006958;GO:0002674;GO:0046872;GO:0045656;GO:0046790;GO:0005576;GO:0048525 | hsa:325 |  | serum amyloid P-component precursor [Homo sapiens] |
| P02745 | 36515.59947 | 33321.85458 | 0.91253752 | -0.132044217 | 0.518656498 | no | no change | GO:0004252;GO:0030449;GO:0005581;GO:0045087;GO:0002376;GO:0005576;GO:0006956;GO:0005515;GO:0010039;GO:0007267;GO:0006508;GO:0006958;GO:0005602 | hsa:712 |  | complement C1q subcomponent subunit A precursor [Homo sapiens] |
| P02748 | 118478.6568 | 134374.9038 | 1.134169709 | 0.181636531 | 0.16556164 | no | no change | GO:0030449;GO:0006955;GO:0019835;GO:0019836;GO:0070062;GO:0016021;GO:0016020;GO:0051260;GO:0072562;GO:0001906;GO:0045087;GO:0006957;GO:0005887;GO:0005886;GO:0006958;GO:0005615;GO:0005829;GO:0044218;GO:0044279;GO:0002376;GO:0005576;GO:0005579 | hsa:735 |  | complement component C9 preproprotein [Homo sapiens] |
| P02750 | 128381.3026 | 136499.81 | 1.063237459 | 0.088463838 | 0.489093419 | no | no change | GO:0043231;GO:0016020;GO:0070062;GO:0005160;GO:0009617;GO:0050873;GO:0045766;GO:0043312;GO:1904813;GO:0001938;GO:1904724;GO:0005576;GO:0003674;GO:0005515;GO:0035580;GO:0008150;GO:0030511;GO:0005615 | hsa:116844 | COG4886 | leucine-rich alpha-2-glycoprotein precursor [Homo sapiens] |
| P02753 | 65298.08789 | 70244.31042 | 1.075748352 | 0.10534063 | 0.552192143 | no | no change | GO:0060347;GO:0032024;GO:0048562;GO:0030324;GO:0060059;GO:0060044;GO:0042593;GO:0051024;GO:0060065;GO:0042572;GO:0001523;GO:0016918;GO:0005615;GO:0048807;GO:0006094;GO:0005501;GO:0032526;GO:0048706;GO:0007601;GO:0045471;GO:0001654;GO:0034633;GO:0070062;GO:0007507;GO:0032991;GO:0060157;GO:0005829;GO:0019841;GO:0048738;GO:0005576;GO:0005515;GO:0060068;GO:0034632;GO:0046982;GO:0050896;GO:0030277 | hsa:5950 |  | retinol-binding protein 4 isoform a precursor [Homo sapiens] |
| P02760 | 410334.5526 | 436345.2333 | 1.06338896 | 0.088669394 | 0.305493949 | no | no change | GO:0005886;GO:0010951;GO:0046329;GO:0070062;GO:0019855;GO:0072562;GO:0010466;GO:0042803;GO:0005515;GO:0020037;GO:0009986;GO:0042167;GO:0018298;GO:0062023;GO:0030163;GO:0030414;GO:0004867;GO:0007155;GO:0005615;GO:0050777;GO:0046904;GO:0019862;GO:0007565;GO:0006898;GO:0043231;GO:0005576;GO:0016032 | hsa:259 |  | protein AMBP preproprotein [Homo sapiens] |
| P02766 | 758885.3474 | 731202.25 | 0.963521371 | -0.05361143 | 0.588755308 | no | no change | GO:0005179;GO:0044267;GO:0032991;GO:0070062;GO:0042562;GO:0070324;GO:0005615;GO:0070327;GO:0005737;GO:0043312;GO:0001523;GO:0035578;GO:0030198;GO:0005515;GO:0042572;GO:0046982;GO:0010469;GO:0006144;GO:0042802;GO:0005576 | hsa:7276 | COG2351 | transthyretin precursor [Homo sapiens] |
| P02775 | 40917.93421 | 40009.81917 | 0.97780643 | -0.032379203 | 0.940780621 | no | no change | GO:0042127;GO:0070098;GO:0005125;GO:0071222;GO:0008009;GO:0042742;GO:0060326;GO:0032496;GO:0061844;GO:0031091;GO:0008083;GO:0031093;GO:0002576;GO:0007186;GO:0005355;GO:0030595;GO:0030593;GO:1904724;GO:0006952;GO:0006954;GO:0006955;GO:0045236;GO:0005615;GO:0090023;GO:0010469;GO:0031640;GO:0043312;GO:0006935;GO:0051781;GO:0005576;GO:1904659;GO:0005515 | hsa:5473 |  | platelet basic protein preproprotein [Homo sapiens] |
| P02790 | 2781504.737 | 2810931.875 | 1.010579575 | 0.015182927 | 0.848389296 | no | no change | GO:0020027;GO:0002925;GO:0015232;GO:0071682;GO:0005615;GO:0051246;GO:0042531;GO:0072562;GO:0046872;GO:0042168;GO:0006879;GO:0016032;GO:0060332;GO:0062023;GO:0060335;GO:0002639;GO:0070062;GO:0015886;GO:0006898;GO:0005623;GO:0005576;GO:0005515 | hsa:3263 |  | hemopexin precursor [Homo sapiens] |
| P03951 | 9396.648474 | 11125.43813 | 1.18397939 | 0.243643967 | 0.149832321 | no | no change | GO:0004252;GO:0016020;GO:0007599;GO:0070062;GO:0016787;GO:0005615;GO:0007597;GO:0007596;GO:0031639;GO:0030193;GO:0005576;GO:0005515;GO:0005886;GO:0070009;GO:0006508;GO:0008233;GO:0008236;GO:0008201;GO:0042802;GO:0051919 | hsa:2160 | COG5640 | coagulation factor XI isoform 1 preproprotein [Homo sapiens] |
| P04003 | 2808926.789 | 2612318.208 | 0.930005801 | -0.10468838 | 0.471247887 | no | no change | GO:0030449;GO:0005615;GO:0045087;GO:0072562;GO:0002376;GO:0045732;GO:0005576;GO:0003723;GO:0005515;GO:0005886;GO:1903027;GO:0006958;GO:0045959;GO:0044216 | hsa:722 |  | C4b-binding protein alpha chain precursor [Homo sapiens] |
| P04004 | 817496.0474 | 919898.8833 | 1.125264014 | 0.170263532 | 0.13447219 | no | no change | GO:0005201;GO:0030449;GO:0005783;GO:0061302;GO:0050840;GO:0014911;GO:0030949;GO:0030247;GO:0031012;GO:0007160;GO:0007155;GO:0090303;GO:0048260;GO:0005737;GO:0032092;GO:0070062;GO:0072562;GO:0005576;GO:0016477;GO:0042802;GO:0033627;GO:0005178;GO:0005796;GO:0008283;GO:0010811;GO:0048709;GO:0048237;GO:0062023;GO:0006955;GO:0010951;GO:0035987;GO:0008201;GO:0005615;GO:0051258;GO:0005604;GO:0043231;GO:0050731;GO:0005044;GO:0006898;GO:0030198;GO:0005515;GO:0097421;GO:0071062;GO:0005518;GO:0030195 | hsa:7448 |  | vitronectin precursor [Homo sapiens] |
| P04114 | 4415674.842 | 4496564.042 | 1.018318649 | 0.026189076 | 0.809856314 | no | no change | GO:0034360;GO:0034374;GO:0034359;GO:0005783;GO:0034371;GO:0006869;GO:0050750;GO:0005788;GO:0005789;GO:0009791;GO:0061024;GO:0050900;GO:0010628;GO:0042157;GO:0034378;GO:0032496;GO:0016042;GO:0001523;GO:0006629;GO:0005737;GO:0043202;GO:0032355;GO:0001701;GO:0031983;GO:0005615;GO:0071682;GO:0071356;GO:0009615;GO:0048844;GO:0005543;GO:0030669;GO:0071379;GO:0070062;GO:0010033;GO:0042953;GO:0010884;GO:0042158;GO:0002224;GO:0010008;GO:0043025;GO:0043687;GO:0070971;GO:0005319;GO:0034361;GO:0034362;GO:0034363;GO:0006642;GO:0005790;GO:0044267;GO:0030317;GO:0008289;GO:0034379;GO:0010744;GO:0007283;GO:0042627;GO:0042159;GO:0017127;GO:0012506;GO:0005886;GO:0042632;GO:0030301;GO:0008201;GO:0008202;GO:0008203;GO:0035473;GO:0031904;GO:0009743;GO:0007399;GO:0034382;GO:0034383;GO:0005829;GO:0006898;GO:0009566;GO:0043231;GO:0010886;GO:0019433;GO:0005576;GO:0045540;GO:0005515;GO:0034447;GO:0010269;GO:0005769;GO:0033344 | hsa:338 |  | RecName: Full=Apolipoprotein B-100; Short=Apo B-100; Contains: RecName: Full=Apolipoprotein B-48; Short=Apo B-48; Flags: Precursor |
| P04180 | 18607.13326 | 19570.84 | 1.051792327 | 0.072849878 | 0.526683582 | no | no change | GO:0034375;GO:0006656;GO:0034372;GO:0043691;GO:0008374;GO:0042158;GO:0090107;GO:0070062;GO:0016740;GO:0016746;GO:0034435;GO:0008203;GO:0006644;GO:0034364;GO:0006629;GO:0042632;GO:0030301;GO:0008202;GO:0005615;GO:0005576;GO:0005515;GO:0034186;GO:0004607;GO:0046470 | hsa:3931 |  | phosphatidylcholine-sterol acyltransferase precursor [Homo sapiens] |
| P04217 | 794323.0421 | 784967.7167 | 0.988222266 | -0.017092533 | 0.820501142 | no | no change | GO:0070062;GO:0031093;GO:0002576;GO:0043312;GO:0072562;GO:1904813;GO:0005576;GO:0003674;GO:0034774;GO:0008150;GO:0062023;GO:0005615 | hsa:1 |  | alpha-1B-glycoprotein precursor [Homo sapiens] |
| P04264 | 224055.9947 | 211548.2708 | 0.944175902 | -0.082872434 | 0.438798224 | no | no change | GO:0018149;GO:0038023;GO:0045095;GO:0030246;GO:0031012;GO:1904813;GO:0061436;GO:0030280;GO:0005856;GO:0050728;GO:0070062;GO:0005634;GO:0016020;GO:0045765;GO:0072562;GO:0005198;GO:0001867;GO:0005882;GO:0051290;GO:0070268;GO:0062023;GO:0005886;GO:0001895;GO:0006979;GO:0001533;GO:0005615;GO:0042730;GO:0031424;GO:0005829;GO:0043312;GO:0005576;GO:0005515;GO:0046982 | hsa:3848 |  | keratin 1 [Homo sapiens] |
| P04275 | 59863.79484 | 65047.3725 | 1.086589527 | 0.119807046 | 0.696185421 | no | no change | GO:0005201;GO:0007599;GO:0005783;GO:0007597;GO:0007596;GO:0031012;GO:0031091;GO:0070062;GO:0031093;GO:0002576;GO:0051087;GO:0051260;GO:0009611;GO:0005576;GO:0002020;GO:0042802;GO:0042803;GO:0005178;GO:0031589;GO:0033093;GO:0062023;GO:0007155;GO:0030168;GO:0047485;GO:0019865;GO:0030198;GO:0005515;GO:0005518 | hsa:7450 |  | RecName: Full=von Willebrand factor; Short=vWF; Contains: RecName: Full=von Willebrand antigen 2; AltName: Full=von Willebrand antigen II; Flags: Precursor |
| P04430 | 78829.74944 | 80451.58348 | 1.020573883 | 0.029380628 | 0.912863026 | no | no change | GO:0038096;GO:0004252;GO:0016020;GO:0030449;GO:0050776;GO:0006898;GO:0038095;GO:0050900;GO:0005576;GO:0006956;GO:0003823;GO:0005886;GO:0006508;GO:0006958;GO:0006955 | hsa:7441 |  | RecName: Full=Immunoglobulin kappa variable 1-16; AltName: Full=Ig kappa chain V-I region BAN; Flags: Precursor |
| P04433 | 102176.9611 | 112058.7604 | 1.096712598 | 0.133185506 | 0.275097037 | no | no change | GO:0038096;GO:0004252;GO:0016020;GO:0030449;GO:0050776;GO:0006898;GO:0038095;GO:0072562;GO:0002376;GO:0050900;GO:0005576;GO:0006956;GO:0002250;GO:0003823;GO:0005886;GO:0006508;GO:0006958;GO:0006955;GO:0070062 | hsa:7441 |  | rheumatoid factor D1 IgG light chain VK3 region, partial [Homo sapiens] |
| P05109 | 8420.225188 | 8955.786278 | 1.063604129 | 0.088961283 | 0.844742267 | no | no change | GO:0045087;GO:0019730;GO:0030307;GO:0032602;GO:0043312;GO:0050786;GO:0008270;GO:0014002;GO:0010043;GO:0032496;GO:0005737;GO:0001816;GO:0045111;GO:0050729;GO:0005615;GO:0005634;GO:0016020;GO:0051493;GO:0050727;GO:0005509;GO:0002224;GO:2001244;GO:0034774;GO:0046872;GO:0035662;GO:0008017;GO:0005856;GO:0030593;GO:0006935;GO:0006919;GO:0032119;GO:0006914;GO:0006915;GO:0018119;GO:0042060;GO:0050832;GO:0045471;GO:0070488;GO:0006954;GO:0005886;GO:0002526;GO:0002523;GO:0070062;GO:0051092;GO:0050544;GO:0005829;GO:0002793;GO:0002544;GO:0002376;GO:0005576;GO:0005515;GO:0042742 | hsa:6279 |  | protein S100-A8 isoform d [Homo sapiens] |
| P05154 | 20698.69611 | 17383.09479 | 0.839815933 | -0.251854935 | 0.063705482 | no | no change | GO:0009897;GO:0006869;GO:0007596;GO:0036024;GO:0002080;GO:0061107;GO:0036029;GO:0031094;GO:0031091;GO:0051346;GO:0007342;GO:0016020;GO:0036025;GO:0032190;GO:0036027;GO:0036026;GO:0010466;GO:0002020;GO:0097183;GO:0097182;GO:0097181;GO:0036028;GO:0007283;GO:0031210;GO:0007338;GO:0030414;GO:0004867;GO:0010951;GO:0008201;GO:0005615;GO:0032991;GO:0070062;GO:0036030;GO:0005539;GO:0005576;GO:0005515;GO:0001972;GO:0045861 | hsa:5104 | COG4826 | plasma serine protease inhibitor preproprotein [Homo sapiens] |
| P05155 | 312577.7684 | 330904.0167 | 1.058629404 | 0.082197631 | 0.497452592 | no | no change | GO:0030449;GO:0007597;GO:0007596;GO:0007599;GO:0005615;GO:0031093;GO:0002576;GO:0072562;GO:0010466;GO:0001869;GO:0045916;GO:0008015;GO:0045087;GO:0030414;GO:0004867;GO:0010951;GO:0006958;GO:0070062;GO:0042730;GO:0007568;GO:0002376;GO:0005576;GO:0005515;GO:0030193 | hsa:710 | COG4826 | unnamed protein product [Homo sapiens] |
| P05160 | 25199.30632 | 24088.64167 | 0.955924793 | -0.065030976 | 0.420343242 | no | no change | GO:0072378;GO:0005576;GO:0007596;GO:0007599;GO:1903363 | hsa:2165 |  | coagulation factor XIII B chain precursor [Homo sapiens] |
| P05546 | 553199.5684 | 483992.8542 | 0.874897382 | -0.192814284 | 0.078658902 | no | no change | GO:0044267;GO:0007599;GO:0006935;GO:0005615;GO:0043687;GO:0007596;GO:0005788;GO:0005576;GO:0030414;GO:0004866;GO:0004867;GO:0010951;GO:0010466;GO:0008201;GO:0070062 | hsa:3053 | COG4826 | heparin cofactor 2 precursor [Homo sapiens] |
| P06312 | 455326.1211 | 385772.4458 | 0.847244267 | -0.239150125 | 0.092048845 | no | no change | GO:0038096;GO:0004252;GO:0016020;GO:0030449;GO:0050776;GO:0006898;GO:0038095;GO:0072562;GO:0002376;GO:0002377;GO:0050900;GO:0005576;GO:0006956;GO:0002250;GO:0003823;GO:0005886;GO:0006508;GO:0006958;GO:0006955 | hsa:7441 |  | immunoglobulin kappa chain, partial [Homo sapiens] |
| P06727 | 898121.5842 | 1004446.754 | 1.118386165 | 0.161418419 | 0.207967732 | no | no change | GO:0034375;GO:0034445;GO:0034372;GO:0034371;GO:0006869;GO:0034380;GO:0045723;GO:0005788;GO:0034378;GO:0051006;GO:0042744;GO:0042157;GO:0042632;GO:0070328;GO:0060228;GO:0001523;GO:0035634;GO:0044267;GO:0042627;GO:0005615;GO:0006982;GO:0032374;GO:0072562;GO:0016042;GO:0033344;GO:0065005;GO:0010898;GO:0002227;GO:0009986;GO:0042802;GO:0033700;GO:0005319;GO:0034361;GO:0034364;GO:0031102;GO:0062023;GO:0016209;GO:0008289;GO:0055088;GO:0031210;GO:0007159;GO:0017127;GO:0010873;GO:0043691;GO:0008203;GO:0030300;GO:0005507;GO:0015485;GO:0070062;GO:0005829;GO:0046470;GO:0019430;GO:0005576;GO:0006695;GO:0005515;GO:0042803;GO:0005769 | hsa:337 |  | RecName: Full=Apolipoprotein A-IV; Short=Apo-AIV; Short=ApoA-IV; AltName: Full=Apolipoprotein A4; Flags: Precursor |
| P07357 | 57584.42 | 55249.77208 | 0.959456952 | -0.059710017 | 0.419629943 | no | no change | GO:0019835;GO:0030449;GO:0070062;GO:0016021;GO:0016020;GO:0072562;GO:0002376;GO:0001848;GO:0005576;GO:0006956;GO:0006957;GO:0005886;GO:0006955;GO:0044877;GO:0006958;GO:0005579;GO:0045087;GO:0005615 | hsa:731 |  | complement component C8 alpha chain preproprotein [Homo sapiens] |
| P07358 | 39937.20684 | 39029.12833 | 0.977262343 | -0.033182194 | 0.69709212 | no | no change | GO:0019835;GO:0030449;GO:0005615;GO:0045087;GO:0016020;GO:0002376;GO:0005576;GO:0006956;GO:0006957;GO:0006955;GO:0044877;GO:0006958;GO:1903561;GO:0005579;GO:0070062 | hsa:732 |  | RecName: Full=Complement component C8 beta chain; AltName: Full=Complement component 8 subunit beta; Flags: Precursor |
| P07996 | 28906.78963 | 29556.91033 | 1.022490242 | 0.032087075 | 0.941127618 | no | no change | GO:0032026;GO:0005783;GO:0050840;GO:0034605;GO:0005788;GO:2001237;GO:0050921;GO:1903671;GO:0048266;GO:0051895;GO:0006986;GO:0043652;GO:0042535;GO:0009612;GO:0030511;GO:0002581;GO:0042327;GO:0051592;GO:0043536;GO:0040037;GO:2001027;GO:0030169;GO:0043236;GO:0045727;GO:0045652;GO:0030198;GO:0043032;GO:0030194;GO:0009897;GO:0018149;GO:0042493;GO:0002605;GO:0071356;GO:0051897;GO:0016477;GO:0002040;GO:0001968;GO:2000379;GO:0030141;GO:0007050;GO:0043394;GO:0006954;GO:0006955;GO:0070052;GO:0090051;GO:0070051;GO:0033574;GO:0043154;GO:0034976;GO:1902043;GO:0031012;GO:0001666;GO:0050431;GO:0005737;GO:0031091;GO:0031093;GO:0005509;GO:0010763;GO:0001786;GO:0051918;GO:0008284;GO:0009986;GO:0017134;GO:2000353;GO:0008201;GO:0071363;GO:0001953;GO:0002544;GO:0016529;GO:0042802;GO:0005515;GO:0032570;GO:0016525;GO:0000187;GO:0005201;GO:0005615;GO:0002576;GO:0010751;GO:0045766;GO:0043066;GO:0010748;GO:0032695;GO:1903588;GO:0005178;GO:0030335;GO:0048661;GO:0032914;GO:0062023;GO:0007155;GO:0070062;GO:0043537;GO:0010595;GO:0010596;GO:0009749;GO:0005577;GO:0005576;GO:0010757;GO:0010754;GO:0010759;GO:0001937;GO:0071636 | hsa:7057 |  | thrombospondin-1 precursor [Homo sapiens] |
| P08185 | 107075.3179 | 93306.80625 | 0.871412834 | -0.198571733 | 0.202457372 | no | no change | GO:0005615;GO:0008289;GO:0010951;GO:0008211;GO:0005576;GO:0004867;GO:0005496;GO:0070062 | hsa:866 | COG4826 | corticosteroid-binding globulin precursor [Homo sapiens] |
| P08571 | 10905.65174 | 10546.54488 | 0.96707149 | -0.048305551 | 0.680798605 | no | no change | GO:0006954;GO:0009897;GO:0045121;GO:0071222;GO:0071223;GO:0097190;GO:0031362;GO:1901224;GO:0007166;GO:0006909;GO:0032496;GO:0045471;GO:0010008;GO:0034612;GO:0016020;GO:0030667;GO:0005615;GO:0071727;GO:0071726;GO:0009617;GO:0071723;GO:0071219;GO:0032729;GO:0002224;GO:0031225;GO:0009986;GO:0001847;GO:0005794;GO:0032760;GO:0070266;GO:0034128;GO:0038124;GO:0045087;GO:0006915;GO:2000484;GO:0035666;GO:0032481;GO:0070891;GO:0034142;GO:0005886;GO:0009408;GO:0001530;GO:0032026;GO:0070062;GO:0051602;GO:0002755;GO:0002756;GO:0016019;GO:0050715;GO:0038123;GO:0031663;GO:0006898;GO:0007249;GO:0043312;GO:0002376;GO:0005576;GO:0002237;GO:0005515;GO:0045807;GO:0046696 | hsa:929 |  | monocyte differentiation antigen CD14 precursor [Homo sapiens] |
| P08603 | 1325699.621 | 1298499.188 | 0.979482205 | -0.029908813 | 0.734881599 | no | no change | GO:1903659;GO:0030449;GO:0070062;GO:0005515;GO:0045087;GO:0072562;GO:0002376;GO:0043395;GO:0005576;GO:0006956;GO:0006957;GO:0016032;GO:0008201;GO:0005615 | hsa:3075 |  | RecName: Full=Complement factor H; AltName: Full=H factor 1; Flags: Precursor |
| P08697 | 217188.5526 | 215477.4083 | 0.992121388 | -0.011411447 | 0.851531928 | no | no change | GO:0030414;GO:0032967;GO:0005615;GO:0031093;GO:0051496;GO:0002576;GO:0030199;GO:0072562;GO:0010466;GO:0048514;GO:0002020;GO:0010757;GO:0051918;GO:0042803;GO:0048661;GO:0009986;GO:0045597;GO:0006953;GO:0046330;GO:0004866;GO:0004867;GO:0010951;GO:0010033;GO:0070062;GO:0042730;GO:2000049;GO:0002034;GO:0005577;GO:0005576;GO:0045944;GO:0005515;GO:0070374;GO:0071636 | hsa:5345 | COG4826 | alpha-2-antiplasmin isoform X1 [Homo sapiens] |
| P09871 | 93659.56368 | 101824.4283 | 1.087175984 | 0.120585492 | 0.198231539 | no | no change | GO:0004252;GO:0006956;GO:0016787;GO:0045087;GO:0072562;GO:0002376;GO:0030449;GO:0005576;GO:0001867;GO:0005509;GO:0005515;GO:0006508;GO:0006958;GO:0046872;GO:0008233;GO:0042802;GO:0008236 | hsa:716 | COG5640 | complement C1s subcomponent isoform 1 preproprotein [Homo sapiens] |
| P0C0L5 | 1776359.432 | 1801409.792 | 1.014102078 | 0.02020288 | 0.885432952 | no | no change | GO:0004252;GO:0030449;GO:0006954;GO:0030246;GO:0030425;GO:0030424;GO:0045202;GO:0032490;GO:0030054;GO:0005615;GO:0072562;GO:0001848;GO:0006508;GO:0045087;GO:0008228;GO:0006956;GO:2000427;GO:0004866;GO:0005886;GO:0010951;GO:0006958;GO:0042995;GO:0070062;GO:0044216;GO:0002376;GO:0005576 | hsa:100293534;hsa:110384692;hsa:720;hsa:721 | | complement C4-B preproprotein [Homo sapiens] |
| P0DJI8 | 33536.79611 | 28121.24417 | 0.838519102 | -0.254084445 | 0.337899825 | no | no change | GO:0000187;GO:0042056;GO:0019221;GO:0001664;GO:0034364;GO:0048246;GO:0048247;GO:0044267;GO:0050728;GO:0050708;GO:0005615;GO:0071682;GO:0045785;GO:0007186;GO:0030593;GO:0045087;GO:0005881;GO:0006953;GO:0050918;GO:0008201;GO:0030168;GO:0070062;GO:0050716;GO:0050715;GO:0006898;GO:0007204;GO:0005576 | hsa:6288 |  | RecName: Full=Serum amyloid A-1 protein; Short=SAA; Contains: RecName: Full=Amyloid protein A; AltName: Full=Amyloid fibril protein AA; Contains: RecName: Full=Serum amyloid protein A(2-104); Contains: RecName: Full=Serum amyloid protein A(3-104); Contains: RecName: Full=Serum amyloid protein A(2-103); Contains: RecName: Full=Serum amyloid protein A(2-102); Contains: RecName: Full=Serum amyloid protein A(4-101); Flags: Precursor |
| P0DOY3 | 7486774.005 | 8981379.125 | 1.199632728 | 0.262592787 | 0.162119368 | no | no change | GO:0005615;GO:0016020;GO:0072562;GO:0002376;GO:0005576;GO:0002250;GO:0003823;GO:0005886;GO:0070062 | hsa:100423062 | | RecName: Full=Immunoglobulin lambda constant 3; AltName: Full=Ig lambda chain C region DOT; AltName: Full=Ig lambda chain C region NEWM; AltName: Full=Ig lambda-3 chain C regions |
| P11021 | 232966.6897 | 214815.3433 | 0.922086087 | -0.117026646 | 0.854798001 | no | no change | GO:1990090;GO:0051087;GO:0005783;GO:0071480;GO:1990440;GO:0035437;GO:0009314;GO:0030433;GO:0019899;GO:0036500;GO:0005789;GO:0042149;GO:0035690;GO:0021762;GO:0045296;GO:0016887;GO:0031204;GO:0034663;GO:0005925;GO:1901998;GO:0019904;GO:0008180;GO:0005737;GO:0005793;GO:0006983;GO:0042623;GO:0043209;GO:0005634;GO:0016020;GO:0071353;GO:0005739;GO:0000166;GO:0031625;GO:0043066;GO:0005509;GO:0031398;GO:0043022;GO:0005524;GO:0016787;GO:0030335;GO:0071236;GO:0030176;GO:0005790;GO:0030182;GO:0042470;GO:0097501;GO:0009986;GO:0071277;GO:0036498;GO:0036499;GO:0051402;GO:0051082;GO:0005788;GO:0005886;GO:0042220;GO:0030512;GO:0001554;GO:0051603;GO:1904313;GO:0032991;GO:0043231;GO:0030968;GO:0071287;GO:0070062;GO:0005829;GO:0071320;GO:0030496;GO:0060904;GO:0090074;GO:0021589;GO:1903897;GO:1903894;GO:1903895;GO:0051787;GO:0005515;GO:1903891;GO:0034976;GO:0034975;GO:0021680;GO:0010976 | hsa:3309 | COG0443 | 78 kDa glucose-regulated protein [Otolemur garnettii] |
| P13473 | 9220.985842 | 8175.757833 | 0.886646826 | -0.173568537 | 0.160090669 | no | no change | GO:0005770;GO:0061684;GO:0101003;GO:0019899;GO:0050821;GO:0044754;GO:0035577;GO:0010008;GO:1990836;GO:0043202;GO:0005615;GO:0097637;GO:0031410;GO:0016021;GO:0016020;GO:0097352;GO:0031902;GO:0046716;GO:0045121;GO:0006914;GO:0017038;GO:0006605;GO:1905146;GO:0061740;GO:0019904;GO:0009267;GO:0005886;GO:0070062;GO:0000421;GO:0030670;GO:0031647;GO:0043312;GO:0031088;GO:0005764;GO:0005765;GO:0005515;GO:0098857;GO:0005768;GO:0002576 | hsa:3920 |  | lysosome-associated membrane glycoprotein 2 isoform A precursor [Homo sapiens] |
| P13645 | 47574.99368 | 51498.59333 | 1.08247189 | 0.114329561 | 0.44488495 | no | no change | GO:0005737;GO:0018149;GO:0045684;GO:0070062;GO:0030216;GO:0031424;GO:0005615;GO:0009986;GO:0016020;GO:0070268;GO:0051290;GO:0005634;GO:0005882;GO:0005576;GO:0046982;GO:0005198;GO:0005829;GO:0001533;GO:0030280 | hsa:3858 |  | Keratin 10 [Homo sapiens] |
| P13647 | 27473.64947 | 24637.18833 | 0.896757031 | -0.157210943 | 0.166085729 | no | no change | GO:0005737;GO:0016020;GO:0005200;GO:0007010;GO:0070062;GO:0031424;GO:0031581;GO:0045095;GO:0070268;GO:0005198;GO:0005634;GO:0005882;GO:0097110;GO:0005515;GO:0005886;GO:0008544;GO:0005829 | hsa:3852 |  | Keratin 5 [Homo sapiens] |
| P13671 | 56036.18947 | 51443.50375 | 0.91804072 | -0.123369948 | 0.110548609 | no | no change | GO:0019835;GO:0001701;GO:0030449;GO:0070062;GO:0045917;GO:0045087;GO:0045766;GO:0002376;GO:0005576;GO:0006956;GO:0001970;GO:0005515;GO:0006955;GO:0006958;GO:0005579 | hsa:729 |  | complement component C6 precursor [Homo sapiens] |
| P14151 | 29660.54053 | 29594.96875 | 0.997789259 | -0.003192956 | 0.969489816 | no | no change | GO:0009897;GO:0016339;GO:0030246;GO:0050900;GO:0050901;GO:0070492;GO:0030667;GO:0043208;GO:0016021;GO:0016020;GO:0005509;GO:0002020;GO:0046872;GO:0009986;GO:0005887;GO:0005886;GO:0007155;GO:0050839;GO:0008201;GO:0050776;GO:0043312;GO:0005515;GO:0033198 | hsa:6402 |  | L-selectin [Homo sapiens] |
| P15814 | 10754431.68 | 9216727.483 | 0.857016694 | -0.222604788 | 0.16666332 | no | no change | GO:0009897;GO:0050853;GO:0045087;GO:0016020;GO:0006910;GO:0006911;GO:0050900;GO:0005576;GO:0042742;GO:0003823;GO:0006955;GO:0042571;GO:0006958;GO:0050871;GO:0034987;GO:0072562 | hsa:3543 |  | immunoglobulin lambda-like polypeptide 1 isoform a precursor [Homo sapiens] |
| P18428 | 15726.31684 | 15829.97208 | 1.006591196 | 0.009477885 | 0.94174906 | no | no change | GO:0006968;GO:0071222;GO:0071223;GO:0006869;GO:0042742;GO:0050829;GO:0032496;GO:0032490;GO:0044130;GO:0019221;GO:0060265;GO:0002281;GO:0070062;GO:0090023;GO:0042535;GO:0016020;GO:0071723;GO:0045919;GO:0002224;GO:0033036;GO:0032722;GO:0009986;GO:0032720;GO:0032760;GO:0015920;GO:0045087;GO:0008289;GO:0006953;GO:0008228;GO:0070891;GO:0034142;GO:0001530;GO:0005615;GO:0034145;GO:0031663;GO:0050830;GO:0002376;GO:0002232;GO:0005576;GO:0005515;GO:0043032;GO:0005102;GO:0032757;GO:0032755 | hsa:3929 |  | lipopolysaccharide-binding protein precursor [Homo sapiens] |
| P19827 | 319962 | 322715.4875 | 1.00860567 | 0.012362241 | 0.872455125 | no | no change | GO:0030212;GO:0070062;GO:0072562;GO:0010466;GO:0005509;GO:0030414;GO:0004867;GO:0010951;GO:0005576 | hsa:3697 | COG2304 | inter-alpha-trypsin inhibitor heavy chain H1 isoform a preproprotein [Homo sapiens] |
| P20851 | 54623.62684 | 49058.4175 | 0.898117176 | -0.155024411 | 0.294707549 | no | no change | GO:0030449;GO:0005615;GO:0045087;GO:0007596;GO:0002376;GO:0045732;GO:0005576;GO:0005515;GO:0005886;GO:1903027;GO:0006958;GO:0045959;GO:0044216 | hsa:725 |  | C4b-binding protein beta chain isoform 1 precursor [Homo sapiens] |
| P22792 | 100444.6111 | 92603.00583 | 0.921931051 | -0.117269236 | 0.282845518 | no | no change | GO:0030449;GO:0070062;GO:0004181;GO:0050790;GO:0072562;GO:0050821;GO:0005576;GO:0006508;GO:0030234 | hsa:1370 | COG4886 | carboxypeptidase N subunit 2 precursor [Homo sapiens] |
| P22891 | 10520.92905 | 10217.00317 | 0.971112258 | -0.042290018 | 0.8297611 | no | no change | GO:0004252;GO:0005796;GO:0006888;GO:0005615;GO:0007596;GO:0005788;GO:0005576;GO:0005509;GO:0006508;GO:0030195;GO:0007599;GO:0070062 | hsa:8858 | COG5640 | vitamin K-dependent protein Z isoform 2 precursor [Homo sapiens] |
| P23083 | 598812.4874 | 630156.8175 | 1.052344149 | 0.073606588 | 0.820499138 | no | no change | GO:0038096;GO:0004252;GO:0016020;GO:0030449;GO:0050776;GO:0006898;GO:0038095;GO:0002376;GO:0050900;GO:0005576;GO:0006956;GO:0002250;GO:0003823;GO:0005886;GO:0006508;GO:0006958;GO:0006955 | hsa:102723407 | | IgM heavy chain VH1 region precursor, partial [Homo sapiens] |
| P23142 | 32198.87526 | 33588.43208 | 1.043155446 | 0.060954157 | 0.613561829 | no | no change | GO:0005201;GO:0008022;GO:0031012;GO:0007162;GO:0044877;GO:0005615;GO:0072378;GO:1900025;GO:0005576;GO:0005509;GO:2000647;GO:0042802;GO:0062023;GO:0071953;GO:0010952;GO:0070062;GO:0005604;GO:0007566;GO:0070051;GO:0005577;GO:0030198;GO:0001968;GO:0016032;GO:2000146;GO:0016504;GO:0001933;GO:0070373;GO:0007229 | hsa:2192 |  | fibulin-1 isoform D precursor [Homo sapiens] |
| P25311 | 349100.7526 | 320440.6208 | 0.917902979 | -0.123586424 | 0.18837224 | no | no change | GO:0008285;GO:0090501;GO:0071806;GO:0070062;GO:0009897;GO:0001580;GO:0005615;GO:0005634;GO:0004540;GO:0008320;GO:0062023;GO:0005515;GO:0005886;GO:0006955;GO:0007155;GO:0001895;GO:0055085;GO:0005576 | hsa:563 |  | zinc-alpha-2-glycoprotein precursor [Homo sapiens] |
| P27169 | 248603.3211 | 252886.7875 | 1.017230126 | 0.024646094 | 0.760941788 | no | no change | GO:0034445;GO:0032411;GO:0016311;GO:0046872;GO:0004064;GO:0046434;GO:0070062;GO:0005615;GO:0005543;GO:0009636;GO:0072562;GO:0005509;GO:0102007;GO:0010875;GO:0019372;GO:0042803;GO:0016787;GO:0034364;GO:0006629;GO:0034366;GO:1902617;GO:0008203;GO:0004063;GO:0043231;GO:0046395;GO:0051099;GO:0031667;GO:0046470;GO:0005576;GO:0019439;GO:0070542 | hsa:5444 |  | serum paraoxonase/arylesterase 1 precursor [Homo sapiens] |
| P35858 | 52673.09368 | 51550.67458 | 0.978690845 | -0.03107489 | 0.816675992 | no | no change | GO:0044267;GO:0005615;GO:0031012;GO:0005654;GO:0007155;GO:0005576;GO:0007165;GO:0042567;GO:0005520;GO:0070062 | hsa:3483 | COG4886 | Insulin-like growth factor binding protein, acid labile subunit [Homo sapiens] |
| P35908 | 86320.11789 | 91746.86042 | 1.062867645 | 0.087961954 | 0.539858112 | no | no change | GO:0005200;GO:0045095;GO:0018149;GO:0008544;GO:0045684;GO:0043616;GO:0051546;GO:0070062;GO:0032980;GO:0005634;GO:0016020;GO:0005198;GO:0030280;GO:0070268;GO:0005882;GO:0001533;GO:0005615;GO:0045109;GO:0008092;GO:0031424;GO:0005829;GO:0003334;GO:0005515 | hsa:3849 |  | keratin, type II cytoskeletal 2 epidermal [Homo sapiens] |
| P36955 | 107954.0368 | 96083.02208 | 0.890036398 | -0.168063759 | 0.135626664 | no | no change | GO:0007614;GO:0030424;GO:0010629;GO:0060041;GO:0060770;GO:0007275;GO:0043203;GO:0005615;GO:0071300;GO:0010447;GO:0001822;GO:0050769;GO:0071333;GO:0048471;GO:0046685;GO:0043025;GO:0071279;GO:0008283;GO:0050728;GO:0062023;GO:0010976;GO:0004867;GO:0010951;GO:1901215;GO:0070062;GO:0042470;GO:0071549;GO:0005604;GO:0007568;GO:0010596;GO:0042698;GO:0005576;GO:0005515;GO:0016525 | hsa:5176 | COG4826 | pigment epithelium-derived factor isoform 1 precursor [Homo sapiens] |
| P41222 | 16649.46113 | 18000.5245 | 1.081147574 | 0.112563461 | 0.336063828 | no | no change | GO:0006633;GO:0006631;GO:0005783;GO:0005789;GO:0005737;GO:0070062;GO:0005634;GO:0016020;GO:0005504;GO:0048471;GO:0005501;GO:0019371;GO:0005794;GO:0005791;GO:0006629;GO:0031965;GO:2000255;GO:0036094;GO:0001516;GO:0045187;GO:0005615;GO:0016853;GO:0004667;GO:0006693;GO:0005576;GO:0005515 | hsa:5730 |  | prostaglandin-H2 D-isomerase precursor [Homo sapiens] |
| P43121 | 5617.6761 | 5122.623125 | 0.911875842 | -0.133090689 | 0.493707777 | no | no change | GO:0009897;GO:0030335;GO:0001525;GO:0005925;GO:0005615;GO:0016021;GO:0016020;GO:0003094;GO:0061042;GO:0005576;GO:0005886;GO:0007155;GO:0009653;GO:0005634 | hsa:4162 |  | cell surface glycoprotein MUC18 precursor [Homo sapiens] |
| P43652 | 149067.1842 | 162630.0425 | 1.090984869 | 0.125631093 | 0.218789176 | no | no change | GO:0008431;GO:0051180;GO:0005615;GO:0072562;GO:0050821;GO:0015031;GO:0005576;GO:0005515;GO:0071693;GO:0046872;GO:0070062 | hsa:173 |  | afamin precursor [Homo sapiens] |
| P48740 | 19459.41895 | 21708.35375 | 1.115570501 | 0.157781691 | 0.219220079 | no | no change | GO:0004252;GO:0006898;GO:0006956;GO:0005615;GO:0016787;GO:0048306;GO:0005829;GO:0005654;GO:0002376;GO:0046872;GO:0005576;GO:0001867;GO:0005509;GO:0005515;GO:0006508;GO:0008233;GO:0042803;GO:0045087;GO:0008236 | hsa:5648 | COG5640 | mannan-binding lectin serine protease 1 isoform 1 precursor [Homo sapiens] |
| P51884 | 83551.94263 | 88452.44458 | 1.058652161 | 0.082228643 | 0.423695269 | no | no change | GO:0005201;GO:0018146;GO:0031012;GO:0014070;GO:0043202;GO:0070062;GO:0005583;GO:0070848;GO:0005576;GO:0005796;GO:0032914;GO:0007601;GO:0030021;GO:0062023;GO:0007409;GO:0005615;GO:0051216;GO:0030199;GO:0030198;GO:0045944;GO:0005515;GO:0005518;GO:0042340 | hsa:4060 | COG4886 | lumican precursor [Homo sapiens] |
| P61626 | 11785.74744 | 9940.730208 | 0.843453523 | -0.245619521 | 0.120078703 | no | no change | GO:0006954;GO:0019730;GO:0016798;GO:0003796;GO:0035578;GO:0042742;GO:0050829;GO:0019835;GO:0044267;GO:0070062;GO:0016998;GO:0035580;GO:0042802;GO:0016787;GO:1904724;GO:0003824;GO:0050830;GO:0008152;GO:0001895;GO:0005615;GO:0031640;GO:0043312;GO:0005576 | hsa:4069 |  | lysozyme C precursor [Homo sapiens] |
| P68871 | 5874401.842 | 6159428.625 | 1.048520137 | 0.068354569 | 0.784446028 | no | no change | GO:0005344;GO:0007596;GO:0015701;GO:1904813;GO:0045429;GO:0042744;GO:0008217;GO:0010942;GO:0050880;GO:0005615;GO:0071682;GO:0072562;GO:0005833;GO:0098869;GO:0046872;GO:0015671;GO:0070293;GO:0030185;GO:1904724;GO:0020037;GO:0031721;GO:0031720;GO:0051291;GO:0070062;GO:0019825;GO:0042542;GO:0030492;GO:0005829;GO:0006898;GO:0043312;GO:0070527;GO:0043177;GO:0005576;GO:0005515;GO:0031838;GO:0004601 | hsa:3043 | COG1018 | PREDICTED: hemoglobin subunit beta [Gorilla gorilla gorilla] |
| P69905 | 836201.7737 | 721367.0333 | 0.862671016 | -0.21311761 | 0.372528049 | no | no change | GO:0005344;GO:0022627;GO:0015701;GO:0042744;GO:0010942;GO:0005615;GO:0071682;GO:0016020;GO:0072562;GO:0005833;GO:0098869;GO:0005506;GO:0046872;GO:0015671;GO:0020037;GO:0031720;GO:0051291;GO:0070062;GO:0019825;GO:0042542;GO:0005829;GO:0006898;GO:0005576;GO:0005515;GO:0031838;GO:0004601 | hsa:3040;hsa:3039 | COG1018 | TPA: globin C1 [Homo sapiens] |
| P80108 | 67531.23053 | 58752.42917 | 0.870003829 | -0.200906344 | 0.07891516 | no | no change | GO:0035774;GO:0051044;GO:0051047;GO:0035690;GO:0031012;GO:0010907;GO:0001503;GO:0032869;GO:0009749;GO:0010867;GO:0005737;GO:0043065;GO:0070062;GO:0002430;GO:0006501;GO:0045919;GO:0005576;GO:0097241;GO:0006507;GO:0010897;GO:0002062;GO:0004630;GO:0035701;GO:0002042;GO:0008285;GO:0008286;GO:0010983;GO:0071397;GO:0071277;GO:0005615;GO:0010595;GO:0043231;GO:0016787;GO:0070633;GO:0071401;GO:0005765;GO:0017080;GO:0071467;GO:1900076;GO:0004621;GO:0046470;GO:0010694;GO:0005622 | hsa:2822 |  | phosphatidylinositol-glycan-specific phospholipase D precursor [Homo sapiens] |
| Q03591 | 159912.5561 | 149517.8025 | 0.934997264 | -0.096965951 | 0.363393597 | no | no change | GO:0032091;GO:0030449;GO:0005615;GO:0072562;GO:0045919;GO:0005576;GO:0006956;GO:0005515;GO:0032991;GO:0046982;GO:0042803 | hsa:3078 |  | complement factor H-related protein 1 precursor [Homo sapiens] |
| Q06033 | 37503.02105 | 34365.64154 | 0.916343286 | -0.126039924 | 0.383645497 | no | no change | GO:0030212;GO:0070062;GO:0002576;GO:0031089;GO:0005576;GO:0030414;GO:0004866;GO:0004867;GO:0010951;GO:0010466 | hsa:3699 | COG2304 | inter-alpha-trypsin inhibitor heavy chain H3 preproprotein [Homo sapiens] |
| Q13103 | 4202.4675 | 3737.351071 | 0.889323016 | -0.169220571 | 0.548085618 | no | no change | GO:0044267;GO:0002576;GO:0005788;GO:0031089;GO:0001501;GO:0005576;GO:0046849;GO:0004866;GO:0010951;GO:0043687;GO:0062023 | hsa:6694 |  | secreted phosphoprotein 24 precursor [Homo sapiens] |
| Q13201 | 46496.28313 | 44074.11227 | 0.94790614 | -0.077183882 | 0.793652619 | no | no change | GO:0005201;GO:0031093;GO:0010811;GO:0002576;GO:0007596;GO:0031012;GO:0005576;GO:0005509;GO:0005515;GO:0007155;GO:0062023 | hsa:22915 |  | multimerin-1 precursor [Homo sapiens] |
| Q13790 | 70172.31053 | 74861.0575 | 1.066817623 | 0.093313562 | 0.394795989 | no | no change | GO:0005319;GO:0034362;GO:0034364;GO:0006629;GO:0006869;GO:0005615;GO:0005576;GO:0005102;GO:0008203;GO:0008202;GO:0015485 | hsa:319 |  | apolipoprotein F preproprotein [Homo sapiens] |
| Q14520 | 96249.33684 | 104873.3896 | 1.089601165 | 0.123800151 | 0.147720993 | no | no change | GO:0004252;GO:0005615;GO:0016787;GO:0005539;GO:0005576;GO:0005509;GO:0007155;GO:0006508;GO:0008233;GO:0008236 | hsa:3026 | COG5640 | hyaluronan-binding protein 2 isoform 1 preproprotein [Homo sapiens] |
| Q14624 | 179441.1722 | 209235.6167 | 1.166040179 | 0.221617502 | 0.126276838 | no | no change | GO:0005737;GO:0030212;GO:0034097;GO:0002576;GO:0004867;GO:0072562;GO:0031089;GO:0006953;GO:0005576;GO:0030414;GO:0005515;GO:0004866;GO:0005886;GO:0010951;GO:0010466;GO:0070062 | hsa:3700 | COG2304 | inter-alpha-trypsin inhibitor heavy chain H4 isoform 1 precursor [Homo sapiens] |
| Q15166 | 27697.90895 | 24672.87967 | 0.890784922 | -0.166850956 | 0.714285075 | no | no change | GO:0016787;GO:0004063;GO:0070062;GO:0005615;GO:0046395;GO:0019372;GO:0009636;GO:0016311;GO:0043231;GO:0019439;GO:0046226;GO:0005576;GO:0010124;GO:0032929;GO:0102007;GO:0046872;GO:0004064;GO:0018733;GO:0042803 | hsa:5446 |  | serum paraoxonase/lactonase 3 [Homo sapiens] |
| Q15582 | 14484.47174 | 15336.56588 | 1.058828113 | 0.082468405 | 0.649952708 | no | no change | GO:0005201;GO:0050840;GO:0031012;GO:0007162;GO:0001525;GO:0044267;GO:0070062;GO:0005576;GO:0002062;GO:0005178;GO:0008283;GO:0007601;GO:0062023;GO:0005886;GO:0007155;GO:0050839;GO:0005615;GO:0005604;GO:0050896;GO:0005802;GO:0030198;GO:0005515;GO:0005518 | hsa:7045 | COG2335 | transforming growth factor-beta-induced protein ig-h3 precursor [Homo sapiens] |
| Q16610 | 32948.33211 | 36623.5975 | 1.111546326 | 0.152568077 | 0.256387295 | no | no change | GO:0008022;GO:0005201;GO:0019899;GO:0031012;GO:0001503;GO:0007165;GO:0030500;GO:0030502;GO:0001525;GO:0005615;GO:0002576;GO:0045766;GO:0010466;GO:2000404;GO:0001960;GO:0002063;GO:0003416;GO:0005576;GO:0005134;GO:0002020;GO:0002828;GO:0062023;GO:0006954;GO:0031214;GO:0070062;GO:0043236;GO:0031089;GO:0001938;GO:0006357;GO:0005515;GO:0043123 | hsa:1893 |  | extracellular matrix protein 1 isoform 1 precursor [Homo sapiens] |
| Q562R1 | 24097.0568 | 21302.47513 | 0.884028091 | -0.177835881 | 0.651452539 | no | no change | GO:0005737;GO:0005856;GO:0005615;GO:0070062;GO:0000166;GO:0003674;GO:0045202;GO:0005515;GO:0008150;GO:0098978;GO:0005524;GO:0015629 | hsa:345651 | COG5277 | beta-actin-like protein 2 [Homo sapiens] |
| Q6EMK4 | 8586.009118 | 8507.862833 | 0.990898416 | -0.013190931 | 0.950221302 | no | no change | GO:0016020;GO:0016021;GO:0070062;GO:0010719;GO:0005615;GO:0009986;GO:0005886;GO:0045296;GO:0031012;GO:0005765;GO:0005739;GO:0071461;GO:0005515;GO:0071456;GO:0005576;GO:0030512;GO:0050431 | hsa:114990 | COG4886 | vasorin precursor [Homo sapiens] |
| Q6UXB8 | 9626.388737 | 10884.05974 | 1.130648267 | 0.177150191 | 0.284673036 | no | no change | GO:0010466;GO:0005576;GO:0030414;GO:0005615;GO:0061052 | hsa:221476 | COG2340 | peptidase inhibitor 16 precursor [Homo sapiens] |
| Q86UD1 | 5391.175643 | 5642.772389 | 1.046668252 | 0.065804244 | 0.752474502 | no | no change |  | hsa:220323 | | out at first protein homolog precursor [Homo sapiens] |
| Q8IV42 | 94837.45412 | 106398.617 | 1.121905032 | 0.165950559 | 0.442348671 | no | no change | GO:0000049;GO:0016310;GO:0016301;GO:0097056;GO:0016740;GO:0001514;GO:0000166;GO:0006412;GO:0005524 | hsa:118672 | | L-seryl-tRNA(Sec) kinase isoform 2 [Homo sapiens] |
| Q8N1N4 | 133954.8405 | 130298.3475 | 0.972703539 | -0.039927928 | 0.75833154 | no | no change | GO:0005615;GO:0031424;GO:0005829;GO:0045095;GO:0070268;GO:0005198;GO:0005882;GO:0070062 | hsa:196374 | | keratin, type II cytoskeletal 78 isoform 1 [Homo sapiens] |
| Q92954 | 41636.00263 | 46914.17583 | 1.126769451 | 0.172192355 | 0.372665373 | no | no change | GO:0008283;GO:0005044;GO:0006898;GO:0030247;GO:0030021;GO:0005576;GO:0006955;GO:0062023 | hsa:10216 |  | unnamed protein product [Homo sapiens] |
| Q96KN2 | 5848.279083 | 6337.689773 | 1.083684565 | 0.115944883 | 0.432915613 | no | no change | GO:0016787;GO:0032268;GO:0005829;GO:0004180;GO:0016805;GO:0005576;GO:0008152;GO:0006508;GO:0008233;GO:0046872;GO:0008237 | hsa:84735 | COG0624 | RecName: Full=Beta-Ala-His dipeptidase; AltName: Full=CNDP dipeptidase 1; AltName: Full=Carnosine dipeptidase 1; AltName: Full=Glutamate carboxypeptidase-like protein 2; AltName: Full=Serum carnosinase; Flags: Precursor |
| Q96PD5 | 195693.1421 | 196772.95 | 1.005517863 | 0.007938711 | 0.917548711 | no | no change | GO:0019730;GO:0008745;GO:0032827;GO:0009253;GO:0008270;GO:0044117;GO:0005615;GO:0016045;GO:0016020;GO:0050727;GO:0046872;GO:0002221;GO:0016787;GO:0045087;GO:0050830;GO:0001519;GO:0070062;GO:0005622;GO:0002376;GO:0005576;GO:0032689;GO:0016019;GO:0042834 | hsa:114770 | | N-acetylmuramoyl-L-alanine amidase isoform 1 precursor [Homo sapiens] |
| Q9HDC9 | 7890.958176 | 6601.896619 | 0.836640681 | -0.257319946 | 0.638304868 | no | no change | GO:0016844;GO:0005783;GO:0009986;GO:0016020;GO:0009058;GO:0008150;GO:0004064;GO:0016021 | hsa:57136 | COG3386 | adipocyte plasma membrane-associated protein [Homo sapiens] |
| Q9UHG3 | 20695.87421 | 22319.38583 | 1.078446149 | 0.108954138 | 0.45757067 | no | no change | GO:0034361;GO:1902476;GO:0070062;GO:0030327;GO:0030328;GO:0030329;GO:0099133;GO:0005774;GO:0005764;GO:0016670;GO:0006821;GO:0055114;GO:0005886;GO:0008555;GO:0001735;GO:0016491 | hsa:51449 |  | prenylcysteine oxidase 1 precursor [Homo sapiens] |
| Q9Y6R7 | 40889.26737 | 41849.04333 | 1.023472564 | 0.03347243 | 0.833937293 | no | no change | GO:0005576;GO:0070062;GO:0005515 | hsa:8857 |  | IgGFc-binding protein precursor [Homo sapiens] |
| A0A075B6I0 | 696118.8067 | 497218.8255 | 0.714272938 | -0.485452632 | 0.095274102 | no | down | GO:0016020;GO:0005615;GO:0006955;GO:0002376;GO:0002377;GO:0005576;GO:0002250;GO:0003823;GO:0005886 | hsa:7441 |  | RecName: Full=Immunoglobulin lambda variable 8-61; Flags: Precursor |
| A0A075B6K5 | 29325.31315 | 21335.71587 | 0.727552874 | -0.458875997 | 0.476006233 | no | down | GO:0016020;GO:0005615;GO:0006955;GO:0002376;GO:0002377;GO:0005576;GO:0002250;GO:0003823;GO:0005886 | hsa:7441 |  | RecName: Full=Immunoglobulin lambda variable 3-9; Flags: Precursor |
| A0A075B6Q5 | 61693.16944 | 42551.92 | 0.689734705 | -0.535886536 | 0.169921381 | no | down | GO:0009897;GO:0050853;GO:0045087;GO:0016020;GO:0006910;GO:0006911;GO:0002376;GO:0005576;GO:0002250;GO:0003823;GO:0005886;GO:0072562;GO:0042571;GO:0006958;GO:0050871;GO:0034987;GO:0042742 | hsa:102723407 | | hCG2036739, partial [Homo sapiens] |
| A0A075B6S9 | 6947.50055 | 5550.555533 | 0.798928405 | -0.323861871 | 0.650267768 | no | down | GO:0002377;GO:0005615;GO:0006955 | hsa:7441 |  | IGKV1D-37 isoform 1, partial [Pan troglodytes] |
| A0A075B7D0 | 351833.2974 | 280345.8871 | 0.79681454 | -0.327684121 | 0.268327103 | no | down | GO:0009897;GO:0050853;GO:0045087;GO:0006910;GO:0006911;GO:0042742;GO:0003823;GO:0034987;GO:0042571;GO:0006958;GO:0050871;GO:0072562 | hsa:102723407 | | hCG1728627 [Homo sapiens] |
| A0A087WSY4 | 27725.29313 | 22516.963 | 0.812145174 | -0.300190458 | 0.094711184 | no | down | GO:0009897;GO:0050853;GO:0045087;GO:0016020;GO:0006910;GO:0006911;GO:0002376;GO:0005576;GO:0002250;GO:0003823;GO:0005886;GO:0072562;GO:0042571;GO:0006958;GO:0050871;GO:0034987;GO:0042742 | hsa:102723407 | | RecName: Full=Immunoglobulin heavy variable 4-30-2; Flags: Precursor |
| A0A0A0MS09 | 49809.89976 | 40204.61665 | 0.807161164 | -0.309071334 | 0.515295281 | no | down | GO:0016021;GO:0016020 | | | immunoglobulin delta-chain, partial [Homo sapiens] |
| A0A0B4J1U3 | 34123.82421 | 27657.68086 | 0.810509417 | -0.303099147 | 0.264093542 | no | down | GO:0016020;GO:0005615;GO:0006955;GO:0002376;GO:0002377;GO:0005576;GO:0002250;GO:0003823;GO:0005886 | hsa:7441 |  | RecName: Full=Immunoglobulin lambda variable 1-36; Flags: Precursor |
| A0A0B4J1V2 | 17139.59753 | 8304.0655 | 0.484495945 | -1.045443504 | 0.242257394 | no | down | GO:0009897;GO:0050853;GO:0045087;GO:0016020;GO:0006910;GO:0006911;GO:0002376;GO:0005576;GO:0002250;GO:0003823;GO:0005886;GO:0072562;GO:0042571;GO:0006958;GO:0050871;GO:0034987;GO:0042742 | hsa:102723407 | | RecName: Full=Immunoglobulin heavy variable 2-26; Flags: Precursor |
| A0A0B4J2D9 | 17281.17761 | 13928.09432 | 0.805969051 | -0.311203654 | 0.132026491 | no | down | GO:0016020;GO:0005615;GO:0006955;GO:0002376;GO:0002377;GO:0005576;GO:0002250;GO:0003823;GO:0005886 | hsa:7441 |  | RecName: Full=Immunoglobulin kappa variable 1D-13; Flags: Precursor |
| A0A0C4DH24 | 50128.34316 | 34415.31354 | 0.686544006 | -0.542575897 | 0.091367527 | no | down | GO:0016020;GO:0005615;GO:0006955;GO:0002376;GO:0002377;GO:0005576;GO:0002250;GO:0003823;GO:0005886 | hsa:7441 |  | RecName: Full=Immunoglobulin kappa variable 6-21; Flags: Precursor |
| A0A0C4DH39 | 34557.78826 | 28223.76042 | 0.816712001 | -0.292100667 | 0.423027921 | no | down | GO:0009897;GO:0050853;GO:0045087;GO:0016020;GO:0006910;GO:0006911;GO:0002376;GO:0005576;GO:0002250;GO:0003823;GO:0005886;GO:0072562;GO:0042571;GO:0006958;GO:0050871;GO:0034987;GO:0042742 | hsa:102723407 | | immunoglobulin heavy chain variable region, partial [Homo sapiens] |
| A0A0C4DH43 | 488918.7659 | 362560.6447 | 0.741556001 | -0.431372449 | 0.344926688 | no | down | GO:0009897;GO:0050853;GO:0045087;GO:0016020;GO:0006910;GO:0006911;GO:0002376;GO:0005576;GO:0002250;GO:0003823;GO:0005886;GO:0072562;GO:0042571;GO:0006958;GO:0050871;GO:0034987;GO:0042742 | hsa:102723407 | | RecName: Full=Immunoglobulin heavy variable 2-70D; Flags: Precursor |
| A0A0J9YXX1 | 260636.5474 | 197626.7463 | 0.758246486 | -0.399261187 | 0.0821272 | no | down | GO:0016020;GO:0002376;GO:0005576;GO:0002250;GO:0003823;GO:0005886 | hsa:102724971 | | RecName: Full=Immunoglobulin heavy variable 5-10-1; Flags: Precursor |
| A0A140T8Y3 | 8465.4935 | 6675.028227 | 0.788498417 | -0.342820236 | 0.12341644 | no | down | GO:0005201;GO:0062023;GO:0030199;GO:0030198;GO:0031012 | hsa:7148 |  | tenascin-X isoform 1 precursor [Homo sapiens] |
| A0A2Q2TTZ9 | 355325.4426 | 256111.95 | 0.720781344 | -0.472366425 | 0.172081852 | no | down |  | hsa:7441 |  | IGKV1D-33 isoform 2, partial [Pan troglodytes] |
| A0A5H1ZRS2 | 1060716.079 | 746996.4417 | 0.704237879 | -0.505865267 | 0.207303873 | no | down |  |  |  | immunoglobulin kappa chain variable region, partial [Homo sapiens] |
| C9JXI5 | 152351.3726 | 100495.5716 | 0.659630234 | -0.600270569 | 0.065322266 | no | down | GO:0090263;GO:0031410;GO:0016021;GO:0016020;GO:0005886 | hsa:130612 | | transmembrane protein 198 [Macaca mulatta] |
| E7ENL6 | 7959.001818 | 6598.1205 | 0.829013569 | -0.27053238 | 0.233373306 | no | down | GO:0010951;GO:0004867 | hsa:1293 |  | collagen alpha-3(VI) chain isoform 4 precursor [Homo sapiens] |
| H9KV75 | 7426.674333 | 4964.479327 | 0.668466006 | -0.5810739 | 0.316086875 | no | down | GO:0019894;GO:0032029;GO:0032391;GO:0030036;GO:0017166;GO:0007041;GO:0042383;GO:0005925;GO:0005923;GO:0030507;GO:0005737;GO:0001725;GO:0097433;GO:0045505;GO:0005815;GO:0003779;GO:0030027;GO:0005509;GO:0042803;GO:0034452;GO:0031941;GO:0051393;GO:0016328;GO:0030486;GO:0048741;GO:1990357;GO:0051017;GO:0031252;GO:0051015;GO:0005915;GO:0045214;GO:0051764;GO:0097381;GO:0007030;GO:0090636;GO:0090637 | hsa:87 | COG5069 | alpha-actinin-1 isoform c [Homo sapiens] |
| P00739 | 54761.93632 | 42310.0025 | 0.772616992 | -0.372174689 | 0.119973925 | no | down | GO:0004252;GO:0070062;GO:0034366;GO:0030492;GO:0006898;GO:0072562;GO:0005576;GO:0010942;GO:0002526;GO:0010033;GO:0005615 | hsa:3250 | COG5640 | haptoglobin-related protein precursor [Homo sapiens] |
| P01619 | 3928846.211 | 2890104.333 | 0.735611469 | -0.442984121 | 0.079914138 | no | down | GO:0004252;GO:0030449;GO:0019731;GO:0006955;GO:0050900;GO:0005615;GO:0016020;GO:0003094;GO:0072562;GO:0071748;GO:0006508;GO:0050776;GO:0006956;GO:0003823;GO:0005886;GO:0006958;GO:0070062;GO:0038096;GO:0038095;GO:0006898;GO:0002376;GO:0005576;GO:0002250;GO:0071751;GO:0071756 | hsa:29802 |  | immunoglobulin light chain variable region, partial [Homo sapiens] |
| P01700 | 1429804.511 | 1160694.1 | 0.811785172 | -0.300830107 | 0.05804878 | no | down | GO:0038096;GO:0004252;GO:0016020;GO:0030449;GO:0050776;GO:0006898;GO:0038095;GO:0072562;GO:0002376;GO:0050900;GO:0005576;GO:0006956;GO:0002250;GO:0003823;GO:0005886;GO:0006508;GO:0006958;GO:0006955 | hsa:7441 |  | RecName: Full=Immunoglobulin lambda variable 1-47; AltName: Full=Ig lambda chain V-I region HA; AltName: Full=Ig lambda chain V-I region WAH; Flags: Precursor |
| P01704 | 38484.2075 | 30804.99432 | 0.800458066 | -0.321102269 | 0.176528951 | no | down | GO:0038096;GO:0004252;GO:0016020;GO:0030449;GO:0050776;GO:0006898;GO:0038095;GO:0002376;GO:0050900;GO:0005576;GO:0006956;GO:0002250;GO:0003823;GO:0005886;GO:0006508;GO:0006958;GO:0006955;GO:0070062 | hsa:7441 |  | RecName: Full=Immunoglobulin lambda variable 2-14; AltName: Full=Ig lambda chain V-II region NIG-84; AltName: Full=Ig lambda chain V-II region TOG; AltName: Full=Ig lambda chain V-II region VIL; Flags: Precursor |
| P01706 | 135250.71 | 111182.1975 | 0.822045204 | -0.282710365 | 0.277308109 | no | down | GO:0038096;GO:0004252;GO:0016020;GO:0030449;GO:0050776;GO:0006898;GO:0038095;GO:0002376;GO:0050900;GO:0005576;GO:0006956;GO:0002250;GO:0003823;GO:0005886;GO:0006508;GO:0006958;GO:0006955 | hsa:7441 |  | hCG2043237, partial [Homo sapiens] |
| P01709 | 156534.4021 | 128485.585 | 0.820813721 | -0.284873248 | 0.156324516 | no | down | GO:0038096;GO:0004252;GO:0016020;GO:0030449;GO:0050776;GO:0006898;GO:0038095;GO:0002376;GO:0050900;GO:0005576;GO:0006956;GO:0002250;GO:0003823;GO:0005886;GO:0006508;GO:0006958;GO:0006955 | hsa:7441 |  | hCG2043240, partial [Homo sapiens] |
| P01718 | 29956.94232 | 18557.02407 | 0.619456548 | -0.690925008 | 0.380812954 | no | down | GO:0038096;GO:0004252;GO:0016020;GO:0030449;GO:0050776;GO:0006898;GO:0038095;GO:0002376;GO:0050900;GO:0005576;GO:0006956;GO:0002250;GO:0003823;GO:0005886;GO:0006508;GO:0006958;GO:0006955 | hsa:29802 |  | immunoglobulin light chain variable region, partial [Homo sapiens] |
| P01833 | 14894.20894 | 11886.09729 | 0.798034816 | -0.325476407 | 0.083864808 | no | down | GO:0043235;GO:0001895;GO:0005615;GO:0002415;GO:0001580;GO:0070062;GO:0016021;GO:0016020;GO:0043312;GO:0005576;GO:0007173;GO:0005887;GO:0005886;GO:0043113;GO:0001792;GO:0035577;GO:0038093 | hsa:5284 |  | polymeric immunoglobulin receptor precursor [Homo sapiens] |
| P01861 | 624816.04 | 474919.1504 | 0.760094364 | -0.395749558 | 0.168573935 | no | down | GO:0004252;GO:0030449;GO:0019221;GO:0009897;GO:0042742;GO:0034987;GO:0042571;GO:0005615;GO:0016020;GO:0072562;GO:0006508;GO:0050853;GO:0045087;GO:0006910;GO:0006911;GO:0006956;GO:0003823;GO:0005886;GO:0006958;GO:0050871;GO:0070062;GO:0038096;GO:0002376;GO:0005576;GO:0002250 | hsa:100423062 | | RecName: Full=Immunoglobulin heavy constant gamma 4; AltName: Full=Ig gamma-4 chain C region |
| P02533 | 84393.37211 | 25924.08125 | 0.307181484 | -1.702836839 | 0.278072671 | no | down | GO:0005200;GO:0045095;GO:0071944;GO:0010043;GO:0008544;GO:0005737;GO:0045110;GO:0010212;GO:0070062;GO:0005634;GO:0045178;GO:0005198;GO:0030855;GO:1990254;GO:0031581;GO:0070268;GO:0005882;GO:0042633;GO:0007568;GO:0031424;GO:0005829;GO:0005622;GO:0005515 | hsa:3861 |  | keratin, type I cytoskeletal 14 [Homo sapiens] |
| P06331 | 708095.7105 | 441931.2375 | 0.624112293 | -0.680122467 | 0.280978893 | no | down | GO:0038096;GO:0004252;GO:0016020;GO:0030449;GO:0050776;GO:0006898;GO:0038095;GO:0002376;GO:0050900;GO:0005576;GO:0006956;GO:0002250;GO:0003823;GO:0005886;GO:0006508;GO:0006958;GO:0006955 | hsa:102724971 | | hCG1793614, partial [Homo sapiens] |
| P08519 | 143288.9263 | 97129.765 | 0.677859535 | -0.560941743 | 0.392662487 | no | down | GO:0004252;GO:0034374;GO:0005515;GO:0006869;GO:0008015;GO:0016787;GO:0034185;GO:0005576;GO:0004866;GO:0010951;GO:0006508;GO:0008233;GO:0008236;GO:0034358;GO:0008201;GO:0001968;GO:0006629 | hsa:4018 | COG5640 | RecName: Full=Apolipoprotein(a); Short=Apo(a); Short=Lp(a); Flags: Precursor |
| P11597 | 7833.990842 | 6168.082 | 0.787348635 | -0.344925497 | 0.054858424 | no | down | GO:0017129;GO:0034375;GO:0034374;GO:0034372;GO:0006869;GO:0015914;GO:0017127;GO:0030301;GO:0034197;GO:0034364;GO:0055091;GO:0005548;GO:0070062;GO:0005615;GO:0008202;GO:0010745;GO:0031982;GO:0005319;GO:0070328;GO:0006629;GO:0006641;GO:0008289;GO:0055088;GO:0031210;GO:0042632;GO:0043691;GO:0010874;GO:0008203;GO:0015485;GO:0005576;GO:0046470 | hsa:1071 |  | cholesteryl ester transfer protein isoform 1 precursor [Homo sapiens] |
| P13796 | 2568131.265 | 1606692.123 | 0.625626947 | -0.67662544 | 0.375443037 | no | down | GO:0032432;GO:0033157;GO:0015629;GO:0030175;GO:0051020;GO:0005925;GO:0030054;GO:0005737;GO:0071803;GO:0001726;GO:0002102;GO:0002286;GO:0005615;GO:0016020;GO:0003779;GO:0005509;GO:0048471;GO:0016477;GO:0022617;GO:0042802;GO:0035722;GO:0005178;GO:0031100;GO:0051017;GO:0051015;GO:0005884;GO:0005886;GO:0051764;GO:0042995;GO:0032587;GO:0001891;GO:0046872;GO:0070062;GO:0005829;GO:0005856;GO:0051639;GO:0044319;GO:0001725;GO:0010737 | hsa:3936 | COG5069 | plastin-2 [Homo sapiens] |
| P19320 | 5751.492059 | 2930.232184 | 0.509473395 | -0.972921286 | 0.135066506 | no | down | GO:0009897;GO:0034113;GO:0005783;GO:0005794;GO:0035094;GO:0030175;GO:0060945;GO:0005902;GO:0050901;GO:0060326;GO:0010043;GO:0032496;GO:0001666;GO:0019221;GO:0045177;GO:0002102;GO:0010212;GO:0005615;GO:0060384;GO:0071356;GO:0016021;GO:0016020;GO:0050839;GO:0035584;GO:1904646;GO:0140039;GO:0022614;GO:0005178;GO:0042383;GO:0009308;GO:0030183;GO:0098609;GO:0007584;GO:0008131;GO:0045471;GO:0007159;GO:0005887;GO:0005886;GO:0007155;GO:0002526;GO:0007157;GO:0042102;GO:0009986;GO:0055114;GO:0070062;GO:0007160;GO:0050776;GO:0007568;GO:0060333;GO:0002544;GO:0005769;GO:0030198;GO:0035924;GO:0071065 | hsa:7412 |  | vascular cell adhesion protein 1 isoform a precursor [Homo sapiens] |
| P20742 | 51671.45238 | 26636.63685 | 0.515500061 | -0.955955497 | 0.17051936 | no | down | GO:0004866;GO:0007565;GO:0070062;GO:0005576;GO:0030414;GO:0072562;GO:0002020;GO:0004867;GO:0010951;GO:0010466;GO:0005615 | hsa:5858 | COG2373 | pregnancy zone protein precursor [Homo sapiens] |
| P26038 | 4686.3125 | 3266.40165 | 0.697008928 | -0.52075096 | 0.24502394 | no | down | GO:0005200;GO:0019899;GO:0030175;GO:0050900;GO:0010628;GO:0005925;GO:0061028;GO:0005737;GO:0071803;GO:0045177;GO:0042995;GO:0070062;GO:1902115;GO:0043209;GO:0005634;GO:0042098;GO:0072562;GO:0003779;GO:2000401;GO:0035722;GO:0022612;GO:0048471;GO:2000643;GO:0022614;GO:1903364;GO:0005856;GO:0005515;GO:0016324;GO:0009986;GO:0071944;GO:0031982;GO:0071394;GO:0016323;GO:0019901;GO:0008361;GO:0008360;GO:0045198;GO:0001771;GO:0005886;GO:0016020;GO:0050839;GO:0072678;GO:0005615;GO:0031528;GO:0007010;GO:0071437;GO:0005829;GO:0008092;GO:0005902;GO:0003725;GO:0016032;GO:0001931;GO:0007159;GO:0005102;GO:0070489;GO:0031143;GO:1902966 | hsa:4478 |  | moesin [Homo sapiens] |
| P55103 | 8468.860111 | 6857.993625 | 0.809789456 | -0.304381236 | 0.058746283 | no | down | GO:0005179;GO:0005125;GO:0008083;GO:0060395;GO:0005160;GO:0010469;GO:0048468;GO:0005576;GO:0005615;GO:0010862;GO:0042981;GO:0043408 | hsa:3626 |  | inhibin beta C chain preproprotein [Homo sapiens] |
| P61224 | 7364.3994 | 5872.168231 | 0.797372319 | -0.326674571 | 0.325802712 | no | down | GO:0005811;GO:0030033;GO:0044877;GO:0007165;GO:0061028;GO:0030054;GO:0005737;GO:0070062;GO:2000114;GO:0016020;GO:0000166;GO:0070382;GO:0045955;GO:0035722;GO:0005525;GO:0008283;GO:0003924;GO:0035577;GO:0032486;GO:0005911;GO:0005886;GO:0045121;GO:0007264;GO:0005829;GO:0071320;GO:0043312;GO:0005622;GO:0019003;GO:0005515;GO:1901888;GO:0070374;GO:2000301 | hsa:5908 | COG1100 | Ras-related protein Rap-1b, partial [Bos mutus] |
| Q13093 | 6661.107222 | 5280.822626 | 0.79278451 | -0.33499932 | 0.099975007 | no | down | GO:0034441;GO:0034440;GO:0034362;GO:0034374;GO:0047499;GO:0016787;GO:0006629;GO:0005615;GO:0005543;GO:0005737;GO:0016788;GO:0016042;GO:0050729;GO:0090026;GO:0003847;GO:0046469;GO:0005576 | hsa:7941 |  | platelet-activating factor acetylhydrolase precursor [Homo sapiens] |
| Q9Y490 | 79157.93965 | 36324.60655 | 0.458887721 | -1.123786891 | 0.415794399 | no | down | GO:0005200;GO:0017166;GO:0007043;GO:0005925;GO:0030054;GO:0005856;GO:0001726;GO:0070062;GO:0002576;GO:0016020;GO:0005737;GO:0003779;GO:0005576;GO:0033622;GO:0001786;GO:0005178;GO:0005515;GO:0006936;GO:0009986;GO:0051015;GO:0036498;GO:0030274;GO:0005886;GO:0007155;GO:0042995;GO:0032587;GO:0007016;GO:0035091;GO:0005829;GO:0030866;GO:0045296;GO:0070527;GO:0007044;GO:0016032;GO:0044877;GO:0007229 | hsa:7094 |  | talin-1 [Homo sapiens] |

| Table S4-3 Proteins identified in S vs M group. | | | | | | | | | | | |
| --- | --- | --- | --- | --- | --- | --- | --- | --- | --- | --- | --- |
| Accession | M | S | FC(S/M) | log2FC(S/M) | Pvalue(S/M) | significant | regulate | GO | KEGG | COG | Description |
| A0A087X0Q4 | 85608.28125 | 126820.3292 | 1.481402585 | 0.56696376 | 0.002569128 | yes | up |  |  |  | RecName: Full=Immunoglobulin kappa variable 2D-40; AltName: Full=Ig kappa chain V-II region Cum; Flags: Precursor |
| A0A0B4J1Y8 | 78605.33 | 124365.8168 | 1.582155012 | 0.661890955 | 0.027812832 | yes | up | GO:0016020;GO:0005615;GO:0006955;GO:0002376;GO:0002377;GO:0005576;GO:0002250;GO:0003823;GO:0005886 | hsa:7441 |  | Unknown (protein for IMAGE:4575521), partial [Homo sapiens] |
| K7ER74 | 428761.3892 | 692444.0838 | 1.614987033 | 0.691522581 | 0.048263623 | yes | up | GO:0034375;GO:0034372;GO:0034371;GO:0034370;GO:0042627;GO:0034378;GO:0051006;GO:0010902;GO:0008047;GO:0048261;GO:0001523;GO:0043085;GO:0055102;GO:0005615;GO:0032375;GO:0005576;GO:0016042;GO:0045833;GO:0016004;GO:0010898;GO:0033700;GO:0034361;GO:0034362;GO:0034363;GO:0070328;GO:0006629;GO:0034366;GO:0060230;GO:0008289;GO:0006869;GO:0042803;GO:0010916;GO:0043274;GO:0042632;GO:0043691;GO:0034382;GO:0042493;GO:0034384;GO:0010518;GO:0033344;GO:0042953;GO:0060697;GO:0045723;GO:0005769 | hsa:344 |  | apolipoprotein C-II isoform X1 [Mesocricetus auratus] |
| P01019 | 153225.3458 | 187123.3125 | 1.2212295 | 0.288334345 | 0.00091984 | yes | up | GO:1903779;GO:0038166;GO:2001238;GO:0035106;GO:0003014;GO:0007166;GO:0050729;GO:0001822;GO:0042127;GO:0032930;GO:0006606;GO:0014824;GO:0019229;GO:0010976;GO:0010873;GO:0050731;GO:0035815;GO:0005829;GO:0007202;GO:0007204;GO:0003081;GO:1904754;GO:0010536;GO:0007565;GO:0005179;GO:0034374;GO:0007200;GO:0071260;GO:0042310;GO:0042311;GO:0048169;GO:0008083;GO:1901201;GO:0072562;GO:0006883;GO:0061098;GO:0007186;GO:2000379;GO:0090190;GO:0048018;GO:0008306;GO:0003331;GO:0033864;GO:0007199;GO:0001974;GO:0062023;GO:0048659;GO:0051969;GO:0016525;GO:0014873;GO:0030308;GO:0048146;GO:0048144;GO:0007267;GO:0045429;GO:1905010;GO:0043085;GO:0005737;GO:0050880;GO:0003051;GO:0002027;GO:0045742;GO:0032270;GO:0070471;GO:0008284;GO:0031701;GO:0031703;GO:0001558;GO:0014068;GO:0004867;GO:0014061;GO:0051092;GO:1904385;GO:0007568;GO:0070062;GO:0002034;GO:2001275;GO:0005515;GO:0070371;GO:0001819;GO:0051387;GO:0010469;GO:0010666;GO:0008217;GO:0061049;GO:0050663;GO:0032355;GO:0005615;GO:0097755;GO:0042981;GO:0010744;GO:0035813;GO:1904707;GO:0046628;GO:0034104;GO:0019216;GO:0051403;GO:0010951;GO:0007263;GO:1905589;GO:0010613;GO:0010595;GO:0002019;GO:0002018;GO:0045777;GO:0002016;GO:0031702;GO:0051924;GO:0005576;GO:1903598;GO:0045893 | hsa:183 | COG4826 | angiotensinogen preproprotein [Homo sapiens] |
| P15169 | 21634.38792 | 26973.77833 | 1.246801085 | 0.318231316 | 0.002083247 | yes | up | GO:0097060;GO:0005794;GO:0051384;GO:0030449;GO:0005615;GO:0016787;GO:0030141;GO:0004181;GO:0004180;GO:0010815;GO:0004185;GO:0005576;GO:0043025;GO:0008270;GO:0016485;GO:0006508;GO:0006518;GO:0046872;GO:0030070;GO:0008233;GO:0008237 | hsa:1369 |  | carboxypeptidase N catalytic chain precursor [Homo sapiens] |
| P55058 | 17563.92375 | 22844.77083 | 1.300664428 | 0.379248794 | 0.002711084 | yes | up | GO:0034375;GO:0006869;GO:0015914;GO:1990050;GO:0035627;GO:0005548;GO:0005615;GO:0019992;GO:0008525;GO:0097001;GO:0010875;GO:0030317;GO:0010189;GO:0005319;GO:0035620;GO:0034364;GO:0006629;GO:0070300;GO:0008289;GO:0031210;GO:0008429;GO:1904121;GO:0005576;GO:1901611 | hsa:5360 |  | phospholipid transfer protein, isoform CRA_c [Homo sapiens] |
| Q08380 | 65083.83583 | 89176.44583 | 1.370178089 | 0.45436342 | 0.008022997 | yes | up | GO:0006968;GO:0070062;GO:0006898;GO:0005044;GO:0002576;GO:0016020;GO:0072562;GO:0031089;GO:0062023;GO:0005615;GO:0007165;GO:0005515;GO:0007155;GO:0005576 | hsa:3959 |  | galectin-3-binding protein precursor [Homo sapiens] |
| Q9NZP8 | 38089.40583 | 65146.30458 | 1.710352345 | 0.774293561 | 0.024160617 | yes | up | GO:0004252;GO:0005615;GO:0016787;GO:0070062;GO:0045087;GO:0031638;GO:0002376;GO:0005576;GO:0006508;GO:0006958;GO:0008233;GO:0008236 | hsa:51279 | COG5640 | complement C1r subcomponent-like protein isoform 1 precursor [Homo sapiens] |
| A0A0G2JL69 | 36451.96917 | 31058.21125 | 0.852031096 | -0.23102201 | 0.003535906 | yes | no change | GO:0004252;GO:0016787;GO:0045087;GO:0005576;GO:0006956;GO:0006508;GO:0006958;GO:0046872;GO:0008233;GO:0008236 | hsa:717 | COG5640 | complement C2 isoform 5 [Homo sapiens] |
| C9JV77 | 2296220.083 | 2053469 | 0.89428231 | -0.161197757 | 0.039617159 | yes | no change | GO:0019210;GO:0006907;GO:0005788;GO:0001501;GO:0030500;GO:0030502;GO:0050766;GO:0044267;GO:0070062;GO:0031093;GO:0002576;GO:0050727;GO:0072562;GO:0005794;GO:0043687;GO:0046627;GO:0006953;GO:0062023;GO:0034774;GO:0010951;GO:0004869;GO:0005615;GO:0043312;GO:0005576 | hsa:197 |  | alpha-2-HS-glycoprotein isoform 1 preproprotein [Homo sapiens] |
| G3XAK1 | 18147.16042 | 15567.44625 | 0.857844748 | -0.221211522 | 0.013070674 | yes | no change | GO:0004252;GO:2000479;GO:0019899;GO:0010628;GO:0005737;GO:0005615;GO:0005773;GO:0033601;GO:0030971;GO:1904036;GO:0006508;GO:0045721;GO:0046425;GO:0030317;GO:0071456;GO:0007283;GO:0062023;GO:0060763;GO:0048012;GO:0007566;GO:0030879;GO:0005576;GO:0010758 | hsa:4485 | COG5640 | hepatocyte growth factor-like protein precursor [Homo sapiens] |
| H0YAC1 | 151327.6013 | 133550.6958 | 0.882527012 | -0.180287658 | 0.028848755 | yes | no change | GO:0004252;GO:0004497;GO:0007597;GO:0008236;GO:0008233;GO:0016491;GO:0070062;GO:0016705;GO:0031639;GO:0031638;GO:0006508;GO:0005506;GO:0046872;GO:0022617;GO:0051919;GO:0016787;GO:0020037;GO:0005886;GO:0055114;GO:0005615;GO:0042730;GO:0002542;GO:0005576 | hsa:3818 | COG5640 | KLKB1 isoform 4, partial [Pan troglodytes] |
| M0R0Q9 | 6022.777783 | 5207.888087 | 0.864698695 | -0.209730583 | 0.025921703 | yes | no change | GO:0006631;GO:0030449;GO:0004866;GO:0005886;GO:0005788;GO:0035578;GO:0007165;GO:0031715;GO:0048260;GO:0010828;GO:0010866;GO:0044267;GO:0070062;GO:0009617;GO:0045766;GO:1905114;GO:0045745;GO:0097242;GO:2000427;GO:0034774;GO:0007186;GO:0009986;GO:0005576;GO:0150064;GO:0043687;GO:0016322;GO:0150062;GO:0006956;GO:0006957;GO:0006954;GO:0006955;GO:0060100;GO:0006958;GO:0005615;GO:0050776;GO:0032991;GO:0010575;GO:0043312;GO:0001798;GO:0010884;GO:0001970;GO:0097278;GO:0005102;GO:0001934;GO:0072562 | hsa:718 |  | C3 isoform 6, partial [Pan troglodytes] |
| P00740 | 34206.12833 | 31076.98208 | 0.908520888 | -0.138408411 | 0.021278176 | yes | no change | GO:0004252;GO:0004175;GO:0006888;GO:0005615;GO:0016787;GO:0070062;GO:0007597;GO:0007596;GO:0031638;GO:0005788;GO:0005796;GO:0005576;GO:0005509;GO:0005515;GO:0005886;GO:0006508;GO:0008233;GO:0046872;GO:0007599;GO:0008236 | hsa:2158 | COG5640 | coagulation factor IX isoform 1 preproprotein [Homo sapiens] |
| P01008 | 459821.625 | 406228.2042 | 0.883447368 | -0.178783908 | 0.018366175 | yes | no change | GO:0007599;GO:0007595;GO:2000266;GO:0007596;GO:0005788;GO:0002438;GO:0070062;GO:0072562;GO:0010466;GO:0002020;GO:0043687;GO:0042802;GO:0044267;GO:0007584;GO:0062023;GO:0030414;GO:0004867;GO:0010951;GO:0005886;GO:0008201;GO:0005615;GO:0005576;GO:0005515;GO:0030193 | hsa:462 | COG4826 | antithrombin-III isoform 1 precursor [Homo sapiens] |
| P01009 | 4974463.417 | 4427646.083 | 0.890075112 | -0.168001007 | 0.048965241 | yes | no change | GO:0048208;GO:0007599;GO:0005783;GO:0007596;GO:0033116;GO:0005788;GO:1904813;GO:0030134;GO:0044267;GO:0006888;GO:0005615;GO:0031093;GO:0002576;GO:0010466;GO:0002020;GO:0042802;GO:0005794;GO:0043687;GO:0006953;GO:0030414;GO:0004867;GO:0010951;GO:0070062;GO:0043231;GO:0043312;GO:0000139;GO:0005576;GO:0005515 | hsa:5265 | COG4826 | alpha-1-antitrypsin precursor [Homo sapiens] |
| P01619 | 3456730.083 | 2890104.333 | 0.836080418 | -0.258286381 | 0.009910237 | yes | no change | GO:0004252;GO:0030449;GO:0019731;GO:0006955;GO:0050900;GO:0005615;GO:0016020;GO:0003094;GO:0072562;GO:0071748;GO:0006508;GO:0050776;GO:0006956;GO:0003823;GO:0005886;GO:0006958;GO:0070062;GO:0038096;GO:0038095;GO:0006898;GO:0002376;GO:0005576;GO:0002250;GO:0071751;GO:0071756 | hsa:29802 |  | immunoglobulin light chain variable region, partial [Homo sapiens] |
| P01834 | 26834204.5 | 22862797.67 | 0.852002066 | -0.231071167 | 0.028022303 | yes | no change | GO:0004252;GO:0030449;GO:0009897;GO:0006955;GO:0050871;GO:0042742;GO:0034987;GO:0042571;GO:0005615;GO:0016020;GO:0072562;GO:0006508;GO:0050853;GO:0045087;GO:0006910;GO:0006911;GO:0050776;GO:0006956;GO:0003823;GO:0005886;GO:0006958;GO:0001895;GO:0070062;GO:0038096;GO:0038095;GO:0006898;GO:0050900;GO:0002376;GO:0005576;GO:0002250 | hsa:100423062 | | light chain kappa Sci, k Sci=Bence Jones protein [human, Peptide, 214 aa] |
| P02768 | 501513014.7 | 445194692 | 0.887703168 | -0.171850749 | 0.001821396 | yes | no change | GO:0034375;GO:0015643;GO:0005788;GO:0008144;GO:0051659;GO:0030170;GO:0003677;GO:0005783;GO:0005737;GO:0044267;GO:0019836;GO:0070062;GO:0031093;GO:0043209;GO:0002576;GO:0051087;GO:0072562;GO:0098869;GO:0043066;GO:0005504;GO:0005507;GO:0043687;GO:0032460;GO:0042802;GO:0005794;GO:0140272;GO:0016209;GO:0008289;GO:0019825;GO:0009267;GO:0001895;GO:0005615;GO:0046872;GO:0032991;GO:0043069;GO:0006898;GO:1903981;GO:0005576;GO:0005515;GO:0005634 | hsa:213 |  | serum albumin preproprotein [Homo sapiens] |
| P04004 | 791457.45 | 919898.8833 | 1.162284698 | 0.216963496 | 0.041901304 | yes | no change | GO:0005201;GO:0030449;GO:0005783;GO:0061302;GO:0050840;GO:0014911;GO:0030949;GO:0030247;GO:0031012;GO:0007160;GO:0007155;GO:0090303;GO:0048260;GO:0005737;GO:0032092;GO:0070062;GO:0072562;GO:0005576;GO:0016477;GO:0042802;GO:0033627;GO:0005178;GO:0005796;GO:0008283;GO:0010811;GO:0048709;GO:0048237;GO:0062023;GO:0006955;GO:0010951;GO:0035987;GO:0008201;GO:0005615;GO:0051258;GO:0005604;GO:0043231;GO:0050731;GO:0005044;GO:0006898;GO:0030198;GO:0005515;GO:0097421;GO:0071062;GO:0005518;GO:0030195 | hsa:7448 |  | vitronectin precursor [Homo sapiens] |
| P05546 | 570904.3792 | 483992.8542 | 0.847765181 | -0.238263381 | 0.022610747 | yes | no change | GO:0044267;GO:0007599;GO:0006935;GO:0005615;GO:0043687;GO:0007596;GO:0005788;GO:0005576;GO:0030414;GO:0004866;GO:0004867;GO:0010951;GO:0010466;GO:0008201;GO:0070062 | hsa:3053 | COG4826 | heparin cofactor 2 precursor [Homo sapiens] |
| P08697 | 244147.6875 | 215477.4083 | 0.882569934 | -0.180217495 | 0.00293186 | yes | no change | GO:0030414;GO:0032967;GO:0005615;GO:0031093;GO:0051496;GO:0002576;GO:0030199;GO:0072562;GO:0010466;GO:0048514;GO:0002020;GO:0010757;GO:0051918;GO:0042803;GO:0048661;GO:0009986;GO:0045597;GO:0006953;GO:0046330;GO:0004866;GO:0004867;GO:0010951;GO:0010033;GO:0070062;GO:0042730;GO:2000049;GO:0002034;GO:0005577;GO:0005576;GO:0045944;GO:0005515;GO:0070374;GO:0071636 | hsa:5345 | COG4826 | alpha-2-antiplasmin isoform X1 [Homo sapiens] |
| P09871 | 89103.85625 | 101824.4283 | 1.142761185 | 0.192523939 | 0.027262272 | yes | no change | GO:0004252;GO:0006956;GO:0016787;GO:0045087;GO:0072562;GO:0002376;GO:0030449;GO:0005576;GO:0001867;GO:0005509;GO:0005515;GO:0006508;GO:0006958;GO:0046872;GO:0008233;GO:0042802;GO:0008236 | hsa:716 | COG5640 | complement C1s subcomponent isoform 1 preproprotein [Homo sapiens] |
| P15814 | 11078226.17 | 9216727.483 | 0.831967803 | -0.265400398 | 0.033423642 | yes | no change | GO:0009897;GO:0050853;GO:0045087;GO:0016020;GO:0006910;GO:0006911;GO:0050900;GO:0005576;GO:0042742;GO:0003823;GO:0006955;GO:0042571;GO:0006958;GO:0050871;GO:0034987;GO:0072562 | hsa:3543 |  | immunoglobulin lambda-like polypeptide 1 isoform a precursor [Homo sapiens] |
| P19827 | 355189.1833 | 322715.4875 | 0.908573523 | -0.138324831 | 0.017277057 | yes | no change | GO:0030212;GO:0070062;GO:0072562;GO:0010466;GO:0005509;GO:0030414;GO:0004867;GO:0010951;GO:0005576 | hsa:3697 | COG2304 | inter-alpha-trypsin inhibitor heavy chain H1 isoform a preproprotein [Homo sapiens] |
| Q96IY4 | 23241.97958 | 19314.74542 | 0.831028413 | -0.26703029 | 0.010882306 | yes | no change | GO:0007599;GO:2000346;GO:0007596;GO:0008270;GO:0030449;GO:0008233;GO:0008237;GO:0005615;GO:0004181;GO:0004180;GO:0071333;GO:0006508;GO:0046872;GO:0051918;GO:0016787;GO:0009408;GO:0070062;GO:0042730;GO:0003331;GO:0097421;GO:0042493;GO:0005623;GO:0005576;GO:0010757 | hsa:1361 | COG2866 | carboxypeptidase B2 isoform 1 preproprotein [Homo sapiens] |
| A0A075B6K4 | 76207.42708 | 49739.26917 | 0.652682699 | -0.615546298 | 0.020017871 | yes | down | GO:0016020;GO:0005615;GO:0006955;GO:0002376;GO:0002377;GO:0005576;GO:0002250;GO:0003823;GO:0005886 | hsa:7441 |  | immunoglobulin light chain variable region, partial [Homo sapiens] |
| A0A0C4DH25 | 544631 | 396296.725 | 0.727642615 | -0.458698057 | 0.009719886 | yes | down | GO:0038096;GO:0004252;GO:0016020;GO:0030449;GO:0005615;GO:0006898;GO:0038095;GO:0050900;GO:0002376;GO:0002377;GO:0050776;GO:0005576;GO:0006956;GO:0002250;GO:0003823;GO:0005886;GO:0006508;GO:0006958;GO:0006955 | hsa:29802 |  | hCG1686089, partial [Homo sapiens] |
| A0A0G2JRQ6 | 296726.0333 | 220779.3208 | 0.744051064 | -0.426526458 | 0.00191363 | yes | down | GO:0002377;GO:0005615;GO:0006955 | hsa:7441 |  | hCG2042707, partial [Homo sapiens] |
| A0A0U1RQV3 | 28724.69542 | 23377.98833 | 0.81386375 | -0.297140803 | 0.001235739 | yes | down | GO:0062023;GO:0007173;GO:0005509;GO:0005006 | hsa:2202 |  | EGF-containing fibulin-like extracellular matrix protein 1 isoform X3 [Homo sapiens] |
| C9JB55 | 14073.08717 | 9811.448136 | 0.697178097 | -0.520400848 | 0.00021968 | yes | down | GO:0055037;GO:1990459;GO:0030139;GO:0005905;GO:0034986;GO:1990712;GO:0004857;GO:0048260;GO:0005615;GO:0005770;GO:0043086;GO:0009925;GO:0048471;GO:0006879;GO:0016324;GO:0008198;GO:0008199;GO:0015091;GO:0031232;GO:0034756;GO:0071281;GO:0005623;GO:0005769 | hsa:7018 |  | serotransferrin isoform 1 precursor [Homo sapiens] |
| D6R934 | 119813.6633 | 96657.57542 | 0.806732494 | -0.309837728 | 0.000364529 | yes | down | GO:0006958;GO:0005576;GO:0005581 | hsa:713 |  | complement C1q subcomponent subunit B precursor [Homo sapiens] |
| J3QRV5 | 384036.1792 | 278678.4542 | 0.725656772 | -0.462640765 | 0.002650154 | yes | down | GO:0005737;GO:0043231;GO:0005829;GO:0006887;GO:0007049;GO:0051301 | hsa:3993 |  | lethal(2) giant larvae protein homolog 2 isoform X4 [Homo sapiens] |
| O00187 | 33615.62133 | 24618.28871 | 0.732346681 | -0.449401337 | 0.037053251 | yes | down | GO:0045087;GO:0004252;GO:0046872;GO:0006956;GO:0005615;GO:0048306;GO:0070062;GO:0016787;GO:0002376;GO:0005576;GO:0001867;GO:0005509;GO:0005515;GO:0006508;GO:0006958;GO:0008236;GO:0008233;GO:0001855 | hsa:10747 | COG5640 | mannan-binding lectin serine protease 2 isoform 1 preproprotein [Homo sapiens] |
| P00748 | 55029.87542 | 40103.85458 | 0.728765135 | -0.456474154 | 0.007780009 | yes | down | GO:0004252;GO:0002542;GO:0007599;GO:0007597;GO:0007596;GO:0008233;GO:0008236;GO:0070062;GO:0031638;GO:0005509;GO:0006508;GO:0051919;GO:0016787;GO:0005791;GO:0045087;GO:0062023;GO:0005886;GO:0016485;GO:0005615;GO:0042730;GO:0016540;GO:0002353;GO:0005576;GO:0051787;GO:0010756;GO:0005515;GO:0030193;GO:0051788;GO:0030194 | hsa:2161 | COG5640 | coagulation factor XII preproprotein [Homo sapiens] |
| P01601 | 98663.38208 | 65756.58375 | 0.666474049 | -0.585379394 | 0.00179868 | yes | down | GO:0038096;GO:0004252;GO:0016020;GO:0030449;GO:0005615;GO:0006898;GO:0038095;GO:0050900;GO:0002376;GO:0002377;GO:0050776;GO:0005576;GO:0006956;GO:0002250;GO:0003823;GO:0005886;GO:0006508;GO:0006958;GO:0006955 | hsa:7441 |  | RecName: Full=Immunoglobulin kappa variable 1D-16; AltName: Full=Ig kappa chain V-I region HK146; AltName: Full=Ig kappa chain V-I region HK189; Flags: Precursor |
| P01700 | 1497228.204 | 1160694.1 | 0.775228584 | -0.367306329 | 0.018793505 | yes | down | GO:0038096;GO:0004252;GO:0016020;GO:0030449;GO:0050776;GO:0006898;GO:0038095;GO:0072562;GO:0002376;GO:0050900;GO:0005576;GO:0006956;GO:0002250;GO:0003823;GO:0005886;GO:0006508;GO:0006958;GO:0006955 | hsa:7441 |  | RecName: Full=Immunoglobulin lambda variable 1-47; AltName: Full=Ig lambda chain V-I region HA; AltName: Full=Ig lambda chain V-I region WAH; Flags: Precursor |
| P01782 | 272726.6167 | 181545.2313 | 0.665667449 | -0.587126472 | 0.000644269 | yes | down | GO:0038096;GO:0004252;GO:0016020;GO:0030449;GO:0050776;GO:0006898;GO:0038095;GO:0002376;GO:0050900;GO:0005576;GO:0006956;GO:0002250;GO:0003823;GO:0005886;GO:0006508;GO:0006958;GO:0006955;GO:0070062 | hsa:102723407 | | hCG2038940, partial [Homo sapiens] |
| P01857 | 9143238.333 | 7511055.083 | 0.8214874 | -0.283689648 | 0.026936541 | yes | down | GO:0004252;GO:0030449;GO:0019221;GO:0009897;GO:0042742;GO:0034987;GO:0042571;GO:0005615;GO:0016020;GO:0072562;GO:0006508;GO:0050853;GO:0045087;GO:0006910;GO:0006911;GO:0006956;GO:0003823;GO:0005886;GO:0006958;GO:0050871;GO:0070062;GO:0038096;GO:0002376;GO:0005576;GO:0002250;GO:0005515 | hsa:100423062 | | IGH@ protein [Homo sapiens] |
| P02747 | 435160.6167 | 317328.5208 | 0.729221599 | -0.455570801 | 0.000165722 | yes | down | GO:0004252;GO:0030449;GO:0005581;GO:0045650;GO:0045087;GO:0072562;GO:0002376;GO:0030853;GO:0005576;GO:0006956;GO:0005515;GO:0006955;GO:0006508;GO:0006958;GO:0005615 | hsa:714 |  | complement C1q subcomponent subunit C isoform X1 [Pongo abelii] |
| P03951 | 13987.7555 | 11125.43813 | 0.795369788 | -0.330302332 | 0.025851695 | yes | down | GO:0004252;GO:0016020;GO:0007599;GO:0070062;GO:0016787;GO:0005615;GO:0007597;GO:0007596;GO:0031639;GO:0030193;GO:0005576;GO:0005515;GO:0005886;GO:0070009;GO:0006508;GO:0008233;GO:0008236;GO:0008201;GO:0042802;GO:0051919 | hsa:2160 | COG5640 | coagulation factor XI isoform 1 preproprotein [Homo sapiens] |
| P08185 | 120878.3283 | 93306.80625 | 0.771906822 | -0.373501387 | 0.000110361 | yes | down | GO:0005615;GO:0008289;GO:0010951;GO:0008211;GO:0005576;GO:0004867;GO:0005496;GO:0070062 | hsa:866 | COG4826 | corticosteroid-binding globulin precursor [Homo sapiens] |
| P0DJI8 | 37156.06125 | 28121.24417 | 0.756841366 | -0.401937151 | 0.033612132 | yes | down | GO:0000187;GO:0042056;GO:0019221;GO:0001664;GO:0034364;GO:0048246;GO:0048247;GO:0044267;GO:0050728;GO:0050708;GO:0005615;GO:0071682;GO:0045785;GO:0007186;GO:0030593;GO:0045087;GO:0005881;GO:0006953;GO:0050918;GO:0008201;GO:0030168;GO:0070062;GO:0050716;GO:0050715;GO:0006898;GO:0007204;GO:0005576 | hsa:6288 |  | RecName: Full=Serum amyloid A-1 protein; Short=SAA; Contains: RecName: Full=Amyloid protein A; AltName: Full=Amyloid fibril protein AA; Contains: RecName: Full=Serum amyloid protein A(2-104); Contains: RecName: Full=Serum amyloid protein A(3-104); Contains: RecName: Full=Serum amyloid protein A(2-103); Contains: RecName: Full=Serum amyloid protein A(2-102); Contains: RecName: Full=Serum amyloid protein A(4-101); Flags: Precursor |
| P19320 | 4600.265593 | 2930.232184 | 0.636970219 | -0.650702172 | 0.039434091 | yes | down | GO:0009897;GO:0034113;GO:0005783;GO:0005794;GO:0035094;GO:0030175;GO:0060945;GO:0005902;GO:0050901;GO:0060326;GO:0010043;GO:0032496;GO:0001666;GO:0019221;GO:0045177;GO:0002102;GO:0010212;GO:0005615;GO:0060384;GO:0071356;GO:0016021;GO:0016020;GO:0050839;GO:0035584;GO:1904646;GO:0140039;GO:0022614;GO:0005178;GO:0042383;GO:0009308;GO:0030183;GO:0098609;GO:0007584;GO:0008131;GO:0045471;GO:0007159;GO:0005887;GO:0005886;GO:0007155;GO:0002526;GO:0007157;GO:0042102;GO:0009986;GO:0055114;GO:0070062;GO:0007160;GO:0050776;GO:0007568;GO:0060333;GO:0002544;GO:0005769;GO:0030198;GO:0035924;GO:0071065 | hsa:7412 |  | vascular cell adhesion protein 1 isoform a precursor [Homo sapiens] |
| P26038 | 7080.574278 | 3266.40165 | 0.461318746 | -1.116164175 | 0.003524202 | yes | down | GO:0005200;GO:0019899;GO:0030175;GO:0050900;GO:0010628;GO:0005925;GO:0061028;GO:0005737;GO:0071803;GO:0045177;GO:0042995;GO:0070062;GO:1902115;GO:0043209;GO:0005634;GO:0042098;GO:0072562;GO:0003779;GO:2000401;GO:0035722;GO:0022612;GO:0048471;GO:2000643;GO:0022614;GO:1903364;GO:0005856;GO:0005515;GO:0016324;GO:0009986;GO:0071944;GO:0031982;GO:0071394;GO:0016323;GO:0019901;GO:0008361;GO:0008360;GO:0045198;GO:0001771;GO:0005886;GO:0016020;GO:0050839;GO:0072678;GO:0005615;GO:0031528;GO:0007010;GO:0071437;GO:0005829;GO:0008092;GO:0005902;GO:0003725;GO:0016032;GO:0001931;GO:0007159;GO:0005102;GO:0070489;GO:0031143;GO:1902966 | hsa:4478 |  | moesin [Homo sapiens] |
| P61224 | 9703.622615 | 5872.168231 | 0.605152165 | -0.724630143 | 0.043365755 | yes | down | GO:0005811;GO:0030033;GO:0044877;GO:0007165;GO:0061028;GO:0030054;GO:0005737;GO:0070062;GO:2000114;GO:0016020;GO:0000166;GO:0070382;GO:0045955;GO:0035722;GO:0005525;GO:0008283;GO:0003924;GO:0035577;GO:0032486;GO:0005911;GO:0005886;GO:0045121;GO:0007264;GO:0005829;GO:0071320;GO:0043312;GO:0005622;GO:0019003;GO:0005515;GO:1901888;GO:0070374;GO:2000301 | hsa:5908 | COG1100 | Ras-related protein Rap-1b, partial [Bos mutus] |
| P80748 | 482747.5292 | 236511.5604 | 0.48992806 | -1.029358173 | 4.60E-05 | yes | down | GO:0038096;GO:0004252;GO:0016020;GO:0030449;GO:0050776;GO:0006898;GO:0038095;GO:0072562;GO:0002376;GO:0050900;GO:0005576;GO:0006956;GO:0002250;GO:0003823;GO:0005886;GO:0006508;GO:0006958;GO:0006955;GO:0070062 | hsa:7441 |  | hCG2040021, partial [Homo sapiens] |
| Q96HR3 | 22188.84 | 15002.38996 | 0.67612322 | -0.5646419 | 0.003515307 | yes | down | GO:0019827;GO:0030521;GO:0038023;GO:0005515;GO:0006355;GO:0005634;GO:0030518;GO:0003712;GO:0000151;GO:0005654;GO:0046966;GO:0045893;GO:0006367;GO:0016567;GO:0016592;GO:0030374;GO:0061630;GO:0042809;GO:0006351 | hsa:90390 |  | mediator of RNA polymerase II transcription subunit 30 isoform 1 [Homo sapiens] |
| Q9Y5Y7 | 11422.03071 | 8747.742478 | 0.765865782 | -0.384836513 | 0.026681148 | yes | down | GO:0038023;GO:0070062;GO:0005540;GO:0016021;GO:0016020;GO:0009611;GO:0007160;GO:0004888;GO:0071944;GO:0005515;GO:0005887;GO:0005886;GO:0007155;GO:0030214;GO:0009653;GO:0006027 | hsa:10894 |  | lymphatic vessel endothelial hyaluronic acid receptor 1 precursor [Homo sapiens] |
| A0A075B6K2 | 50602.50513 | 60776.27556 | 1.201052703 | 0.264299459 | 0.619911913 | no | up | GO:0016020;GO:0005615;GO:0006955;GO:0002376;GO:0002377;GO:0005576;GO:0002250;GO:0003823;GO:0005886 | hsa:7441 |  | hCG2041210, partial [Homo sapiens] |
| A0A075B6K5 | 17224.64667 | 21335.71587 | 1.238673645 | 0.308796128 | 0.642519637 | no | up | GO:0016020;GO:0005615;GO:0006955;GO:0002376;GO:0002377;GO:0005576;GO:0002250;GO:0003823;GO:0005886 | hsa:7441 |  | RecName: Full=Immunoglobulin lambda variable 3-9; Flags: Precursor |
| A0A075B6S9 | 4383.2855 | 5550.555533 | 1.266300252 | 0.340619522 | 0.468318336 | no | up | GO:0002377;GO:0005615;GO:0006955 | hsa:7441 |  | IGKV1D-37 isoform 1, partial [Pan troglodytes] |
| A0A087X1L8 | 2061.326993 | 2639.587619 | 1.280528333 | 0.356739175 | 0.072985964 | no | up | GO:0016021;GO:0016020 | hsa:102723996;hsa:23308 | | ICOS ligand isoform c precursor [Homo sapiens] |
| A0A0B4J1V2 | 6906.333455 | 8304.0655 | 1.202384095 | 0.265897831 | 0.594404282 | no | up | GO:0009897;GO:0050853;GO:0045087;GO:0016020;GO:0006910;GO:0006911;GO:0002376;GO:0005576;GO:0002250;GO:0003823;GO:0005886;GO:0072562;GO:0042571;GO:0006958;GO:0050871;GO:0034987;GO:0042742 | hsa:102723407 | | RecName: Full=Immunoglobulin heavy variable 2-26; Flags: Precursor |
| A0A0B4J231 | 10631262.05 | 13135041 | 1.23551098 | 0.305107832 | 0.093209086 | no | up | GO:0009897;GO:0050853;GO:0070062;GO:0045087;GO:0006910;GO:0006911;GO:0042742;GO:0003823;GO:0034987;GO:0042571;GO:0006958;GO:0050871 | hsa:100423062 | | immunoglobulin lambda-3 surrogate light chain [Homo sapiens] |
| A0A0C4DH32 | 64458.94955 | 79619.69304 | 1.235199978 | 0.304744632 | 0.095708877 | no | up | GO:0009897;GO:0050853;GO:0045087;GO:0016020;GO:0006910;GO:0006911;GO:0002376;GO:0005576;GO:0002250;GO:0003823;GO:0005886;GO:0072562;GO:0042571;GO:0006958;GO:0050871;GO:0034987;GO:0042742 | hsa:102723407 | | RecName: Full=Immunoglobulin heavy variable 3-20; Flags: Precursor |
| A0A2R8Y7X9 | 29958.3093 | 44102.83375 | 1.472140277 | 0.557915149 | 0.068662503 | no | up | GO:0005344;GO:0019825;GO:0020037;GO:0005833;GO:0046872;GO:0015671 | hsa:3048 | COG1018 | hemoglobin subunit gamma-2 [Pan troglodytes] |
| A0A5H1ZRQ7 | 16360.20347 | 19808.58905 | 1.210778893 | 0.275935431 | 0.197834495 | no | up |  | hsa:100423062 | | RecName: Full=Immunoglobulin lambda constant 7; AltName: Full=Ig lambda-7 chain C region |
| C9JXI5 | 76099.971 | 100495.5716 | 1.32057306 | 0.40116412 | 0.394411608 | no | up | GO:0090263;GO:0031410;GO:0016021;GO:0016020;GO:0005886 | hsa:130612 |  | transmembrane protein 198 [Macaca mulatta] |
| D6RE82 | 73169.90329 | 2667021.277 | 36.44970346 | 5.187835172 | 0.255344617 | no | up | GO:0030688;GO:0005730;GO:0005634;GO:0030687;GO:0006364;GO:0003723 | hsa:8568 |  | PREDICTED: ribosomal RNA processing protein 1 homolog A [Callithrix jacchus] |
| H0YJW9 | 782399.1917 | 939131.4021 | 1.200322562 | 0.263422152 | 0.109297613 | no | up |  | hsa:7448 |  | vitronectin, partial [Homo sapiens] |
| I3L1J2 | 6444.674286 | 9190.627538 | 1.426080998 | 0.512055926 | 0.08675887 | no | up | GO:0009897;GO:0034332;GO:0016339;GO:0007043;GO:0044325;GO:0005923;GO:0030054;GO:0050728;GO:0000902;GO:0043184;GO:2000114;GO:0016020;GO:0045766;GO:0005509;GO:0042803;GO:0008285;GO:0016342;GO:0098609;GO:0009986;GO:0008013;GO:0019903;GO:0005913;GO:0005911;GO:0005886;GO:0007155;GO:0044331;GO:0007156;GO:0005102;GO:0007179;GO:0008092;GO:0001955;GO:0045296;GO:1903142;GO:0016021 | hsa:1003 |  | cadherin 5, type 2 preproprotein variant, partial [Homo sapiens] |
| P04040 | 4669.2838 | 19046.50073 | 4.079105394 | 2.028252783 | 0.277220428 | no | up | GO:0016491;GO:0005829;GO:0016020;GO:0005782;GO:0020027;GO:0033189;GO:0051289;GO:0009314;GO:0005886;GO:0019899;GO:0000302;GO:1904813;GO:0009060;GO:0005739;GO:0004601;GO:0009650;GO:0032868;GO:0001666;GO:0055093;GO:0001649;GO:0005783;GO:0050661;GO:0005778;GO:0009636;GO:0070062;GO:0005615;GO:0033591;GO:0005777;GO:0001822;GO:0051262;GO:0006979;GO:0098869;GO:0043066;GO:0046686;GO:0005758;GO:0034774;GO:0046872;GO:0014823;GO:0042802;GO:0042803;GO:0004046;GO:0005794;GO:0005925;GO:0010288;GO:0020037;GO:0006641;GO:0009411;GO:0006625;GO:0016209;GO:0032355;GO:0045471;GO:0014068;GO:0001657;GO:0071363;GO:0034599;GO:0009642;GO:0055114;GO:0008203;GO:0051092;GO:0016684;GO:0007568;GO:0043231;GO:0042542;GO:0070542;GO:0042493;GO:0043312;GO:0032088;GO:0005764;GO:0051781;GO:0042744;GO:0033197;GO:0010193;GO:0004096;GO:0005576;GO:0005102;GO:0014854;GO:0080184 | hsa:847 | COG0753 | catalase [Homo sapiens] |
| P05109 | 6087.032125 | 8955.786278 | 1.471289471 | 0.55708112 | 0.249998057 | no | up | GO:0045087;GO:0019730;GO:0030307;GO:0032602;GO:0043312;GO:0050786;GO:0008270;GO:0014002;GO:0010043;GO:0032496;GO:0005737;GO:0001816;GO:0045111;GO:0050729;GO:0005615;GO:0005634;GO:0016020;GO:0051493;GO:0050727;GO:0005509;GO:0002224;GO:2001244;GO:0034774;GO:0046872;GO:0035662;GO:0008017;GO:0005856;GO:0030593;GO:0006935;GO:0006919;GO:0032119;GO:0006914;GO:0006915;GO:0018119;GO:0042060;GO:0050832;GO:0045471;GO:0070488;GO:0006954;GO:0005886;GO:0002526;GO:0002523;GO:0070062;GO:0051092;GO:0050544;GO:0005829;GO:0002793;GO:0002544;GO:0002376;GO:0005576;GO:0005515;GO:0042742 | hsa:6279 |  | protein S100-A8 isoform d [Homo sapiens] |
| P0DTE1 | 18878.19 | 50622.72045 | 2.681545236 | 1.423064591 | 0.165796424 | no | up |  | hsa:102723407 | | immunoglobulin heavy chain variable region, partial [Homo sapiens] |
| P32119 | 12268.95304 | 15456.33191 | 1.259792246 | 0.333185837 | 0.2316353 | no | up | GO:0000187;GO:0045581;GO:0042744;GO:0008379;GO:0032496;GO:0032088;GO:0042981;GO:0034599;GO:0002536;GO:0016491;GO:0005737;GO:0010310;GO:0070062;GO:0042098;GO:0043066;GO:0048538;GO:2001240;GO:0016209;GO:0045454;GO:0006979;GO:0055114;GO:0048872;GO:0045321;GO:0005829;GO:0005623;GO:0031665;GO:0019430;GO:0005515;GO:0051920;GO:0004601;GO:0030194 | hsa:7001 | COG0450 | peroxiredoxin-2 [Homo sapiens] |
| P59665 | 117345.0063 | 200544.0638 | 1.70901234 | 0.773162814 | 0.244028406 | no | up | GO:0071222;GO:0019730;GO:0019731;GO:0035578;GO:0051673;GO:0042742;GO:0050829;GO:0061844;GO:0005615;GO:0030520;GO:0051852;GO:0044657;GO:0002227;GO:0042803;GO:0010818;GO:0005796;GO:0006935;GO:0006952;GO:0050830;GO:0062023;GO:0006955;GO:0070062;GO:0050832;GO:0051607;GO:0031640;GO:0043312;GO:0005576;GO:0042832 | hsa:1667;hsa:728358;hsa:1668 | | neutrophil defensin 1 preproprotein [Homo sapiens] |
| P68871 | 4757867.333 | 6159428.625 | 1.294577632 | 0.372481482 | 0.183870169 | no | up | GO:0005344;GO:0007596;GO:0015701;GO:1904813;GO:0045429;GO:0042744;GO:0008217;GO:0010942;GO:0050880;GO:0005615;GO:0071682;GO:0072562;GO:0005833;GO:0098869;GO:0046872;GO:0015671;GO:0070293;GO:0030185;GO:1904724;GO:0020037;GO:0031721;GO:0031720;GO:0051291;GO:0070062;GO:0019825;GO:0042542;GO:0030492;GO:0005829;GO:0006898;GO:0043312;GO:0070527;GO:0043177;GO:0005576;GO:0005515;GO:0031838;GO:0004601 | hsa:3043 | COG1018 | PREDICTED: hemoglobin subunit beta [Gorilla gorilla gorilla] |
| P69905 | 554854.3042 | 721367.0333 | 1.300101717 | 0.3786245 | 0.167848277 | no | up | GO:0005344;GO:0022627;GO:0015701;GO:0042744;GO:0010942;GO:0005615;GO:0071682;GO:0016020;GO:0072562;GO:0005833;GO:0098869;GO:0005506;GO:0046872;GO:0015671;GO:0020037;GO:0031720;GO:0051291;GO:0070062;GO:0019825;GO:0042542;GO:0005829;GO:0006898;GO:0005576;GO:0005515;GO:0031838;GO:0004601 | hsa:3040;hsa:3039 | COG1018 | TPA: globin C1 [Homo sapiens] |
| Q15848 | 11252.50825 | 14125.85138 | 1.255351346 | 0.3280912 | 0.141298736 | no | up | GO:0034115;GO:0005783;GO:0010804;GO:0050728;GO:0009967;GO:0009617;GO:0030853;GO:0043124;GO:0033034;GO:0032720;GO:0043123;GO:2000481;GO:0014823;GO:0072659;GO:0010875;GO:0045650;GO:0071320;GO:0031667;GO:0050731;GO:1904753;GO:0045715;GO:0005125;GO:0033691;GO:0031953;GO:0010906;GO:0046326;GO:0070994;GO:0045721;GO:0010739;GO:0007623;GO:2000467;GO:2000279;GO:0070208;GO:0071872;GO:0019395;GO:0042304;GO:0006006;GO:0034383;GO:0006635;GO:2000478;GO:0050805;GO:0043407;GO:0001666;GO:0010642;GO:0050765;GO:0005581;GO:0032270;GO:0042802;GO:0042803;GO:0009986;GO:0050873;GO:0045892;GO:0046888;GO:0045599;GO:0007584;GO:0120162;GO:0120163;GO:0070543;GO:0042493;GO:1900121;GO:0005515;GO:0005102;GO:0045860;GO:0070373;GO:0045923;GO:0051384;GO:0042593;GO:0035690;GO:0032869;GO:0006091;GO:0005615;GO:0051260;GO:2000590;GO:0010745;GO:0010469;GO:1904706;GO:0005179;GO:0030336;GO:2000534;GO:0034612;GO:0090317;GO:0045471;GO:0009744;GO:0032991;GO:0045776;GO:0009749;GO:0071639;GO:0005576;GO:2000584;GO:0032757;GO:0001934 | hsa:9370 |  | TPA: adiponectin D [Homo sapiens] |
| Q9Y490 | 15657.33965 | 36324.60655 | 2.319973083 | 1.214108067 | 0.284722791 | no | up | GO:0005200;GO:0017166;GO:0007043;GO:0005925;GO:0030054;GO:0005856;GO:0001726;GO:0070062;GO:0002576;GO:0016020;GO:0005737;GO:0003779;GO:0005576;GO:0033622;GO:0001786;GO:0005178;GO:0005515;GO:0006936;GO:0009986;GO:0051015;GO:0036498;GO:0030274;GO:0005886;GO:0007155;GO:0042995;GO:0032587;GO:0007016;GO:0035091;GO:0005829;GO:0030866;GO:0045296;GO:0070527;GO:0007044;GO:0016032;GO:0044877;GO:0007229 | hsa:7094 |  | talin-1 [Homo sapiens] |
| A0A075B6R2 | 189176.7156 | 185933.5563 | 0.982856456 | -0.024947366 | 0.915476278 | no | no change | GO:0009897;GO:0050853;GO:0045087;GO:0016020;GO:0006910;GO:0006911;GO:0002376;GO:0005576;GO:0002250;GO:0003823;GO:0005886;GO:0072562;GO:0042571;GO:0006958;GO:0050871;GO:0034987;GO:0042742 | hsa:102724971 | | immunoglobulin heavy chain VDJ region, partial [Homo sapiens] |
| A0A075B6R9 | 150302.1738 | 142817.3904 | 0.950201763 | -0.073694212 | 0.718058235 | no | no change | GO:0002377;GO:0005615;GO:0006955 | hsa:7441 |  | RecName: Full=Immunoglobulin kappa variable 2-24; Flags: Precursor |
| A0A075B6S5 | 62380.625 | 55566.12583 | 0.890759364 | -0.166892351 | 0.287518845 | no | no change | GO:0016020;GO:0005615;GO:0006955;GO:0002376;GO:0002377;GO:0005576;GO:0002250;GO:0003823;GO:0005886 | hsa:7441 |  | monoclonal IgM antibody light chain [Homo sapiens] |
| A0A075B7D0 | 316636.0583 | 280345.8871 | 0.885388381 | -0.175617654 | 0.517392707 | no | no change | GO:0009897;GO:0050853;GO:0045087;GO:0006910;GO:0006911;GO:0042742;GO:0003823;GO:0034987;GO:0042571;GO:0006958;GO:0050871;GO:0072562 | hsa:102723407 | | hCG1728627 [Homo sapiens] |
| A0A075B7F0 | 28762.55174 | 26816.23604 | 0.932331606 | -0.101084921 | 0.738734226 | no | no change | GO:0009897;GO:0050853;GO:0045087;GO:0006910;GO:0006911;GO:0042742;GO:0003823;GO:0034987;GO:0042571;GO:0006958;GO:0050871;GO:0072562 | hsa:102723407 | | IGHV3-13 isoform 1, partial [Pan troglodytes] |
| A0A087WSY4 | 26257.91982 | 22516.963 | 0.857530343 | -0.221740374 | 0.309548022 | no | no change | GO:0009897;GO:0050853;GO:0045087;GO:0016020;GO:0006910;GO:0006911;GO:0002376;GO:0005576;GO:0002250;GO:0003823;GO:0005886;GO:0072562;GO:0042571;GO:0006958;GO:0050871;GO:0034987;GO:0042742 | hsa:102723407 | | RecName: Full=Immunoglobulin heavy variable 4-30-2; Flags: Precursor |
| A0A087WSZ0 | 43621.46442 | 47885.78267 | 1.097757338 | 0.134559179 | 0.767437161 | no | no change | GO:0016020;GO:0005615;GO:0006955;GO:0002376;GO:0002377;GO:0005576;GO:0002250;GO:0003823;GO:0005886 | | | RecName: Full=Immunoglobulin kappa variable 1D-8; Flags: Precursor |
| A0A087WWT3 | 66017.89583 | 72358.50375 | 1.096043775 | 0.132305419 | 0.23743826 | no | no change | GO:0005794;GO:0005615;GO:0005783 | hsa:213 |  | ALB protein [Homo sapiens] |
| A0A087X1J7 | 51128.205 | 43706.02333 | 0.854831953 | -0.226287259 | 0.076394981 | no | no change | GO:0008430;GO:0005615;GO:0051289;GO:0006979;GO:0098869;GO:0055114;GO:0004601;GO:0004602;GO:0016491 | hsa:2878 | COG0386 | glutathione peroxidase 3 isoform 1 precursor [Homo sapiens] |
| A0A096LPE2 | 623219.7375 | 600397.2708 | 0.963379743 | -0.053823506 | 0.669253914 | no | no change | GO:0042056;GO:0034364;GO:0005615;GO:0006953;GO:0005576;GO:0060326;GO:0050918;GO:0070062 | hsa:6291;hsa:100528017 | | SAA2-SAA4 protein precursor [Homo sapiens] |
| A0A0A0MRJ7 | 20650.47167 | 23400.44375 | 1.133167519 | 0.180361154 | 0.126484195 | no | no change | GO:0048208;GO:0044267;GO:1903561;GO:0006888;GO:0005615;GO:0031093;GO:0008015;GO:0002576;GO:0007596;GO:0033116;GO:0005788;GO:0030134;GO:0046872;GO:0005576;GO:0005886;GO:0016020;GO:0005507;GO:0043687;GO:0000139 | hsa:2153 |  | coagulation factor V preproprotein [Homo sapiens] |
| A0A0A0MS15 | 206527.4629 | 203933.7596 | 0.987441364 | -0.018233015 | 0.933704474 | no | no change | GO:0009897;GO:0050853;GO:0045087;GO:0016020;GO:0006910;GO:0006911;GO:0002376;GO:0005576;GO:0002250;GO:0003823;GO:0005886;GO:0072562;GO:0042571;GO:0006958;GO:0050871;GO:0034987;GO:0042742 | hsa:102723407 | | RecName: Full=Immunoglobulin heavy variable 3-49; Flags: Precursor |
| A0A0A0MS51 | 259748.9917 | 246227.2667 | 0.947943109 | -0.077127617 | 0.413367405 | no | no change | GO:0051127;GO:0015629;GO:1902174;GO:0030155;GO:0030041;GO:0042989;GO:0045159;GO:1990000;GO:0005925;GO:1903923;GO:0001726;GO:0071801;GO:0005615;GO:0045010;GO:1903903;GO:0043209;GO:0005634;GO:1903909;GO:0030478;GO:0005509;GO:0048471;GO:1903906;GO:0097284;GO:2001269;GO:0060271;GO:0030027;GO:0006915;GO:0051016;GO:0051015;GO:0051014;GO:0045471;GO:0045335;GO:0002102;GO:0005886;GO:0014003;GO:0032991;GO:0006911;GO:0048015;GO:0051693;GO:0046597;GO:0090527;GO:0007568;GO:0051593;GO:0005829;GO:0071346;GO:0042246;GO:0031648;GO:0016528;GO:0014891;GO:0097017;GO:0071276 | hsa:2934 |  | gelsolin isoform d [Homo sapiens] |
| A0A0A0MT36 | 149523.145 | 129700.0317 | 0.867424449 | -0.205189988 | 0.576047419 | no | no change | GO:0016020;GO:0005615;GO:0006955;GO:0002376;GO:0002377;GO:0005576;GO:0002250;GO:0003823;GO:0005886 | hsa:7441 |  | RecName: Full=Immunoglobulin kappa variable 6D-21; Flags: Precursor |
| A0A0B4J1V1 | 79740.12053 | 78668.15895 | 0.98655681 | -0.019525965 | 0.960934779 | no | no change | GO:0009897;GO:0050853;GO:0045087;GO:0016020;GO:0006910;GO:0006911;GO:0002376;GO:0005576;GO:0002250;GO:0003823;GO:0005886;GO:0072562;GO:0042571;GO:0006958;GO:0050871;GO:0034987;GO:0042742 | hsa:102723407 | | RecName: Full=Immunoglobulin heavy variable 3-21; Flags: Precursor |
| A0A0B4J1X5 | 66294.06174 | 59118.22 | 0.891757398 | -0.165276816 | 0.457013774 | no | no change | GO:0009897;GO:0050853;GO:0045087;GO:0016020;GO:0006910;GO:0006911;GO:0002376;GO:0005576;GO:0002250;GO:0003823;GO:0005886;GO:0072562;GO:0042571;GO:0006958;GO:0050871;GO:0034987;GO:0042742 | hsa:102723407 | | immunoglobulin heavy chain VH3, partial [Homo sapiens] |
| A0A0B4J1X8 | 89254.54208 | 94104.9375 | 1.054343402 | 0.076344832 | 0.719609252 | no | no change | GO:0009897;GO:0050853;GO:0045087;GO:0016020;GO:0006910;GO:0006911;GO:0002376;GO:0005576;GO:0002250;GO:0003823;GO:0005886;GO:0072562;GO:0042571;GO:0006958;GO:0050871;GO:0034987;GO:0042742 | hsa:102723407 | | RecName: Full=Immunoglobulin heavy variable 3-43; Flags: Precursor |
| A0A0C4DGZ8 | 17803.04204 | 15971.72671 | 0.897134696 | -0.156603487 | 0.488845191 | no | no change | GO:0016021;GO:0016020 | hsa:2811 | COG4886 | glycoprotein Ib (platelet), alpha polypeptide [Homo sapiens] |
| A0A0C4DH21 | 40072.155 | 37914.02208 | 0.946143827 | -0.079868585 | 0.682978001 | no | no change |  | hsa:10877 |  | complement factor H-related protein 4 [Homo sapiens] |
| A0A0C4DH24 | 34211.68261 | 34415.31354 | 1.005952088 | 0.008561593 | 0.975470245 | no | no change | GO:0016020;GO:0005615;GO:0006955;GO:0002376;GO:0002377;GO:0005576;GO:0002250;GO:0003823;GO:0005886 | hsa:7441 |  | RecName: Full=Immunoglobulin kappa variable 6-21; Flags: Precursor |
| A0A0C4DH29 | 36525.98045 | 32287.37333 | 0.883956377 | -0.177952921 | 0.312243222 | no | no change | GO:0009897;GO:0050853;GO:0045087;GO:0016020;GO:0006910;GO:0006911;GO:0002376;GO:0005576;GO:0002250;GO:0003823;GO:0005886;GO:0072562;GO:0042571;GO:0006958;GO:0050871;GO:0034987;GO:0042742 | hsa:102723407 | | immunoglobulin heavy chain variable region, partial [Homo sapiens] |
| A0A0C4DH31 | 31057.25 | 31109.19917 | 1.00167269 | 0.002411166 | 0.983373507 | no | no change | GO:0009897;GO:0050853;GO:0045087;GO:0016020;GO:0006910;GO:0006911;GO:0002376;GO:0005576;GO:0002250;GO:0003823;GO:0005886;GO:0072562;GO:0042571;GO:0006958;GO:0050871;GO:0034987;GO:0042742 | hsa:102723407 | | immunoglobulin heavy chain variable region, partial [Homo sapiens] |
| A0A0C4DH33 | 10923.81908 | 10801.41761 | 0.988794993 | -0.016256658 | 0.95201707 | no | no change | GO:0009897;GO:0050853;GO:0045087;GO:0016020;GO:0006910;GO:0006911;GO:0002376;GO:0005576;GO:0002250;GO:0003823;GO:0005886;GO:0072562;GO:0042571;GO:0006958;GO:0050871;GO:0034987;GO:0042742 | hsa:102723407 | | immunoglobulin heavy chain variable region, partial [Homo sapiens] |
| A0A0C4DH36 | 69261.3875 | 62414.52333 | 0.901144571 | -0.150169518 | 0.199968277 | no | no change | GO:0009897;GO:0050853;GO:0045087;GO:0006910;GO:0006911;GO:0042742;GO:0003823;GO:0034987;GO:0042571;GO:0006958;GO:0050871;GO:0072562 | hsa:102723407 | | immunoglobulin heavy chain variable gene IGHV3-38, partial [Homo sapiens] |
| A0A0C4DH39 | 26098.06167 | 28223.76042 | 1.081450446 | 0.112967559 | 0.728975281 | no | no change | GO:0009897;GO:0050853;GO:0045087;GO:0016020;GO:0006910;GO:0006911;GO:0002376;GO:0005576;GO:0002250;GO:0003823;GO:0005886;GO:0072562;GO:0042571;GO:0006958;GO:0050871;GO:0034987;GO:0042742 | hsa:102723407 | | immunoglobulin heavy chain variable region, partial [Homo sapiens] |
| A0A0C4DH55 | 9231465.583 | 8138870.833 | 0.881644497 | -0.181731055 | 0.164260168 | no | no change | GO:0016020;GO:0005615;GO:0006955;GO:0002376;GO:0002377;GO:0005576;GO:0002250;GO:0003823;GO:0005886 | hsa:29802 |  | RecName: Full=Immunoglobulin kappa variable 3D-7; Flags: Precursor |
| A0A0C4DH73 | 86163.89625 | 84374.96458 | 0.979238037 | -0.030268497 | 0.865425143 | no | no change | GO:0038096;GO:0004252;GO:0016020;GO:0030449;GO:0005615;GO:0006898;GO:0038095;GO:0050900;GO:0002376;GO:0002377;GO:0050776;GO:0005576;GO:0006956;GO:0002250;GO:0003823;GO:0005886;GO:0006508;GO:0006958;GO:0006955 | hsa:7441 |  | immunoglobulin light chain variable region, partial [Homo sapiens] |
| A0A0G2JPR0 | 414208.8667 | 441040.4542 | 1.064777917 | 0.090552556 | 0.598098736 | no | no change | GO:0004866;GO:0005576;GO:0006956;GO:0006954;GO:0010951;GO:0005615 | hsa:100293534;hsa:110384692;hsa:720;hsa:721 | | complement C4A (Rodgers blood group)-like preproprotein [Homo sapiens] |
| A0A0G2JSC0 | 41204.08064 | 49210.14461 | 1.194302696 | 0.256168535 | 0.412953199 | no | no change |  | hsa:7441 |  | Lambda-V immunoglobulin light chain variable domain precursor, partial [Homo sapiens] |
| A0A0J9YX35 | 55634.76146 | 62925.73136 | 1.131050619 | 0.177663497 | 0.453624241 | no | no change | GO:0016020;GO:0002376;GO:0005576;GO:0002250;GO:0003823;GO:0005886 | hsa:102723407 | | RecName: Full=Immunoglobulin heavy variable 3-64D; Flags: Precursor |
| A0A0J9YXX1 | 222593.1875 | 197626.7463 | 0.887838251 | -0.171631229 | 0.374590036 | no | no change | GO:0016020;GO:0002376;GO:0005576;GO:0002250;GO:0003823;GO:0005886 | hsa:102724971 | | RecName: Full=Immunoglobulin heavy variable 5-10-1; Flags: Precursor |
| A0A0J9YY99 | 222880.8396 | 212404.6646 | 0.95299652 | -0.069457148 | 0.916715777 | no | no change |  | hsa:102723407 | | immunoglobulin heavy chain VDJ region, partial [Homo sapiens] |
| A0A0S2Z4L3 | 213795.2708 | 199108.0708 | 0.931302503 | -0.102678239 | 0.373867671 | no | no change | GO:0005576;GO:0005509;GO:0030195 | hsa:5627 |  | vitamin K-dependent protein S isoform 1 precursor [Homo sapiens] |
| A0A140T8Y3 | 7673.41365 | 6675.028227 | 0.869890316 | -0.201094591 | 0.192190366 | no | no change | GO:0005201;GO:0062023;GO:0030199;GO:0030198;GO:0031012 | hsa:7148 |  | tenascin-X isoform 1 precursor [Homo sapiens] |
| A0A182DWH7 | 30627.1725 | 32971.73625 | 1.076551753 | 0.106417676 | 0.252849684 | no | no change | GO:0008430 | hsa:6414 |  | Selenoprotein P, plasma, 1 [Homo sapiens] |
| A0A1W2PQU7 | 81805.52458 | 87935.20958 | 1.074929964 | 0.104242665 | 0.381915978 | no | no change | GO:1904714;GO:0005737;GO:0045111;GO:0061564;GO:0045109;GO:0060291;GO:0016020;GO:0044297;GO:0060020;GO:0005198;GO:0051580;GO:0005883;GO:0005882;GO:0010977;GO:0030198;GO:0014002;GO:0097450;GO:0010625;GO:0031102 | hsa:2670 |  | glial fibrillary acidic protein [Mus musculus] |
| A0A286YEY4 | 577230.3292 | 536669.5125 | 0.929732007 | -0.105113172 | 0.458606092 | no | no change | GO:0016021;GO:0016020 | hsa:100423062 | | unnamed protein product [Homo sapiens] |
| A0A2Q2TTZ9 | 269566.5879 | 256111.95 | 0.950087887 | -0.07386712 | 0.721466906 | no | no change |  | hsa:7441 |  | IGKV1D-33 isoform 2, partial [Pan troglodytes] |
| A0A2R8Y3M9 | 151192.2096 | 144395.1796 | 0.955043782 | -0.066361224 | 0.43318993 | no | no change | GO:0004252;GO:0006898;GO:0005044;GO:0016020;GO:0016042;GO:0005576;GO:0005509;GO:0006508;GO:0004623 | hsa:81579;hsa:3426 | COG5640 | complement factor I isoform X2 [Homo sapiens] |
| A0A3B3ISR2 | 264784.65 | 277518.2125 | 1.048090259 | 0.067762964 | 0.546840549 | no | no change | GO:0004252;GO:0005615;GO:0045087;GO:0031638;GO:0005509;GO:0006958 | hsa:715 | COG5640 | complement C1r subcomponent isoform 1 preproprotein [Homo sapiens] |
| A0A4W8ZXM2 | 872953.3875 | 809666.7421 | 0.927502835 | -0.108576403 | 0.589027055 | no | no change |  | hsa:102723407 | | immunoglobulin heavy chain variable region, partial [Homo sapiens] |
| A0A5H1ZRS2 | 792074.7708 | 746996.4417 | 0.943088291 | -0.084535255 | 0.796187233 | no | no change |  |  |  | immunoglobulin kappa chain variable region, partial [Homo sapiens] |
| A6XND0 | 32510.07375 | 29247.19583 | 0.899634866 | -0.15258852 | 0.322293192 | no | no change | GO:0005520;GO:0001558;GO:0005576 | hsa:3486 |  | insulin-like growth factor binding protein 3 [Homo sapiens] |
| B0YIW2 | 1664405.242 | 1884424.854 | 1.132191132 | 0.179117528 | 0.489848597 | no | no change | GO:0034375;GO:0042627;GO:0034371;GO:0006869;GO:0034379;GO:0034378;GO:0051005;GO:0042157;GO:0005576;GO:0048261;GO:0001523;GO:0055102;GO:0070062;GO:0005543;GO:0045833;GO:0010897;GO:0007186;GO:0062023;GO:0033700;GO:0034361;GO:0034363;GO:0070328;GO:0034366;GO:0006641;GO:0060621;GO:0030234;GO:0070653;GO:0008289;GO:0010916;GO:0042632;GO:0032489;GO:0043691;GO:0005615;GO:0015485;GO:0034382;GO:0050995;GO:0019433;GO:0033344;GO:0010989;GO:0010987;GO:0005769;GO:0045717;GO:0010903 | hsa:345 |  | apolipoprotein C-III precursor variant 1 [Homo sapiens] |
| B1AHL2 | 16703.32688 | 14313.19365 | 0.856906756 | -0.222789868 | 0.092922053 | no | no change | GO:0016504;GO:0005576;GO:0005509;GO:0010952;GO:0030198 | hsa:2192 |  | FBLN1 isoform 5 [Pongo abelii] |
| B4E1Z4 | 531061.825 | 496005.725 | 0.933988665 | -0.098523053 | 0.272155682 | no | no change | GO:0004252;GO:0030449;GO:0070062;GO:0016787;GO:0072562;GO:0001848;GO:0005576;GO:0006956;GO:0006957;GO:0005886;GO:0006508;GO:0008233;GO:0008236;GO:0005615 | hsa:629 | COG5640 | unnamed protein product [Homo sapiens] |
| B7ZKJ8 | 489822.0417 | 482377.3375 | 0.984801206 | -0.022095566 | 0.745235829 | no | no change | GO:0005737;GO:0030212;GO:0034097;GO:0004867;GO:0006953;GO:0005886;GO:0010951 | hsa:3700 | COG2304 | ITIH4 protein [Homo sapiens] |
| C9JC84 | 5372966.625 | 5148328.417 | 0.958191029 | -0.061614789 | 0.491528994 | no | no change | GO:0051258;GO:0005102;GO:0007596;GO:0005577;GO:0030168 | hsa:2266 |  | FGG isoform 6 [Pan troglodytes] |
| C9JF17 | 820149.85 | 919032.8292 | 1.120566966 | 0.164228868 | 0.201551496 | no | no change | GO:0022626;GO:0005783;GO:0006869;GO:0042493;GO:0048678;GO:0030425;GO:0000302;GO:0001525;GO:0051895;GO:0010642;GO:0005737;GO:0070062;GO:0014012;GO:0005615;GO:2000405;GO:0048471;GO:0043025;GO:0005319;GO:0048662;GO:0006629;GO:2000098;GO:0008289;GO:0042308;GO:0060588;GO:0007420;GO:1900016;GO:0007568;GO:0015485;GO:0042246;GO:0071638;GO:0005576;GO:0006006 | hsa:347 | COG3040 | APOD isoform 3, partial [Pan troglodytes] |
| C9JPQ9 | 2195261.946 | 2232012.625 | 1.016740908 | 0.02395209 | 0.873340151 | no | no change | GO:0051258;GO:0005102;GO:0007596;GO:0005577;GO:0030168 | hsa:2266 |  | hypothetical protein, partial [Homo sapiens] |
| D6RAR4 | 27468.56833 | 25873.07667 | 0.941915733 | -0.086330098 | 0.462932573 | no | no change | GO:0005737;GO:0004252;GO:0005791;GO:0005615;GO:0016787;GO:0005576;GO:0006508;GO:0008233;GO:0008236 | hsa:3083 | COG5640 | hepatocyte growth factor activator isoform 1 preproprotein [Homo sapiens] |
| D6RD17 | 2585204.783 | 2865348 | 1.108364033 | 0.148431801 | 0.540569001 | no | no change |  | hsa:3512 |  | JCHAIN isoform 4, partial [Pongo abelii] |
| D6RF35 | 2574258.708 | 2487710.75 | 0.966379464 | -0.049338298 | 0.466756779 | no | no change | GO:0051180;GO:0090482;GO:0035461;GO:0005615;GO:0005499 | hsa:2638 |  | GC isoform 4 [Pan troglodytes] |
| E7END6 | 18291.93458 | 16707.21046 | 0.91336487 | -0.130736793 | 0.09512857 | no | no change | GO:0004252;GO:0016787;GO:0007596;GO:0005576;GO:0005509;GO:0006508;GO:0008233;GO:0008236 | hsa:5624 | COG5640 | vitamin K-dependent protein C isoform X5 [Homo sapiens] |
| E7EUT5 | 9330.010583 | 10620.43657 | 1.138309166 | 0.186892448 | 0.296204822 | no | no change | GO:0051287;GO:0050821;GO:0000226;GO:0097718;GO:0006096;GO:0061844;GO:0005737;GO:0004365;GO:0005634;GO:0005811;GO:0016620;GO:0051873;GO:0097452;GO:0042802;GO:0035605;GO:0035606;GO:0015630;GO:0008017;GO:0031965;GO:0050661;GO:0050832;GO:0051402;GO:0052501;GO:0005886;GO:0055114;GO:0043231;GO:0050715;GO:0005829;GO:0071346;GO:1990904;GO:0019828;GO:0017148;GO:0006417;GO:0006006 | hsa:2597 | COG0057 | GAPDH isoform 4 [Pan troglodytes] |
| E9PAQ1 | 27762.39167 | 27897.92625 | 1.004881949 | 0.007026027 | 0.936045562 | no | no change |  | hsa:5199 |  | properdin precursor [Homo sapiens] |
| E9PHK0 | 142086.1017 | 149272.3833 | 1.05057695 | 0.071181837 | 0.241885391 | no | no change | GO:0005737;GO:0036143;GO:0070062;GO:0002576;GO:0001652;GO:0030246;GO:0008201;GO:0071560;GO:0071310;GO:0001503;GO:0005615;GO:0005509;GO:0005576;GO:0031089;GO:0030282;GO:0062023;GO:0010756 | hsa:7123 |  | tetranectin isoform 1precursor [Homo sapiens] |
| F5H8B0 | 6187.351708 | 6175.647174 | 0.998108313 | -0.002731712 | 0.983400455 | no | no change | GO:0004252;GO:0016787;GO:0007596;GO:0005576;GO:0005509;GO:0006508;GO:0008233;GO:0008236 | hsa:2155 | COG5640 | coagulation factor VII isoform c precursor [Homo sapiens] |
| F8WF14 | 26920.89083 | 26077.91 | 0.968686741 | -0.0458979 | 0.695390115 | no | no change | GO:0019899;GO:0051384;GO:0005783;GO:0016787;GO:0007612;GO:0050805;GO:0050783;GO:0014016;GO:0001540;GO:0016021;GO:0016020;GO:0072562;GO:0042802;GO:0008285;GO:0004104;GO:0003824;GO:0005788;GO:0043279;GO:0051593;GO:0019695;GO:0005641;GO:0005576;GO:0033265;GO:0003990 | hsa:590 | COG2272 | unnamed protein product [Homo sapiens] |
| G3V0E5 | 5906.334542 | 5941.735708 | 1.005993763 | 0.00862136 | 0.946354722 | no | no change | GO:0009897;GO:0055037;GO:0035690;GO:0005905;GO:1990712;GO:0010008;GO:0045780;GO:1990830;GO:0070062;GO:0016021;GO:0045830;GO:0004998;GO:0048471;GO:0042803;GO:0006879;GO:0030890;GO:0030316;GO:0042102;GO:0005887;GO:0005886;GO:0016323;GO:0042470;GO:0031623;GO:0006898;GO:0003725;GO:0033570;GO:0033572;GO:0005769 | hsa:7037 | COG2234 | transferrin receptor variant, partial [Homo sapiens] |
| G3V2W1 | 10282.13542 | 9160.647292 | 0.890928481 | -0.16661847 | 0.217873011 | no | no change | GO:0010951;GO:0007596;GO:0005615;GO:0004867 | hsa:51156 | COG4826 | protein Z-dependent protease inhibitor isoform X1 [Homo sapiens] |
| G3XAP6 | 9080.6428 | 9223.943667 | 1.015780917 | 0.022589276 | 0.875808489 | no | no change | GO:0005201;GO:0060173;GO:0062023;GO:0030509;GO:0035264;GO:0050905;GO:0030500;GO:0030282;GO:0050881;GO:0010259;GO:1900047;GO:0005615;GO:0048844;GO:0036122;GO:0030198;GO:0005509;GO:0002020;GO:0002063;GO:0003417;GO:0043588;GO:0005576;GO:0014829;GO:0005178;GO:0009887;GO:1902732;GO:0009306;GO:0006915;GO:0043066;GO:0048747;GO:0006986;GO:0043395;GO:0035988;GO:0007155;GO:0097084;GO:0016485;GO:0031012;GO:0008201;GO:0070062;GO:0010260;GO:0032991;GO:0001501;GO:0035989;GO:0030199;GO:0043394;GO:0005518;GO:0070527 | hsa:1311 |  | unnamed protein product [Homo sapiens] |
| H0Y5E4 | 20060.71042 | 18186.77875 | 0.906586974 | -0.141482663 | 0.102097196 | no | no change | GO:0005540;GO:0007155;GO:0016021;GO:0016020 | hsa:960 |  | CD44 antigen isoform 8 precursor [Homo sapiens] |
| H0Y755 | 7651.50013 | 8253.877304 | 1.078726676 | 0.109329366 | 0.516196558 | no | no change | GO:0016021;GO:0016020 | hsa:2214 |  | low affinity immunoglobulin gamma Fc region receptor III-A isoform b [Homo sapiens] |
| H3BUA5 | 426551.9625 | 405591.1083 | 0.950859787 | -0.072695476 | 0.584954122 | no | no change |  | hsa:10326 |  | LOW QUALITY PROTEIN: T0061165 isoform 1, partial [Pan troglodytes] |
| I3L145 | 10979.04358 | 12641.29492 | 1.151402198 | 0.203391872 | 0.436414148 | no | no change | GO:0005496 | hsa:6462 |  | SHBG protein, partial [Homo sapiens] |
| J3KNB4 | 13648.52625 | 14444.01538 | 1.058283884 | 0.081726682 | 0.630121402 | no | no change | GO:0071224;GO:0071222;GO:0042742;GO:0050829;GO:0044130;GO:0061844;GO:0005737;GO:0042995;GO:0005615;GO:0071354;GO:0071356;GO:0016021;GO:0045766;GO:0051873;GO:0008284;GO:0045087;GO:0006952;GO:0050830;GO:0044140;GO:0001530;GO:0042581;GO:0071347;GO:0005576;GO:0001934 | hsa:820 |  | cathelicidin antimicrobial peptide [Homo sapiens] |
| J3KPA1 | 11406.30075 | 10629.90188 | 0.931932456 | -0.101702699 | 0.56047118 | no | no change | GO:0016020;GO:0016021;GO:0005576 | hsa:10321 | COG2340 | cysteine-rich secretory protein 3 isoform 3 [Homo sapiens] |
| J3KRP0 | 26132.74042 | 29721.22042 | 1.137317401 | 0.185634936 | 0.228632407 | no | no change | GO:0016787;GO:0032268;GO:0005829;GO:0004180;GO:0016805;GO:0005576;GO:0008152;GO:0006508;GO:0046872;GO:0008237 | hsa:84735 | COG0624 | Carnosine dipeptidase 1 (metallopeptidase M20 family) [Homo sapiens] |
| K7ERG9 | 9029.719 | 9928.051708 | 1.099486231 | 0.136829537 | 0.27898689 | no | no change | GO:0004252;GO:0007219;GO:0005615;GO:0016787;GO:0009617;GO:0006957;GO:0006508;GO:0008233;GO:0008236 | hsa:1675 | COG5640 | complement factor D isoform 2 precursor [Homo sapiens] |
| K7ERI9 | 2494084.083 | 2179113.75 | 0.873713025 | -0.194768596 | 0.085323969 | no | no change | GO:0005576;GO:0042157 | hsa:341 |  | apolipoprotein C-I precursor [Homo sapiens] |
| O00391 | 10590.16629 | 9566.577958 | 0.903345396 | -0.146650385 | 0.25544844 | no | no change | GO:0005788;GO:0030173;GO:0071949;GO:0016491;GO:0016971;GO:0044267;GO:0016972;GO:0045171;GO:0070062;GO:0031093;GO:0016021;GO:0016020;GO:0003756;GO:0035580;GO:0016242;GO:0005794;GO:1904724;GO:0043687;GO:0045454;GO:0055114;GO:0005615;GO:0043231;GO:0043312;GO:0000139;GO:0005576;GO:0085029;GO:0002576 | hsa:5768 |  | sulfhydryl oxidase 1 isoform a precursor [Homo sapiens] |
| O14791 | 36310.42333 | 43243.82 | 1.190947833 | 0.252110221 | 0.076989985 | no | no change | GO:0006869;GO:0005788;GO:0042157;GO:0031224;GO:0019835;GO:0044267;GO:0005615;GO:0072562;GO:0005254;GO:0045087;GO:0034361;GO:0034364;GO:0006629;GO:1902476;GO:0043687;GO:0008289;GO:0008202;GO:0008203;GO:0006898;GO:0031640;GO:0005576;GO:0005515 | hsa:8542 |  | apolipoprotein L1 isoform a precursor [Homo sapiens] |
| O43866 | 150706.8717 | 171118.175 | 1.135437111 | 0.183247801 | 0.260015331 | no | no change | GO:0005737;GO:0006898;GO:0005615;GO:0005044;GO:0009986;GO:0016020;GO:0072562;GO:0002376;GO:0005576;GO:0006954;GO:0006968;GO:0006915 | hsa:922 |  | CD5 antigen-like isoform 1 precursor [Homo sapiens] |
| O75460 | 120191.0938 | 120563.645 | 1.003099658 | 0.004464944 | 0.983528773 | no | no change | GO:0005739;GO:0004540;GO:0005783;GO:0016787;GO:0016310;GO:0005161;GO:0019899;GO:0005789;GO:0008152;GO:1990604;GO:0030176;GO:1990597;GO:0034620;GO:1990630;GO:0046777;GO:0036289;GO:0007257;GO:0006986;GO:0098787;GO:0016241;GO:0030544;GO:0005637;GO:0016021;GO:0000287;GO:0004521;GO:0051082;GO:0000166;GO:0004674;GO:0016740;GO:0071333;GO:1901142;GO:0006402;GO:0046872;GO:0051879;GO:0042802;GO:0042803;GO:0005737;GO:0006468;GO:0033120;GO:0006397;GO:1990332;GO:0004672;GO:0007050;GO:0003824;GO:0043531;GO:0016020;GO:0090502;GO:0036498;GO:0006351;GO:0030968;GO:0070059;GO:1904707;GO:0070054;GO:0016301;GO:0006355;GO:1990579;GO:0005524;GO:0005515;GO:0004519;GO:1900103;GO:0006379;GO:0001935;GO:0035924;GO:0006915;GO:0034976 | hsa:2081 | COG0515 | endoplasmic reticulum to nucleus signalling 1 isoform 1 variant, partial [Homo sapiens] |
| O75636 | 54872.66833 | 62773.78417 | 1.143990006 | 0.194074448 | 0.28637559 | no | no change | GO:0004252;GO:0051607;GO:0006956;GO:0003823;GO:1902679;GO:0045087;GO:0030246;GO:0043654;GO:0002376;GO:0046597;GO:0005576;GO:0001867;GO:0072562;GO:0005515;GO:0006508;GO:0046872;GO:0005581 | hsa:8547 |  | ficolin-3 isoform 1 precursor [Homo sapiens] |
| O75882 | 49620.2125 | 44478.78583 | 0.896384429 | -0.157810507 | 0.085325652 | no | no change | GO:0005737;GO:0043473;GO:0042552;GO:0005615;GO:0070062;GO:0016021;GO:0016020;GO:0030246;GO:0006954;GO:0005576;GO:0005887;GO:0005886;GO:0038023;GO:0021549;GO:0006979;GO:0040014 | hsa:8455 |  | attractin isoform 1 preproprotein [Homo sapiens] |
| O95445 | 59763.54667 | 57454.5025 | 0.961363669 | -0.056845811 | 0.526984687 | no | no change | GO:0034375;GO:0034445;GO:0006869;GO:0043691;GO:0042157;GO:0001523;GO:0005615;GO:0005543;GO:0098869;GO:0005576;GO:0005319;GO:0034361;GO:0034362;GO:0034364;GO:0034365;GO:0034366;GO:0016209;GO:0042632;GO:0034380;GO:0034384;GO:0009749;GO:0033344 | hsa:55937 |  | apolipoprotein M isoform 1 [Homo sapiens] |
| P00450 | 625856.0042 | 629645.5833 | 1.006055034 | 0.008709226 | 0.916303977 | no | no change | GO:0016491;GO:0044267;GO:0046872;GO:0006879;GO:0005615;GO:0006825;GO:0004322;GO:0006811;GO:0051087;GO:0070062;GO:0005623;GO:0005765;GO:0055072;GO:0006826;GO:0005788;GO:0005886;GO:0072562;GO:0005507;GO:0043687;GO:0055114;GO:0005576 | hsa:1356 | COG2132 | RecName: Full=Ceruloplasmin; AltName: Full=Ferroxidase; Flags: Precursor |
| P00488 | 24800.94633 | 22051.47217 | 0.889138337 | -0.169520197 | 0.436973533 | no | no change | GO:0005737;GO:0018149;GO:0007599;GO:0019221;GO:0031093;GO:0003810;GO:0072378;GO:0002576;GO:0007596;GO:0072562;GO:0016740;GO:0062023;GO:0016746;GO:0046872;GO:0005576 | hsa:2162 |  | RecName: Full=Coagulation factor XIII A chain; Short=Coagulation factor XIIIa; AltName: Full=Protein-glutamine gamma-glutamyltransferase A chain; AltName: Full=Transglutaminase A chain; Flags: Precursor |
| P00734 | 579945.2667 | 556802.7667 | 0.960095372 | -0.05875037 | 0.199752646 | no | no change | GO:0004252;GO:0048712;GO:0009897;GO:0030307;GO:0007597;GO:0007596;GO:0005788;GO:0051281;GO:0030449;GO:0032967;GO:0008047;GO:0007166;GO:0007599;GO:0008233;GO:0007275;GO:0061844;GO:0044267;GO:0006888;GO:0005615;GO:0005102;GO:0009611;GO:0070062;GO:0001530;GO:0051838;GO:0010544;GO:0005509;GO:0006508;GO:0008236;GO:0007186;GO:0010468;GO:1900738;GO:0008284;GO:0016787;GO:2000379;GO:0046427;GO:0090218;GO:0070945;GO:0006953;GO:0005796;GO:0008360;GO:0014068;GO:0005886;GO:0008201;GO:0030168;GO:0008083;GO:1900016;GO:0042730;GO:1900182;GO:0051480;GO:0010469;GO:0070053;GO:0005576;GO:0051918;GO:0005515;GO:0072378;GO:0030193;GO:0045861;GO:0072562;GO:0001934;GO:0030194 | hsa:2147 | COG5640 | prothrombin isoform 1 preproprotein [Homo sapiens] |
| P00738 | 11543587 | 11548709.83 | 1.000443782 | 0.0006401 | 0.997358436 | no | no change | GO:0042742;GO:0010942;GO:0005615;GO:0071682;GO:2000296;GO:0072562;GO:0098869;GO:0035580;GO:1904724;GO:0016209;GO:0006952;GO:0006953;GO:0070062;GO:0042542;GO:0051354;GO:0006898;GO:0043312;GO:0002376;GO:0030492;GO:0005576;GO:0005515;GO:0031838 | hsa:3240 | COG5640 | haptoglobin isoform 1 preproprotein [Homo sapiens] |
| P00739 | 38869.02583 | 42310.0025 | 1.088527474 | 0.122377821 | 0.600754703 | no | no change | GO:0004252;GO:0070062;GO:0034366;GO:0030492;GO:0006898;GO:0072562;GO:0005576;GO:0010942;GO:0002526;GO:0010033;GO:0005615 | hsa:3250 | COG5640 | haptoglobin-related protein precursor [Homo sapiens] |
| P00742 | 85201.01083 | 77988.13 | 0.915342779 | -0.127615988 | 0.196076269 | no | no change | GO:0004252;GO:0030335;GO:0005543;GO:0006888;GO:0005615;GO:0016787;GO:0007596;GO:0005788;GO:0005796;GO:0005576;GO:0005509;GO:0005515;GO:0005886;GO:0006508;GO:0008233;GO:0008236;GO:0051897;GO:0031233;GO:0007599;GO:0007598 | hsa:2159 | COG5640 | coagulation factor X isoform 1 preproprotein [Homo sapiens] |
| P00747 | 1153996.371 | 1161562.133 | 1.006556141 | 0.009427641 | 0.875635471 | no | no change | GO:0004252;GO:0004175;GO:0007599;GO:0016787;GO:1904854;GO:0048771;GO:0019899;GO:0008233;GO:0008236;GO:0044267;GO:0070062;GO:0043536;GO:0052182;GO:0051087;GO:0072562;GO:1990405;GO:0006508;GO:0022617;GO:0009986;GO:0051918;GO:0051919;GO:0008285;GO:0007596;GO:0010812;GO:0019900;GO:0062023;GO:0019904;GO:0005886;GO:0051702;GO:0031232;GO:0034185;GO:0005615;GO:0042730;GO:0031093;GO:0052213;GO:2000048;GO:0044218;GO:0005576;GO:0005515;GO:0005102;GO:0002576 | hsa:5340 | COG5640 | plasminogen isoform 1 precursor [Homo sapiens] |
| P00915 | 14078.96017 | 14246.79221 | 1.011920769 | 0.017096335 | 0.942693485 | no | no change | GO:0005737;GO:0015701;GO:0046872;GO:0070062;GO:0006730;GO:0016829;GO:0008270;GO:0005515;GO:0016836;GO:0004089;GO:0004064;GO:0005829;GO:0035722 | hsa:759 | COG3338 | carbonic anhydrase 1 isoform a [Homo sapiens] |
| P00918 | 7127.791125 | 6637.175053 | 0.931168568 | -0.102885735 | 0.670436682 | no | no change | 3P3J:A;1IF6:A;1H9N:A;3RYJ:B;5FLQ:A;3P5A:A;1AVN:A;GO:0010043;6BC9:A;3V3H:B;4Q78:A;5JGS:B;5M78:A;1CAM:A;GO:0043209;5TY9:A;3RZ1:B;5EOI:A;5JN3:A;1CNI:A;4ZWY:A;3PJJ:A;3TMJ:A;5LL4:A;4CAC:A;1CNK:A;3T5U:A;6EQU:A;5EH8:A;1G52:A;5JEG:B;1AM6:A;3SBH:A;1CVD:A;4BF1:A;1BNU:A;1I91:A;4PYY:A;3MHL:A;4HEY:A;5FLO:A;5NXP:A;3S78:B;4Q08:A;4K1Q:A;2O4Z:A;2Q38:A;3DD8:A;5CAC:A;4YYT:A;2VVB:X;1CNC:A;2FNN:A;5G0C:A;1CVB:A;1FR7:B;2FOQ:A;3RZ7:A;4KNI:A;6H2Z:A;4QTL:A;4QEF:A;4FVO:A;6BCC:A;3M1Q:A;5LLG:A;6E92:A;3T83:A;5WEX:A;3D8W:A;4YXI:A;5WLV:A;4Q6D:A;5JQT:A;4YXO:A;5TFX:A;2HKK:A;2POU:A;3M1W:A;6MBY:A;5DOH:B;5OGO:A;4QSI:A;1BNV:A;3DBU:A;1IF9:A;3PYK:A;1CAK:A;1G46:A;6D1L:A;5TY8:A;5DRS:A;5E28:A;4Q9Y:A;5LLC:A;6FJI:A;3MNH:A;1I9P:A;1CIL:A;4FIK:A;6BBS:A;3OIL:A;1LG6:A;4BF6:A;6IC2:A;6C7W:A;1CIN:A;1G4O:A;1BNM:A;4YGL:A;4HF3:A;2CBE:A;3KOK:A;GO:0016829;4MDG:A;3HS4:A;5DSM:A;4K0Z:A;2F14:A;5FNL:A;4JSW:A;GO:0045177;5BRU:A;5DSO:A;3DVC:A;2WD2:A;3RG3:A;4KUV:A;4HEZ:A;5LL4:B;1I9M:A;1RZD:A;5DSL:A;3B4F:A;1TE3:X;5BNL:A;4YGK:A;GO:0016323;5U0G:A;5LMD:A;5CA2:A;4DZ9:A;5SZ1:A;1ZSB:A;1YDB:A;1YO0:A;4E49:A;4YVY:A;4KV0:A;2X7S:A;5THN:A;4KUW:A;1YDD:A;1YO2:A;1I8Z:A;2X7U:A;2CBC:A;5JQ0:A;4QSA:A;3S74:B;3P58:A;3M67:A;3IGP:A;3S76:A;3EFT:A;5TYA:A;3IEO:A;4JSZ:A;3V7X:A;5N24:A;3HKU:A;3KKX:A;5ZXW:A;1BN3:A;2EZ7:A;5JDV:B;5FLT:A;4E4A:A;GO:0030424;1CVH:A;GO:0004064;1XEG:A;5G03:A;2AW1:A;3F8E:A;1RAZ:A;5W8B:A;5G01:A;5N1R:A;5L70:B;2ABE:A;2EU3:A;5L70:A;3IBU:A;GO:0005615;6EDA:A;2GEH:A;5N1S:A;4Q7W:A;1CAY:A;5EKM:A;3D93:A;3M2Z:A;5MJN:A;1ZFQ:A;2NNO:A;GO:0038166;3R17:B;3S9T:A;1BV3:A;5VGY:A;4IWZ:A;1G3Z:A;5DSP:A;4Z0Q:A;GO:0045780;1XPZ:A;3RZ5:A;1UGD:A;GO:0016020;5AMD:A;1RAY:A;4M2U:A;1UGF:A;3DV7:A;3S75:B;1RZE:A;4PXX:A;1BNT:A;5DOG:A;3P4V:A;2WEG:A;6EBE:A;3KS3:A;5WLR:A;5SZ2:A;1KWQ:A;1CIM:A;1CNG:A;5AML:A;2NNV:A;GO:0005829;1CCS:A;5OGP:A;GO:0043627;1CVF:A;1TTM:A;4FVN:A;4JSA:A;1TBT:X;1FSQ:A;5SZ3:A;4RFC:A;1FSQ:B;3MNJ:A;2NWP:A;5FNJ:A;1OKM:A;5LLE:A;4ITP:A;2WD3:A;3K7K:A;1G4J:A;2H4N:A;4HBA:A;1ZH9:A;4R5B:A;5T71:A;6ECZ:A;5L6T:A;5L6T:B;2AX2:A;3P44:A;5TI0:A;GO:2001225;2QOA:A;6CA2:A;4G0C:A;1CA2:A;1CAO:A;3V3F:A;4Z1K:A;1FR7:A;4QK3:A;1CAI:A;5N0E:A;4PQ7:A;3HKN:A;1RZC:A;1ZGF:A;4QK1:A;5GMN:A;4E3D:A;1CNW:A;5ULN:A;GO:0048545;2FOV:A;4FRC:A;2FNK:A;2HD6:A;2WEO:A;1TG9:A;3M40:A;5BRV:A;4Q7P:A;4Q8Z:A;2QO8:A;5DSK:A;4RIV:A;3KOI:A;5Y2S:A;4Q8X:A;3HLJ:A;1LZV:A;1G54:A;5C8I:A;3L14:A;3N2P:A;3OYQ:A;3CAJ:A;2POV:A;6EEO:A;4WL4:A;6B4D:A;5YUJ:A;4FPT:A;3GZ0:A;5NXV:A;1OQ5:A;6GOT:A;5J8Z:A;3ZP9:A;4RUY:A;6B5A:A;2NWZ:A;1I9O:A;1THK:A;2EU2:A;5SZ7:A;4L5W:A;4M2W:A;6H6S:A;5JMZ:A;4Q7V:A;2Q1Q:A;4HT0:A;4QSB:A;4QJM:A;1BNN:A;2NXS:A;GO:0005515;3M1K:A;4Q99:A;3M2X:A;1H9Q:A;6G6T:A;4MDM:A;1FQM:A;5FDC:A;1H4N:A;5SZ5:A;4Q6E:A;3SAP:A;1EOU:A;1TEQ:X;5FLP:A;5T75:A;5NXI:A;3K34:A;2HOC:A;6CJV:A;6FJJ:A;1CNJ:A;3P5L:A;1G53:A;3V5G:A;4MDL:A;1ZSC:A;6HX5:A;4XE1:A;1ZSA:A;1TG3:A;6GXE:A;5JEH:B;4MO8:A;2X7T:A;4CA2:A;1IF7:A;6CEH:A;4QY3:A;GO:0046903;1LGD:A;4Q09:A;2CBD:A;1IF5:A;1CNB:A;3FFP:X;3DCW:A;5NXO:A;5LL8:A;3RYY:A;4Z1E:A;3R16:A;3BET:A;GO:0032849;5JEP:B;5G0B:A;4ZAO:A;3ML2:A;1BCD:A;3TVN:X;4Y0J:A;5NXW:A;6EEA:A;1BN4:A;1UGB:A;4K13:A;5DOH:A;3C7P:A;4RFD:A;GO:0001822;1TB0:X;5SZ4:A;5DSQ:A;GO:0046872;5L6K:B;3N3J:A;4Q07:A;3T84:A;1IF8:A;12CA:A;1DCA:A;4QK2:A;5E2K:A;4Q83:A;3SAX:A;5LVS:B;5T74:A;1CAZ:A;6D1M:A;6GXB:A;3RYZ:A;5LVS:A;2FMZ:A;3IBI:A;2QP6:A;1I9Q:A;4E5Q:A;3OIM:A;5NEA:A;6B59:A;6H29:A;2FOU:A;3M5T:A;5THI:A;4GL1:X;4Q87:A;2WEJ:A;1G0F:A;GO:2001150;6H34:A;6G3Q:A;3CYU:A;5O07:A;6QEB:A;3RGE:A;5E2R:A;5YUK:A;5EH5:A;4MLT:A;5EIJ:A;3RZ0:B;5FLS:A;3NB5:A;5EH7:A;1MOO:A;3U3A:X;4Z1J:A;3SBI:A;4Q90:A;2VVA:X;1I90:A;1XQ0:A;5NXG:A;1ZFK:A;3MHO:A;5FNI:A;6H6S:B;2OSM:A;3VBD:A;2FNM:A;3MHM:A;4K0S:A;3MNU:A;5EHV:A;5FNG:A;4E3G:A;3RZ8:A;4YWP:A;5THJ:A;1CRA:A;1RZA:A;2NNS:A;1FR4:A;3T5Z:A;1F2W:A;1CVC:A;3RG4:A;5TH4:A;3PO6:A;5WLU:A;5JN7:A;6E91:A;4ZX0:A;1Z9Y:A;5JN1:A;1HVA:A;GO:0009268;3NI5:A;5A6H:A;3S77:B;GO:0005737;3S71:B;4Q49:A;4ILX:A;3HKT:A;5U0F:A;1BIC:A;3OYS:A;3M96:A;2FMG:A;3KIG:A;5FLR:A;5OGN:B;6GCY:A;2NXT:A;1CAH:A;5SZ0:A;4E3H:A;4ITO:A;4QIY:C;4QIY:B;4QIY:A;4R5A:A;3DCC:A;1CNX:A;1CAJ:A;4MLX:A;2Q1B:A;3M04:A;4L5U:A;1ZGE:A;1LG5:A;5FDI:A;GO:0070062;3D92:A;2H15:A;3MZC:A;3F4X:A;1FQL:A;8CA2:A;5JG3:B;1UGA:A;5E2S:A;4LHI:A;5NY1:A;3M14:A;5LJT:A;3IBN:A;3P55:A;3V3G:B;1CVA:A;3D9Z:A;1HCA:A;5NXM:A;1BNQ:A;GO:0044070;3DVB:A;5JGT:B;4YGN:A;1UGG:A;3CA2:A;1BNW:A;3V2J:A;GO:0010033;1YDA:A;1I9L:A;4ZWZ:A;3RJ7:A;1YDC:A;3DAZ:A;6EEH:A;5WLT:A;6E8P:A;4M2R:A;5L6K:A;1TEU:X;3DCS:A;2CBB:A;3BL0:A;1FSR:B;1FSR:A;1LUG:A;2OSF:A;2ILI:A;1MUA:A;4N16:A;2GD8:A;3MMF:A;5U0D:A;4E3F:A;2HL4:A;3OKV:A;1ZE8:A;5TY1:A;GO:0008270;5JE7:B;3DC9:A;3V3J:A;3S73:B;GO:0032230;3M5S:A;1T9N:A;4Q06:A;1CCU:A;5AMG:A;3MWO:B;7CA2:A;3MWO:A;5T72:A;GO:0004089;5BYI:A;3DC3:A;1CNH:A;2NNG:A;1CA3:A;5EKH:A;5WG7:A;3OY0:A;5NY3:A;1FQR:A;1HEC:A;9CA2:A;2FOS:A;5EKJ:A;3V2M:A;3N4B:A;1HEA:A;2NWO:A;5NEE:A;3K2F:A;6E8X:A;3M2Y:A;3P3H:A;3U7C:A;4FL7:A;4Q81:A;4WW6:A;GO:0015701;3RYV:B;3S8X:A;5U0E:A;GO:0051453;3EFI:A;4RH2:A;5EHW:A;2WEH:A;4Q8Y:A;2POW:A;4Z1N:A;1HEB:A;4RIU:A;1CAL:A;5FNM:A;1I9N:A;4ZWX:A;1G1D:A;1KWR:A;5TXY:A;3MHI:A;5LLH:A;1CCT:A;4RN4:A;3KWA:A;4CQ0:A;4YXU:A;1CVE:A;5SZ6:A;4KAP:A;3QYK:A;4PYX:A;4YGJ:A;2NWY:A;1FSN:B;1FSN:A;4HEW:A;3V3I:B;4N0X:B;1YO1:A;4KNJ:A;3MNK:A;3HFP:A;4FU5:A;5BRW:A;GO:0005886;1G0E:A;1OKN:A;5N25:A;5CLU:A;4JS6:A;GO:0005902;4PZH:A;1OKL:A;5L9E:C;3M2N:A;5LJQ:A;3T82:A;1FQN:A;6MBV:A;4RUZ:A;4DZ7:A;5DSR:A;1A42:A;4RUX:A;3MNA:A;5L3O:B;1CNY:A;4ZWI:A;1CAN:A;3U45:X;3OIK:A;4KUY:A;5NY6:A;3U47:A;5YUI:A;2CBA:A;5N0D:A;5FNH:A;6H33:A;4K0T:A;3N0N:A;3RLD:A;3BL1:A;4ZX1:A;5UMC:A;1RZB:A;3M5E:A;5OGN:A;4LP6:B;4LP6:A;1G45:A;3MNI:A;6GDC:A;3RYX:B;GO:0015670;4YX4:A;1IF4:A;5JG5:B;4JSS:A;3DVD:A;4BCW:A;5WGP:A;5Y2R:A;3TVO:X;3IBL:A;6C7X:A;GO:0002009;3DD0:A;3S72:B;3M3X:A;1UGC:A;4R59:A;3HKQ:A;1UGE:A;3T85:A;4QIY:D;3MYQ:A;4M2V:A;1G48:A;GO:0071498;1XEV:B;2HNC:A;5JES:B;4L5V:A;3NJ9:A;1TH9:A;5L9E:A;1DCB:A;3M98:A;5L9E:B;5L3O:A;5L9E:D;5FNK:A;2CA2:A;GO:0042475;4IDR:X;3KNE:A;5NYA:A;3M1J:A;1HED:A;3MHC:A;2NXR:A;GO:0045672;4Q7S:A;3OKU:A;3KON:A;1XEV:C;5EHE:A;1XEV:A;4MTY:A;1XEV:D;1BN1:A | hsa:760 | COG3338 | carbonic anhydrase 2 isoform 1 [Homo sapiens] |
| P01011 | 729900.2542 | 747812.3792 | 1.024540511 | 0.03497703 | 0.641615419 | no | no change | GO:0006954;GO:0034774;GO:0035578;GO:0003677;GO:0070062;GO:0031093;GO:0002576;GO:0072562;GO:0010466;GO:0019216;GO:0006953;GO:0062023;GO:0030277;GO:0030414;GO:0004867;GO:0010951;GO:0005615;GO:0043312;GO:0005622;GO:0005576;GO:0005515;GO:0005634 | hsa:12 | COG4826 | serpin peptidase inhibitor, clade A (alpha-1 antiproteinase, antitrypsin), member 3, isoform CRA_b [Homo sapiens] |
| P01023 | 8692043.75 | 7746371.708 | 0.891202568 | -0.166174706 | 0.321907622 | no | no change | GO:0051056;GO:0030414;GO:0019959;GO:0007597;GO:0019899;GO:0072562;GO:0048863;GO:0005615;GO:0031093;GO:0002576;GO:0048306;GO:0019838;GO:0070062;GO:0010466;GO:0002020;GO:0001869;GO:0022617;GO:0019966;GO:0005096;GO:0004866;GO:0004867;GO:0010951;GO:0043547;GO:0043120;GO:0005829;GO:0005576;GO:0005515;GO:0005102 | hsa:2 | COG2373 | alpha-2-macroglobulin isoform a precursor [Homo sapiens] |
| P01024 | 7993185.208 | 7683627.625 | 0.961272312 | -0.056982915 | 0.426954571 | no | no change | GO:0045087;GO:0004252;GO:0006631;GO:0030449;GO:0004866;GO:0005886;GO:0005788;GO:0035578;GO:0007165;GO:0031715;GO:0048260;GO:0010828;GO:0010866;GO:0050766;GO:0044267;GO:0070062;GO:0009617;GO:0045766;GO:1905114;GO:0045745;GO:0097242;GO:2000427;GO:0034774;GO:0007186;GO:0009986;GO:0005576;GO:0006629;GO:0150064;GO:0043687;GO:0016322;GO:0006911;GO:0150062;GO:0006956;GO:0006957;GO:0006954;GO:0006955;GO:0010951;GO:0060100;GO:0006958;GO:0006508;GO:0005615;GO:0050776;GO:0032991;GO:0010575;GO:0043312;GO:0002376;GO:0001798;GO:0010884;GO:0001970;GO:0005515;GO:0097278;GO:0005102;GO:0001934;GO:0072562 | hsa:718 |  | complement C3 preproprotein [Homo sapiens] |
| P01031 | 217947.0667 | 216864.45 | 0.995032662 | -0.007184211 | 0.916734871 | no | no change | GO:0000187;GO:0030449;GO:0006954;GO:0008009;GO:0007166;GO:0060326;GO:0019835;GO:0001701;GO:0005615;GO:0045766;GO:0010760;GO:0007186;GO:0045087;GO:0006935;GO:0090197;GO:0006956;GO:0006957;GO:0004866;GO:0010951;GO:0006958;GO:0070062;GO:0010575;GO:0002376;GO:0005576;GO:0005515;GO:0005102;GO:0005579 | hsa:727 |  | complement C5 isoform 1 preproprotein [Homo sapiens] |
| P01034 | 11225.5915 | 11943.61057 | 1.063962694 | 0.089447566 | 0.475631456 | no | no change | GO:0005783;GO:0060009;GO:0048678;GO:0005788;GO:0008584;GO:0042747;GO:0030414;GO:0070301;GO:0001666;GO:0014070;GO:1904724;GO:0005737;GO:0044267;GO:0032355;GO:0042995;GO:0001540;GO:0005615;GO:0007431;GO:2000117;GO:0005771;GO:0009636;GO:0070062;GO:0043067;GO:0010466;GO:0002020;GO:0030424;GO:0048471;GO:0043025;GO:0042802;GO:0008284;GO:0034103;GO:0045740;GO:0060548;GO:0043687;GO:0006915;GO:0031965;GO:0031982;GO:0006952;GO:0001775;GO:0001654;GO:0060311;GO:0004866;GO:0060313;GO:0004869;GO:0034599;GO:0010035;GO:0006979;GO:0009743;GO:1904813;GO:0005604;GO:0007420;GO:0010716;GO:0007566;GO:0042493;GO:0031667;GO:0010711;GO:0005764;GO:0005576;GO:0005515;GO:0043312;GO:0043292;GO:0045861;GO:0097435 | hsa:1471 |  | cystatin-C precursor [Homo sapiens] |
| P01042 | 1178250.821 | 1153652.767 | 0.979123245 | -0.030437628 | 0.581138086 | no | no change | GO:0007599;GO:0030414;GO:0007597;GO:0007596;GO:0045861;GO:0005788;GO:0007162;GO:0008270;GO:0042311;GO:0044267;GO:0050880;GO:0005615;GO:0031093;GO:0002576;GO:0072562;GO:0043065;GO:0010466;GO:0007186;GO:0043687;GO:0062023;GO:0006954;GO:0005886;GO:0010951;GO:0004869;GO:0008201;GO:0070062;GO:0007204;GO:0005576;GO:0005515;GO:0005102;GO:0030195 | hsa:3827 |  | kininogen-1 isoform 1 precursor [Homo sapiens] |
| P01344 | 43611.33875 | 41745.06167 | 0.957206609 | -0.063097737 | 0.495148968 | no | no change | GO:0046628;GO:0045840;GO:0031017;GO:0051146;GO:0008286;GO:0038028;GO:0008284;GO:0001501;GO:0001503;GO:0051147;GO:0031056;GO:0071902;GO:0051897;GO:0007275;GO:0001649;GO:0044267;GO:0006349;GO:0001701;GO:0008083;GO:0031093;GO:0000122;GO:0002576;GO:0043085;GO:0045725;GO:0010469;GO:0005179;GO:0005178;GO:0009887;GO:2000467;GO:0042104;GO:0005159;GO:0005158;GO:0043410;GO:0048018;GO:0060669;GO:0001892;GO:0043539;GO:0005615;GO:0005975;GO:0050731;GO:0040018;GO:0006355;GO:0051781;GO:0005576;GO:0045944;GO:0005515;GO:0006006;GO:0001934 | hsa:3481 |  | insulin-like growth factor II isoform 2 [Homo sapiens] |
| P01597 | 42113.01583 | 41109.17542 | 0.976163179 | -0.034805761 | 0.724804567 | no | no change | GO:0038096;GO:0004252;GO:0016020;GO:0030449;GO:0050776;GO:0006898;GO:0038095;GO:0072562;GO:0002376;GO:0050900;GO:0005576;GO:0006956;GO:0002250;GO:0003823;GO:0005886;GO:0006508;GO:0006958;GO:0006955;GO:0070062 | hsa:7441 |  | immunoglobulin kappa light chain VC region, partial [Homo sapiens] |
| P01602 | 293847.575 | 313495.2904 | 1.06686363 | 0.093375777 | 0.834814163 | no | no change | GO:0038096;GO:0004252;GO:0016020;GO:0030449;GO:0050776;GO:0006898;GO:0038095;GO:0072562;GO:0002376;GO:0002377;GO:0050900;GO:0005576;GO:0006956;GO:0002250;GO:0003823;GO:0005886;GO:0006508;GO:0006958;GO:0006955;GO:0070062 | hsa:7441 |  | hCG2043208, partial [Homo sapiens] |
| P01699 | 152317.1246 | 130471.1858 | 0.856575951 | -0.223346922 | 0.085031403 | no | no change | GO:0038096;GO:0004252;GO:0016020;GO:0030449;GO:0050776;GO:0006898;GO:0038095;GO:0002376;GO:0050900;GO:0005576;GO:0006956;GO:0002250;GO:0003823;GO:0005886;GO:0006508;GO:0006958;GO:0006955 | hsa:7441 |  | hCG2043214, partial [Homo sapiens] |
| P01701 | 462470.4417 | 464909.8917 | 1.005274824 | 0.007589962 | 0.963550237 | no | no change | GO:0038096;GO:0004252;GO:0016020;GO:0030449;GO:0050776;GO:0006898;GO:0038095;GO:0050900;GO:0005576;GO:0006956;GO:0003823;GO:0005886;GO:0006508;GO:0006958;GO:0006955;GO:0070062 | hsa:7441 |  | RecName: Full=Immunoglobulin lambda variable 1-51; AltName: Full=Ig lambda chain V-I region BL2; AltName: Full=Ig lambda chain V-I region EPS; AltName: Full=Ig lambda chain V-I region NEW; AltName: Full=Ig lambda chain V-I region NIG-64; Flags: Precursor |
| P01703 | 134359.7152 | 117343.7004 | 0.873354787 | -0.19536025 | 0.175844462 | no | no change | GO:0038096;GO:0004252;GO:0016020;GO:0030449;GO:0050776;GO:0006898;GO:0038095;GO:0002376;GO:0050900;GO:0005576;GO:0006956;GO:0002250;GO:0003823;GO:0005886;GO:0006508;GO:0006958;GO:0006955 | hsa:7441 |  | Unknown (protein for MGC:31936) [Homo sapiens] |
| P01705 | 119684.0262 | 120145.3343 | 1.003854383 | 0.00555001 | 0.982595027 | no | no change | GO:0038096;GO:0004252;GO:0016020;GO:0030449;GO:0050776;GO:0006898;GO:0038095;GO:0002376;GO:0050900;GO:0005576;GO:0006956;GO:0002250;GO:0003823;GO:0005886;GO:0006508;GO:0006958;GO:0006955 | hsa:7441 |  | immunoglobulin lambda-chain, partial [Homo sapiens] |
| P01706 | 117634.1529 | 111182.1975 | 0.945152362 | -0.081381179 | 0.75234439 | no | no change | GO:0038096;GO:0004252;GO:0016020;GO:0030449;GO:0050776;GO:0006898;GO:0038095;GO:0002376;GO:0050900;GO:0005576;GO:0006956;GO:0002250;GO:0003823;GO:0005886;GO:0006508;GO:0006958;GO:0006955 | hsa:7441 |  | hCG2043237, partial [Homo sapiens] |
| P01715 | 11104.41688 | 10681.48209 | 0.961912922 | -0.056021796 | 0.814030216 | no | no change | GO:0038096;GO:0004252;GO:0016020;GO:0030449;GO:0050776;GO:0006898;GO:0038095;GO:0002376;GO:0050900;GO:0005576;GO:0006956;GO:0002250;GO:0003823;GO:0005886;GO:0006508;GO:0006958;GO:0006955 | hsa:29802 |  | hCG2040023, partial [Homo sapiens] |
| P01717 | 6849.123 | 5749.16281 | 0.839401309 | -0.252567382 | 0.164203661 | no | no change | GO:0038096;GO:0004252;GO:0016020;GO:0030449;GO:0050776;GO:0006898;GO:0038095;GO:0072562;GO:0002376;GO:0050900;GO:0005576;GO:0006956;GO:0002250;GO:0003823;GO:0005886;GO:0006508;GO:0006958;GO:0006955 | hsa:7441 |  | immunoglobulin lambda light chain variable region, partial [Homo sapiens] |
| P01718 | 19821.73289 | 18557.02407 | 0.93619585 | -0.095117725 | 0.847533004 | no | no change | GO:0038096;GO:0004252;GO:0016020;GO:0030449;GO:0050776;GO:0006898;GO:0038095;GO:0002376;GO:0050900;GO:0005576;GO:0006956;GO:0002250;GO:0003823;GO:0005886;GO:0006508;GO:0006958;GO:0006955 | hsa:29802 |  | immunoglobulin light chain variable region, partial [Homo sapiens] |
| P01721 | 102065.1888 | 91913.69292 | 0.900539097 | -0.151139183 | 0.273890323 | no | no change | GO:0038096;GO:0004252;GO:0016020;GO:0030449;GO:0050776;GO:0006898;GO:0038095;GO:0002376;GO:0050900;GO:0005576;GO:0006956;GO:0002250;GO:0003823;GO:0005886;GO:0006508;GO:0006958;GO:0006955 | hsa:7441 |  | RecName: Full=Immunoglobulin lambda variable 6-57; AltName: Full=Ig lambda chain V-VI region AR; AltName: Full=Ig lambda chain V-VI region EB4; AltName: Full=Ig lambda chain V-VI region NIG-48; AltName: Full=Ig lambda chain V-VI region SUT; AltName: Full=Ig lambda chain V-VI region WLT; Flags: Precursor |
| P01742 | 1446239.483 | 1630025.25 | 1.127078378 | 0.172587845 | 0.714301506 | no | no change | GO:0038096;GO:0004252;GO:0016020;GO:0030449;GO:0050776;GO:0006898;GO:0038095;GO:0002376;GO:0050900;GO:0005576;GO:0006956;GO:0002250;GO:0003823;GO:0005886;GO:0006508;GO:0006958;GO:0006955 | hsa:102724971 | | IgM heavy chain VH1 region precursor, partial [Homo sapiens] |
| P01743 | 15038.59167 | 16041.36275 | 1.066679853 | 0.093127239 | 0.551317377 | no | no change | GO:0038096;GO:0004252;GO:0016020;GO:0030449;GO:0050776;GO:0006898;GO:0038095;GO:0002376;GO:0050900;GO:0005576;GO:0006956;GO:0002250;GO:0003823;GO:0005886;GO:0006508;GO:0006958;GO:0006955 | hsa:102723407 | | IgM heavy chain VH1 region precursor, partial [Homo sapiens] |
| P01763 | 33730.68792 | 33351.55525 | 0.988760008 | -0.016307703 | 0.939940431 | no | no change | GO:0038096;GO:0004252;GO:0016020;GO:0030449;GO:0050776;GO:0006898;GO:0038095;GO:0002376;GO:0050900;GO:0005576;GO:0006956;GO:0002250;GO:0003823;GO:0005886;GO:0006508;GO:0006958;GO:0006955 | hsa:102723407 | | immunoglobulin heavy chain variable region, partial [Homo sapiens] |
| P01764 | 44708.084 | 44849.17591 | 1.003155848 | 0.004545757 | 0.983920529 | no | no change | GO:0004252;GO:0030449;GO:0009897;GO:0006955;GO:0050900;GO:0042742;GO:0034987;GO:0042571;GO:0005615;GO:0016020;GO:0072562;GO:0006508;GO:0050853;GO:0045087;GO:0006910;GO:0006911;GO:0050776;GO:0006956;GO:0003823;GO:0005886;GO:0006958;GO:0050871;GO:0070062;GO:0038096;GO:0038095;GO:0006898;GO:0002376;GO:0005576;GO:0002250 | hsa:102723407 | | RecName: Full=Immunoglobulin heavy variable 3-23; AltName: Full=Ig heavy chain V-III region LAY; AltName: Full=Ig heavy chain V-III region POM; AltName: Full=Ig heavy chain V-III region TEI; AltName: Full=Ig heavy chain V-III region TIL; AltName: Full=Ig heavy chain V-III region TUR; AltName: Full=Ig heavy chain V-III region VH26; AltName: Full=Ig heavy chain V-III region WAS; AltName: Full=Ig heavy chain V-III region ZAP; Flags: Precursor |
| P01780 | 1940048.167 | 1684025.971 | 0.868033072 | -0.204178084 | 0.158652692 | no | no change | GO:0038096;GO:0004252;GO:0016020;GO:0030449;GO:0050776;GO:0006898;GO:0038095;GO:0072562;GO:0002376;GO:0050900;GO:0005576;GO:0006956;GO:0002250;GO:0003823;GO:0005886;GO:0006508;GO:0006958;GO:0006955;GO:0070062 | hsa:102723407 | | immunoglobulin heavy chain variable region precursor, partial [Homo sapiens] |
| P01814 | 33749.13708 | 29691.86739 | 0.879781528 | -0.184782784 | 0.198690351 | no | no change | GO:0038096;GO:0004252;GO:0016020;GO:0030449;GO:0050776;GO:0006898;GO:0038095;GO:0002376;GO:0050900;GO:0005576;GO:0006956;GO:0002250;GO:0003823;GO:0005886;GO:0006508;GO:0006958;GO:0006955 | hsa:102723407 | | RecName: Full=Immunoglobulin heavy variable 2-70; AltName: Full=Ig heavy chain V-II region COR; AltName: Full=Ig heavy chain V-II region DAW; AltName: Full=Ig heavy chain V-II region OU; AltName: Full=Ig heavy chain V-II region SESS; Flags: Precursor |
| P01817 | 40830.34333 | 45693.13 | 1.119097374 | 0.162335572 | 0.380665483 | no | no change | GO:0038096;GO:0004252;GO:0016020;GO:0030449;GO:0050776;GO:0006898;GO:0038095;GO:0002376;GO:0050900;GO:0005576;GO:0006956;GO:0002250;GO:0003823;GO:0005886;GO:0006508;GO:0006958;GO:0006955 | hsa:102723407 | | RecName: Full=Immunoglobulin heavy variable 2-5; AltName: Full=Ig heavy chain V-II region HE; AltName: Full=Ig heavy chain V-II region MCE; Flags: Precursor |
| P01833 | 12415.04043 | 11886.09729 | 0.957394973 | -0.062813864 | 0.640357757 | no | no change | GO:0043235;GO:0001895;GO:0005615;GO:0002415;GO:0001580;GO:0070062;GO:0016021;GO:0016020;GO:0043312;GO:0005576;GO:0007173;GO:0005887;GO:0005886;GO:0043113;GO:0001792;GO:0035577;GO:0038093 | hsa:5284 |  | polymeric immunoglobulin receptor precursor [Homo sapiens] |
| P01860 | 2913433.25 | 2605711.083 | 0.894378165 | -0.161043128 | 0.418340136 | no | no change | GO:0004252;GO:0030449;GO:0009897;GO:0050871;GO:0042742;GO:0034987;GO:0042571;GO:0005615;GO:0016020;GO:0072562;GO:0006508;GO:0050853;GO:0045087;GO:0006910;GO:0006911;GO:0006956;GO:0003823;GO:0005886;GO:0006958;GO:0001895;GO:0070062;GO:0038096;GO:0002376;GO:0005576;GO:0002250 | hsa:100423062 | | Unknown (protein for MGC:105008) [Homo sapiens] |
| P01861 | 557342.3079 | 474919.1504 | 0.852113941 | -0.230881741 | 0.481169044 | no | no change | GO:0004252;GO:0030449;GO:0019221;GO:0009897;GO:0042742;GO:0034987;GO:0042571;GO:0005615;GO:0016020;GO:0072562;GO:0006508;GO:0050853;GO:0045087;GO:0006910;GO:0006911;GO:0006956;GO:0003823;GO:0005886;GO:0006958;GO:0050871;GO:0070062;GO:0038096;GO:0002376;GO:0005576;GO:0002250 | hsa:100423062 | | RecName: Full=Immunoglobulin heavy constant gamma 4; AltName: Full=Ig gamma-4 chain C region |
| P01871 | 10420076.83 | 11343463 | 1.088616061 | 0.122495227 | 0.652146055 | no | no change | GO:0009897;GO:0042834;GO:0019731;GO:0050900;GO:0034987;GO:0050829;GO:0005615;GO:0016021;GO:0016020;GO:0072562;GO:0009986;GO:0050853;GO:0045087;GO:0006910;GO:0006911;GO:0031210;GO:0003823;GO:0005886;GO:0006958;GO:0050871;GO:0070062;GO:0003697;GO:0002376;GO:0005576;GO:0002250;GO:0005515;GO:0071756;GO:0071757 | hsa:3543 |  | immunoglobulin heavy chain [Homo sapiens] |
| P01877 | 249862.9646 | 264324.4792 | 1.057877784 | 0.081172963 | 0.84537714 | no | no change | GO:0009897;GO:0019731;GO:0006955;GO:0050900;GO:0034987;GO:0001895;GO:0005615;GO:0016020;GO:0003094;GO:0072562;GO:0071748;GO:0050853;GO:0045087;GO:0006910;GO:0006911;GO:0003823;GO:0005886;GO:0006958;GO:0050871;GO:0060267;GO:0070062;GO:0006898;GO:0002376;GO:0005576;GO:0002250;GO:0071752;GO:0071751 | hsa:55423 |  | RecName: Full=Immunoglobulin heavy constant alpha 2; AltName: Full=Ig alpha-2 chain C region; AltName: Full=Ig alpha-2 chain C region BUT; AltName: Full=Ig alpha-2 chain C region LAN |
| P02042 | 65725.565 | 74112.88667 | 1.127611252 | 0.17326978 | 0.389880864 | no | no change | GO:0005344;GO:0019825;GO:0020037;GO:0043177;GO:0031721;GO:0007596;GO:0072562;GO:0005833;GO:0098869;GO:0042744;GO:0005515;GO:0031838;GO:0046872;GO:0005829;GO:0015671 | hsa:3045 | COG1018 | hemoglobin subunit delta [Homo sapiens] |
| P02100 | 33791.64692 | 36857.37129 | 1.090724326 | 0.125286515 | 0.680657457 | no | no change | GO:0005344;GO:0019825;GO:0020037;GO:0043177;GO:0031721;GO:0007596;GO:0051291;GO:0072562;GO:0005833;GO:0098869;GO:0042744;GO:0015671;GO:0005515;GO:0031838;GO:0046872;GO:0005829;GO:0014070 | hsa:3046 | COG1018 | hemoglobin subunit epsilon [Homo sapiens] |
| P02538 | 12718.27088 | 14020.07567 | 1.102357058 | 0.140591595 | 0.807148012 | no | no change | GO:0008284;GO:0002009;GO:0005200;GO:0007010;GO:0005829;GO:0031424;GO:0070062;GO:2000536;GO:0030154;GO:0045095;GO:0070268;GO:0042060;GO:0005198;GO:0050830;GO:0005882;GO:0005515;GO:0001899;GO:0016020;GO:0005634;GO:0051801;GO:0061844 | hsa:3853 |  | keratin, type II cytoskeletal 6A [Homo sapiens] |
| P02647 | 33872462.75 | 32231965.5 | 0.951568409 | -0.071620718 | 0.348222463 | no | no change | GO:0034115;GO:0010804;GO:0005788;GO:0019915;GO:0034191;GO:0034190;GO:0005548;GO:0050728;GO:0071682;GO:0051496;GO:0005543;GO:0010898;GO:0005319;GO:0034361;GO:0034362;GO:0034363;GO:0034364;GO:0031102;GO:0034366;GO:0031100;GO:0043534;GO:0030300;GO:0034774;GO:0010873;GO:0043691;GO:0007179;GO:0015485;GO:0005829;GO:0018206;GO:0019433;GO:0045499;GO:0070508;GO:0045723;GO:0034375;GO:0034371;GO:0031072;GO:0015914;GO:0034378;GO:0050821;GO:0010903;GO:0042158;GO:0055102;GO:0044267;GO:0042627;GO:0051180;GO:0014012;GO:0072562;GO:0050919;GO:0007186;GO:0033700;GO:0018158;GO:0006629;GO:0060761;GO:0042632;GO:0032489;GO:0050713;GO:0007229;GO:0006898;GO:0034384;GO:0062023;GO:0006695;GO:0060192;GO:0006869;GO:0019899;GO:0030301;GO:0035025;GO:0051006;GO:0002740;GO:0001540;GO:1900026;GO:0031410;GO:0005634;GO:0042802;GO:0008035;GO:0060354;GO:0009986;GO:0008289;GO:0008202;GO:0008203;GO:0071813;GO:0070062;GO:0042493;GO:0005515;GO:0034380;GO:0070371;GO:0042157;GO:0006656;GO:0030325;GO:0006644;GO:0017127;GO:0030139;GO:0008211;GO:0060228;GO:0001523;GO:0055091;GO:0034365;GO:0005615;GO:0002576;GO:0005576;GO:0043687;GO:0070328;GO:0019216;GO:0007584;GO:0031210;GO:0005886;GO:0043627;GO:1903561;GO:0051346;GO:0051345;GO:0033344;GO:0070653;GO:0001932;GO:0001935;GO:0005769 | hsa:335 |  | apolipoprotein A-I isoform 1 preproprotein [Homo sapiens] |
| P02649 | 1879563.25 | 1848625.629 | 0.983539995 | -0.023944375 | 0.852855293 | no | no change | GO:0051044;GO:0005783;GO:0034447;GO:0007616;GO:0005788;GO:0072358;GO:0043524;GO:0010873;GO:0051651;GO:0050728;GO:1905855;GO:0071682;GO:0016020;GO:0008201;GO:0098869;GO:0060999;GO:0045541;GO:0044794;GO:0005319;GO:0034361;GO:0034362;GO:0034363;GO:0034364;GO:0031102;GO:0016209;GO:0071813;GO:0043537;GO:0010977;GO:0030425;GO:0030516;GO:0043691;GO:0010877;GO:0010875;GO:0061771;GO:0015485;GO:0043025;GO:0019433;GO:1990777;GO:0030195;GO:0034375;GO:0034374;GO:0034372;GO:0034371;GO:1903002;GO:0000302;GO:0019934;GO:0010629;GO:0044877;GO:0042157;GO:0042311;GO:0042158;GO:0042159;GO:0048168;GO:0042982;GO:0044267;GO:0042627;GO:0072562;GO:0035641;GO:0010544;GO:0097006;GO:0007186;GO:0062023;GO:0033700;GO:0015909;GO:0006629;GO:1905890;GO:0048156;GO:1902430;GO:0042632;GO:0032489;GO:1901215;GO:0046907;GO:0034382;GO:0034380;GO:0006898;GO:0034384;GO:0006357;GO:0046983;GO:0032805;GO:0005543;GO:0090209;GO:0006869;GO:0051246;GO:0019068;GO:0050807;GO:0031012;GO:0043407;GO:0051000;GO:1902995;GO:0043083;GO:0005737;GO:0001540;GO:0005634;GO:0030669;GO:1900221;GO:0002021;GO:0090181;GO:0042802;GO:0042803;GO:0006641;GO:0006874;GO:0043687;GO:0017038;GO:0008289;GO:0055088;GO:0055089;GO:0046889;GO:0034378;GO:0006979;GO:0008202;GO:0008203;GO:0032269;GO:0070062;GO:1900272;GO:1902952;GO:0005515;GO:1901630;GO:0005794;GO:0017127;GO:0007271;GO:0060228;GO:0001523;GO:0034365;GO:0005615;GO:0048844;GO:0005198;GO:0033344;GO:1901628;GO:0010468;GO:0032462;GO:0090090;GO:0006707;GO:0070326;GO:0046911;GO:0005886;GO:2000822;GO:1903561;GO:0007263;GO:1905908;GO:0050750;GO:0007010;GO:1905860;GO:0097114;GO:0005576;GO:1905906;GO:0097113;GO:0045807;GO:0098978;GO:0070328;GO:0005769;GO:0001937;GO:0010976 | hsa:348 |  | apolipoprotein E [Homo sapiens] |
| P02671 | 6850534.375 | 6592469.667 | 0.962329259 | -0.055397502 | 0.58593089 | no | no change | GO:0045087;GO:0009897;GO:0045907;GO:0007599;GO:0034116;GO:0007596;GO:0005788;GO:0007160;GO:0045921;GO:0045202;GO:0034622;GO:1902042;GO:0044267;GO:0050839;GO:0031091;GO:0005615;GO:0031093;GO:1900026;GO:0072378;GO:0002576;GO:0031639;GO:0072562;GO:0005198;GO:0065003;GO:0030198;GO:0002224;GO:0046872;GO:0043687;GO:0009986;GO:2000352;GO:0051592;GO:0090277;GO:0005938;GO:0005886;GO:1903561;GO:0030168;GO:0070062;GO:0051258;GO:0042730;GO:0072377;GO:0050714;GO:0002376;GO:0005577;GO:0005576;GO:0002250;GO:0005515;GO:0043152;GO:0005102;GO:0070374;GO:0070527 | hsa:2243 |  | fibrinogen alpha chain isoform alpha-E preproprotein [Homo sapiens] |
| P02675 | 14965424.92 | 14246328.08 | 0.951949454 | -0.071043122 | 0.469678862 | no | no change | GO:1902042;GO:0034116;GO:0045921;GO:0007599;GO:0005783;GO:0009897;GO:0007596;GO:0007160;GO:0045907;GO:0045202;GO:0034622;GO:0044320;GO:0005737;GO:1903561;GO:0031091;GO:0005615;GO:0031093;GO:1900026;GO:0072378;GO:0002576;GO:0051087;GO:0031639;GO:0072562;GO:0005198;GO:0030198;GO:0002224;GO:0045087;GO:0009986;GO:2000352;GO:0051592;GO:0090277;GO:0005938;GO:0005886;GO:0050839;GO:0030168;GO:0070062;GO:0051258;GO:0042730;GO:0050714;GO:0071347;GO:0002376;GO:0005577;GO:0005576;GO:0002250;GO:0005515;GO:0043152;GO:0005102;GO:0070374;GO:0070527 | hsa:2244 |  | fibrinogen beta chain isoform 1 preproprotein [Homo sapiens] |
| P02743 | 389385.7333 | 384314.5167 | 0.986976368 | -0.018912554 | 0.872533857 | no | no change | GO:0006457;GO:0030246;GO:0046597;GO:0044871;GO:0072562;GO:0061045;GO:0044267;GO:0070062;GO:0005615;GO:0005634;GO:0051082;GO:0001849;GO:0005509;GO:0030169;GO:0042802;GO:1903016;GO:0051131;GO:0045087;GO:1903019;GO:0006953;GO:0062023;GO:0044869;GO:0006958;GO:0002674;GO:0046872;GO:0045656;GO:0046790;GO:0005576;GO:0048525 | hsa:325 |  | serum amyloid P-component precursor [Homo sapiens] |
| P02745 | 33476.93917 | 33321.85458 | 0.99536742 | -0.006698928 | 0.966160319 | no | no change | GO:0004252;GO:0030449;GO:0005581;GO:0045087;GO:0002376;GO:0005576;GO:0006956;GO:0005515;GO:0010039;GO:0007267;GO:0006508;GO:0006958;GO:0005602 | hsa:712 |  | complement C1q subcomponent subunit A precursor [Homo sapiens] |
| P02748 | 120882.7829 | 134374.9038 | 1.111613255 | 0.152654942 | 0.216895092 | no | no change | GO:0030449;GO:0006955;GO:0019835;GO:0019836;GO:0070062;GO:0016021;GO:0016020;GO:0051260;GO:0072562;GO:0001906;GO:0045087;GO:0006957;GO:0005887;GO:0005886;GO:0006958;GO:0005615;GO:0005829;GO:0044218;GO:0044279;GO:0002376;GO:0005576;GO:0005579 | hsa:735 |  | complement component C9 preproprotein [Homo sapiens] |
| P02749 | 923438.4708 | 912255.625 | 0.987889994 | -0.017577695 | 0.860332716 | no | no change | GO:0034392;GO:0007597;GO:0051006;GO:0034197;GO:0042627;GO:0070062;GO:0002576;GO:0005543;GO:0031639;GO:0051917;GO:0042802;GO:0051918;GO:0034361;GO:0034364;GO:0006641;GO:0009986;GO:0060230;GO:0008289;GO:0062023;GO:0033033;GO:0008201;GO:0005615;GO:0010596;GO:0031089;GO:0005576;GO:0030195;GO:0005515;GO:0030193;GO:0016525;GO:0001937;GO:0030194 | hsa:350 |  | beta-2-glycoprotein 1 precursor [Homo sapiens] |
| P02750 | 128410.7275 | 136499.81 | 1.062993822 | 0.088133212 | 0.465563207 | no | no change | GO:0043231;GO:0016020;GO:0070062;GO:0005160;GO:0009617;GO:0050873;GO:0045766;GO:0043312;GO:1904813;GO:0001938;GO:1904724;GO:0005576;GO:0003674;GO:0005515;GO:0035580;GO:0008150;GO:0030511;GO:0005615 | hsa:116844 | COG4886 | leucine-rich alpha-2-glycoprotein precursor [Homo sapiens] |
| P02751 | 841470.4125 | 905640.4042 | 1.076259356 | 0.106025779 | 0.389729481 | no | no change | GO:0008022;GO:0018149;GO:0019221;GO:0034446;GO:0048146;GO:0005518;GO:0005201;GO:0005788;GO:0062023;GO:0007160;GO:0007161;GO:0050900;GO:0010628;GO:0097718;GO:0001525;GO:0044267;GO:0070062;GO:0031093;GO:0002576;GO:0051087;GO:0009611;GO:0072562;GO:0008201;GO:0005576;GO:0002020;GO:0030198;GO:1904237;GO:0033622;GO:0042802;GO:0008284;GO:0005178;GO:2001202;GO:0008360;GO:0005793;GO:0016324;GO:0043687;GO:0042060;GO:0006953;GO:0043394;GO:0019899;GO:0007155;GO:0051702;GO:0010952;GO:0035987;GO:1901166;GO:0005615;GO:0052047;GO:0005604;GO:0045773;GO:0031012;GO:0005577;GO:0007044;GO:0005515;GO:0016504;GO:0005102;GO:0001932;GO:0070372 | hsa:2335 |  | fibronectin isoform 1 precursor [Homo sapiens] |
| P02753 | 68764.06375 | 70244.31042 | 1.021526457 | 0.03072657 | 0.853602609 | no | no change | GO:0060347;GO:0032024;GO:0048562;GO:0030324;GO:0060059;GO:0060044;GO:0042593;GO:0051024;GO:0060065;GO:0042572;GO:0001523;GO:0016918;GO:0005615;GO:0048807;GO:0006094;GO:0005501;GO:0032526;GO:0048706;GO:0007601;GO:0045471;GO:0001654;GO:0034633;GO:0070062;GO:0007507;GO:0032991;GO:0060157;GO:0005829;GO:0019841;GO:0048738;GO:0005576;GO:0005515;GO:0060068;GO:0034632;GO:0046982;GO:0050896;GO:0030277 | hsa:5950 |  | retinol-binding protein 4 isoform a precursor [Homo sapiens] |
| P02760 | 455763.3417 | 436345.2333 | 0.957394317 | -0.062814852 | 0.487266101 | no | no change | GO:0005886;GO:0010951;GO:0046329;GO:0070062;GO:0019855;GO:0072562;GO:0010466;GO:0042803;GO:0005515;GO:0020037;GO:0009986;GO:0042167;GO:0018298;GO:0062023;GO:0030163;GO:0030414;GO:0004867;GO:0007155;GO:0005615;GO:0050777;GO:0046904;GO:0019862;GO:0007565;GO:0006898;GO:0043231;GO:0005576;GO:0016032 | hsa:259 |  | protein AMBP preproprotein [Homo sapiens] |
| P02763 | 1697016.625 | 1554695.958 | 0.916134783 | -0.126368229 | 0.328483904 | no | no change | GO:1904469;GO:0050716;GO:0031093;GO:0005615;GO:0002576;GO:0050718;GO:0043312;GO:0072562;GO:0006953;GO:0032715;GO:0062023;GO:0002682;GO:0005515;GO:0006954;GO:0035580;GO:0005576;GO:1904724;GO:0032720;GO:0070062 | hsa:5004 |  | RecName: Full=Alpha-1-acid glycoprotein 1; Short=AGP 1; AltName: Full=Orosomucoid-1; Short=OMD 1; Flags: Precursor |
| P02766 | 718233.1167 | 731202.25 | 1.018056997 | 0.025818335 | 0.735880976 | no | no change | GO:0005179;GO:0044267;GO:0032991;GO:0070062;GO:0042562;GO:0070324;GO:0005615;GO:0070327;GO:0005737;GO:0043312;GO:0001523;GO:0035578;GO:0030198;GO:0005515;GO:0042572;GO:0046982;GO:0010469;GO:0006144;GO:0042802;GO:0005576 | hsa:7276 | COG2351 | transthyretin precursor [Homo sapiens] |
| P02775 | 42574.82442 | 40009.81917 | 0.939753005 | -0.089646472 | 0.75371316 | no | no change | GO:0042127;GO:0070098;GO:0005125;GO:0071222;GO:0008009;GO:0042742;GO:0060326;GO:0032496;GO:0061844;GO:0031091;GO:0008083;GO:0031093;GO:0002576;GO:0007186;GO:0005355;GO:0030595;GO:0030593;GO:1904724;GO:0006952;GO:0006954;GO:0006955;GO:0045236;GO:0005615;GO:0090023;GO:0010469;GO:0031640;GO:0043312;GO:0006935;GO:0051781;GO:0005576;GO:1904659;GO:0005515 | hsa:5473 |  | platelet basic protein preproprotein [Homo sapiens] |
| P02776 | 40245.04375 | 44828.62167 | 1.113891736 | 0.155609018 | 0.637493447 | no | no change | GO:0020005;GO:0070098;GO:0005125;GO:0071222;GO:0019221;GO:0008009;GO:0010469;GO:0032760;GO:0010628;GO:0048248;GO:0032496;GO:0045918;GO:0061844;GO:0005737;GO:0045347;GO:0031093;GO:0002576;GO:0007189;GO:0051873;GO:0010744;GO:0007186;GO:2001240;GO:0042127;GO:0030595;GO:0030593;GO:0006935;GO:0043950;GO:0006952;GO:0062023;GO:0006954;GO:0006955;GO:0008201;GO:0030168;GO:0005615;GO:0090023;GO:0045651;GO:0045652;GO:0045653;GO:0031640;GO:0005576;GO:0045944;GO:0005515;GO:0097679;GO:0042832;GO:0016525 | hsa:5196 |  | platelet factor 4 isoform 1 precursor [Homo sapiens] |
| P02787 | 18754505.5 | 17106102.33 | 0.912106285 | -0.132726148 | 0.120532905 | no | no change | GO:0009617;GO:0016020;GO:0055037;GO:1990459;GO:1900390;GO:0005788;GO:0030139;GO:0061024;GO:0055072;GO:0006826;GO:0005905;GO:0034986;GO:1990712;GO:0048260;GO:0010008;GO:0030665;GO:0007257;GO:0045780;GO:0031232;GO:0005615;GO:0031410;GO:0002576;GO:0005770;GO:0045178;GO:0070062;GO:2000147;GO:0009925;GO:0044267;GO:0048471;GO:0046872;GO:0043687;GO:0009986;GO:0006879;GO:0033572;GO:0007015;GO:0016324;GO:0006811;GO:0030316;GO:0031982;GO:0008198;GO:0008199;GO:0045893;GO:0034774;GO:0015091;GO:0001895;GO:0034756;GO:0071281;GO:0042327;GO:0060395;GO:0031647;GO:0005623;GO:0005576;GO:0005515;GO:0005768;GO:0005769;GO:0070371;GO:0072562 | hsa:7018 |  | serotransferrin isoform 1 precursor [Homo sapiens] |
| P02790 | 3059440.5 | 2810931.875 | 0.918773179 | -0.122219353 | 0.103984814 | no | no change | GO:0020027;GO:0002925;GO:0015232;GO:0071682;GO:0005615;GO:0051246;GO:0042531;GO:0072562;GO:0046872;GO:0042168;GO:0006879;GO:0016032;GO:0060332;GO:0062023;GO:0060335;GO:0002639;GO:0070062;GO:0015886;GO:0006898;GO:0005623;GO:0005576;GO:0005515 | hsa:3263 |  | hemopexin precursor [Homo sapiens] |
| P04003 | 2762448.458 | 2612318.208 | 0.945653194 | -0.080616904 | 0.559998622 | no | no change | GO:0030449;GO:0005615;GO:0045087;GO:0072562;GO:0002376;GO:0045732;GO:0005576;GO:0003723;GO:0005515;GO:0005886;GO:1903027;GO:0006958;GO:0045959;GO:0044216 | hsa:722 |  | C4b-binding protein alpha chain precursor [Homo sapiens] |
| P04114 | 3862682.083 | 4496564.042 | 1.164104098 | 0.219220074 | 0.063239613 | no | no change | GO:0034360;GO:0034374;GO:0034359;GO:0005783;GO:0034371;GO:0006869;GO:0050750;GO:0005788;GO:0005789;GO:0009791;GO:0061024;GO:0050900;GO:0010628;GO:0042157;GO:0034378;GO:0032496;GO:0016042;GO:0001523;GO:0006629;GO:0005737;GO:0043202;GO:0032355;GO:0001701;GO:0031983;GO:0005615;GO:0071682;GO:0071356;GO:0009615;GO:0048844;GO:0005543;GO:0030669;GO:0071379;GO:0070062;GO:0010033;GO:0042953;GO:0010884;GO:0042158;GO:0002224;GO:0010008;GO:0043025;GO:0043687;GO:0070971;GO:0005319;GO:0034361;GO:0034362;GO:0034363;GO:0006642;GO:0005790;GO:0044267;GO:0030317;GO:0008289;GO:0034379;GO:0010744;GO:0007283;GO:0042627;GO:0042159;GO:0017127;GO:0012506;GO:0005886;GO:0042632;GO:0030301;GO:0008201;GO:0008202;GO:0008203;GO:0035473;GO:0031904;GO:0009743;GO:0007399;GO:0034382;GO:0034383;GO:0005829;GO:0006898;GO:0009566;GO:0043231;GO:0010886;GO:0019433;GO:0005576;GO:0045540;GO:0005515;GO:0034447;GO:0010269;GO:0005769;GO:0033344 | hsa:338 |  | RecName: Full=Apolipoprotein B-100; Short=Apo B-100; Contains: RecName: Full=Apolipoprotein B-48; Short=Apo B-48; Flags: Precursor |
| P04180 | 21735.335 | 19570.84 | 0.900415844 | -0.151336652 | 0.097818835 | no | no change | GO:0034375;GO:0006656;GO:0034372;GO:0043691;GO:0008374;GO:0042158;GO:0090107;GO:0070062;GO:0016740;GO:0016746;GO:0034435;GO:0008203;GO:0006644;GO:0034364;GO:0006629;GO:0042632;GO:0030301;GO:0008202;GO:0005615;GO:0005576;GO:0005515;GO:0034186;GO:0004607;GO:0046470 | hsa:3931 |  | phosphatidylcholine-sterol acyltransferase precursor [Homo sapiens] |
| P04196 | 646424.2125 | 560602.9292 | 0.867236899 | -0.205501954 | 0.070312864 | no | no change | GO:0002839;GO:0030308;GO:0032956;GO:2000504;GO:0007162;GO:0008270;GO:0051894;GO:0007599;GO:0001525;GO:0061844;GO:0051715;GO:0070062;GO:0031093;GO:0002576;GO:0072562;GO:0043065;GO:0033629;GO:0030168;GO:0005886;GO:0046872;GO:0010468;GO:0051918;GO:0010543;GO:0008285;GO:0007596;GO:0006935;GO:0020037;GO:0009986;GO:0010593;GO:0043537;GO:0043395;GO:0062023;GO:0004867;GO:0010951;GO:0004869;GO:0043254;GO:0008201;GO:2001027;GO:0050832;GO:0042730;GO:0036019;GO:0050730;GO:0015886;GO:0019865;GO:0005576;GO:1900747;GO:0005515;GO:0030193;GO:0005102;GO:0016525 | hsa:3273 |  | histidine-rich glycoprotein precursor [Homo sapiens] |
| P04217 | 780897.2333 | 784967.7167 | 1.005212572 | 0.00750062 | 0.909411167 | no | no change | GO:0070062;GO:0031093;GO:0002576;GO:0043312;GO:0072562;GO:1904813;GO:0005576;GO:0003674;GO:0034774;GO:0008150;GO:0062023;GO:0005615 | hsa:1 |  | alpha-1B-glycoprotein precursor [Homo sapiens] |
| P04264 | 222059.4333 | 211548.2708 | 0.952665094 | -0.069958967 | 0.520898834 | no | no change | GO:0018149;GO:0038023;GO:0045095;GO:0030246;GO:0031012;GO:1904813;GO:0061436;GO:0030280;GO:0005856;GO:0050728;GO:0070062;GO:0005634;GO:0016020;GO:0045765;GO:0072562;GO:0005198;GO:0001867;GO:0005882;GO:0051290;GO:0070268;GO:0062023;GO:0005886;GO:0001895;GO:0006979;GO:0001533;GO:0005615;GO:0042730;GO:0031424;GO:0005829;GO:0043312;GO:0005576;GO:0005515;GO:0046982 | hsa:3848 |  | keratin 1 [Homo sapiens] |
| P04275 | 71404.84208 | 65047.3725 | 0.910965848 | -0.134531127 | 0.514088799 | no | no change | GO:0005201;GO:0007599;GO:0005783;GO:0007597;GO:0007596;GO:0031012;GO:0031091;GO:0070062;GO:0031093;GO:0002576;GO:0051087;GO:0051260;GO:0009611;GO:0005576;GO:0002020;GO:0042802;GO:0042803;GO:0005178;GO:0031589;GO:0033093;GO:0062023;GO:0007155;GO:0030168;GO:0047485;GO:0019865;GO:0030198;GO:0005515;GO:0005518 | hsa:7450 |  | RecName: Full=von Willebrand factor; Short=vWF; Contains: RecName: Full=von Willebrand antigen 2; AltName: Full=von Willebrand antigen II; Flags: Precursor |
| P04433 | 97628.94542 | 112058.7604 | 1.147802631 | 0.198874587 | 0.08024156 | no | no change | GO:0038096;GO:0004252;GO:0016020;GO:0030449;GO:0050776;GO:0006898;GO:0038095;GO:0072562;GO:0002376;GO:0050900;GO:0005576;GO:0006956;GO:0002250;GO:0003823;GO:0005886;GO:0006508;GO:0006958;GO:0006955;GO:0070062 | hsa:7441 |  | rheumatoid factor D1 IgG light chain VK3 region, partial [Homo sapiens] |
| P05154 | 19250.355 | 17383.09479 | 0.903001259 | -0.147200097 | 0.22392762 | no | no change | GO:0009897;GO:0006869;GO:0007596;GO:0036024;GO:0002080;GO:0061107;GO:0036029;GO:0031094;GO:0031091;GO:0051346;GO:0007342;GO:0016020;GO:0036025;GO:0032190;GO:0036027;GO:0036026;GO:0010466;GO:0002020;GO:0097183;GO:0097182;GO:0097181;GO:0036028;GO:0007283;GO:0031210;GO:0007338;GO:0030414;GO:0004867;GO:0010951;GO:0008201;GO:0005615;GO:0032991;GO:0070062;GO:0036030;GO:0005539;GO:0005576;GO:0005515;GO:0001972;GO:0045861 | hsa:5104 | COG4826 | plasma serine protease inhibitor preproprotein [Homo sapiens] |
| P05155 | 333477.4708 | 330904.0167 | 0.992282974 | -0.011176495 | 0.926175273 | no | no change | GO:0030449;GO:0007597;GO:0007596;GO:0007599;GO:0005615;GO:0031093;GO:0002576;GO:0072562;GO:0010466;GO:0001869;GO:0045916;GO:0008015;GO:0045087;GO:0030414;GO:0004867;GO:0010951;GO:0006958;GO:0070062;GO:0042730;GO:0007568;GO:0002376;GO:0005576;GO:0005515;GO:0030193 | hsa:710 | COG4826 | unnamed protein product [Homo sapiens] |
| P05160 | 26736.40458 | 24088.64167 | 0.900967877 | -0.150452426 | 0.129241581 | no | no change | GO:0072378;GO:0005576;GO:0007596;GO:0007599;GO:1903363 | hsa:2165 |  | coagulation factor XIII B chain precursor [Homo sapiens] |
| P05543 | 26070.655 | 27862.02542 | 1.068712137 | 0.095873308 | 0.382152332 | no | no change | GO:0005615;GO:0070327;GO:0005576;GO:0004867;GO:0010951;GO:0070062 | hsa:6906 | COG4826 | thyroxine-binding globulin precursor [Homo sapiens] |
| P06312 | 430701.625 | 385772.4458 | 0.895683748 | -0.158938666 | 0.109212946 | no | no change | GO:0038096;GO:0004252;GO:0016020;GO:0030449;GO:0050776;GO:0006898;GO:0038095;GO:0072562;GO:0002376;GO:0002377;GO:0050900;GO:0005576;GO:0006956;GO:0002250;GO:0003823;GO:0005886;GO:0006508;GO:0006958;GO:0006955 | hsa:7441 |  | immunoglobulin kappa chain, partial [Homo sapiens] |
| P06331 | 520961.7667 | 441931.2375 | 0.848298792 | -0.237355587 | 0.641147236 | no | no change | GO:0038096;GO:0004252;GO:0016020;GO:0030449;GO:0050776;GO:0006898;GO:0038095;GO:0002376;GO:0050900;GO:0005576;GO:0006956;GO:0002250;GO:0003823;GO:0005886;GO:0006508;GO:0006958;GO:0006955 | hsa:102724971 | | hCG1793614, partial [Homo sapiens] |
| P06727 | 964838.4583 | 1004446.754 | 1.041051738 | 0.05804177 | 0.558757153 | no | no change | GO:0034375;GO:0034445;GO:0034372;GO:0034371;GO:0006869;GO:0034380;GO:0045723;GO:0005788;GO:0034378;GO:0051006;GO:0042744;GO:0042157;GO:0042632;GO:0070328;GO:0060228;GO:0001523;GO:0035634;GO:0044267;GO:0042627;GO:0005615;GO:0006982;GO:0032374;GO:0072562;GO:0016042;GO:0033344;GO:0065005;GO:0010898;GO:0002227;GO:0009986;GO:0042802;GO:0033700;GO:0005319;GO:0034361;GO:0034364;GO:0031102;GO:0062023;GO:0016209;GO:0008289;GO:0055088;GO:0031210;GO:0007159;GO:0017127;GO:0010873;GO:0043691;GO:0008203;GO:0030300;GO:0005507;GO:0015485;GO:0070062;GO:0005829;GO:0046470;GO:0019430;GO:0005576;GO:0006695;GO:0005515;GO:0042803;GO:0005769 | hsa:337 |  | RecName: Full=Apolipoprotein A-IV; Short=Apo-AIV; Short=ApoA-IV; AltName: Full=Apolipoprotein A4; Flags: Precursor |
| P07357 | 51566.1375 | 55249.77208 | 1.071435146 | 0.099544527 | 0.197741242 | no | no change | GO:0019835;GO:0030449;GO:0070062;GO:0016021;GO:0016020;GO:0072562;GO:0002376;GO:0001848;GO:0005576;GO:0006956;GO:0006957;GO:0005886;GO:0006955;GO:0044877;GO:0006958;GO:0005579;GO:0045087;GO:0005615 | hsa:731 |  | complement component C8 alpha chain preproprotein [Homo sapiens] |
| P07358 | 38317.925 | 39029.12833 | 1.018560591 | 0.026531804 | 0.74437234 | no | no change | GO:0019835;GO:0030449;GO:0005615;GO:0045087;GO:0016020;GO:0002376;GO:0005576;GO:0006956;GO:0006957;GO:0006955;GO:0044877;GO:0006958;GO:1903561;GO:0005579;GO:0070062 | hsa:732 |  | RecName: Full=Complement component C8 beta chain; AltName: Full=Complement component 8 subunit beta; Flags: Precursor |
| P07360 | 35935.62708 | 34992.18875 | 0.973746435 | -0.038381954 | 0.647068511 | no | no change | GO:0019835;GO:0036094;GO:0030449;GO:0070062;GO:0045087;GO:0019841;GO:0072562;GO:0002376;GO:0001848;GO:0005576;GO:0044877;GO:0006957;GO:0006958;GO:0005579;GO:0005615 | hsa:733 |  | complement component C8 gamma chain precursor [Homo sapiens] |
| P07737 | 15887.21104 | 14944.56517 | 0.940666372 | -0.088244965 | 0.736626654 | no | no change | GO:0017048;GO:0050821;GO:0060074;GO:0045202;GO:0050434;GO:0005925;GO:0005737;GO:0032232;GO:0032233;GO:0070062;GO:0030837;GO:0030838;GO:0051496;GO:0005634;GO:0016020;GO:0005546;GO:0003779;GO:0098794;GO:0043005;GO:0098793;GO:0001843;GO:0005856;GO:0051054;GO:0098685;GO:0005515;GO:0032781;GO:0060071;GO:0098688;GO:0070064;GO:0005938;GO:0000774;GO:0010033;GO:0010634;GO:0071363;GO:0005829;GO:1900029;GO:0098885;GO:0045296;GO:0051497;GO:0006357;GO:0045944;GO:0003723;GO:0030036;GO:0072562;GO:0005102;GO:0098978;GO:0003785 | hsa:5216 |  | PREDICTED: profilin-1 isoform X2 [Nomascus leucogenys] |
| P07996 | 28167.29163 | 29556.91033 | 1.049334481 | 0.069474618 | 0.856821578 | no | no change | GO:0032026;GO:0005783;GO:0050840;GO:0034605;GO:0005788;GO:2001237;GO:0050921;GO:1903671;GO:0048266;GO:0051895;GO:0006986;GO:0043652;GO:0042535;GO:0009612;GO:0030511;GO:0002581;GO:0042327;GO:0051592;GO:0043536;GO:0040037;GO:2001027;GO:0030169;GO:0043236;GO:0045727;GO:0045652;GO:0030198;GO:0043032;GO:0030194;GO:0009897;GO:0018149;GO:0042493;GO:0002605;GO:0071356;GO:0051897;GO:0016477;GO:0002040;GO:0001968;GO:2000379;GO:0030141;GO:0007050;GO:0043394;GO:0006954;GO:0006955;GO:0070052;GO:0090051;GO:0070051;GO:0033574;GO:0043154;GO:0034976;GO:1902043;GO:0031012;GO:0001666;GO:0050431;GO:0005737;GO:0031091;GO:0031093;GO:0005509;GO:0010763;GO:0001786;GO:0051918;GO:0008284;GO:0009986;GO:0017134;GO:2000353;GO:0008201;GO:0071363;GO:0001953;GO:0002544;GO:0016529;GO:0042802;GO:0005515;GO:0032570;GO:0016525;GO:0000187;GO:0005201;GO:0005615;GO:0002576;GO:0010751;GO:0045766;GO:0043066;GO:0010748;GO:0032695;GO:1903588;GO:0005178;GO:0030335;GO:0048661;GO:0032914;GO:0062023;GO:0007155;GO:0070062;GO:0043537;GO:0010595;GO:0010596;GO:0009749;GO:0005577;GO:0005576;GO:0010757;GO:0010754;GO:0010759;GO:0001937;GO:0071636 | hsa:7057 |  | thrombospondin-1 precursor [Homo sapiens] |
| P08571 | 11841.57217 | 10546.54488 | 0.890637217 | -0.167090197 | 0.164896042 | no | no change | GO:0006954;GO:0009897;GO:0045121;GO:0071222;GO:0071223;GO:0097190;GO:0031362;GO:1901224;GO:0007166;GO:0006909;GO:0032496;GO:0045471;GO:0010008;GO:0034612;GO:0016020;GO:0030667;GO:0005615;GO:0071727;GO:0071726;GO:0009617;GO:0071723;GO:0071219;GO:0032729;GO:0002224;GO:0031225;GO:0009986;GO:0001847;GO:0005794;GO:0032760;GO:0070266;GO:0034128;GO:0038124;GO:0045087;GO:0006915;GO:2000484;GO:0035666;GO:0032481;GO:0070891;GO:0034142;GO:0005886;GO:0009408;GO:0001530;GO:0032026;GO:0070062;GO:0051602;GO:0002755;GO:0002756;GO:0016019;GO:0050715;GO:0038123;GO:0031663;GO:0006898;GO:0007249;GO:0043312;GO:0002376;GO:0005576;GO:0002237;GO:0005515;GO:0045807;GO:0046696 | hsa:929 |  | monocyte differentiation antigen CD14 precursor [Homo sapiens] |
| P08603 | 1326576.996 | 1298499.188 | 0.978834392 | -0.030863302 | 0.713842527 | no | no change | GO:1903659;GO:0030449;GO:0070062;GO:0005515;GO:0045087;GO:0072562;GO:0002376;GO:0043395;GO:0005576;GO:0006956;GO:0006957;GO:0016032;GO:0008201;GO:0005615 | hsa:3075 |  | RecName: Full=Complement factor H; AltName: Full=H factor 1; Flags: Precursor |
| P08779 | 109549.4108 | 113683.1733 | 1.037734228 | 0.053437006 | 0.887338197 | no | no change | GO:0005200;GO:0061436;GO:0008544;GO:0005856;GO:0002009;GO:0051546;GO:0070062;GO:0005634;GO:0005198;GO:0030336;GO:0030216;GO:0008283;GO:0045087;GO:0070268;GO:0005882;GO:0006954;GO:0042633;GO:0007568;GO:0007010;GO:0045104;GO:0005829;GO:0031424;GO:0005515 | hsa:3868 |  | keratin, type I cytoskeletal 16 [Homo sapiens] |
| P0C0L5 | 1868176.25 | 1801409.792 | 0.964261157 | -0.052504162 | 0.658103525 | no | no change | GO:0004252;GO:0030449;GO:0006954;GO:0030246;GO:0030425;GO:0030424;GO:0045202;GO:0032490;GO:0030054;GO:0005615;GO:0072562;GO:0001848;GO:0006508;GO:0045087;GO:0008228;GO:0006956;GO:2000427;GO:0004866;GO:0005886;GO:0010951;GO:0006958;GO:0042995;GO:0070062;GO:0044216;GO:0002376;GO:0005576 | hsa:100293534;hsa:110384692;hsa:720;hsa:721 | | complement C4-B preproprotein [Homo sapiens] |
| P0DOY3 | 9987699.083 | 8981379.125 | 0.899244065 | -0.153215362 | 0.289077172 | no | no change | GO:0005615;GO:0016020;GO:0072562;GO:0002376;GO:0005576;GO:0002250;GO:0003823;GO:0005886;GO:0070062 | hsa:100423062 | | RecName: Full=Immunoglobulin lambda constant 3; AltName: Full=Ig lambda chain C region DOT; AltName: Full=Ig lambda chain C region NEWM; AltName: Full=Ig lambda-3 chain C regions |
| P0DP02 | 315646.3906 | 299306.6975 | 0.948234184 | -0.076684692 | 0.703833318 | no | no change | GO:0016020;GO:0002376;GO:0005576;GO:0002250;GO:0003823;GO:0005886 | hsa:102723407 | | immunoglobulin heavy chain [Homo sapiens] |
| P10643 | 94850.89417 | 86292.0675 | 0.909765461 | -0.13643343 | 0.127164037 | no | no change | GO:0019835;GO:0030449;GO:0070062;GO:0045087;GO:0006883;GO:0002376;GO:0005576;GO:0006956;GO:0006957;GO:0006955;GO:0006958;GO:0005579 | hsa:730 |  | complement component C7 precursor [Homo sapiens] |
| P10909 | 913876.8833 | 954563.8167 | 1.044521241 | 0.062841831 | 0.438645527 | no | no change | GO:0032436;GO:0005783;GO:0019730;GO:1903573;GO:0009615;GO:0016020;GO:1902949;GO:0042127;GO:0005794;GO:0034366;GO:0060548;GO:0031966;GO:0043691;GO:0043231;GO:0005829;GO:0002376;GO:0051787;GO:1902004;GO:0051788;GO:0030449;GO:1902230;GO:0050821;GO:0010628;GO:0044877;GO:0099020;GO:0048260;GO:0005856;GO:0000902;GO:0002434;GO:0072562;GO:0048471;GO:0097418;GO:0051131;GO:0032760;GO:0006629;GO:1905895;GO:0048156;GO:0001774;GO:0006956;GO:1905892;GO:1901216;GO:0006958;GO:0061077;GO:2000060;GO:1902430;GO:1902847;GO:0090201;GO:0031012;GO:0045429;GO:0051082;GO:1902998;GO:1901214;GO:0005737;GO:0001540;GO:0031093;GO:0031410;GO:0005634;GO:0051087;GO:0005739;GO:1900221;GO:0009986;GO:0017038;GO:0061740;GO:0061741;GO:0051092;GO:0031625;GO:0070062;GO:0005622;GO:0005743;GO:0005515;GO:0097440;GO:0032286;GO:0016887;GO:0071944;GO:0045202;GO:0061518;GO:0005615;GO:0002576;GO:0043065;GO:0032464;GO:0032463;GO:0045087;GO:0006915;GO:0042583;GO:0062023;GO:0050750;GO:0032991;GO:0001836;GO:0005576;GO:1905907;GO:1905908 | hsa:1191 |  | clusterin preproprotein [Homo sapiens] |
| P11226 | 107708.6689 | 113633.3227 | 1.055006286 | 0.077251595 | 0.732390315 | no | no change | GO:0004252;GO:0048306;GO:0030246;GO:0042742;GO:0044130;GO:0050766;GO:0005581;GO:0001867;GO:0005509;GO:0051873;GO:0006508;GO:0009986;GO:0045087;GO:0006953;GO:0008228;GO:0006956;GO:0006958;GO:0006979;GO:0005615;GO:0050830;GO:0002376;GO:0005576;GO:0005515;GO:0048525;GO:0005102;GO:0005537 | hsa:4153 |  | mannose-binding lectin [Homo sapiens] |
| P13473 | 9403.000792 | 8175.757833 | 0.869483904 | -0.201768775 | 0.244107844 | no | no change | GO:0005770;GO:0061684;GO:0101003;GO:0019899;GO:0050821;GO:0044754;GO:0035577;GO:0010008;GO:1990836;GO:0043202;GO:0005615;GO:0097637;GO:0031410;GO:0016021;GO:0016020;GO:0097352;GO:0031902;GO:0046716;GO:0045121;GO:0006914;GO:0017038;GO:0006605;GO:1905146;GO:0061740;GO:0019904;GO:0009267;GO:0005886;GO:0070062;GO:0000421;GO:0030670;GO:0031647;GO:0043312;GO:0031088;GO:0005764;GO:0005765;GO:0005515;GO:0098857;GO:0005768;GO:0002576 | hsa:3920 |  | lysosome-associated membrane glycoprotein 2 isoform A precursor [Homo sapiens] |
| P13645 | 46754.77875 | 51498.59333 | 1.101461598 | 0.139419197 | 0.253547692 | no | no change | GO:0005737;GO:0018149;GO:0045684;GO:0070062;GO:0030216;GO:0031424;GO:0005615;GO:0009986;GO:0016020;GO:0070268;GO:0051290;GO:0005634;GO:0005882;GO:0005576;GO:0046982;GO:0005198;GO:0005829;GO:0001533;GO:0030280 | hsa:3858 |  | Keratin 10 [Homo sapiens] |
| P13647 | 22368.1385 | 24637.18833 | 1.101441156 | 0.139392422 | 0.257145813 | no | no change | GO:0005737;GO:0016020;GO:0005200;GO:0007010;GO:0070062;GO:0031424;GO:0031581;GO:0045095;GO:0070268;GO:0005198;GO:0005634;GO:0005882;GO:0097110;GO:0005515;GO:0005886;GO:0008544;GO:0005829 | hsa:3852 |  | Keratin 5 [Homo sapiens] |
| P13671 | 54120.68 | 51443.50375 | 0.950533211 | -0.07319106 | 0.303655218 | no | no change | GO:0019835;GO:0001701;GO:0030449;GO:0070062;GO:0045917;GO:0045087;GO:0045766;GO:0002376;GO:0005576;GO:0006956;GO:0001970;GO:0005515;GO:0006955;GO:0006958;GO:0005579 | hsa:729 |  | complement component C6 precursor [Homo sapiens] |
| P13796 | 1360041.786 | 1606692.123 | 1.18135497 | 0.240442526 | 0.783068266 | no | no change | GO:0032432;GO:0033157;GO:0015629;GO:0030175;GO:0051020;GO:0005925;GO:0030054;GO:0005737;GO:0071803;GO:0001726;GO:0002102;GO:0002286;GO:0005615;GO:0016020;GO:0003779;GO:0005509;GO:0048471;GO:0016477;GO:0022617;GO:0042802;GO:0035722;GO:0005178;GO:0031100;GO:0051017;GO:0051015;GO:0005884;GO:0005886;GO:0051764;GO:0042995;GO:0032587;GO:0001891;GO:0046872;GO:0070062;GO:0005829;GO:0005856;GO:0051639;GO:0044319;GO:0001725;GO:0010737 | hsa:3936 | COG5069 | plastin-2 [Homo sapiens] |
| P14151 | 30580.76667 | 29594.96875 | 0.96776412 | -0.047272642 | 0.569633966 | no | no change | GO:0009897;GO:0016339;GO:0030246;GO:0050900;GO:0050901;GO:0070492;GO:0030667;GO:0043208;GO:0016021;GO:0016020;GO:0005509;GO:0002020;GO:0046872;GO:0009986;GO:0005887;GO:0005886;GO:0007155;GO:0050839;GO:0008201;GO:0050776;GO:0043312;GO:0005515;GO:0033198 | hsa:6402 |  | L-selectin [Homo sapiens] |
| P18206 | 8736.734328 | 8700.482158 | 0.995850604 | -0.005998767 | 0.981975499 | no | no change | GO:0048675;GO:0015629;GO:0030336;GO:0034333;GO:0030032;GO:0034774;GO:0007160;GO:0002162;GO:0005925;GO:0030054;GO:0030055;GO:0005737;GO:0002009;GO:0002102;GO:0070062;GO:0002576;GO:0016020;GO:0034394;GO:0005198;GO:0003779;GO:0035580;GO:0005856;GO:0030334;GO:0042383;GO:0006936;GO:0051015;GO:0005916;GO:0005913;GO:0005912;GO:0005911;GO:0005886;GO:0007155;GO:0090136;GO:1903561;GO:1904813;GO:0031625;GO:0032991;GO:0008013;GO:0005829;GO:0045294;GO:0043312;GO:0045296;GO:0070527;GO:0005576;GO:0005515;GO:0043297;GO:0043034 | hsa:7414 |  | vinculin isoform meta-VCL [Homo sapiens] |
| P18428 | 15522.83508 | 15829.97208 | 1.019786141 | 0.028266637 | 0.806733514 | no | no change | GO:0006968;GO:0071222;GO:0071223;GO:0006869;GO:0042742;GO:0050829;GO:0032496;GO:0032490;GO:0044130;GO:0019221;GO:0060265;GO:0002281;GO:0070062;GO:0090023;GO:0042535;GO:0016020;GO:0071723;GO:0045919;GO:0002224;GO:0033036;GO:0032722;GO:0009986;GO:0032720;GO:0032760;GO:0015920;GO:0045087;GO:0008289;GO:0006953;GO:0008228;GO:0070891;GO:0034142;GO:0001530;GO:0005615;GO:0034145;GO:0031663;GO:0050830;GO:0002376;GO:0002232;GO:0005576;GO:0005515;GO:0043032;GO:0005102;GO:0032757;GO:0032755 | hsa:3929 |  | lipopolysaccharide-binding protein precursor [Homo sapiens] |
| P19652 | 828048.6208 | 767476.8583 | 0.926849993 | -0.109592232 | 0.146164901 | no | no change | GO:1904469;GO:0050716;GO:0031093;GO:0005615;GO:0002576;GO:0050718;GO:0043312;GO:0072562;GO:0006953;GO:0035578;GO:0062023;GO:0002682;GO:0035580;GO:0005576;GO:0070062 | hsa:5005 |  | alpha-1-acid glycoprotein 2 precursor [Homo sapiens] |
| P19823 | 1441078.625 | 1357782.583 | 0.942198822 | -0.085896566 | 0.109034196 | no | no change | GO:0044267;GO:0030212;GO:0070062;GO:0043687;GO:0072562;GO:0062023;GO:0030414;GO:0005788;GO:0004866;GO:0004867;GO:0010951;GO:0010466;GO:0005576 | hsa:3698 | COG2304 | inter-alpha-trypsin inhibitor heavy chain H2 precursor [Homo sapiens] |
| P20851 | 54330.14875 | 49058.4175 | 0.902968584 | -0.1472523 | 0.282535579 | no | no change | GO:0030449;GO:0005615;GO:0045087;GO:0007596;GO:0002376;GO:0045732;GO:0005576;GO:0005515;GO:0005886;GO:1903027;GO:0006958;GO:0045959;GO:0044216 | hsa:725 |  | C4b-binding protein beta chain isoform 1 precursor [Homo sapiens] |
| P22792 | 97010.12417 | 92603.00583 | 0.954570532 | -0.067076294 | 0.471523435 | no | no change | GO:0030449;GO:0070062;GO:0004181;GO:0050790;GO:0072562;GO:0050821;GO:0005576;GO:0006508;GO:0030234 | hsa:1370 | COG4886 | carboxypeptidase N subunit 2 precursor [Homo sapiens] |
| P22891 | 10479.47265 | 10217.00317 | 0.974953941 | -0.03659403 | 0.81504168 | no | no change | GO:0004252;GO:0005796;GO:0006888;GO:0005615;GO:0007596;GO:0005788;GO:0005576;GO:0005509;GO:0006508;GO:0030195;GO:0007599;GO:0070062 | hsa:8858 | COG5640 | vitamin K-dependent protein Z isoform 2 precursor [Homo sapiens] |
| P23142 | 34770.50708 | 33588.43208 | 0.966003516 | -0.049899655 | 0.658830335 | no | no change | GO:0005201;GO:0008022;GO:0031012;GO:0007162;GO:0044877;GO:0005615;GO:0072378;GO:1900025;GO:0005576;GO:0005509;GO:2000647;GO:0042802;GO:0062023;GO:0071953;GO:0010952;GO:0070062;GO:0005604;GO:0007566;GO:0070051;GO:0005577;GO:0030198;GO:0001968;GO:0016032;GO:2000146;GO:0016504;GO:0001933;GO:0070373;GO:0007229 | hsa:2192 |  | fibulin-1 isoform D precursor [Homo sapiens] |
| P25311 | 333564.1083 | 320440.6208 | 0.960656776 | -0.057907019 | 0.513651154 | no | no change | GO:0008285;GO:0090501;GO:0071806;GO:0070062;GO:0009897;GO:0001580;GO:0005615;GO:0005634;GO:0004540;GO:0008320;GO:0062023;GO:0005515;GO:0005886;GO:0006955;GO:0007155;GO:0001895;GO:0055085;GO:0005576 | hsa:563 |  | zinc-alpha-2-glycoprotein precursor [Homo sapiens] |
| P27169 | 238200.1833 | 252886.7875 | 1.061656561 | 0.086317139 | 0.285751509 | no | no change | GO:0034445;GO:0032411;GO:0016311;GO:0046872;GO:0004064;GO:0046434;GO:0070062;GO:0005615;GO:0005543;GO:0009636;GO:0072562;GO:0005509;GO:0102007;GO:0010875;GO:0019372;GO:0042803;GO:0016787;GO:0034364;GO:0006629;GO:0034366;GO:1902617;GO:0008203;GO:0004063;GO:0043231;GO:0046395;GO:0051099;GO:0031667;GO:0046470;GO:0005576;GO:0019439;GO:0070542 | hsa:5444 |  | serum paraoxonase/arylesterase 1 precursor [Homo sapiens] |
| P29622 | 69966.39042 | 66413.45667 | 0.949219422 | -0.075186475 | 0.379444279 | no | no change | GO:0005615;GO:0070062;GO:0002576;GO:0031089;GO:0010466;GO:0030414;GO:0004867;GO:0010951;GO:0005576 | hsa:5267 | COG4826 | kallistatin isoform 1 [Homo sapiens] |
| P35527 | 150298.3288 | 152461.3892 | 1.01439178 | 0.020614959 | 0.892968899 | no | no change | GO:0005200;GO:0045109;GO:0031424;GO:0005615;GO:0005829;GO:0016020;GO:0070268;GO:0005198;GO:0007283;GO:0005882;GO:0008544;GO:0005634;GO:0043588;GO:0070062 | hsa:3857 |  | keratin, type I cytoskeletal 9 [Homo sapiens] |
| P35858 | 56035.23375 | 51550.67458 | 0.91996894 | -0.120342942 | 0.305349261 | no | no change | GO:0044267;GO:0005615;GO:0031012;GO:0005654;GO:0007155;GO:0005576;GO:0007165;GO:0042567;GO:0005520;GO:0070062 | hsa:3483 | COG4886 | Insulin-like growth factor binding protein, acid labile subunit [Homo sapiens] |
| P35908 | 90491.12417 | 91746.86042 | 1.0138769 | 0.019882497 | 0.855094385 | no | no change | GO:0005200;GO:0045095;GO:0018149;GO:0008544;GO:0045684;GO:0043616;GO:0051546;GO:0070062;GO:0032980;GO:0005634;GO:0016020;GO:0005198;GO:0030280;GO:0070268;GO:0005882;GO:0001533;GO:0005615;GO:0045109;GO:0008092;GO:0031424;GO:0005829;GO:0003334;GO:0005515 | hsa:3849 |  | keratin, type II cytoskeletal 2 epidermal [Homo sapiens] |
| P36955 | 100758.2771 | 96083.02208 | 0.953599296 | -0.068544924 | 0.428176832 | no | no change | GO:0007614;GO:0030424;GO:0010629;GO:0060041;GO:0060770;GO:0007275;GO:0043203;GO:0005615;GO:0071300;GO:0010447;GO:0001822;GO:0050769;GO:0071333;GO:0048471;GO:0046685;GO:0043025;GO:0071279;GO:0008283;GO:0050728;GO:0062023;GO:0010976;GO:0004867;GO:0010951;GO:1901215;GO:0070062;GO:0042470;GO:0071549;GO:0005604;GO:0007568;GO:0010596;GO:0042698;GO:0005576;GO:0005515;GO:0016525 | hsa:5176 | COG4826 | pigment epithelium-derived factor isoform 1 precursor [Homo sapiens] |
| P41222 | 18677.39048 | 18000.5245 | 0.963760142 | -0.053253958 | 0.631641509 | no | no change | GO:0006633;GO:0006631;GO:0005783;GO:0005789;GO:0005737;GO:0070062;GO:0005634;GO:0016020;GO:0005504;GO:0048471;GO:0005501;GO:0019371;GO:0005794;GO:0005791;GO:0006629;GO:0031965;GO:2000255;GO:0036094;GO:0001516;GO:0045187;GO:0005615;GO:0016853;GO:0004667;GO:0006693;GO:0005576;GO:0005515 | hsa:5730 |  | prostaglandin-H2 D-isomerase precursor [Homo sapiens] |
| P43121 | 5235.567056 | 5122.623125 | 0.978427565 | -0.031463046 | 0.835975685 | no | no change | GO:0009897;GO:0030335;GO:0001525;GO:0005925;GO:0005615;GO:0016021;GO:0016020;GO:0003094;GO:0061042;GO:0005576;GO:0005886;GO:0007155;GO:0009653;GO:0005634 | hsa:4162 |  | cell surface glycoprotein MUC18 precursor [Homo sapiens] |
| P43251 | 33854.93875 | 31156.51167 | 0.920294433 | -0.119832593 | 0.32276526 | no | no change | GO:0016787;GO:0005615;GO:0047708;GO:0070062;GO:0016810;GO:0016811;GO:0006768;GO:0006807;GO:0005759;GO:0007417;GO:0005576 | hsa:686 | COG0388 | RecName: Full=Biotinidase; Short=Biotinase; Flags: Precursor |
| P43652 | 157090.7458 | 162630.0425 | 1.035261763 | 0.049995595 | 0.563299261 | no | no change | GO:0008431;GO:0051180;GO:0005615;GO:0072562;GO:0050821;GO:0015031;GO:0005576;GO:0005515;GO:0071693;GO:0046872;GO:0070062 | hsa:173 |  | afamin precursor [Homo sapiens] |
[truncated: 25,279 more chars]
