# Supplementary material for: Proteomics of extracellular vesicles in plasma reveals the characteristics and residual traces of COVID-19 patients without underlying diseases after 3 months of recovery
Source: Cell Death Dis. 2021 May 25;12(6):541. doi: 10.1038/s41419-021-03816-3 (PMC8146187; doi:10.1038/s41419-021-03816-3)
Supplement: Supplementary file 18 — Table S5 [file 41419_2021_3816_MOESM18_ESM.docx]

| Table S5-1 KEGG pathway enrichment of DEPs in M vs A group. | | | | | | | | | |
| --- | --- | --- | --- | --- | --- | --- | --- | --- | --- |
| pathway | pathway_name | class | all_number_of_accs | all_KO2acc | diff_number_of_accs | diff_KO2acc | p_value | FDR | url |
| hsa05150 | Staphylococcus aureus infection | Human Diseases | 63\|228 | hsa:102723407(A0A0C4DH29;A0A0C4DH32;A0A0C4DH33;A0A0J9YX35;A0A0C4DH31;A0A0C4DH36;A0A0C4DH34;A0A0C4DH38;A0A0C4DH39;A0A0J9YY99;A0A0G2JMI3;A0A0B4J1U7;P01763;P01764;P01743;P01766;A0A0B4J1X5;A0A0C4DH43;P01782;P01780;A0A087WSY4;P01814;A0A0B4J1X8;A0A4W8ZXM2;A0A075B7D0;P0DP02;P0DP01;A0A075B7F0;A0A075B7D8;P01817;A0A0A0MS14;A0A0A0MS15;A0A0B4J1V1;A0A075B7B8;A0A0B4J1V2;P0DTE1;A0A075B6Q5;A0A0J9YVY3;P23083);hsa:5340(P00747);hsa:629(B4E1Z4);hsa:3858(P13645);hsa:727(P01031);hsa:720(A0A0G2JPR0;P0C0L5;P0C0L4);hsa:721(A0A0G2JPR0;P0C0L5;P0C0L4);hsa:1675(K7ERG9);hsa:5648(P48740);hsa:2214(H0Y755);hsa:718(P01024;M0R0Q9);hsa:10747(O00187);hsa:713(D6R934);hsa:712(P02745);hsa:715(A0A3B3ISR2);hsa:714(P02747);hsa:717(A0A0G2JL69);hsa:716(P09871);hsa:4153(P11226);hsa:3426(A0A2R8Y3M9);hsa:2266(C9JPQ9;C9JC84);hsa:3075(P08603) | 6\|28 | hsa:102723407(A0A0C4DH36;A0A0J9YX35);hsa:2266(C9JPQ9);hsa:3426(A0A2R8Y3M9);hsa:714(P02747);hsa:1675(K7ERG9) | 0.138855605 | 0.242408937 | http://www.kegg.jp/kegg-bin/show_pathway?hsa05150+hsa:102723407%09salmon+hsa:102723407%09salmon+hsa:2266%09salmon+hsa:3426%09green+hsa:714%09green+hsa:1675%09green |
| hsa04610 | Complement and coagulation cascades | Organismal Systems | 58\|228 | hsa:3818(H0YAC1);hsa:5345(P08697);hsa:5104(P05154);hsa:5340(P00747);hsa:629(B4E1Z4);hsa:729(P13671);hsa:2(P01023);hsa:725(P20851);hsa:732(P07358);hsa:727(P01031);hsa:720(A0A0G2JPR0;P0C0L5;P0C0L4);hsa:462(P01008);hsa:722(P04003);hsa:1675(K7ERG9);hsa:7450(P04275);hsa:10544(Q9UNN8);hsa:5624(E7END6);hsa:5627(A0A0S2Z4L3);hsa:2147(P00734);hsa:721(A0A0G2JPR0;P0C0L5;P0C0L4);hsa:2162(P00488);hsa:2161(P00748);hsa:2160(P03951);hsa:731(P07357);hsa:2165(P05160);hsa:730(P10643);hsa:735(P02748);hsa:3827(P01042);hsa:5648(P48740);hsa:1361(Q96IY4);hsa:718(P01024;M0R0Q9);hsa:10747(O00187);hsa:4153(P11226);hsa:1191(P10909);hsa:710(P05155);hsa:713(D6R934);hsa:712(P02745);hsa:715(A0A3B3ISR2);hsa:714(P02747);hsa:717(A0A0G2JL69);hsa:716(P09871);hsa:5265(P01009);hsa:3075(P08603);hsa:2243(P02671);hsa:7448(H0YJW9;P04004);hsa:2244(P02675);hsa:3426(A0A2R8Y3M9);hsa:2266(C9JPQ9;C9JC84);hsa:2155(F5H8B0);hsa:2153(A0A0A0MRJ7);hsa:3053(P05546);hsa:733(P07360);hsa:2158(P00740);hsa:2159(P00742) | 6\|28 | hsa:3426(A0A2R8Y3M9);hsa:2266(C9JPQ9);hsa:2160(P03951);hsa:733(P07360);hsa:714(P02747);hsa:1675(K7ERG9) | 0.166878484 | 0.272833078 | http://www.kegg.jp/kegg-bin/show_pathway?hsa04610+hsa:3426%09green+hsa:2266%09salmon+hsa:2160%09salmon+hsa:733%09green+hsa:714%09green+hsa:1675%09green |
| hsa05146 | Amoebiasis | Human Diseases | 47\|228 | hsa:102723407(A0A0C4DH29;A0A0C4DH32;A0A0C4DH33;A0A0J9YX35;A0A0C4DH31;A0A0C4DH36;A0A0C4DH34;A0A0C4DH38;A0A0C4DH39;A0A0J9YY99;A0A0G2JMI3;A0A0B4J1U7;P01763;P01764;P01743;P01766;A0A0B4J1X5;A0A0C4DH43;P01782;P01780;A0A087WSY4;P01814;A0A0B4J1X8;A0A4W8ZXM2;A0A075B7D0;P0DP02;P0DP01;A0A075B7F0;A0A075B7D8;P01817;A0A0A0MS14;A0A0A0MS15;A0A0B4J1V1;A0A075B7B8;A0A0B4J1V2;P0DTE1;A0A075B6Q5;A0A0J9YVY3;P23083);hsa:2335(P02751);hsa:87(H9KV75);hsa:7414(P18206);hsa:735(P02748);hsa:929(P08571);hsa:733(P07360);hsa:732(P07358);hsa:731(P07357) | 5\|28 | hsa:7414(P18206);hsa:102723407(A0A0C4DH36;A0A0J9YX35);hsa:733(P07360);hsa:2335(P02751) | 0.190035464 | 0.296570497 | http://www.kegg.jp/kegg-bin/show_pathway?hsa05146+hsa:7414%09salmon+hsa:102723407%09salmon+hsa:102723407%09salmon+hsa:733%09green+hsa:2335%09salmon |
| hsa04151 | PI3K-Akt signaling pathway | Environmental Information Processing | 51\|228 | hsa:7450(P04275);hsa:102723407(A0A0C4DH29;A0A0C4DH32;A0A0C4DH33;A0A0J9YX35;A0A0C4DH31;A0A0C4DH36;A0A0C4DH34;A0A0C4DH38;A0A0C4DH39;A0A0J9YY99;A0A0G2JMI3;A0A0B4J1U7;P01763;P01764;P01743;P01766;A0A0B4J1X5;A0A0C4DH43;P01782;P01780;A0A087WSY4;P01814;A0A0B4J1X8;A0A4W8ZXM2;A0A075B7D0;P0DP02;P0DP01;A0A075B7F0;A0A075B7D8;P01817;A0A0A0MS14;A0A0A0MS15;A0A0B4J1V1;A0A075B7B8;A0A0B4J1V2;P0DTE1;A0A075B6Q5;A0A0J9YVY3;P23083);hsa:1291(A0A087X0S5);hsa:7534(E7EX29);hsa:1311(G3XAP6);hsa:3481(P01344);hsa:7448(H0YJW9;P04004);hsa:2335(P02751);hsa:3674(P08514);hsa:7057(P07996);hsa:1293(E7ENL6);hsa:7148(A0A140T8Y3) | 5\|28 | hsa:102723407(A0A0C4DH36;A0A0J9YX35);hsa:2335(P02751);hsa:1293(E7ENL6);hsa:7534(E7EX29) | 0.168154928 | 0.270624337 | http://www.kegg.jp/kegg-bin/show_pathway?hsa04151+hsa:102723407%09salmon+hsa:102723407%09salmon+hsa:2335%09salmon+hsa:1293%09green+hsa:7534%09salmon |
| hsa04810 | Regulation of actin cytoskeleton | Cellular Processes | 12\|228 | hsa:3674(P08514);hsa:2934(A0A0A0MS51);hsa:1072(E9PK25);hsa:2147(P00734);hsa:2335(P02751);hsa:5881(P60763);hsa:4627(P35579);hsa:87(H9KV75);hsa:5216(P07737);hsa:7414(P18206);hsa:4478(P26038);hsa:71(P60709);hsa:60(P60709) | 4\|28 | hsa:7414(P18206);hsa:71(P60709);hsa:60(P60709);hsa:5216(P07737);hsa:2335(P02751) | 0.036759682 | 0.540892465 | http://www.kegg.jp/kegg-bin/show_pathway?hsa04810+hsa:7414%09salmon+hsa:71%09salmon+hsa:60%09salmon+hsa:5216%09salmon+hsa:2335%09salmon |
| hsa05143 | African trypanosomiasis | Human Diseases | 45\|228 | hsa:102723407(A0A0C4DH29;A0A0C4DH32;A0A0C4DH33;A0A0J9YX35;A0A0C4DH31;A0A0C4DH36;A0A0C4DH34;A0A0C4DH38;A0A0C4DH39;A0A0J9YY99;A0A0G2JMI3;A0A0B4J1U7;P01763;P01764;P01743;P01766;A0A0B4J1X5;A0A0C4DH43;P01782;P01780;A0A087WSY4;P01814;A0A0B4J1X8;A0A4W8ZXM2;A0A075B7D0;P0DP02;P0DP01;A0A075B7F0;A0A075B7D8;P01817;A0A0A0MS14;A0A0A0MS15;A0A0B4J1V1;A0A075B7B8;A0A0B4J1V2;P0DTE1;A0A075B6Q5;A0A0J9YVY3;P23083);hsa:3039(P69905);hsa:8542(O14791);hsa:335(P02647);hsa:3250(P00739);hsa:3043(P68871);hsa:3040(P69905);hsa:7412(P19320) | 4\|28 | hsa:102723407(A0A0C4DH36;A0A0J9YX35);hsa:3040(P69905);hsa:3250(P00739);hsa:3039(P69905) | 0.161106244 | 0.272031855 | http://www.kegg.jp/kegg-bin/show_pathway?hsa05143+hsa:102723407%09salmon+hsa:102723407%09salmon+hsa:3040%09green+hsa:3250%09green+hsa:3039%09green |
| hsa04145 | Phagosome | Cellular Processes | 56\|228 | hsa:203068(P07437);hsa:3105(A0A0G2JI36);hsa:7278(P0DPH7);hsa:81027(Q9H4B7);hsa:102723407(A0A0C4DH29;A0A0C4DH32;A0A0C4DH33;A0A0J9YX35;A0A0C4DH31;A0A0C4DH36;A0A0C4DH34;A0A0C4DH38;A0A0C4DH39;A0A0J9YY99;A0A0G2JMI3;A0A0B4J1U7;P01763;P01764;P01743;P01766;A0A0B4J1X5;A0A0C4DH43;P01782;P01780;A0A087WSY4;P01814;A0A0B4J1X8;A0A4W8ZXM2;A0A075B7D0;P0DP02;P0DP01;A0A075B7F0;A0A075B7D8;P01817;A0A0A0MS14;A0A0A0MS15;A0A0B4J1V1;A0A075B7B8;A0A0B4J1V2;P0DTE1;A0A075B6Q5;A0A0J9YVY3;P23083);hsa:1311(G3XAP6);hsa:71(P60709);hsa:7277(P68366);hsa:113457(P0DPH7);hsa:3920(P13473);hsa:718(P01024;M0R0Q9);hsa:7057(P07996);hsa:112714(P0DPH7);hsa:2214(H0Y755);hsa:4153(P11226);hsa:78989(Q9BWP8);hsa:7037(G3V0E5);hsa:929(P08571);hsa:715(A0A3B3ISR2);hsa:60(P60709) | 4\|28 | hsa:102723407(A0A0C4DH36;A0A0J9YX35);hsa:71(P60709);hsa:81027(Q9H4B7);hsa:60(P60709) | 0.080687123 | 0.268089474 | http://www.kegg.jp/kegg-bin/show_pathway?hsa04145+hsa:102723407%09salmon+hsa:102723407%09salmon+hsa:71%09salmon+hsa:81027%09salmon+hsa:60%09salmon |
| hsa04510 | Focal adhesion | Cellular Processes | 20\|228 | hsa:7450(P04275);hsa:29780(A0A087WZB5);hsa:7094(Q9Y490);hsa:1291(A0A087X0S5);hsa:1293(E7ENL6);hsa:5908(P61224);hsa:87(H9KV75);hsa:3674(P08514);hsa:7448(H0YJW9;P04004);hsa:3611(A0A0A0MTH3);hsa:5881(P60763);hsa:1311(G3XAP6);hsa:2316(P21333);hsa:7057(P07996);hsa:7791(H0Y2Y8);hsa:60(P60709);hsa:7414(P18206);hsa:2335(P02751);hsa:71(P60709);hsa:7148(A0A140T8Y3) | 4\|28 | hsa:7414(P18206);hsa:1293(E7ENL6);hsa:71(P60709);hsa:60(P60709);hsa:2335(P02751) | 0.137919278 | 0.244925614 | http://www.kegg.jp/kegg-bin/show_pathway?hsa04510+hsa:7414%09salmon+hsa:1293%09green+hsa:71%09salmon+hsa:60%09salmon+hsa:2335%09salmon |
| hsa05414 | Dilated cardiomyopathy (DCM) | Human Diseases | 44\|228 | hsa:3674(P08514);hsa:102723407(A0A0C4DH29;A0A0C4DH32;A0A0C4DH33;A0A0J9YX35;A0A0C4DH31;A0A0C4DH36;A0A0C4DH34;A0A0C4DH38;A0A0C4DH39;A0A0J9YY99;A0A0G2JMI3;A0A0B4J1U7;P01763;P01764;P01743;P01766;A0A0B4J1X5;A0A0C4DH43;P01782;P01780;A0A087WSY4;P01814;A0A0B4J1X8;A0A4W8ZXM2;A0A075B7D0;P0DP02;P0DP01;A0A075B7F0;A0A075B7D8;P01817;A0A0A0MS14;A0A0A0MS15;A0A0B4J1V1;A0A075B7B8;A0A0B4J1V2;P0DTE1;A0A075B6Q5;A0A0J9YVY3;P23083);hsa:7171(A0A087WWU8;A0A2R8Y5V9);hsa:71(P60709);hsa:70(P68032);hsa:60(P60709) | 4\|28 | hsa:102723407(A0A0C4DH36;A0A0J9YX35);hsa:71(P60709);hsa:70(P68032);hsa:60(P60709) | 0.168803542 | 0.26748869 | http://www.kegg.jp/kegg-bin/show_pathway?hsa05414+hsa:102723407%09salmon+hsa:102723407%09salmon+hsa:71%09salmon+hsa:70%09salmon+hsa:60%09salmon |
| hsa05322 | Systemic lupus erythematosus | Human Diseases | 60\|228 | hsa:735(P02748);hsa:102723407(A0A0C4DH29;A0A0C4DH32;A0A0C4DH33;A0A0J9YX35;A0A0C4DH31;A0A0C4DH36;A0A0C4DH34;A0A0C4DH38;A0A0C4DH39;A0A0J9YY99;A0A0G2JMI3;A0A0B4J1U7;P01763;P01764;P01743;P01766;A0A0B4J1X5;A0A0C4DH43;P01782;P01780;A0A087WSY4;P01814;A0A0B4J1X8;A0A4W8ZXM2;A0A075B7D0;P0DP02;P0DP01;A0A075B7F0;A0A075B7D8;P01817;A0A0A0MS14;A0A0A0MS15;A0A0B4J1V1;A0A075B7B8;A0A0B4J1V2;P0DTE1;A0A075B6Q5;A0A0J9YVY3;P23083);hsa:712(P02745);hsa:733(P07360);hsa:128312(A0A2R8Y619);hsa:715(A0A3B3ISR2);hsa:721(A0A0G2JPR0;P0C0L5;P0C0L4);hsa:727(P01031);hsa:718(P01024;M0R0Q9);hsa:732(P07358);hsa:87(H9KV75);hsa:2214(H0Y755);hsa:730(P10643);hsa:731(P07357);hsa:713(D6R934);hsa:716(P09871);hsa:720(A0A0G2JPR0;P0C0L5;P0C0L4);hsa:714(P02747);hsa:717(A0A0G2JL69);hsa:729(P13671) | 4\|28 | hsa:102723407(A0A0C4DH36;A0A0J9YX35);hsa:733(P07360);hsa:714(P02747) | 0.058388823 | 0.353767576 | http://www.kegg.jp/kegg-bin/show_pathway?hsa05322+hsa:102723407%09salmon+hsa:102723407%09salmon+hsa:733%09green+hsa:714%09green |
| hsa05100 | Bacterial invasion of epithelial cells | Human Diseases | 4\|228 | hsa:7414(P18206);hsa:3611(A0A0A0MTH3);hsa:71(P60709);hsa:60(P60709);hsa:2335(P02751) | 3\|28 | hsa:7414(P18206);hsa:71(P60709);hsa:60(P60709);hsa:2335(P02751) | 0.005974939 | 0.615418707 | http://www.kegg.jp/kegg-bin/show_pathway?hsa05100+hsa:7414%09salmon+hsa:71%09salmon+hsa:60%09salmon+hsa:2335%09salmon |
| hsa05130 | Pathogenic Escherichia coli infection | Human Diseases | 7\|228 | hsa:203068(P07437);hsa:7278(P0DPH7);hsa:81027(Q9H4B7);hsa:7534(E7EX29);hsa:7277(P68366);hsa:113457(P0DPH7);hsa:112714(P0DPH7);hsa:929(P08571);hsa:71(P60709);hsa:60(P60709) | 3\|28 | hsa:71(P60709);hsa:81027(Q9H4B7);hsa:60(P60709);hsa:7534(E7EX29) | 0.036594866 | 0.753854236 | http://www.kegg.jp/kegg-bin/show_pathway?hsa05130+hsa:71%09salmon+hsa:81027%09salmon+hsa:60%09salmon+hsa:7534%09salmon |
| hsa05416 | Viral myocarditis | Human Diseases | 42\|228 | hsa:5881(P60763);hsa:102723407(A0A0C4DH29;A0A0C4DH32;A0A0C4DH33;A0A0J9YX35;A0A0C4DH31;A0A0C4DH36;A0A0C4DH34;A0A0C4DH38;A0A0C4DH39;A0A0J9YY99;A0A0G2JMI3;A0A0B4J1U7;P01763;P01764;P01743;P01766;A0A0B4J1X5;A0A0C4DH43;P01782;P01780;A0A087WSY4;P01814;A0A0B4J1X8;A0A4W8ZXM2;A0A075B7D0;P0DP02;P0DP01;A0A075B7F0;A0A075B7D8;P01817;A0A0A0MS14;A0A0A0MS15;A0A0B4J1V1;A0A075B7B8;A0A0B4J1V2;P0DTE1;A0A075B6Q5;A0A0J9YVY3;P23083);hsa:3105(A0A0G2JI36);hsa:71(P60709);hsa:60(P60709) | 3\|28 | hsa:102723407(A0A0C4DH36;A0A0J9YX35);hsa:71(P60709);hsa:60(P60709) | 0.122039156 | 0.349167585 | http://www.kegg.jp/kegg-bin/show_pathway?hsa05416+hsa:102723407%09salmon+hsa:102723407%09salmon+hsa:71%09salmon+hsa:60%09salmon |
| hsa05131 | Shigellosis | Human Diseases | 4\|228 | hsa:7414(P18206);hsa:71(P60709);hsa:960(H0Y5E4);hsa:5216(P07737);hsa:60(P60709) | 3\|28 | hsa:7414(P18206);hsa:71(P60709);hsa:60(P60709);hsa:5216(P07737) | 0.005974939 | 0.615418707 | http://www.kegg.jp/kegg-bin/show_pathway?hsa05131+hsa:7414%09salmon+hsa:71%09salmon+hsa:60%09salmon+hsa:5216%09salmon |
| hsa05165 | Human papillomavirus infection | Human Diseases | 13\|228 | hsa:7450(P04275);hsa:3674(P08514);hsa:3993(J3QRV5);hsa:1291(A0A087X0S5);hsa:1293(E7ENL6);hsa:1311(G3XAP6);hsa:7448(H0YJW9;P04004);hsa:2335(P02751);hsa:7057(P07996);hsa:5315(H3BTN5);hsa:3105(A0A0G2JI36);hsa:7148(A0A140T8Y3) | 3\|28 | hsa:5315(H3BTN5);hsa:1293(E7ENL6);hsa:2335(P02751) | 0.144235916 | 0.247604989 | http://www.kegg.jp/kegg-bin/show_pathway?hsa05165+hsa:5315%09salmon+hsa:1293%09green+hsa:2335%09salmon |
| hsa04670 | Leukocyte transendothelial migration | Organismal Systems | 7\|228 | hsa:5908(P61224);hsa:87(H9KV75);hsa:7412(P19320);hsa:60(P60709);hsa:7414(P18206);hsa:4478(P26038);hsa:71(P60709);hsa:1003(I3L1J2) | 3\|28 | hsa:7414(P18206);hsa:71(P60709);hsa:60(P60709);hsa:1003(I3L1J2) | 0.036594866 | 0.753854236 | http://www.kegg.jp/kegg-bin/show_pathway?hsa04670+hsa:7414%09salmon+hsa:71%09salmon+hsa:60%09salmon+hsa:1003%09salmon |
| hsa04611 | Platelet activation | Organismal Systems | 11\|228 | hsa:7450(P04275);hsa:3674(P08514);hsa:7094(Q9Y490);hsa:2811(A0A0C4DGZ8);hsa:5908(P61224);hsa:2243(P02671);hsa:83706(Q86UX7);hsa:2244(P02675);hsa:2266(C9JPQ9;C9JC84);hsa:71(P60709);hsa:60(P60709) | 3\|28 | hsa:2266(C9JPQ9);hsa:83706(Q86UX7);hsa:71(P60709);hsa:60(P60709) | 0.106358014 | 0.31299644 | http://www.kegg.jp/kegg-bin/show_pathway?hsa04611+hsa:2266%09salmon+hsa:83706%09salmon+hsa:71%09salmon+hsa:60%09salmon |
| hsa05323 | Rheumatoid arthritis | Human Diseases | 39\|228 | hsa:102723407(A0A0C4DH29;A0A0C4DH32;A0A0C4DH33;A0A0J9YX35;A0A0C4DH31;A0A0C4DH36;A0A0C4DH34;A0A0C4DH38;A0A0C4DH39;A0A0J9YY99;A0A0G2JMI3;A0A0B4J1U7;P01763;P01764;P01743;P01766;A0A0B4J1X5;A0A0C4DH43;P01782;P01780;A0A087WSY4;P01814;A0A0B4J1X8;A0A4W8ZXM2;A0A075B7D0;P0DP02;P0DP01;A0A075B7F0;A0A075B7D8;P01817;A0A0A0MS14;A0A0A0MS15;A0A0B4J1V1;A0A075B7B8;A0A0B4J1V2;P0DTE1;A0A075B6Q5;A0A0J9YVY3;P23083) | 2\|28 | hsa:102723407(A0A0C4DH36;A0A0J9YX35) | 0.076236124 | 0.280440028 | http://www.kegg.jp/kegg-bin/show_pathway?hsa05323+hsa:102723407%09salmon+hsa:102723407%09salmon |
| hsa05169 | Epstein-Barr virus infection | Human Diseases | 41\|228 | hsa:102723407(A0A0C4DH29;A0A0C4DH32;A0A0C4DH33;A0A0J9YX35;A0A0C4DH31;A0A0C4DH36;A0A0C4DH34;A0A0C4DH38;A0A0C4DH39;A0A0J9YY99;A0A0G2JMI3;A0A0B4J1U7;P01763;P01764;P01743;P01766;A0A0B4J1X5;A0A0C4DH43;P01782;P01780;A0A087WSY4;P01814;A0A0B4J1X8;A0A4W8ZXM2;A0A075B7D0;P0DP02;P0DP01;A0A075B7F0;A0A075B7D8;P01817;A0A0A0MS14;A0A0A0MS15;A0A0B4J1V1;A0A075B7B8;A0A0B4J1V2;P0DTE1;A0A075B6Q5;A0A0J9YVY3;P23083);hsa:3105(A0A0G2JI36);hsa:960(H0Y5E4) | 2\|28 | hsa:102723407(A0A0C4DH36;A0A0J9YX35) | 0.06269594 | 0.33987799 | http://www.kegg.jp/kegg-bin/show_pathway?hsa05169+hsa:102723407%09salmon+hsa:102723407%09salmon |
| hsa00590 | Arachidonic acid metabolism | Metabolism | 3\|228 | hsa:2878(A0A087X1J7);hsa:5730(P41222);hsa:81579(A0A2R8Y3M9) | 2\|28 | hsa:81579(A0A2R8Y3M9);hsa:2878(A0A087X1J7) | 0.038779652 | 0.443811568 | http://www.kegg.jp/kegg-bin/show_pathway?hsa00590+hsa:81579%09green+hsa:2878%09salmon |
| hsa04072 | Phospholipase D signaling pathway | Environmental Information Processing | 39\|228 | hsa:102723407(A0A0C4DH29;A0A0C4DH32;A0A0C4DH33;A0A0J9YX35;A0A0C4DH31;A0A0C4DH36;A0A0C4DH34;A0A0C4DH38;A0A0C4DH39;A0A0J9YY99;A0A0G2JMI3;A0A0B4J1U7;P01763;P01764;P01743;P01766;A0A0B4J1X5;A0A0C4DH43;P01782;P01780;A0A087WSY4;P01814;A0A0B4J1X8;A0A4W8ZXM2;A0A075B7D0;P0DP02;P0DP01;A0A075B7F0;A0A075B7D8;P01817;A0A0A0MS14;A0A0A0MS15;A0A0B4J1V1;A0A075B7B8;A0A0B4J1V2;P0DTE1;A0A075B6Q5;A0A0J9YVY3;P23083) | 2\|28 | hsa:102723407(A0A0C4DH36;A0A0J9YX35) | 0.076236124 | 0.280440028 | http://www.kegg.jp/kegg-bin/show_pathway?hsa04072+hsa:102723407%09salmon+hsa:102723407%09salmon |
| hsa05203 | Viral carcinogenesis | Human Diseases | 8\|228 | hsa:2934(A0A0A0MS51);hsa:128312(A0A2R8Y619);hsa:7534(E7EX29);hsa:718(P01024;M0R0Q9);hsa:87(H9KV75);hsa:5315(H3BTN5);hsa:3105(A0A0G2JI36) | 2\|28 | hsa:5315(H3BTN5);hsa:7534(E7EX29) | 0.194731051 | 0.29936266 | http://www.kegg.jp/kegg-bin/show_pathway?hsa05203+hsa:5315%09salmon+hsa:7534%09salmon |
| hsa05418 | Fluid shear stress and atherosclerosis | Human Diseases | 5\|228 | hsa:3674(P08514);hsa:5881(P60763);hsa:7412(P19320);hsa:60(P60709);hsa:71(P60709);hsa:1003(I3L1J2) | 2\|28 | hsa:71(P60709);hsa:60(P60709);hsa:1003(I3L1J2) | 0.101057925 | 0.315423221 | http://www.kegg.jp/kegg-bin/show_pathway?hsa05418+hsa:71%09salmon+hsa:60%09salmon+hsa:1003%09salmon |
| hsa05152 | Tuberculosis | Human Diseases | 46\|228 | hsa:102723407(A0A0C4DH29;A0A0C4DH32;A0A0C4DH33;A0A0J9YX35;A0A0C4DH31;A0A0C4DH36;A0A0C4DH34;A0A0C4DH38;A0A0C4DH39;A0A0J9YY99;A0A0G2JMI3;A0A0B4J1U7;P01763;P01764;P01743;P01766;A0A0B4J1X5;A0A0C4DH43;P01782;P01780;A0A087WSY4;P01814;A0A0B4J1X8;A0A4W8ZXM2;A0A075B7D0;P0DP02;P0DP01;A0A075B7F0;A0A075B7D8;P01817;A0A0A0MS14;A0A0A0MS15;A0A0B4J1V1;A0A075B7B8;A0A0B4J1V2;P0DTE1;A0A075B6Q5;A0A0J9YVY3;P23083);hsa:820(J3KNB4);hsa:3920(P13473);hsa:718(P01024;M0R0Q9);hsa:2214(H0Y755);hsa:3929(P18428);hsa:929(P08571) | 2\|28 | hsa:102723407(A0A0C4DH36;A0A0J9YX35) | 0.037108248 | 0.477768688 | http://www.kegg.jp/kegg-bin/show_pathway?hsa05152+hsa:102723407%09salmon+hsa:102723407%09salmon |
| hsa04672 | Intestinal immune network for IgA production | Organismal Systems | 41\|228 | hsa:102723407(A0A0C4DH29;A0A0C4DH32;A0A0C4DH33;A0A0J9YX35;A0A0C4DH31;A0A0C4DH36;A0A0C4DH34;A0A0C4DH38;A0A0C4DH39;A0A0J9YY99;A0A0G2JMI3;A0A0B4J1U7;P01763;P01764;P01743;P01766;A0A0B4J1X5;A0A0C4DH43;P01782;P01780;A0A087WSY4;P01814;A0A0B4J1X8;A0A4W8ZXM2;A0A075B7D0;P0DP02;P0DP01;A0A075B7F0;A0A075B7D8;P01817;A0A0A0MS14;A0A0A0MS15;A0A0B4J1V1;A0A075B7B8;A0A0B4J1V2;P0DTE1;A0A075B6Q5;A0A0J9YVY3;P23083);hsa:102723996(A0A087X1L8);hsa:5284(P01833);hsa:23308(A0A087X1L8) | 2\|28 | hsa:102723407(A0A0C4DH36;A0A0J9YX35) | 0.06269594 | 0.33987799 | http://www.kegg.jp/kegg-bin/show_pathway?hsa04672+hsa:102723407%09salmon+hsa:102723407%09salmon |
| hsa04520 | Adherens junction | Cellular Processes | 4\|228 | hsa:5881(P60763);hsa:7414(P18206);hsa:87(H9KV75);hsa:71(P60709);hsa:60(P60709) | 2\|28 | hsa:7414(P18206);hsa:71(P60709);hsa:60(P60709) | 0.068596895 | 0.336451436 | http://www.kegg.jp/kegg-bin/show_pathway?hsa04520+hsa:7414%09salmon+hsa:71%09salmon+hsa:60%09salmon |
| hsa04064 | NF-kappa B signaling pathway | Environmental Information Processing | 42\|228 | hsa:102723407(A0A0C4DH29;A0A0C4DH32;A0A0C4DH33;A0A0J9YX35;A0A0C4DH31;A0A0C4DH36;A0A0C4DH34;A0A0C4DH38;A0A0C4DH39;A0A0J9YY99;A0A0G2JMI3;A0A0B4J1U7;P01763;P01764;P01743;P01766;A0A0B4J1X5;A0A0C4DH43;P01782;P01780;A0A087WSY4;P01814;A0A0B4J1X8;A0A4W8ZXM2;A0A075B7D0;P0DP02;P0DP01;A0A075B7F0;A0A075B7D8;P01817;A0A0A0MS14;A0A0A0MS15;A0A0B4J1V1;A0A075B7B8;A0A0B4J1V2;P0DTE1;A0A075B6Q5;A0A0J9YVY3;P23083);hsa:929(P08571);hsa:7412(P19320);hsa:3929(P18428) | 2\|28 | hsa:102723407(A0A0C4DH36;A0A0J9YX35) | 0.0566778 | 0.416986673 | http://www.kegg.jp/kegg-bin/show_pathway?hsa04064+hsa:102723407%09salmon+hsa:102723407%09salmon |
| hsa05320 | Autoimmune thyroid disease | Human Diseases | 40\|228 | hsa:102723407(A0A0C4DH29;A0A0C4DH32;A0A0C4DH33;A0A0J9YX35;A0A0C4DH31;A0A0C4DH36;A0A0C4DH34;A0A0C4DH38;A0A0C4DH39;A0A0J9YY99;A0A0G2JMI3;A0A0B4J1U7;P01763;P01764;P01743;P01766;A0A0B4J1X5;A0A0C4DH43;P01782;P01780;A0A087WSY4;P01814;A0A0B4J1X8;A0A4W8ZXM2;A0A075B7D0;P0DP02;P0DP01;A0A075B7F0;A0A075B7D8;P01817;A0A0A0MS14;A0A0A0MS15;A0A0B4J1V1;A0A075B7B8;A0A0B4J1V2;P0DTE1;A0A075B6Q5;A0A0J9YVY3;P23083);hsa:3105(A0A0G2JI36) | 2\|28 | hsa:102723407(A0A0C4DH36;A0A0J9YX35) | 0.069209068 | 0.297022251 | http://www.kegg.jp/kegg-bin/show_pathway?hsa05320+hsa:102723407%09salmon+hsa:102723407%09salmon |
| hsa05202 | Transcriptional misregulation in cancer | Human Diseases | 42\|228 | hsa:102723407(A0A0C4DH29;A0A0C4DH32;A0A0C4DH33;A0A0J9YX35;A0A0C4DH31;A0A0C4DH36;A0A0C4DH34;A0A0C4DH38;A0A0C4DH39;A0A0J9YY99;A0A0G2JMI3;A0A0B4J1U7;P01763;P01764;P01743;P01766;A0A0B4J1X5;A0A0C4DH43;P01782;P01780;A0A087WSY4;P01814;A0A0B4J1X8;A0A4W8ZXM2;A0A075B7D0;P0DP02;P0DP01;A0A075B7F0;A0A075B7D8;P01817;A0A0A0MS14;A0A0A0MS15;A0A0B4J1V1;A0A075B7B8;A0A0B4J1V2;P0DTE1;A0A075B6Q5;A0A0J9YVY3;P23083);hsa:929(P08571);hsa:3486(A6XND0);hsa:1668(P59665) | 2\|28 | hsa:102723407(A0A0C4DH36;A0A0J9YX35) | 0.0566778 | 0.416986673 | http://www.kegg.jp/kegg-bin/show_pathway?hsa05202+hsa:102723407%09salmon+hsa:102723407%09salmon |
| hsa05205 | Proteoglycans in cancer | Human Diseases | 10\|228 | hsa:71(P60709);hsa:3481(P01344);hsa:7448(H0YJW9;P04004);hsa:960(H0Y5E4);hsa:2335(P02751);hsa:2316(P21333);hsa:7057(P07996);hsa:4478(P26038);hsa:4060(P51884);hsa:60(P60709) | 2\|28 | hsa:71(P60709);hsa:60(P60709);hsa:2335(P02751) | 0.243210283 | 0.313133239 | http://www.kegg.jp/kegg-bin/show_pathway?hsa05205+hsa:71%09salmon+hsa:60%09salmon+hsa:2335%09salmon |
| hsa04270 | Vascular smooth muscle contraction | Organismal Systems | 2\|228 | hsa:81579(A0A2R8Y3M9);hsa:59(P68032);hsa:72(P68032) | 2\|28 | hsa:81579(A0A2R8Y3M9);hsa:59(P68032);hsa:72(P68032) | 0.014607002 | 0.501507072 | http://www.kegg.jp/kegg-bin/show_pathway?hsa04270+hsa:81579%09green+hsa:59%09salmon+hsa:72%09salmon |
| hsa04662 | B cell receptor signaling pathway | Organismal Systems | 40\|228 | hsa:5881(P60763);hsa:102723407(A0A0C4DH29;A0A0C4DH32;A0A0C4DH33;A0A0J9YX35;A0A0C4DH31;A0A0C4DH36;A0A0C4DH34;A0A0C4DH38;A0A0C4DH39;A0A0J9YY99;A0A0G2JMI3;A0A0B4J1U7;P01763;P01764;P01743;P01766;A0A0B4J1X5;A0A0C4DH43;P01782;P01780;A0A087WSY4;P01814;A0A0B4J1X8;A0A4W8ZXM2;A0A075B7D0;P0DP02;P0DP01;A0A075B7F0;A0A075B7D8;P01817;A0A0A0MS14;A0A0A0MS15;A0A0B4J1V1;A0A075B7B8;A0A0B4J1V2;P0DTE1;A0A075B6Q5;A0A0J9YVY3;P23083) | 2\|28 | hsa:102723407(A0A0C4DH36;A0A0J9YX35) | 0.069209068 | 0.297022251 | http://www.kegg.jp/kegg-bin/show_pathway?hsa04662+hsa:102723407%09salmon+hsa:102723407%09salmon |
| hsa04512 | ECM-receptor interaction | Environmental Information Processing | 12\|228 | hsa:7450(P04275);hsa:3674(P08514);hsa:1291(A0A087X0S5);hsa:2811(A0A0C4DGZ8);hsa:1293(E7ENL6);hsa:1311(G3XAP6);hsa:7448(H0YJW9;P04004);hsa:960(H0Y5E4);hsa:2335(P02751);hsa:7057(P07996);hsa:7148(A0A140T8Y3) | 2\|28 | hsa:1293(E7ENL6);hsa:2335(P02751) | 0.276523295 | 0.343155414 | http://www.kegg.jp/kegg-bin/show_pathway?hsa04512+hsa:1293%09green+hsa:2335%09salmon |
| hsa04650 | Natural killer cell mediated cytotoxicity | Organismal Systems | 42\|228 | hsa:5881(P60763);hsa:102723407(A0A0C4DH29;A0A0C4DH32;A0A0C4DH33;A0A0J9YX35;A0A0C4DH31;A0A0C4DH36;A0A0C4DH34;A0A0C4DH38;A0A0C4DH39;A0A0J9YY99;A0A0G2JMI3;A0A0B4J1U7;P01763;P01764;P01743;P01766;A0A0B4J1X5;A0A0C4DH43;P01782;P01780;A0A087WSY4;P01814;A0A0B4J1X8;A0A4W8ZXM2;A0A075B7D0;P0DP02;P0DP01;A0A075B7F0;A0A075B7D8;P01817;A0A0A0MS14;A0A0A0MS15;A0A0B4J1V1;A0A075B7B8;A0A0B4J1V2;P0DTE1;A0A075B6Q5;A0A0J9YVY3;P23083);hsa:3105(A0A0G2JI36);hsa:2214(H0Y755) | 2\|28 | hsa:102723407(A0A0C4DH36;A0A0J9YX35) | 0.0566778 | 0.416986673 | http://www.kegg.jp/kegg-bin/show_pathway?hsa04650+hsa:102723407%09salmon+hsa:102723407%09salmon |
| hsa05140 | Leishmaniasis | Human Diseases | 42\|228 | hsa:718(P01024;M0R0Q9);hsa:2214(H0Y755);hsa:102723407(A0A0C4DH29;A0A0C4DH32;A0A0C4DH33;A0A0J9YX35;A0A0C4DH31;A0A0C4DH36;A0A0C4DH34;A0A0C4DH38;A0A0C4DH39;A0A0J9YY99;A0A0G2JMI3;A0A0B4J1U7;P01763;P01764;P01743;P01766;A0A0B4J1X5;A0A0C4DH43;P01782;P01780;A0A087WSY4;P01814;A0A0B4J1X8;A0A4W8ZXM2;A0A075B7D0;P0DP02;P0DP01;A0A075B7F0;A0A075B7D8;P01817;A0A0A0MS14;A0A0A0MS15;A0A0B4J1V1;A0A075B7B8;A0A0B4J1V2;P0DTE1;A0A075B6Q5;A0A0J9YVY3;P23083) | 2\|28 | hsa:102723407(A0A0C4DH36;A0A0J9YX35) | 0.0566778 | 0.416986673 | http://www.kegg.jp/kegg-bin/show_pathway?hsa05140+hsa:102723407%09salmon+hsa:102723407%09salmon |
| hsa04020 | Calcium signaling pathway | Environmental Information Processing | 39\|228 | hsa:102723407(A0A0C4DH29;A0A0C4DH32;A0A0C4DH33;A0A0J9YX35;A0A0C4DH31;A0A0C4DH36;A0A0C4DH34;A0A0C4DH38;A0A0C4DH39;A0A0J9YY99;A0A0G2JMI3;A0A0B4J1U7;P01763;P01764;P01743;P01766;A0A0B4J1X5;A0A0C4DH43;P01782;P01780;A0A087WSY4;P01814;A0A0B4J1X8;A0A4W8ZXM2;A0A075B7D0;P0DP02;P0DP01;A0A075B7F0;A0A075B7D8;P01817;A0A0A0MS14;A0A0A0MS15;A0A0B4J1V1;A0A075B7B8;A0A0B4J1V2;P0DTE1;A0A075B6Q5;A0A0J9YVY3;P23083) | 2\|28 | hsa:102723407(A0A0C4DH36;A0A0J9YX35) | 0.076236124 | 0.280440028 | http://www.kegg.jp/kegg-bin/show_pathway?hsa04020+hsa:102723407%09salmon+hsa:102723407%09salmon |
| hsa05132 | Salmonella infection | Human Diseases | 6\|228 | hsa:4627(P35579);hsa:2316(P21333);hsa:5216(P07737);hsa:3929(P18428);hsa:929(P08571);hsa:71(P60709);hsa:60(P60709) | 2\|28 | hsa:71(P60709);hsa:60(P60709);hsa:5216(P07737) | 0.13391308 | 0.241983286 | http://www.kegg.jp/kegg-bin/show_pathway?hsa05132+hsa:71%09salmon+hsa:60%09salmon+hsa:5216%09salmon |
| hsa05340 | Primary immunodeficiency | Human Diseases | 41\|228 | hsa:102723407(A0A0C4DH29;A0A0C4DH32;A0A0C4DH33;A0A0J9YX35;A0A0C4DH31;A0A0C4DH36;A0A0C4DH34;A0A0C4DH38;A0A0C4DH39;A0A0J9YY99;A0A0G2JMI3;A0A0B4J1U7;P01763;P01764;P01743;P01766;A0A0B4J1X5;A0A0C4DH43;P01782;P01780;A0A087WSY4;P01814;A0A0B4J1X8;A0A4W8ZXM2;A0A075B7D0;P0DP02;P0DP01;A0A075B7F0;A0A075B7D8;P01817;A0A0A0MS14;A0A0A0MS15;A0A0B4J1V1;A0A075B7B8;A0A0B4J1V2;P0DTE1;A0A075B6Q5;A0A0J9YVY3;P23083);hsa:3543(P01871;P15814) | 2\|28 | hsa:102723407(A0A0C4DH36;A0A0J9YX35) | 0.06269594 | 0.33987799 | http://www.kegg.jp/kegg-bin/show_pathway?hsa05340+hsa:102723407%09salmon+hsa:102723407%09salmon |
| hsa05020 | Prion diseases | Human Diseases | 11\|228 | hsa:735(P02748);hsa:712(P02745);hsa:3309(P11021);hsa:714(P02747);hsa:729(P13671);hsa:713(D6R934);hsa:727(P01031);hsa:733(P07360);hsa:732(P07358);hsa:731(P07357);hsa:730(P10643) | 2\|28 | hsa:733(P07360);hsa:714(P02747) | 0.261804341 | 0.328851794 | http://www.kegg.jp/kegg-bin/show_pathway?hsa05020+hsa:733%09green+hsa:714%09green |
| hsa04390 | Hippo signaling pathway | Environmental Information Processing | 3\|228 | hsa:3993(J3QRV5);hsa:71(P60709);hsa:60(P60709);hsa:7534(E7EX29) | 2\|28 | hsa:71(P60709);hsa:60(P60709);hsa:7534(E7EX29) | 0.038779652 | 0.443811568 | http://www.kegg.jp/kegg-bin/show_pathway?hsa04390+hsa:71%09salmon+hsa:60%09salmon+hsa:7534%09salmon |
| hsa04919 | Thyroid hormone signaling pathway | Organismal Systems | 2\|228 | hsa:71(P60709);hsa:60(P60709);hsa:90390(Q96HR3) | 2\|28 | hsa:71(P60709);hsa:60(P60709);hsa:90390(Q96HR3) | 0.014607002 | 0.501507072 | http://www.kegg.jp/kegg-bin/show_pathway?hsa04919+hsa:71%09salmon+hsa:60%09salmon+hsa:90390%09green |
| hsa04015 | Rap1 signaling pathway | Environmental Information Processing | 7\|228 | hsa:3674(P08514);hsa:7094(Q9Y490);hsa:5908(P61224);hsa:5881(P60763);hsa:7057(P07996);hsa:5216(P07737);hsa:71(P60709);hsa:60(P60709) | 2\|28 | hsa:71(P60709);hsa:60(P60709);hsa:5216(P07737) | 0.165521393 | 0.274979088 | http://www.kegg.jp/kegg-bin/show_pathway?hsa04015+hsa:71%09salmon+hsa:60%09salmon+hsa:5216%09salmon |
| hsa01200 | Carbon metabolism | Metabolism | 5\|228 | hsa:5315(H3BTN5);hsa:2023(A0A2R8Y6G6);hsa:847(P04040);hsa:226(J3KPS3);hsa:2597(E7EUT5) | 2\|28 | hsa:5315(H3BTN5);hsa:847(P04040) | 0.101057925 | 0.315423221 | http://www.kegg.jp/kegg-bin/show_pathway?hsa01200+hsa:5315%09salmon+hsa:847%09green |
| hsa05410 | Hypertrophic cardiomyopathy (HCM) | Human Diseases | 5\|228 | hsa:3674(P08514);hsa:7171(A0A087WWU8;A0A2R8Y5V9);hsa:71(P60709);hsa:70(P68032);hsa:60(P60709) | 2\|28 | hsa:71(P60709);hsa:70(P68032);hsa:60(P60709) | 0.101057925 | 0.315423221 | http://www.kegg.jp/kegg-bin/show_pathway?hsa05410+hsa:71%09salmon+hsa:70%09salmon+hsa:60%09salmon |
| hsa05162 | Measles | Human Diseases | 40\|228 | hsa:102723407(A0A0C4DH29;A0A0C4DH32;A0A0C4DH33;A0A0J9YX35;A0A0C4DH31;A0A0C4DH36;A0A0C4DH34;A0A0C4DH38;A0A0C4DH39;A0A0J9YY99;A0A0G2JMI3;A0A0B4J1U7;P01763;P01764;P01743;P01766;A0A0B4J1X5;A0A0C4DH43;P01782;P01780;A0A087WSY4;P01814;A0A0B4J1X8;A0A4W8ZXM2;A0A075B7D0;P0DP02;P0DP01;A0A075B7F0;A0A075B7D8;P01817;A0A0A0MS14;A0A0A0MS15;A0A0B4J1V1;A0A075B7B8;A0A0B4J1V2;P0DTE1;A0A075B6Q5;A0A0J9YVY3;P23083);hsa:4478(P26038) | 2\|28 | hsa:102723407(A0A0C4DH36;A0A0J9YX35) | 0.069209068 | 0.297022251 | http://www.kegg.jp/kegg-bin/show_pathway?hsa05162+hsa:102723407%09salmon+hsa:102723407%09salmon |
| hsa05330 | Allograft rejection | Human Diseases | 40\|228 | hsa:102723407(A0A0C4DH29;A0A0C4DH32;A0A0C4DH33;A0A0J9YX35;A0A0C4DH31;A0A0C4DH36;A0A0C4DH34;A0A0C4DH38;A0A0C4DH39;A0A0J9YY99;A0A0G2JMI3;A0A0B4J1U7;P01763;P01764;P01743;P01766;A0A0B4J1X5;A0A0C4DH43;P01782;P01780;A0A087WSY4;P01814;A0A0B4J1X8;A0A4W8ZXM2;A0A075B7D0;P0DP02;P0DP01;A0A075B7F0;A0A075B7D8;P01817;A0A0A0MS14;A0A0A0MS15;A0A0B4J1V1;A0A075B7B8;A0A0B4J1V2;P0DTE1;A0A075B6Q5;A0A0J9YVY3;P23083);hsa:3105(A0A0G2JI36) | 2\|28 | hsa:102723407(A0A0C4DH36;A0A0J9YX35) | 0.069209068 | 0.297022251 | http://www.kegg.jp/kegg-bin/show_pathway?hsa05330+hsa:102723407%09salmon+hsa:102723407%09salmon |
| hsa04640 | Hematopoietic cell lineage | Organismal Systems | 45\|228 | hsa:3674(P08514);hsa:102723407(A0A0C4DH29;A0A0C4DH32;A0A0C4DH33;A0A0J9YX35;A0A0C4DH31;A0A0C4DH36;A0A0C4DH34;A0A0C4DH38;A0A0C4DH39;A0A0J9YY99;A0A0G2JMI3;A0A0B4J1U7;P01763;P01764;P01743;P01766;A0A0B4J1X5;A0A0C4DH43;P01782;P01780;A0A087WSY4;P01814;A0A0B4J1X8;A0A4W8ZXM2;A0A075B7D0;P0DP02;P0DP01;A0A075B7F0;A0A075B7D8;P01817;A0A0A0MS14;A0A0A0MS15;A0A0B4J1V1;A0A075B7B8;A0A0B4J1V2;P0DTE1;A0A075B6Q5;A0A0J9YVY3;P23083);hsa:2811(A0A0C4DGZ8);hsa:960(H0Y5E4);hsa:290(P15144);hsa:7037(G3V0E5);hsa:929(P08571) | 2\|28 | hsa:102723407(A0A0C4DH36;A0A0J9YX35) | 0.041372973 | 0.387401473 | http://www.kegg.jp/kegg-bin/show_pathway?hsa04640+hsa:102723407%09salmon+hsa:102723407%09salmon |
| hsa04666 | Fc gamma R-mediated phagocytosis | Organismal Systems | 42\|228 | hsa:2934(A0A0A0MS51);hsa:102723407(A0A0C4DH29;A0A0C4DH32;A0A0C4DH33;A0A0J9YX35;A0A0C4DH31;A0A0C4DH36;A0A0C4DH34;A0A0C4DH38;A0A0C4DH39;A0A0J9YY99;A0A0G2JMI3;A0A0B4J1U7;P01763;P01764;P01743;P01766;A0A0B4J1X5;A0A0C4DH43;P01782;P01780;A0A087WSY4;P01814;A0A0B4J1X8;A0A4W8ZXM2;A0A075B7D0;P0DP02;P0DP01;A0A075B7F0;A0A075B7D8;P01817;A0A0A0MS14;A0A0A0MS15;A0A0B4J1V1;A0A075B7B8;A0A0B4J1V2;P0DTE1;A0A075B6Q5;A0A0J9YVY3;P23083);hsa:1072(E9PK25);hsa:2214(H0Y755) | 2\|28 | hsa:102723407(A0A0C4DH36;A0A0J9YX35) | 0.0566778 | 0.416986673 | http://www.kegg.jp/kegg-bin/show_pathway?hsa04666+hsa:102723407%09salmon+hsa:102723407%09salmon |
| hsa05310 | Asthma | Human Diseases | 39\|228 | hsa:102723407(A0A0C4DH29;A0A0C4DH32;A0A0C4DH33;A0A0J9YX35;A0A0C4DH31;A0A0C4DH36;A0A0C4DH34;A0A0C4DH38;A0A0C4DH39;A0A0J9YY99;A0A0G2JMI3;A0A0B4J1U7;P01763;P01764;P01743;P01766;A0A0B4J1X5;A0A0C4DH43;P01782;P01780;A0A087WSY4;P01814;A0A0B4J1X8;A0A4W8ZXM2;A0A075B7D0;P0DP02;P0DP01;A0A075B7F0;A0A075B7D8;P01817;A0A0A0MS14;A0A0A0MS15;A0A0B4J1V1;A0A075B7B8;A0A0B4J1V2;P0DTE1;A0A075B6Q5;A0A0J9YVY3;P23083) | 2\|28 | hsa:102723407(A0A0C4DH36;A0A0J9YX35) | 0.076236124 | 0.280440028 | http://www.kegg.jp/kegg-bin/show_pathway?hsa05310+hsa:102723407%09salmon+hsa:102723407%09salmon |
| hsa04664 | Fc epsilon RI signaling pathway | Organismal Systems | 40\|228 | hsa:5881(P60763);hsa:102723407(A0A0C4DH29;A0A0C4DH32;A0A0C4DH33;A0A0J9YX35;A0A0C4DH31;A0A0C4DH36;A0A0C4DH34;A0A0C4DH38;A0A0C4DH39;A0A0J9YY99;A0A0G2JMI3;A0A0B4J1U7;P01763;P01764;P01743;P01766;A0A0B4J1X5;A0A0C4DH43;P01782;P01780;A0A087WSY4;P01814;A0A0B4J1X8;A0A4W8ZXM2;A0A075B7D0;P0DP02;P0DP01;A0A075B7F0;A0A075B7D8;P01817;A0A0A0MS14;A0A0A0MS15;A0A0B4J1V1;A0A075B7B8;A0A0B4J1V2;P0DTE1;A0A075B6Q5;A0A0J9YVY3;P23083) | 2\|28 | hsa:102723407(A0A0C4DH36;A0A0J9YX35) | 0.069209068 | 0.297022251 | http://www.kegg.jp/kegg-bin/show_pathway?hsa04664+hsa:102723407%09salmon+hsa:102723407%09salmon |
| hsa05014 | Amyotrophic lateral sclerosis (ALS) | Human Diseases | 1\|228 | hsa:847(P04040) | 1\|28 | hsa:847(P04040) | 0.122807018 | 0.274980931 | http://www.kegg.jp/kegg-bin/show_pathway?hsa05014+hsa:847%09green |
| hsa05142 | Chagas disease (American trypanosomiasis) | Human Diseases | 5\|228 | hsa:718(P01024;M0R0Q9);hsa:713(D6R934);hsa:712(P02745);hsa:714(P02747) | 1\|28 | hsa:714(P02747) | 0.368674283 | 0.387484195 | http://www.kegg.jp/kegg-bin/show_pathway?hsa05142+hsa:714%09green |
| hsa04971 | Gastric acid secretion | Organismal Systems | 2\|228 | hsa:760(P00918);hsa:60(P60709) | 1\|28 | hsa:60(P60709) | 0.216400031 | 0.30533155 | http://www.kegg.jp/kegg-bin/show_pathway?hsa04971+hsa:60%09salmon |
| hsa04110 | Cell cycle | Cellular Processes | 1\|228 | hsa:7534(E7EX29) | 1\|28 | hsa:7534(E7EX29) | 0.122807018 | 0.274980931 | http://www.kegg.jp/kegg-bin/show_pathway?hsa04110+hsa:7534%09salmon |
| hsa05200 | Pathways in cancer | Human Diseases | 4\|228 | hsa:5881(P60763);hsa:3674(P08514);hsa:3481(P01344);hsa:2335(P02751) | 1\|28 | hsa:2335(P02751) | 0.335362597 | 0.375460298 | http://www.kegg.jp/kegg-bin/show_pathway?hsa05200+hsa:2335%09salmon |
| hsa00564 | Glycerophospholipid metabolism | Metabolism | 2\|228 | hsa:81579(A0A2R8Y3M9);hsa:3931(P04180) | 1\|28 | hsa:81579(A0A2R8Y3M9) | 0.216400031 | 0.30533155 | http://www.kegg.jp/kegg-bin/show_pathway?hsa00564+hsa:81579%09green |
| hsa00480 | Glutathione metabolism | Metabolism | 2\|228 | hsa:290(P15144);hsa:2878(A0A087X1J7) | 1\|28 | hsa:2878(A0A087X1J7) | 0.216400031 | 0.30533155 | http://www.kegg.jp/kegg-bin/show_pathway?hsa00480+hsa:2878%09salmon |
| hsa04915 | Estrogen signaling pathway | Organismal Systems | 4\|228 | hsa:3858(P13645);hsa:3868(P08779);hsa:3861(P02533);hsa:3857(P35527) | 1\|28 | hsa:3857(P35527) | 0.335362597 | 0.375460298 | http://www.kegg.jp/kegg-bin/show_pathway?hsa04915+hsa:3857%09salmon |
| hsa00565 | Ether lipid metabolism | Metabolism | 3\|228 | hsa:81579(A0A2R8Y3M9);hsa:7368(Q16880);hsa:7941(Q13093) | 1\|28 | hsa:81579(A0A2R8Y3M9) | 0.285820395 | 0.346347067 | http://www.kegg.jp/kegg-bin/show_pathway?hsa00565+hsa:81579%09green |
| hsa00591 | Linoleic acid metabolism | Metabolism | 1\|228 | hsa:81579(A0A2R8Y3M9) | 1\|28 | hsa:81579(A0A2R8Y3M9) | 0.122807018 | 0.274980931 | http://www.kegg.jp/kegg-bin/show_pathway?hsa00591+hsa:81579%09green |
| hsa04979 | Cholesterol metabolism | Organismal Systems | 15\|228 | hsa:255738(A0A669KAY4);hsa:4035(Q07954);hsa:350(P02749);hsa:5360(P55058);hsa:1071(P11597);hsa:4018(P08519);hsa:3931(P04180);hsa:338(P04114);hsa:348(P02649);hsa:344(K7ER74);hsa:345(B0YIW2);hsa:337(P06727);hsa:336(P02652);hsa:335(P02647);hsa:341(K7ERI9) | 1\|28 | hsa:5360(P55058) | 0.295681504 | 0.350059712 | http://www.kegg.jp/kegg-bin/show_pathway?hsa04979+hsa:5360%09salmon |
| hsa05230 | Central carbon metabolism in cancer | Human Diseases | 1\|228 | hsa:5315(H3BTN5) | 1\|28 | hsa:5315(H3BTN5) | 0.122807018 | 0.274980931 | http://www.kegg.jp/kegg-bin/show_pathway?hsa05230+hsa:5315%09salmon |
| hsa04933 | AGE-RAGE signaling pathway in diabetic complications | Human Diseases | 2\|228 | hsa:7412(P19320);hsa:2335(P02751) | 1\|28 | hsa:2335(P02751) | 0.216400031 | 0.30533155 | http://www.kegg.jp/kegg-bin/show_pathway?hsa04933+hsa:2335%09salmon |
| hsa04975 | Fat digestion and absorption | Organismal Systems | 4\|228 | hsa:81579(A0A2R8Y3M9);hsa:338(P04114);hsa:337(P06727);hsa:335(P02647) | 1\|28 | hsa:81579(A0A2R8Y3M9) | 0.335362597 | 0.375460298 | http://www.kegg.jp/kegg-bin/show_pathway?hsa04975+hsa:81579%09green |
| hsa04213 | Longevity regulating pathway - multiple species | Organismal Systems | 1\|228 | hsa:847(P04040) | 1\|28 | hsa:847(P04040) | 0.122807018 | 0.274980931 | http://www.kegg.jp/kegg-bin/show_pathway?hsa04213+hsa:847%09green |
| hsa01230 | Biosynthesis of amino acids | Metabolism | 4\|228 | hsa:5315(H3BTN5);hsa:2023(A0A2R8Y6G6);hsa:226(J3KPS3);hsa:2597(E7EUT5) | 1\|28 | hsa:5315(H3BTN5) | 0.335362597 | 0.375460298 | http://www.kegg.jp/kegg-bin/show_pathway?hsa01230+hsa:5315%09salmon |
| hsa00010 | Glycolysis / Gluconeogenesis | Metabolism | 4\|228 | hsa:5315(H3BTN5);hsa:2023(A0A2R8Y6G6);hsa:226(J3KPS3);hsa:2597(E7EUT5) | 1\|28 | hsa:5315(H3BTN5) | 0.335362597 | 0.375460298 | http://www.kegg.jp/kegg-bin/show_pathway?hsa00010+hsa:5315%09salmon |
| hsa04921 | Oxytocin signaling pathway | Organismal Systems | 1\|228 | hsa:71(P60709);hsa:60(P60709) | 1\|28 | hsa:71(P60709);hsa:60(P60709) | 0.122807018 | 0.274980931 | http://www.kegg.jp/kegg-bin/show_pathway?hsa04921+hsa:71%09salmon+hsa:60%09salmon |
| hsa04514 | Cell adhesion molecules (CAMs) | Environmental Information Processing | 5\|228 | hsa:102723996(A0A087X1L8);hsa:7412(P19320);hsa:6402(P14151);hsa:23308(A0A087X1L8);hsa:3105(A0A0G2JI36);hsa:1003(I3L1J2) | 1\|28 | hsa:1003(I3L1J2) | 0.368674283 | 0.387484195 | http://www.kegg.jp/kegg-bin/show_pathway?hsa04514+hsa:1003%09salmon |
| hsa00592 | alpha-Linolenic acid metabolism | Metabolism | 1\|228 | hsa:81579(A0A2R8Y3M9) | 1\|28 | hsa:81579(A0A2R8Y3M9) | 0.122807018 | 0.274980931 | http://www.kegg.jp/kegg-bin/show_pathway?hsa00592+hsa:81579%09green |
| hsa04068 | FoxO signaling pathway | Environmental Information Processing | 1\|228 | hsa:847(P04040) | 1\|28 | hsa:847(P04040) | 0.122807018 | 0.274980931 | http://www.kegg.jp/kegg-bin/show_pathway?hsa04068+hsa:847%09green |
| hsa04146 | Peroxisome | Cellular Processes | 1\|228 | hsa:847(P04040) | 1\|28 | hsa:847(P04040) | 0.122807018 | 0.274980931 | http://www.kegg.jp/kegg-bin/show_pathway?hsa04146+hsa:847%09green |
| hsa00230 | Purine metabolism | Metabolism | 1\|228 | hsa:5315(H3BTN5) | 1\|28 | hsa:5315(H3BTN5) | 0.122807018 | 0.274980931 | http://www.kegg.jp/kegg-bin/show_pathway?hsa00230+hsa:5315%09salmon |
| hsa04261 | Adrenergic signaling in cardiomyocytes | Organismal Systems | 3\|228 | hsa:7171(A0A087WWU8;A0A2R8Y5V9);hsa:70(P68032) | 1\|28 | hsa:70(P68032) | 0.285820395 | 0.346347067 | http://www.kegg.jp/kegg-bin/show_pathway?hsa04261+hsa:70%09salmon |
| hsa05144 | Malaria | Human Diseases | 6\|228 | hsa:3039(P69905);hsa:4035(Q07954);hsa:1311(G3XAP6);hsa:7057(P07996);hsa:3043(P68871);hsa:3040(P69905);hsa:7412(P19320) | 1\|28 | hsa:3039(P69905);hsa:3040(P69905) | 0.388843908 | 0.396543787 | http://www.kegg.jp/kegg-bin/show_pathway?hsa05144+hsa:3039%09green+hsa:3040%09green |
| hsa05110 | Vibrio cholerae infection | Human Diseases | 1\|228 | hsa:71(P60709);hsa:60(P60709) | 1\|28 | hsa:71(P60709);hsa:60(P60709) | 0.122807018 | 0.274980931 | http://www.kegg.jp/kegg-bin/show_pathway?hsa05110+hsa:71%09salmon+hsa:60%09salmon |
| hsa00630 | Glyoxylate and dicarboxylate metabolism | Metabolism | 2\|228 | hsa:81888(E7EWH8);hsa:847(P04040) | 1\|28 | hsa:847(P04040) | 0.216400031 | 0.30533155 | http://www.kegg.jp/kegg-bin/show_pathway?hsa00630+hsa:847%09green |
| hsa04210 | Apoptosis | Cellular Processes | 4\|228 | hsa:7278(P0DPH7);hsa:2081(O75460);hsa:7277(P68366);hsa:113457(P0DPH7);hsa:112714(P0DPH7);hsa:71(P60709);hsa:60(P60709) | 1\|28 | hsa:71(P60709);hsa:60(P60709) | 0.335362597 | 0.375460298 | http://www.kegg.jp/kegg-bin/show_pathway?hsa04210+hsa:71%09salmon+hsa:60%09salmon |
| hsa00910 | Nitrogen metabolism | Metabolism | 2\|228 | hsa:760(P00918);hsa:759(P00915) | 1\|28 | hsa:759(P00915) | 0.216400031 | 0.30533155 | http://www.kegg.jp/kegg-bin/show_pathway?hsa00910+hsa:759%09green |
| hsa04211 | Longevity regulating pathway | Organismal Systems | 2\|228 | hsa:847(P04040);hsa:9370(Q15848) | 1\|28 | hsa:847(P04040) | 0.216400031 | 0.30533155 | http://www.kegg.jp/kegg-bin/show_pathway?hsa04211+hsa:847%09green |
| hsa05133 | Pertussis | Human Diseases | 17\|228 | hsa:712(P02745);hsa:715(A0A3B3ISR2);hsa:1072(E9PK25);hsa:721(A0A0G2JPR0;P0C0L5;P0C0L4);hsa:727(P01031);hsa:718(P01024;M0R0Q9);hsa:717(A0A0G2JL69);hsa:710(P05155);hsa:725(P20851);hsa:713(D6R934);hsa:929(P08571);hsa:720(A0A0G2JPR0;P0C0L5;P0C0L4);hsa:714(P02747);hsa:722(P04003);hsa:716(P09871) | 1\|28 | hsa:714(P02747) | 0.255358918 | 0.324715661 | http://www.kegg.jp/kegg-bin/show_pathway?hsa05133+hsa:714%09green |
| hsa04114 | Oocyte meiosis | Cellular Processes | 1\|228 | hsa:7534(E7EX29) | 1\|28 | hsa:7534(E7EX29) | 0.122807018 | 0.274980931 | http://www.kegg.jp/kegg-bin/show_pathway?hsa04114+hsa:7534%09salmon |
| hsa05160 | Hepatitis C | Human Diseases | 1\|228 | hsa:7534(E7EX29) | 1\|28 | hsa:7534(E7EX29) | 0.122807018 | 0.274980931 | http://www.kegg.jp/kegg-bin/show_pathway?hsa05160+hsa:7534%09salmon |
| hsa00620 | Pyruvate metabolism | Metabolism | 1\|228 | hsa:5315(H3BTN5) | 1\|28 | hsa:5315(H3BTN5) | 0.122807018 | 0.274980931 | http://www.kegg.jp/kegg-bin/show_pathway?hsa00620+hsa:5315%09salmon |
| hsa03320 | PPAR signaling pathway | Organismal Systems | 6\|228 | hsa:9370(Q15848);hsa:5360(P55058);hsa:3611(A0A0A0MTH3);hsa:345(B0YIW2);hsa:336(P02652);hsa:335(P02647) | 1\|28 | hsa:5360(P55058) | 0.388843908 | 0.396543787 | http://www.kegg.jp/kegg-bin/show_pathway?hsa03320+hsa:5360%09salmon |
| hsa05225 | Hepatocellular carcinoma | Human Diseases | 2\|228 | hsa:3481(P01344);hsa:71(P60709);hsa:60(P60709) | 1\|28 | hsa:71(P60709);hsa:60(P60709) | 0.216400031 | 0.30533155 | http://www.kegg.jp/kegg-bin/show_pathway?hsa05225+hsa:71%09salmon+hsa:60%09salmon |
| hsa04926 | Relaxin signaling pathway | Organismal Systems | 1\|228 | hsa:59(P68032) | 1\|28 | hsa:59(P68032) | 0.122807018 | 0.274980931 | http://www.kegg.jp/kegg-bin/show_pathway?hsa04926+hsa:59%09salmon |
| hsa05412 | Arrhythmogenic right ventricular cardiomyopathy (ARVC) | Human Diseases | 2\|228 | hsa:3674(P08514);hsa:71(P60709);hsa:60(P60709) | 1\|28 | hsa:71(P60709);hsa:60(P60709) | 0.216400031 | 0.30533155 | http://www.kegg.jp/kegg-bin/show_pathway?hsa05412+hsa:71%09salmon+hsa:60%09salmon |
| hsa04014 | Ras signaling pathway | Environmental Information Processing | 4\|228 | hsa:5881(P60763);hsa:81579(A0A2R8Y3M9);hsa:3481(P01344);hsa:5908(P61224) | 1\|28 | hsa:81579(A0A2R8Y3M9) | 0.335362597 | 0.375460298 | http://www.kegg.jp/kegg-bin/show_pathway?hsa04014+hsa:81579%09green |
| hsa04974 | Protein digestion and absorption | Organismal Systems | 4\|228 | hsa:1293(E7ENL6);hsa:1361(Q96IY4);hsa:7373(J3QT83);hsa:1291(A0A087X0S5) | 1\|28 | hsa:1293(E7ENL6) | 0.335362597 | 0.375460298 | http://www.kegg.jp/kegg-bin/show_pathway?hsa04974+hsa:1293%09green |
| hsa04918 | Thyroid hormone synthesis | Organismal Systems | 6\|228 | hsa:2878(A0A087X1J7);hsa:7276(P02766);hsa:213(P02768;A0A087WWT3);hsa:3309(P11021);hsa:6906(P05543) | 1\|28 | hsa:2878(A0A087X1J7) | 0.388843908 | 0.396543787 | http://www.kegg.jp/kegg-bin/show_pathway?hsa04918+hsa:2878%09salmon |
| hsa00380 | Tryptophan metabolism | Metabolism | 1\|228 | hsa:847(P04040) | 1\|28 | hsa:847(P04040) | 0.122807018 | 0.274980931 | http://www.kegg.jp/kegg-bin/show_pathway?hsa00380+hsa:847%09green |
| hsa04972 | Pancreatic secretion | Organismal Systems | 4\|228 | hsa:760(P00918);hsa:81579(A0A2R8Y3M9);hsa:1361(Q96IY4);hsa:5908(P61224) | 1\|28 | hsa:81579(A0A2R8Y3M9) | 0.335362597 | 0.375460298 | http://www.kegg.jp/kegg-bin/show_pathway?hsa04972+hsa:81579%09green |
| hsa04371 | Apelin signaling pathway | Environmental Information Processing | 1\|228 | hsa:59(P68032) | 1\|28 | hsa:59(P68032) | 0.122807018 | 0.274980931 | http://www.kegg.jp/kegg-bin/show_pathway?hsa04371+hsa:59%09salmon |
| hsa04260 | Cardiac muscle contraction | Organismal Systems | 3\|228 | hsa:7171(A0A087WWU8;A0A2R8Y5V9);hsa:70(P68032) | 1\|28 | hsa:70(P68032) | 0.285820395 | 0.346347067 | http://www.kegg.jp/kegg-bin/show_pathway?hsa04260+hsa:70%09salmon |
| hsa04714 | Thermogenesis | Organismal Systems | 1\|228 | hsa:71(P60709);hsa:60(P60709) | 1\|28 | hsa:71(P60709);hsa:60(P60709) | 0.122807018 | 0.274980931 | http://www.kegg.jp/kegg-bin/show_pathway?hsa04714+hsa:71%09salmon+hsa:60%09salmon |
| hsa04922 | Glucagon signaling pathway | Organismal Systems | 1\|228 | hsa:5315(H3BTN5) | 1\|28 | hsa:5315(H3BTN5) | 0.122807018 | 0.274980931 | http://www.kegg.jp/kegg-bin/show_pathway?hsa04922+hsa:5315%09salmon |
| hsa05164 | Influenza A | Human Diseases | 2\|228 | hsa:71(P60709);hsa:60(P60709);hsa:5340(P00747) | 1\|28 | hsa:71(P60709);hsa:60(P60709) | 0.216400031 | 0.30533155 | http://www.kegg.jp/kegg-bin/show_pathway?hsa05164+hsa:71%09salmon+hsa:60%09salmon |
| hsa05161 | Hepatitis B | Human Diseases | 1\|228 | hsa:7534(E7EX29) | 1\|28 | hsa:7534(E7EX29) | 0.122807018 | 0.274980931 | http://www.kegg.jp/kegg-bin/show_pathway?hsa05161+hsa:7534%09salmon |
| hsa04540 | Gap junction | Cellular Processes | 4\|228 | hsa:203068(P07437);hsa:7278(P0DPH7);hsa:81027(Q9H4B7);hsa:7277(P68366);hsa:113457(P0DPH7);hsa:112714(P0DPH7) | 1\|28 | hsa:81027(Q9H4B7) | 0.335362597 | 0.375460298 | http://www.kegg.jp/kegg-bin/show_pathway?hsa04540+hsa:81027%09salmon |
| hsa05222 | Small cell lung cancer | Human Diseases | 2\|228 | hsa:3674(P08514);hsa:2335(P02751) | 1\|28 | hsa:2335(P02751) | 0.216400031 | 0.30533155 | http://www.kegg.jp/kegg-bin/show_pathway?hsa05222+hsa:2335%09salmon |
| hsa04930 | Type II diabetes mellitus | Human Diseases | 2\|228 | hsa:5315(H3BTN5);hsa:9370(Q15848) | 1\|28 | hsa:5315(H3BTN5) | 0.216400031 | 0.30533155 | http://www.kegg.jp/kegg-bin/show_pathway?hsa04930+hsa:5315%09salmon |
| hsa04530 | Tight junction | Cellular Processes | 7\|228 | hsa:3993(J3QRV5);hsa:7278(P0DPH7);hsa:7277(P68366);hsa:113457(P0DPH7);hsa:4627(P35579);hsa:87(H9KV75);hsa:112714(P0DPH7);hsa:4478(P26038);hsa:71(P60709);hsa:60(P60709) | 1\|28 | hsa:71(P60709);hsa:60(P60709) | 0.398477428 | 0.398477428 | http://www.kegg.jp/kegg-bin/show_pathway?hsa04530+hsa:71%09salmon+hsa:60%09salmon |

| Table S5-2 KEGG pathway enrichment of DEPs in S vs A group. | | | | | | | | | |
| --- | --- | --- | --- | --- | --- | --- | --- | --- | --- |
| pathway | pathway_name | class | all_number_of_accs | all_KO2acc | diff_number_of_accs | diff_KO2acc | p_value | FDR | url |
| hsa05152 | Tuberculosis | Human Diseases | 46\|228 | hsa:102723407(A0A0C4DH29;A0A0C4DH32;A0A0C4DH33;A0A0J9YX35;A0A0C4DH31;A0A0C4DH36;A0A0C4DH34;A0A0C4DH38;A0A0C4DH39;A0A0J9YY99;A0A0G2JMI3;A0A0B4J1U7;P01763;P01764;P01743;P01766;A0A0B4J1X5;A0A0C4DH43;P01782;P01780;A0A087WSY4;P01814;A0A0B4J1X8;A0A4W8ZXM2;A0A075B7D0;P0DP02;P0DP01;A0A075B7F0;A0A075B7D8;P01817;A0A0A0MS14;A0A0A0MS15;A0A0B4J1V1;A0A075B7B8;A0A0B4J1V2;P0DTE1;A0A075B6Q5;A0A0J9YVY3;P23083);hsa:820(J3KNB4);hsa:3920(P13473);hsa:718(P01024;M0R0Q9);hsa:2214(H0Y755);hsa:3929(P18428);hsa:929(P08571) | 8\|44 | hsa:102723407(A0A0C4DH32;A0A0C4DH34;A0A0J9YX35;P01780;P01782;P01817;P0DP01);hsa:718(M0R0Q9) | 0.158872387 | 0.571940591 | http://www.kegg.jp/kegg-bin/show_pathway?hsa05152+hsa:102723407%09salmon+hsa:102723407%09green+hsa:102723407%09salmon+hsa:102723407%09green+hsa:102723407%09green+hsa:102723407%09salmon+hsa:102723407%09green+hsa:718%09green |
| hsa04145 | Phagosome | Cellular Processes | 56\|228 | hsa:203068(P07437);hsa:3105(A0A0G2JI36);hsa:7278(P0DPH7);hsa:81027(Q9H4B7);hsa:102723407(A0A0C4DH29;A0A0C4DH32;A0A0C4DH33;A0A0J9YX35;A0A0C4DH31;A0A0C4DH36;A0A0C4DH34;A0A0C4DH38;A0A0C4DH39;A0A0J9YY99;A0A0G2JMI3;A0A0B4J1U7;P01763;P01764;P01743;P01766;A0A0B4J1X5;A0A0C4DH43;P01782;P01780;A0A087WSY4;P01814;A0A0B4J1X8;A0A4W8ZXM2;A0A075B7D0;P0DP02;P0DP01;A0A075B7F0;A0A075B7D8;P01817;A0A0A0MS14;A0A0A0MS15;A0A0B4J1V1;A0A075B7B8;A0A0B4J1V2;P0DTE1;A0A075B6Q5;A0A0J9YVY3;P23083);hsa:1311(G3XAP6);hsa:71(P60709);hsa:7277(P68366);hsa:113457(P0DPH7);hsa:3920(P13473);hsa:718(P01024;M0R0Q9);hsa:7057(P07996);hsa:112714(P0DPH7);hsa:2214(H0Y755);hsa:4153(P11226);hsa:78989(Q9BWP8);hsa:7037(G3V0E5);hsa:929(P08571);hsa:715(A0A3B3ISR2);hsa:60(P60709) | 8\|44 | hsa:102723407(A0A0C4DH32;A0A0C4DH34;A0A0J9YX35;P01780;P01782;P01817;P0DP01);hsa:718(M0R0Q9) | 0.089580534 | 1 | http://www.kegg.jp/kegg-bin/show_pathway?hsa04145+hsa:102723407%09salmon+hsa:102723407%09green+hsa:102723407%09salmon+hsa:102723407%09green+hsa:102723407%09green+hsa:102723407%09salmon+hsa:102723407%09green+hsa:718%09green |
| hsa05140 | Leishmaniasis | Human Diseases | 42\|228 | hsa:718(P01024;M0R0Q9);hsa:2214(H0Y755);hsa:102723407(A0A0C4DH29;A0A0C4DH32;A0A0C4DH33;A0A0J9YX35;A0A0C4DH31;A0A0C4DH36;A0A0C4DH34;A0A0C4DH38;A0A0C4DH39;A0A0J9YY99;A0A0G2JMI3;A0A0B4J1U7;P01763;P01764;P01743;P01766;A0A0B4J1X5;A0A0C4DH43;P01782;P01780;A0A087WSY4;P01814;A0A0B4J1X8;A0A4W8ZXM2;A0A075B7D0;P0DP02;P0DP01;A0A075B7F0;A0A075B7D8;P01817;A0A0A0MS14;A0A0A0MS15;A0A0B4J1V1;A0A075B7B8;A0A0B4J1V2;P0DTE1;A0A075B6Q5;A0A0J9YVY3;P23083) | 8\|44 | hsa:102723407(A0A0C4DH32;A0A0C4DH34;A0A0J9YX35;P01780;P01782;P01817;P0DP01);hsa:718(M0R0Q9) | 0.171241812 | 0.45328715 | http://www.kegg.jp/kegg-bin/show_pathway?hsa05140+hsa:102723407%09salmon+hsa:102723407%09green+hsa:102723407%09salmon+hsa:102723407%09green+hsa:102723407%09green+hsa:102723407%09salmon+hsa:102723407%09green+hsa:718%09green |
| hsa05323 | Rheumatoid arthritis | Human Diseases | 39\|228 | hsa:102723407(A0A0C4DH29;A0A0C4DH32;A0A0C4DH33;A0A0J9YX35;A0A0C4DH31;A0A0C4DH36;A0A0C4DH34;A0A0C4DH38;A0A0C4DH39;A0A0J9YY99;A0A0G2JMI3;A0A0B4J1U7;P01763;P01764;P01743;P01766;A0A0B4J1X5;A0A0C4DH43;P01782;P01780;A0A087WSY4;P01814;A0A0B4J1X8;A0A4W8ZXM2;A0A075B7D0;P0DP02;P0DP01;A0A075B7F0;A0A075B7D8;P01817;A0A0A0MS14;A0A0A0MS15;A0A0B4J1V1;A0A075B7B8;A0A0B4J1V2;P0DTE1;A0A075B6Q5;A0A0J9YVY3;P23083) | 7\|44 | hsa:102723407(A0A0C4DH32;A0A0C4DH34;A0A0J9YX35;P01780;P01782;P01817;P0DP01) | 0.174597701 | 0.436494253 | http://www.kegg.jp/kegg-bin/show_pathway?hsa05323+hsa:102723407%09salmon+hsa:102723407%09green+hsa:102723407%09salmon+hsa:102723407%09green+hsa:102723407%09green+hsa:102723407%09salmon+hsa:102723407%09green |
| hsa05169 | Epstein-Barr virus infection | Human Diseases | 41\|228 | hsa:102723407(A0A0C4DH29;A0A0C4DH32;A0A0C4DH33;A0A0J9YX35;A0A0C4DH31;A0A0C4DH36;A0A0C4DH34;A0A0C4DH38;A0A0C4DH39;A0A0J9YY99;A0A0G2JMI3;A0A0B4J1U7;P01763;P01764;P01743;P01766;A0A0B4J1X5;A0A0C4DH43;P01782;P01780;A0A087WSY4;P01814;A0A0B4J1X8;A0A4W8ZXM2;A0A075B7D0;P0DP02;P0DP01;A0A075B7F0;A0A075B7D8;P01817;A0A0A0MS14;A0A0A0MS15;A0A0B4J1V1;A0A075B7B8;A0A0B4J1V2;P0DTE1;A0A075B6Q5;A0A0J9YVY3;P23083);hsa:3105(A0A0G2JI36);hsa:960(H0Y5E4) | 7\|44 | hsa:102723407(A0A0C4DH32;A0A0C4DH34;A0A0J9YX35;P01780;P01782;P01817;P0DP01) | 0.164850548 | 0.549501826 | http://www.kegg.jp/kegg-bin/show_pathway?hsa05169+hsa:102723407%09salmon+hsa:102723407%09green+hsa:102723407%09salmon+hsa:102723407%09green+hsa:102723407%09green+hsa:102723407%09salmon+hsa:102723407%09green |
| hsa05416 | Viral myocarditis | Human Diseases | 42\|228 | hsa:5881(P60763);hsa:102723407(A0A0C4DH29;A0A0C4DH32;A0A0C4DH33;A0A0J9YX35;A0A0C4DH31;A0A0C4DH36;A0A0C4DH34;A0A0C4DH38;A0A0C4DH39;A0A0J9YY99;A0A0G2JMI3;A0A0B4J1U7;P01763;P01764;P01743;P01766;A0A0B4J1X5;A0A0C4DH43;P01782;P01780;A0A087WSY4;P01814;A0A0B4J1X8;A0A4W8ZXM2;A0A075B7D0;P0DP02;P0DP01;A0A075B7F0;A0A075B7D8;P01817;A0A0A0MS14;A0A0A0MS15;A0A0B4J1V1;A0A075B7B8;A0A0B4J1V2;P0DTE1;A0A075B6Q5;A0A0J9YVY3;P23083);hsa:3105(A0A0G2JI36);hsa:71(P60709);hsa:60(P60709) | 7\|44 | hsa:102723407(A0A0C4DH32;A0A0C4DH34;A0A0J9YX35;P01780;P01782;P01817;P0DP01) | 0.158679672 | 0.649144111 | http://www.kegg.jp/kegg-bin/show_pathway?hsa05416+hsa:102723407%09salmon+hsa:102723407%09green+hsa:102723407%09salmon+hsa:102723407%09green+hsa:102723407%09green+hsa:102723407%09salmon+hsa:102723407%09green |
| hsa05143 | African trypanosomiasis | Human Diseases | 45\|228 | hsa:102723407(A0A0C4DH29;A0A0C4DH32;A0A0C4DH33;A0A0J9YX35;A0A0C4DH31;A0A0C4DH36;A0A0C4DH34;A0A0C4DH38;A0A0C4DH39;A0A0J9YY99;A0A0G2JMI3;A0A0B4J1U7;P01763;P01764;P01743;P01766;A0A0B4J1X5;A0A0C4DH43;P01782;P01780;A0A087WSY4;P01814;A0A0B4J1X8;A0A4W8ZXM2;A0A075B7D0;P0DP02;P0DP01;A0A075B7F0;A0A075B7D8;P01817;A0A0A0MS14;A0A0A0MS15;A0A0B4J1V1;A0A075B7B8;A0A0B4J1V2;P0DTE1;A0A075B6Q5;A0A0J9YVY3;P23083);hsa:3039(P69905);hsa:8542(O14791);hsa:335(P02647);hsa:3250(P00739);hsa:3043(P68871);hsa:3040(P69905);hsa:7412(P19320) | 7\|44 | hsa:102723407(A0A0C4DH32;A0A0C4DH34;A0A0J9YX35;P01780;P01782;P01817;P0DP01) | 0.136656389 | 1 | http://www.kegg.jp/kegg-bin/show_pathway?hsa05143+hsa:102723407%09salmon+hsa:102723407%09green+hsa:102723407%09salmon+hsa:102723407%09green+hsa:102723407%09green+hsa:102723407%09salmon+hsa:102723407%09green |
| hsa04072 | Phospholipase D signaling pathway | Environmental Information Processing | 39\|228 | hsa:102723407(A0A0C4DH29;A0A0C4DH32;A0A0C4DH33;A0A0J9YX35;A0A0C4DH31;A0A0C4DH36;A0A0C4DH34;A0A0C4DH38;A0A0C4DH39;A0A0J9YY99;A0A0G2JMI3;A0A0B4J1U7;P01763;P01764;P01743;P01766;A0A0B4J1X5;A0A0C4DH43;P01782;P01780;A0A087WSY4;P01814;A0A0B4J1X8;A0A4W8ZXM2;A0A075B7D0;P0DP02;P0DP01;A0A075B7F0;A0A075B7D8;P01817;A0A0A0MS14;A0A0A0MS15;A0A0B4J1V1;A0A075B7B8;A0A0B4J1V2;P0DTE1;A0A075B6Q5;A0A0J9YVY3;P23083) | 7\|44 | hsa:102723407(A0A0C4DH32;A0A0C4DH34;A0A0J9YX35;P01780;P01782;P01817;P0DP01) | 0.174597701 | 0.436494253 | http://www.kegg.jp/kegg-bin/show_pathway?hsa04072+hsa:102723407%09salmon+hsa:102723407%09green+hsa:102723407%09salmon+hsa:102723407%09green+hsa:102723407%09green+hsa:102723407%09salmon+hsa:102723407%09green |
| hsa04672 | Intestinal immune network for IgA production | Organismal Systems | 41\|228 | hsa:102723407(A0A0C4DH29;A0A0C4DH32;A0A0C4DH33;A0A0J9YX35;A0A0C4DH31;A0A0C4DH36;A0A0C4DH34;A0A0C4DH38;A0A0C4DH39;A0A0J9YY99;A0A0G2JMI3;A0A0B4J1U7;P01763;P01764;P01743;P01766;A0A0B4J1X5;A0A0C4DH43;P01782;P01780;A0A087WSY4;P01814;A0A0B4J1X8;A0A4W8ZXM2;A0A075B7D0;P0DP02;P0DP01;A0A075B7F0;A0A075B7D8;P01817;A0A0A0MS14;A0A0A0MS15;A0A0B4J1V1;A0A075B7B8;A0A0B4J1V2;P0DTE1;A0A075B6Q5;A0A0J9YVY3;P23083);hsa:102723996(A0A087X1L8);hsa:5284(P01833);hsa:23308(A0A087X1L8) | 7\|44 | hsa:102723407(A0A0C4DH32;A0A0C4DH34;A0A0J9YX35;P01780;P01782;P01817;P0DP01) | 0.164850548 | 0.549501826 | http://www.kegg.jp/kegg-bin/show_pathway?hsa04672+hsa:102723407%09salmon+hsa:102723407%09green+hsa:102723407%09salmon+hsa:102723407%09green+hsa:102723407%09green+hsa:102723407%09salmon+hsa:102723407%09green |
| hsa04064 | NF-kappa B signaling pathway | Environmental Information Processing | 42\|228 | hsa:102723407(A0A0C4DH29;A0A0C4DH32;A0A0C4DH33;A0A0J9YX35;A0A0C4DH31;A0A0C4DH36;A0A0C4DH34;A0A0C4DH38;A0A0C4DH39;A0A0J9YY99;A0A0G2JMI3;A0A0B4J1U7;P01763;P01764;P01743;P01766;A0A0B4J1X5;A0A0C4DH43;P01782;P01780;A0A087WSY4;P01814;A0A0B4J1X8;A0A4W8ZXM2;A0A075B7D0;P0DP02;P0DP01;A0A075B7F0;A0A075B7D8;P01817;A0A0A0MS14;A0A0A0MS15;A0A0B4J1V1;A0A075B7B8;A0A0B4J1V2;P0DTE1;A0A075B6Q5;A0A0J9YVY3;P23083);hsa:929(P08571);hsa:7412(P19320);hsa:3929(P18428) | 7\|44 | hsa:102723407(A0A0C4DH32;A0A0C4DH34;A0A0J9YX35;P01780;P01782;P01817;P0DP01) | 0.158679672 | 0.649144111 | http://www.kegg.jp/kegg-bin/show_pathway?hsa04064+hsa:102723407%09salmon+hsa:102723407%09green+hsa:102723407%09salmon+hsa:102723407%09green+hsa:102723407%09green+hsa:102723407%09salmon+hsa:102723407%09green |
| hsa05320 | Autoimmune thyroid disease | Human Diseases | 40\|228 | hsa:102723407(A0A0C4DH29;A0A0C4DH32;A0A0C4DH33;A0A0J9YX35;A0A0C4DH31;A0A0C4DH36;A0A0C4DH34;A0A0C4DH38;A0A0C4DH39;A0A0J9YY99;A0A0G2JMI3;A0A0B4J1U7;P01763;P01764;P01743;P01766;A0A0B4J1X5;A0A0C4DH43;P01782;P01780;A0A087WSY4;P01814;A0A0B4J1X8;A0A4W8ZXM2;A0A075B7D0;P0DP02;P0DP01;A0A075B7F0;A0A075B7D8;P01817;A0A0A0MS14;A0A0A0MS15;A0A0B4J1V1;A0A075B7B8;A0A0B4J1V2;P0DTE1;A0A075B6Q5;A0A0J9YVY3;P23083);hsa:3105(A0A0G2JI36) | 7\|44 | hsa:102723407(A0A0C4DH32;A0A0C4DH34;A0A0J9YX35;P01780;P01782;P01817;P0DP01) | 0.170202665 | 0.49413677 | http://www.kegg.jp/kegg-bin/show_pathway?hsa05320+hsa:102723407%09salmon+hsa:102723407%09green+hsa:102723407%09salmon+hsa:102723407%09green+hsa:102723407%09green+hsa:102723407%09salmon+hsa:102723407%09green |
| hsa05202 | Transcriptional misregulation in cancer | Human Diseases | 42\|228 | hsa:102723407(A0A0C4DH29;A0A0C4DH32;A0A0C4DH33;A0A0J9YX35;A0A0C4DH31;A0A0C4DH36;A0A0C4DH34;A0A0C4DH38;A0A0C4DH39;A0A0J9YY99;A0A0G2JMI3;A0A0B4J1U7;P01763;P01764;P01743;P01766;A0A0B4J1X5;A0A0C4DH43;P01782;P01780;A0A087WSY4;P01814;A0A0B4J1X8;A0A4W8ZXM2;A0A075B7D0;P0DP02;P0DP01;A0A075B7F0;A0A075B7D8;P01817;A0A0A0MS14;A0A0A0MS15;A0A0B4J1V1;A0A075B7B8;A0A0B4J1V2;P0DTE1;A0A075B6Q5;A0A0J9YVY3;P23083);hsa:929(P08571);hsa:3486(A6XND0);hsa:1668(P59665) | 7\|44 | hsa:102723407(A0A0C4DH32;A0A0C4DH34;A0A0J9YX35;P01780;P01782;P01817;P0DP01) | 0.158679672 | 0.649144111 | http://www.kegg.jp/kegg-bin/show_pathway?hsa05202+hsa:102723407%09salmon+hsa:102723407%09green+hsa:102723407%09salmon+hsa:102723407%09green+hsa:102723407%09green+hsa:102723407%09salmon+hsa:102723407%09green |
| hsa04662 | B cell receptor signaling pathway | Organismal Systems | 40\|228 | hsa:5881(P60763);hsa:102723407(A0A0C4DH29;A0A0C4DH32;A0A0C4DH33;A0A0J9YX35;A0A0C4DH31;A0A0C4DH36;A0A0C4DH34;A0A0C4DH38;A0A0C4DH39;A0A0J9YY99;A0A0G2JMI3;A0A0B4J1U7;P01763;P01764;P01743;P01766;A0A0B4J1X5;A0A0C4DH43;P01782;P01780;A0A087WSY4;P01814;A0A0B4J1X8;A0A4W8ZXM2;A0A075B7D0;P0DP02;P0DP01;A0A075B7F0;A0A075B7D8;P01817;A0A0A0MS14;A0A0A0MS15;A0A0B4J1V1;A0A075B7B8;A0A0B4J1V2;P0DTE1;A0A075B6Q5;A0A0J9YVY3;P23083) | 7\|44 | hsa:102723407(A0A0C4DH32;A0A0C4DH34;A0A0J9YX35;P01780;P01782;P01817;P0DP01) | 0.170202665 | 0.49413677 | http://www.kegg.jp/kegg-bin/show_pathway?hsa04662+hsa:102723407%09salmon+hsa:102723407%09green+hsa:102723407%09salmon+hsa:102723407%09green+hsa:102723407%09green+hsa:102723407%09salmon+hsa:102723407%09green |
| hsa04650 | Natural killer cell mediated cytotoxicity | Organismal Systems | 42\|228 | hsa:5881(P60763);hsa:102723407(A0A0C4DH29;A0A0C4DH32;A0A0C4DH33;A0A0J9YX35;A0A0C4DH31;A0A0C4DH36;A0A0C4DH34;A0A0C4DH38;A0A0C4DH39;A0A0J9YY99;A0A0G2JMI3;A0A0B4J1U7;P01763;P01764;P01743;P01766;A0A0B4J1X5;A0A0C4DH43;P01782;P01780;A0A087WSY4;P01814;A0A0B4J1X8;A0A4W8ZXM2;A0A075B7D0;P0DP02;P0DP01;A0A075B7F0;A0A075B7D8;P01817;A0A0A0MS14;A0A0A0MS15;A0A0B4J1V1;A0A075B7B8;A0A0B4J1V2;P0DTE1;A0A075B6Q5;A0A0J9YVY3;P23083);hsa:3105(A0A0G2JI36);hsa:2214(H0Y755) | 7\|44 | hsa:102723407(A0A0C4DH32;A0A0C4DH34;A0A0J9YX35;P01780;P01782;P01817;P0DP01) | 0.158679672 | 0.649144111 | http://www.kegg.jp/kegg-bin/show_pathway?hsa04650+hsa:102723407%09salmon+hsa:102723407%09green+hsa:102723407%09salmon+hsa:102723407%09green+hsa:102723407%09green+hsa:102723407%09salmon+hsa:102723407%09green |
| hsa04020 | Calcium signaling pathway | Environmental Information Processing | 39\|228 | hsa:102723407(A0A0C4DH29;A0A0C4DH32;A0A0C4DH33;A0A0J9YX35;A0A0C4DH31;A0A0C4DH36;A0A0C4DH34;A0A0C4DH38;A0A0C4DH39;A0A0J9YY99;A0A0G2JMI3;A0A0B4J1U7;P01763;P01764;P01743;P01766;A0A0B4J1X5;A0A0C4DH43;P01782;P01780;A0A087WSY4;P01814;A0A0B4J1X8;A0A4W8ZXM2;A0A075B7D0;P0DP02;P0DP01;A0A075B7F0;A0A075B7D8;P01817;A0A0A0MS14;A0A0A0MS15;A0A0B4J1V1;A0A075B7B8;A0A0B4J1V2;P0DTE1;A0A075B6Q5;A0A0J9YVY3;P23083) | 7\|44 | hsa:102723407(A0A0C4DH32;A0A0C4DH34;A0A0J9YX35;P01780;P01782;P01817;P0DP01) | 0.174597701 | 0.436494253 | http://www.kegg.jp/kegg-bin/show_pathway?hsa04020+hsa:102723407%09salmon+hsa:102723407%09green+hsa:102723407%09salmon+hsa:102723407%09green+hsa:102723407%09green+hsa:102723407%09salmon+hsa:102723407%09green |
| hsa05340 | Primary immunodeficiency | Human Diseases | 41\|228 | hsa:102723407(A0A0C4DH29;A0A0C4DH32;A0A0C4DH33;A0A0J9YX35;A0A0C4DH31;A0A0C4DH36;A0A0C4DH34;A0A0C4DH38;A0A0C4DH39;A0A0J9YY99;A0A0G2JMI3;A0A0B4J1U7;P01763;P01764;P01743;P01766;A0A0B4J1X5;A0A0C4DH43;P01782;P01780;A0A087WSY4;P01814;A0A0B4J1X8;A0A4W8ZXM2;A0A075B7D0;P0DP02;P0DP01;A0A075B7F0;A0A075B7D8;P01817;A0A0A0MS14;A0A0A0MS15;A0A0B4J1V1;A0A075B7B8;A0A0B4J1V2;P0DTE1;A0A075B6Q5;A0A0J9YVY3;P23083);hsa:3543(P01871;P15814) | 7\|44 | hsa:102723407(A0A0C4DH32;A0A0C4DH34;A0A0J9YX35;P01780;P01782;P01817;P0DP01) | 0.164850548 | 0.549501826 | http://www.kegg.jp/kegg-bin/show_pathway?hsa05340+hsa:102723407%09salmon+hsa:102723407%09green+hsa:102723407%09salmon+hsa:102723407%09green+hsa:102723407%09green+hsa:102723407%09salmon+hsa:102723407%09green |
| hsa05414 | Dilated cardiomyopathy (DCM) | Human Diseases | 44\|228 | hsa:3674(P08514);hsa:102723407(A0A0C4DH29;A0A0C4DH32;A0A0C4DH33;A0A0J9YX35;A0A0C4DH31;A0A0C4DH36;A0A0C4DH34;A0A0C4DH38;A0A0C4DH39;A0A0J9YY99;A0A0G2JMI3;A0A0B4J1U7;P01763;P01764;P01743;P01766;A0A0B4J1X5;A0A0C4DH43;P01782;P01780;A0A087WSY4;P01814;A0A0B4J1X8;A0A4W8ZXM2;A0A075B7D0;P0DP02;P0DP01;A0A075B7F0;A0A075B7D8;P01817;A0A0A0MS14;A0A0A0MS15;A0A0B4J1V1;A0A075B7B8;A0A0B4J1V2;P0DTE1;A0A075B6Q5;A0A0J9YVY3;P23083);hsa:7171(A0A087WWU8;A0A2R8Y5V9);hsa:71(P60709);hsa:70(P68032);hsa:60(P60709) | 7\|44 | hsa:102723407(A0A0C4DH32;A0A0C4DH34;A0A0J9YX35;P01780;P01782;P01817;P0DP01) | 0.144444667 | 1 | http://www.kegg.jp/kegg-bin/show_pathway?hsa05414+hsa:102723407%09salmon+hsa:102723407%09green+hsa:102723407%09salmon+hsa:102723407%09green+hsa:102723407%09green+hsa:102723407%09salmon+hsa:102723407%09green |
| hsa05162 | Measles | Human Diseases | 40\|228 | hsa:102723407(A0A0C4DH29;A0A0C4DH32;A0A0C4DH33;A0A0J9YX35;A0A0C4DH31;A0A0C4DH36;A0A0C4DH34;A0A0C4DH38;A0A0C4DH39;A0A0J9YY99;A0A0G2JMI3;A0A0B4J1U7;P01763;P01764;P01743;P01766;A0A0B4J1X5;A0A0C4DH43;P01782;P01780;A0A087WSY4;P01814;A0A0B4J1X8;A0A4W8ZXM2;A0A075B7D0;P0DP02;P0DP01;A0A075B7F0;A0A075B7D8;P01817;A0A0A0MS14;A0A0A0MS15;A0A0B4J1V1;A0A075B7B8;A0A0B4J1V2;P0DTE1;A0A075B6Q5;A0A0J9YVY3;P23083);hsa:4478(P26038) | 7\|44 | hsa:102723407(A0A0C4DH32;A0A0C4DH34;A0A0J9YX35;P01780;P01782;P01817;P0DP01) | 0.170202665 | 0.49413677 | http://www.kegg.jp/kegg-bin/show_pathway?hsa05162+hsa:102723407%09salmon+hsa:102723407%09green+hsa:102723407%09salmon+hsa:102723407%09green+hsa:102723407%09green+hsa:102723407%09salmon+hsa:102723407%09green |
| hsa05330 | Allograft rejection | Human Diseases | 40\|228 | hsa:102723407(A0A0C4DH29;A0A0C4DH32;A0A0C4DH33;A0A0J9YX35;A0A0C4DH31;A0A0C4DH36;A0A0C4DH34;A0A0C4DH38;A0A0C4DH39;A0A0J9YY99;A0A0G2JMI3;A0A0B4J1U7;P01763;P01764;P01743;P01766;A0A0B4J1X5;A0A0C4DH43;P01782;P01780;A0A087WSY4;P01814;A0A0B4J1X8;A0A4W8ZXM2;A0A075B7D0;P0DP02;P0DP01;A0A075B7F0;A0A075B7D8;P01817;A0A0A0MS14;A0A0A0MS15;A0A0B4J1V1;A0A075B7B8;A0A0B4J1V2;P0DTE1;A0A075B6Q5;A0A0J9YVY3;P23083);hsa:3105(A0A0G2JI36) | 7\|44 | hsa:102723407(A0A0C4DH32;A0A0C4DH34;A0A0J9YX35;P01780;P01782;P01817;P0DP01) | 0.170202665 | 0.49413677 | http://www.kegg.jp/kegg-bin/show_pathway?hsa05330+hsa:102723407%09salmon+hsa:102723407%09green+hsa:102723407%09salmon+hsa:102723407%09green+hsa:102723407%09green+hsa:102723407%09salmon+hsa:102723407%09green |
| hsa04640 | Hematopoietic cell lineage | Organismal Systems | 45\|228 | hsa:3674(P08514);hsa:102723407(A0A0C4DH29;A0A0C4DH32;A0A0C4DH33;A0A0J9YX35;A0A0C4DH31;A0A0C4DH36;A0A0C4DH34;A0A0C4DH38;A0A0C4DH39;A0A0J9YY99;A0A0G2JMI3;A0A0B4J1U7;P01763;P01764;P01743;P01766;A0A0B4J1X5;A0A0C4DH43;P01782;P01780;A0A087WSY4;P01814;A0A0B4J1X8;A0A4W8ZXM2;A0A075B7D0;P0DP02;P0DP01;A0A075B7F0;A0A075B7D8;P01817;A0A0A0MS14;A0A0A0MS15;A0A0B4J1V1;A0A075B7B8;A0A0B4J1V2;P0DTE1;A0A075B6Q5;A0A0J9YVY3;P23083);hsa:2811(A0A0C4DGZ8);hsa:960(H0Y5E4);hsa:290(P15144);hsa:7037(G3V0E5);hsa:929(P08571) | 7\|44 | hsa:102723407(A0A0C4DH32;A0A0C4DH34;A0A0J9YX35;P01780;P01782;P01817;P0DP01) | 0.136656389 | 1 | http://www.kegg.jp/kegg-bin/show_pathway?hsa04640+hsa:102723407%09salmon+hsa:102723407%09green+hsa:102723407%09salmon+hsa:102723407%09green+hsa:102723407%09green+hsa:102723407%09salmon+hsa:102723407%09green |
| hsa04666 | Fc gamma R-mediated phagocytosis | Organismal Systems | 42\|228 | hsa:2934(A0A0A0MS51);hsa:102723407(A0A0C4DH29;A0A0C4DH32;A0A0C4DH33;A0A0J9YX35;A0A0C4DH31;A0A0C4DH36;A0A0C4DH34;A0A0C4DH38;A0A0C4DH39;A0A0J9YY99;A0A0G2JMI3;A0A0B4J1U7;P01763;P01764;P01743;P01766;A0A0B4J1X5;A0A0C4DH43;P01782;P01780;A0A087WSY4;P01814;A0A0B4J1X8;A0A4W8ZXM2;A0A075B7D0;P0DP02;P0DP01;A0A075B7F0;A0A075B7D8;P01817;A0A0A0MS14;A0A0A0MS15;A0A0B4J1V1;A0A075B7B8;A0A0B4J1V2;P0DTE1;A0A075B6Q5;A0A0J9YVY3;P23083);hsa:1072(E9PK25);hsa:2214(H0Y755) | 7\|44 | hsa:102723407(A0A0C4DH32;A0A0C4DH34;A0A0J9YX35;P01780;P01782;P01817;P0DP01) | 0.158679672 | 0.649144111 | http://www.kegg.jp/kegg-bin/show_pathway?hsa04666+hsa:102723407%09salmon+hsa:102723407%09green+hsa:102723407%09salmon+hsa:102723407%09green+hsa:102723407%09green+hsa:102723407%09salmon+hsa:102723407%09green |
| hsa05310 | Asthma | Human Diseases | 39\|228 | hsa:102723407(A0A0C4DH29;A0A0C4DH32;A0A0C4DH33;A0A0J9YX35;A0A0C4DH31;A0A0C4DH36;A0A0C4DH34;A0A0C4DH38;A0A0C4DH39;A0A0J9YY99;A0A0G2JMI3;A0A0B4J1U7;P01763;P01764;P01743;P01766;A0A0B4J1X5;A0A0C4DH43;P01782;P01780;A0A087WSY4;P01814;A0A0B4J1X8;A0A4W8ZXM2;A0A075B7D0;P0DP02;P0DP01;A0A075B7F0;A0A075B7D8;P01817;A0A0A0MS14;A0A0A0MS15;A0A0B4J1V1;A0A075B7B8;A0A0B4J1V2;P0DTE1;A0A075B6Q5;A0A0J9YVY3;P23083) | 7\|44 | hsa:102723407(A0A0C4DH32;A0A0C4DH34;A0A0J9YX35;P01780;P01782;P01817;P0DP01) | 0.174597701 | 0.436494253 | http://www.kegg.jp/kegg-bin/show_pathway?hsa05310+hsa:102723407%09salmon+hsa:102723407%09green+hsa:102723407%09salmon+hsa:102723407%09green+hsa:102723407%09green+hsa:102723407%09salmon+hsa:102723407%09green |
| hsa04664 | Fc epsilon RI signaling pathway | Organismal Systems | 40\|228 | hsa:5881(P60763);hsa:102723407(A0A0C4DH29;A0A0C4DH32;A0A0C4DH33;A0A0J9YX35;A0A0C4DH31;A0A0C4DH36;A0A0C4DH34;A0A0C4DH38;A0A0C4DH39;A0A0J9YY99;A0A0G2JMI3;A0A0B4J1U7;P01763;P01764;P01743;P01766;A0A0B4J1X5;A0A0C4DH43;P01782;P01780;A0A087WSY4;P01814;A0A0B4J1X8;A0A4W8ZXM2;A0A075B7D0;P0DP02;P0DP01;A0A075B7F0;A0A075B7D8;P01817;A0A0A0MS14;A0A0A0MS15;A0A0B4J1V1;A0A075B7B8;A0A0B4J1V2;P0DTE1;A0A075B6Q5;A0A0J9YVY3;P23083) | 7\|44 | hsa:102723407(A0A0C4DH32;A0A0C4DH34;A0A0J9YX35;P01780;P01782;P01817;P0DP01) | 0.170202665 | 0.49413677 | http://www.kegg.jp/kegg-bin/show_pathway?hsa04664+hsa:102723407%09salmon+hsa:102723407%09green+hsa:102723407%09salmon+hsa:102723407%09green+hsa:102723407%09green+hsa:102723407%09salmon+hsa:102723407%09green |
| hsa04979 | Cholesterol metabolism | Organismal Systems | 15\|228 | hsa:255738(A0A669KAY4);hsa:4035(Q07954);hsa:350(P02749);hsa:5360(P55058);hsa:1071(P11597);hsa:4018(P08519);hsa:3931(P04180);hsa:338(P04114);hsa:348(P02649);hsa:344(K7ER74);hsa:345(B0YIW2);hsa:337(P06727);hsa:336(P02652);hsa:335(P02647);hsa:341(K7ERI9) | 4\|44 | hsa:5360(P55058);hsa:344(K7ER74);hsa:255738(A0A669KAY4);hsa:341(K7ERI9) | 0.183690111 | 0.423900257 | http://www.kegg.jp/kegg-bin/show_pathway?hsa04979+hsa:5360%09salmon+hsa:344%09salmon+hsa:255738%09salmon+hsa:341%09green |
| hsa04510 | Focal adhesion | Cellular Processes | 20\|228 | hsa:7450(P04275);hsa:29780(A0A087WZB5);hsa:7094(Q9Y490);hsa:1291(A0A087X0S5);hsa:1293(E7ENL6);hsa:5908(P61224);hsa:87(H9KV75);hsa:3674(P08514);hsa:7448(H0YJW9;P04004);hsa:3611(A0A0A0MTH3);hsa:5881(P60763);hsa:1311(G3XAP6);hsa:2316(P21333);hsa:7057(P07996);hsa:7791(H0Y2Y8);hsa:60(P60709);hsa:7414(P18206);hsa:2335(P02751);hsa:71(P60709);hsa:7148(A0A140T8Y3) | 3\|44 | hsa:7414(P18206);hsa:29780(A0A087WZB5);hsa:2335(P02751) | 0.219748636 | 0.380334178 | http://www.kegg.jp/kegg-bin/show_pathway?hsa04510+hsa:7414%09salmon+hsa:29780%09salmon+hsa:2335%09salmon |
| hsa05133 | Pertussis | Human Diseases | 17\|228 | hsa:712(P02745);hsa:715(A0A3B3ISR2);hsa:1072(E9PK25);hsa:721(A0A0G2JPR0;P0C0L5;P0C0L4);hsa:727(P01031);hsa:718(P01024;M0R0Q9);hsa:717(A0A0G2JL69);hsa:710(P05155);hsa:725(P20851);hsa:713(D6R934);hsa:929(P08571);hsa:720(A0A0G2JPR0;P0C0L5;P0C0L4);hsa:714(P02747);hsa:722(P04003);hsa:716(P09871) | 3\|44 | hsa:718(M0R0Q9);hsa:714(P02747);hsa:717(A0A0G2JL69) | 0.251497329 | 0.419162216 | http://www.kegg.jp/kegg-bin/show_pathway?hsa05133+hsa:718%09green+hsa:714%09green+hsa:717%09green |
| hsa04066 | HIF-1 signaling pathway | Environmental Information Processing | 6\|228 | hsa:7018(C9JB55;P02787);hsa:2023(A0A2R8Y6G6);hsa:226(J3KPS3);hsa:2597(E7EUT5);hsa:7037(G3V0E5) | 3\|44 | hsa:7018(C9JB55;P02787);hsa:2597(E7EUT5) | 0.074086005 | 1 | http://www.kegg.jp/kegg-bin/show_pathway?hsa04066+hsa:7018%09green+hsa:7018%09green+hsa:2597%09salmon |
| hsa04972 | Pancreatic secretion | Organismal Systems | 4\|228 | hsa:760(P00918);hsa:81579(A0A2R8Y3M9);hsa:1361(Q96IY4);hsa:5908(P61224) | 3\|44 | hsa:760(P00918);hsa:81579(A0A2R8Y3M9);hsa:1361(Q96IY4) | 0.022222687 | 1 | http://www.kegg.jp/kegg-bin/show_pathway?hsa04972+hsa:760%09green+hsa:81579%09green+hsa:1361%09green |
| hsa05100 | Bacterial invasion of epithelial cells | Human Diseases | 4\|228 | hsa:7414(P18206);hsa:3611(A0A0A0MTH3);hsa:71(P60709);hsa:60(P60709);hsa:2335(P02751) | 2\|44 | hsa:7414(P18206);hsa:2335(P02751) | 0.145241135 | 0.93369301 | http://www.kegg.jp/kegg-bin/show_pathway?hsa05100+hsa:7414%09salmon+hsa:2335%09salmon |
| hsa05142 | Chagas disease (American trypanosomiasis) | Human Diseases | 5\|228 | hsa:718(P01024;M0R0Q9);hsa:713(D6R934);hsa:712(P02745);hsa:714(P02747) | 2\|44 | hsa:718(M0R0Q9);hsa:714(P02747) | 0.196680703 | 0.354025266 | http://www.kegg.jp/kegg-bin/show_pathway?hsa05142+hsa:718%09green+hsa:714%09green |
| hsa04810 | Regulation of actin cytoskeleton | Cellular Processes | 12\|228 | hsa:3674(P08514);hsa:2934(A0A0A0MS51);hsa:1072(E9PK25);hsa:2147(P00734);hsa:2335(P02751);hsa:5881(P60763);hsa:4627(P35579);hsa:87(H9KV75);hsa:5216(P07737);hsa:7414(P18206);hsa:4478(P26038);hsa:71(P60709);hsa:60(P60709) | 2\|44 | hsa:7414(P18206);hsa:2335(P02751) | 0.294591798 | 0.457125203 | http://www.kegg.jp/kegg-bin/show_pathway?hsa04810+hsa:7414%09salmon+hsa:2335%09salmon |
| hsa05200 | Pathways in cancer | Human Diseases | 4\|228 | hsa:5881(P60763);hsa:3674(P08514);hsa:3481(P01344);hsa:2335(P02751) | 2\|44 | hsa:3481(P01344);hsa:2335(P02751) | 0.145241135 | 0.93369301 | http://www.kegg.jp/kegg-bin/show_pathway?hsa05200+hsa:3481%09green+hsa:2335%09salmon |
| hsa05203 | Viral carcinogenesis | Human Diseases | 8\|228 | hsa:2934(A0A0A0MS51);hsa:128312(A0A2R8Y619);hsa:7534(E7EX29);hsa:718(P01024;M0R0Q9);hsa:87(H9KV75);hsa:5315(H3BTN5);hsa:3105(A0A0G2JI36) | 2\|44 | hsa:718(M0R0Q9);hsa:7534(E7EX29) | 0.293544389 | 0.463491141 | http://www.kegg.jp/kegg-bin/show_pathway?hsa05203+hsa:718%09green+hsa:7534%09salmon |
| hsa05165 | Human papillomavirus infection | Human Diseases | 13\|228 | hsa:7450(P04275);hsa:3674(P08514);hsa:3993(J3QRV5);hsa:1291(A0A087X0S5);hsa:1293(E7ENL6);hsa:1311(G3XAP6);hsa:7448(H0YJW9;P04004);hsa:2335(P02751);hsa:7057(P07996);hsa:5315(H3BTN5);hsa:3105(A0A0G2JI36);hsa:7148(A0A140T8Y3) | 2\|44 | hsa:3993(J3QRV5);hsa:2335(P02751) | 0.280457343 | 0.450735015 | http://www.kegg.jp/kegg-bin/show_pathway?hsa05165+hsa:3993%09green+hsa:2335%09salmon |
| hsa05205 | Proteoglycans in cancer | Human Diseases | 10\|228 | hsa:71(P60709);hsa:3481(P01344);hsa:7448(H0YJW9;P04004);hsa:960(H0Y5E4);hsa:2335(P02751);hsa:2316(P21333);hsa:7057(P07996);hsa:4478(P26038);hsa:4060(P51884);hsa:60(P60709) | 2\|44 | hsa:3481(P01344);hsa:2335(P02751) | 0.308499695 | 0.462749543 | http://www.kegg.jp/kegg-bin/show_pathway?hsa05205+hsa:3481%09green+hsa:2335%09salmon |
| hsa00910 | Nitrogen metabolism | Metabolism | 2\|228 | hsa:760(P00918);hsa:759(P00915) | 2\|44 | hsa:760(P00918);hsa:759(P00915) | 0.036556148 | 1 | http://www.kegg.jp/kegg-bin/show_pathway?hsa00910+hsa:760%09green+hsa:759%09green |
| hsa04978 | Mineral absorption | Organismal Systems | 2\|228 | hsa:7018(C9JB55;P02787) | 2\|44 | hsa:7018(C9JB55;P02787) | 0.036556148 | 1 | http://www.kegg.jp/kegg-bin/show_pathway?hsa04978+hsa:7018%09green+hsa:7018%09green |
| hsa05020 | Prion diseases | Human Diseases | 11\|228 | hsa:735(P02748);hsa:712(P02745);hsa:3309(P11021);hsa:714(P02747);hsa:729(P13671);hsa:713(D6R934);hsa:727(P01031);hsa:733(P07360);hsa:732(P07358);hsa:731(P07357);hsa:730(P10643) | 2\|44 | hsa:733(P07360);hsa:714(P02747) | 0.304411524 | 0.464356562 | http://www.kegg.jp/kegg-bin/show_pathway?hsa05020+hsa:733%09green+hsa:714%09green |
| hsa04390 | Hippo signaling pathway | Environmental Information Processing | 3\|228 | hsa:3993(J3QRV5);hsa:71(P60709);hsa:60(P60709);hsa:7534(E7EX29) | 2\|44 | hsa:3993(J3QRV5);hsa:7534(E7EX29) | 0.089287583 | 1 | http://www.kegg.jp/kegg-bin/show_pathway?hsa04390+hsa:3993%09green+hsa:7534%09salmon |
| hsa04014 | Ras signaling pathway | Environmental Information Processing | 4\|228 | hsa:5881(P60763);hsa:81579(A0A2R8Y3M9);hsa:3481(P01344);hsa:5908(P61224) | 2\|44 | hsa:81579(A0A2R8Y3M9);hsa:3481(P01344) | 0.145241135 | 0.93369301 | http://www.kegg.jp/kegg-bin/show_pathway?hsa04014+hsa:81579%09green+hsa:3481%09green |
| hsa04974 | Protein digestion and absorption | Organismal Systems | 4\|228 | hsa:1293(E7ENL6);hsa:1361(Q96IY4);hsa:7373(J3QT83);hsa:1291(A0A087X0S5) | 2\|44 | hsa:1361(Q96IY4);hsa:7373(J3QT83) | 0.145241135 | 0.93369301 | http://www.kegg.jp/kegg-bin/show_pathway?hsa04974+hsa:1361%09green+hsa:7373%09salmon |
| hsa04216 | Ferroptosis | Cellular Processes | 4\|228 | hsa:7018(C9JB55;P02787);hsa:7037(G3V0E5);hsa:1356(P00450) | 2\|44 | hsa:7018(C9JB55;P02787) | 0.145241135 | 0.93369301 | http://www.kegg.jp/kegg-bin/show_pathway?hsa04216+hsa:7018%09green+hsa:7018%09green |
| hsa04670 | Leukocyte transendothelial migration | Organismal Systems | 7\|228 | hsa:5908(P61224);hsa:87(H9KV75);hsa:7412(P19320);hsa:60(P60709);hsa:7414(P18206);hsa:4478(P26038);hsa:71(P60709);hsa:1003(I3L1J2) | 2\|44 | hsa:7414(P18206);hsa:1003(I3L1J2) | 0.271815545 | 0.444789074 | http://www.kegg.jp/kegg-bin/show_pathway?hsa04670+hsa:7414%09salmon+hsa:1003%09salmon |
| hsa05150 | Staphylococcus aureus infection | Human Diseases | 63\|228 | hsa:102723407(A0A0C4DH29;A0A0C4DH32;A0A0C4DH33;A0A0J9YX35;A0A0C4DH31;A0A0C4DH36;A0A0C4DH34;A0A0C4DH38;A0A0C4DH39;A0A0J9YY99;A0A0G2JMI3;A0A0B4J1U7;P01763;P01764;P01743;P01766;A0A0B4J1X5;A0A0C4DH43;P01782;P01780;A0A087WSY4;P01814;A0A0B4J1X8;A0A4W8ZXM2;A0A075B7D0;P0DP02;P0DP01;A0A075B7F0;A0A075B7D8;P01817;A0A0A0MS14;A0A0A0MS15;A0A0B4J1V1;A0A075B7B8;A0A0B4J1V2;P0DTE1;A0A075B6Q5;A0A0J9YVY3;P23083);hsa:5340(P00747);hsa:629(B4E1Z4);hsa:3858(P13645);hsa:727(P01031);hsa:720(A0A0G2JPR0;P0C0L5;P0C0L4);hsa:721(A0A0G2JPR0;P0C0L5;P0C0L4);hsa:1675(K7ERG9);hsa:5648(P48740);hsa:2214(H0Y755);hsa:718(P01024;M0R0Q9);hsa:10747(O00187);hsa:713(D6R934);hsa:712(P02745);hsa:715(A0A3B3ISR2);hsa:714(P02747);hsa:717(A0A0G2JL69);hsa:716(P09871);hsa:4153(P11226);hsa:3426(A0A2R8Y3M9);hsa:2266(C9JPQ9;C9JC84);hsa:3075(P08603) | 14\|44 | hsa:102723407(A0A0C4DH32;A0A0C4DH34;A0A0J9YX35;P01780;P01782;P01817;P0DP01);hsa:2266(C9JPQ9);hsa:3426(A0A2R8Y3M9);hsa:718(M0R0Q9);hsa:10747(O00187);hsa:714(P02747);hsa:717(A0A0G2JL69);hsa:1675(K7ERG9) | 0.114266225 | 1 | http://www.kegg.jp/kegg-bin/show_pathway?hsa05150+hsa:102723407%09salmon+hsa:102723407%09green+hsa:102723407%09salmon+hsa:102723407%09green+hsa:102723407%09green+hsa:102723407%09salmon+hsa:102723407%09green+hsa:2266%09salmon+hsa:3426%09green+hsa:718%09green+hsa:10747%09green+hsa:714%09green+hsa:717%09green+hsa:1675%09green |
| hsa04610 | Complement and coagulation cascades | Organismal Systems | 58\|228 | hsa:3818(H0YAC1);hsa:5345(P08697);hsa:5104(P05154);hsa:5340(P00747);hsa:629(B4E1Z4);hsa:729(P13671);hsa:2(P01023);hsa:725(P20851);hsa:732(P07358);hsa:727(P01031);hsa:720(A0A0G2JPR0;P0C0L5;P0C0L4);hsa:462(P01008);hsa:722(P04003);hsa:1675(K7ERG9);hsa:7450(P04275);hsa:10544(Q9UNN8);hsa:5624(E7END6);hsa:5627(A0A0S2Z4L3);hsa:2147(P00734);hsa:721(A0A0G2JPR0;P0C0L5;P0C0L4);hsa:2162(P00488);hsa:2161(P00748);hsa:2160(P03951);hsa:731(P07357);hsa:2165(P05160);hsa:730(P10643);hsa:735(P02748);hsa:3827(P01042);hsa:5648(P48740);hsa:1361(Q96IY4);hsa:718(P01024;M0R0Q9);hsa:10747(O00187);hsa:4153(P11226);hsa:1191(P10909);hsa:710(P05155);hsa:713(D6R934);hsa:712(P02745);hsa:715(A0A3B3ISR2);hsa:714(P02747);hsa:717(A0A0G2JL69);hsa:716(P09871);hsa:5265(P01009);hsa:3075(P08603);hsa:2243(P02671);hsa:7448(H0YJW9;P04004);hsa:2244(P02675);hsa:3426(A0A2R8Y3M9);hsa:2266(C9JPQ9;C9JC84);hsa:2155(F5H8B0);hsa:2153(A0A0A0MRJ7);hsa:3053(P05546);hsa:733(P07360);hsa:2158(P00740);hsa:2159(P00742) | 13\|44 | hsa:5265(P01009);hsa:10747(O00187);hsa:2266(C9JPQ9);hsa:1361(Q96IY4);hsa:714(P02747);hsa:718(M0R0Q9);hsa:2161(P00748);hsa:5624(E7END6);hsa:3426(A0A2R8Y3M9);hsa:733(P07360);hsa:462(P01008);hsa:717(A0A0G2JL69);hsa:1675(K7ERG9) | 0.116473734 | 1 | http://www.kegg.jp/kegg-bin/show_pathway?hsa04610+hsa:5265%09green+hsa:10747%09green+hsa:2266%09salmon+hsa:1361%09green+hsa:714%09green+hsa:718%09green+hsa:2161%09green+hsa:5624%09green+hsa:3426%09green+hsa:733%09green+hsa:462%09green+hsa:717%09green+hsa:1675%09green |
| hsa05322 | Systemic lupus erythematosus | Human Diseases | 60\|228 | hsa:735(P02748);hsa:102723407(A0A0C4DH29;A0A0C4DH32;A0A0C4DH33;A0A0J9YX35;A0A0C4DH31;A0A0C4DH36;A0A0C4DH34;A0A0C4DH38;A0A0C4DH39;A0A0J9YY99;A0A0G2JMI3;A0A0B4J1U7;P01763;P01764;P01743;P01766;A0A0B4J1X5;A0A0C4DH43;P01782;P01780;A0A087WSY4;P01814;A0A0B4J1X8;A0A4W8ZXM2;A0A075B7D0;P0DP02;P0DP01;A0A075B7F0;A0A075B7D8;P01817;A0A0A0MS14;A0A0A0MS15;A0A0B4J1V1;A0A075B7B8;A0A0B4J1V2;P0DTE1;A0A075B6Q5;A0A0J9YVY3;P23083);hsa:712(P02745);hsa:733(P07360);hsa:128312(A0A2R8Y619);hsa:715(A0A3B3ISR2);hsa:721(A0A0G2JPR0;P0C0L5;P0C0L4);hsa:727(P01031);hsa:718(P01024;M0R0Q9);hsa:732(P07358);hsa:87(H9KV75);hsa:2214(H0Y755);hsa:730(P10643);hsa:731(P07357);hsa:713(D6R934);hsa:716(P09871);hsa:720(A0A0G2JPR0;P0C0L5;P0C0L4);hsa:714(P02747);hsa:717(A0A0G2JL69);hsa:729(P13671) | 11\|44 | hsa:102723407(A0A0C4DH32;A0A0C4DH34;A0A0J9YX35;P01780;P01782;P01817;P0DP01);hsa:733(P07360);hsa:714(P02747);hsa:717(A0A0G2JL69);hsa:718(M0R0Q9) | 0.14901043 | 0.745052152 | http://www.kegg.jp/kegg-bin/show_pathway?hsa05322+hsa:102723407%09salmon+hsa:102723407%09green+hsa:102723407%09salmon+hsa:102723407%09green+hsa:102723407%09green+hsa:102723407%09salmon+hsa:102723407%09green+hsa:733%09green+hsa:714%09green+hsa:717%09green+hsa:718%09green |
| hsa05146 | Amoebiasis | Human Diseases | 47\|228 | hsa:102723407(A0A0C4DH29;A0A0C4DH32;A0A0C4DH33;A0A0J9YX35;A0A0C4DH31;A0A0C4DH36;A0A0C4DH34;A0A0C4DH38;A0A0C4DH39;A0A0J9YY99;A0A0G2JMI3;A0A0B4J1U7;P01763;P01764;P01743;P01766;A0A0B4J1X5;A0A0C4DH43;P01782;P01780;A0A087WSY4;P01814;A0A0B4J1X8;A0A4W8ZXM2;A0A075B7D0;P0DP02;P0DP01;A0A075B7F0;A0A075B7D8;P01817;A0A0A0MS14;A0A0A0MS15;A0A0B4J1V1;A0A075B7B8;A0A0B4J1V2;P0DTE1;A0A075B6Q5;A0A0J9YVY3;P23083);hsa:2335(P02751);hsa:87(H9KV75);hsa:7414(P18206);hsa:735(P02748);hsa:929(P08571);hsa:733(P07360);hsa:732(P07358);hsa:731(P07357) | 10\|44 | hsa:7414(P18206);hsa:102723407(A0A0C4DH32;A0A0C4DH34;A0A0J9YX35;P01780;P01782;P01817;P0DP01);hsa:733(P07360);hsa:2335(P02751) | 0.14848042 | 0.786072811 | http://www.kegg.jp/kegg-bin/show_pathway?hsa05146+hsa:7414%09salmon+hsa:102723407%09salmon+hsa:102723407%09green+hsa:102723407%09salmon+hsa:102723407%09green+hsa:102723407%09green+hsa:102723407%09salmon+hsa:102723407%09green+hsa:733%09green+hsa:2335%09salmon |
| hsa04151 | PI3K-Akt signaling pathway | Environmental Information Processing | 51\|228 | hsa:7450(P04275);hsa:102723407(A0A0C4DH29;A0A0C4DH32;A0A0C4DH33;A0A0J9YX35;A0A0C4DH31;A0A0C4DH36;A0A0C4DH34;A0A0C4DH38;A0A0C4DH39;A0A0J9YY99;A0A0G2JMI3;A0A0B4J1U7;P01763;P01764;P01743;P01766;A0A0B4J1X5;A0A0C4DH43;P01782;P01780;A0A087WSY4;P01814;A0A0B4J1X8;A0A4W8ZXM2;A0A075B7D0;P0DP02;P0DP01;A0A075B7F0;A0A075B7D8;P01817;A0A0A0MS14;A0A0A0MS15;A0A0B4J1V1;A0A075B7B8;A0A0B4J1V2;P0DTE1;A0A075B6Q5;A0A0J9YVY3;P23083);hsa:1291(A0A087X0S5);hsa:7534(E7EX29);hsa:1311(G3XAP6);hsa:3481(P01344);hsa:7448(H0YJW9;P04004);hsa:2335(P02751);hsa:3674(P08514);hsa:7057(P07996);hsa:1293(E7ENL6);hsa:7148(A0A140T8Y3) | 10\|44 | hsa:102723407(A0A0C4DH32;A0A0C4DH34;A0A0J9YX35;P01780;P01782;P01817;P0DP01);hsa:3481(P01344);hsa:7534(E7EX29);hsa:2335(P02751) | 0.158172582 | 0.749238547 | http://www.kegg.jp/kegg-bin/show_pathway?hsa04151+hsa:102723407%09salmon+hsa:102723407%09green+hsa:102723407%09salmon+hsa:102723407%09green+hsa:102723407%09green+hsa:102723407%09salmon+hsa:102723407%09green+hsa:3481%09green+hsa:7534%09salmon+hsa:2335%09salmon |
| hsa04971 | Gastric acid secretion | Organismal Systems | 2\|228 | hsa:760(P00918);hsa:60(P60709) | 1\|44 | hsa:760(P00918) | 0.312852616 | 0.433180545 | http://www.kegg.jp/kegg-bin/show_pathway?hsa04971+hsa:760%09green |
| hsa05130 | Pathogenic Escherichia coli infection | Human Diseases | 7\|228 | hsa:203068(P07437);hsa:7278(P0DPH7);hsa:81027(Q9H4B7);hsa:7534(E7EX29);hsa:7277(P68366);hsa:113457(P0DPH7);hsa:112714(P0DPH7);hsa:929(P08571);hsa:71(P60709);hsa:60(P60709) | 1\|44 | hsa:7534(E7EX29) | 0.377170407 | 0.484933381 | http://www.kegg.jp/kegg-bin/show_pathway?hsa05130+hsa:7534%09salmon |
| hsa04110 | Cell cycle | Cellular Processes | 1\|228 | hsa:7534(E7EX29) | 1\|44 | hsa:7534(E7EX29) | 0.192982456 | 0.394736842 | http://www.kegg.jp/kegg-bin/show_pathway?hsa04110+hsa:7534%09salmon |
| hsa00590 | Arachidonic acid metabolism | Metabolism | 3\|228 | hsa:2878(A0A087X1J7);hsa:5730(P41222);hsa:81579(A0A2R8Y3M9) | 1\|44 | hsa:81579(A0A2R8Y3M9) | 0.379991341 | 0.468482476 | http://www.kegg.jp/kegg-bin/show_pathway?hsa00590+hsa:81579%09green |
| hsa05131 | Shigellosis | Human Diseases | 4\|228 | hsa:7414(P18206);hsa:71(P60709);hsa:960(H0Y5E4);hsa:5216(P07737);hsa:60(P60709) | 1\|44 | hsa:7414(P18206) | 0.409827698 | 0.455364109 | http://www.kegg.jp/kegg-bin/show_pathway?hsa05131+hsa:7414%09salmon |
| hsa00564 | Glycerophospholipid metabolism | Metabolism | 2\|228 | hsa:81579(A0A2R8Y3M9);hsa:3931(P04180) | 1\|44 | hsa:81579(A0A2R8Y3M9) | 0.312852616 | 0.433180545 | http://www.kegg.jp/kegg-bin/show_pathway?hsa00564+hsa:81579%09green |
| hsa04915 | Estrogen signaling pathway | Organismal Systems | 4\|228 | hsa:3858(P13645);hsa:3868(P08779);hsa:3861(P02533);hsa:3857(P35527) | 1\|44 | hsa:3857(P35527) | 0.409827698 | 0.455364109 | http://www.kegg.jp/kegg-bin/show_pathway?hsa04915+hsa:3857%09salmon |
| hsa00350 | Tyrosine metabolism | Metabolism | 2\|228 | hsa:1621(P09172);hsa:2184(P16930) | 1\|44 | hsa:1621(P09172) | 0.312852616 | 0.433180545 | http://www.kegg.jp/kegg-bin/show_pathway?hsa00350+hsa:1621%09green |
| hsa05418 | Fluid shear stress and atherosclerosis | Human Diseases | 5\|228 | hsa:3674(P08514);hsa:5881(P60763);hsa:7412(P19320);hsa:60(P60709);hsa:71(P60709);hsa:1003(I3L1J2) | 1\|44 | hsa:1003(I3L1J2) | 0.413944271 | 0.423352096 | http://www.kegg.jp/kegg-bin/show_pathway?hsa05418+hsa:1003%09salmon |
| hsa04010 | MAPK signaling pathway | Environmental Information Processing | 5\|228 | hsa:5881(P60763);hsa:5908(P61224);hsa:3481(P01344);hsa:929(P08571);hsa:2316(P21333) | 1\|44 | hsa:3481(P01344) | 0.413944271 | 0.423352096 | http://www.kegg.jp/kegg-bin/show_pathway?hsa04010+hsa:3481%09green |
| hsa00565 | Ether lipid metabolism | Metabolism | 3\|228 | hsa:81579(A0A2R8Y3M9);hsa:7368(Q16880);hsa:7941(Q13093) | 1\|44 | hsa:81579(A0A2R8Y3M9) | 0.379991341 | 0.468482476 | http://www.kegg.jp/kegg-bin/show_pathway?hsa00565+hsa:81579%09green |
| hsa00591 | Linoleic acid metabolism | Metabolism | 1\|228 | hsa:81579(A0A2R8Y3M9) | 1\|44 | hsa:81579(A0A2R8Y3M9) | 0.192982456 | 0.394736842 | http://www.kegg.jp/kegg-bin/show_pathway?hsa00591+hsa:81579%09green |
| hsa00780 | Biotin metabolism | Metabolism | 1\|228 | hsa:686(P43251) | 1\|44 | hsa:686(P43251) | 0.192982456 | 0.394736842 | http://www.kegg.jp/kegg-bin/show_pathway?hsa00780+hsa:686%09green |
| hsa04933 | AGE-RAGE signaling pathway in diabetic complications | Human Diseases | 2\|228 | hsa:7412(P19320);hsa:2335(P02751) | 1\|44 | hsa:2335(P02751) | 0.312852616 | 0.433180545 | http://www.kegg.jp/kegg-bin/show_pathway?hsa04933+hsa:2335%09salmon |
| hsa04520 | Adherens junction | Cellular Processes | 4\|228 | hsa:5881(P60763);hsa:7414(P18206);hsa:87(H9KV75);hsa:71(P60709);hsa:60(P60709) | 1\|44 | hsa:7414(P18206) | 0.409827698 | 0.455364109 | http://www.kegg.jp/kegg-bin/show_pathway?hsa04520+hsa:7414%09salmon |
| hsa04975 | Fat digestion and absorption | Organismal Systems | 4\|228 | hsa:81579(A0A2R8Y3M9);hsa:338(P04114);hsa:337(P06727);hsa:335(P02647) | 1\|44 | hsa:81579(A0A2R8Y3M9) | 0.409827698 | 0.455364109 | http://www.kegg.jp/kegg-bin/show_pathway?hsa04975+hsa:81579%09green |
| hsa01230 | Biosynthesis of amino acids | Metabolism | 4\|228 | hsa:5315(H3BTN5);hsa:2023(A0A2R8Y6G6);hsa:226(J3KPS3);hsa:2597(E7EUT5) | 1\|44 | hsa:2597(E7EUT5) | 0.409827698 | 0.455364109 | http://www.kegg.jp/kegg-bin/show_pathway?hsa01230+hsa:2597%09salmon |
| hsa00010 | Glycolysis / Gluconeogenesis | Metabolism | 4\|228 | hsa:5315(H3BTN5);hsa:2023(A0A2R8Y6G6);hsa:226(J3KPS3);hsa:2597(E7EUT5) | 1\|44 | hsa:2597(E7EUT5) | 0.409827698 | 0.455364109 | http://www.kegg.jp/kegg-bin/show_pathway?hsa00010+hsa:2597%09salmon |
| hsa04514 | Cell adhesion molecules (CAMs) | Environmental Information Processing | 5\|228 | hsa:102723996(A0A087X1L8);hsa:7412(P19320);hsa:6402(P14151);hsa:23308(A0A087X1L8);hsa:3105(A0A0G2JI36);hsa:1003(I3L1J2) | 1\|44 | hsa:1003(I3L1J2) | 0.413944271 | 0.423352096 | http://www.kegg.jp/kegg-bin/show_pathway?hsa04514+hsa:1003%09salmon |
| hsa00592 | alpha-Linolenic acid metabolism | Metabolism | 1\|228 | hsa:81579(A0A2R8Y3M9) | 1\|44 | hsa:81579(A0A2R8Y3M9) | 0.192982456 | 0.394736842 | http://www.kegg.jp/kegg-bin/show_pathway?hsa00592+hsa:81579%09green |
| hsa04270 | Vascular smooth muscle contraction | Organismal Systems | 2\|228 | hsa:81579(A0A2R8Y3M9);hsa:59(P68032);hsa:72(P68032) | 1\|44 | hsa:81579(A0A2R8Y3M9) | 0.312852616 | 0.433180545 | http://www.kegg.jp/kegg-bin/show_pathway?hsa04270+hsa:81579%09green |
| hsa04977 | Vitamin digestion and absorption | Organismal Systems | 4\|228 | hsa:686(P43251);hsa:338(P04114);hsa:337(P06727);hsa:335(P02647) | 1\|44 | hsa:686(P43251) | 0.409827698 | 0.455364109 | http://www.kegg.jp/kegg-bin/show_pathway?hsa04977+hsa:686%09green |
| hsa04512 | ECM-receptor interaction | Environmental Information Processing | 12\|228 | hsa:7450(P04275);hsa:3674(P08514);hsa:1291(A0A087X0S5);hsa:2811(A0A0C4DGZ8);hsa:1293(E7ENL6);hsa:1311(G3XAP6);hsa:7448(H0YJW9;P04004);hsa:960(H0Y5E4);hsa:2335(P02751);hsa:7057(P07996);hsa:7148(A0A140T8Y3) | 1\|44 | hsa:2335(P02751) | 0.216739843 | 0.382482075 | http://www.kegg.jp/kegg-bin/show_pathway?hsa04512+hsa:2335%09salmon |
| hsa00630 | Glyoxylate and dicarboxylate metabolism | Metabolism | 2\|228 | hsa:81888(E7EWH8);hsa:847(P04040) | 1\|44 | hsa:81888(E7EWH8) | 0.312852616 | 0.433180545 | http://www.kegg.jp/kegg-bin/show_pathway?hsa00630+hsa:81888%09salmon |
| hsa04114 | Oocyte meiosis | Cellular Processes | 1\|228 | hsa:7534(E7EX29) | 1\|44 | hsa:7534(E7EX29) | 0.192982456 | 0.394736842 | http://www.kegg.jp/kegg-bin/show_pathway?hsa04114+hsa:7534%09salmon |
| hsa05160 | Hepatitis C | Human Diseases | 1\|228 | hsa:7534(E7EX29) | 1\|44 | hsa:7534(E7EX29) | 0.192982456 | 0.394736842 | http://www.kegg.jp/kegg-bin/show_pathway?hsa05160+hsa:7534%09salmon |
| hsa03320 | PPAR signaling pathway | Organismal Systems | 6\|228 | hsa:9370(Q15848);hsa:5360(P55058);hsa:3611(A0A0A0MTH3);hsa:345(B0YIW2);hsa:336(P02652);hsa:335(P02647) | 1\|44 | hsa:5360(P55058) | 0.400950505 | 0.474809808 | http://www.kegg.jp/kegg-bin/show_pathway?hsa03320+hsa:5360%09salmon |
| hsa05225 | Hepatocellular carcinoma | Human Diseases | 2\|228 | hsa:3481(P01344);hsa:71(P60709);hsa:60(P60709) | 1\|44 | hsa:3481(P01344) | 0.312852616 | 0.433180545 | http://www.kegg.jp/kegg-bin/show_pathway?hsa05225+hsa:3481%09green |
| hsa05134 | Legionellosis | Human Diseases | 3\|228 | hsa:718(P01024;M0R0Q9);hsa:929(P08571) | 1\|44 | hsa:718(M0R0Q9) | 0.379991341 | 0.468482476 | http://www.kegg.jp/kegg-bin/show_pathway?hsa05134+hsa:718%09green |
| hsa04919 | Thyroid hormone signaling pathway | Organismal Systems | 2\|228 | hsa:71(P60709);hsa:60(P60709);hsa:90390(Q96HR3) | 1\|44 | hsa:90390(Q96HR3) | 0.312852616 | 0.433180545 | http://www.kegg.jp/kegg-bin/show_pathway?hsa04919+hsa:90390%09green |
| hsa01200 | Carbon metabolism | Metabolism | 5\|228 | hsa:5315(H3BTN5);hsa:2023(A0A2R8Y6G6);hsa:847(P04040);hsa:226(J3KPS3);hsa:2597(E7EUT5) | 1\|44 | hsa:2597(E7EUT5) | 0.413944271 | 0.423352096 | http://www.kegg.jp/kegg-bin/show_pathway?hsa01200+hsa:2597%09salmon |
| hsa04918 | Thyroid hormone synthesis | Organismal Systems | 6\|228 | hsa:2878(A0A087X1J7);hsa:7276(P02766);hsa:213(P02768;A0A087WWT3);hsa:3309(P11021);hsa:6906(P05543) | 1\|44 | hsa:6906(P05543) | 0.400950505 | 0.474809808 | http://www.kegg.jp/kegg-bin/show_pathway?hsa04918+hsa:6906%09salmon |
| hsa05010 | Alzheimer disease | Human Diseases | 4\|228 | hsa:2597(E7EUT5);hsa:4035(Q07954);hsa:348(P02649);hsa:2081(O75460) | 1\|44 | hsa:2597(E7EUT5) | 0.409827698 | 0.455364109 | http://www.kegg.jp/kegg-bin/show_pathway?hsa05010+hsa:2597%09salmon |
| hsa04976 | Bile secretion | Organismal Systems | 1\|228 | hsa:760(P00918) | 1\|44 | hsa:760(P00918) | 0.192982456 | 0.394736842 | http://www.kegg.jp/kegg-bin/show_pathway?hsa04976+hsa:760%09green |
| hsa05168 | Herpes simplex infection | Human Diseases | 5\|228 | hsa:718(P01024;M0R0Q9);hsa:5199(E9PAQ1);hsa:727(P01031);hsa:3105(A0A0G2JI36) | 1\|44 | hsa:718(M0R0Q9) | 0.413944271 | 0.423352096 | http://www.kegg.jp/kegg-bin/show_pathway?hsa05168+hsa:718%09green |
| hsa04964 | Proximal tubule bicarbonate reclamation | Organismal Systems | 1\|228 | hsa:760(P00918) | 1\|44 | hsa:760(P00918) | 0.192982456 | 0.394736842 | http://www.kegg.jp/kegg-bin/show_pathway?hsa04964+hsa:760%09green |
| hsa04966 | Collecting duct acid secretion | Organismal Systems | 1\|228 | hsa:760(P00918) | 1\|44 | hsa:760(P00918) | 0.192982456 | 0.394736842 | http://www.kegg.jp/kegg-bin/show_pathway?hsa04966+hsa:760%09green |
| hsa05161 | Hepatitis B | Human Diseases | 1\|228 | hsa:7534(E7EX29) | 1\|44 | hsa:7534(E7EX29) | 0.192982456 | 0.394736842 | http://www.kegg.jp/kegg-bin/show_pathway?hsa05161+hsa:7534%09salmon |
| hsa05222 | Small cell lung cancer | Human Diseases | 2\|228 | hsa:3674(P08514);hsa:2335(P02751) | 1\|44 | hsa:2335(P02751) | 0.312852616 | 0.433180545 | http://www.kegg.jp/kegg-bin/show_pathway?hsa05222+hsa:2335%09salmon |
| hsa05167 | Kaposi sarcoma-associated herpesvirus infection | Human Diseases | 3\|228 | hsa:718(P01024;M0R0Q9);hsa:3105(A0A0G2JI36) | 1\|44 | hsa:718(M0R0Q9) | 0.379991341 | 0.468482476 | http://www.kegg.jp/kegg-bin/show_pathway?hsa05167+hsa:718%09green |
| hsa04611 | Platelet activation | Organismal Systems | 11\|228 | hsa:7450(P04275);hsa:3674(P08514);hsa:7094(Q9Y490);hsa:2811(A0A0C4DGZ8);hsa:5908(P61224);hsa:2243(P02671);hsa:83706(Q86UX7);hsa:2244(P02675);hsa:2266(C9JPQ9;C9JC84);hsa:71(P60709);hsa:60(P60709) | 1\|44 | hsa:2266(C9JPQ9) | 0.247776822 | 0.420753094 | http://www.kegg.jp/kegg-bin/show_pathway?hsa04611+hsa:2266%09salmon |
| hsa04530 | Tight junction | Cellular Processes | 7\|228 | hsa:3993(J3QRV5);hsa:7278(P0DPH7);hsa:7277(P68366);hsa:113457(P0DPH7);hsa:4627(P35579);hsa:87(H9KV75);hsa:112714(P0DPH7);hsa:4478(P26038);hsa:71(P60709);hsa:60(P60709) | 1\|44 | hsa:3993(J3QRV5) | 0.377170407 | 0.484933381 | http://www.kegg.jp/kegg-bin/show_pathway?hsa04530+hsa:3993%09green |

| Table S5-3 KEGG pathway enrichment of DEPs in S vs M group. | | | | | | | | | |
| --- | --- | --- | --- | --- | --- | --- | --- | --- | --- |
| pathway | pathway_name | class | all_number_of_accs | all_KO2acc | diff_number_of_accs | diff_KO2acc | p_value | FDR | url |
| hsa04610 | Complement and coagulation cascades | Organismal Systems | 58\|228 | hsa:3818(H0YAC1);hsa:5345(P08697);hsa:5104(P05154);hsa:5340(P00747);hsa:629(B4E1Z4);hsa:729(P13671);hsa:2(P01023);hsa:725(P20851);hsa:732(P07358);hsa:727(P01031);hsa:720(A0A0G2JPR0;P0C0L5;P0C0L4);hsa:462(P01008);hsa:722(P04003);hsa:1675(K7ERG9);hsa:7450(P04275);hsa:10544(Q9UNN8);hsa:5624(E7END6);hsa:5627(A0A0S2Z4L3);hsa:2147(P00734);hsa:721(A0A0G2JPR0;P0C0L5;P0C0L4);hsa:2162(P00488);hsa:2161(P00748);hsa:2160(P03951);hsa:731(P07357);hsa:2165(P05160);hsa:730(P10643);hsa:735(P02748);hsa:3827(P01042);hsa:5648(P48740);hsa:1361(Q96IY4);hsa:718(P01024;M0R0Q9);hsa:10747(O00187);hsa:4153(P11226);hsa:1191(P10909);hsa:710(P05155);hsa:713(D6R934);hsa:712(P02745);hsa:715(A0A3B3ISR2);hsa:714(P02747);hsa:717(A0A0G2JL69);hsa:716(P09871);hsa:5265(P01009);hsa:3075(P08603);hsa:2243(P02671);hsa:7448(H0YJW9;P04004);hsa:2244(P02675);hsa:3426(A0A2R8Y3M9);hsa:2266(C9JPQ9;C9JC84);hsa:2155(F5H8B0);hsa:2153(A0A0A0MRJ7);hsa:3053(P05546);hsa:733(P07360);hsa:2158(P00740);hsa:2159(P00742) | 5\|24 | hsa:2161(P00748);hsa:2160(P03951);hsa:713(D6R934);hsa:10747(O00187);hsa:714(P02747) | 0.176983062 | 0.326435425 | http://www.kegg.jp/kegg-bin/show_pathway?hsa04610+hsa:2161%09green+hsa:2160%09green+hsa:713%09green+hsa:10747%09green+hsa:714%09green |
| hsa05150 | Staphylococcus aureus infection | Human Diseases | 63\|228 | hsa:102723407(A0A0C4DH29;A0A0C4DH32;A0A0C4DH33;A0A0J9YX35;A0A0C4DH31;A0A0C4DH36;A0A0C4DH34;A0A0C4DH38;A0A0C4DH39;A0A0J9YY99;A0A0G2JMI3;A0A0B4J1U7;P01763;P01764;P01743;P01766;A0A0B4J1X5;A0A0C4DH43;P01782;P01780;A0A087WSY4;P01814;A0A0B4J1X8;A0A4W8ZXM2;A0A075B7D0;P0DP02;P0DP01;A0A075B7F0;A0A075B7D8;P01817;A0A0A0MS14;A0A0A0MS15;A0A0B4J1V1;A0A075B7B8;A0A0B4J1V2;P0DTE1;A0A075B6Q5;A0A0J9YVY3;P23083);hsa:5340(P00747);hsa:629(B4E1Z4);hsa:3858(P13645);hsa:727(P01031);hsa:720(A0A0G2JPR0;P0C0L5;P0C0L4);hsa:721(A0A0G2JPR0;P0C0L5;P0C0L4);hsa:1675(K7ERG9);hsa:5648(P48740);hsa:2214(H0Y755);hsa:718(P01024;M0R0Q9);hsa:10747(O00187);hsa:713(D6R934);hsa:712(P02745);hsa:715(A0A3B3ISR2);hsa:714(P02747);hsa:717(A0A0G2JL69);hsa:716(P09871);hsa:4153(P11226);hsa:3426(A0A2R8Y3M9);hsa:2266(C9JPQ9;C9JC84);hsa:3075(P08603) | 4\|24 | hsa:102723407(P01782);hsa:10747(O00187);hsa:713(D6R934);hsa:714(P02747) | 0.09216258 | 0.27319622 | http://www.kegg.jp/kegg-bin/show_pathway?hsa05150+hsa:102723407%09green+hsa:10747%09green+hsa:713%09green+hsa:714%09green |
| hsa04979 | Cholesterol metabolism | Organismal Systems | 15\|228 | hsa:255738(A0A669KAY4);hsa:4035(Q07954);hsa:350(P02749);hsa:5360(P55058);hsa:1071(P11597);hsa:4018(P08519);hsa:3931(P04180);hsa:338(P04114);hsa:348(P02649);hsa:344(K7ER74);hsa:345(B0YIW2);hsa:337(P06727);hsa:336(P02652);hsa:335(P02647);hsa:341(K7ERI9) | 3\|24 | hsa:5360(P55058);hsa:344(K7ER74);hsa:255738(A0A669KAY4) | 0.141277239 | 0.266500246 | http://www.kegg.jp/kegg-bin/show_pathway?hsa04979+hsa:5360%09salmon+hsa:344%09salmon+hsa:255738%09salmon |
| hsa05165 | Human papillomavirus infection | Human Diseases | 13\|228 | hsa:7450(P04275);hsa:3674(P08514);hsa:3993(J3QRV5);hsa:1291(A0A087X0S5);hsa:1293(E7ENL6);hsa:1311(G3XAP6);hsa:7448(H0YJW9;P04004);hsa:2335(P02751);hsa:7057(P07996);hsa:5315(H3BTN5);hsa:3105(A0A0G2JI36);hsa:7148(A0A140T8Y3) | 3\|24 | hsa:5315(H3BTN5);hsa:3993(J3QRV5);hsa:1293(E7ENL6) | 0.109123938 | 0.220909435 | http://www.kegg.jp/kegg-bin/show_pathway?hsa05165+hsa:5315%09green+hsa:3993%09green+hsa:1293%09salmon |
| hsa04510 | Focal adhesion | Cellular Processes | 20\|228 | hsa:7450(P04275);hsa:29780(A0A087WZB5);hsa:7094(Q9Y490);hsa:1291(A0A087X0S5);hsa:1293(E7ENL6);hsa:5908(P61224);hsa:87(H9KV75);hsa:3674(P08514);hsa:7448(H0YJW9;P04004);hsa:3611(A0A0A0MTH3);hsa:5881(P60763);hsa:1311(G3XAP6);hsa:2316(P21333);hsa:7057(P07996);hsa:7791(H0Y2Y8);hsa:60(P60709);hsa:7414(P18206);hsa:2335(P02751);hsa:71(P60709);hsa:7148(A0A140T8Y3) | 3\|24 | hsa:5908(P61224);hsa:29780(A0A087WZB5);hsa:1293(E7ENL6) | 0.209560779 | 0.310599011 | http://www.kegg.jp/kegg-bin/show_pathway?hsa04510+hsa:5908%09green+hsa:29780%09salmon+hsa:1293%09salmon |
| hsa04670 | Leukocyte transendothelial migration | Organismal Systems | 7\|228 | hsa:5908(P61224);hsa:87(H9KV75);hsa:7412(P19320);hsa:60(P60709);hsa:7414(P18206);hsa:4478(P26038);hsa:71(P60709);hsa:1003(I3L1J2) | 3\|24 | hsa:5908(P61224);hsa:4478(P26038);hsa:7412(P19320) | 0.024487583 | 0.677489791 | http://www.kegg.jp/kegg-bin/show_pathway?hsa04670+hsa:5908%09green+hsa:4478%09green+hsa:7412%09green |
| hsa05322 | Systemic lupus erythematosus | Human Diseases | 60\|228 | hsa:735(P02748);hsa:102723407(A0A0C4DH29;A0A0C4DH32;A0A0C4DH33;A0A0J9YX35;A0A0C4DH31;A0A0C4DH36;A0A0C4DH34;A0A0C4DH38;A0A0C4DH39;A0A0J9YY99;A0A0G2JMI3;A0A0B4J1U7;P01763;P01764;P01743;P01766;A0A0B4J1X5;A0A0C4DH43;P01782;P01780;A0A087WSY4;P01814;A0A0B4J1X8;A0A4W8ZXM2;A0A075B7D0;P0DP02;P0DP01;A0A075B7F0;A0A075B7D8;P01817;A0A0A0MS14;A0A0A0MS15;A0A0B4J1V1;A0A075B7B8;A0A0B4J1V2;P0DTE1;A0A075B6Q5;A0A0J9YVY3;P23083);hsa:712(P02745);hsa:733(P07360);hsa:128312(A0A2R8Y619);hsa:715(A0A3B3ISR2);hsa:721(A0A0G2JPR0;P0C0L5;P0C0L4);hsa:727(P01031);hsa:718(P01024;M0R0Q9);hsa:732(P07358);hsa:87(H9KV75);hsa:2214(H0Y755);hsa:730(P10643);hsa:731(P07357);hsa:713(D6R934);hsa:716(P09871);hsa:720(A0A0G2JPR0;P0C0L5;P0C0L4);hsa:714(P02747);hsa:717(A0A0G2JL69);hsa:729(P13671) | 3\|24 | hsa:102723407(P01782);hsa:713(D6R934);hsa:714(P02747) | 0.054711284 | 0.189209856 | http://www.kegg.jp/kegg-bin/show_pathway?hsa05322+hsa:102723407%09green+hsa:713%09green+hsa:714%09green |
| hsa05142 | Chagas disease (American trypanosomiasis) | Human Diseases | 5\|228 | hsa:718(P01024;M0R0Q9);hsa:713(D6R934);hsa:712(P02745);hsa:714(P02747) | 2\|24 | hsa:713(D6R934);hsa:714(P02747) | 0.078327988 | 0.260048921 | http://www.kegg.jp/kegg-bin/show_pathway?hsa05142+hsa:713%09green+hsa:714%09green |
| hsa05143 | African trypanosomiasis | Human Diseases | 45\|228 | hsa:102723407(A0A0C4DH29;A0A0C4DH32;A0A0C4DH33;A0A0J9YX35;A0A0C4DH31;A0A0C4DH36;A0A0C4DH34;A0A0C4DH38;A0A0C4DH39;A0A0J9YY99;A0A0G2JMI3;A0A0B4J1U7;P01763;P01764;P01743;P01766;A0A0B4J1X5;A0A0C4DH43;P01782;P01780;A0A087WSY4;P01814;A0A0B4J1X8;A0A4W8ZXM2;A0A075B7D0;P0DP02;P0DP01;A0A075B7F0;A0A075B7D8;P01817;A0A0A0MS14;A0A0A0MS15;A0A0B4J1V1;A0A075B7B8;A0A0B4J1V2;P0DTE1;A0A075B6Q5;A0A0J9YVY3;P23083);hsa:3039(P69905);hsa:8542(O14791);hsa:335(P02647);hsa:3250(P00739);hsa:3043(P68871);hsa:3040(P69905);hsa:7412(P19320) | 2\|24 | hsa:102723407(P01782);hsa:7412(P19320) | 0.078488779 | 0.250560332 | http://www.kegg.jp/kegg-bin/show_pathway?hsa05143+hsa:102723407%09green+hsa:7412%09green |
| hsa04064 | NF-kappa B signaling pathway | Environmental Information Processing | 42\|228 | hsa:102723407(A0A0C4DH29;A0A0C4DH32;A0A0C4DH33;A0A0J9YX35;A0A0C4DH31;A0A0C4DH36;A0A0C4DH34;A0A0C4DH38;A0A0C4DH39;A0A0J9YY99;A0A0G2JMI3;A0A0B4J1U7;P01763;P01764;P01743;P01766;A0A0B4J1X5;A0A0C4DH43;P01782;P01780;A0A087WSY4;P01814;A0A0B4J1X8;A0A4W8ZXM2;A0A075B7D0;P0DP02;P0DP01;A0A075B7F0;A0A075B7D8;P01817;A0A0A0MS14;A0A0A0MS15;A0A0B4J1V1;A0A075B7B8;A0A0B4J1V2;P0DTE1;A0A075B6Q5;A0A0J9YVY3;P23083);hsa:929(P08571);hsa:7412(P19320);hsa:3929(P18428) | 2\|24 | hsa:102723407(P01782);hsa:7412(P19320) | 0.099800234 | 0.285635151 | http://www.kegg.jp/kegg-bin/show_pathway?hsa04064+hsa:102723407%09green+hsa:7412%09green |
| hsa04145 | Phagosome | Cellular Processes | 56\|228 | hsa:203068(P07437);hsa:3105(A0A0G2JI36);hsa:7278(P0DPH7);hsa:81027(Q9H4B7);hsa:102723407(A0A0C4DH29;A0A0C4DH32;A0A0C4DH33;A0A0J9YX35;A0A0C4DH31;A0A0C4DH36;A0A0C4DH34;A0A0C4DH38;A0A0C4DH39;A0A0J9YY99;A0A0G2JMI3;A0A0B4J1U7;P01763;P01764;P01743;P01766;A0A0B4J1X5;A0A0C4DH43;P01782;P01780;A0A087WSY4;P01814;A0A0B4J1X8;A0A4W8ZXM2;A0A075B7D0;P0DP02;P0DP01;A0A075B7F0;A0A075B7D8;P01817;A0A0A0MS14;A0A0A0MS15;A0A0B4J1V1;A0A075B7B8;A0A0B4J1V2;P0DTE1;A0A075B6Q5;A0A0J9YVY3;P23083);hsa:1311(G3XAP6);hsa:71(P60709);hsa:7277(P68366);hsa:113457(P0DPH7);hsa:3920(P13473);hsa:718(P01024;M0R0Q9);hsa:7057(P07996);hsa:112714(P0DPH7);hsa:2214(H0Y755);hsa:4153(P11226);hsa:78989(Q9BWP8);hsa:7037(G3V0E5);hsa:929(P08571);hsa:715(A0A3B3ISR2);hsa:60(P60709) | 2\|24 | hsa:102723407(P01782);hsa:81027(Q9H4B7) | 0.028590113 | 0.474595883 | http://www.kegg.jp/kegg-bin/show_pathway?hsa04145+hsa:102723407%09green+hsa:81027%09green |
| hsa05133 | Pertussis | Human Diseases | 17\|228 | hsa:712(P02745);hsa:715(A0A3B3ISR2);hsa:1072(E9PK25);hsa:721(A0A0G2JPR0;P0C0L5;P0C0L4);hsa:727(P01031);hsa:718(P01024;M0R0Q9);hsa:717(A0A0G2JL69);hsa:710(P05155);hsa:725(P20851);hsa:713(D6R934);hsa:929(P08571);hsa:720(A0A0G2JPR0;P0C0L5;P0C0L4);hsa:714(P02747);hsa:722(P04003);hsa:716(P09871) | 2\|24 | hsa:713(D6R934);hsa:714(P02747) | 0.296175931 | 0.40299348 | http://www.kegg.jp/kegg-bin/show_pathway?hsa05133+hsa:713%09green+hsa:714%09green |
| hsa05414 | Dilated cardiomyopathy (DCM) | Human Diseases | 44\|228 | hsa:3674(P08514);hsa:102723407(A0A0C4DH29;A0A0C4DH32;A0A0C4DH33;A0A0J9YX35;A0A0C4DH31;A0A0C4DH36;A0A0C4DH34;A0A0C4DH38;A0A0C4DH39;A0A0J9YY99;A0A0G2JMI3;A0A0B4J1U7;P01763;P01764;P01743;P01766;A0A0B4J1X5;A0A0C4DH43;P01782;P01780;A0A087WSY4;P01814;A0A0B4J1X8;A0A4W8ZXM2;A0A075B7D0;P0DP02;P0DP01;A0A075B7F0;A0A075B7D8;P01817;A0A0A0MS14;A0A0A0MS15;A0A0B4J1V1;A0A075B7B8;A0A0B4J1V2;P0DTE1;A0A075B6Q5;A0A0J9YVY3;P23083);hsa:7171(A0A087WWU8;A0A2R8Y5V9);hsa:71(P60709);hsa:70(P68032);hsa:60(P60709) | 2\|24 | hsa:102723407(P01782);hsa:70(P68032) | 0.085185627 | 0.261866926 | http://www.kegg.jp/kegg-bin/show_pathway?hsa05414+hsa:102723407%09green+hsa:70%09green |
| hsa05020 | Prion diseases | Human Diseases | 11\|228 | hsa:735(P02748);hsa:712(P02745);hsa:3309(P11021);hsa:714(P02747);hsa:729(P13671);hsa:713(D6R934);hsa:727(P01031);hsa:733(P07360);hsa:732(P07358);hsa:731(P07357);hsa:730(P10643) | 2\|24 | hsa:713(D6R934);hsa:714(P02747) | 0.22928354 | 0.333869015 | http://www.kegg.jp/kegg-bin/show_pathway?hsa05020+hsa:713%09green+hsa:714%09green |
| hsa04151 | PI3K-Akt signaling pathway | Environmental Information Processing | 51\|228 | hsa:7450(P04275);hsa:102723407(A0A0C4DH29;A0A0C4DH32;A0A0C4DH33;A0A0J9YX35;A0A0C4DH31;A0A0C4DH36;A0A0C4DH34;A0A0C4DH38;A0A0C4DH39;A0A0J9YY99;A0A0G2JMI3;A0A0B4J1U7;P01763;P01764;P01743;P01766;A0A0B4J1X5;A0A0C4DH43;P01782;P01780;A0A087WSY4;P01814;A0A0B4J1X8;A0A4W8ZXM2;A0A075B7D0;P0DP02;P0DP01;A0A075B7F0;A0A075B7D8;P01817;A0A0A0MS14;A0A0A0MS15;A0A0B4J1V1;A0A075B7B8;A0A0B4J1V2;P0DTE1;A0A075B6Q5;A0A0J9YVY3;P23083);hsa:1291(A0A087X0S5);hsa:7534(E7EX29);hsa:1311(G3XAP6);hsa:3481(P01344);hsa:7448(H0YJW9;P04004);hsa:2335(P02751);hsa:3674(P08514);hsa:7057(P07996);hsa:1293(E7ENL6);hsa:7148(A0A140T8Y3) | 2\|24 | hsa:102723407(P01782);hsa:1293(E7ENL6) | 0.046340455 | 0.213680986 | http://www.kegg.jp/kegg-bin/show_pathway?hsa04151+hsa:102723407%09green+hsa:1293%09salmon |
| hsa04974 | Protein digestion and absorption | Organismal Systems | 4\|228 | hsa:1293(E7ENL6);hsa:1361(Q96IY4);hsa:7373(J3QT83);hsa:1291(A0A087X0S5) | 2\|24 | hsa:7373(J3QT83);hsa:1293(E7ENL6) | 0.052115256 | 0.188068096 | http://www.kegg.jp/kegg-bin/show_pathway?hsa04974+hsa:7373%09salmon+hsa:1293%09salmon |
| hsa05162 | Measles | Human Diseases | 40\|228 | hsa:102723407(A0A0C4DH29;A0A0C4DH32;A0A0C4DH33;A0A0J9YX35;A0A0C4DH31;A0A0C4DH36;A0A0C4DH34;A0A0C4DH38;A0A0C4DH39;A0A0J9YY99;A0A0G2JMI3;A0A0B4J1U7;P01763;P01764;P01743;P01766;A0A0B4J1X5;A0A0C4DH43;P01782;P01780;A0A087WSY4;P01814;A0A0B4J1X8;A0A4W8ZXM2;A0A075B7D0;P0DP02;P0DP01;A0A075B7F0;A0A075B7D8;P01817;A0A0A0MS14;A0A0A0MS15;A0A0B4J1V1;A0A075B7B8;A0A0B4J1V2;P0DTE1;A0A075B6Q5;A0A0J9YVY3;P23083);hsa:4478(P26038) | 2\|24 | hsa:102723407(P01782);hsa:4478(P26038) | 0.116046069 | 0.229329136 | http://www.kegg.jp/kegg-bin/show_pathway?hsa05162+hsa:102723407%09green+hsa:4478%09green |
| hsa04530 | Tight junction | Cellular Processes | 7\|228 | hsa:3993(J3QRV5);hsa:7278(P0DPH7);hsa:7277(P68366);hsa:113457(P0DPH7);hsa:4627(P35579);hsa:87(H9KV75);hsa:112714(P0DPH7);hsa:4478(P26038);hsa:71(P60709);hsa:60(P60709) | 2\|24 | hsa:3993(J3QRV5);hsa:4478(P26038) | 0.133568634 | 0.257818525 | http://www.kegg.jp/kegg-bin/show_pathway?hsa04530+hsa:3993%09green+hsa:4478%09green |
| hsa05323 | Rheumatoid arthritis | Human Diseases | 39\|228 | hsa:102723407(A0A0C4DH29;A0A0C4DH32;A0A0C4DH33;A0A0J9YX35;A0A0C4DH31;A0A0C4DH36;A0A0C4DH34;A0A0C4DH38;A0A0C4DH39;A0A0J9YY99;A0A0G2JMI3;A0A0B4J1U7;P01763;P01764;P01743;P01766;A0A0B4J1X5;A0A0C4DH43;P01782;P01780;A0A087WSY4;P01814;A0A0B4J1X8;A0A4W8ZXM2;A0A075B7D0;P0DP02;P0DP01;A0A075B7F0;A0A075B7D8;P01817;A0A0A0MS14;A0A0A0MS15;A0A0B4J1V1;A0A075B7B8;A0A0B4J1V2;P0DTE1;A0A075B6Q5;A0A0J9YVY3;P23083) | 1\|24 | hsa:102723407(P01782) | 0.047679798 | 0.197871161 | http://www.kegg.jp/kegg-bin/show_pathway?hsa05323+hsa:102723407%09green |
| hsa05169 | Epstein-Barr virus infection | Human Diseases | 41\|228 | hsa:102723407(A0A0C4DH29;A0A0C4DH32;A0A0C4DH33;A0A0J9YX35;A0A0C4DH31;A0A0C4DH36;A0A0C4DH34;A0A0C4DH38;A0A0C4DH39;A0A0J9YY99;A0A0G2JMI3;A0A0B4J1U7;P01763;P01764;P01743;P01766;A0A0B4J1X5;A0A0C4DH43;P01782;P01780;A0A087WSY4;P01814;A0A0B4J1X8;A0A4W8ZXM2;A0A075B7D0;P0DP02;P0DP01;A0A075B7F0;A0A075B7D8;P01817;A0A0A0MS14;A0A0A0MS15;A0A0B4J1V1;A0A075B7B8;A0A0B4J1V2;P0DTE1;A0A075B6Q5;A0A0J9YVY3;P23083);hsa:3105(A0A0G2JI36);hsa:960(H0Y5E4) | 1\|24 | hsa:102723407(P01782) | 0.038639014 | 0.267253179 | http://www.kegg.jp/kegg-bin/show_pathway?hsa05169+hsa:102723407%09green |
| hsa05130 | Pathogenic Escherichia coli infection | Human Diseases | 7\|228 | hsa:203068(P07437);hsa:7278(P0DPH7);hsa:81027(Q9H4B7);hsa:7534(E7EX29);hsa:7277(P68366);hsa:113457(P0DPH7);hsa:112714(P0DPH7);hsa:929(P08571);hsa:71(P60709);hsa:60(P60709) | 1\|24 | hsa:81027(Q9H4B7) | 0.385219682 | 0.404724476 | http://www.kegg.jp/kegg-bin/show_pathway?hsa05130+hsa:81027%09green |
| hsa05416 | Viral myocarditis | Human Diseases | 42\|228 | hsa:5881(P60763);hsa:102723407(A0A0C4DH29;A0A0C4DH32;A0A0C4DH33;A0A0J9YX35;A0A0C4DH31;A0A0C4DH36;A0A0C4DH34;A0A0C4DH38;A0A0C4DH39;A0A0J9YY99;A0A0G2JMI3;A0A0B4J1U7;P01763;P01764;P01743;P01766;A0A0B4J1X5;A0A0C4DH43;P01782;P01780;A0A087WSY4;P01814;A0A0B4J1X8;A0A4W8ZXM2;A0A075B7D0;P0DP02;P0DP01;A0A075B7F0;A0A075B7D8;P01817;A0A0A0MS14;A0A0A0MS15;A0A0B4J1V1;A0A075B7B8;A0A0B4J1V2;P0DTE1;A0A075B6Q5;A0A0J9YVY3;P23083);hsa:3105(A0A0G2JI36);hsa:71(P60709);hsa:60(P60709) | 1\|24 | hsa:102723407(P01782) | 0.034713125 | 0.360148669 | http://www.kegg.jp/kegg-bin/show_pathway?hsa05416+hsa:102723407%09green |
| hsa04810 | Regulation of actin cytoskeleton | Cellular Processes | 12\|228 | hsa:3674(P08514);hsa:2934(A0A0A0MS51);hsa:1072(E9PK25);hsa:2147(P00734);hsa:2335(P02751);hsa:5881(P60763);hsa:4627(P35579);hsa:87(H9KV75);hsa:5216(P07737);hsa:7414(P18206);hsa:4478(P26038);hsa:71(P60709);hsa:60(P60709) | 1\|24 | hsa:4478(P26038) | 0.379175447 | 0.408721585 | http://www.kegg.jp/kegg-bin/show_pathway?hsa04810+hsa:4478%09green |
| hsa04072 | Phospholipase D signaling pathway | Environmental Information Processing | 39\|228 | hsa:102723407(A0A0C4DH29;A0A0C4DH32;A0A0C4DH33;A0A0J9YX35;A0A0C4DH31;A0A0C4DH36;A0A0C4DH34;A0A0C4DH38;A0A0C4DH39;A0A0J9YY99;A0A0G2JMI3;A0A0B4J1U7;P01763;P01764;P01743;P01766;A0A0B4J1X5;A0A0C4DH43;P01782;P01780;A0A087WSY4;P01814;A0A0B4J1X8;A0A4W8ZXM2;A0A075B7D0;P0DP02;P0DP01;A0A075B7F0;A0A075B7D8;P01817;A0A0A0MS14;A0A0A0MS15;A0A0B4J1V1;A0A075B7B8;A0A0B4J1V2;P0DTE1;A0A075B6Q5;A0A0J9YVY3;P23083) | 1\|24 | hsa:102723407(P01782) | 0.047679798 | 0.197871161 | http://www.kegg.jp/kegg-bin/show_pathway?hsa04072+hsa:102723407%09green |
| hsa05203 | Viral carcinogenesis | Human Diseases | 8\|228 | hsa:2934(A0A0A0MS51);hsa:128312(A0A2R8Y619);hsa:7534(E7EX29);hsa:718(P01024;M0R0Q9);hsa:87(H9KV75);hsa:5315(H3BTN5);hsa:3105(A0A0G2JI36) | 1\|24 | hsa:5315(H3BTN5) | 0.394433081 | 0.399243241 | http://www.kegg.jp/kegg-bin/show_pathway?hsa05203+hsa:5315%09green |
| hsa05211 | Renal cell carcinoma | Human Diseases | 1\|228 | hsa:5908(P61224) | 1\|24 | hsa:5908(P61224) | 0.105263158 | 0.24962406 | http://www.kegg.jp/kegg-bin/show_pathway?hsa05211+hsa:5908%09green |
| hsa00350 | Tyrosine metabolism | Metabolism | 2\|228 | hsa:1621(P09172);hsa:2184(P16930) | 1\|24 | hsa:1621(P09172) | 0.189195456 | 0.314064456 | http://www.kegg.jp/kegg-bin/show_pathway?hsa00350+hsa:1621%09green |
| hsa05418 | Fluid shear stress and atherosclerosis | Human Diseases | 5\|228 | hsa:3674(P08514);hsa:5881(P60763);hsa:7412(P19320);hsa:60(P60709);hsa:71(P60709);hsa:1003(I3L1J2) | 1\|24 | hsa:7412(P19320) | 0.342259253 | 0.400105888 | http://www.kegg.jp/kegg-bin/show_pathway?hsa05418+hsa:7412%09green |
| hsa04010 | MAPK signaling pathway | Environmental Information Processing | 5\|228 | hsa:5881(P60763);hsa:5908(P61224);hsa:3481(P01344);hsa:929(P08571);hsa:2316(P21333) | 1\|24 | hsa:5908(P61224) | 0.342259253 | 0.400105888 | http://www.kegg.jp/kegg-bin/show_pathway?hsa04010+hsa:5908%09green |
| hsa05152 | Tuberculosis | Human Diseases | 46\|228 | hsa:102723407(A0A0C4DH29;A0A0C4DH32;A0A0C4DH33;A0A0J9YX35;A0A0C4DH31;A0A0C4DH36;A0A0C4DH34;A0A0C4DH38;A0A0C4DH39;A0A0J9YY99;A0A0G2JMI3;A0A0B4J1U7;P01763;P01764;P01743;P01766;A0A0B4J1X5;A0A0C4DH43;P01782;P01780;A0A087WSY4;P01814;A0A0B4J1X8;A0A4W8ZXM2;A0A075B7D0;P0DP02;P0DP01;A0A075B7F0;A0A075B7D8;P01817;A0A0A0MS14;A0A0A0MS15;A0A0B4J1V1;A0A075B7B8;A0A0B4J1V2;P0DTE1;A0A075B6Q5;A0A0J9YVY3;P23083);hsa:820(J3KNB4);hsa:3920(P13473);hsa:718(P01024;M0R0Q9);hsa:2214(H0Y755);hsa:3929(P18428);hsa:929(P08571) | 1\|24 | hsa:102723407(P01782) | 0.022320152 | 0.926286296 | http://www.kegg.jp/kegg-bin/show_pathway?hsa05152+hsa:102723407%09green |
| hsa04720 | Long-term potentiation | Organismal Systems | 1\|228 | hsa:5908(P61224) | 1\|24 | hsa:5908(P61224) | 0.105263158 | 0.24962406 | http://www.kegg.jp/kegg-bin/show_pathway?hsa04720+hsa:5908%09green |
| hsa04722 | Neurotrophin signaling pathway | Organismal Systems | 2\|228 | hsa:5908(P61224);hsa:397(H0YGX7) | 1\|24 | hsa:5908(P61224) | 0.189195456 | 0.314064456 | http://www.kegg.jp/kegg-bin/show_pathway?hsa04722+hsa:5908%09green |
| hsa05230 | Central carbon metabolism in cancer | Human Diseases | 1\|228 | hsa:5315(H3BTN5) | 1\|24 | hsa:5315(H3BTN5) | 0.105263158 | 0.24962406 | http://www.kegg.jp/kegg-bin/show_pathway?hsa05230+hsa:5315%09green |
| hsa04672 | Intestinal immune network for IgA production | Organismal Systems | 41\|228 | hsa:102723407(A0A0C4DH29;A0A0C4DH32;A0A0C4DH33;A0A0J9YX35;A0A0C4DH31;A0A0C4DH36;A0A0C4DH34;A0A0C4DH38;A0A0C4DH39;A0A0J9YY99;A0A0G2JMI3;A0A0B4J1U7;P01763;P01764;P01743;P01766;A0A0B4J1X5;A0A0C4DH43;P01782;P01780;A0A087WSY4;P01814;A0A0B4J1X8;A0A4W8ZXM2;A0A075B7D0;P0DP02;P0DP01;A0A075B7F0;A0A075B7D8;P01817;A0A0A0MS14;A0A0A0MS15;A0A0B4J1V1;A0A075B7B8;A0A0B4J1V2;P0DTE1;A0A075B6Q5;A0A0J9YVY3;P23083);hsa:102723996(A0A087X1L8);hsa:5284(P01833);hsa:23308(A0A087X1L8) | 1\|24 | hsa:102723407(P01782) | 0.038639014 | 0.267253179 | http://www.kegg.jp/kegg-bin/show_pathway?hsa04672+hsa:102723407%09green |
| hsa04933 | AGE-RAGE signaling pathway in diabetic complications | Human Diseases | 2\|228 | hsa:7412(P19320);hsa:2335(P02751) | 1\|24 | hsa:7412(P19320) | 0.189195456 | 0.314064456 | http://www.kegg.jp/kegg-bin/show_pathway?hsa04933+hsa:7412%09green |
| hsa01230 | Biosynthesis of amino acids | Metabolism | 4\|228 | hsa:5315(H3BTN5);hsa:2023(A0A2R8Y6G6);hsa:226(J3KPS3);hsa:2597(E7EUT5) | 1\|24 | hsa:5315(H3BTN5) | 0.305138598 | 0.389638517 | http://www.kegg.jp/kegg-bin/show_pathway?hsa01230+hsa:5315%09green |
| hsa05320 | Autoimmune thyroid disease | Human Diseases | 40\|228 | hsa:102723407(A0A0C4DH29;A0A0C4DH32;A0A0C4DH33;A0A0J9YX35;A0A0C4DH31;A0A0C4DH36;A0A0C4DH34;A0A0C4DH38;A0A0C4DH39;A0A0J9YY99;A0A0G2JMI3;A0A0B4J1U7;P01763;P01764;P01743;P01766;A0A0B4J1X5;A0A0C4DH43;P01782;P01780;A0A087WSY4;P01814;A0A0B4J1X8;A0A4W8ZXM2;A0A075B7D0;P0DP02;P0DP01;A0A075B7F0;A0A075B7D8;P01817;A0A0A0MS14;A0A0A0MS15;A0A0B4J1V1;A0A075B7B8;A0A0B4J1V2;P0DTE1;A0A075B6Q5;A0A0J9YVY3;P23083);hsa:3105(A0A0G2JI36) | 1\|24 | hsa:102723407(P01782) | 0.042951276 | 0.237663729 | http://www.kegg.jp/kegg-bin/show_pathway?hsa05320+hsa:102723407%09green |
| hsa05202 | Transcriptional misregulation in cancer | Human Diseases | 42\|228 | hsa:102723407(A0A0C4DH29;A0A0C4DH32;A0A0C4DH33;A0A0J9YX35;A0A0C4DH31;A0A0C4DH36;A0A0C4DH34;A0A0C4DH38;A0A0C4DH39;A0A0J9YY99;A0A0G2JMI3;A0A0B4J1U7;P01763;P01764;P01743;P01766;A0A0B4J1X5;A0A0C4DH43;P01782;P01780;A0A087WSY4;P01814;A0A0B4J1X8;A0A4W8ZXM2;A0A075B7D0;P0DP02;P0DP01;A0A075B7F0;A0A075B7D8;P01817;A0A0A0MS14;A0A0A0MS15;A0A0B4J1V1;A0A075B7B8;A0A0B4J1V2;P0DTE1;A0A075B6Q5;A0A0J9YVY3;P23083);hsa:929(P08571);hsa:3486(A6XND0);hsa:1668(P59665) | 1\|24 | hsa:102723407(P01782) | 0.034713125 | 0.360148669 | http://www.kegg.jp/kegg-bin/show_pathway?hsa05202+hsa:102723407%09green |
| hsa05146 | Amoebiasis | Human Diseases | 47\|228 | hsa:102723407(A0A0C4DH29;A0A0C4DH32;A0A0C4DH33;A0A0J9YX35;A0A0C4DH31;A0A0C4DH36;A0A0C4DH34;A0A0C4DH38;A0A0C4DH39;A0A0J9YY99;A0A0G2JMI3;A0A0B4J1U7;P01763;P01764;P01743;P01766;A0A0B4J1X5;A0A0C4DH43;P01782;P01780;A0A087WSY4;P01814;A0A0B4J1X8;A0A4W8ZXM2;A0A075B7D0;P0DP02;P0DP01;A0A075B7F0;A0A075B7D8;P01817;A0A0A0MS14;A0A0A0MS15;A0A0B4J1V1;A0A075B7B8;A0A0B4J1V2;P0DTE1;A0A075B6Q5;A0A0J9YVY3;P23083);hsa:2335(P02751);hsa:87(H9KV75);hsa:7414(P18206);hsa:735(P02748);hsa:929(P08571);hsa:733(P07360);hsa:732(P07358);hsa:731(P07357) | 1\|24 | hsa:102723407(P01782) | 0.019923375 | 1 | http://www.kegg.jp/kegg-bin/show_pathway?hsa05146+hsa:102723407%09green |
| hsa00010 | Glycolysis / Gluconeogenesis | Metabolism | 4\|228 | hsa:5315(H3BTN5);hsa:2023(A0A2R8Y6G6);hsa:226(J3KPS3);hsa:2597(E7EUT5) | 1\|24 | hsa:5315(H3BTN5) | 0.305138598 | 0.389638517 | http://www.kegg.jp/kegg-bin/show_pathway?hsa00010+hsa:5315%09green |
| hsa05205 | Proteoglycans in cancer | Human Diseases | 10\|228 | hsa:71(P60709);hsa:3481(P01344);hsa:7448(H0YJW9;P04004);hsa:960(H0Y5E4);hsa:2335(P02751);hsa:2316(P21333);hsa:7057(P07996);hsa:4478(P26038);hsa:4060(P51884);hsa:60(P60709) | 1\|24 | hsa:4478(P26038) | 0.395128947 | 0.395128947 | http://www.kegg.jp/kegg-bin/show_pathway?hsa05205+hsa:4478%09green |
| hsa04514 | Cell adhesion molecules (CAMs) | Environmental Information Processing | 5\|228 | hsa:102723996(A0A087X1L8);hsa:7412(P19320);hsa:6402(P14151);hsa:23308(A0A087X1L8);hsa:3105(A0A0G2JI36);hsa:1003(I3L1J2) | 1\|24 | hsa:7412(P19320) | 0.342259253 | 0.400105888 | http://www.kegg.jp/kegg-bin/show_pathway?hsa04514+hsa:7412%09green |
| hsa04062 | Chemokine signaling pathway | Organismal Systems | 4\|228 | hsa:5881(P60763);hsa:5908(P61224);hsa:5473(P02775);hsa:5196(P02776) | 1\|24 | hsa:5908(P61224) | 0.305138598 | 0.389638517 | http://www.kegg.jp/kegg-bin/show_pathway?hsa04062+hsa:5908%09green |
| hsa04270 | Vascular smooth muscle contraction | Organismal Systems | 2\|228 | hsa:81579(A0A2R8Y3M9);hsa:59(P68032);hsa:72(P68032) | 1\|24 | hsa:59(P68032);hsa:72(P68032) | 0.189195456 | 0.314064456 | http://www.kegg.jp/kegg-bin/show_pathway?hsa04270+hsa:59%09green+hsa:72%09green |
| hsa00230 | Purine metabolism | Metabolism | 1\|228 | hsa:5315(H3BTN5) | 1\|24 | hsa:5315(H3BTN5) | 0.105263158 | 0.24962406 | http://www.kegg.jp/kegg-bin/show_pathway?hsa00230+hsa:5315%09green |
| hsa04261 | Adrenergic signaling in cardiomyocytes | Organismal Systems | 3\|228 | hsa:7171(A0A087WWU8;A0A2R8Y5V9);hsa:70(P68032) | 1\|24 | hsa:70(P68032) | 0.254911576 | 0.358604421 | http://www.kegg.jp/kegg-bin/show_pathway?hsa04261+hsa:70%09green |
| hsa04662 | B cell receptor signaling pathway | Organismal Systems | 40\|228 | hsa:5881(P60763);hsa:102723407(A0A0C4DH29;A0A0C4DH32;A0A0C4DH33;A0A0J9YX35;A0A0C4DH31;A0A0C4DH36;A0A0C4DH34;A0A0C4DH38;A0A0C4DH39;A0A0J9YY99;A0A0G2JMI3;A0A0B4J1U7;P01763;P01764;P01743;P01766;A0A0B4J1X5;A0A0C4DH43;P01782;P01780;A0A087WSY4;P01814;A0A0B4J1X8;A0A4W8ZXM2;A0A075B7D0;P0DP02;P0DP01;A0A075B7F0;A0A075B7D8;P01817;A0A0A0MS14;A0A0A0MS15;A0A0B4J1V1;A0A075B7B8;A0A0B4J1V2;P0DTE1;A0A075B6Q5;A0A0J9YVY3;P23083) | 1\|24 | hsa:102723407(P01782) | 0.042951276 | 0.237663729 | http://www.kegg.jp/kegg-bin/show_pathway?hsa04662+hsa:102723407%09green |
| hsa04614 | Renin-angiotensin system | Organismal Systems | 2\|228 | hsa:290(P15144);hsa:183(P01019) | 1\|24 | hsa:183(P01019) | 0.189195456 | 0.314064456 | http://www.kegg.jp/kegg-bin/show_pathway?hsa04614+hsa:183%09salmon |
| hsa05144 | Malaria | Human Diseases | 6\|228 | hsa:3039(P69905);hsa:4035(Q07954);hsa:1311(G3XAP6);hsa:7057(P07996);hsa:3043(P68871);hsa:3040(P69905);hsa:7412(P19320) | 1\|24 | hsa:7412(P19320) | 0.368350766 | 0.407641514 | http://www.kegg.jp/kegg-bin/show_pathway?hsa05144+hsa:7412%09green |
| hsa04668 | TNF signaling pathway | Environmental Information Processing | 1\|228 | hsa:7412(P19320) | 1\|24 | hsa:7412(P19320) | 0.105263158 | 0.24962406 | http://www.kegg.jp/kegg-bin/show_pathway?hsa04668+hsa:7412%09green |
| hsa04512 | ECM-receptor interaction | Environmental Information Processing | 12\|228 | hsa:7450(P04275);hsa:3674(P08514);hsa:1291(A0A087X0S5);hsa:2811(A0A0C4DGZ8);hsa:1293(E7ENL6);hsa:1311(G3XAP6);hsa:7448(H0YJW9;P04004);hsa:960(H0Y5E4);hsa:2335(P02751);hsa:7057(P07996);hsa:7148(A0A140T8Y3) | 1\|24 | hsa:1293(E7ENL6) | 0.379175447 | 0.408721585 | http://www.kegg.jp/kegg-bin/show_pathway?hsa04512+hsa:1293%09salmon |
| hsa00630 | Glyoxylate and dicarboxylate metabolism | Metabolism | 2\|228 | hsa:81888(E7EWH8);hsa:847(P04040) | 1\|24 | hsa:81888(E7EWH8) | 0.189195456 | 0.314064456 | http://www.kegg.jp/kegg-bin/show_pathway?hsa00630+hsa:81888%09salmon |
| hsa04650 | Natural killer cell mediated cytotoxicity | Organismal Systems | 42\|228 | hsa:5881(P60763);hsa:102723407(A0A0C4DH29;A0A0C4DH32;A0A0C4DH33;A0A0J9YX35;A0A0C4DH31;A0A0C4DH36;A0A0C4DH34;A0A0C4DH38;A0A0C4DH39;A0A0J9YY99;A0A0G2JMI3;A0A0B4J1U7;P01763;P01764;P01743;P01766;A0A0B4J1X5;A0A0C4DH43;P01782;P01780;A0A087WSY4;P01814;A0A0B4J1X8;A0A4W8ZXM2;A0A075B7D0;P0DP02;P0DP01;A0A075B7F0;A0A075B7D8;P01817;A0A0A0MS14;A0A0A0MS15;A0A0B4J1V1;A0A075B7B8;A0A0B4J1V2;P0DTE1;A0A075B6Q5;A0A0J9YVY3;P23083);hsa:3105(A0A0G2JI36);hsa:2214(H0Y755) | 1\|24 | hsa:102723407(P01782) | 0.034713125 | 0.360148669 | http://www.kegg.jp/kegg-bin/show_pathway?hsa04650+hsa:102723407%09green |
| hsa05140 | Leishmaniasis | Human Diseases | 42\|228 | hsa:718(P01024;M0R0Q9);hsa:2214(H0Y755);hsa:102723407(A0A0C4DH29;A0A0C4DH32;A0A0C4DH33;A0A0J9YX35;A0A0C4DH31;A0A0C4DH36;A0A0C4DH34;A0A0C4DH38;A0A0C4DH39;A0A0J9YY99;A0A0G2JMI3;A0A0B4J1U7;P01763;P01764;P01743;P01766;A0A0B4J1X5;A0A0C4DH43;P01782;P01780;A0A087WSY4;P01814;A0A0B4J1X8;A0A4W8ZXM2;A0A075B7D0;P0DP02;P0DP01;A0A075B7F0;A0A075B7D8;P01817;A0A0A0MS14;A0A0A0MS15;A0A0B4J1V1;A0A075B7B8;A0A0B4J1V2;P0DTE1;A0A075B6Q5;A0A0J9YVY3;P23083) | 1\|24 | hsa:102723407(P01782) | 0.034713125 | 0.360148669 | http://www.kegg.jp/kegg-bin/show_pathway?hsa05140+hsa:102723407%09green |
| hsa04020 | Calcium signaling pathway | Environmental Information Processing | 39\|228 | hsa:102723407(A0A0C4DH29;A0A0C4DH32;A0A0C4DH33;A0A0J9YX35;A0A0C4DH31;A0A0C4DH36;A0A0C4DH34;A0A0C4DH38;A0A0C4DH39;A0A0J9YY99;A0A0G2JMI3;A0A0B4J1U7;P01763;P01764;P01743;P01766;A0A0B4J1X5;A0A0C4DH43;P01782;P01780;A0A087WSY4;P01814;A0A0B4J1X8;A0A4W8ZXM2;A0A075B7D0;P0DP02;P0DP01;A0A075B7F0;A0A075B7D8;P01817;A0A0A0MS14;A0A0A0MS15;A0A0B4J1V1;A0A075B7B8;A0A0B4J1V2;P0DTE1;A0A075B6Q5;A0A0J9YVY3;P23083) | 1\|24 | hsa:102723407(P01782) | 0.047679798 | 0.197871161 | http://www.kegg.jp/kegg-bin/show_pathway?hsa04020+hsa:102723407%09green |
| hsa04978 | Mineral absorption | Organismal Systems | 2\|228 | hsa:7018(C9JB55;P02787) | 1\|24 | hsa:7018(C9JB55) | 0.189195456 | 0.314064456 | http://www.kegg.jp/kegg-bin/show_pathway?hsa04978+hsa:7018%09green |
| hsa05340 | Primary immunodeficiency | Human Diseases | 41\|228 | hsa:102723407(A0A0C4DH29;A0A0C4DH32;A0A0C4DH33;A0A0J9YX35;A0A0C4DH31;A0A0C4DH36;A0A0C4DH34;A0A0C4DH38;A0A0C4DH39;A0A0J9YY99;A0A0G2JMI3;A0A0B4J1U7;P01763;P01764;P01743;P01766;A0A0B4J1X5;A0A0C4DH43;P01782;P01780;A0A087WSY4;P01814;A0A0B4J1X8;A0A4W8ZXM2;A0A075B7D0;P0DP02;P0DP01;A0A075B7F0;A0A075B7D8;P01817;A0A0A0MS14;A0A0A0MS15;A0A0B4J1V1;A0A075B7B8;A0A0B4J1V2;P0DTE1;A0A075B6Q5;A0A0J9YVY3;P23083);hsa:3543(P01871;P15814) | 1\|24 | hsa:102723407(P01782) | 0.038639014 | 0.267253179 | http://www.kegg.jp/kegg-bin/show_pathway?hsa05340+hsa:102723407%09green |
| hsa00620 | Pyruvate metabolism | Metabolism | 1\|228 | hsa:5315(H3BTN5) | 1\|24 | hsa:5315(H3BTN5) | 0.105263158 | 0.24962406 | http://www.kegg.jp/kegg-bin/show_pathway?hsa00620+hsa:5315%09green |
| hsa03320 | PPAR signaling pathway | Organismal Systems | 6\|228 | hsa:9370(Q15848);hsa:5360(P55058);hsa:3611(A0A0A0MTH3);hsa:345(B0YIW2);hsa:336(P02652);hsa:335(P02647) | 1\|24 | hsa:5360(P55058) | 0.368350766 | 0.407641514 | http://www.kegg.jp/kegg-bin/show_pathway?hsa03320+hsa:5360%09salmon |
| hsa04934 | Cushing syndrome | Human Diseases | 1\|228 | hsa:5908(P61224) | 1\|24 | hsa:5908(P61224) | 0.105263158 | 0.24962406 | http://www.kegg.jp/kegg-bin/show_pathway?hsa04934+hsa:5908%09green |
| hsa04926 | Relaxin signaling pathway | Organismal Systems | 1\|228 | hsa:59(P68032) | 1\|24 | hsa:59(P68032) | 0.105263158 | 0.24962406 | http://www.kegg.jp/kegg-bin/show_pathway?hsa04926+hsa:59%09green |
| hsa04390 | Hippo signaling pathway | Environmental Information Processing | 3\|228 | hsa:3993(J3QRV5);hsa:71(P60709);hsa:60(P60709);hsa:7534(E7EX29) | 1\|24 | hsa:3993(J3QRV5) | 0.254911576 | 0.358604421 | http://www.kegg.jp/kegg-bin/show_pathway?hsa04390+hsa:3993%09green |
| hsa04919 | Thyroid hormone signaling pathway | Organismal Systems | 2\|228 | hsa:71(P60709);hsa:60(P60709);hsa:90390(Q96HR3) | 1\|24 | hsa:90390(Q96HR3) | 0.189195456 | 0.314064456 | http://www.kegg.jp/kegg-bin/show_pathway?hsa04919+hsa:90390%09green |
| hsa04014 | Ras signaling pathway | Environmental Information Processing | 4\|228 | hsa:5881(P60763);hsa:81579(A0A2R8Y3M9);hsa:3481(P01344);hsa:5908(P61224) | 1\|24 | hsa:5908(P61224) | 0.305138598 | 0.389638517 | http://www.kegg.jp/kegg-bin/show_pathway?hsa04014+hsa:5908%09green |
| hsa04015 | Rap1 signaling pathway | Environmental Information Processing | 7\|228 | hsa:3674(P08514);hsa:7094(Q9Y490);hsa:5908(P61224);hsa:5881(P60763);hsa:7057(P07996);hsa:5216(P07737);hsa:71(P60709);hsa:60(P60709) | 1\|24 | hsa:5908(P61224) | 0.385219682 | 0.404724476 | http://www.kegg.jp/kegg-bin/show_pathway?hsa04015+hsa:5908%09green |
| hsa04216 | Ferroptosis | Cellular Processes | 4\|228 | hsa:7018(C9JB55;P02787);hsa:7037(G3V0E5);hsa:1356(P00450) | 1\|24 | hsa:7018(C9JB55) | 0.305138598 | 0.389638517 | http://www.kegg.jp/kegg-bin/show_pathway?hsa04216+hsa:7018%09green |
| hsa01200 | Carbon metabolism | Metabolism | 5\|228 | hsa:5315(H3BTN5);hsa:2023(A0A2R8Y6G6);hsa:847(P04040);hsa:226(J3KPS3);hsa:2597(E7EUT5) | 1\|24 | hsa:5315(H3BTN5) | 0.342259253 | 0.400105888 | http://www.kegg.jp/kegg-bin/show_pathway?hsa01200+hsa:5315%09green |
| hsa04066 | HIF-1 signaling pathway | Environmental Information Processing | 6\|228 | hsa:7018(C9JB55;P02787);hsa:2023(A0A2R8Y6G6);hsa:226(J3KPS3);hsa:2597(E7EUT5);hsa:7037(G3V0E5) | 1\|24 | hsa:7018(C9JB55) | 0.368350766 | 0.407641514 | http://www.kegg.jp/kegg-bin/show_pathway?hsa04066+hsa:7018%09green |
| hsa05410 | Hypertrophic cardiomyopathy (HCM) | Human Diseases | 5\|228 | hsa:3674(P08514);hsa:7171(A0A087WWU8;A0A2R8Y5V9);hsa:71(P60709);hsa:70(P68032);hsa:60(P60709) | 1\|24 | hsa:70(P68032) | 0.342259253 | 0.400105888 | http://www.kegg.jp/kegg-bin/show_pathway?hsa05410+hsa:70%09green |
| hsa04972 | Pancreatic secretion | Organismal Systems | 4\|228 | hsa:760(P00918);hsa:81579(A0A2R8Y3M9);hsa:1361(Q96IY4);hsa:5908(P61224) | 1\|24 | hsa:5908(P61224) | 0.305138598 | 0.389638517 | http://www.kegg.jp/kegg-bin/show_pathway?hsa04972+hsa:5908%09green |
| hsa04371 | Apelin signaling pathway | Environmental Information Processing | 1\|228 | hsa:59(P68032) | 1\|24 | hsa:59(P68032) | 0.105263158 | 0.24962406 | http://www.kegg.jp/kegg-bin/show_pathway?hsa04371+hsa:59%09green |
| hsa04260 | Cardiac muscle contraction | Organismal Systems | 3\|228 | hsa:7171(A0A087WWU8;A0A2R8Y5V9);hsa:70(P68032) | 1\|24 | hsa:70(P68032) | 0.254911576 | 0.358604421 | http://www.kegg.jp/kegg-bin/show_pathway?hsa04260+hsa:70%09green |
| hsa04922 | Glucagon signaling pathway | Organismal Systems | 1\|228 | hsa:5315(H3BTN5) | 1\|24 | hsa:5315(H3BTN5) | 0.105263158 | 0.24962406 | http://www.kegg.jp/kegg-bin/show_pathway?hsa04922+hsa:5315%09green |
| hsa05330 | Allograft rejection | Human Diseases | 40\|228 | hsa:102723407(A0A0C4DH29;A0A0C4DH32;A0A0C4DH33;A0A0J9YX35;A0A0C4DH31;A0A0C4DH36;A0A0C4DH34;A0A0C4DH38;A0A0C4DH39;A0A0J9YY99;A0A0G2JMI3;A0A0B4J1U7;P01763;P01764;P01743;P01766;A0A0B4J1X5;A0A0C4DH43;P01782;P01780;A0A087WSY4;P01814;A0A0B4J1X8;A0A4W8ZXM2;A0A075B7D0;P0DP02;P0DP01;A0A075B7F0;A0A075B7D8;P01817;A0A0A0MS14;A0A0A0MS15;A0A0B4J1V1;A0A075B7B8;A0A0B4J1V2;P0DTE1;A0A075B6Q5;A0A0J9YVY3;P23083);hsa:3105(A0A0G2JI36) | 1\|24 | hsa:102723407(P01782) | 0.042951276 | 0.237663729 | http://www.kegg.jp/kegg-bin/show_pathway?hsa05330+hsa:102723407%09green |
| hsa04640 | Hematopoietic cell lineage | Organismal Systems | 45\|228 | hsa:3674(P08514);hsa:102723407(A0A0C4DH29;A0A0C4DH32;A0A0C4DH33;A0A0J9YX35;A0A0C4DH31;A0A0C4DH36;A0A0C4DH34;A0A0C4DH38;A0A0C4DH39;A0A0J9YY99;A0A0G2JMI3;A0A0B4J1U7;P01763;P01764;P01743;P01766;A0A0B4J1X5;A0A0C4DH43;P01782;P01780;A0A087WSY4;P01814;A0A0B4J1X8;A0A4W8ZXM2;A0A075B7D0;P0DP02;P0DP01;A0A075B7F0;A0A075B7D8;P01817;A0A0A0MS14;A0A0A0MS15;A0A0B4J1V1;A0A075B7B8;A0A0B4J1V2;P0DTE1;A0A075B6Q5;A0A0J9YVY3;P23083);hsa:2811(A0A0C4DGZ8);hsa:960(H0Y5E4);hsa:290(P15144);hsa:7037(G3V0E5);hsa:929(P08571) | 1\|24 | hsa:102723407(P01782) | 0.024973702 | 0.518204324 | http://www.kegg.jp/kegg-bin/show_pathway?hsa04640+hsa:102723407%09green |
| hsa04540 | Gap junction | Cellular Processes | 4\|228 | hsa:203068(P07437);hsa:7278(P0DPH7);hsa:81027(Q9H4B7);hsa:7277(P68366);hsa:113457(P0DPH7);hsa:112714(P0DPH7) | 1\|24 | hsa:81027(Q9H4B7) | 0.305138598 | 0.389638517 | http://www.kegg.jp/kegg-bin/show_pathway?hsa04540+hsa:81027%09green |
| hsa04666 | Fc gamma R-mediated phagocytosis | Organismal Systems | 42\|228 | hsa:2934(A0A0A0MS51);hsa:102723407(A0A0C4DH29;A0A0C4DH32;A0A0C4DH33;A0A0J9YX35;A0A0C4DH31;A0A0C4DH36;A0A0C4DH34;A0A0C4DH38;A0A0C4DH39;A0A0J9YY99;A0A0G2JMI3;A0A0B4J1U7;P01763;P01764;P01743;P01766;A0A0B4J1X5;A0A0C4DH43;P01782;P01780;A0A087WSY4;P01814;A0A0B4J1X8;A0A4W8ZXM2;A0A075B7D0;P0DP02;P0DP01;A0A075B7F0;A0A075B7D8;P01817;A0A0A0MS14;A0A0A0MS15;A0A0B4J1V1;A0A075B7B8;A0A0B4J1V2;P0DTE1;A0A075B6Q5;A0A0J9YVY3;P23083);hsa:1072(E9PK25);hsa:2214(H0Y755) | 1\|24 | hsa:102723407(P01782) | 0.034713125 | 0.360148669 | http://www.kegg.jp/kegg-bin/show_pathway?hsa04666+hsa:102723407%09green |
| hsa04930 | Type II diabetes mellitus | Human Diseases | 2\|228 | hsa:5315(H3BTN5);hsa:9370(Q15848) | 1\|24 | hsa:5315(H3BTN5) | 0.189195456 | 0.314064456 | http://www.kegg.jp/kegg-bin/show_pathway?hsa04930+hsa:5315%09green |
| hsa04924 | Renin secretion | Organismal Systems | 1\|228 | hsa:183(P01019) | 1\|24 | hsa:183(P01019) | 0.105263158 | 0.24962406 | http://www.kegg.jp/kegg-bin/show_pathway?hsa04924+hsa:183%09salmon |
| hsa04611 | Platelet activation | Organismal Systems | 11\|228 | hsa:7450(P04275);hsa:3674(P08514);hsa:7094(Q9Y490);hsa:2811(A0A0C4DGZ8);hsa:5908(P61224);hsa:2243(P02671);hsa:83706(Q86UX7);hsa:2244(P02675);hsa:2266(C9JPQ9;C9JC84);hsa:71(P60709);hsa:60(P60709) | 1\|24 | hsa:5908(P61224) | 0.388785134 | 0.398384767 | http://www.kegg.jp/kegg-bin/show_pathway?hsa04611+hsa:5908%09green |
| hsa04024 | cAMP signaling pathway | Environmental Information Processing | 2\|228 | hsa:5881(P60763);hsa:5908(P61224) | 1\|24 | hsa:5908(P61224) | 0.189195456 | 0.314064456 | http://www.kegg.jp/kegg-bin/show_pathway?hsa04024+hsa:5908%09green |
| hsa05310 | Asthma | Human Diseases | 39\|228 | hsa:102723407(A0A0C4DH29;A0A0C4DH32;A0A0C4DH33;A0A0J9YX35;A0A0C4DH31;A0A0C4DH36;A0A0C4DH34;A0A0C4DH38;A0A0C4DH39;A0A0J9YY99;A0A0G2JMI3;A0A0B4J1U7;P01763;P01764;P01743;P01766;A0A0B4J1X5;A0A0C4DH43;P01782;P01780;A0A087WSY4;P01814;A0A0B4J1X8;A0A4W8ZXM2;A0A075B7D0;P0DP02;P0DP01;A0A075B7F0;A0A075B7D8;P01817;A0A0A0MS14;A0A0A0MS15;A0A0B4J1V1;A0A075B7B8;A0A0B4J1V2;P0DTE1;A0A075B6Q5;A0A0J9YVY3;P23083) | 1\|24 | hsa:102723407(P01782) | 0.047679798 | 0.197871161 | http://www.kegg.jp/kegg-bin/show_pathway?hsa05310+hsa:102723407%09green |
| hsa04664 | Fc epsilon RI signaling pathway | Organismal Systems | 40\|228 | hsa:5881(P60763);hsa:102723407(A0A0C4DH29;A0A0C4DH32;A0A0C4DH33;A0A0J9YX35;A0A0C4DH31;A0A0C4DH36;A0A0C4DH34;A0A0C4DH38;A0A0C4DH39;A0A0J9YY99;A0A0G2JMI3;A0A0B4J1U7;P01763;P01764;P01743;P01766;A0A0B4J1X5;A0A0C4DH43;P01782;P01780;A0A087WSY4;P01814;A0A0B4J1X8;A0A4W8ZXM2;A0A075B7D0;P0DP02;P0DP01;A0A075B7F0;A0A075B7D8;P01817;A0A0A0MS14;A0A0A0MS15;A0A0B4J1V1;A0A075B7B8;A0A0B4J1V2;P0DTE1;A0A075B6Q5;A0A0J9YVY3;P23083) | 1\|24 | hsa:102723407(P01782) | 0.042951276 | 0.237663729 | http://www.kegg.jp/kegg-bin/show_pathway?hsa04664+hsa:102723407%09green |
