# Supplementary material for: Proteomics of extracellular vesicles in plasma reveals the characteristics and residual traces of COVID-19 patients without underlying diseases after 3 months of recovery
Source: Cell Death Dis. 2021 May 25;12(6):541. doi: 10.1038/s41419-021-03816-3 (PMC8146187; doi:10.1038/s41419-021-03816-3)
Supplement: Supplementary file 19 — Table S6 [file 41419_2021_3816_MOESM19_ESM.docx]

| Table S6-1 GO functional enrichment of DEPs in M vs A group. | | | | | | | | | |
| --- | --- | --- | --- | --- | --- | --- | --- | --- | --- |
| GO | name | namespace | all_number_of_accs | all_GO2acc | diff_number_of_accs | diff_GO2acc | p_value | FDR | url |
| GO:0005198 | structural molecule activity | molecular_function | 37|375 | P08779(GO:0005200;GO:0005198);P68366(GO:0005200);P04275(GO:0005201);P51884(GO:0005201;GO:0030021);A0A1W2PQU7(GO:0005198);P23142(GO:0005201);Q9H4B7(GO:0005200);P02649(GO:0005198);Q8N1N4(GO:0005198);Q86YZ3(GO:0030280);P35527(GO:0005200;GO:0005198);P0DPH7(GO:0005200);P02538(GO:0005200;GO:0005198);P02671(GO:0005198);P02751(GO:0005201);P60709(GO:0005200;GO:0098973);A0A140T8Y3(GO:0005201);P02533(GO:0005200;GO:0005198);A0A087X0S5(GO:0030020);P68032(GO:0005200);Q15582(GO:0005201);P02675(GO:0005198);P04264(GO:0030280;GO:0005198);P26038(GO:0005200);P07996(GO:0005201);F8W1S1(GO:0005198);Q13201(GO:0005201);P18206(GO:0005198);P13645(GO:0005198;GO:0030280);P13647(GO:0005200;GO:0005198);P04004(GO:0005201);P07437(GO:0005200;GO:0005198);Q92954(GO:0030021);G3XAP6(GO:0005201);Q9Y490(GO:0005200);Q16610(GO:0005201);P35908(GO:0005200;GO:0005198;GO:0030280) | 8|51 | P35527(GO:0005200;GO:0005198);P13647(GO:0005200;GO:0005198);Q9H4B7(GO:0005200);P02751(GO:0005201);P60709(GO:0005200;GO:0098973);Q16610(GO:0005201);P18206(GO:0005198);P68032(GO:0005200) | 0.06302944 | 0.229197964 | https://www.ebi.ac.uk/QuickGO/term/GO:0005198 |
| GO:0040011 | locomotion | biological_process | 75|375 | P08779(GO:0051546);P04211(GO:0050900);P03950(GO:0016477);P04196(GO:0006935);Q01518(GO:0001667);A0A0C4DH73(GO:0050900);P02647(GO:0043534;GO:0050919);P15814(GO:0050900);P04114(GO:0050900;GO:0030317);P05109(GO:0030593;GO:0006935;GO:0002523);P13796(GO:0016477;GO:0044319);P05546(GO:0006935);P06702(GO:0030595;GO:0030593;GO:0006935;GO:0002523);P59665(GO:0010818;GO:0006935);P19320(GO:0060326);E9PK25(GO:0001755);P01599(GO:0050900);P09172(GO:0050900);P01703(GO:0050900);P01700(GO:0050900);P01701(GO:0050900);P01706(GO:0050900);P01704(GO:0050900);P01705(GO:0050900);P01602(GO:0050900);P01601(GO:0050900);P01709(GO:0050900);P14151(GO:0050900);P01721(GO:0050900);P0DJI8(GO:0048246;GO:0048247;GO:0030593;GO:0050918);P06312(GO:0050900);P55058(GO:0030317);P02751(GO:0050900;GO:1901166);P60709(GO:0048870);P06331(GO:0050900);P60763(GO:0016477);P01763(GO:0050900);P01764(GO:0050900);P01743(GO:0050900);P01766(GO:0050900);P80108(GO:0097241;GO:0035701;GO:0002042);P26038(GO:0050900;GO:0072678);P01782(GO:0050900);P18428(GO:0002232);P01780(GO:0050900);P01742(GO:0050900);P02776(GO:0030595;GO:0030593;GO:0006935);P07996(GO:0016477);P02775(GO:0060326;GO:0030595;GO:0030593;GO:0006935);P01597(GO:0050900);A0A096LPE2(GO:0060326;GO:0050918);P01877(GO:0050900);P04433(GO:0050900);P01871(GO:0050900);P04430(GO:0050900);P01834(GO:0050900);P01031(GO:0060326;GO:0006935);P01817(GO:0050900);P01814(GO:0050900);P35579(GO:0050900;GO:0043534);P01715(GO:0050900);P01619(GO:0050900);P01717(GO:0050900);P23083(GO:0050900);P01718(GO:0050900);C9J8S2(GO:0006935);A0A0A0MTH3(GO:0010761);P04004(GO:0016477);P21333(GO:0044319);P0CF74(GO:0050900);P80748(GO:0050900);A0A0C4DH25(GO:0050900);P35908(GO:0051546);P01699(GO:0050900);G3XAK1(GO:0030317) | 8|51 | P01715(GO:0050900);P01717(GO:0050900);P03950(GO:0016477);A0A0C4DH73(GO:0050900);P55058(GO:0030317);P02751(GO:0050900;GO:1901166);P60709(GO:0048870);P80748(GO:0050900) | 0.112528885 | 0.250064188 | https://www.ebi.ac.uk/QuickGO/term/GO:0040011 |
| GO:0030054 | cell junction | cellular_component | 28|375 | P08514(GO:0005925);H0Y2Y8(GO:0005925);Q07954(GO:0005925);Q86UX7(GO:0030054);Q01518(GO:0005925);P31151(GO:0005925);A0A0A0MTH3(GO:0005925;GO:0030054);P13796(GO:0005925;GO:0030054);E9PK25(GO:0005925;GO:0005911);P61224(GO:0030054;GO:0005911);P21333(GO:0005925;GO:0005911);P0C0L4(GO:0030054);P60709(GO:0005925);Q9UNN8(GO:0005925);P68032(GO:0005925);H9KV75(GO:0005925;GO:0005923;GO:0005915);P0C0L5(GO:0030054);P26038(GO:0005925);I3L1J2(GO:0005923;GO:0030054;GO:0005913;GO:0005911);P06702(GO:0030054);P18206(GO:0005925;GO:0030054;GO:0030055;GO:0005916;GO:0005913;GO:0005912;GO:0005911);P35579(GO:0005925;GO:0005913);P04040(GO:0005925);A0A0A0MS51(GO:0005925);P43121(GO:0005925);P11021(GO:0005925);Q9Y490(GO:0005925;GO:0030054);P07737(GO:0005925) | 7|51 | P04040(GO:0005925);Q86UX7(GO:0030054);I3L1J2(GO:0005923;GO:0030054;GO:0005913;GO:0005911);P60709(GO:0005925);P18206(GO:0005925;GO:0030054;GO:0030055;GO:0005916;GO:0005913;GO:0005912;GO:0005911);P07737(GO:0005925);P68032(GO:0005925) | 0.044590569 | 0.222952845 | https://www.ebi.ac.uk/QuickGO/term/GO:0030054 |
| GO:0098772 | molecular function regulator | molecular_function | 64|375 | C9JB55(GO:0004857);P02649(GO:0060228);P05154(GO:0030414;GO:0004867);B0YIW2(GO:0055102;GO:0030234);P04196(GO:0004867;GO:0004869);P08519(GO:0004866);Q9UGM5(GO:0030414;GO:0008191;GO:0004869;GO:0004857);P02766(GO:0005179);G3V2W1(GO:0004867);P36955(GO:0004867);K7ER74(GO:0008047;GO:0055102;GO:0016004;GO:0060230);P23142(GO:0016504);P08697(GO:0030414;GO:0004866;GO:0004867);P22792(GO:0030234);A0A0G2JPR0(GO:0004866);E7EUT5(GO:0019828);B1AHL2(GO:0016504);P01024(GO:0004866);P01023(GO:0030414;GO:0005096;GO:0004866;GO:0004867);P21333(GO:0015459);P07737(GO:0000774);P08185(GO:0004867);H0YGX7(GO:0005094);P0DJI8(GO:0042056);P01009(GO:0030414;GO:0004867);P01008(GO:0030414;GO:0004867);P02760(GO:0019855;GO:0030414;GO:0004867);Q6UXB8(GO:0030414);P80108(GO:0017080);Q14624(GO:0004867;GO:0030414;GO:0004866);P0C0L5(GO:0004866);P0C0L4(GO:0004866);P02647(GO:0045499;GO:0055102;GO:0060228);Q06033(GO:0030414;GO:0004866;GO:0004867);P02652(GO:0060228;GO:0055102);P02776(GO:0005125;GO:0008009);P01344(GO:0008083;GO:0005179;GO:0048018;GO:0043539);P02775(GO:0005125;GO:0008009;GO:0008083);P05155(GO:0030414;GO:0004867);A0A096LPE2(GO:0042056);P01042(GO:0030414;GO:0004869);P20742(GO:0004866;GO:0030414;GO:0004867);P06727(GO:0060228);Q13103(GO:0004866);P00734(GO:0008047;GO:0008083);P19827(GO:0030414;GO:0004867);P19823(GO:0030414;GO:0004866;GO:0004867);P29622(GO:0030414;GO:0004867);A0A669KAY4(GO:0019871;GO:0030547);P02751(GO:0016504);P05543(GO:0004867);P05546(GO:0030414;GO:0004866;GO:0004867);P01011(GO:0030414;GO:0004867);P55103(GO:0005179;GO:0005125;GO:0008083);E7EQB2(GO:0004869;GO:0043539);E7ENL6(GO:0004867);P01019(GO:0005179;GO:0008083;GO:0048018;GO:0004867);C9JV77(GO:0019210;GO:0004869);P01031(GO:0008009;GO:0004866);P02749(GO:0060230);Q15848(GO:0005125;GO:0005179);P01034(GO:0030414;GO:0004866;GO:0004869);B7ZKJ8(GO:0004867);M0R0Q9(GO:0004866) | 7|51 | Q14624(GO:0004867;GO:0030414;GO:0004866);P19823(GO:0030414;GO:0004866;GO:0004867);P29622(GO:0030414;GO:0004867);Q9UGM5(GO:0030414;GO:0008191;GO:0004869;GO:0004857);E7ENL6(GO:0004867);P07737(GO:0000774);P02751(GO:0016504) | 0.133524021 | 0.205421571 | https://www.ebi.ac.uk/QuickGO/term/GO:0098772 |
| GO:0016209 | antioxidant activity | molecular_function | 13|375 | P02768(GO:0016209);P02649(GO:0016209);A0A087X1J7(GO:0004601;GO:0004602);P04040(GO:0004601;GO:0016209;GO:0004096);P00738(GO:0016209);P32119(GO:0008379;GO:0016209;GO:0051920;GO:0004601);O95445(GO:0016209);P06727(GO:0016209);P30041(GO:0016209;GO:0051920;GO:0004601;GO:0004602);P06702(GO:0016209);Q5SRP5(GO:0016209);P69905(GO:0004601);P68871(GO:0004601) | 6|51 | A0A087X1J7(GO:0004601;GO:0004602);P04040(GO:0004601;GO:0016209;GO:0004096);P32119(GO:0008379;GO:0016209;GO:0051920;GO:0004601);P30041(GO:0016209;GO:0051920;GO:0004601;GO:0004602);Q5SRP5(GO:0016209);P69905(GO:0004601) | 0.003324866 | 0.033248665 | https://www.ebi.ac.uk/QuickGO/term/GO:0016209 |
| GO:0044425 | membrane part | cellular_component | 104|375 | J3KNB4(GO:0016021);P15814(GO:0009897);P08571(GO:0009897;GO:0045121;GO:0031362;GO:0031225;GO:0046696);E9PK25(GO:0031258;GO:0032587);O75460(GO:0005789;GO:1990604;GO:0030176;GO:0016021;GO:1990332);H0Y755(GO:0016021);A0A0G2JMI3(GO:0009897);A0A0B4J1U7(GO:0009897);P01764(GO:0009897);P00918(GO:0016323);P26038(GO:0016324;GO:0016323;GO:0031528);A0A0G2JMB2(GO:0009897);Q9Y5Y7(GO:0016021;GO:0005887);P61224(GO:0045121);P10643(GO:0005579);P02787(GO:0005905;GO:1990712;GO:0031232;GO:0009925;GO:0016324);P01833(GO:0016021;GO:0005887);P01834(GO:0009897);P04004(GO:0071062);O00391(GO:0030173;GO:0016021);A0A0C4DGZ8(GO:0016021);P35579(GO:0032154;GO:0008305;GO:0001772);G3V0E5(GO:0009897;GO:0005905;GO:1990712;GO:0016021;GO:0005887;GO:0016323);P07357(GO:0016021;GO:0005579);P19320(GO:0009897;GO:0016021;GO:0005887;GO:0071065);P07358(GO:0005579);P09172(GO:0016021);H0Y5E4(GO:0016021);P02671(GO:0009897);P02675(GO:0009897);P07996(GO:0009897);P13671(GO:0005579);P0CF74(GO:0009897);Q16880(GO:0016021);P05154(GO:0009897);I3L1J2(GO:0009897;GO:0016342;GO:0016021);A0A075B7F0(GO:0009897);P00734(GO:0009897);A0A0A0MS14(GO:0009897);A0A0A0MS15(GO:0009897);P13473(GO:0097637;GO:0016021;GO:0045121;GO:0098857);A0A286YEY4(GO:0016021);P01857(GO:0009897);A0A075B6Q5(GO:0009897);P43121(GO:0009897;GO:0016021);C9JB55(GO:0005905;GO:1990712;GO:0009925;GO:0016324;GO:0031232);P41222(GO:0005789);P14151(GO:0009897;GO:0016021;GO:0005887);C9JXI5(GO:0016021);P13796(GO:0032587;GO:0001891);P15144(GO:0009897;GO:0016021);A0A0A0MS09(GO:0016021);A0A0B4J231(GO:0009897);P02751(GO:0016324);A0A075B6R2(GO:0009897);Q9UNN8(GO:0016021;GO:0005887);A0A0B4J1X5(GO:0009897);H9KV75(GO:0016328;GO:0097381);A0A087WSY4(GO:0009897);A0A0B4J1X8(GO:0009897);P01877(GO:0009897);P01871(GO:0009897;GO:0016021);A0A075B7D0(GO:0009897);O75882(GO:0016021;GO:0005887);A0A075B7D8(GO:0009897);F8WF14(GO:0016021);P04114(GO:0005789);Q6EMK4(GO:0016021);P18065(GO:0016324);P15169(GO:0097060);P01860(GO:0009897);A0A075B7B8(GO:0009897);P01031(GO:0005579);P02748(GO:0016021;GO:0005887;GO:0005579);P08514(GO:0009897;GO:0016021;GO:0008305);O14791(GO:0031224);Q07954(GO:0005905;GO:0098797;GO:0016021;GO:0005887;GO:0016323);A0A0C4DH32(GO:0009897);A0A0C4DH33(GO:0009897);P01861(GO:0009897);A0A0C4DH31(GO:0009897);A0A0C4DH36(GO:0009897);A0A0C4DH34(GO:0009897);A0A0G2JI36(GO:0071556;GO:0042612;GO:0005887;GO:0016021);A0A087X1L8(GO:0016021);A0A0C4DH43(GO:0009897);A0A0C4DH38(GO:0009897);A0A0C4DH39(GO:0009897);P25311(GO:0009897);O95497(GO:0016021;GO:0031225);P00747(GO:0031232);P00742(GO:0031233);A0A669KAY4(GO:0031232);Q9HDC9(GO:0016021);A0A0C4DH29(GO:0009897);J3KPA1(GO:0016021);P33908(GO:0016021);A0A0B4J1V1(GO:0009897);A0A0B4J1V0(GO:0009897);P07360(GO:0005579);A0A0B4J1V2(GO:0009897);P07437(GO:0045121);P11021(GO:0005789;GO:0030176);Q9Y490(GO:0032587) | 5|51 | P07360(GO:0005579);C9JXI5(GO:0016021);I3L1J2(GO:0009897;GO:0016342;GO:0016021);A0A0C4DH36(GO:0009897);P02751(GO:0016324) | 0.00069545 | 0.027818002 | https://www.ebi.ac.uk/QuickGO/term/GO:0044425 |
| GO:0022610 | biological adhesion | biological_process | 36|375 | P08514(GO:0007160;GO:0070527;GO:0007155);P04275(GO:0031589;GO:0007155);Q86UX7(GO:0034446;GO:0007159;GO:0007155;GO:0070527);P05109(GO:0070488);P68871(GO:0070527);P19320(GO:0034113;GO:0050901;GO:0140039;GO:0098609;GO:0007159;GO:0007155;GO:0007157;GO:0007160);P25311(GO:0007155);P21333(GO:0070527);Q14520(GO:0007155);P14151(GO:0016339;GO:0050901;GO:0007155);O95497(GO:0098609);P02671(GO:0007160;GO:0070527);P02751(GO:0034446;GO:0007160;GO:0007161;GO:0007155);P02675(GO:0007160;GO:0070527);P02760(GO:0007155);A0A087X0S5(GO:0007155);Q08380(GO:0007155);Q15582(GO:0007155);P60709(GO:0070527);P26038(GO:0007159;GO:0070489);P07996(GO:0007155);P35858(GO:0007155);Q13201(GO:0007155);I3L1J2(GO:0016339;GO:0098609;GO:0007155;GO:0044331;GO:0007156);P06727(GO:0007159);P06702(GO:0070488);Q9Y5Y7(GO:0007160;GO:0007155);P18206(GO:0007160;GO:0007155;GO:0090136;GO:0070527);P35579(GO:0007155;GO:0098609;GO:0070527);A0A0A0MTH3(GO:0034446);H0Y5E4(GO:0007155);P04004(GO:0061302;GO:0007160;GO:0007155;GO:0033627);P43121(GO:0007155);G3XAP6(GO:0007155;GO:0070527);Q9Y490(GO:0007155;GO:0070527);A0A087WZB5(GO:0007155) | 5|51 | Q86UX7(GO:0034446;GO:0007159;GO:0007155;GO:0070527);I3L1J2(GO:0016339;GO:0098609;GO:0007155;GO:0044331;GO:0007156);P02751(GO:0034446;GO:0007160;GO:0007161;GO:0007155);P18206(GO:0007160;GO:0007155;GO:0090136;GO:0070527);P60709(GO:0070527) | 0.198675391 | 0.240818656 | https://www.ebi.ac.uk/QuickGO/term/GO:0022610 |
| GO:0098754 | detoxification | biological_process | 16|375 | P02768(GO:0098869);P69892(GO:0098869);P02649(GO:0098869);A0A087X1J7(GO:0098869);P04040(GO:0098869);P32119(GO:0019430);P00738(GO:0098869);O95445(GO:0098869);P06727(GO:0019430);P02100(GO:0098869);P30041(GO:0098869);P06702(GO:0098869);Q15166(GO:0010124);P69905(GO:0098869);P68871(GO:0098869);P02042(GO:0098869) | 5|51 | P30041(GO:0098869);P69905(GO:0098869);P32119(GO:0019430);A0A087X1J7(GO:0098869);P04040(GO:0098869) | 0.038748063 | 0.221417501 | https://www.ebi.ac.uk/QuickGO/term/GO:0098754 |
| GO:0005488 | binding | molecular_function | 317|375 | P08779(GO:0005515);P00488(GO:0046872);P68366(GO:0019899;GO:0000166;GO:0005525;GO:0019901;GO:0005515);P04275(GO:0051087;GO:0002020;GO:0042802;GO:0042803;GO:0005178;GO:0047485;GO:0019865;GO:0005515;GO:0005518);P02745(GO:0005515);O75882(GO:0030246);P03951(GO:0005515;GO:0008201;GO:0042802);P03950(GO:0003676;GO:0003677;GO:0042277;GO:0008201;GO:0003779;GO:0005507;GO:0042803;GO:0019843;GO:0005515;GO:0005102);P23142(GO:0008022;GO:0044877;GO:0005509;GO:0042802;GO:0070051;GO:0001968);P15814(GO:0003823;GO:0034987);P30041(GO:0042803;GO:0031625;GO:0045296;GO:0005515);B1AHL2(GO:0005509);P08571(GO:0071723;GO:0070891;GO:0001530;GO:0005515);P68871(GO:0046872;GO:0020037;GO:0031721;GO:0031720;GO:0019825;GO:0030492;GO:0043177;GO:0005515);P01877(GO:0034987;GO:0003823);E9PK25(GO:0003779;GO:0051015;GO:0005102);O75460(GO:0005161;GO:0019899;GO:0030544;GO:0000287;GO:0051082;GO:0000166;GO:0046872;GO:0051879;GO:0042802;GO:0042803;GO:0043531;GO:0005524;GO:0005515);Q86YZ3(GO:0046914;GO:0005509;GO:0046872);P01597(GO:0003823);P01042(GO:0008270;GO:0008201;GO:0005515;GO:0005102);A0A087X1J7(GO:0008430);P04264(GO:0030246;GO:0005515;GO:0046982);P01602(GO:0003823);A0A0G2JMI3(GO:0003823;GO:0034987);A0A0B4J1U7(GO:0003823;GO:0034987);P59665(GO:0042803);Q6EMK4(GO:0045296;GO:0005515;GO:0050431);G3XAP6(GO:0036122;GO:0005509;GO:0002020;GO:0005178;GO:0043395;GO:0008201;GO:0043394;GO:0005518);P02533(GO:1990254;GO:0005515);Q96KN2(GO:0046872);P06312(GO:0003823);P01743(GO:0003823);A0A0A0MT36(GO:0003823);P01009(GO:0002020;GO:0042802;GO:0005515);P02790(GO:0046872;GO:0005515);P60763(GO:0048306;GO:0000166;GO:0005525;GO:0019901;GO:0005515);A0A075B7D8(GO:0003823;GO:0034987);P01764(GO:0034987;GO:0003823);A0A087X0S5(GO:0048407);P01766(GO:0003823);P33908(GO:0005509);Q15582(GO:0050840;GO:0005178;GO:0050839;GO:0005515;GO:0005518);F8WF14(GO:0019899;GO:0001540;GO:0042802;GO:0033265);P0DOY3(GO:0003823);P02760(GO:0042803;GO:0005515;GO:0020037;GO:0046904;GO:0019862);P26038(GO:0019899;GO:0003779;GO:0005515;GO:0019901;GO:0050839;GO:0008092;GO:0003725;GO:0005102);P01782(GO:0003823);P18428(GO:0071723;GO:0008289;GO:0070891;GO:0001530;GO:0005515;GO:0005102);P01780(GO:0003823);P06331(GO:0003823);P02776(GO:0005125;GO:0008009;GO:0048248;GO:0008201;GO:0005515);P02775(GO:0005125;GO:0008009;GO:0008083;GO:0045236;GO:0005515);H3BTN5(GO:0030955;GO:0000287;GO:0005524);P0DP01(GO:0003823);E7EUT5(GO:0051287;GO:0097718;GO:0042802;GO:0008017;GO:0050661);A0A0G2JMB2(GO:0003823;GO:0034987);A0A0J9YXX1(GO:0003823);P0DP02(GO:0003823);P06702(GO:0050786;GO:0005509;GO:0046872;GO:0035662;GO:0008017;GO:0050544;GO:0005515;GO:0008270);Q9Y5Y7(GO:0005540;GO:0005515);P61224(GO:0044877;GO:0000166;GO:0005525;GO:0019003;GO:0005515);D6RF35(GO:0005499);P02787(GO:1990459;GO:0034986;GO:0046872;GO:0008198;GO:0008199;GO:0005515);P19827(GO:0005509);P35908(GO:0008092;GO:0005515);P01834(GO:0034987;GO:0003823);Q96IY4(GO:0008270;GO:0046872);P04003(GO:0003723;GO:0005515);P01008(GO:0002020;GO:0042802;GO:0008201;GO:0005515);P01718(GO:0003823);E7END6(GO:0005509);P04004(GO:0050840;GO:0030247;GO:0042802;GO:0005178;GO:0008201;GO:0005515;GO:0005518);P00748(GO:0005509;GO:0051787;GO:0005515);Q9UNN8(GO:0005515);O00391(GO:0071949);A6XND0(GO:0005520);K7ER74(GO:0008289;GO:0042803;GO:0043274);P69905(GO:0005506;GO:0046872;GO:0020037;GO:0031720;GO:0019825;GO:0005515);A0A0J9YX35(GO:0003823);G3XAK1(GO:0019899;GO:0030971);G3V0E5(GO:0042803;GO:0003725);P02768(GO:0015643;GO:0008144;GO:0030170;GO:0003677;GO:0051087;GO:0005504;GO:0005507;GO:0042802;GO:0140272;GO:0008289;GO:0019825;GO:0046872;GO:1903981;GO:0005515);O75636(GO:0003823;GO:0030246;GO:0005515;GO:0046872);Q96PD5(GO:0008270;GO:0046872;GO:0042834);Q86UX7(GO:0005178);P02763(GO:0005515);Q01518(GO:0008179;GO:0003779);A0A0C4DH73(GO:0003823);P02766(GO:0005179;GO:0042562;GO:0070324;GO:0005515;GO:0046982;GO:0042802);A0A2R8Y3M9(GO:0005509);Q9H4B7(GO:0000166;GO:0005525);P20742(GO:0002020);A0A0G2JL69(GO:0046872);P05546(GO:0008201);A0A0B4J2D9(GO:0003823);P19320(GO:0050839;GO:0005178);Q15166(GO:0046872;GO:0042803);P07358(GO:0044877);A0A0U1RQV3(GO:0005509);A0A182DWH7(GO:0008430);P09172(GO:0031418;GO:0005507;GO:0046872);P01703(GO:0003823);P01700(GO:0003823);P01701(GO:0003823);P01706(GO:0003823);P20851(GO:0005515);P21333(GO:0017160;GO:0031852;GO:0017048;GO:0034988;GO:0051020;GO:0044877;GO:0045296;GO:0001664;GO:0044325;GO:0003779;GO:0008134;GO:0042803;GO:0051015;GO:0019900;GO:0046332;GO:0031267;GO:0048365;GO:0003723;GO:0005515);H0Y5E4(GO:0005540);A0A0S2Z4L3(GO:0005509);P23083(GO:0003823);P01709(GO:0003823);P13671(GO:0005515);P55103(GO:0005179;GO:0005125;GO:0008083;GO:0005160);P02671(GO:0050839;GO:0046872;GO:0005515;GO:0005102);P02675(GO:0051087;GO:0050839;GO:0005515;GO:0005102);P07996(GO:0050840;GO:0030169;GO:0043236;GO:0001968;GO:0043394;GO:0070052;GO:0070051;GO:0050431;GO:0005509;GO:0001786;GO:0017134;GO:0008201;GO:0042802;GO:0005515;GO:0005178);P08519(GO:0005515;GO:0034185;GO:0008201;GO:0001968);A0A075B6I0(GO:0003823);P01619(GO:0003823);P0CF74(GO:0034987;GO:0003823);P61626(GO:0042802);I3L145(GO:0005496);P04180(GO:0005515;GO:0034186);P08697(GO:0002020;GO:0042803;GO:0005515);P22891(GO:0005509);Q92954(GO:0030247);A0A0C4DH67(GO:0003823);P05155(GO:0005515);P05154(GO:0032190;GO:0002020;GO:0031210;GO:0008201;GO:0005539;GO:0005515;GO:0001972);I3L1J2(GO:0044325;GO:0043184;GO:0005509;GO:0042803;GO:0008013;GO:0019903;GO:0005102;GO:0008092;GO:0045296);M0R0Q9(GO:0031715;GO:0005102);P01031(GO:0008009;GO:0005515;GO:0005102);O95445(GO:0005543);A0A0A0MS51(GO:0045159;GO:0005509;GO:0051015);A0A075B7F0(GO:0003823;GO:0034987);J3KRP0(GO:0046872);P13645(GO:0046982);A0A0A0MS14(GO:0003823;GO:0034987);P35579(GO:0003779;GO:0005524;GO:0042803;GO:0005178;GO:0051015;GO:0043531;GO:0005516;GO:0000166;GO:0043495;GO:0045296;GO:0003723;GO:0005515;GO:0019904);P13473(GO:0019899;GO:0019904;GO:0005515);P01717(GO:0003823);F5H8B0(GO:0005509);P48740(GO:0048306;GO:0046872;GO:0005509;GO:0005515;GO:0042803);Q14520(GO:0005539;GO:0005509);P00738(GO:0030492;GO:0005515);P00739(GO:0030492);E7EQB2(GO:0008201;GO:0005506;GO:0001530);Q9BWP8(GO:0030246;GO:0048029;GO:0003677;GO:0070492;GO:0120153;GO:0005509;GO:0042806;GO:0046872;GO:0005515;GO:0005537);P01019(GO:0005179;GO:0008083;GO:0048018;GO:0031701;GO:0031703;GO:0005515;GO:0031702);Q14624(GO:0005515);A0A0C4DH25(GO:0003823);P11226(GO:0048306;GO:0030246;GO:0005509;GO:0005515;GO:0005102;GO:0005537);P01011(GO:0003677;GO:0005515);H0Y2Y8(GO:0046872);C9JB55(GO:1990459;GO:0034986;GO:0008198;GO:0008199);C9JD84(GO:0005509);P41222(GO:0005504;GO:0005501;GO:0036094;GO:0005515);A0A0C4DH55(GO:0003823);A0A0B4J1Y8(GO:0003823);B0YIW2(GO:0005543;GO:0070653;GO:0008289;GO:0015485);J3KNB4(GO:0001530);C9J5S7(GO:0016018);P31151(GO:0050786;GO:0005509;GO:0046872;GO:0046914;GO:0005515;GO:0008270);Q8IV42(GO:0000049;GO:0000166;GO:0005524);P01721(GO:0003823);A0A0A0MTH3(GO:0005524;GO:0019901);O14791(GO:0008289;GO:0005515);P13796(GO:0051020;GO:0003779;GO:0005509;GO:0042802;GO:0005178;GO:0051015;GO:0046872);P15144(GO:0042277;GO:0046872;GO:0001618;GO:0008270);P36955(GO:0005515);C9JC84(GO:0005102);C9JPQ9(GO:0005102);P09486(GO:0050840;GO:0046872;GO:0005509;GO:0005515;GO:0005518);A0A0B4J231(GO:0003823;GO:0034987);P18206(GO:0002162;GO:0003779;GO:0051015;GO:0031625;GO:0008013;GO:0045294;GO:0045296;GO:0005515);P0DPH7(GO:0000166;GO:0005525);P80748(GO:0003823);Q13093(GO:0005543);P14151(GO:0030246;GO:0070492;GO:0043208;GO:0005509;GO:0002020;GO:0046872;GO:0050839;GO:0008201;GO:0005515);O00187(GO:0046872;GO:0048306;GO:0005509;GO:0005515;GO:0001855);A0A2R8Y6G6(GO:0046872;GO:0000287);A0A075B6K2(GO:0003823);E7EX29(GO:0019904);P55058(GO:0019992;GO:0097001;GO:0070300;GO:0008289;GO:0031210;GO:0008429;GO:1901611);B4E1Z4(GO:0001848);P02751(GO:0008022;GO:0005518;GO:0097718;GO:0051087;GO:0008201;GO:0002020;GO:0042802;GO:0005178;GO:0043394;GO:0019899;GO:0005515;GO:0005102);A0A075B6R2(GO:0003823;GO:0034987);P02753(GO:0016918;GO:0005501;GO:0019841;GO:0005515;GO:0046982);Q03591(GO:0005515;GO:0046982;GO:0042803);P02100(GO:0019825;GO:0020037;GO:0043177;GO:0031721;GO:0005515;GO:0046872);A0A2R8Y7X9(GO:0019825;GO:0020037;GO:0046872);P68032(GO:0017022;GO:0000166;GO:0005524;GO:0043531);P00734(GO:0005102;GO:0001530;GO:0005509;GO:0008201;GO:0008083;GO:0005515);P69892(GO:0019825;GO:0020037;GO:0043177;GO:0046872);H9KV75(GO:0019894;GO:0032029;GO:0017166;GO:0030507;GO:0045505;GO:0003779;GO:0005509;GO:0042803;GO:0034452;GO:0051393;GO:0051015);A0A075B6K5(GO:0003823);A0A075B6J9(GO:0003823);P43652(GO:0008431;GO:0005515;GO:0046872);P08603(GO:0005515;GO:0043395;GO:0008201);A0A669KAY4(GO:0030169;GO:0034190;GO:0070326;GO:0034189;GO:0034185;GO:0043621;GO:0050750;GO:0003723);P01344(GO:0008083;GO:0005179;GO:0005178;GO:0005159;GO:0005158;GO:0048018;GO:0005515);P27169(GO:0046872;GO:0005543;GO:0005509;GO:0042803);P08185(GO:0008289;GO:0005496);A0A0A0MS15(GO:0003823;GO:0034987);Q16610(GO:0008022;GO:0019899;GO:0005134;GO:0002020;GO:0043236;GO:0005515);A0A075B7D0(GO:0003823;GO:0034987);P01715(GO:0003823);P09871(GO:0005509;GO:0005515;GO:0046872;GO:0042802);A0A2R8Y619(GO:0003677;GO:0046982);P00918(GO:0005515;GO:0046872;GO:0008270);A0A0C4DH32(GO:0003823;GO:0034987);A0A0B4J1X5(GO:0003823;GO:0034987);P01704(GO:0003823);A0A0A0MRJ7(GO:0046872;GO:0005507);P04040(GO:0019899;GO:0050661;GO:0046872;GO:0042802;GO:0042803;GO:0020037;GO:0005102);A0A0C4DH33(GO:0003823;GO:0034987);A0A3B3ISR2(GO:0005509);A0A0B4J1U3(GO:0003823);P13647(GO:0097110;GO:0005515);P18065(GO:0019838;GO:0005520;GO:0031994;GO:0031995;GO:0005515;GO:0005102);P00915(GO:0046872;GO:0008270;GO:0005515);A0A075B6S5(GO:0003823);Q562R1(GO:0000166;GO:0005515;GO:0005524);P02743(GO:0030246;GO:0051082;GO:0001849;GO:0005509;GO:0030169;GO:0042802;GO:0046872;GO:0046790);A0A075B6K4(GO:0003823);P02741(GO:0050750;GO:0001849;GO:0005509;GO:0046872;GO:0042802;GO:0030169;GO:0046790;GO:0033265;GO:0005515);P01860(GO:0034987;GO:0003823);P02747(GO:0005515);D6RE82(GO:0003723);P04211(GO:0003823);A0A075B7B8(GO:0003823;GO:0034987);P07357(GO:0001848;GO:0044877);P02749(GO:0005543;GO:0042802;GO:0008289;GO:0008201;GO:0005515);P01034(GO:0001540;GO:0002020;GO:0042802;GO:0005515);A0A087WZB5(GO:0003779);A0A087WSZ0(GO:0003823);P08514(GO:0050840;GO:0046872;GO:0042802;GO:0070051;GO:0005515);P11597(GO:0017129;GO:0008289;GO:0031210;GO:0015485);P02649(GO:0008201;GO:0071813;GO:0015485;GO:0044877;GO:0048156;GO:0046983;GO:0005543;GO:0001540;GO:0042802;GO:0042803;GO:0008289;GO:0005515;GO:0070326;GO:0046911;GO:0050750);P37802(GO:0045296;GO:0005515);Q07954(GO:0044877;GO:0001540;GO:0005509;GO:0002020;GO:0070325;GO:0032050;GO:0043395;GO:0034185;GO:0046872;GO:0003723;GO:0005515);P04196(GO:0008270;GO:0046872;GO:0020037;GO:0043395;GO:0008201;GO:0019865;GO:0005515;GO:0005102);P32119(GO:0005515);P02647(GO:0034191;GO:0034190;GO:0005543;GO:0015485;GO:0045499;GO:0031072;GO:0019899;GO:0001540;GO:0042802;GO:0008035;GO:0008289;GO:0071813;GO:0005515;GO:0031210;GO:0070653);P01861(GO:0034987;GO:0003823);P10909(GO:0051787;GO:0044877;GO:0048156;GO:0051082;GO:0001540;GO:0051087;GO:0031625;GO:0005515;GO:0050750);P04114(GO:0050750;GO:0005543;GO:0008289;GO:0008201;GO:0035473;GO:0005515);P05109(GO:0050786;GO:0008270;GO:0005509;GO:0046872;GO:0035662;GO:0008017;GO:0050544;GO:0005515);P15169(GO:0008270;GO:0046872);A0A0C4DH31(GO:0003823;GO:0034987);A0A0C4DH36(GO:0003823;GO:0034987);A0A0C4DH34(GO:0003823;GO:0034987);A0A0G2JI36(GO:0030881;GO:0046977;GO:0042605);H0YAC1(GO:0005506;GO:0046872;GO:0020037);A0A0C4DH43(GO:0003823;GO:0034987);A0A0C4DH38(GO:0003823;GO:0034987);A0A0C4DH39(GO:0003823;GO:0034987);P25311(GO:0005515);P01599(GO:0003823);A0A096LPE2(GO:0042056);P01857(GO:0034987;GO:0003823;GO:0005515);P01024(GO:0031715;GO:0005515;GO:0005102);P01023(GO:0019959;GO:0019899;GO:0048306;GO:0019838;GO:0002020;GO:0019966;GO:0043120;GO:0005515;GO:0005102);C9J8S2(GO:0005102);P01601(GO:0003823);A0A0C4DH24(GO:0003823);Q9Y6R7(GO:0005515);P02538(GO:0005515);P00747(GO:1904854;GO:0019899;GO:0051087;GO:1990405;GO:0019900;GO:0019904;GO:0034185;GO:0005515;GO:0005102);P00740(GO:0005509;GO:0005515;GO:0046872);P60709(GO:0019894;GO:0030957;GO:0005524;GO:0000980;GO:0000166;GO:0019901;GO:0050998;GO:0031492;GO:0042802;GO:0005515;GO:0000978);P00742(GO:0005543;GO:0005509;GO:0005515);A0A075B6Q5(GO:0003823;GO:0034987);A0A0A0MRZ9(GO:0003823);P01742(GO:0003823);P00450(GO:0046872;GO:0051087;GO:0005507);Q08380(GO:0005515);P16930(GO:0005515;GO:0046872);P0C0L5(GO:0030246;GO:0001848);P0C0L4(GO:0001849);C9JF17(GO:0008289;GO:0015485);A0A087WSY4(GO:0003823;GO:0034987);P02652(GO:0005543;GO:0031072;GO:0034190;GO:0008035;GO:0008289;GO:0031210;GO:0046982;GO:0015485;GO:0005515;GO:0070653;GO:0042803);P35858(GO:0005520);Q15485(GO:0005515;GO:0048306;GO:0030246;GO:2001065;GO:0043394;GO:0003823;GO:0046872);P01763(GO:0003823);E9PHK0(GO:0036143;GO:0030246;GO:0008201;GO:0005509);P04433(GO:0003823);A0A087WSY6(GO:0003823);P06727(GO:0042802;GO:0008289;GO:0031210;GO:0005507;GO:0015485;GO:0005515;GO:0042803);P04430(GO:0003823);A0A0C4DH29(GO:0003823;GO:0034987);P0DJI8(GO:0042056;GO:0001664;GO:0008201);Q5SRP5(GO:0005543);A0A0B4J1X8(GO:0003823;GO:0034987);P01817(GO:0003823);P01814(GO:0003823);P02750(GO:0005160;GO:0005515);P02042(GO:0019825;GO:0020037;GO:0043177;GO:0031721;GO:0005515;GO:0046872);P51884(GO:0005515;GO:0005518);A0A0B4J1V1(GO:0003823;GO:0034987);A0A0B4J1V0(GO:0003823;GO:0034987);P07360(GO:0036094;GO:0019841;GO:0001848;GO:0044877);A0A0B4J1V2(GO:0003823;GO:0034987);Q13201(GO:0005509;GO:0005515);Q5T749(GO:0005515);P07437(GO:0032794;GO:0044877;GO:0000166;GO:0005525;GO:0042288;GO:0031625;GO:0019904;GO:0005515);Q96HR3(GO:0005515;GO:0046966;GO:0042809);Q13790(GO:0005102;GO:0015485);P11021(GO:0051087;GO:0019899;GO:0045296;GO:0019904;GO:0000166;GO:0031625;GO:0005509;GO:0043022;GO:0005524;GO:0051082;GO:0051787;GO:0005515);Q9Y490(GO:0017166;GO:0003779;GO:0001786;GO:0005178;GO:0005515;GO:0051015;GO:0030274;GO:0035091;GO:0045296;GO:0044877);P01705(GO:0003823);Q15848(GO:0005125;GO:0033691;GO:0042802;GO:0042803;GO:0005515;GO:0005102;GO:0005179);A0A0J9YVY3(GO:0003823);P01699(GO:0003823);P07737(GO:0017048;GO:0005546;GO:0003779;GO:0005515;GO:0070064;GO:0000774;GO:0045296;GO:0003723;GO:0005102;GO:0003785);P01871(GO:0042834;GO:0034987;GO:0031210;GO:0003823;GO:0003697;GO:0005515) | 43|51 | O75636(GO:0003823;GO:0030246;GO:0005515;GO:0046872);P80748(GO:0003823);A0A0B4J1Y8(GO:0003823);Q86UX7(GO:0005178);A0A0C4DH73(GO:0003823);P00739(GO:0030492);Q9H4B7(GO:0000166;GO:0005525);A0A0J9YX35(GO:0003823);A0A0C4DH36(GO:0003823;GO:0034987);P37802(GO:0045296;GO:0005515);A0A182DWH7(GO:0008430);C9JPQ9(GO:0005102);A0A087X1J7(GO:0008430);P32119(GO:0005515);A0A2R8Y3M9(GO:0005509);A0A075B6K2(GO:0003823);E7EX29(GO:0019904);P55058(GO:0019992;GO:0097001;GO:0070300;GO:0008289;GO:0031210;GO:0008429;GO:1901611);P02751(GO:0008022;GO:0005518;GO:0097718;GO:0051087;GO:0008201;GO:0002020;GO:0042802;GO:0005178;GO:0043394;GO:0019899;GO:0005515;GO:0005102);P60709(GO:0019894;GO:0030957;GO:0005524;GO:0000980;GO:0000166;GO:0019901;GO:0050998;GO:0031492;GO:0042802;GO:0005515;GO:0000978);P03950(GO:0003676;GO:0003677;GO:0042277;GO:0008201;GO:0003779;GO:0005507;GO:0042803;GO:0019843;GO:0005515;GO:0005102);H3BTN5(GO:0030955;GO:0000287;GO:0005524);Q16610(GO:0008022;GO:0019899;GO:0005134;GO:0002020;GO:0043236;GO:0005515);Q14624(GO:0005515);P0DOY3(GO:0003823);I3L1J2(GO:0044325;GO:0043184;GO:0005509;GO:0042803;GO:0008013;GO:0019903;GO:0005102;GO:0008092;GO:0045296);P30041(GO:0042803;GO:0031625;GO:0045296;GO:0005515);Q5SRP5(GO:0005543);P18206(GO:0002162;GO:0003779;GO:0051015;GO:0031625;GO:0008013;GO:0045294;GO:0045296;GO:0005515);P13647(GO:0097110;GO:0005515);P01715(GO:0003823);P04040(GO:0019899;GO:0050661;GO:0046872;GO:0042802;GO:0042803;GO:0020037;GO:0005102);P01717(GO:0003823);D6RE82(GO:0003723);P03951(GO:0005515;GO:0008201;GO:0042802);P07360(GO:0036094;GO:0019841;GO:0001848;GO:0044877);P00915(GO:0046872;GO:0008270;GO:0005515);P68032(GO:0017022;GO:0000166;GO:0005524;GO:0043531);Q96HR3(GO:0005515;GO:0046966;GO:0042809);P02747(GO:0005515);A0A075B6J9(GO:0003823);P69905(GO:0005506;GO:0046872;GO:0020037;GO:0031720;GO:0019825;GO:0005515);P07737(GO:0017048;GO:0005546;GO:0003779;GO:0005515;GO:0070064;GO:0000774;GO:0045296;GO:0003723;GO:0005102;GO:0003785) | 0.163428855 | 0.225419111 | https://www.ebi.ac.uk/QuickGO/term/GO:0005488 |
| GO:0044421 | extracellular region part | cellular_component | 297|375 | P08779(GO:0070062);P00488(GO:0072562;GO:0062023);P68366(GO:0070062);P04275(GO:0031012;GO:0070062;GO:0062023);A0A0U1RQV3(GO:0062023);J3KNB4(GO:0005615);Q9UHG3(GO:0034361;GO:0070062);P51884(GO:0031012;GO:0070062;GO:0005583;GO:0062023;GO:0005615);P03950(GO:0005615;GO:0005604;GO:0032311);A0A0A0MRZ9(GO:0005615);P23142(GO:0031012;GO:0005615;GO:0062023;GO:0071953;GO:0070062;GO:0005604;GO:0005577);P15814(GO:0042571;GO:0072562);P41222(GO:0070062;GO:0005615);P08571(GO:0005615;GO:0070062);P68871(GO:0005615;GO:0072562;GO:0070062);P22792(GO:0070062;GO:0072562);Q86YZ3(GO:0070062;GO:0062023);P35527(GO:0005615;GO:0070062);P01042(GO:0005615;GO:0072562;GO:0062023;GO:0070062);P01597(GO:0072562;GO:0070062);P43251(GO:0005615;GO:0070062);Q14520(GO:0005615);P01602(GO:0072562;GO:0070062);A0A0G2JMI3(GO:0072562;GO:0042571);A0A0B4J1U7(GO:0072562;GO:0042571);A0A669KAY4(GO:0005615);P59665(GO:0005615;GO:0062023;GO:0070062);P15169(GO:0005615);P0DJI8(GO:0034364;GO:0005615;GO:0070062);P03951(GO:0070062;GO:0005615);P06312(GO:0072562);P43121(GO:0005615);A0A0A0MT36(GO:0005615);P01009(GO:0005615;GO:0070062);P02790(GO:0005615;GO:0072562;GO:0062023;GO:0070062);P60763(GO:0070062);P01764(GO:0042571;GO:0005615;GO:0072562;GO:0070062);A0A087X0S5(GO:0062023;GO:0005615;GO:0031012;GO:0070062);P01766(GO:0005615;GO:0072562);Q15582(GO:0031012;GO:0070062;GO:0062023;GO:0005615;GO:0005604);F8WF14(GO:0072562);C9JB55(GO:0005615);P0DOY3(GO:0005615;GO:0072562;GO:0070062);P26038(GO:0070062;GO:0072562;GO:0005615);P01782(GO:0070062);P18428(GO:0070062;GO:0005615);P01780(GO:0072562;GO:0070062);A0A140T8Y3(GO:0062023;GO:0031012);P02776(GO:0020005;GO:0062023;GO:0005615);P02775(GO:0005615);Q13201(GO:0031012;GO:0062023);G3XAK1(GO:0005615;GO:0062023);P01008(GO:0070062;GO:0072562;GO:0062023;GO:0005615);A0A0G2JMB2(GO:0042571;GO:0072562);P30041(GO:0005615;GO:0070062);P06702(GO:0070062;GO:0062023;GO:0005615);Q9Y5Y7(GO:0070062);P61224(GO:0070062);D6RF35(GO:0005615);P10643(GO:0070062);P02787(GO:0005615;GO:0070062;GO:0072562);P19827(GO:0070062;GO:0072562);P01833(GO:0005615;GO:0070062);P01834(GO:0042571;GO:0005615;GO:0072562;GO:0070062);P19823(GO:0070062;GO:0072562;GO:0062023);Q96IY4(GO:0005615;GO:0070062);P04003(GO:0005615;GO:0072562);P04004(GO:0031012;GO:0070062;GO:0072562;GO:0062023;GO:0005615;GO:0005604);P00450(GO:0005615;GO:0070062;GO:0072562);P20851(GO:0005615);Q92954(GO:0062023);Q6UXB8(GO:0005615);O00391(GO:0045171;GO:0070062;GO:0005615);P13796(GO:0005615;GO:0070062);P69905(GO:0005615;GO:0072562;GO:0070062);A0A075B6H7(GO:0005615);A0A087WSZ0(GO:0005615);Q9Y6R7(GO:0070062);P02768(GO:0070062;GO:0072562;GO:0005615);P08697(GO:0005615;GO:0072562;GO:0070062;GO:0005577);Q96PD5(GO:0005615;GO:0070062);P02760(GO:0070062;GO:0072562;GO:0062023;GO:0005615);P02763(GO:0005615;GO:0072562;GO:0062023;GO:0070062);Q01518(GO:0070062);A0A0C4DH73(GO:0005615);P02766(GO:0070062;GO:0005615);C9JC84(GO:0005577);Q9H4B7(GO:0070062);Q9BWP8(GO:0005615);P07357(GO:0070062;GO:0072562;GO:0005615);A0A075B6S5(GO:0005615);P37802(GO:0070062);A0A0G2JI36(GO:0070062);Q15166(GO:0070062;GO:0005615);J3QT83(GO:0005615;GO:0062023;GO:0031012);P29622(GO:0005615;GO:0070062);P35579(GO:0070062);Q08830(GO:0005577);P01700(GO:0072562);P01701(GO:0070062);Q86UX7(GO:0070062);P01704(GO:0070062);Q8N1N4(GO:0005615;GO:0070062);P02741(GO:0005615);P02671(GO:0005615;GO:0072562;GO:1903561;GO:0070062;GO:0005577);P02675(GO:1903561;GO:0005615;GO:0072562;GO:0070062;GO:0005577);P07996(GO:0031012;GO:0005615;GO:0062023;GO:0070062;GO:0005577);P13671(GO:0070062);A0A075B6I0(GO:0005615);P01619(GO:0005615;GO:0072562;GO:0071748;GO:0070062;GO:0071751;GO:0071756);P0CF74(GO:0042571;GO:0005615;GO:0072562);P61626(GO:0070062;GO:0005615);P04217(GO:0070062;GO:0072562;GO:0062023;GO:0005615);P04180(GO:0070062;GO:0034364;GO:0005615);P31151(GO:0005615;GO:0062023);E9PHK0(GO:0070062;GO:0005615;GO:0062023);P22891(GO:0005615;GO:0070062);Q06033(GO:0070062);C9JF17(GO:0070062;GO:0005615);P00739(GO:0070062;GO:0034366;GO:0072562;GO:0005615);P05155(GO:0005615;GO:0072562;GO:0070062);P05154(GO:0005615;GO:0070062);P27169(GO:0070062;GO:0005615;GO:0072562;GO:0034364;GO:0034366);P20742(GO:0070062;GO:0072562;GO:0005615);A0A0B4J2D9(GO:0005615);O95445(GO:0005615;GO:0034361;GO:0034362;GO:0034364;GO:0034365;GO:0034366);A0A0A0MS51(GO:0005615);A0A075B7F0(GO:0042571;GO:0072562);Q13103(GO:0062023);A0A087X1J7(GO:0005615);P00734(GO:0005615;GO:0070062;GO:0072562);P13645(GO:0070062;GO:0005615);A0A0A0MS14(GO:0072562;GO:0042571);A0A0A0MS15(GO:0072562;GO:0042571);P13473(GO:0005615;GO:0070062);P01717(GO:0072562);A0A075B6J9(GO:0005615);P48740(GO:0005615);P00738(GO:0005615;GO:0072562;GO:0070062);P01857(GO:0042571;GO:0005615;GO:0072562;GO:0070062);M0R0Q9(GO:0070062;GO:0005615;GO:0072562);A0A075B6Q5(GO:0072562;GO:0042571);P80748(GO:0072562;GO:0070062);G3XAP6(GO:0062023;GO:0005615;GO:0031012;GO:0070062);A0A0C4DH25(GO:0005615);P08185(GO:0005615;GO:0070062);P01019(GO:0072562;GO:0062023;GO:0070062;GO:0005615);P09871(GO:0072562);G3V0E5(GO:0070062);P19652(GO:0005615;GO:0072562;GO:0062023;GO:0070062);A0A0C4DH55(GO:0005615);K7ERG9(GO:0005615);A0A0B4J1Y8(GO:0005615);B0YIW2(GO:0042627;GO:0070062;GO:0062023;GO:0034361;GO:0034363;GO:0034366;GO:0005615);O75636(GO:0072562);Q9UGM5(GO:0005615;GO:0070062);G3V2W1(GO:0005615);O14791(GO:0005615;GO:0072562;GO:0034361;GO:0034364);K7ER74(GO:0042627;GO:0005615;GO:0034361;GO:0034362;GO:0034363;GO:0034366);P15144(GO:0070062;GO:0005615);P36955(GO:0005615;GO:0062023;GO:0070062;GO:0005604);A0A0B4J1U3(GO:0005615);A0A0G2JPR0(GO:0005615);C9JPQ9(GO:0005577);P09486(GO:0005615;GO:0062023;GO:0031012;GO:0005604);P18206(GO:0070062;GO:1903561);Q13093(GO:0034362;GO:0005615);P32119(GO:0070062);O00187(GO:0005615;GO:0070062);A0A0B4J231(GO:0070062;GO:0042571);A0A075B6K2(GO:0005615);P16930(GO:0070062);B4E1Z4(GO:0070062;GO:0072562;GO:0005615);P02751(GO:0062023;GO:0070062;GO:0072562;GO:0005615;GO:0005604;GO:0031012;GO:0005577);A0A075B6R2(GO:0072562;GO:0042571);Q9UNN8(GO:0070062;GO:0005615);Q03591(GO:0005615;GO:0072562);P02750(GO:0070062;GO:0005615);P55058(GO:0005615;GO:0034364);P55056(GO:0034361;GO:0034364);E9PK25(GO:0070062;GO:0005615);P80108(GO:0031012;GO:0070062;GO:0005615);A0A0G2JRQ6(GO:0005615);A0A0B4J1X5(GO:0072562;GO:0042571);P0C0L5(GO:0005615;GO:0072562;GO:0070062);A0A0C4DH43(GO:0072562;GO:0042571);A0A075B6K5(GO:0005615);P13647(GO:0070062);P43652(GO:0005615;GO:0072562;GO:0070062);A0A087WWT3(GO:0005615);P02753(GO:0005615;GO:0070062);A0A087WSY4(GO:0072562;GO:0042571);P01344(GO:0005615);P02647(GO:0034361;GO:0034362;GO:0034363;GO:0034364;GO:0034366;GO:0042627;GO:0072562;GO:0062023;GO:0070062;GO:0034365;GO:0005615;GO:1903561);P02042(GO:0072562);P11226(GO:0005615);P01877(GO:0005615;GO:0072562;GO:0071748;GO:0070062;GO:0071752;GO:0071751);P01871(GO:0005615;GO:0072562;GO:0070062;GO:0071756;GO:0071757);A0A075B7D0(GO:0042571;GO:0072562);O75882(GO:0005615;GO:0070062);A0A075B7D8(GO:0042571;GO:0072562);P00918(GO:0005615;GO:0070062);A0A0C4DH32(GO:0072562;GO:0042571);A0A075B6S9(GO:0005615);Q6EMK4(GO:0070062;GO:0005615;GO:0031012);A0A0A0MRJ7(GO:1903561;GO:0005615);P04040(GO:0070062;GO:0005615);P05109(GO:0005615;GO:0070062);A0A3B3ISR2(GO:0005615);P05543(GO:0005615;GO:0070062);P18065(GO:0005615;GO:0070062);P00915(GO:0070062);P05546(GO:0005615;GO:0070062);Q562R1(GO:0005615;GO:0070062);P02743(GO:0072562;GO:0070062;GO:0005615;GO:0062023);A0A075B6K4(GO:0005615);P55103(GO:0005615);P01860(GO:0042571;GO:0005615;GO:0072562;GO:0070062);P02747(GO:0072562;GO:0005615);P02745(GO:0005602);A0A0B4J1V0(GO:0072562;GO:0042571);P01031(GO:0005615;GO:0070062);P02749(GO:0042627;GO:0070062;GO:0034361;GO:0034364;GO:0062023;GO:0005615);P68032(GO:0070062;GO:0072562;GO:0005615);P01034(GO:0005615;GO:0070062;GO:0005604);P01601(GO:0005615);P08514(GO:0070062;GO:0072562);P11597(GO:0034364;GO:0070062;GO:0005615);P02649(GO:0034361;GO:0034362;GO:0034363;GO:0034364;GO:0042627;GO:0072562;GO:0062023;GO:0031012;GO:0070062;GO:0034365;GO:0005615;GO:1903561);P04196(GO:0070062;GO:0072562;GO:0062023);P08519(GO:0034358);P10909(GO:0034366;GO:0072562;GO:0031012;GO:0070062;GO:0005615;GO:0062023);P04114(GO:0034360;GO:0034359;GO:0005615;GO:0070062;GO:0034361;GO:0034362;GO:0034363;GO:0042627);A0A0C4DH33(GO:0072562;GO:0042571);P01861(GO:0042571;GO:0005615;GO:0072562;GO:0070062);A0A0C4DH31(GO:0072562;GO:0042571);A0A0C4DH36(GO:0042571;GO:0072562);A0A0C4DH34(GO:0072562;GO:0042571);P19320(GO:0005615;GO:0070062);H0YAC1(GO:0070062;GO:0005615);Q14624(GO:0072562;GO:0070062);A0A0C4DH38(GO:0072562;GO:0042571);A0A0C4DH39(GO:0072562;GO:0042571);P25311(GO:0070062;GO:0005615;GO:0062023);P01599(GO:0072562;GO:0070062);O43866(GO:0005615;GO:0072562);P07358(GO:0005615;GO:1903561;GO:0070062);P01023(GO:0072562;GO:0005615;GO:0070062);C9J8S2(GO:0005615;GO:0062023);P02748(GO:0070062;GO:0072562;GO:0005615);A0A0C4DH24(GO:0005615);P09172(GO:0005615);P02538(GO:0070062);P00747(GO:0070062;GO:0072562;GO:0062023;GO:0005615);P00740(GO:0005615;GO:0070062);P60709(GO:0005615;GO:0070062;GO:0072562);P00742(GO:0005615);E7EQB2(GO:0005615);P02533(GO:0070062);P00748(GO:0070062;GO:0062023;GO:0005615);Q08380(GO:0070062;GO:0072562;GO:0062023;GO:0005615);Q16610(GO:0031012;GO:0005615;GO:0062023;GO:0070062);P08603(GO:0070062;GO:0072562;GO:0005615);A0A075B6R9(GO:0005615);P04264(GO:0031012;GO:0070062;GO:0072562;GO:0062023;GO:0005615);P0C0L4(GO:0005615;GO:0072562;GO:0070062);C9JV77(GO:0070062;GO:0072562;GO:0062023;GO:0005615);P02652(GO:0042627;GO:0005615;GO:0072562;GO:0034361;GO:0070062;GO:0034364;GO:0034366);P35858(GO:0005615;GO:0031012;GO:0042567;GO:0070062);Q15485(GO:0072562;GO:0070062);P69892(GO:0072562);D6RAR4(GO:0005615);A0A096LPE2(GO:0034364;GO:0005615;GO:0070062);P01024(GO:0070062;GO:0005615;GO:0072562);P04433(GO:0072562;GO:0070062);A0A087WSY6(GO:0005615);P06727(GO:0042627;GO:0005615;GO:0072562;GO:0034361;GO:0034364;GO:0062023;GO:0070062);Q9NZP8(GO:0005615;GO:0070062);A0A0C4DH29(GO:0072562;GO:0042571);Q5SRP5(GO:0034361;GO:0034365;GO:0034366;GO:0034362;GO:0034364);A0A0B4J1X8(GO:0072562;GO:0042571);P02100(GO:0072562);P33908(GO:0070062);Q15848(GO:0005615);P21333(GO:0070062);A0A0B4J1V1(GO:0072562;GO:0042571);A0A075B7B8(GO:0042571;GO:0072562);P07360(GO:0070062;GO:0072562;GO:0005615);A0A0B4J1V2(GO:0072562;GO:0042571);Q5T749(GO:0070062);P07437(GO:0070062);Q13790(GO:0034362;GO:0034364;GO:0005615);P11021(GO:0070062);Q9Y490(GO:0070062);P01011(GO:0070062;GO:0072562;GO:0062023;GO:0005615);P07737(GO:0070062;GO:0072562);P35908(GO:0070062;GO:0005615) | 40|51 | O75636(GO:0072562);P80748(GO:0072562;GO:0070062);A0A0B4J1Y8(GO:0005615);P03950(GO:0005615;GO:0005604;GO:0032311);A0A0C4DH73(GO:0005615);Q9UGM5(GO:0005615;GO:0070062);P00915(GO:0070062);Q9H4B7(GO:0070062);A0A0C4DH36(GO:0042571;GO:0072562);P37802(GO:0070062);Q86UX7(GO:0070062);C9JPQ9(GO:0005577);P35527(GO:0005615;GO:0070062);A0A087X1J7(GO:0005615);A0A075B6K2(GO:0005615);P32119(GO:0070062);P03951(GO:0070062;GO:0005615);P55058(GO:0005615;GO:0034364);P02751(GO:0062023;GO:0070062;GO:0072562;GO:0005615;GO:0005604;GO:0031012;GO:0005577);P60709(GO:0005615;GO:0070062;GO:0072562);K7ERG9(GO:0005615);A0A075B6R9(GO:0005615);Q16610(GO:0031012;GO:0005615;GO:0062023;GO:0070062);Q14624(GO:0072562;GO:0070062);P0DOY3(GO:0005615;GO:0072562;GO:0070062);P29622(GO:0005615;GO:0070062);P30041(GO:0005615;GO:0070062);Q5SRP5(GO:0034361;GO:0034365;GO:0034366;GO:0034362;GO:0034364);P18206(GO:0070062;GO:1903561);P13647(GO:0070062);P19823(GO:0070062;GO:0072562;GO:0062023);P04040(GO:0070062;GO:0005615);P07360(GO:0070062;GO:0072562;GO:0005615);P00739(GO:0070062;GO:0034366;GO:0072562;GO:0005615);P68032(GO:0070062;GO:0072562;GO:0005615);P02747(GO:0072562;GO:0005615);A0A075B6J9(GO:0005615);P69905(GO:0005615;GO:0072562;GO:0070062);P01717(GO:0072562);P07737(GO:0070062;GO:0072562) | 0.143721134 | 0.205315906 | https://www.ebi.ac.uk/QuickGO/term/GO:0044421 |
| GO:0051704 | multi-organism process | biological_process | 90|375 | J3KNB4(GO:0042742;GO:0050829;GO:0061844;GO:0051873;GO:0050830);K7ERG9(GO:0009617);P23142(GO:0016032);P15814(GO:0042742);P08571(GO:0009617);E9PK25(GO:0009615;GO:0044794);A0A0G2JMI3(GO:0042742);A0A0B4J1U7(GO:0042742);P59665(GO:0019730;GO:0019731;GO:0051673;GO:0042742;GO:0050829;GO:0061844;GO:0051852;GO:0044657;GO:0050830;GO:0050832;GO:0051607;GO:0031640;GO:0042832);P02790(GO:0016032);P01764(GO:0042742);P26038(GO:0016032);P18428(GO:0042742;GO:0050829;GO:0050830);P02776(GO:0061844;GO:0051873;GO:0031640;GO:0042832);P02775(GO:0042742;GO:0061844;GO:0031640);E7EUT5(GO:0061844;GO:0051873;GO:0050832;GO:0052501);A0A0G2JMB2(GO:0042742);P06702(GO:0019730;GO:0042742;GO:0061844;GO:0050832;GO:0035821);P02787(GO:0009617);P01834(GO:0042742);P02768(GO:0019836);O75636(GO:0051607);Q96PD5(GO:0019730;GO:0044117;GO:0016045;GO:0050830);P02760(GO:0007565;GO:0016032);A0A0G2JI36(GO:0016045;GO:0016032);P02671(GO:0043152);P02675(GO:0043152);P0CF74(GO:0042742);P61626(GO:0019730;GO:0042742;GO:0050829;GO:0050830;GO:0031640);P20742(GO:0007565);A0A075B7F0(GO:0042742);P00734(GO:0061844;GO:0051838;GO:0070945);A0A0A0MS14(GO:0042742);A0A0A0MS15(GO:0042742);P00738(GO:0042742);P01857(GO:0042742);E7EQB2(GO:0019732;GO:0019731;GO:0051673;GO:0050829;GO:0061844;GO:0044793;GO:0031640);Q9BWP8(GO:0019730);P01019(GO:0007565);P11226(GO:0042742;GO:0051873;GO:0050830);M0R0Q9(GO:0009617);P31151(GO:0019730;GO:0050829;GO:0061844);O14791(GO:0031640);P15144(GO:0046718;GO:0016032);A0A0B4J231(GO:0042742);P02750(GO:0009617);P02751(GO:0051702;GO:0052047);A0A075B6R2(GO:0042742);A0A0B4J1X5(GO:0042742);A0A0C4DH43(GO:0042742);P08603(GO:0016032);A0A087WSY4(GO:0042742);A0A0B4J1X8(GO:0042742);P01877(GO:0019731);P01871(GO:0019731;GO:0050829);A0A075B7D0(GO:0042742);A0A075B7D8(GO:0042742);P04114(GO:0009615);A0A0C4DH33(GO:0042742);P18065(GO:0007565);P02743(GO:0044871;GO:0044869);P02741(GO:0050830);P01860(GO:0042742);A0A075B7B8(GO:0042742);P02748(GO:0019836);P02649(GO:0044794;GO:0019068);P04196(GO:0061844;GO:0051715;GO:0050832);P10909(GO:0019730;GO:0009615);A0A0C4DH32(GO:0042742);P05109(GO:0019730;GO:0050832;GO:0042742);P01861(GO:0042742);A0A0C4DH31(GO:0042742);A0A0C4DH36(GO:0042742);A0A0C4DH34(GO:0042742);A0A0C4DH38(GO:0042742);A0A0C4DH39(GO:0042742);P01024(GO:0009617);C9J8S2(GO:0019732;GO:0050829;GO:0050830;GO:0061760);P02538(GO:0050830;GO:0051801;GO:0061844);P00747(GO:0052182;GO:0051702;GO:0052213);A0A075B6Q5(GO:0042742);P02652(GO:0016032);Q15485(GO:0050830;GO:0050829);A0A0C4DH29(GO:0042742);P01619(GO:0019731);A0A0B4J1V1(GO:0042742);A0A0B4J1V0(GO:0042742);A0A0B4J1V2(GO:0042742);Q9Y490(GO:0016032);Q15848(GO:0009617) | 4|51 | K7ERG9(GO:0009617);O75636(GO:0051607);A0A0C4DH36(GO:0042742);P02751(GO:0051702;GO:0052047) | 0.001231081 | 0.024621629 | https://www.ebi.ac.uk/QuickGO/term/GO:0051704 |
| GO:0099080 | supramolecular complex | cellular_component | 21|375 | F8W1S1(GO:0005882;GO:0045095);P35527(GO:0005882);P13647(GO:0045095;GO:0005882);P08779(GO:0005882);P21333(GO:0005884);P04264(GO:0045095;GO:0005882);Q9H4B7(GO:0005874);P01034(GO:0043292);P07437(GO:0005874);A0A1W2PQU7(GO:0005883;GO:0005882);P23142(GO:0071953);P0DJI8(GO:0005881);P02538(GO:0045095;GO:0005882);P68032(GO:0005884;GO:0042643);P68366(GO:0005874);P13796(GO:0005884);P0DPH7(GO:0005874);P02533(GO:0045095;GO:0005882);Q8N1N4(GO:0045095;GO:0005882);P13645(GO:0005882);P35908(GO:0045095;GO:0005882) | 4|51 | Q9H4B7(GO:0005874);P35527(GO:0005882);P13647(GO:0045095;GO:0005882);P68032(GO:0005884;GO:0042643) | 0.174558029 | 0.225236166 | https://www.ebi.ac.uk/QuickGO/term/GO:0099080 |
| GO:0009987 | cellular process | biological_process | 275|375 | P08779(GO:0051546;GO:0030216;GO:0070268;GO:0007010;GO:0045104);P00488(GO:0018149;GO:0019221;GO:0002576);P68366(GO:0000086;GO:0000226;GO:0097711;GO:0002576;GO:0000278;GO:0007017;GO:0007010);P04275(GO:0002576;GO:0051260;GO:0030168;GO:0030198);A0A0G2JMB2(GO:0050853;GO:0006910;GO:0006911);J3KNB4(GO:0071224;GO:0071222;GO:0071354;GO:0071356;GO:0071347);Q9UHG3(GO:0030327;GO:0030328;GO:0030329);P51884(GO:0018146;GO:0007409;GO:0030199;GO:0030198;GO:0042340);P03950(GO:0006651;GO:0034332;GO:0030154;GO:0090305;GO:0016477;GO:0009303;GO:0090501;GO:0030041;GO:0007154;GO:0001556);G3XAP6(GO:0030509;GO:0030198;GO:0002063;GO:0009306;GO:0006915;GO:0048747;GO:0097084;GO:0030199);P23142(GO:0030198;GO:0007229);P15814(GO:0050853;GO:0006910;GO:0006911;GO:0050900);B7ZKJ8(GO:0030212);A0A0B4J1X8(GO:0050853;GO:0006910;GO:0006911);P19652(GO:0002576;GO:0043312);B1AHL2(GO:0030198);P08571(GO:0071222;GO:0071223;GO:0097190;GO:0007166;GO:0071727;GO:0071726;GO:0071219;GO:0002224;GO:0070266;GO:0038124;GO:0006915;GO:0035666;GO:0034142;GO:0002755;GO:0002756;GO:0038123;GO:0031663;GO:0007249;GO:0043312);P68871(GO:0042744;GO:0098869;GO:0051291;GO:0043312);A0A0B4J1V2(GO:0050853;GO:0006910;GO:0006911);E9PK25(GO:0030010;GO:0030836;GO:0000281;GO:0035722;GO:0006468;GO:0030036;GO:0001755;GO:0007266;GO:0030043;GO:0030042;GO:0007010);O75460(GO:0016310;GO:0034620;GO:0046777;GO:0036289;GO:0098787;GO:0071333;GO:0006402;GO:0006468;GO:0006397;GO:0007050;GO:0090502;GO:0036498;GO:0006351;GO:0030968;GO:0070059;GO:0070054;GO:1990579;GO:0006379;GO:0035924;GO:0006915;GO:0034976);Q86YZ3(GO:0043163;GO:0043312);P35527(GO:0045109;GO:0070268);P01042(GO:0044267;GO:0002576;GO:0007186;GO:0043687;GO:0007204);A0A087X1J7(GO:0051289;GO:0098869);P01602(GO:0038096;GO:0038095;GO:0050900);P01601(GO:0038096;GO:0038095;GO:0050900);A0A0B4J1U7(GO:0050853;GO:0006910;GO:0006911);P59665(GO:0071222;GO:0030520;GO:0010818;GO:0043312);P0DJI8(GO:0019221;GO:0048246;GO:0048247;GO:0044267;GO:0007186;GO:0030593;GO:0030168;GO:0007204);P06312(GO:0038096;GO:0038095;GO:0050900);P02750(GO:0050873;GO:0043312);P02790(GO:0020027;GO:0042168;GO:0006879);P60763(GO:0030031;GO:0030036;GO:0031175;GO:0035556;GO:0030838;GO:0000902;GO:0051932;GO:0016477;GO:0021894;GO:0007266;GO:0007264;GO:0007015;GO:0043652);P01763(GO:0038096;GO:0038095;GO:0050900);P01764(GO:0050900;GO:0050853;GO:0006910;GO:0006911;GO:0038096;GO:0038095);A0A087X0S5(GO:0071230;GO:0070208;GO:0003429;GO:0030198;GO:0035987;GO:0001649);P01766(GO:0038096;GO:0038095;GO:0050900);Q15582(GO:0044267;GO:0002062;GO:0030198);F8WF14(GO:0050783;GO:0014016;GO:0019695);A0A075B7D8(GO:0050853;GO:0006910;GO:0006911);P26038(GO:0050900;GO:0061028;GO:0042098;GO:0035722;GO:0022614;GO:0071394;GO:0008361;GO:0045198;GO:0001771;GO:0072678;GO:0007010);P01782(GO:0038096;GO:0038095;GO:0050900);P18428(GO:0071222;GO:0071223;GO:0019221;GO:0002281;GO:0002224;GO:0034142;GO:0031663;GO:0002232);P01780(GO:0038096;GO:0038095;GO:0050900);A0A140T8Y3(GO:0030199;GO:0030198);P02776(GO:0070098;GO:0071222;GO:0019221;GO:0002576;GO:0007189;GO:0007186;GO:0030595;GO:0030593;GO:0030168);P02775(GO:0070098;GO:0071222;GO:0060326;GO:0002576;GO:0007186;GO:0030595;GO:0030593;GO:0043312);P13671(GO:0019835);Q13201(GO:0002576);P01833(GO:0043312;GO:0007173;GO:0038093);E7EUT5(GO:0000226;GO:0006096;GO:0035606;GO:0051402;GO:0071346);A0A0U1RQV3(GO:0007173);P30041(GO:0042744;GO:0045454;GO:0098869;GO:0034599;GO:0043312;GO:0046475);P06702(GO:0030595;GO:0030593;GO:0031532;GO:0002224;GO:0035606;GO:0018119;GO:0006914;GO:0006915;GO:0007267;GO:0014002;GO:0002523;GO:0043312;GO:0098869);Q9Y5Y7(GO:0030214);P61224(GO:0030033;GO:0007165;GO:0061028;GO:0035722;GO:0032486;GO:0007264;GO:0071320;GO:0043312);P10643(GO:0019835;GO:0006883);P02787(GO:0061024;GO:0002576;GO:0044267;GO:0043687;GO:0006879;GO:0007015;GO:0030316;GO:0071281;GO:0060395;GO:0070371);P19827(GO:0030212);P35908(GO:0018149;GO:0051546;GO:0032980;GO:0070268;GO:0045109;GO:0003334);P01834(GO:0050853;GO:0006910;GO:0006911;GO:0038096;GO:0038095;GO:0050900);P19823(GO:0044267;GO:0030212;GO:0043687);P01008(GO:0043687;GO:0044267);P04004(GO:0016477;GO:0048709;GO:0035987;GO:0051258;GO:0030198);P01743(GO:0038096;GO:0038095;GO:0050900);A0A0B4J231(GO:0050853;GO:0006910;GO:0006911);O00391(GO:0044267;GO:0043687;GO:0045454;GO:0043312;GO:0085029;GO:0002576);K7ER74(GO:0034375;GO:0034372;GO:0034371;GO:0034370;GO:0034378;GO:0001523);P69905(GO:0042744;GO:0098869;GO:0051291);G3XAK1(GO:0030317;GO:0071456;GO:0048012);G3V0E5(GO:0035690;GO:1990830;GO:0006879;GO:0030316;GO:0031623);P02768(GO:0034375;GO:0051659;GO:0044267;GO:0019836;GO:0002576;GO:0098869;GO:0043687;GO:0009267);O75636(GO:0043654);Q96PD5(GO:0002221;GO:0001519);Q86UX7(GO:0034446;GO:0007229;GO:0033622;GO:0002576);P02763(GO:0002576;GO:0043312);A0A087WSY4(GO:0050853;GO:0006910;GO:0006911);Q01518(GO:0008154;GO:0007010;GO:0000902;GO:0007163;GO:0043312;GO:0007165;GO:0030036;GO:0001667);A0A0C4DH73(GO:0038096;GO:0038095;GO:0050900);P02766(GO:0044267;GO:0043312;GO:0001523;GO:0030198;GO:0042572;GO:0006144);Q9H4B7(GO:0007017;GO:0000278;GO:0000226;GO:0051225);Q9BWP8(GO:0097194);P07357(GO:0019835);P37802(GO:0002576;GO:0030855);A0A0G2JI36(GO:0060333;GO:0060337;GO:0036037);Q15166(GO:0046395;GO:0019372;GO:0016311;GO:0019439;GO:0046226;GO:0010124);J3QT83(GO:0003429);P29622(GO:0002576);P09172(GO:0050900;GO:0042423;GO:0042420;GO:0042421;GO:0006589;GO:0007268);P01703(GO:0038096;GO:0038095;GO:0050900);P01700(GO:0038096;GO:0038095;GO:0050900);P01701(GO:0038096;GO:0038095;GO:0050900);P01706(GO:0038096;GO:0038095;GO:0050900);P21333(GO:0030030;GO:0044319;GO:0031532;GO:0002576;GO:0034329;GO:0042789;GO:0060271;GO:0051764;GO:0030168;GO:0071526;GO:0021943;GO:0090307;GO:0097368;GO:0007195);P01705(GO:0038096;GO:0038095;GO:0050900);Q8N1N4(GO:0070268);P23083(GO:0038096;GO:0038095;GO:0050900);P01709(GO:0038096;GO:0038095;GO:0050900);A0A0C4DH38(GO:0050853;GO:0006910;GO:0006911);P02671(GO:0034622;GO:0044267;GO:0002576;GO:0065003;GO:0030198;GO:0002224;GO:0043687;GO:0030168;GO:0051258);P02675(GO:0034622;GO:0044320;GO:0002576;GO:0030198;GO:0002224;GO:0030168;GO:0051258;GO:0071347);P07996(GO:0034605;GO:0043652;GO:0030198;GO:0018149;GO:0071356;GO:0016477;GO:0007050;GO:0034976;GO:0071363;GO:0002576);H3BTN5(GO:0016310;GO:0006096);P0CF74(GO:0050900;GO:0050853;GO:0006910;GO:0006911;GO:0038096;GO:0038095);P61626(GO:0019835;GO:0044267;GO:0016998;GO:0043312);A0A0C4DH43(GO:0050853;GO:0006910;GO:0006911);P04217(GO:0002576;GO:0043312);P04180(GO:0034375;GO:0006656;GO:0034372;GO:0042158;GO:0034435;GO:0006644;GO:0046470);P31151(GO:0030216;GO:0043312);Q16880(GO:0030913;GO:0006682;GO:0006687;GO:0006665;GO:0007010;GO:0048812);Q06033(GO:0030212;GO:0002576);C9JF17(GO:0014012);P05155(GO:0002576);P05154(GO:0007342);I3L1J2(GO:0034332;GO:0007043;GO:0000902;GO:0007179);M0R0Q9(GO:0006631;GO:0007165;GO:0044267;GO:1905114;GO:0007186;GO:0043687;GO:0016322;GO:0150062;GO:0043312);O95445(GO:0034375;GO:0001523;GO:0098869;GO:0034380);Q9UGM5(GO:0007339);A0A075B7F0(GO:0050853;GO:0006910;GO:0006911);P00734(GO:0051281;GO:0007166;GO:0044267;GO:0051838;GO:0007186;GO:0030168;GO:0051480);P13645(GO:0018149;GO:0030216;GO:0070268;GO:0051290);A0A0A0MS14(GO:0050853;GO:0006910;GO:0006911);P13647(GO:0007010;GO:0031581;GO:0070268);P13473(GO:0061684;GO:0097352;GO:0046716;GO:0006914;GO:1905146;GO:0009267;GO:0043312;GO:0002576);P01717(GO:0038096;GO:0038095;GO:0050900);A0A0A0MS15(GO:0050853;GO:0006910;GO:0006911);P01718(GO:0038096;GO:0038095;GO:0050900);P00738(GO:0098869;GO:0043312);P01857(GO:0019221;GO:0050853;GO:0006910;GO:0006911;GO:0038096);P07358(GO:0019835);P16930(GO:0006572;GO:0006559;GO:0006527;GO:0009072;GO:1902000);P01019(GO:0038166;GO:0007166;GO:0007204;GO:0034374;GO:0007200;GO:0071260;GO:0006883;GO:0007186;GO:0007199;GO:0007267;GO:1904385;GO:0070371;GO:0061049;GO:0050663;GO:0051403;GO:0007263);A0A0C4DH25(GO:0038096;GO:0038095;GO:0050900);A0A0G2JMI3(GO:0050853;GO:0006910;GO:0006911);P01011(GO:0002576;GO:0043312);P08514(GO:0002576;GO:0007229;GO:0030198);P41222(GO:0006633;GO:0006631;GO:0019371;GO:0001516;GO:0006693);K7ERG9(GO:0007219);Q96IY4(GO:0071333);P04430(GO:0038096;GO:0038095;GO:0050900);A0A1W2PQU7(GO:0061564;GO:0045109;GO:0060020;GO:0030198;GO:0014002;GO:0031102);Q8IV42(GO:0016310;GO:0097056;GO:0001514;GO:0006412);A0A0A0MTH3(GO:0018105;GO:0034446;GO:0030030;GO:0033209;GO:0045197;GO:0022011;GO:0010761;GO:0006468;GO:0043491;GO:0007229);O14791(GO:0019835;GO:0044267;GO:0043687);P13796(GO:0002286;GO:0016477;GO:0022617;GO:0035722;GO:0051017;GO:0051764;GO:0051639;GO:0044319;GO:0010737);P15144(GO:0030154;GO:0007165;GO:0007267;GO:0043312;GO:0043171);P36955(GO:0071300;GO:0071333;GO:0071279;GO:0071549);C9JC84(GO:0051258;GO:0030168);B0YIW2(GO:0034375;GO:0034371;GO:0034379;GO:0034378;GO:0001523;GO:0007186;GO:0006641;GO:0019433);C9JPQ9(GO:0051258;GO:0030168);P09486(GO:0030198;GO:0071363;GO:0002576);Q16610(GO:0007165;GO:0002576;GO:0002063);P18206(GO:0048675;GO:0034333;GO:0030032;GO:0002576;GO:0043312;GO:0043297);P0DPH7(GO:0007017;GO:0007010;GO:0000278;GO:0000226);Q13093(GO:0034441;GO:0034440;GO:0034374;GO:0046469);P14151(GO:0050900;GO:0043312);P01721(GO:0038096;GO:0038095;GO:0050900);A0A2R8Y6G6(GO:0006096);P55058(GO:0034375;GO:0030317;GO:0010189);P01009(GO:0048208;GO:0044267;GO:0002576;GO:0043687;GO:0043312);P02751(GO:0018149;GO:0019221;GO:0034446;GO:0050900;GO:0044267;GO:0002576;GO:0030198;GO:0033622;GO:0043687;GO:0035987;GO:1901166;GO:0007044);A0A075B6R2(GO:0050853;GO:0006910;GO:0006911);P02760(GO:0042167;GO:0018298);P02100(GO:0051291;GO:0098869;GO:0042744);P55056(GO:0034379);P68032(GO:0030240;GO:0043503;GO:0031032;GO:0071417;GO:0033275;GO:0055003;GO:0048741;GO:0030049;GO:0030048;GO:0070252);J3QRV5(GO:0006887;GO:0007049;GO:0051301);P69892(GO:0098869;GO:0042744);H9KV75(GO:0030036;GO:0048741;GO:0051017;GO:0045214;GO:0051764;GO:0007030);J3KPS3(GO:0051289;GO:0006096);P33908(GO:1904381;GO:0006491;GO:0006486);E9PHK0(GO:0002576;GO:0071560;GO:0071310);P02753(GO:0042572;GO:0001523);A0A669KAY4(GO:0032802;GO:0032869;GO:0044267;GO:0043687;GO:0006644;GO:0030182;GO:0006641;GO:0006915;GO:0009267;GO:0022008);P01344(GO:0051146;GO:0008286;GO:0038028;GO:0001649;GO:0044267;GO:0006349;GO:0002576);P27169(GO:0016311;GO:0046434;GO:0019372;GO:0046395;GO:0046470;GO:0019439);P35579(GO:0031032;GO:0007229;GO:0030224;GO:0030220;GO:0001768;GO:0031532;GO:0045055;GO:0050900;GO:0032796;GO:0032506;GO:0051295;GO:0000212;GO:0006911;GO:0030048;GO:0043534;GO:0007520;GO:0001778;GO:0000904);P01871(GO:0050900;GO:0050853;GO:0006910;GO:0006911);A0A075B7D0(GO:0050853;GO:0006910;GO:0006911);P01715(GO:0038096;GO:0038095;GO:0050900);O75882(GO:0042552);P01619(GO:0050900;GO:0038096;GO:0038095);P00918(GO:0038166;GO:0032849;GO:0051453;GO:0071498);P01597(GO:0038096;GO:0038095;GO:0050900);A0A0B4J1X5(GO:0050853;GO:0006910;GO:0006911);Q6EMK4(GO:0071456);A0A0A0MRJ7(GO:0048208;GO:0044267;GO:0002576;GO:0043687);P04040(GO:0020027;GO:0051289;GO:0009060;GO:0001649;GO:0051262;GO:0098869;GO:0006641;GO:0006625;GO:0071363;GO:0034599;GO:0043312;GO:0042744);A0A0C4DH33(GO:0050853;GO:0006910;GO:0006911);P18065(GO:0007165;GO:0044267;GO:0032870);P00915(GO:0006730;GO:0035722);P05546(GO:0044267;GO:0043687);P02743(GO:0006457;GO:0044267;GO:0051131);P55103(GO:0060395;GO:0048468);P01860(GO:0050853;GO:0006910;GO:0006911;GO:0038096);D6RE82(GO:0006364);P02745(GO:0007267);A0A075B7B8(GO:0050853;GO:0006910;GO:0006911);P01031(GO:0007166;GO:0060326;GO:0019835;GO:0007186);P02749(GO:0002576;GO:0006641);P80108(GO:0035690;GO:0032869;GO:0002430;GO:0006501;GO:0097241;GO:0006507;GO:0002062;GO:0035701;GO:0002042;GO:0008286;GO:0071397;GO:0071277;GO:0071401;GO:0071467;GO:0046470);P01034(GO:0060009;GO:0070301;GO:0044267;GO:0043687;GO:0006915;GO:0001775;GO:0034599;GO:0043312;GO:0097435);A0A087WZB5(GO:0031532);C9JB55(GO:0006879;GO:0071281);P11597(GO:0034375;GO:0034374;GO:0034372;GO:0006641;GO:0046470);A0A0C4DH32(GO:0050853;GO:0006910;GO:0006911);P02649(GO:0051651;GO:0098869;GO:0031102;GO:0019433;GO:0034375;GO:0034374;GO:0034372;GO:0034371;GO:0019934;GO:0042158;GO:0044267;GO:0007186;GO:0034380;GO:0006641;GO:0006874;GO:0043687;GO:0034378;GO:0007271;GO:0001523;GO:0007263;GO:0007010);P04211(GO:0038096;GO:0038095;GO:0050900);P04196(GO:0051715;GO:0002576;GO:0030168);P32119(GO:0042744;GO:0034599;GO:0042098;GO:0045454;GO:0045321;GO:0019430);P02647(GO:0031102;GO:0043534;GO:0007179;GO:0018206;GO:0019433;GO:0034375;GO:0034371;GO:0034378;GO:0042158;GO:0044267;GO:0014012;GO:0007186;GO:0018158;GO:0007229;GO:0034380;GO:0070371;GO:0006656;GO:0006644;GO:0001523;GO:0002576;GO:0043687);P01861(GO:0019221;GO:0050853;GO:0006910;GO:0006911;GO:0038096);P10909(GO:0000902;GO:0051131;GO:0001774;GO:0061077;GO:0032286;GO:0002576;GO:0006915;GO:0001836);P04114(GO:0034374;GO:0034371;GO:0061024;GO:0050900;GO:0034378;GO:0001523;GO:0071356;GO:0071379;GO:0042158;GO:0002224;GO:0043687;GO:0006642;GO:0044267;GO:0030317;GO:0034379;GO:0019433);P05109(GO:0043312;GO:0014002;GO:0002224;GO:0030593;GO:0006914;GO:0006915;GO:0018119;GO:0002523);P15169(GO:0010815;GO:0006518;GO:0030070);A0A0C4DH31(GO:0050853;GO:0006910;GO:0006911);A0A0C4DH36(GO:0050853;GO:0006910;GO:0006911);A0A0C4DH34(GO:0050853;GO:0006910;GO:0006911);P19320(GO:0060945;GO:0060326;GO:0019221;GO:0071356;GO:0035584;GO:1904646;GO:0022614;GO:0030183;GO:0060333;GO:0030198;GO:0035924);P08697(GO:0002576;GO:0030199);Q14624(GO:0030212;GO:0002576);P25311(GO:0090501);A0A0C4DH39(GO:0050853;GO:0006910;GO:0006911);O95497(GO:0015939;GO:0043312);P01599(GO:0038096;GO:0038095;GO:0050900);O43866(GO:0006915);P01024(GO:0006631;GO:0007165;GO:0044267;GO:1905114;GO:0007186;GO:0043687;GO:0016322;GO:0006911;GO:0150062;GO:0043312);P01023(GO:0048863;GO:0002576;GO:0022617);C9J8S2(GO:0030154;GO:0001523;GO:0002576;GO:0008286);P02748(GO:0019835;GO:0019836;GO:0051260);Q07954(GO:0007205;GO:0150094;GO:0001523;GO:0002265;GO:1904646;GO:0031623);P02538(GO:0007010;GO:0030154;GO:0070268;GO:0051801);P00747(GO:0044267;GO:0022617;GO:0002576);Q96HR3(GO:0030521;GO:0030518;GO:0006367;GO:0016567;GO:0006351);P60709(GO:0043044;GO:0072749;GO:0061024;GO:0016579;GO:0048013;GO:0034329;GO:0038096;GO:0048870;GO:0098974);P06331(GO:0038096;GO:0038095;GO:0050900);A0A075B6Q5(GO:0050853;GO:0006910;GO:0006911);P02533(GO:0045110;GO:0030855;GO:0031581;GO:0070268);P01742(GO:0038096;GO:0038095;GO:0050900);P00450(GO:0044267;GO:0006879;GO:0043687);Q08380(GO:0002576;GO:0007165);P80748(GO:0038096;GO:0038095;GO:0050900);P08519(GO:0034374);P43251(GO:0006768);P04264(GO:0018149;GO:0051290;GO:0070268;GO:0043312);P0C0L4(GO:0044267;GO:0043687);C9JV77(GO:0044267;GO:0002576;GO:0043687;GO:0043312);P02652(GO:0034375;GO:0034374;GO:0034371;GO:0006656;GO:0001523;GO:0009395;GO:0044267;GO:0018158;GO:0006641;GO:0043687;GO:0034378;GO:0046340;GO:0018206;GO:0034380;GO:0034370);P35858(GO:0044267;GO:0007165);Q15485(GO:0043654);A0A096LPE2(GO:0060326);P04433(GO:0038096;GO:0038095;GO:0050900);P06727(GO:0034375;GO:0034372;GO:0034371;GO:0034380;GO:0034378;GO:0042744;GO:0001523;GO:0044267;GO:0065005;GO:0031102;GO:0046470;GO:0019430);C9J5S7(GO:0000413;GO:0006457);A0A0C4DH29(GO:0050853;GO:0006910;GO:0006911);Q5SRP5(GO:0034375;GO:0034380);Q13103(GO:0044267;GO:0002576;GO:0043687);P01817(GO:0038096;GO:0038095;GO:0050900);P01814(GO:0038096;GO:0038095;GO:0050900);P01704(GO:0038096;GO:0038095;GO:0050900);A0A0B4J1V1(GO:0050853;GO:0006910;GO:0006911);A0A0B4J1V0(GO:0050853;GO:0006910;GO:0006911);P07360(GO:0019835);A0A0A0MS51(GO:0051127;GO:0030041;GO:0042989;GO:1990000;GO:0045010;GO:0097284;GO:0060271;GO:0006915;GO:0051016;GO:0051014;GO:0014003;GO:0006911;GO:0048015;GO:0051693;GO:0090527;GO:0071346;GO:0071276);P07437(GO:0000086;GO:0051225;GO:0051301;GO:0097711;GO:0000278;GO:0009987;GO:0007017;GO:0043312;GO:0000226);H0YAC1(GO:0022617);P01877(GO:0050900;GO:0050853;GO:0006910;GO:0006911);P11021(GO:1990090;GO:0071480;GO:1990440;GO:0035437;GO:0030433;GO:0036500;GO:0042149;GO:0035690;GO:0006983;GO:0071353;GO:0071236;GO:0030182;GO:0071277;GO:0036498;GO:0036499;GO:0051402;GO:0030968;GO:0071287;GO:0071320;GO:0034976;GO:0034975);Q9Y490(GO:0007043;GO:0002576;GO:0033622;GO:0036498;GO:0007016;GO:0030866;GO:0007044;GO:0007229);Q15848(GO:0071320;GO:0070208;GO:0071872;GO:0019395;GO:0006635;GO:0050873;GO:0035690;GO:0032869;GO:0006091;GO:0051260);P01699(GO:0038096;GO:0038095;GO:0050900);P07737(GO:0060074;GO:0030837;GO:0030838;GO:0060071;GO:0071363;GO:0098885;GO:0030036);P02042(GO:0098869;GO:0042744) | 37|51 | O75636(GO:0043654);P80748(GO:0038096;GO:0038095;GO:0050900);P03950(GO:0006651;GO:0034332;GO:0030154;GO:0090305;GO:0016477;GO:0009303;GO:0090501;GO:0030041;GO:0007154;GO:0001556);A0A0C4DH73(GO:0038096;GO:0038095;GO:0050900);P01717(GO:0038096;GO:0038095;GO:0050900);Q9H4B7(GO:0007017;GO:0000278;GO:0000226;GO:0051225);A0A0C4DH36(GO:0050853;GO:0006910;GO:0006911);P37802(GO:0002576;GO:0030855);Q14624(GO:0030212;GO:0002576);C9JPQ9(GO:0051258;GO:0030168);P35527(GO:0045109;GO:0070268);A0A087X1J7(GO:0051289;GO:0098869);P32119(GO:0042744;GO:0034599;GO:0042098;GO:0045454;GO:0045321;GO:0019430);P55058(GO:0034375;GO:0030317;GO:0010189);P02751(GO:0018149;GO:0019221;GO:0034446;GO:0050900;GO:0044267;GO:0002576;GO:0030198;GO:0033622;GO:0043687;GO:0035987;GO:1901166;GO:0007044);P60709(GO:0043044;GO:0072749;GO:0061024;GO:0016579;GO:0048013;GO:0034329;GO:0038096;GO:0048870;GO:0098974);Q86UX7(GO:0034446;GO:0007229;GO:0033622;GO:0002576);H3BTN5(GO:0016310;GO:0006096);P68032(GO:0030240;GO:0043503;GO:0031032;GO:0071417;GO:0033275;GO:0055003;GO:0048741;GO:0030049;GO:0030048;GO:0070252);Q9UGM5(GO:0007339);K7ERG9(GO:0007219);P29622(GO:0002576);I3L1J2(GO:0034332;GO:0007043;GO:0000902;GO:0007179);Q16610(GO:0007165;GO:0002576;GO:0002063);P30041(GO:0042744;GO:0045454;GO:0098869;GO:0034599;GO:0043312;GO:0046475);Q5SRP5(GO:0034375;GO:0034380);P18206(GO:0048675;GO:0034333;GO:0030032;GO:0002576;GO:0043312;GO:0043297);P13647(GO:0007010;GO:0031581;GO:0070268);P01715(GO:0038096;GO:0038095;GO:0050900);P19823(GO:0044267;GO:0030212;GO:0043687);P04040(GO:0020027;GO:0051289;GO:0009060;GO:0001649;GO:0051262;GO:0098869;GO:0006641;GO:0006625;GO:0071363;GO:0034599;GO:0043312;GO:0042744);P07360(GO:0019835);P00915(GO:0006730;GO:0035722);Q96HR3(GO:0030521;GO:0030518;GO:0006367;GO:0016567;GO:0006351);D6RE82(GO:0006364);P69905(GO:0042744;GO:0098869;GO:0051291);P07737(GO:0060074;GO:0030837;GO:0030838;GO:0060071;GO:0071363;GO:0098885;GO:0030036) | 0.132735558 | 0.212376893 | https://www.ebi.ac.uk/QuickGO/term/GO:0009987 |
| GO:0044464 | cell part | cellular_component | 298|375 | P08779(GO:0005856;GO:0005634;GO:0005882;GO:0005829);P00488(GO:0005737;GO:0031093);P68366(GO:0005874;GO:0005737;GO:0005856;GO:0015630;GO:0005829);P04275(GO:0005783;GO:0031091;GO:0031093;GO:0033093);J3KNB4(GO:0005737;GO:0042995;GO:0042581);P51884(GO:0043202;GO:0005796);P03950(GO:0030426;GO:0005730;GO:0031410;GO:0005634;GO:0043025);A0A075B7D0(GO:0009897);P01704(GO:0005886);A0A0A0MRZ9(GO:0005886);P15814(GO:0009897);B7ZKJ8(GO:0005737;GO:0005886);P0DP02(GO:0005886);A0A0B4J1X8(GO:0009897;GO:0005886);P19652(GO:0031093;GO:0035578;GO:0035580);P08571(GO:0009897;GO:0031362;GO:0010008;GO:0030667;GO:0009986;GO:0005794;GO:0005886);P68871(GO:1904813;GO:0071682;GO:0005833;GO:1904724;GO:0005829);P01877(GO:0009897;GO:0005886);Q8N1N4(GO:0005829;GO:0045095;GO:0005882);O75460(GO:0005739;GO:0005783;GO:0005789;GO:1990604;GO:0030176;GO:0005637;GO:0005737;GO:1990332);Q86YZ3(GO:0005737;GO:0005634;GO:0035578;GO:0048471;GO:0036457;GO:0001533);P35527(GO:0005829;GO:0005882;GO:0005634);P01042(GO:0005788;GO:0031093;GO:0005886);P01597(GO:0005886);P43251(GO:0005759);P01602(GO:0005886);A0A0G2JMI3(GO:0009897;GO:0005886);A0A0B4J1U7(GO:0009897;GO:0005886);P59665(GO:0035578;GO:0005796);P0DJI8(GO:0071682;GO:0005881);Q96KN2(GO:0005829);A0A087WSY4(GO:0009897;GO:0005886);P01743(GO:0005886);A0A0A0MT36(GO:0005886);B0YIW2(GO:0005769);P02790(GO:0071682);P60763(GO:0042995;GO:0030426;GO:0071944;GO:0005737;GO:0043005;GO:0048471;GO:0043025;GO:0031941;GO:0030027;GO:0012505;GO:0005886;GO:0005829;GO:0005856;GO:0005622);A0A075B7D8(GO:0009897);P01764(GO:0009897;GO:0005886);A0A087X0S5(GO:0005788;GO:0005765;GO:0042383);P01766(GO:0005886);A0A087WWU8(GO:0005829;GO:0015629);F8WF14(GO:0005783;GO:0005788;GO:0005641);P07358(GO:0005579);P0DOY3(GO:0005886);P26038(GO:0030175;GO:0005737;GO:0045177;GO:0042995;GO:0043209;GO:0005634;GO:0048471;GO:0005856;GO:0016324;GO:0009986;GO:0071944;GO:0016323;GO:0005886;GO:0031528;GO:0071437;GO:0005829;GO:0005902;GO:0001931;GO:0031143);P29622(GO:0031089);P18428(GO:0009986);P01780(GO:0005886);P06331(GO:0005886);P02776(GO:0005737;GO:0031093);A0A1W2PQU7(GO:0005737;GO:0045111;GO:0044297;GO:0005883;GO:0005882;GO:0097450);P13671(GO:0005579);Q13201(GO:0031093);P01833(GO:0005887;GO:0005886;GO:0035577);G3XAK1(GO:0005737;GO:0005773);E7EUT5(GO:0005737;GO:0005634;GO:0005811;GO:0015630;GO:0031965;GO:0005886;GO:0043231;GO:0005829);A0A0G2JMB2(GO:0009897);A0A0J9YXX1(GO:0005886);P30041(GO:0035578;GO:0005737;GO:0005634;GO:0048471;GO:0005829;GO:0005764);P06702(GO:0005737;GO:0005634;GO:0005654;GO:0034774;GO:0005856;GO:0005886;GO:0005829);Q9Y5Y7(GO:0071944;GO:0005887;GO:0005886);P61224(GO:0005811;GO:0005737;GO:0070382;GO:0035577;GO:0005886;GO:0005829;GO:0005622);P10643(GO:0005579);P02787(GO:0055037;GO:0005788;GO:0030139;GO:0005905;GO:1990712;GO:0010008;GO:0030665;GO:0031232;GO:0031410;GO:0005770;GO:0045178;GO:0009925;GO:0048471;GO:0009986;GO:0016324;GO:0034774;GO:0005768;GO:0005769);P35908(GO:0045095;GO:0005634;GO:0005882;GO:0001533;GO:0005829);P01834(GO:0009897;GO:0005886);P19823(GO:0005788);P04003(GO:0005886);P01718(GO:0005886);P04004(GO:0005783;GO:0005737;GO:0005796;GO:0048237;GO:0043231;GO:0071062);P00748(GO:0005791;GO:0005886);P20851(GO:0005886);Q9UNN8(GO:0005813;GO:0009986;GO:0005887;GO:0005886;GO:0048471);O00391(GO:0005788;GO:0030173;GO:0031093;GO:0035580;GO:0005794;GO:1904724;GO:0043231;GO:0000139);K7ER74(GO:0005769);P69905(GO:0022627;GO:0071682;GO:0005833;GO:0005829);A0A0J9YX35(GO:0005886);P15144(GO:0009897;GO:0030667;GO:0005793;GO:0005886;GO:0005765);G3V0E5(GO:0009897;GO:0055037;GO:0005905;GO:1990712;GO:0010008;GO:0048471;GO:0005887;GO:0005886;GO:0016323;GO:0042470;GO:0005769);P02768(GO:0005788;GO:0005783;GO:0005737;GO:0031093;GO:0043209;GO:0005794;GO:0005634);Q96PD5(GO:0005622);Q86UX7(GO:0002102;GO:0031093;GO:0042995);P02763(GO:0031093;GO:0035580;GO:1904724);A0A087WWT3(GO:0005794;GO:0005783);A0A0C4DH73(GO:0005886);P02766(GO:0005737;GO:0035578);J3KPS3(GO:0031430;GO:0005829;GO:0031674);A0A2R8Y619(GO:0005634;GO:0005694;GO:0000786);P07357(GO:0005886;GO:0005579);A0A075B6S5(GO:0005886);P37802(GO:0005829);A0A0G2JI36(GO:0005783;GO:0055038;GO:0071556;GO:0005886;GO:0031901;GO:0005794;GO:0009986;GO:0042612;GO:0005887;GO:0012507;GO:0030670;GO:0000139);Q15166(GO:0043231);P02748(GO:0005887;GO:0005886;GO:0005829;GO:0005579);J3KRP0(GO:0005829);P09172(GO:0005783;GO:0034774;GO:0005815;GO:0005737;GO:0030667;GO:0031410;GO:0030658;GO:0042584;GO:0043231;GO:0034466);P01703(GO:0005886);P01700(GO:0005886);P01701(GO:0005886);P01706(GO:0005886);P04264(GO:0045095;GO:1904813;GO:0005856;GO:0005634;GO:0005882;GO:0005886;GO:0001533;GO:0005829);P02042(GO:0005833;GO:0005829);P01705(GO:0005886);A0A0C4DH67(GO:0005886);E9PK25(GO:0015629;GO:0005737;GO:0005634;GO:0005622;GO:0031258;GO:0030027;GO:0032587;GO:0016363;GO:0005829;GO:0030864);P23083(GO:0005886);P01709(GO:0005886);P02671(GO:0009897;GO:0005788;GO:0031091;GO:0031093;GO:0009986;GO:0005938;GO:0005886);P02675(GO:0005783;GO:0009897;GO:0005737;GO:0031091;GO:0031093;GO:0009986;GO:0005938;GO:0005886);P07996(GO:0005783;GO:0005788;GO:0009897;GO:0030141;GO:0005737;GO:0031091;GO:0031093;GO:0009986;GO:0016529);H3BTN5(GO:0005737;GO:0005634;GO:0005829);A0A075B6I0(GO:0005886);P01619(GO:0005886);P0CF74(GO:0009897;GO:0005886);P61626(GO:0035578;GO:0035580;GO:1904724);D6RE82(GO:0005730;GO:0005634);P04217(GO:0031093;GO:1904813;GO:0034774);Q16880(GO:0005886;GO:0043231);P22891(GO:0005796;GO:0005788);Q06033(GO:0031089);C9JF17(GO:0022626;GO:0005783;GO:0030425;GO:0005737;GO:0048471;GO:0043025);P05155(GO:0031093);P05154(GO:0009897;GO:0002080;GO:0031094;GO:0031091);F8W1S1(GO:0005882;GO:0045095);B4E1Z4(GO:0005886);I3L1J2(GO:0009897;GO:0016342;GO:0009986;GO:0005886);M0R0Q9(GO:0005886;GO:0005788;GO:0035578;GO:0034774;GO:0009986);P01031(GO:0005579);A0A075B7F0(GO:0009897);H0YGX7(GO:0005737);P00734(GO:0009897;GO:0005788;GO:0005796;GO:0005886);P13645(GO:0005737;GO:0009986;GO:0005634;GO:0005882;GO:0005829;GO:0001533);A0A0A0MS14(GO:0009897;GO:0005886);P35579(GO:0016459;GO:0015629;GO:0005886;GO:0031252;GO:0005903;GO:0001931;GO:0008180;GO:0005737;GO:0001726;GO:0005634;GO:0097513;GO:0005938;GO:0016460;GO:0042641;GO:0001725;GO:0032154;GO:0008305;GO:0001772;GO:0005826;GO:0005829;GO:0005856;GO:0030863;GO:0005819);P13473(GO:0005770;GO:0101003;GO:0044754;GO:0035577;GO:0010008;GO:1990836;GO:0043202;GO:0097637;GO:0031410;GO:0031902;GO:0005886;GO:0000421;GO:0030670;GO:0031088;GO:0005764;GO:0005765;GO:0005768);P01717(GO:0005886);A0A0A0MS15(GO:0009897;GO:0005886);P48740(GO:0005829;GO:0005654);P00738(GO:0071682;GO:0035580;GO:1904724);P01857(GO:0009897;GO:0005886);A0A075B6Q5(GO:0009897;GO:0005886);P16930(GO:0005829);P01019(GO:0005829;GO:0005737);Q14624(GO:0005737;GO:0031089;GO:0005886);Q16610(GO:0031089);P11226(GO:0009986);P01011(GO:0034774;GO:0035578;GO:0031093;GO:0005622;GO:0005634);H0Y2Y8(GO:0005829);Q9UHG3(GO:0005774;GO:0005764;GO:0005886);P41222(GO:0005783;GO:0005789;GO:0005737;GO:0005634;GO:0048471;GO:0005794;GO:0005791;GO:0031965);A0A0C4DH55(GO:0005886);P0DP01(GO:0005886);P14151(GO:0009897;GO:0030667;GO:0009986;GO:0005887;GO:0005886);P04430(GO:0005886);P31151(GO:0005783;GO:0035578;GO:0005737;GO:0005634;GO:0005829);P01721(GO:0005886);A0A0A0MTH3(GO:0005829;GO:0005654;GO:0030027);C9JXI5(GO:0031410;GO:0005886);O14791(GO:0005788);P13796(GO:0032432;GO:0015629;GO:0030175;GO:0005737;GO:0001726;GO:0002102;GO:0048471;GO:0005884;GO:0005886;GO:0042995;GO:0032587;GO:0001891;GO:0005829;GO:0005856;GO:0001725);D6RAR4(GO:0005737;GO:0005791);P36955(GO:0030424;GO:0043203;GO:0048471;GO:0043025;GO:0042470);A0A0B4J1U3(GO:0005886);P09486(GO:0005737;GO:0031091;GO:0031093;GO:0031092;GO:0005634;GO:0005739;GO:0016363;GO:0009986;GO:0005886;GO:0071682;GO:0005622);A0A0B4J231(GO:0009897);P18206(GO:0015629;GO:0034774;GO:0005737;GO:0002102;GO:0035580;GO:0005856;GO:0042383;GO:0005886;GO:1904813;GO:0005829;GO:0043034);P0DPH7(GO:0005737;GO:0005856;GO:0005874);P80748(GO:0005886);A0A075B6K2(GO:0005886);Q13093(GO:0005737);P32119(GO:0005737;GO:0005829);P06312(GO:0005886);A0A2R8Y6G6(GO:0000015);P80108(GO:0005737;GO:0043231;GO:0005765;GO:0005622);P08514(GO:0009897;GO:0031092;GO:0009986;GO:0008305;GO:0005886);P01009(GO:0005783;GO:0033116;GO:0005788;GO:1904813;GO:0030134;GO:0031093;GO:0005794;GO:0043231;GO:0000139);P02751(GO:0005788;GO:0031093;GO:0005793;GO:0016324);A0A075B6R2(GO:0009897;GO:0005886);P02760(GO:0005886;GO:0009986;GO:0043231);P02750(GO:0043231;GO:1904813;GO:1904724;GO:0035580);P02775(GO:0031091;GO:0031093;GO:1904724);A0A2R8Y7X9(GO:0005833);P01008(GO:0005788;GO:0005886);J3QRV5(GO:0005737;GO:0043231;GO:0005829);P69892(GO:0005829;GO:0005833);H9KV75(GO:0032391;GO:0042383;GO:0005737;GO:0001725;GO:0097433;GO:0005815;GO:0030027;GO:0031941;GO:0016328;GO:0030486;GO:1990357;GO:0031252;GO:0097381;GO:0090636;GO:0090637);A0A075B6K5(GO:0005886);A0A075B6J9(GO:0005886);Q01518(GO:0005737;GO:0030864;GO:0035578;GO:0005886);P02753(GO:0005829);A0A669KAY4(GO:0005770;GO:0005788;GO:0005737;GO:0036020;GO:0030134;GO:0005794;GO:0005791;GO:0009986;GO:0048471;GO:0005886;GO:0031232;GO:0005764;GO:0005765;GO:0005783;GO:0005769);P01344(GO:0031093);P27169(GO:0043231);P13647(GO:0005737;GO:0045095;GO:0005634;GO:0005882;GO:0005886;GO:0005829);P01871(GO:0009897;GO:0009986;GO:0005886);P02100(GO:0005833;GO:0005829);P01715(GO:0005886);O75882(GO:0005737;GO:0005887;GO:0005886);A0A0B4J1V2(GO:0009897;GO:0005886);P00918(GO:0043209;GO:0045177;GO:0016323;GO:0030424;GO:0005829;GO:0005737;GO:0005886;GO:0005902);A0A0C4DH32(GO:0009897;GO:0005886);A0A0B4J1X5(GO:0009897;GO:0005886);Q6EMK4(GO:0009986;GO:0005886;GO:0005765;GO:0005739);A0A0A0MRJ7(GO:0031093;GO:0033116;GO:0005788;GO:0030134;GO:0005886;GO:0000139);P04040(GO:0005829;GO:0005782;GO:0005886;GO:1904813;GO:0005739;GO:0005783;GO:0005778;GO:0005777;GO:0005758;GO:0034774;GO:0005794;GO:0043231;GO:0005764);A0A0C4DH33(GO:0009897;GO:0005886);P18065(GO:0031410;GO:0016324);P00915(GO:0005737;GO:0005829);P05546(GO:0005788);Q562R1(GO:0005737;GO:0005856;GO:0015629);P02743(GO:0005634);A0A075B6K4(GO:0005886);P01860(GO:0009897;GO:0005886);C9J8S2(GO:0031089);P04211(GO:0005886);A0A075B7B8(GO:0009897);A0A0B4J2D9(GO:0005886);P02749(GO:0009986;GO:0031089);P68032(GO:0015629;GO:0030175;GO:0030017;GO:0005737;GO:0001725;GO:0031674;GO:0030027;GO:0005856;GO:0044297;GO:0005884;GO:0005865;GO:0005829;GO:0042643);P01034(GO:0005783;GO:0005788;GO:1904724;GO:0005737;GO:0042995;GO:0005771;GO:0030424;GO:0048471;GO:0043025;GO:0031965;GO:1904813;GO:0005764;GO:0043292);A0A087WSZ0(GO:0005886);C9JB55(GO:0055037;GO:0030139;GO:0005905;GO:1990712;GO:0005770;GO:0009925;GO:0048471;GO:0016324;GO:0031232;GO:0005769);Q15582(GO:0005886;GO:0005802);P02649(GO:0005783;GO:0005788;GO:0071682;GO:0030425;GO:0043025;GO:0005737;GO:0005634;GO:0030669;GO:0005794;GO:0005886;GO:0005769);Q07954(GO:0030425;GO:0005905;GO:0005737;GO:0098797;GO:0045177;GO:0030136;GO:0043025;GO:0044295;GO:0030666;GO:0005887;GO:0005886;GO:0016323;GO:0005634;GO:0005765;GO:0005768;GO:0005769);P04196(GO:0031093;GO:0005886;GO:0009986;GO:0036019);A0A0C4DH25(GO:0005886);P02647(GO:0005788;GO:0071682;GO:0034774;GO:0005829;GO:0031410;GO:0005634;GO:0009986;GO:0030139;GO:0005886;GO:0005769);P01861(GO:0009897;GO:0005886);P10909(GO:0005783;GO:0005794;GO:0031966;GO:0043231;GO:0005829;GO:0099020;GO:0005856;GO:0048471;GO:0097418;GO:0005737;GO:0031093;GO:0031410;GO:0005634;GO:0005739;GO:0009986;GO:0005622;GO:0005743;GO:0097440;GO:0071944;GO:0042583);P04114(GO:0005783;GO:0005788;GO:0005789;GO:0005737;GO:0043202;GO:0071682;GO:0030669;GO:0010008;GO:0043025;GO:0070971;GO:0005790;GO:0005886;GO:0031904;GO:0005829;GO:0043231;GO:0005769);P05109(GO:0005737;GO:0045111;GO:0005634;GO:0034774;GO:0005856;GO:0005886;GO:0005829);P15169(GO:0097060;GO:0005794;GO:0030141;GO:0043025);A0A0C4DH31(GO:0009897;GO:0005886);A0A0C4DH36(GO:0009897);A0A0C4DH34(GO:0009897;GO:0005886);P19320(GO:0009897;GO:0005783;GO:0005794;GO:0030175;GO:0005902;GO:0045177;GO:0002102;GO:0042383;GO:0005887;GO:0005886;GO:0009986;GO:0005769;GO:0071065);P08697(GO:0031093;GO:0009986);A0A0C4DH43(GO:0009897;GO:0005886);A0A0C4DH38(GO:0009897;GO:0005886);A0A0C4DH39(GO:0009897;GO:0005886);P25311(GO:0009897;GO:0005634;GO:0005886);P01599(GO:0005886);O43866(GO:0005737;GO:0009986);P01024(GO:0005886;GO:0005788;GO:0035578;GO:0034774;GO:0009986);P01023(GO:0031093;GO:0005829);H0YCR7(GO:0005654;GO:0005829);P01601(GO:0005886);A0A0C4DH24(GO:0005886);O95497(GO:0035577;GO:0005886);A0A0B4J1Y8(GO:0005886);P02538(GO:0005829;GO:0045095;GO:0005882;GO:0005634);P01782(GO:0005886);P00747(GO:0009986;GO:0005886;GO:0031232;GO:0031093);P00740(GO:0005788;GO:0005796;GO:0005886);P60709(GO:0015629;GO:0036464;GO:0043209;GO:0005634;GO:0097433;GO:0098871;GO:0005654;GO:0005856;GO:0000790;GO:0005886;GO:0044305;GO:0030863;GO:0005829;GO:0005737;GO:0098793;GO:0035267);P00742(GO:0005788;GO:0005796;GO:0005886;GO:0031233);E7EQB2(GO:0009986;GO:0042581);P02533(GO:0045095;GO:0071944;GO:0005737;GO:0005634;GO:0045178;GO:0005882;GO:0005829;GO:0005622);P01742(GO:0005886);P00450(GO:0005765;GO:0005788;GO:0005886);Q08380(GO:0031089);P43121(GO:0009897;GO:0005886;GO:0005634);P0C0L5(GO:0030425;GO:0030424;GO:0005886;GO:0042995);P0C0L4(GO:0005788;GO:0030424;GO:0043025;GO:0030425;GO:0005886;GO:0042995);Q9H4B7(GO:0005737;GO:0005856;GO:0005874);C9JV77(GO:0005788;GO:0031093;GO:0005794;GO:0034774);P02652(GO:0005788;GO:0005829;GO:0005769);Q9HDC9(GO:0005783;GO:0009986);P35858(GO:0005654);P01763(GO:0005886);E9PHK0(GO:0005737;GO:0001652;GO:0031089);P04433(GO:0005886);A0A087WSY6(GO:0005886);P06727(GO:0005788;GO:0009986;GO:0005829;GO:0005769);C9J5S7(GO:0005829;GO:0005634);A0A0C4DH29(GO:0009897;GO:0005886);Q13103(GO:0005788;GO:0031089);P33908(GO:0005794;GO:0005829;GO:0005793;GO:0000139;GO:0005783);P01814(GO:0005886);P21333(GO:0098794;GO:0030018;GO:0015629;GO:0005737;GO:0005730;GO:0043025;GO:0005856;GO:0031941;GO:0048471;GO:0005634;GO:0005884;GO:0005886;GO:0097440;GO:0030863;GO:0043198;GO:0005829;GO:0031523;GO:0005938);P03951(GO:0005886);A0A0B4J1V1(GO:0009897;GO:0005886);A0A0B4J1V0(GO:0009897;GO:0005886);P07360(GO:0005579);A0A0A0MS51(GO:0015629;GO:0001726;GO:0043209;GO:0005634;GO:0030478;GO:0048471;GO:0030027;GO:0045335;GO:0002102;GO:0005886;GO:0005829;GO:0016528);Q5T749(GO:0005737);P01817(GO:0005886);P07437(GO:0035578;GO:0036464;GO:0005874;GO:0005737;GO:0005634;GO:0005856;GO:0044297;GO:0045298;GO:0005641;GO:0005829);H0YAC1(GO:0005886);Q96HR3(GO:0005634;GO:0000151;GO:0005654;GO:0016592);P11021(GO:0005783;GO:0005789;GO:0034663;GO:0008180;GO:0005737;GO:0005793;GO:0043209;GO:0005634;GO:0005739;GO:0030176;GO:0005790;GO:0042470;GO:0009986;GO:0005788;GO:0005886;GO:0043231;GO:0005829;GO:0030496);Q9Y490(GO:0005856;GO:0001726;GO:0005737;GO:0009986;GO:0005886;GO:0042995;GO:0032587;GO:0005829);Q15848(GO:0005783;GO:0009986);A0A0J9YVY3(GO:0005886);P01699(GO:0005886);P07737(GO:0005737;GO:0005634;GO:0098794;GO:0043005;GO:0098793;GO:0005856;GO:0005938;GO:0005829) | 37|51 | P80748(GO:0005886);A0A0B4J1Y8(GO:0005886);P03950(GO:0030426;GO:0005730;GO:0031410;GO:0005634;GO:0043025);A0A0C4DH73(GO:0005886);P01717(GO:0005886);P00915(GO:0005737;GO:0005829);Q9H4B7(GO:0005737;GO:0005856;GO:0005874);C9JXI5(GO:0031410;GO:0005886);A0A0C4DH36(GO:0009897);P37802(GO:0005829);P35527(GO:0005829;GO:0005882;GO:0005634);A0A075B6K2(GO:0005886);P32119(GO:0005737;GO:0005829);P03951(GO:0005886);P02751(GO:0005788;GO:0031093;GO:0005793;GO:0016324);P60709(GO:0015629;GO:0036464;GO:0043209;GO:0005634;GO:0097433;GO:0098871;GO:0005654;GO:0005856;GO:0000790;GO:0005886;GO:0044305;GO:0030863;GO:0005829;GO:0005737;GO:0098793;GO:0035267);Q86UX7(GO:0002102;GO:0031093;GO:0042995);H3BTN5(GO:0005737;GO:0005634;GO:0005829);A0A075B6J9(GO:0005886);Q14624(GO:0005737;GO:0031089;GO:0005886);P0DOY3(GO:0005886);P29622(GO:0031089);I3L1J2(GO:0009897;GO:0016342;GO:0009986;GO:0005886);Q16610(GO:0031089);P30041(GO:0035578;GO:0005737;GO:0005634;GO:0048471;GO:0005829;GO:0005764);P18206(GO:0015629;GO:0034774;GO:0005737;GO:0002102;GO:0035580;GO:0005856;GO:0042383;GO:0005886;GO:1904813;GO:0005829;GO:0043034);P13647(GO:0005737;GO:0045095;GO:0005634;GO:0005882;GO:0005886;GO:0005829);P01715(GO:0005886);P19823(GO:0005788);P04040(GO:0005829;GO:0005782;GO:0005886;GO:1904813;GO:0005739;GO:0005783;GO:0005778;GO:0005777;GO:0005758;GO:0034774;GO:0005794;GO:0043231;GO:0005764);P07360(GO:0005579);A0A0J9YX35(GO:0005886);P68032(GO:0015629;GO:0030175;GO:0030017;GO:0005737;GO:0001725;GO:0031674;GO:0030027;GO:0005856;GO:0044297;GO:0005884;GO:0005865;GO:0005829;GO:0042643);Q96HR3(GO:0005634;GO:0000151;GO:0005654;GO:0016592);D6RE82(GO:0005730;GO:0005634);P69905(GO:0022627;GO:0071682;GO:0005833;GO:0005829);P07737(GO:0005737;GO:0005634;GO:0098794;GO:0043005;GO:0098793;GO:0005856;GO:0005938;GO:0005829) | 0.060461178 | 0.268716344 | https://www.ebi.ac.uk/QuickGO/term/GO:0044464 |
| GO:0065007 | biological regulation | biological_process | 291|375 | P08779(GO:0061436;GO:0030336);P00488(GO:0007599;GO:0019221;GO:0007596);P68366(GO:0010389);P04275(GO:0007599;GO:0007596);A0A0U1RQV3(GO:0007173);J3KNB4(GO:0044130;GO:0045766;GO:0008284;GO:0044140;GO:0001934);P01700(GO:0038096;GO:0030449;GO:0050776;GO:0038095;GO:0006956;GO:0006958);P03951(GO:0007599;GO:0007596;GO:0030193;GO:0051919);P03950(GO:0032148;GO:0032431;GO:0042592;GO:0048662;GO:0017148;GO:0042327;GO:0050714;GO:0007202;GO:0001938);G3XAP6(GO:0030509;GO:0030500;GO:1900047;GO:0014829;GO:1902732;GO:0043066);P05160(GO:0007596;GO:0007599;GO:1903363);P23142(GO:0007162;GO:1900025;GO:2000647;GO:0010952;GO:2000146;GO:0001933;GO:0070373;GO:0007229);Q03591(GO:0032091;GO:0030449;GO:0045919;GO:0006956);B7ZKJ8(GO:0010951);P41222(GO:2000255;GO:0045187);B1AHL2(GO:0010952);P08571(GO:0097190;GO:1901224;GO:0007166;GO:0032729;GO:0002224;GO:0032760;GO:0034128;GO:0038124;GO:2000484;GO:0035666;GO:0032481;GO:0034142;GO:0002755;GO:0002756;GO:0050715;GO:0038123;GO:0031663;GO:0007249;GO:0045807);P68871(GO:0007596;GO:0045429;GO:0008217;GO:0010942;GO:0050880);P48740(GO:0006956;GO:0001867);E9PK25(GO:0022604;GO:0061001;GO:0030836;GO:0043066;GO:0035722;GO:0044794;GO:0007266);O75460(GO:0007257;GO:0016241;GO:0071333;GO:0006402;GO:0033120;GO:0007050;GO:0036498;GO:0030968;GO:0070059;GO:1904707;GO:0006355;GO:1900103);Q86YZ3(GO:0061436);P01597(GO:0038096;GO:0030449;GO:0050776;GO:0038095;GO:0006956;GO:0006958);P01042(GO:0007599;GO:0007596;GO:0045861;GO:0007162;GO:0042311;GO:0050880;GO:0043065;GO:0010466;GO:0007186;GO:0010951;GO:0007204;GO:0030195);P14151(GO:0050776);P01602(GO:0038096;GO:0030449;GO:0050776;GO:0038095;GO:0006956;GO:0006958);A0A0G2JMI3(GO:0050853;GO:0006958;GO:0050871);A0A0B4J1U7(GO:0050853;GO:0006958;GO:0050871);P59665(GO:0030520);C9JC84(GO:0007596);P51884(GO:0032914;GO:0045944);P06312(GO:0038096;GO:0030449;GO:0050776;GO:0038095;GO:0006956;GO:0006958);P01743(GO:0038096;GO:0030449;GO:0050776;GO:0038095;GO:0006956;GO:0006958);K7ERG9(GO:0007219;GO:0006957);P02790(GO:0002925;GO:0051246;GO:0042531;GO:0006879;GO:0060332;GO:0060335;GO:0002639);P60763(GO:0022604;GO:0035556;GO:0030838;GO:1900026;GO:0051056;GO:0014041;GO:0007266;GO:0007264;GO:0048873;GO:0033630);A0A075B7D8(GO:0050853;GO:0006958;GO:0050871);P01764(GO:0030449;GO:0050853;GO:0050776;GO:0006956;GO:0006958;GO:0050871;GO:0038096;GO:0038095);P01766(GO:0038096;GO:0030449;GO:0038095;GO:0050776;GO:0006956;GO:0006958);Q15582(GO:0007162);F8WF14(GO:0050805;GO:0008285;GO:0019695);P26038(GO:0010628;GO:0071803;GO:1902115;GO:2000401;GO:0035722;GO:2000643;GO:1903364;GO:0008361;GO:0008360;GO:1902966);P29622(GO:0010466;GO:0010951);P18428(GO:0044130;GO:0019221;GO:0060265;GO:0090023;GO:0042535;GO:0045919;GO:0002224;GO:0032722;GO:0032720;GO:0032760;GO:0034142;GO:0034145;GO:0031663;GO:0043032;GO:0032757;GO:0032755);P01780(GO:0038096;GO:0030449;GO:0050776;GO:0038095;GO:0006956;GO:0006958);P06331(GO:0038096;GO:0030449;GO:0050776;GO:0038095;GO:0006956;GO:0006958);P02776(GO:0070098;GO:0019221;GO:0010469;GO:0032760;GO:0010628;GO:0045918;GO:0045347;GO:0007189;GO:0010744;GO:0007186;GO:2001240;GO:0042127;GO:0043950;GO:0090023;GO:0045651;GO:0045652;GO:0045653;GO:0045944;GO:0016525);D6R934(GO:0006958);P02775(GO:0042127;GO:0070098;GO:0007186;GO:0090023;GO:0010469;GO:0051781);H3BTN5(GO:1903672);Q13201(GO:0010811;GO:0007596);E7EUT5(GO:0050821;GO:0052501;GO:0050715;GO:0017148;GO:0006417);A0A0G2JMB2(GO:0050853;GO:0006958;GO:0050871);P30041(GO:0048026;GO:0045454);P06702(GO:0030307;GO:0050729;GO:0051493;GO:0050727;GO:0045113;GO:0002224;GO:2001244;GO:0006919;GO:0032119;GO:0051092;GO:0002793;GO:0010976;GO:0006417;GO:0030194);P61224(GO:0007165;GO:2000114;GO:0045955;GO:0035722;GO:0032486;GO:0007264;GO:1901888;GO:0070374;GO:2000301);P10643(GO:0030449;GO:0006883;GO:0006956;GO:0006957;GO:0006958);P02787(GO:0055072;GO:0048260;GO:0007257;GO:0045780;GO:2000147;GO:0006879;GO:0045893;GO:0001895;GO:0034756;GO:0042327;GO:0060395;GO:0031647;GO:0070371;GO:0034756);P19827(GO:0010466;GO:0010951);P01833(GO:0001895;GO:0007173;GO:0038093);P01834(GO:0030449;GO:0050871;GO:0050853;GO:0050776;GO:0006956;GO:0006958;GO:0001895;GO:0038096;GO:0038095);P19823(GO:0010951;GO:0010466);P04003(GO:0030449;GO:0045732;GO:1903027;GO:0006958;GO:0045959);E7END6(GO:0007596);P04004(GO:0030449;GO:0014911;GO:0030949;GO:0090303;GO:0048260;GO:0032092;GO:0010811;GO:0010951;GO:0050731;GO:0030195);P00450(GO:0006879;GO:0055072);E7ENL6(GO:0010951);O00391(GO:0016242;GO:0045454);C9JV77(GO:0030500;GO:0030502;GO:0050766;GO:0050727;GO:0046627;GO:0010951);K7ER74(GO:0051006;GO:0010902;GO:0048261;GO:0043085;GO:0032375;GO:0045833;GO:0010898;GO:0070328;GO:0010916;GO:0042632;GO:0010518;GO:0060697;GO:0045723);P69905(GO:0010942);P18206(GO:0030336;GO:0030334);G3XAK1(GO:2000479;GO:0010628;GO:0033601;GO:1904036;GO:0045721;GO:0046425;GO:0048012;GO:0010758);G3V0E5(GO:0045780;GO:0045830;GO:0006879;GO:0030890;GO:0042102);P02768(GO:0051659;GO:0043066;GO:0032460;GO:0001895;GO:0043069);O75636(GO:0006956;GO:1902679;GO:0046597;GO:0001867);O00187(GO:0006956;GO:0001867;GO:0006958);Q96PD5(GO:0032827;GO:0050727;GO:0002221;GO:0032689);A6XND0(GO:0001558);P02763(GO:1904469;GO:0050716;GO:0050718;GO:0032715;GO:0002682;GO:0032720);A0A0B4J1X5(GO:0050853;GO:0006958;GO:0050871);A0A0C4DH73(GO:0038096;GO:0030449;GO:0038095;GO:0050776;GO:0006956;GO:0006958);P00915(GO:0035722);P20742(GO:0010951;GO:0010466);Q9BWP8(GO:0001867;GO:0006956);P07357(GO:0030449;GO:0006956;GO:0006957;GO:0006958);A0A0G2JI36(GO:0060333;GO:0060337;GO:0050776;GO:0001916);Q15166(GO:0010124;GO:0032929);P07358(GO:0030449;GO:0006956;GO:0006957;GO:0006958);P09172(GO:0042127;GO:2001236;GO:0045907;GO:0042593;GO:0006589;GO:0042309;GO:0120162);P01703(GO:0038096;GO:0030449;GO:0050776;GO:0038095;GO:0006956;GO:0006958);Q8IV42(GO:0001514);P01701(GO:0038096;GO:0030449;GO:0050776;GO:0038095;GO:0006956;GO:0006958);P01706(GO:0038096;GO:0030449;GO:0050776;GO:0038095;GO:0006956;GO:0006958);P0C0L5(GO:0030449;GO:0006956;GO:2000427;GO:0010951;GO:0006958);P21333(GO:2000179;GO:0050821;GO:0042177;GO:1905031;GO:0051220;GO:1905000;GO:0043066;GO:0016479;GO:0030334;GO:2001046;GO:0032233;GO:0042307;GO:0043433;GO:0071526;GO:1901381;GO:2001224;GO:1900026;GO:0043123;GO:0007195);P01705(GO:0038096;GO:0030449;GO:0050776;GO:0038095;GO:0006956;GO:0006958);P22792(GO:0030449;GO:0050790;GO:0050821);P23083(GO:0038096;GO:0030449;GO:0050776;GO:0038095;GO:0006956;GO:0006958);P01709(GO:0038096;GO:0030449;GO:0050776;GO:0038095;GO:0006956;GO:0006958);P02741(GO:0010628;GO:0097756;GO:0010745;GO:2000482;GO:0032930;GO:0006958;GO:0010888);P02671(GO:0045907;GO:0007599;GO:0034116;GO:0007596;GO:0045921;GO:1902042;GO:1900026;GO:0002224;GO:2000352;GO:0090277;GO:0042730;GO:0050714;GO:0070374);P02675(GO:1902042;GO:0034116;GO:0045921;GO:0007599;GO:0007596;GO:0045907;GO:1900026;GO:0002224;GO:2000352;GO:0090277;GO:0042730;GO:0050714;GO:0070374);P07996(GO:2001237;GO:0050921;GO:1903671;GO:0051895;GO:0042535;GO:0030511;GO:0002581;GO:0042327;GO:0043536;GO:0040037;GO:2001027;GO:0045727;GO:0045652;GO:0043032;GO:0030194;GO:0002605;GO:0051897;GO:2000379;GO:0007050;GO:0090051;GO:0043154;GO:1902043;GO:0010763;GO:0051918;GO:0008284;GO:2000353;GO:0001953;GO:0016525;GO:0000187;GO:0010751;GO:0045766;GO:0043066;GO:0010748;GO:0032695;GO:1903588;GO:0030335;GO:0048661;GO:0032914;GO:0043537;GO:0010595;GO:0010596;GO:0010757;GO:0010754;GO:0010759;GO:0001937;GO:0071636);P13671(GO:0030449;GO:0045917;GO:0045766;GO:0006956;GO:0001970;GO:0006958);F5H8B0(GO:0007596);P0CF74(GO:0030449;GO:0050853;GO:0050776;GO:0006956;GO:0006958;GO:0050871;GO:0038096;GO:0038095);P61626(GO:0001895);P04180(GO:0090107;GO:0042632);P31151(GO:0010820;GO:0090026;GO:0071624;GO:0051238;GO:0070374);E9PHK0(GO:0010756);O75882(GO:0040014);P22891(GO:0007596;GO:0030195;GO:0007599);Q06033(GO:0010951;GO:0010466);C9JF17(GO:0051895;GO:0010642;GO:2000405;GO:0048662;GO:2000098;GO:0042308;GO:0060588;GO:1900016;GO:0071638);P00739(GO:0010942);P05155(GO:0030449;GO:0007596;GO:0007599;GO:0010466;GO:0001869;GO:0045916;GO:0010951;GO:0006958;GO:0042730;GO:0030193);P05154(GO:0007596;GO:0051346;GO:0010466;GO:0010951;GO:0045861);B4E1Z4(GO:0030449;GO:0006956;GO:0006957);P27169(GO:0034445;GO:0032411;GO:0010875;GO:0051099);I3L1J2(GO:0050728;GO:2000114;GO:0045766;GO:0008285;GO:0007179;GO:1903142);M0R0Q9(GO:0030449;GO:0007165;GO:0048260;GO:0010828;GO:0010866;GO:0045766;GO:1905114;GO:0045745;GO:2000427;GO:0007186;GO:0006956;GO:0006957;GO:0060100;GO:0006958;GO:0050776;GO:0010575;GO:0001798;GO:0010884;GO:0001970;GO:0001934);P01031(GO:0000187;GO:0030449;GO:0007166;GO:0045766;GO:0010760;GO:0007186;GO:0090197;GO:0006956;GO:0006957;GO:0010951;GO:0006958;GO:0010575);O95445(GO:0034445;GO:0042632);A0A075B7F0(GO:0050853;GO:0006958;GO:0050871);P00734(GO:0048712;GO:0030307;GO:0007596;GO:0051281;GO:0030449;GO:0032967;GO:0007166;GO:0007599;GO:0010544;GO:0007186;GO:0010468;GO:1900738;GO:0008284;GO:2000379;GO:0046427;GO:0090218;GO:0008360;GO:0014068;GO:1900016;GO:0042730;GO:1900182;GO:0051480;GO:0010469;GO:0051918;GO:0030193;GO:0045861;GO:0001934;GO:0030194);P13645(GO:0045684);A0A0A0MS14(GO:0050853;GO:0006958;GO:0050871);P35579(GO:0007229;GO:1903923;GO:1903919;GO:1905684;GO:0008360);P01715(GO:0038096;GO:0030449;GO:0050776;GO:0038095;GO:0006956;GO:0006958);P01717(GO:0038096;GO:0030449;GO:0050776;GO:0038095;GO:0006956;GO:0006958);A0A0A0MS15(GO:0050853;GO:0006958;GO:0050871);P01718(GO:0038096;GO:0030449;GO:0050776;GO:0038095;GO:0006956;GO:0006958);P00738(GO:0010942;GO:2000296;GO:0051354);P01857(GO:0030449;GO:0019221;GO:0050853;GO:0006956;GO:0006958;GO:0050871;GO:0038096);E7EQB2(GO:0032680;GO:2000117;GO:0045071;GO:0044793;GO:0045669;GO:1900229;GO:0043123;GO:1902732;GO:0032780;GO:0034145;GO:0051092;GO:0033690;GO:0031665;GO:0001817);P80748(GO:0038096;GO:0030449;GO:0050776;GO:0038095;GO:0006956;GO:0006958);P01019(GO:1903779;GO:0038166;GO:2001238;GO:0007166;GO:0050729;GO:0042127;GO:0032930;GO:0014824;GO:0019229;GO:0010976;GO:0010873;GO:0050731;GO:0035815;GO:0007202;GO:0007204;GO:0003081;GO:1904754;GO:0010536;GO:0007200;GO:0042310;GO:0042311;GO:0048169;GO:1901201;GO:0006883;GO:0061098;GO:0007186;GO:2000379;GO:0090190;GO:0003331;GO:0033864;GO:0007199;GO:0051969;GO:0016525;GO:0030308;GO:0048146;GO:0045429;GO:1905010;GO:0043085;GO:0050880;GO:0002027;GO:0045742;GO:0032270;GO:0008284;GO:0001558;GO:0014068;GO:0014061;GO:0051092;GO:0002034;GO:0070371;GO:0001819;GO:0051387;GO:0010469;GO:0010666;GO:0008217;GO:0097755;GO:0042981;GO:0010744;GO:0035813;GO:1904707;GO:0046628;GO:0034104;GO:0019216;GO:0051403;GO:0010951;GO:0007263;GO:1905589;GO:0010613;GO:0010595;GO:0002019;GO:0002018;GO:0045777;GO:0002016;GO:0051924;GO:1903598;GO:0045893);A0A0S2Z4L3(GO:0030195);A0A0C4DH25(GO:0038096;GO:0030449;GO:0038095;GO:0050776;GO:0006956;GO:0006958);P08185(GO:0010951;GO:0008211);P01011(GO:0010466;GO:0019216;GO:0030277;GO:0010951);P08514(GO:0002687;GO:0007229;GO:0045652);P19652(GO:1904469;GO:0050716;GO:0050718;GO:0002682);P04264(GO:0061436;GO:0050728;GO:0045765;GO:0001867;GO:0001895;GO:0042730);B0YIW2(GO:0051005;GO:0048261;GO:0045833;GO:0010897;GO:0007186;GO:0070328;GO:0060621;GO:0010916;GO:0042632;GO:0032489;GO:0050995;GO:0010989;GO:0010987;GO:0045717;GO:0010903);C9J5S7(GO:0045069);A0A1W2PQU7(GO:1904714;GO:0060291;GO:0051580;GO:0010977;GO:0010625);Q9UGM5(GO:0010951;GO:0010466);H0YGX7(GO:0050790);G3V2W1(GO:0010951;GO:0007596);A0A0A0MTH3(GO:0033209;GO:1901224;GO:0045669;GO:2000178;GO:0008284;GO:0045893;GO:0030513;GO:0043491;GO:0007229;GO:0090263;GO:0001934);C9JXI5(GO:0090263);P07437(GO:0050807;GO:0010389);P13796(GO:0033157;GO:0071803;GO:0035722;GO:0010737);P15144(GO:0008217;GO:0007165);P36955(GO:0010629;GO:0060770;GO:0050769;GO:0071333;GO:0050728;GO:0010976;GO:0010951;GO:1901215;GO:0010596;GO:0016525);P0DJI8(GO:0000187;GO:0019221;GO:0050728;GO:0050708;GO:0045785;GO:0007186;GO:0050716;GO:0050715;GO:0007204);A0A0G2JPR0(GO:0006956;GO:0010951);C9JPQ9(GO:0007596);P09486(GO:0022604;GO:0050807;GO:0042127;GO:0010595;GO:0001937;GO:0016525);P11226(GO:0044130;GO:0050766;GO:0001867;GO:0006956;GO:0006958;GO:0048525);P04211(GO:0038096;GO:0030449;GO:0050776;GO:0038095;GO:0006956;GO:0006958);Q96KN2(GO:0032268);Q13093(GO:0050729;GO:0090026);P43121(GO:0030335);Q96IY4(GO:0007599;GO:2000346;GO:0007596;GO:0030449;GO:0071333;GO:0051918;GO:0042730;GO:0003331;GO:0010757);P01721(GO:0038096;GO:0030449;GO:0050776;GO:0038095;GO:0006956;GO:0006958);A0A0B4J231(GO:0050853;GO:0006958;GO:0050871);P55058(GO:0010875);P01009(GO:0007599;GO:0007596;GO:0010466;GO:0010951);P01008(GO:0007599;GO:0007595;GO:2000266;GO:0007596;GO:0010466;GO:0010951;GO:0030193);A0A075B6R2(GO:0050853;GO:0006958;GO:0050871);Q9UNN8(GO:0007596;GO:0007599;GO:0050819);P02100(GO:0007596);P55056(GO:0070328;GO:0010890);P02751(GO:0019221;GO:0048146;GO:0010628;GO:1904237;GO:0008284;GO:2001202;GO:0008360;GO:0010952;GO:0045773;GO:0001932;GO:0070372);J3KRP0(GO:0032268);P69892(GO:0007596);A0A0C4DH43(GO:0050853;GO:0006958;GO:0050871);P43652(GO:0050821);Q01518(GO:0007165;GO:0007190);P02753(GO:0032024;GO:0060044;GO:0042593;GO:0051024;GO:0042572;GO:0030277);A0A669KAY4(GO:0032805;GO:1905601;GO:0010469;GO:0043523;GO:1905598;GO:0001920;GO:0002092;GO:0042632;GO:2000650;GO:1905596;GO:0010989;GO:0043525);P01344(GO:0046628;GO:0045840;GO:0008286;GO:0038028;GO:0008284;GO:0051147;GO:0031056;GO:0071902;GO:0051897;GO:0006349;GO:0000122;GO:0043085;GO:0045725;GO:0010469;GO:2000467;GO:0042104;GO:0043410;GO:0050731;GO:0040018;GO:0006355;GO:0051781;GO:0045944;GO:0001934);A0A0B4J1X8(GO:0050853;GO:0006958;GO:0050871);P01877(GO:0001895;GO:0050853;GO:0006958;GO:0050871;GO:0060267);P01871(GO:0050853;GO:0006958;GO:0050871);A0A075B7D0(GO:0050853;GO:0006958;GO:0050871);P13473(GO:0050821;GO:0046716;GO:0031647);P02766(GO:0070327;GO:0042572;GO:0010469);P09871(GO:0006956;GO:0030449;GO:0001867;GO:0006958);A0A0B4J1V2(GO:0050853;GO:0006958;GO:0050871);P00918(GO:0038166;GO:0045780;GO:2001225;GO:0032849;GO:2001150;GO:0044070;GO:0032230;GO:0051453;GO:0045672);A0A0C4DH32(GO:0050853;GO:0006958;GO:0050871);P01763(GO:0038096;GO:0030449;GO:0050776;GO:0038095;GO:0006956;GO:0006958);P01704(GO:0038096;GO:0030449;GO:0050776;GO:0038095;GO:0006956;GO:0006958);A0A0A0MRJ7(GO:0007596);P04040(GO:0043066;GO:0014068;GO:0051092;GO:0032088;GO:0051781);A0A0C4DH33(GO:0050853;GO:0006958;GO:0050871);A0A3B3ISR2(GO:0006958);P05543(GO:0070327;GO:0010951);P18065(GO:0007165;GO:0043567;GO:0040008;GO:0090090;GO:0042104;GO:0001558);P01861(GO:0030449;GO:0019221;GO:0050853;GO:0006956;GO:0006958;GO:0050871;GO:0038096);P05546(GO:0007599;GO:0007596;GO:0010951;GO:0010466);P02743(GO:0046597;GO:0044871;GO:0061045;GO:1903016;GO:1903019;GO:0044869;GO:0006958;GO:0002674;GO:0045656;GO:0048525);P55103(GO:0060395;GO:0010469;GO:0010862;GO:0042981;GO:0043408);P01860(GO:0030449;GO:0050871;GO:0050853;GO:0006956;GO:0006958;GO:0001895;GO:0038096);P02747(GO:0030449;GO:0045650;GO:0030853;GO:0006956;GO:0006958);P02745(GO:0030449;GO:0006956;GO:0006958);A0A075B7B8(GO:0050853;GO:0006958;GO:0050871);P01619(GO:0030449;GO:0050776;GO:0006956;GO:0006958;GO:0038096;GO:0038095);P02760(GO:0010951;GO:0046329;GO:0010466;GO:0050777);P02749(GO:0034392;GO:0051006;GO:0051917;GO:0051918;GO:0033033;GO:0010596;GO:0030195;GO:0030193;GO:0016525;GO:0001937;GO:0030194);P80108(GO:0035774;GO:0051044;GO:0051047;GO:0010907;GO:0010867;GO:0043065;GO:0002430;GO:0045919;GO:0010897;GO:0008285;GO:0008286;GO:0010983;GO:0010595;GO:1900076;GO:0010694);P01034(GO:2000117;GO:0043067;GO:0010466;GO:0008284;GO:0034103;GO:0045740;GO:0060548;GO:0060311;GO:0060313;GO:0010716;GO:0010711;GO:0045861);P01601(GO:0038096;GO:0030449;GO:0038095;GO:0050776;GO:0006956;GO:0006958);C9JB55(GO:0048260;GO:0043086;GO:0006879;GO:0034756);P11597(GO:0055091;GO:0010745;GO:0070328;GO:0055088;GO:0042632;GO:0010874);P02649(GO:0051044;GO:0043524;GO:0010873;GO:0051651;GO:0050728;GO:1905855;GO:0060999;GO:0045541;GO:0044794;GO:0043537;GO:0010977;GO:0030516;GO:0010875;GO:0030195;GO:1903002;GO:0019934;GO:0010629;GO:0042311;GO:0048168;GO:0010544;GO:0097006;GO:0007186;GO:1905890;GO:1902430;GO:0042632;GO:0032489;GO:1901215;GO:0006357;GO:0032805;GO:0090209;GO:0051246;GO:0050807;GO:0043407;GO:0051000;GO:1902995;GO:1900221;GO:0090181;GO:0006874;GO:0055088;GO:0055089;GO:0046889;GO:0032269;GO:1900272;GO:1902952;GO:1901630;GO:1901628;GO:0010468;GO:0032462;GO:0090090;GO:2000822;GO:0007263;GO:1905908;GO:1905860;GO:1905906;GO:0045807;GO:0070328;GO:0001937;GO:0010976);Q07954(GO:0051246;GO:0007205;GO:0032370;GO:0010942;GO:0051222;GO:2000587;GO:0045732;GO:0032374;GO:1900223;GO:0014912;GO:1903078;GO:0032429;GO:0032092;GO:1905167;GO:0010977;GO:0010875;GO:0010715;GO:0043524;GO:0030178;GO:0045807;GO:0032956);Q6UXB8(GO:0010466;GO:0061052);P04196(GO:0002839;GO:0030308;GO:0032956;GO:2000504;GO:0007162;GO:0051894;GO:0007599;GO:0043065;GO:0033629;GO:0010468;GO:0051918;GO:0010543;GO:0008285;GO:0007596;GO:0010593;GO:0043537;GO:0010951;GO:0043254;GO:2001027;GO:0042730;GO:0050730;GO:1900747;GO:0030193;GO:0016525);P32119(GO:0000187;GO:0045581;GO:0032088;GO:0042981;GO:0010310;GO:0043066;GO:2001240;GO:0045454;GO:0048872;GO:0031665;GO:0030194);P02647(GO:0034115;GO:0010804;GO:0019915;GO:0050728;GO:0051496;GO:0010898;GO:0030300;GO:0010873;GO:0007179;GO:0045723;GO:0050821;GO:0010903;GO:0007186;GO:0060761;GO:0042632;GO:0032489;GO:0050713;GO:0007229;GO:0060192;GO:0035025;GO:0051006;GO:0002740;GO:1900026;GO:0060354;GO:0070371;GO:0008211;GO:0055091;GO:0070328;GO:0019216;GO:0051346;GO:0051345;GO:0001932);Q9NZP8(GO:0006958);P10909(GO:0032436;GO:1903573;GO:1902949;GO:0042127;GO:0060548;GO:1902004;GO:0030449;GO:1902230;GO:0050821;GO:0010628;GO:0048260;GO:0032760;GO:1905895;GO:0006956;GO:1905892;GO:1901216;GO:0006958;GO:2000060;GO:1902430;GO:1902847;GO:0090201;GO:0045429;GO:1902998;GO:1901214;GO:1900221;GO:0051092;GO:0043065;GO:0032464;GO:0032463;GO:1905907;GO:1905908);P04114(GO:0010628;GO:0010884;GO:0002224;GO:0010744;GO:0042632;GO:0010886;GO:0045540);P05109(GO:0030307;GO:0050729;GO:0051493;GO:0050727;GO:0002224;GO:2001244;GO:0006919;GO:0032119;GO:0051092;GO:0002793);P15169(GO:0030449;GO:0030070);A0A0C4DH31(GO:0050853;GO:0006958;GO:0050871);A0A0C4DH36(GO:0050853;GO:0006958;GO:0050871);A0A0C4DH34(GO:0050853;GO:0006958;GO:0050871);P19320(GO:0019221;GO:0035584;GO:0042102;GO:0050776;GO:0060333);P08697(GO:0032967;GO:0051496;GO:0010466;GO:0010757;GO:0051918;GO:0048661;GO:0045597;GO:0046330;GO:0010951;GO:0042730;GO:2000049;GO:0002034;GO:0045944;GO:0070374;GO:0071636);Q14624(GO:0010951;GO:0010466);P25311(GO:0008285;GO:0001895);A0A0C4DH39(GO:0050853;GO:0006958;GO:0050871);A0A0C4DH38(GO:0050853;GO:0006958;GO:0050871);P01599(GO:0038096;GO:0030449;GO:0050776;GO:0038095;GO:0006956;GO:0006958);P01024(GO:0030449;GO:0007165;GO:0048260;GO:0010828;GO:0010866;GO:0050766;GO:0045766;GO:1905114;GO:0045745;GO:2000427;GO:0007186;GO:0006956;GO:0006957;GO:0010951;GO:0060100;GO:0006958;GO:0050776;GO:0010575;GO:0001798;GO:0010884;GO:0001970;GO:0001934);P01023(GO:0051056;GO:0010466;GO:0001869;GO:0010951;GO:0043547);C9J8S2(GO:0050994;GO:0050921;GO:0045600;GO:0008286;GO:0046626;GO:0010759;GO:0001934);P02748(GO:0030449;GO:0006957;GO:0006958);O95497(GO:0033089;GO:1902176);P02538(GO:0008284;GO:2000536;GO:0001899);P01782(GO:0038096;GO:0030449;GO:0050776;GO:0038095;GO:0006956;GO:0006958);P00747(GO:0007599;GO:0043536;GO:0051918;GO:0051919;GO:0008285;GO:0007596;GO:0010812;GO:0042730;GO:2000048);P00740(GO:0007596;GO:0007599);P60709(GO:0032091;GO:0045815;GO:1903076;GO:0048013;GO:0001895;GO:0038096;GO:0051621;GO:0022898;GO:0051623;GO:0000079);P00742(GO:0030335;GO:0007596;GO:0051897;GO:0007599);P15814(GO:0050853;GO:0006958;GO:0050871);A0A075B6Q5(GO:0050853;GO:0006958;GO:0050871);P01742(GO:0038096;GO:0030449;GO:0050776;GO:0038095;GO:0006956;GO:0006958);P00748(GO:0002542;GO:0007599;GO:0007596;GO:0051919;GO:0042730;GO:0010756;GO:0030193;GO:0030194);Q08380(GO:0007165);Q16610(GO:0007165;GO:0030500;GO:0030502;GO:0045766;GO:0010466;GO:2000404;GO:0001960;GO:0002828;GO:0001938;GO:0006357;GO:0043123);P08519(GO:0010951);P20851(GO:0030449;GO:0007596;GO:0045732;GO:1903027;GO:0006958;GO:0045959);P0C0L4(GO:0030449;GO:2000427;GO:0006956;GO:0010951;GO:0006958);A0A087WSY4(GO:0050853;GO:0006958;GO:0050871);P02652(GO:0060192;GO:0031647;GO:0010903;GO:0010873;GO:0002740;GO:0043085;GO:0032375;GO:0060621;GO:0042632;GO:0050996;GO:0030300;GO:0045416;GO:0019216;GO:0050995;GO:0060695);P35858(GO:0007165);Q15485(GO:0001867;GO:0006956);A0A0G2JL69(GO:0006956;GO:0006958);P04433(GO:0038096;GO:0030449;GO:0050776;GO:0038095;GO:0006956;GO:0006958);P06727(GO:0034445;GO:0045723;GO:0051006;GO:0042632;GO:0070328;GO:0032374;GO:0010898;GO:0055088;GO:0010873;GO:0030300);P04430(GO:0038096;GO:0030449;GO:0050776;GO:0038095;GO:0006956;GO:0006958);A0A0C4DH29(GO:0050853;GO:0006958;GO:0050871);Q6EMK4(GO:0010719;GO:0030512);Q5SRP5(GO:0034445);Q13103(GO:0010951);P01817(GO:0038096;GO:0030449;GO:0050776;GO:0038095;GO:0006956;GO:0006958);P01814(GO:0038096;GO:0030449;GO:0050776;GO:0038095;GO:0006956;GO:0006958);P02750(GO:0045766;GO:0001938;GO:0030511);P02042(GO:0007596);A0A0B4J1V1(GO:0050853;GO:0006958;GO:0050871);A0A0B4J1V0(GO:0050853;GO:0006958;GO:0050871);P07360(GO:0030449;GO:0006957;GO:0006958);A0A0A0MS51(GO:0051127;GO:1902174;GO:0030155;GO:0042989;GO:1903923;GO:0071801;GO:0045010;GO:1903903;GO:1903909;GO:1903906;GO:2001269;GO:0051016;GO:0048015;GO:0051693;GO:0046597;GO:0031648);Q86UX7(GO:0030335;GO:0007229;GO:0033632);P68032(GO:0010628;GO:0043066);H0YAC1(GO:0051919;GO:0042730;GO:0002542);Q96HR3(GO:0030521;GO:0006355;GO:0030518;GO:0045893);P11021(GO:1990440;GO:0035437;GO:0036500;GO:0006983;GO:0043066;GO:0031398;GO:0030335;GO:0036498;GO:0036499;GO:0030512;GO:0030968;GO:0060904;GO:0090074;GO:1903897;GO:1903894;GO:1903895;GO:1903891;GO:0010976);Q9Y490(GO:0036498;GO:0007016;GO:0007229);P08603(GO:1903659;GO:0030449;GO:0006956;GO:0006957);Q15848(GO:0034115;GO:0010804;GO:0050728;GO:0009967;GO:0030853;GO:0043124;GO:0033034;GO:0032720;GO:0043123;GO:2000481;GO:0010875;GO:0045650;GO:0050731;GO:1904753;GO:0045715;GO:0031953;GO:0010906;GO:0046326;GO:0045721;GO:0010739;GO:2000467;GO:2000279;GO:0042304;GO:2000478;GO:0050805;GO:0043407;GO:0010642;GO:0050765;GO:0032270;GO:0045892;GO:0046888;GO:0045599;GO:0120162;GO:0120163;GO:1900121;GO:0045860;GO:0070373;GO:0045923;GO:0042593;GO:2000590;GO:0010745;GO:0010469;GO:1904706;GO:0030336;GO:2000534;GO:0090317;GO:0045776;GO:0071639;GO:2000584;GO:0032757;GO:0001934);P01699(GO:0038096;GO:0030449;GO:0050776;GO:0038095;GO:0006956;GO:0006958);P07737(GO:0050821;GO:0050434;GO:0032232;GO:0032233;GO:0030837;GO:0030838;GO:0051496;GO:0051054;GO:0032781;GO:0060071;GO:0010634;GO:1900029;GO:0051497;GO:0006357;GO:0045944);P35908(GO:0045684) | 36|51 | O75636(GO:0006956;GO:1902679;GO:0046597;GO:0001867);P03951(GO:0007599;GO:0007596;GO:0030193;GO:0051919);P03950(GO:0032148;GO:0032431;GO:0042592;GO:0048662;GO:0017148;GO:0042327;GO:0050714;GO:0007202;GO:0001938);P29622(GO:0010466;GO:0010951);Q9UGM5(GO:0010951;GO:0010466);P00915(GO:0035722);P01717(GO:0038096;GO:0030449;GO:0050776;GO:0038095;GO:0006956;GO:0006958);C9JXI5(GO:0090263);A0A0C4DH36(GO:0050853;GO:0006958;GO:0050871);Q86UX7(GO:0030335;GO:0007229;GO:0033632);C9JPQ9(GO:0007596);E7ENL6(GO:0010951);P32119(GO:0000187;GO:0045581;GO:0032088;GO:0042981;GO:0010310;GO:0043066;GO:2001240;GO:0045454;GO:0048872;GO:0031665;GO:0030194);P55058(GO:0010875);P02751(GO:0019221;GO:0048146;GO:0010628;GO:1904237;GO:0008284;GO:2001202;GO:0008360;GO:0010952;GO:0045773;GO:0001932;GO:0070372);P60709(GO:0032091;GO:0045815;GO:1903076;GO:0048013;GO:0001895;GO:0038096;GO:0051621;GO:0022898;GO:0051623;GO:0000079);K7ERG9(GO:0007219;GO:0006957);H3BTN5(GO:1903672);Q16610(GO:0007165;GO:0030500;GO:0030502;GO:0045766;GO:0010466;GO:2000404;GO:0001960;GO:0002828;GO:0001938;GO:0006357;GO:0043123);Q14624(GO:0010951;GO:0010466);A0A0C4DH73(GO:0038096;GO:0030449;GO:0038095;GO:0050776;GO:0006956;GO:0006958);I3L1J2(GO:0050728;GO:2000114;GO:0045766;GO:0008285;GO:0007179;GO:1903142);P30041(GO:0048026;GO:0045454);Q5SRP5(GO:0034445);P18206(GO:0030336;GO:0030334);P69905(GO:0010942);P01715(GO:0038096;GO:0030449;GO:0050776;GO:0038095;GO:0006956;GO:0006958);P19823(GO:0010951;GO:0010466);P04040(GO:0043066;GO:0014068;GO:0051092;GO:0032088;GO:0051781);P07360(GO:0030449;GO:0006957;GO:0006958);P00739(GO:0010942);Q96HR3(GO:0030521;GO:0006355;GO:0030518;GO:0045893);P02747(GO:0030449;GO:0045650;GO:0030853;GO:0006956;GO:0006958);P80748(GO:0038096;GO:0030449;GO:0050776;GO:0038095;GO:0006956;GO:0006958);P68032(GO:0010628;GO:0043066);P07737(GO:0050821;GO:0050434;GO:0032232;GO:0032233;GO:0030837;GO:0030838;GO:0051496;GO:0051054;GO:0032781;GO:0060071;GO:0010634;GO:1900029;GO:0051497;GO:0006357;GO:0045944) | 0.060535077 | 0.242140306 | https://www.ebi.ac.uk/QuickGO/term/GO:0065007 |
| GO:0043226 | organelle | cellular_component | 213|375 | P08779(GO:0005856;GO:0070062;GO:0005634);P68366(GO:0070062;GO:0005856;GO:0015630);P04275(GO:0005783;GO:0031091;GO:0070062;GO:0033093);J3KNB4(GO:0042581);Q9UHG3(GO:0070062;GO:0005764);P51884(GO:0070062);P03950(GO:0005730;GO:0031410;GO:0005634);G3XAP6(GO:0070062);P23142(GO:0070062);P19652(GO:0070062);P08571(GO:0005794;GO:0070062);P68871(GO:0070062);E9PK25(GO:0015629;GO:0070062;GO:0005634;GO:0031982;GO:0030864);O75460(GO:0005739;GO:0005783);Q86YZ3(GO:0070062;GO:0005634);P35527(GO:0005634;GO:0070062);P01042(GO:0070062);P43251(GO:0070062);P01602(GO:0070062);P08185(GO:0070062);P59665(GO:0070062);P15169(GO:0005794;GO:0030141);P0DJI8(GO:0070062);P03951(GO:0070062);P43121(GO:0005634);P02750(GO:0043231;GO:0070062);P02790(GO:0070062);P60763(GO:0070062;GO:0005856);P01764(GO:0070062);A0A087X0S5(GO:0070062);A0A087WWU8(GO:0015629);P00918(GO:0070062);P0DOY3(GO:0070062);P26038(GO:0070062;GO:0005634;GO:0005856;GO:0031982);P01782(GO:0070062);P18428(GO:0070062);P01780(GO:0070062);P31151(GO:0005783;GO:0005634);H3BTN5(GO:0005634);P01008(GO:0070062);P30041(GO:0005634;GO:0070062;GO:0005764);P06702(GO:0070062;GO:0005634;GO:0005856);Q9Y5Y7(GO:0070062);P61224(GO:0005811;GO:0070062;GO:0070382);P10643(GO:0070062);P02787(GO:0055037;GO:0030139;GO:0031410;GO:0005770;GO:0070062;GO:0031982;GO:0005768;GO:0005769);P19827(GO:0070062);P01833(GO:0070062);P01834(GO:0070062);P19823(GO:0070062);Q96IY4(GO:0070062);P04004(GO:0005783;GO:0070062;GO:0043231);P00450(GO:0070062);O00391(GO:0070062;GO:0005794;GO:0043231);K7ER74(GO:0005769);P69905(GO:0070062);G3XAK1(GO:0005773);G3V0E5(GO:0055037;GO:0070062;GO:0042470;GO:0005769);P02768(GO:0005783;GO:0070062;GO:0005794;GO:0005634);P08697(GO:0070062);Q96PD5(GO:0070062);Q86UX7(GO:0002102;GO:0070062);P02763(GO:0070062);A0A087WWT3(GO:0005794;GO:0005783);P02766(GO:0070062);Q9H4B7(GO:0005856;GO:0070062);A0A2R8Y619(GO:0005634;GO:0005694);P07357(GO:0070062);P37802(GO:0031982;GO:0070062);P19320(GO:0005783;GO:0005794;GO:0002102;GO:0070062;GO:0005769);Q15166(GO:0070062;GO:0043231);P07358(GO:1903561;GO:0070062);P09172(GO:0005783;GO:0031410;GO:0043231);P01701(GO:0070062);P0C0L5(GO:0070062);P21333(GO:0015629;GO:0070062;GO:0005730;GO:0005856;GO:0005634;GO:0030863);P22792(GO:0070062);P02671(GO:0031091;GO:1903561;GO:0070062);P02675(GO:0005783;GO:1903561;GO:0031091;GO:0070062);P07996(GO:0005783;GO:0030141;GO:0031091;GO:0016529;GO:0070062);P13671(GO:0070062);P61626(GO:0070062);P04217(GO:0070062);P04180(GO:0070062);E9PHK0(GO:0070062);P22891(GO:0070062);Q06033(GO:0070062);C9JF17(GO:0022626;GO:0005783;GO:0070062);P00739(GO:0070062);P05155(GO:0070062);P05154(GO:0031094;GO:0031091;GO:0070062);P27169(GO:0070062;GO:0043231);P20742(GO:0070062);P00734(GO:0070062);P13645(GO:0070062;GO:0005634);P35579(GO:0015629;GO:0005634;GO:0070062;GO:0005856;GO:0030863;GO:0005819);P13473(GO:0005770;GO:0044754;GO:0031410;GO:0070062;GO:0005764;GO:0005768);P00738(GO:0070062);P01857(GO:0070062);M0R0Q9(GO:0070062);E7EQB2(GO:0042581);P80748(GO:0070062);P01019(GO:0070062);Q8N1N4(GO:0070062);P01011(GO:0070062;GO:0005634);Q9Y6R7(GO:0070062);P41222(GO:0005783;GO:0070062;GO:0005634;GO:0005794;GO:0005791);P11597(GO:0070062;GO:0031982);B0YIW2(GO:0070062;GO:0005769);C9J5S7(GO:0005634);A0A1W2PQU7(GO:0045111);Q9UGM5(GO:0070062);C9JXI5(GO:0031410);P13796(GO:0015629;GO:0002102;GO:0070062;GO:0005856);P15144(GO:0070062;GO:0005793);P36955(GO:0070062;GO:0042470);P09486(GO:0031091;GO:0005634;GO:0005739;GO:0031982);Q16610(GO:0070062);P18206(GO:0015629;GO:0002102;GO:0070062;GO:0005856;GO:1903561);P0DPH7(GO:0005856);P32119(GO:0070062);O00187(GO:0070062);A0A0B4J231(GO:0070062);P80108(GO:0070062;GO:0043231);P08514(GO:0070062);P01009(GO:0005783;GO:0030134;GO:0005794;GO:0070062;GO:0043231);P02751(GO:0070062;GO:0005793);P02760(GO:0070062;GO:0043231);B4E1Z4(GO:0070062);P68032(GO:0015629;GO:0070062;GO:0005856);J3QRV5(GO:0043231);Q14624(GO:0070062);P13647(GO:0070062;GO:0005634);P43652(GO:0070062);Q01518(GO:0030864;GO:0070062);Q9UNN8(GO:0070062);C9JV77(GO:0070062;GO:0005794);P29622(GO:0070062);P01877(GO:0070062);P01871(GO:0070062);O75882(GO:0070062);P01619(GO:0070062);F8WF14(GO:0005783);P01597(GO:0070062);Q6EMK4(GO:0070062;GO:0005739);A0A0A0MRJ7(GO:1903561;GO:0030134);P04040(GO:0005739;GO:0005783;GO:0005778;GO:0070062;GO:0005777;GO:0005794;GO:0043231;GO:0005764);P05543(GO:0070062);P18065(GO:0031410;GO:0070062);P00915(GO:0070062);P05546(GO:0070062);Q562R1(GO:0005856;GO:0070062;GO:0015629);P02743(GO:0070062;GO:0005634);D6RAR4(GO:0005791);D6RE82(GO:0005730;GO:0005634);P01031(GO:0070062);P02749(GO:0070062);P02748(GO:0070062);P01034(GO:0005783;GO:0005771;GO:0070062;GO:0031982;GO:0005764;GO:0043292);C9JB55(GO:0055037;GO:0030139;GO:0005770;GO:0005769);Q15582(GO:0070062);P02649(GO:0005783;GO:0005634;GO:0070062;GO:0005794;GO:1903561;GO:0005769);Q07954(GO:0030136;GO:0005634;GO:0005768;GO:0005769);P04196(GO:0070062;GO:0036019);P02647(GO:0031410;GO:0005634;GO:0070062;GO:0030139;GO:1903561;GO:0005769);P10909(GO:0005783;GO:0005794;GO:0043231;GO:0005856;GO:0031410;GO:0005634;GO:0005739;GO:0070062;GO:0042583);P04114(GO:0005783;GO:0070062;GO:0005790;GO:0043231;GO:0005769);P05109(GO:0045111;GO:0005634;GO:0005856;GO:0070062);P01861(GO:0070062);P01860(GO:0070062);A0A0G2JI36(GO:0005783;GO:0070062;GO:0005794);H0YAC1(GO:0070062);H9KV75(GO:1990357);P25311(GO:0070062;GO:0005634);P01599(GO:0070062);P01024(GO:0070062);P01023(GO:0070062);P02753(GO:0070062);P02538(GO:0070062;GO:0005634);P00747(GO:0070062);P00740(GO:0070062);P60709(GO:0015629;GO:0036464;GO:0005634;GO:0070062;GO:0098871;GO:0005856;GO:0031982;GO:0030863);P02533(GO:0070062;GO:0005634);P00748(GO:0070062;GO:0005791);Q08380(GO:0070062);P16930(GO:0070062);P08603(GO:0070062);E7EUT5(GO:0005634;GO:0005811;GO:0015630;GO:0043231);P04264(GO:0005856;GO:0070062;GO:0005634);P0C0L4(GO:0070062);A0A669KAY4(GO:0005770;GO:0030134;GO:0005794;GO:0005791;GO:0005764;GO:0005783;GO:0005769);P02652(GO:0070062;GO:0005769);Q9HDC9(GO:0005783);P35858(GO:0070062);Q15485(GO:0070062);A0A096LPE2(GO:0070062);P04433(GO:0070062);P06727(GO:0070062;GO:0005769);Q9NZP8(GO:0070062);P02775(GO:0031091);P33908(GO:0005794;GO:0070062;GO:0005793;GO:0005783);P01704(GO:0070062);P07360(GO:0070062);A0A0A0MS51(GO:0015629;GO:0005634;GO:0045335;GO:0002102);Q5T749(GO:0070062);P07437(GO:0036464;GO:0070062;GO:0005634;GO:0005856);Q96HR3(GO:0005634);P11021(GO:0005783;GO:0005793;GO:0005634;GO:0005739;GO:0005790;GO:0042470;GO:0043231;GO:0070062);Q9Y490(GO:0005856;GO:0070062);Q15848(GO:0005783);Q16880(GO:0043231);P07737(GO:0070062;GO:0005634;GO:0005856);P35908(GO:0070062;GO:0005634) | 30|51 | P03951(GO:0070062);P03950(GO:0005730;GO:0031410;GO:0005634);P29622(GO:0070062);Q9UGM5(GO:0070062);P00915(GO:0070062);Q9H4B7(GO:0005856;GO:0070062);C9JXI5(GO:0031410);P37802(GO:0031982;GO:0070062);P35527(GO:0005634;GO:0070062);P32119(GO:0070062);P02751(GO:0070062;GO:0005793);P60709(GO:0015629;GO:0036464;GO:0005634;GO:0070062;GO:0098871;GO:0005856;GO:0031982;GO:0030863);Q86UX7(GO:0002102;GO:0070062);H3BTN5(GO:0005634);P80748(GO:0070062);Q14624(GO:0070062);P0DOY3(GO:0070062);Q16610(GO:0070062);P30041(GO:0005634;GO:0070062;GO:0005764);P18206(GO:0015629;GO:0002102;GO:0070062;GO:0005856;GO:1903561);P13647(GO:0070062;GO:0005634);P19823(GO:0070062);P04040(GO:0005739;GO:0005783;GO:0005778;GO:0070062;GO:0005777;GO:0005794;GO:0043231;GO:0005764);P07360(GO:0070062);P00739(GO:0070062);P68032(GO:0015629;GO:0070062;GO:0005856);Q96HR3(GO:0005634);D6RE82(GO:0005730;GO:0005634);P69905(GO:0070062);P07737(GO:0070062;GO:0005634;GO:0005856) | 0.115522266 | 0.231044531 | https://www.ebi.ac.uk/QuickGO/term/GO:0043226 |
| GO:0045202 | synapse | cellular_component | 13|375 | P35579(GO:0031594);P10909(GO:0045202);P0C0L5(GO:0045202);P21333(GO:0098978);P0C0L4(GO:0045202);Q562R1(GO:0045202;GO:0098978);P02671(GO:0045202);P02649(GO:0098978);P60709(GO:0098685;GO:0098978);P02675(GO:0045202);P09486(GO:0045202;GO:0098978);P07737(GO:0045202;GO:0098685;GO:0098688;GO:0098978);P68032(GO:0098978;GO:0045202) | 3|51 | P60709(GO:0098685;GO:0098978);P07737(GO:0045202;GO:0098685;GO:0098688;GO:0098978);P68032(GO:0098978;GO:0045202) | 0.168493413 | 0.224657884 | https://www.ebi.ac.uk/QuickGO/term/GO:0045202 |
| GO:0022414 | reproductive process | biological_process | 19|375 | P35527(GO:0007283);P35579(GO:0051295;GO:0000212);P01344(GO:0060669;GO:0001892);P21333(GO:0097368);P03950(GO:0001541;GO:0001556;GO:0001890);P23142(GO:0007566);P11021(GO:0001554);P01019(GO:0007565);Q9UGM5(GO:0007339;GO:0007338);P05154(GO:0061107;GO:0007342;GO:0007283;GO:0007338);P04114(GO:0007283;GO:0009566);P36955(GO:0042698);P20742(GO:0007565);P18065(GO:0007565);G3XAK1(GO:0007283;GO:0007566);P02753(GO:0060065;GO:0048807;GO:0060068);P02760(GO:0007565);P01034(GO:0060009;GO:0008584;GO:0007566);P09172(GO:0042711) | 3|51 | P03950(GO:0001541;GO:0001556;GO:0001890);P35527(GO:0007283);Q9UGM5(GO:0007339;GO:0007338) | 0.241593835 | 0.268437595 | https://www.ebi.ac.uk/QuickGO/term/GO:0022414 |
| GO:0005576 | extracellular region | cellular_component | 274|375 | P00488(GO:0005576);P68366(GO:0005576);P04275(GO:0005576);J3KNB4(GO:0005576);P0C0L5(GO:0005576);P03950(GO:0005576);G3XAP6(GO:0005576);P05160(GO:0005576);P23142(GO:0005576);P15814(GO:0005576);P0DP02(GO:0005576);P41222(GO:0005576);B1AHL2(GO:0005576);P08571(GO:0005576);P68871(GO:0005576);P22792(GO:0005576);Q86YZ3(GO:0005576);P01597(GO:0005576);P01042(GO:0005576);P02787(GO:0005576);Q14520(GO:0005576);P01602(GO:0005576);P08185(GO:0005576);A0A0B4J1U7(GO:0005576);P59665(GO:0005576);P0DJI8(GO:0005576);A0A2R8Y3M9(GO:0005576);Q96KN2(GO:0005576);P06312(GO:0005576);P43121(GO:0005576);A0A0A0MT36(GO:0005576);P01009(GO:0005576);P02790(GO:0005576);P01763(GO:0005576);P01764(GO:0005576);A0A087X0S5(GO:0005576);P01766(GO:0005576);Q15582(GO:0005576);Q08830(GO:0005576);P0DOY3(GO:0005576);P01782(GO:0005576);P18428(GO:0005576);P01780(GO:0005576);P06331(GO:0005576);P02776(GO:0005576);D6R934(GO:0005576);P02775(GO:0005576);Q13201(GO:0005576);A0A0J9YXX1(GO:0005576);P30041(GO:0005576);P06702(GO:0005576);P0DP01(GO:0005576);P10643(GO:0005576);P08697(GO:0005576);P19827(GO:0005576);P01833(GO:0005576);P01834(GO:0005576);P19823(GO:0005576);P04003(GO:0005576);E7END6(GO:0005576);P04004(GO:0005576);P00450(GO:0005576);Q92954(GO:0005576);Q6UXB8(GO:0005576);O00391(GO:0005576);A6XND0(GO:0005576);P69905(GO:0005576);G3XAK1(GO:0005576);Q9Y6R7(GO:0005576);P02768(GO:0005576);O75636(GO:0005576);Q96PD5(GO:0005576);P02760(GO:0005576);P02763(GO:0005576);Q01518(GO:0005576);A0A0C4DH73(GO:0005576);P02766(GO:0005576);P07357(GO:0005576);A0A075B6S5(GO:0005576);P37802(GO:0005576);Q15166(GO:0005576);P07358(GO:0005576);P29622(GO:0005576);P09172(GO:0005576);P01703(GO:0005576);P01700(GO:0005576);P01701(GO:0005576);P01706(GO:0005576);P04264(GO:0005576);P01704(GO:0005576);P01705(GO:0005576);A0A0C4DH67(GO:0005576);P23083(GO:0005576);P01709(GO:0005576);P02741(GO:0005576);P02671(GO:0005576);P03951(GO:0005576);P07996(GO:0005576);P13671(GO:0005576);A0A075B6I0(GO:0005576);F5H8B0(GO:0005576);P0CF74(GO:0005576);P61626(GO:0005576);P04180(GO:0005576);E9PHK0(GO:0005576);P22891(GO:0005576);Q06033(GO:0005576);C9JF17(GO:0005576);P00739(GO:0005576);P05155(GO:0005576);P05154(GO:0005576);P27169(GO:0005576);P20742(GO:0005576);P01031(GO:0005576);O95445(GO:0005576);Q13103(GO:0005576);J3KRP0(GO:0005576);P13645(GO:0005576);A0A0A0MS14(GO:0005576);A0A0A0MS15(GO:0005576);P01715(GO:0005576);P01717(GO:0005576);P48740(GO:0005576);K7ERI9(GO:0005576);P00738(GO:0005576);P01857(GO:0005576);M0R0Q9(GO:0005576);A0A075B6Q5(GO:0005576);P80748(GO:0005576);P01019(GO:0005576);A0A0S2Z4L3(GO:0005576);A0A0C4DH25(GO:0005576);A0A0G2JMI3(GO:0005576);P01011(GO:0005576);P09871(GO:0005576);P19652(GO:0005576);A0A0C4DH55(GO:0005576);A0A0B4J1Y8(GO:0005576);B0YIW2(GO:0005576);P04430(GO:0005576);P31151(GO:0005576);Q9UGM5(GO:0005576);O00187(GO:0005576);O14791(GO:0005576);K7ER74(GO:0005576);A0A087WSZ0(GO:0005576);P36955(GO:0005576);A0A0B4J1U3(GO:0005576);A0A0G2JPR0(GO:0005576);P09486(GO:0005576);Q16610(GO:0005576);P11226(GO:0005576);Q13093(GO:0005576);Q96IY4(GO:0005576);P01721(GO:0005576);Q9NZP8(GO:0005576);A0A075B6K2(GO:0005576);P55058(GO:0005576);B4E1Z4(GO:0005576);P02751(GO:0005576);A0A075B6R2(GO:0005576);Q9UNN8(GO:0005576);Q03591(GO:0005576);P02750(GO:0005576);P55056(GO:0005576);P01008(GO:0005576);P00734(GO:0005576);A0A0B4J1X5(GO:0005576);A0A0C4DH43(GO:0005576);A0A075B6K5(GO:0005576);P43652(GO:0005576);P02753(GO:0005576);C9JV77(GO:0005576);P01814(GO:0005576);P01344(GO:0005576);A0A0B4J1X8(GO:0005576);P01877(GO:0005576);P01871(GO:0005576);P10909(GO:0005576);O75882(GO:0005576);P01619(GO:0005576);F8WF14(GO:0005576);P04114(GO:0005576);Q6EMK4(GO:0005576);A0A0A0MRJ7(GO:0005576);P04040(GO:0005576);A0A0C4DH33(GO:0005576);A0A669KAY4(GO:0005576);P05543(GO:0005576);P18065(GO:0005576);A0A0J9YX35(GO:0005576);P05546(GO:0005576);P02743(GO:0005576);A0A075B6K4(GO:0005576);P55103(GO:0005576);P01860(GO:0005576);D6RAR4(GO:0005576);P02745(GO:0005576);P01718(GO:0005576);A0A0B4J2D9(GO:0005576);P02749(GO:0005576);P02748(GO:0005576);P01034(GO:0005576);P01601(GO:0005576);P04217(GO:0005576);P11597(GO:0005576);P02649(GO:0043083;GO:0005576);P04211(GO:0005576);P04196(GO:0005576);P02647(GO:0005576);P01861(GO:0005576);P02747(GO:0005576);A0A0C4DH32(GO:0005576);P05109(GO:0005576);P15169(GO:0005576);A0A0C4DH31(GO:0005576);A0A0C4DH34(GO:0005576);H0YAC1(GO:0005576);Q14624(GO:0005576);A0A0C4DH38(GO:0005576);A0A0C4DH39(GO:0005576);P25311(GO:0005576);P01599(GO:0005576);O43866(GO:0005576);A0A075B6J9(GO:0005576);P01023(GO:0005576);P01743(GO:0005576);C9J8S2(GO:0005576);A0A0C4DH24(GO:0005576);O95497(GO:0005576);P00747(GO:0005576);P00740(GO:0005576);P00742(GO:0005576);E7EQB2(GO:0005576);A0A0A0MRZ9(GO:0005576);P01742(GO:0005576);P00748(GO:0005576);Q08380(GO:0005576);Q9BWP8(GO:0005576);P08603(GO:0005576);P43251(GO:0005576);P20851(GO:0005576);P0C0L4(GO:0005576);A0A087WSY4(GO:0005576);P02652(GO:0005576);P35858(GO:0005576);Q15485(GO:0005576);A0A0G2JL69(GO:0005576);A0A096LPE2(GO:0005576);P01024(GO:0005576);P04433(GO:0005576);A0A087WSY6(GO:0005576);P06727(GO:0005576);C9J5S7(GO:0005576);A0A0C4DH29(GO:0005576);P18206(GO:0005576);J3KPA1(GO:0005576);P01817(GO:0005576);P08519(GO:0005576);P21333(GO:0005576);P51884(GO:0005576);A0A0B4J1V1(GO:0005576);A0A0B4J1V0(GO:0005576);P07360(GO:0005576);A0A0B4J1V2(GO:0005576);Q86UX7(GO:0005576);P07437(GO:0005576);P80108(GO:0005576);Q13790(GO:0005576);P02675(GO:0005576);Q9Y490(GO:0005576);Q15848(GO:0005576);A0A0J9YVY3(GO:0005576);P01699(GO:0005576) | 29|51 | O75636(GO:0005576);A0A0B4J1Y8(GO:0005576);Q86UX7(GO:0005576);A0A0C4DH73(GO:0005576);Q9UGM5(GO:0005576);A0A0J9YX35(GO:0005576);P37802(GO:0005576);A0A075B6J9(GO:0005576);A0A075B6K2(GO:0005576);A0A2R8Y3M9(GO:0005576);P03951(GO:0005576);P55058(GO:0005576);P02751(GO:0005576);P03950(GO:0005576);Q16610(GO:0005576);Q14624(GO:0005576);P0DOY3(GO:0005576);P29622(GO:0005576);P30041(GO:0005576);P18206(GO:0005576);P19823(GO:0005576);P01715(GO:0005576);P01717(GO:0005576);P04040(GO:0005576);P07360(GO:0005576);P00739(GO:0005576);P02747(GO:0005576);P80748(GO:0005576);P69905(GO:0005576) | 0.003280014 | 0.043733515 | https://www.ebi.ac.uk/QuickGO/term/GO:0005576 |
| GO:0050896 | response to stimulus | biological_process | 252|375 | P08779(GO:0045087;GO:0006954);P00488(GO:0072378);P01857(GO:0042742;GO:0045087;GO:0006956;GO:0006958;GO:0002250);P04275(GO:0007597;GO:0009611);J3KNB4(GO:0071224;GO:0071222;GO:0042742;GO:0050829;GO:0061844;GO:0071354;GO:0071356;GO:0051873;GO:0045087;GO:0006952;GO:0050830;GO:0071347);P03951(GO:0007597);K7ERG9(GO:0009617;GO:0006957);A0A0A0MRZ9(GO:0006955;GO:0002250);P23142(GO:0072378);P15814(GO:0045087;GO:0042742;GO:0006955;GO:0006958);B7ZKJ8(GO:0034097;GO:0006953);P0DP02(GO:0002250);P08571(GO:0006954;GO:0071222;GO:0071223;GO:0032496;GO:0045471;GO:0034612;GO:0071727;GO:0071726;GO:0009617;GO:0071219;GO:0045087;GO:0009408;GO:0032026;GO:0051602;GO:0002237);P68871(GO:0042542);E9PK25(GO:0043200;GO:0009615);O75460(GO:0034620;GO:0006986;GO:0071333;GO:0036498;GO:0030968;GO:0070059;GO:0035924;GO:0034976);P01597(GO:0006956;GO:0002250;GO:0006958;GO:0006955);P01042(GO:0007597;GO:0006954);A0A087X1J7(GO:0006979);P14151(GO:0033198);P01602(GO:0006956;GO:0002250;GO:0006958;GO:0006955);P11226(GO:0042742;GO:0001867;GO:0051873;GO:0045087;GO:0006953;GO:0006956;GO:0006958;GO:0006979;GO:0050830);A0A0B4J1U7(GO:0045087;GO:0002250;GO:0006958;GO:0042742);A0A669KAY4(GO:0032869;GO:0009267);P59665(GO:0071222;GO:0019730;GO:0019731;GO:0042742;GO:0050829;GO:0061844;GO:0051852;GO:0002227;GO:0006952;GO:0050830;GO:0006955;GO:0050832;GO:0051607;GO:0042832);P0DJI8(GO:0045087;GO:0006953);P51884(GO:0014070;GO:0070848);P06312(GO:0006956;GO:0002250;GO:0006958;GO:0006955);A0A0A0MT36(GO:0006955;GO:0002250);P01009(GO:0006953);A0A075B7D8(GO:0045087;GO:0042742;GO:0006958);P01764(GO:0006955;GO:0042742;GO:0045087;GO:0006956;GO:0006958;GO:0002250);A0A087X0S5(GO:0071230);P01766(GO:0006956;GO:0002250;GO:0006958;GO:0006955);B4E1Z4(GO:0006956;GO:0006957);P00918(GO:0010043;GO:0043627;GO:0048545;GO:0009268;GO:0010033;GO:0071498);P0DOY3(GO:0002250);P26038(GO:0071394);P01782(GO:0006956;GO:0002250;GO:0006958;GO:0006955);P18428(GO:0006968;GO:0071222;GO:0071223;GO:0042742;GO:0050829;GO:0032496;GO:0032490;GO:0045087;GO:0006953;GO:0050830);P01780(GO:0006956;GO:0002250;GO:0006958;GO:0006955);P06331(GO:0006956;GO:0002250;GO:0006958;GO:0006955);P02776(GO:0071222;GO:0032496;GO:0061844;GO:0051873;GO:0006952;GO:0006954;GO:0006955;GO:0042832);D6R934(GO:0006958);A0A1W2PQU7(GO:0031102);P0DP01(GO:0002250);E7EUT5(GO:0061844;GO:0051873;GO:0050832;GO:0071346);A0A0G2JMB2(GO:0045087;GO:0042742;GO:0006958);A0A0J9YXX1(GO:0002250);P30041(GO:0034599;GO:0006979);P06702(GO:0045087;GO:0019730;GO:0042742;GO:0061844;GO:0050832;GO:0006954);Q9Y5Y7(GO:0009611);P61224(GO:0071320);P10643(GO:0045087;GO:0006956;GO:0006957;GO:0006955;GO:0006958);P02787(GO:0009617;GO:0071281);P01833(GO:0001580);P01834(GO:0006955;GO:0042742;GO:0045087;GO:0006956;GO:0006958;GO:0002250);P05160(GO:0072378);P04003(GO:0045087;GO:0006958);P01008(GO:0002438;GO:0007584);P01718(GO:0006956;GO:0002250;GO:0006958;GO:0006955);P04004(GO:0006955);P01743(GO:0006956;GO:0002250;GO:0006958;GO:0006955);Q92954(GO:0006955);P69905(GO:0042542);A0A075B6H7(GO:0006955);A0A0J9YX35(GO:0002250);A0A087WSZ0(GO:0006955;GO:0002250);G3V0E5(GO:0035690;GO:1990830);P02768(GO:0009267);O75636(GO:0051607;GO:0006956;GO:0045087;GO:0001867);Q96PD5(GO:0019730;GO:0016045;GO:0045087;GO:0050830);P02763(GO:0006953;GO:0006954);A0A0C4DH73(GO:0006956;GO:0002250;GO:0006958;GO:0006955);P02753(GO:0032526;GO:0045471;GO:0050896);P07357(GO:0006956;GO:0006957;GO:0006955;GO:0006958;GO:0045087);A0A0B4J2D9(GO:0006955;GO:0002250);P19320(GO:0035094;GO:0010043;GO:0032496;GO:0001666;GO:0010212;GO:0071356;GO:1904646;GO:0007584;GO:0045471;GO:0002526;GO:0002544;GO:0035924);Q15166(GO:0009636);P07358(GO:0045087;GO:0006956;GO:0006957;GO:0006955;GO:0006958);P09172(GO:0042596;GO:0008542;GO:0048265;GO:0001975);P01703(GO:0006956;GO:0002250;GO:0006958;GO:0006955);P01700(GO:0006956;GO:0002250;GO:0006958;GO:0006955);P01701(GO:0006956;GO:0006958;GO:0006955);P01706(GO:0006956;GO:0002250;GO:0006958;GO:0006955);P20851(GO:0045087;GO:0006958);P01704(GO:0006956;GO:0002250;GO:0006958;GO:0006955);P01705(GO:0006956;GO:0002250;GO:0006958;GO:0006955);A0A0C4DH67(GO:0002250);P23083(GO:0006956;GO:0002250;GO:0006958;GO:0006955);P01709(GO:0006956;GO:0002250;GO:0006958;GO:0006955);P02671(GO:0045087;GO:0072378;GO:0051592;GO:0072377;GO:0002250;GO:0043152);P02675(GO:0044320;GO:0072378;GO:0045087;GO:0051592;GO:0071347;GO:0002250;GO:0043152);P07996(GO:0032026;GO:0034605;GO:0006986;GO:0009612;GO:0051592;GO:0042493;GO:0071356;GO:0006954;GO:0006955;GO:0033574;GO:0034976;GO:0001666;GO:0071363;GO:0002544;GO:0032570;GO:0009749);P13671(GO:0045087;GO:0006956;GO:0006955;GO:0006958);A0A075B6I0(GO:0006955;GO:0002250);P0CF74(GO:0042742;GO:0045087;GO:0006956;GO:0006958;GO:0002250);P61626(GO:0006954;GO:0019730;GO:0042742;GO:0050829;GO:0050830);P02652(GO:0043627;GO:0002526;GO:0051384;GO:0042493;GO:0009749);E9PHK0(GO:0071560;GO:0071310);C9JF17(GO:0042493;GO:0048678;GO:0000302;GO:0014012);P05155(GO:0007597;GO:0045087;GO:0006958);M0R0Q9(GO:0009617;GO:0006956;GO:0006957;GO:0006954;GO:0006955;GO:0006958);P01031(GO:0006954;GO:0045087;GO:0006956;GO:0006957;GO:0006958);O95445(GO:0009749);A0A075B7F0(GO:0045087;GO:0042742;GO:0006958);P00734(GO:0007597;GO:0061844;GO:0009611;GO:0051838;GO:0070945;GO:0006953;GO:0072378);A0A0A0MS14(GO:0045087;GO:0002250;GO:0006958;GO:0042742);A0A0A0MS15(GO:0045087;GO:0002250;GO:0006958;GO:0042742);P01715(GO:0006956;GO:0002250;GO:0006958;GO:0006955);P01717(GO:0006956;GO:0002250;GO:0006958;GO:0006955);P48740(GO:0006956;GO:0001867;GO:0045087);P00738(GO:0042742;GO:0006952;GO:0006953;GO:0042542);P00739(GO:0002526;GO:0010033);E7EQB2(GO:0019732;GO:0019731;GO:0050829;GO:0061844;GO:0002227);Q9BWP8(GO:0019730;GO:0001867;GO:0045087;GO:0006956);G3XAP6(GO:0006986);A0A0C4DH25(GO:0006956;GO:0002250;GO:0006958;GO:0006955);A0A0G2JMI3(GO:0045087;GO:0002250;GO:0006958;GO:0042742);P01011(GO:0006954;GO:0006953);P19652(GO:0006953);A0A0C4DH55(GO:0006955;GO:0002250);A0A0B4J1Y8(GO:0006955;GO:0002250);Q96IY4(GO:0071333;GO:0009408;GO:0042493);P31151(GO:0019730;GO:0000302;GO:0050829;GO:0032496;GO:0061844;GO:0045087);P01721(GO:0006956;GO:0002250;GO:0006958;GO:0006955);O14791(GO:0045087);K7ER74(GO:0042493);G3XAK1(GO:0071456);P36955(GO:0071300;GO:0010447;GO:0071333;GO:0046685;GO:0071279;GO:0071549);A0A0B4J1U3(GO:0006955;GO:0002250);A0A0G2JPR0(GO:0006956;GO:0006954);P09486(GO:0051384;GO:0032496;GO:0034097;GO:0033591;GO:0046686;GO:0010288;GO:0042060;GO:0051592;GO:0045471;GO:0043434;GO:0009629;GO:0071363;GO:0051591);Q16610(GO:0006954);Q07954(GO:1904646);P32119(GO:0032496;GO:0034599;GO:0006979);O00187(GO:0045087;GO:0006956;GO:0001867;GO:0006958);A0A0B4J231(GO:0045087;GO:0042742;GO:0006958);A0A075B6K2(GO:0006955;GO:0002250);P02750(GO:0009617);P02751(GO:0009611;GO:0042060;GO:0006953);A0A075B6R2(GO:0045087;GO:0002250;GO:0006958;GO:0042742);A0A075B6K4(GO:0006955;GO:0002250);Q03591(GO:0006956);Q9NZP8(GO:0045087;GO:0006958);P02775(GO:0071222;GO:0042742;GO:0032496;GO:0061844;GO:0006952;GO:0006954;GO:0006955);A0A075B6R9(GO:0006955);P68032(GO:0009991;GO:0009612;GO:0043503;GO:0071417;GO:0010226;GO:0045471;GO:0042493;GO:0048545);A0A0G2JRQ6(GO:0006955);A0A0B4J1X5(GO:0045087;GO:0002250;GO:0006958;GO:0042742);P0C0L5(GO:0006954;GO:0032490;GO:0045087;GO:0006956;GO:0006958);A0A0C4DH43(GO:0045087;GO:0002250;GO:0006958;GO:0042742);A0A075B6K5(GO:0006955;GO:0002250);A0A075B6J9(GO:0006955;GO:0002250);P08603(GO:0045087;GO:0006956;GO:0006957);A0A087WSY4(GO:0045087;GO:0002250;GO:0006958;GO:0042742);A0A087WSY6(GO:0006955;GO:0002250);P27169(GO:0009636;GO:1902617;GO:0031667;GO:0070542);P01877(GO:0019731;GO:0006955;GO:0045087;GO:0006958;GO:0002250);P01871(GO:0019731;GO:0050829;GO:0045087;GO:0006958;GO:0002250);P03950(GO:0001666;GO:0009725);A0A075B7D0(GO:0045087;GO:0042742;GO:0006958);P13473(GO:0009267);O75882(GO:0006954;GO:0006979);A0A0B4J1V2(GO:0045087;GO:0002250;GO:0006958;GO:0042742);F8WF14(GO:0051384;GO:0043279;GO:0051593);A0A0C4DH32(GO:0045087;GO:0002250;GO:0006958;GO:0042742);A0A075B6S9(GO:0006955);P01763(GO:0006956;GO:0002250;GO:0006958;GO:0006955);Q6EMK4(GO:0071461;GO:0071456);P04040(GO:0033189;GO:0009314;GO:0000302;GO:0009650;GO:0032868;GO:0001666;GO:0055093;GO:0009636;GO:0033591;GO:0006979;GO:0046686;GO:0014823;GO:0010288;GO:0009411;GO:0032355;GO:0045471;GO:0071363;GO:0034599;GO:0009642;GO:0042542;GO:0070542;GO:0042493;GO:0033197;GO:0010193;GO:0014854;GO:0080184);A0A0C4DH33(GO:0045087;GO:0002250;GO:0006958;GO:0042742);A0A3B3ISR2(GO:0045087;GO:0006958);P18065(GO:0051384;GO:0032355;GO:0009612;GO:0032526;GO:0007584;GO:0032870;GO:0043627;GO:0010226;GO:0042493;GO:0048545);P15169(GO:0051384);A0A075B6S5(GO:0006955;GO:0002250);P02743(GO:0045087;GO:0006953;GO:0006958);P02741(GO:0045087;GO:0006953;GO:0050830;GO:0006954;GO:0006958);P01860(GO:0042742;GO:0045087;GO:0006956;GO:0006958;GO:0002250);P02747(GO:0045087;GO:0006956;GO:0006955;GO:0006958);P02745(GO:0045087;GO:0006956;GO:0010039;GO:0006958);A0A075B7B8(GO:0045087;GO:0042742;GO:0006958);A0A0G2JL69(GO:0045087;GO:0006956;GO:0006958);P02749(GO:0007597);P02748(GO:0006955;GO:0045087;GO:0006957;GO:0006958);P01034(GO:0048678;GO:0070301;GO:0001666;GO:0014070;GO:0032355;GO:0009636;GO:0006952;GO:0034599;GO:0010035;GO:0006979;GO:0009743;GO:0042493;GO:0031667);P01601(GO:0006956;GO:0002250;GO:0006958;GO:0006955);C9JB55(GO:0071281);Q15582(GO:0050896);P02649(GO:0031102;GO:0061771;GO:0000302;GO:0002021;GO:0006979);P04211(GO:0006956;GO:0002250;GO:0006958;GO:0006955);P04196(GO:0061844;GO:0050832);P02647(GO:0031102;GO:0014012;GO:0042493;GO:0007584;GO:0043627);P10909(GO:0019730;GO:0009615;GO:0051788;GO:0006956;GO:0006958;GO:0045087);P04114(GO:0032496;GO:0032355;GO:0071356;GO:0009615;GO:0071379;GO:0010033;GO:0009743;GO:0010269);P05109(GO:0045087;GO:0019730;GO:0010043;GO:0032496;GO:0042060;GO:0050832;GO:0045471;GO:0006954;GO:0002526;GO:0002544;GO:0042742);P01861(GO:0042742;GO:0045087;GO:0006956;GO:0006958;GO:0002250);A0A0C4DH31(GO:0045087;GO:0002250;GO:0006958;GO:0042742);A0A0C4DH36(GO:0045087;GO:0042742;GO:0006958);A0A0C4DH34(GO:0045087;GO:0002250;GO:0006958;GO:0042742);A0A0G2JI36(GO:0006955;GO:0016045;GO:0002419;GO:0001913);P08697(GO:0006953;GO:0010033);Q14624(GO:0034097;GO:0006953);P25311(GO:0001580;GO:0006955);A0A0C4DH39(GO:0045087;GO:0002250;GO:0006958;GO:0042742);A0A0C4DH38(GO:0045087;GO:0002250;GO:0006958;GO:0042742);P01599(GO:0006956;GO:0002250;GO:0006958;GO:0006955);O43866(GO:0006954;GO:0006968);P01024(GO:0045087;GO:0009617;GO:0006956;GO:0006957;GO:0006954;GO:0006955;GO:0006958);P01023(GO:0007597);C9J8S2(GO:0019732;GO:0050829;GO:0045087;GO:0050830;GO:0006954;GO:0061760);O95497(GO:0045087;GO:0002544;GO:0006954;GO:0002526;GO:0006979);P02538(GO:0042060;GO:0050830;GO:0061844);P09871(GO:0006956;GO:0045087;GO:0001867;GO:0006958);P00740(GO:0007597);P60709(GO:0072749);P00742(GO:0007598);A0A075B6Q5(GO:0045087;GO:0002250;GO:0006958;GO:0042742);P02533(GO:0010043;GO:0010212);P01742(GO:0006956;GO:0002250;GO:0006958;GO:0006955);P00748(GO:0007597;GO:0045087;GO:0002353;GO:0051788);Q08380(GO:0006968);P80748(GO:0006956;GO:0002250;GO:0006958;GO:0006955);P04264(GO:0001867;GO:0006979);P0C0L4(GO:0006954;GO:0045087;GO:0006956;GO:0006958);C9JV77(GO:0006953);P01019(GO:0071260;GO:0014873;GO:1904385;GO:0032355;GO:0051403);Q15485(GO:0045087;GO:0050830;GO:0001867;GO:0050829;GO:0006956);A0A096LPE2(GO:0006953);P04433(GO:0006956;GO:0002250;GO:0006958;GO:0006955);A0A0C4DH24(GO:0006955;GO:0002250);P06727(GO:0035634;GO:0006982;GO:0002227;GO:0031102);P04430(GO:0006956;GO:0006958;GO:0006955);A0A0C4DH29(GO:0045087;GO:0002250;GO:0006958;GO:0042742);A0A0B4J1X8(GO:0045087;GO:0002250;GO:0006958;GO:0042742);P02100(GO:0014070);P01817(GO:0006956;GO:0002250;GO:0006958;GO:0006955);P01814(GO:0006956;GO:0002250;GO:0006958;GO:0006955);P01619(GO:0019731;GO:0006955;GO:0006956;GO:0006958;GO:0002250);A0A0B4J1V1(GO:0045087;GO:0002250;GO:0006958;GO:0042742);A0A0B4J1V0(GO:0045087;GO:0002250;GO:0006958;GO:0042742);P07360(GO:0045087;GO:0006957;GO:0006958);A0A0A0MS51(GO:0045471;GO:0051593;GO:0071346;GO:0014891;GO:0071276);P07437(GO:0042267);H0YAC1(GO:0007597);P80108(GO:0035690;GO:0032869;GO:0009749;GO:0071397;GO:0071277;GO:0071401;GO:0071467);P11021(GO:1990090;GO:0071480;GO:1990440;GO:0009314;GO:0030433;GO:0036500;GO:0042149;GO:0035690;GO:0006983;GO:0071353;GO:0071236;GO:0097501;GO:0071277;GO:0036498;GO:0036499;GO:0042220;GO:1904313;GO:0030968;GO:0071287;GO:0071320;GO:0034976);Q9Y490(GO:0036498);Q15848(GO:0009617;GO:0014823;GO:0071320;GO:0031667;GO:0070994;GO:0071872;GO:0001666;GO:0007584;GO:0070543;GO:0042493;GO:0051384;GO:0035690;GO:0032869;GO:0034612;GO:0045471;GO:0009744;GO:0009749);A0A0J9YVY3(GO:0002250);P01699(GO:0006956;GO:0002250;GO:0006958;GO:0006955);P07737(GO:0010033;GO:0071363) | 29|51 | O75636(GO:0051607;GO:0006956;GO:0045087;GO:0001867);P03951(GO:0007597);P03950(GO:0001666;GO:0009725);A0A0C4DH73(GO:0006956;GO:0002250;GO:0006958;GO:0006955);A0A0J9YX35(GO:0002250);A0A0C4DH36(GO:0045087;GO:0042742;GO:0006958);A0A087X1J7(GO:0006979);P32119(GO:0032496;GO:0034599;GO:0006979);A0A0B4J1Y8(GO:0006955;GO:0002250);A0A075B6K2(GO:0006955;GO:0002250);P02751(GO:0009611;GO:0042060;GO:0006953);P60709(GO:0072749);K7ERG9(GO:0009617;GO:0006957);A0A075B6R9(GO:0006955);P80748(GO:0006956;GO:0002250;GO:0006958;GO:0006955);Q14624(GO:0034097;GO:0006953);P0DOY3(GO:0002250);Q16610(GO:0006954);P30041(GO:0034599;GO:0006979);P69905(GO:0042542);P01715(GO:0006956;GO:0002250;GO:0006958;GO:0006955);P01717(GO:0006956;GO:0002250;GO:0006958;GO:0006955);P04040(GO:0033189;GO:0009314;GO:0000302;GO:0009650;GO:0032868;GO:0001666;GO:0055093;GO:0009636;GO:0033591;GO:0006979;GO:0046686;GO:0014823;GO:0010288;GO:0009411;GO:0032355;GO:0045471;GO:0071363;GO:0034599;GO:0009642;GO:0042542;GO:0070542;GO:0042493;GO:0033197;GO:0010193;GO:0014854;GO:0080184);P07360(GO:0045087;GO:0006957;GO:0006958);P00739(GO:0002526;GO:0010033);P02747(GO:0045087;GO:0006956;GO:0006955;GO:0006958);A0A075B6J9(GO:0006955;GO:0002250);P68032(GO:0009991;GO:0009612;GO:0043503;GO:0071417;GO:0010226;GO:0045471;GO:0042493;GO:0048545);P07737(GO:0010033;GO:0071363) | 0.03079983 | 0.2053322 | https://www.ebi.ac.uk/QuickGO/term/GO:0050896 |
| GO:0008152 | metabolic process | biological_process | 197|375 | P00488(GO:0018149;GO:0072378);P04275(GO:0007597);P03951(GO:0007597;GO:0031639;GO:0006508);P03950(GO:0006651;GO:0090305;GO:0009303;GO:0090501);P05160(GO:0072378);P23142(GO:0072378);B7ZKJ8(GO:0030212);P21333(GO:0042789);P68871(GO:0042744);P22792(GO:0006508);O75460(GO:0016310;GO:0008152;GO:0046777;GO:0036289;GO:0098787;GO:1901142;GO:0006402;GO:0006468;GO:0006397;GO:0090502;GO:0006351;GO:0070054;GO:1990579;GO:0006379);P01597(GO:0006508);P01042(GO:0007597;GO:0044267;GO:0043687);A0A087X1J7(GO:0055114);Q14520(GO:0006508);P01602(GO:0006508);P08519(GO:0006508;GO:0006629);P0DJI8(GO:0044267);A0A2R8Y3M9(GO:0016042;GO:0006508);P51884(GO:0018146;GO:0042340);P06312(GO:0006508);P01743(GO:0006508);K7ERG9(GO:0006508);P02790(GO:0020027;GO:0042168);P01763(GO:0006508);P01764(GO:0006508);P01766(GO:0006508);Q15582(GO:0044267);P01782(GO:0006508);P01780(GO:0006508);P06331(GO:0006508);E7EUT5(GO:0006096;GO:0035606;GO:0055114;GO:0006006);P30041(GO:0042744;GO:0016042;GO:0006629;GO:0008152;GO:0055114;GO:0046475);P06702(GO:0035606;GO:0018119;GO:0006914);Q9Y5Y7(GO:0030214;GO:0006027);P02787(GO:0044267;GO:0043687;GO:0070371);P19827(GO:0030212);P01834(GO:0006508);P19823(GO:0044267;GO:0030212;GO:0043687);Q96IY4(GO:0006508);E7END6(GO:0006508);P00748(GO:0007597;GO:0031638;GO:0006508;GO:0016485;GO:0016540;GO:0002353);O00391(GO:0044267;GO:0043687;GO:0055114);P69905(GO:0042744);D6RAR4(GO:0006508);G3V0E5(GO:0031623);P02768(GO:0044267;GO:0043687);O75636(GO:0006508);Q96PD5(GO:0009253;GO:0001519);P02760(GO:0042167;GO:0018298;GO:0030163);A0A0C4DH73(GO:0006508);P02766(GO:0044267;GO:0001523;GO:0042572;GO:0006144);J3KPS3(GO:0006096);Q9BWP8(GO:0006508);A0A0G2JL69(GO:0006508);P19320(GO:0009308;GO:0055114);Q15166(GO:0046395;GO:0019372;GO:0016311;GO:0019439;GO:0046226;GO:0010124);P09172(GO:0042423;GO:0042420;GO:0042421;GO:0006589;GO:0055114);P01703(GO:0006508);P01700(GO:0006508);P01701(GO:0006508);P01706(GO:0006508);P0C0L5(GO:0006508);P02042(GO:0042744);P01705(GO:0006508);E9PK25(GO:0006468);P23083(GO:0006508);P01709(GO:0006508);P02671(GO:0044267;GO:0072378;GO:0031639;GO:0043687;GO:0072377);P02675(GO:0072378;GO:0031639);P07996(GO:0018149);H3BTN5(GO:0016310;GO:0006096);F5H8B0(GO:0006508);P0CF74(GO:0006508);P61626(GO:0044267;GO:0016998;GO:0008152);P02652(GO:0006656;GO:0001523;GO:0009395;GO:0044267;GO:0018158;GO:0006641;GO:0043687;GO:0008203;GO:0046340;GO:0018206;GO:0042157);Q16880(GO:0006682;GO:0006687;GO:0006665;GO:0006629;GO:0008152);P22891(GO:0006508);Q06033(GO:0030212);C9JF17(GO:0006629;GO:0006006);P05155(GO:0007597);M0R0Q9(GO:0006631;GO:0044267;GO:0043687);O95445(GO:0042157;GO:0001523);A0A0A0MS51(GO:1990000);P00734(GO:0007597;GO:0044267;GO:0006508;GO:0072378);P13645(GO:0018149);P35908(GO:0018149);P13473(GO:0061684;GO:0006914;GO:1905146);P01717(GO:0006508);P48740(GO:0006508);K7ERI9(GO:0042157);P01857(GO:0006508);E7EQB2(GO:0006508);P16930(GO:0006572;GO:0006559;GO:0006527;GO:0009072;GO:0008152;GO:1902000);G3XAP6(GO:0016485);A0A0C4DH25(GO:0006508);P11226(GO:0006508);P01019(GO:0070371;GO:0051403);Q9UHG3(GO:0030327;GO:0030328;GO:0030329;GO:0055114);P41222(GO:0006633;GO:0006631;GO:0019371;GO:0006629;GO:0001516;GO:0006693);B0YIW2(GO:0042157;GO:0001523;GO:0006641;GO:0019433);P04430(GO:0006508);Q8IV42(GO:0016310;GO:0097056;GO:0001514;GO:0006412);O00187(GO:0006508);A0A0A0MTH3(GO:0018105;GO:0006468);O14791(GO:0042157;GO:0044267;GO:0006629;GO:0043687;GO:0008202;GO:0008203);K7ER74(GO:0001523;GO:0016042;GO:0006629);G3XAK1(GO:0006508);P04211(GO:0006508);P80748(GO:0006508);Q96KN2(GO:0008152;GO:0006508);Q13093(GO:0034440;GO:0006629;GO:0016042;GO:0046469);P32119(GO:0042744;GO:0002536;GO:0055114;GO:0019430);P01721(GO:0006508);A0A2R8Y6G6(GO:0006096);P55058(GO:0010189;GO:0006629);P01009(GO:0044267;GO:0043687);P02751(GO:0018149;GO:0044267;GO:0043687);P02753(GO:0042572;GO:0001523;GO:0006094);P02100(GO:0042744);P55056(GO:0006629);P01008(GO:0043687;GO:0044267);J3KRP0(GO:0008152;GO:0006508);P69892(GO:0042744);Q14624(GO:0030212);P01817(GO:0006508);A0A669KAY4(GO:0032802;GO:0042157;GO:0044267;GO:0008203;GO:0043687;GO:0006644;GO:0006641;GO:0016540);P01344(GO:0044267;GO:0006349;GO:0005975;GO:0006006);P27169(GO:0016311;GO:0046434;GO:0019372;GO:0006629;GO:0008203;GO:0046395;GO:0046470;GO:0019439);P08185(GO:0008211);P35579(GO:0006509);P01715(GO:0006508);P09871(GO:0006508);P01619(GO:0006508);F8WF14(GO:0050783;GO:0019695);Q9NZP8(GO:0031638;GO:0006508);P15144(GO:0006508;GO:0043171);A0A0A0MRJ7(GO:0044267;GO:0043687);P04040(GO:0020027;GO:0009060;GO:0006641;GO:0055114;GO:0008203;GO:0042744);A0A3B3ISR2(GO:0031638);P18065(GO:0044267);P00915(GO:0006730);P05546(GO:0044267;GO:0043687);P02743(GO:0044267);P02747(GO:0006508);D6RE82(GO:0006364);P02745(GO:0006508);P01718(GO:0006508);P02749(GO:0007597;GO:0031639;GO:0006641);P80108(GO:0006501;GO:0006507;GO:0046470);P01034(GO:0044267;GO:0043687);P01601(GO:0006508);P11597(GO:0008202;GO:0006629;GO:0006641;GO:0008203;GO:0046470);P02649(GO:0019433;GO:0042157;GO:0042158;GO:0042159;GO:0042982;GO:0044267;GO:0006629;GO:0006641;GO:0043687;GO:0008202;GO:0008203;GO:0001523;GO:0006707);Q07954(GO:0150094;GO:0001523;GO:0006629;GO:0031623;GO:0042157);P02647(GO:0018206;GO:0019433;GO:0042158;GO:0044267;GO:0018158;GO:0006629;GO:0006695;GO:0008202;GO:0008203;GO:0070371;GO:0042157;GO:0006656;GO:0006644;GO:0008211;GO:0001523;GO:0043687);P01861(GO:0006508);P10909(GO:0006629);P04114(GO:0042157;GO:0016042;GO:0001523;GO:0006629;GO:0042158;GO:0043687;GO:0006642;GO:0044267;GO:0042159;GO:0008202;GO:0008203;GO:0019433);P05109(GO:0006914;GO:0018119);P15169(GO:0010815;GO:0016485;GO:0006508;GO:0006518;GO:0030070);P01860(GO:0006508);H0YAC1(GO:0007597;GO:0031639;GO:0031638;GO:0006508;GO:0055114);P25311(GO:0090501);P01599(GO:0006508);P01024(GO:0006631;GO:0044267;GO:0006629;GO:0043687;GO:0006508);P01023(GO:0007597);B4E1Z4(GO:0006508);O95497(GO:0015939;GO:0006807);P00747(GO:0044267;GO:0006508);P00740(GO:0007597;GO:0031638;GO:0006508);P60709(GO:0016579);P00742(GO:0006508;GO:0007598);P01742(GO:0006508);P00450(GO:0044267;GO:0043687;GO:0055114);P43251(GO:0006768;GO:0006807);P04264(GO:0018149);P0C0L4(GO:0044267;GO:0006508;GO:0043687);C9JV77(GO:0044267;GO:0043687);P04180(GO:0006656;GO:0042158;GO:0034435;GO:0008203;GO:0006644;GO:0006629;GO:0008202;GO:0046470);Q9HDC9(GO:0009058);P35858(GO:0044267);Q15485(GO:0006508);P04433(GO:0006508);P06727(GO:0042744;GO:0042157;GO:0001523;GO:0044267;GO:0016042;GO:0008203;GO:0046470;GO:0019430;GO:0006695);C9J5S7(GO:0000413);Q5SRP5(GO:0042157);Q13103(GO:0044267;GO:0043687);P33908(GO:1904381;GO:0006491;GO:0006486;GO:0008152);P01814(GO:0006508);P01704(GO:0006508);C9J8S2(GO:0001523);Q96HR3(GO:0006367;GO:0016567;GO:0006351);Q13790(GO:0006629;GO:0008203;GO:0008202);P11021(GO:0030433;GO:0051603);Q15848(GO:0019395;GO:0006006;GO:0006635;GO:0006091);P01699(GO:0006508) | 25|51 | O75636(GO:0006508);P03951(GO:0007597;GO:0031639;GO:0006508);P03950(GO:0006651;GO:0090305;GO:0009303;GO:0090501);A0A0C4DH73(GO:0006508);P01717(GO:0006508);A0A087X1J7(GO:0055114);P32119(GO:0042744;GO:0002536;GO:0055114;GO:0019430);A0A2R8Y3M9(GO:0016042;GO:0006508);P55058(GO:0010189;GO:0006629);P02751(GO:0018149;GO:0044267;GO:0043687);P60709(GO:0016579);K7ERG9(GO:0006508);H3BTN5(GO:0016310;GO:0006096);D6RE82(GO:0006364);Q14624(GO:0030212);P30041(GO:0042744;GO:0016042;GO:0006629;GO:0008152;GO:0055114;GO:0046475);Q5SRP5(GO:0042157);P01715(GO:0006508);P19823(GO:0044267;GO:0030212;GO:0043687);P04040(GO:0020027;GO:0009060;GO:0006641;GO:0055114;GO:0008203;GO:0042744);P00915(GO:0006730);Q96HR3(GO:0006367;GO:0016567;GO:0006351);P02747(GO:0006508);P80748(GO:0006508);P69905(GO:0042744) | 0.103441464 | 0.258603661 | https://www.ebi.ac.uk/QuickGO/term/GO:0008152 |
| GO:0016020 | membrane | cellular_component | 203|375 | P33908(GO:0016020;GO:0000139);P03951(GO:0016020;GO:0005886);P15814(GO:0016020);B7ZKJ8(GO:0005886);P30041(GO:0016020);P08571(GO:0045121;GO:0010008;GO:0016020;GO:0030667;GO:0005886);E9PK25(GO:0016020;GO:0031258;GO:0032587);O75460(GO:0005789;GO:0005637;GO:0016020);Q86YZ3(GO:0001533);P01597(GO:0016020;GO:0005886);P01042(GO:0005886);H0Y755(GO:0016020);P01602(GO:0016020;GO:0005886);A0A0G2JMI3(GO:0016020;GO:0005886);A0A0B4J1U7(GO:0016020;GO:0005886);P15169(GO:0097060);A0A0B4J1U3(GO:0016020;GO:0005886);A0A2R8Y3M9(GO:0016020);P06312(GO:0016020;GO:0005886);P43121(GO:0016020;GO:0005886);A0A0A0MT36(GO:0016020;GO:0005886);P02750(GO:0016020);P60763(GO:0016020;GO:0005886);P01763(GO:0016020;GO:0005886);P01764(GO:0016020;GO:0005886);A0A087X0S5(GO:0016020;GO:0005765;GO:0042383);P01766(GO:0016020;GO:0005886);Q15582(GO:0005886);F8WF14(GO:0016020);C9JB55(GO:0009925;GO:0016324);P0DOY3(GO:0016020;GO:0005886);P26038(GO:0016324;GO:0016323;GO:0005886;GO:0016020;GO:0031528);P20851(GO:0005886);P18428(GO:0016020);P01780(GO:0016020;GO:0005886);P06331(GO:0016020;GO:0005886);P0DP01(GO:0016020;GO:0005886);E7EUT5(GO:0031965;GO:0005886);A0A0J9YXX1(GO:0016020;GO:0005886);P0DP02(GO:0016020;GO:0005886);P06702(GO:0016020;GO:0005886);Q9Y5Y7(GO:0016020;GO:0005886);P61224(GO:0016020;GO:0035577;GO:0005886;GO:0045121);P02787(GO:0016020;GO:0010008;GO:0030665;GO:0009925;GO:0016324);P01833(GO:0016020;GO:0005886;GO:0035577);P01834(GO:0016020;GO:0005886);P04003(GO:0005886);P00450(GO:0005765;GO:0005886);Q16880(GO:0016020;GO:0005886);A0A0C4DGZ8(GO:0016020);P69905(GO:0016020);P15144(GO:0030667;GO:0016020;GO:0005886;GO:0005765);G3V0E5(GO:0010008;GO:0005886;GO:0016323);Q96PD5(GO:0016020);P02760(GO:0005886);Q01518(GO:0016020;GO:0005886);A0A0C4DH73(GO:0016020;GO:0005886);P07357(GO:0016020;GO:0005886);A0A0G2JI36(GO:0055038;GO:0016020;GO:0005886;GO:0031901;GO:0012507;GO:0030670;GO:0000139);P07358(GO:0016020);P35579(GO:0016020;GO:0005886;GO:0032154);P01703(GO:0016020;GO:0005886);P01700(GO:0016020;GO:0005886);P01701(GO:0016020;GO:0005886);P01706(GO:0016020;GO:0005886);P04264(GO:0016020;GO:0005886;GO:0001533);P01704(GO:0016020;GO:0005886);P01705(GO:0016020;GO:0005886);P23083(GO:0016020;GO:0005886);P01709(GO:0016020;GO:0005886);P02671(GO:0005886);P02675(GO:0005886);A0A075B6I0(GO:0016020;GO:0005886);P0CF74(GO:0016020;GO:0005886);O00391(GO:0016020;GO:0000139);A0A0C4DH67(GO:0016020;GO:0005886);P35527(GO:0016020);P05154(GO:0002080;GO:0016020);I3L1J2(GO:0016020;GO:0005886);M0R0Q9(GO:0005886);P00734(GO:0005886);P13645(GO:0016020;GO:0001533);A0A0A0MS14(GO:0016020;GO:0005886);A0A0A0MS15(GO:0016020;GO:0005886);P01715(GO:0016020;GO:0005886);P01717(GO:0016020;GO:0005886);A0A087X1L8(GO:0016020);P01718(GO:0016020;GO:0005886);A0A286YEY4(GO:0016020);P01857(GO:0016020;GO:0005886);A0A075B6Q5(GO:0016020;GO:0005886);P80748(GO:0016020;GO:0005886);A0A0C4DH25(GO:0016020;GO:0005886);P01601(GO:0016020;GO:0005886);Q9UHG3(GO:0005774;GO:0005886);P41222(GO:0005789;GO:0016020;GO:0031965);A0A0C4DH55(GO:0016020;GO:0005886);A0A0B4J1Y8(GO:0016020;GO:0005886);A0A0A0MS09(GO:0016020);A0A1W2PQU7(GO:0016020);C9JXI5(GO:0016020;GO:0005886);P13796(GO:0016020;GO:0005886;GO:0032587);A0A087WSZ0(GO:0016020;GO:0005886);P09486(GO:0031092;GO:0005886);P04211(GO:0016020;GO:0005886);P14151(GO:0030667;GO:0016020;GO:0005886);P01721(GO:0016020;GO:0005886);A0A075B6K2(GO:0016020;GO:0005886);B4E1Z4(GO:0005886);P01008(GO:0005886);A0A075B6R2(GO:0016020;GO:0005886);Q9UNN8(GO:0016020;GO:0005886);P01009(GO:0033116;GO:0000139);P80108(GO:0005765);A0A0B4J1X5(GO:0016020;GO:0005886);A0A0C4DH43(GO:0016020;GO:0005886);A0A075B6K5(GO:0016020;GO:0005886);P13647(GO:0016020;GO:0005886);A0A075B6K4(GO:0016020;GO:0005886);A0A087WSY4(GO:0016020;GO:0005886);A0A087WSY6(GO:0016020;GO:0005886);A0A0B4J1X8(GO:0016020;GO:0005886);P01877(GO:0016020;GO:0005886);P01871(GO:0016020;GO:0005886);P13473(GO:0101003;GO:0035577;GO:0010008;GO:0016020;GO:0031902;GO:0045121;GO:0005886;GO:0000421;GO:0030670;GO:0031088;GO:0005765;GO:0098857);P10909(GO:0016020;GO:0031966;GO:0005743);O75882(GO:0016020;GO:0005886);P01619(GO:0016020;GO:0005886);P00918(GO:0016323;GO:0016020;GO:0005886);P04114(GO:0005789;GO:0030669;GO:0010008;GO:0012506;GO:0005886);Q6EMK4(GO:0016020;GO:0005886;GO:0005765);A0A0A0MRJ7(GO:0033116;GO:0005886;GO:0016020;GO:0000139);P04040(GO:0016020;GO:0005886;GO:0005778);A0A0C4DH33(GO:0016020;GO:0005886);P02751(GO:0016324);P18065(GO:0016324);P01861(GO:0016020;GO:0005886);A0A075B6S5(GO:0016020;GO:0005886);P01860(GO:0016020;GO:0005886);A0A0B4J2D9(GO:0016020;GO:0005886);P02748(GO:0016020;GO:0005886);P01034(GO:0031965);P08514(GO:0031092;GO:0016020;GO:0005886);P02649(GO:0016020;GO:0030669;GO:0005886);Q07954(GO:0016020;GO:0030666;GO:0005886;GO:0016323;GO:0005765);P04196(GO:0005886);P02647(GO:0005886);A0A0A0MS51(GO:0005886);A0A0C4DH32(GO:0016020;GO:0005886);P05109(GO:0016020;GO:0005886);A0A0J9YX35(GO:0016020;GO:0005886);A0A0C4DH31(GO:0016020;GO:0005886);A0A0C4DH34(GO:0016020;GO:0005886);P19320(GO:0016020;GO:0042383;GO:0005886);H0YAC1(GO:0005886);Q14624(GO:0005886);A0A0C4DH38(GO:0016020;GO:0005886);A0A0C4DH39(GO:0016020;GO:0005886);P25311(GO:0005886);P01599(GO:0016020;GO:0005886);O43866(GO:0016020);P01024(GO:0005886);P01743(GO:0016020;GO:0005886);P07437(GO:0045121);P09172(GO:0030667;GO:0016020;GO:0030658;GO:0042584);O95497(GO:0016020;GO:0035577;GO:0005886);P02538(GO:0016020);P01782(GO:0016020;GO:0005886);P00747(GO:0005886);P00740(GO:0005886);P60709(GO:0016020;GO:0005886);P00742(GO:0005886);A0A0A0MRZ9(GO:0016020;GO:0005886);P01742(GO:0016020;GO:0005886);P00748(GO:0005886);Q08380(GO:0016020);A0A075B6J9(GO:0016020;GO:0005886);P0C0L5(GO:0005886);P0C0L4(GO:0005886);A0A669KAY4(GO:0036020;GO:0005886;GO:0005765);Q9HDC9(GO:0016020);H0Y5E4(GO:0016020);P04433(GO:0016020;GO:0005886);A0A0C4DH24(GO:0016020;GO:0005886);P04430(GO:0016020;GO:0005886);A0A0C4DH29(GO:0016020;GO:0005886);P18206(GO:0016020;GO:0042383;GO:0005886);J3KPA1(GO:0016020);P01817(GO:0016020;GO:0005886);P01814(GO:0016020;GO:0005886);P21333(GO:0016020;GO:0005886);A0A0B4J1V1(GO:0016020;GO:0005886);A0A0B4J1V0(GO:0016020;GO:0005886);A0A0B4J1V2(GO:0016020;GO:0005886);Q86UX7(GO:0016020);P68032(GO:0016020);H9KV75(GO:0042383;GO:0097381);P11021(GO:0005789;GO:0016020;GO:0005886);Q9Y490(GO:0016020;GO:0005886;GO:0032587);A0A0J9YVY3(GO:0016020;GO:0005886);P01699(GO:0016020;GO:0005886);P07737(GO:0016020);P35908(GO:0016020;GO:0001533) | 25|51 | A0A0B4J1Y8(GO:0016020;GO:0005886);Q86UX7(GO:0016020);A0A0C4DH73(GO:0016020;GO:0005886);A0A0J9YX35(GO:0016020;GO:0005886);P35527(GO:0016020);A0A2R8Y3M9(GO:0016020);A0A075B6K2(GO:0016020;GO:0005886);P02751(GO:0016324);P03951(GO:0016020;GO:0005886);A0A075B6J9(GO:0016020;GO:0005886);Q14624(GO:0005886);P0DOY3(GO:0016020;GO:0005886);I3L1J2(GO:0016020;GO:0005886);P30041(GO:0016020);P18206(GO:0016020;GO:0042383;GO:0005886);P13647(GO:0016020;GO:0005886);P01715(GO:0016020;GO:0005886);P01717(GO:0016020;GO:0005886);P04040(GO:0016020;GO:0005886;GO:0005778);P60709(GO:0016020;GO:0005886);C9JXI5(GO:0016020;GO:0005886);P68032(GO:0016020);P80748(GO:0016020;GO:0005886);P69905(GO:0016020);P07737(GO:0016020) | 0.087878943 | 0.251082695 | https://www.ebi.ac.uk/QuickGO/term/GO:0016020 |
| GO:0002376 | immune system process | biological_process | 198|375 | P08779(GO:0045087);J3KNB4(GO:0061844;GO:0045087);P0C0L5(GO:0045087;GO:0008228;GO:0006956;GO:0006958;GO:0002376);K7ERG9(GO:0006957);P15814(GO:0050853;GO:0045087;GO:0050900;GO:0006955;GO:0006958);P0DP02(GO:0002376;GO:0002250);P08571(GO:0002224;GO:0038124;GO:0045087;GO:0035666;GO:0034142;GO:0002755;GO:0002756;GO:0038123;GO:0043312;GO:0002376);P68871(GO:0043312);Q86YZ3(GO:0043312);P01597(GO:0038096;GO:0002376;GO:0050900;GO:0006956;GO:0002250;GO:0006958;GO:0006955);P0DP01(GO:0002376;GO:0002250);P01602(GO:0038096;GO:0002376;GO:0002377;GO:0050900;GO:0006956;GO:0002250;GO:0006958;GO:0006955);P11226(GO:0001867;GO:0045087;GO:0008228;GO:0006956;GO:0006958;GO:0002376);A0A0B4J1U7(GO:0050853;GO:0045087;GO:0002376;GO:0002250;GO:0006958);P59665(GO:0019730;GO:0019731;GO:0061844;GO:0002227;GO:0010818;GO:0006955;GO:0051607;GO:0043312);P0DJI8(GO:0048246;GO:0048247;GO:0030593;GO:0045087);P06312(GO:0038096;GO:0002376;GO:0002377;GO:0050900;GO:0006956;GO:0002250;GO:0006958;GO:0006955);A0A0A0MT36(GO:0006955;GO:0002376;GO:0002377;GO:0002250);P01763(GO:0038096;GO:0002376;GO:0050900;GO:0006956;GO:0002250;GO:0006958;GO:0006955);P01764(GO:0006955;GO:0050900;GO:0050853;GO:0045087;GO:0006956;GO:0006958;GO:0038096;GO:0002376;GO:0002250);P01766(GO:0038096;GO:0050900;GO:0002376;GO:0006956;GO:0002250;GO:0006958;GO:0006955);B4E1Z4(GO:0006956;GO:0006957);P0DOY3(GO:0002376;GO:0002250);P26038(GO:0050900;GO:0042098;GO:0072678);P01782(GO:0038096;GO:0002376;GO:0050900;GO:0006956;GO:0002250;GO:0006958;GO:0006955);P18428(GO:0002281;GO:0002224;GO:0045087;GO:0008228;GO:0034142;GO:0002376;GO:0002232);P01780(GO:0038096;GO:0002376;GO:0050900;GO:0006956;GO:0002250;GO:0006958;GO:0006955);P02776(GO:0061844;GO:0030595;GO:0030593;GO:0006955);D6R934(GO:0006958);P02775(GO:0061844;GO:0030595;GO:0030593;GO:0006955;GO:0043312);E7EUT5(GO:0061844);A0A0G2JMB2(GO:0050853;GO:0045087;GO:0006958);A0A0J9YXX1(GO:0002376;GO:0002250);P30041(GO:0043312);P06702(GO:0045087;GO:0019730;GO:0030595;GO:0030593;GO:0061844;GO:0002376;GO:0002224;GO:0002523;GO:0043312);P61224(GO:0043312);P10643(GO:0045087;GO:0002376;GO:0006956;GO:0006957;GO:0006955;GO:0006958);P01833(GO:0043312);P01834(GO:0006955;GO:0050853;GO:0045087;GO:0006956;GO:0006958;GO:0038096;GO:0050900;GO:0002376;GO:0002250);P04003(GO:0045087;GO:0002376;GO:0006958);P01718(GO:0038096;GO:0002376;GO:0050900;GO:0006956;GO:0002250;GO:0006958;GO:0006955);P35579(GO:0050900);P04004(GO:0006955);P01743(GO:0038096;GO:0002376;GO:0050900;GO:0006956;GO:0002250;GO:0006958;GO:0006955);Q92954(GO:0006955);O00391(GO:0043312);P02751(GO:0050900);A0A075B6H7(GO:0002377;GO:0006955);A0A087WSZ0(GO:0006955;GO:0002376;GO:0002377;GO:0002250);O75636(GO:0051607;GO:0006956;GO:0045087;GO:0002376;GO:0001867);Q96PD5(GO:0019730;GO:0002221;GO:0045087;GO:0002376);P02763(GO:0043312);Q01518(GO:0043312);A0A0C4DH73(GO:0038096;GO:0050900;GO:0002376;GO:0002377;GO:0006956;GO:0002250;GO:0006958;GO:0006955);P02766(GO:0043312);P07357(GO:0002376;GO:0006956;GO:0006957;GO:0006955;GO:0006958;GO:0045087);A0A0B4J2D9(GO:0006955;GO:0002376;GO:0002377;GO:0002250);A0A0G2JI36(GO:0006955;GO:0042590;GO:0002474;GO:0002419;GO:0002479;GO:0019885;GO:0002480;GO:0036037;GO:0001913);P07358(GO:0045087;GO:0002376;GO:0006956;GO:0006957;GO:0006955;GO:0006958);P09172(GO:0050900;GO:0002443);P01703(GO:0038096;GO:0002376;GO:0050900;GO:0006956;GO:0002250;GO:0006958;GO:0006955);P01700(GO:0038096;GO:0002376;GO:0050900;GO:0006956;GO:0002250;GO:0006958;GO:0006955);P01701(GO:0038096;GO:0050900;GO:0006956;GO:0006958;GO:0006955);P01706(GO:0038096;GO:0002376;GO:0050900;GO:0006956;GO:0002250;GO:0006958;GO:0006955);P20851(GO:0045087;GO:0002376;GO:0006958);P01704(GO:0038096;GO:0002376;GO:0050900;GO:0006956;GO:0002250;GO:0006958;GO:0006955);P01705(GO:0038096;GO:0002376;GO:0050900;GO:0006956;GO:0002250;GO:0006958;GO:0006955);P23083(GO:0038096;GO:0002376;GO:0050900;GO:0006956;GO:0002250;GO:0006958;GO:0006955);P01709(GO:0038096;GO:0002376;GO:0050900;GO:0006956;GO:0002250;GO:0006958;GO:0006955);P02671(GO:0045087;GO:0002224;GO:0002376;GO:0002250;GO:0043152);P02675(GO:0002224;GO:0045087;GO:0002376;GO:0002250;GO:0043152);P07996(GO:0006955);P13671(GO:0045087;GO:0002376;GO:0006956;GO:0006955;GO:0006958);A0A075B6I0(GO:0006955;GO:0002376;GO:0002377;GO:0002250);P0CF74(GO:0050900;GO:0050853;GO:0045087;GO:0006956;GO:0006958;GO:0038096;GO:0002376;GO:0002250);P61626(GO:0019730;GO:0043312);A0A0C4DH67(GO:0002376;GO:0002250);P05155(GO:0045087;GO:0006958;GO:0002376);M0R0Q9(GO:0006956;GO:0006957;GO:0006955;GO:0006958;GO:0043312);P01031(GO:0045087;GO:0006956;GO:0006957;GO:0006958;GO:0002376);A0A075B7F0(GO:0050853;GO:0045087;GO:0006958);P00734(GO:0061844;GO:0070945);A0A0A0MS14(GO:0050853;GO:0045087;GO:0002376;GO:0002250;GO:0006958);A0A0A0MS15(GO:0050853;GO:0045087;GO:0002376;GO:0002250;GO:0006958);P13473(GO:0043312);P01717(GO:0038096;GO:0002376;GO:0050900;GO:0006956;GO:0002250;GO:0006958;GO:0006955);P48740(GO:0006956;GO:0002376;GO:0001867;GO:0045087);P00738(GO:0043312;GO:0002376);P01857(GO:0050853;GO:0045087;GO:0006956;GO:0006958;GO:0038096;GO:0002376;GO:0002250);A0A075B6Q5(GO:0050853;GO:0045087;GO:0002376;GO:0002250;GO:0006958);Q9BWP8(GO:0019730;GO:0001867;GO:0045087;GO:0006956;GO:0002376);A0A0C4DH25(GO:0038096;GO:0050900;GO:0002376;GO:0002377;GO:0006956;GO:0002250;GO:0006958;GO:0006955);A0A0G2JMI3(GO:0050853;GO:0045087;GO:0002376;GO:0002250;GO:0006958);P01011(GO:0043312);P19652(GO:0043312);A0A0C4DH55(GO:0006955;GO:0002376;GO:0002377;GO:0002250);A0A0B4J1Y8(GO:0006955;GO:0002376;GO:0002377;GO:0002250);P14151(GO:0050900;GO:0043312);P04430(GO:0038096;GO:0050900;GO:0006956;GO:0006958;GO:0006955);P31151(GO:0019730;GO:0061844;GO:0045087;GO:0043312);P01721(GO:0038096;GO:0002376;GO:0050900;GO:0006956;GO:0002250;GO:0006958;GO:0006955);P13796(GO:0002286);P15144(GO:0043312);A0A0B4J1U3(GO:0006955;GO:0002376;GO:0002377;GO:0002250);A0A0G2JPR0(GO:0006956);P04211(GO:0038096;GO:0002376;GO:0050900;GO:0006956;GO:0002250;GO:0006958;GO:0006955);P32119(GO:0002536;GO:0042098;GO:0045321);O00187(GO:0045087;GO:0006956;GO:0002376;GO:0001867;GO:0006958);A0A0B4J231(GO:0050853;GO:0045087;GO:0006958);A0A075B6K2(GO:0006955;GO:0002376;GO:0002377;GO:0002250);P02750(GO:0043312);P01008(GO:0002438);A0A075B6R2(GO:0050853;GO:0045087;GO:0002376;GO:0002250;GO:0006958);A0A075B6K4(GO:0006955;GO:0002376;GO:0002377;GO:0002250);Q03591(GO:0006956);P01009(GO:0043312);A0A075B6R9(GO:0002377;GO:0006955);P80108(GO:0002430);A0A0G2JRQ6(GO:0002377;GO:0006955);A0A0B4J1X5(GO:0050853;GO:0045087;GO:0002376;GO:0002250;GO:0006958);A0A0C4DH43(GO:0050853;GO:0045087;GO:0002376;GO:0002250;GO:0006958);A0A075B6K5(GO:0006955;GO:0002376;GO:0002377;GO:0002250);A0A075B6J9(GO:0006955;GO:0002376;GO:0002377;GO:0002250);P08603(GO:0045087;GO:0002376;GO:0006956;GO:0006957);C9JV77(GO:0043312);A0A087WSY6(GO:0006955;GO:0002376;GO:0002377;GO:0002250);A0A0B4J1X8(GO:0050853;GO:0045087;GO:0002376;GO:0002250;GO:0006958);P01877(GO:0019731;GO:0006955;GO:0050900;GO:0050853;GO:0045087;GO:0006958;GO:0002376;GO:0002250);P01871(GO:0019731;GO:0050900;GO:0050853;GO:0045087;GO:0006958;GO:0002376;GO:0002250);A0A075B7D0(GO:0050853;GO:0045087;GO:0006958);P01715(GO:0038096;GO:0002376;GO:0050900;GO:0006956;GO:0002250;GO:0006958;GO:0006955);P09871(GO:0006956;GO:0045087;GO:0002376;GO:0001867;GO:0006958);A0A075B7D8(GO:0050853;GO:0045087;GO:0006958);P04114(GO:0050900;GO:0002224);A0A075B6S9(GO:0002377;GO:0006955);P04040(GO:0043312);A0A0C4DH33(GO:0050853;GO:0045087;GO:0002376;GO:0002250;GO:0006958);A0A3B3ISR2(GO:0045087;GO:0006958);A0A0J9YX35(GO:0002376;GO:0002250);A0A075B6S5(GO:0006955;GO:0002376;GO:0002377;GO:0002250);P02743(GO:0045087;GO:0006958);P02741(GO:0045087;GO:0006958;GO:0008228);P01860(GO:0050853;GO:0045087;GO:0006956;GO:0006958;GO:0038096;GO:0002376;GO:0002250);P02747(GO:0045087;GO:0002376;GO:0006956;GO:0006955;GO:0006958);P02745(GO:0045087;GO:0002376;GO:0006956;GO:0006958);A0A0B4J1V0(GO:0050853;GO:0045087;GO:0002376;GO:0002250;GO:0006958);A0A0G2JL69(GO:0045087;GO:0006956;GO:0006958);P02748(GO:0006955;GO:0045087;GO:0006957;GO:0006958;GO:0002376);P01034(GO:0043312);P01601(GO:0038096;GO:0050900;GO:0002376;GO:0002377;GO:0006956;GO:0002250;GO:0006958;GO:0006955);P04217(GO:0043312);O14791(GO:0045087);Q07954(GO:0002265);P04196(GO:0061844);P10909(GO:0019730;GO:0002376;GO:0002434;GO:0001774;GO:0006956;GO:0006958;GO:0045087);A0A0C4DH32(GO:0050853;GO:0045087;GO:0002376;GO:0002250;GO:0006958);P05109(GO:0045087;GO:0019730;GO:0043312;GO:0002224;GO:0030593;GO:0002523;GO:0002376);P01861(GO:0050853;GO:0045087;GO:0006956;GO:0006958;GO:0038096;GO:0002376;GO:0002250);A0A0C4DH31(GO:0050853;GO:0045087;GO:0002376;GO:0002250;GO:0006958);A0A0C4DH36(GO:0050853;GO:0045087;GO:0006958);A0A0C4DH34(GO:0050853;GO:0045087;GO:0002376;GO:0002250;GO:0006958);P19320(GO:0030183);A0A0C4DH38(GO:0050853;GO:0045087;GO:0002376;GO:0002250;GO:0006958);A0A0C4DH39(GO:0050853;GO:0045087;GO:0002376;GO:0002250;GO:0006958);P25311(GO:0006955);P01599(GO:0038096;GO:0002376;GO:0050900;GO:0006956;GO:0002250;GO:0006958;GO:0006955);O43866(GO:0002376);P01024(GO:0045087;GO:0006956;GO:0006957;GO:0006955;GO:0006958;GO:0043312;GO:0002376);C9J8S2(GO:0019732;GO:0045087;GO:0061760);O95497(GO:0045087;GO:0043312);P02538(GO:0061844);P60709(GO:0038096);P06331(GO:0038096;GO:0002376;GO:0050900;GO:0006956;GO:0002250;GO:0006958;GO:0006955);E7EQB2(GO:0019732;GO:0019731;GO:0061844;GO:0002227);A0A0A0MRZ9(GO:0006955;GO:0002376;GO:0002377;GO:0002250);P01742(GO:0038096;GO:0002376;GO:0050900;GO:0006956;GO:0002250;GO:0006958;GO:0006955);P00748(GO:0045087);P80748(GO:0038096;GO:0002376;GO:0050900;GO:0006956;GO:0002250;GO:0006958;GO:0006955);P04264(GO:0001867;GO:0043312);P0C0L4(GO:0045087;GO:0006956;GO:0006958;GO:0002376);A0A087WSY4(GO:0050853;GO:0045087;GO:0002376;GO:0002250;GO:0006958);Q15485(GO:0002376;GO:0045087;GO:0008228;GO:0001867;GO:0006956);P04433(GO:0038096;GO:0002376;GO:0050900;GO:0006956;GO:0002250;GO:0006958;GO:0006955);A0A0C4DH24(GO:0006955;GO:0002376;GO:0002377;GO:0002250);P06727(GO:0002227);Q9NZP8(GO:0045087;GO:0002376;GO:0006958);A0A0C4DH29(GO:0050853;GO:0045087;GO:0002376;GO:0002250;GO:0006958);P18206(GO:0043312);P01817(GO:0038096;GO:0002376;GO:0050900;GO:0006956;GO:0002250;GO:0006958;GO:0006955);P01814(GO:0038096;GO:0002376;GO:0050900;GO:0006956;GO:0002250;GO:0006958;GO:0006955);P01619(GO:0019731;GO:0006955;GO:0050900;GO:0006956;GO:0006958;GO:0038096;GO:0002376;GO:0002250);A0A0B4J1V1(GO:0050853;GO:0045087;GO:0002376;GO:0002250;GO:0006958);A0A075B7B8(GO:0050853;GO:0045087;GO:0006958);P07360(GO:0045087;GO:0002376;GO:0006957;GO:0006958);A0A0B4J1V2(GO:0050853;GO:0045087;GO:0002376;GO:0002250;GO:0006958);P07437(GO:0042267;GO:0043312);A0A0J9YVY3(GO:0002376;GO:0002250);P01699(GO:0038096;GO:0002376;GO:0050900;GO:0006956;GO:0002250;GO:0006958;GO:0006955) | 21|51 | O75636(GO:0051607;GO:0006956;GO:0045087;GO:0002376;GO:0001867);P01715(GO:0038096;GO:0002376;GO:0050900;GO:0006956;GO:0002250;GO:0006958;GO:0006955);P04040(GO:0043312);P01717(GO:0038096;GO:0002376;GO:0050900;GO:0006956;GO:0002250;GO:0006958;GO:0006955);A0A0B4J1Y8(GO:0006955;GO:0002376;GO:0002377;GO:0002250);K7ERG9(GO:0006957);P07360(GO:0045087;GO:0002376;GO:0006957;GO:0006958);P32119(GO:0002536;GO:0042098;GO:0045321);A0A0C4DH73(GO:0038096;GO:0050900;GO:0002376;GO:0002377;GO:0006956;GO:0002250;GO:0006958;GO:0006955);P0DOY3(GO:0002376;GO:0002250);A0A075B6K2(GO:0006955;GO:0002376;GO:0002377;GO:0002250);A0A0J9YX35(GO:0002376;GO:0002250);A0A0C4DH36(GO:0050853;GO:0045087;GO:0006958);P80748(GO:0038096;GO:0002376;GO:0050900;GO:0006956;GO:0002250;GO:0006958;GO:0006955);P60709(GO:0038096);P30041(GO:0043312);P02747(GO:0045087;GO:0002376;GO:0006956;GO:0006955;GO:0006958);P02751(GO:0050900);P18206(GO:0043312);A0A075B6R9(GO:0002377;GO:0006955);A0A075B6J9(GO:0006955;GO:0002376;GO:0002377;GO:0002250) | 0.024572029 | 0.196576228 | https://www.ebi.ac.uk/QuickGO/term/GO:0002376 |
| GO:0003824 | catalytic activity | molecular_function | 133|375 | P00488(GO:0003810;GO:0016740;GO:0016746);P68366(GO:0003924);P03951(GO:0004252;GO:0016787;GO:0070009;GO:0008233;GO:0008236);K7ERG9(GO:0004252;GO:0016787;GO:0008233;GO:0008236);J3KPS3(GO:0016829;GO:0003824;GO:0004332);P22792(GO:0004181);O75460(GO:0004540;GO:0016787;GO:0004521;GO:0004674;GO:0016740;GO:0004672;GO:0003824;GO:0016301;GO:0004519);A0A087X1J7(GO:0004601;GO:0004602;GO:0016491);Q14520(GO:0004252;GO:0016787;GO:0008233;GO:0008236);P01602(GO:0004252);P11226(GO:0004252);P15169(GO:0016787;GO:0004181;GO:0004180;GO:0004185;GO:0008233;GO:0008237);A0A2R8Y3M9(GO:0004252;GO:0004623);Q96KN2(GO:0016787;GO:0004180;GO:0016805;GO:0008233;GO:0008237);P06312(GO:0004252);P01743(GO:0004252);P03950(GO:0004540;GO:0016787;GO:0004518;GO:0004519);P60763(GO:0003924);P01763(GO:0004252);P01764(GO:0004252);P01766(GO:0004252);F8WF14(GO:0016787;GO:0004104;GO:0003824;GO:0003990);P01782(GO:0004252);P01780(GO:0004252);P06331(GO:0004252);E7EUT5(GO:0004365;GO:0016620;GO:0035605);A0A0U1RQV3(GO:0005006);P30041(GO:0016491;GO:0047499;GO:0016787;GO:0102568;GO:0003824;GO:0051920;GO:0102567;GO:0004623;GO:0004601;GO:0004602);P61224(GO:0003924);P01834(GO:0004252);E7END6(GO:0004252;GO:0016787;GO:0008233;GO:0008236);P00748(GO:0004252;GO:0008233;GO:0008236;GO:0016787);O00391(GO:0016491;GO:0016971;GO:0016972;GO:0003756);P43251(GO:0016787;GO:0047708;GO:0016810;GO:0016811);D6RAR4(GO:0004252;GO:0016787;GO:0008233;GO:0008236);O75636(GO:0004252);P69905(GO:0004601);P68871(GO:0004601);Q96PD5(GO:0008745;GO:0016787);A0A0C4DH73(GO:0004252);Q9H4B7(GO:0003924);A0A0G2JL69(GO:0004252;GO:0016787;GO:0008233;GO:0008236);E7EWH8(GO:0008903;GO:0016853);P19320(GO:0008131);Q15166(GO:0016787;GO:0004063;GO:0102007;GO:0004064;GO:0018733);P09172(GO:0016491;GO:0004500;GO:0004497;GO:0003824;GO:0016715);P01703(GO:0004252);P01700(GO:0004252);P01701(GO:0004252);P01706(GO:0004252);P01704(GO:0004252);P01705(GO:0004252);P23083(GO:0004252);P01709(GO:0004252);H3BTN5(GO:0016301;GO:0003824;GO:0016740;GO:0004743);F5H8B0(GO:0004252;GO:0016787;GO:0008233;GO:0008236);P0CF74(GO:0004252);P61626(GO:0016798;GO:0003796;GO:0016787;GO:0003824);Q16880(GO:0008489;GO:0016740;GO:0015020;GO:0008194;GO:0016757;GO:0047263;GO:0016758);P22891(GO:0004252);P00734(GO:0004252;GO:0008233;GO:0008236;GO:0016787);P35579(GO:0016887;GO:0000146;GO:0003774;GO:0030898);P01715(GO:0004252);P01717(GO:0004252);P48740(GO:0004252;GO:0016787;GO:0008233;GO:0008236);P00739(GO:0004252);E7EQB2(GO:0004252);Q9BWP8(GO:0004252);A0A0C4DH25(GO:0004252);P01601(GO:0004252);Q9UHG3(GO:0016670;GO:0008555;GO:0001735;GO:0016491);P41222(GO:0016853;GO:0004667);Q96IY4(GO:0008233;GO:0008237;GO:0004181;GO:0004180;GO:0016787);C9J5S7(GO:0016853;GO:0003755);Q8IV42(GO:0016301;GO:0016740);P01721(GO:0004252);A0A0A0MTH3(GO:0004674;GO:0004672);G3XAK1(GO:0004252);P0DPH7(GO:0003924);Q13093(GO:0047499;GO:0016787;GO:0016788;GO:0003847);P32119(GO:0008379;GO:0016491;GO:0051920;GO:0004601);O00187(GO:0004252;GO:0016787;GO:0008236;GO:0008233);A0A2R8Y6G6(GO:0004634);P16930(GO:0016787;GO:0004334;GO:0003824);B4E1Z4(GO:0004252;GO:0016787;GO:0008233;GO:0008236);Q9NZP8(GO:0004252;GO:0016787;GO:0008233;GO:0008236);P80108(GO:0004630;GO:0016787;GO:0004621);J3KRP0(GO:0016787;GO:0004180;GO:0016805;GO:0008237);P33908(GO:0015923;GO:0016798;GO:0003824;GO:0016787;GO:0004571);A0A669KAY4(GO:0004252);P27169(GO:0004064;GO:0102007;GO:0016787;GO:0004063);P10909(GO:0016887);P09871(GO:0004252;GO:0016787;GO:0008233;GO:0008236);P00918(GO:0016829;GO:0004064;GO:0004089);P15144(GO:0004177;GO:0008233;GO:0008237;GO:0070006;GO:0016787);P04040(GO:0016491;GO:0004601;GO:0004046;GO:0016684;GO:0004096);A0A3B3ISR2(GO:0004252);P00915(GO:0016829;GO:0016836;GO:0004089;GO:0004064);P02747(GO:0004252);P02745(GO:0004252);P01718(GO:0004252);P68032(GO:0016887);P04211(GO:0004252);P08519(GO:0004252;GO:0016787;GO:0008233;GO:0008236);P01857(GO:0004252);P01597(GO:0004252);P01861(GO:0004252);P01860(GO:0004252);H0YAC1(GO:0004252;GO:0004497;GO:0008236;GO:0008233;GO:0016491;GO:0016705;GO:0016787);P25311(GO:0004540);P01599(GO:0004252);P01024(GO:0004252);O95497(GO:0016811;GO:0016787;GO:0017159);P00747(GO:0004252;GO:0004175;GO:0016787;GO:0008233;GO:0008236);P00740(GO:0004252;GO:0004175;GO:0016787;GO:0008233;GO:0008236);P00742(GO:0004252;GO:0016787;GO:0008233;GO:0008236);P01742(GO:0004252);P00450(GO:0016491;GO:0004322);P80748(GO:0004252);P0C0L5(GO:0004252);P0C0L4(GO:0004252);P04180(GO:0008374;GO:0016740;GO:0016746;GO:0004607);Q9HDC9(GO:0016844;GO:0004064);Q15485(GO:0004252);P04433(GO:0004252);P04430(GO:0004252);P01817(GO:0004252);P01814(GO:0004252);P01619(GO:0004252);P07437(GO:0003924);Q96HR3(GO:0061630);P11021(GO:0016887;GO:0042623;GO:0016787);P01699(GO:0004252) | 21|51 | O75636(GO:0004252);P01715(GO:0004252);A0A087X1J7(GO:0004601;GO:0004602;GO:0016491);P01717(GO:0004252);Q9H4B7(GO:0003924);P03951(GO:0004252;GO:0016787;GO:0070009;GO:0008233;GO:0008236);K7ERG9(GO:0004252;GO:0016787;GO:0008233;GO:0008236);P04040(GO:0016491;GO:0004601;GO:0004046;GO:0016684;GO:0004096);P32119(GO:0008379;GO:0016491;GO:0051920;GO:0004601);A0A0C4DH73(GO:0004252);P00739(GO:0004252);A0A2R8Y3M9(GO:0004252;GO:0004623);H3BTN5(GO:0016301;GO:0003824;GO:0016740;GO:0004743);Q96HR3(GO:0061630);P02747(GO:0004252);P80748(GO:0004252);P03950(GO:0004540;GO:0016787;GO:0004518;GO:0004519);P00915(GO:0016829;GO:0016836;GO:0004089;GO:0004064);P30041(GO:0016491;GO:0047499;GO:0016787;GO:0102568;GO:0003824;GO:0051920;GO:0102567;GO:0004623;GO:0004601;GO:0004602);P69905(GO:0004601);P68032(GO:0016887) | 0.08085766 | 0.269525535 | https://www.ebi.ac.uk/QuickGO/term/GO:0003824 |
| GO:0051179 | localization | biological_process | 169|375 | P00488(GO:0002576);P68366(GO:0097711;GO:0002576);P04275(GO:0002576);P08571(GO:0006909;GO:0006898;GO:0043312);P68871(GO:0015701;GO:0015671;GO:0030185;GO:0006898;GO:0043312);Q86YZ3(GO:0043312);P01042(GO:0002576);P01602(GO:0006898);P01601(GO:0006898);P59665(GO:0043312);P0DJI8(GO:0006898);A0A2R8Y3M9(GO:0006898);P06312(GO:0006898);P02790(GO:0015886;GO:0006898);P01763(GO:0006898);P01764(GO:0006898);P01766(GO:0006898);P01782(GO:0006898);P18428(GO:0006869;GO:0033036;GO:0015920);P01780(GO:0006898);P00742(GO:0006888);P02776(GO:0002576);P02775(GO:0002576;GO:0043312;GO:1904659);Q13201(GO:0002576);P02751(GO:0002576);P30041(GO:0043312);P06702(GO:0032119;GO:0043312);P61224(GO:0043312);D6RF35(GO:0051180;GO:0035461);P02787(GO:0006826;GO:0002576;GO:0033572;GO:0006811);P01833(GO:0002415;GO:0043312;GO:0043113);P01834(GO:0006898);P04004(GO:0006898);P01743(GO:0006898);Q92954(GO:0006898);Q16880(GO:0002175);P69905(GO:0015701;GO:0015671;GO:0006898);G3V0E5(GO:0031623;GO:0006898;GO:0033572);P02768(GO:0051659;GO:0002576;GO:0006898);P02760(GO:0006898);P02763(GO:0002576;GO:0043312);Q01518(GO:0006898;GO:0043312);A0A0C4DH73(GO:0006898);P02766(GO:0070327;GO:0043312);P37802(GO:0002576);P29622(GO:0002576);E9PHK0(GO:0002576);P01703(GO:0006898);P01700(GO:0006898);P01701(GO:0006898);P01706(GO:0006898);P21333(GO:1902396;GO:0051220;GO:0002576;GO:0034394;GO:0045184;GO:0072659;GO:0043113);P01705(GO:0006898);P23083(GO:0006898);P01709(GO:0006898);P02671(GO:0002576);P02675(GO:0002576);P07996(GO:0002576);P0CF74(GO:0006898);P61626(GO:0043312);P04180(GO:0043691;GO:0030301);O00391(GO:0043312;GO:0002576);P22891(GO:0006888);Q06033(GO:0002576);C9JF17(GO:0006869);P05155(GO:0002576);P05154(GO:0006869);M0R0Q9(GO:0043312);O95445(GO:0006869;GO:0043691;GO:0033344);P00734(GO:0006888);P35579(GO:0032418;GO:0045055;GO:0051295;GO:0015031);P01715(GO:0006898);P01717(GO:0006898);P01718(GO:0006898);P00738(GO:0006898;GO:0043312);P00739(GO:0006898);Q9BWP8(GO:0006898);G3XAP6(GO:0009306);P04433(GO:0006898);P01019(GO:0006606;GO:0050663);Q9UHG3(GO:1902476;GO:0099133;GO:0006821);P19652(GO:0002576;GO:0043312);B0YIW2(GO:0006869;GO:0033700;GO:0043691;GO:0033344);P31151(GO:0051238;GO:0043312);O14791(GO:0006869;GO:1902476;GO:0006898);K7ER74(GO:0033700;GO:0006869;GO:0043691;GO:0033344;GO:0042953);P15144(GO:0043312);P09486(GO:0006898;GO:0002576);Q13103(GO:0002576);P04211(GO:0006898);P14151(GO:0043312);P01721(GO:0006898);P08514(GO:0002576);P55058(GO:0006869;GO:0015914;GO:0035627);P01009(GO:0006888;GO:0002576;GO:0043312);P01008(GO:0007595);Q86UX7(GO:0002576);P02750(GO:0043312);A0A2R8Y7X9(GO:0015671);P55056(GO:0006869);P80108(GO:0070633);J3QRV5(GO:0006887);P69892(GO:0015671);H9KV75(GO:0007041);P43652(GO:0051180;GO:0015031;GO:0071693);P02753(GO:0034633);C9JV77(GO:0006907;GO:0002576;GO:0043312);P01344(GO:0002576);P08519(GO:0006869);P01877(GO:0006898);Q16610(GO:0002576);P02100(GO:0015671);P13473(GO:0017038;GO:0006605;GO:0061740;GO:0043312;GO:0002576);P01619(GO:0006898);P00918(GO:0046903;GO:0015701;GO:0015670);P01597(GO:0006898);A0A0A0MRJ7(GO:0006888;GO:0002576);P04040(GO:0006625;GO:0043312);P05543(GO:0070327);P00915(GO:0015701);P48740(GO:0006898);P02749(GO:0034197;GO:0002576);P01034(GO:0043312);P04217(GO:0002576;GO:0043312);P11597(GO:0006869;GO:0015914;GO:0030301;GO:0034197;GO:0043691);P02649(GO:0051651;GO:0043691;GO:0010877;GO:0033700;GO:0015909;GO:0046907;GO:0006898;GO:0006869;GO:0017038;GO:0033344;GO:0097114;GO:0097113);Q07954(GO:0006909;GO:0043277;GO:0150093;GO:0045056;GO:0006897;GO:0031623;GO:0006898;GO:0042953);P04196(GO:0002576;GO:0015886);P02647(GO:0019915;GO:0043691;GO:0070508;GO:0015914;GO:0051180;GO:0033700;GO:0006898;GO:0006869;GO:0030301;GO:0002576;GO:0033344);P10909(GO:0043691;GO:0017038;GO:0061740;GO:0061741;GO:0002576);P04114(GO:0006869;GO:0042953;GO:0030301;GO:0006898;GO:0033344);P05109(GO:0043312;GO:0032119);P08697(GO:0002576);Q14624(GO:0002576);P25311(GO:0071806;GO:0055085);P01599(GO:0006898);O43866(GO:0006898);P01024(GO:0043312);P01023(GO:0002576);C9J8S2(GO:0002576);O95497(GO:0043312);P00747(GO:0002576);P00740(GO:0006888);P60709(GO:0048488);P06331(GO:0006898);P01742(GO:0006898);P00450(GO:0006825;GO:0006811;GO:0006826);Q08380(GO:0006898;GO:0002576);P80748(GO:0006898);P04264(GO:0043312);A0A669KAY4(GO:0007041);P02652(GO:0006869;GO:0030301;GO:0033344;GO:0033700;GO:0043691);A0A0C4DH25(GO:0006898);P06727(GO:0006869;GO:0033344;GO:0033700;GO:0043691);P04430(GO:0006898);Q5SRP5(GO:0043691;GO:0033344);P18206(GO:0002576;GO:0034394;GO:0043312);P01817(GO:0006898);P01814(GO:0006898);P01704(GO:0006898);A0A0A0MS51(GO:0042989);P01011(GO:0002576;GO:0043312);P07437(GO:0030705;GO:0097711;GO:0043312);Q13790(GO:0006869);P11021(GO:0035437;GO:0031204;GO:1901998);Q9Y490(GO:0002576;GO:0007016);Q15848(GO:0072659);P01699(GO:0006898);P02042(GO:0015671) | 20|51 | Q16610(GO:0002576);P00739(GO:0006898);P01715(GO:0006898);P01717(GO:0006898);P04040(GO:0006625;GO:0043312);P29622(GO:0002576);Q86UX7(GO:0002576);P00915(GO:0015701);P37802(GO:0002576);A0A0C4DH73(GO:0006898);A0A2R8Y3M9(GO:0006898);P55058(GO:0006869;GO:0015914;GO:0035627);P80748(GO:0006898);P60709(GO:0048488);P30041(GO:0043312);Q14624(GO:0002576);Q5SRP5(GO:0043691;GO:0033344);P69905(GO:0015701;GO:0015671;GO:0006898);P18206(GO:0002576;GO:0034394;GO:0043312);P02751(GO:0002576) | 0.08098816 | 0.24919434 | https://www.ebi.ac.uk/QuickGO/term/GO:0051179 |
| GO:0044456 | synapse part | cellular_component | 5|375 | P15169(GO:0097060);P02649(GO:0043083);P07737(GO:0098794;GO:0098793);P60709(GO:0098871;GO:0044305;GO:0098793);P21333(GO:0098794) | 2|51 | P07737(GO:0098794;GO:0098793);P60709(GO:0098871;GO:0044305;GO:0098793) | 0.119018654 | 0.226702198 | https://www.ebi.ac.uk/QuickGO/term/GO:0044456 |
| GO:0005623 | cell | cellular_component | 7|375 | C9JB55(GO:0005623);P02787(GO:0005623);Q96IY4(GO:0005623);P32119(GO:0005623);P02790(GO:0005623);P30041(GO:0005623);P00450(GO:0005623) | 2|51 | P30041(GO:0005623);P32119(GO:0005623) | 0.188044765 | 0.235055957 | https://www.ebi.ac.uk/QuickGO/term/GO:0005623 |
| GO:0005215 | transporter activity | molecular_function | 23|375 | G3V0E5(GO:0033570);P11597(GO:0017127;GO:0005548;GO:0005319);P02649(GO:0005319;GO:0017127);Q07954(GO:0042954);Q9UHG3(GO:0008555);P02647(GO:0005548;GO:0005319;GO:0017127);P04114(GO:0005319;GO:0017127);O14791(GO:0005254);P25311(GO:0008320);P55058(GO:1990050;GO:0005548;GO:0008525;GO:0005319;GO:0035620;GO:1904121);P02753(GO:0034632);P02790(GO:0015232);P55056(GO:0005319);C9JB55(GO:0015091);C9JF17(GO:0005319);P02652(GO:0017127;GO:0005319);P02775(GO:0005355);P06727(GO:0005319;GO:0017127);O95445(GO:0005319);Q5SRP5(GO:0005319);D6RF35(GO:0090482);P02787(GO:0015091);Q13790(GO:0005319) | 2|51 | Q5SRP5(GO:0005319);P55058(GO:1990050;GO:0005548;GO:0008525;GO:0005319;GO:0035620;GO:1904121) | 0.218682553 | 0.249922918 | https://www.ebi.ac.uk/QuickGO/term/GO:0005215 |
| GO:0040007 | growth | biological_process | 8|375 | Q16610(GO:0003416);Q96PD5(GO:0044117);C9JF17(GO:0042246);A0A0A0MS51(GO:0042246);G3XAK1(GO:0060763);G3XAP6(GO:0035264);P18206(GO:0048675);P01019(GO:0061049) | 2|51 | P18206(GO:0048675);Q16610(GO:0003416) | 0.217341595 | 0.255695994 | https://www.ebi.ac.uk/QuickGO/term/GO:0040007 |
| GO:0044422 | organelle part | cellular_component | 136|375 | P08779(GO:0005882);P00488(GO:0031093);P68366(GO:0005874);P04275(GO:0031093);P51884(GO:0043202;GO:0005796);P03950(GO:0005730);P41222(GO:0005789;GO:0031965);P08571(GO:0010008;GO:0030667);P68871(GO:1904813;GO:0071682;GO:1904724);E9PK25(GO:0016363;GO:0030864);O75460(GO:0005789;GO:1990604;GO:0030176;GO:0005637;GO:1990332);Q86YZ3(GO:0035578);P35527(GO:0005882);P01042(GO:0005788;GO:0031093);P43251(GO:0005759);P59665(GO:0035578;GO:0005796);P0DJI8(GO:0071682;GO:0005881);P02790(GO:0071682);P60763(GO:0031941);A0A087X0S5(GO:0005788;GO:0005765);Q15582(GO:0005802);A0A1W2PQU7(GO:0005883;GO:0005882);P02776(GO:0020005;GO:0031093);P02775(GO:0031093;GO:1904724);Q13201(GO:0031093);E7EUT5(GO:0031965);P30041(GO:0035578);P06702(GO:0005654;GO:0034774);P61224(GO:0035577);P02787(GO:0005788;GO:0010008;GO:0030665;GO:0034774);P01833(GO:0035577);P19823(GO:0005788);P01008(GO:0005788);P04004(GO:0005796;GO:0048237);O00391(GO:0005788;GO:0030173;GO:0031093;GO:0035580;GO:1904724;GO:0000139);P69905(GO:0022627;GO:0071682);G3V0E5(GO:0010008);P02768(GO:0005788;GO:0031093);Q86UX7(GO:0002102;GO:0031093);P02763(GO:0031093;GO:0035580;GO:1904724);Q01518(GO:0030864;GO:0035578);P02766(GO:0035578);J3KPS3(GO:0031430;GO:0031674);A0A0G2JI36(GO:0055038;GO:0071556;GO:0031901;GO:0012507;GO:0030670;GO:0000139);P09172(GO:0034774;GO:0005815;GO:0030667;GO:0030658;GO:0042584;GO:0034466);P21333(GO:0030018;GO:0005730;GO:0031941;GO:0005884;GO:0031523);Q8N1N4(GO:0045095;GO:0005882);P02671(GO:0005788;GO:0031093);P02675(GO:0031093);P07996(GO:0005788;GO:0031093);P61626(GO:0035578;GO:0035580;GO:1904724);D6RE82(GO:0005730);E9PHK0(GO:0001652;GO:0031089);P22891(GO:0005796;GO:0005788);Q06033(GO:0031089);P05155(GO:0031093);P05154(GO:0002080);F8W1S1(GO:0005882;GO:0045095);M0R0Q9(GO:0005788;GO:0035578;GO:0034774);P00734(GO:0005788;GO:0005796);P13645(GO:0005882);P35579(GO:0016459;GO:0008180;GO:0097513;GO:0016460;GO:0042641;GO:0001725;GO:0005826;GO:0005819);P13473(GO:0101003;GO:0035577;GO:0010008;GO:1990836;GO:0043202;GO:0097637;GO:0031902;GO:0000421;GO:0030670;GO:0031088;GO:0005765);P48740(GO:0005654);P00738(GO:0071682;GO:0035580;GO:1904724);P01011(GO:0034774;GO:0035578;GO:0031093);Q9UHG3(GO:0005774);P19652(GO:0031093;GO:0035578;GO:0035580);P31151(GO:0035578);A0A0A0MTH3(GO:0005654);O14791(GO:0005788);P13796(GO:0032432;GO:0002102;GO:0005884;GO:0001725);P15144(GO:0030667;GO:0005765);P09486(GO:0031093;GO:0031092;GO:0016363;GO:0071682);P18206(GO:0034774;GO:0002102;GO:0035580;GO:1904813;GO:0043034);P0DPH7(GO:0005874);P14151(GO:0030667);P08514(GO:0031092);P01009(GO:0033116;GO:0005788;GO:1904813;GO:0031093;GO:0000139);P02751(GO:0005788;GO:0031093);Q9UNN8(GO:0005813);P02750(GO:1904813;GO:1904724;GO:0035580);P68032(GO:0030017;GO:0001725;GO:0031674;GO:0005884;GO:0005865;GO:0042643);H9KV75(GO:0032391;GO:0001725;GO:0005815;GO:0031941;GO:0030486;GO:1990357;GO:0097381);A0A669KAY4(GO:0005788;GO:0036020;GO:0005765);P01344(GO:0031093);P29622(GO:0031089);P13647(GO:0045095;GO:0005882);F8WF14(GO:0005788;GO:0005641);Q6EMK4(GO:0005765);A0A0A0MRJ7(GO:0031093;GO:0033116;GO:0005788;GO:0000139);P04040(GO:0005782;GO:1904813;GO:0005778;GO:0005758;GO:0034774);Q9H4B7(GO:0005874);P05546(GO:0005788);C9J8S2(GO:0031089);P02749(GO:0031089);P80108(GO:0005765);P01034(GO:0005788;GO:1904724;GO:0031965;GO:1904813);P04217(GO:0031093;GO:1904813;GO:0034774);P02649(GO:0005788;GO:0071682;GO:0030669);Q07954(GO:0030666;GO:0005765);P04196(GO:0031093);P02647(GO:0005788;GO:0071682;GO:0034774);P10909(GO:0031966;GO:0099020;GO:0031093;GO:0005743);P04114(GO:0005788;GO:0005789;GO:0043202;GO:0031983;GO:0071682;GO:0030669;GO:0010008;GO:0070971;GO:0012506;GO:0031904);P05109(GO:0034774);P19320(GO:0002102);P08697(GO:0031093);Q14624(GO:0031089);P01024(GO:0005788;GO:0035578;GO:0034774);P01023(GO:0031093);H0YCR7(GO:0005654);O95497(GO:0035577);P02538(GO:0045095;GO:0005882);P00747(GO:0031093);P00740(GO:0005788;GO:0005796);P60709(GO:0005654;GO:0000790;GO:0035267);P00742(GO:0005788;GO:0005796);P02533(GO:0045095;GO:0005882);P00450(GO:0005765;GO:0005788);Q08380(GO:0031089);Q16610(GO:0031089);P04264(GO:0045095;GO:1904813;GO:0005882);P0C0L4(GO:0005788);C9JV77(GO:0005788;GO:0031093;GO:0034774);P02652(GO:0005788);P35858(GO:0005654);P06727(GO:0005788);Q13103(GO:0005788;GO:0031089);P33908(GO:0000139);A0A2R8Y619(GO:0000786);A0A0A0MS51(GO:0030478;GO:0002102);P07437(GO:0035578;GO:0005874;GO:0045298;GO:0005641);Q96HR3(GO:0005654;GO:0016592);P11021(GO:0005789;GO:0034663;GO:0008180;GO:0030176;GO:0005788);P35908(GO:0045095;GO:0005882) | 18|51 | Q16610(GO:0031089);P35527(GO:0005882);P69905(GO:0022627;GO:0071682);Q14624(GO:0031089);P19823(GO:0005788);P04040(GO:0005782;GO:1904813;GO:0005778;GO:0005758;GO:0034774);D6RE82(GO:0005730);P03950(GO:0005730);P29622(GO:0031089);Q9H4B7(GO:0005874);Q96HR3(GO:0005654;GO:0016592);P13647(GO:0045095;GO:0005882);P02751(GO:0005788;GO:0031093);P60709(GO:0005654;GO:0000790;GO:0035267);Q86UX7(GO:0002102;GO:0031093);P30041(GO:0035578);P18206(GO:0034774;GO:0002102;GO:0035580;GO:1904813;GO:0043034);P68032(GO:0030017;GO:0001725;GO:0031674;GO:0005884;GO:0005865;GO:0042643) | 0.12343629 | 0.224429618 | https://www.ebi.ac.uk/QuickGO/term/GO:0044422 |
| GO:0071840 | cellular component organization or biogenesis | biological_process | 134|375 | P08779(GO:0007010;GO:0045104);P68366(GO:0000226;GO:0007010);P04275(GO:0051260;GO:0030198);P51884(GO:0007409;GO:0030199;GO:0030198);P03950(GO:0034332;GO:0030041);G3XAP6(GO:0030198;GO:0030199);P23142(GO:0030198);P15814(GO:0006911);A0A0B4J1X8(GO:0006911);B1AHL2(GO:0030198);P68871(GO:0051291);E9PK25(GO:0030836;GO:0030036;GO:0030043;GO:0030042;GO:0007010);Q86YZ3(GO:0043163);P35527(GO:0045109);A0A087X1J7(GO:0051289);A0A0G2JMI3(GO:0006911);A0A0B4J1U7(GO:0006911);C9JC84(GO:0051258);P60763(GO:0030031;GO:0030036;GO:0031175;GO:0030838;GO:0000902;GO:0007015;GO:0043652);A0A075B7D8(GO:0006911);P01764(GO:0006911);A0A087X0S5(GO:0070208;GO:0003429;GO:0030198);Q15582(GO:0030198);P26038(GO:0008361;GO:0007010);A0A140T8Y3(GO:0030199;GO:0030198);E7EUT5(GO:0000226);A0A0G2JMB2(GO:0006911);P06702(GO:0031532);P61224(GO:0030033);P02787(GO:0061024;GO:0007015);P35908(GO:0045109);P01834(GO:0006911);P04004(GO:0051258;GO:0030198);O00391(GO:0085029);P13796(GO:0022617;GO:0051017;GO:0051764;GO:0051639);P69905(GO:0051291);P02768(GO:0034375);Q86UX7(GO:0034446;GO:0033622);Q01518(GO:0008154;GO:0007010;GO:0000902;GO:0030036);P02766(GO:0030198);J3KPS3(GO:0051289);P19320(GO:0030198);J3QT83(GO:0003429);P21333(GO:0030030;GO:0031532;GO:0034329;GO:0060271;GO:0051764;GO:0021943;GO:0090307);P02671(GO:0034622;GO:0065003;GO:0030198;GO:0051258);P02675(GO:0034622;GO:0030198;GO:0051258);P07996(GO:0043652;GO:0030198);P0CF74(GO:0006911);P02652(GO:0034375;GO:0034374;GO:0034371;GO:0034378;GO:0034380;GO:0034370);Q16880(GO:0030913;GO:0007010;GO:0048812);C9JF17(GO:0014012);P05154(GO:0007342);I3L1J2(GO:0034332;GO:0007043;GO:0000902);M0R0Q9(GO:0150062);O95445(GO:0034375;GO:0034380);A0A075B7F0(GO:0006911);P13645(GO:0051290);A0A0A0MS14(GO:0006911);P35579(GO:0031032;GO:0031532;GO:0032796;GO:0051295;GO:0000212;GO:0006911;GO:0001778;GO:0000904);P13473(GO:0097352);A0A0A0MS15(GO:0006911);P01857(GO:0006911);A0A075B6Q5(GO:0006911);P01019(GO:0034374);B0YIW2(GO:0034375;GO:0034371;GO:0034379;GO:0034378);A0A1W2PQU7(GO:0061564;GO:0045109;GO:0030198;GO:0031102);A0A0A0MTH3(GO:0034446;GO:0030030);K7ER74(GO:0034375;GO:0034372;GO:0034371;GO:0034370;GO:0034378);C9JPQ9(GO:0051258);P09486(GO:0030198);P0DPH7(GO:0007010;GO:0000226);Q13093(GO:0034441;GO:0034374);A0A0B4J231(GO:0006911);P55058(GO:0034375);P01009(GO:0048208);P02751(GO:0034446;GO:0030198;GO:0033622;GO:0007044);A0A075B6R2(GO:0006911);P02100(GO:0051291);P55056(GO:0034379);P68032(GO:0030240;GO:0031032;GO:0055003);A0A0B4J1X5(GO:0006911);H9KV75(GO:0030036;GO:0051017;GO:0045214;GO:0051764;GO:0007030);A0A087WSY4(GO:0006911);P08519(GO:0034374);P13647(GO:0007010;GO:0031581);P01871(GO:0006911);A0A075B7D0(GO:0006911);A0A0B4J1V2(GO:0006911);A0A0C4DH32(GO:0006911);A0A0A0MRJ7(GO:0048208);P04040(GO:0051289;GO:0051262;GO:0006625);Q9H4B7(GO:0000226;GO:0051225);P02743(GO:0051131);P01860(GO:0006911);A0A075B7B8(GO:0006911);P02748(GO:0051260);P01034(GO:0097435);A0A087WZB5(GO:0031532);P08514(GO:0030198);P11597(GO:0034375;GO:0034374;GO:0034372);P02649(GO:0031102;GO:0034375;GO:0034374;GO:0034372;GO:0034371;GO:0034380;GO:0034378;GO:0007010);P02647(GO:0031102;GO:0034375;GO:0034371;GO:0034378;GO:0014012;GO:0034380);P10909(GO:0000902;GO:0051131;GO:0032286;GO:0001836);P04114(GO:0034374;GO:0034371;GO:0061024;GO:0034378;GO:0034379);H0YAC1(GO:0022617);P01861(GO:0006911);A0A0C4DH31(GO:0006911);A0A0C4DH36(GO:0006911);A0A0C4DH34(GO:0006911);P08697(GO:0030199);A0A0C4DH43(GO:0006911);A0A0C4DH38(GO:0006911);A0A0C4DH39(GO:0006911);A0A0C4DH33(GO:0006911);P01024(GO:0006911;GO:0150062);P01023(GO:0022617);P02538(GO:0007010);P00747(GO:0022617);P60709(GO:0043044;GO:0061024;GO:0034329;GO:0098974);P02533(GO:0045110;GO:0031581);P04264(GO:0051290);P04180(GO:0034375;GO:0034372);P06727(GO:0034375;GO:0034372;GO:0034371;GO:0034380;GO:0034378;GO:0065005;GO:0031102);A0A0C4DH29(GO:0006911);Q5SRP5(GO:0034375;GO:0034380);P18206(GO:0034333;GO:0030032;GO:0043297);A0A0B4J1V1(GO:0006911);A0A0B4J1V0(GO:0006911);A0A0A0MS51(GO:0051127;GO:0030041;GO:0042989;GO:1990000;GO:0045010;GO:0060271;GO:0051016;GO:0006911;GO:0051693;GO:0090527);P07437(GO:0051225;GO:0000226);P01877(GO:0006911);Q9Y490(GO:0007043;GO:0033622;GO:0007016;GO:0030866;GO:0007044);Q15848(GO:0070208;GO:0051260);P07737(GO:0060074;GO:0030837;GO:0030838;GO:0098885;GO:0030036) | 18|51 | P69905(GO:0051291);P35527(GO:0045109);P13647(GO:0007010;GO:0031581);P60709(GO:0043044;GO:0061024;GO:0034329;GO:0098974);P04040(GO:0051289;GO:0051262;GO:0006625);Q86UX7(GO:0034446;GO:0033622);A0A087X1J7(GO:0051289);Q9H4B7(GO:0000226;GO:0051225);C9JPQ9(GO:0051258);P55058(GO:0034375);A0A0C4DH36(GO:0006911);P02751(GO:0034446;GO:0030198;GO:0033622;GO:0007044);I3L1J2(GO:0034332;GO:0007043;GO:0000902);P03950(GO:0034332;GO:0030041);Q5SRP5(GO:0034375;GO:0034380);P18206(GO:0034333;GO:0030032;GO:0043297);P07737(GO:0060074;GO:0030837;GO:0030838;GO:0098885;GO:0030036);P68032(GO:0030240;GO:0031032;GO:0055003) | 0.124690296 | 0.216852689 | https://www.ebi.ac.uk/QuickGO/term/GO:0071840 |
| GO:0032991 | protein-containing complex | cellular_component | 136|375 | P51884(GO:0005583);P03950(GO:0032311);P23142(GO:0005577);P15814(GO:0042571);A0A0B4J1X8(GO:0042571);P08571(GO:0046696);P68871(GO:0005833;GO:0031838);A0A0B4J1V2(GO:0042571);O75460(GO:1990604;GO:1990597;GO:1990630;GO:1990332);A0A0G2JMI3(GO:0042571);A0A0B4J1U7(GO:0042571);P0DJI8(GO:0034364);P60763(GO:0031941);P01764(GO:0042571);A0A087X0S5(GO:0032991;GO:0005589;GO:0005581);P07358(GO:0005579);D6R934(GO:0005581);E7EUT5(GO:0097452;GO:1990904);A0A0G2JMB2(GO:0042571);Q03591(GO:0032991);P10643(GO:0005579);P02787(GO:1990712);P01833(GO:0043235);P01834(GO:0042571);P04004(GO:0071062);P13796(GO:0002102);P69905(GO:0022627;GO:0005833;GO:0031838);G3V0E5(GO:1990712);P02768(GO:0032991);O75636(GO:0005581);Q86UX7(GO:0002102);A0A0B4J1X5(GO:0042571);P02766(GO:0032991);P07357(GO:0005579);P19320(GO:0002102;GO:0071065);J3QT83(GO:0005581);P21333(GO:0031941;GO:0031523);P02042(GO:0005833;GO:0031838);P02671(GO:0005577);P02675(GO:0005577);P07996(GO:0005577);P13671(GO:0005579);P01619(GO:0071748;GO:0071751;GO:0071756);P0CF74(GO:0042571);P04180(GO:0034364);C9JF17(GO:0022626);P05154(GO:0036024;GO:0036029;GO:0036025;GO:0036027;GO:0036026;GO:0097183;GO:0097182;GO:0097181;GO:0036028;GO:0032991;GO:0036030);P27169(GO:0034364;GO:0034366);I3L1J2(GO:0016342);M0R0Q9(GO:0032991);O95445(GO:0034361;GO:0034362;GO:0034364;GO:0034365;GO:0034366);A0A075B7F0(GO:0042571);A0A0A0MS14(GO:0042571);P35579(GO:0016459;GO:0008180;GO:0097513;GO:0016460;GO:0008305;GO:0032991);A0A0A0MS15(GO:0042571);P00738(GO:0031838);P01857(GO:0042571);A0A075B6Q5(GO:0042571);Q9BWP8(GO:0005581);G3XAP6(GO:0032991);P11226(GO:0005581);Q9UHG3(GO:0034361);B0YIW2(GO:0042627;GO:0034361;GO:0034363;GO:0034366);O14791(GO:0034361;GO:0034364);K7ER74(GO:0042627;GO:0034361;GO:0034362;GO:0034363;GO:0034366);C9JC84(GO:0005577);C9JPQ9(GO:0005577);A0A2R8Y6G6(GO:0000015);Q13093(GO:0034362);A0A0B4J231(GO:0042571);P08514(GO:0008305);P55058(GO:0034364);P02751(GO:0005577);A0A075B6R2(GO:0042571);P02753(GO:0032991);P02100(GO:0005833;GO:0031838);A0A2R8Y7X9(GO:0005833);P55056(GO:0034361;GO:0034364);P69892(GO:0005833;GO:0031838);A0A0C4DH43(GO:0042571);A0A087WSY4(GO:0042571);P02647(GO:0034361;GO:0034362;GO:0034363;GO:0034364;GO:0034366;GO:0042627;GO:0034365);P01877(GO:0071748;GO:0071752;GO:0071751);P01871(GO:0071756;GO:0071757);A0A075B7D0(GO:0042571);P10909(GO:0034366;GO:0032991);A0A075B7D8(GO:0042571);Q08830(GO:0005577);A0A0C4DH32(GO:0042571);P01860(GO:0042571);P02747(GO:0005581);D6RE82(GO:0030688;GO:0030687);P02745(GO:0005581;GO:0005602);A0A075B7B8(GO:0042571);P01031(GO:0005579);P02749(GO:0042627;GO:0034361;GO:0034364);P02748(GO:0005579);C9JB55(GO:1990712);P11597(GO:0034364);P02649(GO:0034361;GO:0034362;GO:0034363;GO:0034364;GO:1990777;GO:0042627;GO:0034365);Q07954(GO:0098797;GO:0043235);P08519(GO:0034358);P00739(GO:0034366);P04114(GO:0034360;GO:0034359;GO:0034361;GO:0034362;GO:0034363;GO:0042627);A0A0C4DH33(GO:0042571);P01861(GO:0042571);A0A0C4DH31(GO:0042571);A0A0C4DH36(GO:0042571);A0A0C4DH34(GO:0042571);A0A0G2JI36(GO:0042612);P08697(GO:0005577);H9KV75(GO:0031941);A0A0C4DH38(GO:0042571);A0A0C4DH39(GO:0042571);P01024(GO:0032991);P60709(GO:0036464;GO:0032991;GO:1990904;GO:0035267);E7EQB2(GO:0032991);A0A669KAY4(GO:1990667;GO:1990666);P02652(GO:0042627;GO:0034361;GO:0034364;GO:0034366);P35858(GO:0042567);Q15485(GO:0005581);A0A096LPE2(GO:0034364);P06727(GO:0042627;GO:0034361;GO:0034364);A0A0C4DH29(GO:0042571);Q5SRP5(GO:0034361;GO:0034365;GO:0034366;GO:0034362;GO:0034364);P18206(GO:0002102;GO:0032991);A0A2R8Y619(GO:0000786);A0A0B4J1V1(GO:0042571);A0A0B4J1V0(GO:0042571);P07360(GO:0005579);A0A0A0MS51(GO:0002102;GO:0032991);P07437(GO:0036464;GO:0045298;GO:0032991);Q96HR3(GO:0000151;GO:0016592);Q13790(GO:0034362;GO:0034364);P11021(GO:0034663;GO:0008180;GO:0032991);Q15848(GO:0005581;GO:0032991) | 17|51 | O75636(GO:0005581);P18206(GO:0002102;GO:0032991);Q86UX7(GO:0002102);P07360(GO:0005579);P00739(GO:0034366);I3L1J2(GO:0016342);Q96HR3(GO:0000151;GO:0016592);A0A0C4DH36(GO:0042571);D6RE82(GO:0030688;GO:0030687);P60709(GO:0036464;GO:0032991;GO:1990904;GO:0035267);P03950(GO:0032311);P02747(GO:0005581);Q5SRP5(GO:0034361;GO:0034365;GO:0034366;GO:0034362;GO:0034364);P69905(GO:0022627;GO:0005833;GO:0031838);P55058(GO:0034364);C9JPQ9(GO:0005577);P02751(GO:0005577) | 0.113124509 | 0.238156861 | https://www.ebi.ac.uk/QuickGO/term/GO:0032991 |
| GO:0032502 | developmental process | biological_process | 93|375 | P08779(GO:0008544;GO:0002009;GO:0030216;GO:0007568);P51884(GO:0007409;GO:0051216);P03950(GO:0030154;GO:0001525;GO:0007275;GO:0001541;GO:0001556;GO:0001890);E9PK25(GO:0001842);Q86YZ3(GO:0007275);P35527(GO:0008544;GO:0043588);G3XAP6(GO:0060173;GO:0035264;GO:0030282;GO:0010259;GO:0048844;GO:0002063;GO:0003417;GO:0043588;GO:0009887;GO:0048747;GO:0097084;GO:0010260;GO:0001501;GO:0035989);P43121(GO:0001525;GO:0061042;GO:0009653);P60763(GO:0000902;GO:0021894);A0A087X0S5(GO:0003429;GO:0035987;GO:0001649);Q15582(GO:0001525;GO:0002062);P00918(GO:0001822;GO:0002009;GO:0042475);P26038(GO:0061028;GO:0022612;GO:0045198);P31151(GO:0008544;GO:0030216;GO:0001525);P06702(GO:0014002);Q9Y5Y7(GO:0009653);P61224(GO:0061028);P02787(GO:0030316);P35908(GO:0008544;GO:0003334);P04004(GO:0048709;GO:0035987;GO:0097421);Q16880(GO:0007417;GO:0030913;GO:0007422;GO:0048812);Q16610(GO:0001525;GO:0002063;GO:0003416;GO:0031214);P15144(GO:0030154;GO:0001525;GO:0007275);G3V0E5(GO:0030316);Q86UX7(GO:0034446);Q01518(GO:0000902);P37802(GO:0030855);P19320(GO:0060945;GO:0030183;GO:0007568);J3QT83(GO:0003429);P21333(GO:0021987;GO:0021943;GO:0097368);P07996(GO:0002040);P13671(GO:0001701);E9PHK0(GO:0030282);C9JF17(GO:0001525;GO:0014012;GO:0007420;GO:0007568;GO:0042246);P05155(GO:0007568);P05154(GO:0061107);I3L1J2(GO:0000902;GO:0001955);M0R0Q9(GO:0016322);P00734(GO:0007275);P13645(GO:0030216);P13647(GO:0008544);E7EQB2(GO:0060349);Q9BWP8(GO:0007275;GO:0032502);P01019(GO:0001822;GO:0007568;GO:0061049);P32119(GO:0048538);A0A1W2PQU7(GO:0060020;GO:0014002;GO:0031102);A0A0A0MTH3(GO:0034446;GO:0003151;GO:0001658;GO:0021675);P13796(GO:0031100);G3XAK1(GO:0060763;GO:0030879);P36955(GO:0060041;GO:0007275;GO:0001822;GO:0007568);P09486(GO:0030324;GO:0060348;GO:0007507;GO:0048839);Q13103(GO:0001501);Q96IY4(GO:0097421);P02750(GO:0050873);P02751(GO:0034446;GO:0001525;GO:0035987);P02753(GO:0060347;GO:0048562;GO:0030324;GO:0060059;GO:0060065;GO:0048807;GO:0048706;GO:0001654;GO:0007507;GO:0060157;GO:0048738;GO:0060068);P80108(GO:0002062);H9KV75(GO:0048741);C9JV77(GO:0001501);P01344(GO:0031017;GO:0051146;GO:0001501;GO:0007275;GO:0001649;GO:0001701;GO:0009887;GO:0060669;GO:0001892);P35579(GO:0030224;GO:0030220;GO:0001525;GO:0001701;GO:0007520;GO:0000904);O75882(GO:0021549);F8WF14(GO:0014016);P04040(GO:0001649;GO:0001822;GO:0001657;GO:0007568);P18065(GO:0007568);P55103(GO:0048468);P01031(GO:0001701);P68032(GO:0055008;GO:0055003;GO:0048741);P01034(GO:0060009;GO:0008584;GO:0007431;GO:0001654;GO:0007420);P02649(GO:0072358;GO:0031102;GO:0048844);Q07954(GO:0035909;GO:0007275;GO:0021987;GO:0007568);P04196(GO:0001525);P02647(GO:0031102;GO:0031100;GO:0014012;GO:0030325);P10909(GO:0000902);P04114(GO:0001701;GO:0048844;GO:0007399);P05109(GO:0014002);P08697(GO:0048514);P01024(GO:0016322);P01023(GO:0048863);C9J8S2(GO:0048566;GO:0030154);P02538(GO:0002009;GO:0030154);P60709(GO:0021762);P02533(GO:0008544;GO:0030855;GO:0007568);P43251(GO:0007417);A0A669KAY4(GO:0001889;GO:0001822;GO:0030182;GO:0022008);P02652(GO:0031100);P06727(GO:0031102);P18206(GO:0048675;GO:0002009);A0A0A0MS51(GO:0014003;GO:0007568;GO:0042246);Q96HR3(GO:0019827);P11021(GO:0021762;GO:0030182;GO:0021589;GO:0021680);Q15848(GO:2000478;GO:0050873);P07737(GO:0060074;GO:0001843) | 15|51 | P35527(GO:0008544;GO:0043588);P13647(GO:0008544);P37802(GO:0030855);P04040(GO:0001649;GO:0001822;GO:0001657;GO:0007568);Q86UX7(GO:0034446);P32119(GO:0048538);P68032(GO:0055008;GO:0055003;GO:0048741);Q96HR3(GO:0019827);I3L1J2(GO:0000902;GO:0001955);P02751(GO:0034446;GO:0001525;GO:0035987);P60709(GO:0021762);P03950(GO:0030154;GO:0001525;GO:0007275;GO:0001541;GO:0001556;GO:0001890);P18206(GO:0048675;GO:0002009);P07737(GO:0060074;GO:0001843);Q16610(GO:0001525;GO:0002063;GO:0003416;GO:0031214) | 0.095553197 | 0.254808524 | https://www.ebi.ac.uk/QuickGO/term/GO:0032502 |
| GO:0032501 | multicellular organismal process | biological_process | 104|375 | P08779(GO:0061436;GO:0042633;GO:0031424);P00488(GO:0007596);P04275(GO:0007596);P03951(GO:0007596);P03950(GO:0007275);P05160(GO:0007596);P68871(GO:0007596;GO:0050880;GO:0070293);Q8N1N4(GO:0031424);Q86YZ3(GO:0031424;GO:0007275;GO:0061436);P35527(GO:0031424);P01042(GO:0007596;GO:0042311;GO:0050880);C9JC84(GO:0007596);P51884(GO:0007601);P43121(GO:0003094);P60763(GO:0050905;GO:0050885);Q15582(GO:0007601);Q13201(GO:0007596);P06702(GO:0032602;GO:0001816);Q96IY4(GO:0007596);E7END6(GO:0007596);P35908(GO:0031424);P02768(GO:0034375);P02760(GO:0007565);P19320(GO:0060384);P09172(GO:0045907;GO:0048149;GO:0007613;GO:0042596;GO:0008542;GO:0008306;GO:0042309;GO:0048265;GO:0001816;GO:0001974);P20851(GO:0007596);P02042(GO:0007596);P02671(GO:0045907;GO:0007596);P02675(GO:0007596;GO:0045907);P13671(GO:0001701);F5H8B0(GO:0007596);P04180(GO:0034375;GO:0034372);E9PHK0(GO:0001503);P22891(GO:0007596);P05155(GO:0007596;GO:0008015);P05154(GO:0007596);P20742(GO:0007565);O95445(GO:0034375;GO:0034380;GO:0034384);P00734(GO:0007596;GO:0007275);P13645(GO:0031424);P35579(GO:0001701);Q9BWP8(GO:0001503;GO:0007275);G3XAP6(GO:0035264;GO:0050905;GO:0050881;GO:0010259;GO:0014829);M0R0Q9(GO:0097242;GO:0150064);B0YIW2(GO:0034375;GO:0034371;GO:0034379;GO:0034378;GO:0034382);G3V2W1(GO:0007596);A0A0A0MTH3(GO:0001658);K7ER74(GO:0034375;GO:0034372;GO:0034371;GO:0034370;GO:0034378;GO:0034382;GO:0034384);P15144(GO:0007275);P36955(GO:0007614;GO:0007275);C9JPQ9(GO:0007596);P09486(GO:0001503);P18206(GO:0006936);Q13093(GO:0034441;GO:0034374);P32119(GO:0002536);P55058(GO:0034375);P01009(GO:0007596);P01008(GO:0007596);Q9UNN8(GO:0007596);P02100(GO:0007596);P55056(GO:0034447;GO:0034379);P68032(GO:0060048;GO:0006936;GO:0090131;GO:0060047);P69892(GO:0007596);P13647(GO:0031424);P02753(GO:0007601;GO:0030277);A0A669KAY4(GO:0034383);P01344(GO:0001503;GO:0007275;GO:0001701);P08519(GO:0034374;GO:0008015);P01877(GO:0003094);F8WF14(GO:0007612);A0A0A0MRJ7(GO:0008015;GO:0007596);P18065(GO:0007565);P05546(GO:0007596);P02741(GO:0097756);P01031(GO:0001701);P80108(GO:0001503);P11597(GO:0034375;GO:0034374;GO:0034372);P02649(GO:0034447;GO:0007616;GO:0034375;GO:0034374;GO:0034372;GO:0034371;GO:0042311;GO:0034382;GO:0034380;GO:0034384;GO:0002021;GO:0034378);Q07954(GO:0150094;GO:0150093;GO:0007275;GO:0097242);P04196(GO:0007596);P02647(GO:0034375;GO:0034371;GO:0034378;GO:0034384;GO:0034380);P04114(GO:0034374;GO:0034371;GO:0009791;GO:0034378;GO:0001701;GO:0034379;GO:0034382;GO:0034383;GO:0034447);P05109(GO:0032602;GO:0001816);P08697(GO:0002034);P01024(GO:0097242;GO:0150064);P02538(GO:0031424);P00747(GO:0048771;GO:0007596);P00740(GO:0007596);P00742(GO:0007596);P02533(GO:0042633;GO:0031424);P00748(GO:0007596);Q16610(GO:0001503;GO:0003416);P04264(GO:0061436;GO:0031424);P02652(GO:0034384;GO:0034375;GO:0034374;GO:0034371;GO:0034378;GO:0034380;GO:0034370);P06727(GO:0034375;GO:0034372;GO:0034371;GO:0034380;GO:0034378);Q5SRP5(GO:0034375;GO:0034384;GO:0034380);Q13103(GO:0046849);Q15848(GO:0034383);P01619(GO:0003094);A0A0A0MS51(GO:0014891;GO:0097017);P01019(GO:0035106;GO:0003014;GO:0014824;GO:0019229;GO:0003081;GO:0007565;GO:0034374;GO:0042310;GO:0042311;GO:0008306;GO:0001974;GO:0050880;GO:0070471;GO:0002034;GO:0097755;GO:0002019;GO:0002018;GO:0002016);Q96HR3(GO:0019827);Q9Y490(GO:0006936);P01011(GO:0030277) | 12|51 | P35527(GO:0031424);P13647(GO:0031424);P03951(GO:0007596);P03950(GO:0007275);P32119(GO:0002536);P68032(GO:0060048;GO:0006936;GO:0090131;GO:0060047);P55058(GO:0034375);Q16610(GO:0001503;GO:0003416);Q5SRP5(GO:0034375;GO:0034384;GO:0034380);P18206(GO:0006936);C9JPQ9(GO:0007596);Q96HR3(GO:0019827) | 0.106807328 | 0.251311361 | https://www.ebi.ac.uk/QuickGO/term/GO:0032501 |
| GO:0031974 | membrane-enclosed lumen | cellular_component | 84|375 | P04217(GO:0031093;GO:1904813;GO:0034774);P02768(GO:0005788;GO:0031093);P00488(GO:0031093);P02649(GO:0005788;GO:0071682);P04275(GO:0031093);P51884(GO:0043202;GO:0005796);Q86UX7(GO:0031093);P02763(GO:0031093;GO:0035580;GO:1904724);Q01518(GO:0035578);P02647(GO:0005788;GO:0071682;GO:0034774);P01023(GO:0031093);P02766(GO:0035578);P04114(GO:0005788;GO:0043202;GO:0031983;GO:0071682;GO:0031904);P08697(GO:0031093);P19652(GO:0031093;GO:0035578;GO:0035580);P05546(GO:0005788);P06702(GO:0034774);P68871(GO:1904813;GO:0071682;GO:1904724);Q14624(GO:0031089);P02776(GO:0031093);P29622(GO:0031089);P09486(GO:0031093;GO:0071682);Q16610(GO:0031089);P01024(GO:0005788;GO:0035578;GO:0034774);P10909(GO:0099020;GO:0031093);P02787(GO:0005788;GO:0034774);C9J8S2(GO:0031089);P09172(GO:0034774;GO:0034466);P59665(GO:0035578;GO:0005796);P0DJI8(GO:0071682);P04196(GO:0031093);P00747(GO:0031093);P02671(GO:0005788;GO:0031093);P01009(GO:0005788;GO:1904813;GO:0031093);P00740(GO:0005788;GO:0005796);P02675(GO:0031093);P00742(GO:0005788;GO:0005796);P02790(GO:0071682);P02750(GO:1904813;GO:1904724;GO:0035580);Q86YZ3(GO:0035578);P61626(GO:0035578;GO:0035580;GO:1904724);Q08380(GO:0031089);P02751(GO:0005788;GO:0031093);F8WF14(GO:0005788;GO:0005641);P31151(GO:0035578);E9PHK0(GO:0031089);P04264(GO:1904813);P0C0L4(GO:0005788);P22891(GO:0005796;GO:0005788);A0A669KAY4(GO:0005788);Q06033(GO:0031089);P02652(GO:0005788);C9JV77(GO:0005788;GO:0031093;GO:0034774);P07996(GO:0005788;GO:0031093);P01344(GO:0031093);P02775(GO:0031093;GO:1904724);P05155(GO:0031093);P43251(GO:0005759);Q13201(GO:0031093);P01042(GO:0005788;GO:0031093);M0R0Q9(GO:0005788;GO:0035578;GO:0034774);P06727(GO:0005788);P30041(GO:0035578);P00450(GO:0005788);P18206(GO:0034774;GO:0035580;GO:1904813);O14791(GO:0005788);Q13103(GO:0005788;GO:0031089);A0A0A0MRJ7(GO:0031093;GO:0005788);P00734(GO:0005788;GO:0005796);P13473(GO:0043202);P19823(GO:0005788);P04040(GO:0005782;GO:1904813;GO:0005758;GO:0034774);P05109(GO:0034774);P01008(GO:0005788);P00738(GO:0071682;GO:0035580;GO:1904724);P04004(GO:0005796;GO:0048237);A0A087X0S5(GO:0005788);P07437(GO:0035578;GO:0005641);O00391(GO:0005788;GO:0031093;GO:0035580;GO:1904724);P11021(GO:0005788);P02749(GO:0031089);P69905(GO:0071682);P01034(GO:0005788;GO:1904724;GO:1904813);P01011(GO:0034774;GO:0035578;GO:0031093) | 10|51 | P18206(GO:0034774;GO:0035580;GO:1904813);Q14624(GO:0031089);P19823(GO:0005788);P04040(GO:0005782;GO:1904813;GO:0005758;GO:0034774);Q86UX7(GO:0031093);P29622(GO:0031089);P02751(GO:0005788;GO:0031093);P30041(GO:0035578);P69905(GO:0071682);Q16610(GO:0031089) | 0.130069393 | 0.216782322 | https://www.ebi.ac.uk/QuickGO/term/GO:0031974 |
| GO:0140110 | transcription regulator activity | molecular_function | 1|375 | Q96HR3(GO:0003712;GO:0030374) | 1|51 | Q96HR3(GO:0003712;GO:0030374) | 0.136 | 0.201481481 | https://www.ebi.ac.uk/QuickGO/term/GO:0140110 |
| GO:0008283 | cell proliferation | biological_process | 16|375 | Q15582(GO:0008283);P08779(GO:0008283);P35908(GO:0043616);Q07954(GO:0008283);P26038(GO:0042098);G3XAP6(GO:0035988);P32119(GO:0042098);P02647(GO:0001935);P04004(GO:0008283);P10909(GO:0061518);Q08830(GO:0072574);Q92954(GO:0008283);P01019(GO:0048659;GO:0048144);P36955(GO:0008283);P61224(GO:0008283);O75460(GO:0001935) | 1|51 | P32119(GO:0042098) | 0.241770429 | 0.261373437 | https://www.ebi.ac.uk/QuickGO/term/GO:0008283 |
| GO:0038024 | cargo receptor activity | molecular_function | 7|375 | G3V0E5(GO:0004998);O43866(GO:0005044);Q07954(GO:0030226;GO:0016964;GO:0005044;GO:0005041);P04004(GO:0005044);A0A2R8Y3M9(GO:0005044);Q92954(GO:0005044);Q08380(GO:0005044) | 1|51 | A0A2R8Y3M9(GO:0005044) | 0.399908535 | 0.399908535 | https://www.ebi.ac.uk/QuickGO/term/GO:0038024 |
| GO:0140104 | molecular carrier activity | molecular_function | 8|375 | C9JB55(GO:0034986);P02787(GO:0034986);P69892(GO:0005344);A0A2R8Y7X9(GO:0005344);P02042(GO:0005344);P68871(GO:0005344);P02100(GO:0005344);P69905(GO:0005344) | 1|51 | P69905(GO:0005344) | 0.394940727 | 0.405067412 | https://www.ebi.ac.uk/QuickGO/term/GO:0140104 |
| GO:0060089 | molecular transducer activity | molecular_function | 12|375 | P00734(GO:0070053);P01833(GO:0001792);Q07954(GO:0038023);P04264(GO:0038023);Q96PD5(GO:0016019);P08571(GO:0001847;GO:0016019);Q96HR3(GO:0038023);P15144(GO:0038023);A0A0U1RQV3(GO:0005006);Q9UNN8(GO:0038023);Q9Y5Y7(GO:0038023;GO:0004888);O75882(GO:0038023) | 1|51 | Q96HR3(GO:0038023) | 0.328903374 | 0.346214078 | https://www.ebi.ac.uk/QuickGO/term/GO:0060089 |

| Table S6-2 GO functional enrichment of DEPs in S vs A group. | | | | | | | | | |
| --- | --- | --- | --- | --- | --- | --- | --- | --- | --- |
| GO | name | namespace | all_number_of_accs | all_GO2acc | diff_number_of_accs | diff_GO2acc | p_value | FDR | url |
| GO:0005488 | binding | molecular_function | 317|375 | P08779(GO:0005515);P00488(GO:0046872);P68366(GO:0019899;GO:0000166;GO:0005525;GO:0019901;GO:0005515);P04275(GO:0051087;GO:0002020;GO:0042802;GO:0042803;GO:0005178;GO:0047485;GO:0019865;GO:0005515;GO:0005518);P02745(GO:0005515);O75882(GO:0030246);P03951(GO:0005515;GO:0008201;GO:0042802);P03950(GO:0003676;GO:0003677;GO:0042277;GO:0008201;GO:0003779;GO:0005507;GO:0042803;GO:0019843;GO:0005515;GO:0005102);P23142(GO:0008022;GO:0044877;GO:0005509;GO:0042802;GO:0070051;GO:0001968);P15814(GO:0003823;GO:0034987);P30041(GO:0042803;GO:0031625;GO:0045296;GO:0005515);B1AHL2(GO:0005509);P08571(GO:0071723;GO:0070891;GO:0001530;GO:0005515);P68871(GO:0046872;GO:0020037;GO:0031721;GO:0031720;GO:0019825;GO:0030492;GO:0043177;GO:0005515);P01877(GO:0034987;GO:0003823);E9PK25(GO:0003779;GO:0051015;GO:0005102);O75460(GO:0005161;GO:0019899;GO:0030544;GO:0000287;GO:0051082;GO:0000166;GO:0046872;GO:0051879;GO:0042802;GO:0042803;GO:0043531;GO:0005524;GO:0005515);Q86YZ3(GO:0046914;GO:0005509;GO:0046872);P01597(GO:0003823);P01042(GO:0008270;GO:0008201;GO:0005515;GO:0005102);A0A087X1J7(GO:0008430);P04264(GO:0030246;GO:0005515;GO:0046982);P01602(GO:0003823);A0A0G2JMI3(GO:0003823;GO:0034987);A0A0B4J1U7(GO:0003823;GO:0034987);P59665(GO:0042803);Q6EMK4(GO:0045296;GO:0005515;GO:0050431);G3XAP6(GO:0036122;GO:0005509;GO:0002020;GO:0005178;GO:0043395;GO:0008201;GO:0043394;GO:0005518);P02533(GO:1990254;GO:0005515);Q96KN2(GO:0046872);P06312(GO:0003823);P01743(GO:0003823);A0A0A0MT36(GO:0003823);P01009(GO:0002020;GO:0042802;GO:0005515);P02790(GO:0046872;GO:0005515);P60763(GO:0048306;GO:0000166;GO:0005525;GO:0019901;GO:0005515);A0A075B7D8(GO:0003823;GO:0034987);P01764(GO:0034987;GO:0003823);A0A087X0S5(GO:0048407);P01766(GO:0003823);P33908(GO:0005509);Q15582(GO:0050840;GO:0005178;GO:0050839;GO:0005515;GO:0005518);F8WF14(GO:0019899;GO:0001540;GO:0042802;GO:0033265);P0DOY3(GO:0003823);P02760(GO:0042803;GO:0005515;GO:0020037;GO:0046904;GO:0019862);P26038(GO:0019899;GO:0003779;GO:0005515;GO:0019901;GO:0050839;GO:0008092;GO:0003725;GO:0005102);P01782(GO:0003823);P18428(GO:0071723;GO:0008289;GO:0070891;GO:0001530;GO:0005515;GO:0005102);P01780(GO:0003823);P06331(GO:0003823);P02776(GO:0005125;GO:0008009;GO:0048248;GO:0008201;GO:0005515);P02775(GO:0005125;GO:0008009;GO:0008083;GO:0045236;GO:0005515);H3BTN5(GO:0030955;GO:0000287;GO:0005524);P0DP01(GO:0003823);E7EUT5(GO:0051287;GO:0097718;GO:0042802;GO:0008017;GO:0050661);A0A0G2JMB2(GO:0003823;GO:0034987);A0A0J9YXX1(GO:0003823);P0DP02(GO:0003823);P06702(GO:0050786;GO:0005509;GO:0046872;GO:0035662;GO:0008017;GO:0050544;GO:0005515;GO:0008270);Q9Y5Y7(GO:0005540;GO:0005515);P61224(GO:0044877;GO:0000166;GO:0005525;GO:0019003;GO:0005515);D6RF35(GO:0005499);P02787(GO:1990459;GO:0034986;GO:0046872;GO:0008198;GO:0008199;GO:0005515);P19827(GO:0005509);P35908(GO:0008092;GO:0005515);P01834(GO:0034987;GO:0003823);Q96IY4(GO:0008270;GO:0046872);P04003(GO:0003723;GO:0005515);P01008(GO:0002020;GO:0042802;GO:0008201;GO:0005515);P01718(GO:0003823);E7END6(GO:0005509);P04004(GO:0050840;GO:0030247;GO:0042802;GO:0005178;GO:0008201;GO:0005515;GO:0005518);P00748(GO:0005509;GO:0051787;GO:0005515);Q9UNN8(GO:0005515);O00391(GO:0071949);A6XND0(GO:0005520);K7ER74(GO:0008289;GO:0042803;GO:0043274);P69905(GO:0005506;GO:0046872;GO:0020037;GO:0031720;GO:0019825;GO:0005515);A0A0J9YX35(GO:0003823);G3XAK1(GO:0019899;GO:0030971);G3V0E5(GO:0042803;GO:0003725);P02768(GO:0015643;GO:0008144;GO:0030170;GO:0003677;GO:0051087;GO:0005504;GO:0005507;GO:0042802;GO:0140272;GO:0008289;GO:0019825;GO:0046872;GO:1903981;GO:0005515);O75636(GO:0003823;GO:0030246;GO:0005515;GO:0046872);Q96PD5(GO:0008270;GO:0046872;GO:0042834);Q86UX7(GO:0005178);P02763(GO:0005515);Q01518(GO:0008179;GO:0003779);A0A0C4DH73(GO:0003823);P02766(GO:0005179;GO:0042562;GO:0070324;GO:0005515;GO:0046982;GO:0042802);A0A2R8Y3M9(GO:0005509);Q9H4B7(GO:0000166;GO:0005525);P20742(GO:0002020);A0A0G2JL69(GO:0046872);P05546(GO:0008201);A0A0B4J2D9(GO:0003823);P19320(GO:0050839;GO:0005178);Q15166(GO:0046872;GO:0042803);P07358(GO:0044877);A0A0U1RQV3(GO:0005509);A0A182DWH7(GO:0008430);P09172(GO:0031418;GO:0005507;GO:0046872);P01703(GO:0003823);P01700(GO:0003823);P01701(GO:0003823);P01706(GO:0003823);P20851(GO:0005515);P21333(GO:0017160;GO:0031852;GO:0017048;GO:0034988;GO:0051020;GO:0044877;GO:0045296;GO:0001664;GO:0044325;GO:0003779;GO:0008134;GO:0042803;GO:0051015;GO:0019900;GO:0046332;GO:0031267;GO:0048365;GO:0003723;GO:0005515);H0Y5E4(GO:0005540);A0A0S2Z4L3(GO:0005509);P23083(GO:0003823);P01709(GO:0003823);P13671(GO:0005515);P55103(GO:0005179;GO:0005125;GO:0008083;GO:0005160);P02671(GO:0050839;GO:0046872;GO:0005515;GO:0005102);P02675(GO:0051087;GO:0050839;GO:0005515;GO:0005102);P07996(GO:0050840;GO:0030169;GO:0043236;GO:0001968;GO:0043394;GO:0070052;GO:0070051;GO:0050431;GO:0005509;GO:0001786;GO:0017134;GO:0008201;GO:0042802;GO:0005515;GO:0005178);P08519(GO:0005515;GO:0034185;GO:0008201;GO:0001968);A0A075B6I0(GO:0003823);P01619(GO:0003823);P0CF74(GO:0034987;GO:0003823);P61626(GO:0042802);I3L145(GO:0005496);P04180(GO:0005515;GO:0034186);P08697(GO:0002020;GO:0042803;GO:0005515);P22891(GO:0005509);Q92954(GO:0030247);A0A0C4DH67(GO:0003823);P05155(GO:0005515);P05154(GO:0032190;GO:0002020;GO:0031210;GO:0008201;GO:0005539;GO:0005515;GO:0001972);I3L1J2(GO:0044325;GO:0043184;GO:0005509;GO:0042803;GO:0008013;GO:0019903;GO:0005102;GO:0008092;GO:0045296);M0R0Q9(GO:0031715;GO:0005102);P01031(GO:0008009;GO:0005515;GO:0005102);O95445(GO:0005543);A0A0A0MS51(GO:0045159;GO:0005509;GO:0051015);A0A075B7F0(GO:0003823;GO:0034987);J3KRP0(GO:0046872);P13645(GO:0046982);A0A0A0MS14(GO:0003823;GO:0034987);P35579(GO:0003779;GO:0005524;GO:0042803;GO:0005178;GO:0051015;GO:0043531;GO:0005516;GO:0000166;GO:0043495;GO:0045296;GO:0003723;GO:0005515;GO:0019904);P13473(GO:0019899;GO:0019904;GO:0005515);P01717(GO:0003823);F5H8B0(GO:0005509);P48740(GO:0048306;GO:0046872;GO:0005509;GO:0005515;GO:0042803);Q14520(GO:0005539;GO:0005509);P00738(GO:0030492;GO:0005515);P00739(GO:0030492);E7EQB2(GO:0008201;GO:0005506;GO:0001530);Q9BWP8(GO:0030246;GO:0048029;GO:0003677;GO:0070492;GO:0120153;GO:0005509;GO:0042806;GO:0046872;GO:0005515;GO:0005537);P01019(GO:0005179;GO:0008083;GO:0048018;GO:0031701;GO:0031703;GO:0005515;GO:0031702);Q14624(GO:0005515);A0A0C4DH25(GO:0003823);P11226(GO:0048306;GO:0030246;GO:0005509;GO:0005515;GO:0005102;GO:0005537);P01011(GO:0003677;GO:0005515);H0Y2Y8(GO:0046872);C9JB55(GO:1990459;GO:0034986;GO:0008198;GO:0008199);C9JD84(GO:0005509);P41222(GO:0005504;GO:0005501;GO:0036094;GO:0005515);A0A0C4DH55(GO:0003823);A0A0B4J1Y8(GO:0003823);B0YIW2(GO:0005543;GO:0070653;GO:0008289;GO:0015485);J3KNB4(GO:0001530);C9J5S7(GO:0016018);P31151(GO:0050786;GO:0005509;GO:0046872;GO:0046914;GO:0005515;GO:0008270);Q8IV42(GO:0000049;GO:0000166;GO:0005524);P01721(GO:0003823);A0A0A0MTH3(GO:0005524;GO:0019901);O14791(GO:0008289;GO:0005515);P13796(GO:0051020;GO:0003779;GO:0005509;GO:0042802;GO:0005178;GO:0051015;GO:0046872);P15144(GO:0042277;GO:0046872;GO:0001618;GO:0008270);P36955(GO:0005515);C9JC84(GO:0005102);C9JPQ9(GO:0005102);P09486(GO:0050840;GO:0046872;GO:0005509;GO:0005515;GO:0005518);A0A0B4J231(GO:0003823;GO:0034987);P18206(GO:0002162;GO:0003779;GO:0051015;GO:0031625;GO:0008013;GO:0045294;GO:0045296;GO:0005515);P0DPH7(GO:0000166;GO:0005525);P80748(GO:0003823);Q13093(GO:0005543);P14151(GO:0030246;GO:0070492;GO:0043208;GO:0005509;GO:0002020;GO:0046872;GO:0050839;GO:0008201;GO:0005515);O00187(GO:0046872;GO:0048306;GO:0005509;GO:0005515;GO:0001855);A0A2R8Y6G6(GO:0046872;GO:0000287);A0A075B6K2(GO:0003823);E7EX29(GO:0019904);P55058(GO:0019992;GO:0097001;GO:0070300;GO:0008289;GO:0031210;GO:0008429;GO:1901611);B4E1Z4(GO:0001848);P02751(GO:0008022;GO:0005518;GO:0097718;GO:0051087;GO:0008201;GO:0002020;GO:0042802;GO:0005178;GO:0043394;GO:0019899;GO:0005515;GO:0005102);A0A075B6R2(GO:0003823;GO:0034987);P02753(GO:0016918;GO:0005501;GO:0019841;GO:0005515;GO:0046982);Q03591(GO:0005515;GO:0046982;GO:0042803);P02100(GO:0019825;GO:0020037;GO:0043177;GO:0031721;GO:0005515;GO:0046872);A0A2R8Y7X9(GO:0019825;GO:0020037;GO:0046872);P68032(GO:0017022;GO:0000166;GO:0005524;GO:0043531);P00734(GO:0005102;GO:0001530;GO:0005509;GO:0008201;GO:0008083;GO:0005515);P69892(GO:0019825;GO:0020037;GO:0043177;GO:0046872);H9KV75(GO:0019894;GO:0032029;GO:0017166;GO:0030507;GO:0045505;GO:0003779;GO:0005509;GO:0042803;GO:0034452;GO:0051393;GO:0051015);A0A075B6K5(GO:0003823);A0A075B6J9(GO:0003823);P43652(GO:0008431;GO:0005515;GO:0046872);P08603(GO:0005515;GO:0043395;GO:0008201);A0A669KAY4(GO:0030169;GO:0034190;GO:0070326;GO:0034189;GO:0034185;GO:0043621;GO:0050750;GO:0003723);P01344(GO:0008083;GO:0005179;GO:0005178;GO:0005159;GO:0005158;GO:0048018;GO:0005515);P27169(GO:0046872;GO:0005543;GO:0005509;GO:0042803);P08185(GO:0008289;GO:0005496);A0A0A0MS15(GO:0003823;GO:0034987);Q16610(GO:0008022;GO:0019899;GO:0005134;GO:0002020;GO:0043236;GO:0005515);A0A075B7D0(GO:0003823;GO:0034987);P01715(GO:0003823);P09871(GO:0005509;GO:0005515;GO:0046872;GO:0042802);A0A2R8Y619(GO:0003677;GO:0046982);P00918(GO:0005515;GO:0046872;GO:0008270);A0A0C4DH32(GO:0003823;GO:0034987);A0A0B4J1X5(GO:0003823;GO:0034987);P01704(GO:0003823);A0A0A0MRJ7(GO:0046872;GO:0005507);P04040(GO:0019899;GO:0050661;GO:0046872;GO:0042802;GO:0042803;GO:0020037;GO:0005102);A0A0C4DH33(GO:0003823;GO:0034987);A0A3B3ISR2(GO:0005509);A0A0B4J1U3(GO:0003823);P13647(GO:0097110;GO:0005515);P18065(GO:0019838;GO:0005520;GO:0031994;GO:0031995;GO:0005515;GO:0005102);P00915(GO:0046872;GO:0008270;GO:0005515);A0A075B6S5(GO:0003823);Q562R1(GO:0000166;GO:0005515;GO:0005524);P02743(GO:0030246;GO:0051082;GO:0001849;GO:0005509;GO:0030169;GO:0042802;GO:0046872;GO:0046790);A0A075B6K4(GO:0003823);P02741(GO:0050750;GO:0001849;GO:0005509;GO:0046872;GO:0042802;GO:0030169;GO:0046790;GO:0033265;GO:0005515);P01860(GO:0034987;GO:0003823);P02747(GO:0005515);D6RE82(GO:0003723);P04211(GO:0003823);A0A075B7B8(GO:0003823;GO:0034987);P07357(GO:0001848;GO:0044877);P02749(GO:0005543;GO:0042802;GO:0008289;GO:0008201;GO:0005515);P01034(GO:0001540;GO:0002020;GO:0042802;GO:0005515);A0A087WZB5(GO:0003779);A0A087WSZ0(GO:0003823);P08514(GO:0050840;GO:0046872;GO:0042802;GO:0070051;GO:0005515);P11597(GO:0017129;GO:0008289;GO:0031210;GO:0015485);P02649(GO:0008201;GO:0071813;GO:0015485;GO:0044877;GO:0048156;GO:0046983;GO:0005543;GO:0001540;GO:0042802;GO:0042803;GO:0008289;GO:0005515;GO:0070326;GO:0046911;GO:0050750);P37802(GO:0045296;GO:0005515);Q07954(GO:0044877;GO:0001540;GO:0005509;GO:0002020;GO:0070325;GO:0032050;GO:0043395;GO:0034185;GO:0046872;GO:0003723;GO:0005515);P04196(GO:0008270;GO:0046872;GO:0020037;GO:0043395;GO:0008201;GO:0019865;GO:0005515;GO:0005102);P32119(GO:0005515);P02647(GO:0034191;GO:0034190;GO:0005543;GO:0015485;GO:0045499;GO:0031072;GO:0019899;GO:0001540;GO:0042802;GO:0008035;GO:0008289;GO:0071813;GO:0005515;GO:0031210;GO:0070653);P01861(GO:0034987;GO:0003823);P10909(GO:0051787;GO:0044877;GO:0048156;GO:0051082;GO:0001540;GO:0051087;GO:0031625;GO:0005515;GO:0050750);P04114(GO:0050750;GO:0005543;GO:0008289;GO:0008201;GO:0035473;GO:0005515);P05109(GO:0050786;GO:0008270;GO:0005509;GO:0046872;GO:0035662;GO:0008017;GO:0050544;GO:0005515);P15169(GO:0008270;GO:0046872);A0A0C4DH31(GO:0003823;GO:0034987);A0A0C4DH36(GO:0003823;GO:0034987);A0A0C4DH34(GO:0003823;GO:0034987);A0A0G2JI36(GO:0030881;GO:0046977;GO:0042605);H0YAC1(GO:0005506;GO:0046872;GO:0020037);A0A0C4DH43(GO:0003823;GO:0034987);A0A0C4DH38(GO:0003823;GO:0034987);A0A0C4DH39(GO:0003823;GO:0034987);P25311(GO:0005515);P01599(GO:0003823);A0A096LPE2(GO:0042056);P01857(GO:0034987;GO:0003823;GO:0005515);P01024(GO:0031715;GO:0005515;GO:0005102);P01023(GO:0019959;GO:0019899;GO:0048306;GO:0019838;GO:0002020;GO:0019966;GO:0043120;GO:0005515;GO:0005102);C9J8S2(GO:0005102);P01601(GO:0003823);A0A0C4DH24(GO:0003823);Q9Y6R7(GO:0005515);P02538(GO:0005515);P00747(GO:1904854;GO:0019899;GO:0051087;GO:1990405;GO:0019900;GO:0019904;GO:0034185;GO:0005515;GO:0005102);P00740(GO:0005509;GO:0005515;GO:0046872);P60709(GO:0019894;GO:0030957;GO:0005524;GO:0000980;GO:0000166;GO:0019901;GO:0050998;GO:0031492;GO:0042802;GO:0005515;GO:0000978);P00742(GO:0005543;GO:0005509;GO:0005515);A0A075B6Q5(GO:0003823;GO:0034987);A0A0A0MRZ9(GO:0003823);P01742(GO:0003823);P00450(GO:0046872;GO:0051087;GO:0005507);Q08380(GO:0005515);P16930(GO:0005515;GO:0046872);P0C0L5(GO:0030246;GO:0001848);P0C0L4(GO:0001849);C9JF17(GO:0008289;GO:0015485);A0A087WSY4(GO:0003823;GO:0034987);P02652(GO:0005543;GO:0031072;GO:0034190;GO:0008035;GO:0008289;GO:0031210;GO:0046982;GO:0015485;GO:0005515;GO:0070653;GO:0042803);P35858(GO:0005520);Q15485(GO:0005515;GO:0048306;GO:0030246;GO:2001065;GO:0043394;GO:0003823;GO:0046872);P01763(GO:0003823);E9PHK0(GO:0036143;GO:0030246;GO:0008201;GO:0005509);P04433(GO:0003823);A0A087WSY6(GO:0003823);P06727(GO:0042802;GO:0008289;GO:0031210;GO:0005507;GO:0015485;GO:0005515;GO:0042803);P04430(GO:0003823);A0A0C4DH29(GO:0003823;GO:0034987);P0DJI8(GO:0042056;GO:0001664;GO:0008201);Q5SRP5(GO:0005543);A0A0B4J1X8(GO:0003823;GO:0034987);P01817(GO:0003823);P01814(GO:0003823);P02750(GO:0005160;GO:0005515);P02042(GO:0019825;GO:0020037;GO:0043177;GO:0031721;GO:0005515;GO:0046872);P51884(GO:0005515;GO:0005518);A0A0B4J1V1(GO:0003823;GO:0034987);A0A0B4J1V0(GO:0003823;GO:0034987);P07360(GO:0036094;GO:0019841;GO:0001848;GO:0044877);A0A0B4J1V2(GO:0003823;GO:0034987);Q13201(GO:0005509;GO:0005515);Q5T749(GO:0005515);P07437(GO:0032794;GO:0044877;GO:0000166;GO:0005525;GO:0042288;GO:0031625;GO:0019904;GO:0005515);Q96HR3(GO:0005515;GO:0046966;GO:0042809);Q13790(GO:0005102;GO:0015485);P11021(GO:0051087;GO:0019899;GO:0045296;GO:0019904;GO:0000166;GO:0031625;GO:0005509;GO:0043022;GO:0005524;GO:0051082;GO:0051787;GO:0005515);Q9Y490(GO:0017166;GO:0003779;GO:0001786;GO:0005178;GO:0005515;GO:0051015;GO:0030274;GO:0035091;GO:0045296;GO:0044877);P01705(GO:0003823);Q15848(GO:0005125;GO:0033691;GO:0042802;GO:0042803;GO:0005515;GO:0005102;GO:0005179);A0A0J9YVY3(GO:0003823);P01699(GO:0003823);P07737(GO:0017048;GO:0005546;GO:0003779;GO:0005515;GO:0070064;GO:0000774;GO:0045296;GO:0003723;GO:0005102;GO:0003785);P01871(GO:0042834;GO:0034987;GO:0031210;GO:0003823;GO:0003697;GO:0005515) | 65|82 | C9JB55(GO:1990459;GO:0034986;GO:0008198;GO:0008199);O75636(GO:0003823;GO:0030246;GO:0005515;GO:0046872);P80748(GO:0003823);P37802(GO:0045296;GO:0005515);P04211(GO:0003823);P32119(GO:0005515);P02763(GO:0005515);A0A0C4DH25(GO:0003823);A0A0C4DH73(GO:0003823);P01857(GO:0034987;GO:0003823;GO:0005515);A0A0C4DH32(GO:0003823;GO:0034987);P15169(GO:0008270;GO:0046872);A0A0G2JL69(GO:0046872);A0A0C4DH34(GO:0003823;GO:0034987);P01721(GO:0003823);Q9Y5Y7(GO:0005540;GO:0005515);P01599(GO:0003823);P09172(GO:0031418;GO:0005507;GO:0046872);P01703(GO:0003823);P0DP01(GO:0003823);P01780(GO:0003823);P01601(GO:0003823);Q96IY4(GO:0008270;GO:0046872);O00187(GO:0046872;GO:0048306;GO:0005509;GO:0005515;GO:0001855);A0A182DWH7(GO:0008430);A0A2R8Y3M9(GO:0005509);A0A075B6K2(GO:0003823);E7EX29(GO:0019904);P55058(GO:0019992;GO:0097001;GO:0070300;GO:0008289;GO:0031210;GO:0008429;GO:1901611);P01009(GO:0002020;GO:0042802;GO:0005515);P02751(GO:0008022;GO:0005518;GO:0097718;GO:0051087;GO:0008201;GO:0002020;GO:0042802;GO:0005178;GO:0043394;GO:0019899;GO:0005515;GO:0005102);A0A075B6K4(GO:0003823);Q86YZ3(GO:0046914;GO:0005509;GO:0046872);P00748(GO:0005509;GO:0051787;GO:0005515);Q08380(GO:0005515);P01008(GO:0002020;GO:0042802;GO:0008201;GO:0005515);C9JPQ9(GO:0005102);P00918(GO:0005515;GO:0046872;GO:0008270);P01782(GO:0003823);A0A0C4DH67(GO:0003823);A0A669KAY4(GO:0030169;GO:0034190;GO:0070326;GO:0034189;GO:0034185;GO:0043621;GO:0050750;GO:0003723);P01344(GO:0008083;GO:0005179;GO:0005178;GO:0005159;GO:0005158;GO:0048018;GO:0005515);I3L1J2(GO:0044325;GO:0043184;GO:0005509;GO:0042803;GO:0008013;GO:0019903;GO:0005102;GO:0008092;GO:0045296);A0A087WSY6(GO:0003823);M0R0Q9(GO:0031715;GO:0005102);P30041(GO:0042803;GO:0031625;GO:0045296;GO:0005515);Q5SRP5(GO:0005543);P18206(GO:0002162;GO:0003779;GO:0051015;GO:0031625;GO:0008013;GO:0045294;GO:0045296;GO:0005515);E7END6(GO:0005509);P02787(GO:1990459;GO:0034986;GO:0046872;GO:0008198;GO:0008199;GO:0005515);P01817(GO:0003823);P01715(GO:0003823);P01717(GO:0003823);A0A0B4J1V0(GO:0003823;GO:0034987);P07360(GO:0036094;GO:0019841;GO:0001848;GO:0044877);P00915(GO:0046872;GO:0008270;GO:0005515);A0A075B6S5(GO:0003823);P01699(GO:0003823);Q96HR3(GO:0005515;GO:0046966;GO:0042809);P02747(GO:0005515);A0A075B6J9(GO:0003823);K7ER74(GO:0008289;GO:0042803;GO:0043274);A0A087WZB5(GO:0003779);A0A0J9YX35(GO:0003823);E7EUT5(GO:0051287;GO:0097718;GO:0042802;GO:0008017;GO:0050661) | 0.044561417 | 0.13368425 | https://www.ebi.ac.uk/QuickGO/term/GO:0005488 |
| GO:0044421 | extracellular region part | cellular_component | 297|375 | P08779(GO:0070062);P00488(GO:0072562;GO:0062023);P68366(GO:0070062);P04275(GO:0031012;GO:0070062;GO:0062023);A0A0U1RQV3(GO:0062023);J3KNB4(GO:0005615);Q9UHG3(GO:0034361;GO:0070062);P51884(GO:0031012;GO:0070062;GO:0005583;GO:0062023;GO:0005615);P03950(GO:0005615;GO:0005604;GO:0032311);A0A0A0MRZ9(GO:0005615);P23142(GO:0031012;GO:0005615;GO:0062023;GO:0071953;GO:0070062;GO:0005604;GO:0005577);P15814(GO:0042571;GO:0072562);P41222(GO:0070062;GO:0005615);P08571(GO:0005615;GO:0070062);P68871(GO:0005615;GO:0072562;GO:0070062);P22792(GO:0070062;GO:0072562);Q86YZ3(GO:0070062;GO:0062023);P35527(GO:0005615;GO:0070062);P01042(GO:0005615;GO:0072562;GO:0062023;GO:0070062);P01597(GO:0072562;GO:0070062);P43251(GO:0005615;GO:0070062);Q14520(GO:0005615);P01602(GO:0072562;GO:0070062);A0A0G2JMI3(GO:0072562;GO:0042571);A0A0B4J1U7(GO:0072562;GO:0042571);A0A669KAY4(GO:0005615);P59665(GO:0005615;GO:0062023;GO:0070062);P15169(GO:0005615);P0DJI8(GO:0034364;GO:0005615;GO:0070062);P03951(GO:0070062;GO:0005615);P06312(GO:0072562);P43121(GO:0005615);A0A0A0MT36(GO:0005615);P01009(GO:0005615;GO:0070062);P02790(GO:0005615;GO:0072562;GO:0062023;GO:0070062);P60763(GO:0070062);P01764(GO:0042571;GO:0005615;GO:0072562;GO:0070062);A0A087X0S5(GO:0062023;GO:0005615;GO:0031012;GO:0070062);P01766(GO:0005615;GO:0072562);Q15582(GO:0031012;GO:0070062;GO:0062023;GO:0005615;GO:0005604);F8WF14(GO:0072562);C9JB55(GO:0005615);P0DOY3(GO:0005615;GO:0072562;GO:0070062);P26038(GO:0070062;GO:0072562;GO:0005615);P01782(GO:0070062);P18428(GO:0070062;GO:0005615);P01780(GO:0072562;GO:0070062);A0A140T8Y3(GO:0062023;GO:0031012);P02776(GO:0020005;GO:0062023;GO:0005615);P02775(GO:0005615);Q13201(GO:0031012;GO:0062023);G3XAK1(GO:0005615;GO:0062023);P01008(GO:0070062;GO:0072562;GO:0062023;GO:0005615);A0A0G2JMB2(GO:0042571;GO:0072562);P30041(GO:0005615;GO:0070062);P06702(GO:0070062;GO:0062023;GO:0005615);Q9Y5Y7(GO:0070062);P61224(GO:0070062);D6RF35(GO:0005615);P10643(GO:0070062);P02787(GO:0005615;GO:0070062;GO:0072562);P19827(GO:0070062;GO:0072562);P01833(GO:0005615;GO:0070062);P01834(GO:0042571;GO:0005615;GO:0072562;GO:0070062);P19823(GO:0070062;GO:0072562;GO:0062023);Q96IY4(GO:0005615;GO:0070062);P04003(GO:0005615;GO:0072562);P04004(GO:0031012;GO:0070062;GO:0072562;GO:0062023;GO:0005615;GO:0005604);P00450(GO:0005615;GO:0070062;GO:0072562);P20851(GO:0005615);Q92954(GO:0062023);Q6UXB8(GO:0005615);O00391(GO:0045171;GO:0070062;GO:0005615);P13796(GO:0005615;GO:0070062);P69905(GO:0005615;GO:0072562;GO:0070062);A0A075B6H7(GO:0005615);A0A087WSZ0(GO:0005615);Q9Y6R7(GO:0070062);P02768(GO:0070062;GO:0072562;GO:0005615);P08697(GO:0005615;GO:0072562;GO:0070062;GO:0005577);Q96PD5(GO:0005615;GO:0070062);P02760(GO:0070062;GO:0072562;GO:0062023;GO:0005615);P02763(GO:0005615;GO:0072562;GO:0062023;GO:0070062);Q01518(GO:0070062);A0A0C4DH73(GO:0005615);P02766(GO:0070062;GO:0005615);C9JC84(GO:0005577);Q9H4B7(GO:0070062);Q9BWP8(GO:0005615);P07357(GO:0070062;GO:0072562;GO:0005615);A0A075B6S5(GO:0005615);P37802(GO:0070062);A0A0G2JI36(GO:0070062);Q15166(GO:0070062;GO:0005615);J3QT83(GO:0005615;GO:0062023;GO:0031012);P29622(GO:0005615;GO:0070062);P35579(GO:0070062);Q08830(GO:0005577);P01700(GO:0072562);P01701(GO:0070062);Q86UX7(GO:0070062);P01704(GO:0070062);Q8N1N4(GO:0005615;GO:0070062);P02741(GO:0005615);P02671(GO:0005615;GO:0072562;GO:1903561;GO:0070062;GO:0005577);P02675(GO:1903561;GO:0005615;GO:0072562;GO:0070062;GO:0005577);P07996(GO:0031012;GO:0005615;GO:0062023;GO:0070062;GO:0005577);P13671(GO:0070062);A0A075B6I0(GO:0005615);P01619(GO:0005615;GO:0072562;GO:0071748;GO:0070062;GO:0071751;GO:0071756);P0CF74(GO:0042571;GO:0005615;GO:0072562);P61626(GO:0070062;GO:0005615);P04217(GO:0070062;GO:0072562;GO:0062023;GO:0005615);P04180(GO:0070062;GO:0034364;GO:0005615);P31151(GO:0005615;GO:0062023);E9PHK0(GO:0070062;GO:0005615;GO:0062023);P22891(GO:0005615;GO:0070062);Q06033(GO:0070062);C9JF17(GO:0070062;GO:0005615);P00739(GO:0070062;GO:0034366;GO:0072562;GO:0005615);P05155(GO:0005615;GO:0072562;GO:0070062);P05154(GO:0005615;GO:0070062);P27169(GO:0070062;GO:0005615;GO:0072562;GO:0034364;GO:0034366);P20742(GO:0070062;GO:0072562;GO:0005615);A0A0B4J2D9(GO:0005615);O95445(GO:0005615;GO:0034361;GO:0034362;GO:0034364;GO:0034365;GO:0034366);A0A0A0MS51(GO:0005615);A0A075B7F0(GO:0042571;GO:0072562);Q13103(GO:0062023);A0A087X1J7(GO:0005615);P00734(GO:0005615;GO:0070062;GO:0072562);P13645(GO:0070062;GO:0005615);A0A0A0MS14(GO:0072562;GO:0042571);A0A0A0MS15(GO:0072562;GO:0042571);P13473(GO:0005615;GO:0070062);P01717(GO:0072562);A0A075B6J9(GO:0005615);P48740(GO:0005615);P00738(GO:0005615;GO:0072562;GO:0070062);P01857(GO:0042571;GO:0005615;GO:0072562;GO:0070062);M0R0Q9(GO:0070062;GO:0005615;GO:0072562);A0A075B6Q5(GO:0072562;GO:0042571);P80748(GO:0072562;GO:0070062);G3XAP6(GO:0062023;GO:0005615;GO:0031012;GO:0070062);A0A0C4DH25(GO:0005615);P08185(GO:0005615;GO:0070062);P01019(GO:0072562;GO:0062023;GO:0070062;GO:0005615);P09871(GO:0072562);G3V0E5(GO:0070062);P19652(GO:0005615;GO:0072562;GO:0062023;GO:0070062);A0A0C4DH55(GO:0005615);K7ERG9(GO:0005615);A0A0B4J1Y8(GO:0005615);B0YIW2(GO:0042627;GO:0070062;GO:0062023;GO:0034361;GO:0034363;GO:0034366;GO:0005615);O75636(GO:0072562);Q9UGM5(GO:0005615;GO:0070062);G3V2W1(GO:0005615);O14791(GO:0005615;GO:0072562;GO:0034361;GO:0034364);K7ER74(GO:0042627;GO:0005615;GO:0034361;GO:0034362;GO:0034363;GO:0034366);P15144(GO:0070062;GO:0005615);P36955(GO:0005615;GO:0062023;GO:0070062;GO:0005604);A0A0B4J1U3(GO:0005615);A0A0G2JPR0(GO:0005615);C9JPQ9(GO:0005577);P09486(GO:0005615;GO:0062023;GO:0031012;GO:0005604);P18206(GO:0070062;GO:1903561);Q13093(GO:0034362;GO:0005615);P32119(GO:0070062);O00187(GO:0005615;GO:0070062);A0A0B4J231(GO:0070062;GO:0042571);A0A075B6K2(GO:0005615);P16930(GO:0070062);B4E1Z4(GO:0070062;GO:0072562;GO:0005615);P02751(GO:0062023;GO:0070062;GO:0072562;GO:0005615;GO:0005604;GO:0031012;GO:0005577);A0A075B6R2(GO:0072562;GO:0042571);Q9UNN8(GO:0070062;GO:0005615);Q03591(GO:0005615;GO:0072562);P02750(GO:0070062;GO:0005615);P55058(GO:0005615;GO:0034364);P55056(GO:0034361;GO:0034364);E9PK25(GO:0070062;GO:0005615);P80108(GO:0031012;GO:0070062;GO:0005615);A0A0G2JRQ6(GO:0005615);A0A0B4J1X5(GO:0072562;GO:0042571);P0C0L5(GO:0005615;GO:0072562;GO:0070062);A0A0C4DH43(GO:0072562;GO:0042571);A0A075B6K5(GO:0005615);P13647(GO:0070062);P43652(GO:0005615;GO:0072562;GO:0070062);A0A087WWT3(GO:0005615);P02753(GO:0005615;GO:0070062);A0A087WSY4(GO:0072562;GO:0042571);P01344(GO:0005615);P02647(GO:0034361;GO:0034362;GO:0034363;GO:0034364;GO:0034366;GO:0042627;GO:0072562;GO:0062023;GO:0070062;GO:0034365;GO:0005615;GO:1903561);P02042(GO:0072562);P11226(GO:0005615);P01877(GO:0005615;GO:0072562;GO:0071748;GO:0070062;GO:0071752;GO:0071751);P01871(GO:0005615;GO:0072562;GO:0070062;GO:0071756;GO:0071757);A0A075B7D0(GO:0042571;GO:0072562);O75882(GO:0005615;GO:0070062);A0A075B7D8(GO:0042571;GO:0072562);P00918(GO:0005615;GO:0070062);A0A0C4DH32(GO:0072562;GO:0042571);A0A075B6S9(GO:0005615);Q6EMK4(GO:0070062;GO:0005615;GO:0031012);A0A0A0MRJ7(GO:1903561;GO:0005615);P04040(GO:0070062;GO:0005615);P05109(GO:0005615;GO:0070062);A0A3B3ISR2(GO:0005615);P05543(GO:0005615;GO:0070062);P18065(GO:0005615;GO:0070062);P00915(GO:0070062);P05546(GO:0005615;GO:0070062);Q562R1(GO:0005615;GO:0070062);P02743(GO:0072562;GO:0070062;GO:0005615;GO:0062023);A0A075B6K4(GO:0005615);P55103(GO:0005615);P01860(GO:0042571;GO:0005615;GO:0072562;GO:0070062);P02747(GO:0072562;GO:0005615);P02745(GO:0005602);A0A0B4J1V0(GO:0072562;GO:0042571);P01031(GO:0005615;GO:0070062);P02749(GO:0042627;GO:0070062;GO:0034361;GO:0034364;GO:0062023;GO:0005615);P68032(GO:0070062;GO:0072562;GO:0005615);P01034(GO:0005615;GO:0070062;GO:0005604);P01601(GO:0005615);P08514(GO:0070062;GO:0072562);P11597(GO:0034364;GO:0070062;GO:0005615);P02649(GO:0034361;GO:0034362;GO:0034363;GO:0034364;GO:0042627;GO:0072562;GO:0062023;GO:0031012;GO:0070062;GO:0034365;GO:0005615;GO:1903561);P04196(GO:0070062;GO:0072562;GO:0062023);P08519(GO:0034358);P10909(GO:0034366;GO:0072562;GO:0031012;GO:0070062;GO:0005615;GO:0062023);P04114(GO:0034360;GO:0034359;GO:0005615;GO:0070062;GO:0034361;GO:0034362;GO:0034363;GO:0042627);A0A0C4DH33(GO:0072562;GO:0042571);P01861(GO:0042571;GO:0005615;GO:0072562;GO:0070062);A0A0C4DH31(GO:0072562;GO:0042571);A0A0C4DH36(GO:0042571;GO:0072562);A0A0C4DH34(GO:0072562;GO:0042571);P19320(GO:0005615;GO:0070062);H0YAC1(GO:0070062;GO:0005615);Q14624(GO:0072562;GO:0070062);A0A0C4DH38(GO:0072562;GO:0042571);A0A0C4DH39(GO:0072562;GO:0042571);P25311(GO:0070062;GO:0005615;GO:0062023);P01599(GO:0072562;GO:0070062);O43866(GO:0005615;GO:0072562);P07358(GO:0005615;GO:1903561;GO:0070062);P01023(GO:0072562;GO:0005615;GO:0070062);C9J8S2(GO:0005615;GO:0062023);P02748(GO:0070062;GO:0072562;GO:0005615);A0A0C4DH24(GO:0005615);P09172(GO:0005615);P02538(GO:0070062);P00747(GO:0070062;GO:0072562;GO:0062023;GO:0005615);P00740(GO:0005615;GO:0070062);P60709(GO:0005615;GO:0070062;GO:0072562);P00742(GO:0005615);E7EQB2(GO:0005615);P02533(GO:0070062);P00748(GO:0070062;GO:0062023;GO:0005615);Q08380(GO:0070062;GO:0072562;GO:0062023;GO:0005615);Q16610(GO:0031012;GO:0005615;GO:0062023;GO:0070062);P08603(GO:0070062;GO:0072562;GO:0005615);A0A075B6R9(GO:0005615);P04264(GO:0031012;GO:0070062;GO:0072562;GO:0062023;GO:0005615);P0C0L4(GO:0005615;GO:0072562;GO:0070062);C9JV77(GO:0070062;GO:0072562;GO:0062023;GO:0005615);P02652(GO:0042627;GO:0005615;GO:0072562;GO:0034361;GO:0070062;GO:0034364;GO:0034366);P35858(GO:0005615;GO:0031012;GO:0042567;GO:0070062);Q15485(GO:0072562;GO:0070062);P69892(GO:0072562);D6RAR4(GO:0005615);A0A096LPE2(GO:0034364;GO:0005615;GO:0070062);P01024(GO:0070062;GO:0005615;GO:0072562);P04433(GO:0072562;GO:0070062);A0A087WSY6(GO:0005615);P06727(GO:0042627;GO:0005615;GO:0072562;GO:0034361;GO:0034364;GO:0062023;GO:0070062);Q9NZP8(GO:0005615;GO:0070062);A0A0C4DH29(GO:0072562;GO:0042571);Q5SRP5(GO:0034361;GO:0034365;GO:0034366;GO:0034362;GO:0034364);A0A0B4J1X8(GO:0072562;GO:0042571);P02100(GO:0072562);P33908(GO:0070062);Q15848(GO:0005615);P21333(GO:0070062);A0A0B4J1V1(GO:0072562;GO:0042571);A0A075B7B8(GO:0042571;GO:0072562);P07360(GO:0070062;GO:0072562;GO:0005615);A0A0B4J1V2(GO:0072562;GO:0042571);Q5T749(GO:0070062);P07437(GO:0070062);Q13790(GO:0034362;GO:0034364;GO:0005615);P11021(GO:0070062);Q9Y490(GO:0070062);P01011(GO:0070062;GO:0072562;GO:0062023;GO:0005615);P07737(GO:0070062;GO:0072562);P35908(GO:0070062;GO:0005615) | 61|82 | C9JB55(GO:0005615);O75636(GO:0072562);P19652(GO:0005615;GO:0072562;GO:0062023;GO:0070062);A0A0C4DH34(GO:0072562;GO:0042571);Q96IY4(GO:0005615;GO:0070062);P02763(GO:0005615;GO:0072562;GO:0062023;GO:0070062);P29622(GO:0005615;GO:0070062);Q9UGM5(GO:0005615;GO:0070062);P00915(GO:0070062);A0A0C4DH32(GO:0072562;GO:0042571);P15169(GO:0005615);K7ER74(GO:0042627;GO:0005615;GO:0034361;GO:0034362;GO:0034363;GO:0034366);P37802(GO:0070062);J3QT83(GO:0005615;GO:0062023;GO:0031012);Q9Y5Y7(GO:0070062);P01599(GO:0072562;GO:0070062);Q86YZ3(GO:0070062;GO:0062023);P35527(GO:0005615;GO:0070062);A0A075B6J9(GO:0005615);A0A0G2JRQ6(GO:0005615);P01601(GO:0005615);P32119(GO:0070062);O00187(GO:0005615;GO:0070062);A0A075B6K2(GO:0005615);C9JPQ9(GO:0005577);P55058(GO:0005615;GO:0034364);P01009(GO:0005615;GO:0070062);P02751(GO:0062023;GO:0070062;GO:0072562;GO:0005615;GO:0005604;GO:0031012;GO:0005577);A0A075B6K4(GO:0005615);Q9NZP8(GO:0005615;GO:0070062);P09172(GO:0005615);P00748(GO:0070062;GO:0062023;GO:0005615);Q08380(GO:0070062;GO:0072562;GO:0062023;GO:0005615);P01008(GO:0070062;GO:0072562;GO:0062023;GO:0005615);P02787(GO:0005615;GO:0070062;GO:0072562);K7ERG9(GO:0005615);A0A075B6R9(GO:0005615);P01782(GO:0070062);P01780(GO:0072562;GO:0070062);A0A669KAY4(GO:0005615);A0A087WSY6(GO:0005615);A0A0C4DH73(GO:0005615);A0A0C4DH25(GO:0005615);M0R0Q9(GO:0070062;GO:0005615;GO:0072562);P30041(GO:0005615;GO:0070062);Q5SRP5(GO:0034361;GO:0034365;GO:0034366;GO:0034362;GO:0034364);P18206(GO:0070062;GO:1903561);P00918(GO:0005615;GO:0070062);P01344(GO:0005615);P19823(GO:0070062;GO:0072562;GO:0062023);P01717(GO:0072562);A0A0B4J1V0(GO:0072562;GO:0042571);P07360(GO:0070062;GO:0072562;GO:0005615);P05543(GO:0005615;GO:0070062);P01857(GO:0042571;GO:0005615;GO:0072562;GO:0070062);A0A075B6S5(GO:0005615);P55056(GO:0034361;GO:0034364);P02747(GO:0072562;GO:0005615);P80748(GO:0072562;GO:0070062);P43251(GO:0005615;GO:0070062);A0A075B6H7(GO:0005615) | 0.057264463 | 0.150319215 | https://www.ebi.ac.uk/QuickGO/term/GO:0044421 |
| GO:0005576 | extracellular region | cellular_component | 274|375 | P00488(GO:0005576);P68366(GO:0005576);P04275(GO:0005576);J3KNB4(GO:0005576);P0C0L5(GO:0005576);P03950(GO:0005576);G3XAP6(GO:0005576);P05160(GO:0005576);P23142(GO:0005576);P15814(GO:0005576);P0DP02(GO:0005576);P41222(GO:0005576);B1AHL2(GO:0005576);P08571(GO:0005576);P68871(GO:0005576);P22792(GO:0005576);Q86YZ3(GO:0005576);P01597(GO:0005576);P01042(GO:0005576);P02787(GO:0005576);Q14520(GO:0005576);P01602(GO:0005576);P08185(GO:0005576);A0A0B4J1U7(GO:0005576);P59665(GO:0005576);P0DJI8(GO:0005576);A0A2R8Y3M9(GO:0005576);Q96KN2(GO:0005576);P06312(GO:0005576);P43121(GO:0005576);A0A0A0MT36(GO:0005576);P01009(GO:0005576);P02790(GO:0005576);P01763(GO:0005576);P01764(GO:0005576);A0A087X0S5(GO:0005576);P01766(GO:0005576);Q15582(GO:0005576);Q08830(GO:0005576);P0DOY3(GO:0005576);P01782(GO:0005576);P18428(GO:0005576);P01780(GO:0005576);P06331(GO:0005576);P02776(GO:0005576);D6R934(GO:0005576);P02775(GO:0005576);Q13201(GO:0005576);A0A0J9YXX1(GO:0005576);P30041(GO:0005576);P06702(GO:0005576);P0DP01(GO:0005576);P10643(GO:0005576);P08697(GO:0005576);P19827(GO:0005576);P01833(GO:0005576);P01834(GO:0005576);P19823(GO:0005576);P04003(GO:0005576);E7END6(GO:0005576);P04004(GO:0005576);P00450(GO:0005576);Q92954(GO:0005576);Q6UXB8(GO:0005576);O00391(GO:0005576);A6XND0(GO:0005576);P69905(GO:0005576);G3XAK1(GO:0005576);Q9Y6R7(GO:0005576);P02768(GO:0005576);O75636(GO:0005576);Q96PD5(GO:0005576);P02760(GO:0005576);P02763(GO:0005576);Q01518(GO:0005576);A0A0C4DH73(GO:0005576);P02766(GO:0005576);P07357(GO:0005576);A0A075B6S5(GO:0005576);P37802(GO:0005576);Q15166(GO:0005576);P07358(GO:0005576);P29622(GO:0005576);P09172(GO:0005576);P01703(GO:0005576);P01700(GO:0005576);P01701(GO:0005576);P01706(GO:0005576);P04264(GO:0005576);P01704(GO:0005576);P01705(GO:0005576);A0A0C4DH67(GO:0005576);P23083(GO:0005576);P01709(GO:0005576);P02741(GO:0005576);P02671(GO:0005576);P03951(GO:0005576);P07996(GO:0005576);P13671(GO:0005576);A0A075B6I0(GO:0005576);F5H8B0(GO:0005576);P0CF74(GO:0005576);P61626(GO:0005576);P04180(GO:0005576);E9PHK0(GO:0005576);P22891(GO:0005576);Q06033(GO:0005576);C9JF17(GO:0005576);P00739(GO:0005576);P05155(GO:0005576);P05154(GO:0005576);P27169(GO:0005576);P20742(GO:0005576);P01031(GO:0005576);O95445(GO:0005576);Q13103(GO:0005576);J3KRP0(GO:0005576);P13645(GO:0005576);A0A0A0MS14(GO:0005576);A0A0A0MS15(GO:0005576);P01715(GO:0005576);P01717(GO:0005576);P48740(GO:0005576);K7ERI9(GO:0005576);P00738(GO:0005576);P01857(GO:0005576);M0R0Q9(GO:0005576);A0A075B6Q5(GO:0005576);P80748(GO:0005576);P01019(GO:0005576);A0A0S2Z4L3(GO:0005576);A0A0C4DH25(GO:0005576);A0A0G2JMI3(GO:0005576);P01011(GO:0005576);P09871(GO:0005576);P19652(GO:0005576);A0A0C4DH55(GO:0005576);A0A0B4J1Y8(GO:0005576);B0YIW2(GO:0005576);P04430(GO:0005576);P31151(GO:0005576);Q9UGM5(GO:0005576);O00187(GO:0005576);O14791(GO:0005576);K7ER74(GO:0005576);A0A087WSZ0(GO:0005576);P36955(GO:0005576);A0A0B4J1U3(GO:0005576);A0A0G2JPR0(GO:0005576);P09486(GO:0005576);Q16610(GO:0005576);P11226(GO:0005576);Q13093(GO:0005576);Q96IY4(GO:0005576);P01721(GO:0005576);Q9NZP8(GO:0005576);A0A075B6K2(GO:0005576);P55058(GO:0005576);B4E1Z4(GO:0005576);P02751(GO:0005576);A0A075B6R2(GO:0005576);Q9UNN8(GO:0005576);Q03591(GO:0005576);P02750(GO:0005576);P55056(GO:0005576);P01008(GO:0005576);P00734(GO:0005576);A0A0B4J1X5(GO:0005576);A0A0C4DH43(GO:0005576);A0A075B6K5(GO:0005576);P43652(GO:0005576);P02753(GO:0005576);C9JV77(GO:0005576);P01814(GO:0005576);P01344(GO:0005576);A0A0B4J1X8(GO:0005576);P01877(GO:0005576);P01871(GO:0005576);P10909(GO:0005576);O75882(GO:0005576);P01619(GO:0005576);F8WF14(GO:0005576);P04114(GO:0005576);Q6EMK4(GO:0005576);A0A0A0MRJ7(GO:0005576);P04040(GO:0005576);A0A0C4DH33(GO:0005576);A0A669KAY4(GO:0005576);P05543(GO:0005576);P18065(GO:0005576);A0A0J9YX35(GO:0005576);P05546(GO:0005576);P02743(GO:0005576);A0A075B6K4(GO:0005576);P55103(GO:0005576);P01860(GO:0005576);D6RAR4(GO:0005576);P02745(GO:0005576);P01718(GO:0005576);A0A0B4J2D9(GO:0005576);P02749(GO:0005576);P02748(GO:0005576);P01034(GO:0005576);P01601(GO:0005576);P04217(GO:0005576);P11597(GO:0005576);P02649(GO:0043083;GO:0005576);P04211(GO:0005576);P04196(GO:0005576);P02647(GO:0005576);P01861(GO:0005576);P02747(GO:0005576);A0A0C4DH32(GO:0005576);P05109(GO:0005576);P15169(GO:0005576);A0A0C4DH31(GO:0005576);A0A0C4DH34(GO:0005576);H0YAC1(GO:0005576);Q14624(GO:0005576);A0A0C4DH38(GO:0005576);A0A0C4DH39(GO:0005576);P25311(GO:0005576);P01599(GO:0005576);O43866(GO:0005576);A0A075B6J9(GO:0005576);P01023(GO:0005576);P01743(GO:0005576);C9J8S2(GO:0005576);A0A0C4DH24(GO:0005576);O95497(GO:0005576);P00747(GO:0005576);P00740(GO:0005576);P00742(GO:0005576);E7EQB2(GO:0005576);A0A0A0MRZ9(GO:0005576);P01742(GO:0005576);P00748(GO:0005576);Q08380(GO:0005576);Q9BWP8(GO:0005576);P08603(GO:0005576);P43251(GO:0005576);P20851(GO:0005576);P0C0L4(GO:0005576);A0A087WSY4(GO:0005576);P02652(GO:0005576);P35858(GO:0005576);Q15485(GO:0005576);A0A0G2JL69(GO:0005576);A0A096LPE2(GO:0005576);P01024(GO:0005576);P04433(GO:0005576);A0A087WSY6(GO:0005576);P06727(GO:0005576);C9J5S7(GO:0005576);A0A0C4DH29(GO:0005576);P18206(GO:0005576);J3KPA1(GO:0005576);P01817(GO:0005576);P08519(GO:0005576);P21333(GO:0005576);P51884(GO:0005576);A0A0B4J1V1(GO:0005576);A0A0B4J1V0(GO:0005576);P07360(GO:0005576);A0A0B4J1V2(GO:0005576);Q86UX7(GO:0005576);P07437(GO:0005576);P80108(GO:0005576);Q13790(GO:0005576);P02675(GO:0005576);Q9Y490(GO:0005576);Q15848(GO:0005576);A0A0J9YVY3(GO:0005576);P01699(GO:0005576) | 61|82 | O75636(GO:0005576);P01857(GO:0005576);P37802(GO:0005576);P04211(GO:0005576);P02763(GO:0005576);A0A0C4DH73(GO:0005576);Q9UGM5(GO:0005576);P01721(GO:0005576);A0A0C4DH32(GO:0005576);A0A0J9YX35(GO:0005576);P19652(GO:0005576);K7ER74(GO:0005576);A0A0C4DH34(GO:0005576);P05543(GO:0005576);P01599(GO:0005576);Q86YZ3(GO:0005576);P01703(GO:0005576);P18206(GO:0005576);P01780(GO:0005576);P80748(GO:0005576);P01601(GO:0005576);Q96IY4(GO:0005576);O00187(GO:0005576);P15169(GO:0005576);A0A2R8Y3M9(GO:0005576);A0A075B6K2(GO:0005576);P55058(GO:0005576);P01009(GO:0005576);P01008(GO:0005576);A0A075B6K4(GO:0005576);Q9NZP8(GO:0005576);P09172(GO:0005576);P00748(GO:0005576);Q08380(GO:0005576);P02751(GO:0005576);P01344(GO:0005576);P01782(GO:0005576);A0A0C4DH67(GO:0005576);A0A669KAY4(GO:0005576);A0A087WSY6(GO:0005576);P29622(GO:0005576);A0A0C4DH25(GO:0005576);M0R0Q9(GO:0005576);P30041(GO:0005576);P0DP01(GO:0005576);P02787(GO:0005576);P01817(GO:0005576);P01715(GO:0005576);P19823(GO:0005576);P01717(GO:0005576);A0A0B4J1V0(GO:0005576);P07360(GO:0005576);K7ERI9(GO:0005576);E7END6(GO:0005576);A0A075B6S5(GO:0005576);P55056(GO:0005576);P02747(GO:0005576);A0A075B6J9(GO:0005576);A0A0G2JL69(GO:0005576);P43251(GO:0005576);P01699(GO:0005576) | 0.107978753 | 0.181404305 | https://www.ebi.ac.uk/QuickGO/term/GO:0005576 |
| GO:0022610 | biological adhesion | biological_process | 36|375 | P08514(GO:0007160;GO:0070527;GO:0007155);P04275(GO:0031589;GO:0007155);Q86UX7(GO:0034446;GO:0007159;GO:0007155;GO:0070527);P05109(GO:0070488);P68871(GO:0070527);P19320(GO:0034113;GO:0050901;GO:0140039;GO:0098609;GO:0007159;GO:0007155;GO:0007157;GO:0007160);P25311(GO:0007155);P21333(GO:0070527);Q14520(GO:0007155);P14151(GO:0016339;GO:0050901;GO:0007155);O95497(GO:0098609);P02671(GO:0007160;GO:0070527);P02751(GO:0034446;GO:0007160;GO:0007161;GO:0007155);P02675(GO:0007160;GO:0070527);P02760(GO:0007155);A0A087X0S5(GO:0007155);Q08380(GO:0007155);Q15582(GO:0007155);P60709(GO:0070527);P26038(GO:0007159;GO:0070489);P07996(GO:0007155);P35858(GO:0007155);Q13201(GO:0007155);I3L1J2(GO:0016339;GO:0098609;GO:0007155;GO:0044331;GO:0007156);P06727(GO:0007159);P06702(GO:0070488);Q9Y5Y7(GO:0007160;GO:0007155);P18206(GO:0007160;GO:0007155;GO:0090136;GO:0070527);P35579(GO:0007155;GO:0098609;GO:0070527);A0A0A0MTH3(GO:0034446);H0Y5E4(GO:0007155);P04004(GO:0061302;GO:0007160;GO:0007155;GO:0033627);P43121(GO:0007155);G3XAP6(GO:0007155;GO:0070527);Q9Y490(GO:0007155;GO:0070527);A0A087WZB5(GO:0007155) | 6|82 | I3L1J2(GO:0016339;GO:0098609;GO:0007155;GO:0044331;GO:0007156);P02751(GO:0034446;GO:0007160;GO:0007161;GO:0007155);Q9Y5Y7(GO:0007160;GO:0007155);P18206(GO:0007160;GO:0007155;GO:0090136;GO:0070527);A0A087WZB5(GO:0007155);Q08380(GO:0007155) | 0.131301673 | 0.190161044 | https://www.ebi.ac.uk/QuickGO/term/GO:0022610 |
| GO:0065007 | biological regulation | biological_process | 291|375 | P08779(GO:0061436;GO:0030336);P00488(GO:0007599;GO:0019221;GO:0007596);P68366(GO:0010389);P04275(GO:0007599;GO:0007596);A0A0U1RQV3(GO:0007173);J3KNB4(GO:0044130;GO:0045766;GO:0008284;GO:0044140;GO:0001934);P01700(GO:0038096;GO:0030449;GO:0050776;GO:0038095;GO:0006956;GO:0006958);P03951(GO:0007599;GO:0007596;GO:0030193;GO:0051919);P03950(GO:0032148;GO:0032431;GO:0042592;GO:0048662;GO:0017148;GO:0042327;GO:0050714;GO:0007202;GO:0001938);G3XAP6(GO:0030509;GO:0030500;GO:1900047;GO:0014829;GO:1902732;GO:0043066);P05160(GO:0007596;GO:0007599;GO:1903363);P23142(GO:0007162;GO:1900025;GO:2000647;GO:0010952;GO:2000146;GO:0001933;GO:0070373;GO:0007229);Q03591(GO:0032091;GO:0030449;GO:0045919;GO:0006956);B7ZKJ8(GO:0010951);P41222(GO:2000255;GO:0045187);B1AHL2(GO:0010952);P08571(GO:0097190;GO:1901224;GO:0007166;GO:0032729;GO:0002224;GO:0032760;GO:0034128;GO:0038124;GO:2000484;GO:0035666;GO:0032481;GO:0034142;GO:0002755;GO:0002756;GO:0050715;GO:0038123;GO:0031663;GO:0007249;GO:0045807);P68871(GO:0007596;GO:0045429;GO:0008217;GO:0010942;GO:0050880);P48740(GO:0006956;GO:0001867);E9PK25(GO:0022604;GO:0061001;GO:0030836;GO:0043066;GO:0035722;GO:0044794;GO:0007266);O75460(GO:0007257;GO:0016241;GO:0071333;GO:0006402;GO:0033120;GO:0007050;GO:0036498;GO:0030968;GO:0070059;GO:1904707;GO:0006355;GO:1900103);Q86YZ3(GO:0061436);P01597(GO:0038096;GO:0030449;GO:0050776;GO:0038095;GO:0006956;GO:0006958);P01042(GO:0007599;GO:0007596;GO:0045861;GO:0007162;GO:0042311;GO:0050880;GO:0043065;GO:0010466;GO:0007186;GO:0010951;GO:0007204;GO:0030195);P14151(GO:0050776);P01602(GO:0038096;GO:0030449;GO:0050776;GO:0038095;GO:0006956;GO:0006958);A0A0G2JMI3(GO:0050853;GO:0006958;GO:0050871);A0A0B4J1U7(GO:0050853;GO:0006958;GO:0050871);P59665(GO:0030520);C9JC84(GO:0007596);P51884(GO:0032914;GO:0045944);P06312(GO:0038096;GO:0030449;GO:0050776;GO:0038095;GO:0006956;GO:0006958);P01743(GO:0038096;GO:0030449;GO:0050776;GO:0038095;GO:0006956;GO:0006958);K7ERG9(GO:0007219;GO:0006957);P02790(GO:0002925;GO:0051246;GO:0042531;GO:0006879;GO:0060332;GO:0060335;GO:0002639);P60763(GO:0022604;GO:0035556;GO:0030838;GO:1900026;GO:0051056;GO:0014041;GO:0007266;GO:0007264;GO:0048873;GO:0033630);A0A075B7D8(GO:0050853;GO:0006958;GO:0050871);P01764(GO:0030449;GO:0050853;GO:0050776;GO:0006956;GO:0006958;GO:0050871;GO:0038096;GO:0038095);P01766(GO:0038096;GO:0030449;GO:0038095;GO:0050776;GO:0006956;GO:0006958);Q15582(GO:0007162);F8WF14(GO:0050805;GO:0008285;GO:0019695);P26038(GO:0010628;GO:0071803;GO:1902115;GO:2000401;GO:0035722;GO:2000643;GO:1903364;GO:0008361;GO:0008360;GO:1902966);P29622(GO:0010466;GO:0010951);P18428(GO:0044130;GO:0019221;GO:0060265;GO:0090023;GO:0042535;GO:0045919;GO:0002224;GO:0032722;GO:0032720;GO:0032760;GO:0034142;GO:0034145;GO:0031663;GO:0043032;GO:0032757;GO:0032755);P01780(GO:0038096;GO:0030449;GO:0050776;GO:0038095;GO:0006956;GO:0006958);P06331(GO:0038096;GO:0030449;GO:0050776;GO:0038095;GO:0006956;GO:0006958);P02776(GO:0070098;GO:0019221;GO:0010469;GO:0032760;GO:0010628;GO:0045918;GO:0045347;GO:0007189;GO:0010744;GO:0007186;GO:2001240;GO:0042127;GO:0043950;GO:0090023;GO:0045651;GO:0045652;GO:0045653;GO:0045944;GO:0016525);D6R934(GO:0006958);P02775(GO:0042127;GO:0070098;GO:0007186;GO:0090023;GO:0010469;GO:0051781);H3BTN5(GO:1903672);Q13201(GO:0010811;GO:0007596);E7EUT5(GO:0050821;GO:0052501;GO:0050715;GO:0017148;GO:0006417);A0A0G2JMB2(GO:0050853;GO:0006958;GO:0050871);P30041(GO:0048026;GO:0045454);P06702(GO:0030307;GO:0050729;GO:0051493;GO:0050727;GO:0045113;GO:0002224;GO:2001244;GO:0006919;GO:0032119;GO:0051092;GO:0002793;GO:0010976;GO:0006417;GO:0030194);P61224(GO:0007165;GO:2000114;GO:0045955;GO:0035722;GO:0032486;GO:0007264;GO:1901888;GO:0070374;GO:2000301);P10643(GO:0030449;GO:0006883;GO:0006956;GO:0006957;GO:0006958);P02787(GO:0055072;GO:0048260;GO:0007257;GO:0045780;GO:2000147;GO:0006879;GO:0045893;GO:0001895;GO:0034756;GO:0042327;GO:0060395;GO:0031647;GO:0070371;GO:0034756);P19827(GO:0010466;GO:0010951);P01833(GO:0001895;GO:0007173;GO:0038093);P01834(GO:0030449;GO:0050871;GO:0050853;GO:0050776;GO:0006956;GO:0006958;GO:0001895;GO:0038096;GO:0038095);P19823(GO:0010951;GO:0010466);P04003(GO:0030449;GO:0045732;GO:1903027;GO:0006958;GO:0045959);E7END6(GO:0007596);P04004(GO:0030449;GO:0014911;GO:0030949;GO:0090303;GO:0048260;GO:0032092;GO:0010811;GO:0010951;GO:0050731;GO:0030195);P00450(GO:0006879;GO:0055072);E7ENL6(GO:0010951);O00391(GO:0016242;GO:0045454);C9JV77(GO:0030500;GO:0030502;GO:0050766;GO:0050727;GO:0046627;GO:0010951);K7ER74(GO:0051006;GO:0010902;GO:0048261;GO:0043085;GO:0032375;GO:0045833;GO:0010898;GO:0070328;GO:0010916;GO:0042632;GO:0010518;GO:0060697;GO:0045723);P69905(GO:0010942);P18206(GO:0030336;GO:0030334);G3XAK1(GO:2000479;GO:0010628;GO:0033601;GO:1904036;GO:0045721;GO:0046425;GO:0048012;GO:0010758);G3V0E5(GO:0045780;GO:0045830;GO:0006879;GO:0030890;GO:0042102);P02768(GO:0051659;GO:0043066;GO:0032460;GO:0001895;GO:0043069);O75636(GO:0006956;GO:1902679;GO:0046597;GO:0001867);O00187(GO:0006956;GO:0001867;GO:0006958);Q96PD5(GO:0032827;GO:0050727;GO:0002221;GO:0032689);A6XND0(GO:0001558);P02763(GO:1904469;GO:0050716;GO:0050718;GO:0032715;GO:0002682;GO:0032720);A0A0B4J1X5(GO:0050853;GO:0006958;GO:0050871);A0A0C4DH73(GO:0038096;GO:0030449;GO:0038095;GO:0050776;GO:0006956;GO:0006958);P00915(GO:0035722);P20742(GO:0010951;GO:0010466);Q9BWP8(GO:0001867;GO:0006956);P07357(GO:0030449;GO:0006956;GO:0006957;GO:0006958);A0A0G2JI36(GO:0060333;GO:0060337;GO:0050776;GO:0001916);Q15166(GO:0010124;GO:0032929);P07358(GO:0030449;GO:0006956;GO:0006957;GO:0006958);P09172(GO:0042127;GO:2001236;GO:0045907;GO:0042593;GO:0006589;GO:0042309;GO:0120162);P01703(GO:0038096;GO:0030449;GO:0050776;GO:0038095;GO:0006956;GO:0006958);Q8IV42(GO:0001514);P01701(GO:0038096;GO:0030449;GO:0050776;GO:0038095;GO:0006956;GO:0006958);P01706(GO:0038096;GO:0030449;GO:0050776;GO:0038095;GO:0006956;GO:0006958);P0C0L5(GO:0030449;GO:0006956;GO:2000427;GO:0010951;GO:0006958);P21333(GO:2000179;GO:0050821;GO:0042177;GO:1905031;GO:0051220;GO:1905000;GO:0043066;GO:0016479;GO:0030334;GO:2001046;GO:0032233;GO:0042307;GO:0043433;GO:0071526;GO:1901381;GO:2001224;GO:1900026;GO:0043123;GO:0007195);P01705(GO:0038096;GO:0030449;GO:0050776;GO:0038095;GO:0006956;GO:0006958);P22792(GO:0030449;GO:0050790;GO:0050821);P23083(GO:0038096;GO:0030449;GO:0050776;GO:0038095;GO:0006956;GO:0006958);P01709(GO:0038096;GO:0030449;GO:0050776;GO:0038095;GO:0006956;GO:0006958);P02741(GO:0010628;GO:0097756;GO:0010745;GO:2000482;GO:0032930;GO:0006958;GO:0010888);P02671(GO:0045907;GO:0007599;GO:0034116;GO:0007596;GO:0045921;GO:1902042;GO:1900026;GO:0002224;GO:2000352;GO:0090277;GO:0042730;GO:0050714;GO:0070374);P02675(GO:1902042;GO:0034116;GO:0045921;GO:0007599;GO:0007596;GO:0045907;GO:1900026;GO:0002224;GO:2000352;GO:0090277;GO:0042730;GO:0050714;GO:0070374);P07996(GO:2001237;GO:0050921;GO:1903671;GO:0051895;GO:0042535;GO:0030511;GO:0002581;GO:0042327;GO:0043536;GO:0040037;GO:2001027;GO:0045727;GO:0045652;GO:0043032;GO:0030194;GO:0002605;GO:0051897;GO:2000379;GO:0007050;GO:0090051;GO:0043154;GO:1902043;GO:0010763;GO:0051918;GO:0008284;GO:2000353;GO:0001953;GO:0016525;GO:0000187;GO:0010751;GO:0045766;GO:0043066;GO:0010748;GO:0032695;GO:1903588;GO:0030335;GO:0048661;GO:0032914;GO:0043537;GO:0010595;GO:0010596;GO:0010757;GO:0010754;GO:0010759;GO:0001937;GO:0071636);P13671(GO:0030449;GO:0045917;GO:0045766;GO:0006956;GO:0001970;GO:0006958);F5H8B0(GO:0007596);P0CF74(GO:0030449;GO:0050853;GO:0050776;GO:0006956;GO:0006958;GO:0050871;GO:0038096;GO:0038095);P61626(GO:0001895);P04180(GO:0090107;GO:0042632);P31151(GO:0010820;GO:0090026;GO:0071624;GO:0051238;GO:0070374);E9PHK0(GO:0010756);O75882(GO:0040014);P22891(GO:0007596;GO:0030195;GO:0007599);Q06033(GO:0010951;GO:0010466);C9JF17(GO:0051895;GO:0010642;GO:2000405;GO:0048662;GO:2000098;GO:0042308;GO:0060588;GO:1900016;GO:0071638);P00739(GO:0010942);P05155(GO:0030449;GO:0007596;GO:0007599;GO:0010466;GO:0001869;GO:0045916;GO:0010951;GO:0006958;GO:0042730;GO:0030193);P05154(GO:0007596;GO:0051346;GO:0010466;GO:0010951;GO:0045861);B4E1Z4(GO:0030449;GO:0006956;GO:0006957);P27169(GO:0034445;GO:0032411;GO:0010875;GO:0051099);I3L1J2(GO:0050728;GO:2000114;GO:0045766;GO:0008285;GO:0007179;GO:1903142);M0R0Q9(GO:0030449;GO:0007165;GO:0048260;GO:0010828;GO:0010866;GO:0045766;GO:1905114;GO:0045745;GO:2000427;GO:0007186;GO:0006956;GO:0006957;GO:0060100;GO:0006958;GO:0050776;GO:0010575;GO:0001798;GO:0010884;GO:0001970;GO:0001934);P01031(GO:0000187;GO:0030449;GO:0007166;GO:0045766;GO:0010760;GO:0007186;GO:0090197;GO:0006956;GO:0006957;GO:0010951;GO:0006958;GO:0010575);O95445(GO:0034445;GO:0042632);A0A075B7F0(GO:0050853;GO:0006958;GO:0050871);P00734(GO:0048712;GO:0030307;GO:0007596;GO:0051281;GO:0030449;GO:0032967;GO:0007166;GO:0007599;GO:0010544;GO:0007186;GO:0010468;GO:1900738;GO:0008284;GO:2000379;GO:0046427;GO:0090218;GO:0008360;GO:0014068;GO:1900016;GO:0042730;GO:1900182;GO:0051480;GO:0010469;GO:0051918;GO:0030193;GO:0045861;GO:0001934;GO:0030194);P13645(GO:0045684);A0A0A0MS14(GO:0050853;GO:0006958;GO:0050871);P35579(GO:0007229;GO:1903923;GO:1903919;GO:1905684;GO:0008360);P01715(GO:0038096;GO:0030449;GO:0050776;GO:0038095;GO:0006956;GO:0006958);P01717(GO:0038096;GO:0030449;GO:0050776;GO:0038095;GO:0006956;GO:0006958);A0A0A0MS15(GO:0050853;GO:0006958;GO:0050871);P01718(GO:0038096;GO:0030449;GO:0050776;GO:0038095;GO:0006956;GO:0006958);P00738(GO:0010942;GO:2000296;GO:0051354);P01857(GO:0030449;GO:0019221;GO:0050853;GO:0006956;GO:0006958;GO:0050871;GO:0038096);E7EQB2(GO:0032680;GO:2000117;GO:0045071;GO:0044793;GO:0045669;GO:1900229;GO:0043123;GO:1902732;GO:0032780;GO:0034145;GO:0051092;GO:0033690;GO:0031665;GO:0001817);P80748(GO:0038096;GO:0030449;GO:0050776;GO:0038095;GO:0006956;GO:0006958);P01019(GO:1903779;GO:0038166;GO:2001238;GO:0007166;GO:0050729;GO:0042127;GO:0032930;GO:0014824;GO:0019229;GO:0010976;GO:0010873;GO:0050731;GO:0035815;GO:0007202;GO:0007204;GO:0003081;GO:1904754;GO:0010536;GO:0007200;GO:0042310;GO:0042311;GO:0048169;GO:1901201;GO:0006883;GO:0061098;GO:0007186;GO:2000379;GO:0090190;GO:0003331;GO:0033864;GO:0007199;GO:0051969;GO:0016525;GO:0030308;GO:0048146;GO:0045429;GO:1905010;GO:0043085;GO:0050880;GO:0002027;GO:0045742;GO:0032270;GO:0008284;GO:0001558;GO:0014068;GO:0014061;GO:0051092;GO:0002034;GO:0070371;GO:0001819;GO:0051387;GO:0010469;GO:0010666;GO:0008217;GO:0097755;GO:0042981;GO:0010744;GO:0035813;GO:1904707;GO:0046628;GO:0034104;GO:0019216;GO:0051403;GO:0010951;GO:0007263;GO:1905589;GO:0010613;GO:0010595;GO:0002019;GO:0002018;GO:0045777;GO:0002016;GO:0051924;GO:1903598;GO:0045893);A0A0S2Z4L3(GO:0030195);A0A0C4DH25(GO:0038096;GO:0030449;GO:0038095;GO:0050776;GO:0006956;GO:0006958);P08185(GO:0010951;GO:0008211);P01011(GO:0010466;GO:0019216;GO:0030277;GO:0010951);P08514(GO:0002687;GO:0007229;GO:0045652);P19652(GO:1904469;GO:0050716;GO:0050718;GO:0002682);P04264(GO:0061436;GO:0050728;GO:0045765;GO:0001867;GO:0001895;GO:0042730);B0YIW2(GO:0051005;GO:0048261;GO:0045833;GO:0010897;GO:0007186;GO:0070328;GO:0060621;GO:0010916;GO:0042632;GO:0032489;GO:0050995;GO:0010989;GO:0010987;GO:0045717;GO:0010903);C9J5S7(GO:0045069);A0A1W2PQU7(GO:1904714;GO:0060291;GO:0051580;GO:0010977;GO:0010625);Q9UGM5(GO:0010951;GO:0010466);H0YGX7(GO:0050790);G3V2W1(GO:0010951;GO:0007596);A0A0A0MTH3(GO:0033209;GO:1901224;GO:0045669;GO:2000178;GO:0008284;GO:0045893;GO:0030513;GO:0043491;GO:0007229;GO:0090263;GO:0001934);C9JXI5(GO:0090263);P07437(GO:0050807;GO:0010389);P13796(GO:0033157;GO:0071803;GO:0035722;GO:0010737);P15144(GO:0008217;GO:0007165);P36955(GO:0010629;GO:0060770;GO:0050769;GO:0071333;GO:0050728;GO:0010976;GO:0010951;GO:1901215;GO:0010596;GO:0016525);P0DJI8(GO:0000187;GO:0019221;GO:0050728;GO:0050708;GO:0045785;GO:0007186;GO:0050716;GO:0050715;GO:0007204);A0A0G2JPR0(GO:0006956;GO:0010951);C9JPQ9(GO:0007596);P09486(GO:0022604;GO:0050807;GO:0042127;GO:0010595;GO:0001937;GO:0016525);P11226(GO:0044130;GO:0050766;GO:0001867;GO:0006956;GO:0006958;GO:0048525);P04211(GO:0038096;GO:0030449;GO:0050776;GO:0038095;GO:0006956;GO:0006958);Q96KN2(GO:0032268);Q13093(GO:0050729;GO:0090026);P43121(GO:0030335);Q96IY4(GO:0007599;GO:2000346;GO:0007596;GO:0030449;GO:0071333;GO:0051918;GO:0042730;GO:0003331;GO:0010757);P01721(GO:0038096;GO:0030449;GO:0050776;GO:0038095;GO:0006956;GO:0006958);A0A0B4J231(GO:0050853;GO:0006958;GO:0050871);P55058(GO:0010875);P01009(GO:0007599;GO:0007596;GO:0010466;GO:0010951);P01008(GO:0007599;GO:0007595;GO:2000266;GO:0007596;GO:0010466;GO:0010951;GO:0030193);A0A075B6R2(GO:0050853;GO:0006958;GO:0050871);Q9UNN8(GO:0007596;GO:0007599;GO:0050819);P02100(GO:0007596);P55056(GO:0070328;GO:0010890);P02751(GO:0019221;GO:0048146;GO:0010628;GO:1904237;GO:0008284;GO:2001202;GO:0008360;GO:0010952;GO:0045773;GO:0001932;GO:0070372);J3KRP0(GO:0032268);P69892(GO:0007596);A0A0C4DH43(GO:0050853;GO:0006958;GO:0050871);P43652(GO:0050821);Q01518(GO:0007165;GO:0007190);P02753(GO:0032024;GO:0060044;GO:0042593;GO:0051024;GO:0042572;GO:0030277);A0A669KAY4(GO:0032805;GO:1905601;GO:0010469;GO:0043523;GO:1905598;GO:0001920;GO:0002092;GO:0042632;GO:2000650;GO:1905596;GO:0010989;GO:0043525);P01344(GO:0046628;GO:0045840;GO:0008286;GO:0038028;GO:0008284;GO:0051147;GO:0031056;GO:0071902;GO:0051897;GO:0006349;GO:0000122;GO:0043085;GO:0045725;GO:0010469;GO:2000467;GO:0042104;GO:0043410;GO:0050731;GO:0040018;GO:0006355;GO:0051781;GO:0045944;GO:0001934);A0A0B4J1X8(GO:0050853;GO:0006958;GO:0050871);P01877(GO:0001895;GO:0050853;GO:0006958;GO:0050871;GO:0060267);P01871(GO:0050853;GO:0006958;GO:0050871);A0A075B7D0(GO:0050853;GO:0006958;GO:0050871);P13473(GO:0050821;GO:0046716;GO:0031647);P02766(GO:0070327;GO:0042572;GO:0010469);P09871(GO:0006956;GO:0030449;GO:0001867;GO:0006958);A0A0B4J1V2(GO:0050853;GO:0006958;GO:0050871);P00918(GO:0038166;GO:0045780;GO:2001225;GO:0032849;GO:2001150;GO:0044070;GO:0032230;GO:0051453;GO:0045672);A0A0C4DH32(GO:0050853;GO:0006958;GO:0050871);P01763(GO:0038096;GO:0030449;GO:0050776;GO:0038095;GO:0006956;GO:0006958);P01704(GO:0038096;GO:0030449;GO:0050776;GO:0038095;GO:0006956;GO:0006958);A0A0A0MRJ7(GO:0007596);P04040(GO:0043066;GO:0014068;GO:0051092;GO:0032088;GO:0051781);A0A0C4DH33(GO:0050853;GO:0006958;GO:0050871);A0A3B3ISR2(GO:0006958);P05543(GO:0070327;GO:0010951);P18065(GO:0007165;GO:0043567;GO:0040008;GO:0090090;GO:0042104;GO:0001558);P01861(GO:0030449;GO:0019221;GO:0050853;GO:0006956;GO:0006958;GO:0050871;GO:0038096);P05546(GO:0007599;GO:0007596;GO:0010951;GO:0010466);P02743(GO:0046597;GO:0044871;GO:0061045;GO:1903016;GO:1903019;GO:0044869;GO:0006958;GO:0002674;GO:0045656;GO:0048525);P55103(GO:0060395;GO:0010469;GO:0010862;GO:0042981;GO:0043408);P01860(GO:0030449;GO:0050871;GO:0050853;GO:0006956;GO:0006958;GO:0001895;GO:0038096);P02747(GO:0030449;GO:0045650;GO:0030853;GO:0006956;GO:0006958);P02745(GO:0030449;GO:0006956;GO:0006958);A0A075B7B8(GO:0050853;GO:0006958;GO:0050871);P01619(GO:0030449;GO:0050776;GO:0006956;GO:0006958;GO:0038096;GO:0038095);P02760(GO:0010951;GO:0046329;GO:0010466;GO:0050777);P02749(GO:0034392;GO:0051006;GO:0051917;GO:0051918;GO:0033033;GO:0010596;GO:0030195;GO:0030193;GO:0016525;GO:0001937;GO:0030194);P80108(GO:0035774;GO:0051044;GO:0051047;GO:0010907;GO:0010867;GO:0043065;GO:0002430;GO:0045919;GO:0010897;GO:0008285;GO:0008286;GO:0010983;GO:0010595;GO:1900076;GO:0010694);P01034(GO:2000117;GO:0043067;GO:0010466;GO:0008284;GO:0034103;GO:0045740;GO:0060548;GO:0060311;GO:0060313;GO:0010716;GO:0010711;GO:0045861);P01601(GO:0038096;GO:0030449;GO:0038095;GO:0050776;GO:0006956;GO:0006958);C9JB55(GO:0048260;GO:0043086;GO:0006879;GO:0034756);P11597(GO:0055091;GO:0010745;GO:0070328;GO:0055088;GO:0042632;GO:0010874);P02649(GO:0051044;GO:0043524;GO:0010873;GO:0051651;GO:0050728;GO:1905855;GO:0060999;GO:0045541;GO:0044794;GO:0043537;GO:0010977;GO:0030516;GO:0010875;GO:0030195;GO:1903002;GO:0019934;GO:0010629;GO:0042311;GO:0048168;GO:0010544;GO:0097006;GO:0007186;GO:1905890;GO:1902430;GO:0042632;GO:0032489;GO:1901215;GO:0006357;GO:0032805;GO:0090209;GO:0051246;GO:0050807;GO:0043407;GO:0051000;GO:1902995;GO:1900221;GO:0090181;GO:0006874;GO:0055088;GO:0055089;GO:0046889;GO:0032269;GO:1900272;GO:1902952;GO:1901630;GO:1901628;GO:0010468;GO:0032462;GO:0090090;GO:2000822;GO:0007263;GO:1905908;GO:1905860;GO:1905906;GO:0045807;GO:0070328;GO:0001937;GO:0010976);Q07954(GO:0051246;GO:0007205;GO:0032370;GO:0010942;GO:0051222;GO:2000587;GO:0045732;GO:0032374;GO:1900223;GO:0014912;GO:1903078;GO:0032429;GO:0032092;GO:1905167;GO:0010977;GO:0010875;GO:0010715;GO:0043524;GO:0030178;GO:0045807;GO:0032956);Q6UXB8(GO:0010466;GO:0061052);P04196(GO:0002839;GO:0030308;GO:0032956;GO:2000504;GO:0007162;GO:0051894;GO:0007599;GO:0043065;GO:0033629;GO:0010468;GO:0051918;GO:0010543;GO:0008285;GO:0007596;GO:0010593;GO:0043537;GO:0010951;GO:0043254;GO:2001027;GO:0042730;GO:0050730;GO:1900747;GO:0030193;GO:0016525);P32119(GO:0000187;GO:0045581;GO:0032088;GO:0042981;GO:0010310;GO:0043066;GO:2001240;GO:0045454;GO:0048872;GO:0031665;GO:0030194);P02647(GO:0034115;GO:0010804;GO:0019915;GO:0050728;GO:0051496;GO:0010898;GO:0030300;GO:0010873;GO:0007179;GO:0045723;GO:0050821;GO:0010903;GO:0007186;GO:0060761;GO:0042632;GO:0032489;GO:0050713;GO:0007229;GO:0060192;GO:0035025;GO:0051006;GO:0002740;GO:1900026;GO:0060354;GO:0070371;GO:0008211;GO:0055091;GO:0070328;GO:0019216;GO:0051346;GO:0051345;GO:0001932);Q9NZP8(GO:0006958);P10909(GO:0032436;GO:1903573;GO:1902949;GO:0042127;GO:0060548;GO:1902004;GO:0030449;GO:1902230;GO:0050821;GO:0010628;GO:0048260;GO:0032760;GO:1905895;GO:0006956;GO:1905892;GO:1901216;GO:0006958;GO:2000060;GO:1902430;GO:1902847;GO:0090201;GO:0045429;GO:1902998;GO:1901214;GO:1900221;GO:0051092;GO:0043065;GO:0032464;GO:0032463;GO:1905907;GO:1905908);P04114(GO:0010628;GO:0010884;GO:0002224;GO:0010744;GO:0042632;GO:0010886;GO:0045540);P05109(GO:0030307;GO:0050729;GO:0051493;GO:0050727;GO:0002224;GO:2001244;GO:0006919;GO:0032119;GO:0051092;GO:0002793);P15169(GO:0030449;GO:0030070);A0A0C4DH31(GO:0050853;GO:0006958;GO:0050871);A0A0C4DH36(GO:0050853;GO:0006958;GO:0050871);A0A0C4DH34(GO:0050853;GO:0006958;GO:0050871);P19320(GO:0019221;GO:0035584;GO:0042102;GO:0050776;GO:0060333);P08697(GO:0032967;GO:0051496;GO:0010466;GO:0010757;GO:0051918;GO:0048661;GO:0045597;GO:0046330;GO:0010951;GO:0042730;GO:2000049;GO:0002034;GO:0045944;GO:0070374;GO:0071636);Q14624(GO:0010951;GO:0010466);P25311(GO:0008285;GO:0001895);A0A0C4DH39(GO:0050853;GO:0006958;GO:0050871);A0A0C4DH38(GO:0050853;GO:0006958;GO:0050871);P01599(GO:0038096;GO:0030449;GO:0050776;GO:0038095;GO:0006956;GO:0006958);P01024(GO:0030449;GO:0007165;GO:0048260;GO:0010828;GO:0010866;GO:0050766;GO:0045766;GO:1905114;GO:0045745;GO:2000427;GO:0007186;GO:0006956;GO:0006957;GO:0010951;GO:0060100;GO:0006958;GO:0050776;GO:0010575;GO:0001798;GO:0010884;GO:0001970;GO:0001934);P01023(GO:0051056;GO:0010466;GO:0001869;GO:0010951;GO:0043547);C9J8S2(GO:0050994;GO:0050921;GO:0045600;GO:0008286;GO:0046626;GO:0010759;GO:0001934);P02748(GO:0030449;GO:0006957;GO:0006958);O95497(GO:0033089;GO:1902176);P02538(GO:0008284;GO:2000536;GO:0001899);P01782(GO:0038096;GO:0030449;GO:0050776;GO:0038095;GO:0006956;GO:0006958);P00747(GO:0007599;GO:0043536;GO:0051918;GO:0051919;GO:0008285;GO:0007596;GO:0010812;GO:0042730;GO:2000048);P00740(GO:0007596;GO:0007599);P60709(GO:0032091;GO:0045815;GO:1903076;GO:0048013;GO:0001895;GO:0038096;GO:0051621;GO:0022898;GO:0051623;GO:0000079);P00742(GO:0030335;GO:0007596;GO:0051897;GO:0007599);P15814(GO:0050853;GO:0006958;GO:0050871);A0A075B6Q5(GO:0050853;GO:0006958;GO:0050871);P01742(GO:0038096;GO:0030449;GO:0050776;GO:0038095;GO:0006956;GO:0006958);P00748(GO:0002542;GO:0007599;GO:0007596;GO:0051919;GO:0042730;GO:0010756;GO:0030193;GO:0030194);Q08380(GO:0007165);Q16610(GO:0007165;GO:0030500;GO:0030502;GO:0045766;GO:0010466;GO:2000404;GO:0001960;GO:0002828;GO:0001938;GO:0006357;GO:0043123);P08519(GO:0010951);P20851(GO:0030449;GO:0007596;GO:0045732;GO:1903027;GO:0006958;GO:0045959);P0C0L4(GO:0030449;GO:2000427;GO:0006956;GO:0010951;GO:0006958);A0A087WSY4(GO:0050853;GO:0006958;GO:0050871);P02652(GO:0060192;GO:0031647;GO:0010903;GO:0010873;GO:0002740;GO:0043085;GO:0032375;GO:0060621;GO:0042632;GO:0050996;GO:0030300;GO:0045416;GO:0019216;GO:0050995;GO:0060695);P35858(GO:0007165);Q15485(GO:0001867;GO:0006956);A0A0G2JL69(GO:0006956;GO:0006958);P04433(GO:0038096;GO:0030449;GO:0050776;GO:0038095;GO:0006956;GO:0006958);P06727(GO:0034445;GO:0045723;GO:0051006;GO:0042632;GO:0070328;GO:0032374;GO:0010898;GO:0055088;GO:0010873;GO:0030300);P04430(GO:0038096;GO:0030449;GO:0050776;GO:0038095;GO:0006956;GO:0006958);A0A0C4DH29(GO:0050853;GO:0006958;GO:0050871);Q6EMK4(GO:0010719;GO:0030512);Q5SRP5(GO:0034445);Q13103(GO:0010951);P01817(GO:0038096;GO:0030449;GO:0050776;GO:0038095;GO:0006956;GO:0006958);P01814(GO:0038096;GO:0030449;GO:0050776;GO:0038095;GO:0006956;GO:0006958);P02750(GO:0045766;GO:0001938;GO:0030511);P02042(GO:0007596);A0A0B4J1V1(GO:0050853;GO:0006958;GO:0050871);A0A0B4J1V0(GO:0050853;GO:0006958;GO:0050871);P07360(GO:0030449;GO:0006957;GO:0006958);A0A0A0MS51(GO:0051127;GO:1902174;GO:0030155;GO:0042989;GO:1903923;GO:0071801;GO:0045010;GO:1903903;GO:1903909;GO:1903906;GO:2001269;GO:0051016;GO:0048015;GO:0051693;GO:0046597;GO:0031648);Q86UX7(GO:0030335;GO:0007229;GO:0033632);P68032(GO:0010628;GO:0043066);H0YAC1(GO:0051919;GO:0042730;GO:0002542);Q96HR3(GO:0030521;GO:0006355;GO:0030518;GO:0045893);P11021(GO:1990440;GO:0035437;GO:0036500;GO:0006983;GO:0043066;GO:0031398;GO:0030335;GO:0036498;GO:0036499;GO:0030512;GO:0030968;GO:0060904;GO:0090074;GO:1903897;GO:1903894;GO:1903895;GO:1903891;GO:0010976);Q9Y490(GO:0036498;GO:0007016;GO:0007229);P08603(GO:1903659;GO:0030449;GO:0006956;GO:0006957);Q15848(GO:0034115;GO:0010804;GO:0050728;GO:0009967;GO:0030853;GO:0043124;GO:0033034;GO:0032720;GO:0043123;GO:2000481;GO:0010875;GO:0045650;GO:0050731;GO:1904753;GO:0045715;GO:0031953;GO:0010906;GO:0046326;GO:0045721;GO:0010739;GO:2000467;GO:2000279;GO:0042304;GO:2000478;GO:0050805;GO:0043407;GO:0010642;GO:0050765;GO:0032270;GO:0045892;GO:0046888;GO:0045599;GO:0120162;GO:0120163;GO:1900121;GO:0045860;GO:0070373;GO:0045923;GO:0042593;GO:2000590;GO:0010745;GO:0010469;GO:1904706;GO:0030336;GO:2000534;GO:0090317;GO:0045776;GO:0071639;GO:2000584;GO:0032757;GO:0001934);P01699(GO:0038096;GO:0030449;GO:0050776;GO:0038095;GO:0006956;GO:0006958);P07737(GO:0050821;GO:0050434;GO:0032232;GO:0032233;GO:0030837;GO:0030838;GO:0051496;GO:0051054;GO:0032781;GO:0060071;GO:0010634;GO:1900029;GO:0051497;GO:0006357;GO:0045944);P35908(GO:0045684) | 59|82 | C9JB55(GO:0048260;GO:0043086;GO:0006879;GO:0034756);O75636(GO:0006956;GO:1902679;GO:0046597;GO:0001867);P19652(GO:1904469;GO:0050716;GO:0050718;GO:0002682);O00187(GO:0006956;GO:0001867;GO:0006958);P04211(GO:0038096;GO:0030449;GO:0050776;GO:0038095;GO:0006956;GO:0006958);K7ERG9(GO:0007219;GO:0006957);P02763(GO:1904469;GO:0050716;GO:0050718;GO:0032715;GO:0002682;GO:0032720);P29622(GO:0010466;GO:0010951);Q9UGM5(GO:0010951;GO:0010466);E7END6(GO:0007596);A0A0C4DH32(GO:0050853;GO:0006958;GO:0050871);P15169(GO:0030449;GO:0030070);K7ER74(GO:0051006;GO:0010902;GO:0048261;GO:0043085;GO:0032375;GO:0045833;GO:0010898;GO:0070328;GO:0010916;GO:0042632;GO:0010518;GO:0060697;GO:0045723);A0A0C4DH34(GO:0050853;GO:0006958;GO:0050871);P32119(GO:0000187;GO:0045581;GO:0032088;GO:0042981;GO:0010310;GO:0043066;GO:2001240;GO:0045454;GO:0048872;GO:0031665;GO:0030194);C9JPQ9(GO:0007596);Q86YZ3(GO:0061436);P01703(GO:0038096;GO:0030449;GO:0050776;GO:0038095;GO:0006956;GO:0006958);P01601(GO:0038096;GO:0030449;GO:0038095;GO:0050776;GO:0006956;GO:0006958);Q96IY4(GO:0007599;GO:2000346;GO:0007596;GO:0030449;GO:0071333;GO:0051918;GO:0042730;GO:0003331;GO:0010757);P01721(GO:0038096;GO:0030449;GO:0050776;GO:0038095;GO:0006956;GO:0006958);P55058(GO:0010875);P01009(GO:0007599;GO:0007596;GO:0010466;GO:0010951);P01008(GO:0007599;GO:0007595;GO:2000266;GO:0007596;GO:0010466;GO:0010951;GO:0030193);Q9NZP8(GO:0006958);P09172(GO:0042127;GO:2001236;GO:0045907;GO:0042593;GO:0006589;GO:0042309;GO:0120162);P55056(GO:0070328;GO:0010890);Q08380(GO:0007165);P02751(GO:0019221;GO:0048146;GO:0010628;GO:1904237;GO:0008284;GO:2001202;GO:0008360;GO:0010952;GO:0045773;GO:0001932;GO:0070372);P02787(GO:0055072;GO:0048260;GO:0007257;GO:0045780;GO:2000147;GO:0006879;GO:0045893;GO:0001895;GO:0034756;GO:0042327;GO:0060395;GO:0031647;GO:0070371;GO:0034756);P01599(GO:0038096;GO:0030449;GO:0050776;GO:0038095;GO:0006956;GO:0006958);P01782(GO:0038096;GO:0030449;GO:0050776;GO:0038095;GO:0006956;GO:0006958);P01780(GO:0038096;GO:0030449;GO:0050776;GO:0038095;GO:0006956;GO:0006958);A0A669KAY4(GO:0032805;GO:1905601;GO:0010469;GO:0043523;GO:1905598;GO:0001920;GO:0002092;GO:0042632;GO:2000650;GO:1905596;GO:0010989;GO:0043525);P01344(GO:0046628;GO:0045840;GO:0008286;GO:0038028;GO:0008284;GO:0051147;GO:0031056;GO:0071902;GO:0051897;GO:0006349;GO:0000122;GO:0043085;GO:0045725;GO:0010469;GO:2000467;GO:0042104;GO:0043410;GO:0050731;GO:0040018;GO:0006355;GO:0051781;GO:0045944;GO:0001934);A0A0C4DH73(GO:0038096;GO:0030449;GO:0038095;GO:0050776;GO:0006956;GO:0006958);I3L1J2(GO:0050728;GO:2000114;GO:0045766;GO:0008285;GO:0007179;GO:1903142);A0A0C4DH25(GO:0038096;GO:0030449;GO:0038095;GO:0050776;GO:0006956;GO:0006958);M0R0Q9(GO:0030449;GO:0007165;GO:0048260;GO:0010828;GO:0010866;GO:0045766;GO:1905114;GO:0045745;GO:2000427;GO:0007186;GO:0006956;GO:0006957;GO:0060100;GO:0006958;GO:0050776;GO:0010575;GO:0001798;GO:0010884;GO:0001970;GO:0001934);P30041(GO:0048026;GO:0045454);Q5SRP5(GO:0034445);P18206(GO:0030336;GO:0030334);P00918(GO:0038166;GO:0045780;GO:2001225;GO:0032849;GO:2001150;GO:0044070;GO:0032230;GO:0051453;GO:0045672);P01817(GO:0038096;GO:0030449;GO:0050776;GO:0038095;GO:0006956;GO:0006958);P01715(GO:0038096;GO:0030449;GO:0050776;GO:0038095;GO:0006956;GO:0006958);P19823(GO:0010951;GO:0010466);P01717(GO:0038096;GO:0030449;GO:0050776;GO:0038095;GO:0006956;GO:0006958);A0A0B4J1V0(GO:0050853;GO:0006958;GO:0050871);P07360(GO:0030449;GO:0006957;GO:0006958);P05543(GO:0070327;GO:0010951);P01857(GO:0030449;GO:0019221;GO:0050853;GO:0006956;GO:0006958;GO:0050871;GO:0038096);P00748(GO:0002542;GO:0007599;GO:0007596;GO:0051919;GO:0042730;GO:0010756;GO:0030193;GO:0030194);Q96HR3(GO:0030521;GO:0006355;GO:0030518;GO:0045893);P02747(GO:0030449;GO:0045650;GO:0030853;GO:0006956;GO:0006958);P80748(GO:0038096;GO:0030449;GO:0050776;GO:0038095;GO:0006956;GO:0006958);P00915(GO:0035722);A0A0G2JL69(GO:0006956;GO:0006958);P01699(GO:0038096;GO:0030449;GO:0050776;GO:0038095;GO:0006956;GO:0006958);E7EUT5(GO:0050821;GO:0052501;GO:0050715;GO:0017148;GO:0006417) | 0.044865412 | 0.125623155 | https://www.ebi.ac.uk/QuickGO/term/GO:0065007 |
| GO:0009987 | cellular process | biological_process | 275|375 | P08779(GO:0051546;GO:0030216;GO:0070268;GO:0007010;GO:0045104);P00488(GO:0018149;GO:0019221;GO:0002576);P68366(GO:0000086;GO:0000226;GO:0097711;GO:0002576;GO:0000278;GO:0007017;GO:0007010);P04275(GO:0002576;GO:0051260;GO:0030168;GO:0030198);A0A0G2JMB2(GO:0050853;GO:0006910;GO:0006911);J3KNB4(GO:0071224;GO:0071222;GO:0071354;GO:0071356;GO:0071347);Q9UHG3(GO:0030327;GO:0030328;GO:0030329);P51884(GO:0018146;GO:0007409;GO:0030199;GO:0030198;GO:0042340);P03950(GO:0006651;GO:0034332;GO:0030154;GO:0090305;GO:0016477;GO:0009303;GO:0090501;GO:0030041;GO:0007154;GO:0001556);G3XAP6(GO:0030509;GO:0030198;GO:0002063;GO:0009306;GO:0006915;GO:0048747;GO:0097084;GO:0030199);P23142(GO:0030198;GO:0007229);P15814(GO:0050853;GO:0006910;GO:0006911;GO:0050900);B7ZKJ8(GO:0030212);A0A0B4J1X8(GO:0050853;GO:0006910;GO:0006911);P19652(GO:0002576;GO:0043312);B1AHL2(GO:0030198);P08571(GO:0071222;GO:0071223;GO:0097190;GO:0007166;GO:0071727;GO:0071726;GO:0071219;GO:0002224;GO:0070266;GO:0038124;GO:0006915;GO:0035666;GO:0034142;GO:0002755;GO:0002756;GO:0038123;GO:0031663;GO:0007249;GO:0043312);P68871(GO:0042744;GO:0098869;GO:0051291;GO:0043312);A0A0B4J1V2(GO:0050853;GO:0006910;GO:0006911);E9PK25(GO:0030010;GO:0030836;GO:0000281;GO:0035722;GO:0006468;GO:0030036;GO:0001755;GO:0007266;GO:0030043;GO:0030042;GO:0007010);O75460(GO:0016310;GO:0034620;GO:0046777;GO:0036289;GO:0098787;GO:0071333;GO:0006402;GO:0006468;GO:0006397;GO:0007050;GO:0090502;GO:0036498;GO:0006351;GO:0030968;GO:0070059;GO:0070054;GO:1990579;GO:0006379;GO:0035924;GO:0006915;GO:0034976);Q86YZ3(GO:0043163;GO:0043312);P35527(GO:0045109;GO:0070268);P01042(GO:0044267;GO:0002576;GO:0007186;GO:0043687;GO:0007204);A0A087X1J7(GO:0051289;GO:0098869);P01602(GO:0038096;GO:0038095;GO:0050900);P01601(GO:0038096;GO:0038095;GO:0050900);A0A0B4J1U7(GO:0050853;GO:0006910;GO:0006911);P59665(GO:0071222;GO:0030520;GO:0010818;GO:0043312);P0DJI8(GO:0019221;GO:0048246;GO:0048247;GO:0044267;GO:0007186;GO:0030593;GO:0030168;GO:0007204);P06312(GO:0038096;GO:0038095;GO:0050900);P02750(GO:0050873;GO:0043312);P02790(GO:0020027;GO:0042168;GO:0006879);P60763(GO:0030031;GO:0030036;GO:0031175;GO:0035556;GO:0030838;GO:0000902;GO:0051932;GO:0016477;GO:0021894;GO:0007266;GO:0007264;GO:0007015;GO:0043652);P01763(GO:0038096;GO:0038095;GO:0050900);P01764(GO:0050900;GO:0050853;GO:0006910;GO:0006911;GO:0038096;GO:0038095);A0A087X0S5(GO:0071230;GO:0070208;GO:0003429;GO:0030198;GO:0035987;GO:0001649);P01766(GO:0038096;GO:0038095;GO:0050900);Q15582(GO:0044267;GO:0002062;GO:0030198);F8WF14(GO:0050783;GO:0014016;GO:0019695);A0A075B7D8(GO:0050853;GO:0006910;GO:0006911);P26038(GO:0050900;GO:0061028;GO:0042098;GO:0035722;GO:0022614;GO:0071394;GO:0008361;GO:0045198;GO:0001771;GO:0072678;GO:0007010);P01782(GO:0038096;GO:0038095;GO:0050900);P18428(GO:0071222;GO:0071223;GO:0019221;GO:0002281;GO:0002224;GO:0034142;GO:0031663;GO:0002232);P01780(GO:0038096;GO:0038095;GO:0050900);A0A140T8Y3(GO:0030199;GO:0030198);P02776(GO:0070098;GO:0071222;GO:0019221;GO:0002576;GO:0007189;GO:0007186;GO:0030595;GO:0030593;GO:0030168);P02775(GO:0070098;GO:0071222;GO:0060326;GO:0002576;GO:0007186;GO:0030595;GO:0030593;GO:0043312);P13671(GO:0019835);Q13201(GO:0002576);P01833(GO:0043312;GO:0007173;GO:0038093);E7EUT5(GO:0000226;GO:0006096;GO:0035606;GO:0051402;GO:0071346);A0A0U1RQV3(GO:0007173);P30041(GO:0042744;GO:0045454;GO:0098869;GO:0034599;GO:0043312;GO:0046475);P06702(GO:0030595;GO:0030593;GO:0031532;GO:0002224;GO:0035606;GO:0018119;GO:0006914;GO:0006915;GO:0007267;GO:0014002;GO:0002523;GO:0043312;GO:0098869);Q9Y5Y7(GO:0030214);P61224(GO:0030033;GO:0007165;GO:0061028;GO:0035722;GO:0032486;GO:0007264;GO:0071320;GO:0043312);P10643(GO:0019835;GO:0006883);P02787(GO:0061024;GO:0002576;GO:0044267;GO:0043687;GO:0006879;GO:0007015;GO:0030316;GO:0071281;GO:0060395;GO:0070371);P19827(GO:0030212);P35908(GO:0018149;GO:0051546;GO:0032980;GO:0070268;GO:0045109;GO:0003334);P01834(GO:0050853;GO:0006910;GO:0006911;GO:0038096;GO:0038095;GO:0050900);P19823(GO:0044267;GO:0030212;GO:0043687);P01008(GO:0043687;GO:0044267);P04004(GO:0016477;GO:0048709;GO:0035987;GO:0051258;GO:0030198);P01743(GO:0038096;GO:0038095;GO:0050900);A0A0B4J231(GO:0050853;GO:0006910;GO:0006911);O00391(GO:0044267;GO:0043687;GO:0045454;GO:0043312;GO:0085029;GO:0002576);K7ER74(GO:0034375;GO:0034372;GO:0034371;GO:0034370;GO:0034378;GO:0001523);P69905(GO:0042744;GO:0098869;GO:0051291);G3XAK1(GO:0030317;GO:0071456;GO:0048012);G3V0E5(GO:0035690;GO:1990830;GO:0006879;GO:0030316;GO:0031623);P02768(GO:0034375;GO:0051659;GO:0044267;GO:0019836;GO:0002576;GO:0098869;GO:0043687;GO:0009267);O75636(GO:0043654);Q96PD5(GO:0002221;GO:0001519);Q86UX7(GO:0034446;GO:0007229;GO:0033622;GO:0002576);P02763(GO:0002576;GO:0043312);A0A087WSY4(GO:0050853;GO:0006910;GO:0006911);Q01518(GO:0008154;GO:0007010;GO:0000902;GO:0007163;GO:0043312;GO:0007165;GO:0030036;GO:0001667);A0A0C4DH73(GO:0038096;GO:0038095;GO:0050900);P02766(GO:0044267;GO:0043312;GO:0001523;GO:0030198;GO:0042572;GO:0006144);Q9H4B7(GO:0007017;GO:0000278;GO:0000226;GO:0051225);Q9BWP8(GO:0097194);P07357(GO:0019835);P37802(GO:0002576;GO:0030855);A0A0G2JI36(GO:0060333;GO:0060337;GO:0036037);Q15166(GO:0046395;GO:0019372;GO:0016311;GO:0019439;GO:0046226;GO:0010124);J3QT83(GO:0003429);P29622(GO:0002576);P09172(GO:0050900;GO:0042423;GO:0042420;GO:0042421;GO:0006589;GO:0007268);P01703(GO:0038096;GO:0038095;GO:0050900);P01700(GO:0038096;GO:0038095;GO:0050900);P01701(GO:0038096;GO:0038095;GO:0050900);P01706(GO:0038096;GO:0038095;GO:0050900);P21333(GO:0030030;GO:0044319;GO:0031532;GO:0002576;GO:0034329;GO:0042789;GO:0060271;GO:0051764;GO:0030168;GO:0071526;GO:0021943;GO:0090307;GO:0097368;GO:0007195);P01705(GO:0038096;GO:0038095;GO:0050900);Q8N1N4(GO:0070268);P23083(GO:0038096;GO:0038095;GO:0050900);P01709(GO:0038096;GO:0038095;GO:0050900);A0A0C4DH38(GO:0050853;GO:0006910;GO:0006911);P02671(GO:0034622;GO:0044267;GO:0002576;GO:0065003;GO:0030198;GO:0002224;GO:0043687;GO:0030168;GO:0051258);P02675(GO:0034622;GO:0044320;GO:0002576;GO:0030198;GO:0002224;GO:0030168;GO:0051258;GO:0071347);P07996(GO:0034605;GO:0043652;GO:0030198;GO:0018149;GO:0071356;GO:0016477;GO:0007050;GO:0034976;GO:0071363;GO:0002576);H3BTN5(GO:0016310;GO:0006096);P0CF74(GO:0050900;GO:0050853;GO:0006910;GO:0006911;GO:0038096;GO:0038095);P61626(GO:0019835;GO:0044267;GO:0016998;GO:0043312);A0A0C4DH43(GO:0050853;GO:0006910;GO:0006911);P04217(GO:0002576;GO:0043312);P04180(GO:0034375;GO:0006656;GO:0034372;GO:0042158;GO:0034435;GO:0006644;GO:0046470);P31151(GO:0030216;GO:0043312);Q16880(GO:0030913;GO:0006682;GO:0006687;GO:0006665;GO:0007010;GO:0048812);Q06033(GO:0030212;GO:0002576);C9JF17(GO:0014012);P05155(GO:0002576);P05154(GO:0007342);I3L1J2(GO:0034332;GO:0007043;GO:0000902;GO:0007179);M0R0Q9(GO:0006631;GO:0007165;GO:0044267;GO:1905114;GO:0007186;GO:0043687;GO:0016322;GO:0150062;GO:0043312);O95445(GO:0034375;GO:0001523;GO:0098869;GO:0034380);Q9UGM5(GO:0007339);A0A075B7F0(GO:0050853;GO:0006910;GO:0006911);P00734(GO:0051281;GO:0007166;GO:0044267;GO:0051838;GO:0007186;GO:0030168;GO:0051480);P13645(GO:0018149;GO:0030216;GO:0070268;GO:0051290);A0A0A0MS14(GO:0050853;GO:0006910;GO:0006911);P13647(GO:0007010;GO:0031581;GO:0070268);P13473(GO:0061684;GO:0097352;GO:0046716;GO:0006914;GO:1905146;GO:0009267;GO:0043312;GO:0002576);P01717(GO:0038096;GO:0038095;GO:0050900);A0A0A0MS15(GO:0050853;GO:0006910;GO:0006911);P01718(GO:0038096;GO:0038095;GO:0050900);P00738(GO:0098869;GO:0043312);P01857(GO:0019221;GO:0050853;GO:0006910;GO:0006911;GO:0038096);P07358(GO:0019835);P16930(GO:0006572;GO:0006559;GO:0006527;GO:0009072;GO:1902000);P01019(GO:0038166;GO:0007166;GO:0007204;GO:0034374;GO:0007200;GO:0071260;GO:0006883;GO:0007186;GO:0007199;GO:0007267;GO:1904385;GO:0070371;GO:0061049;GO:0050663;GO:0051403;GO:0007263);A0A0C4DH25(GO:0038096;GO:0038095;GO:0050900);A0A0G2JMI3(GO:0050853;GO:0006910;GO:0006911);P01011(GO:0002576;GO:0043312);P08514(GO:0002576;GO:0007229;GO:0030198);P41222(GO:0006633;GO:0006631;GO:0019371;GO:0001516;GO:0006693);K7ERG9(GO:0007219);Q96IY4(GO:0071333);P04430(GO:0038096;GO:0038095;GO:0050900);A0A1W2PQU7(GO:0061564;GO:0045109;GO:0060020;GO:0030198;GO:0014002;GO:0031102);Q8IV42(GO:0016310;GO:0097056;GO:0001514;GO:0006412);A0A0A0MTH3(GO:0018105;GO:0034446;GO:0030030;GO:0033209;GO:0045197;GO:0022011;GO:0010761;GO:0006468;GO:0043491;GO:0007229);O14791(GO:0019835;GO:0044267;GO:0043687);P13796(GO:0002286;GO:0016477;GO:0022617;GO:0035722;GO:0051017;GO:0051764;GO:0051639;GO:0044319;GO:0010737);P15144(GO:0030154;GO:0007165;GO:0007267;GO:0043312;GO:0043171);P36955(GO:0071300;GO:0071333;GO:0071279;GO:0071549);C9JC84(GO:0051258;GO:0030168);B0YIW2(GO:0034375;GO:0034371;GO:0034379;GO:0034378;GO:0001523;GO:0007186;GO:0006641;GO:0019433);C9JPQ9(GO:0051258;GO:0030168);P09486(GO:0030198;GO:0071363;GO:0002576);Q16610(GO:0007165;GO:0002576;GO:0002063);P18206(GO:0048675;GO:0034333;GO:0030032;GO:0002576;GO:0043312;GO:0043297);P0DPH7(GO:0007017;GO:0007010;GO:0000278;GO:0000226);Q13093(GO:0034441;GO:0034440;GO:0034374;GO:0046469);P14151(GO:0050900;GO:0043312);P01721(GO:0038096;GO:0038095;GO:0050900);A0A2R8Y6G6(GO:0006096);P55058(GO:0034375;GO:0030317;GO:0010189);P01009(GO:0048208;GO:0044267;GO:0002576;GO:0043687;GO:0043312);P02751(GO:0018149;GO:0019221;GO:0034446;GO:0050900;GO:0044267;GO:0002576;GO:0030198;GO:0033622;GO:0043687;GO:0035987;GO:1901166;GO:0007044);A0A075B6R2(GO:0050853;GO:0006910;GO:0006911);P02760(GO:0042167;GO:0018298);P02100(GO:0051291;GO:0098869;GO:0042744);P55056(GO:0034379);P68032(GO:0030240;GO:0043503;GO:0031032;GO:0071417;GO:0033275;GO:0055003;GO:0048741;GO:0030049;GO:0030048;GO:0070252);J3QRV5(GO:0006887;GO:0007049;GO:0051301);P69892(GO:0098869;GO:0042744);H9KV75(GO:0030036;GO:0048741;GO:0051017;GO:0045214;GO:0051764;GO:0007030);J3KPS3(GO:0051289;GO:0006096);P33908(GO:1904381;GO:0006491;GO:0006486);E9PHK0(GO:0002576;GO:0071560;GO:0071310);P02753(GO:0042572;GO:0001523);A0A669KAY4(GO:0032802;GO:0032869;GO:0044267;GO:0043687;GO:0006644;GO:0030182;GO:0006641;GO:0006915;GO:0009267;GO:0022008);P01344(GO:0051146;GO:0008286;GO:0038028;GO:0001649;GO:0044267;GO:0006349;GO:0002576);P27169(GO:0016311;GO:0046434;GO:0019372;GO:0046395;GO:0046470;GO:0019439);P35579(GO:0031032;GO:0007229;GO:0030224;GO:0030220;GO:0001768;GO:0031532;GO:0045055;GO:0050900;GO:0032796;GO:0032506;GO:0051295;GO:0000212;GO:0006911;GO:0030048;GO:0043534;GO:0007520;GO:0001778;GO:0000904);P01871(GO:0050900;GO:0050853;GO:0006910;GO:0006911);A0A075B7D0(GO:0050853;GO:0006910;GO:0006911);P01715(GO:0038096;GO:0038095;GO:0050900);O75882(GO:0042552);P01619(GO:0050900;GO:0038096;GO:0038095);P00918(GO:0038166;GO:0032849;GO:0051453;GO:0071498);P01597(GO:0038096;GO:0038095;GO:0050900);A0A0B4J1X5(GO:0050853;GO:0006910;GO:0006911);Q6EMK4(GO:0071456);A0A0A0MRJ7(GO:0048208;GO:0044267;GO:0002576;GO:0043687);P04040(GO:0020027;GO:0051289;GO:0009060;GO:0001649;GO:0051262;GO:0098869;GO:0006641;GO:0006625;GO:0071363;GO:0034599;GO:0043312;GO:0042744);A0A0C4DH33(GO:0050853;GO:0006910;GO:0006911);P18065(GO:0007165;GO:0044267;GO:0032870);P00915(GO:0006730;GO:0035722);P05546(GO:0044267;GO:0043687);P02743(GO:0006457;GO:0044267;GO:0051131);P55103(GO:0060395;GO:0048468);P01860(GO:0050853;GO:0006910;GO:0006911;GO:0038096);D6RE82(GO:0006364);P02745(GO:0007267);A0A075B7B8(GO:0050853;GO:0006910;GO:0006911);P01031(GO:0007166;GO:0060326;GO:0019835;GO:0007186);P02749(GO:0002576;GO:0006641);P80108(GO:0035690;GO:0032869;GO:0002430;GO:0006501;GO:0097241;GO:0006507;GO:0002062;GO:0035701;GO:0002042;GO:0008286;GO:0071397;GO:0071277;GO:0071401;GO:0071467;GO:0046470);P01034(GO:0060009;GO:0070301;GO:0044267;GO:0043687;GO:0006915;GO:0001775;GO:0034599;GO:0043312;GO:0097435);A0A087WZB5(GO:0031532);C9JB55(GO:0006879;GO:0071281);P11597(GO:0034375;GO:0034374;GO:0034372;GO:0006641;GO:0046470);A0A0C4DH32(GO:0050853;GO:0006910;GO:0006911);P02649(GO:0051651;GO:0098869;GO:0031102;GO:0019433;GO:0034375;GO:0034374;GO:0034372;GO:0034371;GO:0019934;GO:0042158;GO:0044267;GO:0007186;GO:0034380;GO:0006641;GO:0006874;GO:0043687;GO:0034378;GO:0007271;GO:0001523;GO:0007263;GO:0007010);P04211(GO:0038096;GO:0038095;GO:0050900);P04196(GO:0051715;GO:0002576;GO:0030168);P32119(GO:0042744;GO:0034599;GO:0042098;GO:0045454;GO:0045321;GO:0019430);P02647(GO:0031102;GO:0043534;GO:0007179;GO:0018206;GO:0019433;GO:0034375;GO:0034371;GO:0034378;GO:0042158;GO:0044267;GO:0014012;GO:0007186;GO:0018158;GO:0007229;GO:0034380;GO:0070371;GO:0006656;GO:0006644;GO:0001523;GO:0002576;GO:0043687);P01861(GO:0019221;GO:0050853;GO:0006910;GO:0006911;GO:0038096);P10909(GO:0000902;GO:0051131;GO:0001774;GO:0061077;GO:0032286;GO:0002576;GO:0006915;GO:0001836);P04114(GO:0034374;GO:0034371;GO:0061024;GO:0050900;GO:0034378;GO:0001523;GO:0071356;GO:0071379;GO:0042158;GO:0002224;GO:0043687;GO:0006642;GO:0044267;GO:0030317;GO:0034379;GO:0019433);P05109(GO:0043312;GO:0014002;GO:0002224;GO:0030593;GO:0006914;GO:0006915;GO:0018119;GO:0002523);P15169(GO:0010815;GO:0006518;GO:0030070);A0A0C4DH31(GO:0050853;GO:0006910;GO:0006911);A0A0C4DH36(GO:0050853;GO:0006910;GO:0006911);A0A0C4DH34(GO:0050853;GO:0006910;GO:0006911);P19320(GO:0060945;GO:0060326;GO:0019221;GO:0071356;GO:0035584;GO:1904646;GO:0022614;GO:0030183;GO:0060333;GO:0030198;GO:0035924);P08697(GO:0002576;GO:0030199);Q14624(GO:0030212;GO:0002576);P25311(GO:0090501);A0A0C4DH39(GO:0050853;GO:0006910;GO:0006911);O95497(GO:0015939;GO:0043312);P01599(GO:0038096;GO:0038095;GO:0050900);O43866(GO:0006915);P01024(GO:0006631;GO:0007165;GO:0044267;GO:1905114;GO:0007186;GO:0043687;GO:0016322;GO:0006911;GO:0150062;GO:0043312);P01023(GO:0048863;GO:0002576;GO:0022617);C9J8S2(GO:0030154;GO:0001523;GO:0002576;GO:0008286);P02748(GO:0019835;GO:0019836;GO:0051260);Q07954(GO:0007205;GO:0150094;GO:0001523;GO:0002265;GO:1904646;GO:0031623);P02538(GO:0007010;GO:0030154;GO:0070268;GO:0051801);P00747(GO:0044267;GO:0022617;GO:0002576);Q96HR3(GO:0030521;GO:0030518;GO:0006367;GO:0016567;GO:0006351);P60709(GO:0043044;GO:0072749;GO:0061024;GO:0016579;GO:0048013;GO:0034329;GO:0038096;GO:0048870;GO:0098974);P06331(GO:0038096;GO:0038095;GO:0050900);A0A075B6Q5(GO:0050853;GO:0006910;GO:0006911);P02533(GO:0045110;GO:0030855;GO:0031581;GO:0070268);P01742(GO:0038096;GO:0038095;GO:0050900);P00450(GO:0044267;GO:0006879;GO:0043687);Q08380(GO:0002576;GO:0007165);P80748(GO:0038096;GO:0038095;GO:0050900);P08519(GO:0034374);P43251(GO:0006768);P04264(GO:0018149;GO:0051290;GO:0070268;GO:0043312);P0C0L4(GO:0044267;GO:0043687);C9JV77(GO:0044267;GO:0002576;GO:0043687;GO:0043312);P02652(GO:0034375;GO:0034374;GO:0034371;GO:0006656;GO:0001523;GO:0009395;GO:0044267;GO:0018158;GO:0006641;GO:0043687;GO:0034378;GO:0046340;GO:0018206;GO:0034380;GO:0034370);P35858(GO:0044267;GO:0007165);Q15485(GO:0043654);A0A096LPE2(GO:0060326);P04433(GO:0038096;GO:0038095;GO:0050900);P06727(GO:0034375;GO:0034372;GO:0034371;GO:0034380;GO:0034378;GO:0042744;GO:0001523;GO:0044267;GO:0065005;GO:0031102;GO:0046470;GO:0019430);C9J5S7(GO:0000413;GO:0006457);A0A0C4DH29(GO:0050853;GO:0006910;GO:0006911);Q5SRP5(GO:0034375;GO:0034380);Q13103(GO:0044267;GO:0002576;GO:0043687);P01817(GO:0038096;GO:0038095;GO:0050900);P01814(GO:0038096;GO:0038095;GO:0050900);P01704(GO:0038096;GO:0038095;GO:0050900);A0A0B4J1V1(GO:0050853;GO:0006910;GO:0006911);A0A0B4J1V0(GO:0050853;GO:0006910;GO:0006911);P07360(GO:0019835);A0A0A0MS51(GO:0051127;GO:0030041;GO:0042989;GO:1990000;GO:0045010;GO:0097284;GO:0060271;GO:0006915;GO:0051016;GO:0051014;GO:0014003;GO:0006911;GO:0048015;GO:0051693;GO:0090527;GO:0071346;GO:0071276);P07437(GO:0000086;GO:0051225;GO:0051301;GO:0097711;GO:0000278;GO:0009987;GO:0007017;GO:0043312;GO:0000226);H0YAC1(GO:0022617);P01877(GO:0050900;GO:0050853;GO:0006910;GO:0006911);P11021(GO:1990090;GO:0071480;GO:1990440;GO:0035437;GO:0030433;GO:0036500;GO:0042149;GO:0035690;GO:0006983;GO:0071353;GO:0071236;GO:0030182;GO:0071277;GO:0036498;GO:0036499;GO:0051402;GO:0030968;GO:0071287;GO:0071320;GO:0034976;GO:0034975);Q9Y490(GO:0007043;GO:0002576;GO:0033622;GO:0036498;GO:0007016;GO:0030866;GO:0007044;GO:0007229);Q15848(GO:0071320;GO:0070208;GO:0071872;GO:0019395;GO:0006635;GO:0050873;GO:0035690;GO:0032869;GO:0006091;GO:0051260);P01699(GO:0038096;GO:0038095;GO:0050900);P07737(GO:0060074;GO:0030837;GO:0030838;GO:0060071;GO:0071363;GO:0098885;GO:0030036);P02042(GO:0098869;GO:0042744) | 59|82 | C9JB55(GO:0006879;GO:0071281);O75636(GO:0043654);P19652(GO:0002576;GO:0043312);A0A0C4DH34(GO:0050853;GO:0006910;GO:0006911);P04211(GO:0038096;GO:0038095;GO:0050900);J3QRV5(GO:0006887;GO:0007049;GO:0051301);K7ERG9(GO:0007219);P02763(GO:0002576;GO:0043312);A0A0C4DH73(GO:0038096;GO:0038095;GO:0050900);Q9UGM5(GO:0007339);P00915(GO:0006730;GO:0035722);A0A0C4DH32(GO:0050853;GO:0006910;GO:0006911);P15169(GO:0010815;GO:0006518;GO:0030070);K7ER74(GO:0034375;GO:0034372;GO:0034371;GO:0034370;GO:0034378;GO:0001523);P37802(GO:0002576;GO:0030855);P32119(GO:0042744;GO:0034599;GO:0042098;GO:0045454;GO:0045321;GO:0019430);J3QT83(GO:0003429);Q5SRP5(GO:0034375;GO:0034380);C9JPQ9(GO:0051258;GO:0030168);P09172(GO:0050900;GO:0042423;GO:0042420;GO:0042421;GO:0006589;GO:0007268);P01703(GO:0038096;GO:0038095;GO:0050900);P35527(GO:0045109;GO:0070268);P01008(GO:0043687;GO:0044267);P01601(GO:0038096;GO:0038095;GO:0050900);Q96IY4(GO:0071333);P01721(GO:0038096;GO:0038095;GO:0050900);P55058(GO:0034375;GO:0030317;GO:0010189);P01009(GO:0048208;GO:0044267;GO:0002576;GO:0043687;GO:0043312);P02751(GO:0018149;GO:0019221;GO:0034446;GO:0050900;GO:0044267;GO:0002576;GO:0030198;GO:0033622;GO:0043687;GO:0035987;GO:1901166;GO:0007044);Q86YZ3(GO:0043163;GO:0043312);P55056(GO:0034379);Q08380(GO:0002576;GO:0007165);P43251(GO:0006768);P02787(GO:0061024;GO:0002576;GO:0044267;GO:0043687;GO:0006879;GO:0007015;GO:0030316;GO:0071281;GO:0060395;GO:0070371);P01599(GO:0038096;GO:0038095;GO:0050900);P01782(GO:0038096;GO:0038095;GO:0050900);P01780(GO:0038096;GO:0038095;GO:0050900);A0A669KAY4(GO:0032802;GO:0032869;GO:0044267;GO:0043687;GO:0006644;GO:0030182;GO:0006641;GO:0006915;GO:0009267;GO:0022008);P01344(GO:0051146;GO:0008286;GO:0038028;GO:0001649;GO:0044267;GO:0006349;GO:0002576);P29622(GO:0002576);I3L1J2(GO:0034332;GO:0007043;GO:0000902;GO:0007179);A0A0C4DH25(GO:0038096;GO:0038095;GO:0050900);M0R0Q9(GO:0006631;GO:0007165;GO:0044267;GO:1905114;GO:0007186;GO:0043687;GO:0016322;GO:0150062;GO:0043312);P30041(GO:0042744;GO:0045454;GO:0098869;GO:0034599;GO:0043312;GO:0046475);Q9Y5Y7(GO:0030214);P18206(GO:0048675;GO:0034333;GO:0030032;GO:0002576;GO:0043312;GO:0043297);P00918(GO:0038166;GO:0032849;GO:0051453;GO:0071498);P01817(GO:0038096;GO:0038095;GO:0050900);P01715(GO:0038096;GO:0038095;GO:0050900);P19823(GO:0044267;GO:0030212;GO:0043687);P01717(GO:0038096;GO:0038095;GO:0050900);A0A0B4J1V0(GO:0050853;GO:0006910;GO:0006911);P07360(GO:0019835);P01857(GO:0019221;GO:0050853;GO:0006910;GO:0006911;GO:0038096);A0A087WZB5(GO:0031532);Q96HR3(GO:0030521;GO:0030518;GO:0006367;GO:0016567;GO:0006351);P80748(GO:0038096;GO:0038095;GO:0050900);P01699(GO:0038096;GO:0038095;GO:0050900);E7EUT5(GO:0000226;GO:0006096;GO:0035606;GO:0051402;GO:0071346) | 0.105483831 | 0.184596704 | https://www.ebi.ac.uk/QuickGO/term/GO:0009987 |
| GO:0044464 | cell part | cellular_component | 298|375 | P08779(GO:0005856;GO:0005634;GO:0005882;GO:0005829);P00488(GO:0005737;GO:0031093);P68366(GO:0005874;GO:0005737;GO:0005856;GO:0015630;GO:0005829);P04275(GO:0005783;GO:0031091;GO:0031093;GO:0033093);J3KNB4(GO:0005737;GO:0042995;GO:0042581);P51884(GO:0043202;GO:0005796);P03950(GO:0030426;GO:0005730;GO:0031410;GO:0005634;GO:0043025);A0A075B7D0(GO:0009897);P01704(GO:0005886);A0A0A0MRZ9(GO:0005886);P15814(GO:0009897);B7ZKJ8(GO:0005737;GO:0005886);P0DP02(GO:0005886);A0A0B4J1X8(GO:0009897;GO:0005886);P19652(GO:0031093;GO:0035578;GO:0035580);P08571(GO:0009897;GO:0031362;GO:0010008;GO:0030667;GO:0009986;GO:0005794;GO:0005886);P68871(GO:1904813;GO:0071682;GO:0005833;GO:1904724;GO:0005829);P01877(GO:0009897;GO:0005886);Q8N1N4(GO:0005829;GO:0045095;GO:0005882);O75460(GO:0005739;GO:0005783;GO:0005789;GO:1990604;GO:0030176;GO:0005637;GO:0005737;GO:1990332);Q86YZ3(GO:0005737;GO:0005634;GO:0035578;GO:0048471;GO:0036457;GO:0001533);P35527(GO:0005829;GO:0005882;GO:0005634);P01042(GO:0005788;GO:0031093;GO:0005886);P01597(GO:0005886);P43251(GO:0005759);P01602(GO:0005886);A0A0G2JMI3(GO:0009897;GO:0005886);A0A0B4J1U7(GO:0009897;GO:0005886);P59665(GO:0035578;GO:0005796);P0DJI8(GO:0071682;GO:0005881);Q96KN2(GO:0005829);A0A087WSY4(GO:0009897;GO:0005886);P01743(GO:0005886);A0A0A0MT36(GO:0005886);B0YIW2(GO:0005769);P02790(GO:0071682);P60763(GO:0042995;GO:0030426;GO:0071944;GO:0005737;GO:0043005;GO:0048471;GO:0043025;GO:0031941;GO:0030027;GO:0012505;GO:0005886;GO:0005829;GO:0005856;GO:0005622);A0A075B7D8(GO:0009897);P01764(GO:0009897;GO:0005886);A0A087X0S5(GO:0005788;GO:0005765;GO:0042383);P01766(GO:0005886);A0A087WWU8(GO:0005829;GO:0015629);F8WF14(GO:0005783;GO:0005788;GO:0005641);P07358(GO:0005579);P0DOY3(GO:0005886);P26038(GO:0030175;GO:0005737;GO:0045177;GO:0042995;GO:0043209;GO:0005634;GO:0048471;GO:0005856;GO:0016324;GO:0009986;GO:0071944;GO:0016323;GO:0005886;GO:0031528;GO:0071437;GO:0005829;GO:0005902;GO:0001931;GO:0031143);P29622(GO:0031089);P18428(GO:0009986);P01780(GO:0005886);P06331(GO:0005886);P02776(GO:0005737;GO:0031093);A0A1W2PQU7(GO:0005737;GO:0045111;GO:0044297;GO:0005883;GO:0005882;GO:0097450);P13671(GO:0005579);Q13201(GO:0031093);P01833(GO:0005887;GO:0005886;GO:0035577);G3XAK1(GO:0005737;GO:0005773);E7EUT5(GO:0005737;GO:0005634;GO:0005811;GO:0015630;GO:0031965;GO:0005886;GO:0043231;GO:0005829);A0A0G2JMB2(GO:0009897);A0A0J9YXX1(GO:0005886);P30041(GO:0035578;GO:0005737;GO:0005634;GO:0048471;GO:0005829;GO:0005764);P06702(GO:0005737;GO:0005634;GO:0005654;GO:0034774;GO:0005856;GO:0005886;GO:0005829);Q9Y5Y7(GO:0071944;GO:0005887;GO:0005886);P61224(GO:0005811;GO:0005737;GO:0070382;GO:0035577;GO:0005886;GO:0005829;GO:0005622);P10643(GO:0005579);P02787(GO:0055037;GO:0005788;GO:0030139;GO:0005905;GO:1990712;GO:0010008;GO:0030665;GO:0031232;GO:0031410;GO:0005770;GO:0045178;GO:0009925;GO:0048471;GO:0009986;GO:0016324;GO:0034774;GO:0005768;GO:0005769);P35908(GO:0045095;GO:0005634;GO:0005882;GO:0001533;GO:0005829);P01834(GO:0009897;GO:0005886);P19823(GO:0005788);P04003(GO:0005886);P01718(GO:0005886);P04004(GO:0005783;GO:0005737;GO:0005796;GO:0048237;GO:0043231;GO:0071062);P00748(GO:0005791;GO:0005886);P20851(GO:0005886);Q9UNN8(GO:0005813;GO:0009986;GO:0005887;GO:0005886;GO:0048471);O00391(GO:0005788;GO:0030173;GO:0031093;GO:0035580;GO:0005794;GO:1904724;GO:0043231;GO:0000139);K7ER74(GO:0005769);P69905(GO:0022627;GO:0071682;GO:0005833;GO:0005829);A0A0J9YX35(GO:0005886);P15144(GO:0009897;GO:0030667;GO:0005793;GO:0005886;GO:0005765);G3V0E5(GO:0009897;GO:0055037;GO:0005905;GO:1990712;GO:0010008;GO:0048471;GO:0005887;GO:0005886;GO:0016323;GO:0042470;GO:0005769);P02768(GO:0005788;GO:0005783;GO:0005737;GO:0031093;GO:0043209;GO:0005794;GO:0005634);Q96PD5(GO:0005622);Q86UX7(GO:0002102;GO:0031093;GO:0042995);P02763(GO:0031093;GO:0035580;GO:1904724);A0A087WWT3(GO:0005794;GO:0005783);A0A0C4DH73(GO:0005886);P02766(GO:0005737;GO:0035578);J3KPS3(GO:0031430;GO:0005829;GO:0031674);A0A2R8Y619(GO:0005634;GO:0005694;GO:0000786);P07357(GO:0005886;GO:0005579);A0A075B6S5(GO:0005886);P37802(GO:0005829);A0A0G2JI36(GO:0005783;GO:0055038;GO:0071556;GO:0005886;GO:0031901;GO:0005794;GO:0009986;GO:0042612;GO:0005887;GO:0012507;GO:0030670;GO:0000139);Q15166(GO:0043231);P02748(GO:0005887;GO:0005886;GO:0005829;GO:0005579);J3KRP0(GO:0005829);P09172(GO:0005783;GO:0034774;GO:0005815;GO:0005737;GO:0030667;GO:0031410;GO:0030658;GO:0042584;GO:0043231;GO:0034466);P01703(GO:0005886);P01700(GO:0005886);P01701(GO:0005886);P01706(GO:0005886);P04264(GO:0045095;GO:1904813;GO:0005856;GO:0005634;GO:0005882;GO:0005886;GO:0001533;GO:0005829);P02042(GO:0005833;GO:0005829);P01705(GO:0005886);A0A0C4DH67(GO:0005886);E9PK25(GO:0015629;GO:0005737;GO:0005634;GO:0005622;GO:0031258;GO:0030027;GO:0032587;GO:0016363;GO:0005829;GO:0030864);P23083(GO:0005886);P01709(GO:0005886);P02671(GO:0009897;GO:0005788;GO:0031091;GO:0031093;GO:0009986;GO:0005938;GO:0005886);P02675(GO:0005783;GO:0009897;GO:0005737;GO:0031091;GO:0031093;GO:0009986;GO:0005938;GO:0005886);P07996(GO:0005783;GO:0005788;GO:0009897;GO:0030141;GO:0005737;GO:0031091;GO:0031093;GO:0009986;GO:0016529);H3BTN5(GO:0005737;GO:0005634;GO:0005829);A0A075B6I0(GO:0005886);P01619(GO:0005886);P0CF74(GO:0009897;GO:0005886);P61626(GO:0035578;GO:0035580;GO:1904724);D6RE82(GO:0005730;GO:0005634);P04217(GO:0031093;GO:1904813;GO:0034774);Q16880(GO:0005886;GO:0043231);P22891(GO:0005796;GO:0005788);Q06033(GO:0031089);C9JF17(GO:0022626;GO:0005783;GO:0030425;GO:0005737;GO:0048471;GO:0043025);P05155(GO:0031093);P05154(GO:0009897;GO:0002080;GO:0031094;GO:0031091);F8W1S1(GO:0005882;GO:0045095);B4E1Z4(GO:0005886);I3L1J2(GO:0009897;GO:0016342;GO:0009986;GO:0005886);M0R0Q9(GO:0005886;GO:0005788;GO:0035578;GO:0034774;GO:0009986);P01031(GO:0005579);A0A075B7F0(GO:0009897);H0YGX7(GO:0005737);P00734(GO:0009897;GO:0005788;GO:0005796;GO:0005886);P13645(GO:0005737;GO:0009986;GO:0005634;GO:0005882;GO:0005829;GO:0001533);A0A0A0MS14(GO:0009897;GO:0005886);P35579(GO:0016459;GO:0015629;GO:0005886;GO:0031252;GO:0005903;GO:0001931;GO:0008180;GO:0005737;GO:0001726;GO:0005634;GO:0097513;GO:0005938;GO:0016460;GO:0042641;GO:0001725;GO:0032154;GO:0008305;GO:0001772;GO:0005826;GO:0005829;GO:0005856;GO:0030863;GO:0005819);P13473(GO:0005770;GO:0101003;GO:0044754;GO:0035577;GO:0010008;GO:1990836;GO:0043202;GO:0097637;GO:0031410;GO:0031902;GO:0005886;GO:0000421;GO:0030670;GO:0031088;GO:0005764;GO:0005765;GO:0005768);P01717(GO:0005886);A0A0A0MS15(GO:0009897;GO:0005886);P48740(GO:0005829;GO:0005654);P00738(GO:0071682;GO:0035580;GO:1904724);P01857(GO:0009897;GO:0005886);A0A075B6Q5(GO:0009897;GO:0005886);P16930(GO:0005829);P01019(GO:0005829;GO:0005737);Q14624(GO:0005737;GO:0031089;GO:0005886);Q16610(GO:0031089);P11226(GO:0009986);P01011(GO:0034774;GO:0035578;GO:0031093;GO:0005622;GO:0005634);H0Y2Y8(GO:0005829);Q9UHG3(GO:0005774;GO:0005764;GO:0005886);P41222(GO:0005783;GO:0005789;GO:0005737;GO:0005634;GO:0048471;GO:0005794;GO:0005791;GO:0031965);A0A0C4DH55(GO:0005886);P0DP01(GO:0005886);P14151(GO:0009897;GO:0030667;GO:0009986;GO:0005887;GO:0005886);P04430(GO:0005886);P31151(GO:0005783;GO:0035578;GO:0005737;GO:0005634;GO:0005829);P01721(GO:0005886);A0A0A0MTH3(GO:0005829;GO:0005654;GO:0030027);C9JXI5(GO:0031410;GO:0005886);O14791(GO:0005788);P13796(GO:0032432;GO:0015629;GO:0030175;GO:0005737;GO:0001726;GO:0002102;GO:0048471;GO:0005884;GO:0005886;GO:0042995;GO:0032587;GO:0001891;GO:0005829;GO:0005856;GO:0001725);D6RAR4(GO:0005737;GO:0005791);P36955(GO:0030424;GO:0043203;GO:0048471;GO:0043025;GO:0042470);A0A0B4J1U3(GO:0005886);P09486(GO:0005737;GO:0031091;GO:0031093;GO:0031092;GO:0005634;GO:0005739;GO:0016363;GO:0009986;GO:0005886;GO:0071682;GO:0005622);A0A0B4J231(GO:0009897);P18206(GO:0015629;GO:0034774;GO:0005737;GO:0002102;GO:0035580;GO:0005856;GO:0042383;GO:0005886;GO:1904813;GO:0005829;GO:0043034);P0DPH7(GO:0005737;GO:0005856;GO:0005874);P80748(GO:0005886);A0A075B6K2(GO:0005886);Q13093(GO:0005737);P32119(GO:0005737;GO:0005829);P06312(GO:0005886);A0A2R8Y6G6(GO:0000015);P80108(GO:0005737;GO:0043231;GO:0005765;GO:0005622);P08514(GO:0009897;GO:0031092;GO:0009986;GO:0008305;GO:0005886);P01009(GO:0005783;GO:0033116;GO:0005788;GO:1904813;GO:0030134;GO:0031093;GO:0005794;GO:0043231;GO:0000139);P02751(GO:0005788;GO:0031093;GO:0005793;GO:0016324);A0A075B6R2(GO:0009897;GO:0005886);P02760(GO:0005886;GO:0009986;GO:0043231);P02750(GO:0043231;GO:1904813;GO:1904724;GO:0035580);P02775(GO:0031091;GO:0031093;GO:1904724);A0A2R8Y7X9(GO:0005833);P01008(GO:0005788;GO:0005886);J3QRV5(GO:0005737;GO:0043231;GO:0005829);P69892(GO:0005829;GO:0005833);H9KV75(GO:0032391;GO:0042383;GO:0005737;GO:0001725;GO:0097433;GO:0005815;GO:0030027;GO:0031941;GO:0016328;GO:0030486;GO:1990357;GO:0031252;GO:0097381;GO:0090636;GO:0090637);A0A075B6K5(GO:0005886);A0A075B6J9(GO:0005886);Q01518(GO:0005737;GO:0030864;GO:0035578;GO:0005886);P02753(GO:0005829);A0A669KAY4(GO:0005770;GO:0005788;GO:0005737;GO:0036020;GO:0030134;GO:0005794;GO:0005791;GO:0009986;GO:0048471;GO:0005886;GO:0031232;GO:0005764;GO:0005765;GO:0005783;GO:0005769);P01344(GO:0031093);P27169(GO:0043231);P13647(GO:0005737;GO:0045095;GO:0005634;GO:0005882;GO:0005886;GO:0005829);P01871(GO:0009897;GO:0009986;GO:0005886);P02100(GO:0005833;GO:0005829);P01715(GO:0005886);O75882(GO:0005737;GO:0005887;GO:0005886);A0A0B4J1V2(GO:0009897;GO:0005886);P00918(GO:0043209;GO:0045177;GO:0016323;GO:0030424;GO:0005829;GO:0005737;GO:0005886;GO:0005902);A0A0C4DH32(GO:0009897;GO:0005886);A0A0B4J1X5(GO:0009897;GO:0005886);Q6EMK4(GO:0009986;GO:0005886;GO:0005765;GO:0005739);A0A0A0MRJ7(GO:0031093;GO:0033116;GO:0005788;GO:0030134;GO:0005886;GO:0000139);P04040(GO:0005829;GO:0005782;GO:0005886;GO:1904813;GO:0005739;GO:0005783;GO:0005778;GO:0005777;GO:0005758;GO:0034774;GO:0005794;GO:0043231;GO:0005764);A0A0C4DH33(GO:0009897;GO:0005886);P18065(GO:0031410;GO:0016324);P00915(GO:0005737;GO:0005829);P05546(GO:0005788);Q562R1(GO:0005737;GO:0005856;GO:0015629);P02743(GO:0005634);A0A075B6K4(GO:0005886);P01860(GO:0009897;GO:0005886);C9J8S2(GO:0031089);P04211(GO:0005886);A0A075B7B8(GO:0009897);A0A0B4J2D9(GO:0005886);P02749(GO:0009986;GO:0031089);P68032(GO:0015629;GO:0030175;GO:0030017;GO:0005737;GO:0001725;GO:0031674;GO:0030027;GO:0005856;GO:0044297;GO:0005884;GO:0005865;GO:0005829;GO:0042643);P01034(GO:0005783;GO:0005788;GO:1904724;GO:0005737;GO:0042995;GO:0005771;GO:0030424;GO:0048471;GO:0043025;GO:0031965;GO:1904813;GO:0005764;GO:0043292);A0A087WSZ0(GO:0005886);C9JB55(GO:0055037;GO:0030139;GO:0005905;GO:1990712;GO:0005770;GO:0009925;GO:0048471;GO:0016324;GO:0031232;GO:0005769);Q15582(GO:0005886;GO:0005802);P02649(GO:0005783;GO:0005788;GO:0071682;GO:0030425;GO:0043025;GO:0005737;GO:0005634;GO:0030669;GO:0005794;GO:0005886;GO:0005769);Q07954(GO:0030425;GO:0005905;GO:0005737;GO:0098797;GO:0045177;GO:0030136;GO:0043025;GO:0044295;GO:0030666;GO:0005887;GO:0005886;GO:0016323;GO:0005634;GO:0005765;GO:0005768;GO:0005769);P04196(GO:0031093;GO:0005886;GO:0009986;GO:0036019);A0A0C4DH25(GO:0005886);P02647(GO:0005788;GO:0071682;GO:0034774;GO:0005829;GO:0031410;GO:0005634;GO:0009986;GO:0030139;GO:0005886;GO:0005769);P01861(GO:0009897;GO:0005886);P10909(GO:0005783;GO:0005794;GO:0031966;GO:0043231;GO:0005829;GO:0099020;GO:0005856;GO:0048471;GO:0097418;GO:0005737;GO:0031093;GO:0031410;GO:0005634;GO:0005739;GO:0009986;GO:0005622;GO:0005743;GO:0097440;GO:0071944;GO:0042583);P04114(GO:0005783;GO:0005788;GO:0005789;GO:0005737;GO:0043202;GO:0071682;GO:0030669;GO:0010008;GO:0043025;GO:0070971;GO:0005790;GO:0005886;GO:0031904;GO:0005829;GO:0043231;GO:0005769);P05109(GO:0005737;GO:0045111;GO:0005634;GO:0034774;GO:0005856;GO:0005886;GO:0005829);P15169(GO:0097060;GO:0005794;GO:0030141;GO:0043025);A0A0C4DH31(GO:0009897;GO:0005886);A0A0C4DH36(GO:0009897);A0A0C4DH34(GO:0009897;GO:0005886);P19320(GO:0009897;GO:0005783;GO:0005794;GO:0030175;GO:0005902;GO:0045177;GO:0002102;GO:0042383;GO:0005887;GO:0005886;GO:0009986;GO:0005769;GO:0071065);P08697(GO:0031093;GO:0009986);A0A0C4DH43(GO:0009897;GO:0005886);A0A0C4DH38(GO:0009897;GO:0005886);A0A0C4DH39(GO:0009897;GO:0005886);P25311(GO:0009897;GO:0005634;GO:0005886);P01599(GO:0005886);O43866(GO:0005737;GO:0009986);P01024(GO:0005886;GO:0005788;GO:0035578;GO:0034774;GO:0009986);P01023(GO:0031093;GO:0005829);H0YCR7(GO:0005654;GO:0005829);P01601(GO:0005886);A0A0C4DH24(GO:0005886);O95497(GO:0035577;GO:0005886);A0A0B4J1Y8(GO:0005886);P02538(GO:0005829;GO:0045095;GO:0005882;GO:0005634);P01782(GO:0005886);P00747(GO:0009986;GO:0005886;GO:0031232;GO:0031093);P00740(GO:0005788;GO:0005796;GO:0005886);P60709(GO:0015629;GO:0036464;GO:0043209;GO:0005634;GO:0097433;GO:0098871;GO:0005654;GO:0005856;GO:0000790;GO:0005886;GO:0044305;GO:0030863;GO:0005829;GO:0005737;GO:0098793;GO:0035267);P00742(GO:0005788;GO:0005796;GO:0005886;GO:0031233);E7EQB2(GO:0009986;GO:0042581);P02533(GO:0045095;GO:0071944;GO:0005737;GO:0005634;GO:0045178;GO:0005882;GO:0005829;GO:0005622);P01742(GO:0005886);P00450(GO:0005765;GO:0005788;GO:0005886);Q08380(GO:0031089);P43121(GO:0009897;GO:0005886;GO:0005634);P0C0L5(GO:0030425;GO:0030424;GO:0005886;GO:0042995);P0C0L4(GO:0005788;GO:0030424;GO:0043025;GO:0030425;GO:0005886;GO:0042995);Q9H4B7(GO:0005737;GO:0005856;GO:0005874);C9JV77(GO:0005788;GO:0031093;GO:0005794;GO:0034774);P02652(GO:0005788;GO:0005829;GO:0005769);Q9HDC9(GO:0005783;GO:0009986);P35858(GO:0005654);P01763(GO:0005886);E9PHK0(GO:0005737;GO:0001652;GO:0031089);P04433(GO:0005886);A0A087WSY6(GO:0005886);P06727(GO:0005788;GO:0009986;GO:0005829;GO:0005769);C9J5S7(GO:0005829;GO:0005634);A0A0C4DH29(GO:0009897;GO:0005886);Q13103(GO:0005788;GO:0031089);P33908(GO:0005794;GO:0005829;GO:0005793;GO:0000139;GO:0005783);P01814(GO:0005886);P21333(GO:0098794;GO:0030018;GO:0015629;GO:0005737;GO:0005730;GO:0043025;GO:0005856;GO:0031941;GO:0048471;GO:0005634;GO:0005884;GO:0005886;GO:0097440;GO:0030863;GO:0043198;GO:0005829;GO:0031523;GO:0005938);P03951(GO:0005886);A0A0B4J1V1(GO:0009897;GO:0005886);A0A0B4J1V0(GO:0009897;GO:0005886);P07360(GO:0005579);A0A0A0MS51(GO:0015629;GO:0001726;GO:0043209;GO:0005634;GO:0030478;GO:0048471;GO:0030027;GO:0045335;GO:0002102;GO:0005886;GO:0005829;GO:0016528);Q5T749(GO:0005737);P01817(GO:0005886);P07437(GO:0035578;GO:0036464;GO:0005874;GO:0005737;GO:0005634;GO:0005856;GO:0044297;GO:0045298;GO:0005641;GO:0005829);H0YAC1(GO:0005886);Q96HR3(GO:0005634;GO:0000151;GO:0005654;GO:0016592);P11021(GO:0005783;GO:0005789;GO:0034663;GO:0008180;GO:0005737;GO:0005793;GO:0043209;GO:0005634;GO:0005739;GO:0030176;GO:0005790;GO:0042470;GO:0009986;GO:0005788;GO:0005886;GO:0043231;GO:0005829;GO:0030496);Q9Y490(GO:0005856;GO:0001726;GO:0005737;GO:0009986;GO:0005886;GO:0042995;GO:0032587;GO:0005829);Q15848(GO:0005783;GO:0009986);A0A0J9YVY3(GO:0005886);P01699(GO:0005886);P07737(GO:0005737;GO:0005634;GO:0098794;GO:0043005;GO:0098793;GO:0005856;GO:0005938;GO:0005829) | 58|82 | C9JB55(GO:0055037;GO:0030139;GO:0005905;GO:1990712;GO:0005770;GO:0009925;GO:0048471;GO:0016324;GO:0031232;GO:0005769);P19652(GO:0031093;GO:0035578;GO:0035580);P37802(GO:0005829);P04211(GO:0005886);J3QRV5(GO:0005737;GO:0043231;GO:0005829);P02763(GO:0031093;GO:0035580;GO:1904724);A0A0C4DH73(GO:0005886);P00915(GO:0005737;GO:0005829);A0A0C4DH32(GO:0009897;GO:0005886);A0A0J9YX35(GO:0005886);K7ER74(GO:0005769);A0A0C4DH34(GO:0009897;GO:0005886);P01599(GO:0005886);P09172(GO:0005783;GO:0034774;GO:0005815;GO:0005737;GO:0030667;GO:0031410;GO:0030658;GO:0042584;GO:0043231;GO:0034466);P01703(GO:0005886);P35527(GO:0005829;GO:0005882;GO:0005634);P0DP01(GO:0005886);P01780(GO:0005886);P01601(GO:0005886);P32119(GO:0005737;GO:0005829);P01721(GO:0005886);P15169(GO:0097060;GO:0005794;GO:0030141;GO:0043025);A0A075B6K2(GO:0005886);P01009(GO:0005783;GO:0033116;GO:0005788;GO:1904813;GO:0030134;GO:0031093;GO:0005794;GO:0043231;GO:0000139);P01008(GO:0005788;GO:0005886);A0A075B6K4(GO:0005886);Q86YZ3(GO:0005737;GO:0005634;GO:0035578;GO:0048471;GO:0036457;GO:0001533);P00748(GO:0005791;GO:0005886);Q08380(GO:0031089);P80748(GO:0005886);P00918(GO:0043209;GO:0045177;GO:0016323;GO:0030424;GO:0005829;GO:0005737;GO:0005886;GO:0005902);P01344(GO:0031093);P01782(GO:0005886);A0A0C4DH67(GO:0005886);A0A669KAY4(GO:0005770;GO:0005788;GO:0005737;GO:0036020;GO:0030134;GO:0005794;GO:0005791;GO:0009986;GO:0048471;GO:0005886;GO:0031232;GO:0005764;GO:0005765;GO:0005783;GO:0005769);A0A087WSY6(GO:0005886);P29622(GO:0031089);I3L1J2(GO:0009897;GO:0016342;GO:0009986;GO:0005886);A0A0C4DH25(GO:0005886);E7EUT5(GO:0005737;GO:0005634;GO:0005811;GO:0015630;GO:0031965;GO:0005886;GO:0043231;GO:0005829);P30041(GO:0035578;GO:0005737;GO:0005634;GO:0048471;GO:0005829;GO:0005764);Q9Y5Y7(GO:0071944;GO:0005887;GO:0005886);P18206(GO:0015629;GO:0034774;GO:0005737;GO:0002102;GO:0035580;GO:0005856;GO:0042383;GO:0005886;GO:1904813;GO:0005829;GO:0043034);P02787(GO:0055037;GO:0005788;GO:0030139;GO:0005905;GO:1990712;GO:0010008;GO:0030665;GO:0031232;GO:0031410;GO:0005770;GO:0045178;GO:0009925;GO:0048471;GO:0009986;GO:0016324;GO:0034774;GO:0005768;GO:0005769);P01817(GO:0005886);P01715(GO:0005886);P19823(GO:0005788);P01717(GO:0005886);A0A0B4J1V0(GO:0009897;GO:0005886);P07360(GO:0005579);P01857(GO:0009897;GO:0005886);A0A075B6S5(GO:0005886);Q96HR3(GO:0005634;GO:0000151;GO:0005654;GO:0016592);A0A075B6J9(GO:0005886);P02751(GO:0005788;GO:0031093;GO:0005793;GO:0016324);P43251(GO:0005759);P01699(GO:0005886);M0R0Q9(GO:0005886;GO:0005788;GO:0035578;GO:0034774;GO:0009986) | 0.011398904 | 0.079792325 | https://www.ebi.ac.uk/QuickGO/term/GO:0044464 |
| GO:0050896 | response to stimulus | biological_process | 252|375 | P08779(GO:0045087;GO:0006954);P00488(GO:0072378);P01857(GO:0042742;GO:0045087;GO:0006956;GO:0006958;GO:0002250);P04275(GO:0007597;GO:0009611);J3KNB4(GO:0071224;GO:0071222;GO:0042742;GO:0050829;GO:0061844;GO:0071354;GO:0071356;GO:0051873;GO:0045087;GO:0006952;GO:0050830;GO:0071347);P03951(GO:0007597);K7ERG9(GO:0009617;GO:0006957);A0A0A0MRZ9(GO:0006955;GO:0002250);P23142(GO:0072378);P15814(GO:0045087;GO:0042742;GO:0006955;GO:0006958);B7ZKJ8(GO:0034097;GO:0006953);P0DP02(GO:0002250);P08571(GO:0006954;GO:0071222;GO:0071223;GO:0032496;GO:0045471;GO:0034612;GO:0071727;GO:0071726;GO:0009617;GO:0071219;GO:0045087;GO:0009408;GO:0032026;GO:0051602;GO:0002237);P68871(GO:0042542);E9PK25(GO:0043200;GO:0009615);O75460(GO:0034620;GO:0006986;GO:0071333;GO:0036498;GO:0030968;GO:0070059;GO:0035924;GO:0034976);P01597(GO:0006956;GO:0002250;GO:0006958;GO:0006955);P01042(GO:0007597;GO:0006954);A0A087X1J7(GO:0006979);P14151(GO:0033198);P01602(GO:0006956;GO:0002250;GO:0006958;GO:0006955);P11226(GO:0042742;GO:0001867;GO:0051873;GO:0045087;GO:0006953;GO:0006956;GO:0006958;GO:0006979;GO:0050830);A0A0B4J1U7(GO:0045087;GO:0002250;GO:0006958;GO:0042742);A0A669KAY4(GO:0032869;GO:0009267);P59665(GO:0071222;GO:0019730;GO:0019731;GO:0042742;GO:0050829;GO:0061844;GO:0051852;GO:0002227;GO:0006952;GO:0050830;GO:0006955;GO:0050832;GO:0051607;GO:0042832);P0DJI8(GO:0045087;GO:0006953);P51884(GO:0014070;GO:0070848);P06312(GO:0006956;GO:0002250;GO:0006958;GO:0006955);A0A0A0MT36(GO:0006955;GO:0002250);P01009(GO:0006953);A0A075B7D8(GO:0045087;GO:0042742;GO:0006958);P01764(GO:0006955;GO:0042742;GO:0045087;GO:0006956;GO:0006958;GO:0002250);A0A087X0S5(GO:0071230);P01766(GO:0006956;GO:0002250;GO:0006958;GO:0006955);B4E1Z4(GO:0006956;GO:0006957);P00918(GO:0010043;GO:0043627;GO:0048545;GO:0009268;GO:0010033;GO:0071498);P0DOY3(GO:0002250);P26038(GO:0071394);P01782(GO:0006956;GO:0002250;GO:0006958;GO:0006955);P18428(GO:0006968;GO:0071222;GO:0071223;GO:0042742;GO:0050829;GO:0032496;GO:0032490;GO:0045087;GO:0006953;GO:0050830);P01780(GO:0006956;GO:0002250;GO:0006958;GO:0006955);P06331(GO:0006956;GO:0002250;GO:0006958;GO:0006955);P02776(GO:0071222;GO:0032496;GO:0061844;GO:0051873;GO:0006952;GO:0006954;GO:0006955;GO:0042832);D6R934(GO:0006958);A0A1W2PQU7(GO:0031102);P0DP01(GO:0002250);E7EUT5(GO:0061844;GO:0051873;GO:0050832;GO:0071346);A0A0G2JMB2(GO:0045087;GO:0042742;GO:0006958);A0A0J9YXX1(GO:0002250);P30041(GO:0034599;GO:0006979);P06702(GO:0045087;GO:0019730;GO:0042742;GO:0061844;GO:0050832;GO:0006954);Q9Y5Y7(GO:0009611);P61224(GO:0071320);P10643(GO:0045087;GO:0006956;GO:0006957;GO:0006955;GO:0006958);P02787(GO:0009617;GO:0071281);P01833(GO:0001580);P01834(GO:0006955;GO:0042742;GO:0045087;GO:0006956;GO:0006958;GO:0002250);P05160(GO:0072378);P04003(GO:0045087;GO:0006958);P01008(GO:0002438;GO:0007584);P01718(GO:0006956;GO:0002250;GO:0006958;GO:0006955);P04004(GO:0006955);P01743(GO:0006956;GO:0002250;GO:0006958;GO:0006955);Q92954(GO:0006955);P69905(GO:0042542);A0A075B6H7(GO:0006955);A0A0J9YX35(GO:0002250);A0A087WSZ0(GO:0006955;GO:0002250);G3V0E5(GO:0035690;GO:1990830);P02768(GO:0009267);O75636(GO:0051607;GO:0006956;GO:0045087;GO:0001867);Q96PD5(GO:0019730;GO:0016045;GO:0045087;GO:0050830);P02763(GO:0006953;GO:0006954);A0A0C4DH73(GO:0006956;GO:0002250;GO:0006958;GO:0006955);P02753(GO:0032526;GO:0045471;GO:0050896);P07357(GO:0006956;GO:0006957;GO:0006955;GO:0006958;GO:0045087);A0A0B4J2D9(GO:0006955;GO:0002250);P19320(GO:0035094;GO:0010043;GO:0032496;GO:0001666;GO:0010212;GO:0071356;GO:1904646;GO:0007584;GO:0045471;GO:0002526;GO:0002544;GO:0035924);Q15166(GO:0009636);P07358(GO:0045087;GO:0006956;GO:0006957;GO:0006955;GO:0006958);P09172(GO:0042596;GO:0008542;GO:0048265;GO:0001975);P01703(GO:0006956;GO:0002250;GO:0006958;GO:0006955);P01700(GO:0006956;GO:0002250;GO:0006958;GO:0006955);P01701(GO:0006956;GO:0006958;GO:0006955);P01706(GO:0006956;GO:0002250;GO:0006958;GO:0006955);P20851(GO:0045087;GO:0006958);P01704(GO:0006956;GO:0002250;GO:0006958;GO:0006955);P01705(GO:0006956;GO:0002250;GO:0006958;GO:0006955);A0A0C4DH67(GO:0002250);P23083(GO:0006956;GO:0002250;GO:0006958;GO:0006955);P01709(GO:0006956;GO:0002250;GO:0006958;GO:0006955);P02671(GO:0045087;GO:0072378;GO:0051592;GO:0072377;GO:0002250;GO:0043152);P02675(GO:0044320;GO:0072378;GO:0045087;GO:0051592;GO:0071347;GO:0002250;GO:0043152);P07996(GO:0032026;GO:0034605;GO:0006986;GO:0009612;GO:0051592;GO:0042493;GO:0071356;GO:0006954;GO:0006955;GO:0033574;GO:0034976;GO:0001666;GO:0071363;GO:0002544;GO:0032570;GO:0009749);P13671(GO:0045087;GO:0006956;GO:0006955;GO:0006958);A0A075B6I0(GO:0006955;GO:0002250);P0CF74(GO:0042742;GO:0045087;GO:0006956;GO:0006958;GO:0002250);P61626(GO:0006954;GO:0019730;GO:0042742;GO:0050829;GO:0050830);P02652(GO:0043627;GO:0002526;GO:0051384;GO:0042493;GO:0009749);E9PHK0(GO:0071560;GO:0071310);C9JF17(GO:0042493;GO:0048678;GO:0000302;GO:0014012);P05155(GO:0007597;GO:0045087;GO:0006958);M0R0Q9(GO:0009617;GO:0006956;GO:0006957;GO:0006954;GO:0006955;GO:0006958);P01031(GO:0006954;GO:0045087;GO:0006956;GO:0006957;GO:0006958);O95445(GO:0009749);A0A075B7F0(GO:0045087;GO:0042742;GO:0006958);P00734(GO:0007597;GO:0061844;GO:0009611;GO:0051838;GO:0070945;GO:0006953;GO:0072378);A0A0A0MS14(GO:0045087;GO:0002250;GO:0006958;GO:0042742);A0A0A0MS15(GO:0045087;GO:0002250;GO:0006958;GO:0042742);P01715(GO:0006956;GO:0002250;GO:0006958;GO:0006955);P01717(GO:0006956;GO:0002250;GO:0006958;GO:0006955);P48740(GO:0006956;GO:0001867;GO:0045087);P00738(GO:0042742;GO:0006952;GO:0006953;GO:0042542);P00739(GO:0002526;GO:0010033);E7EQB2(GO:0019732;GO:0019731;GO:0050829;GO:0061844;GO:0002227);Q9BWP8(GO:0019730;GO:0001867;GO:0045087;GO:0006956);G3XAP6(GO:0006986);A0A0C4DH25(GO:0006956;GO:0002250;GO:0006958;GO:0006955);A0A0G2JMI3(GO:0045087;GO:0002250;GO:0006958;GO:0042742);P01011(GO:0006954;GO:0006953);P19652(GO:0006953);A0A0C4DH55(GO:0006955;GO:0002250);A0A0B4J1Y8(GO:0006955;GO:0002250);Q96IY4(GO:0071333;GO:0009408;GO:0042493);P31151(GO:0019730;GO:0000302;GO:0050829;GO:0032496;GO:0061844;GO:0045087);P01721(GO:0006956;GO:0002250;GO:0006958;GO:0006955);O14791(GO:0045087);K7ER74(GO:0042493);G3XAK1(GO:0071456);P36955(GO:0071300;GO:0010447;GO:0071333;GO:0046685;GO:0071279;GO:0071549);A0A0B4J1U3(GO:0006955;GO:0002250);A0A0G2JPR0(GO:0006956;GO:0006954);P09486(GO:0051384;GO:0032496;GO:0034097;GO:0033591;GO:0046686;GO:0010288;GO:0042060;GO:0051592;GO:0045471;GO:0043434;GO:0009629;GO:0071363;GO:0051591);Q16610(GO:0006954);Q07954(GO:1904646);P32119(GO:0032496;GO:0034599;GO:0006979);O00187(GO:0045087;GO:0006956;GO:0001867;GO:0006958);A0A0B4J231(GO:0045087;GO:0042742;GO:0006958);A0A075B6K2(GO:0006955;GO:0002250);P02750(GO:0009617);P02751(GO:0009611;GO:0042060;GO:0006953);A0A075B6R2(GO:0045087;GO:0002250;GO:0006958;GO:0042742);A0A075B6K4(GO:0006955;GO:0002250);Q03591(GO:0006956);Q9NZP8(GO:0045087;GO:0006958);P02775(GO:0071222;GO:0042742;GO:0032496;GO:0061844;GO:0006952;GO:0006954;GO:0006955);A0A075B6R9(GO:0006955);P68032(GO:0009991;GO:0009612;GO:0043503;GO:0071417;GO:0010226;GO:0045471;GO:0042493;GO:0048545);A0A0G2JRQ6(GO:0006955);A0A0B4J1X5(GO:0045087;GO:0002250;GO:0006958;GO:0042742);P0C0L5(GO:0006954;GO:0032490;GO:0045087;GO:0006956;GO:0006958);A0A0C4DH43(GO:0045087;GO:0002250;GO:0006958;GO:0042742);A0A075B6K5(GO:0006955;GO:0002250);A0A075B6J9(GO:0006955;GO:0002250);P08603(GO:0045087;GO:0006956;GO:0006957);A0A087WSY4(GO:0045087;GO:0002250;GO:0006958;GO:0042742);A0A087WSY6(GO:0006955;GO:0002250);P27169(GO:0009636;GO:1902617;GO:0031667;GO:0070542);P01877(GO:0019731;GO:0006955;GO:0045087;GO:0006958;GO:0002250);P01871(GO:0019731;GO:0050829;GO:0045087;GO:0006958;GO:0002250);P03950(GO:0001666;GO:0009725);A0A075B7D0(GO:0045087;GO:0042742;GO:0006958);P13473(GO:0009267);O75882(GO:0006954;GO:0006979);A0A0B4J1V2(GO:0045087;GO:0002250;GO:0006958;GO:0042742);F8WF14(GO:0051384;GO:0043279;GO:0051593);A0A0C4DH32(GO:0045087;GO:0002250;GO:0006958;GO:0042742);A0A075B6S9(GO:0006955);P01763(GO:0006956;GO:0002250;GO:0006958;GO:0006955);Q6EMK4(GO:0071461;GO:0071456);P04040(GO:0033189;GO:0009314;GO:0000302;GO:0009650;GO:0032868;GO:0001666;GO:0055093;GO:0009636;GO:0033591;GO:0006979;GO:0046686;GO:0014823;GO:0010288;GO:0009411;GO:0032355;GO:0045471;GO:0071363;GO:0034599;GO:0009642;GO:0042542;GO:0070542;GO:0042493;GO:0033197;GO:0010193;GO:0014854;GO:0080184);A0A0C4DH33(GO:0045087;GO:0002250;GO:0006958;GO:0042742);A0A3B3ISR2(GO:0045087;GO:0006958);P18065(GO:0051384;GO:0032355;GO:0009612;GO:0032526;GO:0007584;GO:0032870;GO:0043627;GO:0010226;GO:0042493;GO:0048545);P15169(GO:0051384);A0A075B6S5(GO:0006955;GO:0002250);P02743(GO:0045087;GO:0006953;GO:0006958);P02741(GO:0045087;GO:0006953;GO:0050830;GO:0006954;GO:0006958);P01860(GO:0042742;GO:0045087;GO:0006956;GO:0006958;GO:0002250);P02747(GO:0045087;GO:0006956;GO:0006955;GO:0006958);P02745(GO:0045087;GO:0006956;GO:0010039;GO:0006958);A0A075B7B8(GO:0045087;GO:0042742;GO:0006958);A0A0G2JL69(GO:0045087;GO:0006956;GO:0006958);P02749(GO:0007597);P02748(GO:0006955;GO:0045087;GO:0006957;GO:0006958);P01034(GO:0048678;GO:0070301;GO:0001666;GO:0014070;GO:0032355;GO:0009636;GO:0006952;GO:0034599;GO:0010035;GO:0006979;GO:0009743;GO:0042493;GO:0031667);P01601(GO:0006956;GO:0002250;GO:0006958;GO:0006955);C9JB55(GO:0071281);Q15582(GO:0050896);P02649(GO:0031102;GO:0061771;GO:0000302;GO:0002021;GO:0006979);P04211(GO:0006956;GO:0002250;GO:0006958;GO:0006955);P04196(GO:0061844;GO:0050832);P02647(GO:0031102;GO:0014012;GO:0042493;GO:0007584;GO:0043627);P10909(GO:0019730;GO:0009615;GO:0051788;GO:0006956;GO:0006958;GO:0045087);P04114(GO:0032496;GO:0032355;GO:0071356;GO:0009615;GO:0071379;GO:0010033;GO:0009743;GO:0010269);P05109(GO:0045087;GO:0019730;GO:0010043;GO:0032496;GO:0042060;GO:0050832;GO:0045471;GO:0006954;GO:0002526;GO:0002544;GO:0042742);P01861(GO:0042742;GO:0045087;GO:0006956;GO:0006958;GO:0002250);A0A0C4DH31(GO:0045087;GO:0002250;GO:0006958;GO:0042742);A0A0C4DH36(GO:0045087;GO:0042742;GO:0006958);A0A0C4DH34(GO:0045087;GO:0002250;GO:0006958;GO:0042742);A0A0G2JI36(GO:0006955;GO:0016045;GO:0002419;GO:0001913);P08697(GO:0006953;GO:0010033);Q14624(GO:0034097;GO:0006953);P25311(GO:0001580;GO:0006955);A0A0C4DH39(GO:0045087;GO:0002250;GO:0006958;GO:0042742);A0A0C4DH38(GO:0045087;GO:0002250;GO:0006958;GO:0042742);P01599(GO:0006956;GO:0002250;GO:0006958;GO:0006955);O43866(GO:0006954;GO:0006968);P01024(GO:0045087;GO:0009617;GO:0006956;GO:0006957;GO:0006954;GO:0006955;GO:0006958);P01023(GO:0007597);C9J8S2(GO:0019732;GO:0050829;GO:0045087;GO:0050830;GO:0006954;GO:0061760);O95497(GO:0045087;GO:0002544;GO:0006954;GO:0002526;GO:0006979);P02538(GO:0042060;GO:0050830;GO:0061844);P09871(GO:0006956;GO:0045087;GO:0001867;GO:0006958);P00740(GO:0007597);P60709(GO:0072749);P00742(GO:0007598);A0A075B6Q5(GO:0045087;GO:0002250;GO:0006958;GO:0042742);P02533(GO:0010043;GO:0010212);P01742(GO:0006956;GO:0002250;GO:0006958;GO:0006955);P00748(GO:0007597;GO:0045087;GO:0002353;GO:0051788);Q08380(GO:0006968);P80748(GO:0006956;GO:0002250;GO:0006958;GO:0006955);P04264(GO:0001867;GO:0006979);P0C0L4(GO:0006954;GO:0045087;GO:0006956;GO:0006958);C9JV77(GO:0006953);P01019(GO:0071260;GO:0014873;GO:1904385;GO:0032355;GO:0051403);Q15485(GO:0045087;GO:0050830;GO:0001867;GO:0050829;GO:0006956);A0A096LPE2(GO:0006953);P04433(GO:0006956;GO:0002250;GO:0006958;GO:0006955);A0A0C4DH24(GO:0006955;GO:0002250);P06727(GO:0035634;GO:0006982;GO:0002227;GO:0031102);P04430(GO:0006956;GO:0006958;GO:0006955);A0A0C4DH29(GO:0045087;GO:0002250;GO:0006958;GO:0042742);A0A0B4J1X8(GO:0045087;GO:0002250;GO:0006958;GO:0042742);P02100(GO:0014070);P01817(GO:0006956;GO:0002250;GO:0006958;GO:0006955);P01814(GO:0006956;GO:0002250;GO:0006958;GO:0006955);P01619(GO:0019731;GO:0006955;GO:0006956;GO:0006958;GO:0002250);A0A0B4J1V1(GO:0045087;GO:0002250;GO:0006958;GO:0042742);A0A0B4J1V0(GO:0045087;GO:0002250;GO:0006958;GO:0042742);P07360(GO:0045087;GO:0006957;GO:0006958);A0A0A0MS51(GO:0045471;GO:0051593;GO:0071346;GO:0014891;GO:0071276);P07437(GO:0042267);H0YAC1(GO:0007597);P80108(GO:0035690;GO:0032869;GO:0009749;GO:0071397;GO:0071277;GO:0071401;GO:0071467);P11021(GO:1990090;GO:0071480;GO:1990440;GO:0009314;GO:0030433;GO:0036500;GO:0042149;GO:0035690;GO:0006983;GO:0071353;GO:0071236;GO:0097501;GO:0071277;GO:0036498;GO:0036499;GO:0042220;GO:1904313;GO:0030968;GO:0071287;GO:0071320;GO:0034976);Q9Y490(GO:0036498);Q15848(GO:0009617;GO:0014823;GO:0071320;GO:0031667;GO:0070994;GO:0071872;GO:0001666;GO:0007584;GO:0070543;GO:0042493;GO:0051384;GO:0035690;GO:0032869;GO:0034612;GO:0045471;GO:0009744;GO:0009749);A0A0J9YVY3(GO:0002250);P01699(GO:0006956;GO:0002250;GO:0006958;GO:0006955);P07737(GO:0010033;GO:0071363) | 56|82 | C9JB55(GO:0071281);O75636(GO:0051607;GO:0006956;GO:0045087;GO:0001867);P19652(GO:0006953);P04211(GO:0006956;GO:0002250;GO:0006958;GO:0006955);K7ERG9(GO:0009617;GO:0006957);P02763(GO:0006953;GO:0006954);A0A0C4DH73(GO:0006956;GO:0002250;GO:0006958;GO:0006955);P01721(GO:0006956;GO:0002250;GO:0006958;GO:0006955);A0A0C4DH32(GO:0045087;GO:0002250;GO:0006958;GO:0042742);P15169(GO:0051384);A0A0G2JL69(GO:0045087;GO:0006956;GO:0006958);A0A0C4DH34(GO:0045087;GO:0002250;GO:0006958;GO:0042742);P32119(GO:0032496;GO:0034599;GO:0006979);P01599(GO:0006956;GO:0002250;GO:0006958;GO:0006955);P09172(GO:0042596;GO:0008542;GO:0048265;GO:0001975);P01703(GO:0006956;GO:0002250;GO:0006958;GO:0006955);A0A075B6J9(GO:0006955;GO:0002250);A0A0G2JRQ6(GO:0006955);A0A0C4DH67(GO:0002250);P01601(GO:0006956;GO:0002250;GO:0006958;GO:0006955);Q96IY4(GO:0071333;GO:0009408;GO:0042493);O00187(GO:0045087;GO:0006956;GO:0001867;GO:0006958);A0A075B6K2(GO:0006955;GO:0002250);Q9NZP8(GO:0045087;GO:0006958);P01008(GO:0002438;GO:0007584);A0A075B6K4(GO:0006955;GO:0002250);P01009(GO:0006953);P00748(GO:0007597;GO:0045087;GO:0002353;GO:0051788);Q08380(GO:0006968);P02751(GO:0009611;GO:0042060;GO:0006953);P02787(GO:0009617;GO:0071281);A0A075B6R9(GO:0006955);P01782(GO:0006956;GO:0002250;GO:0006958;GO:0006955);P01780(GO:0006956;GO:0002250;GO:0006958;GO:0006955);A0A669KAY4(GO:0032869;GO:0009267);A0A087WSY6(GO:0006955;GO:0002250);A0A0C4DH25(GO:0006956;GO:0002250;GO:0006958;GO:0006955);E7EUT5(GO:0061844;GO:0051873;GO:0050832;GO:0071346);P30041(GO:0034599;GO:0006979);Q9Y5Y7(GO:0009611);P0DP01(GO:0002250);P00918(GO:0010043;GO:0043627;GO:0048545;GO:0009268;GO:0010033;GO:0071498);P01817(GO:0006956;GO:0002250;GO:0006958;GO:0006955);P01715(GO:0006956;GO:0002250;GO:0006958;GO:0006955);P01717(GO:0006956;GO:0002250;GO:0006958;GO:0006955);A0A0B4J1V0(GO:0045087;GO:0002250;GO:0006958;GO:0042742);P07360(GO:0045087;GO:0006957;GO:0006958);P01857(GO:0042742;GO:0045087;GO:0006956;GO:0006958;GO:0002250);A0A075B6S5(GO:0006955;GO:0002250);A0A075B6H7(GO:0006955);P02747(GO:0045087;GO:0006956;GO:0006955;GO:0006958);P80748(GO:0006956;GO:0002250;GO:0006958;GO:0006955);K7ER74(GO:0042493);P01699(GO:0006956;GO:0002250;GO:0006958;GO:0006955);A0A0J9YX35(GO:0002250);M0R0Q9(GO:0009617;GO:0006956;GO:0006957;GO:0006954;GO:0006955;GO:0006958) | 0.103444446 | 0.188898554 | https://www.ebi.ac.uk/QuickGO/term/GO:0050896 |
| GO:0005623 | cell | cellular_component | 7|375 | C9JB55(GO:0005623);P02787(GO:0005623);Q96IY4(GO:0005623);P32119(GO:0005623);P02790(GO:0005623);P30041(GO:0005623);P00450(GO:0005623) | 5|82 | C9JB55(GO:0005623);P02787(GO:0005623);P32119(GO:0005623);P30041(GO:0005623);Q96IY4(GO:0005623) | 0.005967929 | 0.050130607 | https://www.ebi.ac.uk/QuickGO/term/GO:0005623 |
| GO:0005215 | transporter activity | molecular_function | 23|375 | G3V0E5(GO:0033570);P11597(GO:0017127;GO:0005548;GO:0005319);P02649(GO:0005319;GO:0017127);Q07954(GO:0042954);Q9UHG3(GO:0008555);P02647(GO:0005548;GO:0005319;GO:0017127);P04114(GO:0005319;GO:0017127);O14791(GO:0005254);P25311(GO:0008320);P55058(GO:1990050;GO:0005548;GO:0008525;GO:0005319;GO:0035620;GO:1904121);P02753(GO:0034632);P02790(GO:0015232);P55056(GO:0005319);C9JB55(GO:0015091);C9JF17(GO:0005319);P02652(GO:0017127;GO:0005319);P02775(GO:0005355);P06727(GO:0005319;GO:0017127);O95445(GO:0005319);Q5SRP5(GO:0005319);D6RF35(GO:0090482);P02787(GO:0015091);Q13790(GO:0005319) | 5|82 | C9JB55(GO:0015091);P02787(GO:0015091);Q5SRP5(GO:0005319);P55058(GO:1990050;GO:0005548;GO:0008525;GO:0005319;GO:0035620;GO:1904121);P55056(GO:0005319) | 0.204451647 | 0.268342786 | https://www.ebi.ac.uk/QuickGO/term/GO:0005215 |
| GO:0002376 | immune system process | biological_process | 198|375 | P08779(GO:0045087);J3KNB4(GO:0061844;GO:0045087);P0C0L5(GO:0045087;GO:0008228;GO:0006956;GO:0006958;GO:0002376);K7ERG9(GO:0006957);P15814(GO:0050853;GO:0045087;GO:0050900;GO:0006955;GO:0006958);P0DP02(GO:0002376;GO:0002250);P08571(GO:0002224;GO:0038124;GO:0045087;GO:0035666;GO:0034142;GO:0002755;GO:0002756;GO:0038123;GO:0043312;GO:0002376);P68871(GO:0043312);Q86YZ3(GO:0043312);P01597(GO:0038096;GO:0002376;GO:0050900;GO:0006956;GO:0002250;GO:0006958;GO:0006955);P0DP01(GO:0002376;GO:0002250);P01602(GO:0038096;GO:0002376;GO:0002377;GO:0050900;GO:0006956;GO:0002250;GO:0006958;GO:0006955);P11226(GO:0001867;GO:0045087;GO:0008228;GO:0006956;GO:0006958;GO:0002376);A0A0B4J1U7(GO:0050853;GO:0045087;GO:0002376;GO:0002250;GO:0006958);P59665(GO:0019730;GO:0019731;GO:0061844;GO:0002227;GO:0010818;GO:0006955;GO:0051607;GO:0043312);P0DJI8(GO:0048246;GO:0048247;GO:0030593;GO:0045087);P06312(GO:0038096;GO:0002376;GO:0002377;GO:0050900;GO:0006956;GO:0002250;GO:0006958;GO:0006955);A0A0A0MT36(GO:0006955;GO:0002376;GO:0002377;GO:0002250);P01763(GO:0038096;GO:0002376;GO:0050900;GO:0006956;GO:0002250;GO:0006958;GO:0006955);P01764(GO:0006955;GO:0050900;GO:0050853;GO:0045087;GO:0006956;GO:0006958;GO:0038096;GO:0002376;GO:0002250);P01766(GO:0038096;GO:0050900;GO:0002376;GO:0006956;GO:0002250;GO:0006958;GO:0006955);B4E1Z4(GO:0006956;GO:0006957);P0DOY3(GO:0002376;GO:0002250);P26038(GO:0050900;GO:0042098;GO:0072678);P01782(GO:0038096;GO:0002376;GO:0050900;GO:0006956;GO:0002250;GO:0006958;GO:0006955);P18428(GO:0002281;GO:0002224;GO:0045087;GO:0008228;GO:0034142;GO:0002376;GO:0002232);P01780(GO:0038096;GO:0002376;GO:0050900;GO:0006956;GO:0002250;GO:0006958;GO:0006955);P02776(GO:0061844;GO:0030595;GO:0030593;GO:0006955);D6R934(GO:0006958);P02775(GO:0061844;GO:0030595;GO:0030593;GO:0006955;GO:0043312);E7EUT5(GO:0061844);A0A0G2JMB2(GO:0050853;GO:0045087;GO:0006958);A0A0J9YXX1(GO:0002376;GO:0002250);P30041(GO:0043312);P06702(GO:0045087;GO:0019730;GO:0030595;GO:0030593;GO:0061844;GO:0002376;GO:0002224;GO:0002523;GO:0043312);P61224(GO:0043312);P10643(GO:0045087;GO:0002376;GO:0006956;GO:0006957;GO:0006955;GO:0006958);P01833(GO:0043312);P01834(GO:0006955;GO:0050853;GO:0045087;GO:0006956;GO:0006958;GO:0038096;GO:0050900;GO:0002376;GO:0002250);P04003(GO:0045087;GO:0002376;GO:0006958);P01718(GO:0038096;GO:0002376;GO:0050900;GO:0006956;GO:0002250;GO:0006958;GO:0006955);P35579(GO:0050900);P04004(GO:0006955);P01743(GO:0038096;GO:0002376;GO:0050900;GO:0006956;GO:0002250;GO:0006958;GO:0006955);Q92954(GO:0006955);O00391(GO:0043312);P02751(GO:0050900);A0A075B6H7(GO:0002377;GO:0006955);A0A087WSZ0(GO:0006955;GO:0002376;GO:0002377;GO:0002250);O75636(GO:0051607;GO:0006956;GO:0045087;GO:0002376;GO:0001867);Q96PD5(GO:0019730;GO:0002221;GO:0045087;GO:0002376);P02763(GO:0043312);Q01518(GO:0043312);A0A0C4DH73(GO:0038096;GO:0050900;GO:0002376;GO:0002377;GO:0006956;GO:0002250;GO:0006958;GO:0006955);P02766(GO:0043312);P07357(GO:0002376;GO:0006956;GO:0006957;GO:0006955;GO:0006958;GO:0045087);A0A0B4J2D9(GO:0006955;GO:0002376;GO:0002377;GO:0002250);A0A0G2JI36(GO:0006955;GO:0042590;GO:0002474;GO:0002419;GO:0002479;GO:0019885;GO:0002480;GO:0036037;GO:0001913);P07358(GO:0045087;GO:0002376;GO:0006956;GO:0006957;GO:0006955;GO:0006958);P09172(GO:0050900;GO:0002443);P01703(GO:0038096;GO:0002376;GO:0050900;GO:0006956;GO:0002250;GO:0006958;GO:0006955);P01700(GO:0038096;GO:0002376;GO:0050900;GO:0006956;GO:0002250;GO:0006958;GO:0006955);P01701(GO:0038096;GO:0050900;GO:0006956;GO:0006958;GO:0006955);P01706(GO:0038096;GO:0002376;GO:0050900;GO:0006956;GO:0002250;GO:0006958;GO:0006955);P20851(GO:0045087;GO:0002376;GO:0006958);P01704(GO:0038096;GO:0002376;GO:0050900;GO:0006956;GO:0002250;GO:0006958;GO:0006955);P01705(GO:0038096;GO:0002376;GO:0050900;GO:0006956;GO:0002250;GO:0006958;GO:0006955);P23083(GO:0038096;GO:0002376;GO:0050900;GO:0006956;GO:0002250;GO:0006958;GO:0006955);P01709(GO:0038096;GO:0002376;GO:0050900;GO:0006956;GO:0002250;GO:0006958;GO:0006955);P02671(GO:0045087;GO:0002224;GO:0002376;GO:0002250;GO:0043152);P02675(GO:0002224;GO:0045087;GO:0002376;GO:0002250;GO:0043152);P07996(GO:0006955);P13671(GO:0045087;GO:0002376;GO:0006956;GO:0006955;GO:0006958);A0A075B6I0(GO:0006955;GO:0002376;GO:0002377;GO:0002250);P0CF74(GO:0050900;GO:0050853;GO:0045087;GO:0006956;GO:0006958;GO:0038096;GO:0002376;GO:0002250);P61626(GO:0019730;GO:0043312);A0A0C4DH67(GO:0002376;GO:0002250);P05155(GO:0045087;GO:0006958;GO:0002376);M0R0Q9(GO:0006956;GO:0006957;GO:0006955;GO:0006958;GO:0043312);P01031(GO:0045087;GO:0006956;GO:0006957;GO:0006958;GO:0002376);A0A075B7F0(GO:0050853;GO:0045087;GO:0006958);P00734(GO:0061844;GO:0070945);A0A0A0MS14(GO:0050853;GO:0045087;GO:0002376;GO:0002250;GO:0006958);A0A0A0MS15(GO:0050853;GO:0045087;GO:0002376;GO:0002250;GO:0006958);P13473(GO:0043312);P01717(GO:0038096;GO:0002376;GO:0050900;GO:0006956;GO:0002250;GO:0006958;GO:0006955);P48740(GO:0006956;GO:0002376;GO:0001867;GO:0045087);P00738(GO:0043312;GO:0002376);P01857(GO:0050853;GO:0045087;GO:0006956;GO:0006958;GO:0038096;GO:0002376;GO:0002250);A0A075B6Q5(GO:0050853;GO:0045087;GO:0002376;GO:0002250;GO:0006958);Q9BWP8(GO:0019730;GO:0001867;GO:0045087;GO:0006956;GO:0002376);A0A0C4DH25(GO:0038096;GO:0050900;GO:0002376;GO:0002377;GO:0006956;GO:0002250;GO:0006958;GO:0006955);A0A0G2JMI3(GO:0050853;GO:0045087;GO:0002376;GO:0002250;GO:0006958);P01011(GO:0043312);P19652(GO:0043312);A0A0C4DH55(GO:0006955;GO:0002376;GO:0002377;GO:0002250);A0A0B4J1Y8(GO:0006955;GO:0002376;GO:0002377;GO:0002250);P14151(GO:0050900;GO:0043312);P04430(GO:0038096;GO:0050900;GO:0006956;GO:0006958;GO:0006955);P31151(GO:0019730;GO:0061844;GO:0045087;GO:0043312);P01721(GO:0038096;GO:0002376;GO:0050900;GO:0006956;GO:0002250;GO:0006958;GO:0006955);P13796(GO:0002286);P15144(GO:0043312);A0A0B4J1U3(GO:0006955;GO:0002376;GO:0002377;GO:0002250);A0A0G2JPR0(GO:0006956);P04211(GO:0038096;GO:0002376;GO:0050900;GO:0006956;GO:0002250;GO:0006958;GO:0006955);P32119(GO:0002536;GO:0042098;GO:0045321);O00187(GO:0045087;GO:0006956;GO:0002376;GO:0001867;GO:0006958);A0A0B4J231(GO:0050853;GO:0045087;GO:0006958);A0A075B6K2(GO:0006955;GO:0002376;GO:0002377;GO:0002250);P02750(GO:0043312);P01008(GO:0002438);A0A075B6R2(GO:0050853;GO:0045087;GO:0002376;GO:0002250;GO:0006958);A0A075B6K4(GO:0006955;GO:0002376;GO:0002377;GO:0002250);Q03591(GO:0006956);P01009(GO:0043312);A0A075B6R9(GO:0002377;GO:0006955);P80108(GO:0002430);A0A0G2JRQ6(GO:0002377;GO:0006955);A0A0B4J1X5(GO:0050853;GO:0045087;GO:0002376;GO:0002250;GO:0006958);A0A0C4DH43(GO:0050853;GO:0045087;GO:0002376;GO:0002250;GO:0006958);A0A075B6K5(GO:0006955;GO:0002376;GO:0002377;GO:0002250);A0A075B6J9(GO:0006955;GO:0002376;GO:0002377;GO:0002250);P08603(GO:0045087;GO:0002376;GO:0006956;GO:0006957);C9JV77(GO:0043312);A0A087WSY6(GO:0006955;GO:0002376;GO:0002377;GO:0002250);A0A0B4J1X8(GO:0050853;GO:0045087;GO:0002376;GO:0002250;GO:0006958);P01877(GO:0019731;GO:0006955;GO:0050900;GO:0050853;GO:0045087;GO:0006958;GO:0002376;GO:0002250);P01871(GO:0019731;GO:0050900;GO:0050853;GO:0045087;GO:0006958;GO:0002376;GO:0002250);A0A075B7D0(GO:0050853;GO:0045087;GO:0006958);P01715(GO:0038096;GO:0002376;GO:0050900;GO:0006956;GO:0002250;GO:0006958;GO:0006955);P09871(GO:0006956;GO:0045087;GO:0002376;GO:0001867;GO:0006958);A0A075B7D8(GO:0050853;GO:0045087;GO:0006958);P04114(GO:0050900;GO:0002224);A0A075B6S9(GO:0002377;GO:0006955);P04040(GO:0043312);A0A0C4DH33(GO:0050853;GO:0045087;GO:0002376;GO:0002250;GO:0006958);A0A3B3ISR2(GO:0045087;GO:0006958);A0A0J9YX35(GO:0002376;GO:0002250);A0A075B6S5(GO:0006955;GO:0002376;GO:0002377;GO:0002250);P02743(GO:0045087;GO:0006958);P02741(GO:0045087;GO:0006958;GO:0008228);P01860(GO:0050853;GO:0045087;GO:0006956;GO:0006958;GO:0038096;GO:0002376;GO:0002250);P02747(GO:0045087;GO:0002376;GO:0006956;GO:0006955;GO:0006958);P02745(GO:0045087;GO:0002376;GO:0006956;GO:0006958);A0A0B4J1V0(GO:0050853;GO:0045087;GO:0002376;GO:0002250;GO:0006958);A0A0G2JL69(GO:0045087;GO:0006956;GO:0006958);P02748(GO:0006955;GO:0045087;GO:0006957;GO:0006958;GO:0002376);P01034(GO:0043312);P01601(GO:0038096;GO:0050900;GO:0002376;GO:0002377;GO:0006956;GO:0002250;GO:0006958;GO:0006955);P04217(GO:0043312);O14791(GO:0045087);Q07954(GO:0002265);P04196(GO:0061844);P10909(GO:0019730;GO:0002376;GO:0002434;GO:0001774;GO:0006956;GO:0006958;GO:0045087);A0A0C4DH32(GO:0050853;GO:0045087;GO:0002376;GO:0002250;GO:0006958);P05109(GO:0045087;GO:0019730;GO:0043312;GO:0002224;GO:0030593;GO:0002523;GO:0002376);P01861(GO:0050853;GO:0045087;GO:0006956;GO:0006958;GO:0038096;GO:0002376;GO:0002250);A0A0C4DH31(GO:0050853;GO:0045087;GO:0002376;GO:0002250;GO:0006958);A0A0C4DH36(GO:0050853;GO:0045087;GO:0006958);A0A0C4DH34(GO:0050853;GO:0045087;GO:0002376;GO:0002250;GO:0006958);P19320(GO:0030183);A0A0C4DH38(GO:0050853;GO:0045087;GO:0002376;GO:0002250;GO:0006958);A0A0C4DH39(GO:0050853;GO:0045087;GO:0002376;GO:0002250;GO:0006958);P25311(GO:0006955);P01599(GO:0038096;GO:0002376;GO:0050900;GO:0006956;GO:0002250;GO:0006958;GO:0006955);O43866(GO:0002376);P01024(GO:0045087;GO:0006956;GO:0006957;GO:0006955;GO:0006958;GO:0043312;GO:0002376);C9J8S2(GO:0019732;GO:0045087;GO:0061760);O95497(GO:0045087;GO:0043312);P02538(GO:0061844);P60709(GO:0038096);P06331(GO:0038096;GO:0002376;GO:0050900;GO:0006956;GO:0002250;GO:0006958;GO:0006955);E7EQB2(GO:0019732;GO:0019731;GO:0061844;GO:0002227);A0A0A0MRZ9(GO:0006955;GO:0002376;GO:0002377;GO:0002250);P01742(GO:0038096;GO:0002376;GO:0050900;GO:0006956;GO:0002250;GO:0006958;GO:0006955);P00748(GO:0045087);P80748(GO:0038096;GO:0002376;GO:0050900;GO:0006956;GO:0002250;GO:0006958;GO:0006955);P04264(GO:0001867;GO:0043312);P0C0L4(GO:0045087;GO:0006956;GO:0006958;GO:0002376);A0A087WSY4(GO:0050853;GO:0045087;GO:0002376;GO:0002250;GO:0006958);Q15485(GO:0002376;GO:0045087;GO:0008228;GO:0001867;GO:0006956);P04433(GO:0038096;GO:0002376;GO:0050900;GO:0006956;GO:0002250;GO:0006958;GO:0006955);A0A0C4DH24(GO:0006955;GO:0002376;GO:0002377;GO:0002250);P06727(GO:0002227);Q9NZP8(GO:0045087;GO:0002376;GO:0006958);A0A0C4DH29(GO:0050853;GO:0045087;GO:0002376;GO:0002250;GO:0006958);P18206(GO:0043312);P01817(GO:0038096;GO:0002376;GO:0050900;GO:0006956;GO:0002250;GO:0006958;GO:0006955);P01814(GO:0038096;GO:0002376;GO:0050900;GO:0006956;GO:0002250;GO:0006958;GO:0006955);P01619(GO:0019731;GO:0006955;GO:0050900;GO:0006956;GO:0006958;GO:0038096;GO:0002376;GO:0002250);A0A0B4J1V1(GO:0050853;GO:0045087;GO:0002376;GO:0002250;GO:0006958);A0A075B7B8(GO:0050853;GO:0045087;GO:0006958);P07360(GO:0045087;GO:0002376;GO:0006957;GO:0006958);A0A0B4J1V2(GO:0050853;GO:0045087;GO:0002376;GO:0002250;GO:0006958);P07437(GO:0042267;GO:0043312);A0A0J9YVY3(GO:0002376;GO:0002250);P01699(GO:0038096;GO:0002376;GO:0050900;GO:0006956;GO:0002250;GO:0006958;GO:0006955) | 49|82 | O75636(GO:0051607;GO:0006956;GO:0045087;GO:0002376;GO:0001867);P19652(GO:0043312);P04211(GO:0038096;GO:0002376;GO:0050900;GO:0006956;GO:0002250;GO:0006958;GO:0006955);K7ERG9(GO:0006957);P02763(GO:0043312);A0A0C4DH73(GO:0038096;GO:0050900;GO:0002376;GO:0002377;GO:0006956;GO:0002250;GO:0006958;GO:0006955);O00187(GO:0045087;GO:0006956;GO:0002376;GO:0001867;GO:0006958);A0A0C4DH32(GO:0050853;GO:0045087;GO:0002376;GO:0002250;GO:0006958);A0A0J9YX35(GO:0002376;GO:0002250);A0A0G2JL69(GO:0045087;GO:0006956;GO:0006958);A0A0C4DH34(GO:0050853;GO:0045087;GO:0002376;GO:0002250;GO:0006958);P01599(GO:0038096;GO:0002376;GO:0050900;GO:0006956;GO:0002250;GO:0006958;GO:0006955);Q86YZ3(GO:0043312);P01703(GO:0038096;GO:0002376;GO:0050900;GO:0006956;GO:0002250;GO:0006958;GO:0006955);P18206(GO:0043312);P01601(GO:0038096;GO:0050900;GO:0002376;GO:0002377;GO:0006956;GO:0002250;GO:0006958;GO:0006955);P32119(GO:0002536;GO:0042098;GO:0045321);P01721(GO:0038096;GO:0002376;GO:0050900;GO:0006956;GO:0002250;GO:0006958;GO:0006955);A0A075B6K2(GO:0006955;GO:0002376;GO:0002377;GO:0002250);Q9NZP8(GO:0045087;GO:0002376;GO:0006958);P01008(GO:0002438);A0A075B6K4(GO:0006955;GO:0002376;GO:0002377;GO:0002250);P01009(GO:0043312);P09172(GO:0050900;GO:0002443);A0A0C4DH67(GO:0002376;GO:0002250);A0A075B6J9(GO:0006955;GO:0002376;GO:0002377;GO:0002250);A0A075B6R9(GO:0002377;GO:0006955);P01782(GO:0038096;GO:0002376;GO:0050900;GO:0006956;GO:0002250;GO:0006958;GO:0006955);P00748(GO:0045087);P01780(GO:0038096;GO:0002376;GO:0050900;GO:0006956;GO:0002250;GO:0006958;GO:0006955);A0A087WSY6(GO:0006955;GO:0002376;GO:0002377;GO:0002250);A0A0C4DH25(GO:0038096;GO:0050900;GO:0002376;GO:0002377;GO:0006956;GO:0002250;GO:0006958;GO:0006955);M0R0Q9(GO:0006956;GO:0006957;GO:0006955;GO:0006958;GO:0043312);P30041(GO:0043312);P0DP01(GO:0002376;GO:0002250);A0A0G2JRQ6(GO:0002377;GO:0006955);P01817(GO:0038096;GO:0002376;GO:0050900;GO:0006956;GO:0002250;GO:0006958;GO:0006955);P01715(GO:0038096;GO:0002376;GO:0050900;GO:0006956;GO:0002250;GO:0006958;GO:0006955);P01717(GO:0038096;GO:0002376;GO:0050900;GO:0006956;GO:0002250;GO:0006958;GO:0006955);A0A0B4J1V0(GO:0050853;GO:0045087;GO:0002376;GO:0002250;GO:0006958);P07360(GO:0045087;GO:0002376;GO:0006957;GO:0006958);P01857(GO:0050853;GO:0045087;GO:0006956;GO:0006958;GO:0038096;GO:0002376;GO:0002250);A0A075B6S5(GO:0006955;GO:0002376;GO:0002377;GO:0002250);A0A075B6H7(GO:0002377;GO:0006955);P02747(GO:0045087;GO:0002376;GO:0006956;GO:0006955;GO:0006958);P80748(GO:0038096;GO:0002376;GO:0050900;GO:0006956;GO:0002250;GO:0006958;GO:0006955);P02751(GO:0050900);P01699(GO:0038096;GO:0002376;GO:0050900;GO:0006956;GO:0002250;GO:0006958;GO:0006955);E7EUT5(GO:0061844) | 0.036276839 | 0.126968935 | https://www.ebi.ac.uk/QuickGO/term/GO:0002376 |
| GO:0008152 | metabolic process | biological_process | 197|375 | P00488(GO:0018149;GO:0072378);P04275(GO:0007597);P03951(GO:0007597;GO:0031639;GO:0006508);P03950(GO:0006651;GO:0090305;GO:0009303;GO:0090501);P05160(GO:0072378);P23142(GO:0072378);B7ZKJ8(GO:0030212);P21333(GO:0042789);P68871(GO:0042744);P22792(GO:0006508);O75460(GO:0016310;GO:0008152;GO:0046777;GO:0036289;GO:0098787;GO:1901142;GO:0006402;GO:0006468;GO:0006397;GO:0090502;GO:0006351;GO:0070054;GO:1990579;GO:0006379);P01597(GO:0006508);P01042(GO:0007597;GO:0044267;GO:0043687);A0A087X1J7(GO:0055114);Q14520(GO:0006508);P01602(GO:0006508);P08519(GO:0006508;GO:0006629);P0DJI8(GO:0044267);A0A2R8Y3M9(GO:0016042;GO:0006508);P51884(GO:0018146;GO:0042340);P06312(GO:0006508);P01743(GO:0006508);K7ERG9(GO:0006508);P02790(GO:0020027;GO:0042168);P01763(GO:0006508);P01764(GO:0006508);P01766(GO:0006508);Q15582(GO:0044267);P01782(GO:0006508);P01780(GO:0006508);P06331(GO:0006508);E7EUT5(GO:0006096;GO:0035606;GO:0055114;GO:0006006);P30041(GO:0042744;GO:0016042;GO:0006629;GO:0008152;GO:0055114;GO:0046475);P06702(GO:0035606;GO:0018119;GO:0006914);Q9Y5Y7(GO:0030214;GO:0006027);P02787(GO:0044267;GO:0043687;GO:0070371);P19827(GO:0030212);P01834(GO:0006508);P19823(GO:0044267;GO:0030212;GO:0043687);Q96IY4(GO:0006508);E7END6(GO:0006508);P00748(GO:0007597;GO:0031638;GO:0006508;GO:0016485;GO:0016540;GO:0002353);O00391(GO:0044267;GO:0043687;GO:0055114);P69905(GO:0042744);D6RAR4(GO:0006508);G3V0E5(GO:0031623);P02768(GO:0044267;GO:0043687);O75636(GO:0006508);Q96PD5(GO:0009253;GO:0001519);P02760(GO:0042167;GO:0018298;GO:0030163);A0A0C4DH73(GO:0006508);P02766(GO:0044267;GO:0001523;GO:0042572;GO:0006144);J3KPS3(GO:0006096);Q9BWP8(GO:0006508);A0A0G2JL69(GO:0006508);P19320(GO:0009308;GO:0055114);Q15166(GO:0046395;GO:0019372;GO:0016311;GO:0019439;GO:0046226;GO:0010124);P09172(GO:0042423;GO:0042420;GO:0042421;GO:0006589;GO:0055114);P01703(GO:0006508);P01700(GO:0006508);P01701(GO:0006508);P01706(GO:0006508);P0C0L5(GO:0006508);P02042(GO:0042744);P01705(GO:0006508);E9PK25(GO:0006468);P23083(GO:0006508);P01709(GO:0006508);P02671(GO:0044267;GO:0072378;GO:0031639;GO:0043687;GO:0072377);P02675(GO:0072378;GO:0031639);P07996(GO:0018149);H3BTN5(GO:0016310;GO:0006096);F5H8B0(GO:0006508);P0CF74(GO:0006508);P61626(GO:0044267;GO:0016998;GO:0008152);P02652(GO:0006656;GO:0001523;GO:0009395;GO:0044267;GO:0018158;GO:0006641;GO:0043687;GO:0008203;GO:0046340;GO:0018206;GO:0042157);Q16880(GO:0006682;GO:0006687;GO:0006665;GO:0006629;GO:0008152);P22891(GO:0006508);Q06033(GO:0030212);C9JF17(GO:0006629;GO:0006006);P05155(GO:0007597);M0R0Q9(GO:0006631;GO:0044267;GO:0043687);O95445(GO:0042157;GO:0001523);A0A0A0MS51(GO:1990000);P00734(GO:0007597;GO:0044267;GO:0006508;GO:0072378);P13645(GO:0018149);P35908(GO:0018149);P13473(GO:0061684;GO:0006914;GO:1905146);P01717(GO:0006508);P48740(GO:0006508);K7ERI9(GO:0042157);P01857(GO:0006508);E7EQB2(GO:0006508);P16930(GO:0006572;GO:0006559;GO:0006527;GO:0009072;GO:0008152;GO:1902000);G3XAP6(GO:0016485);A0A0C4DH25(GO:0006508);P11226(GO:0006508);P01019(GO:0070371;GO:0051403);Q9UHG3(GO:0030327;GO:0030328;GO:0030329;GO:0055114);P41222(GO:0006633;GO:0006631;GO:0019371;GO:0006629;GO:0001516;GO:0006693);B0YIW2(GO:0042157;GO:0001523;GO:0006641;GO:0019433);P04430(GO:0006508);Q8IV42(GO:0016310;GO:0097056;GO:0001514;GO:0006412);O00187(GO:0006508);A0A0A0MTH3(GO:0018105;GO:0006468);O14791(GO:0042157;GO:0044267;GO:0006629;GO:0043687;GO:0008202;GO:0008203);K7ER74(GO:0001523;GO:0016042;GO:0006629);G3XAK1(GO:0006508);P04211(GO:0006508);P80748(GO:0006508);Q96KN2(GO:0008152;GO:0006508);Q13093(GO:0034440;GO:0006629;GO:0016042;GO:0046469);P32119(GO:0042744;GO:0002536;GO:0055114;GO:0019430);P01721(GO:0006508);A0A2R8Y6G6(GO:0006096);P55058(GO:0010189;GO:0006629);P01009(GO:0044267;GO:0043687);P02751(GO:0018149;GO:0044267;GO:0043687);P02753(GO:0042572;GO:0001523;GO:0006094);P02100(GO:0042744);P55056(GO:0006629);P01008(GO:0043687;GO:0044267);J3KRP0(GO:0008152;GO:0006508);P69892(GO:0042744);Q14624(GO:0030212);P01817(GO:0006508);A0A669KAY4(GO:0032802;GO:0042157;GO:0044267;GO:0008203;GO:0043687;GO:0006644;GO:0006641;GO:0016540);P01344(GO:0044267;GO:0006349;GO:0005975;GO:0006006);P27169(GO:0016311;GO:0046434;GO:0019372;GO:0006629;GO:0008203;GO:0046395;GO:0046470;GO:0019439);P08185(GO:0008211);P35579(GO:0006509);P01715(GO:0006508);P09871(GO:0006508);P01619(GO:0006508);F8WF14(GO:0050783;GO:0019695);Q9NZP8(GO:0031638;GO:0006508);P15144(GO:0006508;GO:0043171);A0A0A0MRJ7(GO:0044267;GO:0043687);P04040(GO:0020027;GO:0009060;GO:0006641;GO:0055114;GO:0008203;GO:0042744);A0A3B3ISR2(GO:0031638);P18065(GO:0044267);P00915(GO:0006730);P05546(GO:0044267;GO:0043687);P02743(GO:0044267);P02747(GO:0006508);D6RE82(GO:0006364);P02745(GO:0006508);P01718(GO:0006508);P02749(GO:0007597;GO:0031639;GO:0006641);P80108(GO:0006501;GO:0006507;GO:0046470);P01034(GO:0044267;GO:0043687);P01601(GO:0006508);P11597(GO:0008202;GO:0006629;GO:0006641;GO:0008203;GO:0046470);P02649(GO:0019433;GO:0042157;GO:0042158;GO:0042159;GO:0042982;GO:0044267;GO:0006629;GO:0006641;GO:0043687;GO:0008202;GO:0008203;GO:0001523;GO:0006707);Q07954(GO:0150094;GO:0001523;GO:0006629;GO:0031623;GO:0042157);P02647(GO:0018206;GO:0019433;GO:0042158;GO:0044267;GO:0018158;GO:0006629;GO:0006695;GO:0008202;GO:0008203;GO:0070371;GO:0042157;GO:0006656;GO:0006644;GO:0008211;GO:0001523;GO:0043687);P01861(GO:0006508);P10909(GO:0006629);P04114(GO:0042157;GO:0016042;GO:0001523;GO:0006629;GO:0042158;GO:0043687;GO:0006642;GO:0044267;GO:0042159;GO:0008202;GO:0008203;GO:0019433);P05109(GO:0006914;GO:0018119);P15169(GO:0010815;GO:0016485;GO:0006508;GO:0006518;GO:0030070);P01860(GO:0006508);H0YAC1(GO:0007597;GO:0031639;GO:0031638;GO:0006508;GO:0055114);P25311(GO:0090501);P01599(GO:0006508);P01024(GO:0006631;GO:0044267;GO:0006629;GO:0043687;GO:0006508);P01023(GO:0007597);B4E1Z4(GO:0006508);O95497(GO:0015939;GO:0006807);P00747(GO:0044267;GO:0006508);P00740(GO:0007597;GO:0031638;GO:0006508);P60709(GO:0016579);P00742(GO:0006508;GO:0007598);P01742(GO:0006508);P00450(GO:0044267;GO:0043687;GO:0055114);P43251(GO:0006768;GO:0006807);P04264(GO:0018149);P0C0L4(GO:0044267;GO:0006508;GO:0043687);C9JV77(GO:0044267;GO:0043687);P04180(GO:0006656;GO:0042158;GO:0034435;GO:0008203;GO:0006644;GO:0006629;GO:0008202;GO:0046470);Q9HDC9(GO:0009058);P35858(GO:0044267);Q15485(GO:0006508);P04433(GO:0006508);P06727(GO:0042744;GO:0042157;GO:0001523;GO:0044267;GO:0016042;GO:0008203;GO:0046470;GO:0019430;GO:0006695);C9J5S7(GO:0000413);Q5SRP5(GO:0042157);Q13103(GO:0044267;GO:0043687);P33908(GO:1904381;GO:0006491;GO:0006486;GO:0008152);P01814(GO:0006508);P01704(GO:0006508);C9J8S2(GO:0001523);Q96HR3(GO:0006367;GO:0016567;GO:0006351);Q13790(GO:0006629;GO:0008203;GO:0008202);P11021(GO:0030433;GO:0051603);Q15848(GO:0019395;GO:0006006;GO:0006635;GO:0006091);P01699(GO:0006508) | 47|82 | O75636(GO:0006508);P01857(GO:0006508);P04211(GO:0006508);K7ERG9(GO:0006508);A0A0C4DH73(GO:0006508);E7END6(GO:0006508);P15169(GO:0010815;GO:0016485;GO:0006508;GO:0006518;GO:0030070);K7ER74(GO:0001523;GO:0016042;GO:0006629);Q9Y5Y7(GO:0030214;GO:0006027);P01599(GO:0006508);P09172(GO:0042423;GO:0042420;GO:0042421;GO:0006589;GO:0055114);P01703(GO:0006508);P80748(GO:0006508);P01601(GO:0006508);P32119(GO:0042744;GO:0002536;GO:0055114;GO:0019430);O00187(GO:0006508);A0A2R8Y3M9(GO:0016042;GO:0006508);P55058(GO:0010189;GO:0006629);Q9NZP8(GO:0031638;GO:0006508);P01008(GO:0043687;GO:0044267);P01009(GO:0044267;GO:0043687);P00748(GO:0007597;GO:0031638;GO:0006508;GO:0016485;GO:0016540;GO:0002353);P02751(GO:0018149;GO:0044267;GO:0043687);P01782(GO:0006508);P01780(GO:0006508);A0A669KAY4(GO:0032802;GO:0042157;GO:0044267;GO:0008203;GO:0043687;GO:0006644;GO:0006641;GO:0016540);P01344(GO:0044267;GO:0006349;GO:0005975;GO:0006006);A0A0C4DH25(GO:0006508);M0R0Q9(GO:0006631;GO:0044267;GO:0043687);P30041(GO:0042744;GO:0016042;GO:0006629;GO:0008152;GO:0055114;GO:0046475);Q5SRP5(GO:0042157);P02787(GO:0044267;GO:0043687;GO:0070371);P01817(GO:0006508);P01715(GO:0006508);P19823(GO:0044267;GO:0030212;GO:0043687);P01717(GO:0006508);Q96IY4(GO:0006508);K7ERI9(GO:0042157);P00915(GO:0006730);P55056(GO:0006629);Q96HR3(GO:0006367;GO:0016567;GO:0006351);P02747(GO:0006508);P01721(GO:0006508);A0A0G2JL69(GO:0006508);P43251(GO:0006768;GO:0006807);P01699(GO:0006508);E7EUT5(GO:0006096;GO:0035606;GO:0055114;GO:0006006) | 0.061879363 | 0.152878426 | https://www.ebi.ac.uk/QuickGO/term/GO:0008152 |
| GO:0016020 | membrane | cellular_component | 203|375 | P33908(GO:0016020;GO:0000139);P03951(GO:0016020;GO:0005886);P15814(GO:0016020);B7ZKJ8(GO:0005886);P30041(GO:0016020);P08571(GO:0045121;GO:0010008;GO:0016020;GO:0030667;GO:0005886);E9PK25(GO:0016020;GO:0031258;GO:0032587);O75460(GO:0005789;GO:0005637;GO:0016020);Q86YZ3(GO:0001533);P01597(GO:0016020;GO:0005886);P01042(GO:0005886);H0Y755(GO:0016020);P01602(GO:0016020;GO:0005886);A0A0G2JMI3(GO:0016020;GO:0005886);A0A0B4J1U7(GO:0016020;GO:0005886);P15169(GO:0097060);A0A0B4J1U3(GO:0016020;GO:0005886);A0A2R8Y3M9(GO:0016020);P06312(GO:0016020;GO:0005886);P43121(GO:0016020;GO:0005886);A0A0A0MT36(GO:0016020;GO:0005886);P02750(GO:0016020);P60763(GO:0016020;GO:0005886);P01763(GO:0016020;GO:0005886);P01764(GO:0016020;GO:0005886);A0A087X0S5(GO:0016020;GO:0005765;GO:0042383);P01766(GO:0016020;GO:0005886);Q15582(GO:0005886);F8WF14(GO:0016020);C9JB55(GO:0009925;GO:0016324);P0DOY3(GO:0016020;GO:0005886);P26038(GO:0016324;GO:0016323;GO:0005886;GO:0016020;GO:0031528);P20851(GO:0005886);P18428(GO:0016020);P01780(GO:0016020;GO:0005886);P06331(GO:0016020;GO:0005886);P0DP01(GO:0016020;GO:0005886);E7EUT5(GO:0031965;GO:0005886);A0A0J9YXX1(GO:0016020;GO:0005886);P0DP02(GO:0016020;GO:0005886);P06702(GO:0016020;GO:0005886);Q9Y5Y7(GO:0016020;GO:0005886);P61224(GO:0016020;GO:0035577;GO:0005886;GO:0045121);P02787(GO:0016020;GO:0010008;GO:0030665;GO:0009925;GO:0016324);P01833(GO:0016020;GO:0005886;GO:0035577);P01834(GO:0016020;GO:0005886);P04003(GO:0005886);P00450(GO:0005765;GO:0005886);Q16880(GO:0016020;GO:0005886);A0A0C4DGZ8(GO:0016020);P69905(GO:0016020);P15144(GO:0030667;GO:0016020;GO:0005886;GO:0005765);G3V0E5(GO:0010008;GO:0005886;GO:0016323);Q96PD5(GO:0016020);P02760(GO:0005886);Q01518(GO:0016020;GO:0005886);A0A0C4DH73(GO:0016020;GO:0005886);P07357(GO:0016020;GO:0005886);A0A0G2JI36(GO:0055038;GO:0016020;GO:0005886;GO:0031901;GO:0012507;GO:0030670;GO:0000139);P07358(GO:0016020);P35579(GO:0016020;GO:0005886;GO:0032154);P01703(GO:0016020;GO:0005886);P01700(GO:0016020;GO:0005886);P01701(GO:0016020;GO:0005886);P01706(GO:0016020;GO:0005886);P04264(GO:0016020;GO:0005886;GO:0001533);P01704(GO:0016020;GO:0005886);P01705(GO:0016020;GO:0005886);P23083(GO:0016020;GO:0005886);P01709(GO:0016020;GO:0005886);P02671(GO:0005886);P02675(GO:0005886);A0A075B6I0(GO:0016020;GO:0005886);P0CF74(GO:0016020;GO:0005886);O00391(GO:0016020;GO:0000139);A0A0C4DH67(GO:0016020;GO:0005886);P35527(GO:0016020);P05154(GO:0002080;GO:0016020);I3L1J2(GO:0016020;GO:0005886);M0R0Q9(GO:0005886);P00734(GO:0005886);P13645(GO:0016020;GO:0001533);A0A0A0MS14(GO:0016020;GO:0005886);A0A0A0MS15(GO:0016020;GO:0005886);P01715(GO:0016020;GO:0005886);P01717(GO:0016020;GO:0005886);A0A087X1L8(GO:0016020);P01718(GO:0016020;GO:0005886);A0A286YEY4(GO:0016020);P01857(GO:0016020;GO:0005886);A0A075B6Q5(GO:0016020;GO:0005886);P80748(GO:0016020;GO:0005886);A0A0C4DH25(GO:0016020;GO:0005886);P01601(GO:0016020;GO:0005886);Q9UHG3(GO:0005774;GO:0005886);P41222(GO:0005789;GO:0016020;GO:0031965);A0A0C4DH55(GO:0016020;GO:0005886);A0A0B4J1Y8(GO:0016020;GO:0005886);A0A0A0MS09(GO:0016020);A0A1W2PQU7(GO:0016020);C9JXI5(GO:0016020;GO:0005886);P13796(GO:0016020;GO:0005886;GO:0032587);A0A087WSZ0(GO:0016020;GO:0005886);P09486(GO:0031092;GO:0005886);P04211(GO:0016020;GO:0005886);P14151(GO:0030667;GO:0016020;GO:0005886);P01721(GO:0016020;GO:0005886);A0A075B6K2(GO:0016020;GO:0005886);B4E1Z4(GO:0005886);P01008(GO:0005886);A0A075B6R2(GO:0016020;GO:0005886);Q9UNN8(GO:0016020;GO:0005886);P01009(GO:0033116;GO:0000139);P80108(GO:0005765);A0A0B4J1X5(GO:0016020;GO:0005886);A0A0C4DH43(GO:0016020;GO:0005886);A0A075B6K5(GO:0016020;GO:0005886);P13647(GO:0016020;GO:0005886);A0A075B6K4(GO:0016020;GO:0005886);A0A087WSY4(GO:0016020;GO:0005886);A0A087WSY6(GO:0016020;GO:0005886);A0A0B4J1X8(GO:0016020;GO:0005886);P01877(GO:0016020;GO:0005886);P01871(GO:0016020;GO:0005886);P13473(GO:0101003;GO:0035577;GO:0010008;GO:0016020;GO:0031902;GO:0045121;GO:0005886;GO:0000421;GO:0030670;GO:0031088;GO:0005765;GO:0098857);P10909(GO:0016020;GO:0031966;GO:0005743);O75882(GO:0016020;GO:0005886);P01619(GO:0016020;GO:0005886);P00918(GO:0016323;GO:0016020;GO:0005886);P04114(GO:0005789;GO:0030669;GO:0010008;GO:0012506;GO:0005886);Q6EMK4(GO:0016020;GO:0005886;GO:0005765);A0A0A0MRJ7(GO:0033116;GO:0005886;GO:0016020;GO:0000139);P04040(GO:0016020;GO:0005886;GO:0005778);A0A0C4DH33(GO:0016020;GO:0005886);P02751(GO:0016324);P18065(GO:0016324);P01861(GO:0016020;GO:0005886);A0A075B6S5(GO:0016020;GO:0005886);P01860(GO:0016020;GO:0005886);A0A0B4J2D9(GO:0016020;GO:0005886);P02748(GO:0016020;GO:0005886);P01034(GO:0031965);P08514(GO:0031092;GO:0016020;GO:0005886);P02649(GO:0016020;GO:0030669;GO:0005886);Q07954(GO:0016020;GO:0030666;GO:0005886;GO:0016323;GO:0005765);P04196(GO:0005886);P02647(GO:0005886);A0A0A0MS51(GO:0005886);A0A0C4DH32(GO:0016020;GO:0005886);P05109(GO:0016020;GO:0005886);A0A0J9YX35(GO:0016020;GO:0005886);A0A0C4DH31(GO:0016020;GO:0005886);A0A0C4DH34(GO:0016020;GO:0005886);P19320(GO:0016020;GO:0042383;GO:0005886);H0YAC1(GO:0005886);Q14624(GO:0005886);A0A0C4DH38(GO:0016020;GO:0005886);A0A0C4DH39(GO:0016020;GO:0005886);P25311(GO:0005886);P01599(GO:0016020;GO:0005886);O43866(GO:0016020);P01024(GO:0005886);P01743(GO:0016020;GO:0005886);P07437(GO:0045121);P09172(GO:0030667;GO:0016020;GO:0030658;GO:0042584);O95497(GO:0016020;GO:0035577;GO:0005886);P02538(GO:0016020);P01782(GO:0016020;GO:0005886);P00747(GO:0005886);P00740(GO:0005886);P60709(GO:0016020;GO:0005886);P00742(GO:0005886);A0A0A0MRZ9(GO:0016020;GO:0005886);P01742(GO:0016020;GO:0005886);P00748(GO:0005886);Q08380(GO:0016020);A0A075B6J9(GO:0016020;GO:0005886);P0C0L5(GO:0005886);P0C0L4(GO:0005886);A0A669KAY4(GO:0036020;GO:0005886;GO:0005765);Q9HDC9(GO:0016020);H0Y5E4(GO:0016020);P04433(GO:0016020;GO:0005886);A0A0C4DH24(GO:0016020;GO:0005886);P04430(GO:0016020;GO:0005886);A0A0C4DH29(GO:0016020;GO:0005886);P18206(GO:0016020;GO:0042383;GO:0005886);J3KPA1(GO:0016020);P01817(GO:0016020;GO:0005886);P01814(GO:0016020;GO:0005886);P21333(GO:0016020;GO:0005886);A0A0B4J1V1(GO:0016020;GO:0005886);A0A0B4J1V0(GO:0016020;GO:0005886);A0A0B4J1V2(GO:0016020;GO:0005886);Q86UX7(GO:0016020);P68032(GO:0016020);H9KV75(GO:0042383;GO:0097381);P11021(GO:0005789;GO:0016020;GO:0005886);Q9Y490(GO:0016020;GO:0005886;GO:0032587);A0A0J9YVY3(GO:0016020;GO:0005886);P01699(GO:0016020;GO:0005886);P07737(GO:0016020);P35908(GO:0016020;GO:0001533) | 46|82 | C9JB55(GO:0009925;GO:0016324);P04211(GO:0016020;GO:0005886);A0A0C4DH73(GO:0016020;GO:0005886);P01857(GO:0016020;GO:0005886);A0A0C4DH32(GO:0016020;GO:0005886);A0A0J9YX35(GO:0016020;GO:0005886);A0A0C4DH34(GO:0016020;GO:0005886);P01599(GO:0016020;GO:0005886);P09172(GO:0030667;GO:0016020;GO:0030658;GO:0042584);P01703(GO:0016020;GO:0005886);P35527(GO:0016020);P0DP01(GO:0016020;GO:0005886);P01601(GO:0016020;GO:0005886);P01721(GO:0016020;GO:0005886);A0A2R8Y3M9(GO:0016020);A0A075B6K2(GO:0016020;GO:0005886);P01009(GO:0033116;GO:0000139);P02751(GO:0016324);A0A075B6K4(GO:0016020;GO:0005886);Q86YZ3(GO:0001533);P01780(GO:0016020;GO:0005886);Q08380(GO:0016020);P80748(GO:0016020;GO:0005886);P00918(GO:0016323;GO:0016020;GO:0005886);P01782(GO:0016020;GO:0005886);A0A0C4DH67(GO:0016020;GO:0005886);A0A669KAY4(GO:0036020;GO:0005886;GO:0005765);A0A087WSY6(GO:0016020;GO:0005886);I3L1J2(GO:0016020;GO:0005886);A0A0C4DH25(GO:0016020;GO:0005886);E7EUT5(GO:0031965;GO:0005886);P30041(GO:0016020);Q9Y5Y7(GO:0016020;GO:0005886);P18206(GO:0016020;GO:0042383;GO:0005886);P02787(GO:0016020;GO:0010008;GO:0030665;GO:0009925;GO:0016324);P01817(GO:0016020;GO:0005886);P01715(GO:0016020;GO:0005886);P01717(GO:0016020;GO:0005886);A0A0B4J1V0(GO:0016020;GO:0005886);P01008(GO:0005886);P15169(GO:0097060);A0A075B6S5(GO:0016020;GO:0005886);P00748(GO:0005886);A0A075B6J9(GO:0016020;GO:0005886);P01699(GO:0016020;GO:0005886);M0R0Q9(GO:0005886) | 0.092170285 | 0.18434057 | https://www.ebi.ac.uk/QuickGO/term/GO:0016020 |
| GO:0043226 | organelle | cellular_component | 213|375 | P08779(GO:0005856;GO:0070062;GO:0005634);P68366(GO:0070062;GO:0005856;GO:0015630);P04275(GO:0005783;GO:0031091;GO:0070062;GO:0033093);J3KNB4(GO:0042581);Q9UHG3(GO:0070062;GO:0005764);P51884(GO:0070062);P03950(GO:0005730;GO:0031410;GO:0005634);G3XAP6(GO:0070062);P23142(GO:0070062);P19652(GO:0070062);P08571(GO:0005794;GO:0070062);P68871(GO:0070062);E9PK25(GO:0015629;GO:0070062;GO:0005634;GO:0031982;GO:0030864);O75460(GO:0005739;GO:0005783);Q86YZ3(GO:0070062;GO:0005634);P35527(GO:0005634;GO:0070062);P01042(GO:0070062);P43251(GO:0070062);P01602(GO:0070062);P08185(GO:0070062);P59665(GO:0070062);P15169(GO:0005794;GO:0030141);P0DJI8(GO:0070062);P03951(GO:0070062);P43121(GO:0005634);P02750(GO:0043231;GO:0070062);P02790(GO:0070062);P60763(GO:0070062;GO:0005856);P01764(GO:0070062);A0A087X0S5(GO:0070062);A0A087WWU8(GO:0015629);P00918(GO:0070062);P0DOY3(GO:0070062);P26038(GO:0070062;GO:0005634;GO:0005856;GO:0031982);P01782(GO:0070062);P18428(GO:0070062);P01780(GO:0070062);P31151(GO:0005783;GO:0005634);H3BTN5(GO:0005634);P01008(GO:0070062);P30041(GO:0005634;GO:0070062;GO:0005764);P06702(GO:0070062;GO:0005634;GO:0005856);Q9Y5Y7(GO:0070062);P61224(GO:0005811;GO:0070062;GO:0070382);P10643(GO:0070062);P02787(GO:0055037;GO:0030139;GO:0031410;GO:0005770;GO:0070062;GO:0031982;GO:0005768;GO:0005769);P19827(GO:0070062);P01833(GO:0070062);P01834(GO:0070062);P19823(GO:0070062);Q96IY4(GO:0070062);P04004(GO:0005783;GO:0070062;GO:0043231);P00450(GO:0070062);O00391(GO:0070062;GO:0005794;GO:0043231);K7ER74(GO:0005769);P69905(GO:0070062);G3XAK1(GO:0005773);G3V0E5(GO:0055037;GO:0070062;GO:0042470;GO:0005769);P02768(GO:0005783;GO:0070062;GO:0005794;GO:0005634);P08697(GO:0070062);Q96PD5(GO:0070062);Q86UX7(GO:0002102;GO:0070062);P02763(GO:0070062);A0A087WWT3(GO:0005794;GO:0005783);P02766(GO:0070062);Q9H4B7(GO:0005856;GO:0070062);A0A2R8Y619(GO:0005634;GO:0005694);P07357(GO:0070062);P37802(GO:0031982;GO:0070062);P19320(GO:0005783;GO:0005794;GO:0002102;GO:0070062;GO:0005769);Q15166(GO:0070062;GO:0043231);P07358(GO:1903561;GO:0070062);P09172(GO:0005783;GO:0031410;GO:0043231);P01701(GO:0070062);P0C0L5(GO:0070062);P21333(GO:0015629;GO:0070062;GO:0005730;GO:0005856;GO:0005634;GO:0030863);P22792(GO:0070062);P02671(GO:0031091;GO:1903561;GO:0070062);P02675(GO:0005783;GO:1903561;GO:0031091;GO:0070062);P07996(GO:0005783;GO:0030141;GO:0031091;GO:0016529;GO:0070062);P13671(GO:0070062);P61626(GO:0070062);P04217(GO:0070062);P04180(GO:0070062);E9PHK0(GO:0070062);P22891(GO:0070062);Q06033(GO:0070062);C9JF17(GO:0022626;GO:0005783;GO:0070062);P00739(GO:0070062);P05155(GO:0070062);P05154(GO:0031094;GO:0031091;GO:0070062);P27169(GO:0070062;GO:0043231);P20742(GO:0070062);P00734(GO:0070062);P13645(GO:0070062;GO:0005634);P35579(GO:0015629;GO:0005634;GO:0070062;GO:0005856;GO:0030863;GO:0005819);P13473(GO:0005770;GO:0044754;GO:0031410;GO:0070062;GO:0005764;GO:0005768);P00738(GO:0070062);P01857(GO:0070062);M0R0Q9(GO:0070062);E7EQB2(GO:0042581);P80748(GO:0070062);P01019(GO:0070062);Q8N1N4(GO:0070062);P01011(GO:0070062;GO:0005634);Q9Y6R7(GO:0070062);P41222(GO:0005783;GO:0070062;GO:0005634;GO:0005794;GO:0005791);P11597(GO:0070062;GO:0031982);B0YIW2(GO:0070062;GO:0005769);C9J5S7(GO:0005634);A0A1W2PQU7(GO:0045111);Q9UGM5(GO:0070062);C9JXI5(GO:0031410);P13796(GO:0015629;GO:0002102;GO:0070062;GO:0005856);P15144(GO:0070062;GO:0005793);P36955(GO:0070062;GO:0042470);P09486(GO:0031091;GO:0005634;GO:0005739;GO:0031982);Q16610(GO:0070062);P18206(GO:0015629;GO:0002102;GO:0070062;GO:0005856;GO:1903561);P0DPH7(GO:0005856);P32119(GO:0070062);O00187(GO:0070062);A0A0B4J231(GO:0070062);P80108(GO:0070062;GO:0043231);P08514(GO:0070062);P01009(GO:0005783;GO:0030134;GO:0005794;GO:0070062;GO:0043231);P02751(GO:0070062;GO:0005793);P02760(GO:0070062;GO:0043231);B4E1Z4(GO:0070062);P68032(GO:0015629;GO:0070062;GO:0005856);J3QRV5(GO:0043231);Q14624(GO:0070062);P13647(GO:0070062;GO:0005634);P43652(GO:0070062);Q01518(GO:0030864;GO:0070062);Q9UNN8(GO:0070062);C9JV77(GO:0070062;GO:0005794);P29622(GO:0070062);P01877(GO:0070062);P01871(GO:0070062);O75882(GO:0070062);P01619(GO:0070062);F8WF14(GO:0005783);P01597(GO:0070062);Q6EMK4(GO:0070062;GO:0005739);A0A0A0MRJ7(GO:1903561;GO:0030134);P04040(GO:0005739;GO:0005783;GO:0005778;GO:0070062;GO:0005777;GO:0005794;GO:0043231;GO:0005764);P05543(GO:0070062);P18065(GO:0031410;GO:0070062);P00915(GO:0070062);P05546(GO:0070062);Q562R1(GO:0005856;GO:0070062;GO:0015629);P02743(GO:0070062;GO:0005634);D6RAR4(GO:0005791);D6RE82(GO:0005730;GO:0005634);P01031(GO:0070062);P02749(GO:0070062);P02748(GO:0070062);P01034(GO:0005783;GO:0005771;GO:0070062;GO:0031982;GO:0005764;GO:0043292);C9JB55(GO:0055037;GO:0030139;GO:0005770;GO:0005769);Q15582(GO:0070062);P02649(GO:0005783;GO:0005634;GO:0070062;GO:0005794;GO:1903561;GO:0005769);Q07954(GO:0030136;GO:0005634;GO:0005768;GO:0005769);P04196(GO:0070062;GO:0036019);P02647(GO:0031410;GO:0005634;GO:0070062;GO:0030139;GO:1903561;GO:0005769);P10909(GO:0005783;GO:0005794;GO:0043231;GO:0005856;GO:0031410;GO:0005634;GO:0005739;GO:0070062;GO:0042583);P04114(GO:0005783;GO:0070062;GO:0005790;GO:0043231;GO:0005769);P05109(GO:0045111;GO:0005634;GO:0005856;GO:0070062);P01861(GO:0070062);P01860(GO:0070062);A0A0G2JI36(GO:0005783;GO:0070062;GO:0005794);H0YAC1(GO:0070062);H9KV75(GO:1990357);P25311(GO:0070062;GO:0005634);P01599(GO:0070062);P01024(GO:0070062);P01023(GO:0070062);P02753(GO:0070062);P02538(GO:0070062;GO:0005634);P00747(GO:0070062);P00740(GO:0070062);P60709(GO:0015629;GO:0036464;GO:0005634;GO:0070062;GO:0098871;GO:0005856;GO:0031982;GO:0030863);P02533(GO:0070062;GO:0005634);P00748(GO:0070062;GO:0005791);Q08380(GO:0070062);P16930(GO:0070062);P08603(GO:0070062);E7EUT5(GO:0005634;GO:0005811;GO:0015630;GO:0043231);P04264(GO:0005856;GO:0070062;GO:0005634);P0C0L4(GO:0070062);A0A669KAY4(GO:0005770;GO:0030134;GO:0005794;GO:0005791;GO:0005764;GO:0005783;GO:0005769);P02652(GO:0070062;GO:0005769);Q9HDC9(GO:0005783);P35858(GO:0070062);Q15485(GO:0070062);A0A096LPE2(GO:0070062);P04433(GO:0070062);P06727(GO:0070062;GO:0005769);Q9NZP8(GO:0070062);P02775(GO:0031091);P33908(GO:0005794;GO:0070062;GO:0005793;GO:0005783);P01704(GO:0070062);P07360(GO:0070062);A0A0A0MS51(GO:0015629;GO:0005634;GO:0045335;GO:0002102);Q5T749(GO:0070062);P07437(GO:0036464;GO:0070062;GO:0005634;GO:0005856);Q96HR3(GO:0005634);P11021(GO:0005783;GO:0005793;GO:0005634;GO:0005739;GO:0005790;GO:0042470;GO:0043231;GO:0070062);Q9Y490(GO:0005856;GO:0070062);Q15848(GO:0005783);Q16880(GO:0043231);P07737(GO:0070062;GO:0005634;GO:0005856);P35908(GO:0070062;GO:0005634) | 40|82 | C9JB55(GO:0055037;GO:0030139;GO:0005770;GO:0005769);P19652(GO:0070062);J3QRV5(GO:0043231);Q96IY4(GO:0070062);P02763(GO:0070062);P29622(GO:0070062);Q9UGM5(GO:0070062);P00915(GO:0070062);P15169(GO:0005794;GO:0030141);K7ER74(GO:0005769);P37802(GO:0031982;GO:0070062);P01599(GO:0070062);P09172(GO:0005783;GO:0031410;GO:0043231);P35527(GO:0005634;GO:0070062);P32119(GO:0070062);O00187(GO:0070062);P01009(GO:0005783;GO:0030134;GO:0005794;GO:0070062;GO:0043231);P01008(GO:0070062);Q9NZP8(GO:0070062);Q86YZ3(GO:0070062;GO:0005634);P00748(GO:0070062;GO:0005791);Q08380(GO:0070062);P02751(GO:0070062;GO:0005793);P00918(GO:0070062);P01782(GO:0070062);P01780(GO:0070062);A0A669KAY4(GO:0005770;GO:0030134;GO:0005794;GO:0005791;GO:0005764;GO:0005783;GO:0005769);M0R0Q9(GO:0070062);P30041(GO:0005634;GO:0070062;GO:0005764);Q9Y5Y7(GO:0070062);P18206(GO:0015629;GO:0002102;GO:0070062;GO:0005856;GO:1903561);P02787(GO:0055037;GO:0030139;GO:0031410;GO:0005770;GO:0070062;GO:0031982;GO:0005768;GO:0005769);P19823(GO:0070062);P07360(GO:0070062);P05543(GO:0070062);P01857(GO:0070062);Q96HR3(GO:0005634);P80748(GO:0070062);P43251(GO:0070062);E7EUT5(GO:0005634;GO:0005811;GO:0015630;GO:0043231) | 0.025551391 | 0.119239823 | https://www.ebi.ac.uk/QuickGO/term/GO:0043226 |
| GO:0005198 | structural molecule activity | molecular_function | 37|375 | P08779(GO:0005200;GO:0005198);P68366(GO:0005200);P04275(GO:0005201);P51884(GO:0005201;GO:0030021);A0A1W2PQU7(GO:0005198);P23142(GO:0005201);Q9H4B7(GO:0005200);P02649(GO:0005198);Q8N1N4(GO:0005198);Q86YZ3(GO:0030280);P35527(GO:0005200;GO:0005198);P0DPH7(GO:0005200);P02538(GO:0005200;GO:0005198);P02671(GO:0005198);P02751(GO:0005201);P60709(GO:0005200;GO:0098973);A0A140T8Y3(GO:0005201);P02533(GO:0005200;GO:0005198);A0A087X0S5(GO:0030020);P68032(GO:0005200);Q15582(GO:0005201);P02675(GO:0005198);P04264(GO:0030280;GO:0005198);P26038(GO:0005200);P07996(GO:0005201);F8W1S1(GO:0005198);Q13201(GO:0005201);P18206(GO:0005198);P13645(GO:0005198;GO:0030280);P13647(GO:0005200;GO:0005198);P04004(GO:0005201);P07437(GO:0005200;GO:0005198);Q92954(GO:0030021);G3XAP6(GO:0005201);Q9Y490(GO:0005200);Q16610(GO:0005201);P35908(GO:0005200;GO:0005198;GO:0030280) | 4|82 | P35527(GO:0005200;GO:0005198);P18206(GO:0005198);P02751(GO:0005201);Q86YZ3(GO:0030280) | 0.039326034 | 0.12705334 | https://www.ebi.ac.uk/QuickGO/term/GO:0005198 |
| GO:0022414 | reproductive process | biological_process | 19|375 | P35527(GO:0007283);P35579(GO:0051295;GO:0000212);P01344(GO:0060669;GO:0001892);P21333(GO:0097368);P03950(GO:0001541;GO:0001556;GO:0001890);P23142(GO:0007566);P11021(GO:0001554);P01019(GO:0007565);Q9UGM5(GO:0007339;GO:0007338);P05154(GO:0061107;GO:0007342;GO:0007283;GO:0007338);P04114(GO:0007283;GO:0009566);P36955(GO:0042698);P20742(GO:0007565);P18065(GO:0007565);G3XAK1(GO:0007283;GO:0007566);P02753(GO:0060065;GO:0048807;GO:0060068);P02760(GO:0007565);P01034(GO:0060009;GO:0008584;GO:0007566);P09172(GO:0042711) | 4|82 | P35527(GO:0007283);P01344(GO:0060669;GO:0001892);Q9UGM5(GO:0007339;GO:0007338);P09172(GO:0042711) | 0.224352177 | 0.277140924 | https://www.ebi.ac.uk/QuickGO/term/GO:0022414 |
| GO:0051179 | localization | biological_process | 169|375 | P00488(GO:0002576);P68366(GO:0097711;GO:0002576);P04275(GO:0002576);P08571(GO:0006909;GO:0006898;GO:0043312);P68871(GO:0015701;GO:0015671;GO:0030185;GO:0006898;GO:0043312);Q86YZ3(GO:0043312);P01042(GO:0002576);P01602(GO:0006898);P01601(GO:0006898);P59665(GO:0043312);P0DJI8(GO:0006898);A0A2R8Y3M9(GO:0006898);P06312(GO:0006898);P02790(GO:0015886;GO:0006898);P01763(GO:0006898);P01764(GO:0006898);P01766(GO:0006898);P01782(GO:0006898);P18428(GO:0006869;GO:0033036;GO:0015920);P01780(GO:0006898);P00742(GO:0006888);P02776(GO:0002576);P02775(GO:0002576;GO:0043312;GO:1904659);Q13201(GO:0002576);P02751(GO:0002576);P30041(GO:0043312);P06702(GO:0032119;GO:0043312);P61224(GO:0043312);D6RF35(GO:0051180;GO:0035461);P02787(GO:0006826;GO:0002576;GO:0033572;GO:0006811);P01833(GO:0002415;GO:0043312;GO:0043113);P01834(GO:0006898);P04004(GO:0006898);P01743(GO:0006898);Q92954(GO:0006898);Q16880(GO:0002175);P69905(GO:0015701;GO:0015671;GO:0006898);G3V0E5(GO:0031623;GO:0006898;GO:0033572);P02768(GO:0051659;GO:0002576;GO:0006898);P02760(GO:0006898);P02763(GO:0002576;GO:0043312);Q01518(GO:0006898;GO:0043312);A0A0C4DH73(GO:0006898);P02766(GO:0070327;GO:0043312);P37802(GO:0002576);P29622(GO:0002576);E9PHK0(GO:0002576);P01703(GO:0006898);P01700(GO:0006898);P01701(GO:0006898);P01706(GO:0006898);P21333(GO:1902396;GO:0051220;GO:0002576;GO:0034394;GO:0045184;GO:0072659;GO:0043113);P01705(GO:0006898);P23083(GO:0006898);P01709(GO:0006898);P02671(GO:0002576);P02675(GO:0002576);P07996(GO:0002576);P0CF74(GO:0006898);P61626(GO:0043312);P04180(GO:0043691;GO:0030301);O00391(GO:0043312;GO:0002576);P22891(GO:0006888);Q06033(GO:0002576);C9JF17(GO:0006869);P05155(GO:0002576);P05154(GO:0006869);M0R0Q9(GO:0043312);O95445(GO:0006869;GO:0043691;GO:0033344);P00734(GO:0006888);P35579(GO:0032418;GO:0045055;GO:0051295;GO:0015031);P01715(GO:0006898);P01717(GO:0006898);P01718(GO:0006898);P00738(GO:0006898;GO:0043312);P00739(GO:0006898);Q9BWP8(GO:0006898);G3XAP6(GO:0009306);P04433(GO:0006898);P01019(GO:0006606;GO:0050663);Q9UHG3(GO:1902476;GO:0099133;GO:0006821);P19652(GO:0002576;GO:0043312);B0YIW2(GO:0006869;GO:0033700;GO:0043691;GO:0033344);P31151(GO:0051238;GO:0043312);O14791(GO:0006869;GO:1902476;GO:0006898);K7ER74(GO:0033700;GO:0006869;GO:0043691;GO:0033344;GO:0042953);P15144(GO:0043312);P09486(GO:0006898;GO:0002576);Q13103(GO:0002576);P04211(GO:0006898);P14151(GO:0043312);P01721(GO:0006898);P08514(GO:0002576);P55058(GO:0006869;GO:0015914;GO:0035627);P01009(GO:0006888;GO:0002576;GO:0043312);P01008(GO:0007595);Q86UX7(GO:0002576);P02750(GO:0043312);A0A2R8Y7X9(GO:0015671);P55056(GO:0006869);P80108(GO:0070633);J3QRV5(GO:0006887);P69892(GO:0015671);H9KV75(GO:0007041);P43652(GO:0051180;GO:0015031;GO:0071693);P02753(GO:0034633);C9JV77(GO:0006907;GO:0002576;GO:0043312);P01344(GO:0002576);P08519(GO:0006869);P01877(GO:0006898);Q16610(GO:0002576);P02100(GO:0015671);P13473(GO:0017038;GO:0006605;GO:0061740;GO:0043312;GO:0002576);P01619(GO:0006898);P00918(GO:0046903;GO:0015701;GO:0015670);P01597(GO:0006898);A0A0A0MRJ7(GO:0006888;GO:0002576);P04040(GO:0006625;GO:0043312);P05543(GO:0070327);P00915(GO:0015701);P48740(GO:0006898);P02749(GO:0034197;GO:0002576);P01034(GO:0043312);P04217(GO:0002576;GO:0043312);P11597(GO:0006869;GO:0015914;GO:0030301;GO:0034197;GO:0043691);P02649(GO:0051651;GO:0043691;GO:0010877;GO:0033700;GO:0015909;GO:0046907;GO:0006898;GO:0006869;GO:0017038;GO:0033344;GO:0097114;GO:0097113);Q07954(GO:0006909;GO:0043277;GO:0150093;GO:0045056;GO:0006897;GO:0031623;GO:0006898;GO:0042953);P04196(GO:0002576;GO:0015886);P02647(GO:0019915;GO:0043691;GO:0070508;GO:0015914;GO:0051180;GO:0033700;GO:0006898;GO:0006869;GO:0030301;GO:0002576;GO:0033344);P10909(GO:0043691;GO:0017038;GO:0061740;GO:0061741;GO:0002576);P04114(GO:0006869;GO:0042953;GO:0030301;GO:0006898;GO:0033344);P05109(GO:0043312;GO:0032119);P08697(GO:0002576);Q14624(GO:0002576);P25311(GO:0071806;GO:0055085);P01599(GO:0006898);O43866(GO:0006898);P01024(GO:0043312);P01023(GO:0002576);C9J8S2(GO:0002576);O95497(GO:0043312);P00747(GO:0002576);P00740(GO:0006888);P60709(GO:0048488);P06331(GO:0006898);P01742(GO:0006898);P00450(GO:0006825;GO:0006811;GO:0006826);Q08380(GO:0006898;GO:0002576);P80748(GO:0006898);P04264(GO:0043312);A0A669KAY4(GO:0007041);P02652(GO:0006869;GO:0030301;GO:0033344;GO:0033700;GO:0043691);A0A0C4DH25(GO:0006898);P06727(GO:0006869;GO:0033344;GO:0033700;GO:0043691);P04430(GO:0006898);Q5SRP5(GO:0043691;GO:0033344);P18206(GO:0002576;GO:0034394;GO:0043312);P01817(GO:0006898);P01814(GO:0006898);P01704(GO:0006898);A0A0A0MS51(GO:0042989);P01011(GO:0002576;GO:0043312);P07437(GO:0030705;GO:0097711;GO:0043312);Q13790(GO:0006869);P11021(GO:0035437;GO:0031204;GO:1901998);Q9Y490(GO:0002576;GO:0007016);Q15848(GO:0072659);P01699(GO:0006898);P02042(GO:0015671) | 38|82 | P19652(GO:0002576;GO:0043312);P04211(GO:0006898);J3QRV5(GO:0006887);P02763(GO:0002576;GO:0043312);P29622(GO:0002576);K7ER74(GO:0033700;GO:0006869;GO:0043691;GO:0033344;GO:0042953);P37802(GO:0002576);P01599(GO:0006898);Q86YZ3(GO:0043312);P01703(GO:0006898);P01601(GO:0006898);P01721(GO:0006898);A0A2R8Y3M9(GO:0006898);P55058(GO:0006869;GO:0015914;GO:0035627);P01009(GO:0006888;GO:0002576;GO:0043312);P01008(GO:0007595);P55056(GO:0006869);Q08380(GO:0006898;GO:0002576);P02751(GO:0002576);P02787(GO:0006826;GO:0002576;GO:0033572;GO:0006811);P01782(GO:0006898);P01780(GO:0006898);A0A669KAY4(GO:0007041);P01344(GO:0002576);A0A0C4DH73(GO:0006898);A0A0C4DH25(GO:0006898);M0R0Q9(GO:0043312);P30041(GO:0043312);Q5SRP5(GO:0043691;GO:0033344);P18206(GO:0002576;GO:0034394;GO:0043312);P00918(GO:0046903;GO:0015701;GO:0015670);P01817(GO:0006898);P01715(GO:0006898);P01717(GO:0006898);P05543(GO:0070327);P00915(GO:0015701);P80748(GO:0006898);P01699(GO:0006898) | 0.096325592 | 0.183894313 | https://www.ebi.ac.uk/QuickGO/term/GO:0051179 |
| GO:0003824 | catalytic activity | molecular_function | 133|375 | P00488(GO:0003810;GO:0016740;GO:0016746);P68366(GO:0003924);P03951(GO:0004252;GO:0016787;GO:0070009;GO:0008233;GO:0008236);K7ERG9(GO:0004252;GO:0016787;GO:0008233;GO:0008236);J3KPS3(GO:0016829;GO:0003824;GO:0004332);P22792(GO:0004181);O75460(GO:0004540;GO:0016787;GO:0004521;GO:0004674;GO:0016740;GO:0004672;GO:0003824;GO:0016301;GO:0004519);A0A087X1J7(GO:0004601;GO:0004602;GO:0016491);Q14520(GO:0004252;GO:0016787;GO:0008233;GO:0008236);P01602(GO:0004252);P11226(GO:0004252);P15169(GO:0016787;GO:0004181;GO:0004180;GO:0004185;GO:0008233;GO:0008237);A0A2R8Y3M9(GO:0004252;GO:0004623);Q96KN2(GO:0016787;GO:0004180;GO:0016805;GO:0008233;GO:0008237);P06312(GO:0004252);P01743(GO:0004252);P03950(GO:0004540;GO:0016787;GO:0004518;GO:0004519);P60763(GO:0003924);P01763(GO:0004252);P01764(GO:0004252);P01766(GO:0004252);F8WF14(GO:0016787;GO:0004104;GO:0003824;GO:0003990);P01782(GO:0004252);P01780(GO:0004252);P06331(GO:0004252);E7EUT5(GO:0004365;GO:0016620;GO:0035605);A0A0U1RQV3(GO:0005006);P30041(GO:0016491;GO:0047499;GO:0016787;GO:0102568;GO:0003824;GO:0051920;GO:0102567;GO:0004623;GO:0004601;GO:0004602);P61224(GO:0003924);P01834(GO:0004252);E7END6(GO:0004252;GO:0016787;GO:0008233;GO:0008236);P00748(GO:0004252;GO:0008233;GO:0008236;GO:0016787);O00391(GO:0016491;GO:0016971;GO:0016972;GO:0003756);P43251(GO:0016787;GO:0047708;GO:0016810;GO:0016811);D6RAR4(GO:0004252;GO:0016787;GO:0008233;GO:0008236);O75636(GO:0004252);P69905(GO:0004601);P68871(GO:0004601);Q96PD5(GO:0008745;GO:0016787);A0A0C4DH73(GO:0004252);Q9H4B7(GO:0003924);A0A0G2JL69(GO:0004252;GO:0016787;GO:0008233;GO:0008236);E7EWH8(GO:0008903;GO:0016853);P19320(GO:0008131);Q15166(GO:0016787;GO:0004063;GO:0102007;GO:0004064;GO:0018733);P09172(GO:0016491;GO:0004500;GO:0004497;GO:0003824;GO:0016715);P01703(GO:0004252);P01700(GO:0004252);P01701(GO:0004252);P01706(GO:0004252);P01704(GO:0004252);P01705(GO:0004252);P23083(GO:0004252);P01709(GO:0004252);H3BTN5(GO:0016301;GO:0003824;GO:0016740;GO:0004743);F5H8B0(GO:0004252;GO:0016787;GO:0008233;GO:0008236);P0CF74(GO:0004252);P61626(GO:0016798;GO:0003796;GO:0016787;GO:0003824);Q16880(GO:0008489;GO:0016740;GO:0015020;GO:0008194;GO:0016757;GO:0047263;GO:0016758);P22891(GO:0004252);P00734(GO:0004252;GO:0008233;GO:0008236;GO:0016787);P35579(GO:0016887;GO:0000146;GO:0003774;GO:0030898);P01715(GO:0004252);P01717(GO:0004252);P48740(GO:0004252;GO:0016787;GO:0008233;GO:0008236);P00739(GO:0004252);E7EQB2(GO:0004252);Q9BWP8(GO:0004252);A0A0C4DH25(GO:0004252);P01601(GO:0004252);Q9UHG3(GO:0016670;GO:0008555;GO:0001735;GO:0016491);P41222(GO:0016853;GO:0004667);Q96IY4(GO:0008233;GO:0008237;GO:0004181;GO:0004180;GO:0016787);C9J5S7(GO:0016853;GO:0003755);Q8IV42(GO:0016301;GO:0016740);P01721(GO:0004252);A0A0A0MTH3(GO:0004674;GO:0004672);G3XAK1(GO:0004252);P0DPH7(GO:0003924);Q13093(GO:0047499;GO:0016787;GO:0016788;GO:0003847);P32119(GO:0008379;GO:0016491;GO:0051920;GO:0004601);O00187(GO:0004252;GO:0016787;GO:0008236;GO:0008233);A0A2R8Y6G6(GO:0004634);P16930(GO:0016787;GO:0004334;GO:0003824);B4E1Z4(GO:0004252;GO:0016787;GO:0008233;GO:0008236);Q9NZP8(GO:0004252;GO:0016787;GO:0008233;GO:0008236);P80108(GO:0004630;GO:0016787;GO:0004621);J3KRP0(GO:0016787;GO:0004180;GO:0016805;GO:0008237);P33908(GO:0015923;GO:0016798;GO:0003824;GO:0016787;GO:0004571);A0A669KAY4(GO:0004252);P27169(GO:0004064;GO:0102007;GO:0016787;GO:0004063);P10909(GO:0016887);P09871(GO:0004252;GO:0016787;GO:0008233;GO:0008236);P00918(GO:0016829;GO:0004064;GO:0004089);P15144(GO:0004177;GO:0008233;GO:0008237;GO:0070006;GO:0016787);P04040(GO:0016491;GO:0004601;GO:0004046;GO:0016684;GO:0004096);A0A3B3ISR2(GO:0004252);P00915(GO:0016829;GO:0016836;GO:0004089;GO:0004064);P02747(GO:0004252);P02745(GO:0004252);P01718(GO:0004252);P68032(GO:0016887);P04211(GO:0004252);P08519(GO:0004252;GO:0016787;GO:0008233;GO:0008236);P01857(GO:0004252);P01597(GO:0004252);P01861(GO:0004252);P01860(GO:0004252);H0YAC1(GO:0004252;GO:0004497;GO:0008236;GO:0008233;GO:0016491;GO:0016705;GO:0016787);P25311(GO:0004540);P01599(GO:0004252);P01024(GO:0004252);O95497(GO:0016811;GO:0016787;GO:0017159);P00747(GO:0004252;GO:0004175;GO:0016787;GO:0008233;GO:0008236);P00740(GO:0004252;GO:0004175;GO:0016787;GO:0008233;GO:0008236);P00742(GO:0004252;GO:0016787;GO:0008233;GO:0008236);P01742(GO:0004252);P00450(GO:0016491;GO:0004322);P80748(GO:0004252);P0C0L5(GO:0004252);P0C0L4(GO:0004252);P04180(GO:0008374;GO:0016740;GO:0016746;GO:0004607);Q9HDC9(GO:0016844;GO:0004064);Q15485(GO:0004252);P04433(GO:0004252);P04430(GO:0004252);P01817(GO:0004252);P01814(GO:0004252);P01619(GO:0004252);P07437(GO:0003924);Q96HR3(GO:0061630);P11021(GO:0016887;GO:0042623;GO:0016787);P01699(GO:0004252) | 36|82 | O75636(GO:0004252);P04211(GO:0004252);K7ERG9(GO:0004252;GO:0016787;GO:0008233;GO:0008236);A0A0C4DH73(GO:0004252);E7END6(GO:0004252;GO:0016787;GO:0008233;GO:0008236);P15169(GO:0016787;GO:0004181;GO:0004180;GO:0004185;GO:0008233;GO:0008237);A0A0G2JL69(GO:0004252;GO:0016787;GO:0008233;GO:0008236);E7EWH8(GO:0008903;GO:0016853);P01599(GO:0004252);P09172(GO:0016491;GO:0004500;GO:0004497;GO:0003824;GO:0016715);P01703(GO:0004252);P01601(GO:0004252);P32119(GO:0008379;GO:0016491;GO:0051920;GO:0004601);O00187(GO:0004252;GO:0016787;GO:0008236;GO:0008233);A0A2R8Y3M9(GO:0004252;GO:0004623);Q9NZP8(GO:0004252;GO:0016787;GO:0008233;GO:0008236);P00748(GO:0004252;GO:0008233;GO:0008236;GO:0016787);P80748(GO:0004252);P01782(GO:0004252);P01780(GO:0004252);A0A669KAY4(GO:0004252);A0A0C4DH25(GO:0004252);E7EUT5(GO:0004365;GO:0016620;GO:0035605);P30041(GO:0016491;GO:0047499;GO:0016787;GO:0102568;GO:0003824;GO:0051920;GO:0102567;GO:0004623;GO:0004601;GO:0004602);P00918(GO:0016829;GO:0004064;GO:0004089);P01817(GO:0004252);P01715(GO:0004252);P01717(GO:0004252);Q96IY4(GO:0008233;GO:0008237;GO:0004181;GO:0004180;GO:0016787);P01857(GO:0004252);Q96HR3(GO:0061630);P02747(GO:0004252);P01721(GO:0004252);P00915(GO:0016829;GO:0016836;GO:0004089;GO:0004064);P43251(GO:0016787;GO:0047708;GO:0016810;GO:0016811);P01699(GO:0004252) | 0.020586211 | 0.108077608 | https://www.ebi.ac.uk/QuickGO/term/GO:0003824 |
| GO:0016209 | antioxidant activity | molecular_function | 13|375 | P02768(GO:0016209);P02649(GO:0016209);A0A087X1J7(GO:0004601;GO:0004602);P04040(GO:0004601;GO:0016209;GO:0004096);P00738(GO:0016209);P32119(GO:0008379;GO:0016209;GO:0051920;GO:0004601);O95445(GO:0016209);P06727(GO:0016209);P30041(GO:0016209;GO:0051920;GO:0004601;GO:0004602);P06702(GO:0016209);Q5SRP5(GO:0016209);P69905(GO:0004601);P68871(GO:0004601) | 3|82 | P30041(GO:0016209;GO:0051920;GO:0004601;GO:0004602);Q5SRP5(GO:0016209);P32119(GO:0008379;GO:0016209;GO:0051920;GO:0004601) | 0.258193514 | 0.309832216 | https://www.ebi.ac.uk/QuickGO/term/GO:0016209 |
| GO:0032991 | protein-containing complex | cellular_component | 136|375 | P51884(GO:0005583);P03950(GO:0032311);P23142(GO:0005577);P15814(GO:0042571);A0A0B4J1X8(GO:0042571);P08571(GO:0046696);P68871(GO:0005833;GO:0031838);A0A0B4J1V2(GO:0042571);O75460(GO:1990604;GO:1990597;GO:1990630;GO:1990332);A0A0G2JMI3(GO:0042571);A0A0B4J1U7(GO:0042571);P0DJI8(GO:0034364);P60763(GO:0031941);P01764(GO:0042571);A0A087X0S5(GO:0032991;GO:0005589;GO:0005581);P07358(GO:0005579);D6R934(GO:0005581);E7EUT5(GO:0097452;GO:1990904);A0A0G2JMB2(GO:0042571);Q03591(GO:0032991);P10643(GO:0005579);P02787(GO:1990712);P01833(GO:0043235);P01834(GO:0042571);P04004(GO:0071062);P13796(GO:0002102);P69905(GO:0022627;GO:0005833;GO:0031838);G3V0E5(GO:1990712);P02768(GO:0032991);O75636(GO:0005581);Q86UX7(GO:0002102);A0A0B4J1X5(GO:0042571);P02766(GO:0032991);P07357(GO:0005579);P19320(GO:0002102;GO:0071065);J3QT83(GO:0005581);P21333(GO:0031941;GO:0031523);P02042(GO:0005833;GO:0031838);P02671(GO:0005577);P02675(GO:0005577);P07996(GO:0005577);P13671(GO:0005579);P01619(GO:0071748;GO:0071751;GO:0071756);P0CF74(GO:0042571);P04180(GO:0034364);C9JF17(GO:0022626);P05154(GO:0036024;GO:0036029;GO:0036025;GO:0036027;GO:0036026;GO:0097183;GO:0097182;GO:0097181;GO:0036028;GO:0032991;GO:0036030);P27169(GO:0034364;GO:0034366);I3L1J2(GO:0016342);M0R0Q9(GO:0032991);O95445(GO:0034361;GO:0034362;GO:0034364;GO:0034365;GO:0034366);A0A075B7F0(GO:0042571);A0A0A0MS14(GO:0042571);P35579(GO:0016459;GO:0008180;GO:0097513;GO:0016460;GO:0008305;GO:0032991);A0A0A0MS15(GO:0042571);P00738(GO:0031838);P01857(GO:0042571);A0A075B6Q5(GO:0042571);Q9BWP8(GO:0005581);G3XAP6(GO:0032991);P11226(GO:0005581);Q9UHG3(GO:0034361);B0YIW2(GO:0042627;GO:0034361;GO:0034363;GO:0034366);O14791(GO:0034361;GO:0034364);K7ER74(GO:0042627;GO:0034361;GO:0034362;GO:0034363;GO:0034366);C9JC84(GO:0005577);C9JPQ9(GO:0005577);A0A2R8Y6G6(GO:0000015);Q13093(GO:0034362);A0A0B4J231(GO:0042571);P08514(GO:0008305);P55058(GO:0034364);P02751(GO:0005577);A0A075B6R2(GO:0042571);P02753(GO:0032991);P02100(GO:0005833;GO:0031838);A0A2R8Y7X9(GO:0005833);P55056(GO:0034361;GO:0034364);P69892(GO:0005833;GO:0031838);A0A0C4DH43(GO:0042571);A0A087WSY4(GO:0042571);P02647(GO:0034361;GO:0034362;GO:0034363;GO:0034364;GO:0034366;GO:0042627;GO:0034365);P01877(GO:0071748;GO:0071752;GO:0071751);P01871(GO:0071756;GO:0071757);A0A075B7D0(GO:0042571);P10909(GO:0034366;GO:0032991);A0A075B7D8(GO:0042571);Q08830(GO:0005577);A0A0C4DH32(GO:0042571);P01860(GO:0042571);P02747(GO:0005581);D6RE82(GO:0030688;GO:0030687);P02745(GO:0005581;GO:0005602);A0A075B7B8(GO:0042571);P01031(GO:0005579);P02749(GO:0042627;GO:0034361;GO:0034364);P02748(GO:0005579);C9JB55(GO:1990712);P11597(GO:0034364);P02649(GO:0034361;GO:0034362;GO:0034363;GO:0034364;GO:1990777;GO:0042627;GO:0034365);Q07954(GO:0098797;GO:0043235);P08519(GO:0034358);P00739(GO:0034366);P04114(GO:0034360;GO:0034359;GO:0034361;GO:0034362;GO:0034363;GO:0042627);A0A0C4DH33(GO:0042571);P01861(GO:0042571);A0A0C4DH31(GO:0042571);A0A0C4DH36(GO:0042571);A0A0C4DH34(GO:0042571);A0A0G2JI36(GO:0042612);P08697(GO:0005577);H9KV75(GO:0031941);A0A0C4DH38(GO:0042571);A0A0C4DH39(GO:0042571);P01024(GO:0032991);P60709(GO:0036464;GO:0032991;GO:1990904;GO:0035267);E7EQB2(GO:0032991);A0A669KAY4(GO:1990667;GO:1990666);P02652(GO:0042627;GO:0034361;GO:0034364;GO:0034366);P35858(GO:0042567);Q15485(GO:0005581);A0A096LPE2(GO:0034364);P06727(GO:0042627;GO:0034361;GO:0034364);A0A0C4DH29(GO:0042571);Q5SRP5(GO:0034361;GO:0034365;GO:0034366;GO:0034362;GO:0034364);P18206(GO:0002102;GO:0032991);A0A2R8Y619(GO:0000786);A0A0B4J1V1(GO:0042571);A0A0B4J1V0(GO:0042571);P07360(GO:0005579);A0A0A0MS51(GO:0002102;GO:0032991);P07437(GO:0036464;GO:0045298;GO:0032991);Q96HR3(GO:0000151;GO:0016592);Q13790(GO:0034362;GO:0034364);P11021(GO:0034663;GO:0008180;GO:0032991);Q15848(GO:0005581;GO:0032991) | 22|82 | C9JB55(GO:1990712);O75636(GO:0005581);A0A0C4DH32(GO:0042571);K7ER74(GO:0042627;GO:0034361;GO:0034362;GO:0034363;GO:0034366);A0A0C4DH34(GO:0042571);J3QT83(GO:0005581);C9JPQ9(GO:0005577);P55058(GO:0034364);P02751(GO:0005577);P55056(GO:0034361;GO:0034364);A0A669KAY4(GO:1990667;GO:1990666);I3L1J2(GO:0016342);M0R0Q9(GO:0032991);Q5SRP5(GO:0034361;GO:0034365;GO:0034366;GO:0034362;GO:0034364);P18206(GO:0002102;GO:0032991);P02787(GO:1990712);A0A0B4J1V0(GO:0042571);P07360(GO:0005579);P01857(GO:0042571);Q96HR3(GO:0000151;GO:0016592);P02747(GO:0005581);E7EUT5(GO:0097452;GO:1990904) | 0.013631678 | 0.081790069 | https://www.ebi.ac.uk/QuickGO/term/GO:0032991 |
| GO:0044422 | organelle part | cellular_component | 136|375 | P08779(GO:0005882);P00488(GO:0031093);P68366(GO:0005874);P04275(GO:0031093);P51884(GO:0043202;GO:0005796);P03950(GO:0005730);P41222(GO:0005789;GO:0031965);P08571(GO:0010008;GO:0030667);P68871(GO:1904813;GO:0071682;GO:1904724);E9PK25(GO:0016363;GO:0030864);O75460(GO:0005789;GO:1990604;GO:0030176;GO:0005637;GO:1990332);Q86YZ3(GO:0035578);P35527(GO:0005882);P01042(GO:0005788;GO:0031093);P43251(GO:0005759);P59665(GO:0035578;GO:0005796);P0DJI8(GO:0071682;GO:0005881);P02790(GO:0071682);P60763(GO:0031941);A0A087X0S5(GO:0005788;GO:0005765);Q15582(GO:0005802);A0A1W2PQU7(GO:0005883;GO:0005882);P02776(GO:0020005;GO:0031093);P02775(GO:0031093;GO:1904724);Q13201(GO:0031093);E7EUT5(GO:0031965);P30041(GO:0035578);P06702(GO:0005654;GO:0034774);P61224(GO:0035577);P02787(GO:0005788;GO:0010008;GO:0030665;GO:0034774);P01833(GO:0035577);P19823(GO:0005788);P01008(GO:0005788);P04004(GO:0005796;GO:0048237);O00391(GO:0005788;GO:0030173;GO:0031093;GO:0035580;GO:1904724;GO:0000139);P69905(GO:0022627;GO:0071682);G3V0E5(GO:0010008);P02768(GO:0005788;GO:0031093);Q86UX7(GO:0002102;GO:0031093);P02763(GO:0031093;GO:0035580;GO:1904724);Q01518(GO:0030864;GO:0035578);P02766(GO:0035578);J3KPS3(GO:0031430;GO:0031674);A0A0G2JI36(GO:0055038;GO:0071556;GO:0031901;GO:0012507;GO:0030670;GO:0000139);P09172(GO:0034774;GO:0005815;GO:0030667;GO:0030658;GO:0042584;GO:0034466);P21333(GO:0030018;GO:0005730;GO:0031941;GO:0005884;GO:0031523);Q8N1N4(GO:0045095;GO:0005882);P02671(GO:0005788;GO:0031093);P02675(GO:0031093);P07996(GO:0005788;GO:0031093);P61626(GO:0035578;GO:0035580;GO:1904724);D6RE82(GO:0005730);E9PHK0(GO:0001652;GO:0031089);P22891(GO:0005796;GO:0005788);Q06033(GO:0031089);P05155(GO:0031093);P05154(GO:0002080);F8W1S1(GO:0005882;GO:0045095);M0R0Q9(GO:0005788;GO:0035578;GO:0034774);P00734(GO:0005788;GO:0005796);P13645(GO:0005882);P35579(GO:0016459;GO:0008180;GO:0097513;GO:0016460;GO:0042641;GO:0001725;GO:0005826;GO:0005819);P13473(GO:0101003;GO:0035577;GO:0010008;GO:1990836;GO:0043202;GO:0097637;GO:0031902;GO:0000421;GO:0030670;GO:0031088;GO:0005765);P48740(GO:0005654);P00738(GO:0071682;GO:0035580;GO:1904724);P01011(GO:0034774;GO:0035578;GO:0031093);Q9UHG3(GO:0005774);P19652(GO:0031093;GO:0035578;GO:0035580);P31151(GO:0035578);A0A0A0MTH3(GO:0005654);O14791(GO:0005788);P13796(GO:0032432;GO:0002102;GO:0005884;GO:0001725);P15144(GO:0030667;GO:0005765);P09486(GO:0031093;GO:0031092;GO:0016363;GO:0071682);P18206(GO:0034774;GO:0002102;GO:0035580;GO:1904813;GO:0043034);P0DPH7(GO:0005874);P14151(GO:0030667);P08514(GO:0031092);P01009(GO:0033116;GO:0005788;GO:1904813;GO:0031093;GO:0000139);P02751(GO:0005788;GO:0031093);Q9UNN8(GO:0005813);P02750(GO:1904813;GO:1904724;GO:0035580);P68032(GO:0030017;GO:0001725;GO:0031674;GO:0005884;GO:0005865;GO:0042643);H9KV75(GO:0032391;GO:0001725;GO:0005815;GO:0031941;GO:0030486;GO:1990357;GO:0097381);A0A669KAY4(GO:0005788;GO:0036020;GO:0005765);P01344(GO:0031093);P29622(GO:0031089);P13647(GO:0045095;GO:0005882);F8WF14(GO:0005788;GO:0005641);Q6EMK4(GO:0005765);A0A0A0MRJ7(GO:0031093;GO:0033116;GO:0005788;GO:0000139);P04040(GO:0005782;GO:1904813;GO:0005778;GO:0005758;GO:0034774);Q9H4B7(GO:0005874);P05546(GO:0005788);C9J8S2(GO:0031089);P02749(GO:0031089);P80108(GO:0005765);P01034(GO:0005788;GO:1904724;GO:0031965;GO:1904813);P04217(GO:0031093;GO:1904813;GO:0034774);P02649(GO:0005788;GO:0071682;GO:0030669);Q07954(GO:0030666;GO:0005765);P04196(GO:0031093);P02647(GO:0005788;GO:0071682;GO:0034774);P10909(GO:0031966;GO:0099020;GO:0031093;GO:0005743);P04114(GO:0005788;GO:0005789;GO:0043202;GO:0031983;GO:0071682;GO:0030669;GO:0010008;GO:0070971;GO:0012506;GO:0031904);P05109(GO:0034774);P19320(GO:0002102);P08697(GO:0031093);Q14624(GO:0031089);P01024(GO:0005788;GO:0035578;GO:0034774);P01023(GO:0031093);H0YCR7(GO:0005654);O95497(GO:0035577);P02538(GO:0045095;GO:0005882);P00747(GO:0031093);P00740(GO:0005788;GO:0005796);P60709(GO:0005654;GO:0000790;GO:0035267);P00742(GO:0005788;GO:0005796);P02533(GO:0045095;GO:0005882);P00450(GO:0005765;GO:0005788);Q08380(GO:0031089);Q16610(GO:0031089);P04264(GO:0045095;GO:1904813;GO:0005882);P0C0L4(GO:0005788);C9JV77(GO:0005788;GO:0031093;GO:0034774);P02652(GO:0005788);P35858(GO:0005654);P06727(GO:0005788);Q13103(GO:0005788;GO:0031089);P33908(GO:0000139);A0A2R8Y619(GO:0000786);A0A0A0MS51(GO:0030478;GO:0002102);P07437(GO:0035578;GO:0005874;GO:0045298;GO:0005641);Q96HR3(GO:0005654;GO:0016592);P11021(GO:0005789;GO:0034663;GO:0008180;GO:0030176;GO:0005788);P35908(GO:0045095;GO:0005882) | 20|82 | P02787(GO:0005788;GO:0010008;GO:0030665;GO:0034774);P35527(GO:0005882);P19652(GO:0031093;GO:0035578;GO:0035580);E7EUT5(GO:0031965);P19823(GO:0005788);P01008(GO:0005788);A0A669KAY4(GO:0005788;GO:0036020;GO:0005765);P29622(GO:0031089);P01344(GO:0031093);P09172(GO:0034774;GO:0005815;GO:0030667;GO:0030658;GO:0042584;GO:0034466);P30041(GO:0035578);Q96HR3(GO:0005654;GO:0016592);P01009(GO:0033116;GO:0005788;GO:1904813;GO:0031093;GO:0000139);P02751(GO:0005788;GO:0031093);P43251(GO:0005759);Q86YZ3(GO:0035578);P18206(GO:0034774;GO:0002102;GO:0035580;GO:1904813;GO:0043034);P02763(GO:0031093;GO:0035580;GO:1904724);Q08380(GO:0031089);M0R0Q9(GO:0005788;GO:0035578;GO:0034774) | 0.003977287 | 0.083523036 | https://www.ebi.ac.uk/QuickGO/term/GO:0044422 |
| GO:0071840 | cellular component organization or biogenesis | biological_process | 134|375 | P08779(GO:0007010;GO:0045104);P68366(GO:0000226;GO:0007010);P04275(GO:0051260;GO:0030198);P51884(GO:0007409;GO:0030199;GO:0030198);P03950(GO:0034332;GO:0030041);G3XAP6(GO:0030198;GO:0030199);P23142(GO:0030198);P15814(GO:0006911);A0A0B4J1X8(GO:0006911);B1AHL2(GO:0030198);P68871(GO:0051291);E9PK25(GO:0030836;GO:0030036;GO:0030043;GO:0030042;GO:0007010);Q86YZ3(GO:0043163);P35527(GO:0045109);A0A087X1J7(GO:0051289);A0A0G2JMI3(GO:0006911);A0A0B4J1U7(GO:0006911);C9JC84(GO:0051258);P60763(GO:0030031;GO:0030036;GO:0031175;GO:0030838;GO:0000902;GO:0007015;GO:0043652);A0A075B7D8(GO:0006911);P01764(GO:0006911);A0A087X0S5(GO:0070208;GO:0003429;GO:0030198);Q15582(GO:0030198);P26038(GO:0008361;GO:0007010);A0A140T8Y3(GO:0030199;GO:0030198);E7EUT5(GO:0000226);A0A0G2JMB2(GO:0006911);P06702(GO:0031532);P61224(GO:0030033);P02787(GO:0061024;GO:0007015);P35908(GO:0045109);P01834(GO:0006911);P04004(GO:0051258;GO:0030198);O00391(GO:0085029);P13796(GO:0022617;GO:0051017;GO:0051764;GO:0051639);P69905(GO:0051291);P02768(GO:0034375);Q86UX7(GO:0034446;GO:0033622);Q01518(GO:0008154;GO:0007010;GO:0000902;GO:0030036);P02766(GO:0030198);J3KPS3(GO:0051289);P19320(GO:0030198);J3QT83(GO:0003429);P21333(GO:0030030;GO:0031532;GO:0034329;GO:0060271;GO:0051764;GO:0021943;GO:0090307);P02671(GO:0034622;GO:0065003;GO:0030198;GO:0051258);P02675(GO:0034622;GO:0030198;GO:0051258);P07996(GO:0043652;GO:0030198);P0CF74(GO:0006911);P02652(GO:0034375;GO:0034374;GO:0034371;GO:0034378;GO:0034380;GO:0034370);Q16880(GO:0030913;GO:0007010;GO:0048812);C9JF17(GO:0014012);P05154(GO:0007342);I3L1J2(GO:0034332;GO:0007043;GO:0000902);M0R0Q9(GO:0150062);O95445(GO:0034375;GO:0034380);A0A075B7F0(GO:0006911);P13645(GO:0051290);A0A0A0MS14(GO:0006911);P35579(GO:0031032;GO:0031532;GO:0032796;GO:0051295;GO:0000212;GO:0006911;GO:0001778;GO:0000904);P13473(GO:0097352);A0A0A0MS15(GO:0006911);P01857(GO:0006911);A0A075B6Q5(GO:0006911);P01019(GO:0034374);B0YIW2(GO:0034375;GO:0034371;GO:0034379;GO:0034378);A0A1W2PQU7(GO:0061564;GO:0045109;GO:0030198;GO:0031102);A0A0A0MTH3(GO:0034446;GO:0030030);K7ER74(GO:0034375;GO:0034372;GO:0034371;GO:0034370;GO:0034378);C9JPQ9(GO:0051258);P09486(GO:0030198);P0DPH7(GO:0007010;GO:0000226);Q13093(GO:0034441;GO:0034374);A0A0B4J231(GO:0006911);P55058(GO:0034375);P01009(GO:0048208);P02751(GO:0034446;GO:0030198;GO:0033622;GO:0007044);A0A075B6R2(GO:0006911);P02100(GO:0051291);P55056(GO:0034379);P68032(GO:0030240;GO:0031032;GO:0055003);A0A0B4J1X5(GO:0006911);H9KV75(GO:0030036;GO:0051017;GO:0045214;GO:0051764;GO:0007030);A0A087WSY4(GO:0006911);P08519(GO:0034374);P13647(GO:0007010;GO:0031581);P01871(GO:0006911);A0A075B7D0(GO:0006911);A0A0B4J1V2(GO:0006911);A0A0C4DH32(GO:0006911);A0A0A0MRJ7(GO:0048208);P04040(GO:0051289;GO:0051262;GO:0006625);Q9H4B7(GO:0000226;GO:0051225);P02743(GO:0051131);P01860(GO:0006911);A0A075B7B8(GO:0006911);P02748(GO:0051260);P01034(GO:0097435);A0A087WZB5(GO:0031532);P08514(GO:0030198);P11597(GO:0034375;GO:0034374;GO:0034372);P02649(GO:0031102;GO:0034375;GO:0034374;GO:0034372;GO:0034371;GO:0034380;GO:0034378;GO:0007010);P02647(GO:0031102;GO:0034375;GO:0034371;GO:0034378;GO:0014012;GO:0034380);P10909(GO:0000902;GO:0051131;GO:0032286;GO:0001836);P04114(GO:0034374;GO:0034371;GO:0061024;GO:0034378;GO:0034379);H0YAC1(GO:0022617);P01861(GO:0006911);A0A0C4DH31(GO:0006911);A0A0C4DH36(GO:0006911);A0A0C4DH34(GO:0006911);P08697(GO:0030199);A0A0C4DH43(GO:0006911);A0A0C4DH38(GO:0006911);A0A0C4DH39(GO:0006911);A0A0C4DH33(GO:0006911);P01024(GO:0006911;GO:0150062);P01023(GO:0022617);P02538(GO:0007010);P00747(GO:0022617);P60709(GO:0043044;GO:0061024;GO:0034329;GO:0098974);P02533(GO:0045110;GO:0031581);P04264(GO:0051290);P04180(GO:0034375;GO:0034372);P06727(GO:0034375;GO:0034372;GO:0034371;GO:0034380;GO:0034378;GO:0065005;GO:0031102);A0A0C4DH29(GO:0006911);Q5SRP5(GO:0034375;GO:0034380);P18206(GO:0034333;GO:0030032;GO:0043297);A0A0B4J1V1(GO:0006911);A0A0B4J1V0(GO:0006911);A0A0A0MS51(GO:0051127;GO:0030041;GO:0042989;GO:1990000;GO:0045010;GO:0060271;GO:0051016;GO:0006911;GO:0051693;GO:0090527);P07437(GO:0051225;GO:0000226);P01877(GO:0006911);Q9Y490(GO:0007043;GO:0033622;GO:0007016;GO:0030866;GO:0007044);Q15848(GO:0070208;GO:0051260);P07737(GO:0060074;GO:0030837;GO:0030838;GO:0098885;GO:0030036) | 20|82 | P02787(GO:0061024;GO:0007015);P35527(GO:0045109);J3QT83(GO:0003429);E7EUT5(GO:0000226);A0A0C4DH34(GO:0006911);A0A0B4J1V0(GO:0006911);P01857(GO:0006911);A0A087WZB5(GO:0031532);A0A0C4DH32(GO:0006911);M0R0Q9(GO:0150062);P55058(GO:0034375);K7ER74(GO:0034375;GO:0034372;GO:0034371;GO:0034370;GO:0034378);P02751(GO:0034446;GO:0030198;GO:0033622;GO:0007044);I3L1J2(GO:0034332;GO:0007043;GO:0000902);P01009(GO:0048208);Q5SRP5(GO:0034375;GO:0034380);P18206(GO:0034333;GO:0030032;GO:0043297);P55056(GO:0034379);C9JPQ9(GO:0051258);Q86YZ3(GO:0043163) | 0.005245965 | 0.055082631 | https://www.ebi.ac.uk/QuickGO/term/GO:0071840 |
| GO:0001906 | cell killing | biological_process | 18|375 | P00734(GO:0051838;GO:0070945);P02768(GO:0019836);O14791(GO:0031640);P01024(GO:0097278);J3KNB4(GO:0051873);P04196(GO:0051715);P02776(GO:0051873;GO:0031640);P59665(GO:0031640);P02775(GO:0031640);P02538(GO:0051801);P07437(GO:0042267);P11226(GO:0051873);E7EQB2(GO:0031640);E7EUT5(GO:0051873);A0A0G2JI36(GO:0002419;GO:0001913);P02748(GO:0019836;GO:0001906);P61626(GO:0031640);M0R0Q9(GO:0097278) | 2|82 | M0R0Q9(GO:0097278);E7EUT5(GO:0051873) | 0.139070138 | 0.194698193 | https://www.ebi.ac.uk/QuickGO/term/GO:0001906 |
| GO:0030054 | cell junction | cellular_component | 28|375 | P08514(GO:0005925);H0Y2Y8(GO:0005925);Q07954(GO:0005925);Q86UX7(GO:0030054);Q01518(GO:0005925);P31151(GO:0005925);A0A0A0MTH3(GO:0005925;GO:0030054);P13796(GO:0005925;GO:0030054);E9PK25(GO:0005925;GO:0005911);P61224(GO:0030054;GO:0005911);P21333(GO:0005925;GO:0005911);P0C0L4(GO:0030054);P60709(GO:0005925);Q9UNN8(GO:0005925);P68032(GO:0005925);H9KV75(GO:0005925;GO:0005923;GO:0005915);P0C0L5(GO:0030054);P26038(GO:0005925);I3L1J2(GO:0005923;GO:0030054;GO:0005913;GO:0005911);P06702(GO:0030054);P18206(GO:0005925;GO:0030054;GO:0030055;GO:0005916;GO:0005913;GO:0005912;GO:0005911);P35579(GO:0005925;GO:0005913);P04040(GO:0005925);A0A0A0MS51(GO:0005925);P43121(GO:0005925);P11021(GO:0005925);Q9Y490(GO:0005925;GO:0030054);P07737(GO:0005925) | 2|82 | P18206(GO:0005925;GO:0030054;GO:0030055;GO:0005916;GO:0005913;GO:0005912;GO:0005911);I3L1J2(GO:0005923;GO:0030054;GO:0005913;GO:0005911) | 0.026182494 | 0.109966474 | https://www.ebi.ac.uk/QuickGO/term/GO:0030054 |
| GO:0038024 | cargo receptor activity | molecular_function | 7|375 | G3V0E5(GO:0004998);O43866(GO:0005044);Q07954(GO:0030226;GO:0016964;GO:0005044;GO:0005041);P04004(GO:0005044);A0A2R8Y3M9(GO:0005044);Q92954(GO:0005044);Q08380(GO:0005044) | 2|82 | A0A2R8Y3M9(GO:0005044);Q08380(GO:0005044) | 0.295257424 | 0.335157076 | https://www.ebi.ac.uk/QuickGO/term/GO:0038024 |
| GO:0140104 | molecular carrier activity | molecular_function | 8|375 | C9JB55(GO:0034986);P02787(GO:0034986);P69892(GO:0005344);A0A2R8Y7X9(GO:0005344);P02042(GO:0005344);P68871(GO:0005344);P02100(GO:0005344);P69905(GO:0005344) | 2|82 | C9JB55(GO:0034986);P02787(GO:0034986) | 0.308094703 | 0.340525724 | https://www.ebi.ac.uk/QuickGO/term/GO:0140104 |
| GO:0060089 | molecular transducer activity | molecular_function | 12|375 | P00734(GO:0070053);P01833(GO:0001792);Q07954(GO:0038023);P04264(GO:0038023);Q96PD5(GO:0016019);P08571(GO:0001847;GO:0016019);Q96HR3(GO:0038023);P15144(GO:0038023);A0A0U1RQV3(GO:0005006);Q9UNN8(GO:0038023);Q9Y5Y7(GO:0038023;GO:0004888);O75882(GO:0038023) | 2|82 | Q9Y5Y7(GO:0038023;GO:0004888);Q96HR3(GO:0038023) | 0.2703584 | 0.315418134 | https://www.ebi.ac.uk/QuickGO/term/GO:0060089 |
| GO:0098754 | detoxification | biological_process | 16|375 | P02768(GO:0098869);P69892(GO:0098869);P02649(GO:0098869);A0A087X1J7(GO:0098869);P04040(GO:0098869);P32119(GO:0019430);P00738(GO:0098869);O95445(GO:0098869);P06727(GO:0019430);P02100(GO:0098869);P30041(GO:0098869);P06702(GO:0098869);Q15166(GO:0010124);P69905(GO:0098869);P68871(GO:0098869);P02042(GO:0098869) | 2|82 | P30041(GO:0098869);P32119(GO:0019430) | 0.180739125 | 0.244872363 | https://www.ebi.ac.uk/QuickGO/term/GO:0098754 |
| GO:0032501 | multicellular organismal process | biological_process | 104|375 | P08779(GO:0061436;GO:0042633;GO:0031424);P00488(GO:0007596);P04275(GO:0007596);P03951(GO:0007596);P03950(GO:0007275);P05160(GO:0007596);P68871(GO:0007596;GO:0050880;GO:0070293);Q8N1N4(GO:0031424);Q86YZ3(GO:0031424;GO:0007275;GO:0061436);P35527(GO:0031424);P01042(GO:0007596;GO:0042311;GO:0050880);C9JC84(GO:0007596);P51884(GO:0007601);P43121(GO:0003094);P60763(GO:0050905;GO:0050885);Q15582(GO:0007601);Q13201(GO:0007596);P06702(GO:0032602;GO:0001816);Q96IY4(GO:0007596);E7END6(GO:0007596);P35908(GO:0031424);P02768(GO:0034375);P02760(GO:0007565);P19320(GO:0060384);P09172(GO:0045907;GO:0048149;GO:0007613;GO:0042596;GO:0008542;GO:0008306;GO:0042309;GO:0048265;GO:0001816;GO:0001974);P20851(GO:0007596);P02042(GO:0007596);P02671(GO:0045907;GO:0007596);P02675(GO:0007596;GO:0045907);P13671(GO:0001701);F5H8B0(GO:0007596);P04180(GO:0034375;GO:0034372);E9PHK0(GO:0001503);P22891(GO:0007596);P05155(GO:0007596;GO:0008015);P05154(GO:0007596);P20742(GO:0007565);O95445(GO:0034375;GO:0034380;GO:0034384);P00734(GO:0007596;GO:0007275);P13645(GO:0031424);P35579(GO:0001701);Q9BWP8(GO:0001503;GO:0007275);G3XAP6(GO:0035264;GO:0050905;GO:0050881;GO:0010259;GO:0014829);M0R0Q9(GO:0097242;GO:0150064);B0YIW2(GO:0034375;GO:0034371;GO:0034379;GO:0034378;GO:0034382);G3V2W1(GO:0007596);A0A0A0MTH3(GO:0001658);K7ER74(GO:0034375;GO:0034372;GO:0034371;GO:0034370;GO:0034378;GO:0034382;GO:0034384);P15144(GO:0007275);P36955(GO:0007614;GO:0007275);C9JPQ9(GO:0007596);P09486(GO:0001503);P18206(GO:0006936);Q13093(GO:0034441;GO:0034374);P32119(GO:0002536);P55058(GO:0034375);P01009(GO:0007596);P01008(GO:0007596);Q9UNN8(GO:0007596);P02100(GO:0007596);P55056(GO:0034447;GO:0034379);P68032(GO:0060048;GO:0006936;GO:0090131;GO:0060047);P69892(GO:0007596);P13647(GO:0031424);P02753(GO:0007601;GO:0030277);A0A669KAY4(GO:0034383);P01344(GO:0001503;GO:0007275;GO:0001701);P08519(GO:0034374;GO:0008015);P01877(GO:0003094);F8WF14(GO:0007612);A0A0A0MRJ7(GO:0008015;GO:0007596);P18065(GO:0007565);P05546(GO:0007596);P02741(GO:0097756);P01031(GO:0001701);P80108(GO:0001503);P11597(GO:0034375;GO:0034374;GO:0034372);P02649(GO:0034447;GO:0007616;GO:0034375;GO:0034374;GO:0034372;GO:0034371;GO:0042311;GO:0034382;GO:0034380;GO:0034384;GO:0002021;GO:0034378);Q07954(GO:0150094;GO:0150093;GO:0007275;GO:0097242);P04196(GO:0007596);P02647(GO:0034375;GO:0034371;GO:0034378;GO:0034384;GO:0034380);P04114(GO:0034374;GO:0034371;GO:0009791;GO:0034378;GO:0001701;GO:0034379;GO:0034382;GO:0034383;GO:0034447);P05109(GO:0032602;GO:0001816);P08697(GO:0002034);P01024(GO:0097242;GO:0150064);P02538(GO:0031424);P00747(GO:0048771;GO:0007596);P00740(GO:0007596);P00742(GO:0007596);P02533(GO:0042633;GO:0031424);P00748(GO:0007596);Q16610(GO:0001503;GO:0003416);P04264(GO:0061436;GO:0031424);P02652(GO:0034384;GO:0034375;GO:0034374;GO:0034371;GO:0034378;GO:0034380;GO:0034370);P06727(GO:0034375;GO:0034372;GO:0034371;GO:0034380;GO:0034378);Q5SRP5(GO:0034375;GO:0034384;GO:0034380);Q13103(GO:0046849);Q15848(GO:0034383);P01619(GO:0003094);A0A0A0MS51(GO:0014891;GO:0097017);P01019(GO:0035106;GO:0003014;GO:0014824;GO:0019229;GO:0003081;GO:0007565;GO:0034374;GO:0042310;GO:0042311;GO:0008306;GO:0001974;GO:0050880;GO:0070471;GO:0002034;GO:0097755;GO:0002019;GO:0002018;GO:0002016);Q96HR3(GO:0019827);Q9Y490(GO:0006936);P01011(GO:0030277) | 19|82 | P35527(GO:0031424);P18206(GO:0006936);Q96HR3(GO:0019827);P32119(GO:0002536);Q96IY4(GO:0007596);E7END6(GO:0007596);P01344(GO:0001503;GO:0007275;GO:0001701);P00748(GO:0007596);M0R0Q9(GO:0097242;GO:0150064);P55058(GO:0034375);K7ER74(GO:0034375;GO:0034372;GO:0034371;GO:0034370;GO:0034378;GO:0034382;GO:0034384);P01008(GO:0007596);A0A669KAY4(GO:0034383);P01009(GO:0007596);Q5SRP5(GO:0034375;GO:0034384;GO:0034380);Q86YZ3(GO:0031424;GO:0007275;GO:0061436);P55056(GO:0034447;GO:0034379);C9JPQ9(GO:0007596);P09172(GO:0045907;GO:0048149;GO:0007613;GO:0042596;GO:0008542;GO:0008306;GO:0042309;GO:0048265;GO:0001816;GO:0001974) | 0.066241828 | 0.154564265 | https://www.ebi.ac.uk/QuickGO/term/GO:0032501 |
| GO:0031974 | membrane-enclosed lumen | cellular_component | 84|375 | P04217(GO:0031093;GO:1904813;GO:0034774);P02768(GO:0005788;GO:0031093);P00488(GO:0031093);P02649(GO:0005788;GO:0071682);P04275(GO:0031093);P51884(GO:0043202;GO:0005796);Q86UX7(GO:0031093);P02763(GO:0031093;GO:0035580;GO:1904724);Q01518(GO:0035578);P02647(GO:0005788;GO:0071682;GO:0034774);P01023(GO:0031093);P02766(GO:0035578);P04114(GO:0005788;GO:0043202;GO:0031983;GO:0071682;GO:0031904);P08697(GO:0031093);P19652(GO:0031093;GO:0035578;GO:0035580);P05546(GO:0005788);P06702(GO:0034774);P68871(GO:1904813;GO:0071682;GO:1904724);Q14624(GO:0031089);P02776(GO:0031093);P29622(GO:0031089);P09486(GO:0031093;GO:0071682);Q16610(GO:0031089);P01024(GO:0005788;GO:0035578;GO:0034774);P10909(GO:0099020;GO:0031093);P02787(GO:0005788;GO:0034774);C9J8S2(GO:0031089);P09172(GO:0034774;GO:0034466);P59665(GO:0035578;GO:0005796);P0DJI8(GO:0071682);P04196(GO:0031093);P00747(GO:0031093);P02671(GO:0005788;GO:0031093);P01009(GO:0005788;GO:1904813;GO:0031093);P00740(GO:0005788;GO:0005796);P02675(GO:0031093);P00742(GO:0005788;GO:0005796);P02790(GO:0071682);P02750(GO:1904813;GO:1904724;GO:0035580);Q86YZ3(GO:0035578);P61626(GO:0035578;GO:0035580;GO:1904724);Q08380(GO:0031089);P02751(GO:0005788;GO:0031093);F8WF14(GO:0005788;GO:0005641);P31151(GO:0035578);E9PHK0(GO:0031089);P04264(GO:1904813);P0C0L4(GO:0005788);P22891(GO:0005796;GO:0005788);A0A669KAY4(GO:0005788);Q06033(GO:0031089);P02652(GO:0005788);C9JV77(GO:0005788;GO:0031093;GO:0034774);P07996(GO:0005788;GO:0031093);P01344(GO:0031093);P02775(GO:0031093;GO:1904724);P05155(GO:0031093);P43251(GO:0005759);Q13201(GO:0031093);P01042(GO:0005788;GO:0031093);M0R0Q9(GO:0005788;GO:0035578;GO:0034774);P06727(GO:0005788);P30041(GO:0035578);P00450(GO:0005788);P18206(GO:0034774;GO:0035580;GO:1904813);O14791(GO:0005788);Q13103(GO:0005788;GO:0031089);A0A0A0MRJ7(GO:0031093;GO:0005788);P00734(GO:0005788;GO:0005796);P13473(GO:0043202);P19823(GO:0005788);P04040(GO:0005782;GO:1904813;GO:0005758;GO:0034774);P05109(GO:0034774);P01008(GO:0005788);P00738(GO:0071682;GO:0035580;GO:1904724);P04004(GO:0005796;GO:0048237);A0A087X0S5(GO:0005788);P07437(GO:0035578;GO:0005641);O00391(GO:0005788;GO:0031093;GO:0035580;GO:1904724);P11021(GO:0005788);P02749(GO:0031089);P69905(GO:0071682);P01034(GO:0005788;GO:1904724;GO:1904813);P01011(GO:0034774;GO:0035578;GO:0031093) | 17|82 | P02787(GO:0005788;GO:0034774);P19652(GO:0031093;GO:0035578;GO:0035580);P19823(GO:0005788);P01008(GO:0005788);P02763(GO:0031093;GO:0035580;GO:1904724);A0A669KAY4(GO:0005788);P29622(GO:0031089);P01344(GO:0031093);P09172(GO:0034774;GO:0034466);P30041(GO:0035578);P01009(GO:0005788;GO:1904813;GO:0031093);P02751(GO:0005788;GO:0031093);P43251(GO:0005759);Q86YZ3(GO:0035578);P18206(GO:0034774;GO:0035580;GO:1904813);Q08380(GO:0031089);M0R0Q9(GO:0005788;GO:0035578;GO:0034774) | 0.111483502 | 0.180088734 | https://www.ebi.ac.uk/QuickGO/term/GO:0031974 |
| GO:0040011 | locomotion | biological_process | 75|375 | P08779(GO:0051546);P04211(GO:0050900);P03950(GO:0016477);P04196(GO:0006935);Q01518(GO:0001667);A0A0C4DH73(GO:0050900);P02647(GO:0043534;GO:0050919);P15814(GO:0050900);P04114(GO:0050900;GO:0030317);P05109(GO:0030593;GO:0006935;GO:0002523);P13796(GO:0016477;GO:0044319);P05546(GO:0006935);P06702(GO:0030595;GO:0030593;GO:0006935;GO:0002523);P59665(GO:0010818;GO:0006935);P19320(GO:0060326);E9PK25(GO:0001755);P01599(GO:0050900);P09172(GO:0050900);P01703(GO:0050900);P01700(GO:0050900);P01701(GO:0050900);P01706(GO:0050900);P01704(GO:0050900);P01705(GO:0050900);P01602(GO:0050900);P01601(GO:0050900);P01709(GO:0050900);P14151(GO:0050900);P01721(GO:0050900);P0DJI8(GO:0048246;GO:0048247;GO:0030593;GO:0050918);P06312(GO:0050900);P55058(GO:0030317);P02751(GO:0050900;GO:1901166);P60709(GO:0048870);P06331(GO:0050900);P60763(GO:0016477);P01763(GO:0050900);P01764(GO:0050900);P01743(GO:0050900);P01766(GO:0050900);P80108(GO:0097241;GO:0035701;GO:0002042);P26038(GO:0050900;GO:0072678);P01782(GO:0050900);P18428(GO:0002232);P01780(GO:0050900);P01742(GO:0050900);P02776(GO:0030595;GO:0030593;GO:0006935);P07996(GO:0016477);P02775(GO:0060326;GO:0030595;GO:0030593;GO:0006935);P01597(GO:0050900);A0A096LPE2(GO:0060326;GO:0050918);P01877(GO:0050900);P04433(GO:0050900);P01871(GO:0050900);P04430(GO:0050900);P01834(GO:0050900);P01031(GO:0060326;GO:0006935);P01817(GO:0050900);P01814(GO:0050900);P35579(GO:0050900;GO:0043534);P01715(GO:0050900);P01619(GO:0050900);P01717(GO:0050900);P23083(GO:0050900);P01718(GO:0050900);C9J8S2(GO:0006935);A0A0A0MTH3(GO:0010761);P04004(GO:0016477);P21333(GO:0044319);P0CF74(GO:0050900);P80748(GO:0050900);A0A0C4DH25(GO:0050900);P35908(GO:0051546);P01699(GO:0050900);G3XAK1(GO:0030317) | 17|82 | P01703(GO:0050900);P01715(GO:0050900);P04211(GO:0050900);P01717(GO:0050900);P01817(GO:0050900);P01782(GO:0050900);P01601(GO:0050900);A0A0C4DH73(GO:0050900);P01721(GO:0050900);P55058(GO:0030317);A0A0C4DH25(GO:0050900);P02751(GO:0050900;GO:1901166);P01780(GO:0050900);P09172(GO:0050900);P01699(GO:0050900);P01599(GO:0050900);P80748(GO:0050900) | 0.120622636 | 0.187635212 | https://www.ebi.ac.uk/QuickGO/term/GO:0040011 |
| GO:0032502 | developmental process | biological_process | 93|375 | P08779(GO:0008544;GO:0002009;GO:0030216;GO:0007568);P51884(GO:0007409;GO:0051216);P03950(GO:0030154;GO:0001525;GO:0007275;GO:0001541;GO:0001556;GO:0001890);E9PK25(GO:0001842);Q86YZ3(GO:0007275);P35527(GO:0008544;GO:0043588);G3XAP6(GO:0060173;GO:0035264;GO:0030282;GO:0010259;GO:0048844;GO:0002063;GO:0003417;GO:0043588;GO:0009887;GO:0048747;GO:0097084;GO:0010260;GO:0001501;GO:0035989);P43121(GO:0001525;GO:0061042;GO:0009653);P60763(GO:0000902;GO:0021894);A0A087X0S5(GO:0003429;GO:0035987;GO:0001649);Q15582(GO:0001525;GO:0002062);P00918(GO:0001822;GO:0002009;GO:0042475);P26038(GO:0061028;GO:0022612;GO:0045198);P31151(GO:0008544;GO:0030216;GO:0001525);P06702(GO:0014002);Q9Y5Y7(GO:0009653);P61224(GO:0061028);P02787(GO:0030316);P35908(GO:0008544;GO:0003334);P04004(GO:0048709;GO:0035987;GO:0097421);Q16880(GO:0007417;GO:0030913;GO:0007422;GO:0048812);Q16610(GO:0001525;GO:0002063;GO:0003416;GO:0031214);P15144(GO:0030154;GO:0001525;GO:0007275);G3V0E5(GO:0030316);Q86UX7(GO:0034446);Q01518(GO:0000902);P37802(GO:0030855);P19320(GO:0060945;GO:0030183;GO:0007568);J3QT83(GO:0003429);P21333(GO:0021987;GO:0021943;GO:0097368);P07996(GO:0002040);P13671(GO:0001701);E9PHK0(GO:0030282);C9JF17(GO:0001525;GO:0014012;GO:0007420;GO:0007568;GO:0042246);P05155(GO:0007568);P05154(GO:0061107);I3L1J2(GO:0000902;GO:0001955);M0R0Q9(GO:0016322);P00734(GO:0007275);P13645(GO:0030216);P13647(GO:0008544);E7EQB2(GO:0060349);Q9BWP8(GO:0007275;GO:0032502);P01019(GO:0001822;GO:0007568;GO:0061049);P32119(GO:0048538);A0A1W2PQU7(GO:0060020;GO:0014002;GO:0031102);A0A0A0MTH3(GO:0034446;GO:0003151;GO:0001658;GO:0021675);P13796(GO:0031100);G3XAK1(GO:0060763;GO:0030879);P36955(GO:0060041;GO:0007275;GO:0001822;GO:0007568);P09486(GO:0030324;GO:0060348;GO:0007507;GO:0048839);Q13103(GO:0001501);Q96IY4(GO:0097421);P02750(GO:0050873);P02751(GO:0034446;GO:0001525;GO:0035987);P02753(GO:0060347;GO:0048562;GO:0030324;GO:0060059;GO:0060065;GO:0048807;GO:0048706;GO:0001654;GO:0007507;GO:0060157;GO:0048738;GO:0060068);P80108(GO:0002062);H9KV75(GO:0048741);C9JV77(GO:0001501);P01344(GO:0031017;GO:0051146;GO:0001501;GO:0007275;GO:0001649;GO:0001701;GO:0009887;GO:0060669;GO:0001892);P35579(GO:0030224;GO:0030220;GO:0001525;GO:0001701;GO:0007520;GO:0000904);O75882(GO:0021549);F8WF14(GO:0014016);P04040(GO:0001649;GO:0001822;GO:0001657;GO:0007568);P18065(GO:0007568);P55103(GO:0048468);P01031(GO:0001701);P68032(GO:0055008;GO:0055003;GO:0048741);P01034(GO:0060009;GO:0008584;GO:0007431;GO:0001654;GO:0007420);P02649(GO:0072358;GO:0031102;GO:0048844);Q07954(GO:0035909;GO:0007275;GO:0021987;GO:0007568);P04196(GO:0001525);P02647(GO:0031102;GO:0031100;GO:0014012;GO:0030325);P10909(GO:0000902);P04114(GO:0001701;GO:0048844;GO:0007399);P05109(GO:0014002);P08697(GO:0048514);P01024(GO:0016322);P01023(GO:0048863);C9J8S2(GO:0048566;GO:0030154);P02538(GO:0002009;GO:0030154);P60709(GO:0021762);P02533(GO:0008544;GO:0030855;GO:0007568);P43251(GO:0007417);A0A669KAY4(GO:0001889;GO:0001822;GO:0030182;GO:0022008);P02652(GO:0031100);P06727(GO:0031102);P18206(GO:0048675;GO:0002009);A0A0A0MS51(GO:0014003;GO:0007568;GO:0042246);Q96HR3(GO:0019827);P11021(GO:0021762;GO:0030182;GO:0021589;GO:0021680);Q15848(GO:2000478;GO:0050873);P07737(GO:0060074;GO:0001843) | 17|82 | P00918(GO:0001822;GO:0002009;GO:0042475);P02787(GO:0030316);P35527(GO:0008544;GO:0043588);Q96HR3(GO:0019827);Q96IY4(GO:0097421);P32119(GO:0048538);A0A669KAY4(GO:0001889;GO:0001822;GO:0030182;GO:0022008);P01344(GO:0031017;GO:0051146;GO:0001501;GO:0007275;GO:0001649;GO:0001701;GO:0009887;GO:0060669;GO:0001892);M0R0Q9(GO:0016322);I3L1J2(GO:0000902;GO:0001955);J3QT83(GO:0003429);P02751(GO:0034446;GO:0001525;GO:0035987);P37802(GO:0030855);Q86YZ3(GO:0007275);Q9Y5Y7(GO:0009653);P18206(GO:0048675;GO:0002009);P43251(GO:0007417) | 0.074598785 | 0.164902578 | https://www.ebi.ac.uk/QuickGO/term/GO:0032502 |
[truncated: 208,720 more chars]
