# Supplementary material for: Proteomics of extracellular vesicles in plasma reveals the characteristics and residual traces of COVID-19 patients without underlying diseases after 3 months of recovery
Source: Cell Death Dis. 2021 May 25;12(6):541. doi: 10.1038/s41419-021-03816-3 (PMC8146187; doi:10.1038/s41419-021-03816-3)
Supplement: Supplementary file 20 — Table S7 [file 41419_2021_3816_MOESM20_ESM.docx]

| Table S7-1 Relationships between these DEPs and clinical parameter in A vs C group | | | | |
| --- | --- | --- | --- | --- |
| Metabolites | Microbes | Correlation_coefficient | P_value | corrected_pvalue |
| Globin | A0A0C4DH34 | 0.586895906 | 0.000107529 | 0.178469435 |
| Globin | A0A075B6S5 | 0.570912544 | 0.000181802 | 0.178469435 |
| Platelets | O75460 | 0.558060646 | 0.000272057 | 0.178469435 |
| RBC | P43251 | 0.511737353 | 0.001023699 | 0.218009541 |
| Total protein | A0A075B6S5 | 0.502627621 | 0.001300239 | 0.218009541 |
| Platelets | P19652 | 0.501258631 | 0.001347049 | 0.218009541 |
| UA | P80108 | 0.500957488 | 0.001357544 | 0.218009541 |
| RBC | P19652 | 0.499151854 | 0.001421996 | 0.218009541 |
| α-HBDH | P43121 | 0.498813631 | 0.001434364 | 0.218009541 |
| Globin | P01717 | 0.498657551 | 0.001440104 | 0.218009541 |
| RBC | P80108 | 0.474965807 | 0.002584984 | 0.260186919 |
| Creatine Kinase | P35908 | 0.473543098 | 0.002673929 | 0.260186919 |
| Total protein | A0A0C4DH34 | 0.473177211 | 0.002697231 | 0.260186919 |
| LDH | P43121 | 0.466818779 | 0.003131527 | 0.260186919 |
| Monocytes | P80108 | 0.463262824 | 0.003400066 | 0.260186919 |
| ALT | P01715 | 0.462787632 | 0.003437429 | 0.260186919 |
| Platelets | A0A2R8Y7X9 | 0.457152249 | 0.003908556 | 0.260475833 |
| ALP | P43121 | 0.455846 | 0.00402545 | 0.260475833 |
| Creatine Kinase | P13647 | 0.454719568 | 0.004128679 | 0.260475833 |
| Platelets | P18206 | 0.453580679 | 0.004235379 | 0.260475833 |
| ALP | P41222 | 0.450325694 | 0.004553637 | 0.267600847 |
| Creatinine | P01717 | 0.44530889 | 0.005084742 | 0.285907777 |
| Total protein | P01717 | 0.442897918 | 0.0053585 | 0.29293136 |
| Creatinine | P80108 | 0.439459431 | 0.005770928 | 0.298873324 |
| UA | A0A0C4DH33 | 0.434887284 | 0.006361571 | 0.32101465 |
| AST | P01715 | 0.428761045 | 0.007234102 | 0.353271748 |
| Creatine Kinase | P04264 | 0.425937078 | 0.00766973 | 0.353271748 |
| Creatinine | P01715 | 0.419617713 | 0.008726772 | 0.365410374 |
| γ-GT | Q15582 | 0.416571297 | 0.009279339 | 0.380452895 |
| γ-GT | O75882 | 0.411749109 | 0.010215034 | 0.410269111 |
| UA | P01715 | 0.406988769 | 0.01121642 | 0.418224098 |
| Creatinine | P68032 | 0.405986122 | 0.011437651 | 0.418224098 |
| Creatine Kinase | A0A1W2PQU7 | 0.403939814 | 0.011900686 | 0.418224098 |
| Platelets | P68032 | 0.401139437 | 0.012560063 | 0.433652718 |
| Eosinophils | A0A5H1ZRQ7 | 0.398263005 | 0.013269328 | 0.450242034 |
| ALT | P43251 | 0.39498351 | 0.014119047 | 0.454046955 |
| Globin | P01782 | 0.393343964 | 0.014560803 | 0.454725392 |
| Creatinine | P41222 | 0.392517689 | 0.014787818 | 0.454725392 |
| UA | P43251 | 0.390326641 | 0.015404299 | 0.462710207 |
| Monocytes | O75882 | 0.387534005 | 0.016221309 | 0.467280259 |
| Globin | A0A075B6J9 | 0.385133333 | 0.016952496 | 0.47660733 |
| RBC | P68032 | 0.381816117 | 0.018008247 | 0.480930359 |
| Creatinine | P43251 | 0.379383926 | 0.018816889 | 0.480930359 |
| Hematocrit | P80108 | 0.377455545 | 0.019479438 | 0.488490917 |
| Globin | A0A075B6S9 | 0.375282591 | 0.020249285 | 0.488490917 |
| Hemoglobin | P80108 | 0.374876937 | 0.02039578 | 0.488490917 |
| WLGG | A0A0C4DH38 | 0.372407605 | 0.021306756 | 0.488490917 |
| Hematocrit | O75460 | 0.370779759 | 0.021925655 | 0.488490917 |
| UA | P01717 | 0.370269138 | 0.022122847 | 0.488490917 |
| BUN | A0A0G2JI36 | 0.367891755 | 0.023060499 | 0.488490917 |
| Basophils | C9J8S2 | 0.36753702 | 0.0232032 | 0.488490917 |
| Monocytes | A0A0C4DH33 | 0.367030478 | 0.023408242 | 0.488490917 |
| RBC | O75460 | 0.363885094 | 0.024715426 | 0.488490917 |
| INR | A0A0G2JI36 | 0.363481485 | 0.024887459 | 0.488490917 |
| Glucose | A0A0C4DH33 | 0.363099146 | 0.025051341 | 0.488490917 |
| CRP | P19652 | 0.361067227 | 0.025937362 | 0.488490917 |
| Hematocrit | P01717 | 0.360339789 | 0.026260801 | 0.488490917 |
| Creatine Kinase | Q8N1N4 | 0.360054724 | 0.026388455 | 0.488490917 |
| AST | A0A0C4DH33 | 0.359963497 | 0.026429416 | 0.488490917 |
| DBIL | A0A1W2PQU7 | 0.359415332 | 0.026676648 | 0.488490917 |
| RBC | P04264 | 0.35808482 | 0.027284694 | 0.488490917 |
| γ-GT | P01715 | 0.357751184 | 0.027438949 | 0.488490917 |
| Eosinophils | P68032 | 0.35678421 | 0.027890096 | 0.488490917 |
| Mg | P01715 | 0.355923646 | 0.02829673 | 0.488490917 |
| ALP | D6RE82 | 0.354003638 | 0.029221619 | 0.500070829 |
| PT | A0A0G2JI36 | 0.353080266 | 0.029675205 | 0.500303774 |
| Ca | P80108 | 0.352941903 | 0.029743669 | 0.500303774 |
| Hematocrit | P68032 | 0.351284865 | 0.030573752 | 0.501397283 |
| Globin | A0A087WSY6 | 0.349553932 | 0.031461097 | 0.501397283 |
| P | Q5SRP5 | 0.348967029 | 0.031766725 | 0.501397283 |
| Creatine Kinase | P13645 | 0.348782494 | 0.031863323 | 0.501397283 |
| Hemoglobin | P01717 | 0.348207442 | 0.032165892 | 0.501397283 |
| AST | P43121 | 0.347993917 | 0.032278838 | 0.501397283 |
| DBIL | Q8N1N4 | 0.347369115 | 0.032611205 | 0.501397283 |
| RBC | A0A2R8Y7X9 | 0.346046516 | 0.033324029 | 0.504474535 |
| Hematocrit | A0A2R8Y7X9 | 0.34429549 | 0.034287342 | 0.511193097 |
| BUN | P13473 | 0.342216147 | 0.035460742 | 0.517789523 |
| TT | P43251 | 0.342096713 | 0.035529125 | 0.517789523 |
| Hematocrit | A0A1W2PQU7 | 0.337948021 | 0.037972142 | 0.517789523 |
| Hemoglobin | P68032 | 0.337721828 | 0.038109174 | 0.517789523 |
| Hematocrit | P43251 | 0.336196995 | 0.039043493 | 0.517789523 |
| CRP | O75460 | 0.335980942 | 0.03917737 | 0.517789523 |
| WBC | P19652 | 0.335649799 | 0.039383286 | 0.517789523 |
| RBC | P01715 | 0.334900118 | 0.039852715 | 0.517789523 |
| Creatine Kinase | F8W1S1 | 0.333849717 | 0.040518083 | 0.517789523 |
| γ-GT | P80108 | 0.333169361 | 0.040953839 | 0.519981647 |
| DBIL | P13647 | 0.329847343 | 0.04313646 | 0.528261063 |
| CK-MB activity | A0A2R8Y7X9 | 0.328976741 | 0.043723757 | 0.528261063 |
| ALT | A0A0C4DH33 | 0.328842311 | 0.043815015 | 0.528261063 |
| Hematocrit | P04264 | 0.328317378 | 0.044172844 | 0.528261063 |
| FIB | P02750 | 0.328145994 | 0.044290181 | 0.528261063 |
| Hemoglobin | P43251 | 0.325922806 | 0.045835212 | 0.536926768 |
| Lymphocytes | Q6ZRK6 | 0.324533123 | 0.046822868 | 0.545250912 |
| ALP | A0A0C4DH73 | 0.323682362 | 0.047435921 | 0.545929197 |
| Eosinophils | P41222 | 0.320082755 | 0.050101657 | 0.558181714 |
| Creatinine | P19652 | 0.318980139 | 0.050941842 | 0.558181714 |
| Mg | A0A075B6R2 | 0.317378278 | 0.052182537 | 0.558181714 |
| ALP | A0A5H1ZRQ7 | 0.317102438 | 0.052398607 | 0.558181714 |
| Hemoglobin | O75460 | 0.316942406 | 0.052524289 | 0.558181714 |
| DBIL | P04264 | 0.316267971 | 0.053056618 | 0.558181714 |
| ALT | P68032 | 0.31623455 | 0.053083109 | 0.558181714 |
| RBC | A0A1W2PQU7 | 0.311463752 | 0.056974567 | 0.569167247 |
| Hematocrit | P41222 | 0.310266393 | 0.057986056 | 0.57634625 |
| CRP | D6RE82 | 0.308922809 | 0.059138026 | 0.578907127 |
| Hemoglobin | P01715 | 0.308287107 | 0.05968937 | 0.578907127 |
| TBIL | A0A1W2PQU7 | 0.307515501 | 0.060364058 | 0.578907127 |
| Monocytes | P01717 | 0.30731255 | 0.060542519 | 0.578907127 |
| TBIL | P13647 | 0.306530225 | 0.061234362 | 0.579054416 |
| Hematocrit | P01715 | 0.306102974 | 0.061614835 | 0.579054416 |
| γ-GT | P43251 | 0.305989756 | 0.061715972 | 0.579054416 |
| TT | P80108 | 0.30522007 | 0.062407012 | 0.579325467 |
| Globin | P08185 | 0.304888099 | 0.062706947 | 0.5793335 |
| Eosinophils | Q6ZRK6 | 0.304568666 | 0.062996631 | 0.5793335 |
| FIB | A0A182DWH7 | 0.303376122 | 0.06408748 | 0.582101457 |
| Hemoglobin | P41222 | 0.301960524 | 0.065401688 | 0.582101457 |
| Creatinine | A0A0C4DH33 | 0.301050558 | 0.066257651 | 0.582101457 |
| α-HBDH | D6RAR4 | 0.298741134 | 0.068469715 | 0.596138448 |
| Eosinophils | O75460 | 0.297506653 | 0.069675764 | 0.601411858 |
| FIB | C9J8S2 | 0.29518705 | 0.071987054 | 0.610649418 |
| Total protein | A0A075B6S9 | 0.295133613 | 0.072040999 | 0.610649418 |
| RBC | P01717 | 0.294823464 | 0.072354725 | 0.610649418 |
| Hematocrit | P19652 | 0.291436392 | 0.075850999 | 0.623787423 |
| Hematocrit | P35908 | 0.291108075 | 0.076196798 | 0.623787423 |
| BUN | A0A0C4DH73 | 0.290998636 | 0.076312337 | 0.623787423 |
| Platelets | P80108 | 0.290795673 | 0.076526976 | 0.623787423 |
| ALT | P80108 | 0.289939365 | 0.077437736 | 0.627150058 |
| Glucose | P0DP01 | 0.288985203 | 0.078462508 | 0.632845144 |
| Eosinophils | C9J8S2 | 0.28677221 | 0.080879938 | 0.641615452 |
| Neutrophils | P19652 | 0.28605822 | 0.081672117 | 0.641615452 |
| TBIL | Q8N1N4 | 0.285401534 | 0.082406025 | 0.641615452 |
| WLGG | P35908 | 0.285351282 | 0.082462397 | 0.641615452 |
| Monocytes | P19652 | 0.28458671 | 0.083323772 | 0.643063468 |
| Ca | A0A0C4DH33 | 0.283059762 | 0.085064921 | 0.650539941 |
| Eosinophils | A0A075B6K2 | 0.282029423 | 0.086255622 | 0.651628433 |
| ALT | P41222 | 0.281817483 | 0.086502138 | 0.651628433 |
| Creatinine | P18206 | 0.281177327 | 0.087250041 | 0.651628433 |
| Ca | P43121 | 0.279864658 | 0.088799268 | 0.65546807 |
| ALT | P19652 | 0.279862137 | 0.088802264 | 0.65546807 |
| Hemoglobin | A0A0C4DH33 | 0.279557621 | 0.089164681 | 0.65546807 |
| Glucose | A0A0C4DH34 | 0.279476939 | 0.089260896 | 0.65546807 |
| RBC | P13647 | 0.276990428 | 0.092265518 | 0.663110325 |
| TT | P01717 | 0.2758891 | 0.093620923 | 0.663110325 |
| CRP | C9J8S2 | 0.274533844 | 0.095309764 | 0.663110325 |
| LDH | A0A075B6K2 | 0.273773419 | 0.096267538 | 0.663110325 |
| TT | A0A0J9YVY3 | 0.273714905 | 0.096341543 | 0.663110325 |
| Hemoglobin | A0A1W2PQU7 | 0.272807026 | 0.097495355 | 0.663110325 |
| Total protein | A0A0C4DH33 | 0.272403821 | 0.098011161 | 0.663110325 |
| ALP | Q86YZ3 | 0.271776325 | 0.098818046 | 0.663110325 |
| P | A0A075B6J9 | 0.270885857 | 0.099971781 | 0.663110325 |
| Lymphocytes | O75460 | 0.270782038 | 0.10010696 | 0.663110325 |
| Glucose | P01715 | 0.270708431 | 0.100202887 | 0.663110325 |
| IBIL | P13647 | 0.269862116 | 0.101310869 | 0.663110325 |
| Total protein | P01782 | 0.269761372 | 0.101443382 | 0.663110325 |
| Hemoglobin | P19652 | 0.269740548 | 0.101470788 | 0.663110325 |
| Hemoglobin | P04264 | 0.26930248 | 0.102048652 | 0.663110325 |
| TBIL | P04264 | 0.268651847 | 0.102911537 | 0.663110325 |
| CRP | P80108 | 0.267952196 | 0.103845619 | 0.663110325 |
| IBIL | A0A1W2PQU7 | 0.267782886 | 0.104072625 | 0.663110325 |
| Basophils | P19652 | 0.267549704 | 0.104385888 | 0.663110325 |
| Eosinophils | O75882 | 0.267361358 | 0.104639439 | 0.663110325 |
| Creatine Kinase | A0A0C4DH38 | 0.267359785 | 0.104641559 | 0.663110325 |
| DBIL | P35908 | 0.266878478 | 0.105291632 | 0.663110325 |
| Total protein | A0A087WSY6 | 0.266805383 | 0.105390626 | 0.663110325 |
| TT | P41222 | 0.266718462 | 0.105508435 | 0.663110325 |
| Hemoglobin | P35908 | 0.266126485 | 0.106313442 | 0.663110325 |
| INR | A0A075B6S9 | 0.265970983 | 0.106525675 | 0.663110325 |
| RBC | P13645 | 0.264623807 | 0.108377812 | 0.664447144 |
| Hemoglobin | A0A2R8Y7X9 | 0.263060007 | 0.110558253 | 0.66802439 |
| TT | P68032 | 0.262544951 | 0.111283621 | 0.66802439 |
| Total protein | P08185 | 0.262535621 | 0.111296794 | 0.66802439 |
| α-HBDH | P13796 | 0.262506867 | 0.111337398 | 0.66802439 |
| α-HBDH | A0A075B6K2 | 0.260932301 | 0.113578029 | 0.669257105 |
| ALP | C9J8S2 | 0.259793854 | 0.115219117 | 0.669742017 |
| Lymphocytes | P13473 | 0.25907077 | 0.116270687 | 0.669742017 |
| Platelets | P41222 | 0.258276275 | 0.117434416 | 0.669742017 |
| BUN | F8W1S1 | 0.257437547 | 0.118672409 | 0.669742017 |
| Platelets | Q9NZP8 | 0.256648797 | 0.11984555 | 0.673874405 |
| RBC | P35908 | 0.256196994 | 0.12052144 | 0.675744144 |
| APTT | Q6ZRK6 | 0.255450445 | 0.121644521 | 0.677975049 |
| Glucose | A0A0J9YVY3 | 0.255246406 | 0.121952829 | 0.677975049 |
| RBC | P41222 | 0.254908799 | 0.122464245 | 0.678900378 |
| ALP | A0A2R8Y7X9 | 0.254501688 | 0.123083081 | 0.679463918 |
| APTT | P13645 | 0.254338471 | 0.123331837 | 0.679463918 |
| ALT | P13796 | 0.254011858 | 0.12383075 | 0.679463918 |
| ALP | Q9NZP8 | 0.253625983 | 0.124422129 | 0.680174306 |
| γ-GT | A0A0C4DH33 | 0.252849925 | 0.125617875 | 0.684808801 |
| WBC | O75460 | 0.251491111 | 0.127732143 | 0.688076211 |
| CO2 | A0A0C4DH38 | 0.251149589 | 0.128267683 | 0.688076211 |
| Basophils | Q5SRP5 | 0.250897117 | 0.128664657 | 0.688076211 |
| ALT | P18206 | 0.249190776 | 0.13137162 | 0.695038293 |
| UA | P19652 | 0.248946764 | 0.131762153 | 0.695038293 |
| IBIL | Q8N1N4 | 0.248850954 | 0.13191573 | 0.695038293 |
| WLC | A0A075B6J9 | 0.248069563 | 0.133173201 | 0.695038293 |
| α-HBDH | C9J8S2 | 0.247477616 | 0.134131703 | 0.695038293 |
| Monocytes | Q15582 | 0.247380812 | 0.134288936 | 0.695038293 |
| PT | A0A075B6S9 | 0.247323565 | 0.134381984 | 0.695038293 |
| DBIL | P13645 | 0.246728441 | 0.135352106 | 0.695038293 |
| Creatine Kinase | P43251 | 0.246566351 | 0.135617228 | 0.695038293 |
| Platelets | A0A0C4DH73 | 0.244719279 | 0.138665554 | 0.704956501 |
| Albumin | D6RAR4 | 0.244046686 | 0.13978803 | 0.705391904 |
| CK-MB activity | Q86YZ3 | 0.243313394 | 0.141019418 | 0.708815856 |
| P | O75882 | 0.242636645 | 0.142162923 | 0.708815856 |
| LDH | D6RAR4 | 0.242556931 | 0.142298063 | 0.708815856 |
| FIB | A0A075B6R2 | 0.242328254 | 0.142686267 | 0.708815856 |
| LDH | P13796 | 0.24146236 | 0.144163272 | 0.709571448 |
| CK-MB activity | P68032 | 0.241083409 | 0.144813188 | 0.709571448 |
| WLGG | D6RE82 | 0.238970847 | 0.148475756 | 0.709571448 |
| Total protein | A0A075B6J9 | 0.238668746 | 0.149004998 | 0.709571448 |
| CRP | O75882 | 0.237936291 | 0.150293884 | 0.709571448 |
| LDH | A0A0C4DH33 | 0.236713927 | 0.152462951 | 0.709571448 |
| Total protein | P01715 | 0.23653034 | 0.152790684 | 0.709571448 |
| APTT | A0A075B6J9 | 0.235725668 | 0.154233209 | 0.709571448 |
| Hematocrit | P13645 | 0.235622438 | 0.154418983 | 0.709571448 |
| WLGG | P02750 | 0.235498516 | 0.154642211 | 0.709571448 |
| DBIL | P43251 | 0.234134668 | 0.157114496 | 0.709571448 |
| γ-GT | P01717 | 0.233913024 | 0.157518971 | 0.709571448 |
| Globin | P0DP01 | 0.233437491 | 0.158389312 | 0.709571448 |
| ALP | D6RAR4 | 0.233375311 | 0.158503373 | 0.709571448 |
| Globin | A0A0C4DH33 | 0.233304161 | 0.158633964 | 0.709571448 |
| Creatinine | A0A1W2PQU7 | 0.233298682 | 0.158644023 | 0.709571448 |
| INR | Q6ZRK6 | 0.232628297 | 0.15987834 | 0.710972812 |
| UA | O75882 | 0.231328993 | 0.162290396 | 0.717724717 |
| PT | A0A087WSY6 | 0.231010411 | 0.162885811 | 0.718457533 |
| WLGG | Q9NZP8 | 0.230786065 | 0.163306049 | 0.718457533 |
| UA | P68032 | 0.230655325 | 0.163551308 | 0.718457533 |
| Albumin | P80108 | 0.230360802 | 0.164104787 | 0.718925797 |
| DBIL | A0A0G2JI36 | 0.230210096 | 0.164388521 | 0.718925797 |
| DBIL | A0A075B6S5 | 0.229644714 | 0.165456117 | 0.720393004 |
| INR | A0A075B6J9 | 0.228449827 | 0.167728811 | 0.726232251 |
| CK-MB activity | P01782 | 0.227414927 | 0.169715277 | 0.726232251 |
| APTT | A0A0C4DH38 | 0.227295164 | 0.169946246 | 0.726232251 |
| AST | P13796 | 0.227205958 | 0.170118429 | 0.726232251 |
| WLL | A0A0C4DH38 | 0.226943227 | 0.170626275 | 0.726823613 |
| P | A0A075B6K2 | 0.22669803 | 0.171101209 | 0.727272526 |
| IBIL | P04264 | 0.226088861 | 0.172285241 | 0.727556982 |
| Albumin | P43251 | 0.225762345 | 0.172922296 | 0.727556982 |
| FIB | P18206 | 0.225172136 | 0.174078106 | 0.727556982 |
| P | A0A0J9YVY3 | 0.224880656 | 0.174650949 | 0.727556982 |
| Lymphocytes | A0A0C4DH38 | 0.22448422 | 0.17543222 | 0.727556982 |
| IBIL | P13796 | 0.224337931 | 0.175721147 | 0.727556982 |
| Ca | P01717 | 0.223478501 | 0.177425421 | 0.730067372 |
| Ca | D6RE82 | 0.223343478 | 0.177694243 | 0.730067372 |
| APTT | P35908 | 0.222149271 | 0.180084482 | 0.730275423 |
| AST | P01717 | 0.221636873 | 0.181117042 | 0.730275423 |
| Total protein | P43121 | 0.221290147 | 0.181818133 | 0.730275423 |
| Creatine Kinase | P80108 | 0.221285913 | 0.181826706 | 0.730275423 |
| CO2 | Q5SRP5 | 0.218429653 | 0.187675785 | 0.747856471 |
| Basophils | Q9NZP8 | 0.217368722 | 0.189881871 | 0.753077147 |
| Neutrophils | A0A075B6R2 | 0.217224777 | 0.190182592 | 0.753077147 |
| WBC | Q6ZRK6 | 0.2166211 | 0.191447413 | 0.753537019 |
| Mg | P01717 | 0.216256267 | 0.192214678 | 0.753676973 |
| DBIL | A0A0C4DH38 | 0.216065341 | 0.192617066 | 0.753676973 |
| P | P02750 | 0.215153884 | 0.194546194 | 0.7563465 |
| Albumin | P43121 | 0.215099719 | 0.194661262 | 0.7563465 |
| WLGG | P13647 | 0.214664524 | 0.195587523 | 0.7563465 |
| Eosinophils | P19652 | 0.214086401 | 0.196822765 | 0.7563465 |
| APTT | P13647 | 0.213718767 | 0.197611109 | 0.7563465 |
| LDH | P43251 | 0.213660257 | 0.197736779 | 0.7563465 |
| Eosinophils | D6RAR4 | 0.213647924 | 0.197763275 | 0.7563465 |
| FIB | D6RE82 | 0.213393537 | 0.198310363 | 0.7563465 |
| Monocytes | P18206 | 0.212597098 | 0.200030044 | 0.757363281 |
| Mg | P19652 | 0.21151223 | 0.20238924 | 0.757525386 |
| PT | Q6ZRK6 | 0.211436049 | 0.202555634 | 0.757525386 |
| Ca | P19652 | 0.211173068 | 0.203130764 | 0.757525386 |
| INR | A0A087WSY6 | 0.210682726 | 0.204206161 | 0.757525386 |
| Albumin | A0A0C4DH33 | 0.210555161 | 0.20448658 | 0.757525386 |
| Lymphocytes | Q9NZP8 | 0.20959887 | 0.206597289 | 0.761579569 |
| γ-GT | P68032 | 0.20781485 | 0.210575326 | 0.765566513 |
| WLC | P08185 | 0.207601827 | 0.211053852 | 0.765566513 |
| UA | P35908 | 0.207364447 | 0.211587975 | 0.765566513 |
| INR | A0A0C4DH34 | 0.207282849 | 0.211771795 | 0.765566513 |
| WLC | A0A075B6K2 | 0.207150894 | 0.212069287 | 0.765566513 |
| Albumin | D6RE82 | 0.206930568 | 0.212566657 | 0.765566513 |
| Neutrophils | A0A075B6S5 | 0.205953164 | 0.214782799 | 0.765566513 |
| PT | A0A075B6J9 | 0.205452279 | 0.215924651 | 0.765566513 |
| INR | A0A075B6S5 | 0.2054184 | 0.216002037 | 0.765566513 |
| Hematocrit | A0A0C4DH33 | 0.204881253 | 0.217231501 | 0.766149809 |
| DBIL | P13796 | 0.203909613 | 0.219467697 | 0.768244548 |
| BUN | P68032 | 0.203775636 | 0.219777277 | 0.768244548 |
| FIB | Q5SRP5 | 0.20278643 | 0.222072317 | 0.773004258 |
| TBIL | P43251 | 0.201872048 | 0.224208331 | 0.773696448 |
| WLC | P80108 | 0.201286053 | 0.225584598 | 0.773696448 |
| CRP | P18206 | 0.200324548 | 0.227855277 | 0.777156301 |
| TBIL | A0A0C4DH38 | 0.199792022 | 0.229119572 | 0.777508352 |
| TBIL | P13796 | 0.199354121 | 0.230162787 | 0.777508352 |
| Globin | P01715 | 0.198670112 | 0.231798774 | 0.777508352 |
| APTT | A0A1W2PQU7 | 0.19860955 | 0.231944003 | 0.777508352 |
| Hemoglobin | P13645 | 0.197349764 | 0.234979048 | 0.777846285 |
| WBC | P80108 | 0.197318744 | 0.235054117 | 0.777846285 |
| AST | P43251 | 0.197270038 | 0.235172022 | 0.777846285 |
| Lymphocytes | A0A0C4DH73 | 0.196136383 | 0.237927641 | 0.784324283 |
| Glucose | P68032 | 0.195799703 | 0.238750211 | 0.784767482 |
| Lymphocytes | A0A2R8Y7X9 | 0.195589128 | 0.239265658 | 0.784767482 |
| ALT | P01717 | 0.19496683 | 0.240793316 | 0.784767482 |
| Neutrophils | D6RE82 | 0.194572118 | 0.241765683 | 0.784767482 |
| CK-MB activity | P41222 | 0.194180655 | 0.242732655 | 0.784767482 |
| TT | Q15582 | 0.194041382 | 0.243077307 | 0.784767482 |
| WLC | P01717 | 0.194013099 | 0.243147338 | 0.784767482 |
| IBIL | A0A0C4DH38 | 0.193587219 | 0.24420349 | 0.784767482 |
| PT | A0A0C4DH34 | 0.192947442 | 0.245795883 | 0.784767482 |
| CRP | A0A0C4DH33 | 0.192813437 | 0.246130299 | 0.784767482 |
| CO2 | P80108 | 0.192796175 | 0.2461734 | 0.784767482 |
| CK-MB activity | A0A5H1ZRQ7 | 0.192172193 | 0.247734786 | 0.784767482 |
| AST | A0A075B6R2 | 0.19200658 | 0.248150312 | 0.784767482 |
| BUN | D6RAR4 | 0.190971275 | 0.250758481 | 0.784899738 |
| Creatinine | A0A2R8Y7X9 | 0.190622094 | 0.251642266 | 0.784899738 |
| TT | A0A0C4DH38 | 0.190200065 | 0.252713201 | 0.784899738 |
| CK-MB activity | P04264 | 0.190153613 | 0.252831264 | 0.784899738 |
| AST | C9J8S2 | 0.190142718 | 0.25285896 | 0.784899738 |
| PT | P35908 | 0.18998577 | 0.253258156 | 0.784900868 |
| UA | P41222 | 0.189305548 | 0.25499316 | 0.786662659 |
| APTT | A0A2R8Y7X9 | 0.188755714 | 0.256401363 | 0.788831984 |
| INR | A0A0J9YVY3 | 0.188705304 | 0.256530727 | 0.788831984 |
| P | A0A075B6S5 | 0.188328082 | 0.257500156 | 0.789022985 |
| APTT | Q5SRP5 | 0.18827321 | 0.257641375 | 0.789022985 |
| IBIL | P43251 | 0.188115563 | 0.258047383 | 0.789022985 |
| WLGG | Q86YZ3 | 0.187558848 | 0.259484554 | 0.789022985 |
| LDH | C9J8S2 | 0.187136263 | 0.260579006 | 0.789022985 |
| WBC | P41222 | 0.187127357 | 0.260602104 | 0.789022985 |
| TBIL | A0A0G2JI36 | 0.185018724 | 0.266109215 | 0.79786156 |
| APTT | A0A087WSY6 | 0.184923666 | 0.266359271 | 0.79786156 |
| Creatinine | P35908 | 0.184713028 | 0.26691392 | 0.797957888 |
| Mg | A0A0C4DH33 | 0.183873992 | 0.269130799 | 0.801777602 |
| ALP | Q6ZRK6 | 0.183557556 | 0.269970011 | 0.801777602 |
| IBIL | C9J8S2 | 0.182986491 | 0.271488864 | 0.802237363 |
| Creatinine | P13647 | 0.182196203 | 0.273600008 | 0.803355228 |
| TBIL | P35908 | 0.181838108 | 0.274560136 | 0.803355228 |
| Lymphocytes | P19652 | 0.18092268 | 0.277024602 | 0.803355228 |
| Lymphocytes | P13647 | 0.180813228 | 0.277320223 | 0.803355228 |
| Glucose | P01717 | 0.180643139 | 0.277780033 | 0.803355228 |
| RBC | A0A0C4DH33 | 0.18020138 | 0.278976578 | 0.803355228 |
| P | D6RAR4 | 0.180116101 | 0.27920795 | 0.803355228 |
| PT | A0A075B6S5 | 0.180113531 | 0.279214927 | 0.803355228 |
| Glucose | P80108 | 0.179569952 | 0.280692682 | 0.806428027 |
| TT | A0A5H1ZRQ7 | 0.178785711 | 0.282833639 | 0.808890307 |
| BUN | P19652 | 0.177948019 | 0.285132189 | 0.8101601 |
| WBC | C9J8S2 | 0.177787876 | 0.285572979 | 0.8101601 |
| α-HBDH | F8W1S1 | 0.177555713 | 0.286212787 | 0.8101601 |
| UA | P13647 | 0.176834273 | 0.288206886 | 0.813071773 |
| Hematocrit | Q9NZP8 | 0.176196993 | 0.289975806 | 0.814163192 |
| Lymphocytes | A0A5H1ZRQ7 | 0.176099938 | 0.290245817 | 0.814163192 |
| Neutrophils | P13796 | 0.175749618 | 0.291221771 | 0.814163192 |
| γ-GT | P18206 | 0.175723152 | 0.291295589 | 0.814163192 |
| ALT | Q15582 | 0.175146598 | 0.292906664 | 0.814163192 |
| Eosinophils | P18206 | 0.175124085 | 0.292969689 | 0.814163192 |
| Lymphocytes | F8W1S1 | 0.17488757 | 0.293632333 | 0.814163192 |
| Globin | Q15582 | 0.174503278 | 0.294711053 | 0.814163192 |
| P | P13796 | 0.174312969 | 0.295246198 | 0.814163192 |
| FIB | P43121 | 0.174203858 | 0.295553295 | 0.814163192 |
| Creatinine | P13645 | 0.173770313 | 0.296775553 | 0.815718281 |
| Basophils | P43251 | 0.172489417 | 0.300405558 | 0.815981856 |
| PT | P02750 | 0.172435122 | 0.300560053 | 0.815981856 |
| WLGG | P13645 | 0.172252469 | 0.301080153 | 0.815981856 |
| Ca | A0A182DWH7 | 0.172140039 | 0.301400578 | 0.815981856 |
| UA | Q15582 | 0.172128905 | 0.301432322 | 0.815981856 |
| ALP | P01019 | 0.171638128 | 0.302833684 | 0.817526325 |
| INR | P02750 | 0.170322891 | 0.30660962 | 0.821776342 |
| WLGG | A0A1W2PQU7 | 0.170268279 | 0.306767049 | 0.821776342 |
| WLL | Q86YZ3 | 0.16995104 | 0.307682564 | 0.822716422 |
| Hematocrit | Q86YZ3 | 0.168875333 | 0.310799811 | 0.825366857 |
| WBC | P18206 | 0.168817459 | 0.310968085 | 0.825366857 |
| WLL | P08185 | 0.168500428 | 0.311890905 | 0.826112115 |
| BUN | P02750 | 0.168207937 | 0.312743826 | 0.827257861 |
| APTT | F8W1S1 | 0.167685452 | 0.314271076 | 0.828557891 |
| Eosinophils | Q9NZP8 | 0.167607838 | 0.314498346 | 0.828557891 |
| RBC | Q8N1N4 | 0.167004106 | 0.316269728 | 0.831826643 |
| DBIL | F8W1S1 | 0.166897071 | 0.316584429 | 0.831826643 |
| ALP | P04264 | 0.166602825 | 0.317450571 | 0.83206456 |
| IBIL | A0A0G2JI36 | 0.166415103 | 0.318003928 | 0.83206456 |
| Ca | P43251 | 0.166109656 | 0.318905599 | 0.83206456 |
| Neutrophils | D6RAR4 | 0.165572336 | 0.320495636 | 0.83206456 |
| Lymphocytes | A0A182DWH7 | 0.165490074 | 0.320739505 | 0.83206456 |
| AST | O75882 | 0.164592733 | 0.323407223 | 0.83206456 |
| Basophils | P41222 | 0.164369326 | 0.324073541 | 0.83206456 |
| PT | P13645 | 0.163659798 | 0.326195401 | 0.83206456 |
| Ca | P08185 | 0.163478216 | 0.326739811 | 0.83206456 |
| Basophils | P68032 | 0.16249474 | 0.329698238 | 0.83206456 |
| FIB | A0A087WSY6 | 0.162428979 | 0.329896647 | 0.83206456 |
| P | P08185 | 0.162268731 | 0.330380443 | 0.83206456 |
| UA | P18206 | 0.162259976 | 0.330406886 | 0.83206456 |
| Monocytes | P43251 | 0.162213327 | 0.330547811 | 0.83206456 |
| Hemoglobin | P18206 | 0.161466677 | 0.332808487 | 0.834352996 |
| Eosinophils | P13796 | 0.161249921 | 0.333466557 | 0.834939166 |
| α-HBDH | P04264 | 0.160700617 | 0.335137839 | 0.83540434 |
| WLL | D6RE82 | 0.160630902 | 0.335350319 | 0.83540434 |
| WLC | O75460 | 0.160116564 | 0.336920506 | 0.837783 |
| PT | A0A0J9YVY3 | 0.159770788 | 0.337978639 | 0.837962529 |
| α-HBDH | A0A0C4DH33 | 0.159559922 | 0.338624928 | 0.837962529 |
| α-HBDH | Q86YZ3 | 0.159311906 | 0.33938605 | 0.837962529 |
| TBIL | A0A075B6S5 | 0.159286242 | 0.339464869 | 0.837962529 |
| CO2 | P19652 | 0.159076004 | 0.340110972 | 0.837962529 |
| LDH | P04264 | 0.159041164 | 0.340218112 | 0.837962529 |
| Albumin | A0A182DWH7 | 0.15820882 | 0.342783961 | 0.842195798 |
| AST | Q15582 | 0.157136167 | 0.346108021 | 0.847751568 |
| Hemoglobin | Q15582 | 0.155733276 | 0.350485019 | 0.852561013 |
| TT | A0A1W2PQU7 | 0.155628213 | 0.350814163 | 0.852561013 |
| Eosinophils | A0A0C4DH73 | 0.155440101 | 0.351403949 | 0.852561013 |
| ALP | F8W1S1 | 0.155057199 | 0.352606317 | 0.852561013 |
| LDH | Q8N1N4 | 0.154334509 | 0.354882451 | 0.854322914 |
| Monocytes | P68032 | 0.154265688 | 0.355099667 | 0.854322914 |
| CK-MB activity | P13796 | 0.153881536 | 0.356313621 | 0.854571513 |
| TBIL | C9J8S2 | 0.153820827 | 0.356505697 | 0.854571513 |
| ALT | O75882 | 0.153239581 | 0.358347838 | 0.855440566 |
| Creatinine | O75460 | 0.153198009 | 0.358479813 | 0.855440566 |
| Basophils | P80108 | 0.152638955 | 0.360257412 | 0.855440566 |
| CO2 | A0A075B6K2 | 0.152597753 | 0.36038863 | 0.855440566 |
| Ca | P02750 | 0.152513882 | 0.360655826 | 0.855440566 |
| Mg | P13647 | 0.152477891 | 0.360770522 | 0.855440566 |
| Neutrophils | P41222 | 0.152431188 | 0.360919389 | 0.855440566 |
| Glucose | P18206 | 0.152422531 | 0.360946986 | 0.855440566 |
| Glucose | A0A075B6S9 | 0.152338483 | 0.361214995 | 0.855440566 |
| Lymphocytes | P18206 | 0.151414063 | 0.364170632 | 0.858558188 |
| Neutrophils | P80108 | 0.150908296 | 0.365793822 | 0.861103159 |
| WLGG | P01019 | 0.150674406 | 0.36654592 | 0.861732166 |
| α-HBDH | Q8N1N4 | 0.150082116 | 0.368454615 | 0.862210087 |
| γ-GT | P41222 | 0.148913427 | 0.372238132 | 0.862336813 |
| Creatine Kinase | A0A0G2JI36 | 0.148776033 | 0.37268444 | 0.862336813 |
| P | A0A0C4DH34 | 0.148472604 | 0.37367122 | 0.862336813 |
| BUN | A0A5H1ZRQ7 | 0.14830734 | 0.374209323 | 0.862336813 |
| Monocytes | O75460 | 0.147835827 | 0.375747107 | 0.862336813 |
| BUN | P13796 | 0.14653899 | 0.379995811 | 0.862336813 |
| Creatine Kinase | P01019 | 0.146429551 | 0.380355645 | 0.862336813 |
| WLGG | A0A5H1ZRQ7 | 0.146411218 | 0.380415941 | 0.862336813 |
| BUN | A0A182DWH7 | 0.146101233 | 0.381436349 | 0.862336813 |
| P | Q6ZRK6 | 0.146087212 | 0.381482541 | 0.862336813 |
| CO2 | A0A075B6J9 | 0.144843464 | 0.385593146 | 0.862336813 |
| APTT | P01717 | 0.143914086 | 0.388681599 | 0.862336813 |
| RBC | P13473 | 0.143912451 | 0.388687045 | 0.862336813 |
| FIB | P0DP01 | 0.143844656 | 0.388912899 | 0.862336813 |
| AST | P41222 | 0.143597246 | 0.389737786 | 0.862336813 |
| Platelets | P13645 | 0.143482547 | 0.39012055 | 0.862336813 |
| WLC | P13796 | 0.1432745 | 0.390815382 | 0.862336813 |
| WLL | Q9NZP8 | 0.143155927 | 0.391211714 | 0.862336813 |
| TBIL | P13645 | 0.14308393 | 0.391452477 | 0.862336813 |
| Eosinophils | P01019 | 0.142833887 | 0.392289311 | 0.862336813 |
| TT | P13796 | 0.142567735 | 0.393181197 | 0.862336813 |
| BUN | O75460 | 0.142489742 | 0.393442777 | 0.862336813 |
| CRP | P01019 | 0.14219213 | 0.394441864 | 0.862336813 |
| Basophils | O75460 | 0.142158898 | 0.394553516 | 0.862336813 |
| Creatine Kinase | A0A0C4DH33 | 0.141621977 | 0.396359973 | 0.862336813 |
| FIB | P08185 | 0.14149515 | 0.396787379 | 0.862336813 |
| WBC | A0A0C4DH73 | 0.141395351 | 0.397123886 | 0.862336813 |
| TT | A0A075B6S5 | 0.140921456 | 0.398724035 | 0.862336813 |
| Hematocrit | A0A0C4DH38 | 0.140738716 | 0.399342066 | 0.862336813 |
| Hemoglobin | A0A075B6K2 | 0.140356489 | 0.400636547 | 0.862336813 |
| AST | A0A075B6S5 | 0.140139582 | 0.401372216 | 0.862336813 |
| LDH | F8W1S1 | 0.139598821 | 0.403209657 | 0.86533981 |
| CRP | A0A2R8Y7X9 | 0.139453453 | 0.403704421 | 0.865457844 |
| APTT | P04264 | 0.138939096 | 0.405457842 | 0.865607403 |
| Lymphocytes | P80108 | 0.138784003 | 0.405987398 | 0.865607403 |
| Hematocrit | P13647 | 0.138659373 | 0.406413232 | 0.865607403 |
| TT | A0A0G2JI36 | 0.13740217 | 0.410723047 | 0.869964662 |
| Hemoglobin | Q86YZ3 | 0.135752472 | 0.416417619 | 0.872981011 |
| RBC | P18206 | 0.135366485 | 0.417756411 | 0.872981011 |
| Glucose | A0A075B6R2 | 0.135251956 | 0.418154121 | 0.872981011 |
| Ca | A0A075B6R2 | 0.134970948 | 0.419130844 | 0.872981011 |
| Ca | A0A075B6S9 | 0.134444826 | 0.420962981 | 0.872981011 |
| Eosinophils | A0A182DWH7 | 0.134283585 | 0.421525379 | 0.872981011 |
| WLGG | Q5SRP5 | 0.1335185 | 0.424199682 | 0.875078589 |
| Ca | O75882 | 0.133107011 | 0.425641932 | 0.87568998 |
| Albumin | P19652 | 0.133026794 | 0.425923411 | 0.87568998 |
| Platelets | P13647 | 0.132975816 | 0.426102343 | 0.87568998 |
| IBIL | P35908 | 0.132523529 | 0.427691705 | 0.87568998 |
| γ-GT | A0A075B6K2 | 0.132256463 | 0.428631738 | 0.875957696 |
| P | A0A075B6R2 | 0.131501178 | 0.431296458 | 0.878795192 |
| ALT | A0A075B6K2 | 0.13124729 | 0.43219426 | 0.878891068 |
| Basophils | O75882 | 0.130692482 | 0.434159784 | 0.878891068 |
| CRP | P43251 | 0.130580138 | 0.434558387 | 0.878891068 |
| Neutrophils | P18206 | 0.130208604 | 0.435878053 | 0.878891068 |
| Creatinine | P04264 | 0.13010888 | 0.436232641 | 0.878891068 |
| INR | P35908 | 0.129743709 | 0.437532443 | 0.878891068 |
| WLL | P35908 | 0.129731442 | 0.437576143 | 0.878891068 |
| IBIL | A0A075B6S5 | 0.129678267 | 0.437765603 | 0.878891068 |
| Creatinine | Q15582 | 0.129452317 | 0.43857116 | 0.878928761 |
| Eosinophils | P13473 | 0.129241099 | 0.439324932 | 0.879025157 |
| TBIL | F8W1S1 | 0.129063512 | 0.439959238 | 0.879025157 |
| WLGG | P08185 | 0.128600295 | 0.441616117 | 0.880547637 |
| APTT | P08185 | 0.127880901 | 0.444196086 | 0.883900806 |
| PT | A0A0C4DH33 | 0.127742803 | 0.44469229 | 0.883994371 |
| Lymphocytes | C9J8S2 | 0.127567505 | 0.445322592 | 0.884354047 |
| WLC | Q5SRP5 | 0.127357055 | 0.446079932 | 0.884532634 |
| CK-MB activity | P01019 | 0.126072944 | 0.450716206 | 0.887009493 |
| INR | P18206 | 0.125680758 | 0.452137383 | 0.888907832 |
| INR | A0A0C4DH33 | 0.125308789 | 0.453487532 | 0.888907832 |
| DBIL | A0A0C4DH33 | 0.124794692 | 0.455357153 | 0.889913484 |
| Hematocrit | F8W1S1 | 0.124513356 | 0.456382048 | 0.890414289 |
| RBC | A0A0C4DH38 | 0.124103969 | 0.457875641 | 0.890414289 |
| BUN | A0A0J9YVY3 | 0.123508745 | 0.460051909 | 0.893474893 |
| LDH | Q86YZ3 | 0.123349018 | 0.46063685 | 0.893474893 |
| WLGG | O75460 | 0.121655631 | 0.46686266 | 0.896542204 |
| Platelets | Q5SRP5 | 0.121632614 | 0.466947589 | 0.896542204 |
| γ-GT | O75460 | 0.121431465 | 0.467690154 | 0.896542204 |
| LDH | O75882 | 0.121059551 | 0.46906476 | 0.896542204 |
| Creatine Kinase | P19652 | 0.120711356 | 0.47035364 | 0.896542204 |
| LDH | A0A075B6R2 | 0.120293351 | 0.471903396 | 0.896542204 |
| Hematocrit | P01782 | 0.12016416 | 0.472382918 | 0.896542204 |
| Glucose | A0A075B6K2 | 0.119655374 | 0.47427389 | 0.896542204 |
| Eosinophils | F8W1S1 | 0.119652473 | 0.474284682 | 0.896542204 |
| APTT | P0DP01 | 0.119471709 | 0.474957484 | 0.896542204 |
| RBC | P01019 | 0.119288647 | 0.475639344 | 0.896542204 |
| FIB | A0A075B6K2 | 0.119259938 | 0.475746327 | 0.896542204 |
| Albumin | P13473 | 0.119231423 | 0.475852599 | 0.896542204 |
| Hematocrit | P18206 | 0.119219735 | 0.475896161 | 0.896542204 |
| Creatine Kinase | Q15582 | 0.119069769 | 0.476455289 | 0.896542204 |
| ALT | P0DP01 | 0.118975821 | 0.476805737 | 0.896542204 |
| Lymphocytes | P35908 | 0.118426098 | 0.478859046 | 0.896542204 |
| Hemoglobin | Q9NZP8 | 0.118387955 | 0.479001687 | 0.896542204 |
| Albumin | P13647 | 0.118355526 | 0.479122977 | 0.896542204 |
| IBIL | D6RAR4 | 0.118078355 | 0.480160307 | 0.896542204 |
| PT | F8W1S1 | 0.117472308 | 0.48243255 | 0.899931051 |
| Mg | D6RE82 | 0.116969341 | 0.484322559 | 0.902601133 |
| ALP | P13645 | 0.116359266 | 0.486620198 | 0.90478404 |
| CK-MB activity | P43121 | 0.11595971 | 0.488128043 | 0.90478404 |
| Platelets | Q6ZRK6 | 0.115811527 | 0.488687867 | 0.90478404 |
| Creatine Kinase | Q86YZ3 | 0.115805189 | 0.48871182 | 0.90478404 |
| AST | A0A075B6K2 | 0.115802624 | 0.488721513 | 0.90478404 |
| Globin | P43121 | 0.115495195 | 0.489884057 | 0.90485492 |
| Mg | P80108 | 0.115430271 | 0.490129748 | 0.90485492 |
| WLL | P02750 | 0.114108119 | 0.495146949 | 0.908196691 |
| Hemoglobin | O75882 | 0.113897755 | 0.49594764 | 0.908196691 |
| Neutrophils | C9J8S2 | 0.113831255 | 0.49620089 | 0.908196691 |
| Eosinophils | A0A0C4DH38 | 0.113784785 | 0.496377901 | 0.908196691 |
| WBC | P68032 | 0.112883748 | 0.499816421 | 0.909501853 |
| γ-GT | P01782 | 0.11288304 | 0.499819126 | 0.909501853 |
| Monocytes | P13796 | 0.112824966 | 0.500041161 | 0.909501853 |
| Total protein | Q15582 | 0.112656027 | 0.500687355 | 0.909737011 |
| WBC | P13796 | 0.111737348 | 0.504208726 | 0.91354215 |
| α-HBDH | P43251 | 0.111548998 | 0.504932226 | 0.91354215 |
| Total protein | P0DP01 | 0.111025883 | 0.506944396 | 0.915290432 |
| Mg | P41222 | 0.110221437 | 0.510046558 | 0.917609994 |
| FIB | O75460 | 0.1096012 | 0.512444837 | 0.917609994 |
| FIB | O75882 | 0.1096012 | 0.512444837 | 0.917609994 |
| Lymphocytes | P01782 | 0.109341656 | 0.51345009 | 0.917774547 |
| IBIL | Q15582 | 0.108885971 | 0.515217395 | 0.918431009 |
| γ-GT | P13796 | 0.108499233 | 0.516719673 | 0.919413649 |
| TBIL | D6RAR4 | 0.108161379 | 0.518033835 | 0.919413649 |
| α-HBDH | A0A075B6R2 | 0.108045987 | 0.518483058 | 0.919413649 |
| Creatine Kinase | A0A5H1ZRQ7 | 0.107470678 | 0.520725623 | 0.919413649 |
| UA | A0A1W2PQU7 | 0.10745746 | 0.520777202 | 0.919413649 |
| DBIL | Q15582 | 0.10743036 | 0.520882963 | 0.919413649 |
| ALP | O75460 | 0.107383293 | 0.52106667 | 0.919413649 |
| WBC | A0A0C4DH38 | 0.107031465 | 0.522440896 | 0.919413649 |
| INR | Q15582 | 0.106821955 | 0.523260083 | 0.919413649 |
| ALP | Q5SRP5 | 0.10612485 | 0.5259903 | 0.919573522 |
| Total protein | A0A075B6R2 | 0.106087163 | 0.526138103 | 0.919573522 |
| Lymphocytes | P68032 | 0.105291705 | 0.529262445 | 0.921836903 |
| Creatine Kinase | P13473 | 0.105280439 | 0.529306758 | 0.921836903 |
| Hematocrit | Q8N1N4 | 0.104842683 | 0.531030063 | 0.923151459 |
| Lymphocytes | P01019 | 0.1038691 | 0.534872485 | 0.926407938 |
| WLL | A0A5H1ZRQ7 | 0.103779562 | 0.535226537 | 0.926407938 |
| BUN | P13647 | 0.103638852 | 0.53578316 | 0.926556467 |
| DBIL | A0A0J9YVY3 | 0.103163747 | 0.537664655 | 0.927640541 |
| Globin | P18206 | 0.103106847 | 0.537890199 | 0.927640541 |
| Mg | P01782 | 0.102787908 | 0.539155284 | 0.927640541 |
| INR | P13645 | 0.102764037 | 0.539250028 | 0.927640541 |
| IBIL | A0A075B6R2 | 0.102648283 | 0.539709563 | 0.927640541 |
| CRP | Q9NZP8 | 0.101988346 | 0.54233304 | 0.928275659 |
| α-HBDH | P13473 | 0.101149435 | 0.545676768 | 0.929019461 |
| γ-GT | C9J8S2 | 0.10101763 | 0.546203005 | 0.929019461 |
| PT | P18206 | 0.100990442 | 0.546311584 | 0.929019461 |
| CK-MB activity | F8W1S1 | 0.100982774 | 0.546342207 | 0.929019461 |
| WLC | P43251 | 0.100935423 | 0.54653134 | 0.929019461 |
| FIB | Q9NZP8 | 0.100833104 | 0.546940135 | 0.929019461 |
| Neutrophils | O75882 | 0.100787919 | 0.547120709 | 0.929019461 |
| Hemoglobin | P01019 | 0.100317638 | 0.549001778 | 0.929820919 |
| CRP | P68032 | 0.09999775 | 0.550283036 | 0.929820919 |
| Glucose | P19652 | 0.09979756 | 0.551085581 | 0.930134153 |
| Basophils | Q15582 | 0.099129015 | 0.553769708 | 0.930877332 |
| Albumin | P01717 | 0.099084793 | 0.553947467 | 0.930877332 |
| Lymphocytes | P01717 | 0.098723531 | 0.555400656 | 0.930877332 |
| Monocytes | P08185 | 0.098557218 | 0.556070254 | 0.930877332 |
| CK-MB activity | P01717 | 0.097821872 | 0.559035365 | 0.933148089 |
| Globin | A0A075B6R2 | 0.097432822 | 0.560607087 | 0.9349786 |
| Mg | P35908 | 0.097291228 | 0.561179617 | 0.93514097 |
| ALP | A0A1W2PQU7 | 0.09665591 | 0.563751846 | 0.936715898 |
| Albumin | P13796 | 0.096458111 | 0.564553787 | 0.936715898 |
| APTT | Q9NZP8 | 0.09612965 | 0.565886638 | 0.936715898 |
| Platelets | A0A182DWH7 | 0.096092812 | 0.566036213 | 0.936715898 |
| RBC | A0A0C4DH73 | 0.095978113 | 0.56650204 | 0.936715898 |
| ALT | P35908 | 0.095952733 | 0.566605143 | 0.936715898 |
| Ca | A0A0C4DH34 | 0.095828277 | 0.567110841 | 0.936715898 |
| PT | Q15582 | 0.095651034 | 0.56783138 | 0.936715898 |
| Hematocrit | Q15582 | 0.095649796 | 0.567836416 | 0.936715898 |
| APTT | O75460 | 0.095144266 | 0.569893845 | 0.937527239 |
| Platelets | A0A075B6K2 | 0.095108301 | 0.570040351 | 0.937527239 |
| Hemoglobin | P01782 | 0.095060818 | 0.570233793 | 0.937527239 |
| Monocytes | P0DP01 | 0.094050605 | 0.574356493 | 0.940238197 |
| Creatinine | P01782 | 0.093997921 | 0.574571868 | 0.940238197 |
| WLGG | F8W1S1 | 0.093813059 | 0.575327882 | 0.940238197 |
| Monocytes | P41222 | 0.093445183 | 0.57683369 | 0.940238197 |
| Hematocrit | P01019 | 0.093132696 | 0.578114169 | 0.940238197 |
| WBC | O75882 | 0.093023257 | 0.578562922 | 0.940238197 |
| ALP | P19652 | 0.092824702 | 0.579377494 | 0.940238197 |
| APTT | Q15582 | 0.09240709 | 0.581092423 | 0.940238197 |
| FIB | A0A0C4DH34 | 0.092393812 | 0.581146986 | 0.940238197 |
| LDH | P01715 | 0.092356884 | 0.581298748 | 0.940238197 |
| IBIL | P41222 | 0.092294253 | 0.581556177 | 0.940238197 |
| Creatinine | P0DP01 | 0.092129096 | 0.582235266 | 0.940238197 |
| Eosinophils | P43121 | 0.091978852 | 0.582853345 | 0.940238197 |
| Creatine Kinase | P13796 | 0.091928866 | 0.583059045 | 0.940238197 |
| γ-GT | P19652 | 0.091840765 | 0.583421671 | 0.940238197 |
| P | C9J8S2 | 0.091369648 | 0.585362506 | 0.940238197 |
| IBIL | F8W1S1 | 0.091249828 | 0.585856579 | 0.940238197 |
| INR | P0DP01 | 0.091238538 | 0.58590314 | 0.940238197 |
| Platelets | P04264 | 0.090839446 | 0.587550174 | 0.940845194 |
| Glucose | P43251 | 0.090386825 | 0.589420599 | 0.942264971 |
| UA | P13645 | 0.090277398 | 0.589873194 | 0.942264971 |
| Monocytes | A0A075B6S5 | 0.089996569 | 0.591035414 | 0.942493602 |
| Hemoglobin | A0A5H1ZRQ7 | 0.089750848 | 0.592053162 | 0.942493602 |
| CK-MB activity | C9J8S2 | 0.089687665 | 0.592314984 | 0.942493602 |
| WLC | P02750 | 0.089239546 | 0.594173388 | 0.942493602 |
| CO2 | P13796 | 0.089227077 | 0.594225133 | 0.942493602 |
| BUN | A0A0C4DH38 | 0.088974009 | 0.595275801 | 0.943238951 |
| Lymphocytes | P41222 | 0.088790765 | 0.596037087 | 0.943345364 |
| INR | A0A075B6K2 | 0.088507486 | 0.597214803 | 0.943345364 |
| Mg | P08185 | 0.088496541 | 0.597260327 | 0.943345364 |
| APTT | A0A0C4DH73 | 0.088356068 | 0.597844731 | 0.943399659 |
| UA | P04264 | 0.087651146 | 0.60078113 | 0.94511372 |
| PT | P0DP01 | 0.087080739 | 0.603161759 | 0.945449941 |
| Ca | P41222 | 0.087074148 | 0.603189291 | 0.945449941 |
| Glucose | O75882 | 0.086885156 | 0.603978968 | 0.945449941 |
| CK-MB activity | P19652 | 0.086613322 | 0.605115574 | 0.945449941 |
| FIB | A0A0C4DH73 | 0.086036942 | 0.607528587 | 0.945449941 |
| RBC | P01782 | 0.085909714 | 0.608061782 | 0.945449941 |
| CRP | A0A075B6K2 | 0.085829451 | 0.608398257 | 0.945449941 |
| TBIL | Q15582 | 0.085718988 | 0.60886146 | 0.945449941 |
| UA | A0A0J9YVY3 | 0.085426133 | 0.610090217 | 0.945449941 |
| WLC | Q15582 | 0.084561195 | 0.613725454 | 0.949967412 |
| Mg | A0A1W2PQU7 | 0.084209131 | 0.615207752 | 0.950597113 |
| WBC | A0A075B6S5 | 0.083283175 | 0.619113491 | 0.950835485 |
| P | P19652 | 0.082995748 | 0.620327981 | 0.950835485 |
| WLL | P01019 | 0.082861474 | 0.620895678 | 0.950835485 |
| AST | D6RAR4 | 0.08246085 | 0.622590769 | 0.950835485 |
| ALP | P13647 | 0.082316245 | 0.623203081 | 0.950835485 |
| TT | A0A0C4DH33 | 0.08226356 | 0.623426232 | 0.950835485 |
| WLC | A0A0C4DH38 | 0.082222019 | 0.623602202 | 0.950835485 |
| TT | Q86YZ3 | 0.081951161 | 0.624750095 | 0.950835485 |
| FIB | Q6ZRK6 | 0.081896584 | 0.624981497 | 0.950835485 |
| ALP | P01717 | 0.081858549 | 0.625142783 | 0.950835485 |
| APTT | A0A075B6S5 | 0.081458382 | 0.626840718 | 0.950835485 |
| Platelets | A0A075B6J9 | 0.081427166 | 0.62697325 | 0.950835485 |
| Creatine Kinase | P68032 | 0.081182716 | 0.628011493 | 0.951214042 |
| CO2 | P01717 | 0.081104791 | 0.628342609 | 0.951214042 |
| ALT | A0A0G2JI36 | 0.080151193 | 0.632400385 | 0.95390421 |
| UA | P01019 | 0.079881819 | 0.633548561 | 0.95390421 |
| TBIL | P41222 | 0.07880188 | 0.638160148 | 0.95390421 |
| Ca | Q9NZP8 | 0.078614273 | 0.638962649 | 0.95390421 |
| Hematocrit | A0A0C4DH73 | 0.078577292 | 0.639120883 | 0.95390421 |
| INR | P08185 | 0.078416527 | 0.639808956 | 0.95390421 |
| IBIL | P13645 | 0.07780696 | 0.642420588 | 0.95390421 |
| CK-MB activity | P80108 | 0.07760026 | 0.643307139 | 0.95390421 |
| CO2 | P43251 | 0.077512602 | 0.643683259 | 0.95390421 |
| WLGG | P43251 | 0.07750741 | 0.643705537 | 0.95390421 |
| CRP | A0A0C4DH73 | 0.077449797 | 0.643952795 | 0.95390421 |
| CRP | P01715 | 0.077430047 | 0.644037563 | 0.95390421 |
| Glucose | A0A075B6J9 | 0.077364994 | 0.644316803 | 0.95390421 |
| Total protein | P18206 | 0.076769886 | 0.646873565 | 0.95390421 |
| Hemoglobin | A0A0C4DH38 | 0.07666195 | 0.647337718 | 0.95390421 |
| WLC | A0A087WSY6 | 0.076607998 | 0.647569777 | 0.95390421 |
| Hematocrit | A0A5H1ZRQ7 | 0.076294302 | 0.648919692 | 0.95390421 |
| Neutrophils | A0A0C4DH33 | 0.07627907 | 0.648985267 | 0.95390421 |
| WBC | A0A075B6K2 | 0.075567773 | 0.652050368 | 0.95390421 |
| γ-GT | P01019 | 0.075291892 | 0.653240708 | 0.95390421 |
| Albumin | O75882 | 0.075217619 | 0.653561316 | 0.95390421 |
| CK-MB activity | A0A1W2PQU7 | 0.075182122 | 0.653714566 | 0.95390421 |
| CO2 | A0A087WSY6 | 0.074447132 | 0.65689083 | 0.95390421 |
| CK-MB activity | Q8N1N4 | 0.074412714 | 0.657039712 | 0.95390421 |
| Creatinine | A0A0C4DH73 | 0.074410461 | 0.657049458 | 0.95390421 |
| α-HBDH | P01019 | 0.074329509 | 0.657399691 | 0.95390421 |
| RBC | Q86YZ3 | 0.073904661 | 0.659238939 | 0.95390421 |
| PT | A0A0C4DH38 | 0.073822415 | 0.659595223 | 0.95390421 |
| Hemoglobin | P13647 | 0.073704989 | 0.660104037 | 0.95390421 |
| WLGG | P13796 | 0.073539031 | 0.660823401 | 0.95390421 |
| APTT | Q8N1N4 | 0.073246852 | 0.662090611 | 0.954128086 |
| TT | P01782 | 0.073204526 | 0.66227426 | 0.954128086 |
| TT | P19652 | 0.073094774 | 0.662750555 | 0.954128086 |
| CO2 | A0A075B6S5 | 0.072366991 | 0.665912244 | 0.956105281 |
| Hemoglobin | P13473 | 0.071843199 | 0.668191269 | 0.957731275 |
| Neutrophils | O75460 | 0.071788138 | 0.668431012 | 0.957731275 |
| Hemoglobin | F8W1S1 | 0.071735566 | 0.668659945 | 0.957731275 |
| Glucose | P13645 | 0.070689938 | 0.673219432 | 0.961264243 |
| CRP | Q5SRP5 | 0.070543337 | 0.673859614 | 0.961264243 |
| Creatine Kinase | P41222 | 0.070530082 | 0.67391751 | 0.961264243 |
| ALT | P43121 | 0.070248327 | 0.675148563 | 0.961427187 |
| CO2 | P08185 | 0.069739446 | 0.677374089 | 0.962507008 |
| Platelets | P35908 | 0.069278758 | 0.679391174 | 0.962548016 |
| WBC | P13473 | 0.068946649 | 0.680846654 | 0.962548016 |
| Monocytes | P01782 | 0.068704697 | 0.681907735 | 0.962548016 |
| RBC | F8W1S1 | 0.068661007 | 0.682099404 | 0.962548016 |
| Hemoglobin | A0A0C4DH73 | 0.068119619 | 0.684476088 | 0.962548016 |
| PT | A0A1W2PQU7 | 0.068118455 | 0.684481202 | 0.962548016 |
| DBIL | A0A087WSY6 | 0.068006375 | 0.684973607 | 0.962548016 |
| α-HBDH | A0A0G2JI36 | 0.067828048 | 0.685757322 | 0.962548016 |
| Mg | A0A075B6S9 | 0.067645238 | 0.686561074 | 0.962548016 |
| RBC | D6RAR4 | 0.06763338 | 0.686613221 | 0.962548016 |
| Glucose | A0A1W2PQU7 | 0.067516551 | 0.68712707 | 0.962548016 |
| CK-MB activity | A0A0C4DH73 | 0.067158299 | 0.688703633 | 0.962548016 |
| WLL | P01717 | 0.067080135 | 0.689047782 | 0.962548016 |
| WLL | A0A1W2PQU7 | 0.066890967 | 0.689880929 | 0.962548016 |
| CK-MB activity | A0A075B6K2 | 0.06673264 | 0.69057852 | 0.962548016 |
| γ-GT | P0DP01 | 0.066706904 | 0.690691936 | 0.962548016 |
| Albumin | P08185 | 0.06656814 | 0.691303572 | 0.962548016 |
| TBIL | A0A0C4DH33 | 0.066345529 | 0.692285194 | 0.962548016 |
| BUN | Q6ZRK6 | 0.066149591 | 0.693149607 | 0.962548016 |
| CK-MB activity | P13647 | 0.065619484 | 0.695490176 | 0.962548016 |
| TBIL | A0A075B6R2 | 0.065575573 | 0.695684178 | 0.962548016 |
| LDH | A0A1W2PQU7 | 0.065455345 | 0.696215458 | 0.962548016 |
| RBC | Q9NZP8 | 0.065335158 | 0.696746698 | 0.962548016 |
| Total protein | A0A075B6K2 | 0.064947663 | 0.698460442 | 0.962548016 |
| Creatinine | A0A075B6S5 | 0.064890299 | 0.698714267 | 0.962548016 |
| CRP | A0A182DWH7 | 0.064742333 | 0.699369137 | 0.962548016 |
| WLL | P13645 | 0.064460672 | 0.70061631 | 0.962548016 |
| Neutrophils | A0A075B6K2 | 0.064451338 | 0.700657653 | 0.962548016 |
| CK-MB activity | P35908 | 0.064410414 | 0.700838928 | 0.962548016 |
| UA | C9J8S2 | 0.064348464 | 0.701113372 | 0.962548016 |
| LDH | P01019 | 0.06414186 | 0.702028916 | 0.962548016 |
| ALP | P02750 | 0.063707519 | 0.703954997 | 0.962548016 |
| CRP | P02750 | 0.063427768 | 0.705196516 | 0.962548016 |
| Hematocrit | A0A182DWH7 | 0.063365254 | 0.705474054 | 0.962548016 |
| Lymphocytes | Q15582 | 0.063153289 | 0.706415372 | 0.962548016 |
| WLL | P13796 | 0.063071932 | 0.706776783 | 0.962548016 |
| ALP | P68032 | 0.063064937 | 0.706807861 | 0.962548016 |
| Lymphocytes | Q8N1N4 | 0.063043838 | 0.706901603 | 0.962548016 |
| Creatinine | F8W1S1 | 0.062991916 | 0.707132302 | 0.962548016 |
| Total protein | P80108 | 0.062951619 | 0.707311369 | 0.962548016 |
| Ca | D6RAR4 | 0.062935276 | 0.707383997 | 0.962548016 |
| Glucose | A0A0G2JI36 | 0.062172974 | 0.710774433 | 0.962548016 |
| IBIL | P68032 | 0.06164402 | 0.713130256 | 0.962548016 |
| TBIL | P68032 | 0.061316598 | 0.714589825 | 0.962548016 |
| Hemoglobin | P0DP01 | 0.061067867 | 0.715699276 | 0.962548016 |
| Eosinophils | P04264 | 0.060948305 | 0.716232782 | 0.962548016 |
| Monocytes | P01715 | 0.060855135 | 0.716648613 | 0.962548016 |
| PT | P08185 | 0.060769121 | 0.717032578 | 0.962548016 |
| BUN | P43251 | 0.060738715 | 0.717168325 | 0.962548016 |
| INR | F8W1S1 | 0.060544455 | 0.718035804 | 0.962548016 |
| BUN | A0A2R8Y7X9 | 0.060519837 | 0.718145762 | 0.962548016 |
| UA | A0A075B6S5 | 0.060403786 | 0.718664184 | 0.962548016 |
| DBIL | P01715 | 0.060328391 | 0.719001059 | 0.962548016 |
| BUN | A0A0C4DH33 | 0.060304258 | 0.719108898 | 0.962548016 |
| ALT | P01782 | 0.060134761 | 0.719866456 | 0.962548016 |
| CK-MB activity | P01715 | 0.06004371 | 0.720273509 | 0.962548016 |
| Hematocrit | A0A075B6K2 | 0.059775746 | 0.72147192 | 0.962548016 |
| Platelets | A0A0C4DH38 | 0.059647588 | 0.722045311 | 0.962548016 |
| Creatine Kinase | C9J8S2 | 0.05949412 | 0.722732138 | 0.962548016 |
| CO2 | C9J8S2 | 0.059459029 | 0.722889211 | 0.962548016 |
| TT | P13647 | 0.058936777 | 0.72522825 | 0.964054943 |
| RBC | D6RE82 | 0.05887825 | 0.725490534 | 0.964054943 |
| Globin | A0A2R8Y7X9 | 0.058678643 | 0.726385279 | 0.96441897 |
| Albumin | P01715 | 0.058461911 | 0.727357191 | 0.96441897 |
| WBC | Q5SRP5 | 0.058300046 | 0.728083338 | 0.96441897 |
| WLL | O75460 | 0.058211343 | 0.728481366 | 0.96441897 |
| WLGG | P01782 | 0.058161561 | 0.728704781 | 0.96441897 |
| ALP | P18206 | 0.057770558 | 0.730460301 | 0.965443836 |
| APTT | P80108 | 0.0575902 | 0.731270528 | 0.96586604 |
| WLC | P01782 | 0.057192841 | 0.733056607 | 0.966601577 |
| WLL | P13647 | 0.05705406 | 0.73368074 | 0.966601577 |
| WLC | D6RE82 | 0.056958924 | 0.734108685 | 0.966601577 |
| α-HBDH | A0A0C4DH73 | 0.056704986 | 0.735251347 | 0.966601577 |
| BUN | P01019 | 0.056470589 | 0.736306581 | 0.966601577 |
| TT | A0A075B6S9 | 0.056226941 | 0.737403963 | 0.966601577 |
| Platelets | P0DP01 | 0.05620688 | 0.737494341 | 0.966601577 |
| ALT | A0A075B6S5 | 0.056081963 | 0.738057188 | 0.966601577 |
| UA | F8W1S1 | 0.056066103 | 0.738128656 | 0.966601577 |
| AST | P80108 | 0.055924246 | 0.738768008 | 0.966601577 |
| Neutrophils | P01717 | 0.055652963 | 0.739991172 | 0.966601577 |
| Mg | Q5SRP5 | 0.055534311 | 0.740526347 | 0.966601577 |
| DBIL | P41222 | 0.055393985 | 0.741159441 | 0.966601577 |
| Lymphocytes | P0DP01 | 0.055223817 | 0.74192739 | 0.966962321 |
| BUN | Q9NZP8 | 0.054829002 | 0.743710101 | 0.967052232 |
| Hematocrit | O75882 | 0.054719563 | 0.744204485 | 0.967052232 |
| UA | P43121 | 0.054622737 | 0.744641975 | 0.967052232 |
| PT | A0A075B6K2 | 0.054520224 | 0.745105246 | 0.967052232 |
| LDH | A0A0G2JI36 | 0.05390221 | 0.747900005 | 0.968056625 |
| WLGG | A0A182DWH7 | 0.053697134 | 0.748828094 | 0.968078142 |
| UA | A0A0G2JI36 | 0.05357122 | 0.749398102 | 0.968078142 |
| Hematocrit | P13473 | 0.053406293 | 0.750144916 | 0.968078142 |
| Hemoglobin | Q8N1N4 | 0.052787229 | 0.752950141 | 0.968078142 |
| P | P13473 | 0.052556674 | 0.753995675 | 0.968078142 |
| DBIL | P68032 | 0.052440585 | 0.754522287 | 0.968078142 |
| AST | A0A075B6S9 | 0.05226949 | 0.755298619 | 0.968078142 |
| CRP | Q15582 | 0.052253965 | 0.755369077 | 0.968078142 |
| WLGG | P04264 | 0.052208992 | 0.755573184 | 0.968078142 |
| Ca | P18206 | 0.052009539 | 0.756478585 | 0.968607583 |
| LDH | P35908 | 0.051663751 | 0.758049023 | 0.969357035 |
| P | P18206 | 0.051475042 | 0.758906468 | 0.969823331 |
| BUN | Q8N1N4 | 0.05132695 | 0.759579562 | 0.970053587 |
| P | P43121 | 0.050910502 | 0.761473292 | 0.971211561 |
| Lymphocytes | P04264 | 0.050566411 | 0.763039026 | 0.97257824 |
| ALT | A0A2R8Y7X9 | 0.049947998 | 0.765855352 | 0.973252685 |
| α-HBDH | A0A1W2PQU7 | 0.049917903 | 0.765992484 | 0.973252685 |
| IBIL | O75882 | 0.049573211 | 0.767563621 | 0.973302323 |
| ALT | P13647 | 0.048085901 | 0.774353333 | 0.977313613 |
| IBIL | A0A0C4DH33 | 0.048043776 | 0.774545885 | 0.977313613 |
| CO2 | Q15582 | 0.04784323 | 0.775462735 | 0.977313613 |
| APTT | A0A5H1ZRQ7 | 0.047553822 | 0.776786378 | 0.977313613 |
| γ-GT | A0A075B6S9 | 0.047419506 | 0.777400896 | 0.977313613 |
| TT | P01019 | 0.047303074 | 0.7779337 | 0.977313613 |
| LDH | P13473 | 0.04717601 | 0.778515277 | 0.977313613 |
| Glucose | Q6ZRK6 | 0.047104002 | 0.77884491 | 0.977313613 |
| Creatine Kinase | Q9NZP8 | 0.046949385 | 0.779552833 | 0.977313613 |
| WLGG | A0A0G2JI36 | 0.046734096 | 0.780538842 | 0.977313613 |
| FIB | P13473 | 0.046361308 | 0.782246983 | 0.977313613 |
| BUN | A0A1W2PQU7 | 0.04629275 | 0.782561228 | 0.977313613 |
| AST | P0DP01 | 0.045776412 | 0.78492904 | 0.977366647 |
| UA | D6RAR4 | 0.045521694 | 0.786097825 | 0.977366647 |
| Monocytes | A0A075B6K2 | 0.04516524 | 0.787734206 | 0.977366647 |
| ALT | C9J8S2 | 0.044949336 | 0.788725796 | 0.977366647 |
| WLC | A0A075B6S9 | 0.044678796 | 0.789968777 | 0.977366647 |
| Creatine Kinase | A0A0C4DH73 | 0.044432285 | 0.791101805 | 0.977366647 |
| WLC | A0A0C4DH33 | 0.044329801 | 0.791572972 | 0.977366647 |
| Glucose | P13473 | 0.044317995 | 0.791627254 | 0.977366647 |
| UA | A0A2R8Y7X9 | 0.044208568 | 0.792130432 | 0.977374727 |
| ALP | A0A075B6R2 | 0.044004163 | 0.793070569 | 0.977921604 |
| Mg | P68032 | 0.0433599 | 0.796035656 | 0.98062035 |
| WLGG | A0A075B6R2 | 0.043280138 | 0.796402942 | 0.98062035 |
| Mg | P43251 | 0.043203901 | 0.796754034 | 0.98062035 |
| CRP | P01782 | 0.042832914 | 0.798463101 | 0.981146196 |
| Basophils | P01019 | 0.042659999 | 0.799260006 | 0.981146196 |
| CO2 | P43121 | 0.042245237 | 0.801172309 | 0.981146196 |
| AST | A0A0G2JI36 | 0.041577354 | 0.804254048 | 0.981146196 |
| Platelets | P13473 | 0.041370254 | 0.805210241 | 0.981146196 |
| Basophils | A0A075B6J9 | 0.041303756 | 0.805517324 | 0.981146196 |
| CK-MB activity | A0A0J9YVY3 | 0.041118388 | 0.806373494 | 0.981411897 |
| CO2 | A0A5H1ZRQ7 | 0.040522862 | 0.809125594 | 0.983544886 |
| CRP | P13473 | 0.039436954 | 0.814149684 | 0.984417279 |
| Lymphocytes | A0A1W2PQU7 | 0.039402398 | 0.814309683 | 0.984417279 |
| APTT | P01782 | 0.039305859 | 0.814756713 | 0.984417279 |
| Glucose | P01782 | 0.039174919 | 0.815363128 | 0.984417279 |
| γ-GT | P08185 | 0.038906291 | 0.816607548 | 0.984417279 |
| PT | Q8N1N4 | 0.03883081 | 0.816957291 | 0.984417279 |
| APTT | P18206 | 0.03873444 | 0.817403877 | 0.984417279 |
| Platelets | D6RE82 | 0.038415236 | 0.818883496 | 0.984417279 |
| Globin | A0A075B6K2 | 0.038381382 | 0.819040456 | 0.984417279 |
| TBIL | A0A0J9YVY3 | 0.038315203 | 0.819347309 | 0.984417279 |
| γ-GT | Q6ZRK6 | 0.037903345 | 0.821257571 | 0.985509086 |
| TT | D6RAR4 | 0.037644906 | 0.82245677 | 0.986346693 |
| Mg | A0A075B6S5 | 0.03704762 | 0.825229791 | 0.986841213 |
| Platelets | F8W1S1 | 0.03672383 | 0.826733931 | 0.986841213 |
| Creatine Kinase | P01717 | 0.036483171 | 0.827852282 | 0.986841213 |
| CK-MB activity | O75460 | 0.036381992 | 0.828322568 | 0.986841213 |
| BUN | P41222 | 0.036172092 | 0.829298378 | 0.986841213 |
| Creatine Kinase | O75882 | 0.036114912 | 0.829564249 | 0.986841213 |
| Albumin | A0A075B6K2 | 0.036045002 | 0.829889333 | 0.986841213 |
| α-HBDH | A0A075B6S9 | 0.035782784 | 0.831108911 | 0.987032552 |
| α-HBDH | O75882 | 0.035686922 | 0.831554863 | 0.987032552 |
| Glucose | D6RAR4 | 0.035563823 | 0.832127596 | 0.987117003 |
| UA | P13473 | 0.035126115 | 0.834164786 | 0.987640188 |
| BUN | P43121 | 0.035093393 | 0.834317123 | 0.987640188 |
| CK-MB activity | P13645 | 0.035063007 | 0.834458591 | 0.987640188 |
| α-HBDH | P01715 | 0.034925394 | 0.835099343 | 0.987665569 |
| ALT | A0A0J9YVY3 | 0.034318937 | 0.837924356 | 0.988319063 |
| WLGG | A0A0C4DH73 | 0.033359189 | 0.84239915 | 0.988319063 |
| Creatine Kinase | Q6ZRK6 | 0.033219479 | 0.843050954 | 0.988319063 |
| DBIL | C9J8S2 | 0.033183406 | 0.843219268 | 0.988319063 |
| WLL | Q5SRP5 | 0.032951077 | 0.844303449 | 0.988319063 |
| LDH | A0A075B6S9 | 0.032853166 | 0.844760441 | 0.988319063 |
| CK-MB activity | A0A0C4DH33 | 0.032371851 | 0.847007676 | 0.988319063 |
| γ-GT | A0A2R8Y7X9 | 0.032111389 | 0.848224259 | 0.988319063 |
| ALT | P01019 | 0.032093779 | 0.848306524 | 0.988319063 |
| Albumin | P02750 | 0.031860738 | 0.849395341 | 0.988319063 |
| Creatinine | A0A0G2JI36 | 0.031687345 | 0.85020565 | 0.988319063 |
| LDH | A0A182DWH7 | 0.031633102 | 0.850459174 | 0.988319063 |
| INR | A0A0C4DH38 | 0.031037591 | 0.85324345 | 0.988319063 |
| α-HBDH | A0A182DWH7 | 0.030870282 | 0.854026008 | 0.988319063 |
| Basophils | P18206 | 0.030709586 | 0.854777766 | 0.988319063 |
| Basophils | A0A0C4DH33 | 0.030332179 | 0.856543825 | 0.988319063 |
| CK-MB activity | A0A0G2JI36 | 0.030176935 | 0.857270481 | 0.988319063 |
| ALP | A0A182DWH7 | 0.029883424 | 0.858644647 | 0.988319063 |
| APTT | A0A0J9YVY3 | 0.029829473 | 0.858897279 | 0.988319063 |
| ALP | Q8N1N4 | 0.029336109 | 0.861208172 | 0.988319063 |
| Ca | P01715 | 0.029300795 | 0.861373622 | 0.988319063 |
| Hematocrit | P0DP01 | 0.029252272 | 0.861600969 | 0.988319063 |
| γ-GT | A0A075B6R2 | 0.028713938 | 0.864124002 | 0.989070215 |
| DBIL | A0A075B6K2 | 0.028678445 | 0.864290398 | 0.989070215 |
| UA | A0A5H1ZRQ7 | 0.028648394 | 0.864431285 | 0.989070215 |
| Eosinophils | A0A2R8Y7X9 | 0.028281767 | 0.866150439 | 0.989539648 |
| CRP | A0A075B6R2 | 0.028263151 | 0.86623775 | 0.989539648 |
| Mg | Q9NZP8 | 0.028142999 | 0.866801296 | 0.989539648 |
| WBC | A0A075B6R2 | 0.027469221 | 0.869962716 | 0.989940284 |
| γ-GT | D6RAR4 | 0.027289201 | 0.870807721 | 0.989940284 |
| Monocytes | C9J8S2 | 0.026930127 | 0.872493622 | 0.989940284 |
| CRP | P01717 | 0.026868454 | 0.872783238 | 0.989940284 |
| Hemoglobin | A0A182DWH7 | 0.026503131 | 0.874499142 | 0.989940284 |
| Lymphocytes | P13645 | 0.026487168 | 0.874574135 | 0.989940284 |
| CO2 | D6RAR4 | 0.025837534 | 0.877626869 | 0.989940284 |
| WLGG | Q15582 | 0.025670454 | 0.878412287 | 0.989940284 |
| AST | P01782 | 0.025440049 | 0.879495576 | 0.989940284 |
| Albumin | P04264 | 0.025401001 | 0.87967919 | 0.989940284 |
| AST | P18206 | 0.025224789 | 0.880507857 | 0.989940284 |
| Platelets | C9J8S2 | 0.025176419 | 0.880735345 | 0.989940284 |
| CO2 | P68032 | 0.02492586 | 0.881913903 | 0.989940284 |
| Mg | A0A182DWH7 | 0.024844992 | 0.882294338 | 0.989940284 |
| Glucose | Q15582 | 0.024621109 | 0.883347705 | 0.990557426 |
| WLC | P0DP01 | 0.024237001 | 0.885155381 | 0.992019242 |
| AST | P68032 | 0.023676245 | 0.887795426 | 0.993284536 |
| Albumin | Q9NZP8 | 0.023430234 | 0.888954027 | 0.993284536 |
| Platelets | A0A5H1ZRQ7 | 0.02327378 | 0.88969097 | 0.993284536 |
| CO2 | O75460 | 0.022991026 | 0.891023058 | 0.993327147 |
| γ-GT | A0A0G2JI36 | 0.022804314 | 0.891902842 | 0.993327147 |
| CK-MB activity | A0A075B6S9 | 0.022524275 | 0.893222618 | 0.993327147 |
| APTT | P01715 | 0.022351432 | 0.894037342 | 0.993327147 |
| CO2 | P41222 | 0.022162524 | 0.894927912 | 0.993327147 |
| DBIL | D6RAR4 | 0.021683192 | 0.897188195 | 0.993327147 |
| P | A0A0G2JI36 | 0.020947764 | 0.900657643 | 0.993327147 |
| Hemoglobin | A0A0G2JI36 | 0.020762455 | 0.901532143 | 0.993327147 |
| RBC | P13796 | 0.020574556 | 0.902418986 | 0.993327147 |
| PT | P13647 | 0.02051232 | 0.902712748 | 0.993327147 |
| APTT | P41222 | 0.020403838 | 0.903224835 | 0.993327147 |
| WLL | Q15582 | 0.020252455 | 0.903939498 | 0.993327147 |
| ALP | P35908 | 0.020250672 | 0.903947917 | 0.993327147 |
| WLL | A0A075B6R2 | 0.020136727 | 0.904485889 | 0.993327147 |
| Creatinine | A0A182DWH7 | 0.020025168 | 0.905012636 | 0.993327147 |
| UA | Q8N1N4 | 0.020025168 | 0.905012636 | 0.993327147 |
| LDH | P19652 | 0.019921192 | 0.905503616 | 0.993327147 |
| UA | A0A075B6K2 | 0.019767115 | 0.90623124 | 0.993327147 |
| BUN | P35908 | 0.019480164 | 0.907586549 | 0.993951213 |
| Glucose | P41222 | 0.019293336 | 0.90846911 | 0.994014271 |
| WLL | P43251 | 0.019095172 | 0.909405331 | 0.994014271 |
| Mg | Q6ZRK6 | 0.018603245 | 0.911729955 | 0.995176648 |
| IBIL | P43121 | 0.018364537 | 0.912858242 | 0.995207188 |
| α-HBDH | P35908 | 0.017405585 | 0.917392543 | 0.996554013 |
| CRP | D6RAR4 | 0.017089347 | 0.918888409 | 0.996554013 |
| Total protein | P13796 | 0.016750605 | 0.920491031 | 0.996554013 |
| WLGG | A0A2R8Y7X9 | 0.016493577 | 0.921707261 | 0.996554013 |
| Mg | F8W1S1 | 0.016345464 | 0.922408198 | 0.996554013 |
| Creatine Kinase | O75460 | 0.015978112 | 0.924146913 | 0.996554013 |
| γ-GT | A0A0J9YVY3 | 0.015733358 | 0.925305556 | 0.996554013 |
| PT | P68032 | 0.015711204 | 0.925410438 | 0.996554013 |
| Ca | O75460 | 0.015678997 | 0.925562916 | 0.996554013 |
| P | P0DP01 | 0.015565057 | 0.926102366 | 0.996554013 |
| Neutrophils | P68032 | 0.01555722 | 0.926139473 | 0.996554013 |
| WLL | A0A2R8Y7X9 | 0.015391866 | 0.9269224 | 0.996554013 |
| Mg | A0A0C4DH34 | 0.015390703 | 0.926927908 | 0.996554013 |
| WLC | Q86YZ3 | 0.015091449 | 0.928345005 | 0.996554013 |
| WLL | A0A0C4DH73 | 0.015044681 | 0.928566493 | 0.996554013 |
| TBIL | P01715 | 0.014768298 | 0.929875512 | 0.996554013 |
| Mg | P04264 | 0.014511234 | 0.931093197 | 0.996554013 |
| WBC | Q15582 | 0.014445965 | 0.931402396 | 0.996554013 |
| Hemoglobin | D6RAR4 | 0.014237219 | 0.932391345 | 0.996554013 |
| Eosinophils | P02750 | 0.013921645 | 0.933886594 | 0.996554013 |
| TBIL | P43121 | 0.013573903 | 0.935534517 | 0.996554013 |
| Glucose | P02750 | 0.013131258 | 0.937632564 | 0.996554013 |
| IBIL | A0A0J9YVY3 | 0.013072634 | 0.937910462 | 0.996554013 |
| P | A0A0C4DH33 | 0.012592392 | 0.940187234 | 0.996554013 |
| INR | A0A1W2PQU7 | 0.012393102 | 0.941132187 | 0.996554013 |
| FIB | P13645 | 0.012384936 | 0.941170908 | 0.996554013 |
| CRP | P41222 | 0.011885836 | 0.943537784 | 0.996554013 |
| WLC | P43121 | 0.011376422 | 0.945954065 | 0.996554013 |
| PT | P04264 | 0.010640081 | 0.949447559 | 0.996554013 |
| Lymphocytes | P43251 | 0.010397855 | 0.950596978 | 0.996554013 |
| Lymphocytes | A0A087WSY6 | 0.010178953 | 0.951635808 | 0.996554013 |
| WBC | P01782 | 0.00995896 | 0.952679891 | 0.996554013 |
| WLL | P01782 | 0.009836907 | 0.95325919 | 0.996554013 |
| Mg | O75882 | 0.009564222 | 0.954553509 | 0.996554013 |
| Lymphocytes | A0A075B6K2 | 0.009476253 | 0.954971086 | 0.996554013 |
| Ca | A0A075B6K2 | 0.0093757 | 0.955448411 | 0.996554013 |
| TBIL | P01019 | 0.009195907 | 0.956301931 | 0.996554013 |
| Eosinophils | A0A0G2JI36 | 0.009123729 | 0.956644589 | 0.996554013 |
| Neutrophils | A0A0C4DH73 | 0.00908295 | 0.956838184 | 0.996554013 |
| FIB | D6RAR4 | 0.008877697 | 0.957812658 | 0.996554013 |
| Albumin | A0A1W2PQU7 | 0.008868453 | 0.957856548 | 0.996554013 |
| UA | O75460 | 0.008863599 | 0.957879593 | 0.996554013 |
| UA | Q6ZRK6 | 0.008853701 | 0.95792659 | 0.996554013 |
| Mg | P02750 | 0.008794687 | 0.958206779 | 0.996554013 |
| Albumin | A0A0C4DH38 | 0.008649479 | 0.958896234 | 0.996554013 |
| FIB | P01715 | 0.00837647 | 0.960192571 | 0.996554013 |
| Mg | Q8N1N4 | 0.008135086 | 0.961338825 | 0.996554013 |
| Total protein | D6RE82 | 0.0081016 | 0.961497845 | 0.996554013 |
| Ca | C9J8S2 | 0.007943486 | 0.962248725 | 0.996554013 |
| Creatinine | Q8N1N4 | 0.007878755 | 0.962556141 | 0.996554013 |
| Neutrophils | A0A075B6S9 | 0.007434525 | 0.964665987 | 0.996554013 |
| FIB | A0A2R8Y7X9 | 0.006795274 | 0.967702483 | 0.996777049 |
| Neutrophils | Q5SRP5 | 0.006589108 | 0.968681889 | 0.996777049 |
| Globin | P80108 | 0.006568505 | 0.968779769 | 0.996777049 |
| PT | A0A0C4DH73 | 0.005813652 | 0.972366134 | 0.996777049 |
| FIB | F8W1S1 | 0.004624552 | 0.978016702 | 0.998173129 |
| WLL | A0A0G2JI36 | 0.004615426 | 0.978060077 | 0.998173129 |
| RBC | A0A0G2JI36 | 0.003985072 | 0.981055947 | 0.998173129 |
| ALP | A0A075B6J9 | 0.003502819 | 0.983348121 | 0.998173129 |
| Albumin | A0A075B6R2 | 0.003175125 | 0.984905741 | 0.998173129 |
| BUN | P01782 | 0.003173735 | 0.98491235 | 0.998173129 |
| WLC | O75882 | 0.003157887 | 0.98498768 | 0.998173129 |
| WBC | A0A2R8Y7X9 | 0.003064296 | 0.985432558 | 0.998173129 |
| γ-GT | P43121 | 0.003045764 | 0.985520644 | 0.998173129 |
| ALT | A0A1W2PQU7 | 0.002957447 | 0.985940456 | 0.998173129 |
| TT | P35908 | 0.002743798 | 0.986956043 | 0.998173129 |
| Eosinophils | Q5SRP5 | 0.002684874 | 0.987236144 | 0.998173129 |
| Basophils | A0A075B6S5 | 0.002589191 | 0.987690985 | 0.998173129 |
| Mg | A0A0C4DH38 | 0.002528473 | 0.987979618 | 0.998173129 |
| CK-MB activity | P13473 | 0.002088392 | 0.990071649 | 0.999210772 |
| LDH | A0A0J9YVY3 | 0.001949855 | 0.990730236 | 0.999362945 |
| Glucose | A0A075B6S5 | 0.001641407 | 0.992196571 | 0.999379904 |
| ALT | P04264 | 0.001423956 | 0.993230332 | 0.999379904 |
| WLGG | Q6ZRK6 | 0.00104928 | 0.995011562 | 0.999379904 |
| TBIL | A0A087WSY6 | 0.000985276 | 0.995315846 | 0.999379904 |
| Mg | A0A2R8Y7X9 | 0.000219867 | 0.998954712 | 0.999975649 |
| Monocytes | A0A075B6S9 | 0 | 1 | 1 |
| Total protein | A0A2R8Y7X9 | -0.000109481 | 0.999479507 | 0.999987631 |
| WBC | D6RE82 | -0.000218878 | 0.998959413 | 0.999975649 |
| Glucose | P04264 | -0.000547136 | 0.997398822 | 0.999430184 |
| TBIL | O75882 | -0.000547375 | 0.997397682 | 0.999430184 |
| CO2 | O75882 | -0.000656886 | 0.99687705 | 0.999430184 |
| CO2 | A0A075B6R2 | -0.000875849 | 0.995836075 | 0.999390819 |
| Creatine Kinase | A0A182DWH7 | -0.000984952 | 0.995317384 | 0.999379904 |
| ALP | P13473 | -0.000985168 | 0.995316359 | 0.999379904 |
| Creatine Kinase | P01782 | -0.00120383 | 0.994276819 | 0.999379904 |
| TT | A0A075B6J9 | -0.001317023 | 0.993738695 | 0.999379904 |
| Total protein | P02750 | -0.001751697 | 0.991672258 | 0.999379904 |
| BUN | P80108 | -0.002188783 | 0.989594413 | 0.999210772 |
| Total protein | A0A182DWH7 | -0.002518065 | 0.988029093 | 0.998173129 |
| WLL | P41222 | -0.002906597 | 0.986182174 | 0.998173129 |
| WBC | P01717 | -0.003177213 | 0.984895816 | 0.998173129 |
| ALT | O75460 | -0.003614658 | 0.982816533 | 0.998173129 |
| RBC | A0A182DWH7 | -0.00372093 | 0.98231141 | 0.998173129 |
| TT | F8W1S1 | -0.003803963 | 0.981916751 | 0.998173129 |
| CRP | P13796 | -0.003834148 | 0.981773281 | 0.998173129 |
| UA | Q9NZP8 | -0.004158232 | 0.980232948 | 0.998173129 |
| UA | A0A0C4DH73 | -0.00459594 | 0.978152681 | 0.998173129 |
| WBC | D6RAR4 | -0.004815322 | 0.977110095 | 0.998173129 |
| ALP | P43251 | -0.005035302 | 0.976064698 | 0.998173129 |
| Albumin | P35908 | -0.005036405 | 0.976059456 | 0.998173129 |
| IBIL | P01715 | -0.005051854 | 0.975986042 | 0.998173129 |
| INR | Q8N1N4 | -0.005593347 | 0.973412928 | 0.997228861 |
| WLL | P0DP01 | -0.005792758 | 0.972465414 | 0.996777049 |
| ALP | P01715 | -0.00595159 | 0.971710739 | 0.996777049 |
| CO2 | P13473 | -0.006021459 | 0.97137877 | 0.996777049 |
| DBIL | A0A075B6R2 | -0.006023109 | 0.971370932 | 0.996777049 |
| WBC | Q9NZP8 | -0.006128591 | 0.970869765 | 0.996777049 |
| Neutrophils | P43251 | -0.006237689 | 0.970351432 | 0.996777049 |
| Eosinophils | P35908 | -0.006248297 | 0.97030103 | 0.996777049 |
| CRP | A0A075B6S5 | -0.007011014 | 0.966677648 | 0.996554013 |
| WLL | A0A075B6K2 | -0.007050923 | 0.966488073 | 0.996554013 |
| Neutrophils | P01715 | -0.007072596 | 0.966385125 | 0.996554013 |
| BUN | A0A075B6S9 | -0.007191163 | 0.965821919 | 0.996554013 |
| Creatinine | Q9NZP8 | -0.007331619 | 0.965154766 | 0.996554013 |
| WLC | P19652 | -0.007368403 | 0.96498005 | 0.996554013 |
| γ-GT | A0A5H1ZRQ7 | -0.007475411 | 0.96447179 | 0.996554013 |
| CK-MB activity | A0A075B6R2 | -0.007584161 | 0.963955269 | 0.996554013 |
| ALT | A0A075B6R2 | -0.007776991 | 0.963039441 | 0.996554013 |
| WLC | A0A075B6S5 | -0.007836238 | 0.962758062 | 0.996554013 |
| LDH | A0A0C4DH73 | -0.007880911 | 0.9625459 | 0.996554013 |
| α-HBDH | P13647 | -0.008100712 | 0.961502059 | 0.996554013 |
| BUN | P04264 | -0.008317374 | 0.960473192 | 0.996554013 |
| Total protein | D6RAR4 | -0.009086929 | 0.956819294 | 0.996554013 |
| ALP | A0A075B6K2 | -0.009243284 | 0.956077017 | 0.996554013 |
| WLL | A0A182DWH7 | -0.009373994 | 0.955456514 | 0.996554013 |
| α-HBDH | A0A0J9YVY3 | -0.009520921 | 0.954759052 | 0.996554013 |
| Platelets | P01019 | -0.009521725 | 0.954755237 | 0.996554013 |
| INR | P80108 | -0.009651265 | 0.954140342 | 0.996554013 |
| α-HBDH | P02750 | -0.009961687 | 0.952666951 | 0.996554013 |
| UA | A0A075B6S9 | -0.010115275 | 0.951938014 | 0.996554013 |
| RBC | Q15582 | -0.010177839 | 0.951641096 | 0.996554013 |
| WLGG | O75882 | -0.010292984 | 0.951094648 | 0.996554013 |
| Creatinine | Q6ZRK6 | -0.010300384 | 0.951059532 | 0.996554013 |
| Basophils | A0A075B6K2 | -0.010543049 | 0.949907987 | 0.996554013 |
| Eosinophils | A0A0C4DH33 | -0.010633649 | 0.949478076 | 0.996554013 |
| α-HBDH | Q9NZP8 | -0.010837439 | 0.948511117 | 0.996554013 |
| Platelets | P01717 | -0.010956507 | 0.94794619 | 0.996554013 |
| IBIL | P01019 | -0.011490479 | 0.94541302 | 0.996554013 |
| Albumin | P18206 | -0.012216692 | 0.941968715 | 0.996554013 |
| TT | P04264 | -0.012731222 | 0.939529008 | 0.996554013 |
| Albumin | A0A0C4DH73 | -0.012809988 | 0.939155579 | 0.996554013 |
| RBC | A0A075B6K2 | -0.013101533 | 0.937773467 | 0.996554013 |
| LDH | Q15582 | -0.013244309 | 0.937096686 | 0.996554013 |
| BUN | P01717 | -0.013585325 | 0.935480387 | 0.996554013 |
| Hematocrit | P43121 | -0.013686423 | 0.935001264 | 0.996554013 |
| Eosinophils | Q86YZ3 | -0.013912539 | 0.933929743 | 0.996554013 |
| UA | P01782 | -0.014006675 | 0.933483684 | 0.996554013 |
| Total protein | P43251 | -0.014670464 | 0.930338923 | 0.996554013 |
| Monocytes | Q6ZRK6 | -0.014858001 | 0.929450638 | 0.996554013 |
| Eosinophils | A0A0J9YVY3 | -0.01504761 | 0.928552622 | 0.996554013 |
| Mg | P13645 | -0.015500636 | 0.926407379 | 0.996554013 |
| FIB | A0A0C4DH33 | -0.015509418 | 0.926365798 | 0.996554013 |
| PT | P01715 | -0.015641465 | 0.92574061 | 0.996554013 |
| DBIL | P01019 | -0.015879105 | 0.924615585 | 0.996554013 |
| Ca | A0A0C4DH73 | -0.016007927 | 0.924005783 | 0.996554013 |
| WLGG | P01717 | -0.016263359 | 0.92279678 | 0.996554013 |
| Hemoglobin | P43121 | -0.016388581 | 0.922204142 | 0.996554013 |
| TT | P01715 | -0.016832271 | 0.92010463 | 0.996554013 |
| RBC | O75882 | -0.017400821 | 0.917415074 | 0.996554013 |
| APTT | O75882 | -0.017627419 | 0.916343391 | 0.996554013 |
| LDH | P68032 | -0.017666575 | 0.916158216 | 0.996554013 |
| Glucose | P35908 | -0.018274334 | 0.913284645 | 0.995207188 |
| Basophils | A0A0C4DH73 | -0.018494219 | 0.912245261 | 0.995176648 |
| WLL | A0A075B6J9 | -0.018516531 | 0.912139804 | 0.995176648 |
| Glucose | P08185 | -0.019040324 | 0.909664482 | 0.994014271 |
| Ca | A0A075B6J9 | -0.019077941 | 0.909486747 | 0.994014271 |
| Albumin | P13645 | -0.019707673 | 0.906511969 | 0.993327147 |
| Lymphocytes | Q5SRP5 | -0.019993967 | 0.905159967 | 0.993327147 |
| Eosinophils | Q8N1N4 | -0.020169943 | 0.904329063 | 0.993327147 |
| CK-MB activity | D6RAR4 | -0.02022443 | 0.904071808 | 0.993327147 |
| Albumin | A0A075B6S5 | -0.020255109 | 0.903926972 | 0.993327147 |
| AST | A0A0J9YVY3 | -0.020338205 | 0.903534673 | 0.993327147 |
| Ca | Q5SRP5 | -0.020812343 | 0.901296706 | 0.993327147 |
| Globin | A0A0J9YVY3 | -0.02099306 | 0.9004439 | 0.993327147 |
| WLC | A0A5H1ZRQ7 | -0.02100006 | 0.900410873 | 0.993327147 |
| TBIL | A0A075B6K2 | -0.021296986 | 0.899009925 | 0.993327147 |
| WLGG | Q8N1N4 | -0.021454051 | 0.898268989 | 0.993327147 |
| CO2 | P13647 | -0.021458291 | 0.898248989 | 0.993327147 |
| APTT | A0A075B6S9 | -0.021887786 | 0.896223337 | 0.993327147 |
| WLC | A0A075B6R2 | -0.022806961 | 0.891890368 | 0.993327147 |
| Hematocrit | D6RAR4 | -0.022872777 | 0.891580229 | 0.993327147 |
| AST | A0A1W2PQU7 | -0.023246942 | 0.889817397 | 0.993284536 |
| WBC | A0A5H1ZRQ7 | -0.023492058 | 0.88866284 | 0.993284536 |
| IBIL | A0A087WSY6 | -0.023528124 | 0.888492979 | 0.993284536 |
| Mg | A0A075B6J9 | -0.023855589 | 0.886950941 | 0.993284536 |
| TT | Q8N1N4 | -0.025023436 | 0.881454903 | 0.989940284 |
| PT | P01717 | -0.025146897 | 0.880874196 | 0.989940284 |
| CO2 | D6RE82 | -0.025290129 | 0.880200571 | 0.989940284 |
| AST | P08185 | -0.025440049 | 0.879495576 | 0.989940284 |
| FIB | A0A075B6S9 | -0.025755637 | 0.878011841 | 0.989940284 |
| Creatinine | A0A087WSY6 | -0.025824807 | 0.877686692 | 0.989940284 |
| Lymphocytes | P01715 | -0.025937109 | 0.877158835 | 0.989940284 |
| FIB | A0A075B6J9 | -0.025975484 | 0.87697847 | 0.989940284 |
| INR | P68032 | -0.026024718 | 0.876747081 | 0.989940284 |
| LDH | A0A075B6S5 | -0.026269704 | 0.875595837 | 0.989940284 |
| DBIL | A0A075B6S9 | -0.026588207 | 0.874099496 | 0.989940284 |
| P | A0A075B6S9 | -0.026644809 | 0.873833623 | 0.989940284 |
| BUN | P18206 | -0.026679756 | 0.873669473 | 0.989940284 |
| CK-MB activity | Q15582 | -0.026709438 | 0.873530059 | 0.989940284 |
| WLGG | P80108 | -0.027158597 | 0.871420861 | 0.989940284 |
| APTT | A0A0C4DH34 | -0.027481255 | 0.869906231 | 0.989940284 |
| ALP | O75882 | -0.028132015 | 0.866852822 | 0.989539648 |
| WBC | P43251 | -0.02878249 | 0.863802643 | 0.989070215 |
| Creatinine | A0A0C4DH38 | -0.029217049 | 0.861766012 | 0.988319063 |
| Globin | P13796 | -0.029229846 | 0.861706047 | 0.988319063 |
| Hematocrit | A0A0G2JI36 | -0.029476884 | 0.86054867 | 0.988319063 |
| Eosinophils | P80108 | -0.029487579 | 0.86049857 | 0.988319063 |
| APTT | A0A075B6K2 | -0.029842453 | 0.858836499 | 0.988319063 |
| Platelets | P43251 | -0.030425742 | 0.856105935 | 0.988319063 |
| P | P13647 | -0.030439074 | 0.856043546 | 0.988319063 |
| TT | O75882 | -0.030511032 | 0.855706805 | 0.988319063 |
| Neutrophils | P43121 | -0.030529581 | 0.855620004 | 0.988319063 |
| TT | O75460 | -0.030620784 | 0.85519325 | 0.988319063 |
| IBIL | A0A075B6K2 | -0.030763524 | 0.854525421 | 0.988319063 |
| AST | A0A087WSY6 | -0.031142129 | 0.852754557 | 0.988319063 |
| BUN | A0A075B6K2 | -0.031350098 | 0.85178212 | 0.988319063 |
| LDH | P13645 | -0.031523644 | 0.850970801 | 0.988319063 |
| INR | P01715 | -0.031671556 | 0.850279445 | 0.988319063 |
| Hemoglobin | Q6ZRK6 | -0.031679438 | 0.850242606 | 0.988319063 |
| γ-GT | P13473 | -0.031892199 | 0.849248335 | 0.988319063 |
| AST | P13647 | -0.032348339 | 0.847117486 | 0.988319063 |
| ALT | D6RAR4 | -0.032422385 | 0.846771683 | 0.988319063 |
| INR | P13647 | -0.032682693 | 0.845556237 | 0.988319063 |
| FIB | A0A0G2JI36 | -0.032687978 | 0.845531563 | 0.988319063 |
| CRP | P08185 | -0.032754582 | 0.845220631 | 0.988319063 |
| RBC | P43121 | -0.032987789 | 0.844132108 | 0.988319063 |
| CK-MB activity | Q6ZRK6 | -0.033305918 | 0.842647668 | 0.988319063 |
| Creatine Kinase | P43121 | -0.033455701 | 0.841948943 | 0.988319063 |
| Ca | P13473 | -0.033550861 | 0.841505091 | 0.988319063 |
| WLL | F8W1S1 | -0.03400718 | 0.839377371 | 0.988319063 |
| WBC | P0DP01 | -0.034072872 | 0.839071154 | 0.988319063 |
| P | P80108 | -0.034161838 | 0.838656483 | 0.988319063 |
| CO2 | P01782 | -0.034705501 | 0.836123418 | 0.988282815 |
| P | D6RE82 | -0.035037783 | 0.834576033 | 0.987640188 |
| TT | P0DP01 | -0.035708466 | 0.831454636 | 0.987032552 |
| α-HBDH | A0A5H1ZRQ7 | -0.036126182 | 0.829511842 | 0.986841213 |
| BUN | P0DP01 | -0.036264053 | 0.828870826 | 0.986841213 |
| Albumin | A0A075B6S9 | -0.036337382 | 0.828529932 | 0.986841213 |
| Creatine Kinase | P01715 | -0.03637534 | 0.828353489 | 0.986841213 |
| DBIL | P43121 | -0.036521122 | 0.827675902 | 0.986841213 |
| WLGG | A0A075B6J9 | -0.036583498 | 0.827386019 | 0.986841213 |
| DBIL | O75882 | -0.037452786 | 0.823348497 | 0.986814763 |
| APTT | A0A182DWH7 | -0.038101501 | 0.820338363 | 0.985006649 |
| APTT | P02750 | -0.038320476 | 0.81932286 | 0.984417279 |
| Ca | P68032 | -0.038323176 | 0.819310341 | 0.984417279 |
| LDH | Q9NZP8 | -0.038638356 | 0.817849193 | 0.984417279 |
| ALP | P80108 | -0.038749934 | 0.817332069 | 0.984417279 |
| γ-GT | P35908 | -0.039015886 | 0.816099796 | 0.984417279 |
| TT | A0A0C4DH73 | -0.039291185 | 0.814824668 | 0.984417279 |
| CK-MB activity | A0A0C4DH34 | -0.039349707 | 0.814553666 | 0.984417279 |
| Albumin | Q15582 | -0.039743808 | 0.812729237 | 0.984417279 |
| IBIL | P13473 | -0.040052528 | 0.81130074 | 0.984417279 |
| Platelets | A0A1W2PQU7 | -0.040604138 | 0.808749859 | 0.983544886 |
| CO2 | A0A075B6S9 | -0.041273591 | 0.805656633 | 0.981146196 |
| CRP | Q6ZRK6 | -0.041420489 | 0.804978276 | 0.981146196 |
| Glucose | O75460 | -0.041472889 | 0.804736331 | 0.981146196 |
| LDH | P13647 | -0.04148424 | 0.804683923 | 0.981146196 |
| Creatinine | A0A0J9YVY3 | -0.041509074 | 0.804569268 | 0.981146196 |
| ALT | P13645 | -0.041623332 | 0.804041804 | 0.981146196 |
| Eosinophils | A0A1W2PQU7 | -0.042203412 | 0.801365209 | 0.981146196 |
| WLC | A0A0C4DH34 | -0.042222118 | 0.801278935 | 0.981146196 |
| Globin | A0A5H1ZRQ7 | -0.042277635 | 0.801022893 | 0.981146196 |
| Platelets | P01782 | -0.042464705 | 0.800160286 | 0.981146196 |
| Creatinine | O75882 | -0.042676588 | 0.799183543 | 0.981146196 |
| Globin | P02750 | -0.044337408 | 0.791538 | 0.977366647 |
| γ-GT | Q9NZP8 | -0.04460524 | 0.790306815 | 0.977366647 |
| INR | P04264 | -0.0446371 | 0.790160392 | 0.977366647 |
| DBIL | Q86YZ3 | -0.044649923 | 0.790101462 | 0.977366647 |
| Glucose | P43121 | -0.044680697 | 0.789960042 | 0.977366647 |
| γ-GT | A0A1W2PQU7 | -0.045482002 | 0.786279997 | 0.977366647 |
| WLL | P80108 | -0.045596956 | 0.785752432 | 0.977366647 |
| INR | A0A0C4DH73 | -0.045624161 | 0.785627594 | 0.977366647 |
| ALP | A0A0C4DH34 | -0.045974498 | 0.784020434 | 0.977366647 |
| WBC | A0A0C4DH33 | -0.046076393 | 0.783553161 | 0.977366647 |
| CK-MB activity | P43251 | -0.046274376 | 0.782645454 | 0.977313613 |
| AST | P04264 | -0.046384228 | 0.782141932 | 0.977313613 |
| Hematocrit | D6RE82 | -0.046511629 | 0.781558081 | 0.977313613 |
| IBIL | Q5SRP5 | -0.046793836 | 0.780265203 | 0.977313613 |
| WLGG | P41222 | -0.047093408 | 0.778893408 | 0.977313613 |
| ALP | A0A075B6S9 | -0.047179486 | 0.778499363 | 0.977313613 |
| α-HBDH | A0A075B6S5 | -0.047509583 | 0.776988762 | 0.977313613 |
| Eosinophils | P01782 | -0.047574756 | 0.776690612 | 0.977313613 |
| BUN | A0A087WSY6 | -0.047934337 | 0.775046177 | 0.977313613 |
| WLL | A0A0C4DH33 | -0.048145614 | 0.774080419 | 0.977313613 |
| WLGG | P0DP01 | -0.04854178 | 0.772270421 | 0.977313613 |
| BUN | Q86YZ3 | -0.048961114 | 0.770355865 | 0.976214 |
| PT | P80108 | -0.049690274 | 0.767029936 | 0.973252685 |
| TBIL | P13473 | -0.049701686 | 0.766977911 | 0.973252685 |
| RBC | A0A0J9YVY3 | -0.049885148 | 0.766141745 | 0.973252685 |
| LDH | P41222 | -0.050033449 | 0.765466021 | 0.973252685 |
| Monocytes | A0A075B6R2 | -0.050376128 | 0.76390528 | 0.973052162 |
| WLL | C9J8S2 | -0.051040392 | 0.760882494 | 0.971087385 |
| FIB | P01019 | -0.051731767 | 0.757740045 | 0.969357035 |
| CRP | P0DP01 | -0.052311235 | 0.75510918 | 0.968078142 |
| TT | A0A2R8Y7X9 | -0.052461414 | 0.754427791 | 0.968078142 |
| LDH | D6RE82 | -0.052539407 | 0.754073994 | 0.968078142 |
| CK-MB activity | A0A0C4DH38 | -0.052759384 | 0.75307639 | 0.968078142 |
| α-HBDH | P13645 | -0.052873568 | 0.752558714 | 0.968078142 |
| APTT | Q86YZ3 | -0.053209193 | 0.751037714 | 0.968078142 |
| AST | Q8N1N4 | -0.053840794 | 0.74817791 | 0.968056625 |
| INR | P01717 | -0.054128278 | 0.746877315 | 0.967646186 |
| Neutrophils | P0DP01 | -0.054338326 | 0.745927484 | 0.967052232 |
| WLC | P41222 | -0.054402507 | 0.745637332 | 0.967052232 |
| WLL | O75882 | -0.054855222 | 0.74359167 | 0.967052232 |
| Hematocrit | Q6ZRK6 | -0.054979975 | 0.743028258 | 0.967052232 |
| LDH | P18206 | -0.055394186 | 0.741158534 | 0.966601577 |
| CO2 | Q9NZP8 | -0.055725868 | 0.739662397 | 0.966601577 |
| α-HBDH | D6RE82 | -0.055938703 | 0.738702846 | 0.966601577 |
| Mg | C9J8S2 | -0.056216681 | 0.737450185 | 0.966601577 |
| WLC | Q9NZP8 | -0.056257171 | 0.737267782 | 0.966601577 |
| AST | A0A0C4DH34 | -0.056362868 | 0.73679169 | 0.966601577 |
| AST | P35908 | -0.057788388 | 0.73038022 | 0.965443836 |
| Basophils | P01715 | -0.05818245 | 0.728611031 | 0.96441897 |
| Eosinophils | Q15582 | -0.059084777 | 0.724565144 | 0.964054943 |
| INR | O75882 | -0.059881712 | 0.720997934 | 0.962548016 |
| Albumin | O75460 | -0.059998916 | 0.720473796 | 0.962548016 |
| Ca | Q15582 | -0.060084549 | 0.720090926 | 0.962548016 |
| CRP | A0A5H1ZRQ7 | -0.060546236 | 0.718027849 | 0.962548016 |
| WLL | A0A075B6S9 | -0.060642038 | 0.717599995 | 0.962548016 |
| WLL | A0A087WSY6 | -0.060757366 | 0.717085056 | 0.962548016 |
| PT | A0A5H1ZRQ7 | -0.060846135 | 0.716688783 | 0.962548016 |
| Ca | P0DP01 | -0.060918747 | 0.716364695 | 0.962548016 |
| Creatine Kinase | A0A2R8Y7X9 | -0.060957593 | 0.71619133 | 0.962548016 |
| LDH | A0A5H1ZRQ7 | -0.061814023 | 0.712372821 | 0.962548016 |
| WLC | C9J8S2 | -0.061850203 | 0.71221166 | 0.962548016 |
| WLGG | A0A075B6K2 | -0.062698311 | 0.708437338 | 0.962548016 |
| FIB | P04264 | -0.062801488 | 0.707978636 | 0.962548016 |
| Glucose | Q8N1N4 | -0.062811184 | 0.707935536 | 0.962548016 |
| Creatinine | P13473 | -0.063030038 | 0.706962915 | 0.962548016 |
| WBC | P13647 | -0.06380301 | 0.703531386 | 0.962548016 |
| α-HBDH | A0A087WSY6 | -0.063929946 | 0.702968419 | 0.962548016 |
| CK-MB activity | P0DP01 | -0.06393083 | 0.7029645 | 0.962548016 |
| UA | P13796 | -0.064452591 | 0.700652104 | 0.962548016 |
| LDH | P02750 | -0.064579688 | 0.700089221 | 0.962548016 |
| TT | A0A075B6K2 | -0.064756395 | 0.699306892 | 0.962548016 |
| Albumin | P41222 | -0.06550127 | 0.6960125 | 0.962548016 |
| Albumin | A0A0C4DH34 | -0.065801731 | 0.694685189 | 0.962548016 |
| WLL | P04264 | -0.066543782 | 0.691410959 | 0.962548016 |
| Globin | C9J8S2 | -0.066692357 | 0.690756045 | 0.962548016 |
| WLL | Q6ZRK6 | -0.066707466 | 0.690689457 | 0.962548016 |
| WLC | A0A2R8Y7X9 | -0.066783461 | 0.690354573 | 0.962548016 |
| BUN | C9J8S2 | -0.066844075 | 0.69008751 | 0.962548016 |
| α-HBDH | P41222 | -0.067030169 | 0.689267812 | 0.962548016 |
| WLGG | P13473 | -0.067586462 | 0.686819565 | 0.962548016 |
| BUN | D6RE82 | -0.068071136 | 0.684689071 | 0.962548016 |
| IBIL | Q6ZRK6 | -0.068113567 | 0.684502671 | 0.962548016 |
| DBIL | P01782 | -0.069210996 | 0.679688051 | 0.962548016 |
| Mg | A0A087WSY6 | -0.069807831 | 0.677074858 | 0.962507008 |
| PT | A0A2R8Y7X9 | -0.069873519 | 0.676787471 | 0.962507008 |
| TT | P43121 | -0.070270074 | 0.675053514 | 0.961427187 |
| α-HBDH | A0A0C4DH34 | -0.070498091 | 0.674057243 | 0.961264243 |
| P | A0A0C4DH73 | -0.071170496 | 0.671122508 | 0.959861261 |
| α-HBDH | P19652 | -0.071373844 | 0.670235932 | 0.95929041 |
| Creatinine | P01019 | -0.072331346 | 0.666067246 | 0.956105281 |
| Glucose | A0A2R8Y7X9 | -0.072440773 | 0.665591457 | 0.956105281 |
| UA | D6RE82 | -0.072878481 | 0.663689593 | 0.95478152 |
| TBIL | Q86YZ3 | -0.0734658 | 0.661140926 | 0.95390421 |
| Lymphocytes | A0A075B6S5 | -0.073551144 | 0.660770887 | 0.95390421 |
| Mg | Q15582 | -0.073655506 | 0.660318495 | 0.95390421 |
| Eosinophils | P43251 | -0.073664138 | 0.660281084 | 0.95390421 |
| Monocytes | P43121 | -0.073672131 | 0.660246441 | 0.95390421 |
| DBIL | A0A5H1ZRQ7 | -0.073708101 | 0.66009055 | 0.95390421 |
| DBIL | A0A0C4DH34 | -0.073919972 | 0.659172618 | 0.95390421 |
| WBC | A0A0G2JI36 | -0.074261506 | 0.657693954 | 0.95390421 |
| Hemoglobin | C9J8S2 | -0.074304787 | 0.657506662 | 0.95390421 |
| Creatinine | D6RE82 | -0.074519888 | 0.656576146 | 0.95390421 |
| Ca | P01782 | -0.074667113 | 0.655939556 | 0.95390421 |
| TT | P13645 | -0.074850805 | 0.655145617 | 0.95390421 |
| INR | A0A5H1ZRQ7 | -0.075467569 | 0.652482619 | 0.95390421 |
| γ-GT | A0A075B6S5 | -0.075620677 | 0.6518222 | 0.95390421 |
| BUN | A0A075B6R2 | -0.075622436 | 0.651814616 | 0.95390421 |
| Basophils | F8W1S1 | -0.075805597 | 0.651024913 | 0.95390421 |
| WLGG | A0A0C4DH33 | -0.076023427 | 0.650086223 | 0.95390421 |
| Neutrophils | P02750 | -0.076274896 | 0.649003234 | 0.95390421 |
| LDH | P80108 | -0.076291598 | 0.648931332 | 0.95390421 |
| CK-MB activity | Q9NZP8 | -0.076391191 | 0.648502634 | 0.95390421 |
| CRP | P43121 | -0.07646198 | 0.648197993 | 0.95390421 |
| TBIL | Q6ZRK6 | -0.077344608 | 0.64440432 | 0.95390421 |
| Neutrophils | Q15582 | -0.077916394 | 0.641951419 | 0.95390421 |
| LDH | P08185 | -0.078042911 | 0.641409173 | 0.95390421 |
| Basophils | Q6ZRK6 | -0.078240984 | 0.64056062 | 0.95390421 |
| LDH | A0A0C4DH34 | -0.078371283 | 0.640002656 | 0.95390421 |
| Creatinine | A0A075B6K2 | -0.078483632 | 0.639521711 | 0.95390421 |
| Basophils | D6RE82 | -0.078538784 | 0.639285672 | 0.95390421 |
| Neutrophils | P01782 | -0.078572992 | 0.639139283 | 0.95390421 |
| WLGG | P19652 | -0.078995553 | 0.637332128 | 0.95390421 |
| Total protein | A0A0C4DH73 | -0.079264299 | 0.636183857 | 0.95390421 |
| Basophils | P01717 | -0.079612303 | 0.634698189 | 0.95390421 |
| ALP | A0A0J9YVY3 | -0.079948419 | 0.633264607 | 0.95390421 |
| INR | P43251 | -0.080500323 | 0.630913517 | 0.95390421 |
| PT | A0A075B6R2 | -0.081391133 | 0.62712625 | 0.950835485 |
| ALT | Q6ZRK6 | -0.081499633 | 0.6266656 | 0.950835485 |
| Platelets | P02750 | -0.081536611 | 0.626508638 | 0.950835485 |
| BUN | P13645 | -0.081751027 | 0.625598819 | 0.950835485 |
| TBIL | Q5SRP5 | -0.081892669 | 0.624998099 | 0.950835485 |
| APTT | P19652 | -0.082005818 | 0.62451839 | 0.950835485 |
| FIB | P35908 | -0.082420103 | 0.622763282 | 0.950835485 |
| Eosinophils | P13645 | -0.082652917 | 0.62177787 | 0.950835485 |
| α-HBDH | Q5SRP5 | -0.083340498 | 0.618871396 | 0.950835485 |
| INR | A0A075B6R2 | -0.083680854 | 0.617434788 | 0.950835485 |
| ALP | A0A0C4DH33 | -0.083908051 | 0.61647659 | 0.950804019 |
| Eosinophils | P13647 | -0.083968348 | 0.616222392 | 0.950804019 |
| γ-GT | P04264 | -0.084169102 | 0.615376383 | 0.950597113 |
| Basophils | A0A0J9YVY3 | -0.084495169 | 0.614003327 | 0.949967412 |
| Mg | P13796 | -0.085418401 | 0.610122676 | 0.945449941 |
| WLGG | A0A087WSY6 | -0.085444169 | 0.610014511 | 0.945449941 |
| α-HBDH | Q15582 | -0.085604825 | 0.609340339 | 0.945449941 |
| γ-GT | A0A0C4DH38 | -0.08581303 | 0.608467105 | 0.945449941 |
| DBIL | P19652 | -0.085856679 | 0.608284103 | 0.945449941 |
| RBC | P0DP01 | -0.086003872 | 0.607667161 | 0.945449941 |
| LDH | P01782 | -0.08603328 | 0.607543934 | 0.945449941 |
| WLL | A0A075B6S5 | -0.086333324 | 0.606287273 | 0.945449941 |
| Hemoglobin | D6RE82 | -0.086956555 | 0.603680584 | 0.945449941 |
| APTT | P68032 | -0.087420832 | 0.601741869 | 0.945449941 |
| Albumin | Q8N1N4 | -0.088027607 | 0.599212182 | 0.943399659 |
| Creatinine | A0A5H1ZRQ7 | -0.088030696 | 0.599199315 | 0.943399659 |
| Ca | P13796 | -0.088153243 | 0.598688971 | 0.943399659 |
| AST | P19652 | -0.088601551 | 0.596823621 | 0.943345364 |
| Lymphocytes | O75882 | -0.089202652 | 0.594326504 | 0.942493602 |
| PT | P41222 | -0.089206223 | 0.594311684 | 0.942493602 |
| AST | Q6ZRK6 | -0.089359539 | 0.593675512 | 0.942493602 |
| WLGG | A0A075B6S9 | -0.089359765 | 0.593674572 | 0.942493602 |
| UA | A0A075B6R2 | -0.090605679 | 0.588515871 | 0.941625393 |
| Creatine Kinase | P18206 | -0.09097739 | 0.586980655 | 0.94069864 |
| Hematocrit | P13796 | -0.091162792 | 0.586215583 | 0.940238197 |
| ALT | Q8N1N4 | -0.091242724 | 0.585885875 | 0.940238197 |
| Total protein | A0A0J9YVY3 | -0.091319078 | 0.585571006 | 0.940238197 |
| PT | O75882 | -0.091482755 | 0.58489628 | 0.940238197 |
| Neutrophils | P08185 | -0.091704969 | 0.583980807 | 0.940238197 |
| UA | P0DP01 | -0.09234819 | 0.581334476 | 0.940238197 |
| Neutrophils | A0A0G2JI36 | -0.092663674 | 0.580038487 | 0.940238197 |
| IBIL | P19652 | -0.0931276 | 0.578135064 | 0.940238197 |
| APTT | D6RE82 | -0.093939909 | 0.574809067 | 0.940238197 |
| Monocytes | A0A5H1ZRQ7 | -0.094016624 | 0.574495406 | 0.940238197 |
| TT | A0A087WSY6 | -0.094496397 | 0.572535542 | 0.939741407 |
| DBIL | P01717 | -0.094502004 | 0.572512655 | 0.939741407 |
| Neutrophils | Q6ZRK6 | -0.095428439 | 0.568736887 | 0.937415573 |
| Albumin | A0A087WSY6 | -0.096458111 | 0.564553787 | 0.936715898 |
| TBIL | P19652 | -0.096666496 | 0.563708942 | 0.936715898 |
| PT | P01019 | -0.096857642 | 0.562934501 | 0.936715898 |
| WBC | A0A087WSY6 | -0.098276335 | 0.55720198 | 0.930877332 |
| TBIL | A0A075B6S9 | -0.098331842 | 0.556978248 | 0.930877332 |
| Neutrophils | A0A075B6J9 | -0.098489823 | 0.5563417 | 0.930877332 |
| CO2 | Q86YZ3 | -0.098538723 | 0.556144741 | 0.930877332 |
| TT | A0A0C4DH34 | -0.09866697 | 0.555628336 | 0.930877332 |
| Basophils | A0A5H1ZRQ7 | -0.099434128 | 0.552543954 | 0.93020231 |
| Albumin | A0A0G2JI36 | -0.099480607 | 0.552357342 | 0.93020231 |
| Lymphocytes | A0A0G2JI36 | -0.09951119 | 0.552234568 | 0.93020231 |
| APTT | P43251 | -0.099961698 | 0.550427526 | 0.929820919 |
| Basophils | D6RAR4 | -0.100115373 | 0.549811753 | 0.929820919 |
| P | A0A182DWH7 | -0.100295653 | 0.549089791 | 0.929820919 |
| WBC | P02750 | -0.100465118 | 0.548411548 | 0.929820919 |
| WLL | A0A0C4DH34 | -0.10172519 | 0.543380874 | 0.928275659 |
| LDH | Q5SRP5 | -0.101762574 | 0.543231956 | 0.928275659 |
| Basophils | P13645 | -0.1018415 | 0.542917631 | 0.928275659 |
| γ-GT | A0A087WSY6 | -0.102252307 | 0.541282972 | 0.927913666 |
| TBIL | A0A5H1ZRQ7 | -0.10234482 | 0.540915176 | 0.927913666 |
| FIB | Q8N1N4 | -0.102477122 | 0.540389399 | 0.927913666 |
| WLGG | C9J8S2 | -0.102698306 | 0.539510949 | 0.927640541 |
| BUN | A0A075B6J9 | -0.103201096 | 0.537516631 | 0.927640541 |
| PT | P43251 | -0.103877901 | 0.534837691 | 0.926407938 |
| UA | A0A182DWH7 | -0.104612354 | 0.531937883 | 0.923151459 |
| IBIL | P80108 | -0.104727512 | 0.531483905 | 0.923151459 |
| Globin | A0A0C4DH73 | -0.105096077 | 0.5300322 | 0.922284146 |
| Eosinophils | A0A075B6S9 | -0.105542254 | 0.528277381 | 0.921675431 |
| ALT | A0A182DWH7 | -0.105701355 | 0.527652322 | 0.921401748 |
| IBIL | A0A075B6S9 | -0.106155264 | 0.525871041 | 0.919573522 |
| ALT | P08185 | -0.106468101 | 0.524645085 | 0.919413649 |
| α-HBDH | P68032 | -0.1064791 | 0.524602006 | 0.919413649 |
| INR | A0A2R8Y7X9 | -0.106492934 | 0.524547827 | 0.919413649 |
| Lymphocytes | P13796 | -0.10682428 | 0.523250987 | 0.919413649 |
| RBC | Q6ZRK6 | -0.106834772 | 0.52320995 | 0.919413649 |
| Basophils | P04264 | -0.107019881 | 0.522486173 | 0.919413649 |
| Neutrophils | A0A0C4DH34 | -0.107353907 | 0.52118138 | 0.919413649 |
| P | Q8N1N4 | -0.107631689 | 0.520097519 | 0.919413649 |
| α-HBDH | P08185 | -0.108264926 | 0.517630891 | 0.919413649 |
| Lymphocytes | A0A075B6R2 | -0.108684949 | 0.515997991 | 0.918990087 |
| PT | C9J8S2 | -0.108995428 | 0.514792607 | 0.918431009 |
| ALP | A0A075B6S5 | -0.109025239 | 0.514676944 | 0.918431009 |
| Basophils | A0A0C4DH38 | -0.109485777 | 0.512891765 | 0.917609994 |
| Albumin | Q5SRP5 | -0.109500146 | 0.512836118 | 0.917609994 |
| Glucose | A0A0C4DH73 | -0.109536576 | 0.512695043 | 0.917609994 |
| Globin | A0A182DWH7 | -0.109803506 | 0.511661962 | 0.917609994 |
| Basophils | P0DP01 | -0.109852635 | 0.511471933 | 0.917609994 |
| Total protein | C9J8S2 | -0.110349474 | 0.509552176 | 0.917609994 |
| CK-MB activity | P18206 | -0.110554724 | 0.508760159 | 0.917609994 |
| TT | P13473 | -0.111398192 | 0.505511891 | 0.91354215 |
| PT | P01782 | -0.111446618 | 0.505325714 | 0.91354215 |
| IBIL | Q86YZ3 | -0.112153011 | 0.502613909 | 0.911653616 |
| CO2 | P18206 | -0.112549516 | 0.50109498 | 0.909737011 |
| WLC | A0A0C4DH73 | -0.112865218 | 0.499887259 | 0.909501853 |
| Monocytes | A0A087WSY6 | -0.113154222 | 0.498782971 | 0.909501853 |
| WLGG | P68032 | -0.113334156 | 0.49809607 | 0.909501853 |
| α-HBDH | P01782 | -0.11373838 | 0.496554695 | 0.908196691 |
| DBIL | Q6ZRK6 | -0.113738585 | 0.496553913 | 0.908196691 |
| Globin | P41222 | -0.11394103 | 0.495782874 | 0.908196691 |
| FIB | P01782 | -0.114752457 | 0.49269859 | 0.907044739 |
| WLL | P01715 | -0.114862969 | 0.49227929 | 0.907044739 |
| Basophils | A0A182DWH7 | -0.115280633 | 0.490696267 | 0.905051785 |
| Platelets | A0A075B6R2 | -0.115683488 | 0.489171859 | 0.90478404 |
| TT | Q6ZRK6 | -0.115845975 | 0.488557695 | 0.90478404 |
| CK-MB activity | A0A075B6S5 | -0.116070644 | 0.487709157 | 0.90478404 |
| Lymphocytes | D6RAR4 | -0.118097744 | 0.480087702 | 0.896542204 |
| ALP | A0A0C4DH38 | -0.118110676 | 0.480039283 | 0.896542204 |
| Ca | A0A075B6S5 | -0.118195518 | 0.479721675 | 0.896542204 |
| Platelets | A0A075B6S5 | -0.11831017 | 0.479292643 | 0.896542204 |
| WBC | A0A182DWH7 | -0.118413134 | 0.478907522 | 0.896542204 |
| WLC | A0A0J9YVY3 | -0.119003966 | 0.476700737 | 0.896542204 |
| ALP | P0DP01 | -0.119007208 | 0.476688642 | 0.896542204 |
| FIB | P13647 | -0.119355707 | 0.475389503 | 0.896542204 |
| RBC | A0A5H1ZRQ7 | -0.119546035 | 0.474680782 | 0.896542204 |
| Globin | Q6ZRK6 | -0.119548366 | 0.474672107 | 0.896542204 |
| TBIL | P01782 | -0.120203638 | 0.47223636 | 0.896542204 |
| TBIL | A0A0C4DH34 | -0.120422588 | 0.471423961 | 0.896542204 |
| TT | Q9NZP8 | -0.1205076 | 0.47110873 | 0.896542204 |
| γ-GT | D6RE82 | -0.121212274 | 0.468500031 | 0.896542204 |
| Platelets | P01715 | -0.12148232 | 0.467502357 | 0.896542204 |
| APTT | A0A075B6R2 | -0.121859112 | 0.466112198 | 0.896542204 |
| WBC | F8W1S1 | -0.121929635 | 0.465852253 | 0.896542204 |
| Albumin | P01782 | -0.121968599 | 0.465708663 | 0.896542204 |
| DBIL | P80108 | -0.122871421 | 0.462388241 | 0.895649664 |
| FIB | Q15582 | -0.12330135 | 0.460811492 | 0.893474893 |
| Mg | P18206 | -0.124118526 | 0.457822485 | 0.890414289 |
| Monocytes | D6RE82 | -0.124129414 | 0.457782733 | 0.890414289 |
| DBIL | A0A0C4DH73 | -0.124404576 | 0.456778661 | 0.890414289 |
| LDH | A0A087WSY6 | -0.125000007 | 0.454609987 | 0.889336435 |
| Total protein | P41222 | -0.125056029 | 0.454406229 | 0.889336435 |
| TBIL | A0A0C4DH73 | -0.125348967 | 0.453341593 | 0.888907832 |
| Neutrophils | A0A2R8Y7X9 | -0.125410375 | 0.453118586 | 0.888907832 |
| Platelets | Q8N1N4 | -0.126518554 | 0.449104382 | 0.884722146 |
| Total protein | P13647 | -0.126669605 | 0.448558724 | 0.884532634 |
| Platelets | D6RAR4 | -0.126737444 | 0.448313779 | 0.884532634 |
| Ca | A0A1W2PQU7 | -0.126857341 | 0.447881047 | 0.884532634 |
| Basophils | P08185 | -0.126870343 | 0.447834134 | 0.884532634 |
| Mg | P13473 | -0.126973298 | 0.447462752 | 0.884532634 |
| Creatinine | D6RAR4 | -0.12704492 | 0.447204495 | 0.884532634 |
| Lymphocytes | A0A0C4DH33 | -0.128064802 | 0.44353578 | 0.883480176 |
| WBC | A0A075B6S9 | -0.128892463 | 0.440570664 | 0.879354024 |
| ALP | P01782 | -0.129166448 | 0.439591509 | 0.879025157 |
| Basophils | Q8N1N4 | -0.129582829 | 0.438105761 | 0.878891068 |
| Globin | D6RE82 | -0.129618495 | 0.437978623 | 0.878891068 |
| INR | P01019 | -0.129743709 | 0.437532443 | 0.878891068 |
| INR | P01782 | -0.129743709 | 0.437532443 | 0.878891068 |
| UA | A0A075B6J9 | -0.129780599 | 0.437401039 | 0.878891068 |
| UA | P08185 | -0.130327735 | 0.435454667 | 0.878891068 |
| WLL | Q8N1N4 | -0.130888725 | 0.433463986 | 0.878891068 |
| Creatine Kinase | A0A075B6K2 | -0.131483246 | 0.431359835 | 0.878795192 |
| γ-GT | P13647 | -0.132062198 | 0.429316249 | 0.876446451 |
| Eosinophils | D6RE82 | -0.132420058 | 0.428055773 | 0.87568998 |
| Ca | Q6ZRK6 | -0.132430086 | 0.428020481 | 0.87568998 |
| Creatine Kinase | A0A0J9YVY3 | -0.132683026 | 0.427130847 | 0.87568998 |
| ALP | A0A0G2JI36 | -0.132864833 | 0.426492037 | 0.87568998 |
| Mg | P0DP01 | -0.133605653 | 0.423894566 | 0.875078589 |
| Creatine Kinase | A0A075B6S5 | -0.134062929 | 0.422295692 | 0.872981011 |
| Hematocrit | C9J8S2 | -0.134093265 | 0.422189744 | 0.872981011 |
| Albumin | C9J8S2 | -0.134152013 | 0.421984604 | 0.872981011 |
| Basophils | A0A075B6S9 | -0.134569099 | 0.420529814 | 0.872981011 |
| DBIL | P13473 | -0.134698616 | 0.420078636 | 0.872981011 |
| FIB | P13796 | -0.134809476 | 0.419692666 | 0.872981011 |
| Basophils | P02750 | -0.134884505 | 0.41943156 | 0.872981011 |
| P | P01715 | -0.135070701 | 0.418783979 | 0.872981011 |
| α-HBDH | Q6ZRK6 | -0.135229874 | 0.418230827 | 0.872981011 |
| TBIL | P80108 | -0.135311199 | 0.417948368 | 0.872981011 |
| Hemoglobin | P13796 | -0.135691652 | 0.416628411 | 0.872981011 |
| Basophils | A0A2R8Y7X9 | -0.136240748 | 0.414727516 | 0.872923799 |
| Neutrophils | A0A087WSY6 | -0.136244256 | 0.414715388 | 0.872923799 |
| IBIL | A0A5H1ZRQ7 | -0.136444068 | 0.414024897 | 0.872923799 |
| Neutrophils | P13473 | -0.136572555 | 0.413581229 | 0.872923799 |
| WLGG | P01715 | -0.136633415 | 0.413371173 | 0.872923799 |
| CO2 | P02750 | -0.13728927 | 0.41111135 | 0.869964662 |
| CRP | P13647 | -0.137372058 | 0.410826593 | 0.869964662 |
| IBIL | P01782 | -0.13744802 | 0.410565414 | 0.869964662 |
| Glucose | A0A0C4DH38 | -0.137878208 | 0.409088094 | 0.869422645 |
| Lymphocytes | P43121 | -0.138166115 | 0.408101078 | 0.868262619 |
| BUN | P01715 | -0.13870905 | 0.406243466 | 0.865607403 |
| INR | P41222 | -0.13899332 | 0.405272787 | 0.865607403 |
| INR | P13796 | -0.1392853 | 0.404277166 | 0.865607403 |
| FIB | Q86YZ3 | -0.140145747 | 0.401351298 | 0.862336813 |
| TBIL | Q9NZP8 | -0.140347053 | 0.400668536 | 0.862336813 |
| CO2 | P0DP01 | -0.140398969 | 0.400492564 | 0.862336813 |
| WBC | P01019 | -0.140629277 | 0.399712456 | 0.862336813 |
| Albumin | P0DP01 | -0.14073548 | 0.399353014 | 0.862336813 |
| AST | Q86YZ3 | -0.140910889 | 0.39875976 | 0.862336813 |
| Lymphocytes | D6RE82 | -0.141301379 | 0.397440896 | 0.862336813 |
| Glucose | P13647 | -0.141598731 | 0.396438293 | 0.862336813 |
| Mg | Q86YZ3 | -0.141791609 | 0.395788735 | 0.862336813 |
| UA | A0A0C4DH34 | -0.141817585 | 0.395701302 | 0.862336813 |
| CO2 | Q6ZRK6 | -0.142076396 | 0.394830782 | 0.862336813 |
| Total protein | A0A5H1ZRQ7 | -0.14210456 | 0.394736116 | 0.862336813 |
| Monocytes | P35908 | -0.142128727 | 0.394654897 | 0.862336813 |
| γ-GT | P13645 | -0.14247374 | 0.393496458 | 0.862336813 |
| Glucose | D6RE82 | -0.142802429 | 0.392394664 | 0.862336813 |
| Eosinophils | P0DP01 | -0.142880695 | 0.392132576 | 0.862336813 |
| Hematocrit | A0A075B6R2 | -0.143036938 | 0.391609669 | 0.862336813 |
| ALT | F8W1S1 | -0.143329665 | 0.390631073 | 0.862336813 |
| Mg | A0A075B6K2 | -0.143357935 | 0.39053664 | 0.862336813 |
| WBC | P35908 | -0.143365255 | 0.390512192 | 0.862336813 |
| WLC | P18206 | -0.143987272 | 0.388437868 | 0.862336813 |
| Platelets | A0A0G2JI36 | -0.144482593 | 0.386790662 | 0.862336813 |
| LDH | P01717 | -0.144532149 | 0.386626087 | 0.862336813 |
| α-HBDH | A0A2R8Y7X9 | -0.144937069 | 0.385282881 | 0.862336813 |
| P | A0A087WSY6 | -0.144968826 | 0.385177653 | 0.862336813 |
| Basophils | A0A087WSY6 | -0.144994678 | 0.385092002 | 0.862336813 |
| TBIL | P01717 | -0.145432653 | 0.383642648 | 0.862336813 |
| ALT | A0A0C4DH73 | -0.145572125 | 0.383181775 | 0.862336813 |
| TT | P08185 | -0.145860292 | 0.382230587 | 0.862336813 |
| CRP | P35908 | -0.146135825 | 0.381322398 | 0.862336813 |
| CK-MB activity | A0A075B6J9 | -0.146297375 | 0.380790503 | 0.862336813 |
| Creatine Kinase | Q5SRP5 | -0.146308544 | 0.380753746 | 0.862336813 |
| Monocytes | A0A182DWH7 | -0.146409052 | 0.380423066 | 0.862336813 |
| ALT | D6RE82 | -0.146557941 | 0.379933519 | 0.862336813 |
| APTT | A0A0G2JI36 | -0.146752881 | 0.379293119 | 0.862336813 |
| Albumin | A0A0J9YVY3 | -0.147655893 | 0.376334923 | 0.862336813 |
| IBIL | A0A0C4DH34 | -0.148172468 | 0.374648813 | 0.862336813 |
| IBIL | Q9NZP8 | -0.148172468 | 0.374648813 | 0.862336813 |
| INR | C9J8S2 | -0.148357812 | 0.374044938 | 0.862336813 |
| Total protein | O75882 | -0.14856582 | 0.373367907 | 0.862336813 |
| TT | P18206 | -0.148754126 | 0.372755634 | 0.862336813 |
| IBIL | P01717 | -0.148882632 | 0.37233814 | 0.862336813 |
| Creatinine | C9J8S2 | -0.149008359 | 0.37192994 | 0.862336813 |
| ALT | A0A087WSY6 | -0.149186783 | 0.371351108 | 0.862336813 |
| Mg | P43121 | -0.150173312 | 0.368160344 | 0.862210087 |
| Albumin | F8W1S1 | -0.150416435 | 0.367376522 | 0.861736584 |
| P | P43251 | -0.150552972 | 0.366936766 | 0.861732166 |
| α-HBDH | P18206 | -0.151381045 | 0.364276467 | 0.858558188 |
| CK-MB activity | O75882 | -0.151463398 | 0.364012529 | 0.858558188 |
| Monocytes | A0A0C4DH73 | -0.151677144 | 0.363328017 | 0.858558188 |
| CK-MB activity | A0A087WSY6 | -0.152452636 | 0.360851018 | 0.855440566 |
| APTT | P13473 | -0.153938825 | 0.356132426 | 0.854571513 |
| IBIL | A0A0C4DH73 | -0.154410156 | 0.354643783 | 0.854322914 |
| Monocytes | A0A0G2JI36 | -0.15491048 | 0.353067696 | 0.852561013 |
| Hemoglobin | A0A0J9YVY3 | -0.155041001 | 0.352657235 | 0.852561013 |
| Mg | D6RAR4 | -0.155226231 | 0.352075224 | 0.852561013 |
| Ca | Q86YZ3 | -0.155274855 | 0.351922539 | 0.852561013 |
| Hemoglobin | A0A075B6R2 | -0.155733276 | 0.350485019 | 0.852561013 |
| Eosinophils | A0A087WSY6 | -0.156097816 | 0.34934444 | 0.852561013 |
| γ-GT | Q86YZ3 | -0.156655567 | 0.347603715 | 0.849793927 |
| Lymphocytes | P02750 | -0.157062338 | 0.346337531 | 0.847751568 |
| Globin | P43251 | -0.157644116 | 0.344531489 | 0.845433878 |
| Creatine Kinase | A0A087WSY6 | -0.158905611 | 0.340635175 | 0.837962529 |
| Creatinine | A0A075B6S9 | -0.159650731 | 0.338346514 | 0.837962529 |
| DBIL | Q5SRP5 | -0.160039364 | 0.337156573 | 0.837783 |
| Globin | A0A0G2JI36 | -0.16072078 | 0.335076404 | 0.83540434 |
| Total protein | A0A0G2JI36 | -0.160792861 | 0.334856822 | 0.83540434 |
| γ-GT | Q8N1N4 | -0.161543302 | 0.332576046 | 0.834352996 |
| WBC | P01715 | -0.161724311 | 0.332027356 | 0.834352996 |
| ALT | A0A075B6S9 | -0.162187027 | 0.330627279 | 0.83206456 |
| FIB | P01717 | -0.162716724 | 0.329029033 | 0.83206456 |
| DBIL | P08185 | -0.16273345 | 0.328978643 | 0.83206456 |
| Ca | F8W1S1 | -0.162747695 | 0.328935733 | 0.83206456 |
| WLL | A0A0J9YVY3 | -0.163106515 | 0.327855989 | 0.83206456 |
| WLC | P13647 | -0.163157492 | 0.327702772 | 0.83206456 |
| Lymphocytes | Q86YZ3 | -0.163395221 | 0.32698883 | 0.83206456 |
| Platelets | O75882 | -0.163511003 | 0.326641468 | 0.83206456 |
| CRP | F8W1S1 | -0.163650353 | 0.326223702 | 0.83206456 |
| APTT | A0A0C4DH33 | -0.163965863 | 0.325279048 | 0.83206456 |
| Ca | A0A0C4DH38 | -0.164136076 | 0.324770129 | 0.83206456 |
| Glucose | P01019 | -0.164250151 | 0.324429334 | 0.83206456 |
| AST | F8W1S1 | -0.164638347 | 0.323271286 | 0.83206456 |
| LDH | Q6ZRK6 | -0.165256427 | 0.321432786 | 0.83206456 |
| Monocytes | A0A0C4DH38 | -0.165396133 | 0.321018136 | 0.83206456 |
| Eosinophils | P01717 | -0.165487011 | 0.320748587 | 0.83206456 |
| FIB | A0A075B6S5 | -0.165607414 | 0.320391684 | 0.83206456 |
| APTT | P01019 | -0.165872916 | 0.31960555 | 0.83206456 |
| AST | P01019 | -0.166237564 | 0.318527822 | 0.83206456 |
| WBC | Q8N1N4 | -0.16777018 | 0.314023093 | 0.828557891 |
| UA | Q86YZ3 | -0.168741117 | 0.311190146 | 0.825366857 |
| Creatinine | Q86YZ3 | -0.169493392 | 0.309006334 | 0.822901848 |
| Lymphocytes | A0A075B6J9 | -0.169539764 | 0.308872035 | 0.822901848 |
| Total protein | P13473 | -0.169586187 | 0.308737629 | 0.822901848 |
| ALT | Q9NZP8 | -0.170217519 | 0.30691342 | 0.821776342 |
| CRP | A0A0G2JI36 | -0.170704039 | 0.305512316 | 0.821377374 |
| IBIL | P08185 | -0.171262859 | 0.303908016 | 0.818181909 |
| Total protein | P19652 | -0.171447365 | 0.303379503 | 0.817877894 |
| PT | P13796 | -0.171886664 | 0.302123498 | 0.816729457 |
| ALT | P13473 | -0.172298686 | 0.300948497 | 0.815981856 |
| P | F8W1S1 | -0.172699627 | 0.299807897 | 0.815981856 |
| Basophils | P43121 | -0.172774212 | 0.299596021 | 0.815981856 |
| Glucose | P13796 | -0.173223177 | 0.298322661 | 0.815981856 |
| CK-MB activity | A0A182DWH7 | -0.173226644 | 0.298312843 | 0.815981856 |
| α-HBDH | A0A0C4DH38 | -0.173289562 | 0.298134672 | 0.815981856 |
| BUN | A0A0C4DH34 | -0.174117649 | 0.295796079 | 0.814163192 |
| WLL | P68032 | -0.174293871 | 0.295299933 | 0.814163192 |
| Albumin | A0A075B6J9 | -0.174412907 | 0.294965095 | 0.814163192 |
| Creatinine | P43121 | -0.174629006 | 0.29435785 | 0.814163192 |
| Glucose | A0A087WSY6 | -0.174974011 | 0.293390038 | 0.814163192 |
| α-HBDH | P80108 | -0.175150537 | 0.292895638 | 0.814163192 |
| Creatinine | P13796 | -0.175958856 | 0.290638604 | 0.814163192 |
| LDH | A0A2R8Y7X9 | -0.176773214 | 0.288376066 | 0.813071773 |
| FIB | A0A1W2PQU7 | -0.177444343 | 0.286520035 | 0.8101601 |
| Creatine Kinase | P08185 | -0.177619701 | 0.286036352 | 0.8101601 |
| TBIL | D6RE82 | -0.177678055 | 0.285875514 | 0.8101601 |
| CRP | Q86YZ3 | -0.177847415 | 0.285409047 | 0.8101601 |
| Basophils | P35908 | -0.178654157 | 0.283193812 | 0.808890307 |
| Mg | A0A0C4DH73 | -0.17875202 | 0.282925851 | 0.808890307 |
| BUN | A0A075B6S5 | -0.17904241 | 0.282131693 | 0.808890307 |
| WLC | A0A0G2JI36 | -0.1802254 | 0.278911431 | 0.803355228 |
| WLGG | A0A075B6S5 | -0.180933299 | 0.276995931 | 0.803355228 |
| CRP | P13645 | -0.180971802 | 0.276891992 | 0.803355228 |
| Platelets | Q86YZ3 | -0.181038709 | 0.276711437 | 0.803355228 |
| Globin | O75882 | -0.181400208 | 0.275737232 | 0.803355228 |
| IBIL | D6RE82 | -0.181659007 | 0.27504117 | 0.803355228 |
| Monocytes | A0A1W2PQU7 | -0.181968672 | 0.274209814 | 0.803355228 |
| LDH | P0DP01 | -0.182445813 | 0.272932052 | 0.80288532 |
| Ca | P13647 | -0.1826658 | 0.272344254 | 0.802355527 |
| Albumin | A0A2R8Y7X9 | -0.182733925 | 0.272162394 | 0.802355527 |
| WLL | P19652 | -0.183197924 | 0.270925867 | 0.801777602 |
| DBIL | Q9NZP8 | -0.183321531 | 0.270597084 | 0.801777602 |
| Glucose | C9J8S2 | -0.18338155 | 0.270437536 | 0.801777602 |
| AST | A0A182DWH7 | -0.183563115 | 0.269955253 | 0.801777602 |
| Creatinine | A0A075B6J9 | -0.1846036 | 0.267202362 | 0.797957888 |
| CO2 | A0A0C4DH73 | -0.185460943 | 0.264947966 | 0.796057399 |
| TBIL | P08185 | -0.185779211 | 0.264114278 | 0.794765901 |
| AST | A0A2R8Y7X9 | -0.1863045 | 0.262742105 | 0.791847568 |
| DBIL | P0DP01 | -0.186372862 | 0.262563873 | 0.791847568 |
| CRP | A0A075B6S9 | -0.186483684 | 0.262275113 | 0.791847568 |
| P | Q15582 | -0.187233151 | 0.260327805 | 0.789022985 |
| Albumin | P68032 | -0.187597851 | 0.259383696 | 0.789022985 |
| Mg | A0A0J9YVY3 | -0.187885718 | 0.25864009 | 0.789022985 |
| IBIL | A0A182DWH7 | -0.187896696 | 0.25861176 | 0.789022985 |
| CO2 | A0A0C4DH34 | -0.189292781 | 0.255025801 | 0.786662659 |
| Glucose | F8W1S1 | -0.189415482 | 0.254712224 | 0.786662659 |
| AST | P13645 | -0.190471404 | 0.252024305 | 0.784899738 |
| IBIL | P0DP01 | -0.190512801 | 0.251919315 | 0.784899738 |
| WLC | D6RAR4 | -0.190759763 | 0.251293574 | 0.784899738 |
| ALP | A0A087WSY6 | -0.191232021 | 0.250099883 | 0.784899738 |
| LDH | A0A0C4DH38 | -0.191331009 | 0.249850161 | 0.784899738 |
| Monocytes | A0A075B6J9 | -0.191736592 | 0.248828714 | 0.784767482 |
| Basophils | P13647 | -0.1918467 | 0.248551894 | 0.784767482 |
| WBC | A0A075B6J9 | -0.191956227 | 0.248276739 | 0.784767482 |
| Hematocrit | A0A0J9YVY3 | -0.192086489 | 0.24794976 | 0.784767482 |
| Lymphocytes | A0A075B6S9 | -0.192232308 | 0.247584072 | 0.784767482 |
| WBC | A0A1W2PQU7 | -0.192722301 | 0.24635791 | 0.784767482 |
| Total protein | P13645 | -0.192796175 | 0.2461734 | 0.784767482 |
| WLGG | A0A0J9YVY3 | -0.192974113 | 0.24572936 | 0.784767482 |
| FIB | A0A5H1ZRQ7 | -0.193381904 | 0.244713759 | 0.784767482 |
| Total protein | Q6ZRK6 | -0.194298445 | 0.242441422 | 0.784767482 |
| Ca | P04264 | -0.194616924 | 0.241655171 | 0.784767482 |
| TBIL | A0A182DWH7 | -0.194756168 | 0.241311949 | 0.784767482 |
| WLC | P68032 | -0.194899362 | 0.240959333 | 0.784767482 |
| WLL | P43121 | -0.19532294 | 0.239918309 | 0.784767482 |
| CRP | A0A087WSY6 | -0.196417942 | 0.237241216 | 0.783373679 |
| UA | A0A0C4DH38 | -0.19729715 | 0.235106387 | 0.777846285 |
| ALT | Q86YZ3 | -0.19752221 | 0.234562012 | 0.777846285 |
| UA | A0A087WSY6 | -0.19806314 | 0.233257111 | 0.777846285 |
| WLGG | A0A0C4DH34 | -0.198294959 | 0.2326994 | 0.777508352 |
| P | P68032 | -0.198375406 | 0.232506071 | 0.777508352 |
| CO2 | A0A182DWH7 | -0.198489191 | 0.232232812 | 0.777508352 |
| PT | P19652 | -0.198651403 | 0.231843632 | 0.777508352 |
| DBIL | A0A2R8Y7X9 | -0.198653081 | 0.231839608 | 0.777508352 |
| Total protein | Q8N1N4 | -0.198708153 | 0.231707581 | 0.777508352 |
| P | P04264 | -0.198839417 | 0.231393103 | 0.777508352 |
| ALT | A0A5H1ZRQ7 | -0.199967451 | 0.228702549 | 0.777508352 |
| P | P35908 | -0.200919785 | 0.226447741 | 0.773696448 |
| LDH | O75460 | -0.20107269 | 0.226087132 | 0.773696448 |
| γ-GT | A0A182DWH7 | -0.201216759 | 0.225747722 | 0.773696448 |
| Monocytes | A0A0J9YVY3 | -0.201260959 | 0.225643661 | 0.773696448 |
| Basophils | Q86YZ3 | -0.201470533 | 0.225150708 | 0.773696448 |
| P | P01782 | -0.20179573 | 0.224387247 | 0.773696448 |
| Albumin | Q86YZ3 | -0.201951757 | 0.224021572 | 0.773696448 |
| Creatinine | A0A075B6R2 | -0.202221371 | 0.223390652 | 0.773696448 |
| RBC | A0A075B6R2 | -0.202681261 | 0.222317281 | 0.773004258 |
| WBC | P08185 | -0.203337896 | 0.220790851 | 0.770419141 |
| TT | C9J8S2 | -0.203833199 | 0.219644229 | 0.768244548 |
| WLL | D6RAR4 | -0.204144749 | 0.218925093 | 0.768244548 |
| CRP | P04264 | -0.204305333 | 0.218555058 | 0.768244548 |
| PT | A0A182DWH7 | -0.205123205 | 0.216677108 | 0.765566513 |
| Hemoglobin | A0A087WSY6 | -0.205234993 | 0.216421293 | 0.765566513 |
| Glucose | A0A182DWH7 | -0.205285332 | 0.216306166 | 0.765566513 |
| TBIL | P0DP01 | -0.205381154 | 0.216087132 | 0.765566513 |
| α-HBDH | P01717 | -0.205479496 | 0.215862498 | 0.765566513 |
| Creatine Kinase | D6RAR4 | -0.205636118 | 0.215505075 | 0.765566513 |
| TT | D6RE82 | -0.205784837 | 0.215166064 | 0.765566513 |
| WLC | P35908 | -0.205964404 | 0.214757224 | 0.765566513 |
| AST | A0A5H1ZRQ7 | -0.206126692 | 0.214388186 | 0.765566513 |
| Platelets | Q15582 | -0.206851269 | 0.212745866 | 0.765566513 |
| γ-GT | Q5SRP5 | -0.207584643 | 0.211092486 | 0.765566513 |
| Globin | D6RAR4 | -0.207674227 | 0.21089113 | 0.765566513 |
| WLL | P13473 | -0.207848055 | 0.210500804 | 0.765566513 |
| Hematocrit | A0A075B6S9 | -0.209275044 | 0.207315452 | 0.76261086 |
| Creatine Kinase | D6RE82 | -0.209575926 | 0.206648115 | 0.761579569 |
| CO2 | P13645 | -0.210422628 | 0.204778204 | 0.757525386 |
| Platelets | A0A0C4DH33 | -0.210583924 | 0.204423328 | 0.757525386 |
| Hemoglobin | A0A075B6S9 | -0.210948733 | 0.203622275 | 0.757525386 |
| Eosinophils | P01715 | -0.21107682 | 0.203341538 | 0.757525386 |
| γ-GT | F8W1S1 | -0.211726817 | 0.201921058 | 0.757525386 |
| WBC | P04264 | -0.212202465 | 0.200885992 | 0.757363281 |
| Basophils | A0A1W2PQU7 | -0.212313636 | 0.200644608 | 0.757363281 |
| Neutrophils | A0A0C4DH38 | -0.212519152 | 0.200198904 | 0.757363281 |
| WLGG | P18206 | -0.212741575 | 0.199717317 | 0.757363281 |
| Albumin | P01019 | -0.212952357 | 0.199261681 | 0.757363281 |
| P | P41222 | -0.213399539 | 0.198297442 | 0.7563465 |
| WLGG | D6RAR4 | -0.213424405 | 0.198243921 | 0.7563465 |
| Globin | Q86YZ3 | -0.213739741 | 0.197566071 | 0.7563465 |
| Albumin | Q6ZRK6 | -0.214052651 | 0.196895045 | 0.7563465 |
| FIB | P41222 | -0.214656311 | 0.195605032 | 0.7563465 |
| WBC | P43121 | -0.216058323 | 0.192631869 | 0.753676973 |
| P | A0A5H1ZRQ7 | -0.216804587 | 0.191062348 | 0.75352846 |
| Creatinine | P08185 | -0.216884609 | 0.190894584 | 0.75352846 |
| CO2 | A0A1W2PQU7 | -0.217757861 | 0.189070587 | 0.75169882 |
| Monocytes | P13473 | -0.218406307 | 0.187724135 | 0.747856471 |
| AST | D6RE82 | -0.219530081 | 0.185406772 | 0.741627086 |
| Platelets | P13796 | -0.219984683 | 0.184475106 | 0.739403277 |
| CRP | Q8N1N4 | -0.22139468 | 0.181606561 | 0.730275423 |
| Monocytes | Q9NZP8 | -0.221698864 | 0.180991898 | 0.730275423 |
| RBC | A0A075B6S9 | -0.221829109 | 0.180729167 | 0.730275423 |
| DBIL | A0A182DWH7 | -0.221869428 | 0.180647888 | 0.730275423 |
| Ca | P35908 | -0.221918115 | 0.180549779 | 0.730275423 |
| Basophils | A0A075B6R2 | -0.222547104 | 0.179285682 | 0.730275423 |
| Neutrophils | A0A0J9YVY3 | -0.222578789 | 0.17922217 | 0.730275423 |
| RBC | A0A075B6S5 | -0.223474695 | 0.177432994 | 0.730067372 |
| WLC | P13645 | -0.224209973 | 0.175974148 | 0.727556982 |
| Monocytes | D6RAR4 | -0.224662166 | 0.175081227 | 0.727556982 |
| P | A0A0C4DH38 | -0.225008261 | 0.174400004 | 0.727556982 |
| WLC | A0A182DWH7 | -0.225145643 | 0.174130118 | 0.727556982 |
| Ca | A0A087WSY6 | -0.225536345 | 0.173364223 | 0.727556982 |
| Platelets | A0A087WSY6 | -0.226004164 | 0.172450328 | 0.727556982 |
| PT | O75460 | -0.226403366 | 0.171673213 | 0.727556982 |
| UA | Q5SRP5 | -0.22736763 | 0.169806465 | 0.726232251 |
| DBIL | D6RE82 | -0.227564004 | 0.169428092 | 0.726232251 |
| P | P01019 | -0.227636094 | 0.16928934 | 0.726232251 |
| Ca | A0A2R8Y7X9 | -0.228058141 | 0.168478666 | 0.726232251 |
| α-HBDH | O75460 | -0.229337734 | 0.166037868 | 0.721330076 |
| INR | P13473 | -0.229765909 | 0.165226848 | 0.720393004 |
| TT | A0A182DWH7 | -0.2323448 | 0.160402403 | 0.710972812 |
| WLC | P01019 | -0.232397087 | 0.160305653 | 0.710972812 |
| Basophils | P13796 | -0.232533982 | 0.160052549 | 0.710972812 |
| γ-GT | A0A0C4DH73 | -0.233328148 | 0.158589928 | 0.709571448 |
| Mg | A0A5H1ZRQ7 | -0.233556013 | 0.158172062 | 0.709571448 |
| WLC | F8W1S1 | -0.233707531 | 0.157894646 | 0.709571448 |
| Hematocrit | A0A087WSY6 | -0.233980851 | 0.157395114 | 0.709571448 |
| Ca | P13645 | -0.234088525 | 0.15719864 | 0.709571448 |
| Total protein | Q86YZ3 | -0.234420263 | 0.15659443 | 0.709571448 |
| Mg | O75460 | -0.234488351 | 0.156470629 | 0.709571448 |
| CRP | A0A0C4DH38 | -0.235964444 | 0.153804129 | 0.709571448 |
| Creatine Kinase | A0A075B6R2 | -0.236169634 | 0.153436097 | 0.709571448 |
| Eosinophils | A0A075B6R2 | -0.236558348 | 0.152740652 | 0.709571448 |
| FIB | A0A0C4DH38 | -0.237176997 | 0.151638572 | 0.709571448 |
| AST | P13473 | -0.237184598 | 0.151625067 | 0.709571448 |
| Neutrophils | Q9NZP8 | -0.237251041 | 0.151507058 | 0.709571448 |
| WLL | P18206 | -0.2374541 | 0.151146817 | 0.709571448 |
| WLC | Q6ZRK6 | -0.237504779 | 0.151057008 | 0.709571448 |
| CO2 | A0A0C4DH33 | -0.237586934 | 0.1509115 | 0.709571448 |
| Glucose | Q5SRP5 | -0.237753283 | 0.150617188 | 0.709571448 |
| Monocytes | Q5SRP5 | -0.238010879 | 0.150162262 | 0.709571448 |
| WLC | A0A1W2PQU7 | -0.239414614 | 0.147700831 | 0.709571448 |
| Creatinine | A0A0C4DH34 | -0.239864311 | 0.146918572 | 0.709571448 |
| γ-GT | A0A075B6J9 | -0.240013454 | 0.146659804 | 0.709571448 |
| Neutrophils | P13647 | -0.240205736 | 0.146326685 | 0.709571448 |
| Monocytes | A0A2R8Y7X9 | -0.240246937 | 0.146255377 | 0.709571448 |
| AST | A0A075B6J9 | -0.240254949 | 0.146241514 | 0.709571448 |
| Monocytes | P13645 | -0.240576193 | 0.145686446 | 0.709571448 |
| INR | P19652 | -0.240623582 | 0.145604695 | 0.709571448 |
| Lymphocytes | P08185 | -0.241011337 | 0.144937038 | 0.709571448 |
| Basophils | P13473 | -0.242150976 | 0.142987751 | 0.708815856 |
| Ca | A0A0J9YVY3 | -0.242882721 | 0.14174634 | 0.708815856 |
| P | P13645 | -0.24318411 | 0.141237344 | 0.708815856 |
| CK-MB activity | Q5SRP5 | -0.244086154 | 0.139721978 | 0.705391904 |
| Monocytes | P13647 | -0.244527262 | 0.138985326 | 0.704956501 |
| P | Q9NZP8 | -0.24460752 | 0.138851604 | 0.704956501 |
| CO2 | P04264 | -0.245785016 | 0.136900602 | 0.699793209 |
| Globin | Q8N1N4 | -0.246647356 | 0.135484684 | 0.695038293 |
| BUN | Q5SRP5 | -0.247049236 | 0.134828531 | 0.695038293 |
| Creatine Kinase | P0DP01 | -0.247165267 | 0.134639523 | 0.695038293 |
| CO2 | P01715 | -0.248042382 | 0.133217102 | 0.695038293 |
| ALT | A0A0C4DH38 | -0.248644639 | 0.132246888 | 0.695038293 |
| Creatine Kinase | A0A075B6S9 | -0.249496805 | 0.130883046 | 0.695038293 |
| WBC | P13645 | -0.249521207 | 0.130844147 | 0.695038293 |
| Glucose | Q9NZP8 | -0.250916453 | 0.128634222 | 0.688076211 |
| FIB | P19652 | -0.251205951 | 0.128179187 | 0.688076211 |
| APTT | C9J8S2 | -0.251223817 | 0.128151144 | 0.688076211 |
| Globin | P13645 | -0.251792685 | 0.127260632 | 0.688076211 |
| PT | P13473 | -0.253935946 | 0.123946924 | 0.679463918 |
| P | O75460 | -0.255337841 | 0.121814596 | 0.677975049 |
| P | P01717 | -0.257371503 | 0.118770307 | 0.669742017 |
| WLC | P01715 | -0.257485017 | 0.118602082 | 0.669742017 |
| FIB | P68032 | -0.257498373 | 0.1185823 | 0.669742017 |
| CRP | A0A1W2PQU7 | -0.257873863 | 0.118027174 | 0.669742017 |
| WLGG | P43121 | -0.258348853 | 0.117327745 | 0.669742017 |
| INR | O75460 | -0.258390683 | 0.1172663 | 0.669742017 |
| α-HBDH | P0DP01 | -0.258411015 | 0.117236443 | 0.669742017 |
| APTT | P13796 | -0.259265389 | 0.115986948 | 0.669742017 |
| INR | A0A182DWH7 | -0.259487418 | 0.115663884 | 0.669742017 |
| Eosinophils | P08185 | -0.260455345 | 0.114263408 | 0.669257105 |
| DBIL | A0A075B6J9 | -0.260526836 | 0.114160479 | 0.669257105 |
| RBC | Q5SRP5 | -0.260786603 | 0.113787065 | 0.669257105 |
| TT | A0A075B6R2 | -0.260990049 | 0.113495256 | 0.669257105 |
| INR | Q5SRP5 | -0.261009242 | 0.113467755 | 0.669257105 |
| γ-GT | A0A0C4DH34 | -0.26127492 | 0.113087605 | 0.669257105 |
| Glucose | Q86YZ3 | -0.262138978 | 0.111857895 | 0.669107409 |
| Neutrophils | Q8N1N4 | -0.262748962 | 0.110995878 | 0.66802439 |
| ALP | Q15582 | -0.263368198 | 0.110125931 | 0.66802439 |
| WLC | P13473 | -0.263508121 | 0.109930074 | 0.66802439 |
| PT | Q86YZ3 | -0.264976645 | 0.107890378 | 0.663525825 |
| Monocytes | P04264 | -0.265270374 | 0.107485871 | 0.663110325 |
| CK-MB activity | P02750 | -0.265335735 | 0.107396016 | 0.663110325 |
| Neutrophils | A0A182DWH7 | -0.265703657 | 0.106891278 | 0.663110325 |
| TBIL | A0A2R8Y7X9 | -0.266024445 | 0.106452671 | 0.663110325 |
| INR | P43121 | -0.266108601 | 0.106337834 | 0.663110325 |
| TT | Q5SRP5 | -0.266348175 | 0.10601143 | 0.663110325 |
| DBIL | P18206 | -0.266625481 | 0.105634568 | 0.663110325 |
| Platelets | P43121 | -0.26766026 | 0.104237274 | 0.663110325 |
| Globin | P13647 | -0.268323422 | 0.103349207 | 0.663110325 |
| ALP | P08185 | -0.26873189 | 0.102805083 | 0.663110325 |
| Monocytes | Q8N1N4 | -0.268892187 | 0.102592148 | 0.663110325 |
| FIB | P80108 | -0.269838155 | 0.101342374 | 0.663110325 |
| Basophils | A0A0G2JI36 | -0.271728122 | 0.098880238 | 0.663110325 |
| Total protein | A0A1W2PQU7 | -0.271732031 | 0.098875193 | 0.663110325 |
| Globin | P19652 | -0.272483475 | 0.097909098 | 0.663110325 |
| PT | Q5SRP5 | -0.272694417 | 0.097639203 | 0.663110325 |
| Total protein | P35908 | -0.273045804 | 0.097190875 | 0.663110325 |
| Creatine Kinase | A0A075B6J9 | -0.273707254 | 0.096351223 | 0.663110325 |
| CRP | A0A075B6J9 | -0.273758192 | 0.096286793 | 0.663110325 |
| Albumin | A0A5H1ZRQ7 | -0.274450346 | 0.095414573 | 0.663110325 |
| CO2 | P35908 | -0.276220755 | 0.093211155 | 0.663110325 |
| Creatine Kinase | P02750 | -0.277209306 | 0.091997951 | 0.663110325 |
| LDH | A0A075B6J9 | -0.278021031 | 0.09101087 | 0.660920265 |
| AST | A0A0C4DH73 | -0.278195712 | 0.090799522 | 0.660920265 |
| Hemoglobin | P08185 | -0.278392397 | 0.090562006 | 0.660920265 |
| Total protein | P04264 | -0.281037923 | 0.08741357 | 0.651628433 |
| P | A0A1W2PQU7 | -0.281397192 | 0.086992609 | 0.651628433 |
| WBC | A0A0J9YVY3 | -0.281650398 | 0.086696866 | 0.651628433 |
| UA | P02750 | -0.28286918 | 0.0852842 | 0.650539941 |
| Total protein | Q9NZP8 | -0.284103393 | 0.083871873 | 0.644765025 |
| ALP | P13796 | -0.285041891 | 0.082810124 | 0.641615452 |
| γ-GT | P02750 | -0.285057075 | 0.082793032 | 0.641615452 |
| RBC | C9J8S2 | -0.285895869 | 0.081853086 | 0.641615452 |
| Globin | P13473 | -0.28594891 | 0.081793928 | 0.641615452 |
| BUN | O75882 | -0.286073875 | 0.081654683 | 0.641615452 |
| P | A0A2R8Y7X9 | -0.286433873 | 0.081254578 | 0.641615452 |
| Lymphocytes | A0A0J9YVY3 | -0.290627134 | 0.076705567 | 0.623787423 |
| INR | D6RAR4 | -0.291402396 | 0.075886749 | 0.623787423 |
| Total protein | A0A0C4DH38 | -0.291438625 | 0.075848652 | 0.623787423 |
| IBIL | A0A2R8Y7X9 | -0.292295909 | 0.074951523 | 0.623787423 |
| Monocytes | P02750 | -0.294574134 | 0.072607705 | 0.610649418 |
| Basophils | A0A0C4DH34 | -0.295660917 | 0.071510065 | 0.610649418 |
| Glucose | A0A5H1ZRQ7 | -0.295813872 | 0.071356632 | 0.610649418 |
| BUN | Q15582 | -0.298440496 | 0.068761904 | 0.596138448 |
| PT | P43121 | -0.300740589 | 0.066551234 | 0.582101457 |
| Mg | P01019 | -0.300778306 | 0.066515456 | 0.582101457 |
| ALT | A0A075B6J9 | -0.300892873 | 0.066406872 | 0.582101457 |
| Monocytes | A0A0C4DH34 | -0.301269001 | 0.06605137 | 0.582101457 |
| Neutrophils | P01019 | -0.302254324 | 0.065127196 | 0.582101457 |
| INR | Q86YZ3 | -0.302341389 | 0.065046028 | 0.582101457 |
| Monocytes | F8W1S1 | -0.302387508 | 0.065003065 | 0.582101457 |
| CRP | A0A0J9YVY3 | -0.304083053 | 0.063439046 | 0.580688568 |
| CO2 | Q8N1N4 | -0.305561684 | 0.062099551 | 0.579203398 |
| Hematocrit | Q5SRP5 | -0.305907711 | 0.061789343 | 0.579054416 |
| WLC | P04264 | -0.307250703 | 0.060596986 | 0.578907127 |
| CRP | A0A0C4DH34 | -0.307498697 | 0.060378818 | 0.578907127 |
| Platelets | A0A0J9YVY3 | -0.308731421 | 0.059303589 | 0.578907127 |
| Globin | O75460 | -0.309267102 | 0.058841116 | 0.578907127 |
| FIB | P43251 | -0.311596212 | 0.056863538 | 0.569167247 |
| RBC | A0A075B6J9 | -0.312229826 | 0.056334821 | 0.568548344 |
| CO2 | A0A0G2JI36 | -0.312410215 | 0.056185014 | 0.568548344 |
| PT | Q9NZP8 | -0.312950002 | 0.055738638 | 0.568360829 |
| AST | Q9NZP8 | -0.313395091 | 0.055372703 | 0.567570208 |
| Eosinophils | A0A075B6J9 | -0.313839921 | 0.055008899 | 0.566793264 |
| CO2 | F8W1S1 | -0.313959925 | 0.054911082 | 0.566793264 |
| DBIL | P02750 | -0.315282371 | 0.0538423 | 0.560643631 |
| Neutrophils | P35908 | -0.315933467 | 0.053322237 | 0.558181714 |
| Ca | Q8N1N4 | -0.316540315 | 0.052841141 | 0.558181714 |
| CK-MB activity | P08185 | -0.316776134 | 0.052655128 | 0.558181714 |
| Monocytes | P01019 | -0.317512284 | 0.052077827 | 0.558181714 |
| Globin | P01019 | -0.318463009 | 0.051339765 | 0.558181714 |
| IBIL | O75460 | -0.319107028 | 0.050844583 | 0.558181714 |
| Hematocrit | P08185 | -0.319452809 | 0.050580299 | 0.558181714 |
| ALT | A0A0C4DH34 | -0.319513837 | 0.050533769 | 0.558181714 |
| Ca | A0A5H1ZRQ7 | -0.322133802 | 0.048568379 | 0.552500408 |
| INR | Q9NZP8 | -0.32254966 | 0.04826215 | 0.55220879 |
| Mg | A0A0G2JI36 | -0.324122202 | 0.047118173 | 0.545462144 |
| α-HBDH | A0A075B6J9 | -0.326217875 | 0.045627683 | 0.536926768 |
| Neutrophils | A0A5H1ZRQ7 | -0.327114499 | 0.045001713 | 0.533514289 |
| Neutrophils | A0A1W2PQU7 | -0.328189978 | 0.044260043 | 0.528261063 |
| TBIL | P18206 | -0.32836723 | 0.04413876 | 0.528261063 |
| IBIL | P18206 | -0.329225221 | 0.04355548 | 0.528261063 |
| Platelets | A0A0C4DH34 | -0.332275372 | 0.041532196 | 0.520607404 |
| CK-MB activity | D6RE82 | -0.332494034 | 0.041390127 | 0.520607404 |
| Eosinophils | A0A075B6S5 | -0.334119483 | 0.040346347 | 0.517789523 |
| CO2 | A0A0J9YVY3 | -0.334301247 | 0.040230968 | 0.517789523 |
| RBC | P08185 | -0.335321482 | 0.039588313 | 0.517789523 |
| Ca | P01019 | -0.335727899 | 0.039334642 | 0.517789523 |
| Neutrophils | P04264 | -0.336506896 | 0.038852113 | 0.517789523 |
| CO2 | A0A2R8Y7X9 | -0.33774912 | 0.038092619 | 0.517789523 |
| Total protein | P01019 | -0.33774912 | 0.038092619 | 0.517789523 |
| CO2 | P01019 | -0.337968082 | 0.037960008 | 0.517789523 |
| TBIL | O75460 | -0.339591699 | 0.036988371 | 0.517789523 |
| TBIL | P02750 | -0.3404675 | 0.036472735 | 0.517789523 |
| Hemoglobin | Q5SRP5 | -0.34077288 | 0.036294324 | 0.517789523 |
| Globin | A0A0C4DH38 | -0.340795925 | 0.036280889 | 0.517789523 |
| Total protein | F8W1S1 | -0.342446742 | 0.035329019 | 0.517789523 |
| Ca | A0A0G2JI36 | -0.345003423 | 0.033895171 | 0.509203791 |
| AST | A0A0C4DH38 | -0.346620673 | 0.033013031 | 0.503640665 |
| RBC | P02750 | -0.347469225 | 0.032557763 | 0.501397283 |
| Total protein | O75460 | -0.347711898 | 0.032428514 | 0.501397283 |
| PT | D6RAR4 | -0.34816099 | 0.032190436 | 0.501397283 |
| FIB | A0A0J9YVY3 | -0.34959833 | 0.031438075 | 0.501397283 |
| Platelets | A0A075B6S9 | -0.356164908 | 0.028182239 | 0.488490917 |
| TBIL | A0A075B6J9 | -0.356560337 | 0.027995414 | 0.488490917 |
| Globin | A0A1W2PQU7 | -0.356888762 | 0.027841024 | 0.488490917 |
| Neutrophils | P13645 | -0.36036332 | 0.026250286 | 0.488490917 |
| Globin | P35908 | -0.361924616 | 0.025560384 | 0.488490917 |
| Hemoglobin | P02750 | -0.362939576 | 0.025120002 | 0.488490917 |
| IBIL | A0A075B6J9 | -0.364412346 | 0.024492177 | 0.488490917 |
| Neutrophils | F8W1S1 | -0.364779431 | 0.024337736 | 0.488490917 |
| Hemoglobin | A0A075B6J9 | -0.366006054 | 0.023827518 | 0.488490917 |
| Hemoglobin | A0A075B6S5 | -0.366663157 | 0.023557869 | 0.488490917 |
| IBIL | P02750 | -0.367038742 | 0.023404885 | 0.488490917 |
| DBIL | O75460 | -0.369709373 | 0.022340713 | 0.488490917 |
| Globin | P68032 | -0.370824997 | 0.021908255 | 0.488490917 |
| Globin | F8W1S1 | -0.37184809 | 0.021517817 | 0.488490917 |
| RBC | A0A087WSY6 | -0.372640224 | 0.021219517 | 0.488490917 |
| Platelets | P08185 | -0.373426735 | 0.020926754 | 0.488490917 |
| Creatine Kinase | A0A0C4DH34 | -0.37942545 | 0.018802833 | 0.480930359 |
| Hematocrit | A0A075B6S5 | -0.37942545 | 0.018802833 | 0.480930359 |
| BUN | P08185 | -0.379753767 | 0.018692001 | 0.480930359 |
| Globin | Q9NZP8 | -0.380644854 | 0.018393948 | 0.480930359 |
| AST | O75460 | -0.383026261 | 0.017616889 | 0.480930359 |
| Globin | P04264 | -0.386994409 | 0.016383302 | 0.467280259 |
| P | Q86YZ3 | -0.387018203 | 0.01637613 | 0.467280259 |
| RBC | A0A0C4DH34 | -0.389931606 | 0.01551772 | 0.462710207 |
| Total protein | Q5SRP5 | -0.394290662 | 0.014304325 | 0.454046955 |
| APTT | D6RAR4 | -0.394700898 | 0.014194375 | 0.454046955 |
| Hematocrit | A0A075B6J9 | -0.396607392 | 0.013692724 | 0.454046955 |
| ALT | Q5SRP5 | -0.404769279 | 0.011711114 | 0.418224098 |
| Creatinine | Q5SRP5 | -0.405152142 | 0.01162448 | 0.418224098 |
| Total protein | P68032 | -0.406186198 | 0.011393211 | 0.418224098 |
| Monocytes | Q86YZ3 | -0.410394234 | 0.010491969 | 0.412963899 |
| WLC | Q8N1N4 | -0.422338138 | 0.008257368 | 0.353271748 |
| Globin | Q5SRP5 | -0.422870055 | 0.00816816 | 0.353271748 |
| Hematocrit | P02750 | -0.422982222 | 0.008149455 | 0.353271748 |
| APTT | P43121 | -0.424465006 | 0.007905604 | 0.353271748 |
| Basophils | P01782 | -0.426846578 | 0.007527036 | 0.353271748 |
| Neutrophils | Q86YZ3 | -0.44126249 | 0.005551369 | 0.295272813 |
| AST | Q5SRP5 | -0.44964076 | 0.004623185 | 0.267600847 |
| PT | D6RE82 | -0.45478118 | 0.004122974 | 0.260475833 |
| INR | D6RE82 | -0.460738226 | 0.003602697 | 0.260475833 |
| WBC | Q86YZ3 | -0.465535702 | 0.003226208 | 0.260186919 |
| TT | P02750 | -0.465787118 | 0.003207461 | 0.260186919 |
| WBC | A0A0C4DH34 | -0.466320116 | 0.003168032 | 0.260186919 |
| Hemoglobin | A0A0C4DH34 | -0.466980795 | 0.003119746 | 0.260186919 |
| Eosinophils | A0A0C4DH34 | -0.467964591 | 0.003049035 | 0.260186919 |
| Lymphocytes | A0A0C4DH34 | -0.475455608 | 0.002554967 | 0.260186919 |
| AST | P02750 | -0.48950164 | 0.001814356 | 0.25504664 |
| Creatinine | P02750 | -0.507194836 | 0.001154292 | 0.218009541 |
| Hematocrit | A0A0C4DH34 | -0.509110814 | 0.001097513 | 0.218009541 |
| ALT | P02750 | -0.523468162 | 0.000744952 | 0.218009541 |

| Table S7-2 Relationships between these DEPs and clinical parameter in M vs C group | | | | |
| --- | --- | --- | --- | --- |
| Metabolites | Microbes | Correlation_coefficient | P_value | corrected_pvalue |
| Ca | Q8N1N4 | -0.612203803 | 1.29E-05 | 0.042154166 |
| Platelets | P43121 | -0.574104906 | 5.69E-05 | 0.093330499 |
| BUN | P55056 | 0.559335476 | 9.65E-05 | 0.105556535 |
| γ-GT | Q16880 | -0.540006226 | 0.000185887 | 0.132603807 |
| Ca | P35908 | -0.536141431 | 0.000210905 | 0.132603807 |
| FIB | A0A2R8Y3M9 | 0.530800363 | 0.000250487 | 0.132603807 |
| CRP | A0A2R8Y3M9 | 0.520997003 | 0.000340963 | 0.132603807 |
| Ca | O75882 | 0.519885556 | 0.000352886 | 0.132603807 |
| AST | Q5SRP5 | -0.516671799 | 0.000389504 | 0.132603807 |
| Ca | P13645 | -0.511795423 | 0.000451614 | 0.132603807 |
| INR | A0A0A0MS15 | -0.504558528 | 0.000560237 | 0.132603807 |
| CK-MB activity | P19823 | -0.50302774 | 0.000586013 | 0.132603807 |
| PT | A0A0A0MS15 | -0.502517154 | 0.000594844 | 0.132603807 |
| α-HBDH | E7ENL6 | 0.502174746 | 0.000600832 | 0.132603807 |
| INR | A0A2R8Y3M9 | -0.500095416 | 0.000638373 | 0.132603807 |
| ALP | D6RE82 | 0.49964122 | 0.000646848 | 0.132603807 |
| Ca | A0A1W2PQU7 | -0.492817635 | 0.000786866 | 0.141318547 |
| Ca | P13647 | -0.490851808 | 0.000831939 | 0.141318547 |
| P | P02766 | -0.49013846 | 0.000848856 | 0.141318547 |
| ALP | C9J8S2 | 0.489605374 | 0.000861698 | 0.141318547 |
| Ca | P04264 | -0.486693328 | 0.000934958 | 0.146031585 |
| INR | P04430 | 0.484472118 | 0.000994519 | 0.148066241 |
| BUN | P08185 | -0.482914861 | 0.001038269 | 0.148066241 |
| Creatinine | K7ERG9 | 0.481123528 | 0.001241583 | 0.169683067 |
| ALP | P01019 | 0.472290528 | 0.001385328 | 0.177076889 |
| TT | P02750 | -0.470711847 | 0.001444852 | 0.177076889 |
| Creatinine | P30041 | 0.470380093 | 0.001457645 | 0.177076889 |
| ALT | Q16880 | -0.467395412 | 0.001577338 | 0.18220835 |
| LDH | P01814 | -0.466412424 | 0.001618622 | 0.18220835 |
| LDH | P43121 | 0.465298949 | 0.00166654 | 0.18220835 |
| WLGG | P01019 | 0.457461458 | 0.002040861 | 0.212357573 |
| BUN | P01019 | 0.454596945 | 0.002195152 | 0.212357573 |
| Hematocrit | P02042 | 0.454572929 | 0.002196488 | 0.212357573 |
| UA | P55056 | 0.454487107 | 0.002201268 | 0.212357573 |
| UA | P03950 | 0.451164283 | 0.002393528 | 0.224307757 |
| CK-MB activity | D6R934 | -0.449785637 | 0.002477543 | 0.225731681 |
| ALT | P30041 | 0.444678937 | 0.002811809 | 0.249263041 |
| AST | Q16880 | -0.440376291 | 0.003123573 | 0.269613646 |
| Glucose | P35527 | -0.437143628 | 0.00337737 | 0.275581786 |
| Glucose | P02745 | 0.43601074 | 0.003470495 | 0.275581786 |
| FIB | P01782 | -0.435547872 | 0.003509186 | 0.275581786 |
| Creatinine | P26038 | 0.435315034 | 0.003528791 | 0.275581786 |
| ALT | P01715 | 0.432076344 | 0.003811609 | 0.279428148 |
| BUN | P01814 | -0.432018126 | 0.003816869 | 0.279428148 |
| TT | P02753 | 0.431833216 | 0.003833618 | 0.279428148 |
| ALP | P04430 | -0.42960367 | 0.004040688 | 0.28811863 |
| Hematocrit | P00915 | 0.426704943 | 0.004324478 | 0.301793379 |
| ALT | Q86YZ3 | -0.425411384 | 0.004456632 | 0.30419577 |
| Hemoglobin | P00915 | 0.422953508 | 0.004717468 | 0.30419577 |
| INR | P01019 | -0.422331373 | 0.004785562 | 0.30419577 |
| Ca | A0A0J9YXX1 | -0.421518612 | 0.00487581 | 0.30419577 |
| DBIL | A0A2R8Y3M9 | -0.420921575 | 0.004943046 | 0.30419577 |
| BUN | P01782 | -0.420464417 | 0.004995072 | 0.30419577 |
| ALT | P03950 | 0.419120297 | 0.00515081 | 0.30419577 |
| CK-MB activity | P01817 | -0.419025221 | 0.005161985 | 0.30419577 |
| Albumin | Q16880 | -0.418202436 | 0.005259571 | 0.30419577 |
| AST | P32119 | 0.417979146 | 0.005286329 | 0.30419577 |
| α-HBDH | P43121 | 0.414916458 | 0.005665475 | 0.304990992 |
| RBC | P01817 | -0.414530083 | 0.005714944 | 0.304990992 |
| Albumin | P35527 | -0.413746242 | 0.005816455 | 0.304990992 |
| Hematocrit | Q86UD1 | -0.412701235 | 0.005954219 | 0.304990992 |
| PT | P04430 | 0.412600537 | 0.005967643 | 0.304990992 |
| WLGG | P02763 | 0.412599906 | 0.005967727 | 0.304990992 |
| α-HBDH | D6R934 | -0.412206857 | 0.006020374 | 0.304990992 |
| Creatinine | E7EX29 | 0.412031226 | 0.006044029 | 0.304990992 |
| WLC | Q8N1N4 | -0.409166906 | 0.006441341 | 0.31675502 |
| CK-MB activity | P01019 | 0.408771749 | 0.006497891 | 0.31675502 |
| WLL | P0DP01 | -0.40798097 | 0.006612348 | 0.31675502 |
| Lymphocytes | E7EX29 | 0.407458875 | 0.00668887 | 0.31675502 |
| Creatine Kinase | K7ERG9 | 0.406978069 | 0.006760016 | 0.31675502 |
| Albumin | P43121 | 0.405990453 | 0.006908212 | 0.318166121 |
| WLGG | P02766 | 0.405491562 | 0.006984134 | 0.318166121 |
| APTT | P13473 | -0.402507179 | 0.007453543 | 0.326806334 |
| ALP | P01782 | -0.401949386 | 0.007544234 | 0.326806334 |
| Basophils | A0A096LPE2 | 0.401607272 | 0.007600328 | 0.326806334 |
| ALP | A0A0C4DH73 | 0.40149606 | 0.00761864 | 0.326806334 |
| AST | P43121 | 0.400552527 | 0.007775537 | 0.326806334 |
| Ca | A0A087X0Q4 | -0.400423779 | 0.007797161 | 0.326806334 |
| Hemoglobin | P02042 | 0.399984972 | 0.00787125 | 0.326806334 |
| CK-MB activity | P08185 | -0.397227099 | 0.008350938 | 0.335297762 |
| ALP | P19823 | -0.396660578 | 0.008452532 | 0.335297762 |
| PT | A0A2R8Y3M9 | -0.396353689 | 0.008508009 | 0.335297762 |
| RBC | H3BTN5 | 0.395987069 | 0.008574694 | 0.335297762 |
| RBC | Q86UD1 | -0.395017616 | 0.008753202 | 0.335297762 |
| BUN | P19823 | -0.394336419 | 0.008880536 | 0.335297762 |
| Hematocrit | P26038 | 0.393592142 | 0.009021479 | 0.335297762 |
| P | P30041 | -0.392669619 | 0.009198841 | 0.335297762 |
| DBIL | H3BTN5 | 0.391919959 | 0.009345168 | 0.335297762 |
| PT | Q5SRP5 | -0.391869221 | 0.009355143 | 0.335297762 |
| Total protein | P35527 | -0.391662271 | 0.009395925 | 0.335297762 |
| Neutrophils | P35527 | -0.391466871 | 0.009434572 | 0.335297762 |
| Monocytes | E7EX29 | 0.391142956 | 0.009498936 | 0.335297762 |
| WLGG | Q5SRP5 | 0.390538764 | 0.009620002 | 0.335297762 |
| Hemoglobin | Q16880 | -0.390314009 | 0.009665374 | 0.335297762 |
| Basophils | P32119 | -0.389377662 | 0.009856379 | 0.335297762 |
| γ-GT | P32119 | 0.389222318 | 0.009888379 | 0.335297762 |
| TT | P01782 | 0.389089458 | 0.009915818 | 0.335297762 |
| Albumin | Q8N1N4 | -0.388595184 | 0.010018472 | 0.335312111 |
| Hematocrit | P30041 | 0.386782753 | 0.010402721 | 0.341449337 |
| Hemoglobin | P32119 | 0.385780745 | 0.010620522 | 0.341449337 |
| Hemoglobin | P69905 | 0.385402973 | 0.010703644 | 0.341449337 |
| CRP | P02763 | 0.38534745 | 0.010715907 | 0.341449337 |
| Lymphocytes | P02750 | -0.385318339 | 0.010722342 | 0.341449337 |
| LDH | A0A087X0Q4 | -0.384624105 | 0.010876778 | 0.343036836 |
| Neutrophils | O75636 | -0.383688883 | 0.011087828 | 0.343906773 |
| P | A0A0J9YVY3 | 0.383329167 | 0.011169932 | 0.343906773 |
| Creatinine | Q16880 | -0.382965871 | 0.011713396 | 0.343906773 |
| Creatine Kinase | P30041 | 0.382905033 | 0.011267407 | 0.343906773 |
| APTT | P02763 | -0.381286819 | 0.011646012 | 0.343906773 |
| Hematocrit | P32119 | 0.381164574 | 0.011675049 | 0.343906773 |
| DBIL | P02750 | -0.380635851 | 0.011801352 | 0.343906773 |
| FIB | P02763 | 0.380481205 | 0.011838513 | 0.343906773 |
| BUN | Q96HR3 | 0.380441762 | 0.011848008 | 0.343906773 |
| CO2 | A0A087X0Q4 | -0.379056404 | 0.012185622 | 0.350603871 |
| CK-MB activity | P04264 | 0.378618947 | 0.012293922 | 0.350633733 |
| Basophils | P69905 | -0.377722597 | 0.012518393 | 0.350633733 |
| Hematocrit | P69905 | 0.376708717 | 0.012776499 | 0.350633733 |
| WLGG | Q9H4B7 | -0.376645976 | 0.012792619 | 0.350633733 |
| WLL | Q5SRP5 | 0.376609954 | 0.012801882 | 0.350633733 |
| ALT | Q86UD1 | -0.376329261 | 0.012874257 | 0.350633733 |
| Creatine Kinase | P80748 | 0.376090329 | 0.012936139 | 0.350633733 |
| BUN | P13473 | 0.375684352 | 0.013041864 | 0.350633733 |
| BUN | P0DP01 | -0.374414742 | 0.01337726 | 0.354540375 |
| APTT | A0A140T8Y3 | -0.374225032 | 0.013428001 | 0.354540375 |
| Total protein | A0A1W2PQU7 | -0.373914367 | 0.013511447 | 0.354540375 |
| ALP | K7ERG9 | 0.372936498 | 0.013776991 | 0.357644007 |
| PT | A0A140T8Y3 | -0.371943128 | 0.014051274 | 0.357644007 |
| BUN | P69905 | 0.371757601 | 0.014103011 | 0.357644007 |
| Hemoglobin | P01817 | -0.371500964 | 0.014174845 | 0.357644007 |
| WBC | P43121 | -0.371500717 | 0.014174915 | 0.357644007 |
| Total protein | Q16880 | -0.371044493 | 0.014303383 | 0.358130495 |
| BUN | A0A0C4DH36 | -0.37054937 | 0.014443921 | 0.358909551 |
| INR | A0A140T8Y3 | -0.369328747 | 0.014795405 | 0.362736102 |
| Mg | P19652 | 0.36924736 | 0.014819097 | 0.362736102 |
| Ca | Q92954 | 0.3685169 | 0.015033176 | 0.365250506 |
| α-HBDH | Q5SRP5 | -0.367780847 | 0.015251541 | 0.367831293 |
| ALT | P02753 | 0.366764015 | 0.015557623 | 0.372474488 |
| Ca | P02753 | 0.366324247 | 0.015691603 | 0.372959846 |
| TT | Q5SRP5 | -0.365332473 | 0.015997347 | 0.377491356 |
| Mg | P01814 | -0.364699978 | 0.016194954 | 0.378230673 |
| Globin | P26038 | -0.364495409 | 0.016259306 | 0.378230673 |
| INR | Q9NZP8 | -0.363403173 | 0.01660657 | 0.379863729 |
| WLGG | P02750 | 0.363315384 | 0.016634752 | 0.379863729 |
| Mg | Q96HR3 | 0.363184184 | 0.016676944 | 0.379863729 |
| RBC | Q16880 | -0.362723263 | 0.01682589 | 0.380505244 |
| WLGG | P0DP01 | -0.362381286 | 0.016937124 | 0.380505244 |
| AST | P00915 | 0.361456622 | 0.017241002 | 0.384697189 |
| Creatinine | P02042 | 0.361069163 | 0.017875016 | 0.388278484 |
| DBIL | A0A075B6S5 | 0.360833112 | 0.017448499 | 0.385684761 |
| CK-MB activity | P19652 | 0.360618407 | 0.017520436 | 0.385684761 |
| Albumin | P19652 | 0.359818748 | 0.017790569 | 0.388278484 |
| ALP | P55056 | 0.359185598 | 0.018006938 | 0.38857076 |
| WLGG | A0A2R8Y3M9 | 0.357154819 | 0.018715954 | 0.399899048 |
| ALT | A0A0B4J1Y8 | -0.356986534 | 0.018775748 | 0.399899048 |
| LDH | E7ENL6 | 0.356505112 | 0.018947694 | 0.40095766 |
| Monocytes | Q9Y5Y7 | -0.355998073 | 0.019130221 | 0.402225163 |
| Hematocrit | H3BTN5 | 0.35548399 | 0.019316792 | 0.402499154 |
| Albumin | P35908 | -0.355287025 | 0.019388679 | 0.402499154 |
| α-HBDH | P19823 | -0.354798535 | 0.019567936 | 0.403665603 |
| BUN | P01718 | 0.353851898 | 0.019919285 | 0.406675794 |
| Hemoglobin | P26038 | 0.353584344 | 0.020019544 | 0.406675794 |
| γ-GT | E7EX29 | 0.353408105 | 0.020085817 | 0.406675794 |
| WLGG | P04430 | -0.352407997 | 0.020465397 | 0.410041915 |
| Ca | A0A0B4J1Y8 | -0.35231213 | 0.020502096 | 0.410041915 |
| BUN | P02766 | 0.350462528 | 0.021221043 | 0.421848605 |
| Lymphocytes | A0A0B4J1V2 | 0.349684686 | 0.021529645 | 0.425356006 |
| RBC | P69905 | 0.348827556 | 0.021874051 | 0.425356006 |
| IBIL | P02751 | -0.348640499 | 0.021949823 | 0.425356006 |
| Mg | A0A075B6S9 | -0.348477915 | 0.022015861 | 0.425356006 |
| α-HBDH | P02751 | -0.348226793 | 0.022118186 | 0.425356006 |
| CK-MB activity | A0A0J9YXX1 | 0.348086386 | 0.022175572 | 0.425356006 |
| α-HBDH | P01814 | -0.347698032 | 0.022334946 | 0.425922218 |
| RBC | E7EX29 | 0.34614237 | 0.022983006 | 0.433322253 |
| BUN | Q15582 | -0.345856147 | 0.023103937 | 0.433322253 |
| Hematocrit | P01817 | -0.345819811 | 0.023119328 | 0.433322253 |
| INR | Q15582 | 0.34456733 | 0.023655082 | 0.436778876 |
| INR | P02763 | -0.344416038 | 0.023720494 | 0.436778876 |
| ALT | E7EX29 | 0.343529643 | 0.024106776 | 0.436778876 |
| INR | A0A075B6S5 | 0.343432641 | 0.024149365 | 0.436778876 |
| Hemoglobin | Q86UD1 | -0.343235283 | 0.02423621 | 0.436778876 |
| Neutrophils | Q9H4B7 | -0.342834749 | 0.024413262 | 0.436778876 |
| RBC | P26038 | 0.342666029 | 0.024488165 | 0.436778876 |
| Platelets | P19652 | 0.342647845 | 0.024496249 | 0.436778876 |
| Lymphocytes | P43121 | -0.342532202 | 0.024547714 | 0.436778876 |
| Creatinine | P03950 | 0.342335646 | 0.024635394 | 0.436778876 |
| BUN | P04430 | -0.341512537 | 0.02500541 | 0.438001495 |
| ALP | C9JV77 | -0.341203652 | 0.025145455 | 0.438001495 |
| UA | A0A0B4J1V2 | 0.341161368 | 0.025164676 | 0.438001495 |
| Glucose | Q16880 | -0.340999219 | 0.025238501 | 0.438001495 |
| Monocytes | O75636 | 0.34056922 | 0.025435148 | 0.439090977 |
| ALP | A0A2R8Y3M9 | 0.339692564 | 0.025840016 | 0.440261922 |
| INR | Q86YZ3 | -0.338916616 | 0.026202836 | 0.440261922 |
| Neutrophils | A0A0J9YXX1 | -0.338682275 | 0.02631324 | 0.440261922 |
| DBIL | P01718 | -0.338591229 | 0.026356238 | 0.440261922 |
| P | Q16880 | 0.338472119 | 0.026412578 | 0.440261922 |
| Hematocrit | E7EX29 | 0.338446872 | 0.026424533 | 0.440261922 |
| LDH | P15169 | 0.338405776 | 0.026444002 | 0.440261922 |
| LDH | P30041 | 0.338126163 | 0.026576787 | 0.440261922 |
| CO2 | A0A0G2JI36 | -0.337631126 | 0.026813232 | 0.441946745 |
| Ca | D6R934 | 0.33661002 | 0.027306472 | 0.447826139 |
| FIB | C9J8S2 | 0.336048351 | 0.02758098 | 0.448225489 |
| BUN | D6R934 | -0.335963755 | 0.027622523 | 0.448225489 |
| Creatinine | A0A0B4J1Y8 | -0.335409824 | 0.027895831 | 0.448225489 |
| CK-MB activity | A0A0B4J1V2 | 0.33533917 | 0.027930853 | 0.448225489 |
| RBC | P13473 | 0.335158409 | 0.028020617 | 0.448225489 |
| BUN | A0A0C4DH73 | 0.334755524 | 0.028221548 | 0.448225489 |
| Creatinine | H3BTN5 | 0.334580414 | 0.028309253 | 0.448225489 |
| WLGG | A0A075B6S5 | -0.333934227 | 0.028634858 | 0.448225489 |
| Mg | K7ERG9 | 0.333929365 | 0.028637319 | 0.448225489 |
| Hemoglobin | H3BTN5 | 0.333461196 | 0.028875174 | 0.448225489 |
| APTT | Q5SRP5 | -0.333447422 | 0.028882197 | 0.448225489 |
| PT | P01019 | -0.332867484 | 0.029179165 | 0.448225489 |
| Ca | A0A2R8Y7X9 | -0.332753975 | 0.029237584 | 0.448225489 |
| CO2 | P35908 | -0.332741559 | 0.02924398 | 0.448225489 |
| WLC | P37802 | 0.331545039 | 0.029865816 | 0.455627335 |
| LDH | P01718 | -0.330544304 | 0.030394269 | 0.459972143 |
| Lymphocytes | A0A0C4DH33 | -0.330425594 | 0.030457465 | 0.459972143 |
| CK-MB activity | P0DP01 | -0.33021224 | 0.030571319 | 0.459972143 |
| Neutrophils | P02763 | 0.329696056 | 0.030848232 | 0.460511704 |
| AST | P02751 | -0.329449892 | 0.030981017 | 0.460511704 |
| Hematocrit | Q16880 | -0.329355797 | 0.031031898 | 0.460511704 |
| TT | A0A2R8Y3M9 | -0.328914735 | 0.031271321 | 0.460511704 |
| ALT | P26038 | 0.32875782 | 0.031356867 | 0.460511704 |
| LDH | P80748 | 0.328588157 | 0.03144958 | 0.460511704 |
| Creatinine | P02750 | -0.327997584 | 0.032277537 | 0.460533311 |
| Basophils | P13647 | -0.327901298 | 0.031827232 | 0.460533311 |
| TT | Q9Y5Y7 | -0.327853719 | 0.03185353 | 0.460533311 |
| Mg | P35527 | -0.3277904 | 0.031888556 | 0.460533311 |
| CK-MB activity | A0A0G2JI36 | 0.327144838 | 0.032247475 | 0.460533311 |
| CK-MB activity | P04430 | -0.327062497 | 0.032293494 | 0.460533311 |
| Platelets | Q9NZP8 | 0.326561446 | 0.03257469 | 0.461657276 |
| CK-MB activity | P13647 | 0.325984457 | 0.032901002 | 0.461657276 |
| CRP | Q92954 | 0.325981888 | 0.03290246 | 0.461657276 |
| RBC | P32119 | 0.325491831 | 0.03318173 | 0.461657276 |
| IBIL | Q9H4B7 | -0.325380248 | 0.03324559 | 0.461657276 |
| BUN | P19652 | 0.325240704 | 0.033325594 | 0.461657276 |
| TT | Q86UD1 | -0.325185039 | 0.033357553 | 0.461657276 |
| INR | A0A5H1ZRS9 | 0.322856941 | 0.034716953 | 0.478452128 |
| Creatinine | P00915 | 0.321806101 | 0.035835956 | 0.482865151 |
| PT | P01782 | 0.321744156 | 0.035382627 | 0.482865151 |
| Neutrophils | P08185 | -0.321162924 | 0.035734469 | 0.482865151 |
| WBC | P01718 | 0.320798464 | 0.035956553 | 0.482865151 |
| BUN | P00915 | 0.320785352 | 0.035964564 | 0.482865151 |
| Mg | P15169 | -0.32066617 | 0.036037446 | 0.482865151 |
| BUN | C9JV77 | -0.320332266 | 0.036242281 | 0.482865151 |
| DBIL | E7EX29 | 0.319631923 | 0.036675014 | 0.482865151 |
| Total protein | P04264 | -0.31961333 | 0.036686559 | 0.482865151 |
| Total protein | P35908 | -0.319537807 | 0.036733488 | 0.482865151 |
| ALP | P19652 | 0.31951954 | 0.036744847 | 0.482865151 |
| Mg | A0A0C4DH36 | -0.319377745 | 0.036833111 | 0.482865151 |
| WBC | P19652 | 0.319024427 | 0.037053801 | 0.482865151 |
| WLGG | C9J8S2 | 0.318737607 | 0.037233751 | 0.482865151 |
| Glucose | D6RE82 | 0.318719093 | 0.037245391 | 0.482865151 |
| ALT | Q5SRP5 | -0.317989033 | 0.03770678 | 0.484126296 |
| ALT | O75882 | 0.317922631 | 0.037748976 | 0.484126296 |
| Total protein | Q5SRP5 | -0.317563683 | 0.037977746 | 0.484126296 |
| Basophils | Q15582 | 0.31755948 | 0.037980432 | 0.484126296 |
| INR | P80748 | -0.31725914 | 0.038172732 | 0.484126296 |
| APTT | P01814 | 0.317172636 | 0.038228265 | 0.484126296 |
| Ca | P08185 | 0.316346883 | 0.038761718 | 0.488993979 |
| CK-MB activity | P13645 | 0.314743614 | 0.039814848 | 0.500355183 |
| CO2 | P0DP01 | 0.312731451 | 0.041169521 | 0.514836391 |
| CK-MB activity | Q9NZP8 | 0.312465064 | 0.041351649 | 0.514836391 |
| Hemoglobin | E7EX29 | 0.312164593 | 0.041557867 | 0.514836391 |
| RBC | P02042 | 0.311747164 | 0.041845746 | 0.514836391 |
| PT | Q86YZ3 | -0.311255584 | 0.042186844 | 0.514836391 |
| Lymphocytes | P02042 | 0.311230278 | 0.042204465 | 0.514836391 |
| Creatinine | P32119 | 0.311084265 | 0.042763886 | 0.514836391 |
| LDH | P00918 | 0.311067326 | 0.042318071 | 0.514836391 |
| PT | A0A0J9YVY3 | 0.311015689 | 0.042354124 | 0.514836391 |
| IBIL | Q15582 | -0.310271917 | 0.042876192 | 0.514836391 |
| BUN | A0A075B6S5 | -0.31021333 | 0.042917537 | 0.514836391 |
| WLGG | Q9NZP8 | 0.309923819 | 0.043122323 | 0.514836391 |
| Albumin | A0A1W2PQU7 | -0.309894274 | 0.043143266 | 0.514836391 |
| PT | A0A5H1ZRS9 | 0.309864139 | 0.043164636 | 0.514836391 |
| INR | A0A0J9YVY3 | 0.308996303 | 0.043783759 | 0.515222707 |
| TBIL | H3BTN5 | 0.308980618 | 0.043795015 | 0.515222707 |
| TT | A0A0C4DH36 | 0.308831231 | 0.043902336 | 0.515222707 |
| Glucose | P19652 | 0.308674155 | 0.044015413 | 0.515222707 |
| APTT | A0A5H1ZRS9 | 0.308563665 | 0.044095094 | 0.515222707 |
| WLGG | P00915 | 0.30850215 | 0.044139506 | 0.515222707 |
| TBIL | P02751 | -0.308110561 | 0.044423082 | 0.515504045 |
| BUN | A0A0B4J1V2 | 0.308035041 | 0.04447794 | 0.515504045 |
| CK-MB activity | Q15582 | -0.307452256 | 0.044903137 | 0.517436353 |
| INR | P01817 | 0.306895644 | 0.045312313 | 0.517436353 |
| Platelets | E7ENL6 | -0.306794697 | 0.045386845 | 0.517436353 |
| WLC | A0A0J9YVY3 | -0.306761711 | 0.04541122 | 0.517436353 |
| Glucose | P04430 | -0.306360302 | 0.045708703 | 0.517436353 |
| BUN | D6RE82 | 0.306211065 | 0.045819704 | 0.517436353 |
| WLGG | A0A2R8Y7X9 | 0.305816776 | 0.046114025 | 0.517436353 |
| AST | E7ENL6 | 0.305813774 | 0.046116272 | 0.517436353 |
| Lymphocytes | A0A0A0MS15 | 0.305641576 | 0.046245296 | 0.517436353 |
| UA | Q16880 | -0.305432854 | 0.046402078 | 0.517436353 |
| ALP | Q9NZP8 | 0.305315314 | 0.046490559 | 0.517436353 |
| CRP | P19652 | 0.305135966 | 0.04662583 | 0.517436353 |
| Monocytes | A0A0B4J1V2 | -0.304882603 | 0.046817468 | 0.517436353 |
| Albumin | P55056 | 0.304531736 | 0.04708391 | 0.517436353 |
| RBC | P02750 | -0.30449723 | 0.047110179 | 0.517436353 |
| Monocytes | P02763 | -0.30335846 | 0.047983805 | 0.517436353 |
| APTT | A0A0A0MS15 | -0.303352971 | 0.047988047 | 0.517436353 |
| Mg | A0A0B4J1V2 | 0.302829131 | 0.048394326 | 0.517436353 |
| WBC | P08185 | -0.302790048 | 0.048424749 | 0.517436353 |
| WBC | Q9H4B7 | -0.302724266 | 0.04847599 | 0.517436353 |
| γ-GT | A0A0B4J1Y8 | -0.302638969 | 0.048542498 | 0.517436353 |
| AST | P35527 | -0.302285787 | 0.048818665 | 0.517436353 |
| WLC | Q5SRP5 | 0.30189081 | 0.049129017 | 0.517436353 |
| Neutrophils | P19652 | 0.301831227 | 0.049175972 | 0.517436353 |
| DBIL | D6RE82 | -0.301727227 | 0.049258018 | 0.517436353 |
| Mg | C9J8S2 | 0.301575291 | 0.049378079 | 0.517436353 |
| BUN | P32119 | 0.301302626 | 0.049594133 | 0.517436353 |
| IBIL | A0A0G2JI36 | 0.301236202 | 0.049646882 | 0.517436353 |
| Globin | H3BTN5 | -0.301171795 | 0.049698071 | 0.517436353 |
| ALP | A0A5H1ZRS9 | -0.301159822 | 0.049707592 | 0.517436353 |
| Platelets | A0A5H1ZRS9 | -0.301034673 | 0.049807197 | 0.517436353 |
| Lymphocytes | P0DJI8 | -0.300883627 | 0.049927627 | 0.517436353 |
| P | P00915 | -0.300838916 | 0.04996332 | 0.517436353 |
| AST | P19823 | -0.300469803 | 0.050258776 | 0.517436353 |
| Platelets | C9JV77 | -0.300430489 | 0.050290328 | 0.517436353 |
| CK-MB activity | P02745 | 0.300388753 | 0.05032384 | 0.517436353 |
| Monocytes | P04430 | 0.299973511 | 0.050658252 | 0.519247079 |
| P | Q86UD1 | 0.299711341 | 0.05087031 | 0.519796313 |
| CRP | P35527 | -0.299169198 | 0.051311095 | 0.520297683 |
| BUN | C9J8S2 | 0.299137221 | 0.05133719 | 0.520297683 |
| Mg | C9JV77 | -0.299066109 | 0.051395259 | 0.520297683 |
| WLL | P01019 | 0.298686292 | 0.051706306 | 0.521162533 |
| Albumin | P0DP01 | 0.29831188 | 0.05201441 | 0.521162533 |
| CRP | P02745 | -0.297960739 | 0.052304706 | 0.521162533 |
| TBIL | P02750 | -0.297915726 | 0.052342013 | 0.521162533 |
| Hematocrit | A0A0B4J1Y8 | -0.297722901 | 0.052502071 | 0.521162533 |
| ALT | P55056 | 0.297660261 | 0.052554151 | 0.521162533 |
| APTT | A0A2R8Y3M9 | -0.297613657 | 0.052592926 | 0.521162533 |
| Ca | P02751 | 0.296688616 | 0.053367337 | 0.525703185 |
| INR | P01782 | 0.29668344 | 0.053371695 | 0.525703185 |
| ALT | P32119 | 0.295996932 | 0.053952358 | 0.526079541 |
| γ-GT | Q8N1N4 | -0.295739322 | 0.054171556 | 0.526079541 |
| Mg | P01782 | -0.295579783 | 0.054307665 | 0.526079541 |
| TT | A0A0J9YVY3 | 0.295519881 | 0.05435884 | 0.526079541 |
| RBC | P02766 | 0.295208252 | 0.054625696 | 0.526079541 |
| Basophils | A0A0B4J1V2 | -0.295163351 | 0.054664232 | 0.526079541 |
| BUN | O75636 | -0.295110442 | 0.05470967 | 0.526079541 |
| CK-MB activity | A0A1W2PQU7 | 0.294844283 | 0.054938702 | 0.526079541 |
| WBC | P35527 | -0.294786122 | 0.054988853 | 0.526079541 |
| TBIL | P00915 | 0.294592965 | 0.05515567 | 0.526079541 |
| ALP | P0DP01 | -0.294144368 | 0.055544661 | 0.526079541 |
| AST | D6R934 | -0.293584194 | 0.056033491 | 0.526079541 |
| INR | Q9Y5Y7 | -0.29350631 | 0.056101728 | 0.526079541 |
| Hematocrit | P02750 | -0.293482373 | 0.056122713 | 0.526079541 |
| Platelets | A0A075B6S9 | -0.293251608 | 0.056325346 | 0.526079541 |
| Lymphocytes | Q86UD1 | -0.293209034 | 0.056362794 | 0.526079541 |
| Neutrophils | I3L1J2 | -0.292851975 | 0.056677648 | 0.526079541 |
| RBC | A0A0A0MS15 | 0.292565047 | 0.056931679 | 0.526079541 |
| Hemoglobin | P30041 | 0.292482946 | 0.057004536 | 0.526079541 |
| BUN | P02763 | 0.292391922 | 0.057085397 | 0.526079541 |
| Albumin | A0A075B6S9 | -0.292262764 | 0.057200291 | 0.526079541 |
| INR | O75636 | 0.291993391 | 0.057440514 | 0.526079541 |
| Monocytes | P13647 | -0.29178682 | 0.057625276 | 0.526079541 |
| RBC | P00915 | 0.291658805 | 0.057740016 | 0.526079541 |
| Neutrophils | A0A5H1ZRS9 | -0.29133472 | 0.058031308 | 0.526079541 |
| ALP | E7EX29 | -0.291302208 | 0.058060595 | 0.526079541 |
| Albumin | P32119 | 0.291163155 | 0.058185988 | 0.526079541 |
| CRP | D6RE82 | 0.291087627 | 0.058254188 | 0.526079541 |
| Eosinophils | P03951 | 0.291065145 | 0.0582745 | 0.526079541 |
| DBIL | A0A0B4J1Y8 | -0.290967639 | 0.058362665 | 0.526079541 |
| P | P04430 | 0.290946273 | 0.058381998 | 0.526079541 |
| γ-GT | Q86UD1 | -0.290440861 | 0.058840817 | 0.528761319 |
| INR | A0A2R8Y7X9 | -0.290102242 | 0.05914983 | 0.530085908 |
| CK-MB activity | P00918 | 0.289739446 | 0.059482342 | 0.531613306 |
| ALP | A0A0J9YX35 | -0.289071119 | 0.060098791 | 0.535197218 |
| γ-GT | Q5SRP5 | -0.288744998 | 0.060401442 | 0.535197218 |
| Platelets | P08185 | -0.288648901 | 0.060490856 | 0.535197218 |
| ALP | P0DJI8 | -0.288466683 | 0.060660689 | 0.535197218 |
| Eosinophils | E7EX29 | 0.288296301 | 0.060819836 | 0.535197218 |
| BUN | O75882 | -0.288238627 | 0.060873781 | 0.535197218 |
| Mg | A0A2R8Y3M9 | 0.288076604 | 0.061025536 | 0.535197218 |
| TBIL | Q9H4B7 | -0.287789827 | 0.061294877 | 0.535223703 |
| Basophils | Q92954 | 0.287683117 | 0.061395342 | 0.535223703 |
| CK-MB activity | A0A0A0MS15 | 0.287552925 | 0.06151809 | 0.535223703 |
| WLGG | A0A0C4DH36 | -0.287177117 | 0.061873511 | 0.536891843 |
| WLGG | O75636 | -0.286071374 | 0.062928765 | 0.544607778 |
| INR | C9J8S2 | -0.285098615 | 0.063868905 | 0.551289498 |
| ALP | P02766 | 0.284764518 | 0.064194363 | 0.551667219 |
| FIB | O75636 | -0.284699953 | 0.064257411 | 0.551667219 |
| γ-GT | O75882 | 0.284536502 | 0.064417239 | 0.551667219 |
| Eosinophils | Q9H4B7 | -0.284106881 | 0.064838846 | 0.553831806 |
| AST | P02753 | 0.283671944 | 0.065267899 | 0.554448747 |
| Glucose | Q5SRP5 | -0.283527754 | 0.065410635 | 0.554448747 |
| IBIL | H3BTN5 | 0.283412321 | 0.065525083 | 0.554448747 |
| WLL | A0A075B6S5 | -0.283253128 | 0.065683177 | 0.554448747 |
| TT | P02763 | -0.283139504 | 0.065796201 | 0.554448747 |
| γ-GT | P26038 | 0.282899299 | 0.066035648 | 0.554448747 |
| Basophils | P02751 | 0.282840519 | 0.066094348 | 0.554448747 |
| γ-GT | A0A1W2PQU7 | -0.282492744 | 0.066442494 | 0.55460552 |
| WLC | Q16880 | -0.282424771 | 0.06651071 | 0.55460552 |
| WLGG | Q6ZRK6 | 0.282315688 | 0.066620297 | 0.55460552 |
| DBIL | O75636 | 0.281848905 | 0.067090857 | 0.557108892 |
| UA | P01019 | 0.281496583 | 0.067447772 | 0.558596632 |
| CO2 | Q15582 | 0.280835689 | 0.068121336 | 0.558596632 |
| P | A0A096LPE2 | 0.280813216 | 0.068144334 | 0.558596632 |
| WLC | A0A075B6S5 | -0.280542462 | 0.068421886 | 0.558596632 |
| INR | O75882 | 0.280495205 | 0.06847042 | 0.558596632 |
| UA | P30041 | 0.280427891 | 0.068539602 | 0.558596632 |
| FIB | H3BTN5 | -0.280188266 | 0.068786324 | 0.558596632 |
| UA | K7ERG9 | 0.280137426 | 0.06883876 | 0.558596632 |
| γ-GT | P69905 | 0.280070513 | 0.068907823 | 0.558596632 |
| ALP | P03950 | 0.279847309 | 0.06913859 | 0.558596632 |
| CRP | H3BTN5 | -0.279842698 | 0.069143364 | 0.558596632 |
| ALT | Q9Y5Y7 | -0.279288038 | 0.069719499 | 0.560529662 |
| AST | P55056 | 0.279283314 | 0.069724421 | 0.560529662 |
| AST | P01715 | 0.2788548 | 0.070172144 | 0.561019996 |
| WLL | C9J8S2 | 0.278680755 | 0.070354638 | 0.561019996 |
| UA | P01715 | 0.278275136 | 0.070781402 | 0.561019996 |
| INR | D6RE82 | -0.278225827 | 0.070833422 | 0.561019996 |
| ALP | P02750 | 0.277964622 | 0.071109483 | 0.561019996 |
| APTT | P01019 | -0.277903643 | 0.071174053 | 0.561019996 |
| LDH | Q6ZRK6 | -0.277655535 | 0.071437247 | 0.561019996 |
| BUN | P35527 | -0.277515577 | 0.071586054 | 0.561019996 |
| BUN | Q9H4B7 | -0.277508249 | 0.071593852 | 0.561019996 |
| BUN | P01817 | -0.277440062 | 0.071666444 | 0.561019996 |
| BUN | A0A0G2JI36 | 0.277310828 | 0.071804188 | 0.561019996 |
| Neutrophils | P13647 | -0.277213519 | 0.071908042 | 0.561019996 |
| DBIL | P30041 | 0.277045121 | 0.072088047 | 0.561019996 |
| P | P01817 | 0.276959213 | 0.072180012 | 0.561019996 |
| P | H3BTN5 | -0.276340807 | 0.072844756 | 0.564345769 |
| Lymphocytes | A0A0J9YXX1 | 0.275961037 | 0.073255368 | 0.564345769 |
| Ca | A0A0C4DH73 | -0.275896218 | 0.073325633 | 0.564345769 |
| INR | P02766 | -0.275653864 | 0.073588818 | 0.564345769 |
| Hematocrit | P01782 | 0.274979238 | 0.07432535 | 0.564345769 |
| BUN | A0A2R8Y3M9 | 0.274872571 | 0.074442335 | 0.564345769 |
| TBIL | A0A0G2JI36 | 0.274498038 | 0.074854241 | 0.564345769 |
| INR | A0A0C4DH33 | 0.274491715 | 0.074861211 | 0.564345769 |
| Platelets | Q5SRP5 | 0.274409761 | 0.074951588 | 0.564345769 |
| UA | E7EX29 | 0.274373344 | 0.074991775 | 0.564345769 |
| WLL | P02766 | 0.274367367 | 0.074998373 | 0.564345769 |
| CRP | A0A0G2JI36 | -0.274154464 | 0.075233674 | 0.564345769 |
| IBIL | A0A140T8Y3 | 0.27406948 | 0.07532776 | 0.564345769 |
| Basophils | P43121 | -0.273961127 | 0.075447853 | 0.564345769 |
| Monocytes | P32119 | -0.273937756 | 0.075473775 | 0.564345769 |
| PT | A0A075B6S5 | 0.273770074 | 0.075659972 | 0.564345769 |
| Eosinophils | O75882 | 0.273587618 | 0.075862984 | 0.564345769 |
| INR | A0A0J9YX35 | 0.273460132 | 0.076005086 | 0.564345769 |
| Creatine Kinase | P02042 | 0.27338293 | 0.076091241 | 0.564345769 |
| UA | P26038 | 0.273000629 | 0.076519006 | 0.564345769 |
| Platelets | A0A0C4DH33 | -0.272988185 | 0.076532962 | 0.564345769 |
| APTT | P01782 | 0.272919502 | 0.076610024 | 0.564345769 |
| FIB | P55056 | 0.272840602 | 0.076698624 | 0.564345769 |
| DBIL | A0A0C4DH36 | 0.272778949 | 0.076767913 | 0.564345769 |
| WLL | P30041 | 0.272241961 | 0.077373487 | 0.564345769 |
| Creatine Kinase | Q86UD1 | -0.272179094 | 0.077444629 | 0.564345769 |
| Creatine Kinase | P13647 | 0.272099087 | 0.077535239 | 0.564345769 |
| CK-MB activity | P02042 | 0.27205879 | 0.077580909 | 0.564345769 |
| Glucose | A0A0C4DH36 | -0.272044117 | 0.077597543 | 0.564345769 |
| CK-MB activity | P30041 | 0.271503395 | 0.078212498 | 0.564794786 |
| UA | P35527 | -0.271378412 | 0.078355182 | 0.564794786 |
| CK-MB activity | P02751 | -0.271299273 | 0.078445635 | 0.564794786 |
| γ-GT | P35527 | -0.271138534 | 0.078629604 | 0.564794786 |
| γ-GT | Q86YZ3 | -0.270942148 | 0.078854831 | 0.564794786 |
| P | P01019 | -0.27083815 | 0.078974306 | 0.564794786 |
| Hemoglobin | P02766 | 0.270786955 | 0.079033173 | 0.564794786 |
| INR | A0A075B6S9 | 0.270018082 | 0.079921393 | 0.564794786 |
| CRP | P01782 | -0.269788532 | 0.080188083 | 0.564794786 |
| BUN | P03951 | -0.269662075 | 0.080335297 | 0.564794786 |
| CRP | Q86UD1 | -0.269544199 | 0.080472712 | 0.564794786 |
| ALT | P02750 | -0.269383671 | 0.080660144 | 0.564794786 |
| CO2 | P13473 | -0.269275867 | 0.080786208 | 0.564794786 |
| WLL | P02763 | 0.269222979 | 0.08084811 | 0.564794786 |
| INR | A0A0J9YXX1 | -0.269148312 | 0.080935566 | 0.564794786 |
| FIB | A0A096LPE2 | 0.269063739 | 0.081034714 | 0.564794786 |
| Lymphocytes | A0A075B6S9 | -0.268905238 | 0.081220787 | 0.564794786 |
| ALT | P00915 | 0.26885443 | 0.081280504 | 0.564794786 |
| IBIL | P00915 | 0.268806659 | 0.081336683 | 0.564794786 |
| Monocytes | P69905 | -0.268643542 | 0.081528736 | 0.564794786 |
| IBIL | P02745 | 0.268504544 | 0.081692672 | 0.564794786 |
| Eosinophils | A0A140T8Y3 | 0.268329669 | 0.081899286 | 0.564794786 |
| ALP | P13647 | 0.268218105 | 0.082031313 | 0.564794786 |
| APTT | P55056 | -0.268010878 | 0.082276989 | 0.564794786 |
| AST | P03950 | 0.267841207 | 0.082478567 | 0.564794786 |
| UA | P01817 | -0.267602975 | 0.082762251 | 0.564794786 |
| Hemoglobin | P19823 | -0.267462561 | 0.08292981 | 0.564794786 |
| ALT | P01718 | -0.267359861 | 0.083052532 | 0.564794786 |
| FIB | Q5SRP5 | 0.26723589 | 0.08320086 | 0.564794786 |
| ALP | A0A087X0Q4 | 0.267160344 | 0.083291351 | 0.564794786 |
| DBIL | P02763 | -0.266656728 | 0.083896554 | 0.564794786 |
| CK-MB activity | A0A0B4J1Y8 | 0.266372473 | 0.084239657 | 0.564794786 |
| Neutrophils | P43121 | -0.266280378 | 0.084351053 | 0.564794786 |
| WLGG | P43121 | -0.266140002 | 0.084521068 | 0.564794786 |
| Albumin | A0A5H1ZRS9 | -0.266087625 | 0.084584571 | 0.564794786 |
| PT | C9J8S2 | -0.265951116 | 0.084750256 | 0.564794786 |
| INR | A0A0G2JI36 | 0.265710204 | 0.085043274 | 0.564794786 |
| TBIL | P02745 | 0.265669841 | 0.085092443 | 0.564794786 |
| BUN | K7ERG9 | 0.26550878 | 0.085288867 | 0.564794786 |
| PT | A0A0G2JI36 | 0.265276438 | 0.085572845 | 0.564794786 |
| ALP | P35527 | -0.265271484 | 0.085578908 | 0.564794786 |
| ALP | Q96HR3 | 0.265271484 | 0.085578908 | 0.564794786 |
| WBC | I3L1J2 | -0.265165368 | 0.085708858 | 0.564794786 |
| UA | P19652 | 0.265111187 | 0.085775268 | 0.564794786 |
| RBC | A0A0B4J1Y8 | -0.26504796 | 0.085852816 | 0.564794786 |
| Albumin | O75882 | 0.265030224 | 0.08587458 | 0.564794786 |
| Basophils | P00915 | -0.26502961 | 0.085875332 | 0.564794786 |
| Total protein | P03950 | 0.264840096 | 0.086108147 | 0.564794786 |
| Eosinophils | P19652 | 0.264735364 | 0.086237018 | 0.564794786 |
| WLL | P00915 | 0.264546262 | 0.086470085 | 0.564794786 |
| APTT | P03951 | -0.264461565 | 0.086574632 | 0.564794786 |
| Mg | P19823 | -0.264430221 | 0.086613347 | 0.564794786 |
| TT | P02745 | 0.26426859 | 0.086813201 | 0.564974803 |
| P | P37802 | -0.263440087 | 0.087843265 | 0.566012113 |
| γ-GT | Q92954 | 0.263266282 | 0.088060554 | 0.566012113 |
| Glucose | A0A0J9YX35 | -0.263207592 | 0.088134021 | 0.566012113 |
| Glucose | Q92954 | 0.263132066 | 0.088228634 | 0.566012113 |
| WLC | P0DP01 | -0.26302339 | 0.088364912 | 0.566012113 |
| APTT | P0DP01 | 0.262998907 | 0.088395637 | 0.566012113 |
| Platelets | P01718 | 0.262748325 | 0.088710571 | 0.566012113 |
| Total protein | Q86YZ3 | -0.262497154 | 0.089027118 | 0.566012113 |
| ALP | P01814 | -0.262475971 | 0.089053855 | 0.566012113 |
| RBC | P19652 | 0.262432509 | 0.089108732 | 0.566012113 |
| Basophils | I3L1J2 | -0.262396113 | 0.089154706 | 0.566012113 |
| γ-GT | P55056 | 0.262206556 | 0.089394448 | 0.566012113 |
| Neutrophils | P13473 | 0.262186145 | 0.089420292 | 0.566012113 |
| TT | P01814 | 0.261994986 | 0.08966262 | 0.566012113 |
| PT | Q9NZP8 | -0.261965726 | 0.089699757 | 0.566012113 |
| Glucose | Q86YZ3 | -0.261939048 | 0.089733628 | 0.566012113 |
| CK-MB activity | P00915 | 0.261729366 | 0.090000185 | 0.566425156 |
| TT | K7ERG9 | -0.261616052 | 0.090144491 | 0.566425156 |
| Total protein | P19652 | 0.261083006 | 0.090825731 | 0.569614526 |
| Neutrophils | P01718 | 0.260938314 | 0.091011336 | 0.569689281 |
| Ca | P03951 | 0.260623257 | 0.091416494 | 0.569891217 |
| WLC | P01817 | -0.2604645 | 0.09162118 | 0.569891217 |
| CK-MB activity | P13473 | 0.260438188 | 0.091655138 | 0.569891217 |
| IBIL | P02750 | -0.260347444 | 0.091772328 | 0.569891217 |
| CRP | A0A1W2PQU7 | -0.260120858 | 0.092065455 | 0.569891217 |
| Basophils | P01782 | -0.260104935 | 0.092086081 | 0.569891217 |
| Creatinine | Q86UD1 | -0.259640597 | 0.092689145 | 0.572543116 |
| CK-MB activity | A0A5H1ZRS9 | -0.259222962 | 0.093234157 | 0.574827132 |
| PT | P02763 | -0.259014639 | 0.093506941 | 0.575081177 |
| α-HBDH | P13647 | 0.258715134 | 0.093900202 | 0.575081177 |
| Albumin | P01817 | -0.258685812 | 0.09393877 | 0.575081177 |
| P | P02745 | -0.258596024 | 0.094056952 | 0.575081177 |
| γ-GT | P01718 | -0.258523866 | 0.09415201 | 0.575081177 |
| DBIL | P02745 | 0.258115852 | 0.094690912 | 0.577285869 |
| TBIL | A0A140T8Y3 | 0.257984456 | 0.094864964 | 0.577285869 |
| BUN | P13647 | 0.257504249 | 0.095503165 | 0.580093299 |
| CRP | P03950 | 0.256907284 | 0.096301149 | 0.582892374 |
| INR | P04264 | -0.256893667 | 0.096319411 | 0.582892374 |
| IBIL | P55056 | 0.256571009 | 0.096752915 | 0.584133691 |
| Platelets | A0A2R8Y7X9 | 0.256476104 | 0.09688071 | 0.584133691 |
| α-HBDH | Q15582 | -0.255618106 | 0.098041952 | 0.588266023 |
| BUN | P15169 | -0.255616388 | 0.098044287 | 0.588266023 |
| ALP | A0A0B4J1Y8 | 0.255572393 | 0.09810412 | 0.588266023 |
| Mg | P08185 | -0.255335459 | 0.098426831 | 0.589124096 |
| Monocytes | P00915 | -0.254954218 | 0.098947803 | 0.589359098 |
| Total protein | P43121 | 0.254795883 | 0.099164791 | 0.589359098 |
| Hematocrit | A0A0A0MS15 | 0.254663552 | 0.099346425 | 0.589359098 |
| CO2 | O75636 | 0.25454276 | 0.099512442 | 0.589359098 |
| APTT | P00918 | -0.254465211 | 0.099619137 | 0.589359098 |
| INR | Q5SRP5 | -0.253956495 | 0.100321237 | 0.589359098 |
| Creatine Kinase | P19823 | -0.253823212 | 0.100505813 | 0.589359098 |
| Monocytes | P01019 | -0.253819743 | 0.10051062 | 0.589359098 |
| Mg | P02766 | 0.253819665 | 0.100510729 | 0.589359098 |
| Glucose | A0A087X0Q4 | 0.253766861 | 0.100583929 | 0.589359098 |
| ALT | P0DP01 | 0.253705033 | 0.100669689 | 0.589359098 |
| Creatine Kinase | A0A075B6S9 | -0.253647585 | 0.100749426 | 0.589359098 |
| ALT | A0A0C4DH36 | 0.25350644 | 0.100945537 | 0.589359098 |
| LDH | P32119 | 0.253294571 | 0.101240464 | 0.589359098 |
| Hemoglobin | A0A0B4J1Y8 | -0.253116782 | 0.10148846 | 0.589359098 |
| Creatinine | Q96HR3 | 0.25294473 | 0.101751483 | 0.589359098 |
| UA | A0A0A0MS15 | 0.252803262 | 0.10192692 | 0.589359098 |
| Total protein | P01715 | 0.252755841 | 0.101993365 | 0.589359098 |
| Creatine Kinase | Q16880 | -0.25261489 | 0.102191058 | 0.589359098 |
| α-HBDH | A0A0J9YX35 | -0.252521078 | 0.102322797 | 0.589359098 |
| α-HBDH | P01817 | -0.252521078 | 0.102322797 | 0.589359098 |
| PT | P01814 | 0.252280107 | 0.102661787 | 0.589359098 |
| APTT | P69905 | -0.252227763 | 0.102735536 | 0.589359098 |
| UA | Q86UD1 | -0.252188306 | 0.102791155 | 0.589359098 |
| ALT | P35527 | -0.252069929 | 0.10295816 | 0.589359098 |
| Hematocrit | P00918 | 0.251650872 | 0.10355103 | 0.589490749 |
| Albumin | Q9H4B7 | -0.2515605 | 0.103679227 | 0.589490749 |
| LDH | I3L1J2 | 0.251452228 | 0.103832976 | 0.589490749 |
| CRP | P69905 | 0.251057413 | 0.104395102 | 0.589490749 |
| Platelets | I3L1J2 | -0.251056011 | 0.104397103 | 0.589490749 |
| PT | A0A0C4DH33 | 0.251003212 | 0.104472454 | 0.589490749 |
| Basophils | P19652 | 0.250665974 | 0.104954717 | 0.589490749 |
| Eosinophils | P15169 | -0.250359908 | 0.105393873 | 0.589490749 |
| Albumin | P04264 | -0.250302126 | 0.105476939 | 0.589490749 |
| Basophils | P02042 | -0.249927273 | 0.106017031 | 0.589490749 |
| ALP | A0A0A0MS15 | 0.249858387 | 0.106116511 | 0.589490749 |
| α-HBDH | P08185 | -0.249575124 | 0.106526329 | 0.589490749 |
| Hemoglobin | A0A0J9YX35 | -0.249480615 | 0.106663332 | 0.589490749 |
| LDH | P55056 | 0.249140963 | 0.107156809 | 0.589490749 |
| WLC | A0A087X0Q4 | -0.249013787 | 0.107342028 | 0.589490749 |
| Hemoglobin | A0A0A0MS15 | 0.24887618 | 0.107542715 | 0.589490749 |
| FIB | P02750 | 0.248744215 | 0.107735443 | 0.589490749 |
| UA | A0A5H1ZRS9 | -0.24872579 | 0.107762371 | 0.589490749 |
| ALP | P80748 | 0.248725071 | 0.107763422 | 0.589490749 |
| Creatine Kinase | P15169 | 0.248612322 | 0.107928327 | 0.589490749 |
| ALP | A0A140T8Y3 | 0.248419729 | 0.108210453 | 0.589490749 |
| Albumin | Q86YZ3 | -0.248397061 | 0.108243696 | 0.589490749 |
| Albumin | A0A0J9YX35 | -0.24833838 | 0.108329789 | 0.589490749 |
| RBC | P30041 | 0.248319189 | 0.108357956 | 0.589490749 |
| ALT | P00918 | 0.248246962 | 0.108464015 | 0.589490749 |
| Creatine Kinase | P00915 | 0.248234721 | 0.108481998 | 0.589490749 |
| Ca | P35527 | -0.248072209 | 0.108720952 | 0.589490749 |
| Neutrophils | P04264 | -0.24798943 | 0.108842824 | 0.589490749 |
| α-HBDH | P32119 | 0.247988842 | 0.108843689 | 0.589490749 |
| INR | P01814 | 0.247967444 | 0.108875209 | 0.589490749 |
| Creatinine | P02753 | 0.247810329 | 0.109071725 | 0.589490749 |
| TT | O75636 | 0.247747066 | 0.109200239 | 0.589490749 |
| CK-MB activity | Q8N1N4 | 0.247678312 | 0.109301794 | 0.589490749 |
| Monocytes | Q86UD1 | -0.247641331 | 0.109356447 | 0.589490749 |
| DBIL | P00915 | 0.247534236 | 0.109514838 | 0.589490749 |
| WLGG | Q16880 | -0.247528351 | 0.109523546 | 0.589490749 |
| Basophils | P00918 | -0.247358485 | 0.109775146 | 0.589490749 |
| Albumin | P00915 | 0.247205449 | 0.110002194 | 0.589490749 |
| Hemoglobin | P01782 | 0.246836211 | 0.110551477 | 0.589490749 |
| Eosinophils | A0A075B6S5 | -0.246803874 | 0.110599681 | 0.589490749 |
| RBC | A0A0J9YX35 | -0.246799838 | 0.110605698 | 0.589490749 |
| FIB | A0A0J9YVY3 | -0.246724168 | 0.110718566 | 0.589490749 |
| CO2 | P01019 | -0.246609548 | 0.110889695 | 0.589490749 |
| CK-MB activity | P02766 | 0.246387134 | 0.111222337 | 0.589490749 |
| α-HBDH | P19652 | 0.246327022 | 0.11131237 | 0.589490749 |
| CK-MB activity | P32119 | 0.246311182 | 0.111336104 | 0.589490749 |
| Neutrophils | Q6ZRK6 | 0.246211976 | 0.111484835 | 0.589490749 |
| Ca | Q86YZ3 | -0.246101313 | 0.111650923 | 0.589490749 |
| DBIL | P26038 | 0.246010356 | 0.111787575 | 0.589490749 |
| ALT | Q8N1N4 | -0.245189796 | 0.113026115 | 0.595065259 |
| INR | E7EX29 | 0.24467829 | 0.113803424 | 0.596590082 |
| Globin | P30041 | -0.24466387 | 0.113825396 | 0.596590082 |
| CK-MB activity | P69905 | 0.244640246 | 0.1138614 | 0.596590082 |
| Hemoglobin | P00918 | 0.244323877 | 0.114344381 | 0.597796124 |
| Globin | P04264 | -0.24416412 | 0.114588861 | 0.597796124 |
| WBC | Q86YZ3 | -0.244112771 | 0.114667525 | 0.597796124 |
| P | Q9NZP8 | -0.243860131 | 0.115055156 | 0.597796124 |
| INR | A0A0C4DH73 | -0.243504333 | 0.115602747 | 0.597796124 |
| Basophils | A0A075B6S5 | -0.243443117 | 0.11569716 | 0.597796124 |
| Platelets | P03951 | -0.243410625 | 0.115747295 | 0.597796124 |
| Lymphocytes | P19652 | 0.243335102 | 0.115863893 | 0.597796124 |
| INR | P0DP01 | 0.24328658 | 0.11593885 | 0.597796124 |
| Ca | P03950 | 0.243208247 | 0.11605994 | 0.597796124 |
| CRP | A0A075B6S5 | -0.242900313 | 0.116536876 | 0.597796124 |
| TBIL | Q15582 | -0.242788101 | 0.116711041 | 0.597796124 |
| INR | P03951 | 0.242747873 | 0.116773527 | 0.597796124 |
| Creatinine | A0A0B4J1V2 | 0.242307547 | 0.117459139 | 0.597796124 |
| γ-GT | P00918 | 0.242091202 | 0.117797115 | 0.597796124 |
| DBIL | P01782 | 0.242016679 | 0.117913704 | 0.597796124 |
| IBIL | Q16880 | -0.24191844 | 0.11806753 | 0.597796124 |
| Ca | A0A075B6S9 | -0.24188458 | 0.118120583 | 0.597796124 |
| CO2 | O75882 | 0.241774067 | 0.118293869 | 0.597796124 |
| UA | P02753 | 0.241703477 | 0.118404654 | 0.597796124 |
| WLC | P13647 | -0.24164141 | 0.118502129 | 0.597796124 |
| Globin | P04430 | 0.241348085 | 0.118963608 | 0.597796124 |
| TT | A0A0G2JI36 | 0.241305916 | 0.119030062 | 0.597796124 |
| Creatine Kinase | P00918 | 0.240996769 | 0.119518104 | 0.597796124 |
| TBIL | Q16880 | -0.240900169 | 0.119670913 | 0.597796124 |
| Monocytes | A0A087X0Q4 | -0.240811103 | 0.119811934 | 0.597796124 |
| BUN | P02753 | -0.240740043 | 0.119924536 | 0.597796124 |
| Globin | A0A075B6S5 | 0.240613466 | 0.120125309 | 0.597796124 |
| FIB | P01019 | 0.240435115 | 0.120408632 | 0.597796124 |
| Monocytes | A0A2R8Y7X9 | -0.240432945 | 0.120412083 | 0.597796124 |
| Glucose | P13473 | 0.240398785 | 0.120466407 | 0.597796124 |
| α-HBDH | P80748 | 0.240359578 | 0.12052878 | 0.597796124 |
| INR | A0A087X0Q4 | -0.240327203 | 0.120580304 | 0.597796124 |
| WLGG | P32119 | 0.240262043 | 0.120684051 | 0.597796124 |
| Lymphocytes | P55056 | 0.240087613 | 0.120962112 | 0.597796124 |
| TT | P01718 | -0.239869265 | 0.121310861 | 0.597796124 |
| APTT | Q9Y5Y7 | -0.239691892 | 0.121594722 | 0.597796124 |
| Creatine Kinase | I3L1J2 | 0.239402968 | 0.122058175 | 0.597796124 |
| RBC | P02745 | 0.239398864 | 0.122064769 | 0.597796124 |
| LDH | A0A0C4DH33 | 0.239370141 | 0.122110917 | 0.597796124 |
| INR | P08185 | 0.239343805 | 0.122153241 | 0.597796124 |
| AST | Q9H4B7 | -0.239333222 | 0.122170252 | 0.597796124 |
| ALP | Q15582 | -0.239280772 | 0.122254586 | 0.597796124 |
| BUN | A0A0J9YVY3 | -0.239276411 | 0.1222616 | 0.597796124 |
| Platelets | P13645 | 0.23925686 | 0.122293048 | 0.597796124 |
| UA | Q96HR3 | 0.239060671 | 0.122608961 | 0.598113718 |
| ALT | A0A1W2PQU7 | -0.238990116 | 0.122722723 | 0.598113718 |
| DBIL | O75882 | 0.238842194 | 0.122961487 | 0.598388245 |
| Mg | A0A0J9YX35 | -0.238661727 | 0.123253254 | 0.598858719 |
| CO2 | P04264 | -0.238298564 | 0.12384197 | 0.598858719 |
| Ca | P55056 | 0.238243076 | 0.123932108 | 0.598858719 |
| Creatinine | P19823 | -0.238145575 | 0.123940417 | 0.598858719 |
| BUN | P00918 | 0.237915605 | 0.124465064 | 0.598858719 |
| WLC | P30041 | 0.237912209 | 0.124470601 | 0.598858719 |
| Globin | P02766 | -0.237742724 | 0.124747121 | 0.598858719 |
| TBIL | P55056 | 0.237728443 | 0.124770443 | 0.598858719 |
| Creatinine | P80748 | 0.23769254 | 0.124673317 | 0.598858719 |
| Creatinine | P00918 | 0.237658961 | 0.124883952 | 0.598858719 |
| FIB | A0A075B6S5 | -0.237338087 | 0.125409163 | 0.60049935 |
| CRP | Q86YZ3 | -0.237036203 | 0.125904804 | 0.601953955 |
| BUN | Q92954 | 0.236737778 | 0.126396206 | 0.601953955 |
| Hemoglobin | P37802 | 0.236715882 | 0.126432318 | 0.601953955 |
| CRP | P02751 | 0.236706959 | 0.126447035 | 0.601953955 |
| LDH | A0A0C4DH36 | -0.236151498 | 0.127365792 | 0.605448981 |
| Neutrophils | A0A0G2JI36 | 0.235990103 | 0.127633681 | 0.605844389 |
| WLC | P13645 | -0.235680765 | 0.128148309 | 0.607408169 |
| ALP | A0A0C4DH36 | -0.235427497 | 0.128570812 | 0.607895206 |
| AST | D6RE82 | 0.235397017 | 0.12862173 | 0.607895206 |
| Ca | P19823 | 0.235218727 | 0.128919868 | 0.608220233 |
| ALT | P02042 | 0.235134217 | 0.129061366 | 0.608220233 |
| Neutrophils | A0A0B4J1Y8 | 0.234934301 | 0.129396557 | 0.608924975 |
| CK-MB activity | P01814 | -0.234690581 | 0.129806071 | 0.60915691 |
| Total protein | A0A5H1ZRS9 | -0.234574434 | 0.130001569 | 0.60915691 |
| PT | P04264 | -0.234573585 | 0.130002999 | 0.60915691 |
| WBC | P13473 | 0.23437913 | 0.1303308 | 0.609821719 |
| Basophils | P01814 | 0.234168312 | 0.130686882 | 0.60994731 |
| Hemoglobin | P15169 | -0.234143073 | 0.130729561 | 0.60994731 |
| Ca | A0A0G2JI36 | -0.233715019 | 0.131454976 | 0.612311047 |
| UA | D6RE82 | 0.233624042 | 0.131609539 | 0.612311047 |
| WLL | Q86YZ3 | 0.233482132 | 0.131850905 | 0.612565111 |
| Platelets | P02042 | 0.233366066 | 0.13204856 | 0.612615666 |
| CRP | O75636 | -0.233232639 | 0.132276052 | 0.61280431 |
| Globin | A0A1W2PQU7 | -0.232983337 | 0.132701895 | 0.613910035 |
| Mg | Q15582 | -0.232219604 | 0.134012818 | 0.6147144 |
| INR | P13473 | -0.232081793 | 0.134250389 | 0.6147144 |
| Lymphocytes | P03951 | -0.232006652 | 0.134380057 | 0.6147144 |
| Platelets | Q92954 | 0.232006652 | 0.134380057 | 0.6147144 |
| ALT | Q6ZRK6 | -0.231970786 | 0.134441982 | 0.6147144 |
| Albumin | P13645 | -0.231948651 | 0.13448021 | 0.6147144 |
| α-HBDH | A0A2R8Y3M9 | 0.231823867 | 0.134695869 | 0.6147144 |
| AST | H3BTN5 | -0.231464866 | 0.135317751 | 0.6147144 |
| Lymphocytes | P01718 | 0.231344293 | 0.135527092 | 0.6147144 |
| CRP | P01715 | 0.231333509 | 0.135545829 | 0.6147144 |
| WLC | I3L1J2 | -0.231309304 | 0.135587887 | 0.6147144 |
| WBC | Q6ZRK6 | 0.230852443 | 0.136383551 | 0.6147144 |
| FIB | P01814 | -0.230615271 | 0.136797973 | 0.6147144 |
| FIB | P02745 | -0.230615271 | 0.136797973 | 0.6147144 |
| Hematocrit | P02766 | 0.230571715 | 0.136874182 | 0.6147144 |
| Ca | P19652 | 0.230530986 | 0.136945472 | 0.6147144 |
| FIB | A0A140T8Y3 | 0.230423104 | 0.13713444 | 0.6147144 |
| RBC | E7ENL6 | -0.230395018 | 0.137183668 | 0.6147144 |
| WBC | P13647 | -0.230301658 | 0.137347399 | 0.6147144 |
| CO2 | A0A0A0MS15 | -0.230289799 | 0.137368208 | 0.6147144 |
| CK-MB activity | A0A087X0Q4 | 0.230285385 | 0.137375952 | 0.6147144 |
| γ-GT | P01715 | 0.230279226 | 0.13738676 | 0.6147144 |
| UA | Q92954 | 0.230226149 | 0.137479929 | 0.6147144 |
| Globin | Q5SRP5 | -0.230172577 | 0.137574014 | 0.6147144 |
| Creatine Kinase | A0A0C4DH33 | 0.230005289 | 0.137868118 | 0.6147144 |
| CRP | P32119 | 0.229984905 | 0.137903987 | 0.6147144 |
| γ-GT | Q9H4B7 | -0.229966764 | 0.137935914 | 0.6147144 |
| Mg | A0A0J9YVY3 | -0.229756947 | 0.138305582 | 0.615525521 |
| Total protein | D6RE82 | 0.229589916 | 0.138600392 | 0.616001743 |
| WLGG | Q86YZ3 | 0.22945828 | 0.138833059 | 0.616028966 |
| PT | A0A2R8Y7X9 | -0.229352431 | 0.139020356 | 0.616028966 |
| APTT | P43121 | -0.229267961 | 0.139169958 | 0.616028966 |
| Monocytes | Q86YZ3 | -0.228745699 | 0.140097571 | 0.616886255 |
| Albumin | A0A2R8Y7X9 | -0.228700917 | 0.140177324 | 0.616886255 |
| WLL | Q16880 | -0.228691436 | 0.140194212 | 0.616886255 |
| APTT | Q86YZ3 | -0.22864331 | 0.140279964 | 0.616886255 |
| ALP | A0A0B4J1V2 | 0.228560676 | 0.140427294 | 0.616886255 |
| TBIL | A0A2R8Y3M9 | -0.228515333 | 0.140508188 | 0.616886255 |
| Monocytes | P01814 | 0.228407516 | 0.140700671 | 0.616886255 |
| Total protein | P26038 | -0.228313748 | 0.140868233 | 0.616886255 |
| Globin | E7EX29 | -0.228161533 | 0.141140551 | 0.617124537 |
| WLC | P00915 | 0.2280731 | 0.141298941 | 0.617124537 |
| γ-GT | P02745 | 0.227916842 | 0.141579132 | 0.617175284 |
| Basophils | Q5SRP5 | 0.227856809 | 0.141686887 | 0.617175284 |
| WBC | E7EX29 | 0.227603317 | 0.14214256 | 0.617628211 |
| Creatine Kinase | P03951 | -0.227391161 | 0.14252476 | 0.617628211 |
| Mg | P01718 | 0.227295228 | 0.142697832 | 0.617628211 |
| WLL | P43121 | -0.227276092 | 0.142732373 | 0.617628211 |
| Creatinine | P02745 | 0.227121715 | 0.142721626 | 0.617628211 |
| P | P02042 | -0.226932747 | 0.143353187 | 0.619497304 |
| WLL | Q9NZP8 | 0.226820749 | 0.143556124 | 0.619558009 |
| ALP | D6R934 | -0.226436524 | 0.144253943 | 0.620111455 |
| WLC | P15169 | 0.22642608 | 0.144272947 | 0.620111455 |
| ALP | P02763 | 0.226285416 | 0.144529068 | 0.620111455 |
| Creatinine | P01019 | 0.226215645 | 0.144354836 | 0.620111455 |
| γ-GT | P03951 | 0.226100168 | 0.144866877 | 0.620111455 |
| P | P32119 | -0.226025923 | 0.145002431 | 0.620111455 |
| PT | P01817 | 0.226022999 | 0.14500777 | 0.620111455 |
| WLL | P01715 | -0.225619569 | 0.145745991 | 0.6211684 |
| Total protein | O75636 | -0.225587197 | 0.145805347 | 0.6211684 |
| γ-GT | C9JV77 | 0.22549461 | 0.145975209 | 0.6211684 |
| Mg | P02763 | 0.225474322 | 0.14601245 | 0.6211684 |
| WLL | P04430 | -0.22532594 | 0.14628503 | 0.621199003 |
| Eosinophils | P13473 | 0.225240691 | 0.146441803 | 0.621199003 |
| WLGG | P69905 | 0.225097575 | 0.146705272 | 0.621199003 |
| α-HBDH | P35527 | -0.225025513 | 0.146838066 | 0.621199003 |
| Ca | Q6ZRK6 | -0.224915034 | 0.147041828 | 0.621199003 |
| WLGG | Q15582 | -0.224781649 | 0.147288112 | 0.621199003 |
| RBC | O75882 | 0.224747956 | 0.147350372 | 0.621199003 |
| Monocytes | A0A0A0MS15 | -0.224550303 | 0.147715992 | 0.621199003 |
| ALT | P43121 | 0.224452405 | 0.147897333 | 0.621199003 |
| Lymphocytes | P08185 | -0.224303306 | 0.148173829 | 0.621199003 |
| PT | P80748 | -0.224282615 | 0.14821223 | 0.621199003 |
| Basophils | P30041 | -0.224239471 | 0.148292323 | 0.621199003 |
| Eosinophils | H3BTN5 | 0.223824095 | 0.149065076 | 0.623639603 |
| Total protein | P32119 | 0.223472553 | 0.149721381 | 0.624897848 |
| WLL | A0A0J9YVY3 | -0.223458927 | 0.149746862 | 0.624897848 |
| DBIL | P0DJI8 | 0.223347684 | 0.149955013 | 0.624971336 |
| Hemoglobin | P19652 | 0.223112131 | 0.150396466 | 0.625945752 |
| Lymphocytes | Q9NZP8 | 0.223019415 | 0.150570487 | 0.625945752 |
| WBC | A0A5H1ZRS9 | -0.222826292 | 0.15093344 | 0.626660359 |
| PT | Q9Y5Y7 | -0.222088216 | 0.152326487 | 0.631644597 |
| WLC | A0A1W2PQU7 | -0.221720307 | 0.153024388 | 0.633258492 |
| RBC | A0A075B6S9 | -0.221467785 | 0.153504761 | 0.633258492 |
| CK-MB activity | P80748 | 0.221399043 | 0.15363572 | 0.633258492 |
| Albumin | P02763 | 0.221374632 | 0.153682243 | 0.633258492 |
| CO2 | A0A140T8Y3 | -0.221313889 | 0.153798058 | 0.633258492 |
| Creatine Kinase | P04264 | 0.221274029 | 0.153874091 | 0.633258492 |
| WLC | A0A0C4DH36 | -0.220543864 | 0.155271744 | 0.638209673 |
| γ-GT | Q9Y5Y7 | -0.220120284 | 0.156086786 | 0.640756768 |
| Mg | E7ENL6 | 0.219771742 | 0.156759782 | 0.642109281 |
| Basophils | A0A0J9YX35 | 0.219722597 | 0.156854844 | 0.642109281 |
| Albumin | A0A0B4J1Y8 | -0.219645765 | 0.15700355 | 0.642109281 |
| Ca | O75636 | 0.219189678 | 0.1578884 | 0.643935235 |
| CK-MB activity | P26038 | 0.218980572 | 0.158295299 | 0.643935235 |
| Creatine Kinase | A0A0J9YVY3 | -0.218977398 | 0.158301481 | 0.643935235 |
| Creatine Kinase | A0A0J9YXX1 | 0.218932904 | 0.158388162 | 0.643935235 |
| Mg | P03950 | 0.218910601 | 0.158431626 | 0.643935235 |
| Platelets | P02751 | 0.218563558 | 0.159109046 | 0.644573066 |
| Mg | Q9NZP8 | 0.218425881 | 0.159378374 | 0.644573066 |
| Ca | A0A0C4DH33 | 0.218366226 | 0.159495174 | 0.644573066 |
| Monocytes | P04264 | -0.21834851 | 0.159529874 | 0.644573066 |
| Globin | O75636 | -0.218327446 | 0.159571137 | 0.644573066 |
| WLC | P01715 | -0.218196308 | 0.159828208 | 0.644817369 |
| TT | P35908 | -0.218038639 | 0.160137685 | 0.645272243 |
| RBC | P01019 | 0.217649062 | 0.160904227 | 0.647187161 |
| CO2 | Q8N1N4 | -0.21759666 | 0.161007538 | 0.647187161 |
| Monocytes | D6R934 | 0.217289667 | 0.161613746 | 0.648828749 |
| Creatine Kinase | P01718 | -0.216789485 | 0.162604989 | 0.650905079 |
| WBC | A0A0C4DH33 | -0.216567243 | 0.163046835 | 0.650905079 |
| Monocytes | P08185 | 0.216533351 | 0.163114294 | 0.650905079 |
| Monocytes | P02042 | -0.216457719 | 0.163264903 | 0.650905079 |
| Monocytes | Q15582 | 0.216306456 | 0.163566424 | 0.650905079 |
| Globin | C9J8S2 | 0.216287 | 0.163605235 | 0.650905079 |
| ALT | Q92954 | 0.216232753 | 0.163713487 | 0.650905079 |
| WLC | P32119 | 0.21623024 | 0.163718503 | 0.650905079 |
| INR | C9JV77 | 0.216044851 | 0.164088849 | 0.651587682 |
| UA | P02042 | 0.215728471 | 0.164722275 | 0.651652692 |
| CRP | P01019 | 0.21570998 | 0.164759351 | 0.651652692 |
| Glucose | P19823 | -0.215626306 | 0.164927199 | 0.651652692 |
| Eosinophils | P0DP01 | -0.21552648 | 0.165127608 | 0.651652692 |
| CO2 | P01718 | -0.215447695 | 0.165285901 | 0.651652692 |
| CK-MB activity | A0A140T8Y3 | 0.215246799 | 0.165690034 | 0.651652692 |
| UA | P69905 | 0.21519991 | 0.165784462 | 0.651652692 |
| Eosinophils | A0A0B4J1V2 | 0.215185996 | 0.165812489 | 0.651652692 |
| WLGG | A0A5H1ZRS9 | -0.215145893 | 0.165893292 | 0.651652692 |
| CO2 | E7ENL6 | 0.214903029 | 0.166383239 | 0.652795483 |
| α-HBDH | P00915 | 0.214676908 | 0.166840351 | 0.653216771 |
| CK-MB activity | K7ERG9 | 0.214563395 | 0.167070164 | 0.653216771 |
| P | A0A0J9YX35 | 0.214539483 | 0.167118603 | 0.653216771 |
| INR | P13647 | -0.214456286 | 0.167287222 | 0.653216771 |
| Platelets | E7EX29 | 0.214217152 | 0.167772564 | 0.654332949 |
| CO2 | P15169 | 0.213894495 | 0.168429039 | 0.655435189 |
| WLL | A0A2R8Y3M9 | 0.213881834 | 0.168454837 | 0.655435189 |
| α-HBDH | P00918 | 0.213706197 | 0.168813004 | 0.656050537 |
| TBIL | P01718 | -0.21344795 | 0.169340633 | 0.657322219 |
| CRP | P02750 | 0.213217532 | 0.169812406 | 0.657436235 |
| UA | P32119 | 0.213161174 | 0.169927944 | 0.657436235 |
| Mg | P01019 | 0.213044813 | 0.170166667 | 0.657436235 |
| RBC | P15169 | -0.213042333 | 0.170171757 | 0.657436235 |
| P | P02751 | -0.212876972 | 0.170511431 | 0.657973521 |
| CO2 | P03950 | -0.212615319 | 0.171049901 | 0.658709062 |
| Creatine Kinase | D6R934 | -0.212589212 | 0.171103695 | 0.658709062 |
| Eosinophils | Q86UD1 | -0.21248614 | 0.171316196 | 0.658753956 |
| Ca | P43121 | 0.21227333 | 0.171755545 | 0.659670009 |
| BUN | P02042 | 0.211893526 | 0.172541672 | 0.660924548 |
| RBC | P00918 | 0.211819849 | 0.172694471 | 0.660924548 |
| Monocytes | P00918 | -0.211749069 | 0.172841351 | 0.660924548 |
| WBC | O75636 | -0.211726507 | 0.17288819 | 0.660924548 |
| Glucose | P01817 | -0.211396858 | 0.173573591 | 0.662462492 |
| WLC | P02763 | 0.211289179 | 0.173797899 | 0.662462492 |
| CRP | C9J8S2 | 0.211181622 | 0.174022159 | 0.662462492 |
| INR | P0DJI8 | 0.211127864 | 0.174134326 | 0.662462492 |
| Neutrophils | P04430 | 0.211039852 | 0.174318074 | 0.662462492 |
| IBIL | P80748 | 0.210951671 | 0.174502315 | 0.662462492 |
| WLL | Q9H4B7 | -0.210547314 | 0.175348954 | 0.664440441 |
| ALP | A0A0J9YVY3 | -0.210443916 | 0.175565921 | 0.664440441 |
| FIB | P02751 | 0.210371284 | 0.175718444 | 0.664440441 |
| ALT | O75636 | 0.21025989 | 0.175952551 | 0.664440441 |
| INR | A0A0C4DH36 | 0.210220112 | 0.176036202 | 0.664440441 |
| Ca | A0A0C4DH36 | -0.210116632 | 0.176253952 | 0.664497658 |
| Neutrophils | P02753 | -0.209779122 | 0.176965506 | 0.666414307 |
| Eosinophils | Q92954 | 0.20957901 | 0.177388362 | 0.666486465 |
| CK-MB activity | P35908 | 0.209474635 | 0.177609205 | 0.666486465 |
| WLGG | P01782 | -0.209459217 | 0.177641843 | 0.666486465 |
| UA | P02750 | -0.209385737 | 0.177797456 | 0.666486465 |
| CRP | A0A2R8Y7X9 | 0.209063454 | 0.178481122 | 0.667074577 |
| Creatine Kinase | P32119 | 0.209039765 | 0.178531447 | 0.667074577 |
| ALT | P37802 | 0.208857712 | 0.178918549 | 0.667074577 |
| PT | P55056 | -0.20877049 | 0.179104224 | 0.667074577 |
| AST | P19652 | 0.208762575 | 0.17912108 | 0.667074577 |
| WLL | P02750 | 0.208737446 | 0.179174604 | 0.667074577 |
| P | P01718 | 0.208551861 | 0.179570239 | 0.667789552 |
| TBIL | A0A075B6S5 | 0.208427732 | 0.179835209 | 0.668017537 |
| CO2 | P0DJI8 | 0.208152361 | 0.180424027 | 0.668690178 |
| Creatinine | P55056 | 0.20794322 | 0.180328627 | 0.668690178 |
| ALP | P01817 | -0.207774589 | 0.181234045 | 0.66954027 |
| Creatine Kinase | P13645 | 0.207529362 | 0.181761248 | 0.66954027 |
| UA | P00918 | 0.207500033 | 0.181824375 | 0.66954027 |
| PT | A0A0J9YXX1 | -0.207484119 | 0.181858634 | 0.66954027 |
| Glucose | A0A0B4J1Y8 | 0.207477347 | 0.181873215 | 0.66954027 |
| Albumin | P01715 | 0.207475048 | 0.181878165 | 0.66954027 |
| Globin | C9JV77 | -0.207297755 | 0.182260177 | 0.670105075 |
| ALT | A0A0C4DH33 | 0.207092262 | 0.182703667 | 0.670105075 |
| PT | Q15582 | 0.207030106 | 0.182837965 | 0.670105075 |
| γ-GT | P04264 | -0.207025095 | 0.182848793 | 0.670105075 |
| CK-MB activity | P03951 | -0.206816327 | 0.183300397 | 0.671010381 |
| Globin | A0A0G2JI36 | -0.206475532 | 0.184039308 | 0.672964249 |
| Hematocrit | P02745 | 0.206253309 | 0.184522273 | 0.673309472 |
| WLL | Q8N1N4 | -0.206243197 | 0.184544273 | 0.673309472 |
| Monocytes | A0A075B6S5 | 0.205718028 | 0.185689327 | 0.67611609 |
| Neutrophils | A0A2R8Y3M9 | 0.205701342 | 0.185725792 | 0.67611609 |
| LDH | O75882 | 0.205490318 | 0.186187384 | 0.677045033 |
| Lymphocytes | A0A0J9YVY3 | -0.205335836 | 0.186525814 | 0.677524552 |
| UA | A0A1W2PQU7 | -0.20500623 | 0.187249361 | 0.678649617 |
| WBC | P04264 | -0.20500623 | 0.187249361 | 0.678649617 |
| TBIL | P01782 | 0.204878419 | 0.187530463 | 0.678918234 |
| IBIL | A0A0J9YX35 | -0.204456203 | 0.188461209 | 0.679005295 |
| Creatine Kinase | P26038 | 0.204402597 | 0.188579612 | 0.679005295 |
| PT | P02753 | 0.204381694 | 0.188625797 | 0.679005295 |
| Hematocrit | A0A075B6S9 | -0.204290477 | 0.188827435 | 0.679005295 |
| FIB | Q8N1N4 | -0.204177228 | 0.189077985 | 0.679005295 |
| TT | O75882 | 0.204093867 | 0.189262564 | 0.679005295 |
| DBIL | A0A0G2JI36 | 0.204093844 | 0.189262614 | 0.679005295 |
| LDH | A0A2R8Y7X9 | -0.204055435 | 0.189347701 | 0.679005295 |
| Ca | P02766 | -0.203992326 | 0.189487568 | 0.679005295 |
| WLGG | Q96HR3 | 0.203930505 | 0.18962465 | 0.679005295 |
| CK-MB activity | A0A075B6S5 | -0.203626358 | 0.19030009 | 0.679195266 |
| Albumin | P03950 | 0.203576606 | 0.190410739 | 0.679195266 |
| Creatinine | P01817 | -0.203412866 | 0.190171403 | 0.679195266 |
| Creatinine | P15169 | -0.203261854 | 0.190505989 | 0.679195266 |
| WBC | P04430 | 0.203236218 | 0.191168997 | 0.680819011 |
| BUN | H3BTN5 | -0.202959421 | 0.191787172 | 0.682279745 |
| Neutrophils | A0A087X0Q4 | 0.202756278 | 0.192241754 | 0.683155961 |
| CK-MB activity | O75882 | -0.20233518 | 0.193186492 | 0.685770232 |
| Lymphocytes | Q86YZ3 | -0.201761723 | 0.194478324 | 0.689132697 |
| Creatinine | A0A2R8Y3M9 | -0.201449713 | 0.194553926 | 0.689132697 |
| Albumin | P13473 | 0.201435055 | 0.195216935 | 0.690031158 |
| Creatinine | C9J8S2 | 0.201403093 | 0.195289307 | 0.690031158 |
| Basophils | A0A0A0MS15 | -0.201337143 | 0.195438703 | 0.690031158 |
| Hematocrit | O75882 | 0.201193269 | 0.195764897 | 0.690439638 |
| Lymphocytes | A0A0B4J1Y8 | -0.200936524 | 0.196347947 | 0.690896672 |
| TBIL | A0A0C4DH36 | 0.200876003 | 0.196485565 | 0.690896672 |
| WLC | P02745 | 0.20085805 | 0.196526401 | 0.690896672 |
| PT | P13473 | -0.200598249 | 0.197118016 | 0.692025054 |
| TT | P80748 | -0.200531887 | 0.197269337 | 0.692025054 |
| γ-GT | P37802 | 0.199960821 | 0.198574874 | 0.69586067 |
| Creatinine | P69905 | 0.19948656 | 0.199008006 | 0.696634215 |
| PT | Q96HR3 | -0.199463216 | 0.19971742 | 0.698297576 |
| AST | P13647 | 0.199379987 | 0.199908971 | 0.698297576 |
| RBC | Q92954 | 0.199222146 | 0.200272597 | 0.698779931 |
| Platelets | A0A075B6S5 | -0.198852056 | 0.201127014 | 0.698779931 |
| α-HBDH | D6RE82 | 0.198814082 | 0.201214827 | 0.698779931 |
| Albumin | Q96HR3 | 0.19879155 | 0.201266945 | 0.698779931 |
| Total protein | Q8N1N4 | -0.198625487 | 0.201651349 | 0.698779931 |
| APTT | Q9H4B7 | 0.198534772 | 0.201861553 | 0.698779931 |
| APTT | P04430 | 0.198533162 | 0.201865285 | 0.698779931 |
| Creatine Kinase | P02753 | 0.198391424 | 0.202194032 | 0.698779931 |
| Monocytes | P37802 | 0.198388766 | 0.202200201 | 0.698779931 |
| WLGG | P19652 | 0.198085866 | 0.202904024 | 0.698779931 |
| Glucose | P80748 | -0.197953256 | 0.203212697 | 0.698779931 |
| Lymphocytes | E7ENL6 | -0.197828835 | 0.20350261 | 0.698779931 |
| Creatinine | P35527 | -0.19782543 | 0.202833037 | 0.698779931 |
| Total protein | P13645 | -0.197794734 | 0.20358212 | 0.698779931 |
| γ-GT | H3BTN5 | 0.197782936 | 0.203609632 | 0.698779931 |
| Monocytes | P02751 | 0.197776707 | 0.203624159 | 0.698779931 |
| Lymphocytes | P26038 | 0.197553707 | 0.204144711 | 0.698779931 |
| INR | Q96HR3 | -0.197435946 | 0.20441998 | 0.698779931 |
| INR | P02753 | 0.1973603 | 0.20459694 | 0.698779931 |
| TT | P04430 | 0.197344521 | 0.204633864 | 0.698779931 |
| WBC | Q92954 | 0.197153321 | 0.205081678 | 0.698779931 |
| WLGG | C9JV77 | -0.197138087 | 0.205117387 | 0.698779931 |
| Hematocrit | P37802 | 0.197090304 | 0.205229421 | 0.698779931 |
| TT | C9J8S2 | -0.196999499 | 0.205442444 | 0.698779931 |
| CRP | P00918 | 0.19690909 | 0.205654691 | 0.698779931 |
| Mg | P37802 | -0.19669094 | 0.206167461 | 0.698779931 |
| Hematocrit | A0A2R8Y3M9 | -0.196661889 | 0.206235815 | 0.698779931 |
| DBIL | A0A0C4DH33 | 0.196636527 | 0.2062955 | 0.698779931 |
| UA | P19823 | -0.196549251 | 0.206500986 | 0.698779931 |
| WLC | C9J8S2 | 0.196485411 | 0.206651382 | 0.698779931 |
| Creatinine | P02766 | 0.196466324 | 0.206001102 | 0.698779931 |
| Monocytes | P13645 | -0.196112811 | 0.207530697 | 0.701030572 |
| Creatine Kinase | P01019 | 0.195748219 | 0.208393639 | 0.703221335 |
| CK-MB activity | Q5SRP5 | -0.19550738 | 0.208965047 | 0.704424824 |
| WLGG | P55056 | 0.195400491 | 0.209218997 | 0.704556788 |
| P | P43121 | 0.195169424 | 0.209768711 | 0.704917478 |
| Ca | A0A140T8Y3 | 0.195105375 | 0.209921265 | 0.704917478 |
| WLGG | D6RE82 | 0.195084565 | 0.209970846 | 0.704917478 |
| Ca | A0A096LPE2 | 0.194616844 | 0.211087393 | 0.707119659 |
| Albumin | Q92954 | 0.194561942 | 0.211218726 | 0.707119659 |
| IBIL | Q96HR3 | 0.194486414 | 0.211399494 | 0.707119659 |
| Monocytes | P03950 | 0.194406938 | 0.211589826 | 0.707119659 |
| Albumin | P15169 | -0.194335356 | 0.211761353 | 0.707119659 |
| DBIL | Q86UD1 | -0.194205169 | 0.212073564 | 0.707119659 |
| Monocytes | A0A140T8Y3 | -0.194179192 | 0.212135898 | 0.707119659 |
| PT | P0DP01 | 0.193644021 | 0.21342295 | 0.710379734 |
| Creatine Kinase | A0A0B4J1Y8 | -0.193414397 | 0.213976843 | 0.710379734 |
| Neutrophils | A0A1W2PQU7 | -0.193392488 | 0.214029744 | 0.710379734 |
| Eosinophils | P26038 | 0.193244907 | 0.21438632 | 0.710379734 |
| AST | A0A0B4J1V2 | 0.193220501 | 0.214445329 | 0.710379734 |
| TT | P00918 | -0.193201515 | 0.21449124 | 0.710379734 |
| γ-GT | P30041 | 0.193144161 | 0.214629975 | 0.710379734 |
| CRP | Q9H4B7 | -0.192990463 | 0.215002064 | 0.710893921 |
| Basophils | H3BTN5 | -0.192806549 | 0.21544789 | 0.711240737 |
| UA | A0A0C4DH73 | 0.192622796 | 0.215893965 | 0.711240737 |
| CO2 | A0A2R8Y3M9 | -0.192588155 | 0.215978131 | 0.711240737 |
| CRP | Q16880 | -0.192447139 | 0.216320987 | 0.711240737 |
| Glucose | I3L1J2 | -0.19239699 | 0.216443006 | 0.711240737 |
| Hemoglobin | P02745 | 0.192361492 | 0.216529406 | 0.711240737 |
| P | A0A0J9YXX1 | -0.192322291 | 0.216624846 | 0.711240737 |
| Eosinophils | Q6ZRK6 | 0.192105539 | 0.21715309 | 0.711422871 |
| BUN | P80748 | 0.191806684 | 0.217882884 | 0.711422871 |
| ALP | P08185 | -0.191757057 | 0.218004237 | 0.711422871 |
| LDH | A0A140T8Y3 | 0.191698242 | 0.218148117 | 0.711422871 |
| Albumin | H3BTN5 | 0.191543579 | 0.218526782 | 0.711422871 |
| ALT | A0A087X0Q4 | -0.191509637 | 0.218609945 | 0.711422871 |
| PT | P03951 | 0.191442313 | 0.218774964 | 0.711422871 |
| ALT | H3BTN5 | 0.191346576 | 0.219009772 | 0.711422871 |
| DBIL | A0A075B6S9 | 0.191300285 | 0.219123368 | 0.711422871 |
| Mg | O75636 | -0.191293172 | 0.219140827 | 0.711422871 |
| Eosinophils | P02751 | 0.19126922 | 0.219199626 | 0.711422871 |
| INR | P26038 | 0.191235228 | 0.219283086 | 0.711422871 |
| TBIL | P80748 | 0.191058755 | 0.219716743 | 0.711949802 |
| TBIL | P32119 | 0.190983237 | 0.219902496 | 0.711949802 |
| FIB | Q96HR3 | 0.190807132 | 0.22033609 | 0.711949802 |
| RBC | A0A5H1ZRS9 | -0.19076389 | 0.220442649 | 0.711949802 |
| Neutrophils | P02751 | -0.190598453 | 0.220850649 | 0.711949802 |
| FIB | A0A1W2PQU7 | -0.190504983 | 0.221081396 | 0.711949802 |
| Creatine Kinase | P35527 | 0.190386289 | 0.221374652 | 0.711949802 |
| ALT | A0A0B4J1V2 | 0.190307128 | 0.221570383 | 0.711949802 |
| ALT | A0A2R8Y7X9 | -0.190299944 | 0.221588154 | 0.711949802 |
| DBIL | P37802 | 0.190146555 | 0.221967778 | 0.711949802 |
| Neutrophils | P01019 | 0.190069852 | 0.222157781 | 0.711949802 |
| α-HBDH | A0A0C4DH36 | -0.189900685 | 0.222577224 | 0.711949802 |
| γ-GT | P00915 | 0.189766696 | 0.222909831 | 0.711949802 |
| α-HBDH | A0A0C4DH73 | 0.18974961 | 0.222952268 | 0.711949802 |
| Glucose | P0DJI8 | 0.189720939 | 0.223023495 | 0.711949802 |
| Hemoglobin | P80748 | 0.189717088 | 0.223033062 | 0.711949802 |
| Eosinophils | A0A2R8Y3M9 | 0.189453373 | 0.223688945 | 0.711949802 |
| RBC | C9J8S2 | -0.189433651 | 0.223738047 | 0.711949802 |
| Albumin | P69905 | 0.189274933 | 0.224133492 | 0.711949802 |
| PT | A0A0C4DH36 | 0.189247915 | 0.224200856 | 0.711949802 |
| WLGG | P01817 | -0.189239926 | 0.224220776 | 0.711949802 |
| Creatinine | P19652 | 0.189066747 | 0.223862716 | 0.711949802 |
| Monocytes | P02750 | -0.189003438 | 0.224811034 | 0.713133649 |
| INR | P55056 | -0.188887953 | 0.225099667 | 0.713172885 |
| TT | A0A2R8Y7X9 | -0.188709145 | 0.225547062 | 0.713172885 |
| PT | O75882 | 0.188642563 | 0.225713812 | 0.713172885 |
| Hematocrit | P19823 | -0.188580928 | 0.225868251 | 0.713172885 |
| APTT | O75636 | 0.188491167 | 0.226093293 | 0.713172885 |
| Lymphocytes | P13473 | 0.188354359 | 0.226436583 | 0.713172885 |
| CK-MB activity | D6RE82 | 0.188284126 | 0.226612956 | 0.713172885 |
| DBIL | P19652 | -0.188277185 | 0.226630392 | 0.713172885 |
| CRP | P01814 | -0.188217531 | 0.22678028 | 0.713172885 |
| WLC | P02750 | 0.187995606 | 0.227338497 | 0.714243554 |
| UA | O75882 | 0.187865746 | 0.227665575 | 0.714586685 |
| CK-MB activity | Q6ZRK6 | -0.187729161 | 0.228009939 | 0.71484051 |
| Ca | C9JV77 | 0.187660842 | 0.228182321 | 0.71484051 |
| INR | P69905 | -0.187450679 | 0.228713166 | 0.715037037 |
| LDH | P0DJI8 | 0.187289963 | 0.229119687 | 0.715037037 |
| DBIL | Q16880 | -0.187219023 | 0.22929928 | 0.715037037 |
| Creatine Kinase | A0A0C4DH36 | -0.187214443 | 0.22931088 | 0.715037037 |
| LDH | P26038 | 0.187204899 | 0.22933505 | 0.715037037 |
| WLL | P02751 | 0.187068659 | 0.229680268 | 0.715071879 |
| WLGG | A0A087X0Q4 | 0.187028441 | 0.229782244 | 0.715071879 |
| Basophils | C9J8S2 | 0.186908869 | 0.230085613 | 0.715337261 |
| FIB | A0A2R8Y7X9 | 0.186199359 | 0.23189137 | 0.719949768 |
| TBIL | A0A0J9YX35 | -0.186074613 | 0.232209855 | 0.719949768 |
| α-HBDH | Q16880 | -0.185972747 | 0.232470149 | 0.719949768 |
| γ-GT | D6RE82 | 0.185906265 | 0.232640136 | 0.719949768 |
| WLGG | P01715 | -0.185895881 | 0.232666693 | 0.719949768 |
| Glucose | A0A0C4DH33 | -0.185611787 | 0.233394098 | 0.720771534 |
| CK-MB activity | A0A0C4DH33 | -0.18559485 | 0.233437515 | 0.720771534 |
| PT | P00918 | -0.185534792 | 0.233591506 | 0.720771534 |
| Hematocrit | A0A0J9YX35 | -0.185031347 | 0.234885102 | 0.722756056 |
| ALP | P03951 | -0.184730498 | 0.235660456 | 0.722756056 |
| IBIL | Q5SRP5 | -0.184580542 | 0.236047574 | 0.722756056 |
| Platelets | P02763 | 0.184578209 | 0.236053602 | 0.722756056 |
| FIB | Q92954 | 0.184537539 | 0.23615867 | 0.722756056 |
| APTT | P00915 | -0.18448875 | 0.236284755 | 0.722756056 |
| AST | P01019 | 0.18447378 | 0.236323453 | 0.722756056 |
| CRP | E7ENL6 | 0.184468195 | 0.236337888 | 0.722756056 |
| Platelets | P01814 | 0.18435164 | 0.236639335 | 0.722756056 |
| LDH | A0A0J9YVY3 | -0.184344218 | 0.236658538 | 0.722756056 |
| Glucose | A0A2R8Y3M9 | 0.184132026 | 0.237208031 | 0.723760316 |
| Creatinine | A0A075B6S5 | 0.184083359 | 0.236482146 | 0.722756056 |
| Total protein | Q9H4B7 | -0.183938226 | 0.237710651 | 0.724619828 |
| Monocytes | P13473 | -0.183709224 | 0.238305501 | 0.725758629 |
| WLGG | A0A075B6S9 | -0.183603444 | 0.238580614 | 0.725896987 |
| Hemoglobin | A0A096LPE2 | -0.183521628 | 0.238793552 | 0.725896987 |
| TBIL | Q96HR3 | 0.183129439 | 0.23981606 | 0.728050999 |
| WLC | P0DJI8 | 0.183054545 | 0.24001166 | 0.728050999 |
| WLGG | P13473 | 0.182921398 | 0.240359666 | 0.728050999 |
| RBC | P55056 | 0.182909794 | 0.24039001 | 0.728050999 |
| WLL | A0A0C4DH36 | -0.182703724 | 0.240929337 | 0.728443857 |
| Hemoglobin | P02750 | -0.182690529 | 0.240963898 | 0.728443857 |
| Basophils | A0A2R8Y3M9 | 0.182541299 | 0.241355013 | 0.728566761 |
| WLC | P02766 | 0.182505538 | 0.241448802 | 0.728566761 |
| WLL | Q86UD1 | -0.182353947 | 0.241846647 | 0.729096509 |
| IBIL | P01782 | 0.181797591 | 0.243310595 | 0.732436706 |
| Ca | E7EX29 | 0.181763224 | 0.243401223 | 0.732436706 |
| UA | Q86YZ3 | -0.181644565 | 0.243714305 | 0.732706618 |
| Neutrophils | A0A0C4DH36 | -0.181310177 | 0.244598054 | 0.734566149 |
| CK-MB activity | Q86YZ3 | 0.181241158 | 0.244780732 | 0.734566149 |
| ALP | Q86YZ3 | 0.180739687 | 0.246110786 | 0.737882429 |
| Creatine Kinase | A0A1W2PQU7 | 0.18064419 | 0.246364625 | 0.737896718 |
| LDH | O75636 | 0.18056867 | 0.246565489 | 0.737896718 |
| IBIL | P01019 | 0.180438075 | 0.246913099 | 0.738263413 |
| P | A0A0B4J1Y8 | 0.180238107 | 0.247446 | 0.738651571 |
| BUN | A0A0C4DH33 | -0.180184263 | 0.247589624 | 0.738651571 |
| CRP | P03951 | 0.18013596 | 0.247718515 | 0.738651571 |
| WLGG | A0A0J9YX35 | -0.179288244 | 0.24998791 | 0.743510852 |
| LDH | P00915 | 0.179209307 | 0.250199939 | 0.743510852 |
| Creatine Kinase | P02750 | -0.179209307 | 0.250199939 | 0.743510852 |
| Lymphocytes | P19823 | -0.179140553 | 0.250384715 | 0.743510852 |
| P | P0DP01 | 0.179104534 | 0.250481552 | 0.743510852 |
| ALT | P0DJI8 | 0.178959065 | 0.250872903 | 0.743978925 |
| Total protein | A0A0B4J1V2 | 0.178845213 | 0.251179481 | 0.743978925 |
| Monocytes | A0A2R8Y3M9 | -0.178793168 | 0.25131971 | 0.743978925 |
| Glucose | A0A075B6S5 | -0.178694164 | 0.251586611 | 0.744097461 |
| FIB | P01718 | 0.178570758 | 0.251919562 | 0.744410959 |
| UA | P15169 | -0.178276136 | 0.252715656 | 0.74493813 |
| DBIL | A0A0C4DH73 | -0.178149067 | 0.253059526 | 0.74493813 |
| AST | P69905 | 0.178117833 | 0.253144097 | 0.74493813 |
| Mg | Q8N1N4 | -0.178105767 | 0.253176775 | 0.74493813 |
| P | A0A075B6S5 | 0.178039811 | 0.253355443 | 0.74493813 |
| Creatine Kinase | O75636 | 0.178000985 | 0.253460656 | 0.74493813 |
| P | P80748 | -0.177737536 | 0.254175352 | 0.7451831 |
| WLC | A0A0B4J1Y8 | -0.177728046 | 0.254201123 | 0.7451831 |
| IBIL | A0A5H1ZRS9 | -0.177719041 | 0.254225576 | 0.7451831 |
| Creatine Kinase | D6RE82 | -0.177094743 | 0.25592481 | 0.748155015 |
| Platelets | P02753 | -0.177025909 | 0.256112629 | 0.748155015 |
| WLC | Q96HR3 | 0.17701547 | 0.25614112 | 0.748155015 |
| INR | A0A096LPE2 | 0.177011537 | 0.256151854 | 0.748155015 |
| WBC | Q16880 | -0.17684147 | 0.256616339 | 0.74851492 |
| Lymphocytes | P01817 | -0.17679934 | 0.256731489 | 0.74851492 |
| IBIL | P32119 | 0.176586111 | 0.257314825 | 0.749549402 |
| Albumin | Q5SRP5 | -0.176471757 | 0.257628029 | 0.74979586 |
| Mg | P0DP01 | -0.17629347 | 0.25811685 | 0.750552543 |
| Hematocrit | P01718 | -0.176112595 | 0.258613396 | 0.750631373 |
| IBIL | D6R934 | -0.175830824 | 0.259388201 | 0.750631373 |
| APTT | A0A075B6S5 | 0.175728744 | 0.259669277 | 0.750631373 |
| APTT | Q92954 | -0.175728744 | 0.259669277 | 0.750631373 |
| α-HBDH | P04264 | 0.175699679 | 0.259749344 | 0.750631373 |
| Albumin | P01814 | -0.175679766 | 0.259804209 | 0.750631373 |
| TT | Q16880 | -0.175673815 | 0.259820609 | 0.750631373 |
| α-HBDH | A0A096LPE2 | -0.175473067 | 0.260374173 | 0.750631373 |
| LDH | D6R934 | -0.1754333 | 0.260483925 | 0.750631373 |
| P | Q96HR3 | -0.175394907 | 0.260589914 | 0.750631373 |
| CO2 | Q96HR3 | -0.175361753 | 0.260681463 | 0.750631373 |
| CO2 | D6R934 | 0.175286198 | 0.260890172 | 0.750631373 |
| ALT | P35908 | -0.175178772 | 0.261187116 | 0.750827117 |
| LDH | Q5SRP5 | -0.174991577 | 0.261705089 | 0.751375391 |
| FIB | A0A0C4DH36 | -0.174944306 | 0.261835998 | 0.751375391 |
| Creatine Kinase | P01817 | -0.174753619 | 0.262364514 | 0.751454832 |
| WBC | A0A1W2PQU7 | -0.174727225 | 0.262437724 | 0.751454832 |
| α-HBDH | P55056 | 0.17456662 | 0.262883495 | 0.751454832 |
| Albumin | D6RE82 | 0.174546836 | 0.262938443 | 0.751454832 |
| DBIL | A0A2R8Y7X9 | -0.174521084 | 0.263009975 | 0.751454832 |
| Creatinine | A0A0J9YVY3 | -0.174348866 | 0.263488692 | 0.751454832 |
| Glucose | A0A2R8Y7X9 | -0.174313665 | 0.263586611 | 0.751454832 |
| WBC | P02753 | -0.174274172 | 0.263696497 | 0.751454832 |
| DBIL | P02753 | 0.174143169 | 0.264061226 | 0.751832547 |
| Mg | P04430 | -0.173977898 | 0.26452184 | 0.751832547 |
| PT | A0A0J9YX35 | 0.173811459 | 0.264986244 | 0.751832547 |
| Albumin | A0A0C4DH36 | -0.173791549 | 0.265041836 | 0.751832547 |
| LDH | P01715 | -0.173772822 | 0.265094128 | 0.751832547 |
| Glucose | P55056 | 0.173709458 | 0.26527112 | 0.751832547 |
| BUN | Q9NZP8 | 0.173607704 | 0.26555551 | 0.751832547 |
| Mg | P04264 | -0.173558385 | 0.26569342 | 0.751832547 |
| Hemoglobin | P01718 | -0.173457186 | 0.265976557 | 0.751832547 |
| AST | Q86YZ3 | -0.173405509 | 0.266121216 | 0.751832547 |
| TT | P03950 | 0.173056408 | 0.267099814 | 0.75394784 |
| CRP | A0A5H1ZRS9 | -0.172809676 | 0.267792892 | 0.755194131 |
| γ-GT | P02763 | 0.172735381 | 0.26800182 | 0.755194131 |
| Monocytes | A0A1W2PQU7 | -0.17236448 | 0.269046464 | 0.756633449 |
| APTT | P32119 | -0.172330466 | 0.2691424 | 0.756633449 |
| Monocytes | C9J8S2 | 0.172308401 | 0.269204645 | 0.756633449 |
| CK-MB activity | P15169 | -0.172106426 | 0.26977486 | 0.757275718 |
| Glucose | P02042 | -0.172047889 | 0.269940269 | 0.757275718 |
| Hematocrit | Q6ZRK6 | -0.171982264 | 0.27012579 | 0.757275718 |
| Creatine Kinase | P35908 | 0.171808333 | 0.270617892 | 0.757677088 |
| PT | P0DJI8 | 0.171768399 | 0.27073096 | 0.757677088 |
| AST | A0A0G2JI36 | 0.171529497 | 0.271408036 | 0.75871719 |
| ALT | Q15582 | 0.171474085 | 0.271565238 | 0.75871719 |
| Albumin | P01718 | -0.171314546 | 0.272018185 | 0.759335869 |
| Total protein | Q15582 | -0.170908546 | 0.273173098 | 0.761911363 |
| WLGG | P02753 | -0.170600267 | 0.274052187 | 0.763450815 |
| CO2 | C9J8S2 | -0.1705518 | 0.274190567 | 0.763450815 |
| Neutrophils | P19823 | -0.170285068 | 0.274952932 | 0.763999623 |
| Ca | P01814 | -0.170119619 | 0.275426514 | 0.763999623 |
| Monocytes | Q96HR3 | -0.169944268 | 0.275929025 | 0.763999623 |
| Hemoglobin | Q8N1N4 | -0.169921836 | 0.275993351 | 0.763999623 |
| AST | P00918 | 0.169798564 | 0.276347032 | 0.763999623 |
| γ-GT | P02766 | 0.169783286 | 0.276390885 | 0.763999623 |
| Creatinine | Q9Y5Y7 | -0.169435216 | 0.276371662 | 0.763999623 |
| Albumin | P02753 | 0.169335355 | 0.277678682 | 0.763999623 |
| PT | A0A075B6S9 | 0.169318921 | 0.277726005 | 0.763999623 |
| Hematocrit | P19652 | 0.16924704 | 0.277933051 | 0.763999623 |
| Glucose | P02750 | 0.169177907 | 0.278132277 | 0.763999623 |
| ALT | P08185 | 0.168979092 | 0.27870574 | 0.763999623 |
| Monocytes | P01715 | -0.168954463 | 0.278776833 | 0.763999623 |
| Ca | Q15582 | 0.16890988 | 0.278905557 | 0.763999623 |
| WBC | A0A0J9YXX1 | -0.168686526 | 0.279551026 | 0.763999623 |
| Total protein | P04430 | 0.168563644 | 0.279906558 | 0.763999623 |
| APTT | P02750 | -0.168479084 | 0.280151385 | 0.763999623 |
| BUN | P03950 | -0.168224957 | 0.280888004 | 0.763999623 |
| IBIL | A0A2R8Y3M9 | -0.168202425 | 0.280953378 | 0.763999623 |
| RBC | P37802 | 0.168081743 | 0.281303685 | 0.763999623 |
| Platelets | A0A0J9YVY3 | -0.168073364 | 0.281328019 | 0.763999623 |
| Lymphocytes | Q16880 | -0.167887626 | 0.281867755 | 0.763999623 |
| FIB | A0A0G2JI36 | -0.167857345 | 0.281955812 | 0.763999623 |
| Eosinophils | A0A0J9YX35 | 0.167738869 | 0.282300517 | 0.763999623 |
| Glucose | P32119 | 0.167591864 | 0.282728606 | 0.763999623 |
| CK-MB activity | A0A0C4DH36 | -0.167549327 | 0.282852556 | 0.763999623 |
| Glucose | P01782 | -0.167440812 | 0.283168922 | 0.763999623 |
| WLC | A0A0A0MS15 | 0.167290207 | 0.283608381 | 0.763999623 |
| LDH | Q8N1N4 | -0.167277124 | 0.283646578 | 0.763999623 |
| UA | A0A0B4J1Y8 | -0.167258174 | 0.283701909 | 0.763999623 |
| Lymphocytes | P15169 | -0.167207919 | 0.283848682 | 0.763999623 |
| Ca | H3BTN5 | 0.167125355 | 0.284089921 | 0.763999623 |
| Neutrophils | O75882 | -0.167113462 | 0.284124684 | 0.763999623 |
| FIB | A0A0A0MS15 | 0.167088431 | 0.284197852 | 0.763999623 |
| Platelets | P37802 | 0.16699661 | 0.284466361 | 0.763999623 |
| APTT | P02753 | 0.166968738 | 0.284547901 | 0.763999623 |
| Globin | Q96HR3 | -0.166956282 | 0.284584344 | 0.763999623 |
| WBC | P19823 | -0.166949825 | 0.28460324 | 0.763999623 |
| Creatine Kinase | P04430 | -0.166893634 | 0.284767689 | 0.763999623 |
| Glucose | Q9Y5Y7 | 0.166610028 | 0.28559865 | 0.763999623 |
| Creatine Kinase | A0A0C4DH73 | -0.166446403 | 0.286078787 | 0.763999623 |
| α-HBDH | P01019 | 0.166408595 | 0.286189801 | 0.763999623 |
| WBC | P15169 | -0.166345755 | 0.286374385 | 0.763999623 |
| Glucose | D6R934 | -0.166307925 | 0.286485541 | 0.763999623 |
| Creatinine | A0A0J9YX35 | -0.166263969 | 0.285562399 | 0.763999623 |
| LDH | P03951 | 0.166219842 | 0.286744464 | 0.763999623 |
| WLL | P32119 | 0.166179326 | 0.286863615 | 0.763999623 |
| α-HBDH | A0A0G2JI36 | 0.166145825 | 0.286962159 | 0.763999623 |
| WBC | A0A0G2JI36 | 0.166125855 | 0.287020911 | 0.763999623 |
| FIB | P02753 | -0.166106446 | 0.28707802 | 0.763999623 |
| TT | P02751 | -0.16604889 | 0.287247419 | 0.763999623 |
| Globin | P00918 | -0.166004957 | 0.287376766 | 0.763999623 |
| α-HBDH | Q9H4B7 | -0.165934064 | 0.287585568 | 0.763999623 |
| Ca | P00918 | -0.165913145 | 0.287647202 | 0.763999623 |
| IBIL | A0A0C4DH36 | 0.165861035 | 0.287800766 | 0.763999623 |
| Globin | A0A087X0Q4 | 0.165823095 | 0.287912606 | 0.763999623 |
| WBC | P02745 | 0.165817193 | 0.287930005 | 0.763999623 |
| ALT | P02745 | 0.165803646 | 0.28796995 | 0.763999623 |
| CK-MB activity | A0A096LPE2 | -0.165726488 | 0.288197514 | 0.763999623 |
| α-HBDH | P01718 | -0.165423283 | 0.289092894 | 0.763999623 |
| Globin | P13647 | -0.16536982 | 0.28925096 | 0.763999623 |
| WBC | P01814 | 0.165364141 | 0.289267753 | 0.763999623 |
| Creatine Kinase | Q9Y5Y7 | -0.1653136 | 0.289417236 | 0.763999623 |
| Albumin | A0A140T8Y3 | 0.165304542 | 0.289444034 | 0.763999623 |
| α-HBDH | Q6ZRK6 | -0.165203099 | 0.289744238 | 0.763999623 |
| FIB | Q86YZ3 | -0.165197494 | 0.289760833 | 0.763999623 |
| Monocytes | A0A0J9YXX1 | -0.165028212 | 0.290262263 | 0.764174888 |
| Lymphocytes | A0A0J9YX35 | 0.165017752 | 0.290293265 | 0.764174888 |
| WLC | E7EX29 | 0.164907945 | 0.290618851 | 0.764418469 |
| TT | A0A075B6S9 | 0.164510487 | 0.29179931 | 0.765485763 |
| WLL | P01782 | -0.16446453 | 0.291936006 | 0.765485763 |
| INR | P32119 | -0.164454309 | 0.291966412 | 0.765485763 |
| Hematocrit | P80748 | 0.164413568 | 0.292087633 | 0.765485763 |
| INR | P19652 | -0.164378663 | 0.292191517 | 0.765485763 |
| Lymphocytes | A0A096LPE2 | 0.164262522 | 0.292537343 | 0.765780115 |
| Neutrophils | Q92954 | 0.164168398 | 0.292817805 | 0.76590303 |
| Hemoglobin | A0A075B6S9 | -0.164005996 | 0.293302125 | 0.766558542 |
| Mg | Q86UD1 | 0.163859752 | 0.293738702 | 0.76708833 |
| ALP | H3BTN5 | -0.163748361 | 0.294071512 | 0.767346508 |
| Monocytes | Q5SRP5 | -0.163628143 | 0.294430971 | 0.767673757 |
| CK-MB activity | P35527 | -0.16352389 | 0.294742922 | 0.767876714 |
| WBC | A0A140T8Y3 | -0.163444794 | 0.294979739 | 0.767883765 |
| Neutrophils | A0A0C4DH33 | -0.163268389 | 0.295508347 | 0.768502869 |
| Globin | P02745 | -0.163178991 | 0.295776466 | 0.768502869 |
| BUN | Q6ZRK6 | 0.163102102 | 0.296007193 | 0.768502869 |
| Ca | P04430 | 0.162963243 | 0.296424174 | 0.768502869 |
| Platelets | A0A0C4DH36 | -0.162903108 | 0.296604872 | 0.768502869 |
| RBC | P02763 | 0.162896956 | 0.296623363 | 0.768502869 |
| IBIL | A0A0J9YVY3 | 0.162712015 | 0.297179552 | 0.76923747 |
| Monocytes | O75882 | 0.162608 | 0.297492659 | 0.76923747 |
| Mg | P55056 | 0.162568881 | 0.297610472 | 0.76923747 |
| Basophils | O75882 | 0.162432208 | 0.298022315 | 0.769695428 |
| UA | P00915 | 0.162343791 | 0.298288939 | 0.769777908 |
| WLGG | P80748 | 0.162070254 | 0.299114777 | 0.771302256 |
| Creatine Kinase | A0A075B6S5 | -0.161990714 | 0.299355193 | 0.771315815 |
| IBIL | I3L1J2 | -0.161851662 | 0.299775783 | 0.771793226 |
| Total protein | A0A2R8Y3M9 | 0.161619217 | 0.30047971 | 0.772998784 |
| Glucose | A0A5H1ZRS9 | -0.161247693 | 0.301607019 | 0.774767658 |
| PT | Q9H4B7 | -0.161236927 | 0.301639725 | 0.774767658 |
| TT | Q96HR3 | -0.161122748 | 0.301986745 | 0.775052052 |
| WLL | P02745 | 0.160879047 | 0.302728269 | 0.776336685 |
| Neutrophils | P69905 | 0.160770248 | 0.303059693 | 0.776336685 |
| IBIL | A0A075B6S5 | 0.160725083 | 0.303197346 | 0.776336685 |
| Globin | P01715 | 0.16041824 | 0.304133579 | 0.778126473 |
| Albumin | P02751 | -0.160196382 | 0.304811659 | 0.778888476 |
| ALP | P15169 | -0.160099765 | 0.305107258 | 0.778888476 |
| RBC | P04430 | -0.160087822 | 0.305143808 | 0.778888476 |
| ALP | A0A2R8Y7X9 | 0.159948656 | 0.30556994 | 0.779232674 |
| CRP | A0A0A0MS15 | 0.159818738 | 0.305968096 | 0.779232674 |
| WLGG | P02042 | 0.159700806 | 0.306329807 | 0.779232674 |
| Lymphocytes | K7ERG9 | 0.159580096 | 0.306700319 | 0.779232674 |
| WBC | P02763 | 0.159474459 | 0.3070248 | 0.779232674 |
| WLGG | P01814 | -0.159384879 | 0.30730013 | 0.779232674 |
| Glucose | Q86UD1 | 0.159328953 | 0.307472105 | 0.779232674 |
| γ-GT | P13647 | -0.159261719 | 0.307678932 | 0.779232674 |
| ALP | Q92954 | -0.158966449 | 0.308588293 | 0.779232674 |
| Glucose | P02763 | 0.158830865 | 0.309006431 | 0.779232674 |
| UA | P03951 | 0.158794881 | 0.309117469 | 0.779232674 |
| Total protein | P13647 | -0.158749343 | 0.309258018 | 0.779232674 |
| Lymphocytes | C9JV77 | -0.158598297 | 0.309724509 | 0.779232674 |
| Total protein | P69905 | 0.158598297 | 0.309724509 | 0.779232674 |
| PT | P02766 | -0.158526342 | 0.309946893 | 0.779232674 |
| UA | C9J8S2 | 0.158522067 | 0.309960109 | 0.779232674 |
| Total protein | E7EX29 | -0.158472007 | 0.310114886 | 0.779232674 |
| CRP | P0DP01 | -0.158465204 | 0.310135923 | 0.779232674 |
| Monocytes | D6RE82 | -0.15844826 | 0.310188325 | 0.779232674 |
| Total protein | P55056 | 0.158447251 | 0.310191446 | 0.779232674 |
| Ca | Q9NZP8 | -0.158324659 | 0.310570752 | 0.779232674 |
| BUN | A0A075B6S9 | -0.158275595 | 0.310722641 | 0.779232674 |
| AST | A0A2R8Y7X9 | -0.158217667 | 0.310902029 | 0.779232674 |
| WLC | P04264 | -0.158192381 | 0.310980357 | 0.779232674 |
| DBIL | Q9NZP8 | -0.15789283 | 0.311909195 | 0.780737031 |
| P | A0A0A0MS15 | -0.157787404 | 0.312236514 | 0.780737031 |
| Hematocrit | O75636 | 0.157767544 | 0.312298199 | 0.780737031 |
| Platelets | O75882 | -0.157692021 | 0.312532842 | 0.780737031 |
| Creatine Kinase | P55056 | 0.157535025 | 0.313020971 | 0.781146979 |
| ALP | Q86UD1 | 0.157484424 | 0.313178403 | 0.781146979 |
| P | P19823 | 0.157409561 | 0.31341141 | 0.781146979 |
| Glucose | P03951 | -0.157093771 | 0.314395498 | 0.783004733 |
| AST | C9JV77 | -0.157007011 | 0.314666208 | 0.783084341 |
| Hematocrit | K7ERG9 | 0.156785745 | 0.315357273 | 0.783244871 |
| WLC | P35908 | -0.156780649 | 0.315373201 | 0.783244871 |
| Lymphocytes | Q92954 | 0.156710222 | 0.315593368 | 0.783244871 |
| WBC | C9JV77 | -0.156680636 | 0.31568589 | 0.783244871 |
| Monocytes | P01718 | -0.156259664 | 0.3170042 | 0.785209206 |
| P | C9J8S2 | -0.156197438 | 0.31719936 | 0.785209206 |
| Globin | Q15582 | -0.156153229 | 0.317338061 | 0.785209206 |
| CO2 | P01782 | 0.156095382 | 0.317519605 | 0.785209206 |
| γ-GT | P03950 | 0.156046018 | 0.317674578 | 0.785209206 |
| Hematocrit | C9J8S2 | -0.155787018 | 0.318488467 | 0.786628142 |
| IBIL | P01718 | -0.155706055 | 0.318743157 | 0.786664827 |
| INR | P02750 | -0.155301148 | 0.320018824 | 0.787780501 |
| CO2 | P00918 | -0.155297742 | 0.320029569 | 0.787780501 |
| APTT | H3BTN5 | -0.155274723 | 0.320102188 | 0.787780501 |
| Neutrophils | P02745 | 0.155257694 | 0.320155917 | 0.787780501 |
| Creatine Kinase | A0A0A0MS15 | 0.155118381 | 0.320595687 | 0.788029195 |
| Ca | Q9Y5Y7 | -0.155073484 | 0.320737493 | 0.788029195 |
| P | A0A0C4DH73 | -0.154840226 | 0.321474873 | 0.789249688 |
| FIB | P0DP01 | -0.15459285 | 0.322258046 | 0.790547902 |
| Hematocrit | Q9Y5Y7 | -0.154444532 | 0.322728182 | 0.790547902 |
| TT | Q6ZRK6 | -0.154386041 | 0.322913707 | 0.790547902 |
| Hematocrit | Q8N1N4 | -0.154369009 | 0.32296774 | 0.790547902 |
| CK-MB activity | P02750 | -0.154105886 | 0.323803235 | 0.791490325 |
| α-HBDH | E7EX29 | -0.154078822 | 0.32388925 | 0.791490325 |
| WLL | P01814 | -0.154019863 | 0.324076679 | 0.791490325 |
| TBIL | P43121 | 0.153711103 | 0.325059329 | 0.793299553 |
| Eosinophils | A0A0B4J1Y8 | -0.153444866 | 0.325908145 | 0.794779714 |
| Creatine Kinase | A0A0G2JI36 | 0.153274679 | 0.326451463 | 0.795513222 |
| Globin | P0DP01 | -0.152683885 | 0.328341947 | 0.798370438 |
| PT | P37802 | -0.152633876 | 0.328502283 | 0.798370438 |
| WLC | D6R934 | 0.152467024 | 0.329037592 | 0.798370438 |
| IBIL | A0A2R8Y7X9 | -0.152416925 | 0.32919843 | 0.798370438 |
| Creatinine | P01718 | -0.152401923 | 0.329246605 | 0.798370438 |
| AST | A0A0C4DH73 | 0.152391383 | 0.32928045 | 0.798370438 |
| UA | P13473 | 0.152376638 | 0.329327806 | 0.798370438 |
| UA | Q8N1N4 | -0.152150111 | 0.330055837 | 0.799544421 |
| ALT | Q9NZP8 | -0.151967774 | 0.33064258 | 0.800045998 |
| Platelets | A0A2R8Y3M9 | 0.15187675 | 0.330935726 | 0.800045998 |
| P | P00918 | -0.151802166 | 0.331176051 | 0.800045998 |
| PT | A0A0C4DH73 | -0.151640472 | 0.33169743 | 0.800045998 |
| α-HBDH | P0DJI8 | 0.151527754 | 0.332061189 | 0.800045998 |
| Albumin | A0A0B4J1V2 | 0.151251941 | 0.33295233 | 0.800045998 |
| Neutrophils | C9JV77 | -0.1511044 | 0.333429639 | 0.800045998 |
| α-HBDH | A0A087X0Q4 | -0.150998993 | 0.333770898 | 0.800045998 |
| ALP | A0A0J9YXX1 | 0.150882129 | 0.334149508 | 0.800045998 |
| WBC | P55056 | 0.150790954 | 0.334445073 | 0.800045998 |
| WLL | P01817 | -0.150746161 | 0.334590341 | 0.800045998 |
| WLL | Q96HR3 | 0.150746161 | 0.334590341 | 0.800045998 |
| WLC | A0A0B4J1V2 | -0.150511968 | 0.335350486 | 0.800045998 |
| CO2 | Q9NZP8 | -0.150504357 | 0.33537521 | 0.800045998 |
| γ-GT | P43121 | 0.150458469 | 0.335524285 | 0.800045998 |
| APTT | A0A0C4DH36 | 0.150354933 | 0.335860791 | 0.800045998 |
| Hemoglobin | O75882 | 0.150353248 | 0.33586627 | 0.800045998 |
| Neutrophils | P37802 | -0.150217559 | 0.3363076 | 0.800045998 |
| Hemoglobin | D6RE82 | 0.150126585 | 0.336603695 | 0.800045998 |
| WLGG | P19823 | -0.15006505 | 0.336804064 | 0.800045998 |
| BUN | E7EX29 | -0.150056719 | 0.336831197 | 0.800045998 |
| ALP | A0A075B6S9 | -0.150015762 | 0.336964609 | 0.800045998 |
| WLGG | A0A0J9YVY3 | -0.149722704 | 0.337920162 | 0.800045998 |
| BUN | Q5SRP5 | 0.149580755 | 0.338383605 | 0.800045998 |
| Mg | P00915 | 0.149533054 | 0.33853943 | 0.800045998 |
| TBIL | P02753 | 0.149524243 | 0.33856822 | 0.800045998 |
| Globin | A0A2R8Y7X9 | 0.149505196 | 0.338630456 | 0.800045998 |
| TT | H3BTN5 | 0.14949181 | 0.338674199 | 0.800045998 |
| APTT | Q6ZRK6 | -0.149488558 | 0.338684828 | 0.800045998 |
| Mg | D6RE82 | 0.149457265 | 0.338787104 | 0.800045998 |
| Neutrophils | Q16880 | -0.149443082 | 0.338833464 | 0.800045998 |
| CRP | P04430 | -0.149328873 | 0.339206931 | 0.800045998 |
| ALP | E7ENL6 | 0.149315741 | 0.339249891 | 0.800045998 |
| Eosinophils | A0A0J9YVY3 | 0.149281555 | 0.33936174 | 0.800045998 |
| INR | A0A1W2PQU7 | -0.149249471 | 0.339466729 | 0.800045998 |
| CO2 | Q92954 | -0.149219932 | 0.339563412 | 0.800045998 |
| IBIL | P43121 | 0.149159132 | 0.339762463 | 0.800045998 |
| Mg | A0A075B6S5 | -0.149078316 | 0.340027155 | 0.800045998 |
| TT | A0A0J9YXX1 | -0.149072646 | 0.340045731 | 0.800045998 |
| TT | C9JV77 | 0.149072646 | 0.340045731 | 0.800045998 |
| RBC | P35527 | -0.148925729 | 0.340527264 | 0.800045998 |
| Neutrophils | A0A0A0MS15 | -0.148914481 | 0.340564147 | 0.800045998 |
| WLL | P37802 | 0.148857419 | 0.340751298 | 0.800045998 |
| Hemoglobin | P35527 | -0.14816217 | 0.343036656 | 0.803725115 |
| Eosinophils | P00915 | 0.147991532 | 0.343598999 | 0.803725115 |
| Eosinophils | A0A096LPE2 | 0.147915872 | 0.343848522 | 0.803725115 |
| WLL | A0A140T8Y3 | 0.147785233 | 0.344279622 | 0.803725115 |
| ALP | P00915 | 0.147784398 | 0.344282378 | 0.803725115 |
| INR | Q8N1N4 | -0.147736552 | 0.34444035 | 0.803725115 |
| DBIL | P32119 | 0.147689129 | 0.344596973 | 0.803725115 |
| RBC | P19823 | -0.147641886 | 0.34475304 | 0.803725115 |
| CO2 | A0A0C4DH33 | 0.147601085 | 0.344887864 | 0.803725115 |
| Glucose | A0A096LPE2 | -0.147577514 | 0.344965767 | 0.803725115 |
| Globin | Q86UD1 | -0.14756301 | 0.345013708 | 0.803725115 |
| TBIL | I3L1J2 | -0.147384873 | 0.345602852 | 0.804076959 |
| WLC | P69905 | 0.147369104 | 0.345655034 | 0.804076959 |
| Total protein | P03951 | 0.147194324 | 0.34623373 | 0.804852328 |
| P | P15169 | -0.146829946 | 0.347442098 | 0.80533948 |
| TBIL | P01019 | 0.14680562 | 0.347522859 | 0.80533948 |
| Total protein | A0A0A0MS15 | 0.146741186 | 0.347736835 | 0.80533948 |
| TBIL | E7EX29 | 0.14673578 | 0.347754795 | 0.80533948 |
| PT | P13647 | -0.146646325 | 0.348052006 | 0.80533948 |
| Creatinine | A0A096LPE2 | -0.146632437 | 0.346880064 | 0.80533948 |
| α-HBDH | P15169 | 0.146542295 | 0.34839784 | 0.80533948 |
| Monocytes | A0A0C4DH36 | 0.146271568 | 0.349298816 | 0.80533948 |
| Albumin | A0A075B6S5 | -0.146223572 | 0.349458698 | 0.80533948 |
| APTT | Q15582 | 0.146125965 | 0.349783974 | 0.80533948 |
| Monocytes | P02766 | -0.146120305 | 0.349802839 | 0.80533948 |
| AST | P30041 | 0.146082977 | 0.34992729 | 0.80533948 |
| TBIL | P0DJI8 | 0.146050447 | 0.350035762 | 0.80533948 |
| Albumin | Q9NZP8 | -0.145845928 | 0.350718223 | 0.80533948 |
| CO2 | Q6ZRK6 | -0.145786012 | 0.35091831 | 0.80533948 |
| FIB | P15169 | 0.145711385 | 0.351167624 | 0.80533948 |
| Basophils | Q96HR3 | -0.145688312 | 0.351244727 | 0.80533948 |
| DBIL | E7ENL6 | -0.145676917 | 0.351282809 | 0.80533948 |
| TT | Q9NZP8 | -0.14566224 | 0.351331865 | 0.80533948 |
| TT | P01817 | -0.145586453 | 0.351585234 | 0.80533948 |
| INR | H3BTN5 | 0.145520954 | 0.351804297 | 0.80533948 |
| Globin | P35908 | -0.145501267 | 0.35187016 | 0.80533948 |
| Albumin | K7ERG9 | -0.145392756 | 0.352233306 | 0.80533948 |
| DBIL | P03950 | 0.145282267 | 0.352603304 | 0.80533948 |
| APTT | E7ENL6 | 0.145222144 | 0.35280474 | 0.80533948 |
| CK-MB activity | Q86UD1 | -0.145215477 | 0.352827083 | 0.80533948 |
| Basophils | Q6ZRK6 | 0.144908171 | 0.353857815 | 0.806689058 |
| DBIL | P35527 | 0.144892559 | 0.35391023 | 0.806689058 |
| AST | Q86UD1 | -0.144745699 | 0.354403502 | 0.807252421 |
| Basophils | P02763 | 0.144621299 | 0.354821665 | 0.807252702 |
| UA | P02763 | 0.144599237 | 0.354895853 | 0.807252702 |
| ALT | P13645 | -0.144331582 | 0.355796687 | 0.808465447 |
| LDH | A0A0G2JI36 | 0.144248456 | 0.356076743 | 0.808465447 |
| Basophils | A0A5H1ZRS9 | 0.144128831 | 0.356479999 | 0.808465447 |
| Hemoglobin | P13473 | 0.144006678 | 0.356892062 | 0.808465447 |
| P | E7EX29 | -0.143993908 | 0.356935157 | 0.808465447 |
| FIB | P13647 | -0.143974028 | 0.357002254 | 0.808465447 |
| PT | P69905 | -0.143922245 | 0.357177056 | 0.808465447 |
| Hematocrit | A0A0B4J1V2 | 0.143612404 | 0.358224058 | 0.808465447 |
| Platelets | P35908 | 0.143569221 | 0.358370129 | 0.808465447 |
| Eosinophils | P01019 | 0.143527575 | 0.358511033 | 0.808465447 |
| Glucose | P08185 | -0.143348066 | 0.359118767 | 0.808465447 |
| Hemoglobin | Q9Y5Y7 | -0.143326689 | 0.359191181 | 0.808465447 |
| WLC | A0A0J9YXX1 | -0.143290768 | 0.359312881 | 0.808465447 |
| γ-GT | P02753 | 0.14329013 | 0.359315044 | 0.808465447 |
| Ca | P02750 | -0.143278524 | 0.359354372 | 0.808465447 |
| ALT | A0A075B6S5 | 0.1432731 | 0.359372751 | 0.808465447 |
| Total protein | P02766 | -0.143116083 | 0.359905077 | 0.808657957 |
| UA | P02766 | 0.143089063 | 0.359996729 | 0.808657957 |
| TBIL | A0A2R8Y7X9 | -0.143029755 | 0.36019795 | 0.808657957 |
| INR | P19823 | 0.142895211 | 0.36065469 | 0.80898953 |
| FIB | Q9Y5Y7 | 0.142840969 | 0.360838928 | 0.80898953 |
| P | P03950 | -0.142758472 | 0.36111924 | 0.809064964 |
| Globin | P03951 | 0.14255498 | 0.361811243 | 0.810062032 |
| Hemoglobin | Q5SRP5 | -0.142394106 | 0.362358882 | 0.810734742 |
| α-HBDH | A0A0C4DH33 | 0.142242041 | 0.362876992 | 0.811340515 |
| ALP | O75882 | -0.142042264 | 0.363558343 | 0.812310194 |
| Monocytes | H3BTN5 | 0.141801064 | 0.364381997 | 0.812592299 |
| Globin | P00915 | -0.141799521 | 0.364387269 | 0.812592299 |
| Hemoglobin | A0A5H1ZRS9 | -0.141740047 | 0.364590538 | 0.812592299 |
| TBIL | A0A0J9YVY3 | 0.141625922 | 0.364980779 | 0.812592299 |
| CO2 | P00915 | 0.141588938 | 0.365107297 | 0.812592299 |
| WLL | P35908 | 0.141548619 | 0.365245253 | 0.812592299 |
| CK-MB activity | A0A2R8Y3M9 | 0.141497913 | 0.365418793 | 0.812592299 |
| APTT | A0A2R8Y7X9 | -0.141292858 | 0.366121101 | 0.812953037 |
| AST | Q15582 | -0.141268477 | 0.366204658 | 0.812953037 |
| WLGG | P08185 | -0.14121911 | 0.366373881 | 0.812953037 |
| Monocytes | A0A5H1ZRS9 | 0.141128618 | 0.366684201 | 0.812953037 |
| TBIL | Q92954 | -0.140990788 | 0.367157153 | 0.812953037 |
| WLL | Q6ZRK6 | 0.140914691 | 0.367418435 | 0.812953037 |
| FIB | D6RE82 | 0.140801462 | 0.367807409 | 0.812953037 |
| Ca | P01019 | -0.140783436 | 0.367869358 | 0.812953037 |
| Globin | A0A0C4DH73 | 0.14074188 | 0.368012194 | 0.812953037 |
| FIB | P00918 | 0.140709062 | 0.368125016 | 0.812953037 |
| Total protein | H3BTN5 | -0.140656029 | 0.368307382 | 0.812953037 |
| Neutrophils | A0A140T8Y3 | -0.140376384 | 0.369269888 | 0.813130243 |
| AST | P02750 | -0.140360485 | 0.369324659 | 0.813130243 |
| BUN | I3L1J2 | -0.140320679 | 0.3694618 | 0.813130243 |
| CRP | P30041 | 0.140292561 | 0.369558692 | 0.813130243 |
| Albumin | A0A0J9YXX1 | -0.140256804 | 0.36968193 | 0.813130243 |
| α-HBDH | I3L1J2 | 0.140200771 | 0.369875098 | 0.813130243 |
| Total protein | A0A0J9YX35 | -0.140095163 | 0.370239341 | 0.813373468 |
| Glucose | P35908 | -0.140024928 | 0.370481696 | 0.813373468 |
| Neutrophils | P32119 | 0.139701719 | 0.371598207 | 0.815279009 |
| UA | A0A075B6S5 | 0.139615661 | 0.371895829 | 0.815386577 |
| Total protein | P02763 | 0.139490979 | 0.37232728 | 0.815614537 |
| Eosinophils | P13647 | -0.139441919 | 0.372497127 | 0.815614537 |
| γ-GT | P01814 | -0.13912692 | 0.37358878 | 0.817016954 |
| Hematocrit | P15169 | -0.139113364 | 0.373635802 | 0.817016954 |
| Globin | P35527 | -0.138702142 | 0.375063891 | 0.81863071 |
| LDH | P37802 | 0.138612866 | 0.375374357 | 0.81863071 |
| FIB | Q9H4B7 | -0.138577044 | 0.375498974 | 0.81863071 |
| TT | P0DJI8 | 0.138462493 | 0.375897639 | 0.81863071 |
| WLL | A0A0A0MS15 | 0.138430808 | 0.376007956 | 0.81863071 |
| AST | P35908 | -0.138241836 | 0.376666289 | 0.81863071 |
| Eosinophils | P69905 | 0.138231354 | 0.376702823 | 0.81863071 |
| Monocytes | P03951 | 0.138103353 | 0.377149164 | 0.81863071 |
| PT | O75636 | 0.138095739 | 0.377175721 | 0.81863071 |
| Basophils | D6R934 | 0.138055065 | 0.377317625 | 0.81863071 |
| BUN | A0A5H1ZRS9 | -0.138040401 | 0.377368791 | 0.81863071 |
| Creatinine | Q9NZP8 | 0.137873754 | 0.376682957 | 0.81863071 |
| γ-GT | P04430 | -0.137871016 | 0.37796012 | 0.819371575 |
| APTT | P80748 | -0.137668028 | 0.378669487 | 0.820102755 |
| Basophils | P04264 | -0.137562597 | 0.379038235 | 0.820102755 |
| Platelets | P19823 | -0.137527381 | 0.379161455 | 0.820102755 |
| Mg | P01817 | -0.137482494 | 0.379318544 | 0.820102755 |
| WLC | A0A0C4DH73 | -0.137408553 | 0.379577398 | 0.820102755 |
| Neutrophils | H3BTN5 | -0.13734568 | 0.379797587 | 0.820102755 |
| AST | A0A140T8Y3 | 0.137170155 | 0.380412696 | 0.820890554 |
| ALT | P02763 | -0.13707342 | 0.380751945 | 0.821082432 |
| Creatinine | Q6ZRK6 | -0.136668675 | 0.382173325 | 0.823606114 |
| ALP | Q9Y5Y7 | 0.136526793 | 0.382672323 | 0.824139999 |
| IBIL | Q92954 | -0.136329311 | 0.383367508 | 0.825095424 |
| Eosinophils | Q5SRP5 | 0.13616465 | 0.383947727 | 0.82570339 |
| ALT | P01782 | 0.136090544 | 0.384209023 | 0.82570339 |
| DBIL | A0A140T8Y3 | 0.136034924 | 0.384405206 | 0.82570339 |
| CK-MB activity | P02753 | 0.13580154 | 0.385229046 | 0.826591985 |
| Neutrophils | P55056 | 0.135774968 | 0.38532291 | 0.826591985 |
| Lymphocytes | Q96HR3 | 0.135639306 | 0.38580234 | 0.826728005 |
| UA | P01814 | -0.135538189 | 0.386159915 | 0.826728005 |
| Eosinophils | A0A0A0MS15 | 0.135431924 | 0.386535905 | 0.826728005 |
| Neutrophils | Q86YZ3 | -0.135412092 | 0.386606098 | 0.826728005 |
| Ca | C9J8S2 | 0.135400657 | 0.386646573 | 0.826728005 |
| γ-GT | P13645 | -0.134963709 | 0.388195114 | 0.828527176 |
| WBC | P0DJI8 | -0.13485861 | 0.388568127 | 0.828527176 |
| ALT | A0A2R8Y3M9 | -0.134729638 | 0.389026154 | 0.828527176 |
| IBIL | P13647 | 0.13466768 | 0.389246304 | 0.828527176 |
| Mg | P13645 | -0.134602486 | 0.38947803 | 0.828527176 |
| CK-MB activity | Q16880 | -0.134586314 | 0.389535525 | 0.828527176 |
| Basophils | A0A0G2JI36 | -0.134550608 | 0.389662483 | 0.828527176 |
| Lymphocytes | A0A5H1ZRS9 | -0.134506461 | 0.389819489 | 0.828527176 |
| γ-GT | P19652 | 0.134433846 | 0.390077817 | 0.828527176 |
| Creatinine | Q8N1N4 | -0.134400483 | 0.388914093 | 0.828527176 |
| α-HBDH | P02750 | 0.134380795 | 0.390266612 | 0.828527176 |
| Neutrophils | Q15582 | -0.13411365 | 0.39121812 | 0.829772704 |
| γ-GT | A0A0A0MS15 | 0.134055372 | 0.391425872 | 0.829772704 |
| FIB | P35527 | -0.134003109 | 0.39161224 | 0.829772704 |
| PT | P08185 | 0.133706943 | 0.392669317 | 0.830825478 |
| Monocytes | A0A075B6S9 | 0.133633634 | 0.39293123 | 0.830825478 |
| DBIL | P04430 | 0.133580571 | 0.393120873 | 0.830825478 |
| CO2 | P13645 | -0.133580172 | 0.393122299 | 0.830825478 |
| FIB | Q6ZRK6 | 0.133360674 | 0.393907336 | 0.831948526 |
| Mg | I3L1J2 | 0.13321849 | 0.394416343 | 0.832327682 |
| Platelets | Q96HR3 | -0.132920478 | 0.39548445 | 0.832327682 |
| P | A0A075B6S9 | 0.132848056 | 0.395744271 | 0.832327682 |
| BUN | A0A087X0Q4 | 0.132829905 | 0.395809407 | 0.832327682 |
| Lymphocytes | P00918 | 0.132717882 | 0.396211533 | 0.832327682 |
| LDH | C9JV77 | 0.132688897 | 0.396315619 | 0.832327682 |
| CO2 | P01814 | -0.132673519 | 0.396370849 | 0.832327682 |
| WBC | A0A0J9YX35 | 0.132668857 | 0.396387595 | 0.832327682 |
| Eosinophils | A0A0J9YXX1 | 0.132556833 | 0.396790069 | 0.832327682 |
| WLGG | P35908 | 0.132531134 | 0.396882432 | 0.832327682 |
| Hemoglobin | Q6ZRK6 | -0.132487964 | 0.397037615 | 0.832327682 |
| Monocytes | A0A0G2JI36 | 0.132380102 | 0.397425498 | 0.832327682 |
| CO2 | P02753 | 0.132220193 | 0.398000957 | 0.832327682 |
| Albumin | E7ENL6 | 0.132196736 | 0.39808541 | 0.832327682 |
| PT | D6RE82 | -0.132117896 | 0.39836934 | 0.832327682 |
| Basophils | K7ERG9 | -0.132063377 | 0.398565754 | 0.832327682 |
| AST | P37802 | -0.131928784 | 0.399050881 | 0.832327682 |
| CRP | P01718 | 0.131914473 | 0.399102481 | 0.832327682 |
| CRP | Q8N1N4 | -0.131797589 | 0.399524087 | 0.832327682 |
| Platelets | A0A087X0Q4 | 0.131787633 | 0.399560012 | 0.832327682 |
| LDH | Q9Y5Y7 | 0.131707135 | 0.399850536 | 0.832327682 |
| WLL | I3L1J2 | -0.131647744 | 0.400064961 | 0.832327682 |
| Lymphocytes | Q9Y5Y7 | -0.131636587 | 0.400105252 | 0.832327682 |
| WBC | A0A0B4J1V2 | 0.131616703 | 0.400177059 | 0.832327682 |
| Total protein | P30041 | -0.131475396 | 0.400687586 | 0.832861396 |
| ALT | A0A140T8Y3 | 0.131302841 | 0.40131152 | 0.83325195 |
| Mg | A0A5H1ZRS9 | -0.13119195 | 0.401712783 | 0.83325195 |
| γ-GT | P02751 | 0.131103278 | 0.402033811 | 0.83325195 |
| Neutrophils | D6RE82 | 0.131093072 | 0.402070769 | 0.83325195 |
| α-HBDH | A0A075B6S9 | -0.131023382 | 0.402323195 | 0.83325195 |
| CRP | P55056 | 0.130966773 | 0.402528302 | 0.83325195 |
| UA | Q9NZP8 | 0.130932155 | 0.402653762 | 0.83325195 |
| Eosinophils | A0A0G2JI36 | 0.13084454 | 0.402971394 | 0.833383462 |
| FIB | P04430 | -0.130663392 | 0.403628564 | 0.834132671 |
| PT | P15169 | -0.130604518 | 0.403842281 | 0.834132671 |
| Lymphocytes | P01019 | 0.130277173 | 0.40503176 | 0.83569773 |
| CO2 | A0A1W2PQU7 | -0.130255778 | 0.405109571 | 0.83569773 |
| DBIL | P55056 | 0.129851547 | 0.406581371 | 0.837238101 |
| WLGG | P02751 | 0.129845759 | 0.406602467 | 0.837238101 |
| RBC | P43121 | -0.129796774 | 0.406781035 | 0.837238101 |
| ALT | A0A075B6S9 | -0.129708648 | 0.407102399 | 0.837238101 |
| DBIL | P03951 | 0.129700381 | 0.407132552 | 0.837238101 |
| Eosinophils | I3L1J2 | -0.129576851 | 0.407583285 | 0.837353768 |
| WLL | P80748 | 0.129545046 | 0.40769938 | 0.837353768 |
| DBIL | P19823 | 0.129473632 | 0.407960128 | 0.837364969 |
| INR | P00918 | -0.129328709 | 0.408489567 | 0.837914612 |
| APTT | A0A087X0Q4 | -0.129210091 | 0.4089232 | 0.837914612 |
| Basophils | P55056 | 0.129190649 | 0.408994297 | 0.837914612 |
| WBC | P02042 | 0.128817911 | 0.410358769 | 0.839587627 |
| LDH | P01817 | -0.12876185 | 0.410564216 | 0.839587627 |
| DBIL | Q9Y5Y7 | -0.128717803 | 0.410725675 | 0.839587627 |
| WLL | A0A2R8Y7X9 | 0.128609703 | 0.41112208 | 0.839587627 |
| CRP | A0A0J9YVY3 | -0.128467176 | 0.411645066 | 0.839587627 |
| Total protein | P02753 | 0.128389098 | 0.411931728 | 0.839587627 |
| Platelets | P15169 | -0.128389098 | 0.411931728 | 0.839587627 |
| Eosinophils | P55056 | 0.128168536 | 0.412742127 | 0.839587627 |
| LDH | P19823 | -0.128157688 | 0.412782006 | 0.839587627 |
| RBC | Q8N1N4 | -0.128157688 | 0.412782006 | 0.839587627 |
| Neutrophils | A0A096LPE2 | -0.128148009 | 0.412817592 | 0.839587627 |
| CRP | I3L1J2 | 0.128093682 | 0.413017363 | 0.839587627 |
| Monocytes | Q92954 | 0.128044346 | 0.413198829 | 0.839587627 |
| Ca | A0A075B6S5 | -0.127929954 | 0.413619758 | 0.839587627 |
| WBC | Q86UD1 | -0.127921605 | 0.41365049 | 0.839587627 |
| TBIL | Q5SRP5 | -0.127761313 | 0.414240748 | 0.83958859 |
| FIB | A0A5H1ZRS9 | -0.127733516 | 0.414343159 | 0.83958859 |
| ALP | P04264 | 0.127686929 | 0.414514825 | 0.83958859 |
| IBIL | P15169 | -0.12764351 | 0.414674853 | 0.83958859 |
| Lymphocytes | P69905 | 0.127482822 | 0.415267408 | 0.839756546 |
| WLGG | A0A0C4DH33 | -0.127481124 | 0.415273672 | 0.839756546 |
| RBC | P01782 | 0.127326967 | 0.4158426 | 0.839756546 |
| Glucose | P13647 | -0.12726106 | 0.41608597 | 0.839756546 |
| P | Q9Y5Y7 | 0.127257658 | 0.416098534 | 0.839756546 |
| α-HBDH | A0A075B6S5 | -0.127204755 | 0.416293946 | 0.839756546 |
| Neutrophils | P01782 | -0.127015292 | 0.416994208 | 0.840652121 |
| γ-GT | A0A0J9YX35 | -0.126788678 | 0.417832663 | 0.841564319 |
| LDH | P02751 | -0.126722806 | 0.418076565 | 0.841564319 |
| Neutrophils | A0A0C4DH73 | 0.12663772 | 0.418391725 | 0.841564319 |
| ALP | P01718 | 0.126615787 | 0.418472989 | 0.841564319 |
| Ca | P02745 | -0.126493388 | 0.418926647 | 0.841926464 |
| Platelets | P13473 | 0.1264255 | 0.419178386 | 0.841926464 |
| Albumin | P13647 | -0.126359522 | 0.419423123 | 0.841926464 |
| CK-MB activity | P0DJI8 | -0.126231633 | 0.419897746 | 0.842363674 |
| PT | Q6ZRK6 | 0.126111859 | 0.420342525 | 0.842524666 |
| FIB | P04264 | -0.126071696 | 0.420491731 | 0.842524666 |
| WBC | P02751 | -0.12587307 | 0.421230062 | 0.84348877 |
| γ-GT | A0A0J9YVY3 | 0.125722486 | 0.421790298 | 0.843974313 |
| WLL | P13647 | -0.125647783 | 0.422068385 | 0.843974313 |
| Mg | P02042 | 0.125583513 | 0.422307714 | 0.843974313 |
| WLL | P69905 | 0.125491892 | 0.422649025 | 0.843974313 |
| UA | P01782 | -0.125420017 | 0.422916885 | 0.843974313 |
| Total protein | A0A0B4J1Y8 | -0.125335148 | 0.423233297 | 0.843974313 |
| Globin | P03950 | 0.125324313 | 0.423273703 | 0.843974313 |
| WLGG | A0A0A0MS15 | 0.125106863 | 0.424085058 | 0.844745511 |
| WBC | A0A2R8Y3M9 | 0.125042474 | 0.424325476 | 0.844745511 |
| Globin | A0A140T8Y3 | -0.124990695 | 0.424518865 | 0.844745511 |
| Hematocrit | P43121 | -0.124839127 | 0.425085241 | 0.844745511 |
| DBIL | A0A087X0Q4 | -0.124711905 | 0.425560971 | 0.844745511 |
| Creatine Kinase | A0A096LPE2 | 0.124683762 | 0.42566625 | 0.844745511 |
| ALP | Q16880 | -0.124664753 | 0.425737365 | 0.844745511 |
| Ca | Q5SRP5 | 0.124645955 | 0.425807698 | 0.844745511 |
| Neutrophils | Q9Y5Y7 | 0.124523316 | 0.426266724 | 0.844745511 |
| BUN | P01715 | -0.12441475 | 0.426673306 | 0.844745511 |
| DBIL | P80748 | 0.124409573 | 0.4266927 | 0.844745511 |
| Neutrophils | K7ERG9 | -0.124145744 | 0.42768168 | 0.844745511 |
| ALP | P37802 | -0.124090599 | 0.427888555 | 0.844745511 |
| WLC | Q9H4B7 | -0.123871748 | 0.428710129 | 0.844745511 |
| BUN | P0DJI8 | -0.123843686 | 0.428815537 | 0.844745511 |
| DBIL | P13645 | -0.123729326 | 0.429245256 | 0.844745511 |
| PT | C9JV77 | 0.123718649 | 0.429285391 | 0.844745511 |
| Globin | P0DJI8 | -0.123668522 | 0.429473833 | 0.844745511 |
| γ-GT | K7ERG9 | -0.12345811 | 0.430265347 | 0.844745511 |
| Basophils | A0A1W2PQU7 | -0.123445195 | 0.430313956 | 0.844745511 |
| Globin | D6RE82 | 0.123441885 | 0.430326415 | 0.844745511 |
| Total protein | A0A0G2JI36 | -0.123420081 | 0.430408487 | 0.844745511 |
| Ca | P01718 | -0.12336944 | 0.430599143 | 0.844745511 |
| Hemoglobin | A0A2R8Y3M9 | -0.123304774 | 0.430842667 | 0.844745511 |
| TT | P01715 | 0.123298779 | 0.430865246 | 0.844745511 |
| ALP | O75636 | -0.123153665 | 0.431412025 | 0.844745511 |
| P | A0A0C4DH33 | 0.123143814 | 0.43144916 | 0.844745511 |
| CO2 | A0A075B6S5 | 0.122851448 | 0.432552001 | 0.844745511 |
| RBC | Q9Y5Y7 | -0.122795758 | 0.432762248 | 0.844745511 |
| LDH | P02753 | 0.122720238 | 0.433047452 | 0.844745511 |
| Total protein | P15169 | -0.12264935 | 0.433315258 | 0.844745511 |
| ALT | P13473 | -0.122632701 | 0.433378168 | 0.844745511 |
| WLGG | P01718 | 0.122573874 | 0.433600498 | 0.844745511 |
| ALP | P43121 | 0.122525079 | 0.433784961 | 0.844745511 |
| Globin | Q86YZ3 | -0.122481592 | 0.433949395 | 0.844745511 |
| Hemoglobin | P01019 | 0.122473676 | 0.43397933 | 0.844745511 |
| DBIL | A0A0A0MS15 | -0.122368833 | 0.434375919 | 0.844745511 |
| DBIL | P43121 | 0.122222215 | 0.43493087 | 0.844745511 |
| Creatine Kinase | E7EX29 | 0.122074823 | 0.435489146 | 0.844745511 |
| Neutrophils | P00915 | 0.121955825 | 0.435940167 | 0.844745511 |
| Creatinine | A0A0J9YXX1 | 0.121866506 | 0.434967283 | 0.844745511 |
| CRP | P37802 | 0.121865276 | 0.436283534 | 0.844745511 |
| ALP | P26038 | -0.121802795 | 0.436520552 | 0.844745511 |
| CO2 | A0A2R8Y7X9 | -0.121793686 | 0.436555114 | 0.844745511 |
| Hematocrit | P02753 | 0.121743074 | 0.43674717 | 0.844745511 |
| TBIL | A0A5H1ZRS9 | -0.121733878 | 0.436782069 | 0.844745511 |
| BUN | A0A2R8Y7X9 | 0.121578253 | 0.437372936 | 0.844745511 |
| WBC | A0A0B4J1Y8 | 0.121573662 | 0.437390374 | 0.844745511 |
| Globin | A0A0C4DH36 | 0.121477693 | 0.437754967 | 0.844745511 |
| Globin | A0A2R8Y3M9 | 0.121477693 | 0.437754967 | 0.844745511 |
| IBIL | P02753 | 0.121450157 | 0.437859612 | 0.844745511 |
| Creatinine | P37802 | 0.121438738 | 0.43790301 | 0.844745511 |
| α-HBDH | A0A0B4J1Y8 | 0.121430738 | 0.437933414 | 0.844745511 |
| CK-MB activity | C9JV77 | -0.121294776 | 0.438450354 | 0.844745511 |
| Total protein | Q92954 | 0.121289936 | 0.438468763 | 0.844745511 |
| Total protein | A0A0J9YXX1 | -0.121214413 | 0.438756059 | 0.844745511 |
| Mg | A0A0A0MS15 | -0.121187711 | 0.438857661 | 0.844745511 |
| Mg | P69905 | 0.121187711 | 0.438857661 | 0.844745511 |
| γ-GT | Q96HR3 | 0.121187268 | 0.438859347 | 0.844745511 |
| ALT | A0A096LPE2 | 0.121120584 | 0.439113139 | 0.844745511 |
| AST | P08185 | -0.120989981 | 0.439610434 | 0.844828642 |
| Ca | P01782 | -0.120973951 | 0.439671491 | 0.844828642 |
| Neutrophils | P15169 | -0.120747594 | 0.440534194 | 0.845204996 |
| WLGG | K7ERG9 | 0.120683893 | 0.440777142 | 0.845204996 |
| Creatine Kinase | A0A5H1ZRS9 | 0.120681194 | 0.440787437 | 0.845204996 |
| Globin | P01718 | 0.120652186 | 0.440898094 | 0.845204996 |
| LDH | A0A0B4J1Y8 | -0.120459181 | 0.441634756 | 0.84554101 |
| DBIL | P02751 | -0.120403676 | 0.441846735 | 0.84554101 |
| DBIL | D6R934 | 0.120403676 | 0.441846735 | 0.84554101 |
| LDH | Q16880 | -0.120228073 | 0.442517741 | 0.845929738 |
| APTT | P04264 | -0.120148015 | 0.44282384 | 0.845929738 |
| WLGG | P26038 | -0.120117642 | 0.442940002 | 0.845929738 |
| Albumin | P02745 | 0.120015111 | 0.443332255 | 0.845929738 |
| ALP | I3L1J2 | -0.119979006 | 0.443470427 | 0.845929738 |
| CO2 | A0A0C4DH73 | -0.119904826 | 0.443754385 | 0.845929738 |
| Total protein | A0A075B6S9 | -0.119878494 | 0.443855207 | 0.845929738 |
| LDH | A0A0J9YX35 | -0.119699432 | 0.444541147 | 0.846150702 |
| Mg | P35908 | -0.119520338 | 0.445227792 | 0.846150702 |
| INR | P35908 | -0.119369319 | 0.44580725 | 0.846150702 |
| Albumin | A0A0A0MS15 | 0.119335353 | 0.445937633 | 0.846150702 |
| Lymphocytes | A0A0C4DH36 | 0.119326338 | 0.445972242 | 0.846150702 |
| WLGG | Q86UD1 | -0.119309537 | 0.446036744 | 0.846150702 |
| Platelets | P0DJI8 | -0.119250815 | 0.446262236 | 0.846150702 |
| Lymphocytes | P30041 | 0.119137895 | 0.446696023 | 0.846150702 |
| CRP | A0A0C4DH36 | -0.119108767 | 0.446807955 | 0.846150702 |
| P | Q8N1N4 | 0.11909624 | 0.446856098 | 0.846150702 |
| PT | A0A1W2PQU7 | -0.119027177 | 0.447121571 | 0.846150702 |
| RBC | P01718 | -0.118982312 | 0.447294073 | 0.846150702 |
| Hematocrit | P13473 | 0.118948723 | 0.447423247 | 0.846150702 |
| CRP | A0A140T8Y3 | 0.11890725 | 0.447582765 | 0.846150702 |
| PT | A0A087X0Q4 | -0.118648833 | 0.44857743 | 0.847261457 |
| Hemoglobin | Q15582 | -0.118620401 | 0.448686936 | 0.847261457 |
| Albumin | A0A087X0Q4 | -0.118277951 | 0.450007083 | 0.848658191 |
| Basophils | P19823 | 0.118274286 | 0.450021224 | 0.848658191 |
| P | Q6ZRK6 | 0.118227223 | 0.450202821 | 0.848658191 |
| APTT | P35908 | -0.118109048 | 0.450658983 | 0.849030134 |
| BUN | P37802 | -0.11794456 | 0.451294341 | 0.849526223 |
| Eosinophils | P01782 | -0.117878736 | 0.45154873 | 0.849526223 |
| RBC | Q9H4B7 | -0.117723981 | 0.452147118 | 0.849526223 |
| TBIL | O75636 | 0.117655945 | 0.45241033 | 0.849526223 |
| ALT | P04264 | -0.117567109 | 0.452754135 | 0.849526223 |
| Mg | P80748 | 0.117474016 | 0.453114564 | 0.849526223 |
| α-HBDH | P04430 | -0.117464688 | 0.453150689 | 0.849526223 |
| CRP | D6R934 | -0.117447135 | 0.453218669 | 0.849526223 |
| INR | P00915 | -0.117326878 | 0.453684563 | 0.849526223 |
| Creatinine | E7ENL6 | -0.117308646 | 0.453755219 | 0.849526223 |
| RBC | A0A140T8Y3 | 0.117304491 | 0.453771324 | 0.849526223 |
| RBC | A0A087X0Q4 | 0.117056227 | 0.454734056 | 0.8507129 |
| γ-GT | P08185 | 0.116948363 | 0.455152683 | 0.8507129 |
| Neutrophils | A0A2R8Y7X9 | 0.116896357 | 0.455354594 | 0.8507129 |
| DBIL | Q6ZRK6 | -0.116853581 | 0.455520708 | 0.8507129 |
| BUN | P02745 | 0.116669814 | 0.456234713 | 0.8507129 |
| CO2 | A0A5H1ZRS9 | 0.116655987 | 0.456288459 | 0.8507129 |
| Mg | P02751 | 0.11664033 | 0.456349326 | 0.8507129 |
| Glucose | E7EX29 | -0.116606695 | 0.456480092 | 0.8507129 |
| UA | A0A2R8Y7X9 | 0.116358969 | 0.457443841 | 0.851797239 |
| Glucose | P00918 | 0.116256985 | 0.457840916 | 0.851797239 |
| TT | P13645 | -0.116256959 | 0.457841016 | 0.851797239 |
| WLGG | H3BTN5 | -0.11616841 | 0.458185934 | 0.851955704 |
| WBC | P01019 | 0.116056934 | 0.458620357 | 0.852042546 |
| CRP | P19823 | -0.115936561 | 0.459089699 | 0.852042546 |
| Albumin | P19823 | -0.115785504 | 0.459679049 | 0.852042546 |
| PT | P01715 | 0.115764281 | 0.459761883 | 0.852042546 |
| INR | P01718 | -0.115651781 | 0.460201109 | 0.852042546 |
| WLC | E7ENL6 | 0.115630571 | 0.460283944 | 0.852042546 |
| Monocytes | Q16880 | -0.115565127 | 0.460539578 | 0.852042546 |
| IBIL | K7ERG9 | -0.115558918 | 0.460563839 | 0.852042546 |
| IBIL | P01817 | -0.115256803 | 0.461744984 | 0.853063574 |
| Creatinine | A0A5H1ZRS9 | -0.115221987 | 0.460570559 | 0.852042546 |
| α-HBDH | P30041 | 0.115213316 | 0.461915135 | 0.853063574 |
| Total protein | E7ENL6 | 0.11520186 | 0.461959963 | 0.853063574 |
| CO2 | P08185 | 0.115144899 | 0.462182894 | 0.853063574 |
| Mg | Q16880 | -0.115048746 | 0.462559346 | 0.853063574 |
| LDH | Q96HR3 | 0.115017183 | 0.462682957 | 0.853063574 |
| PT | P35908 | -0.114865388 | 0.463277676 | 0.853680212 |
| UA | O75636 | 0.114773285 | 0.463638725 | 0.853865815 |
| Lymphocytes | P03950 | 0.114527554 | 0.464602747 | 0.854269361 |
| Monocytes | A0A0J9YX35 | 0.114506285 | 0.464686238 | 0.854269361 |
| Hemoglobin | P03950 | 0.114497171 | 0.464722016 | 0.854269361 |
| Globin | P01814 | 0.114451931 | 0.464899637 | 0.854269361 |
| BUN | P43121 | -0.114358293 | 0.465267395 | 0.854466436 |
| FIB | A0A0J9YX35 | 0.114212345 | 0.465840905 | 0.855040946 |
| Ca | P32119 | -0.114093558 | 0.466307966 | 0.855419534 |
| Albumin | P0DJI8 | 0.113972815 | 0.466782973 | 0.855423249 |
| Globin | O75882 | -0.11392311 | 0.466978587 | 0.855423249 |
| TT | Q92954 | 0.113831781 | 0.46733813 | 0.855423249 |
| DBIL | Q92954 | -0.113827957 | 0.46735319 | 0.855423249 |
| LDH | K7ERG9 | 0.1135823 | 0.468321039 | 0.855789081 |
| FIB | P26038 | -0.113515381 | 0.468584874 | 0.855789081 |
| UA | I3L1J2 | 0.11349743 | 0.468655662 | 0.855789081 |
| WLGG | Q8N1N4 | -0.113417585 | 0.468970588 | 0.855789081 |
| Hemoglobin | P04430 | -0.113368161 | 0.469165586 | 0.855789081 |
| CK-MB activity | A0A075B6S9 | -0.113272827 | 0.469541833 | 0.855789081 |
| Ca | P26038 | 0.113053889 | 0.470406514 | 0.855789081 |
| UA | A0A075B6S9 | -0.113033427 | 0.470487371 | 0.855789081 |
| RBC | P01715 | 0.112964029 | 0.470761654 | 0.855789081 |
| WBC | P69905 | 0.112961075 | 0.470773328 | 0.855789081 |
| DBIL | P08185 | 0.112920961 | 0.470931913 | 0.855789081 |
| WLL | C9JV77 | -0.112864758 | 0.471154153 | 0.855789081 |
| Platelets | A0A0C4DH73 | -0.11283136 | 0.47128624 | 0.855789081 |
| Monocytes | I3L1J2 | -0.112788288 | 0.471456619 | 0.855789081 |
| WLGG | O75882 | -0.112785732 | 0.471466729 | 0.855789081 |
| Total protein | C9J8S2 | 0.11257429 | 0.472303604 | 0.856248872 |
| Eosinophils | A0A087X0Q4 | -0.112506855 | 0.472570676 | 0.856248872 |
| Hematocrit | I3L1J2 | 0.112464005 | 0.472740421 | 0.856248872 |
| Glucose | C9JV77 | 0.112457993 | 0.47276424 | 0.856248872 |
| Basophils | A0A2R8Y7X9 | -0.112364675 | 0.473134027 | 0.856386614 |
| WLL | K7ERG9 | 0.112241195 | 0.473623573 | 0.856386614 |
| Creatinine | P01715 | 0.112241195 | 0.473623573 | 0.856386614 |
| PT | P02750 | -0.111914301 | 0.474920868 | 0.857617914 |
| WLC | P43121 | -0.111847822 | 0.475184921 | 0.857617914 |
| CO2 | P30041 | 0.111831714 | 0.475248912 | 0.857617914 |
| Albumin | C9J8S2 | -0.111792741 | 0.475403763 | 0.857617914 |
| TBIL | P26038 | 0.111705058 | 0.47575224 | 0.857617914 |
| Eosinophils | P02750 | -0.111674592 | 0.475873355 | 0.857617914 |
| ALT | P15169 | 0.11151864 | 0.476493573 | 0.857675339 |
| Monocytes | P02753 | 0.111254125 | 0.47754652 | 0.857675339 |
| APTT | Q16880 | -0.111236975 | 0.477614831 | 0.857675339 |
| AST | P15169 | 0.111229063 | 0.477646345 | 0.857675339 |
| DBIL | I3L1J2 | -0.111213394 | 0.477708764 | 0.857675339 |
| Hematocrit | P01019 | 0.111169854 | 0.47788223 | 0.857675339 |
| AST | Q9Y5Y7 | -0.111153397 | 0.477947804 | 0.857675339 |
| Lymphocytes | P13645 | 0.111018808 | 0.478484266 | 0.857675339 |
| α-HBDH | A0A2R8Y7X9 | -0.110964243 | 0.478701849 | 0.857675339 |
| Hematocrit | A0A0C4DH33 | 0.110947474 | 0.478768727 | 0.857675339 |
| Neutrophils | P00918 | 0.110879239 | 0.47904091 | 0.857675339 |
| P | K7ERG9 | -0.110859254 | 0.479120646 | 0.857675339 |
| FIB | P01817 | 0.110813168 | 0.479304541 | 0.857675339 |
| Hematocrit | P01715 | 0.11070737 | 0.479726847 | 0.857962954 |
| TBIL | A0A0B4J1V2 | 0.110637013 | 0.480007792 | 0.85799758 |
| WBC | E7ENL6 | -0.110421958 | 0.480867077 | 0.859065366 |
| Total protein | K7ERG9 | -0.110339101 | 0.481198356 | 0.859189227 |
| INR | Q86UD1 | -0.110227753 | 0.481643739 | 0.859515128 |
| Hemoglobin | A0A0B4J1V2 | 0.110162468 | 0.481904976 | 0.859515128 |
| IBIL | P08185 | -0.110045322 | 0.482373917 | 0.859883939 |
| Creatine Kinase | Q6ZRK6 | -0.109946341 | 0.482770332 | 0.860123134 |
| Monocytes | A0A096LPE2 | 0.109817124 | 0.483288093 | 0.860578146 |
| DBIL | A0A5H1ZRS9 | 0.109746476 | 0.483571294 | 0.860615216 |
| Globin | P13473 | -0.109616998 | 0.484090552 | 0.861072132 |
| Hematocrit | A0A096LPE2 | -0.109508348 | 0.484526506 | 0.861226 |
| γ-GT | E7ENL6 | 0.109460361 | 0.484719118 | 0.861226 |
| TBIL | P13647 | 0.109349042 | 0.485166084 | 0.861226 |
| IBIL | P35527 | -0.109290035 | 0.485403096 | 0.861226 |
| Eosinophils | P03950 | 0.109268428 | 0.485489901 | 0.861226 |
| WLC | P04430 | -0.109161396 | 0.485920008 | 0.861523041 |
| IBIL | A0A1W2PQU7 | 0.109063449 | 0.486313779 | 0.861755373 |
| Total protein | P01817 | -0.108828641 | 0.487258444 | 0.862570095 |
| Basophils | O75636 | 0.108753247 | 0.487561969 | 0.862570095 |
| Total protein | P00915 | 0.108753118 | 0.487562487 | 0.862570095 |
| TT | A0A0C4DH73 | -0.108526705 | 0.488474576 | 0.862703214 |
| Globin | P13645 | -0.108483811 | 0.488647474 | 0.862703214 |
| Total protein | C9JV77 | -0.108451026 | 0.488779641 | 0.862703214 |
| TT | P43121 | 0.108429185 | 0.488867701 | 0.862703214 |
| Lymphocytes | P32119 | 0.108375503 | 0.489084176 | 0.862703214 |
| Basophils | P15169 | 0.108342857 | 0.489215847 | 0.862703214 |
| WBC | A0A087X0Q4 | 0.108053007 | 0.490385698 | 0.863850959 |
| AST | P02042 | 0.10805109 | 0.490393441 | 0.863850959 |
| CRP | P00915 | 0.107930519 | 0.490880504 | 0.864107415 |
| FIB | A0A0C4DH33 | -0.107833517 | 0.491272534 | 0.864107415 |
| Neutrophils | A0A075B6S9 | 0.107819458 | 0.491329369 | 0.864107415 |
| WLGG | A0A0B4J1V2 | 0.107734977 | 0.49167095 | 0.864244757 |
| BUN | A0A0A0MS15 | 0.107608081 | 0.492184261 | 0.864459413 |
| FIB | A0A087X0Q4 | -0.107413991 | 0.492969911 | 0.864459413 |
| Hemoglobin | P01715 | 0.107399594 | 0.493028213 | 0.864459413 |
| P | E7ENL6 | -0.107389727 | 0.493068174 | 0.864459413 |
| CRP | P04264 | -0.107326289 | 0.493325131 | 0.864459413 |
| RBC | A0A0C4DH73 | -0.107314128 | 0.493374396 | 0.864459413 |
| Total protein | Q86UD1 | -0.107154574 | 0.494021007 | 0.865130221 |
| Glucose | P02766 | 0.106718028 | 0.495792393 | 0.867564538 |
| CO2 | P26038 | -0.106681664 | 0.495940094 | 0.867564538 |
| Eosinophils | A0A0C4DH73 | -0.106454032 | 0.4968652 | 0.867986297 |
| RBC | A0A0B4J1V2 | 0.106449665 | 0.496882954 | 0.867986297 |
| Lymphocytes | A0A2R8Y7X9 | 0.106411905 | 0.4970365 | 0.867986297 |
| Hemoglobin | A0A0C4DH33 | 0.106309049 | 0.497454876 | 0.867986297 |
| WBC | P02750 | -0.106165289 | 0.498039932 | 0.867986297 |
| Basophils | P02750 | -0.106126753 | 0.498196819 | 0.867986297 |
| UA | Q9Y5Y7 | -0.106014271 | 0.498654903 | 0.867986297 |
| APTT | P02042 | 0.105950764 | 0.498913633 | 0.867986297 |
| CO2 | P02766 | -0.105927263 | 0.499009393 | 0.867986297 |
| CRP | Q9Y5Y7 | -0.105891244 | 0.499156182 | 0.867986297 |
| Platelets | A0A140T8Y3 | -0.105880762 | 0.499198902 | 0.867986297 |
| WLC | Q86YZ3 | 0.105795567 | 0.499546197 | 0.867986297 |
| Lymphocytes | P01715 | 0.105717742 | 0.499863555 | 0.867986297 |
| WBC | O75882 | -0.105712236 | 0.499886011 | 0.867986297 |
| CO2 | P02763 | -0.105625045 | 0.500241693 | 0.868144314 |
| α-HBDH | Q96HR3 | 0.10552556 | 0.500647689 | 0.868389434 |
| LDH | Q86YZ3 | -0.105278885 | 0.501655076 | 0.869217459 |
| Creatinine | P04264 | -0.105104198 | 0.501082819 | 0.868684803 |
| CO2 | A0A0B4J1Y8 | -0.105062354 | 0.502540216 | 0.869363596 |
| α-HBDH | H3BTN5 | -0.105060792 | 0.502546604 | 0.869363596 |
| LDH | E7EX29 | 0.105035466 | 0.502650184 | 0.869363596 |
| Basophils | P02753 | 0.104977662 | 0.502886636 | 0.869363596 |
| RBC | P03950 | 0.104934149 | 0.503064666 | 0.869363596 |
| APTT | Q86UD1 | -0.104861908 | 0.503360311 | 0.86941644 |
| WLL | A0A0C4DH73 | -0.104758449 | 0.503783861 | 0.869425668 |
| Glucose | A0A0J9YVY3 | 0.104731116 | 0.503895791 | 0.869425668 |
| WLL | A0A0C4DH33 | -0.104606508 | 0.504406223 | 0.869848798 |
| Lymphocytes | Q15582 | -0.104372784 | 0.505364335 | 0.870542555 |
| Hemoglobin | A0A140T8Y3 | 0.104334772 | 0.505520246 | 0.870542555 |
| Total protein | A0A087X0Q4 | 0.104297261 | 0.505674126 | 0.870542555 |
| Creatine Kinase | Q5SRP5 | -0.104249484 | 0.505870155 | 0.870542555 |
| WLGG | D6R934 | -0.104097756 | 0.506492948 | 0.870836201 |
| CRP | A0A096LPE2 | 0.104078555 | 0.50657179 | 0.870836201 |
| P | P01814 | 0.103982504 | 0.506966278 | 0.870914027 |
| IBIL | P19823 | -0.103851968 | 0.507502646 | 0.870914027 |
| Glucose | P69905 | 0.103848046 | 0.507518769 | 0.870914027 |
| Globin | D6R934 | 0.103724423 | 0.508027004 | 0.870914027 |
| Ca | P0DJI8 | 0.103659555 | 0.508293794 | 0.870914027 |
| Monocytes | P19652 | -0.10361533 | 0.50847572 | 0.870914027 |
| WLC | P02042 | 0.103526992 | 0.508839215 | 0.871081747 |
| Creatinine | P01814 | -0.103443069 | 0.507905077 | 0.870914027 |
| AST | A0A0J9YVY3 | 0.10339288 | 0.50939131 | 0.871130911 |
| Lymphocytes | P35908 | 0.103390985 | 0.509399112 | 0.871130911 |
| α-HBDH | P26038 | -0.103209579 | 0.510146388 | 0.871810872 |
| Monocytes | A0A0B4J1Y8 | -0.103165435 | 0.510328315 | 0.871810872 |
| RBC | C9JV77 | 0.10300948 | 0.510971308 | 0.872362666 |
| TBIL | Q86UD1 | -0.102958097 | 0.511183245 | 0.872362666 |
| WLL | O75636 | -0.102731872 | 0.51211687 | 0.873501473 |
| WBC | P32119 | 0.10246536 | 0.513217855 | 0.874924411 |
| WLC | A0A140T8Y3 | 0.10225324 | 0.514094994 | 0.875964458 |
| TT | A0A0C4DH33 | 0.102126576 | 0.51461912 | 0.87629428 |
| Hematocrit | Q86YZ3 | 0.102037147 | 0.514989328 | 0.87629428 |
| α-HBDH | C9JV77 | -0.101975308 | 0.515245403 | 0.87629428 |
| FIB | P80748 | 0.101899771 | 0.515558287 | 0.87629428 |
| UA | A0A0J9YVY3 | -0.101883818 | 0.515624378 | 0.87629428 |
| CK-MB activity | P37802 | -0.101535033 | 0.517070413 | 0.877385046 |
| Eosinophils | A0A0C4DH33 | -0.101502115 | 0.517206992 | 0.877385046 |
| LDH | D6RE82 | -0.101348036 | 0.517846514 | 0.877385046 |
| Neutrophils | P0DJI8 | -0.101340382 | 0.517878295 | 0.877385046 |
| Creatinine | Q86YZ3 | -0.101324473 | 0.51794435 | 0.877385046 |
| Eosinophils | A0A2R8Y7X9 | -0.101309132 | 0.518008056 | 0.877385046 |
| Glucose | P37802 | -0.10127768 | 0.518138669 | 0.877385046 |
| TBIL | D6R934 | -0.101117657 | 0.518803469 | 0.87760463 |
| α-HBDH | Q9Y5Y7 | 0.100993324 | 0.519320293 | 0.878026063 |
| Platelets | A0A0A0MS15 | 0.100898726 | 0.519713683 | 0.878238475 |
| Creatinine | D6R934 | 0.100875868 | 0.51854103 | 0.87760463 |
| Glucose | P02751 | -0.100827012 | 0.520012012 | 0.878290114 |
| WLL | P15169 | 0.100705295 | 0.520518542 | 0.878340334 |
| FIB | P13473 | 0.100691175 | 0.52057732 | 0.878340334 |
| CRP | A0A087X0Q4 | 0.100604234 | 0.520939296 | 0.878499173 |
| γ-GT | P0DP01 | 0.100299308 | 0.522209842 | 0.88018925 |
| WLGG | A0A140T8Y3 | 0.099727887 | 0.524594923 | 0.883755017 |
| Mg | A0A1W2PQU7 | -0.09966344 | 0.52486426 | 0.883755017 |
| Creatinine | P43121 | -0.099538588 | 0.525386232 | 0.883926739 |
| P | P69905 | -0.099448383 | 0.525763511 | 0.883926739 |
| PT | I3L1J2 | 0.099226183 | 0.526693431 | 0.883926739 |
| LDH | P01019 | 0.099157952 | 0.526979141 | 0.883926739 |
| P | O75882 | 0.099146108 | 0.527028743 | 0.883926739 |
| TT | P08185 | 0.099053354 | 0.527417284 | 0.883926739 |
| Mg | E7EX29 | 0.099018144 | 0.527564814 | 0.883926739 |
| Globin | Q6ZRK6 | -0.098985358 | 0.527702207 | 0.883926739 |
| AST | P02745 | 0.098971166 | 0.527761681 | 0.883926739 |
| IBIL | P0DJI8 | 0.098942603 | 0.5278814 | 0.883926739 |
| Basophils | P01718 | 0.098930857 | 0.527930635 | 0.883926739 |
| Hemoglobin | D6R934 | -0.098674041 | 0.529007666 | 0.885192926 |
| BUN | P04264 | 0.098621862 | 0.529226624 | 0.885192926 |
| P | P01782 | 0.09846599 | 0.529880975 | 0.885487632 |
| γ-GT | A0A075B6S9 | -0.09845128 | 0.52994275 | 0.885487632 |
| BUN | P02750 | 0.098319804 | 0.530495024 | 0.885959104 |
| WLL | E7EX29 | 0.098149591 | 0.531210439 | 0.886300703 |
| PT | Q8N1N4 | -0.098142561 | 0.531239994 | 0.886300703 |
| UA | P13645 | -0.098010345 | 0.531796045 | 0.88677734 |
| AST | P01817 | -0.097836176 | 0.532528964 | 0.887548273 |
| WBC | Q9NZP8 | 0.09770831 | 0.53306735 | 0.8876859 |
| AST | A0A0C4DH33 | 0.097575029 | 0.533628819 | 0.8876859 |
| APTT | P15169 | 0.097568344 | 0.533656986 | 0.8876859 |
| PT | E7EX29 | 0.097559541 | 0.533694084 | 0.8876859 |
| IBIL | A0A0B4J1V2 | 0.097435707 | 0.534216041 | 0.888103707 |
| TBIL | P03950 | 0.097212439 | 0.535157742 | 0.889186082 |
| γ-GT | A0A0C4DH73 | 0.097116343 | 0.535563305 | 0.889186082 |
| CK-MB activity | P01715 | -0.096830707 | 0.536769677 | 0.889186082 |
| Neutrophils | Q96HR3 | 0.096809515 | 0.536859229 | 0.889186082 |
| ALP | A0A096LPE2 | -0.096634072 | 0.537600918 | 0.889186082 |
| Mg | P13647 | -0.096631852 | 0.537610307 | 0.889186082 |
| TT | Q15582 | 0.096628177 | 0.53762585 | 0.889186082 |
| γ-GT | P01817 | -0.09658648 | 0.537802203 | 0.889186082 |
| WLL | P02753 | -0.09649625 | 0.538183911 | 0.889186082 |
| LDH | P69905 | 0.096439227 | 0.53842521 | 0.889186082 |
| PT | P32119 | -0.096402176 | 0.538582021 | 0.889186082 |
| Eosinophils | P04430 | -0.096291551 | 0.539050358 | 0.889186082 |
| APTT | P26038 | -0.096174684 | 0.539545333 | 0.889186082 |
| Globin | P02751 | 0.09616984 | 0.539565852 | 0.889186082 |
| CO2 | P04430 | 0.096081124 | 0.539941753 | 0.889186082 |
| WLC | P00918 | 0.096072314 | 0.539979087 | 0.889186082 |
| ALT | P69905 | 0.096019439 | 0.540203192 | 0.889186082 |
| LDH | A0A5H1ZRS9 | 0.095986106 | 0.540344493 | 0.889186082 |
| Globin | Q16880 | -0.095867657 | 0.540846749 | 0.889186082 |
| WBC | P80748 | -0.095745083 | 0.541366735 | 0.889186082 |
| CK-MB activity | P01718 | 0.095737331 | 0.541399629 | 0.889186082 |
| Creatine Kinase | P02763 | -0.095684025 | 0.541625842 | 0.889186082 |
| TT | P26038 | 0.095681607 | 0.541636106 | 0.889186082 |
| IBIL | A0A0J9YXX1 | 0.095543811 | 0.542221092 | 0.889186082 |
| CO2 | P69905 | -0.095500756 | 0.542403934 | 0.889186082 |
| BUN | P26038 | 0.095451379 | 0.542613663 | 0.889186082 |
| γ-GT | P01782 | -0.095375364 | 0.542936613 | 0.889186082 |
| TT | P0DP01 | 0.095343401 | 0.543072436 | 0.889186082 |
| CK-MB activity | Q9Y5Y7 | 0.095319314 | 0.543174801 | 0.889186082 |
| ALT | A0A0J9YVY3 | 0.095260988 | 0.543422711 | 0.889186082 |
| Creatine Kinase | P01814 | -0.095230905 | 0.5435506 | 0.889186082 |
| TT | A0A140T8Y3 | -0.095162269 | 0.543842435 | 0.889186082 |
| Glucose | P01019 | 0.095011521 | 0.544483669 | 0.889186082 |
| Hemoglobin | Q92954 | 0.094971876 | 0.544652368 | 0.889186082 |
| ALP | Q9H4B7 | 0.094954769 | 0.544725169 | 0.889186082 |
| WLC | A0A0G2JI36 | -0.094939069 | 0.544791985 | 0.889186082 |
| P | A0A2R8Y7X9 | -0.094914262 | 0.544897569 | 0.889186082 |
| Eosinophils | Q8N1N4 | -0.094802347 | 0.54537403 | 0.889276765 |
| TBIL | K7ERG9 | -0.094698687 | 0.54581552 | 0.889276765 |
| UA | P43121 | 0.094619496 | 0.546152912 | 0.889276765 |
| Creatine Kinase | A0A140T8Y3 | 0.094600396 | 0.546234304 | 0.889276765 |
| ALT | C9JV77 | 0.094582928 | 0.546308744 | 0.889276765 |
| FIB | A0A075B6S9 | -0.094370539 | 0.54721424 | 0.890111558 |
| P | Q86YZ3 | -0.094182652 | 0.548015867 | 0.890111558 |
| Hemoglobin | O75636 | 0.094065223 | 0.548517168 | 0.890111558 |
| γ-GT | I3L1J2 | 0.094055108 | 0.548560356 | 0.890111558 |
| Total protein | P0DP01 | 0.094029683 | 0.548668928 | 0.890111558 |
| RBC | P01814 | 0.093947062 | 0.549021813 | 0.890111558 |
| INR | Q6ZRK6 | 0.093938821 | 0.549057017 | 0.890111558 |
| WLC | P01814 | -0.093880158 | 0.549307644 | 0.890111558 |
| Albumin | P00918 | 0.09382094 | 0.549560703 | 0.890111558 |
| WBC | P01782 | -0.093706347 | 0.550050548 | 0.890111558 |
| Glucose | Q9H4B7 | -0.093644816 | 0.550313657 | 0.890111558 |
| WBC | A0A0C4DH73 | 0.093630838 | 0.550373435 | 0.890111558 |
| Lymphocytes | A0A0C4DH73 | 0.093572995 | 0.550620839 | 0.890111558 |
| Lymphocytes | P01814 | 0.093572995 | 0.550620839 | 0.890111558 |
| Platelets | A0A0J9YX35 | 0.093421949 | 0.551267142 | 0.890576741 |
| Platelets | A0A096LPE2 | 0.093346426 | 0.551590428 | 0.890576741 |
| P | P02750 | 0.093251751 | 0.551995823 | 0.890576741 |
| Hemoglobin | Q9H4B7 | -0.093162418 | 0.552378475 | 0.890576741 |
| Lymphocytes | P37802 | 0.09293182 | 0.553366798 | 0.890576741 |
| WLC | Q86UD1 | -0.092850694 | 0.5537147 | 0.890576741 |
| Eosinophils | K7ERG9 | -0.092759519 | 0.554105815 | 0.890576741 |
| IBIL | P04264 | 0.092749249 | 0.554149877 | 0.890576741 |
| RBC | A0A075B6S5 | -0.09273874 | 0.554194969 | 0.890576741 |
| γ-GT | P01019 | 0.092726048 | 0.554249427 | 0.890576741 |
| Eosinophils | P01718 | 0.092599681 | 0.554791789 | 0.890576741 |
| Neutrophils | A0A075B6S5 | -0.092580707 | 0.554873246 | 0.890576741 |
| TBIL | P30041 | 0.092461967 | 0.555383131 | 0.890576741 |
| CRP | O75882 | 0.092447134 | 0.555446843 | 0.890576741 |
| WLC | P01782 | -0.092311568 | 0.556029287 | 0.890576741 |
| Glucose | A0A1W2PQU7 | -0.09229259 | 0.556110844 | 0.890576741 |
| INR | A0A0B4J1V2 | -0.092253726 | 0.556277882 | 0.890576741 |
| Lymphocytes | P13647 | 0.092213581 | 0.556450451 | 0.890576741 |
| Hematocrit | Q92954 | 0.092213581 | 0.556450451 | 0.890576741 |
| WLGG | P30041 | 0.092185557 | 0.556570933 | 0.890576741 |
| Hemoglobin | A0A0C4DH36 | -0.092176363 | 0.556610463 | 0.890576741 |
| Neutrophils | P80748 | -0.091976591 | 0.557469701 | 0.891230277 |
| Total protein | A0A140T8Y3 | 0.091955058 | 0.557562356 | 0.891230277 |
| PT | A0A096LPE2 | 0.091786374 | 0.558288423 | 0.891956174 |
| Basophils | P03951 | -0.091681039 | 0.558742039 | 0.89218883 |
| TT | P01019 | -0.091626248 | 0.558978063 | 0.89218883 |
| AST | A0A0C4DH36 | 0.091404563 | 0.559933481 | 0.892279539 |
| TBIL | A0A0J9YXX1 | 0.091375926 | 0.560056958 | 0.892279539 |
| TT | E7ENL6 | -0.091314197 | 0.560323163 | 0.892279539 |
| Neutrophils | P01814 | 0.091296961 | 0.560397502 | 0.892279539 |
| CRP | A0A0B4J1Y8 | -0.091166588 | 0.56095996 | 0.892279539 |
| α-HBDH | P03950 | -0.091155889 | 0.561006128 | 0.892279539 |
| CRP | P02042 | 0.091087617 | 0.561300784 | 0.892279539 |
| Creatinine | A0A0A0MS15 | 0.090909091 | 0.560867324 | 0.892279539 |
| Eosinophils | P02763 | 0.090868011 | 0.562249074 | 0.892279539 |
| Monocytes | P26038 | 0.090857464 | 0.562294637 | 0.892279539 |
| UA | P04264 | -0.090837014 | 0.562382983 | 0.892279539 |
| ALP | A0A1W2PQU7 | 0.090816384 | 0.562472117 | 0.892279539 |
| Glucose | Q15582 | 0.090782073 | 0.562620369 | 0.892279539 |
| Ca | P01817 | -0.090730464 | 0.562843404 | 0.892279539 |
| Hemoglobin | C9J8S2 | -0.090650099 | 0.563190786 | 0.892330064 |
| DBIL | P13473 | -0.090548401 | 0.563630526 | 0.892330064 |
| γ-GT | A0A0C4DH33 | -0.090534319 | 0.56369143 | 0.892330064 |
| BUN | A0A0J9YXX1 | 0.090466302 | 0.563985637 | 0.892365117 |
| Mg | Q86YZ3 | -0.090264484 | 0.56485902 | 0.892472003 |
| γ-GT | A0A096LPE2 | 0.090228122 | 0.565016448 | 0.892472003 |
| Monocytes | Q6ZRK6 | -0.09022335 | 0.565037111 | 0.892472003 |
| WBC | P00915 | 0.090157436 | 0.565322535 | 0.892472003 |
| CO2 | A0A0C4DH36 | -0.090136394 | 0.565413665 | 0.892472003 |
| WLC | Q9NZP8 | 0.090037111 | 0.565843746 | 0.892721254 |
| Platelets | K7ERG9 | 0.089570276 | 0.567868034 | 0.894623731 |
| Monocytes | Q8N1N4 | -0.089472216 | 0.568293668 | 0.894623731 |
| PT | Q16880 | -0.089440638 | 0.568430764 | 0.894623731 |
| Hematocrit | C9JV77 | 0.08941923 | 0.568523713 | 0.894623731 |
| α-HBDH | A0A140T8Y3 | 0.089398714 | 0.5686128 | 0.894623731 |
| Hematocrit | A0A140T8Y3 | 0.08938183 | 0.568686121 | 0.894623731 |
| Lymphocytes | P80748 | 0.089041615 | 0.570164434 | 0.894855865 |
| DBIL | Q9H4B7 | -0.089013921 | 0.570284852 | 0.894855865 |
| Glucose | P01718 | 0.08894988 | 0.570563347 | 0.894855865 |
| WLC | D6RE82 | -0.088860668 | 0.570951413 | 0.894855865 |
| Creatinine | P35908 | -0.088643914 | 0.570708728 | 0.894855865 |
| Mg | A0A2R8Y7X9 | -0.088598145 | 0.572094064 | 0.894855865 |
| CRP | P13645 | -0.088519642 | 0.57243596 | 0.894855865 |
| Creatine Kinase | P01715 | -0.088484591 | 0.572588641 | 0.894855865 |
| WLGG | I3L1J2 | -0.088422902 | 0.572857405 | 0.894855865 |
| WBC | P37802 | -0.088393279 | 0.572986488 | 0.894855865 |
| Creatine Kinase | O75882 | 0.088358571 | 0.57313774 | 0.894855865 |
| TBIL | P15169 | -0.088355234 | 0.573152283 | 0.894855865 |
| γ-GT | Q15582 | 0.088335754 | 0.573237188 | 0.894855865 |
| BUN | Q9Y5Y7 | 0.088276383 | 0.573495982 | 0.894855865 |
| Glucose | P15169 | -0.087761039 | 0.575744602 | 0.894855865 |
| TBIL | P01817 | -0.087751096 | 0.575788027 | 0.894855865 |
| AST | A0A0J9YXX1 | 0.087696928 | 0.576024621 | 0.894855865 |
| ALP | Q5SRP5 | 0.087689243 | 0.576058193 | 0.894855865 |
| Eosinophils | P19823 | 0.087614619 | 0.576384215 | 0.894855865 |
| LDH | P13647 | 0.08760337 | 0.576433367 | 0.894855865 |
| α-HBDH | P35908 | -0.08754769 | 0.576676687 | 0.894855865 |
| AST | Q96HR3 | 0.08746993 | 0.577016579 | 0.894855865 |
| Platelets | P0DP01 | -0.087458934 | 0.577064648 | 0.894855865 |
| CO2 | I3L1J2 | 0.087427192 | 0.577203426 | 0.894855865 |
| IBIL | P04430 | -0.087388966 | 0.577370571 | 0.894855865 |
| ALT | A0A0J9YX35 | -0.087324766 | 0.577651339 | 0.894855865 |
| AST | P80748 | 0.087015934 | 0.579002817 | 0.894855865 |
| TT | Q86YZ3 | -0.086963286 | 0.579233351 | 0.894855865 |
| Hematocrit | P03950 | 0.086928674 | 0.579384932 | 0.894855865 |
| BUN | P02751 | -0.086917123 | 0.579435523 | 0.894855865 |
| Monocytes | A0A0C4DH33 | 0.086904021 | 0.579492909 | 0.894855865 |
| RBC | A0A2R8Y3M9 | -0.086848168 | 0.579737574 | 0.894855865 |
| APTT | P01817 | 0.086769371 | 0.580082825 | 0.894855865 |
| Neutrophils | A0A0J9YVY3 | 0.086713677 | 0.580326904 | 0.894855865 |
| APTT | I3L1J2 | 0.08665403 | 0.580588362 | 0.894855865 |
| CO2 | Q86UD1 | -0.086627862 | 0.580703083 | 0.894855865 |
| DBIL | A0A0J9YX35 | -0.086618087 | 0.580745939 | 0.894855865 |
| RBC | P80748 | 0.086470568 | 0.581392879 | 0.894855865 |
| Basophils | Q9NZP8 | 0.086428052 | 0.58157939 | 0.894855865 |
| WLL | P03950 | 0.086417766 | 0.581624515 | 0.894855865 |
| FIB | P19823 | 0.086414631 | 0.581638269 | 0.894855865 |
| WBC | Q96HR3 | 0.086381999 | 0.58178145 | 0.894855865 |
| AST | P0DJI8 | 0.08633494 | 0.581987956 | 0.894855865 |
| Ca | P0DP01 | 0.086310612 | 0.582094724 | 0.894855865 |
| IBIL | E7EX29 | 0.086301952 | 0.582132733 | 0.894855865 |
| γ-GT | A0A2R8Y7X9 | -0.086140606 | 0.582841084 | 0.894855865 |
| Mg | A0A140T8Y3 | 0.086127787 | 0.58289738 | 0.894855865 |
| Eosinophils | P01817 | -0.086101413 | 0.583013212 | 0.894855865 |
| Hemoglobin | Q9NZP8 | 0.086056457 | 0.583210677 | 0.894855865 |
| ALT | P19652 | 0.086039466 | 0.583285315 | 0.894855865 |
| TT | A0A5H1ZRS9 | 0.086018024 | 0.583379513 | 0.894855865 |
| TBIL | P02763 | -0.085863164 | 0.584060037 | 0.894855865 |
| TBIL | E7ENL6 | -0.085820544 | 0.584247389 | 0.894855865 |
| α-HBDH | P13645 | 0.085810333 | 0.584292278 | 0.894855865 |
| TBIL | A0A075B6S9 | 0.085669164 | 0.58491306 | 0.894855865 |
| Creatine Kinase | P69905 | 0.085564326 | 0.585374267 | 0.894855865 |
| INR | P13645 | -0.08547993 | 0.585745661 | 0.894855865 |
| WBC | Q15582 | -0.085475894 | 0.585763427 | 0.894855865 |
| BUN | A0A096LPE2 | -0.085406835 | 0.586067414 | 0.894855865 |
| TT | A0A075B6S5 | 0.085335943 | 0.586379542 | 0.894855865 |
| Total protein | P01019 | 0.085189943 | 0.587022595 | 0.894855865 |
| RBC | O75636 | 0.085111205 | 0.587369523 | 0.894855865 |
| APTT | D6RE82 | -0.085107991 | 0.587383687 | 0.894855865 |
| Neutrophils | A0A0J9YX35 | 0.085029262 | 0.587730669 | 0.894855865 |
| IBIL | P13473 | 0.084969792 | 0.587992833 | 0.894855865 |
| TT | P02042 | 0.084957009 | 0.588049195 | 0.894855865 |
| Mg | P02750 | 0.08488445 | 0.588369141 | 0.894855865 |
| WLL | P35527 | 0.084804459 | 0.588721955 | 0.894855865 |
| Basophils | Q8N1N4 | -0.084786493 | 0.588801207 | 0.894855865 |
| α-HBDH | P69905 | 0.084677274 | 0.589283112 | 0.894855865 |
| INR | P02042 | -0.084647825 | 0.589413081 | 0.894855865 |
| TT | P04264 | -0.084578075 | 0.589720961 | 0.894855865 |
| UA | A0A096LPE2 | 0.084569789 | 0.589757539 | 0.894855865 |
| P | P55056 | 0.084561353 | 0.589794781 | 0.894855865 |
| γ-GT | Q9NZP8 | 0.084551017 | 0.589840414 | 0.894855865 |
| INR | K7ERG9 | 0.084496533 | 0.590080979 | 0.894855865 |
| WLC | P01019 | 0.084468614 | 0.590204269 | 0.894855865 |
| Neutrophils | P01715 | -0.084424295 | 0.590400002 | 0.894855865 |
| WLC | Q15582 | 0.084390184 | 0.59055067 | 0.894855865 |
| IBIL | A0A087X0Q4 | 0.084365563 | 0.590659435 | 0.894855865 |
| Lymphocytes | O75882 | -0.084132621 | 0.591688891 | 0.896001644 |
| Glucose | P03950 | -0.083657391 | 0.593791558 | 0.897931342 |
| α-HBDH | A0A5H1ZRS9 | -0.083544215 | 0.594292791 | 0.897931342 |
| Hematocrit | A0A2R8Y7X9 | 0.083452914 | 0.594697286 | 0.897931342 |
| RBC | D6RE82 | 0.083449762 | 0.594711252 | 0.897931342 |
| DBIL | Q96HR3 | 0.083443602 | 0.594738546 | 0.897931342 |
| RBC | A0A0C4DH36 | -0.083374242 | 0.595045924 | 0.897931342 |
| AST | E7EX29 | -0.0832883 | 0.59542688 | 0.897931342 |
| Basophils | P80748 | -0.083227013 | 0.595698614 | 0.897931342 |
| Hematocrit | P04430 | -0.0832231 | 0.595715965 | 0.897931342 |
| Eosinophils | C9J8S2 | 0.083100272 | 0.596260731 | 0.897931342 |
| Lymphocytes | P35527 | -0.083075299 | 0.596371518 | 0.897931342 |
| Hematocrit | D6RE82 | 0.083075299 | 0.596371518 | 0.897931342 |
| CO2 | P01817 | 0.083034281 | 0.596553502 | 0.897931342 |
| TBIL | O75882 | 0.082917989 | 0.597069591 | 0.897931342 |
| APTT | P0DJI8 | 0.082917989 | 0.597069591 | 0.897931342 |
| LDH | P13473 | -0.082845601 | 0.597390941 | 0.897970176 |
| FIB | Q9NZP8 | 0.082788843 | 0.597642955 | 0.897970176 |
| Basophils | A0A087X0Q4 | -0.08257039 | 0.598613358 | 0.898167088 |
| Platelets | P35527 | -0.082471115 | 0.59905458 | 0.898167088 |
| Hemoglobin | K7ERG9 | 0.082429846 | 0.599238038 | 0.898167088 |
| CRP | P13647 | -0.082401816 | 0.599362656 | 0.898167088 |
| Mg | P30041 | 0.082332019 | 0.59967302 | 0.898167088 |
| Glucose | A0A140T8Y3 | -0.082195033 | 0.600282357 | 0.898167088 |
| WLC | K7ERG9 | 0.082194157 | 0.600286252 | 0.898167088 |
| FIB | K7ERG9 | 0.082184545 | 0.60032902 | 0.898167088 |
| INR | D6R934 | 0.082151508 | 0.600476015 | 0.898167088 |
| WLGG | E7EX29 | -0.082143349 | 0.600512325 | 0.898167088 |
| Albumin | A0A0C4DH33 | 0.082065036 | 0.600860852 | 0.898278757 |
| Basophils | E7EX29 | 0.081880286 | 0.601683416 | 0.899098681 |
| Globin | A0A0J9YXX1 | -0.081740587 | 0.602305724 | 0.89961875 |
| UA | H3BTN5 | 0.0815698 | 0.603066897 | 0.900345663 |
| TBIL | A0A1W2PQU7 | 0.081407643 | 0.603789993 | 0.901015095 |
| Hematocrit | A0A0J9YXX1 | 0.081187224 | 0.604773498 | 0.901687127 |
| Eosinophils | P01814 | -0.081183494 | 0.604790146 | 0.901687127 |
| IBIL | O75636 | 0.081117828 | 0.60508328 | 0.901714293 |
| LDH | P02042 | -0.080957597 | 0.605798819 | 0.90237063 |
| Mg | Q5SRP5 | 0.08082816 | 0.606377105 | 0.902822018 |
| Monocytes | K7ERG9 | -0.080698947 | 0.60695463 | 0.903271864 |
| Eosinophils | P00918 | 0.080558055 | 0.607584625 | 0.903799352 |
| WLL | A0A0B4J1V2 | -0.080364612 | 0.608450053 | 0.90400381 |
| INR | Q9H4B7 | -0.080314873 | 0.608672664 | 0.90400381 |
| UA | E7ENL6 | -0.080314336 | 0.608675066 | 0.90400381 |
| Total protein | P19823 | -0.080280948 | 0.608824517 | 0.90400381 |
| CO2 | D6RE82 | -0.080163214 | 0.609351634 | 0.904074607 |
| LDH | P02766 | -0.080126875 | 0.609514369 | 0.904074607 |
| Eosinophils | A0A0C4DH36 | 0.08004859 | 0.609865019 | 0.904074607 |
| Mg | O75882 | -0.079958121 | 0.610270344 | 0.904074607 |
| WLC | A0A2R8Y3M9 | 0.0799197 | 0.610442513 | 0.904074607 |
| Eosinophils | P01715 | 0.079901073 | 0.610525992 | 0.904074607 |
| WLL | E7ENL6 | -0.079772619 | 0.611101803 | 0.904518915 |
| Total protein | O75882 | 0.079601241 | 0.61187039 | 0.905248028 |
| WLC | P02753 | -0.079527553 | 0.612200986 | 0.905328781 |
| α-HBDH | K7ERG9 | -0.079465203 | 0.612480774 | 0.905334358 |
| UA | P0DP01 | -0.079287171 | 0.613279976 | 0.90564468 |
| ALT | K7ERG9 | 0.079159333 | 0.613854123 | 0.90564468 |
| ALP | A0A075B6S5 | -0.079105452 | 0.61409618 | 0.90564468 |
| Ca | A0A5H1ZRS9 | -0.079086721 | 0.614180342 | 0.90564468 |
| Platelets | P03950 | -0.079054418 | 0.61432549 | 0.90564468 |
| Glucose | Q6ZRK6 | -0.079019645 | 0.614481753 | 0.90564468 |
| Neutrophils | P02766 | 0.078988107 | 0.614623493 | 0.90564468 |
| Creatinine | A0A0C4DH33 | 0.078680107 | 0.616008457 | 0.906474307 |
| Basophils | A0A075B6S9 | 0.078646006 | 0.616161881 | 0.906474307 |
| Basophils | Q9H4B7 | -0.078619825 | 0.616279679 | 0.906474307 |
| TT | I3L1J2 | -0.078617091 | 0.616291983 | 0.906474307 |
| WLGG | P15169 | -0.078191789 | 0.618206947 | 0.908883364 |
| Total protein | P02745 | -0.077939735 | 0.61934303 | 0.909537907 |
| Mg | A0A0C4DH33 | -0.077876844 | 0.619626631 | 0.909537907 |
| WLC | Q92954 | 0.077723673 | 0.620317578 | 0.909537907 |
| CK-MB activity | P55056 | 0.077698532 | 0.62043102 | 0.909537907 |
| Basophils | P01817 | 0.077645714 | 0.620669372 | 0.909537907 |
| Platelets | P00915 | -0.077637643 | 0.6207058 | 0.909537907 |
| BUN | A0A0B4J1Y8 | 0.077631778 | 0.620732269 | 0.909537907 |
| Total protein | Q6ZRK6 | -0.077539713 | 0.621147839 | 0.909537907 |
| Creatinine | P13473 | 0.077318031 | 0.62107518 | 0.909537907 |
| Creatine Kinase | C9J8S2 | -0.077258595 | 0.622417499 | 0.910985364 |
| α-HBDH | P01715 | -0.077197887 | 0.622691825 | 0.910985364 |
| IBIL | E7ENL6 | -0.077053219 | 0.623345759 | 0.911535483 |
| Mg | A0A096LPE2 | -0.076926533 | 0.623918638 | 0.911966637 |
| Mg | Q9H4B7 | -0.076831656 | 0.624347821 | 0.912187463 |
| ALP | P02751 | -0.076687712 | 0.6249992 | 0.91273258 |
| WLC | P03950 | 0.076591357 | 0.625435383 | 0.912963087 |
| LDH | A0A096LPE2 | -0.076501908 | 0.625840416 | 0.913147938 |
| Monocytes | P35527 | 0.076312313 | 0.626699285 | 0.913167 |
| Creatinine | A0A140T8Y3 | 0.076271358 | 0.626884875 | 0.913167 |
| UA | P04430 | 0.076100169 | 0.627660874 | 0.913167 |
| WLGG | A0A0G2JI36 | -0.076056382 | 0.627859424 | 0.913167 |
| Platelets | P01817 | -0.07605166 | 0.627880838 | 0.913167 |
| Creatinine | A0A0G2JI36 | 0.076045474 | 0.627908891 | 0.913167 |
| IBIL | Q8N1N4 | 0.075981877 | 0.628197325 | 0.913167 |
| APTT | K7ERG9 | 0.075970398 | 0.628249389 | 0.913167 |
| CRP | Q96HR3 | -0.075906348 | 0.628539944 | 0.913167 |
| TT | A0A087X0Q4 | 0.075786805 | 0.629082379 | 0.913167 |
| Mg | P43121 | -0.07575158 | 0.629242253 | 0.913167 |
| γ-GT | A0A0B4J1V2 | 0.075697591 | 0.629487322 | 0.913167 |
| WBC | P00918 | 0.07557289 | 0.630053519 | 0.913167 |
| Hemoglobin | P02753 | 0.075403287 | 0.630823928 | 0.913167 |
| FIB | I3L1J2 | 0.07527422 | 0.631410463 | 0.913167 |
| Albumin | A0A0J9YVY3 | 0.075236655 | 0.631581218 | 0.913167 |
| Hematocrit | A0A087X0Q4 | -0.075220907 | 0.631652807 | 0.913167 |
| Ca | P00915 | -0.075155067 | 0.631952143 | 0.913167 |
| CRP | Q9NZP8 | 0.075151061 | 0.63197036 | 0.913167 |
| WLC | O75882 | 0.075057069 | 0.632397795 | 0.913167 |
| Mg | P32119 | 0.075031791 | 0.632512769 | 0.913167 |
| Basophils | P02745 | -0.075019221 | 0.632569946 | 0.913167 |
| PT | P01718 | -0.074999091 | 0.632661516 | 0.913167 |
| Basophils | P37802 | -0.074985724 | 0.632722322 | 0.913167 |
| WLL | P00918 | 0.074928974 | 0.632980509 | 0.913167 |
| Monocytes | P19823 | 0.074875312 | 0.633224686 | 0.913167 |
| Lymphocytes | P02745 | 0.074843292 | 0.633370404 | 0.913167 |
| WBC | H3BTN5 | -0.07466774 | 0.634169555 | 0.913674974 |
| P | P35908 | -0.074510719 | 0.634884703 | 0.913674974 |
| P | O75636 | 0.074510719 | 0.634884703 | 0.913674974 |
| TBIL | A0A0B4J1Y8 | -0.074462863 | 0.635102722 | 0.913674974 |
| APTT | C9JV77 | -0.074460052 | 0.635115531 | 0.913674974 |
| Glucose | A0A075B6S9 | -0.07430557 | 0.635819539 | 0.914286755 |
| WLL | A0A096LPE2 | 0.074203901 | 0.636283034 | 0.914381617 |
| Monocytes | P43121 | -0.074040572 | 0.637027922 | 0.914381617 |
| ALT | P03951 | 0.074018135 | 0.637130281 | 0.914381617 |
| RBC | K7ERG9 | 0.074009743 | 0.637168562 | 0.914381617 |
| DBIL | P01715 | 0.073889531 | 0.637717084 | 0.914381617 |
| Hemoglobin | A0A0J9YXX1 | 0.073816645 | 0.638049754 | 0.914381617 |
| CO2 | P19823 | 0.073816645 | 0.638049754 | 0.914381617 |
| WLC | P80748 | 0.073802196 | 0.638115708 | 0.914381617 |
| INR | P30041 | 0.073729448 | 0.638447832 | 0.91445803 |
| Creatine Kinase | P03950 | 0.073516273 | 0.63942146 | 0.915246323 |
| Platelets | P01019 | 0.073408355 | 0.639914577 | 0.915246323 |
| γ-GT | P35908 | -0.073348196 | 0.64018953 | 0.915246323 |
| Creatine Kinase | P02745 | 0.073254542 | 0.64061767 | 0.915246323 |
| UA | A0A2R8Y3M9 | 0.073243478 | 0.640668257 | 0.915246323 |
| CO2 | A0A075B6S9 | -0.073238032 | 0.640693157 | 0.915246323 |
| Hematocrit | E7ENL6 | -0.073108873 | 0.641283825 | 0.915246323 |
| CO2 | P13647 | -0.072985546 | 0.641848022 | 0.915246323 |
| Hematocrit | A0A0C4DH73 | -0.072955217 | 0.641986805 | 0.915246323 |
| TBIL | P01715 | 0.072811883 | 0.642642841 | 0.915246323 |
| WLC | P35527 | -0.072625753 | 0.64349515 | 0.915246323 |
| PT | P26038 | 0.07256997 | 0.643750676 | 0.915246323 |
| Creatinine | O75636 | 0.072485654 | 0.643121442 | 0.915246323 |
| Monocytes | P55056 | -0.0724551 | 0.644276992 | 0.915246323 |
| Creatine Kinase | A0A2R8Y3M9 | -0.0723483 | 0.644766481 | 0.915246323 |
| Creatinine | P0DP01 | -0.072337373 | 0.644816571 | 0.915246323 |
| Creatinine | A0A075B6S9 | -0.072264544 | 0.645150459 | 0.915246323 |
| FIB | P69905 | 0.072213626 | 0.645383941 | 0.915246323 |
| RBC | A0A1W2PQU7 | -0.07219726 | 0.645458991 | 0.915246323 |
| P | P0DJI8 | 0.07216809 | 0.645592768 | 0.915246323 |
| ALT | A0A0C4DH73 | -0.072052382 | 0.646123523 | 0.915246323 |
| CRP | Q6ZRK6 | 0.072047631 | 0.646145319 | 0.915246323 |
| LDH | A0A1W2PQU7 | -0.07204622 | 0.646151796 | 0.915246323 |
| TT | P13647 | -0.071997465 | 0.646375492 | 0.915246323 |
| Creatine Kinase | P01782 | 0.071895179 | 0.646844897 | 0.915246323 |
| Hematocrit | A0A0C4DH36 | -0.071746849 | 0.647525851 | 0.915246323 |
| Total protein | P13473 | 0.071671326 | 0.647872671 | 0.915246323 |
| DBIL | A0A0J9YVY3 | 0.071640017 | 0.648016471 | 0.915246323 |
| LDH | P01782 | -0.071593099 | 0.648231985 | 0.915246323 |
| BUN | A0A0J9YX35 | 0.071587692 | 0.648256824 | 0.915246323 |
| DBIL | P02766 | -0.071577075 | 0.648305594 | 0.915246323 |
| Platelets | P00918 | 0.071528276 | 0.648529791 | 0.915246323 |
| Basophils | E7ENL6 | -0.071517756 | 0.648578126 | 0.915246323 |
| RBC | P03951 | -0.071517579 | 0.648578941 | 0.915246323 |
| Platelets | P04264 | 0.071444757 | 0.648913569 | 0.915246323 |
| CO2 | P37802 | 0.071365744 | 0.649276726 | 0.915246323 |
| UA | A0A0J9YX35 | -0.071355759 | 0.64932262 | 0.915246323 |
| WLC | P02751 | 0.071292451 | 0.64961366 | 0.915263233 |
| Platelets | P32119 | -0.071142665 | 0.650302462 | 0.915801065 |
| TT | P02766 | 0.071088023 | 0.650553805 | 0.915801065 |
| Platelets | Q86UD1 | -0.070827117 | 0.651754467 | 0.916843733 |
| AST | A0A0B4J1Y8 | -0.070750409 | 0.652107638 | 0.916843733 |
| PT | Q86UD1 | -0.070735029 | 0.652178454 | 0.916843733 |
| APTT | P19823 | -0.070684187 | 0.652412584 | 0.916843733 |
| TT | P55056 | -0.070557516 | 0.652996058 | 0.917150526 |
| Eosinophils | P32119 | 0.070515393 | 0.653190131 | 0.917150526 |
| Creatinine | P13645 | -0.070220477 | 0.653563341 | 0.917281882 |
| FIB | P13645 | -0.069947508 | 0.655808731 | 0.920039623 |
| Globin | A0A0A0MS15 | 0.069804346 | 0.656469515 | 0.920572898 |
| Hemoglobin | A0A1W2PQU7 | -0.069736707 | 0.656781802 | 0.920617226 |
| RBC | A0A2R8Y7X9 | 0.069554055 | 0.657625394 | 0.921241231 |
| DBIL | A0A096LPE2 | 0.069385169 | 0.658405777 | 0.921241231 |
| INR | E7ENL6 | -0.069365149 | 0.658498311 | 0.921241231 |
| CK-MB activity | A0A0J9YX35 | -0.069343851 | 0.658596752 | 0.921241231 |
| γ-GT | A0A0J9YXX1 | -0.069336376 | 0.658631307 | 0.921241231 |
| APTT | A0A0J9YX35 | 0.069098324 | 0.659732043 | 0.922387511 |
| BUN | Q86UD1 | 0.068991504 | 0.660226206 | 0.922554041 |
| Globin | P01817 | 0.068897796 | 0.660659824 | 0.922554041 |
| TT | A0A0A0MS15 | -0.068890206 | 0.660694952 | 0.922554041 |
| PT | P30041 | -0.068632468 | 0.661888189 | 0.92371996 |
| Neutrophils | E7ENL6 | -0.068588208 | 0.662093179 | 0.92371996 |
| Glucose | Q96HR3 | -0.06827537 | 0.663542794 | 0.925146424 |
| Creatine Kinase | A0A087X0Q4 | -0.068194692 | 0.66391683 | 0.925146424 |
| Eosinophils | A0A1W2PQU7 | -0.068169924 | 0.664031678 | 0.925146424 |
| WLL | P13473 | 0.06812417 | 0.66424385 | 0.925146424 |
| AST | C9J8S2 | 0.067989091 | 0.664870399 | 0.925626022 |
| TT | A0A096LPE2 | 0.067904978 | 0.665260667 | 0.925752578 |
| CO2 | P19652 | 0.067847847 | 0.665525786 | 0.925752578 |
| UA | P02745 | 0.067580322 | 0.666767805 | 0.926250579 |
| CRP | P13473 | 0.067447133 | 0.667386483 | 0.926250579 |
| γ-GT | P80748 | 0.067444007 | 0.667401005 | 0.926250579 |
| TBIL | A0A0C4DH73 | -0.067436944 | 0.667433821 | 0.926250579 |
| Globin | A0A0J9YX35 | 0.06738688 | 0.667666437 | 0.926250579 |
| WLC | Q9Y5Y7 | 0.067370974 | 0.667740345 | 0.926250579 |
| APTT | C9J8S2 | -0.067302492 | 0.668058601 | 0.926250579 |
| Mg | A0A0C4DH73 | 0.067225453 | 0.668416689 | 0.926250579 |
| LDH | P35527 | -0.06706189 | 0.669177197 | 0.926250579 |
| FIB | A0A0B4J1Y8 | -0.067041854 | 0.669270379 | 0.926250579 |
| Glucose | O75882 | 0.06699143 | 0.66950491 | 0.926250579 |
| CK-MB activity | P01782 | 0.06698935 | 0.669514585 | 0.926250579 |
| UA | A0A0C4DH33 | 0.066827758 | 0.670266393 | 0.926250579 |
| WBC | A0A075B6S5 | -0.066825235 | 0.670278133 | 0.926250579 |
| Total protein | P02751 | -0.066762331 | 0.670570883 | 0.926250579 |
| Total protein | P37802 | -0.066736274 | 0.670692164 | 0.926250579 |
| RBC | P0DP01 | -0.066686807 | 0.670922428 | 0.926250579 |
| ALP | P32119 | 0.066638977 | 0.671145099 | 0.926250579 |
| TT | A0A0J9YX35 | 0.066616602 | 0.671249277 | 0.926250579 |
| ALT | P80748 | -0.066381943 | 0.672342195 | 0.926762648 |
| WBC | A0A0C4DH36 | -0.066372183 | 0.672387668 | 0.926762648 |
| PT | H3BTN5 | 0.066337438 | 0.672549553 | 0.926762648 |
| CRP | A0A0C4DH73 | 0.066238674 | 0.673009797 | 0.926762648 |
| Hematocrit | A0A5H1ZRS9 | -0.06623367 | 0.673033118 | 0.926762648 |
| WBC | P30041 | 0.065995249 | 0.674144671 | 0.927661442 |
| ALP | A0A0G2JI36 | -0.06595127 | 0.674349782 | 0.927661442 |
| TT | P37802 | -0.065874094 | 0.674709773 | 0.927661442 |
| APTT | Q96HR3 | -0.065851081 | 0.674817134 | 0.927661442 |
| Creatine Kinase | P43121 | 0.065790061 | 0.675101831 | 0.927664015 |
| ALP | Q6ZRK6 | 0.065669375 | 0.675665047 | 0.927813521 |
| WLC | O75636 | 0.065645524 | 0.675776373 | 0.927813521 |
| P | P26038 | -0.065309861 | 0.67734385 | 0.929576498 |
| Basophils | P0DJI8 | -0.06516987 | 0.677997978 | 0.930085056 |
| CRP | P35908 | -0.065030214 | 0.678650771 | 0.930383675 |
| Neutrophils | Q9NZP8 | 0.064942421 | 0.679061265 | 0.930383675 |
| Hemoglobin | Q86YZ3 | 0.064941275 | 0.679066621 | 0.930383675 |
| WBC | P0DP01 | 0.064486899 | 0.681192601 | 0.932616786 |
| Albumin | A0A2R8Y3M9 | 0.064274927 | 0.682185228 | 0.932616786 |
| UA | P08185 | -0.064182429 | 0.682618548 | 0.932616786 |
| TBIL | D6RE82 | -0.064114183 | 0.682938322 | 0.932616786 |
| LDH | A0A0B4J1V2 | -0.064081263 | 0.683092588 | 0.932616786 |
| WLC | C9JV77 | -0.063998504 | 0.683480469 | 0.932616786 |
| α-HBDH | A0A0A0MS15 | -0.063980064 | 0.683566908 | 0.932616786 |
| WBC | A0A2R8Y7X9 | 0.063729377 | 0.684742386 | 0.932616786 |
| AST | O75882 | 0.063710797 | 0.684829537 | 0.932616786 |
| Eosinophils | A0A075B6S9 | -0.063635068 | 0.685184791 | 0.932616786 |
| PT | P35527 | -0.063561875 | 0.685528218 | 0.932616786 |
| FIB | Q15582 | -0.06352684 | 0.685692623 | 0.932616786 |
| AST | P04430 | -0.063492084 | 0.685855738 | 0.932616786 |
| RBC | P02751 | 0.063436923 | 0.686114639 | 0.932616786 |
| ALP | P13473 | -0.063390138 | 0.686334256 | 0.932616786 |
| ALT | A0A0J9YXX1 | 0.063282103 | 0.686841491 | 0.932616786 |
| Mg | P03951 | -0.0632086 | 0.687186672 | 0.932616786 |
| TBIL | P13645 | -0.063207975 | 0.687189605 | 0.932616786 |
| Hematocrit | P04264 | 0.063137227 | 0.687521909 | 0.932616786 |
| Hemoglobin | P02763 | 0.062861257 | 0.688818691 | 0.932616786 |
| Creatinine | P0DJI8 | -0.0628209 | 0.688126952 | 0.932616786 |
| Globin | Q9NZP8 | 0.062778584 | 0.689207345 | 0.932616786 |
| Creatine Kinase | Q9NZP8 | 0.062757242 | 0.68930769 | 0.932616786 |
| ALT | D6RE82 | 0.062677256 | 0.689683803 | 0.932616786 |
| α-HBDH | P02766 | 0.062544856 | 0.690306547 | 0.932616786 |
| FIB | P43121 | -0.062470132 | 0.690658097 | 0.932616786 |
| WLGG | A0A1W2PQU7 | 0.062395468 | 0.691009431 | 0.932616786 |
| WBC | P03951 | -0.062370219 | 0.691128255 | 0.932616786 |
| RBC | Q6ZRK6 | -0.062357626 | 0.691187524 | 0.932616786 |
| ALP | P01715 | -0.062318283 | 0.691372696 | 0.932616786 |
| Creatinine | P01782 | -0.062216853 | 0.690977847 | 0.932616786 |
| Lymphocytes | D6RE82 | -0.062155428 | 0.692139385 | 0.932616786 |
| APTT | P03950 | 0.062131767 | 0.6922508 | 0.932616786 |
| Platelets | A0A0B4J1V2 | 0.062082249 | 0.692483996 | 0.932616786 |
| WLL | A0A5H1ZRS9 | -0.062044439 | 0.692662074 | 0.932616786 |
| Creatine Kinase | P19652 | 0.06200204 | 0.692861781 | 0.932616786 |
| APTT | P01718 | -0.061932103 | 0.693191249 | 0.932616786 |
| γ-GT | P02750 | -0.061918292 | 0.693256316 | 0.932616786 |
| Hematocrit | Q5SRP5 | -0.061868046 | 0.693493061 | 0.932616786 |
| CRP | P01817 | -0.061858009 | 0.693540356 | 0.932616786 |
| Albumin | Q9Y5Y7 | -0.061858009 | 0.693540356 | 0.932616786 |
| RBC | D6R934 | -0.061851 | 0.693573384 | 0.932616786 |
| Eosinophils | P35908 | 0.061814459 | 0.693745579 | 0.932616786 |
| CO2 | A0A0B4J1V2 | 0.061768051 | 0.693964297 | 0.932616786 |
| Basophils | A0A0J9YXX1 | -0.061640519 | 0.694565464 | 0.932616786 |
| RBC | P02753 | 0.061624439 | 0.694641276 | 0.932616786 |
| γ-GT | A0A087X0Q4 | -0.061539818 | 0.695040289 | 0.932616786 |
| RBC | P35908 | -0.061473399 | 0.695353529 | 0.932616786 |
| AST | P01718 | -0.061446824 | 0.695478875 | 0.932616786 |
| TBIL | P08185 | -0.06139556 | 0.695720691 | 0.932616786 |
| α-HBDH | A0A0J9YXX1 | 0.061260722 | 0.696356877 | 0.932616786 |
| CO2 | K7ERG9 | -0.061199061 | 0.696647874 | 0.932616786 |
| BUN | Q16880 | -0.061166699 | 0.696800617 | 0.932616786 |
| γ-GT | O75636 | 0.061161344 | 0.696825888 | 0.932616786 |
| Monocytes | C9JV77 | 0.061110355 | 0.697066573 | 0.932616786 |
| Albumin | P26038 | 0.061107997 | 0.697077707 | 0.932616786 |
| Albumin | C9JV77 | 0.060951664 | 0.697815834 | 0.932616786 |
| Creatine Kinase | Q15582 | 0.060944758 | 0.697848449 | 0.932616786 |
| Globin | A0A0C4DH33 | -0.060930012 | 0.697918091 | 0.932616786 |
| P | P02763 | -0.060757219 | 0.698734298 | 0.932616786 |
| Hemoglobin | A0A2R8Y7X9 | 0.060745734 | 0.698788559 | 0.932616786 |
| PT | K7ERG9 | 0.060686456 | 0.69906865 | 0.932616786 |
| IBIL | P0DP01 | 0.060651839 | 0.699232235 | 0.932616786 |
| APTT | P08185 | 0.06056487 | 0.699643275 | 0.932616786 |
| Hemoglobin | P43121 | -0.060506212 | 0.699920558 | 0.932616786 |
| FIB | D6R934 | 0.060505349 | 0.699924634 | 0.932616786 |
| Glucose | A0A0J9YXX1 | -0.060496207 | 0.699967855 | 0.932616786 |
| P | Q5SRP5 | 0.060482796 | 0.700031259 | 0.932616786 |
| AST | Q92954 | 0.060154493 | 0.701583966 | 0.93430589 |
| γ-GT | A0A5H1ZRS9 | -0.060025923 | 0.702192366 | 0.934736591 |
| Basophils | P13645 | -0.059834805 | 0.703097087 | 0.935316455 |
| Creatine Kinase | A0A2R8Y7X9 | 0.059736436 | 0.703562909 | 0.935316455 |
| LDH | P35908 | -0.059736436 | 0.703562909 | 0.935316455 |
| Hemoglobin | A0A075B6S5 | -0.059385755 | 0.705224405 | 0.935316455 |
| UA | P0DJI8 | -0.05934987 | 0.705394503 | 0.935316455 |
| Creatine Kinase | P02751 | -0.059283315 | 0.705710014 | 0.935316455 |
| IBIL | Q86UD1 | -0.059255549 | 0.705841657 | 0.935316455 |
| Mg | D6R934 | -0.059191746 | 0.706144185 | 0.935316455 |
| Neutrophils | Q8N1N4 | -0.05897678 | 0.707163808 | 0.935316455 |
| Hemoglobin | E7ENL6 | 0.058938264 | 0.707346548 | 0.935316455 |
| P | P04264 | -0.058716865 | 0.708397304 | 0.935316455 |
| P | A0A140T8Y3 | -0.058689939 | 0.70852513 | 0.935316455 |
| Monocytes | Q9H4B7 | 0.058649851 | 0.708715454 | 0.935316455 |
| PT | P02745 | 0.058643396 | 0.708746102 | 0.935316455 |
| IBIL | Q9NZP8 | 0.058610275 | 0.708903369 | 0.935316455 |
| Lymphocytes | P02766 | 0.058605847 | 0.708924392 | 0.935316455 |
| TBIL | P35527 | -0.05860142 | 0.708945412 | 0.935316455 |
| Globin | A0A0B4J1Y8 | -0.058588002 | 0.709009127 | 0.935316455 |
| Globin | A0A075B6S9 | 0.058566929 | 0.7091092 | 0.935316455 |
| DBIL | P0DP01 | 0.058541223 | 0.709231278 | 0.935316455 |
| IBIL | P37802 | -0.05847662 | 0.709538104 | 0.935316455 |
| AST | P01814 | -0.058414175 | 0.709834728 | 0.935316455 |
| FIB | P03950 | 0.05840527 | 0.709877028 | 0.935316455 |
| Globin | E7ENL6 | 0.058357039 | 0.710106167 | 0.935316455 |
| Eosinophils | P02745 | 0.058334086 | 0.710215225 | 0.935316455 |
| Ca | Q9H4B7 | 0.058313217 | 0.71031438 | 0.935316455 |
| TBIL | P0DP01 | 0.058263792 | 0.71054924 | 0.935316455 |
| Hematocrit | A0A0J9YVY3 | -0.058011786 | 0.711747143 | 0.935316455 |
| AST | P01782 | 0.057884512 | 0.712352397 | 0.935316455 |
| Globin | I3L1J2 | -0.057872349 | 0.71241025 | 0.935316455 |
| Albumin | P30041 | 0.057870162 | 0.712420652 | 0.935316455 |
| INR | P02751 | 0.057869156 | 0.712425435 | 0.935316455 |
| Lymphocytes | P02763 | -0.057850617 | 0.712513618 | 0.935316455 |
| Albumin | A0A096LPE2 | -0.057779459 | 0.712852114 | 0.935316455 |
| TBIL | P35908 | -0.05777073 | 0.71289364 | 0.935316455 |
| Creatinine | O75882 | 0.0576865 | 0.712493176 | 0.935316455 |
| Creatine Kinase | E7ENL6 | -0.057598755 | 0.713711961 | 0.935554331 |
| Creatine Kinase | P0DP01 | -0.057548524 | 0.713951035 | 0.935554331 |
| IBIL | P03951 | -0.057326287 | 0.71500911 | 0.935554331 |
| ALT | Q96HR3 | -0.057233634 | 0.715450385 | 0.935554331 |
| Creatinine | A0A2R8Y7X9 | 0.057082452 | 0.715379217 | 0.935554331 |
| CRP | A0A0J9YX35 | 0.056948643 | 0.716808284 | 0.935554331 |
| Lymphocytes | P01782 | -0.056868818 | 0.717188781 | 0.935554331 |
| WLC | P26038 | 0.056845901 | 0.71729803 | 0.935554331 |
| Hemoglobin | P08185 | -0.056816906 | 0.717436265 | 0.935554331 |
| Basophils | Q86YZ3 | -0.056747403 | 0.717767652 | 0.935554331 |
| WLL | P02042 | 0.05674416 | 0.717783117 | 0.935554331 |
| IBIL | P26038 | 0.056713373 | 0.717929924 | 0.935554331 |
| PT | P00915 | -0.056676005 | 0.718108132 | 0.935554331 |
| TBIL | P04264 | 0.056637971 | 0.718289528 | 0.935554331 |
| Basophils | P35908 | -0.056633766 | 0.718309582 | 0.935554331 |
| IBIL | P01715 | 0.056605368 | 0.718445032 | 0.935554331 |
| CK-MB activity | Q92954 | -0.056583975 | 0.718547077 | 0.935554331 |
| DBIL | C9JV77 | 0.056536064 | 0.71877563 | 0.935554331 |
| RBC | Q86YZ3 | 0.056513868 | 0.718881519 | 0.935554331 |
| WLC | P08185 | 0.056469268 | 0.719094307 | 0.935554331 |
| Hemoglobin | P0DJI8 | -0.056439134 | 0.719238093 | 0.935554331 |
| Total protein | A0A075B6S5 | 0.05641568 | 0.719350007 | 0.935554331 |
| AST | A0A5H1ZRS9 | 0.056068528 | 0.721007202 | 0.937337939 |
| TT | A0A1W2PQU7 | -0.055930662 | 0.721665678 | 0.937661753 |
| CK-MB activity | H3BTN5 | 0.055777036 | 0.722399667 | 0.937661753 |
| TT | E7EX29 | 0.055777014 | 0.722399771 | 0.937661753 |
| Creatinine | D6RE82 | 0.055723346 | 0.721887242 | 0.937661753 |
| WBC | A0A0A0MS15 | -0.055649942 | 0.723007077 | 0.938078803 |
| α-HBDH | P02753 | 0.05551989 | 0.723628795 | 0.938149975 |
| P | P03951 | 0.055467411 | 0.723879722 | 0.938149975 |
| Total protein | P01718 | -0.055458989 | 0.723919996 | 0.938149975 |
| γ-GT | A0A140T8Y3 | 0.055298494 | 0.724687592 | 0.938513131 |
| LDH | P02763 | -0.055280747 | 0.724772488 | 0.938513131 |
| P | Q15582 | -0.055013999 | 0.726048899 | 0.939794944 |
| Platelets | P13647 | -0.054829697 | 0.726931228 | 0.940233434 |
| BUN | P13645 | 0.054823485 | 0.726960972 | 0.940233434 |
| IBIL | P03950 | 0.054734116 | 0.727388949 | 0.940416143 |
| P | I3L1J2 | 0.054601581 | 0.728023797 | 0.940655184 |
| Mg | P0DJI8 | -0.054568575 | 0.728181921 | 0.940655184 |
| Ca | P01715 | 0.054479896 | 0.728606826 | 0.940655184 |
| TT | Q8N1N4 | -0.054339139 | 0.729281424 | 0.940655184 |
| Globin | P55056 | -0.054317451 | 0.729385386 | 0.940655184 |
| Basophils | A0A140T8Y3 | -0.05428632 | 0.72953462 | 0.940655184 |
| APTT | P02751 | -0.054221417 | 0.72984578 | 0.940655184 |
| CO2 | P80748 | 0.054096947 | 0.730442639 | 0.941025866 |
| Creatinine | A0A0C4DH73 | 0.054062217 | 0.729868123 | 0.940655184 |
| LDH | P02745 | 0.053770344 | 0.732009514 | 0.942674207 |
| α-HBDH | P0DP01 | 0.053633483 | 0.732666423 | 0.94314987 |
| WLGG | P00918 | 0.053339856 | 0.734076419 | 0.943925407 |
| α-HBDH | A0A1W2PQU7 | 0.053329309 | 0.734127081 | 0.943925407 |
| IBIL | P19652 | 0.053247737 | 0.734518955 | 0.943925407 |
| ALT | P13647 | -0.053150918 | 0.734984157 | 0.943925407 |
| γ-GT | P13473 | 0.053137703 | 0.735047662 | 0.943925407 |
| ALP | P35908 | 0.05311474 | 0.73515801 | 0.943925407 |
| TBIL | P02766 | 0.053088658 | 0.735283358 | 0.943925407 |
| P | A0A5H1ZRS9 | 0.052898076 | 0.736199471 | 0.944731716 |
| CRP | E7EX29 | 0.052746844 | 0.73692669 | 0.944986448 |
| CO2 | A0A096LPE2 | 0.052736968 | 0.736974187 | 0.944986448 |
| WBC | P26038 | 0.052494294 | 0.738141616 | 0.945499847 |
| TBIL | P13473 | 0.052409002 | 0.738552066 | 0.945499847 |
| Ca | P02042 | -0.052396843 | 0.738610587 | 0.945499847 |
| Basophils | A0A0B4J1Y8 | -0.05228561 | 0.739145993 | 0.945499847 |
| Eosinophils | P13645 | -0.052205602 | 0.73953118 | 0.945499847 |
| Eosinophils | P37802 | -0.052095812 | 0.740059846 | 0.945499847 |
| α-HBDH | Q8N1N4 | 0.052045176 | 0.740303714 | 0.945499847 |
| CRP | A0A0C4DH33 | 0.052041242 | 0.74032266 | 0.945499847 |
| IBIL | P02766 | 0.051963749 | 0.740695924 | 0.945499847 |
| Lymphocytes | Q6ZRK6 | 0.0519393 | 0.740813698 | 0.945499847 |
| Platelets | P30041 | 0.051883972 | 0.74108025 | 0.945499847 |
| Neutrophils | P02042 | 0.051878422 | 0.741106986 | 0.945499847 |
| WLGG | P04264 | 0.051811933 | 0.741427348 | 0.945499847 |
| Glucose | Q8N1N4 | -0.051810734 | 0.741433125 | 0.945499847 |
| WLL | D6R934 | 0.051755662 | 0.741698508 | 0.945499847 |
| P | P13645 | -0.051688977 | 0.742019894 | 0.945542056 |
| DBIL | P02042 | 0.051472004 | 0.743065885 | 0.946353776 |
| CRP | P08185 | -0.051359519 | 0.743608339 | 0.946353776 |
| Lymphocytes | Q8N1N4 | 0.051355639 | 0.743627052 | 0.946353776 |
| Neutrophils | P02750 | 0.051198792 | 0.744383647 | 0.946353776 |
| Creatine Kinase | Q86YZ3 | -0.051158641 | 0.744577363 | 0.946353776 |
| CK-MB activity | Q96HR3 | 0.051115457 | 0.744785736 | 0.946353776 |
| Globin | Q92954 | 0.05106898 | 0.74501001 | 0.946353776 |
| WLGG | P03951 | -0.051022117 | 0.745236174 | 0.946353776 |
| DBIL | P15169 | 0.051018507 | 0.745253598 | 0.946353776 |
| Platelets | Q9H4B7 | -0.050921427 | 0.745722178 | 0.946510814 |
| AST | A0A1W2PQU7 | -0.050771906 | 0.746444055 | 0.946510814 |
| DBIL | P35908 | -0.050716175 | 0.746713175 | 0.946510814 |
| TT | D6RE82 | -0.050625586 | 0.747150684 | 0.946510814 |
| Creatine Kinase | P37802 | 0.050596035 | 0.747293421 | 0.946510814 |
| Basophils | P35527 | -0.050477922 | 0.747864011 | 0.946510814 |
| Platelets | O75636 | -0.050449363 | 0.748001996 | 0.946510814 |
| Basophils | A0A0C4DH73 | -0.050395844 | 0.748260599 | 0.946510814 |
| CO2 | Q9H4B7 | -0.050265486 | 0.748890602 | 0.946510814 |
| WLC | P13473 | 0.050194905 | 0.749231776 | 0.946510814 |
| Hematocrit | A0A0G2JI36 | -0.050138071 | 0.749506536 | 0.946510814 |
| INR | P01715 | 0.050133694 | 0.749527696 | 0.946510814 |
| Globin | Q8N1N4 | 0.050086885 | 0.749754018 | 0.946510814 |
| Albumin | P03951 | 0.049773416 | 0.751270157 | 0.946510814 |
| P | P02753 | 0.049724192 | 0.751508323 | 0.946510814 |
| Glucose | A0A0G2JI36 | 0.049712149 | 0.751566591 | 0.946510814 |
| Hemoglobin | I3L1J2 | 0.049680076 | 0.751721792 | 0.946510814 |
| Hematocrit | Q9NZP8 | 0.04961861 | 0.752019242 | 0.946510814 |
| LDH | A0A0A0MS15 | -0.049616736 | 0.752028311 | 0.946510814 |
| RBC | P08185 | -0.049541216 | 0.752393825 | 0.946510814 |
| Ca | D6RE82 | -0.049523711 | 0.752478555 | 0.946510814 |
| ALT | P01817 | -0.049521837 | 0.752487629 | 0.946510814 |
| FIB | P02042 | -0.049476909 | 0.752705112 | 0.946510814 |
| ALT | P02766 | -0.049446231 | 0.752853625 | 0.946510814 |
| CO2 | P03951 | 0.049412575 | 0.753016566 | 0.946510814 |
| Hemoglobin | A0A0J9YVY3 | -0.049403112 | 0.753062381 | 0.946510814 |
| FIB | E7ENL6 | -0.049240864 | 0.753848045 | 0.946510814 |
| FIB | P19652 | 0.04917476 | 0.754168217 | 0.946510814 |
| WLL | O75882 | -0.049105523 | 0.754503603 | 0.946510814 |
| Monocytes | A0A0C4DH73 | 0.049084927 | 0.754603383 | 0.946510814 |
| α-HBDH | A0A0B4J1V2 | 0.049063306 | 0.754708126 | 0.946510814 |
| P | A0A0B4J1V2 | 0.049045925 | 0.754792335 | 0.946510814 |
| FIB | P02766 | -0.049023685 | 0.754900088 | 0.946510814 |
| RBC | A0A0C4DH33 | -0.048825618 | 0.755859934 | 0.947352152 |
| Ca | P02763 | 0.048540798 | 0.757240824 | 0.948096503 |
| ALT | I3L1J2 | 0.048536051 | 0.757263843 | 0.948096503 |
| DBIL | A0A1W2PQU7 | 0.048524268 | 0.757320987 | 0.948096503 |
| LDH | P04264 | 0.048408414 | 0.757882916 | 0.948272148 |
| Glucose | A0A0B4J1V2 | 0.048376134 | 0.758039504 | 0.948272148 |
| PT | E7ENL6 | 0.048257928 | 0.758613001 | 0.948384918 |
| Ca | A0A2R8Y3M9 | 0.048238363 | 0.758707934 | 0.948384918 |
| Glucose | E7ENL6 | -0.048084644 | 0.759453947 | 0.948740755 |
| Creatine Kinase | Q8N1N4 | -0.048030813 | 0.759715243 | 0.948740755 |
| P | P01715 | 0.047898198 | 0.760359075 | 0.948752836 |
| RBC | P0DJI8 | -0.047879773 | 0.760448538 | 0.948752836 |
| Creatinine | P02763 | -0.047870734 | 0.759860355 | 0.948740755 |
| INR | P37802 | -0.047790998 | 0.760879632 | 0.948929731 |
| UA | P13647 | 0.047646015 | 0.761583832 | 0.949446967 |
| Lymphocytes | A0A1W2PQU7 | -0.047503966 | 0.762273964 | 0.949946278 |
| WLC | A0A2R8Y7X9 | -0.047293012 | 0.763299202 | 0.950836601 |
| TBIL | P19823 | -0.047198309 | 0.76375959 | 0.950836601 |
| ALT | A0A0A0MS15 | 0.047178055 | 0.763858062 | 0.950836601 |
| Glucose | P0DP01 | 0.047016618 | 0.764643086 | 0.950920238 |
| Monocytes | P35908 | -0.046967241 | 0.764883236 | 0.950920238 |
| γ-GT | P02042 | 0.046930735 | 0.765060805 | 0.950920238 |
| Globin | P43121 | -0.046925779 | 0.765084911 | 0.950920238 |
| CK-MB activity | E7EX29 | 0.046799552 | 0.765698981 | 0.951045984 |
| Albumin | Q6ZRK6 | -0.046773519 | 0.765825641 | 0.951045984 |
| Ca | P15169 | -0.046726189 | 0.766055942 | 0.951045984 |
| Mg | P02745 | 0.046383289 | 0.76772502 | 0.951588888 |
| Ca | P30041 | -0.046370128 | 0.7677891 | 0.951588888 |
| ALT | A0A5H1ZRS9 | 0.046270785 | 0.768272865 | 0.951588888 |
| RBC | A0A0J9YXX1 | 0.046142809 | 0.768896183 | 0.951588888 |
| WLGG | P02745 | 0.046125257 | 0.768981683 | 0.951588888 |
| α-HBDH | Q92954 | -0.046077732 | 0.769213206 | 0.951588888 |
| APTT | Q9NZP8 | -0.04606555 | 0.769272556 | 0.951588888 |
| CO2 | Q16880 | -0.046012627 | 0.769530399 | 0.951588888 |
| IBIL | A0A075B6S9 | 0.046000424 | 0.769589859 | 0.951588888 |
| FIB | P0DJI8 | -0.045926657 | 0.769949303 | 0.951588888 |
| Eosinophils | P02753 | -0.045925798 | 0.769953492 | 0.951588888 |
| Albumin | P08185 | 0.045921452 | 0.769974668 | 0.951588888 |
| Total protein | P08185 | 0.045691414 | 0.7710959 | 0.952119679 |
| Eosinophils | Q9Y5Y7 | -0.045623156 | 0.771428685 | 0.952119679 |
| FIB | E7EX29 | -0.045610902 | 0.771488434 | 0.952119679 |
| Glucose | P43121 | 0.045595143 | 0.771565276 | 0.952119679 |
| WLL | P19823 | -0.04552004 | 0.771931496 | 0.952135648 |
| α-HBDH | P02745 | 0.045473434 | 0.772158788 | 0.952135648 |
| CK-MB activity | A0A2R8Y7X9 | 0.045343132 | 0.772794347 | 0.95239 |
| LDH | Q92954 | 0.045312088 | 0.772945787 | 0.95239 |
| UA | P01718 | 0.045222357 | 0.773383564 | 0.95257157 |
| CK-MB activity | Q9H4B7 | -0.045124447 | 0.77386133 | 0.952647789 |
| IBIL | P02763 | -0.045090636 | 0.774026329 | 0.952647789 |
| Eosinophils | O75636 | -0.044715233 | 0.775859023 | 0.954267132 |
| Globin | P19823 | 0.044647585 | 0.776189403 | 0.954267132 |
| Basophils | Q16880 | 0.044568312 | 0.77657661 | 0.954267132 |
| Globin | A0A0J9YVY3 | -0.0444146 | 0.777327557 | 0.954267132 |
| Hematocrit | A0A075B6S5 | -0.044407523 | 0.777362137 | 0.954267132 |
| TBIL | P02042 | 0.044404169 | 0.777378526 | 0.954267132 |
| Basophils | P08185 | 0.044404156 | 0.777378591 | 0.954267132 |
| CO2 | P32119 | 0.044274876 | 0.778010346 | 0.954596138 |
| Basophils | P26038 | -0.044133949 | 0.778699181 | 0.954596138 |
| Creatinine | Q5SRP5 | -0.044104613 | 0.778842592 | 0.954596138 |
| DBIL | A0A0J9YXX1 | 0.04398929 | 0.779406427 | 0.954596138 |
| ALT | P01814 | 0.043927003 | 0.779711006 | 0.954596138 |
| ALT | P19823 | -0.043927003 | 0.779711006 | 0.954596138 |
| APTT | D6R934 | 0.043875548 | 0.779962644 | 0.954596138 |
| APTT | A0A0C4DH33 | 0.043839444 | 0.780139219 | 0.954596138 |
| DBIL | P00918 | 0.043763468 | 0.780510839 | 0.954596138 |
| WLL | P03951 | 0.043649354 | 0.781069092 | 0.954596138 |
| Ca | A0A0J9YX35 | -0.043626231 | 0.781182222 | 0.954596138 |
| Ca | P13473 | 0.043626231 | 0.781182222 | 0.954596138 |
| Total protein | P00918 | -0.043575577 | 0.78143007 | 0.954596138 |
| AST | P13473 | -0.043356635 | 0.782501586 | 0.955549218 |
| CK-MB activity | P03950 | 0.043201392 | 0.783261593 | 0.955884369 |
| Platelets | P69905 | 0.043123632 | 0.78364235 | 0.955884369 |
| RBC | P13647 | -0.043122004 | 0.783650325 | 0.955884369 |
| Basophils | Q86UD1 | 0.043039487 | 0.784054428 | 0.956021756 |
| RBC | A0A0J9YVY3 | -0.042909058 | 0.784693284 | 0.956105588 |
| APTT | P19652 | -0.042893823 | 0.784767918 | 0.956105588 |
| AST | A0A096LPE2 | 0.042826973 | 0.785095422 | 0.956105588 |
| CO2 | Q5SRP5 | 0.04278743 | 0.785289163 | 0.956105588 |
| Mg | A0A0B4J1Y8 | 0.042671205 | 0.785858678 | 0.956443957 |
| WLC | Q6ZRK6 | -0.0424348 | 0.787017433 | 0.956471429 |
| Ca | E7ENL6 | 0.042387201 | 0.787250798 | 0.956471429 |
| Glucose | P01814 | -0.042370003 | 0.787335124 | 0.956471429 |
| Neutrophils | P35908 | -0.042363602 | 0.787366505 | 0.956471429 |
| Creatinine | P03951 | -0.042283298 | 0.787232818 | 0.956471429 |
| TT | P69905 | -0.042213251 | 0.788103788 | 0.956471429 |
| TBIL | A0A087X0Q4 | 0.04213865 | 0.788469675 | 0.956471429 |
| Lymphocytes | P0DP01 | 0.042030136 | 0.78900198 | 0.956471429 |
| Creatinine | A0A1W2PQU7 | -0.041981275 | 0.78872005 | 0.956471429 |
| Mg | Q92954 | -0.041760118 | 0.790326927 | 0.956471429 |
| Glucose | C9J8S2 | -0.041713313 | 0.790556656 | 0.956471429 |
| INR | I3L1J2 | 0.041612783 | 0.791050128 | 0.956471429 |
| APTT | P02766 | -0.041610029 | 0.79106365 | 0.956471429 |
| Total protein | P01782 | 0.041537649 | 0.791418996 | 0.956471429 |
| Hemoglobin | C9JV77 | 0.041479363 | 0.79170518 | 0.956471429 |
| AST | O75636 | -0.041389318 | 0.792147354 | 0.956471429 |
| LDH | Q9NZP8 | -0.04138504 | 0.792168362 | 0.956471429 |
| TT | A0A0B4J1V2 | 0.041343263 | 0.792373535 | 0.956471429 |
| TBIL | Q8N1N4 | 0.04130796 | 0.792546924 | 0.956471429 |
| CRP | P02766 | 0.041238673 | 0.792887254 | 0.956471429 |
| Neutrophils | P13645 | -0.041230886 | 0.792925504 | 0.956471429 |
| P | A0A0G2JI36 | 0.041093568 | 0.793600108 | 0.956471429 |
| Hematocrit | P55056 | 0.041084511 | 0.793644608 | 0.956471429 |
| Eosinophils | C9JV77 | 0.041007879 | 0.794021154 | 0.956471429 |
| UA | P37802 | 0.041000762 | 0.794056123 | 0.956471429 |
| α-HBDH | P03951 | -0.040941198 | 0.794348837 | 0.956471429 |
| RBC | P04264 | 0.040931919 | 0.794394437 | 0.956471429 |
| Creatine Kinase | P0DJI8 | -0.040931919 | 0.794394437 | 0.956471429 |
| Mg | A0A0J9YXX1 | -0.040926431 | 0.794421407 | 0.956471429 |
| APTT | P30041 | -0.040831138 | 0.794889769 | 0.956471429 |
| Basophils | A0A0C4DH36 | 0.040792727 | 0.795078579 | 0.956471429 |
| Monocytes | P0DJI8 | -0.040765447 | 0.795212679 | 0.956471429 |
| α-HBDH | P02763 | 0.040639049 | 0.795834099 | 0.956573323 |
| Eosinophils | P02042 | 0.040629577 | 0.79588067 | 0.956573323 |
| WLGG | E7ENL6 | -0.040502433 | 0.796505889 | 0.956669668 |
| FIB | P35908 | 0.0403369 | 0.797320073 | 0.956669668 |
| Albumin | O75636 | -0.040332328 | 0.797342562 | 0.956669668 |
| WLL | A0A0B4J1Y8 | -0.040299228 | 0.797505391 | 0.956669668 |
| CK-MB activity | A0A0C4DH73 | 0.040254371 | 0.797726072 | 0.956669668 |
| Total protein | D6R934 | 0.040253758 | 0.797729088 | 0.956669668 |
| UA | Q6ZRK6 | -0.040198187 | 0.798002504 | 0.956669668 |
| Hematocrit | P01814 | 0.039951666 | 0.799215683 | 0.957681796 |
| WLL | P55056 | 0.039907981 | 0.799430719 | 0.957681796 |
| Glucose | P04264 | -0.039651072 | 0.800695604 | 0.958846872 |
| Lymphocytes | A0A140T8Y3 | -0.0395823 | 0.801034285 | 0.958902356 |
| ALP | P13645 | 0.039514949 | 0.801366007 | 0.958949472 |
| Creatine Kinase | Q9H4B7 | -0.039135162 | 0.803237197 | 0.959072412 |
| LDH | A0A075B6S9 | -0.039087506 | 0.803472073 | 0.959072412 |
| Globin | P01019 | 0.039057194 | 0.803621476 | 0.959072412 |
| Hematocrit | P35527 | -0.03904539 | 0.803679654 | 0.959072412 |
| WBC | P01715 | -0.038896161 | 0.804415299 | 0.959072412 |
| ALP | Q8N1N4 | 0.038834959 | 0.804717045 | 0.959072412 |
| Mg | Q6ZRK6 | 0.038783249 | 0.804972022 | 0.959072412 |
| UA | C9JV77 | -0.038735984 | 0.805205094 | 0.959072412 |
| LDH | A0A2R8Y3M9 | 0.038666315 | 0.805548675 | 0.959072412 |
| DBIL | Q15582 | 0.038622899 | 0.805762804 | 0.959072412 |
| Hemoglobin | Q96HR3 | 0.038608296 | 0.805834829 | 0.959072412 |
| WLC | P01718 | -0.03851359 | 0.806301984 | 0.959072412 |
| DBIL | P01817 | 0.038471733 | 0.806508471 | 0.959072412 |
| WLC | P19652 | 0.038430474 | 0.806712021 | 0.959072412 |
| ALP | P30041 | 0.038399296 | 0.806865849 | 0.959072412 |
| PT | P19652 | -0.038288463 | 0.80741273 | 0.959072412 |
| Globin | Q9H4B7 | -0.03827231 | 0.80749244 | 0.959072412 |
| CO2 | P35527 | 0.038230524 | 0.80769865 | 0.959072412 |
| Glucose | O75636 | -0.038216081 | 0.807769931 | 0.959072412 |
| APTT | A0A096LPE2 | 0.038211751 | 0.8077913 | 0.959072412 |
| Globin | P02763 | -0.038075098 | 0.808465786 | 0.959072412 |
| Glucose | P30041 | 0.038052452 | 0.808577572 | 0.959072412 |
| WLC | A0A0C4DH33 | -0.038039763 | 0.808640214 | 0.959072412 |
| Platelets | H3BTN5 | -0.038007722 | 0.808798387 | 0.959072412 |
| RBC | Q15582 | -0.037911113 | 0.809275354 | 0.959072412 |
| Creatine Kinase | P13473 | -0.037835593 | 0.809648253 | 0.959072412 |
| PT | D6R934 | -0.037834449 | 0.809653903 | 0.959072412 |
| WLGG | P13647 | 0.037753207 | 0.810055101 | 0.959072412 |
| P | A0A2R8Y3M9 | -0.037633203 | 0.810647809 | 0.959072412 |
| CRP | Q15582 | -0.037613295 | 0.810746146 | 0.959072412 |
| APTT | A0A0G2JI36 | -0.037600713 | 0.810808295 | 0.959072412 |
| TT | P15169 | 0.037590255 | 0.810859954 | 0.959072412 |
| CO2 | A0A0J9YX35 | -0.03747498 | 0.811429437 | 0.959072412 |
| FIB | A0A0C4DH73 | -0.037466484 | 0.811471417 | 0.959072412 |
| α-HBDH | C9J8S2 | 0.037373823 | 0.811929254 | 0.959072412 |
| CK-MB activity | C9J8S2 | -0.037340503 | 0.812093904 | 0.959072412 |
| WBC | D6R934 | 0.037301318 | 0.812287549 | 0.959072412 |
| AST | I3L1J2 | 0.037233813 | 0.812621166 | 0.95912106 |
| APTT | A0A0B4J1V2 | 0.037118152 | 0.813192856 | 0.959450564 |
| TT | D6R934 | -0.036983961 | 0.813856251 | 0.95955794 |
| IBIL | P35908 | -0.036933536 | 0.814105567 | 0.95955794 |
| Eosinophils | P35527 | -0.036922223 | 0.814161508 | 0.95955794 |
| Monocytes | Q9NZP8 | 0.036832603 | 0.814604672 | 0.959735389 |
| CRP | A0A075B6S9 | -0.036732939 | 0.815097569 | 0.959794194 |
| Total protein | A0A0C4DH73 | 0.036704177 | 0.815239825 | 0.959794194 |
| Ca | P37802 | -0.036606119 | 0.815724869 | 0.960008811 |
| Neutrophils | P01817 | 0.03654899 | 0.816007489 | 0.960008811 |
| APTT | P13645 | 0.036399336 | 0.816747945 | 0.960226621 |
| IBIL | A0A096LPE2 | 0.036253778 | 0.817468284 | 0.960226621 |
| FIB | P32119 | 0.03618235 | 0.817821822 | 0.960226621 |
| Basophils | P02766 | 0.036032208 | 0.818565079 | 0.960226621 |
| APTT | P02745 | 0.036021749 | 0.818616858 | 0.960226621 |
| Hematocrit | Q9H4B7 | -0.035991376 | 0.818767239 | 0.960226621 |
| Globin | A0A0B4J1V2 | 0.035923398 | 0.819103824 | 0.960226621 |
| UA | A0A0G2JI36 | 0.03588558 | 0.81929109 | 0.960226621 |
| Monocytes | P30041 | 0.035776137 | 0.81983308 | 0.960226621 |
| Albumin | P01019 | 0.03557402 | 0.820834238 | 0.960226621 |
| Lymphocytes | O75636 | 0.035420286 | 0.821595922 | 0.960226621 |
| Total protein | Q9NZP8 | -0.035420286 | 0.821595922 | 0.960226621 |
| Creatine Kinase | P02766 | 0.035418949 | 0.82160255 | 0.960226621 |
| TT | A0A0B4J1Y8 | -0.035355879 | 0.821915079 | 0.960226621 |
| γ-GT | P0DJI8 | 0.03534944 | 0.821946988 | 0.960226621 |
| Total protein | P0DJI8 | -0.035344763 | 0.821970165 | 0.960226621 |
| RBC | Q96HR3 | -0.035343428 | 0.821976781 | 0.960226621 |
| AST | P04264 | 0.035336036 | 0.822013417 | 0.960226621 |
| IBIL | A0A0C4DH33 | -0.035235471 | 0.822511818 | 0.960226621 |
| LDH | P13645 | -0.035116868 | 0.823099705 | 0.960226621 |
| BUN | A0A1W2PQU7 | 0.035114216 | 0.823112853 | 0.960226621 |
| WLL | P13645 | -0.035075374 | 0.823305406 | 0.960226621 |
| TBIL | A0A096LPE2 | 0.035040025 | 0.823480652 | 0.960226621 |
| TBIL | Q9Y5Y7 | -0.035040025 | 0.823480652 | 0.960226621 |
| TBIL | Q6ZRK6 | -0.035033817 | 0.823511428 | 0.960226621 |
| WLL | P0DJI8 | 0.034919483 | 0.824078309 | 0.960546145 |
| INR | P15169 | -0.034721494 | 0.825060173 | 0.961070324 |
| Eosinophils | E7ENL6 | -0.034689147 | 0.82522061 | 0.961070324 |
| TT | P35527 | -0.03463457 | 0.825491327 | 0.961070324 |
| γ-GT | P19823 | -0.034592493 | 0.825700053 | 0.961070324 |
| Eosinophils | P0DJI8 | -0.034501093 | 0.826153488 | 0.96119478 |
| CO2 | P02745 | 0.034452805 | 0.826393073 | 0.96119478 |
| Platelets | P02750 | 0.034287441 | 0.827213637 | 0.961619932 |
| FIB | C9JV77 | -0.034218381 | 0.827556381 | 0.961619932 |
| P | P13473 | 0.034157043 | 0.827860825 | 0.961619932 |
| α-HBDH | Q9NZP8 | 0.034142844 | 0.827931307 | 0.961619932 |
| CRP | C9JV77 | -0.034063446 | 0.828325438 | 0.961737145 |
| γ-GT | A0A0C4DH36 | 0.03391124 | 0.829081096 | 0.961918573 |
| Eosinophils | A0A5H1ZRS9 | -0.033895811 | 0.829157707 | 0.961918573 |
| P | P13647 | 0.033854769 | 0.829361501 | 0.961918573 |
| DBIL | P01814 | 0.03355884 | 0.830831254 | 0.962849941 |
| UA | Q9H4B7 | 0.033516612 | 0.83104103 | 0.962849941 |
| ALT | A0A0G2JI36 | 0.033490604 | 0.831170233 | 0.962849941 |
| Platelets | A0A1W2PQU7 | 0.033456688 | 0.831338729 | 0.962849941 |
| Monocytes | P01782 | -0.033277916 | 0.832227009 | 0.963383179 |
| Basophils | P0DP01 | -0.033119692 | 0.833013363 | 0.963383179 |
| PT | P02042 | -0.033067309 | 0.833273737 | 0.963383179 |
| Monocytes | P02745 | -0.033051021 | 0.833354697 | 0.963383179 |
| WLGG | A0A0B4J1Y8 | 0.033015557 | 0.833530986 | 0.963383179 |
| Platelets | P04430 | 0.033009435 | 0.833561422 | 0.963383179 |
| Hemoglobin | P04264 | 0.032790608 | 0.834649383 | 0.963730715 |
| Total protein | I3L1J2 | -0.032781716 | 0.834693599 | 0.963730715 |
| TT | P03951 | 0.0327399 | 0.834901536 | 0.963730715 |
| CK-MB activity | P43121 | -0.032712578 | 0.835037406 | 0.963730715 |
| Hemoglobin | P35908 | -0.032639499 | 0.835400842 | 0.963811031 |
| BUN | P30041 | 0.032397849 | 0.836602861 | 0.964717839 |
| INR | P02745 | 0.032300823 | 0.837085589 | 0.964717839 |
| Mg | P00918 | -0.03227833 | 0.837197505 | 0.964717839 |
| Mg | Q9Y5Y7 | -0.032134828 | 0.8379116 | 0.964717839 |
| CK-MB activity | O75636 | 0.032127546 | 0.83794784 | 0.964717839 |
| FIB | P01715 | 0.032126798 | 0.837951562 | 0.964717839 |
| Basophils | P01019 | -0.03201039 | 0.838530929 | 0.965046122 |
| Basophils | A0A0C4DH33 | -0.031847436 | 0.839342095 | 0.965602226 |
| ALP | P00918 | 0.031795048 | 0.839602911 | 0.965602226 |
| Globin | P69905 | 0.031729248 | 0.839930525 | 0.965640422 |
| CO2 | A0A0J9YXX1 | -0.031581737 | 0.840665068 | 0.965969294 |
| Monocytes | E7ENL6 | 0.031553521 | 0.840805589 | 0.965969294 |
| Mg | P13473 | 0.031376931 | 0.841685143 | 0.966548719 |
| Hemoglobin | P13647 | 0.03127952 | 0.842170405 | 0.966548719 |
| TBIL | Q86YZ3 | -0.031156505 | 0.842783297 | 0.966548719 |
| FIB | A0A0J9YXX1 | -0.031121353 | 0.842958449 | 0.966548719 |
| IBIL | Q86YZ3 | -0.031080064 | 0.843164195 | 0.966548719 |
| Lymphocytes | A0A0G2JI36 | 0.031058225 | 0.843273021 | 0.966548719 |
| γ-GT | C9J8S2 | -0.031038224 | 0.843372693 | 0.966548719 |
| Neutrophils | P03950 | -0.030947779 | 0.843823433 | 0.966652712 |
| CO2 | P02751 | -0.030901748 | 0.844052856 | 0.966652712 |
| Albumin | E7EX29 | 0.030552595 | 0.84579346 | 0.968308044 |
| α-HBDH | Q86YZ3 | -0.0304749 | 0.846180886 | 0.968413575 |
| Platelets | P55056 | 0.030360245 | 0.846752675 | 0.968703843 |
| WBC | P13645 | -0.030203496 | 0.84753452 | 0.968703843 |
| Globin | Q9Y5Y7 | 0.030142786 | 0.847837374 | 0.968703843 |
| Hematocrit | D6R934 | -0.030133676 | 0.847882818 | 0.968703843 |
| WBC | P01817 | -0.030127987 | 0.847911199 | 0.968703843 |
| Platelets | Q6ZRK6 | 0.030031254 | 0.848393807 | 0.968786633 |
| TBIL | P69905 | 0.029904849 | 0.849024533 | 0.968786633 |
| γ-GT | D6R934 | -0.02989942 | 0.849051624 | 0.968786633 |
| BUN | E7ENL6 | 0.029863765 | 0.849229547 | 0.968786633 |
| INR | P35527 | 0.029804507 | 0.849525276 | 0.968786633 |
| Albumin | P02750 | -0.02975831 | 0.849755837 | 0.968786633 |
| Ca | A0A0B4J1V2 | -0.029677543 | 0.850158956 | 0.968875149 |
| IBIL | P30041 | 0.029537463 | 0.850858209 | 0.968875149 |
| LDH | Q86UD1 | -0.029395951 | 0.851564719 | 0.968875149 |
| WBC | P02766 | 0.0293729 | 0.851679817 | 0.968875149 |
| Hemoglobin | A0A0C4DH73 | 0.029315106 | 0.851968402 | 0.968875149 |
| Eosinophils | Q86YZ3 | -0.029223888 | 0.852423917 | 0.968875149 |
| AST | K7ERG9 | 0.029207087 | 0.852507822 | 0.968875149 |
| FIB | A0A0B4J1V2 | -0.029196255 | 0.852561918 | 0.968875149 |
| DBIL | P01019 | -0.029175027 | 0.852667935 | 0.968875149 |
| TBIL | P04430 | -0.029037035 | 0.853357164 | 0.968875149 |
| Eosinophils | P02766 | 0.028826571 | 0.854408565 | 0.968875149 |
| P | P35527 | -0.028791667 | 0.854582958 | 0.968875149 |
| Platelets | C9J8S2 | -0.028755819 | 0.854762077 | 0.968875149 |
| Albumin | P37802 | -0.028692528 | 0.855078328 | 0.968875149 |
| FIB | P03951 | 0.028553086 | 0.855775174 | 0.968875149 |
| Platelets | P02766 | 0.028547694 | 0.855802126 | 0.968875149 |
| BUN | Q86YZ3 | 0.028518484 | 0.85594811 | 0.968875149 |
| UA | A0A0C4DH36 | 0.028391286 | 0.856583887 | 0.968875149 |
| LDH | P03950 | -0.028299477 | 0.857042834 | 0.968875149 |
| CO2 | Q86YZ3 | -0.028168278 | 0.857698769 | 0.968875149 |
| Platelets | P26038 | 0.028108313 | 0.857998601 | 0.968875149 |
| Hemoglobin | P02751 | -0.028106235 | 0.858008989 | 0.968875149 |
| DBIL | Q86YZ3 | -0.028057121 | 0.858254577 | 0.968875149 |
| Hemoglobin | P01814 | 0.028030681 | 0.858386795 | 0.968875149 |
| Hematocrit | P02751 | -0.028019033 | 0.858445045 | 0.968875149 |
| Neutrophils | A0A0B4J1V2 | 0.027979157 | 0.858644454 | 0.968875149 |
| Ca | K7ERG9 | 0.027975226 | 0.858664113 | 0.968875149 |
| WLL | A0A087X0Q4 | -0.027904408 | 0.859018285 | 0.968875149 |
| Globin | P80748 | 0.027876411 | 0.859158312 | 0.968875149 |
| APTT | E7EX29 | 0.027870467 | 0.859188039 | 0.968875149 |
| WBC | Q8N1N4 | -0.027787216 | 0.85960444 | 0.968875149 |
| Lymphocytes | P02751 | 0.027716941 | 0.859955973 | 0.968875149 |
| Platelets | P01715 | -0.027676843 | 0.860156562 | 0.968875149 |
| WLGG | Q9Y5Y7 | 0.027643562 | 0.860323054 | 0.968875149 |
| ALT | P04430 | -0.027632675 | 0.860377521 | 0.968875149 |
| Basophils | P04430 | 0.027614695 | 0.860467472 | 0.968875149 |
| Lymphocytes | P04430 | 0.027451145 | 0.86128577 | 0.969463735 |
| INR | P43121 | -0.027338457 | 0.861849672 | 0.969567263 |
| APTT | A0A0J9YVY3 | -0.027258382 | 0.86225042 | 0.969567263 |
| WLC | P55056 | 0.02721505 | 0.862467291 | 0.969567263 |
| Globin | P19652 | 0.027196498 | 0.862560144 | 0.969567263 |
| P | D6RE82 | -0.02690245 | 0.864032132 | 0.970500574 |
| CK-MB activity | P02763 | 0.02681093 | 0.864490367 | 0.970500574 |
| Hemoglobin | P55056 | 0.026746256 | 0.864814214 | 0.970500574 |
| Hematocrit | P13645 | 0.026735142 | 0.864869871 | 0.970500574 |
| Creatinine | Q15582 | 0.026729085 | 0.86468974 | 0.970500574 |
| Albumin | P02766 | 0.026586104 | 0.865616248 | 0.971005915 |
| Neutrophils | E7EX29 | 0.026165608 | 0.867722704 | 0.972811127 |
| TT | P32119 | -0.026146448 | 0.867818707 | 0.972811127 |
| Albumin | A0A0C4DH73 | -0.026057403 | 0.868264898 | 0.972978772 |
| P | Q92954 | -0.025920057 | 0.868953197 | 0.973162134 |
| Albumin | P02042 | 0.025906346 | 0.869021918 | 0.973162134 |
| Globin | P32119 | 0.025761128 | 0.869749782 | 0.973644807 |
| Monocytes | P01817 | -0.025639122 | 0.870361385 | 0.973997046 |
| TT | P13473 | -0.02538858 | 0.87161756 | 0.97464924 |
| Lymphocytes | C9J8S2 | -0.02535884 | 0.87176669 | 0.97464924 |
| WLGG | A0A0C4DH73 | 0.025274114 | 0.872191577 | 0.97464924 |
| Eosinophils | P80748 | -0.025270538 | 0.872209511 | 0.97464924 |
| Albumin | D6R934 | -0.025226587 | 0.872429929 | 0.97464924 |
| Neutrophils | P03951 | -0.025070795 | 0.873211327 | 0.974885529 |
| TBIL | A0A0C4DH33 | 0.024997168 | 0.873580654 | 0.974885529 |
| INR | A0A0B4J1Y8 | -0.024926284 | 0.87393625 | 0.974885529 |
| LDH | A0A0J9YXX1 | -0.024846128 | 0.874338384 | 0.974885529 |
| WLL | A0A1W2PQU7 | 0.024786597 | 0.874637066 | 0.974885529 |
| WLGG | P37802 | -0.024786117 | 0.874639474 | 0.974885529 |
| APTT | A0A1W2PQU7 | -0.024769673 | 0.874721985 | 0.974885529 |
| TBIL | A0A0A0MS15 | -0.024694155 | 0.875100907 | 0.974976554 |
| Platelets | P02745 | -0.024469452 | 0.876228559 | 0.975901417 |
| CRP | A0A0J9YXX1 | 0.024395772 | 0.876598368 | 0.97598189 |
| PT | P03950 | -0.024293314 | 0.877112657 | 0.9760084 |
| Glucose | A0A0A0MS15 | 0.024243798 | 0.877361221 | 0.9760084 |
| Mg | H3BTN5 | -0.024168368 | 0.877739894 | 0.9760084 |
| Globin | A0A096LPE2 | 0.024099119 | 0.87808756 | 0.9760084 |
| Glucose | P00915 | -0.024092747 | 0.878119557 | 0.9760084 |
| DBIL | K7ERG9 | 0.024035385 | 0.87840756 | 0.9760084 |
| CO2 | P02750 | 0.023875189 | 0.879211966 | 0.976571367 |
| Neutrophils | P0DP01 | 0.023787948 | 0.879650089 | 0.976727249 |
| Ca | A0A0J9YVY3 | -0.023639442 | 0.880395961 | 0.97674711 |
| α-HBDH | A0A0J9YVY3 | -0.023617101 | 0.880508175 | 0.97674711 |
| APTT | Q8N1N4 | -0.023561396 | 0.880787987 | 0.97674711 |
| Ca | Q16880 | 0.023514312 | 0.881024505 | 0.97674711 |
| Albumin | A0A0G2JI36 | 0.023487953 | 0.88115692 | 0.97674711 |
| Glucose | H3BTN5 | -0.023417407 | 0.881511322 | 0.976809844 |
| AST | A0A075B6S5 | -0.023305137 | 0.882075381 | 0.976980883 |
| Globin | P02750 | 0.023268115 | 0.882261395 | 0.976980883 |
| Mg | A0A0G2JI36 | -0.023182735 | 0.882690412 | 0.977038774 |
| RBC | I3L1J2 | 0.023084272 | 0.883185204 | 0.977038774 |
| IBIL | P13645 | -0.023036255 | 0.883426514 | 0.977038774 |
| FIB | O75882 | -0.022963329 | 0.883793025 | 0.977038774 |
| CO2 | P43121 | -0.022961332 | 0.883803062 | 0.977038774 |
| IBIL | O75882 | 0.022885197 | 0.884185721 | 0.977122131 |
| Creatinine | P08185 | -0.022802779 | 0.884474271 | 0.977122131 |
| Hematocrit | Q96HR3 | -0.022732423 | 0.884953664 | 0.977265637 |
| WBC | Q5SRP5 | -0.022667861 | 0.885278225 | 0.977265637 |
| AST | A0A0A0MS15 | 0.022624143 | 0.88549801 | 0.977265637 |
| Creatine Kinase | P08185 | 0.022505004 | 0.886097008 | 0.977269061 |
| RBC | Q9NZP8 | 0.022505004 | 0.886097008 | 0.977269061 |
| Total protein | P02042 | 0.022128239 | 0.887991688 | 0.979029491 |
| Total protein | A0A0C4DH36 | -0.021977193 | 0.888751443 | 0.979537881 |
| APTT | O75882 | 0.021900015 | 0.88913968 | 0.979636597 |
| APTT | P13647 | -0.021597946 | 0.89065947 | 0.979907923 |
| APTT | P37802 | -0.021594102 | 0.890678812 | 0.979907923 |
| CO2 | P55056 | 0.021533003 | 0.890986268 | 0.979907923 |
| APTT | P35527 | 0.021522429 | 0.891039478 | 0.979907923 |
| Total protein | A0A0J9YVY3 | 0.021494563 | 0.891179707 | 0.979907923 |
| INR | Q92954 | 0.021483452 | 0.891235625 | 0.979907923 |
| Eosinophils | P08185 | -0.021411863 | 0.891595898 | 0.979907923 |
| Creatine Kinase | H3BTN5 | -0.021376085 | 0.89177596 | 0.979907923 |
| UA | A0A0J9YXX1 | -0.021293465 | 0.892191789 | 0.980030745 |
| Eosinophils | D6R934 | -0.021184882 | 0.892738331 | 0.980030745 |
| LDH | A0A0C4DH73 | -0.021145641 | 0.89293586 | 0.980030745 |
| ALT | Q9H4B7 | -0.020845289 | 0.894447959 | 0.980030745 |
| AST | A0A0J9YX35 | -0.020808158 | 0.894634919 | 0.980030745 |
| Globin | A0A5H1ZRS9 | -0.020699557 | 0.89518177 | 0.980030745 |
| LDH | Q9H4B7 | -0.02066241 | 0.895368833 | 0.980030745 |
| Albumin | Q15582 | -0.020619336 | 0.895585746 | 0.980030745 |
| Basophils | P01715 | 0.0205893 | 0.895737008 | 0.980030745 |
| Lymphocytes | Q5SRP5 | 0.020558636 | 0.895891437 | 0.980030745 |
| Globin | P08185 | 0.020548466 | 0.895942659 | 0.980030745 |
| Platelets | A0A0B4J1Y8 | -0.020505268 | 0.896160215 | 0.980030745 |
| DBIL | Q8N1N4 | -0.020482986 | 0.896272441 | 0.980030745 |
| DBIL | Q5SRP5 | -0.020344215 | 0.8969714 | 0.980030745 |
| Hematocrit | P0DJI8 | -0.020315687 | 0.897115102 | 0.980030745 |
| Hemoglobin | P0DP01 | -0.020249343 | 0.897449298 | 0.980030745 |
| Globin | P01782 | 0.020246282 | 0.897464714 | 0.980030745 |
| Creatinine | P02751 | 0.020235578 | 0.897449079 | 0.980030745 |
| UA | D6R934 | -0.020085325 | 0.898275582 | 0.980030745 |
| Total protein | P01814 | 0.020013595 | 0.898636977 | 0.980030745 |
| Lymphocytes | P00915 | 0.019938072 | 0.899017502 | 0.980030745 |
| WLC | P03951 | 0.019921103 | 0.899103002 | 0.980030745 |
| RBC | Q5SRP5 | 0.019904619 | 0.899186059 | 0.980030745 |
| Total protein | P80748 | 0.019787026 | 0.899778619 | 0.980030745 |
| Creatinine | Q92954 | -0.019782543 | 0.899741621 | 0.980030745 |
| CRP | P26038 | -0.019700036 | 0.900216997 | 0.980030745 |
| LDH | P0DP01 | 0.019635979 | 0.900539827 | 0.980030745 |
| Mg | P26038 | -0.019616046 | 0.90064029 | 0.980030745 |
| AST | A0A2R8Y3M9 | -0.019597502 | 0.900733752 | 0.980030745 |
| LDH | H3BTN5 | 0.019571865 | 0.900862963 | 0.980030745 |
| LDH | C9J8S2 | 0.01951214 | 0.901163989 | 0.980030745 |
| Hematocrit | P03951 | -0.019409411 | 0.901681799 | 0.980030745 |
| P | A0A0C4DH36 | 0.019345582 | 0.902003549 | 0.980030745 |
| Glucose | P26038 | 0.01920681 | 0.902703133 | 0.980030745 |
| LDH | P02750 | 0.019106597 | 0.903208374 | 0.980030745 |
| Monocytes | P0DP01 | 0.018946438 | 0.904015923 | 0.980030745 |
| CO2 | C9JV77 | -0.018888599 | 0.904307582 | 0.980030745 |
| WLL | P19652 | 0.018862756 | 0.904437899 | 0.980030745 |
| WLL | Q92954 | 0.018862756 | 0.904437899 | 0.980030745 |
| WLC | A0A096LPE2 | 0.018823089 | 0.904637934 | 0.980030745 |
| Creatinine | Q9H4B7 | 0.0187877 | 0.904816402 | 0.980030745 |
| Basophils | D6RE82 | 0.018713766 | 0.905189265 | 0.980030745 |
| Globin | K7ERG9 | -0.01865982 | 0.905461341 | 0.980030745 |
| Total protein | Q9Y5Y7 | 0.018654181 | 0.905489782 | 0.980030745 |
| Mg | P02753 | 0.018644263 | 0.905539801 | 0.980030745 |
| INR | Q16880 | 0.018608905 | 0.905718135 | 0.980030745 |
| Eosinophils | P30041 | 0.018519087 | 0.906171167 | 0.980030745 |
| DBIL | C9J8S2 | 0.018342439 | 0.907062245 | 0.980030745 |
| PT | Q92954 | -0.018311873 | 0.907216441 | 0.980030745 |
| Ca | A0A0A0MS15 | -0.018221701 | 0.907671355 | 0.980030745 |
| Platelets | Q15582 | -0.018201043 | 0.907775581 | 0.980030745 |
| Creatine Kinase | A0A0J9YX35 | 0.018200355 | 0.907779049 | 0.980030745 |
| Eosinophils | Q96HR3 | -0.01815847 | 0.907990371 | 0.980030745 |
| DBIL | A0A0B4J1V2 | 0.01802722 | 0.908652607 | 0.980030745 |
| WLGG | P13645 | 0.018007806 | 0.908750565 | 0.980030745 |
| Globin | P15169 | 0.017979907 | 0.90889134 | 0.980030745 |
| Total protein | P02750 | 0.017974474 | 0.908918758 | 0.980030745 |
| IBIL | Q6ZRK6 | -0.017806761 | 0.909765087 | 0.980371775 |
| WLC | H3BTN5 | -0.017759674 | 0.910002719 | 0.980371775 |
| TT | P19652 | -0.017734112 | 0.910131724 | 0.980371775 |
| IBIL | A0A0C4DH73 | -0.017598188 | 0.910817745 | 0.980788642 |
| α-HBDH | O75636 | 0.017449108 | 0.911570238 | 0.980823456 |
| Eosinophils | D6RE82 | 0.017401867 | 0.911808706 | 0.980823456 |
| Ca | Q96HR3 | 0.017390006 | 0.911868585 | 0.980823456 |
| LDH | P04430 | -0.017354821 | 0.912046202 | 0.980823456 |
| Basophils | P03950 | 0.017200115 | 0.91282722 | 0.98134162 |
| AST | P26038 | -0.017003198 | 0.91382146 | 0.98188542 |
| Neutrophils | Q86UD1 | -0.016981352 | 0.913931765 | 0.98188542 |
| LDH | Q15582 | -0.016538912 | 0.916166158 | 0.983276537 |
| WBC | D6RE82 | 0.016536414 | 0.916178775 | 0.983276537 |
| Hematocrit | P08185 | -0.016464014 | 0.91654447 | 0.983276537 |
| TT | Q9H4B7 | 0.016460446 | 0.916562489 | 0.983276537 |
| WLGG | P0DJI8 | -0.016428174 | 0.916725503 | 0.983276537 |
| AST | Q8N1N4 | 0.016268196 | 0.91753363 | 0.983296723 |
| WBC | A0A096LPE2 | -0.016234379 | 0.917704468 | 0.983296723 |
| LDH | A0A075B6S5 | -0.016161311 | 0.918073605 | 0.983296723 |
| LDH | P19652 | 0.016161311 | 0.918073605 | 0.983296723 |
| Monocytes | A0A0J9YVY3 | 0.01610483 | 0.91835896 | 0.983296723 |
| Eosinophils | Q16880 | 0.016039982 | 0.918686596 | 0.983296723 |
| BUN | P35908 | 0.016009062 | 0.918842822 | 0.983296723 |
| Platelets | D6RE82 | -0.015935353 | 0.919215252 | 0.983374437 |
| WLL | A0A0J9YXX1 | -0.015744945 | 0.920177401 | 0.983688899 |
| Lymphocytes | P02753 | -0.015708784 | 0.920360143 | 0.983688899 |
| Creatinine | A0A087X0Q4 | 0.015705225 | 0.920408912 | 0.983688899 |
| Total protein | A0A096LPE2 | -0.015633261 | 0.920741809 | 0.983724147 |
| FIB | P00915 | 0.015485139 | 0.921490411 | 0.98420337 |
| Creatine Kinase | A0A0B4J1V2 | 0.015406692 | 0.921886913 | 0.984306339 |
| CO2 | P02042 | 0.015337542 | 0.922236431 | 0.984331952 |
| TBIL | Q9NZP8 | 0.015254493 | 0.922656225 | 0.984331952 |
| UA | A0A140T8Y3 | 0.015209446 | 0.922883938 | 0.984331952 |
| WLGG | A0A0J9YXX1 | 0.015164468 | 0.923111306 | 0.984331952 |
| AST | Q9NZP8 | -0.014906208 | 0.924416958 | 0.98498734 |
| ALP | P02042 | -0.014884216 | 0.924528148 | 0.98498734 |
| P | A0A087X0Q4 | -0.014811461 | 0.924896004 | 0.98498734 |
| FIB | P08185 | 0.014805304 | 0.924927136 | 0.98498734 |
| WBC | K7ERG9 | -0.014573187 | 0.926100854 | 0.985917171 |
| Glucose | A0A0C4DH73 | 0.014425438 | 0.926848041 | 0.9861046 |
| ALT | P02751 | -0.014365113 | 0.927153128 | 0.9861046 |
| APTT | A0A0B4J1Y8 | -0.014348828 | 0.927235491 | 0.9861046 |
| Ca | Q86UD1 | 0.014257746 | 0.927696151 | 0.9861046 |
| WLGG | P35527 | -0.014216689 | 0.927903814 | 0.9861046 |
| UA | Q5SRP5 | 0.014138598 | 0.928298802 | 0.9861046 |
| Creatine Kinase | Q92954 | -0.014122267 | 0.928381404 | 0.9861046 |
| Eosinophils | Q9NZP8 | -0.013921494 | 0.92939701 | 0.986863772 |
| Mg | A0A087X0Q4 | -0.013793723 | 0.930043391 | 0.987230525 |
| LDH | P08185 | -0.013669146 | 0.930673657 | 0.987357564 |
| Albumin | P01782 | 0.013595167 | 0.93104796 | 0.987357564 |
| UA | A0A087X0Q4 | 0.013591573 | 0.931066142 | 0.987357564 |
| APTT | A0A075B6S9 | -0.013477941 | 0.931641096 | 0.98748575 |
| ALP | P02745 | 0.013448682 | 0.931789145 | 0.98748575 |
| Monocytes | P15169 | -0.013386798 | 0.932102286 | 0.987498546 |
| Neutrophils | Q5SRP5 | -0.013217513 | 0.932958935 | 0.987828746 |
| γ-GT | A0A0G2JI36 | -0.013206178 | 0.933016297 | 0.987828746 |
| WLL | Q15582 | 0.013094806 | 0.933579931 | 0.987868082 |
| Creatinine | P04430 | 0.013079813 | 0.933655809 | 0.987868082 |
| TBIL | C9J8S2 | 0.01295494 | 0.934287812 | 0.988218002 |
| Lymphocytes | H3BTN5 | 0.012747688 | 0.935336832 | 0.989008642 |
| RBC | P13645 | -0.012611864 | 0.936024371 | 0.989416673 |
| CRP | P15169 | 0.012386708 | 0.937164217 | 0.989682229 |
| CRP | P0DJI8 | -0.012386708 | 0.937164217 | 0.989682229 |
| UA | P02751 | 0.012383433 | 0.937180793 | 0.989682229 |
| DBIL | P04264 | 0.012244442 | 0.937884496 | 0.990106581 |
| IBIL | P69905 | 0.012160121 | 0.938311424 | 0.99023595 |
| IBIL | P00918 | -0.012100986 | 0.938610844 | 0.99023595 |
| α-HBDH | P01782 | 0.012010425 | 0.939069404 | 0.990401172 |
| Albumin | Q86UD1 | 0.011805411 | 0.940107569 | 0.990818479 |
| DBIL | P13647 | 0.011639778 | 0.940946388 | 0.990818479 |
| CRP | A0A0B4J1V2 | 0.011556328 | 0.941369028 | 0.990818479 |
| ALT | E7ENL6 | -0.011417826 | 0.942070522 | 0.990818479 |
| UA | P35908 | -0.01140182 | 0.942151592 | 0.990818479 |
| WLL | P04264 | 0.01122412 | 0.943051686 | 0.990818479 |
| BUN | A0A140T8Y3 | -0.011199841 | 0.943174669 | 0.990818479 |
| AST | A0A087X0Q4 | 0.011198572 | 0.943181095 | 0.990818479 |
| Hemoglobin | A0A0G2JI36 | 0.010956214 | 0.944408827 | 0.990818479 |
| WLL | A0A075B6S9 | -0.010955129 | 0.944414322 | 0.990818479 |
| WLL | A0A0J9YX35 | -0.010912338 | 0.944631101 | 0.990818479 |
| Platelets | P01782 | 0.010875312 | 0.944818684 | 0.990818479 |
| Creatinine | I3L1J2 | 0.010789556 | 0.94525315 | 0.990818479 |
| Glucose | P13645 | 0.010724671 | 0.945581886 | 0.990818479 |
| IBIL | A0A0B4J1Y8 | 0.010574418 | 0.94634317 | 0.990818479 |
| Hematocrit | P13647 | 0.01057322 | 0.94634924 | 0.990818479 |
| WLL | P01718 | 0.010477948 | 0.946831978 | 0.990818479 |
| FIB | Q16880 | 0.010424143 | 0.947104614 | 0.990818479 |
| WBC | P03950 | 0.010289165 | 0.947788586 | 0.990818479 |
| IBIL | Q9Y5Y7 | -0.010196375 | 0.948258799 | 0.990818479 |
| PT | P02751 | 0.010139632 | 0.948546353 | 0.990818479 |
| Neutrophils | P26038 | 0.010075423 | 0.948871751 | 0.990818479 |
| INR | P03950 | -0.009995503 | 0.949276783 | 0.990818479 |
| TT | P00915 | 0.009928071 | 0.949618531 | 0.990818479 |
| ALP | P02753 | -0.009897626 | 0.949772835 | 0.990818479 |
| APTT | A0A0C4DH73 | -0.009817248 | 0.950180211 | 0.990818479 |
| WBC | A0A075B6S9 | -0.009770401 | 0.950417655 | 0.990818479 |
| Total protein | Q96HR3 | 0.009666944 | 0.950942031 | 0.990818479 |
| Eosinophils | Q15582 | -0.009608857 | 0.951236455 | 0.990818479 |
| Platelets | Q9Y5Y7 | -0.009515898 | 0.951707652 | 0.990818479 |
| Platelets | P80748 | 0.009515898 | 0.951707652 | 0.990818479 |
| WBC | P35908 | -0.009514101 | 0.951716759 | 0.990818479 |
| Creatinine | P13647 | 0.009513742 | 0.951888342 | 0.990818479 |
| IBIL | P02042 | 0.009441088 | 0.952086864 | 0.990818479 |
| Glucose | K7ERG9 | -0.009440731 | 0.952088672 | 0.990818479 |
| PT | A0A0B4J1V2 | -0.009383298 | 0.952379811 | 0.990818479 |
| Hematocrit | P35908 | -0.009364852 | 0.952473317 | 0.990818479 |
| Hemoglobin | A0A087X0Q4 | 0.009293191 | 0.952836588 | 0.990818479 |
| WLC | A0A5H1ZRS9 | 0.009254686 | 0.953031785 | 0.990818479 |
| Glucose | P01715 | -0.009199974 | 0.953309144 | 0.990818479 |
| WLGG | A0A096LPE2 | 0.009161866 | 0.953502334 | 0.990818479 |
| CO2 | H3BTN5 | 0.009103671 | 0.953797363 | 0.990818479 |
| Neutrophils | D6R934 | 0.009061733 | 0.954009977 | 0.990818479 |
| P | P19652 | 0.008992673 | 0.954360101 | 0.990818479 |
| α-HBDH | P13473 | -0.008913397 | 0.954762026 | 0.990818479 |
| Hematocrit | Q15582 | 0.008836191 | 0.955153471 | 0.990818479 |
| Creatine Kinase | C9JV77 | -0.008835857 | 0.955155163 | 0.990818479 |
| Basophils | A0A0J9YVY3 | 0.008824001 | 0.955215277 | 0.990818479 |
| WLGG | P03950 | -0.008805594 | 0.955308601 | 0.990818479 |
| Monocytes | P80748 | -0.008697637 | 0.955855979 | 0.990818479 |
| CO2 | E7EX29 | 0.008685896 | 0.955915514 | 0.990818479 |
| Lymphocytes | A0A2R8Y3M9 | -0.008685145 | 0.95591932 | 0.990818479 |
| CRP | K7ERG9 | 0.008610272 | 0.956298962 | 0.990818479 |
| Lymphocytes | Q9H4B7 | -0.008599709 | 0.956352522 | 0.990818479 |
| WLL | D6RE82 | -0.00857398 | 0.956482984 | 0.990818479 |
| AST | P02763 | 0.008550261 | 0.956603254 | 0.990818479 |
| Glucose | Q9NZP8 | -0.008534421 | 0.956683574 | 0.990818479 |
| WLL | H3BTN5 | -0.008339228 | 0.957673371 | 0.99153051 |
| WBC | Q9Y5Y7 | 0.008079435 | 0.95899084 | 0.99236802 |
| P | C9JV77 | -0.007934711 | 0.959724815 | 0.99236802 |
| Creatinine | C9JV77 | -0.007852613 | 0.960348484 | 0.99236802 |
| Globin | P02753 | -0.00778122 | 0.960503289 | 0.99236802 |
| α-HBDH | P02042 | -0.007780338 | 0.960507763 | 0.99236802 |
| P | D6R934 | 0.007708005 | 0.960874633 | 0.99236802 |
| Hematocrit | P0DP01 | -0.007703637 | 0.960896791 | 0.99236802 |
| BUN | Q8N1N4 | 0.007702473 | 0.960902692 | 0.99236802 |
| ALT | D6R934 | 0.007409374 | 0.962389364 | 0.993296957 |
| P | P08185 | 0.007405731 | 0.962407845 | 0.993296957 |
| PT | P19823 | 0.007264214 | 0.963125697 | 0.993644317 |
| Lymphocytes | A0A087X0Q4 | 0.007174685 | 0.963579857 | 0.993644317 |
| FIB | Q86UD1 | -0.007160222 | 0.963653223 | 0.993644317 |
| Globin | P37802 | -0.007096772 | 0.963975098 | 0.993663032 |
| PT | P13645 | -0.007037208 | 0.964277266 | 0.993663032 |
| CRP | P80748 | 0.006873112 | 0.96510974 | 0.994163867 |
| WLL | A0A0G2JI36 | 0.006799411 | 0.965483646 | 0.994163867 |
| ALT | C9J8S2 | 0.006722325 | 0.965874733 | 0.994163867 |
| Ca | I3L1J2 | 0.006701884 | 0.965978437 | 0.994163867 |
| CO2 | P01715 | -0.006629605 | 0.966345148 | 0.994163867 |
| AST | P03951 | 0.006582945 | 0.966581882 | 0.994163867 |
| PT | P43121 | 0.006487084 | 0.967068248 | 0.994352305 |
| γ-GT | A0A2R8Y3M9 | -0.006358358 | 0.967721381 | 0.994475629 |
| Lymphocytes | P04264 | 0.006343932 | 0.967794576 | 0.994475629 |
| Hemoglobin | P03951 | 0.00619546 | 0.968547921 | 0.994938046 |
| Ca | P80748 | -0.006048698 | 0.969292624 | 0.995391298 |
| Glucose | P02753 | 0.005966542 | 0.969709506 | 0.995507725 |
| IBIL | C9JV77 | -0.00581571 | 0.970474898 | 0.995875488 |
| CK-MB activity | E7ENL6 | -0.005776282 | 0.97067498 | 0.995875488 |
| TBIL | P37802 | -0.005690865 | 0.971108443 | 0.995919761 |
| TT | P19823 | -0.005608224 | 0.971527824 | 0.995919761 |
| TBIL | P03951 | 0.00558828 | 0.971629035 | 0.995919761 |
| UA | P80748 | 0.005436629 | 0.972398645 | 0.996397237 |
| Ca | P69905 | -0.00529261 | 0.973129548 | 0.99680063 |
| AST | A0A075B6S9 | 0.005148586 | 0.973860503 | 0.99680063 |
| WBC | C9J8S2 | 0.005133998 | 0.973934538 | 0.99680063 |
| Platelets | D6R934 | -0.005060041 | 0.974309896 | 0.99680063 |
| TBIL | C9JV77 | 0.005059659 | 0.974311835 | 0.99680063 |
| Platelets | Q86YZ3 | 0.004949715 | 0.974869848 | 0.996962955 |
| APTT | A0A0J9YXX1 | 0.004908624 | 0.975078402 | 0.996962955 |
| Platelets | A0A0G2JI36 | -0.004663012 | 0.97632504 | 0.997926498 |
| Creatinine | A0A0C4DH36 | 0.004530353 | 0.977280881 | 0.997943205 |
| Lymphocytes | I3L1J2 | 0.004422286 | 0.97754693 | 0.997943205 |
| Globin | P02042 | 0.004306112 | 0.978136629 | 0.997943205 |
| WLC | A0A075B6S9 | 0.004286797 | 0.978234676 | 0.997943205 |
| Albumin | P04430 | -0.004046136 | 0.979456316 | 0.997943205 |
| CO2 | Q9Y5Y7 | -0.004004383 | 0.979668264 | 0.997943205 |
| α-HBDH | O75882 | 0.004003475 | 0.979672873 | 0.997943205 |
| RBC | A0A0G2JI36 | -0.003935604 | 0.980017408 | 0.997943205 |
| Albumin | I3L1J2 | 0.003854577 | 0.980428735 | 0.997943205 |
| AST | Q6ZRK6 | -0.003781568 | 0.980799361 | 0.997943205 |
| P | Q9H4B7 | -0.00374473 | 0.980986371 | 0.997943205 |
| ALP | P69905 | 0.003702165 | 0.98120245 | 0.997943205 |
| Neutrophils | P30041 | 0.003696678 | 0.981230309 | 0.997943205 |
| RBC | A0A096LPE2 | -0.003624967 | 0.981594353 | 0.997943205 |
| Platelets | Q16880 | -0.003549581 | 0.98197706 | 0.997943205 |
| α-HBDH | P37802 | -0.003508995 | 0.982183102 | 0.997943205 |
| TBIL | P01814 | 0.003473796 | 0.982361798 | 0.997943205 |
| UA | Q15582 | 0.003322385 | 0.983130475 | 0.997943205 |
| APTT | P01715 | 0.003118282 | 0.984166678 | 0.997943205 |
| P | A0A1W2PQU7 | -0.003022747 | 0.984651705 | 0.997943205 |
| Platelets | Q8N1N4 | 0.00302092 | 0.984660982 | 0.997943205 |
| PT | A0A0B4J1Y8 | -0.002989034 | 0.984822865 | 0.997943205 |
| WLL | Q9Y5Y7 | -0.00296192 | 0.984960523 | 0.997943205 |
| Hemoglobin | P13645 | 0.002871067 | 0.985421791 | 0.997943205 |
| CRP | P02753 | 0.002870091 | 0.985426747 | 0.997943205 |
| WBC | A0A0J9YVY3 | 0.002784354 | 0.985862043 | 0.997943205 |
| AST | P0DP01 | 0.002761914 | 0.985975971 | 0.997943205 |
| Albumin | P80748 | -0.002643505 | 0.986577158 | 0.997943205 |
| Mg | P01715 | -0.002425385 | 0.987684607 | 0.997943205 |
| FIB | P37802 | 0.002417308 | 0.987725621 | 0.997943205 |
| IBIL | D6RE82 | 0.002416919 | 0.987727596 | 0.997943205 |
| γ-GT | P15169 | -0.002346537 | 0.988084948 | 0.997943205 |
| IBIL | A0A0A0MS15 | 0.002265861 | 0.988494568 | 0.997943205 |
| Total protein | A0A0C4DH33 | -0.00219025 | 0.988878479 | 0.997943205 |
| Lymphocytes | A0A075B6S5 | -0.002190167 | 0.988878899 | 0.997943205 |
| CK-MB activity | A0A0J9YVY3 | -0.002169545 | 0.988983604 | 0.997943205 |
| Neutrophils | C9J8S2 | -0.002053754 | 0.989571527 | 0.997943205 |
| γ-GT | A0A075B6S5 | 0.002043758 | 0.989622285 | 0.997943205 |
| DBIL | P69905 | 0.00204074 | 0.989637606 | 0.997943205 |
| IBIL | P01814 | -0.002039275 | 0.989645046 | 0.997943205 |
| Hematocrit | P02763 | 0.002039121 | 0.989645828 | 0.997943205 |
| WLL | P26038 | 0.002033035 | 0.989676731 | 0.997943205 |
| FIB | P30041 | 0.001994316 | 0.989873324 | 0.997943205 |
| Hematocrit | A0A1W2PQU7 | -0.001888075 | 0.990412767 | 0.997943205 |
| CO2 | A0A0J9YVY3 | -0.001844278 | 0.990635147 | 0.997943205 |
| AST | P02766 | 0.001815985 | 0.99077881 | 0.997943205 |
| Total protein | A0A2R8Y7X9 | -0.001812552 | 0.99079624 | 0.997943205 |
| WLGG | Q92954 | -0.001737595 | 0.991176838 | 0.997943205 |
| WLL | P08185 | -0.001714796 | 0.991292604 | 0.997943205 |
| ALT | P01019 | -0.001663329 | 0.991553935 | 0.997943205 |
| AST | P13645 | 0.001513321 | 0.992315624 | 0.99840345 |
| CK-MB activity | I3L1J2 | 0.001387258 | 0.992955732 | 0.998741123 |
| Platelets | A0A0J9YXX1 | 0.001283891 | 0.9934806 | 0.998962713 |
| γ-GT | Q6ZRK6 | -0.001151349 | 0.994153617 | 0.998969443 |
| WLC | A0A0J9YX35 | -0.001019584 | 0.99482269 | 0.998969443 |
| CRP | P43121 | -0.001008094 | 0.994881034 | 0.998969443 |
| Eosinophils | P04264 | 0.000907924 | 0.99538968 | 0.998969443 |
| Lymphocytes | D6R934 | -0.00075523 | 0.996165032 | 0.998969443 |
| Creatine Kinase | Q96HR3 | -0.000755201 | 0.996165177 | 0.998969443 |
| Basophils | P13473 | -0.000738701 | 0.996248962 | 0.998969443 |
| CRP | Q5SRP5 | 0.000691745 | 0.996487401 | 0.998969443 |
| α-HBDH | Q86UD1 | 0.000685553 | 0.99651884 | 0.998969443 |
| Basophils | C9JV77 | 0.000574545 | 0.997082522 | 0.998969443 |
| TBIL | P19652 | 0.000528621 | 0.99731572 | 0.998969443 |
| TT | P30041 | 0.000500226 | 0.997459906 | 0.998969443 |
| Basophils | Q9Y5Y7 | -0.000492468 | 0.997499303 | 0.998969443 |
| ALP | A0A0C4DH33 | 0.000415565 | 0.997889806 | 0.998969443 |
| TBIL | P00918 | -0.000382885 | 0.998055751 | 0.998969443 |
| IBIL | C9J8S2 | 0.000316022 | 0.998395274 | 0.999004423 |
| WLC | P19823 | 7.84E-05 | 0.999601743 | 0.999605545 |
| Eosinophils | P43121 | -7.77E-05 | 0.999605545 | 0.999605545 |

| Table S7-3 Relationships between these DEPs and clinical parameter in S vs C group | | | | |
| --- | --- | --- | --- | --- |
| Metabolites | Microbes | Correlation_coefficient | P_value | corrected_pvalue |
| Monocytes | P02649 | -0.540259008 | 0.000184349 | 0.136114025 |
| Total protein | P68032 | -0.528158208 | 0.000272447 | 0.136114025 |
| APTT | P00748 | -0.527472546 | 0.000278422 | 0.136114025 |
| Ca | P15814 | -0.519062883 | 0.000361952 | 0.136114025 |
| Lymphocytes | P08779 | -0.514594277 | 0.000414959 | 0.136114025 |
| Hemoglobin | Q16880 | -0.513128698 | 0.000433805 | 0.136114025 |
| Total protein | P02766 | -0.510347455 | 0.000471696 | 0.136114025 |
| Globin | P68032 | -0.509474694 | 0.000484184 | 0.136114025 |
| RBC | Q16880 | -0.50519242 | 0.000549864 | 0.136114025 |
| DBIL | Q5SRP5 | -0.504856815 | 0.000555334 | 0.136114025 |
| Platelets | P43121 | -0.489827233 | 0.000856333 | 0.16634151 |
| Ca | A0A0C4DH38 | -0.486763283 | 0.000933135 | 0.16634151 |
| γ-GT | Q16880 | -0.481844203 | 0.001069335 | 0.170687993 |
| Globin | P02766 | -0.481401777 | 0.001082412 | 0.170687993 |
| CK-MB activity | P08185 | -0.47304533 | 0.001357645 | 0.174013152 |
| INR | P00748 | -0.47286685 | 0.001364146 | 0.174013152 |
| PT | P00748 | -0.470233082 | 0.001463346 | 0.174013152 |
| ALP | E7EX29 | -0.469069162 | 0.001509187 | 0.174013152 |
| Mg | P15169 | -0.463418689 | 0.001750312 | 0.188849452 |
| Mg | P08185 | -0.458341166 | 0.001995441 | 0.195870474 |
| WBC | Q16880 | -0.457205422 | 0.002054251 | 0.195870474 |
| WBC | P55058 | -0.448295391 | 0.002571269 | 0.212153552 |
| Globin | E7EX29 | -0.447440663 | 0.002626422 | 0.212153552 |
| P | P15814 | -0.447248211 | 0.002638983 | 0.212153552 |
| ALT | A0A0A0MS15 | -0.443445032 | 0.002898289 | 0.220055239 |
| P | P00915 | -0.440524379 | 0.003112358 | 0.232012173 |
| Globin | H0YJW9 | -0.436614947 | 0.003420552 | 0.243610019 |
| WLGG | P19823 | -0.436449693 | 0.003434149 | 0.243610019 |
| APTT | H0YAC1 | -0.436086266 | 0.003464218 | 0.243610019 |
| CO2 | P01019 | -0.435590493 | 0.003505608 | 0.243610019 |
| BUN | Q15582 | -0.428242404 | 0.004171865 | 0.277909928 |
| Glucose | O00187 | -0.426656601 | 0.004329355 | 0.280376972 |
| TBIL | C9JPQ9 | -0.426052485 | 0.004390702 | 0.280376972 |
| IBIL | C9JPQ9 | -0.420826098 | 0.004953872 | 0.305823959 |
| INR | Q86YZ3 | -0.41812959 | 0.005268287 | 0.305823959 |
| Ca | P04211 | -0.416717782 | 0.005439721 | 0.305823959 |
| WBC | D6RD17 | -0.416657228 | 0.005447181 | 0.305823959 |
| ALP | J3QT83 | -0.414388965 | 0.005733105 | 0.312958562 |
| Creatinine | Q16880 | -0.413863405 | 0.005801183 | 0.312958562 |
| Creatinine | P02649 | -0.412655265 | 0.005960344 | 0.317336833 |
| Ca | P04264 | -0.407413682 | 0.006695529 | 0.34314587 |
| Creatinine | D6RAR4 | -0.406841092 | 0.006780404 | 0.343205627 |
| RBC | P02649 | -0.406102503 | 0.006891258 | 0.344562896 |
| WLL | H0YJW9 | -0.404543782 | 0.007130359 | 0.352222548 |
| INR | P01019 | -0.403736923 | 0.007256919 | 0.354206776 |
| Hemoglobin | P02649 | -0.402282018 | 0.007490038 | 0.354768272 |
| Albumin | P04211 | -0.401042495 | 0.007693717 | 0.354768272 |
| WLGG | C9JPQ9 | -0.400998296 | 0.007701067 | 0.354768272 |
| Hematocrit | D6RAR4 | -0.398761433 | 0.00808105 | 0.364091266 |
| WLC | P01817 | -0.397119511 | 0.00837015 | 0.369006619 |
| INR | A0A0G2JRQ6 | -0.393980074 | 0.008947779 | 0.373303318 |
| PT | Q86YZ3 | -0.393160553 | 0.009104086 | 0.373303318 |
| ALT | P01009 | -0.393148094 | 0.00910648 | 0.373303318 |
| WLL | C9JPQ9 | -0.392380615 | 0.009255017 | 0.373303318 |
| TBIL | Q5SRP5 | -0.391758704 | 0.009376903 | 0.373303318 |
| PT | A0A0G2JRQ6 | -0.387321973 | 0.010287105 | 0.389248084 |
| CK-MB activity | E7EX29 | -0.38568563 | 0.010641398 | 0.396633929 |
| APTT | P36955 | -0.384502109 | 0.010904112 | 0.401032516 |
| CK-MB activity | P19823 | -0.380910398 | 0.011735623 | 0.415074605 |
| P | Q86YZ3 | -0.377902537 | 0.012473053 | 0.4171362 |
| PT | P01019 | -0.37786587 | 0.012482281 | 0.4171362 |
| Lymphocytes | P55058 | -0.377147611 | 0.012664218 | 0.4171362 |
| WBC | P15169 | -0.375731491 | 0.013029551 | 0.4171362 |
| ALT | P02649 | -0.373634395 | 0.013587026 | 0.426028123 |
| UA | D6RAR4 | -0.373541738 | 0.013612118 | 0.426028123 |
| Platelets | P08779 | -0.371243019 | 0.014247359 | 0.439204293 |
| Creatinine | P55058 | -0.369766301 | 0.014668584 | 0.447385568 |
| Mg | P55058 | -0.367703594 | 0.015274615 | 0.447385568 |
| Hematocrit | Q16880 | -0.366966251 | 0.015496336 | 0.447385568 |
| APTT | P01601 | -0.366602483 | 0.015606723 | 0.447385568 |
| Globin | P37802 | -0.366252135 | 0.015713666 | 0.447385568 |
| Lymphocytes | Q16880 | -0.366121669 | 0.015753648 | 0.447385568 |
| Neutrophils | P55058 | -0.365263359 | 0.01601884 | 0.447385568 |
| FIB | P30041 | -0.365194079 | 0.016040409 | 0.447385568 |
| TBIL | Q16880 | -0.363299983 | 0.0166397 | 0.450229043 |
| RBC | D6RD17 | -0.363203819 | 0.016670624 | 0.450229043 |
| Mg | P19823 | -0.362550287 | 0.016882076 | 0.450229043 |
| BUN | E7EWH8 | -0.362448506 | 0.016915211 | 0.450229043 |
| ALP | P37802 | -0.362222684 | 0.016988922 | 0.450229043 |
| Monocytes | Q16880 | -0.360021466 | 0.017721758 | 0.452965457 |
| BUN | A0A0J9YX35 | -0.358933697 | 0.018093636 | 0.452965457 |
| AST | P01861 | -0.358670173 | 0.01818471 | 0.452965457 |
| Eosinophils | A0A0C4DH67 | -0.355717512 | 0.019231853 | 0.464453259 |
| AST | A0A0A0MS15 | -0.35556185 | 0.019288437 | 0.464453259 |
| Hematocrit | A0A096LPE2 | -0.354807048 | 0.0195648 | 0.464453259 |
| Ca | J3QT83 | -0.352952822 | 0.020257874 | 0.464453259 |
| Ca | A0A0J9YXX1 | -0.352421156 | 0.020460363 | 0.464453259 |
| P | P32119 | -0.352283515 | 0.020513061 | 0.464453259 |
| IBIL | Q16880 | -0.350147249 | 0.021345677 | 0.47051572 |
| WLGG | Q16880 | -0.34907863 | 0.021772692 | 0.47051572 |
| Ca | A0A1W2PQU7 | -0.349017217 | 0.021797448 | 0.47051572 |
| γ-GT | D6RD17 | -0.349000013 | 0.021804387 | 0.47051572 |
| Creatine Kinase | P19823 | -0.348650182 | 0.021945896 | 0.471089908 |
| TT | P02763 | -0.346554122 | 0.022809967 | 0.482228743 |
| APTT | P55056 | -0.345304192 | 0.023338644 | 0.488206335 |
| RBC | C9J8S2 | -0.344716021 | 0.023590941 | 0.488499285 |
| Hemoglobin | A0A075B6R2 | -0.343194013 | 0.024254403 | 0.491411708 |
| WLGG | H0YJW9 | -0.343075419 | 0.024306748 | 0.491411708 |
| APTT | A0A0G2JI36 | -0.342968224 | 0.024354141 | 0.491411708 |
| DBIL | C9JPQ9 | -0.341948278 | 0.024808956 | 0.491411708 |
| Hematocrit | P02649 | -0.341212908 | 0.025141248 | 0.491411708 |
| Ca | A0A5H1ZRS9 | -0.341150335 | 0.025169694 | 0.491411708 |
| Glucose | A0A0A0MS15 | -0.340872194 | 0.02529646 | 0.491411708 |
| Hemoglobin | D6RAR4 | -0.34062497 | 0.025409581 | 0.491411708 |
| Mg | P04430 | -0.340227076 | 0.025592528 | 0.491679691 |
| IBIL | Q5SRP5 | -0.339814888 | 0.025783203 | 0.491679691 |
| Neutrophils | D6RD17 | -0.338833304 | 0.026242042 | 0.493505582 |
| Mg | A0A5H1ZRS9 | -0.335419643 | 0.027890967 | 0.508991332 |
| Glucose | P00915 | -0.334528981 | 0.028335057 | 0.508991332 |
| TT | A0A2R8Y3M9 | -0.333850414 | 0.028677317 | 0.508991332 |
| P | P01814 | -0.333094149 | 0.029062797 | 0.51129338 |
| Monocytes | D6RAR4 | -0.332955594 | 0.029133885 | 0.51129338 |
| DBIL | A0A0C4DH21 | -0.331973212 | 0.029642047 | 0.51129338 |
| IBIL | A0A669KAY4 | -0.331883758 | 0.029688682 | 0.51129338 |
| APTT | A0A2R8Y3M9 | -0.331105332 | 0.030097065 | 0.51129338 |
| Creatinine | Q5SRP5 | -0.331083249 | 0.030108718 | 0.51129338 |
| RBC | P01817 | -0.330652177 | 0.030336935 | 0.51129338 |
| Lymphocytes | D6RD17 | -0.33002304 | 0.030672578 | 0.51129338 |
| Basophils | P32119 | -0.329807654 | 0.030788189 | 0.51129338 |
| CRP | D6RD17 | -0.329784024 | 0.030800894 | 0.51129338 |
| APTT | P01857 | -0.329594815 | 0.030902786 | 0.51129338 |
| WLGG | A0A075B6S9 | -0.328815303 | 0.031325506 | 0.51129338 |
| WLL | P01817 | -0.328563478 | 0.031463084 | 0.51129338 |
| LDH | P68032 | -0.328496842 | 0.031499572 | 0.51129338 |
| Mg | P01715 | -0.328298204 | 0.031608549 | 0.51129338 |
| Mg | P09172 | -0.327760721 | 0.031904984 | 0.51129338 |
| Globin | P02649 | -0.327631143 | 0.031976792 | 0.51129338 |
| Albumin | A0A0J9YXX1 | -0.327239798 | 0.032194472 | 0.51129338 |
| RBC | D6RAR4 | -0.327177988 | 0.032228964 | 0.51129338 |
| BUN | P04433 | -0.327053051 | 0.032298777 | 0.51129338 |
| γ-GT | A0A0A0MS15 | -0.326808823 | 0.032435608 | 0.511484585 |
| Hematocrit | D6RD17 | -0.325202032 | 0.033347794 | 0.52127784 |
| Hematocrit | A0A075B6R2 | -0.324824417 | 0.033565207 | 0.52127784 |
| APTT | P13473 | -0.324459057 | 0.033776674 | 0.521441587 |
| Neutrophils | J3QT83 | -0.324367027 | 0.033830113 | 0.521441587 |
| WLC | A0A075B6R2 | -0.324140508 | 0.033961941 | 0.521512953 |
| DBIL | Q16880 | -0.323887363 | 0.034109765 | 0.52182849 |
| FIB | Q15582 | -0.322934011 | 0.034671233 | 0.524546334 |
| Ca | A0A0C4DH25 | -0.321861347 | 0.035312032 | 0.529771093 |
| Creatine Kinase | D6RAR4 | -0.321540497 | 0.035505584 | 0.529771093 |
| Hemoglobin | P01817 | -0.321206022 | 0.035708282 | 0.529771093 |
| Glucose | J3QRV5 | -0.321162924 | 0.035734469 | 0.529771093 |
| WBC | P02649 | -0.320534602 | 0.036118043 | 0.529771093 |
| Ca | P35908 | -0.320045913 | 0.036418706 | 0.529771093 |
| Total protein | A0A0C4DH38 | -0.32001512 | 0.036437719 | 0.529771093 |
| Basophils | A0A0G2JI36 | -0.319805893 | 0.036567127 | 0.529771093 |
| RBC | P04430 | -0.319557012 | 0.03672155 | 0.530135055 |
| ALT | P13473 | -0.318950657 | 0.037100016 | 0.530885663 |
| INR | F8W1S1 | -0.318242374 | 0.037546144 | 0.531415034 |
| CK-MB activity | P55058 | -0.31791864 | 0.037751513 | 0.531415034 |
| Basophils | A0A0G2JRQ6 | -0.317467337 | 0.038039344 | 0.531415034 |
| Basophils | P36955 | -0.317298291 | 0.038147619 | 0.531415034 |
| Creatinine | P08185 | -0.316910183 | 0.03839716 | 0.531452747 |
| RBC | Q5SRP5 | -0.316495011 | 0.038665579 | 0.531452747 |
| Albumin | Q6ZRK6 | -0.31644348 | 0.038699002 | 0.531452747 |
| TBIL | A0A669KAY4 | -0.315967357 | 0.039008933 | 0.531483735 |
| Neutrophils | Q16880 | -0.315952428 | 0.039018684 | 0.531483735 |
| Albumin | K7ERG9 | -0.31568216 | 0.039195552 | 0.532125042 |
| Monocytes | P01019 | -0.314886478 | 0.039720068 | 0.535698281 |
| Albumin | D6RD17 | -0.314095817 | 0.040246939 | 0.539256379 |
| Globin | P55058 | -0.313809912 | 0.040438857 | 0.540062905 |
| BUN | P08185 | -0.313082879 | 0.040930253 | 0.542374649 |
| Hematocrit | P01817 | -0.312967307 | 0.041008815 | 0.542374649 |
| AST | P01009 | -0.312045325 | 0.041639955 | 0.542780809 |
| Albumin | A0A0C4DH38 | -0.311980694 | 0.041684493 | 0.542780809 |
| Albumin | P09172 | -0.311956098 | 0.041701452 | 0.542780809 |
| WLL | P19823 | -0.310871598 | 0.042454857 | 0.545122646 |
| WBC | P09172 | -0.310580535 | 0.042658935 | 0.545122646 |
| γ-GT | P02649 | -0.309995021 | 0.043071885 | 0.545122646 |
| INR | A0A0C4DH21 | -0.309798883 | 0.043210941 | 0.545122646 |
| CK-MB activity | P15169 | -0.309418548 | 0.04348163 | 0.546854852 |
| Ca | Q8N1N4 | -0.308926379 | 0.043833957 | 0.549600066 |
| Total protein | P02649 | -0.308534757 | 0.04411596 | 0.550398766 |
| Hemoglobin | Q5SRP5 | -0.307759408 | 0.044678632 | 0.551754184 |
| γ-GT | C9J8S2 | -0.306762335 | 0.045410759 | 0.552141756 |
| Albumin | P04264 | -0.306617345 | 0.045518028 | 0.552141756 |
| AST | A0A0C4DH38 | -0.306056117 | 0.045935185 | 0.552605748 |
| WLGG | P01817 | -0.305758159 | 0.046157911 | 0.552605748 |
| P | P30041 | -0.305655077 | 0.046235169 | 0.552605748 |
| Glucose | A0A0G2JRQ6 | -0.305153863 | 0.046612317 | 0.552605748 |
| CO2 | P13645 | -0.30401452 | 0.047478912 | 0.559675462 |
| TT | P01008 | -0.303981577 | 0.047504161 | 0.559675462 |
| Globin | P61224 | -0.303209313 | 0.04809919 | 0.559950113 |
| WBC | D6RAR4 | -0.302941066 | 0.048307279 | 0.559950113 |
| Hemoglobin | D6RD17 | -0.302391555 | 0.048735829 | 0.559950113 |
| Basophils | P30041 | -0.302269367 | 0.048831536 | 0.559950113 |
| WLC | H0YJW9 | -0.302025659 | 0.049022882 | 0.559950113 |
| BUN | P15169 | -0.301906742 | 0.049116468 | 0.559950113 |
| P | P13645 | -0.301590348 | 0.04936617 | 0.559950113 |
| ALT | C9J8S2 | -0.301577628 | 0.04937623 | 0.559950113 |
| ALT | J3QRV5 | -0.301025282 | 0.049814678 | 0.56109939 |
| Lymphocytes | P15169 | -0.299814981 | 0.050786394 | 0.570477307 |
| WLL | Q16880 | -0.299340284 | 0.051171664 | 0.571835886 |
| Total protein | P61224 | -0.299322325 | 0.051186285 | 0.571835886 |
| P | A0A0G2JRQ6 | -0.298870595 | 0.051555184 | 0.572140225 |
| ALP | P68032 | -0.298754582 | 0.051650269 | 0.572140225 |
| CO2 | Q96HR3 | -0.297367743 | 0.052797909 | 0.576771312 |
| Lymphocytes | P02649 | -0.297171776 | 0.052961717 | 0.576771312 |
| PT | P55056 | -0.295484302 | 0.054389254 | 0.579871041 |
| Monocytes | P68032 | -0.295347956 | 0.054505934 | 0.579871041 |
| ALT | P01861 | -0.295050157 | 0.054761478 | 0.579871041 |
| Glucose | C9J8S2 | -0.295027746 | 0.054780748 | 0.579871041 |
| WBC | P08185 | -0.294937139 | 0.054858711 | 0.579871041 |
| WLC | P01715 | -0.294002037 | 0.05566854 | 0.579871041 |
| AST | P68032 | -0.293927427 | 0.055733565 | 0.579871041 |
| Ca | P01715 | -0.29365749 | 0.055969334 | 0.579871041 |
| IBIL | I3L1J2 | -0.293129681 | 0.056432645 | 0.579871041 |
| RBC | A0A0C4DH21 | -0.2927382 | 0.056778269 | 0.579871041 |
| BUN | O00187 | -0.292240893 | 0.057219766 | 0.580224953 |
| Neutrophils | A0A096LPE2 | -0.291787806 | 0.057624394 | 0.580224953 |
| Hemoglobin | P04430 | -0.291140252 | 0.058206663 | 0.58191419 |
| Basophils | P13647 | -0.290335544 | 0.058936788 | 0.58191419 |
| CO2 | A0A0B4J1U3 | -0.289858362 | 0.059373189 | 0.58191419 |
| Hemoglobin | A0A096LPE2 | -0.288941855 | 0.060218607 | 0.58191419 |
| ALP | O14791 | -0.288865417 | 0.060289548 | 0.58191419 |
| ALT | J3KPA1 | -0.288326883 | 0.060791246 | 0.58191419 |
| CRP | P04196 | -0.288249512 | 0.060863597 | 0.58191419 |
| CO2 | P00918 | -0.288216663 | 0.060894336 | 0.58191419 |
| Neutrophils | P15169 | -0.28793657 | 0.061156938 | 0.58191419 |
| TT | P01009 | -0.287648239 | 0.061428206 | 0.58191419 |
| α-HBDH | P68032 | -0.287627517 | 0.061447739 | 0.58191419 |
| Monocytes | Q5SRP5 | -0.287230041 | 0.06182336 | 0.58191419 |
| WLC | C9JPQ9 | -0.287177117 | 0.061873511 | 0.58191419 |
| TBIL | I3L1J2 | -0.286577285 | 0.06244419 | 0.58191419 |
| UA | Q16880 | -0.286555669 | 0.062464834 | 0.58191419 |
| Total protein | P09172 | -0.286193501 | 0.062811515 | 0.58191419 |
| Total protein | Q5SRP5 | -0.286176013 | 0.062828294 | 0.58191419 |
| ALT | Q16880 | -0.285898383 | 0.063095145 | 0.58191419 |
| DBIL | P02649 | -0.285498474 | 0.063481112 | 0.58191419 |
| UA | P01601 | -0.285347529 | 0.063627281 | 0.58191419 |
| Monocytes | A0A096LPE2 | -0.285098897 | 0.063868631 | 0.58191419 |
| BUN | O14791 | -0.284689449 | 0.064267673 | 0.584251569 |
| α-HBDH | P61224 | -0.284427532 | 0.06452397 | 0.584762605 |
| AST | P13473 | -0.283312288 | 0.06562439 | 0.589309333 |
| PT | Q5SRP5 | -0.282920703 | 0.066014284 | 0.589309333 |
| LDH | P61224 | -0.282243827 | 0.066692568 | 0.589309333 |
| Ca | P01857 | -0.282073082 | 0.066864539 | 0.589558298 |
| Ca | P13647 | -0.28177051 | 0.067170145 | 0.590449058 |
| INR | A0A1W2PQU7 | -0.281511583 | 0.067432546 | 0.590659907 |
| PT | F8W1S1 | -0.281380467 | 0.067565731 | 0.590659907 |
| CO2 | K7ERG9 | -0.281203989 | 0.067745322 | 0.590969828 |
| Total protein | P55058 | -0.281042309 | 0.067910186 | 0.591150238 |
| Neutrophils | P04433 | -0.280611669 | 0.068350856 | 0.59372566 |
| Total protein | H0YJW9 | -0.279682792 | 0.069309073 | 0.595892745 |
| Creatinine | J3QT83 | -0.279132567 | 0.069881668 | 0.595892745 |
| ALT | P02763 | -0.279091279 | 0.069924785 | 0.595892745 |
| Hemoglobin | P55058 | -0.278967921 | 0.070053732 | 0.595892745 |
| TT | P01861 | -0.277817989 | 0.071264828 | 0.600615812 |
| UA | P04196 | -0.277419111 | 0.07168876 | 0.601071403 |
| Globin | A0A087WZB5 | -0.277168081 | 0.071956576 | 0.601428214 |
| APTT | P80748 | -0.277104349 | 0.072024696 | 0.601428214 |
| PT | A0A0C4DH21 | -0.275966906 | 0.073249009 | 0.60426748 |
| APTT | Q9Y5Y7 | -0.275065151 | 0.074231233 | 0.604560747 |
| Total protein | A0A140T8Y3 | -0.274376445 | 0.074988352 | 0.60641468 |
| Creatinine | A0A669KAY4 | -0.274165845 | 0.075221081 | 0.606534529 |
| Mg | A0A075B6S9 | -0.273905087 | 0.075510024 | 0.606534529 |
| PT | A0A140T8Y3 | -0.273828627 | 0.075594913 | 0.606534529 |
| PT | A0A087WZB5 | -0.27295909 | 0.076565599 | 0.607068993 |
| Ca | J3KPA1 | -0.27295518 | 0.076569986 | 0.607068993 |
| Hematocrit | Q9Y5Y7 | -0.272940117 | 0.076586887 | 0.607068993 |
| P | A0A286YEY4 | -0.272428556 | 0.077162636 | 0.607068993 |
| TT | P55056 | -0.272071073 | 0.077566985 | 0.607068993 |
| APTT | J3KPA1 | -0.27196388 | 0.077688555 | 0.607068993 |
| Hematocrit | P04430 | -0.271902459 | 0.077758281 | 0.607068993 |
| Hemoglobin | A0A669KAY4 | -0.270607232 | 0.079240098 | 0.608885722 |
| CO2 | P04264 | -0.270402976 | 0.079475782 | 0.608885722 |
| INR | Q5SRP5 | -0.270269477 | 0.07963012 | 0.608885722 |
| P | P36955 | -0.269784352 | 0.080192946 | 0.608885722 |
| P | P00918 | -0.269732224 | 0.080253607 | 0.608885722 |
| UA | Q9Y5Y7 | -0.269490694 | 0.080535146 | 0.608885722 |
| LDH | P61626 | -0.268736816 | 0.081418872 | 0.612583018 |
| Hematocrit | P55056 | -0.267880076 | 0.082432354 | 0.612583018 |
| APTT | P07360 | -0.267739143 | 0.082600011 | 0.612583018 |
| Lymphocytes | P04430 | -0.267468412 | 0.082922823 | 0.612583018 |
| Ca | A0A0G2JI36 | -0.266632753 | 0.08392545 | 0.614454188 |
| BUN | J3QT83 | -0.265823495 | 0.084905381 | 0.615738916 |
| Basophils | P00748 | -0.265739432 | 0.085007682 | 0.615738916 |
| AST | P02763 | -0.265647915 | 0.085119163 | 0.615738916 |
| Neutrophils | P09172 | -0.265620823 | 0.085152187 | 0.615738916 |
| WBC | P08779 | -0.265488731 | 0.085313344 | 0.615818151 |
| P | F8W1S1 | -0.264309215 | 0.086762936 | 0.622991307 |
| ALT | P01008 | -0.263586208 | 0.08766091 | 0.626149357 |
| INR | A0A140T8Y3 | -0.263255615 | 0.088073903 | 0.626914936 |
| Lymphocytes | P08185 | -0.261450747 | 0.090355329 | 0.635146671 |
| AST | P19652 | -0.261174962 | 0.090707927 | 0.635146671 |
| IBIL | P02751 | -0.261119083 | 0.0907795 | 0.635146671 |
| Globin | Q5SRP5 | -0.260388726 | 0.091719 | 0.636864667 |
| AST | P02649 | -0.260037771 | 0.092173123 | 0.636864667 |
| APTT | P01019 | -0.25995998 | 0.092274015 | 0.636864667 |
| INR | P55056 | -0.259123773 | 0.093363961 | 0.639772235 |
| CO2 | F8W1S1 | -0.258961289 | 0.093576898 | 0.639772235 |
| RBC | A0A075B6R2 | -0.258902618 | 0.093653878 | 0.639772235 |
| CK-MB activity | P04196 | -0.258797461 | 0.093791976 | 0.639772235 |
| α-HBDH | P37802 | -0.258690249 | 0.093932934 | 0.639772235 |
| Globin | P04004 | -0.258600515 | 0.094051037 | 0.639772235 |
| AST | P01008 | -0.257915013 | 0.09495705 | 0.64351059 |
| CO2 | P35908 | -0.257336017 | 0.095727529 | 0.644267024 |
| RBC | P01861 | -0.25724105 | 0.095854362 | 0.644267024 |
| Monocytes | P55056 | -0.257050186 | 0.096109661 | 0.644925712 |
| Ca | Q6ZRK6 | -0.256711883 | 0.09656346 | 0.64691207 |
| INR | P02766 | -0.256098393 | 0.097390604 | 0.650328135 |
| Creatine Kinase | Q16880 | -0.255616388 | 0.098044287 | 0.651762813 |
| Platelets | P55058 | -0.255550524 | 0.098133872 | 0.651762813 |
| INR | P04264 | -0.255493317 | 0.098211733 | 0.651762813 |
| Eosinophils | H0YJW9 | -0.255471586 | 0.098241322 | 0.651762813 |
| Neutrophils | A0A0B4J1U3 | -0.255201843 | 0.098609178 | 0.653146413 |
| γ-GT | A0A140T8Y3 | -0.254898926 | 0.099023535 | 0.653778571 |
| AST | A0A286YEY4 | -0.253821124 | 0.100508706 | 0.656954459 |
| INR | A0A2R8Y3M9 | -0.253602455 | 0.1008121 | 0.656954459 |
| BUN | P19823 | -0.253275441 | 0.101267126 | 0.656954459 |
| Ca | A0A0A0MS15 | -0.25264792 | 0.102144704 | 0.660400387 |
| DBIL | A0A2R8Y3M9 | -0.252248255 | 0.10270666 | 0.660400387 |
| ALP | E7EWH8 | -0.251553741 | 0.103688819 | 0.660952607 |
| P | A0A0J9YXX1 | -0.251426022 | 0.103870216 | 0.660952607 |
| RBC | A0A669KAY4 | -0.251332114 | 0.104003745 | 0.660952607 |
| AST | C9J8S2 | -0.251303004 | 0.104045165 | 0.660952607 |
| INR | P80748 | -0.251106516 | 0.104325064 | 0.660952607 |
| CO2 | P04211 | -0.250613708 | 0.10502961 | 0.662494466 |
| Mg | P01817 | -0.24978381 | 0.10622429 | 0.667138971 |
| Glucose | P04196 | -0.249650747 | 0.106416802 | 0.667138971 |
| Basophils | P01857 | -0.249342024 | 0.106864479 | 0.667960971 |
| Albumin | A0A140T8Y3 | -0.249168442 | 0.10711682 | 0.667960971 |
| FIB | A0A0J9YX35 | -0.248158662 | 0.108593784 | 0.670363306 |
| Mg | J3QT83 | -0.247918129 | 0.108947879 | 0.670363306 |
| CRP | C9JB55 | -0.247139676 | 0.110099887 | 0.672981067 |
| CO2 | P13473 | -0.246761599 | 0.110662724 | 0.672981067 |
| TBIL | P02751 | -0.246479141 | 0.11108464 | 0.672981067 |
| DBIL | I3L1J2 | -0.246470918 | 0.11109694 | 0.672981067 |
| TBIL | D6RD17 | -0.24595054 | 0.11187751 | 0.672981067 |
| CO2 | C9J8S2 | -0.245939532 | 0.111894067 | 0.672981067 |
| FIB | P68032 | -0.245715701 | 0.11223113 | 0.672981067 |
| Albumin | P01715 | -0.245603361 | 0.112400591 | 0.672981067 |
| AST | Q5SRP5 | -0.245341969 | 0.112795644 | 0.672981067 |
| Albumin | Q16880 | -0.245278768 | 0.112891321 | 0.672981067 |
| WBC | P19823 | -0.245176879 | 0.113045694 | 0.672981067 |
| γ-GT | Q5SRP5 | -0.244789979 | 0.113633352 | 0.672981067 |
| ALT | Q9Y5Y7 | -0.243845605 | 0.115077474 | 0.672981067 |
| CRP | P01814 | -0.243467757 | 0.11565915 | 0.672981067 |
| Albumin | P15814 | -0.242937024 | 0.116479938 | 0.672981067 |
| PT | A0A1W2PQU7 | -0.242681423 | 0.116876799 | 0.672981067 |
| γ-GT | K7ERG9 | -0.242512596 | 0.117139489 | 0.672981067 |
| PT | P02766 | -0.242378828 | 0.117347945 | 0.672981067 |
| Glucose | P01009 | -0.242250332 | 0.11754845 | 0.672981067 |
| P | Q92954 | -0.24175579 | 0.118322546 | 0.672981067 |
| Mg | A0A0C4DH33 | -0.241711434 | 0.118392163 | 0.672981067 |
| DBIL | A0A669KAY4 | -0.241631676 | 0.118517422 | 0.672981067 |
| Platelets | P19823 | -0.241579825 | 0.118598907 | 0.672981067 |
| APTT | P00918 | -0.241530421 | 0.118676585 | 0.672981067 |
| WLC | Q16880 | -0.241525749 | 0.118683933 | 0.672981067 |
| APTT | Q96HR3 | -0.24085194 | 0.11974726 | 0.672981067 |
| Creatinine | P43121 | -0.240826992 | 0.119786767 | 0.672981067 |
| Hematocrit | A0A0C4DH67 | -0.240596813 | 0.120151742 | 0.672981067 |
| IBIL | O14791 | -0.24012686 | 0.120899506 | 0.675994408 |
| Lymphocytes | P09172 | -0.239845266 | 0.12134924 | 0.675994408 |
| BUN | A0A140T8Y3 | -0.239561735 | 0.121803339 | 0.677603381 |
| AST | P61224 | -0.238621022 | 0.123319135 | 0.679850942 |
| Creatinine | P04196 | -0.23853211 | 0.123463132 | 0.679850942 |
| INR | A0A087WZB5 | -0.238336342 | 0.123780631 | 0.679850942 |
| Total protein | P01861 | -0.238293062 | 0.123850906 | 0.679850942 |
| TT | Q6ZRK6 | -0.238032636 | 0.124274401 | 0.681183213 |
| INR | C9J8S2 | -0.237646458 | 0.124904387 | 0.681901448 |
| Creatinine | D6RD17 | -0.237021935 | 0.125928264 | 0.685021541 |
| CRP | P15814 | -0.236897752 | 0.126132605 | 0.685021541 |
| Neutrophils | P02649 | -0.236813292 | 0.126271725 | 0.685021541 |
| BUN | P35527 | -0.236586749 | 0.126645446 | 0.685021541 |
| Lymphocytes | A0A0C4DH33 | -0.236187167 | 0.127306643 | 0.68511067 |
| BUN | P55058 | -0.236133662 | 0.127395375 | 0.68511067 |
| Mg | A0A669KAY4 | -0.236117785 | 0.127421715 | 0.68511067 |
| ALP | P30041 | -0.235345423 | 0.128707953 | 0.68511067 |
| Ca | F8W1S1 | -0.234982925 | 0.129314971 | 0.68511067 |
| RBC | C9JPQ9 | -0.234432243 | 0.130241204 | 0.68511067 |
| Creatinine | A0A075B6R2 | -0.23437913 | 0.1303308 | 0.68511067 |
| Hematocrit | P01009 | -0.234272342 | 0.130511079 | 0.68511067 |
| AST | J3QRV5 | -0.234109806 | 0.130785829 | 0.68511067 |
| APTT | P19652 | -0.234054613 | 0.130879226 | 0.68511067 |
| Creatine Kinase | P08185 | -0.234019258 | 0.13093908 | 0.68511067 |
| ALT | D6RAR4 | -0.233256776 | 0.132234877 | 0.68511067 |
| FIB | E7EWH8 | -0.232658912 | 0.133257587 | 0.68511067 |
| PT | P43121 | -0.23263019 | 0.133306867 | 0.68511067 |
| P | Q15582 | -0.232463302 | 0.133593475 | 0.68511067 |
| IBIL | D6RD17 | -0.231971608 | 0.134440562 | 0.68511067 |
| Ca | P02766 | -0.231921713 | 0.134526743 | 0.68511067 |
| P | P07360 | -0.231783364 | 0.134765925 | 0.68511067 |
| Monocytes | D6RD17 | -0.231723182 | 0.134870066 | 0.68511067 |
| Creatine Kinase | Q5SRP5 | -0.231721762 | 0.134872524 | 0.68511067 |
| Creatine Kinase | C9JPQ9 | -0.23167831 | 0.134947754 | 0.68511067 |
| CK-MB activity | P04004 | -0.231627522 | 0.135035725 | 0.68511067 |
| PT | A0A2R8Y3M9 | -0.231409749 | 0.135413417 | 0.68511067 |
| Lymphocytes | A0A075B6S9 | -0.231371342 | 0.135480108 | 0.68511067 |
| UA | P02649 | -0.231132254 | 0.135895825 | 0.68511067 |
| Platelets | A0A0C4DH33 | -0.230966767 | 0.13618412 | 0.68511067 |
| CK-MB activity | P37802 | -0.230842937 | 0.136400143 | 0.68511067 |
| CRP | P02745 | -0.2305543 | 0.136904662 | 0.68511067 |
| ALT | Q5SRP5 | -0.230544424 | 0.136921949 | 0.68511067 |
| Albumin | A0A5H1ZRS9 | -0.230397365 | 0.137179554 | 0.68511067 |
| Lymphocytes | P19823 | -0.230185406 | 0.137551479 | 0.68511067 |
| IBIL | P04430 | -0.229859163 | 0.1381254 | 0.68511067 |
| Lymphocytes | A0A669KAY4 | -0.229550649 | 0.138669767 | 0.68511067 |
| P | P01601 | -0.22913916 | 0.139398304 | 0.68511067 |
| TT | C9JPQ9 | -0.229120443 | 0.139431509 | 0.68511067 |
| BUN | D6RD17 | -0.22895979 | 0.139716762 | 0.68511067 |
| WLGG | P04430 | -0.228708373 | 0.140164042 | 0.68511067 |
| APTT | D6RAR4 | -0.228692278 | 0.140192712 | 0.68511067 |
| CO2 | E7EWH8 | -0.228453514 | 0.140618527 | 0.68511067 |
| PT | P61224 | -0.22826533 | 0.140954813 | 0.68511067 |
| INR | Q9Y5Y7 | -0.2282649 | 0.140955582 | 0.68511067 |
| LDH | J3QRV5 | -0.228232575 | 0.141013405 | 0.68511067 |
| Basophils | P00915 | -0.228042299 | 0.141354139 | 0.685313478 |
| RBC | A0A140T8Y3 | -0.227731406 | 0.141912174 | 0.686942048 |
| Ca | P13473 | -0.227231842 | 0.142812272 | 0.688440559 |
| Ca | Q9Y5Y7 | -0.227231842 | 0.142812272 | 0.688440559 |
| CK-MB activity | A0A096LPE2 | -0.226694433 | 0.143785261 | 0.691085124 |
| Basophils | F8W1S1 | -0.226555105 | 0.144038314 | 0.691085124 |
| Albumin | P68032 | -0.226480484 | 0.144173979 | 0.691085124 |
| AST | H0YJW9 | -0.226301092 | 0.144500508 | 0.691309313 |
| AST | P02766 | -0.225846215 | 0.14533092 | 0.693663295 |
| Creatine Kinase | A0A075B6R2 | -0.225335097 | 0.146268198 | 0.696362099 |
| Eosinophils | D6RD17 | -0.225260216 | 0.146405885 | 0.696362099 |
| TT | A0A096LPE2 | -0.224280935 | 0.148215348 | 0.699452723 |
| RBC | E7EWH8 | -0.224170486 | 0.148420456 | 0.699452723 |
| Creatinine | E7EWH8 | -0.224032662 | 0.148676696 | 0.699603904 |
| INR | Q96IY4 | -0.223878099 | 0.148964441 | 0.699603904 |
| Hemoglobin | P43121 | -0.223763181 | 0.149178647 | 0.699785586 |
| Platelets | P15169 | -0.223304639 | 0.150035613 | 0.702221475 |
| CO2 | A0A0A0MS15 | -0.22304469 | 0.150523034 | 0.702897994 |
| Mg | H0YJW9 | -0.222577382 | 0.151402186 | 0.705118749 |
| γ-GT | A0A286YEY4 | -0.222517804 | 0.151514541 | 0.705118749 |
| Platelets | I3L1J2 | -0.222345769 | 0.151839315 | 0.70547476 |
| Hemoglobin | A0A140T8Y3 | -0.222239026 | 0.152041085 | 0.70547476 |
| α-HBDH | A0A096LPE2 | -0.222113132 | 0.152279308 | 0.70547476 |
| Hemoglobin | A0A0C4DH21 | -0.221844479 | 0.152788581 | 0.706238084 |
| Hemoglobin | C9JPQ9 | -0.221844479 | 0.152788581 | 0.706238084 |
| γ-GT | P01703 | -0.221684687 | 0.153092081 | 0.706844068 |
| Glucose | Q96IY4 | -0.221106288 | 0.154194356 | 0.70953632 |
| TT | Q96HR3 | -0.22050007 | 0.155355868 | 0.7104881 |
| Lymphocytes | D6RAR4 | -0.220292267 | 0.155755485 | 0.7104881 |
| Total protein | J3QRV5 | -0.219939587 | 0.156435427 | 0.711070124 |
| CK-MB activity | A0A669KAY4 | -0.21983847 | 0.156630774 | 0.711169627 |
| FIB | A0A0B4J1U3 | -0.219552745 | 0.157183722 | 0.711904609 |
| Glucose | P04211 | -0.219293941 | 0.1576858 | 0.711904609 |
| CK-MB activity | O14791 | -0.218649702 | 0.158940698 | 0.712742364 |
| WLL | A0A075B6S9 | -0.21834336 | 0.159539962 | 0.712742364 |
| INR | A0A0C4DH73 | -0.217902974 | 0.16040432 | 0.714069177 |
| Albumin | Q9Y5Y7 | -0.217706625 | 0.160790797 | 0.714238645 |
| IBIL | P01703 | -0.217171336 | 0.161847852 | 0.714772857 |
| RBC | A0A096LPE2 | -0.216985771 | 0.162215472 | 0.714772857 |
| Hematocrit | P09172 | -0.216965559 | 0.16225555 | 0.714772857 |
| CO2 | A0A0C4DH21 | -0.216926634 | 0.162332755 | 0.714772857 |
| Eosinophils | P04430 | -0.216536117 | 0.163108786 | 0.715236389 |
| Hematocrit | P01857 | -0.216373391 | 0.163432949 | 0.715892189 |
| TBIL | A0A2R8Y3M9 | -0.216197849 | 0.163783165 | 0.715896563 |
| Globin | P30041 | -0.215839988 | 0.164498804 | 0.716732303 |
| PT | P04264 | -0.215674794 | 0.164829918 | 0.717412594 |
| PT | H0YAC1 | -0.215523496 | 0.165133601 | 0.71797218 |
| UA | A0A669KAY4 | -0.215416021 | 0.165349571 | 0.718149621 |
| Albumin | A0A0A0MS15 | -0.215289341 | 0.165604395 | 0.718232421 |
| Glucose | Q96HR3 | -0.214838589 | 0.166513414 | 0.719394096 |
| CK-MB activity | P02751 | -0.21447555 | 0.167248168 | 0.721198779 |
| Albumin | A0A1W2PQU7 | -0.214458399 | 0.167282936 | 0.721198779 |
| WLL | E7EWH8 | -0.213943645 | 0.168328919 | 0.723426173 |
| BUN | A0A096LPE2 | -0.213101757 | 0.170049811 | 0.724749162 |
| Hemoglobin | A0A5H1ZRS9 | -0.212852826 | 0.170561071 | 0.724749162 |
| PT | P80748 | -0.21272449 | 0.170825084 | 0.724749162 |
| Total protein | P01009 | -0.212688831 | 0.170898492 | 0.724749162 |
| CO2 | P00748 | -0.212319209 | 0.17166076 | 0.726325199 |
| Eosinophils | Q16880 | -0.212009615 | 0.172301114 | 0.726784534 |
| Mg | P35527 | -0.211664497 | 0.173016972 | 0.72755855 |
| P | P01703 | -0.211460768 | 0.173440559 | 0.728592512 |
| WBC | A0A096LPE2 | -0.211273455 | 0.17383067 | 0.728633975 |
| Hemoglobin | P01009 | -0.211114944 | 0.174161292 | 0.728633975 |
| Albumin | P61626 | -0.210915977 | 0.174576934 | 0.72940246 |
| WLGG | E7EWH8 | -0.210771264 | 0.174879686 | 0.729406624 |
| INR | H0YAC1 | -0.210188256 | 0.176103216 | 0.730053776 |
| Glucose | P01008 | -0.209326035 | 0.177923961 | 0.732813729 |
| UA | P01009 | -0.209310228 | 0.177957466 | 0.732813729 |
| γ-GT | P55058 | -0.209112204 | 0.178377586 | 0.732813729 |
| P | P80748 | -0.207758882 | 0.181267779 | 0.742636176 |
| WBC | A0A669KAY4 | -0.207669748 | 0.181459304 | 0.742636176 |
| CK-MB activity | P04430 | -0.207654056 | 0.181493036 | 0.742636176 |
| ALP | P04430 | -0.207405245 | 0.182028501 | 0.743450975 |
| Globin | J3QRV5 | -0.207016359 | 0.182867676 | 0.743473719 |
| Ca | P13645 | -0.206656921 | 0.183645756 | 0.743473719 |
| FIB | J3KPA1 | -0.206313287 | 0.184391833 | 0.743473719 |
| INR | P13647 | -0.206179628 | 0.184682608 | 0.743473719 |
| Neutrophils | O00187 | -0.206078914 | 0.184901926 | 0.743473719 |
| WLGG | P43121 | -0.205987466 | 0.185101227 | 0.743473719 |
| α-HBDH | D6RD17 | -0.205719068 | 0.185687056 | 0.743473719 |
| TBIL | P01703 | -0.205701342 | 0.185725792 | 0.743473719 |
| BUN | P02751 | -0.205474798 | 0.186221364 | 0.743473719 |
| α-HBDH | K7ERG9 | -0.205341324 | 0.186513784 | 0.743473719 |
| WBC | P04430 | -0.205277653 | 0.186653392 | 0.743473719 |
| RBC | P43121 | -0.205186692 | 0.186852967 | 0.743473719 |
| Basophils | P35527 | -0.205052119 | 0.187148506 | 0.743473719 |
| Ca | P61626 | -0.20496269 | 0.187345087 | 0.743473719 |
| Basophils | P35908 | -0.204883074 | 0.187520222 | 0.743473719 |
| CO2 | P02649 | -0.204841584 | 0.187611533 | 0.743473719 |
| DBIL | P55058 | -0.204715555 | 0.187889099 | 0.743473719 |
| WLC | P15814 | -0.204562358 | 0.18822689 | 0.743473719 |
| APTT | P02747 | -0.204524005 | 0.188311522 | 0.743473719 |
| FIB | P61626 | -0.204230424 | 0.188960266 | 0.743473719 |
| CO2 | A0A0C4DH25 | -0.204086269 | 0.189279393 | 0.743473719 |
| CK-MB activity | Q5SRP5 | -0.203803887 | 0.189905632 | 0.743473719 |
| Glucose | K7ERG9 | -0.203737966 | 0.190052036 | 0.743473719 |
| ALP | P15169 | -0.203580622 | 0.190401806 | 0.743473719 |
| ALT | H0YAC1 | -0.203078613 | 0.191520804 | 0.745984403 |
| PT | C9J8S2 | -0.202185701 | 0.193522638 | 0.748530959 |
| Globin | P55056 | -0.202031652 | 0.193869494 | 0.748998676 |
| FIB | J3QT83 | -0.201969782 | 0.194008925 | 0.748998676 |
| Hemoglobin | P01861 | -0.200989889 | 0.196226658 | 0.751851719 |
| WBC | J3QT83 | -0.200638712 | 0.197025793 | 0.751851719 |
| UA | P68032 | -0.200466154 | 0.197419302 | 0.751851719 |
| WLC | P35908 | -0.200455314 | 0.19744404 | 0.751851719 |
| LDH | P37802 | -0.200450445 | 0.197455153 | 0.751851719 |
| ALT | Q96HR3 | -0.200431406 | 0.19749861 | 0.751851719 |
| Albumin | P01019 | -0.200256857 | 0.197897337 | 0.752670763 |
| Hematocrit | P04196 | -0.200060424 | 0.198346732 | 0.753006438 |
| WLGG | A0A087WZB5 | -0.199903998 | 0.198705113 | 0.753645664 |
| FIB | A0A075B6S9 | -0.199397618 | 0.199868383 | 0.754124338 |
| P | A0A0A0MS15 | -0.199297429 | 0.200099106 | 0.754124338 |
| WLGG | P01715 | -0.199009043 | 0.20076427 | 0.754124338 |
| Creatine Kinase | P04004 | -0.198905042 | 0.20100453 | 0.754124338 |
| Ca | A0A0B4J1V2 | -0.198532785 | 0.20186616 | 0.754124338 |
| INR | P61224 | -0.198396282 | 0.20218276 | 0.754124338 |
| RBC | P01009 | -0.19825536 | 0.202509975 | 0.754124338 |
| LDH | P01861 | -0.197862004 | 0.203425294 | 0.754804907 |
| PT | Q96IY4 | -0.197367779 | 0.204579439 | 0.756335168 |
| TT | K7ERG9 | -0.197058704 | 0.205303535 | 0.758328371 |
| Basophils | P04004 | -0.196853415 | 0.205785473 | 0.758345751 |
| Mg | P43121 | -0.196480973 | 0.206661841 | 0.759666216 |
| AST | P15814 | -0.1960518 | 0.207674928 | 0.760238575 |
| Basophils | Q86YZ3 | -0.195645258 | 0.208637788 | 0.761397481 |
| Hematocrit | P08185 | -0.195151429 | 0.209811566 | 0.763967514 |
| ALT | P04196 | -0.195061357 | 0.210026151 | 0.764070293 |
| WBC | A0A075B6R2 | -0.194586023 | 0.211161114 | 0.765417279 |
| TBIL | P04430 | -0.194555908 | 0.211233165 | 0.765417279 |
| Albumin | A0A0B4J1V2 | -0.194409837 | 0.21158288 | 0.765417279 |
| INR | P43121 | -0.194365165 | 0.211689913 | 0.765417279 |
| Hematocrit | Q6ZRK6 | -0.194201415 | 0.212082572 | 0.765417279 |
| P | O14791 | -0.19408457 | 0.212363065 | 0.765417279 |
| TBIL | P55058 | -0.193996603 | 0.212574405 | 0.765417279 |
| CO2 | P01703 | -0.193587382 | 0.21355948 | 0.765417279 |
| Hemoglobin | Q9Y5Y7 | -0.193509438 | 0.213747466 | 0.765417279 |
| TT | P07360 | -0.192824135 | 0.215405232 | 0.769365459 |
| Creatine Kinase | P04430 | -0.192816776 | 0.215423081 | 0.769365459 |
| α-HBDH | E7EX29 | -0.192802553 | 0.215457583 | 0.769365459 |
| PT | Q9Y5Y7 | -0.192601903 | 0.215944726 | 0.769889894 |
| Monocytes | P55058 | -0.192258418 | 0.216780417 | 0.770384197 |
| Monocytes | P02766 | -0.192107212 | 0.217149007 | 0.770729717 |
| TT | C9J8S2 | -0.191503468 | 0.218625064 | 0.772070289 |
| Monocytes | A0A1W2PQU7 | -0.191502388 | 0.218627709 | 0.772070289 |
| TT | P00748 | -0.191236171 | 0.21928077 | 0.773710119 |
| UA | J3KPA1 | -0.190888342 | 0.220136067 | 0.774484517 |
| IBIL | P55058 | -0.190666768 | 0.220682108 | 0.774484517 |
| γ-GT | Q9Y5Y7 | -0.190632169 | 0.220767457 | 0.774484517 |
| Ca | P02745 | -0.190620586 | 0.220796034 | 0.774484517 |
| ALP | A0A0J9YX35 | -0.190557146 | 0.220952603 | 0.774484517 |
| Total protein | P61626 | -0.190339521 | 0.221490275 | 0.774484517 |
| APTT | P01008 | -0.190098568 | 0.222086635 | 0.774484517 |
| Creatinine | Q9Y5Y7 | -0.190055499 | 0.222193347 | 0.774484517 |
| WLL | A0A075B6R2 | -0.189871779 | 0.222648949 | 0.774484517 |
| CO2 | Q9Y5Y7 | -0.189659741 | 0.223175575 | 0.774484517 |
| DBIL | P02763 | -0.189450681 | 0.223695645 | 0.774484517 |
| APTT | P02763 | -0.189343309 | 0.223963074 | 0.774484517 |
| Creatine Kinase | C9JB55 | -0.189246338 | 0.224204788 | 0.774484517 |
| WLC | P19823 | -0.189239926 | 0.224220776 | 0.774484517 |
| RBC | P55058 | -0.189041206 | 0.224716697 | 0.774484517 |
| ALP | P08185 | -0.188925839 | 0.225004949 | 0.774484517 |
| CK-MB activity | A0A0J9YX35 | -0.188830614 | 0.225243068 | 0.774484517 |
| TT | P04196 | -0.1887408 | 0.225467813 | 0.774484517 |
| P | P35908 | -0.188720614 | 0.225518348 | 0.774484517 |
| Mg | P37802 | -0.188657557 | 0.225676255 | 0.774484517 |
| RBC | P01715 | -0.188519951 | 0.226021111 | 0.774484517 |
| Glucose | E7EWH8 | -0.187839547 | 0.2277316 | 0.77792749 |
| α-HBDH | P08185 | -0.187587383 | 0.228367773 | 0.77792749 |
| Hemoglobin | P19823 | -0.18754019 | 0.228486971 | 0.77792749 |
| Lymphocytes | P43121 | -0.187403548 | 0.228832331 | 0.77792749 |
| Hematocrit | A0A5H1ZRS9 | -0.187221514 | 0.229292974 | 0.77792749 |
| UA | A0A0C4DH38 | -0.186959641 | 0.229956766 | 0.77792749 |
| INR | P01857 | -0.186892832 | 0.230126323 | 0.77792749 |
| APTT | P30041 | -0.186774884 | 0.230425876 | 0.77792749 |
| Total protein | P01715 | -0.186770846 | 0.230436137 | 0.77792749 |
| Creatinine | P19823 | -0.186204553 | 0.231878115 | 0.77792749 |
| Neutrophils | Q9Y5Y7 | -0.186143101 | 0.232034964 | 0.77792749 |
| ALT | A0A140T8Y3 | -0.186050115 | 0.232272435 | 0.77792749 |
| Platelets | P01817 | -0.185772544 | 0.2329823 | 0.778573261 |
| P | P19652 | -0.185396471 | 0.233946434 | 0.779441534 |
| Platelets | D6RAR4 | -0.184941854 | 0.235115563 | 0.779932595 |
| ALP | P13473 | -0.184846673 | 0.235360842 | 0.779932595 |
| RBC | P19823 | -0.184811758 | 0.235450858 | 0.779932595 |
| Hematocrit | Q5SRP5 | -0.184287135 | 0.236806276 | 0.781727642 |
| IBIL | A0A2R8Y3M9 | -0.184021748 | 0.237493948 | 0.782737288 |
| Ca | A0A0C4DH73 | -0.183585779 | 0.238626579 | 0.784504381 |
| α-HBDH | P61626 | -0.183439131 | 0.239008388 | 0.784575174 |
| WBC | I3L1J2 | -0.183270916 | 0.23944686 | 0.785385702 |
| DBIL | P01019 | -0.183027344 | 0.240082728 | 0.786213406 |
| FIB | A0A0G2JI36 | -0.182681302 | 0.240988068 | 0.788038417 |
| Hemoglobin | J3QT83 | -0.182600916 | 0.241198712 | 0.788038417 |
| CO2 | A0A2R8Y3M9 | -0.182182117 | 0.242298148 | 0.788430482 |
| PT | A0A0C4DH73 | -0.181481526 | 0.244144928 | 0.790541998 |
| BUN | P30041 | -0.181355698 | 0.24447762 | 0.790541998 |
| CRP | A0A0G2JI36 | -0.181278309 | 0.244682389 | 0.790541998 |
| Platelets | P61626 | -0.180606428 | 0.246465048 | 0.79272903 |
| CRP | P68032 | -0.180285818 | 0.247318782 | 0.793463041 |
| DBIL | P61224 | -0.180203317 | 0.247538792 | 0.793463041 |
| ALP | P55058 | -0.180163185 | 0.247645862 | 0.793463041 |
| Platelets | P01715 | -0.180062344 | 0.247915036 | 0.793463041 |
| LDH | P02766 | -0.180032515 | 0.247994698 | 0.793463041 |
| WBC | P04433 | -0.179937328 | 0.248249017 | 0.793463041 |
| Basophils | P00918 | -0.17988964 | 0.248376494 | 0.793463041 |
| CRP | D6RAR4 | -0.179806678 | 0.248598371 | 0.793463041 |
| Total protein | P01019 | -0.179682788 | 0.248929956 | 0.793463041 |
| CRP | P55058 | -0.179580126 | 0.249204949 | 0.793463041 |
| Total protein | J3QT83 | -0.179484239 | 0.24946198 | 0.793463041 |
| Eosinophils | C9JPQ9 | -0.179450996 | 0.249551131 | 0.793463041 |
| Platelets | A0A075B6R2 | -0.179278057 | 0.250015267 | 0.793463041 |
| P | P04264 | -0.179201479 | 0.250220972 | 0.793463041 |
| Neutrophils | P04211 | -0.1788182 | 0.251252258 | 0.793463041 |
| ALT | A0A0C4DH38 | -0.178799941 | 0.251301458 | 0.793463041 |
| Albumin | P02766 | -0.178727923 | 0.251495581 | 0.793463041 |
| Basophils | P13645 | -0.17868103 | 0.251622033 | 0.793463041 |
| Globin | P01861 | -0.178467587 | 0.252198148 | 0.793734738 |
| WLC | A0A075B6S9 | -0.178289126 | 0.252680518 | 0.793851715 |
| WLC | E7EWH8 | -0.178256024 | 0.25277006 | 0.793851715 |
| Mg | P02751 | -0.177637515 | 0.254447046 | 0.7945686 |
| P | J3KPA1 | -0.177536701 | 0.254721091 | 0.7945686 |
| Globin | J3QT83 | -0.177528312 | 0.254743905 | 0.7945686 |
| Total protein | A0A0A0MS15 | -0.177341398 | 0.255252552 | 0.7945686 |
| Total protein | A0A087WZB5 | -0.177183232 | 0.255683498 | 0.7945686 |
| TT | A0A0C4DH73 | -0.177171352 | 0.255715885 | 0.7945686 |
| INR | P19652 | -0.177135982 | 0.25581233 | 0.7945686 |
| CRP | A0A1W2PQU7 | -0.177012538 | 0.256149124 | 0.794975705 |
| FIB | P00915 | -0.17668834 | 0.257035045 | 0.794975705 |
| TBIL | O14791 | -0.176628281 | 0.257199388 | 0.794975705 |
| RBC | A0A5H1ZRS9 | -0.176428389 | 0.257746878 | 0.794975705 |
| BUN | P37802 | -0.176384594 | 0.257866932 | 0.794975705 |
| DBIL | D6RD17 | -0.175999456 | 0.258924316 | 0.794975705 |
| CRP | P04211 | -0.175955296 | 0.259045742 | 0.794975705 |
| Platelets | P01857 | -0.175955296 | 0.259045742 | 0.794975705 |
| Lymphocytes | H0YJW9 | -0.17565986 | 0.259859062 | 0.796279638 |
| WLL | P01715 | -0.175506919 | 0.26028077 | 0.796296881 |
| CRP | A0A0B4J1V2 | -0.175093457 | 0.2614231 | 0.79808988 |
| TT | P13473 | -0.174751598 | 0.262370118 | 0.799222652 |
| AST | A0A0J9YXX1 | -0.174748414 | 0.262378949 | 0.799222652 |
| Neutrophils | D6RAR4 | -0.173834247 | 0.26492263 | 0.801229841 |
| P | P04004 | -0.173686425 | 0.265335476 | 0.801229841 |
| CO2 | P15814 | -0.173495988 | 0.265867971 | 0.801229841 |
| Platelets | P08185 | -0.173463225 | 0.265959654 | 0.801229841 |
| CO2 | A0A0G2JI36 | -0.173392762 | 0.266156905 | 0.801229841 |
| α-HBDH | P01861 | -0.173384231 | 0.266180794 | 0.801229841 |
| Hematocrit | A0A669KAY4 | -0.173322944 | 0.266352446 | 0.801229841 |
| Globin | P01008 | -0.173180777 | 0.266750911 | 0.801229841 |
| Hematocrit | C9JPQ9 | -0.172721098 | 0.268041998 | 0.802161494 |
| Ca | A0A075B6R2 | -0.172314959 | 0.269186145 | 0.802161494 |
| PT | P02649 | -0.172252369 | 0.269362753 | 0.802161494 |
| FIB | P37802 | -0.171818378 | 0.270589455 | 0.802161494 |
| Neutrophils | A0A669KAY4 | -0.171736528 | 0.270821222 | 0.802161494 |
| P | P02745 | -0.17172216 | 0.270861921 | 0.802161494 |
| DBIL | P55056 | -0.171314198 | 0.272019172 | 0.802161494 |
| CO2 | H0YAC1 | -0.171305573 | 0.272043674 | 0.802161494 |
| UA | P09172 | -0.171280306 | 0.27211546 | 0.802161494 |
| ALT | Q6ZRK6 | -0.171274345 | 0.272132397 | 0.802161494 |
| P | O00187 | -0.171268867 | 0.272147961 | 0.802161494 |
| Glucose | P32119 | -0.170964698 | 0.273013175 | 0.803556366 |
| ALT | P19652 | -0.169950706 | 0.275910565 | 0.809962627 |
| FIB | P32119 | -0.169587568 | 0.276953089 | 0.810497977 |
| Mg | O75636 | -0.169377068 | 0.277558594 | 0.81058835 |
| Hemoglobin | P01715 | -0.169355328 | 0.277621176 | 0.81058835 |
| AST | H0YAC1 | -0.169062457 | 0.278465186 | 0.81058835 |
| γ-GT | E7EWH8 | -0.168839872 | 0.279107766 | 0.81058835 |
| Neutrophils | P08185 | -0.16862375 | 0.279732615 | 0.81058835 |
| LDH | A0A0B4J1V2 | -0.168517703 | 0.280039554 | 0.81058835 |
| α-HBDH | P01814 | -0.16824692 | 0.280824291 | 0.81058835 |
| α-HBDH | P02766 | -0.168095823 | 0.281262801 | 0.81058835 |
| CK-MB activity | P01814 | -0.167952722 | 0.281678516 | 0.81058835 |
| CRP | A0A0C4DH38 | -0.167874945 | 0.281904629 | 0.81058835 |
| TT | Q9Y5Y7 | -0.167870423 | 0.281917778 | 0.81058835 |
| Eosinophils | Q5SRP5 | -0.167818003 | 0.282070248 | 0.81058835 |
| Ca | P01019 | -0.167549444 | 0.282852215 | 0.811542396 |
| CRP | A0A087WZB5 | -0.167229057 | 0.283786942 | 0.811961443 |
| PT | P13647 | -0.166805654 | 0.2850253 | 0.812617395 |
| Eosinophils | P55058 | -0.166654701 | 0.285467654 | 0.812617395 |
| DBIL | Q9Y5Y7 | -0.166477803 | 0.285986607 | 0.812617395 |
| Globin | P01009 | -0.166458976 | 0.286041872 | 0.812617395 |
| Total protein | A0A0J9YXX1 | -0.166087621 | 0.28713342 | 0.813577762 |
| WLC | P04433 | -0.166019334 | 0.287334433 | 0.813585066 |
| AST | P09172 | -0.165589864 | 0.288600748 | 0.814297159 |
| PT | P37802 | -0.165400827 | 0.289159279 | 0.814297159 |
| DBIL | P02751 | -0.165193135 | 0.289773736 | 0.814297159 |
| WLL | P43121 | -0.165146193 | 0.289912731 | 0.814297159 |
| LDH | D6RD17 | -0.164998327 | 0.290350846 | 0.814297159 |
| TT | P02747 | -0.164996966 | 0.29035488 | 0.814297159 |
| ALT | A0A669KAY4 | -0.164947829 | 0.290500565 | 0.814297159 |
| Creatinine | P55056 | -0.164911089 | 0.290609525 | 0.814297159 |
| CK-MB activity | A0A075B6S9 | -0.16484824 | 0.290795978 | 0.814297159 |
| P | A0A1W2PQU7 | -0.164771681 | 0.29102321 | 0.814297159 |
| Albumin | A0A0C4DH21 | -0.164753001 | 0.29107867 | 0.814297159 |
| UA | P55056 | -0.164684562 | 0.291281921 | 0.814297159 |
| Globin | J3KPA1 | -0.164564855 | 0.291637653 | 0.814297159 |
| LDH | Q6ZRK6 | -0.164514414 | 0.291787632 | 0.814297159 |
| Globin | D6RAR4 | -0.164193201 | 0.292743883 | 0.815614594 |
| Glucose | P00918 | -0.164136848 | 0.292911855 | 0.815614594 |
| CO2 | A0A0G2JRQ6 | -0.16390348 | 0.293608117 | 0.816684722 |
| Lymphocytes | Q5SRP5 | -0.163675984 | 0.294287889 | 0.817766976 |
| CRP | A0A0C4DH25 | -0.163494942 | 0.294829579 | 0.817862837 |
| FIB | O75636 | -0.163393279 | 0.295134045 | 0.817939008 |
| WBC | P01715 | -0.163133196 | 0.295913873 | 0.817939008 |
| Ca | P35527 | -0.163086502 | 0.296054021 | 0.817939008 |
| LDH | K7ERG9 | -0.162807415 | 0.296892563 | 0.818719605 |
| WLGG | A0A140T8Y3 | -0.162533975 | 0.297715621 | 0.81976766 |
| Basophils | P43121 | -0.161982714 | 0.29937938 | 0.820557066 |
| Albumin | P01009 | -0.161882476 | 0.299682548 | 0.820557066 |
| RBC | P04196 | -0.161776374 | 0.300003669 | 0.820557066 |
| INR | P02763 | -0.161555277 | 0.30067353 | 0.821423865 |
| Eosinophils | I3L1J2 | -0.161484552 | 0.300888008 | 0.821423865 |
| TT | O00187 | -0.161291718 | 0.301473294 | 0.821423865 |
| Glucose | A0A140T8Y3 | -0.160665062 | 0.303380334 | 0.823178955 |
| CK-MB activity | I3L1J2 | -0.160540548 | 0.303760173 | 0.823178955 |
| Neutrophils | Q15582 | -0.160468191 | 0.303981045 | 0.823178955 |
| WLC | P43121 | -0.160059723 | 0.305229818 | 0.824571058 |
| Basophils | A0A0J9YX35 | -0.159923024 | 0.305648468 | 0.824684208 |
| Total protein | A0A1W2PQU7 | -0.159894267 | 0.305736584 | 0.824684208 |
| ALT | A0A286YEY4 | -0.159437511 | 0.307138344 | 0.82629082 |
| Basophils | P07360 | -0.159071759 | 0.308263766 | 0.82714754 |
| Albumin | P01861 | -0.158332091 | 0.310547749 | 0.829411982 |
| P | P35527 | -0.158274494 | 0.31072605 | 0.829411982 |
| FIB | A0A0C4DH33 | -0.157600491 | 0.312817368 | 0.831745272 |
| UA | P04211 | -0.157133688 | 0.314270996 | 0.834238841 |
| Neutrophils | O75636 | -0.156919012 | 0.314940935 | 0.834238841 |
| APTT | Q86YZ3 | -0.156907774 | 0.314976031 | 0.834238841 |
| Albumin | P13647 | -0.156519128 | 0.316191256 | 0.834896683 |
| AST | Q96HR3 | -0.156022662 | 0.317747918 | 0.835503882 |
| LDH | A0A0C4DH38 | -0.155932485 | 0.318031188 | 0.835503882 |
| WLC | P08779 | -0.155909689 | 0.31810282 | 0.835503882 |
| Ca | D6RD17 | -0.155673479 | 0.318845671 | 0.836174065 |
| ALT | D6RD17 | -0.155580152 | 0.319139474 | 0.836174065 |
| INR | P35908 | -0.155504517 | 0.319377704 | 0.836174065 |
| UA | P55058 | -0.155396987 | 0.319716591 | 0.836527135 |
| Platelets | Q86YZ3 | -0.155029181 | 0.320877463 | 0.837702456 |
| α-HBDH | P02751 | -0.1550259 | 0.320887829 | 0.837702456 |
| Monocytes | P09172 | -0.154818762 | 0.321542779 | 0.837702456 |
| α-HBDH | P55058 | -0.154572608 | 0.322322183 | 0.837702456 |
| Creatinine | P68032 | -0.154547966 | 0.322400273 | 0.837702456 |
| Ca | P01703 | -0.154463189 | 0.322669019 | 0.837702456 |
| Creatine Kinase | P01008 | -0.154124977 | 0.323742569 | 0.837702456 |
| CK-MB activity | J3QT83 | -0.154027444 | 0.324052573 | 0.837702456 |
| ALP | O75636 | -0.154026304 | 0.324056199 | 0.837702456 |
| INR | P02649 | -0.15368929 | 0.325128823 | 0.837702456 |
| Hematocrit | A0A140T8Y3 | -0.153351998 | 0.326204554 | 0.837702456 |
| Creatinine | P15169 | -0.153282743 | 0.326425707 | 0.837702456 |
| Hemoglobin | P55056 | -0.1532359 | 0.326575341 | 0.837702456 |
| LDH | P01715 | -0.153213994 | 0.326645336 | 0.837702456 |
| WLC | J3KPA1 | -0.153157917 | 0.326824548 | 0.837702456 |
| Total protein | E7EX29 | -0.152935087 | 0.327537291 | 0.838273709 |
| BUN | P01814 | -0.152765717 | 0.328079684 | 0.838470813 |
| Hemoglobin | P09172 | -0.152736018 | 0.32817485 | 0.838470813 |
| FIB | C9JB55 | -0.152672346 | 0.32837894 | 0.838470813 |
| ALP | P55056 | -0.152591042 | 0.328639658 | 0.838470813 |
| RBC | J3QT83 | -0.152423298 | 0.329177968 | 0.838856174 |
| Albumin | A0A0G2JI36 | -0.152346769 | 0.329423744 | 0.838856174 |
| INR | I3L1J2 | -0.152267941 | 0.329677019 | 0.838856174 |
| Hematocrit | P01861 | -0.151952273 | 0.330692489 | 0.838882222 |
| APTT | P02649 | -0.151882487 | 0.330917246 | 0.838882222 |
| WLGG | D6RD17 | -0.151783899 | 0.331234927 | 0.838882222 |
| α-HBDH | J3QRV5 | -0.151777307 | 0.331256175 | 0.838882222 |
| Lymphocytes | P01817 | -0.151493414 | 0.332172061 | 0.839128436 |
| FIB | P55058 | -0.151306859 | 0.332774772 | 0.839379532 |
| Glucose | A0A0B4J1V2 | -0.151110105 | 0.333411175 | 0.839403158 |
| Globin | P19823 | -0.150976177 | 0.333844796 | 0.839403158 |
| Neutrophils | A0A075B6R2 | -0.150802342 | 0.334408148 | 0.839704725 |
| APTT | P02766 | -0.150447496 | 0.335559938 | 0.840287718 |
| WBC | P43121 | -0.150080589 | 0.336753457 | 0.840287718 |
| UA | Q96HR3 | -0.149884849 | 0.337391259 | 0.840287718 |
| Total protein | P04264 | -0.149773421 | 0.337754673 | 0.840287718 |
| ALP | Q15582 | -0.149644977 | 0.338173881 | 0.840635009 |
| ALT | P01601 | -0.149605027 | 0.338304333 | 0.840635009 |
| PT | P01857 | -0.149482074 | 0.338706017 | 0.841123363 |
| Creatinine | A0A075B6S9 | -0.149226513 | 0.339541871 | 0.842178871 |
| INR | P02747 | -0.14915122 | 0.339788371 | 0.842280726 |
| TT | P13647 | -0.148890479 | 0.34064286 | 0.842305751 |
| FIB | A0A1W2PQU7 | -0.148511875 | 0.341885962 | 0.842305751 |
| Creatine Kinase | A0A669KAY4 | -0.14840891 | 0.342224518 | 0.842305751 |
| WLGG | A0A669KAY4 | -0.148400176 | 0.342253246 | 0.842305751 |
| P | P02751 | -0.148377616 | 0.342327456 | 0.842305751 |
| CO2 | P19652 | -0.148343979 | 0.342438121 | 0.842305751 |
| Hemoglobin | Q6ZRK6 | -0.148289011 | 0.342619016 | 0.842305751 |
| Platelets | O75636 | -0.148240449 | 0.342778876 | 0.842305751 |
| Ca | A0A0B4J1U3 | -0.147980232 | 0.343636259 | 0.843162723 |
| RBC | Q6ZRK6 | -0.147889444 | 0.343935706 | 0.843162723 |
| Ca | P00918 | -0.147860648 | 0.344030718 | 0.843162723 |
| Creatine Kinase | P09172 | -0.147784033 | 0.344283585 | 0.843227419 |
| CRP | P01601 | -0.147334241 | 0.345770418 | 0.844144089 |
| RBC | P08779 | -0.147124358 | 0.346465554 | 0.844144089 |
| Basophils | Q8N1N4 | -0.14698501 | 0.346927551 | 0.844144089 |
| CO2 | J3QT83 | -0.14697679 | 0.346954815 | 0.844144089 |
| TT | Q96IY4 | -0.146924429 | 0.34712852 | 0.844144089 |
| Total protein | D6RAR4 | -0.146676744 | 0.347950922 | 0.844404882 |
| Hematocrit | P55058 | -0.146665663 | 0.347987741 | 0.844404882 |
| Mg | A0A087WZB5 | -0.146560689 | 0.348336676 | 0.844404882 |
| APTT | P02745 | -0.146520152 | 0.348471478 | 0.844404882 |
| Albumin | A0A0C4DH25 | -0.146321212 | 0.349133497 | 0.845125503 |
| UA | P80748 | -0.14626043 | 0.349335916 | 0.845125503 |
| UA | J3QRV5 | -0.146184921 | 0.349587479 | 0.845125503 |
| APTT | P43121 | -0.146113635 | 0.349825073 | 0.845125503 |
| FIB | P04433 | -0.146094591 | 0.349888565 | 0.845125503 |
| Eosinophils | P01703 | -0.146059481 | 0.350005635 | 0.845125503 |
| Albumin | P02649 | -0.145943511 | 0.350392497 | 0.845303538 |
| Globin | A0A140T8Y3 | -0.145723913 | 0.351125761 | 0.845425068 |
| BUN | E7EX29 | -0.145541552 | 0.3517354 | 0.845425068 |
| APTT | A0A0C4DH67 | -0.145468557 | 0.351979605 | 0.845425068 |
| Eosinophils | A0A075B6S9 | -0.145433116 | 0.352098208 | 0.845425068 |
| α-HBDH | E7EWH8 | -0.145254141 | 0.352697529 | 0.845425068 |
| CRP | P15169 | -0.145219757 | 0.35281274 | 0.845425068 |
| Creatinine | A0A140T8Y3 | -0.145086371 | 0.353259893 | 0.845425068 |
| WLL | J3KPA1 | -0.145076123 | 0.353294261 | 0.845425068 |
| Eosinophils | O14791 | -0.145075151 | 0.353297522 | 0.845425068 |
| γ-GT | O00187 | -0.145037983 | 0.353422191 | 0.845425068 |
| Monocytes | A0A0C4DH21 | -0.145006546 | 0.353527658 | 0.845425068 |
| PT | P02747 | -0.144943144 | 0.35374042 | 0.845425068 |
| INR | Q96HR3 | -0.144915688 | 0.35383258 | 0.845425068 |
| γ-GT | P01861 | -0.144659294 | 0.354693915 | 0.845425068 |
| Creatine Kinase | P01019 | -0.144081557 | 0.356639438 | 0.845425068 |
| Hemoglobin | A0A0C4DH67 | -0.143944411 | 0.357102223 | 0.845425068 |
| CO2 | P55056 | -0.143887617 | 0.357293975 | 0.845425068 |
| Total protein | P13473 | -0.143655596 | 0.358077995 | 0.845425068 |
| AST | Q6ZRK6 | -0.143488976 | 0.35864166 | 0.845425068 |
| Basophils | P01019 | -0.143350533 | 0.359110409 | 0.845425068 |
| Glucose | A0A0C4DH38 | -0.143250898 | 0.359447993 | 0.845425068 |
| ALP | A0A075B6S9 | -0.142931404 | 0.360531793 | 0.845425068 |
| Basophils | P01817 | -0.142843397 | 0.360830679 | 0.845425068 |
| UA | A0A140T8Y3 | -0.142743831 | 0.361169003 | 0.845425068 |
| Monocytes | H0YAC1 | -0.142662853 | 0.361444305 | 0.845425068 |
| LDH | A0A1W2PQU7 | -0.142635916 | 0.361535912 | 0.845425068 |
| Neutrophils | I3L1J2 | -0.142591275 | 0.361687759 | 0.845425068 |
| PT | P02763 | -0.142522382 | 0.361922169 | 0.845425068 |
| BUN | A0A669KAY4 | -0.142489962 | 0.362032511 | 0.845425068 |
| FIB | P02745 | -0.142468665 | 0.362105007 | 0.845425068 |
| γ-GT | D6RAR4 | -0.142462896 | 0.362124647 | 0.845425068 |
| Total protein | Q6ZRK6 | -0.142411282 | 0.362300387 | 0.845425068 |
| Basophils | J3KPA1 | -0.142153862 | 0.363177635 | 0.845425068 |
| LDH | Q5SRP5 | -0.141890684 | 0.364075831 | 0.845425068 |
| FIB | P15169 | -0.141864345 | 0.364165797 | 0.845425068 |
| Hematocrit | A0A0C4DH21 | -0.141832192 | 0.364275639 | 0.845425068 |
| WBC | H0YJW9 | -0.141503379 | 0.365400084 | 0.845425068 |
| Basophils | A0A1W2PQU7 | -0.141491033 | 0.365442343 | 0.845425068 |
| CO2 | A0A1W2PQU7 | -0.141395076 | 0.365770906 | 0.845425068 |
| UA | Q6ZRK6 | -0.141335325 | 0.365975586 | 0.845425068 |
| ALT | P02766 | -0.141209598 | 0.366406493 | 0.845425068 |
| Total protein | J3KPA1 | -0.141158336 | 0.366582272 | 0.845425068 |
| LDH | P08185 | -0.141124942 | 0.366696808 | 0.845425068 |
| WLC | D6RD17 | -0.141061147 | 0.366915677 | 0.845425068 |
| Creatinine | I3L1J2 | -0.139709208 | 0.371572314 | 0.845425068 |
| WLC | Q8N1N4 | -0.139639478 | 0.371813445 | 0.845425068 |
| Lymphocytes | O75636 | -0.139636751 | 0.371822879 | 0.845425068 |
| Eosinophils | P15169 | -0.139623475 | 0.371868798 | 0.845425068 |
| γ-GT | P13473 | -0.139584858 | 0.372002391 | 0.845425068 |
| PT | D6RAR4 | -0.139496429 | 0.372308412 | 0.845425068 |
| Monocytes | A0A0G2JI36 | -0.139233337 | 0.37321977 | 0.845425068 |
| ALT | K7ERG9 | -0.139167467 | 0.373448153 | 0.845425068 |
| ALT | Q86YZ3 | -0.13915651 | 0.373486153 | 0.845425068 |
| BUN | P61224 | -0.139148076 | 0.373515402 | 0.845425068 |
| CK-MB activity | Q16880 | -0.139113122 | 0.37363664 | 0.845425068 |
| WLC | P04211 | -0.138533736 | 0.375649669 | 0.847602684 |
| APTT | A0A087WZB5 | -0.137960911 | 0.377646226 | 0.849997457 |
| INR | A0A0B4J1V2 | -0.137735612 | 0.378433219 | 0.849997457 |
| Neutrophils | E7EWH8 | -0.137093275 | 0.380682299 | 0.85218879 |
| TT | Q16880 | -0.137018562 | 0.380944411 | 0.85218879 |
| Creatinine | P01861 | -0.136897346 | 0.381369894 | 0.85218879 |
| INR | P37802 | -0.136886936 | 0.381406446 | 0.85218879 |
| ALP | P19823 | -0.136803157 | 0.381700705 | 0.85238175 |
| Albumin | C9JPQ9 | -0.136727616 | 0.381966139 | 0.852510163 |
| Monocytes | P19823 | -0.13653901 | 0.38262934 | 0.852744986 |
| CK-MB activity | E7EWH8 | -0.136294602 | 0.383489769 | 0.852923323 |
| Total protein | P37802 | -0.136168007 | 0.383935892 | 0.852923323 |
| Creatine Kinase | P01009 | -0.136077026 | 0.384256699 | 0.852923323 |
| γ-GT | C9JPQ9 | -0.136025179 | 0.384439585 | 0.852923323 |
| LDH | A0A0A0MS15 | -0.135912083 | 0.384838701 | 0.85334704 |
| Creatinine | H0YJW9 | -0.135764715 | 0.385359132 | 0.853577764 |
| Globin | A0A0C4DH38 | -0.135568903 | 0.38605128 | 0.853637363 |
| Albumin | P13473 | -0.135518974 | 0.386227883 | 0.853637363 |
| Hemoglobin | P08185 | -0.135403715 | 0.386635751 | 0.853638437 |
| BUN | K7ERG9 | -0.135321881 | 0.386925487 | 0.853818351 |
| WLGG | A0A5H1ZRS9 | -0.135234505 | 0.387234986 | 0.854041659 |
| IBIL | O75636 | -0.135165748 | 0.387478637 | 0.854119577 |
| APTT | A0A0G2JRQ6 | -0.134662593 | 0.389264382 | 0.856679689 |
| Creatinine | A0A096LPE2 | -0.134556575 | 0.389641264 | 0.856808801 |
| PT | Q96HR3 | -0.134503607 | 0.389829639 | 0.856808801 |
| Glucose | D6RD17 | -0.134340193 | 0.390411138 | 0.856808801 |
| WBC | Q5SRP5 | -0.134177061 | 0.390992141 | 0.856808801 |
| INR | P36955 | -0.13417559 | 0.390997382 | 0.856808801 |
| PT | P19652 | -0.134049714 | 0.391446047 | 0.857333756 |
| WBC | E7EWH8 | -0.133775525 | 0.392424387 | 0.857455298 |
| γ-GT | P01009 | -0.133677306 | 0.392775192 | 0.857496425 |
| Eosinophils | P01817 | -0.133566058 | 0.393172751 | 0.857631122 |
| WLL | A0A5H1ZRS9 | -0.133478913 | 0.393484339 | 0.857631122 |
| AST | K7ERG9 | -0.133278834 | 0.394200269 | 0.857631122 |
| TT | A0A0B4J1V2 | -0.132789006 | 0.395956194 | 0.857631122 |
| Creatine Kinase | A0A140T8Y3 | -0.132703598 | 0.396262826 | 0.857631122 |
| LDH | E7EX29 | -0.132663769 | 0.396405869 | 0.857631122 |
| BUN | A0A0A0MS15 | -0.132603361 | 0.396622874 | 0.857631122 |
| Platelets | A0A0C4DH67 | -0.13251123 | 0.396953976 | 0.857631122 |
| FIB | P09172 | -0.132460847 | 0.397135108 | 0.857631122 |
| Glucose | A0A286YEY4 | -0.132452332 | 0.397165725 | 0.857631122 |
| AST | O00187 | -0.132369081 | 0.397465144 | 0.857631122 |
| TT | A0A075B6R2 | -0.132330289 | 0.397604708 | 0.857631122 |
| WLL | P35908 | -0.132215207 | 0.398018906 | 0.857631122 |
| APTT | P15814 | -0.132094714 | 0.39845285 | 0.857631122 |
| TT | P80748 | -0.132027819 | 0.398693883 | 0.857631122 |
| ALT | A0A075B6S9 | -0.1318359 | 0.399385871 | 0.858138836 |
| Mg | O14791 | -0.131788242 | 0.399557814 | 0.858138836 |
| AST | P37802 | -0.131629963 | 0.400129172 | 0.85877212 |
| Mg | P04433 | -0.13156089 | 0.400378661 | 0.85877212 |
| Creatine Kinase | P02649 | -0.130942044 | 0.402617924 | 0.858950137 |
| α-HBDH | Q5SRP5 | -0.130851311 | 0.402946842 | 0.858950137 |
| WLGG | A0A075B6R2 | -0.13061041 | 0.403820891 | 0.858950137 |
| WLGG | P04433 | -0.13061041 | 0.403820891 | 0.858950137 |
| TT | A0A286YEY4 | -0.130364239 | 0.404715191 | 0.859596373 |
| FIB | P35527 | -0.130155626 | 0.405473938 | 0.859596373 |
| WLL | A0A087WZB5 | -0.130154538 | 0.405477899 | 0.859596373 |
| γ-GT | P04196 | -0.129966151 | 0.406163789 | 0.860160916 |
| γ-GT | J3QRV5 | -0.129890413 | 0.406439727 | 0.86030092 |
| CO2 | P32119 | -0.12946109 | 0.408005933 | 0.861836334 |
| Glucose | A0A087WZB5 | -0.129383716 | 0.408288567 | 0.86198925 |
| APTT | P13645 | -0.128998154 | 0.409698633 | 0.863609342 |
| TT | P19652 | -0.12892751 | 0.409957293 | 0.863609342 |
| WLL | Q8N1N4 | -0.128740017 | 0.410644242 | 0.863626732 |
| Creatine Kinase | P68032 | -0.128677997 | 0.410871617 | 0.863626732 |
| ALP | A0A096LPE2 | -0.128644824 | 0.410993268 | 0.863626732 |
| CK-MB activity | P35527 | -0.128488006 | 0.41156861 | 0.863626732 |
| DBIL | P19652 | -0.128315619 | 0.412201606 | 0.863626732 |
| UA | C9JB55 | -0.128294193 | 0.412280319 | 0.863626732 |
| Total protein | P02745 | -0.127945625 | 0.413562077 | 0.864222486 |
| WLC | P01857 | -0.127792237 | 0.414126836 | 0.864425087 |
| Monocytes | P15169 | -0.12769346 | 0.414490756 | 0.864425087 |
| INR | A0A0B4J1U3 | -0.127690308 | 0.414502371 | 0.864425087 |
| Mg | P68032 | -0.127630868 | 0.414721457 | 0.864442284 |
| APTT | A0A140T8Y3 | -0.127432841 | 0.415451815 | 0.865244329 |
| ALT | P02747 | -0.127292852 | 0.415968564 | 0.865363385 |
| TT | P01019 | -0.127037077 | 0.416913657 | 0.866635275 |
| Hemoglobin | E7EWH8 | -0.126724661 | 0.418069694 | 0.867101284 |
| TBIL | Q9Y5Y7 | -0.126713235 | 0.41811201 | 0.867101284 |
| RBC | A0A0C4DH67 | -0.126395676 | 0.419289004 | 0.868077524 |
| INR | P01601 | -0.126233969 | 0.419889074 | 0.868077524 |
| Mg | Q15582 | -0.126180232 | 0.420088591 | 0.868077524 |
| Platelets | P04430 | -0.126129619 | 0.420276557 | 0.868077524 |
| APTT | P32119 | -0.125826068 | 0.421404883 | 0.868130708 |
| Albumin | A0A0C4DH73 | -0.125774299 | 0.421597486 | 0.868130708 |
| WBC | Q9Y5Y7 | -0.125571035 | 0.422354188 | 0.868130708 |
| Hematocrit | P04433 | -0.125519224 | 0.422547191 | 0.868130708 |
| Hemoglobin | P04196 | -0.12550534 | 0.422598917 | 0.868130708 |
| α-HBDH | J3QT83 | -0.125407039 | 0.422965263 | 0.868130708 |
| TBIL | O75636 | -0.125202946 | 0.423726442 | 0.868130708 |
| Ca | A0A286YEY4 | -0.124962384 | 0.424624623 | 0.868130708 |
| P | A0A0C4DH25 | -0.124957524 | 0.424642781 | 0.868130708 |
| APTT | P01009 | -0.124919758 | 0.424783887 | 0.868130708 |
| CO2 | A0A0C4DH67 | -0.124891391 | 0.424889895 | 0.868130708 |
| WBC | P04211 | -0.12466493 | 0.425736703 | 0.868130708 |
| WBC | O75636 | -0.124589421 | 0.426019265 | 0.868130708 |
| Ca | O00187 | -0.124508526 | 0.426322101 | 0.86831625 |
| Albumin | Q8N1N4 | -0.124036876 | 0.42809015 | 0.870080546 |
| TBIL | P61224 | -0.123635209 | 0.429599093 | 0.871534055 |
| TT | A0A0C4DH21 | -0.123634298 | 0.429602518 | 0.871534055 |
| ALP | A0A075B6R2 | -0.123130395 | 0.431499742 | 0.871755386 |
| UA | P61224 | -0.123002287 | 0.431982819 | 0.871755386 |
| γ-GT | A0A075B6R2 | -0.122998269 | 0.431997977 | 0.871755386 |
| CRP | J3QT83 | -0.122548479 | 0.433696492 | 0.871755386 |
| RBC | P08185 | -0.122427405 | 0.434154333 | 0.871755386 |
| ALT | P09172 | -0.122309102 | 0.434601953 | 0.871755386 |
| Neutrophils | Q5SRP5 | -0.1221497 | 0.435205485 | 0.871942711 |
| α-HBDH | A0A0J9YX35 | -0.121562423 | 0.437433063 | 0.872737498 |
| Albumin | A0A286YEY4 | -0.121468512 | 0.437789855 | 0.873024517 |
| α-HBDH | A0A0B4J1V2 | -0.121260216 | 0.438581806 | 0.873329482 |
| Albumin | O00187 | -0.121015272 | 0.439514112 | 0.874509844 |
| INR | A0A0A0MS15 | -0.12086392 | 0.440090732 | 0.874634998 |
| APTT | C9JB55 | -0.120732631 | 0.440591252 | 0.875062008 |
| UA | Q5SRP5 | -0.120597324 | 0.441107423 | 0.875062008 |
| ALP | A0A286YEY4 | -0.120562031 | 0.441242115 | 0.875062008 |
| UA | P01008 | -0.120511949 | 0.441433283 | 0.875062008 |
| DBIL | A0A140T8Y3 | -0.120471905 | 0.441586169 | 0.875062008 |
| UA | P13473 | -0.119983388 | 0.443453656 | 0.875380095 |
| P | Q96IY4 | -0.119895762 | 0.443789089 | 0.875470971 |
| APTT | A0A1W2PQU7 | -0.119859526 | 0.443927841 | 0.875470971 |
| CRP | P55056 | -0.11946836 | 0.445427185 | 0.875675323 |
| α-HBDH | P02649 | -0.11944247 | 0.445526518 | 0.875675323 |
| Glucose | A0A2R8Y3M9 | -0.119312819 | 0.446024143 | 0.876233343 |
| WLL | P01009 | -0.119104261 | 0.446825272 | 0.876790688 |
| Hematocrit | P43121 | -0.119004571 | 0.447208486 | 0.876790688 |
| Globin | P09172 | -0.118893143 | 0.447637034 | 0.876790688 |
| Total protein | Q16880 | -0.118882181 | 0.447679207 | 0.876790688 |
| DBIL | A0A096LPE2 | -0.118793965 | 0.448018657 | 0.876790688 |
| DBIL | K7ERG9 | -0.118793965 | 0.448018657 | 0.876790688 |
| CO2 | D6RAR4 | -0.118509014 | 0.449116103 | 0.878376006 |
| WLL | P04433 | -0.118472408 | 0.449257191 | 0.878376006 |
| Creatine Kinase | A0A075B6S9 | -0.118376755 | 0.449625978 | 0.878485095 |
| P | P02766 | -0.118233691 | 0.45017786 | 0.878734026 |
| Creatine Kinase | P37802 | -0.117684385 | 0.452300291 | 0.880964938 |
| γ-GT | P35527 | -0.117545143 | 0.452839164 | 0.881177301 |
| PT | P13473 | -0.117482622 | 0.453081239 | 0.881230114 |
| α-HBDH | Q6ZRK6 | -0.117183796 | 0.454239222 | 0.883063448 |
| APTT | A0A0B4J1U3 | -0.11685614 | 0.45551077 | 0.883842698 |
| CO2 | P01008 | -0.11684732 | 0.455545026 | 0.883842698 |
| PT | E7EX29 | -0.116807933 | 0.455698012 | 0.883842698 |
| CRP | Q6ZRK6 | -0.116802809 | 0.455717918 | 0.883842698 |
| γ-GT | A0A0C4DH38 | -0.116484814 | 0.45695412 | 0.883936921 |
| Mg | A0A0J9YX35 | -0.116332714 | 0.457546048 | 0.883936921 |
| ALP | C9JB55 | -0.116298405 | 0.457679625 | 0.883936921 |
| Platelets | P36955 | -0.116296633 | 0.457686523 | 0.883936921 |
| Basophils | H0YAC1 | -0.11621874 | 0.45798987 | 0.883936921 |
| FIB | P15814 | -0.116180704 | 0.458138038 | 0.883936921 |
| γ-GT | P04433 | -0.115954649 | 0.459019158 | 0.883977258 |
| RBC | A0A075B6S9 | -0.115777494 | 0.459710309 | 0.884288927 |
| WLGG | P15169 | -0.115764631 | 0.459760518 | 0.884288927 |
| INR | Q8N1N4 | -0.11564514 | 0.460227045 | 0.884288927 |
| PT | I3L1J2 | -0.115640358 | 0.460245718 | 0.884288927 |
| APTT | Q96IY4 | -0.115479027 | 0.460876022 | 0.884662157 |
| ALP | P09172 | -0.115466968 | 0.460923151 | 0.884662157 |
| Total protein | A0A0G2JI36 | -0.115169559 | 0.462086376 | 0.884892173 |
| Neutrophils | A0A140T8Y3 | -0.11494429 | 0.462968494 | 0.884992806 |
| Platelets | P04004 | -0.114937322 | 0.462995793 | 0.884992806 |
| FIB | P01715 | -0.114935653 | 0.463002334 | 0.884992806 |
| Glucose | Q16880 | -0.114555409 | 0.464493413 | 0.887015834 |
| Albumin | A0A075B6R2 | -0.114443281 | 0.464933602 | 0.887343259 |
| PT | A0A0B4J1V2 | -0.113893603 | 0.467094737 | 0.889910977 |
| WLGG | P02751 | -0.113655394 | 0.468032948 | 0.89010742 |
| Globin | P36955 | -0.113439829 | 0.468882843 | 0.89010742 |
| WLGG | Q8N1N4 | -0.113330896 | 0.469312636 | 0.89010742 |
| Neutrophils | P04264 | -0.113196149 | 0.46984457 | 0.89010742 |
| APTT | A0A0C4DH73 | -0.113137725 | 0.470075308 | 0.89010742 |
| ALT | P43121 | -0.113052333 | 0.470412661 | 0.89010742 |
| WLL | D6RD17 | -0.112943696 | 0.470842033 | 0.89010742 |
| CK-MB activity | C9J8S2 | -0.112881361 | 0.471088493 | 0.89010742 |
| γ-GT | P04430 | -0.112657535 | 0.471974032 | 0.89010742 |
| Basophils | A0A087WZB5 | -0.112507216 | 0.472569245 | 0.890679088 |
| TT | I3L1J2 | -0.112470291 | 0.472715519 | 0.890679088 |
| LDH | P15814 | -0.112416442 | 0.472928872 | 0.890679088 |
| WLC | A0A140T8Y3 | -0.112080576 | 0.474260764 | 0.891880844 |
| Basophils | P04433 | -0.111992604 | 0.474609947 | 0.891880844 |
| P | Q8N1N4 | -0.111812053 | 0.475327028 | 0.891880844 |
| Platelets | A0A5H1ZRS9 | -0.111765596 | 0.475511631 | 0.891880844 |
| IBIL | P01715 | -0.111762291 | 0.475524762 | 0.891880844 |
| FIB | P01814 | -0.111648297 | 0.4759779 | 0.892322538 |
| ALP | A0A0G2JRQ6 | -0.111497216 | 0.476578807 | 0.892459649 |
| APTT | P68032 | -0.111494502 | 0.476589608 | 0.892459649 |
| Creatine Kinase | C9J8S2 | -0.111332773 | 0.477233319 | 0.892459649 |
| Platelets | P35527 | -0.111236975 | 0.477614831 | 0.892459649 |
| Globin | P00918 | -0.111227115 | 0.477654105 | 0.892459649 |
| Eosinophils | P01715 | -0.111225339 | 0.477661179 | 0.892459649 |
| AST | P01601 | -0.111141508 | 0.477995182 | 0.892459649 |
| CRP | A0A075B6S9 | -0.111070665 | 0.47827753 | 0.892459649 |
| Hemoglobin | C9J8S2 | -0.110989635 | 0.478600589 | 0.892459649 |
| Creatinine | P09172 | -0.110850421 | 0.479155889 | 0.892459649 |
| TT | A0A140T8Y3 | -0.110787421 | 0.479407295 | 0.892459649 |
| Mg | A0A0C4DH67 | -0.110651359 | 0.479950501 | 0.892459649 |
| Basophils | A0A0C4DH67 | -0.110536089 | 0.480410949 | 0.892459649 |
| RBC | Q9Y5Y7 | -0.110343269 | 0.48118169 | 0.892459649 |
| UA | D6RD17 | -0.110318269 | 0.481281665 | 0.892459649 |
| PT | P35908 | -0.110220335 | 0.481673421 | 0.892459649 |
| Hemoglobin | A0A075B6S9 | -0.110136177 | 0.482010202 | 0.892459649 |
| CK-MB activity | P01861 | -0.110121735 | 0.482068006 | 0.892459649 |
| CK-MB activity | Q92954 | -0.110121735 | 0.482068006 | 0.892459649 |
| Hematocrit | P01019 | -0.109734917 | 0.483617639 | 0.892459649 |
| TBIL | A0A096LPE2 | -0.109722485 | 0.483667486 | 0.892459649 |
| Hematocrit | C9J8S2 | -0.109704335 | 0.483740264 | 0.892459649 |
| FIB | A0A0C4DH25 | -0.109684254 | 0.483820795 | 0.892459649 |
| Total protein | P19823 | -0.109667679 | 0.483887268 | 0.892459649 |
| Glucose | P01019 | -0.109269398 | 0.485486001 | 0.894201529 |
| WBC | O00187 | -0.109185638 | 0.485822572 | 0.894419643 |
| PT | P01601 | -0.109085602 | 0.486224702 | 0.89455189 |
| CK-MB activity | D6RAR4 | -0.109059223 | 0.486330771 | 0.89455189 |
| IBIL | P61224 | -0.108897771 | 0.486980224 | 0.894658247 |
| CK-MB activity | C9JPQ9 | -0.108679755 | 0.487857929 | 0.895320233 |
| TBIL | P13647 | -0.108514254 | 0.48852476 | 0.895320233 |
| Mg | E7EX29 | -0.108506971 | 0.488554115 | 0.895320233 |
| CRP | Q16880 | -0.108140765 | 0.490031347 | 0.895320233 |
| PT | P04004 | -0.108102168 | 0.490187181 | 0.895320233 |
| Creatine Kinase | J3QRV5 | -0.10775911 | 0.491573362 | 0.895529282 |
| TBIL | A0A0C4DH21 | -0.10775911 | 0.491573362 | 0.895529282 |
| Basophils | A0A0C4DH25 | -0.107681945 | 0.491885436 | 0.895529282 |
| Ca | P61224 | -0.107681846 | 0.49188584 | 0.895529282 |
| CRP | Q9Y5Y7 | -0.107612144 | 0.492167818 | 0.895644942 |
| LDH | A0A096LPE2 | -0.107505778 | 0.492598289 | 0.895714434 |
| Monocytes | J3QRV5 | -0.107356254 | 0.493203748 | 0.895714434 |
| TT | P61224 | -0.107332902 | 0.49329834 | 0.895714434 |
| APTT | P04264 | -0.107095657 | 0.494259886 | 0.896854778 |
| DBIL | A0A0C4DH73 | -0.106929683 | 0.494933149 | 0.897093683 |
| α-HBDH | P01019 | -0.10682584 | 0.495354619 | 0.897223938 |
| Monocytes | P01008 | -0.106675827 | 0.495963805 | 0.897223938 |
| Globin | O75636 | -0.106642502 | 0.496099187 | 0.897223938 |
| WLC | A0A5H1ZRS9 | -0.106467204 | 0.496811643 | 0.897229723 |
| Creatinine | P01009 | -0.106391815 | 0.497118204 | 0.897229723 |
| Globin | P13473 | -0.106264873 | 0.497634619 | 0.897625138 |
| Total protein | P04211 | -0.105966772 | 0.498848408 | 0.898448932 |
| INR | P13645 | -0.105812656 | 0.499476525 | 0.898448932 |
| RBC | P01008 | -0.105811718 | 0.499480348 | 0.898448932 |
| TBIL | P02763 | -0.10572022 | 0.49985345 | 0.898448932 |
| CK-MB activity | A0A087WZB5 | -0.105696567 | 0.499949922 | 0.898448932 |
| Albumin | P01703 | -0.105529547 | 0.500631413 | 0.898448932 |
| Globin | P08185 | -0.105509614 | 0.500712776 | 0.898448932 |
| Platelets | P01601 | -0.105346626 | 0.501378331 | 0.898448932 |
| INR | P13473 | -0.10520758 | 0.501946473 | 0.898538809 |
| FIB | A0A0C4DH38 | -0.105151846 | 0.502174291 | 0.898538809 |
| APTT | F8W1S1 | -0.105072328 | 0.502499428 | 0.898538809 |
[truncated: 646,584 more chars]
